# Role of inflammatory cytokines and the gut microbiome in vascular dementia: insights from Mendelian randomization analysis

**DOI:** 10.3389/fmicb.2024.1398618

**Published:** 2024-08-23

**Authors:** Yihan Yang, Ting Rao, Sheng Wei, Jing Cheng, Ying Zhan, Teng Lin, Jincheng Chen, Xiaoling Zhong, Yijing Jiang, Shanli Yang

**Affiliations:** ^1^The Institution of Rehabilitation Industry, Fujian University of Traditional Chinese Medicine, Fuzhou, China; ^2^Fujian Rehabilitation Hospital, Fujian University of Traditional Chinese Medicine Subsidiary Rehabilitation Hospital, Fuzhou, China; ^3^Department of General Practice, The Second Affiliated Hospital of Wannan Medical College, Anhui, China; ^4^The First Clinical Medical College, Fujian University of Traditional Chinese Medicine, Fuzhou, China; ^5^Guangdong Provincial Hospital of Chinese Medicine, The Second Clinical School of Guangzhou University of Chinese Medicine, Guangzhou, China

**Keywords:** Mendelian randomization study, vascular dementia, inflammatory cytokines, gut microbiome, genome-wide association study

## Abstract

**Background:**

Both inflammatory cytokines and the gut microbiome are susceptibility factors for vascular dementia (VaD). The trends in the overall changes in the dynamics of inflammatory cytokines and in the composition of the gut microbiome are influenced by a variety of factors, making it difficult to fully explain the different effects of both on the different subtypes of VaD. Therefore, this Mendelian randomization (MR) study identified the inflammatory cytokines and gut microbiome members that influence the risk of developing VaD and their causal effects, and investigated whether inflammatory cytokines are gut microbiome mediators affecting VaD.

**Methods:**

We obtained pooled genome-wide association study (GWAS) data for 196 gut microbiota and 41 inflammatory cytokines and used GWAS data for six VaD subtypes, namely, VaD (mixed), VaD (multiple infarctions), VaD (other), VaD (subcortical), VaD (sudden onset), and VaD (undefined). We used the inverse-variance weighted (IVW) method as the primary MR analysis method. We conducted sensitivity analyses and reverse MR analyses to examine reverse causal associations, enhancing the reliability and stability of the conclusions. Finally, we used multivariable MR (MVMR) analysis to assess the direct causal effects of inflammatory cytokines and the gut microbiome on the risk of VaD, and performed mediation MR analysis to explore whether inflammatory factors were potential mediators.

**Results:**

Our two-sample MR study revealed relationships between the risk of six VaD subtypes and inflammatory cytokines and the gut microbiota: 7 inflammatory cytokines and 14 gut microbiota constituents were positively correlated with increased VaD subtype risk, while 2 inflammatory cytokines and 11 gut microbiota constituents were negatively correlated with decreased VaD subtype risk. After Bonferroni correction, interleukin-18 was correlated with an increased risk of VaD (multiple infarctions); macrophage migration inhibitory factor was correlated with an increased risk of VaD (sudden onset); interleukin-4 was correlated with a decreased risk of VaD (other); *Ruminiclostridium 6* and *Bacillales* were positively and negatively correlated with the risk of VaD (undefined), respectively; *Negativicutes* and *Selenomonadales* were correlated with a decreased risk of VaD (mixed); and *Melainabacteria* was correlated with an increased risk of VaD (multiple infarctions). Sensitivity analyses revealed no multilevel effects or heterogeneity and no inverse causality between VaD and inflammatory cytokines or the gut microbiota. The MVMR results further confirmed that the causal effects of *Negativicutes*, *Selenomonadales*, and *Melainabacteria* on VaD remain significant. Mediation MR analysis showed that inflammatory cytokines were not potential mediators.

**Conclusion:**

This study helps us to better understand the pathological mechanisms of VaD and suggests the potential value of targeting increases or decreases in inflammatory cytokines and gut microbiome members for VaD prevention and intervention.

## 1 Introduction

Vasculsar dementia (VaD) is defined as cognitive dysfunction and neurological dysfunction caused by cerebrovascular disease and/or reduced cerebral blood flow (O’Brien and Thomas, 2015; Song et al., 2023). VaD is the second most common form of dementia and accounts for approximately 20% of all dementia cases. An individual’s risk of VaD doubles approximately every 5.3 years (Wolters and Ikram, 2019; Zhang et al., 2024). VaD affects the ability of patients to live independently and creates a significant socioeconomic burden. With increasing life expectancy and a globally aging population, there is an urgent need for an in-depth study of the pathogenesis of VaD to provide a theoretical basis for new therapeutic approaches.

Neuroinflammation plays a crucial role in the pathophysiological process of VaD onset and progression (Tian et al., 2022). When the expression of inflammatory cytokines is elevated *in vivo*, multiple neuropathological pathways for the onset and development of VaD are initiated (Gao et al., 2023; Liu et al., 2023a), and the release of inflammatory mediators in combination with factors such as oxidative stress increases the permeability of the blood–brain barrier (BBB), allowing immune cells to reach the brain (Zhu et al., 2018; Tashiro et al., 2023; Wang et al., 2023a). Microglia are activated in the brain, triggering processes such as oxidative stress, synaptic disruption, and inhibition of neurogenesis, exacerbating the disruption of the BBB and ultimately leading to neuronal destruction and impaired brain function (Tashiro et al., 2023; Liu et al., 2023a; Wang et al., 2023a). Compared to those of controls, autopsy studies of brain tissue from VaD patients who had undergone *in vivo* serum testing revealed increased hippocampal tumor necrosis factor-alpha (TNF-α), interleukin-1β (IL-1β), transforming growth factor beta (TGF-β), inducible nitric oxide synthase, interleukin-23, and interleukin-17 *in vivo* (Belkhelfa et al., 2018; Dubenko et al., 2021). Gao et al. reduced TNF-α, chemokine ligand 9, interleukin-6 (IL-6), and antiangiogenic factors in the cerebrospinal fluid and brain tissues of rats, which attenuated neuronal damage, preserved the integrity of cerebral white matter in VaD rats, and ultimately restored VaD rat cognitive function (Gao et al., 2023). Although the role of inflammatory cytokines in VaD has been partially elucidated, the inflammatory process changes over time, so we need to understand the overall trend of the dynamics of inflammatory cytokines in VaD and its subtypes while analyzing novel inflammatory cytokines critical for VaD and determining their causal relationships.

There is a network of bidirectional communication between the central nervous system and the gut, with the gut microbiome acting as a key node in the communication between the networks (Fung, 2020). Alterations and imbalances in its composition and metabolites (a decrease in dominant genera and an increase in potentially pathogenic bacteria) induce increased intestinal barrier permeability and immune activation, triggering systemic inflammation, which in turn may compromise the BBB, promote apoptosis and neurological damage, and ultimately lead to the development of cognitively dysfunctional diseases (Fung et al., 2017; Angoorani et al., 2022; Pei et al., 2023). The possibility that the gut microbiome is a major risk factor for VaD susceptibility has been confirmed by several studies (Alkasir et al., 2017). Liu et al. reduced neuronal apoptosis in VaD mice by accelerating the rate of butyric acid production in the feces and brain and the content of butyrate in the brain by administering VaD mice a 6-week gavage of *Clostridium butyricum* (Liu et al., 2015). Treatment with probiotics in mice with common carotid artery obstruction not only improved the gut microbiome imbalance but also significantly reduced the number of damaged neuronal cells and apoptotic cells in the hippocampus, ultimately improving spatial learning and memory abilities (Rahmati et al., 2019). However, because of the shortage of randomized controlled trials (RCTs), the existence of mostly basic experimental research, and the presence of limitations such as the difficulty of controlling for confounding factors and the unclear temporal sequence of causal events, evidence for the role of the gut microbiome in VaD and its subtypes is needed.

Mendelian randomization is a method that uses single-nucleotide polymorphisms (SNPs) as instrumental variables (IVs) to infer the causal relationship between exposure and outcome after removing the effects of confounders (Li et al., 2023b). As Mendelian randomization studies can reduce confounding factors, limiting our ability to make causal inferences and interpret the results of observational studies (confounding variables, reverse causal associations, regression dilution bias) and RCTs (representativeness, feasibility, and ethical issues), Mendelian randomization studies have become more common for exploring the underlying biological mechanisms of disease onset, finding new therapeutic targets, and detecting causality between exposures and disease risk, among other goals (Amin et al., 2023; Gong et al., 2023). To date, no animal model or clinical trial has revealed the relationship between the gut microbiome or inflammatory cytokines and the pathogenesis of the various subtypes of VaD. Mendelian randomization studies provide us with an accurate and stable method to do so. Therefore, the present study aimed to investigate the association between inflammatory cytokines and the gut microbiome on the pathogenesis of different subtypes of VaD by using Mendelian randomization based on the existing research and to provide another perspective with new evidence on the etiology and pathological mechanisms of different subtypes of VaD. In addition, this study used mediation MR to determine whether inflammatory cytokines are a mediating factor for the gut microbiome to affect VaD.

## 2 Materials and methods

### 2.1 Study design

**Figure 1:**
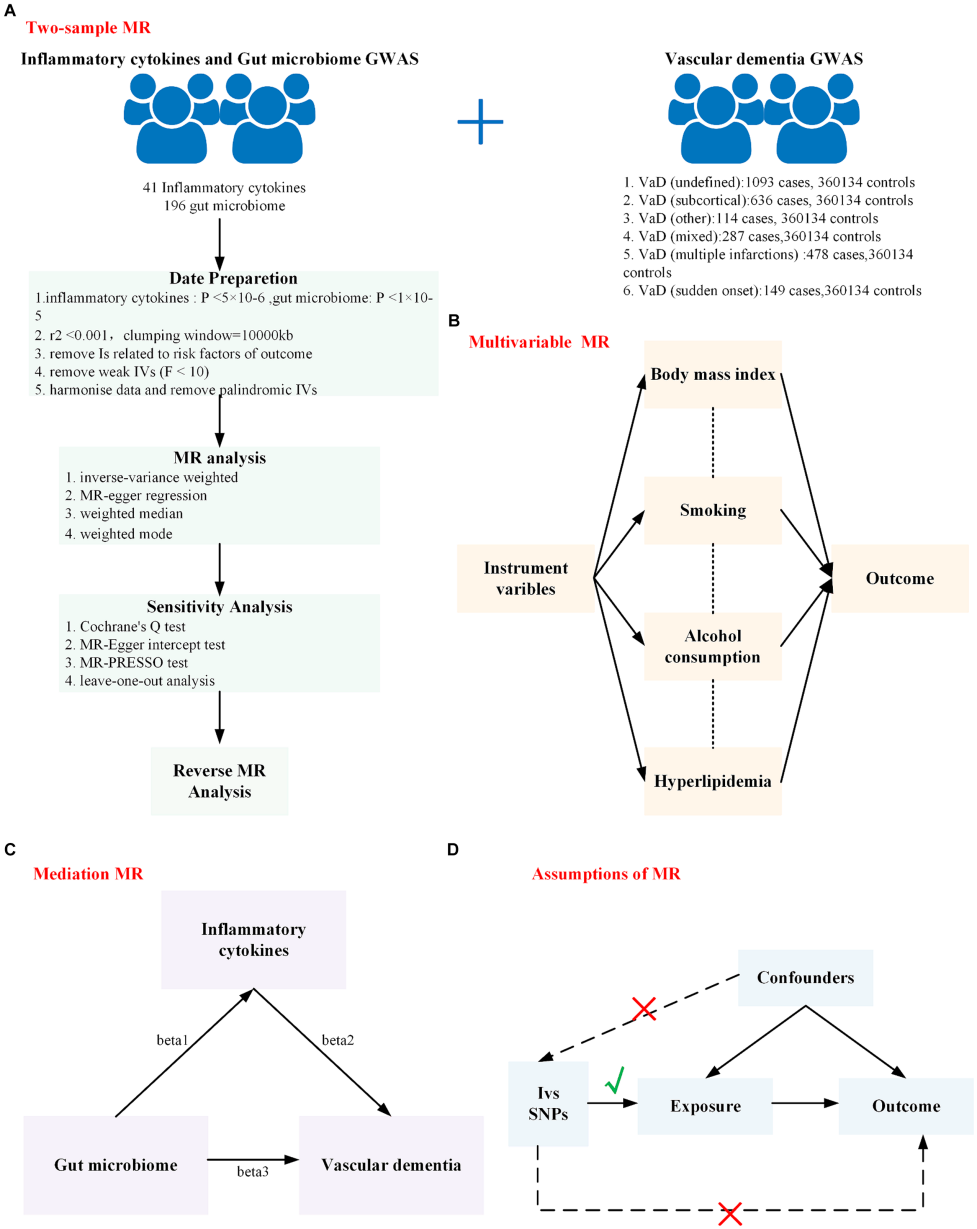
The overall design of this study is shown in this figure. **(A)** Workflow of the two-sample Mendelian randomization study; **(B)** Workflow of the multivariable Mendelian randomization study; **(C)** Workflow of the mediation Mendelian randomization study; **(D)** The assumption of Mendelian randomization: (1) There is a strong correlation between genetic instrumental variables and exposure factors. (2) Instrumental variables are independent of any confounders that may affect the exposure and outcomes. (3) Genetic variation can influence the outcome only through exposure factors and not through other factors. MR, Mendelian randomization; GWAS, genome-wide association study.

The overall design of this study is shown in Figure 1. In this study, the TwoSampleMR (version 0.5.6) and MR-PRESSO (version 1.0) packages in R (version 4.2.1) were used for analyses.

### 2.2 Data sources

Inflammatory cytokine dataset: Inflammatory cytokine summary data were obtained from a meta-analysis of summary statistics of inflammatory cytokine GWASs published by the University of Bristol.^1^ The data contained information on 41 inflammatory cytokines (Ahola-Olli et al., 2017). This is the most recent, largest, and most commonly used dataset.

Gut microbiota dataset: The gut microbiota summary data were obtained from the MiBioGen consortium,^2^ which contains 211 taxa (35 families, 20 orders, 16 phyla, 9 classes, and 131 genera) (Kurilshikov et al., 2021). This dataset is the most widely used dataset of a single gut microbiota that we are aware of.

Vascular dementia dataset: Data for six VaD subtypes were obtained from FinnGen Research.^3^ The Finnish database has a total sample of 377,277 (210,870 females and 166,407 males) with 20,175,454 variants and 2,272 available phenotypes (Kurki et al., 2023). This dataset is the most detailed and largest sample size dataset available for VaD classification. The six subtypes of VaD selected for this study were VaD (undefined), VaD (subcortical), VaD (other), VaD (mixed), VaD (multiple infarctions), and VaD (sudden onset). Except for the diagnostic criteria for VaD (multiple infarctions), which were based on the ICD-9 and ICD-10, the diagnostic criteria for other vascular dementias were based on the ICD-10.

To prevent population stratification bias from confounding the study results, all SNPs and their accompanying summary data in this study were limited to people of European ancestry. In addition, sample-specific information on the inflammatory cytokines, gut microbiota, and VaD datasets used in this study is presented in Table 1.

**Table 1:** Details of genome-wide association study data.

Phenotypes	Date type	Population	Consortium	Sample size (case/control)
Inflammatory cytokine	Exposure	Europeans	University of Bristol	8,293
Gut microbiota	Exposure	Europeans	MiBioGen	18,340
VaD (undefined)	Outcome	Europeans	FinnGen Biobank	1,093/360,143
VaD (subcortical)	Outcome	Europeans	FinnGen Biobank	636/360,143
VaD (other)	Outcome	Europeans	FinnGen Biobank	114/360,143
VaD (mixed)	Outcome	Europeans	FinnGen Biobank	287/360,143
VaD (multiple infarctions)	Outcome	Europeans	FinnGen Biobank	478/360,143
VaD (sudden onset)	Outcome	Europeans	FinnGen Biobank	149/360,143

VaD, vascular dementia.

### 2.3 Selection of instrumental variables

In two-sample MR analysis, the choice of IVs largely affects the reliability of causal relationships. First, we eliminated 3 unknown families and 12 unknown genera in the gut microbiota. Moreover, to identify SNPs that were highly predictive of inflammatory cytokine and gut microbiome levels, we initially used a genome-wide significance threshold of *p* < 5 × 10^−8^. However, the number of SNPs screened was limited; for inflammatory cytokines, we used a locus-wide significance threshold (*p* < 5 × 10^−6^) to rescreen for SNPs associated with exposure; for the gut microbiota, we used a locus-wide significance threshold (*p* < 1 × 10^−5^) to rescreen for exposure-related SNPs. The linkage disequilibrium correlation coefficient was set to *r*^2^ < 0.001, and the clustering distance was set to 10,000 kb to ensure that there was no linkage disequilibrium among the included IVs. To prevent potential multiple effects, we used a PhenoScanner V2^4^ to further exclude IVs associated with confounding factors or risk factors for VaD. Finally, we calculated the F statistic for each SNP and each group of IVs using the formula used to calculate the F statistic in the study of Liu et al. (2023b). Since SNPs with *F* statistics less than 10 did not have sufficient validity, we deleted them.

### 2.4 Statistical analysis

Since the statistical power of the inverse-variance weighted (IVW) method is significantly greater than that of other MR methods, IVW was used as the main method in this study (Lin et al., 2021). In addition, MR–Egger, the weighted median, and the weighted mode served as supplements to the IVW method to provide more stable and accurate results. When more than 50% of the SNPs are invalid instruments, the weighted median and weighted mode methods are more robust than the IVW and MR–Egger methods are (Li et al., 2023a). In this study, the MR method was used to follow a consistent beta value direction. The results of the IVW method were significant, but the results of the other methods were not significant. Moreover, on the premise that no pleiotropy or heterogeneity is found, as long as the beta of other methods is consistent, this can also be regarded as a positive result (Wang et al., 2023b). Finally, the Bonferroni-corrected *p*-value (*p* < 0.05/*N*, *N* = number of test methods) was considered to indicate statistical significance (Wu et al., 2020), and the Bonferroni-corrected *p*-value in this study was 0.0125. The results with *p*-values between 0.0125 and 0.05 are suggestive results.

### 2.5 Sensitivity analysis

First, Cochrane’s *Q* test was used to assess heterogeneity among the IVs. If the test results showed heterogeneity (*p* < 0.05), the random effects IVW model was used; if not, the fixed-effects IVW model was used (Barili et al., 2018). Next, we used the MR–Egger intercept and Mendelian randomization pleiotropy residual sum and outlier (MR-PRESSO) test to measure the level of pleiotropy. A *p*-value <0.05 in the MR–Egger intercept and MR-PRESSO methods indicated the occurrence of horizontal pleiotropy, which needed to be removed before MR analysis. The number of MR-PRESSO cycles was 3,000. Moreover, we conducted leave-one-out sensitivity analyses to analyze the significance of the results and to determine whether there were abnormal IVs that significantly affected the causal effect estimate.

### 2.6 Reverse MR analysis

Finally, a reverse Mendelian randomization analysis was performed to explore the presence of reverse causality. The dataset, correlation methods, and parameters for the reverse MR analysis were the same as those for the forward MR analysis, but with VaD as the exposure and inflammatory cytokines and gut microbiota as the outcome.

### 2.7 Multivariable MR analysis

As the incidence of VaD may be influenced by body mass index (BMI), smoking/smokers in the household, alcohol consumption, and hyperlipidemia (Wolters and Ikram, 2019), two-sample MR may not reflect the direct effects of inflammatory cytokines and the gut microbiota on the incidence of VaD. Therefore, we used multivariable MR (MVMR) analysis to clarify whether the significant effects of the significant inflammatory cytokines and gut microbiota on VaD in the results were direct or indirect effects and whether they were driven by potential confounding factors (Burgess and Thompson, 2015). In addition, pleiotropy was assessed using the Egger regression line, with *p* < 0.05 indicating the presence of pleiotropy. The Cochrane *Q* test was used to assess heterogeneity. The test results showed heterogeneity (*p* < 0.05) (Sanderson et al., 2019). The GWAS data for BMI, smoking/smokers in the household, alcohol consumption, and hyperlipidemia are shown in Table 2.

**Table 2:** Confounders genome-wide association study details.

Phenotypes	Date type	Population	Consortium	Sample size (case/control)	GWAS ID
Body mass index	Confounder	Europeans	UK Biobank	120,286	ieu-a-1089
Alcohol consumption	Confounder	Europeans	UK Biobank	112,117	ieu-a-1283
Smoking/smokers in household	Confounder	Europeans	MRC-IEU	425,516	ukb-b-960
Hyperlipidemia	Confounder	Europeans	MRC-IEU	3,439/459,571	ukb-b-17462

GWAS, genome-wide association study.

### 2.8 Mediation analysis

We performed a mediated MR analysis using a two-step MR approach. First, we calculated the causal effect of the gut microbiota on inflammatory cytokines (beta1); then, we calculated the causal effect of inflammatory cytokines on VaD (beta2). We previously calculated the causal effect of the gut microbiota on VaD (beta3). We used beta3 as the total effect of the gut microbiota on VaD (Figure 1C), beta1 × beta2 as the mediating effect of the gut microbiota on VaD, beta3 − (beta1 × beta2) as the direct effect of the gut microbiota on VaD, and beta1 × beta2/beta3 as the proportion of the mediating effect in the causal relationship (Ahola-Olli et al., 2017).

### 2.9 Ethical approval

This study was conducted by using previously published, publicly available large-scale GWAS summary datasets. The ethics committee approved the data collection, and all participants provided written informed consent for the corresponding original GWAS.

## 3 Results

### 3.1 The two-sample MR analysis

#### 3.1.1 Selection of instrumental variables

IVs were screened according to the conditions described in the Methods section. All SNPs that were included in the analysis of inflammatory cytokines and VaD subtypes after screening are shown in Supplementary Table S1, totaling 331 distinct SNPs with F statistics ranging from 11.16 to 789.146; and all SNPs that were included in the analysis of gut microbiota and VaD subtypes after screening are shown in Supplementary Table S2, comprising 1,658 distinct SNPs with F statistics ranging from 16.913 to 88.429; all SNPs that were included in the reverse Mendelian randomization analysis after screening are shown in Supplementary Tables S3, S4. When inflammatory cytokines were the outcome, there were 40 distinct SNPs in the VaD subtype, with F statistics ranging from 4,772.892 to 77,865.614. When gut microbiota was the outcome, there were 40 distinct SNPs in the VaD subtype, with F statistics ranging from 6,178.734 to 77,865.614. All F statistics were greater than 10, indicating that no weak IVs were found in the IV strength test. After determining that all IVs were valid under the present conditions, we performed a Mendelian randomization analysis.

#### 3.1.2 Inflammatory cytokines genetically predict the risk of vascular dementia subtypes

**Figure 2:**
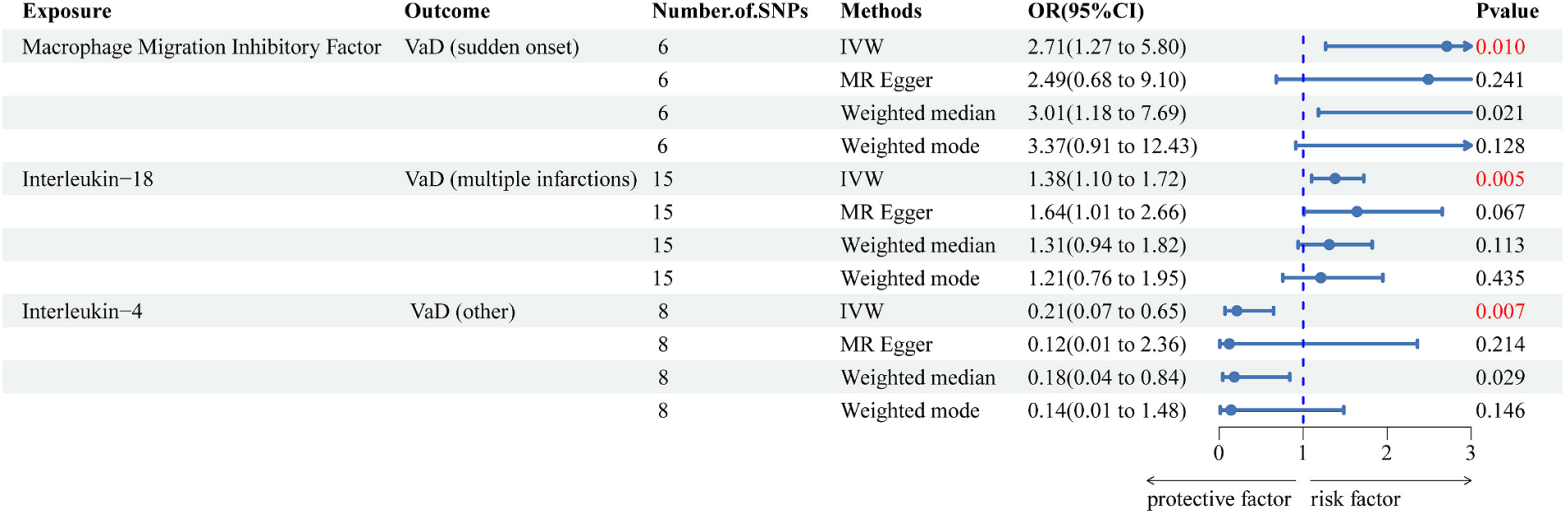
Forest plot of the associations between three genetically significant inflammatory cytokines and the risk of vascular dementia. The main results are from inverse-variance weighted analyses. OR, odds ratios; CI, confidence interval; MR, Mendelian randomization; SNPs, single nucleotide polymorphisms; IVW, inverse-variance weighted; VaD, vascular dementia.

We used the IVW method as the main method for identifying nine inflammatory cytokines related to an increase or decrease in the risk of various VaD subtypes. After the Bonferroni correction test, we detected three significant inflammatory cytokines (Figure 2) and six suggestive inflammatory cytokines (Supplementary Table S5). Interleukin-18 levels (OR = 1.375, 95% CI = 1.099–1.721, *p* = 0.005) were positively correlated with an increased risk of VaD (multiple infarctions), macrophage migration inhibitory factor levels (OR = 2.712, 95% CI = 1.277–5.804, *p* = 0.010) were positively associated with an increased risk of VaD (sudden onset), and interleukin-4 levels (OR = 0.210, 95% CI = 0.068–0.647, *p* = 0.007) were positively associated with a reduced risk of VaD (other).

#### 3.1.3 Effect of the genetically predicted gut microbiome on the risk of vascular dementia subtypes

**Figure 3:**
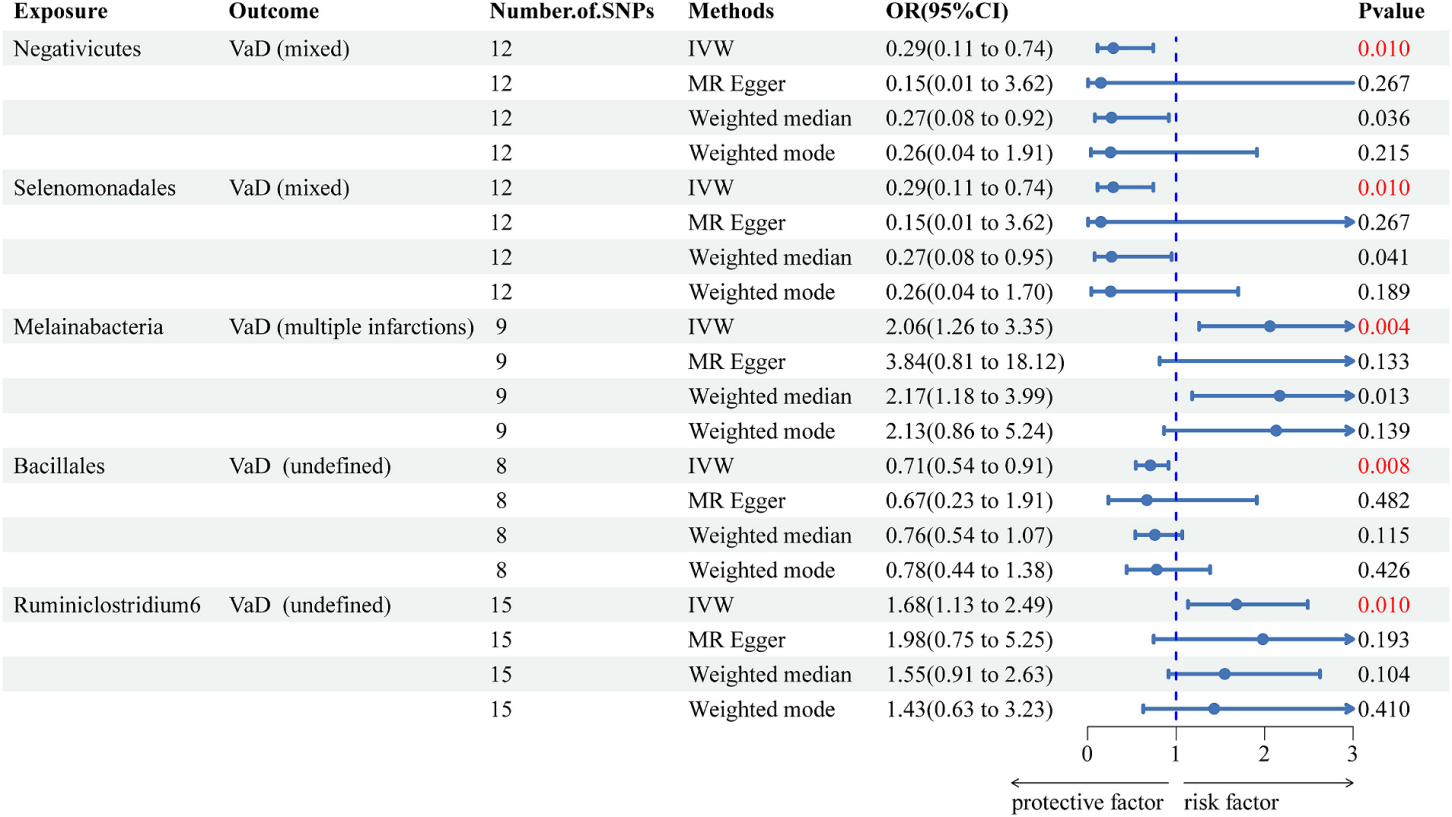
Forest plot of the associations between five genetically significant gut microbiomes and the risk of vascular dementia. The main results are from inverse-variance weighted analyses. OR, odds ratios; CI, confidence interval; MR, Mendelian randomization; SNPs, single nucleotide polymorphisms; IVW, inverse-variance weighted; VaD, vascular dementia.

We used the IVW method as the primary method to determine and identify 26 gut microbiotas related to increased or decreased risk for different subtypes of VaD. After Bonferroni correction, we found 5 gut microbiotas with significant associations (Figure 3) and 21 suggestive gut microbiotas (Supplementary Table S6). *Negativicutes* (OR = 0.289, 95% CI = 0.112–0.742, *p* = 0.010) and *Selenomonadales* (OR = 0.289, 95% CI = 0.112–0.742, *p* = 0.010) were negatively associated with the risk of VaD (mixed). *Melainabacteria* (OR = 2.055, 95% CI = 1.260–3.352, *p* = 0.004) was positively associated with the risk of VaD (multiple infarctions). *Ruminiclostridium 6* (OR = 1.680, 95% CI = 1.134–2.487, *p* = 0.010) and *Bacillales* (OR = 0.705, 95% CI = 0.545–0.914, *p* = 0.008) were positively and negatively associated with the risk of VaD (undefined), respectively.

#### 3.1.4 Sensitivity analyses

According to the heterogeneity test, the *p*-values of Cochran’s *Q* statistic were all greater than 0.05, indicating that there was no heterogeneity between SNPs. MR–Egger regression and MR-PRESSO tests showed no horizontal pleiotropy. The results of Cochran’s *Q* test, MR–Egger regression, and MR-PRESSO test for statistical significance are shown in Tables 3, 4. The results of Cochran’s *Q* test, MR–Egger regression, and MR-PRESSO test for the suggestive knot results are shown in Supplementary Tables S7, S8. The MR-PRESSO test removed the *Melainabacteria* outlier SNP, rs113884518. In addition, all the included IVs showed obvious symmetry in the funnel plot, excluding directional pleiotropy (Supplementary Figures S1, S2). The leave-one-out method showed that the significant results were not driven by a single SNP (Supplementary Figures S3, S4). The forest plot and scatter plot are shown in Supplementary Figures S5–S8.

**Table 3:** Sensitivity analysis for the association between three significant inflammatory cytokines and vascular dementia.

Exposure	Outcome	Pleiotropy				Heterogeneity	
		Egger intercept	Intercept’s se	Egger *p*-value	MR-PRESSO Global *p*-value	Cochran’s Q	Cochran’s Q *p*-value
MIF	VaD (sudden onset)	0.028	0.173	0.880	0.954	1.233	0.942
Interleukin-18	VaD (multiple infarctions)	−0.044	0.056	0.440	0.576	12.270	0.585
Interleukin-4	VaD (other)	0.082	0.211	0.711	0.479	6.909	0.438

MIF, macrophage migration inhibitory factor; MR, Mendelian randomization; VaD, vascular dementia.

**Table 4:** Sensitivity analysis for the association between five significant gut microbiomes and vascular dementia.

Exposure	Outcome	Pleiotropy				Heterogeneity	
		Egger intercept	Intercept’s se	Egger *p*-value	MR-PRESSO Global *p*-value	Cochran’s Q	Cochran’s Q *p*-value
*Negativicutes*	VaD (mixed)	0.045	0.104	0.671	0.945	4.867	0.937
*Selenomonadales*	VaD (mixed)	0.045	0.104	0.671	0.949	4.867	0.937
*Melainabacteria*	VaD (multiple infarctions)	−0.071	0.085	0.433	0.315	6.137	0.632
*Bacillales*	VaD (undefined)	0.008	0.081	0.923	0.595	5.927	0.548
*Ruminiclostridium 6*	VaD (undefined)	−0.015	0.043	0.724	0.770	10.030	0.760

MR, Mendelian randomization; VaD, vascular dementia.

#### 3.1.5 Reverse causality

In the reverse MR analysis results of inflammatory cytokines, there was no *p* < 0.05 for any inflammatory cytokine, and in the reverse Mendelian results of the gut microbiota, there were only two gut microbiotas with *p* < 0.05, genus *Parasutterella* (*p* = 0.030) and *Senegalimassilia* (*p* = 0.41), which were not related to the results shown in Figure 3 and Supplementary Table S6. Thus, the results of the reverse MR analysis did not show a reverse causal relationship between VaD and 9 inflammatory cytokines and 25 gut microbiotas.

### 3.2 Genetic predictors for multivariable MR

**Figure 4:**
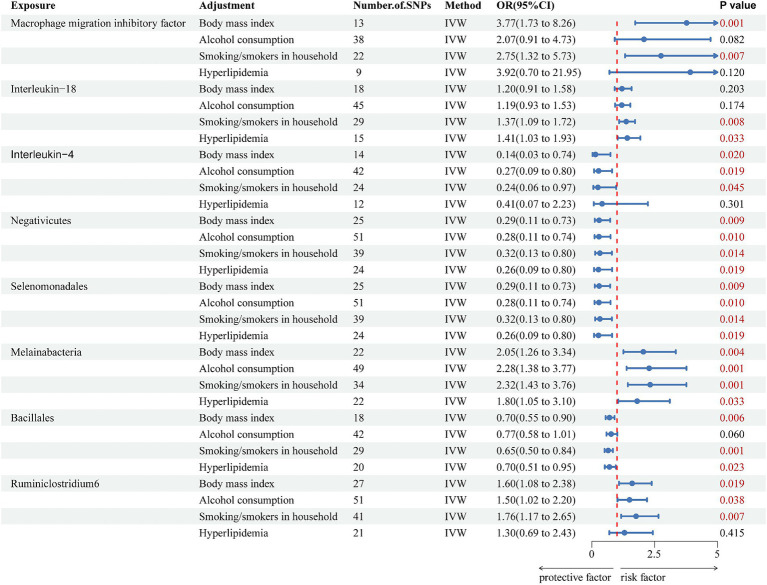
Forest plot of multivariate Mendelian randomization analysis of significant effects of inflammatory cytokines and the gut microbiota on vascular dementia after adjustment for confounders. The main results are from inverse-variance weighted analyses. OR, odds ratios; CI, confidence interval; SNPs, single nucleotide polymorphisms; IVW, inverse-variance weighted.

The genetically predicted effects of *Negativicutes*, *Selenomonadales*, and *Melainabacteria* on VaD persisted after adjustment for BMI, smoking/smokers in the household, alcohol consumption, and hyperlipidemia. For *Negativicutes*, after adjustment for BMI (OR = 0.289, 95% CI = 0.114–0.729, *p* = 0.009), alcohol consumption (OR = 0.281, 95% CI = 0.107–0.743, *p* = 0.010), smoking/smokers in the household (OR = 0.320, 95% CI = 0.129–0.797, *p* = 0.014) and hyperlipidemia (OR = 0.262, 95% CI = 0.086–0.804, *p* = 0.019); and for *Selenomonadales*, after adjustment for BMI (OR = 0.289, 95% CI = 0.114–0.729, *p* = 0.009), alcohol consumption (OR = 0.281, 95% CI = 0.107–0.743, *p* = 0.010), smoking/smokers in the household (OR = 0.320, 95% CI = 0.129–0.797, *p* = 0.014) and hyperlipidemia (OR = 0.262, 95% CI = 0.086–0.804, *p* = 0.019); and for Melainabacteria, after adjustment for BMI (OR = 2.05, 95% CI = 1.26–3.34, *p* = 0.004), alcohol consumption (OR = 2.28, 95% CI = 1.38–3.77, *p* = 0.001), smoking/smokers in the household (OR = 2.32, 95% CI = 1.43–3.76, *p* = 0.001) and hyperlipidemia (OR = 1.80, 95% CI = 1.05–3.10, *p* = 0.033), the above results strongly support the association of *Negativicutes* and *Selenomonadales* with a reduced risk of developing VaD and *Melainabacteria* with an increased risk of developing VaD. However, the remaining association of inflammatory cytokines and the gut microbiome with VaD was partially attenuated (Figure 4). Finally, sensitivity analysis showed no heterogeneity and no pleiotropy (Supplementary Table S9).

### 3.3 Mediating role of inflammatory cytokines between gut microbiome and VaD

We first used two-sample MR to analyze whether the gut microbiome in the outcomes had a causal effect on the inflammatory cytokines in the outcomes. Then, inflammatory cytokines and the gut microbiome with causal effects were included in the mediation analysis (Supplementary Table S10) to explore whether inflammatory cytokines are mediating factors for the incidence of VaD influenced by the gut microbiota. Our results show that inflammatory cytokines associated with each subtype of VaD do not play a mediating role in the causal pathways between the gut microbiota associated with each subtype of VaD and each subtype of VaD (Table 5).

**Table 5:** Mediation analysis of inflammatory cytokines on vascular dementia subtypes.

Outcome	Mediator	Exposure	Total effect	Direct effect	Mediation effect	
			(95% CI)	(95% CI)	(95% CI)	*p*-value
VaD (mixed)	Eotaxin	*Haemophilus*	2.206 (1.116–4.362)	2.385 (1.198–4.749)	0.925 (0.840–1.019)	0.100
VaD (multiple infarctions)	IL-18	*Melainabacteria*	2.055 (1.260–3.352)	1.193 (1.166–3.140)	1.074 (0.993–1.162)	0.064
VaD (other)	MIF	*Actinobacteria*	3.968 (1.202–13.103)	3.259 (0.954–11.126)	1.218 (0.916–1.620)	0.151

VaD, vascular dementia; IL-18, interleukin-18; MIF, macrophage migration inhibitory factor.

Finally, the STROBE-MR checklist was checked and uploaded as Supplementary Table S11.

## 4 Discussion

**Figure 5:**
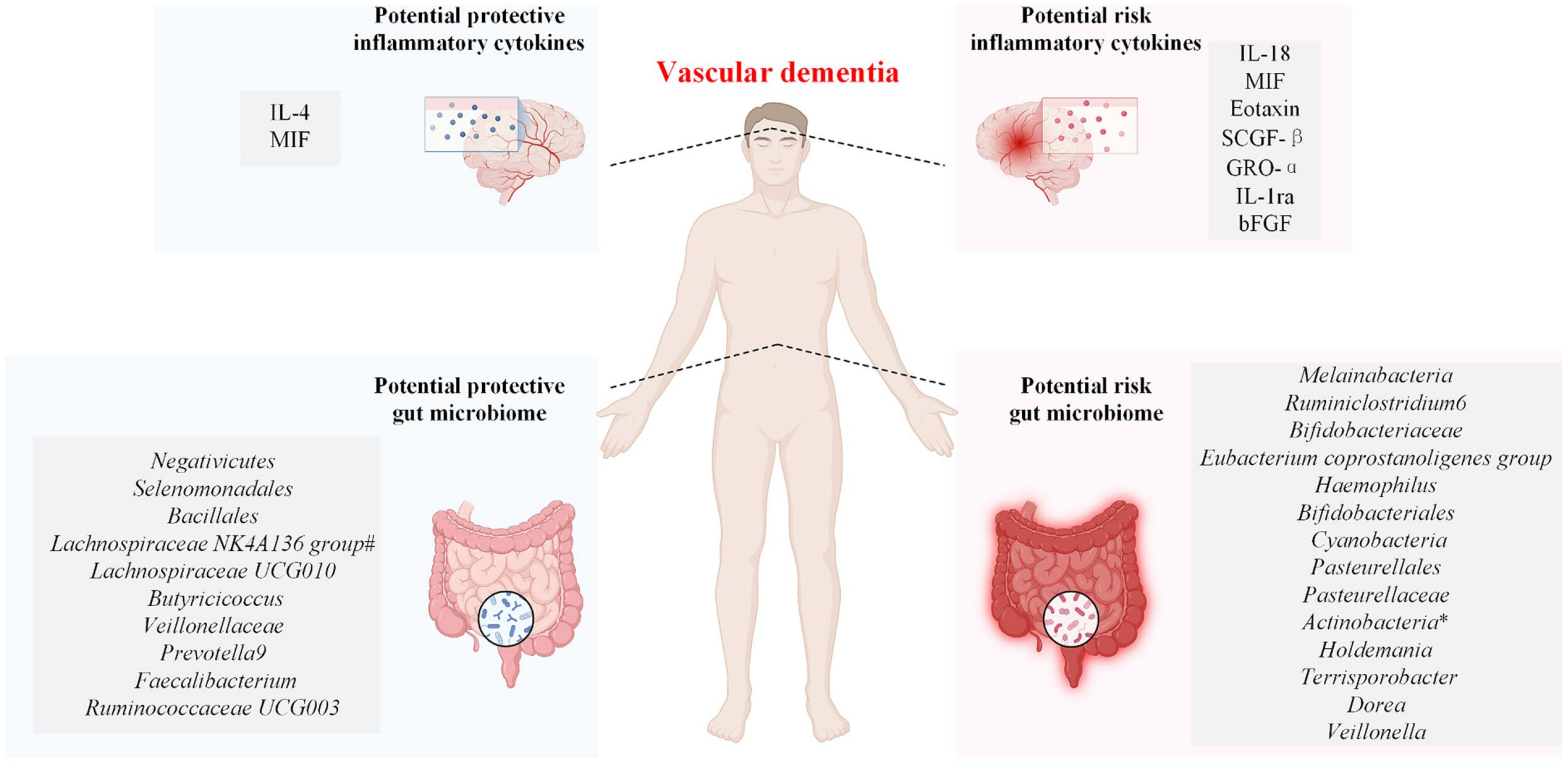
Associations between the genetically determined gut microbiome and inflammatory cytokines and the risk of vascular dementia. IL-4, interleukin-4; IL-18, interleukin-18; SCGF-β, stem cell growth factor beta; MIF, macrophage migration inhibitory factor; GRO-α, growth-regulated protein alpha; IL-1ra, interleukin-1-receptor antagonist; bFGF, fibroblast growth factor basic. The symbol * indicates the phylum *Actinobacteria* and the class *Actinobacteria*. The symbol # indicates that the *Lachnospiraceae NK4A136 group* has a protective effect on both vascular dementia (sudden onset) and vascular dementia (mixed).

Because the changes in inflammatory cytokines and gut microbiota composition are dynamic processes, high (low) values observed at a single time point may not accurately represent the overall trends of change in both. Therefore, this study serves as the first Mendelian randomization study using 41 inflammatory cytokines and 196 gut microbiota datasets with various subtypes of VaD as exposures to predict the inflammatory cytokines and gut microbiota that influence the risk of developing VaD. In addition, this study also used multivariate MR analysis and mediation analysis to determine whether inflammatory cytokines and the gut microbiota act as independent risk factors in the pathogenesis of VaD and to determine the relationships among them. Seven inflammatory cytokines and 15 gut microbiotas were associated with an increased risk of developing VaD subtypes, and two inflammatory cytokines and 11 gut microbiotas were associated with a decreased risk of developing VaD subtypes (Figure 5). Fibroblast growth factor basic and *Veillonella* increase the risk of developing VaD, and the finding that *Prevotella 9* reduces the risk of developing VaD is consistent with the findings of relevant studies (Ji et al., 2024). After the Bonferroni correction test, we obtained the results of the significance study. The inflammatory cytokines interleukin-18 (IL-18) and macrophage migration inhibitory factor (MIF) were associated with increased VaD risk, and interleukin-4 (IL-4) was associated with decreased VaD risk. The gut microbiota: *Ruminiclostridium 6* and *Melainabacteria* were associated with increased VaD risk, and *Negativicutes*, *Selenomonadales*, and *Bacillales* were associated with decreased VaD risk.

Furthermore, the effects of *Negativicutes*, *Selenomonadales*, and *Melainabacteria* on VaD remained significant after adjusting for the confounders BMI, smoking/smokers in the household, alcohol consumption, and hyperlipidemia. Unfortunately, the data on inflammatory cytokines and the gut microbiota analyzed in this study did not reveal the role of inflammatory cytokines as mediators of the role of the gut microbiota in VaD.

### 4.1 Inflammatory cytokines

MIF is a cytokine involved in various inflammatory responses and immune processes and is expressed by various cell types, such as immune cells, neurons, and glial cells, in response to stimuli such as hypoxia or ischemia (Thiele et al., 2022). During MIF expression, MIF can first induce the degradation of connexin and simultaneously change the actin cytoskeleton from cortical actin rings to stress fibers, which further leads to the destabilization of cell–cell junctions, resulting in vascular leakage (Chen et al., 2015). Moreover, MIF receptor expression in endothelial cells can induce endothelial cell autophagy to disrupt tight junctions, leading to BBB injury (Liu et al., 2018). Moreover, MIF stimulates the release of proinflammatory cytokines such as TNF-α and IL-1β (Sumaiya et al., 2022), and when inflammatory cytokines enter the CNS through the damaged BBB and bind to their receptors on microglia, astrocytes, or infiltrating inflammatory cells, they activate an inflammatory cascade response to induce neuronal apoptosis, neuroinflammation, and neurodegeneration, which can lead to disruption of brain homeostasis; this is one of the reasons for the onset of VaD (Liang et al., 2018; Liu et al., 2018; Zhao et al., 2023a). Atherosclerosis is a major cause of chronic hypoxia and hypoperfusion (Nam et al., 2020), whereas MIF is upregulated in atherosclerotic plaques and induces the expression of chemokines and adhesion molecules, which promote monocyte adhesion to the endothelium, thereby facilitating the migration and recruitment of atherosclerotic leukocytes, which can indirectly cause damage to neurons (Sinitski et al., 2019). MIF not only enhances intraplaque inflammation through macrophage secretion of proinflammatory cytokines, including MIF, leading to plaque instability but also accelerates foam cell formation by promoting macrophage uptake of oxidized low-density lipoproteins, which further contributes to atherosclerosis (Asare et al., 2013; Sinitski et al., 2019), ultimately triggering the development of cardiovascular disease, which decreases cerebral blood flow and leads to cerebral ischemia and hypoxia. When MIF levels are increased *in vivo*, inflammation within the white matter and hippocampus is also increased, and executive function and spatial learning are disrupted in mice and subjects (Bancroft et al., 2019; Zhao et al., 2023b). Therefore, MIF impairs cognitive function by increasing endothelial dysfunction, reducing intercellular tight junctions, increasing the permeability of the BBB, promoting atherosclerosis, enhancing reactive oxygen species production, and enhancing inflammatory responses. Our study showed that strategies to reduce MIF levels or block its activity might improve cognitive function, but the results were no longer significant after adjusting for alcohol consumption and hyperlipidemia.

IL-18 is a proinflammatory cytokine that is released mainly by microglia (Zhang et al., 2023c). Clinical and basic studies have shown increased plasma IL-18 levels in VaD patients and mice compared with those in non-demented controls (Malaguarnera et al., 2006; Poh et al., 2021). IL-18 not only promotes microglial activation and induces immune cells to produce IL-1β and TNF-α to promote neuroinflammatory responses but also activates interferon-γ to promote the production of IL-8 and chemokines (Yasuda et al., 2019; Li et al., 2022), which in turn promotes the production of IL-18, leading to the formation of a vicious cycle of severe inflammatory responses (Yasuda et al., 2019). A significant increase in BBB permeability is an important pathological change in VaD (Hussain et al., 2021). When IL-18 crosses the BBB, it can accelerate the disruption of BBB integrity by promoting the expression of proinflammatory cytokines and matrix metalloproteinases, which allows the accelerated infiltration of inflammatory cytokines and immune cells into the brain, exacerbating neuroinflammation and neuronal damage and causing neuronal loss (Weekman and Wilcock, 2016; Qin et al., 2020). Additionally, inflammatory vesicle (e.g., NLRP3)-mediated caspase-1 activation can activate cellular pyroptosis and cleave the precursor of IL-18 to its active and secreted form and sustain its release, thereby amplifying and enhancing the inflammatory response and creating a lingering inflammatory milieu that can hinder neuronal function and sustain cognitive dysfunction (Fu et al., 2019; Ising et al., 2019; Yu et al., 2021). Inhibiting the activation of inflammatory vesicles induced by chronic cerebral insufficiency of cerebral perfusion reduces the release of proinflammatory cytokines, the production of apoptotic substances and cellular pyroptosis and further reduces white matter damage and neuronal cell death to restore cognitive function in VaD mice, which has been proven to be possible by Poh et al. (2021). Finally, high IL-18 levels may be strongly associated with the development of cerebrovascular disease through their association with cardiovascular risk factors (Yasuda et al., 2019). For example, enhanced IL-18 mRNA expression may not only increase the severity of internal carotid artery stenosis (Arapi et al., 2018) but also cause a similar increase in blood pressure (Li et al., 2022). The above findings support our conclusions that elevated peripheral levels of IL-18 may lead to alterations in multiple signaling pathways accompanied by instability of the neurovascular unit, increasing the degree of damage to brain function in VaD patients and indirectly showing that elevated levels of IL-18 increase the likelihood of VaD. In addition, our results also showed that after adjusting for BMI and alcohol consumption, the effect of IL-18 on the increased incidence of VaD was no longer significant.

IL-4 is an anti-inflammatory cytokine (Pu et al., 2021b). It not only inhibits the production of proinflammatory cytokines such as IL-1β and TNF-α but also promotes the production of other anti-inflammatory cytokines such as interleukin-10 (IL-10) and TGF-β (Gärtner et al., 2023). IL-4 induces the differentiation of M2-type macrophages, which have tissue-repairing properties, and M2-type microglia, which have anti-inflammatory effects, to modulate immune cell activity (Dang et al., 2023; Kang et al., 2023). It prevents neuronal loss in the hippocampus, amygdala, and white matter; increases oligodendroglial production or regeneration; improves synaptic connectivity; restores the structural and functional integrity of the brain; and contributes to the restoration of neurological function (Zhang et al., 2019; Pu et al., 2021a,b, 2023). Increasing the expression level of IL-4 in the hippocampus and prefrontal cortex of VaD rats or mice inhibited the release of proinflammatory cytokines and reduced microglia/macrophage activation, stabilizing the anti-inflammatory environment. These findings show that it has the potential to attenuate the deleterious effects of neuroinflammation and improve cognitive function in VaD rats and mice (Zhu et al., 2020; Zhang et al., 2021b; Wang et al., 2023a). IL-4 plays an important role in attenuating the inflammatory response, promoting the release of anti-inflammatory cytokines, regulating the activity of immune cells, promoting neural repair, and reducing apoptosis and oxidative stress; thus, the current evidence indirectly supports our view that elevated IL-4 slows the progression of VaD pathogenesis. Unfortunately, after adjusting for hyperlipidemia, the beneficial effects of IL-4 on VaD were not significant.

### 4.2 The gut microbiome

*Negativicutes* are a class of bacteria in the phylum *Bacillota* (Rands et al., 2019). The reduced abundance of *Negativicutes* in fecal samples from Alzheimer’s disease (AD) patients in a clinical study of 43 patients with AD may indicate a protective role for *Negativicutes* in dementia (Zhuang et al., 2018). In addition, *Negativicutes* can produce propionic acid through synergistic metabolism (Duncan et al., 2023). *Selenomonadales*, on the other hand, is an order of bacteria within *Negativicutes* that can metabolize not only propionic acid but also lactic and acetic acid (Reichardt et al., 2014). Acetic acid and propionic acid have various potential healthful and neuroprotective effects. Propionic acid acts as a mediator of fatty acid metabolism by improving lipid biosynthesis, lowering cholesterol, and reducing cardiovascular disease incidence. It also has some inhibitory effects on neuroinflammation (Hu et al., 2018; Cuevas-Sierra et al., 2021); acetic acid not only enhances intestinal mucosal barrier function but can also be transported to the central nervous system through the circulatory system to promote the repair of the BBB, which in turn can indirectly reduce neuroinflammation and enhance synaptic function (Mirzaei et al., 2021; Zhu et al., 2023). Dai et al. improved the intestinal health status of voles after increasing the abundance of *Negativicutes* and *Selenomonadales* in their gut, which ultimately favored their overall health status (Dai et al., 2022). After Naumova et al. administered probiotic treatment to obese patients, *Selenomonadales* and *Negativicutes* increased in the gut along with a decrease in blood glucose in obese patients, which may indicate their role in the beneficial effects of probiotics on host health (Naumova et al., 2020). Therefore, the above studies not only revealed the indirect protective effect of *Negativicutes* and *Selenomonadales* on this organism, but also supported our findings that *Negativicutes* and *Selenomonadales* have beneficial effects on VaD even after adjusting for BMI, smoking/smokers in the household, alcohol consumption, and hyperlipidemia factors.

Research on the role of *Melainabacteria* in neurological diseases has been scarce. Only a few studies have speculated that *Melainabacteria* may synthesize β-N-methylamino-L-alanine (BMAA), which can cause erroneous protein folding and aggregation in the body, triggering endoplasmic reticulum stress responses and cell apoptosis, thereby causing neurodegeneration (Silva et al., 2020). Moreover, BMAA can also activate innate immune responses in neurons, leading to neuroinflammation. Most importantly, BMAA can cause excessive phosphorylation of the Tau protein and the accumulation of Aβ (Silva et al., 2020). The above results indicate that *Melainabacteria* may indirectly participate in the pathogenesis of neurodegenerative diseases such as Alzheimer’s disease and Parkinson’s disease (Silva et al., 2020; Hu and Rzymski, 2022). Although studies on the role of *Melainabacteria* in neurological disorders are limited, our findings add to the recent evidence that an increase in the abundance of *Melainabacteria* is positively associated with an increase in the incidence of VaD, even after adjusting for four confounders.

*Bacillales* are an order of gram-positive bacteria within *Bacillota* (Parte, 2014). Research has shown that *Bacillales* species exhibit excellent potential probiotic characteristics, lowering cholesterol levels to prevent cardiovascular disease; thus, they could reduce the number of causative factors of VaD and lower the risk of VaD (He et al., 2021; Farnsworth von Cederwald et al., 2022). Therefore, our results suggest that an increase in *Bacillales* abundance may indirectly reduce the incidence of VaD. However, the results were not significant after adjustment for alcohol consumption, the reason for which needs to be investigated in our further research.

*Ruminiclostridium 6* abundance may be positively associated with the risk of developing AD (Ning et al., 2022), implying that *Ruminiclostridium 6* may be pathogenic in individuals with cognitive dysfunction. The *Ruminiclostridium 6* level is positively correlated with the expression level of aconitate decarboxylase 1 (a protein that is abnormally expressed in ulcerative colitis) and simultaneously promotes the release of proinflammatory cytokines, such as IL-6, IL-1β, and TNF-α, which in turn indirectly increase intestinal permeability, exacerbate microbial imbalances, and contribute to the development of inflammatory diseases of the intestinal tract (Ge et al., 2021; Zhang et al., 2023b). A longitudinal study showed that patients with inflammatory bowel disease (IBD) were more than twice as likely to develop dementia as the general population (Zhang et al., 2021a). Similarly, our findings showed that the abundance of *Ruminiclostridium 6* in the gut of VaD patients was positively correlated with the incidence of VaD even after adjusting for BMI, smoking/smokers in the household, and alcohol consumption.

### 4.3 Multivariable MR

Hyperlipidemia is one of the known risk factors for VaD (Shang et al., 2024) and is characterized by elevated blood lipid levels and can lead to an imbalance between pro-and anti-inflammatory responses in the body (Collado et al., 2021). IL-4 reduces adiposity, inhibits adipocyte differentiation, and promotes lipolysis (Tsao et al., 2014; Shiau et al., 2019). However, the presence of hyperlipidemia exacerbates the inflammatory process (Don-Doncow et al., 2021), which may reduce the anti-inflammatory effects of IL-4. In addition, hyperlipidemia can cause atherosclerosis, and MIF is upregulated in atherosclerotic plaques (Sinitski et al., 2019) and accelerates the exacerbation of atherosclerosis (Zhao et al., 2023a). In addition, hyperlipidemia can alter the intestinal environment, affecting the composition, metabolism, and function of the gut microbiome (Liang et al., 2021; Di Vincenzo et al., 2024), so that the beneficial independent effect of *Ruminiclostridium 6* in the context of hyperlipidemia is weakened. For the above reasons, it is possible that adjustment for hyperlipidemia, while reducing the influence of confounding factors, may ultimately weaken the independent effects of MIF, IL-4, and *Ruminiclostridium 6* on the incidence of VaD.

BMI is a measure of body fat that can be based on height and weight (Khanna et al., 2022), and a higher BMI is not only a risk factor for developing VaD (Hakim, 2021) but is also commonly associated with increased adipose tissue in the body (Agrawal et al., 2023). Studies have shown that adipose tissue is not only a major source of inflammation but also secretes inflammatory cytokines such as IL-18 (Ahmad et al., 2017). Therefore, a higher BMI may indicate that the body is often in a low-grade chronic inflammatory state and may affect the expression level of IL-18 in the body, masking the independent effect of IL-18 on VaD and rendering the relationship between it and VaD insignificant.

Studies have shown that excessive alcohol consumption can alter the body’s immune system and inflammatory response (Crews et al., 2017; Petralia et al., 2020), affecting the levels, functions, and pathways of MIF and IL-18 (Kazmi et al., 2022; Zhao et al., 2023a). Therefore, alcohol consumption directly or indirectly interferes with the expression of inflammatory cytokines, which in our findings, interferes with the independent effects of MIF and IL-18 on the pathogenesis of VaD. In addition, excessive alcohol consumption impairs the intestinal barrier function, leads to increased intestinal permeability, alters the intestinal pH and microenvironment (Gorky and Schwaber, 2016; Bishehsari et al., 2017), affects intestinal immune homeostasis, and causes alterations in the abundance (Engen et al., 2015) and function (Couch et al., 2015) of the gut microbiome, ultimately leading to an imbalance in the gut microbiome. This, in turn, allows alcohol to interfere with the potentially beneficial effects of *Bacillales* on VaD and reduces the beneficial effects of *Bacillales* in reducing the incidence of VaD. Finally, the inflammation associated with excessive alcohol consumption leads to brain damage and cognitive dysfunction, which is itself a risk factor for the development of VaD (Rehm et al., 2019). These factors explain why the independent effects of IL-18, MIF, and *Bacillales* on the incidence of VaD were attenuated after adjustment for alcohol consumption.

### 4.4 Gut microbiome-inflammatory cytokines-vascular dementia

The exact relationships among the gut microbiome, inflammatory cytokines, and VaD remain an area of ongoing research. Studies have shown that gut microbiome imbalance is associated with a variety of central nervous system disorders, and there is a link between gut microbiome imbalance and cognitive dysfunction and inflammatory cytokine responses (Song et al., 2024). A metabolically dysregulated gut microbiome or an altered composition of VaD patients may affect the activation of peripheral immune cells, both cellular and humoral, and further lead to elevated levels of proinflammatory factors, such as IL-1β, IL-6, and TNF-α (Parker et al., 2020), as well as further damage to the vasculature and BBB, leading to systemic inflammation and neurodegeneration, which may contribute to the development of VaD (Alkasir et al., 2017; Sun et al., 2021; Kaur et al., 2023). On the other hand, the gut microbiome can produce short-chain fatty acids (SCFAs), including acetic, butyric, and propionic acids (Morrison and Preston, 2016), through innervation of the enteric nervous system. SCFAs can enter the circulation, regulate microglial development and maturation, reduce proinflammatory factors in the host, reduce synaptic dysfunction and neuronal death, regulate gut homeostasis, and ultimately improve cognitive dysfunction (Sarkar et al., 2016; Zhu et al., 2023).

However, our research revealed that the 41 inflammatory cytokines we selected are not the mediators of the 196 common gut microbiomes in increasing or decreasing the incidence of VaD, and our results are in line with the findings of Ji et al. (2024). We believe this is because (1) the amount of GWAS data on inflammatory cytokines and the gut microbiome is limited, which limits our discovery of inflammatory cytokines with mediating effects; (2) Mendelian randomization studies themselves have many gene–environment interactions, in which case the effect of genes on disease may be influenced by environmental factors that change over time or between different groups. This phenomenon deserves further investigation; (3) The gut microbiome does not directly act on inflammatory cytokines to intervene in the pathogenesis and progression of VaD. It is possible that one or more combinations of SCFAs, immune cells, or trimethylamine N-oxide (TMAO) act on inflammatory factors in VaD patients, ultimately enhancing or ameliorating the course of VaD. Research suggests that the gut microbiome can alleviate cognitive dysfunction due to systemic inflammation through acetic and propionic acids (He et al., 2020). According to our results, *Negativicutes* can metabolize propionic acid, and *Selenomonadales* can metabolize propionic acid and acetic acid, among others. Propionate and butyrate inhibit histone deacetylase activity and induce the differentiation of peripheral CD4+ T cells into Treg cells, which produce the anti-inflammatory cytokine IL-10 and suppress the function of Th2 and Th17 cells (Arpaia et al., 2013; Anania et al., 2022). In addition, increasing the abundance of the indole-producing gut microbiome in AD mice can reduce the release of inflammatory factors such as IL-18 and attenuate the inflammatory response, which improves cognitive deficits in AD mice (Zhang et al., 2023a). In addition, trimethylamine N-oxide (TMAO) is another gut microbiome metabolite that not only causes systemic inflammation and neuroinflammation with age but also leads to peripheral and central inflammatory responses and cognitive deficits in mice with elevated circulation levels (Brunt et al., 2021). Studies have also shown that IL-18 and MIF are related to the occurrence of TMAO-mediated inflammatory processes in a variety of diseases (Fang et al., 2021; Zarbock et al., 2022; Constantino-Jonapa et al., 2023), but unfortunately, studies on cognitive dysfunction diseases are still sparse and need to be explored further. Therefore, to obtain more definite evidence on whether inflammatory cytokines act as intermediary factors for the gut microbiome to act on VaD or how the gut microbiome improves or exacerbates VaD through inflammatory cytokines, further research is needed.

To date, our understanding of neurological disorders has evolved from a single-organ, brain-centered view to a more integrated, whole-body view. A good way to control the first site of action in the gut and produce a new generation of safer treatments for central nervous system disorders is to target the gut microbiome (Long-Smith et al., 2020). Research has shown that dietary modulation of the composition and increased diversity of the gut microbiota may be a way to combat VaD (Livingston et al., 2017; Merra et al., 2020) and that probiotic supplementation significantly improves cognitive function in patients with Alzheimer’s disease, mild cognitive dysfunction, and VaD mice (Liu et al., 2015; Xiang et al., 2022). However, recent studies have shown that the gut microbiome has coevolved with the host and is interdependent (Cryan et al., 2019), that there is a high degree of interindividual variability and heterogeneity, and that the composition of the gut microbiome evolves continuously with age (Falony et al., 2016). Both the host genome and the associated microbial genome contribute to genetic variation in organisms, as shown by our findings; inflammatory cytokines and the gut microbiome influence the pathogenesis of VaD. In addition, aging alters the gut microbiota (Xu et al., 2019; Wilmanski et al., 2021), and the beneficial gut microbiome that produces SCFAs is reduced in the gut of older or older mice compared to that in younger or younger mice (Kim et al., 2019; Lee et al., 2020), which in turn contributes to the body’s inflammatory response, which in turn contributes to accelerated aging and may promote the development of age-related diseases (Xia et al., 2016; Yang et al., 2017). On the other hand, changes in the composition of the gut microbiota in elderly individuals have also been suggested to contribute to inflammation (Thevaranjan et al., 2017). Unfortunately, the impact of age-related dysbiosis on VaD has not been extensively studied; therefore, based on the above points, in the future, we need to understand the latest impact of the microbiome on host health in an evolutionary context through macroeconomics, metabolomics scores, brain imaging techniques, and the use of forward and reverse gut microbiome approaches (Peh et al., 2022). Genetic and functional analyses of gut microbes have been performed, and where possible, the direct effects of gut microbiome metabolites on CNS function have been studied in conjunction with other pathways (e.g., immune or neuronal pathways) to clarify the involvement of a wider range of metabolic pathways with a greater number of relevant inflammatory factors (Bonaz et al., 2017). In addition, the inclusion of aging and dysbiosis as independent but interdependent biological variables in related studies to understand the role of coevolution of the host and its microbiota will contribute to new therapeutic insights into neurodevelopment and decline in patients with cerebrovascular disease and provide new directions to combat age-associated neurodegeneration and cognitive decline (Cryan et al., 2019; Boehme et al., 2020).

## 5 Conclusion

We assessed the potential pathogenic role of inflammatory cytokines and the gut microbiome in the etiogenesis of various subtypes of VaD. In addition, *Negativicutes*, *Selenomonadales*, and *Melainabacteria* were identified as playing critical roles in the pathogenesis of vascular dementia (VaD). Thus, the present study may provide new insights into the gut microbiome and inflammatory cytokine-mediated reduction in VaD disease risk.

## 6 Limitations

Our study has the following limitations: (1) Only participants from the European population were included, which may limit the generalization of our results to other ethnicities. In the future, we will include more ethnicities in our studies. (2) Mendelian randomization studies are different from randomized controlled trials. Therefore, the results from MR analysis may differ to some extent from those expected in previous or future RCTs, which can be interpreted as a life-course effect. (3) Our study included datasets for only 196 common gut microbiomes and 41 common inflammatory cytokines, which resulted in an incomplete set of exposure tools. In the future, we will collect more data to increase the comprehensiveness of our study. (4) There was some overlap between the inflammatory cytokine and VaD sample data. Due to the lack of individual-level GWAS data, we were unable to remove overlapping samples. Therefore, we calculated the maximum overlap rate to be 2.3% (the largest sample size for inflammatory cytokines was 8,337, and the smallest sample size for VaD was 360,248). According to the study by Burgess et al. (2016), the estimated bias from a 30% sample overlap rate is less than 0.1%. Therefore, we assume that the sample overlap between exposure and outcome in our study has little impact on the results.

## Data availability statement

The original contributions presented in the study are included in the article/Supplementary material, further inquiries can be directed to the corresponding authors.

## Author contributions

YY: Conceptualization, Data curation, Formal analysis, Writing – original draft, Writing – review & editing. TR: Investigation, Methodology, Writing – review & editing. SW: Conceptualization, Data curation, Formal analysis, Visualization, Writing – review & editing. JCh: Methodology, Supervision, Validation, Visualization, Writing – review & editing. YZ: Conceptualization, Investigation, Software, Writing – review & editing. TL: Investigation, Project administration, Validation, Writing – review & editing. JcC: Formal analysis, Funding acquisition, Resources, Writing – review & editing. XZ: Writing – review & editing. YJ: Writing – review & editing, Writing – original draft. SY: Conceptualization, Data curation, Formal analysis, Funding acquisition, Investigation, Methodology, Project administration, Resources, Software, Supervision, Validation, Visualization, Writing – original draft, Writing – review & editing.

## Footnotes

^1^ https://data.bris.ac.uk/data/dataset/3g3i5smgghp0s2uvm1doflkx9x

^2^ https://mibiogen.gcc.rug.nl/menu/main/home/

^3^ https://r9.finngen.fi

^4^ www.phenoscanner.medschl.cam.ac.uk

## References

[ref1] Agrawal S. Klarqvist M. D. R. Diamant N. Stanley T. L. Ellinor P. T. Mehta N. N.. (2023). BMI-adjusted adipose tissue volumes exhibit depot-specific and divergent associations with cardiometabolic diseases. Nat. Commun. 14:266. doi: 10.1038/s41467-022-35704-5, PMID: 36650173 PMC9844175

[ref2] Ahmad R. Thomas R. Kochumon S. Sindhu S. (2017). Increased adipose tissue expression of IL-18R and its ligand IL-18 associates with inflammation and insulin resistance in obesity. Immun. Inflamm. Dis. 5, 318–335. doi: 10.1002/iid3.170, PMID: 28508444 PMC5569378

[ref3] Ahola-Olli A. V. Würtz P. Havulinna A. S. Aalto K. Pitkänen N. Lehtimäki T.. (2017). Genome-wide association study identifies 27 loci influencing concentrations of circulating cytokines and growth factors. Am. J. Hum. Genet. 100, 40–50. doi: 10.1016/j.ajhg.2016.11.007, PMID: 27989323 PMC5223028

[ref4] Alkasir R. Li J. Li X. Jin M. Zhu B. (2017). Human gut microbiota: the links with dementia development. Protein Cell 8, 90–102. doi: 10.1007/s13238-016-0338-6, PMID: 27866330 PMC5291774

[ref5] Amin N. Liu J. Bonnechere B. Mahmoudian Dehkordi S. Arnold M. Batra R.. (2023). Interplay of metabolome and gut microbiome in individuals with major depressive disorder vs control individuals. JAMA Psychiatry 80, 597–609. doi: 10.1001/jamapsychiatry.2023.0685, PMID: 37074710 PMC10116384

[ref6] Anania C. Brindisi G. Martinelli I. Bonucci E. D’Orsi M. Ialongo S.. (2022). Probiotics function in preventing atopic dermatitis in children. Int. J. Mol. Sci. 23:5049. doi: 10.3390/ijms23105409, PMID: 35628229 PMC9141149

[ref7] Angoorani P. Ejtahed H.-S. Siadat S. D. Sharifi F. Larijani B. (2022). Is there any link between cognitive impairment and gut microbiota? A systematic review. Gerontology 68, 1201–1213. doi: 10.1159/000522381, PMID: 35263739

[ref8] Arapi B. Bayoğlu B. Cengiz M. Dirican A. Deser S. B. Junusbekov Y.. (2018). Increased expression of Interleukin-18 mRNA is associated with carotid artery stenosis. Balk. Med. 35, 250–255. doi: 10.4274/balkanmedj.2017.0323, PMID: 29485097 PMC5981122

[ref9] Arpaia N. Campbell C. Fan X. Dikiy S. van der Veeken J. de Roos P.. (2013). Metabolites produced by commensal bacteria promote peripheral regulatory T-cell generation. Nature 504, 451–455. doi: 10.1038/nature12726, PMID: 24226773 PMC3869884

[ref10] Asare Y. Schmitt M. Bernhagen J. (2013). The vascular biology of macrophage migration inhibitory factor (MIF). Expression and effects in inflammation, atherogenesis and angiogenesis. Thromb. Haemost. 109, 391–398. doi: 10.1160/TH12-11-0831 23329140

[ref11] Bancroft E. Srinivasan R. Shapiro L. A. (2019). Macrophage migration inhibitory factor alters functional properties of CA1 hippocampal neurons in mouse brain slices. Int. J. Mol. Sci. 21:276. doi: 10.3390/ijms21010276, PMID: 31906137 PMC6981710

[ref12] Barili F. Parolari A. Kappetein P. A. Freemantle N. (2018). Statistical primer: heterogeneity, random-or fixed-effects model analyses? Interact. Cardiovasc. Thorac. Surg. 27, 317–321. doi: 10.1093/icvts/ivy163 29868857

[ref13] Belkhelfa M. Beder N. Mouhoub D. Amri M. Hayet R. Tighilt N.. (2018). The involvement of neuroinflammation and necroptosis in the hippocampus during vascular dementia. J. Neuroimmunol. 320, 48–57. doi: 10.1016/j.jneuroim.2018.04.004, PMID: 29759140

[ref14] Bishehsari F. Magno E. Swanson G. Desai V. Voigt R. M. Forsyth C. B.. (2017). Alcohol and gut-derived inflammation. Alcohol Res. 38, 163–171. Available at: https://pubmed.ncbi.nlm.nih.gov/28988571, PMID: 28988571 10.35946/arcr.v38.2.02 PMC5513683

[ref15] Boehme M. van de Wouw M. Bastiaanssen T. F. S. Olavarría-Ramírez L. Lyons K. Fouhy F.. (2020). Mid-life microbiota crises: middle age is associated with pervasive neuroimmune alterations that are reversed by targeting the gut microbiome. Mol. Psychiatry 25, 2567–2583. doi: 10.1038/s41380-019-0425-1, PMID: 31092898

[ref16] Bonaz B. Sinniger V. Pellissier S. (2017). The Vagus nerve in the neuro-immune Axis: implications in the pathology of the gastrointestinal tract. Front. Immunol. 8:1452. doi: 10.3389/fimmu.2017.01452, PMID: 29163522 PMC5673632

[ref17] Brunt V. E. LaRocca T. J. Bazzoni A. E. Sapinsley Z. J. Miyamoto-Ditmon J. Gioscia-Ryan R. A.. (2021). The gut microbiome-derived metabolite trimethylamine N-oxide modulates neuroinflammation and cognitive function with aging. Gero Sci. 43, 377–394. doi: 10.1007/s11357-020-00257-2, PMID: 32862276 PMC8050157

[ref18] Burgess S. Davies N. M. Thompson S. G. (2016). Bias due to participant overlap in two-sample Mendelian randomization. Genet. Epidemiol. 40, 597–608. doi: 10.1002/gepi.21998, PMID: 27625185 PMC5082560

[ref19] Burgess S. Thompson S. G. (2015). Multivariable Mendelian randomization: the use of pleiotropic genetic variants to estimate causal effects. Am. J. Epidemiol. 181, 251–260. doi: 10.1093/aje/kwu283, PMID: 25632051 PMC4325677

[ref20] Chen H.-R. Chuang Y.-C. Chao C.-H. Yeh T.-M. (2015). Macrophage migration inhibitory factor induces vascular leakage via autophagy. Biol. Open. 4, 244–252. doi: 10.1242/bio.201410322, PMID: 25617421 PMC4365493

[ref21] Collado A. Domingo E. Marques P. Perello E. Martínez-Hervás S. Piqueras L.. (2021). Oral unsaturated fat load impairs postprandial systemic inflammation in primary hypercholesterolemia patients. Front. Pharmacol. 12:656244. doi: 10.3389/fphar.2021.656244 33959024 PMC8093814

[ref22] Constantino-Jonapa L. A. Espinoza-Palacios Y. Escalona-Montaño A. R. Hernández-Ruiz P. Amezcua-Guerra L. M. Amedei A.. (2023). Contribution of trimethylamine N-oxide (TMAO) to chronic inflammatory and degenerative diseases. Biomedicines. 11:431. doi: 10.3390/biomedicines11020431, PMID: 36830968 PMC9952918

[ref23] Couch R. D. Dailey A. Zaidi F. Navarro K. Forsyth C. B. Mutlu E.. (2015). Alcohol induced alterations to the human fecal VOC metabolome. PLoS One 10:e0119362. doi: 10.1371/journal.pone.0119362, PMID: 25751150 PMC4353727

[ref24] Crews F. T. Lawrimore C. J. Walter T. J. Coleman L. G. (2017). The role of neuroimmune signaling in alcoholism. Neuropharmacology 122, 56–73. doi: 10.1016/j.neuropharm.2017.01.031, PMID: 28159648 PMC5493978

[ref25] Cryan J. F. O’Riordan K. J. Cowan C. S. M. Sandhu K. V. Bastiaanssen T. F. S. Boehme M.. (2019). The microbiota-gut-brain axis. Physiol. Rev. 99, 1877–2013. doi: 10.1152/physrev.00018.2018 31460832

[ref26] Cuevas-Sierra A. Romo-Hualde A. Aranaz P. Goni L. Cuervo M. Martínez J. A.. (2021). Diet- and sex-related changes of gut microbiota composition and functional profiles after 4 months of weight loss intervention. Eur. J. Nutr. 60, 3279–3301. doi: 10.1007/s00394-021-02508-0, PMID: 33591390

[ref27] Dai X. Chen L. Liu M. Liu Y. Jiang S. Xu T.. (2022). Effect of 6-methoxybenzoxazolinone on the cecal microbiota of adult male Brandt’s vole. Front. Microbiol. 13:847073. doi: 10.3389/fmicb.2022.847073, PMID: 35422782 PMC9002351

[ref28] Dang B. Gao Q. Zhang L. Zhang J. Cai H. Zhu Y.. (2023). The glycolysis/HIF-1α axis defines the inflammatory role of IL-4-primed macrophages. Cell Rep. 42:112471. doi: 10.1016/j.celrep.2023.112471, PMID: 37149865

[ref29] Di Vincenzo F. Del Gaudio A. Petito V. Lopetuso L. R. Scaldaferri F. (2024). Gut microbiota, intestinal permeability, and systemic inflammation: a narrative review. Intern. Emerg. Med. 19, 275–293. doi: 10.1007/s11739-023-03374-w, PMID: 37505311 PMC10954893

[ref30] Don-Doncow N. Vanherle L. Matthes F. Petersen S. K. Matuskova H. Rattik S.. (2021). Simvastatin therapy attenuates memory deficits that associate with brain monocyte infiltration in chronic hypercholesterolemia. NPJ Aging Mech. Dis. 7:19. doi: 10.1038/s41514-021-00071-w, PMID: 34349106 PMC8338939

[ref31] Dubenko O. E. Chyniak O. S. Potapov O. O. (2021). Levels of proinflammatory cytokines IL-17 and IL-23 in patients with Alzheimer’s disease, mild cognitive impairment and vascular dementia. Wiad. Lek. 74, 68–71. doi: 10.36740/WLek202101113, PMID: 33851590

[ref32] Duncan S. H. Conti E. Ricci L. Walker A. W. (2023). Links between diet, intestinal anaerobes, microbial metabolites and health. Biomedicines. 11:1338. doi: 10.3390/biomedicines11051338, PMID: 37239009 PMC10216541

[ref33] Engen P. A. Green S. J. Voigt R. M. Forsyth C. B. Keshavarzian A. (2015). The gastrointestinal microbiome: alcohol effects on the composition of intestinal microbiota. Alcohol Res. 37, 223–236. Available at: https://pubmed.ncbi.nlm.nih.gov/26695747, PMID: 26695747 10.35946/arcr.v37.2.07 PMC4590619

[ref34] Falony G. Joossens M. Vieira-Silva S. Wang J. Darzi Y. Faust K.. (2016). Population-level analysis of gut microbiome variation. Science 352, 560–564. doi: 10.1126/science.aad3503, PMID: 27126039

[ref35] Fang Q. Zheng B. Liu N. Liu J. Liu W. Huang X.. (2021). Trimethylamine N-oxide exacerbates renal inflammation and fibrosis in rats with diabetic kidney disease. Front. Physiol. 12:682482. doi: 10.3389/fphys.2021.682482 34220546 PMC8243655

[ref36] Farnsworth von Cederwald B. Josefsson M. Wåhlin A. Nyberg L. Karalija N. (2022). Association of cardiovascular risk trajectory with cognitive decline and incident dementia. Neurology 98, e2013–e2022. doi: 10.1212/WNL.0000000000200255, PMID: 35444051 PMC9162045

[ref37] Fu Q. Wu J. Zhou X.-Y. Ji M.-H. Mao Q.-H. Li Q.. (2019). NLRP3/Caspase-1 pathway-induced pyroptosis mediated cognitive deficits in a mouse model of sepsis-associated encephalopathy. Inflammation 42, 306–318. doi: 10.1007/s10753-018-0894-4, PMID: 30276509 PMC6394578

[ref38] Fung T. C. (2020). The microbiota-immune axis as a central mediator of gut-brain communication. Neurobiol. Dis. 136:104714. doi: 10.1016/j.nbd.2019.104714, PMID: 31846737

[ref39] Fung T. C. Olson C. A. Hsiao E. Y. (2017). Interactions between the microbiota, immune and nervous systems in health and disease. Nat. Neurosci. 20, 145–155. doi: 10.1038/nn.4476, PMID: 28092661 PMC6960010

[ref40] Gao H. Findeis E. L. Culmone L. Powell B. Landschoot-Ward J. Zacharek A.. (2023). Early therapeutic effects of an Angiopoietin-1 mimetic peptide in middle-aged rats with vascular dementia. Front. Aging Neurosci. 15:1180913. doi: 10.3389/fnagi.2023.1180913, PMID: 37304071 PMC10248134

[ref41] Gärtner Y. Bitar L. Zipp F. Vogelaar C. F. (2023). Interleukin-4 as a therapeutic target. Pharmacol. Ther. 242:108348. doi: 10.1016/j.pharmthera.2023.108348 36657567

[ref42] Ge H. Cai Z. Chai J. Liu J. Liu B. Yu Y.. (2021). Egg white peptides ameliorate dextran sulfate sodium-induced acute colitis symptoms by inhibiting the production of pro-inflammatory cytokines and modulation of gut microbiota composition. Food Chem. 360:129981. doi: 10.1016/j.foodchem.2021.129981, PMID: 34020366

[ref43] Gong W. Guo P. Li Y. Liu L. Yan R. Liu S.. (2023). Role of the gut-brain axis in the shared genetic etiology between gastrointestinal tract diseases and psychiatric disorders: a genome-wide pleiotropic analysis. JAMA Psychiatry 80, 360–370. doi: 10.1001/jamapsychiatry.2022.4974, PMID: 36753304 PMC9909581

[ref44] Gorky J. Schwaber J. (2016). The role of the gut-brain axis in alcohol use disorders. Prog. Neuro-Psychopharmacol. Biol. Psychiatry 65, 234–241. doi: 10.1016/j.pnpbp.2015.06.013, PMID: 26188287 PMC4679635

[ref45] Hakim A. M. (2021). A proposed hypothesis on dementia: inflammation, small vessel disease, and hypoperfusion is the sequence that links all harmful lifestyles to cognitive impairment. Front. Aging Neurosci. 13:679837. doi: 10.3389/fnagi.2021.679837, PMID: 33994998 PMC8116506

[ref46] He Q. Li J. Ma Y. Chen Q. Chen G. (2021). Probiotic potential and cholesterol-lowering capabilities of bacterial strains isolated from Pericarpium Citri Reticulatae “Chachiensis”. Microorganisms. 9:1224. doi: 10.3390/microorganisms9061224, PMID: 34200041 PMC8227569

[ref47] He J. Zhang P. Shen L. Niu L. Tan Y. Chen L.. (2020). Short-chain fatty acids and their association with signalling pathways in inflammation, glucose and lipid metabolism. Int. J. Mol. Sci. 21:6356. doi: 10.3390/ijms21176356, PMID: 32887215 PMC7503625

[ref48] Hu C. Rzymski P. (2022). Non-photosynthetic melainabacteria (cyanobacteria) in human gut: characteristics and association with health. Life (Basel). 12:476. doi: 10.3390/life12040476, PMID: 35454968 PMC9029806

[ref49] Hu M. Zheng P. Xie Y. Boz Z. Yu Y. Tang R.. (2018). Propionate protects haloperidol-induced neurite lesions mediated by neuropeptide Y. Front. Neurosci. 12:743. doi: 10.3389/fnins.2018.00743, PMID: 30374288 PMC6196753

[ref50] Hussain B. Fang C. Chang J. (2021). Blood-brain barrier breakdown: an emerging biomarker of cognitive impairment in normal aging and dementia. Front. Neurosci. 15:688090. doi: 10.3389/fnins.2021.688090, PMID: 34489623 PMC8418300

[ref51] Ising C. Venegas C. Zhang S. Scheiblich H. Schmidt S. V. Vieira-Saecker A.. (2019). NLRP3 inflammasome activation drives tau pathology. Nature 575, 669–673. doi: 10.1038/s41586-019-1769-z, PMID: 31748742 PMC7324015

[ref52] Ji D. Chen W.-Z. Zhang L. Zhang Z.-H. Chen L.-J. (2024). Gut microbiota, circulating cytokines and dementia: a Mendelian randomization study. J. Neuroinflammation 21:2. doi: 10.1186/s12974-023-02999-0, PMID: 38178103 PMC10765696

[ref53] Kang R. Gamdzyk M. Luo Y. Tang H. Huang L. Lenahan C.. (2023). Three days delayed recanalization improved neurological function in pMCAO rats by increasing M2 microglia-possible involvement of the IL-4R/STAT6/PPARγ pathway. Transl. Stroke Res. 14, 250–262. doi: 10.1007/s12975-022-01032-5, PMID: 35867328 PMC11586074

[ref54] Kaur N. LaForce G. Mallela D. P. Saha P. P. Buffa J. Li X. S.. (2023). Exploratory transcriptomic profiling reveals the role of gut microbiota in vascular dementia. Int. J. Mol. Sci. 24:8091. doi: 10.3390/ijms24098091, PMID: 37175797 PMC10178712

[ref55] Kazmi N. Wallen G. R. Yang L. Alkhatib J. Schwandt M. L. Feng D.. (2022). An exploratory study of pro-inflammatory cytokines in individuals with alcohol use disorder: MCP-1 and IL-8 associated with alcohol consumption, sleep quality, anxiety, depression, and liver biomarkers. Front. Psych. 13:931280. doi: 10.3389/fpsyt.2022.931280, PMID: 36032219 PMC9405018

[ref56] Khanna D. Peltzer C. Kahar P. Parmar M. S. (2022). Body mass index (BMI): a screening tool analysis. Cureus 14:e22119. doi: 10.7759/cureus.22119, PMID: 35308730 PMC8920809

[ref57] Kim B.-S. Choi C. W. Shin H. Jin S.-P. Bae J.-S. Han M.. (2019). Comparison of the gut microbiota of centenarians in longevity villages of South Korea with those of other age groups. J. Microbiol. Biotechnol. 29, 429–440. doi: 10.4014/jmb.1811.11023, PMID: 30661321

[ref58] Kurilshikov A. Medina-Gomez C. Bacigalupe R. Radjabzadeh D. Wang J. Demirkan A.. (2021). Large-scale association analyses identify host factors influencing human gut microbiome composition. Nat. Genet. 53, 156–165. doi: 10.1038/s41588-020-00763-1, PMID: 33462485 PMC8515199

[ref59] Kurki M. I. Karjalainen J. Palta P. Sipilä T. P. Kristiansson K. Donner K. M.. (2023). FinnGen provides genetic insights from a well-phenotyped isolated population. Nature 613, 508–518. doi: 10.1038/s41586-022-05473-8, PMID: 36653562 PMC9849126

[ref60] Lee J. Venna V. R. Durgan D. J. Shi H. Hudobenko J. Putluri N.. (2020). Young versus aged microbiota transplants to germ-free mice: increased short-chain fatty acids and improved cognitive performance. Gut Microbes 12, 1814107–1814114. doi: 10.1080/19490976.2020.1814107, PMID: 32897773 PMC7757789

[ref61] Li Y. Fu R. Li R. Zeng J. Liu T. Li X.. (2023a). Causality of gut microbiome and hypertension: a bidirectional Mendelian randomization study. Front. Cardiovasc. Med. 10:1167346. doi: 10.3389/fcvm.2023.1167346, PMID: 37215554 PMC10192878

[ref62] Li W. Ren A. Qin Q. Zhao L. Peng Q. Ma R.. (2023b). Causal associations between human gut microbiota and cholelithiasis: a Mendelian randomization study. Front. Cell. Infect. Microbiol. 13:1169119. doi: 10.3389/fcimb.2023.1169119, PMID: 37305422 PMC10248444

[ref63] Li H. Tian J. Yin Y. Diao S. Zhang X. Zuo T.. (2022). Interleukin-18 mediated inflammatory brain injury after intracerebral hemorrhage in male mice. J. Neurosci. Res. 100, 1359–1369. doi: 10.1002/jnr.25044, PMID: 35316547

[ref64] Liang H. Jiang F. Cheng R. Luo Y. Wang J. Luo Z.. (2021). A high-fat diet and high-fat and high-cholesterol diet may affect glucose and lipid metabolism differentially through gut microbiota in mice. Exp. Anim. 70, 73–83. doi: 10.1538/expanim.20-0094, PMID: 32999215 PMC7887617

[ref65] Liang C.-J. Li J.-H. Zhang Z. Zhang J.-Y. Liu S.-Q. Yang J. (2018). Suppression of MIF-induced neuronal apoptosis may underlie the therapeutic effects of effective components of Fufang Danshen in the treatment of Alzheimer’s disease. Acta Pharmacol. Sin. 39, 1421–1438. doi: 10.1038/aps.2017.210, PMID: 29770796 PMC6289349

[ref66] Lin Z. Deng Y. Pan W. (2021). Combining the strengths of inverse-variance weighting and egger regression in Mendelian randomization using a mixture of regressions model. PLoS Genet. 17:e1009922. doi: 10.1371/journal.pgen.1009922, PMID: 34793444 PMC8639093

[ref67] Liu Q. Pan P. Ling Z. Zhang Z. Zhang X. Li S. (2023a). Acupuncture regulates the Th17/Treg balance and improves cognitive deficits in a rat model of vascular dementia. Heliyon. 9:e13346. doi: 10.1016/j.heliyon.2023.e13346, PMID: 36816326 PMC9929319

[ref68] Liu J. Sun J. Wang F. Yu X. Ling Z. Li H.. (2015). Neuroprotective effects of *Clostridium butyricum* against vascular dementia in mice via metabolic butyrate. Biomed. Res. Int. 2015:412946. doi: 10.1155/2015/412946, PMID: 26523278 PMC4615854

[ref69] Liu Y.-C. Tsai Y.-H. Tang S.-C. Liou H.-C. Kang K.-H. Liou H.-H.. (2018). Cytokine MIF enhances blood-brain barrier permeability: impact for therapy in ischemic stroke. Sci. Rep. 8:743. doi: 10.1038/s41598-017-16927-9, PMID: 29335619 PMC5768806

[ref70] Liu C. Zhu S. Zhang J. Ren K. Li K. Yu J. (2023b). Inflammatory bowel diseases, interleukin-6 and interleukin-6 receptor subunit alpha in causal association with cerebral cortical structure: a Mendelian randomization analysis. Front. Immunol. 14:1154746. doi: 10.3389/fimmu.2023.1154746, PMID: 37153572 PMC10157470

[ref71] Livingston G. Sommerlad A. Orgeta V. Costafreda S. G. Huntley J. Ames D.. (2017). Dementia prevention, intervention, and care. Lancet (London, England) 390, 2673–2734. doi: 10.1016/S0140-6736(17)31363-6 28735855

[ref72] Long-Smith C. O’Riordan K. J. Clarke G. Stanton C. Dinan T. G. Cryan J. F. (2020). Microbiota-gut-brain Axis: new therapeutic opportunities. Annu. Rev. Pharmacol. Toxicol. 60, 477–502. doi: 10.1146/annurev-pharmtox-010919-023628, PMID: 31506009

[ref73] Malaguarnera L. Motta M. Di Rosa M. Anzaldi M. Malaguarnera M. (2006). Interleukin-18 and transforming growth factor-beta 1 plasma levels in Alzheimer’s disease and vascular dementia. Neuropathology 26, 307–312. doi: 10.1111/j.1440-1789.2006.00701.x, PMID: 16961066

[ref74] Merra G. Noce A. Marrone G. Cintoni M. Tarsitano M. G. Capacci A.. (2020). Influence of Mediterranean diet on human gut microbiota. Nutrients 13:7. doi: 10.3390/nu13010007, PMID: 33375042 PMC7822000

[ref75] Mirzaei R. Bouzari B. Hosseini-Fard S. R. Mazaheri M. Ahmadyousefi Y. Abdi M.. (2021). Role of microbiota-derived short-chain fatty acids in nervous system disorders. Biomed. Pharmacother. 139:111661. doi: 10.1016/j.biopha.2021.111661, PMID: 34243604

[ref76] Morrison D. J. Preston T. (2016). Formation of short chain fatty acids by the gut microbiota and their impact on human metabolism. Gut Microbes 7, 189–200. doi: 10.1080/19490976.2015.1134082, PMID: 26963409 PMC4939913

[ref77] Nam K.-W. Kwon H.-M. Lee Y.-S. (2020). Distinct association between cerebral arterial pulsatility and subtypes of cerebral small vessel disease. PLoS One 15:e0236049. doi: 10.1371/journal.pone.0236049, PMID: 32673353 PMC7365409

[ref78] Naumova N. Alikina T. Tupikin A. Kalmykova A. Soldatova G. Vlassov V.. (2020). Human gut microbiome response to short-term bifidobacterium-based probiotic treatment. Indian J. Microbiol. 60, 451–457. doi: 10.1007/s12088-020-00888-1, PMID: 33087994 PMC7539239

[ref79] Ning J. Huang S.-Y. Chen S.-D. Zhang Y.-R. Huang Y.-Y. Yu J.-T. (2022). Investigating casual associations among gut microbiota, metabolites, and neurodegenerative diseases: a Mendelian randomization study. J. Alzheimers Dis. 87, 211–222. doi: 10.3233/JAD-215411, PMID: 35275534

[ref80] O’Brien J. T. Thomas A. (2015). Vascular dementia. Lancet 386, 1698–1706. doi: 10.1016/S0140-6736(15)00463-8 26595643

[ref81] Parker A. Fonseca S. Carding S. R. (2020). Gut microbes and metabolites as modulators of blood-brain barrier integrity and brain health. Gut Microbes 11, 135–157. doi: 10.1080/19490976.2019.1638722, PMID: 31368397 PMC7053956

[ref82] Parte A. C. (2014). LPSN--list of prokaryotic names with standing in nomenclature. Nucleic Acids Res. 42, D613–D616. doi: 10.1093/nar/gkt1111, PMID: 24243842 PMC3965054

[ref83] Peh A. O’Donnell J. A. Broughton B. R. S. Marques F. Z. (2022). Gut microbiota and their metabolites in stroke: a double-edged sword. Stroke 53, 1788–1801. doi: 10.1161/STROKEAHA.121.036800, PMID: 35135325

[ref84] Pei Y. Lu Y. Li H. Jiang C. Wang L. (2023). Gut microbiota and intestinal barrier function in subjects with cognitive impairments: a cross-sectional study. Front. Aging Neurosci. 15:1174599. doi: 10.3389/fnagi.2023.1174599, PMID: 37350810 PMC10282132

[ref85] Petralia M. C. Mazzon E. Mangano K. Fagone P. Di Marco R. Falzone L.. (2020). Transcriptomic analysis reveals moderate modulation of macrophage migration inhibitory factor superfamily genes in alcohol use disorders. Exp. Ther. Med. 19, 1755–1762. doi: 10.3892/etm.2020.8410, PMID: 32104230 PMC7026954

[ref86] Poh L. Fann D. Y. Wong P. Lim H. M. Foo S. L. Kang S.-W.. (2021). AIM2 inflammasome mediates hallmark neuropathological alterations and cognitive impairment in a mouse model of vascular dementia. Mol. Psychiatry 26, 4544–4560. doi: 10.1038/s41380-020-00971-5, PMID: 33299135

[ref87] Pu H. Ma C. Zhao Y. Wang Y. Zhang W. Miao W.. (2021a). Intranasal delivery of interleukin-4 attenuates chronic cognitive deficits via beneficial microglial responses in experimental traumatic brain injury. J. Cereb. Blood Flow Metab. 41, 2870–2886. doi: 10.1177/0271678X211028680, PMID: 34259069 PMC8545055

[ref88] Pu H. Wang Y. Yang T. Leak R. K. Stetler R. A. Yu F.. (2023). Interleukin-4 mitigates anxiety-like behavior and loss of neurons and fiber tracts in limbic structures in a microglial PPARγ-dependent manner after traumatic brain injury. Neurobiol. Dis. 180:106078. doi: 10.1016/j.nbd.2023.106078, PMID: 36914076

[ref89] Pu H. Zheng X. Jiang X. Mu H. Xu F. Zhu W.. (2021b). Interleukin-4 improves white matter integrity and functional recovery after murine traumatic brain injury via oligodendroglial PPARγ. J. Cereb. Blood Flow Metab. 41, 511–529. doi: 10.1177/0271678X20941393, PMID: 32757740 PMC7922743

[ref90] Qin X. Akter F. Qin L. Cheng J. Guo M. Yao S.. (2020). Adaptive immunity regulation and cerebral ischemia. Front. Immunol. 11:689. doi: 10.3389/fimmu.2020.00689, PMID: 32477327 PMC7235404

[ref91] Rahmati H. Momenabadi S. Vafaei A. A. Bandegi A. R. Mazaheri Z. Vakili A. (2019). Probiotic supplementation attenuates hippocampus injury and spatial learning and memory impairments in a cerebral hypoperfusion mouse model. Mol. Biol. Rep. 46, 4985–4995. doi: 10.1007/s11033-019-04949-7, PMID: 31286392

[ref92] Rands C. M. Brüssow H. Zdobnov E. M. (2019). Comparative genomics groups phages of negativicutes and classical firmicutes despite different gram-staining properties. Env. Microbiol. 21, 3989–4001. doi: 10.1111/1462-2920.14746, PMID: 31314945

[ref93] Rehm J. Hasan O. S. M. Black S. E. Shield K. D. Schwarzinger M. (2019). Alcohol use and dementia: a systematic scoping review. Alzheimers Res. Ther. 11:1. doi: 10.1186/s13195-018-0453-0 30611304 PMC6320619

[ref94] Reichardt N. Duncan S. H. Young P. Belenguer A. McWilliam Leitch C. Scott K. P.. (2014). Phylogenetic distribution of three pathways for propionate production within the human gut microbiota. ISME J. 8, 1323–1335. doi: 10.1038/ismej.2014.14, PMID: 24553467 PMC4030238

[ref95] Sanderson E. Davey Smith G. Windmeijer F. Bowden J. (2019). An examination of multivariable Mendelian randomization in the single-sample and two-sample summary data settings. Int. J. Epidemiol. 48, 713–727. doi: 10.1093/ije/dyy262, PMID: 30535378 PMC6734942

[ref96] Sarkar A. Lehto S. M. Harty S. Dinan T. G. Cryan J. F. Burnet P. W. J. (2016). Psychobiotics and the manipulation of bacteria-gut-brain signals. Trends Neurosci. 39, 763–781. doi: 10.1016/j.tins.2016.09.002, PMID: 27793434 PMC5102282

[ref97] Shang G. Shao Q. Lv K. Xu W. Ji J. Fan S.. (2024). Hypercholesterolemia and the increased risk of vascular dementia: a cholesterol perspective. Curr. Atheroscler. Rep. 26, 435–449. doi: 10.1007/s11883-024-01217-3, PMID: 38814418

[ref98] Shiau M.-Y. Chuang P.-H. Yang C.-P. Hsiao C.-W. Chang S.-W. Chang K.-Y.. (2019). Mechanism of Interleukin-4 reducing lipid deposit by regulating hormone-sensitive lipase. Sci. Rep. 9:11974. doi: 10.1038/s41598-019-47908-9, PMID: 31427606 PMC6700157

[ref99] Silva D. F. Candeias E. Esteves A. R. Magalhães J. D. Ferreira I. L. Nunes-Costa D.. (2020). Microbial BMAA elicits mitochondrial dysfunction, innate immunity activation, and Alzheimer’s disease features in cortical neurons. J. Neuroinflammation 17:332. doi: 10.1186/s12974-020-02004-y, PMID: 33153477 PMC7643281

[ref100] Sinitski D. Kontos C. Krammer C. Asare Y. Kapurniotu A. Bernhagen J. (2019). Macrophage migration inhibitory factor (MIF)-based therapeutic concepts in atherosclerosis and inflammation. Thromb. Haemost. 119, 553–566. doi: 10.1055/s-0039-1677803, PMID: 30716779

[ref101] Song J. Li M. Kang N. Jin W. Xiao Y. Li Z.. (2024). Baicalein ameliorates cognitive impairment of vascular dementia rats via suppressing neuroinflammation and regulating intestinal microbiota. Brain Res. Bull. 208:110888. doi: 10.1016/j.brainresbull.2024.110888 38295883

[ref102] Song X. Wang Y. Yang W. Wang Y. Yang C. Chen Z. (2023). Abnormal spontaneous discharges of primary sensory neurons and pain behavior in a rat model of vascular dementia. Int. J. Mol. Sci. 24:10198. doi: 10.3390/ijms241210198, PMID: 37373344 PMC10299503

[ref103] Sumaiya K. Langford D. Natarajaseenivasan K. Shanmughapriya S. (2022). Macrophage migration inhibitory factor (MIF): a multifaceted cytokine regulated by genetic and physiological strategies. Pharmacol. Ther. 233:108024. doi: 10.1016/j.pharmthera.2021.108024, PMID: 34673115

[ref104] Sun P. Su L. Zhu H. Li X. Guo Y. Du X.. (2021). Gut microbiota regulation and their implication in the development of neurodegenerative disease. Microorganisms. 9:2281. doi: 10.3390/microorganisms9112281, PMID: 34835406 PMC8621510

[ref105] Tashiro R. Ozaki D. Bautista-Garrido J. Sun G. Obertas L. Mobley A. S.. (2023). Young astrocytic mitochondria attenuate the elevated level of CCL11 in the aged mice, contributing to cognitive function improvement. Int. J. Mol. Sci. 24:5187. doi: 10.3390/ijms24065187, PMID: 36982260 PMC10049211

[ref106] Thevaranjan N. Puchta A. Schulz C. Naidoo A. Szamosi J. C. Verschoor C. P.. (2017). Age-associated microbial Dysbiosis promotes intestinal permeability, systemic inflammation, and macrophage dysfunction. Cell Host Microbe 21, 455–466.e4. doi: 10.1016/j.chom.2017.03.002, PMID: 28407483 PMC5392495

[ref107] Thiele M. Donnelly S. C. Mitchell R. A. (2022). OxMIF: a druggable isoform of macrophage migration inhibitory factor in cancer and inflammatory diseases. J. Immunother. Cancer 10:e005475. doi: 10.1136/jitc-2022-005475, PMID: 36180072 PMC9528626

[ref108] Tian Z. Ji X. Liu J. (2022). Neuroinflammation in vascular cognitive impairment and dementia: current evidence, advances, and prospects. Int. J. Mol. Sci. 23:6224. doi: 10.3390/ijms23116224, PMID: 35682903 PMC9181710

[ref109] Tsao C.-H. Shiau M.-Y. Chuang P.-H. Chang Y.-H. Hwang J. (2014). Interleukin-4 regulates lipid metabolism by inhibiting adipogenesis and promoting lipolysis. J. Lipid Res. 55, 385–397. doi: 10.1194/jlr.M041392, PMID: 24347527 PMC3934724

[ref110] Wang Y. Zhi H. Zhang X. (2023a). Effect of Huangdisan grain on improving cognitive impairment in VD rats and its mechanism in immune inflammatory response. J. Neuroimmunol. 377:578058. doi: 10.1016/j.jneuroim.2023.578058, PMID: 36871311

[ref111] Wang S. Zhu H. Pan L. Zhang M. Wan X. Xu H.. (2023b). Systemic inflammatory regulators and risk of acute-on-chronic liver failure: a bidirectional Mendelian-randomization study. Front. Cell Dev. Biol. 11:1125233. doi: 10.3389/fcell.2023.1125233, PMID: 36743413 PMC9892464

[ref112] Weekman E. M. Wilcock D. M. (2016). Matrix metalloproteinase in blood-brain barrier breakdown in dementia. J. Alzheimers Dis. 49, 893–903. doi: 10.3233/JAD-150759 26599057

[ref113] Wilmanski T. Diener C. Rappaport N. Patwardhan S. Wiedrick J. Lapidus J.. (2021). Gut microbiome pattern reflects healthy ageing and predicts survival in humans. Nat. Metab. 3, 274–286. doi: 10.1038/s42255-021-00348-0, PMID: 33619379 PMC8169080

[ref114] Wolters F. J. Ikram M. A. (2019). Epidemiology of vascular dementia. Arter. Thromb. Vasc. Biol. 39, 1542–1549. doi: 10.1161/ATVBAHA.119.311908 31294622

[ref115] Wu F. Huang Y. Hu J. Shao Z. (2020). Mendelian randomization study of inflammatory bowel disease and bone mineral density. BMC Med. 18:312. doi: 10.1186/s12916-020-01778-5, PMID: 33167994 PMC7654011

[ref116] Xia S. Zhang X. Zheng S. Khanabdali R. Kalionis B. Wu J.. (2016). An update on inflammaging: mechanisms, prevention, and treatment. J Immunol Res 2016, 1–12. doi: 10.1155/2016/8426874 PMC4963991 27493973

[ref117] Xiang S. Ji J.-L. Li S. Cao X.-P. Xu W. Tan L.. (2022). Efficacy and safety of probiotics for the treatment of Alzheimer’s disease, mild cognitive impairment, and Parkinson’s disease: a systematic review and meta-analysis. Front. Aging Neurosci. 14:730036. doi: 10.3389/fnagi.2022.730036, PMID: 35185522 PMC8851038

[ref118] Xu C. Zhu H. Qiu P. (2019). Aging progression of human gut microbiota. BMC Microbiol. 19:236. doi: 10.1186/s12866-019-1616-2, PMID: 31660868 PMC6819604

[ref119] Yang X.-H. Song S.-Q. Xu Y. (2017). Resveratrol ameliorates chronic unpredictable mild stress-induced depression-like behavior: involvement of the HPA axis, inflammatory markers, BDNF, and Wnt/β-catenin pathway in rats. Neuropsychiatr. Dis. Treat. 13, 2727–2736. doi: 10.2147/NDT.S150028, PMID: 29138567 PMC5667793

[ref120] Yasuda K. Nakanishi K. Tsutsui H. (2019). Interleukin-18 in health and disease. Int. J. Mol. Sci. 20:649. doi: 10.3390/ijms20030649, PMID: 30717382 PMC6387150

[ref121] Yu Q. Zhao T. Liu M. Cao D. Li J. Li Y.. (2021). Targeting NLRP3 Inflammasome in translational treatment of nervous system diseases: an update. Front. Pharmacol. 12:707696. doi: 10.3389/fphar.2021.707696, PMID: 34526897 PMC8435574

[ref122] Zarbock K. R. Han J. H. Singh A. P. Thomas S. P. Bendlin B. B. Denu J. M.. (2022). Trimethylamine N-oxide reduces neurite density and plaque intensity in a murine model of Alzheimer’s disease. J. Alzheimers Dis. 90, 585–597. doi: 10.3233/JAD-220413, PMID: 36155509 PMC9881463

[ref123] Zhang D. Jia N. Hu Z. Keqing Z. Chenxi S. Chunying S.. (2024). Bioinformatics identification of potential biomarkers and therapeutic targets for ischemic stroke and vascular dementia. Exp. Gerontol. 187:112374. doi: 10.1016/j.exger.2024.112374, PMID: 38320734

[ref124] Zhang B. Wang H. E. Bai Y.-M. Tsai S.-J. Su T.-P. Chen T.-J.. (2021a). Inflammatory bowel disease is associated with higher dementia risk: a nationwide longitudinal study. Gut 70, 85–91. doi: 10.1136/gutjnl-2020-320789, PMID: 32576641

[ref125] Zhang T. Wu X. Liu B. Huang H. Zhou C. Liang P. (2023a). The contribution of probiotics for the double-edge effect of cefazolin on postoperative neurocognitive disorders by rebalancing the gut microbiota. Front. Neurosci. 17:1156453. doi: 10.3389/fnins.2023.1156453, PMID: 37179548 PMC10174111

[ref126] Zhang M.-N. Xie R. Wang H.-G. Wen X. Wang J.-Y. He L.. (2023b). Cepharanthine alleviates DSS-induced ulcerative colitis via regulating Aconitate decarboxylase 1 expression and macrophage infiltration. Molecules 28:1060. doi: 10.3390/molecules28031060, PMID: 36770726 PMC9920045

[ref127] Zhang Y. Yang Y. Li H. Feng Q. Ge W. Xu X. (2023c). Investigating the potential mechanisms and therapeutic targets of inflammatory cytokines in post-stroke depression. Mol. Neurobiol. 61, 132–147. doi: 10.1007/s12035-023-03563-w, PMID: 37592185

[ref128] Zhang F. Zhao K. Tang T. Deng Y. Zhang Y. Feng S.. (2021b). Bisindole compound 4ae ameliorated cognitive impairment in rats with vascular dementia by anti-inflammation effect via microglia cells. Eur. J. Pharmacol. 908:174357. doi: 10.1016/j.ejphar.2021.174357, PMID: 34284012

[ref129] Zhang Q. Zhu W. Xu F. Dai X. Shi L. Cai W.. (2019). The interleukin-4/PPARγ signaling axis promotes oligodendrocyte differentiation and remyelination after brain injury. PLoS Biol. 17:e3000330. doi: 10.1371/journal.pbio.3000330, PMID: 31226122 PMC6608986

[ref130] Zhao J. Wang X. Li Q. Lu C. Li S. (2023a). The relevance of serum macrophage migratory inhibitory factor and cognitive dysfunction in patients with cerebral small vascular disease. Front. Aging Neurosci. 15:1083818. doi: 10.3389/fnagi.2023.1083818, PMID: 36824264 PMC9941340

[ref131] Zhao J. Wang X. Yu M. Zhang S. Li Q. Liu H.. (2023b). The relevance of serum macrophage migration inhibitory factor level and executive function in patients with white matter hyperintensity in cerebral small vessel disease. Brain Sci. 13:616. doi: 10.3390/brainsci13040616, PMID: 37190581 PMC10136861

[ref132] Zhu N. Liang X. Zhang M. Yin X. Yang H. Zhi Y.. (2020). Astaxanthin protects cognitive function of vascular dementia. Behav. Brain Funct. 16:10. doi: 10.1186/s12993-020-00172-8, PMID: 33208152 PMC7672991

[ref133] Zhu J.-D. Wang J.-J. Zhang X.-H. Yu Y. Kang Z.-S. (2018). *Panax ginseng* extract attenuates neuronal injury and cognitive deficits in rats with vascular dementia induced by chronic cerebral hypoperfusion. Neural Regen. Res. 13, 664–672. doi: 10.4103/1673-5374.230292, PMID: 29722318 PMC5950676

[ref134] Zhu G. Zhao J. Wang G. Chen W. (2023). *Bifidobacterium breve* HNXY26M4 attenuates cognitive deficits and neuroinflammation by regulating the gut-brain axis in APP/PS1 mice. J. Agric. Food Chem. 71, 4646–4655. doi: 10.1021/acs.jafc.3c00652, PMID: 36888896

[ref135] Zhuang Z.-Q. Shen L.-L. Li W.-W. Fu X. Zeng F. Gui L.. (2018). Gut microbiota is altered in patients with Alzheimer’s disease. J. Alzheimers Dis. 63, 1337–1346. doi: 10.3233/JAD-180176 29758946

