# Supplementary material for: Role of inflammatory cytokines and the gut microbiome in vascular dementia: insights from Mendelian randomization analysis
Source: Front Microbiol. 2024 Aug 23;15:1398618. doi: 10.3389/fmicb.2024.1398618 (PMC11380139; doi:10.3389/fmicb.2024.1398618)
Supplement: Supplementary file 1 [file Data_Sheet_1.zip › Supplementary Table S1.pdf]

Supplementary Table S1 Instrumental Variables related to exposures (inflammatory cytokines) and outcomes (6 subtypes of vascular dementia)

Outcome	Exposure	SNP	Outcome							Exposure								F-statistics
			Effect allele	Other allele	Beta	Pos	Pval	SE	Samplesize	Effect allele	Other allele	Beta	Chr	Pos	Pval	SE	Samplesize	
Vascular dementia (mixed)	CTACK	rs116303454	A	G	0.508	27253164	0.050	0.260	360421	A	G	0.383	3	27294655	3.27E-06	0.082	3631	22.030
Vascular dementia (mixed)	CTACK	rs2070074	G	A	0.095	34649445	0.477	0.134	360421	G	A	-0.447	9	34649442	1.78E-32	0.037	3631	142.656
Vascular dementia (mixed)	CTACK	rs2731674	G	T	0.099	177412889	0.298	0.095	360421	G	T	0.133	5	176839890	5.63E-07	0.027	3631	24.925
Vascular dementia (mixed)	CTACK	rs3766110	C	A	0.081	169545945	0.414	0.098	360421	C	A	0.129	1	169515183	3.85E-06	0.028	3631	21.432
Vascular dementia (mixed)	CTACK	rs55764737	C	T	-0.432	61031215	0.217	0.350	360421	C	T	-0.531	15	61323414	4.62E-08	0.097	3631	29.878
Vascular dementia (mixed)	CTACK	rs57338032	G	A	0.033	78506597	0.770	0.113	360421	G	A	-0.158	15	78798939	6.23E-07	0.032	3631	24.937
Vascular dementia (mixed)	CTACK	rs7333764	T	C	-0.175	33634664	0.432	0.222	360421	T	C	0.277	13	34208801	2.85E-06	0.059	3631	21.867
Vascular dementia (mixed)	CTACK	rs76395525	A	G	0.060	79449049	0.887	0.425	360421	A	G	0.528	15	79741391	9.55E-07	0.108	3631	23.742
Vascular dementia (mixed)	beta-nerve growth factor	rs28637706	T	G	-0.054	33794463	0.559	0.092	360421	T	G	-0.159	19	34285368	1.42E-09	0.026	3531	36.504
Vascular dementia (mixed)	beta-nerve growth factor	rs67476890	T	C	0.199	62499295	0.134	0.133	360421	T	C	0.177	15	62791494	3.13E-06	0.038	3531	21.786
Vascular dementia (mixed)	beta-nerve growth factor	rs71641308	T	C	-0.243	77621033	0.085	0.141	360421	T	C	0.204	1	78086718	2.30E-06	0.043	3531	22.365
Vascular dementia (mixed)	beta-nerve growth factor	rs72780728	A	G	-0.187	17561702	0.205	0.148	360421	A	G	0.188	10	17603701	2.99E-06	0.040	3531	21.832
Vascular dementia (mixed)	beta-nerve growth factor	rs73472576	C	T	0.153	74456947	0.071	0.085	360421	C	T	0.118	18	72124182	2.69E-06	0.025	3531	21.963
Vascular dementia (mixed)	beta-nerve growth factor	rs7970581	G	T	-0.002	112827443	0.979	0.095	360421	G	T	-0.138	12	113265248	9.27E-07	0.028	3531	23.947
Vascular dementia (mixed)	beta-nerve growth factor	rs9436119	A	G	-0.036	150495277	0.677	0.085	360421	A	G	-0.112	1	150467753	3.91E-06	0.025	3531	20.765
Vascular dementia (mixed)	Vascular endothelial growth factor	rs10153304	A	G	0.131	7818613	0.389	0.152	360421	A	G	0.155	17	7721931	1.94E-06	0.033	7118	22.658
Vascular dementia (mixed)	Vascular endothelial growth factor	rs10934631	C	T	-0.041	122978753	0.734	0.120	360421	C	T	0.115	3	122697600	2.47E-06	0.025	7118	22.071
Vascular dementia (mixed)	Vascular endothelial growth factor	rs10967186	C	T	-0.108	2617099	0.203	0.085	360421	C	T	-0.090	9	2617099	1.23E-07	0.017	7118	27.903
Vascular dementia (mixed)	Vascular endothelial growth factor	rs13209117	A	G	-0.114	44184028	0.238	0.097	360421	A	G	0.130	6	44151765	5.28E-11	0.020	7118	41.959
Vascular dementia (mixed)	Vascular endothelial growth factor	rs143479231	A	G	-0.103	193393005	0.628	0.213	360421	A	G	-0.260	3	193110794	1.90E-07	0.049	7118	27.997
Vascular dementia (mixed)	Vascular endothelial growth factor	rs4082730	A	G	-0.175	89980326	0.510	0.265	360421	A	G	0.252	15	90523558	2.64E-06	0.053	7118	22.305
Vascular dementia (mixed)	Vascular endothelial growth factor	rs6921438	A	G	-0.068	43957870	0.422	0.084	360421	A	G	-0.490	6	43925607	########	0.018	7118	784.000
Vascular dementia (mixed)	Vascular endothelial growth factor	rs73418461	A	G	-0.200	118463484	0.431	0.254	360421	A	G	-0.249	10	120222996	1.67E-06	0.052	7118	22.878
Vascular dementia (mixed)	Vascular endothelial growth factor	rs8045833	A	G	-0.130	88509031	0.203	0.102	360421	A	G	0.108	16	88575439	2.83E-07	0.021	7118	26.199
Vascular dementia (mixed)	Vascular endothelial growth factor	rs9472183	G	A	0.035	43972465	0.675	0.084	360421	G	A	0.128	6	43940202	5.19E-14	0.017	7118	56.869
Vascular dementia (mixed)	Macrophage Migration Inhibitory Factor	rs113218956	A	G	-0.344	24828867	0.562	0.593	360421	A	G	-0.895	22	25224834	2.26E-06	0.188	3494	22.678
Vascular dementia (mixed)	Macrophage Migration Inhibitory Factor	rs118055855	C	T	-0.314	29867025	0.520	0.488	360421	C	T	-0.691	11	29888572	4.13E-06	0.150	3494	21.203
Vascular dementia (mixed)	Macrophage Migration Inhibitory Factor	rs12594190	G	A	0.090	24791308	0.325	0.092	360421	G	A	-0.136	15	25036455	3.70E-07	0.027	3494	25.755
Vascular dementia (mixed)	Macrophage Migration Inhibitory Factor	rs13142904	T	C	-0.141	53452247	0.337	0.146	360421	T	C	-0.223	4	54318414	2.56E-07	0.043	3494	27.532
Vascular dementia (mixed)	Macrophage Migration Inhibitory Factor	rs141009259	C	T	0.230	207111559	0.558	0.393	360421	C	T	0.618	2	207976283	2.47E-06	0.132	3494	21.839
Vascular dementia (mixed)	Macrophage Migration Inhibitory Factor	rs78098071	C	T	0.229	163882733	0.493	0.335	360421	C	T	0.487	5	163309739	1.78E-07	0.092	3494	28.108
Vascular dementia (mixed)	TRAIL	rs11618126	G	A	-0.044	50252103	0.964	0.985	360421	G	A	-0.891	13	50826239	1.46E-06	0.191	8186	21.661
Vascular dementia (mixed)	TRAIL	rs11657269	G	A	0.199	6416464	0.149	0.138	360421	G	A	-0.119	17	6319784	4.78E-06	0.026	8186	20.878
Vascular dementia (mixed)	TRAIL	rs11699445	G	T	-0.060	15770145	0.481	0.086	360421	G	T	-0.075	20	15750790	3.27E-06	0.016	8186	21.470
Vascular dementia (mixed)	TRAIL	rs13185784	A	G	-0.083	180267068	0.384	0.096	360421	A	G	0.085	5	179694068	3.90E-06	0.018	8186	21.372
Vascular dementia (mixed)	TRAIL	rs138987090	G	A	-0.252	32786284	0.481	0.358	360421	G	A	0.750	18	30366247	4.50E-23	0.075	8186	99.389
Vascular dementia (mixed)	TRAIL	rs146783010	G	A	0.121	89527045	0.846	0.625	360421	G	A	0.602	11	89260213	4.83E-06	0.135	8186	19.859
Vascular dementia (mixed)	TRAIL	rs193112415	C	T	-0.414	31255157	0.167	0.300	360421	C	T	1.042	18	28835120	2.15E-62	0.062	8186	279.797
Vascular dementia (mixed)	TRAIL	rs57396456	C	T	-0.040	30365911	0.886	0.279	360421	C	T	0.563	18	27945877	1.25E-27	0.052	8186	117.961
Vascular dementia (mixed)	TRAIL	rs62093514	T	C	-0.085	31651014	0.770	0.290	360421	T	C	1.062	18	29230977	6.86E-82	0.055	8186	370.005
Vascular dementia (mixed)	TRAIL	rs73039026	C	A	0.465	172442691	0.130	0.307	360421	C	A	0.300	3	172160481	2.02E-06	0.064	8186	22.305
Vascular dementia (mixed)	TRAIL	rs747324	C	T	-0.093	74222941	0.323	0.094	360421	C	T	0.086	14	74689644	1.61E-06	0.018	8186	23.072
Vascular dementia (mixed)	TRAIL	rs74778900	T	C	0.399	30506300	0.290	0.377	360421	T	C	0.591	18	28086266	2.59E-28	0.053	8186	123.243
Vascular dementia (mixed)	TRAIL	rs75928541	A	G	-0.001	16400148	0.998	0.272	360421	A	G	0.275	4	16401771	4.24E-06	0.059	8186	21.506
Vascular dementia (mixed)	TRAIL	rs79287178	A	G	-0.181	172576710	0.396	0.214	360421	A	G	-0.432	3	172294500	9.12E-25	0.042	8186	105.148
Vascular dementia (mixed)	Tumor necrosis factor beta	rs10925040	T	C	-0.118	247459396	0.183	0.089	360421	T	C	0.176	1	247622698	2.67E-06	0.037	1559	22.138
Vascular dementia (mixed)	Tumor necrosis factor beta	rs753274	T	C	0.085	14325650	0.324	0.086	360421	T	C	-0.174	19	14436462	2.77E-06	0.037	1559	21.895
Vascular dementia (mixed)	Tumor necrosis factor beta	rs7629875	G	A	0.021	174667832	0.904	0.175	360421	G	A	-0.377	3	174385622	1.37E-06	0.077	1559	23.674
Vascular dementia (mixed)	Tumor necrosis factor beta	rs78296352	T	G	-0.093	22495351	0.798	0.364	360421	T	G	1.222	1	22821844	4.76E-21	0.137	1559	79.962
Vascular dementia (mixed)	Tumor necrosis factor alpha	rs10834997	A	G	0.040	26505401	0.657	0.091	360421	A	G	-0.125	11	26526948	1.33E-06	0.026	3454	23.361
Vascular dementia (mixed)	Tumor necrosis factor alpha	rs115669577	A	G	0.170	123440293	0.793	0.651	360421	A	G	0.989	4	124361448	8.28E-07	0.200	3454	24.571
Vascular dementia (mixed)	Tumor necrosis factor alpha	rs79105320	A	G	-0.056	18959850	0.890	0.407	360421	A	G	0.561	8	18817360	3.59E-06	0.118	3454	22.601
Vascular dementia (mixed)	Tumor necrosis factor alpha	rs8121916	A	C	-0.085	12420677	0.381	0.097	360421	A	C	0.131	20	12401325	2.72E-06	0.028	3454	22.070
Vascular dementia (mixed)	Stromal-cell-derived factor 1 alpha	rs10474392	G	A	-0.176	92198776	0.069	0.097	360421	G	A	-0.096	5	91494593	1.24E-06	0.018	5998	29.209
Vascular dementia (mixed)	Stromal-cell-derived factor 1 alpha	rs12407262	A	G	0.239	63354605	0.086	0.139	360421	A	G	0.118	1	63820276	3.99E-06	0.027	5998	19.646
Vascular dementia (mixed)	Stromal-cell-derived factor 1 alpha	rs139840550	A	G	0.289	38688625	0.336	0.300	360421	A	G	0.183	9	38688622	3.79E-06	0.055	5998	11.160
Vascular dementia (mixed)	Stromal-cell-derived factor 1 alpha	rs149893336	G	A	0.173	170311440	0.674	0.412	360421	G	A	0.503	4	171232591	4.52E-06	0.108	5998	21.686
Vascular dementia (mixed)	Stromal-cell-derived factor 1 alpha	rs4581824	G	T	0.155	9074853	0.079	0.088	360421	G	T	0.070	19	9185529	3.05E-06	0.017	5998	16.419
Vascular dementia (mixed)	Stromal-cell-derived factor 1 alpha	rs67689854	A	C	0.063	89558819	0.544	0.104	360421	A	C	-0.068	16	89625227	3.07E-06	0.020	5998	12.196
Vascular dementia (mixed)	Stromal-cell-derived factor 1 alpha	rs9267091	A	G	-0.147	31446032	0.155	0.104	360421	A	G	0.078	6	31413809	3.63E-06	0.020	5998	14.802
Vascular dementia (mixed)	Stem cell growth factor beta	rs112346514	T	C	-0.104	12297173	0.635	0.218	360421	T	C	-0.331	19	12407988	2.37E-06	0.071	3682	21.725

Vascular dementia (mixed)	Stem cell growth factor beta	rs116924815	T	C	-0.068	50727476	0.800	0.268	360421	T	C	0.608	19	51230733	1.74E-16	0.074	3682	67.850
Vascular dementia (mixed)	Stem cell growth factor beta	rs117716477	A	C	-0.054	103847180	0.871	0.331	360421	A	C	0.838	12	104240958	1.34E-23	0.084	3682	99.383
Vascular dementia (mixed)	Stem cell growth factor beta	rs12480722	C	T	0.053	20248260	0.673	0.124	360421	C	T	-0.162	20	20228904	4.72E-06	0.036	3682	20.927
Vascular dementia (mixed)	Stem cell growth factor beta	rs139413256	A	G	0.060	146182552	0.855	0.331	360421	A	G	-0.538	7	145879644	7.04E-07	0.108	3682	24.605
Vascular dementia (mixed)	Stem cell growth factor beta	rs143829871	C	T	-0.013	47555755	0.928	0.144	360421	C	T	0.190	3	47597245	1.90E-06	0.040	3682	22.610
Vascular dementia (mixed)	Stem cell growth factor beta	rs151194174	A	G	0.188	20956159	0.399	0.223	360421	A	G	0.464	7	20995778	1.13E-06	0.094	3682	24.210
Vascular dementia (mixed)	Stem cell growth factor beta	rs17876031	G	A	0.097	177404118	0.275	0.089	360421	G	A	0.151	5	176831119	2.25E-09	0.026	3682	35.251
Vascular dementia (mixed)	Stem cell growth factor beta	rs264162	G	A	-0.004	10944028	0.960	0.084	360421	G	A	-0.110	18	10944026	2.68E-06	0.023	3682	21.978
Vascular dementia (mixed)	Stem cell growth factor beta	rs34911860	A	G	0.349	79885030	0.279	0.323	360421	A	G	-0.368	1	80350715	3.24E-06	0.079	3682	21.695
Vascular dementia (mixed)	Stem cell growth factor beta	rs4656185	A	G	0.195	169507088	0.033	0.091	360421	A	G	0.205	1	169476326	1.16E-15	0.026	3682	64.125
Vascular dementia (mixed)	Stem cell growth factor beta	rs4737732	G	A	0.088	65421393	0.338	0.092	360421	G	A	0.115	8	66333628	4.68E-06	0.025	3682	20.717
Vascular dementia (mixed)	Stem cell growth factor beta	rs7762066	C	T	0.066	94468249	0.507	0.100	360421	C	T	-0.139	6	95177967	3.50E-06	0.030	3682	21.581
Vascular dementia (mixed)	Stem cell growth factor beta	rs78217154	C	T	0.456	100541844	0.132	0.303	360421	C	T	-0.400	8	101554072	3.77E-06	0.086	3682	21.401
Vascular dementia (mixed)	Stem cell factor	rs113127926	A	C	0.083	97971174	0.699	0.215	360421	A	C	0.198	14	98437511	2.27E-06	0.042	8290	22.269
Vascular dementia (mixed)	Stem cell factor	rs13412535	A	G	0.023	224010157	0.828	0.105	360421	A	G	-0.107	2	224874874	6.04E-07	0.021	8290	25.094
Vascular dementia (mixed)	Stem cell factor	rs1557570	T	G	0.179	169538606	0.050	0.091	360421	T	G	0.119	1	169507844	2.74E-12	0.017	8290	48.671
Vascular dementia (mixed)	Stem cell factor	rs1568119	T	C	-0.204	33385679	0.698	0.527	360421	T	C	-0.591	8	33243197	1.24E-07	0.113	8290	27.365
Vascular dementia (mixed)	Stem cell factor	rs1942355	T	C	0.003	71694503	0.972	0.084	360421	T	C	-0.072	18	69361739	4.70E-06	0.016	8290	20.798
Vascular dementia (mixed)	Stem cell factor	rs4841899	C	T	0.019	134532566	0.841	0.096	360421	C	T	0.100	9	137424412	1.78E-08	0.018	8290	31.815
Vascular dementia (mixed)	Stem cell factor	rs635634	T	C	-0.200	133279427	0.056	0.104	360421	T	C	-0.103	9	136155000	6.74E-08	0.019	8290	29.194
Vascular dementia (mixed)	Stem cell factor	rs78666213	G	T	-0.351	179217495	0.228	0.291	360421	G	T	0.274	4	180138649	2.59E-06	0.058	8290	22.695
Vascular dementia (mixed)	Stem cell factor	rs80271436	A	G	-0.131	133022383	0.621	0.265	360421	A	G	-0.237	9	135897770	9.95E-07	0.049	8290	23.879
Vascular dementia (mixed)	Interleukin-16	rs117217798	T	C	-0.198	33156215	0.190	0.151	360421	T	C	-0.204	17	31483233	4.15E-06	0.044	3483	21.028
Vascular dementia (mixed)	Interleukin-16	rs117916513	A	G	-0.167	121393565	0.613	0.329	360421	A	G	-0.502	11	121264274	3.79E-07	0.099	3483	25.921
Vascular dementia (mixed)	Interleukin-16	rs1255143	T	C	0.073	128253936	0.389	0.085	360421	T	C	0.131	10	130052200	7.10E-08	0.024	3483	29.124
Vascular dementia (mixed)	Interleukin-16	rs12765671	A	G	-0.447	104924411	0.320	0.449	360421	A	G	-0.602	10	106684169	4.84E-06	0.132	3483	20.883
Vascular dementia (mixed)	Interleukin-16	rs144691581	A	G	-0.080	96410095	0.786	0.292	360421	A	G	0.488	15	96953325	4.20E-07	0.097	3483	25.488
Vascular dementia (mixed)	Interleukin-16	rs1801020	G	A	0.093	177409531	0.326	0.095	360421	G	A	-0.173	5	176836532	4.53E-10	0.027	3483	40.594
Vascular dementia (mixed)	Interleukin-16	rs4253283	C	T	-0.038	186244057	0.681	0.091	360421	C	T	-0.146	4	187165211	1.75E-08	0.026	3483	31.053
Vascular dementia (mixed)	Interleukin-16	rs4513633	A	C	0.058	112649483	0.710	0.157	360421	A	C	-0.224	4	113570639	7.44E-07	0.045	3483	24.429
Vascular dementia (mixed)	Interleukin-16	rs4778636	A	G	-0.060	81299298	0.768	0.203	360421	A	G	-0.727	15	81591639	1.11E-30	0.063	3483	131.978
Vascular dementia (mixed)	Interleukin-16	rs9706053	T	C	-0.171	65982530	0.602	0.328	360421	T	C	0.458	12	66376310	7.01E-07	0.093	3483	24.170
Vascular dementia (mixed)	RANTES	rs112072646	A	G	-0.272	53217255	0.415	0.333	360421	A	G	0.429	2	53444393	6.48E-07	0.086	3421	24.722
Vascular dementia (mixed)	RANTES	rs147509526	T	C	-0.118	15665520	0.618	0.237	360421	T	C	-0.358	19	15776330	6.93E-07	0.072	3421	24.930
Vascular dementia (mixed)	RANTES	rs4940620	G	A	0.088	64303876	0.643	0.190	360421	G	A	0.249	18	61971111	3.54E-06	0.054	3421	21.331
Vascular dementia (mixed)	RANTES	rs62438851	G	A	-0.012	144909173	0.929	0.138	360421	G	A	0.196	6	145230309	2.33E-06	0.041	3421	22.345
Vascular dementia (mixed)	RANTES	rs7000423	T	C	0.151	110041420	0.083	0.087	360421	T	C	-0.132	8	111053649	1.82E-07	0.025	3421	27.139
Vascular dementia (mixed)	RANTES	rs72793342	A	G	-0.183	30537031	0.076	0.103	360421	A	G	-0.149	16	30548352	1.48E-06	0.031	3421	23.309
Vascular dementia (mixed)	RANTES	rs74472919	T	C	0.080	81626515	0.712	0.217	360421	T	C	0.331	13	82200650	3.97E-08	0.061	3421	29.987
Vascular dementia (mixed)	RANTES	rs75613039	T	C	-0.121	129706688	0.641	0.259	360421	T	C	0.370	11	129576583	4.81E-06	0.081	3421	20.866
Vascular dementia (mixed)	RANTES	rs818452	T	C	0.076	152594661	0.662	0.173	360421	T	C	0.238	6	152915796	2.36E-06	0.051	3421	22.230
Vascular dementia (mixed)	Platelet-derived growth factor BB	rs116445074	T	G	0.329	52238766	0.275	0.302	360421	T	G	0.293	5	51534600	3.11E-07	0.059	8293	24.932
Vascular dementia (mixed)	Platelet-derived growth factor BB	rs11766649	G	A	-0.256	145142154	0.013	0.104	360421	G	A	-0.091	7	144839247	3.53E-06	0.020	8293	21.461
Vascular dementia (mixed)	Platelet-derived growth factor BB	rs11916118	G	A	0.009	117193342	0.932	0.107	360421	G	A	-0.089	3	116912189	4.93E-06	0.019	8293	20.999
Vascular dementia (mixed)	Platelet-derived growth factor BB	rs12289510	G	A	0.013	125077155	0.882	0.085	360421	G	A	0.078	11	124947051	7.69E-07	0.016	8293	24.371
Vascular dementia (mixed)	Platelet-derived growth factor BB	rs13412535	A	G	0.023	224010157	0.828	0.105	360421	A	G	0.335	2	224874874	2.46E-55	0.021	8293	245.347
Vascular dementia (mixed)	Platelet-derived growth factor BB	rs2324229	C	T	-0.037	83208412	0.673	0.088	360421	C	T	-0.089	6	83918131	3.48E-08	0.016	8293	30.834
Vascular dementia (mixed)	Platelet-derived growth factor BB	rs35859699	A	G	0.290	111263595	0.454	0.387	360421	A	G	-0.395	4	112184751	2.07E-06	0.084	8293	22.030
Vascular dementia (mixed)	Platelet-derived growth factor BB	rs4965869	T	C	0.011	101450115	0.912	0.098	360421	T	C	0.184	15	101990320	5.66E-24	0.018	8293	103.342
Vascular dementia (mixed)	Platelet-derived growth factor BB	rs55680718	T	C	0.181	224302160	0.166	0.130	360421	T	C	-0.138	2	225166877	1.86E-08	0.025	8293	31.606
Vascular dementia (mixed)	Platelet-derived growth factor BB	rs72777070	G	T	0.184	9658748	0.077	0.104	360421	G	T	0.107	2	9798877	8.98E-08	0.020	8293	28.569
Vascular dementia (mixed)	Platelet-derived growth factor BB	rs73162807	A	C	-0.014	146757003	0.957	0.269	360421	A	C	-0.239	3	146474790	1.74E-06	0.050	8293	22.959
Vascular dementia (mixed)	Platelet-derived growth factor BB	rs9936075	G	A	-0.084	7271908	0.339	0.088	360421	G	A	0.078	16	7321909	1.76E-06	0.016	8293	22.737
Vascular dementia (mixed)	Platelet-derived growth factor BB	rs9941733	G	A	0.121	393417	0.278	0.111	360421	G	A	-0.116	20	374061	3.31E-07	0.023	8293	25.930
Vascular dementia (mixed)	Macrophage inflammatory protein 1b	rs11130043	A	G	0.055	45069747	0.514	0.085	360421	A	G	-0.073	3	45111239	3.22E-06	0.016	8243	21.679
Vascular dementia (mixed)	Macrophage inflammatory protein 1b	rs113010081	C	T	-0.254	46415921	0.042	0.125	360421	C	T	0.595	3	46457412	########	0.024	8243	636.493
Vascular dementia (mixed)	Macrophage inflammatory protein 1b	rs113877493	T	C	-0.374	36443746	0.002	0.120	360421	T	C	-0.612	17	34812273	########	0.022	8243	789.146
Vascular dementia (mixed)	Macrophage inflammatory protein 1b	rs116237296	A	G	0.584	86579833	0.336	0.607	360421	A	G	0.544	1	87045516	7.23E-07	0.112	8243	23.778
Vascular dementia (mixed)	Macrophage inflammatory protein 1b	rs117453826	G	A	-0.035	36775624	0.904	0.291	360421	G	A	0.577	17	35132809	5.07E-22	0.059	8243	94.808
Vascular dementia (mixed)	Macrophage inflammatory protein 1b	rs141102180	T	G	0.296	36108811	0.170	0.216	360421	T	G	0.323	17	34436204	1.08E-16	0.039	8243	67.340
Vascular dementia (mixed)	Macrophage inflammatory protein 1b	rs17138331	G	A	-0.109	7826737	0.483	0.155	360421	G	A	0.139	7	7866368	2.26E-06	0.030	8243	22.234
Vascular dementia (mixed)	Macrophage inflammatory protein 1b	rs17641689	G	A	-0.138	36668383	0.307	0.135	360421	G	A	0.245	17	35024819	1.28E-16	0.029	8243	69.805
Vascular dementia (mixed)	Macrophage inflammatory protein 1b	rs2079664	G	A	-0.011	34680936	0.908	0.095	360421	G	A	-0.100	17	33007955	1.51E-08	0.018	8243	31.961
Vascular dementia (mixed)	Macrophage inflammatory protein 1b	rs281749	C	T	0.038	107626417	0.682	0.092	360421	C	T	-0.080	8	108638645	3.17E-06	0.017	8243	21.832

Vascular dementia (mixed)	Macrophage inflammatory protein 1b	rs34437725	C	T	-0.253	35499766	0.314	0.251	360421	C	T	0.263	17	33826785	7.67E-08	0.048	8243	29.717
Vascular dementia (mixed)	Macrophage inflammatory protein 1b	rs72791296	T	C	0.009	121614355	0.968	0.224	360421	T	C	0.237	5	120950050	3.78E-07	0.047	8243	25.844
Vascular dementia (mixed)	Macrophage inflammatory protein 1b	rs72799710	T	C	0.019	123825971	0.873	0.116	360421	T	C	-0.101	5	123161665	3.21E-06	0.022	8243	21.635
Vascular dementia (mixed)	Macrophage inflammatory protein 1b	rs74810984	C	T	-0.159	127876202	0.560	0.272	360421	C	T	-0.221	10	129674466	1.96E-06	0.047	8243	21.660
Vascular dementia (mixed)	Macrophage inflammatory protein 1b	rs76582507	A	G	-0.506	37510075	0.242	0.433	360421	A	G	0.318	9	37510072	3.26E-06	0.068	8243	21.994
Vascular dementia (mixed)	Macrophage inflammatory protein 1b	rs76583883	T	G	-0.043	45936445	0.850	0.227	360421	T	G	-0.232	21	47356359	4.99E-06	0.051	8243	20.559
Vascular dementia (mixed)	Macrophage inflammatory protein 1b	rs76776296	G	A	0.154	115488433	0.600	0.294	360421	G	A	-0.300	7	115128487	5.55E-07	0.060	8243	25.117
Vascular dementia (mixed)	Macrophage inflammatory protein 1a	rs10835056	G	T	-0.040	26675470	0.657	0.090	360421	G	T	-0.119	11	26697017	2.60E-06	0.025	3522	22.097
Vascular dementia (mixed)	Macrophage inflammatory protein 1a	rs12690897	A	G	0.033	85716861	0.724	0.093	360421	A	G	0.125	7	85346177	2.11E-06	0.026	3522	22.690
Vascular dementia (mixed)	Macrophage inflammatory protein 1a	rs184154340	A	G	-0.097	80790993	0.664	0.224	360421	A	G	0.331	11	80502036	1.86E-06	0.069	3522	22.813
Vascular dementia (mixed)	Macrophage inflammatory protein 1a	rs34771762	G	A	-0.041	200547932	0.802	0.162	360421	G	A	-0.249	2	201412655	2.13E-06	0.052	3522	22.667
Vascular dementia (mixed)	Macrophage inflammatory protein 1a	rs57786342	A	G	0.040	68793311	0.691	0.100	360421	A	G	0.131	14	69260028	4.05E-06	0.029	3522	21.257
Vascular dementia (mixed)	Macrophage inflammatory protein 1a	rs60198979	A	G	-0.107	43250698	0.501	0.159	360421	A	G	-0.215	22	43646704	2.61E-06	0.046	3522	21.955
Vascular dementia (mixed)	Macrophage inflammatory protein 1a	rs7232268	G	A	0.065	70101678	0.763	0.216	360421	G	A	-0.282	18	67768914	2.55E-06	0.060	3522	22.180
Vascular dementia (mixed)	Monokine induced by gamma interferon	rs111607343	A	G	0.240	897855	0.501	0.356	360421	A	G	-0.521	19	897855	2.83E-06	0.112	3685	21.678
Vascular dementia (mixed)	Monokine induced by gamma interferon	rs11177248	A	G	0.257	68482106	0.232	0.215	360421	A	G	0.307	12	68875886	4.45E-06	0.067	3685	21.037
Vascular dementia (mixed)	Monokine induced by gamma interferon	rs112337562	G	T	0.042	92665225	0.875	0.269	360421	G	T	0.370	14	93131570	2.98E-06	0.080	3685	21.606
Vascular dementia (mixed)	Monokine induced by gamma interferon	rs112861654	G	A	0.362	42179062	0.039	0.175	360421	G	A	0.277	21	43599172	1.81E-07	0.053	3685	27.320
Vascular dementia (mixed)	Monokine induced by gamma interferon	rs117831247	T	C	0.205	66742081	0.745	0.632	360421	T	C	-0.833	10	68501839	2.16E-06	0.175	3685	22.576
Vascular dementia (mixed)	Monokine induced by gamma interferon	rs139010077	T	C	-0.411	170618359	0.209	0.327	360421	T	C	0.432	3	170336148	3.55E-06	0.095	3685	20.698
Vascular dementia (mixed)	Monokine induced by gamma interferon	rs1796086	C	T	0.155	71183729	0.306	0.151	360421	C	T	0.210	7	70648715	2.23E-07	0.040	3685	27.050
Vascular dementia (mixed)	Monokine induced by gamma interferon	rs41272086	A	G	0.293	160587614	0.036	0.140	360421	A	G	-0.223	6	161008646	7.43E-08	0.042	3685	28.771
Vascular dementia (mixed)	Monokine induced by gamma interferon	rs55876513	G	T	-0.029	75962545	0.754	0.093	360421	G	T	-0.166	4	76883698	8.23E-11	0.026	3685	42.378
Vascular dementia (mixed)	Monokine induced by gamma interferon	rs5752128	C	T	0.195	25322656	0.135	0.130	360421	C	T	0.169	22	25718623	4.34E-06	0.037	3685	20.852
Vascular dementia (mixed)	Monokine induced by gamma interferon	rs62562991	A	G	0.039	95973777	0.916	0.370	360421	A	G	0.624	9	98736059	8.40E-07	0.126	3685	24.495
Vascular dementia (mixed)	Monokine induced by gamma interferon	rs6679677	A	C	0.046	113761186	0.701	0.120	360421	A	C	0.162	1	114303808	8.86E-07	0.033	3685	24.246
Vascular dementia (mixed)	Monokine induced by gamma interferon	rs77086208	T	C	0.102	70152774	0.690	0.255	360421	T	C	0.323	14	70619491	3.83E-06	0.070	3685	21.361
Vascular dementia (mixed)	Monokine induced by gamma interferon	rs816960	T	C	0.016	107870173	0.851	0.087	360421	T	C	-0.122	13	108522521	5.01E-07	0.024	3685	25.164
Vascular dementia (mixed)	Macrophage colony stimulating factor	rs116274860	G	T	0.129	148675030	0.724	0.365	360421	G	T	-0.819	3	148392817	2.74E-06	0.174	840	22.129
Vascular dementia (mixed)	Macrophage colony stimulating factor	rs117867915	C	T	-0.192	44630078	0.534	0.308	360421	C	T	-0.527	18	42210043	1.61E-06	0.110	840	23.054
Vascular dementia (mixed)	Macrophage colony stimulating factor	rs12962919	T	C	-0.108	78018752	0.515	0.166	360421	T	C	0.305	18	75778756	4.65E-06	0.066	840	21.255
Vascular dementia (mixed)	Macrophage colony stimulating factor	rs56367447	T	C	0.211	4014005	0.374	0.237	360421	T	C	-0.497	8	3871527	1.72E-08	0.088	840	31.642
Vascular dementia (mixed)	Macrophage colony stimulating factor	rs62294910	A	G	0.059	182480551	0.735	0.174	360421	A	G	0.343	3	182198339	6.82E-07	0.069	840	24.654
Vascular dementia (mixed)	Macrophage colony stimulating factor	rs78296352	T	G	-0.093	22495351	0.798	0.364	360421	T	G	0.527	1	22821844	1.05E-06	0.111	840	22.460
Vascular dementia (mixed)	Macrophage colony stimulating factor	rs9387100	C	T	-0.025	112781752	0.767	0.085	360421	C	T	0.135	6	113102954	4.07E-06	0.029	840	21.438
Vascular dementia (mixed)	Monocyte chemoattractant protein-1	rs10145849	A	G	-0.064	82475647	0.463	0.087	360421	A	G	-0.076	14	82941991	3.41E-06	0.016	8293	21.720
Vascular dementia (mixed)	Monocyte chemoattractant protein-1	rs10744620	C	T	-0.061	3629928	0.483	0.087	360421	C	T	-0.079	12	3739094	9.91E-07	0.016	8293	23.955
Vascular dementia (mixed)	Monocyte chemoattractant protein-1	rs111995966	G	T	0.083	108558513	0.599	0.157	360421	G	T	-0.145	2	109174969	2.53E-06	0.031	8293	21.939
Vascular dementia (mixed)	Monocyte chemoattractant protein-1	rs112313229	A	G	0.003	46323369	0.987	0.162	360421	A	G	-0.165	3	46364860	1.43E-07	0.031	8293	27.655
Vascular dementia (mixed)	Monocyte chemoattractant protein-1	rs12073356	A	G	0.078	207834503	0.638	0.165	360421	A	G	-0.143	1	208007848	4.17E-06	0.031	8293	21.024
Vascular dementia (mixed)	Monocyte chemoattractant protein-1	rs12075	A	G	-0.050	159205564	0.556	0.084	360421	A	G	0.219	1	159175354	1.44E-44	0.016	8293	198.719
Vascular dementia (mixed)	Monocyte chemoattractant protein-1	rs12493471	C	T	0.031	45910186	0.723	0.088	360421	C	T	-0.116	3	45951678	6.81E-13	0.016	8293	51.538
Vascular dementia (mixed)	Monocyte chemoattractant protein-1	rs146522229	T	C	1.033	47295223	0.107	0.641	360421	T	C	-0.598	19	47798480	3.56E-07	0.118	8293	25.779
Vascular dementia (mixed)	Monocyte chemoattractant protein-1	rs2228467	C	T	0.236	42864624	0.135	0.158	360421	C	T	0.264	3	42906116	9.19E-20	0.029	8293	82.117
Vascular dementia (mixed)	Monocyte chemoattractant protein-1	rs2712431	A	C	0.031	128598047	0.741	0.092	360421	A	C	-0.079	3	128316890	4.75E-06	0.017	8293	20.936
Vascular dementia (mixed)	Monocyte chemoattractant protein-1	rs56212190	T	C	-0.307	41702868	0.114	0.194	360421	T	C	0.181	1	42168539	9.85E-07	0.037	8293	23.547
Vascular dementia (mixed)	Monocyte chemoattractant protein-1	rs7197349	G	A	-0.013	78653322	0.900	0.107	360421	G	A	-0.097	16	78687219	2.62E-06	0.021	8293	22.081
Vascular dementia (mixed)	Monocyte chemoattractant protein-1	rs7517040	G	A	0.128	158889343	0.208	0.102	360421	G	A	0.099	1	158859133	2.44E-07	0.019	8293	26.703
Vascular dementia (mixed)	Monocyte chemoattractant protein-1	rs9317045	C	A	-0.089	59055904	0.448	0.118	360421	C	A	-0.113	13	59630038	1.52E-06	0.024	8293	23.089
Vascular dementia (mixed)	Interleukin-12p70	rs13209117	A	G	-0.114	44184028	0.238	0.097	360421	A	G	0.100	6	44151765	5.57E-08	0.019	8270	29.021
Vascular dementia (mixed)	Interleukin-12p70	rs17229494	G	A	0.000	37555798	0.999	0.136	360421	G	A	0.117	21	38928100	4.93E-06	0.026	8270	20.796
Vascular dementia (mixed)	Interleukin-12p70	rs282258	C	T	0.027	224050083	0.752	0.085	360421	C	T	-0.073	2	224914800	3.21E-06	0.016	8270	21.898
Vascular dementia (mixed)	Interleukin-12p70	rs41282644	A	G	-0.054	43785985	0.727	0.156	360421	A	G	0.147	6	43753722	1.05E-06	0.030	8270	23.478
Vascular dementia (mixed)	Interleukin-12p70	rs4349809	G	T	-0.053	43957093	0.532	0.084	360421	G	T	-0.378	6	43924830	########	0.016	8270	564.287
Vascular dementia (mixed)	Interleukin-12p70	rs71361173	G	T	0.028	76000450	0.822	0.126	360421	G	T	-0.111	18	73712405	3.06E-06	0.024	8270	21.570
Vascular dementia (mixed)	Interleukin-12p70	rs72831623	A	G	-0.233	47644927	0.168	0.169	360421	A	G	0.191	17	45722293	2.42E-07	0.037	8270	26.732
Vascular dementia (mixed)	Interleukin-12p70	rs782107	A	G	-0.215	58439747	0.011	0.084	360421	A	G	0.075	12	58833530	1.60E-06	0.016	8270	23.114
Vascular dementia (mixed)	Interleukin-12p70	rs79121401	C	T	1.069	78986084	0.072	0.595	360421	C	T	-0.555	11	78697129	4.24E-06	0.121	8270	21.163
Vascular dementia (mixed)	Interleukin-12p70	rs9472183	G	A	0.035	43972465	0.675	0.084	360421	G	A	0.102	6	43940202	8.61E-11	0.016	8270	42.126
Vascular dementia (mixed)	Interferon gamma-induced protein 10	rs10809307	C	T	0.062	11045908	0.545	0.103	360421	C	T	-0.131	9	11045908	3.64E-06	0.028	3685	21.415
Vascular dementia (mixed)	Interferon gamma-induced protein 10	rs113831257	A	G	0.004	75234311	0.982	0.197	360421	A	G	0.359	4	76159521	2.53E-08	0.064	3685	31.110
Vascular dementia (mixed)	Interferon gamma-induced protein 10	rs11626201	A	C	0.159	36511495	0.066	0.087	360421	A	C	0.116	14	36980700	1.93E-06	0.025	3685	22.495
Vascular dementia (mixed)	Interferon gamma-induced protein 10	rs143799975	G	A	-0.748	75885862	0.142	0.510	360421	G	A	0.798	4	76807015	1.00E-06	0.164	3685	23.787
Vascular dementia (mixed)	Interferon gamma-induced protein 10	rs34383175	T	C	-0.365	144361034	0.110	0.228	360421	T	C	-0.315	8	145584694	1.51E-06	0.066	3685	23.031

Vascular dementia (mixed)	Interferon gamma-induced protein 10	rs75970138	A	G	0.037	119813998	0.923	0.387	360421	A	G	-0.485	9	122576276	1.53E-06	0.104	3685	21.748
Vascular dementia (mixed)	Interferon gamma-induced protein 10	rs7645625	G	T	0.033	146856250	0.696	0.085	360421	G	T	0.109	3	146574037	4.41E-06	0.024	3685	20.997
Vascular dementia (mixed)	Interferon gamma-induced protein 10	rs79848609	C	A	-0.039	86772934	0.840	0.196	360421	C	A	-0.260	15	87316165	8.75E-07	0.054	3685	23.496
Vascular dementia (mixed)	Interferon gamma-induced protein 10	rs8112909	A	G	-0.023	45910150	0.821	0.104	360421	A	G	-0.143	19	46413408	1.94E-06	0.030	3685	22.746
Vascular dementia (mixed)	Interleukin-18	rs10414578	T	C	0.055	54634619	0.654	0.123	360421	T	C	-0.177	19	55146070	4.16E-07	0.035	3636	25.604
Vascular dementia (mixed)	Interleukin-18	rs115267715	T	C	-0.543	69239188	0.087	0.317	360421	T	C	0.451	5	68535015	1.72E-08	0.080	3636	31.753
Vascular dementia (mixed)	Interleukin-18	rs116383510	C	A	-0.204	2545536	0.558	0.348	360421	C	A	0.543	5	2545650	3.00E-07	0.106	3636	26.402
Vascular dementia (mixed)	Interleukin-18	rs117266781	T	C	-0.280	41261422	0.552	0.471	360421	T	C	0.684	7	41301020	3.15E-06	0.147	3636	21.716
Vascular dementia (mixed)	Interleukin-18	rs144841621	T	C	-0.368	69921801	0.374	0.414	360421	T	C	0.518	10	71681557	3.81E-06	0.114	3636	20.610
Vascular dementia (mixed)	Interleukin-18	rs17229943	C	A	0.094	69386709	0.506	0.141	360421	C	A	0.312	5	68682536	1.62E-11	0.046	3636	45.410
Vascular dementia (mixed)	Interleukin-18	rs1852105	C	T	0.142	64265217	0.537	0.231	360421	C	T	-0.304	7	63725595	4.32E-06	0.066	3636	21.096
Vascular dementia (mixed)	Interleukin-18	rs1979967	T	C	0.139	79367271	0.176	0.103	360421	T	C	0.140	15	79659613	9.45E-07	0.029	3636	24.031
Vascular dementia (mixed)	Interleukin-18	rs2729385	A	G	0.161	57495520	0.083	0.093	360421	A	G	0.123	11	57262993	3.79E-06	0.026	3636	22.076
Vascular dementia (mixed)	Interleukin-18	rs385076	C	T	0.136	32264782	0.121	0.088	360421	C	T	0.243	2	32489851	1.66E-22	0.025	3636	96.166
Vascular dementia (mixed)	Interleukin-18	rs4482818	G	A	0.130	65062779	0.132	0.086	360421	G	A	-0.129	4	65928497	1.45E-07	0.024	3636	27.778
Vascular dementia (mixed)	Interleukin-18	rs658805	A	G	0.130	70199369	0.143	0.089	360421	A	G	0.123	6	70909073	4.94E-07	0.024	3636	25.247
Vascular dementia (mixed)	Interleukin-18	rs71478720	T	C	-0.038	112138882	0.702	0.098	360421	T	C	-0.267	11	112009605	3.71E-22	0.028	3636	93.515
Vascular dementia (mixed)	Interleukin-18	rs78623212	T	C	0.121	103667180	0.800	0.479	360421	T	C	0.871	7	103307627	6.71E-07	0.178	3636	23.970
Vascular dementia (mixed)	Interleukin-18	rs78716465	A	G	0.133	42015086	0.559	0.227	360421	A	G	0.327	20	40643726	1.63E-06	0.068	3636	22.919
Vascular dementia (mixed)	Interleukin-17	rs117029961	A	G	0.425	37147653	0.387	0.492	360421	A	G	0.459	10	37436581	4.94E-06	0.102	7760	20.405
Vascular dementia (mixed)	Interleukin-17	rs117556572	T	C	-0.296	104436567	0.281	0.275	360421	T	C	-0.510	13	105088917	3.28E-06	0.110	7760	21.552
Vascular dementia (mixed)	Interleukin-17	rs1530455	C	T	-0.043	123136052	0.627	0.088	360421	C	T	-0.108	3	122854899	4.87E-10	0.017	7760	38.972
Vascular dementia (mixed)	Interleukin-17	rs17106604	T	C	0.009	77912813	0.937	0.119	360421	T	C	0.113	14	78379156	6.37E-07	0.023	7760	25.178
Vascular dementia (mixed)	Interleukin-17	rs17282552	C	T	0.111	207109091	0.564	0.193	360421	C	T	0.200	2	207973815	8.21E-07	0.041	7760	24.411
Vascular dementia (mixed)	Interleukin-17	rs184080173	C	T	0.174	77331424	0.470	0.241	360421	C	T	-0.238	12	77725204	4.19E-07	0.047	7760	25.620
Vascular dementia (mixed)	Interleukin-17	rs187475560	T	C	-0.586	160353411	0.021	0.254	360421	T	C	-0.243	4	161274563	3.29E-06	0.052	7760	21.910
Vascular dementia (mixed)	Interleukin-17	rs62191444	T	G	0.118	393023	0.317	0.118	360421	T	G	-0.114	20	373667	4.22E-06	0.025	7760	21.153
Vascular dementia (mixed)	Interleukin-17	rs78296352	T	G	-0.093	22495351	0.798	0.364	360421	T	G	0.303	1	22821844	4.27E-06	0.065	7760	21.956
Vascular dementia (mixed)	Interleukin-17	rs78612928	C	T	0.183	29812292	0.114	0.115	360421	C	T	-0.104	4	29813914	2.62E-06	0.022	7760	21.820
Vascular dementia (mixed)	Interleukin-13	rs117795020	A	G	-0.125	87469237	0.612	0.247	360421	A	G	-0.352	9	90084152	9.86E-07	0.072	3557	24.197
Vascular dementia (mixed)	Interleukin-13	rs12623722	A	G	-0.118	22955811	0.201	0.092	360421	A	G	-0.119	2	23178683	4.19E-06	0.026	3557	21.096
Vascular dementia (mixed)	Interleukin-13	rs139083458	T	C	-0.562	26160409	0.421	0.699	360421	T	C	0.990	5	26160518	2.81E-06	0.211	3557	22.086
Vascular dementia (mixed)	Interleukin-13	rs142167313	C	T	0.281	44204360	0.191	0.214	360421	C	T	0.313	6	44172097	3.98E-07	0.062	3557	25.735
Vascular dementia (mixed)	Interleukin-13	rs27949	T	C	0.063	59254997	0.487	0.090	360421	T	C	-0.117	5	58550823	3.43E-06	0.025	3557	21.482
Vascular dementia (mixed)	Interleukin-13	rs6799107	C	T	0.170	127338175	0.112	0.107	360421	C	T	0.146	3	127057018	1.25E-06	0.030	3557	23.495
Vascular dementia (mixed)	Interleukin-13	rs7073807	C	T	0.003	67393670	0.979	0.125	360421	C	T	-0.168	10	69153428	2.37E-06	0.036	3557	22.323
Vascular dementia (mixed)	Interleukin-13	rs75995699	A	G	0.025	5140622	0.918	0.237	360421	A	G	0.332	6	5140856	2.64E-06	0.070	3557	22.610
Vascular dementia (mixed)	Interleukin-13	rs9472168	G	A	-0.051	43961248	0.550	0.085	360421	G	A	-0.424	6	43928985	1.08E-65	0.025	3557	292.851
Vascular dementia (mixed)	Interleukin-10	rs10457128	A	G	0.142	105570101	0.106	0.088	360421	A	G	-0.087	6	106017976	5.24E-07	0.017	7681	25.292
Vascular dementia (mixed)	Interleukin-10	rs10493718	A	C	-0.113	82597250	0.327	0.116	360421	A	C	-0.110	1	83062933	7.16E-07	0.022	7681	24.552
Vascular dementia (mixed)	Interleukin-10	rs11206302	T	C	0.097	54208270	0.450	0.129	360421	T	C	-0.119	1	54673943	2.20E-06	0.025	7681	22.440
Vascular dementia (mixed)	Interleukin-10	rs2086656	T	C	-0.041	59632755	0.640	0.089	360421	T	C	-0.079	4	60498473	3.78E-06	0.017	7681	21.289
Vascular dementia (mixed)	Interleukin-10	rs282258	C	T	0.027	224050083	0.752	0.085	360421	C	T	-0.099	2	224914800	1.00E-09	0.016	7681	37.497
Vascular dementia (mixed)	Interleukin-10	rs3025021	C	T	0.011	43781426	0.904	0.089	360421	C	T	-0.095	6	43749163	1.46E-06	0.020	7681	23.585
Vascular dementia (mixed)	Interleukin-10	rs41282660	G	A	-0.141	44229269	0.249	0.122	360421	G	A	0.119	6	44197006	3.72E-06	0.026	7681	21.924
Vascular dementia (mixed)	Interleukin-10	rs4349809	G	T	-0.053	43957093	0.532	0.084	360421	G	T	-0.285	6	43924830	5.77E-67	0.017	7681	298.976
Vascular dementia (mixed)	Interleukin-10	rs465757	A	G	-0.005	15599638	0.956	0.088	360421	A	G	0.084	20	15580283	1.17E-06	0.017	7681	23.306
Vascular dementia (mixed)	Interleukin-10	rs7088799	G	T	0.051	63256414	0.555	0.086	360421	G	T	0.085	10	65016174	3.23E-07	0.017	7681	26.028
Vascular dementia (mixed)	Interleukin-8	rs11634944	C	T	-0.052	24937946	0.557	0.089	360421	C	T	0.121	15	25183093	1.29E-06	0.025	3526	23.208
Vascular dementia (mixed)	Interleukin-8	rs12075	A	G	-0.050	159205564	0.556	0.084	360421	A	G	0.120	1	159175354	3.88E-07	0.024	3526	25.855
Vascular dementia (mixed)	Interleukin-8	rs141926526	C	A	-0.621	32809028	0.172	0.454	360421	C	A	0.615	7	32848640	2.57E-06	0.131	3526	22.100
Vascular dementia (mixed)	Interleukin-8	rs2673604	A	C	-0.130	132399360	0.155	0.091	360421	A	C	-0.127	8	133411607	7.02E-07	0.026	3526	24.648
Vascular dementia (mixed)	Interleukin-6	rs1333040	T	C	-0.068	22083405	0.417	0.084	360421	T	C	0.074	9	22083404	3.17E-06	0.016	8189	21.817
Vascular dementia (mixed)	Interleukin-6	rs13412535	A	G	0.023	224010157	0.828	0.105	360421	A	G	-0.116	2	224874874	7.34E-08	0.022	8189	29.311
Vascular dementia (mixed)	Interleukin-6	rs72831623	A	G	-0.233	47644927	0.168	0.169	360421	A	G	0.197	17	45722293	1.08E-07	0.037	8189	28.130
Vascular dementia (mixed)	Interleukin-6	rs73273528	T	C	-0.041	51814574	0.883	0.278	360421	T	C	0.267	20	50431113	9.58E-07	0.055	8189	23.347
Vascular dementia (mixed)	Interleukin-6	rs76856708	C	T	0.671	80695146	0.077	0.379	360421	C	T	-0.329	16	80729043	2.61E-06	0.070	8189	22.077
Vascular dementia (mixed)	Interleukin-1-receptor antagonist	rs1054402	C	T	0.050	116401230	0.610	0.098	360421	C	T	-0.131	9	119163509	1.13E-06	0.027	3638	23.576
Vascular dementia (mixed)	Interleukin-1-receptor antagonist	rs11627423	C	A	-0.092	32731417	0.291	0.087	360421	C	A	-0.117	14	33200623	2.12E-06	0.025	3638	22.476
Vascular dementia (mixed)	Interleukin-1-receptor antagonist	rs12121840	T	C	0.071	165572405	0.714	0.195	360421	T	C	0.269	1	165541642	2.43E-06	0.057	3638	22.227
Vascular dementia (mixed)	Interleukin-1-receptor antagonist	rs2809154	T	C	-0.011	84153389	0.938	0.138	360421	T	C	-0.179	13	84727524	3.74E-06	0.039	3638	21.188
Vascular dementia (mixed)	Interleukin-1-receptor antagonist	rs61335305	A	C	0.243	66160736	0.437	0.313	360421	A	C	0.445	15	66453074	1.00E-06	0.091	3638	24.051
Vascular dementia (mixed)	Interleukin-1-receptor antagonist	rs9623661	T	C	-0.160	42697370	0.272	0.146	360421	T	C	-0.197	22	43093376	3.86E-06	0.043	3638	21.298
Vascular dementia (mixed)	Interleukin-1-beta	rs143319329	T	C	-0.067	128499405	0.891	0.492	360421	T	C	0.280	7	128139459	2.00E-06	0.072	3309	15.347

Vascular dementia (mixed)	Interleukin-1-beta	rs61335305	A	C	0.243	66160736	0.437	0.313	360421	A	C	0.297	15	66453074	1.90E-06	0.072	3309	16.783
Vascular dementia (mixed)	Interleukin-1-beta	rs62015704	G	A	0.021	7417906	0.868	0.128	360421	G	A	-0.108	16	7467907	2.09E-06	0.028	3309	14.618
Vascular dementia (mixed)	Interleukin-1-beta	rs9898641	C	T	0.128	59493672	0.146	0.088	360421	C	T	0.203	17	57571033	3.59E-06	0.045	3309	20.033
Vascular dementia (mixed)	Hepatocyte growth factor	rs11060254	A	G	0.162	129331024	0.074	0.091	360421	A	G	-0.080	12	129815569	1.58E-06	0.017	8292	22.948
Vascular dementia (mixed)	Hepatocyte growth factor	rs150322232	G	A	0.030	7890743	0.891	0.218	360421	G	A	-0.210	7	7930374	4.89E-06	0.046	8292	20.650
Vascular dementia (mixed)	Hepatocyte growth factor	rs1698249	C	A	-0.210	83889842	0.297	0.202	360421	C	A	0.170	14	84356186	4.09E-06	0.037	8292	20.835
Vascular dementia (mixed)	Hepatocyte growth factor	rs2003620	T	C	0.261	134794733	0.300	0.251	360421	T	C	0.228	7	134479484	2.83E-06	0.049	8292	21.721
Vascular dementia (mixed)	Hepatocyte growth factor	rs3748034	T	G	-0.004	3444364	0.974	0.128	360421	T	G	0.150	4	3446091	1.81E-10	0.023	8292	40.818
Vascular dementia (mixed)	Hepatocyte growth factor	rs5745687	T	C	-0.294	81729735	0.164	0.211	360421	T	C	-0.307	7	81359051	2.75E-14	0.041	8292	57.252
Vascular dementia (mixed)	Hepatocyte growth factor	rs62481625	C	T	0.037	156194766	0.755	0.117	360421	C	T	-0.109	7	155987460	1.18E-06	0.023	8292	23.512
Vascular dementia (mixed)	Interleukin-9	rs41294750	T	C	0.236	53084968	0.342	0.248	360421	T	C	0.351	1	53550640	2.36E-06	0.075	3634	22.070
Vascular dementia (mixed)	Interleukin-9	rs4880409	T	C	-0.334	132516716	0.413	0.408	360421	T	C	-0.336	10	134330220	3.50E-06	0.072	3634	21.533
Vascular dementia (mixed)	Interleukin-9	rs61867538	T	C	-0.058	1503276	0.785	0.212	360421	T	C	0.357	11	1524506	3.93E-06	0.077	3634	21.227
Vascular dementia (mixed)	Interleukin-9	rs7232268	G	A	0.065	70101678	0.763	0.216	360421	G	A	-0.276	18	67768914	2.52E-06	0.059	3634	22.092
Vascular dementia (mixed)	Interleukin-9	rs7242404	A	G	-0.124	12741268	0.184	0.093	360421	A	G	-0.123	18	12741267	3.27E-06	0.026	3634	21.637
Vascular dementia (mixed)	Interleukin-9	rs76963786	T	C	-0.224	31886823	0.266	0.201	360421	T	C	-0.287	12	32039757	4.50E-07	0.056	3634	26.457
Vascular dementia (mixed)	Interleukin-7	rs117509142	C	T	0.103	86121854	0.663	0.237	360421	C	T	0.327	8	87134083	1.99E-06	0.069	3409	22.590
Vascular dementia (mixed)	Interleukin-7	rs141425475	C	T	-0.082	17679056	0.784	0.299	360421	C	T	0.478	5	17679165	2.53E-06	0.102	3409	22.144
Vascular dementia (mixed)	Interleukin-7	rs144701438	A	G	-0.239	66293168	0.513	0.366	360421	A	G	-0.482	18	63960405	9.75E-07	0.099	3409	23.742
Vascular dementia (mixed)	Interleukin-7	rs17091524	C	T	0.545	56482041	0.104	0.335	360421	C	T	-0.492	14	56948759	1.91E-06	0.101	3409	23.627
Vascular dementia (mixed)	Interleukin-7	rs28793375	T	C	0.002	41558099	0.986	0.124	360421	T	C	0.164	8	41415618	4.46E-06	0.036	3409	20.588
Vascular dementia (mixed)	Interleukin-7	rs4320361	T	G	-0.070	43960774	0.410	0.085	360421	T	G	-0.325	6	43928511	6.87E-39	0.025	3409	169.836
Vascular dementia (mixed)	Interleukin-7	rs62006410	T	C	-0.034	102541598	0.728	0.098	360421	T	C	-0.156	14	103007935	3.39E-07	0.030	3409	26.405
Vascular dementia (mixed)	Interleukin-7	rs75904417	C	A	0.002	167796811	0.984	0.119	360421	C	A	0.170	2	168653321	1.16E-06	0.035	3409	23.671
Vascular dementia (mixed)	Interleukin-7	rs77981494	C	T	-0.028	17451009	0.923	0.294	360421	C	T	0.518	16	17544866	1.07E-06	0.106	3409	23.683
Vascular dementia (mixed)	Interleukin-7	rs78346957	A	G	-0.039	125214944	0.924	0.405	360421	A	G	0.459	10	126903513	4.51E-06	0.101	3409	20.758
Vascular dementia (mixed)	Interleukin-5	rs11680908	G	A	-0.224	109460295	0.237	0.189	360421	G	A	-0.263	2	110076751	2.03E-06	0.055	3364	22.605
Vascular dementia (mixed)	Interleukin-5	rs6737109	C	T	-0.069	22956659	0.418	0.085	360421	C	T	-0.116	2	23179531	2.40E-06	0.025	3364	22.056
Vascular dementia (mixed)	Interleukin-5	rs72831687	A	G	0.281	16092129	0.406	0.338	360421	A	G	-0.524	6	16092360	1.69E-06	0.111	3364	22.317
Vascular dementia (mixed)	Interleukin-5	rs73040130	C	T	-0.073	36255288	0.692	0.185	360421	C	T	-0.264	19	36746190	6.00E-07	0.053	3364	24.868
Vascular dementia (mixed)	Interleukin-5	rs7767396	G	A	-0.058	43959313	0.494	0.084	360421	G	A	-0.152	6	43927050	7.69E-10	0.025	3364	37.928
Vascular dementia (mixed)	Interleukin-4	rs10512267	C	T	-0.066	99427847	0.435	0.085	360421	C	T	0.082	9	102190129	2.94E-07	0.016	8124	26.194
Vascular dementia (mixed)	Interleukin-4	rs116705532	G	T	-0.285	113162547	0.568	0.500	360421	G	T	0.468	1	113705169	1.76E-06	0.098	8124	22.879
Vascular dementia (mixed)	Interleukin-4	rs117146485	C	T	-0.144	135932411	0.644	0.312	360421	C	T	0.292	9	138824257	2.71E-06	0.063	8124	21.610
Vascular dementia (mixed)	Interleukin-4	rs17713451	A	G	0.027	151465386	0.836	0.133	360421	A	G	0.127	7	151162472	4.97E-07	0.025	8124	25.357
Vascular dementia (mixed)	Interleukin-4	rs73023729	A	G	-0.036	159232998	0.859	0.204	360421	A	G	-0.180	6	159654030	9.03E-07	0.037	8124	24.080
Vascular dementia (mixed)	Interleukin-4	rs7613691	G	A	0.037	147935804	0.847	0.192	360421	G	A	-0.178	3	147653591	4.05E-06	0.038	8124	21.367
Vascular dementia (mixed)	Interleukin-4	rs9508291	C	T	-0.085	29136483	0.659	0.193	360421	C	T	0.168	13	29710620	3.03E-06	0.036	8124	21.795
Vascular dementia (mixed)	Interleukin-4	rs9941733	G	A	0.121	393417	0.278	0.111	360421	G	A	-0.114	20	374061	6.88E-07	0.023	8124	24.782
Vascular dementia (mixed)	Interleukin-2 receptor antagonist	rs11241559	G	T	0.137	120641005	0.158	0.097	360421	G	T	0.126	5	119976700	2.00E-06	0.027	3677	22.580
Vascular dementia (mixed)	Interleukin-2 receptor antagonist	rs117244812	A	G	0.413	6539990	0.393	0.484	360421	A	G	-0.706	17	6443310	2.10E-06	0.149	3677	22.537
Vascular dementia (mixed)	Interleukin-2 receptor antagonist	rs12722497	A	C	-0.118	6053965	0.485	0.170	360421	A	C	0.628	10	6095928	1.57E-38	0.049	3677	167.609
Vascular dementia (mixed)	Interleukin-2 receptor antagonist	rs185231391	C	T	-0.508	59373953	0.328	0.519	360421	C	T	-0.850	3	59359679	1.47E-06	0.181	3677	22.094
Vascular dementia (mixed)	Interleukin-2 receptor antagonist	rs4733117	C	A	0.018	32280094	0.860	0.104	360421	C	A	-0.137	8	32137610	2.63E-06	0.029	3677	21.981
Vascular dementia (mixed)	Interleukin-2 receptor antagonist	rs61705228	T	C	0.038	100275145	0.865	0.225	360421	T	C	0.330	4	101196302	3.99E-06	0.072	3677	21.281
Vascular dementia (mixed)	Interleukin-2	rs12051139	C	T	-0.016	86885068	0.848	0.085	360421	C	T	0.113	16	86918674	4.76E-06	0.025	3475	20.967
Vascular dementia (mixed)	Interleukin-2	rs13412535	A	G	0.023	224010157	0.828	0.105	360421	A	G	0.176	2	224874874	1.18E-07	0.033	3475	28.231
Vascular dementia (mixed)	Interleukin-2	rs170117	T	C	0.014	54524213	0.905	0.121	360421	T	C	-0.162	4	55390380	3.87E-06	0.035	3475	21.467
Vascular dementia (mixed)	Interleukin-2	rs2807544	G	A	-0.109	14877749	0.208	0.087	360421	G	A	-0.118	1	15204245	3.41E-06	0.025	3475	21.569
Vascular dementia (mixed)	Interleukin-2	rs4634519	G	A	0.090	67727941	0.339	0.094	360421	G	A	0.126	7	67192928	2.77E-06	0.027	3475	21.975
Vascular dementia (mixed)	Interleukin-2	rs61335305	A	C	0.243	66160736	0.437	0.313	360421	A	C	0.451	15	66453074	7.32E-07	0.092	3475	24.179
Vascular dementia (mixed)	Interleukin-2	rs62124990	T	G	0.156	19038882	0.610	0.306	360421	T	G	-0.696	2	19238636	3.22E-06	0.150	3475	21.680
Vascular dementia (mixed)	Interleukin-2	rs7615304	G	A	0.047	156957914	0.575	0.084	360421	G	A	0.117	3	156675703	1.21E-06	0.024	3475	23.454
Vascular dementia (mixed)	Interleukin-2	rs80336398	C	T	0.577	64075258	0.030	0.266	360421	C	T	-0.400	3	64060934	2.82E-06	0.086	3475	21.745
Vascular dementia (mixed)	Interferon gamma	rs10487554	A	G	0.003	149670595	0.978	0.093	360421	A	G	-0.090	7	149367686	1.09E-06	0.018	7701	23.919
Vascular dementia (mixed)	Interferon gamma	rs113600793	A	C	-0.228	47384095	0.211	0.182	360421	A	C	0.183	17	45461461	8.95E-07	0.037	7701	24.044
Vascular dementia (mixed)	Interferon gamma	rs115729819	G	A	0.143	168783516	0.585	0.262	360421	G	A	-0.248	4	169704667	1.38E-06	0.052	7701	23.264
Vascular dementia (mixed)	Interferon gamma	rs11843756	G	T	0.070	48680756	0.737	0.209	360421	G	T	-0.184	13	49254892	3.09E-06	0.039	7701	21.921
Vascular dementia (mixed)	Interferon gamma	rs12420286	C	T	0.149	103907166	0.565	0.259	360421	C	T	-0.238	11	103777894	2.08E-06	0.050	7701	22.491
Vascular dementia (mixed)	Interferon gamma	rs1867282	T	C	0.018	99409865	0.836	0.085	360421	T	C	0.077	9	102172147	3.15E-06	0.017	7701	21.740
Vascular dementia (mixed)	Interferon gamma	rs2073438	A	G	0.026	6996757	0.790	0.096	360421	A	G	0.090	17	6900076	1.68E-06	0.019	7701	22.816
Vascular dementia (mixed)	Interferon gamma	rs74148555	T	C	-0.046	90320085	0.875	0.294	360421	T	C	-0.373	10	92079842	2.64E-06	0.077	7701	23.249
Vascular dementia (mixed)	Interferon gamma	rs78296352	T	G	-0.093	22495351	0.798	0.364	360421	T	G	0.343	1	22821844	1.38E-07	0.065	7701	27.675
Vascular dementia (mixed)	Growth-regulated protein alpha	rs1113500	T	G	-0.078	108052820	0.364	0.085	360421	T	G	0.117	1	108595442	1.57E-06	0.024	3505	23.150

Vascular dementia (mixed)	Growth-regulated protein alpha	rs12075	A	G	-0.050	159205564	0.556	0.084	360421	A	G	0.375	1	159175354	1.24E-55	0.024	3505	250.494
Vascular dementia (mixed)	Growth-regulated protein alpha	rs140734053	A	G	-0.356	5359496	0.452	0.473	360421	A	G	0.726	10	5401459	3.58E-06	0.156	3505	21.613
Vascular dementia (mixed)	Growth-regulated protein alpha	rs185768063	G	A	0.012	16494752	0.963	0.258	360421	G	A	-0.400	6	16494983	1.46E-07	0.076	3505	27.673
Vascular dementia (mixed)	Growth-regulated protein alpha	rs188345231	T	C	0.970	41579831	0.004	0.338	360421	T	C	0.623	8	41437350	4.34E-06	0.132	3505	22.175
Vascular dementia (mixed)	Growth-regulated protein alpha	rs2422841	A	G	-0.100	3099706	0.415	0.122	360421	A	G	-0.166	20	3080352	4.66E-06	0.036	3505	21.068
Vascular dementia (mixed)	Growth-regulated protein alpha	rs508977	G	T	-0.012	73896666	0.905	0.098	360421	G	T	0.380	4	74762383	7.56E-42	0.028	3505	184.378
Vascular dementia (mixed)	Growth-regulated protein alpha	rs62024303	G	A	-0.037	88327931	0.860	0.212	360421	G	A	0.305	15	88871162	4.41E-06	0.067	3505	21.014
Vascular dementia (mixed)	Growth-regulated protein alpha	rs78653452	T	G	0.094	9781407	0.828	0.432	360421	T	G	-0.736	20	9762055	1.21E-06	0.156	3505	22.328
Vascular dementia (mixed)	Granulocyte-colony stimulating factor	rs115256310	G	A	0.314	72103864	0.532	0.502	360421	G	A	0.682	5	71399691	6.73E-07	0.136	7904	25.155
Vascular dementia (mixed)	Granulocyte-colony stimulating factor	rs11903143	G	A	-0.022	29369594	0.812	0.092	360421	G	A	-0.087	2	29592460	6.35E-07	0.018	7904	24.435
Vascular dementia (mixed)	Granulocyte-colony stimulating factor	rs147128865	T	C	0.248	34972769	0.383	0.284	360421	T	C	0.270	9	34972766	4.92E-06	0.059	7904	21.157
Vascular dementia (mixed)	Granulocyte-colony stimulating factor	rs1817411	T	C	-0.148	97586100	0.150	0.103	360421	T	C	0.089	8	98598328	3.10E-06	0.019	7904	21.713
Vascular dementia (mixed)	Granulocyte-colony stimulating factor	rs2671444	A	G	-0.016	101158297	0.853	0.088	360421	A	G	-0.078	12	101552075	2.48E-06	0.017	7904	22.306
Vascular dementia (mixed)	Granulocyte-colony stimulating factor	rs74148555	T	C	-0.046	90320085	0.875	0.294	360421	T	C	-0.372	10	92079842	1.55E-06	0.076	7904	24.212
Vascular dementia (mixed)	Granulocyte-colony stimulating factor	rs77318030	C	T	0.054	54544688	0.800	0.214	360421	C	T	0.205	19	55055897	2.21E-06	0.043	7904	22.830
Vascular dementia (mixed)	Fibroblast growth factor basic	rs13412535	A	G	0.023	224010157	0.828	0.105	360421	A	G	-0.111	2	224874874	7.34E-07	0.023	7565	24.426
Vascular dementia (mixed)	Fibroblast growth factor basic	rs145577605	A	G	-0.056	27642232	0.811	0.234	360421	A	G	0.208	6	27610011	9.64E-07	0.043	7565	23.640
Vascular dementia (mixed)	Fibroblast growth factor basic	rs747334	G	A	-0.143	90984987	0.089	0.084	360421	G	A	-0.075	10	92744744	4.53E-06	0.016	7565	20.970
Vascular dementia (mixed)	Fibroblast growth factor basic	rs75168112	C	T	0.022	73418832	0.837	0.107	360421	C	T	0.100	18	71086067	3.00E-06	0.021	7565	21.880
Vascular dementia (mixed)	Fibroblast growth factor basic	rs9907295	T	C	-0.088	35930309	0.519	0.137	360421	T	C	-0.132	17	34257313	7.95E-07	0.027	7565	24.043
Vascular dementia (mixed)	Eotaxin	rs11087905	A	C	0.121	24133015	0.174	0.089	360421	A	C	0.094	21	25505329	5.48E-07	0.019	8153	24.789
Vascular dementia (mixed)	Eotaxin	rs112347425	T	C	0.336	46419397	0.023	0.148	360421	T	C	0.158	3	46460888	8.65E-09	0.028	8153	32.535
Vascular dementia (mixed)	Eotaxin	rs12075	A	G	-0.050	159205564	0.556	0.084	360421	A	G	0.167	1	159175354	1.33E-26	0.016	8153	114.737
Vascular dementia (mixed)	Eotaxin	rs1476670	C	A	0.008	44042523	0.945	0.114	360421	C	A	0.101	1	44508195	3.51E-06	0.022	8153	21.535
Vascular dementia (mixed)	Eotaxin	rs2024050	G	A	-0.128	75831075	0.437	0.164	360421	G	A	-0.173	7	75460393	1.10E-08	0.030	8153	32.524
Vascular dementia (mixed)	Eotaxin	rs2210755	C	T	0.220	77608907	0.067	0.120	360421	C	T	0.110	9	80223823	4.85E-06	0.024	8153	20.812
Vascular dementia (mixed)	Eotaxin	rs2211994	C	T	-0.052	16675274	0.579	0.094	360421	C	T	-0.089	21	18047593	6.08E-07	0.018	8153	25.000
Vascular dementia (mixed)	Eotaxin	rs2228467	C	T	0.236	42864624	0.135	0.158	360421	C	T	0.416	3	42906116	2.27E-46	0.029	8153	203.258
Vascular dementia (mixed)	Eotaxin	rs2419841	C	T	0.019	113576224	0.901	0.155	360421	C	T	0.128	10	115335983	4.98E-06	0.028	8153	20.949
Vascular dementia (mixed)	Eotaxin	rs5746492	G	A	-0.051	17911167	0.654	0.114	360421	G	A	-0.095	22	18393933	3.96E-06	0.021	8153	21.240
Vascular dementia (mixed)	Eotaxin	rs5754733	A	C	0.116	33873606	0.294	0.111	360421	A	C	-0.104	22	34269594	1.06E-06	0.021	8153	23.709
Vascular dementia (mixed)	Eotaxin	rs59808887	T	C	0.028	31846414	0.869	0.170	360421	T	C	-0.167	5	31846520	2.91E-06	0.036	8153	21.839
Vascular dementia (mixed)	Eotaxin	rs75426604	A	C	0.143	35388508	0.328	0.146	360421	A	C	-0.137	14	35857714	2.53E-06	0.029	8153	22.035
Vascular dementia (mixed)	Eotaxin	rs79722574	T	C	-0.135	34292033	0.266	0.122	360421	T	C	-0.111	17	32619052	1.06E-06	0.023	8153	23.830
Vascular dementia (mixed)	Eotaxin	rs9317045	C	A	-0.089	59055904	0.448	0.118	360421	C	A	-0.118	13	59630038	5.82E-07	0.024	8153	24.874
Vascular dementia (multiple infarctions)	CTACK	rs116303454	A	G	-0.183	27253164	0.355	0.198	360612	A	G	0.383	3	27294655	3.27E-06	0.082	3631	22.030
Vascular dementia (multiple infarctions)	CTACK	rs2070074	G	A	-0.010	34649445	0.922	0.103	360612	G	A	-0.447	9	34649442	1.78E-32	0.037	3631	142.656
Vascular dementia (multiple infarctions)	CTACK	rs2731674	G	T	-0.018	177412889	0.807	0.074	360612	G	T	0.133	5	176839890	5.63E-07	0.027	3631	24.925
Vascular dementia (multiple infarctions)	CTACK	rs3766110	C	A	0.135	169545945	0.077	0.076	360612	C	A	0.129	1	169515183	3.85E-06	0.028	3631	21.432
Vascular dementia (multiple infarctions)	CTACK	rs55764737	C	T	-0.075	61031215	0.782	0.270	360612	C	T	-0.531	15	61323414	4.62E-08	0.097	3631	29.878
Vascular dementia (multiple infarctions)	CTACK	rs57338032	G	A	0.066	78506597	0.448	0.087	360612	G	A	-0.158	15	78798939	6.23E-07	0.032	3631	24.937
Vascular dementia (multiple infarctions)	CTACK	rs7333764	T	C	-0.120	33634664	0.488	0.172	360612	T	C	0.277	13	34208801	2.85E-06	0.059	3631	21.867
Vascular dementia (multiple infarctions)	CTACK	rs76395525	A	G	0.210	79449049	0.533	0.337	360612	A	G	0.528	15	79741391	9.55E-07	0.108	3631	23.742
Vascular dementia (multiple infarctions)	beta-nerve growth factor	rs28637706	T	G	-0.032	33794463	0.650	0.071	360612	T	G	-0.159	19	34285368	1.42E-09	0.026	3531	36.504
Vascular dementia (multiple infarctions)	beta-nerve growth factor	rs67476890	T	C	-0.123	62499295	0.240	0.104	360612	T	C	0.177	15	62791494	3.13E-06	0.038	3531	21.786
Vascular dementia (multiple infarctions)	beta-nerve growth factor	rs71641308	T	C	-0.089	77621033	0.428	0.112	360612	T	C	0.204	1	78086718	2.30E-06	0.043	3531	22.365
Vascular dementia (multiple infarctions)	beta-nerve growth factor	rs72780728	A	G	0.023	17561702	0.840	0.114	360612	A	G	0.188	10	17603701	2.99E-06	0.040	3531	21.832
Vascular dementia (multiple infarctions)	beta-nerve growth factor	rs73472576	C	T	-0.020	74456947	0.756	0.066	360612	C	T	0.118	18	72124182	2.69E-06	0.025	3531	21.963
Vascular dementia (multiple infarctions)	beta-nerve growth factor	rs7970581	G	T	0.091	112827443	0.216	0.074	360612	G	T	-0.138	12	113265248	9.27E-07	0.028	3531	23.947
Vascular dementia (multiple infarctions)	beta-nerve growth factor	rs9436119	A	G	0.026	150495277	0.693	0.067	360612	A	G	-0.112	1	150467753	3.91E-06	0.025	3531	20.765
Vascular dementia (multiple infarctions)	Vascular endothelial growth factor	rs10153304	A	G	0.279	7818613	0.018	0.117	360612	A	G	0.155	17	7721931	1.94E-06	0.033	7118	22.658
Vascular dementia (multiple infarctions)	Vascular endothelial growth factor	rs10934631	C	T	-0.091	122978753	0.326	0.092	360612	C	T	0.115	3	122697600	2.47E-06	0.025	7118	22.071
Vascular dementia (multiple infarctions)	Vascular endothelial growth factor	rs10967186	C	T	-0.034	2617099	0.611	0.066	360612	C	T	-0.090	9	2617099	1.23E-07	0.017	7118	27.903
Vascular dementia (multiple infarctions)	Vascular endothelial growth factor	rs13209117	A	G	0.052	44184028	0.487	0.075	360612	A	G	0.130	6	44151765	5.28E-11	0.020	7118	41.959
Vascular dementia (multiple infarctions)	Vascular endothelial growth factor	rs143479231	A	G	-0.124	193393005	0.456	0.167	360612	A	G	-0.260	3	193110794	1.90E-07	0.049	7118	27.997
Vascular dementia (multiple infarctions)	Vascular endothelial growth factor	rs4082730	A	G	0.254	89980326	0.207	0.201	360612	A	G	0.252	15	90523558	2.64E-06	0.053	7118	22.305
Vascular dementia (multiple infarctions)	Vascular endothelial growth factor	rs6921438	A	G	0.059	43957870	0.365	0.065	360612	A	G	-0.490	6	43925607	########	0.018	7118	784.000
Vascular dementia (multiple infarctions)	Vascular endothelial growth factor	rs73418461	A	G	-0.048	118463484	0.803	0.192	360612	A	G	-0.249	10	120222996	1.67E-06	0.052	7118	22.878
Vascular dementia (multiple infarctions)	Vascular endothelial growth factor	rs8045833	A	G	0.036	88509031	0.648	0.079	360612	A	G	0.108	16	88575439	2.83E-07	0.021	7118	26.199
Vascular dementia (multiple infarctions)	Vascular endothelial growth factor	rs9472183	G	A	0.013	43972465	0.848	0.065	360612	G	A	0.128	6	43940202	5.19E-14	0.017	7118	56.869
Vascular dementia (multiple infarctions)	Macrophage Migration Inhibitory Factor	rs113218956	A	G	0.332	24828867	0.525	0.523	360612	A	G	-0.895	22	25224834	2.26E-06	0.188	3494	22.678
Vascular dementia (multiple infarctions)	Macrophage Migration Inhibitory Factor	rs118055855	C	T	-0.067	29867025	0.856	0.371	360612	C	T	-0.691	11	29888572	4.13E-06	0.150	3494	21.203
Vascular dementia (multiple infarctions)	Macrophage Migration Inhibitory Factor	rs12594190	G	A	0.129	24791308	0.071	0.071	360612	G	A	-0.136	15	25036455	3.70E-07	0.027	3494	25.755
Vascular dementia (multiple infarctions)	Macrophage Migration Inhibitory Factor	rs13142904	T	C	0.064	53452247	0.579	0.114	360612	T	C	-0.223	4	54318414	2.56E-07	0.043	3494	27.532

Vascular dementia (multiple infarctions)	Macrophage Migration Inhibitory Factor	rs141009259	C	T	0.207	207111559	0.491	0.301	360612	C	T	0.618	2	207976283	2.47E-06	0.132	3494	21.839
Vascular dementia (multiple infarctions)	Macrophage Migration Inhibitory Factor	rs78098071	C	T	0.538	163882733	0.036	0.256	360612	C	T	0.487	5	163309739	1.78E-07	0.092	3494	28.108
Vascular dementia (multiple infarctions)	TRAIL	rs11618126	G	A	-1.036	50252103	0.101	0.632	360612	G	A	-0.891	13	50826239	1.46E-06	0.191	8186	21.661
Vascular dementia (multiple infarctions)	TRAIL	rs11657269	G	A	0.078	6416464	0.468	0.107	360612	G	A	-0.119	17	6319784	4.78E-06	0.026	8186	20.878
Vascular dementia (multiple infarctions)	TRAIL	rs11699445	G	T	-0.085	15770145	0.204	0.067	360612	G	T	-0.075	20	15750790	3.27E-06	0.016	8186	21.470
Vascular dementia (multiple infarctions)	TRAIL	rs13185784	A	G	0.152	180267068	0.040	0.074	360612	A	G	0.085	5	179694068	3.90E-06	0.018	8186	21.372
Vascular dementia (multiple infarctions)	TRAIL	rs138987090	G	A	0.313	32786284	0.266	0.281	360612	G	A	0.750	18	30366247	4.50E-23	0.075	8186	99.389
Vascular dementia (multiple infarctions)	TRAIL	rs146783010	G	A	0.053	89527045	0.912	0.474	360612	G	A	0.602	11	89260213	4.83E-06	0.135	8186	19.859
Vascular dementia (multiple infarctions)	TRAIL	rs193112415	C	T	0.285	31255157	0.226	0.236	360612	C	T	1.042	18	28835120	2.15E-62	0.062	8186	279.797
Vascular dementia (multiple infarctions)	TRAIL	rs57396456	C	T	0.272	30365911	0.219	0.221	360612	C	T	0.563	18	27945877	1.25E-27	0.052	8186	117.961
Vascular dementia (multiple infarctions)	TRAIL	rs62093514	T	C	-0.048	31651014	0.830	0.223	360612	T	C	1.062	18	29230977	6.86E-82	0.055	8186	370.005
Vascular dementia (multiple infarctions)	TRAIL	rs73039026	C	A	0.348	172442691	0.136	0.233	360612	C	A	0.300	3	172160481	2.02E-06	0.064	8186	22.305
Vascular dementia (multiple infarctions)	TRAIL	rs747324	C	T	0.000	74222941	0.999	0.073	360612	C	T	0.086	14	74689644	1.61E-06	0.018	8186	23.072
Vascular dementia (multiple infarctions)	TRAIL	rs74778900	T	C	-0.366	30506300	0.207	0.290	360612	T	C	0.591	18	28086266	2.59E-28	0.053	8186	123.243
Vascular dementia (multiple infarctions)	TRAIL	rs75928541	A	G	0.047	16400148	0.827	0.217	360612	A	G	0.275	4	16401771	4.24E-06	0.059	8186	21.506
Vascular dementia (multiple infarctions)	TRAIL	rs79287178	A	G	0.118	172576710	0.476	0.166	360612	A	G	-0.432	3	172294500	9.12E-25	0.042	8186	105.148
Vascular dementia (multiple infarctions)	Tumor necrosis factor beta	rs10925040	T	C	0.079	247459396	0.250	0.069	360612	T	C	0.176	1	247622698	2.67E-06	0.037	1559	22.138
Vascular dementia (multiple infarctions)	Tumor necrosis factor beta	rs753274	T	C	0.096	14325650	0.151	0.067	360612	T	C	-0.174	19	14436462	2.77E-06	0.037	1559	21.895
Vascular dementia (multiple infarctions)	Tumor necrosis factor beta	rs7629875	G	A	-0.028	174667832	0.838	0.137	360612	G	A	-0.377	3	174385622	1.37E-06	0.077	1559	23.674
Vascular dementia (multiple infarctions)	Tumor necrosis factor beta	rs78296352	T	G	-0.597	22495351	0.030	0.275	360612	T	G	1.222	1	22821844	4.76E-21	0.137	1559	79.962
Vascular dementia (multiple infarctions)	Tumor necrosis factor alpha	rs10834997	A	G	-0.126	26505401	0.075	0.071	360612	A	G	-0.125	11	26526948	1.33E-06	0.026	3454	23.361
Vascular dementia (multiple infarctions)	Tumor necrosis factor alpha	rs115669577	A	G	-0.356	123440293	0.468	0.491	360612	A	G	0.989	4	124361448	8.28E-07	0.200	3454	24.571
Vascular dementia (multiple infarctions)	Tumor necrosis factor alpha	rs79105320	A	G	0.073	18959850	0.819	0.319	360612	A	G	0.561	8	18817360	3.59E-06	0.118	3454	22.601
Vascular dementia (multiple infarctions)	Tumor necrosis factor alpha	rs8121916	A	C	-0.019	12420677	0.805	0.075	360612	A	C	0.131	20	12401325	2.72E-06	0.028	3454	22.070
Vascular dementia (multiple infarctions)	Stromal-cell-derived factor 1 alpha	rs10474392	G	A	0.012	92198776	0.875	0.075	360612	G	A	-0.096	5	91494593	1.24E-06	0.018	5998	29.209
Vascular dementia (multiple infarctions)	Stromal-cell-derived factor 1 alpha	rs12407262	A	G	0.079	63354605	0.456	0.106	360612	A	G	0.118	1	63820276	3.99E-06	0.027	5998	19.646
Vascular dementia (multiple infarctions)	Stromal-cell-derived factor 1 alpha	rs139840550	A	G	0.389	38688625	0.085	0.226	360612	A	G	0.183	9	38688622	3.79E-06	0.055	5998	11.160
Vascular dementia (multiple infarctions)	Stromal-cell-derived factor 1 alpha	rs149893336	G	A	-0.430	170311440	0.160	0.306	360612	G	A	0.503	4	171232591	4.52E-06	0.108	5998	21.686
Vascular dementia (multiple infarctions)	Stromal-cell-derived factor 1 alpha	rs4581824	G	T	-0.098	9074853	0.154	0.068	360612	G	T	0.070	19	9185529	3.05E-06	0.017	5998	16.419
Vascular dementia (multiple infarctions)	Stromal-cell-derived factor 1 alpha	rs482700	A	G	-0.031	115146334	0.704	0.082	360612	A	G	-0.089	4	116067490	1.57E-06	0.020	5998	19.351
Vascular dementia (multiple infarctions)	Stromal-cell-derived factor 1 alpha	rs67689854	A	C	-0.118	89558819	0.145	0.081	360612	A	C	-0.068	16	89625227	3.07E-06	0.020	5998	12.196
Vascular dementia (multiple infarctions)	Stromal-cell-derived factor 1 alpha	rs9267091	A	G	-0.014	31446032	0.862	0.080	360612	A	G	0.078	6	31413809	3.63E-06	0.020	5998	14.802
Vascular dementia (multiple infarctions)	Stem cell growth factor beta	rs112346514	T	C	-0.013	12297173	0.939	0.170	360612	T	C	-0.331	19	12407988	2.37E-06	0.071	3682	21.725
Vascular dementia (multiple infarctions)	Stem cell growth factor beta	rs116924815	T	C	0.195	50727476	0.355	0.211	360612	T	C	0.608	19	51230733	1.74E-16	0.074	3682	67.850
Vascular dementia (multiple infarctions)	Stem cell growth factor beta	rs117716477	A	C	0.134	103847180	0.613	0.264	360612	A	C	0.838	12	104240958	1.34E-23	0.084	3682	99.383
Vascular dementia (multiple infarctions)	Stem cell growth factor beta	rs12480722	C	T	-0.119	20248260	0.220	0.097	360612	C	T	-0.162	20	20228904	4.72E-06	0.036	3682	20.927
Vascular dementia (multiple infarctions)	Stem cell growth factor beta	rs139413256	A	G	-0.042	146182552	0.870	0.257	360612	A	G	-0.538	7	145879644	7.04E-07	0.108	3682	24.605
Vascular dementia (multiple infarctions)	Stem cell growth factor beta	rs143829871	C	T	0.113	47555755	0.314	0.113	360612	C	T	0.190	3	47597245	1.90E-06	0.040	3682	22.610
Vascular dementia (multiple infarctions)	Stem cell growth factor beta	rs151194174	A	G	0.060	20956159	0.730	0.174	360612	A	G	0.464	7	20995778	1.13E-06	0.094	3682	24.210
Vascular dementia (multiple infarctions)	Stem cell growth factor beta	rs17876031	G	A	-0.020	177404118	0.777	0.070	360612	G	A	0.151	5	176831119	2.25E-09	0.026	3682	35.251
Vascular dementia (multiple infarctions)	Stem cell growth factor beta	rs264162	G	A	0.020	10944028	0.762	0.065	360612	G	A	-0.110	18	10944026	2.68E-06	0.023	3682	21.978
Vascular dementia (multiple infarctions)	Stem cell growth factor beta	rs34911860	A	G	-0.163	79885030	0.522	0.255	360612	A	G	-0.368	1	80350715	3.24E-06	0.079	3682	21.695
Vascular dementia (multiple infarctions)	Stem cell growth factor beta	rs4656185	A	G	0.115	169507088	0.105	0.071	360612	A	G	0.205	1	169476326	1.16E-15	0.026	3682	64.125
Vascular dementia (multiple infarctions)	Stem cell growth factor beta	rs4737732	G	A	0.067	65421393	0.356	0.072	360612	G	A	0.115	8	66333628	4.68E-06	0.025	3682	20.717
Vascular dementia (multiple infarctions)	Stem cell growth factor beta	rs7762066	C	T	0.040	94468249	0.607	0.078	360612	C	T	-0.139	6	95177967	3.50E-06	0.030	3682	21.581
Vascular dementia (multiple infarctions)	Stem cell growth factor beta	rs78217154	C	T	-0.424	100541844	0.062	0.227	360612	C	T	-0.400	8	101554072	3.77E-06	0.086	3682	21.401
Vascular dementia (multiple infarctions)	Stem cell factor	rs113127926	A	C	-0.132	97971174	0.422	0.164	360612	A	C	0.198	14	98437511	2.27E-06	0.042	8290	22.269
Vascular dementia (multiple infarctions)	Stem cell factor	rs1557570	T	G	0.125	169538606	0.077	0.071	360612	T	G	0.119	1	169507844	2.74E-12	0.017	8290	48.671
Vascular dementia (multiple infarctions)	Stem cell factor	rs1568119	T	C	-0.081	33385679	0.852	0.435	360612	T	C	-0.591	8	33243197	1.24E-07	0.113	8290	27.365
Vascular dementia (multiple infarctions)	Stem cell factor	rs1942355	T	C	0.032	71694503	0.629	0.066	360612	T	C	-0.072	18	69361739	4.70E-06	0.016	8290	20.798
Vascular dementia (multiple infarctions)	Stem cell factor	rs4841899	C	T	-0.014	134532566	0.847	0.074	360612	C	T	0.100	9	137424412	1.78E-08	0.018	8290	31.815
Vascular dementia (multiple infarctions)	Stem cell factor	rs635634	T	C	-0.095	133279427	0.238	0.081	360612	T	C	-0.103	9	136155000	6.74E-08	0.019	8290	29.194
Vascular dementia (multiple infarctions)	Stem cell factor	rs78666213	G	T	0.011	179217495	0.962	0.223	360612	G	T	0.274	4	180138649	2.59E-06	0.058	8290	22.695
Vascular dementia (multiple infarctions)	Stem cell factor	rs80271436	A	G	-0.134	133022383	0.511	0.203	360612	A	G	-0.237	9	135897770	9.95E-07	0.049	8290	23.879
Vascular dementia (multiple infarctions)	Interleukin-16	rs117217798	T	C	0.086	33156215	0.467	0.118	360612	T	C	-0.204	17	31483233	4.15E-06	0.044	3483	21.028
Vascular dementia (multiple infarctions)	Interleukin-16	rs117916513	A	G	-0.040	121393565	0.876	0.258	360612	A	G	-0.502	11	121264274	3.79E-07	0.099	3483	25.921
Vascular dementia (multiple infarctions)	Interleukin-16	rs1255143	T	C	0.134	128253936	0.042	0.066	360612	T	C	0.131	10	130052200	7.10E-08	0.024	3483	29.124
Vascular dementia (multiple infarctions)	Interleukin-16	rs12765671	A	G	0.437	104924411	0.216	0.353	360612	A	G	-0.602	10	106684169	4.84E-06	0.132	3483	20.883
Vascular dementia (multiple infarctions)	Interleukin-16	rs144691581	A	G	-0.139	96410095	0.534	0.223	360612	A	G	0.488	15	96953325	4.20E-07	0.097	3483	25.488
Vascular dementia (multiple infarctions)	Interleukin-16	rs1801020	G	A	-0.016	177409531	0.832	0.074	360612	G	A	-0.173	5	176836532	4.53E-10	0.027	3483	40.594
Vascular dementia (multiple infarctions)	Interleukin-16	rs4253283	C	T	0.049	186244057	0.494	0.071	360612	C	T	-0.146	4	187165211	1.75E-08	0.026	3483	31.053
Vascular dementia (multiple infarctions)	Interleukin-16	rs4513633	A	C	0.064	112649483	0.601	0.123	360612	A	C	-0.224	4	113570639	7.44E-07	0.045	3483	24.429
Vascular dementia (multiple infarctions)	Interleukin-16	rs4778636	A	G	0.133	81299298	0.394	0.157	360612	A	G	-0.727	15	81591639	1.11E-30	0.063	3483	131.978
Vascular dementia (multiple infarctions)	Interleukin-16	rs9706053	T	C	0.157	65982530	0.540	0.256	360612	T	C	0.458	12	66376310	7.01E-07	0.093	3483	24.170

Vascular dementia (multiple infarctions)	RANTES	rs112072646	A	G	-0.143	53217255	0.577	0.257	360612	A	G	0.429	2	53444393	6.48E-07	0.086	3421	24.722
Vascular dementia (multiple infarctions)	RANTES	rs147509526	T	C	0.243	15665520	0.197	0.188	360612	T	C	-0.358	19	15776330	6.93E-07	0.072	3421	24.930
Vascular dementia (multiple infarctions)	RANTES	rs4940620	G	A	-0.020	64303876	0.894	0.147	360612	G	A	0.249	18	61971111	3.54E-06	0.054	3421	21.331
Vascular dementia (multiple infarctions)	RANTES	rs62438851	G	A	0.066	144909173	0.532	0.105	360612	G	A	0.196	6	145230309	2.33E-06	0.041	3421	22.345
Vascular dementia (multiple infarctions)	RANTES	rs7000423	T	C	-0.045	110041420	0.507	0.068	360612	T	C	-0.132	8	111053649	1.82E-07	0.025	3421	27.139
Vascular dementia (multiple infarctions)	RANTES	rs72793342	A	G	0.081	30537031	0.311	0.080	360612	A	G	-0.149	16	30548352	1.48E-06	0.031	3421	23.309
Vascular dementia (multiple infarctions)	RANTES	rs74472919	T	C	0.038	81626515	0.822	0.171	360612	T	C	0.331	13	82200650	3.97E-08	0.061	3421	29.987
Vascular dementia (multiple infarctions)	RANTES	rs75613039	T	C	-0.167	129706688	0.400	0.199	360612	T	C	0.370	11	129576583	4.81E-06	0.081	3421	20.866
Vascular dementia (multiple infarctions)	RANTES	rs818452	T	C	-0.083	152594661	0.542	0.137	360612	T	C	0.238	6	152915796	2.36E-06	0.051	3421	22.230
Vascular dementia (multiple infarctions)	Platelet-derived growth factor BB	rs116445074	T	G	0.047	52238766	0.845	0.238	360612	T	G	0.293	5	51534600	3.11E-07	0.059	8293	24.932
Vascular dementia (multiple infarctions)	Platelet-derived growth factor BB	rs11766649	G	A	-0.112	145142154	0.161	0.080	360612	G	A	-0.091	7	144839247	3.53E-06	0.020	8293	21.461
Vascular dementia (multiple infarctions)	Platelet-derived growth factor BB	rs11916118	G	A	-0.068	117193342	0.413	0.083	360612	G	A	-0.089	3	116912189	4.93E-06	0.019	8293	20.999
Vascular dementia (multiple infarctions)	Platelet-derived growth factor BB	rs12289510	G	A	0.015	125077155	0.823	0.066	360612	G	A	0.078	11	124947051	7.69E-07	0.016	8293	24.371
Vascular dementia (multiple infarctions)	Platelet-derived growth factor BB	rs2324229	C	T	0.107	83208412	0.117	0.068	360612	C	T	-0.089	6	83918131	3.48E-08	0.016	8293	30.834
Vascular dementia (multiple infarctions)	Platelet-derived growth factor BB	rs35859699	A	G	-0.098	111263595	0.752	0.311	360612	A	G	-0.395	4	112184751	2.07E-06	0.084	8293	22.030
Vascular dementia (multiple infarctions)	Platelet-derived growth factor BB	rs4965869	T	C	0.089	101450115	0.238	0.075	360612	T	C	0.184	15	101990320	5.66E-24	0.018	8293	103.342
Vascular dementia (multiple infarctions)	Platelet-derived growth factor BB	rs55680718	T	C	0.168	224302160	0.092	0.100	360612	T	C	-0.138	2	225166877	1.86E-08	0.025	8293	31.606
Vascular dementia (multiple infarctions)	Platelet-derived growth factor BB	rs72777070	G	T	0.172	9658748	0.032	0.081	360612	G	T	0.107	2	9798877	8.98E-08	0.020	8293	28.569
Vascular dementia (multiple infarctions)	Platelet-derived growth factor BB	rs73162807	A	C	-0.099	146757003	0.639	0.211	360612	A	C	-0.239	3	146474790	1.74E-06	0.050	8293	22.959
Vascular dementia (multiple infarctions)	Platelet-derived growth factor BB	rs9936075	G	A	-0.049	7271908	0.474	0.068	360612	G	A	0.078	16	7321909	1.76E-06	0.016	8293	22.737
Vascular dementia (multiple infarctions)	Platelet-derived growth factor BB	rs9941733	G	A	0.030	393417	0.729	0.087	360612	G	A	-0.116	20	374061	3.31E-07	0.023	8293	25.930
Vascular dementia (multiple infarctions)	Macrophage inflammatory protein 1b	rs11130043	A	G	0.077	45069747	0.243	0.066	360612	A	G	-0.073	3	45111239	3.22E-06	0.016	8243	21.679
Vascular dementia (multiple infarctions)	Macrophage inflammatory protein 1b	rs113010081	C	T	-0.150	46415921	0.125	0.098	360612	C	T	0.595	3	46457412	########	0.024	8243	636.493
Vascular dementia (multiple infarctions)	Macrophage inflammatory protein 1b	rs113877493	T	C	0.071	36443746	0.425	0.089	360612	T	C	-0.612	17	34812273	########	0.022	8243	789.146
Vascular dementia (multiple infarctions)	Macrophage inflammatory protein 1b	rs116237296	A	G	0.835	86579833	0.068	0.457	360612	A	G	0.544	1	87045516	7.23E-07	0.112	8243	23.778
Vascular dementia (multiple infarctions)	Macrophage inflammatory protein 1b	rs117453826	G	A	0.034	36775624	0.880	0.223	360612	G	A	0.577	17	35132809	5.07E-22	0.059	8243	94.808
Vascular dementia (multiple infarctions)	Macrophage inflammatory protein 1b	rs141102180	T	G	0.119	36108811	0.485	0.171	360612	T	G	0.323	17	34436204	1.08E-16	0.039	8243	67.340
Vascular dementia (multiple infarctions)	Macrophage inflammatory protein 1b	rs17138331	G	A	-0.002	7826737	0.985	0.121	360612	G	A	0.139	7	7866368	2.26E-06	0.030	8243	22.234
Vascular dementia (multiple infarctions)	Macrophage inflammatory protein 1b	rs17641689	G	A	-0.027	36668383	0.795	0.104	360612	G	A	0.245	17	35024819	1.28E-16	0.029	8243	69.805
Vascular dementia (multiple infarctions)	Macrophage inflammatory protein 1b	rs2079664	G	A	0.088	34680936	0.235	0.074	360612	G	A	-0.100	17	33007955	1.51E-08	0.018	8243	31.961
Vascular dementia (multiple infarctions)	Macrophage inflammatory protein 1b	rs281749	C	T	0.138	107626417	0.051	0.071	360612	C	T	-0.080	8	108638645	3.17E-06	0.017	8243	21.832
Vascular dementia (multiple infarctions)	Macrophage inflammatory protein 1b	rs34437725	C	T	-0.241	35499766	0.220	0.196	360612	C	T	0.263	17	33826785	7.67E-08	0.048	8243	29.717
Vascular dementia (multiple infarctions)	Macrophage inflammatory protein 1b	rs72791296	T	C	0.275	121614355	0.111	0.172	360612	T	C	0.237	5	120950050	3.78E-07	0.047	8243	25.844
Vascular dementia (multiple infarctions)	Macrophage inflammatory protein 1b	rs72799710	T	C	0.115	123825971	0.204	0.090	360612	T	C	-0.101	5	123161665	3.21E-06	0.022	8243	21.635
Vascular dementia (multiple infarctions)	Macrophage inflammatory protein 1b	rs74810984	C	T	0.316	127876202	0.137	0.212	360612	C	T	-0.221	10	129674466	1.96E-06	0.047	8243	21.660
Vascular dementia (multiple infarctions)	Macrophage inflammatory protein 1b	rs76582507	A	G	-0.254	37510075	0.466	0.348	360612	A	G	0.318	9	37510072	3.26E-06	0.068	8243	21.994
Vascular dementia (multiple infarctions)	Macrophage inflammatory protein 1b	rs76583883	T	G	0.077	45936445	0.661	0.176	360612	T	G	-0.232	21	47356359	4.99E-06	0.051	8243	20.559
Vascular dementia (multiple infarctions)	Macrophage inflammatory protein 1b	rs76776296	G	A	0.114	115488433	0.616	0.227	360612	G	A	-0.300	7	115128487	5.55E-07	0.060	8243	25.117
Vascular dementia (multiple infarctions)	Macrophage inflammatory protein 1a	rs10835056	G	T	-0.037	26675470	0.596	0.070	360612	G	T	-0.119	11	26697017	2.60E-06	0.025	3522	22.097
Vascular dementia (multiple infarctions)	Macrophage inflammatory protein 1a	rs12690897	A	G	-0.036	85716861	0.620	0.072	360612	A	G	0.125	7	85346177	2.11E-06	0.026	3522	22.690
Vascular dementia (multiple infarctions)	Macrophage inflammatory protein 1a	rs184154340	A	G	0.113	80790993	0.498	0.167	360612	A	G	0.331	11	80502036	1.86E-06	0.069	3522	22.813
Vascular dementia (multiple infarctions)	Macrophage inflammatory protein 1a	rs34771762	G	A	0.048	200547932	0.703	0.126	360612	G	A	-0.249	2	201412655	2.13E-06	0.052	3522	22.667
Vascular dementia (multiple infarctions)	Macrophage inflammatory protein 1a	rs57786342	A	G	-0.032	68793311	0.675	0.077	360612	A	G	0.131	14	69260028	4.05E-06	0.029	3522	21.257
Vascular dementia (multiple infarctions)	Macrophage inflammatory protein 1a	rs60198979	A	G	-0.037	43250698	0.764	0.124	360612	A	G	-0.215	22	43646704	2.61E-06	0.046	3522	21.955
Vascular dementia (multiple infarctions)	Macrophage inflammatory protein 1a	rs7232268	G	A	-0.118	70101678	0.479	0.167	360612	G	A	-0.282	18	67768914	2.55E-06	0.060	3522	22.180
Vascular dementia (multiple infarctions)	Monokine induced by gamma interferon	rs111607343	A	G	-0.265	897855	0.361	0.289	360612	A	G	-0.521	19	897855	2.83E-06	0.112	3685	21.678
Vascular dementia (multiple infarctions)	Monokine induced by gamma interferon	rs11177248	A	G	-0.231	68482106	0.160	0.165	360612	A	G	0.307	12	68875886	4.45E-06	0.067	3685	21.037
Vascular dementia (multiple infarctions)	Monokine induced by gamma interferon	rs112337562	G	T	-0.303	92665225	0.151	0.211	360612	G	T	0.370	14	93131570	2.98E-06	0.080	3685	21.606
Vascular dementia (multiple infarctions)	Monokine induced by gamma interferon	rs112861654	G	A	0.174	42179062	0.195	0.134	360612	G	A	0.277	21	43599172	1.81E-07	0.053	3685	27.320
Vascular dementia (multiple infarctions)	Monokine induced by gamma interferon	rs117831247	T	C	0.022	66742081	0.965	0.504	360612	T	C	-0.833	10	68501839	2.16E-06	0.175	3685	22.576
Vascular dementia (multiple infarctions)	Monokine induced by gamma interferon	rs139010077	T	C	0.493	170618359	0.050	0.252	360612	T	C	0.432	3	170336148	3.55E-06	0.095	3685	20.698
Vascular dementia (multiple infarctions)	Monokine induced by gamma interferon	rs1796086	C	T	0.227	71183729	0.053	0.117	360612	C	T	0.210	7	70648715	2.23E-07	0.040	3685	27.050
Vascular dementia (multiple infarctions)	Monokine induced by gamma interferon	rs41272086	A	G	0.063	160587614	0.566	0.110	360612	A	G	-0.223	6	161008646	7.43E-08	0.042	3685	28.771
Vascular dementia (multiple infarctions)	Monokine induced by gamma interferon	rs55876513	G	T	0.067	75962545	0.353	0.072	360612	G	T	-0.166	4	76883698	8.23E-11	0.026	3685	42.378
Vascular dementia (multiple infarctions)	Monokine induced by gamma interferon	rs5752128	C	T	0.260	25322656	0.005	0.093	360612	C	T	0.169	22	25718623	4.34E-06	0.037	3685	20.852
Vascular dementia (multiple infarctions)	Monokine induced by gamma interferon	rs62562991	A	G	0.527	95973777	0.049	0.268	360612	A	G	0.624	9	98736059	8.40E-07	0.126	3685	24.495
Vascular dementia (multiple infarctions)	Monokine induced by gamma interferon	rs6679677	A	C	-0.069	113761186	0.460	0.093	360612	A	C	0.162	1	114303808	8.86E-07	0.033	3685	24.246
Vascular dementia (multiple infarctions)	Monokine induced by gamma interferon	rs77086208	T	C	0.025	70152774	0.898	0.196	360612	T	C	0.323	14	70619491	3.83E-06	0.070	3685	21.361
Vascular dementia (multiple infarctions)	Monokine induced by gamma interferon	rs816960	T	C	-0.114	107870173	0.093	0.068	360612	T	C	-0.122	13	108522521	5.01E-07	0.024	3685	25.164
Vascular dementia (multiple infarctions)	Macrophage colony stimulating factor	rs116274860	G	T	-0.222	148675030	0.443	0.289	360612	G	T	-0.819	3	148392817	2.74E-06	0.174	840	22.129
Vascular dementia (multiple infarctions)	Macrophage colony stimulating factor	rs117867915	C	T	-0.234	44630078	0.331	0.240	360612	C	T	-0.527	18	42210043	1.61E-06	0.110	840	23.054
Vascular dementia (multiple infarctions)	Macrophage colony stimulating factor	rs12962919	T	C	-0.058	78018752	0.649	0.127	360612	T	C	0.305	18	75778756	4.65E-06	0.066	840	21.255
Vascular dementia (multiple infarctions)	Macrophage colony stimulating factor	rs56367447	T	C	-0.029	4014005	0.871	0.180	360612	T	C	-0.497	8	3871527	1.72E-08	0.088	840	31.642
Vascular dementia (multiple infarctions)	Macrophage colony stimulating factor	rs62294910	A	G	-0.174	182480551	0.198	0.135	360612	A	G	0.343	3	182198339	6.82E-07	0.069	840	24.654

Vascular dementia (multiple infarctions)	Macrophage colony stimulating factor	rs78296352	T	G	-0.597	22495351	0.030	0.275	360612	T	G	0.527	1	22821844	1.05E-06	0.111	840	22.460
Vascular dementia (multiple infarctions)	Macrophage colony stimulating factor	rs9387100	C	T	-0.094	112781752	0.154	0.066	360612	C	T	0.135	6	113102954	4.07E-06	0.029	840	21.438
Vascular dementia (multiple infarctions)	Monocyte chemoattractant protein-1	rs10145849	A	G	0.089	82475647	0.192	0.068	360612	A	G	-0.076	14	82941991	3.41E-06	0.016	8293	21.720
Vascular dementia (multiple infarctions)	Monocyte chemoattractant protein-1	rs10744620	C	T	-0.041	3629928	0.547	0.068	360612	C	T	-0.079	12	3739094	9.91E-07	0.016	8293	23.955
Vascular dementia (multiple infarctions)	Monocyte chemoattractant protein-1	rs111995966	G	T	-0.017	108558513	0.893	0.123	360612	G	T	-0.145	2	109174969	2.53E-06	0.031	8293	21.939
Vascular dementia (multiple infarctions)	Monocyte chemoattractant protein-1	rs112313229	A	G	0.093	46323369	0.458	0.125	360612	A	G	-0.165	3	46364860	1.43E-07	0.031	8293	27.655
Vascular dementia (multiple infarctions)	Monocyte chemoattractant protein-1	rs12073356	A	G	0.065	207834503	0.614	0.128	360612	A	G	-0.143	1	208007848	4.17E-06	0.031	8293	21.024
Vascular dementia (multiple infarctions)	Monocyte chemoattractant protein-1	rs12075	A	G	-0.031	159205564	0.639	0.066	360612	A	G	0.219	1	159175354	1.44E-44	0.016	8293	198.719
Vascular dementia (multiple infarctions)	Monocyte chemoattractant protein-1	rs12493471	C	T	-0.036	45910186	0.594	0.068	360612	C	T	-0.116	3	45951678	6.81E-13	0.016	8293	51.538
Vascular dementia (multiple infarctions)	Monocyte chemoattractant protein-1	rs146522229	T	C	0.485	47295223	0.335	0.503	360612	T	C	-0.598	19	47798480	3.56E-07	0.118	8293	25.779
Vascular dementia (multiple infarctions)	Monocyte chemoattractant protein-1	rs2228467	C	T	-0.183	42864624	0.137	0.123	360612	C	T	0.264	3	42906116	9.19E-20	0.029	8293	82.117
Vascular dementia (multiple infarctions)	Monocyte chemoattractant protein-1	rs2712431	A	C	0.043	128598047	0.553	0.072	360612	A	C	-0.079	3	128316890	4.75E-06	0.017	8293	20.936
Vascular dementia (multiple infarctions)	Monocyte chemoattractant protein-1	rs56212190	T	C	0.066	41702868	0.669	0.154	360612	T	C	0.181	1	42168539	9.85E-07	0.037	8293	23.547
Vascular dementia (multiple infarctions)	Monocyte chemoattractant protein-1	rs7197349	G	A	-0.170	78653322	0.039	0.082	360612	G	A	-0.097	16	78687219	2.62E-06	0.021	8293	22.081
Vascular dementia (multiple infarctions)	Monocyte chemoattractant protein-1	rs7517040	G	A	-0.107	158889343	0.178	0.079	360612	G	A	0.099	1	158859133	2.44E-07	0.019	8293	26.703
Vascular dementia (multiple infarctions)	Monocyte chemoattractant protein-1	rs9317045	C	A	0.113	59055904	0.222	0.092	360612	C	A	-0.113	13	59630038	1.52E-06	0.024	8293	23.089
Vascular dementia (multiple infarctions)	Interleukin-12p70	rs13209117	A	G	0.052	44184028	0.487	0.075	360612	A	G	0.100	6	44151765	5.57E-08	0.019	8270	29.021
Vascular dementia (multiple infarctions)	Interleukin-12p70	rs17229494	G	A	0.045	37555798	0.665	0.105	360612	G	A	0.117	21	38928100	4.93E-06	0.026	8270	20.796
Vascular dementia (multiple infarctions)	Interleukin-12p70	rs282258	C	T	-0.046	224050083	0.490	0.066	360612	C	T	-0.073	2	224914800	3.21E-06	0.016	8270	21.898
Vascular dementia (multiple infarctions)	Interleukin-12p70	rs41282644	A	G	0.100	43785985	0.412	0.121	360612	A	G	0.147	6	43753722	1.05E-06	0.030	8270	23.478
Vascular dementia (multiple infarctions)	Interleukin-12p70	rs4349809	G	T	0.038	43957093	0.561	0.065	360612	G	T	-0.378	6	43924830	########	0.016	8270	564.287
Vascular dementia (multiple infarctions)	Interleukin-12p70	rs71361173	G	T	-0.191	76000450	0.051	0.098	360612	G	T	-0.111	18	73712405	3.06E-06	0.024	8270	21.570
Vascular dementia (multiple infarctions)	Interleukin-12p70	rs72831623	A	G	-0.205	47644927	0.116	0.130	360612	A	G	0.191	17	45722293	2.42E-07	0.037	8270	26.732
Vascular dementia (multiple infarctions)	Interleukin-12p70	rs782107	A	G	0.005	58439747	0.941	0.065	360612	A	G	0.075	12	58833530	1.60E-06	0.016	8270	23.114
Vascular dementia (multiple infarctions)	Interleukin-12p70	rs79121401	C	T	0.099	78986084	0.836	0.480	360612	C	T	-0.555	11	78697129	4.24E-06	0.121	8270	21.163
Vascular dementia (multiple infarctions)	Interleukin-12p70	rs9472183	G	A	0.013	43972465	0.848	0.065	360612	G	A	0.102	6	43940202	8.61E-11	0.016	8270	42.126
Vascular dementia (multiple infarctions)	Interferon gamma-induced protein 10	rs10809307	C	T	-0.027	11045908	0.731	0.079	360612	C	T	-0.131	9	11045908	3.64E-06	0.028	3685	21.415
Vascular dementia (multiple infarctions)	Interferon gamma-induced protein 10	rs113831257	A	G	-0.065	75234311	0.668	0.151	360612	A	G	0.359	4	76159521	2.53E-08	0.064	3685	31.110
Vascular dementia (multiple infarctions)	Interferon gamma-induced protein 10	rs11626201	A	C	-0.062	36511495	0.357	0.068	360612	A	C	0.116	14	36980700	1.93E-06	0.025	3685	22.495
Vascular dementia (multiple infarctions)	Interferon gamma-induced protein 10	rs143799975	G	A	0.113	75885862	0.762	0.373	360612	G	A	0.798	4	76807015	1.00E-06	0.164	3685	23.787
Vascular dementia (multiple infarctions)	Interferon gamma-induced protein 10	rs34383175	T	C	-0.166	144361034	0.347	0.177	360612	T	C	-0.315	8	145584694	1.51E-06	0.066	3685	23.031
Vascular dementia (multiple infarctions)	Interferon gamma-induced protein 10	rs75970138	A	G	-0.231	119813998	0.437	0.298	360612	A	G	-0.485	9	122576276	1.53E-06	0.104	3685	21.748
Vascular dementia (multiple infarctions)	Interferon gamma-induced protein 10	rs7645625	G	T	0.031	146856250	0.642	0.067	360612	G	T	0.109	3	146574037	4.41E-06	0.024	3685	20.997
Vascular dementia (multiple infarctions)	Interferon gamma-induced protein 10	rs79848609	C	A	-0.023	86772934	0.883	0.155	360612	C	A	-0.260	15	87316165	8.75E-07	0.054	3685	23.496
Vascular dementia (multiple infarctions)	Interferon gamma-induced protein 10	rs8112909	A	G	0.117	45910150	0.149	0.081	360612	A	G	-0.143	19	46413408	1.94E-06	0.030	3685	22.746
Vascular dementia (multiple infarctions)	Interleukin-18	rs10414578	T	C	-0.033	54634619	0.729	0.096	360612	T	C	-0.177	19	55146070	4.16E-07	0.035	3636	25.604
Vascular dementia (multiple infarctions)	Interleukin-18	rs115267715	T	C	0.548	69239188	0.008	0.205	360612	T	C	0.451	5	68535015	1.72E-08	0.080	3636	31.753
Vascular dementia (multiple infarctions)	Interleukin-18	rs116383510	C	A	0.453	2545536	0.097	0.273	360612	C	A	0.543	5	2545650	3.00E-07	0.106	3636	26.402
Vascular dementia (multiple infarctions)	Interleukin-18	rs117266781	T	C	-0.079	41261422	0.827	0.361	360612	T	C	0.684	7	41301020	3.15E-06	0.147	3636	21.716
Vascular dementia (multiple infarctions)	Interleukin-18	rs144841621	T	C	-0.099	69921801	0.773	0.343	360612	T	C	0.518	10	71681557	3.81E-06	0.114	3636	20.610
Vascular dementia (multiple infarctions)	Interleukin-18	rs17229943	C	A	0.080	69386709	0.469	0.111	360612	C	A	0.312	5	68682536	1.62E-11	0.046	3636	45.410
Vascular dementia (multiple infarctions)	Interleukin-18	rs1852105	C	T	-0.055	64265217	0.753	0.175	360612	C	T	-0.304	7	63725595	4.32E-06	0.066	3636	21.096
Vascular dementia (multiple infarctions)	Interleukin-18	rs1979967	T	C	0.065	79367271	0.413	0.080	360612	T	C	0.140	15	79659613	9.45E-07	0.029	3636	24.031
Vascular dementia (multiple infarctions)	Interleukin-18	rs2729385	A	G	-0.004	57495520	0.951	0.072	360612	A	G	0.123	11	57262993	3.79E-06	0.026	3636	22.076
Vascular dementia (multiple infarctions)	Interleukin-18	rs385076	C	T	0.177	32264782	0.009	0.068	360612	C	T	0.243	2	32489851	1.66E-22	0.025	3636	96.166
Vascular dementia (multiple infarctions)	Interleukin-18	rs4482818	G	A	0.027	65062779	0.681	0.067	360612	G	A	-0.129	4	65928497	1.45E-07	0.024	3636	27.778
Vascular dementia (multiple infarctions)	Interleukin-18	rs658805	A	G	-0.026	70199369	0.706	0.069	360612	A	G	0.123	6	70909073	4.94E-07	0.024	3636	25.247
Vascular dementia (multiple infarctions)	Interleukin-18	rs71478720	T	C	-0.088	112138882	0.251	0.076	360612	T	C	-0.267	11	112009605	3.71E-22	0.028	3636	93.515
Vascular dementia (multiple infarctions)	Interleukin-18	rs78623212	T	C	-0.117	103667180	0.745	0.362	360612	T	C	0.871	7	103307627	6.71E-07	0.178	3636	23.970
Vascular dementia (multiple infarctions)	Interleukin-18	rs78716465	A	G	0.028	42015086	0.870	0.173	360612	A	G	0.327	20	40643726	1.63E-06	0.068	3636	22.919
Vascular dementia (multiple infarctions)	Interleukin-17	rs117029961	A	G	0.018	37147653	0.960	0.363	360612	A	G	0.459	10	37436581	4.94E-06	0.102	7760	20.405
Vascular dementia (multiple infarctions)	Interleukin-17	rs117556572	T	C	0.045	104436567	0.835	0.215	360612	T	C	-0.510	13	105088917	3.28E-06	0.110	7760	21.552
Vascular dementia (multiple infarctions)	Interleukin-17	rs1530455	C	T	-0.023	123136052	0.741	0.068	360612	C	T	-0.108	3	122854899	4.87E-10	0.017	7760	38.972
Vascular dementia (multiple infarctions)	Interleukin-17	rs17106604	T	C	0.120	77912813	0.191	0.092	360612	T	C	0.113	14	78379156	6.37E-07	0.023	7760	25.178
Vascular dementia (multiple infarctions)	Interleukin-17	rs17282552	C	T	-0.130	207109091	0.392	0.153	360612	C	T	0.200	2	207973815	8.21E-07	0.041	7760	24.411
Vascular dementia (multiple infarctions)	Interleukin-17	rs184080173	C	T	-0.026	77331424	0.888	0.185	360612	C	T	-0.238	12	77725204	4.19E-07	0.047	7760	25.620
Vascular dementia (multiple infarctions)	Interleukin-17	rs187475560	T	C	-0.319	160353411	0.112	0.200	360612	T	C	-0.243	4	161274563	3.29E-06	0.052	7760	21.910
Vascular dementia (multiple infarctions)	Interleukin-17	rs62191444	T	G	0.020	393023	0.824	0.092	360612	T	G	-0.114	20	373667	4.22E-06	0.025	7760	21.153
Vascular dementia (multiple infarctions)	Interleukin-17	rs78296352	T	G	-0.597	22495351	0.030	0.275	360612	T	G	0.303	1	22821844	4.27E-06	0.065	7760	21.956
Vascular dementia (multiple infarctions)	Interleukin-17	rs78612928	C	T	0.165	29812292	0.063	0.089	360612	C	T	-0.104	4	29813914	2.62E-06	0.022	7760	21.820
Vascular dementia (multiple infarctions)	Interleukin-13	rs117795020	A	G	0.078	87469237	0.683	0.190	360612	A	G	-0.352	9	90084152	9.86E-07	0.072	3557	24.197
Vascular dementia (multiple infarctions)	Interleukin-13	rs12623722	A	G	-0.102	22955811	0.157	0.072	360612	A	G	-0.119	2	23178683	4.19E-06	0.026	3557	21.096
Vascular dementia (multiple infarctions)	Interleukin-13	rs139083458	T	C	-0.810	26160409	0.111	0.509	360612	T	C	0.990	5	26160518	2.81E-06	0.211	3557	22.086
Vascular dementia (multiple infarctions)	Interleukin-13	rs142167313	C	T	-0.049	44204360	0.768	0.165	360612	C	T	0.313	6	44172097	3.98E-07	0.062	3557	25.735

Vascular dementia (multiple infarctions)	Interleukin-13	rs27949	T	C	0.021	59254997	0.764	0.070	360612	T	C	-0.117	5	58550823	3.43E-06	0.025	3557	21.482
Vascular dementia (multiple infarctions)	Interleukin-13	rs6799107	C	T	-0.006	127338175	0.946	0.083	360612	C	T	0.146	3	127057018	1.25E-06	0.030	3557	23.495
Vascular dementia (multiple infarctions)	Interleukin-13	rs7073807	C	T	-0.003	67393670	0.972	0.097	360612	C	T	-0.168	10	69153428	2.37E-06	0.036	3557	22.323
Vascular dementia (multiple infarctions)	Interleukin-13	rs75995699	A	G	0.225	5140622	0.219	0.183	360612	A	G	0.332	6	5140856	2.64E-06	0.070	3557	22.610
Vascular dementia (multiple infarctions)	Interleukin-13	rs9472168	G	A	0.040	43961248	0.543	0.066	360612	G	A	-0.424	6	43928985	1.08E-65	0.025	3557	292.851
Vascular dementia (multiple infarctions)	Interleukin-10	rs10457128	A	G	-0.034	105570101	0.620	0.068	360612	A	G	-0.087	6	106017976	5.24E-07	0.017	7681	25.292
Vascular dementia (multiple infarctions)	Interleukin-10	rs10493718	A	C	0.028	82597250	0.752	0.090	360612	A	C	-0.110	1	83062933	7.16E-07	0.022	7681	24.552
Vascular dementia (multiple infarctions)	Interleukin-10	rs11206302	T	C	-0.077	54208270	0.440	0.100	360612	T	C	-0.119	1	54673943	2.20E-06	0.025	7681	22.440
Vascular dementia (multiple infarctions)	Interleukin-10	rs2086656	T	C	-0.010	59632755	0.887	0.069	360612	T	C	-0.079	4	60498473	3.78E-06	0.017	7681	21.289
Vascular dementia (multiple infarctions)	Interleukin-10	rs282258	C	T	-0.046	224050083	0.490	0.066	360612	C	T	-0.099	2	224914800	1.00E-09	0.016	7681	37.497
Vascular dementia (multiple infarctions)	Interleukin-10	rs3025021	C	T	0.012	43781426	0.857	0.069	360612	C	T	-0.095	6	43749163	1.46E-06	0.020	7681	23.585
Vascular dementia (multiple infarctions)	Interleukin-10	rs41282660	G	A	0.066	44229269	0.490	0.095	360612	G	A	0.119	6	44197006	3.72E-06	0.026	7681	21.924
Vascular dementia (multiple infarctions)	Interleukin-10	rs4349809	G	T	0.038	43957093	0.561	0.065	360612	G	T	-0.285	6	43924830	5.77E-67	0.017	7681	298.976
Vascular dementia (multiple infarctions)	Interleukin-10	rs465757	A	G	0.032	15599638	0.644	0.069	360612	A	G	0.084	20	15580283	1.17E-06	0.017	7681	23.306
Vascular dementia (multiple infarctions)	Interleukin-10	rs7088799	G	T	-0.042	63256414	0.528	0.067	360612	G	T	0.085	10	65016174	3.23E-07	0.017	7681	26.028
Vascular dementia (multiple infarctions)	Interleukin-8	rs11634944	C	T	-0.045	24937946	0.513	0.068	360612	C	T	0.121	15	25183093	1.29E-06	0.025	3526	23.208
Vascular dementia (multiple infarctions)	Interleukin-8	rs12075	A	G	-0.031	159205564	0.639	0.066	360612	A	G	0.120	1	159175354	3.88E-07	0.024	3526	25.855
Vascular dementia (multiple infarctions)	Interleukin-8	rs141926526	C	A	-0.372	32809028	0.306	0.363	360612	C	A	0.615	7	32848640	2.57E-06	0.131	3526	22.100
Vascular dementia (multiple infarctions)	Interleukin-8	rs2673604	A	C	-0.065	132399360	0.360	0.071	360612	A	C	-0.127	8	133411607	7.02E-07	0.026	3526	24.648
Vascular dementia (multiple infarctions)	Interleukin-6	rs1333040	T	C	0.070	22083405	0.281	0.065	360612	T	C	0.074	9	22083404	3.17E-06	0.016	8189	21.817
Vascular dementia (multiple infarctions)	Interleukin-6	rs72831623	A	G	-0.205	47644927	0.116	0.130	360612	A	G	0.197	17	45722293	1.08E-07	0.037	8189	28.130
Vascular dementia (multiple infarctions)	Interleukin-6	rs73273528	T	C	-0.173	51814574	0.411	0.210	360612	T	C	0.267	20	50431113	9.58E-07	0.055	8189	23.347
Vascular dementia (multiple infarctions)	Interleukin-6	rs76856708	C	T	0.357	80695146	0.222	0.292	360612	C	T	-0.329	16	80729043	2.61E-06	0.070	8189	22.077
Vascular dementia (multiple infarctions)	Interleukin-1-receptor antagonist	rs1054402	C	T	0.128	116401230	0.092	0.076	360612	C	T	-0.131	9	119163509	1.13E-06	0.027	3638	23.576
Vascular dementia (multiple infarctions)	Interleukin-1-receptor antagonist	rs11627423	C	A	-0.048	32731417	0.477	0.068	360612	C	A	-0.117	14	33200623	2.12E-06	0.025	3638	22.476
Vascular dementia (multiple infarctions)	Interleukin-1-receptor antagonist	rs12121840	T	C	0.113	165572405	0.443	0.148	360612	T	C	0.269	1	165541642	2.43E-06	0.057	3638	22.227
Vascular dementia (multiple infarctions)	Interleukin-1-receptor antagonist	rs2809154	T	C	-0.010	84153389	0.928	0.108	360612	T	C	-0.179	13	84727524	3.74E-06	0.039	3638	21.188
Vascular dementia (multiple infarctions)	Interleukin-1-receptor antagonist	rs61335305	A	C	-0.184	66160736	0.444	0.240	360612	A	C	0.445	15	66453074	1.00E-06	0.091	3638	24.051
Vascular dementia (multiple infarctions)	Interleukin-1-receptor antagonist	rs9623661	T	C	-0.192	42697370	0.095	0.115	360612	T	C	-0.197	22	43093376	3.86E-06	0.043	3638	21.298
Vascular dementia (multiple infarctions)	Interleukin-1-beta	rs143319329	T	C	-0.026	128499405	0.946	0.380	360612	T	C	0.280	7	128139459	2.00E-06	0.072	3309	15.347
Vascular dementia (multiple infarctions)	Interleukin-1-beta	rs61335305	A	C	-0.184	66160736	0.444	0.240	360612	A	C	0.297	15	66453074	1.90E-06	0.072	3309	16.783
Vascular dementia (multiple infarctions)	Interleukin-1-beta	rs62015704	G	A	0.102	7417906	0.306	0.100	360612	G	A	-0.108	16	7467907	2.09E-06	0.028	3309	14.618
Vascular dementia (multiple infarctions)	Interleukin-1-beta	rs9898641	C	T	-0.021	59493672	0.757	0.068	360612	C	T	0.203	17	57571033	3.59E-06	0.045	3309	20.033
Vascular dementia (multiple infarctions)	Hepatocyte growth factor	rs11060254	A	G	-0.048	129331024	0.496	0.070	360612	A	G	-0.080	12	129815569	1.58E-06	0.017	8292	22.948
Vascular dementia (multiple infarctions)	Hepatocyte growth factor	rs150322232	G	A	0.243	7890743	0.160	0.173	360612	G	A	-0.210	7	7930374	4.89E-06	0.046	8292	20.650
Vascular dementia (multiple infarctions)	Hepatocyte growth factor	rs1698249	C	A	-0.146	83889842	0.347	0.155	360612	C	A	0.170	14	84356186	4.09E-06	0.037	8292	20.835
Vascular dementia (multiple infarctions)	Hepatocyte growth factor	rs2003620	T	C	0.012	134794733	0.950	0.192	360612	T	C	0.228	7	134479484	2.83E-06	0.049	8292	21.721
Vascular dementia (multiple infarctions)	Hepatocyte growth factor	rs3748034	T	G	0.050	3444364	0.615	0.100	360612	T	G	0.150	4	3446091	1.81E-10	0.023	8292	40.818
Vascular dementia (multiple infarctions)	Hepatocyte growth factor	rs5745687	T	C	0.298	81729735	0.064	0.161	360612	T	C	-0.307	7	81359051	2.75E-14	0.041	8292	57.252
Vascular dementia (multiple infarctions)	Hepatocyte growth factor	rs62481625	C	T	0.078	156194766	0.394	0.091	360612	C	T	-0.109	7	155987460	1.18E-06	0.023	8292	23.512
Vascular dementia (multiple infarctions)	Interleukin-9	rs41294750	T	C	-0.404	53084968	0.033	0.190	360612	T	C	0.351	1	53550640	2.36E-06	0.075	3634	22.070
Vascular dementia (multiple infarctions)	Interleukin-9	rs4880409	T	C	0.055	132516716	0.867	0.327	360612	T	C	-0.336	10	134330220	3.50E-06	0.072	3634	21.533
Vascular dementia (multiple infarctions)	Interleukin-9	rs61867538	T	C	0.012	1503276	0.942	0.164	360612	T	C	0.357	11	1524506	3.93E-06	0.077	3634	21.227
Vascular dementia (multiple infarctions)	Interleukin-9	rs7232268	G	A	-0.118	70101678	0.479	0.167	360612	G	A	-0.276	18	67768914	2.52E-06	0.059	3634	22.092
Vascular dementia (multiple infarctions)	Interleukin-9	rs7242404	A	G	0.055	12741268	0.442	0.072	360612	A	G	-0.123	18	12741267	3.27E-06	0.026	3634	21.637
Vascular dementia (multiple infarctions)	Interleukin-9	rs76963786	T	C	-0.057	31886823	0.714	0.157	360612	T	C	-0.287	12	32039757	4.50E-07	0.056	3634	26.457
Vascular dementia (multiple infarctions)	Interleukin-7	rs117509142	C	T	-0.112	86121854	0.547	0.185	360612	C	T	0.327	8	87134083	1.99E-06	0.069	3409	22.590
Vascular dementia (multiple infarctions)	Interleukin-7	rs141425475	C	T	-0.278	17679056	0.232	0.233	360612	C	T	0.478	5	17679165	2.53E-06	0.102	3409	22.144
Vascular dementia (multiple infarctions)	Interleukin-7	rs144701438	A	G	0.270	66293168	0.320	0.271	360612	A	G	-0.482	18	63960405	9.75E-07	0.099	3409	23.742
Vascular dementia (multiple infarctions)	Interleukin-7	rs17091524	C	T	0.040	56482041	0.879	0.261	360612	C	T	-0.492	14	56948759	1.91E-06	0.101	3409	23.627
Vascular dementia (multiple infarctions)	Interleukin-7	rs28793375	T	C	0.044	41558099	0.648	0.096	360612	T	C	0.164	8	41415618	4.46E-06	0.036	3409	20.588
Vascular dementia (multiple infarctions)	Interleukin-7	rs4320361	T	G	0.039	43960774	0.550	0.066	360612	T	G	-0.325	6	43928511	6.87E-39	0.025	3409	169.836
Vascular dementia (multiple infarctions)	Interleukin-7	rs62006410	T	C	0.019	102541598	0.802	0.077	360612	T	C	-0.156	14	103007935	3.39E-07	0.030	3409	26.405
Vascular dementia (multiple infarctions)	Interleukin-7	rs75904417	C	A	-0.120	167796811	0.202	0.094	360612	C	A	0.170	2	168653321	1.16E-06	0.035	3409	23.671
Vascular dementia (multiple infarctions)	Interleukin-7	rs77981494	C	T	-0.208	17451009	0.359	0.227	360612	C	T	0.518	16	17544866	1.07E-06	0.106	3409	23.683
Vascular dementia (multiple infarctions)	Interleukin-7	rs78346957	A	G	0.314	125214944	0.314	0.312	360612	A	G	0.459	10	126903513	4.51E-06	0.101	3409	20.758
Vascular dementia (multiple infarctions)	Interleukin-5	rs11680908	G	A	-0.264	109460295	0.070	0.146	360612	G	A	-0.263	2	110076751	2.03E-06	0.055	3364	22.605
Vascular dementia (multiple infarctions)	Interleukin-5	rs6737109	C	T	-0.063	22956659	0.343	0.066	360612	C	T	-0.116	2	23179531	2.40E-06	0.025	3364	22.056
Vascular dementia (multiple infarctions)	Interleukin-5	rs72831687	A	G	-0.124	16092129	0.616	0.248	360612	A	G	-0.524	6	16092360	1.69E-06	0.111	3364	22.317
Vascular dementia (multiple infarctions)	Interleukin-5	rs73040130	C	T	-0.105	36255288	0.461	0.143	360612	C	T	-0.264	19	36746190	6.00E-07	0.053	3364	24.868
Vascular dementia (multiple infarctions)	Interleukin-5	rs7767396	G	A	0.037	43959313	0.567	0.065	360612	G	A	-0.152	6	43927050	7.69E-10	0.025	3364	37.928
Vascular dementia (multiple infarctions)	Interleukin-4	rs10512267	C	T	0.016	99427847	0.804	0.066	360612	C	T	0.082	9	102190129	2.94E-07	0.016	8124	26.194
Vascular dementia (multiple infarctions)	Interleukin-4	rs116705532	G	T	0.281	113162547	0.485	0.402	360612	G	T	0.468	1	113705169	1.76E-06	0.098	8124	22.879
Vascular dementia (multiple infarctions)	Interleukin-4	rs117146485	C	T	-0.120	135932411	0.619	0.242	360612	C	T	0.292	9	138824257	2.71E-06	0.063	8124	21.610

Vascular dementia (multiple infarctions)	Interleukin-4	rs17713451	A	G	0.153	151465386	0.141	0.104	360612	A	G	0.127	7	151162472	4.97E-07	0.025	8124	25.357
Vascular dementia (multiple infarctions)	Interleukin-4	rs73023729	A	G	-0.185	159232998	0.240	0.158	360612	A	G	-0.180	6	159654030	9.03E-07	0.037	8124	24.080
Vascular dementia (multiple infarctions)	Interleukin-4	rs7613691	G	A	-0.108	147935804	0.465	0.147	360612	G	A	-0.178	3	147653591	4.05E-06	0.038	8124	21.367
Vascular dementia (multiple infarctions)	Interleukin-4	rs9508291	C	T	0.296	29136483	0.046	0.148	360612	C	T	0.168	13	29710620	3.03E-06	0.036	8124	21.795
Vascular dementia (multiple infarctions)	Interleukin-4	rs9941733	G	A	0.030	393417	0.729	0.087	360612	G	A	-0.114	20	374061	6.88E-07	0.023	8124	24.782
Vascular dementia (multiple infarctions)	Interleukin-2 receptor antagonist	rs11241559	G	T	0.059	120641005	0.438	0.076	360612	G	T	0.126	5	119976700	2.00E-06	0.027	3677	22.580
Vascular dementia (multiple infarctions)	Interleukin-2 receptor antagonist	rs117244812	A	G	-0.562	6539990	0.155	0.395	360612	A	G	-0.706	17	6443310	2.10E-06	0.149	3677	22.537
Vascular dementia (multiple infarctions)	Interleukin-2 receptor antagonist	rs12722497	A	C	0.115	6053965	0.379	0.130	360612	A	C	0.628	10	6095928	1.57E-38	0.049	3677	167.609
Vascular dementia (multiple infarctions)	Interleukin-2 receptor antagonist	rs185231391	C	T	-0.078	59373953	0.846	0.404	360612	C	T	-0.850	3	59359679	1.47E-06	0.181	3677	22.094
Vascular dementia (multiple infarctions)	Interleukin-2 receptor antagonist	rs4733117	C	A	0.151	32280094	0.063	0.081	360612	C	A	-0.137	8	32137610	2.63E-06	0.029	3677	21.981
Vascular dementia (multiple infarctions)	Interleukin-2 receptor antagonist	rs61705228	T	C	-0.198	100275145	0.266	0.178	360612	T	C	0.330	4	101196302	3.99E-06	0.072	3677	21.281
Vascular dementia (multiple infarctions)	Interleukin-2	rs12051139	C	T	-0.010	86885068	0.882	0.066	360612	C	T	0.113	16	86918674	4.76E-06	0.025	3475	20.967
Vascular dementia (multiple infarctions)	Interleukin-2	rs170117	T	C	0.009	54524213	0.922	0.094	360612	T	C	-0.162	4	55390380	3.87E-06	0.035	3475	21.467
Vascular dementia (multiple infarctions)	Interleukin-2	rs2807544	G	A	0.002	14877749	0.979	0.068	360612	G	A	-0.118	1	15204245	3.41E-06	0.025	3475	21.569
Vascular dementia (multiple infarctions)	Interleukin-2	rs4634519	G	A	0.029	67727941	0.697	0.073	360612	G	A	0.126	7	67192928	2.77E-06	0.027	3475	21.975
Vascular dementia (multiple infarctions)	Interleukin-2	rs61335305	A	C	-0.184	66160736	0.444	0.240	360612	A	C	0.451	15	66453074	7.32E-07	0.092	3475	24.179
Vascular dementia (multiple infarctions)	Interleukin-2	rs62124990	T	G	0.071	19038882	0.754	0.228	360612	T	G	-0.696	2	19238636	3.22E-06	0.150	3475	21.680
Vascular dementia (multiple infarctions)	Interleukin-2	rs7615304	G	A	-0.081	156957914	0.217	0.066	360612	G	A	0.117	3	156675703	1.21E-06	0.024	3475	23.454
Vascular dementia (multiple infarctions)	Interleukin-2	rs80336398	C	T	-0.024	64075258	0.911	0.211	360612	C	T	-0.400	3	64060934	2.82E-06	0.086	3475	21.745
Vascular dementia (multiple infarctions)	Interferon gamma	rs10487554	A	G	-0.095	149670595	0.187	0.072	360612	A	G	-0.090	7	149367686	1.09E-06	0.018	7701	23.919
Vascular dementia (multiple infarctions)	Interferon gamma	rs113600793	A	C	-0.193	47384095	0.168	0.140	360612	A	C	0.183	17	45461461	8.95E-07	0.037	7701	24.044
Vascular dementia (multiple infarctions)	Interferon gamma	rs115729819	G	A	-0.222	168783516	0.280	0.205	360612	G	A	-0.248	4	169704667	1.38E-06	0.052	7701	23.264
Vascular dementia (multiple infarctions)	Interferon gamma	rs11843756	G	T	-0.042	48680756	0.796	0.164	360612	G	T	-0.184	13	49254892	3.09E-06	0.039	7701	21.921
Vascular dementia (multiple infarctions)	Interferon gamma	rs12420286	C	T	-0.023	103907166	0.907	0.193	360612	C	T	-0.238	11	103777894	2.08E-06	0.050	7701	22.491
Vascular dementia (multiple infarctions)	Interferon gamma	rs1867282	T	C	0.013	99409865	0.850	0.066	360612	T	C	0.077	9	102172147	3.15E-06	0.017	7701	21.740
Vascular dementia (multiple infarctions)	Interferon gamma	rs2073438	A	G	0.063	6996757	0.399	0.074	360612	A	G	0.090	17	6900076	1.68E-06	0.019	7701	22.816
Vascular dementia (multiple infarctions)	Interferon gamma	rs74148555	T	C	-0.248	90320085	0.288	0.234	360612	T	C	-0.373	10	92079842	2.64E-06	0.077	7701	23.249
Vascular dementia (multiple infarctions)	Interferon gamma	rs78296352	T	G	-0.597	22495351	0.030	0.275	360612	T	G	0.343	1	22821844	1.38E-07	0.065	7701	27.675
Vascular dementia (multiple infarctions)	Growth-regulated protein alpha	rs1113500	T	G	-0.036	108052820	0.589	0.067	360612	T	G	0.117	1	108595442	1.57E-06	0.024	3505	23.150
Vascular dementia (multiple infarctions)	Growth-regulated protein alpha	rs12075	A	G	-0.031	159205564	0.639	0.066	360612	A	G	0.375	1	159175354	1.24E-55	0.024	3505	250.494
Vascular dementia (multiple infarctions)	Growth-regulated protein alpha	rs140734053	A	G	0.069	5359496	0.843	0.350	360612	A	G	0.726	10	5401459	3.58E-06	0.156	3505	21.613
Vascular dementia (multiple infarctions)	Growth-regulated protein alpha	rs185768063	G	A	0.034	16494752	0.866	0.202	360612	G	A	-0.400	6	16494983	1.46E-07	0.076	3505	27.673
Vascular dementia (multiple infarctions)	Growth-regulated protein alpha	rs188345231	T	C	-0.274	41579831	0.454	0.366	360612	T	C	0.623	8	41437350	4.34E-06	0.132	3505	22.175
Vascular dementia (multiple infarctions)	Growth-regulated protein alpha	rs2422841	A	G	0.099	3099706	0.299	0.095	360612	A	G	-0.166	20	3080352	4.66E-06	0.036	3505	21.068
Vascular dementia (multiple infarctions)	Growth-regulated protein alpha	rs508977	G	T	0.069	73896666	0.367	0.076	360612	G	T	0.380	4	74762383	7.56E-42	0.028	3505	184.378
Vascular dementia (multiple infarctions)	Growth-regulated protein alpha	rs62024303	G	A	-0.063	88327931	0.698	0.163	360612	G	A	0.305	15	88871162	4.41E-06	0.067	3505	21.014
Vascular dementia (multiple infarctions)	Growth-regulated protein alpha	rs78653452	T	G	0.514	9781407	0.110	0.322	360612	T	G	-0.736	20	9762055	1.21E-06	0.156	3505	22.328
Vascular dementia (multiple infarctions)	Granulocyte-colony stimulating factor	rs115256310	G	A	-0.534	72103864	0.172	0.391	360612	G	A	0.682	5	71399691	6.73E-07	0.136	7904	25.155
Vascular dementia (multiple infarctions)	Granulocyte-colony stimulating factor	rs11903143	G	A	-0.017	29369594	0.811	0.072	360612	G	A	-0.087	2	29592460	6.35E-07	0.018	7904	24.435
Vascular dementia (multiple infarctions)	Granulocyte-colony stimulating factor	rs147128865	T	C	-0.183	34972769	0.403	0.219	360612	T	C	0.270	9	34972766	4.92E-06	0.059	7904	21.157
Vascular dementia (multiple infarctions)	Granulocyte-colony stimulating factor	rs1817411	T	C	0.065	97586100	0.417	0.080	360612	T	C	0.089	8	98598328	3.10E-06	0.019	7904	21.713
Vascular dementia (multiple infarctions)	Granulocyte-colony stimulating factor	rs2671444	A	G	0.036	101158297	0.601	0.068	360612	A	G	-0.078	12	101552075	2.48E-06	0.017	7904	22.306
Vascular dementia (multiple infarctions)	Granulocyte-colony stimulating factor	rs74148555	T	C	-0.248	90320085	0.288	0.234	360612	T	C	-0.372	10	92079842	1.55E-06	0.076	7904	24.212
Vascular dementia (multiple infarctions)	Granulocyte-colony stimulating factor	rs77318030	C	T	-0.054	54544688	0.743	0.164	360612	C	T	0.205	19	55055897	2.21E-06	0.043	7904	22.830
Vascular dementia (multiple infarctions)	Fibroblast growth factor basic	rs145577605	A	G	0.090	27642232	0.614	0.179	360612	A	G	0.208	6	27610011	9.64E-07	0.043	7565	23.640
Vascular dementia (multiple infarctions)	Fibroblast growth factor basic	rs747334	G	A	-0.045	90984987	0.494	0.065	360612	G	A	-0.075	10	92744744	4.53E-06	0.016	7565	20.970
Vascular dementia (multiple infarctions)	Fibroblast growth factor basic	rs75168112	C	T	-0.024	73418832	0.774	0.083	360612	C	T	0.100	18	71086067	3.00E-06	0.021	7565	21.880
Vascular dementia (multiple infarctions)	Fibroblast growth factor basic	rs9907295	T	C	0.051	35930309	0.631	0.105	360612	T	C	-0.132	17	34257313	7.95E-07	0.027	7565	24.043
Vascular dementia (multiple infarctions)	Eotaxin	rs11087905	A	C	0.053	24133015	0.443	0.069	360612	A	C	0.094	21	25505329	5.48E-07	0.019	8153	24.789
Vascular dementia (multiple infarctions)	Eotaxin	rs112347425	T	C	-0.153	46419397	0.187	0.116	360612	T	C	0.158	3	46460888	8.65E-09	0.028	8153	32.535
Vascular dementia (multiple infarctions)	Eotaxin	rs12075	A	G	-0.031	159205564	0.639	0.066	360612	A	G	0.167	1	159175354	1.33E-26	0.016	8153	114.737
Vascular dementia (multiple infarctions)	Eotaxin	rs1476670	C	A	0.114	44042523	0.202	0.089	360612	C	A	0.101	1	44508195	3.51E-06	0.022	8153	21.535
Vascular dementia (multiple infarctions)	Eotaxin	rs2024050	G	A	0.167	75831075	0.190	0.127	360612	G	A	-0.173	7	75460393	1.10E-08	0.030	8153	32.524
Vascular dementia (multiple infarctions)	Eotaxin	rs2210755	C	T	-0.074	77608907	0.434	0.094	360612	C	T	0.110	9	80223823	4.85E-06	0.024	8153	20.812
Vascular dementia (multiple infarctions)	Eotaxin	rs2211994	C	T	0.031	16675274	0.672	0.073	360612	C	T	-0.089	21	18047593	6.08E-07	0.018	8153	25.000
Vascular dementia (multiple infarctions)	Eotaxin	rs2228467	C	T	-0.183	42864624	0.137	0.123	360612	C	T	0.416	3	42906116	2.27E-46	0.029	8153	203.258
Vascular dementia (multiple infarctions)	Eotaxin	rs2419841	C	T	-0.249	113576224	0.038	0.120	360612	C	T	0.128	10	115335983	4.98E-06	0.028	8153	20.949
Vascular dementia (multiple infarctions)	Eotaxin	rs5746492	G	A	-0.155	17911167	0.077	0.088	360612	G	A	-0.095	22	18393933	3.96E-06	0.021	8153	21.240
Vascular dementia (multiple infarctions)	Eotaxin	rs5754733	A	C	-0.030	33873606	0.733	0.087	360612	A	C	-0.104	22	34269594	1.06E-06	0.021	8153	23.709
Vascular dementia (multiple infarctions)	Eotaxin	rs59808887	T	C	-0.175	31846414	0.188	0.133	360612	T	C	-0.167	5	31846520	2.91E-06	0.036	8153	21.839
Vascular dementia (multiple infarctions)	Eotaxin	rs75426604	A	C	-0.139	35388508	0.217	0.112	360612	A	C	-0.137	14	35857714	2.53E-06	0.029	8153	22.035
Vascular dementia (multiple infarctions)	Eotaxin	rs79722574	T	C	-0.013	34292033	0.890	0.094	360612	T	C	-0.111	17	32619052	1.06E-06	0.023	8153	23.830
Vascular dementia (multiple infarctions)	Eotaxin	rs9317045	C	A	0.113	59055904	0.222	0.092	360612	C	A	-0.118	13	59630038	5.82E-07	0.024	8153	24.874
Vascular dementia (other)	CTACK	rs116303454	A	G	0.147	27253164	0.721	0.411	360248	A	G	0.383	3	27294655	3.27E-06	0.082	3631	22.030

Vascular dementia (other)	CTACK	rs2070074	G	A	0.199	34649445	0.336	0.207	360248	G	A	-0.447	9	34649442	1.78E-32	0.037	3631	142.656
Vascular dementia (other)	CTACK	rs2731674	G	T	0.333	177412889	0.028	0.151	360248	G	T	0.133	5	176839890	5.63E-07	0.027	3631	24.925
Vascular dementia (other)	CTACK	rs3766110	C	A	0.023	169545945	0.884	0.156	360248	C	A	0.129	1	169515183	3.85E-06	0.028	3631	21.432
Vascular dementia (other)	CTACK	rs55764737	C	T	1.166	61031215	0.038	0.563	360248	C	T	-0.531	15	61323414	4.62E-08	0.097	3631	29.878
Vascular dementia (other)	CTACK	rs57338032	G	A	0.101	78506597	0.564	0.176	360248	G	A	-0.158	15	78798939	6.23E-07	0.032	3631	24.937
Vascular dementia (other)	CTACK	rs7333764	T	C	-0.050	33634664	0.888	0.353	360248	T	C	0.277	13	34208801	2.85E-06	0.059	3631	21.867
Vascular dementia (other)	CTACK	rs76395525	A	G	0.308	79449049	0.645	0.667	360248	A	G	0.528	15	79741391	9.55E-07	0.108	3631	23.742
Vascular dementia (other)	beta-nerve growth factor	rs28637706	T	G	-0.048	33794463	0.739	0.145	360248	T	G	-0.159	19	34285368	1.42E-09	0.026	3531	36.504
Vascular dementia (other)	beta-nerve growth factor	rs67476890	T	C	0.205	62499295	0.332	0.211	360248	T	C	0.177	15	62791494	3.13E-06	0.038	3531	21.786
Vascular dementia (other)	beta-nerve growth factor	rs71641308	T	C	-0.081	77621033	0.722	0.228	360248	T	C	0.204	1	78086718	2.30E-06	0.043	3531	22.365
Vascular dementia (other)	beta-nerve growth factor	rs72780728	A	G	0.007	17561702	0.976	0.232	360248	A	G	0.188	10	17603701	2.99E-06	0.040	3531	21.832
Vascular dementia (other)	beta-nerve growth factor	rs73472576	C	T	-0.160	74456947	0.235	0.134	360248	C	T	0.118	18	72124182	2.69E-06	0.025	3531	21.963
Vascular dementia (other)	beta-nerve growth factor	rs7970581	G	T	0.164	112827443	0.273	0.149	360248	G	T	-0.138	12	113265248	9.27E-07	0.028	3531	23.947
Vascular dementia (other)	beta-nerve growth factor	rs9436119	A	G	0.324	150495277	0.017	0.136	360248	A	G	-0.112	1	150467753	3.91E-06	0.025	3531	20.765
Vascular dementia (other)	Vascular endothelial growth factor	rs10153304	A	G	0.482	7818613	0.047	0.243	360248	A	G	0.155	17	7721931	1.94E-06	0.033	7118	22.658
Vascular dementia (other)	Vascular endothelial growth factor	rs10934631	C	T	-0.306	122978753	0.106	0.189	360248	C	T	0.115	3	122697600	2.47E-06	0.025	7118	22.071
Vascular dementia (other)	Vascular endothelial growth factor	rs10967186	C	T	-0.242	2617099	0.071	0.134	360248	C	T	-0.090	9	2617099	1.23E-07	0.017	7118	27.903
Vascular dementia (other)	Vascular endothelial growth factor	rs13209117	A	G	0.229	44184028	0.133	0.153	360248	A	G	0.130	6	44151765	5.28E-11	0.020	7118	41.959
Vascular dementia (other)	Vascular endothelial growth factor	rs143479231	A	G	-0.556	193393005	0.105	0.343	360248	A	G	-0.260	3	193110794	1.90E-07	0.049	7118	27.997
Vascular dementia (other)	Vascular endothelial growth factor	rs4082730	A	G	-0.685	89980326	0.100	0.416	360248	A	G	0.252	15	90523558	2.64E-06	0.053	7118	22.305
Vascular dementia (other)	Vascular endothelial growth factor	rs6921438	A	G	0.043	43957870	0.745	0.133	360248	A	G	-0.490	6	43925607	########	0.018	7118	784.000
Vascular dementia (other)	Vascular endothelial growth factor	rs73418461	A	G	0.074	118463484	0.851	0.396	360248	A	G	-0.249	10	120222996	1.67E-06	0.052	7118	22.878
Vascular dementia (other)	Vascular endothelial growth factor	rs8045833	A	G	0.082	88509031	0.608	0.161	360248	A	G	0.108	16	88575439	2.83E-07	0.021	7118	26.199
Vascular dementia (other)	Vascular endothelial growth factor	rs9472183	G	A	0.007	43972465	0.957	0.133	360248	G	A	0.128	6	43940202	5.19E-14	0.017	7118	56.869
Vascular dementia (other)	Macrophage Migration Inhibitory Factor	rs113218956	A	G	1.178	24828867	0.260	1.046	360248	A	G	-0.895	22	25224834	2.26E-06	0.188	3494	22.678
Vascular dementia (other)	Macrophage Migration Inhibitory Factor	rs118055855	C	T	0.206	29867025	0.792	0.780	360248	C	T	-0.691	11	29888572	4.13E-06	0.150	3494	21.203
Vascular dementia (other)	Macrophage Migration Inhibitory Factor	rs12594190	G	A	0.243	24791308	0.097	0.146	360248	G	A	-0.136	15	25036455	3.70E-07	0.027	3494	25.755
Vascular dementia (other)	Macrophage Migration Inhibitory Factor	rs13142904	T	C	0.075	53452247	0.744	0.230	360248	T	C	-0.223	4	54318414	2.56E-07	0.043	3494	27.532
Vascular dementia (other)	Macrophage Migration Inhibitory Factor	rs141009259	C	T	-0.617	207111559	0.326	0.628	360248	C	T	0.618	2	207976283	2.47E-06	0.132	3494	21.839
Vascular dementia (other)	Macrophage Migration Inhibitory Factor	rs78098071	C	T	-0.261	163882733	0.610	0.511	360248	C	T	0.487	5	163309739	1.78E-07	0.092	3494	28.108
Vascular dementia (other)	TRAIL	rs11618126	G	A	1.083	50252103	0.456	1.452	360248	G	A	-0.891	13	50826239	1.46E-06	0.191	8186	21.661
Vascular dementia (other)	TRAIL	rs11657269	G	A	-0.139	6416464	0.522	0.217	360248	G	A	-0.119	17	6319784	4.78E-06	0.026	8186	20.878
Vascular dementia (other)	TRAIL	rs11699445	G	T	0.114	15770145	0.399	0.135	360248	G	T	-0.075	20	15750790	3.27E-06	0.016	8186	21.470
Vascular dementia (other)	TRAIL	rs13185784	A	G	0.363	180267068	0.016	0.151	360248	A	G	0.085	5	179694068	3.90E-06	0.018	8186	21.372
Vascular dementia (other)	TRAIL	rs138987090	G	A	-0.641	32786284	0.262	0.572	360248	G	A	0.750	18	30366247	4.50E-23	0.075	8186	99.389
Vascular dementia (other)	TRAIL	rs146783010	G	A	-0.101	89527045	0.917	0.974	360248	G	A	0.602	11	89260213	4.83E-06	0.135	8186	19.859
Vascular dementia (other)	TRAIL	rs193112415	C	T	-0.328	31255157	0.497	0.484	360248	C	T	1.042	18	28835120	2.15E-62	0.062	8186	279.797
Vascular dementia (other)	TRAIL	rs57396456	C	T	-0.648	30365911	0.144	0.444	360248	C	T	0.563	18	27945877	1.25E-27	0.052	8186	117.961
Vascular dementia (other)	TRAIL	rs62093514	T	C	-0.850	31651014	0.063	0.457	360248	T	C	1.062	18	29230977	6.86E-82	0.055	8186	370.005
Vascular dementia (other)	TRAIL	rs73039026	C	A	-1.045	172442691	0.027	0.471	360248	C	A	0.300	3	172160481	2.02E-06	0.064	8186	22.305
Vascular dementia (other)	TRAIL	rs747324	C	T	0.350	74222941	0.018	0.148	360248	C	T	0.086	14	74689644	1.61E-06	0.018	8186	23.072
Vascular dementia (other)	TRAIL	rs74778900	T	C	0.113	30506300	0.854	0.614	360248	T	C	0.591	18	28086266	2.59E-28	0.053	8186	123.243
Vascular dementia (other)	TRAIL	rs75928541	A	G	-0.190	16400148	0.678	0.458	360248	A	G	0.275	4	16401771	4.24E-06	0.059	8186	21.506
Vascular dementia (other)	TRAIL	rs79287178	A	G	0.273	172576710	0.402	0.325	360248	A	G	-0.432	3	172294500	9.12E-25	0.042	8186	105.148
Vascular dementia (other)	Tumor necrosis factor beta	rs10925040	T	C	-0.025	247459396	0.855	0.139	360248	T	C	0.176	1	247622698	2.67E-06	0.037	1559	22.138
Vascular dementia (other)	Tumor necrosis factor beta	rs753274	T	C	0.051	14325650	0.711	0.137	360248	T	C	-0.174	19	14436462	2.77E-06	0.037	1559	21.895
Vascular dementia (other)	Tumor necrosis factor beta	rs7629875	G	A	-0.375	174667832	0.176	0.277	360248	G	A	-0.377	3	174385622	1.37E-06	0.077	1559	23.674
Vascular dementia (other)	Tumor necrosis factor beta	rs78296352	T	G	0.586	22495351	0.304	0.570	360248	T	G	1.222	1	22821844	4.76E-21	0.137	1559	79.962
Vascular dementia (other)	Tumor necrosis factor alpha	rs10834997	A	G	-0.199	26505401	0.166	0.144	360248	A	G	-0.125	11	26526948	1.33E-06	0.026	3454	23.361
Vascular dementia (other)	Tumor necrosis factor alpha	rs115669577	A	G	0.120	123440293	0.911	1.065	360248	A	G	0.989	4	124361448	8.28E-07	0.200	3454	24.571
Vascular dementia (other)	Tumor necrosis factor alpha	rs79105320	A	G	-0.182	18959850	0.779	0.650	360248	A	G	0.561	8	18817360	3.59E-06	0.118	3454	22.601
Vascular dementia (other)	Tumor necrosis factor alpha	rs8121916	A	C	0.217	12420677	0.151	0.151	360248	A	C	0.131	20	12401325	2.72E-06	0.028	3454	22.070
Vascular dementia (other)	Stromal-cell-derived factor 1 alpha	rs10474392	G	A	0.005	92198776	0.975	0.154	360248	G	A	-0.096	5	91494593	1.24E-06	0.018	5998	29.209
Vascular dementia (other)	Stromal-cell-derived factor 1 alpha	rs12407262	A	G	0.158	63354605	0.464	0.216	360248	A	G	0.118	1	63820276	3.99E-06	0.027	5998	19.646
Vascular dementia (other)	Stromal-cell-derived factor 1 alpha	rs139840550	A	G	-0.232	38688625	0.612	0.457	360248	A	G	0.183	9	38688622	3.79E-06	0.055	5998	11.160
Vascular dementia (other)	Stromal-cell-derived factor 1 alpha	rs149893336	G	A	0.277	170311440	0.671	0.652	360248	G	A	0.503	4	171232591	4.52E-06	0.108	5998	21.686
Vascular dementia (other)	Stromal-cell-derived factor 1 alpha	rs4581824	G	T	0.356	9074853	0.011	0.140	360248	G	T	0.070	19	9185529	3.05E-06	0.017	5998	16.419
Vascular dementia (other)	Stromal-cell-derived factor 1 alpha	rs482700	A	G	-0.266	115146334	0.110	0.166	360248	A	G	-0.089	4	116067490	1.57E-06	0.020	5998	19.351
Vascular dementia (other)	Stromal-cell-derived factor 1 alpha	rs67689854	A	C	-0.093	89558819	0.569	0.164	360248	A	C	-0.068	16	89625227	3.07E-06	0.020	5998	12.196
Vascular dementia (other)	Stromal-cell-derived factor 1 alpha	rs9267091	A	G	0.027	31446032	0.869	0.165	360248	A	G	0.078	6	31413809	3.63E-06	0.020	5998	14.802
Vascular dementia (other)	Stem cell growth factor beta	rs112346514	T	C	0.540	12297173	0.123	0.350	360248	T	C	-0.331	19	12407988	2.37E-06	0.071	3682	21.725
Vascular dementia (other)	Stem cell growth factor beta	rs116924815	T	C	0.379	50727476	0.371	0.423	360248	T	C	0.608	19	51230733	1.74E-16	0.074	3682	67.850
Vascular dementia (other)	Stem cell growth factor beta	rs117716477	A	C	0.197	103847180	0.721	0.552	360248	A	C	0.838	12	104240958	1.34E-23	0.084	3682	99.383
Vascular dementia (other)	Stem cell growth factor beta	rs12480722	C	T	-0.267	20248260	0.178	0.199	360248	C	T	-0.162	20	20228904	4.72E-06	0.036	3682	20.927

Vascular dementia (other)	Stem cell growth factor beta	rs139413256	A	G	0.193	146182552	0.692	0.486	360248	A	G	-0.538	7	145879644	7.04E-07	0.108	3682	24.605
Vascular dementia (other)	Stem cell growth factor beta	rs143829871	C	T	0.211	47555755	0.357	0.229	360248	C	T	0.190	3	47597245	1.90E-06	0.040	3682	22.610
Vascular dementia (other)	Stem cell growth factor beta	rs151194174	A	G	-0.570	20956159	0.105	0.351	360248	A	G	0.464	7	20995778	1.13E-06	0.094	3682	24.210
Vascular dementia (other)	Stem cell growth factor beta	rs17876031	G	A	0.275	177404118	0.052	0.142	360248	G	A	0.151	5	176831119	2.25E-09	0.026	3682	35.251
Vascular dementia (other)	Stem cell growth factor beta	rs264162	G	A	0.036	10944028	0.785	0.133	360248	G	A	-0.110	18	10944026	2.68E-06	0.023	3682	21.978
Vascular dementia (other)	Stem cell growth factor beta	rs34911860	A	G	-0.797	79885030	0.120	0.513	360248	A	G	-0.368	1	80350715	3.24E-06	0.079	3682	21.695
Vascular dementia (other)	Stem cell growth factor beta	rs4656185	A	G	-0.030	169507088	0.837	0.144	360248	A	G	0.205	1	169476326	1.16E-15	0.026	3682	64.125
Vascular dementia (other)	Stem cell growth factor beta	rs4737732	G	A	-0.001	65421393	0.995	0.146	360248	G	A	0.115	8	66333628	4.68E-06	0.025	3682	20.717
Vascular dementia (other)	Stem cell growth factor beta	rs7762066	C	T	-0.011	94468249	0.943	0.157	360248	C	T	-0.139	6	95177967	3.50E-06	0.030	3682	21.581
Vascular dementia (other)	Stem cell growth factor beta	rs78217154	C	T	0.051	100541844	0.914	0.467	360248	C	T	-0.400	8	101554072	3.77E-06	0.086	3682	21.401
Vascular dementia (other)	Stem cell factor	rs113127926	A	C	-0.003	97971174	0.992	0.339	360248	A	C	0.198	14	98437511	2.27E-06	0.042	8290	22.269
Vascular dementia (other)	Stem cell factor	rs13412535	A	G	0.057	224010157	0.731	0.165	360248	A	G	-0.107	2	224874874	6.04E-07	0.021	8290	25.094
Vascular dementia (other)	Stem cell factor	rs1557570	T	G	-0.063	169538606	0.660	0.144	360248	T	G	0.119	1	169507844	2.74E-12	0.017	8290	48.671
Vascular dementia (other)	Stem cell factor	rs1568119	T	C	-1.024	33385679	0.219	0.834	360248	T	C	-0.591	8	33243197	1.24E-07	0.113	8290	27.365
Vascular dementia (other)	Stem cell factor	rs1942355	T	C	-0.163	71694503	0.223	0.134	360248	T	C	-0.072	18	69361739	4.70E-06	0.016	8290	20.798
Vascular dementia (other)	Stem cell factor	rs4841899	C	T	-0.110	134532566	0.466	0.150	360248	C	T	0.100	9	137424412	1.78E-08	0.018	8290	31.815
Vascular dementia (other)	Stem cell factor	rs635634	T	C	-0.114	133279427	0.486	0.164	360248	T	C	-0.103	9	136155000	6.74E-08	0.019	8290	29.194
Vascular dementia (other)	Stem cell factor	rs78666213	G	T	-0.041	179217495	0.927	0.444	360248	G	T	0.274	4	180138649	2.59E-06	0.058	8290	22.695
Vascular dementia (other)	Stem cell factor	rs80271436	A	G	0.013	133022383	0.976	0.418	360248	A	G	-0.237	9	135897770	9.95E-07	0.049	8290	23.879
Vascular dementia (other)	Interleukin-16	rs117217798	T	C	-0.027	33156215	0.909	0.238	360248	T	C	-0.204	17	31483233	4.15E-06	0.044	3483	21.028
Vascular dementia (other)	Interleukin-16	rs117916513	A	G	-0.001	121393565	0.999	0.504	360248	A	G	-0.502	11	121264274	3.79E-07	0.099	3483	25.921
Vascular dementia (other)	Interleukin-16	rs1255143	T	C	0.219	128253936	0.104	0.135	360248	T	C	0.131	10	130052200	7.10E-08	0.024	3483	29.124
Vascular dementia (other)	Interleukin-16	rs12765671	A	G	0.556	104924411	0.450	0.736	360248	A	G	-0.602	10	106684169	4.84E-06	0.132	3483	20.883
Vascular dementia (other)	Interleukin-16	rs144691581	A	G	0.015	96410095	0.973	0.457	360248	A	G	0.488	15	96953325	4.20E-07	0.097	3483	25.488
Vascular dementia (other)	Interleukin-16	rs1801020	G	A	0.313	177409531	0.038	0.151	360248	G	A	-0.173	5	176836532	4.53E-10	0.027	3483	40.594
Vascular dementia (other)	Interleukin-16	rs4253283	C	T	0.270	186244057	0.062	0.145	360248	C	T	-0.146	4	187165211	1.75E-08	0.026	3483	31.053
Vascular dementia (other)	Interleukin-16	rs4513633	A	C	-0.305	112649483	0.215	0.246	360248	A	C	-0.224	4	113570639	7.44E-07	0.045	3483	24.429
Vascular dementia (other)	Interleukin-16	rs4778636	A	G	-0.018	81299298	0.955	0.321	360248	A	G	-0.727	15	81591639	1.11E-30	0.063	3483	131.978
Vascular dementia (other)	Interleukin-16	rs9706053	T	C	-0.462	65982530	0.382	0.528	360248	T	C	0.458	12	66376310	7.01E-07	0.093	3483	24.170
Vascular dementia (other)	RANTES	rs112072646	A	G	-0.507	53217255	0.329	0.520	360248	A	G	0.429	2	53444393	6.48E-07	0.086	3421	24.722
Vascular dementia (other)	RANTES	rs147509526	T	C	0.601	15665520	0.106	0.372	360248	T	C	-0.358	19	15776330	6.93E-07	0.072	3421	24.930
Vascular dementia (other)	RANTES	rs4940620	G	A	0.304	64303876	0.301	0.294	360248	G	A	0.249	18	61971111	3.54E-06	0.054	3421	21.331
Vascular dementia (other)	RANTES	rs62438851	G	A	-0.264	144909173	0.224	0.217	360248	G	A	0.196	6	145230309	2.33E-06	0.041	3421	22.345
Vascular dementia (other)	RANTES	rs7000423	T	C	0.068	110041420	0.620	0.137	360248	T	C	-0.132	8	111053649	1.82E-07	0.025	3421	27.139
Vascular dementia (other)	RANTES	rs72793342	A	G	-0.213	30537031	0.192	0.164	360248	A	G	-0.149	16	30548352	1.48E-06	0.031	3421	23.309
Vascular dementia (other)	RANTES	rs74472919	T	C	0.327	81626515	0.353	0.352	360248	T	C	0.331	13	82200650	3.97E-08	0.061	3421	29.987
Vascular dementia (other)	RANTES	rs75613039	T	C	-0.551	129706688	0.176	0.408	360248	T	C	0.370	11	129576583	4.81E-06	0.081	3421	20.866
Vascular dementia (other)	RANTES	rs818452	T	C	0.168	152594661	0.535	0.271	360248	T	C	0.238	6	152915796	2.36E-06	0.051	3421	22.230
Vascular dementia (other)	Platelet-derived growth factor BB	rs116445074	T	G	0.128	52238766	0.790	0.481	360248	T	G	0.293	5	51534600	3.11E-07	0.059	8293	24.932
Vascular dementia (other)	Platelet-derived growth factor BB	rs11766649	G	A	0.099	145142154	0.545	0.163	360248	G	A	-0.091	7	144839247	3.53E-06	0.020	8293	21.461
Vascular dementia (other)	Platelet-derived growth factor BB	rs11916118	G	A	0.239	117193342	0.154	0.167	360248	G	A	-0.089	3	116912189	4.93E-06	0.019	8293	20.999
Vascular dementia (other)	Platelet-derived growth factor BB	rs12289510	G	A	-0.112	125077155	0.401	0.133	360248	G	A	0.078	11	124947051	7.69E-07	0.016	8293	24.371
Vascular dementia (other)	Platelet-derived growth factor BB	rs13412535	A	G	0.057	224010157	0.731	0.165	360248	A	G	0.335	2	224874874	2.46E-55	0.021	8293	245.347
Vascular dementia (other)	Platelet-derived growth factor BB	rs2324229	C	T	-0.161	83208412	0.242	0.138	360248	C	T	-0.089	6	83918131	3.48E-08	0.016	8293	30.834
Vascular dementia (other)	Platelet-derived growth factor BB	rs35859699	A	G	0.293	111263595	0.644	0.633	360248	A	G	-0.395	4	112184751	2.07E-06	0.084	8293	22.030
Vascular dementia (other)	Platelet-derived growth factor BB	rs4965869	T	C	0.043	101450115	0.778	0.154	360248	T	C	0.184	15	101990320	5.66E-24	0.018	8293	103.342
Vascular dementia (other)	Platelet-derived growth factor BB	rs55680718	T	C	0.169	224302160	0.406	0.203	360248	T	C	-0.138	2	225166877	1.86E-08	0.025	8293	31.606
Vascular dementia (other)	Platelet-derived growth factor BB	rs72777070	G	T	0.046	9658748	0.781	0.165	360248	G	T	0.107	2	9798877	8.98E-08	0.020	8293	28.569
Vascular dementia (other)	Platelet-derived growth factor BB	rs73162807	A	C	0.711	146757003	0.092	0.422	360248	A	C	-0.239	3	146474790	1.74E-06	0.050	8293	22.959
Vascular dementia (other)	Platelet-derived growth factor BB	rs9936075	G	A	0.215	7271908	0.123	0.139	360248	G	A	0.078	16	7321909	1.76E-06	0.016	8293	22.737
Vascular dementia (other)	Platelet-derived growth factor BB	rs9941733	G	A	0.252	393417	0.151	0.176	360248	G	A	-0.116	20	374061	3.31E-07	0.023	8293	25.930
Vascular dementia (other)	Macrophage inflammatory protein 1b	rs11130043	A	G	-0.143	45069747	0.286	0.134	360248	A	G	-0.073	3	45111239	3.22E-06	0.016	8243	21.679
Vascular dementia (other)	Macrophage inflammatory protein 1b	rs113010081	C	T	-0.167	46415921	0.401	0.199	360248	C	T	0.595	3	46457412	########	0.024	8243	636.493
Vascular dementia (other)	Macrophage inflammatory protein 1b	rs113877493	T	C	-0.205	36443746	0.266	0.184	360248	T	C	-0.612	17	34812273	########	0.022	8243	789.146
Vascular dementia (other)	Macrophage inflammatory protein 1b	rs116237296	A	G	-1.039	86579833	0.280	0.962	360248	A	G	0.544	1	87045516	7.23E-07	0.112	8243	23.778
Vascular dementia (other)	Macrophage inflammatory protein 1b	rs117453826	G	A	-0.160	36775624	0.731	0.467	360248	G	A	0.577	17	35132809	5.07E-22	0.059	8243	94.808
Vascular dementia (other)	Macrophage inflammatory protein 1b	rs141102180	T	G	0.666	36108811	0.011	0.264	360248	T	G	0.323	17	34436204	1.08E-16	0.039	8243	67.340
Vascular dementia (other)	Macrophage inflammatory protein 1b	rs17138331	G	A	-0.229	7826737	0.348	0.243	360248	G	A	0.139	7	7866368	2.26E-06	0.030	8243	22.234
Vascular dementia (other)	Macrophage inflammatory protein 1b	rs17641689	G	A	-0.110	36668383	0.606	0.214	360248	G	A	0.245	17	35024819	1.28E-16	0.029	8243	69.805
Vascular dementia (other)	Macrophage inflammatory protein 1b	rs2079664	G	A	0.305	34680936	0.042	0.150	360248	G	A	-0.100	17	33007955	1.51E-08	0.018	8243	31.961
Vascular dementia (other)	Macrophage inflammatory protein 1b	rs281749	C	T	0.119	107626417	0.409	0.144	360248	C	T	-0.080	8	108638645	3.17E-06	0.017	8243	21.832
Vascular dementia (other)	Macrophage inflammatory protein 1b	rs34437725	C	T	0.175	35499766	0.653	0.388	360248	C	T	0.263	17	33826785	7.67E-08	0.048	8243	29.717
Vascular dementia (other)	Macrophage inflammatory protein 1b	rs72791296	T	C	0.237	121614355	0.512	0.361	360248	T	C	0.237	5	120950050	3.78E-07	0.047	8243	25.844
Vascular dementia (other)	Macrophage inflammatory protein 1b	rs72799710	T	C	-0.027	123825971	0.883	0.183	360248	T	C	-0.101	5	123161665	3.21E-06	0.022	8243	21.635

Vascular dementia (other)	Macrophage inflammatory protein 1b	rs74810984	C	T	-0.126	127876202	0.763	0.416	360248	C	T	-0.221	10	129674466	1.96E-06	0.047	8243	21.660
Vascular dementia (other)	Macrophage inflammatory protein 1b	rs76582507	A	G	-0.016	37510075	0.983	0.717	360248	A	G	0.318	9	37510072	3.26E-06	0.068	8243	21.994
Vascular dementia (other)	Macrophage inflammatory protein 1b	rs76583883	T	G	-0.148	45936445	0.681	0.360	360248	T	G	-0.232	21	47356359	4.99E-06	0.051	8243	20.559
Vascular dementia (other)	Macrophage inflammatory protein 1b	rs76776296	G	A	0.283	115488433	0.546	0.469	360248	G	A	-0.300	7	115128487	5.55E-07	0.060	8243	25.117
Vascular dementia (other)	Macrophage inflammatory protein 1a	rs10835056	G	T	0.054	26675470	0.704	0.141	360248	G	T	-0.119	11	26697017	2.60E-06	0.025	3522	22.097
Vascular dementia (other)	Macrophage inflammatory protein 1a	rs12690897	A	G	-0.105	85716861	0.473	0.146	360248	A	G	0.125	7	85346177	2.11E-06	0.026	3522	22.690
Vascular dementia (other)	Macrophage inflammatory protein 1a	rs184154340	A	G	-0.331	80790993	0.342	0.348	360248	A	G	0.331	11	80502036	1.86E-06	0.069	3522	22.813
Vascular dementia (other)	Macrophage inflammatory protein 1a	rs34771762	G	A	0.337	200547932	0.195	0.260	360248	G	A	-0.249	2	201412655	2.13E-06	0.052	3522	22.667
Vascular dementia (other)	Macrophage inflammatory protein 1a	rs57786342	A	G	-0.229	68793311	0.144	0.157	360248	A	G	0.131	14	69260028	4.05E-06	0.029	3522	21.257
Vascular dementia (other)	Macrophage inflammatory protein 1a	rs60198979	A	G	-0.318	43250698	0.209	0.253	360248	A	G	-0.215	22	43646704	2.61E-06	0.046	3522	21.955
Vascular dementia (other)	Macrophage inflammatory protein 1a	rs7232268	G	A	-0.527	70101678	0.128	0.346	360248	G	A	-0.282	18	67768914	2.55E-06	0.060	3522	22.180
Vascular dementia (other)	Monokine induced by gamma interferon	rs111607343	A	G	0.269	897855	0.641	0.576	360248	A	G	-0.521	19	897855	2.83E-06	0.112	3685	21.678
Vascular dementia (other)	Monokine induced by gamma interferon	rs11177248	A	G	0.104	68482106	0.760	0.339	360248	A	G	0.307	12	68875886	4.45E-06	0.067	3685	21.037
Vascular dementia (other)	Monokine induced by gamma interferon	rs112337562	G	T	-0.092	92665225	0.835	0.439	360248	G	T	0.370	14	93131570	2.98E-06	0.080	3685	21.606
Vascular dementia (other)	Monokine induced by gamma interferon	rs112861654	G	A	0.112	42179062	0.690	0.281	360248	G	A	0.277	21	43599172	1.81E-07	0.053	3685	27.320
Vascular dementia (other)	Monokine induced by gamma interferon	rs117831247	T	C	-0.426	66742081	0.671	1.002	360248	T	C	-0.833	10	68501839	2.16E-06	0.175	3685	22.576
Vascular dementia (other)	Monokine induced by gamma interferon	rs139010077	T	C	-0.495	170618359	0.344	0.522	360248	T	C	0.432	3	170336148	3.55E-06	0.095	3685	20.698
Vascular dementia (other)	Monokine induced by gamma interferon	rs1796086	C	T	-0.085	71183729	0.720	0.238	360248	C	T	0.210	7	70648715	2.23E-07	0.040	3685	27.050
Vascular dementia (other)	Monokine induced by gamma interferon	rs41272086	A	G	-0.263	160587614	0.229	0.219	360248	A	G	-0.223	6	161008646	7.43E-08	0.042	3685	28.771
Vascular dementia (other)	Monokine induced by gamma interferon	rs55876513	G	T	0.114	75962545	0.437	0.147	360248	G	T	-0.166	4	76883698	8.23E-11	0.026	3685	42.378
Vascular dementia (other)	Monokine induced by gamma interferon	rs5752128	C	T	0.054	25322656	0.792	0.205	360248	C	T	0.169	22	25718623	4.34E-06	0.037	3685	20.852
Vascular dementia (other)	Monokine induced by gamma interferon	rs62562991	A	G	-0.723	95973777	0.199	0.563	360248	A	G	0.624	9	98736059	8.40E-07	0.126	3685	24.495
Vascular dementia (other)	Monokine induced by gamma interferon	rs6679677	A	C	-0.017	113761186	0.930	0.190	360248	A	C	0.162	1	114303808	8.86E-07	0.033	3685	24.246
Vascular dementia (other)	Monokine induced by gamma interferon	rs77086208	T	C	-0.312	70152774	0.432	0.398	360248	T	C	0.323	14	70619491	3.83E-06	0.070	3685	21.361
Vascular dementia (other)	Monokine induced by gamma interferon	rs816960	T	C	0.247	107870173	0.073	0.138	360248	T	C	-0.122	13	108522521	5.01E-07	0.024	3685	25.164
Vascular dementia (other)	Macrophage colony stimulating factor	rs116274860	G	T	-0.405	148675030	0.475	0.567	360248	G	T	-0.819	3	148392817	2.74E-06	0.174	840	22.129
Vascular dementia (other)	Macrophage colony stimulating factor	rs117867915	C	T	-0.291	44630078	0.555	0.492	360248	C	T	-0.527	18	42210043	1.61E-06	0.110	840	23.054
Vascular dementia (other)	Macrophage colony stimulating factor	rs12962919	T	C	-0.342	78018752	0.180	0.255	360248	T	C	0.305	18	75778756	4.65E-06	0.066	840	21.255
Vascular dementia (other)	Macrophage colony stimulating factor	rs56367447	T	C	-0.086	4014005	0.818	0.374	360248	T	C	-0.497	8	3871527	1.72E-08	0.088	840	31.642
Vascular dementia (other)	Macrophage colony stimulating factor	rs62294910	A	G	0.223	182480551	0.416	0.275	360248	A	G	0.343	3	182198339	6.82E-07	0.069	840	24.654
Vascular dementia (other)	Macrophage colony stimulating factor	rs78296352	T	G	0.586	22495351	0.304	0.570	360248	T	G	0.527	1	22821844	1.05E-06	0.111	840	22.460
Vascular dementia (other)	Macrophage colony stimulating factor	rs9387100	C	T	0.143	112781752	0.288	0.134	360248	C	T	0.135	6	113102954	4.07E-06	0.029	840	21.438
Vascular dementia (other)	Monocyte chemoattractant protein-1	rs10145849	A	G	-0.171	82475647	0.216	0.138	360248	A	G	-0.076	14	82941991	3.41E-06	0.016	8293	21.720
Vascular dementia (other)	Monocyte chemoattractant protein-1	rs10744620	C	T	0.041	3629928	0.764	0.137	360248	C	T	-0.079	12	3739094	9.91E-07	0.016	8293	23.955
Vascular dementia (other)	Monocyte chemoattractant protein-1	rs111995966	G	T	-0.035	108558513	0.890	0.251	360248	G	T	-0.145	2	109174969	2.53E-06	0.031	8293	21.939
Vascular dementia (other)	Monocyte chemoattractant protein-1	rs112313229	A	G	0.394	46323369	0.128	0.258	360248	A	G	-0.165	3	46364860	1.43E-07	0.031	8293	27.655
Vascular dementia (other)	Monocyte chemoattractant protein-1	rs12073356	A	G	-0.187	207834503	0.460	0.253	360248	A	G	-0.143	1	208007848	4.17E-06	0.031	8293	21.024
Vascular dementia (other)	Monocyte chemoattractant protein-1	rs12075	A	G	-0.101	159205564	0.451	0.134	360248	A	G	0.219	1	159175354	1.44E-44	0.016	8293	198.719
Vascular dementia (other)	Monocyte chemoattractant protein-1	rs12493471	C	T	-0.255	45910186	0.064	0.137	360248	C	T	-0.116	3	45951678	6.81E-13	0.016	8293	51.538
Vascular dementia (other)	Monocyte chemoattractant protein-1	rs146522229	T	C	0.001	47295223	0.999	0.999	360248	T	C	-0.598	19	47798480	3.56E-07	0.118	8293	25.779
Vascular dementia (other)	Monocyte chemoattractant protein-1	rs2228467	C	T	-0.064	42864624	0.800	0.252	360248	C	T	0.264	3	42906116	9.19E-20	0.029	8293	82.117
Vascular dementia (other)	Monocyte chemoattractant protein-1	rs2712431	A	C	0.180	128598047	0.216	0.146	360248	A	C	-0.079	3	128316890	4.75E-06	0.017	8293	20.936
Vascular dementia (other)	Monocyte chemoattractant protein-1	rs56212190	T	C	0.115	41702868	0.712	0.313	360248	T	C	0.181	1	42168539	9.85E-07	0.037	8293	23.547
Vascular dementia (other)	Monocyte chemoattractant protein-1	rs7197349	G	A	-0.137	78653322	0.417	0.169	360248	G	A	-0.097	16	78687219	2.62E-06	0.021	8293	22.081
Vascular dementia (other)	Monocyte chemoattractant protein-1	rs7517040	G	A	0.069	158889343	0.668	0.160	360248	G	A	0.099	1	158859133	2.44E-07	0.019	8293	26.703
Vascular dementia (other)	Monocyte chemoattractant protein-1	rs9317045	C	A	-0.147	59055904	0.428	0.186	360248	C	A	-0.113	13	59630038	1.52E-06	0.024	8293	23.089
Vascular dementia (other)	Interleukin-12p70	rs13209117	A	G	0.229	44184028	0.133	0.153	360248	A	G	0.100	6	44151765	5.57E-08	0.019	8270	29.021
Vascular dementia (other)	Interleukin-12p70	rs17229494	G	A	-0.107	37555798	0.616	0.213	360248	G	A	0.117	21	38928100	4.93E-06	0.026	8270	20.796
Vascular dementia (other)	Interleukin-12p70	rs282258	C	T	0.087	224050083	0.517	0.134	360248	C	T	-0.073	2	224914800	3.21E-06	0.016	8270	21.898
Vascular dementia (other)	Interleukin-12p70	rs41282644	A	G	-0.101	43785985	0.678	0.242	360248	A	G	0.147	6	43753722	1.05E-06	0.030	8270	23.478
Vascular dementia (other)	Interleukin-12p70	rs4349809	G	T	-0.005	43957093	0.968	0.133	360248	G	T	-0.378	6	43924830	########	0.016	8270	564.287
Vascular dementia (other)	Interleukin-12p70	rs71361173	G	T	-0.251	76000450	0.211	0.201	360248	G	T	-0.111	18	73712405	3.06E-06	0.024	8270	21.570
Vascular dementia (other)	Interleukin-12p70	rs72831623	A	G	-0.438	47644927	0.101	0.267	360248	A	G	0.191	17	45722293	2.42E-07	0.037	8270	26.732
Vascular dementia (other)	Interleukin-12p70	rs782107	A	G	-0.035	58439747	0.793	0.133	360248	A	G	0.075	12	58833530	1.60E-06	0.016	8270	23.114
Vascular dementia (other)	Interleukin-12p70	rs79121401	C	T	-1.028	78986084	0.321	1.035	360248	C	T	-0.555	11	78697129	4.24E-06	0.121	8270	21.163
Vascular dementia (other)	Interleukin-12p70	rs9472183	G	A	0.007	43972465	0.957	0.133	360248	G	A	0.102	6	43940202	8.61E-11	0.016	8270	42.126
Vascular dementia (other)	Interferon gamma-induced protein 10	rs10809307	C	T	0.048	11045908	0.763	0.161	360248	C	T	-0.131	9	11045908	3.64E-06	0.028	3685	21.415
Vascular dementia (other)	Interferon gamma-induced protein 10	rs113831257	A	G	-0.203	75234311	0.514	0.311	360248	A	G	0.359	4	76159521	2.53E-08	0.064	3685	31.110
Vascular dementia (other)	Interferon gamma-induced protein 10	rs11626201	A	C	0.266	36511495	0.053	0.137	360248	A	C	0.116	14	36980700	1.93E-06	0.025	3685	22.495
Vascular dementia (other)	Interferon gamma-induced protein 10	rs143799975	G	A	-1.011	75885862	0.194	0.779	360248	G	A	0.798	4	76807015	1.00E-06	0.164	3685	23.787
Vascular dementia (other)	Interferon gamma-induced protein 10	rs34383175	T	C	-0.159	144361034	0.655	0.355	360248	T	C	-0.315	8	145584694	1.51E-06	0.066	3685	23.031
Vascular dementia (other)	Interferon gamma-induced protein 10	rs75970138	A	G	-0.145	119813998	0.816	0.626	360248	A	G	-0.485	9	122576276	1.53E-06	0.104	3685	21.748
Vascular dementia (other)	Interferon gamma-induced protein 10	rs7645625	G	T	-0.056	146856250	0.677	0.135	360248	G	T	0.109	3	146574037	4.41E-06	0.024	3685	20.997
Vascular dementia (other)	Interferon gamma-induced protein 10	rs79848609	C	A	-0.186	86772934	0.552	0.313	360248	C	A	-0.260	15	87316165	8.75E-07	0.054	3685	23.496

Vascular dementia (other)	Interferon gamma-induced protein 10	rs8112909	A	G	-0.220	45910150	0.181	0.164	360248	A	G	-0.143	19	46413408	1.94E-06	0.030	3685	22.746
Vascular dementia (other)	Interleukin-18	rs10414578	T	C	0.171	54634619	0.381	0.195	360248	T	C	-0.177	19	55146070	4.16E-07	0.035	3636	25.604
Vascular dementia (other)	Interleukin-18	rs115267715	T	C	0.174	69239188	0.723	0.491	360248	T	C	0.451	5	68535015	1.72E-08	0.080	3636	31.753
Vascular dementia (other)	Interleukin-18	rs116383510	C	A	0.659	2545536	0.241	0.561	360248	C	A	0.543	5	2545650	3.00E-07	0.106	3636	26.402
Vascular dementia (other)	Interleukin-18	rs117266781	T	C	1.247	41261422	0.100	0.757	360248	T	C	0.684	7	41301020	3.15E-06	0.147	3636	21.716
Vascular dementia (other)	Interleukin-18	rs144841621	T	C	-0.112	69921801	0.869	0.676	360248	T	C	0.518	10	71681557	3.81E-06	0.114	3636	20.610
Vascular dementia (other)	Interleukin-18	rs17229943	C	A	-0.093	69386709	0.676	0.224	360248	C	A	0.312	5	68682536	1.62E-11	0.046	3636	45.410
Vascular dementia (other)	Interleukin-18	rs1852105	C	T	-0.256	64265217	0.477	0.360	360248	C	T	-0.304	7	63725595	4.32E-06	0.066	3636	21.096
Vascular dementia (other)	Interleukin-18	rs1979967	T	C	-0.237	79367271	0.147	0.163	360248	T	C	0.140	15	79659613	9.45E-07	0.029	3636	24.031
Vascular dementia (other)	Interleukin-18	rs2729385	A	G	0.068	57495520	0.645	0.147	360248	A	G	0.123	11	57262993	3.79E-06	0.026	3636	22.076
Vascular dementia (other)	Interleukin-18	rs385076	C	T	0.221	32264782	0.111	0.139	360248	C	T	0.243	2	32489851	1.66E-22	0.025	3636	96.166
Vascular dementia (other)	Interleukin-18	rs4482818	G	A	0.053	65062779	0.698	0.136	360248	G	A	-0.129	4	65928497	1.45E-07	0.024	3636	27.778
Vascular dementia (other)	Interleukin-18	rs658805	A	G	0.035	70199369	0.800	0.140	360248	A	G	0.123	6	70909073	4.94E-07	0.024	3636	25.247
Vascular dementia (other)	Interleukin-18	rs71478720	T	C	-0.051	112138882	0.745	0.156	360248	T	C	-0.267	11	112009605	3.71E-22	0.028	3636	93.515
Vascular dementia (other)	Interleukin-18	rs78623212	T	C	0.763	103667180	0.321	0.769	360248	T	C	0.871	7	103307627	6.71E-07	0.178	3636	23.970
Vascular dementia (other)	Interleukin-18	rs78716465	A	G	-0.190	42015086	0.594	0.355	360248	A	G	0.327	20	40643726	1.63E-06	0.068	3636	22.919
Vascular dementia (other)	Interleukin-17	rs117029961	A	G	1.149	37147653	0.013	0.462	360248	A	G	0.459	10	37436581	4.94E-06	0.102	7760	20.405
Vascular dementia (other)	Interleukin-17	rs117556572	T	C	0.148	104436567	0.727	0.424	360248	T	C	-0.510	13	105088917	3.28E-06	0.110	7760	21.552
Vascular dementia (other)	Interleukin-17	rs1530455	C	T	-0.078	123136052	0.569	0.138	360248	C	T	-0.108	3	122854899	4.87E-10	0.017	7760	38.972
Vascular dementia (other)	Interleukin-17	rs17106604	T	C	-0.164	77912813	0.381	0.187	360248	T	C	0.113	14	78379156	6.37E-07	0.023	7760	25.178
Vascular dementia (other)	Interleukin-17	rs17282552	C	T	-0.439	207109091	0.135	0.294	360248	C	T	0.200	2	207973815	8.21E-07	0.041	7760	24.411
Vascular dementia (other)	Interleukin-17	rs184080173	C	T	-0.116	77331424	0.750	0.364	360248	C	T	-0.238	12	77725204	4.19E-07	0.047	7760	25.620
Vascular dementia (other)	Interleukin-17	rs187475560	T	C	0.065	160353411	0.872	0.399	360248	T	C	-0.243	4	161274563	3.29E-06	0.052	7760	21.910
Vascular dementia (other)	Interleukin-17	rs62191444	T	G	0.332	393023	0.074	0.185	360248	T	G	-0.114	20	373667	4.22E-06	0.025	7760	21.153
Vascular dementia (other)	Interleukin-17	rs78296352	T	G	0.586	22495351	0.304	0.570	360248	T	G	0.303	1	22821844	4.27E-06	0.065	7760	21.956
Vascular dementia (other)	Interleukin-17	rs78612928	C	T	0.249	29812292	0.166	0.180	360248	C	T	-0.104	4	29813914	2.62E-06	0.022	7760	21.820
Vascular dementia (other)	Interleukin-13	rs117795020	A	G	0.392	87469237	0.329	0.401	360248	A	G	-0.352	9	90084152	9.86E-07	0.072	3557	24.197
Vascular dementia (other)	Interleukin-13	rs12623722	A	G	-0.105	22955811	0.470	0.145	360248	A	G	-0.119	2	23178683	4.19E-06	0.026	3557	21.096
Vascular dementia (other)	Interleukin-13	rs139083458	T	C	0.098	26160409	0.926	1.066	360248	T	C	0.990	5	26160518	2.81E-06	0.211	3557	22.086
Vascular dementia (other)	Interleukin-13	rs142167313	C	T	0.050	44204360	0.881	0.335	360248	C	T	0.313	6	44172097	3.98E-07	0.062	3557	25.735
Vascular dementia (other)	Interleukin-13	rs27949	T	C	-0.036	59254997	0.802	0.142	360248	T	C	-0.117	5	58550823	3.43E-06	0.025	3557	21.482
Vascular dementia (other)	Interleukin-13	rs6799107	C	T	-0.142	127338175	0.408	0.172	360248	C	T	0.146	3	127057018	1.25E-06	0.030	3557	23.495
Vascular dementia (other)	Interleukin-13	rs7073807	C	T	-0.091	67393670	0.641	0.196	360248	C	T	-0.168	10	69153428	2.37E-06	0.036	3557	22.323
Vascular dementia (other)	Interleukin-13	rs75995699	A	G	0.731	5140622	0.048	0.369	360248	A	G	0.332	6	5140856	2.64E-06	0.070	3557	22.610
Vascular dementia (other)	Interleukin-13	rs9472168	G	A	-0.014	43961248	0.914	0.134	360248	G	A	-0.424	6	43928985	1.08E-65	0.025	3557	292.851
Vascular dementia (other)	Interleukin-10	rs10457128	A	G	-0.294	105570101	0.033	0.138	360248	A	G	-0.087	6	106017976	5.24E-07	0.017	7681	25.292
Vascular dementia (other)	Interleukin-10	rs10493718	A	C	-0.047	82597250	0.800	0.183	360248	A	C	-0.110	1	83062933	7.16E-07	0.022	7681	24.552
Vascular dementia (other)	Interleukin-10	rs11206302	T	C	0.000	54208270	0.999	0.201	360248	T	C	-0.119	1	54673943	2.20E-06	0.025	7681	22.440
Vascular dementia (other)	Interleukin-10	rs2086656	T	C	0.112	59632755	0.429	0.141	360248	T	C	-0.079	4	60498473	3.78E-06	0.017	7681	21.289
Vascular dementia (other)	Interleukin-10	rs282258	C	T	0.087	224050083	0.517	0.134	360248	C	T	-0.099	2	224914800	1.00E-09	0.016	7681	37.497
Vascular dementia (other)	Interleukin-10	rs3025021	C	T	-0.039	43781426	0.781	0.141	360248	C	T	-0.095	6	43749163	1.46E-06	0.020	7681	23.585
Vascular dementia (other)	Interleukin-10	rs41282660	G	A	0.098	44229269	0.612	0.193	360248	G	A	0.119	6	44197006	3.72E-06	0.026	7681	21.924
Vascular dementia (other)	Interleukin-10	rs4349809	G	T	-0.005	43957093	0.968	0.133	360248	G	T	-0.285	6	43924830	5.77E-67	0.017	7681	298.976
Vascular dementia (other)	Interleukin-10	rs465757	A	G	-0.010	15599638	0.944	0.139	360248	A	G	0.084	20	15580283	1.17E-06	0.017	7681	23.306
Vascular dementia (other)	Interleukin-10	rs7088799	G	T	-0.047	63256414	0.728	0.135	360248	G	T	0.085	10	65016174	3.23E-07	0.017	7681	26.028
Vascular dementia (other)	Interleukin-8	rs11634944	C	T	0.014	24937946	0.922	0.139	360248	C	T	0.121	15	25183093	1.29E-06	0.025	3526	23.208
Vascular dementia (other)	Interleukin-8	rs12075	A	G	-0.101	159205564	0.451	0.134	360248	A	G	0.120	1	159175354	3.88E-07	0.024	3526	25.855
Vascular dementia (other)	Interleukin-8	rs141926526	C	A	0.420	32809028	0.583	0.764	360248	C	A	0.615	7	32848640	2.57E-06	0.131	3526	22.100
Vascular dementia (other)	Interleukin-8	rs2673604	A	C	-0.254	132399360	0.077	0.144	360248	A	C	-0.127	8	133411607	7.02E-07	0.026	3526	24.648
Vascular dementia (other)	Interleukin-6	rs1333040	T	C	0.037	22083405	0.781	0.133	360248	T	C	0.074	9	22083404	3.17E-06	0.016	8189	21.817
Vascular dementia (other)	Interleukin-6	rs13412535	A	G	0.057	224010157	0.731	0.165	360248	A	G	-0.116	2	224874874	7.34E-08	0.022	8189	29.311
Vascular dementia (other)	Interleukin-6	rs72831623	A	G	-0.438	47644927	0.101	0.267	360248	A	G	0.197	17	45722293	1.08E-07	0.037	8189	28.130
Vascular dementia (other)	Interleukin-6	rs73273528	T	C	-0.648	51814574	0.137	0.436	360248	T	C	0.267	20	50431113	9.58E-07	0.055	8189	23.347
Vascular dementia (other)	Interleukin-6	rs76856708	C	T	0.366	80695146	0.538	0.595	360248	C	T	-0.329	16	80729043	2.61E-06	0.070	8189	22.077
Vascular dementia (other)	Interleukin-1-receptor antagonist	rs1054402	C	T	-0.059	116401230	0.698	0.153	360248	C	T	-0.131	9	119163509	1.13E-06	0.027	3638	23.576
Vascular dementia (other)	Interleukin-1-receptor antagonist	rs11627423	C	A	-0.342	32731417	0.013	0.137	360248	C	A	-0.117	14	33200623	2.12E-06	0.025	3638	22.476
Vascular dementia (other)	Interleukin-1-receptor antagonist	rs12121840	T	C	0.050	165572405	0.869	0.301	360248	T	C	0.269	1	165541642	2.43E-06	0.057	3638	22.227
Vascular dementia (other)	Interleukin-1-receptor antagonist	rs2809154	T	C	-0.189	84153389	0.385	0.217	360248	T	C	-0.179	13	84727524	3.74E-06	0.039	3638	21.188
Vascular dementia (other)	Interleukin-1-receptor antagonist	rs61335305	A	C	0.266	66160736	0.596	0.502	360248	A	C	0.445	15	66453074	1.00E-06	0.091	3638	24.051
Vascular dementia (other)	Interleukin-1-receptor antagonist	rs9623661	T	C	0.022	42697370	0.926	0.235	360248	T	C	-0.197	22	43093376	3.86E-06	0.043	3638	21.298
Vascular dementia (other)	Interleukin-1-beta	rs143319329	T	C	-0.402	128499405	0.611	0.790	360248	T	C	0.280	7	128139459	2.00E-06	0.072	3309	15.347
Vascular dementia (other)	Interleukin-1-beta	rs61335305	A	C	0.266	66160736	0.596	0.502	360248	A	C	0.297	15	66453074	1.90E-06	0.072	3309	16.783
Vascular dementia (other)	Interleukin-1-beta	rs62015704	G	A	0.015	7417906	0.943	0.203	360248	G	A	-0.108	16	7467907	2.09E-06	0.028	3309	14.618
Vascular dementia (other)	Interleukin-1-beta	rs9898641	C	T	0.232	59493672	0.093	0.139	360248	C	T	0.203	17	57571033	3.59E-06	0.045	3309	20.033

Vascular dementia (other)	Hepatocyte growth factor	rs11060254	A	G	0.050	129331024	0.727	0.143	360248	A	G	-0.080	12	129815569	1.58E-06	0.017	8292	22.948
Vascular dementia (other)	Hepatocyte growth factor	rs150322232	G	A	0.027	7890743	0.938	0.346	360248	G	A	-0.210	7	7930374	4.89E-06	0.046	8292	20.650
Vascular dementia (other)	Hepatocyte growth factor	rs1698249	C	A	0.128	83889842	0.685	0.315	360248	C	A	0.170	14	84356186	4.09E-06	0.037	8292	20.835
Vascular dementia (other)	Hepatocyte growth factor	rs2003620	T	C	0.486	134794733	0.210	0.388	360248	T	C	0.228	7	134479484	2.83E-06	0.049	8292	21.721
Vascular dementia (other)	Hepatocyte growth factor	rs3748034	T	G	0.345	3444364	0.087	0.202	360248	T	G	0.150	4	3446091	1.81E-10	0.023	8292	40.818
Vascular dementia (other)	Hepatocyte growth factor	rs5745687	T	C	-0.043	81729735	0.894	0.326	360248	T	C	-0.307	7	81359051	2.75E-14	0.041	8292	57.252
Vascular dementia (other)	Hepatocyte growth factor	rs62481625	C	T	-0.012	156194766	0.947	0.185	360248	C	T	-0.109	7	155987460	1.18E-06	0.023	8292	23.512
Vascular dementia (other)	Interleukin-9	rs41294750	T	C	-0.247	53084968	0.534	0.397	360248	T	C	0.351	1	53550640	2.36E-06	0.075	3634	22.070
Vascular dementia (other)	Interleukin-9	rs4880409	T	C	0.152	132516716	0.817	0.658	360248	T	C	-0.336	10	134330220	3.50E-06	0.072	3634	21.533
Vascular dementia (other)	Interleukin-9	rs61867538	T	C	-0.183	1503276	0.582	0.333	360248	T	C	0.357	11	1524506	3.93E-06	0.077	3634	21.227
Vascular dementia (other)	Interleukin-9	rs7232268	G	A	-0.527	70101678	0.128	0.346	360248	G	A	-0.276	18	67768914	2.52E-06	0.059	3634	22.092
Vascular dementia (other)	Interleukin-9	rs7242404	A	G	-0.004	12741268	0.976	0.147	360248	A	G	-0.123	18	12741267	3.27E-06	0.026	3634	21.637
Vascular dementia (other)	Interleukin-9	rs76963786	T	C	-0.352	31886823	0.272	0.320	360248	T	C	-0.287	12	32039757	4.50E-07	0.056	3634	26.457
Vascular dementia (other)	Interleukin-7	rs117509142	C	T	0.327	86121854	0.380	0.372	360248	C	T	0.327	8	87134083	1.99E-06	0.069	3409	22.590
Vascular dementia (other)	Interleukin-7	rs141425475	C	T	-0.602	17679056	0.196	0.465	360248	C	T	0.478	5	17679165	2.53E-06	0.102	3409	22.144
Vascular dementia (other)	Interleukin-7	rs144701438	A	G	0.232	66293168	0.681	0.564	360248	A	G	-0.482	18	63960405	9.75E-07	0.099	3409	23.742
Vascular dementia (other)	Interleukin-7	rs17091524	C	T	0.022	56482041	0.967	0.538	360248	C	T	-0.492	14	56948759	1.91E-06	0.101	3409	23.627
Vascular dementia (other)	Interleukin-7	rs28793375	T	C	0.044	41558099	0.822	0.196	360248	T	C	0.164	8	41415618	4.46E-06	0.036	3409	20.588
Vascular dementia (other)	Interleukin-7	rs4320361	T	G	-0.028	43960774	0.836	0.133	360248	T	G	-0.325	6	43928511	6.87E-39	0.025	3409	169.836
Vascular dementia (other)	Interleukin-7	rs62006410	T	C	0.003	102541598	0.986	0.157	360248	T	C	-0.156	14	103007935	3.39E-07	0.030	3409	26.405
Vascular dementia (other)	Interleukin-7	rs75904417	C	A	-0.029	167796811	0.880	0.191	360248	C	A	0.170	2	168653321	1.16E-06	0.035	3409	23.671
Vascular dementia (other)	Interleukin-7	rs77981494	C	T	-0.059	17451009	0.899	0.464	360248	C	T	0.518	16	17544866	1.07E-06	0.106	3409	23.683
Vascular dementia (other)	Interleukin-7	rs78346957	A	G	0.408	125214944	0.514	0.625	360248	A	G	0.459	10	126903513	4.51E-06	0.101	3409	20.758
Vascular dementia (other)	Interleukin-5	rs11680908	G	A	-0.176	109460295	0.556	0.298	360248	G	A	-0.263	2	110076751	2.03E-06	0.055	3364	22.605
Vascular dementia (other)	Interleukin-5	rs6737109	C	T	0.019	22956659	0.887	0.135	360248	C	T	-0.116	2	23179531	2.40E-06	0.025	3364	22.056
Vascular dementia (other)	Interleukin-5	rs72831687	A	G	0.076	16092129	0.886	0.529	360248	A	G	-0.524	6	16092360	1.69E-06	0.111	3364	22.317
Vascular dementia (other)	Interleukin-5	rs73040130	C	T	-0.542	36255288	0.062	0.291	360248	C	T	-0.264	19	36746190	6.00E-07	0.053	3364	24.868
Vascular dementia (other)	Interleukin-5	rs7767396	G	A	-0.005	43959313	0.970	0.133	360248	G	A	-0.152	6	43927050	7.69E-10	0.025	3364	37.928
Vascular dementia (other)	Interleukin-4	rs10512267	C	T	0.074	99427847	0.580	0.134	360248	C	T	0.082	9	102190129	2.94E-07	0.016	8124	26.194
Vascular dementia (other)	Interleukin-4	rs116705532	G	T	0.292	113162547	0.718	0.808	360248	G	T	0.468	1	113705169	1.76E-06	0.098	8124	22.879
Vascular dementia (other)	Interleukin-4	rs117146485	C	T	-0.286	135932411	0.570	0.503	360248	C	T	0.292	9	138824257	2.71E-06	0.063	8124	21.610
Vascular dementia (other)	Interleukin-4	rs17713451	A	G	-0.207	151465386	0.319	0.208	360248	A	G	0.127	7	151162472	4.97E-07	0.025	8124	25.357
Vascular dementia (other)	Interleukin-4	rs73023729	A	G	0.631	159232998	0.010	0.245	360248	A	G	-0.180	6	159654030	9.03E-07	0.037	8124	24.080
Vascular dementia (other)	Interleukin-4	rs7613691	G	A	0.311	147935804	0.308	0.305	360248	G	A	-0.178	3	147653591	4.05E-06	0.038	8124	21.367
Vascular dementia (other)	Interleukin-4	rs9508291	C	T	-0.508	29136483	0.093	0.302	360248	C	T	0.168	13	29710620	3.03E-06	0.036	8124	21.795
Vascular dementia (other)	Interleukin-4	rs9941733	G	A	0.252	393417	0.151	0.176	360248	G	A	-0.114	20	374061	6.88E-07	0.023	8124	24.782
Vascular dementia (other)	Interleukin-2 receptor antagonist	rs11241559	G	T	0.002	120641005	0.989	0.153	360248	G	T	0.126	5	119976700	2.00E-06	0.027	3677	22.580
Vascular dementia (other)	Interleukin-2 receptor antagonist	rs117244812	A	G	-0.383	6539990	0.629	0.793	360248	A	G	-0.706	17	6443310	2.10E-06	0.149	3677	22.537
Vascular dementia (other)	Interleukin-2 receptor antagonist	rs12722497	A	C	0.048	6053965	0.858	0.267	360248	A	C	0.628	10	6095928	1.57E-38	0.049	3677	167.609
Vascular dementia (other)	Interleukin-2 receptor antagonist	rs185231391	C	T	-0.980	59373953	0.236	0.826	360248	C	T	-0.850	3	59359679	1.47E-06	0.181	3677	22.094
Vascular dementia (other)	Interleukin-2 receptor antagonist	rs4733117	C	A	0.056	32280094	0.732	0.163	360248	C	A	-0.137	8	32137610	2.63E-06	0.029	3677	21.981
Vascular dementia (other)	Interleukin-2 receptor antagonist	rs61705228	T	C	0.100	100275145	0.779	0.358	360248	T	C	0.330	4	101196302	3.99E-06	0.072	3677	21.281
Vascular dementia (other)	Interleukin-2	rs12051139	C	T	0.180	86885068	0.182	0.135	360248	C	T	0.113	16	86918674	4.76E-06	0.025	3475	20.967
Vascular dementia (other)	Interleukin-2	rs13412535	A	G	0.057	224010157	0.731	0.165	360248	A	G	0.176	2	224874874	1.18E-07	0.033	3475	28.231
Vascular dementia (other)	Interleukin-2	rs170117	T	C	0.084	54524213	0.655	0.188	360248	T	C	-0.162	4	55390380	3.87E-06	0.035	3475	21.467
Vascular dementia (other)	Interleukin-2	rs2807544	G	A	-0.308	14877749	0.013	0.124	360248	G	A	-0.118	1	15204245	3.41E-06	0.025	3475	21.569
Vascular dementia (other)	Interleukin-2	rs4634519	G	A	0.007	67727941	0.962	0.148	360248	G	A	0.126	7	67192928	2.77E-06	0.027	3475	21.975
Vascular dementia (other)	Interleukin-2	rs61335305	A	C	0.266	66160736	0.596	0.502	360248	A	C	0.451	15	66453074	7.32E-07	0.092	3475	24.179
Vascular dementia (other)	Interleukin-2	rs62124990	T	G	0.732	19038882	0.118	0.469	360248	T	G	-0.696	2	19238636	3.22E-06	0.150	3475	21.680
Vascular dementia (other)	Interleukin-2	rs7615304	G	A	0.064	156957914	0.628	0.133	360248	G	A	0.117	3	156675703	1.21E-06	0.024	3475	23.454
Vascular dementia (other)	Interleukin-2	rs80336398	C	T	0.261	64075258	0.550	0.437	360248	C	T	-0.400	3	64060934	2.82E-06	0.086	3475	21.745
Vascular dementia (other)	Interferon gamma	rs10487554	A	G	-0.059	149670595	0.687	0.146	360248	A	G	-0.090	7	149367686	1.09E-06	0.018	7701	23.919
Vascular dementia (other)	Interferon gamma	rs113600793	A	C	-0.343	47384095	0.229	0.285	360248	A	C	0.183	17	45461461	8.95E-07	0.037	7701	24.044
Vascular dementia (other)	Interferon gamma	rs115729819	G	A	-0.546	168783516	0.187	0.413	360248	G	A	-0.248	4	169704667	1.38E-06	0.052	7701	23.264
Vascular dementia (other)	Interferon gamma	rs11843756	G	T	0.175	48680756	0.598	0.332	360248	G	T	-0.184	13	49254892	3.09E-06	0.039	7701	21.921
Vascular dementia (other)	Interferon gamma	rs12420286	C	T	0.267	103907166	0.511	0.406	360248	C	T	-0.238	11	103777894	2.08E-06	0.050	7701	22.491
Vascular dementia (other)	Interferon gamma	rs1867282	T	C	0.101	99409865	0.452	0.134	360248	T	C	0.077	9	102172147	3.15E-06	0.017	7701	21.740
Vascular dementia (other)	Interferon gamma	rs2073438	A	G	0.055	6996757	0.715	0.151	360248	A	G	0.090	17	6900076	1.68E-06	0.019	7701	22.816
Vascular dementia (other)	Interferon gamma	rs74148555	T	C	-0.082	90320085	0.861	0.465	360248	T	C	-0.373	10	92079842	2.64E-06	0.077	7701	23.249
Vascular dementia (other)	Interferon gamma	rs78296352	T	G	0.586	22495351	0.304	0.570	360248	T	G	0.343	1	22821844	1.38E-07	0.065	7701	27.675
Vascular dementia (other)	Growth-regulated protein alpha	rs1113500	T	G	0.091	108052820	0.497	0.135	360248	T	G	0.117	1	108595442	1.57E-06	0.024	3505	23.150
Vascular dementia (other)	Growth-regulated protein alpha	rs12075	A	G	-0.101	159205564	0.451	0.134	360248	A	G	0.375	1	159175354	1.24E-55	0.024	3505	250.494
Vascular dementia (other)	Growth-regulated protein alpha	rs140734053	A	G	0.653	5359496	0.382	0.748	360248	A	G	0.726	10	5401459	3.58E-06	0.156	3505	21.613
Vascular dementia (other)	Growth-regulated protein alpha	rs185768063	G	A	-0.216	16494752	0.597	0.408	360248	G	A	-0.400	6	16494983	1.46E-07	0.076	3505	27.673

Vascular dementia (other)	Growth-regulated protein alpha	rs188345231	T	C	1.256	41579831	0.098	0.759	360248	T	C	0.623	8	41437350	4.34E-06	0.132	3505	22.175
Vascular dementia (other)	Growth-regulated protein alpha	rs2422841	A	G	0.085	3099706	0.659	0.192	360248	A	G	-0.166	20	3080352	4.66E-06	0.036	3505	21.068
Vascular dementia (other)	Growth-regulated protein alpha	rs508977	G	T	0.035	73896666	0.824	0.156	360248	G	T	0.380	4	74762383	7.56E-42	0.028	3505	184.378
Vascular dementia (other)	Growth-regulated protein alpha	rs62024303	G	A	0.117	88327931	0.728	0.336	360248	G	A	0.305	15	88871162	4.41E-06	0.067	3505	21.014
Vascular dementia (other)	Growth-regulated protein alpha	rs78653452	T	G	-0.583	9781407	0.385	0.671	360248	T	G	-0.736	20	9762055	1.21E-06	0.156	3505	22.328
Vascular dementia (other)	Granulocyte-colony stimulating factor	rs115256310	G	A	-1.053	72103864	0.184	0.793	360248	G	A	0.682	5	71399691	6.73E-07	0.136	7904	25.155
Vascular dementia (other)	Granulocyte-colony stimulating factor	rs11903143	G	A	-0.089	29369594	0.542	0.146	360248	G	A	-0.087	2	29592460	6.35E-07	0.018	7904	24.435
Vascular dementia (other)	Granulocyte-colony stimulating factor	rs147128865	T	C	0.777	34972769	0.018	0.328	360248	T	C	0.270	9	34972766	4.92E-06	0.059	7904	21.157
Vascular dementia (other)	Granulocyte-colony stimulating factor	rs1817411	T	C	-0.210	97586100	0.191	0.161	360248	T	C	0.089	8	98598328	3.10E-06	0.019	7904	21.713
Vascular dementia (other)	Granulocyte-colony stimulating factor	rs2671444	A	G	-0.118	101158297	0.394	0.138	360248	A	G	-0.078	12	101552075	2.48E-06	0.017	7904	22.306
Vascular dementia (other)	Granulocyte-colony stimulating factor	rs74148555	T	C	-0.082	90320085	0.861	0.465	360248	T	C	-0.372	10	92079842	1.55E-06	0.076	7904	24.212
Vascular dementia (other)	Granulocyte-colony stimulating factor	rs77318030	C	T	-0.239	54544688	0.465	0.327	360248	C	T	0.205	19	55055897	2.21E-06	0.043	7904	22.830
Vascular dementia (other)	Fibroblast growth factor basic	rs13412535	A	G	0.057	224010157	0.731	0.165	360248	A	G	-0.111	2	224874874	7.34E-07	0.023	7565	24.426
Vascular dementia (other)	Fibroblast growth factor basic	rs145577605	A	G	-0.326	27642232	0.381	0.372	360248	A	G	0.208	6	27610011	9.64E-07	0.043	7565	23.640
Vascular dementia (other)	Fibroblast growth factor basic	rs747334	G	A	0.092	90984987	0.490	0.133	360248	G	A	-0.075	10	92744744	4.53E-06	0.016	7565	20.970
Vascular dementia (other)	Fibroblast growth factor basic	rs75168112	C	T	-0.174	73418832	0.306	0.170	360248	C	T	0.100	18	71086067	3.00E-06	0.021	7565	21.880
Vascular dementia (other)	Fibroblast growth factor basic	rs9907295	T	C	0.128	35930309	0.548	0.214	360248	T	C	-0.132	17	34257313	7.95E-07	0.027	7565	24.043
Vascular dementia (other)	Eotaxin	rs11087905	A	C	-0.001	24133015	0.995	0.140	360248	A	C	0.094	21	25505329	5.48E-07	0.019	8153	24.789
Vascular dementia (other)	Eotaxin	rs112347425	T	C	-0.198	46419397	0.399	0.235	360248	T	C	0.158	3	46460888	8.65E-09	0.028	8153	32.535
Vascular dementia (other)	Eotaxin	rs12075	A	G	-0.101	159205564	0.451	0.134	360248	A	G	0.167	1	159175354	1.33E-26	0.016	8153	114.737
Vascular dementia (other)	Eotaxin	rs1476670	C	A	-0.122	44042523	0.500	0.181	360248	C	A	0.101	1	44508195	3.51E-06	0.022	8153	21.535
Vascular dementia (other)	Eotaxin	rs2024050	G	A	0.154	75831075	0.555	0.261	360248	G	A	-0.173	7	75460393	1.10E-08	0.030	8153	32.524
Vascular dementia (other)	Eotaxin	rs2210755	C	T	0.085	77608907	0.659	0.192	360248	C	T	0.110	9	80223823	4.85E-06	0.024	8153	20.812
Vascular dementia (other)	Eotaxin	rs2211994	C	T	-0.013	16675274	0.928	0.150	360248	C	T	-0.089	21	18047593	6.08E-07	0.018	8153	25.000
Vascular dementia (other)	Eotaxin	rs2228467	C	T	-0.064	42864624	0.800	0.252	360248	C	T	0.416	3	42906116	2.27E-46	0.029	8153	203.258
Vascular dementia (other)	Eotaxin	rs2419841	C	T	0.432	113576224	0.074	0.242	360248	C	T	0.128	10	115335983	4.98E-06	0.028	8153	20.949
Vascular dementia (other)	Eotaxin	rs5746492	G	A	-0.369	17911167	0.040	0.180	360248	G	A	-0.095	22	18393933	3.96E-06	0.021	8153	21.240
Vascular dementia (other)	Eotaxin	rs5754733	A	C	0.215	33873606	0.221	0.175	360248	A	C	-0.104	22	34269594	1.06E-06	0.021	8153	23.709
Vascular dementia (other)	Eotaxin	rs59808887	T	C	-0.169	31846414	0.539	0.275	360248	T	C	-0.167	5	31846520	2.91E-06	0.036	8153	21.839
Vascular dementia (other)	Eotaxin	rs75426604	A	C	0.336	35388508	0.138	0.227	360248	A	C	-0.137	14	35857714	2.53E-06	0.029	8153	22.035
Vascular dementia (other)	Eotaxin	rs79722574	T	C	-0.212	34292033	0.263	0.189	360248	T	C	-0.111	17	32619052	1.06E-06	0.023	8153	23.830
Vascular dementia (other)	Eotaxin	rs9317045	C	A	-0.147	59055904	0.428	0.186	360248	C	A	-0.118	13	59630038	5.82E-07	0.024	8153	24.874
Vascular dementia (subcortical)	CTACK	rs116303454	A	G	-0.002	27253164	0.990	0.173	360770	A	G	0.383	3	27294655	3.27E-06	0.082	3631	22.030
Vascular dementia (subcortical)	CTACK	rs2070074	G	A	-0.184	34649445	0.041	0.090	360770	G	A	-0.447	9	34649442	1.78E-32	0.037	3631	142.656
Vascular dementia (subcortical)	CTACK	rs2731674	G	T	0.096	177412889	0.138	0.065	360770	G	T	0.133	5	176839890	5.63E-07	0.027	3631	24.925
Vascular dementia (subcortical)	CTACK	rs3766110	C	A	0.019	169545945	0.777	0.066	360770	C	A	0.129	1	169515183	3.85E-06	0.028	3631	21.432
Vascular dementia (subcortical)	CTACK	rs55764737	C	T	0.129	61031215	0.586	0.237	360770	C	T	-0.531	15	61323414	4.62E-08	0.097	3631	29.878
Vascular dementia (subcortical)	CTACK	rs57338032	G	A	0.134	78506597	0.079	0.076	360770	G	A	-0.158	15	78798939	6.23E-07	0.032	3631	24.937
Vascular dementia (subcortical)	CTACK	rs7333764	T	C	0.167	33634664	0.251	0.145	360770	T	C	0.277	13	34208801	2.85E-06	0.059	3631	21.867
Vascular dementia (subcortical)	CTACK	rs76395525	A	G	-0.500	79449049	0.074	0.280	360770	A	G	0.528	15	79741391	9.55E-07	0.108	3631	23.742
Vascular dementia (subcortical)	beta-nerve growth factor	rs28637706	T	G	0.015	33794463	0.814	0.062	360770	T	G	-0.159	19	34285368	1.42E-09	0.026	3531	36.504
Vascular dementia (subcortical)	beta-nerve growth factor	rs67476890	T	C	-0.042	62499295	0.645	0.090	360770	T	C	0.177	15	62791494	3.13E-06	0.038	3531	21.786
Vascular dementia (subcortical)	beta-nerve growth factor	rs71641308	T	C	0.015	77621033	0.877	0.096	360770	T	C	0.204	1	78086718	2.30E-06	0.043	3531	22.365
Vascular dementia (subcortical)	beta-nerve growth factor	rs72780728	A	G	0.035	17561702	0.724	0.099	360770	A	G	0.188	10	17603701	2.99E-06	0.040	3531	21.832
Vascular dementia (subcortical)	beta-nerve growth factor	rs73472576	C	T	0.045	74456947	0.429	0.057	360770	C	T	0.118	18	72124182	2.69E-06	0.025	3531	21.963
Vascular dementia (subcortical)	beta-nerve growth factor	rs7970581	G	T	0.153	112827443	0.017	0.064	360770	G	T	-0.138	12	113265248	9.27E-07	0.028	3531	23.947
Vascular dementia (subcortical)	beta-nerve growth factor	rs9436119	A	G	0.014	150495277	0.815	0.058	360770	A	G	-0.112	1	150467753	3.91E-06	0.025	3531	20.765
Vascular dementia (subcortical)	Vascular endothelial growth factor	rs10153304	A	G	-0.031	7818613	0.766	0.104	360770	A	G	0.155	17	7721931	1.94E-06	0.033	7118	22.658
Vascular dementia (subcortical)	Vascular endothelial growth factor	rs10934631	C	T	-0.172	122978753	0.033	0.081	360770	C	T	0.115	3	122697600	2.47E-06	0.025	7118	22.071
Vascular dementia (subcortical)	Vascular endothelial growth factor	rs10967186	C	T	-0.005	2617099	0.930	0.057	360770	C	T	-0.090	9	2617099	1.23E-07	0.017	7118	27.903
Vascular dementia (subcortical)	Vascular endothelial growth factor	rs13209117	A	G	-0.026	44184028	0.691	0.066	360770	A	G	0.130	6	44151765	5.28E-11	0.020	7118	41.959
Vascular dementia (subcortical)	Vascular endothelial growth factor	rs143479231	A	G	0.149	193393005	0.309	0.146	360770	A	G	-0.260	3	193110794	1.90E-07	0.049	7118	27.997
Vascular dementia (subcortical)	Vascular endothelial growth factor	rs4082730	A	G	0.092	89980326	0.601	0.176	360770	A	G	0.252	15	90523558	2.64E-06	0.053	7118	22.305
Vascular dementia (subcortical)	Vascular endothelial growth factor	rs6921438	A	G	-0.008	43957870	0.890	0.057	360770	A	G	-0.490	6	43925607	########	0.018	7118	784.000
Vascular dementia (subcortical)	Vascular endothelial growth factor	rs73418461	A	G	0.248	118463484	0.150	0.172	360770	A	G	-0.249	10	120222996	1.67E-06	0.052	7118	22.878
Vascular dementia (subcortical)	Vascular endothelial growth factor	rs8045833	A	G	-0.047	88509031	0.496	0.069	360770	A	G	0.108	16	88575439	2.83E-07	0.021	7118	26.199
Vascular dementia (subcortical)	Vascular endothelial growth factor	rs9472183	G	A	0.085	43972465	0.137	0.057	360770	G	A	0.128	6	43940202	5.19E-14	0.017	7118	56.869
Vascular dementia (subcortical)	Macrophage Migration Inhibitory Factor	rs113218956	A	G	0.762	24828867	0.089	0.447	360770	A	G	-0.895	22	25224834	2.26E-06	0.188	3494	22.678
Vascular dementia (subcortical)	Macrophage Migration Inhibitory Factor	rs118055855	C	T	0.399	29867025	0.212	0.320	360770	C	T	-0.691	11	29888572	4.13E-06	0.150	3494	21.203
Vascular dementia (subcortical)	Macrophage Migration Inhibitory Factor	rs12594190	G	A	-0.011	24791308	0.864	0.062	360770	G	A	-0.136	15	25036455	3.70E-07	0.027	3494	25.755
Vascular dementia (subcortical)	Macrophage Migration Inhibitory Factor	rs13142904	T	C	-0.063	53452247	0.529	0.100	360770	T	C	-0.223	4	54318414	2.56E-07	0.043	3494	27.532
Vascular dementia (subcortical)	Macrophage Migration Inhibitory Factor	rs141009259	C	T	0.152	207111559	0.571	0.269	360770	C	T	0.618	2	207976283	2.47E-06	0.132	3494	21.839
Vascular dementia (subcortical)	Macrophage Migration Inhibitory Factor	rs78098071	C	T	-0.019	163882733	0.932	0.222	360770	C	T	0.487	5	163309739	1.78E-07	0.092	3494	28.108
Vascular dementia (subcortical)	TRAIL	rs11618126	G	A	-0.563	50252103	0.381	0.642	360770	G	A	-0.891	13	50826239	1.46E-06	0.191	8186	21.661

Vascular dementia (subcortical)	TRAIL	rs11657269	G	A	0.095	6416464	0.305	0.093	360770	G	A	-0.119	17	6319784	4.78E-06	0.026	8186	20.878
Vascular dementia (subcortical)	TRAIL	rs11699445	G	T	-0.023	15770145	0.687	0.058	360770	G	T	-0.075	20	15750790	3.27E-06	0.016	8186	21.470
Vascular dementia (subcortical)	TRAIL	rs13185784	A	G	-0.004	180267068	0.950	0.064	360770	A	G	0.085	5	179694068	3.90E-06	0.018	8186	21.372
Vascular dementia (subcortical)	TRAIL	rs138987090	G	A	-0.086	32786284	0.718	0.239	360770	G	A	0.750	18	30366247	4.50E-23	0.075	8186	99.389
Vascular dementia (subcortical)	TRAIL	rs146783010	G	A	-0.605	89527045	0.151	0.422	360770	G	A	0.602	11	89260213	4.83E-06	0.135	8186	19.859
Vascular dementia (subcortical)	TRAIL	rs193112415	C	T	-0.188	31255157	0.356	0.204	360770	C	T	1.042	18	28835120	2.15E-62	0.062	8186	279.797
Vascular dementia (subcortical)	TRAIL	rs57396456	C	T	-0.260	30365911	0.163	0.187	360770	C	T	0.563	18	27945877	1.25E-27	0.052	8186	117.961
Vascular dementia (subcortical)	TRAIL	rs62093514	T	C	0.054	31651014	0.780	0.195	360770	T	C	1.062	18	29230977	6.86E-82	0.055	8186	370.005
Vascular dementia (subcortical)	TRAIL	rs73039026	C	A	-0.162	172442691	0.433	0.207	360770	C	A	0.300	3	172160481	2.02E-06	0.064	8186	22.305
Vascular dementia (subcortical)	TRAIL	rs747324	C	T	-0.006	74222941	0.928	0.063	360770	C	T	0.086	14	74689644	1.61E-06	0.018	8186	23.072
Vascular dementia (subcortical)	TRAIL	rs74778900	T	C	-0.207	30506300	0.402	0.247	360770	T	C	0.591	18	28086266	2.59E-28	0.053	8186	123.243
Vascular dementia (subcortical)	TRAIL	rs75928541	A	G	-0.051	16400148	0.793	0.193	360770	A	G	0.275	4	16401771	4.24E-06	0.059	8186	21.506
Vascular dementia (subcortical)	TRAIL	rs79287178	A	G	-0.019	172576710	0.893	0.142	360770	A	G	-0.432	3	172294500	9.12E-25	0.042	8186	105.148
Vascular dementia (subcortical)	Tumor necrosis factor beta	rs10925040	T	C	0.006	247459396	0.924	0.060	360770	T	C	0.176	1	247622698	2.67E-06	0.037	1559	22.138
Vascular dementia (subcortical)	Tumor necrosis factor beta	rs753274	T	C	-0.002	14325650	0.972	0.058	360770	T	C	-0.174	19	14436462	2.77E-06	0.037	1559	21.895
Vascular dementia (subcortical)	Tumor necrosis factor beta	rs7629875	G	A	-0.078	174667832	0.506	0.118	360770	G	A	-0.377	3	174385622	1.37E-06	0.077	1559	23.674
Vascular dementia (subcortical)	Tumor necrosis factor beta	rs78296352	T	G	-0.171	22495351	0.479	0.241	360770	T	G	1.222	1	22821844	4.76E-21	0.137	1559	79.962
Vascular dementia (subcortical)	Tumor necrosis factor alpha	rs10834997	A	G	-0.002	26505401	0.976	0.061	360770	A	G	-0.125	11	26526948	1.33E-06	0.026	3454	23.361
Vascular dementia (subcortical)	Tumor necrosis factor alpha	rs115669577	A	G	-0.283	123440293	0.539	0.460	360770	A	G	0.989	4	124361448	8.28E-07	0.200	3454	24.571
Vascular dementia (subcortical)	Tumor necrosis factor alpha	rs79105320	A	G	0.179	18959850	0.519	0.278	360770	A	G	0.561	8	18817360	3.59E-06	0.118	3454	22.601
Vascular dementia (subcortical)	Tumor necrosis factor alpha	rs8121916	A	C	-0.081	12420677	0.215	0.065	360770	A	C	0.131	20	12401325	2.72E-06	0.028	3454	22.070
Vascular dementia (subcortical)	Stromal-cell-derived factor 1 alpha	rs10474392	G	A	0.039	92198776	0.553	0.065	360770	G	A	-0.096	5	91494593	1.24E-06	0.018	5998	29.209
Vascular dementia (subcortical)	Stromal-cell-derived factor 1 alpha	rs12407262	A	G	-0.063	63354605	0.498	0.093	360770	A	G	0.118	1	63820276	3.99E-06	0.027	5998	19.646
Vascular dementia (subcortical)	Stromal-cell-derived factor 1 alpha	rs139840550	A	G	-0.086	38688625	0.669	0.201	360770	A	G	0.183	9	38688622	3.79E-06	0.055	5998	11.160
Vascular dementia (subcortical)	Stromal-cell-derived factor 1 alpha	rs149893336	G	A	0.285	170311440	0.310	0.281	360770	G	A	0.503	4	171232591	4.52E-06	0.108	5998	21.686
Vascular dementia (subcortical)	Stromal-cell-derived factor 1 alpha	rs4581824	G	T	0.058	9074853	0.333	0.060	360770	G	T	0.070	19	9185529	3.05E-06	0.017	5998	16.419
Vascular dementia (subcortical)	Stromal-cell-derived factor 1 alpha	rs482700	A	G	-0.015	115146334	0.829	0.071	360770	A	G	-0.089	4	116067490	1.57E-06	0.020	5998	19.351
Vascular dementia (subcortical)	Stromal-cell-derived factor 1 alpha	rs67689854	A	C	-0.022	89558819	0.757	0.070	360770	A	C	-0.068	16	89625227	3.07E-06	0.020	5998	12.196
Vascular dementia (subcortical)	Stromal-cell-derived factor 1 alpha	rs9267091	A	G	-0.075	31446032	0.286	0.070	360770	A	G	0.078	6	31413809	3.63E-06	0.020	5998	14.802
Vascular dementia (subcortical)	Stem cell growth factor beta	rs112346514	T	C	0.066	12297173	0.656	0.148	360770	T	C	-0.331	19	12407988	2.37E-06	0.071	3682	21.725
Vascular dementia (subcortical)	Stem cell growth factor beta	rs116924815	T	C	-0.111	50727476	0.539	0.182	360770	T	C	0.608	19	51230733	1.74E-16	0.074	3682	67.850
Vascular dementia (subcortical)	Stem cell growth factor beta	rs117716477	A	C	0.404	103847180	0.069	0.222	360770	A	C	0.838	12	104240958	1.34E-23	0.084	3682	99.383
Vascular dementia (subcortical)	Stem cell growth factor beta	rs12480722	C	T	-0.076	20248260	0.374	0.086	360770	C	T	-0.162	20	20228904	4.72E-06	0.036	3682	20.927
Vascular dementia (subcortical)	Stem cell growth factor beta	rs139413256	A	G	0.191	146182552	0.400	0.227	360770	A	G	-0.538	7	145879644	7.04E-07	0.108	3682	24.605
Vascular dementia (subcortical)	Stem cell growth factor beta	rs143829871	C	T	-0.195	47555755	0.046	0.098	360770	C	T	0.190	3	47597245	1.90E-06	0.040	3682	22.610
Vascular dementia (subcortical)	Stem cell growth factor beta	rs151194174	A	G	-0.009	20956159	0.955	0.152	360770	A	G	0.464	7	20995778	1.13E-06	0.094	3682	24.210
Vascular dementia (subcortical)	Stem cell growth factor beta	rs17876031	G	A	0.048	177404118	0.425	0.061	360770	G	A	0.151	5	176831119	2.25E-09	0.026	3682	35.251
Vascular dementia (subcortical)	Stem cell growth factor beta	rs264162	G	A	-0.070	10944028	0.220	0.057	360770	G	A	-0.110	18	10944026	2.68E-06	0.023	3682	21.978
Vascular dementia (subcortical)	Stem cell growth factor beta	rs34911860	A	G	0.382	79885030	0.078	0.217	360770	A	G	-0.368	1	80350715	3.24E-06	0.079	3682	21.695
Vascular dementia (subcortical)	Stem cell growth factor beta	rs4656185	A	G	-0.010	169507088	0.866	0.061	360770	A	G	0.205	1	169476326	1.16E-15	0.026	3682	64.125
Vascular dementia (subcortical)	Stem cell growth factor beta	rs4737732	G	A	-0.054	65421393	0.386	0.063	360770	G	A	0.115	8	66333628	4.68E-06	0.025	3682	20.717
Vascular dementia (subcortical)	Stem cell growth factor beta	rs7762066	C	T	0.095	94468249	0.159	0.067	360770	C	T	-0.139	6	95177967	3.50E-06	0.030	3682	21.581
Vascular dementia (subcortical)	Stem cell growth factor beta	rs78217154	C	T	-0.200	100541844	0.317	0.200	360770	C	T	-0.400	8	101554072	3.77E-06	0.086	3682	21.401
Vascular dementia (subcortical)	Stem cell factor	rs113127926	A	C	0.064	97971174	0.660	0.145	360770	A	C	0.198	14	98437511	2.27E-06	0.042	8290	22.269
Vascular dementia (subcortical)	Stem cell factor	rs13412535	A	G	-0.021	224010157	0.764	0.071	360770	A	G	-0.107	2	224874874	6.04E-07	0.021	8290	25.094
Vascular dementia (subcortical)	Stem cell factor	rs1557570	T	G	-0.012	169538606	0.844	0.061	360770	T	G	0.119	1	169507844	2.74E-12	0.017	8290	48.671
Vascular dementia (subcortical)	Stem cell factor	rs1568119	T	C	-0.026	33385679	0.944	0.367	360770	T	C	-0.591	8	33243197	1.24E-07	0.113	8290	27.365
Vascular dementia (subcortical)	Stem cell factor	rs1942355	T	C	-0.072	71694503	0.207	0.057	360770	T	C	-0.072	18	69361739	4.70E-06	0.016	8290	20.798
Vascular dementia (subcortical)	Stem cell factor	rs4841899	C	T	-0.072	134532566	0.263	0.064	360770	C	T	0.100	9	137424412	1.78E-08	0.018	8290	31.815
Vascular dementia (subcortical)	Stem cell factor	rs635634	T	C	-0.031	133279427	0.660	0.071	360770	T	C	-0.103	9	136155000	6.74E-08	0.019	8290	29.194
Vascular dementia (subcortical)	Stem cell factor	rs78666213	G	T	-0.378	179217495	0.056	0.198	360770	G	T	0.274	4	180138649	2.59E-06	0.058	8290	22.695
Vascular dementia (subcortical)	Stem cell factor	rs80271436	A	G	0.182	133022383	0.301	0.176	360770	A	G	-0.237	9	135897770	9.95E-07	0.049	8290	23.879
Vascular dementia (subcortical)	Interleukin-16	rs117217798	T	C	0.094	33156215	0.359	0.102	360770	T	C	-0.204	17	31483233	4.15E-06	0.044	3483	21.028
Vascular dementia (subcortical)	Interleukin-16	rs117916513	A	G	-0.053	121393565	0.815	0.224	360770	A	G	-0.502	11	121264274	3.79E-07	0.099	3483	25.921
Vascular dementia (subcortical)	Interleukin-16	rs1255143	T	C	-0.043	128253936	0.448	0.057	360770	T	C	0.131	10	130052200	7.10E-08	0.024	3483	29.124
Vascular dementia (subcortical)	Interleukin-16	rs12765671	A	G	-0.261	104924411	0.415	0.321	360770	A	G	-0.602	10	106684169	4.84E-06	0.132	3483	20.883
Vascular dementia (subcortical)	Interleukin-16	rs144691581	A	G	0.158	96410095	0.412	0.192	360770	A	G	0.488	15	96953325	4.20E-07	0.097	3483	25.488
Vascular dementia (subcortical)	Interleukin-16	rs1801020	G	A	0.099	177409531	0.123	0.064	360770	G	A	-0.173	5	176836532	4.53E-10	0.027	3483	40.594
Vascular dementia (subcortical)	Interleukin-16	rs4253283	C	T	0.054	186244057	0.384	0.062	360770	C	T	-0.146	4	187165211	1.75E-08	0.026	3483	31.053
Vascular dementia (subcortical)	Interleukin-16	rs4513633	A	C	-0.018	112649483	0.869	0.107	360770	A	C	-0.224	4	113570639	7.44E-07	0.045	3483	24.429
Vascular dementia (subcortical)	Interleukin-16	rs4778636	A	G	0.124	81299298	0.366	0.137	360770	A	G	-0.727	15	81591639	1.11E-30	0.063	3483	131.978
Vascular dementia (subcortical)	Interleukin-16	rs9706053	T	C	0.312	65982530	0.162	0.223	360770	T	C	0.458	12	66376310	7.01E-07	0.093	3483	24.170
Vascular dementia (subcortical)	RANTES	rs112072646	A	G	0.488	53217255	0.023	0.215	360770	A	G	0.429	2	53444393	6.48E-07	0.086	3421	24.722
Vascular dementia (subcortical)	RANTES	rs147509526	T	C	-0.068	15665520	0.665	0.157	360770	T	C	-0.358	19	15776330	6.93E-07	0.072	3421	24.930

Vascular dementia (subcortical)	RANTES	rs4940620	G	A	-0.044	64303876	0.729	0.127	360770	G	A	0.249	18	61971111	3.54E-06	0.054	3421	21.331
Vascular dementia (subcortical)	RANTES	rs62438851	G	A	-0.057	144909173	0.544	0.093	360770	G	A	0.196	6	145230309	2.33E-06	0.041	3421	22.345
Vascular dementia (subcortical)	RANTES	rs7000423	T	C	0.117	110041420	0.047	0.059	360770	T	C	-0.132	8	111053649	1.82E-07	0.025	3421	27.139
Vascular dementia (subcortical)	RANTES	rs72793342	A	G	0.041	30537031	0.560	0.070	360770	A	G	-0.149	16	30548352	1.48E-06	0.031	3421	23.309
Vascular dementia (subcortical)	RANTES	rs74472919	T	C	0.242	81626515	0.101	0.148	360770	T	C	0.331	13	82200650	3.97E-08	0.061	3421	29.987
Vascular dementia (subcortical)	RANTES	rs75613039	T	C	0.044	129706688	0.804	0.177	360770	T	C	0.370	11	129576583	4.81E-06	0.081	3421	20.866
Vascular dementia (subcortical)	RANTES	rs818452	T	C	-0.015	152594661	0.897	0.117	360770	T	C	0.238	6	152915796	2.36E-06	0.051	3421	22.230
Vascular dementia (subcortical)	Platelet-derived growth factor BB	rs116445074	T	G	-0.280	52238766	0.186	0.212	360770	T	G	0.293	5	51534600	3.11E-07	0.059	8293	24.932
Vascular dementia (subcortical)	Platelet-derived growth factor BB	rs11766649	G	A	-0.060	145142154	0.394	0.070	360770	G	A	-0.091	7	144839247	3.53E-06	0.020	8293	21.461
Vascular dementia (subcortical)	Platelet-derived growth factor BB	rs11916118	G	A	-0.028	117193342	0.699	0.072	360770	G	A	-0.089	3	116912189	4.93E-06	0.019	8293	20.999
Vascular dementia (subcortical)	Platelet-derived growth factor BB	rs12289510	G	A	0.004	125077155	0.951	0.057	360770	G	A	0.078	11	124947051	7.69E-07	0.016	8293	24.371
Vascular dementia (subcortical)	Platelet-derived growth factor BB	rs13412535	A	G	-0.021	224010157	0.764	0.071	360770	A	G	0.335	2	224874874	2.46E-55	0.021	8293	245.347
Vascular dementia (subcortical)	Platelet-derived growth factor BB	rs2324229	C	T	-0.043	83208412	0.465	0.059	360770	C	T	-0.089	6	83918131	3.48E-08	0.016	8293	30.834
Vascular dementia (subcortical)	Platelet-derived growth factor BB	rs35859699	A	G	0.144	111263595	0.596	0.271	360770	A	G	-0.395	4	112184751	2.07E-06	0.084	8293	22.030
Vascular dementia (subcortical)	Platelet-derived growth factor BB	rs4965869	T	C	0.052	101450115	0.428	0.066	360770	T	C	0.184	15	101990320	5.66E-24	0.018	8293	103.342
Vascular dementia (subcortical)	Platelet-derived growth factor BB	rs55680718	T	C	-0.034	224302160	0.694	0.087	360770	T	C	-0.138	2	225166877	1.86E-08	0.025	8293	31.606
Vascular dementia (subcortical)	Platelet-derived growth factor BB	rs72777070	G	T	0.020	9658748	0.771	0.070	360770	G	T	0.107	2	9798877	8.98E-08	0.020	8293	28.569
Vascular dementia (subcortical)	Platelet-derived growth factor BB	rs73162807	A	C	0.039	146757003	0.832	0.184	360770	A	C	-0.239	3	146474790	1.74E-06	0.050	8293	22.959
Vascular dementia (subcortical)	Platelet-derived growth factor BB	rs9936075	G	A	-0.012	7271908	0.838	0.059	360770	G	A	0.078	16	7321909	1.76E-06	0.016	8293	22.737
Vascular dementia (subcortical)	Platelet-derived growth factor BB	rs9941733	G	A	0.083	393417	0.268	0.075	360770	G	A	-0.116	20	374061	3.31E-07	0.023	8293	25.930
Vascular dementia (subcortical)	Macrophage inflammatory protein 1b	rs11130043	A	G	0.004	45069747	0.948	0.057	360770	A	G	-0.073	3	45111239	3.22E-06	0.016	8243	21.679
Vascular dementia (subcortical)	Macrophage inflammatory protein 1b	rs113010081	C	T	0.096	46415921	0.258	0.085	360770	C	T	0.595	3	46457412	########	0.024	8243	636.493
Vascular dementia (subcortical)	Macrophage inflammatory protein 1b	rs113877493	T	C	-0.074	36443746	0.336	0.077	360770	T	C	-0.612	17	34812273	########	0.022	8243	789.146
Vascular dementia (subcortical)	Macrophage inflammatory protein 1b	rs116237296	A	G	0.844	86579833	0.007	0.314	360770	A	G	0.544	1	87045516	7.23E-07	0.112	8243	23.778
Vascular dementia (subcortical)	Macrophage inflammatory protein 1b	rs117453826	G	A	-0.050	36775624	0.801	0.196	360770	G	A	0.577	17	35132809	5.07E-22	0.059	8243	94.808
Vascular dementia (subcortical)	Macrophage inflammatory protein 1b	rs141102180	T	G	-0.151	36108811	0.309	0.149	360770	T	G	0.323	17	34436204	1.08E-16	0.039	8243	67.340
Vascular dementia (subcortical)	Macrophage inflammatory protein 1b	rs17138331	G	A	0.043	7826737	0.679	0.104	360770	G	A	0.139	7	7866368	2.26E-06	0.030	8243	22.234
Vascular dementia (subcortical)	Macrophage inflammatory protein 1b	rs17641689	G	A	-0.262	36668383	0.006	0.095	360770	G	A	0.245	17	35024819	1.28E-16	0.029	8243	69.805
Vascular dementia (subcortical)	Macrophage inflammatory protein 1b	rs2079664	G	A	-0.033	34680936	0.603	0.064	360770	G	A	-0.100	17	33007955	1.51E-08	0.018	8243	31.961
Vascular dementia (subcortical)	Macrophage inflammatory protein 1b	rs281749	C	T	0.037	107626417	0.552	0.061	360770	C	T	-0.080	8	108638645	3.17E-06	0.017	8243	21.832
Vascular dementia (subcortical)	Macrophage inflammatory protein 1b	rs34437725	C	T	0.096	35499766	0.572	0.171	360770	C	T	0.263	17	33826785	7.67E-08	0.048	8243	29.717
Vascular dementia (subcortical)	Macrophage inflammatory protein 1b	rs72791296	T	C	0.064	121614355	0.675	0.153	360770	T	C	0.237	5	120950050	3.78E-07	0.047	8243	25.844
Vascular dementia (subcortical)	Macrophage inflammatory protein 1b	rs72799710	T	C	-0.035	123825971	0.660	0.079	360770	T	C	-0.101	5	123161665	3.21E-06	0.022	8243	21.635
Vascular dementia (subcortical)	Macrophage inflammatory protein 1b	rs74810984	C	T	-0.211	127876202	0.250	0.183	360770	C	T	-0.221	10	129674466	1.96E-06	0.047	8243	21.660
Vascular dementia (subcortical)	Macrophage inflammatory protein 1b	rs76582507	A	G	0.331	37510075	0.268	0.298	360770	A	G	0.318	9	37510072	3.26E-06	0.068	8243	21.994
Vascular dementia (subcortical)	Macrophage inflammatory protein 1b	rs76583883	T	G	-0.238	45936445	0.123	0.154	360770	T	G	-0.232	21	47356359	4.99E-06	0.051	8243	20.559
Vascular dementia (subcortical)	Macrophage inflammatory protein 1b	rs76776296	G	A	-0.019	115488433	0.922	0.199	360770	G	A	-0.300	7	115128487	5.55E-07	0.060	8243	25.117
Vascular dementia (subcortical)	Macrophage inflammatory protein 1a	rs10835056	G	T	-0.007	26675470	0.904	0.060	360770	G	T	-0.119	11	26697017	2.60E-06	0.025	3522	22.097
Vascular dementia (subcortical)	Macrophage inflammatory protein 1a	rs12690897	A	G	0.057	85716861	0.361	0.062	360770	A	G	0.125	7	85346177	2.11E-06	0.026	3522	22.690
Vascular dementia (subcortical)	Macrophage inflammatory protein 1a	rs184154340	A	G	0.117	80790993	0.433	0.150	360770	A	G	0.331	11	80502036	1.86E-06	0.069	3522	22.813
Vascular dementia (subcortical)	Macrophage inflammatory protein 1a	rs34771762	G	A	0.068	200547932	0.541	0.111	360770	G	A	-0.249	2	201412655	2.13E-06	0.052	3522	22.667
Vascular dementia (subcortical)	Macrophage inflammatory protein 1a	rs57786342	A	G	0.003	68793311	0.969	0.067	360770	A	G	0.131	14	69260028	4.05E-06	0.029	3522	21.257
Vascular dementia (subcortical)	Macrophage inflammatory protein 1a	rs60198979	A	G	0.156	43250698	0.147	0.108	360770	A	G	-0.215	22	43646704	2.61E-06	0.046	3522	21.955
Vascular dementia (subcortical)	Macrophage inflammatory protein 1a	rs7232268	G	A	-0.116	70101678	0.420	0.144	360770	G	A	-0.282	18	67768914	2.55E-06	0.060	3522	22.180
Vascular dementia (subcortical)	Monokine induced by gamma interferon	rs111607343	A	G	0.452	897855	0.073	0.252	360770	A	G	-0.521	19	897855	2.83E-06	0.112	3685	21.678
Vascular dementia (subcortical)	Monokine induced by gamma interferon	rs11177248	A	G	-0.181	68482106	0.210	0.144	360770	A	G	0.307	12	68875886	4.45E-06	0.067	3685	21.037
Vascular dementia (subcortical)	Monokine induced by gamma interferon	rs112337562	G	T	0.089	92665225	0.624	0.181	360770	G	T	0.370	14	93131570	2.98E-06	0.080	3685	21.606
Vascular dementia (subcortical)	Monokine induced by gamma interferon	rs112861654	G	A	0.212	42179062	0.075	0.119	360770	G	A	0.277	21	43599172	1.81E-07	0.053	3685	27.320
Vascular dementia (subcortical)	Monokine induced by gamma interferon	rs117831247	T	C	-0.057	66742081	0.898	0.444	360770	T	C	-0.833	10	68501839	2.16E-06	0.175	3685	22.576
Vascular dementia (subcortical)	Monokine induced by gamma interferon	rs139010077	T	C	-0.179	170618359	0.421	0.222	360770	T	C	0.432	3	170336148	3.55E-06	0.095	3685	20.698
Vascular dementia (subcortical)	Monokine induced by gamma interferon	rs1796086	C	T	0.096	71183729	0.345	0.102	360770	C	T	0.210	7	70648715	2.23E-07	0.040	3685	27.050
Vascular dementia (subcortical)	Monokine induced by gamma interferon	rs41272086	A	G	-0.086	160587614	0.364	0.095	360770	A	G	-0.223	6	161008646	7.43E-08	0.042	3685	28.771
Vascular dementia (subcortical)	Monokine induced by gamma interferon	rs55876513	G	T	0.050	75962545	0.421	0.063	360770	G	T	-0.166	4	76883698	8.23E-11	0.026	3685	42.378
Vascular dementia (subcortical)	Monokine induced by gamma interferon	rs5752128	C	T	0.013	25322656	0.885	0.088	360770	C	T	0.169	22	25718623	4.34E-06	0.037	3685	20.852
Vascular dementia (subcortical)	Monokine induced by gamma interferon	rs62562991	A	G	0.113	95973777	0.637	0.240	360770	A	G	0.624	9	98736059	8.40E-07	0.126	3685	24.495
Vascular dementia (subcortical)	Monokine induced by gamma interferon	rs6679677	A	C	0.013	113761186	0.872	0.082	360770	A	C	0.162	1	114303808	8.86E-07	0.033	3685	24.246
Vascular dementia (subcortical)	Monokine induced by gamma interferon	rs77086208	T	C	-0.378	70152774	0.028	0.172	360770	T	C	0.323	14	70619491	3.83E-06	0.070	3685	21.361
Vascular dementia (subcortical)	Monokine induced by gamma interferon	rs816960	T	C	-0.064	107870173	0.277	0.059	360770	T	C	-0.122	13	108522521	5.01E-07	0.024	3685	25.164
Vascular dementia (subcortical)	Macrophage colony stimulating factor	rs116274860	G	T	0.042	148675030	0.866	0.247	360770	G	T	-0.819	3	148392817	2.74E-06	0.174	840	22.129
Vascular dementia (subcortical)	Macrophage colony stimulating factor	rs117867915	C	T	0.129	44630078	0.520	0.201	360770	C	T	-0.527	18	42210043	1.61E-06	0.110	840	23.054
Vascular dementia (subcortical)	Macrophage colony stimulating factor	rs12962919	T	C	0.055	78018752	0.622	0.111	360770	T	C	0.305	18	75778756	4.65E-06	0.066	840	21.255
Vascular dementia (subcortical)	Macrophage colony stimulating factor	rs56367447	T	C	0.147	4014005	0.364	0.162	360770	T	C	-0.497	8	3871527	1.72E-08	0.088	840	31.642
Vascular dementia (subcortical)	Macrophage colony stimulating factor	rs62294910	A	G	0.060	182480551	0.608	0.116	360770	A	G	0.343	3	182198339	6.82E-07	0.069	840	24.654
Vascular dementia (subcortical)	Macrophage colony stimulating factor	rs78296352	T	G	-0.171	22495351	0.479	0.241	360770	T	G	0.527	1	22821844	1.05E-06	0.111	840	22.460

Vascular dementia (subcortical)	Macrophage colony stimulating factor	rs9387100	C	T	-0.106	112781752	0.066	0.057	360770	C	T	0.135	6	113102954	4.07E-06	0.029	840	21.438
Vascular dementia (subcortical)	Monocyte chemoattractant protein-1	rs10145849	A	G	0.073	82475647	0.221	0.059	360770	A	G	-0.076	14	82941991	3.41E-06	0.016	8293	21.720
Vascular dementia (subcortical)	Monocyte chemoattractant protein-1	rs10744620	C	T	0.085	3629928	0.149	0.059	360770	C	T	-0.079	12	3739094	9.91E-07	0.016	8293	23.955
Vascular dementia (subcortical)	Monocyte chemoattractant protein-1	rs111995966	G	T	-0.058	108558513	0.586	0.106	360770	G	T	-0.145	2	109174969	2.53E-06	0.031	8293	21.939
Vascular dementia (subcortical)	Monocyte chemoattractant protein-1	rs112313229	A	G	0.130	46323369	0.229	0.108	360770	A	G	-0.165	3	46364860	1.43E-07	0.031	8293	27.655
Vascular dementia (subcortical)	Monocyte chemoattractant protein-1	rs12073356	A	G	0.041	207834503	0.710	0.111	360770	A	G	-0.143	1	208007848	4.17E-06	0.031	8293	21.024
Vascular dementia (subcortical)	Monocyte chemoattractant protein-1	rs12075	A	G	0.090	159205564	0.117	0.057	360770	A	G	0.219	1	159175354	1.44E-44	0.016	8293	198.719
Vascular dementia (subcortical)	Monocyte chemoattractant protein-1	rs12493471	C	T	0.037	45910186	0.531	0.059	360770	C	T	-0.116	3	45951678	6.81E-13	0.016	8293	51.538
Vascular dementia (subcortical)	Monocyte chemoattractant protein-1	rs146522229	T	C	0.159	47295223	0.719	0.443	360770	T	C	-0.598	19	47798480	3.56E-07	0.118	8293	25.779
Vascular dementia (subcortical)	Monocyte chemoattractant protein-1	rs2228467	C	T	-0.087	42864624	0.420	0.108	360770	C	T	0.264	3	42906116	9.19E-20	0.029	8293	82.117
Vascular dementia (subcortical)	Monocyte chemoattractant protein-1	rs2712431	A	C	0.059	128598047	0.346	0.063	360770	A	C	-0.079	3	128316890	4.75E-06	0.017	8293	20.936
Vascular dementia (subcortical)	Monocyte chemoattractant protein-1	rs56212190	T	C	0.016	41702868	0.906	0.132	360770	T	C	0.181	1	42168539	9.85E-07	0.037	8293	23.547
Vascular dementia (subcortical)	Monocyte chemoattractant protein-1	rs7197349	G	A	0.134	78653322	0.060	0.071	360770	G	A	-0.097	16	78687219	2.62E-06	0.021	8293	22.081
Vascular dementia (subcortical)	Monocyte chemoattractant protein-1	rs7517040	G	A	-0.051	158889343	0.455	0.069	360770	G	A	0.099	1	158859133	2.44E-07	0.019	8293	26.703
Vascular dementia (subcortical)	Monocyte chemoattractant protein-1	rs9317045	C	A	0.055	59055904	0.489	0.079	360770	C	A	-0.113	13	59630038	1.52E-06	0.024	8293	23.089
Vascular dementia (subcortical)	Interleukin-12p70	rs13209117	A	G	-0.026	44184028	0.691	0.066	360770	A	G	0.100	6	44151765	5.57E-08	0.019	8270	29.021
Vascular dementia (subcortical)	Interleukin-12p70	rs17229494	G	A	-0.035	37555798	0.703	0.091	360770	G	A	0.117	21	38928100	4.93E-06	0.026	8270	20.796
Vascular dementia (subcortical)	Interleukin-12p70	rs282258	C	T	0.089	224050083	0.121	0.057	360770	C	T	-0.073	2	224914800	3.21E-06	0.016	8270	21.898
Vascular dementia (subcortical)	Interleukin-12p70	rs41282644	A	G	-0.111	43785985	0.285	0.104	360770	A	G	0.147	6	43753722	1.05E-06	0.030	8270	23.478
Vascular dementia (subcortical)	Interleukin-12p70	rs4349809	G	T	0.000	43957093	0.996	0.057	360770	G	T	-0.378	6	43924830	########	0.016	8270	564.287
Vascular dementia (subcortical)	Interleukin-12p70	rs71361173	G	T	-0.066	76000450	0.439	0.086	360770	G	T	-0.111	18	73712405	3.06E-06	0.024	8270	21.570
Vascular dementia (subcortical)	Interleukin-12p70	rs72831623	A	G	-0.141	47644927	0.213	0.113	360770	A	G	0.191	17	45722293	2.42E-07	0.037	8270	26.732
Vascular dementia (subcortical)	Interleukin-12p70	rs782107	A	G	0.012	58439747	0.836	0.057	360770	A	G	0.075	12	58833530	1.60E-06	0.016	8270	23.114
Vascular dementia (subcortical)	Interleukin-12p70	rs79121401	C	T	0.605	78986084	0.145	0.415	360770	C	T	-0.555	11	78697129	4.24E-06	0.121	8270	21.163
Vascular dementia (subcortical)	Interleukin-12p70	rs9472183	G	A	0.085	43972465	0.137	0.057	360770	G	A	-0.102	6	43940202	8.61E-11	0.016	8270	42.126
Vascular dementia (subcortical)	Interferon gamma-induced protein 10	rs10809307	C	T	0.025	11045908	0.714	0.069	360770	C	T	-0.131	9	11045908	3.64E-06	0.028	3685	21.415
Vascular dementia (subcortical)	Interferon gamma-induced protein 10	rs113831257	A	G	-0.048	75234311	0.717	0.131	360770	A	G	0.359	4	76159521	2.53E-08	0.064	3685	31.110
Vascular dementia (subcortical)	Interferon gamma-induced protein 10	rs11626201	A	C	0.029	36511495	0.615	0.059	360770	A	C	0.116	14	36980700	1.93E-06	0.025	3685	22.495
Vascular dementia (subcortical)	Interferon gamma-induced protein 10	rs143799975	G	A	-0.317	75885862	0.352	0.341	360770	G	A	0.798	4	76807015	1.00E-06	0.164	3685	23.787
Vascular dementia (subcortical)	Interferon gamma-induced protein 10	rs34383175	T	C	-0.128	144361034	0.406	0.154	360770	T	C	-0.315	8	145584694	1.51E-06	0.066	3685	23.031
Vascular dementia (subcortical)	Interferon gamma-induced protein 10	rs75970138	A	G	0.545	119813998	0.035	0.258	360770	A	G	-0.485	9	122576276	1.53E-06	0.104	3685	21.748
Vascular dementia (subcortical)	Interferon gamma-induced protein 10	rs7645625	G	T	-0.066	146856250	0.256	0.058	360770	G	T	0.109	3	146574037	4.41E-06	0.024	3685	20.997
Vascular dementia (subcortical)	Interferon gamma-induced protein 10	rs79848609	C	A	-0.022	86772934	0.869	0.132	360770	C	A	-0.260	15	87316165	8.75E-07	0.054	3685	23.496
Vascular dementia (subcortical)	Interferon gamma-induced protein 10	rs8112909	A	G	0.036	45910150	0.605	0.070	360770	A	G	-0.143	19	46413408	1.94E-06	0.030	3685	22.746
Vascular dementia (subcortical)	Interleukin-18	rs10414578	T	C	0.104	54634619	0.210	0.083	360770	T	C	-0.177	19	55146070	4.16E-07	0.035	3636	25.604
Vascular dementia (subcortical)	Interleukin-18	rs115267715	T	C	-0.369	69239188	0.088	0.216	360770	T	C	0.451	5	68535015	1.72E-08	0.080	3636	31.753
Vascular dementia (subcortical)	Interleukin-18	rs116383510	C	A	-0.034	2545536	0.889	0.242	360770	C	A	0.543	5	2545650	3.00E-07	0.106	3636	26.402
Vascular dementia (subcortical)	Interleukin-18	rs117266781	T	C	0.007	41261422	0.982	0.312	360770	T	C	0.684	7	41301020	3.15E-06	0.147	3636	21.716
Vascular dementia (subcortical)	Interleukin-18	rs144841621	T	C	0.094	69921801	0.742	0.286	360770	T	C	0.518	10	71681557	3.81E-06	0.114	3636	20.610
Vascular dementia (subcortical)	Interleukin-18	rs17229943	C	A	0.029	69386709	0.764	0.095	360770	C	A	0.312	5	68682536	1.62E-11	0.046	3636	45.410
Vascular dementia (subcortical)	Interleukin-18	rs1852105	C	T	-0.003	64265217	0.987	0.155	360770	C	T	-0.304	7	63725595	4.32E-06	0.066	3636	21.096
Vascular dementia (subcortical)	Interleukin-18	rs1979967	T	C	0.000	79367271	0.999	0.069	360770	T	C	0.140	15	79659613	9.45E-07	0.029	3636	24.031
Vascular dementia (subcortical)	Interleukin-18	rs2729385	A	G	-0.135	57495520	0.031	0.063	360770	A	G	0.123	11	57262993	3.79E-06	0.026	3636	22.076
Vascular dementia (subcortical)	Interleukin-18	rs385076	C	T	0.030	32264782	0.615	0.059	360770	C	T	0.243	2	32489851	1.66E-22	0.025	3636	96.166
Vascular dementia (subcortical)	Interleukin-18	rs4482818	G	A	0.057	65062779	0.325	0.058	360770	G	A	-0.129	4	65928497	1.45E-07	0.024	3636	27.778
Vascular dementia (subcortical)	Interleukin-18	rs658805	A	G	0.038	70199369	0.525	0.060	360770	A	G	0.123	6	70909073	4.94E-07	0.024	3636	25.247
Vascular dementia (subcortical)	Interleukin-18	rs71478720	T	C	0.024	112138882	0.715	0.066	360770	T	C	-0.267	11	112009605	3.71E-22	0.028	3636	93.515
Vascular dementia (subcortical)	Interleukin-18	rs78623212	T	C	0.164	103667180	0.620	0.330	360770	T	C	0.871	7	103307627	6.71E-07	0.178	3636	23.970
Vascular dementia (subcortical)	Interleukin-18	rs78716465	A	G	-0.022	42015086	0.886	0.151	360770	A	G	0.327	20	40643726	1.63E-06	0.068	3636	22.919
Vascular dementia (subcortical)	Interleukin-17	rs117029961	A	G	0.068	37147653	0.831	0.319	360770	A	G	0.459	10	37436581	4.94E-06	0.102	7760	20.405
Vascular dementia (subcortical)	Interleukin-17	rs117556572	T	C	-0.009	104436567	0.961	0.185	360770	T	C	-0.510	13	105088917	3.28E-06	0.110	7760	21.552
Vascular dementia (subcortical)	Interleukin-17	rs1530455	C	T	-0.077	123136052	0.193	0.059	360770	C	T	-0.108	3	122854899	4.87E-10	0.017	7760	38.972
Vascular dementia (subcortical)	Interleukin-17	rs17106604	T	C	-0.017	77912813	0.836	0.080	360770	T	C	0.113	14	78379156	6.37E-07	0.023	7760	25.178
Vascular dementia (subcortical)	Interleukin-17	rs17282552	C	T	-0.091	207109091	0.481	0.129	360770	C	T	0.200	2	207973815	8.21E-07	0.041	7760	24.411
Vascular dementia (subcortical)	Interleukin-17	rs184080173	C	T	0.034	77331424	0.828	0.158	360770	C	T	-0.238	12	77725204	4.19E-07	0.047	7760	25.620
Vascular dementia (subcortical)	Interleukin-17	rs187475560	T	C	-0.140	160353411	0.409	0.169	360770	T	C	-0.243	4	161274563	3.29E-06	0.052	7760	21.910
Vascular dementia (subcortical)	Interleukin-17	rs62191444	T	G	0.089	393023	0.261	0.079	360770	T	G	-0.114	20	373667	4.22E-06	0.025	7760	21.153
Vascular dementia (subcortical)	Interleukin-17	rs78296352	T	G	-0.171	22495351	0.479	0.241	360770	T	G	0.303	1	22821844	4.27E-06	0.065	7760	21.956
Vascular dementia (subcortical)	Interleukin-17	rs78612928	C	T	0.029	29812292	0.709	0.077	360770	C	T	-0.104	4	29813914	2.62E-06	0.022	7760	21.820
Vascular dementia (subcortical)	Interleukin-13	rs117795020	A	G	-0.091	87469237	0.586	0.167	360770	A	G	-0.352	9	90084152	9.86E-07	0.072	3557	24.197
Vascular dementia (subcortical)	Interleukin-13	rs12623722	A	G	-0.088	22955811	0.156	0.062	360770	A	G	-0.119	2	23178683	4.19E-06	0.026	3557	21.096
Vascular dementia (subcortical)	Interleukin-13	rs139083458	T	C	0.267	26160409	0.584	0.487	360770	T	C	0.990	5	26160518	2.81E-06	0.211	3557	22.086
Vascular dementia (subcortical)	Interleukin-13	rs142167313	C	T	0.004	44204360	0.979	0.143	360770	C	T	0.313	6	44172097	3.98E-07	0.062	3557	25.735
Vascular dementia (subcortical)	Interleukin-13	rs27949	T	C	0.042	59254997	0.488	0.061	360770	T	C	-0.117	5	58550823	3.43E-06	0.025	3557	21.482

Vascular dementia (subcortical)	Interleukin-13	rs6799107	C	T	0.063	127338175	0.386	0.073	360770	C	T	0.146	3	127057018	1.25E-06	0.030	3557	23.495
Vascular dementia (subcortical)	Interleukin-13	rs7073807	C	T	0.136	67393670	0.103	0.084	360770	C	T	-0.168	10	69153428	2.37E-06	0.036	3557	22.323
Vascular dementia (subcortical)	Interleukin-13	rs75995699	A	G	0.159	5140622	0.315	0.158	360770	A	G	0.332	6	5140856	2.64E-06	0.070	3557	22.610
Vascular dementia (subcortical)	Interleukin-13	rs9472168	G	A	0.002	43961248	0.973	0.057	360770	G	A	-0.424	6	43928985	1.08E-65	0.025	3557	292.851
Vascular dementia (subcortical)	Interleukin-10	rs10457128	A	G	-0.009	105570101	0.880	0.059	360770	A	G	-0.087	6	106017976	5.24E-07	0.017	7681	25.292
Vascular dementia (subcortical)	Interleukin-10	rs10493718	A	C	0.128	82597250	0.101	0.078	360770	A	C	-0.110	1	83062933	7.16E-07	0.022	7681	24.552
Vascular dementia (subcortical)	Interleukin-10	rs11206302	T	C	-0.003	54208270	0.971	0.087	360770	T	C	-0.119	1	54673943	2.20E-06	0.025	7681	22.440
Vascular dementia (subcortical)	Interleukin-10	rs2086656	T	C	0.009	59632755	0.883	0.060	360770	T	C	-0.079	4	60498473	3.78E-06	0.017	7681	21.289
Vascular dementia (subcortical)	Interleukin-10	rs282258	C	T	0.089	224050083	0.121	0.057	360770	C	T	-0.099	2	224914800	1.00E-09	0.016	7681	37.497
Vascular dementia (subcortical)	Interleukin-10	rs3025021	C	T	-0.016	43781426	0.793	0.060	360770	C	T	-0.095	6	43749163	1.46E-06	0.020	7681	23.585
Vascular dementia (subcortical)	Interleukin-10	rs41282660	G	A	-0.148	44229269	0.073	0.083	360770	G	A	0.119	6	44197006	3.72E-06	0.026	7681	21.924
Vascular dementia (subcortical)	Interleukin-10	rs4349809	G	T	0.000	43957093	0.996	0.057	360770	G	T	-0.285	6	43924830	5.77E-67	0.017	7681	298.976
Vascular dementia (subcortical)	Interleukin-10	rs465757	A	G	-0.006	15599638	0.914	0.059	360770	A	G	0.084	20	15580283	1.17E-06	0.017	7681	23.306
Vascular dementia (subcortical)	Interleukin-10	rs7088799	G	T	-0.076	63256414	0.195	0.058	360770	G	T	0.085	10	65016174	3.23E-07	0.017	7681	26.028
Vascular dementia (subcortical)	Interleukin-8	rs11634944	C	T	0.057	24937946	0.339	0.060	360770	C	T	0.121	15	25183093	1.29E-06	0.025	3526	23.208
Vascular dementia (subcortical)	Interleukin-8	rs12075	A	G	0.090	159205564	0.117	0.057	360770	A	G	0.120	1	159175354	3.88E-07	0.024	3526	25.855
Vascular dementia (subcortical)	Interleukin-8	rs141926526	C	A	-0.335	32809028	0.297	0.321	360770	C	A	0.615	7	32848640	2.57E-06	0.131	3526	22.100
Vascular dementia (subcortical)	Interleukin-8	rs2673604	A	C	-0.125	132399360	0.042	0.061	360770	A	C	-0.127	8	133411607	7.02E-07	0.026	3526	24.648
Vascular dementia (subcortical)	Interleukin-6	rs1333040	T	C	-0.114	22083405	0.044	0.057	360770	T	C	0.074	9	22083404	3.17E-06	0.016	8189	21.817
Vascular dementia (subcortical)	Interleukin-6	rs13412535	A	G	-0.021	224010157	0.764	0.071	360770	A	G	-0.116	2	224874874	7.34E-08	0.022	8189	29.311
Vascular dementia (subcortical)	Interleukin-6	rs72831623	A	G	-0.141	47644927	0.213	0.113	360770	A	G	0.197	17	45722293	1.08E-07	0.037	8189	28.130
Vascular dementia (subcortical)	Interleukin-6	rs73273528	T	C	0.084	51814574	0.656	0.188	360770	T	C	0.267	20	50431113	9.58E-07	0.055	8189	23.347
Vascular dementia (subcortical)	Interleukin-6	rs76856708	C	T	0.121	80695146	0.637	0.257	360770	C	T	-0.329	16	80729043	2.61E-06	0.070	8189	22.077
Vascular dementia (subcortical)	Interleukin-1-receptor antagonist	rs1054402	C	T	0.021	116401230	0.753	0.066	360770	C	T	-0.131	9	119163509	1.13E-06	0.027	3638	23.576
Vascular dementia (subcortical)	Interleukin-1-receptor antagonist	rs11627423	C	A	-0.076	32731417	0.195	0.059	360770	C	A	-0.117	14	33200623	2.12E-06	0.025	3638	22.476
Vascular dementia (subcortical)	Interleukin-1-receptor antagonist	rs12121840	T	C	0.122	165572405	0.343	0.129	360770	T	C	0.269	1	165541642	2.43E-06	0.057	3638	22.227
Vascular dementia (subcortical)	Interleukin-1-receptor antagonist	rs2809154	T	C	0.044	84153389	0.644	0.094	360770	T	C	-0.179	13	84727524	3.74E-06	0.039	3638	21.188
Vascular dementia (subcortical)	Interleukin-1-receptor antagonist	rs61335305	A	C	-0.042	66160736	0.841	0.211	360770	A	C	0.445	15	66453074	1.00E-06	0.091	3638	24.051
Vascular dementia (subcortical)	Interleukin-1-receptor antagonist	rs9623661	T	C	0.049	42697370	0.627	0.100	360770	T	C	-0.197	22	43093376	3.86E-06	0.043	3638	21.298
Vascular dementia (subcortical)	Interleukin-1-beta	rs143319329	T	C	-0.557	128499405	0.109	0.348	360770	T	C	0.280	7	128139459	2.00E-06	0.072	3309	15.347
Vascular dementia (subcortical)	Interleukin-1-beta	rs61335305	A	C	-0.042	66160736	0.841	0.211	360770	A	C	0.297	15	66453074	1.90E-06	0.072	3309	16.783
Vascular dementia (subcortical)	Interleukin-1-beta	rs62015704	G	A	-0.085	7417906	0.327	0.087	360770	G	A	-0.108	16	7467907	2.09E-06	0.028	3309	14.618
Vascular dementia (subcortical)	Interleukin-1-beta	rs9898641	C	T	0.005	59493672	0.936	0.059	360770	C	T	0.203	17	57571033	3.59E-06	0.045	3309	20.033
Vascular dementia (subcortical)	Hepatocyte growth factor	rs11060254	A	G	-0.041	129331024	0.506	0.061	360770	A	G	-0.080	12	129815569	1.58E-06	0.017	8292	22.948
Vascular dementia (subcortical)	Hepatocyte growth factor	rs150322232	G	A	-0.105	7890743	0.485	0.150	360770	G	A	-0.210	7	7930374	4.89E-06	0.046	8292	20.650
Vascular dementia (subcortical)	Hepatocyte growth factor	rs1698249	C	A	-0.178	83889842	0.193	0.137	360770	C	A	0.170	14	84356186	4.09E-06	0.037	8292	20.835
Vascular dementia (subcortical)	Hepatocyte growth factor	rs2003620	T	C	0.035	134794733	0.835	0.167	360770	T	C	0.228	7	134479484	2.83E-06	0.049	8292	21.721
Vascular dementia (subcortical)	Hepatocyte growth factor	rs3748034	T	G	0.031	3444364	0.723	0.086	360770	T	G	0.150	4	3446091	1.81E-10	0.023	8292	40.818
Vascular dementia (subcortical)	Hepatocyte growth factor	rs5745687	T	C	-0.246	81729735	0.085	0.143	360770	T	C	-0.307	7	81359051	2.75E-14	0.041	8292	57.252
Vascular dementia (subcortical)	Hepatocyte growth factor	rs62481625	C	T	-0.059	156194766	0.461	0.080	360770	C	T	-0.109	7	155987460	1.18E-06	0.023	8292	23.512
Vascular dementia (subcortical)	Interleukin-9	rs41294750	T	C	-0.058	53084968	0.728	0.167	360770	T	C	0.351	1	53550640	2.36E-06	0.075	3634	22.070
Vascular dementia (subcortical)	Interleukin-9	rs4880409	T	C	-0.019	132516716	0.946	0.281	360770	T	C	-0.336	10	134330220	3.50E-06	0.072	3634	21.533
Vascular dementia (subcortical)	Interleukin-9	rs61867538	T	C	0.122	1503276	0.388	0.141	360770	T	C	0.357	11	1524506	3.93E-06	0.077	3634	21.227
Vascular dementia (subcortical)	Interleukin-9	rs7232268	G	A	-0.116	70101678	0.420	0.144	360770	G	A	-0.276	18	67768914	2.52E-06	0.059	3634	22.092
Vascular dementia (subcortical)	Interleukin-9	rs7242404	A	G	-0.001	12741268	0.989	0.063	360770	A	G	-0.123	18	12741267	3.27E-06	0.026	3634	21.637
Vascular dementia (subcortical)	Interleukin-9	rs76963786	T	C	0.252	31886823	0.068	0.138	360770	T	C	-0.287	12	32039757	4.50E-07	0.056	3634	26.457
Vascular dementia (subcortical)	Interleukin-7	rs117509142	C	T	0.305	86121854	0.059	0.162	360770	C	T	0.327	8	87134083	1.99E-06	0.069	3409	22.590
Vascular dementia (subcortical)	Interleukin-7	rs141425475	C	T	-0.250	17679056	0.218	0.203	360770	C	T	0.478	5	17679165	2.53E-06	0.102	3409	22.144
Vascular dementia (subcortical)	Interleukin-7	rs144701438	A	G	0.065	66293168	0.780	0.234	360770	A	G	-0.482	18	63960405	9.75E-07	0.099	3409	23.742
Vascular dementia (subcortical)	Interleukin-7	rs17091524	C	T	-0.232	56482041	0.307	0.228	360770	C	T	-0.492	14	56948759	1.91E-06	0.101	3409	23.627
Vascular dementia (subcortical)	Interleukin-7	rs28793375	T	C	0.193	41558099	0.020	0.083	360770	T	C	0.164	8	41415618	4.46E-06	0.036	3409	20.588
Vascular dementia (subcortical)	Interleukin-7	rs4320361	T	G	-0.002	43960774	0.977	0.057	360770	T	G	-0.325	6	43928511	6.87E-39	0.025	3409	169.836
Vascular dementia (subcortical)	Interleukin-7	rs62006410	T	C	0.032	102541598	0.629	0.066	360770	T	C	-0.156	14	103007935	3.39E-07	0.030	3409	26.405
Vascular dementia (subcortical)	Interleukin-7	rs75904417	C	A	0.024	167796811	0.766	0.081	360770	C	A	0.170	2	168653321	1.16E-06	0.035	3409	23.671
Vascular dementia (subcortical)	Interleukin-7	rs77981494	C	T	-0.160	17451009	0.418	0.197	360770	C	T	0.518	16	17544866	1.07E-06	0.106	3409	23.683
Vascular dementia (subcortical)	Interleukin-7	rs78346957	A	G	-0.048	125214944	0.858	0.268	360770	A	G	0.459	10	126903513	4.51E-06	0.101	3409	20.758
Vascular dementia (subcortical)	Interleukin-5	rs11680908	G	A	-0.043	109460295	0.735	0.128	360770	G	A	-0.263	2	110076751	2.03E-06	0.055	3364	22.605
Vascular dementia (subcortical)	Interleukin-5	rs6737109	C	T	-0.036	22956659	0.531	0.057	360770	C	T	-0.116	2	23179531	2.40E-06	0.025	3364	22.056
Vascular dementia (subcortical)	Interleukin-5	rs72831687	A	G	0.237	16092129	0.279	0.219	360770	A	G	-0.524	6	16092360	1.69E-06	0.111	3364	22.317
Vascular dementia (subcortical)	Interleukin-5	rs73040130	C	T	0.142	36255288	0.244	0.122	360770	C	T	-0.264	19	36746190	6.00E-07	0.053	3364	24.868
Vascular dementia (subcortical)	Interleukin-5	rs7767396	G	A	-0.002	43959313	0.975	0.057	360770	G	A	-0.152	6	43927050	7.69E-10	0.025	3364	37.928
Vascular dementia (subcortical)	Interleukin-4	rs10512267	C	T	0.020	99427847	0.726	0.057	360770	C	T	0.082	9	102190129	2.94E-07	0.016	8124	26.194
Vascular dementia (subcortical)	Interleukin-4	rs116705532	G	T	-0.103	113162547	0.764	0.343	360770	G	T	0.468	1	113705169	1.76E-06	0.098	8124	22.879
Vascular dementia (subcortical)	Interleukin-4	rs117146485	C	T	0.168	135932411	0.437	0.215	360770	C	T	0.292	9	138824257	2.71E-06	0.063	8124	21.610

Vascular dementia (subcortical)	Interleukin-4	rs17713451	A	G	0.053	151465386	0.553	0.090	360770	A	G	0.127	7	151162472	4.97E-07	0.025	8124	25.357
Vascular dementia (subcortical)	Interleukin-4	rs73023729	A	G	-0.088	159232998	0.526	0.139	360770	A	G	-0.180	6	159654030	9.03E-07	0.037	8124	24.080
Vascular dementia (subcortical)	Interleukin-4	rs7613691	G	A	0.087	147935804	0.504	0.130	360770	G	A	-0.178	3	147653591	4.05E-06	0.038	8124	21.367
Vascular dementia (subcortical)	Interleukin-4	rs9508291	C	T	-0.137	29136483	0.288	0.129	360770	C	T	0.168	13	29710620	3.03E-06	0.036	8124	21.795
Vascular dementia (subcortical)	Interleukin-4	rs9941733	G	A	0.083	393417	0.268	0.075	360770	G	A	-0.114	20	374061	6.88E-07	0.023	8124	24.782
Vascular dementia (subcortical)	Interleukin-2 receptor antagonist	rs11241559	G	T	-0.033	120641005	0.614	0.065	360770	G	T	0.126	5	119976700	2.00E-06	0.027	3677	22.580
Vascular dementia (subcortical)	Interleukin-2 receptor antagonist	rs117244812	A	G	-0.021	6539990	0.951	0.335	360770	A	G	-0.706	17	6443310	2.10E-06	0.149	3677	22.537
Vascular dementia (subcortical)	Interleukin-2 receptor antagonist	rs12722497	A	C	0.061	6053965	0.590	0.114	360770	A	C	0.628	10	6095928	1.57E-38	0.049	3677	167.609
Vascular dementia (subcortical)	Interleukin-2 receptor antagonist	rs185231391	C	T	0.307	59373953	0.382	0.352	360770	C	T	-0.850	3	59359679	1.47E-06	0.181	3677	22.094
Vascular dementia (subcortical)	Interleukin-2 receptor antagonist	rs4733117	C	A	0.033	32280094	0.635	0.070	360770	C	A	-0.137	8	32137610	2.63E-06	0.029	3677	21.981
Vascular dementia (subcortical)	Interleukin-2 receptor antagonist	rs61705228	T	C	-0.176	100275145	0.253	0.154	360770	T	C	0.330	4	101196302	3.99E-06	0.072	3677	21.281
Vascular dementia (subcortical)	Interleukin-2	rs12051139	C	T	-0.032	86885068	0.574	0.058	360770	C	T	0.113	16	86918674	4.76E-06	0.025	3475	20.967
Vascular dementia (subcortical)	Interleukin-2	rs13412535	A	G	-0.021	224010157	0.764	0.071	360770	A	G	0.176	2	224874874	1.18E-07	0.033	3475	28.231
Vascular dementia (subcortical)	Interleukin-2	rs170117	T	C	-0.071	54524213	0.379	0.081	360770	T	C	-0.162	4	55390380	3.87E-06	0.035	3475	21.467
Vascular dementia (subcortical)	Interleukin-2	rs2807544	G	A	0.073	14877749	0.214	0.059	360770	G	A	-0.118	1	15204245	3.41E-06	0.025	3475	21.569
Vascular dementia (subcortical)	Interleukin-2	rs4634519	G	A	-0.034	67727941	0.595	0.064	360770	G	A	0.126	7	67192928	2.77E-06	0.027	3475	21.975
Vascular dementia (subcortical)	Interleukin-2	rs61335305	A	C	-0.042	66160736	0.841	0.211	360770	A	C	0.451	15	66453074	7.32E-07	0.092	3475	24.179
Vascular dementia (subcortical)	Interleukin-2	rs62124990	T	G	0.002	19038882	0.990	0.204	360770	T	G	-0.696	2	19238636	3.22E-06	0.150	3475	21.680
Vascular dementia (subcortical)	Interleukin-2	rs7615304	G	A	-0.014	156957914	0.804	0.057	360770	G	A	0.117	3	156675703	1.21E-06	0.024	3475	23.454
Vascular dementia (subcortical)	Interleukin-2	rs80336398	C	T	0.072	64075258	0.689	0.181	360770	C	T	-0.400	3	64060934	2.82E-06	0.086	3475	21.745
Vascular dementia (subcortical)	Interferon gamma	rs10487554	A	G	-0.044	149670595	0.481	0.063	360770	A	G	-0.090	7	149367686	1.09E-06	0.018	7701	23.919
Vascular dementia (subcortical)	Interferon gamma	rs113600793	A	C	-0.182	47384095	0.136	0.122	360770	A	C	0.183	17	45461461	8.95E-07	0.037	7701	24.044
Vascular dementia (subcortical)	Interferon gamma	rs115729819	G	A	-0.005	168783516	0.977	0.177	360770	G	A	-0.248	4	169704667	1.38E-06	0.052	7701	23.264
Vascular dementia (subcortical)	Interferon gamma	rs11843756	G	T	0.048	48680756	0.739	0.144	360770	G	T	-0.184	13	49254892	3.09E-06	0.039	7701	21.921
Vascular dementia (subcortical)	Interferon gamma	rs12420286	C	T	0.136	103907166	0.427	0.171	360770	C	T	-0.238	11	103777894	2.08E-06	0.050	7701	22.491
Vascular dementia (subcortical)	Interferon gamma	rs1867282	T	C	-0.006	99409865	0.914	0.058	360770	T	C	0.077	9	102172147	3.15E-06	0.017	7701	21.740
Vascular dementia (subcortical)	Interferon gamma	rs2073438	A	G	0.076	6996757	0.241	0.065	360770	A	G	0.090	17	6900076	1.68E-06	0.019	7701	22.816
Vascular dementia (subcortical)	Interferon gamma	rs74148555	T	C	-0.022	90320085	0.912	0.198	360770	T	C	-0.373	10	92079842	2.64E-06	0.077	7701	23.249
Vascular dementia (subcortical)	Interferon gamma	rs78296352	T	G	-0.171	22495351	0.479	0.241	360770	T	G	0.343	1	22821844	1.38E-07	0.065	7701	27.675
Vascular dementia (subcortical)	Growth-regulated protein alpha	rs1113500	T	G	0.038	108052820	0.505	0.058	360770	T	G	0.117	1	108595442	1.57E-06	0.024	3505	23.150
Vascular dementia (subcortical)	Growth-regulated protein alpha	rs12075	A	G	0.090	159205564	0.117	0.057	360770	A	G	0.375	1	159175354	1.24E-55	0.024	3505	250.494
Vascular dementia (subcortical)	Growth-regulated protein alpha	rs140734053	A	G	-0.028	5359496	0.929	0.317	360770	A	G	0.726	10	5401459	3.58E-06	0.156	3505	21.613
Vascular dementia (subcortical)	Growth-regulated protein alpha	rs185768063	G	A	-0.179	16494752	0.289	0.169	360770	G	A	-0.400	6	16494983	1.46E-07	0.076	3505	27.673
Vascular dementia (subcortical)	Growth-regulated protein alpha	rs188345231	T	C	0.312	41579831	0.333	0.322	360770	T	C	0.623	8	41437350	4.34E-06	0.132	3505	22.175
Vascular dementia (subcortical)	Growth-regulated protein alpha	rs2422841	A	G	-0.005	3099706	0.953	0.082	360770	A	G	-0.166	20	3080352	4.66E-06	0.036	3505	21.068
Vascular dementia (subcortical)	Growth-regulated protein alpha	rs508977	G	T	0.030	73896666	0.654	0.066	360770	G	T	0.380	4	74762383	7.56E-42	0.028	3505	184.378
Vascular dementia (subcortical)	Growth-regulated protein alpha	rs62024303	G	A	-0.031	88327931	0.828	0.141	360770	G	A	0.305	15	88871162	4.41E-06	0.067	3505	21.014
Vascular dementia (subcortical)	Growth-regulated protein alpha	rs78653452	T	G	-0.337	9781407	0.251	0.293	360770	T	G	-0.736	20	9762055	1.21E-06	0.156	3505	22.328
Vascular dementia (subcortical)	Granulocyte-colony stimulating factor	rs115256310	G	A	0.331	72103864	0.336	0.344	360770	G	A	0.682	5	71399691	6.73E-07	0.136	7904	25.155
Vascular dementia (subcortical)	Granulocyte-colony stimulating factor	rs11903143	G	A	0.119	29369594	0.058	0.063	360770	G	A	-0.087	2	29592460	6.35E-07	0.018	7904	24.435
Vascular dementia (subcortical)	Granulocyte-colony stimulating factor	rs147128865	T	C	0.336	34972769	0.079	0.191	360770	T	C	0.270	9	34972766	4.92E-06	0.059	7904	21.157
Vascular dementia (subcortical)	Granulocyte-colony stimulating factor	rs1817411	T	C	-0.109	97586100	0.116	0.069	360770	T	C	0.089	8	98598328	3.10E-06	0.019	7904	21.713
Vascular dementia (subcortical)	Granulocyte-colony stimulating factor	rs2671444	A	G	-0.034	101158297	0.572	0.059	360770	A	G	-0.078	12	101552075	2.48E-06	0.017	7904	22.306
Vascular dementia (subcortical)	Granulocyte-colony stimulating factor	rs74148555	T	C	-0.022	90320085	0.912	0.198	360770	T	C	-0.372	10	92079842	1.55E-06	0.076	7904	24.212
Vascular dementia (subcortical)	Granulocyte-colony stimulating factor	rs77318030	C	T	0.121	54544688	0.393	0.142	360770	C	T	0.205	19	55055897	2.21E-06	0.043	7904	22.830
Vascular dementia (subcortical)	Fibroblast growth factor basic	rs13412535	A	G	-0.021	224010157	0.764	0.071	360770	A	G	-0.111	2	224874874	7.34E-07	0.023	7565	24.426
Vascular dementia (subcortical)	Fibroblast growth factor basic	rs145577605	A	G	0.033	27642232	0.834	0.155	360770	A	G	0.208	6	27610011	9.64E-07	0.043	7565	23.640
Vascular dementia (subcortical)	Fibroblast growth factor basic	rs747334	G	A	-0.070	90984987	0.218	0.057	360770	G	A	-0.075	10	92744744	4.53E-06	0.016	7565	20.970
Vascular dementia (subcortical)	Fibroblast growth factor basic	rs75168112	C	T	0.032	73418832	0.654	0.072	360770	C	T	0.100	18	71086067	3.00E-06	0.021	7565	21.880
Vascular dementia (subcortical)	Fibroblast growth factor basic	rs9907295	T	C	0.071	35930309	0.440	0.092	360770	T	C	-0.132	17	34257313	7.95E-07	0.027	7565	24.043
Vascular dementia (subcortical)	Eotaxin	rs11087905	A	C	0.036	24133015	0.552	0.060	360770	A	C	0.094	21	25505329	5.48E-07	0.019	8153	24.789
Vascular dementia (subcortical)	Eotaxin	rs112347425	T	C	-0.096	46419397	0.342	0.101	360770	T	C	0.158	3	46460888	8.65E-09	0.028	8153	32.535
Vascular dementia (subcortical)	Eotaxin	rs12075	A	G	0.090	159205564	0.117	0.057	360770	A	G	0.167	1	159175354	1.33E-26	0.016	8153	114.737
Vascular dementia (subcortical)	Eotaxin	rs1476670	C	A	0.039	44042523	0.613	0.077	360770	C	A	0.101	1	44508195	3.51E-06	0.022	8153	21.535
Vascular dementia (subcortical)	Eotaxin	rs2024050	G	A	-0.001	75831075	0.993	0.111	360770	G	A	-0.173	7	75460393	1.10E-08	0.030	8153	32.524
Vascular dementia (subcortical)	Eotaxin	rs2210755	C	T	-0.011	77608907	0.896	0.082	360770	C	T	0.110	9	80223823	4.85E-06	0.024	8153	20.812
Vascular dementia (subcortical)	Eotaxin	rs2211994	C	T	-0.037	16675274	0.557	0.064	360770	C	T	-0.089	21	18047593	6.08E-07	0.018	8153	25.000
Vascular dementia (subcortical)	Eotaxin	rs2228467	C	T	-0.087	42864624	0.420	0.108	360770	C	T	0.416	3	42906116	2.27E-46	0.029	8153	203.258
Vascular dementia (subcortical)	Eotaxin	rs2419841	C	T	-0.111	113576224	0.292	0.105	360770	C	T	0.128	10	115335983	4.98E-06	0.028	8153	20.949
Vascular dementia (subcortical)	Eotaxin	rs5746492	G	A	0.001	17911167	0.991	0.076	360770	G	A	-0.095	22	18393933	3.96E-06	0.021	8153	21.240
Vascular dementia (subcortical)	Eotaxin	rs5754733	A	C	-0.017	33873606	0.820	0.075	360770	A	C	-0.104	22	34269594	1.06E-06	0.021	8153	23.709
Vascular dementia (subcortical)	Eotaxin	rs59808887	T	C	0.105	31846414	0.354	0.114	360770	T	C	-0.167	5	31846520	2.91E-06	0.036	8153	21.839
Vascular dementia (subcortical)	Eotaxin	rs75426604	A	C	0.153	35388508	0.118	0.098	360770	A	C	-0.137	14	35857714	2.53E-06	0.029	8153	22.035
Vascular dementia (subcortical)	Eotaxin	rs79722574	T	C	0.018	34292033	0.825	0.082	360770	T	C	-0.111	17	32619052	1.06E-06	0.023	8153	23.830

Vascular dementia (subcortical)	Eotaxin	rs9317045	C	A	0.055	59055904	0.489	0.079	360770	C	A	-0.118	13	59630038	5.82E-07	0.024	8153	24.874
Vascular dementia (sudden onset)	CTACK	rs116303454	A	G	0.144	27253164	0.692	0.363	360283	A	G	0.383	3	27294655	3.27E-06	0.082	3631	22.030
Vascular dementia (sudden onset)	CTACK	rs2070074	G	A	0.256	34649445	0.164	0.184	360283	G	A	-0.447	9	34649442	1.78E-32	0.037	3631	142.656
Vascular dementia (sudden onset)	CTACK	rs2731674	G	T	-0.074	177412889	0.579	0.133	360283	G	T	0.133	5	176839890	5.63E-07	0.027	3631	24.925
Vascular dementia (sudden onset)	CTACK	rs3766110	C	A	-0.125	169545945	0.356	0.135	360283	C	A	0.129	1	169515183	3.85E-06	0.028	3631	21.432
Vascular dementia (sudden onset)	CTACK	rs55764737	C	T	-1.043	61031215	0.034	0.491	360283	C	T	-0.531	15	61323414	4.62E-08	0.097	3631	29.878
Vascular dementia (sudden onset)	CTACK	rs57338032	G	A	-0.046	78506597	0.765	0.155	360283	G	A	-0.158	15	78798939	6.23E-07	0.032	3631	24.937
Vascular dementia (sudden onset)	CTACK	rs7333764	T	C	-0.294	33634664	0.335	0.305	360283	T	C	0.277	13	34208801	2.85E-06	0.059	3631	21.867
Vascular dementia (sudden onset)	CTACK	rs76395525	A	G	1.088	79449049	0.048	0.551	360283	A	G	0.528	15	79741391	9.55E-07	0.108	3631	23.742
Vascular dementia (sudden onset)	beta-nerve growth factor	rs28637706	T	G	-0.255	33794463	0.044	0.126	360283	T	G	-0.159	19	34285368	1.42E-09	0.026	3531	36.504
Vascular dementia (sudden onset)	beta-nerve growth factor	rs67476890	T	C	0.068	62499295	0.717	0.187	360283	T	C	0.177	15	62791494	3.13E-06	0.038	3531	21.786
Vascular dementia (sudden onset)	beta-nerve growth factor	rs71641308	T	C	-0.182	77621033	0.362	0.200	360283	T	C	0.204	1	78086718	2.30E-06	0.043	3531	22.365
Vascular dementia (sudden onset)	beta-nerve growth factor	rs72780728	A	G	0.014	17561702	0.944	0.201	360283	A	G	0.188	10	17603701	2.99E-06	0.040	3531	21.832
Vascular dementia (sudden onset)	beta-nerve growth factor	rs73472576	C	T	-0.049	74456947	0.673	0.117	360283	C	T	0.118	18	72124182	2.69E-06	0.025	3531	21.963
Vascular dementia (sudden onset)	beta-nerve growth factor	rs7970581	G	T	-0.063	112827443	0.630	0.132	360283	G	T	-0.138	12	113265248	9.27E-07	0.028	3531	23.947
Vascular dementia (sudden onset)	beta-nerve growth factor	rs9436119	A	G	0.296	150495277	0.007	0.109	360283	A	G	-0.112	1	150467753	3.91E-06	0.025	3531	20.765
Vascular dementia (sudden onset)	Vascular endothelial growth factor	rs10153304	A	G	-0.126	7818613	0.550	0.210	360283	A	G	0.155	17	7721931	1.94E-06	0.033	7118	22.658
Vascular dementia (sudden onset)	Vascular endothelial growth factor	rs10934631	C	T	0.064	122978753	0.696	0.163	360283	C	T	0.115	3	122697600	2.47E-06	0.025	7118	22.071
Vascular dementia (sudden onset)	Vascular endothelial growth factor	rs10967186	C	T	-0.018	2617099	0.881	0.118	360283	C	T	-0.090	9	2617099	1.23E-07	0.017	7118	27.903
Vascular dementia (sudden onset)	Vascular endothelial growth factor	rs13209117	A	G	-0.144	44184028	0.282	0.134	360283	A	G	0.130	6	44151765	5.28E-11	0.020	7118	41.959
Vascular dementia (sudden onset)	Vascular endothelial growth factor	rs143479231	A	G	0.472	193393005	0.114	0.299	360283	A	G	-0.260	3	193110794	1.90E-07	0.049	7118	27.997
Vascular dementia (sudden onset)	Vascular endothelial growth factor	rs4082730	A	G	-0.134	89980326	0.707	0.356	360283	A	G	0.252	15	90523558	2.64E-06	0.053	7118	22.305
Vascular dementia (sudden onset)	Vascular endothelial growth factor	rs6921438	A	G	0.076	43957870	0.513	0.117	360283	A	G	-0.490	6	43925607	########	0.018	7118	784.000
Vascular dementia (sudden onset)	Vascular endothelial growth factor	rs73418461	A	G	0.280	118463484	0.419	0.346	360283	A	G	-0.249	10	120222996	1.67E-06	0.052	7118	22.878
Vascular dementia (sudden onset)	Vascular endothelial growth factor	rs8045833	A	G	0.069	88509031	0.626	0.142	360283	A	G	0.108	16	88575439	2.83E-07	0.021	7118	26.199
Vascular dementia (sudden onset)	Vascular endothelial growth factor	rs9472183	G	A	0.085	43972465	0.461	0.116	360283	G	A	0.128	6	43940202	5.19E-14	0.017	7118	56.869
Vascular dementia (sudden onset)	Macrophage Migration Inhibitory Factor	rs113218956	A	G	-0.097	24828867	0.919	0.959	360283	A	G	-0.895	22	25224834	2.26E-06	0.188	3494	22.678
Vascular dementia (sudden onset)	Macrophage Migration Inhibitory Factor	rs118055855	C	T	-1.020	29867025	0.114	0.645	360283	C	T	-0.691	11	29888572	4.13E-06	0.150	3494	21.203
Vascular dementia (sudden onset)	Macrophage Migration Inhibitory Factor	rs12594190	G	A	-0.167	24791308	0.190	0.127	360283	G	A	-0.136	15	25036455	3.70E-07	0.027	3494	25.755
Vascular dementia (sudden onset)	Macrophage Migration Inhibitory Factor	rs13142904	T	C	-0.155	53452247	0.447	0.204	360283	T	C	-0.223	4	54318414	2.56E-07	0.043	3494	27.532
Vascular dementia (sudden onset)	Macrophage Migration Inhibitory Factor	rs141009259	C	T	0.600	207111559	0.296	0.574	360283	C	T	0.618	2	207976283	2.47E-06	0.132	3494	21.839
Vascular dementia (sudden onset)	Macrophage Migration Inhibitory Factor	rs78098071	C	T	0.638	163882733	0.164	0.458	360283	C	T	0.487	5	163309739	1.78E-07	0.092	3494	28.108
Vascular dementia (sudden onset)	TRAIL	rs11618126	G	A	-1.022	50252103	0.422	1.274	360283	G	A	-0.891	13	50826239	1.46E-06	0.191	8186	21.661
Vascular dementia (sudden onset)	TRAIL	rs11657269	G	A	-0.136	6416464	0.476	0.190	360283	G	A	-0.119	17	6319784	4.78E-06	0.026	8186	20.878
Vascular dementia (sudden onset)	TRAIL	rs11699445	G	T	-0.119	15770145	0.315	0.118	360283	G	T	-0.075	20	15750790	3.27E-06	0.016	8186	21.470
Vascular dementia (sudden onset)	TRAIL	rs13185784	A	G	-0.011	180267068	0.935	0.133	360283	A	G	0.085	5	179694068	3.90E-06	0.018	8186	21.372
Vascular dementia (sudden onset)	TRAIL	rs138987090	G	A	-0.493	32786284	0.339	0.516	360283	G	A	0.750	18	30366247	4.50E-23	0.075	8186	99.389
Vascular dementia (sudden onset)	TRAIL	rs146783010	G	A	-0.239	89527045	0.789	0.893	360283	G	A	0.602	11	89260213	4.83E-06	0.135	8186	19.859
Vascular dementia (sudden onset)	TRAIL	rs193112415	C	T	-0.374	31255157	0.356	0.405	360283	C	T	1.042	18	28835120	2.15E-62	0.062	8186	279.797
Vascular dementia (sudden onset)	TRAIL	rs57396456	C	T	-0.559	30365911	0.159	0.397	360283	C	T	0.563	18	27945877	1.25E-27	0.052	8186	117.961
Vascular dementia (sudden onset)	TRAIL	rs62093514	T	C	-0.602	31651014	0.125	0.392	360283	T	C	1.062	18	29230977	6.86E-82	0.055	8186	370.005
Vascular dementia (sudden onset)	TRAIL	rs73039026	C	A	0.484	172442691	0.248	0.419	360283	C	A	0.300	3	172160481	2.02E-06	0.064	8186	22.305
Vascular dementia (sudden onset)	TRAIL	rs747324	C	T	0.103	74222941	0.425	0.130	360283	C	T	0.086	14	74689644	1.61E-06	0.018	8186	23.072
Vascular dementia (sudden onset)	TRAIL	rs74778900	T	C	0.101	30506300	0.846	0.520	360283	T	C	0.591	18	28086266	2.59E-28	0.053	8186	123.243
Vascular dementia (sudden onset)	TRAIL	rs75928541	A	G	-0.462	16400148	0.221	0.377	360283	A	G	0.275	4	16401771	4.24E-06	0.059	8186	21.506
Vascular dementia (sudden onset)	TRAIL	rs79287178	A	G	0.236	172576710	0.414	0.289	360283	A	G	-0.432	3	172294500	9.12E-25	0.042	8186	105.148
Vascular dementia (sudden onset)	Tumor necrosis factor beta	rs10925040	T	C	0.016	247459396	0.892	0.122	360283	T	C	0.176	1	247622698	2.67E-06	0.037	1559	22.138
Vascular dementia (sudden onset)	Tumor necrosis factor beta	rs753274	T	C	0.021	14325650	0.859	0.119	360283	T	C	-0.174	19	14436462	2.77E-06	0.037	1559	21.895
Vascular dementia (sudden onset)	Tumor necrosis factor beta	rs7629875	G	A	-0.185	174667832	0.446	0.243	360283	G	A	-0.377	3	174385622	1.37E-06	0.077	1559	23.674
Vascular dementia (sudden onset)	Tumor necrosis factor beta	rs78296352	T	G	0.186	22495351	0.707	0.496	360283	T	G	1.222	1	22821844	4.76E-21	0.137	1559	79.962
Vascular dementia (sudden onset)	Tumor necrosis factor alpha	rs10834997	A	G	-0.228	26505401	0.070	0.126	360283	A	G	-0.125	11	26526948	1.33E-06	0.026	3454	23.361
Vascular dementia (sudden onset)	Tumor necrosis factor alpha	rs115669577	A	G	0.453	123440293	0.600	0.864	360283	A	G	0.989	4	124361448	8.28E-07	0.200	3454	24.571
Vascular dementia (sudden onset)	Tumor necrosis factor alpha	rs79105320	A	G	-0.159	18959850	0.769	0.540	360283	A	G	0.561	8	18817360	3.59E-06	0.118	3454	22.601
Vascular dementia (sudden onset)	Tumor necrosis factor alpha	rs8121916	A	C	-0.084	12420677	0.529	0.133	360283	A	C	0.131	20	12401325	2.72E-06	0.028	3454	22.070
Vascular dementia (sudden onset)	Stromal-cell-derived factor 1 alpha	rs10474392	G	A	-0.226	92198776	0.089	0.133	360283	G	A	-0.096	5	91494593	1.24E-06	0.018	5998	29.209
Vascular dementia (sudden onset)	Stromal-cell-derived factor 1 alpha	rs12407262	A	G	0.058	63354605	0.759	0.190	360283	A	G	0.118	1	63820276	3.99E-06	0.027	5998	19.646
Vascular dementia (sudden onset)	Stromal-cell-derived factor 1 alpha	rs139840550	A	G	-0.218	38688625	0.591	0.405	360283	A	G	0.183	9	38688622	3.79E-06	0.055	5998	11.160
Vascular dementia (sudden onset)	Stromal-cell-derived factor 1 alpha	rs149893336	G	A	-1.047	170311440	0.065	0.567	360283	G	A	0.503	4	171232591	4.52E-06	0.108	5998	21.686
Vascular dementia (sudden onset)	Stromal-cell-derived factor 1 alpha	rs4581824	G	T	0.096	9074853	0.431	0.122	360283	G	T	0.070	19	9185529	3.05E-06	0.017	5998	16.419
Vascular dementia (sudden onset)	Stromal-cell-derived factor 1 alpha	rs482700	A	G	-0.165	115146334	0.258	0.146	360283	A	G	-0.089	4	116067490	1.57E-06	0.020	5998	19.351
Vascular dementia (sudden onset)	Stromal-cell-derived factor 1 alpha	rs67689854	A	C	-0.026	89558819	0.858	0.144	360283	A	C	-0.068	16	89625227	3.07E-06	0.020	5998	12.196
Vascular dementia (sudden onset)	Stromal-cell-derived factor 1 alpha	rs9267091	A	G	0.102	31446032	0.474	0.143	360283	A	G	0.078	6	31413809	3.63E-06	0.020	5998	14.802
Vascular dementia (sudden onset)	Stem cell growth factor beta	rs112346514	T	C	-0.074	12297173	0.804	0.300	360283	T	C	-0.331	19	12407988	2.37E-06	0.071	3682	21.725
Vascular dementia (sudden onset)	Stem cell growth factor beta	rs116924815	T	C	0.032	50727476	0.929	0.359	360283	T	C	0.608	19	51230733	1.74E-16	0.074	3682	67.850

Vascular dementia (sudden onset)	Stem cell growth factor beta	rs117716477	A	C	0.207	103847180	0.651	0.458	360283	A	C	0.838	12	104240958	1.34E-23	0.084	3682	99.383
Vascular dementia (sudden onset)	Stem cell growth factor beta	rs12480722	C	T	-0.030	20248260	0.863	0.173	360283	C	T	-0.162	20	20228904	4.72E-06	0.036	3682	20.927
Vascular dementia (sudden onset)	Stem cell growth factor beta	rs139413256	A	G	-0.044	146182552	0.928	0.490	360283	A	G	-0.538	7	145879644	7.04E-07	0.108	3682	24.605
Vascular dementia (sudden onset)	Stem cell growth factor beta	rs143829871	C	T	0.185	47555755	0.356	0.201	360283	C	T	0.190	3	47597245	1.90E-06	0.040	3682	22.610
Vascular dementia (sudden onset)	Stem cell growth factor beta	rs151194174	A	G	0.220	20956159	0.483	0.313	360283	A	G	0.464	7	20995778	1.13E-06	0.094	3682	24.210
Vascular dementia (sudden onset)	Stem cell growth factor beta	rs17876031	G	A	-0.071	177404118	0.566	0.124	360283	G	A	0.151	5	176831119	2.25E-09	0.026	3682	35.251
Vascular dementia (sudden onset)	Stem cell growth factor beta	rs264162	G	A	0.030	10944028	0.798	0.116	360283	G	A	-0.110	18	10944026	2.68E-06	0.023	3682	21.978
Vascular dementia (sudden onset)	Stem cell growth factor beta	rs34911860	A	G	-0.309	79885030	0.489	0.447	360283	A	G	-0.368	1	80350715	3.24E-06	0.079	3682	21.695
Vascular dementia (sudden onset)	Stem cell growth factor beta	rs4656185	A	G	-0.186	169507088	0.139	0.126	360283	A	G	0.205	1	169476326	1.16E-15	0.026	3682	64.125
Vascular dementia (sudden onset)	Stem cell growth factor beta	rs4737732	G	A	-0.071	65421393	0.581	0.128	360283	G	A	0.115	8	66333628	4.68E-06	0.025	3682	20.717
Vascular dementia (sudden onset)	Stem cell growth factor beta	rs7762066	C	T	-0.003	94468249	0.984	0.138	360283	C	T	-0.139	6	95177967	3.50E-06	0.030	3682	21.581
Vascular dementia (sudden onset)	Stem cell growth factor beta	rs78217154	C	T	-0.730	100541844	0.068	0.400	360283	C	T	-0.400	8	101554072	3.77E-06	0.086	3682	21.401
Vascular dementia (sudden onset)	Stem cell factor	rs113127926	A	C	0.190	97971174	0.528	0.300	360283	A	C	0.198	14	98437511	2.27E-06	0.042	8290	22.269
Vascular dementia (sudden onset)	Stem cell factor	rs13412535	A	G	0.070	224010157	0.628	0.145	360283	A	G	-0.107	2	224874874	6.04E-07	0.021	8290	25.094
Vascular dementia (sudden onset)	Stem cell factor	rs1557570	T	G	-0.201	169538606	0.110	0.126	360283	T	G	0.119	1	169507844	2.74E-12	0.017	8290	48.671
Vascular dementia (sudden onset)	Stem cell factor	rs1568119	T	C	0.865	33385679	0.274	0.792	360283	T	C	-0.591	8	33243197	1.24E-07	0.113	8290	27.365
Vascular dementia (sudden onset)	Stem cell factor	rs1942355	T	C	0.053	71694503	0.653	0.117	360283	T	C	-0.072	18	69361739	4.70E-06	0.016	8290	20.798
Vascular dementia (sudden onset)	Stem cell factor	rs4841899	C	T	0.155	134532566	0.245	0.133	360283	C	T	0.100	9	137424412	1.78E-08	0.018	8290	31.815
Vascular dementia (sudden onset)	Stem cell factor	rs635634	T	C	0.342	133279427	0.018	0.145	360283	T	C	-0.103	9	136155000	6.74E-08	0.019	8290	29.194
Vascular dementia (sudden onset)	Stem cell factor	rs78666213	G	T	-0.380	179217495	0.342	0.400	360283	G	T	0.274	4	180138649	2.59E-06	0.058	8290	22.695
Vascular dementia (sudden onset)	Stem cell factor	rs80271436	A	G	-0.634	133022383	0.078	0.360	360283	A	G	-0.237	9	135897770	9.95E-07	0.049	8290	23.879
Vascular dementia (sudden onset)	Interleukin-16	rs117217798	T	C	0.397	33156215	0.058	0.209	360283	T	C	-0.204	17	31483233	4.15E-06	0.044	3483	21.028
Vascular dementia (sudden onset)	Interleukin-16	rs117916513	A	G	0.680	121393565	0.142	0.464	360283	A	G	-0.502	11	121264274	3.79E-07	0.099	3483	25.921
Vascular dementia (sudden onset)	Interleukin-16	rs1255143	T	C	0.037	128253936	0.748	0.117	360283	T	C	0.131	10	130052200	7.10E-08	0.024	3483	29.124
Vascular dementia (sudden onset)	Interleukin-16	rs12765671	A	G	0.112	104924411	0.863	0.652	360283	A	G	-0.602	10	106684169	4.84E-06	0.132	3483	20.883
Vascular dementia (sudden onset)	Interleukin-16	rs144691581	A	G	0.411	96410095	0.289	0.387	360283	A	G	0.488	15	96953325	4.20E-07	0.097	3483	25.488
Vascular dementia (sudden onset)	Interleukin-16	rs1801020	G	A	-0.070	177409531	0.601	0.133	360283	G	A	-0.173	5	176836532	4.53E-10	0.027	3483	40.594
Vascular dementia (sudden onset)	Interleukin-16	rs4253283	C	T	0.021	186244057	0.869	0.127	360283	C	T	-0.146	4	187165211	1.75E-08	0.026	3483	31.053
Vascular dementia (sudden onset)	Interleukin-16	rs4513633	A	C	-0.158	112649483	0.486	0.227	360283	A	C	-0.224	4	113570639	7.44E-07	0.045	3483	24.429
Vascular dementia (sudden onset)	Interleukin-16	rs4778636	A	G	0.051	81299298	0.854	0.279	360283	A	G	-0.727	15	81591639	1.11E-30	0.063	3483	131.978
Vascular dementia (sudden onset)	Interleukin-16	rs9706053	T	C	0.432	65982530	0.343	0.456	360283	T	C	0.458	12	66376310	7.01E-07	0.093	3483	24.170
Vascular dementia (sudden onset)	RANTES	rs112072646	A	G	0.554	53217255	0.223	0.454	360283	A	G	0.429	2	53444393	6.48E-07	0.086	3421	24.722
Vascular dementia (sudden onset)	RANTES	rs147509526	T	C	-0.385	15665520	0.243	0.330	360283	T	C	-0.358	19	15776330	6.93E-07	0.072	3421	24.930
Vascular dementia (sudden onset)	RANTES	rs4940620	G	A	-0.174	64303876	0.507	0.262	360283	G	A	0.249	18	61971111	3.54E-06	0.054	3421	21.331
Vascular dementia (sudden onset)	RANTES	rs62438851	G	A	-0.073	144909173	0.699	0.190	360283	G	A	0.196	6	145230309	2.33E-06	0.041	3421	22.345
Vascular dementia (sudden onset)	RANTES	rs7000423	T	C	-0.007	110041420	0.955	0.120	360283	T	C	-0.132	8	111053649	1.82E-07	0.025	3421	27.139
Vascular dementia (sudden onset)	RANTES	rs72793342	A	G	0.332	30537031	0.020	0.143	360283	A	G	-0.149	16	30548352	1.48E-06	0.031	3421	23.309
Vascular dementia (sudden onset)	RANTES	rs74472919	T	C	-0.031	81626515	0.919	0.301	360283	T	C	0.331	13	82200650	3.97E-08	0.061	3421	29.987
Vascular dementia (sudden onset)	RANTES	rs75613039	T	C	0.252	129706688	0.469	0.348	360283	T	C	0.370	11	129576583	4.81E-06	0.081	3421	20.866
Vascular dementia (sudden onset)	RANTES	rs818452	T	C	-0.128	152594661	0.602	0.245	360283	T	C	0.238	6	152915796	2.36E-06	0.051	3421	22.230
Vascular dementia (sudden onset)	Platelet-derived growth factor BB	rs116445074	T	G	-0.103	52238766	0.812	0.430	360283	T	G	0.293	5	51534600	3.11E-07	0.059	8293	24.932
Vascular dementia (sudden onset)	Platelet-derived growth factor BB	rs11766649	G	A	-0.116	145142154	0.416	0.143	360283	G	A	-0.091	7	144839247	3.53E-06	0.020	8293	21.461
Vascular dementia (sudden onset)	Platelet-derived growth factor BB	rs11916118	G	A	-0.125	117193342	0.395	0.147	360283	G	A	-0.089	3	116912189	4.93E-06	0.019	8293	20.999
Vascular dementia (sudden onset)	Platelet-derived growth factor BB	rs12289510	G	A	-0.064	125077155	0.585	0.117	360283	G	A	0.078	11	124947051	7.69E-07	0.016	8293	24.371
Vascular dementia (sudden onset)	Platelet-derived growth factor BB	rs13412535	A	G	0.070	224010157	0.628	0.145	360283	A	G	0.335	2	224874874	2.46E-55	0.021	8293	245.347
Vascular dementia (sudden onset)	Platelet-derived growth factor BB	rs2324229	C	T	0.034	83208412	0.779	0.121	360283	C	T	-0.089	6	83918131	3.48E-08	0.016	8293	30.834
Vascular dementia (sudden onset)	Platelet-derived growth factor BB	rs35859699	A	G	0.177	111263595	0.748	0.551	360283	A	G	-0.395	4	112184751	2.07E-06	0.084	8293	22.030
Vascular dementia (sudden onset)	Platelet-derived growth factor BB	rs4965869	T	C	0.159	101450115	0.237	0.135	360283	T	C	0.184	15	101990320	5.66E-24	0.018	8293	103.342
Vascular dementia (sudden onset)	Platelet-derived growth factor BB	rs55680718	T	C	-0.032	224302160	0.856	0.178	360283	T	C	-0.138	2	225166877	1.86E-08	0.025	8293	31.606
Vascular dementia (sudden onset)	Platelet-derived growth factor BB	rs72777070	G	T	0.118	9658748	0.410	0.143	360283	G	T	0.107	2	9798877	8.98E-08	0.020	8293	28.569
Vascular dementia (sudden onset)	Platelet-derived growth factor BB	rs73162807	A	C	0.258	146757003	0.494	0.378	360283	A	C	-0.239	3	146474790	1.74E-06	0.050	8293	22.959
Vascular dementia (sudden onset)	Platelet-derived growth factor BB	rs9936075	G	A	-0.037	7271908	0.762	0.121	360283	G	A	0.078	16	7321909	1.76E-06	0.016	8293	22.737
Vascular dementia (sudden onset)	Platelet-derived growth factor BB	rs9941733	G	A	-0.273	393417	0.076	0.154	360283	G	A	-0.116	20	374061	3.31E-07	0.023	8293	25.930
Vascular dementia (sudden onset)	Macrophage inflammatory protein 1b	rs11130043	A	G	0.022	45069747	0.849	0.117	360283	A	G	-0.073	3	45111239	3.22E-06	0.016	8243	21.679
Vascular dementia (sudden onset)	Macrophage inflammatory protein 1b	rs113010081	C	T	0.257	46415921	0.141	0.174	360283	C	T	0.595	3	46457412	########	0.024	8243	636.493
Vascular dementia (sudden onset)	Macrophage inflammatory protein 1b	rs113877493	T	C	-0.109	36443746	0.491	0.158	360283	T	C	-0.612	17	34812273	########	0.022	8243	789.146
Vascular dementia (sudden onset)	Macrophage inflammatory protein 1b	rs116237296	A	G	0.582	86579833	0.516	0.896	360283	A	G	0.544	1	87045516	7.23E-07	0.112	8243	23.778
Vascular dementia (sudden onset)	Macrophage inflammatory protein 1b	rs117453826	G	A	0.300	36775624	0.437	0.386	360283	G	A	0.577	17	35132809	5.07E-22	0.059	8243	94.808
Vascular dementia (sudden onset)	Macrophage inflammatory protein 1b	rs141102180	T	G	-0.338	36108811	0.259	0.299	360283	T	G	0.323	17	34436204	1.08E-16	0.039	8243	67.340
Vascular dementia (sudden onset)	Macrophage inflammatory protein 1b	rs17138331	G	A	0.020	7826737	0.924	0.212	360283	G	A	0.139	7	7866368	2.26E-06	0.030	8243	22.234
Vascular dementia (sudden onset)	Macrophage inflammatory protein 1b	rs17641689	G	A	0.119	36668383	0.521	0.185	360283	G	A	0.245	17	35024819	1.28E-16	0.029	8243	69.805
Vascular dementia (sudden onset)	Macrophage inflammatory protein 1b	rs2079664	G	A	0.239	34680936	0.068	0.131	360283	G	A	-0.100	17	33007955	1.51E-08	0.018	8243	31.961
Vascular dementia (sudden onset)	Macrophage inflammatory protein 1b	rs281749	C	T	0.045	107626417	0.721	0.126	360283	C	T	-0.080	8	108638645	3.17E-06	0.017	8243	21.832
Vascular dementia (sudden onset)	Macrophage inflammatory protein 1b	rs34437725	C	T	-0.190	35499766	0.586	0.348	360283	C	T	0.263	17	33826785	7.67E-08	0.048	8243	29.717

Vascular dementia (sudden onset)	Macrophage inflammatory protein 1b	rs72791296	T	C	-0.198	121614355	0.530	0.316	360283	T	C	0.237	5	120950050	3.78E-07	0.047	8243	25.844
Vascular dementia (sudden onset)	Macrophage inflammatory protein 1b	rs72799710	T	C	0.117	123825971	0.473	0.163	360283	T	C	-0.101	5	123161665	3.21E-06	0.022	8243	21.635
Vascular dementia (sudden onset)	Macrophage inflammatory protein 1b	rs74810984	C	T	-0.625	127876202	0.099	0.379	360283	C	T	-0.221	10	129674466	1.96E-06	0.047	8243	21.660
Vascular dementia (sudden onset)	Macrophage inflammatory protein 1b	rs76582507	A	G	0.208	37510075	0.744	0.637	360283	A	G	0.318	9	37510072	3.26E-06	0.068	8243	21.994
Vascular dementia (sudden onset)	Macrophage inflammatory protein 1b	rs76583883	T	G	-0.075	45936445	0.810	0.311	360283	T	G	-0.232	21	47356359	4.99E-06	0.051	8243	20.559
Vascular dementia (sudden onset)	Macrophage inflammatory protein 1b	rs76776296	G	A	0.331	115488433	0.419	0.410	360283	G	A	-0.300	7	115128487	5.55E-07	0.060	8243	25.117
Vascular dementia (sudden onset)	Macrophage inflammatory protein 1a	rs10835056	G	T	0.134	26675470	0.277	0.124	360283	G	T	-0.119	11	26697017	2.60E-06	0.025	3522	22.097
Vascular dementia (sudden onset)	Macrophage inflammatory protein 1a	rs12690897	A	G	-0.201	85716861	0.118	0.129	360283	A	G	0.125	7	85346177	2.11E-06	0.026	3522	22.690
Vascular dementia (sudden onset)	Macrophage inflammatory protein 1a	rs184154340	A	G	-0.416	80790993	0.167	0.301	360283	A	G	0.331	11	80502036	1.86E-06	0.069	3522	22.813
Vascular dementia (sudden onset)	Macrophage inflammatory protein 1a	rs34771762	G	A	0.219	200547932	0.327	0.224	360283	G	A	-0.249	2	201412655	2.13E-06	0.052	3522	22.667
Vascular dementia (sudden onset)	Macrophage inflammatory protein 1a	rs57786342	A	G	0.008	68793311	0.951	0.137	360283	A	G	0.131	14	69260028	4.05E-06	0.029	3522	21.257
Vascular dementia (sudden onset)	Macrophage inflammatory protein 1a	rs60198979	A	G	-0.114	43250698	0.607	0.222	360283	A	G	-0.215	22	43646704	2.61E-06	0.046	3522	21.955
Vascular dementia (sudden onset)	Macrophage inflammatory protein 1a	rs7232268	G	A	0.008	70101678	0.978	0.294	360283	G	A	-0.282	18	67768914	2.55E-06	0.060	3522	22.180
Vascular dementia (sudden onset)	Monokine induced by gamma interferon	rs111607343	A	G	-0.794	897855	0.118	0.508	360283	A	G	-0.521	19	897855	2.83E-06	0.112	3685	21.678
Vascular dementia (sudden onset)	Monokine induced by gamma interferon	rs11177248	A	G	0.192	68482106	0.508	0.290	360283	A	G	0.307	12	68875886	4.45E-06	0.067	3685	21.037
Vascular dementia (sudden onset)	Monokine induced by gamma interferon	rs112337562	G	T	0.058	92665225	0.876	0.371	360283	G	T	0.370	14	93131570	2.98E-06	0.080	3685	21.606
Vascular dementia (sudden onset)	Monokine induced by gamma interferon	rs112861654	G	A	0.000	42179062	0.999	0.243	360283	G	A	0.277	21	43599172	1.81E-07	0.053	3685	27.320
Vascular dementia (sudden onset)	Monokine induced by gamma interferon	rs117831247	T	C	-0.154	66742081	0.862	0.885	360283	T	C	-0.833	10	68501839	2.16E-06	0.175	3685	22.576
Vascular dementia (sudden onset)	Monokine induced by gamma interferon	rs139010077	T	C	0.174	170618359	0.698	0.449	360283	T	C	0.432	3	170336148	3.55E-06	0.095	3685	20.698
Vascular dementia (sudden onset)	Monokine induced by gamma interferon	rs1796086	C	T	-0.192	71183729	0.359	0.210	360283	C	T	0.210	7	70648715	2.23E-07	0.040	3685	27.050
Vascular dementia (sudden onset)	Monokine induced by gamma interferon	rs41272086	A	G	0.089	160587614	0.648	0.194	360283	A	G	-0.223	6	161008646	7.43E-08	0.042	3685	28.771
Vascular dementia (sudden onset)	Monokine induced by gamma interferon	rs55876513	G	T	0.043	75962545	0.740	0.129	360283	G	T	-0.166	4	76883698	8.23E-11	0.026	3685	42.378
Vascular dementia (sudden onset)	Monokine induced by gamma interferon	rs5752128	C	T	-0.198	25322656	0.268	0.179	360283	C	T	0.169	22	25718623	4.34E-06	0.037	3685	20.852
Vascular dementia (sudden onset)	Monokine induced by gamma interferon	rs62562991	A	G	0.175	95973777	0.724	0.495	360283	A	G	0.624	9	98736059	8.40E-07	0.126	3685	24.495
Vascular dementia (sudden onset)	Monokine induced by gamma interferon	rs6679677	A	C	-0.132	113761186	0.427	0.166	360283	A	C	0.162	1	114303808	8.86E-07	0.033	3685	24.246
Vascular dementia (sudden onset)	Monokine induced by gamma interferon	rs77086208	T	C	-0.308	70152774	0.377	0.348	360283	T	C	0.323	14	70619491	3.83E-06	0.070	3685	21.361
Vascular dementia (sudden onset)	Monokine induced by gamma interferon	rs816960	T	C	0.002	107870173	0.990	0.121	360283	T	C	-0.122	13	108522521	5.01E-07	0.024	3685	25.164
Vascular dementia (sudden onset)	Macrophage colony stimulating factor	rs116274860	G	T	0.233	148675030	0.648	0.511	360283	G	T	-0.819	3	148392817	2.74E-06	0.174	840	22.129
Vascular dementia (sudden onset)	Macrophage colony stimulating factor	rs117867915	C	T	-0.705	44630078	0.092	0.418	360283	C	T	-0.527	18	42210043	1.61E-06	0.110	840	23.054
Vascular dementia (sudden onset)	Macrophage colony stimulating factor	rs12962919	T	C	-0.218	78018752	0.337	0.227	360283	T	C	0.305	18	75778756	4.65E-06	0.066	840	21.255
Vascular dementia (sudden onset)	Macrophage colony stimulating factor	rs56367447	T	C	0.175	4014005	0.598	0.332	360283	T	C	-0.497	8	3871527	1.72E-08	0.088	840	31.642
Vascular dementia (sudden onset)	Macrophage colony stimulating factor	rs62294910	A	G	-0.571	182480551	0.016	0.237	360283	A	G	0.343	3	182198339	6.82E-07	0.069	840	24.654
Vascular dementia (sudden onset)	Macrophage colony stimulating factor	rs78296352	T	G	0.186	22495351	0.707	0.496	360283	T	G	0.527	1	22821844	1.05E-06	0.111	840	22.460
Vascular dementia (sudden onset)	Macrophage colony stimulating factor	rs9387100	C	T	-0.004	112781752	0.974	0.118	360283	C	T	0.135	6	113102954	4.07E-06	0.029	840	21.438
Vascular dementia (sudden onset)	Monocyte chemoattractant protein-1	rs10145849	A	G	-0.020	82475647	0.868	0.121	360283	A	G	-0.076	14	82941991	3.41E-06	0.016	8293	21.720
Vascular dementia (sudden onset)	Monocyte chemoattractant protein-1	rs10744620	C	T	0.022	3629928	0.858	0.121	360283	C	T	-0.079	12	3739094	9.91E-07	0.016	8293	23.955
Vascular dementia (sudden onset)	Monocyte chemoattractant protein-1	rs111995966	G	T	-0.081	108558513	0.708	0.217	360283	G	T	-0.145	2	109174969	2.53E-06	0.031	8293	21.939
Vascular dementia (sudden onset)	Monocyte chemoattractant protein-1	rs112313229	A	G	-0.044	46323369	0.844	0.223	360283	A	G	-0.165	3	46364860	1.43E-07	0.031	8293	27.655
Vascular dementia (sudden onset)	Monocyte chemoattractant protein-1	rs12073356	A	G	-0.375	207834503	0.100	0.228	360283	A	G	-0.143	1	208007848	4.17E-06	0.031	8293	21.024
Vascular dementia (sudden onset)	Monocyte chemoattractant protein-1	rs12075	A	G	-0.202	159205564	0.082	0.116	360283	A	G	0.219	1	159175354	1.44E-44	0.016	8293	198.719
Vascular dementia (sudden onset)	Monocyte chemoattractant protein-1	rs12493471	C	T	0.024	45910186	0.841	0.121	360283	C	T	-0.116	3	45951678	6.81E-13	0.016	8293	51.538
Vascular dementia (sudden onset)	Monocyte chemoattractant protein-1	rs146522229	T	C	-1.007	47295223	0.267	0.906	360283	T	C	-0.598	19	47798480	3.56E-07	0.118	8293	25.779
Vascular dementia (sudden onset)	Monocyte chemoattractant protein-1	rs2228467	C	T	0.119	42864624	0.590	0.220	360283	C	T	0.264	3	42906116	9.19E-20	0.029	8293	82.117
Vascular dementia (sudden onset)	Monocyte chemoattractant protein-1	rs2712431	A	C	0.131	128598047	0.309	0.129	360283	A	C	-0.079	3	128316890	4.75E-06	0.017	8293	20.936
Vascular dementia (sudden onset)	Monocyte chemoattractant protein-1	rs56212190	T	C	0.050	41702868	0.858	0.277	360283	T	C	0.181	1	42168539	9.85E-07	0.037	8293	23.547
Vascular dementia (sudden onset)	Monocyte chemoattractant protein-1	rs7197349	G	A	-0.395	78653322	0.007	0.146	360283	G	A	-0.097	16	78687219	2.62E-06	0.021	8293	22.081
Vascular dementia (sudden onset)	Monocyte chemoattractant protein-1	rs7517040	G	A	0.055	158889343	0.698	0.141	360283	G	A	0.099	1	158859133	2.44E-07	0.019	8293	26.703
Vascular dementia (sudden onset)	Monocyte chemoattractant protein-1	rs9317045	C	A	-0.005	59055904	0.975	0.164	360283	C	A	-0.113	13	59630038	1.52E-06	0.024	8293	23.089
Vascular dementia (sudden onset)	Interleukin-12p70	rs13209117	A	G	-0.144	44184028	0.282	0.134	360283	A	G	0.100	6	44151765	5.57E-08	0.019	8270	29.021
Vascular dementia (sudden onset)	Interleukin-12p70	rs17229494	G	A	-0.216	37555798	0.257	0.190	360283	G	A	0.117	21	38928100	4.93E-06	0.026	8270	20.796
Vascular dementia (sudden onset)	Interleukin-12p70	rs282258	C	T	0.143	224050083	0.221	0.117	360283	C	T	-0.073	2	224914800	3.21E-06	0.016	8270	21.898
Vascular dementia (sudden onset)	Interleukin-12p70	rs41282644	A	G	0.089	43785985	0.681	0.216	360283	A	G	0.147	6	43753722	1.05E-06	0.030	8270	23.478
Vascular dementia (sudden onset)	Interleukin-12p70	rs4349809	G	T	0.083	43957093	0.479	0.117	360283	G	T	-0.378	6	43924830	########	0.016	8270	564.287
Vascular dementia (sudden onset)	Interleukin-12p70	rs71361173	G	T	-0.296	76000450	0.090	0.175	360283	G	T	-0.111	18	73712405	3.06E-06	0.024	8270	21.570
Vascular dementia (sudden onset)	Interleukin-12p70	rs72831623	A	G	0.170	47644927	0.459	0.230	360283	A	G	0.191	17	45722293	2.42E-07	0.037	8270	26.732
Vascular dementia (sudden onset)	Interleukin-12p70	rs782107	A	G	-0.031	58439747	0.789	0.116	360283	A	G	0.075	12	58833530	1.60E-06	0.016	8270	23.114
Vascular dementia (sudden onset)	Interleukin-12p70	rs79121401	C	T	1.316	78986084	0.022	0.574	360283	C	T	-0.555	11	78697129	4.24E-06	0.121	8270	21.163
Vascular dementia (sudden onset)	Interleukin-12p70	rs9472183	G	A	0.085	43972465	0.461	0.116	360283	G	A	0.102	6	43940202	8.61E-11	0.016	8270	42.126
Vascular dementia (sudden onset)	Interferon gamma-induced protein 10	rs10809307	C	T	-0.122	11045908	0.390	0.142	360283	C	T	-0.131	9	11045908	3.64E-06	0.028	3685	21.415
Vascular dementia (sudden onset)	Interferon gamma-induced protein 10	rs113831257	A	G	0.125	75234311	0.651	0.276	360283	A	G	0.359	4	76159521	2.53E-08	0.064	3685	31.110
Vascular dementia (sudden onset)	Interferon gamma-induced protein 10	rs11626201	A	C	0.187	36511495	0.120	0.120	360283	A	C	0.116	14	36980700	1.93E-06	0.025	3685	22.495
Vascular dementia (sudden onset)	Interferon gamma-induced protein 10	rs143799975	G	A	0.463	75885862	0.507	0.699	360283	G	A	0.798	4	76807015	1.00E-06	0.164	3685	23.787
Vascular dementia (sudden onset)	Interferon gamma-induced protein 10	rs34383175	T	C	-0.337	144361034	0.290	0.319	360283	T	C	-0.315	8	145584694	1.51E-06	0.066	3685	23.031
Vascular dementia (sudden onset)	Interferon gamma-induced protein 10	rs75970138	A	G	-0.238	119813998	0.642	0.512	360283	A	G	-0.485	9	122576276	1.53E-06	0.104	3685	21.748

Vascular dementia (sudden onset)	Interferon gamma-induced protein 10	rs7645625	G	T	0.099	146856250	0.403	0.119	360283	G	T	0.109	3	146574037	4.41E-06	0.024	3685	20.997
Vascular dementia (sudden onset)	Interferon gamma-induced protein 10	rs79848609	C	A	0.239	86772934	0.381	0.272	360283	C	A	-0.260	15	87316165	8.75E-07	0.054	3685	23.496
Vascular dementia (sudden onset)	Interferon gamma-induced protein 10	rs8112909	A	G	0.082	45910150	0.567	0.144	360283	A	G	-0.143	19	46413408	1.94E-06	0.030	3685	22.746
Vascular dementia (sudden onset)	Interleukin-18	rs10414578	T	C	-0.080	54634619	0.635	0.169	360283	T	C	-0.177	19	55146070	4.16E-07	0.035	3636	25.604
Vascular dementia (sudden onset)	Interleukin-18	rs115267715	T	C	0.527	69239188	0.233	0.442	360283	T	C	0.451	5	68535015	1.72E-08	0.080	3636	31.753
Vascular dementia (sudden onset)	Interleukin-18	rs116383510	C	A	0.191	2545536	0.697	0.491	360283	C	A	0.543	5	2545650	3.00E-07	0.106	3636	26.402
Vascular dementia (sudden onset)	Interleukin-18	rs117266781	T	C	-0.222	41261422	0.730	0.645	360283	T	C	0.684	7	41301020	3.15E-06	0.147	3636	21.716
Vascular dementia (sudden onset)	Interleukin-18	rs144841621	T	C	-0.269	69921801	0.647	0.588	360283	T	C	0.518	10	71681557	3.81E-06	0.114	3636	20.610
Vascular dementia (sudden onset)	Interleukin-18	rs17229943	C	A	-0.618	69386709	0.004	0.213	360283	C	A	0.312	5	68682536	1.62E-11	0.046	3636	45.410
Vascular dementia (sudden onset)	Interleukin-18	rs1852105	C	T	0.221	64265217	0.485	0.317	360283	C	T	-0.304	7	63725595	4.32E-06	0.066	3636	21.096
Vascular dementia (sudden onset)	Interleukin-18	rs1979967	T	C	-0.032	79367271	0.822	0.143	360283	T	C	0.140	15	79659613	9.45E-07	0.029	3636	24.031
Vascular dementia (sudden onset)	Interleukin-18	rs2729385	A	G	0.000	57495520	0.999	0.129	360283	A	G	0.123	11	57262993	3.79E-06	0.026	3636	22.076
Vascular dementia (sudden onset)	Interleukin-18	rs385076	C	T	0.004	32264782	0.973	0.122	360283	C	T	0.243	2	32489851	1.66E-22	0.025	3636	96.166
Vascular dementia (sudden onset)	Interleukin-18	rs4482818	G	A	0.033	65062779	0.785	0.119	360283	G	A	-0.129	4	65928497	1.45E-07	0.024	3636	27.778
Vascular dementia (sudden onset)	Interleukin-18	rs658805	A	G	0.001	70199369	0.992	0.123	360283	A	G	0.123	6	70909073	4.94E-07	0.024	3636	25.247
Vascular dementia (sudden onset)	Interleukin-18	rs71478720	T	C	-0.003	112138882	0.983	0.135	360283	T	C	-0.267	11	112009605	3.71E-22	0.028	3636	93.515
Vascular dementia (sudden onset)	Interleukin-18	rs78623212	T	C	-0.574	103667180	0.390	0.667	360283	T	C	0.871	7	103307627	6.71E-07	0.178	3636	23.970
Vascular dementia (sudden onset)	Interleukin-18	rs78716465	A	G	-0.145	42015086	0.636	0.307	360283	A	G	0.327	20	40643726	1.63E-06	0.068	3636	22.919
Vascular dementia (sudden onset)	Interleukin-17	rs117029961	A	G	-0.109	37147653	0.871	0.669	360283	A	G	0.459	10	37436581	4.94E-06	0.102	7760	20.405
Vascular dementia (sudden onset)	Interleukin-17	rs117556572	T	C	0.225	104436567	0.552	0.378	360283	T	C	-0.510	13	105088917	3.28E-06	0.110	7760	21.552
Vascular dementia (sudden onset)	Interleukin-17	rs1530455	C	T	-0.210	123136052	0.085	0.122	360283	C	T	-0.108	3	122854899	4.87E-10	0.017	7760	38.972
Vascular dementia (sudden onset)	Interleukin-17	rs17106604	T	C	-0.195	77912813	0.237	0.165	360283	T	C	0.113	14	78379156	6.37E-07	0.023	7760	25.178
Vascular dementia (sudden onset)	Interleukin-17	rs17282552	C	T	0.134	207109091	0.620	0.270	360283	C	T	0.200	2	207973815	8.21E-07	0.041	7760	24.411
Vascular dementia (sudden onset)	Interleukin-17	rs184080173	C	T	-0.608	77331424	0.066	0.331	360283	C	T	-0.238	12	77725204	4.19E-07	0.047	7760	25.620
Vascular dementia (sudden onset)	Interleukin-17	rs187475560	T	C	0.206	160353411	0.542	0.338	360283	T	C	-0.243	4	161274563	3.29E-06	0.052	7760	21.910
Vascular dementia (sudden onset)	Interleukin-17	rs62191444	T	G	-0.154	393023	0.345	0.163	360283	T	G	-0.114	20	373667	4.22E-06	0.025	7760	21.153
Vascular dementia (sudden onset)	Interleukin-17	rs78296352	T	G	0.186	22495351	0.707	0.496	360283	T	G	0.303	1	22821844	4.27E-06	0.065	7760	21.956
Vascular dementia (sudden onset)	Interleukin-17	rs78612928	C	T	0.069	29812292	0.661	0.157	360283	C	T	-0.104	4	29813914	2.62E-06	0.022	7760	21.820
Vascular dementia (sudden onset)	Interleukin-13	rs117795020	A	G	-0.083	87469237	0.809	0.345	360283	A	G	-0.352	9	90084152	9.86E-07	0.072	3557	24.197
Vascular dementia (sudden onset)	Interleukin-13	rs12623722	A	G	-0.075	22955811	0.552	0.127	360283	A	G	-0.119	2	23178683	4.19E-06	0.026	3557	21.096
Vascular dementia (sudden onset)	Interleukin-13	rs139083458	T	C	-0.256	26160409	0.774	0.890	360283	T	C	0.990	5	26160518	2.81E-06	0.211	3557	22.086
Vascular dementia (sudden onset)	Interleukin-13	rs142167313	C	T	0.039	44204360	0.898	0.302	360283	C	T	0.313	6	44172097	3.98E-07	0.062	3557	25.735
Vascular dementia (sudden onset)	Interleukin-13	rs27949	T	C	-0.069	59254997	0.579	0.125	360283	T	C	-0.117	5	58550823	3.43E-06	0.025	3557	21.482
Vascular dementia (sudden onset)	Interleukin-13	rs6799107	C	T	-0.015	127338175	0.920	0.148	360283	C	T	0.146	3	127057018	1.25E-06	0.030	3557	23.495
Vascular dementia (sudden onset)	Interleukin-13	rs7073807	C	T	0.081	67393670	0.635	0.172	360283	C	T	-0.168	10	69153428	2.37E-06	0.036	3557	22.323
Vascular dementia (sudden onset)	Interleukin-13	rs75995699	A	G	-0.036	5140622	0.910	0.318	360283	A	G	0.332	6	5140856	2.64E-06	0.070	3557	22.610
Vascular dementia (sudden onset)	Interleukin-13	rs9472168	G	A	0.076	43961248	0.517	0.117	360283	G	A	-0.424	6	43928985	1.08E-65	0.025	3557	292.851
Vascular dementia (sudden onset)	Interleukin-10	rs10457128	A	G	0.105	105570101	0.387	0.121	360283	A	G	-0.087	6	106017976	5.24E-07	0.017	7681	25.292
Vascular dementia (sudden onset)	Interleukin-10	rs10493718	A	C	0.191	82597250	0.233	0.161	360283	A	C	-0.110	1	83062933	7.16E-07	0.022	7681	24.552
Vascular dementia (sudden onset)	Interleukin-10	rs11206302	T	C	-0.124	54208270	0.486	0.177	360283	T	C	-0.119	1	54673943	2.20E-06	0.025	7681	22.440
Vascular dementia (sudden onset)	Interleukin-10	rs2086656	T	C	-0.259	59632755	0.034	0.122	360283	T	C	-0.079	4	60498473	3.78E-06	0.017	7681	21.289
Vascular dementia (sudden onset)	Interleukin-10	rs282258	C	T	0.143	224050083	0.221	0.117	360283	C	T	-0.099	2	224914800	1.00E-09	0.016	7681	37.497
Vascular dementia (sudden onset)	Interleukin-10	rs3025021	C	T	0.019	43781426	0.879	0.122	360283	C	T	-0.095	6	43749163	1.46E-06	0.020	7681	23.585
Vascular dementia (sudden onset)	Interleukin-10	rs41282660	G	A	-0.076	44229269	0.654	0.170	360283	G	A	0.119	6	44197006	3.72E-06	0.026	7681	21.924
Vascular dementia (sudden onset)	Interleukin-10	rs4349809	G	T	0.083	43957093	0.479	0.117	360283	G	T	-0.285	6	43924830	5.77E-67	0.017	7681	298.976
Vascular dementia (sudden onset)	Interleukin-10	rs465757	A	G	0.122	15599638	0.319	0.122	360283	A	G	0.084	20	15580283	1.17E-06	0.017	7681	23.306
Vascular dementia (sudden onset)	Interleukin-10	rs7088799	G	T	0.103	63256414	0.390	0.119	360283	G	T	0.085	10	65016174	3.23E-07	0.017	7681	26.028
Vascular dementia (sudden onset)	Interleukin-8	rs11634944	C	T	-0.220	24937946	0.070	0.121	360283	C	T	0.121	15	25183093	1.29E-06	0.025	3526	23.208
Vascular dementia (sudden onset)	Interleukin-8	rs12075	A	G	-0.202	159205564	0.082	0.116	360283	A	G	0.120	1	159175354	3.88E-07	0.024	3526	25.855
Vascular dementia (sudden onset)	Interleukin-8	rs141926526	C	A	-0.187	32809028	0.770	0.639	360283	C	A	0.615	7	32848640	2.57E-06	0.131	3526	22.100
Vascular dementia (sudden onset)	Interleukin-6	rs1333040	T	C	-0.059	22083405	0.610	0.116	360283	T	C	0.074	9	22083404	3.17E-06	0.016	8189	21.817
Vascular dementia (sudden onset)	Interleukin-6	rs13412535	A	G	0.070	224010157	0.628	0.145	360283	A	G	-0.116	2	224874874	7.34E-08	0.022	8189	29.311
Vascular dementia (sudden onset)	Interleukin-6	rs72831623	A	G	0.170	47644927	0.459	0.230	360283	A	G	0.197	17	45722293	1.08E-07	0.037	8189	28.130
Vascular dementia (sudden onset)	Interleukin-6	rs73273528	T	C	0.572	51814574	0.128	0.376	360283	T	C	0.267	20	50431113	9.58E-07	0.055	8189	23.347
Vascular dementia (sudden onset)	Interleukin-6	rs76856708	C	T	0.064	80695146	0.903	0.529	360283	C	T	-0.329	16	80729043	2.61E-06	0.070	8189	22.077
Vascular dementia (sudden onset)	Interleukin-1-receptor antagonist	rs1054402	C	T	-0.234	116401230	0.083	0.135	360283	C	T	-0.131	9	119163509	1.13E-06	0.027	3638	23.576
Vascular dementia (sudden onset)	Interleukin-1-receptor antagonist	rs11627423	C	A	-0.117	32731417	0.331	0.120	360283	C	A	-0.117	14	33200623	2.12E-06	0.025	3638	22.476
Vascular dementia (sudden onset)	Interleukin-1-receptor antagonist	rs12121840	T	C	-0.209	165572405	0.435	0.268	360283	T	C	0.269	1	165541642	2.43E-06	0.057	3638	22.227
Vascular dementia (sudden onset)	Interleukin-1-receptor antagonist	rs2809154	T	C	0.141	84153389	0.464	0.192	360283	T	C	-0.179	13	84727524	3.74E-06	0.039	3638	21.188
Vascular dementia (sudden onset)	Interleukin-1-receptor antagonist	rs61335305	A	C	0.079	66160736	0.853	0.429	360283	A	C	0.445	15	66453074	1.00E-06	0.091	3638	24.051
Vascular dementia (sudden onset)	Interleukin-1-receptor antagonist	rs9623661	T	C	0.010	42697370	0.959	0.205	360283	T	C	-0.197	22	43093376	3.86E-06	0.043	3638	21.298
Vascular dementia (sudden onset)	Interleukin-1-beta	rs143319329	T	C	-0.063	128499405	0.927	0.688	360283	T	C	0.280	7	128139459	2.00E-06	0.072	3309	15.347
Vascular dementia (sudden onset)	Interleukin-1-beta	rs61335305	A	C	0.079	66160736	0.853	0.429	360283	A	C	0.297	15	66453074	1.90E-06	0.072	3309	16.783
Vascular dementia (sudden onset)	Interleukin-1-beta	rs62015704	G	A	-0.117	7417906	0.509	0.178	360283	G	A	-0.108	16	7467907	2.09E-06	0.028	3309	14.618

Vascular dementia (sudden onset)	Interleukin-1-beta	rs9898641	C	T	0.108	59493672	0.375	0.122	360283	C	T	0.203	17	57571033	3.59E-06	0.045	3309	20.033
Vascular dementia (sudden onset)	Hepatocyte growth factor	rs11060254	A	G	-0.016	129331024	0.896	0.125	360283	A	G	-0.080	12	129815569	1.58E-06	0.017	8292	22.948
Vascular dementia (sudden onset)	Hepatocyte growth factor	rs150322232	G	A	0.099	7890743	0.745	0.305	360283	G	A	-0.210	7	7930374	4.89E-06	0.046	8292	20.650
Vascular dementia (sudden onset)	Hepatocyte growth factor	rs1698249	C	A	-0.290	83889842	0.298	0.279	360283	C	A	0.170	14	84356186	4.09E-06	0.037	8292	20.835
Vascular dementia (sudden onset)	Hepatocyte growth factor	rs2003620	T	C	-0.210	134794733	0.542	0.344	360283	T	C	0.228	7	134479484	2.83E-06	0.049	8292	21.721
Vascular dementia (sudden onset)	Hepatocyte growth factor	rs3748034	T	G	0.222	3444364	0.213	0.178	360283	T	G	0.150	4	3446091	1.81E-10	0.023	8292	40.818
Vascular dementia (sudden onset)	Hepatocyte growth factor	rs5745687	T	C	0.188	81729735	0.506	0.283	360283	T	C	-0.307	7	81359051	2.75E-14	0.041	8292	57.252
Vascular dementia (sudden onset)	Hepatocyte growth factor	rs62481625	C	T	-0.016	156194766	0.923	0.165	360283	C	T	-0.109	7	155987460	1.18E-06	0.023	8292	23.512
Vascular dementia (sudden onset)	Interleukin-9	rs41294750	T	C	-0.485	53084968	0.147	0.335	360283	T	C	0.351	1	53550640	2.36E-06	0.075	3634	22.070
Vascular dementia (sudden onset)	Interleukin-9	rs4880409	T	C	-0.315	132516716	0.586	0.580	360283	T	C	-0.336	10	134330220	3.50E-06	0.072	3634	21.533
Vascular dementia (sudden onset)	Interleukin-9	rs61867538	T	C	0.214	1503276	0.450	0.284	360283	T	C	0.357	11	1524506	3.93E-06	0.077	3634	21.227
Vascular dementia (sudden onset)	Interleukin-9	rs7232268	G	A	0.008	70101678	0.978	0.294	360283	G	A	-0.276	18	67768914	2.52E-06	0.059	3634	22.092
Vascular dementia (sudden onset)	Interleukin-9	rs7242404	A	G	-0.104	12741268	0.419	0.128	360283	A	G	-0.123	18	12741267	3.27E-06	0.026	3634	21.637
Vascular dementia (sudden onset)	Interleukin-9	rs76963786	T	C	-0.186	31886823	0.510	0.283	360283	T	C	-0.287	12	32039757	4.50E-07	0.056	3634	26.457
Vascular dementia (sudden onset)	Interleukin-7	rs117509142	C	T	-0.209	86121854	0.527	0.331	360283	C	T	0.327	8	87134083	1.99E-06	0.069	3409	22.590
Vascular dementia (sudden onset)	Interleukin-7	rs141425475	C	T	0.375	17679056	0.376	0.423	360283	C	T	0.478	5	17679165	2.53E-06	0.102	3409	22.144
Vascular dementia (sudden onset)	Interleukin-7	rs144701438	A	G	0.476	66293168	0.337	0.496	360283	A	G	-0.482	18	63960405	9.75E-07	0.099	3409	23.742
Vascular dementia (sudden onset)	Interleukin-7	rs17091524	C	T	-0.401	56482041	0.394	0.471	360283	C	T	-0.492	14	56948759	1.91E-06	0.101	3409	23.627
Vascular dementia (sudden onset)	Interleukin-7	rs28793375	T	C	0.237	41558099	0.157	0.167	360283	T	C	0.164	8	41415618	4.46E-06	0.036	3409	20.588
Vascular dementia (sudden onset)	Interleukin-7	rs4320361	T	G	0.068	43960774	0.560	0.117	360283	T	G	-0.325	6	43928511	6.87E-39	0.025	3409	169.836
Vascular dementia (sudden onset)	Interleukin-7	rs62006410	T	C	0.204	102541598	0.136	0.136	360283	T	C	-0.156	14	103007935	3.39E-07	0.030	3409	26.405
Vascular dementia (sudden onset)	Interleukin-7	rs75904417	C	A	-0.180	167796811	0.272	0.164	360283	C	A	0.170	2	168653321	1.16E-06	0.035	3409	23.671
Vascular dementia (sudden onset)	Interleukin-7	rs77981494	C	T	-0.397	17451009	0.334	0.412	360283	C	T	0.518	16	17544866	1.07E-06	0.106	3409	23.683
Vascular dementia (sudden onset)	Interleukin-7	rs78346957	A	G	-0.839	125214944	0.128	0.552	360283	A	G	0.459	10	126903513	4.51E-06	0.101	3409	20.758
Vascular dementia (sudden onset)	Interleukin-5	rs11680908	G	A	-0.208	109460295	0.421	0.258	360283	G	A	-0.163	2	110076751	2.03E-06	0.055	3364	22.605
Vascular dementia (sudden onset)	Interleukin-5	rs6737109	C	T	0.061	22956659	0.599	0.117	360283	C	T	-0.116	2	23179531	2.40E-06	0.025	3364	22.056
Vascular dementia (sudden onset)	Interleukin-5	rs72831687	A	G	0.026	16092129	0.955	0.457	360283	A	G	-0.524	6	16092360	1.69E-06	0.111	3364	22.317
Vascular dementia (sudden onset)	Interleukin-5	rs73040130	C	T	-0.089	36255288	0.727	0.255	360283	C	T	-0.264	19	36746190	6.00E-07	0.053	3364	24.868
Vascular dementia (sudden onset)	Interleukin-5	rs7767396	G	A	0.085	43959313	0.464	0.117	360283	G	A	-0.152	6	43927050	7.69E-10	0.025	3364	37.928
Vascular dementia (sudden onset)	Interleukin-4	rs10512267	C	T	-0.108	99427847	0.357	0.117	360283	C	T	0.082	9	102190129	2.94E-07	0.016	8124	26.194
Vascular dementia (sudden onset)	Interleukin-4	rs116705532	G	T	-0.448	113162547	0.553	0.754	360283	G	T	0.468	1	113705169	1.76E-06	0.098	8124	22.879
Vascular dementia (sudden onset)	Interleukin-4	rs117146485	C	T	-0.653	135932411	0.136	0.438	360283	C	T	0.292	9	138824257	2.71E-06	0.063	8124	21.610
Vascular dementia (sudden onset)	Interleukin-4	rs17713451	A	G	-0.036	151465386	0.847	0.186	360283	A	G	0.127	7	151162472	4.97E-07	0.025	8124	25.357
Vascular dementia (sudden onset)	Interleukin-4	rs73023729	A	G	-0.168	159232998	0.554	0.283	360283	A	G	-0.180	6	159654030	9.03E-07	0.037	8124	24.080
Vascular dementia (sudden onset)	Interleukin-4	rs7613691	G	A	0.098	147935804	0.708	0.263	360283	G	A	-0.178	3	147653591	4.05E-06	0.038	8124	21.367
Vascular dementia (sudden onset)	Interleukin-4	rs9508291	C	T	-0.309	29136483	0.240	0.263	360283	C	T	0.168	13	29710620	3.03E-06	0.036	8124	21.795
Vascular dementia (sudden onset)	Interleukin-4	rs9941733	G	A	-0.273	393417	0.076	0.154	360283	G	A	-0.114	20	374061	6.88E-07	0.023	8124	24.782
Vascular dementia (sudden onset)	Interleukin-2 receptor antagonist	rs11241559	G	T	0.053	120641005	0.689	0.133	360283	G	T	0.126	5	119976700	2.00E-06	0.027	3677	22.580
Vascular dementia (sudden onset)	Interleukin-2 receptor antagonist	rs117244812	A	G	0.374	6539990	0.596	0.706	360283	A	G	-0.706	17	6443310	2.10E-06	0.149	3677	22.537
Vascular dementia (sudden onset)	Interleukin-2 receptor antagonist	rs12722497	A	C	0.150	6053965	0.524	0.236	360283	A	C	0.628	10	6095928	1.57E-38	0.049	3677	167.609
Vascular dementia (sudden onset)	Interleukin-2 receptor antagonist	rs185231391	C	T	-0.093	59373953	0.903	0.768	360283	C	T	-0.850	3	59359679	1.47E-06	0.181	3677	22.094
Vascular dementia (sudden onset)	Interleukin-2 receptor antagonist	rs4733117	C	A	0.052	32280094	0.717	0.144	360283	C	A	-0.137	8	32137610	2.63E-06	0.029	3677	21.981
Vascular dementia (sudden onset)	Interleukin-2 receptor antagonist	rs61705228	T	C	0.127	100275145	0.683	0.311	360283	T	C	0.330	4	101196302	3.99E-06	0.072	3677	21.281
Vascular dementia (sudden onset)	Interleukin-2	rs12051139	C	T	0.071	86885068	0.549	0.118	360283	C	T	0.113	16	86918674	4.76E-06	0.025	3475	20.967
Vascular dementia (sudden onset)	Interleukin-2	rs13412535	A	G	0.070	224010157	0.628	0.145	360283	A	G	0.176	2	224874874	1.18E-07	0.033	3475	28.231
Vascular dementia (sudden onset)	Interleukin-2	rs170117	T	C	0.079	54524213	0.629	0.164	360283	T	C	-0.162	4	55390380	3.87E-06	0.035	3475	21.467
Vascular dementia (sudden onset)	Interleukin-2	rs2807544	G	A	0.122	14877749	0.307	0.120	360283	G	A	-0.118	1	15204245	3.41E-06	0.025	3475	21.569
Vascular dementia (sudden onset)	Interleukin-2	rs4634519	G	A	-0.052	67727941	0.689	0.130	360283	G	A	0.126	7	67192928	2.77E-06	0.027	3475	21.975
Vascular dementia (sudden onset)	Interleukin-2	rs61335305	A	C	0.079	66160736	0.853	0.429	360283	A	C	0.451	15	66453074	7.32E-07	0.092	3475	24.179
Vascular dementia (sudden onset)	Interleukin-2	rs62124990	T	G	-0.077	19038882	0.849	0.403	360283	T	G	-0.696	2	19238636	3.22E-06	0.150	3475	21.680
Vascular dementia (sudden onset)	Interleukin-2	rs7615304	G	A	0.280	156957914	0.016	0.116	360283	G	A	0.117	3	156675703	1.21E-06	0.024	3475	23.454
Vascular dementia (sudden onset)	Interleukin-2	rs80336398	C	T	-0.641	64075258	0.088	0.376	360283	C	T	-0.400	3	64060934	2.82E-06	0.086	3475	21.745
Vascular dementia (sudden onset)	Interferon gamma	rs10487554	A	G	0.059	149670595	0.646	0.129	360283	A	G	-0.090	7	149367686	1.09E-06	0.018	7701	23.919
Vascular dementia (sudden onset)	Interferon gamma	rs113600793	A	C	0.110	47384095	0.655	0.246	360283	A	C	0.183	17	45461461	8.95E-07	0.037	7701	24.044
Vascular dementia (sudden onset)	Interferon gamma	rs115729819	G	A	-0.364	168783516	0.322	0.368	360283	G	A	-0.248	4	169704667	1.38E-06	0.052	7701	23.264
Vascular dementia (sudden onset)	Interferon gamma	rs11843756	G	T	0.220	48680756	0.450	0.291	360283	G	T	-0.184	13	49254892	3.09E-06	0.039	7701	21.921
Vascular dementia (sudden onset)	Interferon gamma	rs12420286	C	T	0.039	103907166	0.910	0.348	360283	C	T	-0.238	11	103777894	2.08E-06	0.050	7701	22.491
Vascular dementia (sudden onset)	Interferon gamma	rs1867282	T	C	-0.081	99409865	0.490	0.118	360283	T	C	0.077	9	102172147	3.15E-06	0.017	7701	21.740
Vascular dementia (sudden onset)	Interferon gamma	rs2073438	A	G	0.202	6996757	0.127	0.132	360283	A	G	0.090	17	6900076	1.68E-06	0.019	7701	22.816
Vascular dementia (sudden onset)	Interferon gamma	rs74148555	T	C	-0.358	90320085	0.380	0.407	360283	T	C	-0.373	10	92079842	2.64E-06	0.077	7701	23.249
Vascular dementia (sudden onset)	Interferon gamma	rs78296352	T	G	0.186	22495351	0.707	0.496	360283	T	G	0.343	1	22821844	1.38E-07	0.065	7701	27.675
Vascular dementia (sudden onset)	Growth-regulated protein alpha	rs1113500	T	G	0.036	108052820	0.763	0.119	360283	T	G	0.117	1	108595442	1.57E-06	0.024	3505	23.150
Vascular dementia (sudden onset)	Growth-regulated protein alpha	rs12075	A	G	-0.202	159205564	0.082	0.116	360283	A	G	0.375	1	159175354	1.24E-55	0.024	3505	250.494
Vascular dementia (sudden onset)	Growth-regulated protein alpha	rs140734053	A	G	0.965	5359496	0.126	0.631	360283	A	G	0.726	10	5401459	3.58E-06	0.156	3505	21.613

Vascular dementia (sudden onset)	Growth-regulated protein alpha	rs185768063	G	A	0.011	16494752	0.975	0.362	360283	G	A	-0.400	6	16494983	1.46E-07	0.076	3505	27.673
Vascular dementia (sudden onset)	Growth-regulated protein alpha	rs188345231	T	C	-0.582	41579831	0.396	0.686	360283	T	C	0.623	8	41437350	4.34E-06	0.132	3505	22.175
Vascular dementia (sudden onset)	Growth-regulated protein alpha	rs2422841	A	G	0.124	3099706	0.463	0.169	360283	A	G	-0.166	20	3080352	4.66E-06	0.036	3505	21.068
Vascular dementia (sudden onset)	Growth-regulated protein alpha	rs508977	G	T	0.170	73896666	0.211	0.136	360283	G	T	0.380	4	74762383	7.56E-42	0.028	3505	184.378
Vascular dementia (sudden onset)	Growth-regulated protein alpha	rs62024303	G	A	0.401	88327931	0.169	0.291	360283	G	A	0.305	15	88871162	4.41E-06	0.067	3505	21.014
Vascular dementia (sudden onset)	Growth-regulated protein alpha	rs78653452	T	G	-1.014	9781407	0.073	0.567	360283	T	G	-0.736	20	9762055	1.21E-06	0.156	3505	22.328
Vascular dementia (sudden onset)	Granulocyte-colony stimulating factor	rs115256310	G	A	-0.182	72103864	0.782	0.658	360283	G	A	0.682	5	71399691	6.73E-07	0.136	7904	25.155
Vascular dementia (sudden onset)	Granulocyte-colony stimulating factor	rs11903143	G	A	-0.077	29369594	0.551	0.128	360283	G	A	-0.087	2	29592460	6.35E-07	0.018	7904	24.435
Vascular dementia (sudden onset)	Granulocyte-colony stimulating factor	rs147128865	T	C	0.422	34972769	0.265	0.379	360283	T	C	0.270	9	34972766	4.92E-06	0.059	7904	21.157
Vascular dementia (sudden onset)	Granulocyte-colony stimulating factor	rs1817411	T	C	-0.281	97586100	0.048	0.142	360283	T	C	0.089	8	98598328	3.10E-06	0.019	7904	21.713
Vascular dementia (sudden onset)	Granulocyte-colony stimulating factor	rs2671444	A	G	0.132	101158297	0.279	0.122	360283	A	G	-0.078	12	101552075	2.48E-06	0.017	7904	22.306
Vascular dementia (sudden onset)	Granulocyte-colony stimulating factor	rs74148555	T	C	-0.358	90320085	0.380	0.407	360283	T	C	-0.372	10	92079842	1.55E-06	0.076	7904	24.212
Vascular dementia (sudden onset)	Granulocyte-colony stimulating factor	rs77318030	C	T	0.084	54544688	0.772	0.292	360283	C	T	0.205	19	55055897	2.21E-06	0.043	7904	22.830
Vascular dementia (sudden onset)	Fibroblast growth factor basic	rs13412535	A	G	0.070	224010157	0.628	0.145	360283	A	G	-0.111	2	224874874	7.34E-07	0.023	7565	24.426
Vascular dementia (sudden onset)	Fibroblast growth factor basic	rs145577605	A	G	0.079	27642232	0.806	0.320	360283	A	G	0.208	6	27610011	9.64E-07	0.043	7565	23.640
Vascular dementia (sudden onset)	Fibroblast growth factor basic	rs747334	G	A	-0.093	90984987	0.423	0.116	360283	G	A	-0.075	10	92744744	4.53E-06	0.016	7565	20.970
Vascular dementia (sudden onset)	Fibroblast growth factor basic	rs75168112	C	T	-0.170	73418832	0.247	0.147	360283	C	T	0.100	18	71086067	3.00E-06	0.021	7565	21.880
Vascular dementia (sudden onset)	Fibroblast growth factor basic	rs9907295	T	C	0.205	35930309	0.280	0.189	360283	T	C	-0.132	17	34257313	7.95E-07	0.027	7565	24.043
Vascular dementia (sudden onset)	Eotaxin	rs11087905	A	C	0.045	24133015	0.716	0.123	360283	A	C	0.094	21	25505329	5.48E-07	0.019	8153	24.789
Vascular dementia (sudden onset)	Eotaxin	rs112347425	T	C	0.051	46419397	0.805	0.205	360283	T	C	0.158	3	46460888	8.65E-09	0.028	8153	32.535
Vascular dementia (sudden onset)	Eotaxin	rs12075	A	G	-0.202	159205564	0.082	0.116	360283	A	G	0.167	1	159175354	1.33E-26	0.016	8153	114.737
Vascular dementia (sudden onset)	Eotaxin	rs1476670	C	A	0.016	44042523	0.921	0.158	360283	C	A	0.101	1	44508195	3.51E-06	0.022	8153	21.535
Vascular dementia (sudden onset)	Eotaxin	rs2024050	G	A	-0.064	75831075	0.775	0.226	360283	G	A	-0.173	7	75460393	1.10E-08	0.030	8153	32.524
Vascular dementia (sudden onset)	Eotaxin	rs2210755	C	T	-0.057	77608907	0.731	0.166	360283	C	T	0.110	9	80223823	4.85E-06	0.024	8153	20.812
Vascular dementia (sudden onset)	Eotaxin	rs2211994	C	T	-0.119	16675274	0.360	0.130	360283	C	T	-0.089	21	18047593	6.08E-07	0.018	8153	25.000
Vascular dementia (sudden onset)	Eotaxin	rs2228467	C	T	0.119	42864624	0.590	0.220	360283	C	T	0.416	3	42906116	2.27E-46	0.029	8153	203.258
Vascular dementia (sudden onset)	Eotaxin	rs2419841	C	T	0.155	113576224	0.474	0.216	360283	C	T	0.128	10	115335983	4.98E-06	0.028	8153	20.949
Vascular dementia (sudden onset)	Eotaxin	rs5746492	G	A	-0.117	17911167	0.449	0.155	360283	G	A	-0.095	22	18393933	3.96E-06	0.021	8153	21.240
Vascular dementia (sudden onset)	Eotaxin	rs5754733	A	C	0.249	33873606	0.106	0.154	360283	A	C	-0.104	22	34269594	1.06E-06	0.021	8153	23.709
Vascular dementia (sudden onset)	Eotaxin	rs59808887	T	C	0.079	31846414	0.737	0.235	360283	T	C	-0.167	5	31846520	2.91E-06	0.036	8153	21.839
Vascular dementia (sudden onset)	Eotaxin	rs75426604	A	C	0.092	35388508	0.645	0.200	360283	A	C	-0.137	14	35857714	2.53E-06	0.029	8153	22.035
Vascular dementia (sudden onset)	Eotaxin	rs79722574	T	C	0.039	34292033	0.816	0.168	360283	T	C	-0.111	17	32619052	1.06E-06	0.023	8153	23.830
Vascular dementia (sudden onset)	Eotaxin	rs9317045	C	A	-0.005	59055904	0.975	0.164	360283	C	A	-0.118	13	59630038	5.82E-07	0.024	8153	24.874
Vascular dementia (undefined)	CTACK	rs116303454	A	G	-0.186	27253164	0.164	0.134	361227	A	G	0.383	3	27294655	3.27E-06	0.082	3631	22.030
Vascular dementia (undefined)	CTACK	rs2070074	G	A	0.070	34649445	0.308	0.069	361227	G	A	-0.447	9	34649442	1.78E-32	0.037	3631	142.656
Vascular dementia (undefined)	CTACK	rs2731674	G	T	0.019	177412889	0.698	0.050	361227	G	T	0.133	5	176839890	5.63E-07	0.027	3631	24.925
Vascular dementia (undefined)	CTACK	rs3766110	C	A	-0.064	169545945	0.211	0.051	361227	C	A	0.129	1	169515183	3.85E-06	0.028	3631	21.432
Vascular dementia (undefined)	CTACK	rs55764737	C	T	0.016	61031215	0.931	0.184	361227	C	T	-0.531	15	61323414	4.62E-08	0.097	3631	29.878
Vascular dementia (undefined)	CTACK	rs57338032	G	A	0.103	78506597	0.075	0.058	361227	G	A	-0.158	15	78798939	6.23E-07	0.032	3631	24.937
Vascular dementia (undefined)	CTACK	rs7333764	T	C	0.121	33634664	0.298	0.116	361227	T	C	0.277	13	34208801	2.85E-06	0.059	3631	21.867
Vascular dementia (undefined)	CTACK	rs76395525	A	G	-0.052	79449049	0.813	0.219	361227	A	G	0.528	15	79741391	9.55E-07	0.108	3631	23.742
Vascular dementia (undefined)	beta-nerve growth factor	rs28637706	T	G	-0.023	33794463	0.624	0.048	361227	T	G	-0.159	19	34285368	1.42E-09	0.026	3531	36.504
Vascular dementia (undefined)	beta-nerve growth factor	rs67476890	T	C	-0.003	62499295	0.961	0.070	361227	T	C	0.177	15	62791494	3.13E-06	0.038	3531	21.786
Vascular dementia (undefined)	beta-nerve growth factor	rs71641308	T	C	-0.056	77621033	0.459	0.075	361227	T	C	0.204	1	78086718	2.30E-06	0.043	3531	22.365
Vascular dementia (undefined)	beta-nerve growth factor	rs72780728	A	G	-0.039	17561702	0.616	0.077	361227	A	G	0.188	10	17603701	2.99E-06	0.040	3531	21.832
Vascular dementia (undefined)	beta-nerve growth factor	rs73472576	C	T	0.060	74456947	0.173	0.044	361227	C	T	0.118	18	72124182	2.69E-06	0.025	3531	21.963
Vascular dementia (undefined)	beta-nerve growth factor	rs7970581	G	T	0.083	112827443	0.093	0.050	361227	G	T	-0.138	12	113265248	9.27E-07	0.028	3531	23.947
Vascular dementia (undefined)	beta-nerve growth factor	rs9436119	A	G	0.042	150495277	0.343	0.044	361227	A	G	-0.112	1	150467753	3.91E-06	0.025	3531	20.765
Vascular dementia (undefined)	Vascular endothelial growth factor	rs10153304	A	G	0.125	7818613	0.113	0.079	361227	A	G	0.155	17	7721931	1.94E-06	0.033	7118	22.658
Vascular dementia (undefined)	Vascular endothelial growth factor	rs10934631	C	T	-0.037	122978753	0.556	0.062	361227	C	T	0.115	3	122697600	2.47E-06	0.025	7118	22.071
Vascular dementia (undefined)	Vascular endothelial growth factor	rs10967186	C	T	0.014	2617099	0.760	0.044	361227	C	T	-0.090	9	2617099	1.23E-07	0.017	7118	27.903
Vascular dementia (undefined)	Vascular endothelial growth factor	rs13209117	A	G	0.045	44184028	0.369	0.050	361227	A	G	0.130	6	44151765	5.28E-11	0.020	7118	41.959
Vascular dementia (undefined)	Vascular endothelial growth factor	rs143479231	A	G	0.066	193393005	0.563	0.114	361227	A	G	-0.260	3	193110794	1.90E-07	0.049	7118	27.997
Vascular dementia (undefined)	Vascular endothelial growth factor	rs4082730	A	G	-0.001	89980326	0.996	0.134	361227	A	G	0.252	15	90523558	2.64E-06	0.053	7118	22.305
Vascular dementia (undefined)	Vascular endothelial growth factor	rs6921438	A	G	0.019	43957870	0.660	0.044	361227	A	G	-0.490	6	43925607	########	0.018	7118	784.000
Vascular dementia (undefined)	Vascular endothelial growth factor	rs73418461	A	G	-0.127	118463484	0.331	0.130	361227	A	G	-0.249	10	120222996	1.67E-06	0.052	7118	22.878
Vascular dementia (undefined)	Vascular endothelial growth factor	rs8045833	A	G	0.023	88509031	0.668	0.053	361227	A	G	0.108	16	88575439	2.83E-07	0.021	7118	26.199
Vascular dementia (undefined)	Vascular endothelial growth factor	rs9472183	G	A	0.059	43972465	0.176	0.044	361227	G	A	0.128	6	43940202	5.19E-14	0.017	7118	56.869
Vascular dementia (undefined)	Macrophage Migration Inhibitory Factor	rs113218956	A	G	0.116	24828867	0.736	0.342	361227	A	G	-0.895	22	25224834	2.26E-06	0.188	3494	22.678
Vascular dementia (undefined)	Macrophage Migration Inhibitory Factor	rs118055855	C	T	0.323	29867025	0.185	0.244	361227	C	T	-0.691	11	29888572	4.13E-06	0.150	3494	21.203
Vascular dementia (undefined)	Macrophage Migration Inhibitory Factor	rs12594190	G	A	0.050	24791308	0.299	0.048	361227	G	A	-0.136	15	25036455	3.70E-07	0.027	3494	25.755
Vascular dementia (undefined)	Macrophage Migration Inhibitory Factor	rs13142904	T	C	0.150	53452247	0.051	0.077	361227	T	C	-0.223	4	54318414	2.56E-07	0.043	3494	27.532
Vascular dementia (undefined)	Macrophage Migration Inhibitory Factor	rs141009259	C	T	0.203	207111559	0.323	0.205	361227	C	T	0.618	2	207976283	2.47E-06	0.132	3494	21.839
Vascular dementia (undefined)	Macrophage Migration Inhibitory Factor	rs78098071	C	T	-0.041	163882733	0.810	0.172	361227	C	T	0.487	5	163309739	1.78E-07	0.092	3494	28.108

Vascular dementia (undefined)	TRAIL	rs11618126	G	A	-0.463	50252103	0.302	0.448	361227	G	A	-0.891	13	50826239	1.46E-06	0.191	8186	21.661
Vascular dementia (undefined)	TRAIL	rs11657269	G	A	0.023	6416464	0.749	0.072	361227	G	A	-0.119	17	6319784	4.78E-06	0.026	8186	20.878
Vascular dementia (undefined)	TRAIL	rs11699445	G	T	-0.004	15770145	0.920	0.045	361227	G	T	-0.075	20	15750790	3.27E-06	0.016	8186	21.470
Vascular dementia (undefined)	TRAIL	rs13185784	A	G	0.142	180267068	0.003	0.048	361227	A	G	0.085	5	179694068	3.90E-06	0.018	8186	21.372
Vascular dementia (undefined)	TRAIL	rs138987090	G	A	-0.313	32786284	0.101	0.191	361227	G	A	0.750	18	30366247	4.50E-23	0.075	8186	99.389
Vascular dementia (undefined)	TRAIL	rs146783010	G	A	0.162	89527045	0.614	0.322	361227	G	A	0.602	11	89260213	4.83E-06	0.135	8186	19.859
Vascular dementia (undefined)	TRAIL	rs193112415	C	T	-0.214	31255157	0.173	0.157	361227	C	T	1.042	18	28835120	2.15E-62	0.062	8186	279.797
Vascular dementia (undefined)	TRAIL	rs57396456	C	T	-0.152	30365911	0.308	0.149	361227	C	T	0.563	18	27945877	1.25E-27	0.052	8186	117.961
Vascular dementia (undefined)	TRAIL	rs62093514	T	C	-0.274	31651014	0.068	0.150	361227	T	C	1.062	18	29230977	6.86E-82	0.055	8186	370.005
Vascular dementia (undefined)	TRAIL	rs73039026	C	A	0.226	172442691	0.143	0.154	361227	C	A	0.300	3	172160481	2.02E-06	0.064	8186	22.305
Vascular dementia (undefined)	TRAIL	rs747324	C	T	0.013	74222941	0.793	0.049	361227	C	T	0.086	14	74689644	1.61E-06	0.018	8186	23.072
Vascular dementia (undefined)	TRAIL	rs74778900	T	C	-0.026	30506300	0.892	0.195	361227	T	C	0.591	18	28086266	2.59E-28	0.053	8186	123.243
Vascular dementia (undefined)	TRAIL	rs75928541	A	G	-0.247	16400148	0.090	0.145	361227	A	G	0.275	4	16401771	4.24E-06	0.059	8186	21.506
Vascular dementia (undefined)	TRAIL	rs79287178	A	G	-0.062	172576710	0.575	0.110	361227	A	G	-0.432	3	172294500	9.12E-25	0.042	8186	105.148
Vascular dementia (undefined)	Tumor necrosis factor beta	rs10925040	T	C	0.052	247459396	0.259	0.046	361227	T	C	0.176	1	247622698	2.67E-06	0.037	1559	22.138
Vascular dementia (undefined)	Tumor necrosis factor beta	rs753274	T	C	-0.031	14325650	0.491	0.045	361227	T	C	-0.174	19	14436462	2.77E-06	0.037	1559	21.895
Vascular dementia (undefined)	Tumor necrosis factor beta	rs7629875	G	A	0.036	174667832	0.693	0.092	361227	G	A	-0.377	3	174385622	1.37E-06	0.077	1559	23.674
Vascular dementia (undefined)	Tumor necrosis factor beta	rs78296352	T	G	0.142	22495351	0.459	0.192	361227	T	G	1.222	1	22821844	4.76E-21	0.137	1559	79.962
Vascular dementia (undefined)	Tumor necrosis factor alpha	rs10834997	A	G	0.053	26505401	0.265	0.047	361227	A	G	-0.125	11	26526948	1.33E-06	0.026	3454	23.361
Vascular dementia (undefined)	Tumor necrosis factor alpha	rs115669577	A	G	0.040	123440293	0.904	0.330	361227	A	G	0.989	4	124361448	8.28E-07	0.200	3454	24.571
Vascular dementia (undefined)	Tumor necrosis factor alpha	rs79105320	A	G	-0.086	18959850	0.689	0.214	361227	A	G	0.561	8	18817360	3.59E-06	0.118	3454	22.601
Vascular dementia (undefined)	Tumor necrosis factor alpha	rs8121916	A	C	0.008	12420677	0.871	0.050	361227	A	C	0.131	20	12401325	2.72E-06	0.028	3454	22.070
Vascular dementia (undefined)	Stromal-cell-derived factor 1 alpha	rs10474392	G	A	0.031	92198776	0.546	0.051	361227	G	A	-0.096	5	91494593	1.24E-06	0.018	5998	29.209
Vascular dementia (undefined)	Stromal-cell-derived factor 1 alpha	rs12407262	A	G	0.094	63354605	0.185	0.071	361227	A	G	0.118	1	63820276	3.99E-06	0.027	5998	19.646
Vascular dementia (undefined)	Stromal-cell-derived factor 1 alpha	rs139840550	A	G	0.149	38688625	0.328	0.152	361227	A	G	0.183	9	38688622	3.79E-06	0.055	5998	11.160
Vascular dementia (undefined)	Stromal-cell-derived factor 1 alpha	rs149893336	G	A	0.001	170311440	0.997	0.210	361227	G	A	0.503	4	171232591	4.52E-06	0.108	5998	21.686
Vascular dementia (undefined)	Stromal-cell-derived factor 1 alpha	rs4581824	G	T	-0.059	9074853	0.201	0.046	361227	G	T	0.070	19	9185529	3.05E-06	0.017	5998	16.419
Vascular dementia (undefined)	Stromal-cell-derived factor 1 alpha	rs482700	A	G	-0.069	115146334	0.209	0.055	361227	A	G	-0.089	4	116067490	1.57E-06	0.020	5998	19.351
Vascular dementia (undefined)	Stromal-cell-derived factor 1 alpha	rs67689854	A	C	-0.015	89558819	0.788	0.054	361227	A	C	-0.068	16	89625227	3.07E-06	0.020	5998	12.196
Vascular dementia (undefined)	Stromal-cell-derived factor 1 alpha	rs9267091	A	G	0.010	31446032	0.854	0.054	361227	A	G	0.078	6	31413809	3.63E-06	0.020	5998	14.802
Vascular dementia (undefined)	Stem cell growth factor beta	rs112346514	T	C	-0.109	12297173	0.342	0.115	361227	T	C	-0.331	19	12407988	2.37E-06	0.071	3682	21.725
Vascular dementia (undefined)	Stem cell growth factor beta	rs116924815	T	C	0.261	50727476	0.063	0.140	361227	T	C	0.608	19	51230733	1.74E-16	0.074	3682	67.850
Vascular dementia (undefined)	Stem cell growth factor beta	rs117716477	A	C	0.245	103847180	0.174	0.180	361227	A	C	0.838	12	104240958	1.34E-23	0.084	3682	99.383
Vascular dementia (undefined)	Stem cell growth factor beta	rs12480722	C	T	-0.036	20248260	0.586	0.065	361227	C	T	-0.162	20	20228904	4.72E-06	0.036	3682	20.927
Vascular dementia (undefined)	Stem cell growth factor beta	rs139413256	A	G	-0.017	146182552	0.920	0.172	361227	A	G	-0.538	7	145879644	7.04E-07	0.108	3682	24.605
Vascular dementia (undefined)	Stem cell growth factor beta	rs143829871	C	T	-0.073	47555755	0.332	0.076	361227	C	T	0.190	3	47597245	1.90E-06	0.040	3682	22.610
Vascular dementia (undefined)	Stem cell growth factor beta	rs151194174	A	G	-0.082	20956159	0.483	0.117	361227	A	G	0.464	7	20995778	1.13E-06	0.094	3682	24.210
Vascular dementia (undefined)	Stem cell growth factor beta	rs17876031	G	A	0.005	177404118	0.917	0.047	361227	G	A	0.151	5	176831119	2.25E-09	0.026	3682	35.251
Vascular dementia (undefined)	Stem cell growth factor beta	rs264162	G	A	0.002	10944028	0.962	0.044	361227	G	A	-0.110	18	10944026	2.68E-06	0.023	3682	21.978
Vascular dementia (undefined)	Stem cell growth factor beta	rs34911860	A	G	-0.234	79885030	0.186	0.177	361227	A	G	-0.368	1	80350715	3.24E-06	0.079	3682	21.695
Vascular dementia (undefined)	Stem cell growth factor beta	rs4656185	A	G	-0.009	169507088	0.851	0.048	361227	A	G	0.205	1	169476326	1.16E-15	0.026	3682	64.125
Vascular dementia (undefined)	Stem cell growth factor beta	rs4737732	G	A	-0.061	65421393	0.205	0.048	361227	G	A	0.115	8	66333628	4.68E-06	0.025	3682	20.717
Vascular dementia (undefined)	Stem cell growth factor beta	rs7762066	C	T	-0.030	94468249	0.563	0.052	361227	C	T	-0.139	6	95177967	3.50E-06	0.030	3682	21.581
Vascular dementia (undefined)	Stem cell growth factor beta	rs78217154	C	T	0.083	100541844	0.589	0.153	361227	C	T	-0.400	8	101554072	3.77E-06	0.086	3682	21.401
Vascular dementia (undefined)	Stem cell factor	rs113127926	A	C	-0.186	97971174	0.093	0.111	361227	A	C	0.198	14	98437511	2.27E-06	0.042	8290	22.269
Vascular dementia (undefined)	Stem cell factor	rs13412535	A	G	-0.035	224010157	0.525	0.055	361227	A	G	-0.107	2	224874874	6.04E-07	0.021	8290	25.094
Vascular dementia (undefined)	Stem cell factor	rs1557570	T	G	-0.014	169538606	0.771	0.047	361227	T	G	0.119	1	169507844	2.74E-12	0.017	8290	48.671
Vascular dementia (undefined)	Stem cell factor	rs1568119	T	C	-0.271	33385679	0.339	0.283	361227	T	C	-0.591	8	33243197	1.24E-07	0.113	8290	27.365
Vascular dementia (undefined)	Stem cell factor	rs1942355	T	C	-0.028	71694503	0.520	0.044	361227	T	C	-0.072	18	69361739	4.70E-06	0.016	8290	20.798
Vascular dementia (undefined)	Stem cell factor	rs4841899	C	T	-0.121	134532566	0.015	0.050	361227	C	T	0.100	9	137424412	1.78E-08	0.018	8290	31.815
Vascular dementia (undefined)	Stem cell factor	rs635634	T	C	0.023	133279427	0.674	0.054	361227	T	C	-0.103	9	136155000	6.74E-08	0.019	8290	29.194
Vascular dementia (undefined)	Stem cell factor	rs78666213	G	T	-0.143	179217495	0.341	0.150	361227	G	T	0.274	4	180138649	2.59E-06	0.058	8290	22.695
Vascular dementia (undefined)	Stem cell factor	rs80271436	A	G	0.277	133022383	0.041	0.136	361227	A	G	-0.237	9	135897770	9.95E-07	0.049	8290	23.879
Vascular dementia (undefined)	Interleukin-16	rs117217798	T	C	0.026	33156215	0.736	0.079	361227	T	C	-0.204	17	31483233	4.15E-06	0.044	3483	21.028
Vascular dementia (undefined)	Interleukin-16	rs117916513	A	G	-0.104	121393565	0.552	0.174	361227	A	G	-0.502	11	121264274	3.79E-07	0.099	3483	25.921
Vascular dementia (undefined)	Interleukin-16	rs1255143	T	C	-0.014	128253936	0.755	0.044	361227	T	C	0.131	10	130052200	7.10E-08	0.024	3483	29.124
Vascular dementia (undefined)	Interleukin-16	rs12765671	A	G	-0.254	104924411	0.287	0.239	361227	A	G	-0.602	10	106684169	4.84E-06	0.132	3483	20.883
Vascular dementia (undefined)	Interleukin-16	rs144691581	A	G	0.220	96410095	0.143	0.150	361227	A	G	0.488	15	96953325	4.20E-07	0.097	3483	25.488
Vascular dementia (undefined)	Interleukin-16	rs1801020	G	A	0.022	177409531	0.653	0.050	361227	G	A	-0.173	5	176836532	4.53E-10	0.027	3483	40.594
Vascular dementia (undefined)	Interleukin-16	rs4253283	C	T	-0.088	186244057	0.065	0.048	361227	C	T	-0.146	4	187165211	1.75E-08	0.026	3483	31.053
Vascular dementia (undefined)	Interleukin-16	rs4513633	A	C	0.088	112649483	0.279	0.082	361227	A	C	-0.224	4	113570639	7.44E-07	0.045	3483	24.429
Vascular dementia (undefined)	Interleukin-16	rs4778636	A	G	0.182	81299298	0.078	0.103	361227	A	G	-0.727	15	81591639	1.11E-30	0.063	3483	131.978
Vascular dementia (undefined)	Interleukin-16	rs9706053	T	C	0.045	65982530	0.791	0.169	361227	T	C	0.458	12	66376310	7.01E-07	0.093	3483	24.170
Vascular dementia (undefined)	RANTES	rs112072646	A	G	0.281	53217255	0.093	0.167	361227	A	G	0.429	2	53444393	6.48E-07	0.086	3421	24.722

Vascular dementia (undefined)	RANTES	rs147509526	T	C	-0.133	15665520	0.285	0.125	361227	T	C	-0.358	19	15776330	6.93E-07	0.072	3421	24.930
Vascular dementia (undefined)	RANTES	rs4940620	G	A	0.024	64303876	0.804	0.099	361227	G	A	0.249	18	61971111	3.54E-06	0.054	3421	21.331
Vascular dementia (undefined)	RANTES	rs62438851	G	A	-0.022	144909173	0.762	0.071	361227	G	A	0.196	6	145230309	2.33E-06	0.041	3421	22.345
Vascular dementia (undefined)	RANTES	rs7000423	T	C	-0.014	110041420	0.756	0.045	361227	T	C	-0.132	8	111053649	1.82E-07	0.025	3421	27.139
Vascular dementia (undefined)	RANTES	rs72793342	A	G	-0.041	30537031	0.448	0.053	361227	A	G	-0.149	16	30548352	1.48E-06	0.031	3421	23.309
Vascular dementia (undefined)	RANTES	rs74472919	T	C	0.025	81626515	0.824	0.114	361227	T	C	0.331	13	82200650	3.97E-08	0.061	3421	29.987
Vascular dementia (undefined)	RANTES	rs75613039	T	C	-0.093	129706688	0.488	0.134	361227	T	C	0.370	11	129576583	4.81E-06	0.081	3421	20.866
Vascular dementia (undefined)	RANTES	rs818452	T	C	-0.177	152594661	0.052	0.091	361227	T	C	0.238	6	152915796	2.36E-06	0.051	3421	22.230
Vascular dementia (undefined)	Platelet-derived growth factor BB	rs116445074	T	G	0.029	52238766	0.852	0.156	361227	T	G	0.293	5	51534600	3.11E-07	0.059	8293	24.932
Vascular dementia (undefined)	Platelet-derived growth factor BB	rs11766649	G	A	0.042	145142154	0.434	0.054	361227	G	A	-0.091	7	144839247	3.53E-06	0.020	8293	21.461
Vascular dementia (undefined)	Platelet-derived growth factor BB	rs11916118	G	A	-0.074	117193342	0.185	0.056	361227	G	A	-0.089	3	116912189	4.93E-06	0.019	8293	20.999
Vascular dementia (undefined)	Platelet-derived growth factor BB	rs12289510	G	A	0.009	125077155	0.842	0.044	361227	G	A	0.078	11	124947051	7.69E-07	0.016	8293	24.371
Vascular dementia (undefined)	Platelet-derived growth factor BB	rs13412535	A	G	-0.035	224010157	0.525	0.055	361227	A	G	0.335	2	224874874	2.46E-55	0.021	8293	245.347
Vascular dementia (undefined)	Platelet-derived growth factor BB	rs2324229	C	T	0.026	83208412	0.563	0.046	361227	C	T	-0.089	6	83918131	3.48E-08	0.016	8293	30.834
Vascular dementia (undefined)	Platelet-derived growth factor BB	rs35859699	A	G	0.206	111263595	0.305	0.200	361227	A	G	-0.395	4	112184751	2.07E-06	0.084	8293	22.030
Vascular dementia (undefined)	Platelet-derived growth factor BB	rs4965869	T	C	0.028	101450115	0.585	0.051	361227	T	C	0.184	15	101990320	5.66E-24	0.018	8293	103.342
Vascular dementia (undefined)	Platelet-derived growth factor BB	rs55680718	T	C	0.039	224302160	0.559	0.067	361227	T	C	-0.138	2	225166877	1.86E-08	0.025	8293	31.606
Vascular dementia (undefined)	Platelet-derived growth factor BB	rs72777070	G	T	0.038	9658748	0.480	0.054	361227	G	T	0.107	2	9798877	8.98E-08	0.020	8293	28.569
Vascular dementia (undefined)	Platelet-derived growth factor BB	rs73162807	A	C	-0.045	146757003	0.747	0.140	361227	A	C	-0.239	3	146474790	1.74E-06	0.050	8293	22.959
Vascular dementia (undefined)	Platelet-derived growth factor BB	rs9936075	G	A	-0.003	7271908	0.940	0.046	361227	G	A	0.078	16	7321909	1.76E-06	0.016	8293	22.737
Vascular dementia (undefined)	Platelet-derived growth factor BB	rs9941733	G	A	-0.063	393417	0.277	0.058	361227	G	A	-0.116	20	374061	3.31E-07	0.023	8293	25.930
Vascular dementia (undefined)	Macrophage inflammatory protein 1b	rs11130043	A	G	0.003	45069747	0.937	0.044	361227	A	G	-0.073	3	45111239	3.22E-06	0.016	8243	21.679
Vascular dementia (undefined)	Macrophage inflammatory protein 1b	rs113010081	C	T	-0.004	46415921	0.953	0.065	361227	C	T	0.595	3	46457412	########	0.024	8243	636.493
Vascular dementia (undefined)	Macrophage inflammatory protein 1b	rs113877493	T	C	-0.016	36443746	0.785	0.060	361227	T	C	-0.612	17	34812273	########	0.022	8243	789.146
Vascular dementia (undefined)	Macrophage inflammatory protein 1b	rs116237296	A	G	0.350	86579833	0.256	0.308	361227	A	G	0.544	1	87045516	7.23E-07	0.112	8243	23.778
Vascular dementia (undefined)	Macrophage inflammatory protein 1b	rs117453826	G	A	0.063	36775624	0.668	0.147	361227	G	A	0.577	17	35132809	5.07E-22	0.059	8243	94.808
Vascular dementia (undefined)	Macrophage inflammatory protein 1b	rs141102180	T	G	-0.006	36108811	0.955	0.116	361227	T	G	0.323	17	34436204	1.08E-16	0.039	8243	67.340
Vascular dementia (undefined)	Macrophage inflammatory protein 1b	rs17138331	G	A	0.015	7826737	0.854	0.080	361227	G	A	0.139	7	7866368	2.26E-06	0.030	8243	22.234
Vascular dementia (undefined)	Macrophage inflammatory protein 1b	rs17641689	G	A	-0.021	36668383	0.765	0.070	361227	G	A	0.245	17	35024819	1.28E-16	0.029	8243	69.805
Vascular dementia (undefined)	Macrophage inflammatory protein 1b	rs2079664	G	A	0.016	34680936	0.742	0.049	361227	G	A	-0.100	17	33007955	1.51E-08	0.018	8243	31.961
Vascular dementia (undefined)	Macrophage inflammatory protein 1b	rs281749	C	T	-0.085	107626417	0.073	0.047	361227	C	T	-0.080	8	108638645	3.17E-06	0.017	8243	21.832
Vascular dementia (undefined)	Macrophage inflammatory protein 1b	rs34437725	C	T	0.082	35499766	0.533	0.132	361227	C	T	0.263	17	33826785	7.67E-08	0.048	8243	29.717
Vascular dementia (undefined)	Macrophage inflammatory protein 1b	rs72791296	T	C	-0.217	121614355	0.064	0.117	361227	T	C	0.237	5	120950050	3.78E-07	0.047	8243	25.844
Vascular dementia (undefined)	Macrophage inflammatory protein 1b	rs72799710	T	C	0.083	123825971	0.170	0.061	361227	T	C	-0.101	5	123161665	3.21E-06	0.022	8243	21.635
Vascular dementia (undefined)	Macrophage inflammatory protein 1b	rs74810984	C	T	0.074	127876202	0.597	0.141	361227	C	T	-0.221	10	129674466	1.96E-06	0.047	8243	21.660
Vascular dementia (undefined)	Macrophage inflammatory protein 1b	rs76582507	A	G	0.006	37510075	0.980	0.233	361227	A	G	0.318	9	37510072	3.26E-06	0.068	8243	21.994
Vascular dementia (undefined)	Macrophage inflammatory protein 1b	rs76583883	T	G	0.148	45936445	0.212	0.119	361227	T	G	-0.232	21	47356359	4.99E-06	0.051	8243	20.559
Vascular dementia (undefined)	Macrophage inflammatory protein 1b	rs76776296	G	A	0.324	115488433	0.031	0.150	361227	G	A	-0.300	7	115128487	5.55E-07	0.060	8243	25.117
Vascular dementia (undefined)	Macrophage inflammatory protein 1a	rs10835056	G	T	0.031	26675470	0.505	0.047	361227	G	T	-0.119	11	26697017	2.60E-06	0.025	3522	22.097
Vascular dementia (undefined)	Macrophage inflammatory protein 1a	rs12690897	A	G	-0.018	85716861	0.704	0.048	361227	A	G	0.125	7	85346177	2.11E-06	0.026	3522	22.690
Vascular dementia (undefined)	Macrophage inflammatory protein 1a	rs184154340	A	G	0.026	80790993	0.818	0.113	361227	A	G	0.331	11	80502036	1.86E-06	0.069	3522	22.813
Vascular dementia (undefined)	Macrophage inflammatory protein 1a	rs34771762	G	A	0.103	200547932	0.224	0.084	361227	G	A	-0.249	2	201412655	2.13E-06	0.052	3522	22.667
Vascular dementia (undefined)	Macrophage inflammatory protein 1a	rs57786342	A	G	0.024	68793311	0.648	0.052	361227	A	G	0.131	14	69260028	4.05E-06	0.029	3522	21.257
Vascular dementia (undefined)	Macrophage inflammatory protein 1a	rs60198979	A	G	0.174	43250698	0.036	0.083	361227	A	G	-0.215	22	43646704	2.61E-06	0.046	3522	21.955
Vascular dementia (undefined)	Macrophage inflammatory protein 1a	rs7232268	G	A	0.139	70101678	0.220	0.113	361227	G	A	-0.282	18	67768914	2.55E-06	0.060	3522	22.180
Vascular dementia (undefined)	Monokine induced by gamma interferon	rs111607343	A	G	-0.174	897855	0.363	0.191	361227	A	G	-0.521	19	897855	2.83E-06	0.112	3685	21.678
Vascular dementia (undefined)	Monokine induced by gamma interferon	rs11177248	A	G	0.141	68482106	0.201	0.110	361227	A	G	0.307	12	68875886	4.45E-06	0.067	3685	21.037
Vascular dementia (undefined)	Monokine induced by gamma interferon	rs112337562	G	T	-0.196	92665225	0.168	0.142	361227	G	T	0.370	14	93131570	2.98E-06	0.080	3685	21.606
Vascular dementia (undefined)	Monokine induced by gamma interferon	rs112861654	G	A	-0.040	42179062	0.655	0.089	361227	G	A	0.277	21	43599172	1.81E-07	0.053	3685	27.320
Vascular dementia (undefined)	Monokine induced by gamma interferon	rs117831247	T	C	-0.269	66742081	0.429	0.340	361227	T	C	-0.833	10	68501839	2.16E-06	0.175	3685	22.576
Vascular dementia (undefined)	Monokine induced by gamma interferon	rs139010077	T	C	0.187	170618359	0.278	0.172	361227	T	C	0.432	3	170336148	3.55E-06	0.095	3685	20.698
Vascular dementia (undefined)	Monokine induced by gamma interferon	rs1796086	C	T	0.070	71183729	0.373	0.079	361227	C	T	0.210	7	70648715	2.23E-07	0.040	3685	27.050
Vascular dementia (undefined)	Monokine induced by gamma interferon	rs41272086	A	G	-0.118	160587614	0.109	0.073	361227	A	G	-0.223	6	161008646	7.43E-08	0.042	3685	28.771
Vascular dementia (undefined)	Monokine induced by gamma interferon	rs55876513	G	T	0.001	75962545	0.981	0.049	361227	G	T	-0.166	4	76883698	8.23E-11	0.026	3685	42.378
Vascular dementia (undefined)	Monokine induced by gamma interferon	rs5752128	C	T	-0.104	25322656	0.125	0.068	361227	C	T	0.169	22	25718623	4.34E-06	0.037	3685	20.852
Vascular dementia (undefined)	Monokine induced by gamma interferon	rs62562991	A	G	0.019	95973777	0.918	0.184	361227	A	G	0.624	9	98736059	8.40E-07	0.126	3685	24.495
Vascular dementia (undefined)	Monokine induced by gamma interferon	rs6679677	A	C	-0.014	113761186	0.818	0.063	361227	A	C	0.162	1	114303808	8.86E-07	0.033	3685	24.246
Vascular dementia (undefined)	Monokine induced by gamma interferon	rs77086208	T	C	-0.020	70152774	0.882	0.132	361227	T	C	0.323	14	70619491	3.83E-06	0.070	3685	21.361
Vascular dementia (undefined)	Monokine induced by gamma interferon	rs816960	T	C	0.034	107870173	0.450	0.046	361227	T	C	-0.122	13	108522521	5.01E-07	0.024	3685	25.164
Vascular dementia (undefined)	Macrophage colony stimulating factor	rs116274860	G	T	-0.142	148675030	0.456	0.190	361227	G	T	-0.819	3	148392817	2.74E-06	0.174	840	22.129
Vascular dementia (undefined)	Macrophage colony stimulating factor	rs117867915	C	T	0.134	44630078	0.401	0.160	361227	C	T	-0.527	18	42210043	1.61E-06	0.110	840	23.054
Vascular dementia (undefined)	Macrophage colony stimulating factor	rs12962919	T	C	-0.162	78018752	0.056	0.084	361227	T	C	0.305	18	75778756	4.65E-06	0.066	840	21.255
Vascular dementia (undefined)	Macrophage colony stimulating factor	rs56367447	T	C	-0.025	4014005	0.837	0.123	361227	T	C	-0.497	8	3871527	1.72E-08	0.088	840	31.642
Vascular dementia (undefined)	Macrophage colony stimulating factor	rs62294910	A	G	-0.004	182480551	0.964	0.090	361227	A	G	0.343	3	182198339	6.82E-07	0.069	840	24.654

Vascular dementia (undefined)	Macrophage colony stimulating factor	rs78296352	T	G	0.142	22495351	0.459	0.192	361227	T	G	0.527	1	22821844	1.05E-06	0.111	840	22.460
Vascular dementia (undefined)	Macrophage colony stimulating factor	rs9387100	C	T	-0.043	112781752	0.334	0.044	361227	C	T	0.135	6	113102954	4.07E-06	0.029	840	21.438
Vascular dementia (undefined)	Monocyte chemoattractant protein-1	rs10145849	A	G	-0.039	82475647	0.393	0.046	361227	A	G	-0.076	14	82941991	3.41E-06	0.016	8293	21.720
Vascular dementia (undefined)	Monocyte chemoattractant protein-1	rs10744620	C	T	0.079	3629928	0.083	0.045	361227	C	T	-0.079	12	3739094	9.91E-07	0.016	8293	23.955
Vascular dementia (undefined)	Monocyte chemoattractant protein-1	rs111995966	G	T	-0.012	108558513	0.880	0.083	361227	G	T	-0.145	2	109174969	2.53E-06	0.031	8293	21.939
Vascular dementia (undefined)	Monocyte chemoattractant protein-1	rs112313229	A	G	0.039	46323369	0.638	0.084	361227	A	G	-0.165	3	46364860	1.43E-07	0.031	8293	27.655
Vascular dementia (undefined)	Monocyte chemoattractant protein-1	rs12073356	A	G	-0.032	207834503	0.707	0.086	361227	A	G	-0.143	1	208007848	4.17E-06	0.031	8293	21.024
Vascular dementia (undefined)	Monocyte chemoattractant protein-1	rs12075	A	G	0.011	159205564	0.802	0.044	361227	A	G	0.219	1	159175354	1.44E-44	0.016	8293	198.719
Vascular dementia (undefined)	Monocyte chemoattractant protein-1	rs12493471	C	T	0.043	45910186	0.350	0.046	361227	C	T	-0.116	3	45951678	6.81E-13	0.016	8293	51.538
Vascular dementia (undefined)	Monocyte chemoattractant protein-1	rs146522229	T	C	0.192	47295223	0.573	0.341	361227	T	C	-0.598	19	47798480	3.56E-07	0.118	8293	25.779
Vascular dementia (undefined)	Monocyte chemoattractant protein-1	rs2228467	C	T	0.001	42864624	0.989	0.083	361227	C	T	0.264	3	42906116	9.19E-20	0.029	8293	82.117
Vascular dementia (undefined)	Monocyte chemoattractant protein-1	rs2712431	A	C	0.022	128598047	0.645	0.048	361227	A	C	-0.079	3	128316890	4.75E-06	0.017	8293	20.936
Vascular dementia (undefined)	Monocyte chemoattractant protein-1	rs56212190	T	C	-0.025	41702868	0.806	0.103	361227	T	C	0.181	1	42168539	9.85E-07	0.037	8293	23.547
Vascular dementia (undefined)	Monocyte chemoattractant protein-1	rs7197349	G	A	0.031	78653322	0.576	0.055	361227	G	A	-0.097	16	78687219	2.62E-06	0.021	8293	22.081
Vascular dementia (undefined)	Monocyte chemoattractant protein-1	rs7517040	G	A	-0.033	158889343	0.530	0.053	361227	G	A	0.099	1	158859133	2.44E-07	0.019	8293	26.703
Vascular dementia (undefined)	Monocyte chemoattractant protein-1	rs9317045	C	A	0.005	59055904	0.940	0.061	361227	C	A	-0.113	13	59630038	1.52E-06	0.024	8293	23.089
Vascular dementia (undefined)	Interleukin-12p70	rs13209117	A	G	0.045	44184028	0.369	0.050	361227	A	G	0.100	6	44151765	5.57E-08	0.019	8270	29.021
Vascular dementia (undefined)	Interleukin-12p70	rs17229494	G	A	-0.061	37555798	0.383	0.071	361227	G	A	0.117	21	38928100	4.93E-06	0.026	8270	20.796
Vascular dementia (undefined)	Interleukin-12p70	rs282258	C	T	-0.057	224050083	0.199	0.044	361227	C	T	-0.073	2	224914800	3.21E-06	0.016	8270	21.898
Vascular dementia (undefined)	Interleukin-12p70	rs41282644	A	G	0.030	43785985	0.719	0.082	361227	A	G	0.147	6	43753722	1.05E-06	0.030	8270	23.478
Vascular dementia (undefined)	Interleukin-12p70	rs4349809	G	T	0.009	43957093	0.834	0.044	361227	G	T	-0.378	6	43924830	########	0.016	8270	564.287
Vascular dementia (undefined)	Interleukin-12p70	rs71361173	G	T	0.001	76000450	0.984	0.065	361227	G	T	-0.111	18	73712405	3.06E-06	0.024	8270	21.570
Vascular dementia (undefined)	Interleukin-12p70	rs72831623	A	G	-0.071	47644927	0.414	0.087	361227	A	G	0.191	17	45722293	2.42E-07	0.037	8270	26.732
Vascular dementia (undefined)	Interleukin-12p70	rs782107	A	G	0.057	58439747	0.196	0.044	361227	A	G	0.075	12	58833530	1.60E-06	0.016	8270	23.114
Vascular dementia (undefined)	Interleukin-12p70	rs79121401	C	T	-0.371	78986084	0.239	0.315	361227	C	T	-0.555	11	78697129	4.24E-06	0.121	8270	21.163
Vascular dementia (undefined)	Interleukin-12p70	rs9472183	G	A	0.059	43972465	0.176	0.044	361227	G	A	0.102	6	43940202	8.61E-11	0.016	8270	42.126
Vascular dementia (undefined)	Interferon gamma-induced protein 10	rs10809307	C	T	-0.049	11045908	0.355	0.053	361227	C	T	-0.131	9	11045908	3.64E-06	0.028	3685	21.415
Vascular dementia (undefined)	Interferon gamma-induced protein 10	rs113831257	A	G	-0.051	75234311	0.623	0.103	361227	A	G	0.359	4	76159521	2.53E-08	0.064	3685	31.110
Vascular dementia (undefined)	Interferon gamma-induced protein 10	rs11626201	A	C	0.056	36511495	0.220	0.045	361227	A	C	0.116	14	36980700	1.93E-06	0.025	3685	22.495
Vascular dementia (undefined)	Interferon gamma-induced protein 10	rs143799975	G	A	-0.030	75885862	0.906	0.251	361227	G	A	0.798	4	76807015	1.00E-06	0.164	3685	23.787
Vascular dementia (undefined)	Interferon gamma-induced protein 10	rs34383175	T	C	-0.239	144361034	0.042	0.118	361227	T	C	-0.315	8	145584694	1.51E-06	0.066	3685	23.031
Vascular dementia (undefined)	Interferon gamma-induced protein 10	rs75970138	A	G	-0.009	119813998	0.964	0.202	361227	A	G	-0.485	9	122576276	1.53E-06	0.104	3685	21.748
Vascular dementia (undefined)	Interferon gamma-induced protein 10	rs7645625	G	T	-0.026	146856250	0.556	0.045	361227	G	T	0.109	3	146574037	4.41E-06	0.024	3685	20.997
Vascular dementia (undefined)	Interferon gamma-induced protein 10	rs79848609	C	A	0.132	86772934	0.201	0.103	361227	C	A	-0.260	15	87316165	8.75E-07	0.054	3685	23.496
Vascular dementia (undefined)	Interferon gamma-induced protein 10	rs8112909	A	G	0.116	45910150	0.032	0.054	361227	A	G	-0.143	19	46413408	1.94E-06	0.030	3685	22.746
Vascular dementia (undefined)	Interleukin-18	rs10414578	T	C	0.018	54634619	0.777	0.063	361227	T	C	-0.177	19	55146070	4.16E-07	0.035	3636	25.604
Vascular dementia (undefined)	Interleukin-18	rs115267715	T	C	-0.046	69239188	0.786	0.171	361227	T	C	0.451	5	68535015	1.72E-08	0.080	3636	31.753
Vascular dementia (undefined)	Interleukin-18	rs116383510	C	A	0.188	2545536	0.314	0.187	361227	C	A	0.543	5	2545650	3.00E-07	0.106	3636	26.402
Vascular dementia (undefined)	Interleukin-18	rs117266781	T	C	0.058	41261422	0.805	0.234	361227	T	C	0.684	7	41301020	3.15E-06	0.147	3636	21.716
Vascular dementia (undefined)	Interleukin-18	rs144841621	T	C	0.212	69921801	0.357	0.230	361227	T	C	0.518	10	71681557	3.81E-06	0.114	3636	20.610
Vascular dementia (undefined)	Interleukin-18	rs17229943	C	A	0.060	69386709	0.417	0.074	361227	C	A	0.312	5	68682536	1.62E-11	0.046	3636	45.410
Vascular dementia (undefined)	Interleukin-18	rs1852105	C	T	0.012	64265217	0.917	0.118	361227	C	T	-0.304	7	63725595	4.32E-06	0.066	3636	21.096
Vascular dementia (undefined)	Interleukin-18	rs1979967	T	C	0.054	79367271	0.316	0.053	361227	T	C	0.140	15	79659613	9.45E-07	0.029	3636	24.031
Vascular dementia (undefined)	Interleukin-18	rs2729385	A	G	0.000	57495520	0.992	0.048	361227	A	G	0.123	11	57262993	3.79E-06	0.026	3636	22.076
Vascular dementia (undefined)	Interleukin-18	rs385076	C	T	0.086	32264782	0.060	0.046	361227	C	T	0.243	2	32489851	1.66E-22	0.025	3636	96.166
Vascular dementia (undefined)	Interleukin-18	rs4482818	G	A	0.016	65062779	0.720	0.045	361227	G	A	-0.129	4	65928497	1.45E-07	0.024	3636	27.778
Vascular dementia (undefined)	Interleukin-18	rs658805	A	G	-0.046	70199369	0.318	0.046	361227	A	G	0.123	6	70909073	4.94E-07	0.024	3636	25.247
Vascular dementia (undefined)	Interleukin-18	rs71478720	T	C	0.033	112138882	0.522	0.051	361227	T	C	-0.267	11	112009605	3.71E-22	0.028	3636	93.515
Vascular dementia (undefined)	Interleukin-18	rs78623212	T	C	0.182	103667180	0.463	0.248	361227	T	C	0.871	7	103307627	6.71E-07	0.178	3636	23.970
Vascular dementia (undefined)	Interleukin-18	rs78716465	A	G	0.093	42015086	0.409	0.113	361227	A	G	0.327	20	40643726	1.63E-06	0.068	3636	22.919
Vascular dementia (undefined)	Interleukin-17	rs117029961	A	G	0.103	37147653	0.671	0.242	361227	A	G	0.459	10	37436581	4.94E-06	0.102	7760	20.405
Vascular dementia (undefined)	Interleukin-17	rs117556572	T	C	0.157	104436567	0.265	0.141	361227	T	C	-0.510	13	105088917	3.28E-06	0.110	7760	21.552
Vascular dementia (undefined)	Interleukin-17	rs1530455	C	T	-0.056	123136052	0.224	0.046	361227	C	T	-0.108	3	122854899	4.87E-10	0.017	7760	38.972
Vascular dementia (undefined)	Interleukin-17	rs17106604	T	C	0.061	77912813	0.328	0.062	361227	T	C	0.113	14	78379156	6.37E-07	0.023	7760	25.178
Vascular dementia (undefined)	Interleukin-17	rs17282552	C	T	-0.249	207109091	0.015	0.102	361227	C	T	0.200	2	207973815	8.21E-07	0.041	7760	24.411
Vascular dementia (undefined)	Interleukin-17	rs184080173	C	T	0.033	77331424	0.788	0.123	361227	C	T	-0.238	12	77725204	4.19E-07	0.047	7760	25.620
Vascular dementia (undefined)	Interleukin-17	rs187475560	T	C	-0.046	160353411	0.729	0.132	361227	T	C	-0.243	4	161274563	3.29E-06	0.052	7760	21.910
Vascular dementia (undefined)	Interleukin-17	rs62191444	T	G	-0.090	393023	0.142	0.061	361227	T	G	-0.114	20	373667	4.22E-06	0.025	7760	21.153
Vascular dementia (undefined)	Interleukin-17	rs78296352	T	G	0.142	22495351	0.459	0.192	361227	T	G	0.303	1	22821844	4.27E-06	0.065	7760	21.956
Vascular dementia (undefined)	Interleukin-17	rs78612928	C	T	-0.108	29812292	0.070	0.060	361227	C	T	-0.104	4	29813914	2.62E-06	0.022	7760	21.820
Vascular dementia (undefined)	Interleukin-13	rs117795020	A	G	-0.092	87469237	0.477	0.129	361227	A	G	-0.352	9	90084152	9.86E-07	0.072	3557	24.197
Vascular dementia (undefined)	Interleukin-13	rs12623722	A	G	-0.010	22955811	0.831	0.048	361227	A	G	-0.119	2	23178683	4.19E-06	0.026	3557	21.096
Vascular dementia (undefined)	Interleukin-13	rs139083458	T	C	0.042	26160409	0.899	0.335	361227	T	C	0.990	5	26160518	2.81E-06	0.211	3557	22.086
Vascular dementia (undefined)	Interleukin-13	rs142167313	C	T	0.252	44204360	0.025	0.112	361227	C	T	0.313	6	44172097	3.98E-07	0.062	3557	25.735

Vascular dementia (undefined)	Interleukin-13	rs27949	T	C	0.041	59254997	0.384	0.047	361227	T	C	-0.117	5	58550823	3.43E-06	0.025	3557	21.482
Vascular dementia (undefined)	Interleukin-13	rs6799107	C	T	-0.095	127338175	0.088	0.056	361227	C	T	0.146	3	127057018	1.25E-06	0.030	3557	23.495
Vascular dementia (undefined)	Interleukin-13	rs7073807	C	T	0.010	67393670	0.873	0.065	361227	C	T	-0.168	10	69153428	2.37E-06	0.036	3557	22.323
Vascular dementia (undefined)	Interleukin-13	rs75995699	A	G	0.071	5140622	0.559	0.121	361227	A	G	0.332	6	5140856	2.64E-06	0.070	3557	22.610
Vascular dementia (undefined)	Interleukin-13	rs9472168	G	A	0.008	43961248	0.850	0.044	361227	G	A	-0.424	6	43928985	1.08E-65	0.025	3557	292.851
Vascular dementia (undefined)	Interleukin-10	rs10457128	A	G	0.015	105570101	0.741	0.045	361227	A	G	-0.087	6	106017976	5.24E-07	0.017	7681	25.292
Vascular dementia (undefined)	Interleukin-10	rs10493718	A	C	-0.020	82597250	0.741	0.060	361227	A	C	-0.110	1	83062933	7.16E-07	0.022	7681	24.552
Vascular dementia (undefined)	Interleukin-10	rs11206302	T	C	0.042	54208270	0.530	0.067	361227	T	C	-0.119	1	54673943	2.20E-06	0.025	7681	22.440
Vascular dementia (undefined)	Interleukin-10	rs2086656	T	C	-0.061	59632755	0.187	0.046	361227	T	C	-0.079	4	60498473	3.78E-06	0.017	7681	21.289
Vascular dementia (undefined)	Interleukin-10	rs282258	C	T	-0.057	224050083	0.199	0.044	361227	C	T	-0.099	2	224914800	1.00E-09	0.016	7681	37.497
Vascular dementia (undefined)	Interleukin-10	rs3025021	C	T	0.039	43781426	0.403	0.046	361227	C	T	-0.095	6	43749163	1.46E-06	0.020	7681	23.585
Vascular dementia (undefined)	Interleukin-10	rs41282660	G	A	0.025	44229269	0.693	0.064	361227	G	A	0.119	6	44197006	3.72E-06	0.026	7681	21.924
Vascular dementia (undefined)	Interleukin-10	rs4349809	G	T	0.009	43957093	0.834	0.044	361227	G	T	-0.285	6	43924830	5.77E-67	0.017	7681	298.976
Vascular dementia (undefined)	Interleukin-10	rs465757	A	G	0.040	15599638	0.386	0.046	361227	A	G	0.084	20	15580283	1.17E-06	0.017	7681	23.306
Vascular dementia (undefined)	Interleukin-10	rs7088799	G	T	0.012	63256414	0.791	0.045	361227	G	T	0.085	10	65016174	3.23E-07	0.017	7681	26.028
Vascular dementia (undefined)	Interleukin-8	rs11634944	C	T	0.062	24937946	0.177	0.046	361227	C	T	0.121	15	25183093	1.29E-06	0.025	3526	23.208
Vascular dementia (undefined)	Interleukin-8	rs12075	A	G	0.011	159205564	0.802	0.044	361227	A	G	0.120	1	159175354	3.88E-07	0.024	3526	25.855
Vascular dementia (undefined)	Interleukin-8	rs141926526	C	A	0.114	32809028	0.629	0.235	361227	C	A	0.615	7	32848640	2.57E-06	0.131	3526	22.100
Vascular dementia (undefined)	Interleukin-8	rs2673604	A	C	-0.034	132399360	0.477	0.047	361227	A	C	-0.127	8	133411607	7.02E-07	0.026	3526	24.648
Vascular dementia (undefined)	Interleukin-6	rs1333040	T	C	0.019	22083405	0.667	0.044	361227	T	C	0.074	9	22083404	3.17E-06	0.016	8189	21.817
Vascular dementia (undefined)	Interleukin-6	rs13412535	A	G	-0.035	224010157	0.525	0.055	361227	A	G	-0.116	2	224874874	7.34E-08	0.022	8189	29.311
Vascular dementia (undefined)	Interleukin-6	rs72831623	A	G	-0.071	47644927	0.414	0.087	361227	A	G	0.197	17	45722293	1.08E-07	0.037	8189	28.130
Vascular dementia (undefined)	Interleukin-6	rs73273528	T	C	0.043	51814574	0.761	0.142	361227	T	C	0.267	20	50431113	9.58E-07	0.055	8189	23.347
Vascular dementia (undefined)	Interleukin-6	rs76856708	C	T	0.183	80695146	0.346	0.194	361227	C	T	-0.329	16	80729043	2.61E-06	0.070	8189	22.077
Vascular dementia (undefined)	Interleukin-1-receptor antagonist	rs1054402	C	T	-0.112	116401230	0.028	0.051	361227	C	T	-0.131	9	119163509	1.13E-06	0.027	3638	23.576
Vascular dementia (undefined)	Interleukin-1-receptor antagonist	rs11627423	C	A	-0.034	32731417	0.450	0.045	361227	C	A	-0.117	14	33200623	2.12E-06	0.025	3638	22.476
Vascular dementia (undefined)	Interleukin-1-receptor antagonist	rs12121840	T	C	0.131	165572405	0.183	0.099	361227	T	C	0.269	1	165541642	2.43E-06	0.057	3638	22.227
Vascular dementia (undefined)	Interleukin-1-receptor antagonist	rs2809154	T	C	0.017	84153389	0.813	0.072	361227	T	C	-0.179	13	84727524	3.74E-06	0.039	3638	21.188
Vascular dementia (undefined)	Interleukin-1-receptor antagonist	rs61335305	A	C	0.142	66160736	0.376	0.160	361227	A	C	0.445	15	66453074	1.00E-06	0.091	3638	24.051
Vascular dementia (undefined)	Interleukin-1-receptor antagonist	rs9623661	T	C	-0.081	42697370	0.292	0.077	361227	T	C	-0.197	22	43093376	3.86E-06	0.043	3638	21.298
Vascular dementia (undefined)	Interleukin-1-beta	rs143319329	T	C	-0.143	128499405	0.574	0.254	361227	T	C	0.280	7	128139459	2.00E-06	0.072	3309	15.347
Vascular dementia (undefined)	Interleukin-1-beta	rs61335305	A	C	0.142	66160736	0.376	0.160	361227	A	C	0.297	15	66453074	1.90E-06	0.072	3309	16.783
Vascular dementia (undefined)	Interleukin-1-beta	rs62015704	G	A	-0.005	7417906	0.942	0.067	361227	G	A	-0.108	16	7467907	2.09E-06	0.028	3309	14.618
Vascular dementia (undefined)	Interleukin-1-beta	rs9898641	C	T	-0.043	59493672	0.350	0.046	361227	C	T	0.203	17	57571033	3.59E-06	0.045	3309	20.033
Vascular dementia (undefined)	Hepatocyte growth factor	rs11060254	A	G	0.032	129331024	0.491	0.047	361227	A	G	-0.080	12	129815569	1.58E-06	0.017	8292	22.948
Vascular dementia (undefined)	Hepatocyte growth factor	rs150322232	G	A	0.112	7890743	0.330	0.115	361227	G	A	-0.210	7	7930374	4.89E-06	0.046	8292	20.650
Vascular dementia (undefined)	Hepatocyte growth factor	rs1698249	C	A	0.143	83889842	0.167	0.104	361227	C	A	0.170	14	84356186	4.09E-06	0.037	8292	20.835
Vascular dementia (undefined)	Hepatocyte growth factor	rs2003620	T	C	0.147	134794733	0.252	0.128	361227	T	C	0.228	7	134479484	2.83E-06	0.049	8292	21.721
Vascular dementia (undefined)	Hepatocyte growth factor	rs3748034	T	G	0.011	3444364	0.875	0.067	361227	T	G	0.150	4	3446091	1.81E-10	0.023	8292	40.818
Vascular dementia (undefined)	Hepatocyte growth factor	rs5745687	T	C	-0.078	81729735	0.468	0.108	361227	T	C	-0.307	7	81359051	2.75E-14	0.041	8292	57.252
Vascular dementia (undefined)	Hepatocyte growth factor	rs62481625	C	T	0.048	156194766	0.431	0.061	361227	C	T	-0.109	7	155987460	1.18E-06	0.023	8292	23.512
Vascular dementia (undefined)	Interleukin-9	rs41294750	T	C	-0.213	53084968	0.094	0.127	361227	T	C	0.351	1	53550640	2.36E-06	0.075	3634	22.070
Vascular dementia (undefined)	Interleukin-9	rs4880409	T	C	0.241	132516716	0.267	0.217	361227	T	C	-0.336	10	134330220	3.50E-06	0.072	3634	21.533
Vascular dementia (undefined)	Interleukin-9	rs61867538	T	C	0.030	1503276	0.781	0.109	361227	T	C	0.357	11	1524506	3.93E-06	0.077	3634	21.227
Vascular dementia (undefined)	Interleukin-9	rs7232268	G	A	0.139	70101678	0.220	0.113	361227	G	A	-0.276	18	67768914	2.52E-06	0.059	3634	22.092
Vascular dementia (undefined)	Interleukin-9	rs7242404	A	G	-0.031	12741268	0.518	0.049	361227	A	G	-0.123	18	12741267	3.27E-06	0.026	3634	21.637
Vascular dementia (undefined)	Interleukin-9	rs76963786	T	C	0.135	31886823	0.194	0.104	361227	T	C	-0.287	12	32039757	4.50E-07	0.056	3634	26.457
Vascular dementia (undefined)	Interleukin-7	rs117509142	C	T	0.137	86121854	0.282	0.127	361227	C	T	0.327	8	87134083	1.99E-06	0.069	3409	22.590
Vascular dementia (undefined)	Interleukin-7	rs141425475	C	T	0.096	17679056	0.533	0.154	361227	C	T	0.478	5	17679165	2.53E-06	0.102	3409	22.144
Vascular dementia (undefined)	Interleukin-7	rs144701438	A	G	0.244	66293168	0.186	0.184	361227	A	G	-0.482	18	63960405	9.75E-07	0.099	3409	23.742
Vascular dementia (undefined)	Interleukin-7	rs17091524	C	T	-0.126	56482041	0.476	0.177	361227	C	T	-0.492	14	56948759	1.91E-06	0.101	3409	23.627
Vascular dementia (undefined)	Interleukin-7	rs28793375	T	C	-0.045	41558099	0.482	0.064	361227	T	C	0.164	8	41415618	4.46E-06	0.036	3409	20.588
Vascular dementia (undefined)	Interleukin-7	rs4320361	T	G	0.006	43960774	0.889	0.044	361227	T	G	-0.325	6	43928511	6.87E-39	0.025	3409	169.836
Vascular dementia (undefined)	Interleukin-7	rs62006410	T	C	-0.012	102541598	0.811	0.051	361227	T	C	-0.156	14	103007935	3.39E-07	0.030	3409	26.405
Vascular dementia (undefined)	Interleukin-7	rs75904417	C	A	0.013	167796811	0.839	0.062	361227	C	A	0.170	2	168653321	1.16E-06	0.035	3409	23.671
Vascular dementia (undefined)	Interleukin-7	rs77981494	C	T	-0.109	17451009	0.475	0.152	361227	C	T	0.518	16	17544866	1.07E-06	0.106	3409	23.683
Vascular dementia (undefined)	Interleukin-7	rs78346957	A	G	-0.238	125214944	0.240	0.203	361227	A	G	0.459	10	126903513	4.51E-06	0.101	3409	20.758
Vascular dementia (undefined)	Interleukin-5	rs11680908	G	A	-0.030	109460295	0.754	0.097	361227	G	A	-0.263	2	110076751	2.03E-06	0.055	3364	22.605
Vascular dementia (undefined)	Interleukin-5	rs6737109	C	T	0.010	22956659	0.828	0.044	361227	C	T	-0.116	2	23179531	2.40E-06	0.025	3364	22.056
Vascular dementia (undefined)	Interleukin-5	rs72831687	A	G	-0.023	16092129	0.893	0.168	361227	A	G	-0.524	6	16092360	1.69E-06	0.111	3364	22.317
Vascular dementia (undefined)	Interleukin-5	rs73040130	C	T	-0.039	36255288	0.685	0.096	361227	C	T	-0.264	19	36746190	6.00E-07	0.053	3364	24.868
Vascular dementia (undefined)	Interleukin-5	rs7767396	G	A	0.006	43959313	0.885	0.044	361227	G	A	-0.152	6	43927050	7.69E-10	0.025	3364	37.928
Vascular dementia (undefined)	Interleukin-4	rs10512267	C	T	0.031	99427847	0.484	0.044	361227	C	T	0.082	9	102190129	2.94E-07	0.016	8124	26.194
Vascular dementia (undefined)	Interleukin-4	rs116705532	G	T	0.256	113162547	0.339	0.268	361227	G	T	0.468	1	113705169	1.76E-06	0.098	8124	22.879

Vascular dementia (undefined)	Interleukin-4	rs117146485	C	T	-0.254	135932411	0.123	0.165	361227	C	T	0.292	9	138824257	2.71E-06	0.063	8124	21.610
Vascular dementia (undefined)	Interleukin-4	rs17713451	A	G	-0.090	151465386	0.194	0.069	361227	A	G	0.127	7	151162472	4.97E-07	0.025	8124	25.357
Vascular dementia (undefined)	Interleukin-4	rs73023729	A	G	-0.077	159232998	0.473	0.107	361227	A	G	-0.180	6	159654030	9.03E-07	0.037	8124	24.080
Vascular dementia (undefined)	Interleukin-4	rs7613691	G	A	-0.080	147935804	0.418	0.099	361227	G	A	-0.178	3	147653591	4.05E-06	0.038	8124	21.367
Vascular dementia (undefined)	Interleukin-4	rs9508291	C	T	0.066	29136483	0.509	0.100	361227	C	T	0.168	13	29710620	3.03E-06	0.036	8124	21.795
Vascular dementia (undefined)	Interleukin-4	rs9941733	G	A	-0.063	393417	0.277	0.058	361227	G	A	-0.114	20	374061	6.88E-07	0.023	8124	24.782
Vascular dementia (undefined)	Interleukin-2 receptor antagonist	rs11241559	G	T	0.021	120641005	0.682	0.051	361227	G	T	0.126	5	119976700	2.00E-06	0.027	3677	22.580
Vascular dementia (undefined)	Interleukin-2 receptor antagonist	rs117244812	A	G	-0.134	6539990	0.614	0.265	361227	A	G	-0.706	17	6443310	2.10E-06	0.149	3677	22.537
Vascular dementia (undefined)	Interleukin-2 receptor antagonist	rs12722497	A	C	-0.102	6053965	0.245	0.088	361227	A	C	0.628	10	6095928	1.57E-38	0.049	3677	167.609
Vascular dementia (undefined)	Interleukin-2 receptor antagonist	rs185231391	C	T	-0.123	59373953	0.631	0.256	361227	C	T	-0.850	3	59359679	1.47E-06	0.181	3677	22.094
Vascular dementia (undefined)	Interleukin-2 receptor antagonist	rs4733117	C	A	0.044	32280094	0.414	0.054	361227	C	A	-0.137	8	32137610	2.63E-06	0.029	3677	21.981
Vascular dementia (undefined)	Interleukin-2 receptor antagonist	rs61705228	T	C	-0.014	100275145	0.909	0.119	361227	T	C	0.330	4	101196302	3.99E-06	0.072	3677	21.281
Vascular dementia (undefined)	Interleukin-2	rs12051139	C	T	-0.024	86885068	0.587	0.044	361227	C	T	0.113	16	86918674	4.76E-06	0.025	3475	20.967
Vascular dementia (undefined)	Interleukin-2	rs13412535	A	G	-0.035	224010157	0.525	0.055	361227	A	G	0.176	2	224874874	1.18E-07	0.033	3475	28.231
Vascular dementia (undefined)	Interleukin-2	rs170117	T	C	-0.002	54524213	0.970	0.062	361227	T	C	-0.162	4	55390380	3.87E-06	0.035	3475	21.467
Vascular dementia (undefined)	Interleukin-2	rs2807544	G	A	0.042	14877749	0.356	0.045	361227	G	A	-0.118	1	15204245	3.41E-06	0.025	3475	21.569
Vascular dementia (undefined)	Interleukin-2	rs4634519	G	A	0.023	67727941	0.633	0.049	361227	G	A	0.126	7	67192928	2.77E-06	0.027	3475	21.975
Vascular dementia (undefined)	Interleukin-2	rs61335305	A	C	0.142	66160736	0.376	0.160	361227	A	C	0.451	15	66453074	7.32E-07	0.092	3475	24.179
Vascular dementia (undefined)	Interleukin-2	rs62124990	T	G	0.012	19038882	0.939	0.153	361227	T	G	-0.696	2	19238636	3.22E-06	0.150	3475	21.680
Vascular dementia (undefined)	Interleukin-2	rs7615304	G	A	0.038	156957914	0.387	0.044	361227	G	A	0.117	3	156675703	1.21E-06	0.024	3475	23.454
Vascular dementia (undefined)	Interleukin-2	rs80336398	C	T	-0.038	64075258	0.783	0.140	361227	C	T	-0.400	3	64060934	2.82E-06	0.086	3475	21.745
Vascular dementia (undefined)	Interferon gamma	rs10487554	A	G	0.042	149670595	0.383	0.048	361227	A	G	-0.090	7	149367686	1.09E-06	0.018	7701	23.919
Vascular dementia (undefined)	Interferon gamma	rs113600793	A	C	-0.060	47384095	0.523	0.093	361227	A	C	0.183	17	45461461	8.95E-07	0.037	7701	24.044
Vascular dementia (undefined)	Interferon gamma	rs115729819	G	A	-0.008	168783516	0.955	0.137	361227	G	A	-0.248	4	169704667	1.38E-06	0.052	7701	23.264
Vascular dementia (undefined)	Interferon gamma	rs11843756	G	T	-0.049	48680756	0.662	0.111	361227	G	T	-0.184	13	49254892	3.09E-06	0.039	7701	21.921
Vascular dementia (undefined)	Interferon gamma	rs12420286	C	T	-0.153	103907166	0.246	0.132	361227	C	T	-0.238	11	103777894	2.08E-06	0.050	7701	22.491
Vascular dementia (undefined)	Interferon gamma	rs1867282	T	C	0.064	99409865	0.150	0.044	361227	T	C	0.077	9	102172147	3.15E-06	0.017	7701	21.740
Vascular dementia (undefined)	Interferon gamma	rs2073438	A	G	-0.061	6996757	0.225	0.050	361227	A	G	0.090	17	6900076	1.68E-06	0.019	7701	22.816
Vascular dementia (undefined)	Interferon gamma	rs74148555	T	C	0.000	90320085	0.999	0.154	361227	T	C	-0.373	10	92079842	2.64E-06	0.077	7701	23.249
Vascular dementia (undefined)	Interferon gamma	rs78296352	T	G	0.142	22495351	0.459	0.192	361227	T	G	0.343	1	22821844	1.38E-07	0.065	7701	27.675
Vascular dementia (undefined)	Growth-regulated protein alpha	rs1113500	T	G	-0.017	108052820	0.709	0.045	361227	T	G	0.117	1	108595442	1.57E-06	0.024	3505	23.150
Vascular dementia (undefined)	Growth-regulated protein alpha	rs12075	A	G	0.011	159205564	0.802	0.044	361227	A	G	0.375	1	159175354	1.24E-55	0.024	3505	250.494
Vascular dementia (undefined)	Growth-regulated protein alpha	rs140734053	A	G	-0.306	5359496	0.204	0.241	361227	A	G	0.726	10	5401459	3.58E-06	0.156	3505	21.613
Vascular dementia (undefined)	Growth-regulated protein alpha	rs185768063	G	A	-0.007	16494752	0.960	0.136	361227	G	A	-0.400	6	16494983	1.46E-07	0.076	3505	27.673
Vascular dementia (undefined)	Growth-regulated protein alpha	rs188345231	T	C	-0.406	41579831	0.102	0.248	361227	T	C	0.623	8	41437350	4.34E-06	0.132	3505	22.175
Vascular dementia (undefined)	Growth-regulated protein alpha	rs2422841	A	G	0.178	3099706	0.007	0.066	361227	A	G	-0.166	20	3080352	4.66E-06	0.036	3505	21.068
Vascular dementia (undefined)	Growth-regulated protein alpha	rs508977	G	T	0.047	73896666	0.358	0.051	361227	G	T	0.380	4	74762383	7.56E-42	0.028	3505	184.378
Vascular dementia (undefined)	Growth-regulated protein alpha	rs62024303	G	A	0.144	88327931	0.181	0.108	361227	G	A	0.305	15	88871162	4.41E-06	0.067	3505	21.014
Vascular dementia (undefined)	Growth-regulated protein alpha	rs78653452	T	G	0.103	9781407	0.639	0.220	361227	T	G	-0.736	20	9762055	1.21E-06	0.156	3505	22.328
Vascular dementia (undefined)	Granulocyte-colony stimulating factor	rs115256310	G	A	-0.299	72103864	0.250	0.260	361227	G	A	0.682	5	71399691	6.73E-07	0.136	7904	25.155
Vascular dementia (undefined)	Granulocyte-colony stimulating factor	rs11903143	G	A	0.043	29369594	0.374	0.048	361227	G	A	-0.087	2	29592460	6.35E-07	0.018	7904	24.435
Vascular dementia (undefined)	Granulocyte-colony stimulating factor	rs147128865	T	C	0.026	34972769	0.862	0.149	361227	T	C	0.270	9	34972766	4.92E-06	0.059	7904	21.157
Vascular dementia (undefined)	Granulocyte-colony stimulating factor	rs1817411	T	C	0.030	97586100	0.575	0.053	361227	T	C	0.089	8	98598328	3.10E-06	0.019	7904	21.713
Vascular dementia (undefined)	Granulocyte-colony stimulating factor	rs2671444	A	G	-0.023	101158297	0.610	0.046	361227	A	G	-0.078	12	101552075	2.48E-06	0.017	7904	22.306
Vascular dementia (undefined)	Granulocyte-colony stimulating factor	rs74148555	T	C	0.000	90320085	0.999	0.154	361227	T	C	-0.372	10	92079842	1.55E-06	0.076	7904	24.212
Vascular dementia (undefined)	Granulocyte-colony stimulating factor	rs77318030	C	T	0.085	54544688	0.438	0.110	361227	C	T	0.205	19	55055897	2.21E-06	0.043	7904	22.830
Vascular dementia (undefined)	Fibroblast growth factor basic	rs13412535	A	G	-0.035	224010157	0.525	0.055	361227	A	G	-0.111	2	224874874	7.34E-07	0.023	7565	24.426
Vascular dementia (undefined)	Fibroblast growth factor basic	rs145577605	A	G	0.153	27642232	0.218	0.124	361227	A	G	0.208	6	27610011	9.64E-07	0.043	7565	23.640
Vascular dementia (undefined)	Fibroblast growth factor basic	rs747334	G	A	-0.065	90984987	0.139	0.044	361227	G	A	-0.075	10	92744744	4.53E-06	0.016	7565	20.970
Vascular dementia (undefined)	Fibroblast growth factor basic	rs75168112	C	T	-0.014	73418832	0.799	0.056	361227	C	T	0.100	18	71086067	3.00E-06	0.021	7565	21.880
Vascular dementia (undefined)	Fibroblast growth factor basic	rs9907295	T	C	-0.100	35930309	0.157	0.071	361227	T	C	-0.132	17	34257313	7.95E-07	0.027	7565	24.043
Vascular dementia (undefined)	Eotaxin	rs11087905	A	C	-0.056	24133015	0.225	0.046	361227	A	C	0.094	21	25505329	5.48E-07	0.019	8153	24.789
Vascular dementia (undefined)	Eotaxin	rs112347425	T	C	-0.076	46419397	0.321	0.077	361227	T	C	0.158	3	46460888	8.65E-09	0.028	8153	32.535
Vascular dementia (undefined)	Eotaxin	rs12075	A	G	0.011	159205564	0.802	0.044	361227	A	G	0.167	1	159175354	1.33E-26	0.016	8153	114.737
Vascular dementia (undefined)	Eotaxin	rs1476670	C	A	-0.043	44042523	0.471	0.059	361227	C	A	0.101	1	44508195	3.51E-06	0.022	8153	21.535
Vascular dementia (undefined)	Eotaxin	rs2024050	G	A	-0.089	75831075	0.296	0.086	361227	G	A	-0.173	7	75460393	1.10E-08	0.030	8153	32.524
Vascular dementia (undefined)	Eotaxin	rs2210755	C	T	0.034	77608907	0.593	0.063	361227	C	T	0.110	9	80223823	4.85E-06	0.024	8153	20.812
Vascular dementia (undefined)	Eotaxin	rs2211994	C	T	0.042	16675274	0.390	0.049	361227	C	T	-0.089	21	18047593	6.08E-07	0.018	8153	25.000
Vascular dementia (undefined)	Eotaxin	rs2228467	C	T	0.001	42864624	0.989	0.083	361227	C	T	0.416	3	42906116	2.27E-46	0.029	8153	203.258
Vascular dementia (undefined)	Eotaxin	rs2419841	C	T	0.147	113576224	0.069	0.081	361227	C	T	0.128	10	115335983	4.98E-06	0.028	8153	20.949
Vascular dementia (undefined)	Eotaxin	rs5746492	G	A	-0.044	17911167	0.461	0.059	361227	G	A	-0.095	22	18393933	3.96E-06	0.021	8153	21.240
Vascular dementia (undefined)	Eotaxin	rs5754733	A	C	0.068	33873606	0.239	0.058	361227	A	C	-0.104	22	34269594	1.06E-06	0.021	8153	23.709
Vascular dementia (undefined)	Eotaxin	rs59808887	T	C	0.099	31846414	0.261	0.088	361227	T	C	-0.167	5	31846520	2.91E-06	0.036	8153	21.839
Vascular dementia (undefined)	Eotaxin	rs75426604	A	C	0.030	35388508	0.688	0.075	361227	A	C	-0.137	14	35857714	2.53E-06	0.029	8153	22.035

Vascular dementia (undefined)	Eotaxin	rs79722574	T	C	-0.083	34292033	0.187	0.063	361227	T	C	-0.111	17	32619052	1.06E-06	0.023	8153	23.830
Vascular dementia (undefined)	Eotaxin	rs9317045	C	A	0.005	59055904	0.940	0.061	361227	C	A	-0.118	13	59630038	5.82E-07	0.024	8153	24.874

SE=Standard Error
