# Supplementary material for: Role of inflammatory cytokines and the gut microbiome in vascular dementia: insights from Mendelian randomization analysis
Source: Front Microbiol. 2024 Aug 23;15:1398618. doi: 10.3389/fmicb.2024.1398618 (PMC11380139; doi:10.3389/fmicb.2024.1398618)
Supplement: Supplementary file 1 [file Data_Sheet_1.zip › Supplementary Table S10.docx]

Supplementary Table S10. Associations between genetically determined 26 gut microbiomes with the risk of 9 inflammatory.

|  | Exposure | Outcome | Method | No. of SNP | MR | | | |
| --- | --- | --- | --- | --- | --- | --- | --- | --- |
|  |  |  |  |  | OR | OR_Lci95 | OR_Uci95 | P value |
| VaD (mixed) | Eotaxin | *Bifidobacteriaceae* | IVW | 11 | 1.04 | 0.90 | 1.21 | 0.60 |
|  |  | *Eubacterium coprostanoligenes group* | IVW | 13 | 0.96 | 0.78 | 1.18 | 0.68 |
|  |  | *Haemophilus* | IVW | 9 | 0.84 | 0.73 | 0.95 | 0.01 |
|  |  | *Lachnospiraceae NK4A136 group* | IVW | 15 | 0.90 | 0.79 | 1.04 | 0.14 |
|  |  | *Bifidobacteriales* | IVW | 11 | 1.04 | 0.90 | 1.21 | 0.60 |
|  |  | *Negativicutes* | IVW | 12 | 0.95 | 0.79 | 1.14 | 0.59 |
|  |  | *Selenomonadales* | IVW | 12 | 0.95 | 0.79 | 1.14 | 0.59 |
| VaD (multiple infarctions) | SCGF-β | *Cyanobacteria* | IVW | 8 | 0.87 | 0.70 | 1.08 | 0.20 |
|  |  | *Pasteurellales* | IVW | 13 | 0.93 | 0.78 | 1.11 | 0.40 |
|  |  | *Pasteurellaceae* | IVW | 13 | 0.93 | 0.78 | 1.11 | 0.40 |
|  |  | *Lachnospiraceae UCG010* | IVW | 10 | 0.91 | 0.70 | 1.18 | 0.48 |
|  |  | *Melainabacteria* | IVW | 9 | 0.99 | 0.84 | 1.18 | 0.95 |
|  | Interleukin-18 | *Cyanobacteria* | IVW | 8 | 1.08 | 0.88 | 1.33 | 0.46 |
|  |  | *Pasteurellales* | IVW | 12 | 0.84 | 0.69 | 1.03 | 0.10 |
|  |  | *Pasteurellaceae* | IVW | 12 | 0.84 | 0.69 | 1.03 | 0.10 |
|  |  | *Lachnospiraceae UCG010* | IVW | 9 | 0.97 | 0.73 | 1.27 | 0.81 |
|  |  | *Melainabacteria* | IVW | 9 | 1.25 | 1.05 | 1.49 | 0.01 |
| VaD (other) | MIF | *phylum Actinobacteria* | IVW | 15 | 0.80 | 0.63 | 1.01 | 0.06 |
|  |  | *class Actinobacteria* | IVW | 15 | 0.80 | 0.64 | 0.99 | 0.04 |
|  |  | *Butyricicoccus* | IVW | 8 | 1.23 | 0.89 | 1.69 | 0.20 |
|  | Interleukin-4 | *phylum Actinobacteria* | IVW | 15 | 1.02 | 0.87 | 1.20 | 0.82 |
|  |  | *class Actinobacteria* | IVW | 15 | 1.08 | 0.93 | 1.24 | 0.32 |
|  |  | *Butyricicoccus* | IVW | 8 | 1.00 | 0.82 | 1.21 | 1.00 |
| VaD (subcortical) | GRO-α | *Veillonellaceae* | IVW | 18 | 1.02 | 0.84 | 1.23 | 0.87 |
|  |  | *Prevotella9* | IVW | 15 | 1.07 | 0.91 | 1.27 | 0.42 |
| VaD (sudden onset) | MIF | *Faecalibacterium* | IVW | 10 | 1.07 | 0.85 | 1.36 | 0.55 |
|  |  | *Holdemania* | IVW | 15 | 0.90 | 0.75 | 1.09 | 0.29 |
|  |  | *Lachnospiraceae NK4A136 group* | IVW | 14 | 0.98 | 0.79 | 1.21 | 0.83 |
|  |  | *Terrisporobacter* | IVW | 5 | 0.98 | 0.67 | 1.44 | 0.93 |
| VaD (undefined) | IL-1ra | *Ruminococcaceae UCG003* | IVW | 12 | 1.02 | 0.81 | 1.28 | 0.87 |
|  |  | *Dorea* | IVW | 10 | 0.90 | 0.65 | 1.23 | 0.50 |
|  |  | *Veillonella* | IVW | 7 | 1.06 | 0.82 | 1.37 | 0.68 |
|  |  | *Bacillales* | IVW | 9 | 0.93 | 0.82 | 1.06 | 0.28 |
|  |  | *Ruminiclostridium6* | IVW | 14 | 1.01 | 0.81 | 1.26 | 0.90 |
|  | bFGF | *Ruminococcaceae UCG003* | IVW | 12 | 1.01 | 0.81 | 1.26 | 0.93 |
|  |  | *Dorea* | IVW | 10 | 1.11 | 0.85 | 1.44 | 0.44 |
|  |  | *Veillonella* | IVW | 7 | 1.07 | 0.87 | 1.31 | 0.53 |
|  |  | *Bacillales* | IVW | 9 | 0.99 | 0.89 | 1.10 | 0.81 |
|  |  | *Ruminiclostridium6* | IVW | 15 | 1.01 | 0.86 | 1.18 | 0.90 |

VaD=vascular dementia; IVW=inverse variance-weighted; MR=Mendelian randomization; SCGF-β=stem cell growth factor beta; MIF=macrophage migration inhibitory factor; GRO-α=growth-regulated protein alpha; IL-1ra=interleukin-1-receptor antagonist; bFGF=fibroblast growth factor basic; OR=odds ratios; No. of SNP=number of single nucleotide polymorphisms; OR_Lci95=lower confidence interval of 95%; OR_Uci95=upper confidence interval of 95%.
