# Supplementary material for: Role of inflammatory cytokines and the gut microbiome in vascular dementia: insights from Mendelian randomization analysis
Source: Front Microbiol. 2024 Aug 23;15:1398618. doi: 10.3389/fmicb.2024.1398618 (PMC11380139; doi:10.3389/fmicb.2024.1398618)
Supplement: Supplementary file 1 [file Data_Sheet_1.zip › Supplementary Table S10.pdf]

Supplementary Table S10. ~~The a~~Associations between genetically determined 26 gut microbiomes with the risk of 9 inflammatory.

	Exposure	Outcome	Method	No. of SNP	MR			
					OR	OR_Lci95	OR_Uci95	P value
VaD (mixed)	Eotaxin	*Bifidobacteriaceae*	IVW	11	1.04	0.90	1.21	0.60
		Eubacterium coprostanoligenes group	IVW	13	0.96	0.78	1.18	0.68
		Haemophilus	IVW	9	0.84	0.73	0.95	0.01
		Lachnospiraceae NK4A136 group	IVW	15	0.90	0.79	1.04	0.14
		Bifidobacteriales	IVW	11	1.04	0.90	1.21	0.60
		Negativicutes	IVW	12	0.95	0.79	1.14	0.59
		Selenomonadales	IVW	12	0.95	0.79	1.14	0.59
VaD (multiple infarctions)	SCGF-β	*Cyanobacteria*	IVW	8	0.87	0.70	1.08	0.20
		Pasteurellales	IVW	13	0.93	0.78	1.11	0.40
		Pasteurellaceae	IVW	13	0.93	0.78	1.11	0.40
		Lachnospiraceae UCG010	IVW	10	0.91	0.70	1.18	0.48
		Melainabacteria	IVW	9	0.99	0.84	1.18	0.95
	Interleukin-18	*Cyanobacteria*	IVW	8	1.08	0.88	1.33	0.46
		Pasteurellales	IVW	12	0.84	0.69	1.03	0.10
		Pasteurellaceae	IVW	12	0.84	0.69	1.03	0.10
		Lachnospiraceae UCG010	IVW	9	0.97	0.73	1.27	0.81
		Melainabacteria	IVW	9	1.25	1.05	1.49	0.01
VaD (other)	MIF	*phylum Actinobacteria*	IVW	15	0.80	0.63	1.01	0.06
		class Actinobacteria	IVW	15	0.80	0.64	0.99	0.04
		Butyricicoccus	IVW	8	1.23	0.89	1.69	0.20
	Interleukin-4	*phylum Actinobacteria*	IVW	15	1.02	0.87	1.20	0.82
		class Actinobacteria	IVW	15	1.08	0.93	1.24	0.32
		Butyricicoccus	IVW	8	1.00	0.82	1.21	1.00

VaD (subcortical)	GRO-α	*Veillonellaceae*	IVW	18	1.02	0.84	1.23	0.87
		Prevotella9	IVW	15	1.07	0.91	1.27	0.42
VaD (sudden onset)	MIF	*Faecalibacterium*	IVW	10	1.07	0.85	1.36	0.55
		Holdemania	IVW	15	0.90	0.75	1.09	0.29
		Lachnospiraceae NK4A136 group	IVW	14	0.98	0.79	1.21	0.83
		Terrisporobacter	IVW	5	0.98	0.67	1.44	0.93
VaD (undefined)	IL-1ra	*Ruminococcaceae UCG003*	IVW	12	1.02	0.81	1.28	0.87
		Dorea	IVW	10	0.90	0.65	1.23	0.50
		Veillonella	IVW	7	1.06	0.82	1.37	0.68
		Bacillales	IVW	9	0.93	0.82	1.06	0.28
		Ruminiclostridium6	IVW	14	1.01	0.81	1.26	0.90
	bFGF	*Ruminococcaceae UCG003*	IVW	12	1.01	0.81	1.26	0.93
		Dorea	IVW	10	1.11	0.85	1.44	0.44
		Veillonella	IVW	7	1.07	0.87	1.31	0.53
		Bacillales	IVW	9	0.99	0.89	1.10	0.81
		Ruminiclostridium6	IVW	15	1.01	0.86	1.18	0.90

VaD=vascular dementia; IVW=inverse variance-weighted; MR=Mendelian randomization; SCGF-β=stem cell growth factor beta; MIF=macrophage migration inhibitory factor; GRO-α=growth-regulated protein alpha; IL-1ra=interleukin-1-receptor antagonist; bFGF=fibroblast growth factor basic; OR=odds ratios; No. of SNP=number of single nucleotide polymorphisms; OR_Lci95=lower confidence interval of 95%; OR_Uci95=upper confidence interval of 95%.
