# Supplementary material for: Role of inflammatory cytokines and the gut microbiome in vascular dementia: insights from Mendelian randomization analysis
Source: Front Microbiol. 2024 Aug 23;15:1398618. doi: 10.3389/fmicb.2024.1398618 (PMC11380139; doi:10.3389/fmicb.2024.1398618)
Supplement: Supplementary file 1 [file Data_Sheet_1.zip › Supplementary Table S2.pdf]

Supplementary Table S2 Instrumental Variables related to exposures (gut microbiome) and outcomes (6 subtypes of vascular dementia)

Outcome	Exposure	SNP	Outcome							Exposure								F-statistics
			Effect allele	Other allele	Beta	Pos	Pval	SE	Samplesize	Effect allele	Other allele	Beta	Chr	Pos	Pval	SE	Samplesize	
Vascular dementia (mixed)	class Actinobacteria	rs11655079	T	C	0.072	77352593	0.508	0.108	360421	T	C	-0.056	17	75348675	5.93E-06	0.012	14306	20.351
Vascular dementia (mixed)	class Actinobacteria	rs11745923	G	T	-0.055	475293	0.522	0.086	360421	G	T	0.056	5	475408	1.58E-06	0.012	14306	23.812
Vascular dementia (mixed)	class Actinobacteria	rs12049045	A	G	0.208	114122863	0.016	0.086	360421	A	G	0.051	1	114665485	8.63E-06	0.011	14306	19.936
Vascular dementia (mixed)	class Actinobacteria	rs134366	A	G	0.067	35171362	0.683	0.163	360421	A	G	-0.112	22	35567355	1.50E-06	0.024	14306	22.663
Vascular dementia (mixed)	class Actinobacteria	rs1376754	G	A	-0.096	160755458	0.254	0.084	360421	G	A	0.051	2	161611969	6.71E-06	0.011	14306	20.489
Vascular dementia (mixed)	class Actinobacteria	rs1515761	C	T	0.229	124421463	0.155	0.161	360421	C	T	-0.076	10	126110032	4.96E-06	0.017	14306	20.136
Vascular dementia (mixed)	class Actinobacteria	rs182549	T	C	-0.114	135859184	0.187	0.087	360421	T	C	-0.111	2	136616754	3.79E-20	0.012	14306	85.376
Vascular dementia (mixed)	class Actinobacteria	rs4945008	G	A	0.032	71510202	0.712	0.086	360421	G	A	-0.054	11	71221248	5.39E-06	0.012	14306	19.842
Vascular dementia (mixed)	class Actinobacteria	rs6660520	G	A	0.129	206830208	0.171	0.094	360421	G	A	-0.071	1	207003553	1.11E-07	0.013	14306	27.984
Vascular dementia (mixed)	class Actinobacteria	rs72767435	T	C	-0.341	94309566	0.065	0.185	360421	T	C	-0.126	15	94852795	2.57E-06	0.027	14306	21.323
Vascular dementia (mixed)	class Actinobacteria	rs7322849	T	C	0.097	112205515	0.512	0.147	360421	T	C	0.094	13	112859829	6.21E-07	0.019	14306	23.953
Vascular dementia (mixed)	class Actinobacteria	rs80083040	T	G	-0.049	64742287	0.812	0.206	360421	T	G	0.156	8	65654844	8.62E-06	0.035	14306	20.038
Vascular dementia (mixed)	class Actinobacteria	rs857444	C	T	0.083	14617360	0.341	0.087	360421	C	T	0.051	6	14617591	8.92E-06	0.012	14306	19.500
Vascular dementia (mixed)	class Actinobacteria	rs961091	G	A	-0.014	96025406	0.871	0.087	360421	G	A	0.050	7	95654718	8.68E-06	0.011	14306	19.835
Vascular dementia (mixed)	class Alphaproteobacteria	rs140912403	C	T	-0.061	92755525	0.737	0.180	360421	C	T	-0.161	9	95517807	6.20E-07	0.032	14306	25.577
Vascular dementia (mixed)	class Alphaproteobacteria	rs34569731	G	A	-0.023	75403218	0.788	0.087	360421	G	A	-0.071	2	75630344	7.38E-06	0.016	14306	20.028
Vascular dementia (mixed)	class Alphaproteobacteria	rs62285697	C	T	0.124	176690123	0.202	0.097	360421	C	T	0.081	3	176407911	9.76E-06	0.018	14306	19.812
Vascular dementia (mixed)	class Alphaproteobacteria	rs76784716	A	G	0.002	168176830	0.986	0.135	360421	A	G	0.133	2	169033340	5.09E-07	0.027	14306	24.777
Vascular dementia (mixed)	class Alphaproteobacteria	rs7960664	A	G	0.262	89077458	0.080	0.150	360421	A	G	-0.097	12	89471235	8.84E-06	0.022	14306	20.305
Vascular dementia (mixed)	class Alphaproteobacteria	rs9813022	A	G	0.020	13685237	0.817	0.087	360421	A	G	-0.075	3	13726736	1.05E-06	0.015	14306	23.840
Vascular dementia (mixed)	class Bacilli	rs11110282	A	G	0.122	100191781	0.538	0.199	360421	A	G	-0.101	12	100585559	4.85E-06	0.022	14306	21.669
Vascular dementia (mixed)	class Bacilli	rs11730038	G	A	-0.134	97128348	0.146	0.093	360421	G	A	-0.063	4	98049499	1.96E-06	0.013	14306	24.013
Vascular dementia (mixed)	class Bacilli	rs12797734	T	C	-0.067	8310803	0.495	0.098	360421	T	C	0.057	11	8332350	7.21E-06	0.013	14306	20.363
Vascular dementia (mixed)	class Bacilli	rs13068444	A	G	0.004	64413187	0.969	0.110	360421	A	G	0.060	3	64398863	9.53E-06	0.014	14306	19.415
Vascular dementia (mixed)	class Bacilli	rs1595463	C	A	-0.040	230858942	0.642	0.085	360421	C	A	0.048	2	231723657	7.97E-06	0.011	14306	19.300
Vascular dementia (mixed)	class Bacilli	rs28564647	T	G	0.285	98338890	0.011	0.112	360421	T	G	-0.061	9	101101172	7.81E-06	0.014	14306	19.855
Vascular dementia (mixed)	class Bacilli	rs2952251	G	A	-0.092	10285654	0.359	0.101	360421	G	A	0.060	8	10143164	1.08E-06	0.012	14306	23.361
Vascular dementia (mixed)	class Bacilli	rs34989881	A	G	-0.098	51456601	0.626	0.202	360421	A	G	0.111	19	51959855	6.55E-06	0.025	14306	20.374
Vascular dementia (mixed)	class Bacilli	rs35344081	G	A	0.121	941253	0.203	0.095	360421	G	A	0.062	16	991253	1.01E-06	0.013	14306	23.772
Vascular dementia (mixed)	class Bacilli	rs4028634	C	T	0.031	42683631	0.724	0.087	360421	C	T	-0.052	17	40835649	2.21E-06	0.011	14306	22.464
Vascular dementia (mixed)	class Bacilli	rs4459992	T	C	-0.024	7429760	0.793	0.090	360421	T	C	0.054	4	7431487	4.30E-06	0.012	14306	21.207
Vascular dementia (mixed)	class Bacilli	rs57872228	C	T	-0.078	200449677	0.547	0.130	360421	C	T	-0.071	1	200418805	9.22E-07	0.015	14306	23.757
Vascular dementia (mixed)	class Bacilli	rs694949	A	G	0.139	58445876	0.333	0.144	360421	A	G	-0.081	15	58738075	7.60E-06	0.018	14306	20.314
Vascular dementia (mixed)	class Bacilli	rs74663707	C	T	0.144	184653436	0.410	0.175	360421	C	T	0.098	3	184371224	8.46E-06	0.022	14306	19.095
Vascular dementia (mixed)	class Bacilli	rs7666190	A	C	-0.041	150766268	0.757	0.132	360421	A	C	0.104	4	151687420	8.47E-06	0.025	14306	17.610
Vascular dementia (mixed)	class Bacilli	rs77558518	A	G	0.077	174746168	0.590	0.143	360421	A	G	-0.107	5	174173171	1.34E-06	0.022	14306	23.158
Vascular dementia (mixed)	class Bacilli	rs78938557	T	C	0.022	36309977	0.931	0.257	360421	T	C	0.108	7	36349586	1.07E-06	0.023	14306	21.514
Vascular dementia (mixed)	class Bacilli	rs9581006	T	C	0.056	24399371	0.804	0.224	360421	T	C	-0.225	13	24973509	1.79E-06	0.047	14306	23.207
Vascular dementia (mixed)	class Bacteroidia	rs11146701	A	G	0.017	38769138	0.848	0.089	360421	A	G	0.047	10	39062269	7.08E-06	0.011	14306	20.186
Vascular dementia (mixed)	class Bacteroidia	rs17343978	A	C	-0.072	27037922	0.492	0.105	360421	A	C	-0.055	22	27433885	8.36E-06	0.012	14306	21.067
Vascular dementia (mixed)	class Bacteroidia	rs2032750	C	T	-0.055	53603889	0.514	0.085	360421	C	T	0.051	2	53831026	1.92E-06	0.011	14306	22.657
Vascular dementia (mixed)	class Bacteroidia	rs2363574	T	C	-0.039	200143435	0.859	0.218	360421	T	C	0.223	1	200112563	9.93E-06	0.051	14306	19.221
Vascular dementia (mixed)	class Bacteroidia	rs4916508	A	G	0.192	196209918	0.024	0.085	360421	A	G	0.047	3	195936789	8.47E-06	0.011	14306	19.641
Vascular dementia (mixed)	class Bacteroidia	rs55773148	G	A	0.143	69948897	0.436	0.183	360421	G	A	-0.122	13	70523029	3.90E-07	0.024	14306	26.341
Vascular dementia (mixed)	class Bacteroidia	rs62531359	T	G	0.000	70003946	0.997	0.111	360421	T	G	0.066	8	70916181	9.09E-06	0.015	14306	19.138
Vascular dementia (mixed)	class Bacteroidia	rs62575403	C	T	-0.184	133628698	0.380	0.210	360421	C	T	0.140	9	136493820	7.06E-06	0.031	14306	20.264
Vascular dementia (mixed)	class Bacteroidia	rs72706335	T	C	-0.032	157525648	0.904	0.269	360421	T	C	-0.222	1	157495438	7.66E-06	0.049	14306	20.315
Vascular dementia (mixed)	class Bacteroidia	rs73975615	G	A	0.114	6557880	0.817	0.490	360421	G	A	-0.207	17	6461200	1.22E-06	0.044	14306	21.874
Vascular dementia (mixed)	class Bacteroidia	rs7631304	G	A	-0.020	89290377	0.865	0.119	360421	G	A	-0.065	3	89339527	8.37E-07	0.013	14306	23.590
Vascular dementia (mixed)	class Bacteroidia	rs79585701	A	C	-0.148	13252676	0.224	0.122	360421	A	C	0.065	8	13110185	9.99E-06	0.015	14306	18.687
Vascular dementia (mixed)	class Bacteroidia	rs929878	T	C	-0.214	74256742	0.039	0.104	360421	T	C	0.055	16	74290641	4.73E-06	0.012	14306	20.372
Vascular dementia (mixed)	class Betaproteobacteria	rs11128180	A	G	-0.027	70543064	0.789	0.100	360421	A	G	0.059	3	70592215	3.67E-06	0.013	14306	21.203
Vascular dementia (mixed)	class Betaproteobacteria	rs1511453	A	G	-0.074	23586839	0.679	0.179	360421	A	G	0.092	4	23588462	4.76E-06	0.020	14306	21.502
Vascular dementia (mixed)	class Betaproteobacteria	rs1928341	G	A	0.036	153267537	0.675	0.085	360421	G	A	-0.053	1	153240013	2.02E-06	0.011	14306	22.661
Vascular dementia (mixed)	class Betaproteobacteria	rs2321387	G	A	-0.151	58115206	0.076	0.085	360421	G	A	-0.049	13	58689340	5.80E-06	0.011	14306	20.430
Vascular dementia (mixed)	class Betaproteobacteria	rs2613606	T	C	0.101	111644969	0.239	0.085	360421	T	C	0.051	7	111285025	2.20E-06	0.011	14306	22.123
Vascular dementia (mixed)	class Betaproteobacteria	rs320161	G	A	-0.120	102136835	0.228	0.100	360421	G	A	-0.057	9	104899117	7.33E-06	0.013	14306	20.564
Vascular dementia (mixed)	class Betaproteobacteria	rs4033856	T	C	0.009	45640468	0.951	0.149	360421	T	C	-0.083	4	45642485	5.17E-07	0.017	14306	24.776
Vascular dementia (mixed)	class Betaproteobacteria	rs6087811	T	G	-0.010	32008327	0.943	0.141	360421	T	G	-0.098	20	30596130	7.44E-07	0.020	14306	24.278
Vascular dementia (mixed)	class Betaproteobacteria	rs62395635	T	C	-0.314	174070793	0.077	0.178	360421	T	C	0.110	5	173497796	2.94E-06	0.024	14306	21.623
Vascular dementia (mixed)	class Betaproteobacteria	rs75242906	C	T	0.028	56494209	0.853	0.150	360421	C	T	-0.121	15	56786407	9.27E-06	0.028	14306	18.601
Vascular dementia (mixed)	class Clostridia	rs10774377	G	A	-0.142	5833353	0.094	0.085	360421	G	A	-0.053	12	5942519	3.24E-06	0.011	14306	21.390
Vascular dementia (mixed)	class Clostridia	rs112334273	G	A	-0.109	39331325	0.246	0.094	360421	G	A	0.064	21	40703251	3.81E-07	0.013	14306	25.314
Vascular dementia (mixed)	class Clostridia	rs13105690	C	T	0.146	7418457	0.119	0.094	360421	C	T	0.053	4	7420184	8.78E-06	0.012	14306	20.044

Vascular dementia (mixed)	class Clostridia	rs13179700	C	T	0.080	149698225	0.369	0.089	360421	C	T	-0.051	5	149077788	3.37E-06	0.011	14306	21.830
Vascular dementia (mixed)	class Clostridia	rs1842454	G	A	0.115	105724661	0.288	0.108	360421	G	A	-0.055	5	105060362	8.72E-06	0.013	14306	18.466
Vascular dementia (mixed)	class Clostridia	rs2273429	A	G	0.011	52027354	0.935	0.137	360421	A	G	-0.072	14	52494072	4.52E-06	0.015	14306	22.364
Vascular dementia (mixed)	class Clostridia	rs6797343	T	G	0.007	89219564	0.950	0.107	360421	T	G	-0.059	3	89268714	9.36E-06	0.013	14306	19.375
Vascular dementia (mixed)	class Clostridia	rs6814436	C	T	0.015	160586149	0.903	0.120	360421	C	T	-0.074	4	161507301	9.65E-07	0.015	14306	24.075
Vascular dementia (mixed)	class Clostridia	rs6815608	C	T	-0.109	151210592	0.358	0.118	360421	C	T	-0.104	4	152131744	4.02E-07	0.021	14306	24.229
Vascular dementia (mixed)	class Clostridia	rs72738886	T	C	0.071	35770448	0.652	0.157	360421	T	C	0.087	5	35770550	8.24E-06	0.019	14306	20.697
Vascular dementia (mixed)	class Clostridia	rs992074	T	C	-0.245	17195484	0.391	0.285	360421	T	C	-0.256	21	18567802	8.78E-07	0.051	14306	25.245
Vascular dementia (mixed)	class Coriobacteriia	rs11073596	G	T	-0.188	85890348	0.032	0.087	360421	G	T	-0.051	15	86433579	8.14E-06	0.011	14306	19.912
Vascular dementia (mixed)	class Coriobacteriia	rs11250875	T	C	0.093	1880537	0.365	0.102	360421	T	C	0.061	10	1922731	4.83E-06	0.013	14306	21.526
Vascular dementia (mixed)	class Coriobacteriia	rs11656361	A	C	-0.175	8218014	0.110	0.110	360421	A	C	0.077	17	8121332	8.02E-06	0.018	14306	19.394
Vascular dementia (mixed)	class Coriobacteriia	rs12974142	G	A	0.028	52391913	0.867	0.165	360421	G	A	0.079	19	52895166	8.51E-06	0.018	14306	19.865
Vascular dementia (mixed)	class Coriobacteriia	rs13307134	T	C	0.021	105444233	0.856	0.113	360421	T	C	-0.057	7	105084680	7.80E-06	0.013	14306	20.072
Vascular dementia (mixed)	class Coriobacteriia	rs1397793	A	G	-0.012	91175634	0.893	0.092	360421	A	G	0.050	5	90471451	9.77E-06	0.011	14306	19.682
Vascular dementia (mixed)	class Coriobacteriia	rs1816223	G	A	0.118	11341087	0.267	0.106	360421	G	A	0.059	12	11494021	4.84E-06	0.013	14306	20.652
Vascular dementia (mixed)	class Coriobacteriia	rs240104	T	C	-0.017	176602295	0.859	0.094	360421	T	C	-0.060	1	176571431	1.52E-06	0.013	14306	22.630
Vascular dementia (mixed)	class Coriobacteriia	rs2442778	A	G	0.169	11612938	0.365	0.186	360421	A	G	0.116	3	11654412	9.03E-06	0.026	14306	20.272
Vascular dementia (mixed)	class Coriobacteriia	rs3025411	A	G	0.009	133647784	0.947	0.135	360421	A	G	0.093	9	136512906	8.27E-06	0.021	14306	19.566
Vascular dementia (mixed)	class Coriobacteriia	rs34739816	G	T	0.111	39220432	0.534	0.179	360421	G	T	0.097	17	37376685	3.88E-06	0.021	14306	21.594
Vascular dementia (mixed)	class Coriobacteriia	rs67561917	A	G	-0.006	63440724	0.953	0.110	360421	A	G	-0.071	20	62072077	5.39E-06	0.015	14306	21.486
Vascular dementia (mixed)	class Coriobacteriia	rs719099	A	G	-0.161	64039457	0.258	0.142	360421	A	G	0.078	10	65799217	5.43E-07	0.016	14306	24.957
Vascular dementia (mixed)	class Coriobacteriia	rs8010111	A	G	0.182	39191305	0.236	0.154	360421	A	G	0.103	14	39660509	6.90E-06	0.023	14306	20.328
Vascular dementia (mixed)	class Deltaproteobacteria	rs1035691	G	A	0.059	10637188	0.491	0.086	360421	G	A	0.055	11	10658735	9.65E-06	0.012	14306	20.588
Vascular dementia (mixed)	class Deltaproteobacteria	rs11599763	C	T	-0.134	11813600	0.122	0.086	360421	C	T	0.054	10	11855599	3.94E-06	0.012	14306	21.490
Vascular dementia (mixed)	class Deltaproteobacteria	rs17084793	G	A	-0.048	71645678	0.687	0.120	360421	G	A	-0.071	18	69312914	5.69E-06	0.016	14306	19.850
Vascular dementia (mixed)	class Deltaproteobacteria	rs17791387	A	G	-0.246	79219511	0.087	0.144	360421	A	G	-0.074	9	81834426	1.60E-06	0.015	14306	22.761
Vascular dementia (mixed)	class Deltaproteobacteria	rs2692012	G	A	-0.034	204022477	0.853	0.185	360421	G	A	-0.110	1	203991605	3.14E-06	0.025	14306	18.968
Vascular dementia (mixed)	class Deltaproteobacteria	rs2838334	G	A	0.074	43645080	0.406	0.089	360421	G	A	0.056	21	45064961	5.45E-06	0.012	14306	20.480
Vascular dementia (mixed)	class Deltaproteobacteria	rs3935584	C	T	-0.055	233064573	0.517	0.084	360421	C	T	-0.052	2	233929283	7.50E-06	0.012	14306	20.485
Vascular dementia (mixed)	class Deltaproteobacteria	rs4506934	C	T	-0.072	2953368	0.579	0.130	360421	C	T	-0.094	17	2856662	3.59E-06	0.020	14306	21.666
Vascular dementia (mixed)	class Deltaproteobacteria	rs55744759	A	G	0.000	56955112	0.998	0.132	360421	A	G	-0.078	8	57867671	7.31E-06	0.017	14306	20.854
Vascular dementia (mixed)	class Deltaproteobacteria	rs6058181	C	T	0.052	35106998	0.643	0.113	360421	C	T	0.083	20	33694801	3.40E-07	0.017	14306	24.755
Vascular dementia (mixed)	class Deltaproteobacteria	rs62020470	A	G	0.167	95617836	0.130	0.110	360421	A	G	-0.059	15	96161065	4.85E-06	0.013	14306	20.480
Vascular dementia (mixed)	class Deltaproteobacteria	rs9928243	C	A	0.048	71507738	0.566	0.085	360421	C	A	-0.054	16	71541641	5.02E-06	0.012	14306	20.923
Vascular dementia (mixed)	class Erysipelotrichia	rs1074800	G	A	0.077	3002432	0.369	0.086	360421	G	A	-0.049	5	3002546	6.15E-06	0.011	14306	20.459
Vascular dementia (mixed)	class Erysipelotrichia	rs10781552	C	T	0.002	132083729	0.987	0.093	360421	C	T	-0.055	10	133897233	2.33E-06	0.012	14306	22.633
Vascular dementia (mixed)	class Erysipelotrichia	rs17530232	A	G	-0.011	39811320	0.952	0.185	360421	A	G	0.103	13	40385457	2.79E-06	0.022	14306	21.042
Vascular dementia (mixed)	class Erysipelotrichia	rs1884466	C	T	0.001	63673525	0.990	0.084	360421	C	T	-0.048	1	64139196	9.53E-06	0.011	14306	19.760
Vascular dementia (mixed)	class Erysipelotrichia	rs2300774	A	G	-0.264	196066841	0.001	0.081	360421	A	G	-0.052	3	195793712	8.95E-07	0.011	14306	24.094
Vascular dementia (mixed)	class Erysipelotrichia	rs290833	T	G	0.028	96991871	0.740	0.084	360421	T	G	-0.050	1	97457427	8.03E-06	0.011	14306	19.943
Vascular dementia (mixed)	class Erysipelotrichia	rs35161940	T	C	0.026	72331083	0.852	0.137	360421	T	C	-0.081	17	70327224	1.85E-06	0.017	14306	23.118
Vascular dementia (mixed)	class Erysipelotrichia	rs4078432	T	C	0.002	48528003	0.984	0.112	360421	T	C	0.061	14	48997206	4.23E-06	0.013	14306	20.723
Vascular dementia (mixed)	class Erysipelotrichia	rs56970041	T	G	-0.119	79891267	0.501	0.177	360421	T	G	0.072	14	80357610	5.40E-06	0.016	14306	19.385
Vascular dementia (mixed)	class Erysipelotrichia	rs62504403	C	T	-0.062	38946033	0.553	0.105	360421	C	T	0.068	8	38803551	1.12E-07	0.013	14306	28.371
Vascular dementia (mixed)	class Erysipelotrichia	rs7234058	T	C	0.231	5830508	0.111	0.145	360421	T	C	-0.095	18	5830507	9.12E-07	0.019	14306	23.744
Vascular dementia (mixed)	class Erysipelotrichia	rs7826267	G	T	0.301	3097430	0.074	0.168	360421	G	T	0.084	8	2954952	9.28E-06	0.020	14306	17.755
Vascular dementia (mixed)	class Erysipelotrichia	rs8003149	C	T	0.045	55689786	0.617	0.089	360421	C	T	0.054	14	56156504	4.08E-06	0.012	14306	21.248
Vascular dementia (mixed)	class Gammaproteobacteria	rs11181912	G	A	0.099	43179149	0.268	0.090	360421	G	A	-0.058	12	43572952	9.95E-07	0.012	14306	23.767
Vascular dementia (mixed)	class Gammaproteobacteria	rs12404135	A	G	-0.104	186533562	0.516	0.160	360421	A	G	-0.079	1	186502694	8.89E-06	0.017	14306	20.933
Vascular dementia (mixed)	class Gammaproteobacteria	rs6706173	A	C	-0.210	167130482	0.082	0.121	360421	A	C	0.074	2	167986992	1.99E-07	0.015	14306	25.726
Vascular dementia (mixed)	class Gammaproteobacteria	rs75101789	C	T	0.106	18574411	0.474	0.148	360421	C	T	0.073	3	18615903	8.79E-06	0.016	14306	20.002
Vascular dementia (mixed)	class Gammaproteobacteria	rs79795896	A	G	0.102	50658878	0.610	0.199	360421	A	G	-0.159	18	48185248	7.92E-06	0.035	14306	20.531
Vascular dementia (mixed)	class Gammaproteobacteria	rs9494710	C	T	-0.136	137291417	0.129	0.090	360421	C	T	-0.055	6	137612554	4.55E-06	0.012	14306	20.663
Vascular dementia (mixed)	class Lentisphaeria	rs1002941	A	G	0.248	100702485	0.011	0.098	360421	A	G	-0.105	15	101242690	8.15E-06	0.023	14306	20.234
Vascular dementia (mixed)	class Lentisphaeria	rs11770843	C	T	-0.228	147098287	0.011	0.090	360421	C	T	0.109	7	146795379	1.91E-06	0.023	14306	21.707
Vascular dementia (mixed)	class Lentisphaeria	rs17114848	G	A	-0.007	24917388	0.959	0.142	360421	G	A	0.152	15	25162535	4.06E-06	0.032	14306	22.073
Vascular dementia (mixed)	class Lentisphaeria	rs2031282	A	G	-0.069	20113040	0.533	0.111	360421	A	G	0.122	13	20687179	4.38E-06	0.027	14306	20.490
Vascular dementia (mixed)	class Lentisphaeria	rs2825714	A	G	0.058	19651652	0.603	0.111	360421	A	G	-0.137	21	21023966	1.72E-06	0.029	14306	22.568
Vascular dementia (mixed)	class Lentisphaeria	rs62570196	C	T	-0.267	108323890	0.207	0.212	360421	C	T	-0.216	9	111086170	1.08E-06	0.044	14306	24.192
Vascular dementia (mixed)	class Lentisphaeria	rs72640280	A	G	-0.108	11883735	0.551	0.182	360421	A	G	0.220	1	11943792	5.18E-06	0.049	14306	20.513
Vascular dementia (mixed)	class Lentisphaeria	rs77599476	A	G	-0.347	62762910	0.064	0.187	360421	A	G	0.230	20	61394262	1.86E-06	0.048	14306	23.002
Vascular dementia (mixed)	class Melainabacteria	rs10148250	A	G	-0.114	106605442	0.203	0.089	360421	A	G	-0.086	14	107061448	8.67E-06	0.019	14306	19.866
Vascular dementia (mixed)	class Melainabacteria	rs10738747	A	G	0.156	26184580	0.068	0.085	360421	A	G	-0.081	9	26184578	9.96E-06	0.018	14306	19.514
Vascular dementia (mixed)	class Melainabacteria	rs11150282	T	C	-0.177	80459808	0.045	0.088	360421	T	C	0.099	16	80493705	6.03E-07	0.020	14306	25.235
Vascular dementia (mixed)	class Melainabacteria	rs113884518	T	C	0.059	24648999	0.828	0.272	360421	T	C	-0.205	9	24648997	8.06E-06	0.045	14306	20.371
Vascular dementia (mixed)	class Melainabacteria	rs28678345	T	C	0.261	55828967	0.180	0.195	360421	T	C	0.215	17	53906328	6.69E-06	0.047	14306	20.818
Vascular dementia (mixed)	class Melainabacteria	rs367480	A	G	-0.098	2916401	0.269	0.088	360421	A	G	0.084	11	2937631	8.20E-06	0.019	14306	20.323

Vascular dementia (mixed)	class Melainabacteria	rs4129395	G	A	0.012	113213109	0.884	0.084	360421	G	A	0.090	9	115975389	1.48E-06	0.019	14306	23.440
Vascular dementia (mixed)	class Melainabacteria	rs789069	A	C	0.004	1008277	0.973	0.122	360421	A	C	-0.104	18	1008278	6.85E-06	0.023	14306	19.530
Vascular dementia (mixed)	class Melainabacteria	rs79790072	T	C	0.113	100207478	0.642	0.244	360421	T	C	0.227	15	100747683	3.29E-06	0.049	14306	21.599
Vascular dementia (mixed)	class Melainabacteria	rs9864379	T	C	-0.074	14265449	0.535	0.119	360421	T	C	-0.160	3	14306949	5.36E-08	0.029	14306	29.812
Vascular dementia (mixed)	class Methanobacteria	rs10202904	G	T	0.028	124682691	0.745	0.086	360421	G	T	0.122	2	125440268	3.01E-07	0.024	14306	26.762
Vascular dementia (mixed)	class Methanobacteria	rs10424197	A	G	-0.140	45936063	0.149	0.097	360421	A	G	0.111	19	46439321	9.28E-06	0.025	14306	20.211
Vascular dementia (mixed)	class Methanobacteria	rs4257531	G	A	0.032	2044483	0.818	0.140	360421	G	A	0.164	3	2086167	7.44E-06	0.036	14306	20.316
Vascular dementia (mixed)	class Methanobacteria	rs6508769	C	T	0.065	28336853	0.583	0.118	360421	C	T	-0.154	19	28827760	8.23E-06	0.034	14306	19.856
Vascular dementia (mixed)	class Methanobacteria	rs6776814	T	C	0.412	15011576	0.158	0.292	360421	T	C	-0.200	3	15053083	1.63E-06	0.041	14306	23.483
Vascular dementia (mixed)	class Methanobacteria	rs73068003	G	T	0.007	10734305	0.961	0.143	360421	G	T	-0.158	7	10773932	8.45E-06	0.035	14306	20.206
Vascular dementia (mixed)	class Methanobacteria	rs73457410	A	G	0.109	41382045	0.524	0.170	360421	A	G	0.215	13	41956181	1.41E-06	0.044	14306	24.316
Vascular dementia (mixed)	class Methanobacteria	rs75208022	C	T	-0.016	21185927	0.909	0.144	360421	C	T	-0.227	12	21338861	5.92E-06	0.049	14306	21.717
Vascular dementia (mixed)	class Methanobacteria	rs894996	C	A	-0.011	103497150	0.945	0.163	360421	C	A	0.217	4	104418307	1.88E-06	0.045	14306	23.349
Vascular dementia (mixed)	class Mollicutes	rs10108398	G	A	0.111	58528265	0.237	0.094	360421	G	A	0.077	8	59440824	1.09E-06	0.015	14306	24.960
Vascular dementia (mixed)	class Mollicutes	rs11890098	A	G	-0.164	156676037	0.082	0.094	360421	A	G	0.074	2	157532549	9.57E-07	0.015	14306	23.551
Vascular dementia (mixed)	class Mollicutes	rs12566890	T	G	0.261	61385192	0.036	0.125	360421	T	G	-0.101	1	61850864	3.65E-06	0.023	14306	19.176
Vascular dementia (mixed)	class Mollicutes	rs17214486	C	A	0.044	96693337	0.630	0.091	360421	C	A	0.061	14	97159674	6.61E-06	0.014	14306	20.223
Vascular dementia (mixed)	class Mollicutes	rs2464826	A	C	-0.156	79860934	0.243	0.134	360421	A	C	0.094	7	79490250	8.39E-06	0.021	14306	19.874
Vascular dementia (mixed)	class Mollicutes	rs28537087	G	A	0.058	94766447	0.553	0.098	360421	G	A	0.082	15	95309676	8.07E-06	0.019	14306	19.002
Vascular dementia (mixed)	class Mollicutes	rs3768491	G	A	0.017	109423364	0.856	0.093	360421	G	A	0.068	1	109965986	4.23E-06	0.015	14306	20.875
Vascular dementia (mixed)	class Mollicutes	rs4885016	C	T	-0.082	72596351	0.510	0.124	360421	C	T	0.082	13	73170489	7.27E-06	0.018	14306	20.363
Vascular dementia (mixed)	class Mollicutes	rs6043847	T	C	-0.218	16278879	0.229	0.181	360421	T	C	-0.115	20	16259524	4.55E-06	0.025	14306	21.375
Vascular dementia (mixed)	class Mollicutes	rs72901605	T	C	-0.100	47082326	0.445	0.131	360421	T	C	-0.084	11	47103877	3.26E-06	0.018	14306	22.338
Vascular dementia (mixed)	class Mollicutes	rs74603314	T	C	0.252	46050515	0.247	0.218	360421	T	C	0.222	14	46519718	1.56E-06	0.046	14306	22.924
Vascular dementia (mixed)	class Mollicutes	rs78169027	A	G	0.037	108568360	0.835	0.177	360421	A	G	-0.108	11	108439087	5.88E-06	0.024	14306	20.824
Vascular dementia (mixed)	class Negativicutes	rs1135612	G	A	-0.017	75980359	0.872	0.103	360421	G	A	0.053	7	75609677	9.26E-06	0.012	14306	19.761
Vascular dementia (mixed)	class Negativicutes	rs13086907	G	A	-0.105	142416275	0.302	0.102	360421	G	A	0.063	3	142135117	1.95E-06	0.013	14306	22.532
Vascular dementia (mixed)	class Negativicutes	rs1643968	T	C	0.047	165839623	0.597	0.088	360421	T	C	-0.057	5	165266628	4.15E-07	0.011	14306	25.339
Vascular dementia (mixed)	class Negativicutes	rs1649999	A	G	-0.241	78326315	0.094	0.144	360421	A	G	0.075	10	80086072	7.58E-06	0.017	14306	20.246
Vascular dementia (mixed)	class Negativicutes	rs2834062	A	G	-0.061	33005177	0.508	0.092	360421	A	G	0.049	21	34377485	8.44E-06	0.011	14306	20.190
Vascular dementia (mixed)	class Negativicutes	rs4463806	C	T	-0.180	113838234	0.092	0.107	360421	C	T	0.054	10	115597993	7.81E-06	0.013	14306	17.681
Vascular dementia (mixed)	class Negativicutes	rs4722181	T	G	-0.045	22777952	0.592	0.084	360421	T	G	0.050	7	22817571	2.00E-06	0.011	14306	22.452
Vascular dementia (mixed)	class Negativicutes	rs60274479	T	C	0.138	21238604	0.194	0.106	360421	T	C	-0.066	16	21249925	1.16E-06	0.013	14306	24.182
Vascular dementia (mixed)	class Negativicutes	rs61249479	A	C	0.016	122150629	0.891	0.116	360421	A	C	0.078	9	124912908	2.95E-06	0.017	14306	21.236
Vascular dementia (mixed)	class Negativicutes	rs71405394	G	A	0.175	100704883	0.293	0.166	360421	G	A	-0.114	15	101245088	2.17E-06	0.024	14306	22.539
Vascular dementia (mixed)	class Negativicutes	rs73232831	G	A	0.208	17411803	0.350	0.222	360421	G	A	-0.152	4	17413426	1.87E-06	0.031	14306	23.242
Vascular dementia (mixed)	class Negativicutes	rs9423647	G	A	0.027	5537855	0.754	0.085	360421	G	A	0.048	10	5579818	6.06E-06	0.011	14306	20.628
Vascular dementia (mixed)	class Verrucomicrobiae	rs111862613	T	C	0.068	129825125	0.548	0.113	360421	T	C	0.091	12	130309670	3.74E-06	0.020	14306	21.252
Vascular dementia (mixed)	class Verrucomicrobiae	rs117107102	A	G	-0.188	51947265	0.344	0.198	360421	A	G	0.205	18	49473635	2.92E-06	0.043	14306	22.493
Vascular dementia (mixed)	class Verrucomicrobiae	rs11729256	T	C	0.127	94106121	0.258	0.112	360421	T	C	0.075	4	95027272	6.73E-07	0.015	14306	24.928
Vascular dementia (mixed)	class Verrucomicrobiae	rs12908520	G	A	0.144	97027427	0.092	0.085	360421	G	A	0.062	15	97570657	2.17E-06	0.013	14306	22.341
Vascular dementia (mixed)	class Verrucomicrobiae	rs2602429	T	C	0.023	81029544	0.810	0.096	360421	T	C	-0.075	16	81063149	2.58E-06	0.016	14306	22.863
Vascular dementia (mixed)	class Verrucomicrobiae	rs4242783	A	G	0.105	5022135	0.262	0.094	360421	A	G	-0.069	10	5064327	2.64E-06	0.015	14306	21.781
Vascular dementia (mixed)	class Verrucomicrobiae	rs4936098	G	A	0.002	130410772	0.985	0.088	360421	G	A	-0.065	11	130280667	1.12E-06	0.014	14306	22.786
Vascular dementia (mixed)	class Verrucomicrobiae	rs61779207	G	A	-0.010	40608800	0.923	0.102	360421	G	A	-0.076	1	41074472	6.72E-06	0.017	14306	20.432
Vascular dementia (mixed)	class Verrucomicrobiae	rs74542928	T	C	0.226	99623031	0.244	0.194	360421	T	C	0.112	4	100544188	1.63E-06	0.024	14306	22.508
Vascular dementia (mixed)	class Verrucomicrobiae	rs9349825	A	G	-0.041	56476683	0.701	0.107	360421	A	G	-0.070	6	56341481	2.54E-06	0.015	14306	22.898
Vascular dementia (mixed)	class Verrucomicrobiae	rs941682	G	A	-0.058	33280034	0.538	0.095	360421	G	A	-0.063	20	31867840	9.61E-06	0.014	14306	19.290
Vascular dementia (mixed)	family Acidaminococcaceae	rs262812	T	C	0.073	158226967	0.422	0.091	360421	T	C	-0.066	6	158647999	3.25E-06	0.014	14306	21.313
Vascular dementia (mixed)	family Acidaminococcaceae	rs2933324	G	A	-0.084	104355372	0.404	0.101	360421	G	A	0.066	9	107117653	2.24E-06	0.014	14306	22.315
Vascular dementia (mixed)	family Acidaminococcaceae	rs45497800	T	C	-0.170	63360481	0.160	0.121	360421	T	C	-0.118	20	61991833	5.86E-06	0.026	14306	20.975
Vascular dementia (mixed)	family Acidaminococcaceae	rs6589457	G	A	0.153	114830566	0.422	0.191	360421	G	A	-0.166	11	114701288	2.32E-06	0.035	14306	22.533
Vascular dementia (mixed)	family Acidaminococcaceae	rs6923842	T	C	-0.046	5722948	0.731	0.133	360421	T	C	-0.080	6	5723181	2.21E-06	0.017	14306	22.140
Vascular dementia (mixed)	family Acidaminococcaceae	rs74540770	G	A	-0.218	186835600	0.163	0.156	360421	G	A	-0.109	3	186553389	7.09E-06	0.024	14306	20.021
Vascular dementia (mixed)	family Acidaminococcaceae	rs78702810	T	C	-0.097	18421039	0.485	0.139	360421	T	C	-0.144	3	18462531	9.16E-06	0.032	14306	19.778
Vascular dementia (mixed)	family Actinomycetaceae	rs2889192	T	G	0.025	73779652	0.835	0.118	360421	T	G	-0.089	9	76394568	3.64E-06	0.020	14306	20.714
Vascular dementia (mixed)	family Actinomycetaceae	rs34583783	G	T	0.035	66497478	0.842	0.176	360421	G	T	0.124	6	67207371	5.48E-06	0.026	14306	21.930
Vascular dementia (mixed)	family Actinomycetaceae	rs35011108	A	G	-0.135	132686341	0.414	0.166	360421	A	G	0.242	6	133007480	1.83E-06	0.050	14306	23.041
Vascular dementia (mixed)	family Actinomycetaceae	rs4073240	G	A	-0.114	168824686	0.187	0.087	360421	G	A	0.075	6	169224781	6.05E-06	0.016	14306	20.605
Vascular dementia (mixed)	family Alcaligenaceae	rs112135816	T	G	-0.316	79870412	0.070	0.174	360421	T	G	-0.078	9	82485327	5.28E-06	0.017	14306	20.411
Vascular dementia (mixed)	family Alcaligenaceae	rs1153990	A	G	0.226	104897760	0.013	0.091	360421	A	G	-0.059	5	104233461	5.97E-06	0.013	14306	20.950
Vascular dementia (mixed)	family Alcaligenaceae	rs147968	C	T	0.020	85912233	0.810	0.084	360421	C	T	0.049	16	85945839	9.13E-06	0.011	14306	19.685
Vascular dementia (mixed)	family Alcaligenaceae	rs28480294	C	T	-0.188	71230170	0.033	0.088	360421	C	T	0.052	15	71522509	6.61E-06	0.012	14306	20.033
Vascular dementia (mixed)	family Alcaligenaceae	rs4033856	T	C	0.009	45640468	0.951	0.149	360421	T	C	-0.082	4	45642485	1.03E-06	0.017	14306	23.467
Vascular dementia (mixed)	family Alcaligenaceae	rs62191117	A	G	0.085	238979079	0.419	0.105	360421	A	G	0.068	2	239900775	2.76E-07	0.013	14306	26.221
Vascular dementia (mixed)	family Alcaligenaceae	rs62395635	T	C	-0.314	174070793	0.077	0.178	360421	T	C	0.111	5	173497796	3.35E-06	0.024	14306	21.496
Vascular dementia (mixed)	family Alcaligenaceae	rs6969323	A	C	-0.091	104574040	0.370	0.101	360421	A	C	-0.059	7	104214487	3.89E-06	0.013	14306	21.127

Vascular dementia (mixed)	family Alcaligenaceae	rs74776516	T	G	0.240	20741821	0.153	0.168	360421	T	G	-0.094	11	20763367	6.85E-06	0.021	14306	19.591
Vascular dementia (mixed)	family Alcaligenaceae	rs7638039	T	C	-0.133	70539788	0.176	0.098	360421	T	C	0.060	3	70588939	2.70E-06	0.013	14306	22.295
Vascular dementia (mixed)	family Alcaligenaceae	rs9537886	A	C	-0.146	57971112	0.086	0.085	360421	A	C	-0.057	13	58545246	2.35E-07	0.011	14306	26.604
Vascular dementia (mixed)	family Bacteroidaceae	rs11585893	A	G	-0.115	10584294	0.244	0.098	360421	A	G	-0.074	1	10644351	1.80E-06	0.015	14306	25.175
Vascular dementia (mixed)	family Bacteroidaceae	rs13207588	A	G	-0.056	41551692	0.602	0.107	360421	A	G	-0.059	6	41519430	7.48E-06	0.013	14306	20.365
Vascular dementia (mixed)	family Bacteroidaceae	rs1340391	T	C	-0.078	102495433	0.530	0.124	360421	T	C	-0.059	1	102960989	6.73E-06	0.013	14306	20.040
Vascular dementia (mixed)	family Bacteroidaceae	rs17619981	T	G	-0.021	24159448	0.861	0.122	360421	T	G	0.088	19	24342250	2.69E-06	0.019	14306	22.194
Vascular dementia (mixed)	family Bacteroidaceae	rs2023437	T	C	-0.128	21577814	0.320	0.129	360421	T	C	-0.078	14	22045949	5.02E-06	0.017	14306	21.780
Vascular dementia (mixed)	family Bacteroidaceae	rs66710942	T	C	0.022	77166176	0.792	0.084	360421	T	C	-0.049	3	77215327	5.86E-06	0.011	14306	20.644
Vascular dementia (mixed)	family Bacteroidaceae	rs6795673	C	T	0.048	10551540	0.568	0.084	360421	C	T	0.054	3	10593224	3.38E-07	0.011	14306	26.183
Vascular dementia (mixed)	family Bacteroidaceae	rs9507307	C	T	0.009	24336338	0.925	0.098	360421	C	T	0.060	13	24910476	2.13E-06	0.013	14306	21.912
Vascular dementia (mixed)	family Bacteroidales S24 7group	rs10872669	G	A	-0.077	151194037	0.599	0.146	360421	G	A	0.123	6	151515172	9.49E-06	0.028	14306	19.932
Vascular dementia (mixed)	family Bacteroidales S24 7group	rs12748533	G	T	0.042	242603950	0.652	0.093	360421	G	T	-0.082	1	242767252	2.59E-06	0.017	14306	22.605
Vascular dementia (mixed)	family Bacteroidales S24 7group	rs17043785	T	C	0.074	52912224	0.620	0.149	360421	T	C	-0.176	2	53139362	5.12E-07	0.035	14306	25.761
Vascular dementia (mixed)	family Bacteroidales S24 7group	rs1850003	A	G	0.167	47048773	0.094	0.100	360421	A	G	0.084	15	47340971	2.41E-06	0.018	14306	22.344
Vascular dementia (mixed)	family Bacteroidales S24 7group	rs61508842	T	C	-0.241	158935756	0.105	0.149	360421	T	C	0.123	3	158653545	7.83E-06	0.027	14306	20.250
Vascular dementia (mixed)	family Bacteroidales S24 7group	rs738193	T	C	0.029	25547355	0.744	0.088	360421	T	C	0.085	22	25943322	3.82E-07	0.017	14306	26.100
Vascular dementia (mixed)	family Bacteroidales S24 7group	rs78609301	A	G	-0.025	62526842	0.787	0.092	360421	A	G	-0.087	6	63236747	7.09E-06	0.020	14306	19.628
Vascular dementia (mixed)	family Bacteroidales S24 7group	rs941000	T	C	0.083	91085352	0.342	0.087	360421	T	C	-0.085	7	90714667	3.16E-07	0.016	14306	27.083
Vascular dementia (mixed)	family Bifidobacteriaceae	rs10831953	G	A	-0.057	13076504	0.535	0.091	360421	G	A	0.054	11	13098051	9.95E-06	0.012	14306	18.869
Vascular dementia (mixed)	family Bifidobacteriaceae	rs12446429	T	C	0.148	848055	0.177	0.109	360421	T	C	0.081	16	898055	8.53E-06	0.019	14306	18.040
Vascular dementia (mixed)	family Bifidobacteriaceae	rs13020688	G	A	0.045	192013806	0.619	0.091	360421	G	A	0.058	2	192878532	1.57E-06	0.012	14306	22.887
Vascular dementia (mixed)	family Bifidobacteriaceae	rs182549	T	C	-0.114	135859184	0.187	0.087	360421	T	C	-0.117	2	136616754	5.94E-20	0.013	14306	85.372
Vascular dementia (mixed)	family Bifidobacteriaceae	rs4957061	T	C	0.013	520981	0.881	0.086	360421	T	C	0.057	5	521096	1.15E-06	0.012	14306	23.762
Vascular dementia (mixed)	family Bifidobacteriaceae	rs540489	T	G	0.037	74901626	0.740	0.111	360421	T	G	-0.063	17	72897722	5.37E-06	0.014	14306	20.956
Vascular dementia (mixed)	family Bifidobacteriaceae	rs55888705	A	G	0.072	1516099	0.438	0.093	360421	A	G	0.054	4	1517826	8.66E-06	0.012	14306	19.812
Vascular dementia (mixed)	family Bifidobacteriaceae	rs6899771	A	G	-0.161	96958344	0.251	0.140	360421	A	G	-0.091	6	97406220	7.28E-06	0.020	14306	20.365
Vascular dementia (mixed)	family Bifidobacteriaceae	rs7174549	T	C	-0.099	91920073	0.256	0.087	360421	T	C	-0.055	15	92463303	6.87E-06	0.012	14306	19.590
Vascular dementia (mixed)	family Bifidobacteriaceae	rs7322849	T	C	0.097	112205515	0.512	0.147	360421	T	C	0.111	13	112859829	1.74E-08	0.020	14306	30.320
Vascular dementia (mixed)	family Bifidobacteriaceae	rs857444	C	T	0.083	14617360	0.341	0.087	360421	C	T	0.055	6	14617591	3.82E-06	0.012	14306	21.075
Vascular dementia (mixed)	family Clostridiaceae1	rs10875374	T	C	0.046	101339253	0.587	0.084	360421	T	C	0.054	1	101804809	8.10E-06	0.012	14306	20.197
Vascular dementia (mixed)	family Clostridiaceae1	rs12186080	G	A	0.048	132877783	0.675	0.113	360421	G	A	0.075	3	132596627	5.34E-06	0.016	14306	21.212
Vascular dementia (mixed)	family Clostridiaceae1	rs12341505	G	A	-0.130	133845759	0.373	0.145	360421	G	A	0.081	9	136710881	4.54E-06	0.018	14306	20.627
Vascular dementia (mixed)	family Clostridiaceae1	rs2795528	G	A	0.080	42774816	0.661	0.183	360421	G	A	-0.181	10	43270264	3.81E-06	0.039	14306	21.432
Vascular dementia (mixed)	family Clostridiaceae1	rs2817172	C	T	0.072	3124955	0.405	0.086	360421	C	T	0.056	1	3041519	5.27E-06	0.012	14306	20.668
Vascular dementia (mixed)	family Clostridiaceae1	rs4723021	T	C	-0.121	30895044	0.478	0.170	360421	T	C	-0.106	7	30934659	7.42E-06	0.024	14306	19.331
Vascular dementia (mixed)	family Clostridiaceae1	rs550843	T	C	0.076	165309343	0.423	0.094	360421	T	C	-0.073	6	165722832	7.09E-06	0.017	14306	19.043
Vascular dementia (mixed)	family Clostridiaceae1	rs56188186	A	G	0.086	87677084	0.661	0.196	360421	A	G	0.097	16	87710690	8.24E-06	0.022	14306	19.804
Vascular dementia (mixed)	family Clostridiaceae1	rs62397761	A	G	0.021	48125278	0.818	0.090	360421	A	G	0.062	6	48093014	9.08E-06	0.014	14306	20.443
Vascular dementia (mixed)	family Clostridiaceae1	rs881532	G	A	-0.023	47411024	0.782	0.084	360421	G	A	0.053	22	47806774	7.90E-06	0.012	14306	20.060
Vascular dementia (mixed)	family Clostridiales vadin BB60 group	rs10517600	G	T	0.003	154711806	0.972	0.087	360421	G	T	-0.063	4	155632958	6.83E-06	0.014	14306	20.241
Vascular dementia (mixed)	family Clostridiales vadin BB60 group	rs10904722	C	T	-0.025	6672462	0.797	0.099	360421	C	T	-0.067	10	6714424	5.05E-06	0.015	14306	20.883
Vascular dementia (mixed)	family Clostridiales vadin BB60 group	rs118104867	C	T	0.097	122965298	0.560	0.166	360421	C	T	0.214	8	123977538	3.44E-06	0.046	14306	22.207
Vascular dementia (mixed)	family Clostridiales vadin BB60 group	rs13409132	A	G	-0.076	204751623	0.736	0.224	360421	A	G	-0.165	2	205616346	4.37E-06	0.035	14306	22.065
Vascular dementia (mixed)	family Clostridiales vadin BB60 group	rs17121075	G	A	0.097	85390452	0.342	0.103	360421	G	A	0.077	14	85856796	7.91E-06	0.017	14306	19.948
Vascular dementia (mixed)	family Clostridiales vadin BB60 group	rs2191834	T	G	-0.048	229078235	0.621	0.098	360421	T	G	-0.075	2	229942951	2.50E-06	0.016	14306	21.998
Vascular dementia (mixed)	family Clostridiales vadin BB60 group	rs28691777	C	T	0.390	60072044	0.061	0.208	360421	C	T	0.137	17	58149405	6.96E-07	0.027	14306	26.380
Vascular dementia (mixed)	family Clostridiales vadin BB60 group	rs34088226	A	G	-0.052	4308833	0.768	0.177	360421	A	G	-0.118	5	4308946	7.66E-06	0.027	14306	19.145
Vascular dementia (mixed)	family Clostridiales vadin BB60 group	rs55682560	C	T	0.093	86849059	0.549	0.155	360421	C	T	-0.132	15	87392290	4.97E-07	0.026	14306	25.330
Vascular dementia (mixed)	family Clostridiales vadin BB60 group	rs6588624	A	G	0.063	56383867	0.452	0.084	360421	A	G	0.066	1	56849539	1.79E-06	0.014	14306	22.985
Vascular dementia (mixed)	family Clostridiales vadin BB60 group	rs66714985	A	C	0.021	3447600	0.882	0.139	360421	A	C	0.117	8	3305122	4.85E-06	0.025	14306	21.446
Vascular dementia (mixed)	family Clostridiales vadin BB60 group	rs7226487	A	G	0.025	76661168	0.764	0.084	360421	A	G	-0.064	18	74373125	3.58E-06	0.014	14306	21.537
Vascular dementia (mixed)	family Clostridiales vadin BB60 group	rs7538034	T	G	-0.022	70926011	0.851	0.115	360421	T	G	-0.079	1	71391694	2.37E-06	0.017	14306	22.423
Vascular dementia (mixed)	family Clostridiales vadin BB60 group	rs7725895	A	G	0.083	142865333	0.523	0.129	360421	A	G	-0.116	5	142244898	3.94E-06	0.024	14306	23.380
Vascular dementia (mixed)	family Clostridiales vadin BB60 group	rs989682	A	G	-0.019	15522946	0.847	0.100	360421	A	G	0.070	3	15564453	6.85E-06	0.016	14306	20.413
Vascular dementia (mixed)	family Coriobacteriaceae	rs11073596	G	T	-0.188	85890348	0.032	0.087	360421	G	T	-0.051	15	86433579	8.14E-06	0.011	14306	19.912
Vascular dementia (mixed)	family Coriobacteriaceae	rs11250875	T	C	0.093	1880537	0.365	0.102	360421	T	C	0.061	10	1922731	4.83E-06	0.013	14306	21.526
Vascular dementia (mixed)	family Coriobacteriaceae	rs11656361	A	C	-0.175	8218014	0.110	0.110	360421	A	C	0.077	17	8121332	8.02E-06	0.018	14306	19.394
Vascular dementia (mixed)	family Coriobacteriaceae	rs12974142	G	A	0.028	52391913	0.867	0.165	360421	G	A	0.079	19	52895166	8.51E-06	0.018	14306	19.865
Vascular dementia (mixed)	family Coriobacteriaceae	rs13307134	T	C	0.021	105444233	0.856	0.113	360421	T	C	-0.057	7	105084680	7.80E-06	0.013	14306	20.072
Vascular dementia (mixed)	family Coriobacteriaceae	rs1397793	A	G	-0.012	91175634	0.893	0.092	360421	A	G	0.050	5	90471451	9.77E-06	0.011	14306	19.682
Vascular dementia (mixed)	family Coriobacteriaceae	rs1816223	G	A	0.118	11341087	0.267	0.106	360421	G	A	0.059	12	11494021	4.84E-06	0.013	14306	20.652
Vascular dementia (mixed)	family Coriobacteriaceae	rs240104	T	C	-0.017	176602295	0.859	0.094	360421	T	C	-0.060	1	176571431	1.52E-06	0.013	14306	22.630
Vascular dementia (mixed)	family Coriobacteriaceae	rs2442778	A	G	0.169	11612938	0.365	0.186	360421	A	G	0.116	3	11654412	9.03E-06	0.026	14306	20.272
Vascular dementia (mixed)	family Coriobacteriaceae	rs3025411	A	G	0.009	133647784	0.947	0.135	360421	A	G	0.093	9	136512906	8.27E-06	0.021	14306	19.566
Vascular dementia (mixed)	family Coriobacteriaceae	rs34739816	G	T	0.111	39220432	0.534	0.179	360421	G	T	0.097	17	37376685	3.88E-06	0.021	14306	21.594
Vascular dementia (mixed)	family Coriobacteriaceae	rs67561917	A	G	-0.006	63440724	0.953	0.110	360421	A	G	-0.071	20	62072077	5.39E-06	0.015	14306	21.486

Vascular dementia (mixed)	family Coriobacteriaceae	rs719099	A	G	-0.161	64039457	0.258	0.142	360421	A	G	0.078	10	65799217	5.43E-07	0.016	14306	24.957
Vascular dementia (mixed)	family Coriobacteriaceae	rs8010111	A	G	0.182	39191305	0.236	0.154	360421	A	G	0.103	14	39660509	6.90E-06	0.023	14306	20.328
Vascular dementia (mixed)	family Defluviitaleaceae	rs112893842	T	C	0.011	8786663	0.938	0.147	360421	T	C	0.111	9	8786663	2.75E-06	0.023	14306	22.686
Vascular dementia (mixed)	family Defluviitaleaceae	rs1582238	C	T	0.119	118181062	0.174	0.088	360421	C	T	-0.080	1	118723685	1.69E-06	0.017	14306	23.042
Vascular dementia (mixed)	family Defluviitaleaceae	rs17051335	C	T	0.062	121281755	0.656	0.139	360421	C	T	-0.134	4	122202910	4.58E-06	0.029	14306	21.129
Vascular dementia (mixed)	family Defluviitaleaceae	rs1908593	T	C	0.159	61358915	0.063	0.086	360421	T	C	0.070	18	59026148	7.86E-06	0.016	14306	20.108
Vascular dementia (mixed)	family Defluviitaleaceae	rs4344384	T	G	0.110	64647609	0.190	0.084	360421	T	G	-0.071	10	66407366	5.86E-06	0.016	14306	20.612
Vascular dementia (mixed)	family Defluviitaleaceae	rs4677103	A	G	0.234	72158643	0.036	0.111	360421	A	G	0.098	3	72207794	9.42E-07	0.020	14306	24.598
Vascular dementia (mixed)	family Defluviitaleaceae	rs540220	C	T	0.063	90569026	0.669	0.148	360421	C	T	0.124	9	93331308	9.48E-06	0.029	14306	18.293
Vascular dementia (mixed)	family Defluviitaleaceae	rs55658617	T	C	-0.140	40439076	0.549	0.233	360421	T	C	0.177	21	41811003	1.41E-06	0.036	14306	24.013
Vascular dementia (mixed)	family Defluviitaleaceae	rs72731813	C	T	0.037	146493591	0.851	0.197	360421	C	T	-0.150	4	147414743	2.76E-07	0.029	14306	26.048
Vascular dementia (mixed)	family Defluviitaleaceae	rs9608282	T	G	0.131	24408113	0.583	0.238	360421	T	G	0.139	22	24804081	4.61E-06	0.030	14306	21.554
Vascular dementia (mixed)	family Defluviitaleaceae	rs9725395	A	G	-0.091	84739949	0.493	0.133	360421	A	G	-0.138	1	85205632	3.41E-06	0.030	14306	21.969
Vascular dementia (mixed)	family Desulfovibrionaceae	rs11599763	C	T	-0.134	11813600	0.122	0.086	360421	C	T	0.056	10	11855599	2.50E-06	0.012	14306	22.388
Vascular dementia (mixed)	family Desulfovibrionaceae	rs17791387	A	G	-0.246	79219511	0.087	0.144	360421	A	G	-0.073	9	81834426	2.10E-06	0.015	14306	22.313
Vascular dementia (mixed)	family Desulfovibrionaceae	rs2692012	G	A	-0.034	204022477	0.853	0.185	360421	G	A	-0.114	1	203991605	1.56E-06	0.025	14306	20.295
Vascular dementia (mixed)	family Desulfovibrionaceae	rs2838334	G	A	0.074	43645080	0.406	0.089	360421	G	A	0.057	21	45064961	3.82E-06	0.012	14306	21.157
Vascular dementia (mixed)	family Desulfovibrionaceae	rs3935584	C	T	-0.055	233064573	0.517	0.084	360421	C	T	-0.053	2	233929283	6.78E-06	0.012	14306	20.635
Vascular dementia (mixed)	family Desulfovibrionaceae	rs4506934	C	T	-0.072	2953368	0.579	0.130	360421	C	T	-0.094	17	2856662	3.16E-06	0.020	14306	21.945
Vascular dementia (mixed)	family Desulfovibrionaceae	rs6058181	C	T	0.052	35106998	0.643	0.113	360421	C	T	0.083	20	33694801	2.70E-07	0.017	14306	25.253
Vascular dementia (mixed)	family Desulfovibrionaceae	rs72647048	T	C	0.000	56952819	0.998	0.132	360421	T	C	-0.077	8	57865378	9.61E-06	0.017	14306	20.307
Vascular dementia (mixed)	family Desulfovibrionaceae	rs9928243	C	A	0.048	71507738	0.566	0.085	360421	C	A	-0.054	16	71541641	4.48E-06	0.012	14306	21.142
Vascular dementia (mixed)	family Enterobacteriaceae	rs11026530	T	C	0.075	22357551	0.522	0.118	360421	T	C	0.082	11	22379097	9.43E-06	0.019	14306	19.471
Vascular dementia (mixed)	family Enterobacteriaceae	rs2374342	C	A	0.022	41906402	0.798	0.086	360421	C	A	0.058	2	42133542	4.52E-06	0.013	14306	21.338
Vascular dementia (mixed)	family Enterobacteriaceae	rs35673018	G	A	-0.238	54293833	0.104	0.146	360421	G	A	0.090	16	54327745	7.63E-06	0.020	14306	19.653
Vascular dementia (mixed)	family Enterobacteriaceae	rs504442	T	G	-0.187	57478315	0.169	0.136	360421	T	G	0.084	18	55145547	5.17E-06	0.019	14306	19.728
Vascular dementia (mixed)	family Enterobacteriaceae	rs62210023	A	G	-0.026	56765036	0.771	0.089	360421	A	G	0.061	20	55340092	3.13E-06	0.013	14306	21.742
Vascular dementia (mixed)	family Enterobacteriaceae	rs78143293	A	G	-0.190	60005103	0.138	0.128	360421	A	G	-0.085	18	57672335	1.20E-06	0.017	14306	24.792
Vascular dementia (mixed)	family Enterobacteriaceae	rs79757635	C	A	-0.068	110188071	0.581	0.124	360421	C	A	0.076	13	110840418	9.32E-06	0.017	14306	19.615
Vascular dementia (mixed)	family Erysipelotrichaceae	rs1074800	G	A	0.077	3002432	0.369	0.086	360421	G	A	-0.049	5	3002546	6.15E-06	0.011	14306	20.459
Vascular dementia (mixed)	family Erysipelotrichaceae	rs10781552	C	T	0.002	132083729	0.987	0.093	360421	C	T	-0.055	10	133897233	2.33E-06	0.012	14306	22.633
Vascular dementia (mixed)	family Erysipelotrichaceae	rs17530232	A	G	-0.011	39811320	0.952	0.185	360421	A	G	0.103	13	40385457	2.79E-06	0.022	14306	21.042
Vascular dementia (mixed)	family Erysipelotrichaceae	rs1884466	C	T	0.001	63673525	0.990	0.084	360421	C	T	-0.048	1	64139196	9.53E-06	0.011	14306	19.760
Vascular dementia (mixed)	family Erysipelotrichaceae	rs2300774	A	G	-0.264	196066841	0.001	0.081	360421	A	G	-0.052	3	195793712	8.95E-07	0.011	14306	24.094
Vascular dementia (mixed)	family Erysipelotrichaceae	rs290833	T	G	0.028	96991871	0.740	0.084	360421	T	G	-0.050	1	97457427	8.03E-06	0.011	14306	19.943
Vascular dementia (mixed)	family Erysipelotrichaceae	rs35161940	T	C	0.026	72331083	0.852	0.137	360421	T	C	-0.081	17	70327224	1.85E-06	0.017	14306	23.118
Vascular dementia (mixed)	family Erysipelotrichaceae	rs4078432	T	C	0.002	48528003	0.984	0.112	360421	T	C	0.061	14	48997206	4.23E-06	0.013	14306	20.723
Vascular dementia (mixed)	family Erysipelotrichaceae	rs56970041	T	G	-0.119	79891267	0.501	0.177	360421	T	G	0.072	14	80357610	5.40E-06	0.016	14306	19.385
Vascular dementia (mixed)	family Erysipelotrichaceae	rs62504403	C	T	-0.062	38946033	0.553	0.105	360421	C	T	0.068	8	38803551	1.12E-07	0.013	14306	28.371
Vascular dementia (mixed)	family Erysipelotrichaceae	rs7234058	T	C	0.231	5830508	0.111	0.145	360421	T	C	-0.095	18	5830507	9.12E-07	0.019	14306	23.744
Vascular dementia (mixed)	family Erysipelotrichaceae	rs7826267	G	T	0.301	3097430	0.074	0.168	360421	G	T	0.084	8	2954952	9.28E-06	0.020	14306	17.755
Vascular dementia (mixed)	family Erysipelotrichaceae	rs8003149	C	T	0.045	55689786	0.617	0.089	360421	C	T	0.054	14	56156504	4.08E-06	0.012	14306	21.248
Vascular dementia (mixed)	family Family XI	rs10759623	C	T	-0.051	113059205	0.617	0.101	360421	C	T	-0.162	9	115821485	5.78E-07	0.032	14306	25.425
Vascular dementia (mixed)	family Family XI	rs11547158	A	G	-0.078	149224640	0.531	0.125	360421	A	G	-0.178	7	148921732	2.70E-06	0.037	14306	22.696
Vascular dementia (mixed)	family Family XI	rs17379710	T	C	-0.060	35313121	0.482	0.085	360421	T	C	-0.116	11	35334668	3.97E-06	0.025	14306	21.308
Vascular dementia (mixed)	family Family XI	rs2155352	A	G	0.109	95624045	0.269	0.099	360421	A	G	-0.151	11	95357209	6.63E-07	0.030	14306	24.795
Vascular dementia (mixed)	family Family XI	rs2156611	T	C	-0.051	45765899	0.542	0.084	360421	T	C	-0.112	18	43345864	9.43E-06	0.025	14306	20.068
Vascular dementia (mixed)	family Family XI	rs3733511	A	G	-0.030	119034632	0.741	0.092	360421	A	G	0.128	4	119955787	3.39E-06	0.027	14306	21.795
Vascular dementia (mixed)	family Family XI	rs488164	G	T	-0.040	239832151	0.648	0.088	360421	G	T	-0.118	1	239995451	4.80E-06	0.026	14306	21.386
Vascular dementia (mixed)	family Family XI	rs697771	A	G	0.002	54081288	0.986	0.085	360421	A	G	-0.118	16	54115200	3.19E-06	0.025	14306	21.895
Vascular dementia (mixed)	family Family XIII	rs10404377	A	C	-0.102	16063847	0.241	0.087	360421	A	C	0.050	19	16174657	6.99E-06	0.011	14306	20.311
Vascular dementia (mixed)	family Family XIII	rs118170811	A	G	0.154	100607139	0.529	0.244	360421	A	G	0.152	10	102366896	1.80E-06	0.032	14306	22.860
Vascular dementia (mixed)	family Family XIII	rs482905	G	T	0.051	166587965	0.603	0.098	360421	G	T	0.060	1	166557202	3.72E-06	0.013	14306	22.004
Vascular dementia (mixed)	family Family XIII	rs6501525	A	G	0.007	72222486	0.939	0.090	360421	A	G	0.056	17	70218627	1.24E-06	0.012	14306	23.603
Vascular dementia (mixed)	family Family XIII	rs66753613	G	A	-0.024	38746349	0.827	0.110	360421	G	A	0.065	1	39212021	8.08E-06	0.014	14306	20.375
Vascular dementia (mixed)	family Family XIII	rs6797051	C	T	0.083	88946068	0.559	0.143	360421	C	T	-0.081	3	88995218	4.89E-06	0.017	14306	22.122
Vascular dementia (mixed)	family Family XIII	rs7514702	T	C	-0.066	186944891	0.577	0.119	360421	T	C	-0.066	1	186914023	3.92E-06	0.014	14306	21.931
Vascular dementia (mixed)	family Lachnospiraceae	rs10402491	C	T	0.148	13314985	0.188	0.112	360421	C	T	0.066	19	13425799	7.58E-06	0.015	14306	19.852
Vascular dementia (mixed)	family Lachnospiraceae	rs11139361	C	T	-0.072	69564931	0.423	0.090	360421	C	T	-0.049	9	72179847	4.26E-06	0.011	14306	20.023
Vascular dementia (mixed)	family Lachnospiraceae	rs112040820	A	G	0.028	82511188	0.774	0.097	360421	A	G	0.055	17	80469064	2.42E-06	0.012	14306	22.032
Vascular dementia (mixed)	family Lachnospiraceae	rs11841382	G	T	0.199	37430535	0.193	0.153	360421	G	T	-0.072	13	38004672	9.58E-06	0.017	14306	16.913
Vascular dementia (mixed)	family Lachnospiraceae	rs11979110	T	C	0.018	130751700	0.827	0.084	360421	T	C	-0.050	7	130436459	1.82E-06	0.011	14306	22.730
Vascular dementia (mixed)	family Lachnospiraceae	rs1205443	A	G	-0.199	38248466	0.025	0.088	360421	A	G	0.050	20	36876868	7.29E-06	0.011	14306	19.987
Vascular dementia (mixed)	family Lachnospiraceae	rs12760724	A	C	-0.016	213858626	0.855	0.089	360421	A	C	-0.048	1	214031969	7.27E-06	0.011	14306	20.155
Vascular dementia (mixed)	family Lachnospiraceae	rs13005175	G	A	-0.086	231605011	0.676	0.207	360421	G	A	-0.099	2	232469722	8.37E-06	0.022	14306	20.694
Vascular dementia (mixed)	family Lachnospiraceae	rs2910921	C	T	-0.547	32348487	0.030	0.252	360421	C	T	-0.160	5	32348593	8.42E-06	0.036	14306	20.014
Vascular dementia (mixed)	family Lachnospiraceae	rs3127230	C	T	-0.116	101632746	0.206	0.091	360421	C	T	-0.050	10	103392503	6.20E-06	0.011	14306	20.140

Vascular dementia (mixed)	family Lachnospiraceae	rs35524804	T	C	0.077	97351401	0.452	0.102	360421	T	C	-0.061	9	100113683	2.45E-06	0.013	14306	23.537
Vascular dementia (mixed)	family Lachnospiraceae	rs7359994	C	T	0.166	28917951	0.052	0.086	360421	C	T	0.050	19	29408858	5.36E-06	0.011	14306	20.009
Vascular dementia (mixed)	family Lachnospiraceae	rs79086868	T	C	-0.001	130916218	0.995	0.139	360421	T	C	0.078	9	133791605	3.01E-06	0.016	14306	22.302
Vascular dementia (mixed)	family Lachnospiraceae	rs959845	T	C	-0.007	185985647	0.939	0.087	360421	T	C	0.049	4	186906801	5.17E-06	0.011	14306	20.971
Vascular dementia (mixed)	family Lachnospiraceae	rs9929145	G	A	0.112	76525287	0.563	0.194	360421	G	A	-0.126	16	76559184	2.84E-07	0.025	14306	26.282
Vascular dementia (mixed)	family Lactobacillaceae	rs1530559	G	A	-0.076	134998059	0.371	0.085	360421	G	A	0.077	2	135755629	9.65E-06	0.018	14306	19.063
Vascular dementia (mixed)	family Lactobacillaceae	rs16861661	G	A	-0.110	18174965	0.517	0.169	360421	G	A	-0.193	1	18501459	2.70E-07	0.038	14306	25.969
Vascular dementia (mixed)	family Lactobacillaceae	rs62314653	C	A	0.158	108975306	0.375	0.178	360421	C	A	0.177	4	109896462	6.59E-06	0.039	14306	20.379
Vascular dementia (mixed)	family Lactobacillaceae	rs74599091	A	G	0.365	179751534	0.248	0.316	360421	A	G	0.192	1	179720669	7.70E-06	0.043	14306	20.323
Vascular dementia (mixed)	family Lactobacillaceae	rs768253	T	G	0.013	68097868	0.878	0.085	360421	T	G	-0.079	8	69010103	3.61E-06	0.017	14306	21.570
Vascular dementia (mixed)	family Lactobacillaceae	rs77478751	A	G	-0.170	173433875	0.197	0.132	360421	A	G	-0.219	3	173151665	5.96E-06	0.047	14306	21.421
Vascular dementia (mixed)	family Lactobacillaceae	rs921925	A	C	-0.051	6928006	0.616	0.103	360421	A	C	0.100	19	6928017	5.77E-07	0.020	14306	24.555
Vascular dementia (mixed)	family Lactobacillaceae	rs9345899	A	G	-0.136	66849430	0.317	0.136	360421	A	G	-0.124	6	67559323	9.45E-06	0.028	14306	19.774
Vascular dementia (mixed)	family Methanobacteriaceae	rs10202904	G	T	0.028	124682691	0.745	0.086	360421	G	T	0.122	2	125440268	3.01E-07	0.024	14306	26.762
Vascular dementia (mixed)	family Methanobacteriaceae	rs10424197	A	G	-0.140	45936063	0.149	0.097	360421	A	G	0.111	19	46439321	9.28E-06	0.025	14306	20.211
Vascular dementia (mixed)	family Methanobacteriaceae	rs4257531	G	A	0.032	2044483	0.818	0.140	360421	G	A	0.164	3	2086167	7.44E-06	0.036	14306	20.316
Vascular dementia (mixed)	family Methanobacteriaceae	rs6508769	C	T	0.065	28336853	0.583	0.118	360421	C	T	-0.154	19	28827760	8.23E-06	0.034	14306	19.856
Vascular dementia (mixed)	family Methanobacteriaceae	rs6776814	T	C	0.412	15011576	0.158	0.292	360421	T	C	-0.200	3	15053083	1.63E-06	0.041	14306	23.483
Vascular dementia (mixed)	family Methanobacteriaceae	rs73068003	G	T	0.007	10734305	0.961	0.143	360421	G	T	-0.158	7	10773932	8.45E-06	0.035	14306	20.206
Vascular dementia (mixed)	family Methanobacteriaceae	rs73457410	A	G	0.109	41382045	0.524	0.170	360421	A	G	0.215	13	41956181	1.41E-06	0.044	14306	24.316
Vascular dementia (mixed)	family Methanobacteriaceae	rs75208022	C	T	-0.016	21185927	0.909	0.144	360421	C	T	-0.227	12	21338861	5.92E-06	0.049	14306	21.717
Vascular dementia (mixed)	family Methanobacteriaceae	rs894996	C	A	-0.011	103497150	0.945	0.163	360421	C	A	0.217	4	104418307	1.88E-06	0.045	14306	23.349
Vascular dementia (mixed)	family Oxalobacteraceae	rs111966731	T	C	-0.139	93398708	0.356	0.151	360421	T	C	0.204	15	93941937	4.56E-06	0.045	14306	20.952
Vascular dementia (mixed)	family Oxalobacteraceae	rs11246212	C	T	0.105	610277	0.386	0.121	360421	C	T	-0.136	11	610277	4.51E-06	0.029	14306	21.745
Vascular dementia (mixed)	family Oxalobacteraceae	rs12002250	A	C	-0.218	19682560	0.289	0.205	360421	A	C	0.196	9	19682558	5.53E-06	0.045	14306	19.408
Vascular dementia (mixed)	family Oxalobacteraceae	rs1569853	T	C	0.082	38582525	0.525	0.130	360421	T	C	-0.140	6	38550301	7.45E-07	0.028	14306	24.714
Vascular dementia (mixed)	family Oxalobacteraceae	rs17138946	G	T	0.177	5934557	0.294	0.169	360421	G	T	-0.189	16	5984558	8.09E-06	0.043	14306	19.388
Vascular dementia (mixed)	family Oxalobacteraceae	rs36057338	G	T	-0.334	189014160	0.154	0.234	360421	G	T	0.182	4	189935314	6.26E-06	0.040	14306	20.743
Vascular dementia (mixed)	family Oxalobacteraceae	rs4428215	G	A	-0.051	172229645	0.601	0.097	360421	G	A	0.126	3	171947435	4.88E-08	0.023	14306	29.812
Vascular dementia (mixed)	family Oxalobacteraceae	rs561239	A	G	0.083	18640649	0.424	0.104	360421	A	G	0.106	12	18793583	7.19E-06	0.024	14306	19.864
Vascular dementia (mixed)	family Oxalobacteraceae	rs6000536	C	T	0.218	37025428	0.050	0.111	360421	C	T	-0.118	22	37421469	7.39E-07	0.024	14306	24.058
Vascular dementia (mixed)	family Oxalobacteraceae	rs62435498	C	A	0.085	1754627	0.540	0.139	360421	C	A	0.182	7	1794263	7.46E-06	0.040	14306	20.531
Vascular dementia (mixed)	family Oxalobacteraceae	rs736744	T	C	0.026	84899492	0.756	0.085	360421	T	C	-0.106	9	87514407	1.49E-07	0.020	14306	27.721
Vascular dementia (mixed)	family Oxalobacteraceae	rs7993559	A	C	0.067	22869488	0.435	0.085	360421	A	C	-0.092	13	23443627	5.04E-06	0.020	14306	20.974
Vascular dementia (mixed)	family Oxalobacteraceae	rs80330081	A	C	-0.105	66561674	0.446	0.138	360421	A	C	-0.188	4	67427392	6.64E-06	0.042	14306	19.597
Vascular dementia (mixed)	family Oxalobacteraceae	rs934049	G	A	-0.045	15916656	0.661	0.103	360421	G	A	0.110	2	16056779	4.21E-06	0.024	14306	21.152
Vascular dementia (mixed)	family Pasteurellaceae	rs10965428	C	A	0.391	22718482	0.032	0.182	360421	C	A	-0.120	9	22718481	4.29E-06	0.026	14306	21.561
Vascular dementia (mixed)	family Pasteurellaceae	rs111582866	G	A	-0.120	48708578	0.418	0.148	360421	G	A	-0.114	16	48742489	7.07E-06	0.026	14306	19.753
Vascular dementia (mixed)	family Pasteurellaceae	rs12050685	A	G	-0.116	73185141	0.211	0.093	360421	A	G	-0.067	15	73477482	9.19E-06	0.015	14306	19.385
Vascular dementia (mixed)	family Pasteurellaceae	rs16970009	A	G	-0.013	34535582	0.967	0.306	360421	A	G	0.187	17	32862601	7.32E-06	0.043	14306	19.027
Vascular dementia (mixed)	family Pasteurellaceae	rs4822728	T	C	0.072	26495842	0.389	0.084	360421	T	C	0.069	22	26891808	4.72E-06	0.015	14306	21.156
Vascular dementia (mixed)	family Pasteurellaceae	rs6972479	A	G	0.010	117278006	0.925	0.105	360421	A	G	-0.078	7	116918060	7.75E-06	0.018	14306	19.878
Vascular dementia (mixed)	family Pasteurellaceae	rs72756943	G	A	0.147	26531799	0.387	0.169	360421	G	A	0.140	5	26531908	3.35E-06	0.030	14306	21.308
Vascular dementia (mixed)	family Pasteurellaceae	rs73139353	A	C	-0.100	98253370	0.505	0.150	360421	A	C	-0.223	3	97972214	8.71E-06	0.048	14306	21.092
Vascular dementia (mixed)	family Pasteurellaceae	rs76022354	C	T	0.485	92546628	0.014	0.197	360421	C	T	0.243	10	94306385	1.83E-06	0.050	14306	23.560
Vascular dementia (mixed)	family Pasteurellaceae	rs78909003	T	C	-0.199	102887960	0.274	0.182	360421	T	C	-0.241	9	105650242	2.05E-06	0.050	14306	23.415
Vascular dementia (mixed)	family Pasteurellaceae	rs9382510	C	T	-0.055	55583693	0.567	0.096	360421	C	T	-0.088	6	55448491	2.48E-07	0.017	14306	26.921
Vascular dementia (mixed)	family Pasteurellaceae	rs9895850	T	C	-0.004	66538895	0.985	0.207	360421	T	C	-0.176	17	64535013	9.08E-06	0.041	14306	18.497
Vascular dementia (mixed)	family Pasteurellaceae	rs9938097	C	T	-0.161	84943783	0.063	0.087	360421	C	T	0.071	16	84977389	8.23E-06	0.016	14306	20.209
Vascular dementia (mixed)	family Peptococcaceae	rs117452796	A	G	-0.556	9423378	0.021	0.240	360421	A	G	-0.258	9	9423378	3.15E-06	0.055	14306	22.036
Vascular dementia (mixed)	family Peptococcaceae	rs12144792	C	T	-0.040	27091734	0.650	0.087	360421	C	T	0.064	1	27418225	5.82E-06	0.014	14306	20.788
Vascular dementia (mixed)	family Peptococcaceae	rs12634826	T	G	0.036	183266260	0.684	0.088	360421	T	G	-0.074	3	182984048	1.01E-06	0.015	14306	24.031
Vascular dementia (mixed)	family Peptococcaceae	rs12992764	T	G	-0.110	188031016	0.196	0.085	360421	T	G	0.068	2	188895743	1.46E-06	0.014	14306	23.502
Vascular dementia (mixed)	family Peptococcaceae	rs150600492	A	C	-0.322	127377698	0.074	0.180	360421	A	C	0.136	10	129175962	2.31E-06	0.029	14306	21.957
Vascular dementia (mixed)	family Peptococcaceae	rs35703006	G	T	-0.069	28756700	0.481	0.098	360421	G	T	0.081	8	28614217	4.95E-07	0.016	14306	24.448
Vascular dementia (mixed)	family Peptococcaceae	rs4990837	G	A	0.039	3723235	0.714	0.107	360421	G	A	-0.091	8	3580757	1.74E-06	0.019	14306	23.990
Vascular dementia (mixed)	family Peptococcaceae	rs75430375	C	T	-0.087	89957067	0.668	0.203	360421	C	T	-0.148	5	89252884	3.41E-06	0.032	14306	21.712
Vascular dementia (mixed)	family Peptococcaceae	rs75898026	A	G	0.057	112906768	0.581	0.104	360421	A	G	-0.082	13	113561082	2.02E-06	0.017	14306	22.424
Vascular dementia (mixed)	family Peptostreptococcaceae	rs10805326	A	G	-0.059	14322999	0.520	0.092	360421	A	G	-0.057	4	14324623	4.03E-06	0.012	14306	21.306
Vascular dementia (mixed)	family Peptostreptococcaceae	rs117020988	C	T	0.146	46671379	0.342	0.153	360421	C	T	0.182	7	46710977	1.03E-06	0.037	14306	24.028
Vascular dementia (mixed)	family Peptostreptococcaceae	rs12377846	C	A	-0.141	16786786	0.584	0.257	360421	C	A	-0.252	9	16786784	7.26E-07	0.051	14306	24.260
Vascular dementia (mixed)	family Peptostreptococcaceae	rs12986312	T	G	-0.031	17303329	0.739	0.092	360421	T	G	0.057	19	17414138	5.77E-06	0.013	14306	20.613
Vascular dementia (mixed)	family Peptostreptococcaceae	rs1467258	G	A	-0.141	40060234	0.198	0.109	360421	G	A	0.073	17	38216487	7.90E-06	0.016	14306	19.996
Vascular dementia (mixed)	family Peptostreptococcaceae	rs1520207	T	C	-0.106	152063386	0.210	0.085	360421	T	C	-0.053	3	151781175	3.17E-06	0.011	14306	21.842
Vascular dementia (mixed)	family Peptostreptococcaceae	rs4692811	C	T	0.088	170259039	0.320	0.089	360421	C	T	0.064	4	171180190	4.21E-07	0.013	14306	25.595
Vascular dementia (mixed)	family Peptostreptococcaceae	rs59865771	C	T	-0.019	89180262	0.830	0.088	360421	C	T	-0.057	16	89246670	7.69E-06	0.013	14306	20.349
Vascular dementia (mixed)	family Peptostreptococcaceae	rs61841503	G	A	0.048	16977560	0.704	0.127	360421	G	A	0.092	10	17019559	9.80E-09	0.016	14306	32.495

Vascular dementia (mixed)	family Peptostreptococcaceae	rs6721459	G	A	0.050	67625704	0.560	0.085	360421	G	A	-0.051	2	67852836	5.08E-06	0.011	14306	20.730
Vascular dementia (mixed)	family Peptostreptococcaceae	rs76982728	T	C	0.020	44273225	0.944	0.287	360421	T	C	0.124	7	44312824	3.24E-06	0.027	14306	21.652
Vascular dementia (mixed)	family Peptostreptococcaceae	rs77540684	T	G	-0.021	14608536	0.881	0.143	360421	T	G	0.107	10	14650535	8.14E-06	0.025	14306	18.877
Vascular dementia (mixed)	family Peptostreptococcaceae	rs9573937	A	G	0.075	76777061	0.511	0.114	360421	A	G	-0.069	13	77351196	1.71E-06	0.014	14306	23.559
Vascular dementia (mixed)	family Porphyromonadaceae	rs10762312	A	G	-0.076	69812107	0.406	0.091	360421	A	G	0.052	10	71571863	8.70E-06	0.012	14306	19.427
Vascular dementia (mixed)	family Porphyromonadaceae	rs10858364	G	T	0.018	135184235	0.853	0.100	360421	G	T	0.055	9	138076081	4.31E-06	0.012	14306	20.951
Vascular dementia (mixed)	family Porphyromonadaceae	rs17065783	A	G	0.057	62049912	0.621	0.115	360421	A	G	-0.059	3	62035586	1.79E-06	0.012	14306	23.403
Vascular dementia (mixed)	family Porphyromonadaceae	rs1980561	A	G	0.003	62919798	0.976	0.084	360421	A	G	-0.049	14	63386516	8.95E-06	0.011	14306	19.719
Vascular dementia (mixed)	family Porphyromonadaceae	rs35233670	T	C	-0.002	65754785	0.981	0.084	360421	T	C	-0.047	17	63750903	7.91E-06	0.011	14306	19.953
Vascular dementia (mixed)	family Porphyromonadaceae	rs35961441	A	C	0.086	240766474	0.693	0.217	360421	A	C	0.092	1	240929774	8.37E-06	0.021	14306	19.492
Vascular dementia (mixed)	family Porphyromonadaceae	rs6953849	A	G	0.101	69786706	0.356	0.110	360421	A	G	0.072	7	69251692	2.44E-06	0.015	14306	22.702
Vascular dementia (mixed)	family Porphyromonadaceae	rs7330827	T	C	-0.124	22957663	0.485	0.177	360421	T	C	-0.104	13	23531802	8.05E-06	0.024	14306	19.140
Vascular dementia (mixed)	family Porphyromonadaceae	rs864093	A	C	-0.137	148904825	0.187	0.104	360421	A	C	-0.053	4	149825977	9.60E-06	0.012	14306	20.188
Vascular dementia (mixed)	family Prevotellaceae	rs12057990	C	T	-0.056	99004713	0.549	0.094	360421	C	T	0.059	1	99470269	8.97E-06	0.013	14306	19.889
Vascular dementia (mixed)	family Prevotellaceae	rs12118202	T	C	0.133	210508026	0.223	0.109	360421	T	C	-0.075	1	210681370	5.54E-07	0.015	14306	26.075
Vascular dementia (mixed)	family Prevotellaceae	rs13069367	A	C	-0.015	71752679	0.860	0.086	360421	A	C	-0.054	3	71801830	7.39E-06	0.012	14306	20.339
Vascular dementia (mixed)	family Prevotellaceae	rs148376875	T	G	-0.088	170408919	0.458	0.119	360421	T	G	0.085	3	170126707	2.08E-06	0.018	14306	22.252
Vascular dementia (mixed)	family Prevotellaceae	rs2206482	T	G	0.084	9790461	0.330	0.086	360421	T	G	-0.057	20	9771109	1.30E-06	0.012	14306	23.465
Vascular dementia (mixed)	family Prevotellaceae	rs2278540	G	A	-0.025	32367424	0.773	0.088	360421	G	A	0.055	3	32408916	8.44E-06	0.012	14306	20.218
Vascular dementia (mixed)	family Prevotellaceae	rs34660375	A	G	-0.191	180856881	0.109	0.119	360421	A	G	-0.081	5	180283881	7.40E-06	0.018	14306	20.431
Vascular dementia (mixed)	family Prevotellaceae	rs3758087	C	T	-0.179	23857096	0.057	0.094	360421	C	T	-0.056	8	23714609	8.61E-06	0.012	14306	20.485
Vascular dementia (mixed)	family Prevotellaceae	rs3860225	A	G	-0.151	110619055	0.296	0.145	360421	A	G	0.084	1	111161677	5.50E-07	0.017	14306	24.981
Vascular dementia (mixed)	family Prevotellaceae	rs4493272	T	C	-0.007	118153336	0.933	0.084	360421	T	C	-0.060	2	118910912	3.02E-07	0.012	14306	26.275
Vascular dementia (mixed)	family Prevotellaceae	rs4685827	T	C	-0.057	4823193	0.564	0.098	360421	T	C	-0.068	3	4864877	2.77E-06	0.015	14306	21.840
Vascular dementia (mixed)	family Prevotellaceae	rs7252711	G	A	0.094	17350190	0.480	0.134	360421	G	A	0.074	19	17460999	5.57E-06	0.016	14306	20.934
Vascular dementia (mixed)	family Prevotellaceae	rs7975087	C	A	-0.004	21251898	0.969	0.111	360421	C	A	-0.060	12	21404832	7.59E-06	0.014	14306	19.716
Vascular dementia (mixed)	family Prevotellaceae	rs912860	A	G	0.060	33237702	0.834	0.286	360421	A	G	0.229	14	33706908	9.30E-07	0.048	14306	22.476
Vascular dementia (mixed)	family Prevotellaceae	rs9586501	G	A	-0.007	104423473	0.940	0.095	360421	G	A	0.059	13	105075823	2.59E-06	0.013	14306	21.666
Vascular dementia (mixed)	family Prevotellaceae	rs9958960	G	A	0.070	65351747	0.546	0.116	360421	G	A	-0.091	18	63018983	1.06E-07	0.017	14306	27.765
Vascular dementia (mixed)	family Rhodospirillaceae	rs1035406	G	A	-0.048	120037042	0.714	0.132	360421	G	A	-0.114	5	119372737	5.84E-06	0.025	14306	20.484
Vascular dementia (mixed)	family Rhodospirillaceae	rs11591293	G	T	0.027	111660039	0.754	0.085	360421	G	T	0.074	10	113419797	2.67E-06	0.016	14306	21.923
Vascular dementia (mixed)	family Rhodospirillaceae	rs13336560	C	T	0.055	88487835	0.522	0.086	360421	C	T	-0.070	16	88554243	9.17E-06	0.016	14306	19.710
Vascular dementia (mixed)	family Rhodospirillaceae	rs1549633	A	C	0.138	27945538	0.299	0.132	360421	A	C	0.100	5	27945645	4.70E-06	0.022	14306	20.891
Vascular dementia (mixed)	family Rhodospirillaceae	rs1923415	A	G	-0.087	88558996	0.558	0.148	360421	A	G	-0.100	6	89268715	9.64E-06	0.023	14306	19.321
Vascular dementia (mixed)	family Rhodospirillaceae	rs3754624	C	T	0.111	224769095	0.309	0.109	360421	C	T	0.097	2	225633812	1.71E-06	0.020	14306	23.575
Vascular dementia (mixed)	family Rhodospirillaceae	rs4278423	T	C	0.231	2628361	0.190	0.177	360421	T	C	0.108	10	2670553	3.12E-06	0.024	14306	20.808
Vascular dementia (mixed)	family Rhodospirillaceae	rs61933850	G	A	-0.088	72745618	0.471	0.122	360421	G	A	0.165	12	73139398	7.23E-06	0.036	14306	20.883
Vascular dementia (mixed)	family Rhodospirillaceae	rs6679026	T	C	0.018	78153828	0.900	0.141	360421	T	C	0.112	1	78619512	9.95E-06	0.025	14306	19.891
Vascular dementia (mixed)	family Rhodospirillaceae	rs7001029	C	T	-0.041	130946157	0.780	0.147	360421	C	T	0.117	8	131958403	5.35E-06	0.026	14306	20.171
Vascular dementia (mixed)	family Rhodospirillaceae	rs72714493	A	G	-0.001	91740661	0.993	0.118	360421	A	G	0.082	1	92206218	7.35E-06	0.018	14306	20.415
Vascular dementia (mixed)	family Rhodospirillaceae	rs74354280	C	T	-0.096	133271763	0.304	0.094	360421	C	T	-0.091	4	134192918	6.67E-06	0.020	14306	19.805
Vascular dementia (mixed)	family Rhodospirillaceae	rs76784716	A	G	0.002	168176830	0.986	0.135	360421	A	G	0.136	2	169033340	1.49E-06	0.029	14306	22.666
Vascular dementia (mixed)	family Rhodospirillaceae	rs9813022	A	G	0.020	13685237	0.817	0.087	360421	A	G	-0.084	3	13726736	2.52E-07	0.016	14306	26.522
Vascular dementia (mixed)	family Rikenellaceae	rs10217435	C	T	0.007	83437740	0.956	0.121	360421	C	T	-0.088	9	86052655	6.51E-06	0.020	14306	20.022
Vascular dementia (mixed)	family Rikenellaceae	rs10832801	A	C	-0.004	17567582	0.968	0.094	360421	A	C	-0.053	11	17589129	7.50E-06	0.012	14306	19.005
Vascular dementia (mixed)	family Rikenellaceae	rs1939881	G	A	-0.250	95571670	0.166	0.181	360421	G	A	-0.106	11	95304834	5.64E-07	0.021	14306	26.218
Vascular dementia (mixed)	family Rikenellaceae	rs2447496	A	G	-0.084	98176696	0.377	0.096	360421	A	G	0.055	8	99188924	6.09E-06	0.012	14306	20.297
Vascular dementia (mixed)	family Rikenellaceae	rs2833282	G	A	-0.094	31124392	0.427	0.119	360421	G	A	0.071	21	32496710	4.31E-06	0.016	14306	20.526
Vascular dementia (mixed)	family Rikenellaceae	rs36021379	A	G	0.143	27143593	0.217	0.116	360421	A	G	-0.066	21	28515912	7.20E-06	0.014	14306	20.496
Vascular dementia (mixed)	family Rikenellaceae	rs4264350	T	C	0.115	71366303	0.175	0.084	360421	T	C	-0.053	15	71658642	1.35E-06	0.011	14306	23.514
Vascular dementia (mixed)	family Rikenellaceae	rs59663348	G	A	-0.169	4069710	0.084	0.098	360421	G	A	0.057	18	4069710	6.12E-06	0.013	14306	20.844
Vascular dementia (mixed)	family Rikenellaceae	rs62532512	A	C	-0.025	14158855	0.767	0.085	360421	A	C	0.050	9	14158854	2.76E-06	0.011	14306	22.045
Vascular dementia (mixed)	family Rikenellaceae	rs6744030	C	T	-0.004	173392399	0.972	0.103	360421	C	T	0.070	2	174257127	9.32E-06	0.016	14306	19.669
Vascular dementia (mixed)	family Rikenellaceae	rs6837275	A	G	0.090	186921078	0.329	0.092	360421	A	G	0.057	4	187842232	1.45E-06	0.012	14306	23.022
Vascular dementia (mixed)	family Rikenellaceae	rs74474130	T	G	0.042	89804477	0.856	0.230	360421	T	G	0.138	14	90270821	3.61E-06	0.030	14306	21.702
Vascular dementia (mixed)	family Rikenellaceae	rs77885767	C	T	0.119	21080475	0.557	0.202	360421	C	T	-0.156	14	21548634	2.85E-06	0.034	14306	21.552
Vascular dementia (mixed)	family Rikenellaceae	rs9389714	C	T	-0.022	99796791	0.882	0.146	360421	C	T	-0.064	6	100244667	8.79E-06	0.014	14306	19.718
Vascular dementia (mixed)	family Rikenellaceae	rs9578457	G	A	0.155	22293217	0.432	0.197	360421	G	A	-0.141	13	22867356	3.99E-06	0.032	14306	20.064
Vascular dementia (mixed)	family Rikenellaceae	rs9603208	G	T	0.299	37470552	0.034	0.141	360421	G	T	0.082	13	38044689	1.92E-07	0.016	14306	26.474
Vascular dementia (mixed)	family Ruminococcaceae	rs10093275	T	C	-0.071	68820652	0.423	0.089	360421	T	C	-0.053	8	69732887	5.35E-06	0.012	14306	20.960
Vascular dementia (mixed)	family Ruminococcaceae	rs10166469	C	T	-0.097	29606301	0.315	0.096	360421	C	T	0.053	2	29829167	8.52E-06	0.012	14306	19.686
Vascular dementia (mixed)	family Ruminococcaceae	rs1158100	G	A	-0.096	4698162	0.258	0.085	360421	G	A	0.049	8	4555684	8.61E-06	0.011	14306	19.936
Vascular dementia (mixed)	family Ruminococcaceae	rs1612733	T	C	-0.018	107118984	0.924	0.183	360421	T	C	0.109	1	107661606	4.22E-06	0.024	14306	20.862
Vascular dementia (mixed)	family Ruminococcaceae	rs17376049	T	C	-0.064	60959595	0.634	0.135	360421	T	C	0.085	1	61425267	7.30E-07	0.017	14306	24.306
Vascular dementia (mixed)	family Ruminococcaceae	rs2113833	C	T	-0.101	217352078	0.667	0.234	360421	C	T	-0.169	2	218216801	1.14E-06	0.036	14306	22.701
Vascular dementia (mixed)	family Ruminococcaceae	rs3009418	A	C	0.316	148272338	0.112	0.199	360421	A	C	-0.093	1	147744468	8.69E-06	0.021	14306	19.489
Vascular dementia (mixed)	family Ruminococcaceae	rs56199908	T	C	0.391	2801371	0.037	0.187	360421	T	C	-0.199	9	2801371	1.66E-06	0.041	14306	23.584

Vascular dementia (mixed)	family Ruminococcaceae	rs76724913	T	G	-0.027	24147179	0.849	0.143	360421	T	G	0.090	1	24473669	9.60E-06	0.020	14306	19.682
Vascular dementia (mixed)	family Streptococcaceae	rs10028567	C	T	-0.109	52791410	0.399	0.129	360421	C	T	-0.093	4	53657577	3.72E-06	0.019	14306	24.079
Vascular dementia (mixed)	family Streptococcaceae	rs11110281	T	C	0.123	100190236	0.537	0.199	360421	T	C	-0.131	12	100584014	1.40E-08	0.023	14306	33.387
Vascular dementia (mixed)	family Streptococcaceae	rs16950051	A	G	0.291	120291702	0.096	0.175	360421	A	G	0.107	12	120729505	5.34E-06	0.024	14306	20.391
Vascular dementia (mixed)	family Streptococcaceae	rs2370083	G	T	0.039	97060413	0.822	0.172	360421	G	T	-0.084	14	97526750	4.26E-06	0.018	14306	20.862
Vascular dementia (mixed)	family Streptococcaceae	rs2952251	G	A	-0.092	10285654	0.359	0.101	360421	G	A	0.064	8	10143164	3.72E-07	0.013	14306	25.530
Vascular dementia (mixed)	family Streptococcaceae	rs35344081	G	A	0.121	941253	0.203	0.095	360421	G	A	0.061	16	991253	2.64E-06	0.013	14306	22.072
Vascular dementia (mixed)	family Streptococcaceae	rs57646748	G	A	-0.153	37451236	0.481	0.218	360421	G	A	-0.088	4	37452858	7.88E-06	0.020	14306	19.612
Vascular dementia (mixed)	family Streptococcaceae	rs6806351	T	C	-0.199	132339879	0.051	0.102	360421	T	C	-0.062	3	132058723	6.94E-06	0.014	14306	20.808
Vascular dementia (mixed)	family Streptococcaceae	rs77968078	G	A	-0.105	240278466	0.551	0.177	360421	G	A	-0.099	1	240441766	7.93E-06	0.022	14306	19.515
Vascular dementia (mixed)	family Streptococcaceae	rs7916711	A	G	-0.030	28299340	0.807	0.122	360421	A	G	0.096	10	28588269	6.33E-06	0.022	14306	19.839
Vascular dementia (mixed)	family Streptococcaceae	rs957755	T	G	0.191	46739055	0.114	0.121	360421	T	G	-0.064	7	46778653	7.42E-06	0.014	14306	20.268
Vascular dementia (mixed)	family Veillonellaceae	rs111810795	C	T	0.057	102431593	0.684	0.140	360421	C	T	-0.087	14	102897930	1.73E-06	0.018	14306	23.059
Vascular dementia (mixed)	family Veillonellaceae	rs114889439	A	G	-0.233	61023491	0.305	0.227	360421	A	G	-0.254	13	61597625	6.19E-06	0.054	14306	22.160
Vascular dementia (mixed)	family Veillonellaceae	rs12186441	G	A	0.340	133310611	0.097	0.205	360421	G	A	0.208	5	132646303	4.53E-06	0.045	14306	21.010
Vascular dementia (mixed)	family Veillonellaceae	rs12668619	A	G	0.098	21598192	0.275	0.089	360421	A	G	0.055	7	21637810	2.57E-06	0.012	14306	22.093
Vascular dementia (mixed)	family Veillonellaceae	rs12741784	C	T	-0.038	49623147	0.692	0.096	360421	C	T	-0.062	1	50088819	1.28E-07	0.012	14306	27.151
Vascular dementia (mixed)	family Veillonellaceae	rs1442060	A	G	-0.145	46364050	0.088	0.085	360421	A	G	0.051	4	46366067	4.51E-06	0.011	14306	21.017
Vascular dementia (mixed)	family Veillonellaceae	rs1693340	T	C	-0.096	32628728	0.557	0.163	360421	T	C	0.082	18	30208691	9.25E-06	0.018	14306	20.147
Vascular dementia (mixed)	family Veillonellaceae	rs2175069	G	A	0.015	23315501	0.859	0.087	360421	G	A	0.053	4	23317124	4.64E-06	0.011	14306	21.022
Vascular dementia (mixed)	family Veillonellaceae	rs2561116	T	G	0.389	38348097	0.014	0.158	360421	T	G	-0.084	5	38348199	7.89E-06	0.019	14306	19.960
Vascular dementia (mixed)	family Veillonellaceae	rs2585520	G	T	0.080	78211006	0.713	0.219	360421	G	T	-0.090	13	78785141	5.27E-06	0.020	14306	20.387
Vascular dementia (mixed)	family Veillonellaceae	rs4263802	A	G	-0.042	137307265	0.641	0.089	360421	A	G	-0.051	8	138319508	7.45E-06	0.011	14306	19.650
Vascular dementia (mixed)	family Veillonellaceae	rs4461038	G	A	-0.033	78254654	0.723	0.093	360421	G	A	0.055	15	78546996	3.73E-06	0.012	14306	21.579
Vascular dementia (mixed)	family Veillonellaceae	rs4797169	T	C	-0.008	462180	0.935	0.099	360421	T	C	0.059	18	462180	4.49E-06	0.013	14306	20.991
Vascular dementia (mixed)	family Veillonellaceae	rs61264131	A	C	0.209	138867397	0.144	0.143	360421	A	C	0.202	6	139188534	6.75E-06	0.046	14306	18.990
Vascular dementia (mixed)	family Veillonellaceae	rs6692542	G	A	0.190	244228316	0.032	0.089	360421	G	A	-0.053	1	244391618	8.68E-06	0.012	14306	20.422
Vascular dementia (mixed)	family Veillonellaceae	rs6909981	C	T	-0.124	74824436	0.332	0.128	360421	C	T	-0.064	6	75534152	5.48E-06	0.014	14306	20.298
Vascular dementia (mixed)	family Veillonellaceae	rs79535861	A	C	-0.004	37752555	0.979	0.146	360421	A	C	0.101	13	38326692	1.58E-06	0.021	14306	23.646
Vascular dementia (mixed)	family Veillonellaceae	rs9345168	A	C	-0.121	91671204	0.153	0.085	360421	A	C	-0.051	6	92380922	8.49E-06	0.011	14306	20.207
Vascular dementia (mixed)	family Verrucomicrobiaceae	rs111862613	T	C	0.068	129825125	0.548	0.113	360421	T	C	0.091	12	130309670	3.73E-06	0.020	14306	21.255
Vascular dementia (mixed)	family Verrucomicrobiaceae	rs117107102	A	G	-0.188	51947265	0.344	0.198	360421	A	G	0.205	18	49473635	2.92E-06	0.043	14306	22.493
Vascular dementia (mixed)	family Verrucomicrobiaceae	rs11729256	T	C	0.127	94106121	0.258	0.112	360421	T	C	0.075	4	95027272	6.73E-07	0.015	14306	24.928
Vascular dementia (mixed)	family Verrucomicrobiaceae	rs12908520	G	A	0.144	97027427	0.092	0.085	360421	G	A	0.062	15	97570657	2.15E-06	0.013	14306	22.353
Vascular dementia (mixed)	family Verrucomicrobiaceae	rs2602429	T	C	0.023	81029544	0.810	0.096	360421	T	C	-0.075	16	81063149	2.70E-06	0.016	14306	22.781
Vascular dementia (mixed)	family Verrucomicrobiaceae	rs4242783	A	G	0.105	5022135	0.262	0.094	360421	A	G	-0.069	10	5064327	2.75E-06	0.015	14306	21.699
Vascular dementia (mixed)	family Verrucomicrobiaceae	rs4936098	G	A	0.002	130410772	0.985	0.088	360421	G	A	-0.065	11	130280667	1.13E-06	0.014	14306	22.775
Vascular dementia (mixed)	family Verrucomicrobiaceae	rs61779207	G	A	-0.010	40608800	0.923	0.102	360421	G	A	-0.076	1	41074472	6.63E-06	0.017	14306	20.459
Vascular dementia (mixed)	family Verrucomicrobiaceae	rs74542928	T	C	0.226	99623031	0.244	0.194	360421	T	C	0.112	4	100544188	1.65E-06	0.024	14306	22.492
Vascular dementia (mixed)	family Verrucomicrobiaceae	rs9349825	A	G	-0.041	56476683	0.701	0.107	360421	A	G	-0.070	6	56341481	2.51E-06	0.015	14306	22.919
Vascular dementia (mixed)	family Verrucomicrobiaceae	rs941682	G	A	-0.058	33280034	0.538	0.095	360421	G	A	-0.063	20	31867840	9.58E-06	0.014	14306	19.296
Vascular dementia (mixed)	family Victivallaceae	rs11671100	A	C	-0.145	711637	0.164	0.104	360421	A	C	-0.160	19	711637	4.08E-06	0.035	14306	20.970
Vascular dementia (mixed)	family Victivallaceae	rs11764871	G	T	-0.146	147111885	0.111	0.092	360421	G	T	0.127	7	146808977	7.49E-07	0.026	14306	24.542
Vascular dementia (mixed)	family Victivallaceae	rs2944282	T	C	-0.094	57530097	0.314	0.094	360421	T	C	-0.124	7	57589803	1.57E-06	0.026	14306	23.349
Vascular dementia (mixed)	family Victivallaceae	rs34962571	A	C	0.249	130618711	0.079	0.142	360421	A	C	-0.187	12	131103256	6.25E-06	0.042	14306	19.963
Vascular dementia (mixed)	family Victivallaceae	rs4396289	C	T	-0.316	9295183	0.011	0.125	360421	C	T	-0.153	11	9316730	1.54E-07	0.029	14306	28.090
Vascular dementia (mixed)	family Victivallaceae	rs61702987	T	C	-0.187	30988791	0.162	0.134	360421	T	C	0.146	2	31211657	3.08E-06	0.030	14306	23.522
Vascular dementia (mixed)	family Victivallaceae	rs62570196	C	T	-0.267	108323890	0.207	0.212	360421	C	T	-0.246	9	111086170	2.70E-07	0.048	14306	25.953
Vascular dementia (mixed)	family Victivallaceae	rs6545794	A	G	-0.185	60257774	0.152	0.129	360421	A	G	-0.198	2	60484909	5.97E-07	0.041	14306	23.215
Vascular dementia (mixed)	family Victivallaceae	rs7077363	G	A	-0.114	93527197	0.317	0.113	360421	G	A	0.149	10	95286954	2.83E-06	0.032	14306	21.887
Vascular dementia (mixed)	family Victivallaceae	rs7314815	G	A	-0.112	102131269	0.184	0.085	360421	G	A	0.101	12	102525047	6.40E-06	0.023	14306	20.079
Vascular dementia (mixed)	family Victivallaceae	rs7627405	C	T	-0.094	9926871	0.377	0.106	360421	C	T	-0.134	3	9968555	8.19E-06	0.030	14306	19.809
Vascular dementia (mixed)	genus Actinomyces	rs2715439	T	C	-0.063	98949084	0.458	0.084	360421	T	C	-0.075	15	99492313	6.27E-06	0.016	14306	20.523
Vascular dementia (mixed)	genus Actinomyces	rs34583783	G	T	0.035	66497478	0.842	0.176	360421	G	T	0.127	6	67207371	4.49E-06	0.027	14306	22.237
Vascular dementia (mixed)	genus Actinomyces	rs35011108	A	G	-0.135	132686341	0.414	0.166	360421	A	G	0.233	6	133007480	6.34E-06	0.051	14306	20.641
Vascular dementia (mixed)	genus Actinomyces	rs4073240	G	A	-0.114	168824686	0.187	0.087	360421	G	A	0.075	6	169224781	7.94E-06	0.017	14306	20.064
Vascular dementia (mixed)	genus Actinomyces	rs4146653	G	A	0.023	4740649	0.849	0.120	360421	G	A	0.099	10	4782841	4.50E-06	0.021	14306	21.159
Vascular dementia (mixed)	genus Actinomyces	rs71315246	A	G	0.061	101633595	0.618	0.123	360421	A	G	-0.097	3	101352439	9.83E-06	0.022	14306	19.566
Vascular dementia (mixed)	genus Actinomyces	rs7915461	C	T	0.082	125843552	0.629	0.170	360421	C	T	-0.188	10	127532121	5.92E-06	0.040	14306	21.855
Vascular dementia (mixed)	genus Adlercreutzia	rs11604400	C	T	0.184	98515355	0.178	0.137	360421	C	T	-0.103	11	98386085	9.74E-06	0.023	14306	19.060
Vascular dementia (mixed)	genus Adlercreutzia	rs13231526	C	A	-0.024	48804555	0.878	0.156	360421	C	A	0.143	7	48844151	4.81E-06	0.031	14306	21.123
Vascular dementia (mixed)	genus Adlercreutzia	rs2717140	C	T	0.052	77297814	0.713	0.141	360421	C	T	-0.119	18	75009770	2.05E-06	0.025	14306	22.548
Vascular dementia (mixed)	genus Adlercreutzia	rs55719207	G	A	0.060	105637127	0.490	0.087	360421	G	A	-0.070	3	105355971	9.61E-06	0.016	14306	19.577
Vascular dementia (mixed)	genus Adlercreutzia	rs6664405	T	C	0.298	68978480	0.006	0.109	360421	T	C	-0.095	1	69444163	5.23E-06	0.021	14306	20.451
Vascular dementia (mixed)	genus Adlercreutzia	rs7680684	T	C	0.050	170360208	0.575	0.089	360421	T	C	0.083	4	171281359	9.77E-07	0.017	14306	24.371
Vascular dementia (mixed)	genus Adlercreutzia	rs9490822	C	T	-0.098	123587938	0.245	0.084	360421	C	T	-0.073	6	123909083	2.54E-06	0.016	14306	22.229
Vascular dementia (mixed)	genus Adlercreutzia	rs9915817	C	T	-0.099	50786818	0.290	0.093	360421	C	T	-0.075	17	48864179	8.22E-06	0.017	14306	19.810

Vascular dementia (mixed)	genus Akkermansia	rs111862613	T	C	0.068	129825125	0.548	0.113	360421	T	C	0.091	12	130309670	3.39E-06	0.020	14306	21.449
Vascular dementia (mixed)	genus Akkermansia	rs117107102	A	G	-0.188	51947265	0.344	0.198	360421	A	G	0.204	18	49473635	3.01E-06	0.043	14306	22.427
Vascular dementia (mixed)	genus Akkermansia	rs11729256	T	C	0.127	94106121	0.258	0.112	360421	T	C	0.075	4	95027272	6.58E-07	0.015	14306	24.970
Vascular dementia (mixed)	genus Akkermansia	rs12908520	G	A	0.144	97027427	0.092	0.085	360421	G	A	0.062	15	97570657	2.26E-06	0.013	14306	22.251
Vascular dementia (mixed)	genus Akkermansia	rs2602429	T	C	0.023	81029544	0.810	0.096	360421	T	C	-0.075	16	81063149	2.72E-06	0.016	14306	22.770
Vascular dementia (mixed)	genus Akkermansia	rs4242783	A	G	0.105	5022135	0.262	0.094	360421	A	G	-0.069	10	5064327	3.00E-06	0.015	14306	21.537
Vascular dementia (mixed)	genus Akkermansia	rs4936098	G	A	0.002	130410772	0.985	0.088	360421	G	A	-0.065	11	130280667	1.10E-06	0.014	14306	22.810
Vascular dementia (mixed)	genus Akkermansia	rs61779207	G	A	-0.010	40608800	0.923	0.102	360421	G	A	-0.076	1	41074472	6.32E-06	0.017	14306	20.550
Vascular dementia (mixed)	genus Akkermansia	rs74542928	T	C	0.226	99623031	0.244	0.194	360421	T	C	0.113	4	100544188	1.48E-06	0.024	14306	22.690
Vascular dementia (mixed)	genus Akkermansia	rs9349825	A	G	-0.041	56476683	0.701	0.107	360421	A	G	-0.070	6	56341481	2.60E-06	0.015	14306	22.856
Vascular dementia (mixed)	genus Akkermansia	rs941682	G	A	-0.058	33280034	0.538	0.095	360421	G	A	-0.063	20	31867840	9.17E-06	0.014	14306	19.381
Vascular dementia (mixed)	genus Alistipes	rs1107244	G	A	0.351	37483220	0.025	0.157	360421	G	A	0.076	13	38057357	3.59E-06	0.017	14306	19.636
Vascular dementia (mixed)	genus Alistipes	rs11769002	G	A	0.001	62983120	0.993	0.086	360421	G	A	-0.053	7	62443498	1.45E-06	0.011	14306	23.368
Vascular dementia (mixed)	genus Alistipes	rs11958296	A	G	-0.012	178328178	0.952	0.206	360421	A	G	-0.098	5	177755179	9.30E-06	0.022	14306	20.209
Vascular dementia (mixed)	genus Alistipes	rs12990744	C	T	0.273	177136221	0.047	0.137	360421	C	T	-0.078	2	178000949	8.21E-06	0.017	14306	20.155
Vascular dementia (mixed)	genus Alistipes	rs1689282	A	C	-0.032	14080130	0.724	0.090	360421	A	C	-0.052	9	14080129	5.28E-06	0.011	14306	20.832
Vascular dementia (mixed)	genus Alistipes	rs2290844	C	T	-0.064	126098573	0.635	0.135	360421	C	T	0.081	10	127787142	9.10E-06	0.019	14306	18.042
Vascular dementia (mixed)	genus Alistipes	rs2450745	A	C	0.008	143346550	0.961	0.166	360421	A	C	-0.081	8	144428720	7.12E-06	0.018	14306	19.007
Vascular dementia (mixed)	genus Alistipes	rs2875322	C	T	-0.055	131017657	0.623	0.113	360421	C	T	0.058	11	130887552	8.78E-06	0.013	14306	19.537
Vascular dementia (mixed)	genus Alistipes	rs34417064	A	G	-0.022	54699118	0.790	0.084	360421	A	G	-0.048	17	52776479	7.01E-06	0.011	14306	20.344
Vascular dementia (mixed)	genus Alistipes	rs4810359	A	G	-0.014	42562695	0.912	0.127	360421	A	G	-0.065	20	41191335	7.50E-06	0.015	14306	19.927
Vascular dementia (mixed)	genus Alistipes	rs7129639	A	C	-0.060	17489880	0.505	0.090	360421	A	C	0.052	11	17511427	1.78E-06	0.011	14306	22.948
Vascular dementia (mixed)	genus Alistipes	rs8130320	A	G	-0.102	39208332	0.230	0.085	360421	A	G	-0.049	21	40580258	4.84E-06	0.011	14306	20.906
Vascular dementia (mixed)	genus Allisonella	rs1901739	G	T	-0.014	114951010	0.870	0.084	360421	G	T	-0.116	5	114286707	3.59E-06	0.025	14306	21.682
Vascular dementia (mixed)	genus Allisonella	rs35110698	T	C	-0.141	27866065	0.237	0.119	360421	T	C	-0.146	12	28018998	5.72E-06	0.032	14306	20.797
Vascular dementia (mixed)	genus Allisonella	rs35778461	C	T	-0.093	97388274	0.353	0.100	360421	C	T	0.147	9	100150556	1.21E-06	0.030	14306	24.360
Vascular dementia (mixed)	genus Allisonella	rs594561	T	C	-0.088	88899804	0.296	0.084	360421	T	C	-0.112	11	88632972	9.41E-06	0.025	14306	19.885
Vascular dementia (mixed)	genus Allisonella	rs602075	G	A	-0.027	76495244	0.776	0.095	360421	G	A	-0.169	9	79110160	3.57E-08	0.030	14306	32.374
Vascular dementia (mixed)	genus Allisonella	rs6742198	A	G	-0.008	33382004	0.933	0.098	360421	A	G	-0.149	2	33607071	3.35E-06	0.032	14306	22.210
Vascular dementia (mixed)	genus Allisonella	rs76904847	G	A	0.166	138283265	0.139	0.112	360421	G	A	0.149	7	137968010	6.09E-06	0.033	14306	19.673
Vascular dementia (mixed)	genus Allisonella	rs7898615	T	G	-0.091	120653939	0.462	0.124	360421	T	G	0.168	10	122413451	8.87E-06	0.037	14306	20.214
Vascular dementia (mixed)	genus Alloprevotella	rs2154444	G	T	-0.099	34681344	0.298	0.095	360421	G	T	-0.138	21	36053643	8.37E-06	0.031	14306	20.018
Vascular dementia (mixed)	genus Alloprevotella	rs34619204	G	A	0.105	39621081	0.344	0.111	360421	G	A	-0.156	21	40993008	8.84E-06	0.034	14306	20.528
Vascular dementia (mixed)	genus Alloprevotella	rs4364940	A	G	0.014	233990770	0.876	0.091	360421	A	G	0.126	1	234126516	8.58E-06	0.028	14306	20.067
Vascular dementia (mixed)	genus Alloprevotella	rs4680035	G	A	-0.011	153087811	0.899	0.087	360421	G	A	0.120	3	152805600	4.99E-06	0.026	14306	21.250
Vascular dementia (mixed)	genus Alloprevotella	rs58212166	A	G	-0.062	188155551	0.572	0.109	360421	A	G	-0.162	4	189076705	7.94E-06	0.036	14306	20.227
Vascular dementia (mixed)	genus Anaerofilum	rs10794359	C	T	-0.165	1051715	0.054	0.086	360421	C	T	0.095	11	1051715	2.23E-06	0.020	14306	22.594
Vascular dementia (mixed)	genus Anaerofilum	rs1563175	A	C	-0.112	3791350	0.183	0.084	360421	A	C	0.092	2	3838940	5.54E-06	0.020	14306	20.884
Vascular dementia (mixed)	genus Anaerofilum	rs17012738	T	G	0.092	76471016	0.274	0.084	360421	T	G	0.090	2	76698142	7.24E-06	0.020	14306	20.355
Vascular dementia (mixed)	genus Anaerofilum	rs17096874	C	T	0.096	30522152	0.353	0.104	360421	C	T	-0.126	14	30991358	2.86E-06	0.027	14306	22.071
Vascular dementia (mixed)	genus Anaerofilum	rs4244069	A	G	0.057	66773681	0.649	0.126	360421	A	G	0.147	12	67167461	9.81E-06	0.033	14306	20.197
Vascular dementia (mixed)	genus Anaerofilum	rs4506496	A	G	-0.050	246982388	0.586	0.092	360421	A	G	-0.103	1	247145690	1.49E-06	0.021	14306	23.455
Vascular dementia (mixed)	genus Anaerofilum	rs712981	A	C	-0.134	129967591	0.123	0.087	360421	A	C	0.101	3	129686434	6.83E-07	0.020	14306	24.661
Vascular dementia (mixed)	genus Anaerofilum	rs79598899	C	T	-0.383	190274425	0.060	0.204	360421	C	T	0.183	2	191139151	3.75E-07	0.036	14306	26.116
Vascular dementia (mixed)	genus Anaerofilum	rs816292	T	C	-0.074	117373604	0.427	0.093	360421	T	C	-0.113	12	117811409	2.64E-07	0.022	14306	26.288
Vascular dementia (mixed)	genus Anaerofilum	rs9299345	T	C	-0.260	101577530	0.070	0.143	360421	T	C	-0.136	9	104339812	8.04E-06	0.030	14306	20.349
Vascular dementia (mixed)	genus Anaerostipes	rs10502061	A	G	0.110	105744527	0.403	0.132	360421	A	G	0.084	11	105615253	7.94E-06	0.019	14306	18.944
Vascular dementia (mixed)	genus Anaerostipes	rs2014785	T	C	-0.124	171382313	0.147	0.085	360421	T	C	0.052	3	171100102	4.68E-06	0.011	14306	21.125
Vascular dementia (mixed)	genus Anaerostipes	rs2396460	C	T	0.007	227153955	0.939	0.085	360421	C	T	0.051	2	228018671	2.91E-06	0.011	14306	21.897
Vascular dementia (mixed)	genus Anaerostipes	rs2804244	G	A	-0.101	115623674	0.246	0.087	360421	G	A	0.053	10	117383184	2.04E-06	0.011	14306	22.882
Vascular dementia (mixed)	genus Anaerostipes	rs3900776	G	A	-0.414	13525083	0.103	0.254	360421	G	A	-0.110	9	13525082	2.75E-06	0.024	14306	21.675
Vascular dementia (mixed)	genus Anaerostipes	rs60983350	G	A	0.070	2947443	0.442	0.091	360421	G	A	-0.054	17	2850737	4.42E-06	0.012	14306	21.450
Vascular dementia (mixed)	genus Anaerostipes	rs62157625	T	C	0.131	142016764	0.318	0.131	360421	T	C	0.089	2	142774333	1.45E-06	0.019	14306	22.787
Vascular dementia (mixed)	genus Anaerostipes	rs62215703	G	A	-0.094	24501310	0.359	0.103	360421	G	A	0.064	21	25873624	1.98E-06	0.014	14306	22.260
Vascular dementia (mixed)	genus Anaerostipes	rs6474958	G	A	0.061	1582701	0.502	0.091	360421	G	A	0.050	9	1582701	6.74E-06	0.011	14306	19.935
Vascular dementia (mixed)	genus Anaerostipes	rs6726833	C	A	-0.006	39124428	0.967	0.155	360421	C	A	-0.088	2	39351569	3.32E-06	0.019	14306	21.463
Vascular dementia (mixed)	genus Anaerostipes	rs6854026	C	T	0.129	168769663	0.130	0.085	360421	C	T	0.051	4	169690814	3.20E-06	0.011	14306	21.732
Vascular dementia (mixed)	genus Anaerostipes	rs7193624	T	C	0.167	77540123	0.281	0.154	360421	T	C	-0.075	16	77574020	5.35E-07	0.015	14306	24.803
Vascular dementia (mixed)	genus Anaerostipes	rs78735375	A	C	-0.162	1497548	0.459	0.219	360421	A	C	-0.137	19	1497547	5.33E-06	0.031	14306	20.262
Vascular dementia (mixed)	genus Anaerotruncus	rs10150232	A	G	-0.069	29948802	0.516	0.106	360421	A	G	0.057	14	30418008	6.68E-06	0.012	14306	20.622
Vascular dementia (mixed)	genus Anaerotruncus	rs11018566	A	G	-0.299	89307058	0.114	0.189	360421	A	G	-0.156	11	89040226	6.14E-06	0.037	14306	18.272
Vascular dementia (mixed)	genus Anaerotruncus	rs115414803	A	C	0.312	87242091	0.075	0.175	360421	A	C	-0.144	4	88163243	6.83E-06	0.032	14306	20.669
Vascular dementia (mixed)	genus Anaerotruncus	rs1272208	T	G	0.055	76015978	0.581	0.099	360421	T	G	0.061	9	78630894	4.28E-06	0.013	14306	22.201
Vascular dementia (mixed)	genus Anaerotruncus	rs1431492	C	T	-0.015	151137584	0.897	0.115	360421	C	T	-0.065	3	150855371	7.36E-06	0.015	14306	20.075
Vascular dementia (mixed)	genus Anaerotruncus	rs17734739	T	C	-0.151	210798978	0.217	0.123	360421	T	C	0.066	2	211663702	7.43E-06	0.015	14306	19.603
Vascular dementia (mixed)	genus Anaerotruncus	rs34449434	A	C	-0.099	76129875	0.262	0.088	360421	A	C	-0.050	12	76523655	9.85E-06	0.011	14306	19.208
Vascular dementia (mixed)	genus Anaerotruncus	rs4669806	G	T	-0.025	12060626	0.807	0.102	360421	G	T	0.058	2	12200752	2.42E-06	0.012	14306	21.962

Vascular dementia (mixed)	genus Anaerotruncus	rs6494922	A	G	-0.213	33167666	0.255	0.187	360421	A	G	0.090	15	33459867	6.62E-06	0.020	14306	19.937
Vascular dementia (mixed)	genus Anaerotruncus	rs6563550	T	C	0.351	37484276	0.025	0.157	360421	T	C	0.088	13	38058413	2.35E-07	0.018	14306	24.629
Vascular dementia (mixed)	genus Anaerotruncus	rs7155595	C	A	0.192	77036203	0.037	0.092	360421	C	A	0.054	14	77502546	7.55E-06	0.012	14306	20.575
Vascular dementia (mixed)	genus Anaerotruncus	rs8005030	C	T	-0.080	30137993	0.369	0.089	360421	C	T	0.055	14	30607199	2.28E-06	0.012	14306	22.133
Vascular dementia (mixed)	genus Anaerotruncus	rs9347879	T	C	0.206	164594228	0.015	0.084	360421	T	C	0.051	6	165015261	4.22E-06	0.011	14306	20.988
Vascular dementia (mixed)	genus Bacteroides	rs11585893	A	G	-0.115	10584294	0.244	0.098	360421	A	G	-0.074	1	10644351	1.80E-06	0.015	14306	25.175
Vascular dementia (mixed)	genus Bacteroides	rs13207588	A	G	-0.056	41551692	0.602	0.107	360421	A	G	-0.059	6	41519430	7.48E-06	0.013	14306	20.365
Vascular dementia (mixed)	genus Bacteroides	rs1340391	T	C	-0.078	102495433	0.530	0.124	360421	T	C	-0.059	1	102960989	6.73E-06	0.013	14306	20.040
Vascular dementia (mixed)	genus Bacteroides	rs17619981	T	G	-0.021	24159448	0.861	0.122	360421	T	G	0.088	19	24342250	2.69E-06	0.019	14306	22.194
Vascular dementia (mixed)	genus Bacteroides	rs2023437	T	C	-0.128	21577814	0.320	0.129	360421	T	C	-0.078	14	22045949	5.02E-06	0.017	14306	21.780
Vascular dementia (mixed)	genus Bacteroides	rs66710942	T	C	0.022	77166176	0.792	0.084	360421	T	C	-0.049	3	77215327	5.86E-06	0.011	14306	20.644
Vascular dementia (mixed)	genus Bacteroides	rs6795673	C	T	0.048	10551540	0.568	0.084	360421	C	T	0.054	3	10593224	3.38E-07	0.011	14306	26.183
Vascular dementia (mixed)	genus Bacteroides	rs9507307	C	T	0.009	24336338	0.925	0.098	360421	C	T	0.060	13	24910476	2.13E-06	0.013	14306	21.912
Vascular dementia (mixed)	genus Barnesiella	rs11155559	T	C	-0.028	148280769	0.848	0.145	360421	T	C	0.096	6	148601905	8.92E-06	0.021	14306	20.153
Vascular dementia (mixed)	genus Barnesiella	rs12909713	C	T	0.037	86602682	0.662	0.084	360421	C	T	-0.055	15	87145913	4.95E-06	0.012	14306	21.048
Vascular dementia (mixed)	genus Barnesiella	rs13242616	C	T	0.011	122381464	0.900	0.091	360421	C	T	0.058	7	122021518	2.29E-06	0.012	14306	22.459
Vascular dementia (mixed)	genus Barnesiella	rs199035	G	A	0.001	23435187	0.989	0.085	360421	G	A	0.056	6	23435415	3.00E-06	0.012	14306	21.842
Vascular dementia (mixed)	genus Barnesiella	rs2276875	A	G	-0.086	5729227	0.376	0.097	360421	A	G	-0.070	4	5730954	4.65E-07	0.014	14306	24.964
Vascular dementia (mixed)	genus Barnesiella	rs2428166	G	A	0.009	110509945	0.980	0.364	360421	G	A	-0.166	6	110831148	8.51E-07	0.034	14306	24.178
Vascular dementia (mixed)	genus Barnesiella	rs35177866	A	G	0.066	181871367	0.685	0.162	360421	A	G	0.092	3	181589155	2.95E-06	0.019	14306	23.267
Vascular dementia (mixed)	genus Barnesiella	rs62251337	G	A	-0.131	44285937	0.272	0.120	360421	G	A	0.069	3	44327429	4.24E-06	0.015	14306	21.385
Vascular dementia (mixed)	genus Barnesiella	rs72684847	T	C	0.207	101695712	0.197	0.160	360421	T	C	-0.114	4	102616869	6.76E-06	0.025	14306	20.295
Vascular dementia (mixed)	genus Barnesiella	rs76181748	C	T	0.117	104446188	0.244	0.100	360421	C	T	-0.078	8	105458416	6.78E-06	0.017	14306	20.575
Vascular dementia (mixed)	genus Barnesiella	rs77455852	T	G	-0.017	54811208	0.887	0.119	360421	T	G	-0.089	5	54107036	3.16E-06	0.020	14306	20.773
Vascular dementia (mixed)	genus Barnesiella	rs79795328	A	G	-0.074	113625439	0.539	0.121	360421	A	G	-0.082	4	114546595	4.23E-06	0.018	14306	21.527
Vascular dementia (mixed)	genus Bifidobacterium	rs12022129	A	G	0.114	206830029	0.225	0.094	360421	A	G	-0.062	1	207003374	8.00E-06	0.014	14306	19.872
Vascular dementia (mixed)	genus Bifidobacterium	rs182549	T	C	-0.114	135859184	0.187	0.087	360421	T	C	-0.120	2	136616754	1.28E-20	0.013	14306	88.429
Vascular dementia (mixed)	genus Bifidobacterium	rs2491158	A	G	0.127	124401134	0.307	0.124	360421	A	G	-0.071	10	126089703	8.05E-06	0.016	14306	19.879
Vascular dementia (mixed)	genus Bifidobacterium	rs2686790	C	T	0.036	48051149	0.763	0.118	360421	C	T	-0.071	7	48090746	7.50E-06	0.016	14306	20.065
Vascular dementia (mixed)	genus Bifidobacterium	rs4957061	T	C	0.013	520981	0.881	0.086	360421	T	C	0.053	5	521096	5.78E-06	0.012	14306	20.697
Vascular dementia (mixed)	genus Bifidobacterium	rs540489	T	G	0.037	74901626	0.740	0.111	360421	T	G	-0.064	17	72897722	5.19E-06	0.014	14306	21.121
Vascular dementia (mixed)	genus Bifidobacterium	rs55888705	A	G	0.072	1516099	0.438	0.093	360421	A	G	0.055	4	1517826	6.67E-06	0.012	14306	20.339
Vascular dementia (mixed)	genus Bifidobacterium	rs5746486	T	C	-0.071	17871506	0.412	0.087	360421	T	C	-0.054	22	18354272	9.00E-06	0.012	14306	19.703
Vascular dementia (mixed)	genus Bifidobacterium	rs62181700	G	A	-0.143	188941058	0.143	0.098	360421	G	A	-0.062	2	189805784	2.17E-06	0.013	14306	22.665
Vascular dementia (mixed)	genus Bifidobacterium	rs7322849	T	C	0.097	112205515	0.512	0.147	360421	T	C	0.112	13	112859829	1.08E-08	0.020	14306	31.035
Vascular dementia (mixed)	genus Bifidobacterium	rs75344046	C	T	0.100	30489472	0.626	0.204	360421	C	T	0.232	21	31861790	4.86E-06	0.051	14306	21.088
Vascular dementia (mixed)	genus Bifidobacterium	rs857444	C	T	0.083	14617360	0.341	0.087	360421	C	T	0.056	6	14617591	3.57E-06	0.012	14306	21.208
Vascular dementia (mixed)	genus Bilophila	rs11069458	C	T	0.018	101750335	0.866	0.109	360421	C	T	0.068	13	102402685	7.72E-06	0.016	14306	19.293
Vascular dementia (mixed)	genus Bilophila	rs1241171	G	A	0.155	102834365	0.196	0.120	360421	G	A	-0.069	1	103299921	4.24E-06	0.015	14306	21.281
Vascular dementia (mixed)	genus Bilophila	rs1571225	T	C	0.150	4940871	0.186	0.113	360421	T	C	-0.083	9	4940871	1.12E-06	0.017	14306	23.484
Vascular dementia (mixed)	genus Bilophila	rs1969927	A	G	-0.055	100393069	0.536	0.090	360421	A	G	-0.056	12	100786847	9.07E-06	0.013	14306	19.783
Vascular dementia (mixed)	genus Bilophila	rs2728491	T	G	0.057	47172134	0.565	0.099	360421	T	G	0.063	7	47211732	6.33E-06	0.014	14306	20.241
Vascular dementia (mixed)	genus Bilophila	rs3827020	C	T	-0.192	63349639	0.071	0.106	360421	C	T	0.077	20	61980991	1.79E-06	0.016	14306	22.766
Vascular dementia (mixed)	genus Bilophila	rs4798126	G	A	0.172	3765773	0.106	0.107	360421	G	A	0.073	18	3765773	7.15E-06	0.017	14306	19.033
Vascular dementia (mixed)	genus Bilophila	rs542415	T	C	-0.128	49719357	0.140	0.086	360421	T	C	-0.061	15	50011554	4.71E-06	0.013	14306	21.147
Vascular dementia (mixed)	genus Bilophila	rs60178956	G	A	-0.034	89866110	0.739	0.101	360421	G	A	-0.062	8	90878338	8.06E-06	0.014	14306	19.502
Vascular dementia (mixed)	genus Bilophila	rs6793291	A	C	0.160	194698802	0.405	0.192	360421	A	C	-0.113	3	194419531	3.11E-06	0.024	14306	21.765
Vascular dementia (mixed)	genus Bilophila	rs72676854	T	C	-0.296	108978710	0.123	0.192	360421	T	C	0.123	8	109990939	5.62E-06	0.027	14306	21.007
Vascular dementia (mixed)	genus Bilophila	rs7802841	A	C	-0.010	138899094	0.913	0.092	360421	A	C	-0.067	7	138583840	1.77E-06	0.014	14306	23.681
Vascular dementia (mixed)	genus Bilophila	rs9899990	A	G	0.002	9218262	0.991	0.153	360421	A	G	-0.103	17	9121579	9.07E-06	0.023	14306	19.283
Vascular dementia (mixed)	genus Butyricicoccus	rs10084203	G	A	-0.020	190439423	0.872	0.125	360421	G	A	-0.055	2	191304149	8.59E-06	0.012	14306	19.791
Vascular dementia (mixed)	genus Butyricicoccus	rs12034718	G	A	-0.076	66913912	0.456	0.101	360421	G	A	-0.070	1	67379595	9.58E-06	0.016	14306	19.643
Vascular dementia (mixed)	genus Butyricicoccus	rs12585793	T	C	0.302	26444103	0.239	0.256	360421	T	C	-0.262	13	27018240	5.79E-06	0.056	14306	21.558
Vascular dementia (mixed)	genus Butyricicoccus	rs2017189	T	G	0.168	7458426	0.046	0.084	360421	T	G	0.051	4	7460153	3.87E-06	0.011	14306	21.148
Vascular dementia (mixed)	genus Butyricicoccus	rs4962426	T	G	0.031	125219639	0.771	0.106	360421	T	G	-0.061	10	126908208	7.38E-06	0.014	14306	20.403
Vascular dementia (mixed)	genus Butyricicoccus	rs56221232	T	C	-0.065	152596896	0.646	0.141	360421	T	C	0.083	2	153453410	7.62E-07	0.017	14306	24.467
Vascular dementia (mixed)	genus Butyricicoccus	rs62478070	T	G	0.093	157895458	0.721	0.261	360421	T	G	0.224	7	157688150	5.94E-06	0.049	14306	20.488
Vascular dementia (mixed)	genus Butyricicoccus	rs7322368	C	T	0.013	99561736	0.931	0.146	360421	C	T	-0.082	13	100213990	5.52E-06	0.018	14306	19.834
Vascular dementia (mixed)	genus Butyricimonas	rs11228830	A	G	-0.007	56897486	0.963	0.152	360421	A	G	0.135	11	56664962	6.55E-06	0.030	14306	20.547
Vascular dementia (mixed)	genus Butyricimonas	rs113054641	G	A	-0.309	15178069	0.132	0.206	360421	G	A	-0.145	21	16550389	1.74E-07	0.027	14306	27.842
Vascular dementia (mixed)	genus Butyricimonas	rs12304031	G	A	0.114	128954226	0.374	0.128	360421	G	A	-0.086	12	129438771	6.70E-06	0.020	14306	19.211
Vascular dementia (mixed)	genus Butyricimonas	rs12458763	A	C	0.228	36511655	0.263	0.204	360421	A	C	0.122	18	34091618	6.37E-06	0.027	14306	20.492
Vascular dementia (mixed)	genus Butyricimonas	rs1862649	G	A	-0.030	22161545	0.855	0.165	360421	G	A	0.113	16	22172866	4.76E-06	0.025	14306	20.777
Vascular dementia (mixed)	genus Butyricimonas	rs2114713	G	T	0.080	80236031	0.340	0.084	360421	G	T	0.063	15	80528373	6.88E-06	0.014	14306	20.361
Vascular dementia (mixed)	genus Butyricimonas	rs62130338	A	G	0.056	48659244	0.523	0.088	360421	A	G	0.073	19	49162501	3.90E-06	0.016	14306	21.412
Vascular dementia (mixed)	genus Butyricimonas	rs62390301	T	C	-0.139	160440640	0.183	0.105	360421	T	C	-0.087	5	159867647	7.42E-07	0.017	14306	24.915
Vascular dementia (mixed)	genus Butyricimonas	rs7083431	A	C	0.028	70951399	0.765	0.094	360421	A	C	0.070	10	72711156	8.85E-07	0.014	14306	23.725

Vascular dementia (mixed)	genus Butyricimonas	rs71428626	G	T	0.023	82866140	0.923	0.233	360421	G	T	-0.133	2	83093264	4.80E-06	0.029	14306	21.107
Vascular dementia (mixed)	genus Butyricimonas	rs72814525	A	G	-0.168	70683314	0.086	0.098	360421	A	G	0.066	10	72443070	8.25E-06	0.015	14306	19.666
Vascular dementia (mixed)	genus Butyricimonas	rs78453362	A	G	-0.397	78690859	0.153	0.278	360421	A	G	-0.149	3	78740009	4.06E-06	0.033	14306	20.887
Vascular dementia (mixed)	genus Butyricimonas	rs9657374	C	T	0.102	4976884	0.269	0.092	360421	C	T	0.068	8	4834406	4.50E-06	0.015	14306	21.106
Vascular dementia (mixed)	genus Butyrivibrio	rs1007475	G	T	-0.128	148082269	0.172	0.094	360421	G	T	0.118	6	148403405	7.92E-06	0.026	14306	20.420
Vascular dementia (mixed)	genus Butyrivibrio	rs11761679	T	C	0.097	150899841	0.423	0.121	360421	T	C	0.155	7	150596929	2.20E-06	0.032	14306	23.170
Vascular dementia (mixed)	genus Butyrivibrio	rs142855850	A	G	-0.227	22298971	0.111	0.143	360421	A	G	0.205	10	22587900	6.86E-06	0.046	14306	20.113
Vascular dementia (mixed)	genus Butyrivibrio	rs16934069	T	C	0.166	116930885	0.130	0.110	360421	T	C	-0.134	9	119693164	8.86E-06	0.030	14306	19.961
Vascular dementia (mixed)	genus Butyrivibrio	rs16941336	C	T	0.039	20672384	0.693	0.100	360421	C	T	0.127	17	20575697	1.53E-06	0.027	14306	22.640
Vascular dementia (mixed)	genus Butyrivibrio	rs17163238	G	A	0.026	129640328	0.810	0.106	360421	G	A	0.141	5	128976021	5.51E-06	0.031	14306	20.802
Vascular dementia (mixed)	genus Butyrivibrio	rs4537857	T	C	0.097	26718529	0.277	0.090	360421	T	C	-0.125	13	27292666	1.80E-06	0.026	14306	22.788
Vascular dementia (mixed)	genus Butyrivibrio	rs486484	A	G	0.030	830864	0.726	0.086	360421	A	G	-0.108	20	811507	6.61E-06	0.024	14306	20.380
Vascular dementia (mixed)	genus Butyrivibrio	rs4928024	G	A	-0.112	54254476	0.308	0.110	360421	G	A	0.175	3	54288503	8.19E-06	0.039	14306	20.137
Vascular dementia (mixed)	genus Butyrivibrio	rs72723662	C	T	0.003	75599220	0.983	0.121	360421	C	T	0.224	14	76065563	7.86E-07	0.045	14306	24.869
Vascular dementia (mixed)	genus Butyrivibrio	rs74622183	A	G	0.240	33681771	0.112	0.151	360421	A	G	-0.201	14	34150977	2.46E-06	0.043	14306	22.040
Vascular dementia (mixed)	genus Butyrivibrio	rs77356209	T	C	-0.178	10267736	0.374	0.200	360421	T	C	0.217	18	10267733	6.66E-06	0.048	14306	20.136
Vascular dementia (mixed)	genus Butyrivibrio	rs7752361	G	A	0.121	111459502	0.152	0.085	360421	G	A	0.119	6	111780705	7.69E-07	0.024	14306	24.685
Vascular dementia (mixed)	genus Butyrivibrio	rs7763512	A	G	0.065	21959860	0.448	0.085	360421	A	G	-0.120	6	21960091	3.11E-06	0.025	14306	22.374
Vascular dementia (mixed)	genus Butyrivibrio	rs9349693	G	A	-0.001	54074926	0.992	0.092	360421	G	A	-0.118	6	53939724	5.55E-06	0.026	14306	20.610
Vascular dementia (mixed)	genus Candidatus Soleaferrea	rs10090365	G	A	0.126	137625956	0.135	0.084	360421	G	A	0.083	8	138638199	4.17E-06	0.018	14306	21.255
Vascular dementia (mixed)	genus Candidatus Soleaferrea	rs10809135	C	T	-0.030	10611282	0.723	0.085	360421	C	T	-0.083	9	10611282	5.47E-06	0.018	14306	20.944
Vascular dementia (mixed)	genus Candidatus Soleaferrea	rs36155147	T	C	0.008	312402	0.928	0.092	360421	T	C	-0.105	7	352368	5.41E-06	0.024	14306	18.971
Vascular dementia (mixed)	genus Candidatus Soleaferrea	rs4294381	C	T	0.088	224500405	0.445	0.115	360421	C	T	-0.112	1	224688107	1.37E-06	0.023	14306	23.416
Vascular dementia (mixed)	genus Candidatus Soleaferrea	rs4678258	T	C	-0.115	138226350	0.244	0.098	360421	T	C	0.099	3	137945192	5.53E-06	0.022	14306	20.912
Vascular dementia (mixed)	genus Candidatus Soleaferrea	rs6489992	A	G	-0.076	114914964	0.386	0.087	360421	A	G	-0.084	12	115352769	7.89E-06	0.019	14306	20.192
Vascular dementia (mixed)	genus Candidatus Soleaferrea	rs6494306	A	G	-0.122	62102341	0.177	0.090	360421	A	G	-0.097	15	62394540	5.80E-06	0.021	14306	20.474
Vascular dementia (mixed)	genus Candidatus Soleaferrea	rs7400877	C	T	0.010	75937199	0.926	0.103	360421	C	T	0.095	14	76403542	9.29E-06	0.021	14306	19.976
Vascular dementia (mixed)	genus Candidatus Soleaferrea	rs9973954	G	A	0.028	19686146	0.750	0.088	360421	G	A	-0.089	2	19885907	5.95E-06	0.020	14306	20.842
Vascular dementia (mixed)	genus Catenibacterium	rs12404911	C	T	0.032	239955143	0.767	0.109	360421	C	T	0.141	1	240118443	2.80E-06	0.030	14306	21.406
Vascular dementia (mixed)	genus Catenibacterium	rs212393	A	G	-0.132	159064710	0.203	0.104	360421	A	G	0.135	6	159485742	3.62E-06	0.029	14306	22.332
Vascular dementia (mixed)	genus Catenibacterium	rs73128290	A	G	0.007	57296613	0.941	0.092	360421	A	G	0.130	7	57364320	4.29E-06	0.028	14306	20.782
Vascular dementia (mixed)	genus Catenibacterium	rs7742829	C	T	-0.107	104641790	0.210	0.085	360421	C	T	0.114	6	105089665	5.61E-06	0.025	14306	20.652
Vascular dementia (mixed)	genus Christensenellaceae R 7group	rs10461257	A	G	0.091	155209852	0.309	0.090	360421	A	G	-0.055	4	156131004	6.51E-06	0.012	14306	20.446
Vascular dementia (mixed)	genus Christensenellaceae R 7group	rs17081797	A	G	-0.091	69888324	0.594	0.170	360421	A	G	-0.090	18	67555560	3.34E-06	0.020	14306	19.603
Vascular dementia (mixed)	genus Christensenellaceae R 7group	rs62132810	A	G	-0.185	48775970	0.130	0.122	360421	A	G	-0.083	19	49279227	5.67E-06	0.018	14306	21.293
Vascular dementia (mixed)	genus Christensenellaceae R 7group	rs62190261	A	C	-0.027	230078383	0.856	0.151	360421	A	C	0.096	2	230943099	8.74E-06	0.021	14306	19.922
Vascular dementia (mixed)	genus Christensenellaceae R 7group	rs62467127	C	T	-0.113	118456871	0.673	0.267	360421	C	T	0.114	7	118096925	3.25E-06	0.025	14306	20.506
Vascular dementia (mixed)	genus Christensenellaceae R 7group	rs73952017	C	T	-0.193	1779608	0.161	0.138	360421	C	T	-0.086	18	1779609	8.46E-06	0.019	14306	19.679
Vascular dementia (mixed)	genus Christensenellaceae R 7group	rs78521377	C	T	0.085	124759805	0.721	0.239	360421	C	T	0.125	10	126448374	5.61E-06	0.027	14306	20.689
Vascular dementia (mixed)	genus Christensenellaceae R 7group	rs892686	A	G	-0.053	80428461	0.533	0.085	360421	A	G	0.051	9	83043376	3.97E-06	0.011	14306	21.313
Vascular dementia (mixed)	genus Clostridium innocuum group	rs10506058	A	G	-0.007	30115552	0.934	0.085	360421	A	G	0.100	12	30268485	8.92E-06	0.022	14306	20.184
Vascular dementia (mixed)	genus Clostridium innocuum group	rs1942371	G	A	0.026	71607072	0.843	0.130	360421	G	A	-0.158	18	69274308	4.06E-06	0.034	14306	21.343
Vascular dementia (mixed)	genus Clostridium innocuum group	rs40656	C	T	-0.082	9368046	0.435	0.106	360421	C	T	0.143	5	9368158	8.62E-06	0.031	14306	21.040
Vascular dementia (mixed)	genus Clostridium innocuum group	rs4869133	G	A	-0.002	96381915	0.986	0.111	360421	G	A	-0.181	5	95717619	7.24E-06	0.041	14306	19.448
Vascular dementia (mixed)	genus Clostridium innocuum group	rs61267978	T	C	-0.051	6348228	0.697	0.130	360421	T	C	0.147	18	6348227	5.59E-06	0.032	14306	21.010
Vascular dementia (mixed)	genus Clostridium innocuum group	rs6577484	G	A	-0.052	8359360	0.705	0.138	360421	G	A	0.160	1	8419420	8.41E-06	0.036	14306	19.764
Vascular dementia (mixed)	genus Clostridium innocuum group	rs6890185	C	T	0.128	71890799	0.156	0.091	360421	C	T	-0.113	5	71186626	1.12E-06	0.023	14306	23.669
Vascular dementia (mixed)	genus Clostridium innocuum group	rs77845139	A	G	0.016	59412009	0.869	0.097	360421	A	G	-0.115	15	59704208	8.41E-06	0.026	14306	19.992
Vascular dementia (mixed)	genus Clostridium sensustricto1	rs11264403	G	A	-0.045	155706701	0.780	0.160	360421	G	A	-0.139	1	155676492	7.76E-06	0.033	14306	17.288
Vascular dementia (mixed)	genus Clostridium sensustricto1	rs116847295	C	T	0.092	43091212	0.468	0.127	360421	C	T	0.110	12	43485015	4.58E-06	0.025	14306	19.997
Vascular dementia (mixed)	genus Clostridium sensustricto1	rs12341505	G	A	-0.130	133845759	0.373	0.145	360421	G	A	0.081	9	136710881	4.82E-06	0.018	14306	20.259
Vascular dementia (mixed)	genus Clostridium sensustricto1	rs2795528	G	A	0.080	42774816	0.661	0.183	360421	G	A	-0.184	10	43270264	2.72E-06	0.039	14306	22.085
Vascular dementia (mixed)	genus Clostridium sensustricto1	rs2817172	C	T	0.072	3124955	0.405	0.086	360421	C	T	0.058	1	3041519	2.77E-06	0.012	14306	21.810
Vascular dementia (mixed)	genus Clostridium sensustricto1	rs550843	T	C	0.076	165309343	0.423	0.094	360421	T	C	-0.078	6	165722832	2.05E-06	0.017	14306	21.426
Vascular dementia (mixed)	genus Collinsella	rs10890671	C	T	-0.071	107252473	0.405	0.085	360421	C	T	0.054	11	107123199	6.52E-06	0.012	14306	20.446
Vascular dementia (mixed)	genus Collinsella	rs11597285	G	T	0.001	8547850	0.993	0.086	360421	G	T	-0.054	10	8589813	9.38E-06	0.012	14306	19.914
Vascular dementia (mixed)	genus Collinsella	rs1496626	T	C	-0.023	31269935	0.855	0.124	360421	T	C	-0.072	19	31760841	6.78E-06	0.016	14306	19.978
Vascular dementia (mixed)	genus Collinsella	rs149807560	C	A	0.032	56015274	0.846	0.164	360421	C	A	-0.104	19	56526640	7.10E-06	0.024	14306	19.545
Vascular dementia (mixed)	genus Collinsella	rs2103510	G	A	-0.034	27983148	0.795	0.130	360421	G	A	0.079	21	29355467	2.42E-06	0.017	14306	21.863
Vascular dementia (mixed)	genus Collinsella	rs62448871	C	A	0.027	24425405	0.753	0.085	360421	C	A	-0.054	7	24465024	6.78E-06	0.012	14306	20.164
Vascular dementia (mixed)	genus Collinsella	rs73052258	G	A	0.063	194874587	0.689	0.157	360421	G	A	0.093	2	195739311	1.72E-06	0.020	14306	21.067
Vascular dementia (mixed)	genus Collinsella	rs75672793	A	G	-0.176	148897142	0.381	0.200	360421	A	G	-0.109	4	149818294	6.14E-06	0.024	14306	20.503
Vascular dementia (mixed)	genus Collinsella	rs9541268	C	A	-0.169	68035929	0.260	0.150	360421	C	A	0.096	13	68610061	8.79E-07	0.020	14306	23.657
Vascular dementia (mixed)	genus Coprobacter	rs11532348	C	T	0.034	97896018	0.776	0.118	360421	C	T	-0.104	12	98289796	5.71E-06	0.023	14306	20.994
Vascular dementia (mixed)	genus Coprobacter	rs12684609	T	C	0.054	134893022	0.611	0.106	360421	T	C	0.101	9	137784868	6.10E-06	0.022	14306	20.935
Vascular dementia (mixed)	genus Coprobacter	rs12996055	A	C	-0.050	136465888	0.600	0.096	360421	A	C	0.092	2	137223458	8.08E-06	0.021	14306	19.378
Vascular dementia (mixed)	genus Coprobacter	rs143662916	C	T	-0.163	103679246	0.504	0.244	360421	C	T	0.253	11	103549974	3.07E-06	0.054	14306	21.985

Vascular dementia (mixed)	genus Coprobacter	rs189356	G	A	0.069	58999796	0.418	0.085	360421	G	A	0.078	20	57574851	6.26E-06	0.017	14306	20.645
Vascular dementia (mixed)	genus Coprobacter	rs213863	T	C	0.130	96787063	0.137	0.087	360421	T	C	0.089	6	97234939	2.35E-06	0.019	14306	22.192
Vascular dementia (mixed)	genus Coprobacter	rs28402691	T	C	0.027	95468089	0.819	0.119	360421	T	C	0.111	4	96389240	9.56E-06	0.025	14306	19.419
Vascular dementia (mixed)	genus Coprobacter	rs305411	A	G	0.059	87767445	0.671	0.140	360421	A	G	0.129	1	88233128	1.01E-06	0.026	14306	23.864
Vascular dementia (mixed)	genus Coprobacter	rs3828477	G	T	-0.048	46442226	0.591	0.089	360421	G	T	-0.091	3	46483717	2.89E-06	0.020	14306	21.728
Vascular dementia (mixed)	genus Coprobacter	rs72821405	T	C	0.049	4714563	0.742	0.150	360421	T	C	-0.147	6	4714797	4.76E-06	0.032	14306	21.210
Vascular dementia (mixed)	genus Coprobacter	rs74919520	G	A	-0.160	32017383	0.237	0.135	360421	G	A	0.126	2	32242452	5.76E-06	0.028	14306	20.699
Vascular dementia (mixed)	genus Coprococcus1	rs1010560	C	A	-0.077	29927454	0.417	0.095	360421	C	A	0.058	1	30400301	1.96E-06	0.012	14306	22.354
Vascular dementia (mixed)	genus Coprococcus1	rs12794898	G	T	0.098	124684165	0.433	0.125	360421	G	T	0.090	11	124554061	4.92E-06	0.020	14306	20.980
Vascular dementia (mixed)	genus Coprococcus1	rs1519491	T	C	-0.050	21856898	0.562	0.086	360421	T	C	0.050	2	22079770	8.95E-06	0.011	14306	19.325
Vascular dementia (mixed)	genus Coprococcus1	rs1576241	A	G	0.112	71819574	0.194	0.086	360421	A	G	-0.051	6	72529277	3.33E-06	0.011	14306	21.708
Vascular dementia (mixed)	genus Coprococcus1	rs2907920	G	A	0.172	2600719	0.067	0.094	360421	G	A	-0.056	19	2600717	7.65E-06	0.013	14306	19.579
Vascular dementia (mixed)	genus Coprococcus1	rs4277593	G	A	0.146	4338773	0.087	0.085	360421	G	A	-0.059	20	4319420	1.14E-07	0.011	14306	28.390
Vascular dementia (mixed)	genus Coprococcus1	rs56405618	A	G	-0.097	173547165	0.468	0.133	360421	A	G	-0.090	4	174468316	1.57E-06	0.019	14306	23.095
Vascular dementia (mixed)	genus Coprococcus1	rs73031725	T	C	-0.168	134752388	0.489	0.243	360421	T	C	0.168	11	134622282	1.98E-06	0.036	14306	22.259
Vascular dementia (mixed)	genus Coprococcus1	rs73167075	T	C	0.063	165975355	0.541	0.103	360421	T	C	0.057	3	165693143	8.57E-06	0.013	14306	20.184
Vascular dementia (mixed)	genus Coprococcus1	rs74101919	T	C	0.070	94958765	0.609	0.136	360421	T	C	-0.072	1	95424321	1.03E-06	0.014	14306	24.706
Vascular dementia (mixed)	genus Coprococcus1	rs946513	T	C	-0.217	15871821	0.290	0.205	360421	T	C	-0.206	10	15913820	8.62E-06	0.046	14306	20.034
Vascular dementia (mixed)	genus Coprococcus2	rs10070053	A	G	-0.052	34794684	0.540	0.085	360421	A	G	0.059	5	34794789	7.65E-06	0.014	14306	19.273
Vascular dementia (mixed)	genus Coprococcus2	rs12634070	T	C	0.067	180827092	0.484	0.096	360421	T	C	0.074	3	180544880	9.95E-06	0.016	14306	19.942
Vascular dementia (mixed)	genus Coprococcus2	rs2482516	C	T	-0.127	25554070	0.207	0.101	360421	C	T	0.075	9	25554068	4.72E-06	0.016	14306	21.002
Vascular dementia (mixed)	genus Coprococcus2	rs35890118	A	G	0.023	127917495	0.816	0.097	360421	A	G	-0.067	10	129715759	8.26E-06	0.015	14306	20.304
Vascular dementia (mixed)	genus Coprococcus2	rs61823518	A	C	-0.128	223514894	0.352	0.138	360421	A	C	-0.096	1	223688236	6.68E-06	0.022	14306	19.614
Vascular dementia (mixed)	genus Coprococcus2	rs6677933	C	T	0.089	111596386	0.458	0.120	360421	C	T	-0.080	1	112139008	1.19E-06	0.016	14306	23.995
Vascular dementia (mixed)	genus Coprococcus2	rs72680320	T	C	0.058	130204631	0.516	0.089	360421	T	C	-0.065	4	131125786	2.27E-06	0.014	14306	21.766
Vascular dementia (mixed)	genus Coprococcus2	rs9426473	A	G	0.071	4150395	0.459	0.096	360421	A	G	0.073	1	4210455	6.31E-06	0.016	14306	20.243
Vascular dementia (mixed)	genus Coprococcus3	rs10810043	G	A	0.019	13799810	0.831	0.089	360421	G	A	-0.052	9	13799809	9.27E-06	0.012	14306	19.776
Vascular dementia (mixed)	genus Coprococcus3	rs11077359	C	T	0.056	78347880	0.618	0.112	360421	C	T	0.065	17	76343961	9.64E-06	0.015	14306	18.807
Vascular dementia (mixed)	genus Coprococcus3	rs11080344	C	T	-0.015	27777485	0.858	0.085	360421	C	T	0.052	17	26104511	4.79E-06	0.011	14306	20.906
Vascular dementia (mixed)	genus Coprococcus3	rs13247359	G	A	-0.090	76728409	0.286	0.085	360421	G	A	0.051	7	76357726	7.33E-06	0.011	14306	20.527
Vascular dementia (mixed)	genus Coprococcus3	rs178271	C	T	-0.477	20977267	0.154	0.335	360421	C	T	-0.145	22	21331556	7.81E-07	0.029	14306	24.371
Vascular dementia (mixed)	genus Coprococcus3	rs4575475	A	G	-0.052	98675414	0.602	0.100	360421	A	G	-0.062	14	99141751	7.04E-06	0.014	14306	20.216
Vascular dementia (mixed)	genus Coprococcus3	rs7521171	A	G	0.021	150026539	0.819	0.092	360421	A	G	0.060	1	149998497	4.32E-06	0.013	14306	21.285
Vascular dementia (mixed)	genus Coprococcus3	rs8100692	T	C	0.070	39541492	0.408	0.085	360421	T	C	0.058	19	40032132	4.16E-07	0.011	14306	25.892
Vascular dementia (mixed)	genus Defluviitaleaceae UCG011	rs112893842	T	C	0.011	8786663	0.938	0.147	360421	T	C	0.114	9	8786663	1.45E-06	0.023	14306	23.899
Vascular dementia (mixed)	genus Defluviitaleaceae UCG011	rs1582238	C	T	0.119	118181062	0.174	0.088	360421	C	T	-0.081	1	118723685	1.57E-06	0.017	14306	23.167
Vascular dementia (mixed)	genus Defluviitaleaceae UCG011	rs2892880	G	A	-0.118	119820571	0.226	0.098	360421	G	A	0.082	4	120741726	6.83E-06	0.018	14306	20.258
Vascular dementia (mixed)	genus Defluviitaleaceae UCG011	rs4344384	T	G	0.110	64647609	0.190	0.084	360421	T	G	-0.072	10	66407366	4.83E-06	0.016	14306	20.981
Vascular dementia (mixed)	genus Defluviitaleaceae UCG011	rs4677103	A	G	0.234	72158643	0.036	0.111	360421	A	G	0.098	3	72207794	9.60E-07	0.020	14306	24.576
Vascular dementia (mixed)	genus Defluviitaleaceae UCG011	rs55658617	T	C	-0.140	40439076	0.549	0.233	360421	T	C	0.174	21	41811003	2.15E-06	0.036	14306	23.173
Vascular dementia (mixed)	genus Defluviitaleaceae UCG011	rs72731813	C	T	0.037	146493591	0.851	0.197	360421	C	T	-0.147	4	147414743	4.33E-07	0.029	14306	25.165
Vascular dementia (mixed)	genus Defluviitaleaceae UCG011	rs9608282	T	G	0.131	24408113	0.583	0.238	360421	T	G	0.143	22	24804081	2.52E-06	0.030	14306	22.733
Vascular dementia (mixed)	genus Defluviitaleaceae UCG011	rs9725395	A	G	-0.091	84739949	0.493	0.133	360421	A	G	-0.138	1	85205632	3.52E-06	0.030	14306	21.911
Vascular dementia (mixed)	genus Desulfovibrio	rs12031543	T	C	0.158	68198632	0.196	0.122	360421	T	C	-0.127	1	68664315	6.55E-06	0.028	14306	20.357
Vascular dementia (mixed)	genus Desulfovibrio	rs13066142	G	A	0.217	67428549	0.137	0.146	360421	G	A	0.119	3	67478973	3.79E-06	0.025	14306	22.550
Vascular dementia (mixed)	genus Desulfovibrio	rs16863365	A	G	0.397	197225187	0.052	0.204	360421	A	G	0.109	2	198089911	1.79E-06	0.023	14306	23.235
Vascular dementia (mixed)	genus Desulfovibrio	rs2032031	G	A	0.096	128372006	0.253	0.084	360421	G	A	0.065	10	130170270	9.14E-06	0.015	14306	19.404
Vascular dementia (mixed)	genus Desulfovibrio	rs2590913	A	G	-0.208	63690313	0.282	0.193	360421	A	G	-0.154	13	64264446	6.65E-06	0.034	14306	20.721
Vascular dementia (mixed)	genus Desulfovibrio	rs2853179	T	C	0.185	104449819	0.068	0.101	360421	T	C	-0.081	8	105462047	2.42E-06	0.017	14306	21.718
Vascular dementia (mixed)	genus Desulfovibrio	rs4797774	A	G	0.320	13447996	0.129	0.211	360421	A	G	-0.213	18	13447995	5.64E-06	0.047	14306	20.454
Vascular dementia (mixed)	genus Desulfovibrio	rs6580353	T	C	0.035	140031102	0.740	0.105	360421	T	C	0.077	5	139410687	4.94E-06	0.017	14306	20.632
Vascular dementia (mixed)	genus Desulfovibrio	rs72647089	T	G	-0.085	57060357	0.581	0.154	360421	T	G	-0.107	8	57972916	8.30E-06	0.024	14306	19.836
Vascular dementia (mixed)	genus Dialister	rs10138457	T	C	-0.011	101991715	0.941	0.144	360421	T	C	-0.113	14	102458052	7.88E-06	0.026	14306	18.629
Vascular dementia (mixed)	genus Dialister	rs10938938	A	G	-0.003	23292736	0.977	0.116	360421	A	G	0.077	4	23294359	7.37E-06	0.017	14306	20.482
Vascular dementia (mixed)	genus Dialister	rs11071887	T	C	0.191	32662894	0.036	0.091	360421	T	C	0.066	15	32955095	5.91E-06	0.015	14306	20.496
Vascular dementia (mixed)	genus Dialister	rs11166701	G	A	0.015	137283482	0.857	0.084	360421	G	A	-0.066	8	138295725	5.51E-07	0.013	14306	24.698
Vascular dementia (mixed)	genus Dialister	rs2314294	T	C	-0.042	9243005	0.730	0.123	360421	T	C	0.087	16	9336862	8.08E-06	0.019	14306	19.981
Vascular dementia (mixed)	genus Dialister	rs2435610	A	C	0.012	151192947	0.903	0.096	360421	A	C	0.065	7	150890034	5.93E-06	0.014	14306	20.390
Vascular dementia (mixed)	genus Dialister	rs4747450	C	A	0.047	22910724	0.637	0.101	360421	C	A	0.067	10	23199653	5.84E-06	0.015	14306	20.490
Vascular dementia (mixed)	genus Dialister	rs4753063	A	G	-0.091	92533963	0.286	0.085	360421	A	G	0.060	11	92267129	4.86E-06	0.013	14306	21.023
Vascular dementia (mixed)	genus Dialister	rs75416973	A	G	0.111	172840493	0.273	0.102	360421	A	G	0.073	1	172809633	9.46E-06	0.016	14306	19.541
Vascular dementia (mixed)	genus Dialister	rs764177	C	A	-0.169	45767957	0.054	0.088	360421	C	A	-0.060	3	45809449	9.61E-06	0.014	14306	19.742
Vascular dementia (mixed)	genus Dialister	rs76680460	G	A	-0.219	25447082	0.292	0.208	360421	G	A	-0.161	9	25447080	8.19E-06	0.036	14306	19.600
Vascular dementia (mixed)	genus Dorea	rs11150408	G	T	0.110	81769892	0.194	0.084	360421	G	T	-0.049	16	81803497	7.06E-06	0.011	14306	20.042
Vascular dementia (mixed)	genus Dorea	rs12537781	T	C	0.200	155186473	0.040	0.098	360421	T	C	-0.056	7	154978183	9.15E-06	0.013	14306	19.675
Vascular dementia (mixed)	genus Dorea	rs13279148	G	A	0.131	126893620	0.329	0.134	360421	G	A	0.072	8	127905865	2.25E-06	0.015	14306	22.477
Vascular dementia (mixed)	genus Dorea	rs1899291	T	C	-0.120	60126622	0.301	0.116	360421	T	C	-0.070	4	60992340	4.57E-06	0.015	14306	21.523

Vascular dementia (mixed)	genus Dorea	rs3005511	G	A	-0.019	73896090	0.832	0.091	360421	G	A	-0.052	6	74605806	5.29E-06	0.011	14306	20.917
Vascular dementia (mixed)	genus Dorea	rs345219	G	T	0.143	6784833	0.093	0.085	360421	G	T	0.050	3	6826520	8.80E-06	0.011	14306	19.504
Vascular dementia (mixed)	genus Dorea	rs3752849	G	A	0.373	8707510	0.051	0.191	360421	G	A	0.164	11	8729057	7.68E-06	0.037	14306	20.032
Vascular dementia (mixed)	genus Dorea	rs4793307	C	T	-0.015	72737384	0.878	0.099	360421	C	T	0.057	17	70733523	4.01E-06	0.012	14306	21.979
Vascular dementia (mixed)	genus Dorea	rs62503162	A	G	0.317	15911148	0.124	0.206	360421	A	G	-0.097	8	15768657	7.47E-07	0.019	14306	25.113
Vascular dementia (mixed)	genus Dorea	rs73729431	C	T	0.250	25077822	0.382	0.286	360421	C	T	-0.137	6	25078050	3.17E-06	0.030	14306	20.994
Vascular dementia (mixed)	genus Eggerthella	rs112205261	T	C	0.082	9076080	0.595	0.155	360421	T	C	-0.189	1	9136139	3.35E-06	0.040	14306	21.839
Vascular dementia (mixed)	genus Eggerthella	rs13070736	A	C	0.066	20765962	0.557	0.113	360421	A	C	-0.121	3	20807454	7.62E-06	0.027	14306	19.894
Vascular dementia (mixed)	genus Eggerthella	rs1784446	A	G	-0.086	102590868	0.306	0.084	360421	A	G	-0.091	11	102461599	5.23E-06	0.020	14306	20.997
Vascular dementia (mixed)	genus Eggerthella	rs2223081	A	G	0.123	28405711	0.190	0.094	360421	A	G	-0.103	21	29778032	3.89E-06	0.022	14306	21.520
Vascular dementia (mixed)	genus Eggerthella	rs2240838	G	A	0.003	38296353	0.975	0.085	360421	G	A	-0.098	7	38335954	7.36E-07	0.020	14306	24.613
Vascular dementia (mixed)	genus Eggerthella	rs3851328	G	T	-0.093	45226666	0.359	0.101	360421	G	T	0.108	2	45453805	4.18E-06	0.024	14306	20.762
Vascular dementia (mixed)	genus Eggerthella	rs6430926	T	C	0.037	140579885	0.659	0.085	360421	T	C	-0.088	2	141337454	8.37E-06	0.020	14306	19.916
Vascular dementia (mixed)	genus Eggerthella	rs67490567	T	C	-0.139	65398986	0.150	0.097	360421	T	C	0.108	15	65691324	8.94E-06	0.025	14306	19.537
Vascular dementia (mixed)	genus Eggerthella	rs76663501	C	T	-0.098	57072813	0.612	0.193	360421	C	T	0.175	20	55647869	4.83E-06	0.038	14306	21.454
Vascular dementia (mixed)	genus Eisenbergiella	rs11027642	C	T	-0.048	23980294	0.692	0.120	360421	C	T	0.129	11	24001840	4.92E-06	0.028	14306	20.512
Vascular dementia (mixed)	genus Eisenbergiella	rs11079158	T	C	-0.358	55290280	0.001	0.104	360421	T	C	0.101	17	53367641	7.35E-06	0.023	14306	19.920
Vascular dementia (mixed)	genus Eisenbergiella	rs11938607	C	T	0.010	188139277	0.917	0.097	360421	C	T	-0.098	4	189060431	8.22E-06	0.022	14306	20.386
Vascular dementia (mixed)	genus Eisenbergiella	rs12257723	A	C	0.110	107657343	0.218	0.089	360421	A	C	-0.095	10	109417101	8.85E-06	0.021	14306	20.279
Vascular dementia (mixed)	genus Eisenbergiella	rs12710729	C	A	-0.089	19773449	0.326	0.090	360421	C	A	0.089	2	19973210	9.84E-06	0.020	14306	20.140
Vascular dementia (mixed)	genus Eisenbergiella	rs13258851	A	G	0.116	54418254	0.336	0.120	360421	A	G	0.137	8	55330814	7.75E-06	0.030	14306	20.563
Vascular dementia (mixed)	genus Eisenbergiella	rs1508033	A	C	0.001	53083004	0.992	0.091	360421	A	C	0.092	15	53375201	3.23E-06	0.020	14306	21.863
Vascular dementia (mixed)	genus Eisenbergiella	rs1553971	T	G	-0.172	111700639	0.091	0.102	360421	T	G	0.121	3	111419486	5.27E-06	0.026	14306	21.146
Vascular dementia (mixed)	genus Eisenbergiella	rs2683098	T	C	0.169	35886364	0.103	0.103	360421	T	C	-0.107	15	36178565	2.24E-06	0.023	14306	22.720
Vascular dementia (mixed)	genus Eisenbergiella	rs3812426	A	G	0.064	49910116	0.581	0.117	360421	A	G	-0.106	8	50822676	2.72E-06	0.022	14306	22.550
Vascular dementia (mixed)	genus Eisenbergiella	rs4462860	G	A	0.006	20237941	0.947	0.086	360421	G	A	0.094	21	21610254	4.16E-06	0.020	14306	21.805
Vascular dementia (mixed)	genus Enterorhabdus	rs10098492	T	C	-0.009	112097527	0.961	0.180	360421	T	C	0.132	8	113109756	6.41E-06	0.029	14306	20.300
Vascular dementia (mixed)	genus Enterorhabdus	rs114731706	T	G	0.047	19412192	0.849	0.245	360421	T	G	0.182	2	19611953	2.17E-06	0.038	14306	22.741
Vascular dementia (mixed)	genus Enterorhabdus	rs2051957	C	T	0.090	90080569	0.397	0.106	360421	C	T	0.084	7	89709883	8.90E-06	0.019	14306	19.727
Vascular dementia (mixed)	genus Enterorhabdus	rs3017103	G	A	-0.093	62406721	0.385	0.107	360421	G	A	-0.098	11	62174193	2.94E-06	0.021	14306	22.028
Vascular dementia (mixed)	genus Enterorhabdus	rs73331712	T	C	0.293	69121322	0.157	0.207	360421	T	C	0.262	12	69515102	4.85E-06	0.055	14306	22.589
Vascular dementia (mixed)	genus Enterorhabdus	rs77655283	G	A	0.054	235531235	0.747	0.166	360421	G	A	0.133	2	236439879	5.88E-06	0.030	14306	19.875
Vascular dementia (mixed)	genus Erysipelatoclostridium	rs1434153	G	A	-0.112	34370466	0.188	0.085	360421	G	A	-0.068	2	34595533	6.85E-06	0.015	14306	20.163
Vascular dementia (mixed)	genus Erysipelatoclostridium	rs16936671	C	T	-0.031	36105681	0.799	0.121	360421	C	T	-0.097	10	36394609	6.04E-06	0.022	14306	19.739
Vascular dementia (mixed)	genus Erysipelatoclostridium	rs17804233	C	T	0.087	78519233	0.300	0.084	360421	C	T	0.066	5	77815056	4.59E-06	0.014	14306	21.133
Vascular dementia (mixed)	genus Erysipelatoclostridium	rs2901723	A	C	0.030	36849973	0.727	0.085	360421	A	C	-0.064	11	36871523	8.79E-06	0.014	14306	19.755
Vascular dementia (mixed)	genus Erysipelatoclostridium	rs340991	A	G	0.083	34891937	0.378	0.095	360421	A	G	-0.074	5	34892042	3.75E-06	0.016	14306	21.719
Vascular dementia (mixed)	genus Erysipelatoclostridium	rs3804326	A	G	0.086	24291957	0.655	0.193	360421	A	G	0.141	6	24292185	9.85E-06	0.034	14306	17.691
Vascular dementia (mixed)	genus Erysipelatoclostridium	rs45480394	T	G	0.036	55346409	0.679	0.088	360421	T	G	-0.069	19	55857777	7.66E-06	0.015	14306	20.480
Vascular dementia (mixed)	genus Erysipelatoclostridium	rs4697572	A	G	-0.068	25445165	0.522	0.105	360421	A	G	-0.081	4	25446787	7.59E-07	0.016	14306	24.640
Vascular dementia (mixed)	genus Erysipelatoclostridium	rs58236560	G	T	-0.064	122006205	0.621	0.129	360421	G	T	-0.111	11	121876913	2.16E-06	0.023	14306	22.483
Vascular dementia (mixed)	genus Erysipelatoclostridium	rs61806970	C	T	0.183	167849359	0.267	0.164	360421	C	T	0.143	1	167818597	9.09E-06	0.032	14306	19.759
Vascular dementia (mixed)	genus Erysipelatoclostridium	rs622418	A	G	-0.038	87971128	0.654	0.084	360421	A	G	-0.067	9	90586043	3.68E-06	0.014	14306	21.756
Vascular dementia (mixed)	genus Erysipelatoclostridium	rs6474512	C	A	-0.045	38923088	0.605	0.086	360421	C	A	-0.067	8	38780606	3.02E-06	0.014	14306	21.900
Vascular dementia (mixed)	genus Erysipelatoclostridium	rs710230	C	T	-0.041	41867960	0.800	0.162	360421	C	T	-0.143	1	42333631	6.33E-07	0.028	14306	25.941
Vascular dementia (mixed)	genus Erysipelatoclostridium	rs7221249	G	A	-0.067	10274391	0.432	0.085	360421	G	A	-0.084	17	10177708	4.31E-09	0.014	14306	34.619
Vascular dementia (mixed)	genus Erysipelatoclostridium	rs9590927	A	G	-0.045	45797930	0.596	0.084	360421	A	G	0.065	13	46372065	6.39E-06	0.014	14306	20.272
Vascular dementia (mixed)	genus Escherichia Shigella	rs112767262	T	C	-0.091	3700622	0.370	0.102	360421	T	C	0.073	16	3750623	8.21E-06	0.016	14306	20.075
Vascular dementia (mixed)	genus Escherichia Shigella	rs113127095	A	G	0.171	22679253	0.415	0.210	360421	A	G	0.151	13	23253392	3.33E-06	0.032	14306	21.799
Vascular dementia (mixed)	genus Escherichia Shigella	rs113513883	A	G	0.241	140978399	0.315	0.240	360421	A	G	0.172	7	140678199	5.28E-06	0.038	14306	20.511
Vascular dementia (mixed)	genus Escherichia Shigella	rs1154904	G	A	-0.076	134904951	0.368	0.084	360421	G	A	0.061	11	134774845	3.04E-06	0.013	14306	22.043
Vascular dementia (mixed)	genus Escherichia Shigella	rs118526	A	C	-0.034	80567725	0.708	0.092	360421	A	C	0.059	5	79863544	8.00E-06	0.014	14306	19.108
Vascular dementia (mixed)	genus Escherichia Shigella	rs2798105	A	G	0.162	48496231	0.259	0.143	360421	A	G	-0.101	1	48961903	8.24E-06	0.022	14306	20.629
Vascular dementia (mixed)	genus Escherichia Shigella	rs4731451	G	A	-0.079	128414046	0.384	0.090	360421	G	A	-0.061	7	128054100	7.47E-06	0.014	14306	20.362
Vascular dementia (mixed)	genus Escherichia Shigella	rs57024273	T	C	0.080	235606511	0.405	0.097	360421	T	C	0.063	2	236515155	9.70E-06	0.014	14306	20.028
Vascular dementia (mixed)	genus Escherichia Shigella	rs592299	T	C	-0.026	133303865	0.763	0.085	360421	T	C	-0.059	9	136179347	4.77E-06	0.013	14306	20.903
Vascular dementia (mixed)	genus Escherichia Shigella	rs73208162	A	G	-0.325	37952181	0.181	0.243	360421	A	G	-0.119	21	39324484	2.19E-06	0.025	14306	23.067
Vascular dementia (mixed)	genus Eubacterium brachy group	rs112617308	T	C	0.065	92425456	0.657	0.146	360421	T	C	-0.171	10	94185213	2.38E-06	0.036	14306	22.177
Vascular dementia (mixed)	genus Eubacterium brachy group	rs12151423	G	A	-0.020	217372558	0.812	0.084	360421	G	A	-0.101	2	218237281	9.27E-06	0.023	14306	19.869
Vascular dementia (mixed)	genus Eubacterium brachy group	rs13139592	T	C	-0.065	143744025	0.601	0.124	360421	T	C	-0.146	4	144665178	7.97E-06	0.033	14306	19.911
Vascular dementia (mixed)	genus Eubacterium brachy group	rs1384962	G	A	-0.056	22591645	0.536	0.091	360421	G	A	-0.121	14	23060552	6.99E-06	0.027	14306	20.613
Vascular dementia (mixed)	genus Eubacterium brachy group	rs2913110	T	C	0.009	22603738	0.919	0.089	360421	T	C	-0.105	10	22892667	4.56E-06	0.023	14306	21.004
Vascular dementia (mixed)	genus Eubacterium brachy group	rs4862235	A	G	0.054	183707778	0.528	0.085	360421	A	G	-0.105	4	184628931	3.73E-06	0.023	14306	21.553
Vascular dementia (mixed)	genus Eubacterium brachy group	rs62348779	T	C	0.376	17459987	0.018	0.160	360421	T	C	-0.201	5	17460096	3.78E-06	0.043	14306	21.666
Vascular dementia (mixed)	genus Eubacterium brachy group	rs6591893	A	G	0.193	80525789	0.030	0.089	360421	A	G	-0.108	11	80236833	7.34E-06	0.024	14306	20.281
Vascular dementia (mixed)	genus Eubacterium brachy group	rs720439	G	A	0.044	47794849	0.656	0.098	360421	G	A	0.112	22	48190598	7.03E-06	0.025	14306	19.845
Vascular dementia (mixed)	genus Eubacterium brachy group	rs73199919	T	C	-0.382	6854837	0.052	0.197	360421	T	C	-0.237	4	6856564	8.16E-06	0.053	14306	19.848

Vascular dementia (mixed)	genus Eubacterium coprostanoligenes group	rs1020520	T	G	0.006	33563534	0.963	0.119	360421	T	G	-0.059	7	33603146	8.89E-06	0.013	14306	19.747
Vascular dementia (mixed)	genus Eubacterium coprostanoligenes group	rs10444197	A	G	-0.039	2173737	0.653	0.088	360421	A	G	-0.051	10	2215931	5.98E-06	0.011	14306	19.877
Vascular dementia (mixed)	genus Eubacterium coprostanoligenes group	rs11052069	C	T	0.034	32560985	0.685	0.085	360421	C	T	-0.048	12	32713919	9.38E-06	0.011	14306	19.636
Vascular dementia (mixed)	genus Eubacterium coprostanoligenes group	rs11720857	C	T	0.140	114075660	0.203	0.110	360421	C	T	0.063	3	113794507	9.26E-06	0.014	14306	19.059
Vascular dementia (mixed)	genus Eubacterium coprostanoligenes group	rs12906958	C	T	-0.060	36619397	0.519	0.092	360421	C	T	-0.053	15	36911598	4.35E-06	0.012	14306	21.149
Vascular dementia (mixed)	genus Eubacterium coprostanoligenes group	rs17159861	C	T	0.068	31045547	0.618	0.137	360421	C	T	0.096	7	31085162	1.04E-08	0.017	14306	32.654
Vascular dementia (mixed)	genus Eubacterium coprostanoligenes group	rs2644213	A	G	-0.079	82746340	0.387	0.091	360421	A	G	-0.054	10	84506096	9.86E-06	0.012	14306	19.751
Vascular dementia (mixed)	genus Eubacterium coprostanoligenes group	rs4076415	G	T	-0.035	85897766	0.687	0.088	360421	G	T	-0.052	15	86440997	1.99E-06	0.011	14306	21.816
Vascular dementia (mixed)	genus Eubacterium coprostanoligenes group	rs62024432	C	T	-0.089	97326063	0.540	0.144	360421	C	T	-0.077	15	97869293	7.50E-06	0.017	14306	20.002
Vascular dementia (mixed)	genus Eubacterium coprostanoligenes group	rs6762473	A	C	-0.078	127420731	0.380	0.089	360421	A	C	-0.052	3	127139574	4.26E-06	0.011	14306	21.552
Vascular dementia (mixed)	genus Eubacterium coprostanoligenes group	rs76898927	G	A	0.114	81546326	0.542	0.186	360421	G	A	0.123	3	81595477	4.79E-06	0.027	14306	21.341
Vascular dementia (mixed)	genus Eubacterium coprostanoligenes group	rs9648214	T	C	-0.168	16341181	0.261	0.150	360421	T	C	-0.083	7	16380806	2.52E-07	0.016	14306	25.438
Vascular dementia (mixed)	genus Eubacterium eligens group	rs182318	G	A	0.072	31077144	0.638	0.154	360421	G	A	-0.082	11	31098691	8.40E-06	0.020	14306	17.784
Vascular dementia (mixed)	genus Eubacterium eligens group	rs2200429	A	G	0.301	106911110	0.033	0.141	360421	A	G	-0.089	13	107563458	5.30E-06	0.020	14306	20.054
Vascular dementia (mixed)	genus Eubacterium eligens group	rs265534	T	G	-0.070	81332251	0.407	0.085	360421	T	G	-0.056	10	83092007	2.27E-06	0.012	14306	22.024
Vascular dementia (mixed)	genus Eubacterium eligens group	rs4583233	A	C	0.007	81786881	0.936	0.093	360421	A	C	0.067	16	81820486	2.84E-07	0.013	14306	27.363
Vascular dementia (mixed)	genus Eubacterium eligens group	rs56080211	T	C	-0.321	109503416	0.042	0.158	360421	T	C	-0.123	2	110260993	9.14E-06	0.028	14306	18.953
Vascular dementia (mixed)	genus Eubacterium eligens group	rs6923695	T	G	0.036	70247112	0.831	0.168	360421	T	G	0.103	6	70956815	4.87E-06	0.023	14306	20.243
Vascular dementia (mixed)	genus Eubacterium fissicatena group	rs10147907	T	G	0.224	89018029	0.164	0.161	360421	T	G	0.172	14	89484373	8.27E-06	0.040	14306	18.922
Vascular dementia (mixed)	genus Eubacterium fissicatena group	rs11818408	G	A	-0.124	94998710	0.147	0.086	360421	G	A	0.106	10	96758467	8.20E-06	0.024	14306	19.928
Vascular dementia (mixed)	genus Eubacterium fissicatena group	rs11876297	T	C	-0.061	48226901	0.523	0.095	360421	T	C	0.131	18	45753272	2.67E-06	0.028	14306	21.779
Vascular dementia (mixed)	genus Eubacterium fissicatena group	rs151257695	A	G	-0.271	73629231	0.097	0.164	360421	A	G	0.210	7	73043561	3.10E-06	0.045	14306	21.217
Vascular dementia (mixed)	genus Eubacterium fissicatena group	rs1768152	C	T	0.110	39563463	0.425	0.137	360421	C	T	-0.139	3	39604954	8.70E-06	0.032	14306	19.462
Vascular dementia (mixed)	genus Eubacterium fissicatena group	rs2733072	G	A	-0.041	5576177	0.622	0.084	360421	G	A	0.110	8	5433699	1.49E-06	0.023	14306	23.064
Vascular dementia (mixed)	genus Eubacterium fissicatena group	rs3771393	T	C	0.017	70918116	0.874	0.106	360421	T	C	-0.131	2	71145246	7.38E-07	0.027	14306	24.074
Vascular dementia (mixed)	genus Eubacterium fissicatena group	rs6934739	A	G	0.035	39972698	0.697	0.090	360421	A	G	0.111	6	39940437	9.75E-06	0.025	14306	19.442
Vascular dementia (mixed)	genus Eubacterium fissicatena group	rs7104872	G	A	0.009	115294391	0.947	0.134	360421	G	A	0.139	11	115165111	2.73E-06	0.029	14306	22.548
Vascular dementia (mixed)	genus Eubacterium hallii group	rs10501370	C	T	0.075	58273149	0.670	0.175	360421	C	T	-0.116	11	58040621	5.42E-06	0.025	14306	20.958
Vascular dementia (mixed)	genus Eubacterium hallii group	rs10798999	C	T	-0.055	33843316	0.562	0.095	360421	C	T	0.060	1	34308917	2.61E-06	0.013	14306	22.532
Vascular dementia (mixed)	genus Eubacterium hallii group	rs117748144	T	C	-0.190	11750090	0.320	0.191	360421	T	C	-0.127	11	11771637	7.86E-06	0.029	14306	19.436
Vascular dementia (mixed)	genus Eubacterium hallii group	rs13116360	T	C	-0.080	110964275	0.636	0.170	360421	T	C	0.154	4	111885431	2.94E-07	0.030	14306	26.896
Vascular dementia (mixed)	genus Eubacterium hallii group	rs17074066	T	C	0.144	182788383	0.626	0.295	360421	T	C	-0.081	4	183709536	9.35E-06	0.019	14306	18.495
Vascular dementia (mixed)	genus Eubacterium hallii group	rs17474256	G	A	0.233	103982054	0.098	0.140	360421	G	A	0.081	1	104524676	9.45E-06	0.018	14306	19.297
Vascular dementia (mixed)	genus Eubacterium hallii group	rs281379	A	G	0.068	48711017	0.424	0.085	360421	A	G	-0.050	19	49214274	9.33E-06	0.011	14306	19.838
Vascular dementia (mixed)	genus Eubacterium hallii group	rs28584818	A	G	-0.316	64678780	0.042	0.155	360421	A	G	0.126	3	64664456	4.43E-06	0.027	14306	22.041
Vascular dementia (mixed)	genus Eubacterium hallii group	rs60254196	G	A	-0.027	149159628	0.751	0.084	360421	G	A	0.052	7	148856720	2.70E-06	0.011	14306	21.844
Vascular dementia (mixed)	genus Eubacterium hallii group	rs630939	C	T	-0.021	50858093	0.804	0.085	360421	C	T	-0.051	18	48384463	9.16E-06	0.011	14306	19.806
Vascular dementia (mixed)	genus Eubacterium hallii group	rs6550770	C	T	-0.283	23621925	0.182	0.212	360421	C	T	0.198	3	23663416	4.82E-06	0.044	14306	19.945
Vascular dementia (mixed)	genus Eubacterium hallii group	rs74018587	C	T	-0.061	61721961	0.774	0.214	360421	C	T	0.209	15	62014160	3.70E-06	0.044	14306	22.734
Vascular dementia (mixed)	genus Eubacterium hallii group	rs78056098	G	T	0.066	123919170	0.452	0.087	360421	G	T	-0.051	11	123789877	8.29E-06	0.011	14306	19.896
Vascular dementia (mixed)	genus Eubacterium hallii group	rs949971	T	G	-0.071	110564542	0.429	0.090	360421	T	G	-0.054	3	110283389	3.29E-06	0.012	14306	21.646
Vascular dementia (mixed)	genus Eubacterium nodatum group	rs10263623	C	T	-0.190	65429224	0.374	0.214	360421	C	T	0.193	7	64894137	8.91E-06	0.044	14306	19.425
Vascular dementia (mixed)	genus Eubacterium nodatum group	rs10458299	T	C	-0.356	135367596	0.025	0.158	360421	T	C	-0.188	7	135052348	8.37E-06	0.042	14306	20.013
Vascular dementia (mixed)	genus Eubacterium nodatum group	rs11006576	A	G	0.009	59562257	0.917	0.085	360421	A	G	-0.110	10	61322015	7.99E-06	0.025	14306	20.067
Vascular dementia (mixed)	genus Eubacterium nodatum group	rs113893692	C	T	-0.133	28245520	0.302	0.129	360421	C	T	-0.185	9	28245518	5.76E-06	0.040	14306	21.038
Vascular dementia (mixed)	genus Eubacterium nodatum group	rs34297067	A	G	0.049	51719170	0.677	0.118	360421	A	G	-0.187	14	52185888	6.60E-08	0.034	14306	29.959
Vascular dementia (mixed)	genus Eubacterium nodatum group	rs61841040	G	T	0.141	9617786	0.181	0.105	360421	G	T	0.161	10	9659749	3.56E-06	0.034	14306	22.108
Vascular dementia (mixed)	genus Eubacterium nodatum group	rs6818880	G	A	-0.029	93791596	0.730	0.084	360421	G	A	0.110	4	94712747	7.83E-06	0.025	14306	20.030
Vascular dementia (mixed)	genus Eubacterium nodatum group	rs77910827	C	T	0.201	93835703	0.142	0.137	360421	C	T	0.202	9	96597985	9.05E-07	0.041	14306	23.807
Vascular dementia (mixed)	genus Eubacterium nodatum group	rs7827125	C	T	-0.047	5097390	0.617	0.093	360421	C	T	0.122	8	4954912	7.17E-06	0.027	14306	20.317
Vascular dementia (mixed)	genus Eubacterium nodatum group	rs7880204	T	C	0.043	171091680	0.657	0.097	360421	T	C	-0.125	1	171060821	6.84E-06	0.028	14306	20.766
Vascular dementia (mixed)	genus Eubacterium nodatum group	rs9425984	T	C	0.146	34064135	0.151	0.102	360421	T	C	-0.130	1	34529736	7.21E-06	0.029	14306	19.845
Vascular dementia (mixed)	genus Eubacterium oxidoreducens group	rs12129908	A	C	0.020	195094889	0.816	0.086	360421	A	C	-0.089	1	195064019	5.80E-06	0.020	14306	20.285
Vascular dementia (mixed)	genus Eubacterium oxidoreducens group	rs12423772	G	T	-0.048	94121564	0.689	0.119	360421	G	T	0.141	12	94515340	2.63E-06	0.030	14306	22.834
Vascular dementia (mixed)	genus Eubacterium oxidoreducens group	rs2973294	G	T	0.037	37525057	0.668	0.085	360421	G	T	0.092	4	37526679	2.39E-06	0.020	14306	22.331
Vascular dementia (mixed)	genus Eubacterium oxidoreducens group	rs34561138	G	A	0.043	86446749	0.843	0.217	360421	G	A	0.216	16	86480355	2.51E-06	0.046	14306	22.086
Vascular dementia (mixed)	genus Eubacterium oxidoreducens group	rs440215	T	C	0.017	107546693	0.846	0.085	360421	T	C	-0.093	5	106882394	1.65E-06	0.020	14306	22.812
Vascular dementia (mixed)	genus Eubacterium rectale group	rs10248854	C	A	0.145	121641184	0.091	0.086	360421	C	A	-0.053	7	121281238	4.21E-06	0.011	14306	21.640
Vascular dementia (mixed)	genus Eubacterium rectale group	rs10797540	A	G	0.078	234394624	0.361	0.085	360421	A	G	0.050	1	234530370	3.53E-06	0.011	14306	21.551
Vascular dementia (mixed)	genus Eubacterium rectale group	rs143694765	T	C	-0.307	115540288	0.029	0.141	360421	T	C	0.087	1	116082909	9.75E-06	0.020	14306	19.349
Vascular dementia (mixed)	genus Eubacterium rectale group	rs2884897	A	G	0.113	8107990	0.628	0.233	360421	A	G	-0.129	11	8129537	6.44E-06	0.029	14306	20.034
Vascular dementia (mixed)	genus Eubacterium rectale group	rs314726	T	C	-0.054	124795578	0.522	0.085	360421	T	C	0.053	2	125553155	1.38E-06	0.011	14306	23.329
Vascular dementia (mixed)	genus Eubacterium rectale group	rs35398954	A	G	-0.097	92463535	0.394	0.114	360421	A	G	-0.090	15	93006765	5.40E-07	0.017	14306	26.653
Vascular dementia (mixed)	genus Eubacterium rectale group	rs59427698	A	G	-0.274	126663466	0.010	0.107	360421	A	G	-0.058	9	129425745	5.37E-06	0.013	14306	19.379
Vascular dementia (mixed)	genus Eubacterium rectale group	rs62547233	A	G	0.178	89398504	0.055	0.093	360421	A	G	0.054	9	92013419	9.90E-06	0.012	14306	19.908
Vascular dementia (mixed)	genus Eubacterium ruminantium group	rs10131724	C	A	-0.096	51752348	0.503	0.143	360421	C	A	0.200	14	52219066	2.39E-06	0.041	14306	23.234
Vascular dementia (mixed)	genus Eubacterium ruminantium group	rs10923018	G	A	-0.037	88057716	0.665	0.085	360421	G	A	0.073	1	88523399	6.80E-06	0.016	14306	20.378

Vascular dementia (mixed)	genus Eubacterium ruminantium group	rs11637981	T	G	-0.085	60997423	0.314	0.084	360421	T	G	0.073	15	61289622	5.44E-06	0.016	14306	20.733
Vascular dementia (mixed)	genus Eubacterium ruminantium group	rs13025464	C	T	0.077	200745922	0.369	0.086	360421	C	T	0.074	2	201610645	6.97E-06	0.016	14306	20.252
Vascular dementia (mixed)	genus Eubacterium ruminantium group	rs139749	C	T	0.025	24907088	0.778	0.089	360421	C	T	-0.085	22	25303055	8.59E-07	0.017	14306	24.219
Vascular dementia (mixed)	genus Eubacterium ruminantium group	rs16891896	G	A	-0.227	33897484	0.133	0.151	360421	G	A	-0.175	5	33897589	2.38E-06	0.039	14306	20.027
Vascular dementia (mixed)	genus Eubacterium ruminantium group	rs17519472	C	T	-0.063	30208021	0.605	0.121	360421	C	T	0.108	12	30360954	4.70E-06	0.023	14306	21.227
Vascular dementia (mixed)	genus Eubacterium ruminantium group	rs209813	G	A	0.125	11953400	0.295	0.119	360421	G	A	-0.103	6	11953633	9.23E-06	0.024	14306	19.165
Vascular dementia (mixed)	genus Eubacterium ruminantium group	rs2116427	A	G	0.046	121847301	0.632	0.096	360421	A	G	0.091	5	121182996	4.67E-07	0.018	14306	24.983
Vascular dementia (mixed)	genus Eubacterium ruminantium group	rs2229917	A	G	-0.265	128218658	0.203	0.208	360421	A	G	0.154	9	130980937	2.16E-06	0.032	14306	22.467
Vascular dementia (mixed)	genus Eubacterium ruminantium group	rs2418654	C	T	0.113	71020448	0.192	0.087	360421	C	T	-0.075	2	71247578	6.17E-06	0.017	14306	20.388
Vascular dementia (mixed)	genus Eubacterium ruminantium group	rs2817174	C	T	0.043	3127617	0.615	0.086	360421	C	T	-0.073	1	3044181	7.87E-06	0.016	14306	20.125
Vascular dementia (mixed)	genus Eubacterium ruminantium group	rs57340348	T	C	0.033	129708969	0.756	0.105	360421	T	C	-0.098	6	130030114	4.93E-06	0.021	14306	21.311
Vascular dementia (mixed)	genus Eubacterium ruminantium group	rs606117	G	A	-0.028	2539358	0.764	0.094	360421	G	A	-0.083	9	2539358	4.82E-06	0.018	14306	21.296
Vascular dementia (mixed)	genus Eubacterium ruminantium group	rs6676699	T	G	0.045	198009151	0.627	0.093	360421	T	G	0.089	1	197978281	6.38E-06	0.020	14306	20.439
Vascular dementia (mixed)	genus Eubacterium ruminantium group	rs7000472	G	A	-0.014	137614025	0.869	0.085	360421	G	A	0.076	8	138626268	4.07E-06	0.017	14306	21.284
Vascular dementia (mixed)	genus Eubacterium ruminantium group	rs72836424	C	T	0.171	127239189	0.200	0.134	360421	C	T	-0.140	10	129037453	2.62E-06	0.030	14306	21.624
Vascular dementia (mixed)	genus Eubacterium ruminantium group	rs73139629	A	C	-0.234	62912223	0.107	0.145	360421	A	C	-0.115	12	63306003	5.36E-06	0.025	14306	21.555
Vascular dementia (mixed)	genus Eubacterium ventriosum group	rs11617697	A	G	0.021	98133646	0.909	0.186	360421	A	G	-0.143	13	98785900	7.22E-07	0.029	14306	25.080
Vascular dementia (mixed)	genus Eubacterium ventriosum group	rs12964517	G	A	-0.071	24880821	0.454	0.094	360421	G	A	0.059	18	22460785	2.08E-06	0.012	14306	22.624
Vascular dementia (mixed)	genus Eubacterium ventriosum group	rs13082419	C	T	-0.090	108017587	0.499	0.133	360421	C	T	-0.072	3	107736434	9.56E-06	0.016	14306	19.661
Vascular dementia (mixed)	genus Eubacterium ventriosum group	rs16884680	G	T	0.071	113432826	0.611	0.139	360421	G	T	-0.091	8	114445055	1.74E-06	0.019	14306	22.309
Vascular dementia (mixed)	genus Eubacterium ventriosum group	rs35179274	C	T	0.026	51493907	0.808	0.109	360421	C	T	-0.063	14	51960625	5.76E-06	0.014	14306	20.703
Vascular dementia (mixed)	genus Eubacterium ventriosum group	rs3809430	T	C	0.055	44506307	0.550	0.092	360421	T	C	-0.055	14	44975510	3.55E-06	0.012	14306	21.426
Vascular dementia (mixed)	genus Eubacterium ventriosum group	rs57199565	T	C	-0.103	190505943	0.342	0.108	360421	T	C	0.078	3	190223732	7.97E-07	0.016	14306	23.896
Vascular dementia (mixed)	genus Eubacterium ventriosum group	rs66746423	C	T	0.057	108855642	0.631	0.117	360421	C	T	0.075	1	109398264	6.11E-06	0.016	14306	20.749
Vascular dementia (mixed)	genus Eubacterium ventriosum group	rs6704822	G	A	-0.011	202797056	0.932	0.125	360421	G	A	-0.074	2	203661779	6.62E-06	0.017	14306	19.661
Vascular dementia (mixed)	genus Eubacterium ventriosum group	rs72783037	C	A	0.080	60314477	0.443	0.104	360421	C	A	0.066	16	60348381	6.55E-06	0.014	14306	21.012
Vascular dementia (mixed)	genus Eubacterium ventriosum group	rs73615400	T	C	-0.075	51524380	0.608	0.145	360421	T	C	-0.096	20	50140919	9.54E-07	0.019	14306	24.443
Vascular dementia (mixed)	genus Eubacterium ventriosum group	rs73849225	T	C	0.191	125450659	0.198	0.149	360421	T	C	0.098	4	126371814	5.21E-06	0.022	14306	18.908
Vascular dementia (mixed)	genus Eubacterium ventriosum group	rs78250280	G	A	0.152	147819813	0.211	0.122	360421	G	A	0.075	1	147291928	3.36E-06	0.016	14306	20.798
Vascular dementia (mixed)	genus Eubacterium ventriosum group	rs876734	T	C	0.024	52030212	0.800	0.094	360421	T	C	0.062	12	52423996	2.89E-06	0.013	14306	21.793
Vascular dementia (mixed)	genus Eubacterium ventriosum group	rs9316536	T	G	-0.095	51364440	0.435	0.122	360421	T	G	-0.082	13	51938576	7.84E-06	0.018	14306	19.911
Vascular dementia (mixed)	genus Eubacterium xylanophilum group	rs10140184	A	C	-0.141	72715075	0.098	0.085	360421	A	C	0.058	14	73181783	4.96E-06	0.013	14306	20.959
Vascular dementia (mixed)	genus Eubacterium xylanophilum group	rs10917203	A	C	0.064	22300773	0.460	0.086	360421	A	C	0.061	1	22627266	3.15E-06	0.013	14306	21.898
Vascular dementia (mixed)	genus Eubacterium xylanophilum group	rs112176119	C	T	0.171	73662872	0.239	0.145	360421	C	T	-0.113	16	73696771	3.33E-06	0.025	14306	21.312
Vascular dementia (mixed)	genus Eubacterium xylanophilum group	rs13239072	G	A	0.076	43526745	0.419	0.094	360421	G	A	0.069	7	43566344	1.82E-06	0.014	14306	23.197
Vascular dementia (mixed)	genus Eubacterium xylanophilum group	rs17830032	G	A	-0.148	59420451	0.340	0.156	360421	G	A	-0.161	20	57995506	2.39E-07	0.031	14306	26.749
Vascular dementia (mixed)	genus Eubacterium xylanophilum group	rs1999224	G	T	0.021	127735841	0.884	0.142	360421	G	T	-0.095	9	130498120	3.75E-06	0.020	14306	21.749
Vascular dementia (mixed)	genus Eubacterium xylanophilum group	rs2012708	G	A	0.025	84756953	0.779	0.088	360421	G	A	-0.057	16	84790559	6.53E-06	0.013	14306	20.401
Vascular dementia (mixed)	genus Eubacterium xylanophilum group	rs2213117	T	G	0.040	131139765	0.732	0.116	360421	T	G	0.088	11	131009660	4.21E-06	0.019	14306	21.568
Vascular dementia (mixed)	genus Eubacterium xylanophilum group	rs75586835	A	G	-0.378	86970786	0.020	0.162	360421	A	G	-0.114	4	87891938	9.39E-06	0.026	14306	18.888
Vascular dementia (mixed)	genus Faecalibacterium	rs10927394	G	T	-0.055	245104037	0.862	0.314	360421	G	T	-0.232	1	245267339	7.02E-06	0.051	14306	20.549
Vascular dementia (mixed)	genus Faecalibacterium	rs114946999	C	T	-0.070	36898092	0.580	0.127	360421	C	T	-0.086	2	37125235	5.70E-06	0.019	14306	20.649
Vascular dementia (mixed)	genus Faecalibacterium	rs11776390	T	C	0.212	38736127	0.224	0.174	360421	T	C	-0.078	8	38593645	6.40E-06	0.017	14306	20.793
Vascular dementia (mixed)	genus Faecalibacterium	rs1271565	C	T	-0.200	63577379	0.037	0.096	360421	C	T	-0.058	14	64044097	1.30E-06	0.012	14306	23.196
Vascular dementia (mixed)	genus Faecalibacterium	rs12753492	A	C	0.087	13875051	0.518	0.134	360421	A	C	0.064	1	14201546	8.80E-06	0.015	14306	18.295
Vascular dementia (mixed)	genus Faecalibacterium	rs2835874	T	C	-0.262	37655599	0.255	0.230	360421	T	C	-0.087	21	39027901	7.54E-06	0.020	14306	19.440
Vascular dementia (mixed)	genus Faecalibacterium	rs6910935	G	A	-0.163	131266017	0.353	0.176	360421	G	A	-0.135	6	131587157	1.38E-06	0.028	14306	23.699
Vascular dementia (mixed)	genus Faecalibacterium	rs75499067	C	T	0.032	6049862	0.839	0.160	360421	C	T	0.228	16	6099863	1.76E-06	0.047	14306	23.900
Vascular dementia (mixed)	genus Faecalibacterium	rs79656633	T	C	-0.083	110494205	0.554	0.141	360421	T	C	0.146	1	111036827	8.14E-06	0.032	14306	20.326
Vascular dementia (mixed)	genus Faecalibacterium	rs9536330	T	C	0.140	52992882	0.095	0.084	360421	T	C	-0.048	13	53567017	5.33E-06	0.011	14306	20.026
Vascular dementia (mixed)	genus Family XIII AD3011 group	rs11126423	T	C	0.028	73968424	0.849	0.147	360421	T	C	-0.090	2	74195551	5.91E-06	0.020	14306	21.226
Vascular dementia (mixed)	genus Family XIII AD3011 group	rs11736617	G	A	-0.184	38916209	0.310	0.182	360421	G	A	-0.076	4	38917830	9.02E-06	0.017	14306	19.475
Vascular dementia (mixed)	genus Family XIII AD3011 group	rs12812672	T	C	-0.138	17974663	0.399	0.163	360421	T	C	-0.096	12	18127597	2.56E-06	0.021	14306	21.273
Vascular dementia (mixed)	genus Family XIII AD3011 group	rs149302	T	C	-0.109	14159816	0.269	0.099	360421	T	C	-0.065	5	14159925	7.48E-06	0.014	14306	20.321
Vascular dementia (mixed)	genus Family XIII AD3011 group	rs16840310	G	A	-0.107	240530863	0.212	0.086	360421	G	A	0.061	1	240694163	6.75E-07	0.012	14306	24.780
Vascular dementia (mixed)	genus Family XIII AD3011 group	rs16940167	C	T	0.028	58391498	0.792	0.107	360421	C	T	0.073	15	58683697	3.91E-06	0.016	14306	20.989
Vascular dementia (mixed)	genus Family XIII AD3011 group	rs17156849	G	A	-0.349	28564367	0.051	0.178	360421	G	A	-0.113	7	28603985	4.19E-06	0.025	14306	21.181
Vascular dementia (mixed)	genus Family XIII AD3011 group	rs62029761	A	G	0.179	15912385	0.343	0.188	360421	A	G	0.129	16	16006242	3.89E-06	0.028	14306	21.762
Vascular dementia (mixed)	genus Family XIII AD3011 group	rs62200412	C	T	-0.081	4637993	0.401	0.096	360421	C	T	-0.080	20	4618639	5.80E-07	0.016	14306	23.894
Vascular dementia (mixed)	genus Family XIII AD3011 group	rs72730932	C	A	0.184	192050705	0.204	0.145	360421	C	A	-0.090	1	192019835	6.89E-07	0.018	14306	25.798
Vascular dementia (mixed)	genus Family XIII AD3011 group	rs739451	C	T	-0.157	133813832	0.127	0.103	360421	C	T	0.065	9	136678954	7.88E-06	0.015	14306	19.386
Vascular dementia (mixed)	genus Family XIII AD3011 group	rs9276029	A	G	-0.137	32727559	0.193	0.106	360421	A	G	-0.081	6	32695336	8.93E-06	0.019	14306	19.097
Vascular dementia (mixed)	genus Family XIII AD3011 group	rs9837139	A	G	-0.021	29770374	0.888	0.150	360421	A	G	0.108	3	29811865	8.71E-06	0.024	14306	19.990
Vascular dementia (mixed)	genus Family XIII UCG001	rs112362903	A	G	-0.282	35434008	0.232	0.236	360421	A	G	-0.149	17	33761027	7.88E-06	0.033	14306	20.001
Vascular dementia (mixed)	genus Family XIII UCG001	rs12049454	T	C	0.123	84762642	0.155	0.087	360421	T	C	-0.065	1	85228325	1.17E-06	0.013	14306	23.312
Vascular dementia (mixed)	genus Family XIII UCG001	rs1426266	C	T	0.113	188506308	0.231	0.095	360421	C	T	0.067	3	188224096	1.25E-06	0.014	14306	23.546
Vascular dementia (mixed)	genus Family XIII UCG001	rs3842897	G	A	0.097	183534529	0.510	0.148	360421	G	A	-0.113	1	183503664	5.20E-06	0.024	14306	21.531

Vascular dementia (mixed)	genus Family XIII UCG001	rs62414802	C	T	-0.124	75888918	0.199	0.097	360421	C	T	-0.061	6	76598635	4.29E-06	0.013	14306	20.676
Vascular dementia (mixed)	genus Family XIII UCG001	rs7119679	G	A	-0.040	94948621	0.685	0.099	360421	G	A	-0.081	11	94681786	3.52E-06	0.017	14306	21.429
Vascular dementia (mixed)	genus Family XIII UCG001	rs76463770	A	G	-0.483	45437598	0.043	0.239	360421	A	G	0.193	3	45479090	3.77E-06	0.042	14306	21.158
Vascular dementia (mixed)	genus Family XIII UCG001	rs8076666	G	A	-0.048	80250018	0.710	0.130	360421	G	A	-0.089	17	78223817	8.02E-06	0.020	14306	20.029
Vascular dementia (mixed)	genus Flavonifractor	rs114873521	C	T	0.012	168791398	0.944	0.166	360421	C	T	-0.130	5	168218403	7.13E-06	0.029	14306	19.561
Vascular dementia (mixed)	genus Flavonifractor	rs11811696	T	C	0.059	237188054	0.698	0.152	360421	T	C	-0.116	1	237351354	2.07E-06	0.024	14306	23.191
Vascular dementia (mixed)	genus Flavonifractor	rs12030302	G	A	0.038	77422150	0.651	0.084	360421	G	A	0.069	1	77887835	5.61E-07	0.014	14306	25.361
Vascular dementia (mixed)	genus Flavonifractor	rs34066017	A	G	0.054	44828138	0.602	0.104	360421	A	G	0.076	11	44849689	1.52E-06	0.016	14306	22.878
Vascular dementia (mixed)	genus Flavonifractor	rs806808	C	T	0.069	32092258	0.418	0.085	360421	C	T	-0.067	10	32381186	1.18E-06	0.014	14306	23.877
Vascular dementia (mixed)	genus Fusicatenibacter	rs10439674	A	G	-0.204	41309465	0.054	0.106	360421	A	G	-0.057	21	42681392	7.68E-06	0.013	14306	19.367
Vascular dementia (mixed)	genus Fusicatenibacter	rs167879	T	C	-0.092	57362310	0.436	0.118	360421	T	C	0.066	20	55937366	5.87E-06	0.015	14306	19.656
Vascular dementia (mixed)	genus Fusicatenibacter	rs1864685	A	C	0.033	72725643	0.701	0.086	360421	A	C	-0.049	17	70721782	4.96E-06	0.011	14306	20.949
Vascular dementia (mixed)	genus Fusicatenibacter	rs2025938	G	A	-0.197	110417361	0.243	0.169	360421	G	A	-0.097	10	112177119	2.99E-06	0.021	14306	22.172
Vascular dementia (mixed)	genus Fusicatenibacter	rs206581	A	G	-0.209	10370599	0.041	0.103	360421	A	G	-0.057	18	10370596	8.96E-06	0.013	14306	19.748
Vascular dementia (mixed)	genus Fusicatenibacter	rs2132128	G	A	-0.105	15503209	0.449	0.139	360421	G	A	-0.077	8	15360718	1.08E-06	0.016	14306	23.176
Vascular dementia (mixed)	genus Fusicatenibacter	rs3303	T	C	0.204	118687917	0.232	0.171	360421	T	C	-0.095	10	120447429	3.94E-06	0.020	14306	21.839
Vascular dementia (mixed)	genus Fusicatenibacter	rs4378146	A	C	0.018	24601134	0.854	0.097	360421	A	C	-0.062	1	24927625	7.20E-07	0.013	14306	24.239
Vascular dementia (mixed)	genus Fusicatenibacter	rs60254196	G	A	-0.027	149159628	0.751	0.084	360421	G	A	0.049	7	148856720	5.47E-06	0.011	14306	20.273
Vascular dementia (mixed)	genus Fusicatenibacter	rs62187631	T	C	0.002	225784854	0.988	0.106	360421	T	C	-0.071	2	226649570	4.55E-06	0.016	14306	19.912
Vascular dementia (mixed)	genus Fusicatenibacter	rs62353480	A	G	0.136	29594893	0.226	0.112	360421	A	G	-0.070	5	29595000	1.57E-06	0.015	14306	23.210
Vascular dementia (mixed)	genus Fusicatenibacter	rs6515626	G	A	0.052	25276365	0.757	0.170	360421	G	A	0.142	20	25257001	7.29E-06	0.031	14306	20.386
Vascular dementia (mixed)	genus Fusicatenibacter	rs704418	C	T	-0.133	64267127	0.298	0.128	360421	C	T	-0.074	3	64252803	7.77E-07	0.015	14306	23.937
Vascular dementia (mixed)	genus Fusicatenibacter	rs73103914	A	G	-0.035	58264528	0.770	0.118	360421	A	G	-0.060	12	58658311	8.30E-06	0.013	14306	19.735
Vascular dementia (mixed)	genus Fusicatenibacter	rs792108	C	T	-0.078	5392660	0.360	0.085	360421	C	T	0.051	2	5532793	8.50E-06	0.011	14306	19.939
Vascular dementia (mixed)	genus Fusicatenibacter	rs8028026	G	A	0.035	87809984	0.808	0.144	360421	G	A	0.079	15	88353215	8.06E-06	0.018	14306	19.259
Vascular dementia (mixed)	genus Fusicatenibacter	rs8063430	T	C	-0.159	73787906	0.409	0.192	360421	T	C	-0.104	16	73821805	4.93E-06	0.022	14306	21.924
Vascular dementia (mixed)	genus Fusicatenibacter	rs9905659	G	A	0.136	72701205	0.207	0.108	360421	G	A	-0.062	17	70697344	7.31E-06	0.014	14306	20.354
Vascular dementia (mixed)	genus Gordonibacter	rs13412653	A	C	-0.042	29935628	0.634	0.087	360421	A	C	0.108	2	30158494	8.61E-06	0.024	14306	20.229
Vascular dementia (mixed)	genus Gordonibacter	rs16955299	G	A	-0.039	54879849	0.787	0.143	360421	G	A	-0.196	17	52957210	6.37E-06	0.043	14306	20.527
Vascular dementia (mixed)	genus Gordonibacter	rs322296	G	A	0.042	137252442	0.792	0.160	360421	G	A	0.179	7	136937189	4.02E-06	0.038	14306	22.439
Vascular dementia (mixed)	genus Gordonibacter	rs35042269	C	A	-0.045	8140880	0.739	0.134	360421	C	A	-0.180	4	8142607	8.11E-06	0.040	14306	19.985
Vascular dementia (mixed)	genus Gordonibacter	rs3765837	T	G	0.258	210455105	0.117	0.164	360421	T	G	-0.191	1	210628449	7.17E-06	0.043	14306	19.348
Vascular dementia (mixed)	genus Gordonibacter	rs4596722	G	A	-0.037	86172229	0.661	0.084	360421	G	A	-0.103	9	88787144	9.06E-06	0.023	14306	19.748
Vascular dementia (mixed)	genus Gordonibacter	rs71545975	A	G	-0.101	47104907	0.363	0.111	360421	A	G	-0.154	7	47144505	7.04E-06	0.034	14306	20.639
Vascular dementia (mixed)	genus Gordonibacter	rs72714787	C	A	0.071	135847346	0.567	0.125	360421	C	A	0.181	4	136768501	1.43E-06	0.038	14306	23.141
Vascular dementia (mixed)	genus Gordonibacter	rs72939513	A	G	-0.488	82670413	0.011	0.192	360421	A	G	-0.214	1	83136096	7.98E-06	0.049	14306	19.026
Vascular dementia (mixed)	genus Gordonibacter	rs7294633	T	C	-0.015	28871601	0.874	0.094	360421	T	C	-0.129	12	29024534	3.44E-07	0.025	14306	26.501
Vascular dementia (mixed)	genus Gordonibacter	rs768830	A	G	-0.136	18930984	0.249	0.118	360421	A	G	-0.150	7	18970607	7.76E-06	0.033	14306	20.212
Vascular dementia (mixed)	genus Haemophilus	rs10781340	A	G	-0.001	76137254	0.992	0.127	360421	A	G	-0.095	9	78752170	4.32E-06	0.020	14306	21.803
Vascular dementia (mixed)	genus Haemophilus	rs111582866	G	A	-0.120	48708578	0.418	0.148	360421	G	A	-0.124	16	48742489	1.27E-06	0.026	14306	22.815
Vascular dementia (mixed)	genus Haemophilus	rs35509	G	A	0.181	115055532	0.388	0.210	360421	G	A	0.128	12	115493337	2.01E-06	0.027	14306	22.768
Vascular dementia (mixed)	genus Haemophilus	rs4822728	T	C	0.072	26495842	0.389	0.084	360421	T	C	0.071	22	26891808	3.48E-06	0.015	14306	21.740
Vascular dementia (mixed)	genus Haemophilus	rs76022354	C	T	0.485	92546628	0.014	0.197	360421	C	T	0.245	10	94306385	1.83E-06	0.051	14306	23.421
Vascular dementia (mixed)	genus Haemophilus	rs78909003	T	C	-0.199	102887960	0.274	0.182	360421	T	C	-0.246	9	105650242	1.67E-06	0.050	14306	23.881
Vascular dementia (mixed)	genus Haemophilus	rs9328464	T	C	-0.051	8350684	0.549	0.085	360421	T	C	0.072	6	8350917	1.42E-06	0.015	14306	23.615
Vascular dementia (mixed)	genus Haemophilus	rs9382510	C	T	-0.055	55583693	0.567	0.096	360421	C	T	-0.094	6	55448491	7.12E-08	0.017	14306	29.342
Vascular dementia (mixed)	genus Haemophilus	rs9895850	T	C	-0.004	66538895	0.985	0.207	360421	T	C	-0.193	17	64535013	2.14E-06	0.042	14306	21.437
Vascular dementia (mixed)	genus Holdemanella	rs12513188	G	A	-0.131	70135074	0.168	0.095	360421	G	A	0.090	4	71000791	4.65E-06	0.020	14306	21.426
Vascular dementia (mixed)	genus Holdemanella	rs17586763	T	C	-0.146	50357927	0.438	0.189	360421	T	C	-0.227	13	50932063	7.72E-06	0.051	14306	19.850
Vascular dementia (mixed)	genus Holdemanella	rs1926302	G	A	0.033	64687657	0.742	0.102	360421	G	A	-0.108	1	65153340	7.50E-06	0.023	14306	21.768
Vascular dementia (mixed)	genus Holdemanella	rs34187114	C	A	0.014	137611390	0.918	0.134	360421	C	A	-0.105	8	138623633	5.13E-06	0.023	14306	21.381
Vascular dementia (mixed)	genus Holdemanella	rs35228298	G	A	0.169	85343732	0.143	0.115	360421	G	A	0.093	3	85392882	7.30E-06	0.020	14306	21.231
Vascular dementia (mixed)	genus Holdemanella	rs4541991	T	C	-0.021	102242553	0.815	0.090	360421	T	C	-0.093	9	105004835	2.10E-06	0.019	14306	22.747
Vascular dementia (mixed)	genus Holdemanella	rs62113381	T	C	-0.287	7603339	0.023	0.126	360421	T	C	-0.105	19	7668225	5.54E-06	0.023	14306	20.653
Vascular dementia (mixed)	genus Holdemanella	rs73011279	T	C	-0.143	14909283	0.156	0.101	360421	T	C	-0.096	19	15020095	1.36E-06	0.020	14306	23.274
Vascular dementia (mixed)	genus Holdemanella	rs75764681	T	C	-0.096	4452438	0.640	0.205	360421	T	C	-0.283	10	4494630	1.94E-06	0.060	14306	22.338
Vascular dementia (mixed)	genus Holdemanella	rs8113760	G	A	0.053	43347783	0.557	0.091	360421	G	A	0.079	19	43851935	4.62E-06	0.017	14306	20.756
Vascular dementia (mixed)	genus Holdemania	rs10885477	T	C	0.100	113572654	0.607	0.195	360421	T	C	-0.135	10	115332413	8.60E-06	0.030	14306	20.037
Vascular dementia (mixed)	genus Holdemania	rs11080063	G	A	0.060	28462253	0.487	0.086	360421	G	A	-0.067	17	26789271	6.67E-06	0.015	14306	19.711
Vascular dementia (mixed)	genus Holdemania	rs111745969	A	G	-0.036	92566992	0.769	0.122	360421	A	G	0.121	15	93110222	3.71E-06	0.027	14306	20.616
Vascular dementia (mixed)	genus Holdemania	rs113593397	A	G	-0.301	122961600	0.038	0.145	360421	A	G	-0.129	8	123973840	9.36E-06	0.028	14306	20.827
Vascular dementia (mixed)	genus Holdemania	rs116500994	G	T	-0.059	81111408	0.769	0.200	360421	G	T	-0.138	13	81685543	2.34E-06	0.029	14306	21.981
Vascular dementia (mixed)	genus Holdemania	rs12701617	A	G	-0.033	38253256	0.693	0.084	360421	A	G	-0.066	7	38292857	9.52E-06	0.015	14306	19.545
Vascular dementia (mixed)	genus Holdemania	rs1867876	T	C	0.055	18746521	0.550	0.092	360421	T	C	0.084	11	18768068	2.74E-07	0.016	14306	27.009
Vascular dementia (mixed)	genus Holdemania	rs4146507	C	T	-0.067	106873392	0.496	0.098	360421	C	T	0.079	5	106209093	7.23E-06	0.018	14306	20.167
Vascular dementia (mixed)	genus Holdemania	rs73139538	G	A	-0.008	63862767	0.976	0.256	360421	G	A	-0.149	7	63323145	7.77E-06	0.033	14306	20.590
Vascular dementia (mixed)	genus Holdemania	rs77293403	A	G	-0.073	78092582	0.749	0.227	360421	A	G	0.165	5	77388406	1.77E-06	0.034	14306	23.182

Vascular dementia (mixed)	genus Holdemania	rs80149660	C	T	0.159	128110018	0.437	0.205	360421	C	T	-0.233	10	129908282	6.04E-06	0.052	14306	20.138
Vascular dementia (mixed)	genus Holdemania	rs9500080	C	T	0.007	105289447	0.952	0.111	360421	C	T	0.093	6	105737322	4.09E-07	0.018	14306	26.840
Vascular dementia (mixed)	genus Holdemania	rs9529719	C	T	-0.094	70282384	0.294	0.089	360421	C	T	-0.074	13	70856516	5.97E-06	0.016	14306	21.281
Vascular dementia (mixed)	genus Holdemania	rs967319	T	C	-0.044	60629947	0.651	0.098	360421	T	C	0.079	3	60615680	8.38E-06	0.018	14306	19.910
Vascular dementia (mixed)	genus Howardella	rs10048062	C	T	-0.160	97933690	0.280	0.148	360421	C	T	-0.147	15	98476920	8.59E-06	0.034	14306	19.172
Vascular dementia (mixed)	genus Howardella	rs12452946	A	G	0.043	17349974	0.607	0.084	360421	A	G	-0.106	17	17253288	3.80E-06	0.023	14306	21.370
Vascular dementia (mixed)	genus Howardella	rs1484873	G	A	0.191	45627020	0.107	0.118	360421	G	A	0.228	18	43206985	2.56E-06	0.046	14306	24.177
Vascular dementia (mixed)	genus Howardella	rs17167098	G	A	-0.070	133469602	0.569	0.122	360421	G	A	-0.169	7	133154356	1.12E-06	0.035	14306	23.142
Vascular dementia (mixed)	genus Howardella	rs2154047	A	C	0.068	94986460	0.646	0.147	360421	A	C	0.193	14	95452797	9.97E-06	0.042	14306	21.010
Vascular dementia (mixed)	genus Howardella	rs36081916	T	C	-0.009	93898102	0.950	0.150	360421	T	C	-0.181	7	93527414	4.70E-06	0.040	14306	20.225
Vascular dementia (mixed)	genus Howardella	rs3791893	A	G	0.070	217954673	0.562	0.121	360421	A	G	0.147	2	218819396	9.50E-06	0.034	14306	18.677
Vascular dementia (mixed)	genus Howardella	rs609430	T	G	-0.072	168257164	0.413	0.088	360421	T	G	-0.112	4	169178315	3.34E-06	0.024	14306	21.918
Vascular dementia (mixed)	genus Howardella	rs672217	G	A	0.061	62457901	0.563	0.105	360421	G	A	0.164	18	60125134	3.52E-06	0.035	14306	21.996
Vascular dementia (mixed)	genus Hungatella	rs10044993	A	C	-0.019	59128613	0.899	0.151	360421	A	C	-0.140	5	58424440	8.07E-06	0.032	14306	19.409
Vascular dementia (mixed)	genus Hungatella	rs13128780	T	C	-0.002	165137846	0.984	0.108	360421	T	C	-0.150	4	166058998	1.75E-06	0.031	14306	22.915
Vascular dementia (mixed)	genus Hungatella	rs13249325	T	G	-0.075	14996016	0.370	0.084	360421	T	G	-0.100	8	14853525	9.69E-06	0.023	14306	19.608
Vascular dementia (mixed)	genus Hungatella	rs17092615	G	A	-0.052	95507282	0.667	0.122	360421	G	A	0.152	14	95973619	7.38E-06	0.034	14306	20.302
Vascular dementia (mixed)	genus Hungatella	rs72759041	G	T	-0.129	89034145	0.214	0.104	360421	G	T	-0.126	15	89577376	3.86E-06	0.028	14306	19.937
Vascular dementia (mixed)	genus Intestinibacter	rs10805326	A	G	-0.059	14322999	0.520	0.092	360421	A	G	-0.078	4	14324623	3.55E-08	0.014	14306	30.803
Vascular dementia (mixed)	genus Intestinibacter	rs11109097	T	C	-0.077	97534659	0.365	0.085	360421	T	C	-0.062	12	97928437	5.49E-06	0.014	14306	20.305
Vascular dementia (mixed)	genus Intestinibacter	rs118030283	G	A	0.133	5943075	0.506	0.200	360421	G	A	-0.152	16	5993076	2.67E-06	0.032	14306	21.896
Vascular dementia (mixed)	genus Intestinibacter	rs16938435	T	C	0.199	21502924	0.161	0.142	360421	T	C	-0.112	9	21502923	1.80E-06	0.024	14306	22.706
Vascular dementia (mixed)	genus Intestinibacter	rs2098844	T	C	0.112	127833265	0.199	0.087	360421	T	C	0.058	11	127703160	6.79E-06	0.013	14306	20.070
Vascular dementia (mixed)	genus Intestinibacter	rs2702387	G	A	0.081	178440108	0.345	0.086	360421	G	A	-0.061	4	179361262	4.26E-06	0.013	14306	21.208
Vascular dementia (mixed)	genus Intestinibacter	rs4327025	G	A	-0.082	91903453	0.447	0.108	360421	G	A	-0.081	15	92446683	1.64E-07	0.015	14306	27.546
Vascular dementia (mixed)	genus Intestinibacter	rs447950	A	G	-0.041	149466863	0.635	0.087	360421	A	G	0.063	5	148846426	5.64E-06	0.014	14306	21.143
Vascular dementia (mixed)	genus Intestinibacter	rs478972	C	T	-0.162	125793289	0.268	0.146	360421	C	T	0.143	11	125663184	1.82E-06	0.030	14306	23.061
Vascular dementia (mixed)	genus Intestinibacter	rs6062862	A	G	0.155	62693871	0.314	0.154	360421	A	G	0.092	20	61325223	6.68E-06	0.020	14306	20.406
Vascular dementia (mixed)	genus Intestinibacter	rs62430350	T	C	-0.192	170609106	0.395	0.226	360421	T	C	0.151	6	170918194	6.84E-06	0.035	14306	18.481
Vascular dementia (mixed)	genus Intestinibacter	rs68093214	C	T	-0.073	70608634	0.456	0.098	360421	C	T	0.066	3	70657785	9.26E-06	0.015	14306	19.525
Vascular dementia (mixed)	genus Intestinibacter	rs6875660	C	T	-0.244	160259249	0.169	0.178	360421	C	T	0.089	5	159686256	3.06E-06	0.019	14306	21.089
Vascular dementia (mixed)	genus Intestinibacter	rs893394	G	A	0.095	19855210	0.266	0.085	360421	G	A	0.058	2	20054971	7.85E-06	0.013	14306	19.910
Vascular dementia (mixed)	genus Intestinibacter	rs9348442	C	T	0.197	10303712	0.116	0.126	360421	C	T	0.099	6	10303945	6.26E-06	0.022	14306	19.987
Vascular dementia (mixed)	genus Intestinimonas	rs10262702	T	C	0.133	67425856	0.299	0.128	360421	T	C	0.092	7	66890843	2.06E-06	0.019	14306	22.189
Vascular dementia (mixed)	genus Intestinimonas	rs11258178	A	G	-0.147	13082513	0.082	0.085	360421	A	G	0.066	10	13124513	6.98E-07	0.013	14306	24.264
Vascular dementia (mixed)	genus Intestinimonas	rs12226153	A	G	-0.111	94631127	0.749	0.345	360421	A	G	-0.151	11	94364293	5.12E-07	0.031	14306	24.250
Vascular dementia (mixed)	genus Intestinimonas	rs17067892	C	T	0.031	3906018	0.834	0.150	360421	C	T	0.107	8	3763540	6.38E-06	0.025	14306	18.383
Vascular dementia (mixed)	genus Intestinimonas	rs1859797	G	A	-0.100	21967685	0.238	0.085	360421	G	A	0.060	7	22007303	4.12E-06	0.013	14306	20.981
Vascular dementia (mixed)	genus Intestinimonas	rs2276760	A	G	-0.043	150903169	0.661	0.098	360421	A	G	-0.069	3	150620956	7.84E-06	0.015	14306	20.178
Vascular dementia (mixed)	genus Intestinimonas	rs2731794	C	T	-0.263	17209282	0.257	0.232	360421	C	T	0.121	5	17209391	1.92E-06	0.026	14306	21.942
Vascular dementia (mixed)	genus Intestinimonas	rs2930225	T	G	0.086	85294296	0.387	0.100	360421	T	G	-0.073	16	85327902	1.35E-06	0.015	14306	22.751
Vascular dementia (mixed)	genus Intestinimonas	rs4113676	A	C	0.717	6238275	0.056	0.375	360421	A	C	-0.219	20	6218922	7.42E-06	0.049	14306	19.873
Vascular dementia (mixed)	genus Intestinimonas	rs4784055	T	C	0.069	58784866	0.714	0.188	360421	T	C	-0.175	16	58818770	8.72E-07	0.039	14306	20.631
Vascular dementia (mixed)	genus Intestinimonas	rs62240188	G	A	-0.149	10569458	0.311	0.147	360421	G	A	0.130	3	10611142	2.20E-06	0.027	14306	23.702
Vascular dementia (mixed)	genus Intestinimonas	rs6934519	C	T	-0.135	66490909	0.159	0.096	360421	C	T	0.069	6	67200802	8.57E-06	0.015	14306	20.982
Vascular dementia (mixed)	genus Intestinimonas	rs716604	A	G	-0.042	6373956	0.677	0.100	360421	A	G	0.082	2	6514088	8.57E-07	0.017	14306	24.289
Vascular dementia (mixed)	genus Intestinimonas	rs7170984	T	C	0.032	93276506	0.731	0.094	360421	T	C	-0.066	15	93819735	2.98E-06	0.014	14306	21.858
Vascular dementia (mixed)	genus Intestinimonas	rs72982915	C	T	0.187	140140519	0.309	0.183	360421	C	T	0.183	2	140898088	4.91E-06	0.040	14306	20.682
Vascular dementia (mixed)	genus Intestinimonas	rs9823439	C	T	-0.162	17146010	0.055	0.085	360421	C	T	0.058	3	17187502	9.86E-06	0.013	14306	19.598
Vascular dementia (mixed)	genus Lachnoclostridium	rs1031599	T	G	-0.070	66675401	0.679	0.170	360421	T	G	0.079	3	66725825	6.31E-06	0.018	14306	20.039
Vascular dementia (mixed)	genus Lachnoclostridium	rs12566975	T	C	0.012	185122219	0.889	0.085	360421	T	C	-0.047	1	185091351	9.57E-06	0.011	14306	19.580
Vascular dementia (mixed)	genus Lachnoclostridium	rs1528479	A	G	-0.025	166387541	0.769	0.087	360421	A	G	0.050	2	167244051	9.64E-06	0.011	14306	19.783
Vascular dementia (mixed)	genus Lachnoclostridium	rs1997204	C	T	0.201	101652817	0.312	0.199	360421	C	T	0.108	12	102046595	5.97E-06	0.024	14306	19.941
Vascular dementia (mixed)	genus Lachnoclostridium	rs2385421	A	G	-0.138	22163494	0.297	0.132	360421	A	G	0.075	18	19743455	7.14E-06	0.018	14306	17.046
Vascular dementia (mixed)	genus Lachnoclostridium	rs3821998	C	A	-0.227	38692945	0.096	0.136	360421	C	A	-0.086	4	38694566	6.72E-06	0.019	14306	20.144
Vascular dementia (mixed)	genus Lachnoclostridium	rs4738679	A	G	0.015	58457761	0.862	0.086	360421	A	G	0.052	8	59370320	4.42E-06	0.011	14306	20.813
Vascular dementia (mixed)	genus Lachnoclostridium	rs6112314	A	C	-0.069	19320202	0.440	0.089	360421	A	C	-0.056	20	19300846	2.43E-07	0.011	14306	26.964
Vascular dementia (mixed)	genus Lachnoclostridium	rs615997	T	C	-0.135	22996295	0.108	0.084	360421	T	C	0.051	3	23037786	2.03E-06	0.011	14306	23.094
Vascular dementia (mixed)	genus Lachnoclostridium	rs62285313	A	G	0.058	177752244	0.682	0.141	360421	A	G	0.086	3	177470032	1.58E-06	0.018	14306	22.655
Vascular dementia (mixed)	genus Lachnoclostridium	rs72829893	G	T	-0.168	48617179	0.225	0.139	360421	G	T	0.117	17	46694541	5.58E-06	0.027	14306	19.198
Vascular dementia (mixed)	genus Lachnoclostridium	rs78068103	A	G	-0.010	13912842	0.940	0.132	360421	A	G	0.089	17	13816159	3.67E-06	0.019	14306	20.814
Vascular dementia (mixed)	genus Lachnoclostridium	rs789029	C	T	0.043	1053251	0.721	0.121	360421	C	T	-0.064	18	1053252	3.75E-06	0.014	14306	21.603
Vascular dementia (mixed)	genus Lachnospiraceae FCS020 group	rs10093861	G	A	0.024	120232167	0.775	0.085	360421	G	A	-0.057	8	121244406	3.06E-06	0.012	14306	22.048
Vascular dementia (mixed)	genus Lachnospiraceae FCS020 group	rs1254846	A	G	-0.069	43850646	0.564	0.120	360421	A	G	-0.106	10	44346094	5.60E-06	0.023	14306	20.771
Vascular dementia (mixed)	genus Lachnospiraceae FCS020 group	rs1363769	C	T	0.245	17754496	0.307	0.240	360421	C	T	0.201	19	17865305	1.58E-06	0.045	14306	19.933
Vascular dementia (mixed)	genus Lachnospiraceae FCS020 group	rs2322265	C	T	0.053	165666933	0.579	0.096	360421	C	T	-0.067	4	166588085	5.21E-06	0.014	14306	22.149
Vascular dementia (mixed)	genus Lachnospiraceae FCS020 group	rs2862811	C	T	-0.081	166278627	0.381	0.092	360421	C	T	-0.056	3	165996415	3.92E-06	0.012	14306	21.535

Vascular dementia (mixed)	genus Lachnospiraceae FCS020 group	rs35035870	T	C	0.336	3227646	0.116	0.214	360421	T	C	-0.191	11	3248876	2.62E-06	0.041	14306	21.158
Vascular dementia (mixed)	genus Lachnospiraceae FCS020 group	rs3999074	G	T	-0.143	63803887	0.091	0.084	360421	G	T	-0.055	10	65563647	6.55E-06	0.012	14306	20.418
Vascular dementia (mixed)	genus Lachnospiraceae FCS020 group	rs4452603	T	G	-0.087	71256565	0.363	0.095	360421	T	G	0.060	6	71966268	8.98E-06	0.014	14306	19.748
Vascular dementia (mixed)	genus Lachnospiraceae FCS020 group	rs7249113	G	A	0.031	2571234	0.738	0.092	360421	G	A	0.068	19	2571232	3.72E-07	0.013	14306	25.907
Vascular dementia (mixed)	genus Lachnospiraceae FCS020 group	rs72793667	A	G	-0.086	53719294	0.688	0.213	360421	A	G	-0.117	2	53946431	1.63E-06	0.025	14306	22.467
Vascular dementia (mixed)	genus Lachnospiraceae FCS020 group	rs9308097	G	A	-0.149	164865326	0.076	0.084	360421	G	A	-0.055	4	165786478	7.47E-06	0.012	14306	20.057
Vascular dementia (mixed)	genus Lachnospiraceae FCS020 group	rs9788306	C	T	-0.019	39845177	0.838	0.093	360421	C	T	-0.063	13	40419314	1.39E-06	0.013	14306	23.074
Vascular dementia (mixed)	genus Lachnospiraceae NC2004 group	rs117467633	T	C	0.227	21254094	0.285	0.213	360421	T	C	-0.170	17	21157406	9.13E-06	0.038	14306	19.612
Vascular dementia (mixed)	genus Lachnospiraceae NC2004 group	rs12127733	G	A	-0.034	234021975	0.758	0.109	360421	G	A	0.115	1	234157721	3.11E-06	0.025	14306	21.922
Vascular dementia (mixed)	genus Lachnospiraceae NC2004 group	rs12208226	C	A	0.043	22725679	0.755	0.138	360421	C	A	-0.155	6	22725908	9.75E-06	0.034	14306	20.668
Vascular dementia (mixed)	genus Lachnospiraceae NC2004 group	rs12863463	G	A	-0.036	46938056	0.822	0.158	360421	G	A	-0.156	13	47512191	6.04E-06	0.035	14306	20.498
Vascular dementia (mixed)	genus Lachnospiraceae NC2004 group	rs17067076	G	A	0.054	60561853	0.683	0.133	360421	G	A	-0.155	18	58229086	5.61E-06	0.035	14306	19.277
Vascular dementia (mixed)	genus Lachnospiraceae NC2004 group	rs1928659	T	C	0.076	29334139	0.470	0.105	360421	T	C	0.103	9	29334137	6.17E-06	0.023	14306	20.498
Vascular dementia (mixed)	genus Lachnospiraceae NC2004 group	rs1929743	T	C	0.122	76255426	0.170	0.089	360421	T	C	0.084	13	76829562	9.06E-06	0.019	14306	19.351
Vascular dementia (mixed)	genus Lachnospiraceae NC2004 group	rs3756315	A	G	0.086	150165159	0.346	0.092	360421	A	G	-0.088	5	149544722	3.33E-06	0.019	14306	21.990
Vascular dementia (mixed)	genus Lachnospiraceae NC2004 group	rs6116753	G	A	0.221	5350054	0.043	0.109	360421	G	A	0.099	20	5330700	2.92E-06	0.021	14306	22.623
Vascular dementia (mixed)	genus Lachnospiraceae ND3007 group	rs2861203	G	A	0.108	173232763	0.244	0.093	360421	G	A	0.057	3	172950553	7.37E-06	0.013	14306	20.218
Vascular dementia (mixed)	genus Lachnospiraceae ND3007 group	rs72776675	T	C	-0.034	14095664	0.765	0.113	360421	T	C	-0.065	10	14137663	8.72E-06	0.015	14306	19.123
Vascular dementia (mixed)	genus Lachnospiraceae ND3007 group	rs9932954	A	G	-0.054	1050633	0.538	0.088	360421	A	G	-0.056	16	1100633	1.25E-06	0.012	14306	23.467
Vascular dementia (mixed)	genus Lachnospiraceae NK4A136 group	rs10952110	G	T	-0.012	8458292	0.885	0.085	360421	G	T	0.049	7	8497922	9.08E-06	0.011	14306	19.797
Vascular dementia (mixed)	genus Lachnospiraceae NK4A136 group	rs11263806	A	G	0.046	36882840	0.603	0.088	360421	A	G	-0.052	17	35240097	5.07E-06	0.012	14306	20.189
Vascular dementia (mixed)	genus Lachnospiraceae NK4A136 group	rs12611395	G	A	-0.185	21623323	0.177	0.137	360421	G	A	0.090	19	21806125	5.83E-06	0.020	14306	20.435
Vascular dementia (mixed)	genus Lachnospiraceae NK4A136 group	rs160061	G	A	0.052	6116546	0.534	0.084	360421	G	A	-0.051	5	6116659	2.12E-06	0.011	14306	22.596
Vascular dementia (mixed)	genus Lachnospiraceae NK4A136 group	rs28540839	A	C	-0.087	83822845	0.302	0.084	360421	A	C	0.051	8	84735080	9.34E-06	0.011	14306	21.124
Vascular dementia (mixed)	genus Lachnospiraceae NK4A136 group	rs2880566	T	C	-0.143	31689634	0.228	0.119	360421	T	C	0.060	17	30016653	5.61E-06	0.013	14306	19.815
Vascular dementia (mixed)	genus Lachnospiraceae NK4A136 group	rs4955932	T	C	-0.064	55147348	0.463	0.087	360421	T	C	-0.049	3	55181375	7.05E-06	0.011	14306	20.253
Vascular dementia (mixed)	genus Lachnospiraceae NK4A136 group	rs59805249	T	C	-0.049	90994180	0.738	0.145	360421	T	C	0.094	5	90289997	9.45E-06	0.021	14306	20.261
Vascular dementia (mixed)	genus Lachnospiraceae NK4A136 group	rs68104925	T	C	-0.160	99711344	0.081	0.092	360421	T	C	-0.055	14	100177681	2.37E-06	0.012	14306	22.647
Vascular dementia (mixed)	genus Lachnospiraceae NK4A136 group	rs7073658	G	T	-0.026	60415266	0.757	0.084	360421	G	T	0.050	10	62175024	5.27E-06	0.011	14306	20.745
Vascular dementia (mixed)	genus Lachnospiraceae NK4A136 group	rs73044693	A	G	-0.021	50752863	0.901	0.165	360421	A	G	-0.108	19	51256120	3.57E-06	0.023	14306	21.900
Vascular dementia (mixed)	genus Lachnospiraceae NK4A136 group	rs7616165	G	T	0.567	190697356	0.009	0.216	360421	G	T	-0.231	3	190415145	2.77E-06	0.048	14306	22.739
Vascular dementia (mixed)	genus Lachnospiraceae NK4A136 group	rs76193507	A	G	0.163	162032193	0.277	0.150	360421	A	G	-0.230	3	161749981	2.93E-06	0.050	14306	21.129
Vascular dementia (mixed)	genus Lachnospiraceae NK4A136 group	rs7832116	A	G	0.145	4985407	0.250	0.126	360421	A	G	-0.071	8	4842929	3.57E-06	0.015	14306	22.199
Vascular dementia (mixed)	genus Lachnospiraceae NK4A136 group	rs954878	A	G	-0.015	54112728	0.859	0.087	360421	A	G	-0.052	1	54578401	1.78E-06	0.011	14306	22.782
Vascular dementia (mixed)	genus Lachnospiraceae UCG001	rs12131224	C	T	0.067	166230125	0.621	0.135	360421	C	T	0.117	1	166199362	7.40E-06	0.026	14306	20.424
Vascular dementia (mixed)	genus Lachnospiraceae UCG001	rs2050911	G	A	0.070	81918723	0.431	0.088	360421	G	A	0.075	1	82384407	1.11E-06	0.015	14306	23.831
Vascular dementia (mixed)	genus Lachnospiraceae UCG001	rs2371284	C	T	-0.076	55862478	0.447	0.100	360421	C	T	0.076	12	56256262	7.56E-06	0.017	14306	20.056
Vascular dementia (mixed)	genus Lachnospiraceae UCG001	rs437876	T	C	0.098	42526948	0.267	0.089	360421	T	C	0.078	3	42568440	7.17E-08	0.014	14306	29.376
Vascular dementia (mixed)	genus Lachnospiraceae UCG001	rs4981345	T	C	-0.011	20987814	0.900	0.089	360421	T	C	-0.068	14	21455973	6.09E-06	0.015	14306	20.717
Vascular dementia (mixed)	genus Lachnospiraceae UCG001	rs573933	T	C	-0.158	14477853	0.244	0.135	360421	T	C	-0.108	9	14477851	3.11E-06	0.023	14306	21.565
Vascular dementia (mixed)	genus Lachnospiraceae UCG001	rs62496417	T	G	0.042	97336192	0.682	0.102	360421	T	G	-0.075	7	96965504	5.88E-06	0.017	14306	20.410
Vascular dementia (mixed)	genus Lachnospiraceae UCG001	rs7341608	T	C	0.178	55926941	0.156	0.125	360421	T	C	-0.078	8	56839500	9.48E-06	0.018	14306	19.514
Vascular dementia (mixed)	genus Lachnospiraceae UCG001	rs74034332	G	A	0.025	79058462	0.888	0.175	360421	G	A	0.168	16	79092359	3.33E-06	0.038	14306	19.288
Vascular dementia (mixed)	genus Lachnospiraceae UCG001	rs78848836	A	G	-0.012	53307576	0.931	0.138	360421	A	G	-0.119	1	53773248	3.38E-06	0.026	14306	20.942
Vascular dementia (mixed)	genus Lachnospiraceae UCG001	rs8104225	A	G	-0.124	13852110	0.227	0.103	360421	A	G	0.089	19	13962924	8.04E-06	0.020	14306	20.369
Vascular dementia (mixed)	genus Lachnospiraceae UCG001	rs9403580	C	T	0.071	144685644	0.569	0.125	360421	C	T	0.108	6	145006780	3.47E-06	0.023	14306	22.011
Vascular dementia (mixed)	genus Lachnospiraceae UCG001	rs985416	T	C	-0.220	148551296	0.043	0.109	360421	T	C	-0.097	3	148269083	1.46E-07	0.018	14306	28.481
Vascular dementia (mixed)	genus Lachnospiraceae UCG004	rs11128180	A	G	-0.027	70543064	0.789	0.100	360421	A	G	0.065	3	70592215	4.52E-06	0.014	14306	21.404
Vascular dementia (mixed)	genus Lachnospiraceae UCG004	rs12072562	T	C	0.507	104744847	0.020	0.218	360421	T	C	0.133	1	105287469	7.07E-06	0.030	14306	19.225
Vascular dementia (mixed)	genus Lachnospiraceae UCG004	rs12673420	G	A	0.074	71838022	0.377	0.084	360421	G	A	0.055	7	71303007	2.98E-06	0.012	14306	21.931
Vascular dementia (mixed)	genus Lachnospiraceae UCG004	rs12747809	A	G	-0.026	240195789	0.781	0.094	360421	A	G	0.062	1	240359089	8.65E-07	0.013	14306	24.489
Vascular dementia (mixed)	genus Lachnospiraceae UCG004	rs12894272	G	A	0.053	40067277	0.550	0.089	360421	G	A	-0.058	14	40536481	4.34E-06	0.013	14306	21.436
Vascular dementia (mixed)	genus Lachnospiraceae UCG004	rs233486	G	A	0.017	14817353	0.888	0.122	360421	G	A	0.080	6	14817584	6.28E-06	0.018	14306	20.231
Vascular dementia (mixed)	genus Lachnospiraceae UCG004	rs2444793	T	C	0.032	80609119	0.709	0.086	360421	T	C	0.054	6	81318836	4.77E-06	0.012	14306	21.064
Vascular dementia (mixed)	genus Lachnospiraceae UCG004	rs2726805	A	G	-0.021	182326448	0.799	0.084	360421	A	G	0.055	4	183247601	6.30E-06	0.012	14306	20.594
Vascular dementia (mixed)	genus Lachnospiraceae UCG004	rs2882478	G	A	-0.250	49677451	0.002	0.081	360421	G	A	-0.058	2	49904589	1.21E-06	0.012	14306	23.782
Vascular dementia (mixed)	genus Lachnospiraceae UCG004	rs35182105	A	G	0.146	24419403	0.433	0.186	360421	A	G	-0.110	12	24572337	4.87E-06	0.024	14306	20.522
Vascular dementia (mixed)	genus Lachnospiraceae UCG004	rs6656451	T	C	-0.139	65524739	0.100	0.084	360421	T	C	0.054	1	65990422	5.57E-06	0.012	14306	20.703
Vascular dementia (mixed)	genus Lachnospiraceae UCG004	rs7629954	A	G	-0.025	154524617	0.902	0.202	360421	A	G	0.108	3	154242406	5.77E-06	0.024	14306	20.717
Vascular dementia (mixed)	genus Lachnospiraceae UCG008	rs10741777	T	C	0.014	19551010	0.879	0.091	360421	T	C	-0.097	11	19572557	7.69E-07	0.019	14306	24.994
Vascular dementia (mixed)	genus Lachnospiraceae UCG008	rs10793103	T	C	-0.032	74680730	0.702	0.085	360421	T	C	-0.097	11	74391775	9.35E-08	0.018	14306	28.889
Vascular dementia (mixed)	genus Lachnospiraceae UCG008	rs10801803	G	A	-0.027	90353314	0.819	0.120	360421	G	A	-0.117	1	90818872	1.40E-06	0.024	14306	23.178
Vascular dementia (mixed)	genus Lachnospiraceae UCG008	rs13024781	T	C	-0.050	167772361	0.550	0.084	360421	T	C	-0.080	2	168628871	2.29E-06	0.017	14306	22.380
Vascular dementia (mixed)	genus Lachnospiraceae UCG008	rs57091572	A	G	0.131	80505086	0.291	0.124	360421	A	G	-0.110	6	81214803	2.86E-06	0.024	14306	21.937
Vascular dementia (mixed)	genus Lachnospiraceae UCG008	rs61944774	A	G	-0.109	129055273	0.565	0.189	360421	A	G	0.180	12	129539818	6.34E-06	0.039	14306	20.855
Vascular dementia (mixed)	genus Lachnospiraceae UCG008	rs62277846	C	T	0.060	227235424	0.573	0.107	360421	C	T	0.102	2	228100140	1.59E-06	0.021	14306	23.212
Vascular dementia (mixed)	genus Lachnospiraceae UCG008	rs67078837	T	C	0.117	113299491	0.170	0.085	360421	T	C	-0.085	4	114220647	7.68E-07	0.017	14306	24.556

Vascular dementia (mixed)	genus Lachnospiraceae UCG008	rs75356640	G	A	0.060	31405081	0.639	0.127	360421	G	A	0.137	15	31697284	9.83E-06	0.030	14306	20.284
Vascular dementia (mixed)	genus Lachnospiraceae UCG008	rs955844	A	C	-0.161	84958708	0.184	0.122	360421	A	C	0.112	16	84992314	1.81E-06	0.023	14306	24.080
Vascular dementia (mixed)	genus Lachnospiraceae UCG010	rs10414815	C	T	0.292	42082093	0.144	0.200	360421	C	T	-0.105	19	42586245	4.24E-06	0.023	14306	20.610
Vascular dementia (mixed)	genus Lachnospiraceae UCG010	rs11192447	A	G	0.055	82088641	0.777	0.194	360421	A	G	0.127	10	83848397	4.69E-07	0.024	14306	27.039
Vascular dementia (mixed)	genus Lachnospiraceae UCG010	rs12346653	C	T	-0.169	89652324	0.103	0.103	360421	C	T	0.066	9	92267239	2.70E-06	0.014	14306	22.205
Vascular dementia (mixed)	genus Lachnospiraceae UCG010	rs17730011	G	A	0.146	84063126	0.151	0.102	360421	G	A	-0.070	6	84772845	7.85E-06	0.016	14306	19.998
Vascular dementia (mixed)	genus Lachnospiraceae UCG010	rs2833528	T	C	0.071	31821255	0.417	0.087	360421	T	C	0.056	21	33193567	9.92E-06	0.013	14306	19.359
Vascular dementia (mixed)	genus Lachnospiraceae UCG010	rs336138	G	T	0.065	8146258	0.621	0.131	360421	G	T	0.078	5	8146371	7.48E-06	0.017	14306	20.573
Vascular dementia (mixed)	genus Lachnospiraceae UCG010	rs4576377	C	A	-0.004	81780947	0.966	0.087	360421	C	A	0.057	7	81410263	7.63E-06	0.013	14306	20.272
Vascular dementia (mixed)	genus Lachnospiraceae UCG010	rs72894957	G	A	0.091	184997864	0.742	0.276	360421	G	A	0.222	2	185862591	5.68E-06	0.049	14306	20.879
Vascular dementia (mixed)	genus Lachnospiraceae UCG010	rs74315802	G	T	-0.131	29403135	0.228	0.109	360421	G	T	0.087	14	29872341	3.19E-06	0.018	14306	22.343
Vascular dementia (mixed)	genus Lachnospiraceae UCG010	rs9981767	A	C	0.133	42659765	0.171	0.097	360421	A	C	0.066	21	44079875	9.96E-07	0.013	14306	24.630
Vascular dementia (mixed)	genus Lactobacillus	rs12693845	C	T	0.056	198447960	0.519	0.087	360421	C	T	-0.081	2	199312684	8.96E-06	0.018	14306	20.607
Vascular dementia (mixed)	genus Lactobacillus	rs1530559	G	A	-0.076	134998059	0.371	0.085	360421	G	A	0.080	2	135755629	4.93E-06	0.018	14306	20.355
Vascular dementia (mixed)	genus Lactobacillus	rs16861661	G	A	-0.110	18174965	0.517	0.169	360421	G	A	-0.183	1	18501459	1.28E-06	0.038	14306	23.049
Vascular dementia (mixed)	genus Lactobacillus	rs62314653	C	A	0.158	108975306	0.375	0.178	360421	C	A	0.188	4	109896462	2.24E-06	0.039	14306	22.626
Vascular dementia (mixed)	genus Lactobacillus	rs7399658	G	A	-0.199	23260829	0.068	0.109	360421	G	A	-0.107	13	23834968	3.12E-06	0.022	14306	23.313
Vascular dementia (mixed)	genus Lactobacillus	rs768253	T	G	0.013	68097868	0.878	0.085	360421	T	G	-0.079	8	69010103	4.25E-06	0.017	14306	21.252
Vascular dementia (mixed)	genus Lactobacillus	rs77478751	A	G	-0.170	173433875	0.197	0.132	360421	A	G	-0.220	3	173151665	7.33E-06	0.048	14306	21.361
Vascular dementia (mixed)	genus Lactobacillus	rs921925	A	C	-0.051	6928006	0.616	0.103	360421	A	C	0.099	19	6928017	9.72E-07	0.020	14306	23.495
Vascular dementia (mixed)	genus Lactococcus	rs10417872	G	T	0.046	28276446	0.620	0.093	360421	G	T	-0.118	19	28767353	1.29E-06	0.025	14306	23.276
Vascular dementia (mixed)	genus Lactococcus	rs123059	C	T	0.032	2796641	0.754	0.102	360421	C	T	0.137	17	2699935	1.27E-06	0.027	14306	24.769
Vascular dementia (mixed)	genus Lactococcus	rs12621813	G	A	-0.120	31043183	0.209	0.096	360421	G	A	0.108	2	31266049	6.61E-06	0.024	14306	20.413
Vascular dementia (mixed)	genus Lactococcus	rs17168302	G	A	0.086	14610596	0.529	0.137	360421	G	A	0.192	7	14650221	6.29E-06	0.042	14306	20.402
Vascular dementia (mixed)	genus Lactococcus	rs2293361	C	T	0.158	53887727	0.400	0.188	360421	C	T	-0.199	2	54114864	1.40E-06	0.043	14306	21.369
Vascular dementia (mixed)	genus Lactococcus	rs4766997	C	T	0.029	112723633	0.734	0.085	360421	C	T	0.115	12	113161438	2.06E-06	0.024	14306	23.109
Vascular dementia (mixed)	genus Lactococcus	rs55910161	C	T	-0.097	69758887	0.471	0.134	360421	C	T	0.146	10	71518643	2.36E-06	0.031	14306	22.695
Vascular dementia (mixed)	genus Lactococcus	rs6674304	C	T	-0.172	116345120	0.441	0.224	360421	C	T	0.201	1	116887742	6.18E-06	0.044	14306	20.619
Vascular dementia (mixed)	genus Marvinbryantia	rs11620597	T	C	0.081	85504955	0.767	0.275	360421	T	C	0.119	13	86079090	7.80E-06	0.027	14306	19.339
Vascular dementia (mixed)	genus Marvinbryantia	rs1187983	C	T	0.161	57976188	0.245	0.139	360421	C	T	-0.094	1	58441860	2.02E-06	0.019	14306	23.450
Vascular dementia (mixed)	genus Marvinbryantia	rs146541147	G	A	-0.196	4542085	0.405	0.235	360421	G	A	0.119	19	4542097	6.86E-06	0.027	14306	19.603
Vascular dementia (mixed)	genus Marvinbryantia	rs2724813	G	A	0.132	12474925	0.183	0.099	360421	G	A	0.084	10	12516924	6.28E-07	0.017	14306	25.180
Vascular dementia (mixed)	genus Marvinbryantia	rs2842896	C	T	-0.033	132560525	0.704	0.086	360421	C	T	-0.065	6	132881664	7.25E-07	0.013	14306	24.519
Vascular dementia (mixed)	genus Marvinbryantia	rs2863363	G	A	-0.081	166321977	0.412	0.098	360421	G	A	-0.063	3	166039765	3.11E-06	0.014	14306	21.688
Vascular dementia (mixed)	genus Marvinbryantia	rs3125832	A	C	-0.079	211226838	0.434	0.101	360421	A	C	0.068	1	211400180	5.03E-06	0.015	14306	20.477
Vascular dementia (mixed)	genus Marvinbryantia	rs61884471	G	A	0.119	45805010	0.378	0.135	360421	G	A	0.124	11	45826561	1.01E-06	0.025	14306	25.085
Vascular dementia (mixed)	genus Marvinbryantia	rs72948274	A	C	-0.280	79807639	0.103	0.172	360421	A	C	-0.126	11	79518683	3.26E-06	0.027	14306	21.546
Vascular dementia (mixed)	genus Marvinbryantia	rs8006832	G	T	0.052	21109678	0.726	0.148	360421	G	T	-0.095	14	21577837	6.58E-06	0.022	14306	19.317
Vascular dementia (mixed)	genus Methanobrevibacter	rs10202904	G	T	0.028	124682691	0.745	0.086	360421	G	T	0.113	2	125440268	3.09E-06	0.024	14306	22.260
Vascular dementia (mixed)	genus Methanobrevibacter	rs1334944	T	C	0.082	110506288	0.387	0.094	360421	T	C	0.115	10	112266046	7.61E-06	0.026	14306	20.330
Vascular dementia (mixed)	genus Methanobrevibacter	rs4802933	G	A	0.096	52422567	0.335	0.100	360421	G	A	0.136	19	52925820	9.74E-06	0.031	14306	19.373
Vascular dementia (mixed)	genus Methanobrevibacter	rs6776814	T	C	0.412	15011576	0.158	0.292	360421	T	C	-0.189	3	15053083	8.05E-06	0.042	14306	20.250
Vascular dementia (mixed)	genus Methanobrevibacter	rs76029318	T	C	0.120	41389655	0.484	0.171	360421	T	C	0.223	13	41963791	1.08E-06	0.045	14306	24.060
Vascular dementia (mixed)	genus Methanobrevibacter	rs894996	C	A	-0.011	103497150	0.945	0.163	360421	C	A	0.214	4	104418307	3.82E-06	0.046	14306	22.064
Vascular dementia (mixed)	genus Odoribacter	rs10093869	A	G	0.143	1318658	0.092	0.085	360421	A	G	-0.058	8	1266824	3.67E-06	0.013	14306	21.234
Vascular dementia (mixed)	genus Odoribacter	rs10423795	T	C	-0.199	49019831	0.023	0.087	360421	T	C	-0.055	19	49523088	6.58E-06	0.012	14306	20.657
Vascular dementia (mixed)	genus Odoribacter	rs28417404	A	G	-0.183	70477507	0.200	0.143	360421	A	G	-0.073	14	70944224	3.68E-06	0.016	14306	20.290
Vascular dementia (mixed)	genus Odoribacter	rs4793970	A	G	0.048	48686600	0.586	0.088	360421	A	G	-0.058	17	46763962	6.03E-06	0.013	14306	19.912
Vascular dementia (mixed)	genus Odoribacter	rs6856150	A	G	0.069	11905935	0.584	0.126	360421	A	G	-0.088	4	11907559	6.06E-06	0.019	14306	20.635
Vascular dementia (mixed)	genus Odoribacter	rs74553962	T	G	-0.290	68765245	0.072	0.161	360421	T	G	0.121	8	69677480	9.49E-06	0.026	14306	21.146
Vascular dementia (mixed)	genus Odoribacter	rs77779484	G	A	0.091	67262163	0.600	0.174	360421	G	A	-0.133	12	67655943	6.56E-07	0.027	14306	24.713
Vascular dementia (mixed)	genus Olsenella	rs1035588	A	G	0.162	149220676	0.063	0.087	360421	A	G	-0.108	2	150077190	4.86E-06	0.024	14306	20.850
Vascular dementia (mixed)	genus Olsenella	rs17148768	G	A	-0.132	10735122	0.240	0.113	360421	G	A	0.140	10	10777085	2.20E-06	0.030	14306	22.570
Vascular dementia (mixed)	genus Olsenella	rs2759329	A	G	-0.047	231824606	0.588	0.087	360421	A	G	0.111	1	231960352	3.43E-06	0.024	14306	21.947
Vascular dementia (mixed)	genus Olsenella	rs35225860	A	G	0.149	247478968	0.489	0.216	360421	A	G	-0.224	1	247642270	3.87E-06	0.048	14306	21.486
Vascular dementia (mixed)	genus Olsenella	rs61090148	A	G	-0.146	173889347	0.086	0.085	360421	A	G	-0.105	4	174810498	6.44E-06	0.023	14306	20.515
Vascular dementia (mixed)	genus Olsenella	rs62112538	C	T	0.160	4925006	0.232	0.134	360421	C	T	-0.199	19	4925018	1.19E-06	0.041	14306	24.006
Vascular dementia (mixed)	genus Olsenella	rs72691585	C	A	-0.024	21884426	0.848	0.123	360421	C	A	-0.249	9	21884425	2.95E-06	0.052	14306	22.872
Vascular dementia (mixed)	genus Olsenella	rs7540303	C	T	0.001	179660056	0.993	0.087	360421	C	T	0.108	1	179629191	5.32E-06	0.024	14306	20.892
Vascular dementia (mixed)	genus Olsenella	rs8066522	A	G	0.077	61555512	0.389	0.090	360421	A	G	0.107	17	59632873	9.70E-06	0.024	14306	19.640
Vascular dementia (mixed)	genus Olsenella	rs9460691	C	A	0.120	10421974	0.267	0.108	360421	C	A	0.120	6	10422207	7.28E-06	0.027	14306	19.942
Vascular dementia (mixed)	genus Oscillibacter	rs11627628	T	C	-0.209	21011446	0.188	0.159	360421	T	C	0.144	14	21479605	1.01E-06	0.029	14306	24.605
Vascular dementia (mixed)	genus Oscillibacter	rs11990279	T	C	0.059	11258805	0.574	0.104	360421	T	C	-0.082	8	11116314	4.94E-06	0.018	14306	20.897
Vascular dementia (mixed)	genus Oscillibacter	rs12649930	T	G	0.148	3654564	0.291	0.140	360421	T	G	0.122	4	3656291	4.09E-06	0.026	14306	21.935
Vascular dementia (mixed)	genus Oscillibacter	rs133832	A	C	0.118	44438827	0.208	0.094	360421	A	C	-0.080	22	44834707	1.15E-06	0.016	14306	23.993
Vascular dementia (mixed)	genus Oscillibacter	rs16866406	A	G	0.157	178592420	0.178	0.116	360421	A	G	0.099	2	179457147	3.08E-06	0.021	14306	22.426
Vascular dementia (mixed)	genus Oscillibacter	rs16934185	A	G	-0.029	1798324	0.834	0.139	360421	A	G	-0.130	9	1798324	4.38E-06	0.028	14306	21.175

Vascular dementia (mixed)	genus Oscillibacter	rs234108	A	G	-0.111	184973539	0.195	0.086	360421	A	G	0.075	1	184942671	9.16E-07	0.015	14306	24.116
Vascular dementia (mixed)	genus Oscillibacter	rs36095275	C	T	-0.111	31800923	0.199	0.086	360421	C	T	-0.075	14	32270129	1.40E-06	0.016	14306	23.005
Vascular dementia (mixed)	genus Oscillibacter	rs4506202	G	A	-0.013	21740565	0.882	0.085	360421	G	A	0.071	8	21598077	3.21E-06	0.015	14306	21.825
Vascular dementia (mixed)	genus Oscillibacter	rs61883564	A	G	-0.143	79302798	0.246	0.124	360421	A	G	-0.101	11	79013843	3.39E-06	0.022	14306	21.029
Vascular dementia (mixed)	genus Oscillibacter	rs75453768	G	T	0.075	114710047	0.605	0.145	360421	G	T	0.122	10	116469806	5.35E-06	0.027	14306	20.667
Vascular dementia (mixed)	genus Oscillibacter	rs761240	G	T	-0.020	50891355	0.918	0.197	360421	G	T	0.177	20	49507892	2.04E-06	0.039	14306	20.639
Vascular dementia (mixed)	genus Oscillibacter	rs9393920	G	A	-0.008	28612816	0.927	0.086	360421	G	A	0.074	6	28580593	9.92E-07	0.015	14306	24.294
Vascular dementia (mixed)	genus Oscillospira	rs12206468	G	A	0.305	18093460	0.052	0.157	360421	G	A	-0.133	6	18093691	1.04E-06	0.027	14306	24.319
Vascular dementia (mixed)	genus Oscillospira	rs12925026	T	C	-0.103	89726448	0.566	0.180	360421	T	C	0.136	16	89792856	9.31E-06	0.031	14306	19.534
Vascular dementia (mixed)	genus Oscillospira	rs1954532	C	T	-0.042	27682209	0.693	0.106	360421	C	T	0.083	14	28151415	2.27E-06	0.018	14306	22.228
Vascular dementia (mixed)	genus Oscillospira	rs28889936	A	C	-0.009	88562149	0.952	0.144	360421	A	C	0.114	4	89483300	3.37E-06	0.025	14306	20.348
Vascular dementia (mixed)	genus Oscillospira	rs62422654	C	T	0.006	170159406	0.950	0.104	360421	C	T	0.090	6	170474630	6.47E-06	0.020	14306	20.572
Vascular dementia (mixed)	genus Oscillospira	rs72866977	A	C	0.104	56295685	0.504	0.156	360421	A	C	-0.131	6	56160483	5.63E-06	0.028	14306	21.488
Vascular dementia (mixed)	genus Oscillospira	rs751183	C	T	0.022	76337408	0.845	0.110	360421	C	T	0.077	1	76803093	6.85E-06	0.017	14306	20.216
Vascular dementia (mixed)	genus Oscillospira	rs8076323	A	G	-0.131	14978543	0.144	0.090	360421	A	G	0.072	17	14881860	5.61E-06	0.016	14306	20.885
Vascular dementia (mixed)	genus Oxalobacter	rs10464997	G	A	-0.117	21045182	0.284	0.109	360421	G	A	0.138	8	20902693	3.30E-06	0.029	14306	21.814
Vascular dementia (mixed)	genus Oxalobacter	rs11108500	A	G	-0.203	96425426	0.176	0.150	360421	A	G	-0.199	12	96819204	3.74E-06	0.043	14306	21.708
Vascular dementia (mixed)	genus Oxalobacter	rs111966731	T	C	-0.139	93398708	0.356	0.151	360421	T	C	0.213	15	93941937	7.30E-06	0.047	14306	20.419
Vascular dementia (mixed)	genus Oxalobacter	rs12002250	A	C	-0.218	19682560	0.289	0.205	360421	A	C	0.217	9	19682558	1.42E-06	0.047	14306	21.679
Vascular dementia (mixed)	genus Oxalobacter	rs1569853	T	C	0.082	38582525	0.525	0.130	360421	T	C	-0.138	6	38550301	3.65E-06	0.030	14306	21.617
Vascular dementia (mixed)	genus Oxalobacter	rs36057338	G	T	-0.334	189014160	0.154	0.234	360421	G	T	0.208	4	189935314	8.80E-07	0.042	14306	24.323
Vascular dementia (mixed)	genus Oxalobacter	rs3862635	C	T	0.153	126712683	0.297	0.146	360421	C	T	-0.172	11	126582578	9.19E-06	0.039	14306	19.086
Vascular dementia (mixed)	genus Oxalobacter	rs4428215	G	A	-0.051	172229645	0.601	0.097	360421	G	A	0.130	3	171947435	7.51E-08	0.024	14306	28.931
Vascular dementia (mixed)	genus Oxalobacter	rs6000536	C	T	0.218	37025428	0.050	0.111	360421	C	T	-0.131	22	37421469	2.06E-07	0.025	14306	26.637
Vascular dementia (mixed)	genus Oxalobacter	rs6993398	G	A	0.109	114548460	0.316	0.109	360421	G	A	0.127	8	115560689	7.13E-06	0.028	14306	20.813
Vascular dementia (mixed)	genus Oxalobacter	rs736744	T	C	0.026	84899492	0.756	0.085	360421	T	C	-0.118	9	87514407	2.57E-08	0.021	14306	31.135
Vascular dementia (mixed)	genus Parabacteroides	rs115602804	G	A	0.034	191854576	0.807	0.138	360421	G	A	0.103	3	191572365	1.93E-06	0.022	14306	21.417
Vascular dementia (mixed)	genus Parabacteroides	rs4236095	A	G	-0.034	47400951	0.807	0.139	360421	A	G	-0.076	6	47368687	1.93E-06	0.016	14306	23.541
Vascular dementia (mixed)	genus Parabacteroides	rs60884758	C	T	-0.054	2217340	0.630	0.111	360421	C	T	-0.070	9	2217340	5.71E-07	0.014	14306	24.401
Vascular dementia (mixed)	genus Parabacteroides	rs6657302	T	C	-0.087	85119926	0.619	0.176	360421	T	C	-0.105	1	85585609	9.76E-06	0.023	14306	21.480
Vascular dementia (mixed)	genus Parabacteroides	rs7298818	C	T	-0.028	56003169	0.843	0.141	360421	C	T	0.089	12	56396953	8.54E-06	0.020	14306	19.574
Vascular dementia (mixed)	genus Paraprevotella	rs10842464	C	T	-0.021	25096809	0.822	0.091	360421	C	T	0.076	12	25249743	6.60E-06	0.017	14306	19.305
Vascular dementia (mixed)	genus Paraprevotella	rs140997932	T	C	0.264	149581005	0.145	0.181	360421	T	C	-0.162	3	149298792	2.11E-06	0.035	14306	21.018
Vascular dementia (mixed)	genus Paraprevotella	rs145020347	A	G	-0.225	114655957	0.060	0.120	360421	A	G	-0.125	11	114526679	4.03E-06	0.026	14306	22.579
Vascular dementia (mixed)	genus Paraprevotella	rs17109926	A	G	-0.096	71584948	0.314	0.095	360421	A	G	-0.099	12	71978728	6.75E-06	0.022	14306	20.903
Vascular dementia (mixed)	genus Paraprevotella	rs17785622	A	G	0.116	82749597	0.593	0.217	360421	A	G	0.248	6	83459314	1.93E-06	0.052	14306	22.385
Vascular dementia (mixed)	genus Paraprevotella	rs2081023	A	G	-0.113	175179258	0.354	0.122	360421	A	G	-0.123	5	174606261	2.64E-07	0.024	14306	26.854
Vascular dementia (mixed)	genus Paraprevotella	rs3008582	T	C	-0.040	196002043	0.709	0.108	360421	T	C	0.106	1	195971173	4.36E-06	0.023	14306	21.643
Vascular dementia (mixed)	genus Paraprevotella	rs3801748	G	A	-0.035	82130192	0.690	0.087	360421	G	A	0.078	7	81759508	5.20E-06	0.017	14306	20.624
Vascular dementia (mixed)	genus Paraprevotella	rs4756632	G	T	0.009	41058153	0.944	0.125	360421	G	T	-0.139	11	41079703	3.82E-06	0.029	14306	22.959
Vascular dementia (mixed)	genus Paraprevotella	rs4767113	C	T	-0.062	113694173	0.490	0.090	360421	C	T	0.088	12	114131978	2.14E-06	0.018	14306	23.047
Vascular dementia (mixed)	genus Paraprevotella	rs7240324	T	G	-0.157	71956568	0.108	0.097	360421	T	G	-0.102	18	69623804	5.96E-06	0.023	14306	20.315
Vascular dementia (mixed)	genus Paraprevotella	rs9602779	A	C	0.028	85588285	0.775	0.098	360421	A	C	-0.107	13	86162420	6.93E-07	0.022	14306	23.463
Vascular dementia (mixed)	genus Paraprevotella	rs9900242	A	G	-0.156	71139490	0.079	0.089	360421	A	G	-0.085	17	69135631	1.14E-06	0.018	14306	23.699
Vascular dementia (mixed)	genus Parasutterella	rs10899911	A	G	-0.195	43798391	0.050	0.099	360421	A	G	-0.072	10	44293839	1.15E-06	0.015	14306	23.429
Vascular dementia (mixed)	genus Parasutterella	rs11715853	G	A	-0.207	30122198	0.024	0.092	360421	G	A	-0.066	3	30163689	6.23E-06	0.015	14306	20.586
Vascular dementia (mixed)	genus Parasutterella	rs2090816	C	A	-0.088	137294455	0.421	0.109	360421	C	A	-0.084	6	137615592	2.90E-06	0.018	14306	22.494
Vascular dementia (mixed)	genus Parasutterella	rs35055552	T	C	0.011	113791795	0.925	0.121	360421	T	C	0.110	8	114804024	3.35E-06	0.024	14306	21.653
Vascular dementia (mixed)	genus Parasutterella	rs55877868	A	C	-0.027	14789550	0.849	0.142	360421	A	C	-0.104	17	14692867	2.87E-06	0.023	14306	20.974
Vascular dementia (mixed)	genus Parasutterella	rs62273907	A	G	-0.250	156832626	0.140	0.169	360421	A	G	0.229	3	156550415	5.88E-06	0.050	14306	20.873
Vascular dementia (mixed)	genus Parasutterella	rs6809952	G	A	-0.140	194178920	0.141	0.095	360421	G	A	-0.068	3	193896709	8.13E-06	0.015	14306	20.606
Vascular dementia (mixed)	genus Parasutterella	rs6828768	C	T	-0.025	64691207	0.765	0.084	360421	C	T	0.064	4	65556925	1.78E-06	0.013	14306	23.051
Vascular dementia (mixed)	genus Parasutterella	rs7303158	C	T	-0.122	5166374	0.147	0.084	360421	C	T	0.065	12	5275540	1.33E-06	0.013	14306	23.214
Vascular dementia (mixed)	genus Parasutterella	rs7311004	C	T	-0.024	52866926	0.775	0.085	360421	C	T	0.062	12	53260710	5.92E-06	0.014	14306	20.485
Vascular dementia (mixed)	genus Parasutterella	rs7572229	A	G	0.056	72008314	0.506	0.085	360421	A	G	-0.066	2	72235444	6.32E-07	0.013	14306	24.928
Vascular dementia (mixed)	genus Parasutterella	rs78383039	T	C	-0.040	178089981	0.850	0.214	360421	T	C	-0.146	2	178954708	1.57E-06	0.030	14306	24.250
Vascular dementia (mixed)	genus Parasutterella	rs8039785	G	T	0.039	67023969	0.646	0.084	360421	G	T	-0.062	15	67316307	3.62E-06	0.013	14306	21.615
Vascular dementia (mixed)	genus Parasutterella	rs823424	G	A	-0.044	16817017	0.654	0.097	360421	G	A	-0.071	8	16674526	4.95E-06	0.016	14306	20.661
Vascular dementia (mixed)	genus Peptococcus	rs10031059	C	T	0.198	35356972	0.045	0.099	360421	C	T	0.121	4	35358594	1.24E-07	0.023	14306	28.784
Vascular dementia (mixed)	genus Peptococcus	rs11001941	G	A	-0.119	76956272	0.429	0.150	360421	G	A	-0.196	10	78716030	1.33E-06	0.039	14306	24.873
Vascular dementia (mixed)	genus Peptococcus	rs12069354	C	T	0.002	216050440	0.990	0.176	360421	C	T	0.168	1	216223782	9.28E-06	0.038	14306	19.511
Vascular dementia (mixed)	genus Peptococcus	rs2054133	A	G	-0.001	33466742	0.989	0.089	360421	A	G	-0.090	2	33691809	2.14E-06	0.019	14306	22.606
Vascular dementia (mixed)	genus Peptococcus	rs36121075	A	G	-0.212	44489376	0.061	0.113	360421	A	G	-0.141	20	43118017	6.99E-06	0.031	14306	21.094
Vascular dementia (mixed)	genus Peptococcus	rs413827	G	A	0.035	57518394	0.722	0.099	360421	G	A	0.110	14	57985112	3.30E-06	0.024	14306	21.537
Vascular dementia (mixed)	genus Peptococcus	rs5770862	T	C	0.102	50534684	0.484	0.145	360421	T	C	0.162	22	50973113	3.22E-06	0.036	14306	20.618
Vascular dementia (mixed)	genus Peptococcus	rs6918730	A	G	0.112	98572413	0.523	0.176	360421	A	G	-0.135	6	99020289	1.15E-06	0.029	14306	21.809
Vascular dementia (mixed)	genus Peptococcus	rs7033353	G	T	0.082	101833566	0.327	0.084	360421	G	T	-0.090	9	104595848	2.22E-06	0.019	14306	22.525

Vascular dementia (mixed)	genus Peptococcus	rs72850165	T	C	-0.052	968356	0.745	0.158	360421	T	C	-0.134	11	968356	5.74E-06	0.030	14306	19.985
Vascular dementia (mixed)	genus Peptococcus	rs74592222	G	A	-0.131	75506015	0.323	0.132	360421	G	A	0.138	1	75971700	8.55E-06	0.030	14306	20.735
Vascular dementia (mixed)	genus Peptococcus	rs77681628	C	T	0.110	90530302	0.476	0.155	360421	C	T	0.200	13	91182556	2.69E-07	0.039	14306	26.744
Vascular dementia (mixed)	genus Phascolarctobacterium	rs12618201	A	G	0.143	173571805	0.093	0.085	360421	A	G	0.064	2	174436533	3.38E-06	0.014	14306	21.556
Vascular dementia (mixed)	genus Phascolarctobacterium	rs1264476	G	T	-0.107	101422048	0.320	0.108	360421	G	T	-0.077	8	102434276	4.30E-06	0.017	14306	21.357
Vascular dementia (mixed)	genus Phascolarctobacterium	rs56069061	G	A	-0.067	168621629	0.685	0.167	360421	G	A	-0.111	3	168339417	1.87E-06	0.023	14306	23.279
Vascular dementia (mixed)	genus Phascolarctobacterium	rs56157888	A	C	0.047	182331683	0.647	0.102	360421	A	C	0.095	4	183252836	1.09E-06	0.019	14306	24.232
Vascular dementia (mixed)	genus Phascolarctobacterium	rs74540770	G	A	-0.218	186835600	0.163	0.156	360421	G	A	-0.121	3	186553389	3.60E-06	0.026	14306	21.895
Vascular dementia (mixed)	genus Phascolarctobacterium	rs75882962	T	C	-0.036	52704958	0.777	0.127	360421	T	C	0.097	12	53098742	3.19E-07	0.019	14306	25.835
Vascular dementia (mixed)	genus Phascolarctobacterium	rs7982713	G	A	0.012	70802190	0.898	0.094	360421	G	A	0.073	13	71376322	9.72E-06	0.016	14306	19.839
Vascular dementia (mixed)	genus Prevotella7	rs118038478	A	G	-0.059	47203698	0.718	0.163	360421	A	G	0.206	16	47237609	7.85E-06	0.047	14306	19.239
Vascular dementia (mixed)	genus Prevotella7	rs12124567	A	G	-0.020	3427238	0.849	0.106	360421	A	G	-0.121	1	3343802	9.49E-06	0.028	14306	19.448
Vascular dementia (mixed)	genus Prevotella7	rs12195431	T	C	0.052	90309935	0.720	0.144	360421	T	C	0.197	6	91019654	8.73E-06	0.044	14306	19.741
Vascular dementia (mixed)	genus Prevotella7	rs2240542	C	T	0.038	241126899	0.686	0.095	360421	C	T	0.121	2	242066314	4.84E-06	0.026	14306	21.312
Vascular dementia (mixed)	genus Prevotella7	rs2918132	T	C	0.019	131202057	0.827	0.087	360421	T	C	0.115	10	133000320	6.42E-06	0.025	14306	20.227
Vascular dementia (mixed)	genus Prevotella7	rs430270	A	C	-0.060	60488646	0.580	0.109	360421	A	C	0.139	3	60474379	2.87E-06	0.030	14306	21.944
Vascular dementia (mixed)	genus Prevotella7	rs57404562	C	A	0.312	11961636	0.012	0.125	360421	C	A	0.155	2	12101762	6.22E-07	0.032	14306	24.202
Vascular dementia (mixed)	genus Prevotella7	rs79263163	A	C	-0.086	39800223	0.412	0.104	360421	A	C	-0.144	11	39821773	7.51E-06	0.032	14306	20.891
Vascular dementia (mixed)	genus Prevotella7	rs9426434	T	C	0.111	29409704	0.207	0.088	360421	T	C	-0.124	1	29736216	9.72E-06	0.028	14306	19.713
Vascular dementia (mixed)	genus Prevotella7	rs9608249	A	G	0.095	24217984	0.476	0.133	360421	A	G	-0.158	22	24613952	2.07E-06	0.034	14306	22.130
Vascular dementia (mixed)	genus Prevotella7	rs9959718	G	A	-0.068	73775429	0.517	0.105	360421	G	A	0.133	18	71442664	1.90E-06	0.028	14306	23.333
Vascular dementia (mixed)	genus Prevotella9	rs111509883	T	C	0.005	639161	0.971	0.137	360421	T	C	0.171	19	639161	1.24E-06	0.035	14306	24.235
Vascular dementia (mixed)	genus Prevotella9	rs11685699	C	T	-0.133	11092559	0.397	0.157	360421	C	T	-0.141	2	11232685	2.03E-06	0.030	14306	22.858
Vascular dementia (mixed)	genus Prevotella9	rs117271932	A	G	0.131	43840580	0.474	0.183	360421	A	G	0.208	22	44236460	2.82E-06	0.044	14306	22.326
Vascular dementia (mixed)	genus Prevotella9	rs12648235	T	C	-0.136	160194592	0.173	0.100	360421	T	C	0.079	4	161115744	7.39E-06	0.018	14306	19.565
Vascular dementia (mixed)	genus Prevotella9	rs1304512	G	A	0.014	64440198	0.884	0.095	360421	G	A	0.076	5	63736025	5.29E-06	0.017	14306	21.078
Vascular dementia (mixed)	genus Prevotella9	rs2104588	C	T	-0.344	12455582	0.063	0.185	360421	C	T	-0.106	10	12497581	8.13E-06	0.024	14306	19.716
Vascular dementia (mixed)	genus Prevotella9	rs2495052	A	G	0.107	13834756	0.370	0.119	360421	A	G	0.084	1	14161251	8.97E-06	0.019	14306	19.805
Vascular dementia (mixed)	genus Prevotella9	rs2683313	G	A	0.098	19258094	0.281	0.091	360421	G	A	0.072	8	19115604	1.69E-06	0.015	14306	22.843
Vascular dementia (mixed)	genus Prevotella9	rs4968431	G	T	-0.177	61344417	0.044	0.088	360421	G	T	0.064	17	59421778	8.58E-06	0.014	14306	19.716
Vascular dementia (mixed)	genus Prevotella9	rs7237249	C	T	0.241	74325267	0.025	0.107	360421	C	T	-0.082	18	71992502	8.93E-06	0.018	14306	20.458
Vascular dementia (mixed)	genus Prevotella9	rs72815774	T	C	0.421	91766476	0.008	0.159	360421	T	C	-0.176	2	91954502	8.78E-06	0.039	14306	20.091
Vascular dementia (mixed)	genus Prevotella9	rs746764	T	C	0.081	21490075	0.429	0.103	360421	T	C	-0.092	20	21470713	2.04E-06	0.019	14306	22.457
Vascular dementia (mixed)	genus Prevotella9	rs7976209	T	C	-0.084	1685304	0.468	0.115	360421	T	C	-0.087	12	1794470	7.28E-06	0.020	14306	19.351
Vascular dementia (mixed)	genus Prevotella9	rs9428102	A	G	-0.167	118310194	0.102	0.102	360421	A	G	-0.078	1	118852817	4.62E-06	0.018	14306	19.568
Vascular dementia (mixed)	genus Prevotella9	rs9613013	G	A	0.003	25756771	0.984	0.129	360421	G	A	0.092	22	26152738	6.10E-06	0.020	14306	20.493
Vascular dementia (mixed)	genus Rikenellaceae RC9 gut group	rs12501673	A	G	-0.062	162909364	0.513	0.095	360421	A	G	0.116	4	163830516	6.29E-06	0.026	14306	19.698
Vascular dementia (mixed)	genus Rikenellaceae RC9 gut group	rs17032291	T	C	-0.143	154944519	0.265	0.129	360421	T	C	-0.170	4	155865671	6.61E-06	0.037	14306	21.206
Vascular dementia (mixed)	genus Rikenellaceae RC9 gut group	rs17582787	A	G	0.160	148641486	0.151	0.112	360421	A	G	-0.158	4	149562638	3.55E-06	0.034	14306	21.533
Vascular dementia (mixed)	genus Rikenellaceae RC9 gut group	rs2074881	T	C	-0.242	1970022	0.054	0.126	360421	T	C	-0.142	19	1970021	9.45E-06	0.032	14306	19.283
Vascular dementia (mixed)	genus Rikenellaceae RC9 gut group	rs2900503	T	G	0.016	109895263	0.888	0.114	360421	T	G	0.172	9	112657543	1.55E-07	0.033	14306	27.829
Vascular dementia (mixed)	genus Rikenellaceae RC9 gut group	rs2998141	C	T	0.046	133196694	0.638	0.099	360421	C	T	0.136	10	135010198	4.42E-06	0.029	14306	21.644
Vascular dementia (mixed)	genus Rikenellaceae RC9 gut group	rs4270579	A	G	0.005	83777035	0.959	0.090	360421	A	G	0.118	4	84698188	5.46E-06	0.027	14306	18.960
Vascular dementia (mixed)	genus Rikenellaceae RC9 gut group	rs4717843	G	T	0.063	73920657	0.460	0.085	360421	G	T	-0.119	7	73334987	4.72E-06	0.026	14306	20.992
Vascular dementia (mixed)	genus Rikenellaceae RC9 gut group	rs7712231	A	G	-0.092	31406178	0.458	0.124	360421	A	G	0.156	5	31406285	7.97E-06	0.035	14306	19.886
Vascular dementia (mixed)	genus Rikenellaceae RC9 gut group	rs80309088	G	A	-0.115	165255243	0.374	0.129	360421	G	A	0.174	6	165668732	4.56E-06	0.038	14306	20.606
Vascular dementia (mixed)	genus Rikenellaceae RC9 gut group	rs9887954	G	A	0.021	164831636	0.810	0.086	360421	G	A	-0.115	1	164800873	4.81E-06	0.025	14306	21.272
Vascular dementia (mixed)	genus Romboutsia	rs10279978	A	G	-0.007	5279756	0.935	0.091	360421	A	G	-0.062	7	5319387	1.17E-06	0.013	14306	23.749
Vascular dementia (mixed)	genus Romboutsia	rs11221428	T	C	0.034	99794319	0.739	0.101	360421	T	C	-0.073	11	99665050	6.49E-06	0.016	14306	21.089
Vascular dementia (mixed)	genus Romboutsia	rs16843578	C	T	0.049	171934745	0.796	0.188	360421	C	T	-0.088	1	171903855	5.08E-06	0.020	14306	19.785
Vascular dementia (mixed)	genus Romboutsia	rs28603357	T	C	0.526	106766378	0.083	0.303	360421	T	C	-0.215	7	106406824	8.52E-06	0.047	14306	20.493
Vascular dementia (mixed)	genus Romboutsia	rs34302036	G	A	-0.063	78507408	0.459	0.085	360421	G	A	-0.055	7	78136725	5.88E-06	0.012	14306	20.737
Vascular dementia (mixed)	genus Romboutsia	rs61841503	G	A	0.048	16977560	0.704	0.127	360421	G	A	0.093	10	17019559	4.00E-08	0.017	14306	29.351
Vascular dementia (mixed)	genus Romboutsia	rs62504452	A	G	0.221	146063205	0.071	0.122	360421	A	G	-0.071	7	145760298	4.66E-06	0.016	14306	20.544
Vascular dementia (mixed)	genus Romboutsia	rs7109293	A	G	0.108	133964762	0.409	0.131	360421	A	G	0.092	11	133834657	6.98E-06	0.021	14306	19.958
Vascular dementia (mixed)	genus Romboutsia	rs75200530	T	G	0.334	5230532	0.181	0.250	360421	T	G	-0.191	8	5088054	5.07E-06	0.042	14306	20.500
Vascular dementia (mixed)	genus Romboutsia	rs75987356	G	A	0.076	15926583	0.635	0.159	360421	G	A	-0.130	2	16066705	6.71E-06	0.028	14306	21.347
Vascular dementia (mixed)	genus Romboutsia	rs77702691	A	G	-0.276	88073285	0.066	0.150	360421	A	G	-0.094	13	88725540	7.37E-06	0.021	14306	20.493
Vascular dementia (mixed)	genus Romboutsia	rs9389266	T	G	-0.022	135090599	0.844	0.111	360421	T	G	0.072	6	135411737	9.38E-06	0.016	14306	19.821
Vascular dementia (mixed)	genus Romboutsia	rs9567264	C	T	-0.098	32146619	0.271	0.089	360421	C	T	0.058	13	32720756	5.76E-06	0.013	14306	20.696
Vascular dementia (mixed)	genus Roseburia	rs12740451	T	C	0.154	94713418	0.215	0.124	360421	T	C	0.070	1	95178974	7.34E-06	0.015	14306	20.621
Vascular dementia (mixed)	genus Roseburia	rs16910295	T	C	-0.062	11988022	0.740	0.186	360421	T	C	-0.098	11	12009569	2.91E-06	0.021	14306	21.887
Vascular dementia (mixed)	genus Roseburia	rs2160994	C	T	0.004	50256274	0.960	0.089	360421	C	T	-0.055	12	50650057	9.70E-07	0.011	14306	23.969
Vascular dementia (mixed)	genus Roseburia	rs2943022	T	C	0.080	90303097	0.352	0.086	360421	T	C	0.049	5	89598914	4.11E-06	0.011	14306	21.391
Vascular dementia (mixed)	genus Roseburia	rs302266	T	C	0.039	194214520	0.756	0.127	360421	T	C	-0.078	1	194183650	8.13E-06	0.017	14306	20.192
Vascular dementia (mixed)	genus Roseburia	rs329182	T	C	-0.048	125740801	0.672	0.114	360421	T	C	0.069	5	125076494	5.90E-06	0.015	14306	20.389
Vascular dementia (mixed)	genus Roseburia	rs55858165	A	C	-0.402	85969578	0.072	0.223	360421	A	C	0.179	15	86512809	9.99E-06	0.040	14306	19.601

Vascular dementia (mixed)	genus Roseburia	rs57466170	C	T	-0.016	37566864	0.921	0.161	360421	C	T	0.074	15	37859065	8.30E-06	0.017	14306	18.667
Vascular dementia (mixed)	genus Roseburia	rs6445851	A	G	-0.052	57082200	0.549	0.087	360421	A	G	0.050	3	57116228	3.53E-06	0.011	14306	21.144
Vascular dementia (mixed)	genus Roseburia	rs6930661	C	T	-0.152	12774379	0.395	0.178	360421	C	T	-0.096	6	12774611	2.48E-06	0.020	14306	22.008
Vascular dementia (mixed)	genus Roseburia	rs75326254	C	T	-0.005	165757348	0.979	0.175	360421	C	T	-0.105	6	166170836	7.50E-06	0.023	14306	20.533
Vascular dementia (mixed)	genus Roseburia	rs78753150	A	C	-0.069	144756092	0.624	0.140	360421	A	C	0.097	5	144135655	9.98E-06	0.021	14306	20.479
Vascular dementia (mixed)	genus Roseburia	rs9300744	C	T	0.087	102465136	0.429	0.110	360421	C	T	-0.059	13	103117486	4.75E-06	0.013	14306	21.733
Vascular dementia (mixed)	genus Ruminiclostridium5	rs10827477	A	G	-0.012	34973660	0.888	0.088	360421	A	G	-0.055	10	35262588	2.19E-06	0.012	14306	22.592
Vascular dementia (mixed)	genus Ruminiclostridium5	rs113753996	T	C	0.064	32477558	0.554	0.109	360421	T	C	0.082	5	32477664	3.99E-06	0.017	14306	22.128
Vascular dementia (mixed)	genus Ruminiclostridium5	rs1223978	C	T	-0.022	108122814	0.798	0.085	360421	C	T	-0.048	13	108775162	8.16E-06	0.011	14306	20.004
Vascular dementia (mixed)	genus Ruminiclostridium5	rs1492620	T	C	0.090	50438207	0.475	0.127	360421	T	C	-0.083	6	50405920	3.53E-06	0.018	14306	21.271
Vascular dementia (mixed)	genus Ruminiclostridium5	rs2482038	C	A	0.208	12793086	0.015	0.086	360421	C	A	0.052	10	12835085	1.70E-06	0.011	14306	22.764
Vascular dementia (mixed)	genus Ruminiclostridium5	rs2791343	T	C	0.154	130303784	0.072	0.086	360421	T	C	0.052	8	131316030	5.54E-06	0.011	14306	20.810
Vascular dementia (mixed)	genus Ruminiclostridium5	rs2833828	G	A	0.044	32439578	0.607	0.086	360421	G	A	0.049	21	33811886	6.82E-06	0.011	14306	20.288
Vascular dementia (mixed)	genus Ruminiclostridium5	rs4955951	A	G	0.064	55209503	0.626	0.131	360421	A	G	-0.071	3	55243531	9.96E-06	0.017	14306	18.526
Vascular dementia (mixed)	genus Ruminiclostridium5	rs6121460	G	A	-0.188	61718449	0.225	0.155	360421	G	A	0.093	20	60293505	2.64E-06	0.020	14306	21.934
Vascular dementia (mixed)	genus Ruminiclostridium5	rs79968837	A	G	-0.122	42549316	0.522	0.191	360421	A	G	-0.095	20	41177956	1.15E-06	0.019	14306	24.118
Vascular dementia (mixed)	genus Ruminiclostridium5	rs8053158	G	A	0.111	86736303	0.386	0.128	360421	G	A	0.074	16	86769909	5.90E-06	0.016	14306	21.651
Vascular dementia (mixed)	genus Ruminiclostridium6	rs10829821	T	C	0.297	130853030	0.040	0.145	360421	T	C	-0.098	10	132651293	3.47E-06	0.022	14306	20.406
Vascular dementia (mixed)	genus Ruminiclostridium6	rs116969552	A	G	-0.293	126525834	0.243	0.251	360421	A	G	-0.167	10	128214403	9.16E-06	0.038	14306	19.614
Vascular dementia (mixed)	genus Ruminiclostridium6	rs11992182	A	C	0.054	78854264	0.591	0.101	360421	A	C	0.063	8	79766499	4.65E-06	0.014	14306	20.568
Vascular dementia (mixed)	genus Ruminiclostridium6	rs2548459	C	T	0.087	48706082	0.305	0.085	360421	C	T	0.055	19	49209339	6.40E-06	0.012	14306	20.431
Vascular dementia (mixed)	genus Ruminiclostridium6	rs35362464	C	A	0.015	36476389	0.902	0.122	360421	C	A	0.072	4	36478011	8.99E-06	0.017	14306	18.956
Vascular dementia (mixed)	genus Ruminiclostridium6	rs61060922	T	G	-0.274	72102255	0.246	0.236	360421	T	G	0.159	16	72136154	1.09E-06	0.032	14306	24.380
Vascular dementia (mixed)	genus Ruminiclostridium6	rs663262	C	T	-0.282	86468034	0.234	0.236	360421	C	T	0.135	11	86179076	3.39E-06	0.031	14306	18.872
Vascular dementia (mixed)	genus Ruminiclostridium6	rs67479537	T	C	-0.156	10004839	0.428	0.196	360421	T	C	0.119	19	10115515	9.30E-06	0.026	14306	20.183
Vascular dementia (mixed)	genus Ruminiclostridium6	rs71414120	T	G	0.099	56472234	0.606	0.193	360421	T	G	0.201	14	56938952	1.08E-06	0.041	14306	24.432
Vascular dementia (mixed)	genus Ruminiclostridium6	rs72991535	T	G	0.248	78258244	0.281	0.230	360421	T	G	0.136	18	76018244	4.95E-06	0.030	14306	21.105
Vascular dementia (mixed)	genus Ruminiclostridium6	rs73176030	T	C	0.019	101628002	0.840	0.094	360421	T	C	0.059	7	101271282	7.29E-06	0.013	14306	19.728
Vascular dementia (mixed)	genus Ruminiclostridium6	rs77193512	A	G	-0.064	40267513	0.508	0.097	360421	A	G	0.074	11	40289063	1.30E-06	0.015	14306	23.125
Vascular dementia (mixed)	genus Ruminiclostridium6	rs792058	G	A	0.025	5408472	0.771	0.086	360421	G	A	0.055	2	5548605	8.58E-06	0.013	14306	19.527
Vascular dementia (mixed)	genus Ruminiclostridium6	rs79968172	G	A	-0.211	240340526	0.238	0.179	360421	G	A	0.116	1	240503826	1.66E-06	0.024	14306	22.827
Vascular dementia (mixed)	genus Ruminiclostridium6	rs9555756	A	C	-0.061	111050902	0.684	0.150	360421	A	C	-0.080	13	111703249	7.10E-06	0.018	14306	20.712
Vascular dementia (mixed)	genus Ruminiclostridium9	rs12040548	G	T	0.006	247546983	0.951	0.094	360421	G	T	0.057	1	247710285	3.15E-06	0.012	14306	21.733
Vascular dementia (mixed)	genus Ruminiclostridium9	rs6082461	C	A	-0.161	2229884	0.120	0.103	360421	C	A	-0.059	20	2210530	4.87E-06	0.013	14306	20.038
Vascular dementia (mixed)	genus Ruminiclostridium9	rs7137760	C	T	0.071	10549815	0.398	0.085	360421	C	T	0.051	12	10702414	7.07E-06	0.011	14306	20.504
Vascular dementia (mixed)	genus Ruminiclostridium9	rs74303178	T	C	-0.040	14840035	0.655	0.090	360421	T	C	0.053	8	14697544	7.92E-06	0.012	14306	19.950
Vascular dementia (mixed)	genus Ruminiclostridium9	rs78191726	T	C	0.380	66945427	0.020	0.163	360421	T	C	0.094	6	67655320	7.58E-06	0.021	14306	20.210
Vascular dementia (mixed)	genus Ruminiclostridium9	rs918449	G	A	-0.183	33880293	0.267	0.165	360421	G	A	0.095	19	34371198	2.56E-06	0.020	14306	23.258
Vascular dementia (mixed)	genus Ruminiclostridium9	rs9522712	T	C	0.045	89789324	0.705	0.118	360421	T	C	0.070	13	90441578	4.66E-06	0.015	14306	20.396
Vascular dementia (mixed)	genus Ruminiclostridium9	rs9809789	C	T	0.080	29138492	0.464	0.110	360421	C	T	-0.072	3	29179983	8.72E-06	0.016	14306	20.189
Vascular dementia (mixed)	genus Ruminococcaceae NK4A214 group	rs11241747	T	C	0.074	124510625	0.424	0.093	360421	T	C	-0.053	5	123846318	6.59E-06	0.012	14306	19.780
Vascular dementia (mixed)	genus Ruminococcaceae NK4A214 group	rs11586410	G	A	0.120	157369098	0.301	0.116	360421	G	A	-0.086	1	157338888	3.66E-07	0.017	14306	25.815
Vascular dementia (mixed)	genus Ruminococcaceae NK4A214 group	rs12642039	C	T	0.002	159010870	0.979	0.087	360421	C	T	0.055	4	159932022	3.43E-06	0.012	14306	21.452
Vascular dementia (mixed)	genus Ruminococcaceae NK4A214 group	rs12731	A	G	0.030	238179271	0.732	0.087	360421	A	G	-0.053	2	239087912	4.87E-06	0.012	14306	21.035
Vascular dementia (mixed)	genus Ruminococcaceae NK4A214 group	rs13087692	G	T	0.171	84675888	0.063	0.092	360421	G	T	-0.057	3	84725039	8.69E-06	0.013	14306	20.818
Vascular dementia (mixed)	genus Ruminococcaceae NK4A214 group	rs136761	A	G	0.079	49402365	0.370	0.088	360421	A	G	0.059	22	49796014	8.15E-07	0.012	14306	24.312
Vascular dementia (mixed)	genus Ruminococcaceae NK4A214 group	rs147475196	A	G	-0.030	26467796	0.827	0.138	360421	A	G	-0.134	3	26509287	4.72E-06	0.030	14306	20.535
Vascular dementia (mixed)	genus Ruminococcaceae NK4A214 group	rs35559912	T	C	-0.289	35288836	0.024	0.128	360421	T	C	-0.093	5	35288938	4.89E-06	0.020	14306	20.629
Vascular dementia (mixed)	genus Ruminococcaceae NK4A214 group	rs4814689	C	T	-0.093	18049713	0.653	0.206	360421	C	T	-0.108	20	18030357	4.55E-06	0.023	14306	22.036
Vascular dementia (mixed)	genus Ruminococcaceae NK4A214 group	rs5994253	A	G	-0.027	17312122	0.825	0.121	360421	A	G	-0.081	22	17793012	2.35E-07	0.016	14306	26.493
Vascular dementia (mixed)	genus Ruminococcaceae NK4A214 group	rs62027366	T	C	0.149	24062485	0.153	0.104	360421	T	C	0.062	16	24073806	6.58E-06	0.014	14306	19.998
Vascular dementia (mixed)	genus Ruminococcaceae NK4A214 group	rs6681678	T	C	-0.469	99783035	0.015	0.194	360421	T	C	0.100	1	100248591	9.05E-06	0.024	14306	17.422
Vascular dementia (mixed)	genus Ruminococcaceae NK4A214 group	rs7573569	T	C	-0.111	141139473	0.526	0.176	360421	T	C	0.108	2	141897042	3.23E-06	0.023	14306	21.265
Vascular dementia (mixed)	genus Ruminococcaceae UCG002	rs10916131	C	T	0.126	227375425	0.272	0.115	360421	C	T	-0.069	1	227563126	2.87E-06	0.015	14306	22.321
Vascular dementia (mixed)	genus Ruminococcaceae UCG002	rs10927423	C	A	0.027	14405962	0.809	0.110	360421	C	A	-0.071	1	14732458	8.50E-07	0.015	14306	23.340
Vascular dementia (mixed)	genus Ruminococcaceae UCG002	rs10964441	G	A	0.190	20131748	0.169	0.138	360421	G	A	-0.149	9	20131746	7.45E-06	0.034	14306	18.683
Vascular dementia (mixed)	genus Ruminococcaceae UCG002	rs113147300	A	G	0.254	111304390	0.040	0.123	360421	A	G	-0.076	9	114066670	7.69E-06	0.016	14306	21.240
Vascular dementia (mixed)	genus Ruminococcaceae UCG002	rs11607472	A	G	-0.057	43323201	0.735	0.168	360421	A	G	-0.078	11	43344751	7.19E-06	0.018	14306	19.580
Vascular dementia (mixed)	genus Ruminococcaceae UCG002	rs11750293	G	T	-0.024	124486421	0.783	0.088	360421	G	T	-0.058	5	123822114	1.76E-06	0.012	14306	23.028
Vascular dementia (mixed)	genus Ruminococcaceae UCG002	rs12463378	A	G	0.031	53972554	0.736	0.092	360421	A	G	-0.052	19	54475808	2.96E-06	0.011	14306	21.694
Vascular dementia (mixed)	genus Ruminococcaceae UCG002	rs15256	C	T	0.003	72060790	0.984	0.130	360421	C	T	0.073	10	73820548	9.46E-06	0.017	14306	18.928
Vascular dementia (mixed)	genus Ruminococcaceae UCG002	rs57079348	T	G	0.160	87268195	0.379	0.182	360421	T	G	-0.077	13	87920450	7.22E-06	0.017	14306	19.632
Vascular dementia (mixed)	genus Ruminococcaceae UCG002	rs6542556	G	A	-0.174	120000277	0.045	0.087	360421	G	A	-0.051	2	120757853	7.86E-06	0.011	14306	19.972
Vascular dementia (mixed)	genus Ruminococcaceae UCG002	rs6793778	T	C	0.031	24003962	0.749	0.096	360421	T	C	0.056	3	24045453	9.81E-06	0.013	14306	19.896
Vascular dementia (mixed)	genus Ruminococcaceae UCG002	rs7120052	A	C	-0.073	86624417	0.496	0.107	360421	A	C	0.062	11	86335459	1.97E-06	0.014	14306	21.254
Vascular dementia (mixed)	genus Ruminococcaceae UCG002	rs7155595	C	A	0.192	77036203	0.037	0.092	360421	C	A	0.057	14	77502546	1.15E-06	0.012	14306	23.734
Vascular dementia (mixed)	genus Ruminococcaceae UCG002	rs7249614	G	A	0.146	43133738	0.094	0.087	360421	G	A	0.049	19	43637890	9.07E-06	0.011	14306	19.777

Vascular dementia (mixed)	genus Ruminococcaceae UCG002	rs76847269	A	G	-0.407	141635384	0.125	0.265	360421	A	G	0.164	5	141014951	5.17E-06	0.036	14306	21.077
Vascular dementia (mixed)	genus Ruminococcaceae UCG002	rs77564310	A	C	0.004	80420842	0.971	0.103	360421	A	C	-0.071	15	80713184	3.29E-07	0.014	14306	25.630
Vascular dementia (mixed)	genus Ruminococcaceae UCG002	rs79016051	C	T	-0.221	238775197	0.080	0.126	360421	C	T	-0.089	1	238938497	2.34E-06	0.019	14306	21.964
Vascular dementia (mixed)	genus Ruminococcaceae UCG002	rs882348	A	G	0.108	23323417	0.404	0.130	360421	A	G	-0.080	4	23325040	5.45E-06	0.018	14306	20.057
Vascular dementia (mixed)	genus Ruminococcaceae UCG003	rs10490280	C	T	0.003	37678833	0.975	0.108	360421	C	T	-0.067	2	37905976	4.16E-06	0.014	14306	21.994
Vascular dementia (mixed)	genus Ruminococcaceae UCG003	rs11243416	T	C	-0.014	131541583	0.930	0.166	360421	T	C	-0.093	9	134416970	1.67E-06	0.019	14306	23.437
Vascular dementia (mixed)	genus Ruminococcaceae UCG003	rs11613919	G	T	-0.051	75102683	0.611	0.100	360421	G	T	0.073	12	75496463	1.63E-06	0.016	14306	21.834
Vascular dementia (mixed)	genus Ruminococcaceae UCG003	rs16959793	A	C	0.013	34779517	0.880	0.086	360421	A	C	-0.063	15	35071718	2.22E-06	0.013	14306	22.692
Vascular dementia (mixed)	genus Ruminococcaceae UCG003	rs2523124	C	T	-0.052	97719124	0.542	0.086	360421	C	T	0.055	7	97348436	5.78E-06	0.012	14306	20.468
Vascular dementia (mixed)	genus Ruminococcaceae UCG003	rs3013089	G	A	0.085	13468126	0.330	0.087	360421	G	A	-0.055	1	13794594	4.38E-06	0.012	14306	20.998
Vascular dementia (mixed)	genus Ruminococcaceae UCG003	rs4452755	A	C	-0.055	81114617	0.536	0.089	360421	A	C	-0.063	8	82026852	3.29E-06	0.013	14306	22.172
Vascular dementia (mixed)	genus Ruminococcaceae UCG003	rs4532474	G	A	0.028	105333663	0.802	0.113	360421	G	A	0.077	6	105781538	4.82E-06	0.017	14306	20.367
Vascular dementia (mixed)	genus Ruminococcaceae UCG003	rs646327	G	A	0.077	48706594	0.364	0.085	360421	G	A	0.059	19	49209851	7.83E-07	0.012	14306	24.567
Vascular dementia (mixed)	genus Ruminococcaceae UCG003	rs6759615	A	G	0.018	204373993	0.901	0.145	360421	A	G	0.103	2	205238716	7.86E-07	0.020	14306	26.224
Vascular dementia (mixed)	genus Ruminococcaceae UCG003	rs73341549	T	C	0.377	51473771	0.040	0.184	360421	T	C	-0.170	7	51541468	1.51E-07	0.032	14306	28.393
Vascular dementia (mixed)	genus Ruminococcaceae UCG003	rs78720113	A	G	-0.226	41940901	0.150	0.157	360421	A	G	-0.115	3	41982393	7.59E-06	0.025	14306	21.356
Vascular dementia (mixed)	genus Ruminococcaceae UCG004	rs10976229	T	G	0.100	7317307	0.427	0.126	360421	T	G	0.096	9	7317307	7.04E-06	0.021	14306	20.010
Vascular dementia (mixed)	genus Ruminococcaceae UCG004	rs11961899	G	A	0.043	132566782	0.644	0.093	360421	G	A	-0.071	6	132887921	9.18E-06	0.016	14306	19.237
Vascular dementia (mixed)	genus Ruminococcaceae UCG004	rs12125734	G	T	-0.138	103266658	0.346	0.146	360421	G	T	0.134	1	103732214	2.09E-07	0.026	14306	27.087
Vascular dementia (mixed)	genus Ruminococcaceae UCG004	rs2248146	T	C	-0.134	24752379	0.128	0.088	360421	T	C	0.069	7	24791998	8.20E-06	0.015	14306	20.135
Vascular dementia (mixed)	genus Ruminococcaceae UCG004	rs3800154	A	C	0.033	2119095	0.733	0.096	360421	A	C	-0.080	6	2119329	6.12E-06	0.018	14306	20.178
Vascular dementia (mixed)	genus Ruminococcaceae UCG004	rs511258	G	A	0.029	170248934	0.791	0.108	360421	G	A	-0.076	3	169966722	4.52E-06	0.016	14306	21.735
Vascular dementia (mixed)	genus Ruminococcaceae UCG004	rs550351	A	C	-0.019	18349905	0.824	0.085	360421	A	C	0.079	1	18676399	9.43E-06	0.018	14306	19.055
Vascular dementia (mixed)	genus Ruminococcaceae UCG004	rs6769553	A	G	-0.137	54393768	0.153	0.096	360421	A	G	0.085	3	54427795	7.91E-08	0.016	14306	29.126
Vascular dementia (mixed)	genus Ruminococcaceae UCG004	rs7569771	A	G	-0.090	238924641	0.359	0.098	360421	A	G	-0.076	2	239846337	8.12E-06	0.017	14306	19.869
Vascular dementia (mixed)	genus Ruminococcaceae UCG004	rs872501	G	A	0.155	138240821	0.311	0.153	360421	G	A	0.116	8	139253064	5.81E-06	0.026	14306	20.031
Vascular dementia (mixed)	genus Ruminococcaceae UCG004	rs9818949	T	G	0.142	197956880	0.179	0.106	360421	T	G	-0.086	3	197683751	5.39E-06	0.019	14306	20.774
Vascular dementia (mixed)	genus Ruminococcaceae UCG005	rs10873449	C	T	0.004	94164394	0.968	0.107	360421	C	T	-0.065	14	94630731	4.11E-06	0.014	14306	20.688
Vascular dementia (mixed)	genus Ruminococcaceae UCG005	rs10937802	A	G	-0.042	7300617	0.749	0.130	360421	A	G	-0.076	4	7302344	8.17E-06	0.017	14306	20.186
Vascular dementia (mixed)	genus Ruminococcaceae UCG005	rs10950694	C	T	0.045	17986981	0.603	0.087	360421	C	T	-0.058	7	18026604	4.30E-07	0.011	14306	25.620
Vascular dementia (mixed)	genus Ruminococcaceae UCG005	rs114279581	A	G	-0.177	197257633	0.266	0.159	360421	A	G	-0.147	2	198122357	3.22E-06	0.032	14306	21.519
Vascular dementia (mixed)	genus Ruminococcaceae UCG005	rs12288512	A	G	-0.185	27726124	0.061	0.098	360421	A	G	0.067	11	27747671	3.10E-06	0.014	14306	21.308
Vascular dementia (mixed)	genus Ruminococcaceae UCG005	rs2893871	G	A	-0.048	60903023	0.721	0.133	360421	G	A	-0.074	10	62662781	3.54E-06	0.016	14306	22.435
Vascular dementia (mixed)	genus Ruminococcaceae UCG005	rs72776570	C	A	-0.206	4198714	0.146	0.142	360421	C	A	0.087	10	4240906	5.36E-06	0.020	14306	19.498
Vascular dementia (mixed)	genus Ruminococcaceae UCG005	rs7449320	C	A	0.185	154623531	0.063	0.099	360421	C	A	0.060	5	154003091	4.81E-06	0.013	14306	20.972
Vascular dementia (mixed)	genus Ruminococcaceae UCG005	rs7555878	G	A	-0.164	187936903	0.089	0.096	360421	G	A	-0.059	1	187906034	2.81E-06	0.013	14306	21.931
Vascular dementia (mixed)	genus Ruminococcaceae UCG005	rs7586445	G	A	-0.075	238600389	0.548	0.125	360421	G	A	0.078	2	239509030	8.81E-06	0.018	14306	19.657
Vascular dementia (mixed)	genus Ruminococcaceae UCG005	rs898577	C	T	0.023	68413358	0.900	0.183	360421	C	T	0.123	15	68705697	7.46E-06	0.029	14306	18.413
Vascular dementia (mixed)	genus Ruminococcaceae UCG009	rs12508214	C	T	0.117	7425210	0.191	0.090	360421	C	T	-0.077	4	7426937	4.75E-06	0.017	14306	21.026
Vascular dementia (mixed)	genus Ruminococcaceae UCG009	rs138460696	A	G	-0.149	66169644	0.351	0.160	360421	A	G	0.139	2	66396776	9.81E-06	0.032	14306	19.451
Vascular dementia (mixed)	genus Ruminococcaceae UCG009	rs1550196	A	G	0.024	34459936	0.865	0.141	360421	A	G	-0.131	17	32786955	1.13E-06	0.026	14306	24.849
Vascular dementia (mixed)	genus Ruminococcaceae UCG009	rs2058609	G	A	-0.028	12817858	0.766	0.094	360421	G	A	-0.082	12	12970792	3.12E-06	0.017	14306	21.837
Vascular dementia (mixed)	genus Ruminococcaceae UCG009	rs2192926	A	G	-0.040	75285542	0.657	0.090	360421	A	G	-0.089	2	75512668	4.88E-06	0.019	14306	21.290
Vascular dementia (mixed)	genus Ruminococcaceae UCG009	rs4079028	C	T	0.054	202329957	0.587	0.099	360421	C	T	0.092	1	202299085	3.28E-06	0.020	14306	21.094
Vascular dementia (mixed)	genus Ruminococcaceae UCG009	rs4708333	T	G	-0.060	77396984	0.502	0.089	360421	T	G	-0.084	6	78106701	1.56E-06	0.017	14306	23.122
Vascular dementia (mixed)	genus Ruminococcaceae UCG009	rs758191	T	G	-0.004	1929830	0.980	0.141	360421	T	G	0.177	16	1979831	9.01E-06	0.038	14306	22.270
Vascular dementia (mixed)	genus Ruminococcaceae UCG009	rs78410648	A	G	-0.031	51896148	0.821	0.136	360421	A	G	0.121	19	52399401	9.67E-06	0.028	14306	19.034
Vascular dementia (mixed)	genus Ruminococcaceae UCG009	rs9558661	T	C	-0.215	105997744	0.040	0.105	360421	T	C	-0.090	13	106650093	7.01E-06	0.020	14306	20.004
Vascular dementia (mixed)	genus Ruminococcaceae UCG010	rs12597105	A	G	0.232	5183940	0.029	0.107	360421	A	G	-0.067	16	5233941	4.87E-06	0.014	14306	21.579
Vascular dementia (mixed)	genus Ruminococcaceae UCG010	rs2820282	C	A	-0.043	104280743	0.620	0.087	360421	C	A	0.059	6	104728618	2.85E-06	0.013	14306	22.127
Vascular dementia (mixed)	genus Ruminococcaceae UCG010	rs682403	A	G	0.038	133093170	0.648	0.084	360421	A	G	-0.059	9	135968557	2.37E-06	0.012	14306	22.257
Vascular dementia (mixed)	genus Ruminococcaceae UCG010	rs6958419	C	T	-0.115	16310239	0.173	0.085	360421	C	T	-0.059	7	16349864	2.84E-06	0.012	14306	21.958
Vascular dementia (mixed)	genus Ruminococcaceae UCG010	rs73218807	G	A	0.143	31055860	0.342	0.150	360421	G	A	-0.166	4	31057482	6.43E-06	0.037	14306	20.407
Vascular dementia (mixed)	genus Ruminococcaceae UCG010	rs7441445	T	C	0.043	40705781	0.606	0.084	360421	T	C	0.057	4	40707798	6.80E-06	0.013	14306	20.267
Vascular dementia (mixed)	genus Ruminococcaceae UCG011	rs10274562	C	T	-0.014	11182894	0.871	0.086	360421	C	T	0.111	7	11222521	6.50E-06	0.024	14306	20.570
Vascular dementia (mixed)	genus Ruminococcaceae UCG011	rs12636310	G	A	-0.104	185751703	0.286	0.097	360421	G	A	0.133	3	185469491	2.81E-06	0.028	14306	22.146
Vascular dementia (mixed)	genus Ruminococcaceae UCG011	rs12724320	C	T	-0.003	179401364	0.973	0.086	360421	C	T	-0.121	1	179370499	1.52E-06	0.025	14306	23.524
Vascular dementia (mixed)	genus Ruminococcaceae UCG011	rs1416041	A	C	0.007	104625801	0.942	0.103	360421	A	C	-0.182	6	105073676	7.04E-08	0.034	14306	28.778
Vascular dementia (mixed)	genus Ruminococcaceae UCG011	rs2729556	T	C	-0.094	112123933	0.269	0.085	360421	T	C	0.109	7	111763988	3.19E-06	0.023	14306	21.793
Vascular dementia (mixed)	genus Ruminococcaceae UCG011	rs4490371	T	C	0.028	76155956	0.746	0.086	360421	T	C	-0.112	3	76205107	7.75E-06	0.025	14306	20.172
Vascular dementia (mixed)	genus Ruminococcaceae UCG011	rs79113084	C	T	-0.010	29481986	0.941	0.137	360421	C	T	-0.152	12	29634919	2.06E-06	0.032	14306	22.967
Vascular dementia (mixed)	genus Ruminococcaceae UCG011	rs9729514	G	A	-0.065	219727713	0.653	0.145	360421	G	A	-0.185	1	219901055	2.37E-06	0.039	14306	21.963
Vascular dementia (mixed)	genus Ruminococcaceae UCG013	rs11581881	C	T	-0.252	9301517	0.013	0.101	360421	C	T	0.066	1	9361576	4.73E-06	0.014	14306	20.870
Vascular dementia (mixed)	genus Ruminococcaceae UCG013	rs12189346	G	A	0.047	142015575	0.663	0.108	360421	G	A	0.068	5	141395140	1.68E-06	0.015	14306	22.137
Vascular dementia (mixed)	genus Ruminococcaceae UCG013	rs12336782	T	C	0.085	13609120	0.597	0.161	360421	T	C	-0.086	9	13609119	8.60E-06	0.019	14306	20.448
Vascular dementia (mixed)	genus Ruminococcaceae UCG013	rs12485353	G	A	-0.096	197323125	0.322	0.097	360421	G	A	-0.061	3	197049996	4.19E-06	0.013	14306	21.584
Vascular dementia (mixed)	genus Ruminococcaceae UCG013	rs12781711	C	T	-0.041	2177736	0.672	0.098	360421	C	T	-0.066	10	2219930	2.55E-08	0.012	14306	31.194

Vascular dementia (mixed)	genus Ruminococcaceae UCG013	rs16918863	A	C	-0.103	19491534	0.550	0.172	360421	A	C	0.111	10	19780463	4.15E-06	0.024	14306	21.552
Vascular dementia (mixed)	genus Ruminococcaceae UCG013	rs2730183	G	A	-0.019	40454366	0.824	0.086	360421	G	A	-0.049	8	40311885	8.44E-06	0.011	14306	19.771
Vascular dementia (mixed)	genus Ruminococcaceae UCG013	rs4385846	T	G	-0.189	113890519	0.076	0.107	360421	T	G	-0.060	10	115650278	6.46E-06	0.013	14306	20.612
Vascular dementia (mixed)	genus Ruminococcaceae UCG013	rs75088940	T	C	-0.121	30092624	0.461	0.164	360421	T	C	-0.094	12	30245557	2.55E-06	0.020	14306	22.072
Vascular dementia (mixed)	genus Ruminococcaceae UCG013	rs76973485	G	T	-0.249	9492973	0.238	0.211	360421	G	T	0.195	3	9534657	3.35E-06	0.042	14306	21.735
Vascular dementia (mixed)	genus Ruminococcaceae UCG013	rs9313055	T	C	0.127	3628768	0.415	0.156	360421	T	C	0.105	5	3628882	9.55E-06	0.023	14306	20.089
Vascular dementia (mixed)	genus Ruminococcaceae UCG014	rs10495392	T	C	-0.082	237259689	0.613	0.162	360421	T	C	0.082	1	237422989	9.96E-06	0.019	14306	19.417
Vascular dementia (mixed)	genus Ruminococcaceae UCG014	rs10791168	G	A	0.016	131789106	0.881	0.110	360421	G	A	0.066	11	131659000	9.76E-06	0.015	14306	19.614
Vascular dementia (mixed)	genus Ruminococcaceae UCG014	rs10941294	C	T	-0.055	36435495	0.764	0.183	360421	C	T	-0.122	5	36435597	2.40E-06	0.026	14306	22.035
Vascular dementia (mixed)	genus Ruminococcaceae UCG014	rs115777838	T	C	0.255	26110517	0.055	0.133	360421	T	C	-0.188	5	26110626	4.62E-07	0.039	14306	23.731
Vascular dementia (mixed)	genus Ruminococcaceae UCG014	rs12638134	T	G	0.051	101615765	0.550	0.085	360421	T	G	0.058	3	101334609	1.21E-06	0.012	14306	23.702
Vascular dementia (mixed)	genus Ruminococcaceae UCG014	rs34402072	C	T	0.109	3779026	0.364	0.120	360421	C	T	-0.069	8	3636548	9.80E-06	0.016	14306	19.431
Vascular dementia (mixed)	genus Ruminococcaceae UCG014	rs56105232	G	A	-0.282	14363770	0.114	0.179	360421	G	A	0.139	9	14363769	2.91E-06	0.030	14306	21.678
Vascular dementia (mixed)	genus Ruminococcaceae UCG014	rs72809222	T	C	0.061	56978719	0.551	0.103	360421	T	C	0.067	2	57205854	2.41E-06	0.014	14306	23.078
Vascular dementia (mixed)	genus Ruminococcaceae UCG014	rs853612	A	G	-0.095	118168902	0.267	0.086	360421	A	G	-0.053	10	119928413	9.75E-06	0.012	14306	19.585
Vascular dementia (mixed)	genus Ruminococcaceae UCG014	rs995642	C	T	-0.105	134097088	0.292	0.099	360421	C	T	0.060	2	134854659	1.90E-06	0.013	14306	22.562
Vascular dementia (mixed)	genus Ruminococcus1	rs10167839	A	G	-0.052	181018152	0.556	0.088	360421	A	G	0.052	2	181882879	8.09E-06	0.012	14306	19.952
Vascular dementia (mixed)	genus Ruminococcus1	rs11783695	G	T	-0.043	143514733	0.707	0.115	360421	G	T	-0.073	8	144596903	4.73E-06	0.016	14306	20.689
Vascular dementia (mixed)	genus Ruminococcus1	rs17781867	C	T	-0.272	73259879	0.096	0.164	360421	C	T	0.100	17	71256018	1.96E-06	0.021	14306	22.275
Vascular dementia (mixed)	genus Ruminococcus1	rs3819978	C	T	-0.049	241626455	0.762	0.161	360421	C	T	-0.115	1	241789757	8.74E-06	0.026	14306	19.557
Vascular dementia (mixed)	genus Ruminococcus1	rs6105066	T	C	0.073	13277981	0.439	0.095	360421	T	C	-0.061	20	13258628	5.06E-06	0.013	14306	20.447
Vascular dementia (mixed)	genus Ruminococcus1	rs6493760	T	C	0.141	55146984	0.111	0.088	360421	T	C	-0.054	15	55439182	3.38E-06	0.012	14306	21.334
Vascular dementia (mixed)	genus Ruminococcus1	rs7117576	A	G	0.076	114152851	0.603	0.146	360421	A	G	0.083	11	114023573	6.48E-07	0.017	14306	23.561
Vascular dementia (mixed)	genus Ruminococcus1	rs7583465	C	T	-0.019	10393795	0.826	0.085	360421	C	T	0.053	2	10533921	2.56E-06	0.011	14306	21.952
Vascular dementia (mixed)	genus Ruminococcus1	rs78572139	G	A	0.005	15944478	0.970	0.139	360421	G	A	0.125	5	15944587	5.23E-06	0.028	14306	20.026
Vascular dementia (mixed)	genus Ruminococcus1	rs78613526	G	A	-0.038	65698078	0.843	0.190	360421	G	A	0.167	2	65925212	5.11E-06	0.037	14306	20.757
Vascular dementia (mixed)	genus Ruminococcus2	rs12406309	A	C	-0.120	107531823	0.239	0.102	360421	A	C	-0.063	1	108074445	9.79E-06	0.014	14306	19.728
Vascular dementia (mixed)	genus Ruminococcus2	rs12986628	T	C	-0.112	237194796	0.291	0.106	360421	T	C	-0.067	2	238103439	2.14E-06	0.014	14306	22.572
Vascular dementia (mixed)	genus Ruminococcus2	rs1819812	G	T	0.089	151738636	0.636	0.187	360421	G	T	0.084	7	151435722	5.28E-06	0.018	14306	20.756
Vascular dementia (mixed)	genus Ruminococcus2	rs2368224	T	G	0.084	181791689	0.668	0.196	360421	T	G	0.200	2	182656416	3.63E-06	0.044	14306	20.734
Vascular dementia (mixed)	genus Ruminococcus2	rs2846589	T	G	-0.052	862476	0.542	0.086	360421	T	G	-0.052	18	862477	7.59E-06	0.012	14306	20.124
Vascular dementia (mixed)	genus Ruminococcus2	rs2997412	G	A	-0.031	61493648	0.742	0.094	360421	G	A	0.057	13	62067781	4.22E-06	0.012	14306	21.594
Vascular dementia (mixed)	genus Ruminococcus2	rs4400279	A	G	-0.155	16764953	0.082	0.089	360421	A	G	0.055	7	16804578	5.80E-06	0.012	14306	20.685
Vascular dementia (mixed)	genus Ruminococcus2	rs4799823	C	T	-0.069	35876464	0.524	0.109	360421	C	T	0.084	18	33456427	5.40E-06	0.018	14306	21.089
Vascular dementia (mixed)	genus Ruminococcus2	rs55707116	C	A	0.158	77304469	0.312	0.156	360421	C	A	0.087	9	79919385	8.01E-06	0.019	14306	20.940
Vascular dementia (mixed)	genus Ruminococcus2	rs58681734	A	G	-0.057	134666044	0.584	0.103	360421	A	G	0.072	9	137557890	4.18E-06	0.016	14306	20.269
Vascular dementia (mixed)	genus Ruminococcus2	rs61791565	T	C	0.002	24653840	0.982	0.085	360421	T	C	-0.052	4	24655463	6.79E-06	0.012	14306	19.979
Vascular dementia (mixed)	genus Ruminococcus2	rs75140805	T	G	-0.065	12127041	0.557	0.111	360421	T	G	0.084	11	12148588	3.95E-06	0.018	14306	22.493
Vascular dementia (mixed)	genus Ruminococcus2	rs7635831	G	A	0.024	173323134	0.788	0.087	360421	G	A	0.062	3	173040924	1.98E-06	0.013	14306	22.965
Vascular dementia (mixed)	genus Ruminococcus2	rs7693984	G	A	0.289	4266595	0.155	0.203	360421	G	A	-0.103	4	4268322	9.42E-06	0.024	14306	19.130
Vascular dementia (mixed)	genus Ruminococcus2	rs78120384	A	G	-0.121	106945516	0.410	0.146	360421	A	G	-0.193	3	106664363	3.31E-07	0.039	14306	24.189
Vascular dementia (mixed)	genus Ruminococcus gauvreauii group	rs10931481	A	G	0.002	191090126	0.981	0.093	360421	A	G	-0.061	2	191954852	3.38E-06	0.013	14306	21.862
Vascular dementia (mixed)	genus Ruminococcus gauvreauii group	rs12079579	A	G	0.339	161910692	0.011	0.133	360421	A	G	0.096	1	161880482	5.04E-06	0.021	14306	20.034
Vascular dementia (mixed)	genus Ruminococcus gauvreauii group	rs12539819	C	T	0.001	153790311	0.997	0.166	360421	C	T	0.111	7	153487396	4.49E-06	0.024	14306	21.137
Vascular dementia (mixed)	genus Ruminococcus gauvreauii group	rs1391597	C	T	0.100	60725485	0.248	0.086	360421	C	T	0.059	12	61119266	1.86E-06	0.012	14306	22.345
Vascular dementia (mixed)	genus Ruminococcus gauvreauii group	rs2047242	A	G	-0.048	28912931	0.635	0.100	360421	A	G	-0.068	10	29201860	2.46E-07	0.013	14306	25.552
Vascular dementia (mixed)	genus Ruminococcus gauvreauii group	rs2166943	C	A	-0.012	136078607	0.889	0.085	360421	C	A	-0.057	8	137090850	5.28E-06	0.012	14306	21.077
Vascular dementia (mixed)	genus Ruminococcus gauvreauii group	rs289410	A	G	-0.128	85020252	0.173	0.094	360421	A	G	0.065	15	85563483	2.27E-06	0.014	14306	22.168
Vascular dementia (mixed)	genus Ruminococcus gauvreauii group	rs431418	G	A	0.061	166992547	0.675	0.145	360421	G	A	0.095	5	166419552	5.54E-06	0.021	14306	20.321
Vascular dementia (mixed)	genus Ruminococcus gauvreauii group	rs71386687	T	G	-0.084	2717893	0.525	0.133	360421	T	G	0.121	16	2767894	2.91E-07	0.024	14306	25.733
Vascular dementia (mixed)	genus Ruminococcus gauvreauii group	rs73802842	C	A	0.012	18823237	0.901	0.099	360421	C	A	0.074	4	18824860	7.48E-06	0.017	14306	18.859
Vascular dementia (mixed)	genus Ruminococcus gauvreauii group	rs9870933	G	A	-0.013	112653470	0.882	0.086	360421	G	A	-0.062	3	112372317	8.49E-07	0.013	14306	24.313
Vascular dementia (mixed)	genus Ruminococcus gnavus group	rs11597105	A	G	-0.004	6470619	0.972	0.107	360421	A	G	0.115	10	6512581	6.95E-06	0.025	14306	20.930
Vascular dementia (mixed)	genus Ruminococcus gnavus group	rs11864644	T	C	-0.204	383078	0.122	0.132	360421	T	C	-0.140	16	433078	5.01E-06	0.032	14306	19.297
Vascular dementia (mixed)	genus Ruminococcus gnavus group	rs12136548	C	T	-0.062	114625476	0.501	0.093	360421	C	T	0.090	1	115168097	3.10E-06	0.020	14306	21.074
Vascular dementia (mixed)	genus Ruminococcus gnavus group	rs12989336	G	A	0.004	36032198	0.963	0.094	360421	G	A	-0.085	2	36259341	7.12E-06	0.019	14306	20.305
Vascular dementia (mixed)	genus Ruminococcus gnavus group	rs13163520	G	A	-0.192	18662577	0.076	0.108	360421	G	A	-0.127	5	18662686	5.61E-08	0.023	14306	29.661
Vascular dementia (mixed)	genus Ruminococcus gnavus group	rs2909242	A	C	0.051	128212434	0.569	0.089	360421	A	C	0.091	8	129224680	7.41E-07	0.018	14306	24.588
Vascular dementia (mixed)	genus Ruminococcus gnavus group	rs3124783	G	A	-0.018	132963204	0.885	0.125	360421	G	A	0.116	9	135838591	2.67E-06	0.025	14306	21.682
Vascular dementia (mixed)	genus Ruminococcus gnavus group	rs4388134	T	C	0.038	189189556	0.683	0.094	360421	T	C	0.090	4	190110710	9.12E-06	0.020	14306	19.769
Vascular dementia (mixed)	genus Ruminococcus gnavus group	rs62167033	T	C	-0.080	143942440	0.705	0.211	360421	T	C	0.185	2	144700007	3.50E-06	0.040	14306	21.861
Vascular dementia (mixed)	genus Ruminococcus gnavus group	rs78399089	T	C	0.113	146572523	0.393	0.132	360421	T	C	0.144	3	146290310	6.63E-06	0.033	14306	19.558
Vascular dementia (mixed)	genus Ruminococcus gnavus group	rs934940	A	C	-0.183	121338442	0.141	0.124	360421	A	C	-0.105	2	122096018	2.74E-06	0.023	14306	20.934
Vascular dementia (mixed)	genus Ruminococcus torques group	rs10904297	A	G	0.095	4597570	0.744	0.291	360421	A	G	-0.168	10	4639762	2.69E-06	0.039	14306	18.531
Vascular dementia (mixed)	genus Ruminococcus torques group	rs10967781	C	A	0.111	27225260	0.231	0.093	360421	C	A	0.051	9	27225258	8.37E-06	0.011	14306	20.095
Vascular dementia (mixed)	genus Ruminococcus torques group	rs12434631	A	G	-0.030	25661198	0.824	0.137	360421	A	G	0.075	14	26130404	2.77E-06	0.015	14306	23.711
Vascular dementia (mixed)	genus Ruminococcus torques group	rs1475330	C	T	-0.085	66552209	0.389	0.099	360421	C	T	-0.052	6	67262102	8.13E-06	0.012	14306	19.615

Vascular dementia (mixed)	genus Ruminococcus torques group	rs35866622	T	C	-0.003	48714803	0.976	0.088	360421	T	C	-0.061	19	49218060	2.21E-08	0.011	14306	31.285
Vascular dementia (mixed)	genus Ruminococcus torques group	rs4073731	T	C	-0.013	132793252	0.907	0.114	360421	T	C	0.065	11	132663147	4.05E-06	0.014	14306	21.014
Vascular dementia (mixed)	genus Ruminococcus torques group	rs77034621	T	G	0.023	75857608	0.945	0.326	360421	T	G	-0.152	8	76769843	6.07E-06	0.034	14306	20.359
Vascular dementia (mixed)	genus Sellimonas	rs113379006	T	C	0.065	41966694	0.557	0.111	360421	T	C	-0.163	3	42008186	7.21E-06	0.036	14306	20.782
Vascular dementia (mixed)	genus Sellimonas	rs13417181	T	C	0.016	173443666	0.873	0.100	360421	T	C	0.167	2	174308394	7.62E-07	0.034	14306	24.337
Vascular dementia (mixed)	genus Sellimonas	rs2016057	C	A	-0.105	51813085	0.223	0.086	360421	C	A	0.126	15	52105282	1.03E-06	0.026	14306	24.149
Vascular dementia (mixed)	genus Sellimonas	rs2187447	A	C	0.027	79621928	0.879	0.180	360421	A	C	0.243	11	79332972	3.98E-06	0.053	14306	21.285
Vascular dementia (mixed)	genus Sellimonas	rs2371572	A	C	-0.093	212349450	0.271	0.084	360421	A	C	0.127	2	213214174	4.46E-07	0.025	14306	25.770
Vascular dementia (mixed)	genus Sellimonas	rs41816	A	G	0.142	106609718	0.122	0.092	360421	A	G	0.132	7	106250164	8.39E-06	0.029	14306	20.626
Vascular dementia (mixed)	genus Sellimonas	rs4600608	G	A	-0.174	179413837	0.093	0.103	360421	G	A	0.137	2	180278564	4.95E-06	0.030	14306	20.666
Vascular dementia (mixed)	genus Sellimonas	rs553697	C	T	0.010	93020675	0.925	0.109	360421	C	T	0.154	6	93730393	6.13E-06	0.034	14306	20.562
Vascular dementia (mixed)	genus Sellimonas	rs56203279	T	C	-0.055	111861468	0.535	0.089	360421	T	C	-0.124	7	111501524	3.72E-06	0.027	14306	21.246
Vascular dementia (mixed)	genus Senegalimassilia	rs10036909	C	T	-0.126	128525999	0.563	0.217	360421	C	T	0.186	5	127861692	8.05E-06	0.040	14306	21.416
Vascular dementia (mixed)	genus Senegalimassilia	rs11787826	C	A	0.145	34332384	0.092	0.086	360421	C	A	0.081	9	34332382	2.63E-06	0.017	14306	22.579
Vascular dementia (mixed)	genus Senegalimassilia	rs1990708	A	C	-0.019	205789399	0.906	0.157	360421	A	C	-0.110	2	206654123	8.91E-06	0.025	14306	19.571
Vascular dementia (mixed)	genus Senegalimassilia	rs2017373	C	T	0.007	33962790	0.935	0.088	360421	C	T	0.078	14	34431996	9.50E-06	0.018	14306	19.567
Vascular dementia (mixed)	genus Senegalimassilia	rs7225245	A	G	-0.115	50302254	0.178	0.085	360421	A	G	-0.079	17	48379615	4.18E-06	0.017	14306	21.583
Vascular dementia (mixed)	genus Slackia	rs10409783	G	A	0.034	4555774	0.720	0.094	360421	G	A	-0.095	19	4555786	7.70E-06	0.021	14306	20.261
Vascular dementia (mixed)	genus Slackia	rs12440440	A	G	-0.075	33749695	0.394	0.088	360421	A	G	0.090	15	34041896	2.63E-06	0.019	14306	22.397
Vascular dementia (mixed)	genus Slackia	rs16894137	C	T	-0.038	95934063	0.763	0.125	360421	C	T	-0.123	8	96946291	2.71E-06	0.026	14306	21.791
Vascular dementia (mixed)	genus Slackia	rs35156985	T	C	0.050	99854092	0.813	0.210	360421	T	C	-0.156	7	99451715	8.06E-06	0.035	14306	20.010
Vascular dementia (mixed)	genus Slackia	rs4492265	G	A	0.092	13484058	0.316	0.092	360421	G	A	0.091	7	13523683	2.41E-06	0.019	14306	22.334
Vascular dementia (mixed)	genus Slackia	rs8901	C	T	-0.121	76270929	0.190	0.093	360421	C	T	0.093	17	74267010	6.07E-07	0.019	14306	25.028
Vascular dementia (mixed)	genus Streptococcus	rs10028567	C	T	-0.109	52791410	0.399	0.129	360421	C	T	-0.092	4	53657577	7.30E-06	0.019	14306	23.047
Vascular dementia (mixed)	genus Streptococcus	rs10448310	A	G	0.093	90793892	0.286	0.087	360421	A	G	-0.052	9	93556174	3.31E-06	0.011	14306	21.646
Vascular dementia (mixed)	genus Streptococcus	rs11110281	T	C	0.123	100190236	0.537	0.199	360421	T	C	-0.138	12	100584014	2.58E-09	0.023	14306	36.572
Vascular dementia (mixed)	genus Streptococcus	rs11720390	G	A	0.010	94384747	0.956	0.174	360421	G	A	0.107	3	94103591	3.59E-06	0.023	14306	22.011
Vascular dementia (mixed)	genus Streptococcus	rs11764382	A	G	0.191	46735298	0.113	0.121	360421	A	G	-0.070	7	46774896	1.29E-06	0.014	14306	23.424
Vascular dementia (mixed)	genus Streptococcus	rs17708276	A	G	0.229	10342038	0.090	0.135	360421	A	G	-0.079	8	10199548	3.04E-06	0.017	14306	21.652
Vascular dementia (mixed)	genus Streptococcus	rs1918540	A	G	-0.042	131779552	0.701	0.109	360421	A	G	-0.060	11	131649446	2.44E-06	0.013	14306	21.659
Vascular dementia (mixed)	genus Streptococcus	rs2370083	G	T	0.039	97060413	0.822	0.172	360421	G	T	-0.082	14	97526750	9.75E-06	0.019	14306	19.317
Vascular dementia (mixed)	genus Streptococcus	rs57646748	G	A	-0.153	37451236	0.481	0.218	360421	G	A	-0.091	4	37452858	5.48E-06	0.020	14306	20.527
Vascular dementia (mixed)	genus Streptococcus	rs6806351	T	C	-0.199	132339879	0.051	0.102	360421	T	C	-0.063	3	132058723	4.94E-06	0.014	14306	21.515
Vascular dementia (mixed)	genus Streptococcus	rs71481756	T	G	-0.004	8060441	0.983	0.170	360421	T	G	0.093	10	8102404	6.51E-06	0.021	14306	20.046
Vascular dementia (mixed)	genus Streptococcus	rs7916711	A	G	-0.030	28299340	0.807	0.122	360421	A	G	0.103	10	28588269	2.72E-06	0.022	14306	22.407
Vascular dementia (mixed)	genus Subdoligranulum	rs10065321	T	C	-0.056	142477850	0.511	0.085	360421	T	C	-0.051	5	141857415	2.10E-06	0.011	14306	22.504
Vascular dementia (mixed)	genus Subdoligranulum	rs10497836	T	C	0.157	199423995	0.125	0.102	360421	T	C	0.052	2	200288718	8.38E-06	0.012	14306	19.494
Vascular dementia (mixed)	genus Subdoligranulum	rs1667315	G	A	-0.121	231544032	0.162	0.086	360421	G	A	0.049	2	232408743	6.72E-06	0.011	14306	20.374
Vascular dementia (mixed)	genus Subdoligranulum	rs2114677	C	T	0.030	123713421	0.821	0.133	360421	C	T	-0.104	10	125472937	2.72E-06	0.023	14306	20.368
Vascular dementia (mixed)	genus Subdoligranulum	rs2171249	C	T	-0.074	153372894	0.649	0.163	360421	C	T	0.107	6	153694029	4.51E-06	0.023	14306	20.950
Vascular dementia (mixed)	genus Subdoligranulum	rs3761728	G	T	0.055	48988868	0.572	0.097	360421	G	T	0.054	4	48990885	3.87E-06	0.012	14306	20.903
Vascular dementia (mixed)	genus Subdoligranulum	rs4347804	G	A	-0.020	217351124	0.934	0.237	360421	G	A	-0.166	2	218215847	2.18E-06	0.036	14306	21.579
Vascular dementia (mixed)	genus Subdoligranulum	rs6555306	C	T	-0.045	4671270	0.712	0.121	360421	C	T	0.074	5	4671383	2.81E-06	0.016	14306	22.705
Vascular dementia (mixed)	genus Subdoligranulum	rs75158211	T	C	-0.120	29153787	0.309	0.118	360421	T	C	-0.072	19	29644694	7.52E-06	0.016	14306	20.616
Vascular dementia (mixed)	genus Subdoligranulum	rs76528319	G	T	-0.007	118463753	0.965	0.151	360421	G	T	-0.143	5	117799448	7.41E-06	0.031	14306	21.271
Vascular dementia (mixed)	genus Sutterella	rs1145877	G	A	-0.266	81677104	0.030	0.123	360421	G	A	0.074	6	82386821	7.20E-06	0.016	14306	20.507
Vascular dementia (mixed)	genus Sutterella	rs11591622	T	G	0.032	100760930	0.778	0.114	360421	T	G	-0.069	10	102520687	6.50E-06	0.015	14306	20.680
Vascular dementia (mixed)	genus Sutterella	rs13173038	A	G	0.033	59203357	0.734	0.098	360421	A	G	-0.072	5	58499183	2.73E-06	0.015	14306	22.428
Vascular dementia (mixed)	genus Sutterella	rs143438747	T	C	-0.216	62812878	0.177	0.160	360421	T	C	-0.146	1	63278549	3.28E-06	0.031	14306	22.572
Vascular dementia (mixed)	genus Sutterella	rs2050185	A	G	0.015	147615645	0.865	0.087	360421	A	G	-0.058	6	147936781	7.97E-06	0.013	14306	19.950
Vascular dementia (mixed)	genus Sutterella	rs2321387	G	A	-0.151	58115206	0.076	0.085	360421	G	A	-0.059	13	58689340	1.87E-06	0.012	14306	22.674
Vascular dementia (mixed)	genus Sutterella	rs2613606	T	C	0.101	111644969	0.239	0.085	360421	T	C	0.056	7	111285025	7.20E-06	0.012	14306	20.125
Vascular dementia (mixed)	genus Sutterella	rs607327	T	C	0.049	111824985	0.572	0.087	360421	T	C	-0.058	11	111695709	6.63E-06	0.013	14306	20.083
Vascular dementia (mixed)	genus Sutterella	rs62501473	G	A	-0.039	150429663	0.688	0.096	360421	G	A	0.069	7	150126751	5.52E-06	0.015	14306	21.588
Vascular dementia (mixed)	genus Sutterella	rs7499539	A	G	-0.114	85004459	0.231	0.095	360421	A	G	0.062	16	85038065	2.36E-06	0.013	14306	22.218
Vascular dementia (mixed)	genus Sutterella	rs7638039	T	C	-0.133	70539788	0.176	0.098	360421	T	C	0.065	3	70588939	8.66E-06	0.014	14306	20.133
Vascular dementia (mixed)	genus Sutterella	rs9350083	T	G	-0.065	18583647	0.460	0.088	360421	T	G	-0.059	6	18583878	8.23E-06	0.013	14306	19.609
Vascular dementia (mixed)	genus Terrisporobacter	rs1883097	C	T	-0.096	8917272	0.669	0.225	360421	C	T	0.226	11	8938819	4.16E-07	0.045	14306	24.798
Vascular dementia (mixed)	genus Terrisporobacter	rs2569953	C	A	0.056	144430063	0.514	0.085	360421	C	A	0.078	3	144148905	8.95E-06	0.017	14306	19.723
Vascular dementia (mixed)	genus Terrisporobacter	rs2872237	A	C	0.021	16916130	0.803	0.086	360421	A	C	0.081	19	17026940	3.97E-06	0.018	14306	21.431
Vascular dementia (mixed)	genus Terrisporobacter	rs58405430	G	T	0.020	20599497	0.909	0.178	360421	G	T	0.135	11	20621043	7.94E-06	0.030	14306	19.978
Vascular dementia (mixed)	genus Terrisporobacter	rs7184125	T	C	0.001	15826537	0.994	0.094	360421	T	C	0.091	16	15920394	8.48E-06	0.021	14306	19.721
Vascular dementia (mixed)	genus Turicibacter	rs11054680	T	C	-0.008	12089521	0.943	0.112	360421	T	C	-0.105	12	12242455	2.31E-06	0.023	14306	21.295
Vascular dementia (mixed)	genus Turicibacter	rs11666533	C	T	0.002	11754117	0.992	0.157	360421	C	T	-0.112	19	11864932	7.37E-06	0.025	14306	20.211
Vascular dementia (mixed)	genus Turicibacter	rs149744580	A	G	-0.077	63139827	0.683	0.188	360421	A	G	0.170	2	63366962	7.01E-08	0.032	14306	28.998
Vascular dementia (mixed)	genus Turicibacter	rs2834977	T	C	0.026	35557345	0.823	0.117	360421	T	C	-0.096	21	36929643	3.96E-06	0.021	14306	21.248
Vascular dementia (mixed)	genus Turicibacter	rs2952020	A	G	0.161	26170326	0.099	0.098	360421	A	G	0.076	8	26027842	5.63E-06	0.017	14306	20.966

Vascular dementia (mixed)	genus Turicibacter	rs3734633	G	A	-0.298	125790735	0.094	0.178	360421	G	A	-0.121	6	126111881	5.32E-06	0.027	14306	20.325
Vascular dementia (mixed)	genus Turicibacter	rs4869133	G	A	-0.002	96381915	0.986	0.111	360421	G	A	0.131	5	95717619	2.55E-06	0.027	14306	23.267
Vascular dementia (mixed)	genus Turicibacter	rs55756211	T	C	0.354	131287420	0.012	0.141	360421	T	C	-0.115	7	130972179	2.81E-06	0.024	14306	22.871
Vascular dementia (mixed)	genus Turicibacter	rs7199484	G	A	-0.039	49792388	0.669	0.091	360421	G	A	-0.073	16	49826299	5.77E-06	0.016	14306	20.853
Vascular dementia (mixed)	genus Tyzzerella3	rs10898797	C	T	0.115	87877806	0.394	0.135	360421	C	T	0.122	11	87588698	8.85E-06	0.027	14306	19.850
Vascular dementia (mixed)	genus Tyzzerella3	rs112102233	A	G	-0.028	45602838	0.888	0.199	360421	A	G	-0.216	10	46098286	6.18E-06	0.048	14306	20.522
Vascular dementia (mixed)	genus Tyzzerella3	rs1232220	T	G	-0.045	102232382	0.749	0.139	360421	T	G	0.144	6	102680257	7.91E-06	0.032	14306	20.432
Vascular dementia (mixed)	genus Tyzzerella3	rs17706273	T	C	0.085	16388150	0.589	0.157	360421	T	C	-0.140	5	16388259	5.88E-07	0.027	14306	26.109
Vascular dementia (mixed)	genus Tyzzerella3	rs191093	G	A	0.292	76996549	0.033	0.137	360421	G	A	0.159	12	77390329	6.76E-06	0.035	14306	20.255
Vascular dementia (mixed)	genus Tyzzerella3	rs4904512	T	C	-0.077	89129601	0.542	0.126	360421	T	C	-0.117	14	89595945	3.09E-06	0.025	14306	21.905
Vascular dementia (mixed)	genus Tyzzerella3	rs55799124	A	G	0.042	3835487	0.662	0.095	360421	A	G	-0.114	17	3738781	1.34E-06	0.024	14306	22.968
Vascular dementia (mixed)	genus Tyzzerella3	rs67476743	T	G	0.164	1030321	0.085	0.095	360421	T	G	0.132	19	1030320	3.74E-09	0.022	14306	35.417
Vascular dementia (mixed)	genus Tyzzerella3	rs7019909	T	C	-0.026	33113324	0.842	0.129	360421	T	C	0.144	9	33113322	1.76E-06	0.030	14306	22.842
Vascular dementia (mixed)	genus Tyzzerella3	rs7333521	T	C	-0.168	81018881	0.474	0.234	360421	T	C	-0.207	13	81593016	4.88E-06	0.045	14306	20.908
Vascular dementia (mixed)	genus Tyzzerella3	rs75091807	G	T	-0.210	34449554	0.239	0.178	360421	G	T	-0.185	13	35023691	1.71E-06	0.038	14306	23.320
Vascular dementia (mixed)	genus Tyzzerella3	rs7561370	C	T	0.301	57583271	0.011	0.118	360421	C	T	-0.131	2	57810406	1.52E-06	0.029	14306	21.047
Vascular dementia (mixed)	genus Veillonella	rs1882878	A	G	-0.011	28638351	0.902	0.092	360421	A	G	-0.077	21	30010673	2.98E-06	0.016	14306	22.010
Vascular dementia (mixed)	genus Veillonella	rs2013594	C	T	0.009	44280604	0.916	0.086	360421	C	T	0.072	11	44302154	3.42E-06	0.016	14306	21.577
Vascular dementia (mixed)	genus Veillonella	rs62376424	C	T	-0.027	120578590	0.764	0.091	360421	C	T	-0.076	5	119914285	3.65E-06	0.016	14306	21.733
Vascular dementia (mixed)	genus Veillonella	rs6656807	G	A	-0.084	178975908	0.341	0.088	360421	G	A	-0.070	1	178945043	5.50E-06	0.015	14306	20.855
Vascular dementia (mixed)	genus Veillonella	rs742016	A	G	-0.073	45208919	0.412	0.089	360421	A	G	-0.069	22	45604800	4.66E-06	0.015	14306	21.137
Vascular dementia (mixed)	genus Victivallis	rs11899949	G	A	-0.048	37821926	0.597	0.091	360421	G	A	0.131	2	38049069	2.77E-06	0.028	1531	22.341
Vascular dementia (mixed)	genus Victivallis	rs12512543	A	C	0.120	9606821	0.434	0.154	360421	A	C	-0.178	4	9608445	2.54E-06	0.037	1531	22.619
Vascular dementia (mixed)	genus Victivallis	rs173120	C	T	0.196	72302743	0.066	0.106	360421	C	T	-0.134	13	72876881	7.65E-06	0.029	1531	21.272
Vascular dementia (mixed)	genus Victivallis	rs1882775	A	G	-0.001	39033681	0.996	0.110	360421	A	G	-0.138	21	40405606	8.73E-06	0.031	1531	19.549
Vascular dementia (mixed)	genus Victivallis	rs2546432	C	T	-0.029	181152462	0.734	0.084	360421	C	T	0.111	5	180579462	9.93E-06	0.025	1531	19.698
Vascular dementia (mixed)	genus Victivallis	rs342302	A	G	-0.157	106737783	0.200	0.123	360421	A	G	-0.153	7	106378229	8.16E-06	0.035	1531	18.878
Vascular dementia (mixed)	genus Victivallis	rs4764863	G	A	-0.111	102120406	0.190	0.085	360421	G	A	0.122	12	102514184	8.22E-07	0.025	1531	24.407
Vascular dementia (mixed)	genus Victivallis	rs4895919	C	T	-0.028	131309179	0.740	0.084	360421	C	T	0.117	6	131630319	2.75E-06	0.025	1531	22.313
Vascular dementia (mixed)	genus Victivallis	rs911666	T	C	0.224	95604788	0.009	0.085	360421	T	C	-0.119	14	96071125	7.65E-06	0.026	1531	20.305
Vascular dementia (mixed)	order Actinomycetales	rs2889192	T	G	0.025	73779652	0.835	0.118	360421	T	G	-0.088	9	76394568	3.97E-06	0.019	14306	20.554
Vascular dementia (mixed)	order Actinomycetales	rs34583783	G	T	0.035	66497478	0.842	0.176	360421	G	T	0.124	6	67207371	5.54E-06	0.026	14306	21.908
Vascular dementia (mixed)	order Actinomycetales	rs35011108	A	G	-0.135	132686341	0.414	0.166	360421	A	G	0.242	6	133007480	1.88E-06	0.050	14306	22.987
Vascular dementia (mixed)	order Actinomycetales	rs4073240	G	A	-0.114	168824686	0.187	0.087	360421	G	A	0.075	6	169224781	5.68E-06	0.016	14306	20.729
Vascular dementia (mixed)	order Bacillales	rs10233278	T	C	0.069	117856090	0.421	0.085	360421	T	C	-0.116	7	117496144	3.51E-06	0.025	14306	21.906
Vascular dementia (mixed)	order Bacillales	rs10410917	C	T	-0.005	14859629	0.954	0.087	360421	C	T	-0.115	19	14970441	5.57E-06	0.025	14306	21.049
Vascular dementia (mixed)	order Bacillales	rs11034576	A	G	0.006	38064161	0.966	0.128	360421	A	G	0.206	11	38085711	8.86E-06	0.045	14306	20.604
Vascular dementia (mixed)	order Bacillales	rs11207728	A	G	-0.117	61361862	0.305	0.114	360421	A	G	0.145	1	61827534	5.73E-06	0.032	14306	20.805
Vascular dementia (mixed)	order Bacillales	rs11844714	A	G	0.023	48532023	0.829	0.105	360421	A	G	-0.143	14	49001226	5.06E-06	0.032	14306	20.004
Vascular dementia (mixed)	order Bacillales	rs1287018	G	A	-0.028	5845828	0.793	0.107	360421	G	A	0.141	20	5826474	9.87E-06	0.032	14306	19.534
Vascular dementia (mixed)	order Bacillales	rs4617108	G	A	0.305	49384236	0.041	0.149	360421	G	A	-0.249	7	49423832	1.98E-06	0.053	14306	22.390
Vascular dementia (mixed)	order Bacillales	rs74420793	A	G	0.104	126845554	0.448	0.137	360421	A	G	-0.164	4	127766709	3.07E-06	0.035	14306	21.600
Vascular dementia (mixed)	order Bacteroidales	rs11146701	A	G	0.017	38769138	0.848	0.089	360421	A	G	0.047	10	39062269	7.08E-06	0.011	14306	20.186
Vascular dementia (mixed)	order Bacteroidales	rs17343978	A	C	-0.072	27037922	0.492	0.105	360421	A	C	-0.055	22	27433885	8.36E-06	0.012	14306	21.067
Vascular dementia (mixed)	order Bacteroidales	rs2032750	C	T	-0.055	53603889	0.514	0.085	360421	C	T	0.051	2	53831026	1.92E-06	0.011	14306	22.657
Vascular dementia (mixed)	order Bacteroidales	rs2363574	T	C	-0.039	200143435	0.859	0.218	360421	T	C	0.223	1	200112563	9.93E-06	0.051	14306	19.221
Vascular dementia (mixed)	order Bacteroidales	rs4916508	A	G	0.192	196209918	0.024	0.085	360421	A	G	0.047	3	195936789	8.47E-06	0.011	14306	19.641
Vascular dementia (mixed)	order Bacteroidales	rs55773148	G	A	0.143	69948897	0.436	0.183	360421	G	A	-0.122	13	70523029	3.90E-07	0.024	14306	26.341
Vascular dementia (mixed)	order Bacteroidales	rs62531359	T	G	0.000	70003946	0.997	0.111	360421	T	G	0.066	8	70916181	9.09E-06	0.015	14306	19.138
Vascular dementia (mixed)	order Bacteroidales	rs62575403	C	T	-0.184	133628698	0.380	0.210	360421	C	T	0.140	9	136493820	7.06E-06	0.031	14306	20.264
Vascular dementia (mixed)	order Bacteroidales	rs72706335	T	C	-0.032	157525648	0.904	0.269	360421	T	C	-0.222	1	157495438	7.66E-06	0.049	14306	20.315
Vascular dementia (mixed)	order Bacteroidales	rs73975615	G	A	0.114	6557880	0.817	0.490	360421	G	A	-0.207	17	6461200	1.22E-06	0.044	14306	21.874
Vascular dementia (mixed)	order Bacteroidales	rs7631304	G	A	-0.020	89290377	0.865	0.119	360421	G	A	-0.065	3	89339527	8.37E-07	0.013	14306	23.590
Vascular dementia (mixed)	order Bacteroidales	rs79585701	A	C	-0.148	13252676	0.224	0.122	360421	A	C	0.065	8	13110185	9.99E-06	0.015	14306	18.687
Vascular dementia (mixed)	order Bacteroidales	rs929878	T	C	-0.214	74256742	0.039	0.104	360421	T	C	0.055	16	74290641	4.73E-06	0.012	14306	20.372
Vascular dementia (mixed)	order Bifidobacteriales	rs10831953	G	A	-0.057	13076504	0.535	0.091	360421	G	A	0.054	11	13098051	9.95E-06	0.012	14306	18.869
Vascular dementia (mixed)	order Bifidobacteriales	rs12446429	T	C	0.148	848055	0.177	0.109	360421	T	C	0.081	16	898055	8.53E-06	0.019	14306	18.040
Vascular dementia (mixed)	order Bifidobacteriales	rs13020688	G	A	0.045	192013806	0.619	0.091	360421	G	A	0.058	2	192878532	1.57E-06	0.012	14306	22.887
Vascular dementia (mixed)	order Bifidobacteriales	rs182549	T	C	-0.114	135859184	0.187	0.087	360421	T	C	-0.117	2	136616754	5.94E-20	0.013	14306	85.372
Vascular dementia (mixed)	order Bifidobacteriales	rs4957061	T	C	0.013	520981	0.881	0.086	360421	T	C	0.057	5	521096	1.15E-06	0.012	14306	23.762
Vascular dementia (mixed)	order Bifidobacteriales	rs540489	T	G	0.037	74901626	0.740	0.111	360421	T	G	-0.063	17	72897722	5.37E-06	0.014	14306	20.956
Vascular dementia (mixed)	order Bifidobacteriales	rs55888705	A	G	0.072	1516099	0.438	0.093	360421	A	G	0.054	4	1517826	8.66E-06	0.012	14306	19.812
Vascular dementia (mixed)	order Bifidobacteriales	rs6899771	A	G	-0.161	96958344	0.251	0.140	360421	A	G	-0.091	6	97406220	7.28E-06	0.020	14306	20.365
Vascular dementia (mixed)	order Bifidobacteriales	rs7174549	T	C	-0.099	91920073	0.256	0.087	360421	T	C	-0.055	15	92463303	6.87E-06	0.012	14306	19.590
Vascular dementia (mixed)	order Bifidobacteriales	rs7322849	T	C	0.097	112205515	0.512	0.147	360421	T	C	0.111	13	112859829	1.74E-08	0.020	14306	30.320
Vascular dementia (mixed)	order Bifidobacteriales	rs857444	C	T	0.083	14617360	0.341	0.087	360421	C	T	0.055	6	14617591	3.82E-06	0.012	14306	21.075
Vascular dementia (mixed)	order Burkholderiales	rs1511453	A	G	-0.074	23586839	0.679	0.179	360421	A	G	0.091	4	23588462	8.00E-06	0.020	14306	20.857

Vascular dementia (mixed)	order Burkholderiales	rs1928341	G	A	0.036	153267537	0.675	0.085	360421	G	A	-0.051	1	153240013	4.52E-06	0.011	14306	21.084
Vascular dementia (mixed)	order Burkholderiales	rs2321387	G	A	-0.151	58115206	0.076	0.085	360421	G	A	-0.051	13	58689340	3.26E-06	0.011	14306	21.532
Vascular dementia (mixed)	order Burkholderiales	rs2613606	T	C	0.101	111644969	0.239	0.085	360421	T	C	0.050	7	111285025	4.13E-06	0.011	14306	20.904
Vascular dementia (mixed)	order Burkholderiales	rs4033856	T	C	0.009	45640468	0.951	0.149	360421	T	C	-0.083	4	45642485	5.67E-07	0.017	14306	24.762
Vascular dementia (mixed)	order Burkholderiales	rs6087811	T	G	-0.010	32008327	0.943	0.141	360421	T	G	-0.102	20	30596130	2.88E-07	0.020	14306	26.108
Vascular dementia (mixed)	order Burkholderiales	rs62191117	A	G	0.085	238979079	0.419	0.105	360421	A	G	0.068	2	239900775	2.79E-07	0.013	14306	26.349
Vascular dementia (mixed)	order Burkholderiales	rs62395635	T	C	-0.314	174070793	0.077	0.178	360421	T	C	0.110	5	173497796	2.90E-06	0.024	14306	21.621
Vascular dementia (mixed)	order Burkholderiales	rs75242906	C	T	0.028	56494209	0.853	0.150	360421	C	T	-0.121	15	56786407	9.75E-06	0.028	14306	18.498
Vascular dementia (mixed)	order Burkholderiales	rs7638039	T	C	-0.133	70539788	0.176	0.098	360421	T	C	0.058	3	70588939	4.84E-06	0.013	14306	21.017
Vascular dementia (mixed)	order Clostridiales	rs10774377	G	A	-0.142	5833353	0.094	0.085	360421	G	A	-0.052	12	5942519	3.81E-06	0.011	14306	21.100
Vascular dementia (mixed)	order Clostridiales	rs112334273	G	A	-0.109	39331325	0.246	0.094	360421	G	A	0.064	21	40703251	4.07E-07	0.013	14306	25.186
Vascular dementia (mixed)	order Clostridiales	rs13105690	C	T	0.146	7418457	0.119	0.094	360421	C	T	0.053	4	7420184	9.37E-06	0.012	14306	19.919
Vascular dementia (mixed)	order Clostridiales	rs13179700	C	T	0.080	149698225	0.369	0.089	360421	C	T	-0.051	5	149077788	3.52E-06	0.011	14306	21.746
Vascular dementia (mixed)	order Clostridiales	rs1842454	G	A	0.115	105724661	0.288	0.108	360421	G	A	-0.054	5	105060362	9.92E-06	0.013	14306	18.228
Vascular dementia (mixed)	order Clostridiales	rs2273429	A	G	0.011	52027354	0.935	0.137	360421	A	G	-0.073	14	52494072	4.17E-06	0.015	14306	22.497
Vascular dementia (mixed)	order Clostridiales	rs290772	G	A	-0.028	101509758	0.879	0.184	360421	G	A	0.084	2	102126220	1.00E-05	0.020	14306	18.423
Vascular dementia (mixed)	order Clostridiales	rs6442336	T	C	0.015	12829387	0.876	0.097	360421	T	C	0.055	3	12870886	9.63E-06	0.012	14306	19.458
Vascular dementia (mixed)	order Clostridiales	rs6814436	C	T	0.015	160586149	0.903	0.120	360421	C	T	-0.074	4	161507301	9.06E-07	0.015	14306	24.197
Vascular dementia (mixed)	order Clostridiales	rs6815608	C	T	-0.109	151210592	0.358	0.118	360421	C	T	-0.104	4	152131744	3.72E-07	0.021	14306	24.363
Vascular dementia (mixed)	order Clostridiales	rs72738886	T	C	0.071	35770448	0.652	0.157	360421	T	C	0.087	5	35770550	8.42E-06	0.019	14306	20.657
Vascular dementia (mixed)	order Clostridiales	rs992074	T	C	-0.245	17195484	0.391	0.285	360421	T	C	-0.255	21	18567802	8.95E-07	0.051	14306	25.206
Vascular dementia (mixed)	order Coriobacteriales	rs11073596	G	T	-0.188	85890348	0.032	0.087	360421	G	T	-0.051	15	86433579	8.14E-06	0.011	14306	19.912
Vascular dementia (mixed)	order Coriobacteriales	rs11250875	T	C	0.093	1880537	0.365	0.102	360421	T	C	0.061	10	1922731	4.83E-06	0.013	14306	21.526
Vascular dementia (mixed)	order Coriobacteriales	rs11656361	A	C	-0.175	8218014	0.110	0.110	360421	A	C	0.077	17	8121332	8.02E-06	0.018	14306	19.394
Vascular dementia (mixed)	order Coriobacteriales	rs12974142	G	A	0.028	52391913	0.867	0.165	360421	G	A	0.079	19	52895166	8.51E-06	0.018	14306	19.865
Vascular dementia (mixed)	order Coriobacteriales	rs13307134	T	C	0.021	105444233	0.856	0.113	360421	T	C	-0.057	7	105084680	7.80E-06	0.013	14306	20.072
Vascular dementia (mixed)	order Coriobacteriales	rs1397793	A	G	-0.012	91175634	0.893	0.092	360421	A	G	0.050	5	90471451	9.77E-06	0.011	14306	19.682
Vascular dementia (mixed)	order Coriobacteriales	rs1816223	G	A	0.118	11341087	0.267	0.106	360421	G	A	0.059	12	11494021	4.84E-06	0.013	14306	20.652
Vascular dementia (mixed)	order Coriobacteriales	rs240104	T	C	-0.017	176602295	0.859	0.094	360421	T	C	-0.060	1	176571431	1.52E-06	0.013	14306	22.630
Vascular dementia (mixed)	order Coriobacteriales	rs2442778	A	G	0.169	11612938	0.365	0.186	360421	A	G	0.116	3	11654412	9.03E-06	0.026	14306	20.272
Vascular dementia (mixed)	order Coriobacteriales	rs3025411	A	G	0.009	133647784	0.947	0.135	360421	A	G	0.093	9	136512906	8.27E-06	0.021	14306	19.566
Vascular dementia (mixed)	order Coriobacteriales	rs34739816	G	T	0.111	39220432	0.534	0.179	360421	G	T	0.097	17	37376685	3.88E-06	0.021	14306	21.594
Vascular dementia (mixed)	order Coriobacteriales	rs67561917	A	G	-0.006	63440724	0.953	0.110	360421	A	G	-0.071	20	62072077	5.39E-06	0.015	14306	21.486
Vascular dementia (mixed)	order Coriobacteriales	rs719099	A	G	-0.161	64039457	0.258	0.142	360421	A	G	0.078	10	65799217	5.43E-07	0.016	14306	24.957
Vascular dementia (mixed)	order Coriobacteriales	rs8010111	A	G	0.182	39191305	0.236	0.154	360421	A	G	0.103	14	39660509	6.90E-06	0.023	14306	20.328
Vascular dementia (mixed)	order Desulfovibrionales	rs11599763	C	T	-0.134	11813600	0.122	0.086	360421	C	T	0.055	10	11855599	2.61E-06	0.012	14306	22.305
Vascular dementia (mixed)	order Desulfovibrionales	rs17791387	A	G	-0.246	79219511	0.087	0.144	360421	A	G	-0.073	9	81834426	2.25E-06	0.015	14306	22.210
Vascular dementia (mixed)	order Desulfovibrionales	rs186073	T	C	-0.094	31091909	0.276	0.086	360421	T	C	0.053	3	31133401	8.74E-06	0.012	14306	19.931
Vascular dementia (mixed)	order Desulfovibrionales	rs2692012	G	A	-0.034	204022477	0.853	0.185	360421	G	A	-0.112	1	203991605	2.27E-06	0.025	14306	19.597
Vascular dementia (mixed)	order Desulfovibrionales	rs2838334	G	A	0.074	43645080	0.406	0.089	360421	G	A	0.057	21	45064961	4.17E-06	0.012	14306	20.982
Vascular dementia (mixed)	order Desulfovibrionales	rs3935584	C	T	-0.055	233064573	0.517	0.084	360421	C	T	-0.052	2	233929283	7.20E-06	0.012	14306	20.531
Vascular dementia (mixed)	order Desulfovibrionales	rs4506934	C	T	-0.072	2953368	0.579	0.130	360421	C	T	-0.095	17	2856662	2.43E-06	0.020	14306	22.416
Vascular dementia (mixed)	order Desulfovibrionales	rs6058181	C	T	0.052	35106998	0.643	0.113	360421	C	T	0.084	20	33694801	2.53E-07	0.017	14306	25.379
Vascular dementia (mixed)	order Desulfovibrionales	rs62020470	A	G	0.167	95617836	0.130	0.110	360421	A	G	-0.057	15	96161065	7.51E-06	0.013	14306	19.633
Vascular dementia (mixed)	order Desulfovibrionales	rs72647048	T	C	0.000	56952819	0.998	0.132	360421	T	C	-0.077	8	57865378	9.00E-06	0.017	14306	20.436
Vascular dementia (mixed)	order Desulfovibrionales	rs9928243	C	A	0.048	71507738	0.566	0.085	360421	C	A	-0.054	16	71541641	3.97E-06	0.012	14306	21.378
Vascular dementia (mixed)	order Enterobacteriales	rs11026530	T	C	0.075	22357551	0.522	0.118	360421	T	C	0.082	11	22379097	9.43E-06	0.019	14306	19.471
Vascular dementia (mixed)	order Enterobacteriales	rs2374342	C	A	0.022	41906402	0.798	0.086	360421	C	A	0.058	2	42133542	4.52E-06	0.013	14306	21.338
Vascular dementia (mixed)	order Enterobacteriales	rs35673018	G	A	-0.238	54293833	0.104	0.146	360421	G	A	0.090	16	54327745	7.63E-06	0.020	14306	19.653
Vascular dementia (mixed)	order Enterobacteriales	rs504442	T	G	-0.187	57478315	0.169	0.136	360421	T	G	0.084	18	55145547	5.17E-06	0.019	14306	19.728
Vascular dementia (mixed)	order Enterobacteriales	rs62210023	A	G	-0.026	56765036	0.771	0.089	360421	A	G	0.061	20	55340092	3.13E-06	0.013	14306	21.742
Vascular dementia (mixed)	order Enterobacteriales	rs78143293	A	G	-0.190	60005103	0.138	0.128	360421	A	G	-0.085	18	57672335	1.20E-06	0.017	14306	24.792
Vascular dementia (mixed)	order Enterobacteriales	rs79757635	C	A	-0.068	110188071	0.581	0.124	360421	C	A	0.076	13	110840418	9.32E-06	0.017	14306	19.615
Vascular dementia (mixed)	order Erysipelotrichales	rs1074800	G	A	0.077	3002432	0.369	0.086	360421	G	A	-0.049	5	3002546	6.15E-06	0.011	14306	20.459
Vascular dementia (mixed)	order Erysipelotrichales	rs10781552	C	T	0.002	132083729	0.987	0.093	360421	C	T	-0.055	10	133897233	2.33E-06	0.012	14306	22.633
Vascular dementia (mixed)	order Erysipelotrichales	rs17530232	A	G	-0.011	39811320	0.952	0.185	360421	A	G	0.103	13	40385457	2.79E-06	0.022	14306	21.042
Vascular dementia (mixed)	order Erysipelotrichales	rs1884466	C	T	0.001	63673525	0.990	0.084	360421	C	T	-0.048	1	64139196	9.53E-06	0.011	14306	19.760
Vascular dementia (mixed)	order Erysipelotrichales	rs2300774	A	G	-0.264	196066841	0.001	0.081	360421	A	G	-0.052	3	195793712	8.95E-07	0.011	14306	24.094
Vascular dementia (mixed)	order Erysipelotrichales	rs290833	T	G	0.028	96991871	0.740	0.084	360421	T	G	-0.050	1	97457427	8.03E-06	0.011	14306	19.943
Vascular dementia (mixed)	order Erysipelotrichales	rs35161940	T	C	0.026	72331083	0.852	0.137	360421	T	C	-0.081	17	70327224	1.85E-06	0.017	14306	23.118
Vascular dementia (mixed)	order Erysipelotrichales	rs4078432	T	C	0.002	48528003	0.984	0.112	360421	T	C	0.061	14	48997206	4.23E-06	0.013	14306	20.723
Vascular dementia (mixed)	order Erysipelotrichales	rs56970041	T	G	-0.119	79891267	0.501	0.177	360421	T	G	0.072	14	80357610	5.40E-06	0.016	14306	19.385
Vascular dementia (mixed)	order Erysipelotrichales	rs62504403	C	T	-0.062	38946033	0.553	0.105	360421	C	T	0.068	8	38803551	1.12E-07	0.013	14306	28.371
Vascular dementia (mixed)	order Erysipelotrichales	rs7234058	T	C	0.231	5830508	0.111	0.145	360421	T	C	-0.095	18	5830507	9.12E-07	0.019	14306	23.744
Vascular dementia (mixed)	order Erysipelotrichales	rs7826267	G	T	0.301	3097430	0.074	0.168	360421	G	T	0.084	8	2954952	9.28E-06	0.020	14306	17.755
Vascular dementia (mixed)	order Erysipelotrichales	rs8003149	C	T	0.045	55689786	0.617	0.089	360421	C	T	0.054	14	56156504	4.08E-06	0.012	14306	21.248
Vascular dementia (mixed)	order Gastranaerophilales	rs11150282	T	C	-0.177	80459808	0.045	0.088	360421	T	C	0.098	16	80493705	7.36E-07	0.020	14306	24.834

Vascular dementia (mixed)	order Gastranaerophilales	rs113884518	T	C	0.059	24648999	0.828	0.272	360421	T	C	-0.206	9	24648997	7.74E-06	0.046	14306	20.446
Vascular dementia (mixed)	order Gastranaerophilales	rs28678345	T	C	0.261	55828967	0.180	0.195	360421	T	C	0.213	17	53906328	8.06E-06	0.047	14306	20.434
Vascular dementia (mixed)	order Gastranaerophilales	rs367480	A	G	-0.098	2916401	0.269	0.088	360421	A	G	0.084	11	2937631	7.52E-06	0.019	14306	20.487
Vascular dementia (mixed)	order Gastranaerophilales	rs4129395	G	A	0.012	113213109	0.884	0.084	360421	G	A	0.090	9	115975389	1.22E-06	0.019	14306	23.826
Vascular dementia (mixed)	order Gastranaerophilales	rs789069	A	C	0.004	1008277	0.973	0.122	360421	A	C	-0.104	18	1008278	6.50E-06	0.023	14306	19.725
Vascular dementia (mixed)	order Gastranaerophilales	rs79790072	T	C	0.113	100207478	0.642	0.244	360421	T	C	0.226	15	100747683	3.54E-06	0.049	14306	21.466
Vascular dementia (mixed)	order Gastranaerophilales	rs8028558	A	G	-0.086	61763554	0.327	0.088	360421	A	G	0.083	15	62055753	9.78E-06	0.019	14306	19.603
Vascular dementia (mixed)	order Gastranaerophilales	rs9864379	T	C	-0.074	14265449	0.535	0.119	360421	T	C	-0.161	3	14306949	4.66E-08	0.029	14306	30.065
Vascular dementia (mixed)	order Lactobacillales	rs11110282	A	G	0.122	100191781	0.538	0.199	360421	A	G	-0.102	12	100585559	3.96E-06	0.022	14306	22.090
Vascular dementia (mixed)	order Lactobacillales	rs11627423	C	A	-0.092	32731417	0.291	0.087	360421	C	A	0.050	14	33200623	5.09E-06	0.011	14306	20.697
Vascular dementia (mixed)	order Lactobacillales	rs11730038	G	A	-0.134	97128348	0.146	0.093	360421	G	A	-0.061	4	98049499	5.10E-06	0.013	14306	22.006
Vascular dementia (mixed)	order Lactobacillales	rs12797734	T	C	-0.067	8310803	0.495	0.098	360421	T	C	0.057	11	8332350	7.77E-06	0.013	14306	20.198
Vascular dementia (mixed)	order Lactobacillales	rs1595463	C	A	-0.040	230858942	0.642	0.085	360421	C	A	0.048	2	231723657	7.44E-06	0.011	14306	19.443
Vascular dementia (mixed)	order Lactobacillales	rs2370083	G	T	0.039	97060413	0.822	0.172	360421	G	T	-0.081	14	97526750	8.33E-06	0.018	14306	19.802
Vascular dementia (mixed)	order Lactobacillales	rs2952251	G	A	-0.092	10285654	0.359	0.101	360421	G	A	0.063	8	10143164	3.36E-07	0.012	14306	25.684
Vascular dementia (mixed)	order Lactobacillales	rs34989881	A	G	-0.098	51456601	0.626	0.202	360421	A	G	0.113	19	51959855	4.09E-06	0.025	14306	21.201
Vascular dementia (mixed)	order Lactobacillales	rs35344081	G	A	0.121	941253	0.203	0.095	360421	G	A	0.064	16	991253	4.16E-07	0.013	14306	25.446
Vascular dementia (mixed)	order Lactobacillales	rs4028634	C	T	0.031	42683631	0.724	0.087	360421	C	T	-0.053	17	40835649	1.35E-06	0.011	14306	23.428
Vascular dementia (mixed)	order Lactobacillales	rs57872228	C	T	-0.078	200449677	0.547	0.130	360421	C	T	-0.069	1	200418805	2.58E-06	0.015	14306	21.933
Vascular dementia (mixed)	order Lactobacillales	rs74663707	C	T	0.144	184653436	0.410	0.175	360421	C	T	0.098	3	184371224	8.40E-06	0.022	14306	19.137
Vascular dementia (mixed)	order Lactobacillales	rs77558518	A	G	0.077	174746168	0.590	0.143	360421	A	G	-0.106	5	174173171	1.67E-06	0.022	14306	22.695
Vascular dementia (mixed)	order Lactobacillales	rs78938557	T	C	0.022	36309977	0.931	0.257	360421	T	C	0.106	7	36349586	2.31E-06	0.023	14306	20.412
Vascular dementia (mixed)	order Lactobacillales	rs9581006	T	C	0.056	24399371	0.804	0.224	360421	T	C	-0.226	13	24973509	1.77E-06	0.047	14306	23.163
Vascular dementia (mixed)	order Methanobacteriales	rs10202904	G	T	0.028	124682691	0.745	0.086	360421	G	T	0.122	2	125440268	3.01E-07	0.024	14306	26.762
Vascular dementia (mixed)	order Methanobacteriales	rs10424197	A	G	-0.140	45936063	0.149	0.097	360421	A	G	0.111	19	46439321	9.28E-06	0.025	14306	20.211
Vascular dementia (mixed)	order Methanobacteriales	rs4257531	G	A	0.032	2044483	0.818	0.140	360421	G	A	0.164	3	2086167	7.44E-06	0.036	14306	20.316
Vascular dementia (mixed)	order Methanobacteriales	rs6508769	C	T	0.065	28336853	0.583	0.118	360421	C	T	-0.154	19	28827760	8.23E-06	0.034	14306	19.856
Vascular dementia (mixed)	order Methanobacteriales	rs6776814	T	C	0.412	15011576	0.158	0.292	360421	T	C	-0.200	3	15053083	1.63E-06	0.041	14306	23.483
Vascular dementia (mixed)	order Methanobacteriales	rs73068003	G	T	0.007	10734305	0.961	0.143	360421	G	T	-0.158	7	10773932	8.45E-06	0.035	14306	20.206
Vascular dementia (mixed)	order Methanobacteriales	rs73457410	A	G	0.109	41382045	0.524	0.170	360421	A	G	0.215	13	41956181	1.41E-06	0.044	14306	24.316
Vascular dementia (mixed)	order Methanobacteriales	rs75208022	C	T	-0.016	21185927	0.909	0.144	360421	C	T	-0.227	12	21338861	5.92E-06	0.049	14306	21.717
Vascular dementia (mixed)	order Methanobacteriales	rs894996	C	A	-0.011	103497150	0.945	0.163	360421	C	A	0.217	4	104418307	1.88E-06	0.045	14306	23.349
Vascular dementia (mixed)	order Mollicutes RF9	rs11779863	G	A	0.123	15549321	0.295	0.117	360421	G	A	-0.077	8	15406830	6.69E-06	0.017	14306	20.146
Vascular dementia (mixed)	order Mollicutes RF9	rs12566890	T	G	0.261	61385192	0.036	0.125	360421	T	G	-0.103	1	61850864	8.11E-06	0.024	14306	18.196
Vascular dementia (mixed)	order Mollicutes RF9	rs13100746	C	T	0.142	166128718	0.094	0.085	360421	C	T	0.064	3	165846506	7.29E-06	0.014	14306	20.055
Vascular dementia (mixed)	order Mollicutes RF9	rs17235252	T	C	0.171	82906721	0.189	0.131	360421	T	C	-0.122	7	82536037	2.16E-06	0.026	14306	22.808
Vascular dementia (mixed)	order Mollicutes RF9	rs3932485	C	T	-0.033	184475694	0.708	0.087	360421	C	T	0.063	3	184193482	9.93E-06	0.014	14306	19.551
Vascular dementia (mixed)	order Mollicutes RF9	rs515984	C	T	-0.218	3479267	0.098	0.132	360421	C	T	0.088	10	3521459	6.61E-06	0.019	14306	21.078
Vascular dementia (mixed)	order Mollicutes RF9	rs638542	A	G	0.023	109302630	0.807	0.093	360421	A	G	0.071	1	109845252	5.17E-06	0.016	14306	20.204
Vascular dementia (mixed)	order Mollicutes RF9	rs74603314	T	C	0.252	46050515	0.247	0.218	360421	T	C	0.231	14	46519718	2.28E-06	0.049	14306	22.207
Vascular dementia (mixed)	order Mollicutes RF9	rs7706512	A	G	0.093	17443545	0.271	0.084	360421	A	G	-0.066	5	17443654	2.27E-06	0.014	14306	22.372
Vascular dementia (mixed)	order Mollicutes RF9	rs7801843	A	G	0.140	4066073	0.231	0.117	360421	A	G	-0.087	7	4105705	9.47E-06	0.019	14306	19.983
Vascular dementia (mixed)	order Mollicutes RF9	rs7853673	A	G	0.005	113948961	0.951	0.084	360421	A	G	0.062	9	116711241	6.73E-06	0.014	14306	19.938
Vascular dementia (mixed)	order Mollicutes RF9	rs949341	A	G	0.085	112516062	0.365	0.093	360421	A	G	-0.066	11	112386785	7.73E-06	0.015	14306	19.893
Vascular dementia (mixed)	order NB1n	rs11251024	G	A	0.017	2053532	0.859	0.096	360421	G	A	0.104	10	2095726	6.63E-07	0.021	14306	25.404
Vascular dementia (mixed)	order NB1n	rs11606187	A	G	-0.030	91856434	0.804	0.120	360421	A	G	-0.155	11	91589600	3.31E-06	0.033	14306	22.432
Vascular dementia (mixed)	order NB1n	rs13385922	T	C	0.061	233172678	0.493	0.089	360421	T	C	0.093	2	234081324	3.97E-06	0.020	14306	21.311
Vascular dementia (mixed)	order NB1n	rs166849	A	G	0.130	199914143	0.130	0.086	360421	A	G	-0.091	2	200778866	7.74E-06	0.020	14306	20.294
Vascular dementia (mixed)	order NB1n	rs2172426	T	C	-0.115	18371511	0.181	0.086	360421	T	C	0.102	8	18229020	3.17E-07	0.020	14306	26.348
Vascular dementia (mixed)	order NB1n	rs267959	G	A	-0.043	10737690	0.645	0.094	360421	G	A	-0.099	5	10737802	2.62E-06	0.021	14306	22.231
Vascular dementia (mixed)	order NB1n	rs4383094	C	T	-0.100	81402631	0.409	0.121	360421	C	T	-0.149	15	81694972	4.28E-06	0.032	14306	21.646
Vascular dementia (mixed)	order NB1n	rs60775321	T	C	-0.099	96775441	0.283	0.093	360421	T	C	-0.096	15	97318671	7.10E-06	0.021	14306	20.256
Vascular dementia (mixed)	order NB1n	rs72671304	T	C	-0.046	38956767	0.776	0.160	360421	T	C	0.172	14	39425971	3.80E-06	0.037	14306	21.688
Vascular dementia (mixed)	order NB1n	rs7911787	G	T	0.443	96859811	0.063	0.238	360421	G	T	-0.223	10	98619568	3.39E-06	0.047	14306	22.504
Vascular dementia (mixed)	order NB1n	rs8126061	T	C	-0.386	3424592	0.004	0.135	360421	T	C	-0.159	20	3405239	7.36E-06	0.035	14306	20.450
Vascular dementia (mixed)	order NB1n	rs9542068	T	C	0.017	69732699	0.850	0.089	360421	T	C	0.099	13	70306831	6.52E-06	0.022	14306	20.625
Vascular dementia (mixed)	order Pasteurellales	rs10965428	C	A	0.391	22718482	0.032	0.182	360421	C	A	-0.120	9	22718481	4.29E-06	0.026	14306	21.561
Vascular dementia (mixed)	order Pasteurellales	rs111582866	G	A	-0.120	48708578	0.418	0.148	360421	G	A	-0.114	16	48742489	7.07E-06	0.026	14306	19.753
Vascular dementia (mixed)	order Pasteurellales	rs12050685	A	G	-0.116	73185141	0.211	0.093	360421	A	G	-0.067	15	73477482	9.19E-06	0.015	14306	19.385
Vascular dementia (mixed)	order Pasteurellales	rs16970009	A	G	-0.013	34535582	0.967	0.306	360421	A	G	0.187	17	32862601	7.32E-06	0.043	14306	19.027
Vascular dementia (mixed)	order Pasteurellales	rs4822728	T	C	0.072	26495842	0.389	0.084	360421	T	C	0.069	22	26891808	4.72E-06	0.015	14306	21.156
Vascular dementia (mixed)	order Pasteurellales	rs6972479	A	G	0.010	117278006	0.925	0.105	360421	A	G	-0.078	7	116918060	7.75E-06	0.018	14306	19.878
Vascular dementia (mixed)	order Pasteurellales	rs72756943	G	A	0.147	26531799	0.387	0.169	360421	G	A	0.140	5	26531908	3.35E-06	0.030	14306	21.308
Vascular dementia (mixed)	order Pasteurellales	rs73139353	A	C	-0.100	98253370	0.505	0.150	360421	A	C	-0.223	3	97972214	8.71E-06	0.048	14306	21.092
Vascular dementia (mixed)	order Pasteurellales	rs76022354	C	T	0.485	92546628	0.014	0.197	360421	C	T	0.243	10	94306385	1.83E-06	0.050	14306	23.560
Vascular dementia (mixed)	order Pasteurellales	rs78909003	T	C	-0.199	102887960	0.274	0.182	360421	T	C	-0.241	9	105650242	2.05E-06	0.050	14306	23.415
Vascular dementia (mixed)	order Pasteurellales	rs9382510	C	T	-0.055	55583693	0.567	0.096	360421	C	T	-0.088	6	55448491	2.48E-07	0.017	14306	26.921

Vascular dementia (mixed)	order Pasteurellales	rs9895850	T	C	-0.004	66538895	0.985	0.207	360421	T	C	-0.176	17	64535013	9.08E-06	0.041	14306	18.497
Vascular dementia (mixed)	order Pasteurellales	rs9938097	C	T	-0.161	84943783	0.063	0.087	360421	C	T	0.071	16	84977389	8.23E-06	0.016	14306	20.209
Vascular dementia (mixed)	order Rhodospirillales	rs1035406	G	A	-0.048	120037042	0.714	0.132	360421	G	A	-0.115	5	119372737	4.07E-06	0.025	14306	21.401
Vascular dementia (mixed)	order Rhodospirillales	rs11591293	G	T	0.027	111660039	0.754	0.085	360421	G	T	0.072	10	113419797	4.69E-06	0.016	14306	20.876
Vascular dementia (mixed)	order Rhodospirillales	rs11630875	T	C	0.143	61483535	0.258	0.126	360421	T	C	0.095	15	61775734	3.70E-06	0.020	14306	21.865
Vascular dementia (mixed)	order Rhodospirillales	rs13336560	C	T	0.055	88487835	0.522	0.086	360421	C	T	-0.070	16	88554243	9.75E-06	0.016	14306	19.598
Vascular dementia (mixed)	order Rhodospirillales	rs1549633	A	C	0.138	27945538	0.299	0.132	360421	A	C	0.100	5	27945645	3.88E-06	0.022	14306	21.163
Vascular dementia (mixed)	order Rhodospirillales	rs3730086	A	G	0.080	68281223	0.412	0.097	360421	A	G	0.080	5	67577051	7.98E-06	0.018	14306	20.051
Vascular dementia (mixed)	order Rhodospirillales	rs3754624	C	T	0.111	224769095	0.309	0.109	360421	C	T	0.094	2	225633812	2.68E-06	0.020	14306	22.511
Vascular dementia (mixed)	order Rhodospirillales	rs4278423	T	C	0.231	2628361	0.190	0.177	360421	T	C	0.105	10	2670553	3.98E-06	0.023	14306	20.256
Vascular dementia (mixed)	order Rhodospirillales	rs61933850	G	A	-0.088	72745618	0.471	0.122	360421	G	A	0.165	12	73139398	7.00E-06	0.036	14306	20.888
Vascular dementia (mixed)	order Rhodospirillales	rs7001029	C	T	-0.041	130946157	0.780	0.147	360421	C	T	0.121	8	131958403	2.83E-06	0.026	14306	21.397
Vascular dementia (mixed)	order Rhodospirillales	rs76784716	A	G	0.002	168176830	0.986	0.135	360421	A	G	0.136	2	169033340	1.31E-06	0.028	14306	22.881
Vascular dementia (mixed)	order Rhodospirillales	rs77304857	C	A	-0.106	133326860	0.319	0.106	360421	C	A	-0.100	4	134248015	6.02E-06	0.022	14306	20.157
Vascular dementia (mixed)	order Rhodospirillales	rs9813022	A	G	0.020	13685237	0.817	0.087	360421	A	G	-0.083	3	13726736	3.07E-07	0.016	14306	26.093
Vascular dementia (mixed)	order Selenomonadales	rs1135612	G	A	-0.017	75980359	0.872	0.103	360421	G	A	0.053	7	75609677	9.26E-06	0.012	14306	19.761
Vascular dementia (mixed)	order Selenomonadales	rs13086907	G	A	-0.105	142416275	0.302	0.102	360421	G	A	0.063	3	142135117	1.95E-06	0.013	14306	22.532
Vascular dementia (mixed)	order Selenomonadales	rs1643968	T	C	0.047	165839623	0.597	0.088	360421	T	C	-0.057	5	165266628	4.15E-07	0.011	14306	25.339
Vascular dementia (mixed)	order Selenomonadales	rs1649999	A	G	-0.241	78326315	0.094	0.144	360421	A	G	0.075	10	80086072	7.58E-06	0.017	14306	20.246
Vascular dementia (mixed)	order Selenomonadales	rs2834062	A	G	-0.061	33005177	0.508	0.092	360421	A	G	0.049	21	34377485	8.44E-06	0.011	14306	20.190
Vascular dementia (mixed)	order Selenomonadales	rs4463806	C	T	-0.180	113838234	0.092	0.107	360421	C	T	0.054	10	115597993	7.81E-06	0.013	14306	17.681
Vascular dementia (mixed)	order Selenomonadales	rs4722181	T	G	-0.045	22777952	0.592	0.084	360421	T	G	0.050	7	22817571	2.00E-06	0.011	14306	22.452
Vascular dementia (mixed)	order Selenomonadales	rs60274479	T	C	0.138	21238604	0.194	0.106	360421	T	C	-0.066	16	21249925	1.16E-06	0.013	14306	24.182
Vascular dementia (mixed)	order Selenomonadales	rs61249479	A	C	0.016	122150629	0.891	0.116	360421	A	C	0.078	9	124912908	2.95E-06	0.017	14306	21.236
Vascular dementia (mixed)	order Selenomonadales	rs71405394	G	A	0.175	100704883	0.293	0.166	360421	G	A	-0.114	15	101245088	2.17E-06	0.024	14306	22.539
Vascular dementia (mixed)	order Selenomonadales	rs73232831	G	A	0.208	17411803	0.350	0.222	360421	G	A	-0.152	4	17413426	1.87E-06	0.031	14306	23.242
Vascular dementia (mixed)	order Selenomonadales	rs9423647	G	A	0.027	5537855	0.754	0.085	360421	G	A	0.048	10	5579818	6.06E-06	0.011	14306	20.628
Vascular dementia (mixed)	order Verrucomicrobiales	rs111862613	T	C	0.068	129825125	0.548	0.113	360421	T	C	0.091	12	130309670	3.74E-06	0.020	14306	21.252
Vascular dementia (mixed)	order Verrucomicrobiales	rs117107102	A	G	-0.188	51947265	0.344	0.198	360421	A	G	0.205	18	49473635	2.92E-06	0.043	14306	22.493
Vascular dementia (mixed)	order Verrucomicrobiales	rs11729256	T	C	0.127	94106121	0.258	0.112	360421	T	C	0.075	4	95027272	6.73E-07	0.015	14306	24.928
Vascular dementia (mixed)	order Verrucomicrobiales	rs12908520	G	A	0.144	97027427	0.092	0.085	360421	G	A	0.062	15	97570657	2.17E-06	0.013	14306	22.341
Vascular dementia (mixed)	order Verrucomicrobiales	rs2602429	T	C	0.023	81029544	0.810	0.096	360421	T	C	-0.075	16	81063149	2.58E-06	0.016	14306	22.863
Vascular dementia (mixed)	order Verrucomicrobiales	rs4242783	A	G	0.105	5022135	0.262	0.094	360421	A	G	-0.069	10	5064327	2.64E-06	0.015	14306	21.781
Vascular dementia (mixed)	order Verrucomicrobiales	rs4936098	G	A	0.002	130410772	0.985	0.088	360421	G	A	-0.065	11	130280667	1.12E-06	0.014	14306	22.786
Vascular dementia (mixed)	order Verrucomicrobiales	rs61779207	G	A	-0.010	40608800	0.923	0.102	360421	G	A	-0.076	1	41074472	6.72E-06	0.017	14306	20.432
Vascular dementia (mixed)	order Verrucomicrobiales	rs74542928	T	C	0.226	99623031	0.244	0.194	360421	T	C	0.112	4	100544188	1.63E-06	0.024	14306	22.508
Vascular dementia (mixed)	order Verrucomicrobiales	rs9349825	A	G	-0.041	56476683	0.701	0.107	360421	A	G	-0.070	6	56341481	2.54E-06	0.015	14306	22.898
Vascular dementia (mixed)	order Verrucomicrobiales	rs941682	G	A	-0.058	33280034	0.538	0.095	360421	G	A	-0.063	20	31867840	9.61E-06	0.014	14306	19.290
Vascular dementia (mixed)	order Victivallales	rs1002941	A	G	0.248	100702485	0.011	0.098	360421	A	G	-0.105	15	101242690	8.15E-06	0.023	14306	20.234
Vascular dementia (mixed)	order Victivallales	rs11770843	C	T	-0.228	147098287	0.011	0.090	360421	C	T	0.109	7	146795379	1.91E-06	0.023	14306	21.707
Vascular dementia (mixed)	order Victivallales	rs17114848	G	A	-0.007	24917388	0.959	0.142	360421	G	A	0.152	15	25162535	4.06E-06	0.032	14306	22.073
Vascular dementia (mixed)	order Victivallales	rs2031282	A	G	-0.069	20113040	0.533	0.111	360421	A	G	0.122	13	20687179	4.38E-06	0.027	14306	20.490
Vascular dementia (mixed)	order Victivallales	rs2825714	A	G	0.058	19651652	0.603	0.111	360421	A	G	-0.137	21	21023966	1.72E-06	0.029	14306	22.568
Vascular dementia (mixed)	order Victivallales	rs62570196	C	T	-0.267	108323890	0.207	0.212	360421	C	T	-0.216	9	111086170	1.08E-06	0.044	14306	24.192
Vascular dementia (mixed)	order Victivallales	rs72640280	A	G	-0.108	11883735	0.551	0.182	360421	A	G	0.220	1	11943792	5.18E-06	0.049	14306	20.513
Vascular dementia (mixed)	order Victivallales	rs77599476	A	G	-0.347	62762910	0.064	0.187	360421	A	G	0.230	20	61394262	1.86E-06	0.048	14306	23.002
Vascular dementia (mixed)	phylum Actinobacteria	rs11766971	C	T	-0.150	155211037	0.076	0.085	360421	C	T	-0.048	7	155002747	9.40E-06	0.011	14306	20.023
Vascular dementia (mixed)	phylum Actinobacteria	rs12528285	C	T	0.085	92065492	0.529	0.135	360421	C	T	0.081	6	92775210	5.69E-06	0.018	14306	20.048
Vascular dementia (mixed)	phylum Actinobacteria	rs13192624	T	C	-0.001	112808407	0.995	0.097	360421	T	C	-0.052	6	113129609	9.33E-06	0.012	14306	19.720
Vascular dementia (mixed)	phylum Actinobacteria	rs1397793	A	G	-0.012	91175634	0.893	0.092	360421	A	G	0.052	5	90471451	3.74E-06	0.011	14306	21.832
Vascular dementia (mixed)	phylum Actinobacteria	rs4429415	C	T	0.030	212891467	0.721	0.085	360421	C	T	0.058	2	213756191	2.05E-07	0.011	14306	27.314
Vascular dementia (mixed)	phylum Actinobacteria	rs55888705	A	G	0.072	1516099	0.438	0.093	360421	A	G	0.053	4	1517826	1.31E-06	0.011	14306	23.597
Vascular dementia (mixed)	phylum Actinobacteria	rs6496870	C	T	-0.099	91924192	0.259	0.087	360421	C	T	-0.051	15	92467422	4.62E-06	0.011	14306	20.142
Vascular dementia (mixed)	phylum Actinobacteria	rs6743026	T	C	-0.013	101633121	0.901	0.107	360421	T	C	0.059	2	102249583	9.88E-06	0.013	14306	19.171
Vascular dementia (mixed)	phylum Actinobacteria	rs74037001	G	A	0.061	23377985	0.684	0.150	360421	G	A	-0.082	14	23847194	6.71E-07	0.017	14306	24.562
Vascular dementia (mixed)	phylum Actinobacteria	rs75211493	G	A	-0.016	5300454	0.924	0.163	360421	G	A	0.084	19	5300465	9.27E-06	0.018	14306	20.714
Vascular dementia (mixed)	phylum Actinobacteria	rs7570971	A	C	0.118	135080336	0.172	0.086	360421	A	C	0.087	2	135837906	1.41E-14	0.011	14306	58.161
Vascular dementia (mixed)	phylum Actinobacteria	rs80124826	T	C	-0.364	239014688	0.159	0.258	360421	T	C	-0.124	2	239936384	8.75E-06	0.028	14306	19.876
Vascular dementia (mixed)	phylum Actinobacteria	rs857444	C	T	0.083	14617360	0.341	0.087	360421	C	T	0.051	6	14617591	3.80E-06	0.011	14306	21.315
Vascular dementia (mixed)	phylum Actinobacteria	rs9833771	C	T	0.133	32321506	0.115	0.085	360421	C	T	-0.049	3	32362998	4.07E-06	0.011	14306	21.150
Vascular dementia (mixed)	phylum Bacteroidetes	rs17343978	A	C	-0.072	27037922	0.492	0.105	360421	A	C	-0.056	22	27433885	7.22E-06	0.012	14306	21.374
Vascular dementia (mixed)	phylum Bacteroidetes	rs2032750	C	T	-0.055	53603889	0.514	0.085	360421	C	T	0.051	2	53831026	1.71E-06	0.011	14306	22.878
Vascular dementia (mixed)	phylum Bacteroidetes	rs62531359	T	G	0.000	70003946	0.997	0.111	360421	T	G	0.066	8	70916181	8.42E-06	0.015	14306	19.260
Vascular dementia (mixed)	phylum Bacteroidetes	rs62575403	C	T	-0.184	133628698	0.380	0.210	360421	C	T	0.145	9	136493820	2.96E-06	0.031	14306	21.854
Vascular dementia (mixed)	phylum Bacteroidetes	rs6586324	T	C	-0.061	42522025	0.477	0.085	360421	T	C	0.048	21	43942135	7.37E-06	0.011	14306	20.535
Vascular dementia (mixed)	phylum Bacteroidetes	rs72706335	T	C	-0.032	157525648	0.904	0.269	360421	T	C	-0.223	1	157495438	7.13E-06	0.049	14306	20.464
Vascular dementia (mixed)	phylum Bacteroidetes	rs73512608	G	A	0.143	69948551	0.436	0.183	360421	G	A	-0.123	13	70522683	2.54E-07	0.024	14306	27.045

Vascular dementia (mixed)	phylum Bacteroidetes	rs73846128	A	G	-0.020	89291104	0.864	0.119	360421	A	G	-0.066	3	89340254	4.78E-07	0.013	14306	24.765
Vascular dementia (mixed)	phylum Bacteroidetes	rs73975615	G	A	0.114	6557880	0.817	0.490	360421	G	A	-0.207	17	6461200	1.20E-06	0.044	14306	21.905
Vascular dementia (mixed)	phylum Bacteroidetes	rs929878	T	C	-0.214	74256742	0.039	0.104	360421	T	C	0.054	16	74290641	6.51E-06	0.012	14306	19.745
Vascular dementia (mixed)	phylum Cyanobacteria	rs12555298	G	A	-0.119	107727270	0.288	0.112	360421	G	A	0.097	9	110489551	8.09E-06	0.022	14306	19.730
Vascular dementia (mixed)	phylum Cyanobacteria	rs2585223	T	C	-0.091	100340683	0.479	0.128	360421	T	C	0.111	15	100880888	8.86E-06	0.025	14306	20.206
Vascular dementia (mixed)	phylum Cyanobacteria	rs584122	T	C	-0.186	48320720	0.299	0.179	360421	T	C	0.152	6	48288456	4.23E-06	0.033	14306	21.565
Vascular dementia (mixed)	phylum Cyanobacteria	rs61972390	T	C	-0.122	99914504	0.322	0.123	360421	T	C	0.107	13	100566758	9.11E-06	0.024	14306	19.600
Vascular dementia (mixed)	phylum Cyanobacteria	rs7148504	T	G	-0.085	99701961	0.323	0.086	360421	T	G	-0.080	14	100168298	6.62E-06	0.018	14306	20.537
Vascular dementia (mixed)	phylum Cyanobacteria	rs76531781	T	C	-0.317	21608117	0.142	0.216	360421	T	C	-0.232	7	21647735	2.87E-06	0.049	14306	22.085
Vascular dementia (mixed)	phylum Cyanobacteria	rs789068	G	A	0.004	1008238	0.973	0.122	360421	G	A	-0.111	18	1008239	1.57E-07	0.021	14306	27.366
Vascular dementia (mixed)	phylum Cyanobacteria	rs9864379	T	C	-0.074	14265449	0.535	0.119	360421	T	C	-0.139	3	14306949	2.03E-07	0.027	14306	26.866
Vascular dementia (mixed)	phylum Euryarchaeota	rs10202904	G	T	0.028	124682691	0.745	0.086	360421	G	T	0.116	2	125440268	6.19E-07	0.023	14306	25.371
Vascular dementia (mixed)	phylum Euryarchaeota	rs11022995	A	G	-0.150	13872120	0.075	0.084	360421	A	G	0.104	11	13893667	7.73E-06	0.023	14306	20.538
Vascular dementia (mixed)	phylum Euryarchaeota	rs34928225	T	C	0.047	146519740	0.744	0.144	360421	T	C	0.200	6	146840876	4.33E-06	0.043	14306	22.051
Vascular dementia (mixed)	phylum Euryarchaeota	rs45498998	G	A	-0.071	25996679	0.541	0.116	360421	G	A	-0.132	21	27368994	5.32E-06	0.029	14306	20.358
Vascular dementia (mixed)	phylum Euryarchaeota	rs6064552	T	C	0.145	57467810	0.184	0.109	360421	T	C	-0.124	20	56042866	9.34E-06	0.028	14306	20.095
Vascular dementia (mixed)	phylum Euryarchaeota	rs6508769	C	T	0.065	28336853	0.583	0.118	360421	C	T	-0.151	19	28827760	8.12E-06	0.034	14306	19.941
Vascular dementia (mixed)	phylum Euryarchaeota	rs7015093	G	A	-0.108	57106294	0.261	0.096	360421	G	A	-0.118	8	58018853	7.20E-06	0.026	14306	19.957
Vascular dementia (mixed)	phylum Euryarchaeota	rs76029318	T	C	0.120	41389655	0.484	0.171	360421	T	C	0.215	13	41963791	1.05E-06	0.044	14306	24.017
Vascular dementia (mixed)	phylum Euryarchaeota	rs7635189	A	G	-0.144	15550100	0.125	0.094	360421	A	G	-0.120	3	15591607	4.64E-06	0.026	14306	21.402
Vascular dementia (mixed)	phylum Euryarchaeota	rs77658038	A	C	-0.125	16284554	0.236	0.105	360421	A	C	-0.160	6	16284785	4.75E-06	0.034	14306	22.078
Vascular dementia (mixed)	phylum Euryarchaeota	rs894996	C	A	-0.011	103497150	0.945	0.163	360421	C	A	0.204	4	104418307	5.12E-06	0.044	14306	21.404
Vascular dementia (mixed)	phylum Firmicutes	rs112334273	G	A	-0.109	39331325	0.246	0.094	360421	G	A	0.063	21	40703251	9.26E-07	0.013	14306	24.251
Vascular dementia (mixed)	phylum Firmicutes	rs2273429	A	G	0.011	52027354	0.935	0.137	360421	A	G	-0.070	14	52494072	9.26E-06	0.015	14306	21.041
Vascular dementia (mixed)	phylum Firmicutes	rs2332027	A	G	-0.066	170750887	0.447	0.086	360421	A	G	0.048	4	171672038	4.05E-06	0.010	14306	21.201
Vascular dementia (mixed)	phylum Firmicutes	rs2547978	G	A	0.171	98684594	0.050	0.087	360421	G	A	-0.047	5	98020298	8.57E-06	0.011	14306	19.652
Vascular dementia (mixed)	phylum Firmicutes	rs3792064	G	A	-0.001	230813698	0.996	0.188	360421	G	A	0.090	2	231678413	6.75E-07	0.018	14306	24.168
Vascular dementia (mixed)	phylum Firmicutes	rs3852931	T	C	0.101	55471425	0.252	0.088	360421	T	C	0.048	20	54087963	4.53E-06	0.011	14306	21.080
Vascular dementia (mixed)	phylum Firmicutes	rs4750583	G	A	-0.160	15012766	0.138	0.108	360421	G	A	-0.062	10	15054765	5.79E-06	0.014	14306	19.944
Vascular dementia (mixed)	phylum Firmicutes	rs56199908	T	C	0.391	2801371	0.037	0.187	360421	T	C	-0.186	9	2801371	8.67E-06	0.041	14306	20.572
Vascular dementia (mixed)	phylum Firmicutes	rs6814436	C	T	0.015	160586149	0.903	0.120	360421	C	T	-0.068	4	161507301	6.80E-06	0.015	14306	20.334
Vascular dementia (mixed)	phylum Firmicutes	rs6815608	C	T	-0.109	151210592	0.358	0.118	360421	C	T	-0.094	4	152131744	7.24E-06	0.021	14306	19.693
Vascular dementia (mixed)	phylum Firmicutes	rs7247191	T	C	-0.060	22716783	0.671	0.142	360421	T	C	-0.071	19	22899585	4.73E-06	0.016	14306	20.515
Vascular dementia (mixed)	phylum Firmicutes	rs72738886	T	C	0.071	35770448	0.652	0.157	360421	T	C	0.086	5	35770550	7.68E-06	0.019	14306	20.621
Vascular dementia (mixed)	phylum Firmicutes	rs72771021	C	T	0.015	13799631	0.930	0.168	360421	C	T	-0.141	10	13841631	5.12E-06	0.031	14306	21.022
Vascular dementia (mixed)	phylum Firmicutes	rs8085381	A	G	-0.259	31748342	0.017	0.109	360421	A	G	-0.065	18	29328305	8.67E-06	0.015	14306	19.597
Vascular dementia (mixed)	phylum Firmicutes	rs992074	T	C	-0.245	17195484	0.391	0.285	360421	T	C	-0.233	21	18567802	8.52E-06	0.051	14306	21.042
Vascular dementia (mixed)	phylum Lentisphaerae	rs1002941	A	G	0.248	100702485	0.011	0.098	360421	A	G	-0.108	15	101242690	4.31E-06	0.023	14306	21.281
Vascular dementia (mixed)	phylum Lentisphaerae	rs11770843	C	T	-0.228	147098287	0.011	0.090	360421	C	T	0.112	7	146795379	1.14E-06	0.023	14306	22.715
Vascular dementia (mixed)	phylum Lentisphaerae	rs17114848	G	A	-0.007	24917388	0.959	0.142	360421	G	A	0.149	15	25162535	6.77E-06	0.032	14306	21.120
Vascular dementia (mixed)	phylum Lentisphaerae	rs2031282	A	G	-0.069	20113040	0.533	0.111	360421	A	G	0.120	13	20687179	5.86E-06	0.027	14306	19.850
Vascular dementia (mixed)	phylum Lentisphaerae	rs2825714	A	G	0.058	19651652	0.603	0.111	360421	A	G	-0.138	21	21023966	1.50E-06	0.029	14306	22.861
Vascular dementia (mixed)	phylum Lentisphaerae	rs60995569	T	G	0.030	52170325	0.824	0.134	360421	T	G	-0.161	10	53930085	9.19E-06	0.034	14306	22.743
Vascular dementia (mixed)	phylum Lentisphaerae	rs62570196	C	T	-0.267	108323890	0.207	0.212	360421	C	T	-0.217	9	111086170	9.64E-07	0.044	14306	24.410
Vascular dementia (mixed)	phylum Lentisphaerae	rs72640280	A	G	-0.108	11883735	0.551	0.182	360421	A	G	0.220	1	11943792	5.19E-06	0.049	14306	20.530
Vascular dementia (mixed)	phylum Lentisphaerae	rs77599476	A	G	-0.347	62762910	0.064	0.187	360421	A	G	0.230	20	61394262	1.90E-06	0.048	14306	22.964
Vascular dementia (mixed)	phylum Proteobacteria	rs10750258	C	A	-0.017	124126791	0.843	0.087	360421	C	A	0.049	11	123997498	8.72E-06	0.011	14306	20.598
Vascular dementia (mixed)	phylum Proteobacteria	rs11126162	T	C	-0.319	67858902	0.037	0.153	360421	T	C	-0.077	2	68086034	9.26E-06	0.019	14306	16.971
Vascular dementia (mixed)	phylum Proteobacteria	rs11715072	G	A	0.089	53686766	0.334	0.092	360421	G	A	-0.052	3	53720793	6.90E-06	0.012	14306	20.303
Vascular dementia (mixed)	phylum Proteobacteria	rs12150865	C	T	-0.072	43001168	0.398	0.086	360421	C	T	0.051	19	43505320	1.54E-06	0.011	14306	23.118
Vascular dementia (mixed)	phylum Proteobacteria	rs12467198	C	T	-0.021	124115104	0.805	0.085	360421	C	T	0.050	2	124872681	6.31E-06	0.011	14306	20.013
Vascular dementia (mixed)	phylum Proteobacteria	rs2347697	G	T	-0.153	134703488	0.092	0.091	360421	G	T	0.050	7	134388240	4.27E-06	0.011	14306	21.247
Vascular dementia (mixed)	phylum Proteobacteria	rs2532663	A	G	-0.121	117487723	0.389	0.141	360421	A	G	0.126	10	119247234	7.47E-07	0.026	14306	23.685
Vascular dementia (mixed)	phylum Proteobacteria	rs3890996	G	T	0.082	20314277	0.330	0.084	360421	G	T	0.047	22	20301800	6.95E-06	0.011	14306	20.183
Vascular dementia (mixed)	phylum Proteobacteria	rs4340090	C	T	0.133	129847219	0.310	0.131	360421	C	T	-0.067	12	130331764	9.99E-06	0.015	14306	18.975
Vascular dementia (mixed)	phylum Proteobacteria	rs6707783	C	T	-0.049	51826753	0.734	0.144	360421	C	T	0.085	2	52053891	8.09E-06	0.019	14306	20.470
Vascular dementia (mixed)	phylum Proteobacteria	rs72771021	C	T	0.015	13799631	0.930	0.168	360421	C	T	0.142	10	13841631	7.18E-06	0.031	14306	21.004
Vascular dementia (mixed)	phylum Proteobacteria	rs922773	C	T	-0.038	66267618	0.785	0.138	360421	C	T	-0.080	3	66318042	3.68E-07	0.016	14306	25.929
Vascular dementia (mixed)	phylum Tenericutes	rs10108398	G	A	0.111	58528265	0.237	0.094	360421	G	A	0.077	8	59440824	1.09E-06	0.015	14306	24.960
Vascular dementia (mixed)	phylum Tenericutes	rs11890098	A	G	-0.164	156676037	0.082	0.094	360421	A	G	0.074	2	157532549	9.57E-07	0.015	14306	23.551
Vascular dementia (mixed)	phylum Tenericutes	rs12566890	T	G	0.261	61385192	0.036	0.125	360421	T	G	-0.101	1	61850864	3.65E-06	0.023	14306	19.176
Vascular dementia (mixed)	phylum Tenericutes	rs17214486	C	A	0.044	96693337	0.630	0.091	360421	C	A	0.061	14	97159674	6.61E-06	0.014	14306	20.223
Vascular dementia (mixed)	phylum Tenericutes	rs2464826	A	C	-0.156	79860934	0.243	0.134	360421	A	C	0.094	7	79490250	8.39E-06	0.021	14306	19.874
Vascular dementia (mixed)	phylum Tenericutes	rs28537087	G	A	0.058	94766447	0.553	0.098	360421	G	A	0.082	15	95309676	8.07E-06	0.019	14306	19.002
Vascular dementia (mixed)	phylum Tenericutes	rs3768491	G	A	0.017	109423364	0.856	0.093	360421	G	A	0.068	1	109965986	4.23E-06	0.015	14306	20.875
Vascular dementia (mixed)	phylum Tenericutes	rs4885016	C	T	-0.082	72596351	0.510	0.124	360421	C	T	0.082	13	73170489	7.27E-06	0.018	14306	20.363
Vascular dementia (mixed)	phylum Tenericutes	rs6043847	T	C	-0.218	16278879	0.229	0.181	360421	T	C	-0.115	20	16259524	4.55E-06	0.025	14306	21.375

Vascular dementia (mixed)	phylum Tenericutes	rs72901605	T	C	-0.100	47082326	0.445	0.131	360421	T	C	-0.084	11	47103877	3.26E-06	0.018	14306	22.338
Vascular dementia (mixed)	phylum Tenericutes	rs74603314	T	C	0.252	46050515	0.247	0.218	360421	T	C	0.222	14	46519718	1.56E-06	0.046	14306	22.924
Vascular dementia (mixed)	phylum Tenericutes	rs78169027	A	G	0.037	108568360	0.835	0.177	360421	A	G	-0.108	11	108439087	5.88E-06	0.024	14306	20.824
Vascular dementia (mixed)	phylum Verrucomicrobia	rs11252894	A	C	-0.130	5030858	0.188	0.098	360421	A	C	0.078	10	5073050	1.11E-06	0.016	14306	23.203
Vascular dementia (mixed)	phylum Verrucomicrobia	rs117107102	A	G	-0.188	51947265	0.344	0.198	360421	A	G	0.204	18	49473635	2.68E-06	0.043	14306	22.828
Vascular dementia (mixed)	phylum Verrucomicrobia	rs11729256	T	C	0.127	94106121	0.258	0.112	360421	T	C	0.070	4	95027272	2.23E-06	0.015	14306	22.495
Vascular dementia (mixed)	phylum Verrucomicrobia	rs12512971	A	C	-0.148	16547352	0.349	0.158	360421	A	C	0.171	4	16548975	9.81E-06	0.040	14306	18.467
Vascular dementia (mixed)	phylum Verrucomicrobia	rs12908520	G	A	0.144	97027427	0.092	0.085	360421	G	A	0.059	15	97570657	3.40E-06	0.013	14306	21.524
Vascular dementia (mixed)	phylum Verrucomicrobia	rs2602429	T	C	0.023	81029544	0.810	0.096	360421	T	C	-0.076	16	81063149	8.71E-07	0.015	14306	24.786
Vascular dementia (mixed)	phylum Verrucomicrobia	rs3995795	C	T	-0.098	11347615	0.259	0.086	360421	C	T	0.061	10	11389614	9.72E-06	0.014	14306	19.430
Vascular dementia (mixed)	phylum Verrucomicrobia	rs45598138	C	A	-0.119	55056410	0.653	0.265	360421	C	A	-0.144	1	55522083	2.19E-06	0.031	14306	22.202
Vascular dementia (mixed)	phylum Verrucomicrobia	rs61779207	G	A	-0.010	40608800	0.923	0.102	360421	G	A	-0.076	1	41074472	5.28E-06	0.016	14306	21.109
Vascular dementia (mixed)	phylum Verrucomicrobia	rs74542928	T	C	0.226	99623031	0.244	0.194	360421	T	C	0.116	4	100544188	4.08E-07	0.023	14306	25.144
Vascular dementia (mixed)	phylum Verrucomicrobia	rs76430504	T	C	-0.158	40438056	0.422	0.197	360421	T	C	-0.118	5	40438158	3.50E-06	0.025	14306	21.313
Vascular dementia (mixed)	phylum Verrucomicrobia	rs9349825	A	G	-0.041	56476683	0.701	0.107	360421	A	G	-0.066	6	56341481	6.27E-06	0.014	14306	21.048
Vascular dementia (multiple infarctions)	class Actinobacteria	rs11655079	T	C	0.060	77352593	0.478	0.084	360612	T	C	-0.056	17	75348675	5.93E-06	0.012	14306	20.351
Vascular dementia (multiple infarctions)	class Actinobacteria	rs11745923	G	T	0.013	475293	0.842	0.067	360612	G	T	0.056	5	475408	1.58E-06	0.012	14306	23.812
Vascular dementia (multiple infarctions)	class Actinobacteria	rs12049045	A	G	-0.007	114122863	0.915	0.067	360612	A	G	0.051	1	114665485	8.63E-06	0.011	14306	19.936
Vascular dementia (multiple infarctions)	class Actinobacteria	rs134366	A	G	-0.035	35171362	0.787	0.128	360612	A	G	-0.112	22	35567355	1.50E-06	0.024	14306	22.663
Vascular dementia (multiple infarctions)	class Actinobacteria	rs1376754	G	A	-0.035	160755458	0.592	0.065	360612	G	A	0.051	2	161611969	6.71E-06	0.011	14306	20.489
Vascular dementia (multiple infarctions)	class Actinobacteria	rs1515761	C	T	0.041	124421463	0.734	0.122	360612	C	T	-0.076	10	126110032	4.96E-06	0.017	14306	20.136
Vascular dementia (multiple infarctions)	class Actinobacteria	rs182549	T	C	-0.125	135859184	0.065	0.068	360612	T	C	-0.111	2	136616754	3.79E-20	0.012	14306	85.376
Vascular dementia (multiple infarctions)	class Actinobacteria	rs4945008	G	A	-0.033	71510202	0.621	0.067	360612	G	A	-0.054	11	71221248	5.39E-06	0.012	14306	19.842
Vascular dementia (multiple infarctions)	class Actinobacteria	rs6660520	G	A	0.148	206830208	0.044	0.073	360612	G	A	-0.071	1	207003553	1.11E-07	0.013	14306	27.984
Vascular dementia (multiple infarctions)	class Actinobacteria	rs72767435	T	C	0.165	94309566	0.254	0.145	360612	T	C	-0.126	15	94852795	2.57E-06	0.027	14306	21.323
Vascular dementia (multiple infarctions)	class Actinobacteria	rs7322849	T	C	-0.075	112205515	0.511	0.114	360612	T	C	0.094	13	112859829	6.21E-07	0.019	14306	23.953
Vascular dementia (multiple infarctions)	class Actinobacteria	rs80083040	T	G	-0.235	64742287	0.132	0.156	360612	T	G	0.156	8	65654844	8.62E-06	0.035	14306	20.038
Vascular dementia (multiple infarctions)	class Actinobacteria	rs857444	C	T	-0.109	14617360	0.107	0.068	360612	C	T	0.051	6	14617591	8.92E-06	0.012	14306	19.500
Vascular dementia (multiple infarctions)	class Actinobacteria	rs961091	G	A	-0.085	96025406	0.208	0.068	360612	G	A	0.050	7	95654718	8.68E-06	0.011	14306	19.835
Vascular dementia (multiple infarctions)	class Alphaproteobacteria	rs140912403	C	T	0.020	92755525	0.889	0.140	360612	C	T	-0.161	9	95517807	6.20E-07	0.032	14306	25.577
Vascular dementia (multiple infarctions)	class Alphaproteobacteria	rs34569731	G	A	-0.005	75403218	0.938	0.068	360612	G	A	-0.071	2	75630344	7.38E-06	0.016	14306	20.028
Vascular dementia (multiple infarctions)	class Alphaproteobacteria	rs62285697	C	T	0.038	176690123	0.615	0.076	360612	C	T	0.081	3	176407911	9.76E-06	0.018	14306	19.812
Vascular dementia (multiple infarctions)	class Alphaproteobacteria	rs76784716	A	G	-0.160	168176830	0.129	0.105	360612	A	G	0.133	2	169033340	5.09E-07	0.027	14306	24.777
Vascular dementia (multiple infarctions)	class Alphaproteobacteria	rs7960664	A	G	-0.146	89077458	0.206	0.115	360612	A	G	-0.097	12	89471235	8.84E-06	0.022	14306	20.305
Vascular dementia (multiple infarctions)	class Alphaproteobacteria	rs9813022	A	G	-0.189	13685237	0.005	0.067	360612	A	G	-0.075	3	13726736	1.05E-06	0.015	14306	23.840
Vascular dementia (multiple infarctions)	class Bacilli	rs11110282	A	G	-0.106	100191781	0.475	0.148	360612	A	G	-0.101	12	100585559	4.85E-06	0.022	14306	21.669
Vascular dementia (multiple infarctions)	class Bacilli	rs11730038	G	A	0.077	97128348	0.283	0.072	360612	G	A	-0.063	4	98049499	1.96E-06	0.013	14306	24.013
Vascular dementia (multiple infarctions)	class Bacilli	rs12797734	T	C	-0.003	8310803	0.969	0.076	360612	T	C	0.057	11	8332350	7.21E-06	0.013	14306	20.363
Vascular dementia (multiple infarctions)	class Bacilli	rs13068444	A	G	-0.001	64413187	0.990	0.086	360612	A	G	0.060	3	64398863	9.53E-06	0.014	14306	19.415
Vascular dementia (multiple infarctions)	class Bacilli	rs1595463	C	A	-0.041	230858942	0.532	0.066	360612	C	A	0.048	2	231723657	7.97E-06	0.011	14306	19.300
Vascular dementia (multiple infarctions)	class Bacilli	rs28564647	T	G	0.115	98338890	0.187	0.087	360612	T	G	-0.061	9	101101172	7.81E-06	0.014	14306	19.855
Vascular dementia (multiple infarctions)	class Bacilli	rs2952251	G	A	-0.079	10285654	0.310	0.078	360612	G	A	0.060	8	10143164	1.08E-06	0.012	14306	23.361
Vascular dementia (multiple infarctions)	class Bacilli	rs34989881	A	G	0.050	51456601	0.748	0.157	360612	A	G	0.111	19	51959855	6.55E-06	0.025	14306	20.374
Vascular dementia (multiple infarctions)	class Bacilli	rs35344081	G	A	-0.120	941253	0.105	0.074	360612	G	A	0.062	16	991253	1.01E-06	0.013	14306	23.772
Vascular dementia (multiple infarctions)	class Bacilli	rs4028634	C	T	0.053	42683631	0.435	0.068	360612	C	T	-0.052	17	40835649	2.21E-06	0.011	14306	22.464
Vascular dementia (multiple infarctions)	class Bacilli	rs4459992	T	C	0.030	7429760	0.665	0.070	360612	T	C	0.054	4	7431487	4.30E-06	0.012	14306	21.207
Vascular dementia (multiple infarctions)	class Bacilli	rs57872228	C	T	-0.039	200449677	0.695	0.100	360612	C	T	-0.071	1	200418805	9.22E-07	0.015	14306	23.757
Vascular dementia (multiple infarctions)	class Bacilli	rs694949	A	G	0.093	58445876	0.397	0.110	360612	A	G	-0.081	15	58738075	7.60E-06	0.018	14306	20.314
Vascular dementia (multiple infarctions)	class Bacilli	rs74663707	C	T	0.015	184653436	0.912	0.134	360612	C	T	0.098	3	184371224	8.46E-06	0.022	14306	19.095
Vascular dementia (multiple infarctions)	class Bacilli	rs7666190	A	C	-0.186	150766268	0.072	0.103	360612	A	C	0.104	4	151687420	8.47E-06	0.025	14306	17.610
Vascular dementia (multiple infarctions)	class Bacilli	rs77558518	A	G	0.065	174746168	0.567	0.113	360612	A	G	-0.107	5	174173171	1.34E-06	0.022	14306	23.158
Vascular dementia (multiple infarctions)	class Bacilli	rs78938557	T	C	-0.314	36309977	0.108	0.196	360612	T	C	0.108	7	36349586	1.07E-06	0.023	14306	21.514
Vascular dementia (multiple infarctions)	class Bacilli	rs9581006	T	C	-0.190	24399371	0.279	0.176	360612	T	C	-0.225	13	24973509	1.79E-06	0.047	14306	23.207
Vascular dementia (multiple infarctions)	class Bacteroidia	rs11146701	A	G	-0.037	38769138	0.587	0.069	360612	A	G	0.047	10	39062269	7.08E-06	0.011	14306	20.186
Vascular dementia (multiple infarctions)	class Bacteroidia	rs17343978	A	C	0.048	27037922	0.557	0.081	360612	A	C	-0.055	22	27433885	8.36E-06	0.012	14306	21.067
Vascular dementia (multiple infarctions)	class Bacteroidia	rs2032750	C	T	-0.010	53603889	0.878	0.066	360612	C	T	0.051	2	53831026	1.92E-06	0.011	14306	22.657
Vascular dementia (multiple infarctions)	class Bacteroidia	rs2363574	T	C	0.049	200143435	0.774	0.172	360612	T	C	0.223	1	200112563	9.93E-06	0.051	14306	19.221
Vascular dementia (multiple infarctions)	class Bacteroidia	rs4916508	A	G	0.019	196209918	0.771	0.066	360612	A	G	0.047	3	195936789	8.47E-06	0.011	14306	19.641
Vascular dementia (multiple infarctions)	class Bacteroidia	rs55773148	G	A	0.252	69948897	0.081	0.145	360612	G	A	-0.122	13	70523029	3.90E-07	0.024	14306	26.341
Vascular dementia (multiple infarctions)	class Bacteroidia	rs62531359	T	G	-0.130	70003946	0.130	0.086	360612	T	G	0.066	8	70916181	9.09E-06	0.015	14306	19.138
Vascular dementia (multiple infarctions)	class Bacteroidia	rs62575403	C	T	-0.087	133628698	0.593	0.162	360612	C	T	0.140	9	136493820	7.06E-06	0.031	14306	20.264
Vascular dementia (multiple infarctions)	class Bacteroidia	rs72706335	T	C	-0.231	157525648	0.278	0.213	360612	T	C	-0.222	1	157495438	7.66E-06	0.049	14306	20.315
Vascular dementia (multiple infarctions)	class Bacteroidia	rs73975615	G	A	-0.114	6557880	0.772	0.393	360612	G	A	-0.207	17	6461200	1.22E-06	0.044	14306	21.874
Vascular dementia (multiple infarctions)	class Bacteroidia	rs7631304	G	A	-0.179	89290377	0.054	0.093	360612	G	A	-0.065	3	89339527	8.37E-07	0.013	14306	23.590
Vascular dementia (multiple infarctions)	class Bacteroidia	rs79585701	A	C	0.071	13252676	0.445	0.093	360612	A	C	0.065	8	13110185	9.99E-06	0.015	14306	18.687
Vascular dementia (multiple infarctions)	class Bacteroidia	rs929878	T	C	-0.117	74256742	0.143	0.080	360612	T	C	0.055	16	74290641	4.73E-06	0.012	14306	20.372
Vascular dementia (multiple infarctions)	class Betaproteobacteria	rs11128180	A	G	-0.012	70543064	0.878	0.077	360612	A	G	0.059	3	70592215	3.67E-06	0.013	14306	21.203

Vascular dementia (multiple infarctions)	class Betaproteobacteria	rs1511453	A	G	-0.225	23586839	0.108	0.140	360612	A	G	0.092	4	23588462	4.76E-06	0.020	14306	21.502
Vascular dementia (multiple infarctions)	class Betaproteobacteria	rs1928341	G	A	-0.036	153267537	0.586	0.067	360612	G	A	-0.053	1	153240013	2.02E-06	0.011	14306	22.661
Vascular dementia (multiple infarctions)	class Betaproteobacteria	rs2321387	G	A	0.067	58115206	0.306	0.066	360612	G	A	-0.049	13	58689340	5.80E-06	0.011	14306	20.430
Vascular dementia (multiple infarctions)	class Betaproteobacteria	rs320161	G	A	-0.102	102136835	0.188	0.078	360612	G	A	-0.057	9	104899117	7.33E-06	0.013	14306	20.564
Vascular dementia (multiple infarctions)	class Betaproteobacteria	rs4033856	T	C	0.036	45640468	0.754	0.116	360612	T	C	-0.083	4	45642485	5.17E-07	0.017	14306	24.776
Vascular dementia (multiple infarctions)	class Betaproteobacteria	rs6087811	T	G	-0.047	32008327	0.670	0.109	360612	T	G	-0.098	20	30596130	7.44E-07	0.020	14306	24.278
Vascular dementia (multiple infarctions)	class Betaproteobacteria	rs62395635	T	C	0.131	174070793	0.337	0.137	360612	T	C	0.110	5	173497796	2.94E-06	0.024	14306	21.623
Vascular dementia (multiple infarctions)	class Clostridia	rs10774377	G	A	0.064	5833353	0.334	0.067	360612	G	A	-0.053	12	5942519	3.24E-06	0.011	14306	21.390
Vascular dementia (multiple infarctions)	class Clostridia	rs112334273	G	A	-0.001	39331325	0.991	0.073	360612	G	A	0.064	21	40703251	3.81E-07	0.013	14306	25.314
Vascular dementia (multiple infarctions)	class Clostridia	rs13105690	C	T	-0.012	7418457	0.870	0.073	360612	C	T	0.053	4	7420184	8.78E-06	0.012	14306	20.044
Vascular dementia (multiple infarctions)	class Clostridia	rs13179700	C	T	-0.036	149698225	0.601	0.069	360612	C	T	-0.051	5	149077788	3.37E-06	0.011	14306	21.830
Vascular dementia (multiple infarctions)	class Clostridia	rs1842454	G	A	-0.100	105724661	0.230	0.083	360612	G	A	-0.055	5	105060362	8.72E-06	0.013	14306	18.466
Vascular dementia (multiple infarctions)	class Clostridia	rs2273429	A	G	0.168	52027354	0.113	0.106	360612	A	G	-0.072	14	52494072	4.52E-06	0.015	14306	22.364
Vascular dementia (multiple infarctions)	class Clostridia	rs6797343	T	G	0.068	89219564	0.415	0.083	360612	T	G	-0.059	3	89268714	9.36E-06	0.013	14306	19.375
Vascular dementia (multiple infarctions)	class Clostridia	rs6814436	C	T	0.133	160586149	0.153	0.093	360612	C	T	-0.074	4	161507301	9.65E-07	0.015	14306	24.075
Vascular dementia (multiple infarctions)	class Clostridia	rs6815608	C	T	-0.001	151210592	0.994	0.091	360612	C	T	-0.104	4	152131744	4.02E-07	0.021	14306	24.229
Vascular dementia (multiple infarctions)	class Clostridia	rs72738886	T	C	0.121	35770448	0.330	0.124	360612	T	C	0.087	5	35770550	8.24E-06	0.019	14306	20.697
Vascular dementia (multiple infarctions)	class Clostridia	rs992074	T	C	0.183	17195484	0.402	0.219	360612	T	C	-0.256	21	18567802	8.78E-07	0.051	14306	25.245
Vascular dementia (multiple infarctions)	class Coriobacteriia	rs11073596	G	T	0.045	85890348	0.503	0.068	360612	G	T	-0.051	15	86433579	8.14E-06	0.011	14306	19.912
Vascular dementia (multiple infarctions)	class Coriobacteriia	rs11250875	T	C	0.028	1880537	0.728	0.080	360612	T	C	0.061	10	1922731	4.83E-06	0.013	14306	21.526
Vascular dementia (multiple infarctions)	class Coriobacteriia	rs11656361	A	C	-0.129	8218014	0.130	0.085	360612	A	C	0.077	17	8121332	8.02E-06	0.018	14306	19.394
Vascular dementia (multiple infarctions)	class Coriobacteriia	rs12974142	G	A	-0.138	52391913	0.280	0.128	360612	G	A	0.079	19	52895166	8.51E-06	0.018	14306	19.865
Vascular dementia (multiple infarctions)	class Coriobacteriia	rs13307134	T	C	-0.202	105444233	0.021	0.088	360612	T	C	-0.057	7	105084680	7.80E-06	0.013	14306	20.072
Vascular dementia (multiple infarctions)	class Coriobacteriia	rs1397793	A	G	0.091	91175634	0.203	0.072	360612	A	G	0.050	5	90471451	9.77E-06	0.011	14306	19.682
Vascular dementia (multiple infarctions)	class Coriobacteriia	rs1816223	G	A	0.150	11341087	0.068	0.082	360612	G	A	0.059	12	11494021	4.84E-06	0.013	14306	20.652
Vascular dementia (multiple infarctions)	class Coriobacteriia	rs240104	T	C	-0.183	176602295	0.012	0.073	360612	T	C	-0.060	1	176571431	1.52E-06	0.013	14306	22.630
Vascular dementia (multiple infarctions)	class Coriobacteriia	rs2442778	A	G	-0.136	11612938	0.358	0.148	360612	A	G	0.116	3	11654412	9.03E-06	0.026	14306	20.272
Vascular dementia (multiple infarctions)	class Coriobacteriia	rs3025411	A	G	-0.026	133647784	0.808	0.107	360612	A	G	0.093	9	136512906	8.27E-06	0.021	14306	19.566
Vascular dementia (multiple infarctions)	class Coriobacteriia	rs34739816	G	T	-0.119	39220432	0.387	0.137	360612	G	T	0.097	17	37376685	3.88E-06	0.021	14306	21.594
Vascular dementia (multiple infarctions)	class Coriobacteriia	rs67561917	A	G	-0.033	63440724	0.703	0.086	360612	A	G	-0.071	20	62072077	5.39E-06	0.015	14306	21.486
Vascular dementia (multiple infarctions)	class Coriobacteriia	rs719099	A	G	0.094	64039457	0.389	0.109	360612	A	G	0.078	10	65799217	5.43E-07	0.016	14306	24.957
Vascular dementia (multiple infarctions)	class Coriobacteriia	rs8010111	A	G	0.061	39191305	0.599	0.117	360612	A	G	0.103	14	39660509	6.90E-06	0.023	14306	20.328
Vascular dementia (multiple infarctions)	class Deltaproteobacteria	rs1035691	G	A	0.082	10637188	0.219	0.067	360612	G	A	0.055	11	10658735	9.65E-06	0.012	14306	20.588
Vascular dementia (multiple infarctions)	class Deltaproteobacteria	rs11599763	C	T	0.090	11813600	0.180	0.067	360612	C	T	0.054	10	11855599	3.94E-06	0.012	14306	21.490
Vascular dementia (multiple infarctions)	class Deltaproteobacteria	rs17084793	G	A	-0.087	71645678	0.351	0.093	360612	G	A	-0.071	18	69312914	5.69E-06	0.016	14306	19.850
Vascular dementia (multiple infarctions)	class Deltaproteobacteria	rs17791387	A	G	0.240	79219511	0.033	0.113	360612	A	G	-0.074	9	81834426	1.60E-06	0.015	14306	22.761
Vascular dementia (multiple infarctions)	class Deltaproteobacteria	rs2692012	G	A	-0.064	204022477	0.658	0.145	360612	G	A	-0.110	1	203991605	3.14E-06	0.025	14306	18.968
Vascular dementia (multiple infarctions)	class Deltaproteobacteria	rs2838334	G	A	0.066	43645080	0.333	0.069	360612	G	A	0.056	21	45064961	5.45E-06	0.012	14306	20.480
Vascular dementia (multiple infarctions)	class Deltaproteobacteria	rs3935584	C	T	-0.128	233064573	0.050	0.066	360612	C	T	-0.052	2	233929283	7.50E-06	0.012	14306	20.485
Vascular dementia (multiple infarctions)	class Deltaproteobacteria	rs4506934	C	T	0.094	2953368	0.355	0.102	360612	C	T	-0.094	17	2856662	3.59E-06	0.020	14306	21.666
Vascular dementia (multiple infarctions)	class Deltaproteobacteria	rs55744759	A	G	-0.115	56955112	0.265	0.103	360612	A	G	-0.078	8	57867671	7.31E-06	0.017	14306	20.854
Vascular dementia (multiple infarctions)	class Deltaproteobacteria	rs6058181	C	T	-0.145	35106998	0.098	0.087	360612	C	T	0.083	20	33694801	3.40E-07	0.017	14306	24.755
Vascular dementia (multiple infarctions)	class Deltaproteobacteria	rs62020470	A	G	0.002	95617836	0.977	0.086	360612	A	G	-0.059	15	96161065	4.85E-06	0.013	14306	20.480
Vascular dementia (multiple infarctions)	class Deltaproteobacteria	rs9928243	C	A	-0.026	71507738	0.692	0.066	360612	C	A	-0.054	16	71541641	5.02E-06	0.012	14306	20.923
Vascular dementia (multiple infarctions)	class Erysipelotrichia	rs1074800	G	A	0.013	3002432	0.842	0.066	360612	G	A	-0.049	5	3002546	6.15E-06	0.011	14306	20.459
Vascular dementia (multiple infarctions)	class Erysipelotrichia	rs10781552	C	T	-0.140	132083729	0.055	0.073	360612	C	T	-0.055	10	133897233	2.33E-06	0.012	14306	22.633
Vascular dementia (multiple infarctions)	class Erysipelotrichia	rs17530232	A	G	0.003	39811320	0.984	0.145	360612	A	G	0.103	13	40385457	2.79E-06	0.022	14306	21.042
Vascular dementia (multiple infarctions)	class Erysipelotrichia	rs1884466	C	T	-0.043	63673525	0.518	0.066	360612	C	T	-0.048	1	64139196	9.53E-06	0.011	14306	19.760
Vascular dementia (multiple infarctions)	class Erysipelotrichia	rs2300774	A	G	0.037	196066841	0.575	0.065	360612	A	G	-0.052	3	195793712	8.95E-07	0.011	14306	24.094
Vascular dementia (multiple infarctions)	class Erysipelotrichia	rs290833	T	G	0.039	96991871	0.556	0.066	360612	T	G	-0.050	1	97457427	8.03E-06	0.011	14306	19.943
Vascular dementia (multiple infarctions)	class Erysipelotrichia	rs35161940	T	C	-0.012	72331083	0.907	0.107	360612	T	C	-0.081	17	70327224	1.85E-06	0.017	14306	23.118
Vascular dementia (multiple infarctions)	class Erysipelotrichia	rs4078432	T	C	0.140	48528003	0.110	0.088	360612	T	C	0.061	14	48997206	4.23E-06	0.013	14306	20.723
Vascular dementia (multiple infarctions)	class Erysipelotrichia	rs56970041	T	G	-0.042	79891267	0.760	0.136	360612	T	G	0.072	14	80357610	5.40E-06	0.016	14306	19.385
Vascular dementia (multiple infarctions)	class Erysipelotrichia	rs62504403	C	T	0.043	38946033	0.600	0.082	360612	C	T	0.068	8	38803551	1.12E-07	0.013	14306	28.371
Vascular dementia (multiple infarctions)	class Erysipelotrichia	rs7234058	T	C	-0.136	5830508	0.229	0.113	360612	T	C	-0.095	18	5830507	9.12E-07	0.019	14306	23.744
Vascular dementia (multiple infarctions)	class Erysipelotrichia	rs7826267	G	T	0.000	3097430	0.998	0.132	360612	G	T	0.084	8	2954952	9.28E-06	0.020	14306	17.755
Vascular dementia (multiple infarctions)	class Erysipelotrichia	rs8003149	C	T	0.142	55689786	0.040	0.069	360612	C	T	0.054	14	56156504	4.08E-06	0.012	14306	21.248
Vascular dementia (multiple infarctions)	class Gammaproteobacteria	rs11181912	G	A	0.016	43179149	0.819	0.070	360612	G	A	-0.058	12	43572952	9.95E-07	0.012	14306	23.767
Vascular dementia (multiple infarctions)	class Gammaproteobacteria	rs12404135	A	G	-0.072	186533562	0.560	0.123	360612	A	G	-0.079	1	186502694	8.89E-06	0.017	14306	20.933
Vascular dementia (multiple infarctions)	class Gammaproteobacteria	rs6706173	A	C	0.076	167130482	0.419	0.094	360612	A	C	0.074	2	167986992	1.99E-07	0.015	14306	25.726
Vascular dementia (multiple infarctions)	class Gammaproteobacteria	rs75101789	C	T	0.090	18574411	0.433	0.115	360612	C	T	0.073	3	18615903	8.79E-06	0.016	14306	20.002
Vascular dementia (multiple infarctions)	class Gammaproteobacteria	rs79795896	A	G	-0.046	50658878	0.764	0.153	360612	A	G	-0.159	18	48185248	7.92E-06	0.035	14306	20.531
Vascular dementia (multiple infarctions)	class Gammaproteobacteria	rs9494710	C	T	-0.028	137291417	0.691	0.070	360612	C	T	-0.055	6	137612554	4.55E-06	0.012	14306	20.663
Vascular dementia (multiple infarctions)	class Lentisphaeria	rs1002941	A	G	0.017	100702485	0.818	0.076	360612	A	G	-0.105	15	101242690	8.15E-06	0.023	14306	20.234
Vascular dementia (multiple infarctions)	class Lentisphaeria	rs11770843	C	T	0.018	147098287	0.797	0.070	360612	C	T	0.109	7	146795379	1.91E-06	0.023	14306	21.707
Vascular dementia (multiple infarctions)	class Lentisphaeria	rs17114848	G	A	0.129	24917388	0.246	0.111	360612	G	A	0.152	15	25162535	4.06E-06	0.032	14306	22.073
Vascular dementia (multiple infarctions)	class Lentisphaeria	rs2031282	A	G	-0.035	20113040	0.688	0.086	360612	A	G	0.122	13	20687179	4.38E-06	0.027	14306	20.490

Vascular dementia (multiple infarctions)	class Lentisphaeria	rs2825714	A	G	0.100	19651652	0.252	0.087	360612	A	G	-0.137	21	21023966	1.72E-06	0.029	14306	22.568
Vascular dementia (multiple infarctions)	class Lentisphaeria	rs62570196	C	T	-0.166	108323890	0.307	0.163	360612	C	T	-0.216	9	111086170	1.08E-06	0.044	14306	24.192
Vascular dementia (multiple infarctions)	class Lentisphaeria	rs72640280	A	G	0.102	11883735	0.473	0.142	360612	A	G	0.220	1	11943792	5.18E-06	0.049	14306	20.513
Vascular dementia (multiple infarctions)	class Lentisphaeria	rs77599476	A	G	-0.105	62762910	0.472	0.146	360612	A	G	0.230	20	61394262	1.86E-06	0.048	14306	23.002
Vascular dementia (multiple infarctions)	class Melainabacteria	rs10148250	A	G	-0.083	106605442	0.228	0.069	360612	A	G	-0.086	14	107061448	8.67E-06	0.019	14306	19.866
Vascular dementia (multiple infarctions)	class Melainabacteria	rs10738747	A	G	-0.051	26184580	0.438	0.066	360612	A	G	-0.081	9	26184578	9.96E-06	0.018	14306	19.514
Vascular dementia (multiple infarctions)	class Melainabacteria	rs11150282	T	C	0.078	80459808	0.257	0.068	360612	T	C	0.099	16	80493705	6.03E-07	0.020	14306	25.235
Vascular dementia (multiple infarctions)	class Melainabacteria	rs28678345	T	C	0.086	55828967	0.575	0.153	360612	T	C	0.215	17	53906328	6.69E-06	0.047	14306	20.818
Vascular dementia (multiple infarctions)	class Melainabacteria	rs367480	A	G	-0.067	2916401	0.325	0.068	360612	A	G	0.084	11	2937631	8.20E-06	0.019	14306	20.323
Vascular dementia (multiple infarctions)	class Melainabacteria	rs4129395	G	A	0.055	113213109	0.400	0.065	360612	G	A	0.090	9	115975389	1.48E-06	0.019	14306	23.440
Vascular dementia (multiple infarctions)	class Melainabacteria	rs789069	A	C	-0.169	1008277	0.071	0.094	360612	A	C	-0.104	18	1008278	6.85E-06	0.023	14306	19.530
Vascular dementia (multiple infarctions)	class Melainabacteria	rs79790072	T	C	0.385	100207478	0.050	0.197	360612	T	C	0.227	15	100747683	3.29E-06	0.049	14306	21.599
Vascular dementia (multiple infarctions)	class Melainabacteria	rs9864379	T	C	-0.132	14265449	0.152	0.092	360612	T	C	-0.160	3	14306949	5.36E-08	0.029	14306	29.812
Vascular dementia (multiple infarctions)	class Methanobacteria	rs10202904	G	T	-0.010	124682691	0.886	0.067	360612	G	T	0.122	2	125440268	3.01E-07	0.024	14306	26.762
Vascular dementia (multiple infarctions)	class Methanobacteria	rs10424197	A	G	0.005	45936063	0.948	0.075	360612	A	G	0.111	19	46439321	9.28E-06	0.025	14306	20.211
Vascular dementia (multiple infarctions)	class Methanobacteria	rs4257531	G	A	0.080	2044483	0.457	0.107	360612	G	A	0.164	3	2086167	7.44E-06	0.036	14306	20.316
Vascular dementia (multiple infarctions)	class Methanobacteria	rs6508769	C	T	-0.054	28336853	0.557	0.092	360612	C	T	-0.154	19	28827760	8.23E-06	0.034	14306	19.856
Vascular dementia (multiple infarctions)	class Methanobacteria	rs6776814	T	C	0.044	15011576	0.848	0.228	360612	T	C	-0.200	3	15053083	1.63E-06	0.041	14306	23.483
Vascular dementia (multiple infarctions)	class Methanobacteria	rs73068003	G	T	-0.094	10734305	0.391	0.110	360612	G	T	-0.158	7	10773932	8.45E-06	0.035	14306	20.206
Vascular dementia (multiple infarctions)	class Methanobacteria	rs73457410	A	G	-0.069	41382045	0.603	0.133	360612	A	G	0.215	13	41956181	1.41E-06	0.044	14306	24.316
Vascular dementia (multiple infarctions)	class Methanobacteria	rs75208022	C	T	-0.008	21185927	0.945	0.112	360612	C	T	-0.227	12	21338861	5.92E-06	0.049	14306	21.717
Vascular dementia (multiple infarctions)	class Methanobacteria	rs894996	C	A	-0.197	103497150	0.122	0.127	360612	C	A	0.217	4	104418307	1.88E-06	0.045	14306	23.349
Vascular dementia (multiple infarctions)	class Mollicutes	rs10108398	G	A	0.080	58528265	0.275	0.073	360612	G	A	0.077	8	59440824	1.09E-06	0.015	14306	24.960
Vascular dementia (multiple infarctions)	class Mollicutes	rs11890098	A	G	-0.049	156676037	0.502	0.073	360612	A	G	0.074	2	157532549	9.57E-07	0.015	14306	23.551
Vascular dementia (multiple infarctions)	class Mollicutes	rs12566890	T	G	-0.023	61385192	0.815	0.097	360612	T	G	-0.101	1	61850864	3.65E-06	0.023	14306	19.176
Vascular dementia (multiple infarctions)	class Mollicutes	rs17214486	C	A	-0.059	96693337	0.399	0.070	360612	C	A	0.061	14	97159674	6.61E-06	0.014	14306	20.223
Vascular dementia (multiple infarctions)	class Mollicutes	rs2464826	A	C	-0.106	79860934	0.306	0.104	360612	A	C	0.094	7	79490250	8.39E-06	0.021	14306	19.874
Vascular dementia (multiple infarctions)	class Mollicutes	rs28537087	G	A	0.033	94766447	0.664	0.077	360612	G	A	0.082	15	95309676	8.07E-06	0.019	14306	19.002
Vascular dementia (multiple infarctions)	class Mollicutes	rs3768491	G	A	-0.045	109423364	0.527	0.072	360612	G	A	0.068	1	109965986	4.23E-06	0.015	14306	20.875
Vascular dementia (multiple infarctions)	class Mollicutes	rs4885016	C	T	-0.073	72596351	0.451	0.097	360612	C	T	0.082	13	73170489	7.27E-06	0.018	14306	20.363
Vascular dementia (multiple infarctions)	class Mollicutes	rs6043847	T	C	0.105	16278879	0.450	0.139	360612	T	C	-0.115	20	16259524	4.55E-06	0.025	14306	21.375
Vascular dementia (multiple infarctions)	class Mollicutes	rs72901605	T	C	0.056	47082326	0.579	0.101	360612	T	C	-0.084	11	47103877	3.26E-06	0.018	14306	22.338
Vascular dementia (multiple infarctions)	class Mollicutes	rs74603314	T	C	-0.241	46050515	0.147	0.166	360612	T	C	0.222	14	46519718	1.56E-06	0.046	14306	22.924
Vascular dementia (multiple infarctions)	class Mollicutes	rs78169027	A	G	-0.270	108568360	0.047	0.136	360612	A	G	-0.108	11	108439087	5.88E-06	0.024	14306	20.824
Vascular dementia (multiple infarctions)	class Negativicutes	rs1135612	G	A	0.049	75980359	0.542	0.080	360612	G	A	0.053	7	75609677	9.26E-06	0.012	14306	19.761
Vascular dementia (multiple infarctions)	class Negativicutes	rs13086907	G	A	-0.134	142416275	0.090	0.079	360612	G	A	0.063	3	142135117	1.95E-06	0.013	14306	22.532
Vascular dementia (multiple infarctions)	class Negativicutes	rs1643968	T	C	0.006	165839623	0.934	0.068	360612	T	C	-0.057	5	165266628	4.15E-07	0.011	14306	25.339
Vascular dementia (multiple infarctions)	class Negativicutes	rs1649999	A	G	-0.207	78326315	0.064	0.112	360612	A	G	0.075	10	80086072	7.58E-06	0.017	14306	20.246
Vascular dementia (multiple infarctions)	class Negativicutes	rs2834062	A	G	0.128	33005177	0.075	0.072	360612	A	G	0.049	21	34377485	8.44E-06	0.011	14306	20.190
Vascular dementia (multiple infarctions)	class Negativicutes	rs4463806	C	T	0.060	113838234	0.466	0.082	360612	C	T	0.054	10	115597993	7.81E-06	0.013	14306	17.681
Vascular dementia (multiple infarctions)	class Negativicutes	rs4722181	T	G	-0.024	22777952	0.712	0.065	360612	T	G	0.050	7	22817571	2.00E-06	0.011	14306	22.452
Vascular dementia (multiple infarctions)	class Negativicutes	rs60274479	T	C	0.125	21238604	0.129	0.082	360612	T	C	-0.066	16	21249925	1.16E-06	0.013	14306	24.182
Vascular dementia (multiple infarctions)	class Negativicutes	rs61249479	A	C	0.024	122150629	0.796	0.091	360612	A	C	0.078	9	124912908	2.95E-06	0.017	14306	21.236
Vascular dementia (multiple infarctions)	class Negativicutes	rs71405394	G	A	-0.127	100704883	0.320	0.128	360612	G	A	-0.114	15	101245088	2.17E-06	0.024	14306	22.539
Vascular dementia (multiple infarctions)	class Negativicutes	rs73232831	G	A	0.130	17411803	0.449	0.172	360612	G	A	-0.152	4	17413426	1.87E-06	0.031	14306	23.242
Vascular dementia (multiple infarctions)	class Negativicutes	rs9423647	G	A	-0.060	5537855	0.360	0.066	360612	G	A	0.048	10	5579818	6.06E-06	0.011	14306	20.628
Vascular dementia (multiple infarctions)	class Verrucomicrobiae	rs111862613	T	C	0.018	129825125	0.841	0.088	360612	T	C	0.091	12	130309670	3.74E-06	0.020	14306	21.252
Vascular dementia (multiple infarctions)	class Verrucomicrobiae	rs117107102	A	G	-0.112	51947265	0.471	0.155	360612	A	G	0.205	18	49473635	2.92E-06	0.043	14306	22.493
Vascular dementia (multiple infarctions)	class Verrucomicrobiae	rs11729256	T	C	0.095	94106121	0.275	0.087	360612	T	C	0.075	4	95027272	6.73E-07	0.015	14306	24.928
Vascular dementia (multiple infarctions)	class Verrucomicrobiae	rs12908520	G	A	0.119	97027427	0.070	0.066	360612	G	A	0.062	15	97570657	2.17E-06	0.013	14306	22.341
Vascular dementia (multiple infarctions)	class Verrucomicrobiae	rs2602429	T	C	-0.122	81029544	0.104	0.075	360612	T	C	-0.075	16	81063149	2.58E-06	0.016	14306	22.863
Vascular dementia (multiple infarctions)	class Verrucomicrobiae	rs4242783	A	G	-0.054	5022135	0.462	0.073	360612	A	G	-0.069	10	5064327	2.64E-06	0.015	14306	21.781
Vascular dementia (multiple infarctions)	class Verrucomicrobiae	rs4936098	G	A	0.069	130410772	0.311	0.069	360612	G	A	-0.065	11	130280667	1.12E-06	0.014	14306	22.786
Vascular dementia (multiple infarctions)	class Verrucomicrobiae	rs61779207	G	A	-0.187	40608800	0.019	0.079	360612	G	A	-0.076	1	41074472	6.72E-06	0.017	14306	20.432
Vascular dementia (multiple infarctions)	class Verrucomicrobiae	rs74542928	T	C	-0.146	99623031	0.332	0.151	360612	T	C	0.112	4	100544188	1.63E-06	0.024	14306	22.508
Vascular dementia (multiple infarctions)	class Verrucomicrobiae	rs9349825	A	G	0.081	56476683	0.333	0.084	360612	A	G	-0.070	6	56341481	2.54E-06	0.015	14306	22.898
Vascular dementia (multiple infarctions)	class Verrucomicrobiae	rs941682	G	A	0.115	33280034	0.117	0.073	360612	G	A	-0.063	20	31867840	9.61E-06	0.014	14306	19.290
Vascular dementia (multiple infarctions)	family Acidaminococcaceae	rs262812	T	C	0.101	158226967	0.158	0.071	360612	T	C	-0.066	6	158647999	3.25E-06	0.014	14306	21.313
Vascular dementia (multiple infarctions)	family Acidaminococcaceae	rs2933324	G	A	-0.046	104355372	0.556	0.078	360612	G	A	0.066	9	107117653	2.24E-06	0.014	14306	22.315
Vascular dementia (multiple infarctions)	family Acidaminococcaceae	rs45497800	T	C	-0.089	63360481	0.345	0.094	360612	T	C	-0.118	20	61991833	5.86E-06	0.026	14306	20.975
Vascular dementia (multiple infarctions)	family Acidaminococcaceae	rs6589457	G	A	0.254	114830566	0.095	0.152	360612	G	A	-0.166	11	114701288	2.32E-06	0.035	14306	22.533
Vascular dementia (multiple infarctions)	family Acidaminococcaceae	rs6923842	T	C	0.081	5722948	0.429	0.103	360612	T	C	-0.080	6	5723181	2.21E-06	0.017	14306	22.140
Vascular dementia (multiple infarctions)	family Acidaminococcaceae	rs74540770	G	A	-0.005	186835600	0.967	0.123	360612	G	A	-0.109	3	186553389	7.09E-06	0.024	14306	20.021
Vascular dementia (multiple infarctions)	family Acidaminococcaceae	rs78702810	T	C	0.044	18421039	0.683	0.107	360612	T	C	-0.144	3	18462531	9.16E-06	0.032	14306	19.778
Vascular dementia (multiple infarctions)	family Actinomycetaceae	rs2889192	T	G	0.088	73779652	0.341	0.092	360612	T	G	-0.089	9	76394568	3.64E-06	0.020	14306	20.714
Vascular dementia (multiple infarctions)	family Actinomycetaceae	rs34583783	G	T	-0.097	66497478	0.478	0.137	360612	G	T	0.124	6	67207371	5.48E-06	0.026	14306	21.930
Vascular dementia (multiple infarctions)	family Actinomycetaceae	rs35011108	A	G	0.063	132686341	0.624	0.129	360612	A	G	0.242	6	133007480	1.83E-06	0.050	14306	23.041

Vascular dementia (multiple infarctions)	family Actinomycetaceae	rs4073240	G	A	-0.076	168824686	0.259	0.067	360612	G	A	0.075	6	169224781	6.05E-06	0.016	14306	20.605
Vascular dementia (multiple infarctions)	family Alcaligenaceae	rs112135816	T	G	-0.154	79870412	0.253	0.135	360612	T	G	-0.078	9	82485327	5.28E-06	0.017	14306	20.411
Vascular dementia (multiple infarctions)	family Alcaligenaceae	rs1153990	A	G	0.047	104897760	0.507	0.071	360612	A	G	-0.059	5	104233461	5.97E-06	0.013	14306	20.950
Vascular dementia (multiple infarctions)	family Alcaligenaceae	rs147968	C	T	0.068	85912233	0.297	0.065	360612	C	T	0.049	16	85945839	9.13E-06	0.011	14306	19.685
Vascular dementia (multiple infarctions)	family Alcaligenaceae	rs28480294	C	T	-0.114	71230170	0.096	0.069	360612	C	T	0.052	15	71522509	6.61E-06	0.012	14306	20.033
Vascular dementia (multiple infarctions)	family Alcaligenaceae	rs4033856	T	C	0.036	45640468	0.754	0.116	360612	T	C	-0.082	4	45642485	1.03E-06	0.017	14306	23.467
Vascular dementia (multiple infarctions)	family Alcaligenaceae	rs62191117	A	G	-0.040	238979079	0.619	0.081	360612	A	G	0.068	2	239900775	2.76E-07	0.013	14306	26.221
Vascular dementia (multiple infarctions)	family Alcaligenaceae	rs62395635	T	C	0.131	174070793	0.337	0.137	360612	T	C	0.111	5	173497796	3.35E-06	0.024	14306	21.496
Vascular dementia (multiple infarctions)	family Alcaligenaceae	rs6969323	A	C	0.132	104574040	0.092	0.078	360612	A	C	-0.059	7	104214487	3.89E-06	0.013	14306	21.127
Vascular dementia (multiple infarctions)	family Alcaligenaceae	rs74776516	T	G	-0.094	20741821	0.469	0.130	360612	T	G	-0.094	11	20763367	6.85E-06	0.021	14306	19.591
Vascular dementia (multiple infarctions)	family Alcaligenaceae	rs7638039	T	C	-0.046	70539788	0.544	0.076	360612	T	C	0.060	3	70588939	2.70E-06	0.013	14306	22.295
Vascular dementia (multiple infarctions)	family Alcaligenaceae	rs9537886	A	C	0.063	57971112	0.335	0.066	360612	A	C	-0.057	13	58545246	2.35E-07	0.011	14306	26.604
Vascular dementia (multiple infarctions)	family Bacteroidaceae	rs11585893	A	G	0.016	10584294	0.838	0.076	360612	A	G	-0.074	1	10644351	1.80E-06	0.015	14306	25.175
Vascular dementia (multiple infarctions)	family Bacteroidaceae	rs13207588	A	G	-0.082	41551692	0.326	0.083	360612	A	G	-0.059	6	41519430	7.48E-06	0.013	14306	20.365
Vascular dementia (multiple infarctions)	family Bacteroidaceae	rs1340391	T	C	0.030	102495433	0.755	0.095	360612	T	C	-0.059	1	102960989	6.73E-06	0.013	14306	20.040
Vascular dementia (multiple infarctions)	family Bacteroidaceae	rs17619981	T	G	-0.058	24159448	0.544	0.096	360612	T	G	0.088	19	24342250	2.69E-06	0.019	14306	22.194
Vascular dementia (multiple infarctions)	family Bacteroidaceae	rs2023437	T	C	-0.196	21577814	0.050	0.100	360612	T	C	-0.078	14	22045949	5.02E-06	0.017	14306	21.780
Vascular dementia (multiple infarctions)	family Bacteroidaceae	rs66710942	T	C	-0.028	77166176	0.668	0.065	360612	T	C	-0.049	3	77215327	5.86E-06	0.011	14306	20.644
Vascular dementia (multiple infarctions)	family Bacteroidaceae	rs6795673	C	T	0.027	10551540	0.677	0.066	360612	C	T	0.054	3	10593224	3.38E-07	0.011	14306	26.183
Vascular dementia (multiple infarctions)	family Bacteroidaceae	rs9507307	C	T	-0.029	24336338	0.699	0.076	360612	C	T	0.060	13	24910476	2.13E-06	0.013	14306	21.912
Vascular dementia (multiple infarctions)	family Bacteroidales S24 7group	rs10872669	G	A	-0.098	151194037	0.384	0.112	360612	G	A	0.123	6	151515172	9.49E-06	0.028	14306	19.932
Vascular dementia (multiple infarctions)	family Bacteroidales S24 7group	rs12748533	G	T	-0.014	242603950	0.851	0.072	360612	G	T	-0.082	1	242767252	2.59E-06	0.017	14306	22.605
Vascular dementia (multiple infarctions)	family Bacteroidales S24 7group	rs17043785	T	C	-0.036	52912224	0.760	0.117	360612	T	C	-0.176	2	53139362	5.12E-07	0.035	14306	25.761
Vascular dementia (multiple infarctions)	family Bacteroidales S24 7group	rs1850003	A	G	-0.096	47048773	0.216	0.077	360612	A	G	0.084	15	47340971	2.41E-06	0.018	14306	22.344
Vascular dementia (multiple infarctions)	family Bacteroidales S24 7group	rs61508842	T	C	-0.070	158935756	0.548	0.117	360612	T	C	0.123	3	158653545	7.83E-06	0.027	14306	20.250
Vascular dementia (multiple infarctions)	family Bacteroidales S24 7group	rs738193	T	C	0.048	25547355	0.481	0.069	360612	T	C	0.085	22	25943322	3.82E-07	0.017	14306	26.100
Vascular dementia (multiple infarctions)	family Bacteroidales S24 7group	rs78609301	A	G	0.058	62526842	0.416	0.072	360612	A	G	-0.087	6	63236747	7.09E-06	0.020	14306	19.628
Vascular dementia (multiple infarctions)	family Bacteroidales S24 7group	rs941000	T	C	0.020	91085352	0.773	0.068	360612	T	C	-0.085	7	90714667	3.16E-07	0.016	14306	27.083
Vascular dementia (multiple infarctions)	family Bifidobacteriaceae	rs10831953	G	A	0.033	13076504	0.645	0.071	360612	G	A	0.054	11	13098051	9.95E-06	0.012	14306	18.869
Vascular dementia (multiple infarctions)	family Bifidobacteriaceae	rs12446429	T	C	-0.099	848055	0.240	0.085	360612	T	C	0.081	16	898055	8.53E-06	0.019	14306	18.040
Vascular dementia (multiple infarctions)	family Bifidobacteriaceae	rs13020688	G	A	0.032	192013806	0.656	0.071	360612	G	A	0.058	2	192878532	1.57E-06	0.012	14306	22.887
Vascular dementia (multiple infarctions)	family Bifidobacteriaceae	rs182549	T	C	-0.125	135859184	0.065	0.068	360612	T	C	-0.117	2	136616754	5.94E-20	0.013	14306	85.372
Vascular dementia (multiple infarctions)	family Bifidobacteriaceae	rs4957061	T	C	0.107	520981	0.107	0.067	360612	T	C	0.057	5	521096	1.15E-06	0.012	14306	23.762
Vascular dementia (multiple infarctions)	family Bifidobacteriaceae	rs540489	T	G	-0.123	74901626	0.152	0.086	360612	T	G	-0.063	17	72897722	5.37E-06	0.014	14306	20.956
Vascular dementia (multiple infarctions)	family Bifidobacteriaceae	rs55888705	A	G	0.029	1516099	0.687	0.072	360612	A	G	0.054	4	1517826	8.66E-06	0.012	14306	19.812
Vascular dementia (multiple infarctions)	family Bifidobacteriaceae	rs6899771	A	G	-0.119	96958344	0.276	0.109	360612	A	G	-0.091	6	97406220	7.28E-06	0.020	14306	20.365
Vascular dementia (multiple infarctions)	family Bifidobacteriaceae	rs7174549	T	C	0.084	91920073	0.217	0.068	360612	T	C	-0.055	15	92463303	6.87E-06	0.012	14306	19.590
Vascular dementia (multiple infarctions)	family Bifidobacteriaceae	rs7322849	T	C	-0.075	112205515	0.511	0.114	360612	T	C	0.111	13	112859829	1.74E-08	0.020	14306	30.320
Vascular dementia (multiple infarctions)	family Bifidobacteriaceae	rs857444	C	T	-0.109	14617360	0.107	0.068	360612	C	T	0.055	6	14617591	3.82E-06	0.012	14306	21.075
Vascular dementia (multiple infarctions)	family Clostridiaceae1	rs10875374	T	C	-0.016	101339253	0.803	0.065	360612	T	C	0.054	1	101804809	8.10E-06	0.012	14306	20.197
Vascular dementia (multiple infarctions)	family Clostridiaceae1	rs12186080	G	A	-0.019	132877783	0.832	0.088	360612	G	A	0.075	3	132596627	5.34E-06	0.016	14306	21.212
Vascular dementia (multiple infarctions)	family Clostridiaceae1	rs12341505	G	A	0.094	133845759	0.407	0.113	360612	G	A	0.081	9	136710881	4.54E-06	0.018	14306	20.627
Vascular dementia (multiple infarctions)	family Clostridiaceae1	rs2795528	G	A	-0.278	42774816	0.054	0.145	360612	G	A	-0.181	10	43270264	3.81E-06	0.039	14306	21.432
Vascular dementia (multiple infarctions)	family Clostridiaceae1	rs2817172	C	T	-0.020	3124955	0.767	0.067	360612	C	T	0.056	1	3041519	5.27E-06	0.012	14306	20.668
Vascular dementia (multiple infarctions)	family Clostridiaceae1	rs4723021	T	C	-0.112	30895044	0.397	0.132	360612	T	C	-0.106	7	30934659	7.42E-06	0.024	14306	19.331
Vascular dementia (multiple infarctions)	family Clostridiaceae1	rs550843	T	C	0.040	165309343	0.583	0.073	360612	T	C	-0.073	6	165722832	7.09E-06	0.017	14306	19.043
Vascular dementia (multiple infarctions)	family Clostridiaceae1	rs56188186	A	G	-0.080	87677084	0.598	0.151	360612	A	G	0.097	16	87710690	8.24E-06	0.022	14306	19.804
Vascular dementia (multiple infarctions)	family Clostridiaceae1	rs62397761	A	G	-0.075	48125278	0.280	0.070	360612	A	G	0.062	6	48093014	9.08E-06	0.014	14306	20.443
Vascular dementia (multiple infarctions)	family Clostridiaceae1	rs881532	G	A	-0.016	47411024	0.807	0.065	360612	G	A	0.053	22	47806774	7.90E-06	0.012	14306	20.060
Vascular dementia (multiple infarctions)	family Clostridiales vadin BB60 group	rs10517600	G	T	-0.069	154711806	0.302	0.067	360612	G	T	-0.063	4	155632958	6.83E-06	0.014	14306	20.241
Vascular dementia (multiple infarctions)	family Clostridiales vadin BB60 group	rs10904722	C	T	-0.035	6672462	0.651	0.077	360612	C	T	-0.067	10	6714424	5.05E-06	0.015	14306	20.883
Vascular dementia (multiple infarctions)	family Clostridiales vadin BB60 group	rs118104867	C	T	0.169	122965298	0.194	0.130	360612	C	T	0.214	8	123977538	3.44E-06	0.046	14306	22.207
Vascular dementia (multiple infarctions)	family Clostridiales vadin BB60 group	rs13409132	A	G	0.144	204751623	0.405	0.172	360612	A	G	-0.165	2	205616346	4.37E-06	0.035	14306	22.065
Vascular dementia (multiple infarctions)	family Clostridiales vadin BB60 group	rs17121075	G	A	0.052	85390452	0.519	0.080	360612	G	A	0.077	14	85856796	7.91E-06	0.017	14306	19.948
Vascular dementia (multiple infarctions)	family Clostridiales vadin BB60 group	rs2191834	T	G	0.074	229078235	0.332	0.076	360612	T	G	-0.075	2	229942951	2.50E-06	0.016	14306	21.998
Vascular dementia (multiple infarctions)	family Clostridiales vadin BB60 group	rs28691777	C	T	-0.270	60072044	0.091	0.160	360612	C	T	0.137	17	58149405	6.96E-07	0.027	14306	26.380
Vascular dementia (multiple infarctions)	family Clostridiales vadin BB60 group	rs34088226	A	G	-0.001	4308833	0.996	0.140	360612	A	G	-0.118	5	4308946	7.66E-06	0.027	14306	19.145
Vascular dementia (multiple infarctions)	family Clostridiales vadin BB60 group	rs55682560	C	T	-0.123	86849059	0.309	0.121	360612	C	T	-0.132	15	87392290	4.97E-07	0.026	14306	25.330
Vascular dementia (multiple infarctions)	family Clostridiales vadin BB60 group	rs6588624	A	G	-0.153	56383867	0.019	0.065	360612	A	G	0.066	1	56849539	1.79E-06	0.014	14306	22.985
Vascular dementia (multiple infarctions)	family Clostridiales vadin BB60 group	rs66714985	A	C	0.003	3447600	0.979	0.107	360612	A	C	0.117	8	3305122	4.85E-06	0.025	14306	21.446
Vascular dementia (multiple infarctions)	family Clostridiales vadin BB60 group	rs7226487	A	G	-0.005	76661168	0.940	0.065	360612	A	G	-0.064	18	74373125	3.58E-06	0.014	14306	21.537
Vascular dementia (multiple infarctions)	family Clostridiales vadin BB60 group	rs7538034	T	G	0.045	70926011	0.608	0.088	360612	T	G	-0.079	1	71391694	2.37E-06	0.017	14306	22.423
Vascular dementia (multiple infarctions)	family Clostridiales vadin BB60 group	rs7725895	A	G	0.004	142865333	0.969	0.102	360612	A	G	-0.116	5	142244898	3.94E-06	0.024	14306	23.380
Vascular dementia (multiple infarctions)	family Clostridiales vadin BB60 group	rs989682	A	G	0.046	15522946	0.553	0.078	360612	A	G	0.070	3	15564453	6.85E-06	0.016	14306	20.413
Vascular dementia (multiple infarctions)	family Coriobacteriaceae	rs11073596	G	T	0.045	85890348	0.503	0.068	360612	G	T	-0.051	15	86433579	8.14E-06	0.011	14306	19.912
Vascular dementia (multiple infarctions)	family Coriobacteriaceae	rs11250875	T	C	0.028	1880537	0.728	0.080	360612	T	C	0.061	10	1922731	4.83E-06	0.013	14306	21.526
Vascular dementia (multiple infarctions)	family Coriobacteriaceae	rs11656361	A	C	-0.129	8218014	0.130	0.085	360612	A	C	0.077	17	8121332	8.02E-06	0.018	14306	19.394

Vascular dementia (multiple infarctions)	family Coriobacteriaceae	rs12974142	G	A	-0.138	52391913	0.280	0.128	360612	G	A	0.079	19	52895166	8.51E-06	0.018	14306	19.865
Vascular dementia (multiple infarctions)	family Coriobacteriaceae	rs13307134	T	C	-0.202	105444233	0.021	0.088	360612	T	C	-0.057	7	105084680	7.80E-06	0.013	14306	20.072
Vascular dementia (multiple infarctions)	family Coriobacteriaceae	rs1397793	A	G	0.091	91175634	0.203	0.072	360612	A	G	0.050	5	90471451	9.77E-06	0.011	14306	19.682
Vascular dementia (multiple infarctions)	family Coriobacteriaceae	rs1816223	G	A	0.150	11341087	0.068	0.082	360612	G	A	0.059	12	11494021	4.84E-06	0.013	14306	20.652
Vascular dementia (multiple infarctions)	family Coriobacteriaceae	rs240104	T	C	-0.183	176602295	0.012	0.073	360612	T	C	-0.060	1	176571431	1.52E-06	0.013	14306	22.630
Vascular dementia (multiple infarctions)	family Coriobacteriaceae	rs2442778	A	G	-0.136	11612938	0.358	0.148	360612	A	G	0.116	3	11654412	9.03E-06	0.026	14306	20.272
Vascular dementia (multiple infarctions)	family Coriobacteriaceae	rs3025411	A	G	-0.026	133647784	0.808	0.107	360612	A	G	0.093	9	136512906	8.27E-06	0.021	14306	19.566
Vascular dementia (multiple infarctions)	family Coriobacteriaceae	rs34739816	G	T	-0.119	39220432	0.387	0.137	360612	G	T	0.097	17	37376685	3.88E-06	0.021	14306	21.594
Vascular dementia (multiple infarctions)	family Coriobacteriaceae	rs67561917	A	G	-0.033	63440724	0.703	0.086	360612	A	G	-0.071	20	62072077	5.39E-06	0.015	14306	21.486
Vascular dementia (multiple infarctions)	family Coriobacteriaceae	rs719099	A	G	0.094	64039457	0.389	0.109	360612	A	G	0.078	10	65799217	5.43E-07	0.016	14306	24.957
Vascular dementia (multiple infarctions)	family Coriobacteriaceae	rs8010111	A	G	0.061	39191305	0.599	0.117	360612	A	G	0.103	14	39660509	6.90E-06	0.023	14306	20.328
Vascular dementia (multiple infarctions)	family Defluviitaleaceae	rs112893842	T	C	-0.167	8786663	0.137	0.113	360612	T	C	0.111	9	8786663	2.75E-06	0.023	14306	22.686
Vascular dementia (multiple infarctions)	family Defluviitaleaceae	rs1582238	C	T	0.148	118181062	0.030	0.068	360612	C	T	-0.080	1	118723685	1.69E-06	0.017	14306	23.042
Vascular dementia (multiple infarctions)	family Defluviitaleaceae	rs17051335	C	T	0.056	121281755	0.600	0.107	360612	C	T	-0.134	4	122202910	4.58E-06	0.029	14306	21.129
Vascular dementia (multiple infarctions)	family Defluviitaleaceae	rs1908593	T	C	-0.048	61358915	0.473	0.067	360612	T	C	0.070	18	59026148	7.86E-06	0.016	14306	20.108
Vascular dementia (multiple infarctions)	family Defluviitaleaceae	rs4344384	T	G	0.141	64647609	0.031	0.065	360612	T	G	-0.071	10	66407366	5.86E-06	0.016	14306	20.612
Vascular dementia (multiple infarctions)	family Defluviitaleaceae	rs4677103	A	G	0.089	72158643	0.307	0.087	360612	A	G	0.098	3	72207794	9.42E-07	0.020	14306	24.598
Vascular dementia (multiple infarctions)	family Defluviitaleaceae	rs540220	C	T	-0.076	90569026	0.507	0.115	360612	C	T	0.124	9	93331308	9.48E-06	0.029	14306	18.293
Vascular dementia (multiple infarctions)	family Defluviitaleaceae	rs55658617	T	C	0.136	40439076	0.435	0.174	360612	T	C	0.177	21	41811003	1.41E-06	0.036	14306	24.013
Vascular dementia (multiple infarctions)	family Defluviitaleaceae	rs72731813	C	T	-0.107	146493591	0.485	0.154	360612	C	T	-0.150	4	147414743	2.76E-07	0.029	14306	26.048
Vascular dementia (multiple infarctions)	family Defluviitaleaceae	rs9608282	T	G	0.004	24408113	0.982	0.189	360612	T	G	0.139	22	24804081	4.61E-06	0.030	14306	21.554
Vascular dementia (multiple infarctions)	family Defluviitaleaceae	rs9725395	A	G	0.142	84739949	0.168	0.103	360612	A	G	-0.138	1	85205632	3.41E-06	0.030	14306	21.969
Vascular dementia (multiple infarctions)	family Desulfovibrionaceae	rs11599763	C	T	0.090	11813600	0.180	0.067	360612	C	T	0.056	10	11855599	2.50E-06	0.012	14306	22.388
Vascular dementia (multiple infarctions)	family Desulfovibrionaceae	rs17791387	A	G	0.240	79219511	0.033	0.113	360612	A	G	-0.073	9	81834426	2.10E-06	0.015	14306	22.313
Vascular dementia (multiple infarctions)	family Desulfovibrionaceae	rs2692012	G	A	-0.064	204022477	0.658	0.145	360612	G	A	-0.114	1	203991605	1.56E-06	0.025	14306	20.295
Vascular dementia (multiple infarctions)	family Desulfovibrionaceae	rs2838334	G	A	0.066	43645080	0.333	0.069	360612	G	A	0.057	21	45064961	3.82E-06	0.012	14306	21.157
Vascular dementia (multiple infarctions)	family Desulfovibrionaceae	rs3935584	C	T	-0.128	233064573	0.050	0.066	360612	C	T	-0.053	2	233929283	6.78E-06	0.012	14306	20.635
Vascular dementia (multiple infarctions)	family Desulfovibrionaceae	rs4506934	C	T	0.094	2953368	0.355	0.102	360612	C	T	-0.094	17	2856662	3.16E-06	0.020	14306	21.945
Vascular dementia (multiple infarctions)	family Desulfovibrionaceae	rs6058181	C	T	-0.145	35106998	0.098	0.087	360612	C	T	0.083	20	33694801	2.70E-07	0.017	14306	25.253
Vascular dementia (multiple infarctions)	family Desulfovibrionaceae	rs72647048	T	C	-0.114	56952819	0.266	0.103	360612	T	C	-0.077	8	57865378	9.61E-06	0.017	14306	20.307
Vascular dementia (multiple infarctions)	family Desulfovibrionaceae	rs9928243	C	A	-0.026	71507738	0.692	0.066	360612	C	A	-0.054	16	71541641	4.48E-06	0.012	14306	21.142
Vascular dementia (multiple infarctions)	family Enterobacteriaceae	rs11026530	T	C	0.022	22357551	0.815	0.092	360612	T	C	0.082	11	22379097	9.43E-06	0.019	14306	19.471
Vascular dementia (multiple infarctions)	family Enterobacteriaceae	rs2374342	C	A	0.022	41906402	0.737	0.066	360612	C	A	0.058	2	42133542	4.52E-06	0.013	14306	21.338
Vascular dementia (multiple infarctions)	family Enterobacteriaceae	rs35673018	G	A	-0.118	54293833	0.295	0.113	360612	G	A	0.090	16	54327745	7.63E-06	0.020	14306	19.653
Vascular dementia (multiple infarctions)	family Enterobacteriaceae	rs504442	T	G	0.049	57478315	0.647	0.107	360612	T	G	0.084	18	55145547	5.17E-06	0.019	14306	19.728
Vascular dementia (multiple infarctions)	family Enterobacteriaceae	rs62210023	A	G	-0.051	56765036	0.453	0.068	360612	A	G	0.061	20	55340092	3.13E-06	0.013	14306	21.742
Vascular dementia (multiple infarctions)	family Enterobacteriaceae	rs78143293	A	G	0.039	60005103	0.692	0.099	360612	A	G	-0.085	18	57672335	1.20E-06	0.017	14306	24.792
Vascular dementia (multiple infarctions)	family Enterobacteriaceae	rs79757635	C	A	-0.036	110188071	0.708	0.097	360612	C	A	0.076	13	110840418	9.32E-06	0.017	14306	19.615
Vascular dementia (multiple infarctions)	family Erysipelotrichaceae	rs1074800	G	A	0.013	3002432	0.842	0.066	360612	G	A	-0.049	5	3002546	6.15E-06	0.011	14306	20.459
Vascular dementia (multiple infarctions)	family Erysipelotrichaceae	rs10781552	C	T	-0.140	132083729	0.055	0.073	360612	C	T	-0.055	10	133897233	2.33E-06	0.012	14306	22.633
Vascular dementia (multiple infarctions)	family Erysipelotrichaceae	rs17530232	A	G	0.003	39811320	0.984	0.145	360612	A	G	0.103	13	40385457	2.79E-06	0.022	14306	21.042
Vascular dementia (multiple infarctions)	family Erysipelotrichaceae	rs1884466	C	T	-0.043	63673525	0.518	0.066	360612	C	T	-0.048	1	64139196	9.53E-06	0.011	14306	19.760
Vascular dementia (multiple infarctions)	family Erysipelotrichaceae	rs2300774	A	G	0.037	196066841	0.575	0.065	360612	A	G	-0.052	3	195793712	8.95E-07	0.011	14306	24.094
Vascular dementia (multiple infarctions)	family Erysipelotrichaceae	rs290833	T	G	0.039	96991871	0.556	0.066	360612	T	G	-0.050	1	97457427	8.03E-06	0.011	14306	19.943
Vascular dementia (multiple infarctions)	family Erysipelotrichaceae	rs35161940	T	C	-0.012	72331083	0.907	0.107	360612	T	C	-0.081	17	70327224	1.85E-06	0.017	14306	23.118
Vascular dementia (multiple infarctions)	family Erysipelotrichaceae	rs4078432	T	C	0.140	48528003	0.110	0.088	360612	T	C	0.061	14	48997206	4.23E-06	0.013	14306	20.723
Vascular dementia (multiple infarctions)	family Erysipelotrichaceae	rs56970041	T	G	-0.042	79891267	0.760	0.136	360612	T	G	0.072	14	80357610	5.40E-06	0.016	14306	19.385
Vascular dementia (multiple infarctions)	family Erysipelotrichaceae	rs62504403	C	T	0.043	38946033	0.600	0.082	360612	C	T	0.068	8	38803551	1.12E-07	0.013	14306	28.371
Vascular dementia (multiple infarctions)	family Erysipelotrichaceae	rs7234058	T	C	-0.136	5830508	0.229	0.113	360612	T	C	-0.095	18	5830507	9.12E-07	0.019	14306	23.744
Vascular dementia (multiple infarctions)	family Erysipelotrichaceae	rs7826267	G	T	0.000	3097430	0.998	0.132	360612	G	T	0.084	8	2954952	9.28E-06	0.020	14306	17.755
Vascular dementia (multiple infarctions)	family Erysipelotrichaceae	rs8003149	C	T	0.142	55689786	0.040	0.069	360612	C	T	0.054	14	56156504	4.08E-06	0.012	14306	21.248
Vascular dementia (multiple infarctions)	family Family XI	rs10759623	C	T	0.018	113059205	0.817	0.078	360612	C	T	-0.162	9	115821485	5.78E-07	0.032	14306	25.425
Vascular dementia (multiple infarctions)	family Family XI	rs11547158	A	G	-0.018	149224640	0.854	0.096	360612	A	G	-0.178	7	148921732	2.70E-06	0.037	14306	22.696
Vascular dementia (multiple infarctions)	family Family XI	rs17379710	T	C	0.053	35313121	0.421	0.066	360612	T	C	-0.116	11	35334668	3.97E-06	0.025	14306	21.308
Vascular dementia (multiple infarctions)	family Family XI	rs2155352	A	G	-0.043	95624045	0.573	0.077	360612	A	G	-0.151	11	95357209	6.63E-07	0.030	14306	24.795
Vascular dementia (multiple infarctions)	family Family XI	rs2156611	T	C	-0.061	45765899	0.348	0.065	360612	T	C	-0.112	18	43345864	9.43E-06	0.025	14306	20.068
Vascular dementia (multiple infarctions)	family Family XI	rs3733511	A	G	-0.044	119034632	0.536	0.071	360612	A	G	0.128	4	119955787	3.39E-06	0.027	14306	21.795
Vascular dementia (multiple infarctions)	family Family XI	rs488164	G	T	-0.043	239832151	0.527	0.068	360612	G	T	-0.118	1	239995451	4.80E-06	0.026	14306	21.386
Vascular dementia (multiple infarctions)	family Family XI	rs697771	A	G	0.000	54081288	0.997	0.065	360612	A	G	-0.118	16	54115200	3.19E-06	0.025	14306	21.895
Vascular dementia (multiple infarctions)	family Family XIII	rs10404377	A	C	-0.094	16063847	0.161	0.067	360612	A	C	0.050	19	16174657	6.99E-06	0.011	14306	20.311
Vascular dementia (multiple infarctions)	family Family XIII	rs118170811	A	G	-0.157	100607139	0.404	0.188	360612	A	G	0.152	10	102366896	1.80E-06	0.032	14306	22.860
Vascular dementia (multiple infarctions)	family Family XIII	rs482905	G	T	-0.108	166587965	0.155	0.076	360612	G	T	0.060	1	166557202	3.72E-06	0.013	14306	22.004
Vascular dementia (multiple infarctions)	family Family XIII	rs6501525	A	G	0.019	72222486	0.789	0.070	360612	A	G	0.056	17	70218627	1.24E-06	0.012	14306	23.603
Vascular dementia (multiple infarctions)	family Family XIII	rs66753613	G	A	0.037	38746349	0.661	0.085	360612	G	A	0.065	1	39212021	8.08E-06	0.014	14306	20.375
Vascular dementia (multiple infarctions)	family Family XIII	rs6797051	C	T	0.048	88946068	0.663	0.111	360612	C	T	-0.081	3	88995218	4.89E-06	0.017	14306	22.122
Vascular dementia (multiple infarctions)	family Family XIII	rs7514702	T	C	0.057	186944891	0.537	0.093	360612	T	C	-0.066	1	186914023	3.92E-06	0.014	14306	21.931
Vascular dementia (multiple infarctions)	family Lachnospiraceae	rs10402491	C	T	0.157	13314985	0.071	0.087	360612	C	T	0.066	19	13425799	7.58E-06	0.015	14306	19.852

Vascular dementia (multiple infarctions)	family Lachnospiraceae	rs11139361	C	T	0.003	69564931	0.966	0.070	360612	C	T	-0.049	9	72179847	4.26E-06	0.011	14306	20.023
Vascular dementia (multiple infarctions)	family Lachnospiraceae	rs112040820	A	G	0.025	82511188	0.743	0.075	360612	A	G	0.055	17	80469064	2.42E-06	0.012	14306	22.032
Vascular dementia (multiple infarctions)	family Lachnospiraceae	rs11841382	G	T	-0.023	37430535	0.843	0.117	360612	G	T	-0.072	13	38004672	9.58E-06	0.017	14306	16.913
Vascular dementia (multiple infarctions)	family Lachnospiraceae	rs11979110	T	C	-0.033	130751700	0.614	0.065	360612	T	C	-0.050	7	130436459	1.82E-06	0.011	14306	22.730
Vascular dementia (multiple infarctions)	family Lachnospiraceae	rs1205443	A	G	-0.126	38248466	0.069	0.069	360612	A	G	0.050	20	36876868	7.29E-06	0.011	14306	19.987
Vascular dementia (multiple infarctions)	family Lachnospiraceae	rs12760724	A	C	0.029	213858626	0.677	0.069	360612	A	C	-0.048	1	214031969	7.27E-06	0.011	14306	20.155
Vascular dementia (multiple infarctions)	family Lachnospiraceae	rs13005175	G	A	0.151	231605011	0.344	0.160	360612	G	A	-0.099	2	232469722	8.37E-06	0.022	14306	20.694
Vascular dementia (multiple infarctions)	family Lachnospiraceae	rs2159863	A	G	-0.122	10243730	0.152	0.085	360612	A	G	-0.059	4	10245354	3.70E-06	0.013	14306	20.706
Vascular dementia (multiple infarctions)	family Lachnospiraceae	rs2910921	C	T	0.059	32348487	0.762	0.196	360612	C	T	-0.160	5	32348593	8.42E-06	0.036	14306	20.014
Vascular dementia (multiple infarctions)	family Lachnospiraceae	rs3127230	C	T	-0.054	101632746	0.446	0.071	360612	C	T	-0.050	10	103392503	6.20E-06	0.011	14306	20.140
Vascular dementia (multiple infarctions)	family Lachnospiraceae	rs35524804	T	C	-0.058	97351401	0.466	0.079	360612	T	C	-0.061	9	100113683	2.45E-06	0.013	14306	23.537
Vascular dementia (multiple infarctions)	family Lachnospiraceae	rs7359994	C	T	-0.007	28917951	0.920	0.067	360612	C	T	0.050	19	29408858	5.36E-06	0.011	14306	20.009
Vascular dementia (multiple infarctions)	family Lachnospiraceae	rs79086868	T	C	0.009	130916218	0.935	0.108	360612	T	C	0.078	9	133791605	3.01E-06	0.016	14306	22.302
Vascular dementia (multiple infarctions)	family Lachnospiraceae	rs959845	T	C	-0.117	185985647	0.082	0.067	360612	T	C	0.049	4	186906801	5.17E-06	0.011	14306	20.971
Vascular dementia (multiple infarctions)	family Lachnospiraceae	rs9929145	G	A	-0.184	76525287	0.218	0.150	360612	G	A	-0.126	16	76559184	2.84E-07	0.025	14306	26.282
Vascular dementia (multiple infarctions)	family Lactobacillaceae	rs1530559	G	A	0.123	134998059	0.064	0.066	360612	G	A	0.077	2	135755629	9.65E-06	0.018	14306	19.063
Vascular dementia (multiple infarctions)	family Lactobacillaceae	rs16861661	G	A	-0.030	18174965	0.824	0.133	360612	G	A	-0.193	1	18501459	2.70E-07	0.038	14306	25.969
Vascular dementia (multiple infarctions)	family Lactobacillaceae	rs62314653	C	A	0.237	108975306	0.087	0.138	360612	C	A	0.177	4	109896462	6.59E-06	0.039	14306	20.379
Vascular dementia (multiple infarctions)	family Lactobacillaceae	rs74599091	A	G	-0.347	179751534	0.153	0.243	360612	A	G	0.192	1	179720669	7.70E-06	0.043	14306	20.323
Vascular dementia (multiple infarctions)	family Lactobacillaceae	rs768253	T	G	0.094	68097868	0.156	0.066	360612	T	G	-0.079	8	69010103	3.61E-06	0.017	14306	21.570
Vascular dementia (multiple infarctions)	family Lactobacillaceae	rs77478751	A	G	-0.090	173433875	0.379	0.103	360612	A	G	-0.219	3	173151665	5.96E-06	0.047	14306	21.421
Vascular dementia (multiple infarctions)	family Lactobacillaceae	rs921925	A	C	-0.031	6928006	0.695	0.079	360612	A	C	0.100	19	6928017	5.77E-07	0.020	14306	24.555
Vascular dementia (multiple infarctions)	family Lactobacillaceae	rs9345899	A	G	-0.105	66849430	0.320	0.105	360612	A	G	-0.124	6	67559323	9.45E-06	0.028	14306	19.774
Vascular dementia (multiple infarctions)	family Methanobacteriaceae	rs10202904	G	T	-0.010	124682691	0.886	0.067	360612	G	T	0.122	2	125440268	3.01E-07	0.024	14306	26.762
Vascular dementia (multiple infarctions)	family Methanobacteriaceae	rs10424197	A	G	0.005	45936063	0.948	0.075	360612	A	G	0.111	19	46439321	9.28E-06	0.025	14306	20.211
Vascular dementia (multiple infarctions)	family Methanobacteriaceae	rs4257531	G	A	0.080	2044483	0.457	0.107	360612	G	A	0.164	3	2086167	7.44E-06	0.036	14306	20.316
Vascular dementia (multiple infarctions)	family Methanobacteriaceae	rs6508769	C	T	-0.054	28336853	0.557	0.092	360612	C	T	-0.154	19	28827760	8.23E-06	0.034	14306	19.856
Vascular dementia (multiple infarctions)	family Methanobacteriaceae	rs6776814	T	C	0.044	15011576	0.848	0.228	360612	T	C	-0.200	3	15053083	1.63E-06	0.041	14306	23.483
Vascular dementia (multiple infarctions)	family Methanobacteriaceae	rs73068003	G	T	-0.094	10734305	0.391	0.110	360612	G	T	-0.158	7	10773932	8.45E-06	0.035	14306	20.206
Vascular dementia (multiple infarctions)	family Methanobacteriaceae	rs73457410	A	G	-0.069	41382045	0.603	0.133	360612	A	G	0.215	13	41956181	1.41E-06	0.044	14306	24.316
Vascular dementia (multiple infarctions)	family Methanobacteriaceae	rs75208022	C	T	-0.008	21185927	0.945	0.112	360612	C	T	-0.227	12	21338861	5.92E-06	0.049	14306	21.717
Vascular dementia (multiple infarctions)	family Methanobacteriaceae	rs894996	C	A	-0.197	103497150	0.122	0.127	360612	C	A	0.217	4	104418307	1.88E-06	0.045	14306	23.349
Vascular dementia (multiple infarctions)	family Oxalobacteraceae	rs111966731	T	C	0.212	93398708	0.070	0.117	360612	T	C	0.204	15	93941937	4.56E-06	0.045	14306	20.952
Vascular dementia (multiple infarctions)	family Oxalobacteraceae	rs11246212	C	T	-0.034	610277	0.713	0.094	360612	C	T	-0.136	11	610277	4.51E-06	0.029	14306	21.745
Vascular dementia (multiple infarctions)	family Oxalobacteraceae	rs12002250	A	C	-0.100	19682560	0.538	0.163	360612	A	C	0.196	9	19682558	5.53E-06	0.045	14306	19.408
Vascular dementia (multiple infarctions)	family Oxalobacteraceae	rs1569853	T	C	-0.042	38582525	0.678	0.101	360612	T	C	-0.140	6	38550301	7.45E-07	0.028	14306	24.714
Vascular dementia (multiple infarctions)	family Oxalobacteraceae	rs17138946	G	T	0.150	5934557	0.246	0.130	360612	G	T	-0.189	16	5984558	8.09E-06	0.043	14306	19.388
Vascular dementia (multiple infarctions)	family Oxalobacteraceae	rs36057338	G	T	0.117	189014160	0.525	0.184	360612	G	T	0.182	4	189935314	6.26E-06	0.040	14306	20.743
Vascular dementia (multiple infarctions)	family Oxalobacteraceae	rs4428215	G	A	-0.019	172229645	0.798	0.075	360612	G	A	0.126	3	171947435	4.88E-08	0.023	14306	29.812
Vascular dementia (multiple infarctions)	family Oxalobacteraceae	rs561239	A	G	0.069	18640649	0.394	0.081	360612	A	G	0.106	12	18793583	7.19E-06	0.024	14306	19.864
Vascular dementia (multiple infarctions)	family Oxalobacteraceae	rs6000536	C	T	0.077	37025428	0.380	0.088	360612	C	T	-0.118	22	37421469	7.39E-07	0.024	14306	24.058
Vascular dementia (multiple infarctions)	family Oxalobacteraceae	rs62435498	C	A	0.058	1754627	0.590	0.108	360612	C	A	0.182	7	1794263	7.46E-06	0.040	14306	20.531
Vascular dementia (multiple infarctions)	family Oxalobacteraceae	rs736744	T	C	-0.022	84899492	0.743	0.066	360612	T	C	-0.106	9	87514407	1.49E-07	0.020	14306	27.721
Vascular dementia (multiple infarctions)	family Oxalobacteraceae	rs7993559	A	C	-0.072	22869488	0.279	0.066	360612	A	C	-0.092	13	23443627	5.04E-06	0.020	14306	20.974
Vascular dementia (multiple infarctions)	family Oxalobacteraceae	rs80330081	A	C	-0.018	66561674	0.867	0.107	360612	A	C	-0.188	4	67427392	6.64E-06	0.042	14306	19.597
Vascular dementia (multiple infarctions)	family Oxalobacteraceae	rs934049	G	A	0.046	15916656	0.567	0.081	360612	G	A	0.110	2	16056779	4.21E-06	0.024	14306	21.152
Vascular dementia (multiple infarctions)	family Pasteurellaceae	rs10965428	C	A	-0.305	22718482	0.031	0.142	360612	C	A	-0.120	9	22718481	4.29E-06	0.026	14306	21.561
Vascular dementia (multiple infarctions)	family Pasteurellaceae	rs111582866	G	A	0.052	48708578	0.650	0.114	360612	G	A	-0.114	16	48742489	7.07E-06	0.026	14306	19.753
Vascular dementia (multiple infarctions)	family Pasteurellaceae	rs12050685	A	G	-0.091	73185141	0.209	0.072	360612	A	G	-0.067	15	73477482	9.19E-06	0.015	14306	19.385
Vascular dementia (multiple infarctions)	family Pasteurellaceae	rs16970009	A	G	0.153	34535582	0.514	0.234	360612	A	G	0.187	17	32862601	7.32E-06	0.043	14306	19.027
Vascular dementia (multiple infarctions)	family Pasteurellaceae	rs4822728	T	C	-0.004	26495842	0.946	0.065	360612	T	C	0.069	22	26891808	4.72E-06	0.015	14306	21.156
Vascular dementia (multiple infarctions)	family Pasteurellaceae	rs6972479	A	G	-0.002	117278006	0.983	0.081	360612	A	G	-0.078	7	116918060	7.75E-06	0.018	14306	19.878
Vascular dementia (multiple infarctions)	family Pasteurellaceae	rs72756943	G	A	-0.006	26531799	0.962	0.130	360612	G	A	0.140	5	26531908	3.35E-06	0.030	14306	21.308
Vascular dementia (multiple infarctions)	family Pasteurellaceae	rs73139353	A	C	-0.171	98253370	0.135	0.115	360612	A	C	-0.223	3	97972214	8.71E-06	0.048	14306	21.092
Vascular dementia (multiple infarctions)	family Pasteurellaceae	rs76022354	C	T	-0.050	92546628	0.735	0.149	360612	C	T	0.243	10	94306385	1.83E-06	0.050	14306	23.560
Vascular dementia (multiple infarctions)	family Pasteurellaceae	rs78909003	T	C	-0.189	102887960	0.186	0.143	360612	T	C	-0.241	9	105650242	2.05E-06	0.050	14306	23.415
Vascular dementia (multiple infarctions)	family Pasteurellaceae	rs9382510	C	T	-0.044	55583693	0.560	0.075	360612	C	T	-0.088	6	55448491	2.48E-07	0.017	14306	26.921
Vascular dementia (multiple infarctions)	family Pasteurellaceae	rs9895850	T	C	-0.066	66538895	0.679	0.159	360612	T	C	-0.176	17	64535013	9.08E-06	0.041	14306	18.497
Vascular dementia (multiple infarctions)	family Pasteurellaceae	rs9938097	C	T	0.010	84943783	0.886	0.067	360612	C	T	0.071	16	84977389	8.23E-06	0.016	14306	20.209
Vascular dementia (multiple infarctions)	family Peptococcaceae	rs117452796	A	G	-0.011	9423378	0.955	0.186	360612	A	G	-0.258	9	9423378	3.15E-06	0.055	14306	22.036
Vascular dementia (multiple infarctions)	family Peptococcaceae	rs12144792	C	T	-0.074	27091734	0.274	0.068	360612	C	T	0.064	1	27418225	5.82E-06	0.014	14306	20.788
Vascular dementia (multiple infarctions)	family Peptococcaceae	rs12634826	T	G	-0.027	183266260	0.697	0.069	360612	T	G	-0.074	3	182984048	1.01E-06	0.015	14306	24.031
Vascular dementia (multiple infarctions)	family Peptococcaceae	rs12992764	T	G	0.039	188031016	0.557	0.066	360612	T	G	0.068	2	188895743	1.46E-06	0.014	14306	23.502
Vascular dementia (multiple infarctions)	family Peptococcaceae	rs150600492	A	C	-0.006	127377698	0.964	0.136	360612	A	C	0.136	10	129175962	2.31E-06	0.029	14306	21.957
Vascular dementia (multiple infarctions)	family Peptococcaceae	rs35703006	G	T	-0.050	28756700	0.516	0.076	360612	G	T	0.081	8	28614217	4.95E-07	0.016	14306	24.448
Vascular dementia (multiple infarctions)	family Peptococcaceae	rs4990837	G	A	0.030	3723235	0.713	0.083	360612	G	A	-0.091	8	3580757	1.74E-06	0.019	14306	23.990
Vascular dementia (multiple infarctions)	family Peptococcaceae	rs75430375	C	T	0.017	89957067	0.916	0.156	360612	C	T	-0.148	5	89252884	3.41E-06	0.032	14306	21.712

Vascular dementia (multiple infarctions)	family Peptococcaceae	rs75898026	A	G	-0.010	112906768	0.901	0.080	360612	A	G	-0.082	13	113561082	2.02E-06	0.017	14306	22.424
Vascular dementia (multiple infarctions)	family Peptostreptococcaceae	rs10805326	A	G	0.020	14322999	0.778	0.072	360612	A	G	-0.057	4	14324623	4.03E-06	0.012	14306	21.306
Vascular dementia (multiple infarctions)	family Peptostreptococcaceae	rs117020988	C	T	0.260	46671379	0.030	0.120	360612	C	T	0.182	7	46710977	1.03E-06	0.037	14306	24.028
Vascular dementia (multiple infarctions)	family Peptostreptococcaceae	rs12377846	C	A	-0.100	16786786	0.618	0.200	360612	C	A	-0.252	9	16786784	7.26E-07	0.051	14306	24.260
Vascular dementia (multiple infarctions)	family Peptostreptococcaceae	rs12986312	T	G	0.113	17303329	0.113	0.071	360612	T	G	0.057	19	17414138	5.77E-06	0.013	14306	20.613
Vascular dementia (multiple infarctions)	family Peptostreptococcaceae	rs1467258	G	A	-0.036	40060234	0.671	0.085	360612	G	A	0.073	17	38216487	7.90E-06	0.016	14306	19.996
Vascular dementia (multiple infarctions)	family Peptostreptococcaceae	rs1520207	T	C	0.038	152063386	0.568	0.066	360612	T	C	-0.053	3	151781175	3.17E-06	0.011	14306	21.842
Vascular dementia (multiple infarctions)	family Peptostreptococcaceae	rs4692811	C	T	0.055	170259039	0.430	0.069	360612	C	T	0.064	4	171180190	4.21E-07	0.013	14306	25.595
Vascular dementia (multiple infarctions)	family Peptostreptococcaceae	rs59865771	C	T	0.024	89180262	0.726	0.069	360612	C	T	-0.057	16	89246670	7.69E-06	0.013	14306	20.349
Vascular dementia (multiple infarctions)	family Peptostreptococcaceae	rs61841503	G	A	0.007	16977560	0.941	0.098	360612	G	A	0.092	10	17019559	9.80E-09	0.016	14306	32.495
Vascular dementia (multiple infarctions)	family Peptostreptococcaceae	rs6721459	G	A	0.067	67625704	0.317	0.067	360612	G	A	-0.051	2	67852836	5.08E-06	0.011	14306	20.730
Vascular dementia (multiple infarctions)	family Peptostreptococcaceae	rs76982728	T	C	0.052	44273225	0.817	0.225	360612	T	C	0.124	7	44312824	3.24E-06	0.027	14306	21.652
Vascular dementia (multiple infarctions)	family Peptostreptococcaceae	rs77540684	T	G	0.090	14608536	0.426	0.112	360612	T	G	0.107	10	14650535	8.14E-06	0.025	14306	18.877
Vascular dementia (multiple infarctions)	family Peptostreptococcaceae	rs9573937	A	G	-0.006	76777061	0.946	0.089	360612	A	G	-0.069	13	77351196	1.71E-06	0.014	14306	23.559
Vascular dementia (multiple infarctions)	family Porphyromonadaceae	rs10762312	A	G	0.026	69812107	0.716	0.071	360612	A	G	0.052	10	71571863	8.70E-06	0.012	14306	19.427
Vascular dementia (multiple infarctions)	family Porphyromonadaceae	rs10858364	G	T	0.147	135184235	0.057	0.077	360612	G	T	0.055	9	138076081	4.31E-06	0.012	14306	20.951
Vascular dementia (multiple infarctions)	family Porphyromonadaceae	rs17065783	A	G	0.003	62049912	0.970	0.088	360612	A	G	-0.059	3	62035586	1.79E-06	0.012	14306	23.403
Vascular dementia (multiple infarctions)	family Porphyromonadaceae	rs1980561	A	G	-0.044	62919798	0.505	0.066	360612	A	G	-0.049	14	63386516	8.95E-06	0.011	14306	19.719
Vascular dementia (multiple infarctions)	family Porphyromonadaceae	rs35233670	T	C	-0.055	65754785	0.401	0.065	360612	T	C	-0.047	17	63750903	7.91E-06	0.011	14306	19.953
Vascular dementia (multiple infarctions)	family Porphyromonadaceae	rs35961441	A	C	0.039	240766474	0.820	0.170	360612	A	C	0.092	1	240929774	8.37E-06	0.021	14306	19.492
Vascular dementia (multiple infarctions)	family Porphyromonadaceae	rs6953849	A	G	-0.077	69786706	0.364	0.085	360612	A	G	0.072	7	69251692	2.44E-06	0.015	14306	22.702
Vascular dementia (multiple infarctions)	family Porphyromonadaceae	rs7330827	T	C	-0.278	22957663	0.046	0.139	360612	T	C	-0.104	13	23531802	8.05E-06	0.024	14306	19.140
Vascular dementia (multiple infarctions)	family Porphyromonadaceae	rs864093	A	C	0.113	148904825	0.155	0.080	360612	A	C	-0.053	4	149825977	9.60E-06	0.012	14306	20.188
Vascular dementia (multiple infarctions)	family Prevotellaceae	rs12057990	C	T	-0.173	99004713	0.018	0.073	360612	C	T	0.059	1	99470269	8.97E-06	0.013	14306	19.889
Vascular dementia (multiple infarctions)	family Prevotellaceae	rs12118202	T	C	-0.016	210508026	0.849	0.085	360612	T	C	-0.075	1	210681370	5.54E-07	0.015	14306	26.075
Vascular dementia (multiple infarctions)	family Prevotellaceae	rs13069367	A	C	0.004	71752679	0.957	0.067	360612	A	C	-0.054	3	71801830	7.39E-06	0.012	14306	20.339
Vascular dementia (multiple infarctions)	family Prevotellaceae	rs148376875	T	G	0.117	170408919	0.202	0.092	360612	T	G	0.085	3	170126707	2.08E-06	0.018	14306	22.252
Vascular dementia (multiple infarctions)	family Prevotellaceae	rs2206482	T	G	0.005	9790461	0.943	0.067	360612	T	G	-0.057	20	9771109	1.30E-06	0.012	14306	23.465
Vascular dementia (multiple infarctions)	family Prevotellaceae	rs2278540	G	A	-0.029	32367424	0.671	0.068	360612	G	A	0.055	3	32408916	8.44E-06	0.012	14306	20.218
Vascular dementia (multiple infarctions)	family Prevotellaceae	rs34660375	A	G	-0.219	180856881	0.019	0.093	360612	A	G	-0.081	5	180283881	7.40E-06	0.018	14306	20.431
Vascular dementia (multiple infarctions)	family Prevotellaceae	rs3758087	C	T	-0.031	23857096	0.667	0.073	360612	C	T	-0.056	8	23714609	8.61E-06	0.012	14306	20.485
Vascular dementia (multiple infarctions)	family Prevotellaceae	rs3860225	A	G	0.087	110619055	0.442	0.113	360612	A	G	0.084	1	111161677	5.50E-07	0.017	14306	24.981
Vascular dementia (multiple infarctions)	family Prevotellaceae	rs4493272	T	C	-0.012	118153336	0.853	0.065	360612	T	C	-0.060	2	118910912	3.02E-07	0.012	14306	26.275
Vascular dementia (multiple infarctions)	family Prevotellaceae	rs4685827	T	C	-0.148	4823193	0.053	0.076	360612	T	C	-0.068	3	4864877	2.77E-06	0.015	14306	21.840
Vascular dementia (multiple infarctions)	family Prevotellaceae	rs7252711	G	A	0.071	17350190	0.500	0.105	360612	G	A	0.074	19	17460999	5.57E-06	0.016	14306	20.934
Vascular dementia (multiple infarctions)	family Prevotellaceae	rs7975087	C	A	0.103	21251898	0.230	0.086	360612	C	A	-0.060	12	21404832	7.59E-06	0.014	14306	19.716
Vascular dementia (multiple infarctions)	family Prevotellaceae	rs912860	A	G	0.020	33237702	0.925	0.216	360612	A	G	0.229	14	33706908	9.30E-07	0.048	14306	22.476
Vascular dementia (multiple infarctions)	family Prevotellaceae	rs9586501	G	A	-0.015	104423473	0.837	0.075	360612	G	A	0.059	13	105075823	2.59E-06	0.013	14306	21.666
Vascular dementia (multiple infarctions)	family Prevotellaceae	rs9958960	G	A	0.051	65351747	0.577	0.091	360612	G	A	-0.091	18	63018983	1.06E-07	0.017	14306	27.765
Vascular dementia (multiple infarctions)	family Rhodospirillaceae	rs1035406	G	A	0.090	120037042	0.374	0.102	360612	G	A	-0.114	5	119372737	5.84E-06	0.025	14306	20.484
Vascular dementia (multiple infarctions)	family Rhodospirillaceae	rs11591293	G	T	0.007	111660039	0.916	0.066	360612	G	T	0.074	10	113419797	2.67E-06	0.016	14306	21.923
Vascular dementia (multiple infarctions)	family Rhodospirillaceae	rs13336560	C	T	-0.017	88487835	0.799	0.066	360612	C	T	-0.070	16	88554243	9.17E-06	0.016	14306	19.710
Vascular dementia (multiple infarctions)	family Rhodospirillaceae	rs1549633	A	C	0.002	27945538	0.981	0.103	360612	A	C	0.100	5	27945645	4.70E-06	0.022	14306	20.891
Vascular dementia (multiple infarctions)	family Rhodospirillaceae	rs1923415	A	G	0.071	88558996	0.534	0.115	360612	A	G	-0.100	6	89268715	9.64E-06	0.023	14306	19.321
Vascular dementia (multiple infarctions)	family Rhodospirillaceae	rs3754624	C	T	-0.075	224769095	0.381	0.086	360612	C	T	0.097	2	225633812	1.71E-06	0.020	14306	23.575
Vascular dementia (multiple infarctions)	family Rhodospirillaceae	rs4278423	T	C	-0.072	2628361	0.592	0.134	360612	T	C	0.108	10	2670553	3.12E-06	0.024	14306	20.808
Vascular dementia (multiple infarctions)	family Rhodospirillaceae	rs61933850	G	A	-0.047	72745618	0.620	0.095	360612	G	A	0.165	12	73139398	7.23E-06	0.036	14306	20.883
Vascular dementia (multiple infarctions)	family Rhodospirillaceae	rs6679026	T	C	0.010	78153828	0.928	0.111	360612	T	C	0.112	1	78619512	9.95E-06	0.025	14306	19.891
Vascular dementia (multiple infarctions)	family Rhodospirillaceae	rs7001029	C	T	-0.103	130946157	0.367	0.114	360612	C	T	0.117	8	131958403	5.35E-06	0.026	14306	20.171
Vascular dementia (multiple infarctions)	family Rhodospirillaceae	rs72714493	A	G	-0.006	91740661	0.952	0.092	360612	A	G	0.082	1	92206218	7.35E-06	0.018	14306	20.415
Vascular dementia (multiple infarctions)	family Rhodospirillaceae	rs74354280	C	T	-0.066	133271763	0.365	0.073	360612	C	T	-0.091	4	134192918	6.67E-06	0.020	14306	19.805
Vascular dementia (multiple infarctions)	family Rhodospirillaceae	rs76784716	A	G	-0.160	168176830	0.129	0.105	360612	A	G	0.136	2	169033340	1.49E-06	0.029	14306	22.666
Vascular dementia (multiple infarctions)	family Rhodospirillaceae	rs9813022	A	G	-0.189	13685237	0.005	0.067	360612	A	G	-0.084	3	13726736	2.53E-07	0.016	14306	26.522
Vascular dementia (multiple infarctions)	family Rikenellaceae	rs10217435	C	T	-0.027	83437740	0.774	0.094	360612	C	T	-0.088	9	86052655	6.51E-06	0.020	14306	20.022
Vascular dementia (multiple infarctions)	family Rikenellaceae	rs10832801	A	C	-0.024	17567582	0.741	0.073	360612	A	C	-0.053	11	17589129	7.50E-06	0.012	14306	19.005
Vascular dementia (multiple infarctions)	family Rikenellaceae	rs1939881	G	A	0.133	95571670	0.351	0.142	360612	G	A	-0.106	11	95304834	5.64E-07	0.021	14306	26.218
Vascular dementia (multiple infarctions)	family Rikenellaceae	rs2447496	A	G	-0.019	98176696	0.802	0.074	360612	A	G	0.055	8	99188924	6.09E-06	0.012	14306	20.297
Vascular dementia (multiple infarctions)	family Rikenellaceae	rs2833282	G	A	0.085	31124392	0.355	0.092	360612	G	A	0.071	21	32496710	4.31E-06	0.016	14306	20.526
Vascular dementia (multiple infarctions)	family Rikenellaceae	rs36021379	A	G	0.106	27143593	0.241	0.090	360612	A	G	-0.066	21	28515912	7.20E-06	0.014	14306	20.496
Vascular dementia (multiple infarctions)	family Rikenellaceae	rs4264350	T	C	0.057	71366303	0.384	0.066	360612	T	C	-0.053	15	71658642	1.35E-06	0.011	14306	23.514
Vascular dementia (multiple infarctions)	family Rikenellaceae	rs59663348	G	A	0.141	4069710	0.063	0.076	360612	G	A	0.057	18	4069710	6.12E-06	0.013	14306	20.844
Vascular dementia (multiple infarctions)	family Rikenellaceae	rs62532512	A	C	-0.034	14158855	0.611	0.066	360612	A	C	0.050	9	14158854	2.76E-06	0.011	14306	22.045
Vascular dementia (multiple infarctions)	family Rikenellaceae	rs6744030	C	T	-0.041	173392399	0.613	0.080	360612	C	T	0.070	2	174257127	9.32E-06	0.016	14306	19.669
Vascular dementia (multiple infarctions)	family Rikenellaceae	rs6837275	A	G	0.013	186921078	0.854	0.072	360612	A	G	0.057	4	187842232	1.45E-06	0.012	14306	23.022
Vascular dementia (multiple infarctions)	family Rikenellaceae	rs74474130	T	G	0.141	89804477	0.418	0.175	360612	T	G	0.138	14	90270821	3.61E-06	0.030	14306	21.702
Vascular dementia (multiple infarctions)	family Rikenellaceae	rs77885767	C	T	0.084	21080475	0.592	0.156	360612	C	T	-0.156	14	21548634	2.85E-06	0.034	14306	21.552
Vascular dementia (multiple infarctions)	family Rikenellaceae	rs9389714	C	T	0.084	99796791	0.466	0.115	360612	C	T	-0.064	6	100244667	8.79E-06	0.014	14306	19.718

Vascular dementia (multiple infarctions)	family Rikenellaceae	rs9578457	G	A	0.129	22293217	0.396	0.152	360612	G	A	-0.141	13	22867356	3.99E-06	0.032	14306	20.064
Vascular dementia (multiple infarctions)	family Rikenellaceae	rs9603208	G	T	-0.015	37470552	0.887	0.109	360612	G	T	0.082	13	38044689	1.92E-07	0.016	14306	26.474
Vascular dementia (multiple infarctions)	family Ruminococcaceae	rs10093275	T	C	0.094	68820652	0.177	0.070	360612	T	C	-0.053	8	69732887	5.35E-06	0.012	14306	20.960
Vascular dementia (multiple infarctions)	family Ruminococcaceae	rs10166469	C	T	-0.003	29606301	0.971	0.075	360612	C	T	0.053	2	29829167	8.52E-06	0.012	14306	19.686
Vascular dementia (multiple infarctions)	family Ruminococcaceae	rs1158100	G	A	0.000	4698162	0.995	0.066	360612	G	A	0.049	8	4555684	8.61E-06	0.011	14306	19.936
Vascular dementia (multiple infarctions)	family Ruminococcaceae	rs1612733	T	C	0.251	107118984	0.081	0.144	360612	T	C	0.109	1	107661606	4.22E-06	0.024	14306	20.862
Vascular dementia (multiple infarctions)	family Ruminococcaceae	rs17376049	T	C	-0.229	60959595	0.029	0.105	360612	T	C	0.085	1	61425267	7.30E-07	0.017	14306	24.306
Vascular dementia (multiple infarctions)	family Ruminococcaceae	rs2113833	C	T	-0.328	217352078	0.069	0.180	360612	C	T	-0.169	2	218216801	1.14E-06	0.036	14306	22.701
Vascular dementia (multiple infarctions)	family Ruminococcaceae	rs3009418	A	C	0.137	148272338	0.381	0.157	360612	A	C	-0.093	1	147744468	8.69E-06	0.021	14306	19.489
Vascular dementia (multiple infarctions)	family Ruminococcaceae	rs55793120	T	C	0.046	46990335	0.741	0.140	360612	T	C	0.138	12	47384118	1.44E-07	0.027	14306	26.970
Vascular dementia (multiple infarctions)	family Ruminococcaceae	rs56199908	T	C	0.039	2801371	0.786	0.143	360612	T	C	-0.199	9	2801371	1.66E-06	0.041	14306	23.584
Vascular dementia (multiple infarctions)	family Ruminococcaceae	rs76724913	T	G	0.083	24147179	0.457	0.111	360612	T	G	0.090	1	24473669	9.60E-06	0.020	14306	19.682
Vascular dementia (multiple infarctions)	family Streptococcaceae	rs10028567	C	T	-0.024	52791410	0.814	0.101	360612	C	T	-0.093	4	53657577	3.72E-06	0.019	14306	24.079
Vascular dementia (multiple infarctions)	family Streptococcaceae	rs11110281	T	C	-0.106	100190236	0.476	0.149	360612	T	C	-0.131	12	100584014	1.40E-08	0.023	14306	33.387
Vascular dementia (multiple infarctions)	family Streptococcaceae	rs16950051	A	G	0.143	120291702	0.296	0.136	360612	A	G	0.107	12	120729505	5.34E-06	0.024	14306	20.391
Vascular dementia (multiple infarctions)	family Streptococcaceae	rs2370083	G	T	-0.121	97060413	0.366	0.134	360612	G	T	-0.084	14	97526750	4.26E-06	0.018	14306	20.862
Vascular dementia (multiple infarctions)	family Streptococcaceae	rs2952251	G	A	-0.079	10285654	0.310	0.078	360612	G	A	0.064	8	10143164	3.72E-07	0.013	14306	25.530
Vascular dementia (multiple infarctions)	family Streptococcaceae	rs35344081	G	A	-0.120	941253	0.105	0.074	360612	G	A	0.061	16	991253	2.64E-06	0.013	14306	22.072
Vascular dementia (multiple infarctions)	family Streptococcaceae	rs57646748	G	A	-0.007	37451236	0.969	0.171	360612	G	A	-0.088	4	37452858	7.88E-06	0.020	14306	19.612
Vascular dementia (multiple infarctions)	family Streptococcaceae	rs6806351	T	C	0.006	132339879	0.944	0.079	360612	T	C	-0.062	3	132058723	6.94E-06	0.014	14306	20.808
Vascular dementia (multiple infarctions)	family Streptococcaceae	rs77968078	G	A	-0.197	240278466	0.152	0.138	360612	G	A	-0.099	1	240441766	7.93E-06	0.022	14306	19.515
Vascular dementia (multiple infarctions)	family Streptococcaceae	rs7916711	A	G	-0.060	28299340	0.525	0.095	360612	A	G	0.096	10	28588269	6.33E-06	0.022	14306	19.839
Vascular dementia (multiple infarctions)	family Streptococcaceae	rs957755	T	G	-0.139	46739055	0.140	0.094	360612	T	G	-0.064	7	46778653	7.42E-06	0.014	14306	20.268
Vascular dementia (multiple infarctions)	family Veillonellaceae	rs111810795	C	T	0.246	102431593	0.024	0.109	360612	C	T	-0.087	14	102897930	1.73E-06	0.018	14306	23.059
Vascular dementia (multiple infarctions)	family Veillonellaceae	rs114889439	A	G	0.023	61023491	0.895	0.177	360612	A	G	-0.254	13	61597625	6.19E-06	0.054	14306	22.160
Vascular dementia (multiple infarctions)	family Veillonellaceae	rs12186441	G	A	-0.179	133310611	0.258	0.158	360612	G	A	0.208	5	132646303	4.53E-06	0.045	14306	21.010
Vascular dementia (multiple infarctions)	family Veillonellaceae	rs12668619	A	G	-0.056	21598192	0.423	0.069	360612	A	G	0.055	7	21637810	2.57E-06	0.012	14306	22.093
Vascular dementia (multiple infarctions)	family Veillonellaceae	rs12741784	C	T	0.001	49623147	0.990	0.075	360612	C	T	-0.062	1	50088819	1.28E-07	0.012	14306	27.151
Vascular dementia (multiple infarctions)	family Veillonellaceae	rs1442060	A	G	-0.032	46364050	0.623	0.066	360612	A	G	0.051	4	46366067	4.51E-06	0.011	14306	21.017
Vascular dementia (multiple infarctions)	family Veillonellaceae	rs1693340	T	C	-0.135	32628728	0.285	0.126	360612	T	C	0.082	18	30208691	9.25E-06	0.018	14306	20.147
Vascular dementia (multiple infarctions)	family Veillonellaceae	rs2175069	G	A	-0.059	23315501	0.386	0.068	360612	G	A	0.053	4	23317124	4.64E-06	0.011	14306	21.022
Vascular dementia (multiple infarctions)	family Veillonellaceae	rs2561116	T	G	-0.049	38348097	0.728	0.141	360612	T	G	-0.084	5	38348199	7.89E-06	0.019	14306	19.960
Vascular dementia (multiple infarctions)	family Veillonellaceae	rs2585520	G	T	0.187	78211006	0.268	0.169	360612	G	T	-0.090	13	78785141	5.27E-06	0.020	14306	20.387
Vascular dementia (multiple infarctions)	family Veillonellaceae	rs4263802	A	G	0.141	137307265	0.042	0.069	360612	A	G	-0.051	8	138319508	7.45E-06	0.011	14306	19.650
Vascular dementia (multiple infarctions)	family Veillonellaceae	rs4461038	G	A	-0.178	78254654	0.010	0.069	360612	G	A	0.055	15	78546996	3.73E-06	0.012	14306	21.579
Vascular dementia (multiple infarctions)	family Veillonellaceae	rs4797169	T	C	0.058	462180	0.448	0.076	360612	T	C	0.059	18	462180	4.49E-06	0.013	14306	20.991
Vascular dementia (multiple infarctions)	family Veillonellaceae	rs61264131	A	C	0.084	138867397	0.452	0.112	360612	A	C	0.202	6	139188534	6.75E-06	0.046	14306	18.990
Vascular dementia (multiple infarctions)	family Veillonellaceae	rs6692542	G	A	-0.160	244228316	0.020	0.069	360612	G	A	-0.053	1	244391618	8.68E-06	0.012	14306	20.422
Vascular dementia (multiple infarctions)	family Veillonellaceae	rs6909981	C	T	0.065	74824436	0.507	0.099	360612	C	T	-0.064	6	75534152	5.48E-06	0.014	14306	20.298
Vascular dementia (multiple infarctions)	family Veillonellaceae	rs79535861	A	C	0.160	37752555	0.162	0.115	360612	A	C	0.101	13	38326692	1.58E-06	0.021	14306	23.646
Vascular dementia (multiple infarctions)	family Veillonellaceae	rs9345168	A	C	-0.072	91671204	0.276	0.066	360612	A	C	-0.051	6	92380922	8.49E-06	0.011	14306	20.207
Vascular dementia (multiple infarctions)	family Verrucomicrobiaceae	rs111862613	T	C	0.018	129825125	0.841	0.088	360612	T	C	0.091	12	130309670	3.73E-06	0.020	14306	21.255
Vascular dementia (multiple infarctions)	family Verrucomicrobiaceae	rs117107102	A	G	-0.112	51947265	0.471	0.155	360612	A	G	0.205	18	49473635	2.92E-06	0.043	14306	22.493
Vascular dementia (multiple infarctions)	family Verrucomicrobiaceae	rs11729256	T	C	0.095	94106121	0.275	0.087	360612	T	C	0.075	4	95027272	6.73E-07	0.015	14306	24.928
Vascular dementia (multiple infarctions)	family Verrucomicrobiaceae	rs12908520	G	A	0.119	97027427	0.070	0.066	360612	G	A	0.062	15	97570657	2.15E-06	0.013	14306	22.353
Vascular dementia (multiple infarctions)	family Verrucomicrobiaceae	rs2602429	T	C	-0.122	81029544	0.104	0.075	360612	T	C	-0.075	16	81063149	2.70E-06	0.016	14306	22.781
Vascular dementia (multiple infarctions)	family Verrucomicrobiaceae	rs4242783	A	G	-0.054	5022135	0.462	0.073	360612	A	G	-0.069	10	5064327	2.75E-06	0.015	14306	21.699
Vascular dementia (multiple infarctions)	family Verrucomicrobiaceae	rs4936098	G	A	0.069	130410772	0.311	0.069	360612	G	A	-0.065	11	130280667	1.13E-06	0.014	14306	22.775
Vascular dementia (multiple infarctions)	family Verrucomicrobiaceae	rs61779207	G	A	-0.187	40608800	0.019	0.079	360612	G	A	-0.076	1	41074472	6.63E-06	0.017	14306	20.459
Vascular dementia (multiple infarctions)	family Verrucomicrobiaceae	rs74542928	T	C	-0.146	99623031	0.332	0.151	360612	T	C	0.112	4	100544188	1.65E-06	0.024	14306	22.492
Vascular dementia (multiple infarctions)	family Verrucomicrobiaceae	rs9349825	A	G	0.081	56476683	0.333	0.084	360612	A	G	-0.070	6	56341481	2.51E-06	0.015	14306	22.919
Vascular dementia (multiple infarctions)	family Verrucomicrobiaceae	rs941682	G	A	0.115	33280034	0.117	0.073	360612	G	A	-0.063	20	31867840	9.58E-06	0.014	14306	19.296
Vascular dementia (multiple infarctions)	family Victivallaceae	rs11671100	A	C	-0.146	711637	0.073	0.081	360612	A	C	-0.160	19	711637	4.08E-06	0.035	14306	20.970
Vascular dementia (multiple infarctions)	family Victivallaceae	rs11764871	G	T	-0.008	147111885	0.912	0.071	360612	G	T	0.127	7	146808977	7.49E-07	0.026	14306	24.542
Vascular dementia (multiple infarctions)	family Victivallaceae	rs2944282	T	C	-0.033	57530097	0.653	0.073	360612	T	C	-0.124	7	57589803	1.57E-06	0.026	14306	23.349
Vascular dementia (multiple infarctions)	family Victivallaceae	rs34962571	A	C	-0.130	130618711	0.242	0.111	360612	A	C	-0.187	12	131103256	6.25E-06	0.042	14306	19.963
Vascular dementia (multiple infarctions)	family Victivallaceae	rs4396289	C	T	0.013	9295183	0.890	0.096	360612	C	T	-0.153	11	9316730	1.54E-07	0.029	14306	28.090
Vascular dementia (multiple infarctions)	family Victivallaceae	rs61702987	T	C	-0.003	30988791	0.979	0.104	360612	T	C	0.146	2	31211657	3.08E-06	0.030	14306	23.522
Vascular dementia (multiple infarctions)	family Victivallaceae	rs62570196	C	T	-0.166	108323890	0.307	0.163	360612	C	T	-0.246	9	111086170	2.70E-07	0.048	14306	25.953
Vascular dementia (multiple infarctions)	family Victivallaceae	rs6545794	A	G	0.053	60257774	0.601	0.100	360612	A	G	-0.198	2	60484909	5.97E-07	0.041	14306	23.215
Vascular dementia (multiple infarctions)	family Victivallaceae	rs7077363	G	A	-0.075	93527197	0.392	0.088	360612	G	A	0.149	10	95286954	2.83E-06	0.032	14306	21.887
Vascular dementia (multiple infarctions)	family Victivallaceae	rs7314815	G	A	-0.068	102131269	0.301	0.065	360612	G	A	0.101	12	102525047	6.40E-06	0.023	14306	20.079
Vascular dementia (multiple infarctions)	family Victivallaceae	rs7627405	C	T	0.088	9926871	0.284	0.082	360612	C	T	-0.134	3	9968555	8.19E-06	0.030	14306	19.809
Vascular dementia (multiple infarctions)	genus Actinomyces	rs2715439	T	C	0.124	98949084	0.059	0.066	360612	T	C	-0.075	15	99492313	6.27E-06	0.016	14306	20.523
Vascular dementia (multiple infarctions)	genus Actinomyces	rs34583783	G	T	-0.097	66497478	0.478	0.137	360612	G	T	0.127	6	67207371	4.49E-06	0.027	14306	22.237
Vascular dementia (multiple infarctions)	genus Actinomyces	rs35011108	A	G	0.063	132686341	0.624	0.129	360612	A	G	0.233	6	133007480	6.34E-06	0.051	14306	20.641
Vascular dementia (multiple infarctions)	genus Actinomyces	rs4073240	G	A	-0.076	168824686	0.259	0.067	360612	G	A	0.075	6	169224781	7.94E-06	0.017	14306	20.064

Vascular dementia (multiple infarctions)	genus Actinomyces	rs4146653	G	A	-0.107	4740649	0.253	0.094	360612	G	A	0.099	10	4782841	4.50E-06	0.021	14306	21.159
Vascular dementia (multiple infarctions)	genus Actinomyces	rs71315246	A	G	0.103	101633595	0.281	0.096	360612	A	G	-0.097	3	101352439	9.83E-06	0.022	14306	19.566
Vascular dementia (multiple infarctions)	genus Actinomyces	rs7915461	C	T	0.067	125843552	0.618	0.134	360612	C	T	-0.188	10	127532121	5.92E-06	0.040	14306	21.855
Vascular dementia (multiple infarctions)	genus Adlercreutzia	rs11604400	C	T	-0.205	98515355	0.054	0.107	360612	C	T	-0.103	11	98386085	9.74E-06	0.023	14306	19.060
Vascular dementia (multiple infarctions)	genus Adlercreutzia	rs13231526	C	A	0.098	48804555	0.420	0.122	360612	C	A	0.143	7	48844151	4.81E-06	0.031	14306	21.123
Vascular dementia (multiple infarctions)	genus Adlercreutzia	rs2717140	C	T	-0.063	77297814	0.564	0.110	360612	C	T	-0.119	18	75009770	2.05E-06	0.025	14306	22.548
Vascular dementia (multiple infarctions)	genus Adlercreutzia	rs55719207	G	A	-0.018	105637127	0.786	0.067	360612	G	A	-0.070	3	105355971	9.61E-06	0.016	14306	19.577
Vascular dementia (multiple infarctions)	genus Adlercreutzia	rs6664405	T	C	-0.071	68978480	0.447	0.093	360612	T	C	-0.095	1	69444163	5.23E-06	0.021	14306	20.451
Vascular dementia (multiple infarctions)	genus Adlercreutzia	rs7680684	T	C	0.019	170360208	0.783	0.069	360612	T	C	0.083	4	171281359	9.77E-07	0.017	14306	24.371
Vascular dementia (multiple infarctions)	genus Adlercreutzia	rs9490822	C	T	-0.001	123587938	0.982	0.066	360612	C	T	-0.073	6	123909083	2.54E-06	0.016	14306	22.229
Vascular dementia (multiple infarctions)	genus Adlercreutzia	rs9915817	C	T	-0.041	50786818	0.576	0.073	360612	C	T	-0.075	17	48864179	8.22E-06	0.017	14306	19.810
Vascular dementia (multiple infarctions)	genus Akkermansia	rs111862613	T	C	0.018	129825125	0.841	0.088	360612	T	C	0.091	12	130309670	3.39E-06	0.020	14306	21.449
Vascular dementia (multiple infarctions)	genus Akkermansia	rs117107102	A	G	-0.112	51947265	0.471	0.155	360612	A	G	0.204	18	49473635	3.01E-06	0.043	14306	22.427
Vascular dementia (multiple infarctions)	genus Akkermansia	rs11729256	T	C	0.095	94106121	0.275	0.087	360612	T	C	0.075	4	95027272	6.58E-07	0.015	14306	24.970
Vascular dementia (multiple infarctions)	genus Akkermansia	rs12908520	G	A	0.119	97027427	0.070	0.066	360612	G	A	0.062	15	97570657	2.26E-06	0.013	14306	22.251
Vascular dementia (multiple infarctions)	genus Akkermansia	rs2602429	T	C	-0.122	81029544	0.104	0.075	360612	T	C	-0.075	16	81063149	2.72E-06	0.016	14306	22.770
Vascular dementia (multiple infarctions)	genus Akkermansia	rs4242783	A	G	-0.054	5022135	0.462	0.073	360612	A	G	-0.069	10	5064327	3.00E-06	0.015	14306	21.537
Vascular dementia (multiple infarctions)	genus Akkermansia	rs4936098	G	A	0.069	130410772	0.311	0.069	360612	G	A	-0.065	11	130280667	1.10E-06	0.014	14306	22.810
Vascular dementia (multiple infarctions)	genus Akkermansia	rs61779207	G	A	-0.187	40608800	0.019	0.079	360612	G	A	-0.076	1	41074472	6.32E-06	0.017	14306	20.550
Vascular dementia (multiple infarctions)	genus Akkermansia	rs74542928	T	C	-0.146	99623031	0.332	0.151	360612	T	C	0.113	4	100544188	1.48E-06	0.024	14306	22.690
Vascular dementia (multiple infarctions)	genus Akkermansia	rs9349825	A	G	0.081	56476683	0.333	0.084	360612	A	G	-0.070	6	56341481	2.60E-06	0.015	14306	22.856
Vascular dementia (multiple infarctions)	genus Akkermansia	rs941682	G	A	0.115	33280034	0.117	0.073	360612	G	A	-0.063	20	31867840	9.17E-06	0.014	14306	19.381
Vascular dementia (multiple infarctions)	genus Alistipes	rs1107244	G	A	0.048	37483220	0.691	0.121	360612	G	A	0.076	13	38057357	3.59E-06	0.017	14306	19.636
Vascular dementia (multiple infarctions)	genus Alistipes	rs11769002	G	A	0.065	62983120	0.332	0.067	360612	G	A	-0.053	7	62443498	1.45E-06	0.011	14306	23.368
Vascular dementia (multiple infarctions)	genus Alistipes	rs11958296	A	G	0.047	178328178	0.767	0.157	360612	A	G	-0.098	5	177755179	9.30E-06	0.022	14306	20.209
Vascular dementia (multiple infarctions)	genus Alistipes	rs12990744	C	T	0.017	177136221	0.873	0.105	360612	C	T	-0.078	2	178000949	8.21E-06	0.017	14306	20.155
Vascular dementia (multiple infarctions)	genus Alistipes	rs1689282	A	C	-0.022	14080130	0.753	0.070	360612	A	C	-0.052	9	14080129	5.28E-06	0.011	14306	20.832
Vascular dementia (multiple infarctions)	genus Alistipes	rs2290844	C	T	-0.152	126098573	0.149	0.105	360612	C	T	0.081	10	127787142	9.10E-06	0.019	14306	18.042
Vascular dementia (multiple infarctions)	genus Alistipes	rs2450745	A	C	0.191	143346550	0.135	0.128	360612	A	C	-0.081	8	144428720	7.12E-06	0.018	14306	19.007
Vascular dementia (multiple infarctions)	genus Alistipes	rs2875322	C	T	0.010	131017657	0.905	0.087	360612	C	T	0.058	11	130887552	8.78E-06	0.013	14306	19.537
Vascular dementia (multiple infarctions)	genus Alistipes	rs34417064	A	G	-0.068	54699118	0.302	0.066	360612	A	G	-0.048	17	52776479	7.01E-06	0.011	14306	20.344
Vascular dementia (multiple infarctions)	genus Alistipes	rs4810359	A	G	-0.030	42562695	0.765	0.099	360612	A	G	-0.065	20	41191335	7.50E-06	0.015	14306	19.927
Vascular dementia (multiple infarctions)	genus Alistipes	rs7129639	A	C	0.055	17489880	0.427	0.069	360612	A	C	0.052	11	17511427	1.78E-06	0.011	14306	22.948
Vascular dementia (multiple infarctions)	genus Alistipes	rs8130320	A	G	0.010	39208332	0.881	0.066	360612	A	G	-0.049	21	40580258	4.84E-06	0.011	14306	20.906
Vascular dementia (multiple infarctions)	genus Allisonella	rs1901739	G	T	0.041	114951010	0.528	0.066	360612	G	T	-0.116	5	114286707	3.59E-06	0.025	14306	21.682
Vascular dementia (multiple infarctions)	genus Allisonella	rs35110698	T	C	-0.011	27866065	0.903	0.093	360612	T	C	-0.146	12	28018998	5.72E-06	0.032	14306	20.797
Vascular dementia (multiple infarctions)	genus Allisonella	rs35778461	C	T	0.017	97388274	0.828	0.078	360612	C	T	0.147	9	100150556	1.21E-06	0.030	14306	24.360
Vascular dementia (multiple infarctions)	genus Allisonella	rs594561	T	C	-0.001	88899804	0.982	0.065	360612	T	C	-0.112	11	88632972	9.41E-06	0.025	14306	19.885
Vascular dementia (multiple infarctions)	genus Allisonella	rs602075	G	A	-0.013	76495244	0.857	0.074	360612	G	A	-0.169	9	79110160	3.57E-08	0.030	14306	32.374
Vascular dementia (multiple infarctions)	genus Allisonella	rs6742198	A	G	-0.108	33382004	0.159	0.077	360612	A	G	-0.149	2	33607071	3.35E-06	0.032	14306	22.210
Vascular dementia (multiple infarctions)	genus Allisonella	rs76904847	G	A	0.064	138283265	0.463	0.087	360612	G	A	0.149	7	137968010	6.09E-06	0.033	14306	19.673
Vascular dementia (multiple infarctions)	genus Allisonella	rs7898615	T	G	-0.053	120653939	0.585	0.096	360612	T	G	0.168	10	122413451	8.87E-06	0.037	14306	20.214
Vascular dementia (multiple infarctions)	genus Alloprevotella	rs2154444	G	T	0.039	34681344	0.594	0.074	360612	G	T	-0.138	21	36053643	8.37E-06	0.031	14306	20.018
Vascular dementia (multiple infarctions)	genus Alloprevotella	rs34619204	G	A	-0.001	39621081	0.987	0.086	360612	G	A	-0.156	21	40993008	8.84E-06	0.034	14306	20.528
Vascular dementia (multiple infarctions)	genus Alloprevotella	rs4364940	A	G	-0.064	233990770	0.363	0.071	360612	A	G	0.126	1	234126516	8.58E-06	0.028	14306	20.067
Vascular dementia (multiple infarctions)	genus Alloprevotella	rs4680035	G	A	0.101	153087811	0.132	0.067	360612	G	A	0.120	3	152805600	4.99E-06	0.026	14306	21.250
Vascular dementia (multiple infarctions)	genus Alloprevotella	rs58212166	A	G	0.053	188155551	0.532	0.085	360612	A	G	-0.162	4	189076705	7.94E-06	0.036	14306	20.227
Vascular dementia (multiple infarctions)	genus Anaerofilum	rs10794359	C	T	-0.013	1051715	0.849	0.067	360612	C	T	0.095	11	1051715	2.23E-06	0.020	14306	22.594
Vascular dementia (multiple infarctions)	genus Anaerofilum	rs1563175	A	C	0.021	3791350	0.744	0.066	360612	A	C	0.092	2	3838940	5.54E-06	0.020	14306	20.884
Vascular dementia (multiple infarctions)	genus Anaerofilum	rs17012738	T	G	-0.030	76471016	0.648	0.065	360612	T	G	0.090	2	76698142	7.24E-06	0.020	14306	20.355
Vascular dementia (multiple infarctions)	genus Anaerofilum	rs17096874	C	T	-0.040	30522152	0.615	0.080	360612	C	T	-0.126	14	30991358	2.86E-06	0.027	14306	22.071
Vascular dementia (multiple infarctions)	genus Anaerofilum	rs4244069	A	G	-0.104	66773681	0.293	0.099	360612	A	G	0.147	12	67167461	9.81E-06	0.033	14306	20.197
Vascular dementia (multiple infarctions)	genus Anaerofilum	rs4506496	A	G	-0.124	246982388	0.085	0.072	360612	A	G	-0.103	1	247145690	1.49E-06	0.021	14306	23.455
Vascular dementia (multiple infarctions)	genus Anaerofilum	rs712981	A	C	-0.130	129967591	0.054	0.067	360612	A	C	0.101	3	129686434	6.83E-07	0.020	14306	24.661
Vascular dementia (multiple infarctions)	genus Anaerofilum	rs79598899	C	T	-0.125	190274425	0.419	0.154	360612	C	T	0.183	2	191139151	3.75E-07	0.036	14306	26.116
Vascular dementia (multiple infarctions)	genus Anaerofilum	rs816292	T	C	0.127	117373604	0.078	0.072	360612	T	C	-0.113	12	117811409	2.64E-07	0.022	14306	26.288
Vascular dementia (multiple infarctions)	genus Anaerofilum	rs9299345	T	C	0.036	101577530	0.744	0.110	360612	T	C	-0.136	9	104339812	8.04E-06	0.030	14306	20.349
Vascular dementia (multiple infarctions)	genus Anaerostipes	rs10502061	A	G	-0.039	105744527	0.704	0.102	360612	A	G	0.084	11	105615253	7.94E-06	0.019	14306	18.944
Vascular dementia (multiple infarctions)	genus Anaerostipes	rs2014785	T	C	0.004	171382313	0.951	0.066	360612	T	C	0.052	3	171100102	4.68E-06	0.011	14306	21.125
Vascular dementia (multiple infarctions)	genus Anaerostipes	rs2396460	C	T	-0.152	227153955	0.021	0.066	360612	C	T	0.051	2	228018671	2.91E-06	0.011	14306	21.897
Vascular dementia (multiple infarctions)	genus Anaerostipes	rs2804244	G	A	-0.099	115623674	0.143	0.067	360612	G	A	0.053	10	117383184	2.04E-06	0.011	14306	22.882
Vascular dementia (multiple infarctions)	genus Anaerostipes	rs3900776	G	A	-0.050	13525083	0.796	0.193	360612	G	A	-0.110	9	13525082	2.75E-06	0.024	14306	21.675
Vascular dementia (multiple infarctions)	genus Anaerostipes	rs60983350	G	A	0.010	2947443	0.884	0.070	360612	G	A	-0.054	17	2850737	4.42E-06	0.012	14306	21.450
Vascular dementia (multiple infarctions)	genus Anaerostipes	rs62157625	T	C	0.053	142016764	0.601	0.102	360612	T	C	0.089	2	142774333	1.45E-06	0.019	14306	22.787
Vascular dementia (multiple infarctions)	genus Anaerostipes	rs62215703	G	A	0.016	24501310	0.842	0.080	360612	G	A	0.064	21	25873624	1.98E-06	0.014	14306	22.260
Vascular dementia (multiple infarctions)	genus Anaerostipes	rs6474958	G	A	-0.141	1582701	0.047	0.071	360612	G	A	0.050	9	1582701	6.74E-06	0.011	14306	19.935
Vascular dementia (multiple infarctions)	genus Anaerostipes	rs6726833	C	A	-0.064	39124428	0.598	0.121	360612	C	A	-0.088	2	39351569	3.32E-06	0.019	14306	21.463

Vascular dementia (multiple infarctions)	genus Anaerostipes	rs6854026	C	T	0.060	168769663	0.367	0.066	360612	C	T	0.051	4	169690814	3.20E-06	0.011	14306	21.732
Vascular dementia (multiple infarctions)	genus Anaerostipes	rs7193624	T	C	-0.099	77540123	0.402	0.118	360612	T	C	-0.075	16	77574020	5.35E-07	0.015	14306	24.803
Vascular dementia (multiple infarctions)	genus Anaerostipes	rs78735375	A	C	0.187	1497548	0.263	0.167	360612	A	C	-0.137	19	1497547	5.33E-06	0.031	14306	20.262
Vascular dementia (multiple infarctions)	genus Anaerotruncus	rs10150232	A	G	-0.112	29948802	0.170	0.081	360612	A	G	0.057	14	30418008	6.68E-06	0.012	14306	20.622
Vascular dementia (multiple infarctions)	genus Anaerotruncus	rs11018566	A	G	-0.063	89307058	0.665	0.146	360612	A	G	-0.156	11	89040226	6.14E-06	0.037	14306	18.272
Vascular dementia (multiple infarctions)	genus Anaerotruncus	rs115414803	A	C	-0.005	87242091	0.973	0.136	360612	A	C	-0.144	4	88163243	6.83E-06	0.032	14306	20.669
Vascular dementia (multiple infarctions)	genus Anaerotruncus	rs1272208	T	G	-0.043	76015978	0.574	0.077	360612	T	G	0.061	9	78630894	4.28E-06	0.013	14306	22.201
Vascular dementia (multiple infarctions)	genus Anaerotruncus	rs1431492	C	T	-0.010	151137584	0.911	0.090	360612	C	T	-0.065	3	150855371	7.36E-06	0.015	14306	20.075
Vascular dementia (multiple infarctions)	genus Anaerotruncus	rs17734739	T	C	0.042	210798978	0.653	0.094	360612	T	C	0.066	2	211663702	7.43E-06	0.015	14306	19.603
Vascular dementia (multiple infarctions)	genus Anaerotruncus	rs34449434	A	C	-0.027	76129875	0.691	0.068	360612	A	C	-0.050	12	76523655	9.85E-06	0.011	14306	19.208
Vascular dementia (multiple infarctions)	genus Anaerotruncus	rs4669806	G	T	-0.065	12060626	0.414	0.079	360612	G	T	0.058	2	12200752	2.42E-06	0.012	14306	21.962
Vascular dementia (multiple infarctions)	genus Anaerotruncus	rs6494922	A	G	0.089	33167666	0.534	0.143	360612	A	G	0.090	15	33459867	6.62E-06	0.020	14306	19.937
Vascular dementia (multiple infarctions)	genus Anaerotruncus	rs6563550	T	C	0.048	37484276	0.691	0.121	360612	T	C	0.088	13	38058413	2.35E-07	0.018	14306	24.629
Vascular dementia (multiple infarctions)	genus Anaerotruncus	rs7155595	C	A	0.120	77036203	0.095	0.072	360612	C	A	0.054	14	77502546	7.55E-06	0.012	14306	20.575
Vascular dementia (multiple infarctions)	genus Anaerotruncus	rs8005030	C	T	-0.066	30137993	0.339	0.069	360612	C	T	0.055	14	30607199	2.28E-06	0.012	14306	22.133
Vascular dementia (multiple infarctions)	genus Anaerotruncus	rs9347879	T	C	0.130	164594228	0.047	0.066	360612	T	C	0.051	6	165015261	4.22E-06	0.011	14306	20.988
Vascular dementia (multiple infarctions)	genus Bacteroides	rs11585893	A	G	0.016	10584294	0.838	0.076	360612	A	G	-0.074	1	10644351	1.80E-06	0.015	14306	25.175
Vascular dementia (multiple infarctions)	genus Bacteroides	rs13207588	A	G	-0.082	41551692	0.326	0.083	360612	A	G	-0.059	6	41519430	7.48E-06	0.013	14306	20.365
Vascular dementia (multiple infarctions)	genus Bacteroides	rs1340391	T	C	0.030	102495433	0.755	0.095	360612	T	C	-0.059	1	102960989	6.73E-06	0.013	14306	20.040
Vascular dementia (multiple infarctions)	genus Bacteroides	rs17619981	T	G	-0.058	24159448	0.544	0.096	360612	T	G	0.088	19	24342250	2.69E-06	0.019	14306	22.194
Vascular dementia (multiple infarctions)	genus Bacteroides	rs2023437	T	C	-0.196	21577814	0.050	0.100	360612	T	C	-0.078	14	22045949	5.02E-06	0.017	14306	21.780
Vascular dementia (multiple infarctions)	genus Bacteroides	rs66710942	T	C	-0.028	77166176	0.668	0.065	360612	T	C	-0.049	3	77215327	5.86E-06	0.011	14306	20.644
Vascular dementia (multiple infarctions)	genus Bacteroides	rs6795673	C	T	0.027	10551540	0.677	0.066	360612	C	T	0.054	3	10593224	3.38E-07	0.011	14306	26.183
Vascular dementia (multiple infarctions)	genus Bacteroides	rs9507307	C	T	-0.029	24336338	0.699	0.076	360612	C	T	0.060	13	24910476	2.13E-06	0.013	14306	21.912
Vascular dementia (multiple infarctions)	genus Barnesiella	rs11155559	T	C	-0.313	148280769	0.010	0.121	360612	T	C	0.096	6	148601905	8.92E-06	0.021	14306	20.153
Vascular dementia (multiple infarctions)	genus Barnesiella	rs12909713	C	T	-0.034	86602682	0.598	0.065	360612	C	T	-0.055	15	87145913	4.95E-06	0.012	14306	21.048
Vascular dementia (multiple infarctions)	genus Barnesiella	rs13242616	C	T	0.066	122381464	0.349	0.071	360612	C	T	0.058	7	122021518	2.29E-06	0.012	14306	22.459
Vascular dementia (multiple infarctions)	genus Barnesiella	rs199035	G	A	-0.110	23435187	0.094	0.066	360612	G	A	0.056	6	23435415	3.00E-06	0.012	14306	21.842
Vascular dementia (multiple infarctions)	genus Barnesiella	rs2428166	G	A	0.626	110509945	0.020	0.269	360612	G	A	-0.166	6	110831148	8.51E-07	0.034	14306	24.178
Vascular dementia (multiple infarctions)	genus Barnesiella	rs35177866	A	G	-0.106	181871367	0.396	0.125	360612	A	G	0.092	3	181589155	2.95E-06	0.019	14306	23.267
Vascular dementia (multiple infarctions)	genus Barnesiella	rs62251337	G	A	-0.116	44285937	0.215	0.093	360612	G	A	0.069	3	44327429	4.24E-06	0.015	14306	21.385
Vascular dementia (multiple infarctions)	genus Barnesiella	rs72684847	T	C	0.212	101695712	0.087	0.124	360612	T	C	-0.114	4	102616869	6.76E-06	0.025	14306	20.295
Vascular dementia (multiple infarctions)	genus Barnesiella	rs76181748	C	T	0.015	104446188	0.850	0.078	360612	C	T	-0.078	8	105458416	6.78E-06	0.017	14306	20.575
Vascular dementia (multiple infarctions)	genus Barnesiella	rs77455852	T	G	-0.087	54811208	0.352	0.093	360612	T	G	-0.089	5	54107036	3.16E-06	0.020	14306	20.773
Vascular dementia (multiple infarctions)	genus Barnesiella	rs79795328	A	G	0.008	113625439	0.930	0.095	360612	A	G	-0.082	4	114546595	4.23E-06	0.018	14306	21.527
Vascular dementia (multiple infarctions)	genus Bifidobacterium	rs12022129	A	G	0.144	206830029	0.049	0.073	360612	A	G	-0.062	1	207003374	8.00E-06	0.014	14306	19.872
Vascular dementia (multiple infarctions)	genus Bifidobacterium	rs182549	T	C	-0.125	135859184	0.065	0.068	360612	T	C	-0.120	2	136616754	1.28E-20	0.013	14306	88.429
Vascular dementia (multiple infarctions)	genus Bifidobacterium	rs2491158	A	G	0.018	124401134	0.846	0.094	360612	A	G	-0.071	10	126089703	8.05E-06	0.016	14306	19.879
Vascular dementia (multiple infarctions)	genus Bifidobacterium	rs4957061	T	C	0.107	520981	0.107	0.067	360612	T	C	0.053	5	521096	5.78E-06	0.012	14306	20.697
Vascular dementia (multiple infarctions)	genus Bifidobacterium	rs540489	T	G	-0.123	74901626	0.152	0.086	360612	T	G	-0.064	17	72897722	5.19E-06	0.014	14306	21.121
Vascular dementia (multiple infarctions)	genus Bifidobacterium	rs55888705	A	G	0.029	1516099	0.687	0.072	360612	A	G	0.055	4	1517826	6.67E-06	0.012	14306	20.339
Vascular dementia (multiple infarctions)	genus Bifidobacterium	rs5746486	T	C	-0.033	17871506	0.627	0.067	360612	T	C	-0.054	22	18354272	9.00E-06	0.012	14306	19.703
Vascular dementia (multiple infarctions)	genus Bifidobacterium	rs62181700	G	A	-0.209	188941058	0.006	0.077	360612	G	A	-0.062	2	189805784	2.17E-06	0.013	14306	22.665
Vascular dementia (multiple infarctions)	genus Bifidobacterium	rs7322849	T	C	-0.075	112205515	0.511	0.114	360612	T	C	0.112	13	112859829	1.08E-08	0.020	14306	31.035
Vascular dementia (multiple infarctions)	genus Bifidobacterium	rs75344046	C	T	-0.067	30489472	0.668	0.157	360612	C	T	0.232	21	31861790	4.86E-06	0.051	14306	21.088
Vascular dementia (multiple infarctions)	genus Bifidobacterium	rs857444	C	T	-0.109	14617360	0.107	0.068	360612	C	T	0.056	6	14617591	3.57E-06	0.012	14306	21.208
Vascular dementia (multiple infarctions)	genus Bilophila	rs11069458	C	T	-0.007	101750335	0.935	0.085	360612	C	T	0.068	13	102402685	7.72E-06	0.016	14306	19.293
Vascular dementia (multiple infarctions)	genus Bilophila	rs1241171	G	A	-0.053	102834365	0.566	0.093	360612	G	A	-0.069	1	103299921	4.24E-06	0.015	14306	21.281
Vascular dementia (multiple infarctions)	genus Bilophila	rs1571225	T	C	-0.073	4940871	0.402	0.087	360612	T	C	-0.083	9	4940871	1.12E-06	0.017	14306	23.484
Vascular dementia (multiple infarctions)	genus Bilophila	rs1969927	A	G	0.004	100393069	0.950	0.069	360612	A	G	-0.056	12	100786847	9.07E-06	0.013	14306	19.783
Vascular dementia (multiple infarctions)	genus Bilophila	rs2728491	T	G	0.068	47172134	0.376	0.076	360612	T	G	0.063	7	47211732	6.33E-06	0.014	14306	20.241
Vascular dementia (multiple infarctions)	genus Bilophila	rs3827020	C	T	0.041	63349639	0.617	0.082	360612	C	T	0.077	20	61980991	1.79E-06	0.016	14306	22.766
Vascular dementia (multiple infarctions)	genus Bilophila	rs4798126	G	A	-0.013	3765773	0.880	0.083	360612	G	A	0.073	18	3765773	7.15E-06	0.017	14306	19.033
Vascular dementia (multiple infarctions)	genus Bilophila	rs542415	T	C	0.016	49719357	0.812	0.067	360612	T	C	-0.061	15	50011554	4.71E-06	0.013	14306	21.147
Vascular dementia (multiple infarctions)	genus Bilophila	rs60178956	G	A	0.065	89866110	0.403	0.078	360612	G	A	-0.062	8	90878338	8.06E-06	0.014	14306	19.502
Vascular dementia (multiple infarctions)	genus Bilophila	rs6793291	A	C	0.044	194698802	0.766	0.147	360612	A	C	-0.113	3	194419531	3.11E-06	0.024	14306	21.765
Vascular dementia (multiple infarctions)	genus Bilophila	rs72676854	T	C	0.043	108978710	0.777	0.150	360612	T	C	0.123	8	109990939	5.62E-06	0.027	14306	21.007
Vascular dementia (multiple infarctions)	genus Bilophila	rs7802841	A	C	-0.061	138899094	0.394	0.071	360612	A	C	-0.067	7	138583840	1.77E-06	0.014	14306	23.681
Vascular dementia (multiple infarctions)	genus Bilophila	rs9899990	A	G	0.062	9218262	0.606	0.120	360612	A	G	-0.103	17	9121579	9.07E-06	0.023	14306	19.283
Vascular dementia (multiple infarctions)	genus Butyricicoccus	rs10084203	G	A	0.076	190439423	0.431	0.097	360612	G	A	-0.055	2	191304149	8.59E-06	0.012	14306	19.791
Vascular dementia (multiple infarctions)	genus Butyricicoccus	rs12034718	G	A	-0.060	66913912	0.446	0.078	360612	G	A	-0.070	1	67379595	9.58E-06	0.016	14306	19.643
Vascular dementia (multiple infarctions)	genus Butyricicoccus	rs12585793	T	C	0.168	26444103	0.400	0.200	360612	T	C	-0.262	13	27018240	5.79E-06	0.056	14306	21.558
Vascular dementia (multiple infarctions)	genus Butyricicoccus	rs2017189	T	G	-0.080	7458426	0.220	0.065	360612	T	G	0.051	4	7460153	3.87E-06	0.011	14306	21.148
Vascular dementia (multiple infarctions)	genus Butyricicoccus	rs4962426	T	G	-0.073	125219639	0.376	0.082	360612	T	G	-0.061	10	126908208	7.38E-06	0.014	14306	20.403
Vascular dementia (multiple infarctions)	genus Butyricicoccus	rs56221232	T	C	-0.299	152596896	0.009	0.114	360612	T	C	0.083	2	153453410	7.62E-07	0.017	14306	24.467
Vascular dementia (multiple infarctions)	genus Butyricicoccus	rs62478070	T	G	-0.359	157895458	0.081	0.206	360612	T	G	0.224	7	157688150	5.94E-06	0.049	14306	20.488
Vascular dementia (multiple infarctions)	genus Butyricicoccus	rs7322368	C	T	-0.027	99561736	0.815	0.114	360612	C	T	-0.082	13	100213990	5.52E-06	0.018	14306	19.834

Vascular dementia (multiple infarctions)	genus Butyricimonas	rs11228830	A	G	0.083	56897486	0.485	0.118	360612	A	G	0.135	11	56664962	6.55E-06	0.030	14306	20.547
Vascular dementia (multiple infarctions)	genus Butyricimonas	rs113054641	G	A	-0.194	15178069	0.226	0.161	360612	G	A	-0.145	21	16550389	1.74E-07	0.027	14306	27.842
Vascular dementia (multiple infarctions)	genus Butyricimonas	rs12304031	G	A	0.018	128954226	0.859	0.100	360612	G	A	-0.086	12	129438771	6.70E-06	0.020	14306	19.211
Vascular dementia (multiple infarctions)	genus Butyricimonas	rs12458763	A	C	-0.036	36511655	0.820	0.158	360612	A	C	0.122	18	34091618	6.37E-06	0.027	14306	20.492
Vascular dementia (multiple infarctions)	genus Butyricimonas	rs1862649	G	A	-0.013	22161545	0.915	0.127	360612	G	A	0.113	16	22172866	4.76E-06	0.025	14306	20.777
Vascular dementia (multiple infarctions)	genus Butyricimonas	rs2114713	G	T	-0.084	80236031	0.202	0.066	360612	G	T	0.063	15	80528373	6.88E-06	0.014	14306	20.361
Vascular dementia (multiple infarctions)	genus Butyricimonas	rs62130338	A	G	0.075	48659244	0.276	0.069	360612	A	G	0.073	19	49162501	3.90E-06	0.016	14306	21.412
Vascular dementia (multiple infarctions)	genus Butyricimonas	rs62390301	T	C	-0.007	160440640	0.934	0.081	360612	T	C	-0.087	5	159867647	7.42E-07	0.017	14306	24.915
Vascular dementia (multiple infarctions)	genus Butyricimonas	rs7083431	A	C	0.120	70951399	0.101	0.073	360612	A	C	0.070	10	72711156	8.85E-07	0.014	14306	23.725
Vascular dementia (multiple infarctions)	genus Butyricimonas	rs71428626	G	T	-0.259	82866140	0.152	0.181	360612	G	T	-0.133	2	83093264	4.80E-06	0.029	14306	21.107
Vascular dementia (multiple infarctions)	genus Butyricimonas	rs72814525	A	G	0.166	70683314	0.028	0.076	360612	A	G	0.066	10	72443070	8.25E-06	0.015	14306	19.666
Vascular dementia (multiple infarctions)	genus Butyricimonas	rs78453362	A	G	0.426	78690859	0.042	0.209	360612	A	G	-0.149	3	78740009	4.06E-06	0.033	14306	20.887
Vascular dementia (multiple infarctions)	genus Butyricimonas	rs9657374	C	T	0.142	4976884	0.048	0.072	360612	C	T	0.068	8	4834406	4.50E-06	0.015	14306	21.106
Vascular dementia (multiple infarctions)	genus Butyrivibrio	rs1007475	G	T	-0.022	148082269	0.761	0.073	360612	G	T	0.118	6	148403405	7.92E-06	0.026	14306	20.420
Vascular dementia (multiple infarctions)	genus Butyrivibrio	rs11761679	T	C	-0.046	150899841	0.621	0.093	360612	T	C	0.155	7	150596929	2.20E-06	0.032	14306	23.170
Vascular dementia (multiple infarctions)	genus Butyrivibrio	rs142855850	A	G	-0.013	22298971	0.903	0.111	360612	A	G	0.205	10	22587900	6.86E-06	0.046	14306	20.113
Vascular dementia (multiple infarctions)	genus Butyrivibrio	rs16934069	T	C	0.004	116930885	0.965	0.086	360612	T	C	-0.134	9	119693164	8.86E-06	0.030	14306	19.961
Vascular dementia (multiple infarctions)	genus Butyrivibrio	rs16941336	C	T	-0.009	20672384	0.912	0.077	360612	C	T	0.127	17	20575697	1.53E-06	0.027	14306	22.640
Vascular dementia (multiple infarctions)	genus Butyrivibrio	rs17163238	G	A	0.053	129640328	0.522	0.083	360612	G	A	0.141	5	128976021	5.51E-06	0.031	14306	20.802
Vascular dementia (multiple infarctions)	genus Butyrivibrio	rs4537857	T	C	-0.073	26718529	0.295	0.070	360612	T	C	-0.125	13	27292666	1.80E-06	0.026	14306	22.788
Vascular dementia (multiple infarctions)	genus Butyrivibrio	rs486484	A	G	0.036	830864	0.586	0.066	360612	A	G	-0.108	20	811507	6.61E-06	0.024	14306	20.380
Vascular dementia (multiple infarctions)	genus Butyrivibrio	rs4928024	G	A	-0.046	54254476	0.589	0.086	360612	G	A	0.175	3	54288503	8.19E-06	0.039	14306	20.137
Vascular dementia (multiple infarctions)	genus Butyrivibrio	rs72723662	C	T	0.046	75599220	0.634	0.096	360612	C	T	0.224	14	76065563	7.86E-07	0.045	14306	24.869
Vascular dementia (multiple infarctions)	genus Butyrivibrio	rs74622183	A	G	0.028	33681771	0.808	0.116	360612	A	G	-0.201	14	34150977	2.46E-06	0.043	14306	22.040
Vascular dementia (multiple infarctions)	genus Butyrivibrio	rs77356209	T	C	-0.082	10267736	0.598	0.155	360612	T	C	0.217	18	10267733	6.66E-06	0.048	14306	20.136
Vascular dementia (multiple infarctions)	genus Butyrivibrio	rs7752361	G	A	0.079	111459502	0.228	0.065	360612	G	A	0.119	6	111780705	7.69E-07	0.024	14306	24.685
Vascular dementia (multiple infarctions)	genus Butyrivibrio	rs7763512	A	G	-0.144	21959860	0.029	0.066	360612	A	G	-0.120	6	21960091	3.11E-06	0.025	14306	22.374
Vascular dementia (multiple infarctions)	genus Butyrivibrio	rs9349693	G	A	-0.034	54074926	0.640	0.072	360612	G	A	-0.118	6	53939724	5.55E-06	0.026	14306	20.610
Vascular dementia (multiple infarctions)	genus Candidatus Soleaferrea	rs10090365	G	A	0.006	137625956	0.927	0.065	360612	G	A	0.083	8	138638199	4.17E-06	0.018	14306	21.255
Vascular dementia (multiple infarctions)	genus Candidatus Soleaferrea	rs10809135	C	T	0.107	10611282	0.107	0.066	360612	C	T	-0.083	9	10611282	5.47E-06	0.018	14306	20.944
Vascular dementia (multiple infarctions)	genus Candidatus Soleaferrea	rs36155147	T	C	-0.028	312402	0.692	0.070	360612	T	C	-0.105	7	352368	5.41E-06	0.024	14306	18.971
Vascular dementia (multiple infarctions)	genus Candidatus Soleaferrea	rs4294381	C	T	0.036	224500405	0.689	0.090	360612	C	T	-0.112	1	224688107	1.37E-06	0.023	14306	23.416
Vascular dementia (multiple infarctions)	genus Candidatus Soleaferrea	rs4678258	T	C	-0.046	138226350	0.551	0.076	360612	T	C	0.099	3	137945192	5.53E-06	0.022	14306	20.912
Vascular dementia (multiple infarctions)	genus Candidatus Soleaferrea	rs6489992	A	G	0.054	114914964	0.425	0.068	360612	A	G	-0.084	12	115352769	7.89E-06	0.019	14306	20.192
Vascular dementia (multiple infarctions)	genus Candidatus Soleaferrea	rs6494306	A	G	-0.061	62102341	0.380	0.070	360612	A	G	-0.097	15	62394540	5.80E-06	0.021	14306	20.474
Vascular dementia (multiple infarctions)	genus Candidatus Soleaferrea	rs7400877	C	T	0.028	75937199	0.724	0.080	360612	C	T	0.095	14	76403542	9.29E-06	0.021	14306	19.976
Vascular dementia (multiple infarctions)	genus Candidatus Soleaferrea	rs9973954	G	A	-0.079	19686146	0.248	0.068	360612	G	A	-0.089	2	19885907	5.95E-06	0.020	14306	20.842
Vascular dementia (multiple infarctions)	genus Catenibacterium	rs12404911	C	T	0.005	239955143	0.951	0.084	360612	C	T	0.141	1	240118443	2.80E-06	0.030	14306	21.406
Vascular dementia (multiple infarctions)	genus Catenibacterium	rs212393	A	G	-0.084	159064710	0.297	0.081	360612	A	G	0.135	6	159485742	3.62E-06	0.029	14306	22.332
Vascular dementia (multiple infarctions)	genus Catenibacterium	rs73128290	A	G	0.081	57296613	0.259	0.072	360612	A	G	0.130	7	57364320	4.29E-06	0.028	14306	20.782
Vascular dementia (multiple infarctions)	genus Catenibacterium	rs7742829	C	T	0.001	104641790	0.991	0.066	360612	C	T	0.114	6	105089665	5.61E-06	0.025	14306	20.652
Vascular dementia (multiple infarctions)	genus Christensenellaceae R 7group	rs10461257	A	G	0.009	155209852	0.900	0.070	360612	A	G	-0.055	4	156131004	6.51E-06	0.012	14306	20.446
Vascular dementia (multiple infarctions)	genus Christensenellaceae R 7group	rs17081797	A	G	0.004	69888324	0.973	0.133	360612	A	G	-0.090	18	67555560	3.34E-06	0.020	14306	19.603
Vascular dementia (multiple infarctions)	genus Christensenellaceae R 7group	rs62132810	A	G	-0.045	48775970	0.630	0.094	360612	A	G	-0.083	19	49279227	5.67E-06	0.018	14306	21.293
Vascular dementia (multiple infarctions)	genus Christensenellaceae R 7group	rs62190261	A	C	-0.162	230078383	0.163	0.116	360612	A	C	0.096	2	230943099	8.74E-06	0.021	14306	19.922
Vascular dementia (multiple infarctions)	genus Christensenellaceae R 7group	rs62467127	C	T	-0.297	118456871	0.156	0.209	360612	C	T	0.114	7	118096925	3.25E-06	0.025	14306	20.506
Vascular dementia (multiple infarctions)	genus Christensenellaceae R 7group	rs73952017	C	T	0.065	1779608	0.545	0.107	360612	C	T	-0.086	18	1779609	8.46E-06	0.019	14306	19.679
Vascular dementia (multiple infarctions)	genus Christensenellaceae R 7group	rs78521377	C	T	-0.101	124759805	0.591	0.188	360612	C	T	0.125	10	126448374	5.61E-06	0.027	14306	20.689
Vascular dementia (multiple infarctions)	genus Christensenellaceae R 7group	rs892686	A	G	-0.027	80428461	0.684	0.066	360612	A	G	0.051	9	83043376	3.97E-06	0.011	14306	21.313
Vascular dementia (multiple infarctions)	genus Clostridium innocuum group	rs10506058	A	G	-0.054	30115552	0.418	0.066	360612	A	G	0.100	12	30268485	8.92E-06	0.022	14306	20.184
Vascular dementia (multiple infarctions)	genus Clostridium innocuum group	rs1942371	G	A	0.221	71607072	0.026	0.100	360612	G	A	-0.158	18	69274308	4.06E-06	0.034	14306	21.343
Vascular dementia (multiple infarctions)	genus Clostridium innocuum group	rs40656	C	T	-0.166	9368046	0.044	0.082	360612	C	T	0.143	5	9368158	8.62E-06	0.031	14306	21.040
Vascular dementia (multiple infarctions)	genus Clostridium innocuum group	rs4869133	G	A	0.022	96381915	0.799	0.087	360612	G	A	-0.181	5	95717619	7.24E-06	0.041	14306	19.448
Vascular dementia (multiple infarctions)	genus Clostridium innocuum group	rs61267978	T	C	-0.046	6348228	0.648	0.101	360612	T	C	0.147	18	6348227	5.59E-06	0.032	14306	21.010
Vascular dementia (multiple infarctions)	genus Clostridium innocuum group	rs6577484	G	A	0.047	8359360	0.659	0.107	360612	G	A	0.160	1	8419420	8.41E-06	0.036	14306	19.764
Vascular dementia (multiple infarctions)	genus Clostridium innocuum group	rs6890185	C	T	-0.043	71890799	0.539	0.070	360612	C	T	-0.113	5	71186626	1.12E-06	0.023	14306	23.669
Vascular dementia (multiple infarctions)	genus Clostridium innocuum group	rs77845139	A	G	0.038	59412009	0.612	0.076	360612	A	G	-0.115	15	59704208	8.41E-06	0.026	14306	19.992
Vascular dementia (multiple infarctions)	genus Clostridium sensustricto1	rs11264403	G	A	-0.035	155706701	0.780	0.125	360612	G	A	-0.139	1	155676492	7.76E-06	0.033	14306	17.288
Vascular dementia (multiple infarctions)	genus Clostridium sensustricto1	rs116847295	C	T	0.071	43091212	0.468	0.098	360612	C	T	0.110	12	43485015	4.58E-06	0.025	14306	19.997
Vascular dementia (multiple infarctions)	genus Clostridium sensustricto1	rs12341505	G	A	0.094	133845759	0.407	0.113	360612	G	A	0.081	9	136710881	4.82E-06	0.018	14306	20.259
Vascular dementia (multiple infarctions)	genus Clostridium sensustricto1	rs2795528	G	A	-0.278	42774816	0.054	0.145	360612	G	A	-0.184	10	43270264	2.72E-06	0.039	14306	22.085
Vascular dementia (multiple infarctions)	genus Clostridium sensustricto1	rs2817172	C	T	-0.020	3124955	0.767	0.067	360612	C	T	0.058	1	3041519	2.77E-06	0.012	14306	21.810
Vascular dementia (multiple infarctions)	genus Clostridium sensustricto1	rs550843	T	C	0.040	165309343	0.583	0.073	360612	T	C	-0.078	6	165722832	2.05E-06	0.017	14306	21.426
Vascular dementia (multiple infarctions)	genus Collinsella	rs10890671	C	T	0.020	107252473	0.764	0.066	360612	C	T	0.054	11	107123199	6.52E-06	0.012	14306	20.446
Vascular dementia (multiple infarctions)	genus Collinsella	rs11597285	G	T	-0.003	8547850	0.964	0.067	360612	G	T	-0.054	10	8589813	9.38E-06	0.012	14306	19.914
Vascular dementia (multiple infarctions)	genus Collinsella	rs1496626	T	C	-0.001	31269935	0.989	0.096	360612	T	C	-0.072	19	31760841	6.78E-06	0.016	14306	19.978
Vascular dementia (multiple infarctions)	genus Collinsella	rs149807560	C	A	-0.153	56015274	0.233	0.128	360612	C	A	-0.104	19	56526640	7.10E-06	0.024	14306	19.545

Vascular dementia (multiple infarctions)	genus Collinsella	rs2103510	G	A	0.074	27983148	0.465	0.102	360612	G	A	0.079	21	29355467	2.42E-06	0.017	14306	21.863
Vascular dementia (multiple infarctions)	genus Collinsella	rs62448871	C	A	-0.092	24425405	0.161	0.066	360612	C	A	-0.054	7	24465024	6.78E-06	0.012	14306	20.164
Vascular dementia (multiple infarctions)	genus Collinsella	rs73052258	G	A	-0.012	194874587	0.923	0.122	360612	G	A	0.093	2	195739311	1.72E-06	0.020	14306	21.067
Vascular dementia (multiple infarctions)	genus Collinsella	rs75672793	A	G	-0.221	148897142	0.145	0.152	360612	A	G	-0.109	4	149818294	6.14E-06	0.024	14306	20.503
Vascular dementia (multiple infarctions)	genus Collinsella	rs9541268	C	A	-0.071	68035929	0.537	0.114	360612	C	A	0.096	13	68610061	8.79E-07	0.020	14306	23.657
Vascular dementia (multiple infarctions)	genus Coprobacter	rs11532348	C	T	-0.046	97896018	0.615	0.092	360612	C	T	-0.104	12	98289796	5.71E-06	0.023	14306	20.994
Vascular dementia (multiple infarctions)	genus Coprobacter	rs12684609	T	C	0.066	134893022	0.423	0.082	360612	T	C	0.101	9	137784868	6.10E-06	0.022	14306	20.935
Vascular dementia (multiple infarctions)	genus Coprobacter	rs12996055	A	C	-0.005	136465888	0.941	0.075	360612	A	C	0.092	2	137223458	8.08E-06	0.021	14306	19.378
Vascular dementia (multiple infarctions)	genus Coprobacter	rs143662916	C	T	0.043	103679246	0.824	0.194	360612	C	T	0.253	11	103549974	3.07E-06	0.054	14306	21.985
Vascular dementia (multiple infarctions)	genus Coprobacter	rs189356	G	A	0.030	58999796	0.651	0.066	360612	G	A	0.078	20	57574851	6.26E-06	0.017	14306	20.645
Vascular dementia (multiple infarctions)	genus Coprobacter	rs213863	T	C	0.043	96787063	0.526	0.068	360612	T	C	0.089	6	97234939	2.35E-06	0.019	14306	22.192
Vascular dementia (multiple infarctions)	genus Coprobacter	rs28402691	T	C	-0.115	95468089	0.212	0.092	360612	T	C	0.111	4	96389240	9.56E-06	0.025	14306	19.419
Vascular dementia (multiple infarctions)	genus Coprobacter	rs305411	A	G	0.097	87767445	0.367	0.108	360612	A	G	0.129	1	88233128	1.01E-06	0.026	14306	23.864
Vascular dementia (multiple infarctions)	genus Coprobacter	rs3828477	G	T	0.035	46442226	0.616	0.069	360612	G	T	-0.091	3	46483717	2.89E-06	0.020	14306	21.728
Vascular dementia (multiple infarctions)	genus Coprobacter	rs72821405	T	C	0.045	4714563	0.696	0.115	360612	T	C	-0.147	6	4714797	4.76E-06	0.032	14306	21.210
Vascular dementia (multiple infarctions)	genus Coprobacter	rs74919520	G	A	-0.115	32017383	0.271	0.105	360612	G	A	0.126	2	32242452	5.76E-06	0.028	14306	20.699
Vascular dementia (multiple infarctions)	genus Coprococcus1	rs1010560	C	A	0.072	29927454	0.331	0.074	360612	C	A	0.058	1	30400301	1.96E-06	0.012	14306	22.354
Vascular dementia (multiple infarctions)	genus Coprococcus1	rs12794898	G	T	0.040	124684165	0.679	0.098	360612	G	T	0.090	11	124554061	4.92E-06	0.020	14306	20.980
Vascular dementia (multiple infarctions)	genus Coprococcus1	rs1519491	T	C	-0.064	21856898	0.343	0.067	360612	T	C	0.050	2	22079770	8.95E-06	0.011	14306	19.325
Vascular dementia (multiple infarctions)	genus Coprococcus1	rs1576241	A	G	0.014	71819574	0.837	0.067	360612	A	G	-0.051	6	72529277	3.33E-06	0.011	14306	21.708
Vascular dementia (multiple infarctions)	genus Coprococcus1	rs2907920	G	A	-0.129	2600719	0.078	0.073	360612	G	A	-0.056	19	2600717	7.65E-06	0.013	14306	19.579
Vascular dementia (multiple infarctions)	genus Coprococcus1	rs4277593	G	A	-0.009	4338773	0.895	0.066	360612	G	A	-0.059	20	4319420	1.14E-07	0.011	14306	28.390
Vascular dementia (multiple infarctions)	genus Coprococcus1	rs56405618	A	G	0.040	173547165	0.704	0.104	360612	A	G	-0.090	4	174468316	1.57E-06	0.019	14306	23.095
Vascular dementia (multiple infarctions)	genus Coprococcus1	rs73031725	T	C	0.164	134752388	0.382	0.188	360612	T	C	0.168	11	134622282	1.98E-06	0.036	14306	22.259
Vascular dementia (multiple infarctions)	genus Coprococcus1	rs73167075	T	C	-0.049	165975355	0.536	0.080	360612	T	C	0.057	3	165693143	8.57E-06	0.013	14306	20.184
Vascular dementia (multiple infarctions)	genus Coprococcus1	rs74101919	T	C	-0.122	94958765	0.244	0.105	360612	T	C	-0.072	1	95424321	1.03E-06	0.014	14306	24.706
Vascular dementia (multiple infarctions)	genus Coprococcus1	rs946513	T	C	0.140	15871821	0.377	0.158	360612	T	C	-0.206	10	15913820	8.62E-06	0.046	14306	20.034
Vascular dementia (multiple infarctions)	genus Coprococcus2	rs10070053	A	G	0.107	34794684	0.107	0.066	360612	A	G	0.059	5	34794789	7.65E-06	0.014	14306	19.273
Vascular dementia (multiple infarctions)	genus Coprococcus2	rs12634070	T	C	-0.067	180827092	0.368	0.075	360612	T	C	0.074	3	180544880	9.95E-06	0.016	14306	19.942
Vascular dementia (multiple infarctions)	genus Coprococcus2	rs2482516	C	T	0.077	25554070	0.327	0.079	360612	C	T	0.075	9	25554068	4.72E-06	0.016	14306	21.002
Vascular dementia (multiple infarctions)	genus Coprococcus2	rs35890118	A	G	0.073	127917495	0.329	0.075	360612	A	G	-0.067	10	129715759	8.26E-06	0.015	14306	20.304
Vascular dementia (multiple infarctions)	genus Coprococcus2	rs61823518	A	C	-0.162	223514894	0.128	0.107	360612	A	C	-0.096	1	223688236	6.68E-06	0.022	14306	19.614
Vascular dementia (multiple infarctions)	genus Coprococcus2	rs6677933	C	T	0.033	111596386	0.722	0.092	360612	C	T	-0.080	1	112139008	1.19E-06	0.016	14306	23.995
Vascular dementia (multiple infarctions)	genus Coprococcus2	rs72680320	T	C	0.015	130204631	0.833	0.069	360612	T	C	-0.065	4	131125786	2.27E-06	0.014	14306	21.766
Vascular dementia (multiple infarctions)	genus Coprococcus2	rs9426473	A	G	-0.089	4150395	0.233	0.075	360612	A	G	0.073	1	4210455	6.31E-06	0.016	14306	20.243
Vascular dementia (multiple infarctions)	genus Coprococcus3	rs10810043	G	A	0.043	13799810	0.540	0.070	360612	G	A	-0.052	9	13799809	9.27E-06	0.012	14306	19.776
Vascular dementia (multiple infarctions)	genus Coprococcus3	rs11077359	C	T	0.124	78347880	0.155	0.087	360612	C	T	0.065	17	76343961	9.64E-06	0.015	14306	18.807
Vascular dementia (multiple infarctions)	genus Coprococcus3	rs11080344	C	T	0.010	27777485	0.881	0.066	360612	C	T	0.052	17	26104511	4.79E-06	0.011	14306	20.906
Vascular dementia (multiple infarctions)	genus Coprococcus3	rs13247359	G	A	-0.029	76728409	0.665	0.066	360612	G	A	0.051	7	76357726	7.33E-06	0.011	14306	20.527
Vascular dementia (multiple infarctions)	genus Coprococcus3	rs178271	C	T	0.169	20977267	0.509	0.256	360612	C	T	-0.145	22	21331556	7.81E-07	0.029	14306	24.371
Vascular dementia (multiple infarctions)	genus Coprococcus3	rs4575475	A	G	-0.167	98675414	0.031	0.078	360612	A	G	-0.062	14	99141751	7.04E-06	0.014	14306	20.216
Vascular dementia (multiple infarctions)	genus Coprococcus3	rs7521171	A	G	0.027	150026539	0.704	0.071	360612	A	G	0.060	1	149998497	4.32E-06	0.013	14306	21.285
Vascular dementia (multiple infarctions)	genus Coprococcus3	rs8100692	T	C	-0.007	39541492	0.916	0.066	360612	T	C	0.058	19	40032132	4.16E-07	0.011	14306	25.892
Vascular dementia (multiple infarctions)	genus Defluviitaleaceae UCG011	rs112893842	T	C	-0.167	8786663	0.137	0.113	360612	T	C	0.114	9	8786663	1.45E-06	0.023	14306	23.899
Vascular dementia (multiple infarctions)	genus Defluviitaleaceae UCG011	rs1582238	C	T	0.148	118181062	0.030	0.068	360612	C	T	-0.081	1	118723685	1.57E-06	0.017	14306	23.167
Vascular dementia (multiple infarctions)	genus Defluviitaleaceae UCG011	rs2892880	G	A	0.017	119820571	0.823	0.075	360612	G	A	0.082	4	120741726	6.83E-06	0.018	14306	20.258
Vascular dementia (multiple infarctions)	genus Defluviitaleaceae UCG011	rs4344384	T	G	0.141	64647609	0.031	0.065	360612	T	G	-0.072	10	66407366	4.83E-06	0.016	14306	20.981
Vascular dementia (multiple infarctions)	genus Defluviitaleaceae UCG011	rs4677103	A	G	0.089	72158643	0.307	0.087	360612	A	G	0.098	3	72207794	9.60E-07	0.020	14306	24.576
Vascular dementia (multiple infarctions)	genus Defluviitaleaceae UCG011	rs55658617	T	C	0.136	40439076	0.435	0.174	360612	T	C	0.174	21	41811003	2.15E-06	0.036	14306	23.173
Vascular dementia (multiple infarctions)	genus Defluviitaleaceae UCG011	rs72731813	C	T	-0.107	146493591	0.485	0.154	360612	C	T	-0.147	4	147414743	4.33E-07	0.029	14306	25.165
Vascular dementia (multiple infarctions)	genus Defluviitaleaceae UCG011	rs9608282	T	G	0.004	24408113	0.982	0.189	360612	T	G	0.143	22	24804081	2.52E-06	0.030	14306	22.733
Vascular dementia (multiple infarctions)	genus Defluviitaleaceae UCG011	rs9725395	A	G	0.142	84739949	0.168	0.103	360612	A	G	-0.138	1	85205632	3.52E-06	0.030	14306	21.911
Vascular dementia (multiple infarctions)	genus Desulfovibrio	rs12031543	T	C	0.105	68198632	0.268	0.095	360612	T	C	-0.127	1	68664315	6.55E-06	0.028	14306	20.357
Vascular dementia (multiple infarctions)	genus Desulfovibrio	rs13066142	G	A	0.114	67428549	0.316	0.113	360612	G	A	0.119	3	67478973	3.79E-06	0.025	14306	22.550
Vascular dementia (multiple infarctions)	genus Desulfovibrio	rs16863365	A	G	-0.080	197225187	0.613	0.157	360612	A	G	0.109	2	198089911	1.79E-06	0.023	14306	23.235
Vascular dementia (multiple infarctions)	genus Desulfovibrio	rs2032031	G	A	-0.164	128372006	0.012	0.065	360612	G	A	0.065	10	130170270	9.14E-06	0.015	14306	19.404
Vascular dementia (multiple infarctions)	genus Desulfovibrio	rs2590913	A	G	0.102	63690313	0.497	0.150	360612	A	G	-0.154	13	64264446	6.65E-06	0.034	14306	20.721
Vascular dementia (multiple infarctions)	genus Desulfovibrio	rs2853179	T	C	0.043	104449819	0.582	0.078	360612	T	C	-0.081	8	105462047	2.42E-06	0.017	14306	21.718
Vascular dementia (multiple infarctions)	genus Desulfovibrio	rs4797774	A	G	0.215	13447996	0.197	0.166	360612	A	G	-0.213	18	13447995	5.64E-06	0.047	14306	20.454
Vascular dementia (multiple infarctions)	genus Desulfovibrio	rs6580353	T	C	-0.194	140031102	0.018	0.082	360612	T	C	0.077	5	139410687	4.94E-06	0.017	14306	20.632
Vascular dementia (multiple infarctions)	genus Desulfovibrio	rs72647089	T	G	0.020	57060357	0.869	0.120	360612	T	G	-0.107	8	57972916	8.30E-06	0.024	14306	19.836
Vascular dementia (multiple infarctions)	genus Dialister	rs10138457	T	C	0.041	101991715	0.712	0.112	360612	T	C	-0.113	14	102458052	7.88E-06	0.026	14306	18.629
Vascular dementia (multiple infarctions)	genus Dialister	rs10938938	A	G	-0.051	23292736	0.571	0.089	360612	A	G	0.077	4	23294359	7.37E-06	0.017	14306	20.482
Vascular dementia (multiple infarctions)	genus Dialister	rs11071887	T	C	-0.036	32662894	0.608	0.071	360612	T	C	0.066	15	32955095	5.91E-06	0.015	14306	20.496
Vascular dementia (multiple infarctions)	genus Dialister	rs11166701	G	A	0.128	137283482	0.050	0.065	360612	G	A	-0.066	8	138295725	5.51E-07	0.013	14306	24.698
Vascular dementia (multiple infarctions)	genus Dialister	rs2314294	T	C	0.071	9243005	0.457	0.095	360612	T	C	0.087	16	9336862	8.08E-06	0.019	14306	19.981
Vascular dementia (multiple infarctions)	genus Dialister	rs2435610	A	C	-0.042	151192947	0.581	0.075	360612	A	C	0.065	7	150890034	5.93E-06	0.014	14306	20.390

Vascular dementia (multiple infarctions)	genus Dialister	rs4747450	C	A	0.023	22910724	0.772	0.078	360612	C	A	0.067	10	23199653	5.84E-06	0.015	14306	20.490
Vascular dementia (multiple infarctions)	genus Dialister	rs4753063	A	G	-0.056	92533963	0.393	0.066	360612	A	G	0.060	11	92267129	4.86E-06	0.013	14306	21.023
Vascular dementia (multiple infarctions)	genus Dialister	rs75416973	A	G	0.080	172840493	0.311	0.079	360612	A	G	0.073	1	172809633	9.46E-06	0.016	14306	19.541
Vascular dementia (multiple infarctions)	genus Dialister	rs764177	C	A	-0.070	45767957	0.307	0.068	360612	C	A	-0.060	3	45809449	9.61E-06	0.014	14306	19.742
Vascular dementia (multiple infarctions)	genus Dialister	rs76680460	G	A	-0.120	25447082	0.463	0.163	360612	G	A	-0.161	9	25447080	8.19E-06	0.036	14306	19.600
Vascular dementia (multiple infarctions)	genus Dorea	rs11150408	G	T	-0.011	81769892	0.871	0.066	360612	G	T	-0.049	16	81803497	7.06E-06	0.011	14306	20.042
Vascular dementia (multiple infarctions)	genus Dorea	rs12537781	T	C	-0.080	155186473	0.297	0.076	360612	T	C	-0.056	7	154978183	9.15E-06	0.013	14306	19.675
Vascular dementia (multiple infarctions)	genus Dorea	rs13279148	G	A	0.039	126893620	0.710	0.104	360612	G	A	0.072	8	127905865	2.25E-06	0.015	14306	22.477
Vascular dementia (multiple infarctions)	genus Dorea	rs1899291	T	C	-0.025	60126622	0.782	0.091	360612	T	C	-0.070	4	60992340	4.57E-06	0.015	14306	21.523
Vascular dementia (multiple infarctions)	genus Dorea	rs3005511	G	A	0.092	73896090	0.196	0.071	360612	G	A	-0.052	6	74605806	5.29E-06	0.011	14306	20.917
Vascular dementia (multiple infarctions)	genus Dorea	rs345219	G	T	0.060	6784833	0.361	0.066	360612	G	T	0.050	3	6826520	8.80E-06	0.011	14306	19.504
Vascular dementia (multiple infarctions)	genus Dorea	rs3752849	G	A	-0.100	8707510	0.509	0.151	360612	G	A	0.164	11	8729057	7.68E-06	0.037	14306	20.032
Vascular dementia (multiple infarctions)	genus Dorea	rs4793307	C	T	-0.007	72737384	0.924	0.077	360612	C	T	0.057	17	70733523	4.01E-06	0.012	14306	21.979
Vascular dementia (multiple infarctions)	genus Dorea	rs62503162	A	G	0.102	15911148	0.525	0.160	360612	A	G	-0.097	8	15768657	7.47E-07	0.019	14306	25.113
Vascular dementia (multiple infarctions)	genus Dorea	rs73729431	C	T	0.107	25077822	0.645	0.233	360612	C	T	-0.137	6	25078050	3.17E-06	0.030	14306	20.994
Vascular dementia (multiple infarctions)	genus Eggerthella	rs112205261	T	C	-0.045	9076080	0.708	0.121	360612	T	C	-0.189	1	9136139	3.35E-06	0.040	14306	21.839
Vascular dementia (multiple infarctions)	genus Eggerthella	rs13070736	A	C	0.098	20765962	0.266	0.088	360612	A	C	-0.121	3	20807454	7.62E-06	0.027	14306	19.894
Vascular dementia (multiple infarctions)	genus Eggerthella	rs1784446	A	G	0.005	102590868	0.943	0.065	360612	A	G	-0.091	11	102461599	5.23E-06	0.020	14306	20.997
Vascular dementia (multiple infarctions)	genus Eggerthella	rs2223081	A	G	0.126	28405711	0.085	0.073	360612	A	G	-0.103	21	29778032	3.89E-06	0.022	14306	21.520
Vascular dementia (multiple infarctions)	genus Eggerthella	rs2240838	G	A	-0.018	38296353	0.790	0.066	360612	G	A	-0.098	7	38335954	7.36E-07	0.020	14306	24.613
Vascular dementia (multiple infarctions)	genus Eggerthella	rs3851328	G	T	-0.076	45226666	0.333	0.079	360612	G	T	0.108	2	45453805	4.18E-06	0.024	14306	20.762
Vascular dementia (multiple infarctions)	genus Eggerthella	rs6430926	T	C	-0.070	140579885	0.292	0.066	360612	T	C	-0.088	2	141337454	8.37E-06	0.020	14306	19.916
Vascular dementia (multiple infarctions)	genus Eggerthella	rs67490567	T	C	-0.109	65398986	0.149	0.075	360612	T	C	0.108	15	65691324	8.94E-06	0.025	14306	19.537
Vascular dementia (multiple infarctions)	genus Eggerthella	rs76663501	C	T	0.163	57072813	0.272	0.149	360612	C	T	0.175	20	55647869	4.83E-06	0.038	14306	21.454
Vascular dementia (multiple infarctions)	genus Eisenbergiella	rs11027642	C	T	0.032	23980294	0.733	0.093	360612	C	T	0.129	11	24001840	4.92E-06	0.028	14306	20.512
Vascular dementia (multiple infarctions)	genus Eisenbergiella	rs11079158	T	C	0.009	55290280	0.909	0.079	360612	T	C	0.101	17	53367641	7.35E-06	0.023	14306	19.920
Vascular dementia (multiple infarctions)	genus Eisenbergiella	rs11938607	C	T	-0.113	188139277	0.133	0.075	360612	C	T	-0.098	4	189060431	8.22E-06	0.022	14306	20.386
Vascular dementia (multiple infarctions)	genus Eisenbergiella	rs12257723	A	C	-0.020	107657343	0.779	0.069	360612	A	C	-0.095	10	109417101	8.85E-06	0.021	14306	20.279
Vascular dementia (multiple infarctions)	genus Eisenbergiella	rs12710729	C	A	0.061	19773449	0.383	0.070	360612	C	A	0.089	2	19973210	9.84E-06	0.020	14306	20.140
Vascular dementia (multiple infarctions)	genus Eisenbergiella	rs13258851	A	G	0.077	54418254	0.406	0.093	360612	A	G	0.137	8	55330814	7.75E-06	0.030	14306	20.563
Vascular dementia (multiple infarctions)	genus Eisenbergiella	rs1508033	A	C	0.035	53083004	0.629	0.072	360612	A	C	0.092	15	53375201	3.23E-06	0.020	14306	21.863
Vascular dementia (multiple infarctions)	genus Eisenbergiella	rs1553971	T	G	-0.042	111700639	0.592	0.078	360612	T	G	0.121	3	111419486	5.27E-06	0.026	14306	21.146
Vascular dementia (multiple infarctions)	genus Eisenbergiella	rs2683098	T	C	0.062	35886364	0.438	0.080	360612	T	C	-0.107	15	36178565	2.24E-06	0.023	14306	22.720
Vascular dementia (multiple infarctions)	genus Eisenbergiella	rs3812426	A	G	-0.030	49910116	0.742	0.091	360612	A	G	-0.106	8	50822676	2.72E-06	0.022	14306	22.550
Vascular dementia (multiple infarctions)	genus Eisenbergiella	rs4462860	G	A	0.002	20237941	0.977	0.067	360612	G	A	0.094	21	21610254	4.16E-06	0.020	14306	21.805
Vascular dementia (multiple infarctions)	genus Enterorhabdus	rs10098492	T	C	-0.154	112097527	0.261	0.137	360612	T	C	0.132	8	113109756	6.41E-06	0.029	14306	20.300
Vascular dementia (multiple infarctions)	genus Enterorhabdus	rs114731706	T	G	-0.104	19412192	0.583	0.190	360612	T	G	0.182	2	19611953	2.17E-06	0.038	14306	22.741
Vascular dementia (multiple infarctions)	genus Enterorhabdus	rs2051957	C	T	0.046	90080569	0.578	0.082	360612	C	T	0.084	7	89709883	8.90E-06	0.019	14306	19.727
Vascular dementia (multiple infarctions)	genus Enterorhabdus	rs3017103	G	A	0.097	62406721	0.249	0.084	360612	G	A	-0.098	11	62174193	2.94E-06	0.021	14306	22.028
Vascular dementia (multiple infarctions)	genus Enterorhabdus	rs73331712	T	C	0.050	69121322	0.765	0.166	360612	T	C	0.262	12	69515102	4.85E-06	0.055	14306	22.589
Vascular dementia (multiple infarctions)	genus Enterorhabdus	rs77655283	G	A	0.009	235531235	0.943	0.129	360612	G	A	0.133	2	236439879	5.88E-06	0.030	14306	19.875
Vascular dementia (multiple infarctions)	genus Erysipelatoclostridium	rs1434153	G	A	-0.063	34370466	0.342	0.066	360612	G	A	-0.068	2	34595533	6.85E-06	0.015	14306	20.163
Vascular dementia (multiple infarctions)	genus Erysipelatoclostridium	rs16936671	C	T	-0.048	36105681	0.610	0.095	360612	C	T	-0.097	10	36394609	6.04E-06	0.022	14306	19.739
Vascular dementia (multiple infarctions)	genus Erysipelatoclostridium	rs17804233	C	T	-0.007	78519233	0.915	0.065	360612	C	T	0.066	5	77815056	4.59E-06	0.014	14306	21.133
Vascular dementia (multiple infarctions)	genus Erysipelatoclostridium	rs2901723	A	C	0.023	36849973	0.729	0.066	360612	A	C	-0.064	11	36871523	8.79E-06	0.014	14306	19.755
Vascular dementia (multiple infarctions)	genus Erysipelatoclostridium	rs340991	A	G	-0.162	34891937	0.028	0.074	360612	A	G	-0.074	5	34892042	3.75E-06	0.016	14306	21.719
Vascular dementia (multiple infarctions)	genus Erysipelatoclostridium	rs3804326	A	G	-0.217	24291957	0.151	0.151	360612	A	G	0.141	6	24292185	9.85E-06	0.034	14306	17.691
Vascular dementia (multiple infarctions)	genus Erysipelatoclostridium	rs45480394	T	G	-0.032	55346409	0.634	0.068	360612	T	G	-0.069	19	55857777	7.66E-06	0.015	14306	20.480
Vascular dementia (multiple infarctions)	genus Erysipelatoclostridium	rs4697572	A	G	0.120	25445165	0.142	0.082	360612	A	G	-0.081	4	25446787	7.59E-07	0.016	14306	24.640
Vascular dementia (multiple infarctions)	genus Erysipelatoclostridium	rs58236560	G	T	0.015	122006205	0.880	0.100	360612	G	T	-0.111	11	121876913	2.16E-06	0.023	14306	22.483
Vascular dementia (multiple infarctions)	genus Erysipelatoclostridium	rs61806970	C	T	0.039	167849359	0.761	0.129	360612	C	T	0.143	1	167818597	9.09E-06	0.032	14306	19.759
Vascular dementia (multiple infarctions)	genus Erysipelatoclostridium	rs622418	A	G	-0.085	87971128	0.195	0.065	360612	A	G	-0.067	9	90586043	3.68E-06	0.014	14306	21.756
Vascular dementia (multiple infarctions)	genus Erysipelatoclostridium	rs6474512	C	A	-0.180	38923088	0.005	0.065	360612	C	A	-0.067	8	38780606	3.02E-06	0.014	14306	21.900
Vascular dementia (multiple infarctions)	genus Erysipelatoclostridium	rs710230	C	T	0.107	41867960	0.394	0.126	360612	C	T	-0.143	1	42333631	6.33E-07	0.028	14306	25.941
Vascular dementia (multiple infarctions)	genus Erysipelatoclostridium	rs7221249	G	A	0.024	10274391	0.718	0.065	360612	G	A	-0.084	17	10177708	4.31E-09	0.014	14306	34.619
Vascular dementia (multiple infarctions)	genus Erysipelatoclostridium	rs9590927	A	G	0.008	45797930	0.897	0.065	360612	A	G	0.065	13	46372065	6.39E-06	0.014	14306	20.272
Vascular dementia (multiple infarctions)	genus Escherichia Shigella	rs112767262	T	C	-0.050	3700622	0.525	0.079	360612	T	C	0.073	16	3750623	8.21E-06	0.016	14306	20.075
Vascular dementia (multiple infarctions)	genus Escherichia Shigella	rs113127095	A	G	0.119	22679253	0.467	0.163	360612	A	G	0.151	13	23253392	3.33E-06	0.032	14306	21.799
Vascular dementia (multiple infarctions)	genus Escherichia Shigella	rs113513883	A	G	-0.100	140978399	0.584	0.183	360612	A	G	0.172	7	140678199	5.28E-06	0.038	14306	20.511
Vascular dementia (multiple infarctions)	genus Escherichia Shigella	rs1154904	G	A	-0.001	134904951	0.982	0.065	360612	G	A	0.061	11	134774845	3.04E-06	0.013	14306	22.043
Vascular dementia (multiple infarctions)	genus Escherichia Shigella	rs118526	A	C	-0.167	80567725	0.020	0.072	360612	A	C	0.059	5	79863544	8.00E-06	0.014	14306	19.108
Vascular dementia (multiple infarctions)	genus Escherichia Shigella	rs2798105	A	G	0.013	48496231	0.909	0.110	360612	A	G	-0.101	1	48961903	8.24E-06	0.022	14306	20.629
Vascular dementia (multiple infarctions)	genus Escherichia Shigella	rs4731451	G	A	0.121	128414046	0.089	0.071	360612	G	A	-0.061	7	128054100	7.47E-06	0.014	14306	20.362
Vascular dementia (multiple infarctions)	genus Escherichia Shigella	rs57024273	T	C	-0.123	235606511	0.101	0.075	360612	T	C	0.063	2	236515155	9.70E-06	0.014	14306	20.028
Vascular dementia (multiple infarctions)	genus Escherichia Shigella	rs592299	T	C	0.014	133303865	0.829	0.066	360612	T	C	-0.059	9	136179347	4.77E-06	0.013	14306	20.903
Vascular dementia (multiple infarctions)	genus Escherichia Shigella	rs73208162	A	G	0.050	37952181	0.784	0.181	360612	A	G	-0.119	21	39324484	2.19E-06	0.025	14306	23.067
Vascular dementia (multiple infarctions)	genus Eubacterium brachy group	rs112617308	T	C	0.229	92425456	0.044	0.114	360612	T	C	-0.171	10	94185213	2.38E-06	0.036	14306	22.177

Vascular dementia (multiple infarctions)	genus Eubacterium brachy group	rs12151423	G	A	-0.015	217372558	0.823	0.065	360612	G	A	-0.101	2	218237281	9.27E-06	0.023	14306	19.869
Vascular dementia (multiple infarctions)	genus Eubacterium brachy group	rs13139592	T	C	-0.108	143744025	0.262	0.096	360612	T	C	-0.146	4	144665178	7.97E-06	0.033	14306	19.911
Vascular dementia (multiple infarctions)	genus Eubacterium brachy group	rs1384962	G	A	-0.066	22591645	0.348	0.071	360612	G	A	-0.121	14	23060552	6.99E-06	0.027	14306	20.613
Vascular dementia (multiple infarctions)	genus Eubacterium brachy group	rs2913110	T	C	0.013	22603738	0.852	0.069	360612	T	C	-0.105	10	22892667	4.56E-06	0.023	14306	21.004
Vascular dementia (multiple infarctions)	genus Eubacterium brachy group	rs4862235	A	G	-0.049	183707778	0.459	0.066	360612	A	G	-0.105	4	184628931	3.73E-06	0.023	14306	21.553
Vascular dementia (multiple infarctions)	genus Eubacterium brachy group	rs62348779	T	C	-0.116	17459987	0.350	0.124	360612	T	C	-0.201	5	17460096	3.78E-06	0.043	14306	21.666
Vascular dementia (multiple infarctions)	genus Eubacterium brachy group	rs6591893	A	G	-0.111	80525789	0.109	0.069	360612	A	G	-0.108	11	80236833	7.34E-06	0.024	14306	20.281
Vascular dementia (multiple infarctions)	genus Eubacterium brachy group	rs720439	G	A	-0.016	47794849	0.832	0.077	360612	G	A	0.112	22	48190598	7.03E-06	0.025	14306	19.845
Vascular dementia (multiple infarctions)	genus Eubacterium brachy group	rs73199919	T	C	0.132	6854837	0.381	0.151	360612	T	C	-0.237	4	6856564	8.16E-06	0.053	14306	19.848
Vascular dementia (multiple infarctions)	genus Eubacterium coprostanoligenes group	rs1020520	T	G	0.131	33563534	0.152	0.092	360612	T	G	-0.059	7	33603146	8.89E-06	0.013	14306	19.747
Vascular dementia (multiple infarctions)	genus Eubacterium coprostanoligenes group	rs10444197	A	G	-0.001	2173737	0.985	0.069	360612	A	G	-0.051	10	2215931	5.98E-06	0.011	14306	19.877
Vascular dementia (multiple infarctions)	genus Eubacterium coprostanoligenes group	rs11052069	C	T	-0.070	32560985	0.290	0.066	360612	C	T	-0.048	12	32713919	9.38E-06	0.011	14306	19.636
Vascular dementia (multiple infarctions)	genus Eubacterium coprostanoligenes group	rs11720857	C	T	-0.010	114075660	0.909	0.086	360612	C	T	0.063	3	113794507	9.26E-06	0.014	14306	19.059
Vascular dementia (multiple infarctions)	genus Eubacterium coprostanoligenes group	rs12906958	C	T	-0.075	36619397	0.294	0.072	360612	C	T	-0.053	15	36911598	4.35E-06	0.012	14306	21.149
Vascular dementia (multiple infarctions)	genus Eubacterium coprostanoligenes group	rs17159861	C	T	-0.097	31045547	0.360	0.106	360612	C	T	0.096	7	31085162	1.04E-08	0.017	14306	32.654
Vascular dementia (multiple infarctions)	genus Eubacterium coprostanoligenes group	rs2644213	A	G	0.023	82746340	0.743	0.071	360612	A	G	-0.054	10	84506096	9.86E-06	0.012	14306	19.751
Vascular dementia (multiple infarctions)	genus Eubacterium coprostanoligenes group	rs4076415	G	T	0.002	85897766	0.971	0.068	360612	G	T	-0.052	15	86440997	1.99E-06	0.011	14306	21.816
Vascular dementia (multiple infarctions)	genus Eubacterium coprostanoligenes group	rs62024432	C	T	0.005	97326063	0.966	0.113	360612	C	T	-0.077	15	97869293	7.50E-06	0.017	14306	20.002
Vascular dementia (multiple infarctions)	genus Eubacterium coprostanoligenes group	rs6762473	A	C	0.043	127420731	0.530	0.069	360612	A	C	-0.052	3	127139574	4.26E-06	0.011	14306	21.552
Vascular dementia (multiple infarctions)	genus Eubacterium coprostanoligenes group	rs76898927	G	A	0.266	81546326	0.068	0.146	360612	G	A	0.123	3	81595477	4.79E-06	0.027	14306	21.341
Vascular dementia (multiple infarctions)	genus Eubacterium coprostanoligenes group	rs9648214	T	C	-0.209	16341181	0.070	0.116	360612	T	C	-0.083	7	16380806	2.52E-07	0.016	14306	25.438
Vascular dementia (multiple infarctions)	genus Eubacterium eligens group	rs182318	G	A	-0.089	31077144	0.455	0.119	360612	G	A	-0.082	11	31098691	8.40E-06	0.020	14306	17.784
Vascular dementia (multiple infarctions)	genus Eubacterium eligens group	rs2200429	A	G	-0.060	106911110	0.583	0.110	360612	A	G	-0.089	13	107563458	5.30E-06	0.020	14306	20.054
Vascular dementia (multiple infarctions)	genus Eubacterium eligens group	rs265534	T	G	0.028	81332251	0.670	0.066	360612	T	G	-0.056	10	83092007	2.27E-06	0.012	14306	22.024
Vascular dementia (multiple infarctions)	genus Eubacterium eligens group	rs4583233	A	C	-0.049	81786881	0.500	0.072	360612	A	C	0.067	16	81820486	2.84E-07	0.013	14306	27.363
Vascular dementia (multiple infarctions)	genus Eubacterium eligens group	rs56080211	T	C	0.099	109503416	0.422	0.123	360612	T	C	-0.123	2	110260993	9.14E-06	0.028	14306	18.953
Vascular dementia (multiple infarctions)	genus Eubacterium eligens group	rs6923695	T	G	0.016	70247112	0.905	0.132	360612	T	G	0.103	6	70956815	4.87E-06	0.023	14306	20.243
Vascular dementia (multiple infarctions)	genus Eubacterium fissicatena group	rs10147907	T	G	0.128	89018029	0.299	0.124	360612	T	G	0.172	14	89484373	8.27E-06	0.040	14306	18.922
Vascular dementia (multiple infarctions)	genus Eubacterium fissicatena group	rs11818408	G	A	-0.020	94998710	0.759	0.067	360612	G	A	0.106	10	96758467	8.20E-06	0.024	14306	19.928
Vascular dementia (multiple infarctions)	genus Eubacterium fissicatena group	rs11876297	T	C	-0.040	48226901	0.586	0.074	360612	T	C	0.131	18	45753272	2.67E-06	0.028	14306	21.779
Vascular dementia (multiple infarctions)	genus Eubacterium fissicatena group	rs151257695	A	G	-0.118	73629231	0.345	0.125	360612	A	G	0.210	7	73043561	3.10E-06	0.045	14306	21.217
Vascular dementia (multiple infarctions)	genus Eubacterium fissicatena group	rs1768152	C	T	-0.181	39563463	0.090	0.107	360612	C	T	-0.139	3	39604954	8.70E-06	0.032	14306	19.462
Vascular dementia (multiple infarctions)	genus Eubacterium fissicatena group	rs2733072	G	A	0.038	5576177	0.568	0.066	360612	G	A	0.110	8	5433699	1.49E-06	0.023	14306	23.064
Vascular dementia (multiple infarctions)	genus Eubacterium fissicatena group	rs3771393	T	C	-0.009	70918116	0.909	0.083	360612	T	C	-0.131	2	71145246	7.38E-07	0.027	14306	24.074
Vascular dementia (multiple infarctions)	genus Eubacterium fissicatena group	rs6934739	A	G	-0.119	39972698	0.085	0.069	360612	A	G	0.111	6	39940437	9.75E-06	0.025	14306	19.442
Vascular dementia (multiple infarctions)	genus Eubacterium fissicatena group	rs7104872	G	A	0.026	115294391	0.803	0.103	360612	G	A	0.139	11	115165111	2.73E-06	0.029	14306	22.548
Vascular dementia (multiple infarctions)	genus Eubacterium hallii group	rs10501370	C	T	-0.069	58273149	0.612	0.136	360612	C	T	-0.116	11	58040621	5.42E-06	0.025	14306	20.958
Vascular dementia (multiple infarctions)	genus Eubacterium hallii group	rs10798999	C	T	0.004	33843316	0.952	0.074	360612	C	T	0.060	1	34308917	2.61E-06	0.013	14306	22.532
Vascular dementia (multiple infarctions)	genus Eubacterium hallii group	rs117748144	T	C	-0.068	11750090	0.652	0.150	360612	T	C	-0.127	11	11771637	7.86E-06	0.029	14306	19.436
Vascular dementia (multiple infarctions)	genus Eubacterium hallii group	rs13116360	T	C	0.069	110964275	0.599	0.131	360612	T	C	0.154	4	111885431	2.94E-07	0.030	14306	26.896
Vascular dementia (multiple infarctions)	genus Eubacterium hallii group	rs17074066	T	C	0.206	182788383	0.357	0.223	360612	T	C	-0.081	4	183709536	9.35E-06	0.019	14306	18.495
Vascular dementia (multiple infarctions)	genus Eubacterium hallii group	rs17474256	G	A	0.172	103982054	0.115	0.109	360612	G	A	0.081	1	104524676	9.45E-06	0.018	14306	19.297
Vascular dementia (multiple infarctions)	genus Eubacterium hallii group	rs281379	A	G	0.076	48711017	0.249	0.066	360612	A	G	-0.050	19	49214274	9.33E-06	0.011	14306	19.838
Vascular dementia (multiple infarctions)	genus Eubacterium hallii group	rs28584818	A	G	-0.067	64678780	0.577	0.120	360612	A	G	0.126	3	64664456	4.43E-06	0.027	14306	22.041
Vascular dementia (multiple infarctions)	genus Eubacterium hallii group	rs60254196	G	A	0.040	149159628	0.543	0.066	360612	G	A	0.052	7	148856720	2.70E-06	0.011	14306	21.844
Vascular dementia (multiple infarctions)	genus Eubacterium hallii group	rs630939	C	T	-0.046	50858093	0.484	0.066	360612	C	T	-0.051	18	48384463	9.16E-06	0.011	14306	19.806
Vascular dementia (multiple infarctions)	genus Eubacterium hallii group	rs6550770	C	T	0.146	23621925	0.367	0.162	360612	C	T	0.198	3	23663416	4.82E-06	0.044	14306	19.945
Vascular dementia (multiple infarctions)	genus Eubacterium hallii group	rs74018587	C	T	-0.085	61721961	0.611	0.166	360612	C	T	0.209	15	62014160	3.70E-06	0.044	14306	22.734
Vascular dementia (multiple infarctions)	genus Eubacterium hallii group	rs78056098	G	T	0.051	123919170	0.457	0.068	360612	G	T	-0.051	11	123789877	8.29E-06	0.011	14306	19.896
Vascular dementia (multiple infarctions)	genus Eubacterium hallii group	rs949971	T	G	-0.084	110564542	0.230	0.070	360612	T	G	-0.054	3	110283389	3.29E-06	0.012	14306	21.646
Vascular dementia (multiple infarctions)	genus Eubacterium nodatum group	rs10263623	C	T	-0.110	65429224	0.513	0.168	360612	C	T	0.193	7	64894137	8.91E-06	0.044	14306	19.425
Vascular dementia (multiple infarctions)	genus Eubacterium nodatum group	rs10458299	T	C	-0.062	135367596	0.616	0.123	360612	T	C	-0.188	7	135052348	8.37E-06	0.042	14306	20.013
Vascular dementia (multiple infarctions)	genus Eubacterium nodatum group	rs11006576	A	G	-0.049	59562257	0.454	0.066	360612	A	G	-0.110	10	61322015	7.99E-06	0.025	14306	20.067
Vascular dementia (multiple infarctions)	genus Eubacterium nodatum group	rs113893692	C	T	-0.013	28245520	0.897	0.100	360612	C	T	-0.185	9	28245518	5.76E-06	0.040	14306	21.038
Vascular dementia (multiple infarctions)	genus Eubacterium nodatum group	rs34297067	A	G	-0.013	51719170	0.884	0.092	360612	A	G	-0.187	14	52185888	6.60E-08	0.034	14306	29.959
Vascular dementia (multiple infarctions)	genus Eubacterium nodatum group	rs61841040	G	T	0.006	9617786	0.940	0.082	360612	G	T	0.161	10	9659749	3.56E-06	0.034	14306	22.108
Vascular dementia (multiple infarctions)	genus Eubacterium nodatum group	rs6818880	G	A	-0.028	93791596	0.667	0.066	360612	G	A	0.110	4	94712747	7.83E-06	0.025	14306	20.030
Vascular dementia (multiple infarctions)	genus Eubacterium nodatum group	rs77910827	C	T	0.118	93835703	0.262	0.106	360612	C	T	0.202	9	96597985	9.05E-07	0.041	14306	23.807
Vascular dementia (multiple infarctions)	genus Eubacterium nodatum group	rs7827125	C	T	0.079	5097390	0.276	0.073	360612	C	T	0.122	8	4954912	7.17E-06	0.027	14306	20.317
Vascular dementia (multiple infarctions)	genus Eubacterium nodatum group	rs7880204	T	C	-0.063	171091680	0.405	0.076	360612	T	C	-0.125	1	171060821	6.84E-06	0.028	14306	20.766
Vascular dementia (multiple infarctions)	genus Eubacterium nodatum group	rs9425984	T	C	-0.038	34064135	0.631	0.079	360612	T	C	-0.130	1	34529736	7.21E-06	0.029	14306	19.845
Vascular dementia (multiple infarctions)	genus Eubacterium oxidoreducens group	rs12129908	A	C	0.134	195094889	0.044	0.066	360612	A	C	-0.089	1	195064019	5.80E-06	0.020	14306	20.285
Vascular dementia (multiple infarctions)	genus Eubacterium oxidoreducens group	rs12423772	G	T	0.230	94121564	0.007	0.086	360612	G	T	0.141	12	94515340	2.63E-06	0.030	14306	22.834
Vascular dementia (multiple infarctions)	genus Eubacterium oxidoreducens group	rs2973294	G	T	0.068	37525057	0.307	0.066	360612	G	T	0.092	4	37526679	2.39E-06	0.020	14306	22.331
Vascular dementia (multiple infarctions)	genus Eubacterium oxidoreducens group	rs34561138	G	A	0.050	86446749	0.760	0.164	360612	G	A	0.216	16	86480355	2.51E-06	0.046	14306	22.086
Vascular dementia (multiple infarctions)	genus Eubacterium oxidoreducens group	rs440215	T	C	-0.067	107546693	0.312	0.066	360612	T	C	-0.093	5	106882394	1.65E-06	0.020	14306	22.812
Vascular dementia (multiple infarctions)	genus Eubacterium rectale group	rs10248854	C	A	-0.019	121641184	0.781	0.067	360612	C	A	-0.053	7	121281238	4.21E-06	0.011	14306	21.640

Vascular dementia (multiple infarctions)	genus Eubacterium rectale group	rs10797540	A	G	0.125	234394624	0.059	0.066	360612	A	G	0.050	1	234530370	3.53E-06	0.011	14306	21.551
Vascular dementia (multiple infarctions)	genus Eubacterium rectale group	rs143694765	T	C	-0.106	115540288	0.330	0.109	360612	T	C	0.087	1	116082909	9.75E-06	0.020	14306	19.349
Vascular dementia (multiple infarctions)	genus Eubacterium rectale group	rs2884897	A	G	-0.034	8107990	0.853	0.181	360612	A	G	-0.129	11	8129537	6.44E-06	0.029	14306	20.034
Vascular dementia (multiple infarctions)	genus Eubacterium rectale group	rs314726	T	C	-0.054	124795578	0.413	0.066	360612	T	C	0.053	2	125553155	1.38E-06	0.011	14306	23.329
Vascular dementia (multiple infarctions)	genus Eubacterium rectale group	rs35398954	A	G	-0.073	92463535	0.410	0.089	360612	A	G	-0.090	15	93006765	5.40E-07	0.017	14306	26.653
Vascular dementia (multiple infarctions)	genus Eubacterium rectale group	rs59427698	A	G	-0.007	126663466	0.938	0.083	360612	A	G	-0.058	9	129425745	5.37E-06	0.013	14306	19.379
Vascular dementia (multiple infarctions)	genus Eubacterium rectale group	rs62547233	A	G	0.035	89398504	0.627	0.073	360612	A	G	0.054	9	92013419	9.90E-06	0.012	14306	19.908
Vascular dementia (multiple infarctions)	genus Eubacterium ruminantium group	rs10131724	C	A	-0.050	51752348	0.655	0.112	360612	C	A	0.200	14	52219066	2.39E-06	0.041	14306	23.234
Vascular dementia (multiple infarctions)	genus Eubacterium ruminantium group	rs10923018	G	A	-0.010	88057716	0.876	0.066	360612	G	A	0.073	1	88523399	6.80E-06	0.016	14306	20.378
Vascular dementia (multiple infarctions)	genus Eubacterium ruminantium group	rs11637981	T	G	0.063	60997423	0.339	0.066	360612	T	G	0.073	15	61289622	5.44E-06	0.016	14306	20.733
Vascular dementia (multiple infarctions)	genus Eubacterium ruminantium group	rs13025464	C	T	0.044	200745922	0.507	0.067	360612	C	T	0.074	2	201610645	6.97E-06	0.016	14306	20.252
Vascular dementia (multiple infarctions)	genus Eubacterium ruminantium group	rs139749	C	T	0.017	24907088	0.802	0.069	360612	C	T	-0.085	22	25303055	8.59E-07	0.017	14306	24.219
Vascular dementia (multiple infarctions)	genus Eubacterium ruminantium group	rs16891896	G	A	0.112	33897484	0.335	0.117	360612	G	A	-0.175	5	33897589	2.38E-06	0.039	14306	20.027
Vascular dementia (multiple infarctions)	genus Eubacterium ruminantium group	rs17519472	C	T	0.059	30208021	0.531	0.095	360612	C	T	0.108	12	30360954	4.70E-06	0.023	14306	21.227
Vascular dementia (multiple infarctions)	genus Eubacterium ruminantium group	rs209813	G	A	0.009	11953400	0.923	0.094	360612	G	A	-0.103	6	11953633	9.23E-06	0.024	14306	19.165
Vascular dementia (multiple infarctions)	genus Eubacterium ruminantium group	rs2116427	A	G	0.016	121847301	0.834	0.075	360612	A	G	0.091	5	121182996	4.67E-07	0.018	14306	24.983
Vascular dementia (multiple infarctions)	genus Eubacterium ruminantium group	rs2229917	A	G	-0.181	128218658	0.271	0.164	360612	A	G	0.154	9	130980937	2.16E-06	0.032	14306	22.467
Vascular dementia (multiple infarctions)	genus Eubacterium ruminantium group	rs2418654	C	T	0.048	71020448	0.469	0.067	360612	C	T	-0.075	2	71247578	6.17E-06	0.017	14306	20.388
Vascular dementia (multiple infarctions)	genus Eubacterium ruminantium group	rs2817174	C	T	0.015	3127617	0.829	0.067	360612	C	T	-0.073	1	3044181	7.87E-06	0.016	14306	20.125
Vascular dementia (multiple infarctions)	genus Eubacterium ruminantium group	rs57340348	T	C	0.110	129708969	0.179	0.082	360612	T	C	-0.098	6	130030114	4.93E-06	0.021	14306	21.311
Vascular dementia (multiple infarctions)	genus Eubacterium ruminantium group	rs606117	G	A	-0.068	2539358	0.351	0.073	360612	G	A	-0.083	9	2539358	4.82E-06	0.018	14306	21.296
Vascular dementia (multiple infarctions)	genus Eubacterium ruminantium group	rs6676699	T	G	-0.025	198009151	0.733	0.072	360612	T	G	0.089	1	197978281	6.38E-06	0.020	14306	20.439
Vascular dementia (multiple infarctions)	genus Eubacterium ruminantium group	rs7000472	G	A	0.131	137614025	0.049	0.067	360612	G	A	0.076	8	138626268	4.07E-06	0.017	14306	21.284
Vascular dementia (multiple infarctions)	genus Eubacterium ruminantium group	rs72836424	C	T	-0.006	127239189	0.956	0.104	360612	C	T	-0.140	10	129037453	2.62E-06	0.030	14306	21.624
Vascular dementia (multiple infarctions)	genus Eubacterium ruminantium group	rs73139629	A	C	-0.453	62912223	0.000	0.122	360612	A	C	-0.115	12	63306003	5.36E-06	0.025	14306	21.555
Vascular dementia (multiple infarctions)	genus Eubacterium ventriosum group	rs11617697	A	G	0.106	98133646	0.464	0.145	360612	A	G	-0.143	13	98785900	7.22E-07	0.029	14306	25.080
Vascular dementia (multiple infarctions)	genus Eubacterium ventriosum group	rs12964517	G	A	0.035	24880821	0.630	0.073	360612	G	A	0.059	18	22460785	2.08E-06	0.012	14306	22.624
Vascular dementia (multiple infarctions)	genus Eubacterium ventriosum group	rs13082419	C	T	0.100	108017587	0.333	0.103	360612	C	T	-0.072	3	107736434	9.56E-06	0.016	14306	19.661
Vascular dementia (multiple infarctions)	genus Eubacterium ventriosum group	rs16884680	G	T	0.234	113432826	0.030	0.108	360612	G	T	-0.091	8	114445055	1.74E-06	0.019	14306	22.309
Vascular dementia (multiple infarctions)	genus Eubacterium ventriosum group	rs35179274	C	T	-0.092	51493907	0.272	0.084	360612	C	T	-0.063	14	51960625	5.76E-06	0.014	14306	20.703
Vascular dementia (multiple infarctions)	genus Eubacterium ventriosum group	rs57199565	T	C	-0.112	190505943	0.187	0.085	360612	T	C	0.078	3	190223732	7.97E-07	0.016	14306	23.896
Vascular dementia (multiple infarctions)	genus Eubacterium ventriosum group	rs66746423	C	T	0.158	108855642	0.082	0.091	360612	C	T	0.075	1	109398264	6.11E-06	0.016	14306	20.749
Vascular dementia (multiple infarctions)	genus Eubacterium ventriosum group	rs6704822	G	A	0.237	202797056	0.016	0.099	360612	G	A	-0.074	2	203661779	6.62E-06	0.017	14306	19.661
Vascular dementia (multiple infarctions)	genus Eubacterium ventriosum group	rs72783037	C	A	-0.107	60314477	0.180	0.080	360612	C	A	0.066	16	60348381	6.55E-06	0.014	14306	21.012
Vascular dementia (multiple infarctions)	genus Eubacterium ventriosum group	rs73615400	T	C	0.209	51524380	0.062	0.112	360612	T	C	-0.096	20	50140919	9.54E-07	0.019	14306	24.443
Vascular dementia (multiple infarctions)	genus Eubacterium ventriosum group	rs73849225	T	C	0.190	125450659	0.104	0.117	360612	T	C	0.098	4	126371814	5.21E-06	0.022	14306	18.908
Vascular dementia (multiple infarctions)	genus Eubacterium ventriosum group	rs78250280	G	A	0.037	147819813	0.693	0.093	360612	G	A	0.075	1	147291928	3.36E-06	0.016	14306	20.798
Vascular dementia (multiple infarctions)	genus Eubacterium ventriosum group	rs876734	T	C	0.088	52030212	0.226	0.073	360612	T	C	0.062	12	52423996	2.89E-06	0.013	14306	21.793
Vascular dementia (multiple infarctions)	genus Eubacterium ventriosum group	rs9316536	T	G	-0.180	51364440	0.060	0.096	360612	T	G	-0.082	13	51938576	7.84E-06	0.018	14306	19.911
Vascular dementia (multiple infarctions)	genus Eubacterium xylanophilum group	rs10140184	A	C	-0.072	72715075	0.276	0.066	360612	A	C	0.058	14	73181783	4.96E-06	0.013	14306	20.959
Vascular dementia (multiple infarctions)	genus Eubacterium xylanophilum group	rs10917203	A	C	0.011	22300773	0.873	0.067	360612	A	C	0.061	1	22627266	3.15E-06	0.013	14306	21.898
Vascular dementia (multiple infarctions)	genus Eubacterium xylanophilum group	rs112176119	C	T	0.181	73662872	0.107	0.112	360612	C	T	-0.113	16	73696771	3.33E-06	0.025	14306	21.312
Vascular dementia (multiple infarctions)	genus Eubacterium xylanophilum group	rs13239072	G	A	0.012	43526745	0.868	0.073	360612	G	A	0.069	7	43566344	1.82E-06	0.014	14306	23.197
Vascular dementia (multiple infarctions)	genus Eubacterium xylanophilum group	rs17830032	G	A	0.020	59420451	0.868	0.122	360612	G	A	-0.161	20	57995506	2.39E-07	0.031	14306	26.749
Vascular dementia (multiple infarctions)	genus Eubacterium xylanophilum group	rs1999224	G	T	0.211	127735841	0.054	0.109	360612	G	T	-0.095	9	130498120	3.75E-06	0.020	14306	21.749
Vascular dementia (multiple infarctions)	genus Eubacterium xylanophilum group	rs2012708	G	A	-0.030	84756953	0.661	0.069	360612	G	A	-0.057	16	84790559	6.53E-06	0.013	14306	20.401
Vascular dementia (multiple infarctions)	genus Eubacterium xylanophilum group	rs2213117	T	G	-0.131	131139765	0.144	0.090	360612	T	G	0.088	11	131009660	4.21E-06	0.019	14306	21.568
Vascular dementia (multiple infarctions)	genus Eubacterium xylanophilum group	rs75586835	A	G	0.047	86970786	0.711	0.126	360612	A	G	-0.114	4	87891938	9.39E-06	0.026	14306	18.888
Vascular dementia (multiple infarctions)	genus Faecalibacterium	rs10927394	G	T	-0.529	245104037	0.030	0.245	360612	G	T	-0.232	1	245267339	7.02E-06	0.051	14306	20.549
Vascular dementia (multiple infarctions)	genus Faecalibacterium	rs114946999	C	T	0.035	36898092	0.723	0.099	360612	C	T	-0.086	2	37125235	5.70E-06	0.019	14306	20.649
Vascular dementia (multiple infarctions)	genus Faecalibacterium	rs11776390	T	C	-0.246	38736127	0.065	0.133	360612	T	C	-0.078	8	38593645	6.40E-06	0.017	14306	20.793
Vascular dementia (multiple infarctions)	genus Faecalibacterium	rs1271565	C	T	-0.017	63577379	0.821	0.075	360612	C	T	-0.058	14	64044097	1.30E-06	0.012	14306	23.196
Vascular dementia (multiple infarctions)	genus Faecalibacterium	rs12753492	A	C	-0.031	13875051	0.767	0.103	360612	A	C	0.064	1	14201546	8.80E-06	0.015	14306	18.295
Vascular dementia (multiple infarctions)	genus Faecalibacterium	rs2835874	T	C	-0.251	37655599	0.144	0.172	360612	T	C	-0.087	21	39027901	7.54E-06	0.020	14306	19.440
Vascular dementia (multiple infarctions)	genus Faecalibacterium	rs6910935	G	A	0.005	131266017	0.970	0.135	360612	G	A	-0.135	6	131587157	1.38E-06	0.028	14306	23.699
Vascular dementia (multiple infarctions)	genus Faecalibacterium	rs75499067	C	T	0.001	6049862	0.995	0.124	360612	C	T	0.228	16	6099863	1.76E-06	0.047	14306	23.900
Vascular dementia (multiple infarctions)	genus Faecalibacterium	rs79656633	T	C	0.112	110494205	0.307	0.110	360612	T	C	0.146	1	111036827	8.14E-06	0.032	14306	20.326
Vascular dementia (multiple infarctions)	genus Faecalibacterium	rs9536330	T	C	0.017	52992882	0.797	0.065	360612	T	C	-0.048	13	53567017	5.33E-06	0.011	14306	20.026
Vascular dementia (multiple infarctions)	genus Family XIII AD3011 group	rs11126423	T	C	-0.078	73968424	0.493	0.114	360612	T	C	-0.090	2	74195551	5.91E-06	0.020	14306	21.226
Vascular dementia (multiple infarctions)	genus Family XIII AD3011 group	rs11736617	G	A	-0.154	38916209	0.280	0.143	360612	G	A	-0.076	4	38917830	9.02E-06	0.017	14306	19.475
Vascular dementia (multiple infarctions)	genus Family XIII AD3011 group	rs12812672	T	C	-0.187	17974663	0.139	0.126	360612	T	C	-0.096	12	18127597	2.56E-06	0.021	14306	21.273
Vascular dementia (multiple infarctions)	genus Family XIII AD3011 group	rs149302	T	C	-0.112	14159816	0.146	0.077	360612	T	C	-0.065	5	14159925	7.48E-06	0.014	14306	20.321
Vascular dementia (multiple infarctions)	genus Family XIII AD3011 group	rs16840310	G	A	-0.028	240530863	0.672	0.067	360612	G	A	0.061	1	240694163	6.75E-07	0.012	14306	24.780
Vascular dementia (multiple infarctions)	genus Family XIII AD3011 group	rs16940167	C	T	-0.059	58391498	0.483	0.083	360612	C	T	0.073	15	58683697	3.91E-06	0.016	14306	20.989
Vascular dementia (multiple infarctions)	genus Family XIII AD3011 group	rs17156849	G	A	-0.085	28564367	0.539	0.139	360612	G	A	-0.113	7	28603985	4.19E-06	0.025	14306	21.181
Vascular dementia (multiple infarctions)	genus Family XIII AD3011 group	rs62029761	A	G	0.255	15912385	0.085	0.148	360612	A	G	0.129	16	16006242	3.89E-06	0.028	14306	21.762
Vascular dementia (multiple infarctions)	genus Family XIII AD3011 group	rs62200412	C	T	0.010	4637993	0.893	0.076	360612	C	T	-0.080	20	4618639	5.80E-07	0.016	14306	23.894

Vascular dementia (multiple infarctions)	genus Family XIII AD3011 group	rs72730932	C	A	0.197	192050705	0.076	0.111	360612	C	A	-0.090	1	192019835	6.89E-07	0.018	14306	25.798
Vascular dementia (multiple infarctions)	genus Family XIII AD3011 group	rs739451	C	T	-0.152	133813832	0.058	0.080	360612	C	T	0.065	9	136678954	7.88E-06	0.015	14306	19.386
Vascular dementia (multiple infarctions)	genus Family XIII AD3011 group	rs9276029	A	G	-0.037	32727559	0.648	0.082	360612	A	G	-0.081	6	32695336	8.93E-06	0.019	14306	19.097
Vascular dementia (multiple infarctions)	genus Family XIII AD3011 group	rs9837139	A	G	0.263	29770374	0.025	0.117	360612	A	G	0.108	3	29811865	8.71E-06	0.024	14306	19.990
Vascular dementia (multiple infarctions)	genus Family XIII UCG001	rs112362903	A	G	0.027	35434008	0.878	0.176	360612	A	G	-0.149	17	33761027	7.88E-06	0.033	14306	20.001
Vascular dementia (multiple infarctions)	genus Family XIII UCG001	rs12049454	T	C	-0.089	84762642	0.186	0.067	360612	T	C	-0.065	1	85228325	1.17E-06	0.013	14306	23.312
Vascular dementia (multiple infarctions)	genus Family XIII UCG001	rs1426266	C	T	-0.025	188506308	0.734	0.073	360612	C	T	0.067	3	188224096	1.25E-06	0.014	14306	23.546
Vascular dementia (multiple infarctions)	genus Family XIII UCG001	rs3842897	G	A	0.056	183534529	0.624	0.115	360612	G	A	-0.113	1	183503664	5.20E-06	0.024	14306	21.531
Vascular dementia (multiple infarctions)	genus Family XIII UCG001	rs62414802	C	T	0.011	75888918	0.883	0.076	360612	C	T	-0.061	6	76598635	4.29E-06	0.013	14306	20.676
Vascular dementia (multiple infarctions)	genus Family XIII UCG001	rs7119679	G	A	-0.095	94948621	0.221	0.078	360612	G	A	-0.081	11	94681786	3.52E-06	0.017	14306	21.429
Vascular dementia (multiple infarctions)	genus Family XIII UCG001	rs76463770	A	G	-0.116	45437598	0.526	0.183	360612	A	G	0.193	3	45479090	3.77E-06	0.042	14306	21.158
Vascular dementia (multiple infarctions)	genus Family XIII UCG001	rs8076666	G	A	0.151	80250018	0.135	0.101	360612	G	A	-0.089	17	78223817	8.02E-06	0.020	14306	20.029
Vascular dementia (multiple infarctions)	genus Flavonifractor	rs114873521	C	T	0.225	168791398	0.080	0.129	360612	C	T	-0.130	5	168218403	7.13E-06	0.029	14306	19.561
Vascular dementia (multiple infarctions)	genus Flavonifractor	rs11811696	T	C	0.021	237188054	0.862	0.120	360612	T	C	-0.116	1	237351354	2.07E-06	0.024	14306	23.191
Vascular dementia (multiple infarctions)	genus Flavonifractor	rs12030302	G	A	-0.014	77422150	0.835	0.065	360612	G	A	0.069	1	77887835	5.61E-07	0.014	14306	25.361
Vascular dementia (multiple infarctions)	genus Flavonifractor	rs34066017	A	G	0.008	44828138	0.925	0.081	360612	A	G	0.076	11	44849689	1.52E-06	0.016	14306	22.878
Vascular dementia (multiple infarctions)	genus Flavonifractor	rs806808	C	T	0.013	32092258	0.849	0.066	360612	C	T	-0.067	10	32381186	1.18E-06	0.014	14306	23.877
Vascular dementia (multiple infarctions)	genus Fusicatenibacter	rs10439674	A	G	-0.056	41309465	0.494	0.081	360612	A	G	-0.057	21	42681392	7.68E-06	0.013	14306	19.367
Vascular dementia (multiple infarctions)	genus Fusicatenibacter	rs167879	T	C	0.066	57362310	0.473	0.092	360612	T	C	0.066	20	55937366	5.87E-06	0.015	14306	19.656
Vascular dementia (multiple infarctions)	genus Fusicatenibacter	rs1864685	A	C	0.035	72725643	0.605	0.067	360612	A	C	-0.049	17	70721782	4.96E-06	0.011	14306	20.949
Vascular dementia (multiple infarctions)	genus Fusicatenibacter	rs2025938	G	A	-0.083	110417361	0.530	0.132	360612	G	A	-0.097	10	112177119	2.99E-06	0.021	14306	22.172
Vascular dementia (multiple infarctions)	genus Fusicatenibacter	rs206581	A	G	-0.165	10370599	0.038	0.080	360612	A	G	-0.057	18	10370596	8.96E-06	0.013	14306	19.748
Vascular dementia (multiple infarctions)	genus Fusicatenibacter	rs2132128	G	A	-0.185	15503209	0.085	0.107	360612	G	A	-0.077	8	15360718	1.08E-06	0.016	14306	23.176
Vascular dementia (multiple infarctions)	genus Fusicatenibacter	rs3303	T	C	0.152	118687917	0.254	0.134	360612	T	C	-0.095	10	120447429	3.94E-06	0.020	14306	21.839
Vascular dementia (multiple infarctions)	genus Fusicatenibacter	rs4378146	A	C	-0.071	24601134	0.345	0.075	360612	A	C	-0.062	1	24927625	7.20E-07	0.013	14306	24.239
Vascular dementia (multiple infarctions)	genus Fusicatenibacter	rs60254196	G	A	0.040	149159628	0.543	0.066	360612	G	A	0.049	7	148856720	5.47E-06	0.011	14306	20.273
Vascular dementia (multiple infarctions)	genus Fusicatenibacter	rs62187631	T	C	0.007	225784854	0.931	0.082	360612	T	C	-0.071	2	226649570	4.55E-06	0.016	14306	19.912
Vascular dementia (multiple infarctions)	genus Fusicatenibacter	rs62353480	A	G	-0.116	29594893	0.183	0.087	360612	A	G	-0.070	5	29595000	1.57E-06	0.015	14306	23.210
Vascular dementia (multiple infarctions)	genus Fusicatenibacter	rs6515626	G	A	0.136	25276365	0.306	0.133	360612	G	A	0.142	20	25257001	7.29E-06	0.031	14306	20.386
Vascular dementia (multiple infarctions)	genus Fusicatenibacter	rs704418	C	T	0.089	64267127	0.367	0.099	360612	C	T	-0.074	3	64252803	7.77E-07	0.015	14306	23.937
Vascular dementia (multiple infarctions)	genus Fusicatenibacter	rs73103914	A	G	0.130	58264528	0.158	0.092	360612	A	G	-0.060	12	58658311	8.30E-06	0.013	14306	19.735
Vascular dementia (multiple infarctions)	genus Fusicatenibacter	rs792108	C	T	0.004	5392660	0.956	0.066	360612	C	T	0.051	2	5532793	8.50E-06	0.011	14306	19.939
Vascular dementia (multiple infarctions)	genus Fusicatenibacter	rs8028026	G	A	0.097	87809984	0.388	0.112	360612	G	A	0.079	15	88353215	8.06E-06	0.018	14306	19.259
Vascular dementia (multiple infarctions)	genus Fusicatenibacter	rs8063430	T	C	-0.029	73787906	0.845	0.149	360612	T	C	-0.104	16	73821805	4.93E-06	0.022	14306	21.924
Vascular dementia (multiple infarctions)	genus Fusicatenibacter	rs9905659	G	A	0.008	72701205	0.927	0.084	360612	G	A	-0.062	17	70697344	7.31E-06	0.014	14306	20.354
Vascular dementia (multiple infarctions)	genus Gordonibacter	rs13412653	A	C	0.003	29935628	0.961	0.068	360612	A	C	0.108	2	30158494	8.61E-06	0.024	14306	20.229
Vascular dementia (multiple infarctions)	genus Gordonibacter	rs16955299	G	A	-0.010	54879849	0.928	0.111	360612	G	A	-0.196	17	52957210	6.37E-06	0.043	14306	20.527
Vascular dementia (multiple infarctions)	genus Gordonibacter	rs322296	G	A	0.028	137252442	0.822	0.125	360612	G	A	0.179	7	136937189	4.02E-06	0.038	14306	22.439
Vascular dementia (multiple infarctions)	genus Gordonibacter	rs35042269	C	A	0.094	8140880	0.364	0.103	360612	C	A	-0.180	4	8142607	8.11E-06	0.040	14306	19.985
Vascular dementia (multiple infarctions)	genus Gordonibacter	rs3765837	T	G	-0.062	210455105	0.625	0.126	360612	T	G	-0.191	1	210628449	7.17E-06	0.043	14306	19.348
Vascular dementia (multiple infarctions)	genus Gordonibacter	rs4596722	G	A	-0.119	86172229	0.068	0.065	360612	G	A	-0.103	9	88787144	9.06E-06	0.023	14306	19.748
Vascular dementia (multiple infarctions)	genus Gordonibacter	rs71545975	A	G	0.058	47104907	0.502	0.087	360612	A	G	-0.154	7	47144505	7.04E-06	0.034	14306	20.639
Vascular dementia (multiple infarctions)	genus Gordonibacter	rs72714787	C	A	0.080	135847346	0.412	0.098	360612	C	A	0.181	4	136768501	1.43E-06	0.038	14306	23.141
Vascular dementia (multiple infarctions)	genus Gordonibacter	rs72939513	A	G	-0.220	82670413	0.142	0.150	360612	A	G	-0.214	1	83136096	7.98E-06	0.049	14306	19.026
Vascular dementia (multiple infarctions)	genus Gordonibacter	rs7294633	T	C	-0.098	28871601	0.182	0.073	360612	T	C	-0.129	12	29024534	3.44E-07	0.025	14306	26.501
Vascular dementia (multiple infarctions)	genus Gordonibacter	rs768830	A	G	-0.054	18930984	0.549	0.090	360612	A	G	-0.150	7	18970607	7.76E-06	0.033	14306	20.212
Vascular dementia (multiple infarctions)	genus Haemophilus	rs10781340	A	G	0.029	76137254	0.770	0.100	360612	A	G	-0.095	9	78752170	4.32E-06	0.020	14306	21.803
Vascular dementia (multiple infarctions)	genus Haemophilus	rs111582866	G	A	0.052	48708578	0.650	0.114	360612	G	A	-0.124	16	48742489	1.27E-06	0.026	14306	22.815
Vascular dementia (multiple infarctions)	genus Haemophilus	rs35509	G	A	0.194	115055532	0.232	0.163	360612	G	A	0.128	12	115493337	2.01E-06	0.027	14306	22.768
Vascular dementia (multiple infarctions)	genus Haemophilus	rs4822728	T	C	-0.004	26495842	0.946	0.065	360612	T	C	0.071	22	26891808	3.48E-06	0.015	14306	21.740
Vascular dementia (multiple infarctions)	genus Haemophilus	rs76022354	C	T	-0.050	92546628	0.735	0.149	360612	C	T	0.245	10	94306385	1.83E-06	0.051	14306	23.421
Vascular dementia (multiple infarctions)	genus Haemophilus	rs78909003	T	C	-0.189	102887960	0.186	0.143	360612	T	C	-0.246	9	105650242	1.67E-06	0.050	14306	23.881
Vascular dementia (multiple infarctions)	genus Haemophilus	rs9328464	T	C	0.023	8350684	0.731	0.066	360612	T	C	0.072	6	8350917	1.42E-06	0.015	14306	23.615
Vascular dementia (multiple infarctions)	genus Haemophilus	rs9382510	C	T	-0.044	55583693	0.560	0.075	360612	C	T	-0.094	6	55448491	7.12E-08	0.017	14306	29.342
Vascular dementia (multiple infarctions)	genus Haemophilus	rs9895850	T	C	-0.066	66538895	0.679	0.159	360612	T	C	-0.193	17	64535013	2.14E-06	0.042	14306	21.437
Vascular dementia (multiple infarctions)	genus Holdemanella	rs12513188	G	A	0.041	70135074	0.580	0.074	360612	G	A	0.090	4	71000791	4.65E-06	0.020	14306	21.426
Vascular dementia (multiple infarctions)	genus Holdemanella	rs17586763	T	C	-0.226	50357927	0.125	0.147	360612	T	C	-0.227	13	50932063	7.72E-06	0.051	14306	19.850
Vascular dementia (multiple infarctions)	genus Holdemanella	rs1926302	G	A	-0.027	64687657	0.730	0.079	360612	G	A	-0.108	1	65153340	7.50E-06	0.023	14306	21.768
Vascular dementia (multiple infarctions)	genus Holdemanella	rs34187114	C	A	0.305	137611390	0.001	0.093	360612	C	A	-0.105	8	138623633	5.13E-06	0.023	14306	21.381
Vascular dementia (multiple infarctions)	genus Holdemanella	rs35228298	G	A	0.029	85343732	0.750	0.090	360612	G	A	0.093	3	85392882	7.30E-06	0.020	14306	21.231
Vascular dementia (multiple infarctions)	genus Holdemanella	rs4541991	T	C	-0.081	102242553	0.249	0.070	360612	T	C	-0.093	9	105004835	2.10E-06	0.019	14306	22.747
Vascular dementia (multiple infarctions)	genus Holdemanella	rs607782	C	T	0.034	4148375	0.615	0.068	360612	C	T	0.085	6	4148609	7.19E-07	0.017	14306	24.518
Vascular dementia (multiple infarctions)	genus Holdemanella	rs62113381	T	C	-0.041	7603339	0.669	0.096	360612	T	C	-0.105	19	7668225	5.54E-06	0.023	14306	20.653
Vascular dementia (multiple infarctions)	genus Holdemanella	rs73011279	T	C	-0.004	14909283	0.959	0.078	360612	T	C	-0.096	19	15020095	1.36E-06	0.020	14306	23.274
Vascular dementia (multiple infarctions)	genus Holdemanella	rs75764681	T	C	0.164	4452438	0.305	0.160	360612	T	C	-0.283	10	4494630	1.94E-06	0.060	14306	22.338
Vascular dementia (multiple infarctions)	genus Holdemanella	rs8113760	G	A	0.107	43347783	0.128	0.071	360612	G	A	0.079	19	43851935	4.62E-06	0.017	14306	20.756
Vascular dementia (multiple infarctions)	genus Holdemania	rs10885477	T	C	0.008	113572654	0.958	0.152	360612	T	C	-0.135	10	115332413	8.60E-06	0.030	14306	20.037

Vascular dementia (multiple infarctions)	genus Holdemania	rs11080063	G	A	0.060	28462253	0.366	0.066	360612	G	A	-0.067	17	26789271	6.67E-06	0.015	14306	19.711
Vascular dementia (multiple infarctions)	genus Holdemania	rs111745969	A	G	-0.023	92566992	0.812	0.097	360612	A	G	0.121	15	93110222	3.71E-06	0.027	14306	20.616
Vascular dementia (multiple infarctions)	genus Holdemania	rs113593397	A	G	-0.117	122961600	0.290	0.111	360612	A	G	-0.129	8	123973840	9.36E-06	0.028	14306	20.827
Vascular dementia (multiple infarctions)	genus Holdemania	rs116500994	G	T	0.098	81111408	0.532	0.156	360612	G	T	-0.138	13	81685543	2.34E-06	0.029	14306	21.981
Vascular dementia (multiple infarctions)	genus Holdemania	rs12701617	A	G	0.026	38253256	0.688	0.065	360612	A	G	-0.066	7	38292857	9.52E-06	0.015	14306	19.545
Vascular dementia (multiple infarctions)	genus Holdemania	rs1867876	T	C	-0.014	18746521	0.848	0.072	360612	T	C	0.084	11	18768068	2.74E-07	0.016	14306	27.009
Vascular dementia (multiple infarctions)	genus Holdemania	rs4146507	C	T	-0.073	106873392	0.341	0.076	360612	C	T	0.079	5	106209093	7.23E-06	0.018	14306	20.167
Vascular dementia (multiple infarctions)	genus Holdemania	rs73139538	G	A	-0.286	63862767	0.148	0.197	360612	G	A	-0.149	7	63323145	7.77E-06	0.033	14306	20.590
Vascular dementia (multiple infarctions)	genus Holdemania	rs77293403	A	G	-0.068	78092582	0.700	0.176	360612	A	G	0.165	5	77388406	1.77E-06	0.034	14306	23.182
Vascular dementia (multiple infarctions)	genus Holdemania	rs80149660	C	T	0.201	128110018	0.194	0.155	360612	C	T	-0.233	10	129908282	6.04E-06	0.052	14306	20.138
Vascular dementia (multiple infarctions)	genus Holdemania	rs9500080	C	T	0.034	105289447	0.697	0.086	360612	C	T	0.093	6	105737322	4.09E-07	0.018	14306	26.840
Vascular dementia (multiple infarctions)	genus Holdemania	rs9529719	C	T	-0.177	70282384	0.011	0.070	360612	C	T	-0.074	13	70856516	5.97E-06	0.016	14306	21.281
Vascular dementia (multiple infarctions)	genus Holdemania	rs967319	T	C	-0.023	60629947	0.764	0.077	360612	T	C	0.079	3	60615680	8.38E-06	0.018	14306	19.910
Vascular dementia (multiple infarctions)	genus Howardella	rs10048062	C	T	-0.123	97933690	0.281	0.114	360612	C	T	-0.147	15	98476920	8.59E-06	0.034	14306	19.172
Vascular dementia (multiple infarctions)	genus Howardella	rs12452946	A	G	0.073	17349974	0.260	0.065	360612	A	G	-0.106	17	17253288	3.80E-06	0.023	14306	21.370
Vascular dementia (multiple infarctions)	genus Howardella	rs1484873	G	A	-0.037	45627020	0.684	0.092	360612	G	A	0.228	18	43206985	2.56E-06	0.046	14306	24.177
Vascular dementia (multiple infarctions)	genus Howardella	rs17167098	G	A	-0.043	133469602	0.653	0.095	360612	G	A	-0.169	7	133154356	1.12E-06	0.035	14306	23.142
Vascular dementia (multiple infarctions)	genus Howardella	rs2154047	A	C	-0.020	94986460	0.858	0.114	360612	A	C	0.193	14	95452797	9.97E-06	0.042	14306	21.010
Vascular dementia (multiple infarctions)	genus Howardella	rs36081916	T	C	-0.060	93898102	0.611	0.118	360612	T	C	-0.181	7	93527414	4.70E-06	0.040	14306	20.225
Vascular dementia (multiple infarctions)	genus Howardella	rs3791893	A	G	0.111	217954673	0.238	0.094	360612	A	G	0.147	2	218819396	9.50E-06	0.034	14306	18.677
Vascular dementia (multiple infarctions)	genus Howardella	rs609430	T	G	-0.132	168257164	0.056	0.069	360612	T	G	-0.112	4	169178315	3.34E-06	0.024	14306	21.918
Vascular dementia (multiple infarctions)	genus Howardella	rs672217	G	A	0.022	62457901	0.790	0.083	360612	G	A	0.164	18	60125134	3.52E-06	0.035	14306	21.996
Vascular dementia (multiple infarctions)	genus Hungatella	rs10044993	A	C	-0.197	59128613	0.093	0.118	360612	A	C	-0.140	5	58424440	8.07E-06	0.032	14306	19.409
Vascular dementia (multiple infarctions)	genus Hungatella	rs13128780	T	C	0.109	165137846	0.193	0.084	360612	T	C	-0.150	4	166058998	1.75E-06	0.031	14306	22.915
Vascular dementia (multiple infarctions)	genus Hungatella	rs13249325	T	G	0.057	14996016	0.380	0.065	360612	T	G	-0.100	8	14853525	9.69E-06	0.023	14306	19.608
Vascular dementia (multiple infarctions)	genus Hungatella	rs17092615	G	A	-0.027	95507282	0.775	0.096	360612	G	A	0.152	14	95973619	7.38E-06	0.034	14306	20.302
Vascular dementia (multiple infarctions)	genus Hungatella	rs72759041	G	T	-0.098	89034145	0.224	0.080	360612	G	T	-0.126	15	89577376	3.86E-06	0.028	14306	19.937
Vascular dementia (multiple infarctions)	genus Intestinibacter	rs10805326	A	G	0.020	14322999	0.778	0.072	360612	A	G	-0.078	4	14324623	3.55E-08	0.014	14306	30.803
Vascular dementia (multiple infarctions)	genus Intestinibacter	rs11109097	T	C	0.043	97534659	0.514	0.066	360612	T	C	-0.062	12	97928437	5.49E-06	0.014	14306	20.305
Vascular dementia (multiple infarctions)	genus Intestinibacter	rs118030283	G	A	0.231	5943075	0.134	0.154	360612	G	A	-0.152	16	5993076	2.67E-06	0.032	14306	21.896
Vascular dementia (multiple infarctions)	genus Intestinibacter	rs16938435	T	C	0.003	21502924	0.978	0.110	360612	T	C	-0.112	9	21502923	1.80E-06	0.024	14306	22.706
Vascular dementia (multiple infarctions)	genus Intestinibacter	rs2098844	T	C	-0.012	127833265	0.860	0.067	360612	T	C	0.058	11	127703160	6.79E-06	0.013	14306	20.070
Vascular dementia (multiple infarctions)	genus Intestinibacter	rs2702387	G	A	-0.023	178440108	0.731	0.067	360612	G	A	-0.061	4	179361262	4.26E-06	0.013	14306	21.208
Vascular dementia (multiple infarctions)	genus Intestinibacter	rs4327025	G	A	0.074	91903453	0.379	0.084	360612	G	A	-0.081	15	92446683	1.64E-07	0.015	14306	27.546
Vascular dementia (multiple infarctions)	genus Intestinibacter	rs447950	A	G	0.023	149466863	0.731	0.067	360612	A	G	0.063	5	148846426	5.64E-06	0.014	14306	21.143
Vascular dementia (multiple infarctions)	genus Intestinibacter	rs478972	C	T	0.090	125793289	0.426	0.113	360612	C	T	0.143	11	125663184	1.82E-06	0.030	14306	23.061
Vascular dementia (multiple infarctions)	genus Intestinibacter	rs6062862	A	G	0.133	62693871	0.261	0.119	360612	A	G	0.092	20	61325223	6.68E-06	0.020	14306	20.406
Vascular dementia (multiple infarctions)	genus Intestinibacter	rs62430350	T	C	0.287	170609106	0.090	0.170	360612	T	C	0.151	6	170918194	6.84E-06	0.035	14306	18.481
Vascular dementia (multiple infarctions)	genus Intestinibacter	rs68093214	C	T	-0.018	70608634	0.810	0.076	360612	C	T	0.066	3	70657785	9.26E-06	0.015	14306	19.525
Vascular dementia (multiple infarctions)	genus Intestinibacter	rs6875660	C	T	0.024	160259249	0.859	0.134	360612	C	T	0.089	5	159686256	3.06E-06	0.019	14306	21.089
Vascular dementia (multiple infarctions)	genus Intestinibacter	rs893394	G	A	0.080	19855210	0.226	0.066	360612	G	A	0.058	2	20054971	7.85E-06	0.013	14306	19.910
Vascular dementia (multiple infarctions)	genus Intestinibacter	rs9348442	C	T	-0.105	10303712	0.287	0.098	360612	C	T	0.099	6	10303945	6.26E-06	0.022	14306	19.987
Vascular dementia (multiple infarctions)	genus Intestinimonas	rs10262702	T	C	0.009	67425856	0.931	0.100	360612	T	C	0.092	7	66890843	2.06E-06	0.019	14306	22.189
Vascular dementia (multiple infarctions)	genus Intestinimonas	rs11258178	A	G	-0.030	13082513	0.643	0.066	360612	A	G	0.066	10	13124513	6.98E-07	0.013	14306	24.264
Vascular dementia (multiple infarctions)	genus Intestinimonas	rs12226153	A	G	0.666	94631127	0.013	0.267	360612	A	G	-0.151	11	94364293	5.12E-07	0.031	14306	24.250
Vascular dementia (multiple infarctions)	genus Intestinimonas	rs17067892	C	T	-0.026	3906018	0.821	0.116	360612	C	T	0.107	8	3763540	6.38E-06	0.025	14306	18.383
Vascular dementia (multiple infarctions)	genus Intestinimonas	rs1859797	G	A	-0.021	21967685	0.752	0.066	360612	G	A	0.060	7	22007303	4.12E-06	0.013	14306	20.981
Vascular dementia (multiple infarctions)	genus Intestinimonas	rs2276760	A	G	0.064	150903169	0.398	0.076	360612	A	G	-0.069	3	150620956	7.84E-06	0.015	14306	20.178
Vascular dementia (multiple infarctions)	genus Intestinimonas	rs2731794	C	T	0.211	17209282	0.218	0.172	360612	C	T	0.121	5	17209391	1.92E-06	0.026	14306	21.942
Vascular dementia (multiple infarctions)	genus Intestinimonas	rs2930225	T	G	0.078	85294296	0.314	0.077	360612	T	G	-0.073	16	85327902	1.35E-06	0.015	14306	22.751
Vascular dementia (multiple infarctions)	genus Intestinimonas	rs4113676	A	C	0.253	6238275	0.388	0.293	360612	A	C	-0.219	20	6218922	7.42E-06	0.049	14306	19.873
Vascular dementia (multiple infarctions)	genus Intestinimonas	rs4784055	T	C	-0.168	58784866	0.249	0.146	360612	T	C	-0.175	16	58818770	8.72E-07	0.039	14306	20.631
Vascular dementia (multiple infarctions)	genus Intestinimonas	rs62240188	G	A	0.098	10569458	0.392	0.114	360612	G	A	0.130	3	10611142	2.20E-06	0.027	14306	23.702
Vascular dementia (multiple infarctions)	genus Intestinimonas	rs6934519	C	T	-0.060	66490909	0.424	0.074	360612	C	T	0.069	6	67200802	8.57E-06	0.015	14306	20.982
Vascular dementia (multiple infarctions)	genus Intestinimonas	rs716604	A	G	0.148	6373956	0.055	0.077	360612	A	G	0.082	2	6514088	8.57E-07	0.017	14306	24.289
Vascular dementia (multiple infarctions)	genus Intestinimonas	rs7170984	T	C	-0.082	93276506	0.258	0.073	360612	T	C	-0.066	15	93819735	2.98E-06	0.014	14306	21.858
Vascular dementia (multiple infarctions)	genus Intestinimonas	rs72982915	C	T	-0.163	140140519	0.248	0.141	360612	C	T	0.183	2	140898088	4.91E-06	0.040	14306	20.682
Vascular dementia (multiple infarctions)	genus Intestinimonas	rs9823439	C	T	0.048	17146010	0.469	0.066	360612	C	T	0.058	3	17187502	9.86E-06	0.013	14306	19.598
Vascular dementia (multiple infarctions)	genus Lachnoclostridium	rs1031599	T	G	-0.005	66675401	0.972	0.132	360612	T	G	0.079	3	66725825	6.31E-06	0.018	14306	20.039
Vascular dementia (multiple infarctions)	genus Lachnoclostridium	rs12566975	T	C	0.082	185122219	0.212	0.066	360612	T	C	-0.047	1	185091351	9.57E-06	0.011	14306	19.580
Vascular dementia (multiple infarctions)	genus Lachnoclostridium	rs1528479	A	G	-0.106	166387541	0.117	0.068	360612	A	G	0.050	2	167244051	9.64E-06	0.011	14306	19.783
Vascular dementia (multiple infarctions)	genus Lachnoclostridium	rs1997204	C	T	0.172	101652817	0.270	0.156	360612	C	T	0.108	12	102046595	5.97E-06	0.024	14306	19.941
Vascular dementia (multiple infarctions)	genus Lachnoclostridium	rs2385421	A	G	0.051	22163494	0.616	0.102	360612	A	G	0.075	18	19743455	7.14E-06	0.018	14306	17.046
Vascular dementia (multiple infarctions)	genus Lachnoclostridium	rs3821998	C	A	-0.017	38692945	0.878	0.107	360612	C	A	-0.086	4	38694566	6.72E-06	0.019	14306	20.144
Vascular dementia (multiple infarctions)	genus Lachnoclostridium	rs4738679	A	G	0.054	58457761	0.422	0.067	360612	A	G	0.052	8	59370320	4.42E-06	0.011	14306	20.813
Vascular dementia (multiple infarctions)	genus Lachnoclostridium	rs6112314	A	C	-0.019	19320202	0.780	0.069	360612	A	C	-0.056	20	19300846	2.43E-07	0.011	14306	26.964
Vascular dementia (multiple infarctions)	genus Lachnoclostridium	rs615997	T	C	0.020	22996295	0.754	0.065	360612	T	C	0.051	3	23037786	2.03E-06	0.011	14306	23.094

Vascular dementia (multiple infarctions)	genus Lachnoclostridium	rs62285313	A	G	-0.031	177752244	0.782	0.111	360612	A	G	0.086	3	177470032	1.58E-06	0.018	14306	22.655
Vascular dementia (multiple infarctions)	genus Lachnoclostridium	rs72829893	G	T	0.083	48617179	0.441	0.107	360612	G	T	0.117	17	46694541	5.58E-06	0.027	14306	19.198
Vascular dementia (multiple infarctions)	genus Lachnoclostridium	rs78068103	A	G	0.097	13912842	0.346	0.103	360612	A	G	0.089	17	13816159	3.67E-06	0.019	14306	20.814
Vascular dementia (multiple infarctions)	genus Lachnoclostridium	rs789029	C	T	0.127	1053251	0.184	0.095	360612	C	T	-0.064	18	1053252	3.75E-06	0.014	14306	21.603
Vascular dementia (multiple infarctions)	genus Lachnospiraceae FCS020 group	rs10093861	G	A	0.093	120232167	0.162	0.067	360612	G	A	-0.057	8	121244406	3.06E-06	0.012	14306	22.048
Vascular dementia (multiple infarctions)	genus Lachnospiraceae FCS020 group	rs1254846	A	G	0.022	43850646	0.816	0.093	360612	A	G	-0.106	10	44346094	5.60E-06	0.023	14306	20.771
Vascular dementia (multiple infarctions)	genus Lachnospiraceae FCS020 group	rs1363769	C	T	-0.006	17754496	0.972	0.183	360612	C	T	0.201	19	17865305	1.58E-06	0.045	14306	19.933
Vascular dementia (multiple infarctions)	genus Lachnospiraceae FCS020 group	rs2322265	C	T	0.138	165666933	0.063	0.074	360612	C	T	-0.067	4	166588085	5.21E-06	0.014	14306	22.149
Vascular dementia (multiple infarctions)	genus Lachnospiraceae FCS020 group	rs2862811	C	T	0.048	166278627	0.502	0.071	360612	C	T	-0.056	3	165996415	3.92E-06	0.012	14306	21.535
Vascular dementia (multiple infarctions)	genus Lachnospiraceae FCS020 group	rs35035870	T	C	-0.118	3227646	0.482	0.167	360612	T	C	-0.191	11	3248876	2.62E-06	0.041	14306	21.158
Vascular dementia (multiple infarctions)	genus Lachnospiraceae FCS020 group	rs3999074	G	T	0.023	63803887	0.732	0.066	360612	G	T	-0.055	10	65563647	6.55E-06	0.012	14306	20.418
Vascular dementia (multiple infarctions)	genus Lachnospiraceae FCS020 group	rs4452603	T	G	0.030	71256565	0.682	0.074	360612	T	G	0.060	6	71966268	8.98E-06	0.014	14306	19.748
Vascular dementia (multiple infarctions)	genus Lachnospiraceae FCS020 group	rs7249113	G	A	-0.028	2571234	0.699	0.072	360612	G	A	0.068	19	2571232	3.72E-07	0.013	14306	25.907
Vascular dementia (multiple infarctions)	genus Lachnospiraceae FCS020 group	rs72793667	A	G	0.198	53719294	0.241	0.169	360612	A	G	-0.117	2	53946431	1.63E-06	0.025	14306	22.467
Vascular dementia (multiple infarctions)	genus Lachnospiraceae FCS020 group	rs9308097	G	A	-0.056	164865326	0.394	0.066	360612	G	A	-0.055	4	165786478	7.47E-06	0.012	14306	20.057
Vascular dementia (multiple infarctions)	genus Lachnospiraceae FCS020 group	rs9788306	C	T	0.005	39845177	0.942	0.072	360612	C	T	-0.063	13	40419314	1.39E-06	0.013	14306	23.074
Vascular dementia (multiple infarctions)	genus Lachnospiraceae NC2004 group	rs117467633	T	C	0.188	21254094	0.257	0.166	360612	T	C	-0.170	17	21157406	9.13E-06	0.038	14306	19.612
Vascular dementia (multiple infarctions)	genus Lachnospiraceae NC2004 group	rs12127733	G	A	-0.053	234021975	0.530	0.084	360612	G	A	0.115	1	234157721	3.11E-06	0.025	14306	21.922
Vascular dementia (multiple infarctions)	genus Lachnospiraceae NC2004 group	rs12208226	C	A	0.029	22725679	0.788	0.109	360612	C	A	-0.155	6	22725908	9.75E-06	0.034	14306	20.668
Vascular dementia (multiple infarctions)	genus Lachnospiraceae NC2004 group	rs12863463	G	A	-0.123	46938056	0.315	0.122	360612	G	A	-0.156	13	47512191	6.04E-06	0.035	14306	20.498
Vascular dementia (multiple infarctions)	genus Lachnospiraceae NC2004 group	rs17067076	G	A	-0.194	60561853	0.060	0.103	360612	G	A	-0.155	18	58229086	5.61E-06	0.035	14306	19.277
Vascular dementia (multiple infarctions)	genus Lachnospiraceae NC2004 group	rs1928659	T	C	0.023	29334139	0.777	0.082	360612	T	C	0.103	9	29334137	6.17E-06	0.023	14306	20.498
Vascular dementia (multiple infarctions)	genus Lachnospiraceae NC2004 group	rs1929743	T	C	-0.084	76255426	0.223	0.069	360612	T	C	0.084	13	76829562	9.06E-06	0.019	14306	19.351
Vascular dementia (multiple infarctions)	genus Lachnospiraceae NC2004 group	rs3756315	A	G	0.024	150165159	0.737	0.071	360612	A	G	-0.088	5	149544722	3.33E-06	0.019	14306	21.990
Vascular dementia (multiple infarctions)	genus Lachnospiraceae NC2004 group	rs6116753	G	A	0.022	5350054	0.792	0.085	360612	G	A	0.099	20	5330700	2.92E-06	0.021	14306	22.623
Vascular dementia (multiple infarctions)	genus Lachnospiraceae ND3007 group	rs2861203	G	A	0.085	173232763	0.234	0.071	360612	G	A	0.057	3	172950553	7.37E-06	0.013	14306	20.218
Vascular dementia (multiple infarctions)	genus Lachnospiraceae ND3007 group	rs72776675	T	C	0.023	14095664	0.798	0.088	360612	T	C	-0.065	10	14137663	8.72E-06	0.015	14306	19.123
Vascular dementia (multiple infarctions)	genus Lachnospiraceae ND3007 group	rs9932954	A	G	0.026	1050633	0.707	0.069	360612	A	G	-0.056	16	1100633	1.25E-06	0.012	14306	23.467
Vascular dementia (multiple infarctions)	genus Lachnospiraceae NK4A136 group	rs10952110	G	T	-0.083	8458292	0.209	0.066	360612	G	T	0.049	7	8497922	9.08E-06	0.011	14306	19.797
Vascular dementia (multiple infarctions)	genus Lachnospiraceae NK4A136 group	rs11263806	A	G	-0.043	36882840	0.534	0.069	360612	A	G	-0.052	17	35240097	5.07E-06	0.012	14306	20.189
Vascular dementia (multiple infarctions)	genus Lachnospiraceae NK4A136 group	rs12611395	G	A	0.061	21623323	0.571	0.107	360612	G	A	0.090	19	21806125	5.83E-06	0.020	14306	20.435
Vascular dementia (multiple infarctions)	genus Lachnospiraceae NK4A136 group	rs160061	G	A	0.110	6116546	0.094	0.065	360612	G	A	-0.051	5	6116659	2.12E-06	0.011	14306	22.596
Vascular dementia (multiple infarctions)	genus Lachnospiraceae NK4A136 group	rs28540839	A	C	-0.030	83822845	0.652	0.065	360612	A	C	0.051	8	84735080	9.34E-06	0.011	14306	21.124
Vascular dementia (multiple infarctions)	genus Lachnospiraceae NK4A136 group	rs2880566	T	C	-0.026	31689634	0.779	0.092	360612	T	C	0.060	17	30016653	5.61E-06	0.013	14306	19.815
Vascular dementia (multiple infarctions)	genus Lachnospiraceae NK4A136 group	rs4955932	T	C	0.032	55147348	0.634	0.067	360612	T	C	-0.049	3	55181375	7.05E-06	0.011	14306	20.253
Vascular dementia (multiple infarctions)	genus Lachnospiraceae NK4A136 group	rs59805249	T	C	-0.211	90994180	0.067	0.115	360612	T	C	0.094	5	90289997	9.45E-06	0.021	14306	20.261
Vascular dementia (multiple infarctions)	genus Lachnospiraceae NK4A136 group	rs68104925	T	C	0.034	99711344	0.634	0.071	360612	T	C	-0.055	14	100177681	2.37E-06	0.012	14306	22.647
Vascular dementia (multiple infarctions)	genus Lachnospiraceae NK4A136 group	rs7073658	G	T	0.007	60415266	0.910	0.066	360612	G	T	0.050	10	62175024	5.27E-06	0.011	14306	20.745
Vascular dementia (multiple infarctions)	genus Lachnospiraceae NK4A136 group	rs73044693	A	G	0.098	50752863	0.438	0.126	360612	A	G	-0.108	19	51256120	3.57E-06	0.023	14306	21.900
Vascular dementia (multiple infarctions)	genus Lachnospiraceae NK4A136 group	rs7616165	G	T	0.176	190697356	0.380	0.200	360612	G	T	-0.231	3	190415145	2.77E-06	0.048	14306	22.739
Vascular dementia (multiple infarctions)	genus Lachnospiraceae NK4A136 group	rs76193507	A	G	-0.011	162032193	0.925	0.118	360612	A	G	-0.230	3	161749981	2.93E-06	0.050	14306	21.129
Vascular dementia (multiple infarctions)	genus Lachnospiraceae NK4A136 group	rs7832116	A	G	0.084	4985407	0.388	0.098	360612	A	G	-0.071	8	4842929	3.57E-06	0.015	14306	22.199
Vascular dementia (multiple infarctions)	genus Lachnospiraceae NK4A136 group	rs954878	A	G	0.065	54112728	0.333	0.067	360612	A	G	-0.052	1	54578401	1.78E-06	0.011	14306	22.782
Vascular dementia (multiple infarctions)	genus Lachnospiraceae UCG001	rs12131224	C	T	0.123	166230125	0.241	0.105	360612	C	T	0.117	1	166199362	7.40E-06	0.026	14306	20.424
Vascular dementia (multiple infarctions)	genus Lachnospiraceae UCG001	rs2050911	G	A	-0.093	81918723	0.177	0.069	360612	G	A	0.075	1	82384407	1.11E-06	0.015	14306	23.831
Vascular dementia (multiple infarctions)	genus Lachnospiraceae UCG001	rs2371284	C	T	-0.025	55862478	0.745	0.078	360612	C	T	0.076	12	56256262	7.56E-06	0.017	14306	20.056
Vascular dementia (multiple infarctions)	genus Lachnospiraceae UCG001	rs437876	T	C	0.073	42526948	0.293	0.069	360612	T	C	0.078	3	42568440	7.17E-08	0.014	14306	29.376
Vascular dementia (multiple infarctions)	genus Lachnospiraceae UCG001	rs4981345	T	C	-0.052	20987814	0.458	0.070	360612	T	C	-0.068	14	21455973	6.09E-06	0.015	14306	20.717
Vascular dementia (multiple infarctions)	genus Lachnospiraceae UCG001	rs573933	T	C	0.091	14477853	0.386	0.105	360612	T	C	-0.108	9	14477851	3.11E-06	0.023	14306	21.565
Vascular dementia (multiple infarctions)	genus Lachnospiraceae UCG001	rs62496417	T	G	0.024	97336192	0.757	0.079	360612	T	G	-0.075	7	96965504	5.88E-06	0.017	14306	20.410
Vascular dementia (multiple infarctions)	genus Lachnospiraceae UCG001	rs7341608	T	C	-0.004	55926941	0.970	0.096	360612	T	C	-0.078	8	56839500	9.48E-06	0.018	14306	19.514
Vascular dementia (multiple infarctions)	genus Lachnospiraceae UCG001	rs74034332	G	A	0.186	79058462	0.176	0.137	360612	G	A	0.168	16	79092359	3.33E-06	0.038	14306	19.288
Vascular dementia (multiple infarctions)	genus Lachnospiraceae UCG001	rs78848836	A	G	0.117	53307576	0.276	0.107	360612	A	G	-0.119	1	53773248	3.38E-06	0.026	14306	20.942
Vascular dementia (multiple infarctions)	genus Lachnospiraceae UCG001	rs8104225	A	G	0.036	13852110	0.653	0.080	360612	A	G	0.089	19	13962924	8.04E-06	0.020	14306	20.369
Vascular dementia (multiple infarctions)	genus Lachnospiraceae UCG001	rs9403580	C	T	-0.167	144685644	0.086	0.098	360612	C	T	0.108	6	145006780	3.47E-06	0.023	14306	22.011
Vascular dementia (multiple infarctions)	genus Lachnospiraceae UCG001	rs985416	T	C	0.062	148551296	0.464	0.085	360612	T	C	-0.097	3	148269083	1.46E-07	0.018	14306	28.481
Vascular dementia (multiple infarctions)	genus Lachnospiraceae UCG004	rs11128180	A	G	-0.012	70543064	0.878	0.077	360612	A	G	0.065	3	70592215	4.52E-06	0.014	14306	21.404
Vascular dementia (multiple infarctions)	genus Lachnospiraceae UCG004	rs12072562	T	C	-0.064	104744847	0.703	0.169	360612	T	C	0.133	1	105287469	7.07E-06	0.030	14306	19.225
Vascular dementia (multiple infarctions)	genus Lachnospiraceae UCG004	rs12673420	G	A	-0.007	71838022	0.915	0.065	360612	G	A	0.055	7	71303007	2.98E-06	0.012	14306	21.931
Vascular dementia (multiple infarctions)	genus Lachnospiraceae UCG004	rs12747809	A	G	0.108	240195789	0.139	0.073	360612	A	G	0.062	1	240359089	8.65E-07	0.013	14306	24.489
Vascular dementia (multiple infarctions)	genus Lachnospiraceae UCG004	rs12894272	G	A	0.064	40067277	0.349	0.069	360612	G	A	-0.058	14	40536481	4.34E-06	0.013	14306	21.436
Vascular dementia (multiple infarctions)	genus Lachnospiraceae UCG004	rs233486	G	A	0.166	14817353	0.077	0.094	360612	G	A	0.080	6	14817584	6.28E-06	0.018	14306	20.231
Vascular dementia (multiple infarctions)	genus Lachnospiraceae UCG004	rs2444793	T	C	-0.121	80609119	0.071	0.067	360612	T	C	0.054	6	81318836	4.77E-06	0.012	14306	21.064
Vascular dementia (multiple infarctions)	genus Lachnospiraceae UCG004	rs2726805	A	G	0.036	182326448	0.586	0.066	360612	A	G	0.055	4	183247601	6.30E-06	0.012	14306	20.594
Vascular dementia (multiple infarctions)	genus Lachnospiraceae UCG004	rs2882478	G	A	-0.018	49677451	0.778	0.065	360612	G	A	-0.058	2	49904589	1.21E-06	0.012	14306	23.782
Vascular dementia (multiple infarctions)	genus Lachnospiraceae UCG004	rs35182105	A	G	0.140	24419403	0.336	0.146	360612	A	G	-0.110	12	24572337	4.87E-06	0.024	14306	20.522
Vascular dementia (multiple infarctions)	genus Lachnospiraceae UCG004	rs6656451	T	C	-0.004	65524739	0.953	0.066	360612	T	C	0.054	1	65990422	5.57E-06	0.012	14306	20.703

Vascular dementia (multiple infarctions)	genus Lachnospiraceae UCG004	rs7629954	A	G	-0.077	154524617	0.624	0.157	360612	A	G	0.108	3	154242406	5.77E-06	0.024	14306	20.717
Vascular dementia (multiple infarctions)	genus Lachnospiraceae UCG008	rs10741777	T	C	0.068	19551010	0.342	0.071	360612	T	C	-0.097	11	19572557	7.69E-07	0.019	14306	24.994
Vascular dementia (multiple infarctions)	genus Lachnospiraceae UCG008	rs10793103	T	C	-0.049	74680730	0.456	0.065	360612	T	C	-0.097	11	74391775	9.35E-08	0.018	14306	28.889
Vascular dementia (multiple infarctions)	genus Lachnospiraceae UCG008	rs10801803	G	A	0.115	90353314	0.224	0.094	360612	G	A	-0.117	1	90818872	1.40E-06	0.024	14306	23.178
Vascular dementia (multiple infarctions)	genus Lachnospiraceae UCG008	rs13024781	T	C	-0.077	167772361	0.243	0.066	360612	T	C	-0.080	2	168628871	2.29E-06	0.017	14306	22.380
Vascular dementia (multiple infarctions)	genus Lachnospiraceae UCG008	rs57091572	A	G	0.045	80505086	0.641	0.096	360612	A	G	-0.110	6	81214803	2.86E-06	0.024	14306	21.937
Vascular dementia (multiple infarctions)	genus Lachnospiraceae UCG008	rs61944774	A	G	-0.118	129055273	0.424	0.147	360612	A	G	0.180	12	129539818	6.34E-06	0.039	14306	20.855
Vascular dementia (multiple infarctions)	genus Lachnospiraceae UCG008	rs62277846	C	T	0.004	227235424	0.965	0.082	360612	C	T	0.102	2	228100140	1.59E-06	0.021	14306	23.212
Vascular dementia (multiple infarctions)	genus Lachnospiraceae UCG008	rs67078837	T	C	0.031	113299491	0.635	0.066	360612	T	C	-0.085	4	114220647	7.68E-07	0.017	14306	24.556
Vascular dementia (multiple infarctions)	genus Lachnospiraceae UCG008	rs75356640	G	A	0.123	31405081	0.221	0.101	360612	G	A	0.137	15	31697284	9.83E-06	0.030	14306	20.284
Vascular dementia (multiple infarctions)	genus Lachnospiraceae UCG008	rs955844	A	C	0.057	84958708	0.539	0.093	360612	A	C	0.112	16	84992314	1.81E-06	0.023	14306	24.080
Vascular dementia (multiple infarctions)	genus Lachnospiraceae UCG010	rs10414815	C	T	-0.025	42082093	0.872	0.157	360612	C	T	-0.105	19	42586245	4.24E-06	0.023	14306	20.610
Vascular dementia (multiple infarctions)	genus Lachnospiraceae UCG010	rs11192447	A	G	-0.114	82088641	0.448	0.151	360612	A	G	0.127	10	83848397	4.69E-07	0.024	14306	27.039
Vascular dementia (multiple infarctions)	genus Lachnospiraceae UCG010	rs12346653	C	T	-0.039	89652324	0.624	0.080	360612	C	T	0.066	9	92267239	2.70E-06	0.014	14306	22.205
Vascular dementia (multiple infarctions)	genus Lachnospiraceae UCG010	rs17730011	G	A	-0.006	84063126	0.935	0.080	360612	G	A	-0.070	6	84772845	7.85E-06	0.016	14306	19.998
Vascular dementia (multiple infarctions)	genus Lachnospiraceae UCG010	rs2833528	T	C	-0.051	31821255	0.448	0.067	360612	T	C	0.056	21	33193567	9.92E-06	0.013	14306	19.359
Vascular dementia (multiple infarctions)	genus Lachnospiraceae UCG010	rs336138	G	T	0.069	8146258	0.499	0.102	360612	G	T	0.078	5	8146371	7.48E-06	0.017	14306	20.573
Vascular dementia (multiple infarctions)	genus Lachnospiraceae UCG010	rs4576377	C	A	-0.047	81780947	0.491	0.068	360612	C	A	0.057	7	81410263	7.63E-06	0.013	14306	20.272
Vascular dementia (multiple infarctions)	genus Lachnospiraceae UCG010	rs72894957	G	A	-0.399	184997864	0.064	0.216	360612	G	A	0.222	2	185862591	5.68E-06	0.049	14306	20.879
Vascular dementia (multiple infarctions)	genus Lachnospiraceae UCG010	rs74315802	G	T	-0.189	29403135	0.026	0.085	360612	G	T	0.087	14	29872341	3.19E-06	0.018	14306	22.343
Vascular dementia (multiple infarctions)	genus Lachnospiraceae UCG010	rs9981767	A	C	-0.034	42659765	0.649	0.075	360612	A	C	0.066	21	44079875	9.96E-07	0.013	14306	24.630
Vascular dementia (multiple infarctions)	genus Lactobacillus	rs12693845	C	T	-0.001	198447960	0.990	0.067	360612	C	T	-0.081	2	199312684	8.96E-06	0.018	14306	20.607
Vascular dementia (multiple infarctions)	genus Lactobacillus	rs1530559	G	A	0.123	134998059	0.064	0.066	360612	G	A	0.080	2	135755629	4.93E-06	0.018	14306	20.355
Vascular dementia (multiple infarctions)	genus Lactobacillus	rs16861661	G	A	-0.030	18174965	0.824	0.133	360612	G	A	-0.183	1	18501459	1.28E-06	0.038	14306	23.049
Vascular dementia (multiple infarctions)	genus Lactobacillus	rs62314653	C	A	0.237	108975306	0.087	0.138	360612	C	A	0.188	4	109896462	2.24E-06	0.039	14306	22.626
Vascular dementia (multiple infarctions)	genus Lactobacillus	rs7399658	G	A	0.099	23260829	0.248	0.086	360612	G	A	-0.107	13	23834968	3.12E-06	0.022	14306	23.313
Vascular dementia (multiple infarctions)	genus Lactobacillus	rs768253	T	G	0.094	68097868	0.156	0.066	360612	T	G	-0.079	8	69010103	4.25E-06	0.017	14306	21.252
Vascular dementia (multiple infarctions)	genus Lactobacillus	rs77478751	A	G	-0.090	173433875	0.379	0.103	360612	A	G	-0.220	3	173151665	7.33E-06	0.048	14306	21.361
Vascular dementia (multiple infarctions)	genus Lactobacillus	rs921925	A	C	-0.031	6928006	0.695	0.079	360612	A	C	0.099	19	6928017	9.72E-07	0.020	14306	23.495
Vascular dementia (multiple infarctions)	genus Lactococcus	rs10417872	G	T	0.008	28276446	0.915	0.072	360612	G	T	-0.118	19	28767353	1.29E-06	0.025	14306	23.276
Vascular dementia (multiple infarctions)	genus Lactococcus	rs123059	C	T	0.005	2796641	0.953	0.079	360612	C	T	0.137	17	2699935	1.27E-06	0.027	14306	24.769
Vascular dementia (multiple infarctions)	genus Lactococcus	rs12621813	G	A	0.124	31043183	0.092	0.074	360612	G	A	0.108	2	31266049	6.61E-06	0.024	14306	20.413
Vascular dementia (multiple infarctions)	genus Lactococcus	rs17168302	G	A	0.054	14610596	0.614	0.106	360612	G	A	0.192	7	14650221	6.29E-06	0.042	14306	20.402
Vascular dementia (multiple infarctions)	genus Lactococcus	rs2293361	C	T	0.224	53887727	0.125	0.146	360612	C	T	-0.199	2	54114864	1.40E-06	0.043	14306	21.369
Vascular dementia (multiple infarctions)	genus Lactococcus	rs4766997	C	T	0.065	112723633	0.324	0.066	360612	C	T	0.115	12	113161438	2.06E-06	0.024	14306	23.109
Vascular dementia (multiple infarctions)	genus Lactococcus	rs55910161	C	T	0.215	69758887	0.040	0.104	360612	C	T	0.146	10	71518643	2.36E-06	0.031	14306	22.695
Vascular dementia (multiple infarctions)	genus Lactococcus	rs6674304	C	T	0.240	116345120	0.151	0.167	360612	C	T	0.201	1	116887742	6.18E-06	0.044	14306	20.619
Vascular dementia (multiple infarctions)	genus Marvinbryantia	rs11620597	T	C	-0.054	85504955	0.799	0.210	360612	T	C	0.119	13	86079090	7.80E-06	0.027	14306	19.339
Vascular dementia (multiple infarctions)	genus Marvinbryantia	rs1187983	C	T	0.127	57976188	0.235	0.107	360612	C	T	-0.094	1	58441860	2.02E-06	0.019	14306	23.450
Vascular dementia (multiple infarctions)	genus Marvinbryantia	rs146541147	G	A	0.282	4542085	0.126	0.184	360612	G	A	0.119	19	4542097	6.86E-06	0.027	14306	19.603
Vascular dementia (multiple infarctions)	genus Marvinbryantia	rs2724813	G	A	-0.047	12474925	0.544	0.077	360612	G	A	0.084	10	12516924	6.28E-07	0.017	14306	25.180
Vascular dementia (multiple infarctions)	genus Marvinbryantia	rs2842896	C	T	-0.080	132560525	0.232	0.067	360612	C	T	-0.065	6	132881664	7.25E-07	0.013	14306	24.519
Vascular dementia (multiple infarctions)	genus Marvinbryantia	rs2863363	G	A	-0.019	166321977	0.802	0.076	360612	G	A	-0.063	3	166039765	3.11E-06	0.014	14306	21.688
Vascular dementia (multiple infarctions)	genus Marvinbryantia	rs3125832	A	C	0.053	211226838	0.502	0.078	360612	A	C	0.068	1	211400180	5.03E-06	0.015	14306	20.477
Vascular dementia (multiple infarctions)	genus Marvinbryantia	rs61884471	G	A	-0.104	45805010	0.315	0.103	360612	G	A	0.124	11	45826561	1.01E-06	0.025	14306	25.085
Vascular dementia (multiple infarctions)	genus Marvinbryantia	rs72948274	A	C	0.136	79807639	0.310	0.133	360612	A	C	-0.126	11	79518683	3.26E-06	0.027	14306	21.546
Vascular dementia (multiple infarctions)	genus Marvinbryantia	rs8006832	G	T	-0.116	21109678	0.306	0.113	360612	G	T	-0.095	14	21577837	6.58E-06	0.022	14306	19.317
Vascular dementia (multiple infarctions)	genus Methanobrevibacter	rs10202904	G	T	-0.010	124682691	0.886	0.067	360612	G	T	0.113	2	125440268	3.09E-06	0.024	14306	22.260
Vascular dementia (multiple infarctions)	genus Methanobrevibacter	rs1334944	T	C	0.013	110506288	0.855	0.073	360612	T	C	0.115	10	112266046	7.61E-06	0.026	14306	20.330
Vascular dementia (multiple infarctions)	genus Methanobrevibacter	rs4802933	G	A	0.002	52422567	0.978	0.078	360612	G	A	0.136	19	52925820	9.74E-06	0.031	14306	19.373
Vascular dementia (multiple infarctions)	genus Methanobrevibacter	rs6776814	T	C	0.044	15011576	0.848	0.228	360612	T	C	-0.189	3	15053083	8.05E-06	0.042	14306	20.250
Vascular dementia (multiple infarctions)	genus Methanobrevibacter	rs76029318	T	C	-0.057	41389655	0.669	0.134	360612	T	C	0.223	13	41963791	1.08E-06	0.045	14306	24.060
Vascular dementia (multiple infarctions)	genus Methanobrevibacter	rs894996	C	A	-0.197	103497150	0.122	0.127	360612	C	A	0.214	4	104418307	3.82E-06	0.046	14306	22.064
Vascular dementia (multiple infarctions)	genus Odoribacter	rs10093869	A	G	0.017	1318658	0.799	0.066	360612	A	G	-0.058	8	1266824	3.67E-06	0.013	14306	21.234
Vascular dementia (multiple infarctions)	genus Odoribacter	rs10423795	T	C	-0.019	49019831	0.774	0.068	360612	T	C	-0.055	19	49523088	6.58E-06	0.012	14306	20.657
Vascular dementia (multiple infarctions)	genus Odoribacter	rs28417404	A	G	0.068	70477507	0.538	0.110	360612	A	G	-0.073	14	70944224	3.68E-06	0.016	14306	20.290
Vascular dementia (multiple infarctions)	genus Odoribacter	rs4793970	A	G	0.070	48686600	0.309	0.068	360612	A	G	-0.058	17	46763962	6.03E-06	0.013	14306	19.912
Vascular dementia (multiple infarctions)	genus Odoribacter	rs6856150	A	G	-0.069	11905935	0.482	0.098	360612	A	G	-0.088	4	11907559	6.06E-06	0.019	14306	20.635
Vascular dementia (multiple infarctions)	genus Odoribacter	rs74553962	T	G	0.202	68765245	0.104	0.124	360612	T	G	0.121	8	69677480	9.49E-06	0.026	14306	21.146
Vascular dementia (multiple infarctions)	genus Odoribacter	rs77779484	G	A	0.010	67262163	0.941	0.134	360612	G	A	-0.133	12	67655943	6.56E-07	0.027	14306	24.713
Vascular dementia (multiple infarctions)	genus Olsenella	rs1035588	A	G	0.158	149220676	0.020	0.068	360612	A	G	-0.108	2	150077190	4.86E-06	0.024	14306	20.850
Vascular dementia (multiple infarctions)	genus Olsenella	rs17148768	G	A	-0.004	10735122	0.965	0.087	360612	G	A	0.140	10	10777085	2.20E-06	0.030	14306	22.570
Vascular dementia (multiple infarctions)	genus Olsenella	rs2759329	A	G	-0.118	231824606	0.081	0.068	360612	A	G	0.111	1	231960352	3.43E-06	0.024	14306	21.947
Vascular dementia (multiple infarctions)	genus Olsenella	rs35225860	A	G	0.068	247478968	0.680	0.165	360612	A	G	-0.224	1	247642270	3.87E-06	0.048	14306	21.486
Vascular dementia (multiple infarctions)	genus Olsenella	rs61090148	A	G	-0.001	173889347	0.987	0.066	360612	A	G	-0.105	4	174810498	6.44E-06	0.023	14306	20.515
Vascular dementia (multiple infarctions)	genus Olsenella	rs62112538	C	T	-0.142	4925006	0.183	0.107	360612	C	T	-0.199	19	4925018	1.19E-06	0.041	14306	24.006
Vascular dementia (multiple infarctions)	genus Olsenella	rs72691585	C	A	-0.105	21884426	0.277	0.097	360612	C	A	-0.249	9	21884425	2.95E-06	0.052	14306	22.872

Vascular dementia (multiple infarctions)	genus Olsenella	rs7540303	C	T	-0.042	179660056	0.531	0.068	360612	C	T	0.108	1	179629191	5.32E-06	0.024	14306	20.892
Vascular dementia (multiple infarctions)	genus Olsenella	rs8066522	A	G	0.012	61555512	0.866	0.069	360612	A	G	0.107	17	59632873	9.70E-06	0.024	14306	19.640
Vascular dementia (multiple infarctions)	genus Olsenella	rs9460691	C	A	0.072	10421974	0.392	0.084	360612	C	A	0.120	6	10422207	7.28E-06	0.027	14306	19.942
Vascular dementia (multiple infarctions)	genus Oscillibacter	rs11627628	T	C	0.174	21011446	0.164	0.125	360612	T	C	0.144	14	21479605	1.01E-06	0.029	14306	24.605
Vascular dementia (multiple infarctions)	genus Oscillibacter	rs11990279	T	C	-0.110	11258805	0.178	0.082	360612	T	C	-0.082	8	11116314	4.94E-06	0.018	14306	20.897
Vascular dementia (multiple infarctions)	genus Oscillibacter	rs12649930	T	G	-0.020	3654564	0.852	0.108	360612	T	G	0.122	4	3656291	4.09E-06	0.026	14306	21.935
Vascular dementia (multiple infarctions)	genus Oscillibacter	rs133832	A	C	-0.023	44438827	0.757	0.073	360612	A	C	-0.080	22	44834707	1.15E-06	0.016	14306	23.993
Vascular dementia (multiple infarctions)	genus Oscillibacter	rs16866406	A	G	-0.034	178592420	0.707	0.091	360612	A	G	0.099	2	179457147	3.08E-06	0.021	14306	22.426
Vascular dementia (multiple infarctions)	genus Oscillibacter	rs16934185	A	G	-0.007	1798324	0.950	0.109	360612	A	G	-0.130	9	1798324	4.38E-06	0.028	14306	21.175
Vascular dementia (multiple infarctions)	genus Oscillibacter	rs234108	A	G	0.008	184973539	0.904	0.066	360612	A	G	0.075	1	184942671	9.16E-07	0.015	14306	24.116
Vascular dementia (multiple infarctions)	genus Oscillibacter	rs36095275	C	T	0.037	31800923	0.576	0.067	360612	C	T	-0.075	14	32270129	1.40E-06	0.016	14306	23.005
Vascular dementia (multiple infarctions)	genus Oscillibacter	rs4506202	G	A	-0.012	21740565	0.855	0.066	360612	G	A	0.071	8	21598077	3.21E-06	0.015	14306	21.825
Vascular dementia (multiple infarctions)	genus Oscillibacter	rs61883564	A	G	-0.149	79302798	0.123	0.096	360612	A	G	-0.101	11	79013843	3.39E-06	0.022	14306	21.029
Vascular dementia (multiple infarctions)	genus Oscillibacter	rs75453768	G	T	-0.036	114710047	0.745	0.112	360612	G	T	0.122	10	116469806	5.35E-06	0.027	14306	20.667
Vascular dementia (multiple infarctions)	genus Oscillibacter	rs761240	G	T	-0.072	50891355	0.645	0.156	360612	G	T	0.177	20	49507892	2.04E-06	0.039	14306	20.639
Vascular dementia (multiple infarctions)	genus Oscillibacter	rs9393920	G	A	-0.023	28612816	0.727	0.067	360612	G	A	0.074	6	28580593	9.92E-07	0.015	14306	24.294
Vascular dementia (multiple infarctions)	genus Oscillospira	rs12206468	G	A	0.028	18093460	0.820	0.124	360612	G	A	-0.133	6	18093691	1.04E-06	0.027	14306	24.319
Vascular dementia (multiple infarctions)	genus Oscillospira	rs12925026	T	C	-0.253	89726448	0.072	0.141	360612	T	C	0.136	16	89792856	9.31E-06	0.031	14306	19.534
Vascular dementia (multiple infarctions)	genus Oscillospira	rs1954532	C	T	-0.053	27682209	0.515	0.081	360612	C	T	0.083	14	28151415	2.27E-06	0.018	14306	22.228
Vascular dementia (multiple infarctions)	genus Oscillospira	rs28889936	A	C	-0.012	88562149	0.912	0.111	360612	A	C	0.114	4	89483300	3.37E-06	0.025	14306	20.348
Vascular dementia (multiple infarctions)	genus Oscillospira	rs62422654	C	T	-0.092	170159406	0.251	0.080	360612	C	T	0.090	6	170474630	6.47E-06	0.020	14306	20.572
Vascular dementia (multiple infarctions)	genus Oscillospira	rs72866977	A	C	-0.037	56295685	0.760	0.122	360612	A	C	-0.131	6	56160483	5.63E-06	0.028	14306	21.488
Vascular dementia (multiple infarctions)	genus Oscillospira	rs751183	C	T	-0.047	76337408	0.581	0.085	360612	C	T	0.077	1	76803093	6.85E-06	0.017	14306	20.216
Vascular dementia (multiple infarctions)	genus Oscillospira	rs8076323	A	G	0.055	14978543	0.431	0.070	360612	A	G	0.072	17	14881860	5.61E-06	0.016	14306	20.885
Vascular dementia (multiple infarctions)	genus Oxalobacter	rs10464997	G	A	-0.090	21045182	0.286	0.085	360612	G	A	0.138	8	20902693	3.30E-06	0.029	14306	21.814
Vascular dementia (multiple infarctions)	genus Oxalobacter	rs11108500	A	G	-0.101	96425426	0.389	0.117	360612	A	G	-0.199	12	96819204	3.74E-06	0.043	14306	21.708
Vascular dementia (multiple infarctions)	genus Oxalobacter	rs111966731	T	C	0.212	93398708	0.070	0.117	360612	T	C	0.213	15	93941937	7.30E-06	0.047	14306	20.419
Vascular dementia (multiple infarctions)	genus Oxalobacter	rs12002250	A	C	-0.100	19682560	0.538	0.163	360612	A	C	0.217	9	19682558	1.42E-06	0.047	14306	21.679
Vascular dementia (multiple infarctions)	genus Oxalobacter	rs1569853	T	C	-0.042	38582525	0.678	0.101	360612	T	C	-0.138	6	38550301	3.65E-06	0.030	14306	21.617
Vascular dementia (multiple infarctions)	genus Oxalobacter	rs36057338	G	T	0.117	189014160	0.525	0.184	360612	G	T	0.208	4	189935314	8.80E-07	0.042	14306	24.323
Vascular dementia (multiple infarctions)	genus Oxalobacter	rs3862635	C	T	0.016	126712683	0.887	0.115	360612	C	T	-0.172	11	126582578	9.19E-06	0.039	14306	19.086
Vascular dementia (multiple infarctions)	genus Oxalobacter	rs4428215	G	A	-0.019	172229645	0.798	0.075	360612	G	A	0.130	3	171947435	7.51E-08	0.024	14306	28.931
Vascular dementia (multiple infarctions)	genus Oxalobacter	rs6000536	C	T	0.077	37025428	0.380	0.088	360612	C	T	-0.131	22	37421469	2.06E-07	0.025	14306	26.637
Vascular dementia (multiple infarctions)	genus Oxalobacter	rs6993398	G	A	0.012	114548460	0.884	0.084	360612	G	A	0.127	8	115560689	7.13E-06	0.028	14306	20.813
Vascular dementia (multiple infarctions)	genus Oxalobacter	rs736744	T	C	-0.022	84899492	0.743	0.066	360612	T	C	-0.118	9	87514407	2.57E-08	0.021	14306	31.135
Vascular dementia (multiple infarctions)	genus Parabacteroides	rs115602804	G	A	-0.056	191854576	0.596	0.106	360612	G	A	0.103	3	191572365	1.93E-06	0.022	14306	21.417
Vascular dementia (multiple infarctions)	genus Parabacteroides	rs4236095	A	G	0.013	47400951	0.906	0.108	360612	A	G	-0.076	6	47368687	1.93E-06	0.016	14306	23.541
Vascular dementia (multiple infarctions)	genus Parabacteroides	rs60884758	C	T	-0.055	2217340	0.527	0.086	360612	C	T	-0.070	9	2217340	5.71E-07	0.014	14306	24.401
Vascular dementia (multiple infarctions)	genus Parabacteroides	rs6657302	T	C	0.010	85119926	0.939	0.136	360612	T	C	-0.105	1	85585609	9.76E-06	0.023	14306	21.480
Vascular dementia (multiple infarctions)	genus Parabacteroides	rs7298818	C	T	-0.113	56003169	0.300	0.109	360612	C	T	0.089	12	56396953	8.54E-06	0.020	14306	19.574
Vascular dementia (multiple infarctions)	genus Paraprevotella	rs10842464	C	T	0.066	25096809	0.352	0.071	360612	C	T	0.076	12	25249743	6.60E-06	0.017	14306	19.305
Vascular dementia (multiple infarctions)	genus Paraprevotella	rs140997932	T	C	0.152	149581005	0.279	0.141	360612	T	C	-0.162	3	149298792	2.11E-06	0.035	14306	21.018
Vascular dementia (multiple infarctions)	genus Paraprevotella	rs145020347	A	G	-0.014	114655957	0.878	0.092	360612	A	G	-0.125	11	114526679	4.03E-06	0.026	14306	22.579
Vascular dementia (multiple infarctions)	genus Paraprevotella	rs17109926	A	G	-0.076	71584948	0.303	0.074	360612	A	G	-0.099	12	71978728	6.75E-06	0.022	14306	20.903
Vascular dementia (multiple infarctions)	genus Paraprevotella	rs17785622	A	G	-0.089	82749597	0.606	0.172	360612	A	G	0.248	6	83459314	1.93E-06	0.052	14306	22.385
Vascular dementia (multiple infarctions)	genus Paraprevotella	rs2081023	A	G	-0.117	175179258	0.213	0.094	360612	A	G	-0.123	5	174606261	2.64E-07	0.024	14306	26.854
Vascular dementia (multiple infarctions)	genus Paraprevotella	rs3008582	T	C	-0.048	196002043	0.570	0.084	360612	T	C	0.106	1	195971173	4.36E-06	0.023	14306	21.643
Vascular dementia (multiple infarctions)	genus Paraprevotella	rs3801748	G	A	-0.025	82130192	0.717	0.068	360612	G	A	0.078	7	81759508	5.20E-06	0.017	14306	20.624
Vascular dementia (multiple infarctions)	genus Paraprevotella	rs4756632	G	T	-0.240	41058153	0.014	0.098	360612	G	T	-0.139	11	41079703	3.82E-06	0.029	14306	22.959
Vascular dementia (multiple infarctions)	genus Paraprevotella	rs4767113	C	T	0.017	113694173	0.808	0.070	360612	C	T	0.088	12	114131978	2.14E-06	0.018	14306	23.047
Vascular dementia (multiple infarctions)	genus Paraprevotella	rs7240324	T	G	-0.043	71956568	0.572	0.076	360612	T	G	-0.102	18	69623804	5.96E-06	0.023	14306	20.315
Vascular dementia (multiple infarctions)	genus Paraprevotella	rs9602779	A	C	0.063	85588285	0.408	0.076	360612	A	C	-0.107	13	86162420	6.93E-07	0.022	14306	23.463
Vascular dementia (multiple infarctions)	genus Paraprevotella	rs9900242	A	G	-0.075	71139490	0.279	0.069	360612	A	G	-0.085	17	69135631	1.14E-06	0.018	14306	23.699
Vascular dementia (multiple infarctions)	genus Parasutterella	rs10899911	A	G	0.023	43798391	0.766	0.078	360612	A	G	-0.072	10	44293839	1.15E-06	0.015	14306	23.429
Vascular dementia (multiple infarctions)	genus Parasutterella	rs11715853	G	A	-0.028	30122198	0.693	0.072	360612	G	A	-0.066	3	30163689	6.23E-06	0.015	14306	20.586
Vascular dementia (multiple infarctions)	genus Parasutterella	rs2090816	C	A	-0.191	137294455	0.024	0.085	360612	C	A	-0.084	6	137615592	2.90E-06	0.018	14306	22.494
Vascular dementia (multiple infarctions)	genus Parasutterella	rs35055552	T	C	-0.113	113791795	0.234	0.095	360612	T	C	0.110	8	114804024	3.35E-06	0.024	14306	21.653
Vascular dementia (multiple infarctions)	genus Parasutterella	rs55877868	A	C	0.102	14789550	0.349	0.109	360612	A	C	-0.104	17	14692877	2.87E-06	0.023	14306	20.974
Vascular dementia (multiple infarctions)	genus Parasutterella	rs62273907	A	G	-0.079	156832626	0.547	0.132	360612	A	G	0.229	3	156550415	5.88E-06	0.050	14306	20.873
Vascular dementia (multiple infarctions)	genus Parasutterella	rs6809952	G	A	0.026	194178920	0.732	0.075	360612	G	A	-0.068	3	193896709	8.13E-06	0.015	14306	20.606
Vascular dementia (multiple infarctions)	genus Parasutterella	rs6828768	C	T	0.057	64691207	0.381	0.065	360612	C	T	0.064	4	65556925	1.78E-06	0.013	14306	23.051
Vascular dementia (multiple infarctions)	genus Parasutterella	rs7303158	C	T	-0.007	5166374	0.912	0.066	360612	C	T	0.065	12	5275540	1.33E-06	0.013	14306	23.214
Vascular dementia (multiple infarctions)	genus Parasutterella	rs7311004	C	T	-0.020	52866926	0.759	0.066	360612	C	T	0.062	12	53260710	5.92E-06	0.014	14306	20.485
Vascular dementia (multiple infarctions)	genus Parasutterella	rs7572229	A	G	-0.103	72008314	0.118	0.066	360612	A	G	-0.066	2	72235444	6.32E-07	0.013	14306	24.928
Vascular dementia (multiple infarctions)	genus Parasutterella	rs78383039	T	C	-0.251	178089981	0.141	0.170	360612	T	C	-0.146	2	178954708	1.57E-06	0.030	14306	24.250
Vascular dementia (multiple infarctions)	genus Parasutterella	rs8039785	G	T	0.102	67023969	0.115	0.065	360612	G	T	-0.062	15	67316307	3.62E-06	0.013	14306	21.615
Vascular dementia (multiple infarctions)	genus Parasutterella	rs823424	G	A	0.032	16817017	0.672	0.075	360612	G	A	-0.071	8	16674526	4.95E-06	0.016	14306	20.661

Vascular dementia (multiple infarctions)	genus Peptococcus	rs10031059	C	T	0.050	35356972	0.513	0.077	360612	C	T	0.121	4	35358594	1.24E-07	0.023	14306	28.784
Vascular dementia (multiple infarctions)	genus Peptococcus	rs11001941	G	A	-0.161	76956272	0.167	0.117	360612	G	A	-0.196	10	78716030	1.33E-06	0.039	14306	24.873
Vascular dementia (multiple infarctions)	genus Peptococcus	rs12069354	C	T	-0.214	216050440	0.108	0.133	360612	C	T	0.168	1	216223782	9.28E-06	0.038	14306	19.511
Vascular dementia (multiple infarctions)	genus Peptococcus	rs2054133	A	G	-0.040	33466742	0.563	0.069	360612	A	G	-0.090	2	33691809	2.14E-06	0.019	14306	22.606
Vascular dementia (multiple infarctions)	genus Peptococcus	rs36121075	A	G	0.005	44489376	0.954	0.088	360612	A	G	-0.141	20	43118017	6.99E-06	0.031	14306	21.094
Vascular dementia (multiple infarctions)	genus Peptococcus	rs413827	G	A	-0.038	57518394	0.618	0.077	360612	G	A	0.110	14	57985112	3.30E-06	0.024	14306	21.537
Vascular dementia (multiple infarctions)	genus Peptococcus	rs5770862	T	C	-0.092	50534684	0.414	0.113	360612	T	C	0.162	22	50973113	3.22E-06	0.036	14306	20.618
Vascular dementia (multiple infarctions)	genus Peptococcus	rs6918730	A	G	0.015	98572413	0.912	0.137	360612	A	G	-0.135	6	99020289	1.15E-06	0.029	14306	21.809
Vascular dementia (multiple infarctions)	genus Peptococcus	rs7033353	G	T	0.073	101833566	0.264	0.065	360612	G	T	-0.090	9	104595848	2.22E-06	0.019	14306	22.525
Vascular dementia (multiple infarctions)	genus Peptococcus	rs72850165	T	C	-0.102	968356	0.401	0.121	360612	T	C	-0.134	11	968356	5.74E-06	0.030	14306	19.985
Vascular dementia (multiple infarctions)	genus Peptococcus	rs74592222	G	A	-0.077	75506015	0.454	0.102	360612	G	A	0.138	1	75971700	8.55E-06	0.030	14306	20.735
Vascular dementia (multiple infarctions)	genus Peptococcus	rs77681628	C	T	-0.178	90530302	0.142	0.122	360612	C	T	0.200	13	91182556	2.69E-07	0.039	14306	26.744
Vascular dementia (multiple infarctions)	genus Phascolarctobacterium	rs12618201	A	G	-0.023	173571805	0.727	0.066	360612	A	G	0.064	2	174436533	3.38E-06	0.014	14306	21.556
Vascular dementia (multiple infarctions)	genus Phascolarctobacterium	rs1264476	G	T	0.047	101422048	0.575	0.084	360612	G	T	-0.077	8	102434276	4.30E-06	0.017	14306	21.357
Vascular dementia (multiple infarctions)	genus Phascolarctobacterium	rs56069061	G	A	-0.109	168621629	0.395	0.128	360612	G	A	-0.111	3	168339417	1.87E-06	0.023	14306	23.279
Vascular dementia (multiple infarctions)	genus Phascolarctobacterium	rs56157888	A	C	-0.062	182331683	0.441	0.080	360612	A	C	0.095	4	183252836	1.09E-06	0.019	14306	24.232
Vascular dementia (multiple infarctions)	genus Phascolarctobacterium	rs74540770	G	A	-0.005	186835600	0.967	0.123	360612	G	A	-0.121	3	186553389	3.60E-06	0.026	14306	21.895
Vascular dementia (multiple infarctions)	genus Phascolarctobacterium	rs75882962	T	C	-0.016	52704958	0.872	0.099	360612	T	C	0.097	12	53098742	3.19E-07	0.019	14306	25.835
Vascular dementia (multiple infarctions)	genus Phascolarctobacterium	rs7982713	G	A	0.056	70802190	0.444	0.073	360612	G	A	0.073	13	71376322	9.72E-06	0.016	14306	19.839
Vascular dementia (multiple infarctions)	genus Prevotella7	rs118038478	A	G	0.165	47203698	0.190	0.126	360612	A	G	0.206	16	47237609	7.85E-06	0.047	14306	19.239
Vascular dementia (multiple infarctions)	genus Prevotella7	rs12124567	A	G	-0.043	3427238	0.597	0.082	360612	A	G	-0.121	1	3343802	9.49E-06	0.028	14306	19.448
Vascular dementia (multiple infarctions)	genus Prevotella7	rs12195431	T	C	-0.117	90309935	0.290	0.111	360612	T	C	0.197	6	91019654	8.73E-06	0.044	14306	19.741
Vascular dementia (multiple infarctions)	genus Prevotella7	rs2240542	C	T	-0.127	241126899	0.087	0.074	360612	C	T	0.121	2	242066314	4.84E-06	0.026	14306	21.312
Vascular dementia (multiple infarctions)	genus Prevotella7	rs2918132	T	C	-0.008	131202057	0.906	0.068	360612	T	C	0.115	10	133000320	6.42E-06	0.025	14306	20.227
Vascular dementia (multiple infarctions)	genus Prevotella7	rs430270	A	C	-0.013	60488646	0.881	0.084	360612	A	C	0.139	3	60474379	2.87E-06	0.030	14306	21.944
Vascular dementia (multiple infarctions)	genus Prevotella7	rs57404562	C	A	0.028	11961636	0.771	0.096	360612	C	A	0.155	2	12101762	6.22E-07	0.032	14306	24.202
Vascular dementia (multiple infarctions)	genus Prevotella7	rs79263163	A	C	0.065	39800223	0.422	0.081	360612	A	C	-0.144	11	39821773	7.51E-06	0.032	14306	20.891
Vascular dementia (multiple infarctions)	genus Prevotella7	rs9426434	T	C	0.100	29409704	0.145	0.069	360612	T	C	-0.124	1	29736216	9.72E-06	0.028	14306	19.713
Vascular dementia (multiple infarctions)	genus Prevotella7	rs9608249	A	G	0.016	24217984	0.879	0.102	360612	A	G	-0.158	22	24613952	2.07E-06	0.034	14306	22.130
Vascular dementia (multiple infarctions)	genus Prevotella7	rs9959718	G	A	0.024	73775429	0.770	0.081	360612	G	A	0.133	18	71442664	1.90E-06	0.028	14306	23.333
Vascular dementia (multiple infarctions)	genus Prevotella9	rs111509883	T	C	-0.200	639161	0.061	0.107	360612	T	C	0.171	19	639161	1.24E-06	0.035	14306	24.235
Vascular dementia (multiple infarctions)	genus Prevotella9	rs11685699	C	T	0.031	11092559	0.801	0.121	360612	C	T	-0.141	2	11232685	2.03E-06	0.030	14306	22.858
Vascular dementia (multiple infarctions)	genus Prevotella9	rs117271932	A	G	-0.025	43840580	0.858	0.142	360612	A	G	0.208	22	44236460	2.82E-06	0.044	14306	22.326
Vascular dementia (multiple infarctions)	genus Prevotella9	rs12648235	T	C	0.022	160194592	0.773	0.078	360612	T	C	0.079	4	161115744	7.39E-06	0.018	14306	19.565
Vascular dementia (multiple infarctions)	genus Prevotella9	rs1304512	G	A	0.090	64440198	0.223	0.074	360612	G	A	0.076	5	63736025	5.29E-06	0.017	14306	21.078
Vascular dementia (multiple infarctions)	genus Prevotella9	rs2104588	C	T	-0.073	12455582	0.611	0.144	360612	C	T	-0.106	10	12497581	8.13E-06	0.024	14306	19.716
Vascular dementia (multiple infarctions)	genus Prevotella9	rs2495052	A	G	-0.129	13834756	0.167	0.093	360612	A	G	0.084	1	14161251	8.97E-06	0.019	14306	19.805
Vascular dementia (multiple infarctions)	genus Prevotella9	rs2683313	G	A	0.043	19258094	0.536	0.070	360612	G	A	0.072	8	19115604	1.69E-06	0.015	14306	22.843
Vascular dementia (multiple infarctions)	genus Prevotella9	rs4968431	G	T	-0.032	61344417	0.642	0.069	360612	G	T	0.064	17	59421778	8.58E-06	0.014	14306	19.716
Vascular dementia (multiple infarctions)	genus Prevotella9	rs7237249	C	T	-0.064	74325267	0.443	0.083	360612	C	T	-0.082	18	71992502	8.93E-06	0.018	14306	20.458
Vascular dementia (multiple infarctions)	genus Prevotella9	rs72815774	T	C	-0.073	91766476	0.600	0.140	360612	T	C	-0.176	2	91954502	8.78E-06	0.039	14306	20.091
Vascular dementia (multiple infarctions)	genus Prevotella9	rs746764	T	C	0.021	21490075	0.794	0.079	360612	T	C	-0.092	20	21470713	2.04E-06	0.019	14306	22.457
Vascular dementia (multiple infarctions)	genus Prevotella9	rs7976209	T	C	0.226	1685304	0.007	0.084	360612	T	C	-0.087	12	1794470	7.28E-06	0.020	14306	19.351
Vascular dementia (multiple infarctions)	genus Prevotella9	rs9428102	A	G	-0.023	118310194	0.770	0.080	360612	A	G	-0.078	1	118852817	4.62E-06	0.018	14306	19.568
Vascular dementia (multiple infarctions)	genus Prevotella9	rs9613013	G	A	0.078	25756771	0.435	0.100	360612	G	A	0.092	22	26152738	6.10E-06	0.020	14306	20.493
Vascular dementia (multiple infarctions)	genus Rikenellaceae RC9 gut group	rs12501673	A	G	0.017	162909364	0.819	0.073	360612	A	G	0.116	4	163830516	6.29E-06	0.026	14306	19.698
Vascular dementia (multiple infarctions)	genus Rikenellaceae RC9 gut group	rs17032291	T	C	-0.051	154944519	0.607	0.099	360612	T	C	-0.170	4	155865671	6.61E-06	0.037	14306	21.206
Vascular dementia (multiple infarctions)	genus Rikenellaceae RC9 gut group	rs17582787	A	G	-0.041	148641486	0.639	0.087	360612	A	G	-0.158	4	149562638	3.55E-06	0.034	14306	21.533
Vascular dementia (multiple infarctions)	genus Rikenellaceae RC9 gut group	rs2074881	T	C	-0.043	1970022	0.659	0.098	360612	T	C	-0.142	19	1970021	9.45E-06	0.032	14306	19.283
Vascular dementia (multiple infarctions)	genus Rikenellaceae RC9 gut group	rs2900503	T	G	-0.002	109895263	0.985	0.088	360612	T	G	0.172	9	112657543	1.55E-07	0.033	14306	27.829
Vascular dementia (multiple infarctions)	genus Rikenellaceae RC9 gut group	rs2998141	C	T	0.071	133196694	0.349	0.076	360612	C	T	0.136	10	135010198	4.42E-06	0.029	14306	21.644
Vascular dementia (multiple infarctions)	genus Rikenellaceae RC9 gut group	rs4270579	A	G	0.025	83777035	0.719	0.070	360612	A	G	0.118	4	84698188	5.46E-06	0.027	14306	18.960
Vascular dementia (multiple infarctions)	genus Rikenellaceae RC9 gut group	rs4717843	G	T	-0.011	73920657	0.868	0.066	360612	G	T	-0.119	7	73334987	4.72E-06	0.026	14306	20.992
Vascular dementia (multiple infarctions)	genus Rikenellaceae RC9 gut group	rs7712231	A	G	-0.122	31406178	0.204	0.096	360612	A	G	0.156	5	31406285	7.97E-06	0.035	14306	19.886
Vascular dementia (multiple infarctions)	genus Rikenellaceae RC9 gut group	rs80309088	G	A	0.072	165255243	0.473	0.100	360612	G	A	0.174	6	165668732	4.56E-06	0.038	14306	20.606
Vascular dementia (multiple infarctions)	genus Rikenellaceae RC9 gut group	rs9887954	G	A	0.092	164831636	0.168	0.067	360612	G	A	-0.115	1	164800873	4.81E-06	0.025	14306	21.272
Vascular dementia (multiple infarctions)	genus Romboutsia	rs10279978	A	G	0.160	5279756	0.024	0.071	360612	A	G	-0.062	7	5319387	1.17E-06	0.013	14306	23.749
Vascular dementia (multiple infarctions)	genus Romboutsia	rs11221428	T	C	0.135	99794319	0.084	0.078	360612	T	C	-0.073	11	99665050	6.49E-06	0.016	14306	21.089
Vascular dementia (multiple infarctions)	genus Romboutsia	rs16843578	C	T	-0.012	171934745	0.937	0.147	360612	C	T	-0.088	1	171903885	5.08E-06	0.020	14306	19.785
Vascular dementia (multiple infarctions)	genus Romboutsia	rs28603357	T	C	-0.231	106766378	0.325	0.235	360612	T	C	-0.215	7	106406824	8.52E-06	0.047	14306	20.493
Vascular dementia (multiple infarctions)	genus Romboutsia	rs34302036	G	A	0.116	78507408	0.081	0.066	360612	G	A	-0.055	7	78136725	5.88E-06	0.012	14306	20.737
Vascular dementia (multiple infarctions)	genus Romboutsia	rs61841503	G	A	0.007	16977560	0.941	0.098	360612	G	A	0.093	10	17019559	4.00E-08	0.017	14306	29.351
Vascular dementia (multiple infarctions)	genus Romboutsia	rs62504452	A	G	-0.013	146063205	0.890	0.093	360612	A	G	-0.071	7	145760298	4.66E-06	0.016	14306	20.544
Vascular dementia (multiple infarctions)	genus Romboutsia	rs7109293	A	G	-0.022	133964762	0.834	0.103	360612	A	G	0.092	11	133834657	6.98E-06	0.021	14306	19.958
Vascular dementia (multiple infarctions)	genus Romboutsia	rs75200530	T	G	0.086	5230532	0.654	0.192	360612	T	G	-0.191	8	5088054	5.07E-06	0.042	14306	20.500
Vascular dementia (multiple infarctions)	genus Romboutsia	rs75987356	G	A	-0.143	15926583	0.244	0.123	360612	G	A	-0.130	2	16066705	6.71E-06	0.028	14306	21.347
Vascular dementia (multiple infarctions)	genus Romboutsia	rs77702691	A	G	0.006	88073285	0.958	0.115	360612	A	G	-0.094	13	88725540	7.37E-06	0.021	14306	20.493

Vascular dementia (multiple infarctions)	genus Romboutsia	rs9389266	T	G	-0.038	135090599	0.664	0.086	360612	T	G	0.072	6	135411737	9.38E-06	0.016	14306	19.821
Vascular dementia (multiple infarctions)	genus Romboutsia	rs9567264	C	T	-0.008	32146619	0.909	0.069	360612	C	T	0.058	13	32720756	5.76E-06	0.013	14306	20.696
Vascular dementia (multiple infarctions)	genus Roseburia	rs12740451	T	C	0.020	94713418	0.833	0.096	360612	T	C	0.070	1	95178974	7.34E-06	0.015	14306	20.621
Vascular dementia (multiple infarctions)	genus Roseburia	rs16910295	T	C	0.032	11988022	0.820	0.141	360612	T	C	-0.098	11	12009569	2.91E-06	0.021	14306	21.887
Vascular dementia (multiple infarctions)	genus Roseburia	rs2160994	C	T	0.082	50256274	0.236	0.069	360612	C	T	-0.055	12	50650057	9.70E-07	0.011	14306	23.969
Vascular dementia (multiple infarctions)	genus Roseburia	rs2943022	T	C	-0.067	90303097	0.312	0.066	360612	T	C	0.049	5	89598914	4.11E-06	0.011	14306	21.391
Vascular dementia (multiple infarctions)	genus Roseburia	rs302266	T	C	-0.040	194214520	0.683	0.099	360612	T	C	-0.078	1	194183650	8.13E-06	0.017	14306	20.192
Vascular dementia (multiple infarctions)	genus Roseburia	rs329182	T	C	-0.052	125740801	0.560	0.089	360612	T	C	0.069	5	125076494	5.90E-06	0.015	14306	20.389
Vascular dementia (multiple infarctions)	genus Roseburia	rs55858165	A	C	-0.046	85969578	0.789	0.174	360612	A	C	0.179	15	86512809	9.99E-06	0.040	14306	19.601
Vascular dementia (multiple infarctions)	genus Roseburia	rs57466170	C	T	-0.210	37566864	0.095	0.126	360612	C	T	0.074	15	37859065	8.30E-06	0.017	14306	18.667
Vascular dementia (multiple infarctions)	genus Roseburia	rs6445851	A	G	-0.117	57082200	0.081	0.067	360612	A	G	0.050	3	57116228	3.53E-06	0.011	14306	21.144
Vascular dementia (multiple infarctions)	genus Roseburia	rs6930661	C	T	-0.135	12774379	0.333	0.139	360612	C	T	-0.096	6	12774611	2.48E-06	0.020	14306	22.008
Vascular dementia (multiple infarctions)	genus Roseburia	rs75326254	C	T	0.033	165757348	0.802	0.133	360612	C	T	-0.105	6	166170836	7.50E-06	0.023	14306	20.533
Vascular dementia (multiple infarctions)	genus Roseburia	rs78753150	A	C	-0.192	144756092	0.079	0.109	360612	A	C	0.097	5	144135655	9.98E-06	0.021	14306	20.479
Vascular dementia (multiple infarctions)	genus Roseburia	rs9300744	C	T	-0.048	102465136	0.575	0.085	360612	C	T	-0.059	13	103117486	4.75E-06	0.013	14306	21.733
Vascular dementia (multiple infarctions)	genus Ruminiclostridium5	rs10827477	A	G	0.120	34973660	0.080	0.068	360612	A	G	-0.055	10	35262588	2.19E-06	0.012	14306	22.592
Vascular dementia (multiple infarctions)	genus Ruminiclostridium5	rs113753996	T	C	0.065	32477558	0.446	0.085	360612	T	C	0.082	5	32477664	3.99E-06	0.017	14306	22.128
Vascular dementia (multiple infarctions)	genus Ruminiclostridium5	rs1223978	C	T	0.030	108122814	0.646	0.066	360612	C	T	-0.048	13	108775162	8.16E-06	0.011	14306	20.004
Vascular dementia (multiple infarctions)	genus Ruminiclostridium5	rs1492620	T	C	-0.111	50438207	0.258	0.098	360612	T	C	-0.083	6	50405920	3.53E-06	0.018	14306	21.271
Vascular dementia (multiple infarctions)	genus Ruminiclostridium5	rs2482038	C	A	0.010	12793086	0.887	0.067	360612	C	A	0.052	10	12835085	1.70E-06	0.011	14306	22.764
Vascular dementia (multiple infarctions)	genus Ruminiclostridium5	rs2791343	T	C	0.028	130303784	0.669	0.067	360612	T	C	0.052	8	131316030	5.54E-06	0.011	14306	20.810
Vascular dementia (multiple infarctions)	genus Ruminiclostridium5	rs2833828	G	A	0.059	32439578	0.378	0.067	360612	G	A	0.049	21	33811886	6.82E-06	0.011	14306	20.288
Vascular dementia (multiple infarctions)	genus Ruminiclostridium5	rs4955951	A	G	-0.019	55209503	0.851	0.101	360612	A	G	-0.071	3	55243531	9.96E-06	0.017	14306	18.526
Vascular dementia (multiple infarctions)	genus Ruminiclostridium5	rs6121460	G	A	-0.025	61718449	0.839	0.121	360612	G	A	0.093	20	60293505	2.64E-06	0.020	14306	21.934
Vascular dementia (multiple infarctions)	genus Ruminiclostridium5	rs79968837	A	G	-0.172	42549316	0.241	0.147	360612	A	G	-0.095	20	41177956	1.15E-06	0.019	14306	24.118
Vascular dementia (multiple infarctions)	genus Ruminiclostridium5	rs8053158	G	A	0.145	86736303	0.144	0.099	360612	G	A	0.074	16	86769909	5.90E-06	0.016	14306	21.651
Vascular dementia (multiple infarctions)	genus Ruminiclostridium6	rs10829821	T	C	0.081	130853030	0.469	0.112	360612	T	C	-0.098	10	132651293	3.47E-06	0.022	14306	20.406
Vascular dementia (multiple infarctions)	genus Ruminiclostridium6	rs116969552	A	G	-0.259	126525834	0.184	0.195	360612	A	G	-0.167	10	128214403	9.16E-06	0.038	14306	19.614
Vascular dementia (multiple infarctions)	genus Ruminiclostridium6	rs11992182	A	C	0.105	78854264	0.187	0.079	360612	A	C	0.063	8	79766499	4.65E-06	0.014	14306	20.568
Vascular dementia (multiple infarctions)	genus Ruminiclostridium6	rs2548459	C	T	0.073	48706082	0.272	0.066	360612	C	T	0.055	19	49209339	6.40E-06	0.012	14306	20.431
Vascular dementia (multiple infarctions)	genus Ruminiclostridium6	rs35362464	C	A	0.079	36476389	0.401	0.094	360612	C	A	0.072	4	36478011	8.99E-06	0.017	14306	18.956
Vascular dementia (multiple infarctions)	genus Ruminiclostridium6	rs61060922	T	G	0.028	72102255	0.884	0.189	360612	T	G	0.159	16	72136154	1.09E-06	0.032	14306	24.380
Vascular dementia (multiple infarctions)	genus Ruminiclostridium6	rs663262	C	T	-0.043	86468034	0.810	0.179	360612	C	T	0.135	11	86179076	3.39E-06	0.031	14306	18.872
Vascular dementia (multiple infarctions)	genus Ruminiclostridium6	rs67479537	T	C	0.027	10004839	0.862	0.154	360612	T	C	0.119	19	10115515	9.30E-06	0.026	14306	20.183
Vascular dementia (multiple infarctions)	genus Ruminiclostridium6	rs71414120	T	G	0.327	56472234	0.012	0.130	360612	T	G	0.201	14	56938952	1.08E-06	0.041	14306	24.432
Vascular dementia (multiple infarctions)	genus Ruminiclostridium6	rs72991535	T	G	-0.224	78258244	0.213	0.180	360612	T	G	0.136	18	76018244	4.95E-06	0.030	14306	21.105
Vascular dementia (multiple infarctions)	genus Ruminiclostridium6	rs73176030	T	C	-0.061	101628002	0.404	0.073	360612	T	C	0.059	7	101271282	7.29E-06	0.013	14306	19.728
Vascular dementia (multiple infarctions)	genus Ruminiclostridium6	rs77193512	A	G	0.105	40267513	0.165	0.075	360612	A	G	0.074	11	40289063	1.30E-06	0.015	14306	23.125
Vascular dementia (multiple infarctions)	genus Ruminiclostridium6	rs792058	G	A	0.018	5408472	0.792	0.066	360612	G	A	0.055	2	5548605	8.58E-06	0.013	14306	19.527
Vascular dementia (multiple infarctions)	genus Ruminiclostridium6	rs79968172	G	A	-0.241	240340526	0.084	0.139	360612	G	A	0.116	1	240503826	1.66E-06	0.024	14306	22.827
Vascular dementia (multiple infarctions)	genus Ruminiclostridium6	rs9555756	A	C	0.020	111050902	0.863	0.117	360612	A	C	-0.080	13	111703249	7.10E-06	0.018	14306	20.712
Vascular dementia (multiple infarctions)	genus Ruminiclostridium9	rs12040548	G	T	0.102	247546983	0.164	0.073	360612	G	T	0.057	1	247710285	3.15E-06	0.012	14306	21.733
Vascular dementia (multiple infarctions)	genus Ruminiclostridium9	rs6082461	C	A	-0.156	2229884	0.056	0.082	360612	C	A	-0.059	20	2210530	4.87E-06	0.013	14306	20.038
Vascular dementia (multiple infarctions)	genus Ruminiclostridium9	rs7137760	C	T	0.027	10549815	0.680	0.066	360612	C	T	0.051	12	10702414	7.07E-06	0.011	14306	20.504
Vascular dementia (multiple infarctions)	genus Ruminiclostridium9	rs74303178	T	C	-0.053	14840035	0.454	0.070	360612	T	C	0.053	8	14697544	7.92E-06	0.012	14306	19.950
Vascular dementia (multiple infarctions)	genus Ruminiclostridium9	rs78191726	T	C	-0.101	66945427	0.425	0.126	360612	T	C	0.094	6	67655320	7.58E-06	0.021	14306	20.210
Vascular dementia (multiple infarctions)	genus Ruminiclostridium9	rs918449	G	A	0.148	33880293	0.254	0.130	360612	G	A	0.095	19	34371198	2.56E-06	0.020	14306	23.258
Vascular dementia (multiple infarctions)	genus Ruminiclostridium9	rs9522712	T	C	-0.061	89789324	0.513	0.093	360612	T	C	0.070	13	90441578	4.66E-06	0.015	14306	20.396
Vascular dementia (multiple infarctions)	genus Ruminiclostridium9	rs9809789	C	T	0.009	29138492	0.914	0.085	360612	C	T	-0.072	3	29179983	8.72E-06	0.016	14306	20.189
Vascular dementia (multiple infarctions)	genus Ruminococcaceae NK4A214 group	rs11241747	T	C	-0.072	124510625	0.316	0.072	360612	T	C	-0.053	5	123846318	6.59E-06	0.012	14306	19.780
Vascular dementia (multiple infarctions)	genus Ruminococcaceae NK4A214 group	rs11586410	G	A	-0.026	157369098	0.777	0.090	360612	G	A	-0.086	1	157338888	3.66E-07	0.017	14306	25.815
Vascular dementia (multiple infarctions)	genus Ruminococcaceae NK4A214 group	rs12642039	C	T	-0.005	159010870	0.938	0.068	360612	C	T	0.055	4	159932022	3.43E-06	0.012	14306	21.452
Vascular dementia (multiple infarctions)	genus Ruminococcaceae NK4A214 group	rs12731	A	G	0.121	238179271	0.071	0.067	360612	A	G	-0.053	2	239087912	4.87E-06	0.012	14306	21.035
Vascular dementia (multiple infarctions)	genus Ruminococcaceae NK4A214 group	rs13087692	G	T	0.004	84675888	0.958	0.072	360612	G	T	-0.057	3	84725039	8.69E-06	0.013	14306	20.818
Vascular dementia (multiple infarctions)	genus Ruminococcaceae NK4A214 group	rs136761	A	G	0.085	49402365	0.211	0.068	360612	A	G	0.059	22	49796014	8.15E-07	0.012	14306	24.312
Vascular dementia (multiple infarctions)	genus Ruminococcaceae NK4A214 group	rs147475196	A	G	0.022	26467796	0.840	0.107	360612	A	G	-0.134	3	26509287	4.72E-06	0.030	14306	20.535
Vascular dementia (multiple infarctions)	genus Ruminococcaceae NK4A214 group	rs35559912	T	C	0.043	35288836	0.666	0.101	360612	T	C	-0.093	5	35288938	4.89E-06	0.020	14306	20.629
Vascular dementia (multiple infarctions)	genus Ruminococcaceae NK4A214 group	rs4814689	C	T	-0.099	18049713	0.529	0.157	360612	C	T	-0.108	20	18030357	4.55E-06	0.023	14306	22.036
Vascular dementia (multiple infarctions)	genus Ruminococcaceae NK4A214 group	rs5994253	A	G	-0.114	17312122	0.223	0.094	360612	A	G	-0.081	22	17793012	2.35E-07	0.016	14306	26.493
Vascular dementia (multiple infarctions)	genus Ruminococcaceae NK4A214 group	rs62027366	T	C	-0.061	24062485	0.447	0.081	360612	T	C	0.062	16	24073806	6.58E-06	0.014	14306	19.998
Vascular dementia (multiple infarctions)	genus Ruminococcaceae NK4A214 group	rs6681678	T	C	-0.112	99783035	0.539	0.182	360612	T	C	0.100	1	100248591	9.05E-06	0.024	14306	17.422
Vascular dementia (multiple infarctions)	genus Ruminococcaceae NK4A214 group	rs7573569	T	C	0.089	141139473	0.518	0.138	360612	T	C	0.108	2	141897042	3.23E-06	0.023	14306	21.265
Vascular dementia (multiple infarctions)	genus Ruminococcaceae UCG002	rs10916131	C	T	-0.091	227375425	0.306	0.089	360612	C	T	-0.069	1	227563126	2.87E-06	0.015	14306	22.321
Vascular dementia (multiple infarctions)	genus Ruminococcaceae UCG002	rs10927423	C	A	0.045	14405962	0.602	0.086	360612	C	A	-0.071	1	14732458	8.50E-07	0.015	14306	23.340
Vascular dementia (multiple infarctions)	genus Ruminococcaceae UCG002	rs10964441	G	A	0.082	20131748	0.450	0.109	360612	G	A	-0.149	9	20131746	7.45E-06	0.034	14306	18.683
Vascular dementia (multiple infarctions)	genus Ruminococcaceae UCG002	rs113147300	A	G	0.024	111304390	0.803	0.095	360612	A	G	-0.076	9	114066670	7.69E-06	0.016	14306	21.240
Vascular dementia (multiple infarctions)	genus Ruminococcaceae UCG002	rs11607472	A	G	0.169	43323201	0.193	0.130	360612	A	G	-0.078	11	43344751	7.19E-06	0.018	14306	19.580

Vascular dementia (multiple infarctions)	genus Ruminococcaceae UCG002	rs116974815	C	A	-0.036	111842219	0.778	0.129	360612	C	A	-0.190	11	111712942	2.03E-06	0.040	14306	22.890
Vascular dementia (multiple infarctions)	genus Ruminococcaceae UCG002	rs11750293	G	T	-0.023	124486421	0.731	0.068	360612	G	T	-0.058	5	123822114	1.76E-06	0.012	14306	23.028
Vascular dementia (multiple infarctions)	genus Ruminococcaceae UCG002	rs12463378	A	G	-0.017	53972554	0.812	0.071	360612	A	G	-0.052	19	54475808	2.96E-06	0.011	14306	21.694
Vascular dementia (multiple infarctions)	genus Ruminococcaceae UCG002	rs15256	C	T	0.070	72060790	0.490	0.102	360612	C	T	0.073	10	73820548	9.46E-06	0.017	14306	18.928
Vascular dementia (multiple infarctions)	genus Ruminococcaceae UCG002	rs55793120	T	C	0.046	46990335	0.741	0.140	360612	T	C	0.137	12	47384118	4.81E-07	0.027	14306	25.119
Vascular dementia (multiple infarctions)	genus Ruminococcaceae UCG002	rs57079348	T	G	-0.162	87268195	0.252	0.141	360612	T	G	-0.077	13	87920450	7.22E-06	0.017	14306	19.632
Vascular dementia (multiple infarctions)	genus Ruminococcaceae UCG002	rs6542556	G	A	-0.027	120000277	0.685	0.067	360612	G	A	-0.051	2	120757853	7.86E-06	0.011	14306	19.972
Vascular dementia (multiple infarctions)	genus Ruminococcaceae UCG002	rs6793778	T	C	-0.028	24003962	0.710	0.075	360612	T	C	0.056	3	24045453	9.81E-06	0.013	14306	19.896
Vascular dementia (multiple infarctions)	genus Ruminococcaceae UCG002	rs7120052	A	C	-0.057	86624417	0.497	0.083	360612	A	C	0.062	11	86335459	1.97E-06	0.014	14306	21.254
Vascular dementia (multiple infarctions)	genus Ruminococcaceae UCG002	rs7155595	C	A	0.120	77036203	0.095	0.072	360612	C	A	0.057	14	77502546	1.15E-06	0.012	14306	23.734
Vascular dementia (multiple infarctions)	genus Ruminococcaceae UCG002	rs7249614	G	A	0.020	43133738	0.765	0.068	360612	G	A	0.049	19	43637890	9.07E-06	0.011	14306	19.777
Vascular dementia (multiple infarctions)	genus Ruminococcaceae UCG002	rs76847269	A	G	-0.060	141635384	0.768	0.204	360612	A	G	0.164	5	141014951	5.17E-06	0.036	14306	21.077
Vascular dementia (multiple infarctions)	genus Ruminococcaceae UCG002	rs77564310	A	C	0.096	80420842	0.233	0.080	360612	A	C	-0.071	15	80713184	3.29E-07	0.014	14306	25.630
Vascular dementia (multiple infarctions)	genus Ruminococcaceae UCG002	rs79016051	C	T	0.012	238775197	0.902	0.097	360612	C	T	-0.089	1	238938497	2.34E-06	0.019	14306	21.964
Vascular dementia (multiple infarctions)	genus Ruminococcaceae UCG002	rs882348	A	G	-0.021	23323417	0.837	0.102	360612	A	G	-0.080	4	23325040	5.45E-06	0.018	14306	20.057
Vascular dementia (multiple infarctions)	genus Ruminococcaceae UCG003	rs10490280	C	T	-0.008	37678833	0.920	0.084	360612	C	T	-0.067	2	37905976	4.16E-06	0.014	14306	21.994
Vascular dementia (multiple infarctions)	genus Ruminococcaceae UCG003	rs11243416	T	C	0.140	131541583	0.269	0.127	360612	T	C	-0.093	9	134416970	1.67E-06	0.019	14306	23.437
Vascular dementia (multiple infarctions)	genus Ruminococcaceae UCG003	rs11613919	G	T	-0.077	75102683	0.328	0.079	360612	G	T	0.073	12	75496463	1.63E-06	0.016	14306	21.834
Vascular dementia (multiple infarctions)	genus Ruminococcaceae UCG003	rs16959793	A	C	0.063	34779517	0.343	0.067	360612	A	C	-0.063	15	35071718	2.22E-06	0.013	14306	22.692
Vascular dementia (multiple infarctions)	genus Ruminococcaceae UCG003	rs2523124	C	T	-0.022	97719124	0.743	0.067	360612	C	T	0.055	7	97348436	5.78E-06	0.012	14306	20.468
Vascular dementia (multiple infarctions)	genus Ruminococcaceae UCG003	rs3013089	G	A	-0.012	13468126	0.855	0.068	360612	G	A	-0.055	1	13794594	4.38E-06	0.012	14306	20.998
Vascular dementia (multiple infarctions)	genus Ruminococcaceae UCG003	rs4452755	A	C	-0.006	81114617	0.927	0.069	360612	A	C	-0.063	8	82026852	3.29E-06	0.013	14306	22.172
Vascular dementia (multiple infarctions)	genus Ruminococcaceae UCG003	rs4532474	G	A	0.007	105333663	0.936	0.087	360612	G	A	0.077	6	105781538	4.82E-06	0.017	14306	20.367
Vascular dementia (multiple infarctions)	genus Ruminococcaceae UCG003	rs646327	G	A	0.067	48706594	0.310	0.066	360612	G	A	0.059	19	49209851	7.83E-07	0.012	14306	24.567
Vascular dementia (multiple infarctions)	genus Ruminococcaceae UCG003	rs6759615	A	G	0.080	204373993	0.471	0.111	360612	A	G	0.103	2	205238716	7.86E-07	0.020	14306	26.224
Vascular dementia (multiple infarctions)	genus Ruminococcaceae UCG003	rs73341549	T	C	0.241	51473771	0.088	0.142	360612	T	C	-0.170	7	51541468	1.51E-07	0.032	14306	28.393
Vascular dementia (multiple infarctions)	genus Ruminococcaceae UCG003	rs78720113	A	G	0.091	41940901	0.451	0.120	360612	A	G	-0.115	3	41982393	7.59E-06	0.025	14306	21.356
Vascular dementia (multiple infarctions)	genus Ruminococcaceae UCG004	rs10976229	T	G	0.034	7317307	0.725	0.098	360612	T	G	0.096	9	7317307	7.04E-06	0.021	14306	20.010
Vascular dementia (multiple infarctions)	genus Ruminococcaceae UCG004	rs11961899	G	A	0.062	132566782	0.396	0.073	360612	G	A	-0.071	6	132887921	9.18E-06	0.016	14306	19.237
Vascular dementia (multiple infarctions)	genus Ruminococcaceae UCG004	rs12125734	G	T	0.061	103266658	0.593	0.115	360612	G	T	0.134	1	103732214	2.09E-07	0.026	14306	27.087
Vascular dementia (multiple infarctions)	genus Ruminococcaceae UCG004	rs2248146	T	C	0.017	24752379	0.807	0.068	360612	T	C	0.069	7	24791998	8.20E-06	0.015	14306	20.135
Vascular dementia (multiple infarctions)	genus Ruminococcaceae UCG004	rs3800154	A	C	0.018	2119095	0.815	0.075	360612	A	C	-0.080	6	2119329	6.12E-06	0.018	14306	20.178
Vascular dementia (multiple infarctions)	genus Ruminococcaceae UCG004	rs511258	G	A	0.047	170248934	0.574	0.084	360612	G	A	-0.076	3	169966722	4.52E-06	0.016	14306	21.735
Vascular dementia (multiple infarctions)	genus Ruminococcaceae UCG004	rs550351	A	C	-0.039	18349905	0.555	0.066	360612	A	C	0.079	1	18676399	9.43E-06	0.018	14306	19.055
Vascular dementia (multiple infarctions)	genus Ruminococcaceae UCG004	rs6769553	A	G	-0.114	54393768	0.122	0.074	360612	A	G	0.085	3	54427795	7.91E-08	0.016	14306	29.126
Vascular dementia (multiple infarctions)	genus Ruminococcaceae UCG004	rs7569771	A	G	0.019	238924641	0.801	0.076	360612	A	G	-0.076	2	239846337	8.12E-06	0.017	14306	19.869
Vascular dementia (multiple infarctions)	genus Ruminococcaceae UCG004	rs872501	G	A	-0.033	138240821	0.783	0.122	360612	G	A	0.116	8	139253064	5.81E-06	0.026	14306	20.031
Vascular dementia (multiple infarctions)	genus Ruminococcaceae UCG004	rs9818949	T	G	0.027	197956880	0.740	0.083	360612	T	G	-0.086	3	197683751	5.39E-06	0.019	14306	20.774
Vascular dementia (multiple infarctions)	genus Ruminococcaceae UCG005	rs10873449	C	T	-0.086	94164394	0.299	0.083	360612	C	T	-0.065	14	94630731	4.11E-06	0.014	14306	20.688
Vascular dementia (multiple infarctions)	genus Ruminococcaceae UCG005	rs10937802	A	G	-0.029	7300617	0.772	0.101	360612	A	G	-0.076	4	7302344	8.17E-06	0.017	14306	20.186
Vascular dementia (multiple infarctions)	genus Ruminococcaceae UCG005	rs10950694	C	T	0.112	17986981	0.101	0.068	360612	C	T	-0.058	7	18026604	4.30E-07	0.011	14306	25.620
Vascular dementia (multiple infarctions)	genus Ruminococcaceae UCG005	rs114279581	A	G	-0.082	197257633	0.508	0.124	360612	A	G	-0.147	2	198122357	3.22E-06	0.032	14306	21.519
Vascular dementia (multiple infarctions)	genus Ruminococcaceae UCG005	rs12288512	A	G	-0.176	27726124	0.021	0.076	360612	A	G	0.067	11	27747671	3.10E-06	0.014	14306	21.308
Vascular dementia (multiple infarctions)	genus Ruminococcaceae UCG005	rs12458218	T	C	-0.003	24995907	0.973	0.085	360612	T	C	0.068	18	22575871	2.41E-06	0.014	14306	21.964
Vascular dementia (multiple infarctions)	genus Ruminococcaceae UCG005	rs2893871	G	A	0.153	60903023	0.142	0.105	360612	G	A	-0.074	10	62662781	3.54E-06	0.016	14306	22.435
Vascular dementia (multiple infarctions)	genus Ruminococcaceae UCG005	rs34781347	G	A	0.002	16332206	0.988	0.129	360612	G	A	0.189	20	16312851	6.05E-07	0.039	14306	23.835
Vascular dementia (multiple infarctions)	genus Ruminococcaceae UCG005	rs55793120	T	C	0.046	46990335	0.741	0.140	360612	T	C	0.122	12	47384118	7.37E-06	0.028	14306	18.906
Vascular dementia (multiple infarctions)	genus Ruminococcaceae UCG005	rs72776570	C	A	0.089	4198714	0.423	0.111	360612	C	A	0.087	10	4240906	5.36E-06	0.020	14306	19.498
Vascular dementia (multiple infarctions)	genus Ruminococcaceae UCG005	rs7449320	C	A	-0.061	154623531	0.427	0.077	360612	C	A	0.060	5	154003091	4.81E-06	0.013	14306	20.972
Vascular dementia (multiple infarctions)	genus Ruminococcaceae UCG005	rs7555878	G	A	-0.083	187936903	0.274	0.076	360612	G	A	-0.059	1	187906034	2.81E-06	0.013	14306	21.931
Vascular dementia (multiple infarctions)	genus Ruminococcaceae UCG005	rs7586445	G	A	-0.051	238600389	0.594	0.096	360612	G	A	0.078	2	239509030	8.81E-06	0.018	14306	19.657
Vascular dementia (multiple infarctions)	genus Ruminococcaceae UCG005	rs898577	C	T	0.137	68413358	0.333	0.141	360612	C	T	0.123	15	68705697	7.46E-06	0.029	14306	18.413
Vascular dementia (multiple infarctions)	genus Ruminococcaceae UCG009	rs12508214	C	T	0.044	7425210	0.528	0.069	360612	C	T	-0.077	4	7426937	4.75E-06	0.017	14306	21.026
Vascular dementia (multiple infarctions)	genus Ruminococcaceae UCG009	rs138460696	A	G	0.045	66169644	0.724	0.126	360612	A	G	0.139	2	66396776	9.81E-06	0.032	14306	19.451
Vascular dementia (multiple infarctions)	genus Ruminococcaceae UCG009	rs1550196	A	G	0.134	34459936	0.225	0.110	360612	A	G	-0.131	17	32786955	1.13E-06	0.026	14306	24.849
Vascular dementia (multiple infarctions)	genus Ruminococcaceae UCG009	rs2058609	G	A	0.063	12817858	0.391	0.073	360612	G	A	-0.082	12	12970792	3.12E-06	0.017	14306	21.837
Vascular dementia (multiple infarctions)	genus Ruminococcaceae UCG009	rs2192926	A	G	-0.089	75285542	0.203	0.070	360612	A	G	-0.089	2	75512668	4.88E-06	0.019	14306	21.290
Vascular dementia (multiple infarctions)	genus Ruminococcaceae UCG009	rs4079028	C	T	0.054	202329957	0.479	0.077	360612	C	T	0.092	1	202299085	3.28E-06	0.020	14306	21.094
Vascular dementia (multiple infarctions)	genus Ruminococcaceae UCG009	rs4708333	T	G	0.094	77396984	0.174	0.069	360612	T	G	-0.084	6	78106701	1.56E-06	0.017	14306	23.122
Vascular dementia (multiple infarctions)	genus Ruminococcaceae UCG009	rs6952765	G	A	-0.031	31962098	0.664	0.070	360612	G	A	0.073	7	32001710	8.13E-06	0.017	14306	19.269
Vascular dementia (multiple infarctions)	genus Ruminococcaceae UCG009	rs758191	T	G	0.030	1929830	0.782	0.110	360612	T	G	0.177	16	1979831	9.01E-06	0.038	14306	22.270
Vascular dementia (multiple infarctions)	genus Ruminococcaceae UCG009	rs78410648	A	G	0.128	51896148	0.225	0.106	360612	A	G	0.121	19	52399401	9.67E-06	0.028	14306	19.034
Vascular dementia (multiple infarctions)	genus Ruminococcaceae UCG009	rs9558661	T	C	-0.125	105997744	0.131	0.083	360612	T	C	-0.090	13	106650093	7.01E-06	0.020	14306	20.004
Vascular dementia (multiple infarctions)	genus Ruminococcaceae UCG010	rs12597105	A	G	0.028	5183940	0.731	0.083	360612	A	G	-0.067	16	5233941	4.87E-06	0.014	14306	21.579
Vascular dementia (multiple infarctions)	genus Ruminococcaceae UCG010	rs2820282	C	A	0.023	104280743	0.738	0.067	360612	C	A	0.059	6	104728618	2.85E-06	0.013	14306	22.127
Vascular dementia (multiple infarctions)	genus Ruminococcaceae UCG010	rs682403	A	G	0.022	133093170	0.739	0.066	360612	A	G	-0.059	9	135968557	2.37E-06	0.012	14306	22.257
Vascular dementia (multiple infarctions)	genus Ruminococcaceae UCG010	rs6958419	C	T	-0.006	16310239	0.931	0.066	360612	C	T	-0.059	7	16349864	2.84E-06	0.012	14306	21.958

Vascular dementia (multiple infarctions)	genus Ruminococcaceae UCG010	rs73218807	G	A	-0.026	31055860	0.823	0.118	360612	G	A	-0.166	4	31057482	6.43E-06	0.037	14306	20.407
Vascular dementia (multiple infarctions)	genus Ruminococcaceae UCG010	rs7441445	T	C	-0.021	40705781	0.747	0.065	360612	T	C	0.057	4	40707798	6.80E-06	0.013	14306	20.267
Vascular dementia (multiple infarctions)	genus Ruminococcaceae UCG011	rs10274562	C	T	-0.056	11182894	0.407	0.068	360612	C	T	0.111	7	11222521	6.50E-06	0.024	14306	20.570
Vascular dementia (multiple infarctions)	genus Ruminococcaceae UCG011	rs12636310	G	A	-0.047	185751703	0.537	0.076	360612	G	A	0.133	3	185469491	2.81E-06	0.028	14306	22.146
Vascular dementia (multiple infarctions)	genus Ruminococcaceae UCG011	rs12724320	C	T	0.030	179401364	0.662	0.067	360612	C	T	-0.121	1	179370499	1.52E-06	0.025	14306	23.524
Vascular dementia (multiple infarctions)	genus Ruminococcaceae UCG011	rs1416041	A	C	-0.090	104625801	0.268	0.081	360612	A	C	-0.182	6	105073676	7.04E-08	0.034	14306	28.778
Vascular dementia (multiple infarctions)	genus Ruminococcaceae UCG011	rs2729556	T	C	0.019	112123933	0.767	0.066	360612	T	C	0.109	7	111763988	3.19E-06	0.023	14306	21.793
Vascular dementia (multiple infarctions)	genus Ruminococcaceae UCG011	rs4490371	T	C	0.001	76155956	0.982	0.066	360612	T	C	-0.112	3	76205107	7.75E-06	0.025	14306	20.172
Vascular dementia (multiple infarctions)	genus Ruminococcaceae UCG011	rs79113084	C	T	0.048	29481986	0.651	0.106	360612	C	T	-0.152	12	29634919	2.06E-06	0.032	14306	22.967
Vascular dementia (multiple infarctions)	genus Ruminococcaceae UCG011	rs9729514	G	A	-0.254	219727713	0.026	0.114	360612	G	A	-0.185	1	219901055	2.37E-06	0.039	14306	21.963
Vascular dementia (multiple infarctions)	genus Ruminococcaceae UCG013	rs11581881	C	T	-0.057	9301517	0.468	0.078	360612	C	T	0.066	1	9361576	4.73E-06	0.014	14306	20.870
Vascular dementia (multiple infarctions)	genus Ruminococcaceae UCG013	rs12189346	G	A	0.112	142015575	0.182	0.084	360612	G	A	0.068	5	141395140	1.68E-06	0.015	14306	22.137
Vascular dementia (multiple infarctions)	genus Ruminococcaceae UCG013	rs12336782	T	C	-0.191	13609120	0.119	0.123	360612	T	C	-0.086	9	13609119	8.60E-06	0.019	14306	20.448
Vascular dementia (multiple infarctions)	genus Ruminococcaceae UCG013	rs12485353	G	A	0.038	197323125	0.617	0.075	360612	G	A	-0.061	3	197049996	4.19E-06	0.013	14306	21.584
Vascular dementia (multiple infarctions)	genus Ruminococcaceae UCG013	rs12781711	C	T	0.059	2177736	0.434	0.075	360612	C	T	-0.066	10	2219930	2.55E-08	0.012	14306	31.194
Vascular dementia (multiple infarctions)	genus Ruminococcaceae UCG013	rs16918863	A	C	-0.033	19491534	0.805	0.135	360612	A	C	0.111	10	19780463	4.15E-06	0.024	14306	21.552
Vascular dementia (multiple infarctions)	genus Ruminococcaceae UCG013	rs2730183	G	A	0.103	40454366	0.124	0.067	360612	G	A	-0.049	8	40311885	8.44E-06	0.011	14306	19.771
Vascular dementia (multiple infarctions)	genus Ruminococcaceae UCG013	rs4385846	T	G	0.057	113890519	0.488	0.082	360612	T	G	-0.060	10	115650278	6.46E-06	0.013	14306	20.612
Vascular dementia (multiple infarctions)	genus Ruminococcaceae UCG013	rs75088940	T	C	-0.052	30092624	0.681	0.127	360612	T	C	-0.094	12	30245557	2.55E-06	0.020	14306	22.072
Vascular dementia (multiple infarctions)	genus Ruminococcaceae UCG013	rs76973485	G	T	0.075	9492973	0.653	0.167	360612	G	T	0.195	3	9534657	3.35E-06	0.042	14306	21.735
Vascular dementia (multiple infarctions)	genus Ruminococcaceae UCG013	rs9313055	T	C	-0.135	3628768	0.258	0.119	360612	T	C	0.105	5	3628882	9.55E-06	0.023	14306	20.089
Vascular dementia (multiple infarctions)	genus Ruminococcaceae UCG014	rs10495392	T	C	-0.129	237259689	0.305	0.126	360612	T	C	0.082	1	237422989	9.96E-06	0.019	14306	19.417
Vascular dementia (multiple infarctions)	genus Ruminococcaceae UCG014	rs10791168	G	A	0.007	131789106	0.936	0.085	360612	G	A	0.066	11	131659000	9.76E-06	0.015	14306	19.614
Vascular dementia (multiple infarctions)	genus Ruminococcaceae UCG014	rs10941294	C	T	-0.062	36435495	0.667	0.144	360612	C	T	-0.122	5	36435597	2.40E-06	0.026	14306	22.035
Vascular dementia (multiple infarctions)	genus Ruminococcaceae UCG014	rs115777838	T	C	-0.103	26110517	0.327	0.105	360612	T	C	-0.188	5	26110626	4.62E-07	0.039	14306	23.731
Vascular dementia (multiple infarctions)	genus Ruminococcaceae UCG014	rs12638134	T	G	0.040	101615765	0.543	0.066	360612	T	G	0.058	3	101334609	1.21E-06	0.012	14306	23.702
Vascular dementia (multiple infarctions)	genus Ruminococcaceae UCG014	rs34402072	C	T	0.055	3779026	0.558	0.093	360612	C	T	-0.069	8	3636548	9.80E-06	0.016	14306	19.431
Vascular dementia (multiple infarctions)	genus Ruminococcaceae UCG014	rs56105232	G	A	-0.066	14363770	0.636	0.139	360612	G	A	0.139	9	14363769	2.91E-06	0.030	14306	21.678
Vascular dementia (multiple infarctions)	genus Ruminococcaceae UCG014	rs72809222	T	C	0.077	56978719	0.334	0.080	360612	T	C	0.067	2	57205854	2.41E-06	0.014	14306	23.078
Vascular dementia (multiple infarctions)	genus Ruminococcaceae UCG014	rs853612	A	G	0.022	118168902	0.739	0.066	360612	A	G	-0.053	10	119928413	9.75E-06	0.012	14306	19.585
Vascular dementia (multiple infarctions)	genus Ruminococcaceae UCG014	rs995642	C	T	0.065	134097088	0.393	0.076	360612	C	T	0.060	2	134854659	1.90E-06	0.013	14306	22.562
Vascular dementia (multiple infarctions)	genus Ruminococcus1	rs10167839	A	G	-0.096	181018152	0.161	0.068	360612	A	G	0.052	2	181882879	8.09E-06	0.012	14306	19.952
Vascular dementia (multiple infarctions)	genus Ruminococcus1	rs11783695	G	T	-0.119	143514733	0.178	0.089	360612	G	T	-0.073	8	144596903	4.73E-06	0.016	14306	20.689
Vascular dementia (multiple infarctions)	genus Ruminococcus1	rs17781867	C	T	0.001	73259879	0.991	0.129	360612	C	T	0.100	17	71256018	1.96E-06	0.021	14306	22.275
Vascular dementia (multiple infarctions)	genus Ruminococcus1	rs3819978	C	T	0.107	241626455	0.391	0.125	360612	C	T	-0.115	1	241789757	8.74E-06	0.026	14306	19.557
Vascular dementia (multiple infarctions)	genus Ruminococcus1	rs6105066	T	C	-0.142	13277981	0.054	0.074	360612	T	C	-0.061	20	13258628	5.06E-06	0.013	14306	20.447
Vascular dementia (multiple infarctions)	genus Ruminococcus1	rs6493760	T	C	0.022	55146984	0.747	0.069	360612	T	C	-0.054	15	55439182	3.38E-06	0.012	14306	21.334
Vascular dementia (multiple infarctions)	genus Ruminococcus1	rs7117576	A	G	-0.200	114152851	0.077	0.113	360612	A	G	0.083	11	114023573	6.48E-07	0.017	14306	23.561
Vascular dementia (multiple infarctions)	genus Ruminococcus1	rs7583465	C	T	0.034	10393795	0.608	0.065	360612	C	T	0.053	2	10533921	2.56E-06	0.011	14306	21.952
Vascular dementia (multiple infarctions)	genus Ruminococcus1	rs78572139	G	A	-0.154	15944478	0.159	0.109	360612	G	A	0.125	5	15944587	5.23E-06	0.028	14306	20.026
Vascular dementia (multiple infarctions)	genus Ruminococcus1	rs78613526	G	A	-0.058	65698078	0.696	0.149	360612	G	A	0.167	2	65925212	5.11E-06	0.037	14306	20.757
Vascular dementia (multiple infarctions)	genus Ruminococcus2	rs12406309	A	C	-0.020	107531823	0.802	0.079	360612	A	C	-0.063	1	108074445	9.79E-06	0.014	14306	19.728
Vascular dementia (multiple infarctions)	genus Ruminococcus2	rs12986628	T	C	-0.009	237194796	0.912	0.082	360612	T	C	-0.067	2	238103439	2.14E-06	0.014	14306	22.572
Vascular dementia (multiple infarctions)	genus Ruminococcus2	rs1819812	G	T	-0.252	151738636	0.080	0.144	360612	G	T	0.084	7	151435722	5.28E-06	0.018	14306	20.756
Vascular dementia (multiple infarctions)	genus Ruminococcus2	rs2368224	T	G	-0.022	181791689	0.882	0.151	360612	T	G	0.200	2	182656416	3.63E-06	0.044	14306	20.734
Vascular dementia (multiple infarctions)	genus Ruminococcus2	rs2846589	T	G	-0.020	862476	0.767	0.067	360612	T	G	-0.052	18	862477	7.59E-06	0.012	14306	20.124
Vascular dementia (multiple infarctions)	genus Ruminococcus2	rs2997412	G	A	0.034	61493648	0.644	0.073	360612	G	A	0.057	13	62067781	4.22E-06	0.012	14306	21.594
Vascular dementia (multiple infarctions)	genus Ruminococcus2	rs4400279	A	G	-0.118	16764953	0.089	0.070	360612	A	G	0.055	7	16804578	5.80E-06	0.012	14306	20.685
Vascular dementia (multiple infarctions)	genus Ruminococcus2	rs4799823	C	T	-0.075	35876464	0.381	0.085	360612	C	T	0.084	18	33456427	5.40E-06	0.018	14306	21.089
Vascular dementia (multiple infarctions)	genus Ruminococcus2	rs55707116	C	A	0.138	77304469	0.264	0.124	360612	C	A	0.087	9	79919385	8.01E-06	0.019	14306	20.940
Vascular dementia (multiple infarctions)	genus Ruminococcus2	rs58681734	A	G	-0.004	134666044	0.958	0.081	360612	A	G	0.072	9	137557890	4.18E-06	0.016	14306	20.269
Vascular dementia (multiple infarctions)	genus Ruminococcus2	rs61791565	T	C	0.011	24653840	0.872	0.065	360612	T	C	-0.052	4	24655463	6.79E-06	0.012	14306	19.979
Vascular dementia (multiple infarctions)	genus Ruminococcus2	rs75140805	T	G	0.041	12127041	0.636	0.086	360612	T	G	0.084	11	12148588	3.95E-06	0.018	14306	22.493
Vascular dementia (multiple infarctions)	genus Ruminococcus2	rs7635831	G	A	0.020	173323134	0.772	0.068	360612	G	A	0.062	3	173040924	1.98E-06	0.013	14306	22.965
Vascular dementia (multiple infarctions)	genus Ruminococcus2	rs7693984	G	A	-0.319	4266595	0.043	0.157	360612	G	A	-0.103	4	4268322	9.42E-06	0.024	14306	19.130
Vascular dementia (multiple infarctions)	genus Ruminococcus2	rs78120384	A	G	-0.014	106945516	0.900	0.114	360612	A	G	-0.193	3	106664363	3.31E-07	0.039	14306	24.189
Vascular dementia (multiple infarctions)	genus Ruminococcus gauvreauii group	rs10931481	A	G	-0.018	191090126	0.799	0.072	360612	A	G	-0.061	2	191954852	3.38E-06	0.013	14306	21.862
Vascular dementia (multiple infarctions)	genus Ruminococcus gauvreauii group	rs12079579	A	G	0.072	161910692	0.542	0.118	360612	A	G	0.096	1	161880482	5.04E-06	0.021	14306	20.034
Vascular dementia (multiple infarctions)	genus Ruminococcus gauvreauii group	rs12539819	C	T	-0.101	153790311	0.428	0.128	360612	C	T	0.111	7	153487396	4.49E-06	0.024	14306	21.137
Vascular dementia (multiple infarctions)	genus Ruminococcus gauvreauii group	rs1391597	C	T	-0.013	60725485	0.840	0.067	360612	C	T	0.059	12	61119266	1.86E-06	0.012	14306	22.345
Vascular dementia (multiple infarctions)	genus Ruminococcus gauvreauii group	rs2047242	A	G	-0.091	28912931	0.241	0.077	360612	A	G	-0.068	10	29201860	2.46E-07	0.013	14306	25.552
Vascular dementia (multiple infarctions)	genus Ruminococcus gauvreauii group	rs2166943	C	A	0.019	136078607	0.772	0.066	360612	C	A	-0.057	8	137090850	5.28E-06	0.012	14306	21.077
Vascular dementia (multiple infarctions)	genus Ruminococcus gauvreauii group	rs289410	A	G	-0.074	85020252	0.309	0.073	360612	A	G	0.065	15	85563483	2.27E-06	0.014	14306	22.168
Vascular dementia (multiple infarctions)	genus Ruminococcus gauvreauii group	rs431418	G	A	0.077	166992547	0.499	0.114	360612	G	A	0.095	5	166419552	5.54E-06	0.021	14306	20.321
Vascular dementia (multiple infarctions)	genus Ruminococcus gauvreauii group	rs71386687	T	G	0.109	2717893	0.291	0.103	360612	T	G	0.121	16	2767894	2.91E-07	0.024	14306	25.733
Vascular dementia (multiple infarctions)	genus Ruminococcus gauvreauii group	rs73802842	C	A	0.091	18823237	0.236	0.077	360612	C	A	0.074	4	18824860	7.48E-06	0.017	14306	18.859
Vascular dementia (multiple infarctions)	genus Ruminococcus gauvreauii group	rs9870933	G	A	-0.033	112653470	0.616	0.067	360612	G	A	-0.062	3	112372317	8.49E-07	0.013	14306	24.313

Vascular dementia (multiple infarctions)	genus Ruminococcus gnavus group	rs11597105	A	G	-0.027	6470619	0.742	0.082	360612	A	G	0.115	10	6512581	6.95E-06	0.025	14306	20.930
Vascular dementia (multiple infarctions)	genus Ruminococcus gnavus group	rs11864644	T	C	-0.051	383078	0.617	0.103	360612	T	C	-0.140	16	433078	5.01E-06	0.032	14306	19.297
Vascular dementia (multiple infarctions)	genus Ruminococcus gnavus group	rs12136548	C	T	0.046	114625476	0.524	0.072	360612	C	T	0.090	1	115168097	3.10E-06	0.020	14306	21.074
Vascular dementia (multiple infarctions)	genus Ruminococcus gnavus group	rs12989336	G	A	0.111	36032198	0.127	0.073	360612	G	A	-0.085	2	36259341	7.12E-06	0.019	14306	20.305
Vascular dementia (multiple infarctions)	genus Ruminococcus gnavus group	rs13163520	G	A	-0.113	18662577	0.178	0.084	360612	G	A	-0.127	5	18662686	5.61E-08	0.023	14306	29.661
Vascular dementia (multiple infarctions)	genus Ruminococcus gnavus group	rs2909242	A	C	0.059	128212434	0.394	0.070	360612	A	C	0.091	8	129224680	7.41E-07	0.018	14306	24.588
Vascular dementia (multiple infarctions)	genus Ruminococcus gnavus group	rs3124783	G	A	0.028	132963204	0.775	0.097	360612	G	A	0.116	9	135838591	2.67E-06	0.025	14306	21.682
Vascular dementia (multiple infarctions)	genus Ruminococcus gnavus group	rs4388134	T	C	0.094	189189556	0.197	0.073	360612	T	C	0.090	4	190110710	9.12E-06	0.020	14306	19.769
Vascular dementia (multiple infarctions)	genus Ruminococcus gnavus group	rs62167033	T	C	-0.131	143942440	0.434	0.167	360612	T	C	0.185	2	144700007	3.50E-06	0.040	14306	21.861
Vascular dementia (multiple infarctions)	genus Ruminococcus gnavus group	rs78399089	T	C	-0.208	146572523	0.042	0.102	360612	T	C	0.144	3	146290310	6.63E-06	0.033	14306	19.558
Vascular dementia (multiple infarctions)	genus Ruminococcus gnavus group	rs934940	A	C	-0.046	121338442	0.633	0.096	360612	A	C	-0.105	2	122096018	2.74E-06	0.023	14306	20.934
Vascular dementia (multiple infarctions)	genus Ruminococcus torques group	rs10904297	A	G	-0.440	4597570	0.051	0.225	360612	A	G	-0.168	10	4639762	2.69E-06	0.039	14306	18.531
Vascular dementia (multiple infarctions)	genus Ruminococcus torques group	rs10967781	C	A	0.181	27225260	0.011	0.072	360612	C	A	0.051	9	27225258	8.37E-06	0.011	14306	20.095
Vascular dementia (multiple infarctions)	genus Ruminococcus torques group	rs12434631	A	G	0.081	25661198	0.445	0.105	360612	A	G	0.075	14	26130404	2.77E-06	0.015	14306	23.711
Vascular dementia (multiple infarctions)	genus Ruminococcus torques group	rs1475330	C	T	-0.017	66552209	0.823	0.076	360612	C	T	-0.052	6	67262102	8.13E-06	0.012	14306	19.615
Vascular dementia (multiple infarctions)	genus Ruminococcus torques group	rs35866622	T	C	0.097	48714803	0.156	0.068	360612	T	C	-0.061	19	49218060	2.21E-08	0.011	14306	31.285
Vascular dementia (multiple infarctions)	genus Ruminococcus torques group	rs4073731	T	C	0.007	132793252	0.938	0.089	360612	T	C	0.065	11	132663147	4.05E-06	0.014	14306	21.014
Vascular dementia (multiple infarctions)	genus Ruminococcus torques group	rs77034621	T	G	-0.435	75857608	0.080	0.248	360612	T	G	-0.152	8	76769843	6.07E-06	0.034	14306	20.359
Vascular dementia (multiple infarctions)	genus Sellimonas	rs113379006	T	C	0.042	41966694	0.631	0.087	360612	T	C	-0.163	3	42008186	7.21E-06	0.036	14306	20.782
Vascular dementia (multiple infarctions)	genus Sellimonas	rs13417181	T	C	-0.090	173443666	0.252	0.078	360612	T	C	0.167	2	174308394	7.62E-07	0.034	14306	24.337
Vascular dementia (multiple infarctions)	genus Sellimonas	rs2016057	C	A	-0.121	51813085	0.072	0.067	360612	C	A	0.126	15	52105282	1.03E-06	0.026	14306	24.149
Vascular dementia (multiple infarctions)	genus Sellimonas	rs2187447	A	C	-0.036	79621928	0.792	0.135	360612	A	C	0.243	11	79332972	3.98E-06	0.053	14306	21.285
Vascular dementia (multiple infarctions)	genus Sellimonas	rs2371572	A	C	0.003	212349450	0.964	0.066	360612	A	C	0.127	2	213214174	4.46E-07	0.025	14306	25.770
Vascular dementia (multiple infarctions)	genus Sellimonas	rs41816	A	G	0.003	106609718	0.966	0.072	360612	A	G	0.132	7	106250164	8.39E-06	0.029	14306	20.626
Vascular dementia (multiple infarctions)	genus Sellimonas	rs4600608	G	A	-0.052	179413837	0.521	0.081	360612	G	A	0.137	2	180278564	4.95E-06	0.030	14306	20.666
Vascular dementia (multiple infarctions)	genus Sellimonas	rs553697	C	T	-0.043	93020675	0.616	0.085	360612	C	T	0.154	6	93730393	6.13E-06	0.034	14306	20.562
Vascular dementia (multiple infarctions)	genus Sellimonas	rs56203279	T	C	0.072	111861468	0.293	0.069	360612	T	C	-0.124	7	111501524	3.72E-06	0.027	14306	21.246
Vascular dementia (multiple infarctions)	genus Senegalimassilia	rs10036909	C	T	-0.013	128525999	0.937	0.165	360612	C	T	0.186	5	127861692	8.05E-06	0.040	14306	21.416
Vascular dementia (multiple infarctions)	genus Senegalimassilia	rs11787826	C	A	0.143	34332384	0.032	0.067	360612	C	A	0.081	9	34332382	2.63E-06	0.017	14306	22.579
Vascular dementia (multiple infarctions)	genus Senegalimassilia	rs1990708	A	C	0.124	205789399	0.318	0.124	360612	A	C	-0.110	2	206654123	8.91E-06	0.025	14306	19.571
Vascular dementia (multiple infarctions)	genus Senegalimassilia	rs2017373	C	T	-0.081	33962790	0.234	0.068	360612	C	T	0.078	14	34431996	9.50E-06	0.018	14306	19.567
Vascular dementia (multiple infarctions)	genus Senegalimassilia	rs7225245	A	G	-0.096	50302254	0.145	0.066	360612	A	G	-0.079	17	48379615	4.18E-06	0.017	14306	21.583
Vascular dementia (multiple infarctions)	genus Slackia	rs10409783	G	A	-0.045	4555774	0.534	0.072	360612	G	A	-0.095	19	4555786	7.70E-06	0.021	14306	20.261
Vascular dementia (multiple infarctions)	genus Slackia	rs12440440	A	G	0.066	33749695	0.339	0.069	360612	A	G	0.090	15	34041896	2.63E-06	0.019	14306	22.397
Vascular dementia (multiple infarctions)	genus Slackia	rs16894137	C	T	-0.060	95934063	0.538	0.097	360612	C	T	-0.123	8	96946291	2.71E-06	0.026	14306	21.791
Vascular dementia (multiple infarctions)	genus Slackia	rs35156985	T	C	0.200	99854092	0.226	0.165	360612	T	C	-0.156	7	99451715	8.06E-06	0.035	14306	20.010
Vascular dementia (multiple infarctions)	genus Slackia	rs4492265	G	A	0.103	13484058	0.148	0.071	360612	G	A	0.091	7	13523683	2.41E-06	0.019	14306	22.334
Vascular dementia (multiple infarctions)	genus Slackia	rs8901	C	T	0.013	76270929	0.857	0.072	360612	C	T	0.093	17	74267010	6.07E-07	0.019	14306	25.028
Vascular dementia (multiple infarctions)	genus Streptococcus	rs10028567	C	T	-0.024	52791410	0.814	0.101	360612	C	T	-0.092	4	53657577	7.30E-06	0.019	14306	23.047
Vascular dementia (multiple infarctions)	genus Streptococcus	rs10448310	A	G	0.027	90793892	0.693	0.068	360612	A	G	-0.052	9	93556174	3.31E-06	0.011	14306	21.646
Vascular dementia (multiple infarctions)	genus Streptococcus	rs11110281	T	C	-0.106	100190236	0.476	0.149	360612	T	C	-0.138	12	100584014	2.58E-09	0.023	14306	36.572
Vascular dementia (multiple infarctions)	genus Streptococcus	rs11720390	G	A	0.048	94384747	0.726	0.136	360612	G	A	0.107	3	94103591	3.59E-06	0.023	14306	22.011
Vascular dementia (multiple infarctions)	genus Streptococcus	rs11764382	A	G	-0.139	46735298	0.140	0.094	360612	A	G	-0.070	7	46774896	1.29E-06	0.014	14306	23.424
Vascular dementia (multiple infarctions)	genus Streptococcus	rs17708276	A	G	0.116	10342038	0.277	0.107	360612	A	G	-0.079	8	10199548	3.04E-06	0.017	14306	21.652
Vascular dementia (multiple infarctions)	genus Streptococcus	rs1918540	A	G	-0.025	131779552	0.766	0.084	360612	A	G	-0.060	11	131649446	2.44E-06	0.013	14306	21.659
Vascular dementia (multiple infarctions)	genus Streptococcus	rs2370083	G	T	-0.121	97060413	0.366	0.134	360612	G	T	-0.082	14	97526750	9.75E-06	0.019	14306	19.317
Vascular dementia (multiple infarctions)	genus Streptococcus	rs57646748	G	A	-0.007	37451236	0.969	0.171	360612	G	A	-0.091	4	37452858	5.48E-06	0.020	14306	20.527
Vascular dementia (multiple infarctions)	genus Streptococcus	rs6806351	T	C	0.006	132339879	0.944	0.079	360612	T	C	-0.063	3	132058723	4.94E-06	0.014	14306	21.515
Vascular dementia (multiple infarctions)	genus Streptococcus	rs71481756	T	G	-0.031	8060441	0.811	0.132	360612	T	G	0.093	10	8102404	6.51E-06	0.021	14306	20.046
Vascular dementia (multiple infarctions)	genus Streptococcus	rs7916711	A	G	-0.060	28299340	0.525	0.095	360612	A	G	0.103	10	28588269	2.72E-06	0.022	14306	22.407
Vascular dementia (multiple infarctions)	genus Subdoligranulum	rs10065321	T	C	0.023	142477850	0.731	0.066	360612	T	C	-0.051	5	141857415	2.10E-06	0.011	14306	22.504
Vascular dementia (multiple infarctions)	genus Subdoligranulum	rs10497836	T	C	-0.110	199423995	0.167	0.079	360612	T	C	0.052	2	200288718	8.38E-06	0.012	14306	19.494
Vascular dementia (multiple infarctions)	genus Subdoligranulum	rs1667315	G	A	-0.057	231544032	0.394	0.067	360612	G	A	0.049	2	232408743	6.72E-06	0.011	14306	20.374
Vascular dementia (multiple infarctions)	genus Subdoligranulum	rs2114677	C	T	-0.008	123713421	0.938	0.104	360612	C	T	-0.104	10	125472937	2.72E-06	0.023	14306	20.368
Vascular dementia (multiple infarctions)	genus Subdoligranulum	rs2171249	C	T	-0.262	153372894	0.036	0.125	360612	C	T	0.107	6	153694029	4.51E-06	0.023	14306	20.950
Vascular dementia (multiple infarctions)	genus Subdoligranulum	rs3761728	G	T	-0.033	48988868	0.659	0.075	360612	G	T	0.054	4	48990885	3.87E-06	0.012	14306	20.903
Vascular dementia (multiple infarctions)	genus Subdoligranulum	rs4347804	G	A	-0.299	217351124	0.101	0.182	360612	G	A	-0.166	2	218215847	2.18E-06	0.036	14306	21.579
Vascular dementia (multiple infarctions)	genus Subdoligranulum	rs6555306	C	T	-0.004	4671270	0.965	0.094	360612	C	T	0.074	5	4671383	2.81E-06	0.016	14306	22.705
Vascular dementia (multiple infarctions)	genus Subdoligranulum	rs75158211	T	C	-0.037	29153787	0.685	0.092	360612	T	C	-0.072	19	29644694	7.52E-06	0.016	14306	20.616
Vascular dementia (multiple infarctions)	genus Subdoligranulum	rs76528319	G	T	0.124	118463753	0.288	0.116	360612	G	T	-0.143	5	117799448	7.41E-06	0.031	14306	21.271
Vascular dementia (multiple infarctions)	genus Sutterella	rs1145877	G	A	0.005	81677104	0.957	0.095	360612	G	A	0.074	6	82386821	7.20E-06	0.016	14306	20.507
Vascular dementia (multiple infarctions)	genus Sutterella	rs11591622	T	G	0.068	100760930	0.439	0.088	360612	T	G	-0.069	10	102520687	6.50E-06	0.015	14306	20.680
Vascular dementia (multiple infarctions)	genus Sutterella	rs13173038	A	G	-0.028	59203357	0.712	0.076	360612	A	G	-0.072	5	58499183	2.73E-06	0.015	14306	22.428
Vascular dementia (multiple infarctions)	genus Sutterella	rs143438747	T	C	-0.085	62812878	0.491	0.123	360612	T	C	-0.146	1	63278549	3.28E-06	0.031	14306	22.572
Vascular dementia (multiple infarctions)	genus Sutterella	rs2050185	A	G	-0.052	147615645	0.439	0.067	360612	A	G	-0.058	6	147936781	7.97E-06	0.013	14306	19.950
Vascular dementia (multiple infarctions)	genus Sutterella	rs2321387	G	A	0.067	58115206	0.306	0.066	360612	G	A	-0.059	13	58689340	1.87E-06	0.012	14306	22.674
Vascular dementia (multiple infarctions)	genus Sutterella	rs607327	T	C	0.088	111824985	0.190	0.067	360612	T	C	-0.058	11	111695709	6.63E-06	0.013	14306	20.083

Vascular dementia (multiple infarctions)	genus Sutterella	rs62501473	G	A	-0.009	150429663	0.906	0.074	360612	G	A	0.069	7	150126751	5.52E-06	0.015	14306	21.588
Vascular dementia (multiple infarctions)	genus Sutterella	rs7499539	A	G	0.023	85004459	0.753	0.074	360612	A	G	0.062	16	85038065	2.36E-06	0.013	14306	22.218
Vascular dementia (multiple infarctions)	genus Sutterella	rs7638039	T	C	-0.046	70539788	0.544	0.076	360612	T	C	0.065	3	70588939	8.66E-06	0.014	14306	20.133
Vascular dementia (multiple infarctions)	genus Sutterella	rs9350083	T	G	-0.064	18583647	0.352	0.068	360612	T	G	-0.059	6	18583878	8.23E-06	0.013	14306	19.609
Vascular dementia (multiple infarctions)	genus Terrisporobacter	rs1883097	C	T	0.038	8917272	0.827	0.173	360612	C	T	0.226	11	8938819	4.16E-07	0.045	14306	24.798
Vascular dementia (multiple infarctions)	genus Terrisporobacter	rs2569953	C	A	-0.023	144430063	0.726	0.066	360612	C	A	0.078	3	144148905	8.95E-06	0.017	14306	19.723
Vascular dementia (multiple infarctions)	genus Terrisporobacter	rs2872237	A	C	-0.015	16916130	0.827	0.067	360612	A	C	0.081	19	17026940	3.97E-06	0.018	14306	21.431
Vascular dementia (multiple infarctions)	genus Terrisporobacter	rs58405430	G	T	-0.029	20599497	0.829	0.136	360612	G	T	0.135	11	20621043	7.94E-06	0.030	14306	19.978
Vascular dementia (multiple infarctions)	genus Terrisporobacter	rs7184125	T	C	-0.066	15826537	0.364	0.073	360612	T	C	0.091	16	15920394	8.48E-06	0.021	14306	19.721
Vascular dementia (multiple infarctions)	genus Turicibacter	rs11054680	T	C	0.124	12089521	0.154	0.087	360612	T	C	-0.105	12	12242455	2.31E-06	0.023	14306	21.295
Vascular dementia (multiple infarctions)	genus Turicibacter	rs11666533	C	T	0.079	11754117	0.507	0.119	360612	C	T	-0.112	19	11864932	7.37E-06	0.025	14306	20.211
Vascular dementia (multiple infarctions)	genus Turicibacter	rs149744580	A	G	0.113	63139827	0.441	0.147	360612	A	G	0.170	2	63366962	7.01E-08	0.032	14306	28.998
Vascular dementia (multiple infarctions)	genus Turicibacter	rs2834977	T	C	-0.029	35557345	0.747	0.090	360612	T	C	-0.096	21	36929643	3.96E-06	0.021	14306	21.248
Vascular dementia (multiple infarctions)	genus Turicibacter	rs2952020	A	G	0.076	26170326	0.319	0.077	360612	A	G	0.076	8	26027842	5.63E-06	0.017	14306	20.966
Vascular dementia (multiple infarctions)	genus Turicibacter	rs3734633	G	A	0.214	125790735	0.127	0.140	360612	G	A	-0.121	6	126111881	5.32E-06	0.027	14306	20.325
Vascular dementia (multiple infarctions)	genus Turicibacter	rs4869133	G	A	0.022	96381915	0.799	0.087	360612	G	A	0.131	5	95717619	2.55E-06	0.027	14306	23.267
Vascular dementia (multiple infarctions)	genus Turicibacter	rs55756211	T	C	0.105	131287420	0.401	0.125	360612	T	C	-0.115	7	130972179	2.81E-06	0.024	14306	22.871
Vascular dementia (multiple infarctions)	genus Turicibacter	rs7199484	G	A	-0.010	49792388	0.891	0.071	360612	G	A	-0.073	16	49826299	5.77E-06	0.016	14306	20.853
Vascular dementia (multiple infarctions)	genus Tyzzerella3	rs10898797	C	T	0.067	87877806	0.516	0.104	360612	C	T	0.122	11	87588698	8.85E-06	0.027	14306	19.850
Vascular dementia (multiple infarctions)	genus Tyzzerella3	rs112102233	A	G	0.216	45602838	0.161	0.154	360612	A	G	-0.216	10	46098286	6.18E-06	0.048	14306	20.522
Vascular dementia (multiple infarctions)	genus Tyzzerella3	rs1232220	T	G	-0.051	102232382	0.640	0.109	360612	T	G	0.144	6	102680257	7.91E-06	0.032	14306	20.432
Vascular dementia (multiple infarctions)	genus Tyzzerella3	rs17706273	T	C	0.054	16388150	0.653	0.121	360612	T	C	-0.140	5	16388259	5.88E-07	0.027	14306	26.109
Vascular dementia (multiple infarctions)	genus Tyzzerella3	rs191093	G	A	0.102	76996549	0.344	0.107	360612	G	A	0.159	12	77390329	6.76E-06	0.035	14306	20.255
Vascular dementia (multiple infarctions)	genus Tyzzerella3	rs4904512	T	C	-0.178	89129601	0.067	0.097	360612	T	C	-0.117	14	89595945	3.09E-06	0.025	14306	21.905
Vascular dementia (multiple infarctions)	genus Tyzzerella3	rs55799124	A	G	0.144	3835487	0.053	0.074	360612	A	G	-0.114	17	3738781	1.34E-06	0.024	14306	22.968
Vascular dementia (multiple infarctions)	genus Tyzzerella3	rs67476743	T	G	0.127	1030321	0.087	0.074	360612	T	G	0.132	19	1030320	3.74E-09	0.022	14306	35.417
Vascular dementia (multiple infarctions)	genus Tyzzerella3	rs7019909	T	C	-0.030	33113324	0.760	0.099	360612	T	C	0.144	9	33113322	1.76E-06	0.030	14306	22.842
Vascular dementia (multiple infarctions)	genus Tyzzerella3	rs7333521	T	C	0.038	81018881	0.833	0.180	360612	T	C	-0.207	13	81593016	4.88E-06	0.045	14306	20.908
Vascular dementia (multiple infarctions)	genus Tyzzerella3	rs75091807	G	T	0.167	34449554	0.219	0.136	360612	G	T	-0.185	13	35023691	1.71E-06	0.038	14306	23.320
Vascular dementia (multiple infarctions)	genus Tyzzerella3	rs7561370	C	T	-0.019	57583271	0.838	0.091	360612	C	T	-0.131	2	57810406	1.52E-06	0.029	14306	21.047
Vascular dementia (multiple infarctions)	genus Veillonella	rs1882878	A	G	-0.131	28638351	0.067	0.071	360612	A	G	-0.077	21	30010673	2.98E-06	0.016	14306	22.010
Vascular dementia (multiple infarctions)	genus Veillonella	rs2013594	C	T	0.048	44280604	0.473	0.067	360612	C	T	0.072	11	44302154	3.42E-06	0.016	14306	21.577
Vascular dementia (multiple infarctions)	genus Veillonella	rs62376424	C	T	-0.004	120578590	0.953	0.071	360612	C	T	-0.076	5	119914285	3.65E-06	0.016	14306	21.733
Vascular dementia (multiple infarctions)	genus Veillonella	rs6656807	G	A	0.135	178975908	0.047	0.068	360612	G	A	-0.070	1	178945043	5.50E-06	0.015	14306	20.855
Vascular dementia (multiple infarctions)	genus Veillonella	rs742016	A	G	-0.094	45208919	0.173	0.069	360612	A	G	-0.069	22	45604800	4.66E-06	0.015	14306	21.137
Vascular dementia (multiple infarctions)	genus Victivallis	rs11899949	G	A	0.029	37821926	0.687	0.071	360612	G	A	0.131	2	38049069	2.77E-06	0.028	1531	22.341
Vascular dementia (multiple infarctions)	genus Victivallis	rs12512543	A	C	0.096	9606821	0.419	0.119	360612	A	C	-0.178	4	9608445	2.54E-06	0.037	1531	22.619
Vascular dementia (multiple infarctions)	genus Victivallis	rs173120	C	T	-0.037	72302743	0.655	0.083	360612	C	T	-0.134	13	72876881	7.65E-06	0.029	1531	21.272
Vascular dementia (multiple infarctions)	genus Victivallis	rs1882775	A	G	0.106	39033681	0.215	0.085	360612	A	G	-0.138	21	40405606	8.73E-06	0.031	1531	19.549
Vascular dementia (multiple infarctions)	genus Victivallis	rs2546432	C	T	-0.006	181152462	0.930	0.066	360612	C	T	0.111	5	180579462	9.93E-06	0.025	1531	19.698
Vascular dementia (multiple infarctions)	genus Victivallis	rs342302	A	G	0.030	106737783	0.757	0.096	360612	A	G	-0.153	7	106378229	8.16E-06	0.035	1531	18.878
Vascular dementia (multiple infarctions)	genus Victivallis	rs4764863	G	A	-0.066	102120406	0.314	0.065	360612	G	A	0.122	12	102514184	8.22E-07	0.025	1531	24.407
Vascular dementia (multiple infarctions)	genus Victivallis	rs4895919	C	T	-0.098	131309179	0.135	0.066	360612	C	T	0.117	6	131630319	2.75E-06	0.025	1531	22.313
Vascular dementia (multiple infarctions)	genus Victivallis	rs56349194	A	G	0.015	9291249	0.880	0.096	360612	A	G	-0.159	11	9312796	6.26E-07	0.032	1531	25.282
Vascular dementia (multiple infarctions)	genus Victivallis	rs911666	T	C	0.088	95604788	0.217	0.071	360612	T	C	-0.119	14	96071125	7.65E-06	0.026	1531	20.305
Vascular dementia (multiple infarctions)	order Actinomycetales	rs2889192	T	G	0.088	73779652	0.341	0.092	360612	T	G	-0.088	9	76394568	3.97E-06	0.019	14306	20.554
Vascular dementia (multiple infarctions)	order Actinomycetales	rs34583783	G	T	-0.097	66497478	0.478	0.137	360612	G	T	0.124	6	67207371	5.54E-06	0.026	14306	21.908
Vascular dementia (multiple infarctions)	order Actinomycetales	rs35011108	A	G	0.063	132686341	0.624	0.129	360612	A	G	0.242	6	133007480	1.88E-06	0.050	14306	22.987
Vascular dementia (multiple infarctions)	order Actinomycetales	rs4073240	G	A	-0.076	168824686	0.259	0.067	360612	G	A	0.075	6	169224781	5.68E-06	0.016	14306	20.729
Vascular dementia (multiple infarctions)	order Bacillales	rs10233278	T	C	0.019	117856090	0.775	0.066	360612	T	C	-0.116	7	117496144	3.51E-06	0.025	14306	21.906
Vascular dementia (multiple infarctions)	order Bacillales	rs10410917	C	T	0.000	14859629	0.997	0.067	360612	C	T	-0.115	19	14970441	5.57E-06	0.025	14306	21.049
Vascular dementia (multiple infarctions)	order Bacillales	rs11034576	A	G	0.003	38064161	0.978	0.099	360612	A	G	0.206	11	38085711	8.86E-06	0.045	14306	20.604
Vascular dementia (multiple infarctions)	order Bacillales	rs11207728	A	G	0.079	61361862	0.364	0.087	360612	A	G	0.145	1	61827534	5.73E-06	0.032	14306	20.805
Vascular dementia (multiple infarctions)	order Bacillales	rs11844714	A	G	-0.104	48532023	0.204	0.081	360612	A	G	-0.143	14	49001226	5.06E-06	0.032	14306	20.004
Vascular dementia (multiple infarctions)	order Bacillales	rs1287018	G	A	-0.181	5845828	0.031	0.084	360612	G	A	0.141	20	5826474	9.87E-06	0.032	14306	19.534
Vascular dementia (multiple infarctions)	order Bacillales	rs4617108	G	A	0.017	49384236	0.881	0.117	360612	G	A	-0.249	7	49423832	1.98E-06	0.053	14306	22.390
Vascular dementia (multiple infarctions)	order Bacillales	rs74420793	A	G	-0.057	126845554	0.591	0.106	360612	A	G	-0.164	4	127766709	3.07E-06	0.035	14306	21.600
Vascular dementia (multiple infarctions)	order Bacteroidales	rs11146701	A	G	-0.037	38769138	0.587	0.069	360612	A	G	0.047	10	39062269	7.08E-06	0.011	14306	20.186
Vascular dementia (multiple infarctions)	order Bacteroidales	rs17343978	A	C	0.048	27037922	0.557	0.081	360612	A	C	-0.055	22	27433885	8.36E-06	0.012	14306	21.067
Vascular dementia (multiple infarctions)	order Bacteroidales	rs2032750	C	T	-0.010	53603889	0.878	0.066	360612	C	T	0.051	2	53831026	1.92E-06	0.011	14306	22.657
Vascular dementia (multiple infarctions)	order Bacteroidales	rs2363574	T	C	0.049	200143435	0.774	0.172	360612	T	C	0.223	1	200112563	9.93E-06	0.051	14306	19.221
Vascular dementia (multiple infarctions)	order Bacteroidales	rs4916508	A	G	0.019	196209918	0.771	0.066	360612	A	G	0.047	3	195936789	8.47E-06	0.011	14306	19.641
Vascular dementia (multiple infarctions)	order Bacteroidales	rs55773148	G	A	0.252	69948897	0.081	0.145	360612	G	A	-0.122	13	70523029	3.90E-07	0.024	14306	26.341
Vascular dementia (multiple infarctions)	order Bacteroidales	rs62531359	T	G	-0.130	70003946	0.130	0.086	360612	T	G	0.066	8	70916181	9.09E-06	0.015	14306	19.138
Vascular dementia (multiple infarctions)	order Bacteroidales	rs62575403	C	T	-0.087	133628698	0.593	0.162	360612	C	T	0.140	9	136493820	7.06E-06	0.031	14306	20.264
Vascular dementia (multiple infarctions)	order Bacteroidales	rs72706335	T	C	-0.231	157525648	0.278	0.213	360612	T	C	-0.222	1	157495438	7.66E-06	0.049	14306	20.315
Vascular dementia (multiple infarctions)	order Bacteroidales	rs73975615	G	A	-0.114	6557880	0.772	0.393	360612	G	A	-0.207	17	6461200	1.22E-06	0.044	14306	21.874

Vascular dementia (multiple infarctions)	order Bacteroidales	rs7631304	G	A	-0.179	89290377	0.054	0.093	360612	G	A	-0.065	3	89339527	8.37E-07	0.013	14306	23.590
Vascular dementia (multiple infarctions)	order Bacteroidales	rs79585701	A	C	0.071	13252676	0.445	0.093	360612	A	C	0.065	8	13110185	9.99E-06	0.015	14306	18.687
Vascular dementia (multiple infarctions)	order Bacteroidales	rs929878	T	C	-0.117	74256742	0.143	0.080	360612	T	C	0.055	16	74290641	4.73E-06	0.012	14306	20.372
Vascular dementia (multiple infarctions)	order Bifidobacteriales	rs10831953	G	A	0.033	13076504	0.645	0.071	360612	G	A	0.054	11	13098051	9.95E-06	0.012	14306	18.869
Vascular dementia (multiple infarctions)	order Bifidobacteriales	rs12446429	T	C	-0.099	848055	0.240	0.085	360612	T	C	0.081	16	898055	8.53E-06	0.019	14306	18.040
Vascular dementia (multiple infarctions)	order Bifidobacteriales	rs13020688	G	A	0.032	192013806	0.656	0.071	360612	G	A	0.058	2	192878532	1.57E-06	0.012	14306	22.887
Vascular dementia (multiple infarctions)	order Bifidobacteriales	rs182549	T	C	-0.125	135859184	0.065	0.068	360612	T	C	-0.117	2	136616754	5.94E-20	0.013	14306	85.372
Vascular dementia (multiple infarctions)	order Bifidobacteriales	rs4957061	T	C	0.107	520981	0.107	0.067	360612	T	C	0.057	5	521096	1.15E-06	0.012	14306	23.762
Vascular dementia (multiple infarctions)	order Bifidobacteriales	rs540489	T	G	-0.123	74901626	0.152	0.086	360612	T	G	-0.063	17	72897722	5.37E-06	0.014	14306	20.956
Vascular dementia (multiple infarctions)	order Bifidobacteriales	rs55888705	A	G	0.029	1516099	0.687	0.072	360612	A	G	0.054	4	1517826	8.66E-06	0.012	14306	19.812
Vascular dementia (multiple infarctions)	order Bifidobacteriales	rs6899771	A	G	-0.119	96958344	0.276	0.109	360612	A	G	-0.091	6	97406220	7.28E-06	0.020	14306	20.365
Vascular dementia (multiple infarctions)	order Bifidobacteriales	rs7174549	T	C	0.084	91920073	0.217	0.068	360612	T	C	-0.055	15	92463303	6.87E-06	0.012	14306	19.590
Vascular dementia (multiple infarctions)	order Bifidobacteriales	rs7322849	T	C	-0.075	112205515	0.511	0.114	360612	T	C	0.111	13	112859829	1.74E-08	0.020	14306	30.320
Vascular dementia (multiple infarctions)	order Bifidobacteriales	rs857444	C	T	-0.109	14617360	0.107	0.068	360612	C	T	0.055	6	14617591	3.82E-06	0.012	14306	21.075
Vascular dementia (multiple infarctions)	order Burkholderiales	rs1511453	A	G	-0.225	23586839	0.108	0.140	360612	A	G	0.091	4	23588462	8.00E-06	0.020	14306	20.857
Vascular dementia (multiple infarctions)	order Burkholderiales	rs1928341	G	A	-0.036	153267537	0.586	0.067	360612	G	A	-0.051	1	153240013	4.52E-06	0.011	14306	21.084
Vascular dementia (multiple infarctions)	order Burkholderiales	rs2321387	G	A	0.067	58115206	0.306	0.066	360612	G	A	-0.051	13	58689340	3.26E-06	0.011	14306	21.532
Vascular dementia (multiple infarctions)	order Burkholderiales	rs4033856	T	C	0.036	45640468	0.754	0.116	360612	T	C	-0.083	4	45642485	5.67E-07	0.017	14306	24.762
Vascular dementia (multiple infarctions)	order Burkholderiales	rs6087811	T	G	-0.047	32008327	0.670	0.109	360612	T	G	-0.102	20	30596130	2.88E-07	0.020	14306	26.108
Vascular dementia (multiple infarctions)	order Burkholderiales	rs62191117	A	G	-0.040	238979079	0.619	0.081	360612	A	G	0.068	2	239900775	2.79E-07	0.013	14306	26.349
Vascular dementia (multiple infarctions)	order Burkholderiales	rs62395635	T	C	0.131	174070793	0.337	0.137	360612	T	C	0.110	5	173497796	2.90E-06	0.024	14306	21.621
Vascular dementia (multiple infarctions)	order Burkholderiales	rs7638039	T	C	-0.046	70539788	0.544	0.076	360612	T	C	0.058	3	70588939	4.84E-06	0.013	14306	21.017
Vascular dementia (multiple infarctions)	order Clostridiales	rs10774377	G	A	0.064	5833353	0.334	0.067	360612	G	A	-0.052	12	5942519	3.81E-06	0.011	14306	21.100
Vascular dementia (multiple infarctions)	order Clostridiales	rs112334273	G	A	-0.001	39331325	0.991	0.073	360612	G	A	0.064	21	40703251	4.07E-07	0.013	14306	25.186
Vascular dementia (multiple infarctions)	order Clostridiales	rs13105690	C	T	-0.012	7418457	0.870	0.073	360612	C	T	0.053	4	7420184	9.37E-06	0.012	14306	19.919
Vascular dementia (multiple infarctions)	order Clostridiales	rs13179700	C	T	-0.036	149698225	0.601	0.069	360612	C	T	-0.051	5	149077788	3.52E-06	0.011	14306	21.746
Vascular dementia (multiple infarctions)	order Clostridiales	rs1842454	G	A	-0.100	105724661	0.230	0.083	360612	G	A	-0.054	5	105060362	9.92E-06	0.013	14306	18.228
Vascular dementia (multiple infarctions)	order Clostridiales	rs2273429	A	G	0.168	52027354	0.113	0.106	360612	A	G	-0.073	14	52494072	4.17E-06	0.015	14306	22.497
Vascular dementia (multiple infarctions)	order Clostridiales	rs290772	G	A	0.006	101509758	0.965	0.142	360612	G	A	0.084	2	102126220	1.00E-05	0.020	14306	18.423
Vascular dementia (multiple infarctions)	order Clostridiales	rs6442336	T	C	-0.001	12829387	0.991	0.075	360612	T	C	0.055	3	12870886	9.63E-06	0.012	14306	19.458
Vascular dementia (multiple infarctions)	order Clostridiales	rs6814436	C	T	0.133	160586149	0.153	0.093	360612	C	T	-0.074	4	161507301	9.06E-07	0.015	14306	24.197
Vascular dementia (multiple infarctions)	order Clostridiales	rs6815608	C	T	-0.001	151210592	0.994	0.091	360612	C	T	-0.104	4	152131744	3.72E-07	0.021	14306	24.363
Vascular dementia (multiple infarctions)	order Clostridiales	rs72738886	T	C	0.121	35770448	0.330	0.124	360612	T	C	0.087	5	35770550	8.42E-06	0.019	14306	20.657
Vascular dementia (multiple infarctions)	order Clostridiales	rs992074	T	C	0.183	17195484	0.402	0.219	360612	T	C	-0.255	21	18567802	8.95E-07	0.051	14306	25.206
Vascular dementia (multiple infarctions)	order Coriobacteriales	rs11073596	G	T	0.045	85890348	0.503	0.068	360612	G	T	-0.051	15	86433579	8.14E-06	0.011	14306	19.912
Vascular dementia (multiple infarctions)	order Coriobacteriales	rs11250875	T	C	0.028	1880537	0.728	0.080	360612	T	C	0.061	10	1922731	4.83E-06	0.013	14306	21.526
Vascular dementia (multiple infarctions)	order Coriobacteriales	rs11656361	A	C	-0.129	8218014	0.130	0.085	360612	A	C	0.077	17	8121332	8.02E-06	0.018	14306	19.394
Vascular dementia (multiple infarctions)	order Coriobacteriales	rs12974142	G	A	-0.138	52391913	0.280	0.128	360612	G	A	0.079	19	52895166	8.51E-06	0.018	14306	19.865
Vascular dementia (multiple infarctions)	order Coriobacteriales	rs13307134	T	C	-0.202	105444233	0.021	0.088	360612	T	C	-0.057	7	105084680	7.80E-06	0.013	14306	20.072
Vascular dementia (multiple infarctions)	order Coriobacteriales	rs1397793	A	G	0.091	91175634	0.203	0.072	360612	A	G	0.050	5	90471451	9.77E-06	0.011	14306	19.682
Vascular dementia (multiple infarctions)	order Coriobacteriales	rs1816223	G	A	0.150	11341087	0.068	0.082	360612	G	A	0.059	12	11494021	4.84E-06	0.013	14306	20.652
Vascular dementia (multiple infarctions)	order Coriobacteriales	rs240104	T	C	-0.183	176602295	0.012	0.073	360612	T	C	-0.060	1	176571431	1.52E-06	0.013	14306	22.630
Vascular dementia (multiple infarctions)	order Coriobacteriales	rs2442778	A	G	-0.136	11612938	0.358	0.148	360612	A	G	0.116	3	11654412	9.03E-06	0.026	14306	20.272
Vascular dementia (multiple infarctions)	order Coriobacteriales	rs3025411	A	G	-0.026	133647784	0.808	0.107	360612	A	G	0.093	9	136512906	8.27E-06	0.021	14306	19.566
Vascular dementia (multiple infarctions)	order Coriobacteriales	rs34739816	G	T	-0.119	39220432	0.387	0.137	360612	G	T	0.097	17	37376685	3.88E-06	0.021	14306	21.594
Vascular dementia (multiple infarctions)	order Coriobacteriales	rs67561917	A	G	-0.033	63440724	0.703	0.086	360612	A	G	-0.071	20	62072077	5.39E-06	0.015	14306	21.486
Vascular dementia (multiple infarctions)	order Coriobacteriales	rs719099	A	G	0.094	64039457	0.389	0.109	360612	A	G	0.078	10	65799217	5.43E-07	0.016	14306	24.957
Vascular dementia (multiple infarctions)	order Coriobacteriales	rs8010111	A	G	0.061	39191305	0.599	0.117	360612	A	G	0.103	14	39660509	6.90E-06	0.023	14306	20.328
Vascular dementia (multiple infarctions)	order Desulfovibrionales	rs11599763	C	T	0.090	11813600	0.180	0.067	360612	C	T	0.055	10	11855599	2.61E-06	0.012	14306	22.305
Vascular dementia (multiple infarctions)	order Desulfovibrionales	rs17791387	A	G	0.240	79219511	0.033	0.113	360612	A	G	-0.073	9	81834426	2.25E-06	0.015	14306	22.210
Vascular dementia (multiple infarctions)	order Desulfovibrionales	rs186073	T	C	0.006	31091909	0.925	0.067	360612	T	C	0.053	3	31133401	8.74E-06	0.012	14306	19.931
Vascular dementia (multiple infarctions)	order Desulfovibrionales	rs2692012	G	A	-0.064	204022477	0.658	0.145	360612	G	A	-0.112	1	203991605	2.27E-06	0.025	14306	19.597
Vascular dementia (multiple infarctions)	order Desulfovibrionales	rs2838334	G	A	0.066	43645080	0.333	0.069	360612	G	A	0.057	21	45064961	4.17E-06	0.012	14306	20.982
Vascular dementia (multiple infarctions)	order Desulfovibrionales	rs3935584	C	T	-0.128	233064573	0.050	0.066	360612	C	T	-0.052	2	233929283	7.20E-06	0.012	14306	20.531
Vascular dementia (multiple infarctions)	order Desulfovibrionales	rs4506934	C	T	0.094	2953368	0.355	0.102	360612	C	T	-0.095	17	2856662	2.43E-06	0.020	14306	22.416
Vascular dementia (multiple infarctions)	order Desulfovibrionales	rs6058181	C	T	-0.145	35106998	0.098	0.087	360612	C	T	0.084	20	33694801	2.53E-07	0.017	14306	25.379
Vascular dementia (multiple infarctions)	order Desulfovibrionales	rs62020470	A	G	0.002	95617836	0.977	0.086	360612	A	G	-0.057	15	96161065	7.51E-06	0.013	14306	19.633
Vascular dementia (multiple infarctions)	order Desulfovibrionales	rs72647048	T	C	-0.114	56952819	0.266	0.103	360612	T	C	-0.077	8	57865378	9.00E-06	0.017	14306	20.436
Vascular dementia (multiple infarctions)	order Desulfovibrionales	rs9928243	C	A	-0.026	71507738	0.692	0.066	360612	C	A	-0.054	16	71541641	3.97E-06	0.012	14306	21.378
Vascular dementia (multiple infarctions)	order Enterobacteriales	rs11026530	T	C	0.022	22357551	0.815	0.092	360612	T	C	0.082	11	22379097	9.43E-06	0.019	14306	19.471
Vascular dementia (multiple infarctions)	order Enterobacteriales	rs2374342	C	A	0.022	41906402	0.737	0.066	360612	C	A	0.058	2	42133542	4.52E-06	0.013	14306	21.338
Vascular dementia (multiple infarctions)	order Enterobacteriales	rs35673018	G	A	-0.118	54293833	0.295	0.113	360612	G	A	0.090	16	54327745	7.63E-06	0.020	14306	19.653
Vascular dementia (multiple infarctions)	order Enterobacteriales	rs504442	T	G	0.049	57478315	0.647	0.107	360612	T	G	0.084	18	55145547	5.17E-06	0.019	14306	19.728
Vascular dementia (multiple infarctions)	order Enterobacteriales	rs62210023	A	G	-0.051	56765036	0.453	0.068	360612	A	G	0.061	20	55340092	3.13E-06	0.013	14306	21.742
Vascular dementia (multiple infarctions)	order Enterobacteriales	rs78143293	A	G	0.039	60005103	0.692	0.099	360612	A	G	-0.085	18	57672335	1.20E-06	0.017	14306	24.792
Vascular dementia (multiple infarctions)	order Enterobacteriales	rs79757635	C	A	-0.036	110188071	0.708	0.097	360612	C	A	0.076	13	110840418	9.32E-06	0.017	14306	19.615
Vascular dementia (multiple infarctions)	order Erysipelotrichales	rs1074800	G	A	0.013	3002432	0.842	0.066	360612	G	A	-0.049	5	3002546	6.15E-06	0.011	14306	20.459

Vascular dementia (multiple infarctions)	order Erysipelotrichales	rs10781552	C	T	-0.140	132083729	0.055	0.073	360612	C	T	-0.055	10	133897233	2.33E-06	0.012	14306	22.633
Vascular dementia (multiple infarctions)	order Erysipelotrichales	rs17530232	A	G	0.003	39811320	0.984	0.145	360612	A	G	0.103	13	40385457	2.79E-06	0.022	14306	21.042
Vascular dementia (multiple infarctions)	order Erysipelotrichales	rs1884466	C	T	-0.043	63673525	0.518	0.066	360612	C	T	-0.048	1	64139196	9.53E-06	0.011	14306	19.760
Vascular dementia (multiple infarctions)	order Erysipelotrichales	rs2300774	A	G	0.037	196066841	0.575	0.065	360612	A	G	-0.052	3	195793712	8.95E-07	0.011	14306	24.094
Vascular dementia (multiple infarctions)	order Erysipelotrichales	rs290833	T	G	0.039	96991871	0.556	0.066	360612	T	G	-0.050	1	97457427	8.03E-06	0.011	14306	19.943
Vascular dementia (multiple infarctions)	order Erysipelotrichales	rs35161940	T	C	-0.012	72331083	0.907	0.107	360612	T	C	-0.081	17	70327224	1.85E-06	0.017	14306	23.118
Vascular dementia (multiple infarctions)	order Erysipelotrichales	rs4078432	T	C	0.140	48528003	0.110	0.088	360612	T	C	0.061	14	48997206	4.23E-06	0.013	14306	20.723
Vascular dementia (multiple infarctions)	order Erysipelotrichales	rs56970041	T	G	-0.042	79891267	0.760	0.136	360612	T	G	0.072	14	80357610	5.40E-06	0.016	14306	19.385
Vascular dementia (multiple infarctions)	order Erysipelotrichales	rs62504403	C	T	0.043	38946033	0.600	0.082	360612	C	T	0.068	8	38803551	1.12E-07	0.013	14306	28.371
Vascular dementia (multiple infarctions)	order Erysipelotrichales	rs7234058	T	C	-0.136	5830508	0.229	0.113	360612	T	C	-0.095	18	5830507	9.12E-07	0.019	14306	23.744
Vascular dementia (multiple infarctions)	order Erysipelotrichales	rs7826267	G	T	0.000	3097430	0.998	0.132	360612	G	T	0.084	8	2954952	9.28E-06	0.020	14306	17.755
Vascular dementia (multiple infarctions)	order Erysipelotrichales	rs8003149	C	T	0.142	55689786	0.040	0.069	360612	C	T	0.054	14	56156504	4.08E-06	0.012	14306	21.248
Vascular dementia (multiple infarctions)	order Gastranaerophilales	rs11150282	T	C	0.078	80459808	0.257	0.068	360612	T	C	0.098	16	80493705	7.36E-07	0.020	14306	24.834
Vascular dementia (multiple infarctions)	order Gastranaerophilales	rs28678345	T	C	0.086	55828967	0.575	0.153	360612	T	C	0.213	17	53906328	8.06E-06	0.047	14306	20.434
Vascular dementia (multiple infarctions)	order Gastranaerophilales	rs367480	A	G	-0.067	2916401	0.325	0.068	360612	A	G	0.084	11	2937631	7.52E-06	0.019	14306	20.487
Vascular dementia (multiple infarctions)	order Gastranaerophilales	rs4129395	G	A	0.055	113213109	0.400	0.065	360612	G	A	0.090	9	115975389	1.22E-06	0.019	14306	23.826
Vascular dementia (multiple infarctions)	order Gastranaerophilales	rs789069	A	C	-0.169	1008277	0.071	0.094	360612	A	C	-0.104	18	1008278	6.50E-06	0.023	14306	19.725
Vascular dementia (multiple infarctions)	order Gastranaerophilales	rs79790072	T	C	0.385	100207478	0.050	0.197	360612	T	C	0.226	15	100747683	3.54E-06	0.049	14306	21.466
Vascular dementia (multiple infarctions)	order Gastranaerophilales	rs8028558	A	G	-0.065	61763554	0.343	0.068	360612	A	G	0.083	15	62055753	9.78E-06	0.019	14306	19.603
Vascular dementia (multiple infarctions)	order Gastranaerophilales	rs9864379	T	C	-0.132	14265449	0.152	0.092	360612	T	C	-0.161	3	14306949	4.66E-08	0.029	14306	30.065
Vascular dementia (multiple infarctions)	order Lactobacillales	rs11110282	A	G	-0.106	100191781	0.475	0.148	360612	A	G	-0.102	12	100585559	3.96E-06	0.022	14306	22.090
Vascular dementia (multiple infarctions)	order Lactobacillales	rs11627423	C	A	-0.048	32731417	0.477	0.068	360612	C	A	0.050	14	33200623	5.09E-06	0.011	14306	20.697
Vascular dementia (multiple infarctions)	order Lactobacillales	rs11730038	G	A	0.077	97128348	0.283	0.072	360612	G	A	-0.061	4	98049499	5.10E-06	0.013	14306	22.006
Vascular dementia (multiple infarctions)	order Lactobacillales	rs12797734	T	C	-0.003	8310803	0.969	0.076	360612	T	C	0.057	11	8332350	7.77E-06	0.013	14306	20.198
Vascular dementia (multiple infarctions)	order Lactobacillales	rs1595463	C	A	-0.041	230858942	0.532	0.066	360612	C	A	0.048	2	231723657	7.44E-06	0.011	14306	19.443
Vascular dementia (multiple infarctions)	order Lactobacillales	rs2370083	G	T	-0.121	97060413	0.366	0.134	360612	G	T	-0.081	14	97526750	8.33E-06	0.018	14306	19.802
Vascular dementia (multiple infarctions)	order Lactobacillales	rs2952251	G	A	-0.079	10285654	0.310	0.078	360612	G	A	0.063	8	10143164	3.36E-07	0.012	14306	25.684
Vascular dementia (multiple infarctions)	order Lactobacillales	rs34989881	A	G	0.050	51456601	0.748	0.157	360612	A	G	0.113	19	51959855	4.09E-06	0.025	14306	21.201
Vascular dementia (multiple infarctions)	order Lactobacillales	rs35344081	G	A	-0.120	941253	0.105	0.074	360612	G	A	0.064	16	991253	4.16E-07	0.013	14306	25.446
Vascular dementia (multiple infarctions)	order Lactobacillales	rs4028634	C	T	0.053	42683631	0.435	0.068	360612	C	T	-0.053	17	40835649	1.35E-06	0.011	14306	23.428
Vascular dementia (multiple infarctions)	order Lactobacillales	rs57872228	C	T	-0.039	200449677	0.695	0.100	360612	C	T	-0.069	1	200418805	2.58E-06	0.015	14306	21.933
Vascular dementia (multiple infarctions)	order Lactobacillales	rs74663707	C	T	0.015	184653436	0.912	0.134	360612	C	T	0.098	3	184371224	8.40E-06	0.022	14306	19.137
Vascular dementia (multiple infarctions)	order Lactobacillales	rs77558518	A	G	0.065	174746168	0.567	0.113	360612	A	G	-0.106	5	174173171	1.67E-06	0.022	14306	22.695
Vascular dementia (multiple infarctions)	order Lactobacillales	rs78938557	T	C	-0.314	36309977	0.108	0.196	360612	T	C	0.106	7	36349586	2.31E-06	0.023	14306	20.412
Vascular dementia (multiple infarctions)	order Lactobacillales	rs9581006	T	C	-0.190	24399371	0.279	0.176	360612	T	C	-0.226	13	24973509	1.77E-06	0.047	14306	23.163
Vascular dementia (multiple infarctions)	order Methanobacteriales	rs10202904	G	T	-0.010	124682691	0.886	0.067	360612	G	T	0.122	2	125440268	3.01E-07	0.024	14306	26.762
Vascular dementia (multiple infarctions)	order Methanobacteriales	rs10424197	A	G	0.005	45936063	0.948	0.075	360612	A	G	0.111	19	46439321	9.28E-06	0.025	14306	20.211
Vascular dementia (multiple infarctions)	order Methanobacteriales	rs4257531	G	A	0.080	2044483	0.457	0.107	360612	G	A	0.164	3	2086167	7.44E-06	0.036	14306	20.316
Vascular dementia (multiple infarctions)	order Methanobacteriales	rs6508769	C	T	-0.054	28336853	0.557	0.092	360612	C	T	-0.154	19	28827760	8.23E-06	0.034	14306	19.856
Vascular dementia (multiple infarctions)	order Methanobacteriales	rs6776814	T	C	0.044	15011576	0.848	0.228	360612	T	C	-0.200	3	15053083	1.63E-06	0.041	14306	23.483
Vascular dementia (multiple infarctions)	order Methanobacteriales	rs73068003	G	T	-0.094	10734305	0.391	0.110	360612	G	T	-0.158	7	10773932	8.45E-06	0.035	14306	20.206
Vascular dementia (multiple infarctions)	order Methanobacteriales	rs73457410	A	G	-0.069	41382045	0.603	0.133	360612	A	G	0.215	13	41956181	1.41E-06	0.044	14306	24.316
Vascular dementia (multiple infarctions)	order Methanobacteriales	rs75208022	C	T	-0.008	21185927	0.945	0.112	360612	C	T	-0.227	12	21338861	5.92E-06	0.049	14306	21.717
Vascular dementia (multiple infarctions)	order Methanobacteriales	rs894996	C	A	-0.197	103497150	0.122	0.127	360612	C	A	0.217	4	104418307	1.88E-06	0.045	14306	23.349
Vascular dementia (multiple infarctions)	order Mollicutes RF9	rs11779863	G	A	0.128	15549321	0.156	0.090	360612	G	A	-0.077	8	15406830	6.69E-06	0.017	14306	20.146
Vascular dementia (multiple infarctions)	order Mollicutes RF9	rs12566890	T	G	-0.023	61385192	0.815	0.097	360612	T	G	-0.103	1	61850864	8.11E-06	0.024	14306	18.196
Vascular dementia (multiple infarctions)	order Mollicutes RF9	rs13100746	C	T	0.024	166128718	0.719	0.065	360612	C	T	0.064	3	165846506	7.29E-06	0.014	14306	20.055
Vascular dementia (multiple infarctions)	order Mollicutes RF9	rs17235252	T	C	-0.068	82906721	0.503	0.101	360612	T	C	-0.122	7	82536037	2.16E-06	0.026	14306	22.808
Vascular dementia (multiple infarctions)	order Mollicutes RF9	rs3932485	C	T	0.100	184475694	0.134	0.067	360612	C	T	0.063	3	184193482	9.93E-06	0.014	14306	19.551
Vascular dementia (multiple infarctions)	order Mollicutes RF9	rs515984	C	T	0.056	3479267	0.578	0.101	360612	C	T	0.088	10	3521459	6.61E-06	0.019	14306	21.078
Vascular dementia (multiple infarctions)	order Mollicutes RF9	rs638542	A	G	-0.047	109302630	0.512	0.072	360612	A	G	0.071	1	109845252	5.17E-06	0.016	14306	20.204
Vascular dementia (multiple infarctions)	order Mollicutes RF9	rs74603314	T	C	-0.241	46050515	0.147	0.166	360612	T	C	0.231	14	46519718	2.28E-06	0.049	14306	22.207
Vascular dementia (multiple infarctions)	order Mollicutes RF9	rs7706512	A	G	-0.007	17443545	0.919	0.066	360612	A	G	-0.066	5	17443654	2.27E-06	0.014	14306	22.372
Vascular dementia (multiple infarctions)	order Mollicutes RF9	rs7801843	A	G	0.042	4066073	0.648	0.091	360612	A	G	-0.087	7	4105705	9.47E-06	0.019	14306	19.983
Vascular dementia (multiple infarctions)	order Mollicutes RF9	rs7853673	A	G	0.116	113948961	0.079	0.066	360612	A	G	0.062	9	116711241	6.73E-06	0.014	14306	19.938
Vascular dementia (multiple infarctions)	order Mollicutes RF9	rs949341	A	G	0.068	112516062	0.352	0.073	360612	A	G	-0.066	11	112386785	7.73E-06	0.015	14306	19.893
Vascular dementia (multiple infarctions)	order NB1n	rs11251024	G	A	-0.075	2053532	0.309	0.074	360612	G	A	0.104	10	2095726	6.63E-07	0.021	14306	25.404
Vascular dementia (multiple infarctions)	order NB1n	rs11606187	A	G	-0.139	91856434	0.143	0.095	360612	A	G	-0.155	11	91589600	3.31E-06	0.033	14306	22.432
Vascular dementia (multiple infarctions)	order NB1n	rs13385922	T	C	0.043	233172678	0.534	0.069	360612	T	C	0.093	2	234081324	3.97E-06	0.020	14306	21.311
Vascular dementia (multiple infarctions)	order NB1n	rs166849	A	G	0.063	199914143	0.337	0.066	360612	A	G	-0.091	2	200778866	7.74E-06	0.020	14306	20.294
Vascular dementia (multiple infarctions)	order NB1n	rs2172426	T	C	-0.047	18371511	0.485	0.067	360612	T	C	0.102	8	18229020	3.17E-07	0.020	14306	26.348
Vascular dementia (multiple infarctions)	order NB1n	rs267959	G	A	-0.019	10737690	0.800	0.073	360612	G	A	-0.099	5	10737802	2.62E-06	0.021	14306	22.231
Vascular dementia (multiple infarctions)	order NB1n	rs4383094	C	T	-0.127	81402631	0.180	0.095	360612	C	T	-0.149	15	81694972	4.28E-06	0.032	14306	21.646
Vascular dementia (multiple infarctions)	order NB1n	rs60775321	T	C	0.129	96775441	0.074	0.072	360612	T	C	-0.096	15	97318671	7.10E-06	0.021	14306	20.256
Vascular dementia (multiple infarctions)	order NB1n	rs72671304	T	C	-0.107	38956767	0.395	0.125	360612	T	C	0.172	14	39425971	3.80E-06	0.037	14306	21.688
Vascular dementia (multiple infarctions)	order NB1n	rs7911787	G	T	0.162	96859811	0.367	0.180	360612	G	T	-0.223	10	98619568	3.39E-06	0.047	14306	22.504
Vascular dementia (multiple infarctions)	order NB1n	rs8126061	T	C	-0.057	3424592	0.566	0.099	360612	T	C	-0.159	20	3405239	7.36E-06	0.035	14306	20.450

Vascular dementia (multiple infarctions)	order NB1n	rs9542068	T	C	0.062	69732699	0.371	0.069	360612	T	C	0.099	13	70306831	6.52E-06	0.022	14306	20.625
Vascular dementia (multiple infarctions)	order Pasteurellales	rs10965428	C	A	-0.305	22718482	0.031	0.142	360612	C	A	-0.120	9	22718481	4.29E-06	0.026	14306	21.561
Vascular dementia (multiple infarctions)	order Pasteurellales	rs111582866	G	A	0.052	48708578	0.650	0.114	360612	G	A	-0.114	16	48742489	7.07E-06	0.026	14306	19.753
Vascular dementia (multiple infarctions)	order Pasteurellales	rs12050685	A	G	-0.091	73185141	0.209	0.072	360612	A	G	-0.067	15	73477482	9.19E-06	0.015	14306	19.385
Vascular dementia (multiple infarctions)	order Pasteurellales	rs16970009	A	G	0.153	34535582	0.514	0.234	360612	A	G	0.187	17	32862601	7.32E-06	0.043	14306	19.027
Vascular dementia (multiple infarctions)	order Pasteurellales	rs4822728	T	C	-0.004	26495842	0.946	0.065	360612	T	C	0.069	22	26891808	4.72E-06	0.015	14306	21.156
Vascular dementia (multiple infarctions)	order Pasteurellales	rs6972479	A	G	-0.002	117278006	0.983	0.081	360612	A	G	-0.078	7	116918060	7.75E-06	0.018	14306	19.878
Vascular dementia (multiple infarctions)	order Pasteurellales	rs72756943	G	A	-0.006	26531799	0.962	0.130	360612	G	A	0.140	5	26531908	3.35E-06	0.030	14306	21.308
Vascular dementia (multiple infarctions)	order Pasteurellales	rs73139353	A	C	-0.171	98253370	0.135	0.115	360612	A	C	-0.223	3	97972214	8.71E-06	0.048	14306	21.092
Vascular dementia (multiple infarctions)	order Pasteurellales	rs76022354	C	T	-0.050	92546628	0.735	0.149	360612	C	T	0.243	10	94306385	1.83E-06	0.050	14306	23.560
Vascular dementia (multiple infarctions)	order Pasteurellales	rs78909003	T	C	-0.189	102887960	0.186	0.143	360612	T	C	-0.241	9	105650242	2.05E-06	0.050	14306	23.415
Vascular dementia (multiple infarctions)	order Pasteurellales	rs9382510	C	T	-0.044	55583693	0.560	0.075	360612	C	T	-0.088	6	55448491	2.48E-07	0.017	14306	26.921
Vascular dementia (multiple infarctions)	order Pasteurellales	rs9895850	T	C	-0.066	66538895	0.679	0.159	360612	T	C	-0.176	17	64535013	9.08E-06	0.041	14306	18.497
Vascular dementia (multiple infarctions)	order Pasteurellales	rs9938097	C	T	0.010	84943783	0.886	0.067	360612	C	T	0.071	16	84977389	8.23E-06	0.016	14306	20.209
Vascular dementia (multiple infarctions)	order Rhodospirillales	rs1035406	G	A	0.090	120037042	0.374	0.102	360612	G	A	-0.115	5	119372737	4.07E-06	0.025	14306	21.401
Vascular dementia (multiple infarctions)	order Rhodospirillales	rs11591293	G	T	0.007	111660039	0.916	0.066	360612	G	T	0.072	10	113419797	4.69E-06	0.016	14306	20.876
Vascular dementia (multiple infarctions)	order Rhodospirillales	rs11630875	T	C	-0.064	61483535	0.510	0.098	360612	T	C	0.095	15	61775734	3.70E-06	0.020	14306	21.865
Vascular dementia (multiple infarctions)	order Rhodospirillales	rs13336560	C	T	-0.017	88487835	0.799	0.066	360612	C	T	-0.070	16	88554243	9.75E-06	0.016	14306	19.598
Vascular dementia (multiple infarctions)	order Rhodospirillales	rs1549633	A	C	0.002	27945538	0.981	0.103	360612	A	C	0.100	5	27945645	3.88E-06	0.022	14306	21.163
Vascular dementia (multiple infarctions)	order Rhodospirillales	rs3730086	A	G	-0.091	68281223	0.223	0.075	360612	A	G	0.080	5	67577051	7.98E-06	0.018	14306	20.051
Vascular dementia (multiple infarctions)	order Rhodospirillales	rs3754624	C	T	-0.075	224769095	0.381	0.086	360612	C	T	0.094	2	225633812	2.68E-06	0.020	14306	22.511
Vascular dementia (multiple infarctions)	order Rhodospirillales	rs4278423	T	C	-0.072	2628361	0.592	0.134	360612	T	C	0.105	10	2670553	3.98E-06	0.023	14306	20.256
Vascular dementia (multiple infarctions)	order Rhodospirillales	rs61933850	G	A	-0.047	72745618	0.620	0.095	360612	G	A	0.165	12	73139398	7.00E-06	0.036	14306	20.888
Vascular dementia (multiple infarctions)	order Rhodospirillales	rs7001029	C	T	-0.103	130946157	0.367	0.114	360612	C	T	0.121	8	131958403	2.83E-06	0.026	14306	21.397
Vascular dementia (multiple infarctions)	order Rhodospirillales	rs76784716	A	G	-0.160	168176830	0.129	0.105	360612	A	G	0.136	2	169033340	1.31E-06	0.028	14306	22.881
Vascular dementia (multiple infarctions)	order Rhodospirillales	rs77304857	C	A	-0.132	133326860	0.109	0.083	360612	C	A	-0.100	4	134248015	6.02E-06	0.022	14306	20.157
Vascular dementia (multiple infarctions)	order Rhodospirillales	rs9813022	A	G	-0.189	13685237	0.005	0.067	360612	A	G	-0.083	3	13726736	3.07E-07	0.016	14306	26.093
Vascular dementia (multiple infarctions)	order Selenomonadales	rs1135612	G	A	0.049	75980359	0.542	0.080	360612	G	A	0.053	7	75609677	9.26E-06	0.012	14306	19.761
Vascular dementia (multiple infarctions)	order Selenomonadales	rs13086907	G	A	-0.134	142416275	0.090	0.079	360612	G	A	0.063	3	142135117	1.95E-06	0.013	14306	22.532
Vascular dementia (multiple infarctions)	order Selenomonadales	rs1643968	T	C	0.006	165839623	0.934	0.068	360612	T	C	-0.057	5	165266628	4.15E-07	0.011	14306	25.339
Vascular dementia (multiple infarctions)	order Selenomonadales	rs1649999	A	G	-0.207	78326315	0.064	0.112	360612	A	G	0.075	10	80086072	7.58E-06	0.017	14306	20.246
Vascular dementia (multiple infarctions)	order Selenomonadales	rs2834062	A	G	0.128	33005177	0.075	0.072	360612	A	G	0.049	21	34377485	8.44E-06	0.011	14306	20.190
Vascular dementia (multiple infarctions)	order Selenomonadales	rs4463806	C	T	0.060	113838234	0.466	0.082	360612	C	T	0.054	10	115597993	7.81E-06	0.013	14306	17.681
Vascular dementia (multiple infarctions)	order Selenomonadales	rs4722181	T	G	-0.024	22777952	0.712	0.065	360612	T	G	0.050	7	22817571	2.00E-06	0.011	14306	22.452
Vascular dementia (multiple infarctions)	order Selenomonadales	rs60274479	T	C	0.125	21238604	0.129	0.082	360612	T	C	-0.066	16	21249925	1.16E-06	0.013	14306	24.182
Vascular dementia (multiple infarctions)	order Selenomonadales	rs61249479	A	C	0.024	122150629	0.796	0.091	360612	A	C	0.078	9	124912908	2.95E-06	0.017	14306	21.236
Vascular dementia (multiple infarctions)	order Selenomonadales	rs71405394	G	A	-0.127	100704883	0.320	0.128	360612	G	A	-0.114	15	101245088	2.17E-06	0.024	14306	22.539
Vascular dementia (multiple infarctions)	order Selenomonadales	rs73232831	G	A	0.130	17411803	0.449	0.172	360612	G	A	-0.152	4	17413426	1.87E-06	0.031	14306	23.242
Vascular dementia (multiple infarctions)	order Selenomonadales	rs9423647	G	A	-0.060	5537855	0.360	0.066	360612	G	A	0.048	10	5579818	6.06E-06	0.011	14306	20.628
Vascular dementia (multiple infarctions)	order Verrucomicrobiales	rs111862613	T	C	0.018	129825125	0.841	0.088	360612	T	C	0.091	12	130309670	3.74E-06	0.020	14306	21.252
Vascular dementia (multiple infarctions)	order Verrucomicrobiales	rs117107102	A	G	-0.112	51947265	0.471	0.155	360612	A	G	0.205	18	49473635	2.92E-06	0.043	14306	22.493
Vascular dementia (multiple infarctions)	order Verrucomicrobiales	rs11729256	T	C	0.095	94106121	0.275	0.087	360612	T	C	0.075	4	95027272	6.73E-07	0.015	14306	24.928
Vascular dementia (multiple infarctions)	order Verrucomicrobiales	rs12908520	G	A	0.119	97027427	0.070	0.066	360612	G	A	0.062	15	97570657	2.17E-06	0.013	14306	22.341
Vascular dementia (multiple infarctions)	order Verrucomicrobiales	rs2602429	T	C	-0.122	81029544	0.104	0.075	360612	T	C	-0.075	16	81063149	2.58E-06	0.016	14306	22.863
Vascular dementia (multiple infarctions)	order Verrucomicrobiales	rs4242783	A	G	-0.054	5022135	0.462	0.073	360612	A	G	-0.069	10	5064327	2.64E-06	0.015	14306	21.781
Vascular dementia (multiple infarctions)	order Verrucomicrobiales	rs4936098	G	A	0.069	130410772	0.311	0.069	360612	G	A	-0.065	11	130280667	1.12E-06	0.014	14306	22.786
Vascular dementia (multiple infarctions)	order Verrucomicrobiales	rs61779207	G	A	-0.187	40608800	0.019	0.079	360612	G	A	-0.076	1	41074472	6.72E-06	0.017	14306	20.432
Vascular dementia (multiple infarctions)	order Verrucomicrobiales	rs74542928	T	C	-0.146	99623031	0.332	0.151	360612	T	C	0.112	4	100544188	1.63E-06	0.024	14306	22.508
Vascular dementia (multiple infarctions)	order Verrucomicrobiales	rs9349825	A	G	0.081	56476683	0.333	0.084	360612	A	G	-0.070	6	56341481	2.54E-06	0.015	14306	22.898
Vascular dementia (multiple infarctions)	order Verrucomicrobiales	rs941682	G	A	0.115	33280034	0.117	0.073	360612	G	A	-0.063	20	31867840	9.61E-06	0.014	14306	19.290
Vascular dementia (multiple infarctions)	order Victivallales	rs1002941	A	G	0.017	100702485	0.818	0.076	360612	A	G	-0.105	15	101242690	8.15E-06	0.023	14306	20.234
Vascular dementia (multiple infarctions)	order Victivallales	rs11770843	C	T	0.018	147098287	0.797	0.070	360612	C	T	0.109	7	146795379	1.91E-06	0.023	14306	21.707
Vascular dementia (multiple infarctions)	order Victivallales	rs17114848	G	A	0.129	24917388	0.246	0.111	360612	G	A	0.152	15	25162535	4.06E-06	0.032	14306	22.073
Vascular dementia (multiple infarctions)	order Victivallales	rs2031282	A	G	-0.035	20113040	0.688	0.086	360612	A	G	0.122	13	20687179	4.38E-06	0.027	14306	20.490
Vascular dementia (multiple infarctions)	order Victivallales	rs2825714	A	G	0.100	19651652	0.252	0.087	360612	A	G	-0.137	21	21023966	1.72E-06	0.029	14306	22.568
Vascular dementia (multiple infarctions)	order Victivallales	rs62570196	C	T	-0.166	108323890	0.307	0.163	360612	C	T	-0.216	9	111086170	1.08E-06	0.044	14306	24.192
Vascular dementia (multiple infarctions)	order Victivallales	rs72640280	A	G	0.102	11883735	0.473	0.142	360612	A	G	0.220	1	11943792	5.18E-06	0.049	14306	20.513
Vascular dementia (multiple infarctions)	order Victivallales	rs77599476	A	G	-0.105	62762910	0.472	0.146	360612	A	G	0.230	20	61394262	1.86E-06	0.048	14306	23.002
Vascular dementia (multiple infarctions)	phylum Actinobacteria	rs11766971	C	T	-0.045	155211037	0.497	0.066	360612	C	T	-0.048	7	155002747	9.40E-06	0.011	14306	20.023
Vascular dementia (multiple infarctions)	phylum Actinobacteria	rs12528285	C	T	0.026	92065492	0.805	0.106	360612	C	T	0.081	6	92775210	5.69E-06	0.018	14306	20.048
Vascular dementia (multiple infarctions)	phylum Actinobacteria	rs13192624	T	C	-0.068	112808407	0.366	0.075	360612	T	C	-0.052	6	113129609	9.33E-06	0.012	14306	19.720
Vascular dementia (multiple infarctions)	phylum Actinobacteria	rs1397793	A	G	0.091	91175634	0.203	0.072	360612	A	G	0.052	5	90471451	3.74E-06	0.011	14306	21.832
Vascular dementia (multiple infarctions)	phylum Actinobacteria	rs4429415	C	T	0.014	212891467	0.828	0.066	360612	C	T	0.058	2	213756191	2.05E-07	0.011	14306	27.314
Vascular dementia (multiple infarctions)	phylum Actinobacteria	rs55888705	A	G	0.029	1516099	0.687	0.072	360612	A	G	0.053	4	1517826	1.31E-06	0.011	14306	23.597
Vascular dementia (multiple infarctions)	phylum Actinobacteria	rs6496870	C	T	0.075	91924192	0.270	0.068	360612	C	T	-0.051	15	92467422	4.62E-06	0.011	14306	20.142
Vascular dementia (multiple infarctions)	phylum Actinobacteria	rs6743026	T	C	0.042	101633121	0.618	0.084	360612	T	C	0.059	2	102249583	9.88E-06	0.013	14306	19.171
Vascular dementia (multiple infarctions)	phylum Actinobacteria	rs74037001	G	A	-0.008	23377985	0.941	0.114	360612	G	A	-0.082	14	23847194	6.71E-07	0.017	14306	24.562

Vascular dementia (multiple infarctions)	phylum Actinobacteria	rs75211493	G	A	0.065	5300454	0.599	0.125	360612	G	A	0.084	19	5300465	9.27E-06	0.018	14306	20.714
Vascular dementia (multiple infarctions)	phylum Actinobacteria	rs7570971	A	C	0.126	135080336	0.062	0.067	360612	A	C	0.087	2	135837906	1.41E-14	0.011	14306	58.161
Vascular dementia (multiple infarctions)	phylum Actinobacteria	rs80124826	T	C	0.127	239014688	0.523	0.199	360612	T	C	-0.124	2	239936384	8.75E-06	0.028	14306	19.876
Vascular dementia (multiple infarctions)	phylum Actinobacteria	rs857444	C	T	-0.109	14617360	0.107	0.068	360612	C	T	0.051	6	14617591	3.80E-06	0.011	14306	21.315
Vascular dementia (multiple infarctions)	phylum Actinobacteria	rs9833771	C	T	0.064	32321506	0.335	0.066	360612	C	T	-0.049	3	32362998	4.07E-06	0.011	14306	21.150
Vascular dementia (multiple infarctions)	phylum Bacteroidetes	rs17343978	A	C	0.048	27037922	0.557	0.081	360612	A	C	-0.056	22	27433885	7.22E-06	0.012	14306	21.374
Vascular dementia (multiple infarctions)	phylum Bacteroidetes	rs2032750	C	T	-0.010	53603889	0.878	0.066	360612	C	T	0.051	2	53831026	1.71E-06	0.011	14306	22.878
Vascular dementia (multiple infarctions)	phylum Bacteroidetes	rs62531359	T	G	-0.130	70003946	0.130	0.086	360612	T	G	0.066	8	70916181	8.42E-06	0.015	14306	19.260
Vascular dementia (multiple infarctions)	phylum Bacteroidetes	rs62575403	C	T	-0.087	133628698	0.593	0.162	360612	C	T	0.145	9	136493820	2.96E-06	0.031	14306	21.854
Vascular dementia (multiple infarctions)	phylum Bacteroidetes	rs6586324	T	C	-0.022	42522025	0.743	0.066	360612	T	C	0.048	21	43942135	7.37E-06	0.011	14306	20.535
Vascular dementia (multiple infarctions)	phylum Bacteroidetes	rs72706335	T	C	-0.231	157525648	0.278	0.213	360612	T	C	-0.223	1	157495438	7.13E-06	0.049	14306	20.464
Vascular dementia (multiple infarctions)	phylum Bacteroidetes	rs73512608	G	A	0.252	69948551	0.081	0.145	360612	G	A	-0.123	13	70522683	2.54E-07	0.024	14306	27.045
Vascular dementia (multiple infarctions)	phylum Bacteroidetes	rs73846128	A	G	-0.180	89291104	0.054	0.093	360612	A	G	-0.066	3	89340254	4.78E-07	0.013	14306	24.765
Vascular dementia (multiple infarctions)	phylum Bacteroidetes	rs73975615	G	A	-0.114	6557880	0.772	0.393	360612	G	A	-0.207	17	6461200	1.20E-06	0.044	14306	21.905
Vascular dementia (multiple infarctions)	phylum Bacteroidetes	rs929878	T	C	-0.117	74256742	0.143	0.080	360612	T	C	0.054	16	74290641	6.51E-06	0.012	14306	19.745
Vascular dementia (multiple infarctions)	phylum Cyanobacteria	rs12555298	G	A	0.007	107727270	0.937	0.087	360612	G	A	0.097	9	110489551	8.09E-06	0.022	14306	19.730
Vascular dementia (multiple infarctions)	phylum Cyanobacteria	rs2585223	T	C	-0.119	100340683	0.237	0.101	360612	T	C	0.111	15	100880888	8.86E-06	0.025	14306	20.206
Vascular dementia (multiple infarctions)	phylum Cyanobacteria	rs584122	T	C	0.156	48320720	0.259	0.138	360612	T	C	0.152	6	48288456	4.23E-06	0.033	14306	21.565
Vascular dementia (multiple infarctions)	phylum Cyanobacteria	rs61972390	T	C	0.061	99914504	0.525	0.095	360612	T	C	0.107	13	100566758	9.11E-06	0.024	14306	19.600
Vascular dementia (multiple infarctions)	phylum Cyanobacteria	rs7148504	T	G	-0.125	99701961	0.061	0.067	360612	T	G	-0.080	14	100168298	6.62E-06	0.018	14306	20.537
Vascular dementia (multiple infarctions)	phylum Cyanobacteria	rs76531781	T	C	-0.116	21608117	0.484	0.166	360612	T	C	-0.232	7	21647735	2.87E-06	0.049	14306	22.085
Vascular dementia (multiple infarctions)	phylum Cyanobacteria	rs789068	G	A	-0.169	1008238	0.071	0.094	360612	G	A	-0.111	18	1008239	1.57E-07	0.021	14306	27.366
Vascular dementia (multiple infarctions)	phylum Cyanobacteria	rs9864379	T	C	-0.132	14265449	0.152	0.092	360612	T	C	-0.139	3	14306949	2.03E-07	0.027	14306	26.866
Vascular dementia (multiple infarctions)	phylum Euryarchaeota	rs10202904	G	T	-0.010	124682691	0.886	0.067	360612	G	T	0.116	2	125440268	6.19E-07	0.023	14306	25.371
Vascular dementia (multiple infarctions)	phylum Euryarchaeota	rs11022995	A	G	-0.080	13872120	0.222	0.065	360612	A	G	0.104	11	13893667	7.73E-06	0.023	14306	20.538
Vascular dementia (multiple infarctions)	phylum Euryarchaeota	rs34928225	T	C	-0.101	146519740	0.366	0.112	360612	T	C	0.200	6	146840876	4.33E-06	0.043	14306	22.051
Vascular dementia (multiple infarctions)	phylum Euryarchaeota	rs45498998	G	A	-0.130	25996679	0.152	0.090	360612	G	A	-0.132	21	27368994	5.32E-06	0.029	14306	20.358
Vascular dementia (multiple infarctions)	phylum Euryarchaeota	rs6064552	T	C	-0.145	57467810	0.087	0.084	360612	T	C	-0.124	20	56042866	9.34E-06	0.028	14306	20.095
Vascular dementia (multiple infarctions)	phylum Euryarchaeota	rs6508769	C	T	-0.054	28336853	0.557	0.092	360612	C	T	-0.151	19	28827760	8.12E-06	0.034	14306	19.941
Vascular dementia (multiple infarctions)	phylum Euryarchaeota	rs7015093	G	A	-0.022	57106294	0.770	0.075	360612	G	A	-0.118	8	58018853	7.20E-06	0.026	14306	19.957
Vascular dementia (multiple infarctions)	phylum Euryarchaeota	rs76029318	T	C	-0.057	41389655	0.669	0.134	360612	T	C	0.215	13	41963791	1.05E-06	0.044	14306	24.017
Vascular dementia (multiple infarctions)	phylum Euryarchaeota	rs7635189	A	G	0.011	15550100	0.878	0.073	360612	A	G	-0.120	3	15591607	4.64E-06	0.026	14306	21.402
Vascular dementia (multiple infarctions)	phylum Euryarchaeota	rs77658038	A	C	-0.019	16284554	0.816	0.081	360612	A	C	-0.160	6	16284785	4.75E-06	0.034	14306	22.078
Vascular dementia (multiple infarctions)	phylum Euryarchaeota	rs894996	C	A	-0.197	103497150	0.122	0.127	360612	C	A	0.204	4	104418307	5.12E-06	0.044	14306	21.404
Vascular dementia (multiple infarctions)	phylum Firmicutes	rs112334273	G	A	-0.001	39331325	0.991	0.073	360612	G	A	0.063	21	40703251	9.26E-07	0.013	14306	24.251
Vascular dementia (multiple infarctions)	phylum Firmicutes	rs2273429	A	G	0.168	52027354	0.113	0.106	360612	A	G	-0.070	14	52494072	9.26E-06	0.015	14306	21.041
Vascular dementia (multiple infarctions)	phylum Firmicutes	rs2332027	A	G	-0.028	170750887	0.678	0.067	360612	A	G	0.048	4	171672038	4.05E-06	0.010	14306	21.201
Vascular dementia (multiple infarctions)	phylum Firmicutes	rs2547978	G	A	-0.048	98684594	0.474	0.068	360612	G	A	-0.047	5	98020298	8.57E-06	0.011	14306	19.652
Vascular dementia (multiple infarctions)	phylum Firmicutes	rs3792064	G	A	0.269	230813698	0.067	0.147	360612	G	A	0.090	2	231678413	6.75E-07	0.018	14306	24.168
Vascular dementia (multiple infarctions)	phylum Firmicutes	rs3852931	T	C	0.001	55471425	0.985	0.068	360612	T	C	0.048	20	54087963	4.53E-06	0.011	14306	21.080
Vascular dementia (multiple infarctions)	phylum Firmicutes	rs4750583	G	A	-0.012	15012766	0.883	0.084	360612	G	A	-0.062	10	15054765	5.79E-06	0.014	14306	19.944
Vascular dementia (multiple infarctions)	phylum Firmicutes	rs56199908	T	C	0.039	2801371	0.786	0.143	360612	T	C	-0.186	9	2801371	8.67E-06	0.041	14306	20.572
Vascular dementia (multiple infarctions)	phylum Firmicutes	rs6814436	C	T	0.133	160586149	0.153	0.093	360612	C	T	-0.068	4	161507301	6.80E-06	0.015	14306	20.334
Vascular dementia (multiple infarctions)	phylum Firmicutes	rs6815608	C	T	-0.001	151210592	0.994	0.091	360612	C	T	-0.094	4	152131744	7.24E-06	0.021	14306	19.693
Vascular dementia (multiple infarctions)	phylum Firmicutes	rs7247191	T	C	0.089	22716783	0.418	0.109	360612	T	C	-0.071	19	22899585	4.73E-06	0.016	14306	20.515
Vascular dementia (multiple infarctions)	phylum Firmicutes	rs72738886	T	C	0.121	35770448	0.330	0.124	360612	T	C	0.086	5	35770550	7.68E-06	0.019	14306	20.621
Vascular dementia (multiple infarctions)	phylum Firmicutes	rs72771021	C	T	0.086	13799631	0.513	0.131	360612	C	T	-0.141	10	13841631	5.12E-06	0.031	14306	21.022
Vascular dementia (multiple infarctions)	phylum Firmicutes	rs8085381	A	G	0.080	31748342	0.350	0.085	360612	A	G	-0.065	18	29328305	8.67E-06	0.015	14306	19.597
Vascular dementia (multiple infarctions)	phylum Firmicutes	rs992074	T	C	0.183	17195484	0.402	0.219	360612	T	C	-0.233	21	18567802	8.52E-06	0.051	14306	21.042
Vascular dementia (multiple infarctions)	phylum Lentisphaerae	rs1002941	A	G	0.017	100702485	0.818	0.076	360612	A	G	-0.108	15	101242690	4.31E-06	0.023	14306	21.281
Vascular dementia (multiple infarctions)	phylum Lentisphaerae	rs11770843	C	T	0.018	147098287	0.797	0.070	360612	C	T	0.112	7	146795379	1.14E-06	0.023	14306	22.715
Vascular dementia (multiple infarctions)	phylum Lentisphaerae	rs17114848	G	A	0.129	24917388	0.246	0.111	360612	G	A	0.149	15	25162535	6.77E-06	0.032	14306	21.120
Vascular dementia (multiple infarctions)	phylum Lentisphaerae	rs2031282	A	G	-0.035	20113040	0.688	0.086	360612	A	G	0.120	13	20687179	5.86E-06	0.027	14306	19.850
Vascular dementia (multiple infarctions)	phylum Lentisphaerae	rs2825714	A	G	0.100	19651652	0.252	0.087	360612	A	G	-0.138	21	21023966	1.50E-06	0.029	14306	22.861
Vascular dementia (multiple infarctions)	phylum Lentisphaerae	rs60995569	T	G	0.152	52170325	0.148	0.105	360612	T	G	-0.161	10	53930085	9.19E-06	0.034	14306	22.743
Vascular dementia (multiple infarctions)	phylum Lentisphaerae	rs62570196	C	T	-0.166	108323890	0.307	0.163	360612	C	T	-0.217	9	111086170	9.64E-07	0.044	14306	24.410
Vascular dementia (multiple infarctions)	phylum Lentisphaerae	rs72640280	A	G	0.102	11883735	0.473	0.142	360612	A	G	0.220	1	11943792	5.19E-06	0.049	14306	20.530
Vascular dementia (multiple infarctions)	phylum Lentisphaerae	rs77599476	A	G	-0.105	62762910	0.472	0.146	360612	A	G	0.230	20	61394262	1.90E-06	0.048	14306	22.964
Vascular dementia (multiple infarctions)	phylum Proteobacteria	rs10750258	C	A	0.015	124126791	0.822	0.068	360612	C	A	0.049	11	123997498	8.72E-06	0.011	14306	20.598
Vascular dementia (multiple infarctions)	phylum Proteobacteria	rs11126162	T	C	0.043	67858902	0.723	0.120	360612	T	C	-0.077	2	68086034	9.26E-06	0.019	14306	16.971
Vascular dementia (multiple infarctions)	phylum Proteobacteria	rs11715072	G	A	0.041	53686766	0.562	0.071	360612	G	A	-0.052	3	53720793	6.90E-06	0.012	14306	20.303
Vascular dementia (multiple infarctions)	phylum Proteobacteria	rs12150865	C	T	0.138	43001168	0.039	0.067	360612	C	T	0.051	19	43505320	1.54E-06	0.011	14306	23.118
Vascular dementia (multiple infarctions)	phylum Proteobacteria	rs12467198	C	T	0.010	124115104	0.879	0.066	360612	C	T	0.050	2	124872681	6.31E-06	0.011	14306	20.013
Vascular dementia (multiple infarctions)	phylum Proteobacteria	rs2347697	G	T	0.039	134703488	0.581	0.070	360612	G	T	0.050	7	134388240	4.27E-06	0.011	14306	21.247
Vascular dementia (multiple infarctions)	phylum Proteobacteria	rs2532663	A	G	-0.161	117487723	0.139	0.109	360612	A	G	0.126	10	119247234	7.47E-07	0.026	14306	23.685
Vascular dementia (multiple infarctions)	phylum Proteobacteria	rs3890996	G	T	-0.030	20314277	0.651	0.065	360612	G	T	0.047	22	20301800	6.95E-06	0.011	14306	20.183
Vascular dementia (multiple infarctions)	phylum Proteobacteria	rs4340090	C	T	0.147	129847219	0.144	0.101	360612	C	T	-0.067	12	130331764	9.99E-06	0.015	14306	18.975

Vascular dementia (multiple infarctions)	phylum Proteobacteria	rs6707783	C	T	-0.204	51826753	0.070	0.113	360612	C	T	0.085	2	52053891	8.09E-06	0.019	14306	20.470
Vascular dementia (multiple infarctions)	phylum Proteobacteria	rs72771021	C	T	0.086	13799631	0.513	0.131	360612	C	T	0.142	10	13841631	7.18E-06	0.031	14306	21.004
Vascular dementia (multiple infarctions)	phylum Proteobacteria	rs922773	C	T	-0.116	66267618	0.289	0.110	360612	C	T	-0.080	3	66318042	3.68E-07	0.016	14306	25.929
Vascular dementia (multiple infarctions)	phylum Tenericutes	rs10108398	G	A	0.080	58528265	0.275	0.073	360612	G	A	0.077	8	59440824	1.09E-06	0.015	14306	24.960
Vascular dementia (multiple infarctions)	phylum Tenericutes	rs11890098	A	G	-0.049	156676037	0.502	0.073	360612	A	G	0.074	2	157532549	9.57E-07	0.015	14306	23.551
Vascular dementia (multiple infarctions)	phylum Tenericutes	rs12566890	T	G	-0.023	61385192	0.815	0.097	360612	T	G	-0.101	1	61850864	3.65E-06	0.023	14306	19.176
Vascular dementia (multiple infarctions)	phylum Tenericutes	rs17214486	C	A	-0.059	96693337	0.399	0.070	360612	C	A	0.061	14	97159674	6.61E-06	0.014	14306	20.223
Vascular dementia (multiple infarctions)	phylum Tenericutes	rs2464826	A	C	-0.106	79860934	0.306	0.104	360612	A	C	0.094	7	79490250	8.39E-06	0.021	14306	19.874
Vascular dementia (multiple infarctions)	phylum Tenericutes	rs28537087	G	A	0.033	94766447	0.664	0.077	360612	G	A	0.082	15	95309676	8.07E-06	0.019	14306	19.002
Vascular dementia (multiple infarctions)	phylum Tenericutes	rs3768491	G	A	-0.045	109423364	0.527	0.072	360612	G	A	0.068	1	109965986	4.23E-06	0.015	14306	20.875
Vascular dementia (multiple infarctions)	phylum Tenericutes	rs4885016	C	T	-0.073	72596351	0.451	0.097	360612	C	T	0.082	13	73170489	7.27E-06	0.018	14306	20.363
Vascular dementia (multiple infarctions)	phylum Tenericutes	rs6043847	T	C	0.105	16278879	0.450	0.139	360612	T	C	-0.115	20	16259524	4.55E-06	0.025	14306	21.375
Vascular dementia (multiple infarctions)	phylum Tenericutes	rs72901605	T	C	0.056	47082326	0.579	0.101	360612	T	C	-0.084	11	47103877	3.26E-06	0.018	14306	22.338
Vascular dementia (multiple infarctions)	phylum Tenericutes	rs74603314	T	C	-0.241	46050515	0.147	0.166	360612	T	C	0.222	14	46519718	1.56E-06	0.046	14306	22.924
Vascular dementia (multiple infarctions)	phylum Tenericutes	rs78169027	A	G	-0.270	108568360	0.047	0.136	360612	A	G	-0.108	11	108439087	5.88E-06	0.024	14306	20.824
Vascular dementia (multiple infarctions)	phylum Verrucomicrobia	rs11252894	A	C	0.030	5030858	0.691	0.077	360612	A	C	0.078	10	5073050	1.11E-06	0.016	14306	23.203
Vascular dementia (multiple infarctions)	phylum Verrucomicrobia	rs117107102	A	G	-0.112	51947265	0.471	0.155	360612	A	G	0.204	18	49473635	2.68E-06	0.043	14306	22.828
Vascular dementia (multiple infarctions)	phylum Verrucomicrobia	rs11729256	T	C	0.095	94106121	0.275	0.087	360612	T	C	0.070	4	95027272	2.23E-06	0.015	14306	22.495
Vascular dementia (multiple infarctions)	phylum Verrucomicrobia	rs12512971	A	C	-0.054	16547352	0.657	0.123	360612	A	C	0.171	4	16548975	9.81E-06	0.040	14306	18.467
Vascular dementia (multiple infarctions)	phylum Verrucomicrobia	rs12908520	G	A	0.119	97027427	0.070	0.066	360612	G	A	0.059	15	97570657	3.40E-06	0.013	14306	21.524
Vascular dementia (multiple infarctions)	phylum Verrucomicrobia	rs2602429	T	C	-0.122	81029544	0.104	0.075	360612	T	C	-0.076	16	81063149	8.71E-07	0.015	14306	24.786
Vascular dementia (multiple infarctions)	phylum Verrucomicrobia	rs3995795	C	T	-0.046	11347615	0.492	0.067	360612	C	T	0.061	10	11389614	9.72E-06	0.014	14306	19.430
Vascular dementia (multiple infarctions)	phylum Verrucomicrobia	rs45598138	C	A	-0.034	55056410	0.870	0.206	360612	C	A	-0.144	1	55522083	2.19E-06	0.031	14306	22.202
Vascular dementia (multiple infarctions)	phylum Verrucomicrobia	rs61779207	G	A	-0.187	40608800	0.019	0.079	360612	G	A	-0.076	1	41074472	5.28E-06	0.016	14306	21.109
Vascular dementia (multiple infarctions)	phylum Verrucomicrobia	rs74542928	T	C	-0.146	99623031	0.332	0.151	360612	T	C	0.116	4	100544188	4.08E-07	0.023	14306	25.144
Vascular dementia (multiple infarctions)	phylum Verrucomicrobia	rs76430504	T	C	0.168	40438056	0.260	0.149	360612	T	C	-0.118	5	40438158	3.50E-06	0.025	14306	21.313
Vascular dementia (multiple infarctions)	phylum Verrucomicrobia	rs9349825	A	G	0.081	56476683	0.333	0.084	360612	A	G	-0.066	6	56341481	6.27E-06	0.014	14306	21.048
gen_samplesize_Vascular dementia (other)	class Actinobacteria	rs11655079	T	C	-0.121	77352593	0.477	0.171	360248	T	C	-0.056	17	75348675	5.93E-06	0.012	14306	20.351
gen_samplesize_Vascular dementia (other)	class Actinobacteria	rs11745923	G	T	0.025	475293	0.853	0.135	360248	G	T	0.056	5	475408	1.58E-06	0.012	14306	23.812
gen_samplesize_Vascular dementia (other)	class Actinobacteria	rs12049045	A	G	0.026	114122863	0.847	0.136	360248	A	G	0.051	1	114665485	8.63E-06	0.011	14306	19.936
gen_samplesize_Vascular dementia (other)	class Actinobacteria	rs134366	A	G	-0.136	35171362	0.600	0.259	360248	A	G	-0.112	22	35567355	1.50E-06	0.024	14306	22.663
gen_samplesize_Vascular dementia (other)	class Actinobacteria	rs1376754	G	A	-0.035	160755458	0.794	0.132	360248	G	A	0.051	2	161611969	6.71E-06	0.011	14306	20.489
gen_samplesize_Vascular dementia (other)	class Actinobacteria	rs1515761	C	T	-0.231	124421463	0.352	0.248	360248	C	T	-0.076	10	126110032	4.96E-06	0.017	14306	20.136
gen_samplesize_Vascular dementia (other)	class Actinobacteria	rs182549	T	C	-0.269	135859184	0.052	0.139	360248	T	C	-0.111	2	136616754	3.79E-20	0.012	14306	85.376
gen_samplesize_Vascular dementia (other)	class Actinobacteria	rs4945008	G	A	0.031	71510202	0.819	0.136	360248	G	A	-0.054	11	71221248	5.39E-06	0.012	14306	19.842
gen_samplesize_Vascular dementia (other)	class Actinobacteria	rs6660520	G	A	0.061	206830208	0.683	0.149	360248	G	A	-0.071	1	207003553	1.11E-07	0.013	14306	27.984
gen_samplesize_Vascular dementia (other)	class Actinobacteria	rs72767435	T	C	-0.996	94309566	0.005	0.352	360248	T	C	-0.126	15	94852795	2.57E-06	0.027	14306	21.323
gen_samplesize_Vascular dementia (other)	class Actinobacteria	rs7322849	T	C	-0.051	112205515	0.824	0.229	360248	T	C	0.094	13	112859829	6.21E-07	0.019	14306	23.953
gen_samplesize_Vascular dementia (other)	class Actinobacteria	rs80083040	T	G	0.219	64742287	0.490	0.318	360248	T	G	0.156	8	65654844	8.62E-06	0.035	14306	20.038
gen_samplesize_Vascular dementia (other)	class Actinobacteria	rs857444	C	T	0.144	14617360	0.294	0.137	360248	C	T	0.051	6	14617591	8.92E-06	0.012	14306	19.500
gen_samplesize_Vascular dementia (other)	class Actinobacteria	rs961091	G	A	-0.002	96025406	0.986	0.138	360248	G	A	0.050	7	95654718	8.68E-06	0.011	14306	19.835
gen_samplesize_Vascular dementia (other)	class Alphaproteobacteria	rs140912403	C	T	0.093	92755525	0.748	0.288	360248	C	T	-0.161	9	95517807	6.20E-07	0.032	14306	25.577
gen_samplesize_Vascular dementia (other)	class Alphaproteobacteria	rs34569731	G	A	0.102	75403218	0.461	0.138	360248	G	A	-0.071	2	75630344	7.38E-06	0.016	14306	20.028
gen_samplesize_Vascular dementia (other)	class Alphaproteobacteria	rs62285697	C	T	0.209	176690123	0.173	0.153	360248	C	T	0.081	3	176407911	9.76E-06	0.018	14306	19.812
gen_samplesize_Vascular dementia (other)	class Alphaproteobacteria	rs76784716	A	G	-0.067	168176830	0.753	0.212	360248	A	G	0.133	2	169033340	5.09E-07	0.027	14306	24.777
gen_samplesize_Vascular dementia (other)	class Alphaproteobacteria	rs7960664	A	G	-0.157	89077458	0.499	0.232	360248	A	G	-0.097	12	89471235	8.84E-06	0.022	14306	20.305
gen_samplesize_Vascular dementia (other)	class Alphaproteobacteria	rs9813022	A	G	0.130	13685237	0.343	0.137	360248	A	G	-0.075	3	13726736	1.05E-06	0.015	14306	23.840
gen_samplesize_Vascular dementia (other)	class Bacilli	rs11110282	A	G	0.024	100191781	0.939	0.312	360248	A	G	-0.101	12	100585559	4.85E-06	0.022	14306	21.669
gen_samplesize_Vascular dementia (other)	class Bacilli	rs11730038	G	A	0.027	97128348	0.852	0.146	360248	G	A	-0.063	4	98049499	1.96E-06	0.013	14306	24.013
gen_samplesize_Vascular dementia (other)	class Bacilli	rs12797734	T	C	0.052	8310803	0.735	0.155	360248	T	C	0.057	11	8332350	7.21E-06	0.013	14306	20.363
gen_samplesize_Vascular dementia (other)	class Bacilli	rs13068444	A	G	0.233	64413187	0.178	0.173	360248	A	G	0.060	3	64398863	9.53E-06	0.014	14306	19.415
gen_samplesize_Vascular dementia (other)	class Bacilli	rs1595463	C	A	-0.337	230858942	0.012	0.134	360248	C	A	0.048	2	231723657	7.97E-06	0.011	14306	19.300
gen_samplesize_Vascular dementia (other)	class Bacilli	rs28564647	T	G	-0.050	98338890	0.778	0.177	360248	T	G	-0.061	9	101101172	7.81E-06	0.014	14306	19.855
gen_samplesize_Vascular dementia (other)	class Bacilli	rs2952251	G	A	0.110	10285654	0.485	0.158	360248	G	A	0.060	8	10143164	1.08E-06	0.012	14306	23.361
gen_samplesize_Vascular dementia (other)	class Bacilli	rs34989881	A	G	-0.390	51456601	0.231	0.326	360248	A	G	0.111	19	51959855	6.55E-06	0.025	14306	20.374
gen_samplesize_Vascular dementia (other)	class Bacilli	rs35344081	G	A	0.086	941253	0.565	0.150	360248	G	A	0.062	16	991253	1.01E-06	0.013	14306	23.772
gen_samplesize_Vascular dementia (other)	class Bacilli	rs4028634	C	T	0.040	42683631	0.772	0.138	360248	C	T	-0.052	17	40835649	2.21E-06	0.011	14306	22.464
gen_samplesize_Vascular dementia (other)	class Bacilli	rs4459992	T	C	-0.024	7429760	0.869	0.142	360248	T	C	0.054	4	7431487	4.30E-06	0.012	14306	21.207
gen_samplesize_Vascular dementia (other)	class Bacilli	rs57872228	C	T	0.271	200449677	0.181	0.202	360248	C	T	-0.071	1	200418805	9.22E-07	0.015	14306	23.757
gen_samplesize_Vascular dementia (other)	class Bacilli	rs694949	A	G	0.001	58445876	0.995	0.225	360248	A	G	-0.081	15	58738075	7.60E-06	0.018	14306	20.314
gen_samplesize_Vascular dementia (other)	class Bacilli	rs74663707	C	T	0.072	184653436	0.789	0.271	360248	C	T	0.098	3	184371224	8.46E-06	0.022	14306	19.095
gen_samplesize_Vascular dementia (other)	class Bacilli	rs7666190	A	C	0.258	150766268	0.218	0.209	360248	A	C	0.104	4	151687420	8.47E-06	0.025	14306	17.610
gen_samplesize_Vascular dementia (other)	class Bacilli	rs77558518	A	G	-0.210	174746168	0.356	0.228	360248	A	G	-0.107	5	174173171	1.34E-06	0.022	14306	23.158
gen_samplesize_Vascular dementia (other)	class Bacilli	rs78938557	T	C	-0.005	36309977	0.990	0.396	360248	T	C	0.108	7	36349586	1.07E-06	0.023	14306	21.514
gen_samplesize_Vascular dementia (other)	class Bacilli	rs9581006	T	C	0.148	24399371	0.679	0.359	360248	T	C	-0.225	13	24973509	1.79E-06	0.047	14306	23.207
gen_samplesize_Vascular dementia (other)	class Bacteroidia	rs11146701	A	G	0.112	38769138	0.419	0.139	360248	A	G	0.047	10	39062269	7.08E-06	0.011	14306	20.186
gen_samplesize_Vascular dementia (other)	class Bacteroidia	rs17343978	A	C	0.115	27037922	0.482	0.164	360248	A	C	-0.055	22	27433885	8.36E-06	0.012	14306	21.067

gen_samplesize_Vascular dementia (other)	class Bacteroidia	rs2032750	C	T	-0.323	53603889	0.015	0.133	360248	C	T	0.051	2	53831026	1.92E-06	0.011	14306	22.657
gen_samplesize_Vascular dementia (other)	class Bacteroidia	rs2363574	T	C	0.089	200143435	0.800	0.350	360248	T	C	0.223	1	200112563	9.93E-06	0.051	14306	19.221
gen_samplesize_Vascular dementia (other)	class Bacteroidia	rs4916508	A	G	0.299	196209918	0.027	0.135	360248	A	G	0.047	3	195936789	8.47E-06	0.011	14306	19.641
gen_samplesize_Vascular dementia (other)	class Bacteroidia	rs55773148	G	A	0.058	69948897	0.842	0.291	360248	G	A	-0.122	13	70523029	3.90E-07	0.024	14306	26.341
gen_samplesize_Vascular dementia (other)	class Bacteroidia	rs62531359	T	G	0.295	70003946	0.089	0.174	360248	T	G	0.066	8	70916181	9.09E-06	0.015	14306	19.138
gen_samplesize_Vascular dementia (other)	class Bacteroidia	rs62575403	C	T	-0.216	133628698	0.509	0.327	360248	C	T	0.140	9	136493820	7.06E-06	0.031	14306	20.264
gen_samplesize_Vascular dementia (other)	class Bacteroidia	rs72706335	T	C	-0.489	157525648	0.256	0.431	360248	T	C	-0.222	1	157495438	7.66E-06	0.049	14306	20.315
gen_samplesize_Vascular dementia (other)	class Bacteroidia	rs73975615	G	A	0.303	6557880	0.710	0.816	360248	G	A	-0.207	17	6461200	1.22E-06	0.044	14306	21.874
gen_samplesize_Vascular dementia (other)	class Bacteroidia	rs7631304	G	A	-0.123	89290377	0.513	0.188	360248	G	A	-0.065	3	89339527	8.37E-07	0.013	14306	23.590
gen_samplesize_Vascular dementia (other)	class Bacteroidia	rs79585701	A	C	-0.036	13252676	0.849	0.190	360248	A	C	0.065	8	13110185	9.99E-06	0.015	14306	18.687
gen_samplesize_Vascular dementia (other)	class Bacteroidia	rs929878	T	C	-0.080	74256742	0.623	0.164	360248	T	C	0.055	16	74290641	4.73E-06	0.012	14306	20.372
gen_samplesize_Vascular dementia (other)	class Betaproteobacteria	rs11128180	A	G	0.076	70543064	0.627	0.157	360248	A	G	0.059	3	70592215	3.67E-06	0.013	14306	21.203
gen_samplesize_Vascular dementia (other)	class Betaproteobacteria	rs1511453	A	G	-0.401	23586839	0.162	0.287	360248	A	G	0.092	4	23588462	4.76E-06	0.020	14306	21.502
gen_samplesize_Vascular dementia (other)	class Betaproteobacteria	rs1928341	G	A	-0.171	153267537	0.208	0.136	360248	G	A	-0.053	1	153240013	2.02E-06	0.011	14306	22.661
gen_samplesize_Vascular dementia (other)	class Betaproteobacteria	rs2321387	G	A	0.125	58115206	0.346	0.133	360248	G	A	-0.049	13	58689340	5.80E-06	0.011	14306	20.430
gen_samplesize_Vascular dementia (other)	class Betaproteobacteria	rs2613606	T	C	-0.080	111644969	0.549	0.134	360248	T	C	0.051	7	111285025	2.20E-06	0.011	14306	22.123
gen_samplesize_Vascular dementia (other)	class Betaproteobacteria	rs320161	G	A	-0.280	102136835	0.076	0.158	360248	G	A	-0.057	9	104899117	7.33E-06	0.013	14306	20.564
gen_samplesize_Vascular dementia (other)	class Betaproteobacteria	rs4033856	T	C	-0.075	45640468	0.751	0.237	360248	T	C	-0.083	4	45642485	5.17E-07	0.017	14306	24.776
gen_samplesize_Vascular dementia (other)	class Betaproteobacteria	rs6087811	T	G	0.032	32008327	0.883	0.221	360248	T	G	-0.098	20	30596130	7.44E-07	0.020	14306	24.278
gen_samplesize_Vascular dementia (other)	class Betaproteobacteria	rs62395635	T	C	0.404	174070793	0.149	0.280	360248	T	C	0.110	5	173497796	2.94E-06	0.024	14306	21.623
gen_samplesize_Vascular dementia (other)	class Betaproteobacteria	rs75242906	C	T	0.034	56494209	0.887	0.239	360248	C	T	-0.121	15	56786407	9.27E-06	0.028	14306	18.601
gen_samplesize_Vascular dementia (other)	class Clostridia	rs10774377	G	A	0.068	5833353	0.613	0.135	360248	G	A	-0.053	12	5942519	3.24E-06	0.011	14306	21.390
gen_samplesize_Vascular dementia (other)	class Clostridia	rs112334273	G	A	0.017	39331325	0.906	0.148	360248	G	A	0.064	21	40703251	3.81E-07	0.013	14306	25.314
gen_samplesize_Vascular dementia (other)	class Clostridia	rs13105690	C	T	-0.085	7418457	0.562	0.147	360248	C	T	0.053	4	7420184	8.78E-06	0.012	14306	20.044
gen_samplesize_Vascular dementia (other)	class Clostridia	rs13179700	C	T	0.034	149698225	0.807	0.140	360248	C	T	-0.051	5	149077788	3.37E-06	0.011	14306	21.830
gen_samplesize_Vascular dementia (other)	class Clostridia	rs1842454	G	A	-0.089	105724661	0.599	0.169	360248	G	A	-0.055	5	105060362	8.72E-06	0.013	14306	18.466
gen_samplesize_Vascular dementia (other)	class Clostridia	rs2273429	A	G	0.054	52027354	0.800	0.213	360248	A	G	-0.072	14	52494072	4.52E-06	0.015	14306	22.364
gen_samplesize_Vascular dementia (other)	class Clostridia	rs6797343	T	G	0.228	89219564	0.177	0.169	360248	T	G	-0.059	3	89268714	9.36E-06	0.013	14306	19.375
gen_samplesize_Vascular dementia (other)	class Clostridia	rs6814436	C	T	-0.014	160586149	0.941	0.190	360248	C	T	-0.074	4	161507301	9.65E-07	0.015	14306	24.075
gen_samplesize_Vascular dementia (other)	class Clostridia	rs6815608	C	T	0.150	151210592	0.422	0.187	360248	C	T	-0.104	4	152131744	4.02E-07	0.021	14306	24.229
gen_samplesize_Vascular dementia (other)	class Clostridia	rs72738886	T	C	-0.307	35770448	0.226	0.253	360248	T	C	0.087	5	35770550	8.24E-06	0.019	14306	20.697
gen_samplesize_Vascular dementia (other)	class Clostridia	rs992074	T	C	-0.169	17195484	0.706	0.450	360248	T	C	-0.256	21	18567802	8.78E-07	0.051	14306	25.245
gen_samplesize_Vascular dementia (other)	class Coriobacteriia	rs11073596	G	T	-0.263	85890348	0.057	0.138	360248	G	T	-0.051	15	86433579	8.14E-06	0.011	14306	19.912
gen_samplesize_Vascular dementia (other)	class Coriobacteriia	rs11250875	T	C	-0.149	1880537	0.357	0.161	360248	T	C	0.061	10	1922731	4.83E-06	0.013	14306	21.526
gen_samplesize_Vascular dementia (other)	class Coriobacteriia	rs11656361	A	C	-0.271	8218014	0.116	0.173	360248	A	C	0.077	17	8121332	8.02E-06	0.018	14306	19.394
gen_samplesize_Vascular dementia (other)	class Coriobacteriia	rs12974142	G	A	0.233	52391913	0.376	0.264	360248	G	A	0.079	19	52895166	8.51E-06	0.018	14306	19.865
gen_samplesize_Vascular dementia (other)	class Coriobacteriia	rs13307134	T	C	0.272	105444233	0.130	0.180	360248	T	C	-0.057	7	105084680	7.80E-06	0.013	14306	20.072
gen_samplesize_Vascular dementia (other)	class Coriobacteriia	rs1397793	A	G	-0.061	91175634	0.672	0.144	360248	A	G	0.050	5	90471451	9.77E-06	0.011	14306	19.682
gen_samplesize_Vascular dementia (other)	class Coriobacteriia	rs1816223	G	A	0.266	11341087	0.111	0.167	360248	G	A	0.059	12	11494021	4.84E-06	0.013	14306	20.652
gen_samplesize_Vascular dementia (other)	class Coriobacteriia	rs240104	T	C	0.099	176602295	0.508	0.149	360248	T	C	-0.060	1	176571431	1.52E-06	0.013	14306	22.630
gen_samplesize_Vascular dementia (other)	class Coriobacteriia	rs2442778	A	G	0.298	11612938	0.329	0.305	360248	A	G	0.116	3	11654412	9.03E-06	0.026	14306	20.272
gen_samplesize_Vascular dementia (other)	class Coriobacteriia	rs3025411	A	G	-0.176	133647784	0.408	0.213	360248	A	G	0.093	9	136512906	8.27E-06	0.021	14306	19.566
gen_samplesize_Vascular dementia (other)	class Coriobacteriia	rs34739816	G	T	-0.283	39220432	0.316	0.282	360248	G	T	0.097	17	37376685	3.88E-06	0.021	14306	21.594
gen_samplesize_Vascular dementia (other)	class Coriobacteriia	rs67561917	A	G	-0.234	63440724	0.180	0.174	360248	A	G	-0.071	20	62072077	5.39E-06	0.015	14306	21.486
gen_samplesize_Vascular dementia (other)	class Coriobacteriia	rs719099	A	G	0.120	64039457	0.591	0.223	360248	A	G	0.078	10	65799217	5.43E-07	0.016	14306	24.957
gen_samplesize_Vascular dementia (other)	class Coriobacteriia	rs8010111	A	G	-0.021	39191305	0.930	0.241	360248	A	G	0.103	14	39660509	6.90E-06	0.023	14306	20.328
gen_samplesize_Vascular dementia (other)	class Deltaproteobacteria	rs1035691	G	A	-0.331	10637188	0.015	0.136	360248	G	A	0.055	11	10658735	9.65E-06	0.012	14306	20.588
gen_samplesize_Vascular dementia (other)	class Deltaproteobacteria	rs11599763	C	T	0.088	11813600	0.520	0.137	360248	C	T	0.054	10	11855599	3.94E-06	0.012	14306	21.490
gen_samplesize_Vascular dementia (other)	class Deltaproteobacteria	rs17084793	G	A	-0.201	71645678	0.286	0.188	360248	G	A	-0.071	18	69312914	5.69E-06	0.016	14306	19.850
gen_samplesize_Vascular dementia (other)	class Deltaproteobacteria	rs17791387	A	G	-0.048	79219511	0.835	0.231	360248	A	G	-0.074	9	81834426	1.60E-06	0.015	14306	22.761
gen_samplesize_Vascular dementia (other)	class Deltaproteobacteria	rs2692012	G	A	0.209	204022477	0.478	0.294	360248	G	A	-0.110	1	203991605	3.14E-06	0.025	14306	18.968
gen_samplesize_Vascular dementia (other)	class Deltaproteobacteria	rs2838334	G	A	0.315	43645080	0.025	0.140	360248	G	A	0.056	21	45064961	5.45E-06	0.012	14306	20.480
gen_samplesize_Vascular dementia (other)	class Deltaproteobacteria	rs3935584	C	T	-0.116	233064573	0.383	0.134	360248	C	T	-0.052	2	233929283	7.50E-06	0.012	14306	20.485
gen_samplesize_Vascular dementia (other)	class Deltaproteobacteria	rs4506934	C	T	-0.003	2953368	0.987	0.204	360248	C	T	-0.094	17	2856662	3.59E-06	0.020	14306	21.666
gen_samplesize_Vascular dementia (other)	class Deltaproteobacteria	rs55744759	A	G	-0.017	56955112	0.936	0.210	360248	A	G	-0.078	8	57867671	7.31E-06	0.017	14306	20.854
gen_samplesize_Vascular dementia (other)	class Deltaproteobacteria	rs6058181	C	T	0.207	35106998	0.247	0.178	360248	C	T	0.083	20	33694801	3.40E-07	0.017	14306	24.755
gen_samplesize_Vascular dementia (other)	class Deltaproteobacteria	rs62020470	A	G	0.107	95617836	0.538	0.174	360248	A	G	-0.059	15	96161065	4.85E-06	0.013	14306	20.480
gen_samplesize_Vascular dementia (other)	class Deltaproteobacteria	rs9928243	C	A	0.037	71507738	0.779	0.133	360248	C	A	-0.054	16	71541641	5.02E-06	0.012	14306	20.923
gen_samplesize_Vascular dementia (other)	class Erysipelotrichia	rs1074800	G	A	0.054	3002432	0.688	0.135	360248	G	A	-0.049	5	3002546	6.15E-06	0.011	14306	20.459
gen_samplesize_Vascular dementia (other)	class Erysipelotrichia	rs10781552	C	T	-0.190	132083729	0.196	0.147	360248	C	T	-0.055	10	133897233	2.33E-06	0.012	14306	22.633
gen_samplesize_Vascular dementia (other)	class Erysipelotrichia	rs17530232	A	G	-0.294	39811320	0.317	0.293	360248	A	G	0.103	13	40385457	2.79E-06	0.022	14306	21.042
gen_samplesize_Vascular dementia (other)	class Erysipelotrichia	rs1884466	C	T	-0.152	63673525	0.256	0.134	360248	C	T	-0.048	1	64139196	9.53E-06	0.011	14306	19.760
gen_samplesize_Vascular dementia (other)	class Erysipelotrichia	rs2300774	A	G	-0.043	196066841	0.745	0.133	360248	A	G	-0.052	3	195793712	8.95E-07	0.011	14306	24.094
gen_samplesize_Vascular dementia (other)	class Erysipelotrichia	rs290833	T	G	-0.264	96991871	0.047	0.133	360248	T	G	-0.050	1	97457427	8.03E-06	0.011	14306	19.943
gen_samplesize_Vascular dementia (other)	class Erysipelotrichia	rs35161940	T	C	-0.021	72331083	0.922	0.218	360248	T	C	-0.081	17	70327224	1.85E-06	0.017	14306	23.118
gen_samplesize_Vascular dementia (other)	class Erysipelotrichia	rs4078432	T	C	0.018	48528003	0.919	0.177	360248	T	C	0.061	14	48997206	4.23E-06	0.013	14306	20.723
gen_samplesize_Vascular dementia (other)	class Erysipelotrichia	rs56970041	T	G	0.127	79891267	0.644	0.275	360248	T	G	0.072	14	80357610	5.40E-06	0.016	14306	19.385

gen_samplesize_Vascular dementia (other)	class Erysipelotrichia	rs62504403	C	T	0.099	38946033	0.551	0.166	360248	C	T	0.068	8	38803551	1.12E-07	0.013	14306	28.371
gen_samplesize_Vascular dementia (other)	class Erysipelotrichia	rs7234058	T	C	-0.110	5830508	0.629	0.228	360248	T	C	-0.095	18	5830507	9.12E-07	0.019	14306	23.744
gen_samplesize_Vascular dementia (other)	class Erysipelotrichia	rs7826267	G	T	0.117	3097430	0.658	0.263	360248	G	T	0.084	8	2954952	9.28E-06	0.020	14306	17.755
gen_samplesize_Vascular dementia (other)	class Erysipelotrichia	rs8003149	C	T	-0.097	55689786	0.493	0.142	360248	C	T	0.054	14	56156504	4.08E-06	0.012	14306	21.248
gen_samplesize_Vascular dementia (other)	class Gammaproteobacteria	rs11181912	G	A	0.044	43179149	0.757	0.142	360248	G	A	-0.058	12	43572952	9.95E-07	0.012	14306	23.767
gen_samplesize_Vascular dementia (other)	class Gammaproteobacteria	rs12404135	A	G	-0.090	186533562	0.719	0.250	360248	A	G	-0.079	1	186502694	8.89E-06	0.017	14306	20.933
gen_samplesize_Vascular dementia (other)	class Gammaproteobacteria	rs6706173	A	C	-0.055	167130482	0.771	0.190	360248	A	C	0.074	2	167986992	1.99E-07	0.015	14306	25.726
gen_samplesize_Vascular dementia (other)	class Gammaproteobacteria	rs75101789	C	T	-0.056	18574411	0.813	0.236	360248	C	T	0.073	3	18615903	8.79E-06	0.016	14306	20.002
gen_samplesize_Vascular dementia (other)	class Gammaproteobacteria	rs79795896	A	G	-0.041	50658878	0.897	0.317	360248	A	G	-0.159	18	48185248	7.92E-06	0.035	14306	20.531
gen_samplesize_Vascular dementia (other)	class Gammaproteobacteria	rs9494710	C	T	-0.035	137291417	0.807	0.143	360248	C	T	-0.055	6	137612554	4.55E-06	0.012	14306	20.663
gen_samplesize_Vascular dementia (other)	class Lentisphaeria	rs1002941	A	G	-0.036	100702485	0.814	0.153	360248	A	G	-0.105	15	101242690	8.15E-06	0.023	14306	20.234
gen_samplesize_Vascular dementia (other)	class Lentisphaeria	rs11770843	C	T	-0.034	147098287	0.810	0.140	360248	C	T	0.109	7	146795379	1.91E-06	0.023	14306	21.707
gen_samplesize_Vascular dementia (other)	class Lentisphaeria	rs17114848	G	A	-0.077	24917388	0.738	0.231	360248	G	A	0.152	15	25162535	4.06E-06	0.032	14306	22.073
gen_samplesize_Vascular dementia (other)	class Lentisphaeria	rs2031282	A	G	-0.329	20113040	0.062	0.177	360248	A	G	0.122	13	20687179	4.38E-06	0.027	14306	20.490
gen_samplesize_Vascular dementia (other)	class Lentisphaeria	rs2825714	A	G	-0.201	19651652	0.257	0.177	360248	A	G	-0.137	21	21023966	1.72E-06	0.029	14306	22.568
gen_samplesize_Vascular dementia (other)	class Lentisphaeria	rs62570196	C	T	0.390	108323890	0.238	0.330	360248	C	T	-0.216	9	111086170	1.08E-06	0.044	14306	24.192
gen_samplesize_Vascular dementia (other)	class Lentisphaeria	rs72640280	A	G	0.100	11883735	0.727	0.288	360248	A	G	0.220	1	11943792	5.18E-06	0.049	14306	20.513
gen_samplesize_Vascular dementia (other)	class Lentisphaeria	rs77599476	A	G	-0.198	62762910	0.489	0.286	360248	A	G	0.230	20	61394262	1.86E-06	0.048	14306	23.002
gen_samplesize_Vascular dementia (other)	class Melainabacteria	rs10148250	A	G	-0.049	106605442	0.729	0.142	360248	A	G	-0.086	14	107061448	8.67E-06	0.019	14306	19.866
gen_samplesize_Vascular dementia (other)	class Melainabacteria	rs10738747	A	G	-0.084	26184580	0.534	0.134	360248	A	G	-0.081	9	26184578	9.96E-06	0.018	14306	19.514
gen_samplesize_Vascular dementia (other)	class Melainabacteria	rs11150282	T	C	0.112	80459808	0.425	0.140	360248	T	C	0.099	16	80493705	6.03E-07	0.020	14306	25.235
gen_samplesize_Vascular dementia (other)	class Melainabacteria	rs113884518	T	C	-0.300	24648999	0.486	0.430	360248	T	C	-0.205	9	24648997	8.06E-06	0.045	14306	20.371
gen_samplesize_Vascular dementia (other)	class Melainabacteria	rs28678345	T	C	0.466	55828967	0.127	0.306	360248	T	C	0.215	17	53906328	6.69E-06	0.047	14306	20.818
gen_samplesize_Vascular dementia (other)	class Melainabacteria	rs367480	A	G	-0.030	2916401	0.831	0.139	360248	A	G	0.084	11	2937631	8.20E-06	0.019	14306	20.323
gen_samplesize_Vascular dementia (other)	class Melainabacteria	rs4129395	G	A	0.118	113213109	0.378	0.134	360248	G	A	0.090	9	115975389	1.48E-06	0.019	14306	23.440
gen_samplesize_Vascular dementia (other)	class Melainabacteria	rs789069	A	C	-0.197	1008277	0.309	0.193	360248	A	C	-0.104	18	1008278	6.85E-06	0.023	14306	19.530
gen_samplesize_Vascular dementia (other)	class Melainabacteria	rs79790072	T	C	-0.217	100207478	0.574	0.386	360248	T	C	0.227	15	100747683	3.29E-06	0.049	14306	21.599
gen_samplesize_Vascular dementia (other)	class Melainabacteria	rs9864379	T	C	-0.122	14265449	0.517	0.188	360248	T	C	-0.160	3	14306949	5.36E-08	0.029	14306	29.812
gen_samplesize_Vascular dementia (other)	class Methanobacteria	rs10202904	G	T	-0.109	124682691	0.423	0.136	360248	G	T	0.122	2	125440268	3.01E-07	0.024	14306	26.762
gen_samplesize_Vascular dementia (other)	class Methanobacteria	rs10424197	A	G	0.112	45936063	0.466	0.154	360248	A	G	0.111	19	46439321	9.28E-06	0.025	14306	20.211
gen_samplesize_Vascular dementia (other)	class Methanobacteria	rs4257531	G	A	0.097	2044483	0.657	0.219	360248	G	A	0.164	3	2086167	7.44E-06	0.036	14306	20.316
gen_samplesize_Vascular dementia (other)	class Methanobacteria	rs6508769	C	T	0.056	28336853	0.767	0.188	360248	C	T	-0.154	19	28827760	8.23E-06	0.034	14306	19.856
gen_samplesize_Vascular dementia (other)	class Methanobacteria	rs6776814	T	C	0.088	15011576	0.851	0.471	360248	T	C	-0.200	3	15053083	1.63E-06	0.041	14306	23.483
gen_samplesize_Vascular dementia (other)	class Methanobacteria	rs73068003	G	T	-0.346	10734305	0.125	0.226	360248	G	T	-0.158	7	10773932	8.45E-06	0.035	14306	20.206
gen_samplesize_Vascular dementia (other)	class Methanobacteria	rs73457410	A	G	-0.101	41382045	0.712	0.274	360248	A	G	0.215	13	41956181	1.41E-06	0.044	14306	24.316
gen_samplesize_Vascular dementia (other)	class Methanobacteria	rs75208022	C	T	0.004	21185927	0.985	0.225	360248	C	T	-0.227	12	21338861	5.92E-06	0.049	14306	21.717
gen_samplesize_Vascular dementia (other)	class Methanobacteria	rs894996	C	A	0.105	103497150	0.682	0.255	360248	C	A	0.217	4	104418307	1.88E-06	0.045	14306	23.349
gen_samplesize_Vascular dementia (other)	class Mollicutes	rs10108398	G	A	-0.118	58528265	0.424	0.148	360248	G	A	0.077	8	59440824	1.09E-06	0.015	14306	24.960
gen_samplesize_Vascular dementia (other)	class Mollicutes	rs11890098	A	G	-0.307	156676037	0.038	0.148	360248	A	G	0.074	2	157532549	9.57E-07	0.015	14306	23.551
gen_samplesize_Vascular dementia (other)	class Mollicutes	rs12566890	T	G	0.075	61385192	0.706	0.200	360248	T	G	-0.101	1	61850864	3.65E-06	0.023	14306	19.176
gen_samplesize_Vascular dementia (other)	class Mollicutes	rs17214486	C	A	-0.105	96693337	0.458	0.142	360248	C	A	0.061	14	97159674	6.61E-06	0.014	14306	20.223
gen_samplesize_Vascular dementia (other)	class Mollicutes	rs2464826	A	C	0.144	79860934	0.495	0.211	360248	A	C	0.094	7	79490250	8.39E-06	0.021	14306	19.874
gen_samplesize_Vascular dementia (other)	class Mollicutes	rs28537087	G	A	0.041	94766447	0.789	0.154	360248	G	A	0.082	15	95309676	8.07E-06	0.019	14306	19.002
gen_samplesize_Vascular dementia (other)	class Mollicutes	rs3768491	G	A	0.089	109423364	0.541	0.145	360248	G	A	0.068	1	109965986	4.23E-06	0.015	14306	20.875
gen_samplesize_Vascular dementia (other)	class Mollicutes	rs4885016	C	T	-0.016	72596351	0.934	0.195	360248	C	T	0.082	13	73170489	7.27E-06	0.018	14306	20.363
gen_samplesize_Vascular dementia (other)	class Mollicutes	rs6043847	T	C	-0.147	16278879	0.599	0.279	360248	T	C	-0.115	20	16259524	4.55E-06	0.025	14306	21.375
gen_samplesize_Vascular dementia (other)	class Mollicutes	rs72901605	T	C	-0.142	47082326	0.491	0.206	360248	T	C	-0.084	11	47103877	3.26E-06	0.018	14306	22.338
gen_samplesize_Vascular dementia (other)	class Mollicutes	rs74603314	T	C	0.101	46050515	0.769	0.343	360248	T	C	0.222	14	46519718	1.56E-06	0.046	14306	22.924
gen_samplesize_Vascular dementia (other)	class Mollicutes	rs78169027	A	G	-0.067	108568360	0.812	0.284	360248	A	G	-0.108	11	108439087	5.88E-06	0.024	14306	20.824
gen_samplesize_Vascular dementia (other)	class Negativicutes	rs1135612	G	A	0.182	75980359	0.260	0.161	360248	G	A	0.053	7	75609677	9.26E-06	0.012	14306	19.761
gen_samplesize_Vascular dementia (other)	class Negativicutes	rs13086907	G	A	-0.030	142416275	0.851	0.161	360248	G	A	0.063	3	142135117	1.95E-06	0.013	14306	22.532
gen_samplesize_Vascular dementia (other)	class Negativicutes	rs1643968	T	C	0.086	165839623	0.535	0.139	360248	T	C	-0.057	5	165266628	4.15E-07	0.011	14306	25.339
gen_samplesize_Vascular dementia (other)	class Negativicutes	rs1649999	A	G	-0.260	78326315	0.249	0.225	360248	A	G	0.075	10	80086072	7.58E-06	0.017	14306	20.246
gen_samplesize_Vascular dementia (other)	class Negativicutes	rs2834062	A	G	0.126	33005177	0.391	0.147	360248	A	G	0.049	21	34377485	8.44E-06	0.011	14306	20.190
gen_samplesize_Vascular dementia (other)	class Negativicutes	rs4463806	C	T	0.105	113838234	0.527	0.167	360248	C	T	0.054	10	115597993	7.81E-06	0.013	14306	17.681
gen_samplesize_Vascular dementia (other)	class Negativicutes	rs4722181	T	G	-0.105	22777952	0.428	0.132	360248	T	G	0.050	7	22817571	2.00E-06	0.011	14306	22.452
gen_samplesize_Vascular dementia (other)	class Negativicutes	rs60274479	T	C	-0.090	21238604	0.591	0.168	360248	T	C	-0.066	16	21249925	1.16E-06	0.013	14306	24.182
gen_samplesize_Vascular dementia (other)	class Negativicutes	rs61249479	A	C	0.132	122150629	0.478	0.186	360248	A	C	0.078	9	124912908	2.95E-06	0.017	14306	21.236
gen_samplesize_Vascular dementia (other)	class Negativicutes	rs71405394	G	A	0.257	100704883	0.327	0.262	360248	G	A	-0.114	15	101245088	2.17E-06	0.024	14306	22.539
gen_samplesize_Vascular dementia (other)	class Negativicutes	rs73232831	G	A	0.117	17411803	0.735	0.346	360248	G	A	-0.152	4	17413426	1.87E-06	0.031	14306	23.242
gen_samplesize_Vascular dementia (other)	class Negativicutes	rs9423647	G	A	0.082	5537855	0.541	0.134	360248	G	A	0.048	10	5579818	6.06E-06	0.011	14306	20.628
gen_samplesize_Vascular dementia (other)	class Verrucomicrobiae	rs111862613	T	C	0.109	129825125	0.542	0.179	360248	T	C	0.091	12	130309670	3.74E-06	0.020	14306	21.252
gen_samplesize_Vascular dementia (other)	class Verrucomicrobiae	rs117107102	A	G	-0.510	51947265	0.106	0.315	360248	A	G	0.205	18	49473635	2.92E-06	0.043	14306	22.493
gen_samplesize_Vascular dementia (other)	class Verrucomicrobiae	rs11729256	T	C	-0.231	94106121	0.192	0.177	360248	T	C	0.075	4	95027272	6.73E-07	0.015	14306	24.928
gen_samplesize_Vascular dementia (other)	class Verrucomicrobiae	rs12908520	G	A	0.088	97027427	0.513	0.134	360248	G	A	0.062	15	97570657	2.17E-06	0.013	14306	22.341
gen_samplesize_Vascular dementia (other)	class Verrucomicrobiae	rs2602429	T	C	-0.031	81029544	0.839	0.152	360248	T	C	-0.075	16	81063149	2.58E-06	0.016	14306	22.863
gen_samplesize_Vascular dementia (other)	class Verrucomicrobiae	rs4242783	A	G	-0.137	5022135	0.356	0.149	360248	A	G	-0.069	10	5064327	2.64E-06	0.015	14306	21.781

gen_samplesize_Vascular dementia (other)	class Verrucomicrobiae	rs4936098	G	A	0.049	130410772	0.723	0.139	360248	G	A	-0.065	11	130280667	1.12E-06	0.014	14306	22.786
gen_samplesize_Vascular dementia (other)	class Verrucomicrobiae	rs61779207	G	A	-0.037	40608800	0.817	0.162	360248	G	A	-0.076	1	41074472	6.72E-06	0.017	14306	20.432
gen_samplesize_Vascular dementia (other)	class Verrucomicrobiae	rs74542928	T	C	-0.380	99623031	0.214	0.306	360248	T	C	0.112	4	100544188	1.63E-06	0.024	14306	22.508
gen_samplesize_Vascular dementia (other)	class Verrucomicrobiae	rs9349825	A	G	-0.366	56476683	0.032	0.171	360248	A	G	-0.070	6	56341481	2.54E-06	0.015	14306	22.898
gen_samplesize_Vascular dementia (other)	class Verrucomicrobiae	rs941682	G	A	0.084	33280034	0.573	0.149	360248	G	A	-0.063	20	31867840	9.61E-06	0.014	14306	19.290
gen_samplesize_Vascular dementia (other)	family Acidaminococcaceae	rs262812	T	C	-0.014	158226967	0.925	0.144	360248	T	C	-0.066	6	158647999	3.25E-06	0.014	14306	21.313
gen_samplesize_Vascular dementia (other)	family Acidaminococcaceae	rs2933324	G	A	0.209	104355372	0.187	0.159	360248	G	A	0.066	9	107117653	2.24E-06	0.014	14306	22.315
gen_samplesize_Vascular dementia (other)	family Acidaminococcaceae	rs45497800	T	C	0.447	63360481	0.020	0.192	360248	T	C	-0.118	20	61991833	5.86E-06	0.026	14306	20.975
gen_samplesize_Vascular dementia (other)	family Acidaminococcaceae	rs6589457	G	A	-0.531	114830566	0.085	0.308	360248	G	A	-0.166	11	114701288	2.32E-06	0.035	14306	22.533
gen_samplesize_Vascular dementia (other)	family Acidaminococcaceae	rs6923842	T	C	-0.107	5722948	0.606	0.208	360248	T	C	-0.080	6	5723181	2.21E-06	0.017	14306	22.140
gen_samplesize_Vascular dementia (other)	family Acidaminococcaceae	rs74540770	G	A	-0.079	186835600	0.747	0.246	360248	G	A	-0.109	3	186553389	7.09E-06	0.024	14306	20.021
gen_samplesize_Vascular dementia (other)	family Acidaminococcaceae	rs78702810	T	C	0.197	18421039	0.361	0.216	360248	T	C	-0.144	3	18462531	9.16E-06	0.032	14306	19.778
gen_samplesize_Vascular dementia (other)	family Actinomycetaceae	rs2889192	T	G	-0.213	73779652	0.254	0.187	360248	T	G	-0.089	9	76394568	3.64E-06	0.020	14306	20.714
gen_samplesize_Vascular dementia (other)	family Actinomycetaceae	rs34583783	G	T	0.111	66497478	0.689	0.278	360248	G	T	0.124	6	67207371	5.48E-06	0.026	14306	21.930
gen_samplesize_Vascular dementia (other)	family Actinomycetaceae	rs35011108	A	G	-0.030	132686341	0.910	0.266	360248	A	G	0.242	6	133007480	1.83E-06	0.050	14306	23.041
gen_samplesize_Vascular dementia (other)	family Actinomycetaceae	rs4073240	G	A	-0.111	168824686	0.419	0.137	360248	G	A	0.075	6	169224781	6.05E-06	0.016	14306	20.605
gen_samplesize_Vascular dementia (other)	family Alcaligenaceae	rs112135816	T	G	0.166	79870412	0.541	0.271	360248	T	G	-0.078	9	82485327	5.28E-06	0.017	14306	20.411
gen_samplesize_Vascular dementia (other)	family Alcaligenaceae	rs1153990	A	G	0.219	104897760	0.129	0.144	360248	A	G	-0.059	5	104233461	5.97E-06	0.013	14306	20.950
gen_samplesize_Vascular dementia (other)	family Alcaligenaceae	rs147968	C	T	0.015	85912233	0.910	0.133	360248	C	T	0.049	16	85945839	9.13E-06	0.011	14306	19.685
gen_samplesize_Vascular dementia (other)	family Alcaligenaceae	rs28480294	C	T	-0.141	71230170	0.312	0.140	360248	C	T	0.052	15	71522509	6.61E-06	0.012	14306	20.033
gen_samplesize_Vascular dementia (other)	family Alcaligenaceae	rs4033856	T	C	-0.075	45640468	0.751	0.237	360248	T	C	-0.082	4	45642485	1.03E-06	0.017	14306	23.467
gen_samplesize_Vascular dementia (other)	family Alcaligenaceae	rs62191117	A	G	-0.047	238979079	0.775	0.165	360248	A	G	0.068	2	239900775	2.76E-07	0.013	14306	26.221
gen_samplesize_Vascular dementia (other)	family Alcaligenaceae	rs62395635	T	C	0.404	174070793	0.149	0.280	360248	T	C	0.111	5	173497796	3.35E-06	0.024	14306	21.496
gen_samplesize_Vascular dementia (other)	family Alcaligenaceae	rs6969323	A	C	0.026	104574040	0.869	0.159	360248	A	C	-0.059	7	104214487	3.89E-06	0.013	14306	21.127
gen_samplesize_Vascular dementia (other)	family Alcaligenaceae	rs74776516	T	G	-0.134	20741821	0.623	0.272	360248	T	G	-0.094	11	20763367	6.85E-06	0.021	14306	19.591
gen_samplesize_Vascular dementia (other)	family Alcaligenaceae	rs7638039	T	C	0.097	70539788	0.528	0.155	360248	T	C	0.060	3	70588939	2.70E-06	0.013	14306	22.295
gen_samplesize_Vascular dementia (other)	family Alcaligenaceae	rs9537886	A	C	0.130	57971112	0.330	0.133	360248	A	C	-0.057	13	58545246	2.35E-07	0.011	14306	26.604
gen_samplesize_Vascular dementia (other)	family Bacteroidaceae	rs11585893	A	G	-0.315	10584294	0.042	0.155	360248	A	G	-0.074	1	10644351	1.80E-06	0.015	14306	25.175
gen_samplesize_Vascular dementia (other)	family Bacteroidaceae	rs13207588	A	G	0.029	41551692	0.865	0.170	360248	A	G	-0.059	6	41519430	7.48E-06	0.013	14306	20.365
gen_samplesize_Vascular dementia (other)	family Bacteroidaceae	rs1340391	T	C	0.419	102495433	0.031	0.195	360248	T	C	-0.059	1	102960989	6.73E-06	0.013	14306	20.040
gen_samplesize_Vascular dementia (other)	family Bacteroidaceae	rs17619981	T	G	-0.361	24159448	0.060	0.192	360248	T	G	0.088	19	24342250	2.69E-06	0.019	14306	22.194
gen_samplesize_Vascular dementia (other)	family Bacteroidaceae	rs2023437	T	C	-0.129	21577814	0.522	0.202	360248	T	C	-0.078	14	22045949	5.02E-06	0.017	14306	21.780
gen_samplesize_Vascular dementia (other)	family Bacteroidaceae	rs66710942	T	C	-0.026	77166176	0.845	0.133	360248	T	C	-0.049	3	77215327	5.86E-06	0.011	14306	20.644
gen_samplesize_Vascular dementia (other)	family Bacteroidaceae	rs6795673	C	T	0.016	10551540	0.902	0.133	360248	C	T	0.054	3	10593224	3.38E-07	0.011	14306	26.183
gen_samplesize_Vascular dementia (other)	family Bacteroidaceae	rs9507307	C	T	0.108	24336338	0.485	0.155	360248	C	T	0.060	13	24910476	2.13E-06	0.013	14306	21.912
gen_samplesize_Vascular dementia (other)	family Bacteroidales S24 7group	rs10872669	G	A	0.267	151194037	0.247	0.231	360248	G	A	0.123	6	151515172	9.49E-06	0.028	14306	19.932
gen_samplesize_Vascular dementia (other)	family Bacteroidales S24 7group	rs12748533	G	T	-0.028	242603950	0.848	0.147	360248	G	T	-0.082	1	242767252	2.59E-06	0.017	14306	22.605
gen_samplesize_Vascular dementia (other)	family Bacteroidales S24 7group	rs17043785	T	C	0.114	52912224	0.632	0.239	360248	T	C	-0.176	2	53139362	5.12E-07	0.035	14306	25.761
gen_samplesize_Vascular dementia (other)	family Bacteroidales S24 7group	rs1850003	A	G	-0.184	47048773	0.239	0.156	360248	A	G	0.084	15	47340971	2.41E-06	0.018	14306	22.344
gen_samplesize_Vascular dementia (other)	family Bacteroidales S24 7group	rs61508842	T	C	0.331	158935756	0.152	0.231	360248	T	C	0.123	3	158653545	7.83E-06	0.027	14306	20.250
gen_samplesize_Vascular dementia (other)	family Bacteroidales S24 7group	rs738193	T	C	-0.154	25547355	0.266	0.138	360248	T	C	0.085	22	25943322	3.82E-07	0.017	14306	26.100
gen_samplesize_Vascular dementia (other)	family Bacteroidales S24 7group	rs78609301	A	G	-0.076	62526842	0.600	0.145	360248	A	G	-0.087	6	63236747	7.09E-06	0.020	14306	19.628
gen_samplesize_Vascular dementia (other)	family Bacteroidales S24 7group	rs941000	T	C	-0.099	91085352	0.475	0.139	360248	T	C	-0.085	7	90714667	3.16E-07	0.016	14306	27.083
gen_samplesize_Vascular dementia (other)	family Bifidobacteriaceae	rs10831953	G	A	0.001	13076504	0.995	0.144	360248	G	A	0.054	11	13098051	9.95E-06	0.012	14306	18.869
gen_samplesize_Vascular dementia (other)	family Bifidobacteriaceae	rs12446429	T	C	-0.175	848055	0.307	0.171	360248	T	C	0.081	16	898055	8.53E-06	0.019	14306	18.040
gen_samplesize_Vascular dementia (other)	family Bifidobacteriaceae	rs13020688	G	A	-0.087	192013806	0.545	0.143	360248	G	A	0.058	2	192878532	1.57E-06	0.012	14306	22.887
gen_samplesize_Vascular dementia (other)	family Bifidobacteriaceae	rs182549	T	C	-0.269	135859184	0.052	0.139	360248	T	C	-0.117	2	136616754	5.94E-20	0.013	14306	85.372
gen_samplesize_Vascular dementia (other)	family Bifidobacteriaceae	rs4957061	T	C	-0.254	520981	0.061	0.136	360248	T	C	0.057	5	521096	1.15E-06	0.012	14306	23.762
gen_samplesize_Vascular dementia (other)	family Bifidobacteriaceae	rs540489	T	G	-0.121	74901626	0.495	0.177	360248	T	G	-0.063	17	72897722	5.37E-06	0.014	14306	20.956
gen_samplesize_Vascular dementia (other)	family Bifidobacteriaceae	rs55888705	A	G	0.223	1516099	0.130	0.148	360248	A	G	0.054	4	1517826	8.66E-06	0.012	14306	19.812
gen_samplesize_Vascular dementia (other)	family Bifidobacteriaceae	rs6899771	A	G	-0.090	96958344	0.685	0.223	360248	A	G	-0.091	6	97406220	7.28E-06	0.020	14306	20.365
gen_samplesize_Vascular dementia (other)	family Bifidobacteriaceae	rs7174549	T	C	-0.280	91920073	0.045	0.139	360248	T	C	-0.055	15	92463303	6.87E-06	0.012	14306	19.590
gen_samplesize_Vascular dementia (other)	family Bifidobacteriaceae	rs7322849	T	C	-0.051	112205515	0.824	0.229	360248	T	C	0.111	13	112859829	1.74E-08	0.020	14306	30.320
gen_samplesize_Vascular dementia (other)	family Bifidobacteriaceae	rs857444	C	T	0.144	14617360	0.294	0.137	360248	C	T	0.055	6	14617591	3.82E-06	0.012	14306	21.075
gen_samplesize_Vascular dementia (other)	family Clostridiaceae1	rs10875374	T	C	0.081	101339253	0.539	0.132	360248	T	C	0.054	1	101804809	8.10E-06	0.012	14306	20.197
gen_samplesize_Vascular dementia (other)	family Clostridiaceae1	rs12186080	G	A	0.058	132877783	0.746	0.178	360248	G	A	0.075	3	132596627	5.34E-06	0.016	14306	21.212
gen_samplesize_Vascular dementia (other)	family Clostridiaceae1	rs12341505	G	A	-0.172	133845759	0.461	0.233	360248	G	A	0.081	9	136710881	4.54E-06	0.018	14306	20.627
gen_samplesize_Vascular dementia (other)	family Clostridiaceae1	rs2795528	G	A	-0.138	42774816	0.635	0.290	360248	G	A	-0.181	10	43270264	3.81E-06	0.039	14306	21.432
gen_samplesize_Vascular dementia (other)	family Clostridiaceae1	rs2817172	C	T	-0.056	3124955	0.677	0.136	360248	C	T	0.056	1	3041519	5.27E-06	0.012	14306	20.668
gen_samplesize_Vascular dementia (other)	family Clostridiaceae1	rs4723021	T	C	-0.161	30895044	0.548	0.268	360248	T	C	-0.106	7	30934659	7.42E-06	0.024	14306	19.331
gen_samplesize_Vascular dementia (other)	family Clostridiaceae1	rs550843	T	C	-0.087	165309343	0.563	0.150	360248	T	C	-0.073	6	165722832	7.09E-06	0.017	14306	19.043
gen_samplesize_Vascular dementia (other)	family Clostridiaceae1	rs56188186	A	G	-0.032	87677084	0.915	0.303	360248	A	G	0.097	16	87710690	8.24E-06	0.022	14306	19.804
gen_samplesize_Vascular dementia (other)	family Clostridiaceae1	rs62397761	A	G	0.152	48125278	0.287	0.143	360248	A	G	0.062	6	48093014	9.08E-06	0.014	14306	20.443
gen_samplesize_Vascular dementia (other)	family Clostridiaceae1	rs881532	G	A	-0.014	47411024	0.918	0.133	360248	G	A	0.053	22	47806774	7.90E-06	0.012	14306	20.060
gen_samplesize_Vascular dementia (other)	family Clostridiales vadin BB60 group	rs10517600	G	T	-0.304	154711806	0.027	0.137	360248	G	T	-0.063	4	155632958	6.83E-06	0.014	14306	20.241
gen_samplesize_Vascular dementia (other)	family Clostridiales vadin BB60 group	rs10904722	C	T	0.196	6672462	0.208	0.155	360248	C	T	-0.067	10	6714424	5.05E-06	0.015	14306	20.883
gen_samplesize_Vascular dementia (other)	family Clostridiales vadin BB60 group	rs118104867	C	T	0.040	122965298	0.876	0.257	360248	C	T	0.214	8	123977538	3.44E-06	0.046	14306	22.207

gen_samplesize_Vascular dementia (other)	family Clostridiales vadin BB60 group	rs13409132	A	G	-0.256	204751623	0.480	0.362	360248	A	G	-0.165	2	205616346	4.37E-06	0.035	14306	22.065
gen_samplesize_Vascular dementia (other)	family Clostridiales vadin BB60 group	rs17121075	G	A	0.062	85390452	0.701	0.162	360248	G	A	0.077	14	85856796	7.91E-06	0.017	14306	19.948
gen_samplesize_Vascular dementia (other)	family Clostridiales vadin BB60 group	rs2191834	T	G	0.001	229078235	0.994	0.156	360248	T	G	-0.075	2	229942951	2.50E-06	0.016	14306	21.998
gen_samplesize_Vascular dementia (other)	family Clostridiales vadin BB60 group	rs28691777	C	T	0.438	60072044	0.176	0.324	360248	C	T	0.137	17	58149405	6.96E-07	0.027	14306	26.380
gen_samplesize_Vascular dementia (other)	family Clostridiales vadin BB60 group	rs34088226	A	G	0.048	4308833	0.866	0.284	360248	A	G	-0.118	5	4308946	7.66E-06	0.027	14306	19.145
gen_samplesize_Vascular dementia (other)	family Clostridiales vadin BB60 group	rs55682560	C	T	-0.188	86849059	0.445	0.247	360248	C	T	-0.132	15	87392290	4.97E-07	0.026	14306	25.330
gen_samplesize_Vascular dementia (other)	family Clostridiales vadin BB60 group	rs6588624	A	G	0.042	56383867	0.750	0.133	360248	A	G	0.066	1	56849539	1.79E-06	0.014	14306	22.985
gen_samplesize_Vascular dementia (other)	family Clostridiales vadin BB60 group	rs66714985	A	C	-0.145	3447600	0.510	0.221	360248	A	C	0.117	8	3305122	4.85E-06	0.025	14306	21.446
gen_samplesize_Vascular dementia (other)	family Clostridiales vadin BB60 group	rs7226487	A	G	0.174	76661168	0.191	0.133	360248	A	G	-0.064	18	74373125	3.58E-06	0.014	14306	21.537
gen_samplesize_Vascular dementia (other)	family Clostridiales vadin BB60 group	rs7538034	T	G	-0.264	70926011	0.138	0.178	360248	T	G	-0.079	1	71391694	2.37E-06	0.017	14306	22.423
gen_samplesize_Vascular dementia (other)	family Clostridiales vadin BB60 group	rs7725895	A	G	-0.042	142865333	0.835	0.204	360248	A	G	-0.116	5	142244898	3.94E-06	0.024	14306	23.380
gen_samplesize_Vascular dementia (other)	family Clostridiales vadin BB60 group	rs989682	A	G	-0.063	15522946	0.690	0.157	360248	A	G	0.070	3	15564453	6.85E-06	0.016	14306	20.413
gen_samplesize_Vascular dementia (other)	family Coriobacteriaceae	rs11073596	G	T	-0.263	85890348	0.057	0.138	360248	G	T	-0.051	15	86433579	8.14E-06	0.011	14306	19.912
gen_samplesize_Vascular dementia (other)	family Coriobacteriaceae	rs11250875	T	C	-0.149	1880537	0.357	0.161	360248	T	C	0.061	10	1922731	4.83E-06	0.013	14306	21.526
gen_samplesize_Vascular dementia (other)	family Coriobacteriaceae	rs11656361	A	C	-0.271	8218014	0.116	0.173	360248	A	C	0.077	17	8121332	8.02E-06	0.018	14306	19.394
gen_samplesize_Vascular dementia (other)	family Coriobacteriaceae	rs12974142	G	A	0.233	52391913	0.376	0.264	360248	G	A	0.079	19	52895166	8.51E-06	0.018	14306	19.865
gen_samplesize_Vascular dementia (other)	family Coriobacteriaceae	rs13307134	T	C	0.272	105444233	0.130	0.180	360248	T	C	-0.057	7	105084680	7.80E-06	0.013	14306	20.072
gen_samplesize_Vascular dementia (other)	family Coriobacteriaceae	rs1397793	A	G	-0.061	91175634	0.672	0.144	360248	A	G	0.050	5	90471451	9.77E-06	0.011	14306	19.682
gen_samplesize_Vascular dementia (other)	family Coriobacteriaceae	rs1816223	G	A	0.266	11341087	0.111	0.167	360248	G	A	0.059	12	11494021	4.84E-06	0.013	14306	20.652
gen_samplesize_Vascular dementia (other)	family Coriobacteriaceae	rs240104	T	C	0.099	176602295	0.508	0.149	360248	T	C	-0.060	1	176571431	1.52E-06	0.013	14306	22.630
gen_samplesize_Vascular dementia (other)	family Coriobacteriaceae	rs2442778	A	G	0.298	11612938	0.329	0.305	360248	A	G	0.116	3	11654412	9.03E-06	0.026	14306	20.272
gen_samplesize_Vascular dementia (other)	family Coriobacteriaceae	rs3025411	A	G	-0.176	133647784	0.408	0.213	360248	A	G	0.093	9	136512906	8.27E-06	0.021	14306	19.566
gen_samplesize_Vascular dementia (other)	family Coriobacteriaceae	rs34739816	G	T	-0.283	39220432	0.316	0.282	360248	G	T	0.097	17	37376685	3.88E-06	0.021	14306	21.594
gen_samplesize_Vascular dementia (other)	family Coriobacteriaceae	rs67561917	A	G	-0.234	63440724	0.180	0.174	360248	A	G	-0.071	20	62072077	5.39E-06	0.015	14306	21.486
gen_samplesize_Vascular dementia (other)	family Coriobacteriaceae	rs719099	A	G	0.120	64039457	0.591	0.223	360248	A	G	0.078	10	65799217	5.43E-07	0.016	14306	24.957
gen_samplesize_Vascular dementia (other)	family Coriobacteriaceae	rs8010111	A	G	-0.021	39191305	0.930	0.241	360248	A	G	0.103	14	39660509	6.90E-06	0.023	14306	20.328
gen_samplesize_Vascular dementia (other)	family Defluviitaleaceae	rs112893842	T	C	-0.023	8786663	0.920	0.230	360248	T	C	0.111	9	8786663	2.75E-06	0.023	14306	22.686
gen_samplesize_Vascular dementia (other)	family Defluviitaleaceae	rs1582238	C	T	0.013	118181062	0.925	0.139	360248	C	T	-0.080	1	118723685	1.69E-06	0.017	14306	23.042
gen_samplesize_Vascular dementia (other)	family Defluviitaleaceae	rs17051335	C	T	-0.191	121281755	0.372	0.214	360248	C	T	-0.134	4	122202910	4.58E-06	0.029	14306	21.129
gen_samplesize_Vascular dementia (other)	family Defluviitaleaceae	rs1908593	T	C	-0.053	61358915	0.694	0.136	360248	T	C	0.070	18	59026148	7.86E-06	0.016	14306	20.108
gen_samplesize_Vascular dementia (other)	family Defluviitaleaceae	rs4344384	T	G	-0.187	64647609	0.158	0.132	360248	T	G	-0.071	10	66407366	5.86E-06	0.016	14306	20.612
gen_samplesize_Vascular dementia (other)	family Defluviitaleaceae	rs4677103	A	G	-0.080	72158643	0.652	0.177	360248	A	G	0.098	3	72207794	9.42E-07	0.020	14306	24.598
gen_samplesize_Vascular dementia (other)	family Defluviitaleaceae	rs540220	C	T	-0.287	90569026	0.221	0.235	360248	C	T	0.124	9	93331308	9.48E-06	0.029	14306	18.293
gen_samplesize_Vascular dementia (other)	family Defluviitaleaceae	rs55658617	T	C	-0.604	40439076	0.099	0.366	360248	T	C	0.177	21	41811003	1.41E-06	0.036	14306	24.013
gen_samplesize_Vascular dementia (other)	family Defluviitaleaceae	rs72731813	C	T	0.325	146493591	0.296	0.311	360248	C	T	-0.150	4	147414743	2.76E-07	0.029	14306	26.048
gen_samplesize_Vascular dementia (other)	family Defluviitaleaceae	rs9608282	T	G	0.082	24408113	0.828	0.377	360248	T	G	0.139	22	24804081	4.61E-06	0.030	14306	21.554
gen_samplesize_Vascular dementia (other)	family Defluviitaleaceae	rs9725395	A	G	0.153	84739949	0.467	0.210	360248	A	G	-0.138	1	85205632	3.41E-06	0.030	14306	21.969
gen_samplesize_Vascular dementia (other)	family Desulfovibrionaceae	rs11599763	C	T	0.088	11813600	0.520	0.137	360248	C	T	0.056	10	11855599	2.50E-06	0.012	14306	22.388
gen_samplesize_Vascular dementia (other)	family Desulfovibrionaceae	rs17791387	A	G	-0.048	79219511	0.835	0.231	360248	A	G	-0.073	9	81834426	2.10E-06	0.015	14306	22.313
gen_samplesize_Vascular dementia (other)	family Desulfovibrionaceae	rs2692012	G	A	0.209	204022477	0.478	0.294	360248	G	A	-0.114	1	203991605	1.56E-06	0.025	14306	20.295
gen_samplesize_Vascular dementia (other)	family Desulfovibrionaceae	rs2838334	G	A	0.315	43645080	0.025	0.140	360248	G	A	0.057	21	45064961	3.82E-06	0.012	14306	21.157
gen_samplesize_Vascular dementia (other)	family Desulfovibrionaceae	rs3935584	C	T	-0.116	233064573	0.383	0.134	360248	C	T	-0.053	2	233929283	6.78E-06	0.012	14306	20.635
gen_samplesize_Vascular dementia (other)	family Desulfovibrionaceae	rs4506934	C	T	-0.003	2953368	0.987	0.204	360248	C	T	-0.094	17	2856662	3.16E-06	0.020	14306	21.945
gen_samplesize_Vascular dementia (other)	family Desulfovibrionaceae	rs6058181	C	T	0.207	35106998	0.247	0.178	360248	C	T	0.083	20	33694801	2.70E-07	0.017	14306	25.253
gen_samplesize_Vascular dementia (other)	family Desulfovibrionaceae	rs72647048	T	C	-0.017	56952819	0.936	0.210	360248	T	C	-0.077	8	57865378	9.61E-06	0.017	14306	20.307
gen_samplesize_Vascular dementia (other)	family Desulfovibrionaceae	rs9928243	C	A	0.037	71507738	0.779	0.133	360248	C	A	-0.054	16	71541641	4.48E-06	0.012	14306	21.142
gen_samplesize_Vascular dementia (other)	family Enterobacteriaceae	rs11026530	T	C	-0.197	22357551	0.292	0.188	360248	T	C	0.082	11	22379097	9.43E-06	0.019	14306	19.471
gen_samplesize_Vascular dementia (other)	family Enterobacteriaceae	rs2374342	C	A	-0.349	41906402	0.005	0.125	360248	C	A	0.058	2	42133542	4.52E-06	0.013	14306	21.338
gen_samplesize_Vascular dementia (other)	family Enterobacteriaceae	rs35673018	G	A	-0.089	54293833	0.700	0.232	360248	G	A	0.090	16	54327745	7.63E-06	0.020	14306	19.653
gen_samplesize_Vascular dementia (other)	family Enterobacteriaceae	rs504442	T	G	-0.098	57478315	0.655	0.219	360248	T	G	0.084	18	55145547	5.17E-06	0.019	14306	19.728
gen_samplesize_Vascular dementia (other)	family Enterobacteriaceae	rs62210023	A	G	0.206	56765036	0.143	0.141	360248	A	G	0.061	20	55340092	3.13E-06	0.013	14306	21.742
gen_samplesize_Vascular dementia (other)	family Enterobacteriaceae	rs78143293	A	G	-0.130	60005103	0.520	0.202	360248	A	G	-0.085	18	57672335	1.20E-06	0.017	14306	24.792
gen_samplesize_Vascular dementia (other)	family Enterobacteriaceae	rs79757635	C	A	-0.219	110188071	0.260	0.194	360248	C	A	0.076	13	110840418	9.32E-06	0.017	14306	19.615
gen_samplesize_Vascular dementia (other)	family Erysipelotrichaceae	rs1074800	G	A	0.054	3002432	0.688	0.135	360248	G	A	-0.049	5	3002546	6.15E-06	0.011	14306	20.459
gen_samplesize_Vascular dementia (other)	family Erysipelotrichaceae	rs10781552	C	T	-0.190	132083729	0.196	0.147	360248	C	T	-0.055	10	133897233	2.33E-06	0.012	14306	22.633
gen_samplesize_Vascular dementia (other)	family Erysipelotrichaceae	rs17530232	A	G	-0.294	39811320	0.317	0.293	360248	A	G	0.103	13	40385457	2.79E-06	0.022	14306	21.042
gen_samplesize_Vascular dementia (other)	family Erysipelotrichaceae	rs1884466	C	T	-0.152	63673525	0.256	0.134	360248	C	T	-0.048	1	64139196	9.53E-06	0.011	14306	19.760
gen_samplesize_Vascular dementia (other)	family Erysipelotrichaceae	rs2300774	A	G	-0.043	196066841	0.745	0.133	360248	A	G	-0.052	3	195793712	8.95E-07	0.011	14306	24.094
gen_samplesize_Vascular dementia (other)	family Erysipelotrichaceae	rs290833	T	G	-0.264	96991871	0.047	0.133	360248	T	G	-0.050	1	97457427	8.03E-06	0.011	14306	19.943
gen_samplesize_Vascular dementia (other)	family Erysipelotrichaceae	rs35161940	T	C	-0.021	72331083	0.922	0.218	360248	T	C	-0.081	17	70327224	1.85E-06	0.017	14306	23.118
gen_samplesize_Vascular dementia (other)	family Erysipelotrichaceae	rs4078432	T	C	0.018	48528003	0.919	0.177	360248	T	C	0.061	14	48997206	4.23E-06	0.013	14306	20.723
gen_samplesize_Vascular dementia (other)	family Erysipelotrichaceae	rs56970041	T	G	0.127	79891267	0.644	0.275	360248	T	G	0.072	14	80357610	5.40E-06	0.016	14306	19.385
gen_samplesize_Vascular dementia (other)	family Erysipelotrichaceae	rs62504403	C	T	0.099	38946033	0.551	0.166	360248	C	T	0.068	8	38803551	1.12E-07	0.013	14306	28.371
gen_samplesize_Vascular dementia (other)	family Erysipelotrichaceae	rs7234058	T	C	-0.110	5830508	0.629	0.228	360248	T	C	-0.095	18	5830507	9.12E-07	0.019	14306	23.744
gen_samplesize_Vascular dementia (other)	family Erysipelotrichaceae	rs7826267	G	T	0.117	3097430	0.658	0.263	360248	G	T	0.084	8	2954952	9.28E-06	0.020	14306	17.755
gen_samplesize_Vascular dementia (other)	family Erysipelotrichaceae	rs8003149	C	T	-0.097	55689786	0.493	0.142	360248	C	T	0.054	14	56156504	4.08E-06	0.012	14306	21.248
gen_samplesize_Vascular dementia (other)	family Family XI	rs10759623	C	T	0.324	113059205	0.044	0.161	360248	C	T	-0.162	9	115821485	5.78E-07	0.032	14306	25.425

gen_samplesize_Vascular dementia (other)	family Family XI	rs11547158	A	G	0.200	149224640	0.306	0.195	360248	A	G	-0.178	7	148921732	2.70E-06	0.037	14306	22.696
gen_samplesize_Vascular dementia (other)	family Family XI	rs17379710	T	C	-0.232	35313121	0.085	0.135	360248	T	C	-0.116	11	35334668	3.97E-06	0.025	14306	21.308
gen_samplesize_Vascular dementia (other)	family Family XI	rs2155352	A	G	0.167	95624045	0.286	0.156	360248	A	G	-0.151	11	95357209	6.63E-07	0.030	14306	24.795
gen_samplesize_Vascular dementia (other)	family Family XI	rs2156611	T	C	0.028	45765899	0.832	0.132	360248	T	C	-0.112	18	43345864	9.43E-06	0.025	14306	20.068
gen_samplesize_Vascular dementia (other)	family Family XI	rs3733511	A	G	0.160	119034632	0.273	0.146	360248	A	G	0.128	4	119955787	3.39E-06	0.027	14306	21.795
gen_samplesize_Vascular dementia (other)	family Family XI	rs488164	G	T	0.065	239832151	0.636	0.138	360248	G	T	-0.118	1	239995451	4.80E-06	0.026	14306	21.386
gen_samplesize_Vascular dementia (other)	family Family XI	rs697771	A	G	-0.093	54081288	0.486	0.134	360248	A	G	-0.118	16	54115200	3.19E-06	0.025	14306	21.895
gen_samplesize_Vascular dementia (other)	family Family XIII	rs10404377	A	C	-0.151	16063847	0.268	0.136	360248	A	C	0.050	19	16174657	6.99E-06	0.011	14306	20.311
gen_samplesize_Vascular dementia (other)	family Family XIII	rs118170811	A	G	-0.013	100607139	0.973	0.382	360248	A	G	0.152	10	102366896	1.80E-06	0.032	14306	22.860
gen_samplesize_Vascular dementia (other)	family Family XIII	rs482905	G	T	0.047	166587965	0.759	0.154	360248	G	T	0.060	1	166557202	3.72E-06	0.013	14306	22.004
gen_samplesize_Vascular dementia (other)	family Family XIII	rs6501525	A	G	0.099	72222486	0.486	0.141	360248	A	G	0.056	17	70218627	1.24E-06	0.012	14306	23.603
gen_samplesize_Vascular dementia (other)	family Family XIII	rs66753613	G	A	-0.251	38746349	0.148	0.174	360248	G	A	0.065	1	39212021	8.08E-06	0.014	14306	20.375
gen_samplesize_Vascular dementia (other)	family Family XIII	rs6797051	C	T	-0.250	88946068	0.264	0.224	360248	C	T	-0.081	3	88995218	4.89E-06	0.017	14306	22.122
gen_samplesize_Vascular dementia (other)	family Family XIII	rs7514702	T	C	0.142	186944891	0.446	0.187	360248	T	C	-0.066	1	186914023	3.92E-06	0.014	14306	21.931
gen_samplesize_Vascular dementia (other)	family Lachnospiraceae	rs10402491	C	T	0.055	13314985	0.754	0.177	360248	C	T	0.066	19	13425799	7.58E-06	0.015	14306	19.852
gen_samplesize_Vascular dementia (other)	family Lachnospiraceae	rs11139361	C	T	-0.047	69564931	0.740	0.142	360248	C	T	-0.049	9	72179847	4.26E-06	0.011	14306	20.023
gen_samplesize_Vascular dementia (other)	family Lachnospiraceae	rs112040820	A	G	-0.143	82511188	0.347	0.152	360248	A	G	0.055	17	80469064	2.42E-06	0.012	14306	22.032
gen_samplesize_Vascular dementia (other)	family Lachnospiraceae	rs11841382	G	T	-0.053	37430535	0.825	0.239	360248	G	T	-0.072	13	38004672	9.58E-06	0.017	14306	16.913
gen_samplesize_Vascular dementia (other)	family Lachnospiraceae	rs11979110	T	C	-0.032	130751700	0.808	0.133	360248	T	C	-0.050	7	130436459	1.82E-06	0.011	14306	22.730
gen_samplesize_Vascular dementia (other)	family Lachnospiraceae	rs1205443	A	G	0.022	38248466	0.877	0.141	360248	A	G	0.050	20	36876868	7.29E-06	0.011	14306	19.987
gen_samplesize_Vascular dementia (other)	family Lachnospiraceae	rs12760724	A	C	-0.181	213858626	0.200	0.141	360248	A	C	-0.048	1	214031969	7.27E-06	0.011	14306	20.155
gen_samplesize_Vascular dementia (other)	family Lachnospiraceae	rs13005175	G	A	0.438	231605011	0.173	0.322	360248	G	A	-0.099	2	232469722	8.37E-06	0.022	14306	20.694
gen_samplesize_Vascular dementia (other)	family Lachnospiraceae	rs2159863	A	G	-0.181	10243730	0.296	0.173	360248	A	G	-0.059	4	10245354	3.70E-06	0.013	14306	20.706
gen_samplesize_Vascular dementia (other)	family Lachnospiraceae	rs2910921	C	T	-0.229	32348487	0.566	0.398	360248	C	T	-0.160	5	32348593	8.42E-06	0.036	14306	20.014
gen_samplesize_Vascular dementia (other)	family Lachnospiraceae	rs3127230	C	T	-0.030	101632746	0.836	0.144	360248	C	T	-0.050	10	103392503	6.20E-06	0.011	14306	20.140
gen_samplesize_Vascular dementia (other)	family Lachnospiraceae	rs35524804	T	C	-0.197	97351401	0.227	0.163	360248	T	C	-0.061	9	100113683	2.45E-06	0.013	14306	23.537
gen_samplesize_Vascular dementia (other)	family Lachnospiraceae	rs7359994	C	T	-0.015	28917951	0.909	0.135	360248	C	T	0.050	19	29408858	5.36E-06	0.011	14306	20.009
gen_samplesize_Vascular dementia (other)	family Lachnospiraceae	rs79086868	T	C	-0.330	130916218	0.131	0.218	360248	T	C	0.078	9	133791605	3.01E-06	0.016	14306	22.302
gen_samplesize_Vascular dementia (other)	family Lachnospiraceae	rs959845	T	C	-0.112	185985647	0.408	0.136	360248	T	C	0.049	4	186906801	5.17E-06	0.011	14306	20.971
gen_samplesize_Vascular dementia (other)	family Lachnospiraceae	rs9929145	G	A	-0.029	76525287	0.924	0.302	360248	G	A	-0.126	16	76559184	2.84E-07	0.025	14306	26.282
gen_samplesize_Vascular dementia (other)	family Lactobacillaceae	rs1530559	G	A	0.097	134998059	0.469	0.135	360248	G	A	0.077	2	135755629	9.65E-06	0.018	14306	19.063
gen_samplesize_Vascular dementia (other)	family Lactobacillaceae	rs16861661	G	A	-0.255	18174965	0.346	0.271	360248	G	A	-0.193	1	18501459	2.70E-07	0.038	14306	25.969
gen_samplesize_Vascular dementia (other)	family Lactobacillaceae	rs62314653	C	A	0.022	108975306	0.937	0.283	360248	C	A	0.177	4	109896462	6.59E-06	0.039	14306	20.379
gen_samplesize_Vascular dementia (other)	family Lactobacillaceae	rs74599091	A	G	-0.078	179751534	0.874	0.489	360248	A	G	0.192	1	179720669	7.70E-06	0.043	14306	20.323
gen_samplesize_Vascular dementia (other)	family Lactobacillaceae	rs768253	T	G	-0.085	68097868	0.525	0.134	360248	T	G	-0.079	8	69010103	3.61E-06	0.017	14306	21.570
gen_samplesize_Vascular dementia (other)	family Lactobacillaceae	rs77478751	A	G	0.074	173433875	0.724	0.208	360248	A	G	-0.219	3	173151665	5.96E-06	0.047	14306	21.421
gen_samplesize_Vascular dementia (other)	family Lactobacillaceae	rs921925	A	C	-0.062	6928006	0.701	0.160	360248	A	C	0.100	19	6928017	5.77E-07	0.020	14306	24.555
gen_samplesize_Vascular dementia (other)	family Lactobacillaceae	rs9345899	A	G	-0.267	66849430	0.213	0.214	360248	A	G	-0.124	6	67559323	9.45E-06	0.028	14306	19.774
gen_samplesize_Vascular dementia (other)	family Methanobacteriaceae	rs10202904	G	T	-0.109	124682691	0.423	0.136	360248	G	T	0.122	2	125440268	3.01E-07	0.024	14306	26.762
gen_samplesize_Vascular dementia (other)	family Methanobacteriaceae	rs10424197	A	G	0.112	45936063	0.466	0.154	360248	A	G	0.111	19	46439321	9.28E-06	0.025	14306	20.211
gen_samplesize_Vascular dementia (other)	family Methanobacteriaceae	rs4257531	G	A	0.097	2044483	0.657	0.219	360248	G	A	0.164	3	2086167	7.44E-06	0.036	14306	20.316
gen_samplesize_Vascular dementia (other)	family Methanobacteriaceae	rs6508769	C	T	0.056	28336853	0.767	0.188	360248	C	T	-0.154	19	28827760	8.23E-06	0.034	14306	19.856
gen_samplesize_Vascular dementia (other)	family Methanobacteriaceae	rs6776814	T	C	0.088	15011576	0.851	0.471	360248	T	C	-0.200	3	15053083	1.63E-06	0.041	14306	23.483
gen_samplesize_Vascular dementia (other)	family Methanobacteriaceae	rs73068003	G	T	-0.346	10734305	0.125	0.226	360248	G	T	-0.158	7	10773932	8.45E-06	0.035	14306	20.206
gen_samplesize_Vascular dementia (other)	family Methanobacteriaceae	rs73457410	A	G	-0.101	41382045	0.712	0.274	360248	A	G	0.215	13	41956181	1.41E-06	0.044	14306	24.316
gen_samplesize_Vascular dementia (other)	family Methanobacteriaceae	rs75208022	C	T	0.004	21185927	0.985	0.225	360248	C	T	-0.227	12	21338861	5.92E-06	0.049	14306	21.717
gen_samplesize_Vascular dementia (other)	family Methanobacteriaceae	rs894996	C	A	0.105	103497150	0.682	0.255	360248	C	A	0.217	4	104418307	1.88E-06	0.045	14306	23.349
gen_samplesize_Vascular dementia (other)	family Oxalobacteraceae	rs111966731	T	C	0.019	93398708	0.934	0.237	360248	T	C	0.204	15	93941937	4.56E-06	0.045	14306	20.952
gen_samplesize_Vascular dementia (other)	family Oxalobacteraceae	rs11246212	C	T	0.048	610277	0.803	0.191	360248	C	T	-0.136	11	610277	4.51E-06	0.029	14306	21.745
gen_samplesize_Vascular dementia (other)	family Oxalobacteraceae	rs12002250	A	C	-0.102	19682560	0.754	0.325	360248	A	C	0.196	9	19682558	5.53E-06	0.045	14306	19.408
gen_samplesize_Vascular dementia (other)	family Oxalobacteraceae	rs1569853	T	C	0.007	38582525	0.971	0.206	360248	T	C	-0.140	6	38550301	7.45E-07	0.028	14306	24.714
gen_samplesize_Vascular dementia (other)	family Oxalobacteraceae	rs17138946	G	T	-0.146	5934557	0.581	0.264	360248	G	T	-0.189	16	5984558	8.09E-06	0.043	14306	19.388
gen_samplesize_Vascular dementia (other)	family Oxalobacteraceae	rs36057338	G	T	0.187	189014160	0.614	0.371	360248	G	T	0.182	4	189935314	6.26E-06	0.040	14306	20.743
gen_samplesize_Vascular dementia (other)	family Oxalobacteraceae	rs4428215	G	A	0.025	172229645	0.868	0.152	360248	G	A	0.126	3	171947435	4.88E-08	0.023	14306	29.812
gen_samplesize_Vascular dementia (other)	family Oxalobacteraceae	rs561239	A	G	0.050	18640649	0.763	0.165	360248	A	G	0.106	12	18793583	7.19E-06	0.024	14306	19.864
gen_samplesize_Vascular dementia (other)	family Oxalobacteraceae	rs6000536	C	T	-0.079	37025428	0.654	0.176	360248	C	T	-0.118	22	37421469	7.39E-07	0.024	14306	24.058
gen_samplesize_Vascular dementia (other)	family Oxalobacteraceae	rs62435498	C	A	0.070	1754627	0.751	0.220	360248	C	A	0.182	7	1794263	7.46E-06	0.040	14306	20.531
gen_samplesize_Vascular dementia (other)	family Oxalobacteraceae	rs736744	T	C	-0.157	84899492	0.244	0.135	360248	T	C	-0.106	9	87514407	1.49E-07	0.020	14306	27.721
gen_samplesize_Vascular dementia (other)	family Oxalobacteraceae	rs7993559	A	C	-0.090	22869488	0.504	0.134	360248	A	C	-0.092	13	23443627	5.04E-06	0.020	14306	20.974
gen_samplesize_Vascular dementia (other)	family Oxalobacteraceae	rs80330081	A	C	-0.251	66561674	0.255	0.220	360248	A	C	-0.188	4	67427392	6.64E-06	0.042	14306	19.597
gen_samplesize_Vascular dementia (other)	family Oxalobacteraceae	rs934049	G	A	0.000	15916656	0.999	0.163	360248	G	A	0.110	2	16056779	4.21E-06	0.024	14306	21.152
gen_samplesize_Vascular dementia (other)	family Pasteurellaceae	rs10965428	C	A	0.011	22718482	0.969	0.288	360248	C	A	-0.120	9	22718481	4.29E-06	0.026	14306	21.561
gen_samplesize_Vascular dementia (other)	family Pasteurellaceae	rs111582866	G	A	0.153	48708578	0.516	0.236	360248	G	A	-0.114	16	48742489	7.07E-06	0.026	14306	19.753
gen_samplesize_Vascular dementia (other)	family Pasteurellaceae	rs12050685	A	G	-0.206	73185141	0.158	0.146	360248	A	G	-0.067	15	73477482	9.19E-06	0.015	14306	19.385
gen_samplesize_Vascular dementia (other)	family Pasteurellaceae	rs16970009	A	G	-0.051	34535582	0.917	0.493	360248	A	G	0.187	17	32862601	7.32E-06	0.043	14306	19.027
gen_samplesize_Vascular dementia (other)	family Pasteurellaceae	rs4822728	T	C	-0.228	26495842	0.086	0.133	360248	T	C	0.069	22	26891808	4.72E-06	0.015	14306	21.156
gen_samplesize_Vascular dementia (other)	family Pasteurellaceae	rs6972479	A	G	-0.163	117278006	0.323	0.165	360248	A	G	-0.078	7	116918060	7.75E-06	0.018	14306	19.878

gen_samplesize_Vascular dementia (other)	family Pasteurellaceae	rs72756943	G	A	-0.032	26531799	0.906	0.267	360248	G	A	0.140	5	26531908	3.35E-06	0.030	14306	21.308
gen_samplesize_Vascular dementia (other)	family Pasteurellaceae	rs73139353	A	C	0.175	98253370	0.454	0.234	360248	A	C	-0.223	3	97972214	8.71E-06	0.048	14306	21.092
gen_samplesize_Vascular dementia (other)	family Pasteurellaceae	rs76022354	C	T	-0.496	92546628	0.105	0.306	360248	C	T	0.243	10	94306385	1.83E-06	0.050	14306	23.560
gen_samplesize_Vascular dementia (other)	family Pasteurellaceae	rs78909003	T	C	-0.158	102887960	0.586	0.290	360248	T	C	-0.241	9	105650242	2.05E-06	0.050	14306	23.415
gen_samplesize_Vascular dementia (other)	family Pasteurellaceae	rs9382510	C	T	-0.173	55583693	0.255	0.152	360248	C	T	-0.088	6	55448491	2.48E-07	0.017	14306	26.921
gen_samplesize_Vascular dementia (other)	family Pasteurellaceae	rs9895850	T	C	-0.175	66538895	0.585	0.320	360248	T	C	-0.176	17	64535013	9.08E-06	0.041	14306	18.497
gen_samplesize_Vascular dementia (other)	family Pasteurellaceae	rs9938097	C	T	0.070	84943783	0.611	0.137	360248	C	T	0.071	16	84977389	8.23E-06	0.016	14306	20.209
gen_samplesize_Vascular dementia (other)	family Peptococcaceae	rs117452796	A	G	0.634	9423378	0.093	0.377	360248	A	G	-0.258	9	9423378	3.15E-06	0.055	14306	22.036
gen_samplesize_Vascular dementia (other)	family Peptococcaceae	rs12144792	C	T	0.010	27091734	0.945	0.138	360248	C	T	0.064	1	27418225	5.82E-06	0.014	14306	20.788
gen_samplesize_Vascular dementia (other)	family Peptococcaceae	rs12634826	T	G	0.067	183266260	0.629	0.140	360248	T	G	-0.074	3	182984048	1.01E-06	0.015	14306	24.031
gen_samplesize_Vascular dementia (other)	family Peptococcaceae	rs12992764	T	G	-0.126	188031016	0.346	0.134	360248	T	G	0.068	2	188895743	1.46E-06	0.014	14306	23.502
gen_samplesize_Vascular dementia (other)	family Peptococcaceae	rs150600492	A	C	-0.374	127377698	0.180	0.279	360248	A	C	0.136	10	129175962	2.31E-06	0.029	14306	21.957
gen_samplesize_Vascular dementia (other)	family Peptococcaceae	rs35703006	G	T	0.130	28756700	0.402	0.155	360248	G	T	0.081	8	28614217	4.95E-07	0.016	14306	24.448
gen_samplesize_Vascular dementia (other)	family Peptococcaceae	rs4990837	G	A	-0.001	3723235	0.997	0.166	360248	G	A	-0.091	8	3580757	1.74E-06	0.019	14306	23.990
gen_samplesize_Vascular dementia (other)	family Peptococcaceae	rs75430375	C	T	0.064	89957067	0.842	0.321	360248	C	T	-0.148	5	89252884	3.41E-06	0.032	14306	21.712
gen_samplesize_Vascular dementia (other)	family Peptococcaceae	rs75898026	A	G	-0.155	112906768	0.339	0.162	360248	A	G	-0.082	13	113561082	2.02E-06	0.017	14306	22.424
gen_samplesize_Vascular dementia (other)	family Peptostreptococcaceae	rs10805326	A	G	-0.055	14322999	0.709	0.147	360248	A	G	-0.057	4	14324623	4.03E-06	0.012	14306	21.306
gen_samplesize_Vascular dementia (other)	family Peptostreptococcaceae	rs117020988	C	T	-0.257	46671379	0.283	0.239	360248	C	T	0.182	7	46710977	1.03E-06	0.037	14306	24.028
gen_samplesize_Vascular dementia (other)	family Peptostreptococcaceae	rs12377846	C	A	0.007	16786786	0.987	0.402	360248	C	A	-0.252	9	16786784	7.26E-07	0.051	14306	24.260
gen_samplesize_Vascular dementia (other)	family Peptostreptococcaceae	rs12986312	T	G	-0.022	17303329	0.878	0.144	360248	T	G	0.057	19	17414138	5.77E-06	0.013	14306	20.613
gen_samplesize_Vascular dementia (other)	family Peptostreptococcaceae	rs1467258	G	A	-0.151	40060234	0.385	0.174	360248	G	A	0.073	17	38216487	7.90E-06	0.016	14306	19.996
gen_samplesize_Vascular dementia (other)	family Peptostreptococcaceae	rs1520207	T	C	-0.071	152063386	0.595	0.134	360248	T	C	-0.053	3	151781175	3.17E-06	0.011	14306	21.842
gen_samplesize_Vascular dementia (other)	family Peptostreptococcaceae	rs4692811	C	T	-0.066	170259039	0.640	0.141	360248	C	T	0.064	4	171180190	4.21E-07	0.013	14306	25.595
gen_samplesize_Vascular dementia (other)	family Peptostreptococcaceae	rs59865771	C	T	0.217	89180262	0.117	0.139	360248	C	T	-0.057	16	89246670	7.69E-06	0.013	14306	20.349
gen_samplesize_Vascular dementia (other)	family Peptostreptococcaceae	rs61841503	G	A	-0.019	16977560	0.923	0.200	360248	G	A	0.092	10	17019559	9.80E-09	0.016	14306	32.495
gen_samplesize_Vascular dementia (other)	family Peptostreptococcaceae	rs6721459	G	A	-0.002	67625704	0.987	0.135	360248	G	A	-0.051	2	67852836	5.08E-06	0.011	14306	20.730
gen_samplesize_Vascular dementia (other)	family Peptostreptococcaceae	rs76982728	T	C	0.015	44273225	0.974	0.457	360248	T	C	0.124	7	44312824	3.24E-06	0.027	14306	21.652
gen_samplesize_Vascular dementia (other)	family Peptostreptococcaceae	rs77540684	T	G	-0.175	14608536	0.447	0.230	360248	T	G	0.107	10	14650535	8.14E-06	0.025	14306	18.877
gen_samplesize_Vascular dementia (other)	family Peptostreptococcaceae	rs9573937	A	G	-0.008	76777061	0.963	0.180	360248	A	G	-0.069	13	77351196	1.71E-06	0.014	14306	23.559
gen_samplesize_Vascular dementia (other)	family Porphyromonadaceae	rs10762312	A	G	-0.252	69812107	0.080	0.144	360248	A	G	0.052	10	71571863	8.70E-06	0.012	14306	19.427
gen_samplesize_Vascular dementia (other)	family Porphyromonadaceae	rs10858364	G	T	-0.228	135184235	0.148	0.158	360248	G	T	0.055	9	138076081	4.31E-06	0.012	14306	20.951
gen_samplesize_Vascular dementia (other)	family Porphyromonadaceae	rs17065783	A	G	0.054	62049912	0.765	0.182	360248	A	G	-0.059	3	62035586	1.79E-06	0.012	14306	23.403
gen_samplesize_Vascular dementia (other)	family Porphyromonadaceae	rs1980561	A	G	0.001	62919798	0.994	0.132	360248	A	G	-0.049	14	63386516	8.95E-06	0.011	14306	19.719
gen_samplesize_Vascular dementia (other)	family Porphyromonadaceae	rs35233670	T	C	-0.215	65754785	0.105	0.133	360248	T	C	-0.047	17	63750903	7.91E-06	0.011	14306	19.953
gen_samplesize_Vascular dementia (other)	family Porphyromonadaceae	rs35961441	A	C	-0.189	240766474	0.589	0.349	360248	A	C	0.092	1	240929774	8.37E-06	0.021	14306	19.492
gen_samplesize_Vascular dementia (other)	family Porphyromonadaceae	rs6953849	A	G	0.171	69786706	0.323	0.173	360248	A	G	0.072	7	69251692	2.44E-06	0.015	14306	22.702
gen_samplesize_Vascular dementia (other)	family Porphyromonadaceae	rs7330827	T	C	-0.224	22957663	0.417	0.275	360248	T	C	-0.104	13	23531802	8.05E-06	0.024	14306	19.140
gen_samplesize_Vascular dementia (other)	family Porphyromonadaceae	rs864093	A	C	0.158	148904825	0.333	0.163	360248	A	C	-0.053	4	149825977	9.60E-06	0.012	14306	20.188
gen_samplesize_Vascular dementia (other)	family Prevotellaceae	rs12057990	C	T	-0.001	99004713	0.993	0.148	360248	C	T	0.059	1	99470269	8.97E-06	0.013	14306	19.889
gen_samplesize_Vascular dementia (other)	family Prevotellaceae	rs12118202	T	C	0.019	210508026	0.912	0.174	360248	T	C	-0.075	1	210681370	5.54E-07	0.015	14306	26.075
gen_samplesize_Vascular dementia (other)	family Prevotellaceae	rs13069367	A	C	0.078	71752679	0.564	0.135	360248	A	C	-0.054	3	71801830	7.39E-06	0.012	14306	20.339
gen_samplesize_Vascular dementia (other)	family Prevotellaceae	rs148376875	T	G	0.111	170408919	0.552	0.187	360248	T	G	0.085	3	170126707	2.08E-06	0.018	14306	22.252
gen_samplesize_Vascular dementia (other)	family Prevotellaceae	rs2206482	T	G	0.234	9790461	0.088	0.137	360248	T	G	-0.057	20	9771109	1.30E-06	0.012	14306	23.465
gen_samplesize_Vascular dementia (other)	family Prevotellaceae	rs2278540	G	A	-0.256	32367424	0.066	0.139	360248	G	A	0.055	3	32408916	8.44E-06	0.012	14306	20.218
gen_samplesize_Vascular dementia (other)	family Prevotellaceae	rs34660375	A	G	0.230	180856881	0.228	0.191	360248	A	G	-0.081	5	180283881	7.40E-06	0.018	14306	20.431
gen_samplesize_Vascular dementia (other)	family Prevotellaceae	rs3758087	C	T	0.050	23857096	0.739	0.149	360248	C	T	-0.056	8	23714609	8.61E-06	0.012	14306	20.485
gen_samplesize_Vascular dementia (other)	family Prevotellaceae	rs3860225	A	G	-0.211	110619055	0.360	0.230	360248	A	G	0.084	1	111161677	5.50E-07	0.017	14306	24.981
gen_samplesize_Vascular dementia (other)	family Prevotellaceae	rs4493272	T	C	-0.055	118153336	0.679	0.133	360248	T	C	-0.060	2	118910912	3.02E-07	0.012	14306	26.275
gen_samplesize_Vascular dementia (other)	family Prevotellaceae	rs4685827	T	C	0.395	4823193	0.011	0.155	360248	T	C	-0.068	3	4864877	2.77E-06	0.015	14306	21.840
gen_samplesize_Vascular dementia (other)	family Prevotellaceae	rs7252711	G	A	-0.285	17350190	0.178	0.212	360248	G	A	0.074	19	17460999	5.57E-06	0.016	14306	20.934
gen_samplesize_Vascular dementia (other)	family Prevotellaceae	rs7975087	C	A	0.339	21251898	0.053	0.175	360248	C	A	-0.060	12	21404832	7.59E-06	0.014	14306	19.716
gen_samplesize_Vascular dementia (other)	family Prevotellaceae	rs912860	A	G	0.103	33237702	0.812	0.432	360248	A	G	0.229	14	33706908	9.30E-07	0.048	14306	22.476
gen_samplesize_Vascular dementia (other)	family Prevotellaceae	rs9586501	G	A	-0.231	104423473	0.125	0.151	360248	G	A	0.059	13	105075823	2.59E-06	0.013	14306	21.666
gen_samplesize_Vascular dementia (other)	family Prevotellaceae	rs9958960	G	A	-0.225	65351747	0.221	0.184	360248	G	A	-0.091	18	63018983	1.06E-07	0.017	14306	27.765
gen_samplesize_Vascular dementia (other)	family Rhodospirillaceae	rs1035406	G	A	-0.102	120037042	0.623	0.208	360248	G	A	-0.114	5	119372737	5.84E-06	0.025	14306	20.484
gen_samplesize_Vascular dementia (other)	family Rhodospirillaceae	rs11591293	G	T	-0.182	111660039	0.175	0.134	360248	G	T	0.074	10	113419797	2.67E-06	0.016	14306	21.923
gen_samplesize_Vascular dementia (other)	family Rhodospirillaceae	rs13336560	C	T	0.050	88487835	0.711	0.135	360248	C	T	-0.070	16	88554243	9.17E-06	0.016	14306	19.710
gen_samplesize_Vascular dementia (other)	family Rhodospirillaceae	rs1549633	A	C	-0.255	27945538	0.224	0.209	360248	A	C	0.100	5	27945645	4.70E-06	0.022	14306	20.891
gen_samplesize_Vascular dementia (other)	family Rhodospirillaceae	rs1923415	A	G	0.176	88558996	0.457	0.236	360248	A	G	-0.100	6	89268715	9.64E-06	0.023	14306	19.321
gen_samplesize_Vascular dementia (other)	family Rhodospirillaceae	rs3754624	C	T	0.138	224769095	0.428	0.174	360248	C	T	0.097	2	225633812	1.71E-06	0.020	14306	23.575
gen_samplesize_Vascular dementia (other)	family Rhodospirillaceae	rs4278423	T	C	-0.225	2628361	0.420	0.278	360248	T	C	0.108	10	2670553	3.12E-06	0.024	14306	20.808
gen_samplesize_Vascular dementia (other)	family Rhodospirillaceae	rs61933850	G	A	0.368	72745618	0.059	0.195	360248	G	A	0.165	12	73139398	7.23E-06	0.036	14306	20.883
gen_samplesize_Vascular dementia (other)	family Rhodospirillaceae	rs6679026	T	C	0.181	78153828	0.418	0.224	360248	T	C	0.112	1	78619512	9.95E-06	0.025	14306	19.891
gen_samplesize_Vascular dementia (other)	family Rhodospirillaceae	rs7001029	C	T	0.072	130946157	0.753	0.227	360248	C	T	0.117	8	131958403	5.35E-06	0.026	14306	20.171
gen_samplesize_Vascular dementia (other)	family Rhodospirillaceae	rs72714493	A	G	0.161	91740661	0.389	0.187	360248	A	G	0.082	1	92206218	7.35E-06	0.018	14306	20.415
gen_samplesize_Vascular dementia (other)	family Rhodospirillaceae	rs74354280	C	T	0.075	133271763	0.611	0.148	360248	C	T	-0.091	4	134192918	6.67E-06	0.020	14306	19.805
gen_samplesize_Vascular dementia (other)	family Rhodospirillaceae	rs76784716	A	G	-0.067	168176830	0.753	0.212	360248	A	G	0.136	2	169033340	1.49E-06	0.029	14306	22.666

gen_samplesize_Vascular dementia (other)	family Rhodospirillaceae	rs9813022	A	G	0.130	13685237	0.343	0.137	360248	A	G	-0.084	3	13726736	2.53E-07	0.016	14306	26.522
gen_samplesize_Vascular dementia (other)	family Rikenellaceae	rs10217435	C	T	0.035	83437740	0.855	0.191	360248	C	T	-0.088	9	86052655	6.51E-06	0.020	14306	20.022
gen_samplesize_Vascular dementia (other)	family Rikenellaceae	rs10832801	A	C	0.062	17567582	0.676	0.148	360248	A	C	-0.053	11	17589129	7.50E-06	0.012	14306	19.005
gen_samplesize_Vascular dementia (other)	family Rikenellaceae	rs1939881	G	A	-0.059	95571670	0.839	0.289	360248	G	A	-0.106	11	95304834	5.64E-07	0.021	14306	26.218
gen_samplesize_Vascular dementia (other)	family Rikenellaceae	rs2447496	A	G	0.003	98176696	0.986	0.151	360248	A	G	0.055	8	99188924	6.09E-06	0.012	14306	20.297
gen_samplesize_Vascular dementia (other)	family Rikenellaceae	rs2833282	G	A	0.253	31124392	0.178	0.188	360248	G	A	0.071	21	32496710	4.31E-06	0.016	14306	20.526
gen_samplesize_Vascular dementia (other)	family Rikenellaceae	rs36021379	A	G	0.107	27143593	0.557	0.182	360248	A	G	-0.066	21	28515912	7.20E-06	0.014	14306	20.496
gen_samplesize_Vascular dementia (other)	family Rikenellaceae	rs4264350	T	C	0.035	71366303	0.791	0.134	360248	T	C	-0.053	15	71658642	1.35E-06	0.011	14306	23.514
gen_samplesize_Vascular dementia (other)	family Rikenellaceae	rs59663348	G	A	0.338	4069710	0.029	0.155	360248	G	A	0.057	18	4069710	6.12E-06	0.013	14306	20.844
gen_samplesize_Vascular dementia (other)	family Rikenellaceae	rs62532512	A	C	-0.077	14158855	0.569	0.135	360248	A	C	0.050	9	14158854	2.76E-06	0.011	14306	22.045
gen_samplesize_Vascular dementia (other)	family Rikenellaceae	rs6744030	C	T	-0.006	173392399	0.971	0.164	360248	C	T	0.070	2	174257127	9.32E-06	0.016	14306	19.669
gen_samplesize_Vascular dementia (other)	family Rikenellaceae	rs6837275	A	G	0.018	186921078	0.904	0.146	360248	A	G	0.057	4	187842232	1.45E-06	0.012	14306	23.022
gen_samplesize_Vascular dementia (other)	family Rikenellaceae	rs74474130	T	G	0.244	89804477	0.506	0.367	360248	T	G	0.138	14	90270821	3.61E-06	0.030	14306	21.702
gen_samplesize_Vascular dementia (other)	family Rikenellaceae	rs77885767	C	T	0.074	21080475	0.815	0.318	360248	C	T	-0.156	14	21548634	2.85E-06	0.034	14306	21.552
gen_samplesize_Vascular dementia (other)	family Rikenellaceae	rs9389714	C	T	-0.240	99796791	0.305	0.234	360248	C	T	-0.064	6	100244667	8.79E-06	0.014	14306	19.718
gen_samplesize_Vascular dementia (other)	family Rikenellaceae	rs9578457	G	A	-0.479	22293217	0.122	0.310	360248	G	A	-0.141	13	22867356	3.99E-06	0.032	14306	20.064
gen_samplesize_Vascular dementia (other)	family Rikenellaceae	rs9603208	G	T	-0.143	37470552	0.517	0.221	360248	G	T	0.082	13	38044689	1.92E-07	0.016	14306	26.474
gen_samplesize_Vascular dementia (other)	family Ruminococcaceae	rs10093275	T	C	0.122	68820652	0.383	0.140	360248	T	C	-0.053	8	69732887	5.35E-06	0.012	14306	20.960
gen_samplesize_Vascular dementia (other)	family Ruminococcaceae	rs10166469	C	T	-0.102	29606301	0.496	0.151	360248	C	T	0.053	2	29829167	8.52E-06	0.012	14306	19.686
gen_samplesize_Vascular dementia (other)	family Ruminococcaceae	rs1158100	G	A	0.065	4698162	0.625	0.134	360248	G	A	0.049	8	4555684	8.61E-06	0.011	14306	19.936
gen_samplesize_Vascular dementia (other)	family Ruminococcaceae	rs1612733	T	C	0.210	107118984	0.477	0.295	360248	T	C	0.109	1	107661606	4.22E-06	0.024	14306	20.862
gen_samplesize_Vascular dementia (other)	family Ruminococcaceae	rs17376049	T	C	-0.106	60959595	0.620	0.215	360248	T	C	0.085	1	61425267	7.30E-07	0.017	14306	24.306
gen_samplesize_Vascular dementia (other)	family Ruminococcaceae	rs2113833	C	T	0.494	217352078	0.185	0.373	360248	C	T	-0.169	2	218216801	1.14E-06	0.036	14306	22.701
gen_samplesize_Vascular dementia (other)	family Ruminococcaceae	rs3009418	A	C	-0.420	148272338	0.191	0.321	360248	A	C	-0.093	1	147744468	8.69E-06	0.021	14306	19.489
gen_samplesize_Vascular dementia (other)	family Ruminococcaceae	rs55793120	T	C	0.481	46990335	0.088	0.282	360248	T	C	0.138	12	47384118	1.44E-07	0.027	14306	26.970
gen_samplesize_Vascular dementia (other)	family Ruminococcaceae	rs56199908	T	C	-0.259	2801371	0.364	0.285	360248	T	C	-0.199	9	2801371	1.66E-06	0.041	14306	23.584
gen_samplesize_Vascular dementia (other)	family Ruminococcaceae	rs76724913	T	G	-0.198	24147179	0.380	0.225	360248	T	G	0.090	1	24473669	9.60E-06	0.020	14306	19.682
gen_samplesize_Vascular dementia (other)	family Streptococcaceae	rs10028567	C	T	-0.162	52791410	0.426	0.203	360248	C	T	-0.093	4	53657577	3.72E-06	0.019	14306	24.079
gen_samplesize_Vascular dementia (other)	family Streptococcaceae	rs11110281	T	C	0.025	100190236	0.937	0.312	360248	T	C	-0.131	12	100584014	1.40E-08	0.023	14306	33.387
gen_samplesize_Vascular dementia (other)	family Streptococcaceae	rs16950051	A	G	0.341	120291702	0.225	0.281	360248	A	G	0.107	12	120729505	5.34E-06	0.024	14306	20.391
gen_samplesize_Vascular dementia (other)	family Streptococcaceae	rs2370083	G	T	-0.281	97060413	0.299	0.270	360248	G	T	-0.084	14	97526750	4.26E-06	0.018	14306	20.862
gen_samplesize_Vascular dementia (other)	family Streptococcaceae	rs2952251	G	A	0.110	10285654	0.485	0.158	360248	G	A	0.064	8	10143164	3.72E-07	0.013	14306	25.530
gen_samplesize_Vascular dementia (other)	family Streptococcaceae	rs35344081	G	A	0.086	941253	0.565	0.150	360248	G	A	0.061	16	991253	2.64E-06	0.013	14306	22.072
gen_samplesize_Vascular dementia (other)	family Streptococcaceae	rs57646748	G	A	0.051	37451236	0.882	0.343	360248	G	A	-0.088	4	37452858	7.88E-06	0.020	14306	19.612
gen_samplesize_Vascular dementia (other)	family Streptococcaceae	rs6806351	T	C	-0.072	132339879	0.653	0.160	360248	T	C	-0.062	3	132058723	6.94E-06	0.014	14306	20.808
gen_samplesize_Vascular dementia (other)	family Streptococcaceae	rs77968078	G	A	-0.157	240278466	0.572	0.277	360248	G	A	-0.099	1	240441766	7.93E-06	0.022	14306	19.515
gen_samplesize_Vascular dementia (other)	family Streptococcaceae	rs7916711	A	G	-0.092	28299340	0.633	0.192	360248	A	G	0.096	10	28588269	6.33E-06	0.022	14306	19.839
gen_samplesize_Vascular dementia (other)	family Streptococcaceae	rs957755	T	G	-0.193	46739055	0.315	0.193	360248	T	G	-0.064	7	46778653	7.42E-06	0.014	14306	20.268
gen_samplesize_Vascular dementia (other)	family Veillonellaceae	rs111810795	C	T	-0.002	102431593	0.993	0.222	360248	C	T	-0.087	14	102897930	1.73E-06	0.018	14306	23.059
gen_samplesize_Vascular dementia (other)	family Veillonellaceae	rs114889439	A	G	0.222	61023491	0.537	0.359	360248	A	G	-0.254	13	61597625	6.19E-06	0.054	14306	22.160
gen_samplesize_Vascular dementia (other)	family Veillonellaceae	rs12186441	G	A	-0.235	133310611	0.466	0.322	360248	G	A	0.208	5	132646303	4.53E-06	0.045	14306	21.010
gen_samplesize_Vascular dementia (other)	family Veillonellaceae	rs12668619	A	G	0.005	21598192	0.975	0.141	360248	A	G	0.055	7	21637810	2.57E-06	0.012	14306	22.093
gen_samplesize_Vascular dementia (other)	family Veillonellaceae	rs12741784	C	T	-0.167	49623147	0.273	0.152	360248	C	T	-0.062	1	50088819	1.28E-07	0.012	14306	27.151
gen_samplesize_Vascular dementia (other)	family Veillonellaceae	rs1442060	A	G	0.131	46364050	0.329	0.134	360248	A	G	0.051	4	46366067	4.51E-06	0.011	14306	21.017
gen_samplesize_Vascular dementia (other)	family Veillonellaceae	rs1693340	T	C	-0.121	32628728	0.638	0.257	360248	T	C	0.082	18	30208691	9.25E-06	0.018	14306	20.147
gen_samplesize_Vascular dementia (other)	family Veillonellaceae	rs2175069	G	A	-0.057	23315501	0.681	0.138	360248	G	A	0.053	4	23317124	4.64E-06	0.011	14306	21.022
gen_samplesize_Vascular dementia (other)	family Veillonellaceae	rs2561116	T	G	-0.233	38348097	0.418	0.288	360248	T	G	-0.084	5	38348199	7.89E-06	0.019	14306	19.960
gen_samplesize_Vascular dementia (other)	family Veillonellaceae	rs2585520	G	T	-0.004	78211006	0.990	0.341	360248	G	T	-0.090	13	78785141	5.27E-06	0.020	14306	20.387
gen_samplesize_Vascular dementia (other)	family Veillonellaceae	rs4263802	A	G	0.081	137307265	0.564	0.141	360248	A	G	-0.051	8	138319508	7.45E-06	0.011	14306	19.650
gen_samplesize_Vascular dementia (other)	family Veillonellaceae	rs4461038	G	A	0.150	78254654	0.310	0.148	360248	G	A	0.055	15	78546996	3.73E-06	0.012	14306	21.579
gen_samplesize_Vascular dementia (other)	family Veillonellaceae	rs4797169	T	C	0.011	462180	0.944	0.158	360248	T	C	0.059	18	462180	4.49E-06	0.013	14306	20.991
gen_samplesize_Vascular dementia (other)	family Veillonellaceae	rs61264131	A	C	0.284	138867397	0.217	0.231	360248	A	C	0.202	6	139188534	6.75E-06	0.046	14306	18.990
gen_samplesize_Vascular dementia (other)	family Veillonellaceae	rs6692542	G	A	-0.117	244228316	0.405	0.140	360248	G	A	-0.053	1	244391618	8.68E-06	0.012	14306	20.422
gen_samplesize_Vascular dementia (other)	family Veillonellaceae	rs6909981	C	T	0.135	74824436	0.502	0.201	360248	C	T	-0.064	6	75534152	5.48E-06	0.014	14306	20.298
gen_samplesize_Vascular dementia (other)	family Veillonellaceae	rs79535861	A	C	0.342	37752555	0.133	0.228	360248	A	C	0.101	13	38326692	1.58E-06	0.021	14306	23.646
gen_samplesize_Vascular dementia (other)	family Veillonellaceae	rs9345168	A	C	0.187	91671204	0.163	0.134	360248	A	C	-0.051	6	92380922	8.49E-06	0.011	14306	20.207
gen_samplesize_Vascular dementia (other)	family Verrucomicrobiaceae	rs111862613	T	C	0.109	129825125	0.542	0.179	360248	T	C	0.091	12	130309670	3.73E-06	0.020	14306	21.255
gen_samplesize_Vascular dementia (other)	family Verrucomicrobiaceae	rs117107102	A	G	-0.510	51947265	0.106	0.315	360248	A	G	0.205	18	49473635	2.92E-06	0.043	14306	22.493
gen_samplesize_Vascular dementia (other)	family Verrucomicrobiaceae	rs11729256	T	C	-0.231	94106121	0.192	0.177	360248	T	C	0.075	4	95027272	6.73E-07	0.015	14306	24.928
gen_samplesize_Vascular dementia (other)	family Verrucomicrobiaceae	rs12908520	G	A	0.088	97027427	0.513	0.134	360248	G	A	0.062	15	97570657	2.15E-06	0.013	14306	22.353
gen_samplesize_Vascular dementia (other)	family Verrucomicrobiaceae	rs2602429	T	C	-0.031	81029544	0.839	0.152	360248	T	C	-0.075	16	81063149	2.70E-06	0.016	14306	22.781
gen_samplesize_Vascular dementia (other)	family Verrucomicrobiaceae	rs4242783	A	G	-0.137	5022135	0.356	0.149	360248	A	G	-0.069	10	5064327	2.75E-06	0.015	14306	21.699
gen_samplesize_Vascular dementia (other)	family Verrucomicrobiaceae	rs4936098	G	A	0.049	130410772	0.723	0.139	360248	G	A	-0.065	11	130280667	1.13E-06	0.014	14306	22.775
gen_samplesize_Vascular dementia (other)	family Verrucomicrobiaceae	rs61779207	G	A	-0.037	40608800	0.817	0.162	360248	G	A	-0.076	1	41074472	6.63E-06	0.017	14306	20.459
gen_samplesize_Vascular dementia (other)	family Verrucomicrobiaceae	rs74542928	T	C	-0.380	99623031	0.214	0.306	360248	T	C	0.112	4	100544188	1.65E-06	0.024	14306	22.492
gen_samplesize_Vascular dementia (other)	family Verrucomicrobiaceae	rs9349825	A	G	-0.366	56476683	0.032	0.171	360248	A	G	-0.070	6	56341481	2.51E-06	0.015	14306	22.919
gen_samplesize_Vascular dementia (other)	family Verrucomicrobiaceae	rs941682	G	A	0.084	33280034	0.573	0.149	360248	G	A	-0.063	20	31867840	9.58E-06	0.014	14306	19.296

gen_samplesize_Vascular dementia (other)	family Victivallaceae	rs11671100	A	C	-0.049	711637	0.766	0.164	360248	A	C	-0.160	19	711637	4.08E-06	0.035	14306	20.970
gen_samplesize_Vascular dementia (other)	family Victivallaceae	rs11764871	G	T	-0.164	147111885	0.253	0.143	360248	G	T	0.127	7	146808977	7.49E-07	0.026	14306	24.542
gen_samplesize_Vascular dementia (other)	family Victivallaceae	rs2944282	T	C	-0.150	57530097	0.314	0.149	360248	T	C	-0.124	7	57589803	1.57E-06	0.026	14306	23.349
gen_samplesize_Vascular dementia (other)	family Victivallaceae	rs34962571	A	C	-0.301	130618711	0.181	0.225	360248	A	C	-0.187	12	131103256	6.25E-06	0.042	14306	19.963
gen_samplesize_Vascular dementia (other)	family Victivallaceae	rs4396289	C	T	0.072	9295183	0.713	0.195	360248	C	T	-0.153	11	9316730	1.54E-07	0.029	14306	28.090
gen_samplesize_Vascular dementia (other)	family Victivallaceae	rs61702987	T	C	0.216	30988791	0.307	0.211	360248	T	C	0.146	2	31211657	3.08E-06	0.030	14306	23.522
gen_samplesize_Vascular dementia (other)	family Victivallaceae	rs62570196	C	T	0.390	108323890	0.238	0.330	360248	C	T	-0.246	9	111086170	2.70E-07	0.048	14306	25.953
gen_samplesize_Vascular dementia (other)	family Victivallaceae	rs6545794	A	G	0.033	60257774	0.875	0.207	360248	A	G	-0.198	2	60484909	5.97E-07	0.041	14306	23.215
gen_samplesize_Vascular dementia (other)	family Victivallaceae	rs7077363	G	A	-0.012	93527197	0.945	0.179	360248	G	A	0.149	10	95286954	2.83E-06	0.032	14306	21.887
gen_samplesize_Vascular dementia (other)	family Victivallaceae	rs7314815	G	A	0.089	102131269	0.502	0.133	360248	G	A	0.101	12	102525047	6.40E-06	0.023	14306	20.079
gen_samplesize_Vascular dementia (other)	family Victivallaceae	rs7627405	C	T	0.028	9926871	0.870	0.168	360248	C	T	-0.134	3	9968555	8.19E-06	0.030	14306	19.809
gen_samplesize_Vascular dementia (other)	genus Actinomyces	rs2715439	T	C	-0.321	98949084	0.016	0.133	360248	T	C	-0.075	15	99492313	6.27E-06	0.016	14306	20.523
gen_samplesize_Vascular dementia (other)	genus Actinomyces	rs34583783	G	T	0.111	66497478	0.689	0.278	360248	G	T	0.127	6	67207371	4.49E-06	0.027	14306	22.237
gen_samplesize_Vascular dementia (other)	genus Actinomyces	rs35011108	A	G	-0.030	132686341	0.910	0.266	360248	A	G	0.233	6	133007480	6.34E-06	0.051	14306	20.641
gen_samplesize_Vascular dementia (other)	genus Actinomyces	rs4073240	G	A	-0.111	168824686	0.419	0.137	360248	G	A	0.075	6	169224781	7.94E-06	0.017	14306	20.064
gen_samplesize_Vascular dementia (other)	genus Actinomyces	rs4146653	G	A	-0.021	4740649	0.911	0.192	360248	G	A	0.099	10	4782841	4.50E-06	0.021	14306	21.159
gen_samplesize_Vascular dementia (other)	genus Actinomyces	rs71315246	A	G	-0.028	101633595	0.887	0.195	360248	A	G	-0.097	3	101352439	9.83E-06	0.022	14306	19.566
gen_samplesize_Vascular dementia (other)	genus Actinomyces	rs7915461	C	T	-0.280	125843552	0.298	0.269	360248	C	T	-0.188	10	127532121	5.92E-06	0.040	14306	21.855
gen_samplesize_Vascular dementia (other)	genus Adlercreutzia	rs11604400	C	T	-0.116	98515355	0.594	0.218	360248	C	T	-0.103	11	98386085	9.74E-06	0.023	14306	19.060
gen_samplesize_Vascular dementia (other)	genus Adlercreutzia	rs13231526	C	A	0.236	48804555	0.347	0.251	360248	C	A	0.143	7	48844151	4.81E-06	0.031	14306	21.123
gen_samplesize_Vascular dementia (other)	genus Adlercreutzia	rs2717140	C	T	0.336	77297814	0.132	0.223	360248	C	T	-0.119	18	75009770	2.05E-06	0.025	14306	22.548
gen_samplesize_Vascular dementia (other)	genus Adlercreutzia	rs55719207	G	A	-0.122	105637127	0.372	0.137	360248	G	A	-0.070	3	105355971	9.61E-06	0.016	14306	19.577
gen_samplesize_Vascular dementia (other)	genus Adlercreutzia	rs6664405	T	C	0.096	68978480	0.613	0.189	360248	T	C	-0.095	1	69444163	5.23E-06	0.021	14306	20.451
gen_samplesize_Vascular dementia (other)	genus Adlercreutzia	rs7680684	T	C	0.062	170360208	0.656	0.139	360248	T	C	0.083	4	171281359	9.77E-07	0.017	14306	24.371
gen_samplesize_Vascular dementia (other)	genus Adlercreutzia	rs9490822	C	T	0.004	123587938	0.977	0.133	360248	C	T	-0.073	6	123909083	2.54E-06	0.016	14306	22.229
gen_samplesize_Vascular dementia (other)	genus Adlercreutzia	rs9915817	C	T	-0.227	50786818	0.125	0.148	360248	C	T	-0.075	17	48864179	8.22E-06	0.017	14306	19.810
gen_samplesize_Vascular dementia (other)	genus Akkermansia	rs111862613	T	C	0.109	129825125	0.542	0.179	360248	T	C	0.091	12	130309670	3.39E-06	0.020	14306	21.449
gen_samplesize_Vascular dementia (other)	genus Akkermansia	rs117107102	A	G	-0.510	51947265	0.106	0.315	360248	A	G	0.204	18	49473635	3.01E-06	0.043	14306	22.427
gen_samplesize_Vascular dementia (other)	genus Akkermansia	rs11729256	T	C	-0.231	94106121	0.192	0.177	360248	T	C	0.075	4	95027272	6.58E-07	0.015	14306	24.970
gen_samplesize_Vascular dementia (other)	genus Akkermansia	rs12908520	G	A	0.088	97027427	0.513	0.134	360248	G	A	0.062	15	97570657	2.26E-06	0.013	14306	22.251
gen_samplesize_Vascular dementia (other)	genus Akkermansia	rs2602429	T	C	-0.031	81029544	0.839	0.152	360248	T	C	-0.075	16	81063149	2.72E-06	0.016	14306	22.770
gen_samplesize_Vascular dementia (other)	genus Akkermansia	rs4242783	A	G	-0.137	5022135	0.356	0.149	360248	A	G	-0.069	10	5064327	3.00E-06	0.015	14306	21.537
gen_samplesize_Vascular dementia (other)	genus Akkermansia	rs4936098	G	A	0.049	130410772	0.723	0.139	360248	G	A	-0.065	11	130280667	1.10E-06	0.014	14306	22.810
gen_samplesize_Vascular dementia (other)	genus Akkermansia	rs61779207	G	A	-0.037	40608800	0.817	0.162	360248	G	A	-0.076	1	41074472	6.32E-06	0.017	14306	20.550
gen_samplesize_Vascular dementia (other)	genus Akkermansia	rs74542928	T	C	-0.380	99623031	0.214	0.306	360248	T	C	0.113	4	100544188	1.48E-06	0.024	14306	22.690
gen_samplesize_Vascular dementia (other)	genus Akkermansia	rs9349825	A	G	-0.366	56476683	0.032	0.171	360248	A	G	-0.070	6	56341481	2.60E-06	0.015	14306	22.856
gen_samplesize_Vascular dementia (other)	genus Akkermansia	rs941682	G	A	0.084	33280034	0.573	0.149	360248	G	A	-0.063	20	31867840	9.17E-06	0.014	14306	19.381
gen_samplesize_Vascular dementia (other)	genus Alistipes	rs1107244	G	A	-0.018	37483220	0.942	0.245	360248	G	A	0.076	13	38057357	3.59E-06	0.017	14306	19.636
gen_samplesize_Vascular dementia (other)	genus Alistipes	rs11769002	G	A	0.284	62983120	0.036	0.135	360248	G	A	-0.053	7	62443498	1.45E-06	0.011	14306	23.368
gen_samplesize_Vascular dementia (other)	genus Alistipes	rs11958296	A	G	-0.071	178328178	0.824	0.319	360248	A	G	-0.098	5	177755179	9.30E-06	0.022	14306	20.209
gen_samplesize_Vascular dementia (other)	genus Alistipes	rs12990744	C	T	0.136	177136221	0.535	0.219	360248	C	T	-0.078	2	178000949	8.21E-06	0.017	14306	20.155
gen_samplesize_Vascular dementia (other)	genus Alistipes	rs1689282	A	C	-0.052	14080130	0.717	0.142	360248	A	C	-0.052	9	14080129	5.28E-06	0.011	14306	20.832
gen_samplesize_Vascular dementia (other)	genus Alistipes	rs2290844	C	T	0.164	126098573	0.440	0.212	360248	C	T	0.081	10	127787142	9.10E-06	0.019	14306	18.042
gen_samplesize_Vascular dementia (other)	genus Alistipes	rs2450745	A	C	0.025	143346550	0.925	0.262	360248	A	C	-0.081	8	144428720	7.12E-06	0.018	14306	19.007
gen_samplesize_Vascular dementia (other)	genus Alistipes	rs2875322	C	T	-0.013	131017657	0.942	0.178	360248	C	T	0.058	11	130887552	8.78E-06	0.013	14306	19.537
gen_samplesize_Vascular dementia (other)	genus Alistipes	rs34417064	A	G	-0.170	54699118	0.198	0.132	360248	A	G	-0.048	17	52776479	7.01E-06	0.011	14306	20.344
gen_samplesize_Vascular dementia (other)	genus Alistipes	rs4810359	A	G	0.139	42562695	0.491	0.203	360248	A	G	-0.065	20	41191335	7.50E-06	0.015	14306	19.927
gen_samplesize_Vascular dementia (other)	genus Alistipes	rs7129639	A	C	-0.034	17489880	0.811	0.142	360248	A	C	0.052	11	17511427	1.78E-06	0.011	14306	22.948
gen_samplesize_Vascular dementia (other)	genus Alistipes	rs8130320	A	G	0.144	39208332	0.283	0.134	360248	A	G	-0.049	21	40580258	4.84E-06	0.011	14306	20.906
gen_samplesize_Vascular dementia (other)	genus Allisonella	rs1901739	G	T	0.034	114951010	0.797	0.134	360248	G	T	-0.116	5	114286707	3.59E-06	0.025	14306	21.682
gen_samplesize_Vascular dementia (other)	genus Allisonella	rs35110698	T	C	0.226	27866065	0.228	0.188	360248	T	C	-0.146	12	28018998	5.72E-06	0.032	14306	20.797
gen_samplesize_Vascular dementia (other)	genus Allisonella	rs35778461	C	T	0.017	97388274	0.916	0.158	360248	C	T	0.147	9	100150556	1.21E-06	0.030	14306	24.360
gen_samplesize_Vascular dementia (other)	genus Allisonella	rs594561	T	C	0.172	88899804	0.193	0.133	360248	T	C	-0.112	11	88632972	9.41E-06	0.025	14306	19.885
gen_samplesize_Vascular dementia (other)	genus Allisonella	rs602075	G	A	-0.242	76495244	0.109	0.151	360248	G	A	-0.169	9	79110160	3.57E-08	0.030	14306	32.374
gen_samplesize_Vascular dementia (other)	genus Allisonella	rs6742198	A	G	0.235	33382004	0.126	0.154	360248	A	G	-0.149	2	33607071	3.35E-06	0.032	14306	22.210
gen_samplesize_Vascular dementia (other)	genus Allisonella	rs76904847	G	A	0.165	138283265	0.348	0.176	360248	G	A	0.149	7	137968010	6.09E-06	0.033	14306	19.673
gen_samplesize_Vascular dementia (other)	genus Allisonella	rs7898615	T	G	0.024	120653939	0.902	0.195	360248	T	G	0.168	10	122413451	8.87E-06	0.037	14306	20.214
gen_samplesize_Vascular dementia (other)	genus Alloprevotella	rs2154444	G	T	0.050	34681344	0.738	0.151	360248	G	T	-0.138	21	36053643	8.37E-06	0.031	14306	20.018
gen_samplesize_Vascular dementia (other)	genus Alloprevotella	rs34619204	G	A	0.202	39621081	0.251	0.176	360248	G	A	-0.156	21	40993008	8.84E-06	0.034	14306	20.528
gen_samplesize_Vascular dementia (other)	genus Alloprevotella	rs4364940	A	G	-0.413	233990770	0.003	0.138	360248	A	G	0.126	1	234126516	8.58E-06	0.028	14306	20.067
gen_samplesize_Vascular dementia (other)	genus Alloprevotella	rs4680035	G	A	0.095	153087811	0.486	0.137	360248	G	A	0.120	3	152805600	4.99E-06	0.026	14306	21.250
gen_samplesize_Vascular dementia (other)	genus Alloprevotella	rs58212166	A	G	0.184	188155551	0.283	0.171	360248	A	G	-0.162	4	189076705	7.94E-06	0.036	14306	20.227
gen_samplesize_Vascular dementia (other)	genus Anaerofilum	rs10794359	C	T	-0.042	1051715	0.756	0.134	360248	C	T	0.095	11	1051715	2.23E-06	0.020	14306	22.594
gen_samplesize_Vascular dementia (other)	genus Anaerofilum	rs1563175	A	C	-0.073	3791350	0.580	0.133	360248	A	C	0.092	2	3838940	5.54E-06	0.020	14306	20.884
gen_samplesize_Vascular dementia (other)	genus Anaerofilum	rs17012738	T	G	0.028	76471016	0.836	0.133	360248	T	G	0.090	2	76698142	7.24E-06	0.020	14306	20.355
gen_samplesize_Vascular dementia (other)	genus Anaerofilum	rs17096874	C	T	-0.078	30522152	0.631	0.163	360248	C	T	-0.126	14	30991358	2.86E-06	0.027	14306	22.071
gen_samplesize_Vascular dementia (other)	genus Anaerofilum	rs4244069	A	G	-0.065	66773681	0.743	0.197	360248	A	G	0.147	12	67167461	9.81E-06	0.033	14306	20.197

gen_samplesize_Vascular dementia (other)	genus Anaerofilum	rs4506496	A	G	-0.027	246982388	0.855	0.146	360248	A	G	-0.103	1	247145690	1.49E-06	0.021	14306	23.455
gen_samplesize_Vascular dementia (other)	genus Anaerofilum	rs712981	A	C	0.103	129967591	0.454	0.137	360248	A	C	0.101	3	129686434	6.83E-07	0.020	14306	24.661
gen_samplesize_Vascular dementia (other)	genus Anaerofilum	rs79598899	C	T	-0.533	190274425	0.097	0.321	360248	C	T	0.183	2	191139151	3.75E-07	0.036	14306	26.116
gen_samplesize_Vascular dementia (other)	genus Anaerofilum	rs816292	T	C	-0.006	117373604	0.968	0.146	360248	T	C	-0.113	12	117811409	2.64E-07	0.022	14306	26.288
gen_samplesize_Vascular dementia (other)	genus Anaerofilum	rs9299345	T	C	-0.132	101577530	0.555	0.223	360248	T	C	-0.136	9	104339812	8.04E-06	0.030	14306	20.349
gen_samplesize_Vascular dementia (other)	genus Anaerostipes	rs10502061	A	G	0.423	105744527	0.042	0.208	360248	A	G	0.084	11	105615253	7.94E-06	0.019	14306	18.944
gen_samplesize_Vascular dementia (other)	genus Anaerostipes	rs2014785	T	C	0.087	171382313	0.516	0.135	360248	T	C	0.052	3	171100102	4.68E-06	0.011	14306	21.125
gen_samplesize_Vascular dementia (other)	genus Anaerostipes	rs2396460	C	T	-0.176	227153955	0.187	0.133	360248	C	T	0.051	2	228018671	2.91E-06	0.011	14306	21.897
gen_samplesize_Vascular dementia (other)	genus Anaerostipes	rs2804244	G	A	0.148	115623674	0.278	0.136	360248	G	A	0.053	10	117383184	2.04E-06	0.011	14306	22.882
gen_samplesize_Vascular dementia (other)	genus Anaerostipes	rs3900776	G	A	0.494	13525083	0.228	0.410	360248	G	A	-0.110	9	13525082	2.75E-06	0.024	14306	21.675
gen_samplesize_Vascular dementia (other)	genus Anaerostipes	rs60983350	G	A	0.066	2947443	0.648	0.144	360248	G	A	-0.054	17	2850737	4.42E-06	0.012	14306	21.450
gen_samplesize_Vascular dementia (other)	genus Anaerostipes	rs62157625	T	C	0.138	142016764	0.498	0.204	360248	T	C	0.089	2	142774333	1.45E-06	0.019	14306	22.787
gen_samplesize_Vascular dementia (other)	genus Anaerostipes	rs62215703	G	A	-0.353	24501310	0.028	0.161	360248	G	A	0.064	21	25873624	1.98E-06	0.014	14306	22.260
gen_samplesize_Vascular dementia (other)	genus Anaerostipes	rs6474958	G	A	0.156	1582701	0.277	0.144	360248	G	A	0.050	9	1582701	6.74E-06	0.011	14306	19.935
gen_samplesize_Vascular dementia (other)	genus Anaerostipes	rs6726833	C	A	0.092	39124428	0.705	0.244	360248	C	A	-0.088	2	39351569	3.32E-06	0.019	14306	21.463
gen_samplesize_Vascular dementia (other)	genus Anaerostipes	rs6854026	C	T	0.075	168769663	0.577	0.135	360248	C	T	0.051	4	169690814	3.20E-06	0.011	14306	21.732
gen_samplesize_Vascular dementia (other)	genus Anaerostipes	rs7193624	T	C	-0.074	77540123	0.761	0.242	360248	T	C	-0.075	16	77574020	5.35E-07	0.015	14306	24.803
gen_samplesize_Vascular dementia (other)	genus Anaerostipes	rs78735375	A	C	0.259	1497548	0.449	0.343	360248	A	C	-0.137	19	1497547	5.33E-06	0.031	14306	20.262
gen_samplesize_Vascular dementia (other)	genus Anaerotruncus	rs10150232	A	G	0.013	29948802	0.940	0.168	360248	A	G	0.057	14	30418008	6.68E-06	0.012	14306	20.622
gen_samplesize_Vascular dementia (other)	genus Anaerotruncus	rs11018566	A	G	-0.444	89307058	0.137	0.299	360248	A	G	-0.156	11	89040226	6.14E-06	0.037	14306	18.272
gen_samplesize_Vascular dementia (other)	genus Anaerotruncus	rs115414803	A	C	-0.197	87242091	0.483	0.281	360248	A	C	-0.144	4	88163243	6.83E-06	0.032	14306	20.669
gen_samplesize_Vascular dementia (other)	genus Anaerotruncus	rs1272208	T	G	0.016	76015978	0.919	0.155	360248	T	G	0.061	9	78630894	4.28E-06	0.013	14306	22.201
gen_samplesize_Vascular dementia (other)	genus Anaerotruncus	rs1431492	C	T	-0.152	151137584	0.407	0.183	360248	C	T	-0.065	3	150855371	7.36E-06	0.015	14306	20.075
gen_samplesize_Vascular dementia (other)	genus Anaerotruncus	rs17734739	T	C	-0.169	210798978	0.376	0.191	360248	T	C	0.066	2	211663702	7.43E-06	0.015	14306	19.603
gen_samplesize_Vascular dementia (other)	genus Anaerotruncus	rs34449434	A	C	0.155	76129875	0.264	0.139	360248	A	C	-0.050	12	76523655	9.85E-06	0.011	14306	19.208
gen_samplesize_Vascular dementia (other)	genus Anaerotruncus	rs4669806	G	T	0.120	12060626	0.456	0.161	360248	G	T	0.058	2	12200752	2.42E-06	0.012	14306	21.962
gen_samplesize_Vascular dementia (other)	genus Anaerotruncus	rs6494922	A	G	-0.483	33167666	0.092	0.287	360248	A	G	0.090	15	33459867	6.62E-06	0.020	14306	19.937
gen_samplesize_Vascular dementia (other)	genus Anaerotruncus	rs6563550	T	C	-0.018	37484276	0.942	0.245	360248	T	C	0.088	13	38058413	2.35E-07	0.018	14306	24.629
gen_samplesize_Vascular dementia (other)	genus Anaerotruncus	rs7155595	C	A	0.145	77036203	0.316	0.145	360248	C	A	0.054	14	77502546	7.55E-06	0.012	14306	20.575
gen_samplesize_Vascular dementia (other)	genus Anaerotruncus	rs8005030	C	T	-0.279	30137993	0.048	0.141	360248	C	T	0.055	14	30607199	2.28E-06	0.012	14306	22.133
gen_samplesize_Vascular dementia (other)	genus Anaerotruncus	rs9347879	T	C	0.029	164594228	0.827	0.133	360248	T	C	0.051	6	165015261	4.22E-06	0.011	14306	20.988
gen_samplesize_Vascular dementia (other)	genus Bacteroides	rs11585893	A	G	-0.315	10584294	0.042	0.155	360248	A	G	-0.074	1	10644351	1.80E-06	0.015	14306	25.175
gen_samplesize_Vascular dementia (other)	genus Bacteroides	rs13207588	A	G	0.029	41551692	0.865	0.170	360248	A	G	-0.059	6	41519430	7.48E-06	0.013	14306	20.365
gen_samplesize_Vascular dementia (other)	genus Bacteroides	rs1340391	T	C	0.419	102495433	0.031	0.195	360248	T	C	-0.059	1	102960989	6.73E-06	0.013	14306	20.040
gen_samplesize_Vascular dementia (other)	genus Bacteroides	rs17619981	T	G	-0.361	24159448	0.060	0.192	360248	T	G	0.088	19	24342250	2.69E-06	0.019	14306	22.194
gen_samplesize_Vascular dementia (other)	genus Bacteroides	rs2023437	T	C	-0.129	21577814	0.522	0.202	360248	T	C	-0.078	14	22045949	5.02E-06	0.017	14306	21.780
gen_samplesize_Vascular dementia (other)	genus Bacteroides	rs66710942	T	C	-0.026	77166176	0.845	0.133	360248	T	C	-0.049	3	77215327	5.86E-06	0.011	14306	20.644
gen_samplesize_Vascular dementia (other)	genus Bacteroides	rs6795673	C	T	0.016	10551540	0.902	0.133	360248	C	T	0.054	3	10593224	3.38E-07	0.011	14306	26.183
gen_samplesize_Vascular dementia (other)	genus Bacteroides	rs9507307	C	T	0.108	24336338	0.485	0.155	360248	C	T	0.060	13	24910476	2.13E-06	0.013	14306	21.912
gen_samplesize_Vascular dementia (other)	genus Barnesiella	rs11155559	T	C	-0.228	148280769	0.312	0.226	360248	T	C	0.096	6	148601905	8.92E-06	0.021	14306	20.153
gen_samplesize_Vascular dementia (other)	genus Barnesiella	rs12909713	C	T	-0.121	86602682	0.364	0.133	360248	C	T	-0.055	15	87145913	4.95E-06	0.012	14306	21.048
gen_samplesize_Vascular dementia (other)	genus Barnesiella	rs13242616	C	T	-0.011	122381464	0.941	0.144	360248	C	T	0.058	7	122021518	2.29E-06	0.012	14306	22.459
gen_samplesize_Vascular dementia (other)	genus Barnesiella	rs199035	G	A	-0.133	23435187	0.322	0.134	360248	G	A	0.056	6	23435415	3.00E-06	0.012	14306	21.842
gen_samplesize_Vascular dementia (other)	genus Barnesiella	rs2276875	A	G	-0.160	5729227	0.298	0.154	360248	A	G	-0.070	4	5730954	4.65E-07	0.014	14306	24.964
gen_samplesize_Vascular dementia (other)	genus Barnesiella	rs2428166	G	A	0.555	110509945	0.328	0.567	360248	G	A	-0.166	6	110831148	8.51E-07	0.034	14306	24.178
gen_samplesize_Vascular dementia (other)	genus Barnesiella	rs35177866	A	G	0.473	181871367	0.064	0.255	360248	A	G	0.092	3	181589155	2.95E-06	0.019	14306	23.267
gen_samplesize_Vascular dementia (other)	genus Barnesiella	rs62251337	G	A	0.059	44285937	0.754	0.189	360248	G	A	0.069	3	44327429	4.24E-06	0.015	14306	21.385
gen_samplesize_Vascular dementia (other)	genus Barnesiella	rs72684847	T	C	-0.293	101695712	0.253	0.256	360248	T	C	-0.114	4	102616869	6.76E-06	0.025	14306	20.295
gen_samplesize_Vascular dementia (other)	genus Barnesiella	rs76181748	C	T	0.011	104446188	0.943	0.158	360248	C	T	-0.078	8	105458416	6.78E-06	0.017	14306	20.575
gen_samplesize_Vascular dementia (other)	genus Barnesiella	rs77455852	T	G	-0.286	54811208	0.128	0.188	360248	T	G	-0.089	5	54107036	3.16E-06	0.020	14306	20.773
gen_samplesize_Vascular dementia (other)	genus Barnesiella	rs79795328	A	G	-0.398	113625439	0.038	0.192	360248	A	G	-0.082	4	114546595	4.23E-06	0.018	14306	21.527
gen_samplesize_Vascular dementia (other)	genus Bifidobacterium	rs12022129	A	G	0.080	206830029	0.591	0.148	360248	A	G	-0.062	1	207003374	8.00E-06	0.014	14306	19.872
gen_samplesize_Vascular dementia (other)	genus Bifidobacterium	rs182549	T	C	-0.269	135859184	0.052	0.139	360248	T	C	-0.120	2	136616754	1.28E-20	0.013	14306	88.429
gen_samplesize_Vascular dementia (other)	genus Bifidobacterium	rs2491158	A	G	-0.122	124401134	0.524	0.192	360248	A	G	-0.071	10	126089703	8.05E-06	0.016	14306	19.879
gen_samplesize_Vascular dementia (other)	genus Bifidobacterium	rs2686790	C	T	0.113	48051149	0.545	0.187	360248	C	T	-0.071	7	48090746	7.50E-06	0.016	14306	20.065
gen_samplesize_Vascular dementia (other)	genus Bifidobacterium	rs4957061	T	C	-0.254	520981	0.061	0.136	360248	T	C	0.053	5	521096	5.78E-06	0.012	14306	20.697
gen_samplesize_Vascular dementia (other)	genus Bifidobacterium	rs540489	T	G	-0.121	74901626	0.495	0.177	360248	T	G	-0.064	17	72897722	5.19E-06	0.014	14306	21.121
gen_samplesize_Vascular dementia (other)	genus Bifidobacterium	rs55888705	A	G	0.223	1516099	0.130	0.148	360248	A	G	0.055	4	1517826	6.67E-06	0.012	14306	20.339
gen_samplesize_Vascular dementia (other)	genus Bifidobacterium	rs5746486	T	C	-0.057	17871506	0.674	0.136	360248	T	C	-0.054	22	18354272	9.00E-06	0.012	14306	19.703
gen_samplesize_Vascular dementia (other)	genus Bifidobacterium	rs62181700	G	A	0.159	188941058	0.300	0.154	360248	G	A	-0.062	2	189805784	2.17E-06	0.013	14306	22.665
gen_samplesize_Vascular dementia (other)	genus Bifidobacterium	rs7322849	T	C	-0.051	112205515	0.824	0.229	360248	T	C	0.112	13	112859829	1.08E-08	0.020	14306	31.035
gen_samplesize_Vascular dementia (other)	genus Bifidobacterium	rs75344046	C	T	-0.175	30489472	0.579	0.314	360248	C	T	0.232	21	31861790	4.86E-06	0.051	14306	21.088
gen_samplesize_Vascular dementia (other)	genus Bifidobacterium	rs857444	C	T	0.144	14617360	0.294	0.137	360248	C	T	0.056	6	14617591	3.57E-06	0.012	14306	21.208
gen_samplesize_Vascular dementia (other)	genus Bilophila	rs11069458	C	T	0.002	101750335	0.989	0.172	360248	C	T	0.068	13	102402685	7.72E-06	0.016	14306	19.293
gen_samplesize_Vascular dementia (other)	genus Bilophila	rs1241171	G	A	0.113	102834365	0.545	0.187	360248	G	A	-0.069	1	103299921	4.24E-06	0.015	14306	21.281
gen_samplesize_Vascular dementia (other)	genus Bilophila	rs1571225	T	C	-0.045	4940871	0.801	0.178	360248	T	C	-0.083	9	4940871	1.12E-06	0.017	14306	23.484
gen_samplesize_Vascular dementia (other)	genus Bilophila	rs1969927	A	G	-0.090	100393069	0.522	0.140	360248	A	G	-0.056	12	100786847	9.07E-06	0.013	14306	19.783

gen_samplesize_Vascular dementia (other)	genus Bilophila	rs2728491	T	G	0.059	47172134	0.702	0.154	360248	T	G	0.063	7	47211732	6.33E-06	0.014	14306	20.241
gen_samplesize_Vascular dementia (other)	genus Bilophila	rs3827020	C	T	0.008	63349639	0.963	0.169	360248	C	T	0.077	20	61980991	1.79E-06	0.016	14306	22.766
gen_samplesize_Vascular dementia (other)	genus Bilophila	rs4798126	G	A	0.345	3765773	0.041	0.169	360248	G	A	0.073	18	3765773	7.15E-06	0.017	14306	19.033
gen_samplesize_Vascular dementia (other)	genus Bilophila	rs542415	T	C	-0.037	49719357	0.786	0.137	360248	T	C	-0.061	15	50011554	4.71E-06	0.013	14306	21.147
gen_samplesize_Vascular dementia (other)	genus Bilophila	rs60178956	G	A	-0.148	89866110	0.347	0.158	360248	G	A	-0.062	8	90878338	8.06E-06	0.014	14306	19.502
gen_samplesize_Vascular dementia (other)	genus Bilophila	rs6793291	A	C	0.192	194698802	0.526	0.302	360248	A	C	-0.113	3	194419531	3.11E-06	0.024	14306	21.765
gen_samplesize_Vascular dementia (other)	genus Bilophila	rs72676854	T	C	-0.059	108978710	0.847	0.304	360248	T	C	0.123	8	109990939	5.62E-06	0.027	14306	21.007
gen_samplesize_Vascular dementia (other)	genus Bilophila	rs7802841	A	C	-0.015	138899094	0.918	0.144	360248	A	C	-0.067	7	138583840	1.77E-06	0.014	14306	23.681
gen_samplesize_Vascular dementia (other)	genus Bilophila	rs9899990	A	G	0.176	9218262	0.473	0.246	360248	A	G	-0.103	17	9121579	9.07E-06	0.023	14306	19.283
gen_samplesize_Vascular dementia (other)	genus Butyricicoccus	rs10084203	G	A	0.135	190439423	0.497	0.198	360248	G	A	-0.055	2	191304149	8.59E-06	0.012	14306	19.791
gen_samplesize_Vascular dementia (other)	genus Butyricicoccus	rs12034718	G	A	0.108	66913912	0.495	0.159	360248	G	A	-0.070	1	67379595	9.58E-06	0.016	14306	19.643
gen_samplesize_Vascular dementia (other)	genus Butyricicoccus	rs12585793	T	C	0.741	26444103	0.066	0.404	360248	T	C	-0.262	13	27018240	5.79E-06	0.056	14306	21.558
gen_samplesize_Vascular dementia (other)	genus Butyricicoccus	rs2017189	T	G	-0.149	7458426	0.262	0.133	360248	T	G	0.051	4	7460153	3.87E-06	0.011	14306	21.148
gen_samplesize_Vascular dementia (other)	genus Butyricicoccus	rs4962426	T	G	-0.056	125219639	0.737	0.167	360248	T	G	-0.061	10	126908208	7.38E-06	0.014	14306	20.403
gen_samplesize_Vascular dementia (other)	genus Butyricicoccus	rs56221232	T	C	-0.141	152596896	0.520	0.220	360248	T	C	0.083	2	153453410	7.62E-07	0.017	14306	24.467
gen_samplesize_Vascular dementia (other)	genus Butyricicoccus	rs62478070	T	G	-0.327	157895458	0.438	0.423	360248	T	G	0.224	7	157688150	5.94E-06	0.049	14306	20.488
gen_samplesize_Vascular dementia (other)	genus Butyricicoccus	rs7322368	C	T	0.155	99561736	0.495	0.228	360248	C	T	-0.082	13	100213990	5.52E-06	0.018	14306	19.834
gen_samplesize_Vascular dementia (other)	genus Butyricimonas	rs11228830	A	G	0.134	56897486	0.572	0.237	360248	A	G	0.135	11	56664962	6.55E-06	0.030	14306	20.547
gen_samplesize_Vascular dementia (other)	genus Butyricimonas	rs113054641	G	A	0.231	15178069	0.483	0.330	360248	G	A	-0.145	21	16550389	1.74E-07	0.027	14306	27.842
gen_samplesize_Vascular dementia (other)	genus Butyricimonas	rs12304031	G	A	-0.024	128954226	0.904	0.201	360248	G	A	-0.086	12	129438771	6.70E-06	0.020	14306	19.211
gen_samplesize_Vascular dementia (other)	genus Butyricimonas	rs12458763	A	C	-0.314	36511655	0.332	0.323	360248	A	C	0.122	18	34091618	6.37E-06	0.027	14306	20.492
gen_samplesize_Vascular dementia (other)	genus Butyricimonas	rs1862649	G	A	-0.403	22161545	0.122	0.260	360248	G	A	0.113	16	22172866	4.76E-06	0.025	14306	20.777
gen_samplesize_Vascular dementia (other)	genus Butyricimonas	rs2114713	G	T	0.130	80236031	0.329	0.133	360248	G	T	0.063	15	80528373	6.88E-06	0.014	14306	20.361
gen_samplesize_Vascular dementia (other)	genus Butyricimonas	rs62130338	A	G	-0.045	48659244	0.749	0.141	360248	A	G	0.073	19	49162501	3.90E-06	0.016	14306	21.412
gen_samplesize_Vascular dementia (other)	genus Butyricimonas	rs62390301	T	C	0.039	160440640	0.811	0.164	360248	T	C	-0.087	5	159867647	7.42E-07	0.017	14306	24.915
gen_samplesize_Vascular dementia (other)	genus Butyricimonas	rs7083431	A	C	0.006	70951399	0.967	0.149	360248	A	C	0.070	10	72711156	8.85E-07	0.014	14306	23.725
gen_samplesize_Vascular dementia (other)	genus Butyricimonas	rs71428626	G	T	0.395	82866140	0.297	0.379	360248	G	T	-0.133	2	83093264	4.80E-06	0.029	14306	21.107
gen_samplesize_Vascular dementia (other)	genus Butyricimonas	rs72814525	A	G	0.322	70683314	0.017	0.135	360248	A	G	0.066	10	72443070	8.25E-06	0.015	14306	19.666
gen_samplesize_Vascular dementia (other)	genus Butyricimonas	rs78453362	A	G	-0.619	78690859	0.156	0.437	360248	A	G	-0.149	3	78740009	4.06E-06	0.033	14306	20.887
gen_samplesize_Vascular dementia (other)	genus Butyricimonas	rs9657374	C	T	-0.068	4976884	0.639	0.145	360248	C	T	0.068	8	4834406	4.50E-06	0.015	14306	21.106
gen_samplesize_Vascular dementia (other)	genus Butyrivibrio	rs1007475	G	T	-0.214	148082269	0.150	0.148	360248	G	T	0.118	6	148403405	7.92E-06	0.026	14306	20.420
gen_samplesize_Vascular dementia (other)	genus Butyrivibrio	rs11761679	T	C	-0.034	150899841	0.858	0.192	360248	T	C	0.155	7	150596929	2.20E-06	0.032	14306	23.170
gen_samplesize_Vascular dementia (other)	genus Butyrivibrio	rs142855850	A	G	0.190	22298971	0.400	0.225	360248	A	G	0.205	10	22587900	6.86E-06	0.046	14306	20.113
gen_samplesize_Vascular dementia (other)	genus Butyrivibrio	rs16934069	T	C	0.398	116930885	0.023	0.175	360248	T	C	-0.134	9	119693164	8.86E-06	0.030	14306	19.961
gen_samplesize_Vascular dementia (other)	genus Butyrivibrio	rs16941336	C	T	-0.080	20672384	0.604	0.155	360248	C	T	0.127	17	20575697	1.53E-06	0.027	14306	22.640
gen_samplesize_Vascular dementia (other)	genus Butyrivibrio	rs17163238	G	A	0.109	129640328	0.516	0.168	360248	G	A	0.141	5	128976021	5.51E-06	0.031	14306	20.802
gen_samplesize_Vascular dementia (other)	genus Butyrivibrio	rs4537857	T	C	-0.069	26718529	0.627	0.142	360248	T	C	-0.125	13	27292666	1.80E-06	0.026	14306	22.788
gen_samplesize_Vascular dementia (other)	genus Butyrivibrio	rs486484	A	G	0.003	830864	0.985	0.134	360248	A	G	-0.108	20	811507	6.61E-06	0.024	14306	20.380
gen_samplesize_Vascular dementia (other)	genus Butyrivibrio	rs4928024	G	A	0.198	54254476	0.252	0.173	360248	G	A	0.175	3	54288503	8.19E-06	0.039	14306	20.137
gen_samplesize_Vascular dementia (other)	genus Butyrivibrio	rs74622183	A	G	0.424	33681771	0.074	0.238	360248	A	G	-0.201	14	34150977	2.46E-06	0.043	14306	22.040
gen_samplesize_Vascular dementia (other)	genus Butyrivibrio	rs77356209	T	C	-0.043	10267736	0.892	0.318	360248	T	C	0.217	18	10267733	6.66E-06	0.048	14306	20.136
gen_samplesize_Vascular dementia (other)	genus Butyrivibrio	rs7752361	G	A	-0.069	111459502	0.609	0.134	360248	G	A	0.119	6	111780705	7.69E-07	0.024	14306	24.685
gen_samplesize_Vascular dementia (other)	genus Butyrivibrio	rs7763512	A	G	0.251	21959860	0.063	0.135	360248	A	G	-0.120	6	21960091	3.11E-06	0.025	14306	22.374
gen_samplesize_Vascular dementia (other)	genus Butyrivibrio	rs9349693	G	A	-0.123	54074926	0.397	0.145	360248	G	A	-0.118	6	53939724	5.55E-06	0.026	14306	20.610
gen_samplesize_Vascular dementia (other)	genus Candidatus Soleaferrea	rs10090365	G	A	0.167	137625956	0.206	0.132	360248	G	A	0.083	8	138638199	4.17E-06	0.018	14306	21.255
gen_samplesize_Vascular dementia (other)	genus Candidatus Soleaferrea	rs10809135	C	T	0.079	10611282	0.560	0.135	360248	C	T	-0.083	9	10611282	5.47E-06	0.018	14306	20.944
gen_samplesize_Vascular dementia (other)	genus Candidatus Soleaferrea	rs36155147	T	C	-0.187	312402	0.201	0.146	360248	T	C	-0.105	7	352368	5.41E-06	0.024	14306	18.971
gen_samplesize_Vascular dementia (other)	genus Candidatus Soleaferrea	rs4294381	C	T	0.193	224500405	0.291	0.182	360248	C	T	-0.112	1	224688107	1.37E-06	0.023	14306	23.416
gen_samplesize_Vascular dementia (other)	genus Candidatus Soleaferrea	rs4678258	T	C	0.062	138226350	0.689	0.155	360248	T	C	0.099	3	137945192	5.53E-06	0.022	14306	20.912
gen_samplesize_Vascular dementia (other)	genus Candidatus Soleaferrea	rs6489992	A	G	0.000	114914964	0.998	0.137	360248	A	G	-0.084	12	115352769	7.89E-06	0.019	14306	20.192
gen_samplesize_Vascular dementia (other)	genus Candidatus Soleaferrea	rs6494306	A	G	0.031	62102341	0.827	0.141	360248	A	G	-0.097	15	62394540	5.80E-06	0.021	14306	20.474
gen_samplesize_Vascular dementia (other)	genus Candidatus Soleaferrea	rs7400877	C	T	0.287	75937199	0.080	0.163	360248	C	T	0.095	14	76403542	9.29E-06	0.021	14306	19.976
gen_samplesize_Vascular dementia (other)	genus Candidatus Soleaferrea	rs9973954	G	A	0.026	19686146	0.852	0.139	360248	G	A	-0.089	2	19885907	5.95E-06	0.020	14306	20.842
gen_samplesize_Vascular dementia (other)	genus Catenibacterium	rs12404911	C	T	0.048	239955143	0.779	0.171	360248	C	T	0.141	1	240118443	2.80E-06	0.030	14306	21.406
gen_samplesize_Vascular dementia (other)	genus Catenibacterium	rs212393	A	G	0.145	159064710	0.376	0.164	360248	A	G	0.135	6	159485742	3.62E-06	0.029	14306	22.332
gen_samplesize_Vascular dementia (other)	genus Catenibacterium	rs73128290	A	G	0.048	57296613	0.744	0.146	360248	A	G	0.130	7	57364320	4.29E-06	0.028	14306	20.782
gen_samplesize_Vascular dementia (other)	genus Catenibacterium	rs7742829	C	T	-0.137	104641790	0.309	0.134	360248	C	T	0.114	6	105089665	5.61E-06	0.025	14306	20.652
gen_samplesize_Vascular dementia (other)	genus Christensenellaceae R 7group	rs10461257	A	G	-0.045	155209852	0.753	0.142	360248	A	G	-0.055	4	156131004	6.51E-06	0.012	14306	20.446
gen_samplesize_Vascular dementia (other)	genus Christensenellaceae R 7group	rs17081797	A	G	0.239	69888324	0.376	0.270	360248	A	G	-0.090	18	67555560	3.34E-06	0.020	14306	19.603
gen_samplesize_Vascular dementia (other)	genus Christensenellaceae R 7group	rs62132810	A	G	-0.016	48775970	0.934	0.192	360248	A	G	-0.083	19	49279227	5.67E-06	0.018	14306	21.293
gen_samplesize_Vascular dementia (other)	genus Christensenellaceae R 7group	rs62190261	A	C	-0.057	230078383	0.809	0.238	360248	A	C	0.096	2	230943099	8.74E-06	0.021	14306	19.922
gen_samplesize_Vascular dementia (other)	genus Christensenellaceae R 7group	rs62467127	C	T	-0.044	118456871	0.915	0.409	360248	C	T	0.114	7	118096925	3.25E-06	0.025	14306	20.506
gen_samplesize_Vascular dementia (other)	genus Christensenellaceae R 7group	rs73952017	C	T	-0.056	1779608	0.797	0.219	360248	C	T	-0.086	18	1779609	8.46E-06	0.019	14306	19.679
gen_samplesize_Vascular dementia (other)	genus Christensenellaceae R 7group	rs78521377	C	T	0.815	124759805	0.031	0.378	360248	C	T	0.125	10	126448374	5.61E-06	0.027	14306	20.689
gen_samplesize_Vascular dementia (other)	genus Christensenellaceae R 7group	rs892686	A	G	-0.262	80428461	0.049	0.133	360248	A	G	0.051	9	83043376	3.97E-06	0.011	14306	21.313
gen_samplesize_Vascular dementia (other)	genus Clostridium innocuum group	rs10506058	A	G	-0.008	30115552	0.954	0.134	360248	A	G	0.100	12	30268485	8.92E-06	0.022	14306	20.184
gen_samplesize_Vascular dementia (other)	genus Clostridium innocuum group	rs1942371	G	A	-0.056	71607072	0.780	0.201	360248	G	A	-0.158	18	69274308	4.06E-06	0.034	14306	21.343

gen_samplesize_Vascular dementia (other)	genus Clostridium innocuum group	rs40656	C	T	0.099	9368046	0.554	0.167	360248	C	T	0.143	5	9368158	8.62E-06	0.031	14306	21.040
gen_samplesize_Vascular dementia (other)	genus Clostridium innocuum group	rs4869133	G	A	0.324	96381915	0.066	0.176	360248	G	A	-0.181	5	95717619	7.24E-06	0.041	14306	19.448
gen_samplesize_Vascular dementia (other)	genus Clostridium innocuum group	rs61267978	T	C	0.118	6348228	0.565	0.205	360248	T	C	0.147	18	6348227	5.59E-06	0.032	14306	21.010
gen_samplesize_Vascular dementia (other)	genus Clostridium innocuum group	rs6577484	G	A	0.028	8359360	0.898	0.216	360248	G	A	0.160	1	8419420	8.41E-06	0.036	14306	19.764
gen_samplesize_Vascular dementia (other)	genus Clostridium innocuum group	rs6890185	C	T	0.076	71890799	0.595	0.143	360248	C	T	-0.113	5	71186626	1.12E-06	0.023	14306	23.669
gen_samplesize_Vascular dementia (other)	genus Clostridium innocuum group	rs77845139	A	G	0.087	59412009	0.570	0.153	360248	A	G	-0.115	15	59704208	8.41E-06	0.026	14306	19.992
gen_samplesize_Vascular dementia (other)	genus Clostridium sensustricto1	rs11264403	G	A	-0.195	155706701	0.441	0.253	360248	G	A	-0.139	1	155676492	7.76E-06	0.033	14306	17.288
gen_samplesize_Vascular dementia (other)	genus Clostridium sensustricto1	rs116847295	C	T	-0.370	43091212	0.061	0.198	360248	C	T	0.110	12	43485015	4.58E-06	0.025	14306	19.997
gen_samplesize_Vascular dementia (other)	genus Clostridium sensustricto1	rs12341505	G	A	-0.172	133845759	0.461	0.233	360248	G	A	0.081	9	136710881	4.82E-06	0.018	14306	20.259
gen_samplesize_Vascular dementia (other)	genus Clostridium sensustricto1	rs2795528	G	A	-0.138	42774816	0.635	0.290	360248	G	A	-0.184	10	43270264	2.72E-06	0.039	14306	22.085
gen_samplesize_Vascular dementia (other)	genus Clostridium sensustricto1	rs2817172	C	T	-0.056	3124955	0.677	0.136	360248	C	T	0.058	1	3041519	2.77E-06	0.012	14306	21.810
gen_samplesize_Vascular dementia (other)	genus Clostridium sensustricto1	rs550843	T	C	-0.087	165309343	0.563	0.150	360248	T	C	-0.078	6	165722832	2.05E-06	0.017	14306	21.426
gen_samplesize_Vascular dementia (other)	genus Collinsella	rs10890671	C	T	-0.095	107252473	0.477	0.134	360248	C	T	0.054	11	107123199	6.52E-06	0.012	14306	20.446
gen_samplesize_Vascular dementia (other)	genus Collinsella	rs11597285	G	T	0.104	8547850	0.442	0.135	360248	G	T	-0.054	10	8589813	9.38E-06	0.012	14306	19.914
gen_samplesize_Vascular dementia (other)	genus Collinsella	rs1496626	T	C	0.254	31269935	0.197	0.197	360248	T	C	-0.072	19	31760841	6.78E-06	0.016	14306	19.978
gen_samplesize_Vascular dementia (other)	genus Collinsella	rs149807560	C	A	-0.037	56015274	0.887	0.263	360248	C	A	-0.104	19	56526640	7.10E-06	0.024	14306	19.545
gen_samplesize_Vascular dementia (other)	genus Collinsella	rs2103510	G	A	0.043	27983148	0.833	0.206	360248	G	A	0.079	21	29355467	2.42E-06	0.017	14306	21.863
gen_samplesize_Vascular dementia (other)	genus Collinsella	rs62448871	C	A	-0.199	24425405	0.134	0.133	360248	C	A	-0.054	7	24465024	6.78E-06	0.012	14306	20.164
gen_samplesize_Vascular dementia (other)	genus Collinsella	rs73052258	G	A	-0.090	194874587	0.721	0.252	360248	G	A	0.093	2	195739311	1.72E-06	0.020	14306	21.067
gen_samplesize_Vascular dementia (other)	genus Collinsella	rs75672793	A	G	-0.261	148897142	0.408	0.316	360248	A	G	-0.109	4	149818294	6.14E-06	0.024	14306	20.503
gen_samplesize_Vascular dementia (other)	genus Collinsella	rs9541268	C	A	0.061	68035929	0.790	0.229	360248	C	A	0.096	13	68610061	8.79E-07	0.020	14306	23.657
gen_samplesize_Vascular dementia (other)	genus Coprobacter	rs11532348	C	T	0.344	97896018	0.068	0.188	360248	C	T	-0.104	12	98289796	5.71E-06	0.023	14306	20.994
gen_samplesize_Vascular dementia (other)	genus Coprobacter	rs12684609	T	C	-0.043	134893022	0.798	0.167	360248	T	C	0.101	9	137784868	6.10E-06	0.022	14306	20.935
gen_samplesize_Vascular dementia (other)	genus Coprobacter	rs12996055	A	C	-0.106	136465888	0.482	0.151	360248	A	C	0.092	2	137223458	8.08E-06	0.021	14306	19.378
gen_samplesize_Vascular dementia (other)	genus Coprobacter	rs143662916	C	T	0.671	103679246	0.088	0.394	360248	C	T	0.253	11	103549974	3.07E-06	0.054	14306	21.985
gen_samplesize_Vascular dementia (other)	genus Coprobacter	rs189356	G	A	0.134	58999796	0.319	0.135	360248	G	A	0.078	20	57574851	6.26E-06	0.017	14306	20.645
gen_samplesize_Vascular dementia (other)	genus Coprobacter	rs213863	T	C	-0.279	96787063	0.044	0.139	360248	T	C	0.089	6	97234939	2.35E-06	0.019	14306	22.192
gen_samplesize_Vascular dementia (other)	genus Coprobacter	rs28402691	T	C	0.166	95468089	0.377	0.188	360248	T	C	0.111	4	96389240	9.56E-06	0.025	14306	19.419
gen_samplesize_Vascular dementia (other)	genus Coprobacter	rs305411	A	G	-0.097	87767445	0.660	0.221	360248	A	G	0.129	1	88233128	1.01E-06	0.026	14306	23.864
gen_samplesize_Vascular dementia (other)	genus Coprobacter	rs3828477	G	T	0.255	46442226	0.070	0.141	360248	G	T	-0.091	3	46483717	2.89E-06	0.020	14306	21.728
gen_samplesize_Vascular dementia (other)	genus Coprobacter	rs72821405	T	C	0.160	4714563	0.502	0.239	360248	T	C	-0.147	6	4714797	4.76E-06	0.032	14306	21.210
gen_samplesize_Vascular dementia (other)	genus Coprobacter	rs74919520	G	A	-0.112	32017383	0.603	0.215	360248	G	A	0.126	2	32242452	5.76E-06	0.028	14306	20.699
gen_samplesize_Vascular dementia (other)	genus Coprococcus1	rs1010560	C	A	0.191	29927454	0.205	0.151	360248	C	A	0.058	1	30400301	1.96E-06	0.012	14306	22.354
gen_samplesize_Vascular dementia (other)	genus Coprococcus1	rs12794898	G	T	-0.051	124684165	0.801	0.201	360248	G	T	0.090	11	124554061	4.92E-06	0.020	14306	20.980
gen_samplesize_Vascular dementia (other)	genus Coprococcus1	rs1519491	T	C	-0.017	21856898	0.898	0.136	360248	T	C	0.050	2	22079770	8.95E-06	0.011	14306	19.325
gen_samplesize_Vascular dementia (other)	genus Coprococcus1	rs1576241	A	G	-0.038	71819574	0.782	0.137	360248	A	G	-0.051	6	72529277	3.33E-06	0.011	14306	21.708
gen_samplesize_Vascular dementia (other)	genus Coprococcus1	rs2907920	G	A	-0.078	2600719	0.597	0.148	360248	G	A	-0.056	19	2600717	7.65E-06	0.013	14306	19.579
gen_samplesize_Vascular dementia (other)	genus Coprococcus1	rs4277593	G	A	0.011	4338773	0.933	0.134	360248	G	A	-0.059	20	4319420	1.14E-07	0.011	14306	28.390
gen_samplesize_Vascular dementia (other)	genus Coprococcus1	rs56405618	A	G	0.114	173547165	0.589	0.212	360248	A	G	-0.090	4	174468316	1.57E-06	0.019	14306	23.095
gen_samplesize_Vascular dementia (other)	genus Coprococcus1	rs73031725	T	C	0.278	134752388	0.468	0.384	360248	T	C	0.168	11	134622282	1.98E-06	0.036	14306	22.259
gen_samplesize_Vascular dementia (other)	genus Coprococcus1	rs73167075	T	C	-0.405	165975355	0.012	0.161	360248	T	C	0.057	3	165693143	8.57E-06	0.013	14306	20.184
gen_samplesize_Vascular dementia (other)	genus Coprococcus1	rs74101919	T	C	0.343	94958765	0.112	0.216	360248	T	C	-0.072	1	95424321	1.03E-06	0.014	14306	24.706
gen_samplesize_Vascular dementia (other)	genus Coprococcus1	rs946513	T	C	-0.011	15871821	0.973	0.325	360248	T	C	-0.206	10	15913820	8.62E-06	0.046	14306	20.034
gen_samplesize_Vascular dementia (other)	genus Coprococcus2	rs10070053	A	G	0.097	34794684	0.473	0.135	360248	A	G	0.059	5	34794789	7.65E-06	0.014	14306	19.273
gen_samplesize_Vascular dementia (other)	genus Coprococcus2	rs12634070	T	C	-0.267	180827092	0.078	0.152	360248	T	C	0.074	3	180544880	9.95E-06	0.016	14306	19.942
gen_samplesize_Vascular dementia (other)	genus Coprococcus2	rs2482516	C	T	-0.044	25554070	0.785	0.160	360248	C	T	0.075	9	25554068	4.72E-06	0.016	14306	21.002
gen_samplesize_Vascular dementia (other)	genus Coprococcus2	rs35890118	A	G	-0.205	127917495	0.177	0.152	360248	A	G	-0.067	10	129715759	8.26E-06	0.015	14306	20.304
gen_samplesize_Vascular dementia (other)	genus Coprococcus2	rs61823518	A	C	-0.374	223514894	0.085	0.217	360248	A	C	-0.096	1	223688236	6.68E-06	0.022	14306	19.614
gen_samplesize_Vascular dementia (other)	genus Coprococcus2	rs6677933	C	T	0.122	111596386	0.516	0.188	360248	C	T	-0.080	1	112139008	1.19E-06	0.016	14306	23.995
gen_samplesize_Vascular dementia (other)	genus Coprococcus2	rs72680320	T	C	0.056	130204631	0.690	0.140	360248	T	C	-0.065	4	131125786	2.27E-06	0.014	14306	21.766
gen_samplesize_Vascular dementia (other)	genus Coprococcus2	rs9426473	A	G	0.051	4150395	0.733	0.151	360248	A	G	0.073	1	4210455	6.31E-06	0.016	14306	20.243
gen_samplesize_Vascular dementia (other)	genus Coprococcus3	rs10810043	G	A	0.129	13799810	0.359	0.141	360248	G	A	-0.052	9	13799809	9.27E-06	0.012	14306	19.776
gen_samplesize_Vascular dementia (other)	genus Coprococcus3	rs11077359	C	T	0.223	78347880	0.214	0.180	360248	C	T	0.065	17	76343961	9.64E-06	0.015	14306	18.807
gen_samplesize_Vascular dementia (other)	genus Coprococcus3	rs11080344	C	T	0.009	27777485	0.947	0.134	360248	C	T	0.052	17	26104511	4.79E-06	0.011	14306	20.906
gen_samplesize_Vascular dementia (other)	genus Coprococcus3	rs13247359	G	A	0.100	76728409	0.456	0.135	360248	G	A	0.051	7	76357726	7.33E-06	0.011	14306	20.527
gen_samplesize_Vascular dementia (other)	genus Coprococcus3	rs178271	C	T	-0.350	20977267	0.506	0.526	360248	C	T	-0.145	22	21331556	7.81E-07	0.029	14306	24.371
gen_samplesize_Vascular dementia (other)	genus Coprococcus3	rs4575475	A	G	-0.070	98675414	0.660	0.158	360248	A	G	-0.062	14	99141751	7.04E-06	0.014	14306	20.216
gen_samplesize_Vascular dementia (other)	genus Coprococcus3	rs7521171	A	G	0.081	150026539	0.581	0.146	360248	A	G	0.060	1	149998497	4.32E-06	0.013	14306	21.285
gen_samplesize_Vascular dementia (other)	genus Coprococcus3	rs8100692	T	C	0.166	39541492	0.214	0.134	360248	T	C	0.058	19	40032132	4.16E-07	0.011	14306	25.892
gen_samplesize_Vascular dementia (other)	genus Defluviitaleaceae UCG011	rs112893842	T	C	-0.023	8786663	0.920	0.230	360248	T	C	0.114	9	8786663	1.45E-06	0.023	14306	23.899
gen_samplesize_Vascular dementia (other)	genus Defluviitaleaceae UCG011	rs1582238	C	T	0.013	118181062	0.925	0.139	360248	C	T	-0.081	1	118723685	1.57E-06	0.017	14306	23.167
gen_samplesize_Vascular dementia (other)	genus Defluviitaleaceae UCG011	rs2892880	G	A	0.172	119820571	0.266	0.155	360248	G	A	0.082	4	120741726	6.83E-06	0.018	14306	20.258
gen_samplesize_Vascular dementia (other)	genus Defluviitaleaceae UCG011	rs4344384	T	G	-0.187	64647609	0.158	0.132	360248	T	G	-0.072	10	66407366	4.83E-06	0.016	14306	20.981
gen_samplesize_Vascular dementia (other)	genus Defluviitaleaceae UCG011	rs4677103	A	G	-0.080	72158643	0.652	0.177	360248	A	G	0.098	3	72207794	9.60E-07	0.020	14306	24.576
gen_samplesize_Vascular dementia (other)	genus Defluviitaleaceae UCG011	rs55658617	T	C	-0.604	40439076	0.099	0.366	360248	T	C	0.174	21	41811003	2.15E-06	0.036	14306	23.173
gen_samplesize_Vascular dementia (other)	genus Defluviitaleaceae UCG011	rs72731813	C	T	0.325	146493591	0.296	0.311	360248	C	T	-0.147	4	147414743	4.33E-07	0.029	14306	25.165
gen_samplesize_Vascular dementia (other)	genus Defluviitaleaceae UCG011	rs9608282	T	G	0.082	24408113	0.828	0.377	360248	T	G	0.143	22	24804081	2.52E-06	0.030	14306	22.733

gen_samplesize_Vascular dementia (other)	genus Defluviitaleaceae UCG011	rs9725395	A	G	0.153	84739949	0.467	0.210	360248	A	G	-0.138	1	85205632	3.52E-06	0.030	14306	21.911
gen_samplesize_Vascular dementia (other)	genus Desulfovibrio	rs12031543	T	C	-0.146	68198632	0.450	0.193	360248	T	C	-0.127	1	68664315	6.55E-06	0.028	14306	20.357
gen_samplesize_Vascular dementia (other)	genus Desulfovibrio	rs13066142	G	A	-0.212	67428549	0.356	0.230	360248	G	A	0.119	3	67478973	3.79E-06	0.025	14306	22.550
gen_samplesize_Vascular dementia (other)	genus Desulfovibrio	rs16863365	A	G	-0.049	197225187	0.877	0.316	360248	A	G	0.109	2	198089911	1.79E-06	0.023	14306	23.235
gen_samplesize_Vascular dementia (other)	genus Desulfovibrio	rs2032031	G	A	0.072	128372006	0.589	0.133	360248	G	A	0.065	10	130170270	9.14E-06	0.015	14306	19.404
gen_samplesize_Vascular dementia (other)	genus Desulfovibrio	rs2590913	A	G	0.068	63690313	0.827	0.312	360248	A	G	-0.154	13	64264446	6.65E-06	0.034	14306	20.721
gen_samplesize_Vascular dementia (other)	genus Desulfovibrio	rs2853179	T	C	0.073	104449819	0.649	0.160	360248	T	C	-0.081	8	105462047	2.42E-06	0.017	14306	21.718
gen_samplesize_Vascular dementia (other)	genus Desulfovibrio	rs4797774	A	G	-0.059	13447996	0.860	0.335	360248	A	G	-0.213	18	13447995	5.64E-06	0.047	14306	20.454
gen_samplesize_Vascular dementia (other)	genus Desulfovibrio	rs6580353	T	C	0.067	140031102	0.688	0.166	360248	T	C	0.077	5	139410687	4.94E-06	0.017	14306	20.632
gen_samplesize_Vascular dementia (other)	genus Desulfovibrio	rs72647089	T	G	0.075	57060357	0.763	0.248	360248	T	G	-0.107	8	57972916	8.30E-06	0.024	14306	19.836
gen_samplesize_Vascular dementia (other)	genus Dialister	rs10138457	T	C	-0.346	101991715	0.125	0.225	360248	T	C	-0.113	14	102458052	7.88E-06	0.026	14306	18.629
gen_samplesize_Vascular dementia (other)	genus Dialister	rs10938938	A	G	0.317	23292736	0.084	0.184	360248	A	G	0.077	4	23294359	7.37E-06	0.017	14306	20.482
gen_samplesize_Vascular dementia (other)	genus Dialister	rs11071887	T	C	0.256	32662894	0.075	0.144	360248	T	C	0.066	15	32955095	5.91E-06	0.015	14306	20.496
gen_samplesize_Vascular dementia (other)	genus Dialister	rs11166701	G	A	0.012	137283482	0.927	0.133	360248	G	A	-0.066	8	138295725	5.51E-07	0.013	14306	24.698
gen_samplesize_Vascular dementia (other)	genus Dialister	rs2314294	T	C	-0.046	9243005	0.813	0.195	360248	T	C	0.087	16	9336862	8.08E-06	0.019	14306	19.981
gen_samplesize_Vascular dementia (other)	genus Dialister	rs2435610	A	C	-0.034	151192947	0.827	0.153	360248	A	C	0.065	7	150890034	5.93E-06	0.014	14306	20.390
gen_samplesize_Vascular dementia (other)	genus Dialister	rs4747450	C	A	-0.053	22910724	0.739	0.160	360248	C	A	0.067	10	23199653	5.84E-06	0.015	14306	20.490
gen_samplesize_Vascular dementia (other)	genus Dialister	rs4753063	A	G	0.032	92533963	0.813	0.134	360248	A	G	0.060	11	92267129	4.86E-06	0.013	14306	21.023
gen_samplesize_Vascular dementia (other)	genus Dialister	rs75416973	A	G	0.289	172840493	0.068	0.159	360248	A	G	0.073	1	172809633	9.46E-06	0.016	14306	19.541
gen_samplesize_Vascular dementia (other)	genus Dialister	rs764177	C	A	0.056	45767957	0.685	0.138	360248	C	A	-0.060	3	45809449	9.61E-06	0.014	14306	19.742
gen_samplesize_Vascular dementia (other)	genus Dialister	rs76680460	G	A	0.282	25447082	0.378	0.321	360248	G	A	-0.161	9	25447080	8.19E-06	0.036	14306	19.600
gen_samplesize_Vascular dementia (other)	genus Dorea	rs11150408	G	T	-0.107	81769892	0.425	0.134	360248	G	T	-0.049	16	81803497	7.06E-06	0.011	14306	20.042
gen_samplesize_Vascular dementia (other)	genus Dorea	rs12537781	T	C	0.067	155186473	0.667	0.155	360248	T	C	-0.056	7	154978183	9.15E-06	0.013	14306	19.675
gen_samplesize_Vascular dementia (other)	genus Dorea	rs13279148	G	A	-0.360	126893620	0.090	0.213	360248	G	A	0.072	8	127905865	2.25E-06	0.015	14306	22.477
gen_samplesize_Vascular dementia (other)	genus Dorea	rs1899291	T	C	-0.115	60126622	0.534	0.185	360248	T	C	-0.070	4	60992340	4.57E-06	0.015	14306	21.523
gen_samplesize_Vascular dementia (other)	genus Dorea	rs3005511	G	A	0.063	73896090	0.661	0.145	360248	G	A	-0.052	6	74605806	5.29E-06	0.011	14306	20.917
gen_samplesize_Vascular dementia (other)	genus Dorea	rs345219	G	T	-0.040	6784833	0.762	0.133	360248	G	T	0.050	3	6826520	8.80E-06	0.011	14306	19.504
gen_samplesize_Vascular dementia (other)	genus Dorea	rs3752849	G	A	-0.489	8707510	0.109	0.306	360248	G	A	0.164	11	8729057	7.68E-06	0.037	14306	20.032
gen_samplesize_Vascular dementia (other)	genus Dorea	rs4793307	C	T	-0.159	72737384	0.310	0.157	360248	C	T	0.057	17	70733523	4.01E-06	0.012	14306	21.979
gen_samplesize_Vascular dementia (other)	genus Dorea	rs62503162	A	G	-0.292	15911148	0.367	0.323	360248	A	G	-0.097	8	15768657	7.47E-07	0.019	14306	25.113
gen_samplesize_Vascular dementia (other)	genus Dorea	rs73729431	C	T	0.679	25077822	0.136	0.456	360248	C	T	-0.137	6	25078050	3.17E-06	0.030	14306	20.994
gen_samplesize_Vascular dementia (other)	genus Eggerthella	rs112205261	T	C	-0.490	9076080	0.045	0.244	360248	T	C	-0.189	1	9136139	3.35E-06	0.040	14306	21.839
gen_samplesize_Vascular dementia (other)	genus Eggerthella	rs13070736	A	C	-0.208	20765962	0.248	0.180	360248	A	C	-0.121	3	20807454	7.62E-06	0.027	14306	19.894
gen_samplesize_Vascular dementia (other)	genus Eggerthella	rs1784446	A	G	0.071	102590868	0.591	0.133	360248	A	G	-0.091	11	102461599	5.23E-06	0.020	14306	20.997
gen_samplesize_Vascular dementia (other)	genus Eggerthella	rs2223081	A	G	-0.102	28405711	0.493	0.148	360248	A	G	-0.103	21	29778032	3.89E-06	0.022	14306	21.520
gen_samplesize_Vascular dementia (other)	genus Eggerthella	rs2240838	G	A	0.159	38296353	0.238	0.134	360248	G	A	-0.098	7	38335954	7.36E-07	0.020	14306	24.613
gen_samplesize_Vascular dementia (other)	genus Eggerthella	rs3851328	G	T	-0.072	45226666	0.654	0.160	360248	G	T	0.108	2	45453805	4.18E-06	0.024	14306	20.762
gen_samplesize_Vascular dementia (other)	genus Eggerthella	rs6430926	T	C	-0.066	140579885	0.620	0.134	360248	T	C	-0.088	2	141337454	8.37E-06	0.020	14306	19.916
gen_samplesize_Vascular dementia (other)	genus Eggerthella	rs67490567	T	C	0.073	65398986	0.635	0.154	360248	T	C	0.108	15	65691324	8.94E-06	0.025	14306	19.537
gen_samplesize_Vascular dementia (other)	genus Eggerthella	rs76663501	C	T	0.086	57072813	0.783	0.312	360248	C	T	0.175	20	55647869	4.83E-06	0.038	14306	21.454
gen_samplesize_Vascular dementia (other)	genus Eisenbergiella	rs11027642	C	T	0.145	23980294	0.448	0.191	360248	C	T	0.129	11	24001840	4.92E-06	0.028	14306	20.512
gen_samplesize_Vascular dementia (other)	genus Eisenbergiella	rs11079158	T	C	-0.030	55290280	0.851	0.160	360248	T	C	0.101	17	53367641	7.35E-06	0.023	14306	19.920
gen_samplesize_Vascular dementia (other)	genus Eisenbergiella	rs11938607	C	T	-0.180	188139277	0.235	0.152	360248	C	T	-0.098	4	189060431	8.22E-06	0.022	14306	20.386
gen_samplesize_Vascular dementia (other)	genus Eisenbergiella	rs12257723	A	C	0.072	107657343	0.614	0.142	360248	A	C	-0.095	10	109417101	8.85E-06	0.021	14306	20.279
gen_samplesize_Vascular dementia (other)	genus Eisenbergiella	rs12710729	C	A	0.064	19773449	0.653	0.142	360248	C	A	0.089	2	19973210	9.84E-06	0.020	14306	20.140
gen_samplesize_Vascular dementia (other)	genus Eisenbergiella	rs13258851	A	G	0.108	54418254	0.569	0.189	360248	A	G	0.137	8	55330814	7.75E-06	0.030	14306	20.563
gen_samplesize_Vascular dementia (other)	genus Eisenbergiella	rs1508033	A	C	0.206	53083004	0.154	0.145	360248	A	C	0.092	15	53375201	3.23E-06	0.020	14306	21.863
gen_samplesize_Vascular dementia (other)	genus Eisenbergiella	rs1553971	T	G	-0.354	111700639	0.027	0.160	360248	T	G	0.121	3	111419486	5.27E-06	0.026	14306	21.146
gen_samplesize_Vascular dementia (other)	genus Eisenbergiella	rs2683098	T	C	0.137	35886364	0.396	0.162	360248	T	C	-0.107	15	36178565	2.24E-06	0.023	14306	22.720
gen_samplesize_Vascular dementia (other)	genus Eisenbergiella	rs3812426	A	G	-0.011	49910116	0.950	0.182	360248	A	G	-0.106	8	50822676	2.72E-06	0.022	14306	22.550
gen_samplesize_Vascular dementia (other)	genus Eisenbergiella	rs4462860	G	A	-0.094	20237941	0.487	0.135	360248	G	A	0.094	21	21610254	4.16E-06	0.020	14306	21.805
gen_samplesize_Vascular dementia (other)	genus Enterorhabdus	rs10098492	T	C	-0.344	112097527	0.224	0.283	360248	T	C	0.132	8	113109756	6.41E-06	0.029	14306	20.300
gen_samplesize_Vascular dementia (other)	genus Enterorhabdus	rs114731706	T	G	-0.346	19412192	0.376	0.391	360248	T	G	0.182	2	19611953	2.17E-06	0.038	14306	22.741
gen_samplesize_Vascular dementia (other)	genus Enterorhabdus	rs2051957	C	T	-0.149	90080569	0.375	0.168	360248	C	T	0.084	7	89709883	8.90E-06	0.019	14306	19.727
gen_samplesize_Vascular dementia (other)	genus Enterorhabdus	rs3017103	G	A	-0.131	62406721	0.438	0.169	360248	G	A	-0.098	11	62174193	2.94E-06	0.021	14306	22.028
gen_samplesize_Vascular dementia (other)	genus Enterorhabdus	rs73331712	T	C	0.102	69121322	0.762	0.336	360248	T	C	0.262	12	69515102	4.85E-06	0.055	14306	22.589
gen_samplesize_Vascular dementia (other)	genus Enterorhabdus	rs77655283	G	A	0.332	235531235	0.200	0.259	360248	G	A	0.133	2	236439879	5.88E-06	0.030	14306	19.875
gen_samplesize_Vascular dementia (other)	genus Erysipelatoclostridium	rs1434153	G	A	-0.147	34370466	0.273	0.134	360248	G	A	-0.068	2	34595533	6.85E-06	0.015	14306	20.163
gen_samplesize_Vascular dementia (other)	genus Erysipelatoclostridium	rs16936671	C	T	-0.005	36105681	0.979	0.193	360248	C	T	-0.097	10	36394609	6.04E-06	0.022	14306	19.739
gen_samplesize_Vascular dementia (other)	genus Erysipelatoclostridium	rs17804233	C	T	-0.003	78519233	0.985	0.132	360248	C	T	0.066	5	77815056	4.59E-06	0.014	14306	21.133
gen_samplesize_Vascular dementia (other)	genus Erysipelatoclostridium	rs2901723	A	C	0.138	36849973	0.303	0.134	360248	A	C	-0.064	11	36871523	8.79E-06	0.014	14306	19.755
gen_samplesize_Vascular dementia (other)	genus Erysipelatoclostridium	rs340991	A	G	0.086	34891937	0.566	0.150	360248	A	G	-0.074	5	34892042	3.75E-06	0.016	14306	21.719
gen_samplesize_Vascular dementia (other)	genus Erysipelatoclostridium	rs3804326	A	G	-0.212	24291957	0.494	0.311	360248	A	G	0.141	6	24292185	9.85E-06	0.034	14306	17.691
gen_samplesize_Vascular dementia (other)	genus Erysipelatoclostridium	rs45480394	T	G	-0.109	55346409	0.432	0.138	360248	T	G	-0.069	19	55857777	7.66E-06	0.015	14306	20.480
gen_samplesize_Vascular dementia (other)	genus Erysipelatoclostridium	rs4697572	A	G	0.024	25445165	0.887	0.167	360248	A	G	-0.081	4	25446787	7.59E-07	0.016	14306	24.640
gen_samplesize_Vascular dementia (other)	genus Erysipelatoclostridium	rs58236560	G	T	-0.069	122006205	0.737	0.206	360248	G	T	-0.111	11	121876913	2.16E-06	0.023	14306	22.483
gen_samplesize_Vascular dementia (other)	genus Erysipelatoclostridium	rs61806970	C	T	-0.026	167849359	0.923	0.266	360248	C	T	0.143	1	167818597	9.09E-06	0.032	14306	19.759

gen_samplesize_Vascular dementia (other)	genus Erysipelatoclostridium	rs622418	A	G	-0.056	87971128	0.676	0.133	360248	A	G	-0.067	9	90586043	3.68E-06	0.014	14306	21.756
gen_samplesize_Vascular dementia (other)	genus Erysipelatoclostridium	rs6474512	C	A	0.027	38923088	0.844	0.137	360248	C	A	-0.067	8	38780606	3.02E-06	0.014	14306	21.900
gen_samplesize_Vascular dementia (other)	genus Erysipelatoclostridium	rs710230	C	T	-0.187	41867960	0.462	0.255	360248	C	T	-0.143	1	42333631	6.33E-07	0.028	14306	25.941
gen_samplesize_Vascular dementia (other)	genus Erysipelatoclostridium	rs7221249	G	A	0.076	10274391	0.567	0.133	360248	G	A	-0.084	17	10177708	4.31E-09	0.014	14306	34.619
gen_samplesize_Vascular dementia (other)	genus Erysipelatoclostridium	rs9590927	A	G	0.108	45797930	0.415	0.133	360248	A	G	0.065	13	46372065	6.39E-06	0.014	14306	20.272
gen_samplesize_Vascular dementia (other)	genus Escherichia Shigella	rs112767262	T	C	0.064	3700622	0.692	0.162	360248	T	C	0.073	16	3750623	8.21E-06	0.016	14306	20.075
gen_samplesize_Vascular dementia (other)	genus Escherichia Shigella	rs113127095	A	G	0.004	22679253	0.989	0.329	360248	A	G	0.151	13	23253392	3.33E-06	0.032	14306	21.799
gen_samplesize_Vascular dementia (other)	genus Escherichia Shigella	rs113513883	A	G	-0.234	140978399	0.538	0.381	360248	A	G	0.172	7	140678199	5.28E-06	0.038	14306	20.511
gen_samplesize_Vascular dementia (other)	genus Escherichia Shigella	rs1154904	G	A	-0.203	134904951	0.124	0.132	360248	G	A	0.061	11	134774845	3.04E-06	0.013	14306	22.043
gen_samplesize_Vascular dementia (other)	genus Escherichia Shigella	rs118526	A	C	-0.141	80567725	0.332	0.145	360248	A	C	0.059	5	79863544	8.00E-06	0.014	14306	19.108
gen_samplesize_Vascular dementia (other)	genus Escherichia Shigella	rs2798105	A	G	0.124	48496231	0.581	0.224	360248	A	G	-0.101	1	48961903	8.24E-06	0.022	14306	20.629
gen_samplesize_Vascular dementia (other)	genus Escherichia Shigella	rs4731451	G	A	0.340	128414046	0.017	0.142	360248	G	A	-0.061	7	128054100	7.47E-06	0.014	14306	20.362
gen_samplesize_Vascular dementia (other)	genus Escherichia Shigella	rs57024273	T	C	-0.302	235606511	0.047	0.152	360248	T	C	0.063	2	236515155	9.70E-06	0.014	14306	20.028
gen_samplesize_Vascular dementia (other)	genus Escherichia Shigella	rs592299	T	C	0.023	133303865	0.864	0.134	360248	T	C	-0.059	9	136179347	4.77E-06	0.013	14306	20.903
gen_samplesize_Vascular dementia (other)	genus Escherichia Shigella	rs73208162	A	G	0.227	37952181	0.548	0.378	360248	A	G	-0.119	21	39324484	2.19E-06	0.025	14306	23.067
gen_samplesize_Vascular dementia (other)	genus Eubacterium brachy group	rs112617308	T	C	0.175	92425456	0.449	0.231	360248	T	C	-0.171	10	94185213	2.38E-06	0.036	14306	22.177
gen_samplesize_Vascular dementia (other)	genus Eubacterium brachy group	rs12151423	G	A	0.072	217372558	0.587	0.132	360248	G	A	-0.101	2	218237281	9.27E-06	0.023	14306	19.869
gen_samplesize_Vascular dementia (other)	genus Eubacterium brachy group	rs13139592	T	C	0.120	143744025	0.539	0.196	360248	T	C	-0.146	4	144665178	7.97E-06	0.033	14306	19.911
gen_samplesize_Vascular dementia (other)	genus Eubacterium brachy group	rs1384962	G	A	-0.023	22591645	0.875	0.144	360248	G	A	-0.121	14	23060552	6.99E-06	0.027	14306	20.613
gen_samplesize_Vascular dementia (other)	genus Eubacterium brachy group	rs2913110	T	C	-0.067	22603738	0.633	0.140	360248	T	C	-0.105	10	22892667	4.56E-06	0.023	14306	21.004
gen_samplesize_Vascular dementia (other)	genus Eubacterium brachy group	rs4862235	A	G	0.187	183707778	0.163	0.134	360248	A	G	-0.105	4	184628931	3.73E-06	0.023	14306	21.553
gen_samplesize_Vascular dementia (other)	genus Eubacterium brachy group	rs62348779	T	C	0.000	17459987	0.999	0.252	360248	T	C	-0.201	5	17460096	3.78E-06	0.043	14306	21.666
gen_samplesize_Vascular dementia (other)	genus Eubacterium brachy group	rs6591893	A	G	0.132	80525789	0.347	0.141	360248	A	G	-0.108	11	80236833	7.34E-06	0.024	14306	20.281
gen_samplesize_Vascular dementia (other)	genus Eubacterium brachy group	rs720439	G	A	-0.016	47794849	0.919	0.155	360248	G	A	0.112	22	48190598	7.03E-06	0.025	14306	19.845
gen_samplesize_Vascular dementia (other)	genus Eubacterium brachy group	rs73199919	T	C	-0.171	6854837	0.578	0.307	360248	T	C	-0.237	4	6856564	8.16E-06	0.053	14306	19.848
gen_samplesize_Vascular dementia (other)	genus Eubacterium coprostanoligenes group	rs1020520	T	G	0.252	33563534	0.179	0.188	360248	T	G	-0.059	7	33603146	8.89E-06	0.013	14306	19.747
gen_samplesize_Vascular dementia (other)	genus Eubacterium coprostanoligenes group	rs10444197	A	G	0.230	2173737	0.096	0.138	360248	A	G	-0.051	10	2215931	5.98E-06	0.011	14306	19.877
gen_samplesize_Vascular dementia (other)	genus Eubacterium coprostanoligenes group	rs11052069	C	T	0.308	32560985	0.021	0.133	360248	C	T	-0.048	12	32713919	9.38E-06	0.011	14306	19.636
gen_samplesize_Vascular dementia (other)	genus Eubacterium coprostanoligenes group	rs11720857	C	T	-0.149	114075660	0.392	0.174	360248	C	T	0.063	3	113794507	9.26E-06	0.014	14306	19.059
gen_samplesize_Vascular dementia (other)	genus Eubacterium coprostanoligenes group	rs12906958	C	T	0.049	36619397	0.737	0.145	360248	C	T	-0.053	15	36911598	4.35E-06	0.012	14306	21.149
gen_samplesize_Vascular dementia (other)	genus Eubacterium coprostanoligenes group	rs17159861	C	T	0.092	31045547	0.668	0.216	360248	C	T	0.096	7	31085162	1.04E-08	0.017	14306	32.654
gen_samplesize_Vascular dementia (other)	genus Eubacterium coprostanoligenes group	rs2644213	A	G	0.172	82746340	0.232	0.144	360248	A	G	-0.054	10	84506096	9.86E-06	0.012	14306	19.751
gen_samplesize_Vascular dementia (other)	genus Eubacterium coprostanoligenes group	rs4076415	G	T	-0.330	85897766	0.008	0.124	360248	G	T	-0.052	15	86440997	1.99E-06	0.011	14306	21.816
gen_samplesize_Vascular dementia (other)	genus Eubacterium coprostanoligenes group	rs62024432	C	T	0.018	97326063	0.936	0.226	360248	C	T	-0.077	15	97869293	7.50E-06	0.017	14306	20.002
gen_samplesize_Vascular dementia (other)	genus Eubacterium coprostanoligenes group	rs6762473	A	C	0.034	127420731	0.808	0.140	360248	A	C	-0.052	3	127139574	4.26E-06	0.011	14306	21.552
gen_samplesize_Vascular dementia (other)	genus Eubacterium coprostanoligenes group	rs76898927	G	A	-0.392	81546326	0.178	0.291	360248	G	A	0.123	3	81595477	4.79E-06	0.027	14306	21.341
gen_samplesize_Vascular dementia (other)	genus Eubacterium coprostanoligenes group	rs9648214	T	C	0.017	16341181	0.943	0.237	360248	T	C	-0.083	7	16380806	2.52E-07	0.016	14306	25.438
gen_samplesize_Vascular dementia (other)	genus Eubacterium eligens group	rs182318	G	A	0.219	31077144	0.371	0.244	360248	G	A	-0.082	11	31098691	8.40E-06	0.020	14306	17.784
gen_samplesize_Vascular dementia (other)	genus Eubacterium eligens group	rs2200429	A	G	0.129	106911110	0.563	0.223	360248	A	G	-0.089	13	107563458	5.30E-06	0.020	14306	20.054
gen_samplesize_Vascular dementia (other)	genus Eubacterium eligens group	rs265534	T	G	0.123	81332251	0.359	0.134	360248	T	G	-0.056	10	83092007	2.27E-06	0.012	14306	22.024
gen_samplesize_Vascular dementia (other)	genus Eubacterium eligens group	rs4583233	A	C	0.166	81786881	0.255	0.146	360248	A	C	0.067	16	81820486	2.84E-07	0.013	14306	27.363
gen_samplesize_Vascular dementia (other)	genus Eubacterium eligens group	rs56080211	T	C	-0.086	109503416	0.728	0.246	360248	T	C	-0.123	2	110260993	9.14E-06	0.028	14306	18.953
gen_samplesize_Vascular dementia (other)	genus Eubacterium eligens group	rs6923695	T	G	-0.221	70247112	0.407	0.267	360248	T	G	0.103	6	70956815	4.87E-06	0.023	14306	20.243
gen_samplesize_Vascular dementia (other)	genus Eubacterium fissicatena group	rs10147907	T	G	0.064	89018029	0.805	0.259	360248	T	G	0.172	14	89484373	8.27E-06	0.040	14306	18.922
gen_samplesize_Vascular dementia (other)	genus Eubacterium fissicatena group	rs11818408	G	A	0.088	94998710	0.519	0.136	360248	G	A	0.106	10	96758467	8.20E-06	0.024	14306	19.928
gen_samplesize_Vascular dementia (other)	genus Eubacterium fissicatena group	rs11876297	T	C	0.133	48226901	0.372	0.150	360248	T	C	0.131	18	45753272	2.67E-06	0.028	14306	21.779
gen_samplesize_Vascular dementia (other)	genus Eubacterium fissicatena group	rs151257695	A	G	-0.087	73629231	0.737	0.257	360248	A	G	0.210	7	73043561	3.10E-06	0.045	14306	21.217
gen_samplesize_Vascular dementia (other)	genus Eubacterium fissicatena group	rs1768152	C	T	0.142	39563463	0.515	0.219	360248	C	T	-0.139	3	39604954	8.70E-06	0.032	14306	19.462
gen_samplesize_Vascular dementia (other)	genus Eubacterium fissicatena group	rs2733072	G	A	0.093	5576177	0.484	0.132	360248	G	A	0.110	8	5433699	1.49E-06	0.023	14306	23.064
gen_samplesize_Vascular dementia (other)	genus Eubacterium fissicatena group	rs3771393	T	C	-0.031	70918116	0.853	0.166	360248	T	C	-0.131	2	71145246	7.38E-07	0.027	14306	24.074
gen_samplesize_Vascular dementia (other)	genus Eubacterium fissicatena group	rs6934739	A	G	-0.161	39972698	0.252	0.141	360248	A	G	0.111	6	39940437	9.75E-06	0.025	14306	19.442
gen_samplesize_Vascular dementia (other)	genus Eubacterium fissicatena group	rs7104872	G	A	-0.065	115294391	0.760	0.211	360248	G	A	0.139	11	115165111	2.73E-06	0.029	14306	22.548
gen_samplesize_Vascular dementia (other)	genus Eubacterium hallii group	rs10501370	C	T	-0.284	58273149	0.306	0.278	360248	C	T	-0.116	11	58040621	5.42E-06	0.025	14306	20.958
gen_samplesize_Vascular dementia (other)	genus Eubacterium hallii group	rs10798999	C	T	0.128	33843316	0.395	0.150	360248	C	T	0.060	1	34308917	2.61E-06	0.013	14306	22.532
gen_samplesize_Vascular dementia (other)	genus Eubacterium hallii group	rs117748144	T	C	-0.467	11750090	0.132	0.310	360248	T	C	-0.127	11	11771637	7.86E-06	0.029	14306	19.436
gen_samplesize_Vascular dementia (other)	genus Eubacterium hallii group	rs13116360	T	C	-0.058	110964275	0.828	0.267	360248	T	C	0.154	4	111885431	2.94E-07	0.030	14306	26.896
gen_samplesize_Vascular dementia (other)	genus Eubacterium hallii group	rs17074066	T	C	0.990	182788383	0.040	0.481	360248	T	C	-0.081	4	183709536	9.35E-06	0.019	14306	18.495
gen_samplesize_Vascular dementia (other)	genus Eubacterium hallii group	rs17474256	G	A	-0.142	103982054	0.519	0.221	360248	G	A	0.081	1	104524676	9.45E-06	0.018	14306	19.297
gen_samplesize_Vascular dementia (other)	genus Eubacterium hallii group	rs281379	A	G	0.120	48711017	0.372	0.135	360248	A	G	-0.050	19	49214274	9.33E-06	0.011	14306	19.838
gen_samplesize_Vascular dementia (other)	genus Eubacterium hallii group	rs28584818	A	G	0.347	64678780	0.148	0.240	360248	A	G	0.126	3	64664456	4.43E-06	0.027	14306	22.041
gen_samplesize_Vascular dementia (other)	genus Eubacterium hallii group	rs60254196	G	A	-0.016	149159628	0.906	0.134	360248	G	A	0.052	7	148856720	2.70E-06	0.011	14306	21.844
gen_samplesize_Vascular dementia (other)	genus Eubacterium hallii group	rs630939	C	T	0.129	50858093	0.340	0.135	360248	C	T	-0.051	18	48384463	9.16E-06	0.011	14306	19.806
gen_samplesize_Vascular dementia (other)	genus Eubacterium hallii group	rs6550770	C	T	-0.486	23621925	0.147	0.335	360248	C	T	0.198	3	23663416	4.82E-06	0.044	14306	19.945
gen_samplesize_Vascular dementia (other)	genus Eubacterium hallii group	rs74018587	C	T	-0.265	61721961	0.426	0.333	360248	C	T	0.209	15	62014160	3.70E-06	0.044	14306	22.734
gen_samplesize_Vascular dementia (other)	genus Eubacterium hallii group	rs78056098	G	T	0.044	123919170	0.751	0.138	360248	G	T	-0.051	11	123789877	8.29E-06	0.011	14306	19.896
gen_samplesize_Vascular dementia (other)	genus Eubacterium hallii group	rs949971	T	G	0.276	110564542	0.051	0.141	360248	T	G	-0.054	3	110283389	3.29E-06	0.012	14306	21.646
gen_samplesize_Vascular dementia (other)	genus Eubacterium nodatum group	rs10263623	C	T	-0.133	65429224	0.698	0.343	360248	C	T	0.193	7	64894137	8.91E-06	0.044	14306	19.425

gen_samplesize_Vascular dementia (other)	genus Eubacterium nodatum group	rs10458299	T	C	0.058	135367596	0.821	0.254	360248	T	C	-0.188	7	135052348	8.37E-06	0.042	14306	20.013
gen_samplesize_Vascular dementia (other)	genus Eubacterium nodatum group	rs11006576	A	G	-0.189	59562257	0.158	0.134	360248	A	G	-0.110	10	61322015	7.99E-06	0.025	14306	20.067
gen_samplesize_Vascular dementia (other)	genus Eubacterium nodatum group	rs113893692	C	T	0.165	28245520	0.421	0.206	360248	C	T	-0.185	9	28245518	5.76E-06	0.040	14306	21.038
gen_samplesize_Vascular dementia (other)	genus Eubacterium nodatum group	rs34297067	A	G	0.020	51719170	0.913	0.186	360248	A	G	-0.187	14	52185888	6.60E-08	0.034	14306	29.959
gen_samplesize_Vascular dementia (other)	genus Eubacterium nodatum group	rs61841040	G	T	-0.080	9617786	0.634	0.167	360248	G	T	0.161	10	9659749	3.56E-06	0.034	14306	22.108
gen_samplesize_Vascular dementia (other)	genus Eubacterium nodatum group	rs6818880	G	A	0.000	93791596	1.000	0.134	360248	G	A	0.110	4	94712747	7.83E-06	0.025	14306	20.030
gen_samplesize_Vascular dementia (other)	genus Eubacterium nodatum group	rs77910827	C	T	0.406	93835703	0.062	0.217	360248	C	T	0.202	9	96597985	9.05E-07	0.041	14306	23.807
gen_samplesize_Vascular dementia (other)	genus Eubacterium nodatum group	rs7827125	C	T	0.107	5097390	0.467	0.148	360248	C	T	0.122	8	4954912	7.17E-06	0.027	14306	20.317
gen_samplesize_Vascular dementia (other)	genus Eubacterium nodatum group	rs7880204	T	C	-0.053	171091680	0.730	0.154	360248	T	C	-0.125	1	171060821	6.84E-06	0.028	14306	20.766
gen_samplesize_Vascular dementia (other)	genus Eubacterium nodatum group	rs9425984	T	C	-0.009	34064135	0.954	0.160	360248	T	C	-0.130	1	34529736	7.21E-06	0.029	14306	19.845
gen_samplesize_Vascular dementia (other)	genus Eubacterium oxidoreducens group	rs12129908	A	C	0.203	195094889	0.133	0.135	360248	A	C	-0.089	1	195064019	5.80E-06	0.020	14306	20.285
gen_samplesize_Vascular dementia (other)	genus Eubacterium oxidoreducens group	rs12423772	G	T	-0.121	94121564	0.517	0.188	360248	G	T	0.141	12	94515340	2.63E-06	0.030	14306	22.834
gen_samplesize_Vascular dementia (other)	genus Eubacterium oxidoreducens group	rs2973294	G	T	0.238	37525057	0.076	0.134	360248	G	T	0.092	4	37526679	2.39E-06	0.020	14306	22.331
gen_samplesize_Vascular dementia (other)	genus Eubacterium oxidoreducens group	rs34561138	G	A	-0.258	86446749	0.448	0.340	360248	G	A	0.216	16	86480355	2.51E-06	0.046	14306	22.086
gen_samplesize_Vascular dementia (other)	genus Eubacterium oxidoreducens group	rs440215	T	C	-0.057	107546693	0.674	0.134	360248	T	C	-0.093	5	106882394	1.65E-06	0.020	14306	22.812
gen_samplesize_Vascular dementia (other)	genus Eubacterium rectale group	rs10248854	C	A	0.027	121641184	0.845	0.135	360248	C	A	-0.053	7	121281238	4.21E-06	0.011	14306	21.640
gen_samplesize_Vascular dementia (other)	genus Eubacterium rectale group	rs10797540	A	G	-0.112	234394624	0.406	0.135	360248	A	G	0.050	1	234530370	3.53E-06	0.011	14306	21.551
gen_samplesize_Vascular dementia (other)	genus Eubacterium rectale group	rs143694765	T	C	0.378	115540288	0.089	0.222	360248	T	C	0.087	1	116082909	9.75E-06	0.020	14306	19.349
gen_samplesize_Vascular dementia (other)	genus Eubacterium rectale group	rs2884897	A	G	-0.344	8107990	0.352	0.370	360248	A	G	-0.129	11	8129537	6.44E-06	0.029	14306	20.034
gen_samplesize_Vascular dementia (other)	genus Eubacterium rectale group	rs314726	T	C	-0.163	124795578	0.221	0.133	360248	T	C	0.053	2	125553155	1.38E-06	0.011	14306	23.329
gen_samplesize_Vascular dementia (other)	genus Eubacterium rectale group	rs35398954	A	G	-0.185	92463535	0.310	0.182	360248	A	G	-0.090	15	93006765	5.40E-07	0.017	14306	26.653
gen_samplesize_Vascular dementia (other)	genus Eubacterium rectale group	rs59427698	A	G	-0.185	126663466	0.271	0.168	360248	A	G	-0.058	9	129425745	5.37E-06	0.013	14306	19.379
gen_samplesize_Vascular dementia (other)	genus Eubacterium rectale group	rs62547233	A	G	-0.002	89398504	0.988	0.146	360248	A	G	0.054	9	92013419	9.90E-06	0.012	14306	19.908
gen_samplesize_Vascular dementia (other)	genus Eubacterium ruminantium group	rs10131724	C	A	-0.031	51752348	0.891	0.230	360248	C	A	0.200	14	52219066	2.39E-06	0.041	14306	23.234
gen_samplesize_Vascular dementia (other)	genus Eubacterium ruminantium group	rs10923018	G	A	-0.080	88057716	0.551	0.134	360248	G	A	0.073	1	88523399	6.80E-06	0.016	14306	20.378
gen_samplesize_Vascular dementia (other)	genus Eubacterium ruminantium group	rs11637981	T	G	-0.043	60997423	0.747	0.133	360248	T	G	0.073	15	61289622	5.44E-06	0.016	14306	20.733
gen_samplesize_Vascular dementia (other)	genus Eubacterium ruminantium group	rs13025464	C	T	-0.115	200745922	0.394	0.135	360248	C	T	0.074	2	201610645	6.97E-06	0.016	14306	20.252
gen_samplesize_Vascular dementia (other)	genus Eubacterium ruminantium group	rs139749	C	T	0.168	24907088	0.232	0.141	360248	C	T	-0.085	22	25303055	8.59E-07	0.017	14306	24.219
gen_samplesize_Vascular dementia (other)	genus Eubacterium ruminantium group	rs16891896	G	A	-0.357	33897484	0.137	0.240	360248	G	A	-0.175	5	33897589	2.38E-06	0.039	14306	20.027
gen_samplesize_Vascular dementia (other)	genus Eubacterium ruminantium group	rs17519472	C	T	0.225	30208021	0.243	0.193	360248	C	T	0.108	12	30360954	4.70E-06	0.023	14306	21.227
gen_samplesize_Vascular dementia (other)	genus Eubacterium ruminantium group	rs209813	G	A	0.340	11953400	0.073	0.190	360248	G	A	-0.103	6	11953633	9.23E-06	0.024	14306	19.165
gen_samplesize_Vascular dementia (other)	genus Eubacterium ruminantium group	rs2116427	A	G	-0.015	121847301	0.922	0.152	360248	A	G	0.091	5	121182996	4.67E-07	0.018	14306	24.983
gen_samplesize_Vascular dementia (other)	genus Eubacterium ruminantium group	rs2229917	A	G	-0.170	128218658	0.609	0.333	360248	A	G	0.154	9	130980937	2.16E-06	0.032	14306	22.467
gen_samplesize_Vascular dementia (other)	genus Eubacterium ruminantium group	rs2418654	C	T	0.157	71020448	0.245	0.135	360248	C	T	-0.075	2	71247578	6.17E-06	0.017	14306	20.388
gen_samplesize_Vascular dementia (other)	genus Eubacterium ruminantium group	rs2817174	C	T	-0.073	3127617	0.593	0.136	360248	C	T	-0.073	1	3044181	7.87E-06	0.016	14306	20.125
gen_samplesize_Vascular dementia (other)	genus Eubacterium ruminantium group	rs57340348	T	C	-0.131	129708969	0.429	0.166	360248	T	C	-0.098	6	130030114	4.93E-06	0.021	14306	21.311
gen_samplesize_Vascular dementia (other)	genus Eubacterium ruminantium group	rs606117	G	A	0.093	2539358	0.532	0.148	360248	G	A	-0.083	9	2539358	4.82E-06	0.018	14306	21.296
gen_samplesize_Vascular dementia (other)	genus Eubacterium ruminantium group	rs6676699	T	G	0.030	198009151	0.836	0.146	360248	T	G	0.089	1	197978281	6.38E-06	0.020	14306	20.439
gen_samplesize_Vascular dementia (other)	genus Eubacterium ruminantium group	rs7000472	G	A	-0.174	137614025	0.196	0.135	360248	G	A	0.076	8	138626268	4.07E-06	0.017	14306	21.284
gen_samplesize_Vascular dementia (other)	genus Eubacterium ruminantium group	rs72836424	C	T	-0.157	127239189	0.461	0.213	360248	C	T	-0.140	10	129037453	2.62E-06	0.030	14306	21.624
gen_samplesize_Vascular dementia (other)	genus Eubacterium ruminantium group	rs73139629	A	C	0.125	62912223	0.580	0.225	360248	A	C	-0.115	12	63306003	5.36E-06	0.025	14306	21.555
gen_samplesize_Vascular dementia (other)	genus Eubacterium ventriosum group	rs11617697	A	G	0.015	98133646	0.959	0.294	360248	A	G	-0.143	13	98785900	7.22E-07	0.029	14306	25.080
gen_samplesize_Vascular dementia (other)	genus Eubacterium ventriosum group	rs12964517	G	A	-0.180	24880821	0.227	0.149	360248	G	A	0.059	18	22460785	2.08E-06	0.012	14306	22.624
gen_samplesize_Vascular dementia (other)	genus Eubacterium ventriosum group	rs13082419	C	T	-0.363	108017587	0.085	0.211	360248	C	T	-0.072	3	107736434	9.56E-06	0.016	14306	19.661
gen_samplesize_Vascular dementia (other)	genus Eubacterium ventriosum group	rs16884680	G	T	-0.093	113432826	0.673	0.221	360248	G	T	-0.091	8	114445055	1.74E-06	0.019	14306	22.309
gen_samplesize_Vascular dementia (other)	genus Eubacterium ventriosum group	rs35179274	C	T	-0.086	51493907	0.618	0.173	360248	C	T	-0.063	14	51960625	5.76E-06	0.014	14306	20.703
gen_samplesize_Vascular dementia (other)	genus Eubacterium ventriosum group	rs3809430	T	C	-0.151	44506307	0.293	0.144	360248	T	C	-0.055	14	44975510	3.55E-06	0.012	14306	21.426
gen_samplesize_Vascular dementia (other)	genus Eubacterium ventriosum group	rs57199565	T	C	-0.055	190505943	0.752	0.173	360248	T	C	0.078	3	190223732	7.97E-07	0.016	14306	23.896
gen_samplesize_Vascular dementia (other)	genus Eubacterium ventriosum group	rs66746423	C	T	-0.244	108855642	0.186	0.184	360248	C	T	0.075	1	109398264	6.11E-06	0.016	14306	20.749
gen_samplesize_Vascular dementia (other)	genus Eubacterium ventriosum group	rs6704822	G	A	-0.210	202797056	0.287	0.197	360248	G	A	-0.074	2	203661779	6.62E-06	0.017	14306	19.661
gen_samplesize_Vascular dementia (other)	genus Eubacterium ventriosum group	rs72783037	C	A	0.016	60314477	0.921	0.163	360248	C	A	0.066	16	60348381	6.55E-06	0.014	14306	21.012
gen_samplesize_Vascular dementia (other)	genus Eubacterium ventriosum group	rs73615400	T	C	0.129	51524380	0.570	0.228	360248	T	C	-0.096	20	50140919	9.54E-07	0.019	14306	24.443
gen_samplesize_Vascular dementia (other)	genus Eubacterium ventriosum group	rs73849225	T	C	0.218	125450659	0.351	0.233	360248	T	C	0.098	4	126371814	5.21E-06	0.022	14306	18.908
gen_samplesize_Vascular dementia (other)	genus Eubacterium ventriosum group	rs78250280	G	A	0.345	147819813	0.073	0.192	360248	G	A	0.075	1	147291928	3.36E-06	0.016	14306	20.798
gen_samplesize_Vascular dementia (other)	genus Eubacterium ventriosum group	rs876734	T	C	0.076	52030212	0.614	0.151	360248	T	C	0.062	12	52423996	2.89E-06	0.013	14306	21.793
gen_samplesize_Vascular dementia (other)	genus Eubacterium ventriosum group	rs9316536	T	G	0.410	51364440	0.033	0.192	360248	T	G	-0.082	13	51938576	7.84E-06	0.018	14306	19.911
gen_samplesize_Vascular dementia (other)	genus Eubacterium xylanophilum group	rs10140184	A	C	-0.233	72715075	0.084	0.134	360248	A	C	0.058	14	73181783	4.96E-06	0.013	14306	20.959
gen_samplesize_Vascular dementia (other)	genus Eubacterium xylanophilum group	rs10917203	A	C	-0.106	22300773	0.436	0.135	360248	A	C	0.061	1	22627266	3.15E-06	0.013	14306	21.898
gen_samplesize_Vascular dementia (other)	genus Eubacterium xylanophilum group	rs112176119	C	T	0.071	73662872	0.751	0.225	360248	C	T	-0.113	16	73696771	3.33E-06	0.025	14306	21.312
gen_samplesize_Vascular dementia (other)	genus Eubacterium xylanophilum group	rs13239072	G	A	-0.291	43526745	0.051	0.149	360248	G	A	0.069	7	43566344	1.82E-06	0.014	14306	23.197
gen_samplesize_Vascular dementia (other)	genus Eubacterium xylanophilum group	rs17830032	G	A	-0.152	59420451	0.538	0.247	360248	G	A	-0.161	20	57995506	2.39E-07	0.031	14306	26.749
gen_samplesize_Vascular dementia (other)	genus Eubacterium xylanophilum group	rs1999224	G	T	0.053	127735841	0.815	0.225	360248	G	T	-0.095	9	130498120	3.75E-06	0.020	14306	21.749
gen_samplesize_Vascular dementia (other)	genus Eubacterium xylanophilum group	rs2012708	G	A	-0.127	84756953	0.363	0.139	360248	G	A	-0.057	16	84790559	6.53E-06	0.013	14306	20.401
gen_samplesize_Vascular dementia (other)	genus Eubacterium xylanophilum group	rs2213117	T	G	0.051	131139765	0.780	0.182	360248	T	G	0.088	11	131009660	4.21E-06	0.019	14306	21.568
gen_samplesize_Vascular dementia (other)	genus Eubacterium xylanophilum group	rs75586835	A	G	-0.141	86970786	0.580	0.255	360248	A	G	-0.114	4	87891938	9.39E-06	0.026	14306	18.888
gen_samplesize_Vascular dementia (other)	genus Faecalibacterium	rs10927394	G	T	0.228	245104037	0.647	0.497	360248	G	T	-0.232	1	245267339	7.02E-06	0.051	14306	20.549
gen_samplesize_Vascular dementia (other)	genus Faecalibacterium	rs114946999	C	T	0.196	36898092	0.327	0.200	360248	C	T	-0.086	2	37125235	5.70E-06	0.019	14306	20.649

gen_samplesize_Vascular dementia (other)	genus Faecalibacterium	rs11776390	T	C	0.387	38736127	0.154	0.272	360248	T	C	-0.078	8	38593645	6.40E-06	0.017	14306	20.793
gen_samplesize_Vascular dementia (other)	genus Faecalibacterium	rs1271565	C	T	-0.100	63577379	0.507	0.151	360248	C	T	-0.058	14	64044097	1.30E-06	0.012	14306	23.196
gen_samplesize_Vascular dementia (other)	genus Faecalibacterium	rs12753492	A	C	-0.226	13875051	0.286	0.212	360248	A	C	0.064	1	14201546	8.80E-06	0.015	14306	18.295
gen_samplesize_Vascular dementia (other)	genus Faecalibacterium	rs2835874	T	C	0.084	37655599	0.813	0.355	360248	T	C	-0.087	21	39027901	7.54E-06	0.020	14306	19.440
gen_samplesize_Vascular dementia (other)	genus Faecalibacterium	rs6910935	G	A	-0.342	131266017	0.212	0.274	360248	G	A	-0.135	6	131587157	1.38E-06	0.028	14306	23.699
gen_samplesize_Vascular dementia (other)	genus Faecalibacterium	rs75499067	C	T	-0.390	6049862	0.123	0.253	360248	C	T	0.228	16	6099863	1.76E-06	0.047	14306	23.900
gen_samplesize_Vascular dementia (other)	genus Faecalibacterium	rs79656633	T	C	0.053	110494205	0.812	0.225	360248	T	C	0.146	1	111036827	8.14E-06	0.032	14306	20.326
gen_samplesize_Vascular dementia (other)	genus Faecalibacterium	rs9536330	T	C	-0.131	52992882	0.323	0.133	360248	T	C	-0.048	13	53567017	5.33E-06	0.011	14306	20.026
gen_samplesize_Vascular dementia (other)	genus Family XIII AD3011 group	rs11126423	T	C	0.222	73968424	0.339	0.232	360248	T	C	-0.090	2	74195551	5.91E-06	0.020	14306	21.226
gen_samplesize_Vascular dementia (other)	genus Family XIII AD3011 group	rs11736617	G	A	0.502	38916209	0.081	0.288	360248	G	A	-0.076	4	38917830	9.02E-06	0.017	14306	19.475
gen_samplesize_Vascular dementia (other)	genus Family XIII AD3011 group	rs12812672	T	C	0.040	17974663	0.875	0.256	360248	T	C	-0.096	12	18127597	2.56E-06	0.021	14306	21.273
gen_samplesize_Vascular dementia (other)	genus Family XIII AD3011 group	rs149302	T	C	-0.088	14159816	0.570	0.155	360248	T	C	-0.065	5	14159925	7.48E-06	0.014	14306	20.321
gen_samplesize_Vascular dementia (other)	genus Family XIII AD3011 group	rs16840310	G	A	-0.035	240530863	0.794	0.135	360248	G	A	0.061	1	240694163	6.75E-07	0.012	14306	24.780
gen_samplesize_Vascular dementia (other)	genus Family XIII AD3011 group	rs16940167	C	T	0.158	58391498	0.347	0.168	360248	C	T	0.073	15	58683697	3.91E-06	0.016	14306	20.989
gen_samplesize_Vascular dementia (other)	genus Family XIII AD3011 group	rs17156849	G	A	-0.434	28564367	0.126	0.283	360248	G	A	-0.113	7	28603985	4.19E-06	0.025	14306	21.181
gen_samplesize_Vascular dementia (other)	genus Family XIII AD3011 group	rs62029761	A	G	-0.122	15912385	0.681	0.297	360248	A	G	0.129	16	16006242	3.89E-06	0.028	14306	21.762
gen_samplesize_Vascular dementia (other)	genus Family XIII AD3011 group	rs62200412	C	T	0.213	4637993	0.163	0.152	360248	C	T	-0.080	20	4618639	5.80E-07	0.016	14306	23.894
gen_samplesize_Vascular dementia (other)	genus Family XIII AD3011 group	rs72730932	C	A	-0.177	192050705	0.440	0.230	360248	C	A	-0.090	1	192019835	6.89E-07	0.018	14306	25.798
gen_samplesize_Vascular dementia (other)	genus Family XIII AD3011 group	rs739451	C	T	-0.170	133813832	0.298	0.163	360248	C	T	0.065	9	136678954	7.88E-06	0.015	14306	19.386
gen_samplesize_Vascular dementia (other)	genus Family XIII AD3011 group	rs9276029	A	G	-0.124	32727559	0.458	0.167	360248	A	G	-0.081	6	32695336	8.93E-06	0.019	14306	19.097
gen_samplesize_Vascular dementia (other)	genus Family XIII AD3011 group	rs9837139	A	G	0.077	29770374	0.749	0.240	360248	A	G	0.108	3	29811865	8.71E-06	0.024	14306	19.990
gen_samplesize_Vascular dementia (other)	genus Family XIII UCG001	rs112362903	A	G	-0.500	35434008	0.164	0.359	360248	A	G	-0.149	17	33761027	7.88E-06	0.033	14306	20.001
gen_samplesize_Vascular dementia (other)	genus Family XIII UCG001	rs12049454	T	C	-0.165	84762642	0.233	0.138	360248	T	C	-0.065	1	85228325	1.17E-06	0.013	14306	23.312
gen_samplesize_Vascular dementia (other)	genus Family XIII UCG001	rs1426266	C	T	-0.245	188506308	0.100	0.149	360248	C	T	0.067	3	188224096	1.25E-06	0.014	14306	23.546
gen_samplesize_Vascular dementia (other)	genus Family XIII UCG001	rs3842897	G	A	-0.546	183534529	0.021	0.236	360248	G	A	-0.113	1	183503664	5.20E-06	0.024	14306	21.531
gen_samplesize_Vascular dementia (other)	genus Family XIII UCG001	rs62414802	C	T	-0.313	75888918	0.039	0.152	360248	C	T	-0.061	6	76598635	4.29E-06	0.013	14306	20.676
gen_samplesize_Vascular dementia (other)	genus Family XIII UCG001	rs7119679	G	A	0.224	94948621	0.155	0.157	360248	G	A	-0.081	11	94681786	3.52E-06	0.017	14306	21.429
gen_samplesize_Vascular dementia (other)	genus Family XIII UCG001	rs76463770	A	G	-0.241	45437598	0.514	0.369	360248	A	G	0.193	3	45479090	3.77E-06	0.042	14306	21.158
gen_samplesize_Vascular dementia (other)	genus Family XIII UCG001	rs8076666	G	A	0.143	80250018	0.484	0.204	360248	G	A	-0.089	17	78223817	8.02E-06	0.020	14306	20.029
gen_samplesize_Vascular dementia (other)	genus Flavonifractor	rs114873521	C	T	-0.197	168791398	0.443	0.257	360248	C	T	-0.130	5	168218403	7.13E-06	0.029	14306	19.561
gen_samplesize_Vascular dementia (other)	genus Flavonifractor	rs11811696	T	C	-0.399	237188054	0.100	0.243	360248	T	C	-0.116	1	237351354	2.07E-06	0.024	14306	23.191
gen_samplesize_Vascular dementia (other)	genus Flavonifractor	rs12030302	G	A	-0.242	77422150	0.069	0.133	360248	G	A	0.069	1	77887835	5.61E-07	0.014	14306	25.361
gen_samplesize_Vascular dementia (other)	genus Flavonifractor	rs34066017	A	G	0.008	44828138	0.960	0.163	360248	A	G	0.076	11	44849689	1.52E-06	0.016	14306	22.878
gen_samplesize_Vascular dementia (other)	genus Flavonifractor	rs806808	C	T	-0.047	32092258	0.726	0.134	360248	C	T	-0.067	10	32381186	1.18E-06	0.014	14306	23.877
gen_samplesize_Vascular dementia (other)	genus Fusicatenibacter	rs10439674	A	G	0.075	41309465	0.653	0.167	360248	A	G	-0.057	21	42681392	7.68E-06	0.013	14306	19.367
gen_samplesize_Vascular dementia (other)	genus Fusicatenibacter	rs167879	T	C	-0.231	57362310	0.219	0.188	360248	T	C	0.066	20	55937366	5.87E-06	0.015	14306	19.656
gen_samplesize_Vascular dementia (other)	genus Fusicatenibacter	rs1864685	A	C	-0.032	72725643	0.812	0.135	360248	A	C	-0.049	17	70721782	4.96E-06	0.011	14306	20.949
gen_samplesize_Vascular dementia (other)	genus Fusicatenibacter	rs2025938	G	A	-0.212	110417361	0.423	0.265	360248	G	A	-0.097	10	112177119	2.99E-06	0.021	14306	22.172
gen_samplesize_Vascular dementia (other)	genus Fusicatenibacter	rs206581	A	G	-0.066	10370599	0.679	0.161	360248	A	G	-0.057	18	10370596	8.96E-06	0.013	14306	19.748
gen_samplesize_Vascular dementia (other)	genus Fusicatenibacter	rs2132128	G	A	-0.020	15503209	0.925	0.219	360248	G	A	-0.077	8	15360718	1.08E-06	0.016	14306	23.176
gen_samplesize_Vascular dementia (other)	genus Fusicatenibacter	rs3303	T	C	0.144	118687917	0.597	0.273	360248	T	C	-0.095	10	120447429	3.94E-06	0.020	14306	21.839
gen_samplesize_Vascular dementia (other)	genus Fusicatenibacter	rs4378146	A	C	0.338	24601134	0.011	0.133	360248	A	C	-0.062	1	24927625	7.20E-07	0.013	14306	24.239
gen_samplesize_Vascular dementia (other)	genus Fusicatenibacter	rs60254196	G	A	-0.016	149159628	0.906	0.134	360248	G	A	0.049	7	148856720	5.47E-06	0.011	14306	20.273
gen_samplesize_Vascular dementia (other)	genus Fusicatenibacter	rs62187631	T	C	0.401	225784854	0.017	0.168	360248	T	C	-0.071	2	226649570	4.55E-06	0.016	14306	19.912
gen_samplesize_Vascular dementia (other)	genus Fusicatenibacter	rs62353480	A	G	0.072	29594893	0.685	0.177	360248	A	G	-0.070	5	29595000	1.57E-06	0.015	14306	23.210
gen_samplesize_Vascular dementia (other)	genus Fusicatenibacter	rs704418	C	T	0.080	64267127	0.690	0.200	360248	C	T	-0.074	3	64252803	7.77E-07	0.015	14306	23.937
gen_samplesize_Vascular dementia (other)	genus Fusicatenibacter	rs73103914	A	G	0.379	58264528	0.017	0.159	360248	A	G	-0.060	12	58658311	8.30E-06	0.013	14306	19.735
gen_samplesize_Vascular dementia (other)	genus Fusicatenibacter	rs792108	C	T	0.039	5392660	0.770	0.135	360248	C	T	0.051	2	5532793	8.50E-06	0.011	14306	19.939
gen_samplesize_Vascular dementia (other)	genus Fusicatenibacter	rs8028026	G	A	0.290	87809984	0.202	0.227	360248	G	A	0.079	15	88353215	8.06E-06	0.018	14306	19.259
gen_samplesize_Vascular dementia (other)	genus Fusicatenibacter	rs8063430	T	C	-0.053	73787906	0.862	0.307	360248	T	C	-0.104	16	73821805	4.93E-06	0.022	14306	21.924
gen_samplesize_Vascular dementia (other)	genus Fusicatenibacter	rs9905659	G	A	-0.052	72701205	0.762	0.173	360248	G	A	-0.062	17	70697344	7.31E-06	0.014	14306	20.354
gen_samplesize_Vascular dementia (other)	genus Gordonibacter	rs13412653	A	C	0.095	29935628	0.490	0.137	360248	A	C	0.108	2	30158494	8.61E-06	0.024	14306	20.229
gen_samplesize_Vascular dementia (other)	genus Gordonibacter	rs16955299	G	A	0.110	54879849	0.626	0.226	360248	G	A	-0.196	17	52957210	6.37E-06	0.043	14306	20.527
gen_samplesize_Vascular dementia (other)	genus Gordonibacter	rs322296	G	A	-0.389	137252442	0.125	0.254	360248	G	A	0.179	7	136937189	4.02E-06	0.038	14306	22.439
gen_samplesize_Vascular dementia (other)	genus Gordonibacter	rs35042269	C	A	-0.132	8140880	0.529	0.210	360248	C	A	-0.180	4	8142607	8.11E-06	0.040	14306	19.985
gen_samplesize_Vascular dementia (other)	genus Gordonibacter	rs3765837	T	G	0.618	210455105	0.014	0.252	360248	T	G	-0.191	1	210628449	7.17E-06	0.043	14306	19.348
gen_samplesize_Vascular dementia (other)	genus Gordonibacter	rs4596722	G	A	-0.211	86172229	0.112	0.133	360248	G	A	-0.103	9	88787144	9.06E-06	0.023	14306	19.748
gen_samplesize_Vascular dementia (other)	genus Gordonibacter	rs71545975	A	G	0.091	47104907	0.605	0.176	360248	A	G	-0.154	7	47144505	7.04E-06	0.034	14306	20.639
gen_samplesize_Vascular dementia (other)	genus Gordonibacter	rs72714787	C	A	0.233	135847346	0.241	0.199	360248	C	A	0.181	4	136768501	1.43E-06	0.038	14306	23.141
gen_samplesize_Vascular dementia (other)	genus Gordonibacter	rs72939513	A	G	0.042	82670413	0.890	0.303	360248	A	G	-0.214	1	83136096	7.98E-06	0.049	14306	19.026
gen_samplesize_Vascular dementia (other)	genus Gordonibacter	rs7294633	T	C	-0.242	28871601	0.106	0.150	360248	T	C	-0.129	12	29024534	3.44E-07	0.025	14306	26.501
gen_samplesize_Vascular dementia (other)	genus Gordonibacter	rs768830	A	G	0.127	18930984	0.488	0.183	360248	A	G	-0.150	7	18970607	7.76E-06	0.033	14306	20.212
gen_samplesize_Vascular dementia (other)	genus Haemophilus	rs10781340	A	G	-0.139	76137254	0.492	0.202	360248	A	G	-0.095	9	78752170	4.32E-06	0.020	14306	21.803
gen_samplesize_Vascular dementia (other)	genus Haemophilus	rs111582866	G	A	0.153	48708578	0.516	0.236	360248	G	A	-0.124	16	48742489	1.27E-06	0.026	14306	22.815
gen_samplesize_Vascular dementia (other)	genus Haemophilus	rs35509	G	A	0.081	115055532	0.811	0.338	360248	G	A	0.128	12	115493337	2.01E-06	0.027	14306	22.768
gen_samplesize_Vascular dementia (other)	genus Haemophilus	rs4822728	T	C	-0.228	26495842	0.086	0.133	360248	T	C	0.071	22	26891808	3.48E-06	0.015	14306	21.740
gen_samplesize_Vascular dementia (other)	genus Haemophilus	rs76022354	C	T	-0.496	92546628	0.105	0.306	360248	C	T	0.245	10	94306385	1.83E-06	0.051	14306	23.421

gen_samplesize_Vascular dementia (other)	genus Haemophilus	rs78909003	T	C	-0.158	102887960	0.586	0.290	360248	T	C	-0.246	9	105650242	1.67E-06	0.050	14306	23.881
gen_samplesize_Vascular dementia (other)	genus Haemophilus	rs9328464	T	C	-0.131	8350684	0.328	0.134	360248	T	C	0.072	6	8350917	1.42E-06	0.015	14306	23.615
gen_samplesize_Vascular dementia (other)	genus Haemophilus	rs9382510	C	T	-0.173	55583693	0.255	0.152	360248	C	T	-0.094	6	55448491	7.12E-08	0.017	14306	29.342
gen_samplesize_Vascular dementia (other)	genus Haemophilus	rs9895850	T	C	-0.175	66538895	0.585	0.320	360248	T	C	-0.193	17	64535013	2.14E-06	0.042	14306	21.437
gen_samplesize_Vascular dementia (other)	genus Holdemanella	rs12513188	G	A	0.032	70135074	0.828	0.149	360248	G	A	0.090	4	71000791	4.65E-06	0.020	14306	21.426
gen_samplesize_Vascular dementia (other)	genus Holdemanella	rs17586763	T	C	-0.308	50357927	0.305	0.300	360248	T	C	-0.227	13	50932063	7.72E-06	0.051	14306	19.850
gen_samplesize_Vascular dementia (other)	genus Holdemanella	rs1926302	G	A	-0.011	64687657	0.948	0.161	360248	G	A	-0.108	1	65153340	7.50E-06	0.023	14306	21.768
gen_samplesize_Vascular dementia (other)	genus Holdemanella	rs34187114	C	A	0.102	137611390	0.625	0.209	360248	C	A	-0.105	8	138623633	5.13E-06	0.023	14306	21.381
gen_samplesize_Vascular dementia (other)	genus Holdemanella	rs35228298	G	A	-0.037	85343732	0.840	0.182	360248	G	A	0.093	3	85392882	7.30E-06	0.020	14306	21.231
gen_samplesize_Vascular dementia (other)	genus Holdemanella	rs4541991	T	C	0.016	102242553	0.908	0.142	360248	T	C	-0.093	9	105004835	2.10E-06	0.019	14306	22.747
gen_samplesize_Vascular dementia (other)	genus Holdemanella	rs607782	C	T	-0.016	4148375	0.906	0.137	360248	C	T	0.085	6	4148609	7.19E-07	0.017	14306	24.518
gen_samplesize_Vascular dementia (other)	genus Holdemanella	rs62113381	T	C	-0.216	7603339	0.268	0.195	360248	T	C	-0.105	19	7668225	5.54E-06	0.023	14306	20.653
gen_samplesize_Vascular dementia (other)	genus Holdemanella	rs73011279	T	C	0.011	14909283	0.945	0.158	360248	T	C	-0.096	19	15020095	1.36E-06	0.020	14306	23.274
gen_samplesize_Vascular dementia (other)	genus Holdemanella	rs75764681	T	C	-0.080	4452438	0.800	0.318	360248	T	C	-0.283	10	4494630	1.94E-06	0.060	14306	22.338
gen_samplesize_Vascular dementia (other)	genus Holdemanella	rs8113760	G	A	0.288	43347783	0.044	0.143	360248	G	A	0.079	19	43851935	4.62E-06	0.017	14306	20.756
gen_samplesize_Vascular dementia (other)	genus Holdemania	rs10885477	T	C	0.219	113572654	0.468	0.302	360248	T	C	-0.135	10	115332413	8.60E-06	0.030	14306	20.037
gen_samplesize_Vascular dementia (other)	genus Holdemania	rs11080063	G	A	-0.095	28462253	0.486	0.136	360248	G	A	-0.067	17	26789271	6.67E-06	0.015	14306	19.711
gen_samplesize_Vascular dementia (other)	genus Holdemania	rs111745969	A	G	0.446	92566992	0.022	0.194	360248	A	G	0.121	15	93110222	3.71E-06	0.027	14306	20.616
gen_samplesize_Vascular dementia (other)	genus Holdemania	rs113593397	A	G	-0.157	122961600	0.483	0.224	360248	A	G	-0.129	8	123973840	9.36E-06	0.028	14306	20.827
gen_samplesize_Vascular dementia (other)	genus Holdemania	rs116500994	G	T	0.687	81111408	0.029	0.314	360248	G	T	-0.138	13	81685543	2.34E-06	0.029	14306	21.981
gen_samplesize_Vascular dementia (other)	genus Holdemania	rs12701617	A	G	-0.046	38253256	0.729	0.133	360248	A	G	-0.066	7	38292857	9.52E-06	0.015	14306	19.545
gen_samplesize_Vascular dementia (other)	genus Holdemania	rs1867876	T	C	-0.095	18746521	0.511	0.145	360248	T	C	0.084	11	18768068	2.74E-07	0.016	14306	27.009
gen_samplesize_Vascular dementia (other)	genus Holdemania	rs4146507	C	T	-0.173	106873392	0.266	0.155	360248	C	T	0.079	5	106209093	7.23E-06	0.018	14306	20.167
gen_samplesize_Vascular dementia (other)	genus Holdemania	rs73139538	G	A	0.199	63862767	0.616	0.395	360248	G	A	-0.149	7	63323145	7.77E-06	0.033	14306	20.590
gen_samplesize_Vascular dementia (other)	genus Holdemania	rs77293403	A	G	0.419	78092582	0.231	0.350	360248	A	G	0.165	5	77388406	1.77E-06	0.034	14306	23.182
gen_samplesize_Vascular dementia (other)	genus Holdemania	rs80149660	C	T	0.304	128110018	0.335	0.316	360248	C	T	-0.233	10	129908282	6.04E-06	0.052	14306	20.138
gen_samplesize_Vascular dementia (other)	genus Holdemania	rs9500080	C	T	0.015	105289447	0.930	0.175	360248	C	T	0.093	6	105737322	4.09E-07	0.018	14306	26.840
gen_samplesize_Vascular dementia (other)	genus Holdemania	rs9529719	C	T	-0.114	70282384	0.419	0.141	360248	C	T	-0.074	13	70856516	5.97E-06	0.016	14306	21.281
gen_samplesize_Vascular dementia (other)	genus Holdemania	rs967319	T	C	-0.147	60629947	0.346	0.156	360248	T	C	0.079	3	60615680	8.38E-06	0.018	14306	19.910
gen_samplesize_Vascular dementia (other)	genus Howardella	rs10048062	C	T	0.030	97933690	0.898	0.234	360248	C	T	-0.147	15	98476920	8.59E-06	0.034	14306	19.172
gen_samplesize_Vascular dementia (other)	genus Howardella	rs12452946	A	G	-0.009	17349974	0.946	0.134	360248	A	G	-0.106	17	17253288	3.80E-06	0.023	14306	21.370
gen_samplesize_Vascular dementia (other)	genus Howardella	rs17167098	G	A	0.222	133469602	0.250	0.193	360248	G	A	-0.169	7	133154356	1.12E-06	0.035	14306	23.142
gen_samplesize_Vascular dementia (other)	genus Howardella	rs2154047	A	C	-0.287	94986460	0.214	0.231	360248	A	C	0.193	14	95452797	9.97E-06	0.042	14306	21.010
gen_samplesize_Vascular dementia (other)	genus Howardella	rs36081916	T	C	-0.003	93898102	0.988	0.236	360248	T	C	-0.181	7	93527414	4.70E-06	0.040	14306	20.225
gen_samplesize_Vascular dementia (other)	genus Howardella	rs3791893	A	G	0.215	217954673	0.256	0.189	360248	A	G	0.147	2	218819396	9.50E-06	0.034	14306	18.677
gen_samplesize_Vascular dementia (other)	genus Howardella	rs609430	T	G	-0.157	168257164	0.261	0.139	360248	T	G	-0.112	4	169178315	3.34E-06	0.024	14306	21.918
gen_samplesize_Vascular dementia (other)	genus Howardella	rs672217	G	A	-0.346	62457901	0.039	0.168	360248	G	A	0.164	18	60125134	3.52E-06	0.035	14306	21.996
gen_samplesize_Vascular dementia (other)	genus Hungatella	rs10044993	A	C	-0.026	59128613	0.915	0.241	360248	A	C	-0.140	5	58424440	8.07E-06	0.032	14306	19.409
gen_samplesize_Vascular dementia (other)	genus Hungatella	rs13128780	T	C	-0.102	165137846	0.550	0.171	360248	T	C	-0.150	4	166058998	1.75E-06	0.031	14306	22.915
gen_samplesize_Vascular dementia (other)	genus Hungatella	rs13249325	T	G	-0.104	14996016	0.430	0.132	360248	T	G	-0.100	8	14853525	9.69E-06	0.023	14306	19.608
gen_samplesize_Vascular dementia (other)	genus Hungatella	rs17092615	G	A	-0.103	95507282	0.593	0.193	360248	G	A	0.152	14	95973619	7.38E-06	0.034	14306	20.302
gen_samplesize_Vascular dementia (other)	genus Hungatella	rs72759041	G	T	0.140	89034145	0.392	0.163	360248	G	T	-0.126	15	89577376	3.86E-06	0.028	14306	19.937
gen_samplesize_Vascular dementia (other)	genus Intestinibacter	rs10805326	A	G	-0.055	14322999	0.709	0.147	360248	A	G	-0.078	4	14324623	3.55E-08	0.014	14306	30.803
gen_samplesize_Vascular dementia (other)	genus Intestinibacter	rs11109097	T	C	0.005	97534659	0.971	0.135	360248	T	C	-0.062	12	97928437	5.49E-06	0.014	14306	20.305
gen_samplesize_Vascular dementia (other)	genus Intestinibacter	rs118030283	G	A	-0.444	5943075	0.160	0.316	360248	G	A	-0.152	16	5993076	2.67E-06	0.032	14306	21.896
gen_samplesize_Vascular dementia (other)	genus Intestinibacter	rs16938435	T	C	-0.124	21502924	0.577	0.222	360248	T	C	-0.112	9	21502923	1.80E-06	0.024	14306	22.706
gen_samplesize_Vascular dementia (other)	genus Intestinibacter	rs2098844	T	C	0.163	127833265	0.233	0.137	360248	T	C	0.058	11	127703160	6.79E-06	0.013	14306	20.070
gen_samplesize_Vascular dementia (other)	genus Intestinibacter	rs2702387	G	A	-0.210	178440108	0.122	0.136	360248	G	A	-0.061	4	179361262	4.26E-06	0.013	14306	21.208
gen_samplesize_Vascular dementia (other)	genus Intestinibacter	rs4327025	G	A	-0.095	91903453	0.576	0.170	360248	G	A	-0.081	15	92446683	1.64E-07	0.015	14306	27.546
gen_samplesize_Vascular dementia (other)	genus Intestinibacter	rs447950	A	G	0.142	149466863	0.300	0.137	360248	A	G	0.063	5	148846426	5.64E-06	0.014	14306	21.143
gen_samplesize_Vascular dementia (other)	genus Intestinibacter	rs478972	C	T	0.158	125793289	0.491	0.230	360248	C	T	0.143	11	125663184	1.82E-06	0.030	14306	23.061
gen_samplesize_Vascular dementia (other)	genus Intestinibacter	rs6062862	A	G	0.240	62693871	0.319	0.241	360248	A	G	0.092	20	61325223	6.68E-06	0.020	14306	20.406
gen_samplesize_Vascular dementia (other)	genus Intestinibacter	rs62430350	T	C	0.634	170609106	0.068	0.348	360248	T	C	0.151	6	170918194	6.84E-06	0.035	14306	18.481
gen_samplesize_Vascular dementia (other)	genus Intestinibacter	rs68093214	C	T	-0.270	70608634	0.083	0.156	360248	C	T	0.066	3	70657785	9.26E-06	0.015	14306	19.525
gen_samplesize_Vascular dementia (other)	genus Intestinibacter	rs6875660	C	T	-0.051	160259249	0.855	0.279	360248	C	T	0.089	5	159686256	3.06E-06	0.019	14306	21.089
gen_samplesize_Vascular dementia (other)	genus Intestinibacter	rs893394	G	A	-0.098	19855210	0.470	0.135	360248	G	A	0.058	2	20054971	7.85E-06	0.013	14306	19.910
gen_samplesize_Vascular dementia (other)	genus Intestinibacter	rs9348442	C	T	-0.285	10303712	0.153	0.200	360248	C	T	0.099	6	10303945	6.26E-06	0.022	14306	19.987
gen_samplesize_Vascular dementia (other)	genus Intestinimonas	rs10262702	T	C	-0.021	67425856	0.915	0.200	360248	T	C	0.092	7	66890843	2.06E-06	0.019	14306	22.189
gen_samplesize_Vascular dementia (other)	genus Intestinimonas	rs11258178	A	G	-0.128	13082513	0.337	0.134	360248	A	G	0.066	10	13124513	6.98E-07	0.013	14306	24.264
gen_samplesize_Vascular dementia (other)	genus Intestinimonas	rs12226153	A	G	1.128	94631127	0.002	0.366	360248	A	G	-0.151	11	94364293	5.12E-07	0.031	14306	24.250
gen_samplesize_Vascular dementia (other)	genus Intestinimonas	rs17067892	C	T	-0.167	3906018	0.478	0.236	360248	C	T	0.107	8	3763540	6.38E-06	0.025	14306	18.383
gen_samplesize_Vascular dementia (other)	genus Intestinimonas	rs1859797	G	A	-0.299	21967685	0.025	0.133	360248	G	A	0.060	7	22007303	4.12E-06	0.013	14306	20.981
gen_samplesize_Vascular dementia (other)	genus Intestinimonas	rs2276760	A	G	0.103	150903169	0.502	0.153	360248	A	G	-0.069	3	150620956	7.84E-06	0.015	14306	20.178
gen_samplesize_Vascular dementia (other)	genus Intestinimonas	rs2731794	C	T	-0.067	17209282	0.854	0.362	360248	C	T	0.121	5	17209391	1.92E-06	0.026	14306	21.942
gen_samplesize_Vascular dementia (other)	genus Intestinimonas	rs2930225	T	G	0.038	85294296	0.812	0.158	360248	T	G	-0.073	16	85327902	1.35E-06	0.015	14306	22.751
gen_samplesize_Vascular dementia (other)	genus Intestinimonas	rs4113676	A	C	-1.003	6238275	0.092	0.595	360248	A	C	-0.219	20	6218922	7.42E-06	0.049	14306	19.873
gen_samplesize_Vascular dementia (other)	genus Intestinimonas	rs4784055	T	C	-0.407	58784866	0.177	0.302	360248	T	C	-0.175	16	58818770	8.72E-07	0.039	14306	20.631

gen_samplesize_Vascular dementia (other)	genus Intestinimonas	rs62240188	G	A	-0.019	10569458	0.935	0.234	360248	G	A	0.130	3	10611142	2.20E-06	0.027	14306	23.702
gen_samplesize_Vascular dementia (other)	genus Intestinimonas	rs6934519	C	T	0.222	66490909	0.143	0.152	360248	C	T	0.069	6	67200802	8.57E-06	0.015	14306	20.982
gen_samplesize_Vascular dementia (other)	genus Intestinimonas	rs716604	A	G	-0.064	6373956	0.685	0.157	360248	A	G	0.082	2	6514088	8.57E-07	0.017	14306	24.289
gen_samplesize_Vascular dementia (other)	genus Intestinimonas	rs7170984	T	C	-0.102	93276506	0.490	0.148	360248	T	C	-0.066	15	93819735	2.98E-06	0.014	14306	21.858
gen_samplesize_Vascular dementia (other)	genus Intestinimonas	rs72982915	C	T	-0.006	140140519	0.983	0.286	360248	C	T	0.183	2	140898088	4.91E-06	0.040	14306	20.682
gen_samplesize_Vascular dementia (other)	genus Intestinimonas	rs9823439	C	T	0.083	17146010	0.533	0.134	360248	C	T	0.058	3	17187502	9.86E-06	0.013	14306	19.598
gen_samplesize_Vascular dementia (other)	genus Lachnoclostridium	rs1031599	T	G	-0.095	66675401	0.726	0.272	360248	T	G	0.079	3	66725825	6.31E-06	0.018	14306	20.039
gen_samplesize_Vascular dementia (other)	genus Lachnoclostridium	rs12566975	T	C	0.087	185122219	0.512	0.133	360248	T	C	-0.047	1	185091351	9.57E-06	0.011	14306	19.580
gen_samplesize_Vascular dementia (other)	genus Lachnoclostridium	rs1528479	A	G	0.123	166387541	0.372	0.137	360248	A	G	0.050	2	167244051	9.64E-06	0.011	14306	19.783
gen_samplesize_Vascular dementia (other)	genus Lachnoclostridium	rs1997204	C	T	0.142	101652817	0.657	0.319	360248	C	T	0.108	12	102046595	5.97E-06	0.024	14306	19.941
gen_samplesize_Vascular dementia (other)	genus Lachnoclostridium	rs2385421	A	G	0.097	22163494	0.636	0.205	360248	A	G	0.075	18	19743455	7.14E-06	0.018	14306	17.046
gen_samplesize_Vascular dementia (other)	genus Lachnoclostridium	rs3821998	C	A	0.017	38692945	0.938	0.218	360248	C	A	-0.086	4	38694566	6.72E-06	0.019	14306	20.144
gen_samplesize_Vascular dementia (other)	genus Lachnoclostridium	rs4738679	A	G	0.052	58457761	0.701	0.136	360248	A	G	0.052	8	59370320	4.42E-06	0.011	14306	20.813
gen_samplesize_Vascular dementia (other)	genus Lachnoclostridium	rs6112314	A	C	0.034	19320202	0.810	0.142	360248	A	C	-0.056	20	19300846	2.43E-07	0.011	14306	26.964
gen_samplesize_Vascular dementia (other)	genus Lachnoclostridium	rs615997	T	C	0.177	22996295	0.182	0.132	360248	T	C	0.051	3	23037786	2.03E-06	0.011	14306	23.094
gen_samplesize_Vascular dementia (other)	genus Lachnoclostridium	rs62285313	A	G	-0.256	177752244	0.255	0.224	360248	A	G	0.086	3	177470032	1.58E-06	0.018	14306	22.655
gen_samplesize_Vascular dementia (other)	genus Lachnoclostridium	rs72829893	G	T	-0.291	48617179	0.184	0.219	360248	G	T	0.117	17	46694541	5.58E-06	0.027	14306	19.198
gen_samplesize_Vascular dementia (other)	genus Lachnoclostridium	rs78068103	A	G	0.055	13912842	0.789	0.207	360248	A	G	0.089	17	13816159	3.67E-06	0.019	14306	20.814
gen_samplesize_Vascular dementia (other)	genus Lachnoclostridium	rs789029	C	T	0.013	1053251	0.945	0.189	360248	C	T	-0.064	18	1053252	3.75E-06	0.014	14306	21.603
gen_samplesize_Vascular dementia (other)	genus Lachnospiraceae FCS020 group	rs10093861	G	A	0.100	120232167	0.459	0.135	360248	G	A	-0.057	8	121244406	3.06E-06	0.012	14306	22.048
gen_samplesize_Vascular dementia (other)	genus Lachnospiraceae FCS020 group	rs1254846	A	G	0.027	43850646	0.884	0.188	360248	A	G	-0.106	10	44346094	5.60E-06	0.023	14306	20.771
gen_samplesize_Vascular dementia (other)	genus Lachnospiraceae FCS020 group	rs1363769	C	T	-0.251	17754496	0.507	0.378	360248	C	T	0.201	19	17865305	1.58E-06	0.045	14306	19.933
gen_samplesize_Vascular dementia (other)	genus Lachnospiraceae FCS020 group	rs2322265	C	T	-0.152	165666933	0.314	0.151	360248	C	T	-0.067	4	166588085	5.21E-06	0.014	14306	22.149
gen_samplesize_Vascular dementia (other)	genus Lachnospiraceae FCS020 group	rs2862811	C	T	0.081	166278627	0.577	0.144	360248	C	T	-0.056	3	165996415	3.92E-06	0.012	14306	21.535
gen_samplesize_Vascular dementia (other)	genus Lachnospiraceae FCS020 group	rs35035870	T	C	-0.144	3227646	0.670	0.337	360248	T	C	-0.191	11	3248876	2.62E-06	0.041	14306	21.158
gen_samplesize_Vascular dementia (other)	genus Lachnospiraceae FCS020 group	rs3999074	G	T	0.039	63803887	0.770	0.134	360248	G	T	-0.055	10	65563647	6.55E-06	0.012	14306	20.418
gen_samplesize_Vascular dementia (other)	genus Lachnospiraceae FCS020 group	rs4452603	T	G	0.330	71256565	0.027	0.150	360248	T	G	0.060	6	71966268	8.98E-06	0.014	14306	19.748
gen_samplesize_Vascular dementia (other)	genus Lachnospiraceae FCS020 group	rs7249113	G	A	0.076	2571234	0.600	0.145	360248	G	A	0.068	19	2571232	3.72E-07	0.013	14306	25.907
gen_samplesize_Vascular dementia (other)	genus Lachnospiraceae FCS020 group	rs72793667	A	G	0.201	53719294	0.556	0.341	360248	A	G	-0.117	2	53946431	1.63E-06	0.025	14306	22.467
gen_samplesize_Vascular dementia (other)	genus Lachnospiraceae FCS020 group	rs9308097	G	A	0.099	164865326	0.454	0.133	360248	G	A	-0.055	4	165786478	7.47E-06	0.012	14306	20.057
gen_samplesize_Vascular dementia (other)	genus Lachnospiraceae FCS020 group	rs9788306	C	T	-0.108	39845177	0.462	0.147	360248	C	T	-0.063	13	40419314	1.39E-06	0.013	14306	23.074
gen_samplesize_Vascular dementia (other)	genus Lachnospiraceae NC2004 group	rs117467633	T	C	-0.417	21254094	0.226	0.344	360248	T	C	-0.170	17	21157406	9.13E-06	0.038	14306	19.612
gen_samplesize_Vascular dementia (other)	genus Lachnospiraceae NC2004 group	rs12127733	G	A	-0.205	234021975	0.236	0.173	360248	G	A	0.115	1	234157721	3.11E-06	0.025	14306	21.922
gen_samplesize_Vascular dementia (other)	genus Lachnospiraceae NC2004 group	rs12208226	C	A	0.312	22725679	0.155	0.220	360248	C	A	-0.155	6	22725908	9.75E-06	0.034	14306	20.668
gen_samplesize_Vascular dementia (other)	genus Lachnospiraceae NC2004 group	rs12863463	G	A	-0.086	46938056	0.732	0.250	360248	G	A	-0.156	13	47512191	6.04E-06	0.035	14306	20.498
gen_samplesize_Vascular dementia (other)	genus Lachnospiraceae NC2004 group	rs17067076	G	A	-0.508	60561853	0.014	0.206	360248	G	A	-0.155	18	58229086	5.61E-06	0.035	14306	19.277
gen_samplesize_Vascular dementia (other)	genus Lachnospiraceae NC2004 group	rs1928659	T	C	0.114	29334139	0.495	0.166	360248	T	C	0.103	9	29334137	6.17E-06	0.023	14306	20.498
gen_samplesize_Vascular dementia (other)	genus Lachnospiraceae NC2004 group	rs1929743	T	C	-0.018	76255426	0.896	0.141	360248	T	C	0.084	13	76829562	9.06E-06	0.019	14306	19.351
gen_samplesize_Vascular dementia (other)	genus Lachnospiraceae NC2004 group	rs3756315	A	G	-0.030	150165159	0.833	0.145	360248	A	G	-0.088	5	149544722	3.33E-06	0.019	14306	21.990
gen_samplesize_Vascular dementia (other)	genus Lachnospiraceae NC2004 group	rs6116753	G	A	0.114	5350054	0.514	0.174	360248	G	A	0.099	20	5330700	2.92E-06	0.021	14306	22.623
gen_samplesize_Vascular dementia (other)	genus Lachnospiraceae ND3007 group	rs2861203	G	A	-0.082	173232763	0.573	0.145	360248	G	A	0.057	3	172950553	7.37E-06	0.013	14306	20.218
gen_samplesize_Vascular dementia (other)	genus Lachnospiraceae ND3007 group	rs72776675	T	C	0.111	14095664	0.535	0.179	360248	T	C	-0.065	10	14137663	8.72E-06	0.015	14306	19.123
gen_samplesize_Vascular dementia (other)	genus Lachnospiraceae ND3007 group	rs9932954	A	G	-0.177	1050633	0.205	0.140	360248	A	G	-0.056	16	1100633	1.25E-06	0.012	14306	23.467
gen_samplesize_Vascular dementia (other)	genus Lachnospiraceae NK4A136 group	rs10952110	G	T	0.178	8458292	0.183	0.133	360248	G	T	0.049	7	8497922	9.08E-06	0.011	14306	19.797
gen_samplesize_Vascular dementia (other)	genus Lachnospiraceae NK4A136 group	rs11263806	A	G	-0.156	36882840	0.264	0.140	360248	A	G	-0.052	17	35240097	5.07E-06	0.012	14306	20.189
gen_samplesize_Vascular dementia (other)	genus Lachnospiraceae NK4A136 group	rs12611395	G	A	0.164	21623323	0.454	0.220	360248	G	A	0.090	19	21806125	5.83E-06	0.020	14306	20.435
gen_samplesize_Vascular dementia (other)	genus Lachnospiraceae NK4A136 group	rs160061	G	A	0.209	6116546	0.116	0.133	360248	G	A	-0.051	5	6116659	2.12E-06	0.011	14306	22.596
gen_samplesize_Vascular dementia (other)	genus Lachnospiraceae NK4A136 group	rs28540839	A	C	-0.200	83822845	0.132	0.133	360248	A	C	0.051	8	84735080	9.34E-06	0.011	14306	21.124
gen_samplesize_Vascular dementia (other)	genus Lachnospiraceae NK4A136 group	rs2880566	T	C	-0.079	31689634	0.668	0.185	360248	T	C	0.060	17	30016653	5.61E-06	0.013	14306	19.815
gen_samplesize_Vascular dementia (other)	genus Lachnospiraceae NK4A136 group	rs4955932	T	C	-0.070	55147348	0.609	0.137	360248	T	C	-0.049	3	55181375	7.05E-06	0.011	14306	20.253
gen_samplesize_Vascular dementia (other)	genus Lachnospiraceae NK4A136 group	rs59805249	T	C	-0.068	90994180	0.766	0.230	360248	T	C	0.094	5	90289997	9.45E-06	0.021	14306	20.261
gen_samplesize_Vascular dementia (other)	genus Lachnospiraceae NK4A136 group	rs68104925	T	C	0.186	99711344	0.199	0.145	360248	T	C	-0.055	14	100177681	2.37E-06	0.012	14306	22.647
gen_samplesize_Vascular dementia (other)	genus Lachnospiraceae NK4A136 group	rs7073658	G	T	-0.087	60415266	0.515	0.133	360248	G	T	0.050	10	62175024	5.27E-06	0.011	14306	20.745
gen_samplesize_Vascular dementia (other)	genus Lachnospiraceae NK4A136 group	rs73044693	A	G	-0.101	50752863	0.697	0.259	360248	A	G	-0.108	19	51256120	3.57E-06	0.023	14306	21.900
gen_samplesize_Vascular dementia (other)	genus Lachnospiraceae NK4A136 group	rs7616165	G	T	1.021	190697356	0.018	0.432	360248	G	T	-0.231	3	190415145	2.77E-06	0.048	14306	22.739
gen_samplesize_Vascular dementia (other)	genus Lachnospiraceae NK4A136 group	rs76193507	A	G	0.349	162032193	0.145	0.240	360248	A	G	-0.230	3	161749981	2.93E-06	0.050	14306	21.129
gen_samplesize_Vascular dementia (other)	genus Lachnospiraceae NK4A136 group	rs7832116	A	G	0.039	4985407	0.842	0.197	360248	A	G	-0.071	8	4842929	3.57E-06	0.015	14306	22.199
gen_samplesize_Vascular dementia (other)	genus Lachnospiraceae NK4A136 group	rs954878	A	G	-0.116	54112728	0.399	0.137	360248	A	G	-0.052	1	54578401	1.78E-06	0.011	14306	22.782
gen_samplesize_Vascular dementia (other)	genus Lachnospiraceae UCG001	rs12131224	C	T	0.009	166230125	0.967	0.213	360248	C	T	0.117	1	166199362	7.40E-06	0.026	14306	20.424
gen_samplesize_Vascular dementia (other)	genus Lachnospiraceae UCG001	rs2050911	G	A	0.046	81918723	0.745	0.141	360248	G	A	0.075	1	82384407	1.11E-06	0.015	14306	23.831
gen_samplesize_Vascular dementia (other)	genus Lachnospiraceae UCG001	rs2371284	C	T	-0.030	55862478	0.847	0.158	360248	C	T	0.076	12	56256262	7.56E-06	0.017	14306	20.056
gen_samplesize_Vascular dementia (other)	genus Lachnospiraceae UCG001	rs437876	T	C	0.053	42526948	0.708	0.141	360248	T	C	0.078	3	42568440	7.17E-08	0.014	14306	29.376
gen_samplesize_Vascular dementia (other)	genus Lachnospiraceae UCG001	rs4981345	T	C	-0.072	20987814	0.614	0.142	360248	T	C	-0.068	14	21455973	6.09E-06	0.015	14306	20.717
gen_samplesize_Vascular dementia (other)	genus Lachnospiraceae UCG001	rs573933	T	C	0.214	14477853	0.320	0.215	360248	T	C	-0.108	9	14477851	3.11E-06	0.023	14306	21.565
gen_samplesize_Vascular dementia (other)	genus Lachnospiraceae UCG001	rs62496417	T	G	0.093	97336192	0.565	0.161	360248	T	G	-0.075	7	96965504	5.88E-06	0.017	14306	20.410
gen_samplesize_Vascular dementia (other)	genus Lachnospiraceae UCG001	rs7341608	T	C	-0.240	55926941	0.218	0.195	360248	T	C	-0.078	8	56839500	9.48E-06	0.018	14306	19.514
gen_samplesize_Vascular dementia (other)	genus Lachnospiraceae UCG001	rs74034332	G	A	0.094	79058462	0.737	0.279	360248	G	A	0.168	16	79092359	3.33E-06	0.038	14306	19.288

gen_samplesize_Vascular dementia (other)	genus Lachnospiraceae UCG001	rs78848836	A	G	-0.299	53307576	0.174	0.219	360248	A	G	-0.119	1	53773248	3.38E-06	0.026	14306	20.942
gen_samplesize_Vascular dementia (other)	genus Lachnospiraceae UCG001	rs8104225	A	G	-0.079	13852110	0.624	0.162	360248	A	G	0.089	19	13962924	8.04E-06	0.020	14306	20.369
gen_samplesize_Vascular dementia (other)	genus Lachnospiraceae UCG001	rs9403580	C	T	0.135	144685644	0.495	0.198	360248	C	T	0.108	6	145006780	3.47E-06	0.023	14306	22.011
gen_samplesize_Vascular dementia (other)	genus Lachnospiraceae UCG001	rs985416	T	C	0.060	148551296	0.726	0.171	360248	T	C	-0.097	3	148269083	1.46E-07	0.018	14306	28.481
gen_samplesize_Vascular dementia (other)	genus Lachnospiraceae UCG004	rs11128180	A	G	0.076	70543064	0.627	0.157	360248	A	G	0.065	3	70592215	4.52E-06	0.014	14306	21.404
gen_samplesize_Vascular dementia (other)	genus Lachnospiraceae UCG004	rs12072562	T	C	0.023	104744847	0.948	0.347	360248	T	C	0.133	1	105287469	7.07E-06	0.030	14306	19.225
gen_samplesize_Vascular dementia (other)	genus Lachnospiraceae UCG004	rs12673420	G	A	-0.320	71838022	0.016	0.132	360248	G	A	0.055	7	71303007	2.98E-06	0.012	14306	21.931
gen_samplesize_Vascular dementia (other)	genus Lachnospiraceae UCG004	rs12747809	A	G	0.043	240195789	0.774	0.148	360248	A	G	0.062	1	240359089	8.65E-07	0.013	14306	24.489
gen_samplesize_Vascular dementia (other)	genus Lachnospiraceae UCG004	rs12894272	G	A	0.094	40067277	0.501	0.140	360248	G	A	-0.058	14	40536481	4.34E-06	0.013	14306	21.436
gen_samplesize_Vascular dementia (other)	genus Lachnospiraceae UCG004	rs233486	G	A	0.167	14817353	0.386	0.193	360248	G	A	0.080	6	14817584	6.28E-06	0.018	14306	20.231
gen_samplesize_Vascular dementia (other)	genus Lachnospiraceae UCG004	rs2444793	T	C	0.061	80609119	0.656	0.136	360248	T	C	0.054	6	81318836	4.77E-06	0.012	14306	21.064
gen_samplesize_Vascular dementia (other)	genus Lachnospiraceae UCG004	rs2726805	A	G	0.339	182326448	0.012	0.134	360248	A	G	0.055	4	183247601	6.30E-06	0.012	14306	20.594
gen_samplesize_Vascular dementia (other)	genus Lachnospiraceae UCG004	rs2882478	G	A	0.144	49677451	0.282	0.133	360248	G	A	-0.058	2	49904589	1.21E-06	0.012	14306	23.782
gen_samplesize_Vascular dementia (other)	genus Lachnospiraceae UCG004	rs35182105	A	G	-0.016	24419403	0.956	0.288	360248	A	G	-0.110	12	24572337	4.87E-06	0.024	14306	20.522
gen_samplesize_Vascular dementia (other)	genus Lachnospiraceae UCG004	rs6656451	T	C	-0.261	65524739	0.047	0.132	360248	T	C	0.054	1	65990422	5.57E-06	0.012	14306	20.703
gen_samplesize_Vascular dementia (other)	genus Lachnospiraceae UCG004	rs7629954	A	G	0.069	154524617	0.829	0.318	360248	A	G	0.108	3	154242406	5.77E-06	0.024	14306	20.717
gen_samplesize_Vascular dementia (other)	genus Lachnospiraceae UCG008	rs10741777	T	C	-0.021	19551010	0.886	0.144	360248	T	C	-0.097	11	19572557	7.69E-07	0.019	14306	24.994
gen_samplesize_Vascular dementia (other)	genus Lachnospiraceae UCG008	rs10793103	T	C	-0.143	74680730	0.282	0.133	360248	T	C	-0.097	11	74391775	9.35E-08	0.018	14306	28.889
gen_samplesize_Vascular dementia (other)	genus Lachnospiraceae UCG008	rs10801803	G	A	0.363	90353314	0.057	0.191	360248	G	A	-0.117	1	90818872	1.40E-06	0.024	14306	23.178
gen_samplesize_Vascular dementia (other)	genus Lachnospiraceae UCG008	rs13024781	T	C	-0.017	167772361	0.901	0.133	360248	T	C	-0.080	2	168628871	2.29E-06	0.017	14306	22.380
gen_samplesize_Vascular dementia (other)	genus Lachnospiraceae UCG008	rs57091572	A	G	0.080	80505086	0.683	0.197	360248	A	G	-0.110	6	81214803	2.86E-06	0.024	14306	21.937
gen_samplesize_Vascular dementia (other)	genus Lachnospiraceae UCG008	rs61944774	A	G	0.253	129055273	0.390	0.294	360248	A	G	0.180	12	129539818	6.34E-06	0.039	14306	20.855
gen_samplesize_Vascular dementia (other)	genus Lachnospiraceae UCG008	rs62277846	C	T	-0.068	227235424	0.685	0.168	360248	C	T	0.102	2	228100140	1.59E-06	0.021	14306	23.212
gen_samplesize_Vascular dementia (other)	genus Lachnospiraceae UCG008	rs67078837	T	C	0.149	113299491	0.270	0.135	360248	T	C	-0.085	4	114220647	7.68E-07	0.017	14306	24.556
gen_samplesize_Vascular dementia (other)	genus Lachnospiraceae UCG008	rs75356640	G	A	0.024	31405081	0.907	0.202	360248	G	A	0.137	15	31697284	9.83E-06	0.030	14306	20.284
gen_samplesize_Vascular dementia (other)	genus Lachnospiraceae UCG008	rs955844	A	C	0.217	84958708	0.258	0.192	360248	A	C	0.112	16	84992314	1.81E-06	0.023	14306	24.080
gen_samplesize_Vascular dementia (other)	genus Lachnospiraceae UCG010	rs10414815	C	T	-0.632	42082093	0.010	0.247	360248	C	T	-0.105	19	42586245	4.24E-06	0.023	14306	20.610
gen_samplesize_Vascular dementia (other)	genus Lachnospiraceae UCG010	rs11192447	A	G	-0.177	82088641	0.556	0.301	360248	A	G	0.127	10	83848397	4.69E-07	0.024	14306	27.039
gen_samplesize_Vascular dementia (other)	genus Lachnospiraceae UCG010	rs12346653	C	T	-0.017	89652324	0.916	0.165	360248	C	T	0.066	9	92267239	2.70E-06	0.014	14306	22.205
gen_samplesize_Vascular dementia (other)	genus Lachnospiraceae UCG010	rs2833528	T	C	0.044	31821255	0.750	0.137	360248	T	C	0.056	21	33193567	9.92E-06	0.013	14306	19.359
gen_samplesize_Vascular dementia (other)	genus Lachnospiraceae UCG010	rs336138	G	T	-0.229	8146258	0.268	0.207	360248	G	T	0.078	5	8146371	7.48E-06	0.017	14306	20.573
gen_samplesize_Vascular dementia (other)	genus Lachnospiraceae UCG010	rs4576377	C	A	0.088	81780947	0.526	0.138	360248	C	A	0.057	7	81410263	7.63E-06	0.013	14306	20.272
gen_samplesize_Vascular dementia (other)	genus Lachnospiraceae UCG010	rs72894957	G	A	0.746	184997864	0.093	0.444	360248	G	A	0.222	2	185862591	5.68E-06	0.049	14306	20.879
gen_samplesize_Vascular dementia (other)	genus Lachnospiraceae UCG010	rs74315802	G	T	0.277	29403135	0.112	0.174	360248	G	T	0.087	14	29872341	3.19E-06	0.018	14306	22.343
gen_samplesize_Vascular dementia (other)	genus Lachnospiraceae UCG010	rs9981767	A	C	0.150	42659765	0.328	0.153	360248	A	C	0.066	21	44079875	9.96E-07	0.013	14306	24.630
gen_samplesize_Vascular dementia (other)	genus Lactobacillus	rs12693845	C	T	0.082	198447960	0.549	0.136	360248	C	T	-0.081	2	199312684	8.96E-06	0.018	14306	20.607
gen_samplesize_Vascular dementia (other)	genus Lactobacillus	rs1530559	G	A	0.097	134998059	0.469	0.135	360248	G	A	0.080	2	135755629	4.93E-06	0.018	14306	20.355
gen_samplesize_Vascular dementia (other)	genus Lactobacillus	rs16861661	G	A	-0.255	18174965	0.346	0.271	360248	G	A	-0.183	1	18501459	1.28E-06	0.038	14306	23.049
gen_samplesize_Vascular dementia (other)	genus Lactobacillus	rs62314653	C	A	0.022	108975306	0.937	0.283	360248	C	A	0.188	4	109896462	2.24E-06	0.039	14306	22.626
gen_samplesize_Vascular dementia (other)	genus Lactobacillus	rs7399658	G	A	0.088	23260829	0.610	0.172	360248	G	A	-0.107	13	23834968	3.12E-06	0.022	14306	23.313
gen_samplesize_Vascular dementia (other)	genus Lactobacillus	rs768253	T	G	-0.085	68097868	0.525	0.134	360248	T	G	-0.079	8	69010103	4.25E-06	0.017	14306	21.252
gen_samplesize_Vascular dementia (other)	genus Lactobacillus	rs77478751	A	G	0.074	173433875	0.724	0.208	360248	A	G	-0.220	3	173151665	7.33E-06	0.048	14306	21.361
gen_samplesize_Vascular dementia (other)	genus Lactobacillus	rs921925	A	C	-0.062	6928006	0.701	0.160	360248	A	C	0.099	19	6928017	9.72E-07	0.020	14306	23.495
gen_samplesize_Vascular dementia (other)	genus Lactococcus	rs10417872	G	T	0.123	28276446	0.400	0.146	360248	G	T	-0.118	19	28767353	1.29E-06	0.025	14306	23.276
gen_samplesize_Vascular dementia (other)	genus Lactococcus	rs123059	C	T	0.233	2796641	0.145	0.160	360248	C	T	0.137	17	2699935	1.27E-06	0.027	14306	24.769
gen_samplesize_Vascular dementia (other)	genus Lactococcus	rs12621813	G	A	0.053	31043183	0.722	0.149	360248	G	A	0.108	2	31266049	6.61E-06	0.024	14306	20.413
gen_samplesize_Vascular dementia (other)	genus Lactococcus	rs17168302	G	A	-0.294	14610596	0.177	0.218	360248	G	A	0.192	7	14650221	6.29E-06	0.042	14306	20.402
gen_samplesize_Vascular dementia (other)	genus Lactococcus	rs2293361	C	T	-0.124	53887727	0.675	0.295	360248	C	T	-0.199	2	54114864	1.40E-06	0.043	14306	21.369
gen_samplesize_Vascular dementia (other)	genus Lactococcus	rs4766997	C	T	-0.022	112723633	0.872	0.134	360248	C	T	0.115	12	113161438	2.06E-06	0.024	14306	23.109
gen_samplesize_Vascular dementia (other)	genus Lactococcus	rs55910161	C	T	-0.056	69758887	0.791	0.212	360248	C	T	0.146	10	71518643	2.36E-06	0.031	14306	22.695
gen_samplesize_Vascular dementia (other)	genus Lactococcus	rs6674304	C	T	-0.287	116345120	0.407	0.345	360248	C	T	0.201	1	116887742	6.18E-06	0.044	14306	20.619
gen_samplesize_Vascular dementia (other)	genus Marvinbryantia	rs11620597	T	C	-0.056	85504955	0.899	0.442	360248	T	C	0.119	13	86079090	7.80E-06	0.027	14306	19.339
gen_samplesize_Vascular dementia (other)	genus Marvinbryantia	rs1187983	C	T	-0.415	57976188	0.055	0.217	360248	C	T	-0.094	1	58441860	2.02E-06	0.019	14306	23.450
gen_samplesize_Vascular dementia (other)	genus Marvinbryantia	rs146541147	G	A	0.004	4542085	0.990	0.367	360248	G	A	0.119	19	4542097	6.86E-06	0.027	14306	19.603
gen_samplesize_Vascular dementia (other)	genus Marvinbryantia	rs2724813	G	A	0.004	12474925	0.978	0.155	360248	G	A	0.084	10	12516924	6.28E-07	0.017	14306	25.180
gen_samplesize_Vascular dementia (other)	genus Marvinbryantia	rs2842896	C	T	0.075	132560525	0.581	0.136	360248	C	T	-0.065	6	132881664	7.25E-07	0.013	14306	24.519
gen_samplesize_Vascular dementia (other)	genus Marvinbryantia	rs2863363	G	A	-0.031	166321977	0.842	0.154	360248	G	A	-0.063	3	166039765	3.11E-06	0.014	14306	21.688
gen_samplesize_Vascular dementia (other)	genus Marvinbryantia	rs3125832	A	C	-0.288	211226838	0.069	0.159	360248	A	C	0.068	1	211400180	5.03E-06	0.015	14306	20.477
gen_samplesize_Vascular dementia (other)	genus Marvinbryantia	rs61884471	G	A	-0.030	45805010	0.889	0.212	360248	G	A	0.124	11	45826561	1.01E-06	0.025	14306	25.085
gen_samplesize_Vascular dementia (other)	genus Marvinbryantia	rs72948274	A	C	0.161	79807639	0.559	0.275	360248	A	C	-0.126	11	79518683	3.26E-06	0.027	14306	21.546
gen_samplesize_Vascular dementia (other)	genus Marvinbryantia	rs8006832	G	T	0.291	21109678	0.205	0.229	360248	G	T	-0.095	14	21577837	6.58E-06	0.022	14306	19.317
gen_samplesize_Vascular dementia (other)	genus Methanobrevibacter	rs10202904	G	T	-0.109	124682691	0.423	0.136	360248	G	T	0.113	2	125440268	3.09E-06	0.024	14306	22.260
gen_samplesize_Vascular dementia (other)	genus Methanobrevibacter	rs1334944	T	C	-0.225	110506288	0.129	0.148	360248	T	C	0.115	10	112266046	7.61E-06	0.026	14306	20.330
gen_samplesize_Vascular dementia (other)	genus Methanobrevibacter	rs4802933	G	A	0.020	52422567	0.902	0.159	360248	G	A	0.136	19	52925820	9.74E-06	0.031	14306	19.373
gen_samplesize_Vascular dementia (other)	genus Methanobrevibacter	rs6776814	T	C	0.088	15011576	0.851	0.471	360248	T	C	-0.189	3	15053083	8.05E-06	0.042	14306	20.250
gen_samplesize_Vascular dementia (other)	genus Methanobrevibacter	rs76029318	T	C	-0.086	41389655	0.754	0.276	360248	T	C	0.223	13	41963791	1.08E-06	0.045	14306	24.060
gen_samplesize_Vascular dementia (other)	genus Methanobrevibacter	rs894996	C	A	0.105	103497150	0.682	0.255	360248	C	A	0.214	4	104418307	3.82E-06	0.046	14306	22.064

gen_samplesize_Vascular dementia (other)	genus Odoribacter	rs10093869	A	G	0.271	1318658	0.044	0.135	360248	A	G	-0.058	8	1266824	3.67E-06	0.013	14306	21.234
gen_samplesize_Vascular dementia (other)	genus Odoribacter	rs10423795	T	C	-0.050	49019831	0.718	0.138	360248	T	C	-0.055	19	49523088	6.58E-06	0.012	14306	20.657
gen_samplesize_Vascular dementia (other)	genus Odoribacter	rs28417404	A	G	-0.375	70477507	0.094	0.224	360248	A	G	-0.073	14	70944224	3.68E-06	0.016	14306	20.290
gen_samplesize_Vascular dementia (other)	genus Odoribacter	rs4793970	A	G	0.018	48686600	0.899	0.139	360248	A	G	-0.058	17	46763962	6.03E-06	0.013	14306	19.912
gen_samplesize_Vascular dementia (other)	genus Odoribacter	rs6856150	A	G	0.112	11905935	0.571	0.199	360248	A	G	-0.088	4	11907559	6.06E-06	0.019	14306	20.635
gen_samplesize_Vascular dementia (other)	genus Odoribacter	rs74553962	T	G	0.079	68765245	0.758	0.256	360248	T	G	0.121	8	69677480	9.49E-06	0.026	14306	21.146
gen_samplesize_Vascular dementia (other)	genus Odoribacter	rs77779484	G	A	-0.158	67262163	0.567	0.276	360248	G	A	-0.133	12	67655943	6.56E-07	0.027	14306	24.713
gen_samplesize_Vascular dementia (other)	genus Olsenella	rs1035588	A	G	0.041	149220676	0.762	0.137	360248	A	G	-0.108	2	150077190	4.86E-06	0.024	14306	20.850
gen_samplesize_Vascular dementia (other)	genus Olsenella	rs17148768	G	A	0.036	10735122	0.839	0.179	360248	G	A	0.140	10	10777085	2.20E-06	0.030	14306	22.570
gen_samplesize_Vascular dementia (other)	genus Olsenella	rs2759329	A	G	0.075	231824606	0.585	0.138	360248	A	G	0.111	1	231960352	3.43E-06	0.024	14306	21.947
gen_samplesize_Vascular dementia (other)	genus Olsenella	rs35225860	A	G	-0.367	247478968	0.279	0.339	360248	A	G	-0.224	1	247642270	3.87E-06	0.048	14306	21.486
gen_samplesize_Vascular dementia (other)	genus Olsenella	rs61090148	A	G	-0.151	173889347	0.264	0.135	360248	A	G	-0.105	4	174810498	6.44E-06	0.023	14306	20.515
gen_samplesize_Vascular dementia (other)	genus Olsenella	rs62112538	C	T	0.020	4925006	0.926	0.214	360248	C	T	-0.199	19	4925018	1.19E-06	0.041	14306	24.006
gen_samplesize_Vascular dementia (other)	genus Olsenella	rs72691585	C	A	-0.028	21884426	0.883	0.193	360248	C	A	-0.249	9	21884425	2.95E-06	0.052	14306	22.872
gen_samplesize_Vascular dementia (other)	genus Olsenella	rs7540303	C	T	-0.205	179660056	0.138	0.138	360248	C	T	0.108	1	179629191	5.32E-06	0.024	14306	20.892
gen_samplesize_Vascular dementia (other)	genus Olsenella	rs8066522	A	G	0.282	61555512	0.047	0.142	360248	A	G	0.107	17	59632873	9.70E-06	0.024	14306	19.640
gen_samplesize_Vascular dementia (other)	genus Olsenella	rs9460691	C	A	0.090	10421974	0.597	0.170	360248	C	A	0.120	6	10422207	7.28E-06	0.027	14306	19.942
gen_samplesize_Vascular dementia (other)	genus Oscillibacter	rs11627628	T	C	-0.204	21011446	0.408	0.247	360248	T	C	0.144	14	21479605	1.01E-06	0.029	14306	24.605
gen_samplesize_Vascular dementia (other)	genus Oscillibacter	rs11990279	T	C	0.253	11258805	0.128	0.166	360248	T	C	-0.082	8	11116314	4.94E-06	0.018	14306	20.897
gen_samplesize_Vascular dementia (other)	genus Oscillibacter	rs12649930	T	G	0.094	3654564	0.670	0.220	360248	T	G	0.122	4	3656291	4.09E-06	0.026	14306	21.935
gen_samplesize_Vascular dementia (other)	genus Oscillibacter	rs133832	A	C	0.129	44438827	0.383	0.148	360248	A	C	-0.080	22	44834707	1.15E-06	0.016	14306	23.993
gen_samplesize_Vascular dementia (other)	genus Oscillibacter	rs16866406	A	G	0.106	178592420	0.566	0.185	360248	A	G	0.099	2	179457147	3.08E-06	0.021	14306	22.426
gen_samplesize_Vascular dementia (other)	genus Oscillibacter	rs16934185	A	G	0.009	1798324	0.966	0.218	360248	A	G	-0.130	9	1798324	4.38E-06	0.028	14306	21.175
gen_samplesize_Vascular dementia (other)	genus Oscillibacter	rs234108	A	G	-0.228	184973539	0.091	0.135	360248	A	G	0.075	1	184942671	9.16E-07	0.015	14306	24.116
gen_samplesize_Vascular dementia (other)	genus Oscillibacter	rs36095275	C	T	-0.028	31800923	0.838	0.135	360248	C	T	-0.075	14	32270129	1.40E-06	0.016	14306	23.005
gen_samplesize_Vascular dementia (other)	genus Oscillibacter	rs4506202	G	A	-0.003	21740565	0.984	0.135	360248	G	A	0.071	8	21598077	3.21E-06	0.015	14306	21.825
gen_samplesize_Vascular dementia (other)	genus Oscillibacter	rs61883564	A	G	-0.157	79302798	0.415	0.192	360248	A	G	-0.101	11	79013843	3.39E-06	0.022	14306	21.029
gen_samplesize_Vascular dementia (other)	genus Oscillibacter	rs75453768	G	T	0.246	114710047	0.286	0.231	360248	G	T	0.122	10	116469806	5.35E-06	0.027	14306	20.667
gen_samplesize_Vascular dementia (other)	genus Oscillibacter	rs761240	G	T	-0.056	50891355	0.859	0.315	360248	G	T	0.177	20	49507892	2.04E-06	0.039	14306	20.639
gen_samplesize_Vascular dementia (other)	genus Oscillibacter	rs9393920	G	A	0.072	28612816	0.601	0.137	360248	G	A	0.074	6	28580593	9.92E-07	0.015	14306	24.294
gen_samplesize_Vascular dementia (other)	genus Oscillospira	rs12206468	G	A	0.488	18093460	0.014	0.200	360248	G	A	-0.133	6	18093691	1.04E-06	0.027	14306	24.319
gen_samplesize_Vascular dementia (other)	genus Oscillospira	rs12925026	T	C	-0.294	89726448	0.316	0.293	360248	T	C	0.136	16	89792856	9.31E-06	0.031	14306	19.534
gen_samplesize_Vascular dementia (other)	genus Oscillospira	rs1954532	C	T	-0.072	27682209	0.661	0.164	360248	C	T	0.083	14	28151415	2.27E-06	0.018	14306	22.228
gen_samplesize_Vascular dementia (other)	genus Oscillospira	rs28889936	A	C	0.166	88562149	0.461	0.225	360248	A	C	0.114	4	89483300	3.37E-06	0.025	14306	20.348
gen_samplesize_Vascular dementia (other)	genus Oscillospira	rs62422654	C	T	0.004	170159406	0.979	0.164	360248	C	T	0.090	6	170474630	6.47E-06	0.020	14306	20.572
gen_samplesize_Vascular dementia (other)	genus Oscillospira	rs72866977	A	C	-0.127	56295685	0.607	0.247	360248	A	C	-0.131	6	56160483	5.63E-06	0.028	14306	21.488
gen_samplesize_Vascular dementia (other)	genus Oscillospira	rs751183	C	T	0.117	76337408	0.502	0.174	360248	C	T	0.077	1	76803093	6.85E-06	0.017	14306	20.216
gen_samplesize_Vascular dementia (other)	genus Oscillospira	rs8076323	A	G	-0.306	14978543	0.030	0.141	360248	A	G	0.072	17	14881860	5.61E-06	0.016	14306	20.885
gen_samplesize_Vascular dementia (other)	genus Oxalobacter	rs10464997	G	A	0.084	21045182	0.625	0.172	360248	G	A	0.138	8	20902693	3.30E-06	0.029	14306	21.814
gen_samplesize_Vascular dementia (other)	genus Oxalobacter	rs11108500	A	G	0.356	96425426	0.136	0.239	360248	A	G	-0.199	12	96819204	3.74E-06	0.043	14306	21.708
gen_samplesize_Vascular dementia (other)	genus Oxalobacter	rs111966731	T	C	0.019	93398708	0.934	0.237	360248	T	C	0.213	15	93941937	7.30E-06	0.047	14306	20.419
gen_samplesize_Vascular dementia (other)	genus Oxalobacter	rs12002250	A	C	-0.102	19682560	0.754	0.325	360248	A	C	0.217	9	19682558	1.42E-06	0.047	14306	21.679
gen_samplesize_Vascular dementia (other)	genus Oxalobacter	rs1569853	T	C	0.007	38582525	0.971	0.206	360248	T	C	-0.138	6	38550301	3.65E-06	0.030	14306	21.617
gen_samplesize_Vascular dementia (other)	genus Oxalobacter	rs36057338	G	T	0.187	189014160	0.614	0.371	360248	G	T	0.208	4	189935314	8.80E-07	0.042	14306	24.323
gen_samplesize_Vascular dementia (other)	genus Oxalobacter	rs3862635	C	T	0.011	126712683	0.962	0.231	360248	C	T	-0.172	11	126582578	9.19E-06	0.039	14306	19.086
gen_samplesize_Vascular dementia (other)	genus Oxalobacter	rs4428215	G	A	0.025	172229645	0.868	0.152	360248	G	A	0.130	3	171947435	7.51E-08	0.024	14306	28.931
gen_samplesize_Vascular dementia (other)	genus Oxalobacter	rs6000536	C	T	-0.079	37025428	0.654	0.176	360248	C	T	-0.131	22	37421469	2.06E-07	0.025	14306	26.637
gen_samplesize_Vascular dementia (other)	genus Oxalobacter	rs6993398	G	A	0.332	114548460	0.054	0.172	360248	G	A	0.127	8	115560689	7.13E-06	0.028	14306	20.813
gen_samplesize_Vascular dementia (other)	genus Oxalobacter	rs736744	T	C	-0.157	84899492	0.244	0.135	360248	T	C	-0.118	9	87514407	2.57E-08	0.021	14306	31.135
gen_samplesize_Vascular dementia (other)	genus Parabacteroides	rs115602804	G	A	-0.455	191854576	0.036	0.217	360248	G	A	0.103	3	191572365	1.93E-06	0.022	14306	21.417
gen_samplesize_Vascular dementia (other)	genus Parabacteroides	rs4236095	A	G	-0.114	47400951	0.601	0.219	360248	A	G	-0.076	6	47368687	1.93E-06	0.016	14306	23.541
gen_samplesize_Vascular dementia (other)	genus Parabacteroides	rs60884758	C	T	0.303	2217340	0.082	0.174	360248	C	T	-0.070	9	2217340	5.71E-07	0.014	14306	24.401
gen_samplesize_Vascular dementia (other)	genus Parabacteroides	rs6657302	T	C	0.174	85119926	0.534	0.279	360248	T	C	-0.105	1	85585609	9.76E-06	0.023	14306	21.480
gen_samplesize_Vascular dementia (other)	genus Parabacteroides	rs7298818	C	T	0.097	56003169	0.660	0.220	360248	C	T	0.089	12	56396953	8.54E-06	0.020	14306	19.574
gen_samplesize_Vascular dementia (other)	genus Paraprevotella	rs10842464	C	T	0.271	25096809	0.061	0.144	360248	C	T	0.076	12	25249743	6.60E-06	0.017	14306	19.305
gen_samplesize_Vascular dementia (other)	genus Paraprevotella	rs140997932	T	C	-0.370	149581005	0.191	0.283	360248	T	C	-0.162	3	149298792	2.11E-06	0.035	14306	21.018
gen_samplesize_Vascular dementia (other)	genus Paraprevotella	rs145020347	A	G	-0.080	114655957	0.673	0.189	360248	A	G	-0.125	11	114526679	4.03E-06	0.026	14306	22.579
gen_samplesize_Vascular dementia (other)	genus Paraprevotella	rs17109926	A	G	0.032	71584948	0.832	0.151	360248	A	G	-0.099	12	71978728	6.75E-06	0.022	14306	20.903
gen_samplesize_Vascular dementia (other)	genus Paraprevotella	rs17785622	A	G	-0.300	82749597	0.394	0.352	360248	A	G	0.248	6	83459314	1.93E-06	0.052	14306	22.385
gen_samplesize_Vascular dementia (other)	genus Paraprevotella	rs2081023	A	G	0.041	175179258	0.830	0.192	360248	A	G	-0.123	5	174606261	2.64E-07	0.024	14306	26.854
gen_samplesize_Vascular dementia (other)	genus Paraprevotella	rs3008582	T	C	-0.219	196002043	0.193	0.168	360248	T	C	0.106	1	195971173	4.36E-06	0.023	14306	21.643
gen_samplesize_Vascular dementia (other)	genus Paraprevotella	rs3801748	G	A	0.206	82130192	0.135	0.138	360248	G	A	0.078	7	81759508	5.20E-06	0.017	14306	20.624
gen_samplesize_Vascular dementia (other)	genus Paraprevotella	rs4756632	G	T	-0.137	41058153	0.490	0.198	360248	G	T	-0.139	11	41079703	3.82E-06	0.029	14306	22.959
gen_samplesize_Vascular dementia (other)	genus Paraprevotella	rs4767113	C	T	-0.016	113694173	0.908	0.141	360248	C	T	0.088	12	114131978	2.14E-06	0.018	14306	23.047
gen_samplesize_Vascular dementia (other)	genus Paraprevotella	rs7240324	T	G	0.260	71956568	0.093	0.155	360248	T	G	-0.102	18	69623804	5.96E-06	0.023	14306	20.315
gen_samplesize_Vascular dementia (other)	genus Paraprevotella	rs9602779	A	C	0.155	85588285	0.316	0.155	360248	A	C	-0.107	13	86162420	6.93E-07	0.022	14306	23.463
gen_samplesize_Vascular dementia (other)	genus Paraprevotella	rs9900242	A	G	0.146	71139490	0.297	0.140	360248	A	G	-0.085	17	69135631	1.14E-06	0.018	14306	23.699

gen_samplesize_Vascular dementia (other)	genus Parasutterella	rs10899911	A	G	-0.025	43798391	0.874	0.157	360248	A	G	-0.072	10	44293839	1.15E-06	0.015	14306	23.429
gen_samplesize_Vascular dementia (other)	genus Parasutterella	rs11715853	G	A	-0.093	30122198	0.527	0.146	360248	G	A	-0.066	3	30163689	6.23E-06	0.015	14306	20.586
gen_samplesize_Vascular dementia (other)	genus Parasutterella	rs2090816	C	A	-0.304	137294455	0.077	0.172	360248	C	A	-0.084	6	137615592	2.90E-06	0.018	14306	22.494
gen_samplesize_Vascular dementia (other)	genus Parasutterella	rs35055552	T	C	0.087	113791795	0.651	0.192	360248	T	C	0.110	8	114804024	3.35E-06	0.024	14306	21.653
gen_samplesize_Vascular dementia (other)	genus Parasutterella	rs55877868	A	C	0.027	14789550	0.902	0.222	360248	A	C	-0.104	17	14692867	2.87E-06	0.023	14306	20.974
gen_samplesize_Vascular dementia (other)	genus Parasutterella	rs62273907	A	G	-0.144	156832626	0.589	0.267	360248	A	G	0.229	3	156550415	5.88E-06	0.050	14306	20.873
gen_samplesize_Vascular dementia (other)	genus Parasutterella	rs6809952	G	A	0.259	194178920	0.088	0.152	360248	G	A	-0.068	3	193896709	8.13E-06	0.015	14306	20.606
gen_samplesize_Vascular dementia (other)	genus Parasutterella	rs6828768	C	T	0.034	64691207	0.795	0.133	360248	C	T	0.064	4	65556925	1.78E-06	0.013	14306	23.051
gen_samplesize_Vascular dementia (other)	genus Parasutterella	rs7303158	C	T	-0.211	5166374	0.113	0.133	360248	C	T	0.065	12	5275540	1.33E-06	0.013	14306	23.214
gen_samplesize_Vascular dementia (other)	genus Parasutterella	rs7311004	C	T	0.047	52866926	0.728	0.134	360248	C	T	0.062	12	53260710	5.92E-06	0.014	14306	20.485
gen_samplesize_Vascular dementia (other)	genus Parasutterella	rs7572229	A	G	-0.090	72008314	0.498	0.133	360248	A	G	-0.066	2	72235444	6.32E-07	0.013	14306	24.928
gen_samplesize_Vascular dementia (other)	genus Parasutterella	rs78383039	T	C	-0.192	178089981	0.579	0.347	360248	T	C	-0.146	2	178954708	1.57E-06	0.030	14306	24.250
gen_samplesize_Vascular dementia (other)	genus Parasutterella	rs8039785	G	T	-0.154	67023969	0.245	0.132	360248	G	T	-0.062	15	67316307	3.62E-06	0.013	14306	21.615
gen_samplesize_Vascular dementia (other)	genus Parasutterella	rs823424	G	A	-0.146	16817017	0.340	0.153	360248	G	A	-0.071	8	16674526	4.95E-06	0.016	14306	20.661
gen_samplesize_Vascular dementia (other)	genus Peptococcus	rs10031059	C	T	0.030	35356972	0.845	0.155	360248	C	T	0.121	4	35358594	1.24E-07	0.023	14306	28.784
gen_samplesize_Vascular dementia (other)	genus Peptococcus	rs11001941	G	A	0.120	76956272	0.610	0.236	360248	G	A	-0.196	10	78716030	1.33E-06	0.039	14306	24.873
gen_samplesize_Vascular dementia (other)	genus Peptococcus	rs12069354	C	T	-0.113	216050440	0.669	0.265	360248	C	T	0.168	1	216223782	9.28E-06	0.038	14306	19.511
gen_samplesize_Vascular dementia (other)	genus Peptococcus	rs2054133	A	G	-0.064	33466742	0.646	0.139	360248	A	G	-0.090	2	33691809	2.14E-06	0.019	14306	22.606
gen_samplesize_Vascular dementia (other)	genus Peptococcus	rs36121075	A	G	0.385	44489376	0.032	0.179	360248	A	G	-0.141	20	43118017	6.99E-06	0.031	14306	21.094
gen_samplesize_Vascular dementia (other)	genus Peptococcus	rs413827	G	A	-0.101	57518394	0.518	0.156	360248	G	A	0.110	14	57985112	3.30E-06	0.024	14306	21.537
gen_samplesize_Vascular dementia (other)	genus Peptococcus	rs5770862	T	C	0.262	50534684	0.255	0.230	360248	T	C	0.162	22	50973113	3.22E-06	0.036	14306	20.618
gen_samplesize_Vascular dementia (other)	genus Peptococcus	rs6918730	A	G	0.245	98572413	0.376	0.276	360248	A	G	-0.135	6	99020289	1.15E-06	0.029	14306	21.809
gen_samplesize_Vascular dementia (other)	genus Peptococcus	rs7033353	G	T	-0.113	101833566	0.398	0.133	360248	G	T	-0.090	9	104595848	2.22E-06	0.019	14306	22.525
gen_samplesize_Vascular dementia (other)	genus Peptococcus	rs74592222	G	A	-0.571	75506015	0.009	0.219	360248	G	A	0.138	1	75971700	8.55E-06	0.030	14306	20.735
gen_samplesize_Vascular dementia (other)	genus Peptococcus	rs77681628	C	T	-0.204	90530302	0.404	0.244	360248	C	T	0.200	13	91182556	2.69E-07	0.039	14306	26.744
gen_samplesize_Vascular dementia (other)	genus Phascolarctobacterium	rs12618201	A	G	-0.004	173571805	0.974	0.134	360248	A	G	0.064	2	174436533	3.38E-06	0.014	14306	21.556
gen_samplesize_Vascular dementia (other)	genus Phascolarctobacterium	rs1264476	G	T	0.060	101422048	0.722	0.169	360248	G	T	-0.077	8	102434276	4.30E-06	0.017	14306	21.357
gen_samplesize_Vascular dementia (other)	genus Phascolarctobacterium	rs56069061	G	A	-0.105	168621629	0.686	0.261	360248	G	A	-0.111	3	168339417	1.87E-06	0.023	14306	23.279
gen_samplesize_Vascular dementia (other)	genus Phascolarctobacterium	rs56157888	A	C	0.301	182331683	0.061	0.161	360248	A	C	0.095	4	183252836	1.09E-06	0.019	14306	24.232
gen_samplesize_Vascular dementia (other)	genus Phascolarctobacterium	rs74540770	G	A	-0.079	186835600	0.747	0.246	360248	G	A	-0.121	3	186553389	3.60E-06	0.026	14306	21.895
gen_samplesize_Vascular dementia (other)	genus Phascolarctobacterium	rs75882962	T	C	0.137	52704958	0.500	0.203	360248	T	C	0.097	12	53098742	3.19E-07	0.019	14306	25.835
gen_samplesize_Vascular dementia (other)	genus Phascolarctobacterium	rs7982713	G	A	-0.274	70802190	0.064	0.148	360248	G	A	0.073	13	71376322	9.72E-06	0.016	14306	19.839
gen_samplesize_Vascular dementia (other)	genus Prevotella7	rs118038478	A	G	-0.549	47203698	0.034	0.259	360248	A	G	0.206	16	47237609	7.85E-06	0.047	14306	19.239
gen_samplesize_Vascular dementia (other)	genus Prevotella7	rs12124567	A	G	0.131	3427238	0.431	0.167	360248	A	G	-0.121	1	3343802	9.49E-06	0.028	14306	19.448
gen_samplesize_Vascular dementia (other)	genus Prevotella7	rs12195431	T	C	-0.141	90309935	0.533	0.227	360248	T	C	0.197	6	91019654	8.73E-06	0.044	14306	19.741
gen_samplesize_Vascular dementia (other)	genus Prevotella7	rs2240542	C	T	-0.179	241126899	0.237	0.151	360248	C	T	0.121	2	242066314	4.84E-06	0.026	14306	21.312
gen_samplesize_Vascular dementia (other)	genus Prevotella7	rs2918132	T	C	0.070	131202057	0.613	0.138	360248	T	C	0.115	10	133000320	6.42E-06	0.025	14306	20.227
gen_samplesize_Vascular dementia (other)	genus Prevotella7	rs430270	A	C	0.289	60488646	0.091	0.171	360248	A	C	0.139	3	60474379	2.87E-06	0.030	14306	21.944
gen_samplesize_Vascular dementia (other)	genus Prevotella7	rs57404562	C	A	-0.276	11961636	0.166	0.199	360248	C	A	0.155	2	12101762	6.22E-07	0.032	14306	24.202
gen_samplesize_Vascular dementia (other)	genus Prevotella7	rs79263163	A	C	0.113	39800223	0.496	0.166	360248	A	C	-0.144	11	39821773	7.51E-06	0.032	14306	20.891
gen_samplesize_Vascular dementia (other)	genus Prevotella7	rs9426434	T	C	0.013	29409704	0.929	0.140	360248	T	C	-0.124	1	29736216	9.72E-06	0.028	14306	19.713
gen_samplesize_Vascular dementia (other)	genus Prevotella7	rs9608249	A	G	0.074	24217984	0.722	0.208	360248	A	G	-0.158	22	24613952	2.07E-06	0.034	14306	22.130
gen_samplesize_Vascular dementia (other)	genus Prevotella7	rs9959718	G	A	0.271	73775429	0.100	0.165	360248	G	A	0.133	18	71442664	1.90E-06	0.028	14306	23.333
gen_samplesize_Vascular dementia (other)	genus Prevotella9	rs111509883	T	C	-0.190	639161	0.380	0.217	360248	T	C	0.171	19	639161	1.24E-06	0.035	14306	24.235
gen_samplesize_Vascular dementia (other)	genus Prevotella9	rs11685699	C	T	-0.127	11092559	0.605	0.246	360248	C	T	-0.141	2	11232685	2.03E-06	0.030	14306	22.858
gen_samplesize_Vascular dementia (other)	genus Prevotella9	rs117271932	A	G	0.093	43840580	0.747	0.288	360248	A	G	0.208	22	44236460	2.82E-06	0.044	14306	22.326
gen_samplesize_Vascular dementia (other)	genus Prevotella9	rs12648235	T	C	0.068	160194592	0.669	0.158	360248	T	C	0.079	4	161115744	7.39E-06	0.018	14306	19.565
gen_samplesize_Vascular dementia (other)	genus Prevotella9	rs1304512	G	A	0.050	64440198	0.737	0.149	360248	G	A	0.076	5	63736025	5.29E-06	0.017	14306	21.078
gen_samplesize_Vascular dementia (other)	genus Prevotella9	rs2104588	C	T	-0.400	12455582	0.169	0.291	360248	C	T	-0.106	10	12497581	8.13E-06	0.024	14306	19.716
gen_samplesize_Vascular dementia (other)	genus Prevotella9	rs2495052	A	G	0.202	13834756	0.280	0.187	360248	A	G	0.084	1	14161251	8.87E-06	0.019	14306	19.805
gen_samplesize_Vascular dementia (other)	genus Prevotella9	rs2683313	G	A	0.033	19258094	0.815	0.143	360248	G	A	0.072	8	19115604	1.69E-06	0.015	14306	22.843
gen_samplesize_Vascular dementia (other)	genus Prevotella9	rs4968431	G	T	0.172	61344417	0.218	0.140	360248	G	T	0.064	17	59421778	8.58E-06	0.014	14306	19.716
gen_samplesize_Vascular dementia (other)	genus Prevotella9	rs7237249	C	T	-0.036	74325267	0.830	0.169	360248	C	T	-0.082	18	71992502	8.93E-06	0.018	14306	20.458
gen_samplesize_Vascular dementia (other)	genus Prevotella9	rs72815774	T	C	-0.088	91766476	0.760	0.289	360248	T	C	-0.176	2	91954502	8.78E-06	0.039	14306	20.091
gen_samplesize_Vascular dementia (other)	genus Prevotella9	rs746764	T	C	0.188	21490075	0.245	0.162	360248	T	C	-0.092	20	21470713	2.04E-06	0.019	14306	22.457
gen_samplesize_Vascular dementia (other)	genus Prevotella9	rs7976209	T	C	0.380	1685304	0.038	0.183	360248	T	C	-0.087	12	1794470	7.28E-06	0.020	14306	19.351
gen_samplesize_Vascular dementia (other)	genus Prevotella9	rs9428102	A	G	0.073	118310194	0.649	0.160	360248	A	G	-0.078	1	118852817	4.62E-06	0.018	14306	19.568
gen_samplesize_Vascular dementia (other)	genus Prevotella9	rs9613013	G	A	0.039	25756771	0.846	0.203	360248	G	A	0.092	22	26152738	6.10E-06	0.020	14306	20.493
gen_samplesize_Vascular dementia (other)	genus Rikenellaceae RC9 gut group	rs12501673	A	G	-0.125	162909364	0.402	0.149	360248	A	G	0.116	4	163830516	6.29E-06	0.026	14306	19.698
gen_samplesize_Vascular dementia (other)	genus Rikenellaceae RC9 gut group	rs17032291	T	C	0.076	154944519	0.707	0.201	360248	T	C	-0.170	4	155865671	6.61E-06	0.037	14306	21.206
gen_samplesize_Vascular dementia (other)	genus Rikenellaceae RC9 gut group	rs17582787	A	G	0.193	148641486	0.273	0.176	360248	A	G	-0.158	4	149562638	3.55E-06	0.034	14306	21.533
gen_samplesize_Vascular dementia (other)	genus Rikenellaceae RC9 gut group	rs2074881	T	C	-0.040	1970022	0.841	0.198	360248	T	C	-0.142	19	1970021	9.45E-06	0.032	14306	19.283
gen_samplesize_Vascular dementia (other)	genus Rikenellaceae RC9 gut group	rs2900503	T	G	-0.194	109895263	0.278	0.179	360248	T	G	0.172	9	112657543	1.55E-07	0.033	14306	27.829
gen_samplesize_Vascular dementia (other)	genus Rikenellaceae RC9 gut group	rs2998141	C	T	0.193	133196694	0.215	0.155	360248	C	T	0.136	10	135010198	4.42E-06	0.029	14306	21.644
gen_samplesize_Vascular dementia (other)	genus Rikenellaceae RC9 gut group	rs4270579	A	G	0.263	83777035	0.065	0.143	360248	A	G	0.118	4	84698188	5.46E-06	0.027	14306	18.960
gen_samplesize_Vascular dementia (other)	genus Rikenellaceae RC9 gut group	rs4717843	G	T	-0.014	73920657	0.917	0.135	360248	G	T	-0.119	7	73334987	4.72E-06	0.026	14306	20.992
gen_samplesize_Vascular dementia (other)	genus Rikenellaceae RC9 gut group	rs7712231	A	G	-0.186	31406178	0.341	0.195	360248	A	G	0.156	5	31406285	7.97E-06	0.035	14306	19.886

gen_samplesize_Vascular dementia (other)	genus Rikenellaceae RC9 gut group	rs80309088	G	A	0.517	165255243	0.011	0.204	360248	G	A	0.174	6	165668732	4.56E-06	0.038	14306	20.606
gen_samplesize_Vascular dementia (other)	genus Rikenellaceae RC9 gut group	rs9887954	G	A	-0.257	164831636	0.058	0.135	360248	G	A	-0.115	1	164800873	4.81E-06	0.025	14306	21.272
gen_samplesize_Vascular dementia (other)	genus Romboutsia	rs10279978	A	G	0.120	5279756	0.402	0.143	360248	A	G	-0.062	7	5319387	1.17E-06	0.013	14306	23.749
gen_samplesize_Vascular dementia (other)	genus Romboutsia	rs11221428	T	C	-0.067	99794319	0.674	0.159	360248	T	C	-0.073	11	99665050	6.49E-06	0.016	14306	21.089
gen_samplesize_Vascular dementia (other)	genus Romboutsia	rs16843578	C	T	0.223	171934745	0.459	0.301	360248	C	T	-0.088	1	171903885	5.08E-06	0.020	14306	19.785
gen_samplesize_Vascular dementia (other)	genus Romboutsia	rs28603357	T	C	-0.109	106766378	0.821	0.481	360248	T	C	-0.215	7	106406824	8.52E-06	0.047	14306	20.493
gen_samplesize_Vascular dementia (other)	genus Romboutsia	rs34302036	G	A	-0.171	78507408	0.205	0.135	360248	G	A	-0.055	7	78136725	5.88E-06	0.012	14306	20.737
gen_samplesize_Vascular dementia (other)	genus Romboutsia	rs61841503	G	A	-0.019	16977560	0.923	0.200	360248	G	A	0.093	10	17019559	4.00E-08	0.017	14306	29.351
gen_samplesize_Vascular dementia (other)	genus Romboutsia	rs62504452	A	G	-0.428	146063205	0.024	0.190	360248	A	G	-0.071	7	145760298	4.66E-06	0.016	14306	20.544
gen_samplesize_Vascular dementia (other)	genus Romboutsia	rs7109293	A	G	0.009	133964762	0.967	0.206	360248	A	G	0.092	11	133834657	6.98E-06	0.021	14306	19.958
gen_samplesize_Vascular dementia (other)	genus Romboutsia	rs75200530	T	G	0.345	5230532	0.391	0.402	360248	T	G	-0.191	8	5088054	5.07E-06	0.042	14306	20.500
gen_samplesize_Vascular dementia (other)	genus Romboutsia	rs75987356	G	A	-0.018	15926583	0.944	0.253	360248	G	A	-0.130	2	16066705	6.71E-06	0.028	14306	21.347
gen_samplesize_Vascular dementia (other)	genus Romboutsia	rs77702691	A	G	0.186	88073285	0.431	0.237	360248	A	G	-0.094	13	88725540	7.37E-06	0.021	14306	20.493
gen_samplesize_Vascular dementia (other)	genus Romboutsia	rs9389266	T	G	-0.097	135090599	0.581	0.177	360248	T	G	0.072	6	135411737	9.38E-06	0.016	14306	19.821
gen_samplesize_Vascular dementia (other)	genus Romboutsia	rs9567264	C	T	-0.109	32146619	0.437	0.140	360248	C	T	0.058	13	32720756	5.76E-06	0.013	14306	20.696
gen_samplesize_Vascular dementia (other)	genus Roseburia	rs12740451	T	C	0.079	94713418	0.687	0.196	360248	T	C	0.070	1	95178974	7.34E-06	0.015	14306	20.621
gen_samplesize_Vascular dementia (other)	genus Roseburia	rs16910295	T	C	0.455	11988022	0.123	0.295	360248	T	C	-0.098	11	12009569	2.91E-06	0.021	14306	21.887
gen_samplesize_Vascular dementia (other)	genus Roseburia	rs2160994	C	T	0.224	50256274	0.111	0.141	360248	C	T	-0.055	12	50650057	9.70E-07	0.011	14306	23.969
gen_samplesize_Vascular dementia (other)	genus Roseburia	rs2943022	T	C	-0.121	90303097	0.374	0.136	360248	T	C	0.049	5	89598914	4.11E-06	0.011	14306	21.391
gen_samplesize_Vascular dementia (other)	genus Roseburia	rs302266	T	C	0.066	194214520	0.743	0.201	360248	T	C	-0.078	1	194183650	8.13E-06	0.017	14306	20.192
gen_samplesize_Vascular dementia (other)	genus Roseburia	rs329182	T	C	0.070	125740801	0.697	0.179	360248	T	C	0.069	5	125076494	5.90E-06	0.015	14306	20.389
gen_samplesize_Vascular dementia (other)	genus Roseburia	rs55858165	A	C	-0.297	85969578	0.401	0.354	360248	A	C	0.179	15	86512809	9.99E-06	0.040	14306	19.601
gen_samplesize_Vascular dementia (other)	genus Roseburia	rs57466170	C	T	-0.256	37566864	0.311	0.253	360248	C	T	0.074	15	37859065	8.30E-06	0.017	14306	18.667
gen_samplesize_Vascular dementia (other)	genus Roseburia	rs6445851	A	G	0.164	57082200	0.231	0.137	360248	A	G	0.050	3	57116228	3.53E-06	0.011	14306	21.144
gen_samplesize_Vascular dementia (other)	genus Roseburia	rs6930661	C	T	-0.158	12774379	0.570	0.279	360248	C	T	-0.096	6	12774611	2.48E-06	0.020	14306	22.008
gen_samplesize_Vascular dementia (other)	genus Roseburia	rs75326254	C	T	0.441	165757348	0.108	0.274	360248	C	T	-0.105	6	166170836	7.50E-06	0.023	14306	20.533
gen_samplesize_Vascular dementia (other)	genus Roseburia	rs78753150	A	C	0.029	144756092	0.896	0.223	360248	A	C	0.097	5	144135655	9.98E-06	0.021	14306	20.479
gen_samplesize_Vascular dementia (other)	genus Roseburia	rs9300744	C	T	0.171	102465136	0.321	0.173	360248	C	T	-0.059	13	103117486	4.75E-06	0.013	14306	21.733
gen_samplesize_Vascular dementia (other)	genus Ruminiclostridium5	rs10827477	A	G	-0.199	34973660	0.150	0.138	360248	A	G	-0.055	10	35262588	2.19E-06	0.012	14306	22.592
gen_samplesize_Vascular dementia (other)	genus Ruminiclostridium5	rs113753996	T	C	-0.086	32477558	0.621	0.173	360248	T	C	0.082	5	32477664	3.99E-06	0.017	14306	22.128
gen_samplesize_Vascular dementia (other)	genus Ruminiclostridium5	rs1223978	C	T	0.136	108122814	0.309	0.134	360248	C	T	-0.048	13	108775162	8.16E-06	0.011	14306	20.004
gen_samplesize_Vascular dementia (other)	genus Ruminiclostridium5	rs1492620	T	C	0.018	50438207	0.929	0.200	360248	T	C	-0.083	6	50405920	3.53E-06	0.018	14306	21.271
gen_samplesize_Vascular dementia (other)	genus Ruminiclostridium5	rs2482038	C	A	-0.045	12793086	0.740	0.136	360248	C	A	0.052	10	12835085	1.70E-06	0.011	14306	22.764
gen_samplesize_Vascular dementia (other)	genus Ruminiclostridium5	rs2791343	T	C	0.023	130303784	0.867	0.135	360248	T	C	0.052	8	131316030	5.54E-06	0.011	14306	20.810
gen_samplesize_Vascular dementia (other)	genus Ruminiclostridium5	rs2833828	G	A	0.363	32439578	0.003	0.123	360248	G	A	0.049	21	33811886	6.82E-06	0.011	14306	20.288
gen_samplesize_Vascular dementia (other)	genus Ruminiclostridium5	rs4955951	A	G	-0.001	55209503	0.997	0.204	360248	A	G	-0.071	3	55243531	9.96E-06	0.017	14306	18.526
gen_samplesize_Vascular dementia (other)	genus Ruminiclostridium5	rs6121460	G	A	-0.321	61718449	0.187	0.243	360248	G	A	0.093	20	60293505	2.64E-06	0.020	14306	21.934
gen_samplesize_Vascular dementia (other)	genus Ruminiclostridium5	rs79968837	A	G	-0.033	42549316	0.915	0.305	360248	A	G	-0.095	20	41177956	1.15E-06	0.019	14306	24.118
gen_samplesize_Vascular dementia (other)	genus Ruminiclostridium5	rs8053158	G	A	-0.040	86736303	0.843	0.203	360248	G	A	0.074	16	86769909	5.90E-06	0.016	14306	21.651
gen_samplesize_Vascular dementia (other)	genus Ruminiclostridium6	rs10829821	T	C	-0.366	130853030	0.111	0.230	360248	T	C	-0.098	10	132651293	3.47E-06	0.022	14306	20.406
gen_samplesize_Vascular dementia (other)	genus Ruminiclostridium6	rs116969552	A	G	0.319	126525834	0.438	0.412	360248	A	G	-0.167	10	128214403	9.16E-06	0.038	14306	19.614
gen_samplesize_Vascular dementia (other)	genus Ruminiclostridium6	rs11992182	A	C	-0.063	78854264	0.695	0.161	360248	A	C	0.063	8	79766499	4.65E-06	0.014	14306	20.568
gen_samplesize_Vascular dementia (other)	genus Ruminiclostridium6	rs2548459	C	T	0.130	48706082	0.333	0.134	360248	C	T	0.055	19	49209339	6.40E-06	0.012	14306	20.431
gen_samplesize_Vascular dementia (other)	genus Ruminiclostridium6	rs35362464	C	A	0.148	36476389	0.439	0.191	360248	C	A	0.072	4	36478011	8.99E-06	0.017	14306	18.956
gen_samplesize_Vascular dementia (other)	genus Ruminiclostridium6	rs61060922	T	G	0.153	72102255	0.694	0.387	360248	T	G	0.159	16	72136154	1.09E-06	0.032	14306	24.380
gen_samplesize_Vascular dementia (other)	genus Ruminiclostridium6	rs663262	C	T	-0.325	86468034	0.382	0.372	360248	C	T	0.135	11	86179076	3.39E-06	0.031	14306	18.872
gen_samplesize_Vascular dementia (other)	genus Ruminiclostridium6	rs67479537	T	C	0.528	10004839	0.089	0.310	360248	T	C	0.119	19	10115515	9.30E-06	0.026	14306	20.183
gen_samplesize_Vascular dementia (other)	genus Ruminiclostridium6	rs71414120	T	G	0.160	56472234	0.594	0.301	360248	T	G	0.201	14	56938952	1.08E-06	0.041	14306	24.432
gen_samplesize_Vascular dementia (other)	genus Ruminiclostridium6	rs72991535	T	G	-0.335	78258244	0.367	0.371	360248	T	G	0.136	18	76018244	4.95E-06	0.030	14306	21.105
gen_samplesize_Vascular dementia (other)	genus Ruminiclostridium6	rs73176030	T	C	-0.082	101628002	0.579	0.148	360248	T	C	0.059	7	101271282	7.29E-06	0.013	14306	19.728
gen_samplesize_Vascular dementia (other)	genus Ruminiclostridium6	rs77193512	A	G	-0.240	40267513	0.121	0.155	360248	A	G	0.074	11	40289063	1.30E-06	0.015	14306	23.125
gen_samplesize_Vascular dementia (other)	genus Ruminiclostridium6	rs792058	G	A	0.083	5408472	0.535	0.135	360248	G	A	0.055	2	5548605	8.58E-06	0.013	14306	19.527
gen_samplesize_Vascular dementia (other)	genus Ruminiclostridium6	rs79968172	G	A	-0.129	240340526	0.646	0.282	360248	G	A	0.116	1	240503826	1.66E-06	0.024	14306	22.827
gen_samplesize_Vascular dementia (other)	genus Ruminiclostridium6	rs9555756	A	C	0.136	111050902	0.565	0.237	360248	A	C	-0.080	13	111703249	7.10E-06	0.018	14306	20.712
gen_samplesize_Vascular dementia (other)	genus Ruminiclostridium9	rs12040548	G	T	0.119	247546983	0.428	0.150	360248	G	T	0.057	1	247710285	3.15E-06	0.012	14306	21.733
gen_samplesize_Vascular dementia (other)	genus Ruminiclostridium9	rs6082461	C	A	0.343	2229884	0.038	0.165	360248	C	A	-0.059	20	2210530	4.87E-06	0.013	14306	20.038
gen_samplesize_Vascular dementia (other)	genus Ruminiclostridium9	rs7137760	C	T	0.012	10549815	0.928	0.134	360248	C	T	0.051	12	10702414	7.07E-06	0.011	14306	20.504
gen_samplesize_Vascular dementia (other)	genus Ruminiclostridium9	rs74303178	T	C	0.018	14840035	0.897	0.142	360248	T	C	0.053	8	14697544	7.92E-06	0.012	14306	19.950
gen_samplesize_Vascular dementia (other)	genus Ruminiclostridium9	rs78191726	T	C	-0.348	66945427	0.179	0.258	360248	T	C	0.094	6	67655320	7.58E-06	0.021	14306	20.210
gen_samplesize_Vascular dementia (other)	genus Ruminiclostridium9	rs918449	G	A	0.083	33880293	0.746	0.256	360248	G	A	0.095	19	34371198	2.56E-06	0.020	14306	23.258
gen_samplesize_Vascular dementia (other)	genus Ruminiclostridium9	rs9522712	T	C	0.148	89789324	0.429	0.187	360248	T	C	0.070	13	90441578	4.66E-06	0.015	14306	20.396
gen_samplesize_Vascular dementia (other)	genus Ruminiclostridium9	rs9809789	C	T	-0.233	29138492	0.180	0.174	360248	C	T	-0.072	3	29179983	8.72E-06	0.016	14306	20.189
gen_samplesize_Vascular dementia (other)	genus Ruminococcaceae NK4A214 group	rs11241747	T	C	-0.023	124510625	0.874	0.145	360248	T	C	-0.053	5	123846318	6.59E-06	0.012	14306	19.780
gen_samplesize_Vascular dementia (other)	genus Ruminococcaceae NK4A214 group	rs11586410	G	A	-0.040	157369098	0.826	0.182	360248	G	A	-0.086	1	157338888	3.66E-07	0.017	14306	25.815
gen_samplesize_Vascular dementia (other)	genus Ruminococcaceae NK4A214 group	rs12642039	C	T	0.037	159010870	0.789	0.138	360248	C	T	0.055	4	159932022	3.43E-06	0.012	14306	21.452
gen_samplesize_Vascular dementia (other)	genus Ruminococcaceae NK4A214 group	rs12731	A	G	0.148	238179271	0.283	0.137	360248	A	G	-0.053	2	239087912	4.87E-06	0.012	14306	21.035
gen_samplesize_Vascular dementia (other)	genus Ruminococcaceae NK4A214 group	rs13087692	G	T	0.182	84675888	0.211	0.146	360248	G	T	-0.057	3	84725039	8.69E-06	0.013	14306	20.818

gen_samplesize_Vascular dementia (other)	genus Ruminococcaceae NK4A214 group	rs136761	A	G	0.113	49402365	0.412	0.138	360248	A	G	0.059	22	49796014	8.15E-07	0.012	14306	24.312
gen_samplesize_Vascular dementia (other)	genus Ruminococcaceae NK4A214 group	rs147475196	A	G	-0.001	26467796	0.995	0.217	360248	A	G	-0.134	3	26509287	4.72E-06	0.030	14306	20.535
gen_samplesize_Vascular dementia (other)	genus Ruminococcaceae NK4A214 group	rs35559912	T	C	0.015	35288836	0.942	0.206	360248	T	C	-0.093	5	35288938	4.89E-06	0.020	14306	20.629
gen_samplesize_Vascular dementia (other)	genus Ruminococcaceae NK4A214 group	rs4814689	C	T	0.079	18049713	0.807	0.322	360248	C	T	-0.108	20	18030357	4.55E-06	0.023	14306	22.036
gen_samplesize_Vascular dementia (other)	genus Ruminococcaceae NK4A214 group	rs5994253	A	G	0.034	17312122	0.860	0.193	360248	A	G	-0.081	22	17793012	2.35E-07	0.016	14306	26.493
gen_samplesize_Vascular dementia (other)	genus Ruminococcaceae NK4A214 group	rs62027366	T	C	0.303	24062485	0.064	0.164	360248	T	C	0.062	16	24073806	6.58E-06	0.014	14306	19.998
gen_samplesize_Vascular dementia (other)	genus Ruminococcaceae NK4A214 group	rs6681678	T	C	0.020	99783035	0.957	0.375	360248	T	C	0.100	1	100248591	9.05E-06	0.024	14306	17.422
gen_samplesize_Vascular dementia (other)	genus Ruminococcaceae NK4A214 group	rs7573569	T	C	0.238	141139473	0.387	0.275	360248	T	C	0.108	2	141897042	3.23E-06	0.023	14306	21.265
gen_samplesize_Vascular dementia (other)	genus Ruminococcaceae UCG002	rs10916131	C	T	-0.438	227375425	0.015	0.179	360248	C	T	-0.069	1	227563126	2.87E-06	0.015	14306	22.321
gen_samplesize_Vascular dementia (other)	genus Ruminococcaceae UCG002	rs10927423	C	A	-0.169	14405962	0.335	0.175	360248	C	A	-0.071	1	14732458	8.50E-07	0.015	14306	23.340
gen_samplesize_Vascular dementia (other)	genus Ruminococcaceae UCG002	rs10964441	G	A	0.018	20131748	0.934	0.218	360248	G	A	-0.149	9	20131746	7.45E-06	0.034	14306	18.683
gen_samplesize_Vascular dementia (other)	genus Ruminococcaceae UCG002	rs113147300	A	G	-0.090	111304390	0.644	0.194	360248	A	G	-0.076	9	114066670	7.69E-06	0.016	14306	21.240
gen_samplesize_Vascular dementia (other)	genus Ruminococcaceae UCG002	rs11607472	A	G	-0.367	43323201	0.166	0.265	360248	A	G	-0.078	11	43344751	7.19E-06	0.018	14306	19.580
gen_samplesize_Vascular dementia (other)	genus Ruminococcaceae UCG002	rs116974815	C	A	0.034	111842219	0.897	0.264	360248	C	A	-0.190	11	111712942	2.03E-06	0.040	14306	22.890
gen_samplesize_Vascular dementia (other)	genus Ruminococcaceae UCG002	rs11750293	G	T	-0.142	124486421	0.307	0.139	360248	G	T	-0.058	5	123822114	1.76E-06	0.012	14306	23.028
gen_samplesize_Vascular dementia (other)	genus Ruminococcaceae UCG002	rs12463378	A	G	-0.097	53972554	0.503	0.145	360248	A	G	-0.052	19	54475808	2.96E-06	0.011	14306	21.694
gen_samplesize_Vascular dementia (other)	genus Ruminococcaceae UCG002	rs15256	C	T	-0.124	72060790	0.553	0.209	360248	C	T	0.073	10	73820548	9.46E-06	0.017	14306	18.928
gen_samplesize_Vascular dementia (other)	genus Ruminococcaceae UCG002	rs55793120	T	C	0.481	46990335	0.088	0.282	360248	T	C	0.137	12	47384118	4.81E-07	0.027	14306	25.119
gen_samplesize_Vascular dementia (other)	genus Ruminococcaceae UCG002	rs57079348	T	G	-0.045	87268195	0.874	0.283	360248	T	G	-0.077	13	87920450	7.22E-06	0.017	14306	19.632
gen_samplesize_Vascular dementia (other)	genus Ruminococcaceae UCG002	rs6542556	G	A	-0.006	120000277	0.963	0.138	360248	G	A	-0.051	2	120757853	7.86E-06	0.011	14306	19.972
gen_samplesize_Vascular dementia (other)	genus Ruminococcaceae UCG002	rs6793778	T	C	-0.104	24003962	0.490	0.151	360248	T	C	0.056	3	24045453	9.81E-06	0.013	14306	19.896
gen_samplesize_Vascular dementia (other)	genus Ruminococcaceae UCG002	rs7120052	A	C	0.243	86624417	0.150	0.169	360248	A	C	0.062	11	86335459	1.97E-06	0.014	14306	21.254
gen_samplesize_Vascular dementia (other)	genus Ruminococcaceae UCG002	rs7155595	C	A	0.145	77036203	0.316	0.145	360248	C	A	0.057	14	77502546	1.15E-06	0.012	14306	23.734
gen_samplesize_Vascular dementia (other)	genus Ruminococcaceae UCG002	rs7249614	G	A	-0.095	43133738	0.487	0.137	360248	G	A	0.049	19	43637890	9.07E-06	0.011	14306	19.777
gen_samplesize_Vascular dementia (other)	genus Ruminococcaceae UCG002	rs76847269	A	G	0.032	141635384	0.939	0.420	360248	A	G	0.164	5	141014951	5.17E-06	0.036	14306	21.077
gen_samplesize_Vascular dementia (other)	genus Ruminococcaceae UCG002	rs77564310	A	C	-0.260	80420842	0.112	0.164	360248	A	C	-0.071	15	80713184	3.29E-07	0.014	14306	25.630
gen_samplesize_Vascular dementia (other)	genus Ruminococcaceae UCG002	rs79016051	C	T	0.040	238775197	0.840	0.198	360248	C	T	-0.089	1	238938497	2.34E-06	0.019	14306	21.964
gen_samplesize_Vascular dementia (other)	genus Ruminococcaceae UCG002	rs882348	A	G	0.119	23323417	0.566	0.208	360248	A	G	-0.080	4	23325040	5.45E-06	0.018	14306	20.057
gen_samplesize_Vascular dementia (other)	genus Ruminococcaceae UCG003	rs10490280	C	T	0.313	37678833	0.067	0.171	360248	C	T	-0.067	2	37905976	4.16E-06	0.014	14306	21.994
gen_samplesize_Vascular dementia (other)	genus Ruminococcaceae UCG003	rs11243416	T	C	0.056	131541583	0.828	0.258	360248	T	C	-0.093	9	134416970	1.67E-06	0.019	14306	23.437
gen_samplesize_Vascular dementia (other)	genus Ruminococcaceae UCG003	rs11613919	G	T	0.141	75102683	0.377	0.159	360248	G	T	0.073	12	75496463	1.63E-06	0.016	14306	21.834
gen_samplesize_Vascular dementia (other)	genus Ruminococcaceae UCG003	rs16959793	A	C	-0.012	34779517	0.930	0.134	360248	A	C	-0.063	15	35071718	2.22E-06	0.013	14306	22.692
gen_samplesize_Vascular dementia (other)	genus Ruminococcaceae UCG003	rs2523124	C	T	0.029	97719124	0.829	0.135	360248	C	T	0.055	7	97348436	5.78E-06	0.012	14306	20.468
gen_samplesize_Vascular dementia (other)	genus Ruminococcaceae UCG003	rs3013089	G	A	-0.073	13468126	0.597	0.138	360248	G	A	-0.055	1	13794594	4.38E-06	0.012	14306	20.998
gen_samplesize_Vascular dementia (other)	genus Ruminococcaceae UCG003	rs4452755	A	C	-0.009	81114617	0.950	0.140	360248	A	C	-0.063	8	82026852	3.29E-06	0.013	14306	22.172
gen_samplesize_Vascular dementia (other)	genus Ruminococcaceae UCG003	rs4532474	G	A	0.408	105333663	0.022	0.177	360248	G	A	0.077	6	105781538	4.82E-06	0.017	14306	20.367
gen_samplesize_Vascular dementia (other)	genus Ruminococcaceae UCG003	rs646327	G	A	0.133	48706594	0.324	0.134	360248	G	A	0.059	19	49209851	7.83E-07	0.012	14306	24.567
gen_samplesize_Vascular dementia (other)	genus Ruminococcaceae UCG003	rs6759615	A	G	0.075	204373993	0.733	0.221	360248	A	G	0.103	2	205238716	7.86E-07	0.020	14306	26.224
gen_samplesize_Vascular dementia (other)	genus Ruminococcaceae UCG003	rs73341549	T	C	0.240	51473771	0.414	0.294	360248	T	C	-0.170	7	51541468	1.51E-07	0.032	14306	28.393
gen_samplesize_Vascular dementia (other)	genus Ruminococcaceae UCG003	rs78720113	A	G	0.411	41940901	0.094	0.245	360248	A	G	-0.115	3	41982393	7.59E-06	0.025	14306	21.356
gen_samplesize_Vascular dementia (other)	genus Ruminococcaceae UCG004	rs10976229	T	G	0.484	7317307	0.015	0.200	360248	T	G	0.096	9	7317307	7.04E-06	0.021	14306	20.010
gen_samplesize_Vascular dementia (other)	genus Ruminococcaceae UCG004	rs11961899	G	A	0.164	132566782	0.270	0.149	360248	G	A	-0.071	6	132887921	9.18E-06	0.016	14306	19.237
gen_samplesize_Vascular dementia (other)	genus Ruminococcaceae UCG004	rs12125734	G	T	-0.281	103266658	0.219	0.228	360248	G	T	0.134	1	103732214	2.09E-07	0.026	14306	27.087
gen_samplesize_Vascular dementia (other)	genus Ruminococcaceae UCG004	rs2248146	T	C	-0.023	24752379	0.867	0.139	360248	T	C	0.069	7	24791998	8.20E-06	0.015	14306	20.135
gen_samplesize_Vascular dementia (other)	genus Ruminococcaceae UCG004	rs3800154	A	C	-0.020	2119095	0.895	0.149	360248	A	C	-0.080	6	2119329	6.12E-06	0.018	14306	20.178
gen_samplesize_Vascular dementia (other)	genus Ruminococcaceae UCG004	rs511258	G	A	-0.185	170248934	0.281	0.171	360248	G	A	-0.076	3	169966722	4.52E-06	0.016	14306	21.735
gen_samplesize_Vascular dementia (other)	genus Ruminococcaceae UCG004	rs6769553	A	G	0.135	54393768	0.371	0.151	360248	A	G	0.085	3	54427795	7.91E-08	0.016	14306	29.126
gen_samplesize_Vascular dementia (other)	genus Ruminococcaceae UCG004	rs7569771	A	G	-0.277	238924641	0.074	0.155	360248	A	G	-0.076	2	239846337	8.12E-06	0.017	14306	19.869
gen_samplesize_Vascular dementia (other)	genus Ruminococcaceae UCG004	rs872501	G	A	0.047	138240821	0.847	0.243	360248	G	A	0.116	8	139253064	5.81E-06	0.026	14306	20.031
gen_samplesize_Vascular dementia (other)	genus Ruminococcaceae UCG004	rs9818949	T	G	0.071	197956880	0.670	0.167	360248	T	G	-0.086	3	197683751	5.39E-06	0.019	14306	20.774
gen_samplesize_Vascular dementia (other)	genus Ruminococcaceae UCG005	rs10873449	C	T	-0.065	94164394	0.698	0.167	360248	C	T	-0.065	14	94630731	4.11E-06	0.014	14306	20.688
gen_samplesize_Vascular dementia (other)	genus Ruminococcaceae UCG005	rs10937802	A	G	-0.143	7300617	0.490	0.207	360248	A	G	-0.076	4	7302344	8.17E-06	0.017	14306	20.186
gen_samplesize_Vascular dementia (other)	genus Ruminococcaceae UCG005	rs10950694	C	T	-0.142	17986981	0.307	0.139	360248	C	T	-0.058	7	18026604	4.30E-07	0.011	14306	25.620
gen_samplesize_Vascular dementia (other)	genus Ruminococcaceae UCG005	rs114279581	A	G	-0.021	197257633	0.935	0.255	360248	A	G	-0.147	2	198122357	3.22E-06	0.032	14306	21.519
gen_samplesize_Vascular dementia (other)	genus Ruminococcaceae UCG005	rs12288512	A	G	-0.107	27726124	0.492	0.156	360248	A	G	0.067	11	27747671	3.10E-06	0.014	14306	21.308
gen_samplesize_Vascular dementia (other)	genus Ruminococcaceae UCG005	rs12458218	T	C	0.257	24995907	0.139	0.173	360248	T	C	0.068	18	22575871	2.41E-06	0.014	14306	21.964
gen_samplesize_Vascular dementia (other)	genus Ruminococcaceae UCG005	rs2893871	G	A	-0.068	60903023	0.747	0.212	360248	G	A	-0.074	10	62662781	3.54E-06	0.016	14306	22.435
gen_samplesize_Vascular dementia (other)	genus Ruminococcaceae UCG005	rs34781347	G	A	-0.087	16332206	0.737	0.259	360248	G	A	0.189	20	16312851	6.05E-07	0.039	14306	23.835
gen_samplesize_Vascular dementia (other)	genus Ruminococcaceae UCG005	rs55793120	T	C	0.481	46990335	0.088	0.282	360248	T	C	0.122	12	47384118	7.37E-06	0.028	14306	18.906
gen_samplesize_Vascular dementia (other)	genus Ruminococcaceae UCG005	rs72776570	C	A	0.252	4198714	0.266	0.227	360248	C	A	0.087	10	4240906	5.36E-06	0.020	14306	19.498
gen_samplesize_Vascular dementia (other)	genus Ruminococcaceae UCG005	rs7449320	C	A	-0.277	154623531	0.077	0.157	360248	C	A	0.060	5	154003091	4.81E-06	0.013	14306	20.972
gen_samplesize_Vascular dementia (other)	genus Ruminococcaceae UCG005	rs7555878	G	A	-0.074	187936903	0.630	0.153	360248	G	A	-0.059	1	187906034	2.81E-06	0.013	14306	21.931
gen_samplesize_Vascular dementia (other)	genus Ruminococcaceae UCG005	rs7586445	G	A	-0.017	238600389	0.931	0.194	360248	G	A	0.078	2	239509030	8.81E-06	0.018	14306	19.657
gen_samplesize_Vascular dementia (other)	genus Ruminococcaceae UCG005	rs898577	C	T	-0.358	68413358	0.218	0.290	360248	C	T	0.123	15	68705697	7.46E-06	0.029	14306	18.413
gen_samplesize_Vascular dementia (other)	genus Ruminococcaceae UCG009	rs12508214	C	T	0.019	7425210	0.890	0.141	360248	C	T	-0.077	4	7426937	4.75E-06	0.017	14306	21.026
gen_samplesize_Vascular dementia (other)	genus Ruminococcaceae UCG009	rs138460696	A	G	0.171	66169644	0.497	0.252	360248	A	G	0.139	2	66396776	9.81E-06	0.032	14306	19.451
gen_samplesize_Vascular dementia (other)	genus Ruminococcaceae UCG009	rs1550196	A	G	0.227	34459936	0.306	0.221	360248	A	G	-0.131	17	32786955	1.13E-06	0.026	14306	24.849

gen_samplesize_Vascular dementia (other)	genus Ruminococcaceae UCG009	rs2058609	G	A	0.100	12817858	0.502	0.149	360248	G	A	-0.082	12	12970792	3.12E-06	0.017	14306	21.837
gen_samplesize_Vascular dementia (other)	genus Ruminococcaceae UCG009	rs2192926	A	G	0.110	75285542	0.438	0.142	360248	A	G	-0.089	2	75512668	4.88E-06	0.019	14306	21.290
gen_samplesize_Vascular dementia (other)	genus Ruminococcaceae UCG009	rs4079028	C	T	-0.015	202329957	0.924	0.156	360248	C	T	0.092	1	202299085	3.28E-06	0.020	14306	21.094
gen_samplesize_Vascular dementia (other)	genus Ruminococcaceae UCG009	rs4708333	T	G	-0.076	77396984	0.591	0.141	360248	T	G	-0.084	6	78106701	1.56E-06	0.017	14306	23.122
gen_samplesize_Vascular dementia (other)	genus Ruminococcaceae UCG009	rs6952765	G	A	-0.070	31962098	0.624	0.143	360248	G	A	0.073	7	32001710	8.13E-06	0.017	14306	19.269
gen_samplesize_Vascular dementia (other)	genus Ruminococcaceae UCG009	rs758191	T	G	0.066	1929830	0.770	0.225	360248	T	G	0.177	16	1979831	9.01E-06	0.038	14306	22.270
gen_samplesize_Vascular dementia (other)	genus Ruminococcaceae UCG009	rs78410648	A	G	0.030	51896148	0.890	0.215	360248	A	G	0.121	19	52399401	9.67E-06	0.028	14306	19.034
gen_samplesize_Vascular dementia (other)	genus Ruminococcaceae UCG009	rs9558661	T	C	0.212	105997744	0.200	0.165	360248	T	C	-0.090	13	106650093	7.01E-06	0.020	14306	20.004
gen_samplesize_Vascular dementia (other)	genus Ruminococcaceae UCG010	rs12597105	A	G	-0.150	5183940	0.373	0.169	360248	A	G	-0.067	16	5233941	4.87E-06	0.014	14306	21.579
gen_samplesize_Vascular dementia (other)	genus Ruminococcaceae UCG010	rs2820282	C	A	-0.012	104280743	0.929	0.137	360248	C	A	0.059	6	104728618	2.85E-06	0.013	14306	22.127
gen_samplesize_Vascular dementia (other)	genus Ruminococcaceae UCG010	rs682403	A	G	-0.146	133093170	0.271	0.133	360248	A	G	-0.059	9	135968557	2.37E-06	0.012	14306	22.257
gen_samplesize_Vascular dementia (other)	genus Ruminococcaceae UCG010	rs6958419	C	T	0.144	16310239	0.282	0.134	360248	C	T	-0.059	7	16349864	2.84E-06	0.012	14306	21.958
gen_samplesize_Vascular dementia (other)	genus Ruminococcaceae UCG010	rs73218807	G	A	0.168	31055860	0.485	0.240	360248	G	A	-0.166	4	31057482	6.43E-06	0.037	14306	20.407
gen_samplesize_Vascular dementia (other)	genus Ruminococcaceae UCG010	rs7441445	T	C	0.209	40705781	0.116	0.133	360248	T	C	0.057	4	40707798	6.80E-06	0.013	14306	20.267
gen_samplesize_Vascular dementia (other)	genus Ruminococcaceae UCG011	rs10274562	C	T	-0.280	11182894	0.040	0.136	360248	C	T	0.111	7	11222521	6.50E-06	0.024	14306	20.570
gen_samplesize_Vascular dementia (other)	genus Ruminococcaceae UCG011	rs12636310	G	A	0.190	185751703	0.218	0.155	360248	G	A	0.133	3	185469491	2.81E-06	0.028	14306	22.146
gen_samplesize_Vascular dementia (other)	genus Ruminococcaceae UCG011	rs12724320	C	T	0.035	179401364	0.795	0.136	360248	C	T	-0.121	1	179370499	1.52E-06	0.025	14306	23.524
gen_samplesize_Vascular dementia (other)	genus Ruminococcaceae UCG011	rs1416041	A	C	-0.034	104625801	0.836	0.163	360248	A	C	-0.182	6	105073676	7.04E-08	0.034	14306	28.778
gen_samplesize_Vascular dementia (other)	genus Ruminococcaceae UCG011	rs2729556	T	C	0.082	112123933	0.542	0.134	360248	T	C	0.109	7	111763988	3.19E-06	0.023	14306	21.793
gen_samplesize_Vascular dementia (other)	genus Ruminococcaceae UCG011	rs4490371	T	C	0.096	76155956	0.477	0.135	360248	T	C	-0.112	3	76205107	7.75E-06	0.025	14306	20.172
gen_samplesize_Vascular dementia (other)	genus Ruminococcaceae UCG011	rs79113084	C	T	-0.236	29481986	0.284	0.220	360248	C	T	-0.152	12	29634919	2.06E-06	0.032	14306	22.967
gen_samplesize_Vascular dementia (other)	genus Ruminococcaceae UCG011	rs9729514	G	A	0.216	219727713	0.344	0.228	360248	G	A	-0.185	1	219901055	2.37E-06	0.039	14306	21.963
gen_samplesize_Vascular dementia (other)	genus Ruminococcaceae UCG013	rs11581881	C	T	-0.154	9301517	0.334	0.160	360248	C	T	0.066	1	9361576	4.73E-06	0.014	14306	20.870
gen_samplesize_Vascular dementia (other)	genus Ruminococcaceae UCG013	rs12189346	G	A	-0.145	142015575	0.396	0.172	360248	G	A	0.068	5	141395140	1.68E-06	0.015	14306	22.137
gen_samplesize_Vascular dementia (other)	genus Ruminococcaceae UCG013	rs12336782	T	C	-0.174	13609120	0.485	0.250	360248	T	C	-0.086	9	13609119	8.60E-06	0.019	14306	20.448
gen_samplesize_Vascular dementia (other)	genus Ruminococcaceae UCG013	rs12485353	G	A	0.047	197323125	0.759	0.152	360248	G	A	-0.061	3	197049996	4.19E-06	0.013	14306	21.584
gen_samplesize_Vascular dementia (other)	genus Ruminococcaceae UCG013	rs12781711	C	T	-0.085	2177736	0.578	0.153	360248	C	T	-0.066	10	2219930	2.55E-08	0.012	14306	31.194
gen_samplesize_Vascular dementia (other)	genus Ruminococcaceae UCG013	rs16918863	A	C	0.565	19491534	0.038	0.272	360248	A	C	0.111	10	19780463	4.15E-06	0.024	14306	21.552
gen_samplesize_Vascular dementia (other)	genus Ruminococcaceae UCG013	rs2730183	G	A	0.065	40454366	0.632	0.136	360248	G	A	-0.049	8	40311885	8.44E-06	0.011	14306	19.771
gen_samplesize_Vascular dementia (other)	genus Ruminococcaceae UCG013	rs4385846	T	G	0.112	113890519	0.499	0.166	360248	T	G	-0.060	10	115650278	6.46E-06	0.013	14306	20.612
gen_samplesize_Vascular dementia (other)	genus Ruminococcaceae UCG013	rs75088940	T	C	-0.415	30092624	0.114	0.262	360248	T	C	-0.094	12	30245557	2.55E-06	0.020	14306	22.072
gen_samplesize_Vascular dementia (other)	genus Ruminococcaceae UCG013	rs76973485	G	T	0.109	9492973	0.749	0.339	360248	G	T	0.195	3	9534657	3.35E-06	0.042	14306	21.735
gen_samplesize_Vascular dementia (other)	genus Ruminococcaceae UCG013	rs9313055	T	C	0.121	3628768	0.617	0.241	360248	T	C	0.105	5	3628882	9.55E-06	0.023	14306	20.089
gen_samplesize_Vascular dementia (other)	genus Ruminococcaceae UCG014	rs10495392	T	C	-0.168	237259689	0.513	0.257	360248	T	C	0.082	1	237422989	9.96E-06	0.019	14306	19.417
gen_samplesize_Vascular dementia (other)	genus Ruminococcaceae UCG014	rs10791168	G	A	0.067	131789106	0.699	0.174	360248	G	A	0.066	11	131659000	9.76E-06	0.015	14306	19.614
gen_samplesize_Vascular dementia (other)	genus Ruminococcaceae UCG014	rs10941294	C	T	0.355	36435495	0.225	0.293	360248	C	T	-0.122	5	36435597	2.40E-06	0.026	14306	22.035
gen_samplesize_Vascular dementia (other)	genus Ruminococcaceae UCG014	rs115777838	T	C	0.188	26110517	0.378	0.213	360248	T	C	-0.188	5	26110626	4.62E-07	0.039	14306	23.731
gen_samplesize_Vascular dementia (other)	genus Ruminococcaceae UCG014	rs12638134	T	G	0.264	101615765	0.049	0.134	360248	T	G	0.058	3	101334609	1.21E-06	0.012	14306	23.702
gen_samplesize_Vascular dementia (other)	genus Ruminococcaceae UCG014	rs34402072	C	T	-0.357	3779026	0.061	0.190	360248	C	T	-0.069	8	3636548	9.80E-06	0.016	14306	19.431
gen_samplesize_Vascular dementia (other)	genus Ruminococcaceae UCG014	rs56105232	G	A	-0.062	14363770	0.830	0.287	360248	G	A	0.139	9	14363769	2.91E-06	0.030	14306	21.678
gen_samplesize_Vascular dementia (other)	genus Ruminococcaceae UCG014	rs72809222	T	C	0.131	56978719	0.419	0.162	360248	T	C	0.067	2	57205854	2.41E-06	0.014	14306	23.078
gen_samplesize_Vascular dementia (other)	genus Ruminococcaceae UCG014	rs853612	A	G	-0.071	118168902	0.601	0.136	360248	A	G	-0.053	10	119928413	9.75E-06	0.012	14306	19.585
gen_samplesize_Vascular dementia (other)	genus Ruminococcaceae UCG014	rs995642	C	T	-0.010	134097088	0.951	0.156	360248	C	T	0.060	2	134854659	1.90E-06	0.013	14306	22.562
gen_samplesize_Vascular dementia (other)	genus Ruminococcus1	rs10167839	A	G	-0.158	181018152	0.258	0.139	360248	A	G	0.052	2	181882879	8.09E-06	0.012	14306	19.952
gen_samplesize_Vascular dementia (other)	genus Ruminococcus1	rs11783695	G	T	-0.005	143514733	0.977	0.181	360248	G	T	-0.073	8	144596903	4.73E-06	0.016	14306	20.689
gen_samplesize_Vascular dementia (other)	genus Ruminococcus1	rs17781867	C	T	0.011	73259879	0.966	0.265	360248	C	T	0.100	17	71256018	1.96E-06	0.021	14306	22.275
gen_samplesize_Vascular dementia (other)	genus Ruminococcus1	rs3819978	C	T	0.237	241626455	0.361	0.259	360248	C	T	-0.115	1	241789757	8.74E-06	0.026	14306	19.557
gen_samplesize_Vascular dementia (other)	genus Ruminococcus1	rs6105066	T	C	0.173	13277981	0.248	0.150	360248	T	C	-0.061	20	13258628	5.06E-06	0.013	14306	20.447
gen_samplesize_Vascular dementia (other)	genus Ruminococcus1	rs6493760	T	C	-0.191	55146984	0.169	0.139	360248	T	C	-0.054	15	55439182	3.38E-06	0.012	14306	21.334
gen_samplesize_Vascular dementia (other)	genus Ruminococcus1	rs7117576	A	G	0.074	114152851	0.746	0.229	360248	A	G	0.083	11	114023573	6.48E-07	0.017	14306	23.561
gen_samplesize_Vascular dementia (other)	genus Ruminococcus1	rs7583465	C	T	0.091	10393795	0.494	0.133	360248	C	T	0.053	2	10533921	2.56E-06	0.011	14306	21.952
gen_samplesize_Vascular dementia (other)	genus Ruminococcus1	rs78572139	G	A	0.100	15944478	0.648	0.218	360248	G	A	0.125	5	15944587	5.23E-06	0.028	14306	20.026
gen_samplesize_Vascular dementia (other)	genus Ruminococcus1	rs78613526	G	A	-0.024	65698078	0.936	0.299	360248	G	A	0.167	2	65925212	5.11E-06	0.037	14306	20.757
gen_samplesize_Vascular dementia (other)	genus Ruminococcus2	rs12406309	A	C	-0.044	107531823	0.784	0.161	360248	A	C	-0.063	1	108074445	9.79E-06	0.014	14306	19.728
gen_samplesize_Vascular dementia (other)	genus Ruminococcus2	rs12986628	T	C	-0.085	237194796	0.609	0.166	360248	T	C	-0.067	2	238103439	2.14E-06	0.014	14306	22.572
gen_samplesize_Vascular dementia (other)	genus Ruminococcus2	rs1819812	G	T	-0.282	151738636	0.338	0.295	360248	G	T	0.084	7	151435722	5.28E-06	0.018	14306	20.756
gen_samplesize_Vascular dementia (other)	genus Ruminococcus2	rs2368224	T	G	0.091	181791689	0.768	0.308	360248	T	G	0.200	2	182656416	3.63E-06	0.044	14306	20.734
gen_samplesize_Vascular dementia (other)	genus Ruminococcus2	rs2846589	T	G	0.004	862476	0.974	0.135	360248	T	G	-0.052	18	862477	7.59E-06	0.012	14306	20.124
gen_samplesize_Vascular dementia (other)	genus Ruminococcus2	rs2997412	G	A	0.167	61493648	0.263	0.149	360248	G	A	0.057	13	62067781	4.22E-06	0.012	14306	21.594
gen_samplesize_Vascular dementia (other)	genus Ruminococcus2	rs4400279	A	G	0.145	16764953	0.308	0.142	360248	A	G	0.055	7	16804578	5.80E-06	0.012	14306	20.685
gen_samplesize_Vascular dementia (other)	genus Ruminococcus2	rs4799823	C	T	-0.301	35876464	0.081	0.173	360248	C	T	0.084	18	33456427	5.40E-06	0.018	14306	21.089
gen_samplesize_Vascular dementia (other)	genus Ruminococcus2	rs55707116	C	A	-0.142	77304469	0.572	0.252	360248	C	A	0.087	9	79919385	8.01E-06	0.019	14306	20.940
gen_samplesize_Vascular dementia (other)	genus Ruminococcus2	rs58681734	A	G	-0.071	134666044	0.668	0.164	360248	A	G	0.072	9	137557890	4.18E-06	0.016	14306	20.269
gen_samplesize_Vascular dementia (other)	genus Ruminococcus2	rs61791565	T	C	0.028	24653840	0.832	0.134	360248	T	C	-0.052	4	24655463	6.79E-06	0.012	14306	19.979
gen_samplesize_Vascular dementia (other)	genus Ruminococcus2	rs75140805	T	G	0.137	12127041	0.433	0.175	360248	T	G	0.084	11	12148588	3.95E-06	0.018	14306	22.493
gen_samplesize_Vascular dementia (other)	genus Ruminococcus2	rs7635831	G	A	-0.238	173323134	0.085	0.138	360248	G	A	0.062	3	173040924	1.98E-06	0.013	14306	22.965
gen_samplesize_Vascular dementia (other)	genus Ruminococcus2	rs7693984	G	A	-0.034	4266595	0.914	0.321	360248	G	A	-0.103	4	4268322	9.42E-06	0.024	14306	19.130

gen_samplesize_Vascular dementia (other)	genus Ruminococcus2	rs78120384	A	G	0.078	106945516	0.740	0.234	360248	A	G	-0.193	3	106664363	3.31E-07	0.039	14306	24.189
gen_samplesize_Vascular dementia (other)	genus Ruminococcus gauvreauii group	rs10931481	A	G	-0.061	191090126	0.677	0.146	360248	A	G	-0.061	2	191954852	3.38E-06	0.013	14306	21.862
gen_samplesize_Vascular dementia (other)	genus Ruminococcus gauvreauii group	rs12079579	A	G	-0.176	161910692	0.463	0.240	360248	A	G	0.096	1	161880482	5.04E-06	0.021	14306	20.034
gen_samplesize_Vascular dementia (other)	genus Ruminococcus gauvreauii group	rs12539819	C	T	-0.006	153790311	0.982	0.261	360248	C	T	0.111	7	153487396	4.49E-06	0.024	14306	21.137
gen_samplesize_Vascular dementia (other)	genus Ruminococcus gauvreauii group	rs1391597	C	T	0.130	60725485	0.337	0.136	360248	C	T	0.059	12	61119266	1.86E-06	0.012	14306	22.345
gen_samplesize_Vascular dementia (other)	genus Ruminococcus gauvreauii group	rs2047242	A	G	0.234	28912931	0.139	0.158	360248	A	G	-0.068	10	29201860	2.46E-07	0.013	14306	25.552
gen_samplesize_Vascular dementia (other)	genus Ruminococcus gauvreauii group	rs2166943	C	A	-0.125	136078607	0.350	0.134	360248	C	A	-0.057	8	137090850	5.28E-06	0.012	14306	21.077
gen_samplesize_Vascular dementia (other)	genus Ruminococcus gauvreauii group	rs289410	A	G	-0.111	85020252	0.456	0.149	360248	A	G	0.065	15	85563483	2.27E-06	0.014	14306	22.168
gen_samplesize_Vascular dementia (other)	genus Ruminococcus gauvreauii group	rs431418	G	A	0.260	166992547	0.248	0.225	360248	G	A	0.095	5	166419552	5.54E-06	0.021	14306	20.321
gen_samplesize_Vascular dementia (other)	genus Ruminococcus gauvreauii group	rs71386687	T	G	0.211	2717893	0.316	0.211	360248	T	G	0.121	16	2767894	2.91E-07	0.024	14306	25.733
gen_samplesize_Vascular dementia (other)	genus Ruminococcus gauvreauii group	rs73802842	C	A	0.043	18823237	0.780	0.155	360248	C	A	0.074	4	18824860	7.48E-06	0.017	14306	18.859
gen_samplesize_Vascular dementia (other)	genus Ruminococcus gauvreauii group	rs9870933	G	A	0.195	112653470	0.150	0.135	360248	G	A	-0.062	3	112372317	8.49E-07	0.013	14306	24.313
gen_samplesize_Vascular dementia (other)	genus Ruminococcus gnavus group	rs11597105	A	G	0.075	6470619	0.651	0.166	360248	A	G	0.115	10	6512581	6.95E-06	0.025	14306	20.930
gen_samplesize_Vascular dementia (other)	genus Ruminococcus gnavus group	rs11864644	T	C	-0.243	383078	0.244	0.209	360248	T	C	-0.140	16	433078	5.01E-06	0.032	14306	19.297
gen_samplesize_Vascular dementia (other)	genus Ruminococcus gnavus group	rs12136548	C	T	0.024	114625476	0.867	0.146	360248	C	T	0.090	1	115168097	3.10E-06	0.020	14306	21.074
gen_samplesize_Vascular dementia (other)	genus Ruminococcus gnavus group	rs12989336	G	A	0.080	36032198	0.586	0.148	360248	G	A	-0.085	2	36259341	7.12E-06	0.019	14306	20.305
gen_samplesize_Vascular dementia (other)	genus Ruminococcus gnavus group	rs13163520	G	A	-0.209	18662577	0.218	0.169	360248	G	A	-0.127	5	18662686	5.61E-08	0.023	14306	29.661
gen_samplesize_Vascular dementia (other)	genus Ruminococcus gnavus group	rs2909242	A	C	-0.036	128212434	0.796	0.141	360248	A	C	0.091	8	129224680	7.41E-07	0.018	14306	24.588
gen_samplesize_Vascular dementia (other)	genus Ruminococcus gnavus group	rs3124783	G	A	0.040	132963204	0.840	0.200	360248	G	A	0.116	9	135838591	2.67E-06	0.025	14306	21.682
gen_samplesize_Vascular dementia (other)	genus Ruminococcus gnavus group	rs4388134	T	C	0.223	189189556	0.133	0.148	360248	T	C	0.090	4	190110710	9.12E-06	0.020	14306	19.769
gen_samplesize_Vascular dementia (other)	genus Ruminococcus gnavus group	rs62167033	T	C	0.233	143942440	0.493	0.340	360248	T	C	0.185	2	144700007	3.50E-06	0.040	14306	21.861
gen_samplesize_Vascular dementia (other)	genus Ruminococcus gnavus group	rs78399089	T	C	-0.068	146572523	0.747	0.211	360248	T	C	0.144	3	146290310	6.63E-06	0.033	14306	19.558
gen_samplesize_Vascular dementia (other)	genus Ruminococcus gnavus group	rs934940	A	C	0.025	121338442	0.899	0.195	360248	A	C	-0.105	2	122096018	2.74E-06	0.023	14306	20.934
gen_samplesize_Vascular dementia (other)	genus Ruminococcus torques group	rs10904297	A	G	0.259	4597570	0.577	0.466	360248	A	G	-0.168	10	4639762	2.69E-06	0.039	14306	18.531
gen_samplesize_Vascular dementia (other)	genus Ruminococcus torques group	rs10967781	C	A	-0.077	27225260	0.594	0.145	360248	C	A	0.051	9	27225258	8.37E-06	0.011	14306	20.095
gen_samplesize_Vascular dementia (other)	genus Ruminococcus torques group	rs12434631	A	G	-0.121	25661198	0.577	0.216	360248	A	G	0.075	14	26130404	2.77E-06	0.015	14306	23.711
gen_samplesize_Vascular dementia (other)	genus Ruminococcus torques group	rs1475330	C	T	0.177	66552209	0.253	0.155	360248	C	T	-0.052	6	67262102	8.13E-06	0.012	14306	19.615
gen_samplesize_Vascular dementia (other)	genus Ruminococcus torques group	rs35866622	T	C	0.091	48714803	0.512	0.140	360248	T	C	-0.061	19	49218060	2.21E-08	0.011	14306	31.285
gen_samplesize_Vascular dementia (other)	genus Ruminococcus torques group	rs4073731	T	C	-0.079	132793252	0.658	0.178	360248	T	C	0.065	11	132663147	4.05E-06	0.014	14306	21.014
gen_samplesize_Vascular dementia (other)	genus Ruminococcus torques group	rs77034621	T	G	-0.777	75857608	0.125	0.506	360248	T	G	-0.152	8	76769843	6.07E-06	0.034	14306	20.359
gen_samplesize_Vascular dementia (other)	genus Sellimonas	rs113379006	T	C	0.139	41966694	0.427	0.175	360248	T	C	-0.163	3	42008186	7.21E-06	0.036	14306	20.782
gen_samplesize_Vascular dementia (other)	genus Sellimonas	rs13417181	T	C	0.033	173443666	0.837	0.159	360248	T	C	0.167	2	174308394	7.62E-07	0.034	14306	24.337
gen_samplesize_Vascular dementia (other)	genus Sellimonas	rs2016057	C	A	0.104	51813085	0.446	0.136	360248	C	A	0.126	15	52105282	1.03E-06	0.026	14306	24.149
gen_samplesize_Vascular dementia (other)	genus Sellimonas	rs2187447	A	C	-0.032	79621928	0.906	0.273	360248	A	C	0.243	11	79332972	3.98E-06	0.053	14306	21.285
gen_samplesize_Vascular dementia (other)	genus Sellimonas	rs2371572	A	C	0.004	212349450	0.978	0.133	360248	A	C	0.127	2	213214174	4.46E-07	0.025	14306	25.770
gen_samplesize_Vascular dementia (other)	genus Sellimonas	rs41816	A	G	-0.004	106609718	0.977	0.145	360248	A	G	0.132	7	106250164	8.39E-06	0.029	14306	20.626
gen_samplesize_Vascular dementia (other)	genus Sellimonas	rs4600608	G	A	-0.302	179413837	0.066	0.165	360248	G	A	0.137	2	180278564	4.95E-06	0.030	14306	20.666
gen_samplesize_Vascular dementia (other)	genus Sellimonas	rs553697	C	T	0.280	93020675	0.105	0.173	360248	C	T	0.154	6	93730393	6.13E-06	0.034	14306	20.562
gen_samplesize_Vascular dementia (other)	genus Sellimonas	rs56203279	T	C	0.010	111861468	0.945	0.140	360248	T	C	-0.124	7	111501524	3.72E-06	0.027	14306	21.246
gen_samplesize_Vascular dementia (other)	genus Senegalimassilia	rs10036909	C	T	0.077	128525999	0.817	0.332	360248	C	T	0.186	5	127861692	8.05E-06	0.040	14306	21.416
gen_samplesize_Vascular dementia (other)	genus Senegalimassilia	rs11787826	C	A	0.048	34332384	0.724	0.136	360248	C	A	0.081	9	34332382	2.63E-06	0.017	14306	22.579
gen_samplesize_Vascular dementia (other)	genus Senegalimassilia	rs1990708	A	C	-0.103	205789399	0.679	0.249	360248	A	C	-0.110	2	206654123	8.91E-06	0.025	14306	19.571
gen_samplesize_Vascular dementia (other)	genus Senegalimassilia	rs2017373	C	T	0.131	33962790	0.345	0.139	360248	C	T	0.078	14	34431996	9.50E-06	0.018	14306	19.567
gen_samplesize_Vascular dementia (other)	genus Senegalimassilia	rs7225245	A	G	0.045	50302254	0.741	0.135	360248	A	G	-0.079	17	48379615	4.18E-06	0.017	14306	21.583
gen_samplesize_Vascular dementia (other)	genus Slackia	rs10409783	G	A	-0.083	4555774	0.572	0.147	360248	G	A	-0.095	19	4555786	7.70E-06	0.021	14306	20.261
gen_samplesize_Vascular dementia (other)	genus Slackia	rs12440440	A	G	-0.025	33749695	0.859	0.140	360248	A	G	0.090	15	34041896	2.63E-06	0.019	14306	22.397
gen_samplesize_Vascular dementia (other)	genus Slackia	rs16894137	C	T	0.016	95934063	0.936	0.199	360248	C	T	-0.123	8	96946291	2.71E-06	0.026	14306	21.791
gen_samplesize_Vascular dementia (other)	genus Slackia	rs35156985	T	C	-0.426	99854092	0.197	0.331	360248	T	C	-0.156	7	99451715	8.06E-06	0.035	14306	20.010
gen_samplesize_Vascular dementia (other)	genus Slackia	rs4492265	G	A	0.097	13484058	0.502	0.145	360248	G	A	0.091	7	13523683	2.41E-06	0.019	14306	22.334
gen_samplesize_Vascular dementia (other)	genus Slackia	rs8901	C	T	0.226	76270929	0.125	0.147	360248	C	T	0.093	17	74267010	6.07E-07	0.019	14306	25.028
gen_samplesize_Vascular dementia (other)	genus Streptococcus	rs10028567	C	T	-0.162	52791410	0.426	0.203	360248	C	T	-0.092	4	53657577	7.30E-06	0.019	14306	23.047
gen_samplesize_Vascular dementia (other)	genus Streptococcus	rs10448310	A	G	-0.035	90793892	0.799	0.139	360248	A	G	-0.052	9	93556174	3.31E-06	0.011	14306	21.646
gen_samplesize_Vascular dementia (other)	genus Streptococcus	rs11110281	T	C	0.025	100190236	0.937	0.312	360248	T	C	-0.138	12	100584014	2.58E-09	0.023	14306	36.572
gen_samplesize_Vascular dementia (other)	genus Streptococcus	rs11720390	G	A	-0.073	94384747	0.790	0.272	360248	G	A	0.107	3	94103591	3.59E-06	0.023	14306	22.011
gen_samplesize_Vascular dementia (other)	genus Streptococcus	rs11764382	A	G	-0.193	46735298	0.315	0.193	360248	A	G	-0.070	7	46774896	1.29E-06	0.014	14306	23.424
gen_samplesize_Vascular dementia (other)	genus Streptococcus	rs17708276	A	G	-0.095	10342038	0.660	0.217	360248	A	G	-0.079	8	10199548	3.04E-06	0.017	14306	21.652
gen_samplesize_Vascular dementia (other)	genus Streptococcus	rs1918540	A	G	-0.109	131779552	0.528	0.173	360248	A	G	-0.060	11	131649446	2.44E-06	0.013	14306	21.659
gen_samplesize_Vascular dementia (other)	genus Streptococcus	rs2370083	G	T	-0.281	97060413	0.299	0.270	360248	G	T	-0.082	14	97526750	9.75E-06	0.019	14306	19.317
gen_samplesize_Vascular dementia (other)	genus Streptococcus	rs57646748	G	A	0.051	37451236	0.882	0.343	360248	G	A	-0.091	4	37452858	5.48E-06	0.020	14306	20.527
gen_samplesize_Vascular dementia (other)	genus Streptococcus	rs6806351	T	C	-0.072	132339879	0.653	0.160	360248	T	C	-0.063	3	132058723	4.94E-06	0.014	14306	21.515
gen_samplesize_Vascular dementia (other)	genus Streptococcus	rs71481756	T	G	0.249	8060441	0.362	0.273	360248	T	G	0.093	10	8102404	6.51E-06	0.021	14306	20.046
gen_samplesize_Vascular dementia (other)	genus Streptococcus	rs7916711	A	G	-0.092	28299340	0.633	0.192	360248	A	G	0.103	10	28588269	2.72E-06	0.022	14306	22.407
gen_samplesize_Vascular dementia (other)	genus Subdoligranulum	rs10065321	T	C	0.018	142477850	0.893	0.134	360248	T	C	-0.051	5	141857415	2.10E-06	0.011	14306	22.504
gen_samplesize_Vascular dementia (other)	genus Subdoligranulum	rs10497836	T	C	-0.051	199423995	0.752	0.161	360248	T	C	0.052	2	200288718	8.38E-06	0.012	14306	19.494
gen_samplesize_Vascular dementia (other)	genus Subdoligranulum	rs1667315	G	A	0.085	231544032	0.536	0.137	360248	G	A	0.049	2	232408743	6.72E-06	0.011	14306	20.374
gen_samplesize_Vascular dementia (other)	genus Subdoligranulum	rs2114677	C	T	-0.342	123713421	0.103	0.210	360248	C	T	-0.104	10	125472937	2.72E-06	0.023	14306	20.368
gen_samplesize_Vascular dementia (other)	genus Subdoligranulum	rs2171249	C	T	0.069	153372894	0.789	0.258	360248	C	T	0.107	6	153694029	4.51E-06	0.023	14306	20.950

gen_samplesize_Vascular dementia (other)	genus Subdoligranulum	rs3761728	G	T	-0.045	48988868	0.769	0.154	360248	G	T	0.054	4	48990885	3.87E-06	0.012	14306	20.903
gen_samplesize_Vascular dementia (other)	genus Subdoligranulum	rs4347804	G	A	0.475	217351124	0.209	0.379	360248	G	A	-0.166	2	218215847	2.18E-06	0.036	14306	21.579
gen_samplesize_Vascular dementia (other)	genus Subdoligranulum	rs6555306	C	T	-0.030	4671270	0.877	0.192	360248	C	T	0.074	5	4671383	2.81E-06	0.016	14306	22.705
gen_samplesize_Vascular dementia (other)	genus Subdoligranulum	rs75158211	T	C	-0.123	29153787	0.513	0.188	360248	T	C	-0.072	19	29644694	7.52E-06	0.016	14306	20.616
gen_samplesize_Vascular dementia (other)	genus Subdoligranulum	rs76528319	G	T	-0.201	118463753	0.408	0.244	360248	G	T	-0.143	5	117799448	7.41E-06	0.031	14306	21.271
gen_samplesize_Vascular dementia (other)	genus Sutterella	rs1145877	G	A	0.252	81677104	0.192	0.193	360248	G	A	0.074	6	82386821	7.20E-06	0.016	14306	20.507
gen_samplesize_Vascular dementia (other)	genus Sutterella	rs11591622	T	G	0.082	100760930	0.652	0.181	360248	T	G	-0.069	10	102520687	6.50E-06	0.015	14306	20.680
gen_samplesize_Vascular dementia (other)	genus Sutterella	rs13173038	A	G	-0.053	59203357	0.731	0.154	360248	A	G	-0.072	5	58499183	2.73E-06	0.015	14306	22.428
gen_samplesize_Vascular dementia (other)	genus Sutterella	rs143438747	T	C	0.255	62812878	0.315	0.253	360248	T	C	-0.146	1	63278549	3.28E-06	0.031	14306	22.572
gen_samplesize_Vascular dementia (other)	genus Sutterella	rs2050185	A	G	-0.056	147615645	0.686	0.138	360248	A	G	-0.058	6	147936781	7.97E-06	0.013	14306	19.950
gen_samplesize_Vascular dementia (other)	genus Sutterella	rs2321387	G	A	0.125	58115206	0.346	0.133	360248	G	A	-0.059	13	58689340	1.87E-06	0.012	14306	22.674
gen_samplesize_Vascular dementia (other)	genus Sutterella	rs2613606	T	C	-0.080	111644969	0.549	0.134	360248	T	C	0.056	7	111285025	7.20E-06	0.012	14306	20.125
gen_samplesize_Vascular dementia (other)	genus Sutterella	rs607327	T	C	-0.020	111824985	0.883	0.137	360248	T	C	-0.058	11	111695709	6.63E-06	0.013	14306	20.083
gen_samplesize_Vascular dementia (other)	genus Sutterella	rs62501473	G	A	0.011	150429663	0.941	0.151	360248	G	A	0.069	7	150126751	5.52E-06	0.015	14306	21.588
gen_samplesize_Vascular dementia (other)	genus Sutterella	rs7499539	A	G	-0.020	85004459	0.894	0.150	360248	A	G	0.062	16	85038065	2.36E-06	0.013	14306	22.218
gen_samplesize_Vascular dementia (other)	genus Sutterella	rs7638039	T	C	0.097	70539788	0.528	0.155	360248	T	C	0.065	3	70588939	8.66E-06	0.014	14306	20.133
gen_samplesize_Vascular dementia (other)	genus Sutterella	rs9350083	T	G	0.170	18583647	0.221	0.139	360248	T	G	-0.059	6	18583878	8.23E-06	0.013	14306	19.609
gen_samplesize_Vascular dementia (other)	genus Terrisporobacter	rs1883097	C	T	0.415	8917272	0.234	0.349	360248	C	T	0.226	11	8938819	4.16E-07	0.045	14306	24.798
gen_samplesize_Vascular dementia (other)	genus Terrisporobacter	rs2569953	C	A	0.059	144430063	0.662	0.134	360248	C	A	0.078	3	144148905	8.95E-06	0.017	14306	19.723
gen_samplesize_Vascular dementia (other)	genus Terrisporobacter	rs2872237	A	C	0.041	16916130	0.762	0.136	360248	A	C	0.081	19	17026940	3.97E-06	0.018	14306	21.431
gen_samplesize_Vascular dementia (other)	genus Terrisporobacter	rs58405430	G	T	0.456	20599497	0.100	0.278	360248	G	T	0.135	11	20621043	7.94E-06	0.030	14306	19.978
gen_samplesize_Vascular dementia (other)	genus Terrisporobacter	rs7184125	T	C	-0.061	15826537	0.682	0.148	360248	T	C	0.091	16	15920394	8.48E-06	0.021	14306	19.721
gen_samplesize_Vascular dementia (other)	genus Turicibacter	rs11054680	T	C	-0.047	12089521	0.790	0.177	360248	T	C	-0.105	12	12242455	2.31E-06	0.023	14306	21.295
gen_samplesize_Vascular dementia (other)	genus Turicibacter	rs11666533	C	T	0.565	11754117	0.020	0.243	360248	C	T	-0.112	19	11864932	7.37E-06	0.025	14306	20.211
gen_samplesize_Vascular dementia (other)	genus Turicibacter	rs149744580	A	G	-0.431	63139827	0.152	0.301	360248	A	G	0.170	2	63366962	7.01E-08	0.032	14306	28.998
gen_samplesize_Vascular dementia (other)	genus Turicibacter	rs2834977	T	C	0.013	35557345	0.943	0.184	360248	T	C	-0.096	21	36929643	3.96E-06	0.021	14306	21.248
gen_samplesize_Vascular dementia (other)	genus Turicibacter	rs2952020	A	G	0.065	26170326	0.674	0.155	360248	A	G	0.076	8	26027842	5.63E-06	0.017	14306	20.966
gen_samplesize_Vascular dementia (other)	genus Turicibacter	rs3734633	G	A	0.163	125790735	0.571	0.289	360248	G	A	-0.121	6	126111881	5.32E-06	0.027	14306	20.325
gen_samplesize_Vascular dementia (other)	genus Turicibacter	rs4869133	G	A	0.324	96381915	0.066	0.176	360248	G	A	0.131	5	95717619	2.55E-06	0.027	14306	23.267
gen_samplesize_Vascular dementia (other)	genus Turicibacter	rs55756211	T	C	-0.352	131287420	0.169	0.256	360248	T	C	-0.115	7	130972179	2.81E-06	0.024	14306	22.871
gen_samplesize_Vascular dementia (other)	genus Turicibacter	rs7199484	G	A	0.127	49792388	0.379	0.144	360248	G	A	-0.073	16	49826299	5.77E-06	0.016	14306	20.853
gen_samplesize_Vascular dementia (other)	genus Tyzzerella3	rs10898797	C	T	0.058	87877806	0.780	0.209	360248	C	T	0.122	11	87588698	8.85E-06	0.027	14306	19.850
gen_samplesize_Vascular dementia (other)	genus Tyzzerella3	rs112102233	A	G	0.088	45602838	0.778	0.311	360248	A	G	-0.216	10	46098286	6.18E-06	0.048	14306	20.522
gen_samplesize_Vascular dementia (other)	genus Tyzzerella3	rs1232220	T	G	-0.152	102232382	0.497	0.223	360248	T	G	0.144	6	102680257	7.91E-06	0.032	14306	20.432
gen_samplesize_Vascular dementia (other)	genus Tyzzerella3	rs17706273	T	C	-0.075	16388150	0.764	0.250	360248	T	C	-0.140	5	16388259	5.88E-07	0.027	14306	26.109
gen_samplesize_Vascular dementia (other)	genus Tyzzerella3	rs191093	G	A	-0.273	76996549	0.208	0.217	360248	G	A	0.159	12	77390329	6.76E-06	0.035	14306	20.255
gen_samplesize_Vascular dementia (other)	genus Tyzzerella3	rs4904512	T	C	-0.080	89129601	0.689	0.199	360248	T	C	-0.117	14	89595945	3.09E-06	0.025	14306	21.905
gen_samplesize_Vascular dementia (other)	genus Tyzzerella3	rs55799124	A	G	0.093	3835487	0.538	0.152	360248	A	G	-0.114	17	3738781	1.34E-06	0.024	14306	22.968
gen_samplesize_Vascular dementia (other)	genus Tyzzerella3	rs67476743	T	G	-0.137	1030321	0.366	0.151	360248	T	G	0.132	19	1030320	3.74E-09	0.022	14306	35.417
gen_samplesize_Vascular dementia (other)	genus Tyzzerella3	rs7019909	T	C	0.113	33113324	0.578	0.204	360248	T	C	0.144	9	33113322	1.76E-06	0.030	14306	22.842
gen_samplesize_Vascular dementia (other)	genus Tyzzerella3	rs7333521	T	C	0.330	81018881	0.369	0.368	360248	T	C	-0.207	13	81593016	4.88E-06	0.045	14306	20.908
gen_samplesize_Vascular dementia (other)	genus Tyzzerella3	rs75091807	G	T	-0.529	34449554	0.060	0.281	360248	G	T	-0.185	13	35023691	1.71E-06	0.038	14306	23.320
gen_samplesize_Vascular dementia (other)	genus Tyzzerella3	rs7561370	C	T	-0.163	57583271	0.386	0.187	360248	C	T	-0.131	2	57810406	1.52E-06	0.029	14306	21.047
gen_samplesize_Vascular dementia (other)	genus Veillonella	rs1882878	A	G	-0.212	28638351	0.146	0.146	360248	A	G	-0.077	21	30010673	2.98E-06	0.016	14306	22.010
gen_samplesize_Vascular dementia (other)	genus Veillonella	rs2013594	C	T	0.017	44280604	0.902	0.137	360248	C	T	0.072	11	44302154	3.42E-06	0.016	14306	21.577
gen_samplesize_Vascular dementia (other)	genus Veillonella	rs62376424	C	T	0.100	120578590	0.486	0.143	360248	C	T	-0.076	5	119914285	3.65E-06	0.016	14306	21.733
gen_samplesize_Vascular dementia (other)	genus Veillonella	rs6656807	G	A	0.020	178975908	0.887	0.138	360248	G	A	-0.070	1	178945043	5.50E-06	0.015	14306	20.855
gen_samplesize_Vascular dementia (other)	genus Veillonella	rs742016	A	G	-0.175	45208919	0.212	0.141	360248	A	G	-0.069	22	45604800	4.66E-06	0.015	14306	21.137
gen_samplesize_Vascular dementia (other)	genus Victivallis	rs11899949	G	A	-0.103	37821926	0.475	0.144	360248	G	A	0.131	2	38049069	2.77E-06	0.028	1531	22.341
gen_samplesize_Vascular dementia (other)	genus Victivallis	rs12512543	A	C	-0.236	9606821	0.331	0.242	360248	A	C	-0.178	4	9608445	2.54E-06	0.037	1531	22.619
gen_samplesize_Vascular dementia (other)	genus Victivallis	rs173120	C	T	-0.079	72302743	0.639	0.168	360248	C	T	-0.134	13	72876881	7.65E-06	0.029	1531	21.272
gen_samplesize_Vascular dementia (other)	genus Victivallis	rs1882775	A	G	0.163	39033681	0.346	0.173	360248	A	G	-0.138	21	40405606	8.73E-06	0.031	1531	19.549
gen_samplesize_Vascular dementia (other)	genus Victivallis	rs2546432	C	T	-0.091	181152462	0.493	0.133	360248	C	T	0.111	5	180579462	9.93E-06	0.025	1531	19.698
gen_samplesize_Vascular dementia (other)	genus Victivallis	rs342302	A	G	-0.182	106737783	0.354	0.196	360248	A	G	-0.153	7	106378229	8.16E-06	0.035	1531	18.878
gen_samplesize_Vascular dementia (other)	genus Victivallis	rs4764863	G	A	0.091	102120406	0.495	0.133	360248	G	A	0.122	12	102514184	8.22E-07	0.025	1531	24.407
gen_samplesize_Vascular dementia (other)	genus Victivallis	rs4895919	C	T	-0.168	131309179	0.208	0.134	360248	C	T	0.117	6	131630319	2.75E-06	0.025	1531	22.313
gen_samplesize_Vascular dementia (other)	genus Victivallis	rs56349194	A	G	0.072	9291249	0.711	0.195	360248	A	G	-0.159	11	9312796	6.26E-07	0.032	1531	25.282
gen_samplesize_Vascular dementia (other)	genus Victivallis	rs911666	T	C	0.229	95604788	0.114	0.145	360248	T	C	-0.119	14	96071125	7.65E-06	0.026	1531	20.305
gen_samplesize_Vascular dementia (other)	order Actinomycetales	rs2889192	T	G	-0.213	73779652	0.254	0.187	360248	T	G	-0.088	9	76394568	3.97E-06	0.019	14306	20.554
gen_samplesize_Vascular dementia (other)	order Actinomycetales	rs34583783	G	T	0.111	66497478	0.689	0.278	360248	G	T	0.124	6	67207371	5.54E-06	0.026	14306	21.908
gen_samplesize_Vascular dementia (other)	order Actinomycetales	rs35011108	A	G	-0.030	132686341	0.910	0.266	360248	A	G	0.242	6	133007480	1.88E-06	0.050	14306	22.987
gen_samplesize_Vascular dementia (other)	order Actinomycetales	rs4073240	G	A	-0.111	168824686	0.419	0.137	360248	G	A	0.075	6	169224781	5.68E-06	0.016	14306	20.729
gen_samplesize_Vascular dementia (other)	order Bacillales	rs10233278	T	C	-0.161	117856090	0.232	0.135	360248	T	C	-0.116	7	117496144	3.51E-06	0.025	14306	21.906
gen_samplesize_Vascular dementia (other)	order Bacillales	rs10410917	C	T	0.289	14859629	0.034	0.136	360248	C	T	-0.115	19	14970441	5.57E-06	0.025	14306	21.049
gen_samplesize_Vascular dementia (other)	order Bacillales	rs11034576	A	G	-0.023	38064161	0.908	0.200	360248	A	G	0.206	11	38085711	8.86E-06	0.045	14306	20.604
gen_samplesize_Vascular dementia (other)	order Bacillales	rs11207728	A	G	0.064	61361862	0.717	0.177	360248	A	G	0.145	1	61827534	5.73E-06	0.032	14306	20.805
gen_samplesize_Vascular dementia (other)	order Bacillales	rs11844714	A	G	-0.279	48532023	0.087	0.163	360248	A	G	-0.143	14	49001226	5.06E-06	0.032	14306	20.004

gen_samplesize_Vascular dementia (other)	order Bacillales	rs1287018	G	A	-0.212	5845828	0.213	0.170	360248	G	A	0.141	20	5826474	9.87E-06	0.032	14306	19.534
gen_samplesize_Vascular dementia (other)	order Bacillales	rs4617108	G	A	0.093	49384236	0.698	0.241	360248	G	A	-0.249	7	49423832	1.98E-06	0.053	14306	22.390
gen_samplesize_Vascular dementia (other)	order Bacillales	rs74420793	A	G	-0.198	126845554	0.355	0.214	360248	A	G	-0.164	4	127766709	3.07E-06	0.035	14306	21.600
gen_samplesize_Vascular dementia (other)	order Bacteroidales	rs11146701	A	G	0.112	38769138	0.419	0.139	360248	A	G	0.047	10	39062269	7.08E-06	0.011	14306	20.186
gen_samplesize_Vascular dementia (other)	order Bacteroidales	rs17343978	A	C	0.115	27037922	0.482	0.164	360248	A	C	-0.055	22	27433885	8.36E-06	0.012	14306	21.067
gen_samplesize_Vascular dementia (other)	order Bacteroidales	rs2032750	C	T	-0.323	53603889	0.015	0.133	360248	C	T	0.051	2	53831026	1.92E-06	0.011	14306	22.657
gen_samplesize_Vascular dementia (other)	order Bacteroidales	rs2363574	T	C	0.089	200143435	0.800	0.350	360248	T	C	0.223	1	200112563	9.93E-06	0.051	14306	19.221
gen_samplesize_Vascular dementia (other)	order Bacteroidales	rs4916508	A	G	0.299	196209918	0.027	0.135	360248	A	G	0.047	3	195936789	8.47E-06	0.011	14306	19.641
gen_samplesize_Vascular dementia (other)	order Bacteroidales	rs55773148	G	A	0.058	69948897	0.842	0.291	360248	G	A	-0.122	13	70523029	3.90E-07	0.024	14306	26.341
gen_samplesize_Vascular dementia (other)	order Bacteroidales	rs62531359	T	G	0.295	70003946	0.089	0.174	360248	T	G	0.066	8	70916181	9.09E-06	0.015	14306	19.138
gen_samplesize_Vascular dementia (other)	order Bacteroidales	rs62575403	C	T	-0.216	133628698	0.509	0.327	360248	C	T	0.140	9	136493820	7.06E-06	0.031	14306	20.264
gen_samplesize_Vascular dementia (other)	order Bacteroidales	rs72706335	T	C	-0.489	157525648	0.256	0.431	360248	T	C	-0.222	1	157495438	7.66E-06	0.049	14306	20.315
gen_samplesize_Vascular dementia (other)	order Bacteroidales	rs73975615	G	A	0.303	6557880	0.710	0.816	360248	G	A	-0.207	17	6461200	1.22E-06	0.044	14306	21.874
gen_samplesize_Vascular dementia (other)	order Bacteroidales	rs7631304	G	A	-0.123	89290377	0.513	0.188	360248	G	A	-0.065	3	89339527	8.37E-07	0.013	14306	23.590
gen_samplesize_Vascular dementia (other)	order Bacteroidales	rs79585701	A	C	-0.036	13252676	0.849	0.190	360248	A	C	0.065	8	13110185	9.99E-06	0.015	14306	18.687
gen_samplesize_Vascular dementia (other)	order Bacteroidales	rs929878	T	C	-0.080	74256742	0.623	0.164	360248	T	C	0.055	16	74290641	4.73E-06	0.012	14306	20.372
gen_samplesize_Vascular dementia (other)	order Bifidobacteriales	rs10831953	G	A	0.001	13076504	0.995	0.144	360248	G	A	0.054	11	13098051	9.95E-06	0.012	14306	18.869
gen_samplesize_Vascular dementia (other)	order Bifidobacteriales	rs12446429	T	C	-0.175	848055	0.307	0.171	360248	T	C	0.081	16	898055	8.53E-06	0.019	14306	18.040
gen_samplesize_Vascular dementia (other)	order Bifidobacteriales	rs13020688	G	A	-0.087	192013806	0.545	0.143	360248	G	A	0.058	2	192878532	1.57E-06	0.012	14306	22.887
gen_samplesize_Vascular dementia (other)	order Bifidobacteriales	rs182549	T	C	-0.269	135859184	0.052	0.139	360248	T	C	-0.117	2	136616754	5.94E-20	0.013	14306	85.372
gen_samplesize_Vascular dementia (other)	order Bifidobacteriales	rs4957061	T	C	-0.254	520981	0.061	0.136	360248	T	C	0.057	5	521096	1.15E-06	0.012	14306	23.762
gen_samplesize_Vascular dementia (other)	order Bifidobacteriales	rs540489	T	G	-0.121	74901626	0.495	0.177	360248	T	G	-0.063	17	72897722	5.37E-06	0.014	14306	20.956
gen_samplesize_Vascular dementia (other)	order Bifidobacteriales	rs55888705	A	G	0.223	1516099	0.130	0.148	360248	A	G	0.054	4	1517826	8.66E-06	0.012	14306	19.812
gen_samplesize_Vascular dementia (other)	order Bifidobacteriales	rs6899771	A	G	-0.090	96958344	0.685	0.223	360248	A	G	-0.091	6	97406220	7.28E-06	0.020	14306	20.365
gen_samplesize_Vascular dementia (other)	order Bifidobacteriales	rs7174549	T	C	-0.280	91920073	0.045	0.139	360248	T	C	-0.055	15	92463303	6.87E-06	0.012	14306	19.590
gen_samplesize_Vascular dementia (other)	order Bifidobacteriales	rs7322849	T	C	-0.051	112205515	0.824	0.229	360248	T	C	0.111	13	112859829	1.74E-08	0.020	14306	30.320
gen_samplesize_Vascular dementia (other)	order Bifidobacteriales	rs857444	C	T	0.144	14617360	0.294	0.137	360248	C	T	0.055	6	14617591	3.82E-06	0.012	14306	21.075
gen_samplesize_Vascular dementia (other)	order Burkholderiales	rs1511453	A	G	-0.401	23586839	0.162	0.287	360248	A	G	0.091	4	23588462	8.00E-06	0.020	14306	20.857
gen_samplesize_Vascular dementia (other)	order Burkholderiales	rs1928341	G	A	-0.171	153267537	0.208	0.136	360248	G	A	-0.051	1	153240013	4.52E-06	0.011	14306	21.084
gen_samplesize_Vascular dementia (other)	order Burkholderiales	rs2321387	G	A	0.125	58115206	0.346	0.133	360248	G	A	-0.051	13	58689340	3.26E-06	0.011	14306	21.532
gen_samplesize_Vascular dementia (other)	order Burkholderiales	rs2613606	T	C	-0.080	111644969	0.549	0.134	360248	T	C	0.050	7	111285025	4.13E-06	0.011	14306	20.904
gen_samplesize_Vascular dementia (other)	order Burkholderiales	rs4033856	T	C	-0.075	45640468	0.751	0.237	360248	T	C	-0.083	4	45642485	5.67E-07	0.017	14306	24.762
gen_samplesize_Vascular dementia (other)	order Burkholderiales	rs6087811	T	G	0.032	32008327	0.883	0.221	360248	T	G	-0.102	20	30596130	2.88E-07	0.020	14306	26.108
gen_samplesize_Vascular dementia (other)	order Burkholderiales	rs62191117	A	G	-0.047	238979079	0.775	0.165	360248	A	G	0.068	2	239900775	2.79E-07	0.013	14306	26.349
gen_samplesize_Vascular dementia (other)	order Burkholderiales	rs62395635	T	C	0.404	174070793	0.149	0.280	360248	T	C	0.110	5	173497796	2.90E-06	0.024	14306	21.621
gen_samplesize_Vascular dementia (other)	order Burkholderiales	rs75242906	C	T	0.034	56494209	0.887	0.239	360248	C	T	-0.121	15	56786407	9.75E-06	0.028	14306	18.498
gen_samplesize_Vascular dementia (other)	order Burkholderiales	rs7638039	T	C	0.097	70539788	0.528	0.155	360248	T	C	0.058	3	70588939	4.84E-06	0.013	14306	21.017
gen_samplesize_Vascular dementia (other)	order Clostridiales	rs10774377	G	A	0.068	5833353	0.613	0.135	360248	G	A	-0.052	12	5942519	3.81E-06	0.011	14306	21.100
gen_samplesize_Vascular dementia (other)	order Clostridiales	rs112334273	G	A	0.017	39331325	0.906	0.148	360248	G	A	0.064	21	40703251	4.07E-07	0.013	14306	25.186
gen_samplesize_Vascular dementia (other)	order Clostridiales	rs13105690	C	T	-0.085	7418457	0.562	0.147	360248	C	T	0.053	4	7420184	9.37E-06	0.012	14306	19.919
gen_samplesize_Vascular dementia (other)	order Clostridiales	rs13179700	C	T	0.034	149698225	0.807	0.140	360248	C	T	-0.051	5	149077788	3.52E-06	0.011	14306	21.746
gen_samplesize_Vascular dementia (other)	order Clostridiales	rs1842454	G	A	-0.089	105724661	0.599	0.169	360248	G	A	-0.054	5	105060362	9.92E-06	0.013	14306	18.228
gen_samplesize_Vascular dementia (other)	order Clostridiales	rs2273429	A	G	0.054	52027354	0.800	0.213	360248	A	G	-0.073	14	52494072	4.17E-06	0.015	14306	22.497
gen_samplesize_Vascular dementia (other)	order Clostridiales	rs290772	G	A	0.014	101509758	0.962	0.285	360248	G	A	0.084	2	102126220	1.00E-05	0.020	14306	18.423
gen_samplesize_Vascular dementia (other)	order Clostridiales	rs6442336	T	C	-0.057	12829387	0.711	0.153	360248	T	C	0.055	3	12870886	9.63E-06	0.012	14306	19.458
gen_samplesize_Vascular dementia (other)	order Clostridiales	rs6814436	C	T	-0.014	160586149	0.941	0.190	360248	C	T	-0.074	4	161507301	9.06E-07	0.015	14306	24.197
gen_samplesize_Vascular dementia (other)	order Clostridiales	rs6815608	C	T	0.150	151210592	0.422	0.187	360248	C	T	-0.104	4	152131744	3.72E-07	0.021	14306	24.363
gen_samplesize_Vascular dementia (other)	order Clostridiales	rs72738886	T	C	-0.307	35770448	0.226	0.253	360248	T	C	0.087	5	35770550	8.42E-06	0.019	14306	20.657
gen_samplesize_Vascular dementia (other)	order Clostridiales	rs992074	T	C	-0.169	17195484	0.706	0.450	360248	T	C	-0.255	21	18567802	8.95E-07	0.051	14306	25.206
gen_samplesize_Vascular dementia (other)	order Coriobacteriales	rs11073596	G	T	-0.263	85890348	0.057	0.138	360248	G	T	-0.051	15	86433579	8.14E-06	0.011	14306	19.912
gen_samplesize_Vascular dementia (other)	order Coriobacteriales	rs11250875	T	C	-0.149	1880537	0.357	0.161	360248	T	C	0.061	10	1922731	4.83E-06	0.013	14306	21.526
gen_samplesize_Vascular dementia (other)	order Coriobacteriales	rs11656361	A	C	-0.271	8218014	0.116	0.173	360248	A	C	0.077	17	8121332	8.02E-06	0.018	14306	19.394
gen_samplesize_Vascular dementia (other)	order Coriobacteriales	rs12974142	G	A	0.233	52391913	0.376	0.264	360248	G	A	0.079	19	52895166	8.51E-06	0.018	14306	19.865
gen_samplesize_Vascular dementia (other)	order Coriobacteriales	rs13307134	T	C	0.272	105444233	0.130	0.180	360248	T	C	-0.057	7	105084680	7.80E-06	0.013	14306	20.072
gen_samplesize_Vascular dementia (other)	order Coriobacteriales	rs1397793	A	G	-0.061	91175634	0.672	0.144	360248	A	G	0.050	5	90471451	9.77E-06	0.011	14306	19.682
gen_samplesize_Vascular dementia (other)	order Coriobacteriales	rs1816223	G	A	0.266	11341087	0.111	0.167	360248	G	A	0.059	12	11494021	4.84E-06	0.013	14306	20.652
gen_samplesize_Vascular dementia (other)	order Coriobacteriales	rs240104	T	C	0.099	176602295	0.508	0.149	360248	T	C	-0.060	1	176571431	1.52E-06	0.013	14306	22.630
gen_samplesize_Vascular dementia (other)	order Coriobacteriales	rs2442778	A	G	0.298	11612938	0.329	0.305	360248	A	G	0.116	3	11654412	9.03E-06	0.026	14306	20.272
gen_samplesize_Vascular dementia (other)	order Coriobacteriales	rs3025411	A	G	-0.176	133647784	0.408	0.213	360248	A	G	0.093	9	136512906	8.27E-06	0.021	14306	19.566
gen_samplesize_Vascular dementia (other)	order Coriobacteriales	rs34739816	G	T	-0.283	39220432	0.316	0.282	360248	G	T	0.097	17	37376685	3.88E-06	0.021	14306	21.594
gen_samplesize_Vascular dementia (other)	order Coriobacteriales	rs67561917	A	G	-0.234	63440724	0.180	0.174	360248	A	G	-0.071	20	62072077	5.39E-06	0.015	14306	21.486
gen_samplesize_Vascular dementia (other)	order Coriobacteriales	rs719099	A	G	0.120	64039457	0.591	0.223	360248	A	G	0.078	10	65799217	5.43E-07	0.016	14306	24.957
gen_samplesize_Vascular dementia (other)	order Coriobacteriales	rs8010111	A	G	-0.021	39191305	0.930	0.241	360248	A	G	0.103	14	39660509	6.90E-06	0.023	14306	20.328
gen_samplesize_Vascular dementia (other)	order Desulfovibrionales	rs11599763	C	T	0.088	11813600	0.520	0.137	360248	C	T	0.055	10	11855599	2.61E-06	0.012	14306	22.305
gen_samplesize_Vascular dementia (other)	order Desulfovibrionales	rs17791387	A	G	-0.048	79219511	0.835	0.231	360248	A	G	-0.073	9	81834426	2.25E-06	0.015	14306	22.210
gen_samplesize_Vascular dementia (other)	order Desulfovibrionales	rs186073	T	C	-0.183	31091909	0.177	0.136	360248	T	C	0.053	3	31133401	8.74E-06	0.012	14306	19.931
gen_samplesize_Vascular dementia (other)	order Desulfovibrionales	rs2692012	G	A	0.209	204022477	0.478	0.294	360248	G	A	-0.112	1	203991605	2.27E-06	0.025	14306	19.597

gen_samplesize_Vascular dementia (other)	order Desulfovibrionales	rs2838334	G	A	0.315	43645080	0.025	0.140	360248	G	A	0.057	21	45064961	4.17E-06	0.012	14306	20.982
gen_samplesize_Vascular dementia (other)	order Desulfovibrionales	rs3935584	C	T	-0.116	233064573	0.383	0.134	360248	C	T	-0.052	2	233929283	7.20E-06	0.012	14306	20.531
gen_samplesize_Vascular dementia (other)	order Desulfovibrionales	rs4506934	C	T	-0.003	2953368	0.987	0.204	360248	C	T	-0.095	17	2856662	2.43E-06	0.020	14306	22.416
gen_samplesize_Vascular dementia (other)	order Desulfovibrionales	rs6058181	C	T	0.207	35106998	0.247	0.178	360248	C	T	0.084	20	33694801	2.53E-07	0.017	14306	25.379
gen_samplesize_Vascular dementia (other)	order Desulfovibrionales	rs62020470	A	G	0.107	95617836	0.538	0.174	360248	A	G	-0.057	15	96161065	7.51E-06	0.013	14306	19.633
gen_samplesize_Vascular dementia (other)	order Desulfovibrionales	rs72647048	T	C	-0.017	56952819	0.936	0.210	360248	T	C	-0.077	8	57865378	9.00E-06	0.017	14306	20.436
gen_samplesize_Vascular dementia (other)	order Desulfovibrionales	rs9928243	C	A	0.037	71507738	0.779	0.133	360248	C	A	-0.054	16	71541641	3.97E-06	0.012	14306	21.378
gen_samplesize_Vascular dementia (other)	order Enterobacteriales	rs11026530	T	C	-0.197	22357551	0.292	0.188	360248	T	C	0.082	11	22379097	9.43E-06	0.019	14306	19.471
gen_samplesize_Vascular dementia (other)	order Enterobacteriales	rs2374342	C	A	-0.349	41906402	0.005	0.125	360248	C	A	0.058	2	42133542	4.52E-06	0.013	14306	21.338
gen_samplesize_Vascular dementia (other)	order Enterobacteriales	rs35673018	G	A	-0.089	54293833	0.700	0.232	360248	G	A	0.090	16	54327745	7.63E-06	0.020	14306	19.653
gen_samplesize_Vascular dementia (other)	order Enterobacteriales	rs504442	T	G	-0.098	57478315	0.655	0.219	360248	T	G	0.084	18	55145547	5.17E-06	0.019	14306	19.728
gen_samplesize_Vascular dementia (other)	order Enterobacteriales	rs62210023	A	G	0.206	56765036	0.143	0.141	360248	A	G	0.061	20	55340092	3.13E-06	0.013	14306	21.742
gen_samplesize_Vascular dementia (other)	order Enterobacteriales	rs78143293	A	G	-0.130	60005103	0.520	0.202	360248	A	G	-0.085	18	57672335	1.20E-06	0.017	14306	24.792
gen_samplesize_Vascular dementia (other)	order Enterobacteriales	rs79757635	C	A	-0.219	110188071	0.260	0.194	360248	C	A	0.076	13	110840418	9.32E-06	0.017	14306	19.615
gen_samplesize_Vascular dementia (other)	order Erysipelotrichales	rs1074800	G	A	0.054	3002432	0.688	0.135	360248	G	A	-0.049	5	3002546	6.15E-06	0.011	14306	20.459
gen_samplesize_Vascular dementia (other)	order Erysipelotrichales	rs10781552	C	T	-0.190	132083729	0.196	0.147	360248	C	T	-0.055	10	133897233	2.33E-06	0.012	14306	22.633
gen_samplesize_Vascular dementia (other)	order Erysipelotrichales	rs17530232	A	G	-0.294	39811320	0.317	0.293	360248	A	G	0.103	13	40385457	2.79E-06	0.022	14306	21.042
gen_samplesize_Vascular dementia (other)	order Erysipelotrichales	rs1884466	C	T	-0.152	63673525	0.256	0.134	360248	C	T	-0.048	1	64139196	9.53E-06	0.011	14306	19.760
gen_samplesize_Vascular dementia (other)	order Erysipelotrichales	rs2300774	A	G	-0.043	196066841	0.745	0.133	360248	A	G	-0.052	3	195793712	8.95E-07	0.011	14306	24.094
gen_samplesize_Vascular dementia (other)	order Erysipelotrichales	rs290833	T	G	-0.264	96991871	0.047	0.133	360248	T	G	-0.050	1	97457427	8.03E-06	0.011	14306	19.943
gen_samplesize_Vascular dementia (other)	order Erysipelotrichales	rs35161940	T	C	-0.021	72331083	0.922	0.218	360248	T	C	-0.081	17	70327224	1.85E-06	0.017	14306	23.118
gen_samplesize_Vascular dementia (other)	order Erysipelotrichales	rs4078432	T	C	0.018	48528003	0.919	0.177	360248	T	C	0.061	14	48997206	4.23E-06	0.013	14306	20.723
gen_samplesize_Vascular dementia (other)	order Erysipelotrichales	rs56970041	T	G	0.127	79891267	0.644	0.275	360248	T	G	0.072	14	80357610	5.40E-06	0.016	14306	19.385
gen_samplesize_Vascular dementia (other)	order Erysipelotrichales	rs62504403	C	T	0.099	38946033	0.551	0.166	360248	C	T	0.068	8	38803551	1.12E-07	0.013	14306	28.371
gen_samplesize_Vascular dementia (other)	order Erysipelotrichales	rs7234058	T	C	-0.110	5830508	0.629	0.228	360248	T	C	-0.095	18	5830507	9.12E-07	0.019	14306	23.744
gen_samplesize_Vascular dementia (other)	order Erysipelotrichales	rs7826267	G	T	0.117	3097430	0.658	0.263	360248	G	T	0.084	8	2954952	9.28E-06	0.020	14306	17.755
gen_samplesize_Vascular dementia (other)	order Erysipelotrichales	rs8003149	C	T	-0.097	55689786	0.493	0.142	360248	C	T	0.054	14	56156504	4.08E-06	0.012	14306	21.248
gen_samplesize_Vascular dementia (other)	order Gastranaerophilales	rs11150282	T	C	0.112	80459808	0.425	0.140	360248	T	C	0.098	16	80493705	7.36E-07	0.020	14306	24.834
gen_samplesize_Vascular dementia (other)	order Gastranaerophilales	rs113884518	T	C	-0.300	24648999	0.486	0.430	360248	T	C	-0.206	9	24648997	7.74E-06	0.046	14306	20.446
gen_samplesize_Vascular dementia (other)	order Gastranaerophilales	rs28678345	T	C	0.466	55828967	0.127	0.306	360248	T	C	0.213	17	53906328	8.06E-06	0.047	14306	20.434
gen_samplesize_Vascular dementia (other)	order Gastranaerophilales	rs367480	A	G	-0.030	2916401	0.831	0.139	360248	A	G	0.084	11	2937631	7.52E-06	0.019	14306	20.487
gen_samplesize_Vascular dementia (other)	order Gastranaerophilales	rs4129395	G	A	0.118	113213109	0.378	0.134	360248	G	A	0.090	9	115975389	1.22E-06	0.019	14306	23.826
gen_samplesize_Vascular dementia (other)	order Gastranaerophilales	rs789069	A	C	-0.197	1008277	0.309	0.193	360248	A	C	-0.104	18	1008278	6.50E-06	0.023	14306	19.725
gen_samplesize_Vascular dementia (other)	order Gastranaerophilales	rs79790072	T	C	-0.217	100207478	0.574	0.386	360248	T	C	0.226	15	100747683	3.54E-06	0.049	14306	21.466
gen_samplesize_Vascular dementia (other)	order Gastranaerophilales	rs8028558	A	G	-0.115	61763554	0.404	0.138	360248	A	G	0.083	15	62055753	9.78E-06	0.019	14306	19.603
gen_samplesize_Vascular dementia (other)	order Gastranaerophilales	rs9864379	T	C	-0.122	14265449	0.517	0.188	360248	T	C	-0.161	3	14306949	4.66E-08	0.029	14306	30.065
gen_samplesize_Vascular dementia (other)	order Lactobacillales	rs11110282	A	G	0.024	100191781	0.939	0.312	360248	A	G	-0.102	12	100585559	3.96E-06	0.022	14306	22.090
gen_samplesize_Vascular dementia (other)	order Lactobacillales	rs11627423	C	A	-0.342	32731417	0.013	0.137	360248	C	A	0.050	14	33200623	5.09E-06	0.011	14306	20.697
gen_samplesize_Vascular dementia (other)	order Lactobacillales	rs11730038	G	A	0.027	97128348	0.852	0.146	360248	G	A	-0.061	4	98049499	5.10E-06	0.013	14306	22.006
gen_samplesize_Vascular dementia (other)	order Lactobacillales	rs12797734	T	C	0.052	8310803	0.735	0.155	360248	T	C	0.057	11	8332350	7.77E-06	0.013	14306	20.198
gen_samplesize_Vascular dementia (other)	order Lactobacillales	rs1595463	C	A	-0.337	230858942	0.012	0.134	360248	C	A	0.048	2	231723657	7.44E-06	0.011	14306	19.443
gen_samplesize_Vascular dementia (other)	order Lactobacillales	rs2370083	G	T	-0.281	97060413	0.299	0.270	360248	G	T	-0.081	14	97526750	8.33E-06	0.018	14306	19.802
gen_samplesize_Vascular dementia (other)	order Lactobacillales	rs2952251	G	A	0.110	10285654	0.485	0.158	360248	G	A	0.063	8	10143164	3.36E-07	0.012	14306	25.684
gen_samplesize_Vascular dementia (other)	order Lactobacillales	rs34989881	A	G	-0.390	51456601	0.231	0.326	360248	A	G	0.113	19	51959855	4.09E-06	0.025	14306	21.201
gen_samplesize_Vascular dementia (other)	order Lactobacillales	rs35344081	G	A	0.086	941253	0.565	0.150	360248	G	A	0.064	16	991253	4.16E-07	0.013	14306	25.446
gen_samplesize_Vascular dementia (other)	order Lactobacillales	rs4028634	C	T	0.040	42683631	0.772	0.138	360248	C	T	-0.053	17	40835649	1.35E-06	0.011	14306	23.428
gen_samplesize_Vascular dementia (other)	order Lactobacillales	rs57872228	C	T	0.271	200449677	0.181	0.202	360248	C	T	-0.069	1	200418805	2.58E-06	0.015	14306	21.933
gen_samplesize_Vascular dementia (other)	order Lactobacillales	rs74663707	C	T	0.072	184653436	0.789	0.271	360248	C	T	0.098	3	184371224	8.40E-06	0.022	14306	19.137
gen_samplesize_Vascular dementia (other)	order Lactobacillales	rs77558518	A	G	-0.210	174746168	0.356	0.228	360248	A	G	-0.106	5	174173171	1.67E-06	0.022	14306	22.695
gen_samplesize_Vascular dementia (other)	order Lactobacillales	rs78938557	T	C	-0.005	36309977	0.990	0.396	360248	T	C	0.106	7	36349586	2.31E-06	0.023	14306	20.412
gen_samplesize_Vascular dementia (other)	order Lactobacillales	rs9581006	T	C	0.148	24399371	0.679	0.359	360248	T	C	-0.226	13	24973509	1.77E-06	0.047	14306	23.163
gen_samplesize_Vascular dementia (other)	order Methanobacteriales	rs10202904	G	T	-0.109	124682691	0.423	0.136	360248	G	T	0.122	2	125440268	3.01E-07	0.024	14306	26.762
gen_samplesize_Vascular dementia (other)	order Methanobacteriales	rs10424197	A	G	0.112	45936063	0.466	0.154	360248	A	G	0.111	19	46439321	9.28E-06	0.025	14306	20.211
gen_samplesize_Vascular dementia (other)	order Methanobacteriales	rs4257531	G	A	0.097	2044483	0.657	0.219	360248	G	A	0.164	3	2086167	7.44E-06	0.036	14306	20.316
gen_samplesize_Vascular dementia (other)	order Methanobacteriales	rs6508769	C	T	0.056	28336853	0.767	0.188	360248	C	T	-0.154	19	28827760	8.23E-06	0.034	14306	19.856
gen_samplesize_Vascular dementia (other)	order Methanobacteriales	rs6776814	T	C	0.088	15011576	0.851	0.471	360248	T	C	-0.200	3	15053083	1.63E-06	0.041	14306	23.483
gen_samplesize_Vascular dementia (other)	order Methanobacteriales	rs73068003	G	T	-0.346	10734305	0.125	0.226	360248	G	T	-0.158	7	10773932	8.45E-06	0.035	14306	20.206
gen_samplesize_Vascular dementia (other)	order Methanobacteriales	rs73457410	A	G	-0.101	41382045	0.712	0.274	360248	A	G	0.215	13	41956181	1.41E-06	0.044	14306	24.316
gen_samplesize_Vascular dementia (other)	order Methanobacteriales	rs75208022	C	T	0.004	21185927	0.985	0.225	360248	C	T	-0.227	12	21338861	5.92E-06	0.049	14306	21.717
gen_samplesize_Vascular dementia (other)	order Methanobacteriales	rs894996	C	A	0.105	103497150	0.682	0.255	360248	C	A	0.217	4	104418307	1.88E-06	0.045	14306	23.349
gen_samplesize_Vascular dementia (other)	order Mollicutes RF9	rs11779863	G	A	0.238	15549321	0.189	0.181	360248	G	A	-0.077	8	15406830	6.69E-06	0.017	14306	20.146
gen_samplesize_Vascular dementia (other)	order Mollicutes RF9	rs12566890	T	G	0.075	61385192	0.706	0.200	360248	T	G	-0.103	1	61850864	8.11E-06	0.024	14306	18.196
gen_samplesize_Vascular dementia (other)	order Mollicutes RF9	rs13100746	C	T	-0.140	166128718	0.294	0.133	360248	C	T	0.064	3	165846506	7.29E-06	0.014	14306	20.055
gen_samplesize_Vascular dementia (other)	order Mollicutes RF9	rs17235252	T	C	-0.083	82906721	0.687	0.207	360248	T	C	-0.122	7	82536037	2.16E-06	0.026	14306	22.808
gen_samplesize_Vascular dementia (other)	order Mollicutes RF9	rs3932485	C	T	0.351	184475694	0.010	0.137	360248	C	T	0.063	3	184193482	9.93E-06	0.014	14306	19.551
gen_samplesize_Vascular dementia (other)	order Mollicutes RF9	rs515984	C	T	0.371	3479267	0.073	0.207	360248	C	T	0.088	10	3521459	6.61E-06	0.019	14306	21.078
gen_samplesize_Vascular dementia (other)	order Mollicutes RF9	rs638542	A	G	0.084	109302630	0.563	0.146	360248	A	G	0.071	1	109845252	5.17E-06	0.016	14306	20.204

gen_samplesize_Vascular dementia (other)	order Mollicutes RF9	rs74603314	T	C	0.101	46050515	0.769	0.343	360248	T	C	0.231	14	46519718	2.28E-06	0.049	14306	22.207
gen_samplesize_Vascular dementia (other)	order Mollicutes RF9	rs7706512	A	G	-0.224	17443545	0.095	0.134	360248	A	G	-0.066	5	17443654	2.27E-06	0.014	14306	22.372
gen_samplesize_Vascular dementia (other)	order Mollicutes RF9	rs7801843	A	G	0.048	4066073	0.796	0.185	360248	A	G	-0.087	7	4105705	9.47E-06	0.019	14306	19.983
gen_samplesize_Vascular dementia (other)	order Mollicutes RF9	rs7853673	A	G	0.064	113948961	0.634	0.134	360248	A	G	0.062	9	116711241	6.73E-06	0.014	14306	19.938
gen_samplesize_Vascular dementia (other)	order Mollicutes RF9	rs949341	A	G	-0.223	112516062	0.132	0.148	360248	A	G	-0.066	11	112386785	7.73E-06	0.015	14306	19.893
gen_samplesize_Vascular dementia (other)	order NB1n	rs11251024	G	A	0.177	2053532	0.239	0.150	360248	G	A	0.104	10	2095726	6.63E-07	0.021	14306	25.404
gen_samplesize_Vascular dementia (other)	order NB1n	rs11606187	A	G	0.100	91856434	0.594	0.188	360248	A	G	-0.155	11	91589600	3.31E-06	0.033	14306	22.432
gen_samplesize_Vascular dementia (other)	order NB1n	rs13385922	T	C	-0.010	233172678	0.945	0.140	360248	T	C	0.093	2	234081324	3.97E-06	0.020	14306	21.311
gen_samplesize_Vascular dementia (other)	order NB1n	rs166849	A	G	0.018	199914143	0.895	0.134	360248	A	G	-0.091	2	200778866	7.74E-06	0.020	14306	20.294
gen_samplesize_Vascular dementia (other)	order NB1n	rs2172426	T	C	-0.004	18371511	0.974	0.136	360248	T	C	0.102	8	18229020	3.17E-07	0.020	14306	26.348
gen_samplesize_Vascular dementia (other)	order NB1n	rs267959	G	A	-0.065	10737690	0.661	0.149	360248	G	A	-0.099	5	10737802	2.62E-06	0.021	14306	22.231
gen_samplesize_Vascular dementia (other)	order NB1n	rs4383094	C	T	-0.121	81402631	0.533	0.194	360248	C	T	-0.149	15	81694972	4.28E-06	0.032	14306	21.646
gen_samplesize_Vascular dementia (other)	order NB1n	rs60775321	T	C	0.074	96775441	0.615	0.147	360248	T	C	-0.096	15	97318671	7.10E-06	0.021	14306	20.256
gen_samplesize_Vascular dementia (other)	order NB1n	rs72671304	T	C	0.085	38956767	0.729	0.247	360248	T	C	0.172	14	39425971	3.80E-06	0.037	14306	21.688
gen_samplesize_Vascular dementia (other)	order NB1n	rs7911787	G	T	-0.625	96859811	0.092	0.372	360248	G	T	-0.223	10	98619568	3.39E-06	0.047	14306	22.504
gen_samplesize_Vascular dementia (other)	order NB1n	rs8126061	T	C	0.196	3424592	0.329	0.201	360248	T	C	-0.159	20	3405239	7.36E-06	0.035	14306	20.450
gen_samplesize_Vascular dementia (other)	order NB1n	rs9542068	T	C	-0.153	69732699	0.273	0.139	360248	T	C	0.099	13	70306831	6.52E-06	0.022	14306	20.625
gen_samplesize_Vascular dementia (other)	order Pasteurellales	rs10965428	C	A	0.011	22718482	0.969	0.288	360248	C	A	-0.120	9	22718481	4.29E-06	0.026	14306	21.561
gen_samplesize_Vascular dementia (other)	order Pasteurellales	rs111582866	G	A	0.153	48708578	0.516	0.236	360248	G	A	-0.114	16	48742489	7.07E-06	0.026	14306	19.753
gen_samplesize_Vascular dementia (other)	order Pasteurellales	rs12050685	A	G	-0.206	73185141	0.158	0.146	360248	A	G	-0.067	15	73477482	9.19E-06	0.015	14306	19.385
gen_samplesize_Vascular dementia (other)	order Pasteurellales	rs16970009	A	G	-0.051	34535582	0.917	0.493	360248	A	G	0.187	17	32862601	7.32E-06	0.043	14306	19.027
gen_samplesize_Vascular dementia (other)	order Pasteurellales	rs4822728	T	C	-0.228	26495842	0.086	0.133	360248	T	C	0.069	22	26891808	4.72E-06	0.015	14306	21.156
gen_samplesize_Vascular dementia (other)	order Pasteurellales	rs6972479	A	G	-0.163	117278006	0.323	0.165	360248	A	G	-0.078	7	116918060	7.75E-06	0.018	14306	19.878
gen_samplesize_Vascular dementia (other)	order Pasteurellales	rs72756943	G	A	-0.032	26531799	0.906	0.267	360248	G	A	0.140	5	26531908	3.35E-06	0.030	14306	21.308
gen_samplesize_Vascular dementia (other)	order Pasteurellales	rs73139353	A	C	0.175	98253370	0.454	0.234	360248	A	C	-0.223	3	97972214	8.71E-06	0.048	14306	21.092
gen_samplesize_Vascular dementia (other)	order Pasteurellales	rs76022354	C	T	-0.496	92546628	0.105	0.306	360248	C	T	0.243	10	94306385	1.83E-06	0.050	14306	23.560
gen_samplesize_Vascular dementia (other)	order Pasteurellales	rs78909003	T	C	-0.158	102887960	0.586	0.290	360248	T	C	-0.241	9	105650242	2.05E-06	0.050	14306	23.415
gen_samplesize_Vascular dementia (other)	order Pasteurellales	rs9382510	C	T	-0.173	55583693	0.255	0.152	360248	C	T	-0.088	6	55448491	2.48E-07	0.017	14306	26.921
gen_samplesize_Vascular dementia (other)	order Pasteurellales	rs9895850	T	C	-0.175	66538895	0.585	0.320	360248	T	C	-0.176	17	64535013	9.08E-06	0.041	14306	18.497
gen_samplesize_Vascular dementia (other)	order Pasteurellales	rs9938097	C	T	0.070	84943783	0.611	0.137	360248	C	T	0.071	16	84977389	8.23E-06	0.016	14306	20.209
gen_samplesize_Vascular dementia (other)	order Rhodospirillales	rs1035406	G	A	-0.102	120037042	0.623	0.208	360248	G	A	-0.115	5	119372737	4.07E-06	0.025	14306	21.401
gen_samplesize_Vascular dementia (other)	order Rhodospirillales	rs11591293	G	T	-0.182	111660039	0.175	0.134	360248	G	T	0.072	10	113419797	4.69E-06	0.016	14306	20.876
gen_samplesize_Vascular dementia (other)	order Rhodospirillales	rs11630875	T	C	-0.122	61483535	0.545	0.202	360248	T	C	0.095	15	61775734	3.70E-06	0.020	14306	21.865
gen_samplesize_Vascular dementia (other)	order Rhodospirillales	rs13336560	C	T	0.050	88487835	0.711	0.135	360248	C	T	-0.070	16	88554243	9.75E-06	0.016	14306	19.598
gen_samplesize_Vascular dementia (other)	order Rhodospirillales	rs1549633	A	C	-0.255	27945538	0.224	0.209	360248	A	C	0.100	5	27945645	3.88E-06	0.022	14306	21.163
gen_samplesize_Vascular dementia (other)	order Rhodospirillales	rs3730086	A	G	0.085	68281223	0.579	0.154	360248	A	G	0.080	5	67577051	7.98E-06	0.018	14306	20.051
gen_samplesize_Vascular dementia (other)	order Rhodospirillales	rs3754624	C	T	0.138	224769095	0.428	0.174	360248	C	T	0.094	2	225633812	2.68E-06	0.020	14306	22.511
gen_samplesize_Vascular dementia (other)	order Rhodospirillales	rs4278423	T	C	-0.225	2628361	0.420	0.278	360248	T	C	0.105	10	2670553	3.98E-06	0.023	14306	20.256
gen_samplesize_Vascular dementia (other)	order Rhodospirillales	rs61933850	G	A	0.368	72745618	0.059	0.195	360248	G	A	0.165	12	73139398	7.00E-06	0.036	14306	20.888
gen_samplesize_Vascular dementia (other)	order Rhodospirillales	rs7001029	C	T	0.072	130946157	0.753	0.227	360248	C	T	0.121	8	131958403	2.83E-06	0.026	14306	21.397
gen_samplesize_Vascular dementia (other)	order Rhodospirillales	rs76784716	A	G	-0.067	168176830	0.753	0.212	360248	A	G	0.136	2	169033340	1.31E-06	0.028	14306	22.881
gen_samplesize_Vascular dementia (other)	order Rhodospirillales	rs77304857	C	A	0.108	133326860	0.520	0.167	360248	C	A	-0.100	4	134248015	6.02E-06	0.022	14306	20.157
gen_samplesize_Vascular dementia (other)	order Rhodospirillales	rs9813022	A	G	0.130	13685237	0.343	0.137	360248	A	G	-0.083	3	13726736	3.07E-07	0.016	14306	26.093
gen_samplesize_Vascular dementia (other)	order Selenomonadales	rs1135612	G	A	0.182	75980359	0.260	0.161	360248	G	A	0.053	7	75609677	9.26E-06	0.012	14306	19.761
gen_samplesize_Vascular dementia (other)	order Selenomonadales	rs13086907	G	A	-0.030	142416275	0.851	0.161	360248	G	A	0.063	3	142135117	1.95E-06	0.013	14306	22.532
gen_samplesize_Vascular dementia (other)	order Selenomonadales	rs1643968	T	C	0.086	165839623	0.535	0.139	360248	T	C	-0.057	5	165266628	4.15E-07	0.011	14306	25.339
gen_samplesize_Vascular dementia (other)	order Selenomonadales	rs1649999	A	G	-0.260	78326315	0.249	0.225	360248	A	G	0.075	10	80086072	7.58E-06	0.017	14306	20.246
gen_samplesize_Vascular dementia (other)	order Selenomonadales	rs2834062	A	G	0.126	33005177	0.391	0.147	360248	A	G	0.049	21	34377485	8.44E-06	0.011	14306	20.190
gen_samplesize_Vascular dementia (other)	order Selenomonadales	rs4463806	C	T	0.105	113838234	0.527	0.167	360248	C	T	0.054	10	115597993	7.81E-06	0.013	14306	17.681
gen_samplesize_Vascular dementia (other)	order Selenomonadales	rs4722181	T	G	-0.105	22777952	0.428	0.132	360248	T	G	0.050	7	22817571	2.00E-06	0.011	14306	22.452
gen_samplesize_Vascular dementia (other)	order Selenomonadales	rs60274479	T	C	-0.090	21238604	0.591	0.168	360248	T	C	-0.066	16	21249925	1.16E-06	0.013	14306	24.182
gen_samplesize_Vascular dementia (other)	order Selenomonadales	rs61249479	A	C	0.132	122150629	0.478	0.186	360248	A	C	0.078	9	124912908	2.95E-06	0.017	14306	21.236
gen_samplesize_Vascular dementia (other)	order Selenomonadales	rs71405394	G	A	0.257	100704883	0.327	0.262	360248	G	A	-0.114	15	101245088	2.17E-06	0.024	14306	22.539
gen_samplesize_Vascular dementia (other)	order Selenomonadales	rs73232831	G	A	0.117	17411803	0.735	0.346	360248	G	A	-0.152	4	17413426	1.87E-06	0.031	14306	23.242
gen_samplesize_Vascular dementia (other)	order Selenomonadales	rs9423647	G	A	0.082	5537855	0.541	0.134	360248	G	A	0.048	10	5579818	6.06E-06	0.011	14306	20.628
gen_samplesize_Vascular dementia (other)	order Verrucomicrobiales	rs111862613	T	C	0.109	129825125	0.542	0.179	360248	T	C	0.091	12	130309670	3.74E-06	0.020	14306	21.252
gen_samplesize_Vascular dementia (other)	order Verrucomicrobiales	rs117107102	A	G	-0.510	51947265	0.106	0.315	360248	A	G	0.205	18	49473635	2.92E-06	0.043	14306	22.493
gen_samplesize_Vascular dementia (other)	order Verrucomicrobiales	rs11729256	T	C	-0.231	94106121	0.192	0.177	360248	T	C	0.075	4	95027272	6.73E-07	0.015	14306	24.928
gen_samplesize_Vascular dementia (other)	order Verrucomicrobiales	rs12908520	G	A	0.088	97027427	0.513	0.134	360248	G	A	0.062	15	97570657	2.17E-06	0.013	14306	22.341
gen_samplesize_Vascular dementia (other)	order Verrucomicrobiales	rs2602429	T	C	-0.031	81029544	0.839	0.152	360248	T	C	-0.075	16	81063149	2.58E-06	0.016	14306	22.863
gen_samplesize_Vascular dementia (other)	order Verrucomicrobiales	rs4242783	A	G	-0.137	5022135	0.356	0.149	360248	A	G	-0.069	10	5064327	2.64E-06	0.015	14306	21.781
gen_samplesize_Vascular dementia (other)	order Verrucomicrobiales	rs4936098	G	A	0.049	130410772	0.723	0.139	360248	G	A	-0.065	11	130280667	1.12E-06	0.014	14306	22.786
gen_samplesize_Vascular dementia (other)	order Verrucomicrobiales	rs61779207	G	A	-0.037	40608800	0.817	0.162	360248	G	A	-0.076	1	41074472	6.72E-06	0.017	14306	20.432
gen_samplesize_Vascular dementia (other)	order Verrucomicrobiales	rs74542928	T	C	-0.380	99623031	0.214	0.306	360248	T	C	0.112	4	100544188	1.63E-06	0.024	14306	22.508
gen_samplesize_Vascular dementia (other)	order Verrucomicrobiales	rs9349825	A	G	-0.366	56476683	0.032	0.171	360248	A	G	-0.070	6	56341481	2.54E-06	0.015	14306	22.898
gen_samplesize_Vascular dementia (other)	order Verrucomicrobiales	rs941682	G	A	0.084	33280034	0.573	0.149	360248	G	A	-0.063	20	31867840	9.61E-06	0.014	14306	19.290
gen_samplesize_Vascular dementia (other)	order Victivallales	rs1002941	A	G	-0.036	100702485	0.814	0.153	360248	A	G	-0.105	15	101242690	8.15E-06	0.023	14306	20.234

gen_samplesize_Vascular dementia (other)	order Victivallales	rs11770843	C	T	-0.034	147098287	0.810	0.140	360248	C	T	0.109	7	146795379	1.91E-06	0.023	14306	21.707
gen_samplesize_Vascular dementia (other)	order Victivallales	rs17114848	G	A	-0.077	24917388	0.738	0.231	360248	G	A	0.152	15	25162535	4.06E-06	0.032	14306	22.073
gen_samplesize_Vascular dementia (other)	order Victivallales	rs2031282	A	G	-0.329	20113040	0.062	0.177	360248	A	G	0.122	13	20687179	4.38E-06	0.027	14306	20.490
gen_samplesize_Vascular dementia (other)	order Victivallales	rs2825714	A	G	-0.201	19651652	0.257	0.177	360248	A	G	-0.137	21	21023966	1.72E-06	0.029	14306	22.568
gen_samplesize_Vascular dementia (other)	order Victivallales	rs62570196	C	T	0.390	108323890	0.238	0.330	360248	C	T	-0.216	9	111086170	1.08E-06	0.044	14306	24.192
gen_samplesize_Vascular dementia (other)	order Victivallales	rs72640280	A	G	0.100	11883735	0.727	0.288	360248	A	G	0.220	1	11943792	5.18E-06	0.049	14306	20.513
gen_samplesize_Vascular dementia (other)	order Victivallales	rs77599476	A	G	-0.198	62762910	0.489	0.286	360248	A	G	0.230	20	61394262	1.86E-06	0.048	14306	23.002
gen_samplesize_Vascular dementia (other)	phylum Actinobacteria	rs11766971	C	T	0.043	155211037	0.751	0.134	360248	C	T	-0.048	7	155002747	9.40E-06	0.011	14306	20.023
gen_samplesize_Vascular dementia (other)	phylum Actinobacteria	rs12528285	C	T	-0.106	92065492	0.623	0.215	360248	C	T	0.081	6	92775210	5.69E-06	0.018	14306	20.048
gen_samplesize_Vascular dementia (other)	phylum Actinobacteria	rs13192624	T	C	-0.043	112808407	0.780	0.154	360248	T	C	-0.052	6	113129609	9.33E-06	0.012	14306	19.720
gen_samplesize_Vascular dementia (other)	phylum Actinobacteria	rs1397793	A	G	-0.061	91175634	0.672	0.144	360248	A	G	0.052	5	90471451	3.74E-06	0.011	14306	21.832
gen_samplesize_Vascular dementia (other)	phylum Actinobacteria	rs4429415	C	T	0.103	212891467	0.440	0.133	360248	C	T	0.058	2	213756191	2.05E-07	0.011	14306	27.314
gen_samplesize_Vascular dementia (other)	phylum Actinobacteria	rs55888705	A	G	0.223	1516099	0.130	0.148	360248	A	G	0.053	4	1517826	1.31E-06	0.011	14306	23.597
gen_samplesize_Vascular dementia (other)	phylum Actinobacteria	rs6496870	C	T	-0.279	91924192	0.045	0.139	360248	C	T	-0.051	15	92467422	4.62E-06	0.011	14306	20.142
gen_samplesize_Vascular dementia (other)	phylum Actinobacteria	rs6743026	T	C	0.246	101633121	0.145	0.169	360248	T	C	0.059	2	102249583	9.88E-06	0.013	14306	19.171
gen_samplesize_Vascular dementia (other)	phylum Actinobacteria	rs74037001	G	A	0.111	23377985	0.634	0.233	360248	G	A	-0.082	14	23847194	6.71E-07	0.017	14306	24.562
gen_samplesize_Vascular dementia (other)	phylum Actinobacteria	rs75211493	G	A	-0.140	5300454	0.582	0.254	360248	G	A	0.084	19	5300465	9.27E-06	0.018	14306	20.714
gen_samplesize_Vascular dementia (other)	phylum Actinobacteria	rs7570971	A	C	0.270	135080336	0.050	0.138	360248	A	C	0.087	2	135837906	1.41E-14	0.011	14306	58.161
gen_samplesize_Vascular dementia (other)	phylum Actinobacteria	rs80124826	T	C	-0.314	239014688	0.436	0.402	360248	T	C	-0.124	2	239936384	8.75E-06	0.028	14306	19.876
gen_samplesize_Vascular dementia (other)	phylum Actinobacteria	rs857444	C	T	0.144	14617360	0.294	0.137	360248	C	T	0.051	6	14617591	3.80E-06	0.011	14306	21.315
gen_samplesize_Vascular dementia (other)	phylum Actinobacteria	rs9833771	C	T	-0.003	32321506	0.983	0.134	360248	C	T	-0.049	3	32362998	4.07E-06	0.011	14306	21.150
gen_samplesize_Vascular dementia (other)	phylum Bacteroidetes	rs17343978	A	C	0.115	27037922	0.482	0.164	360248	A	C	-0.056	22	27433885	7.22E-06	0.012	14306	21.374
gen_samplesize_Vascular dementia (other)	phylum Bacteroidetes	rs2032750	C	T	-0.323	53603889	0.015	0.133	360248	C	T	0.051	2	53831026	1.71E-06	0.011	14306	22.878
gen_samplesize_Vascular dementia (other)	phylum Bacteroidetes	rs62531359	T	G	0.295	70003946	0.089	0.174	360248	T	G	0.066	8	70916181	8.42E-06	0.015	14306	19.260
gen_samplesize_Vascular dementia (other)	phylum Bacteroidetes	rs62575403	C	T	-0.216	133628698	0.509	0.327	360248	C	T	0.145	9	136493820	2.96E-06	0.031	14306	21.854
gen_samplesize_Vascular dementia (other)	phylum Bacteroidetes	rs6586324	T	C	0.125	42522025	0.352	0.134	360248	T	C	0.048	21	43942135	7.37E-06	0.011	14306	20.535
gen_samplesize_Vascular dementia (other)	phylum Bacteroidetes	rs72706335	T	C	-0.489	157525648	0.256	0.431	360248	T	C	-0.223	1	157495438	7.13E-06	0.049	14306	20.464
gen_samplesize_Vascular dementia (other)	phylum Bacteroidetes	rs73512608	G	A	0.058	69948551	0.842	0.291	360248	G	A	-0.123	13	70522683	2.54E-07	0.024	14306	27.045
gen_samplesize_Vascular dementia (other)	phylum Bacteroidetes	rs73846128	A	G	-0.123	89291104	0.512	0.188	360248	A	G	-0.066	3	89340254	4.78E-07	0.013	14306	24.765
gen_samplesize_Vascular dementia (other)	phylum Bacteroidetes	rs73975615	G	A	0.303	6557880	0.710	0.816	360248	G	A	-0.207	17	6461200	1.20E-06	0.044	14306	21.905
gen_samplesize_Vascular dementia (other)	phylum Bacteroidetes	rs929878	T	C	-0.080	74256742	0.623	0.164	360248	T	C	0.054	16	74290641	6.51E-06	0.012	14306	19.745
gen_samplesize_Vascular dementia (other)	phylum Cyanobacteria	rs12555298	G	A	-0.211	107727270	0.232	0.177	360248	G	A	0.097	9	110489551	8.09E-06	0.022	14306	19.730
gen_samplesize_Vascular dementia (other)	phylum Cyanobacteria	rs2585223	T	C	-0.160	100340683	0.432	0.204	360248	T	C	0.111	15	100880888	8.86E-06	0.025	14306	20.206
gen_samplesize_Vascular dementia (other)	phylum Cyanobacteria	rs584122	T	C	-0.572	48320720	0.010	0.222	360248	T	C	0.152	6	48288456	4.23E-06	0.033	14306	21.565
gen_samplesize_Vascular dementia (other)	phylum Cyanobacteria	rs61972390	T	C	-0.118	99914504	0.541	0.192	360248	T	C	0.107	13	100566758	9.11E-06	0.024	14306	19.600
gen_samplesize_Vascular dementia (other)	phylum Cyanobacteria	rs7148504	T	G	0.229	99701961	0.092	0.136	360248	T	G	-0.080	14	100168298	6.62E-06	0.018	14306	20.537
gen_samplesize_Vascular dementia (other)	phylum Cyanobacteria	rs76531781	T	C	-0.018	21608117	0.959	0.339	360248	T	C	-0.232	7	21647735	2.87E-06	0.049	14306	22.085
gen_samplesize_Vascular dementia (other)	phylum Cyanobacteria	rs789068	G	A	-0.197	1008238	0.309	0.193	360248	G	A	-0.111	18	1008239	1.57E-07	0.021	14306	27.366
gen_samplesize_Vascular dementia (other)	phylum Cyanobacteria	rs9864379	T	C	-0.122	14265449	0.517	0.188	360248	T	C	-0.139	3	14306949	2.03E-07	0.027	14306	26.866
gen_samplesize_Vascular dementia (other)	phylum Euryarchaeota	rs10202904	G	T	-0.109	124682691	0.423	0.136	360248	G	T	0.116	2	125440268	6.19E-07	0.023	14306	25.371
gen_samplesize_Vascular dementia (other)	phylum Euryarchaeota	rs11022995	A	G	0.114	13872120	0.391	0.133	360248	A	G	0.104	11	13893667	7.73E-06	0.023	14306	20.538
gen_samplesize_Vascular dementia (other)	phylum Euryarchaeota	rs34928225	T	C	0.090	146519740	0.692	0.228	360248	T	C	0.200	6	146840876	4.33E-06	0.043	14306	22.051
gen_samplesize_Vascular dementia (other)	phylum Euryarchaeota	rs45498998	G	A	-0.122	25996679	0.504	0.182	360248	G	A	-0.132	21	27368994	5.32E-06	0.029	14306	20.358
gen_samplesize_Vascular dementia (other)	phylum Euryarchaeota	rs6064552	T	C	-0.132	57467810	0.444	0.172	360248	T	C	-0.124	20	56042866	9.34E-06	0.028	14306	20.095
gen_samplesize_Vascular dementia (other)	phylum Euryarchaeota	rs6508769	C	T	0.056	28336853	0.767	0.188	360248	C	T	-0.151	19	28827760	8.12E-06	0.034	14306	19.941
gen_samplesize_Vascular dementia (other)	phylum Euryarchaeota	rs7015093	G	A	0.081	57106294	0.595	0.152	360248	G	A	-0.118	8	58018853	7.20E-06	0.026	14306	19.957
gen_samplesize_Vascular dementia (other)	phylum Euryarchaeota	rs76029318	T	C	-0.086	41389655	0.754	0.276	360248	T	C	0.215	13	41963791	1.05E-06	0.044	14306	24.017
gen_samplesize_Vascular dementia (other)	phylum Euryarchaeota	rs7635189	A	G	0.275	15550100	0.062	0.148	360248	A	G	-0.120	3	15591607	4.64E-06	0.026	14306	21.402
gen_samplesize_Vascular dementia (other)	phylum Euryarchaeota	rs77658038	A	C	-0.063	16284554	0.702	0.165	360248	A	C	-0.160	6	16284785	4.75E-06	0.034	14306	22.078
gen_samplesize_Vascular dementia (other)	phylum Euryarchaeota	rs894996	C	A	0.105	103497150	0.682	0.255	360248	C	A	0.204	4	104418307	5.12E-06	0.044	14306	21.404
gen_samplesize_Vascular dementia (other)	phylum Firmicutes	rs112334273	G	A	0.017	39331325	0.906	0.148	360248	G	A	0.063	21	40703251	9.26E-07	0.013	14306	24.251
gen_samplesize_Vascular dementia (other)	phylum Firmicutes	rs2273429	A	G	0.054	52027354	0.800	0.213	360248	A	G	-0.070	14	52494072	9.26E-06	0.015	14306	21.041
gen_samplesize_Vascular dementia (other)	phylum Firmicutes	rs2332027	A	G	0.089	170750887	0.513	0.136	360248	A	G	0.048	4	171672038	4.05E-06	0.010	14306	21.201
gen_samplesize_Vascular dementia (other)	phylum Firmicutes	rs2547978	G	A	-0.138	98684594	0.316	0.137	360248	G	A	-0.047	5	98020298	8.57E-06	0.011	14306	19.652
gen_samplesize_Vascular dementia (other)	phylum Firmicutes	rs3792064	G	A	0.084	230813698	0.785	0.307	360248	G	A	0.090	2	231678413	6.75E-07	0.018	14306	24.168
gen_samplesize_Vascular dementia (other)	phylum Firmicutes	rs3852931	T	C	0.103	55471425	0.464	0.141	360248	T	C	0.048	20	54087963	4.53E-06	0.011	14306	21.080
gen_samplesize_Vascular dementia (other)	phylum Firmicutes	rs4750583	G	A	0.207	15012766	0.220	0.169	360248	G	A	-0.062	10	15054765	5.79E-06	0.014	14306	19.944
gen_samplesize_Vascular dementia (other)	phylum Firmicutes	rs56199908	T	C	-0.259	2801371	0.364	0.285	360248	T	C	-0.186	9	2801371	8.67E-06	0.041	14306	20.572
gen_samplesize_Vascular dementia (other)	phylum Firmicutes	rs6814436	C	T	-0.014	160586149	0.941	0.190	360248	C	T	-0.068	4	161507301	6.80E-06	0.015	14306	20.334
gen_samplesize_Vascular dementia (other)	phylum Firmicutes	rs6815608	C	T	0.150	151210592	0.422	0.187	360248	C	T	-0.094	4	152131744	7.24E-06	0.021	14306	19.693
gen_samplesize_Vascular dementia (other)	phylum Firmicutes	rs7247191	T	C	0.340	22716783	0.123	0.221	360248	T	C	-0.071	19	22899585	4.73E-06	0.016	14306	20.515
gen_samplesize_Vascular dementia (other)	phylum Firmicutes	rs72738886	T	C	-0.307	35770448	0.226	0.253	360248	T	C	0.086	5	35770550	7.68E-06	0.019	14306	20.621
gen_samplesize_Vascular dementia (other)	phylum Firmicutes	rs72771021	C	T	0.525	13799631	0.050	0.268	360248	C	T	-0.141	10	13841631	5.12E-06	0.031	14306	21.022
gen_samplesize_Vascular dementia (other)	phylum Firmicutes	rs8085381	A	G	0.023	31748342	0.893	0.172	360248	A	G	-0.065	18	29328305	8.67E-06	0.015	14306	19.597
gen_samplesize_Vascular dementia (other)	phylum Firmicutes	rs992074	T	C	-0.169	17195484	0.706	0.450	360248	T	C	-0.233	21	18567802	8.52E-06	0.051	14306	21.042
gen_samplesize_Vascular dementia (other)	phylum Lentisphaerae	rs1002941	A	G	-0.036	100702485	0.814	0.153	360248	A	G	-0.108	15	101242690	4.31E-06	0.023	14306	21.281
gen_samplesize_Vascular dementia (other)	phylum Lentisphaerae	rs11770843	C	T	-0.034	147098287	0.810	0.140	360248	C	T	0.112	7	146795379	1.14E-06	0.023	14306	22.715

gen_samplesize_Vascular dementia (other)	phylum Lentisphaerae	rs17114848	G	A	-0.077	24917388	0.738	0.231	360248	G	A	0.149	15	25162535	6.77E-06	0.032	14306	21.120
gen_samplesize_Vascular dementia (other)	phylum Lentisphaerae	rs2031282	A	G	-0.329	20113040	0.062	0.177	360248	A	G	0.120	13	20687179	5.86E-06	0.027	14306	19.850
gen_samplesize_Vascular dementia (other)	phylum Lentisphaerae	rs2825714	A	G	-0.201	19651652	0.257	0.177	360248	A	G	-0.138	21	21023966	1.50E-06	0.029	14306	22.861
gen_samplesize_Vascular dementia (other)	phylum Lentisphaerae	rs60995569	T	G	0.128	52170325	0.542	0.211	360248	T	G	-0.161	10	53930085	9.19E-06	0.034	14306	22.743
gen_samplesize_Vascular dementia (other)	phylum Lentisphaerae	rs62570196	C	T	0.390	108323890	0.238	0.330	360248	C	T	-0.217	9	111086170	9.64E-07	0.044	14306	24.410
gen_samplesize_Vascular dementia (other)	phylum Lentisphaerae	rs72640280	A	G	0.100	11883735	0.727	0.288	360248	A	G	0.220	1	11943792	5.19E-06	0.049	14306	20.530
gen_samplesize_Vascular dementia (other)	phylum Lentisphaerae	rs77599476	A	G	-0.198	62762910	0.489	0.286	360248	A	G	0.230	20	61394262	1.90E-06	0.048	14306	22.964
gen_samplesize_Vascular dementia (other)	phylum Proteobacteria	rs10750258	C	A	0.109	124126791	0.432	0.139	360248	C	A	0.049	11	123997498	8.72E-06	0.011	14306	20.598
gen_samplesize_Vascular dementia (other)	phylum Proteobacteria	rs11126162	T	C	-0.072	67858902	0.769	0.245	360248	T	C	-0.077	2	68086034	9.26E-06	0.019	14306	16.971
gen_samplesize_Vascular dementia (other)	phylum Proteobacteria	rs11715072	G	A	-0.022	53686766	0.881	0.144	360248	G	A	-0.052	3	53720793	6.90E-06	0.012	14306	20.303
gen_samplesize_Vascular dementia (other)	phylum Proteobacteria	rs12150865	C	T	-0.008	43001168	0.951	0.135	360248	C	T	0.051	19	43505320	1.54E-06	0.011	14306	23.118
gen_samplesize_Vascular dementia (other)	phylum Proteobacteria	rs12467198	C	T	0.116	124115104	0.386	0.134	360248	C	T	0.050	2	124872681	6.31E-06	0.011	14306	20.013
gen_samplesize_Vascular dementia (other)	phylum Proteobacteria	rs2347697	G	T	0.229	134703488	0.111	0.144	360248	G	T	0.050	7	134388240	4.27E-06	0.011	14306	21.247
gen_samplesize_Vascular dementia (other)	phylum Proteobacteria	rs2532663	A	G	0.086	117487723	0.697	0.222	360248	A	G	0.126	10	119247234	7.47E-07	0.026	14306	23.685
gen_samplesize_Vascular dementia (other)	phylum Proteobacteria	rs3890996	G	T	-0.221	20314277	0.096	0.133	360248	G	T	0.047	22	20301800	6.95E-06	0.011	14306	20.183
gen_samplesize_Vascular dementia (other)	phylum Proteobacteria	rs4340090	C	T	-0.195	129847219	0.347	0.207	360248	C	T	-0.067	12	130331764	9.99E-06	0.015	14306	18.975
gen_samplesize_Vascular dementia (other)	phylum Proteobacteria	rs6707783	C	T	-0.054	51826753	0.813	0.230	360248	C	T	0.085	2	52053891	8.09E-06	0.019	14306	20.470
gen_samplesize_Vascular dementia (other)	phylum Proteobacteria	rs72771021	C	T	0.525	13799631	0.050	0.268	360248	C	T	0.142	10	13841631	7.18E-06	0.031	14306	21.004
gen_samplesize_Vascular dementia (other)	phylum Proteobacteria	rs922773	C	T	0.191	66267618	0.389	0.221	360248	C	T	-0.080	3	66318042	3.68E-07	0.016	14306	25.929
gen_samplesize_Vascular dementia (other)	phylum Tenericutes	rs10108398	G	A	-0.118	58528265	0.424	0.148	360248	G	A	0.077	8	59440824	1.09E-06	0.015	14306	24.960
gen_samplesize_Vascular dementia (other)	phylum Tenericutes	rs11890098	A	G	-0.307	156676037	0.038	0.148	360248	A	G	0.074	2	157532549	9.57E-07	0.015	14306	23.551
gen_samplesize_Vascular dementia (other)	phylum Tenericutes	rs12566890	T	G	0.075	61385192	0.706	0.200	360248	T	G	-0.101	1	61850864	3.65E-06	0.023	14306	19.176
gen_samplesize_Vascular dementia (other)	phylum Tenericutes	rs17214486	C	A	-0.105	96693337	0.458	0.142	360248	C	A	0.061	14	97159674	6.61E-06	0.014	14306	20.223
gen_samplesize_Vascular dementia (other)	phylum Tenericutes	rs2464826	A	C	0.144	79860934	0.458	0.211	360248	A	C	0.094	7	79490250	8.39E-06	0.021	14306	19.874
gen_samplesize_Vascular dementia (other)	phylum Tenericutes	rs28537087	G	A	0.041	94766447	0.789	0.154	360248	G	A	0.082	15	95309676	8.07E-06	0.019	14306	19.002
gen_samplesize_Vascular dementia (other)	phylum Tenericutes	rs3768491	G	A	0.089	109423364	0.541	0.145	360248	G	A	0.068	1	109965986	4.23E-06	0.015	14306	20.875
gen_samplesize_Vascular dementia (other)	phylum Tenericutes	rs4885016	C	T	-0.016	72596351	0.934	0.195	360248	C	T	0.082	13	73170489	7.27E-06	0.018	14306	20.363
gen_samplesize_Vascular dementia (other)	phylum Tenericutes	rs6043847	T	C	-0.147	16278879	0.599	0.279	360248	T	C	-0.115	20	16259524	4.55E-06	0.025	14306	21.375
gen_samplesize_Vascular dementia (other)	phylum Tenericutes	rs72901605	T	C	-0.142	47082326	0.491	0.206	360248	T	C	-0.084	11	47103877	3.26E-06	0.018	14306	22.338
gen_samplesize_Vascular dementia (other)	phylum Tenericutes	rs74603314	T	C	0.101	46050515	0.769	0.343	360248	T	C	0.222	14	46519718	1.56E-06	0.046	14306	22.924
gen_samplesize_Vascular dementia (other)	phylum Tenericutes	rs78169027	A	G	-0.067	108568360	0.812	0.284	360248	A	G	-0.108	11	108439087	5.88E-06	0.024	14306	20.824
gen_samplesize_Vascular dementia (other)	phylum Verrucomicrobia	rs11252894	A	C	0.268	5030858	0.086	0.156	360248	A	C	0.078	10	5073050	1.11E-06	0.016	14306	23.203
gen_samplesize_Vascular dementia (other)	phylum Verrucomicrobia	rs117107102	A	G	-0.510	51947265	0.106	0.315	360248	A	G	0.204	18	49473635	2.68E-06	0.043	14306	22.828
gen_samplesize_Vascular dementia (other)	phylum Verrucomicrobia	rs11729256	T	C	-0.231	94106121	0.192	0.177	360248	T	C	0.070	4	95027272	2.23E-06	0.015	14306	22.495
gen_samplesize_Vascular dementia (other)	phylum Verrucomicrobia	rs12512971	A	C	-0.222	16547352	0.372	0.248	360248	A	C	0.171	4	16548975	9.81E-06	0.040	14306	18.467
gen_samplesize_Vascular dementia (other)	phylum Verrucomicrobia	rs12908520	G	A	0.088	97027427	0.513	0.134	360248	G	A	0.059	15	97570657	3.40E-06	0.013	14306	21.524
gen_samplesize_Vascular dementia (other)	phylum Verrucomicrobia	rs2602429	T	C	-0.031	81029544	0.839	0.152	360248	T	C	-0.076	16	81063149	8.71E-07	0.015	14306	24.786
gen_samplesize_Vascular dementia (other)	phylum Verrucomicrobia	rs3995795	C	T	-0.213	11347615	0.118	0.136	360248	C	T	0.061	10	11389614	9.72E-06	0.014	14306	19.430
gen_samplesize_Vascular dementia (other)	phylum Verrucomicrobia	rs45598138	C	A	-0.154	55056410	0.714	0.421	360248	C	A	-0.144	1	55522083	2.19E-06	0.031	14306	22.202
gen_samplesize_Vascular dementia (other)	phylum Verrucomicrobia	rs61779207	G	A	-0.037	40608800	0.817	0.162	360248	G	A	-0.076	1	41074472	5.28E-06	0.016	14306	21.109
gen_samplesize_Vascular dementia (other)	phylum Verrucomicrobia	rs74542928	T	C	-0.380	99623031	0.214	0.306	360248	T	C	0.116	4	100544188	4.08E-07	0.023	14306	25.144
gen_samplesize_Vascular dementia (other)	phylum Verrucomicrobia	rs76430504	T	C	0.752	40438056	0.015	0.310	360248	T	C	-0.118	5	40438158	3.50E-06	0.025	14306	21.313
gen_samplesize_Vascular dementia (other)	phylum Verrucomicrobia	rs9349825	A	G	-0.366	56476683	0.032	0.171	360248	A	G	-0.066	6	56341481	6.27E-06	0.014	14306	21.048
Vascular dementia (subcortical)	class Actinobacteria	rs11655079	T	C	0.021	77352593	0.768	0.073	360770	T	C	-0.056	17	75348675	5.93E-06	0.012	14306	20.351
Vascular dementia (subcortical)	class Actinobacteria	rs11745923	G	T	-0.094	475293	0.106	0.058	360770	G	T	0.056	5	475408	1.58E-06	0.012	14306	23.812
Vascular dementia (subcortical)	class Actinobacteria	rs12049045	A	G	0.074	114122863	0.203	0.058	360770	A	G	0.051	1	114665485	8.63E-06	0.011	14306	19.936
Vascular dementia (subcortical)	class Actinobacteria	rs134366	A	G	0.044	35171362	0.695	0.111	360770	A	G	-0.112	22	35567355	1.50E-06	0.024	14306	22.663
Vascular dementia (subcortical)	class Actinobacteria	rs1376754	G	A	0.100	160755458	0.078	0.057	360770	G	A	0.051	2	161611969	6.71E-06	0.011	14306	20.489
Vascular dementia (subcortical)	class Actinobacteria	rs1515761	C	T	0.125	124421463	0.251	0.109	360770	C	T	-0.076	10	126110032	4.96E-06	0.017	14306	20.136
Vascular dementia (subcortical)	class Actinobacteria	rs182549	T	C	-0.005	135859184	0.936	0.059	360770	T	C	-0.111	2	136616754	3.79E-20	0.012	14306	85.376
Vascular dementia (subcortical)	class Actinobacteria	rs4945008	G	A	0.060	71510202	0.304	0.058	360770	G	A	-0.054	11	71221248	5.39E-06	0.012	14306	19.842
Vascular dementia (subcortical)	class Actinobacteria	rs6660520	G	A	-0.117	206830208	0.066	0.064	360770	G	A	-0.071	1	207003553	1.11E-07	0.013	14306	27.984
Vascular dementia (subcortical)	class Actinobacteria	rs72767435	T	C	0.060	94309566	0.627	0.123	360770	T	C	-0.126	15	94852795	2.57E-06	0.027	14306	21.323
Vascular dementia (subcortical)	class Actinobacteria	rs7322849	T	C	0.119	112205515	0.231	0.099	360770	T	C	0.094	13	112859829	6.21E-07	0.019	14306	23.953
Vascular dementia (subcortical)	class Actinobacteria	rs80083040	T	G	-0.084	64742287	0.541	0.138	360770	T	G	0.156	8	65654844	8.62E-06	0.035	14306	20.038
Vascular dementia (subcortical)	class Actinobacteria	rs857444	C	T	0.056	14617360	0.344	0.059	360770	C	T	0.051	6	14617591	8.92E-06	0.012	14306	19.500
Vascular dementia (subcortical)	class Actinobacteria	rs961091	G	A	-0.012	96025406	0.843	0.059	360770	G	A	0.050	7	95654718	8.68E-06	0.011	14306	19.835
Vascular dementia (subcortical)	class Alphaproteobacteria	rs140912403	C	T	0.033	92755525	0.789	0.125	360770	C	T	-0.161	9	95517807	6.20E-07	0.032	14306	25.577
Vascular dementia (subcortical)	class Alphaproteobacteria	rs34569731	G	A	0.050	75403218	0.400	0.059	360770	G	A	-0.071	2	75630344	7.38E-06	0.016	14306	20.028
Vascular dementia (subcortical)	class Alphaproteobacteria	rs62285697	C	T	-0.086	176690123	0.189	0.066	360770	C	T	0.081	3	176407911	9.76E-06	0.018	14306	19.812
Vascular dementia (subcortical)	class Alphaproteobacteria	rs76784716	A	G	-0.020	168176830	0.825	0.090	360770	A	G	0.133	2	169033340	5.09E-07	0.027	14306	24.777
Vascular dementia (subcortical)	class Alphaproteobacteria	rs7960664	A	G	0.027	89077458	0.787	0.101	360770	A	G	-0.097	12	89471235	8.84E-06	0.022	14306	20.305
Vascular dementia (subcortical)	class Alphaproteobacteria	rs9813022	A	G	0.029	13685237	0.616	0.059	360770	A	G	-0.075	3	13726736	1.05E-06	0.015	14306	23.840
Vascular dementia (subcortical)	class Bacilli	rs11110282	A	G	0.160	100191781	0.232	0.134	360770	A	G	-0.101	12	100585559	4.85E-06	0.022	14306	21.669
Vascular dementia (subcortical)	class Bacilli	rs11730038	G	A	-0.030	97128348	0.635	0.063	360770	G	A	-0.063	4	98049499	1.96E-06	0.013	14306	24.013
Vascular dementia (subcortical)	class Bacilli	rs12797734	T	C	0.162	8310803	0.014	0.066	360770	T	C	0.057	11	8332350	7.21E-06	0.013	14306	20.363
Vascular dementia (subcortical)	class Bacilli	rs13068444	A	G	-0.014	64413187	0.852	0.074	360770	A	G	0.060	3	64398863	9.53E-06	0.014	14306	19.415

Vascular dementia (subcortical)	class Bacilli	rs1595463	C	A	0.043	230858942	0.458	0.057	360770	C	A	0.048	2	231723657	7.97E-06	0.011	14306	19.300
Vascular dementia (subcortical)	class Bacilli	rs28564647	T	G	0.027	98338890	0.717	0.076	360770	T	G	-0.061	9	101101172	7.81E-06	0.014	14306	19.855
Vascular dementia (subcortical)	class Bacilli	rs2952251	G	A	0.003	10285654	0.963	0.068	360770	G	A	0.060	8	10143164	1.08E-06	0.012	14306	23.361
Vascular dementia (subcortical)	class Bacilli	rs34989881	A	G	0.279	51456601	0.041	0.136	360770	A	G	0.111	19	51959855	6.55E-06	0.025	14306	20.374
Vascular dementia (subcortical)	class Bacilli	rs35344081	G	A	-0.104	941253	0.107	0.065	360770	G	A	0.062	16	991253	1.01E-06	0.013	14306	23.772
Vascular dementia (subcortical)	class Bacilli	rs4028634	C	T	0.081	42683631	0.171	0.059	360770	C	T	-0.052	17	40835649	2.21E-06	0.011	14306	22.464
Vascular dementia (subcortical)	class Bacilli	rs4459992	T	C	-0.096	7429760	0.113	0.061	360770	T	C	0.054	4	7431487	4.30E-06	0.012	14306	21.207
Vascular dementia (subcortical)	class Bacilli	rs57872228	C	T	-0.114	200449677	0.195	0.088	360770	C	T	-0.071	1	200418805	9.22E-07	0.015	14306	23.757
Vascular dementia (subcortical)	class Bacilli	rs694949	A	G	0.099	58445876	0.304	0.096	360770	A	G	-0.081	15	58738075	7.60E-06	0.018	14306	20.314
Vascular dementia (subcortical)	class Bacilli	rs74663707	C	T	0.040	184653436	0.734	0.118	360770	C	T	0.098	3	184371224	8.46E-06	0.022	14306	19.095
Vascular dementia (subcortical)	class Bacilli	rs7666190	A	C	0.063	150766268	0.481	0.089	360770	A	C	0.104	4	151687420	8.47E-06	0.025	14306	17.610
Vascular dementia (subcortical)	class Bacilli	rs77558518	A	G	0.167	174746168	0.089	0.098	360770	A	G	-0.107	5	174173171	1.34E-06	0.022	14306	23.158
Vascular dementia (subcortical)	class Bacilli	rs78938557	T	C	-0.232	36309977	0.174	0.171	360770	T	C	0.108	7	36349586	1.07E-06	0.023	14306	21.514
Vascular dementia (subcortical)	class Bacilli	rs9581006	T	C	-0.064	24399371	0.673	0.152	360770	T	C	-0.225	13	24973509	1.79E-06	0.047	14306	23.207
Vascular dementia (subcortical)	class Bacteroidia	rs11146701	A	G	0.009	38769138	0.886	0.060	360770	A	G	0.047	10	39062269	7.08E-06	0.011	14306	20.186
Vascular dementia (subcortical)	class Bacteroidia	rs17343978	A	C	-0.037	27037922	0.601	0.070	360770	A	C	-0.055	22	27433885	8.36E-06	0.012	14306	21.067
Vascular dementia (subcortical)	class Bacteroidia	rs2032750	C	T	-0.072	53603889	0.209	0.057	360770	C	T	0.051	2	53831026	1.92E-06	0.011	14306	22.657
Vascular dementia (subcortical)	class Bacteroidia	rs2363574	T	C	0.155	200143435	0.302	0.150	360770	T	C	0.223	1	200112563	9.93E-06	0.051	14306	19.221
Vascular dementia (subcortical)	class Bacteroidia	rs4916508	A	G	0.003	196209918	0.953	0.057	360770	A	G	0.047	3	195936789	8.47E-06	0.011	14306	19.641
Vascular dementia (subcortical)	class Bacteroidia	rs55773148	G	A	-0.001	69948897	0.995	0.124	360770	G	A	-0.122	13	70523029	3.90E-07	0.024	14306	26.341
Vascular dementia (subcortical)	class Bacteroidia	rs62531359	T	G	-0.041	70003946	0.585	0.075	360770	T	G	0.066	8	70916181	9.09E-06	0.015	14306	19.138
Vascular dementia (subcortical)	class Bacteroidia	rs62575403	C	T	0.043	133628698	0.760	0.141	360770	C	T	0.140	9	136493820	7.06E-06	0.031	14306	20.264
Vascular dementia (subcortical)	class Bacteroidia	rs72706335	T	C	-0.229	157525648	0.218	0.186	360770	T	C	-0.222	1	157495438	7.66E-06	0.049	14306	20.315
Vascular dementia (subcortical)	class Bacteroidia	rs73975615	G	A	0.069	6557880	0.834	0.330	360770	G	A	-0.207	17	6461200	1.22E-06	0.044	14306	21.874
Vascular dementia (subcortical)	class Bacteroidia	rs7631304	G	A	-0.026	89290377	0.743	0.080	360770	G	A	-0.065	3	89339527	8.37E-07	0.013	14306	23.590
Vascular dementia (subcortical)	class Bacteroidia	rs79585701	A	C	0.054	13252676	0.508	0.082	360770	A	C	0.065	8	13110185	9.99E-06	0.015	14306	18.687
Vascular dementia (subcortical)	class Bacteroidia	rs929878	T	C	-0.092	74256742	0.188	0.070	360770	T	C	0.055	16	74290641	4.73E-06	0.012	14306	20.372
Vascular dementia (subcortical)	class Betaproteobacteria	rs11128180	A	G	-0.027	70543064	0.687	0.067	360770	A	G	0.059	3	70592215	3.67E-06	0.013	14306	21.203
Vascular dementia (subcortical)	class Betaproteobacteria	rs1511453	A	G	0.170	23586839	0.156	0.120	360770	A	G	0.092	4	23588462	4.76E-06	0.020	14306	21.502
Vascular dementia (subcortical)	class Betaproteobacteria	rs1928341	G	A	0.045	153267537	0.433	0.058	360770	G	A	-0.053	1	153240013	2.02E-06	0.011	14306	22.661
Vascular dementia (subcortical)	class Betaproteobacteria	rs2321387	G	A	0.028	58115206	0.623	0.057	360770	G	A	-0.049	13	58689340	5.80E-06	0.011	14306	20.430
Vascular dementia (subcortical)	class Betaproteobacteria	rs2613606	T	C	-0.050	111644969	0.385	0.057	360770	T	C	0.051	7	111285025	2.20E-06	0.011	14306	22.123
Vascular dementia (subcortical)	class Betaproteobacteria	rs320161	G	A	-0.019	102136835	0.778	0.068	360770	G	A	-0.057	9	104899117	7.33E-06	0.013	14306	20.564
Vascular dementia (subcortical)	class Betaproteobacteria	rs4033856	T	C	-0.095	45640468	0.350	0.101	360770	T	C	-0.083	4	45642485	5.17E-07	0.017	14306	24.776
Vascular dementia (subcortical)	class Betaproteobacteria	rs6087811	T	G	-0.114	32008327	0.225	0.094	360770	T	G	-0.098	20	30596130	7.44E-07	0.020	14306	24.278
Vascular dementia (subcortical)	class Betaproteobacteria	rs62395635	T	C	-0.073	174070793	0.536	0.118	360770	T	C	0.110	5	173497796	2.94E-06	0.024	14306	21.623
Vascular dementia (subcortical)	class Betaproteobacteria	rs75242906	C	T	0.120	56494209	0.247	0.104	360770	C	T	-0.121	15	56786407	9.27E-06	0.028	14306	18.601
Vascular dementia (subcortical)	class Clostridia	rs10774377	G	A	0.054	5833353	0.351	0.058	360770	G	A	-0.053	12	5942519	3.24E-06	0.011	14306	21.390
Vascular dementia (subcortical)	class Clostridia	rs112334273	G	A	0.041	39331325	0.521	0.064	360770	G	A	0.064	21	40703251	3.81E-07	0.013	14306	25.314
Vascular dementia (subcortical)	class Clostridia	rs13105690	C	T	0.016	7418457	0.800	0.064	360770	C	T	0.053	4	7420184	8.78E-06	0.012	14306	20.044
Vascular dementia (subcortical)	class Clostridia	rs13179700	C	T	-0.114	149698225	0.058	0.060	360770	C	T	-0.051	5	149077788	3.37E-06	0.011	14306	21.830
Vascular dementia (subcortical)	class Clostridia	rs1842454	G	A	0.012	105724661	0.868	0.073	360770	G	A	-0.055	5	105060362	8.72E-06	0.013	14306	18.466
Vascular dementia (subcortical)	class Clostridia	rs2273429	A	G	0.141	52027354	0.122	0.091	360770	A	G	-0.072	14	52494072	4.52E-06	0.015	14306	22.364
Vascular dementia (subcortical)	class Clostridia	rs6797343	T	G	0.112	89219564	0.119	0.072	360770	T	G	-0.059	3	89268714	9.36E-06	0.013	14306	19.375
Vascular dementia (subcortical)	class Clostridia	rs6814436	C	T	0.001	160586149	0.995	0.082	360770	C	T	-0.074	4	161507301	9.65E-07	0.015	14306	24.075
Vascular dementia (subcortical)	class Clostridia	rs6815608	C	T	-0.023	151210592	0.768	0.079	360770	C	T	-0.104	4	152131744	4.02E-07	0.021	14306	24.229
Vascular dementia (subcortical)	class Clostridia	rs72738886	T	C	0.154	35770448	0.152	0.108	360770	T	C	0.087	5	35770550	8.24E-06	0.019	14306	20.697
Vascular dementia (subcortical)	class Clostridia	rs992074	T	C	0.040	17195484	0.836	0.194	360770	T	C	-0.256	21	18567802	8.78E-07	0.051	14306	25.245
Vascular dementia (subcortical)	class Coriobacteriia	rs11073596	G	T	-0.027	85890348	0.641	0.059	360770	G	T	-0.051	15	86433579	8.14E-06	0.011	14306	19.912
Vascular dementia (subcortical)	class Coriobacteriia	rs11250875	T	C	-0.054	1880537	0.435	0.069	360770	T	C	0.061	10	1922731	4.83E-06	0.013	14306	21.526
Vascular dementia (subcortical)	class Coriobacteriia	rs11656361	A	C	0.131	8218014	0.076	0.074	360770	A	C	0.077	17	8121332	8.02E-06	0.018	14306	19.394
Vascular dementia (subcortical)	class Coriobacteriia	rs12974142	G	A	-0.066	52391913	0.550	0.111	360770	G	A	0.079	19	52895166	8.51E-06	0.018	14306	19.865
Vascular dementia (subcortical)	class Coriobacteriia	rs13307134	T	C	-0.082	105444233	0.287	0.077	360770	T	C	-0.057	7	105084680	7.80E-06	0.013	14306	20.072
Vascular dementia (subcortical)	class Coriobacteriia	rs1397793	A	G	0.048	91175634	0.441	0.062	360770	A	G	0.050	5	90471451	9.77E-06	0.011	14306	19.682
Vascular dementia (subcortical)	class Coriobacteriia	rs1816223	G	A	0.124	11341087	0.082	0.071	360770	G	A	0.059	12	11494021	4.84E-06	0.013	14306	20.652
Vascular dementia (subcortical)	class Coriobacteriia	rs240104	T	C	0.078	176602295	0.216	0.063	360770	T	C	-0.060	1	176571431	1.52E-06	0.013	14306	22.630
Vascular dementia (subcortical)	class Coriobacteriia	rs2442778	A	G	0.010	11612938	0.940	0.127	360770	A	G	0.116	3	11654412	9.03E-06	0.026	14306	20.272
Vascular dementia (subcortical)	class Coriobacteriia	rs3025411	A	G	0.062	133647784	0.497	0.091	360770	A	G	0.093	9	136512906	8.27E-06	0.021	14306	19.566
Vascular dementia (subcortical)	class Coriobacteriia	rs34739816	G	T	-0.139	39220432	0.249	0.121	360770	G	T	0.097	17	37376685	3.88E-06	0.021	14306	21.594
Vascular dementia (subcortical)	class Coriobacteriia	rs67561917	A	G	-0.071	63440724	0.336	0.074	360770	A	G	-0.071	20	62072077	5.39E-06	0.015	14306	21.486
Vascular dementia (subcortical)	class Coriobacteriia	rs719099	A	G	0.048	64039457	0.616	0.096	360770	A	G	0.078	10	65799217	5.43E-07	0.016	14306	24.957
Vascular dementia (subcortical)	class Coriobacteriia	rs8010111	A	G	0.034	39191305	0.744	0.104	360770	A	G	0.103	14	39660509	6.90E-06	0.023	14306	20.328
Vascular dementia (subcortical)	class Deltaproteobacteria	rs1035691	G	A	-0.085	10637188	0.145	0.058	360770	G	A	0.055	11	10658735	9.65E-06	0.012	14306	20.588
Vascular dementia (subcortical)	class Deltaproteobacteria	rs11599763	C	T	0.095	11813600	0.103	0.058	360770	C	T	0.054	10	11855599	3.94E-06	0.012	14306	21.490
Vascular dementia (subcortical)	class Deltaproteobacteria	rs17084793	G	A	0.037	71645678	0.650	0.081	360770	G	A	-0.071	18	69312914	5.69E-06	0.016	14306	19.850
Vascular dementia (subcortical)	class Deltaproteobacteria	rs17791387	A	G	-0.034	79219511	0.732	0.098	360770	A	G	-0.074	9	81834426	1.60E-06	0.015	14306	22.761
Vascular dementia (subcortical)	class Deltaproteobacteria	rs2692012	G	A	-0.235	204022477	0.061	0.126	360770	G	A	-0.110	1	203991605	3.14E-06	0.025	14306	18.968

Vascular dementia (subcortical)	class Deltaproteobacteria	rs2838334	G	A	0.045	43645080	0.456	0.060	360770	G	A	0.056	21	45064961	5.45E-06	0.012	14306	20.480
Vascular dementia (subcortical)	class Deltaproteobacteria	rs3935584	C	T	-0.066	233064573	0.246	0.057	360770	C	T	-0.052	2	233929283	7.50E-06	0.012	14306	20.485
Vascular dementia (subcortical)	class Deltaproteobacteria	rs4506934	C	T	-0.095	2953368	0.282	0.088	360770	C	T	-0.094	17	2856662	3.59E-06	0.020	14306	21.666
Vascular dementia (subcortical)	class Deltaproteobacteria	rs55744759	A	G	-0.025	56955112	0.784	0.090	360770	A	G	-0.078	8	57867671	7.31E-06	0.017	14306	20.854
Vascular dementia (subcortical)	class Deltaproteobacteria	rs6058181	C	T	-0.084	35106998	0.271	0.076	360770	C	T	0.083	20	33694801	3.40E-07	0.017	14306	24.755
Vascular dementia (subcortical)	class Deltaproteobacteria	rs62020470	A	G	0.058	95617836	0.433	0.075	360770	A	G	-0.059	15	96161065	4.85E-06	0.013	14306	20.480
Vascular dementia (subcortical)	class Deltaproteobacteria	rs9928243	C	A	0.039	71507738	0.491	0.057	360770	C	A	-0.054	16	71541641	5.02E-06	0.012	14306	20.923
Vascular dementia (subcortical)	class Erysipelotrichia	rs1074800	G	A	0.060	3002432	0.294	0.058	360770	G	A	-0.049	5	3002546	6.15E-06	0.011	14306	20.459
Vascular dementia (subcortical)	class Erysipelotrichia	rs10781552	C	T	-0.006	132083729	0.921	0.063	360770	C	T	-0.055	10	133897233	2.33E-06	0.012	14306	22.633
Vascular dementia (subcortical)	class Erysipelotrichia	rs17530232	A	G	-0.076	39811320	0.545	0.125	360770	A	G	0.103	13	40385457	2.79E-06	0.022	14306	21.042
Vascular dementia (subcortical)	class Erysipelotrichia	rs1884466	C	T	0.044	63673525	0.442	0.057	360770	C	T	-0.048	1	64139196	9.53E-06	0.011	14306	19.760
Vascular dementia (subcortical)	class Erysipelotrichia	rs2300774	A	G	0.057	196066841	0.319	0.057	360770	A	G	-0.052	3	195793712	8.95E-07	0.011	14306	24.094
Vascular dementia (subcortical)	class Erysipelotrichia	rs290833	T	G	-0.055	96991871	0.337	0.057	360770	T	G	-0.050	1	97457427	8.03E-06	0.011	14306	19.943
Vascular dementia (subcortical)	class Erysipelotrichia	rs35161940	T	C	-0.053	72331083	0.571	0.093	360770	T	C	-0.081	17	70327224	1.85E-06	0.017	14306	23.118
Vascular dementia (subcortical)	class Erysipelotrichia	rs4078432	T	C	-0.078	48528003	0.305	0.076	360770	T	C	0.061	14	48997206	4.23E-06	0.013	14306	20.723
Vascular dementia (subcortical)	class Erysipelotrichia	rs56970041	T	G	0.075	79891267	0.515	0.116	360770	T	G	0.072	14	80357610	5.40E-06	0.016	14306	19.385
Vascular dementia (subcortical)	class Erysipelotrichia	rs62504403	C	T	-0.107	38946033	0.135	0.072	360770	C	T	0.068	8	38803551	1.12E-07	0.013	14306	28.371
Vascular dementia (subcortical)	class Erysipelotrichia	rs7234058	T	C	0.090	5830508	0.362	0.098	360770	T	C	-0.095	18	5830507	9.12E-07	0.019	14306	23.744
Vascular dementia (subcortical)	class Erysipelotrichia	rs7826267	G	T	0.004	3097430	0.973	0.114	360770	G	T	0.084	8	2954952	9.28E-06	0.020	14306	17.755
Vascular dementia (subcortical)	class Erysipelotrichia	rs8003149	C	T	-0.049	55689786	0.422	0.060	360770	C	T	0.054	14	56156504	4.08E-06	0.012	14306	21.248
Vascular dementia (subcortical)	class Gammaproteobacteria	rs11181912	G	A	0.009	43179149	0.888	0.060	360770	G	A	-0.058	12	43572952	9.95E-07	0.012	14306	23.767
Vascular dementia (subcortical)	class Gammaproteobacteria	rs12404135	A	G	0.007	186533562	0.950	0.108	360770	A	G	-0.079	1	186502694	8.89E-06	0.017	14306	20.933
Vascular dementia (subcortical)	class Gammaproteobacteria	rs6706173	A	C	0.045	167130482	0.581	0.082	360770	A	C	0.074	2	167986992	1.99E-07	0.015	14306	25.726
Vascular dementia (subcortical)	class Gammaproteobacteria	rs75101789	C	T	0.041	18574411	0.680	0.100	360770	C	T	0.073	3	18615903	8.79E-06	0.016	14306	20.002
Vascular dementia (subcortical)	class Gammaproteobacteria	rs79795896	A	G	0.045	50658878	0.743	0.136	360770	A	G	-0.159	18	48185248	7.92E-06	0.035	14306	20.531
Vascular dementia (subcortical)	class Gammaproteobacteria	rs9494710	C	T	-0.036	137291417	0.554	0.061	360770	C	T	-0.055	6	137612554	4.55E-06	0.012	14306	20.663
Vascular dementia (subcortical)	class Lentisphaeria	rs1002941	A	G	0.050	100702485	0.446	0.066	360770	A	G	-0.105	15	101242690	8.15E-06	0.023	14306	20.234
Vascular dementia (subcortical)	class Lentisphaeria	rs11770843	C	T	0.027	147098287	0.658	0.061	360770	C	T	0.109	7	146795379	1.91E-06	0.023	14306	21.707
Vascular dementia (subcortical)	class Lentisphaeria	rs17114848	G	A	0.161	24917388	0.097	0.097	360770	G	A	0.152	15	25162535	4.06E-06	0.032	14306	22.073
Vascular dementia (subcortical)	class Lentisphaeria	rs2031282	A	G	-0.041	20113040	0.584	0.075	360770	A	G	0.122	13	20687179	4.38E-06	0.027	14306	20.490
Vascular dementia (subcortical)	class Lentisphaeria	rs2825714	A	G	0.078	19651652	0.302	0.075	360770	A	G	-0.137	21	21023966	1.72E-06	0.029	14306	22.568
Vascular dementia (subcortical)	class Lentisphaeria	rs62570196	C	T	-0.061	108323890	0.666	0.142	360770	C	T	-0.216	9	111086170	1.08E-06	0.044	14306	24.192
Vascular dementia (subcortical)	class Lentisphaeria	rs72640280	A	G	-0.027	11883735	0.828	0.122	360770	A	G	0.220	1	11943792	5.18E-06	0.049	14306	20.513
Vascular dementia (subcortical)	class Lentisphaeria	rs77599476	A	G	0.019	62762910	0.882	0.125	360770	A	G	0.230	20	61394262	1.86E-06	0.048	14306	23.002
Vascular dementia (subcortical)	class Melainabacteria	rs10148250	A	G	0.018	106605442	0.767	0.060	360770	A	G	-0.086	14	107061448	8.67E-06	0.019	14306	19.866
Vascular dementia (subcortical)	class Melainabacteria	rs10738747	A	G	-0.009	26184580	0.871	0.057	360770	A	G	-0.081	9	26184578	9.96E-06	0.018	14306	19.514
Vascular dementia (subcortical)	class Melainabacteria	rs11150282	T	C	-0.032	80459808	0.596	0.060	360770	T	C	0.099	16	80493705	6.03E-07	0.020	14306	25.235
Vascular dementia (subcortical)	class Melainabacteria	rs113884518	T	C	0.410	24648999	0.028	0.186	360770	T	C	-0.205	9	24648997	8.06E-06	0.045	14306	20.371
Vascular dementia (subcortical)	class Melainabacteria	rs28678345	T	C	0.081	55828967	0.545	0.134	360770	T	C	0.215	17	53906328	6.69E-06	0.047	14306	20.818
Vascular dementia (subcortical)	class Melainabacteria	rs367480	A	G	-0.022	2916401	0.715	0.060	360770	A	G	0.084	11	2937631	8.20E-06	0.019	14306	20.323
Vascular dementia (subcortical)	class Melainabacteria	rs4129395	G	A	-0.036	113213109	0.531	0.057	360770	G	A	0.090	9	115975389	1.48E-06	0.019	14306	23.440
Vascular dementia (subcortical)	class Melainabacteria	rs789069	A	C	-0.080	1008277	0.329	0.082	360770	A	C	-0.104	18	1008278	6.85E-06	0.023	14306	19.530
Vascular dementia (subcortical)	class Melainabacteria	rs79790072	T	C	-0.099	100207478	0.547	0.165	360770	T	C	0.227	15	100747683	3.29E-06	0.049	14306	21.599
Vascular dementia (subcortical)	class Melainabacteria	rs9864379	T	C	0.010	14265449	0.900	0.080	360770	T	C	-0.160	3	14306949	5.36E-08	0.029	14306	29.812
Vascular dementia (subcortical)	class Methanobacteria	rs10202904	G	T	0.027	124682691	0.644	0.058	360770	G	T	0.122	2	125440268	3.01E-07	0.024	14306	26.762
Vascular dementia (subcortical)	class Methanobacteria	rs10424197	A	G	-0.017	45936063	0.799	0.066	360770	A	G	0.111	19	46439321	9.28E-06	0.025	14306	20.211
Vascular dementia (subcortical)	class Methanobacteria	rs4257531	G	A	-0.191	2044483	0.041	0.093	360770	G	A	0.164	3	2086167	7.44E-06	0.036	14306	20.316
Vascular dementia (subcortical)	class Methanobacteria	rs6508769	C	T	-0.034	28336853	0.671	0.080	360770	C	T	-0.154	19	28827760	8.23E-06	0.034	14306	19.856
Vascular dementia (subcortical)	class Methanobacteria	rs6776814	T	C	-0.246	15011576	0.220	0.200	360770	T	C	-0.200	3	15053083	1.63E-06	0.041	14306	23.483
Vascular dementia (subcortical)	class Methanobacteria	rs73068003	G	T	0.204	10734305	0.035	0.096	360770	G	T	-0.158	7	10773932	8.45E-06	0.035	14306	20.206
Vascular dementia (subcortical)	class Methanobacteria	rs73457410	A	G	0.108	41382045	0.347	0.115	360770	A	G	0.215	13	41956181	1.41E-06	0.044	14306	24.316
Vascular dementia (subcortical)	class Methanobacteria	rs75208022	C	T	0.082	21185927	0.401	0.098	360770	C	T	-0.227	12	21338861	5.92E-06	0.049	14306	21.717
Vascular dementia (subcortical)	class Methanobacteria	rs894996	C	A	0.070	103497150	0.527	0.110	360770	C	A	0.217	4	104418307	1.88E-06	0.045	14306	23.349
Vascular dementia (subcortical)	class Mollicutes	rs10108398	G	A	0.099	58528265	0.118	0.063	360770	G	A	0.077	8	59440824	1.09E-06	0.015	14306	24.960
Vascular dementia (subcortical)	class Mollicutes	rs11890098	A	G	-0.006	156676037	0.929	0.063	360770	A	G	0.074	2	157532549	9.57E-07	0.015	14306	23.551
Vascular dementia (subcortical)	class Mollicutes	rs12566890	T	G	-0.025	61385192	0.767	0.085	360770	T	G	-0.101	1	61850864	3.65E-06	0.023	14306	19.176
Vascular dementia (subcortical)	class Mollicutes	rs17214486	C	A	0.118	96693337	0.053	0.061	360770	C	A	0.061	14	97159674	6.61E-06	0.014	14306	20.223
Vascular dementia (subcortical)	class Mollicutes	rs2464826	A	C	0.073	79860934	0.416	0.090	360770	A	C	0.094	7	79490250	8.39E-06	0.021	14306	19.874
Vascular dementia (subcortical)	class Mollicutes	rs28537087	G	A	0.013	94766447	0.844	0.066	360770	G	A	0.082	15	95309676	8.07E-06	0.019	14306	19.002
Vascular dementia (subcortical)	class Mollicutes	rs3768491	G	A	0.051	109423364	0.411	0.062	360770	G	A	0.068	1	109965986	4.23E-06	0.015	14306	20.875
Vascular dementia (subcortical)	class Mollicutes	rs4885016	C	T	-0.006	72596351	0.942	0.083	360770	C	T	0.082	13	73170489	7.27E-06	0.018	14306	20.363
Vascular dementia (subcortical)	class Mollicutes	rs6043847	T	C	0.029	16278879	0.808	0.120	360770	T	C	-0.115	20	16259524	4.55E-06	0.025	14306	21.375
Vascular dementia (subcortical)	class Mollicutes	rs72901605	T	C	-0.050	47082326	0.569	0.088	360770	T	C	-0.084	11	47103877	3.26E-06	0.018	14306	22.338
Vascular dementia (subcortical)	class Mollicutes	rs74603314	T	C	0.203	46050515	0.169	0.147	360770	T	C	0.222	14	46519718	1.56E-06	0.046	14306	22.924
Vascular dementia (subcortical)	class Mollicutes	rs78169027	A	G	0.176	108568360	0.141	0.119	360770	A	G	-0.108	11	108439087	5.88E-06	0.024	14306	20.824
Vascular dementia (subcortical)	class Negativicutes	rs1135612	G	A	-0.022	75980359	0.755	0.069	360770	G	A	0.053	7	75609677	9.26E-06	0.012	14306	19.761
Vascular dementia (subcortical)	class Negativicutes	rs13086907	G	A	-0.077	142416275	0.263	0.069	360770	G	A	0.063	3	142135117	1.95E-06	0.013	14306	22.532

Vascular dementia (subcortical)	class Negativicutes	rs1643968	T	C	-0.021	165839623	0.725	0.059	360770	T	C	-0.057	5	165266628	4.15E-07	0.011	14306	25.339
Vascular dementia (subcortical)	class Negativicutes	rs1649999	A	G	-0.097	78326315	0.318	0.097	360770	A	G	0.075	10	80086072	7.58E-06	0.017	14306	20.246
Vascular dementia (subcortical)	class Negativicutes	rs2834062	A	G	0.075	33005177	0.231	0.062	360770	A	G	0.049	21	34377485	8.44E-06	0.011	14306	20.190
Vascular dementia (subcortical)	class Negativicutes	rs4463806	C	T	0.087	113838234	0.229	0.072	360770	C	T	0.054	10	115597993	7.81E-06	0.013	14306	17.681
Vascular dementia (subcortical)	class Negativicutes	rs4722181	T	G	-0.088	22777952	0.122	0.057	360770	T	G	0.050	7	22817571	2.00E-06	0.011	14306	22.452
Vascular dementia (subcortical)	class Negativicutes	rs60274479	T	C	0.058	21238604	0.422	0.072	360770	T	C	-0.066	16	21249925	1.16E-06	0.013	14306	24.182
Vascular dementia (subcortical)	class Negativicutes	rs61249479	A	C	-0.055	122150629	0.485	0.079	360770	A	C	0.078	9	124912908	2.95E-06	0.017	14306	21.236
Vascular dementia (subcortical)	class Negativicutes	rs71405394	G	A	0.130	100704883	0.252	0.114	360770	G	A	-0.114	15	101245088	2.17E-06	0.024	14306	22.539
Vascular dementia (subcortical)	class Negativicutes	rs73232831	G	A	0.109	17411803	0.484	0.156	360770	G	A	-0.152	4	17413426	1.87E-06	0.031	14306	23.242
Vascular dementia (subcortical)	class Negativicutes	rs9423647	G	A	-0.020	5537855	0.730	0.057	360770	G	A	0.048	10	5579818	6.06E-06	0.011	14306	20.628
Vascular dementia (subcortical)	class Verrucomicrobiae	rs111862613	T	C	0.047	129825125	0.536	0.076	360770	T	C	0.091	12	130309670	3.74E-06	0.020	14306	21.252
Vascular dementia (subcortical)	class Verrucomicrobiae	rs117107102	A	G	-0.057	51947265	0.671	0.134	360770	A	G	0.205	18	49473635	2.92E-06	0.043	14306	22.493
Vascular dementia (subcortical)	class Verrucomicrobiae	rs11729256	T	C	-0.064	94106121	0.395	0.076	360770	T	C	0.075	4	95027272	6.73E-07	0.015	14306	24.928
Vascular dementia (subcortical)	class Verrucomicrobiae	rs12908520	G	A	0.100	97027427	0.081	0.057	360770	G	A	0.062	15	97570657	2.17E-06	0.013	14306	22.341
Vascular dementia (subcortical)	class Verrucomicrobiae	rs2602429	T	C	0.035	81029544	0.587	0.065	360770	T	C	-0.075	16	81063149	2.58E-06	0.016	14306	22.863
Vascular dementia (subcortical)	class Verrucomicrobiae	rs4242783	A	G	-0.058	5022135	0.363	0.064	360770	A	G	-0.069	10	5064327	2.64E-06	0.015	14306	21.781
Vascular dementia (subcortical)	class Verrucomicrobiae	rs4936098	G	A	0.056	130410772	0.348	0.059	360770	G	A	-0.065	11	130280667	1.12E-06	0.014	14306	22.786
Vascular dementia (subcortical)	class Verrucomicrobiae	rs61779207	G	A	-0.077	40608800	0.265	0.069	360770	G	A	-0.076	1	41074472	6.72E-06	0.017	14306	20.432
Vascular dementia (subcortical)	class Verrucomicrobiae	rs74542928	T	C	0.043	99623031	0.746	0.133	360770	T	C	0.112	4	100544188	1.63E-06	0.024	14306	22.508
Vascular dementia (subcortical)	class Verrucomicrobiae	rs9349825	A	G	-0.064	56476683	0.376	0.072	360770	A	G	-0.070	6	56341481	2.54E-06	0.015	14306	22.898
Vascular dementia (subcortical)	class Verrucomicrobiae	rs941682	G	A	0.065	33280034	0.305	0.063	360770	G	A	-0.063	20	31867840	9.61E-06	0.014	14306	19.290
Vascular dementia (subcortical)	family Acidaminococcaceae	rs262812	T	C	-0.071	158226967	0.249	0.062	360770	T	C	-0.066	6	158647999	3.25E-06	0.014	14306	21.313
Vascular dementia (subcortical)	family Acidaminococcaceae	rs2933324	G	A	0.090	104355372	0.184	0.068	360770	G	A	0.066	9	107117653	2.24E-06	0.014	14306	22.315
Vascular dementia (subcortical)	family Acidaminococcaceae	rs45497800	T	C	0.034	63360481	0.680	0.082	360770	T	C	-0.118	20	61991833	5.86E-06	0.026	14306	20.975
Vascular dementia (subcortical)	family Acidaminococcaceae	rs6589457	G	A	-0.189	114830566	0.146	0.130	360770	G	A	-0.166	11	114701288	2.32E-06	0.035	14306	22.533
Vascular dementia (subcortical)	family Acidaminococcaceae	rs6923842	T	C	0.057	5722948	0.524	0.089	360770	T	C	-0.080	6	5723181	2.21E-06	0.017	14306	22.140
Vascular dementia (subcortical)	family Acidaminococcaceae	rs74540770	G	A	-0.037	186835600	0.721	0.105	360770	G	A	-0.109	3	186553389	7.09E-06	0.024	14306	20.021
Vascular dementia (subcortical)	family Acidaminococcaceae	rs78702810	T	C	-0.035	18421039	0.704	0.093	360770	T	C	-0.144	3	18462531	9.16E-06	0.032	14306	19.778
Vascular dementia (subcortical)	family Actinomycetaceae	rs2889192	T	G	0.116	73779652	0.149	0.081	360770	T	G	-0.089	9	76394568	3.64E-06	0.020	14306	20.714
Vascular dementia (subcortical)	family Actinomycetaceae	rs34583783	G	T	-0.036	66497478	0.763	0.120	360770	G	T	0.124	6	67207371	5.48E-06	0.026	14306	21.930
Vascular dementia (subcortical)	family Actinomycetaceae	rs35011108	A	G	-0.263	132686341	0.020	0.113	360770	A	G	0.242	6	133007480	1.83E-06	0.050	14306	23.041
Vascular dementia (subcortical)	family Actinomycetaceae	rs4073240	G	A	0.065	168824686	0.265	0.058	360770	G	A	0.075	6	169224781	6.05E-06	0.016	14306	20.605
Vascular dementia (subcortical)	family Alcaligenaceae	rs112135816	T	G	-0.103	79870412	0.385	0.119	360770	T	G	-0.078	9	82485327	5.28E-06	0.017	14306	20.411
Vascular dementia (subcortical)	family Alcaligenaceae	rs1153990	A	G	0.051	104897760	0.408	0.061	360770	A	G	-0.059	5	104233461	5.97E-06	0.013	14306	20.950
Vascular dementia (subcortical)	family Alcaligenaceae	rs147968	C	T	-0.020	85912233	0.729	0.057	360770	C	T	0.049	16	85945839	9.13E-06	0.011	14306	19.685
Vascular dementia (subcortical)	family Alcaligenaceae	rs28480294	C	T	-0.068	71230170	0.249	0.059	360770	C	T	0.052	15	71522509	6.61E-06	0.012	14306	20.033
Vascular dementia (subcortical)	family Alcaligenaceae	rs4033856	T	C	-0.095	45640468	0.350	0.101	360770	T	C	-0.082	4	45642485	1.03E-06	0.017	14306	23.467
Vascular dementia (subcortical)	family Alcaligenaceae	rs62191117	A	G	0.022	238979079	0.752	0.071	360770	A	G	0.068	2	239900775	2.76E-07	0.013	14306	26.221
Vascular dementia (subcortical)	family Alcaligenaceae	rs62395635	T	C	-0.073	174070793	0.536	0.118	360770	T	C	0.111	5	173497796	3.35E-06	0.024	14306	21.496
Vascular dementia (subcortical)	family Alcaligenaceae	rs6969323	A	C	0.141	104574040	0.040	0.068	360770	A	C	-0.059	7	104214487	3.89E-06	0.013	14306	21.127
Vascular dementia (subcortical)	family Alcaligenaceae	rs74776516	T	G	0.037	20741821	0.750	0.116	360770	T	G	-0.094	11	20763367	6.85E-06	0.021	14306	19.591
Vascular dementia (subcortical)	family Alcaligenaceae	rs7638039	T	C	0.022	70539788	0.733	0.066	360770	T	C	0.060	3	70588939	2.70E-06	0.013	14306	22.295
Vascular dementia (subcortical)	family Alcaligenaceae	rs9537886	A	C	0.022	57971112	0.707	0.057	360770	A	C	-0.057	13	58545246	2.35E-07	0.011	14306	26.604
Vascular dementia (subcortical)	family Bacteroidaceae	rs11585893	A	G	0.064	10584294	0.335	0.066	360770	A	G	-0.074	1	10644351	1.80E-06	0.015	14306	25.175
Vascular dementia (subcortical)	family Bacteroidaceae	rs13207588	A	G	0.000	41551692	0.995	0.072	360770	A	G	-0.059	6	41519430	7.48E-06	0.013	14306	20.365
Vascular dementia (subcortical)	family Bacteroidaceae	rs1340391	T	C	0.125	102495433	0.132	0.083	360770	T	C	-0.059	1	102960989	6.73E-06	0.013	14306	20.040
Vascular dementia (subcortical)	family Bacteroidaceae	rs17619981	T	G	-0.032	24159448	0.695	0.082	360770	T	G	0.088	19	24342250	2.69E-06	0.019	14306	22.194
Vascular dementia (subcortical)	family Bacteroidaceae	rs2023437	T	C	-0.005	21577814	0.953	0.087	360770	T	C	-0.078	14	22045949	5.02E-06	0.017	14306	21.780
Vascular dementia (subcortical)	family Bacteroidaceae	rs66710942	T	C	-0.018	77166176	0.753	0.057	360770	T	C	-0.049	3	77215327	5.86E-06	0.011	14306	20.644
Vascular dementia (subcortical)	family Bacteroidaceae	rs6795673	C	T	0.059	10551540	0.299	0.057	360770	C	T	0.054	3	10593224	3.38E-07	0.011	14306	26.183
Vascular dementia (subcortical)	family Bacteroidaceae	rs9507307	C	T	-0.119	24336338	0.071	0.066	360770	C	T	0.060	13	24910476	2.13E-06	0.013	14306	21.912
Vascular dementia (subcortical)	family Bacteroidales S24 7group	rs10872669	G	A	0.005	151194037	0.961	0.100	360770	G	A	0.123	6	151515172	9.49E-06	0.028	14306	19.932
Vascular dementia (subcortical)	family Bacteroidales S24 7group	rs12748533	G	T	-0.053	242603950	0.397	0.063	360770	G	T	-0.082	1	242767252	2.59E-06	0.017	14306	22.605
Vascular dementia (subcortical)	family Bacteroidales S24 7group	rs17043785	T	C	0.132	52912224	0.193	0.101	360770	T	C	-0.176	2	53139362	5.12E-07	0.035	14306	25.761
Vascular dementia (subcortical)	family Bacteroidales S24 7group	rs1850003	A	G	0.123	47048773	0.066	0.067	360770	A	G	0.084	15	47340971	2.41E-06	0.018	14306	22.344
Vascular dementia (subcortical)	family Bacteroidales S24 7group	rs61508842	T	C	0.031	158935756	0.753	0.100	360770	T	C	0.123	3	158653545	7.83E-06	0.027	14306	20.250
Vascular dementia (subcortical)	family Bacteroidales S24 7group	rs738193	T	C	-0.094	25547355	0.112	0.059	360770	T	C	0.085	22	25943322	3.82E-07	0.017	14306	26.100
Vascular dementia (subcortical)	family Bacteroidales S24 7group	rs78609301	A	G	-0.088	62526842	0.158	0.062	360770	A	G	-0.087	6	63236747	7.09E-06	0.020	14306	19.628
Vascular dementia (subcortical)	family Bacteroidales S24 7group	rs941000	T	C	-0.063	91085352	0.289	0.059	360770	T	C	-0.085	7	90714667	3.16E-07	0.016	14306	27.083
Vascular dementia (subcortical)	family Bifidobacteriaceae	rs10831953	G	A	-0.054	13076504	0.383	0.062	360770	G	A	0.054	11	13098051	9.95E-06	0.012	14306	18.869
Vascular dementia (subcortical)	family Bifidobacteriaceae	rs12446429	T	C	0.044	848055	0.545	0.073	360770	T	C	0.081	16	898055	8.53E-06	0.019	14306	18.040
Vascular dementia (subcortical)	family Bifidobacteriaceae	rs13020688	G	A	0.037	192013806	0.549	0.062	360770	G	A	0.058	2	192878532	1.57E-06	0.012	14306	22.887
Vascular dementia (subcortical)	family Bifidobacteriaceae	rs182549	T	C	-0.005	135859184	0.936	0.059	360770	T	C	-0.117	2	136616754	5.94E-20	0.013	14306	85.372
Vascular dementia (subcortical)	family Bifidobacteriaceae	rs4957061	T	C	0.066	520981	0.255	0.058	360770	T	C	0.057	5	521096	1.15E-06	0.012	14306	23.762
Vascular dementia (subcortical)	family Bifidobacteriaceae	rs540489	T	G	-0.157	74901626	0.038	0.075	360770	T	G	-0.063	17	72897722	5.37E-06	0.014	14306	20.956
Vascular dementia (subcortical)	family Bifidobacteriaceae	rs55888705	A	G	-0.012	1516099	0.850	0.063	360770	A	G	0.054	4	1517826	8.66E-06	0.012	14306	19.812
Vascular dementia (subcortical)	family Bifidobacteriaceae	rs6899771	A	G	0.114	96958344	0.228	0.095	360770	A	G	-0.091	6	97406220	7.28E-06	0.020	14306	20.365

Vascular dementia (subcortical)	family Bifidobacteriaceae	rs7174549	T	C	0.054	91920073	0.360	0.059	360770	T	C	-0.055	15	92463303	6.87E-06	0.012	14306	19.590
Vascular dementia (subcortical)	family Bifidobacteriaceae	rs7322849	T	C	0.119	112205515	0.231	0.099	360770	T	C	0.111	13	112859829	1.74E-08	0.020	14306	30.320
Vascular dementia (subcortical)	family Bifidobacteriaceae	rs857444	C	T	0.056	14617360	0.344	0.059	360770	C	T	0.055	6	14617591	3.82E-06	0.012	14306	21.075
Vascular dementia (subcortical)	family Clostridiaceae1	rs10875374	T	C	0.051	101339253	0.365	0.057	360770	T	C	0.054	1	101804809	8.10E-06	0.012	14306	20.197
Vascular dementia (subcortical)	family Clostridiaceae1	rs12186080	G	A	0.071	132877783	0.350	0.076	360770	G	A	0.075	3	132596627	5.34E-06	0.016	14306	21.212
Vascular dementia (subcortical)	family Clostridiaceae1	rs12341505	G	A	0.079	133845759	0.419	0.098	360770	G	A	0.081	9	136710881	4.54E-06	0.018	14306	20.627
Vascular dementia (subcortical)	family Clostridiaceae1	rs2795528	G	A	-0.176	42774816	0.156	0.124	360770	G	A	-0.181	10	43270264	3.81E-06	0.039	14306	21.432
Vascular dementia (subcortical)	family Clostridiaceae1	rs2817172	C	T	-0.020	3124955	0.730	0.058	360770	C	T	0.056	1	3041519	5.27E-06	0.012	14306	20.668
Vascular dementia (subcortical)	family Clostridiaceae1	rs4723021	T	C	-0.069	30895044	0.546	0.115	360770	T	C	-0.106	7	30934659	7.42E-06	0.024	14306	19.331
Vascular dementia (subcortical)	family Clostridiaceae1	rs550843	T	C	-0.013	165309343	0.838	0.064	360770	T	C	-0.073	6	165722832	7.09E-06	0.017	14306	19.043
Vascular dementia (subcortical)	family Clostridiaceae1	rs56188186	A	G	-0.099	87677084	0.457	0.133	360770	A	G	0.097	16	87710690	8.24E-06	0.022	14306	19.804
Vascular dementia (subcortical)	family Clostridiaceae1	rs62397761	A	G	0.108	48125278	0.075	0.061	360770	A	G	0.062	6	48093014	9.08E-06	0.014	14306	20.443
Vascular dementia (subcortical)	family Clostridiaceae1	rs881532	G	A	-0.055	47411024	0.338	0.057	360770	G	A	0.053	22	47806774	7.90E-06	0.012	14306	20.060
Vascular dementia (subcortical)	family Clostridiales vadin BB60 group	rs10517600	G	T	0.026	154711806	0.658	0.058	360770	G	T	-0.063	4	155632958	6.83E-06	0.014	14306	20.241
Vascular dementia (subcortical)	family Clostridiales vadin BB60 group	rs10904722	C	T	0.003	6672462	0.964	0.067	360770	C	T	-0.067	10	6714424	5.05E-06	0.015	14306	20.883
Vascular dementia (subcortical)	family Clostridiales vadin BB60 group	rs118104867	C	T	-0.092	122965298	0.411	0.112	360770	C	T	0.214	8	123977538	3.44E-06	0.046	14306	22.207
Vascular dementia (subcortical)	family Clostridiales vadin BB60 group	rs13409132	A	G	-0.058	204751623	0.709	0.154	360770	A	G	-0.165	2	205616346	4.37E-06	0.035	14306	22.065
Vascular dementia (subcortical)	family Clostridiales vadin BB60 group	rs17121075	G	A	0.066	85390452	0.337	0.069	360770	G	A	0.077	14	85856796	7.91E-06	0.017	14306	19.948
Vascular dementia (subcortical)	family Clostridiales vadin BB60 group	rs2191834	T	G	-0.126	229078235	0.056	0.066	360770	T	G	-0.075	2	229942951	2.50E-06	0.016	14306	21.998
Vascular dementia (subcortical)	family Clostridiales vadin BB60 group	rs28691777	C	T	-0.062	60072044	0.657	0.141	360770	C	T	0.137	17	58149405	6.96E-07	0.027	14306	26.380
Vascular dementia (subcortical)	family Clostridiales vadin BB60 group	rs34088226	A	G	-0.025	4308833	0.831	0.118	360770	A	G	-0.118	5	4308946	7.66E-06	0.027	14306	19.145
Vascular dementia (subcortical)	family Clostridiales vadin BB60 group	rs55682560	C	T	-0.083	86849059	0.426	0.105	360770	C	T	-0.132	15	87392290	4.97E-07	0.026	14306	25.330
Vascular dementia (subcortical)	family Clostridiales vadin BB60 group	rs6588624	A	G	0.029	56383867	0.609	0.057	360770	A	G	0.066	1	56849539	1.79E-06	0.014	14306	22.985
Vascular dementia (subcortical)	family Clostridiales vadin BB60 group	rs66714985	A	C	0.001	3447600	0.994	0.094	360770	A	C	0.117	8	3305122	4.85E-06	0.025	14306	21.446
Vascular dementia (subcortical)	family Clostridiales vadin BB60 group	rs7226487	A	G	0.012	76661168	0.839	0.057	360770	A	G	-0.064	18	74373125	3.58E-06	0.014	14306	21.537
Vascular dementia (subcortical)	family Clostridiales vadin BB60 group	rs7538034	T	G	0.084	70926011	0.279	0.077	360770	T	G	-0.079	1	71391694	2.37E-06	0.017	14306	22.423
Vascular dementia (subcortical)	family Clostridiales vadin BB60 group	rs7725895	A	G	0.138	142865333	0.109	0.086	360770	A	G	-0.116	5	142244898	3.94E-06	0.024	14306	23.380
Vascular dementia (subcortical)	family Clostridiales vadin BB60 group	rs989682	A	G	0.122	15522946	0.070	0.067	360770	A	G	0.070	3	15564453	6.85E-06	0.016	14306	20.413
Vascular dementia (subcortical)	family Coriobacteriaceae	rs11073596	G	T	-0.027	85890348	0.641	0.059	360770	G	T	-0.051	15	86433579	8.14E-06	0.011	14306	19.912
Vascular dementia (subcortical)	family Coriobacteriaceae	rs11250875	T	C	-0.054	1880537	0.435	0.069	360770	T	C	0.061	10	1922731	4.83E-06	0.013	14306	21.526
Vascular dementia (subcortical)	family Coriobacteriaceae	rs11656361	A	C	0.131	8218014	0.076	0.074	360770	A	C	0.077	17	8121332	8.02E-06	0.018	14306	19.394
Vascular dementia (subcortical)	family Coriobacteriaceae	rs12974142	G	A	-0.066	52391913	0.550	0.111	360770	G	A	0.079	19	52895166	8.51E-06	0.018	14306	19.865
Vascular dementia (subcortical)	family Coriobacteriaceae	rs13307134	T	C	-0.082	105444233	0.287	0.077	360770	T	C	-0.057	7	105084680	7.80E-06	0.013	14306	20.072
Vascular dementia (subcortical)	family Coriobacteriaceae	rs1397793	A	G	0.048	91175634	0.441	0.062	360770	A	G	0.050	5	90471451	9.77E-06	0.011	14306	19.682
Vascular dementia (subcortical)	family Coriobacteriaceae	rs1816223	G	A	0.124	11341087	0.082	0.071	360770	G	A	0.059	12	11494021	4.84E-06	0.013	14306	20.652
Vascular dementia (subcortical)	family Coriobacteriaceae	rs240104	T	C	0.078	176602295	0.216	0.063	360770	T	C	-0.060	1	176571431	1.52E-06	0.013	14306	22.630
Vascular dementia (subcortical)	family Coriobacteriaceae	rs2442778	A	G	0.010	11612938	0.940	0.127	360770	A	G	0.116	3	11654412	9.03E-06	0.026	14306	20.272
Vascular dementia (subcortical)	family Coriobacteriaceae	rs3025411	A	G	0.062	133647784	0.497	0.091	360770	A	G	0.093	9	136512906	8.27E-06	0.021	14306	19.566
Vascular dementia (subcortical)	family Coriobacteriaceae	rs34739816	G	T	-0.139	39220432	0.249	0.121	360770	G	T	0.097	17	37376685	3.88E-06	0.021	14306	21.594
Vascular dementia (subcortical)	family Coriobacteriaceae	rs67561917	A	G	-0.071	63440724	0.336	0.074	360770	A	G	-0.071	20	62072077	5.39E-06	0.015	14306	21.486
Vascular dementia (subcortical)	family Coriobacteriaceae	rs719099	A	G	0.048	64039457	0.616	0.096	360770	A	G	0.078	10	65799217	5.43E-07	0.016	14306	24.957
Vascular dementia (subcortical)	family Coriobacteriaceae	rs8010111	A	G	0.034	39191305	0.744	0.104	360770	A	G	0.103	14	39660509	6.90E-06	0.023	14306	20.328
Vascular dementia (subcortical)	family Defluviitaleaceae	rs112893842	T	C	0.046	8786663	0.641	0.099	360770	T	C	0.111	9	8786663	2.75E-06	0.023	14306	22.686
Vascular dementia (subcortical)	family Defluviitaleaceae	rs1582238	C	T	-0.080	118181062	0.178	0.059	360770	C	T	-0.080	1	118723685	1.69E-06	0.017	14306	23.042
Vascular dementia (subcortical)	family Defluviitaleaceae	rs17051335	C	T	0.034	121281755	0.713	0.093	360770	C	T	-0.134	4	122202910	4.58E-06	0.029	14306	21.129
Vascular dementia (subcortical)	family Defluviitaleaceae	rs1908593	T	C	-0.064	61358915	0.265	0.058	360770	T	C	0.070	18	59026148	7.86E-06	0.016	14306	20.108
Vascular dementia (subcortical)	family Defluviitaleaceae	rs4344384	T	G	-0.052	64647609	0.361	0.057	360770	T	G	-0.071	10	66407366	5.86E-06	0.016	14306	20.612
Vascular dementia (subcortical)	family Defluviitaleaceae	rs4677103	A	G	0.096	72158643	0.203	0.075	360770	A	G	0.098	3	72207794	9.42E-07	0.020	14306	24.598
Vascular dementia (subcortical)	family Defluviitaleaceae	rs540220	C	T	0.089	90569026	0.376	0.100	360770	C	T	0.124	9	93331308	9.48E-06	0.029	14306	18.293
Vascular dementia (subcortical)	family Defluviitaleaceae	rs55658617	T	C	-0.006	40439076	0.971	0.157	360770	T	C	0.177	21	41811003	1.41E-06	0.036	14306	24.013
Vascular dementia (subcortical)	family Defluviitaleaceae	rs72731813	C	T	0.124	146493591	0.355	0.134	360770	C	T	-0.150	4	147414743	2.76E-07	0.029	14306	26.048
Vascular dementia (subcortical)	family Defluviitaleaceae	rs9608282	T	G	-0.047	24408113	0.772	0.161	360770	T	G	0.139	22	24804081	4.61E-06	0.030	14306	21.554
Vascular dementia (subcortical)	family Defluviitaleaceae	rs9725395	A	G	-0.014	84739949	0.875	0.090	360770	A	G	-0.138	1	85205632	3.41E-06	0.030	14306	21.969
Vascular dementia (subcortical)	family Desulfovibrionaceae	rs11599763	C	T	0.095	11813600	0.103	0.058	360770	C	T	0.056	10	11855599	2.50E-06	0.012	14306	22.388
Vascular dementia (subcortical)	family Desulfovibrionaceae	rs17791387	A	G	-0.034	79219511	0.732	0.098	360770	A	G	-0.073	9	81834426	2.10E-06	0.015	14306	22.313
Vascular dementia (subcortical)	family Desulfovibrionaceae	rs2692012	G	A	-0.235	204022477	0.061	0.126	360770	G	A	-0.114	1	203991605	1.56E-06	0.025	14306	20.295
Vascular dementia (subcortical)	family Desulfovibrionaceae	rs2838334	G	A	0.045	43645080	0.456	0.060	360770	G	A	0.057	21	45064961	3.82E-06	0.012	14306	21.157
Vascular dementia (subcortical)	family Desulfovibrionaceae	rs3935584	C	T	-0.066	233064573	0.246	0.057	360770	C	T	-0.053	2	233929283	6.78E-06	0.012	14306	20.635
Vascular dementia (subcortical)	family Desulfovibrionaceae	rs4506934	C	T	-0.095	2953368	0.282	0.088	360770	C	T	-0.094	17	2856662	3.16E-06	0.020	14306	21.945
Vascular dementia (subcortical)	family Desulfovibrionaceae	rs6058181	C	T	-0.084	35106998	0.271	0.076	360770	C	T	0.083	20	33694801	2.70E-07	0.017	14306	25.253
Vascular dementia (subcortical)	family Desulfovibrionaceae	rs72647048	T	C	-0.024	56952819	0.787	0.090	360770	T	C	-0.077	8	57865378	9.61E-06	0.017	14306	20.307
Vascular dementia (subcortical)	family Desulfovibrionaceae	rs9928243	C	A	0.039	71507738	0.491	0.057	360770	C	A	-0.054	16	71541641	4.48E-06	0.012	14306	21.142
Vascular dementia (subcortical)	family Enterobacteriaceae	rs11026530	T	C	-0.021	22357551	0.793	0.080	360770	T	C	0.082	11	22379097	9.43E-06	0.019	14306	19.471
Vascular dementia (subcortical)	family Enterobacteriaceae	rs2374342	C	A	-0.031	41906402	0.586	0.058	360770	C	A	0.058	2	42133542	4.52E-06	0.013	14306	21.338
Vascular dementia (subcortical)	family Enterobacteriaceae	rs35673018	G	A	-0.068	54293833	0.491	0.099	360770	G	A	0.090	16	54327745	7.63E-06	0.020	14306	19.653
Vascular dementia (subcortical)	family Enterobacteriaceae	rs504442	T	G	0.055	57478315	0.555	0.093	360770	T	G	0.084	18	55145547	5.17E-06	0.019	14306	19.728
Vascular dementia (subcortical)	family Enterobacteriaceae	rs62210023	A	G	0.041	56765036	0.490	0.060	360770	A	G	0.061	20	55340092	3.13E-06	0.013	14306	21.742

Vascular dementia (subcortical)	family Enterobacteriaceae	rs78143293	A	G	-0.005	60005103	0.957	0.087	360770	A	G	-0.085	18	57672335	1.20E-06	0.017	14306	24.792
Vascular dementia (subcortical)	family Enterobacteriaceae	rs79757635	C	A	-0.166	110188071	0.047	0.083	360770	C	A	0.076	13	110840418	9.32E-06	0.017	14306	19.615
Vascular dementia (subcortical)	family Erysipelotrichaceae	rs1074800	G	A	0.060	3002432	0.294	0.058	360770	G	A	-0.049	5	3002546	6.15E-06	0.011	14306	20.459
Vascular dementia (subcortical)	family Erysipelotrichaceae	rs10781552	C	T	-0.006	132083729	0.921	0.063	360770	C	T	-0.055	10	133897233	2.33E-06	0.012	14306	22.633
Vascular dementia (subcortical)	family Erysipelotrichaceae	rs17530232	A	G	-0.076	39811320	0.545	0.125	360770	A	G	0.103	13	40385457	2.79E-06	0.022	14306	21.042
Vascular dementia (subcortical)	family Erysipelotrichaceae	rs1884466	C	T	0.044	63673525	0.442	0.057	360770	C	T	-0.048	1	64139196	9.53E-06	0.011	14306	19.760
Vascular dementia (subcortical)	family Erysipelotrichaceae	rs2300774	A	G	0.057	196066841	0.319	0.057	360770	A	G	-0.052	3	195793712	8.95E-07	0.011	14306	24.094
Vascular dementia (subcortical)	family Erysipelotrichaceae	rs290833	T	G	-0.055	96991871	0.337	0.057	360770	T	G	-0.050	1	97457427	8.03E-06	0.011	14306	19.943
Vascular dementia (subcortical)	family Erysipelotrichaceae	rs35161940	T	C	-0.053	72331083	0.571	0.093	360770	T	C	-0.081	17	70327224	1.85E-06	0.017	14306	23.118
Vascular dementia (subcortical)	family Erysipelotrichaceae	rs4078432	T	C	-0.078	48528003	0.305	0.076	360770	T	C	0.061	14	48997206	4.23E-06	0.013	14306	20.723
Vascular dementia (subcortical)	family Erysipelotrichaceae	rs56970041	T	G	0.075	79891267	0.515	0.116	360770	T	G	0.072	14	80357610	5.40E-06	0.016	14306	19.385
Vascular dementia (subcortical)	family Erysipelotrichaceae	rs62504403	C	T	-0.107	38946033	0.135	0.072	360770	C	T	0.068	8	38803551	1.12E-07	0.013	14306	28.371
Vascular dementia (subcortical)	family Erysipelotrichaceae	rs7234058	T	C	0.090	5830508	0.362	0.098	360770	T	C	-0.095	18	5830507	9.12E-07	0.019	14306	23.744
Vascular dementia (subcortical)	family Erysipelotrichaceae	rs7826267	G	T	0.004	3097430	0.973	0.114	360770	G	T	0.084	8	2954952	9.28E-06	0.020	14306	17.755
Vascular dementia (subcortical)	family Erysipelotrichaceae	rs8003149	C	T	-0.049	55689786	0.422	0.060	360770	C	T	0.054	14	56156504	4.08E-06	0.012	14306	21.248
Vascular dementia (subcortical)	family Family XI	rs10759623	C	T	0.084	113059205	0.219	0.068	360770	C	T	-0.162	9	115821485	5.78E-07	0.032	14306	25.425
Vascular dementia (subcortical)	family Family XI	rs11547158	A	G	-0.155	149224640	0.063	0.083	360770	A	G	-0.178	7	148921732	2.70E-06	0.037	14306	22.696
Vascular dementia (subcortical)	family Family XI	rs17379710	T	C	-0.062	35313121	0.283	0.057	360770	T	C	-0.116	11	35334668	3.97E-06	0.025	14306	21.308
Vascular dementia (subcortical)	family Family XI	rs2155352	A	G	0.113	95624045	0.091	0.067	360770	A	G	-0.151	11	95357209	6.63E-07	0.030	14306	24.795
Vascular dementia (subcortical)	family Family XI	rs2156611	T	C	-0.065	45765899	0.249	0.057	360770	T	C	-0.112	18	43345864	9.43E-06	0.025	14306	20.068
Vascular dementia (subcortical)	family Family XI	rs3733511	A	G	0.022	119034632	0.723	0.062	360770	A	G	0.128	4	119955787	3.39E-06	0.027	14306	21.795
Vascular dementia (subcortical)	family Family XI	rs488164	G	T	0.059	239832151	0.316	0.059	360770	G	T	-0.118	1	239995451	4.80E-06	0.026	14306	21.386
Vascular dementia (subcortical)	family Family XI	rs697771	A	G	-0.122	54081288	0.033	0.057	360770	A	G	-0.118	16	54115200	3.19E-06	0.025	14306	21.895
Vascular dementia (subcortical)	family Family XIII	rs10404377	A	C	-0.037	16063847	0.520	0.058	360770	A	C	0.050	19	16174657	6.99E-06	0.011	14306	20.311
Vascular dementia (subcortical)	family Family XIII	rs118170811	A	G	-0.285	100607139	0.084	0.165	360770	A	G	0.152	10	102366896	1.80E-06	0.032	14306	22.860
Vascular dementia (subcortical)	family Family XIII	rs482905	G	T	-0.120	166587965	0.069	0.066	360770	G	T	0.060	1	166557202	3.72E-06	0.013	14306	22.004
Vascular dementia (subcortical)	family Family XIII	rs6501525	A	G	0.039	72222486	0.513	0.060	360770	A	G	0.056	17	70218627	1.24E-06	0.012	14306	23.603
Vascular dementia (subcortical)	family Family XIII	rs66753613	G	A	0.027	38746349	0.710	0.074	360770	G	A	0.065	1	39212021	8.08E-06	0.014	14306	20.375
Vascular dementia (subcortical)	family Family XIII	rs6797051	C	T	0.022	88946068	0.820	0.097	360770	C	T	-0.081	3	88995218	4.89E-06	0.017	14306	22.122
Vascular dementia (subcortical)	family Family XIII	rs7514702	T	C	-0.070	186944891	0.377	0.080	360770	T	C	-0.066	1	186914023	3.92E-06	0.014	14306	21.931
Vascular dementia (subcortical)	family Lachnospiraceae	rs10402491	C	T	0.012	13314985	0.871	0.075	360770	C	T	0.066	19	13425799	7.58E-06	0.015	14306	19.852
Vascular dementia (subcortical)	family Lachnospiraceae	rs11139361	C	T	0.091	69564931	0.134	0.061	360770	C	T	-0.049	9	72179847	4.26E-06	0.011	14306	20.023
Vascular dementia (subcortical)	family Lachnospiraceae	rs112040820	A	G	0.050	82511188	0.445	0.065	360770	A	G	0.055	17	80469064	2.42E-06	0.012	14306	22.032
Vascular dementia (subcortical)	family Lachnospiraceae	rs11841382	G	T	0.146	37430535	0.156	0.103	360770	G	T	-0.072	13	38004672	9.58E-06	0.017	14306	16.913
Vascular dementia (subcortical)	family Lachnospiraceae	rs11979110	T	C	-0.006	130751700	0.918	0.057	360770	T	C	-0.050	7	130436459	1.82E-06	0.011	14306	22.730
Vascular dementia (subcortical)	family Lachnospiraceae	rs1205443	A	G	0.045	38248466	0.456	0.060	360770	A	G	0.050	20	36876868	7.29E-06	0.011	14306	19.987
Vascular dementia (subcortical)	family Lachnospiraceae	rs12760724	A	C	0.057	213858626	0.346	0.061	360770	A	C	-0.048	1	214031969	7.27E-06	0.011	14306	20.155
Vascular dementia (subcortical)	family Lachnospiraceae	rs13005175	G	A	0.252	231605011	0.073	0.140	360770	G	A	-0.099	2	232469722	8.37E-06	0.022	14306	20.694
Vascular dementia (subcortical)	family Lachnospiraceae	rs2159863	A	G	-0.097	10243730	0.191	0.074	360770	A	G	-0.059	4	10245354	3.70E-06	0.013	14306	20.706
Vascular dementia (subcortical)	family Lachnospiraceae	rs2910921	C	T	0.144	32348487	0.392	0.169	360770	C	T	-0.160	5	32348593	8.42E-06	0.036	14306	20.014
Vascular dementia (subcortical)	family Lachnospiraceae	rs3127230	C	T	-0.028	101632746	0.656	0.062	360770	C	T	-0.050	10	103392503	6.20E-06	0.011	14306	20.140
Vascular dementia (subcortical)	family Lachnospiraceae	rs35524804	T	C	-0.032	97351401	0.642	0.070	360770	T	C	-0.061	9	100113683	2.45E-06	0.013	14306	23.537
Vascular dementia (subcortical)	family Lachnospiraceae	rs7359994	C	T	0.056	28917951	0.337	0.058	360770	C	T	0.050	19	29408858	5.36E-06	0.011	14306	20.009
Vascular dementia (subcortical)	family Lachnospiraceae	rs79086868	T	C	0.052	130916218	0.579	0.094	360770	T	C	0.078	9	133791605	3.01E-06	0.016	14306	22.302
Vascular dementia (subcortical)	family Lachnospiraceae	rs959845	T	C	-0.056	185985647	0.342	0.058	360770	T	C	0.049	4	186906801	5.17E-06	0.011	14306	20.971
Vascular dementia (subcortical)	family Lachnospiraceae	rs9929145	G	A	0.167	76525287	0.197	0.129	360770	G	A	-0.126	16	76559184	2.84E-07	0.025	14306	26.282
Vascular dementia (subcortical)	family Lactobacillaceae	rs1530559	G	A	0.078	134998059	0.172	0.057	360770	G	A	0.077	2	135755629	9.65E-06	0.018	14306	19.063
Vascular dementia (subcortical)	family Lactobacillaceae	rs16861661	G	A	0.219	18174965	0.059	0.116	360770	G	A	-0.193	1	18501459	2.70E-07	0.038	14306	25.969
Vascular dementia (subcortical)	family Lactobacillaceae	rs62314653	C	A	0.094	108975306	0.436	0.120	360770	C	A	0.177	4	109896462	6.59E-06	0.039	14306	20.379
Vascular dementia (subcortical)	family Lactobacillaceae	rs74599091	A	G	0.447	179751534	0.036	0.213	360770	A	G	0.192	1	179720669	7.70E-06	0.043	14306	20.323
Vascular dementia (subcortical)	family Lactobacillaceae	rs768253	T	G	-0.080	68097868	0.164	0.057	360770	T	G	-0.079	8	69010103	3.61E-06	0.017	14306	21.570
Vascular dementia (subcortical)	family Lactobacillaceae	rs77478751	A	G	0.144	173433875	0.105	0.089	360770	A	G	-0.219	3	173151665	5.96E-06	0.047	14306	21.421
Vascular dementia (subcortical)	family Lactobacillaceae	rs921925	A	C	-0.042	6928006	0.540	0.069	360770	A	C	0.100	19	6928017	5.77E-07	0.020	14306	24.555
Vascular dementia (subcortical)	family Lactobacillaceae	rs9345899	A	G	0.154	66849430	0.095	0.092	360770	A	G	-0.124	6	67559323	9.45E-06	0.028	14306	19.774
Vascular dementia (subcortical)	family Methanobacteriaceae	rs10202904	G	T	0.027	124682691	0.644	0.058	360770	G	T	0.122	2	125440268	3.01E-07	0.024	14306	26.762
Vascular dementia (subcortical)	family Methanobacteriaceae	rs10424197	A	G	-0.017	45936063	0.799	0.066	360770	A	G	0.111	19	46439321	9.28E-06	0.025	14306	20.211
Vascular dementia (subcortical)	family Methanobacteriaceae	rs4257531	G	A	-0.191	2044483	0.041	0.093	360770	G	A	0.164	3	2086167	7.44E-06	0.036	14306	20.316
Vascular dementia (subcortical)	family Methanobacteriaceae	rs6508769	C	T	-0.034	28336853	0.671	0.080	360770	C	T	-0.154	19	28827760	8.23E-06	0.034	14306	19.856
Vascular dementia (subcortical)	family Methanobacteriaceae	rs6776814	T	C	-0.246	15011576	0.220	0.200	360770	T	C	-0.200	3	15053083	1.63E-06	0.041	14306	23.483
Vascular dementia (subcortical)	family Methanobacteriaceae	rs73068003	G	T	0.204	10734305	0.035	0.096	360770	G	T	-0.158	7	10773932	8.45E-06	0.035	14306	20.206
Vascular dementia (subcortical)	family Methanobacteriaceae	rs73457410	A	G	0.108	41382045	0.347	0.115	360770	A	G	0.215	13	41956181	1.41E-06	0.044	14306	24.316
Vascular dementia (subcortical)	family Methanobacteriaceae	rs75208022	C	T	0.082	21185927	0.401	0.098	360770	C	T	-0.227	12	21338861	5.92E-06	0.049	14306	21.717
Vascular dementia (subcortical)	family Methanobacteriaceae	rs894996	C	A	0.070	103497150	0.527	0.110	360770	C	A	0.217	4	104418307	1.88E-06	0.045	14306	23.349
Vascular dementia (subcortical)	family Oxalobacteraceae	rs111966731	T	C	-0.029	93398708	0.777	0.102	360770	T	C	0.204	15	93941937	4.56E-06	0.045	14306	20.952
Vascular dementia (subcortical)	family Oxalobacteraceae	rs11246212	C	T	-0.039	610277	0.627	0.081	360770	C	T	-0.136	11	610277	4.51E-06	0.029	14306	21.745
Vascular dementia (subcortical)	family Oxalobacteraceae	rs12002250	A	C	0.027	19682560	0.846	0.137	360770	A	C	0.196	9	19682558	5.53E-06	0.045	14306	19.408
Vascular dementia (subcortical)	family Oxalobacteraceae	rs1569853	T	C	0.120	38582525	0.172	0.088	360770	T	C	-0.140	6	38550301	7.45E-07	0.028	14306	24.714

Vascular dementia (subcortical)	family Oxalobacteraceae	rs17138946	G	T	-0.014	5934557	0.905	0.114	360770	G	T	-0.189	16	5984558	8.09E-06	0.043	14306	19.388
Vascular dementia (subcortical)	family Oxalobacteraceae	rs36057338	G	T	-0.098	189014160	0.545	0.162	360770	G	T	0.182	4	189935314	6.26E-06	0.040	14306	20.743
Vascular dementia (subcortical)	family Oxalobacteraceae	rs4428215	G	A	-0.018	172229645	0.777	0.065	360770	G	A	0.126	3	171947435	4.88E-08	0.023	14306	29.812
Vascular dementia (subcortical)	family Oxalobacteraceae	rs561239	A	G	-0.053	18640649	0.448	0.070	360770	A	G	0.106	12	18793583	7.19E-06	0.024	14306	19.864
Vascular dementia (subcortical)	family Oxalobacteraceae	rs6000536	C	T	-0.108	37025428	0.154	0.076	360770	C	T	-0.118	22	37421469	7.39E-07	0.024	14306	24.058
Vascular dementia (subcortical)	family Oxalobacteraceae	rs62435498	C	A	0.084	1754627	0.375	0.094	360770	C	A	0.182	7	1794263	7.46E-06	0.040	14306	20.531
Vascular dementia (subcortical)	family Oxalobacteraceae	rs736744	T	C	0.046	84899492	0.426	0.057	360770	T	C	-0.106	9	87514407	1.49E-07	0.020	14306	27.721
Vascular dementia (subcortical)	family Oxalobacteraceae	rs7993559	A	C	-0.057	22869488	0.319	0.058	360770	A	C	-0.092	13	23443627	5.04E-06	0.020	14306	20.974
Vascular dementia (subcortical)	family Oxalobacteraceae	rs80330081	A	C	-0.012	66561674	0.893	0.093	360770	A	C	-0.188	4	67427392	6.64E-06	0.042	14306	19.597
Vascular dementia (subcortical)	family Oxalobacteraceae	rs934049	G	A	-0.009	15916656	0.896	0.070	360770	G	A	0.110	2	16056779	4.21E-06	0.024	14306	21.152
Vascular dementia (subcortical)	family Pasteurellaceae	rs10965428	C	A	0.148	22718482	0.232	0.124	360770	C	A	-0.120	9	22718481	4.29E-06	0.026	14306	21.561
Vascular dementia (subcortical)	family Pasteurellaceae	rs111582866	G	A	-0.002	48708578	0.983	0.101	360770	G	A	-0.114	16	48742489	7.07E-06	0.026	14306	19.753
Vascular dementia (subcortical)	family Pasteurellaceae	rs12050685	A	G	0.023	73185141	0.720	0.063	360770	A	G	-0.067	15	73477482	9.19E-06	0.015	14306	19.385
Vascular dementia (subcortical)	family Pasteurellaceae	rs16970009	A	G	0.345	34535582	0.098	0.208	360770	A	G	0.187	17	32862601	7.32E-06	0.043	14306	19.027
Vascular dementia (subcortical)	family Pasteurellaceae	rs4822728	T	C	-0.085	26495842	0.135	0.057	360770	T	C	0.069	22	26891808	4.72E-06	0.015	14306	21.156
Vascular dementia (subcortical)	family Pasteurellaceae	rs6972479	A	G	-0.064	117278006	0.363	0.070	360770	A	G	-0.078	7	116918060	7.75E-06	0.018	14306	19.878
Vascular dementia (subcortical)	family Pasteurellaceae	rs72756943	G	A	-0.023	26531799	0.844	0.115	360770	G	A	0.140	5	26531908	3.35E-06	0.030	14306	21.308
Vascular dementia (subcortical)	family Pasteurellaceae	rs73139353	A	C	-0.088	98253370	0.379	0.100	360770	A	C	-0.223	3	97972214	8.71E-06	0.048	14306	21.092
Vascular dementia (subcortical)	family Pasteurellaceae	rs76022354	C	T	-0.127	92546628	0.334	0.131	360770	C	T	0.243	10	94306385	1.83E-06	0.050	14306	23.560
Vascular dementia (subcortical)	family Pasteurellaceae	rs78909003	T	C	0.109	102887960	0.372	0.122	360770	T	C	-0.241	9	105650242	2.05E-06	0.050	14306	23.415
Vascular dementia (subcortical)	family Pasteurellaceae	rs9382510	C	T	-0.026	55583693	0.684	0.065	360770	C	T	-0.088	6	55448491	2.48E-07	0.017	14306	26.921
Vascular dementia (subcortical)	family Pasteurellaceae	rs9895850	T	C	0.113	66538895	0.416	0.139	360770	T	C	-0.176	17	64535013	9.08E-06	0.041	14306	18.497
Vascular dementia (subcortical)	family Pasteurellaceae	rs9938097	C	T	0.013	84943783	0.823	0.059	360770	C	T	0.071	16	84977389	8.23E-06	0.016	14306	20.209
Vascular dementia (subcortical)	family Peptococcaceae	rs117452796	A	G	-0.346	9423378	0.034	0.163	360770	A	G	-0.258	9	9423378	3.15E-06	0.055	14306	22.036
Vascular dementia (subcortical)	family Peptococcaceae	rs12144792	C	T	-0.028	27091734	0.629	0.059	360770	C	T	0.064	1	27418225	5.82E-06	0.014	14306	20.788
Vascular dementia (subcortical)	family Peptococcaceae	rs12992764	T	G	0.109	188031016	0.057	0.057	360770	T	G	0.068	2	188895743	1.46E-06	0.014	14306	23.502
Vascular dementia (subcortical)	family Peptococcaceae	rs150600492	A	C	0.091	127377698	0.452	0.121	360770	A	C	0.136	10	129175962	2.31E-06	0.029	14306	21.957
Vascular dementia (subcortical)	family Peptococcaceae	rs35703006	G	T	0.165	28756700	0.012	0.066	360770	G	T	0.081	8	28614217	4.95E-07	0.016	14306	24.448
Vascular dementia (subcortical)	family Peptococcaceae	rs4990837	G	A	0.030	3723235	0.680	0.072	360770	G	A	-0.091	8	3580757	1.74E-06	0.019	14306	23.990
Vascular dementia (subcortical)	family Peptococcaceae	rs75430375	C	T	0.282	89957067	0.042	0.139	360770	C	T	-0.148	5	89252884	3.41E-06	0.032	14306	21.712
Vascular dementia (subcortical)	family Peptococcaceae	rs75898026	A	G	-0.018	112906768	0.799	0.070	360770	A	G	-0.082	13	113561082	2.02E-06	0.017	14306	22.424
Vascular dementia (subcortical)	family Peptostreptococcaceae	rs10805326	A	G	0.004	14322999	0.946	0.063	360770	A	G	-0.057	4	14324623	4.03E-06	0.012	14306	21.306
Vascular dementia (subcortical)	family Peptostreptococcaceae	rs117020988	C	T	0.091	46671379	0.376	0.103	360770	C	T	0.182	7	46710977	1.03E-06	0.037	14306	24.028
Vascular dementia (subcortical)	family Peptostreptococcaceae	rs12377846	C	A	0.176	16786786	0.319	0.177	360770	C	A	-0.252	9	16786784	7.26E-07	0.051	14306	24.260
Vascular dementia (subcortical)	family Peptostreptococcaceae	rs12986312	T	G	0.002	17303329	0.968	0.062	360770	T	G	0.057	19	17414138	5.77E-06	0.013	14306	20.613
Vascular dementia (subcortical)	family Peptostreptococcaceae	rs1467258	G	A	-0.080	40060234	0.278	0.074	360770	G	A	0.073	17	38216487	7.90E-06	0.016	14306	19.996
Vascular dementia (subcortical)	family Peptostreptococcaceae	rs1520207	T	C	-0.038	152063386	0.511	0.057	360770	T	C	-0.053	3	151781175	3.17E-06	0.011	14306	21.842
Vascular dementia (subcortical)	family Peptostreptococcaceae	rs4692811	C	T	-0.043	170259039	0.468	0.060	360770	C	T	0.064	4	171180190	4.21E-07	0.013	14306	25.595
Vascular dementia (subcortical)	family Peptostreptococcaceae	rs59865771	C	T	-0.059	89180262	0.324	0.059	360770	C	T	-0.057	16	89246670	7.69E-06	0.013	14306	20.349
Vascular dementia (subcortical)	family Peptostreptococcaceae	rs61841503	G	A	-0.097	16977560	0.258	0.086	360770	G	A	0.092	10	17019559	9.80E-09	0.016	14306	32.495
Vascular dementia (subcortical)	family Peptostreptococcaceae	rs6721459	G	A	0.000	67625704	0.993	0.058	360770	G	A	-0.051	2	67852836	5.08E-06	0.011	14306	20.730
Vascular dementia (subcortical)	family Peptostreptococcaceae	rs76982728	T	C	-0.338	44273225	0.090	0.199	360770	T	C	0.124	7	44312824	3.24E-06	0.027	14306	21.652
Vascular dementia (subcortical)	family Peptostreptococcaceae	rs77540684	T	G	-0.058	14608536	0.550	0.097	360770	T	G	0.107	10	14650535	8.14E-06	0.025	14306	18.877
Vascular dementia (subcortical)	family Peptostreptococcaceae	rs9573937	A	G	0.042	76777061	0.581	0.077	360770	A	G	-0.069	13	77351196	1.71E-06	0.014	14306	23.559
Vascular dementia (subcortical)	family Porphyromonadaceae	rs10762312	A	G	-0.014	69812107	0.818	0.062	360770	A	G	0.052	10	71571863	8.70E-06	0.012	14306	19.427
Vascular dementia (subcortical)	family Porphyromonadaceae	rs10858364	G	T	0.049	135184235	0.466	0.067	360770	G	T	0.055	9	138076081	4.31E-06	0.012	14306	20.951
Vascular dementia (subcortical)	family Porphyromonadaceae	rs17065783	A	G	0.076	62049912	0.329	0.078	360770	A	G	-0.059	3	62035586	1.79E-06	0.012	14306	23.403
Vascular dementia (subcortical)	family Porphyromonadaceae	rs1980561	A	G	0.059	62919798	0.303	0.057	360770	A	G	-0.049	14	63386516	8.95E-06	0.011	14306	19.719
Vascular dementia (subcortical)	family Porphyromonadaceae	rs35233670	T	C	-0.006	65754785	0.917	0.057	360770	T	C	-0.047	17	63750903	7.91E-06	0.011	14306	19.953
Vascular dementia (subcortical)	family Porphyromonadaceae	rs35961441	A	C	-0.028	240766474	0.854	0.150	360770	A	C	0.092	1	240929774	8.37E-06	0.021	14306	19.492
Vascular dementia (subcortical)	family Porphyromonadaceae	rs6953849	A	G	0.059	69786706	0.423	0.074	360770	A	G	0.072	7	69251692	2.44E-06	0.015	14306	22.702
Vascular dementia (subcortical)	family Porphyromonadaceae	rs7330827	T	C	-0.163	22957663	0.174	0.120	360770	T	C	-0.104	13	23531802	8.05E-06	0.024	14306	19.140
Vascular dementia (subcortical)	family Porphyromonadaceae	rs864093	A	C	-0.111	148904825	0.111	0.069	360770	A	C	-0.053	4	149825977	9.60E-06	0.012	14306	20.188
Vascular dementia (subcortical)	family Prevotellaceae	rs12057990	C	T	-0.009	99004713	0.885	0.064	360770	C	T	0.059	1	99470269	8.97E-06	0.013	14306	19.889
Vascular dementia (subcortical)	family Prevotellaceae	rs12118202	T	C	-0.030	210508026	0.680	0.074	360770	T	C	-0.075	1	210681370	5.54E-07	0.015	14306	26.075
Vascular dementia (subcortical)	family Prevotellaceae	rs13069367	A	C	-0.041	71752679	0.479	0.058	360770	A	C	-0.054	3	71801830	7.39E-06	0.012	14306	20.339
Vascular dementia (subcortical)	family Prevotellaceae	rs148376875	T	G	0.139	170408919	0.083	0.080	360770	T	G	0.085	3	170126707	2.08E-06	0.018	14306	22.252
Vascular dementia (subcortical)	family Prevotellaceae	rs2206482	T	G	0.014	9790461	0.816	0.058	360770	T	G	-0.057	20	9771109	1.30E-06	0.012	14306	23.465
Vascular dementia (subcortical)	family Prevotellaceae	rs2278540	G	A	-0.076	32367424	0.199	0.059	360770	G	A	0.055	3	32408916	8.44E-06	0.012	14306	20.218
Vascular dementia (subcortical)	family Prevotellaceae	rs34660375	A	G	0.051	180856881	0.528	0.081	360770	A	G	-0.081	5	180283881	7.40E-06	0.018	14306	20.431
Vascular dementia (subcortical)	family Prevotellaceae	rs3758087	C	T	-0.078	23857096	0.223	0.064	360770	C	T	-0.056	8	23714609	8.61E-06	0.012	14306	20.485
Vascular dementia (subcortical)	family Prevotellaceae	rs3860225	A	G	-0.130	110619055	0.185	0.098	360770	A	G	0.084	1	111161677	5.50E-07	0.017	14306	24.981
Vascular dementia (subcortical)	family Prevotellaceae	rs4493272	T	C	-0.080	118153336	0.159	0.057	360770	T	C	-0.060	2	118910912	3.02E-07	0.012	14306	26.275
Vascular dementia (subcortical)	family Prevotellaceae	rs4685827	T	C	-0.004	4823193	0.955	0.066	360770	T	C	-0.068	3	4864877	2.77E-06	0.015	14306	21.840
Vascular dementia (subcortical)	family Prevotellaceae	rs7252711	G	A	0.136	17350190	0.134	0.091	360770	G	A	0.074	19	17460999	5.57E-06	0.016	14306	20.934
Vascular dementia (subcortical)	family Prevotellaceae	rs7975087	C	A	-0.072	21251898	0.339	0.075	360770	C	A	-0.060	12	21404832	7.59E-06	0.014	14306	19.716
Vascular dementia (subcortical)	family Prevotellaceae	rs912860	A	G	-0.197	33237702	0.297	0.189	360770	A	G	0.229	14	33706908	9.30E-07	0.048	14306	22.476

Vascular dementia (subcortical)	family Prevotellaceae	rs9586501	G	A	-0.057	104423473	0.378	0.064	360770	G	A	0.059	13	105075823	2.59E-06	0.013	14306	21.666
Vascular dementia (subcortical)	family Prevotellaceae	rs9958960	G	A	-0.033	65351747	0.670	0.078	360770	G	A	-0.091	18	63018983	1.06E-07	0.017	14306	27.765
Vascular dementia (subcortical)	family Rhodospirillaceae	rs1035406	G	A	0.024	120037042	0.786	0.089	360770	G	A	-0.114	5	119372737	5.84E-06	0.025	14306	20.484
Vascular dementia (subcortical)	family Rhodospirillaceae	rs11591293	G	T	0.065	111660039	0.259	0.057	360770	G	T	0.074	10	113419797	2.67E-06	0.016	14306	21.923
Vascular dementia (subcortical)	family Rhodospirillaceae	rs13336560	C	T	-0.015	88487835	0.796	0.058	360770	C	T	-0.070	16	88554243	9.17E-06	0.016	14306	19.710
Vascular dementia (subcortical)	family Rhodospirillaceae	rs1549633	A	C	-0.066	27945538	0.461	0.089	360770	A	C	0.100	5	27945645	4.70E-06	0.022	14306	20.891
Vascular dementia (subcortical)	family Rhodospirillaceae	rs1923415	A	G	0.095	88558996	0.345	0.101	360770	A	G	-0.100	6	89268715	9.64E-06	0.023	14306	19.321
Vascular dementia (subcortical)	family Rhodospirillaceae	rs3754624	C	T	0.041	224769095	0.576	0.074	360770	C	T	0.097	2	225633812	1.71E-06	0.020	14306	23.575
Vascular dementia (subcortical)	family Rhodospirillaceae	rs4278423	T	C	-0.052	2628361	0.665	0.119	360770	T	C	0.108	10	2670553	3.12E-06	0.024	14306	20.808
Vascular dementia (subcortical)	family Rhodospirillaceae	rs61933850	G	A	0.010	72745618	0.903	0.083	360770	G	A	0.165	12	73139398	7.23E-06	0.036	14306	20.883
Vascular dementia (subcortical)	family Rhodospirillaceae	rs6679026	T	C	-0.094	78153828	0.324	0.095	360770	T	C	0.112	1	78619512	9.95E-06	0.025	14306	19.891
Vascular dementia (subcortical)	family Rhodospirillaceae	rs7001029	C	T	-0.064	130946157	0.520	0.099	360770	C	T	0.117	8	131958403	5.35E-06	0.026	14306	20.171
Vascular dementia (subcortical)	family Rhodospirillaceae	rs72714493	A	G	-0.038	91740661	0.633	0.080	360770	A	G	0.082	1	92206218	7.35E-06	0.018	14306	20.415
Vascular dementia (subcortical)	family Rhodospirillaceae	rs74354280	C	T	0.020	133271763	0.755	0.063	360770	C	T	-0.091	4	134192918	6.67E-06	0.020	14306	19.805
Vascular dementia (subcortical)	family Rhodospirillaceae	rs76784716	A	G	-0.020	168176830	0.825	0.090	360770	A	G	0.136	2	169033340	1.49E-06	0.029	14306	22.666
Vascular dementia (subcortical)	family Rhodospirillaceae	rs9813022	A	G	0.029	13685237	0.616	0.059	360770	A	G	-0.084	3	13726736	2.53E-07	0.016	14306	26.522
Vascular dementia (subcortical)	family Rikenellaceae	rs10217435	C	T	-0.052	83437740	0.530	0.082	360770	C	T	-0.088	9	86052655	6.51E-06	0.020	14306	20.022
Vascular dementia (subcortical)	family Rikenellaceae	rs10832801	A	C	0.033	17567582	0.598	0.063	360770	A	C	-0.053	11	17589129	7.50E-06	0.012	14306	19.005
Vascular dementia (subcortical)	family Rikenellaceae	rs1939881	G	A	0.078	95571670	0.526	0.122	360770	G	A	-0.106	11	95304834	5.64E-07	0.021	14306	26.218
Vascular dementia (subcortical)	family Rikenellaceae	rs2447496	A	G	-0.064	98176696	0.323	0.064	360770	A	G	0.055	8	99188924	6.09E-06	0.012	14306	20.297
Vascular dementia (subcortical)	family Rikenellaceae	rs2833282	G	A	0.119	31124392	0.137	0.080	360770	G	A	0.071	21	32496710	4.31E-06	0.016	14306	20.526
Vascular dementia (subcortical)	family Rikenellaceae	rs36021379	A	G	-0.113	27143593	0.151	0.079	360770	A	G	-0.066	21	28515912	7.20E-06	0.014	14306	20.496
Vascular dementia (subcortical)	family Rikenellaceae	rs4264350	T	C	0.005	71366303	0.935	0.057	360770	T	C	-0.053	15	71658642	1.35E-06	0.011	14306	23.514
Vascular dementia (subcortical)	family Rikenellaceae	rs59663348	G	A	-0.163	4069710	0.013	0.066	360770	G	A	0.057	18	4069710	6.12E-06	0.013	14306	20.844
Vascular dementia (subcortical)	family Rikenellaceae	rs62532512	A	C	-0.035	14158855	0.545	0.058	360770	A	C	0.050	9	14158854	2.76E-06	0.011	14306	22.045
Vascular dementia (subcortical)	family Rikenellaceae	rs6744030	C	T	-0.026	173392399	0.709	0.070	360770	C	T	0.070	2	174257127	9.32E-06	0.016	14306	19.669
Vascular dementia (subcortical)	family Rikenellaceae	rs6837275	A	G	0.094	186921078	0.133	0.062	360770	A	G	0.057	4	187842232	1.45E-06	0.012	14306	23.022
Vascular dementia (subcortical)	family Rikenellaceae	rs74474130	T	G	0.024	89804477	0.877	0.155	360770	T	G	0.138	14	90270821	3.61E-06	0.030	14306	21.702
Vascular dementia (subcortical)	family Rikenellaceae	rs77885767	C	T	0.111	21080475	0.411	0.135	360770	C	T	-0.156	14	21548634	2.85E-06	0.034	14306	21.552
Vascular dementia (subcortical)	family Rikenellaceae	rs9389714	C	T	0.051	99796791	0.616	0.101	360770	C	T	-0.064	6	100244667	8.79E-06	0.014	14306	19.718
Vascular dementia (subcortical)	family Rikenellaceae	rs9578457	G	A	-0.044	22293217	0.741	0.132	360770	G	A	-0.141	13	22867356	3.99E-06	0.032	14306	20.064
Vascular dementia (subcortical)	family Rikenellaceae	rs9603208	G	T	0.186	37470552	0.050	0.095	360770	G	T	0.082	13	38044689	1.92E-07	0.016	14306	26.474
Vascular dementia (subcortical)	family Ruminococcaceae	rs10093275	T	C	-0.076	68820652	0.207	0.060	360770	T	C	-0.053	8	69732887	5.35E-06	0.012	14306	20.960
Vascular dementia (subcortical)	family Ruminococcaceae	rs10166469	C	T	0.020	29606301	0.761	0.065	360770	C	T	0.053	2	29829167	8.52E-06	0.012	14306	19.686
Vascular dementia (subcortical)	family Ruminococcaceae	rs1158100	G	A	0.035	4698162	0.542	0.057	360770	G	A	0.049	8	4555684	8.61E-06	0.011	14306	19.936
Vascular dementia (subcortical)	family Ruminococcaceae	rs1612733	T	C	-0.022	107118984	0.861	0.125	360770	T	C	0.109	1	107661606	4.22E-06	0.024	14306	20.862
Vascular dementia (subcortical)	family Ruminococcaceae	rs17376049	T	C	-0.070	60959595	0.441	0.091	360770	T	C	0.085	1	61425267	7.30E-07	0.017	14306	24.306
Vascular dementia (subcortical)	family Ruminococcaceae	rs2113833	C	T	-0.024	217352078	0.879	0.159	360770	C	T	-0.169	2	218216801	1.14E-06	0.036	14306	22.701
Vascular dementia (subcortical)	family Ruminococcaceae	rs3009418	A	C	0.155	148272338	0.256	0.136	360770	A	C	-0.093	1	147744468	8.69E-06	0.021	14306	19.489
Vascular dementia (subcortical)	family Ruminococcaceae	rs55793120	T	C	0.117	46990335	0.334	0.121	360770	T	C	0.138	12	47384118	1.44E-07	0.027	14306	26.970
Vascular dementia (subcortical)	family Ruminococcaceae	rs56199908	T	C	-0.142	2801371	0.255	0.125	360770	T	C	-0.199	9	2801371	1.66E-06	0.041	14306	23.584
Vascular dementia (subcortical)	family Ruminococcaceae	rs76724913	T	G	0.049	24147179	0.609	0.096	360770	T	G	0.090	1	24473669	9.60E-06	0.020	14306	19.682
Vascular dementia (subcortical)	family Streptococcaceae	rs10028567	C	T	0.014	52791410	0.873	0.087	360770	C	T	-0.093	4	53657577	3.72E-06	0.019	14306	24.079
Vascular dementia (subcortical)	family Streptococcaceae	rs11110281	T	C	0.162	100190236	0.229	0.134	360770	T	C	-0.131	12	100584014	1.40E-08	0.023	14306	33.387
Vascular dementia (subcortical)	family Streptococcaceae	rs16950051	A	G	0.163	120291702	0.171	0.119	360770	A	G	0.107	12	120729505	5.34E-06	0.024	14306	20.391
Vascular dementia (subcortical)	family Streptococcaceae	rs2370083	G	T	-0.079	97060413	0.488	0.114	360770	G	T	-0.084	14	97526750	4.26E-06	0.018	14306	20.862
Vascular dementia (subcortical)	family Streptococcaceae	rs2952251	G	A	0.003	10285654	0.963	0.068	360770	G	A	0.064	8	10143164	3.72E-07	0.013	14306	25.530
Vascular dementia (subcortical)	family Streptococcaceae	rs35344081	G	A	-0.104	941253	0.107	0.065	360770	G	A	0.061	16	991253	2.64E-06	0.013	14306	22.072
Vascular dementia (subcortical)	family Streptococcaceae	rs57646748	G	A	0.332	37451236	0.012	0.132	360770	G	A	-0.088	4	37452858	7.88E-06	0.020	14306	19.612
Vascular dementia (subcortical)	family Streptococcaceae	rs6806351	T	C	-0.054	132339879	0.435	0.069	360770	T	C	-0.062	3	132058723	6.94E-06	0.014	14306	20.808
Vascular dementia (subcortical)	family Streptococcaceae	rs77968078	G	A	-0.048	240278466	0.687	0.118	360770	G	A	-0.099	1	240441766	7.93E-06	0.022	14306	19.515
Vascular dementia (subcortical)	family Streptococcaceae	rs7916711	A	G	0.079	28299340	0.332	0.082	360770	A	G	0.096	10	28588269	6.33E-06	0.022	14306	19.839
Vascular dementia (subcortical)	family Streptococcaceae	rs957755	T	G	-0.066	46739055	0.421	0.082	360770	T	G	-0.064	7	46778653	7.42E-06	0.014	14306	20.268
Vascular dementia (subcortical)	family Veillonellaceae	rs111810795	C	T	-0.054	102431593	0.576	0.096	360770	C	T	-0.087	14	102897930	1.73E-06	0.018	14306	23.059
Vascular dementia (subcortical)	family Veillonellaceae	rs114889439	A	G	0.063	61023491	0.686	0.156	360770	A	G	-0.254	13	61597625	6.19E-06	0.054	14306	22.160
Vascular dementia (subcortical)	family Veillonellaceae	rs12186441	G	A	-0.182	133310611	0.187	0.138	360770	G	A	0.208	5	132646303	4.53E-06	0.045	14306	21.010
Vascular dementia (subcortical)	family Veillonellaceae	rs12668619	A	G	-0.018	21598192	0.771	0.060	360770	A	G	0.055	7	21637810	2.57E-06	0.012	14306	22.093
Vascular dementia (subcortical)	family Veillonellaceae	rs12741784	C	T	0.003	49623147	0.959	0.065	360770	C	T	-0.062	1	50088819	1.28E-07	0.012	14306	27.151
Vascular dementia (subcortical)	family Veillonellaceae	rs1442060	A	G	-0.074	46364050	0.198	0.057	360770	A	G	0.051	4	46366067	4.51E-06	0.011	14306	21.017
Vascular dementia (subcortical)	family Veillonellaceae	rs1693340	T	C	0.024	32628728	0.826	0.109	360770	T	C	0.082	18	30208691	9.25E-06	0.018	14306	20.147
Vascular dementia (subcortical)	family Veillonellaceae	rs2175069	G	A	-0.064	23315501	0.278	0.059	360770	G	A	0.053	4	23317124	4.64E-06	0.011	14306	21.022
Vascular dementia (subcortical)	family Veillonellaceae	rs2561116	T	G	0.010	38348097	0.937	0.123	360770	T	G	-0.084	5	38348199	7.89E-06	0.019	14306	19.960
Vascular dementia (subcortical)	family Veillonellaceae	rs2585520	G	T	0.027	78211006	0.856	0.149	360770	G	T	-0.090	13	78785141	5.27E-06	0.020	14306	20.387
Vascular dementia (subcortical)	family Veillonellaceae	rs4263802	A	G	-0.010	137307265	0.873	0.060	360770	A	G	-0.051	8	138319508	7.45E-06	0.011	14306	19.650
Vascular dementia (subcortical)	family Veillonellaceae	rs4461038	G	A	-0.081	78254654	0.197	0.063	360770	G	A	0.055	15	78546996	3.73E-06	0.012	14306	21.579
Vascular dementia (subcortical)	family Veillonellaceae	rs4797169	T	C	-0.022	462180	0.744	0.067	360770	T	C	0.059	18	462180	4.49E-06	0.013	14306	20.991
Vascular dementia (subcortical)	family Veillonellaceae	rs61264131	A	C	-0.128	138867397	0.184	0.096	360770	A	C	0.202	6	139188534	6.75E-06	0.046	14306	18.990

Vascular dementia (subcortical)	family Veillonellaceae	rs6692542	G	A	0.079	244228316	0.187	0.060	360770	G	A	-0.053	1	244391618	8.68E-06	0.012	14306	20.422
Vascular dementia (subcortical)	family Veillonellaceae	rs6909981	C	T	-0.063	74824436	0.470	0.087	360770	C	T	-0.064	6	75534152	5.48E-06	0.014	14306	20.298
Vascular dementia (subcortical)	family Veillonellaceae	rs79535861	A	C	0.001	37752555	0.991	0.098	360770	A	C	0.101	13	38326692	1.58E-06	0.021	14306	23.646
Vascular dementia (subcortical)	family Veillonellaceae	rs9345168	A	C	0.053	91671204	0.351	0.057	360770	A	C	-0.051	6	92380922	8.49E-06	0.011	14306	20.207
Vascular dementia (subcortical)	family Verrucomicrobiaceae	rs111862613	T	C	0.047	129825125	0.536	0.076	360770	T	C	0.091	12	130309670	3.73E-06	0.020	14306	21.255
Vascular dementia (subcortical)	family Verrucomicrobiaceae	rs117107102	A	G	-0.057	51947265	0.671	0.134	360770	A	G	0.205	18	49473635	2.92E-06	0.043	14306	22.493
Vascular dementia (subcortical)	family Verrucomicrobiaceae	rs11729256	T	C	-0.064	94106121	0.395	0.076	360770	T	C	0.075	4	95027272	6.73E-07	0.015	14306	24.928
Vascular dementia (subcortical)	family Verrucomicrobiaceae	rs12908520	G	A	0.100	97027427	0.081	0.057	360770	G	A	0.062	15	97570657	2.15E-06	0.013	14306	22.353
Vascular dementia (subcortical)	family Verrucomicrobiaceae	rs2602429	T	C	0.035	81029544	0.587	0.065	360770	T	C	-0.075	16	81063149	2.70E-06	0.016	14306	22.781
Vascular dementia (subcortical)	family Verrucomicrobiaceae	rs4242783	A	G	-0.058	5022135	0.363	0.064	360770	A	G	-0.069	10	5064327	2.75E-06	0.015	14306	21.699
Vascular dementia (subcortical)	family Verrucomicrobiaceae	rs4936098	G	A	0.056	130410772	0.348	0.059	360770	G	A	-0.065	11	130280667	1.13E-06	0.014	14306	22.775
Vascular dementia (subcortical)	family Verrucomicrobiaceae	rs61779207	G	A	-0.077	40608800	0.265	0.069	360770	G	A	-0.076	1	41074472	6.63E-06	0.017	14306	20.459
Vascular dementia (subcortical)	family Verrucomicrobiaceae	rs74542928	T	C	0.043	99623031	0.746	0.133	360770	T	C	0.112	4	100544188	1.65E-06	0.024	14306	22.492
Vascular dementia (subcortical)	family Verrucomicrobiaceae	rs9349825	A	G	-0.064	56476683	0.376	0.072	360770	A	G	-0.070	6	56341481	2.51E-06	0.015	14306	22.919
Vascular dementia (subcortical)	family Verrucomicrobiaceae	rs941682	G	A	0.065	33280034	0.305	0.063	360770	G	A	-0.063	20	31867840	9.58E-06	0.014	14306	19.296
Vascular dementia (subcortical)	family Victivallaceae	rs11671100	A	C	-0.032	711637	0.650	0.071	360770	A	C	-0.160	19	711637	4.08E-06	0.035	14306	20.970
Vascular dementia (subcortical)	family Victivallaceae	rs11764871	G	T	0.047	147111885	0.450	0.062	360770	G	T	0.127	7	146808977	7.49E-07	0.026	14306	24.542
Vascular dementia (subcortical)	family Victivallaceae	rs2944282	T	C	0.036	57530097	0.566	0.063	360770	T	C	-0.124	7	57589803	1.57E-06	0.026	14306	23.349
Vascular dementia (subcortical)	family Victivallaceae	rs34962571	A	C	0.044	130618711	0.650	0.096	360770	A	C	-0.187	12	131103256	6.25E-06	0.042	14306	19.963
Vascular dementia (subcortical)	family Victivallaceae	rs4396289	C	T	0.082	9295183	0.336	0.085	360770	C	T	-0.153	11	9316730	1.54E-07	0.029	14306	28.090
Vascular dementia (subcortical)	family Victivallaceae	rs61702987	T	C	-0.027	30988791	0.768	0.092	360770	T	C	0.146	2	31211657	3.08E-06	0.030	14306	23.522
Vascular dementia (subcortical)	family Victivallaceae	rs62570196	C	T	-0.061	108323890	0.666	0.142	360770	C	T	-0.246	9	111086170	2.70E-07	0.048	14306	25.953
Vascular dementia (subcortical)	family Victivallaceae	rs6545794	A	G	0.137	60257774	0.119	0.088	360770	A	G	-0.198	2	60484909	5.97E-07	0.041	14306	23.215
Vascular dementia (subcortical)	family Victivallaceae	rs7077363	G	A	0.181	93527197	0.019	0.077	360770	G	A	0.149	10	95286954	2.83E-06	0.032	14306	21.887
Vascular dementia (subcortical)	family Victivallaceae	rs7314815	G	A	0.095	102131269	0.095	0.057	360770	G	A	0.101	12	102525047	6.40E-06	0.023	14306	20.079
Vascular dementia (subcortical)	family Victivallaceae	rs7627405	C	T	0.026	9926871	0.714	0.072	360770	C	T	-0.134	3	9968555	8.19E-06	0.030	14306	19.809
Vascular dementia (subcortical)	genus Actinomyces	rs2715439	T	C	-0.074	98949084	0.192	0.057	360770	T	C	-0.075	15	99492313	6.27E-06	0.016	14306	20.523
Vascular dementia (subcortical)	genus Actinomyces	rs34583783	G	T	-0.036	66497478	0.763	0.120	360770	G	T	0.127	6	67207371	4.49E-06	0.027	14306	22.237
Vascular dementia (subcortical)	genus Actinomyces	rs35011108	A	G	-0.263	132686341	0.020	0.113	360770	A	G	0.233	6	133007480	6.34E-06	0.051	14306	20.641
Vascular dementia (subcortical)	genus Actinomyces	rs4073240	G	A	0.065	168824686	0.265	0.058	360770	G	A	0.075	6	169224781	7.94E-06	0.017	14306	20.064
Vascular dementia (subcortical)	genus Actinomyces	rs4146653	G	A	-0.015	4740649	0.852	0.081	360770	G	A	0.099	10	4782841	4.50E-06	0.021	14306	21.159
Vascular dementia (subcortical)	genus Actinomyces	rs71315246	A	G	-0.130	101633595	0.117	0.083	360770	A	G	-0.097	3	101352439	9.83E-06	0.022	14306	19.566
Vascular dementia (subcortical)	genus Actinomyces	rs7915461	C	T	0.081	125843552	0.481	0.115	360770	C	T	-0.188	10	127532121	5.92E-06	0.040	14306	21.855
Vascular dementia (subcortical)	genus Adlercreutzia	rs11604400	C	T	-0.134	98515355	0.151	0.093	360770	C	T	-0.103	11	98386085	9.74E-06	0.023	14306	19.060
Vascular dementia (subcortical)	genus Adlercreutzia	rs13231526	C	A	-0.019	48804555	0.857	0.106	360770	C	A	0.143	7	48844151	4.81E-06	0.031	14306	21.123
Vascular dementia (subcortical)	genus Adlercreutzia	rs2717140	C	T	-0.007	77297814	0.939	0.095	360770	C	T	-0.119	18	75009770	2.05E-06	0.025	14306	22.548
Vascular dementia (subcortical)	genus Adlercreutzia	rs55719207	G	A	-0.005	105637127	0.932	0.059	360770	G	A	-0.070	3	105355971	9.61E-06	0.016	14306	19.577
Vascular dementia (subcortical)	genus Adlercreutzia	rs6664405	T	C	0.079	68978480	0.328	0.081	360770	T	C	-0.095	1	69444163	5.23E-06	0.021	14306	20.451
Vascular dementia (subcortical)	genus Adlercreutzia	rs7680684	T	C	-0.042	170360208	0.481	0.060	360770	T	C	0.083	4	171281359	9.77E-07	0.017	14306	24.371
Vascular dementia (subcortical)	genus Adlercreutzia	rs9490822	C	T	-0.108	123587938	0.057	0.057	360770	C	T	-0.073	6	123909083	2.54E-06	0.016	14306	22.229
Vascular dementia (subcortical)	genus Adlercreutzia	rs9915817	C	T	-0.118	50786818	0.060	0.063	360770	C	T	-0.075	17	48864179	8.22E-06	0.017	14306	19.810
Vascular dementia (subcortical)	genus Akkermansia	rs111862613	T	C	0.047	129825125	0.536	0.076	360770	T	C	0.091	12	130309670	3.39E-06	0.020	14306	21.449
Vascular dementia (subcortical)	genus Akkermansia	rs117107102	A	G	-0.057	51947265	0.671	0.134	360770	A	G	0.204	18	49473635	3.01E-06	0.043	14306	22.427
Vascular dementia (subcortical)	genus Akkermansia	rs11729256	T	C	-0.064	94106121	0.395	0.076	360770	T	C	0.075	4	95027272	6.58E-07	0.015	14306	24.970
Vascular dementia (subcortical)	genus Akkermansia	rs12908520	G	A	0.100	97027427	0.081	0.057	360770	G	A	0.062	15	97570657	2.26E-06	0.013	14306	22.251
Vascular dementia (subcortical)	genus Akkermansia	rs2602429	T	C	0.035	81029544	0.587	0.065	360770	T	C	-0.075	16	81063149	2.72E-06	0.016	14306	22.770
Vascular dementia (subcortical)	genus Akkermansia	rs4242783	A	G	-0.058	5022135	0.363	0.064	360770	A	G	-0.069	10	5064327	3.00E-06	0.015	14306	21.537
Vascular dementia (subcortical)	genus Akkermansia	rs4936098	G	A	0.056	130410772	0.348	0.059	360770	G	A	-0.065	11	130280667	1.10E-06	0.014	14306	22.810
Vascular dementia (subcortical)	genus Akkermansia	rs61779207	G	A	-0.077	40608800	0.265	0.069	360770	G	A	-0.076	1	41074472	6.32E-06	0.017	14306	20.550
Vascular dementia (subcortical)	genus Akkermansia	rs74542928	T	C	0.043	99623031	0.746	0.133	360770	T	C	0.113	4	100544188	1.48E-06	0.024	14306	22.690
Vascular dementia (subcortical)	genus Akkermansia	rs9349825	A	G	-0.064	56476683	0.376	0.072	360770	A	G	-0.070	6	56341481	2.60E-06	0.015	14306	22.856
Vascular dementia (subcortical)	genus Akkermansia	rs941682	G	A	0.065	33280034	0.305	0.063	360770	G	A	-0.063	20	31867840	9.17E-06	0.014	14306	19.381
Vascular dementia (subcortical)	genus Alistipes	rs1107244	G	A	0.151	37483220	0.154	0.106	360770	G	A	0.076	13	38057357	3.59E-06	0.017	14306	19.636
Vascular dementia (subcortical)	genus Alistipes	rs11769002	G	A	0.025	62983120	0.660	0.058	360770	G	A	-0.053	7	62443498	1.45E-06	0.011	14306	23.368
Vascular dementia (subcortical)	genus Alistipes	rs11958296	A	G	-0.024	178328178	0.866	0.141	360770	A	G	-0.098	5	177755179	9.30E-06	0.022	14306	20.209
Vascular dementia (subcortical)	genus Alistipes	rs12990744	C	T	-0.083	177136221	0.373	0.094	360770	C	T	-0.078	2	178000949	8.21E-06	0.017	14306	20.155
Vascular dementia (subcortical)	genus Alistipes	rs1689282	A	C	-0.003	14080130	0.959	0.061	360770	A	C	-0.052	9	14080129	5.28E-06	0.011	14306	20.832
Vascular dementia (subcortical)	genus Alistipes	rs2290844	C	T	-0.095	126098573	0.293	0.090	360770	C	T	0.081	10	127787142	9.10E-06	0.019	14306	18.042
Vascular dementia (subcortical)	genus Alistipes	rs2450745	A	C	0.140	143346550	0.206	0.111	360770	A	C	-0.081	8	144428720	7.12E-06	0.018	14306	19.007
Vascular dementia (subcortical)	genus Alistipes	rs2875322	C	T	-0.020	131017657	0.796	0.076	360770	C	T	0.058	11	130887552	8.78E-06	0.013	14306	19.537
Vascular dementia (subcortical)	genus Alistipes	rs34417064	A	G	-0.090	54699118	0.112	0.057	360770	A	G	-0.048	17	52776479	7.01E-06	0.011	14306	20.344
Vascular dementia (subcortical)	genus Alistipes	rs4810359	A	G	0.101	42562695	0.244	0.087	360770	A	G	-0.065	20	41191335	7.50E-06	0.015	14306	19.927
Vascular dementia (subcortical)	genus Alistipes	rs7129639	A	C	-0.192	17489880	0.001	0.058	360770	A	C	0.052	11	17511427	1.78E-06	0.011	14306	22.948
Vascular dementia (subcortical)	genus Alistipes	rs8130320	A	G	-0.009	39208332	0.877	0.057	360770	A	G	-0.049	21	40580258	4.84E-06	0.011	14306	20.906
Vascular dementia (subcortical)	genus Allisonella	rs1901739	G	T	0.084	114951010	0.140	0.057	360770	G	T	-0.116	5	114286707	3.59E-06	0.025	14306	21.682
Vascular dementia (subcortical)	genus Allisonella	rs35110698	T	C	0.010	27866065	0.898	0.081	360770	T	C	-0.146	12	28018998	5.72E-06	0.032	14306	20.797
Vascular dementia (subcortical)	genus Allisonella	rs35778461	C	T	0.008	97388274	0.904	0.067	360770	C	T	0.147	9	100150556	1.21E-06	0.030	14306	24.360

Vascular dementia (subcortical)	genus Allisonella	rs594561	T	C	0.054	88899804	0.339	0.057	360770	T	C	-0.112	11	88632972	9.41E-06	0.025	14306	19.885
Vascular dementia (subcortical)	genus Allisonella	rs602075	G	A	-0.079	76495244	0.220	0.065	360770	G	A	-0.169	9	79110160	3.57E-08	0.030	14306	32.374
Vascular dementia (subcortical)	genus Allisonella	rs6742198	A	G	-0.006	33382004	0.923	0.066	360770	A	G	-0.149	2	33607071	3.35E-06	0.032	14306	22.210
Vascular dementia (subcortical)	genus Allisonella	rs76904847	G	A	0.021	138283265	0.778	0.075	360770	G	A	0.149	7	137968010	6.09E-06	0.033	14306	19.673
Vascular dementia (subcortical)	genus Allisonella	rs7898615	T	G	0.014	120653939	0.869	0.083	360770	T	G	0.168	10	122413451	8.87E-06	0.037	14306	20.214
Vascular dementia (subcortical)	genus Alloprevotella	rs2154444	G	T	0.086	34681344	0.176	0.064	360770	G	T	-0.138	21	36053643	8.37E-06	0.031	14306	20.018
Vascular dementia (subcortical)	genus Alloprevotella	rs34619204	G	A	-0.070	39621081	0.353	0.075	360770	G	A	-0.156	21	40993008	8.84E-06	0.034	14306	20.528
Vascular dementia (subcortical)	genus Alloprevotella	rs4364940	A	G	0.013	233990770	0.833	0.061	360770	A	G	0.126	1	234126516	8.58E-06	0.028	14306	20.067
Vascular dementia (subcortical)	genus Alloprevotella	rs4680035	G	A	0.011	153087811	0.850	0.058	360770	G	A	0.120	3	152805600	4.99E-06	0.026	14306	21.250
Vascular dementia (subcortical)	genus Alloprevotella	rs58212166	A	G	-0.048	188155551	0.518	0.073	360770	A	G	-0.162	4	189076705	7.94E-06	0.036	14306	20.227
Vascular dementia (subcortical)	genus Anaerofilum	rs10794359	C	T	0.118	1051715	0.039	0.057	360770	C	T	0.095	11	1051715	2.23E-06	0.020	14306	22.594
Vascular dementia (subcortical)	genus Anaerofilum	rs1563175	A	C	-0.038	3791350	0.509	0.057	360770	A	C	0.092	2	3838940	5.54E-06	0.020	14306	20.884
Vascular dementia (subcortical)	genus Anaerofilum	rs17012738	T	G	0.093	76471016	0.100	0.057	360770	T	G	0.090	2	76698142	7.24E-06	0.020	14306	20.355
Vascular dementia (subcortical)	genus Anaerofilum	rs17096874	C	T	-0.013	30522152	0.849	0.070	360770	C	T	-0.126	14	30991358	2.86E-06	0.027	14306	22.071
Vascular dementia (subcortical)	genus Anaerofilum	rs4244069	A	G	-0.114	66773681	0.183	0.085	360770	A	G	0.147	12	67167461	9.81E-06	0.033	14306	20.197
Vascular dementia (subcortical)	genus Anaerofilum	rs4506496	A	G	-0.037	246982388	0.555	0.063	360770	A	G	-0.103	1	247145690	1.49E-06	0.021	14306	23.455
Vascular dementia (subcortical)	genus Anaerofilum	rs712981	A	C	0.032	129967591	0.582	0.059	360770	A	C	0.101	3	129686434	6.83E-07	0.020	14306	24.661
Vascular dementia (subcortical)	genus Anaerofilum	rs79598899	C	T	0.208	190274425	0.128	0.136	360770	C	T	0.183	2	191139151	3.75E-07	0.036	14306	26.116
Vascular dementia (subcortical)	genus Anaerofilum	rs816292	T	C	0.062	117373604	0.326	0.063	360770	T	C	-0.113	12	117811409	2.64E-07	0.022	14306	26.288
Vascular dementia (subcortical)	genus Anaerofilum	rs9299345	T	C	-0.115	101577530	0.230	0.096	360770	T	C	-0.136	9	104339812	8.04E-06	0.030	14306	20.349
Vascular dementia (subcortical)	genus Anaerostipes	rs10502061	A	G	-0.001	105744527	0.988	0.089	360770	A	G	0.084	11	105615253	7.94E-06	0.019	14306	18.944
Vascular dementia (subcortical)	genus Anaerostipes	rs2014785	T	C	-0.027	171382313	0.636	0.057	360770	T	C	0.052	3	171100102	4.68E-06	0.011	14306	21.125
Vascular dementia (subcortical)	genus Anaerostipes	rs2396460	C	T	0.006	227153955	0.922	0.057	360770	C	T	0.051	2	228018671	2.91E-06	0.011	14306	21.897
Vascular dementia (subcortical)	genus Anaerostipes	rs2804244	G	A	0.092	115623674	0.117	0.059	360770	G	A	0.053	10	117383184	2.04E-06	0.011	14306	22.882
Vascular dementia (subcortical)	genus Anaerostipes	rs3900776	G	A	-0.160	13525083	0.362	0.176	360770	G	A	-0.110	9	13525082	2.75E-06	0.024	14306	21.675
Vascular dementia (subcortical)	genus Anaerostipes	rs60983350	G	A	0.090	2947443	0.142	0.061	360770	G	A	-0.054	17	2850737	4.42E-06	0.012	14306	21.450
Vascular dementia (subcortical)	genus Anaerostipes	rs62157625	T	C	-0.148	142016764	0.090	0.088	360770	T	C	0.089	2	142774333	1.45E-06	0.019	14306	22.787
Vascular dementia (subcortical)	genus Anaerostipes	rs62215703	G	A	-0.120	24501310	0.080	0.069	360770	G	A	0.064	21	25873624	1.98E-06	0.014	14306	22.260
Vascular dementia (subcortical)	genus Anaerostipes	rs6474958	G	A	-0.087	1582701	0.159	0.061	360770	G	A	0.050	9	1582701	6.74E-06	0.011	14306	19.935
Vascular dementia (subcortical)	genus Anaerostipes	rs6726833	C	A	0.059	39124428	0.574	0.105	360770	C	A	-0.088	2	39351569	3.32E-06	0.019	14306	21.463
Vascular dementia (subcortical)	genus Anaerostipes	rs6854026	C	T	-0.006	168769663	0.922	0.057	360770	C	T	0.051	4	169690814	3.20E-06	0.011	14306	21.732
Vascular dementia (subcortical)	genus Anaerostipes	rs7193624	T	C	0.072	77540123	0.484	0.103	360770	T	C	-0.075	16	77574020	5.35E-07	0.015	14306	24.803
Vascular dementia (subcortical)	genus Anaerostipes	rs78735375	A	C	-0.130	1497548	0.375	0.147	360770	A	C	-0.137	19	1497547	5.33E-06	0.031	14306	20.262
Vascular dementia (subcortical)	genus Anaerotruncus	rs10150232	A	G	-0.098	29948802	0.169	0.072	360770	A	G	0.057	14	30418008	6.68E-06	0.012	14306	20.622
Vascular dementia (subcortical)	genus Anaerotruncus	rs11018566	A	G	0.287	89307058	0.026	0.128	360770	A	G	-0.156	11	89040226	6.14E-06	0.037	14306	18.272
Vascular dementia (subcortical)	genus Anaerotruncus	rs115414803	A	C	0.068	87242091	0.572	0.119	360770	A	C	-0.144	4	88163243	6.83E-06	0.032	14306	20.669
Vascular dementia (subcortical)	genus Anaerotruncus	rs1272208	T	G	0.048	76015978	0.475	0.067	360770	T	G	0.061	9	78630894	4.28E-06	0.013	14306	22.201
Vascular dementia (subcortical)	genus Anaerotruncus	rs1431492	C	T	0.166	151137584	0.035	0.079	360770	C	T	-0.065	3	150855371	7.36E-06	0.015	14306	20.075
Vascular dementia (subcortical)	genus Anaerotruncus	rs17734739	T	C	0.008	210798978	0.925	0.083	360770	T	C	0.066	2	211663702	7.43E-06	0.015	14306	19.603
Vascular dementia (subcortical)	genus Anaerotruncus	rs34449434	A	C	-0.094	76129875	0.113	0.059	360770	A	C	-0.050	12	76523655	9.85E-06	0.011	14306	19.208
Vascular dementia (subcortical)	genus Anaerotruncus	rs4669806	G	T	0.006	12060626	0.934	0.069	360770	G	T	0.058	2	12200752	2.42E-06	0.012	14306	21.962
Vascular dementia (subcortical)	genus Anaerotruncus	rs6494922	A	G	-0.087	33167666	0.478	0.123	360770	A	G	0.090	15	33459867	6.62E-06	0.020	14306	19.937
Vascular dementia (subcortical)	genus Anaerotruncus	rs6563550	T	C	0.151	37484276	0.154	0.106	360770	T	C	0.088	13	38058413	2.35E-07	0.018	14306	24.629
Vascular dementia (subcortical)	genus Anaerotruncus	rs7155595	C	A	0.085	77036203	0.170	0.062	360770	C	A	0.054	14	77502546	7.55E-06	0.012	14306	20.575
Vascular dementia (subcortical)	genus Anaerotruncus	rs8005030	C	T	0.004	30137993	0.946	0.060	360770	C	T	0.055	14	30607199	2.28E-06	0.012	14306	22.133
Vascular dementia (subcortical)	genus Anaerotruncus	rs9347879	T	C	0.060	164594228	0.296	0.057	360770	T	C	0.051	6	165015261	4.22E-06	0.011	14306	20.988
Vascular dementia (subcortical)	genus Bacteroides	rs11585893	A	G	0.064	10584294	0.335	0.066	360770	A	G	-0.074	1	10644351	1.80E-06	0.015	14306	25.175
Vascular dementia (subcortical)	genus Bacteroides	rs13207588	A	G	0.000	41551692	0.995	0.072	360770	A	G	-0.059	6	41519430	7.48E-06	0.013	14306	20.365
Vascular dementia (subcortical)	genus Bacteroides	rs1340391	T	C	0.125	102495433	0.132	0.083	360770	T	C	-0.059	1	102960989	6.73E-06	0.013	14306	20.040
Vascular dementia (subcortical)	genus Bacteroides	rs17619981	T	G	-0.032	24159448	0.695	0.082	360770	T	G	0.088	19	24342250	2.69E-06	0.019	14306	22.194
Vascular dementia (subcortical)	genus Bacteroides	rs2023437	T	C	-0.005	21577814	0.953	0.087	360770	T	C	-0.078	14	22045949	5.02E-06	0.017	14306	21.780
Vascular dementia (subcortical)	genus Bacteroides	rs66710942	T	C	-0.018	77166176	0.753	0.057	360770	T	C	-0.049	3	77215327	5.86E-06	0.011	14306	20.644
Vascular dementia (subcortical)	genus Bacteroides	rs6795673	C	T	0.059	10551540	0.299	0.057	360770	C	T	0.054	3	10593224	3.38E-07	0.011	14306	26.183
Vascular dementia (subcortical)	genus Bacteroides	rs9507307	C	T	-0.119	24336338	0.071	0.066	360770	C	T	0.060	13	24910476	2.13E-06	0.013	14306	21.912
Vascular dementia (subcortical)	genus Barnesiella	rs11155559	T	C	0.056	148280769	0.561	0.097	360770	T	C	0.096	6	148601905	8.92E-06	0.021	14306	20.153
Vascular dementia (subcortical)	genus Barnesiella	rs12909713	C	T	-0.044	86602682	0.435	0.057	360770	C	T	-0.055	15	87145913	4.95E-06	0.012	14306	21.048
Vascular dementia (subcortical)	genus Barnesiella	rs13242616	C	T	-0.003	122381464	0.964	0.061	360770	C	T	0.058	7	122021518	2.29E-06	0.012	14306	22.459
Vascular dementia (subcortical)	genus Barnesiella	rs199035	G	A	-0.061	23435187	0.289	0.057	360770	G	A	0.056	6	23435415	3.00E-06	0.012	14306	21.842
Vascular dementia (subcortical)	genus Barnesiella	rs2276875	A	G	0.038	5729227	0.567	0.066	360770	A	G	-0.070	4	5730954	4.65E-07	0.014	14306	24.964
Vascular dementia (subcortical)	genus Barnesiella	rs2428166	G	A	-0.268	110509945	0.288	0.252	360770	G	A	-0.166	6	110831148	8.51E-07	0.034	14306	24.178
Vascular dementia (subcortical)	genus Barnesiella	rs35177866	A	G	0.133	181871367	0.224	0.110	360770	A	G	0.092	3	181589155	2.95E-06	0.019	14306	23.267
Vascular dementia (subcortical)	genus Barnesiella	rs62251337	G	A	0.043	44285937	0.593	0.081	360770	G	A	0.069	3	44327429	4.24E-06	0.015	14306	21.385
Vascular dementia (subcortical)	genus Barnesiella	rs72684847	T	C	0.261	101695712	0.016	0.108	360770	T	C	-0.114	4	102616869	6.76E-06	0.025	14306	20.295
Vascular dementia (subcortical)	genus Barnesiella	rs76181748	C	T	0.035	104446188	0.605	0.067	360770	C	T	-0.078	8	105458416	6.78E-06	0.017	14306	20.575
Vascular dementia (subcortical)	genus Barnesiella	rs77455852	T	G	0.093	54811208	0.242	0.080	360770	T	G	-0.089	5	54107036	3.16E-06	0.020	14306	20.773
Vascular dementia (subcortical)	genus Barnesiella	rs79795328	A	G	-0.064	113625439	0.438	0.082	360770	A	G	-0.082	4	114546595	4.23E-06	0.018	14306	21.527
Vascular dementia (subcortical)	genus Bifidobacterium	rs12022129	A	G	-0.117	206830029	0.065	0.063	360770	A	G	-0.062	1	207003374	8.00E-06	0.014	14306	19.872

Vascular dementia (subcortical)	genus Bifidobacterium	rs182549	T	C	-0.005	135859184	0.936	0.059	360770	T	C	-0.120	2	136616754	1.28E-20	0.013	14306	88.429
Vascular dementia (subcortical)	genus Bifidobacterium	rs2491158	A	G	0.175	124401134	0.036	0.084	360770	A	G	-0.071	10	126089703	8.05E-06	0.016	14306	19.879
Vascular dementia (subcortical)	genus Bifidobacterium	rs2686790	C	T	-0.092	48051149	0.247	0.080	360770	C	T	-0.071	7	48090746	7.50E-06	0.016	14306	20.065
Vascular dementia (subcortical)	genus Bifidobacterium	rs4957061	T	C	0.066	520981	0.255	0.058	360770	T	C	0.053	5	521096	5.78E-06	0.012	14306	20.697
Vascular dementia (subcortical)	genus Bifidobacterium	rs540489	T	G	-0.157	74901626	0.038	0.075	360770	T	G	-0.064	17	72897722	5.19E-06	0.014	14306	21.121
Vascular dementia (subcortical)	genus Bifidobacterium	rs55888705	A	G	-0.012	1516099	0.850	0.063	360770	A	G	0.055	4	1517826	6.67E-06	0.012	14306	20.339
Vascular dementia (subcortical)	genus Bifidobacterium	rs5746486	T	C	0.033	17871506	0.567	0.058	360770	T	C	-0.054	22	18354272	9.00E-06	0.012	14306	19.703
Vascular dementia (subcortical)	genus Bifidobacterium	rs62181700	G	A	-0.008	188941058	0.899	0.066	360770	G	A	-0.062	2	189805784	2.17E-06	0.013	14306	22.665
Vascular dementia (subcortical)	genus Bifidobacterium	rs7322849	T	C	0.119	112205515	0.231	0.099	360770	T	C	0.112	13	112859829	1.08E-08	0.020	14306	31.035
Vascular dementia (subcortical)	genus Bifidobacterium	rs75344046	C	T	-0.101	30489472	0.459	0.137	360770	C	T	0.232	21	31861790	4.86E-06	0.051	14306	21.088
Vascular dementia (subcortical)	genus Bifidobacterium	rs857444	C	T	0.056	14617360	0.344	0.059	360770	C	T	0.056	6	14617591	3.57E-06	0.012	14306	21.208
Vascular dementia (subcortical)	genus Bilophila	rs11069458	C	T	0.039	101750335	0.597	0.073	360770	C	T	0.068	13	102402685	7.72E-06	0.016	14306	19.293
Vascular dementia (subcortical)	genus Bilophila	rs1241171	G	A	-0.068	102834365	0.397	0.081	360770	G	A	-0.069	1	103299921	4.24E-06	0.015	14306	21.281
Vascular dementia (subcortical)	genus Bilophila	rs1571225	T	C	0.101	4940871	0.185	0.076	360770	T	C	-0.083	9	4940871	1.12E-06	0.017	14306	23.484
Vascular dementia (subcortical)	genus Bilophila	rs1969927	A	G	0.003	100393069	0.958	0.060	360770	A	G	-0.056	12	100786847	9.07E-06	0.013	14306	19.783
Vascular dementia (subcortical)	genus Bilophila	rs2728491	T	G	-0.115	47172134	0.085	0.067	360770	T	G	0.063	7	47211732	6.33E-06	0.014	14306	20.241
Vascular dementia (subcortical)	genus Bilophila	rs3827020	C	T	0.011	63349639	0.877	0.072	360770	C	T	0.077	20	61980991	1.79E-06	0.016	14306	22.766
Vascular dementia (subcortical)	genus Bilophila	rs4798126	G	A	0.041	3765773	0.568	0.072	360770	G	A	0.073	18	3765773	7.15E-06	0.017	14306	19.033
Vascular dementia (subcortical)	genus Bilophila	rs542415	T	C	-0.120	49719357	0.040	0.058	360770	T	C	-0.061	15	50011554	4.71E-06	0.013	14306	21.147
Vascular dementia (subcortical)	genus Bilophila	rs60178956	G	A	-0.039	89866110	0.562	0.068	360770	G	A	-0.062	8	90878338	8.06E-06	0.014	14306	19.502
Vascular dementia (subcortical)	genus Bilophila	rs6793291	A	C	-0.191	194698802	0.135	0.128	360770	A	C	-0.113	3	194419531	3.11E-06	0.024	14306	21.765
Vascular dementia (subcortical)	genus Bilophila	rs72676854	T	C	-0.037	108978710	0.774	0.129	360770	T	C	0.123	8	109990939	5.62E-06	0.027	14306	21.007
Vascular dementia (subcortical)	genus Bilophila	rs7802841	A	C	-0.071	138899094	0.252	0.062	360770	A	C	-0.067	7	138583840	1.77E-06	0.014	14306	23.681
Vascular dementia (subcortical)	genus Bilophila	rs9899990	A	G	0.087	9218262	0.403	0.104	360770	A	G	-0.103	17	9121579	9.07E-06	0.023	14306	19.283
Vascular dementia (subcortical)	genus Butyricicoccus	rs10084203	G	A	-0.051	190439423	0.544	0.084	360770	G	A	-0.055	2	191304149	8.59E-06	0.012	14306	19.791
Vascular dementia (subcortical)	genus Butyricicoccus	rs12034718	G	A	0.014	66913912	0.840	0.068	360770	G	A	-0.070	1	67379595	9.58E-06	0.016	14306	19.643
Vascular dementia (subcortical)	genus Butyricicoccus	rs12585793	T	C	-0.147	26444103	0.402	0.175	360770	T	C	-0.262	13	27018240	5.79E-06	0.056	14306	21.558
Vascular dementia (subcortical)	genus Butyricicoccus	rs2017189	T	G	-0.047	7458426	0.408	0.057	360770	T	G	0.051	4	7460153	3.87E-06	0.011	14306	21.148
Vascular dementia (subcortical)	genus Butyricicoccus	rs4962426	T	G	-0.085	125219639	0.235	0.071	360770	T	G	-0.061	10	126908208	7.38E-06	0.014	14306	20.403
Vascular dementia (subcortical)	genus Butyricicoccus	rs56221232	T	C	0.117	152596896	0.217	0.094	360770	T	C	0.083	2	153453410	7.62E-07	0.017	14306	24.467
Vascular dementia (subcortical)	genus Butyricicoccus	rs62478070	T	G	-0.197	157895458	0.275	0.181	360770	T	G	0.224	7	157688150	5.94E-06	0.049	14306	20.488
Vascular dementia (subcortical)	genus Butyricicoccus	rs7322368	C	T	-0.157	99561736	0.108	0.098	360770	C	T	-0.082	13	100213990	5.52E-06	0.018	14306	19.834
Vascular dementia (subcortical)	genus Butyricimonas	rs11228830	A	G	0.071	56897486	0.489	0.102	360770	A	G	0.135	11	56664962	6.55E-06	0.030	14306	20.547
Vascular dementia (subcortical)	genus Butyricimonas	rs113054641	G	A	0.227	15178069	0.106	0.141	360770	G	A	-0.145	21	16550389	1.74E-07	0.027	14306	27.842
Vascular dementia (subcortical)	genus Butyricimonas	rs12304031	G	A	-0.063	128954226	0.471	0.087	360770	G	A	-0.086	12	129438771	6.70E-06	0.020	14306	19.211
Vascular dementia (subcortical)	genus Butyricimonas	rs12458763	A	C	-0.054	36511655	0.697	0.138	360770	A	C	0.122	18	34091618	6.37E-06	0.027	14306	20.492
Vascular dementia (subcortical)	genus Butyricimonas	rs1862649	G	A	-0.051	22161545	0.646	0.111	360770	G	A	0.113	16	22172866	4.76E-06	0.025	14306	20.777
Vascular dementia (subcortical)	genus Butyricimonas	rs2114713	G	T	0.044	80236031	0.443	0.057	360770	G	T	0.063	15	80528373	6.88E-06	0.014	14306	20.361
Vascular dementia (subcortical)	genus Butyricimonas	rs62130338	A	G	0.032	48659244	0.597	0.060	360770	A	G	0.073	19	49162501	3.90E-06	0.016	14306	21.412
Vascular dementia (subcortical)	genus Butyricimonas	rs62390301	T	C	0.069	160440640	0.331	0.071	360770	T	C	-0.087	5	159867647	7.42E-07	0.017	14306	24.915
Vascular dementia (subcortical)	genus Butyricimonas	rs7083431	A	C	0.040	70951399	0.530	0.064	360770	A	C	0.070	10	72711156	8.85E-07	0.014	14306	23.725
Vascular dementia (subcortical)	genus Butyricimonas	rs71428626	G	T	-0.200	82866140	0.218	0.162	360770	G	T	-0.133	2	83093264	4.80E-06	0.029	14306	21.107
Vascular dementia (subcortical)	genus Butyricimonas	rs72814525	A	G	-0.036	70683314	0.588	0.066	360770	A	G	0.066	10	72443070	8.25E-06	0.015	14306	19.666
Vascular dementia (subcortical)	genus Butyricimonas	rs78453362	A	G	-0.135	78690859	0.469	0.186	360770	A	G	-0.149	3	78740009	4.06E-06	0.033	14306	20.887
Vascular dementia (subcortical)	genus Butyricimonas	rs9657374	C	T	0.000	4976884	0.996	0.062	360770	C	T	0.068	8	4834406	4.50E-06	0.015	14306	21.106
Vascular dementia (subcortical)	genus Butyrivibrio	rs1007475	G	T	0.030	148082269	0.644	0.064	360770	G	T	0.118	6	148403405	7.92E-06	0.026	14306	20.420
Vascular dementia (subcortical)	genus Butyrivibrio	rs11761679	T	C	0.104	150899841	0.203	0.082	360770	T	C	0.155	7	150596929	2.20E-06	0.032	14306	23.170
Vascular dementia (subcortical)	genus Butyrivibrio	rs142855850	A	G	-0.078	22298971	0.424	0.097	360770	A	G	0.205	10	22587900	6.86E-06	0.046	14306	20.113
Vascular dementia (subcortical)	genus Butyrivibrio	rs16934069	T	C	0.113	116930885	0.129	0.074	360770	T	C	-0.134	9	119693164	8.86E-06	0.030	14306	19.961
Vascular dementia (subcortical)	genus Butyrivibrio	rs16941336	C	T	-0.031	20672384	0.641	0.067	360770	C	T	0.127	17	20575697	1.53E-06	0.027	14306	22.640
Vascular dementia (subcortical)	genus Butyrivibrio	rs17163238	G	A	0.018	129640328	0.807	0.072	360770	G	A	0.141	5	128976021	5.51E-06	0.031	14306	20.802
Vascular dementia (subcortical)	genus Butyrivibrio	rs4537857	T	C	0.021	26718529	0.729	0.061	360770	T	C	-0.125	13	27292666	1.80E-06	0.026	14306	22.788
Vascular dementia (subcortical)	genus Butyrivibrio	rs486484	A	G	-0.090	830864	0.120	0.058	360770	A	G	-0.108	20	811507	6.61E-06	0.024	14306	20.380
Vascular dementia (subcortical)	genus Butyrivibrio	rs4928024	G	A	-0.021	54254476	0.776	0.073	360770	G	A	0.175	3	54288503	8.19E-06	0.039	14306	20.137
Vascular dementia (subcortical)	genus Butyrivibrio	rs72723662	C	T	0.116	75599220	0.163	0.083	360770	C	T	0.224	14	76065563	7.86E-07	0.045	14306	24.869
Vascular dementia (subcortical)	genus Butyrivibrio	rs74622183	A	G	-0.095	33681771	0.346	0.101	360770	A	G	-0.201	14	34150977	2.46E-06	0.043	14306	22.040
Vascular dementia (subcortical)	genus Butyrivibrio	rs77356209	T	C	-0.147	10267736	0.279	0.135	360770	T	C	0.217	18	10267733	6.66E-06	0.048	14306	20.136
Vascular dementia (subcortical)	genus Butyrivibrio	rs7752361	G	A	-0.010	111459502	0.866	0.057	360770	G	A	0.119	6	111780705	7.69E-07	0.024	14306	24.685
Vascular dementia (subcortical)	genus Butyrivibrio	rs7763512	A	G	-0.032	21959860	0.577	0.057	360770	A	G	-0.120	6	21960091	3.11E-06	0.025	14306	22.374
Vascular dementia (subcortical)	genus Butyrivibrio	rs9349693	G	A	-0.020	54074926	0.743	0.062	360770	G	A	-0.118	6	53939724	5.55E-06	0.026	14306	20.610
Vascular dementia (subcortical)	genus Candidatus Soleaferrea	rs10090365	G	A	-0.006	137625956	0.910	0.057	360770	G	A	0.083	8	138638199	4.17E-06	0.018	14306	21.255
Vascular dementia (subcortical)	genus Candidatus Soleaferrea	rs10809135	C	T	-0.014	10611282	0.802	0.058	360770	C	T	-0.083	9	10611282	5.47E-06	0.018	14306	20.944
Vascular dementia (subcortical)	genus Candidatus Soleaferrea	rs36155147	T	C	0.026	312402	0.678	0.062	360770	T	C	-0.105	7	352368	5.41E-06	0.024	14306	18.971
Vascular dementia (subcortical)	genus Candidatus Soleaferrea	rs4294381	C	T	-0.038	224500405	0.628	0.078	360770	C	T	-0.112	1	224688107	1.37E-06	0.023	14306	23.416
Vascular dementia (subcortical)	genus Candidatus Soleaferrea	rs4678258	T	C	-0.039	138226350	0.561	0.066	360770	T	C	0.099	3	137945192	5.53E-06	0.022	14306	20.912
Vascular dementia (subcortical)	genus Candidatus Soleaferrea	rs6489992	A	G	0.011	114914964	0.848	0.059	360770	A	G	-0.084	12	115352769	7.89E-06	0.019	14306	20.192
Vascular dementia (subcortical)	genus Candidatus Soleaferrea	rs6494306	A	G	-0.025	62102341	0.679	0.061	360770	A	G	-0.097	15	62394540	5.80E-06	0.021	14306	20.474

Vascular dementia (subcortical)	genus Candidatus Soleaferrea	rs7400877	C	T	0.019	75937199	0.788	0.070	360770	C	T	0.095	14	76403542	9.29E-06	0.021	14306	19.976
Vascular dementia (subcortical)	genus Candidatus Soleaferrea	rs9973954	G	A	-0.065	19686146	0.274	0.059	360770	G	A	-0.089	2	19885907	5.95E-06	0.020	14306	20.842
Vascular dementia (subcortical)	genus Catenibacterium	rs12404911	C	T	0.048	239955143	0.513	0.073	360770	C	T	0.141	1	240118443	2.80E-06	0.030	14306	21.406
Vascular dementia (subcortical)	genus Catenibacterium	rs212393	A	G	-0.124	159064710	0.078	0.070	360770	A	G	0.135	6	159485742	3.62E-06	0.029	14306	22.332
Vascular dementia (subcortical)	genus Catenibacterium	rs73128290	A	G	0.035	57296613	0.574	0.062	360770	A	G	0.130	7	57364320	4.29E-06	0.028	14306	20.782
Vascular dementia (subcortical)	genus Catenibacterium	rs7742829	C	T	0.002	104641790	0.966	0.058	360770	C	T	0.114	6	105089665	5.61E-06	0.025	14306	20.652
Vascular dementia (subcortical)	genus Christensenellaceae R 7group	rs10461257	A	G	0.060	155209852	0.326	0.061	360770	A	G	-0.055	4	156131004	6.51E-06	0.012	14306	20.446
Vascular dementia (subcortical)	genus Christensenellaceae R 7group	rs17081797	A	G	0.124	69888324	0.281	0.115	360770	A	G	-0.090	18	67555560	3.34E-06	0.020	14306	19.603
Vascular dementia (subcortical)	genus Christensenellaceae R 7group	rs62132810	A	G	0.007	48775970	0.931	0.082	360770	A	G	-0.083	19	49279227	5.67E-06	0.018	14306	21.293
Vascular dementia (subcortical)	genus Christensenellaceae R 7group	rs62190261	A	C	-0.080	230078383	0.433	0.102	360770	A	C	0.096	2	230943099	8.74E-06	0.021	14306	19.922
Vascular dementia (subcortical)	genus Christensenellaceae R 7group	rs62467127	C	T	0.349	118456871	0.054	0.181	360770	C	T	0.114	7	118096925	3.25E-06	0.025	14306	20.506
Vascular dementia (subcortical)	genus Christensenellaceae R 7group	rs73952017	C	T	-0.223	1779608	0.017	0.093	360770	C	T	-0.086	18	1779609	8.46E-06	0.019	14306	19.679
Vascular dementia (subcortical)	genus Christensenellaceae R 7group	rs78521377	C	T	-0.003	124759805	0.985	0.165	360770	C	T	0.125	10	126448374	5.61E-06	0.027	14306	20.689
Vascular dementia (subcortical)	genus Christensenellaceae R 7group	rs892686	A	G	0.075	80428461	0.187	0.057	360770	A	G	0.051	9	83043376	3.97E-06	0.011	14306	21.313
Vascular dementia (subcortical)	genus Clostridium innocuum group	rs10506058	A	G	0.010	30115552	0.861	0.058	360770	A	G	0.100	12	30268485	8.92E-06	0.022	14306	20.184
Vascular dementia (subcortical)	genus Clostridium innocuum group	rs1942371	G	A	-0.049	71607072	0.570	0.087	360770	G	A	-0.158	18	69274308	4.06E-06	0.034	14306	21.343
Vascular dementia (subcortical)	genus Clostridium innocuum group	rs40656	C	T	0.020	9368046	0.773	0.071	360770	C	T	0.143	5	9368158	8.62E-06	0.031	14306	21.040
Vascular dementia (subcortical)	genus Clostridium innocuum group	rs4869133	G	A	-0.011	96381915	0.886	0.074	360770	G	A	-0.181	5	95717619	7.24E-06	0.041	14306	19.448
Vascular dementia (subcortical)	genus Clostridium innocuum group	rs61267978	T	C	-0.041	6348228	0.641	0.087	360770	T	C	0.147	18	6348227	5.59E-06	0.032	14306	21.010
Vascular dementia (subcortical)	genus Clostridium innocuum group	rs6890185	C	T	-0.030	71890799	0.618	0.061	360770	C	T	-0.113	5	71186626	1.12E-06	0.023	14306	23.669
Vascular dementia (subcortical)	genus Clostridium sensustricto1	rs11264403	G	A	0.053	155706701	0.625	0.108	360770	G	A	-0.139	1	155676492	7.76E-06	0.033	14306	17.288
Vascular dementia (subcortical)	genus Clostridium sensustricto1	rs116847295	C	T	0.162	43091212	0.059	0.086	360770	C	T	0.110	12	43485015	4.58E-06	0.025	14306	19.997
Vascular dementia (subcortical)	genus Clostridium sensustricto1	rs12341505	G	A	0.079	133845759	0.419	0.098	360770	G	A	0.081	9	136710881	4.82E-06	0.018	14306	20.259
Vascular dementia (subcortical)	genus Clostridium sensustricto1	rs2795528	G	A	-0.176	42774816	0.156	0.124	360770	G	A	-0.184	10	43270264	2.72E-06	0.039	14306	22.085
Vascular dementia (subcortical)	genus Clostridium sensustricto1	rs2817172	C	T	-0.020	3124955	0.730	0.058	360770	C	T	0.058	1	3041519	2.77E-06	0.012	14306	21.810
Vascular dementia (subcortical)	genus Clostridium sensustricto1	rs550843	T	C	-0.013	165309343	0.838	0.064	360770	T	C	-0.078	6	165722832	2.05E-06	0.017	14306	21.426
Vascular dementia (subcortical)	genus Collinsella	rs10890671	C	T	0.020	107252473	0.721	0.057	360770	C	T	0.054	11	107123199	6.52E-06	0.012	14306	20.446
Vascular dementia (subcortical)	genus Collinsella	rs11597285	G	T	0.007	8547850	0.902	0.058	360770	G	T	-0.054	10	8589813	9.38E-06	0.012	14306	19.914
Vascular dementia (subcortical)	genus Collinsella	rs1496626	T	C	0.137	31269935	0.101	0.084	360770	T	C	-0.072	19	31760841	6.78E-06	0.016	14306	19.978
Vascular dementia (subcortical)	genus Collinsella	rs149807560	C	A	-0.154	56015274	0.166	0.111	360770	C	A	-0.104	19	56526640	7.10E-06	0.024	14306	19.545
Vascular dementia (subcortical)	genus Collinsella	rs2103510	G	A	-0.025	27983148	0.779	0.088	360770	G	A	0.079	21	29355467	2.42E-06	0.017	14306	21.863
Vascular dementia (subcortical)	genus Collinsella	rs62448871	C	A	-0.134	24425405	0.020	0.057	360770	C	A	-0.054	7	24465024	6.78E-06	0.012	14306	20.164
Vascular dementia (subcortical)	genus Collinsella	rs75672793	A	G	-0.063	148897142	0.638	0.134	360770	A	G	-0.109	4	149818294	6.14E-06	0.024	14306	20.503
Vascular dementia (subcortical)	genus Collinsella	rs9541268	C	A	0.015	68035929	0.884	0.100	360770	C	A	0.096	13	68610061	8.79E-07	0.020	14306	23.657
Vascular dementia (subcortical)	genus Coprobacter	rs11532348	C	T	-0.020	97896018	0.801	0.081	360770	C	T	-0.104	12	98289796	5.71E-06	0.023	14306	20.994
Vascular dementia (subcortical)	genus Coprobacter	rs12684609	T	C	0.020	134893022	0.775	0.072	360770	T	C	0.101	9	137784868	6.10E-06	0.022	14306	20.935
Vascular dementia (subcortical)	genus Coprobacter	rs12996055	A	C	0.050	136465888	0.445	0.065	360770	A	C	0.092	2	137223458	8.08E-06	0.021	14306	19.378
Vascular dementia (subcortical)	genus Coprobacter	rs143662916	C	T	0.236	103679246	0.159	0.168	360770	C	T	0.253	11	103549974	3.07E-06	0.054	14306	21.985
Vascular dementia (subcortical)	genus Coprobacter	rs189356	G	A	0.074	58999796	0.198	0.057	360770	G	A	0.078	20	57574851	6.26E-06	0.017	14306	20.645
Vascular dementia (subcortical)	genus Coprobacter	rs213863	T	C	-0.005	96787063	0.938	0.059	360770	T	C	0.089	6	97234939	2.35E-06	0.019	14306	22.192
Vascular dementia (subcortical)	genus Coprobacter	rs28402691	T	C	-0.086	95468089	0.279	0.080	360770	T	C	0.111	4	96389240	9.56E-06	0.025	14306	19.419
Vascular dementia (subcortical)	genus Coprobacter	rs305411	A	G	-0.019	87767445	0.838	0.093	360770	A	G	0.129	1	88233128	1.01E-06	0.026	14306	23.864
Vascular dementia (subcortical)	genus Coprobacter	rs3828477	G	T	-0.027	46442226	0.652	0.060	360770	G	T	-0.091	3	46483717	2.89E-06	0.020	14306	21.728
Vascular dementia (subcortical)	genus Coprobacter	rs72821405	T	C	0.090	4714563	0.371	0.100	360770	T	C	-0.147	6	4714797	4.76E-06	0.032	14306	21.210
Vascular dementia (subcortical)	genus Coprobacter	rs74919520	G	A	0.081	32017383	0.377	0.092	360770	G	A	0.126	2	32242452	5.76E-06	0.028	14306	20.699
Vascular dementia (subcortical)	genus Coprococcus1	rs1010560	C	A	-0.023	29927454	0.722	0.064	360770	C	A	0.058	1	30400301	1.96E-06	0.012	14306	22.354
Vascular dementia (subcortical)	genus Coprococcus1	rs12794898	G	T	0.081	124684165	0.343	0.085	360770	G	T	0.090	11	124554061	4.92E-06	0.020	14306	20.980
Vascular dementia (subcortical)	genus Coprococcus1	rs1519491	T	C	0.044	21856898	0.448	0.058	360770	T	C	0.050	2	22079770	8.95E-06	0.011	14306	19.325
Vascular dementia (subcortical)	genus Coprococcus1	rs1576241	A	G	0.020	71819574	0.734	0.059	360770	A	G	-0.051	6	72529277	3.33E-06	0.011	14306	21.708
Vascular dementia (subcortical)	genus Coprococcus1	rs2907920	G	A	-0.058	2600719	0.355	0.063	360770	G	A	-0.056	19	2600717	7.65E-06	0.013	14306	19.579
Vascular dementia (subcortical)	genus Coprococcus1	rs4277593	G	A	0.051	4338773	0.372	0.058	360770	G	A	-0.059	20	4319420	1.14E-07	0.011	14306	28.390
Vascular dementia (subcortical)	genus Coprococcus1	rs56405618	A	G	0.115	173547165	0.198	0.089	360770	A	G	-0.090	4	174468316	1.57E-06	0.019	14306	23.095
Vascular dementia (subcortical)	genus Coprococcus1	rs73031725	T	C	-0.043	134752388	0.796	0.166	360770	T	C	0.168	11	134622282	1.98E-06	0.036	14306	22.259
Vascular dementia (subcortical)	genus Coprococcus1	rs73167075	T	C	-0.045	165975355	0.520	0.070	360770	T	C	0.057	3	165693143	8.57E-06	0.013	14306	20.184
Vascular dementia (subcortical)	genus Coprococcus1	rs74101919	T	C	0.039	94958765	0.669	0.092	360770	T	C	-0.072	1	95424321	1.03E-06	0.014	14306	24.706
Vascular dementia (subcortical)	genus Coprococcus1	rs946513	T	C	0.018	15871821	0.900	0.140	360770	T	C	-0.206	10	15913820	8.62E-06	0.046	14306	20.034
Vascular dementia (subcortical)	genus Coprococcus2	rs10070053	A	G	0.074	34794684	0.196	0.057	360770	A	G	0.059	5	34794789	7.65E-06	0.014	14306	19.273
Vascular dementia (subcortical)	genus Coprococcus2	rs12634070	T	C	0.008	180827092	0.903	0.065	360770	T	C	0.074	3	180544880	9.95E-06	0.016	14306	19.942
Vascular dementia (subcortical)	genus Coprococcus2	rs2482516	C	T	0.060	25554070	0.382	0.068	360770	C	T	0.075	9	25554068	4.72E-06	0.016	14306	21.002
Vascular dementia (subcortical)	genus Coprococcus2	rs35890118	A	G	-0.046	127917495	0.480	0.065	360770	A	G	-0.067	10	129715759	8.26E-06	0.015	14306	20.304
Vascular dementia (subcortical)	genus Coprococcus2	rs61823518	A	C	-0.055	223514894	0.558	0.093	360770	A	C	-0.096	1	223688236	6.68E-06	0.022	14306	19.614
Vascular dementia (subcortical)	genus Coprococcus2	rs6677933	C	T	-0.041	111596386	0.613	0.080	360770	C	T	-0.080	1	112139008	1.19E-06	0.016	14306	23.995
Vascular dementia (subcortical)	genus Coprococcus2	rs72680320	T	C	-0.025	130204631	0.674	0.060	360770	T	C	-0.065	4	131125786	2.27E-06	0.014	14306	21.766
Vascular dementia (subcortical)	genus Coprococcus2	rs9426473	A	G	-0.005	4150395	0.935	0.065	360770	A	G	0.073	1	4210455	6.31E-06	0.016	14306	20.243
Vascular dementia (subcortical)	genus Coprococcus3	rs10810043	G	A	-0.054	13799810	0.369	0.060	360770	G	A	-0.052	9	13799809	9.27E-06	0.012	14306	19.776
Vascular dementia (subcortical)	genus Coprococcus3	rs11077359	C	T	0.069	78347880	0.360	0.076	360770	C	T	0.065	17	76343961	9.64E-06	0.015	14306	18.807
Vascular dementia (subcortical)	genus Coprococcus3	rs11080344	C	T	-0.024	27777485	0.679	0.057	360770	C	T	0.052	17	26104511	4.79E-06	0.011	14306	20.906

Vascular dementia (subcortical)	genus Coprococcus3	rs13247359	G	A	0.005	76728409	0.930	0.057	360770	G	A	0.051	7	76357726	7.33E-06	0.011	14306	20.527
Vascular dementia (subcortical)	genus Coprococcus3	rs178271	C	T	0.346	20977267	0.126	0.226	360770	C	T	-0.145	22	21331556	7.81E-07	0.029	14306	24.371
Vascular dementia (subcortical)	genus Coprococcus3	rs4575475	A	G	0.091	98675414	0.176	0.068	360770	A	G	-0.062	14	99141751	7.04E-06	0.014	14306	20.216
Vascular dementia (subcortical)	genus Coprococcus3	rs7521171	A	G	0.010	150026539	0.867	0.062	360770	A	G	0.060	1	149998497	4.32E-06	0.013	14306	21.285
Vascular dementia (subcortical)	genus Coprococcus3	rs8100692	T	C	-0.061	39541492	0.286	0.057	360770	T	C	0.058	19	40032132	4.16E-07	0.011	14306	25.892
Vascular dementia (subcortical)	genus Defluviitaleaceae UCG011	rs112893842	T	C	0.046	8786663	0.641	0.099	360770	T	C	0.114	9	8786663	1.45E-06	0.023	14306	23.899
Vascular dementia (subcortical)	genus Defluviitaleaceae UCG011	rs1582238	C	T	-0.080	118181062	0.178	0.059	360770	C	T	-0.081	1	118723685	1.57E-06	0.017	14306	23.167
Vascular dementia (subcortical)	genus Defluviitaleaceae UCG011	rs2892880	G	A	0.039	119820571	0.549	0.066	360770	G	A	0.082	4	120741726	6.83E-06	0.018	14306	20.258
Vascular dementia (subcortical)	genus Defluviitaleaceae UCG011	rs4344384	T	G	-0.052	64647609	0.361	0.057	360770	T	G	-0.072	10	66407366	4.83E-06	0.016	14306	20.981
Vascular dementia (subcortical)	genus Defluviitaleaceae UCG011	rs4677103	A	G	0.096	72158643	0.203	0.075	360770	A	G	0.098	3	72207794	9.60E-07	0.020	14306	24.576
Vascular dementia (subcortical)	genus Defluviitaleaceae UCG011	rs55658617	T	C	-0.006	40439076	0.971	0.157	360770	T	C	0.174	21	41811003	2.15E-06	0.036	14306	23.173
Vascular dementia (subcortical)	genus Defluviitaleaceae UCG011	rs72731813	C	T	0.124	146493591	0.355	0.134	360770	C	T	-0.147	4	147414743	4.33E-07	0.029	14306	25.165
Vascular dementia (subcortical)	genus Defluviitaleaceae UCG011	rs9608282	T	G	-0.047	24408113	0.772	0.161	360770	T	G	0.143	22	24804081	2.52E-06	0.030	14306	22.733
Vascular dementia (subcortical)	genus Defluviitaleaceae UCG011	rs9725395	A	G	-0.014	84739949	0.875	0.090	360770	A	G	-0.138	1	85205632	3.52E-06	0.030	14306	21.911
Vascular dementia (subcortical)	genus Desulfovibrio	rs12031543	T	C	-0.046	68198632	0.575	0.083	360770	T	C	-0.127	1	68664315	6.55E-06	0.028	14306	20.357
Vascular dementia (subcortical)	genus Desulfovibrio	rs13066142	G	A	0.039	67428549	0.688	0.098	360770	G	A	0.119	3	67478973	3.79E-06	0.025	14306	22.550
Vascular dementia (subcortical)	genus Desulfovibrio	rs16863365	A	G	0.234	197225187	0.089	0.137	360770	A	G	0.109	2	198089911	1.79E-06	0.023	14306	23.235
Vascular dementia (subcortical)	genus Desulfovibrio	rs2032031	G	A	0.040	128372006	0.483	0.056	360770	G	A	0.065	10	130170270	9.14E-06	0.015	14306	19.404
Vascular dementia (subcortical)	genus Desulfovibrio	rs2590913	A	G	-0.102	63690313	0.437	0.131	360770	A	G	-0.154	13	64264446	6.65E-06	0.034	14306	20.721
Vascular dementia (subcortical)	genus Desulfovibrio	rs2853179	T	C	0.060	104449819	0.376	0.068	360770	T	C	-0.081	8	105462047	2.42E-06	0.017	14306	21.718
Vascular dementia (subcortical)	genus Desulfovibrio	rs4797774	A	G	-0.294	13447996	0.043	0.145	360770	A	G	-0.213	18	13447995	5.64E-06	0.047	14306	20.454
Vascular dementia (subcortical)	genus Desulfovibrio	rs6580353	T	C	-0.017	140031102	0.807	0.071	360770	T	C	0.077	5	139410687	4.94E-06	0.017	14306	20.632
Vascular dementia (subcortical)	genus Desulfovibrio	rs72647089	T	G	-0.062	57060357	0.555	0.105	360770	T	G	-0.107	8	57972916	8.30E-06	0.024	14306	19.836
Vascular dementia (subcortical)	genus Dialister	rs10138457	T	C	-0.067	101991715	0.490	0.097	360770	T	C	-0.113	14	102458052	7.88E-06	0.026	14306	18.629
Vascular dementia (subcortical)	genus Dialister	rs10938938	A	G	-0.137	23292736	0.081	0.078	360770	A	G	0.077	4	23294359	7.37E-06	0.017	14306	20.482
Vascular dementia (subcortical)	genus Dialister	rs11071887	T	C	0.067	32662894	0.277	0.061	360770	T	C	0.066	15	32955095	5.91E-06	0.015	14306	20.496
Vascular dementia (subcortical)	genus Dialister	rs11166701	G	A	0.034	137283482	0.553	0.057	360770	G	A	-0.066	8	138295725	5.51E-07	0.013	14306	24.698
Vascular dementia (subcortical)	genus Dialister	rs2314294	T	C	0.087	9243005	0.298	0.084	360770	T	C	0.087	16	9336862	8.08E-06	0.019	14306	19.981
Vascular dementia (subcortical)	genus Dialister	rs2435610	A	C	0.009	151192947	0.887	0.066	360770	A	C	0.065	7	150890034	5.93E-06	0.014	14306	20.390
Vascular dementia (subcortical)	genus Dialister	rs4747450	C	A	0.038	22910724	0.579	0.068	360770	C	A	0.067	10	23199653	5.84E-06	0.015	14306	20.490
Vascular dementia (subcortical)	genus Dialister	rs4753063	A	G	-0.062	92533963	0.281	0.057	360770	A	G	0.060	11	92267129	4.86E-06	0.013	14306	21.023
Vascular dementia (subcortical)	genus Dialister	rs75416973	A	G	-0.047	172840493	0.495	0.069	360770	A	G	0.073	1	172809633	9.46E-06	0.016	14306	19.541
Vascular dementia (subcortical)	genus Dialister	rs764177	C	A	-0.039	45767957	0.512	0.059	360770	C	A	-0.060	3	45809449	9.61E-06	0.014	14306	19.742
Vascular dementia (subcortical)	genus Dialister	rs76680460	G	A	-0.026	25447082	0.853	0.139	360770	G	A	-0.161	9	25447080	8.19E-06	0.036	14306	19.600
Vascular dementia (subcortical)	genus Dorea	rs11150408	G	T	0.156	81769892	0.006	0.056	360770	G	T	-0.049	16	81803497	7.06E-06	0.011	14306	20.042
Vascular dementia (subcortical)	genus Dorea	rs12537781	T	C	0.015	155186473	0.827	0.066	360770	T	C	-0.056	7	154978183	9.15E-06	0.013	14306	19.675
Vascular dementia (subcortical)	genus Dorea	rs13279148	G	A	0.069	126893620	0.449	0.092	360770	G	A	0.072	8	127905865	2.25E-06	0.015	14306	22.477
Vascular dementia (subcortical)	genus Dorea	rs1899291	T	C	0.165	60126622	0.034	0.078	360770	T	C	-0.070	4	60992340	4.57E-06	0.015	14306	21.523
Vascular dementia (subcortical)	genus Dorea	rs3005511	G	A	0.068	73896090	0.271	0.062	360770	G	A	-0.052	6	74605806	5.29E-06	0.011	14306	20.917
Vascular dementia (subcortical)	genus Dorea	rs345219	G	T	0.042	6784833	0.457	0.057	360770	G	T	0.050	3	6826520	8.80E-06	0.011	14306	19.504
Vascular dementia (subcortical)	genus Dorea	rs3752849	G	A	-0.002	8707510	0.990	0.128	360770	G	A	0.164	11	8729057	7.68E-06	0.037	14306	20.032
Vascular dementia (subcortical)	genus Dorea	rs4793307	C	T	0.000	72737384	0.999	0.068	360770	C	T	0.057	17	70733523	4.01E-06	0.012	14306	21.979
Vascular dementia (subcortical)	genus Dorea	rs62503162	A	G	-0.033	15911148	0.814	0.140	360770	A	G	-0.097	8	15768657	7.47E-07	0.019	14306	25.113
Vascular dementia (subcortical)	genus Dorea	rs73729431	C	T	-0.046	25077822	0.821	0.201	360770	C	T	-0.137	6	25078050	3.17E-06	0.030	14306	20.994
Vascular dementia (subcortical)	genus Eggerthella	rs112205261	T	C	-0.057	9076080	0.585	0.103	360770	T	C	-0.189	1	9136139	3.35E-06	0.040	14306	21.839
Vascular dementia (subcortical)	genus Eggerthella	rs13070736	A	C	-0.075	20765962	0.326	0.077	360770	A	C	-0.121	3	20807454	7.62E-06	0.027	14306	19.894
Vascular dementia (subcortical)	genus Eggerthella	rs1784446	A	G	0.018	102590868	0.747	0.056	360770	A	G	-0.091	11	102461599	5.23E-06	0.020	14306	20.997
Vascular dementia (subcortical)	genus Eggerthella	rs2223081	A	G	0.008	28405711	0.897	0.063	360770	A	G	-0.103	21	29778032	3.89E-06	0.022	14306	21.520
Vascular dementia (subcortical)	genus Eggerthella	rs2240838	G	A	0.073	38296353	0.204	0.057	360770	G	A	-0.098	7	38335954	7.36E-07	0.020	14306	24.613
Vascular dementia (subcortical)	genus Eggerthella	rs3851328	G	T	0.081	45226666	0.235	0.069	360770	G	T	0.108	2	45453805	4.18E-06	0.024	14306	20.762
Vascular dementia (subcortical)	genus Eggerthella	rs6430926	T	C	-0.102	140579885	0.076	0.057	360770	T	C	-0.088	2	141337454	8.37E-06	0.020	14306	19.916
Vascular dementia (subcortical)	genus Eggerthella	rs67490567	T	C	-0.007	65398986	0.914	0.065	360770	T	C	0.108	15	65691324	8.94E-06	0.025	14306	19.537
Vascular dementia (subcortical)	genus Eggerthella	rs76663501	C	T	-0.136	57072813	0.299	0.131	360770	C	T	0.175	20	55647869	4.83E-06	0.038	14306	21.454
Vascular dementia (subcortical)	genus Eisenbergiella	rs11027642	C	T	-0.009	23980294	0.911	0.081	360770	C	T	0.129	11	24001840	4.92E-06	0.028	14306	20.512
Vascular dementia (subcortical)	genus Eisenbergiella	rs11079158	T	C	0.026	55290280	0.705	0.069	360770	T	C	0.101	17	53367641	7.35E-06	0.023	14306	19.920
Vascular dementia (subcortical)	genus Eisenbergiella	rs11938607	C	T	0.014	188139277	0.825	0.065	360770	C	T	-0.098	4	189060431	8.22E-06	0.022	14306	20.386
Vascular dementia (subcortical)	genus Eisenbergiella	rs12257723	A	C	0.019	107657343	0.755	0.061	360770	A	C	-0.095	10	109417101	8.85E-06	0.021	14306	20.279
Vascular dementia (subcortical)	genus Eisenbergiella	rs12710729	C	A	-0.001	19773449	0.981	0.061	360770	C	A	0.089	2	19973210	9.84E-06	0.020	14306	20.140
Vascular dementia (subcortical)	genus Eisenbergiella	rs13258851	A	G	0.033	54418254	0.684	0.082	360770	A	G	0.137	8	55330814	7.75E-06	0.030	14306	20.563
Vascular dementia (subcortical)	genus Eisenbergiella	rs1508033	A	C	-0.035	53083004	0.575	0.062	360770	A	C	0.092	15	53375201	3.23E-06	0.020	14306	21.863
Vascular dementia (subcortical)	genus Eisenbergiella	rs1553971	T	G	0.009	111700639	0.898	0.068	360770	T	G	0.121	3	111419486	5.27E-06	0.026	14306	21.146
Vascular dementia (subcortical)	genus Eisenbergiella	rs2683098	T	C	0.016	35886364	0.822	0.069	360770	T	C	-0.107	15	36178565	2.24E-06	0.023	14306	22.720
Vascular dementia (subcortical)	genus Eisenbergiella	rs3812426	A	G	-0.047	49910116	0.550	0.078	360770	A	G	-0.106	8	50822676	2.72E-06	0.022	14306	22.550
Vascular dementia (subcortical)	genus Eisenbergiella	rs4462860	G	A	0.001	20237941	0.985	0.058	360770	G	A	0.094	21	21610254	4.16E-06	0.020	14306	21.805
Vascular dementia (subcortical)	genus Enterorhabdus	rs10098492	T	C	-0.063	112097527	0.605	0.122	360770	T	C	0.132	8	113109756	6.41E-06	0.029	14306	20.300
Vascular dementia (subcortical)	genus Enterorhabdus	rs114731706	T	G	0.260	19412192	0.116	0.166	360770	T	G	0.182	2	19611953	2.17E-06	0.038	14306	22.741
Vascular dementia (subcortical)	genus Enterorhabdus	rs2051957	C	T	0.022	90080569	0.761	0.071	360770	C	T	0.084	7	89709883	8.90E-06	0.019	14306	19.727

Vascular dementia (subcortical)	genus Enterorhabdus	rs3017103	G	A	0.054	62406721	0.455	0.072	360770	G	A	-0.098	11	62174193	2.94E-06	0.021	14306	22.028
Vascular dementia (subcortical)	genus Enterorhabdus	rs73331712	T	C	-0.072	69121322	0.608	0.141	360770	T	C	0.262	12	69515102	4.85E-06	0.055	14306	22.589
Vascular dementia (subcortical)	genus Enterorhabdus	rs77655283	G	A	-0.061	235531235	0.586	0.112	360770	G	A	0.133	2	236439879	5.88E-06	0.030	14306	19.875
Vascular dementia (subcortical)	genus Erysipelatoclostridium	rs1434153	G	A	0.132	34370466	0.021	0.057	360770	G	A	-0.068	2	34595533	6.85E-06	0.015	14306	20.163
Vascular dementia (subcortical)	genus Erysipelatoclostridium	rs16936671	C	T	-0.007	36105681	0.930	0.083	360770	C	T	-0.097	10	36394609	6.04E-06	0.022	14306	19.739
Vascular dementia (subcortical)	genus Erysipelatoclostridium	rs17804233	C	T	0.119	78519233	0.037	0.057	360770	C	T	0.066	5	77815056	4.59E-06	0.014	14306	21.133
Vascular dementia (subcortical)	genus Erysipelatoclostridium	rs2901723	A	C	-0.105	36849973	0.067	0.057	360770	A	C	-0.064	11	36871523	8.79E-06	0.014	14306	19.755
Vascular dementia (subcortical)	genus Erysipelatoclostridium	rs340991	A	G	0.072	34891937	0.264	0.064	360770	A	G	-0.074	5	34892042	3.75E-06	0.016	14306	21.719
Vascular dementia (subcortical)	genus Erysipelatoclostridium	rs3804326	A	G	-0.138	24291957	0.292	0.131	360770	A	G	0.141	6	24292185	9.85E-06	0.034	14306	17.691
Vascular dementia (subcortical)	genus Erysipelatoclostridium	rs45480394	T	G	0.043	55346409	0.468	0.059	360770	T	G	-0.069	19	55857777	7.66E-06	0.015	14306	20.480
Vascular dementia (subcortical)	genus Erysipelatoclostridium	rs4697572	A	G	0.032	25445165	0.655	0.071	360770	A	G	-0.081	4	25446787	7.59E-07	0.016	14306	24.640
Vascular dementia (subcortical)	genus Erysipelatoclostridium	rs58236560	G	T	0.011	122006205	0.898	0.088	360770	G	T	-0.111	11	121876913	2.16E-06	0.023	14306	22.483
Vascular dementia (subcortical)	genus Erysipelatoclostridium	rs61806970	C	T	-0.007	167849359	0.946	0.112	360770	C	T	0.143	1	167818597	9.09E-06	0.032	14306	19.759
Vascular dementia (subcortical)	genus Erysipelatoclostridium	rs622418	A	G	0.004	87971128	0.939	0.057	360770	A	G	-0.067	9	90586043	3.68E-06	0.014	14306	21.756
Vascular dementia (subcortical)	genus Erysipelatoclostridium	rs6474512	C	A	0.020	38923088	0.726	0.058	360770	C	A	-0.067	8	38780606	3.02E-06	0.014	14306	21.900
Vascular dementia (subcortical)	genus Erysipelatoclostridium	rs710230	C	T	0.030	41867960	0.785	0.109	360770	C	T	-0.143	1	42333631	6.33E-07	0.028	14306	25.941
Vascular dementia (subcortical)	genus Erysipelatoclostridium	rs7221249	G	A	-0.080	10274391	0.160	0.057	360770	G	A	-0.084	17	10177708	4.31E-09	0.014	14306	34.619
Vascular dementia (subcortical)	genus Erysipelatoclostridium	rs9590927	A	G	0.055	45797930	0.338	0.057	360770	A	G	0.065	13	46372065	6.39E-06	0.014	14306	20.272
Vascular dementia (subcortical)	genus Escherichia Shigella	rs112767262	T	C	-0.042	3700622	0.544	0.069	360770	T	C	0.073	16	3750623	8.21E-06	0.016	14306	20.075
Vascular dementia (subcortical)	genus Escherichia Shigella	rs113127095	A	G	0.094	22679253	0.514	0.144	360770	A	G	0.151	13	23253392	3.33E-06	0.032	14306	21.799
Vascular dementia (subcortical)	genus Escherichia Shigella	rs113513883	A	G	-0.151	140978399	0.356	0.163	360770	A	G	0.172	7	140678199	5.28E-06	0.038	14306	20.511
Vascular dementia (subcortical)	genus Escherichia Shigella	rs1154904	G	A	0.009	134904951	0.870	0.057	360770	G	A	0.061	11	134774845	3.04E-06	0.013	14306	22.043
Vascular dementia (subcortical)	genus Escherichia Shigella	rs118526	A	C	0.021	80567725	0.734	0.062	360770	A	C	0.059	5	79863544	8.00E-06	0.014	14306	19.108
Vascular dementia (subcortical)	genus Escherichia Shigella	rs2798105	A	G	0.072	48496231	0.453	0.097	360770	A	G	-0.101	1	48961903	8.24E-06	0.022	14306	20.629
Vascular dementia (subcortical)	genus Escherichia Shigella	rs4731451	G	A	-0.026	128414046	0.673	0.061	360770	G	A	-0.061	7	128054100	7.47E-06	0.014	14306	20.362
Vascular dementia (subcortical)	genus Escherichia Shigella	rs57024273	T	C	0.104	235606511	0.113	0.065	360770	T	C	0.063	2	236515155	9.70E-06	0.014	14306	20.028
Vascular dementia (subcortical)	genus Escherichia Shigella	rs592299	T	C	-0.124	133303865	0.031	0.057	360770	T	C	-0.059	9	136179347	4.77E-06	0.013	14306	20.903
Vascular dementia (subcortical)	genus Escherichia Shigella	rs73208162	A	G	0.007	37952181	0.967	0.163	360770	A	G	-0.119	21	39324484	2.19E-06	0.025	14306	23.067
Vascular dementia (subcortical)	genus Eubacterium brachy group	rs112617308	T	C	0.179	92425456	0.067	0.098	360770	T	C	-0.171	10	94185213	2.38E-06	0.036	14306	22.177
Vascular dementia (subcortical)	genus Eubacterium brachy group	rs12151423	G	A	0.005	217372558	0.934	0.057	360770	G	A	-0.101	2	218237281	9.27E-06	0.023	14306	19.869
Vascular dementia (subcortical)	genus Eubacterium brachy group	rs13139592	T	C	-0.109	143744025	0.195	0.084	360770	T	C	-0.146	4	144665178	7.97E-06	0.033	14306	19.911
Vascular dementia (subcortical)	genus Eubacterium brachy group	rs1384962	G	A	-0.139	22591645	0.024	0.061	360770	G	A	-0.121	14	23060552	6.99E-06	0.027	14306	20.613
Vascular dementia (subcortical)	genus Eubacterium brachy group	rs2913110	T	C	0.097	22603738	0.105	0.059	360770	T	C	-0.105	10	22892667	4.56E-06	0.023	14306	21.004
Vascular dementia (subcortical)	genus Eubacterium brachy group	rs4862235	A	G	-0.008	183707778	0.895	0.058	360770	A	G	-0.105	4	184628931	3.73E-06	0.023	14306	21.553
Vascular dementia (subcortical)	genus Eubacterium brachy group	rs62348779	T	C	-0.059	17459987	0.582	0.106	360770	T	C	-0.201	5	17460096	3.78E-06	0.043	14306	21.666
Vascular dementia (subcortical)	genus Eubacterium brachy group	rs6591893	A	G	-0.023	80525789	0.706	0.060	360770	A	G	-0.108	11	80236833	7.34E-06	0.024	14306	20.281
Vascular dementia (subcortical)	genus Eubacterium brachy group	rs720439	G	A	-0.058	47794849	0.381	0.066	360770	G	A	0.112	22	48190598	7.03E-06	0.025	14306	19.845
Vascular dementia (subcortical)	genus Eubacterium brachy group	rs73199919	T	C	-0.327	6854837	0.013	0.132	360770	T	C	-0.237	4	6856564	8.16E-06	0.053	14306	19.848
Vascular dementia (subcortical)	genus Eubacterium coprostanoligenes group	rs1020520	T	G	-0.035	33563534	0.665	0.080	360770	T	G	-0.059	7	33603146	8.89E-06	0.013	14306	19.747
Vascular dementia (subcortical)	genus Eubacterium coprostanoligenes group	rs10444197	A	G	-0.036	2173737	0.547	0.060	360770	A	G	-0.051	10	2215931	5.98E-06	0.011	14306	19.877
Vascular dementia (subcortical)	genus Eubacterium coprostanoligenes group	rs11052069	C	T	-0.028	32560985	0.628	0.057	360770	C	T	-0.048	12	32713919	9.38E-06	0.011	14306	19.636
Vascular dementia (subcortical)	genus Eubacterium coprostanoligenes group	rs11720857	C	T	0.015	114075660	0.841	0.074	360770	C	T	0.063	3	113794507	9.26E-06	0.014	14306	19.059
Vascular dementia (subcortical)	genus Eubacterium coprostanoligenes group	rs12906958	C	T	-0.003	36619397	0.959	0.063	360770	C	T	-0.053	15	36911598	4.35E-06	0.012	14306	21.149
Vascular dementia (subcortical)	genus Eubacterium coprostanoligenes group	rs17159861	C	T	0.042	31045547	0.649	0.092	360770	C	T	0.096	7	31085162	1.04E-08	0.017	14306	32.654
Vascular dementia (subcortical)	genus Eubacterium coprostanoligenes group	rs2644213	A	G	0.071	82746340	0.250	0.062	360770	A	G	-0.054	10	84506096	9.86E-06	0.012	14306	19.751
Vascular dementia (subcortical)	genus Eubacterium coprostanoligenes group	rs4076415	G	T	-0.017	85897766	0.771	0.059	360770	G	T	-0.052	15	86440997	1.99E-06	0.011	14306	21.816
Vascular dementia (subcortical)	genus Eubacterium coprostanoligenes group	rs62024432	C	T	-0.114	97326063	0.239	0.097	360770	C	T	-0.077	15	97869293	7.50E-06	0.017	14306	20.002
Vascular dementia (subcortical)	genus Eubacterium coprostanoligenes group	rs6762473	A	C	0.022	127420731	0.718	0.060	360770	A	C	-0.052	3	127139574	4.26E-06	0.011	14306	21.552
Vascular dementia (subcortical)	genus Eubacterium coprostanoligenes group	rs76898927	G	A	0.080	81546326	0.523	0.125	360770	G	A	0.123	3	81595477	4.79E-06	0.027	14306	21.341
Vascular dementia (subcortical)	genus Eubacterium coprostanoligenes group	rs9648214	T	C	-0.006	16341181	0.949	0.102	360770	T	C	-0.083	7	16380806	2.52E-07	0.016	14306	25.438
Vascular dementia (subcortical)	genus Eubacterium eligens group	rs182318	G	A	-0.084	31077144	0.420	0.105	360770	G	A	-0.082	11	31098691	8.40E-06	0.020	14306	17.784
Vascular dementia (subcortical)	genus Eubacterium eligens group	rs2200429	A	G	0.041	106911110	0.666	0.095	360770	A	G	-0.089	13	107563458	5.30E-06	0.020	14306	20.054
Vascular dementia (subcortical)	genus Eubacterium eligens group	rs265534	T	G	-0.017	81332251	0.770	0.057	360770	T	G	-0.056	10	83092007	2.27E-06	0.012	14306	22.024
Vascular dementia (subcortical)	genus Eubacterium eligens group	rs4583233	A	C	-0.012	81786881	0.853	0.063	360770	A	C	0.067	16	81820486	2.84E-07	0.013	14306	27.363
Vascular dementia (subcortical)	genus Eubacterium eligens group	rs56080211	T	C	-0.117	109503416	0.277	0.108	360770	T	C	-0.123	2	110260993	9.14E-06	0.028	14306	18.953
Vascular dementia (subcortical)	genus Eubacterium eligens group	rs6923695	T	G	0.191	70247112	0.096	0.115	360770	T	G	0.103	6	70956815	4.87E-06	0.023	14306	20.243
Vascular dementia (subcortical)	genus Eubacterium fissicatena group	rs10147907	T	G	-0.105	89018029	0.339	0.110	360770	T	G	0.172	14	89484373	8.27E-06	0.040	14306	18.922
Vascular dementia (subcortical)	genus Eubacterium fissicatena group	rs11818408	G	A	0.013	94998710	0.824	0.058	360770	G	A	0.106	10	96758467	8.20E-06	0.024	14306	19.928
Vascular dementia (subcortical)	genus Eubacterium fissicatena group	rs11876297	T	C	0.031	48226901	0.624	0.064	360770	T	C	0.131	18	45753272	2.67E-06	0.028	14306	21.779
Vascular dementia (subcortical)	genus Eubacterium fissicatena group	rs151257695	A	G	-0.223	73629231	0.042	0.110	360770	A	G	0.210	7	73043561	3.10E-06	0.045	14306	21.217
Vascular dementia (subcortical)	genus Eubacterium fissicatena group	rs1768152	C	T	-0.061	39563463	0.510	0.093	360770	C	T	-0.139	3	39604954	8.70E-06	0.032	14306	19.462
Vascular dementia (subcortical)	genus Eubacterium fissicatena group	rs2733072	G	A	0.003	5576177	0.959	0.057	360770	G	A	0.110	8	5433699	1.49E-06	0.023	14306	23.064
Vascular dementia (subcortical)	genus Eubacterium fissicatena group	rs3771393	T	C	-0.077	70918116	0.285	0.072	360770	T	C	-0.131	2	71145246	7.38E-07	0.027	14306	24.074
Vascular dementia (subcortical)	genus Eubacterium fissicatena group	rs6934739	A	G	-0.006	39972698	0.915	0.060	360770	A	G	0.111	6	39940437	9.75E-06	0.025	14306	19.442
Vascular dementia (subcortical)	genus Eubacterium fissicatena group	rs7104872	G	A	0.036	115294391	0.693	0.090	360770	G	A	0.139	11	115165111	2.73E-06	0.029	14306	22.548
Vascular dementia (subcortical)	genus Eubacterium hallii group	rs10501370	C	T	0.001	58273149	0.993	0.119	360770	C	T	-0.116	11	58040621	5.42E-06	0.025	14306	20.958
Vascular dementia (subcortical)	genus Eubacterium hallii group	rs10798999	C	T	0.000	33843316	0.995	0.064	360770	C	T	0.060	1	34308917	2.61E-06	0.013	14306	22.532

Vascular dementia (subcortical)	genus Eubacterium hallii group	rs117748144	T	C	0.006	11750090	0.964	0.132	360770	T	C	-0.127	11	11771637	7.86E-06	0.029	14306	19.436
Vascular dementia (subcortical)	genus Eubacterium hallii group	rs13116360	T	C	-0.055	110964275	0.629	0.114	360770	T	C	0.154	4	111885431	2.94E-07	0.030	14306	26.896
Vascular dementia (subcortical)	genus Eubacterium hallii group	rs17074066	T	C	0.105	182788383	0.609	0.206	360770	T	C	-0.081	4	183709536	9.35E-06	0.019	14306	18.495
Vascular dementia (subcortical)	genus Eubacterium hallii group	rs17474256	G	A	-0.093	103982054	0.330	0.095	360770	G	A	0.081	1	104524676	9.45E-06	0.018	14306	19.297
Vascular dementia (subcortical)	genus Eubacterium hallii group	rs281379	A	G	0.093	48711017	0.106	0.058	360770	A	G	-0.050	19	49214274	9.33E-06	0.011	14306	19.838
Vascular dementia (subcortical)	genus Eubacterium hallii group	rs28584818	A	G	0.118	64678780	0.253	0.103	360770	A	G	0.126	3	64664456	4.43E-06	0.027	14306	22.041
Vascular dementia (subcortical)	genus Eubacterium hallii group	rs60254196	G	A	0.060	149159628	0.289	0.057	360770	G	A	0.052	7	148856720	2.70E-06	0.011	14306	21.844
Vascular dementia (subcortical)	genus Eubacterium hallii group	rs630939	C	T	0.042	50858093	0.469	0.057	360770	C	T	-0.051	18	48384463	9.16E-06	0.011	14306	19.806
Vascular dementia (subcortical)	genus Eubacterium hallii group	rs6550770	C	T	0.046	23621925	0.746	0.143	360770	C	T	0.198	3	23663416	4.82E-06	0.044	14306	19.945
Vascular dementia (subcortical)	genus Eubacterium hallii group	rs74018587	C	T	-0.056	61721961	0.700	0.145	360770	C	T	0.209	15	62014160	3.70E-06	0.044	14306	22.734
Vascular dementia (subcortical)	genus Eubacterium hallii group	rs78056098	G	T	-0.022	123919170	0.715	0.059	360770	G	T	-0.051	11	123789877	8.29E-06	0.011	14306	19.896
Vascular dementia (subcortical)	genus Eubacterium hallii group	rs949971	T	G	0.092	110564542	0.130	0.061	360770	T	G	-0.054	3	110283389	3.29E-06	0.012	14306	21.646
Vascular dementia (subcortical)	genus Eubacterium nodatum group	rs10263623	C	T	-0.114	65429224	0.436	0.146	360770	C	T	0.193	7	64894137	8.91E-06	0.044	14306	19.425
Vascular dementia (subcortical)	genus Eubacterium nodatum group	rs10458299	T	C	0.192	135367596	0.076	0.108	360770	T	C	-0.188	7	135052348	8.37E-06	0.042	14306	20.013
Vascular dementia (subcortical)	genus Eubacterium nodatum group	rs11006576	A	G	0.029	59562257	0.617	0.057	360770	A	G	-0.110	10	61322015	7.99E-06	0.025	14306	20.067
Vascular dementia (subcortical)	genus Eubacterium nodatum group	rs113893692	C	T	-0.089	28245520	0.302	0.087	360770	C	T	-0.185	9	28245518	5.76E-06	0.040	14306	21.038
Vascular dementia (subcortical)	genus Eubacterium nodatum group	rs34297067	A	G	-0.073	51719170	0.359	0.080	360770	A	G	-0.187	14	52185888	6.60E-08	0.034	14306	29.959
Vascular dementia (subcortical)	genus Eubacterium nodatum group	rs61841040	G	T	0.087	9617786	0.223	0.072	360770	G	T	0.161	10	9659749	3.56E-06	0.034	14306	22.108
Vascular dementia (subcortical)	genus Eubacterium nodatum group	rs6818880	G	A	-0.036	93791596	0.529	0.057	360770	G	A	0.110	4	94712747	7.83E-06	0.025	14306	20.030
Vascular dementia (subcortical)	genus Eubacterium nodatum group	rs77910827	C	T	0.036	93835703	0.696	0.092	360770	C	T	0.202	9	96597985	9.05E-07	0.041	14306	23.807
Vascular dementia (subcortical)	genus Eubacterium nodatum group	rs7827125	C	T	-0.025	5097390	0.696	0.063	360770	C	T	0.122	8	4954912	7.17E-06	0.027	14306	20.317
Vascular dementia (subcortical)	genus Eubacterium nodatum group	rs7880204	T	C	0.083	171091680	0.208	0.066	360770	T	C	-0.125	1	171060821	6.84E-06	0.028	14306	20.766
Vascular dementia (subcortical)	genus Eubacterium nodatum group	rs9425984	T	C	-0.107	34064135	0.119	0.068	360770	T	C	-0.130	1	34529736	7.21E-06	0.029	14306	19.845
Vascular dementia (subcortical)	genus Eubacterium oxidoreducens group	rs12129908	A	C	-0.007	195094889	0.898	0.058	360770	A	C	-0.089	1	195064019	5.80E-06	0.020	14306	20.285
Vascular dementia (subcortical)	genus Eubacterium oxidoreducens group	rs12423772	G	T	-0.113	94121564	0.161	0.081	360770	G	T	0.141	12	94515340	2.63E-06	0.030	14306	22.834
Vascular dementia (subcortical)	genus Eubacterium oxidoreducens group	rs2973294	G	T	0.002	37525057	0.977	0.058	360770	G	T	0.092	4	37526679	2.39E-06	0.020	14306	22.331
Vascular dementia (subcortical)	genus Eubacterium oxidoreducens group	rs34561138	G	A	-0.059	86446749	0.688	0.146	360770	G	A	0.216	16	86480355	2.51E-06	0.046	14306	22.086
Vascular dementia (subcortical)	genus Eubacterium oxidoreducens group	rs440215	T	C	0.006	107546693	0.920	0.057	360770	T	C	-0.093	5	106882394	1.65E-06	0.020	14306	22.812
Vascular dementia (subcortical)	genus Eubacterium rectale group	rs10248854	C	A	-0.009	121641184	0.870	0.058	360770	C	A	-0.053	7	121281238	4.21E-06	0.011	14306	21.640
Vascular dementia (subcortical)	genus Eubacterium rectale group	rs10797540	A	G	0.058	234394624	0.312	0.058	360770	A	G	0.050	1	234530370	3.53E-06	0.011	14306	21.551
Vascular dementia (subcortical)	genus Eubacterium rectale group	rs143694765	T	C	-0.082	115540288	0.383	0.094	360770	T	C	0.087	1	116082909	9.75E-06	0.020	14306	19.349
Vascular dementia (subcortical)	genus Eubacterium rectale group	rs2884897	A	G	0.031	8107990	0.846	0.158	360770	A	G	-0.129	11	8129537	6.44E-06	0.029	14306	20.034
Vascular dementia (subcortical)	genus Eubacterium rectale group	rs314726	T	C	-0.142	124795578	0.013	0.057	360770	T	C	0.053	2	125553155	1.38E-06	0.011	14306	23.329
Vascular dementia (subcortical)	genus Eubacterium rectale group	rs35398954	A	G	-0.003	92463535	0.969	0.078	360770	A	G	-0.090	15	93006765	5.40E-07	0.017	14306	26.653
Vascular dementia (subcortical)	genus Eubacterium rectale group	rs59427698	A	G	-0.051	126663466	0.476	0.072	360770	A	G	-0.058	9	129425745	5.37E-06	0.013	14306	19.379
Vascular dementia (subcortical)	genus Eubacterium rectale group	rs62547233	A	G	-0.021	89398504	0.741	0.063	360770	A	G	0.054	9	92013419	9.90E-06	0.012	14306	19.908
Vascular dementia (subcortical)	genus Eubacterium ruminantium group	rs10131724	C	A	0.030	51752348	0.757	0.096	360770	C	A	0.200	14	52219066	2.39E-06	0.041	14306	23.234
Vascular dementia (subcortical)	genus Eubacterium ruminantium group	rs10923018	G	A	0.070	88057716	0.222	0.058	360770	G	A	0.073	1	88523399	6.80E-06	0.016	14306	20.378
Vascular dementia (subcortical)	genus Eubacterium ruminantium group	rs11637981	T	G	-0.047	60997423	0.408	0.057	360770	T	G	0.073	15	61289622	5.44E-06	0.016	14306	20.733
Vascular dementia (subcortical)	genus Eubacterium ruminantium group	rs13025464	C	T	0.070	200745922	0.226	0.058	360770	C	T	0.074	2	201610645	6.97E-06	0.016	14306	20.252
Vascular dementia (subcortical)	genus Eubacterium ruminantium group	rs139749	C	T	0.004	24907088	0.952	0.060	360770	C	T	-0.085	22	25303055	8.59E-07	0.017	14306	24.219
Vascular dementia (subcortical)	genus Eubacterium ruminantium group	rs16891896	G	A	-0.161	33897484	0.114	0.102	360770	G	A	-0.175	5	33897589	2.38E-06	0.039	14306	20.027
Vascular dementia (subcortical)	genus Eubacterium ruminantium group	rs17519472	C	T	-0.029	30208021	0.722	0.082	360770	C	T	0.108	12	30360954	4.70E-06	0.023	14306	21.227
Vascular dementia (subcortical)	genus Eubacterium ruminantium group	rs209813	G	A	0.090	11953400	0.263	0.081	360770	G	A	-0.103	6	11953633	9.23E-06	0.024	14306	19.165
Vascular dementia (subcortical)	genus Eubacterium ruminantium group	rs2116427	A	G	-0.023	121847301	0.724	0.065	360770	A	G	0.091	5	121182996	4.67E-07	0.018	14306	24.983
Vascular dementia (subcortical)	genus Eubacterium ruminantium group	rs2229917	A	G	-0.068	128218658	0.628	0.140	360770	A	G	0.154	9	130980937	2.16E-06	0.032	14306	22.467
Vascular dementia (subcortical)	genus Eubacterium ruminantium group	rs2418654	C	T	-0.088	71020448	0.132	0.058	360770	C	T	-0.075	2	71247578	6.17E-06	0.017	14306	20.388
Vascular dementia (subcortical)	genus Eubacterium ruminantium group	rs2817174	C	T	-0.001	3127617	0.981	0.058	360770	C	T	-0.073	1	3044181	7.87E-06	0.016	14306	20.125
Vascular dementia (subcortical)	genus Eubacterium ruminantium group	rs57340348	T	C	-0.007	129708969	0.918	0.071	360770	T	C	-0.098	6	130030114	4.93E-06	0.021	14306	21.311
Vascular dementia (subcortical)	genus Eubacterium ruminantium group	rs606117	G	A	0.004	2539358	0.952	0.064	360770	G	A	-0.083	9	2539358	4.82E-06	0.018	14306	21.296
Vascular dementia (subcortical)	genus Eubacterium ruminantium group	rs6676699	T	G	0.083	198009151	0.182	0.062	360770	T	G	0.089	1	197978281	6.38E-06	0.020	14306	20.439
Vascular dementia (subcortical)	genus Eubacterium ruminantium group	rs7000472	G	A	0.038	137614025	0.513	0.058	360770	G	A	0.076	8	138626268	4.07E-06	0.017	14306	21.284
Vascular dementia (subcortical)	genus Eubacterium ruminantium group	rs72836424	C	T	-0.022	127239189	0.806	0.091	360770	C	T	-0.140	10	129037453	2.62E-06	0.030	14306	21.624
Vascular dementia (subcortical)	genus Eubacterium ruminantium group	rs73139629	A	C	-0.059	62912223	0.546	0.097	360770	A	C	-0.115	12	63306003	5.36E-06	0.025	14306	21.555
Vascular dementia (subcortical)	genus Eubacterium ventriosum group	rs11617697	A	G	0.050	98133646	0.685	0.123	360770	A	G	-0.143	13	98785900	7.22E-07	0.029	14306	25.080
Vascular dementia (subcortical)	genus Eubacterium ventriosum group	rs12964517	G	A	-0.051	24880821	0.425	0.064	360770	G	A	0.059	18	22460785	2.08E-06	0.012	14306	22.624
Vascular dementia (subcortical)	genus Eubacterium ventriosum group	rs13082419	C	T	-0.041	108017587	0.647	0.090	360770	C	T	-0.072	3	107736434	9.56E-06	0.016	14306	19.661
Vascular dementia (subcortical)	genus Eubacterium ventriosum group	rs16884680	G	T	0.064	113432826	0.493	0.094	360770	G	T	-0.091	8	114445055	1.74E-06	0.019	14306	22.309
Vascular dementia (subcortical)	genus Eubacterium ventriosum group	rs35179274	C	T	-0.004	51493907	0.954	0.074	360770	C	T	-0.063	14	51960625	5.76E-06	0.014	14306	20.703
Vascular dementia (subcortical)	genus Eubacterium ventriosum group	rs3809430	T	C	0.213	44506307	0.000	0.059	360770	T	C	-0.055	14	44975510	3.55E-06	0.012	14306	21.426
Vascular dementia (subcortical)	genus Eubacterium ventriosum group	rs57199565	T	C	0.019	190505943	0.799	0.073	360770	T	C	0.078	3	190223732	7.97E-07	0.016	14306	23.896
Vascular dementia (subcortical)	genus Eubacterium ventriosum group	rs66746423	C	T	-0.025	108855642	0.749	0.079	360770	C	T	0.075	1	109398264	6.11E-06	0.016	14306	20.749
Vascular dementia (subcortical)	genus Eubacterium ventriosum group	rs6704822	G	A	0.179	202797056	0.035	0.085	360770	G	A	-0.074	2	203661779	6.62E-06	0.017	14306	19.661
Vascular dementia (subcortical)	genus Eubacterium ventriosum group	rs72783037	C	A	-0.176	60314477	0.012	0.070	360770	C	A	0.066	16	60348381	6.55E-06	0.014	14306	21.012
Vascular dementia (subcortical)	genus Eubacterium ventriosum group	rs73615400	T	C	-0.063	51524380	0.513	0.097	360770	T	C	-0.096	20	50140919	9.54E-07	0.019	14306	24.443
Vascular dementia (subcortical)	genus Eubacterium ventriosum group	rs73849225	T	C	0.059	125450659	0.559	0.102	360770	T	C	0.098	4	126371814	5.21E-06	0.022	14306	18.908
Vascular dementia (subcortical)	genus Eubacterium ventriosum group	rs78250280	G	A	-0.032	147819813	0.700	0.082	360770	G	A	0.075	1	147291928	3.36E-06	0.016	14306	20.798

Vascular dementia (subcortical)	genus Eubacterium ventriosum group	rs876734	T	C	-0.031	52030212	0.629	0.064	360770	T	C	0.062	12	52423996	2.89E-06	0.013	14306	21.793
Vascular dementia (subcortical)	genus Eubacterium ventriosum group	rs9316536	T	G	-0.046	51364440	0.573	0.082	360770	T	G	-0.082	13	51938576	7.84E-06	0.018	14306	19.911
Vascular dementia (subcortical)	genus Eubacterium xylanophilum group	rs10140184	A	C	-0.064	72715075	0.270	0.058	360770	A	C	0.058	14	73181783	4.96E-06	0.013	14306	20.959
Vascular dementia (subcortical)	genus Eubacterium xylanophilum group	rs10917203	A	C	-0.073	22300773	0.207	0.058	360770	A	C	0.061	1	22627266	3.15E-06	0.013	14306	21.898
Vascular dementia (subcortical)	genus Eubacterium xylanophilum group	rs112176119	C	T	0.056	73662872	0.569	0.098	360770	C	T	-0.113	16	73696771	3.33E-06	0.025	14306	21.312
Vascular dementia (subcortical)	genus Eubacterium xylanophilum group	rs13239072	G	A	-0.041	43526745	0.522	0.064	360770	G	A	0.069	7	43566344	1.82E-06	0.014	14306	23.197
Vascular dementia (subcortical)	genus Eubacterium xylanophilum group	rs17830032	G	A	-0.051	59420451	0.633	0.106	360770	G	A	-0.161	20	57995506	2.39E-07	0.031	14306	26.749
Vascular dementia (subcortical)	genus Eubacterium xylanophilum group	rs1999224	G	T	-0.164	127735841	0.091	0.097	360770	G	T	-0.095	9	130498120	3.75E-06	0.020	14306	21.749
Vascular dementia (subcortical)	genus Eubacterium xylanophilum group	rs2012708	G	A	0.013	84756953	0.830	0.060	360770	G	A	-0.057	16	84790559	6.53E-06	0.013	14306	20.401
Vascular dementia (subcortical)	genus Eubacterium xylanophilum group	rs2213117	T	G	-0.055	131139765	0.483	0.078	360770	T	G	0.088	11	131009660	4.21E-06	0.019	14306	21.568
Vascular dementia (subcortical)	genus Faecalibacterium	rs10927394	G	T	0.103	245104037	0.621	0.209	360770	G	T	-0.232	1	245267339	7.02E-06	0.051	14306	20.549
Vascular dementia (subcortical)	genus Faecalibacterium	rs114946999	C	T	-0.040	36898092	0.641	0.086	360770	C	T	-0.086	2	37125235	5.70E-06	0.019	14306	20.649
Vascular dementia (subcortical)	genus Faecalibacterium	rs11776390	T	C	0.206	38736127	0.080	0.118	360770	T	C	-0.078	8	38593645	6.40E-06	0.017	14306	20.793
Vascular dementia (subcortical)	genus Faecalibacterium	rs1271565	C	T	0.043	63577379	0.510	0.065	360770	C	T	-0.058	14	64044097	1.30E-06	0.012	14306	23.196
Vascular dementia (subcortical)	genus Faecalibacterium	rs12753492	A	C	0.104	13875051	0.251	0.090	360770	A	C	0.064	1	14201546	8.80E-06	0.015	14306	18.295
Vascular dementia (subcortical)	genus Faecalibacterium	rs2835874	T	C	0.078	37655599	0.611	0.154	360770	T	C	-0.087	21	39027901	7.54E-06	0.020	14306	19.440
Vascular dementia (subcortical)	genus Faecalibacterium	rs6910935	G	A	-0.134	131266017	0.259	0.119	360770	G	A	-0.135	6	131587157	1.38E-06	0.028	14306	23.699
Vascular dementia (subcortical)	genus Faecalibacterium	rs75499067	C	T	-0.117	6049862	0.284	0.109	360770	C	T	0.228	16	6099863	1.76E-06	0.047	14306	23.900
Vascular dementia (subcortical)	genus Faecalibacterium	rs79656633	T	C	0.017	110494205	0.857	0.096	360770	T	C	0.146	1	111036827	8.14E-06	0.032	14306	20.326
Vascular dementia (subcortical)	genus Faecalibacterium	rs9536330	T	C	0.045	52992882	0.425	0.057	360770	T	C	-0.048	13	53567017	5.33E-06	0.011	14306	20.026
Vascular dementia (subcortical)	genus Family XIII AD3011 group	rs11126423	T	C	-0.102	73968424	0.301	0.099	360770	T	C	-0.090	2	74195551	5.91E-06	0.020	14306	21.226
Vascular dementia (subcortical)	genus Family XIII AD3011 group	rs11736617	G	A	-0.106	38916209	0.393	0.124	360770	G	A	-0.076	4	38917830	9.02E-06	0.017	14306	19.475
Vascular dementia (subcortical)	genus Family XIII AD3011 group	rs12812672	T	C	-0.088	17974663	0.424	0.110	360770	T	C	-0.096	12	18127597	2.56E-06	0.021	14306	21.273
Vascular dementia (subcortical)	genus Family XIII AD3011 group	rs149302	T	C	0.036	14159816	0.591	0.067	360770	T	C	-0.065	5	14159925	7.48E-06	0.014	14306	20.321
Vascular dementia (subcortical)	genus Family XIII AD3011 group	rs16840310	G	A	-0.057	240530863	0.324	0.058	360770	G	A	0.061	1	240694163	6.75E-07	0.012	14306	24.780
Vascular dementia (subcortical)	genus Family XIII AD3011 group	rs16940167	C	T	0.049	58391498	0.496	0.072	360770	C	T	0.073	15	58683697	3.91E-06	0.016	14306	20.989
Vascular dementia (subcortical)	genus Family XIII AD3011 group	rs17156849	G	A	0.135	28564367	0.258	0.120	360770	G	A	-0.113	7	28603985	4.19E-06	0.025	14306	21.181
Vascular dementia (subcortical)	genus Family XIII AD3011 group	rs62029761	A	G	0.072	15912385	0.567	0.126	360770	A	G	0.129	16	16006242	3.89E-06	0.028	14306	21.762
Vascular dementia (subcortical)	genus Family XIII AD3011 group	rs62200412	C	T	-0.081	4637993	0.214	0.065	360770	C	T	-0.080	20	4618639	5.80E-07	0.016	14306	23.894
Vascular dementia (subcortical)	genus Family XIII AD3011 group	rs72730932	C	A	-0.083	192050705	0.393	0.097	360770	C	A	-0.090	1	192019835	6.89E-07	0.018	14306	25.798
Vascular dementia (subcortical)	genus Family XIII AD3011 group	rs739451	C	T	-0.026	133813832	0.709	0.069	360770	C	T	0.065	9	136678954	7.88E-06	0.015	14306	19.386
Vascular dementia (subcortical)	genus Family XIII AD3011 group	rs9276029	A	G	-0.118	32727559	0.097	0.071	360770	A	G	-0.081	6	32695336	8.93E-06	0.019	14306	19.097
Vascular dementia (subcortical)	genus Family XIII AD3011 group	rs9837139	A	G	0.080	29770374	0.432	0.102	360770	A	G	0.108	3	29811865	8.71E-06	0.024	14306	19.990
Vascular dementia (subcortical)	genus Family XIII UCG001	rs112362903	A	G	-0.188	35434008	0.238	0.159	360770	A	G	-0.149	17	33761027	7.88E-06	0.033	14306	20.001
Vascular dementia (subcortical)	genus Family XIII UCG001	rs12049454	T	C	-0.033	84762642	0.571	0.059	360770	T	C	-0.065	1	85228325	1.17E-06	0.013	14306	23.312
Vascular dementia (subcortical)	genus Family XIII UCG001	rs1426266	C	T	-0.048	188506308	0.457	0.064	360770	C	T	0.067	3	188224096	1.25E-06	0.014	14306	23.546
Vascular dementia (subcortical)	genus Family XIII UCG001	rs3842897	G	A	0.014	183534529	0.885	0.099	360770	G	A	-0.113	1	183503664	5.20E-06	0.024	14306	21.531
Vascular dementia (subcortical)	genus Family XIII UCG001	rs62414802	C	T	0.105	75888918	0.110	0.066	360770	C	T	-0.061	6	76598635	4.29E-06	0.013	14306	20.676
Vascular dementia (subcortical)	genus Family XIII UCG001	rs7119679	G	A	0.141	94948621	0.037	0.067	360770	G	A	-0.081	11	94681786	3.52E-06	0.017	14306	21.429
Vascular dementia (subcortical)	genus Family XIII UCG001	rs76463770	A	G	-0.241	45437598	0.133	0.160	360770	A	G	0.193	3	45479090	3.77E-06	0.042	14306	21.158
Vascular dementia (subcortical)	genus Family XIII UCG001	rs8076666	G	A	0.047	80250018	0.593	0.088	360770	G	A	-0.089	17	78223817	8.02E-06	0.020	14306	20.029
Vascular dementia (subcortical)	genus Flavonifractor	rs114873521	C	T	0.263	168791398	0.018	0.111	360770	C	T	-0.130	5	168218403	7.13E-06	0.029	14306	19.561
Vascular dementia (subcortical)	genus Flavonifractor	rs11811696	T	C	-0.165	237188054	0.114	0.104	360770	T	C	-0.116	1	237351354	2.07E-06	0.024	14306	23.191
Vascular dementia (subcortical)	genus Flavonifractor	rs12030302	G	A	0.031	77422150	0.585	0.057	360770	G	A	0.069	1	77887835	5.61E-07	0.014	14306	25.361
Vascular dementia (subcortical)	genus Flavonifractor	rs34066017	A	G	0.050	44828138	0.477	0.070	360770	A	G	0.076	11	44849689	1.52E-06	0.016	14306	22.878
Vascular dementia (subcortical)	genus Flavonifractor	rs806808	C	T	0.062	32092258	0.284	0.058	360770	C	T	-0.067	10	32381186	1.18E-06	0.014	14306	23.877
Vascular dementia (subcortical)	genus Fusicatenibacter	rs10439674	A	G	0.015	41309465	0.828	0.071	360770	A	G	-0.057	21	42681392	7.68E-06	0.013	14306	19.367
Vascular dementia (subcortical)	genus Fusicatenibacter	rs167879	T	C	0.125	57362310	0.118	0.080	360770	T	C	0.066	20	55937366	5.87E-06	0.015	14306	19.656
Vascular dementia (subcortical)	genus Fusicatenibacter	rs1864685	A	C	-0.044	72725643	0.452	0.058	360770	A	C	-0.049	17	70721782	4.96E-06	0.011	14306	20.949
Vascular dementia (subcortical)	genus Fusicatenibacter	rs2025938	G	A	-0.204	110417361	0.076	0.115	360770	G	A	-0.097	10	112177119	2.99E-06	0.021	14306	22.172
Vascular dementia (subcortical)	genus Fusicatenibacter	rs206581	A	G	0.078	10370599	0.257	0.069	360770	A	G	-0.057	18	10370596	8.96E-06	0.013	14306	19.748
Vascular dementia (subcortical)	genus Fusicatenibacter	rs2132128	G	A	-0.041	15503209	0.661	0.094	360770	G	A	-0.077	8	15360718	1.08E-06	0.016	14306	23.176
Vascular dementia (subcortical)	genus Fusicatenibacter	rs3303	T	C	-0.018	118687917	0.876	0.117	360770	T	C	-0.095	10	120447429	3.94E-06	0.020	14306	21.839
Vascular dementia (subcortical)	genus Fusicatenibacter	rs4378146	A	C	0.176	24601134	0.005	0.063	360770	A	C	-0.062	1	24927625	7.20E-07	0.013	14306	24.239
Vascular dementia (subcortical)	genus Fusicatenibacter	rs60254196	G	A	0.060	149159628	0.289	0.057	360770	G	A	0.049	7	148856720	5.47E-06	0.011	14306	20.273
Vascular dementia (subcortical)	genus Fusicatenibacter	rs62187631	T	C	-0.068	225784854	0.345	0.072	360770	T	C	-0.071	2	226649570	4.55E-06	0.016	14306	19.912
Vascular dementia (subcortical)	genus Fusicatenibacter	rs62353480	A	G	-0.003	29594893	0.971	0.076	360770	A	G	-0.070	5	29595000	1.57E-06	0.015	14306	23.210
Vascular dementia (subcortical)	genus Fusicatenibacter	rs6515626	G	A	0.204	25276365	0.075	0.114	360770	G	A	0.142	20	25257001	7.29E-06	0.031	14306	20.386
Vascular dementia (subcortical)	genus Fusicatenibacter	rs704418	C	T	0.104	64267127	0.232	0.087	360770	C	T	-0.074	3	64252803	7.77E-07	0.015	14306	23.937
Vascular dementia (subcortical)	genus Fusicatenibacter	rs73103914	A	G	-0.046	58264528	0.565	0.080	360770	A	G	-0.060	12	58658311	8.30E-06	0.013	14306	19.735
Vascular dementia (subcortical)	genus Fusicatenibacter	rs792108	C	T	0.034	5392660	0.551	0.058	360770	C	T	0.051	2	5532793	8.50E-06	0.011	14306	19.939
Vascular dementia (subcortical)	genus Fusicatenibacter	rs8028026	G	A	0.043	87809984	0.660	0.097	360770	G	A	0.079	15	88353215	8.06E-06	0.018	14306	19.259
Vascular dementia (subcortical)	genus Fusicatenibacter	rs8063430	T	C	0.055	73787906	0.674	0.131	360770	T	C	-0.104	16	73821805	4.93E-06	0.022	14306	21.924
Vascular dementia (subcortical)	genus Fusicatenibacter	rs9905659	G	A	-0.071	72701205	0.335	0.073	360770	G	A	-0.062	17	70697344	7.31E-06	0.014	14306	20.354
Vascular dementia (subcortical)	genus Gordonibacter	rs13412653	A	C	0.008	29935628	0.898	0.059	360770	A	C	0.108	2	30158494	8.61E-06	0.024	14306	20.229
Vascular dementia (subcortical)	genus Gordonibacter	rs16955299	G	A	0.001	54879849	0.994	0.096	360770	G	A	-0.196	17	52957210	6.37E-06	0.043	14306	20.527
Vascular dementia (subcortical)	genus Gordonibacter	rs322296	G	A	-0.228	137252442	0.036	0.109	360770	G	A	0.179	7	136937189	4.02E-06	0.038	14306	22.439

Vascular dementia (subcortical)	genus Gordonibacter	rs35042269	C	A	-0.089	8140880	0.328	0.091	360770	C	A	-0.180	4	8142607	8.11E-06	0.040	14306	19.985
Vascular dementia (subcortical)	genus Gordonibacter	rs3765837	T	G	0.052	210455105	0.636	0.110	360770	T	G	-0.191	1	210628449	7.17E-06	0.043	14306	19.348
Vascular dementia (subcortical)	genus Gordonibacter	rs4596722	G	A	0.047	86172229	0.408	0.057	360770	G	A	-0.103	9	88787144	9.06E-06	0.023	14306	19.748
Vascular dementia (subcortical)	genus Gordonibacter	rs71545975	A	G	0.133	47104907	0.077	0.075	360770	A	G	-0.154	7	47144505	7.04E-06	0.034	14306	20.639
Vascular dementia (subcortical)	genus Gordonibacter	rs72714787	C	A	0.017	135847346	0.842	0.085	360770	C	A	0.181	4	136768501	1.43E-06	0.038	14306	23.141
Vascular dementia (subcortical)	genus Gordonibacter	rs72939513	A	G	0.136	82670413	0.289	0.128	360770	A	G	-0.214	1	83136096	7.98E-06	0.049	14306	19.026
Vascular dementia (subcortical)	genus Gordonibacter	rs7294633	T	C	0.025	28871601	0.695	0.064	360770	T	C	-0.129	12	29024534	3.44E-07	0.025	14306	26.501
Vascular dementia (subcortical)	genus Gordonibacter	rs768830	A	G	0.006	18930984	0.937	0.079	360770	A	G	-0.150	7	18970607	7.76E-06	0.033	14306	20.212
Vascular dementia (subcortical)	genus Haemophilus	rs10781340	A	G	-0.124	76137254	0.151	0.086	360770	A	G	-0.095	9	78752170	4.32E-06	0.020	14306	21.803
Vascular dementia (subcortical)	genus Haemophilus	rs111582866	G	A	-0.002	48708578	0.983	0.101	360770	G	A	-0.124	16	48742489	1.27E-06	0.026	14306	22.815
Vascular dementia (subcortical)	genus Haemophilus	rs35509	G	A	-0.092	115055532	0.525	0.145	360770	G	A	0.128	12	115493337	2.01E-06	0.027	14306	22.768
Vascular dementia (subcortical)	genus Haemophilus	rs4822728	T	C	-0.085	26495842	0.135	0.057	360770	T	C	0.071	22	26891808	3.48E-06	0.015	14306	21.740
Vascular dementia (subcortical)	genus Haemophilus	rs76022354	C	T	-0.127	92546628	0.334	0.131	360770	C	T	0.245	10	94306385	1.83E-06	0.051	14306	23.421
Vascular dementia (subcortical)	genus Haemophilus	rs78909003	T	C	0.109	102887960	0.372	0.122	360770	T	C	-0.246	9	105650242	1.67E-06	0.050	14306	23.881
Vascular dementia (subcortical)	genus Haemophilus	rs9328464	T	C	-0.112	8350684	0.051	0.057	360770	T	C	0.072	6	8350917	1.42E-06	0.015	14306	23.615
Vascular dementia (subcortical)	genus Haemophilus	rs9382510	C	T	-0.026	55583693	0.684	0.065	360770	C	T	-0.094	6	55448491	7.12E-08	0.017	14306	29.342
Vascular dementia (subcortical)	genus Haemophilus	rs9895850	T	C	0.113	66538895	0.416	0.139	360770	T	C	-0.193	17	64535013	2.14E-06	0.042	14306	21.437
Vascular dementia (subcortical)	genus Holdemanella	rs12513188	G	A	0.008	70135074	0.901	0.064	360770	G	A	0.090	4	71000791	4.65E-06	0.020	14306	21.426
Vascular dementia (subcortical)	genus Holdemanella	rs17586763	T	C	-0.112	50357927	0.377	0.127	360770	T	C	-0.227	13	50932063	7.72E-06	0.051	14306	19.850
Vascular dementia (subcortical)	genus Holdemanella	rs1926302	G	A	-0.015	64687657	0.831	0.068	360770	G	A	-0.108	1	65153340	7.50E-06	0.023	14306	21.768
Vascular dementia (subcortical)	genus Holdemanella	rs34187114	C	A	-0.001	137611390	0.990	0.091	360770	C	A	-0.105	8	138623633	5.13E-06	0.023	14306	21.381
Vascular dementia (subcortical)	genus Holdemanella	rs35228298	G	A	0.112	85343732	0.153	0.078	360770	G	A	0.093	3	85392882	7.30E-06	0.020	14306	21.231
Vascular dementia (subcortical)	genus Holdemanella	rs4541991	T	C	0.027	102242553	0.660	0.061	360770	T	C	-0.093	9	105004835	2.10E-06	0.019	14306	22.747
Vascular dementia (subcortical)	genus Holdemanella	rs607782	C	T	-0.036	4148375	0.546	0.059	360770	C	T	0.085	6	4148609	7.19E-07	0.017	14306	24.518
Vascular dementia (subcortical)	genus Holdemanella	rs62113381	T	C	-0.030	7603339	0.720	0.084	360770	T	C	-0.105	19	7668225	5.54E-06	0.023	14306	20.653
Vascular dementia (subcortical)	genus Holdemanella	rs73011279	T	C	0.124	14909283	0.068	0.068	360770	T	C	-0.096	19	15020095	1.36E-06	0.020	14306	23.274
Vascular dementia (subcortical)	genus Holdemanella	rs75764681	T	C	-0.054	4452438	0.698	0.139	360770	T	C	-0.283	10	4494630	1.94E-06	0.060	14306	22.338
Vascular dementia (subcortical)	genus Holdemanella	rs8113760	G	A	0.104	43347783	0.090	0.061	360770	G	A	0.079	19	43851935	4.62E-06	0.017	14306	20.756
Vascular dementia (subcortical)	genus Holdemania	rs10885477	T	C	-0.034	113572654	0.796	0.130	360770	T	C	-0.135	10	115332413	8.60E-06	0.030	14306	20.037
Vascular dementia (subcortical)	genus Holdemania	rs11080063	G	A	0.049	28462253	0.394	0.058	360770	G	A	-0.067	17	26789271	6.67E-06	0.015	14306	19.711
Vascular dementia (subcortical)	genus Holdemania	rs111745969	A	G	0.106	92566992	0.201	0.083	360770	A	G	0.121	15	93110222	3.71E-06	0.027	14306	20.616
Vascular dementia (subcortical)	genus Holdemania	rs113593397	A	G	-0.023	122961600	0.809	0.097	360770	A	G	-0.129	8	123973840	9.36E-06	0.028	14306	20.827
Vascular dementia (subcortical)	genus Holdemania	rs116500994	G	T	0.019	81111408	0.884	0.134	360770	G	T	-0.138	13	81685543	2.34E-06	0.029	14306	21.981
Vascular dementia (subcortical)	genus Holdemania	rs12701617	A	G	0.028	38253256	0.618	0.057	360770	A	G	-0.066	7	38292857	9.52E-06	0.015	14306	19.545
Vascular dementia (subcortical)	genus Holdemania	rs1867876	T	C	-0.080	18746521	0.197	0.062	360770	T	C	0.084	11	18768068	2.74E-07	0.016	14306	27.009
Vascular dementia (subcortical)	genus Holdemania	rs4146507	C	T	0.016	106873392	0.809	0.066	360770	C	T	0.079	5	106209093	7.23E-06	0.018	14306	20.167
Vascular dementia (subcortical)	genus Holdemania	rs73139538	G	A	0.328	63862767	0.057	0.172	360770	G	A	-0.149	7	63323145	7.77E-06	0.033	14306	20.590
Vascular dementia (subcortical)	genus Holdemania	rs77293403	A	G	0.154	78092582	0.315	0.154	360770	A	G	0.165	5	77388406	1.77E-06	0.034	14306	23.182
Vascular dementia (subcortical)	genus Holdemania	rs80149660	C	T	0.038	128110018	0.786	0.139	360770	C	T	-0.233	10	129908282	6.04E-06	0.052	14306	20.138
Vascular dementia (subcortical)	genus Holdemania	rs9500080	C	T	0.052	105289447	0.487	0.075	360770	C	T	0.093	6	105737322	4.09E-07	0.018	14306	26.840
Vascular dementia (subcortical)	genus Holdemania	rs9529719	C	T	-0.021	70282384	0.729	0.060	360770	C	T	-0.074	13	70856516	5.97E-06	0.016	14306	21.281
Vascular dementia (subcortical)	genus Holdemania	rs967319	T	C	-0.028	60629947	0.673	0.066	360770	T	C	0.079	3	60615680	8.38E-06	0.018	14306	19.910
Vascular dementia (subcortical)	genus Howardella	rs10048062	C	T	0.115	97933690	0.246	0.099	360770	C	T	-0.147	15	98476920	8.59E-06	0.034	14306	19.172
Vascular dementia (subcortical)	genus Howardella	rs12452946	A	G	0.050	17349974	0.375	0.057	360770	A	G	-0.106	17	17253288	3.80E-06	0.023	14306	21.370
Vascular dementia (subcortical)	genus Howardella	rs1484873	G	A	-0.011	45627020	0.895	0.079	360770	G	A	0.228	18	43206985	2.56E-06	0.046	14306	24.177
Vascular dementia (subcortical)	genus Howardella	rs17167098	G	A	0.079	133469602	0.332	0.082	360770	G	A	-0.169	7	133154356	1.12E-06	0.035	14306	23.142
Vascular dementia (subcortical)	genus Howardella	rs2154047	A	C	0.038	94986460	0.696	0.098	360770	A	C	0.193	14	95452797	9.97E-06	0.042	14306	21.010
Vascular dementia (subcortical)	genus Howardella	rs36081916	T	C	0.079	93898102	0.436	0.101	360770	T	C	-0.181	7	93527414	4.70E-06	0.040	14306	20.225
Vascular dementia (subcortical)	genus Howardella	rs3791893	A	G	-0.031	217954673	0.701	0.082	360770	A	G	0.147	2	218819396	9.50E-06	0.034	14306	18.677
Vascular dementia (subcortical)	genus Howardella	rs609430	T	G	0.021	168257164	0.727	0.060	360770	T	G	-0.112	4	169178315	3.34E-06	0.024	14306	21.918
Vascular dementia (subcortical)	genus Howardella	rs672217	G	A	-0.008	62457901	0.906	0.071	360770	G	A	0.164	18	60125134	3.52E-06	0.035	14306	21.996
Vascular dementia (subcortical)	genus Hungatella	rs10044993	A	C	0.011	59128613	0.911	0.103	360770	A	C	-0.140	5	58424440	8.07E-06	0.032	14306	19.409
Vascular dementia (subcortical)	genus Hungatella	rs13128780	T	C	0.077	165137846	0.286	0.072	360770	T	C	-0.150	4	166058998	1.75E-06	0.031	14306	22.915
Vascular dementia (subcortical)	genus Hungatella	rs13249325	T	G	-0.048	14996016	0.398	0.057	360770	T	G	-0.100	8	14853525	9.69E-06	0.023	14306	19.608
Vascular dementia (subcortical)	genus Hungatella	rs17092615	G	A	-0.152	95507282	0.062	0.081	360770	G	A	0.152	14	95973619	7.38E-06	0.034	14306	20.302
Vascular dementia (subcortical)	genus Hungatella	rs72759041	G	T	0.036	89034145	0.606	0.070	360770	G	T	-0.126	15	89577376	3.86E-06	0.028	14306	19.937
Vascular dementia (subcortical)	genus Intestinibacter	rs10805326	A	G	0.004	14322999	0.946	0.063	360770	A	G	-0.078	4	14324623	3.55E-08	0.014	14306	30.803
Vascular dementia (subcortical)	genus Intestinibacter	rs11109097	T	C	-0.070	97534659	0.224	0.057	360770	T	C	-0.062	12	97928437	5.49E-06	0.014	14306	20.305
Vascular dementia (subcortical)	genus Intestinibacter	rs118030283	G	A	0.134	5943075	0.319	0.135	360770	G	A	-0.152	16	5993076	2.67E-06	0.032	14306	21.896
Vascular dementia (subcortical)	genus Intestinibacter	rs16938435	T	C	-0.024	21502924	0.803	0.096	360770	T	C	-0.112	9	21502923	1.80E-06	0.024	14306	22.706
Vascular dementia (subcortical)	genus Intestinibacter	rs2098844	T	C	0.017	127833265	0.768	0.059	360770	T	C	0.058	11	127703160	6.79E-06	0.013	14306	20.070
Vascular dementia (subcortical)	genus Intestinibacter	rs2702387	G	A	-0.017	178440108	0.765	0.058	360770	G	A	-0.061	4	179361262	4.26E-06	0.013	14306	21.208
Vascular dementia (subcortical)	genus Intestinibacter	rs4327025	G	A	0.008	91903453	0.915	0.073	360770	G	A	-0.081	15	92446683	1.64E-07	0.015	14306	27.546
Vascular dementia (subcortical)	genus Intestinibacter	rs447950	A	G	0.007	149466863	0.909	0.059	360770	A	G	0.063	5	148846426	5.64E-06	0.014	14306	21.143
Vascular dementia (subcortical)	genus Intestinibacter	rs478972	C	T	0.193	125793289	0.049	0.098	360770	C	T	0.143	11	125663184	1.82E-06	0.030	14306	23.061
Vascular dementia (subcortical)	genus Intestinibacter	rs6062862	A	G	-0.024	62693871	0.821	0.104	360770	A	G	0.092	20	61325223	6.68E-06	0.020	14306	20.406
Vascular dementia (subcortical)	genus Intestinibacter	rs62430350	T	C	0.092	170609106	0.538	0.149	360770	T	C	0.151	6	170918194	6.84E-06	0.035	14306	18.481

Vascular dementia (subcortical)	genus Intestinibacter	rs68093214	C	T	-0.060	70608634	0.367	0.066	360770	C	T	0.066	3	70657785	9.26E-06	0.015	14306	19.525
Vascular dementia (subcortical)	genus Intestinibacter	rs6875660	C	T	-0.047	160259249	0.696	0.119	360770	C	T	0.089	5	159686256	3.06E-06	0.019	14306	21.089
Vascular dementia (subcortical)	genus Intestinibacter	rs893394	G	A	0.077	19855210	0.183	0.058	360770	G	A	0.058	2	20054971	7.85E-06	0.013	14306	19.910
Vascular dementia (subcortical)	genus Intestinibacter	rs9348442	C	T	-0.023	10303712	0.789	0.085	360770	C	T	0.099	6	10303945	6.26E-06	0.022	14306	19.987
Vascular dementia (subcortical)	genus Intestinimonas	rs10262702	T	C	-0.046	67425856	0.593	0.087	360770	T	C	0.092	7	66890843	2.06E-06	0.019	14306	22.189
Vascular dementia (subcortical)	genus Intestinimonas	rs11258178	A	G	0.058	13082513	0.306	0.057	360770	A	G	0.066	10	13124513	6.98E-07	0.013	14306	24.264
Vascular dementia (subcortical)	genus Intestinimonas	rs12226153	A	G	-0.269	94631127	0.259	0.238	360770	A	G	-0.151	11	94364293	5.12E-07	0.031	14306	24.250
Vascular dementia (subcortical)	genus Intestinimonas	rs17067892	C	T	-0.133	3906018	0.184	0.100	360770	C	T	0.107	8	3763540	6.38E-06	0.025	14306	18.383
Vascular dementia (subcortical)	genus Intestinimonas	rs1859797	G	A	-0.147	21967685	0.008	0.056	360770	G	A	0.060	7	22007303	4.12E-06	0.013	14306	20.981
Vascular dementia (subcortical)	genus Intestinimonas	rs2276760	A	G	0.099	150903169	0.135	0.066	360770	A	G	-0.069	3	150620956	7.84E-06	0.015	14306	20.178
Vascular dementia (subcortical)	genus Intestinimonas	rs2731794	C	T	-0.277	17209282	0.075	0.155	360770	C	T	0.121	5	17209391	1.92E-06	0.026	14306	21.942
Vascular dementia (subcortical)	genus Intestinimonas	rs2930225	T	G	0.008	85294296	0.909	0.068	360770	T	G	-0.073	16	85327902	1.35E-06	0.015	14306	22.751
Vascular dementia (subcortical)	genus Intestinimonas	rs4113676	A	C	-0.047	6238275	0.856	0.261	360770	A	C	-0.219	20	6218922	7.42E-06	0.049	14306	19.873
Vascular dementia (subcortical)	genus Intestinimonas	rs4784055	T	C	0.129	58784866	0.323	0.130	360770	T	C	-0.175	16	58818770	8.72E-07	0.039	14306	20.631
Vascular dementia (subcortical)	genus Intestinimonas	rs62240188	G	A	0.180	10569458	0.073	0.100	360770	G	A	0.130	3	10611142	2.20E-06	0.027	14306	23.702
Vascular dementia (subcortical)	genus Intestinimonas	rs6934519	C	T	-0.016	66490909	0.801	0.065	360770	C	T	0.069	6	67200802	8.57E-06	0.015	14306	20.982
Vascular dementia (subcortical)	genus Intestinimonas	rs716604	A	G	-0.126	6373956	0.061	0.067	360770	A	G	0.082	2	6514088	8.57E-07	0.017	14306	24.289
Vascular dementia (subcortical)	genus Intestinimonas	rs7170984	T	C	0.024	93276506	0.704	0.063	360770	T	C	-0.066	15	93819735	2.98E-06	0.014	14306	21.858
Vascular dementia (subcortical)	genus Intestinimonas	rs72982915	C	T	-0.118	140140519	0.327	0.121	360770	C	T	0.183	2	140898088	4.91E-06	0.040	14306	20.682
Vascular dementia (subcortical)	genus Intestinimonas	rs9823439	C	T	0.022	17146010	0.695	0.057	360770	C	T	0.058	3	17187502	9.86E-06	0.013	14306	19.598
Vascular dementia (subcortical)	genus Lachnoclostridium	rs1031599	T	G	-0.073	66675401	0.528	0.116	360770	T	G	0.079	3	66725825	6.31E-06	0.018	14306	20.039
Vascular dementia (subcortical)	genus Lachnoclostridium	rs12566975	T	C	-0.037	185122219	0.518	0.057	360770	T	C	-0.047	1	185091351	9.57E-06	0.011	14306	19.580
Vascular dementia (subcortical)	genus Lachnoclostridium	rs1528479	A	G	-0.010	166387541	0.859	0.059	360770	A	G	0.050	2	167244051	9.64E-06	0.011	14306	19.783
Vascular dementia (subcortical)	genus Lachnoclostridium	rs1997204	C	T	0.078	101652817	0.569	0.136	360770	C	T	0.108	12	102046595	5.97E-06	0.024	14306	19.941
Vascular dementia (subcortical)	genus Lachnoclostridium	rs2385421	A	G	0.086	22163494	0.330	0.088	360770	A	G	0.075	18	19743455	7.14E-06	0.018	14306	17.046
Vascular dementia (subcortical)	genus Lachnoclostridium	rs3821998	C	A	0.047	38692945	0.614	0.093	360770	C	A	-0.086	4	38694566	6.72E-06	0.019	14306	20.144
Vascular dementia (subcortical)	genus Lachnoclostridium	rs4738679	A	G	-0.036	58457761	0.538	0.058	360770	A	G	0.052	8	59370320	4.42E-06	0.011	14306	20.813
Vascular dementia (subcortical)	genus Lachnoclostridium	rs6112314	A	C	-0.069	19320202	0.252	0.060	360770	A	C	-0.056	20	19300846	2.43E-07	0.011	14306	26.964
Vascular dementia (subcortical)	genus Lachnoclostridium	rs615997	T	C	-0.031	22996295	0.583	0.057	360770	T	C	0.051	3	23037786	2.03E-06	0.011	14306	23.094
Vascular dementia (subcortical)	genus Lachnoclostridium	rs62285313	A	G	0.095	177752244	0.322	0.096	360770	A	G	0.086	3	177470032	1.58E-06	0.018	14306	22.655
Vascular dementia (subcortical)	genus Lachnoclostridium	rs72829893	G	T	0.240	48617179	0.005	0.086	360770	G	T	0.117	17	46694541	5.58E-06	0.027	14306	19.198
Vascular dementia (subcortical)	genus Lachnoclostridium	rs78068103	A	G	0.058	13912842	0.517	0.089	360770	A	G	0.089	17	13816159	3.67E-06	0.019	14306	20.814
Vascular dementia (subcortical)	genus Lachnoclostridium	rs789029	C	T	0.107	1053251	0.187	0.081	360770	C	T	-0.064	18	1053252	3.75E-06	0.014	14306	21.603
Vascular dementia (subcortical)	genus Lachnospiraceae FCS020 group	rs10093861	G	A	-0.086	120232167	0.134	0.058	360770	G	A	-0.057	8	121244406	3.06E-06	0.012	14306	22.048
Vascular dementia (subcortical)	genus Lachnospiraceae FCS020 group	rs1254846	A	G	0.019	43850646	0.814	0.081	360770	A	G	-0.106	10	44346094	5.60E-06	0.023	14306	20.771
Vascular dementia (subcortical)	genus Lachnospiraceae FCS020 group	rs1363769	C	T	-0.075	17754496	0.645	0.163	360770	C	T	0.201	19	17865305	1.58E-06	0.045	14306	19.933
Vascular dementia (subcortical)	genus Lachnospiraceae FCS020 group	rs2322265	C	T	0.087	165666933	0.180	0.065	360770	C	T	-0.067	4	166588085	5.21E-06	0.014	14306	22.149
Vascular dementia (subcortical)	genus Lachnospiraceae FCS020 group	rs2862811	C	T	0.104	166278627	0.091	0.062	360770	C	T	-0.056	3	165996415	3.92E-06	0.012	14306	21.535
Vascular dementia (subcortical)	genus Lachnospiraceae FCS020 group	rs35035870	T	C	0.018	3227646	0.899	0.145	360770	T	C	-0.191	11	3248876	2.62E-06	0.041	14306	21.158
Vascular dementia (subcortical)	genus Lachnospiraceae FCS020 group	rs3999074	G	T	0.016	63803887	0.781	0.057	360770	G	T	-0.055	10	65563647	6.55E-06	0.012	14306	20.418
Vascular dementia (subcortical)	genus Lachnospiraceae FCS020 group	rs4452603	T	G	-0.014	71256565	0.834	0.064	360770	T	G	0.060	6	71966268	8.98E-06	0.014	14306	19.748
Vascular dementia (subcortical)	genus Lachnospiraceae FCS020 group	rs7249113	G	A	-0.065	2571234	0.295	0.062	360770	G	A	0.068	19	2571232	3.72E-07	0.013	14306	25.907
Vascular dementia (subcortical)	genus Lachnospiraceae FCS020 group	rs72793667	A	G	-0.051	53719294	0.723	0.144	360770	A	G	-0.117	2	53946431	1.63E-06	0.025	14306	22.467
Vascular dementia (subcortical)	genus Lachnospiraceae FCS020 group	rs9308097	G	A	0.012	164865326	0.830	0.057	360770	G	A	-0.055	4	165786478	7.47E-06	0.012	14306	20.057
Vascular dementia (subcortical)	genus Lachnospiraceae FCS020 group	rs9788306	C	T	-0.135	39845177	0.032	0.063	360770	C	T	-0.063	13	40419314	1.39E-06	0.013	14306	23.074
Vascular dementia (subcortical)	genus Lachnospiraceae NC2004 group	rs117467633	T	C	0.087	21254094	0.550	0.145	360770	T	C	-0.170	17	21157406	9.13E-06	0.038	14306	19.612
Vascular dementia (subcortical)	genus Lachnospiraceae NC2004 group	rs12127733	G	A	0.079	234021975	0.287	0.074	360770	G	A	0.115	1	234157721	3.11E-06	0.025	14306	21.922
Vascular dementia (subcortical)	genus Lachnospiraceae NC2004 group	rs12208226	C	A	-0.047	22725679	0.622	0.095	360770	C	A	-0.155	6	22725908	9.75E-06	0.034	14306	20.668
Vascular dementia (subcortical)	genus Lachnospiraceae NC2004 group	rs12863463	G	A	-0.114	46938056	0.281	0.106	360770	G	A	-0.156	13	47512191	6.04E-06	0.035	14306	20.498
Vascular dementia (subcortical)	genus Lachnospiraceae NC2004 group	rs17067076	G	A	-0.108	60561853	0.226	0.089	360770	G	A	-0.155	18	58229086	5.61E-06	0.035	14306	19.277
Vascular dementia (subcortical)	genus Lachnospiraceae NC2004 group	rs1928659	T	C	-0.068	29334139	0.334	0.070	360770	T	C	0.103	9	29334137	6.17E-06	0.023	14306	20.498
Vascular dementia (subcortical)	genus Lachnospiraceae NC2004 group	rs1929743	T	C	0.042	76255426	0.487	0.060	360770	T	C	0.084	13	76829562	9.06E-06	0.019	14306	19.351
Vascular dementia (subcortical)	genus Lachnospiraceae NC2004 group	rs3756315	A	G	-0.077	150165159	0.211	0.062	360770	A	G	-0.088	5	149544722	3.33E-06	0.019	14306	21.990
Vascular dementia (subcortical)	genus Lachnospiraceae NC2004 group	rs6116753	G	A	0.060	5350054	0.418	0.074	360770	G	A	0.099	20	5330700	2.92E-06	0.021	14306	22.623
Vascular dementia (subcortical)	genus Lachnospiraceae ND3007 group	rs2861203	G	A	-0.002	173232763	0.977	0.062	360770	G	A	0.057	3	172950553	7.37E-06	0.013	14306	20.218
Vascular dementia (subcortical)	genus Lachnospiraceae ND3007 group	rs72776675	T	C	0.031	14095664	0.689	0.077	360770	T	C	-0.065	10	14137663	8.72E-06	0.015	14306	19.123
Vascular dementia (subcortical)	genus Lachnospiraceae ND3007 group	rs9932954	A	G	-0.032	1050633	0.593	0.060	360770	A	G	-0.056	16	1100633	1.25E-06	0.012	14306	23.467
Vascular dementia (subcortical)	genus Lachnospiraceae NK4A136 group	rs10952110	G	T	0.104	8458292	0.070	0.057	360770	G	T	0.049	7	8497922	9.08E-06	0.011	14306	19.797
Vascular dementia (subcortical)	genus Lachnospiraceae NK4A136 group	rs11263806	A	G	-0.061	36882840	0.304	0.060	360770	A	G	-0.052	17	35240097	5.07E-06	0.012	14306	20.189
Vascular dementia (subcortical)	genus Lachnospiraceae NK4A136 group	rs12611395	G	A	-0.013	21623323	0.888	0.093	360770	G	A	0.090	19	21806125	5.83E-06	0.020	14306	20.435
Vascular dementia (subcortical)	genus Lachnospiraceae NK4A136 group	rs160061	G	A	-0.074	6116546	0.194	0.057	360770	G	A	-0.051	5	6116659	2.12E-06	0.011	14306	22.596
Vascular dementia (subcortical)	genus Lachnospiraceae NK4A136 group	rs28540839	A	C	-0.020	83822845	0.724	0.057	360770	A	C	0.051	8	84735080	9.34E-06	0.011	14306	21.124
Vascular dementia (subcortical)	genus Lachnospiraceae NK4A136 group	rs2880566	T	C	0.014	31689634	0.856	0.080	360770	T	C	0.060	17	30016653	5.61E-06	0.013	14306	19.815
Vascular dementia (subcortical)	genus Lachnospiraceae NK4A136 group	rs4955932	T	C	-0.033	55147348	0.572	0.059	360770	T	C	-0.049	3	55181375	7.05E-06	0.011	14306	20.253
Vascular dementia (subcortical)	genus Lachnospiraceae NK4A136 group	rs59805249	T	C	-0.119	90994180	0.219	0.097	360770	T	C	0.094	5	90289997	9.45E-06	0.021	14306	20.261
Vascular dementia (subcortical)	genus Lachnospiraceae NK4A136 group	rs68104925	T	C	0.041	99711344	0.504	0.062	360770	T	C	-0.055	14	100177681	2.37E-06	0.012	14306	22.647
Vascular dementia (subcortical)	genus Lachnospiraceae NK4A136 group	rs7073658	G	T	0.140	60415266	0.014	0.057	360770	G	T	0.050	10	62175024	5.27E-06	0.011	14306	20.745

Vascular dementia (subcortical)	genus Lachnospiraceae NK4A136 group	rs73044693	A	G	-0.114	50752863	0.306	0.112	360770	A	G	-0.108	19	51256120	3.57E-06	0.023	14306	21.900
Vascular dementia (subcortical)	genus Lachnospiraceae NK4A136 group	rs7616165	G	T	0.033	190697356	0.858	0.185	360770	G	T	-0.231	3	190415145	2.77E-06	0.048	14306	22.739
Vascular dementia (subcortical)	genus Lachnospiraceae NK4A136 group	rs76193507	A	G	-0.093	162032193	0.359	0.101	360770	A	G	-0.230	3	161749981	2.93E-06	0.050	14306	21.129
Vascular dementia (subcortical)	genus Lachnospiraceae NK4A136 group	rs7832116	A	G	0.012	4985407	0.890	0.086	360770	A	G	-0.071	8	4842929	3.57E-06	0.015	14306	22.199
Vascular dementia (subcortical)	genus Lachnospiraceae NK4A136 group	rs954878	A	G	-0.068	54112728	0.247	0.059	360770	A	G	-0.052	1	54578401	1.78E-06	0.011	14306	22.782
Vascular dementia (subcortical)	genus Lachnospiraceae UCG001	rs12131224	C	T	-0.055	166230125	0.549	0.092	360770	C	T	0.117	1	166199362	7.40E-06	0.026	14306	20.424
Vascular dementia (subcortical)	genus Lachnospiraceae UCG001	rs2050911	G	A	0.045	81918723	0.449	0.060	360770	G	A	0.075	1	82384407	1.11E-06	0.015	14306	23.831
Vascular dementia (subcortical)	genus Lachnospiraceae UCG001	rs2371284	C	T	-0.069	55862478	0.309	0.068	360770	C	T	0.076	12	56256262	7.56E-06	0.017	14306	20.056
Vascular dementia (subcortical)	genus Lachnospiraceae UCG001	rs437876	T	C	0.048	42526948	0.419	0.060	360770	T	C	0.078	3	42568440	7.17E-08	0.014	14306	29.376
Vascular dementia (subcortical)	genus Lachnospiraceae UCG001	rs4981345	T	C	-0.034	20987814	0.575	0.061	360770	T	C	-0.068	14	21455973	6.09E-06	0.015	14306	20.717
Vascular dementia (subcortical)	genus Lachnospiraceae UCG001	rs573933	T	C	-0.001	14477853	0.992	0.092	360770	T	C	-0.108	9	14477851	3.11E-06	0.023	14306	21.565
Vascular dementia (subcortical)	genus Lachnospiraceae UCG001	rs62496417	T	G	0.049	97336192	0.478	0.069	360770	T	G	-0.075	7	96965504	5.88E-06	0.017	14306	20.410
Vascular dementia (subcortical)	genus Lachnospiraceae UCG001	rs7341608	T	C	0.256	55926941	0.001	0.077	360770	T	C	-0.078	8	56839500	9.48E-06	0.018	14306	19.514
Vascular dementia (subcortical)	genus Lachnospiraceae UCG001	rs74034332	G	A	-0.046	79058462	0.697	0.118	360770	G	A	0.168	16	79092359	3.33E-06	0.038	14306	19.288
Vascular dementia (subcortical)	genus Lachnospiraceae UCG001	rs78848836	A	G	-0.041	53307576	0.657	0.093	360770	A	G	-0.119	1	53773248	3.38E-06	0.026	14306	20.942
Vascular dementia (subcortical)	genus Lachnospiraceae UCG001	rs8104225	A	G	0.066	13852110	0.340	0.069	360770	A	G	0.089	19	13962924	8.04E-06	0.020	14306	20.369
Vascular dementia (subcortical)	genus Lachnospiraceae UCG001	rs9403580	C	T	0.050	144685644	0.559	0.085	360770	C	T	0.108	6	145006780	3.47E-06	0.023	14306	22.011
Vascular dementia (subcortical)	genus Lachnospiraceae UCG001	rs985416	T	C	0.154	148551296	0.037	0.074	360770	T	C	-0.097	3	148269083	1.46E-07	0.018	14306	28.481
Vascular dementia (subcortical)	genus Lachnospiraceae UCG004	rs11128180	A	G	-0.027	70543064	0.687	0.067	360770	A	G	0.065	3	70592215	4.52E-06	0.014	14306	21.404
Vascular dementia (subcortical)	genus Lachnospiraceae UCG004	rs12072562	T	C	-0.116	104744847	0.427	0.146	360770	T	C	0.133	1	105287469	7.07E-06	0.030	14306	19.225
Vascular dementia (subcortical)	genus Lachnospiraceae UCG004	rs12673420	G	A	0.042	71838022	0.462	0.057	360770	G	A	0.055	7	71303007	2.98E-06	0.012	14306	21.931
Vascular dementia (subcortical)	genus Lachnospiraceae UCG004	rs12747809	A	G	-0.002	240195789	0.971	0.063	360770	A	G	0.062	1	240359089	8.65E-07	0.013	14306	24.489
Vascular dementia (subcortical)	genus Lachnospiraceae UCG004	rs12894272	G	A	0.011	40067277	0.859	0.060	360770	G	A	-0.058	14	40536481	4.34E-06	0.013	14306	21.436
Vascular dementia (subcortical)	genus Lachnospiraceae UCG004	rs233486	G	A	0.033	14817353	0.692	0.082	360770	G	A	0.080	6	14817584	6.28E-06	0.018	14306	20.231
Vascular dementia (subcortical)	genus Lachnospiraceae UCG004	rs2444793	T	C	0.041	80609119	0.478	0.058	360770	T	C	0.054	6	81318836	4.77E-06	0.012	14306	21.064
Vascular dementia (subcortical)	genus Lachnospiraceae UCG004	rs2726805	A	G	-0.017	182326448	0.766	0.057	360770	A	G	0.055	4	183247601	6.30E-06	0.012	14306	20.594
Vascular dementia (subcortical)	genus Lachnospiraceae UCG004	rs2882478	G	A	-0.054	49677451	0.344	0.057	360770	G	A	-0.058	2	49904589	1.21E-06	0.012	14306	23.782
Vascular dementia (subcortical)	genus Lachnospiraceae UCG004	rs35182105	A	G	0.071	24419403	0.572	0.125	360770	A	G	-0.110	12	24572337	4.87E-06	0.024	14306	20.522
Vascular dementia (subcortical)	genus Lachnospiraceae UCG004	rs6656451	T	C	0.132	65524739	0.020	0.057	360770	T	C	0.054	1	65990422	5.57E-06	0.012	14306	20.703
Vascular dementia (subcortical)	genus Lachnospiraceae UCG004	rs7629954	A	G	-0.172	154524617	0.210	0.137	360770	A	G	0.108	3	154242406	5.77E-06	0.024	14306	20.717
Vascular dementia (subcortical)	genus Lachnospiraceae UCG008	rs10741777	T	C	0.077	19551010	0.212	0.062	360770	T	C	-0.097	11	19572557	7.69E-07	0.019	14306	24.994
Vascular dementia (subcortical)	genus Lachnospiraceae UCG008	rs10793103	T	C	-0.014	74680730	0.799	0.057	360770	T	C	-0.097	11	74391775	9.35E-08	0.018	14306	28.889
Vascular dementia (subcortical)	genus Lachnospiraceae UCG008	rs10801803	G	A	0.037	90353314	0.645	0.081	360770	G	A	-0.117	1	90818872	1.40E-06	0.024	14306	23.178
Vascular dementia (subcortical)	genus Lachnospiraceae UCG008	rs13024781	T	C	-0.085	167772361	0.135	0.057	360770	T	C	-0.080	2	168628871	2.29E-06	0.017	14306	22.380
Vascular dementia (subcortical)	genus Lachnospiraceae UCG008	rs57091572	A	G	-0.074	80505086	0.379	0.084	360770	A	G	-0.110	6	81214803	2.86E-06	0.024	14306	21.937
Vascular dementia (subcortical)	genus Lachnospiraceae UCG008	rs61944774	A	G	-0.106	129055273	0.411	0.129	360770	A	G	0.180	12	129539818	6.34E-06	0.039	14306	20.855
Vascular dementia (subcortical)	genus Lachnospiraceae UCG008	rs62277846	C	T	0.078	227235424	0.274	0.071	360770	C	T	0.102	2	228100140	1.59E-06	0.021	14306	23.212
Vascular dementia (subcortical)	genus Lachnospiraceae UCG008	rs67078837	T	C	-0.098	113299491	0.090	0.058	360770	T	C	-0.085	4	114220647	7.68E-07	0.017	14306	24.556
Vascular dementia (subcortical)	genus Lachnospiraceae UCG008	rs75356640	G	A	-0.126	31405081	0.151	0.088	360770	G	A	0.137	15	31697284	9.83E-06	0.030	14306	20.284
Vascular dementia (subcortical)	genus Lachnospiraceae UCG008	rs955844	A	C	0.051	84958708	0.536	0.082	360770	A	C	0.112	16	84992314	1.81E-06	0.023	14306	24.080
Vascular dementia (subcortical)	genus Lachnospiraceae UCG010	rs10414815	C	T	-0.253	42082093	0.065	0.137	360770	C	T	-0.105	19	42586245	4.24E-06	0.023	14306	20.610
Vascular dementia (subcortical)	genus Lachnospiraceae UCG010	rs11192447	A	G	-0.045	82088641	0.728	0.130	360770	A	G	0.127	10	83848397	4.69E-07	0.024	14306	27.039
Vascular dementia (subcortical)	genus Lachnospiraceae UCG010	rs12346653	C	T	0.082	89652324	0.241	0.070	360770	C	T	0.066	9	92267239	2.70E-06	0.014	14306	22.205
Vascular dementia (subcortical)	genus Lachnospiraceae UCG010	rs17730011	G	A	0.027	84063126	0.700	0.069	360770	G	A	-0.070	6	84772845	7.85E-06	0.016	14306	19.998
Vascular dementia (subcortical)	genus Lachnospiraceae UCG010	rs2833528	T	C	-0.012	31821255	0.838	0.059	360770	T	C	0.056	21	33193567	9.92E-06	0.013	14306	19.359
Vascular dementia (subcortical)	genus Lachnospiraceae UCG010	rs336138	G	T	-0.046	8146258	0.602	0.088	360770	G	T	0.078	5	8146371	7.48E-06	0.017	14306	20.573
Vascular dementia (subcortical)	genus Lachnospiraceae UCG010	rs4576377	C	A	-0.043	81780947	0.463	0.059	360770	C	A	0.057	7	81410263	7.63E-06	0.013	14306	20.272
Vascular dementia (subcortical)	genus Lachnospiraceae UCG010	rs72894957	G	A	0.104	184997864	0.583	0.190	360770	G	A	0.222	2	185862591	5.68E-06	0.049	14306	20.879
Vascular dementia (subcortical)	genus Lachnospiraceae UCG010	rs74315802	G	T	-0.017	29403135	0.816	0.074	360770	G	T	0.087	14	29872341	3.19E-06	0.018	14306	22.343
Vascular dementia (subcortical)	genus Lachnospiraceae UCG010	rs9981767	A	C	0.033	42659765	0.618	0.066	360770	A	C	0.066	21	44079875	9.96E-07	0.013	14306	24.630
Vascular dementia (subcortical)	genus Lactobacillus	rs12693845	C	T	-0.071	198447960	0.225	0.059	360770	C	T	-0.081	2	199312684	8.96E-06	0.018	14306	20.607
Vascular dementia (subcortical)	genus Lactobacillus	rs1530559	G	A	0.078	134998059	0.172	0.057	360770	G	A	0.080	2	135755629	4.93E-06	0.018	14306	20.355
Vascular dementia (subcortical)	genus Lactobacillus	rs16861661	G	A	0.219	18174965	0.059	0.116	360770	G	A	-0.183	1	18501459	1.28E-06	0.038	14306	23.049
Vascular dementia (subcortical)	genus Lactobacillus	rs62314653	C	A	0.094	108975306	0.436	0.120	360770	C	A	0.188	4	109896462	2.24E-06	0.039	14306	22.626
Vascular dementia (subcortical)	genus Lactobacillus	rs7399658	G	A	-0.040	23260829	0.589	0.074	360770	G	A	-0.107	13	23834968	3.12E-06	0.022	14306	23.313
Vascular dementia (subcortical)	genus Lactobacillus	rs768253	T	G	-0.080	68097868	0.164	0.057	360770	T	G	-0.079	8	69010103	4.25E-06	0.017	14306	21.252
Vascular dementia (subcortical)	genus Lactobacillus	rs77478751	A	G	0.144	173433875	0.105	0.089	360770	A	G	-0.220	3	173151665	7.33E-06	0.048	14306	21.361
Vascular dementia (subcortical)	genus Lactobacillus	rs921925	A	C	-0.042	6928006	0.540	0.069	360770	A	C	0.099	19	6928017	9.72E-07	0.020	14306	23.495
Vascular dementia (subcortical)	genus Lactococcus	rs10417872	G	T	0.024	28276446	0.697	0.063	360770	G	T	-0.118	19	28767353	1.29E-06	0.025	14306	23.276
Vascular dementia (subcortical)	genus Lactococcus	rs123059	C	T	0.037	2796641	0.589	0.068	360770	C	T	0.137	17	2699935	1.27E-06	0.027	14306	24.769
Vascular dementia (subcortical)	genus Lactococcus	rs12621813	G	A	-0.010	31043183	0.872	0.064	360770	G	A	0.108	2	31266049	6.61E-06	0.024	14306	20.413
Vascular dementia (subcortical)	genus Lactococcus	rs17168302	G	A	-0.037	14610596	0.686	0.091	360770	G	A	0.192	7	14650221	6.29E-06	0.042	14306	20.402
Vascular dementia (subcortical)	genus Lactococcus	rs2293361	C	T	0.050	53887727	0.687	0.125	360770	C	T	-0.199	2	54114864	1.40E-06	0.043	14306	21.369
Vascular dementia (subcortical)	genus Lactococcus	rs4766997	C	T	-0.011	112723633	0.844	0.057	360770	C	T	0.115	12	113161438	2.06E-06	0.024	14306	23.109
Vascular dementia (subcortical)	genus Lactococcus	rs55910161	C	T	0.077	69758887	0.395	0.091	360770	C	T	0.146	10	71518643	2.36E-06	0.031	14306	22.695
Vascular dementia (subcortical)	genus Lactococcus	rs6674304	C	T	0.028	116345120	0.849	0.149	360770	C	T	0.201	1	116887742	6.18E-06	0.044	14306	20.619
Vascular dementia (subcortical)	genus Marvinbryantia	rs11620597	T	C	0.034	85504955	0.855	0.186	360770	T	C	0.119	13	86079090	7.80E-06	0.027	14306	19.339

Vascular dementia (subcortical)	genus Marvinbryantia	rs1187983	C	T	-0.038	57976188	0.681	0.093	360770	C	T	-0.094	1	58441860	2.02E-06	0.019	14306	23.450
Vascular dementia (subcortical)	genus Marvinbryantia	rs146541147	G	A	0.139	4542085	0.378	0.157	360770	G	A	0.119	19	4542097	6.86E-06	0.027	14306	19.603
Vascular dementia (subcortical)	genus Marvinbryantia	rs2724813	G	A	-0.064	12474925	0.335	0.067	360770	G	A	0.084	10	12516924	6.28E-07	0.017	14306	25.180
Vascular dementia (subcortical)	genus Marvinbryantia	rs2842896	C	T	-0.061	132560525	0.290	0.058	360770	C	T	-0.065	6	132881664	7.25E-07	0.013	14306	24.519
Vascular dementia (subcortical)	genus Marvinbryantia	rs2863363	G	A	0.081	166321977	0.220	0.066	360770	G	A	-0.063	3	166039765	3.11E-06	0.014	14306	21.688
Vascular dementia (subcortical)	genus Marvinbryantia	rs3125832	A	C	0.088	211226838	0.196	0.068	360770	A	C	0.068	1	211400180	5.03E-06	0.015	14306	20.477
Vascular dementia (subcortical)	genus Marvinbryantia	rs61884471	G	A	0.160	45805010	0.079	0.091	360770	G	A	0.124	11	45826561	1.01E-06	0.025	14306	25.085
Vascular dementia (subcortical)	genus Marvinbryantia	rs72948274	A	C	-0.032	79807639	0.788	0.117	360770	A	C	-0.126	11	79518683	3.26E-06	0.027	14306	21.546
Vascular dementia (subcortical)	genus Marvinbryantia	rs8006832	G	T	0.187	21109678	0.059	0.099	360770	G	T	-0.095	14	21577837	6.58E-06	0.022	14306	19.317
Vascular dementia (subcortical)	genus Methanobrevibacter	rs10202904	G	T	0.027	124682691	0.644	0.058	360770	G	T	0.113	2	125440268	3.09E-06	0.024	14306	22.260
Vascular dementia (subcortical)	genus Methanobrevibacter	rs1334944	T	C	-0.034	110506288	0.599	0.064	360770	T	C	0.115	10	112266046	7.61E-06	0.026	14306	20.330
Vascular dementia (subcortical)	genus Methanobrevibacter	rs4802933	G	A	0.073	52422567	0.278	0.067	360770	G	A	0.136	19	52925820	9.74E-06	0.031	14306	19.373
Vascular dementia (subcortical)	genus Methanobrevibacter	rs6776814	T	C	-0.246	15011576	0.220	0.200	360770	T	C	-0.189	3	15053083	8.05E-06	0.042	14306	20.250
Vascular dementia (subcortical)	genus Methanobrevibacter	rs76029318	T	C	0.120	41389655	0.302	0.116	360770	T	C	0.223	13	41963791	1.08E-06	0.045	14306	24.060
Vascular dementia (subcortical)	genus Methanobrevibacter	rs894996	C	A	0.070	103497150	0.527	0.110	360770	C	A	0.214	4	104418307	3.82E-06	0.046	14306	22.064
Vascular dementia (subcortical)	genus Odoribacter	rs10093869	A	G	0.045	1318658	0.438	0.058	360770	A	G	-0.058	8	1266824	3.67E-06	0.013	14306	21.234
Vascular dementia (subcortical)	genus Odoribacter	rs10423795	T	C	0.054	49019831	0.356	0.059	360770	T	C	-0.055	19	49523088	6.58E-06	0.012	14306	20.657
Vascular dementia (subcortical)	genus Odoribacter	rs28417404	A	G	-0.234	70477507	0.015	0.096	360770	A	G	-0.073	14	70944224	3.68E-06	0.016	14306	20.290
Vascular dementia (subcortical)	genus Odoribacter	rs4793970	A	G	0.062	48686600	0.290	0.059	360770	A	G	-0.058	17	46763962	6.03E-06	0.013	14306	19.912
Vascular dementia (subcortical)	genus Odoribacter	rs6856150	A	G	-0.084	11905935	0.326	0.086	360770	A	G	-0.088	4	11907559	6.06E-06	0.019	14306	20.635
Vascular dementia (subcortical)	genus Odoribacter	rs74553962	T	G	0.115	68765245	0.286	0.108	360770	T	G	0.121	8	69677480	9.49E-06	0.026	14306	21.146
Vascular dementia (subcortical)	genus Odoribacter	rs77779484	G	A	-0.014	67262163	0.905	0.119	360770	G	A	-0.133	12	67655943	6.56E-07	0.027	14306	24.713
Vascular dementia (subcortical)	genus Olsenella	rs1035588	A	G	-0.007	149220676	0.910	0.059	360770	A	G	-0.108	2	150077190	4.86E-06	0.024	14306	20.850
Vascular dementia (subcortical)	genus Olsenella	rs17148768	G	A	-0.011	10735122	0.890	0.076	360770	G	A	0.140	10	10777085	2.20E-06	0.030	14306	22.570
Vascular dementia (subcortical)	genus Olsenella	rs2759329	A	G	-0.079	231824606	0.183	0.059	360770	A	G	0.111	1	231960352	3.43E-06	0.024	14306	21.947
Vascular dementia (subcortical)	genus Olsenella	rs35225860	A	G	0.051	247478968	0.731	0.148	360770	A	G	-0.224	1	247642270	3.87E-06	0.048	14306	21.486
Vascular dementia (subcortical)	genus Olsenella	rs61090148	A	G	0.066	173889347	0.251	0.057	360770	A	G	-0.105	4	174810498	6.44E-06	0.023	14306	20.515
Vascular dementia (subcortical)	genus Olsenella	rs62112538	C	T	-0.124	4925006	0.176	0.091	360770	C	T	-0.199	19	4925018	1.19E-06	0.041	14306	24.006
Vascular dementia (subcortical)	genus Olsenella	rs72691585	C	A	-0.010	21884426	0.902	0.083	360770	C	A	-0.249	9	21884425	2.95E-06	0.052	14306	22.872
Vascular dementia (subcortical)	genus Olsenella	rs7540303	C	T	0.025	179660056	0.665	0.059	360770	C	T	0.108	1	179629191	5.32E-06	0.024	14306	20.892
Vascular dementia (subcortical)	genus Olsenella	rs8066522	A	G	-0.054	61555512	0.373	0.061	360770	A	G	0.107	17	59632873	9.70E-06	0.024	14306	19.640
Vascular dementia (subcortical)	genus Olsenella	rs9460691	C	A	-0.033	10421974	0.645	0.073	360770	C	A	0.120	6	10422207	7.28E-06	0.027	14306	19.942
Vascular dementia (subcortical)	genus Oscillibacter	rs11627628	T	C	0.004	21011446	0.972	0.108	360770	T	C	0.144	14	21479605	1.01E-06	0.029	14306	24.605
Vascular dementia (subcortical)	genus Oscillibacter	rs11990279	T	C	-0.006	11258805	0.935	0.070	360770	T	C	-0.082	8	11116314	4.94E-06	0.018	14306	20.897
Vascular dementia (subcortical)	genus Oscillibacter	rs12649930	T	G	0.001	3654564	0.991	0.094	360770	T	G	0.122	4	3656291	4.09E-06	0.026	14306	21.935
Vascular dementia (subcortical)	genus Oscillibacter	rs133832	A	C	0.038	44438827	0.552	0.063	360770	A	C	-0.080	22	44834707	1.15E-06	0.016	14306	23.993
Vascular dementia (subcortical)	genus Oscillibacter	rs16866406	A	G	0.191	178592420	0.015	0.079	360770	A	G	0.099	2	179457147	3.08E-06	0.021	14306	22.426
Vascular dementia (subcortical)	genus Oscillibacter	rs16934185	A	G	0.055	1798324	0.554	0.093	360770	A	G	-0.130	9	1798324	4.38E-06	0.028	14306	21.175
Vascular dementia (subcortical)	genus Oscillibacter	rs234108	A	G	-0.120	184973539	0.038	0.058	360770	A	G	0.075	1	184942671	9.16E-07	0.015	14306	24.116
Vascular dementia (subcortical)	genus Oscillibacter	rs36095275	C	T	0.012	31800923	0.843	0.058	360770	C	T	-0.075	14	32270129	1.40E-06	0.016	14306	23.005
Vascular dementia (subcortical)	genus Oscillibacter	rs4506202	G	A	0.049	21740565	0.387	0.057	360770	G	A	0.071	8	21598077	3.21E-06	0.015	14306	21.825
Vascular dementia (subcortical)	genus Oscillibacter	rs61883564	A	G	0.014	79302798	0.869	0.083	360770	A	G	-0.101	11	79013843	3.39E-06	0.022	14306	21.029
Vascular dementia (subcortical)	genus Oscillibacter	rs75453768	G	T	0.046	114710047	0.637	0.098	360770	G	T	0.122	10	116469806	5.35E-06	0.027	14306	20.667
Vascular dementia (subcortical)	genus Oscillibacter	rs761240	G	T	-0.198	50891355	0.140	0.134	360770	G	T	0.177	20	49507892	2.04E-06	0.039	14306	20.639
Vascular dementia (subcortical)	genus Oscillibacter	rs9393920	G	A	0.048	28612816	0.413	0.058	360770	G	A	0.074	6	28580593	9.92E-07	0.015	14306	24.294
Vascular dementia (subcortical)	genus Oscillospira	rs12206468	G	A	0.147	18093460	0.171	0.108	360770	G	A	-0.133	6	18093691	1.04E-06	0.027	14306	24.319
Vascular dementia (subcortical)	genus Oscillospira	rs12925026	T	C	0.039	89726448	0.751	0.123	360770	T	C	0.136	16	89792856	9.31E-06	0.031	14306	19.534
Vascular dementia (subcortical)	genus Oscillospira	rs1954532	C	T	0.006	27682209	0.930	0.071	360770	C	T	0.083	14	28151415	2.27E-06	0.018	14306	22.228
Vascular dementia (subcortical)	genus Oscillospira	rs28889936	A	C	0.037	88562149	0.698	0.096	360770	A	C	0.114	4	89483300	3.37E-06	0.025	14306	20.348
Vascular dementia (subcortical)	genus Oscillospira	rs62422654	C	T	-0.109	170159406	0.118	0.070	360770	C	T	0.090	6	170474630	6.47E-06	0.020	14306	20.572
Vascular dementia (subcortical)	genus Oscillospira	rs72866977	A	C	-0.006	56295685	0.957	0.106	360770	A	C	-0.131	6	56160483	5.63E-06	0.028	14306	21.488
Vascular dementia (subcortical)	genus Oscillospira	rs751183	C	T	-0.102	76337408	0.173	0.075	360770	C	T	0.077	1	76803093	6.85E-06	0.017	14306	20.216
Vascular dementia (subcortical)	genus Oscillospira	rs8076323	A	G	0.044	14978543	0.470	0.060	360770	A	G	0.072	17	14881860	5.61E-06	0.016	14306	20.885
Vascular dementia (subcortical)	genus Oxalobacter	rs10464997	G	A	0.065	21045182	0.377	0.074	360770	G	A	0.138	8	20902693	3.30E-06	0.029	14306	21.814
Vascular dementia (subcortical)	genus Oxalobacter	rs11108500	A	G	-0.127	96425426	0.211	0.102	360770	A	G	-0.199	12	96819204	3.74E-06	0.043	14306	21.708
Vascular dementia (subcortical)	genus Oxalobacter	rs111966731	T	C	-0.029	93398708	0.777	0.102	360770	T	C	0.213	15	93941937	7.30E-06	0.047	14306	20.419
Vascular dementia (subcortical)	genus Oxalobacter	rs12002250	A	C	0.027	19682560	0.846	0.137	360770	A	C	0.217	9	19682558	1.42E-06	0.047	14306	21.679
Vascular dementia (subcortical)	genus Oxalobacter	rs1569853	T	C	0.120	38582525	0.172	0.088	360770	T	C	-0.138	6	38550301	3.65E-06	0.030	14306	21.617
Vascular dementia (subcortical)	genus Oxalobacter	rs36057338	G	T	-0.098	189014160	0.545	0.162	360770	G	T	0.208	4	189935314	8.80E-07	0.042	14306	24.323
Vascular dementia (subcortical)	genus Oxalobacter	rs3862635	C	T	0.055	126712683	0.583	0.099	360770	C	T	-0.172	11	126582578	9.19E-06	0.039	14306	19.086
Vascular dementia (subcortical)	genus Oxalobacter	rs4428215	G	A	-0.018	172229645	0.777	0.065	360770	G	A	0.130	3	171947435	7.51E-08	0.024	14306	28.931
Vascular dementia (subcortical)	genus Oxalobacter	rs6000536	C	T	-0.108	37025428	0.154	0.076	360770	C	T	-0.131	22	37421469	2.06E-07	0.025	14306	26.637
Vascular dementia (subcortical)	genus Oxalobacter	rs6993398	G	A	-0.033	114548460	0.651	0.073	360770	G	A	0.127	8	115560689	7.13E-06	0.028	14306	20.813
Vascular dementia (subcortical)	genus Oxalobacter	rs736744	T	C	0.046	84899492	0.426	0.057	360770	T	C	-0.118	9	87514407	2.57E-08	0.021	14306	31.135
Vascular dementia (subcortical)	genus Parabacteroides	rs115602804	G	A	0.096	191854576	0.297	0.092	360770	G	A	0.103	3	191572365	1.93E-06	0.022	14306	21.417
Vascular dementia (subcortical)	genus Parabacteroides	rs4236095	A	G	0.105	47400951	0.265	0.094	360770	A	G	-0.076	6	47368687	1.93E-06	0.016	14306	23.541
Vascular dementia (subcortical)	genus Parabacteroides	rs60884758	C	T	0.136	2217340	0.066	0.074	360770	C	T	-0.070	9	2217340	5.71E-07	0.014	14306	24.401

Vascular dementia (subcortical)	genus Parabacteroides	rs6657302	T	C	0.117	85119926	0.318	0.117	360770	T	C	-0.105	1	85585609	9.76E-06	0.023	14306	21.480
Vascular dementia (subcortical)	genus Parabacteroides	rs7298818	C	T	-0.006	56003169	0.952	0.095	360770	C	T	0.089	12	56396953	8.54E-06	0.020	14306	19.574
Vascular dementia (subcortical)	genus Paraprevotella	rs10842464	C	T	-0.071	25096809	0.253	0.062	360770	C	T	0.076	12	25249743	6.60E-06	0.017	14306	19.305
Vascular dementia (subcortical)	genus Paraprevotella	rs140997932	T	C	0.214	149581005	0.077	0.121	360770	T	C	-0.162	3	149298792	2.11E-06	0.035	14306	21.018
Vascular dementia (subcortical)	genus Paraprevotella	rs145020347	A	G	-0.133	114655957	0.100	0.081	360770	A	G	-0.125	11	114526679	4.03E-06	0.026	14306	22.579
Vascular dementia (subcortical)	genus Paraprevotella	rs17109926	A	G	-0.013	71584948	0.843	0.064	360770	A	G	-0.099	12	71978728	6.75E-06	0.022	14306	20.903
Vascular dementia (subcortical)	genus Paraprevotella	rs17785622	A	G	0.054	82749597	0.723	0.151	360770	A	G	0.248	6	83459314	1.93E-06	0.052	14306	22.385
Vascular dementia (subcortical)	genus Paraprevotella	rs2081023	A	G	-0.009	175179258	0.909	0.082	360770	A	G	-0.123	5	174606261	2.64E-07	0.024	14306	26.854
Vascular dementia (subcortical)	genus Paraprevotella	rs3008582	T	C	0.057	196002043	0.428	0.072	360770	T	C	0.106	1	195971173	4.36E-06	0.023	14306	21.643
Vascular dementia (subcortical)	genus Paraprevotella	rs3801748	G	A	-0.012	82130192	0.846	0.059	360770	G	A	0.078	7	81759508	5.20E-06	0.017	14306	20.624
Vascular dementia (subcortical)	genus Paraprevotella	rs4756632	G	T	-0.054	41058153	0.525	0.084	360770	G	T	-0.139	11	41079703	3.82E-06	0.029	14306	22.959
Vascular dementia (subcortical)	genus Paraprevotella	rs4767113	C	T	0.075	113694173	0.217	0.060	360770	C	T	0.088	12	114131978	2.14E-06	0.018	14306	23.047
Vascular dementia (subcortical)	genus Paraprevotella	rs7240324	T	G	0.031	71956568	0.634	0.066	360770	T	G	-0.102	18	69623804	5.96E-06	0.023	14306	20.315
Vascular dementia (subcortical)	genus Paraprevotella	rs9602779	A	C	-0.038	85588285	0.562	0.066	360770	A	C	-0.107	13	86162420	6.93E-07	0.022	14306	23.463
Vascular dementia (subcortical)	genus Paraprevotella	rs9900242	A	G	-0.008	71139490	0.896	0.060	360770	A	G	-0.085	17	69135631	1.14E-06	0.018	14306	23.699
Vascular dementia (subcortical)	genus Parasutterella	rs10899911	A	G	-0.085	43798391	0.207	0.067	360770	A	G	-0.072	10	44293839	1.15E-06	0.015	14306	23.429
Vascular dementia (subcortical)	genus Parasutterella	rs11715853	G	A	-0.042	30122198	0.502	0.062	360770	G	A	-0.066	3	30163689	6.23E-06	0.015	14306	20.586
Vascular dementia (subcortical)	genus Parasutterella	rs2090816	C	A	-0.028	137294455	0.709	0.075	360770	C	A	-0.084	6	137615592	2.90E-06	0.018	14306	22.494
Vascular dementia (subcortical)	genus Parasutterella	rs35055552	T	C	-0.054	113791795	0.512	0.082	360770	T	C	0.110	8	114804024	3.35E-06	0.024	14306	21.653
Vascular dementia (subcortical)	genus Parasutterella	rs55877868	A	C	0.052	14789550	0.581	0.094	360770	A	C	-0.104	17	14692867	2.87E-06	0.023	14306	20.974
Vascular dementia (subcortical)	genus Parasutterella	rs62273907	A	G	0.085	156832626	0.449	0.113	360770	A	G	0.229	3	156550415	5.88E-06	0.050	14306	20.873
Vascular dementia (subcortical)	genus Parasutterella	rs6809952	G	A	0.058	194178920	0.372	0.065	360770	G	A	-0.068	3	193896709	8.13E-06	0.015	14306	20.606
Vascular dementia (subcortical)	genus Parasutterella	rs6828768	C	T	-0.028	64691207	0.616	0.057	360770	C	T	0.064	4	65556925	1.78E-06	0.013	14306	23.051
Vascular dementia (subcortical)	genus Parasutterella	rs7303158	C	T	0.048	5166374	0.396	0.057	360770	C	T	0.065	12	5275540	1.33E-06	0.013	14306	23.214
Vascular dementia (subcortical)	genus Parasutterella	rs7311004	C	T	-0.033	52866926	0.566	0.057	360770	C	T	0.062	12	53260710	5.92E-06	0.014	14306	20.485
Vascular dementia (subcortical)	genus Parasutterella	rs7572229	A	G	-0.004	72008314	0.950	0.057	360770	A	G	-0.066	2	72235444	6.32E-07	0.013	14306	24.928
Vascular dementia (subcortical)	genus Parasutterella	rs78383039	T	C	0.091	178089981	0.534	0.145	360770	T	C	-0.146	2	178954708	1.57E-06	0.030	14306	24.250
Vascular dementia (subcortical)	genus Parasutterella	rs8039785	G	T	0.057	67023969	0.316	0.057	360770	G	T	-0.062	15	67316307	3.62E-06	0.013	14306	21.615
Vascular dementia (subcortical)	genus Parasutterella	rs823424	G	A	0.111	16817017	0.086	0.065	360770	G	A	-0.071	8	16674526	4.95E-06	0.016	14306	20.661
Vascular dementia (subcortical)	genus Peptococcus	rs10031059	C	T	0.038	35356972	0.567	0.067	360770	C	T	0.121	4	35358594	1.24E-07	0.023	14306	28.784
Vascular dementia (subcortical)	genus Peptococcus	rs11001941	G	A	0.022	76956272	0.825	0.101	360770	G	A	-0.196	10	78716030	1.33E-06	0.039	14306	24.873
Vascular dementia (subcortical)	genus Peptococcus	rs12069354	C	T	-0.031	216050440	0.790	0.116	360770	C	T	0.168	1	216223782	9.28E-06	0.038	14306	19.511
Vascular dementia (subcortical)	genus Peptococcus	rs2054133	A	G	0.001	33466742	0.993	0.060	360770	A	G	-0.090	2	33691809	2.14E-06	0.019	14306	22.606
Vascular dementia (subcortical)	genus Peptococcus	rs36121075	A	G	-0.022	44489376	0.777	0.076	360770	A	G	-0.141	20	43118017	6.99E-06	0.031	14306	21.094
Vascular dementia (subcortical)	genus Peptococcus	rs413827	G	A	-0.005	57518394	0.942	0.067	360770	G	A	0.110	14	57985112	3.30E-06	0.024	14306	21.537
Vascular dementia (subcortical)	genus Peptococcus	rs5770862	T	C	-0.115	50534684	0.236	0.097	360770	T	C	0.162	22	50973113	3.22E-06	0.036	14306	20.618
Vascular dementia (subcortical)	genus Peptococcus	rs6918730	A	G	0.070	98572413	0.554	0.118	360770	A	G	-0.135	6	99020289	1.15E-06	0.029	14306	21.809
Vascular dementia (subcortical)	genus Peptococcus	rs7033353	G	T	-0.112	101833566	0.049	0.057	360770	G	T	-0.090	9	104595848	2.22E-06	0.019	14306	22.525
Vascular dementia (subcortical)	genus Peptococcus	rs72850165	T	C	0.029	968356	0.784	0.105	360770	T	C	-0.134	11	968356	5.74E-06	0.030	14306	19.985
Vascular dementia (subcortical)	genus Peptococcus	rs74592222	G	A	0.008	75506015	0.927	0.089	360770	G	A	0.138	1	75971700	8.55E-06	0.030	14306	20.735
Vascular dementia (subcortical)	genus Peptococcus	rs77681628	C	T	0.156	90530302	0.136	0.105	360770	C	T	0.200	13	91182556	2.69E-07	0.039	14306	26.744
Vascular dementia (subcortical)	genus Phascolarctobacterium	rs12618201	A	G	-0.012	173571805	0.835	0.057	360770	A	G	0.064	2	174436533	3.38E-06	0.014	14306	21.556
Vascular dementia (subcortical)	genus Phascolarctobacterium	rs1264476	G	T	0.007	101422048	0.919	0.072	360770	G	T	-0.077	8	102434276	4.30E-06	0.017	14306	21.357
Vascular dementia (subcortical)	genus Phascolarctobacterium	rs56069061	G	A	-0.070	168621629	0.534	0.113	360770	G	A	-0.111	3	168339417	1.87E-06	0.023	14306	23.279
Vascular dementia (subcortical)	genus Phascolarctobacterium	rs56157888	A	C	0.007	182331683	0.923	0.069	360770	A	C	0.095	4	183252836	1.09E-06	0.019	14306	24.232
Vascular dementia (subcortical)	genus Phascolarctobacterium	rs74540770	G	A	-0.037	186835600	0.721	0.105	360770	G	A	-0.121	3	186553389	3.60E-06	0.026	14306	21.895
Vascular dementia (subcortical)	genus Phascolarctobacterium	rs75882962	T	C	0.016	52704958	0.851	0.086	360770	T	C	0.097	12	53098742	3.19E-07	0.019	14306	25.835
Vascular dementia (subcortical)	genus Phascolarctobacterium	rs7982713	G	A	-0.131	70802190	0.036	0.063	360770	G	A	0.073	13	71376322	9.72E-06	0.016	14306	19.839
Vascular dementia (subcortical)	genus Prevotella7	rs118038478	A	G	-0.095	47203698	0.395	0.112	360770	A	G	0.206	16	47237609	7.85E-06	0.047	14306	19.239
Vascular dementia (subcortical)	genus Prevotella7	rs12124567	A	G	0.026	3427238	0.720	0.072	360770	A	G	-0.121	1	3343802	9.49E-06	0.028	14306	19.448
Vascular dementia (subcortical)	genus Prevotella7	rs12195431	T	C	-0.044	90309935	0.649	0.096	360770	T	C	0.197	6	91019654	8.73E-06	0.044	14306	19.741
Vascular dementia (subcortical)	genus Prevotella7	rs2240542	C	T	-0.012	241126899	0.848	0.065	360770	C	T	0.121	2	242066314	4.84E-06	0.026	14306	21.312
Vascular dementia (subcortical)	genus Prevotella7	rs2918132	T	C	-0.102	131202057	0.082	0.059	360770	T	C	0.115	10	133000320	6.42E-06	0.025	14306	20.227
Vascular dementia (subcortical)	genus Prevotella7	rs430270	A	C	0.091	60488646	0.211	0.073	360770	A	C	0.139	3	60474379	2.87E-06	0.030	14306	21.944
Vascular dementia (subcortical)	genus Prevotella7	rs57404562	C	A	0.061	11961636	0.475	0.085	360770	C	A	0.155	2	12101762	6.22E-07	0.032	14306	24.202
Vascular dementia (subcortical)	genus Prevotella7	rs79263163	A	C	0.097	39800223	0.170	0.071	360770	A	C	-0.144	11	39821773	7.51E-06	0.032	14306	20.891
Vascular dementia (subcortical)	genus Prevotella7	rs9426434	T	C	0.060	29409704	0.316	0.060	360770	T	C	-0.124	1	29736216	9.72E-06	0.028	14306	19.713
Vascular dementia (subcortical)	genus Prevotella7	rs9608249	A	G	0.037	24217984	0.679	0.089	360770	A	G	-0.158	22	24613952	2.07E-06	0.034	14306	22.130
Vascular dementia (subcortical)	genus Prevotella7	rs9959718	G	A	-0.016	73775429	0.817	0.071	360770	G	A	0.133	18	71442664	1.90E-06	0.028	14306	23.333
Vascular dementia (subcortical)	genus Prevotella9	rs111509883	T	C	-0.062	639161	0.502	0.092	360770	T	C	0.171	19	639161	1.24E-06	0.035	14306	24.235
Vascular dementia (subcortical)	genus Prevotella9	rs11685699	C	T	0.112	11092559	0.288	0.105	360770	C	T	-0.141	2	11232685	2.03E-06	0.030	14306	22.858
Vascular dementia (subcortical)	genus Prevotella9	rs117271932	A	G	-0.135	43840580	0.268	0.122	360770	A	G	0.208	22	44236460	2.82E-06	0.044	14306	22.326
Vascular dementia (subcortical)	genus Prevotella9	rs12648235	T	C	-0.011	160194592	0.868	0.067	360770	T	C	0.079	4	161115744	7.39E-06	0.018	14306	19.565
Vascular dementia (subcortical)	genus Prevotella9	rs1304512	G	A	0.077	64440198	0.230	0.064	360770	G	A	0.076	5	63736025	5.29E-06	0.017	14306	21.078
Vascular dementia (subcortical)	genus Prevotella9	rs2104588	C	T	0.016	12455582	0.899	0.125	360770	C	T	-0.106	10	12497581	8.13E-06	0.024	14306	19.716
Vascular dementia (subcortical)	genus Prevotella9	rs2495052	A	G	-0.121	13834756	0.128	0.080	360770	A	G	0.084	1	14161251	8.97E-06	0.019	14306	19.805
Vascular dementia (subcortical)	genus Prevotella9	rs2683313	G	A	-0.114	19258094	0.062	0.061	360770	G	A	0.072	8	19115604	1.69E-06	0.015	14306	22.843

Vascular dementia (subcortical)	genus Prevotella9	rs4968431	G	T	-0.050	61344417	0.400	0.060	360770	G	T	0.064	17	59421778	8.58E-06	0.014	14306	19.716
Vascular dementia (subcortical)	genus Prevotella9	rs7237249	C	T	-0.050	74325267	0.487	0.072	360770	C	T	-0.082	18	71992502	8.93E-06	0.018	14306	20.458
Vascular dementia (subcortical)	genus Prevotella9	rs72815774	T	C	0.090	91766476	0.467	0.124	360770	T	C	-0.176	2	91954502	8.78E-06	0.039	14306	20.091
Vascular dementia (subcortical)	genus Prevotella9	rs746764	T	C	0.105	21490075	0.130	0.069	360770	T	C	-0.092	20	21470713	2.04E-06	0.019	14306	22.457
Vascular dementia (subcortical)	genus Prevotella9	rs7976209	T	C	0.095	1685304	0.227	0.078	360770	T	C	-0.087	12	1794470	7.28E-06	0.020	14306	19.351
Vascular dementia (subcortical)	genus Prevotella9	rs9428102	A	G	0.036	118310194	0.606	0.069	360770	A	G	-0.078	1	118852817	4.62E-06	0.018	14306	19.568
Vascular dementia (subcortical)	genus Prevotella9	rs9613013	G	A	0.071	25756771	0.413	0.087	360770	G	A	0.092	22	26152738	6.10E-06	0.020	14306	20.493
Vascular dementia (subcortical)	genus Rikenellaceae RC9 gut group	rs12501673	A	G	-0.112	162909364	0.080	0.064	360770	A	G	0.116	4	163830516	6.29E-06	0.026	14306	19.698
Vascular dementia (subcortical)	genus Rikenellaceae RC9 gut group	rs17032291	T	C	-0.062	154944519	0.475	0.086	360770	T	C	-0.170	4	155865671	6.61E-06	0.037	14306	21.206
Vascular dementia (subcortical)	genus Rikenellaceae RC9 gut group	rs17582787	A	G	-0.051	148641486	0.504	0.076	360770	A	G	-0.158	4	149562638	3.55E-06	0.034	14306	21.533
Vascular dementia (subcortical)	genus Rikenellaceae RC9 gut group	rs2074881	T	C	-0.101	1970022	0.233	0.084	360770	T	C	-0.142	19	1970021	9.45E-06	0.032	14306	19.283
Vascular dementia (subcortical)	genus Rikenellaceae RC9 gut group	rs2900503	T	G	0.023	109895263	0.764	0.077	360770	T	G	0.172	9	112657543	1.55E-07	0.033	14306	27.829
Vascular dementia (subcortical)	genus Rikenellaceae RC9 gut group	rs2998141	C	T	0.058	133196694	0.379	0.066	360770	C	T	0.136	10	135010198	4.42E-06	0.029	14306	21.644
Vascular dementia (subcortical)	genus Rikenellaceae RC9 gut group	rs4270579	A	G	-0.013	83777035	0.827	0.061	360770	A	G	0.118	4	84698188	5.46E-06	0.027	14306	18.960
Vascular dementia (subcortical)	genus Rikenellaceae RC9 gut group	rs4717843	G	T	0.010	73920657	0.858	0.057	360770	G	T	-0.119	7	73334987	4.72E-06	0.026	14306	20.992
Vascular dementia (subcortical)	genus Rikenellaceae RC9 gut group	rs7712231	A	G	0.050	31406178	0.550	0.084	360770	A	G	0.156	5	31406285	7.97E-06	0.035	14306	19.886
Vascular dementia (subcortical)	genus Rikenellaceae RC9 gut group	rs80309088	G	A	0.025	165255243	0.772	0.087	360770	G	A	0.174	6	165668732	4.56E-06	0.038	14306	20.606
Vascular dementia (subcortical)	genus Rikenellaceae RC9 gut group	rs9887954	G	A	-0.023	164831636	0.690	0.058	360770	G	A	-0.115	1	164800873	4.81E-06	0.025	14306	21.272
Vascular dementia (subcortical)	genus Romboutsia	rs10279978	A	G	-0.087	5279756	0.155	0.061	360770	A	G	-0.062	7	5319387	1.17E-06	0.013	14306	23.749
Vascular dementia (subcortical)	genus Romboutsia	rs11221428	T	C	0.016	99794319	0.808	0.068	360770	T	C	-0.073	11	99665050	6.49E-06	0.016	14306	21.089
Vascular dementia (subcortical)	genus Romboutsia	rs16843578	C	T	-0.113	171934745	0.379	0.128	360770	C	T	-0.088	1	171903885	5.08E-06	0.020	14306	19.785
Vascular dementia (subcortical)	genus Romboutsia	rs28603357	T	C	0.014	106766378	0.943	0.204	360770	T	C	-0.215	7	106406824	8.52E-06	0.047	14306	20.493
Vascular dementia (subcortical)	genus Romboutsia	rs34302036	G	A	-0.008	78507408	0.884	0.058	360770	G	A	-0.055	7	78136725	5.88E-06	0.012	14306	20.737
Vascular dementia (subcortical)	genus Romboutsia	rs61841503	G	A	-0.097	16977560	0.258	0.086	360770	G	A	0.093	10	17019559	4.00E-08	0.017	14306	29.351
Vascular dementia (subcortical)	genus Romboutsia	rs62504452	A	G	-0.085	146063205	0.299	0.082	360770	A	G	-0.071	7	145760298	4.66E-06	0.016	14306	20.544
Vascular dementia (subcortical)	genus Romboutsia	rs7109293	A	G	-0.005	133964762	0.959	0.089	360770	A	G	0.092	11	133834657	6.98E-06	0.021	14306	19.958
Vascular dementia (subcortical)	genus Romboutsia	rs75200530	T	G	-0.244	5230532	0.145	0.168	360770	T	G	-0.191	8	5088054	5.07E-06	0.042	14306	20.500
Vascular dementia (subcortical)	genus Romboutsia	rs75987356	G	A	0.173	15926583	0.104	0.106	360770	G	A	-0.130	2	16066705	6.71E-06	0.028	14306	21.347
Vascular dementia (subcortical)	genus Romboutsia	rs77702691	A	G	-0.146	88073285	0.148	0.101	360770	A	G	-0.094	13	88725540	7.37E-06	0.021	14306	20.493
Vascular dementia (subcortical)	genus Romboutsia	rs9389266	T	G	0.109	135090599	0.143	0.075	360770	T	G	0.072	6	135411737	9.38E-06	0.016	14306	19.821
Vascular dementia (subcortical)	genus Romboutsia	rs9567264	C	T	0.093	32146619	0.121	0.060	360770	C	T	0.058	13	32720756	5.76E-06	0.013	14306	20.696
Vascular dementia (subcortical)	genus Roseburia	rs12740451	T	C	0.225	94713418	0.004	0.078	360770	T	C	0.070	1	95178974	7.34E-06	0.015	14306	20.621
Vascular dementia (subcortical)	genus Roseburia	rs16910295	T	C	0.025	11988022	0.843	0.125	360770	T	C	-0.098	11	12009569	2.91E-06	0.021	14306	21.887
Vascular dementia (subcortical)	genus Roseburia	rs2160994	C	T	0.006	50256274	0.916	0.060	360770	C	T	-0.055	12	50650057	9.70E-07	0.011	14306	23.969
Vascular dementia (subcortical)	genus Roseburia	rs2943022	T	C	-0.024	90303097	0.676	0.058	360770	T	C	0.049	5	89598914	4.11E-06	0.011	14306	21.391
Vascular dementia (subcortical)	genus Roseburia	rs302266	T	C	0.076	194214520	0.382	0.086	360770	T	C	-0.078	1	194183650	8.13E-06	0.017	14306	20.192
Vascular dementia (subcortical)	genus Roseburia	rs329182	T	C	0.056	125740801	0.465	0.077	360770	T	C	0.069	5	125076494	5.90E-06	0.015	14306	20.389
Vascular dementia (subcortical)	genus Roseburia	rs55858165	A	C	-0.044	85969578	0.768	0.148	360770	A	C	0.179	15	86512809	9.99E-06	0.040	14306	19.601
Vascular dementia (subcortical)	genus Roseburia	rs57466170	C	T	0.103	37566864	0.344	0.108	360770	C	T	0.074	15	37859065	8.30E-06	0.017	14306	18.667
Vascular dementia (subcortical)	genus Roseburia	rs6445851	A	G	-0.013	57082200	0.828	0.059	360770	A	G	0.050	3	57116228	3.53E-06	0.011	14306	21.144
Vascular dementia (subcortical)	genus Roseburia	rs6930661	C	T	-0.145	12774379	0.223	0.119	360770	C	T	-0.096	6	12774611	2.48E-06	0.020	14306	22.008
Vascular dementia (subcortical)	genus Roseburia	rs75326254	C	T	-0.099	165757348	0.403	0.119	360770	C	T	-0.105	6	166170836	7.50E-06	0.023	14306	20.533
Vascular dementia (subcortical)	genus Roseburia	rs78753150	A	C	0.095	144756092	0.320	0.095	360770	A	C	0.097	5	144135655	9.98E-06	0.021	14306	20.479
Vascular dementia (subcortical)	genus Roseburia	rs9300744	C	T	-0.013	102465136	0.858	0.075	360770	C	T	-0.059	13	103117486	4.75E-06	0.013	14306	21.733
Vascular dementia (subcortical)	genus Ruminiclostridium5	rs10827477	A	G	-0.086	34973660	0.149	0.060	360770	A	G	-0.055	10	35262588	2.19E-06	0.012	14306	22.592
Vascular dementia (subcortical)	genus Ruminiclostridium5	rs113753996	T	C	-0.001	32477558	0.985	0.073	360770	T	C	0.082	5	32477664	3.99E-06	0.017	14306	22.128
Vascular dementia (subcortical)	genus Ruminiclostridium5	rs1223978	C	T	0.009	108122814	0.876	0.057	360770	C	T	-0.048	13	108775162	8.16E-06	0.011	14306	20.004
Vascular dementia (subcortical)	genus Ruminiclostridium5	rs1492620	T	C	-0.051	50438207	0.555	0.086	360770	T	C	-0.083	6	50405920	3.53E-06	0.018	14306	21.271
Vascular dementia (subcortical)	genus Ruminiclostridium5	rs2482038	C	A	0.048	12793086	0.403	0.058	360770	C	A	0.052	10	12835085	1.70E-06	0.011	14306	22.764
Vascular dementia (subcortical)	genus Ruminiclostridium5	rs2791343	T	C	0.057	130303784	0.325	0.058	360770	T	C	0.052	8	131316030	5.54E-06	0.011	14306	20.810
Vascular dementia (subcortical)	genus Ruminiclostridium5	rs2833828	G	A	0.045	32439578	0.435	0.058	360770	G	A	0.049	21	33811886	6.82E-06	0.011	14306	20.288
Vascular dementia (subcortical)	genus Ruminiclostridium5	rs4955951	A	G	-0.066	55209503	0.457	0.088	360770	A	G	-0.071	3	55243531	9.96E-06	0.017	14306	18.526
Vascular dementia (subcortical)	genus Ruminiclostridium5	rs6121460	G	A	-0.064	61718449	0.533	0.103	360770	G	A	0.093	20	60293505	2.64E-06	0.020	14306	21.934
Vascular dementia (subcortical)	genus Ruminiclostridium5	rs79968837	A	G	0.068	42549316	0.600	0.130	360770	A	G	-0.095	20	41177956	1.15E-06	0.019	14306	24.118
Vascular dementia (subcortical)	genus Ruminiclostridium5	rs8053158	G	A	0.019	86736303	0.824	0.087	360770	G	A	0.074	16	86769909	5.90E-06	0.016	14306	21.651
Vascular dementia (subcortical)	genus Ruminiclostridium6	rs10829821	T	C	0.229	130853030	0.019	0.097	360770	T	C	-0.098	10	132651293	3.47E-06	0.022	14306	20.406
Vascular dementia (subcortical)	genus Ruminiclostridium6	rs116969552	A	G	0.175	126525834	0.301	0.170	360770	A	G	-0.167	10	128214403	9.16E-06	0.038	14306	19.614
Vascular dementia (subcortical)	genus Ruminiclostridium6	rs11992182	A	C	0.006	78854264	0.930	0.068	360770	A	C	0.063	8	79766499	4.65E-06	0.014	14306	20.568
Vascular dementia (subcortical)	genus Ruminiclostridium6	rs2548459	C	T	0.098	48706082	0.089	0.058	360770	C	T	0.055	19	49209339	6.40E-06	0.012	14306	20.431
Vascular dementia (subcortical)	genus Ruminiclostridium6	rs35362464	C	A	0.033	36476389	0.688	0.083	360770	C	A	0.072	4	36478011	8.99E-06	0.017	14306	18.956
Vascular dementia (subcortical)	genus Ruminiclostridium6	rs61060922	T	G	0.097	72102255	0.553	0.164	360770	T	G	0.159	16	72136154	1.09E-06	0.032	14306	24.380
Vascular dementia (subcortical)	genus Ruminiclostridium6	rs663262	C	T	-0.120	86468034	0.451	0.159	360770	C	T	0.135	11	86179076	3.39E-06	0.031	14306	18.872
Vascular dementia (subcortical)	genus Ruminiclostridium6	rs67479537	T	C	0.114	10004839	0.390	0.133	360770	T	C	0.119	19	10115515	9.30E-06	0.026	14306	20.183
Vascular dementia (subcortical)	genus Ruminiclostridium6	rs71414120	T	G	0.020	56472234	0.875	0.128	360770	T	G	0.201	14	56938952	1.08E-06	0.041	14306	24.432
Vascular dementia (subcortical)	genus Ruminiclostridium6	rs72991535	T	G	0.307	78258244	0.051	0.157	360770	T	G	0.136	18	76018244	4.95E-06	0.030	14306	21.105
Vascular dementia (subcortical)	genus Ruminiclostridium6	rs73176030	T	C	-0.068	101628002	0.288	0.064	360770	T	C	0.059	7	101271282	7.29E-06	0.013	14306	19.728
Vascular dementia (subcortical)	genus Ruminiclostridium6	rs77193512	A	G	0.076	40267513	0.247	0.066	360770	A	G	0.074	11	40289063	1.30E-06	0.015	14306	23.125

Vascular dementia (subcortical)	genus Ruminiclostridium6	rs792058	G	A	-0.058	5408472	0.314	0.058	360770	G	A	0.055	2	5548605	8.58E-06	0.013	14306	19.527
Vascular dementia (subcortical)	genus Ruminiclostridium6	rs79968172	G	A	-0.144	240340526	0.229	0.120	360770	G	A	0.116	1	240503826	1.66E-06	0.024	14306	22.827
Vascular dementia (subcortical)	genus Ruminiclostridium6	rs9555756	A	C	0.013	111050902	0.899	0.101	360770	A	C	-0.080	13	111703249	7.10E-06	0.018	14306	20.712
Vascular dementia (subcortical)	genus Ruminiclostridium9	rs12040548	G	T	0.012	247546983	0.857	0.064	360770	G	T	0.057	1	247710285	3.15E-06	0.012	14306	21.733
Vascular dementia (subcortical)	genus Ruminiclostridium9	rs6082461	C	A	0.011	2229884	0.878	0.070	360770	C	A	-0.059	20	2210530	4.87E-06	0.013	14306	20.038
Vascular dementia (subcortical)	genus Ruminiclostridium9	rs7137760	C	T	-0.007	10549815	0.905	0.057	360770	C	T	0.051	12	10702414	7.07E-06	0.011	14306	20.504
Vascular dementia (subcortical)	genus Ruminiclostridium9	rs74303178	T	C	0.003	14840035	0.964	0.061	360770	T	C	0.053	8	14697544	7.92E-06	0.012	14306	19.950
Vascular dementia (subcortical)	genus Ruminiclostridium9	rs78191726	T	C	0.152	66945427	0.167	0.110	360770	T	C	0.094	6	67655320	7.58E-06	0.021	14306	20.210
Vascular dementia (subcortical)	genus Ruminiclostridium9	rs918449	G	A	-0.072	33880293	0.509	0.110	360770	G	A	0.095	19	34371198	2.56E-06	0.020	14306	23.258
Vascular dementia (subcortical)	genus Ruminiclostridium9	rs9522712	T	C	0.092	89789324	0.256	0.081	360770	T	C	0.070	13	90441578	4.66E-06	0.015	14306	20.396
Vascular dementia (subcortical)	genus Ruminiclostridium9	rs9809789	C	T	-0.032	29138492	0.662	0.074	360770	C	T	-0.072	3	29179983	8.72E-06	0.016	14306	20.189
Vascular dementia (subcortical)	genus Ruminococcaceae NK4A214 group	rs11241747	T	C	-0.021	124510625	0.738	0.063	360770	T	C	-0.053	5	123846318	6.59E-06	0.012	14306	19.780
Vascular dementia (subcortical)	genus Ruminococcaceae NK4A214 group	rs11586410	G	A	0.042	157369098	0.592	0.078	360770	G	A	-0.086	1	157338888	3.66E-07	0.017	14306	25.815
Vascular dementia (subcortical)	genus Ruminococcaceae NK4A214 group	rs12642039	C	T	-0.014	159010870	0.809	0.059	360770	C	T	0.055	4	159932022	3.43E-06	0.012	14306	21.452
Vascular dementia (subcortical)	genus Ruminococcaceae NK4A214 group	rs12731	A	G	-0.025	238179271	0.677	0.059	360770	A	G	-0.053	2	239087912	4.87E-06	0.012	14306	21.035
Vascular dementia (subcortical)	genus Ruminococcaceae NK4A214 group	rs13087692	G	T	0.015	84675888	0.805	0.062	360770	G	T	-0.057	3	84725039	8.69E-06	0.013	14306	20.818
Vascular dementia (subcortical)	genus Ruminococcaceae NK4A214 group	rs136761	A	G	0.052	49402365	0.385	0.059	360770	A	G	0.059	22	49796014	8.15E-07	0.012	14306	24.312
Vascular dementia (subcortical)	genus Ruminococcaceae NK4A214 group	rs147475196	A	G	-0.074	26467796	0.422	0.093	360770	A	G	-0.134	3	26509287	4.72E-06	0.030	14306	20.535
Vascular dementia (subcortical)	genus Ruminococcaceae NK4A214 group	rs35559912	T	C	0.071	35288836	0.419	0.088	360770	T	C	-0.093	5	35288938	4.89E-06	0.020	14306	20.629
Vascular dementia (subcortical)	genus Ruminococcaceae NK4A214 group	rs4814689	C	T	0.257	18049713	0.060	0.137	360770	C	T	-0.108	20	18030357	4.55E-06	0.023	14306	22.036
Vascular dementia (subcortical)	genus Ruminococcaceae NK4A214 group	rs5994253	A	G	-0.015	17312122	0.857	0.082	360770	A	G	-0.081	22	17793012	2.35E-07	0.016	14306	26.493
Vascular dementia (subcortical)	genus Ruminococcaceae NK4A214 group	rs62027366	T	C	-0.085	24062485	0.224	0.070	360770	T	C	0.062	16	24073806	6.58E-06	0.014	14306	19.998
Vascular dementia (subcortical)	genus Ruminococcaceae NK4A214 group	rs6681678	T	C	-0.020	99783035	0.899	0.161	360770	T	C	0.100	1	100248591	9.05E-06	0.024	14306	17.422
Vascular dementia (subcortical)	genus Ruminococcaceae NK4A214 group	rs7573569	T	C	-0.005	141139473	0.966	0.119	360770	T	C	0.108	2	141897042	3.23E-06	0.023	14306	21.265
Vascular dementia (subcortical)	genus Ruminococcaceae UCG002	rs10916131	C	T	-0.073	227375425	0.344	0.078	360770	C	T	-0.069	1	227563126	2.87E-06	0.015	14306	22.321
Vascular dementia (subcortical)	genus Ruminococcaceae UCG002	rs10927423	C	A	0.047	14405962	0.531	0.075	360770	C	A	-0.071	1	14732458	8.50E-07	0.015	14306	23.340
Vascular dementia (subcortical)	genus Ruminococcaceae UCG002	rs10964441	G	A	0.076	20131748	0.409	0.093	360770	G	A	-0.149	9	20131746	7.45E-06	0.034	14306	18.683
Vascular dementia (subcortical)	genus Ruminococcaceae UCG002	rs113147300	A	G	-0.019	111304390	0.822	0.083	360770	A	G	-0.076	9	114066670	7.69E-06	0.016	14306	21.240
Vascular dementia (subcortical)	genus Ruminococcaceae UCG002	rs11607472	A	G	-0.085	43323201	0.455	0.114	360770	A	G	-0.078	11	43344751	7.19E-06	0.018	14306	19.580
Vascular dementia (subcortical)	genus Ruminococcaceae UCG002	rs116974815	C	A	0.052	111842219	0.641	0.111	360770	C	A	-0.190	11	111712942	2.03E-06	0.040	14306	22.890
Vascular dementia (subcortical)	genus Ruminococcaceae UCG002	rs11750293	G	T	-0.012	124486421	0.842	0.059	360770	G	T	-0.058	5	123822114	1.76E-06	0.012	14306	23.028
Vascular dementia (subcortical)	genus Ruminococcaceae UCG002	rs12463378	A	G	0.056	53972554	0.367	0.062	360770	A	G	-0.052	19	54475808	2.96E-06	0.011	14306	21.694
Vascular dementia (subcortical)	genus Ruminococcaceae UCG002	rs15256	C	T	-0.019	72060790	0.828	0.088	360770	C	T	0.073	10	73820548	9.46E-06	0.017	14306	18.928
Vascular dementia (subcortical)	genus Ruminococcaceae UCG002	rs55793120	T	C	0.117	46990335	0.334	0.121	360770	T	C	0.137	12	47384118	4.81E-07	0.027	14306	25.119
Vascular dementia (subcortical)	genus Ruminococcaceae UCG002	rs57079348	T	G	-0.152	87268195	0.212	0.122	360770	T	G	-0.077	13	87920450	7.22E-06	0.017	14306	19.632
Vascular dementia (subcortical)	genus Ruminococcaceae UCG002	rs6542556	G	A	0.019	120000277	0.743	0.059	360770	G	A	-0.051	2	120757853	7.86E-06	0.011	14306	19.972
Vascular dementia (subcortical)	genus Ruminococcaceae UCG002	rs6793778	T	C	-0.028	24003962	0.661	0.064	360770	T	C	0.056	3	24045453	9.81E-06	0.013	14306	19.896
Vascular dementia (subcortical)	genus Ruminococcaceae UCG002	rs7120052	A	C	0.026	86624417	0.715	0.072	360770	A	C	0.062	11	86335459	1.97E-06	0.014	14306	21.254
Vascular dementia (subcortical)	genus Ruminococcaceae UCG002	rs7155595	C	A	0.085	77036203	0.170	0.062	360770	C	A	0.057	14	77502546	1.15E-06	0.012	14306	23.734
Vascular dementia (subcortical)	genus Ruminococcaceae UCG002	rs7249614	G	A	-0.046	43133738	0.436	0.059	360770	G	A	0.049	19	43637890	9.07E-06	0.011	14306	19.777
Vascular dementia (subcortical)	genus Ruminococcaceae UCG002	rs76847269	A	G	-0.306	141635384	0.083	0.176	360770	A	G	0.164	5	141014951	5.17E-06	0.036	14306	21.077
Vascular dementia (subcortical)	genus Ruminococcaceae UCG002	rs77564310	A	C	0.101	80420842	0.147	0.070	360770	A	C	-0.071	15	80713184	3.29E-07	0.014	14306	25.630
Vascular dementia (subcortical)	genus Ruminococcaceae UCG002	rs79016051	C	T	-0.028	238775197	0.742	0.085	360770	C	T	-0.089	1	238938497	2.34E-06	0.019	14306	21.964
Vascular dementia (subcortical)	genus Ruminococcaceae UCG002	rs882348	A	G	-0.007	23323417	0.940	0.089	360770	A	G	-0.080	4	23325040	5.45E-06	0.018	14306	20.057
Vascular dementia (subcortical)	genus Ruminococcaceae UCG003	rs10490280	C	T	0.069	37678833	0.343	0.073	360770	C	T	-0.067	2	37905976	4.16E-06	0.014	14306	21.994
Vascular dementia (subcortical)	genus Ruminococcaceae UCG003	rs11243416	T	C	-0.091	131541583	0.410	0.111	360770	T	C	-0.093	9	134416970	1.67E-06	0.019	14306	23.437
Vascular dementia (subcortical)	genus Ruminococcaceae UCG003	rs11613919	G	T	0.012	75102683	0.863	0.068	360770	G	T	0.073	12	75496463	1.63E-06	0.016	14306	21.834
Vascular dementia (subcortical)	genus Ruminococcaceae UCG003	rs16959793	A	C	0.044	34779517	0.447	0.058	360770	A	C	-0.063	15	35071718	2.22E-06	0.013	14306	22.692
Vascular dementia (subcortical)	genus Ruminococcaceae UCG003	rs2523124	C	T	-0.006	97719124	0.920	0.058	360770	C	T	0.055	7	97348436	5.78E-06	0.012	14306	20.468
Vascular dementia (subcortical)	genus Ruminococcaceae UCG003	rs3013089	G	A	0.054	13468126	0.359	0.059	360770	G	A	-0.055	1	13794594	4.38E-06	0.012	14306	20.998
Vascular dementia (subcortical)	genus Ruminococcaceae UCG003	rs4452755	A	C	0.067	81114617	0.267	0.060	360770	A	C	-0.063	8	82026852	3.29E-06	0.013	14306	22.172
Vascular dementia (subcortical)	genus Ruminococcaceae UCG003	rs4532474	G	A	-0.026	105333663	0.732	0.076	360770	G	A	0.077	6	105781538	4.82E-06	0.017	14306	20.367
Vascular dementia (subcortical)	genus Ruminococcaceae UCG003	rs646327	G	A	0.097	48706594	0.091	0.058	360770	G	A	0.059	19	49209851	7.83E-07	0.012	14306	24.567
Vascular dementia (subcortical)	genus Ruminococcaceae UCG003	rs6759615	A	G	0.165	204373993	0.087	0.097	360770	A	G	0.103	2	205238716	7.86E-07	0.020	14306	26.224
Vascular dementia (subcortical)	genus Ruminococcaceae UCG003	rs73341549	T	C	0.241	51473771	0.052	0.124	360770	T	C	-0.170	7	51541468	1.51E-07	0.032	14306	28.393
Vascular dementia (subcortical)	genus Ruminococcaceae UCG003	rs78720113	A	G	-0.174	41940901	0.103	0.107	360770	A	G	-0.115	3	41982393	7.59E-06	0.025	14306	21.356
Vascular dementia (subcortical)	genus Ruminococcaceae UCG004	rs10976229	T	G	-0.137	7317307	0.104	0.085	360770	T	G	0.096	9	7317307	7.04E-06	0.021	14306	20.010
Vascular dementia (subcortical)	genus Ruminococcaceae UCG004	rs11961899	G	A	-0.039	132566782	0.535	0.063	360770	G	A	-0.071	6	132887921	9.18E-06	0.016	14306	19.237
Vascular dementia (subcortical)	genus Ruminococcaceae UCG004	rs12125734	G	T	0.053	103266658	0.590	0.099	360770	G	T	0.134	1	103732214	2.09E-07	0.026	14306	27.087
Vascular dementia (subcortical)	genus Ruminococcaceae UCG004	rs2248146	T	C	-0.045	24752379	0.446	0.059	360770	T	C	0.069	7	24791998	8.20E-06	0.015	14306	20.135
Vascular dementia (subcortical)	genus Ruminococcaceae UCG004	rs3800154	A	C	-0.023	2119095	0.719	0.064	360770	A	C	-0.080	6	2119329	6.12E-06	0.018	14306	20.178
Vascular dementia (subcortical)	genus Ruminococcaceae UCG004	rs511258	G	A	-0.061	170248934	0.402	0.073	360770	G	A	-0.076	3	169966722	4.52E-06	0.016	14306	21.735
Vascular dementia (subcortical)	genus Ruminococcaceae UCG004	rs550351	A	C	0.014	18349905	0.805	0.058	360770	A	C	0.079	1	18676399	9.43E-06	0.018	14306	19.055
Vascular dementia (subcortical)	genus Ruminococcaceae UCG004	rs6769553	A	G	0.093	54393768	0.152	0.065	360770	A	G	0.085	3	54427795	7.91E-08	0.016	14306	29.126
Vascular dementia (subcortical)	genus Ruminococcaceae UCG004	rs7569771	A	G	-0.042	238924641	0.527	0.066	360770	A	G	-0.076	2	239846337	8.12E-06	0.017	14306	19.869
Vascular dementia (subcortical)	genus Ruminococcaceae UCG004	rs872501	G	A	0.074	138240821	0.478	0.104	360770	G	A	0.116	8	139253064	5.81E-06	0.026	14306	20.031
Vascular dementia (subcortical)	genus Ruminococcaceae UCG004	rs9818949	T	G	-0.020	197956880	0.775	0.071	360770	T	G	-0.086	3	197683751	5.39E-06	0.019	14306	20.774

Vascular dementia (subcortical)	genus Ruminococcaceae UCG005	rs10873449	C	T	0.067	94164394	0.351	0.072	360770	C	T	-0.065	14	94630731	4.11E-06	0.014	14306	20.688
Vascular dementia (subcortical)	genus Ruminococcaceae UCG005	rs10937802	A	G	-0.127	7300617	0.145	0.087	360770	A	G	-0.076	4	7302344	8.17E-06	0.017	14306	20.186
Vascular dementia (subcortical)	genus Ruminococcaceae UCG005	rs10950694	C	T	0.094	17986981	0.112	0.059	360770	C	T	-0.058	7	18026604	4.30E-07	0.011	14306	25.620
Vascular dementia (subcortical)	genus Ruminococcaceae UCG005	rs114279581	A	G	-0.088	197257633	0.415	0.108	360770	A	G	-0.147	2	198122357	3.22E-06	0.032	14306	21.519
Vascular dementia (subcortical)	genus Ruminococcaceae UCG005	rs12288512	A	G	-0.021	27726124	0.752	0.066	360770	A	G	0.067	11	27747671	3.10E-06	0.014	14306	21.308
Vascular dementia (subcortical)	genus Ruminococcaceae UCG005	rs12458218	T	C	-0.064	24995907	0.384	0.074	360770	T	C	0.068	18	22575871	2.41E-06	0.014	14306	21.964
Vascular dementia (subcortical)	genus Ruminococcaceae UCG005	rs2893871	G	A	0.046	60903023	0.614	0.091	360770	G	A	-0.074	10	62662781	3.54E-06	0.016	14306	22.435
Vascular dementia (subcortical)	genus Ruminococcaceae UCG005	rs34781347	G	A	-0.117	16332206	0.289	0.110	360770	G	A	0.189	20	16312851	6.05E-07	0.039	14306	23.835
Vascular dementia (subcortical)	genus Ruminococcaceae UCG005	rs55793120	T	C	0.117	46990335	0.334	0.121	360770	T	C	0.122	12	47384118	7.37E-06	0.028	14306	18.906
Vascular dementia (subcortical)	genus Ruminococcaceae UCG005	rs72776570	C	A	0.010	4198714	0.916	0.096	360770	C	A	0.087	10	4240906	5.36E-06	0.020	14306	19.498
Vascular dementia (subcortical)	genus Ruminococcaceae UCG005	rs7449320	C	A	-0.064	154623531	0.337	0.067	360770	C	A	0.060	5	154003091	4.81E-06	0.013	14306	20.972
Vascular dementia (subcortical)	genus Ruminococcaceae UCG005	rs7555878	G	A	0.121	187936903	0.066	0.066	360770	G	A	-0.059	1	187906034	2.81E-06	0.013	14306	21.931
Vascular dementia (subcortical)	genus Ruminococcaceae UCG005	rs7586445	G	A	0.089	238600389	0.287	0.083	360770	G	A	0.078	2	239509030	8.81E-06	0.018	14306	19.657
Vascular dementia (subcortical)	genus Ruminococcaceae UCG005	rs898577	C	T	0.042	68413358	0.732	0.122	360770	C	T	0.123	15	68705697	7.46E-06	0.029	14306	18.413
Vascular dementia (subcortical)	genus Ruminococcaceae UCG009	rs12508214	C	T	-0.060	7425210	0.321	0.060	360770	C	T	-0.077	4	7426937	4.75E-06	0.017	14306	21.026
Vascular dementia (subcortical)	genus Ruminococcaceae UCG009	rs138460696	A	G	-0.003	66169644	0.981	0.109	360770	A	G	0.139	2	66396776	9.81E-06	0.032	14306	19.451
Vascular dementia (subcortical)	genus Ruminococcaceae UCG009	rs1550196	A	G	-0.052	34459936	0.584	0.095	360770	A	G	-0.131	17	32786955	1.13E-06	0.026	14306	24.849
Vascular dementia (subcortical)	genus Ruminococcaceae UCG009	rs2058609	G	A	0.022	12817858	0.728	0.064	360770	G	A	-0.082	12	12970792	3.12E-06	0.017	14306	21.837
Vascular dementia (subcortical)	genus Ruminococcaceae UCG009	rs2192926	A	G	-0.042	75285542	0.489	0.061	360770	A	G	-0.089	2	75512668	4.88E-06	0.019	14306	21.290
Vascular dementia (subcortical)	genus Ruminococcaceae UCG009	rs4079028	C	T	0.008	202329957	0.904	0.067	360770	C	T	0.092	1	202299085	3.28E-06	0.020	14306	21.094
Vascular dementia (subcortical)	genus Ruminococcaceae UCG009	rs4708333	T	G	0.029	77396984	0.633	0.060	360770	T	G	-0.084	6	78106701	1.56E-06	0.017	14306	23.122
Vascular dementia (subcortical)	genus Ruminococcaceae UCG009	rs6952765	G	A	-0.055	31962098	0.365	0.061	360770	G	A	0.073	7	32001710	8.13E-06	0.017	14306	19.269
Vascular dementia (subcortical)	genus Ruminococcaceae UCG009	rs758191	T	G	-0.080	1929830	0.400	0.095	360770	T	G	0.177	16	1979831	9.01E-06	0.038	14306	22.270
Vascular dementia (subcortical)	genus Ruminococcaceae UCG009	rs78410648	A	G	0.049	51896148	0.591	0.091	360770	A	G	0.121	19	52399401	9.67E-06	0.028	14306	19.034
Vascular dementia (subcortical)	genus Ruminococcaceae UCG009	rs9558661	T	C	0.069	105997744	0.333	0.071	360770	T	C	-0.090	13	106650093	7.01E-06	0.020	14306	20.004
Vascular dementia (subcortical)	genus Ruminococcaceae UCG010	rs12597105	A	G	-0.033	5183940	0.645	0.073	360770	A	G	-0.067	16	5233941	4.87E-06	0.014	14306	21.579
Vascular dementia (subcortical)	genus Ruminococcaceae UCG010	rs2820282	C	A	-0.150	104280743	0.009	0.057	360770	C	A	0.059	6	104728618	2.85E-06	0.013	14306	22.127
Vascular dementia (subcortical)	genus Ruminococcaceae UCG010	rs682403	A	G	-0.096	133093170	0.093	0.057	360770	A	G	-0.059	9	135968557	2.37E-06	0.012	14306	22.257
Vascular dementia (subcortical)	genus Ruminococcaceae UCG010	rs6958419	C	T	0.041	16310239	0.475	0.057	360770	C	T	-0.059	7	16349864	2.84E-06	0.012	14306	21.958
Vascular dementia (subcortical)	genus Ruminococcaceae UCG010	rs73218807	G	A	0.011	31055860	0.914	0.101	360770	G	A	-0.166	4	31057482	6.43E-06	0.037	14306	20.407
Vascular dementia (subcortical)	genus Ruminococcaceae UCG010	rs7441445	T	C	0.023	40705781	0.692	0.057	360770	T	C	0.057	4	40707798	6.80E-06	0.013	14306	20.267
Vascular dementia (subcortical)	genus Ruminococcaceae UCG011	rs10274562	C	T	-0.060	11182894	0.308	0.058	360770	C	T	0.111	7	11222521	6.50E-06	0.024	14306	20.570
Vascular dementia (subcortical)	genus Ruminococcaceae UCG011	rs12636310	G	A	0.100	185751703	0.129	0.066	360770	G	A	0.133	3	185469491	2.81E-06	0.028	14306	22.146
Vascular dementia (subcortical)	genus Ruminococcaceae UCG011	rs12724320	C	T	-0.007	179401364	0.906	0.058	360770	C	T	-0.121	1	179370499	1.52E-06	0.025	14306	23.524
Vascular dementia (subcortical)	genus Ruminococcaceae UCG011	rs1416041	A	C	0.068	104625801	0.330	0.070	360770	A	C	-0.182	6	105073676	7.04E-08	0.034	14306	28.778
Vascular dementia (subcortical)	genus Ruminococcaceae UCG011	rs2729556	T	C	0.037	112123933	0.521	0.057	360770	T	C	0.109	7	111763988	3.19E-06	0.023	14306	21.793
Vascular dementia (subcortical)	genus Ruminococcaceae UCG011	rs4490371	T	C	0.113	76155956	0.051	0.058	360770	T	C	-0.112	3	76205107	7.75E-06	0.025	14306	20.172
Vascular dementia (subcortical)	genus Ruminococcaceae UCG011	rs79113084	C	T	0.032	29481986	0.728	0.093	360770	C	T	-0.152	12	29634919	2.06E-06	0.032	14306	22.967
Vascular dementia (subcortical)	genus Ruminococcaceae UCG011	rs9729514	G	A	0.005	219727713	0.958	0.099	360770	G	A	-0.185	1	219901055	2.37E-06	0.039	14306	21.963
Vascular dementia (subcortical)	genus Ruminococcaceae UCG013	rs11581881	C	T	0.101	9301517	0.138	0.068	360770	C	T	0.066	1	9361576	4.73E-06	0.014	14306	20.870
Vascular dementia (subcortical)	genus Ruminococcaceae UCG013	rs12189346	G	A	0.133	142015575	0.068	0.073	360770	G	A	0.068	5	141395140	1.68E-06	0.015	14306	22.137
Vascular dementia (subcortical)	genus Ruminococcaceae UCG013	rs12336782	T	C	0.110	13609120	0.308	0.108	360770	T	C	-0.086	9	13609119	8.60E-06	0.019	14306	20.448
Vascular dementia (subcortical)	genus Ruminococcaceae UCG013	rs12485353	G	A	-0.021	197323125	0.749	0.065	360770	G	A	-0.061	3	197049996	4.19E-06	0.013	14306	21.584
Vascular dementia (subcortical)	genus Ruminococcaceae UCG013	rs12781711	C	T	-0.039	2177736	0.551	0.066	360770	C	T	-0.066	10	2219930	2.55E-08	0.012	14306	31.194
Vascular dementia (subcortical)	genus Ruminococcaceae UCG013	rs16918863	A	C	0.264	19491534	0.021	0.114	360770	A	C	0.111	10	19780463	4.15E-06	0.024	14306	21.552
Vascular dementia (subcortical)	genus Ruminococcaceae UCG013	rs2730183	G	A	0.076	40454366	0.192	0.058	360770	G	A	-0.049	8	40311885	8.44E-06	0.011	14306	19.771
Vascular dementia (subcortical)	genus Ruminococcaceae UCG013	rs4385846	T	G	0.091	113890519	0.207	0.072	360770	T	G	-0.060	10	115650278	6.46E-06	0.013	14306	20.612
Vascular dementia (subcortical)	genus Ruminococcaceae UCG013	rs75088940	T	C	0.047	30092624	0.671	0.112	360770	T	C	-0.094	12	30245557	2.55E-06	0.020	14306	22.072
Vascular dementia (subcortical)	genus Ruminococcaceae UCG013	rs76973485	G	T	0.010	9492973	0.946	0.144	360770	G	T	0.195	3	9534657	3.35E-06	0.042	14306	21.735
Vascular dementia (subcortical)	genus Ruminococcaceae UCG013	rs9313055	T	C	0.010	3628768	0.923	0.105	360770	T	C	0.105	5	3628882	9.55E-06	0.023	14306	20.089
Vascular dementia (subcortical)	genus Ruminococcaceae UCG014	rs10495392	T	C	-0.003	237259689	0.981	0.110	360770	T	C	0.082	1	237422989	9.96E-06	0.019	14306	19.417
Vascular dementia (subcortical)	genus Ruminococcaceae UCG014	rs10791168	G	A	0.088	131789106	0.231	0.073	360770	G	A	0.066	11	131659000	9.76E-06	0.015	14306	19.614
Vascular dementia (subcortical)	genus Ruminococcaceae UCG014	rs10941294	C	T	-0.105	36435495	0.403	0.126	360770	C	T	-0.122	5	36435597	2.40E-06	0.026	14306	22.035
Vascular dementia (subcortical)	genus Ruminococcaceae UCG014	rs115777838	T	C	0.107	26110517	0.239	0.091	360770	T	C	-0.188	5	26110626	4.62E-07	0.039	14306	23.731
Vascular dementia (subcortical)	genus Ruminococcaceae UCG014	rs12638134	T	G	0.055	101615765	0.342	0.057	360770	T	G	0.058	3	101334609	1.21E-06	0.012	14306	23.702
Vascular dementia (subcortical)	genus Ruminococcaceae UCG014	rs34402072	C	T	-0.063	3779026	0.433	0.081	360770	C	T	-0.069	8	3636548	9.80E-06	0.016	14306	19.431
Vascular dementia (subcortical)	genus Ruminococcaceae UCG014	rs56105232	G	A	0.079	14363770	0.516	0.122	360770	G	A	0.139	9	14363769	2.91E-06	0.030	14306	21.678
Vascular dementia (subcortical)	genus Ruminococcaceae UCG014	rs72809222	T	C	0.079	56978719	0.259	0.070	360770	T	C	0.067	2	57205854	2.41E-06	0.014	14306	23.078
Vascular dementia (subcortical)	genus Ruminococcaceae UCG014	rs853612	A	G	-0.007	118168902	0.909	0.058	360770	A	G	-0.053	10	119928413	9.75E-06	0.012	14306	19.585
Vascular dementia (subcortical)	genus Ruminococcaceae UCG014	rs995642	C	T	0.007	134097088	0.920	0.067	360770	C	T	0.060	2	134854659	1.90E-06	0.013	14306	22.562
Vascular dementia (subcortical)	genus Ruminococcus1	rs10167839	A	G	0.039	181018152	0.507	0.059	360770	A	G	0.052	2	181882879	8.09E-06	0.012	14306	19.952
Vascular dementia (subcortical)	genus Ruminococcus1	rs11783695	G	T	-0.021	143514733	0.788	0.078	360770	G	T	-0.073	8	144596903	4.73E-06	0.016	14306	20.689
Vascular dementia (subcortical)	genus Ruminococcus1	rs17781867	C	T	-0.002	73259879	0.986	0.114	360770	C	T	0.100	17	71256018	1.96E-06	0.021	14306	22.275
Vascular dementia (subcortical)	genus Ruminococcus1	rs3819978	C	T	0.203	241626455	0.061	0.109	360770	C	T	-0.115	1	241789757	8.74E-06	0.026	14306	19.557
Vascular dementia (subcortical)	genus Ruminococcus1	rs6105066	T	C	0.104	13277981	0.105	0.064	360770	T	C	-0.061	20	13258628	5.06E-06	0.013	14306	20.447
Vascular dementia (subcortical)	genus Ruminococcus1	rs6493760	T	C	-0.056	55146984	0.342	0.059	360770	T	C	-0.054	15	55439182	3.38E-06	0.012	14306	21.334
Vascular dementia (subcortical)	genus Ruminococcus1	rs7117576	A	G	-0.059	114152851	0.555	0.099	360770	A	G	0.083	11	114023573	6.48E-07	0.017	14306	23.561

Vascular dementia (subcortical)	genus Ruminococcus1	rs7583465	C	T	0.038	10393795	0.500	0.057	360770	C	T	0.053	2	10533921	2.56E-06	0.011	14306	21.952
Vascular dementia (subcortical)	genus Ruminococcus1	rs78572139	G	A	-0.129	15944478	0.170	0.094	360770	G	A	0.125	5	15944587	5.23E-06	0.028	14306	20.026
Vascular dementia (subcortical)	genus Ruminococcus1	rs78613526	G	A	-0.128	65698078	0.320	0.129	360770	G	A	0.167	2	65925212	5.11E-06	0.037	14306	20.757
Vascular dementia (subcortical)	genus Ruminococcus2	rs12406309	A	C	-0.109	107531823	0.112	0.069	360770	A	C	-0.063	1	108074445	9.79E-06	0.014	14306	19.728
Vascular dementia (subcortical)	genus Ruminococcus2	rs12986628	T	C	-0.115	237194796	0.108	0.071	360770	T	C	-0.067	2	238103439	2.14E-06	0.014	14306	22.572
Vascular dementia (subcortical)	genus Ruminococcus2	rs1819812	G	T	-0.027	151738636	0.833	0.127	360770	G	T	0.084	7	151435722	5.28E-06	0.018	14306	20.756
Vascular dementia (subcortical)	genus Ruminococcus2	rs2368224	T	G	0.002	181791689	0.986	0.131	360770	T	G	0.200	2	182656416	3.63E-06	0.044	14306	20.734
Vascular dementia (subcortical)	genus Ruminococcus2	rs2846589	T	G	-0.027	862476	0.646	0.058	360770	T	G	-0.052	18	862477	7.59E-06	0.012	14306	20.124
Vascular dementia (subcortical)	genus Ruminococcus2	rs2997412	G	A	0.001	61493648	0.985	0.064	360770	G	A	0.057	13	62067781	4.22E-06	0.012	14306	21.594
Vascular dementia (subcortical)	genus Ruminococcus2	rs4400279	A	G	0.080	16764953	0.189	0.061	360770	A	G	0.055	7	16804578	5.80E-06	0.012	14306	20.685
Vascular dementia (subcortical)	genus Ruminococcus2	rs4799823	C	T	-0.048	35876464	0.512	0.074	360770	C	T	0.084	18	33456427	5.40E-06	0.018	14306	21.089
Vascular dementia (subcortical)	genus Ruminococcus2	rs55707116	C	A	0.064	77304469	0.555	0.109	360770	C	A	0.087	9	79919385	8.01E-06	0.019	14306	20.940
Vascular dementia (subcortical)	genus Ruminococcus2	rs58681734	A	G	0.031	134666044	0.661	0.070	360770	A	G	0.072	9	137557890	4.18E-06	0.016	14306	20.269
Vascular dementia (subcortical)	genus Ruminococcus2	rs61791565	T	C	0.047	24653840	0.411	0.057	360770	T	C	-0.052	4	24655463	6.79E-06	0.012	14306	19.979
Vascular dementia (subcortical)	genus Ruminococcus2	rs75140805	T	G	0.039	12127041	0.611	0.076	360770	T	G	0.084	11	12148588	3.95E-06	0.018	14306	22.493
Vascular dementia (subcortical)	genus Ruminococcus2	rs7635831	G	A	-0.062	173323134	0.290	0.059	360770	G	A	0.062	3	173040924	1.98E-06	0.013	14306	22.965
Vascular dementia (subcortical)	genus Ruminococcus2	rs7693984	G	A	0.039	4266595	0.774	0.136	360770	G	A	-0.103	4	4268322	9.42E-06	0.024	14306	19.130
Vascular dementia (subcortical)	genus Ruminococcus2	rs78120384	A	G	0.010	106945516	0.921	0.099	360770	A	G	-0.193	3	106664363	3.31E-07	0.039	14306	24.189
Vascular dementia (subcortical)	genus Ruminococcus gauvreauii group	rs10931481	A	G	0.014	191090126	0.820	0.063	360770	A	G	-0.061	2	191954852	3.38E-06	0.013	14306	21.862
Vascular dementia (subcortical)	genus Ruminococcus gauvreauii group	rs12079579	A	G	0.237	161910692	0.012	0.094	360770	A	G	0.096	1	161880482	5.04E-06	0.021	14306	20.034
Vascular dementia (subcortical)	genus Ruminococcus gauvreauii group	rs12539819	C	T	0.131	153790311	0.241	0.112	360770	C	T	0.111	7	153487396	4.49E-06	0.024	14306	21.137
Vascular dementia (subcortical)	genus Ruminococcus gauvreauii group	rs1391597	C	T	0.023	60725485	0.689	0.058	360770	C	T	0.059	12	61119266	1.86E-06	0.012	14306	22.345
Vascular dementia (subcortical)	genus Ruminococcus gauvreauii group	rs2047242	A	G	-0.012	28912931	0.858	0.068	360770	A	G	-0.068	10	29201860	2.46E-07	0.013	14306	25.552
Vascular dementia (subcortical)	genus Ruminococcus gauvreauii group	rs2166943	C	A	0.020	136078607	0.732	0.058	360770	C	A	-0.057	8	137090850	5.28E-06	0.012	14306	21.077
Vascular dementia (subcortical)	genus Ruminococcus gauvreauii group	rs289410	A	G	0.007	85020252	0.912	0.063	360770	A	G	0.065	15	85563483	2.27E-06	0.014	14306	22.168
Vascular dementia (subcortical)	genus Ruminococcus gauvreauii group	rs431418	G	A	-0.201	166992547	0.038	0.097	360770	G	A	0.095	5	166419552	5.54E-06	0.021	14306	20.321
Vascular dementia (subcortical)	genus Ruminococcus gauvreauii group	rs71386687	T	G	0.194	2717893	0.030	0.089	360770	T	G	0.121	16	2767894	2.91E-07	0.024	14306	25.733
Vascular dementia (subcortical)	genus Ruminococcus gauvreauii group	rs73802842	C	A	0.115	18823237	0.086	0.067	360770	C	A	0.074	4	18824860	7.48E-06	0.017	14306	18.859
Vascular dementia (subcortical)	genus Ruminococcus gauvreauii group	rs9870933	G	A	0.001	112653470	0.992	0.058	360770	G	A	-0.062	3	112372317	8.49E-07	0.013	14306	24.313
Vascular dementia (subcortical)	genus Ruminococcus gnavus group	rs11597105	A	G	-0.012	6470619	0.867	0.071	360770	A	G	0.115	10	6512581	6.95E-06	0.025	14306	20.930
Vascular dementia (subcortical)	genus Ruminococcus gnavus group	rs11864644	T	C	-0.105	383078	0.240	0.089	360770	T	C	-0.140	16	433078	5.01E-06	0.032	14306	19.297
Vascular dementia (subcortical)	genus Ruminococcus gnavus group	rs12136548	C	T	0.022	114625476	0.730	0.063	360770	C	T	0.090	1	115168097	3.10E-06	0.020	14306	21.074
Vascular dementia (subcortical)	genus Ruminococcus gnavus group	rs12989336	G	A	0.038	36032198	0.557	0.064	360770	G	A	-0.085	2	36259341	7.12E-06	0.019	14306	20.305
Vascular dementia (subcortical)	genus Ruminococcus gnavus group	rs13163520	G	A	-0.145	18662577	0.046	0.073	360770	G	A	-0.127	5	18662686	5.61E-08	0.023	14306	29.661
Vascular dementia (subcortical)	genus Ruminococcus gnavus group	rs2909242	A	C	0.064	128212434	0.286	0.060	360770	A	C	0.091	8	129224680	7.41E-07	0.018	14306	24.588
Vascular dementia (subcortical)	genus Ruminococcus gnavus group	rs3124783	G	A	0.015	132963204	0.860	0.085	360770	G	A	0.116	9	135838591	2.67E-06	0.025	14306	21.682
Vascular dementia (subcortical)	genus Ruminococcus gnavus group	rs4388134	T	C	-0.013	189189556	0.844	0.064	360770	T	C	0.090	4	190110710	9.12E-06	0.020	14306	19.769
Vascular dementia (subcortical)	genus Ruminococcus gnavus group	rs62167033	T	C	0.018	143942440	0.899	0.144	360770	T	C	0.185	2	144700007	3.50E-06	0.040	14306	21.861
Vascular dementia (subcortical)	genus Ruminococcus gnavus group	rs78399089	T	C	-0.010	146572523	0.914	0.090	360770	T	C	0.144	3	146290310	6.63E-06	0.033	14306	19.558
Vascular dementia (subcortical)	genus Ruminococcus gnavus group	rs934940	A	C	0.049	121338442	0.556	0.084	360770	A	C	-0.105	2	122096018	2.74E-06	0.023	14306	20.934
Vascular dementia (subcortical)	genus Ruminococcus torques group	rs10904297	A	G	-0.129	4597570	0.511	0.196	360770	A	G	-0.168	10	4639762	2.69E-06	0.039	14306	18.531
Vascular dementia (subcortical)	genus Ruminococcus torques group	rs10967781	C	A	0.061	27225260	0.330	0.062	360770	C	A	0.051	9	27225258	8.37E-06	0.011	14306	20.095
Vascular dementia (subcortical)	genus Ruminococcus torques group	rs12434631	A	G	-0.155	25661198	0.097	0.094	360770	A	G	0.075	14	26130404	2.77E-06	0.015	14306	23.711
Vascular dementia (subcortical)	genus Ruminococcus torques group	rs1475330	C	T	0.144	66552209	0.029	0.066	360770	C	T	-0.052	6	67262102	8.13E-06	0.012	14306	19.615
Vascular dementia (subcortical)	genus Ruminococcus torques group	rs35866622	T	C	0.022	48714803	0.709	0.060	360770	T	C	-0.061	19	49218060	2.21E-08	0.011	14306	31.285
Vascular dementia (subcortical)	genus Ruminococcus torques group	rs4073731	T	C	-0.044	132793252	0.565	0.077	360770	T	C	0.065	11	132663147	4.05E-06	0.014	14306	21.014
Vascular dementia (subcortical)	genus Ruminococcus torques group	rs77034621	T	G	-0.136	75857608	0.547	0.225	360770	T	G	-0.152	8	76769843	6.07E-06	0.034	14306	20.359
Vascular dementia (subcortical)	genus Sellimonas	rs113379006	T	C	-0.014	41966694	0.849	0.074	360770	T	C	-0.163	3	42008186	7.21E-06	0.036	14306	20.782
Vascular dementia (subcortical)	genus Sellimonas	rs13417181	T	C	-0.104	173443666	0.124	0.068	360770	T	C	0.167	2	174308394	7.62E-07	0.034	14306	24.337
Vascular dementia (subcortical)	genus Sellimonas	rs2016057	C	A	0.051	51813085	0.375	0.058	360770	C	A	0.126	15	52105282	1.03E-06	0.026	14306	24.149
Vascular dementia (subcortical)	genus Sellimonas	rs2187447	A	C	0.007	79621928	0.951	0.119	360770	A	C	0.243	11	79332972	3.98E-06	0.053	14306	21.285
Vascular dementia (subcortical)	genus Sellimonas	rs2371572	A	C	-0.041	212349450	0.475	0.057	360770	A	C	0.127	2	213214174	4.46E-07	0.025	14306	25.770
Vascular dementia (subcortical)	genus Sellimonas	rs41816	A	G	-0.086	106609718	0.167	0.062	360770	A	G	0.132	7	106250164	8.39E-06	0.029	14306	20.626
Vascular dementia (subcortical)	genus Sellimonas	rs4600608	G	A	-0.092	179413837	0.195	0.071	360770	G	A	0.137	2	180278564	4.95E-06	0.030	14306	20.666
Vascular dementia (subcortical)	genus Sellimonas	rs553697	C	T	-0.082	93020675	0.265	0.074	360770	C	T	0.154	6	93730393	6.13E-06	0.034	14306	20.562
Vascular dementia (subcortical)	genus Sellimonas	rs56203279	T	C	-0.052	111861468	0.386	0.060	360770	T	C	-0.124	7	111501524	3.72E-06	0.027	14306	21.246
Vascular dementia (subcortical)	genus Senegalimassilia	rs10036909	C	T	-0.134	128525999	0.354	0.145	360770	C	T	0.186	5	127861692	8.05E-06	0.040	14306	21.416
Vascular dementia (subcortical)	genus Senegalimassilia	rs11787826	C	A	-0.037	34332384	0.522	0.058	360770	C	A	0.081	9	34332382	2.63E-06	0.017	14306	22.579
Vascular dementia (subcortical)	genus Senegalimassilia	rs1990708	A	C	-0.212	205789399	0.049	0.108	360770	A	C	-0.110	2	206654123	8.91E-06	0.025	14306	19.571
Vascular dementia (subcortical)	genus Senegalimassilia	rs2017373	C	T	-0.094	33962790	0.111	0.059	360770	C	T	0.078	14	34431996	9.50E-06	0.018	14306	19.567
Vascular dementia (subcortical)	genus Senegalimassilia	rs7225245	A	G	-0.025	50302254	0.668	0.058	360770	A	G	-0.079	17	48379615	4.18E-06	0.017	14306	21.583
Vascular dementia (subcortical)	genus Slackia	rs10409783	G	A	0.064	4555774	0.313	0.063	360770	G	A	-0.095	19	4555786	7.70E-06	0.021	14306	20.261
Vascular dementia (subcortical)	genus Slackia	rs12440440	A	G	0.073	33749695	0.219	0.060	360770	A	G	0.090	15	34041896	2.63E-06	0.019	14306	22.397
Vascular dementia (subcortical)	genus Slackia	rs16894137	C	T	-0.038	95934063	0.659	0.086	360770	C	T	-0.123	8	96946291	2.71E-06	0.026	14306	21.791
Vascular dementia (subcortical)	genus Slackia	rs35156985	T	C	-0.232	99854092	0.104	0.142	360770	T	C	-0.156	7	99451715	8.06E-06	0.035	14306	20.010
Vascular dementia (subcortical)	genus Slackia	rs4492265	G	A	-0.041	13484058	0.509	0.062	360770	G	A	0.091	7	13523683	2.41E-06	0.019	14306	22.334
Vascular dementia (subcortical)	genus Slackia	rs8901	C	T	0.039	76270929	0.536	0.063	360770	C	T	0.093	17	74267010	6.07E-07	0.019	14306	25.028

Vascular dementia (subcortical)	genus Streptococcus	rs10028567	C	T	0.014	52791410	0.873	0.087	360770	C	T	-0.092	4	53657577	7.30E-06	0.019	14306	23.047
Vascular dementia (subcortical)	genus Streptococcus	rs10448310	A	G	0.022	90793892	0.710	0.059	360770	A	G	-0.052	9	93556174	3.31E-06	0.011	14306	21.646
Vascular dementia (subcortical)	genus Streptococcus	rs11110281	T	C	0.162	100190236	0.229	0.134	360770	T	C	-0.138	12	100584014	2.58E-09	0.023	14306	36.572
Vascular dementia (subcortical)	genus Streptococcus	rs11720390	G	A	-0.098	94384747	0.408	0.118	360770	G	A	0.107	3	94103591	3.59E-06	0.023	14306	22.011
Vascular dementia (subcortical)	genus Streptococcus	rs11764382	A	G	-0.066	46735298	0.421	0.082	360770	A	G	-0.070	7	46774896	1.29E-06	0.014	14306	23.424
Vascular dementia (subcortical)	genus Streptococcus	rs17708276	A	G	0.078	10342038	0.399	0.093	360770	A	G	-0.079	8	10199548	3.04E-06	0.017	14306	21.652
Vascular dementia (subcortical)	genus Streptococcus	rs1918540	A	G	0.034	131779552	0.644	0.074	360770	A	G	-0.060	11	131649446	2.44E-06	0.013	14306	21.659
Vascular dementia (subcortical)	genus Streptococcus	rs2370083	G	T	-0.079	97060413	0.488	0.114	360770	G	T	-0.082	14	97526750	9.75E-06	0.019	14306	19.317
Vascular dementia (subcortical)	genus Streptococcus	rs57646748	G	A	0.332	37451236	0.012	0.132	360770	G	A	-0.091	4	37452858	5.48E-06	0.020	14306	20.527
Vascular dementia (subcortical)	genus Streptococcus	rs6806351	T	C	-0.054	132339879	0.435	0.069	360770	T	C	-0.063	3	132058723	4.94E-06	0.014	14306	21.515
Vascular dementia (subcortical)	genus Streptococcus	rs71481756	T	G	-0.042	8060441	0.719	0.117	360770	T	G	0.093	10	8102404	6.51E-06	0.021	14306	20.046
Vascular dementia (subcortical)	genus Streptococcus	rs7916711	A	G	0.079	28299340	0.332	0.082	360770	A	G	0.103	10	28588269	2.72E-06	0.022	14306	22.407
Vascular dementia (subcortical)	genus Subdoligranulum	rs10065321	T	C	0.024	142477850	0.671	0.057	360770	T	C	-0.051	5	141857415	2.10E-06	0.011	14306	22.504
Vascular dementia (subcortical)	genus Subdoligranulum	rs10497836	T	C	-0.018	199423995	0.798	0.069	360770	T	C	0.052	2	200288718	8.38E-06	0.012	14306	19.494
Vascular dementia (subcortical)	genus Subdoligranulum	rs1667315	G	A	-0.079	231544032	0.176	0.058	360770	G	A	0.049	2	232408743	6.72E-06	0.011	14306	20.374
Vascular dementia (subcortical)	genus Subdoligranulum	rs2114677	C	T	0.037	123713421	0.677	0.089	360770	C	T	-0.104	10	125472937	2.72E-06	0.023	14306	20.368
Vascular dementia (subcortical)	genus Subdoligranulum	rs2171249	C	T	0.059	153372894	0.587	0.109	360770	C	T	0.107	6	153694029	4.51E-06	0.023	14306	20.950
Vascular dementia (subcortical)	genus Subdoligranulum	rs3761728	G	T	-0.034	48988868	0.605	0.066	360770	G	T	0.054	4	48990885	3.87E-06	0.012	14306	20.903
Vascular dementia (subcortical)	genus Subdoligranulum	rs4347804	G	A	0.001	217351124	0.996	0.161	360770	G	A	-0.166	2	218215847	2.18E-06	0.036	14306	21.579
Vascular dementia (subcortical)	genus Subdoligranulum	rs6555306	C	T	0.026	4671270	0.750	0.081	360770	C	T	0.074	5	4671383	2.81E-06	0.016	14306	22.705
Vascular dementia (subcortical)	genus Subdoligranulum	rs75158211	T	C	-0.049	29153787	0.536	0.080	360770	T	C	-0.072	19	29644694	7.52E-06	0.016	14306	20.616
Vascular dementia (subcortical)	genus Subdoligranulum	rs76528319	G	T	-0.148	118463753	0.153	0.104	360770	G	T	-0.143	5	117799448	7.41E-06	0.031	14306	21.271
Vascular dementia (subcortical)	genus Sutterella	rs1145877	G	A	-0.016	81677104	0.843	0.082	360770	G	A	0.074	6	82386821	7.20E-06	0.016	14306	20.507
Vascular dementia (subcortical)	genus Sutterella	rs11591622	T	G	-0.040	100760930	0.606	0.077	360770	T	G	-0.069	10	102520687	6.50E-06	0.015	14306	20.680
Vascular dementia (subcortical)	genus Sutterella	rs13173038	A	G	-0.145	59203357	0.028	0.066	360770	A	G	-0.072	5	58499183	2.73E-06	0.015	14306	22.428
Vascular dementia (subcortical)	genus Sutterella	rs143438747	T	C	-0.041	62812878	0.700	0.107	360770	T	C	-0.146	1	63278549	3.28E-06	0.031	14306	22.572
Vascular dementia (subcortical)	genus Sutterella	rs2050185	A	G	0.003	147615645	0.957	0.059	360770	A	G	-0.058	6	147936781	7.97E-06	0.013	14306	19.950
Vascular dementia (subcortical)	genus Sutterella	rs2321387	G	A	0.028	58115206	0.623	0.057	360770	G	A	-0.059	13	58689340	1.87E-06	0.012	14306	22.674
Vascular dementia (subcortical)	genus Sutterella	rs2613606	T	C	-0.050	111644969	0.385	0.057	360770	T	C	0.056	7	111285025	7.20E-06	0.012	14306	20.125
Vascular dementia (subcortical)	genus Sutterella	rs607327	T	C	0.003	111824985	0.953	0.058	360770	T	C	-0.058	11	111695709	6.63E-06	0.013	14306	20.083
Vascular dementia (subcortical)	genus Sutterella	rs62501473	G	A	0.161	150429663	0.013	0.065	360770	G	A	0.069	7	150126751	5.52E-06	0.015	14306	21.588
Vascular dementia (subcortical)	genus Sutterella	rs7499539	A	G	-0.037	85004459	0.569	0.064	360770	A	G	0.062	16	85038065	2.36E-06	0.013	14306	22.218
Vascular dementia (subcortical)	genus Sutterella	rs7638039	T	C	0.022	70539788	0.733	0.066	360770	T	C	0.065	3	70588939	8.66E-06	0.014	14306	20.133
Vascular dementia (subcortical)	genus Sutterella	rs9350083	T	G	-0.003	18583647	0.966	0.059	360770	T	G	-0.059	6	18583878	8.23E-06	0.013	14306	19.609
Vascular dementia (subcortical)	genus Terrisporobacter	rs1883097	C	T	-0.061	8917272	0.688	0.153	360770	C	T	0.226	11	8938819	4.16E-07	0.045	14306	24.798
Vascular dementia (subcortical)	genus Terrisporobacter	rs2569953	C	A	0.000	144430063	0.998	0.058	360770	C	A	0.078	3	144148905	8.95E-06	0.017	14306	19.723
Vascular dementia (subcortical)	genus Terrisporobacter	rs2872237	A	C	0.016	16916130	0.782	0.058	360770	A	C	0.081	19	17026940	3.97E-06	0.018	14306	21.431
Vascular dementia (subcortical)	genus Terrisporobacter	rs58405430	G	T	0.256	20599497	0.032	0.119	360770	G	T	0.135	11	20621043	7.94E-06	0.030	14306	19.978
Vascular dementia (subcortical)	genus Terrisporobacter	rs7184125	T	C	0.016	15826537	0.806	0.063	360770	T	C	0.091	16	15920394	8.48E-06	0.021	14306	19.721
Vascular dementia (subcortical)	genus Turicibacter	rs11054680	T	C	-0.015	12089521	0.845	0.075	360770	T	C	-0.105	12	12242455	2.31E-06	0.023	14306	21.295
Vascular dementia (subcortical)	genus Turicibacter	rs11666533	C	T	-0.250	11754117	0.018	0.106	360770	C	T	-0.112	19	11864932	7.37E-06	0.025	14306	20.211
Vascular dementia (subcortical)	genus Turicibacter	rs149744580	A	G	-0.156	63139827	0.217	0.127	360770	A	G	0.170	2	63366962	7.01E-08	0.032	14306	28.998
Vascular dementia (subcortical)	genus Turicibacter	rs2834977	T	C	-0.025	35557345	0.753	0.079	360770	T	C	-0.096	21	36929643	3.96E-06	0.021	14306	21.248
Vascular dementia (subcortical)	genus Turicibacter	rs2952020	A	G	0.075	26170326	0.257	0.066	360770	A	G	0.076	8	26027842	5.63E-06	0.017	14306	20.966
Vascular dementia (subcortical)	genus Turicibacter	rs3734633	G	A	-0.025	125790735	0.837	0.123	360770	G	A	-0.121	6	126111881	5.32E-06	0.027	14306	20.325
Vascular dementia (subcortical)	genus Turicibacter	rs4869133	G	A	-0.011	96381915	0.886	0.074	360770	G	A	0.131	5	95717619	2.55E-06	0.027	14306	23.267
Vascular dementia (subcortical)	genus Turicibacter	rs55756211	T	C	0.000	131287420	0.998	0.109	360770	T	C	-0.115	7	130972179	2.81E-06	0.024	14306	22.871
Vascular dementia (subcortical)	genus Turicibacter	rs7199484	G	A	-0.111	49792388	0.072	0.061	360770	G	A	-0.073	16	49826299	5.77E-06	0.016	14306	20.853
Vascular dementia (subcortical)	genus Tyzzerella3	rs10898797	C	T	0.118	87877806	0.192	0.090	360770	C	T	0.122	11	87588698	8.85E-06	0.027	14306	19.850
Vascular dementia (subcortical)	genus Tyzzerella3	rs112102233	A	G	-0.034	45602838	0.796	0.132	360770	A	G	-0.216	10	46098286	6.18E-06	0.048	14306	20.522
Vascular dementia (subcortical)	genus Tyzzerella3	rs1232220	T	G	-0.007	102232382	0.943	0.095	360770	T	G	0.144	6	102680257	7.91E-06	0.032	14306	20.432
Vascular dementia (subcortical)	genus Tyzzerella3	rs17706273	T	C	0.101	16388150	0.343	0.107	360770	T	C	-0.140	5	16388259	5.88E-07	0.027	14306	26.109
Vascular dementia (subcortical)	genus Tyzzerella3	rs191093	G	A	0.030	76996549	0.747	0.092	360770	G	A	0.159	12	77390329	6.76E-06	0.035	14306	20.255
Vascular dementia (subcortical)	genus Tyzzerella3	rs4904512	T	C	-0.085	89129601	0.315	0.084	360770	T	C	-0.117	14	89595945	3.09E-06	0.025	14306	21.905
Vascular dementia (subcortical)	genus Tyzzerella3	rs55799124	A	G	0.020	3835487	0.756	0.064	360770	A	G	-0.114	17	3738781	1.34E-06	0.024	14306	22.968
Vascular dementia (subcortical)	genus Tyzzerella3	rs67476743	T	G	-0.005	1030321	0.944	0.064	360770	T	G	0.132	19	1030320	3.74E-09	0.022	14306	35.417
Vascular dementia (subcortical)	genus Tyzzerella3	rs7019909	T	C	-0.022	33113324	0.801	0.087	360770	T	C	0.144	9	33113322	1.76E-06	0.030	14306	22.842
Vascular dementia (subcortical)	genus Tyzzerella3	rs7333521	T	C	-0.125	81018881	0.429	0.158	360770	T	C	-0.207	13	81593016	4.88E-06	0.045	14306	20.908
Vascular dementia (subcortical)	genus Tyzzerella3	rs75091807	G	T	-0.047	34449554	0.695	0.120	360770	G	T	-0.185	13	35023691	1.71E-06	0.038	14306	23.320
Vascular dementia (subcortical)	genus Tyzzerella3	rs7561370	C	T	0.120	57583271	0.132	0.080	360770	C	T	-0.131	2	57810406	1.52E-06	0.029	14306	21.047
Vascular dementia (subcortical)	genus Veillonella	rs1882878	A	G	0.088	28638351	0.156	0.062	360770	A	G	-0.077	21	30010673	2.98E-06	0.016	14306	22.010
Vascular dementia (subcortical)	genus Veillonella	rs2013594	C	T	-0.012	44280604	0.838	0.058	360770	C	T	0.072	11	44302154	3.42E-06	0.016	14306	21.577
Vascular dementia (subcortical)	genus Veillonella	rs62376424	C	T	-0.010	120578590	0.871	0.062	360770	C	T	-0.076	5	119914285	3.65E-06	0.016	14306	21.733
Vascular dementia (subcortical)	genus Veillonella	rs6656807	G	A	-0.043	178975908	0.474	0.059	360770	G	A	-0.070	1	178945043	5.50E-06	0.015	14306	20.855
Vascular dementia (subcortical)	genus Veillonella	rs742016	A	G	0.018	45208919	0.765	0.060	360770	A	G	-0.069	22	45604800	4.66E-06	0.015	14306	21.137
Vascular dementia (subcortical)	genus Victivallis	rs11899949	G	A	-0.072	37821926	0.241	0.061	360770	G	A	0.131	2	38049069	2.77E-06	0.028	1531	22.341
Vascular dementia (subcortical)	genus Victivallis	rs12512543	A	C	0.108	9606821	0.297	0.104	360770	A	C	-0.178	4	9608445	2.54E-06	0.037	1531	22.619

Vascular dementia (subcortical)	genus Victivallis	rs173120	C	T	-0.014	72302743	0.842	0.072	360770	C	T	-0.134	13	72876881	7.65E-06	0.029	1531	21.272
Vascular dementia (subcortical)	genus Victivallis	rs1882775	A	G	0.093	39033681	0.208	0.074	360770	A	G	-0.138	21	40405606	8.73E-06	0.031	1531	19.549
Vascular dementia (subcortical)	genus Victivallis	rs2546432	C	T	0.097	181152462	0.086	0.057	360770	C	T	0.111	5	180579462	9.93E-06	0.025	1531	19.698
Vascular dementia (subcortical)	genus Victivallis	rs342302	A	G	0.062	106737783	0.462	0.084	360770	A	G	-0.153	7	106378229	8.16E-06	0.035	1531	18.878
Vascular dementia (subcortical)	genus Victivallis	rs4764863	G	A	0.091	102120406	0.109	0.057	360770	G	A	0.122	12	102514184	8.22E-07	0.025	1531	24.407
Vascular dementia (subcortical)	genus Victivallis	rs4895919	C	T	0.017	131309179	0.771	0.057	360770	C	T	0.117	6	131630319	2.75E-06	0.025	1531	22.313
Vascular dementia (subcortical)	genus Victivallis	rs56349194	A	G	0.082	9291249	0.333	0.085	360770	A	G	-0.159	11	9312796	6.26E-07	0.032	1531	25.282
Vascular dementia (subcortical)	genus Victivallis	rs911666	T	C	-0.026	95604788	0.674	0.062	360770	T	C	-0.119	14	96071125	7.65E-06	0.026	1531	20.305
Vascular dementia (subcortical)	order Actinomycetales	rs2889192	T	G	0.116	73779652	0.149	0.081	360770	T	G	-0.088	9	76394568	3.97E-06	0.019	14306	20.554
Vascular dementia (subcortical)	order Actinomycetales	rs34583783	G	T	-0.036	66497478	0.763	0.120	360770	G	T	0.124	6	67207371	5.54E-06	0.026	14306	21.908
Vascular dementia (subcortical)	order Actinomycetales	rs35011108	A	G	-0.263	132686341	0.020	0.113	360770	A	G	0.242	6	133007480	1.88E-06	0.050	14306	22.987
Vascular dementia (subcortical)	order Actinomycetales	rs4073240	G	A	0.065	168824686	0.265	0.058	360770	G	A	0.075	6	169224781	5.68E-06	0.016	14306	20.729
Vascular dementia (subcortical)	order Bacillales	rs10233278	T	C	0.116	117856090	0.044	0.058	360770	T	C	-0.116	7	117496144	3.51E-06	0.025	14306	21.906
Vascular dementia (subcortical)	order Bacillales	rs10410917	C	T	-0.066	14859629	0.258	0.059	360770	C	T	-0.115	19	14970441	5.57E-06	0.025	14306	21.049
Vascular dementia (subcortical)	order Bacillales	rs11034576	A	G	-0.036	38064161	0.674	0.085	360770	A	G	0.206	11	38085711	8.86E-06	0.045	14306	20.604
Vascular dementia (subcortical)	order Bacillales	rs11207728	A	G	0.006	61361862	0.936	0.076	360770	A	G	0.145	1	61827534	5.73E-06	0.032	14306	20.805
Vascular dementia (subcortical)	order Bacillales	rs11844714	A	G	0.069	48532023	0.324	0.070	360770	A	G	-0.143	14	49001226	5.06E-06	0.032	14306	20.004
Vascular dementia (subcortical)	order Bacillales	rs1287018	G	A	0.011	5845828	0.881	0.073	360770	G	A	0.141	20	5826474	9.87E-06	0.032	14306	19.534
Vascular dementia (subcortical)	order Bacillales	rs4617108	G	A	0.022	49384236	0.833	0.102	360770	G	A	-0.249	7	49423832	1.98E-06	0.053	14306	22.390
Vascular dementia (subcortical)	order Bacillales	rs74420793	A	G	0.059	126845554	0.522	0.093	360770	A	G	-0.164	4	127766709	3.07E-06	0.035	14306	21.600
Vascular dementia (subcortical)	order Bacteroidales	rs11146701	A	G	0.009	38769138	0.886	0.060	360770	A	G	0.047	10	39062269	7.08E-06	0.011	14306	20.186
Vascular dementia (subcortical)	order Bacteroidales	rs17343978	A	C	-0.037	27037922	0.601	0.070	360770	A	C	-0.055	22	27433885	8.36E-06	0.012	14306	21.067
Vascular dementia (subcortical)	order Bacteroidales	rs2032750	C	T	-0.072	53603889	0.209	0.057	360770	C	T	0.051	2	53831026	1.92E-06	0.011	14306	22.657
Vascular dementia (subcortical)	order Bacteroidales	rs2363574	T	C	0.155	200143435	0.302	0.150	360770	T	C	0.223	1	200112563	9.93E-06	0.051	14306	19.221
Vascular dementia (subcortical)	order Bacteroidales	rs4916508	A	G	0.003	196209918	0.953	0.057	360770	A	G	0.047	3	195936789	8.47E-06	0.011	14306	19.641
Vascular dementia (subcortical)	order Bacteroidales	rs55773148	G	A	-0.001	69948897	0.995	0.124	360770	G	A	-0.122	13	70523029	3.90E-07	0.024	14306	26.341
Vascular dementia (subcortical)	order Bacteroidales	rs62531359	T	G	-0.041	70003946	0.585	0.075	360770	T	G	0.066	8	70916181	9.09E-06	0.015	14306	19.138
Vascular dementia (subcortical)	order Bacteroidales	rs62575403	C	T	0.043	133628698	0.760	0.141	360770	C	T	0.140	9	136493820	7.06E-06	0.031	14306	20.264
Vascular dementia (subcortical)	order Bacteroidales	rs72706335	T	C	-0.229	157525648	0.218	0.186	360770	T	C	-0.222	1	157495438	7.66E-06	0.049	14306	20.315
Vascular dementia (subcortical)	order Bacteroidales	rs73975615	G	A	0.069	6557880	0.834	0.330	360770	G	A	-0.207	17	6461200	1.22E-06	0.044	14306	21.874
Vascular dementia (subcortical)	order Bacteroidales	rs7631304	G	A	-0.026	89290377	0.743	0.080	360770	G	A	-0.065	3	89339527	8.37E-07	0.013	14306	23.590
Vascular dementia (subcortical)	order Bacteroidales	rs79585701	A	C	0.054	13252676	0.508	0.082	360770	A	C	0.065	8	13110185	9.99E-06	0.015	14306	18.687
Vascular dementia (subcortical)	order Bacteroidales	rs929878	T	C	-0.092	74256742	0.188	0.070	360770	T	C	0.055	16	74290641	4.73E-06	0.012	14306	20.372
Vascular dementia (subcortical)	order Bifidobacteriales	rs10831953	G	A	-0.054	13076504	0.383	0.062	360770	G	A	0.054	11	13098051	9.95E-06	0.012	14306	18.869
Vascular dementia (subcortical)	order Bifidobacteriales	rs12446429	T	C	0.044	848055	0.545	0.073	360770	T	C	0.081	16	898055	8.53E-06	0.019	14306	18.040
Vascular dementia (subcortical)	order Bifidobacteriales	rs13020688	G	A	0.037	192013806	0.549	0.062	360770	G	A	0.058	2	192878532	1.57E-06	0.012	14306	22.887
Vascular dementia (subcortical)	order Bifidobacteriales	rs182549	T	C	-0.005	135859184	0.936	0.059	360770	T	C	-0.117	2	136616754	5.94E-20	0.013	14306	85.372
Vascular dementia (subcortical)	order Bifidobacteriales	rs4957061	T	C	0.066	520981	0.255	0.058	360770	T	C	0.057	5	521096	1.15E-06	0.012	14306	23.762
Vascular dementia (subcortical)	order Bifidobacteriales	rs540489	T	G	-0.157	74901626	0.038	0.075	360770	T	G	-0.063	17	72897722	5.37E-06	0.014	14306	20.956
Vascular dementia (subcortical)	order Bifidobacteriales	rs55888705	A	G	-0.012	1516099	0.850	0.063	360770	A	G	0.054	4	1517826	8.66E-06	0.012	14306	19.812
Vascular dementia (subcortical)	order Bifidobacteriales	rs6899771	A	G	0.114	96958344	0.228	0.095	360770	A	G	-0.091	6	97406220	7.28E-06	0.020	14306	20.365
Vascular dementia (subcortical)	order Bifidobacteriales	rs7174549	T	C	0.054	91920073	0.360	0.059	360770	T	C	-0.055	15	92463303	6.87E-06	0.012	14306	19.590
Vascular dementia (subcortical)	order Bifidobacteriales	rs7322849	T	C	0.119	112205515	0.231	0.099	360770	T	C	0.111	13	112859829	1.74E-08	0.020	14306	30.320
Vascular dementia (subcortical)	order Bifidobacteriales	rs857444	C	T	0.056	14617360	0.344	0.059	360770	C	T	0.055	6	14617591	3.82E-06	0.012	14306	21.075
Vascular dementia (subcortical)	order Burkholderiales	rs1511453	A	G	0.170	23586839	0.156	0.120	360770	A	G	0.091	4	23588462	8.00E-06	0.020	14306	20.857
Vascular dementia (subcortical)	order Burkholderiales	rs1928341	G	A	0.045	153267537	0.433	0.058	360770	G	A	-0.051	1	153240013	4.52E-06	0.011	14306	21.084
Vascular dementia (subcortical)	order Burkholderiales	rs2321387	G	A	0.028	58115206	0.623	0.057	360770	G	A	-0.051	13	58689340	3.26E-06	0.011	14306	21.532
Vascular dementia (subcortical)	order Burkholderiales	rs2613606	T	C	-0.050	111644969	0.385	0.057	360770	T	C	0.050	7	111285025	4.13E-06	0.011	14306	20.904
Vascular dementia (subcortical)	order Burkholderiales	rs4033856	T	C	-0.095	45640468	0.350	0.101	360770	T	C	-0.083	4	45642485	5.67E-07	0.017	14306	24.762
Vascular dementia (subcortical)	order Burkholderiales	rs6087811	T	G	-0.114	32008327	0.225	0.094	360770	T	G	-0.102	20	30596130	2.88E-07	0.020	14306	26.108
Vascular dementia (subcortical)	order Burkholderiales	rs62191117	A	G	0.022	238979079	0.752	0.071	360770	A	G	0.068	2	239900775	2.79E-07	0.013	14306	26.349
Vascular dementia (subcortical)	order Burkholderiales	rs62395635	T	C	-0.073	174070793	0.536	0.118	360770	T	C	0.110	5	173497796	2.90E-06	0.024	14306	21.621
Vascular dementia (subcortical)	order Burkholderiales	rs75242906	C	T	0.120	56494209	0.247	0.104	360770	C	T	-0.121	15	56786407	9.75E-06	0.028	14306	18.498
Vascular dementia (subcortical)	order Burkholderiales	rs7638039	T	C	0.022	70539788	0.733	0.066	360770	T	C	0.058	3	70588939	4.84E-06	0.013	14306	21.017
Vascular dementia (subcortical)	order Clostridiales	rs10774377	G	A	0.054	5833353	0.351	0.058	360770	G	A	-0.052	12	5942519	3.81E-06	0.011	14306	21.100
Vascular dementia (subcortical)	order Clostridiales	rs112334273	G	A	0.041	39331325	0.521	0.064	360770	G	A	0.064	21	40703251	4.07E-07	0.013	14306	25.186
Vascular dementia (subcortical)	order Clostridiales	rs13105690	C	T	0.016	7418457	0.800	0.064	360770	C	T	0.053	4	7420184	9.37E-06	0.012	14306	19.919
Vascular dementia (subcortical)	order Clostridiales	rs13179700	C	T	-0.114	149698225	0.058	0.060	360770	C	T	-0.051	5	149077788	3.52E-06	0.011	14306	21.746
Vascular dementia (subcortical)	order Clostridiales	rs1842454	G	A	0.012	105724661	0.868	0.073	360770	G	A	-0.054	5	105060362	9.92E-06	0.013	14306	18.228
Vascular dementia (subcortical)	order Clostridiales	rs2273429	A	G	0.141	52027354	0.122	0.091	360770	A	G	-0.073	14	52494072	4.17E-06	0.015	14306	22.497
Vascular dementia (subcortical)	order Clostridiales	rs290772	G	A	0.034	101509758	0.782	0.124	360770	G	A	0.084	2	102126220	1.00E-05	0.020	14306	18.423
Vascular dementia (subcortical)	order Clostridiales	rs6442336	T	C	-0.017	12829387	0.798	0.066	360770	T	C	0.055	3	12870886	9.63E-06	0.012	14306	19.458
Vascular dementia (subcortical)	order Clostridiales	rs6814436	C	T	0.001	160586149	0.995	0.082	360770	C	T	-0.074	4	161507301	9.06E-07	0.015	14306	24.197
Vascular dementia (subcortical)	order Clostridiales	rs6815608	C	T	-0.023	151210592	0.768	0.079	360770	C	T	-0.104	4	152131744	3.72E-07	0.021	14306	24.363
Vascular dementia (subcortical)	order Clostridiales	rs72738886	T	C	0.154	35770448	0.152	0.108	360770	T	C	0.087	5	35770550	8.42E-06	0.019	14306	20.657
Vascular dementia (subcortical)	order Clostridiales	rs992074	T	C	0.040	17195484	0.836	0.194	360770	T	C	-0.255	21	18567802	8.95E-07	0.051	14306	25.206
Vascular dementia (subcortical)	order Coriobacteriales	rs11073596	G	T	-0.027	85890348	0.641	0.059	360770	G	T	-0.051	15	86433579	8.14E-06	0.011	14306	19.912

Vascular dementia (subcortical)	order Coriobacteriales	rs11250875	T	C	-0.054	1880537	0.435	0.069	360770	T	C	0.061	10	1922731	4.83E-06	0.013	14306	21.526
Vascular dementia (subcortical)	order Coriobacteriales	rs11656361	A	C	0.131	8218014	0.076	0.074	360770	A	C	0.077	17	8121332	8.02E-06	0.018	14306	19.394
Vascular dementia (subcortical)	order Coriobacteriales	rs12974142	G	A	-0.066	52391913	0.550	0.111	360770	G	A	0.079	19	52895166	8.51E-06	0.018	14306	19.865
Vascular dementia (subcortical)	order Coriobacteriales	rs13307134	T	C	-0.082	105444233	0.287	0.077	360770	T	C	-0.057	7	105084680	7.80E-06	0.013	14306	20.072
Vascular dementia (subcortical)	order Coriobacteriales	rs1397793	A	G	0.048	91175634	0.441	0.062	360770	A	G	0.050	5	90471451	9.77E-06	0.011	14306	19.682
Vascular dementia (subcortical)	order Coriobacteriales	rs1816223	G	A	0.124	11341087	0.082	0.071	360770	G	A	0.059	12	11494021	4.84E-06	0.013	14306	20.652
Vascular dementia (subcortical)	order Coriobacteriales	rs240104	T	C	0.078	176602295	0.216	0.063	360770	T	C	-0.060	1	176571431	1.52E-06	0.013	14306	22.630
Vascular dementia (subcortical)	order Coriobacteriales	rs2442778	A	G	0.010	11612938	0.940	0.127	360770	A	G	0.116	3	11654412	9.03E-06	0.026	14306	20.272
Vascular dementia (subcortical)	order Coriobacteriales	rs3025411	A	G	0.062	133647784	0.497	0.091	360770	A	G	0.093	9	136512906	8.27E-06	0.021	14306	19.566
Vascular dementia (subcortical)	order Coriobacteriales	rs34739816	G	T	-0.139	39220432	0.249	0.121	360770	G	T	0.097	17	37376685	3.88E-06	0.021	14306	21.594
Vascular dementia (subcortical)	order Coriobacteriales	rs67561917	A	G	-0.071	63440724	0.336	0.074	360770	A	G	-0.071	20	62072077	5.39E-06	0.015	14306	21.486
Vascular dementia (subcortical)	order Coriobacteriales	rs719099	A	G	0.048	64039457	0.616	0.096	360770	A	G	0.078	10	65799217	5.43E-07	0.016	14306	24.957
Vascular dementia (subcortical)	order Coriobacteriales	rs8010111	A	G	0.034	39191305	0.744	0.104	360770	A	G	0.103	14	39660509	6.90E-06	0.023	14306	20.328
Vascular dementia (subcortical)	order Desulfovibrionales	rs11599763	C	T	0.095	11813600	0.103	0.058	360770	C	T	0.055	10	11855599	2.61E-06	0.012	14306	22.305
Vascular dementia (subcortical)	order Desulfovibrionales	rs17791387	A	G	-0.034	79219511	0.732	0.098	360770	A	G	-0.073	9	81834426	2.25E-06	0.015	14306	22.210
Vascular dementia (subcortical)	order Desulfovibrionales	rs186073	T	C	0.047	31091909	0.419	0.058	360770	T	C	0.053	3	31133401	8.74E-06	0.012	14306	19.931
Vascular dementia (subcortical)	order Desulfovibrionales	rs2692012	G	A	-0.235	204022477	0.061	0.126	360770	G	A	-0.112	1	203991605	2.27E-06	0.025	14306	19.597
Vascular dementia (subcortical)	order Desulfovibrionales	rs2838334	G	A	0.045	43645080	0.456	0.060	360770	G	A	0.057	21	45064961	4.17E-06	0.012	14306	20.982
Vascular dementia (subcortical)	order Desulfovibrionales	rs3935584	C	T	-0.066	233064573	0.246	0.057	360770	C	T	-0.052	2	233929283	7.20E-06	0.012	14306	20.531
Vascular dementia (subcortical)	order Desulfovibrionales	rs4506934	C	T	-0.095	2953368	0.282	0.088	360770	C	T	-0.095	17	2856662	2.43E-06	0.020	14306	22.416
Vascular dementia (subcortical)	order Desulfovibrionales	rs6058181	C	T	-0.084	35106998	0.271	0.076	360770	C	T	0.084	20	33694801	2.53E-07	0.017	14306	25.379
Vascular dementia (subcortical)	order Desulfovibrionales	rs62020470	A	G	0.058	95617836	0.433	0.075	360770	A	G	-0.057	15	96161065	7.51E-06	0.013	14306	19.633
Vascular dementia (subcortical)	order Desulfovibrionales	rs72647048	T	C	-0.024	56952819	0.787	0.090	360770	T	C	-0.077	8	57865378	9.00E-06	0.017	14306	20.436
Vascular dementia (subcortical)	order Desulfovibrionales	rs9928243	C	A	0.039	71507738	0.491	0.057	360770	C	A	-0.054	16	71541641	3.97E-06	0.012	14306	21.378
Vascular dementia (subcortical)	order Enterobacteriales	rs11026530	T	C	-0.021	22357551	0.793	0.080	360770	T	C	0.082	11	22379097	9.43E-06	0.019	14306	19.471
Vascular dementia (subcortical)	order Enterobacteriales	rs2374342	C	A	-0.031	41906402	0.586	0.058	360770	C	A	0.058	2	42133542	4.52E-06	0.013	14306	21.338
Vascular dementia (subcortical)	order Enterobacteriales	rs35673018	G	A	-0.068	54293833	0.491	0.099	360770	G	A	0.090	16	54327745	7.63E-06	0.020	14306	19.653
Vascular dementia (subcortical)	order Enterobacteriales	rs504442	T	G	0.055	57478315	0.555	0.093	360770	T	G	0.084	18	55145547	5.17E-06	0.019	14306	19.728
Vascular dementia (subcortical)	order Enterobacteriales	rs62210023	A	G	0.041	56765036	0.490	0.060	360770	A	G	0.061	20	55340092	3.13E-06	0.013	14306	21.742
Vascular dementia (subcortical)	order Enterobacteriales	rs78143293	A	G	-0.005	60005103	0.957	0.087	360770	A	G	-0.085	18	57672335	1.20E-06	0.017	14306	24.792
Vascular dementia (subcortical)	order Enterobacteriales	rs79757635	C	A	-0.166	110188071	0.047	0.083	360770	C	A	0.076	13	110840418	9.32E-06	0.017	14306	19.615
Vascular dementia (subcortical)	order Erysipelotrichales	rs1074800	G	A	0.060	3002432	0.294	0.058	360770	G	A	-0.049	5	3002546	6.15E-06	0.011	14306	20.459
Vascular dementia (subcortical)	order Erysipelotrichales	rs10781552	C	T	-0.006	132083729	0.921	0.063	360770	C	T	-0.055	10	133897233	2.33E-06	0.012	14306	22.633
Vascular dementia (subcortical)	order Erysipelotrichales	rs17530232	A	G	-0.076	39811320	0.545	0.125	360770	A	G	0.103	13	40385457	2.79E-06	0.022	14306	21.042
Vascular dementia (subcortical)	order Erysipelotrichales	rs1884466	C	T	0.044	63673525	0.442	0.057	360770	C	T	-0.048	1	64139196	9.53E-06	0.011	14306	19.760
Vascular dementia (subcortical)	order Erysipelotrichales	rs2300774	A	G	0.057	196066841	0.319	0.057	360770	A	G	-0.052	3	195793712	8.95E-07	0.011	14306	24.094
Vascular dementia (subcortical)	order Erysipelotrichales	rs290833	T	G	-0.055	96991871	0.337	0.057	360770	T	G	-0.050	1	97457427	8.03E-06	0.011	14306	19.943
Vascular dementia (subcortical)	order Erysipelotrichales	rs35161940	T	C	-0.053	72331083	0.571	0.093	360770	T	C	-0.081	17	70327224	1.85E-06	0.017	14306	23.118
Vascular dementia (subcortical)	order Erysipelotrichales	rs4078432	T	C	-0.078	48528003	0.305	0.076	360770	T	C	0.061	14	48997206	4.23E-06	0.013	14306	20.723
Vascular dementia (subcortical)	order Erysipelotrichales	rs56970041	T	G	0.075	79891267	0.515	0.116	360770	T	G	0.072	14	80357610	5.40E-06	0.016	14306	19.385
Vascular dementia (subcortical)	order Erysipelotrichales	rs62504403	C	T	-0.107	38946033	0.135	0.072	360770	C	T	0.068	8	38803551	1.12E-07	0.013	14306	28.371
Vascular dementia (subcortical)	order Erysipelotrichales	rs7234058	T	C	0.090	5830508	0.362	0.098	360770	T	C	-0.095	18	5830507	9.12E-07	0.019	14306	23.744
Vascular dementia (subcortical)	order Erysipelotrichales	rs7826267	G	T	0.004	3097430	0.973	0.114	360770	G	T	0.084	8	2954952	9.28E-06	0.020	14306	17.755
Vascular dementia (subcortical)	order Erysipelotrichales	rs8003149	C	T	-0.049	55689786	0.422	0.060	360770	C	T	0.054	14	56156504	4.08E-06	0.012	14306	21.248
Vascular dementia (subcortical)	order Gastranaerophilales	rs11150282	T	C	-0.032	80459808	0.596	0.060	360770	T	C	0.098	16	80493705	7.36E-07	0.020	14306	24.834
Vascular dementia (subcortical)	order Gastranaerophilales	rs113884518	T	C	0.410	24648999	0.028	0.186	360770	T	C	-0.206	9	24648997	7.74E-06	0.046	14306	20.446
Vascular dementia (subcortical)	order Gastranaerophilales	rs28678345	T	C	0.081	55828967	0.545	0.134	360770	T	C	0.213	17	53906328	8.06E-06	0.047	14306	20.434
Vascular dementia (subcortical)	order Gastranaerophilales	rs367480	A	G	-0.022	2916401	0.715	0.060	360770	A	G	0.084	11	2937631	7.52E-06	0.019	14306	20.487
Vascular dementia (subcortical)	order Gastranaerophilales	rs4129395	G	A	-0.036	113213109	0.531	0.057	360770	G	A	0.090	9	115975389	1.22E-06	0.019	14306	23.826
Vascular dementia (subcortical)	order Gastranaerophilales	rs789069	A	C	-0.080	1008277	0.329	0.082	360770	A	C	-0.104	18	1008278	6.50E-06	0.023	14306	19.725
Vascular dementia (subcortical)	order Gastranaerophilales	rs79790072	T	C	-0.099	100207478	0.547	0.165	360770	T	C	0.226	15	100747683	3.54E-06	0.049	14306	21.466
Vascular dementia (subcortical)	order Gastranaerophilales	rs8028558	A	G	-0.049	61763554	0.409	0.059	360770	A	G	0.083	15	62055753	9.78E-06	0.019	14306	19.603
Vascular dementia (subcortical)	order Gastranaerophilales	rs9864379	T	C	0.010	14265449	0.900	0.080	360770	T	C	-0.161	3	14306949	4.66E-08	0.029	14306	30.065
Vascular dementia (subcortical)	order Lactobacillales	rs11110282	A	G	0.160	100191781	0.232	0.134	360770	A	G	-0.102	12	100585559	3.96E-06	0.022	14306	22.090
Vascular dementia (subcortical)	order Lactobacillales	rs11627423	C	A	-0.076	32731417	0.195	0.059	360770	C	A	0.050	14	33200623	5.09E-06	0.011	14306	20.697
Vascular dementia (subcortical)	order Lactobacillales	rs11730038	G	A	-0.030	97128348	0.635	0.063	360770	G	A	-0.061	4	98049499	5.10E-06	0.013	14306	22.006
Vascular dementia (subcortical)	order Lactobacillales	rs12797734	T	C	0.162	8310803	0.014	0.066	360770	T	C	0.057	11	8332350	7.77E-06	0.013	14306	20.198
Vascular dementia (subcortical)	order Lactobacillales	rs1595463	C	A	0.043	230858942	0.458	0.057	360770	C	A	0.048	2	231723657	7.44E-06	0.011	14306	19.443
Vascular dementia (subcortical)	order Lactobacillales	rs2370083	G	T	-0.079	97060413	0.488	0.114	360770	G	T	-0.081	14	97526750	8.33E-06	0.018	14306	19.802
Vascular dementia (subcortical)	order Lactobacillales	rs2952251	G	A	0.003	10285654	0.963	0.068	360770	G	A	0.063	8	10143164	3.36E-07	0.012	14306	25.684
Vascular dementia (subcortical)	order Lactobacillales	rs34989881	A	G	0.279	51456601	0.041	0.136	360770	A	G	0.113	19	51959855	4.09E-06	0.025	14306	21.201
Vascular dementia (subcortical)	order Lactobacillales	rs35344081	G	A	-0.104	941253	0.107	0.065	360770	G	A	0.064	16	991253	4.16E-07	0.013	14306	25.446
Vascular dementia (subcortical)	order Lactobacillales	rs4028634	C	T	0.081	42683631	0.171	0.059	360770	C	T	-0.053	17	40835649	1.35E-06	0.011	14306	23.428
Vascular dementia (subcortical)	order Lactobacillales	rs57872228	C	T	-0.114	200449677	0.195	0.088	360770	C	T	-0.069	1	200418805	2.58E-06	0.015	14306	21.933
Vascular dementia (subcortical)	order Lactobacillales	rs74663707	C	T	0.040	184653436	0.734	0.118	360770	C	T	0.098	3	184371224	8.40E-06	0.022	14306	19.137
Vascular dementia (subcortical)	order Lactobacillales	rs77558518	A	G	0.167	174746168	0.089	0.098	360770	A	G	-0.106	5	174173171	1.67E-06	0.022	14306	22.695
Vascular dementia (subcortical)	order Lactobacillales	rs78938557	T	C	-0.232	36309977	0.174	0.171	360770	T	C	0.106	7	36349586	2.31E-06	0.023	14306	20.412

Vascular dementia (subcortical)	order Lactobacillales	rs9581006	T	C	-0.064	24399371	0.673	0.152	360770	T	C	-0.226	13	24973509	1.77E-06	0.047	14306	23.163
Vascular dementia (subcortical)	order Methanobacteriales	rs10202904	G	T	0.027	124682691	0.644	0.058	360770	G	T	0.122	2	125440268	3.01E-07	0.024	14306	26.762
Vascular dementia (subcortical)	order Methanobacteriales	rs10424197	A	G	-0.017	45936063	0.799	0.066	360770	A	G	0.111	19	46439321	9.28E-06	0.025	14306	20.211
Vascular dementia (subcortical)	order Methanobacteriales	rs4257531	G	A	-0.191	2044483	0.041	0.093	360770	G	A	0.164	3	2086167	7.44E-06	0.036	14306	20.316
Vascular dementia (subcortical)	order Methanobacteriales	rs6508769	C	T	-0.034	28336853	0.671	0.080	360770	C	T	-0.154	19	28827760	8.23E-06	0.034	14306	19.856
Vascular dementia (subcortical)	order Methanobacteriales	rs6776814	T	C	-0.246	15011576	0.220	0.200	360770	T	C	-0.200	3	15053083	1.63E-06	0.041	14306	23.483
Vascular dementia (subcortical)	order Methanobacteriales	rs73068003	G	T	0.204	10734305	0.035	0.096	360770	G	T	-0.158	7	10773932	8.45E-06	0.035	14306	20.206
Vascular dementia (subcortical)	order Methanobacteriales	rs73457410	A	G	0.108	41382045	0.347	0.115	360770	A	G	0.215	13	41956181	1.41E-06	0.044	14306	24.316
Vascular dementia (subcortical)	order Methanobacteriales	rs75208022	C	T	0.082	21185927	0.401	0.098	360770	C	T	-0.227	12	21338861	5.92E-06	0.049	14306	21.717
Vascular dementia (subcortical)	order Methanobacteriales	rs894996	C	A	0.070	103497150	0.527	0.110	360770	C	A	0.217	4	104418307	1.88E-06	0.045	14306	23.349
Vascular dementia (subcortical)	order Mollicutes RF9	rs11779863	G	A	0.113	15549321	0.151	0.079	360770	G	A	-0.077	8	15406830	6.69E-06	0.017	14306	20.146
Vascular dementia (subcortical)	order Mollicutes RF9	rs12566890	T	G	-0.025	61385192	0.767	0.085	360770	T	G	-0.103	1	61850864	8.11E-06	0.024	14306	18.196
Vascular dementia (subcortical)	order Mollicutes RF9	rs13100746	C	T	-0.002	166128718	0.969	0.057	360770	C	T	0.064	3	165846506	7.29E-06	0.014	14306	20.055
Vascular dementia (subcortical)	order Mollicutes RF9	rs17235252	T	C	0.005	82906721	0.953	0.088	360770	T	C	-0.122	7	82536037	2.16E-06	0.026	14306	22.808
Vascular dementia (subcortical)	order Mollicutes RF9	rs3932485	C	T	0.018	184475694	0.758	0.058	360770	C	T	0.063	3	184193482	9.93E-06	0.014	14306	19.551
Vascular dementia (subcortical)	order Mollicutes RF9	rs515984	C	T	0.083	3479267	0.351	0.089	360770	C	T	0.088	10	3521459	6.61E-06	0.019	14306	21.078
Vascular dementia (subcortical)	order Mollicutes RF9	rs638542	A	G	0.052	109302630	0.404	0.062	360770	A	G	0.071	1	109845252	5.17E-06	0.016	14306	20.204
Vascular dementia (subcortical)	order Mollicutes RF9	rs74603314	T	C	0.203	46050515	0.169	0.147	360770	T	C	0.231	14	46519718	2.28E-06	0.049	14306	22.207
Vascular dementia (subcortical)	order Mollicutes RF9	rs7706512	A	G	-0.028	17443545	0.623	0.057	360770	A	G	-0.066	5	17443654	2.27E-06	0.014	14306	22.372
Vascular dementia (subcortical)	order Mollicutes RF9	rs7801843	A	G	0.122	4066073	0.124	0.079	360770	A	G	-0.087	7	4105705	9.47E-06	0.019	14306	19.983
Vascular dementia (subcortical)	order Mollicutes RF9	rs7853673	A	G	0.005	113948961	0.927	0.057	360770	A	G	0.062	9	116711241	6.73E-06	0.014	14306	19.938
Vascular dementia (subcortical)	order Mollicutes RF9	rs949341	A	G	0.046	112516062	0.465	0.063	360770	A	G	-0.066	11	112386785	7.73E-06	0.015	14306	19.893
Vascular dementia (subcortical)	order NB1n	rs11251024	G	A	0.022	2053532	0.733	0.065	360770	G	A	0.104	10	2095726	6.63E-07	0.021	14306	25.404
Vascular dementia (subcortical)	order NB1n	rs11606187	A	G	0.118	91856434	0.146	0.081	360770	A	G	-0.155	11	91589600	3.31E-06	0.033	14306	22.432
Vascular dementia (subcortical)	order NB1n	rs13385922	T	C	0.030	233172678	0.610	0.060	360770	T	C	0.093	2	234081324	3.97E-06	0.020	14306	21.311
Vascular dementia (subcortical)	order NB1n	rs166849	A	G	-0.124	199914143	0.032	0.058	360770	A	G	-0.091	2	200778866	7.74E-06	0.020	14306	20.294
Vascular dementia (subcortical)	order NB1n	rs2172426	T	C	-0.107	18371511	0.064	0.058	360770	T	C	0.102	8	18229020	3.17E-07	0.020	14306	26.348
Vascular dementia (subcortical)	order NB1n	rs267959	G	A	0.042	10737690	0.513	0.063	360770	G	A	-0.099	5	10737802	2.62E-06	0.021	14306	22.231
Vascular dementia (subcortical)	order NB1n	rs4383094	C	T	-0.080	81402631	0.334	0.082	360770	C	T	-0.149	15	81694972	4.28E-06	0.032	14306	21.646
Vascular dementia (subcortical)	order NB1n	rs60775321	T	C	-0.028	96775441	0.660	0.063	360770	T	C	-0.096	15	97318671	7.10E-06	0.021	14306	20.256
Vascular dementia (subcortical)	order NB1n	rs72671304	T	C	0.129	38956767	0.226	0.107	360770	T	C	0.172	14	39425971	3.80E-06	0.037	14306	21.688
Vascular dementia (subcortical)	order NB1n	rs7911787	G	T	0.206	96859811	0.194	0.159	360770	G	T	-0.223	10	98619568	3.39E-06	0.047	14306	22.504
Vascular dementia (subcortical)	order NB1n	rs8126061	T	C	0.001	3424592	0.988	0.086	360770	T	C	-0.159	20	3405239	7.36E-06	0.035	14306	20.450
Vascular dementia (subcortical)	order NB1n	rs9542068	T	C	0.030	69732699	0.621	0.060	360770	T	C	0.099	13	70306831	6.52E-06	0.022	14306	20.625
Vascular dementia (subcortical)	order Pasteurellales	rs10965428	C	A	0.148	22718482	0.232	0.124	360770	C	A	-0.120	9	22718481	4.29E-06	0.026	14306	21.561
Vascular dementia (subcortical)	order Pasteurellales	rs111582866	G	A	-0.002	48708578	0.983	0.101	360770	G	A	-0.114	16	48742489	7.07E-06	0.026	14306	19.753
Vascular dementia (subcortical)	order Pasteurellales	rs12050685	A	G	0.023	73185141	0.720	0.063	360770	A	G	-0.067	15	73477482	9.19E-06	0.015	14306	19.385
Vascular dementia (subcortical)	order Pasteurellales	rs16970009	A	G	0.345	34535582	0.098	0.208	360770	A	G	0.187	17	32862601	7.32E-06	0.043	14306	19.027
Vascular dementia (subcortical)	order Pasteurellales	rs4822728	T	C	-0.085	26495842	0.135	0.057	360770	T	C	0.069	22	26891808	4.72E-06	0.015	14306	21.156
Vascular dementia (subcortical)	order Pasteurellales	rs6972479	A	G	-0.064	117278006	0.363	0.070	360770	A	G	-0.078	7	116918060	7.75E-06	0.018	14306	19.878
Vascular dementia (subcortical)	order Pasteurellales	rs72756943	G	A	-0.023	26531799	0.844	0.115	360770	G	A	0.140	5	26531908	3.35E-06	0.030	14306	21.308
Vascular dementia (subcortical)	order Pasteurellales	rs73139353	A	C	-0.088	98253370	0.379	0.100	360770	A	C	-0.223	3	97972214	8.71E-06	0.048	14306	21.092
Vascular dementia (subcortical)	order Pasteurellales	rs76022354	C	T	-0.127	92546628	0.334	0.131	360770	C	T	0.243	10	94306385	1.83E-06	0.050	14306	23.560
Vascular dementia (subcortical)	order Pasteurellales	rs78909003	T	C	0.109	102887960	0.372	0.122	360770	T	C	-0.241	9	105650242	2.05E-06	0.050	14306	23.415
Vascular dementia (subcortical)	order Pasteurellales	rs9382510	C	T	-0.026	55583693	0.684	0.065	360770	C	T	-0.088	6	55448491	2.48E-07	0.017	14306	26.921
Vascular dementia (subcortical)	order Pasteurellales	rs9895850	T	C	0.113	66538895	0.416	0.139	360770	T	C	-0.176	17	64535013	9.08E-06	0.041	14306	18.497
Vascular dementia (subcortical)	order Pasteurellales	rs9938097	C	T	0.013	84943783	0.823	0.059	360770	C	T	0.071	16	84977389	8.23E-06	0.016	14306	20.209
Vascular dementia (subcortical)	order Rhodospirillales	rs1035406	G	A	0.024	120037042	0.786	0.089	360770	G	A	-0.115	5	119372737	4.07E-06	0.025	14306	21.401
Vascular dementia (subcortical)	order Rhodospirillales	rs11591293	G	T	0.065	111660039	0.259	0.057	360770	G	T	0.072	10	113419797	4.69E-06	0.016	14306	20.876
Vascular dementia (subcortical)	order Rhodospirillales	rs11630875	T	C	0.041	61483535	0.637	0.086	360770	T	C	0.095	15	61775734	3.70E-06	0.020	14306	21.865
Vascular dementia (subcortical)	order Rhodospirillales	rs13336560	C	T	-0.015	88487835	0.796	0.058	360770	C	T	-0.070	16	88554243	9.75E-06	0.016	14306	19.598
Vascular dementia (subcortical)	order Rhodospirillales	rs1549633	A	C	-0.066	27945538	0.461	0.089	360770	A	C	0.100	5	27945645	3.88E-06	0.022	14306	21.163
Vascular dementia (subcortical)	order Rhodospirillales	rs3730086	A	G	0.068	68281223	0.297	0.065	360770	A	G	0.080	5	67577051	7.98E-06	0.018	14306	20.051
Vascular dementia (subcortical)	order Rhodospirillales	rs3754624	C	T	0.041	224769095	0.576	0.074	360770	C	T	0.094	2	225633812	2.68E-06	0.020	14306	22.511
Vascular dementia (subcortical)	order Rhodospirillales	rs4278423	T	C	-0.052	2628361	0.665	0.119	360770	T	C	0.105	10	2670553	3.98E-06	0.023	14306	20.256
Vascular dementia (subcortical)	order Rhodospirillales	rs61933850	G	A	0.010	72745618	0.903	0.083	360770	G	A	0.165	12	73139398	7.00E-06	0.036	14306	20.888
Vascular dementia (subcortical)	order Rhodospirillales	rs7001029	C	T	-0.064	130946157	0.520	0.099	360770	C	T	0.121	8	131958403	2.83E-06	0.026	14306	21.397
Vascular dementia (subcortical)	order Rhodospirillales	rs76784716	A	G	-0.020	168176830	0.825	0.090	360770	A	G	0.136	2	169033340	1.31E-06	0.028	14306	22.881
Vascular dementia (subcortical)	order Rhodospirillales	rs77304857	C	A	-0.005	133326860	0.940	0.071	360770	C	A	-0.100	4	134248015	6.02E-06	0.022	14306	20.157
Vascular dementia (subcortical)	order Rhodospirillales	rs9813022	A	G	0.029	13685237	0.616	0.059	360770	A	G	-0.083	3	13726736	3.07E-07	0.016	14306	26.093
Vascular dementia (subcortical)	order Selenomonadales	rs1135612	G	A	-0.022	75980359	0.755	0.069	360770	G	A	0.053	7	75609677	9.26E-06	0.012	14306	19.761
Vascular dementia (subcortical)	order Selenomonadales	rs13086907	G	A	-0.077	142416275	0.263	0.069	360770	G	A	0.063	3	142135117	1.95E-06	0.013	14306	22.532
Vascular dementia (subcortical)	order Selenomonadales	rs1643968	T	C	-0.021	165839623	0.725	0.059	360770	T	C	-0.057	5	165266628	4.15E-07	0.011	14306	25.339
Vascular dementia (subcortical)	order Selenomonadales	rs1649999	A	G	-0.097	78326315	0.318	0.097	360770	A	G	0.075	10	80086072	7.58E-06	0.017	14306	20.246
Vascular dementia (subcortical)	order Selenomonadales	rs2834062	A	G	0.075	33005177	0.231	0.062	360770	A	G	0.049	21	34377485	8.44E-06	0.011	14306	20.190
Vascular dementia (subcortical)	order Selenomonadales	rs4463806	C	T	0.087	113838234	0.229	0.072	360770	C	T	0.054	10	115597993	7.81E-06	0.013	14306	17.681
Vascular dementia (subcortical)	order Selenomonadales	rs4722181	T	G	-0.088	22777952	0.122	0.057	360770	T	G	0.050	7	22817571	2.00E-06	0.011	14306	22.452

Vascular dementia (subcortical)	order Selenomonadales	rs60274479	T	C	0.058	21238604	0.422	0.072	360770	T	C	-0.066	16	21249925	1.16E-06	0.013	14306	24.182
Vascular dementia (subcortical)	order Selenomonadales	rs61249479	A	C	-0.055	122150629	0.485	0.079	360770	A	C	0.078	9	124912908	2.95E-06	0.017	14306	21.236
Vascular dementia (subcortical)	order Selenomonadales	rs71405394	G	A	0.130	100704883	0.252	0.114	360770	G	A	-0.114	15	101245088	2.17E-06	0.024	14306	22.539
Vascular dementia (subcortical)	order Selenomonadales	rs73232831	G	A	0.109	17411803	0.484	0.156	360770	G	A	-0.152	4	17413426	1.87E-06	0.031	14306	23.242
Vascular dementia (subcortical)	order Selenomonadales	rs9423647	G	A	-0.020	5537855	0.730	0.057	360770	G	A	0.048	10	5579818	6.06E-06	0.011	14306	20.628
Vascular dementia (subcortical)	order Verrucomicrobiales	rs111862613	T	C	0.047	129825125	0.536	0.076	360770	T	C	0.091	12	130309670	3.74E-06	0.020	14306	21.252
Vascular dementia (subcortical)	order Verrucomicrobiales	rs117107102	A	G	-0.057	51947265	0.671	0.134	360770	A	G	0.205	18	49473635	2.92E-06	0.043	14306	22.493
Vascular dementia (subcortical)	order Verrucomicrobiales	rs11729256	T	C	-0.064	94106121	0.395	0.076	360770	T	C	0.075	4	95027272	6.73E-07	0.015	14306	24.928
Vascular dementia (subcortical)	order Verrucomicrobiales	rs12908520	G	A	0.100	97027427	0.081	0.057	360770	G	A	0.062	15	97570657	2.17E-06	0.013	14306	22.341
Vascular dementia (subcortical)	order Verrucomicrobiales	rs2602429	T	C	0.035	81029544	0.587	0.065	360770	T	C	-0.075	16	81063149	2.58E-06	0.016	14306	22.863
Vascular dementia (subcortical)	order Verrucomicrobiales	rs4242783	A	G	-0.058	5022135	0.363	0.064	360770	A	G	-0.069	10	5064327	2.64E-06	0.015	14306	21.781
Vascular dementia (subcortical)	order Verrucomicrobiales	rs4936098	G	A	0.056	130410772	0.348	0.059	360770	G	A	-0.065	11	130280667	1.12E-06	0.014	14306	22.786
Vascular dementia (subcortical)	order Verrucomicrobiales	rs61779207	G	A	-0.077	40608800	0.265	0.069	360770	G	A	-0.076	1	41074472	6.72E-06	0.017	14306	20.432
Vascular dementia (subcortical)	order Verrucomicrobiales	rs74542928	T	C	0.043	99623031	0.746	0.133	360770	T	C	0.112	4	100544188	1.63E-06	0.024	14306	22.508
Vascular dementia (subcortical)	order Verrucomicrobiales	rs9349825	A	G	-0.064	56476683	0.376	0.072	360770	A	G	-0.070	6	56341481	2.54E-06	0.015	14306	22.898
Vascular dementia (subcortical)	order Verrucomicrobiales	rs941682	G	A	0.065	33280034	0.305	0.063	360770	G	A	-0.063	20	31867840	9.61E-06	0.014	14306	19.290
Vascular dementia (subcortical)	order Victivallales	rs1002941	A	G	0.050	100702485	0.446	0.066	360770	A	G	-0.105	15	101242690	8.15E-06	0.023	14306	20.234
Vascular dementia (subcortical)	order Victivallales	rs11770843	C	T	0.027	147098287	0.658	0.061	360770	C	T	0.109	7	146795379	1.91E-06	0.023	14306	21.707
Vascular dementia (subcortical)	order Victivallales	rs17114848	G	A	0.161	24917388	0.097	0.097	360770	G	A	0.152	15	25162535	4.06E-06	0.032	14306	22.073
Vascular dementia (subcortical)	order Victivallales	rs2031282	A	G	-0.041	20113040	0.584	0.075	360770	A	G	0.122	13	20687179	4.38E-06	0.027	14306	20.490
Vascular dementia (subcortical)	order Victivallales	rs2825714	A	G	0.078	19651652	0.302	0.075	360770	A	G	-0.137	21	21023966	1.72E-06	0.029	14306	22.568
Vascular dementia (subcortical)	order Victivallales	rs62570196	C	T	-0.061	108323890	0.666	0.142	360770	C	T	-0.216	9	111086170	1.08E-06	0.044	14306	24.192
Vascular dementia (subcortical)	order Victivallales	rs72640280	A	G	-0.027	11883735	0.828	0.122	360770	A	G	0.220	1	11943792	5.18E-06	0.049	14306	20.513
Vascular dementia (subcortical)	order Victivallales	rs77599476	A	G	0.019	62762910	0.882	0.125	360770	A	G	0.230	20	61394262	1.86E-06	0.048	14306	23.002
Vascular dementia (subcortical)	phylum Actinobacteria	rs11766971	C	T	0.001	155211037	0.993	0.057	360770	C	T	-0.048	7	155002747	9.40E-06	0.011	14306	20.023
Vascular dementia (subcortical)	phylum Actinobacteria	rs12528285	C	T	-0.048	92065492	0.600	0.091	360770	C	T	0.081	6	92775210	5.69E-06	0.018	14306	20.048
Vascular dementia (subcortical)	phylum Actinobacteria	rs13192624	T	C	-0.089	112808407	0.179	0.066	360770	T	C	-0.052	6	113129609	9.33E-06	0.012	14306	19.720
Vascular dementia (subcortical)	phylum Actinobacteria	rs1397793	A	G	0.048	91175634	0.441	0.062	360770	A	G	0.052	5	90471451	3.74E-06	0.011	14306	21.832
Vascular dementia (subcortical)	phylum Actinobacteria	rs4429415	C	T	0.103	212891467	0.073	0.057	360770	C	T	0.058	2	213756191	2.05E-07	0.011	14306	27.314
Vascular dementia (subcortical)	phylum Actinobacteria	rs55888705	A	G	-0.012	1516099	0.850	0.063	360770	A	G	0.053	4	1517826	1.31E-06	0.011	14306	23.597
Vascular dementia (subcortical)	phylum Actinobacteria	rs6496870	C	T	0.053	91924192	0.369	0.059	360770	C	T	-0.051	15	92467422	4.62E-06	0.011	14306	20.142
Vascular dementia (subcortical)	phylum Actinobacteria	rs6743026	T	C	0.035	101633121	0.631	0.072	360770	T	C	0.059	2	102249583	9.88E-06	0.013	14306	19.171
Vascular dementia (subcortical)	phylum Actinobacteria	rs74037001	G	A	-0.017	23377985	0.868	0.102	360770	G	A	-0.082	14	23847194	6.71E-07	0.017	14306	24.562
Vascular dementia (subcortical)	phylum Actinobacteria	rs75211493	G	A	0.038	5300454	0.727	0.110	360770	G	A	0.084	19	5300465	9.27E-06	0.018	14306	20.714
Vascular dementia (subcortical)	phylum Actinobacteria	rs7570971	A	C	-0.004	135080336	0.939	0.058	360770	A	C	0.087	2	135837906	1.41E-14	0.011	14306	58.161
Vascular dementia (subcortical)	phylum Actinobacteria	rs80124826	T	C	0.083	239014688	0.639	0.176	360770	T	C	-0.124	2	239936384	8.75E-06	0.028	14306	19.876
Vascular dementia (subcortical)	phylum Actinobacteria	rs857444	C	T	0.056	14617360	0.344	0.059	360770	C	T	0.051	6	14617591	3.80E-06	0.011	14306	21.315
Vascular dementia (subcortical)	phylum Actinobacteria	rs9833771	C	T	0.000	32321506	0.995	0.057	360770	C	T	-0.049	3	32362998	4.07E-06	0.011	14306	21.150
Vascular dementia (subcortical)	phylum Bacteroidetes	rs17343978	A	C	-0.037	27037922	0.601	0.070	360770	A	C	-0.056	22	27433885	7.22E-06	0.012	14306	21.374
Vascular dementia (subcortical)	phylum Bacteroidetes	rs2032750	C	T	-0.072	53603889	0.209	0.057	360770	C	T	0.051	2	53831026	1.71E-06	0.011	14306	22.878
Vascular dementia (subcortical)	phylum Bacteroidetes	rs62531359	T	G	-0.041	70003946	0.585	0.075	360770	T	G	0.066	8	70916181	8.42E-06	0.015	14306	19.260
Vascular dementia (subcortical)	phylum Bacteroidetes	rs62575403	C	T	0.043	133628698	0.760	0.141	360770	C	T	0.145	9	136493820	2.96E-06	0.031	14306	21.854
Vascular dementia (subcortical)	phylum Bacteroidetes	rs6586324	T	C	0.011	42522025	0.850	0.057	360770	T	C	0.048	21	43942135	7.37E-06	0.011	14306	20.535
Vascular dementia (subcortical)	phylum Bacteroidetes	rs72706335	T	C	-0.229	157525648	0.218	0.186	360770	T	C	-0.223	1	157495438	7.13E-06	0.049	14306	20.464
Vascular dementia (subcortical)	phylum Bacteroidetes	rs73512608	G	A	-0.001	69948551	0.995	0.124	360770	G	A	-0.123	13	70522683	2.54E-07	0.024	14306	27.045
Vascular dementia (subcortical)	phylum Bacteroidetes	rs73846128	A	G	-0.027	89291104	0.740	0.080	360770	A	G	-0.066	3	89340254	4.78E-07	0.013	14306	24.765
Vascular dementia (subcortical)	phylum Bacteroidetes	rs73975615	G	A	0.069	6557880	0.834	0.330	360770	G	A	-0.207	17	6461200	1.20E-06	0.044	14306	21.905
Vascular dementia (subcortical)	phylum Bacteroidetes	rs929878	T	C	-0.092	74256742	0.188	0.070	360770	T	C	0.054	16	74290641	6.51E-06	0.012	14306	19.745
Vascular dementia (subcortical)	phylum Cyanobacteria	rs12555298	G	A	0.014	107727270	0.852	0.075	360770	G	A	0.097	9	110489551	8.09E-06	0.022	14306	19.730
Vascular dementia (subcortical)	phylum Cyanobacteria	rs2585223	T	C	-0.057	100340683	0.512	0.087	360770	T	C	0.111	15	100880888	8.86E-06	0.025	14306	20.206
Vascular dementia (subcortical)	phylum Cyanobacteria	rs584122	T	C	0.059	48320720	0.628	0.121	360770	T	C	0.152	6	48288456	4.23E-06	0.033	14306	21.565
Vascular dementia (subcortical)	phylum Cyanobacteria	rs61972390	T	C	-0.045	99914504	0.586	0.083	360770	T	C	0.107	13	100566758	9.11E-06	0.024	14306	19.600
Vascular dementia (subcortical)	phylum Cyanobacteria	rs7148504	T	G	0.025	99701961	0.670	0.058	360770	T	G	-0.080	14	100168298	6.62E-06	0.018	14306	20.537
Vascular dementia (subcortical)	phylum Cyanobacteria	rs76531781	T	C	-0.026	21608117	0.860	0.147	360770	T	C	-0.232	7	21647735	2.87E-06	0.049	14306	22.085
Vascular dementia (subcortical)	phylum Cyanobacteria	rs789068	G	A	-0.080	1008238	0.329	0.082	360770	G	A	-0.111	18	1008239	1.57E-07	0.021	14306	27.366
Vascular dementia (subcortical)	phylum Cyanobacteria	rs9864379	T	C	0.010	14265449	0.900	0.080	360770	T	C	-0.139	3	14306949	2.03E-07	0.027	14306	26.866
Vascular dementia (subcortical)	phylum Euryarchaeota	rs10202904	G	T	0.027	124682691	0.644	0.058	360770	G	T	0.116	2	125440268	6.19E-07	0.023	14306	25.371
Vascular dementia (subcortical)	phylum Euryarchaeota	rs11022995	A	G	0.007	13872120	0.900	0.057	360770	A	G	0.104	11	13893667	7.73E-06	0.023	14306	20.538
Vascular dementia (subcortical)	phylum Euryarchaeota	rs34928225	T	C	-0.171	146519740	0.076	0.096	360770	T	C	0.200	6	146840876	4.33E-06	0.043	14306	22.051
Vascular dementia (subcortical)	phylum Euryarchaeota	rs45498998	G	A	-0.142	25996679	0.071	0.079	360770	G	A	-0.132	21	27368994	5.32E-06	0.029	14306	20.358
Vascular dementia (subcortical)	phylum Euryarchaeota	rs6064552	T	C	-0.080	57467810	0.274	0.074	360770	T	C	-0.124	20	56042866	9.34E-06	0.028	14306	20.095
Vascular dementia (subcortical)	phylum Euryarchaeota	rs6508769	C	T	-0.034	28336853	0.671	0.080	360770	C	T	-0.151	19	28827760	8.12E-06	0.034	14306	19.941
Vascular dementia (subcortical)	phylum Euryarchaeota	rs7015093	G	A	0.069	57106294	0.284	0.065	360770	G	A	-0.118	8	58018853	7.20E-06	0.026	14306	19.957
Vascular dementia (subcortical)	phylum Euryarchaeota	rs76029318	T	C	0.120	41389655	0.302	0.116	360770	T	C	0.215	13	41963791	1.05E-06	0.044	14306	24.017
Vascular dementia (subcortical)	phylum Euryarchaeota	rs7635189	A	G	0.025	15550100	0.693	0.063	360770	A	G	-0.120	3	15591607	4.64E-06	0.026	14306	21.402
Vascular dementia (subcortical)	phylum Euryarchaeota	rs77658038	A	C	0.029	16284554	0.684	0.071	360770	A	C	-0.160	6	16284785	4.75E-06	0.034	14306	22.078
Vascular dementia (subcortical)	phylum Euryarchaeota	rs894996	C	A	0.070	103497150	0.527	0.110	360770	C	A	0.204	4	104418307	5.12E-06	0.044	14306	21.404

Vascular dementia (subcortical)	phylum Firmicutes	rs112334273	G	A	0.041	39331325	0.521	0.064	360770	G	A	0.063	21	40703251	9.26E-07	0.013	14306	24.251
Vascular dementia (subcortical)	phylum Firmicutes	rs2273429	A	G	0.141	52027354	0.122	0.091	360770	A	G	-0.070	14	52494072	9.26E-06	0.015	14306	21.041
Vascular dementia (subcortical)	phylum Firmicutes	rs2332027	A	G	-0.072	170750887	0.217	0.058	360770	A	G	0.048	4	171672038	4.05E-06	0.010	14306	21.201
Vascular dementia (subcortical)	phylum Firmicutes	rs3792064	G	A	-0.143	230813698	0.264	0.128	360770	G	A	0.090	2	231678413	6.75E-07	0.018	14306	24.168
Vascular dementia (subcortical)	phylum Firmicutes	rs3852931	T	C	-0.106	55471425	0.077	0.060	360770	T	C	0.048	20	54087963	4.53E-06	0.011	14306	21.080
Vascular dementia (subcortical)	phylum Firmicutes	rs4750583	G	A	-0.005	15012766	0.949	0.073	360770	G	A	-0.062	10	15054765	5.79E-06	0.014	14306	19.944
Vascular dementia (subcortical)	phylum Firmicutes	rs56199908	T	C	-0.142	2801371	0.255	0.125	360770	T	C	-0.186	9	2801371	8.67E-06	0.041	14306	20.572
Vascular dementia (subcortical)	phylum Firmicutes	rs6814436	C	T	0.001	160586149	0.995	0.082	360770	C	T	-0.068	4	161507301	6.80E-06	0.015	14306	20.334
Vascular dementia (subcortical)	phylum Firmicutes	rs6815608	C	T	-0.023	151210592	0.768	0.079	360770	C	T	-0.094	4	152131744	7.24E-06	0.021	14306	19.693
Vascular dementia (subcortical)	phylum Firmicutes	rs7247191	T	C	0.029	22716783	0.762	0.095	360770	T	C	-0.071	19	22899585	4.73E-06	0.016	14306	20.515
Vascular dementia (subcortical)	phylum Firmicutes	rs72738886	T	C	0.154	35770448	0.152	0.108	360770	T	C	0.086	5	35770550	7.68E-06	0.019	14306	20.621
Vascular dementia (subcortical)	phylum Firmicutes	rs72771021	C	T	0.102	13799631	0.375	0.116	360770	C	T	-0.141	10	13841631	5.12E-06	0.031	14306	21.022
Vascular dementia (subcortical)	phylum Firmicutes	rs8085381	A	G	-0.101	31748342	0.168	0.073	360770	A	G	-0.065	18	29328305	8.67E-06	0.015	14306	19.597
Vascular dementia (subcortical)	phylum Firmicutes	rs992074	T	C	0.040	17195484	0.836	0.194	360770	T	C	-0.233	21	18567802	8.52E-06	0.051	14306	21.042
Vascular dementia (subcortical)	phylum Lentisphaerae	rs1002941	A	G	0.050	100702485	0.446	0.066	360770	A	G	-0.108	15	101242690	4.31E-06	0.023	14306	21.281
Vascular dementia (subcortical)	phylum Lentisphaerae	rs11770843	C	T	0.027	147098287	0.658	0.061	360770	C	T	0.112	7	146795379	1.14E-06	0.023	14306	22.715
Vascular dementia (subcortical)	phylum Lentisphaerae	rs17114848	G	A	0.161	24917388	0.097	0.097	360770	G	A	0.149	15	25162535	6.77E-06	0.032	14306	21.120
Vascular dementia (subcortical)	phylum Lentisphaerae	rs2031282	A	G	-0.041	20113040	0.584	0.075	360770	A	G	0.120	13	20687179	5.86E-06	0.027	14306	19.850
Vascular dementia (subcortical)	phylum Lentisphaerae	rs2825714	A	G	0.078	19651652	0.302	0.075	360770	A	G	-0.138	21	21023966	1.50E-06	0.029	14306	22.861
Vascular dementia (subcortical)	phylum Lentisphaerae	rs60995569	T	G	0.133	52170325	0.140	0.090	360770	T	G	-0.161	10	53930085	9.19E-06	0.034	14306	22.743
Vascular dementia (subcortical)	phylum Lentisphaerae	rs62570196	C	T	-0.061	108323890	0.666	0.142	360770	C	T	-0.217	9	111086170	9.64E-07	0.044	14306	24.410
Vascular dementia (subcortical)	phylum Lentisphaerae	rs72640280	A	G	-0.027	11883735	0.828	0.122	360770	A	G	0.220	1	11943792	5.19E-06	0.049	14306	20.530
Vascular dementia (subcortical)	phylum Lentisphaerae	rs77599476	A	G	0.019	62762910	0.882	0.125	360770	A	G	0.230	20	61394262	1.90E-06	0.048	14306	22.964
Vascular dementia (subcortical)	phylum Proteobacteria	rs10750258	C	A	-0.046	124126791	0.438	0.059	360770	C	A	0.049	11	123997498	8.72E-06	0.011	14306	20.598
Vascular dementia (subcortical)	phylum Proteobacteria	rs11126162	T	C	-0.034	67858902	0.740	0.102	360770	T	C	-0.077	2	68086034	9.26E-06	0.019	14306	16.971
Vascular dementia (subcortical)	phylum Proteobacteria	rs11715072	G	A	0.032	53686766	0.611	0.062	360770	G	A	-0.052	3	53720793	6.90E-06	0.012	14306	20.303
Vascular dementia (subcortical)	phylum Proteobacteria	rs12150865	C	T	-0.066	43001168	0.250	0.058	360770	C	T	0.051	19	43505320	1.54E-06	0.011	14306	23.118
Vascular dementia (subcortical)	phylum Proteobacteria	rs12467198	C	T	0.025	124115104	0.659	0.057	360770	C	T	0.050	2	124872681	6.31E-06	0.011	14306	20.013
Vascular dementia (subcortical)	phylum Proteobacteria	rs2347697	G	T	-0.085	134703488	0.164	0.061	360770	G	T	0.050	7	134388240	4.27E-06	0.011	14306	21.247
Vascular dementia (subcortical)	phylum Proteobacteria	rs2532663	A	G	-0.016	117487723	0.863	0.095	360770	A	G	0.126	10	119247234	7.47E-07	0.026	14306	23.685
Vascular dementia (subcortical)	phylum Proteobacteria	rs3890996	G	T	0.067	20314277	0.236	0.057	360770	G	T	0.047	22	20301800	6.95E-06	0.011	14306	20.183
Vascular dementia (subcortical)	phylum Proteobacteria	rs4340090	C	T	0.072	129847219	0.410	0.088	360770	C	T	-0.067	12	130331764	9.99E-06	0.015	14306	18.975
Vascular dementia (subcortical)	phylum Proteobacteria	rs6707783	C	T	-0.019	51826753	0.849	0.098	360770	C	T	0.085	2	52053891	8.09E-06	0.019	14306	20.470
Vascular dementia (subcortical)	phylum Proteobacteria	rs72771021	C	T	0.102	13799631	0.375	0.116	360770	C	T	0.142	10	13841631	7.18E-06	0.031	14306	21.004
Vascular dementia (subcortical)	phylum Proteobacteria	rs922773	C	T	-0.006	66267618	0.948	0.095	360770	C	T	-0.080	3	66318042	3.68E-07	0.016	14306	25.929
Vascular dementia (subcortical)	phylum Tenericutes	rs10108398	G	A	0.099	58528265	0.118	0.063	360770	G	A	0.077	8	59440824	1.09E-06	0.015	14306	24.960
Vascular dementia (subcortical)	phylum Tenericutes	rs11890098	A	G	-0.006	156676037	0.929	0.063	360770	A	G	0.074	2	157532549	9.57E-07	0.015	14306	23.551
Vascular dementia (subcortical)	phylum Tenericutes	rs12566890	T	G	-0.025	61385192	0.767	0.085	360770	T	G	-0.101	1	61850864	3.65E-06	0.023	14306	19.176
Vascular dementia (subcortical)	phylum Tenericutes	rs17214486	C	A	0.118	96693337	0.053	0.061	360770	C	A	0.061	14	97159674	6.61E-06	0.014	14306	20.223
Vascular dementia (subcortical)	phylum Tenericutes	rs2464826	A	C	0.073	79860934	0.416	0.090	360770	A	C	0.094	7	79490250	8.39E-06	0.021	14306	19.874
Vascular dementia (subcortical)	phylum Tenericutes	rs28537087	G	A	0.013	94766447	0.844	0.066	360770	G	A	0.082	15	95309676	8.07E-06	0.019	14306	19.002
Vascular dementia (subcortical)	phylum Tenericutes	rs3768491	G	A	0.051	109423364	0.411	0.062	360770	G	A	0.068	1	109965986	4.23E-06	0.015	14306	20.875
Vascular dementia (subcortical)	phylum Tenericutes	rs4885016	C	T	-0.006	72596351	0.942	0.083	360770	C	T	0.082	13	73170489	7.27E-06	0.018	14306	20.363
Vascular dementia (subcortical)	phylum Tenericutes	rs6043847	T	C	0.029	16278879	0.808	0.120	360770	T	C	-0.115	20	16259524	4.55E-06	0.025	14306	21.375
Vascular dementia (subcortical)	phylum Tenericutes	rs72901605	T	C	-0.050	47082326	0.569	0.088	360770	T	C	-0.084	11	47103877	3.26E-06	0.018	14306	22.338
Vascular dementia (subcortical)	phylum Tenericutes	rs74603314	T	C	0.203	46050515	0.169	0.147	360770	T	C	0.222	14	46519718	1.56E-06	0.046	14306	22.924
Vascular dementia (subcortical)	phylum Tenericutes	rs78169027	A	G	0.176	108568360	0.141	0.119	360770	A	G	-0.108	11	108439087	5.88E-06	0.024	14306	20.824
Vascular dementia (subcortical)	phylum Verrucomicrobia	rs11252894	A	C	0.074	5030858	0.267	0.067	360770	A	C	0.078	10	5073050	1.11E-06	0.016	14306	23.203
Vascular dementia (subcortical)	phylum Verrucomicrobia	rs117107102	A	G	-0.057	51947265	0.671	0.134	360770	A	G	0.204	18	49473635	2.68E-06	0.043	14306	22.828
Vascular dementia (subcortical)	phylum Verrucomicrobia	rs11729256	T	C	-0.064	94106121	0.395	0.076	360770	T	C	0.070	4	95027272	2.23E-06	0.015	14306	22.495
Vascular dementia (subcortical)	phylum Verrucomicrobia	rs12512971	A	C	-0.063	16547352	0.551	0.106	360770	A	C	0.171	4	16548975	9.81E-06	0.040	14306	18.467
Vascular dementia (subcortical)	phylum Verrucomicrobia	rs12908520	G	A	0.100	97027427	0.081	0.057	360770	G	A	0.059	15	97570657	3.40E-06	0.013	14306	21.524
Vascular dementia (subcortical)	phylum Verrucomicrobia	rs2602429	T	C	0.035	81029544	0.587	0.065	360770	T	C	-0.076	16	81063149	8.71E-07	0.015	14306	24.786
Vascular dementia (subcortical)	phylum Verrucomicrobia	rs3995795	C	T	-0.131	11347615	0.025	0.058	360770	C	T	0.061	10	11389614	9.72E-06	0.014	14306	19.430
Vascular dementia (subcortical)	phylum Verrucomicrobia	rs45598138	C	A	-0.273	55056410	0.133	0.181	360770	C	A	-0.144	1	55522083	2.19E-06	0.031	14306	22.202
Vascular dementia (subcortical)	phylum Verrucomicrobia	rs61779207	G	A	-0.077	40608800	0.265	0.069	360770	G	A	-0.076	1	41074472	5.28E-06	0.016	14306	21.109
Vascular dementia (subcortical)	phylum Verrucomicrobia	rs74542928	T	C	0.043	99623031	0.746	0.133	360770	T	C	0.116	4	100544188	4.08E-07	0.023	14306	25.144
Vascular dementia (subcortical)	phylum Verrucomicrobia	rs76430504	T	C	0.136	40438056	0.306	0.132	360770	T	C	-0.118	5	40438158	3.50E-06	0.025	14306	21.313
Vascular dementia (subcortical)	phylum Verrucomicrobia	rs9349825	A	G	-0.064	56476683	0.376	0.072	360770	A	G	-0.066	6	56341481	6.27E-06	0.014	14306	21.048
Vascular dementia (sudden onset)	class Actinobacteria	rs11655079	T	C	0.000	77352593	1.000	0.150	360283	T	C	-0.056	17	75348675	5.93E-06	0.012	14306	20.351
Vascular dementia (sudden onset)	class Actinobacteria	rs11745923	G	T	0.037	475293	0.756	0.119	360283	G	T	0.056	5	475408	1.58E-06	0.012	14306	23.812
Vascular dementia (sudden onset)	class Actinobacteria	rs12049045	A	G	0.106	114122863	0.370	0.118	360283	A	G	0.051	1	114665485	8.63E-06	0.011	14306	19.936
Vascular dementia (sudden onset)	class Actinobacteria	rs134366	A	G	0.135	35171362	0.557	0.230	360283	A	G	-0.112	22	35567355	1.50E-06	0.024	14306	22.663
Vascular dementia (sudden onset)	class Actinobacteria	rs1376754	G	A	0.068	160755458	0.558	0.116	360283	G	A	0.051	2	161611969	6.71E-06	0.011	14306	20.489
Vascular dementia (sudden onset)	class Actinobacteria	rs1515761	C	T	-0.243	124421463	0.269	0.220	360283	C	T	-0.076	10	126110032	4.96E-06	0.017	14306	20.136
Vascular dementia (sudden onset)	class Actinobacteria	rs182549	T	C	0.090	135859184	0.454	0.120	360283	T	C	-0.111	2	136616754	3.79E-20	0.012	14306	85.376
Vascular dementia (sudden onset)	class Actinobacteria	rs4945008	G	A	0.157	71510202	0.187	0.119	360283	G	A	-0.054	11	71221248	5.39E-06	0.012	14306	19.842

Vascular dementia (sudden onset)	class Actinobacteria	rs6660520	G	A	0.030	206830208	0.815	0.130	360283	G	A	-0.071	1	207003553	1.11E-07	0.013	14306	27.984
Vascular dementia (sudden onset)	class Actinobacteria	rs72767435	T	C	-0.043	94309566	0.869	0.259	360283	T	C	-0.126	15	94852795	2.57E-06	0.027	14306	21.323
Vascular dementia (sudden onset)	class Actinobacteria	rs7322849	T	C	0.011	112205515	0.957	0.203	360283	T	C	0.094	13	112859829	6.21E-07	0.019	14306	23.953
Vascular dementia (sudden onset)	class Actinobacteria	rs80083040	T	G	-0.195	64742287	0.484	0.279	360283	T	G	0.156	8	65654844	8.62E-06	0.035	14306	20.038
Vascular dementia (sudden onset)	class Actinobacteria	rs857444	C	T	-0.124	14617360	0.303	0.120	360283	C	T	0.051	6	14617591	8.92E-06	0.012	14306	19.500
Vascular dementia (sudden onset)	class Actinobacteria	rs961091	G	A	-0.033	96025406	0.783	0.121	360283	G	A	0.050	7	95654718	8.68E-06	0.011	14306	19.835
Vascular dementia (sudden onset)	class Alphaproteobacteria	rs140912403	C	T	-0.147	92755525	0.554	0.249	360283	C	T	-0.161	9	95517807	6.20E-07	0.032	14306	25.577
Vascular dementia (sudden onset)	class Alphaproteobacteria	rs34569731	G	A	-0.016	75403218	0.893	0.121	360283	G	A	-0.071	2	75630344	7.38E-06	0.016	14306	20.028
Vascular dementia (sudden onset)	class Alphaproteobacteria	rs62285697	C	T	0.036	176690123	0.788	0.134	360283	C	T	0.081	3	176407911	9.76E-06	0.018	14306	19.812
Vascular dementia (sudden onset)	class Alphaproteobacteria	rs76784716	A	G	-0.097	168176830	0.601	0.186	360283	A	G	0.133	2	169033340	5.09E-07	0.027	14306	24.777
Vascular dementia (sudden onset)	class Alphaproteobacteria	rs7960664	A	G	-0.040	89077458	0.848	0.207	360283	A	G	-0.097	12	89471235	8.84E-06	0.022	14306	20.305
Vascular dementia (sudden onset)	class Alphaproteobacteria	rs9813022	A	G	-0.181	13685237	0.132	0.120	360283	A	G	-0.075	3	13726736	1.05E-06	0.015	14306	23.840
Vascular dementia (sudden onset)	class Bacilli	rs11110282	A	G	-0.403	100191781	0.136	0.271	360283	A	G	-0.101	12	100585559	4.85E-06	0.022	14306	21.669
Vascular dementia (sudden onset)	class Bacilli	rs11730038	G	A	-0.211	97128348	0.100	0.128	360283	G	A	-0.063	4	98049499	1.96E-06	0.013	14306	24.013
Vascular dementia (sudden onset)	class Bacilli	rs12797734	T	C	-0.033	8310803	0.804	0.135	360283	T	C	0.057	11	8332350	7.21E-06	0.013	14306	20.363
Vascular dementia (sudden onset)	class Bacilli	rs13068444	A	G	0.199	64413187	0.190	0.152	360283	A	G	0.060	3	64398863	9.53E-06	0.014	14306	19.415
Vascular dementia (sudden onset)	class Bacilli	rs1595463	C	A	0.026	230858942	0.824	0.117	360283	C	A	0.048	2	231723657	7.97E-06	0.011	14306	19.300
Vascular dementia (sudden onset)	class Bacilli	rs28564647	T	G	-0.275	98338890	0.080	0.157	360283	T	G	-0.061	9	101101172	7.81E-06	0.014	14306	19.855
Vascular dementia (sudden onset)	class Bacilli	rs2952251	G	A	-0.067	10285654	0.633	0.140	360283	G	A	0.060	8	10143164	1.08E-06	0.012	14306	23.361
Vascular dementia (sudden onset)	class Bacilli	rs34989881	A	G	0.537	51456601	0.055	0.280	360283	A	G	0.111	19	51959855	6.55E-06	0.025	14306	20.374
Vascular dementia (sudden onset)	class Bacilli	rs35344081	G	A	-0.017	941253	0.897	0.132	360283	G	A	0.062	16	991253	1.01E-06	0.013	14306	23.772
Vascular dementia (sudden onset)	class Bacilli	rs4028634	C	T	-0.004	42683631	0.972	0.121	360283	C	T	-0.052	17	40835649	2.21E-06	0.011	14306	22.464
Vascular dementia (sudden onset)	class Bacilli	rs4459992	T	C	0.094	7429760	0.450	0.124	360283	T	C	0.054	4	7431487	4.30E-06	0.012	14306	21.207
Vascular dementia (sudden onset)	class Bacilli	rs57872228	C	T	0.154	200449677	0.394	0.181	360283	C	T	-0.071	1	200418805	9.22E-07	0.015	14306	23.757
Vascular dementia (sudden onset)	class Bacilli	rs694949	A	G	0.254	58445876	0.188	0.193	360283	A	G	-0.081	15	58738075	7.60E-06	0.018	14306	20.314
Vascular dementia (sudden onset)	class Bacilli	rs74663707	C	T	-0.039	184653436	0.871	0.238	360283	C	T	0.098	3	184371224	8.46E-06	0.022	14306	19.095
Vascular dementia (sudden onset)	class Bacilli	rs7666190	A	C	0.029	150766268	0.870	0.180	360283	A	C	0.104	4	151687420	8.47E-06	0.025	14306	17.610
Vascular dementia (sudden onset)	class Bacilli	rs77558518	A	G	0.027	174746168	0.892	0.202	360283	A	G	-0.107	5	174173171	1.34E-06	0.022	14306	23.158
Vascular dementia (sudden onset)	class Bacilli	rs78938557	T	C	0.571	36309977	0.106	0.353	360283	T	C	0.108	7	36349586	1.07E-06	0.023	14306	21.514
Vascular dementia (sudden onset)	class Bacilli	rs9581006	T	C	0.202	24399371	0.510	0.306	360283	T	C	-0.225	13	24973509	1.79E-06	0.047	14306	23.207
Vascular dementia (sudden onset)	class Bacteroidia	rs11146701	A	G	-0.061	38769138	0.620	0.123	360283	A	G	0.047	10	39062269	7.08E-06	0.011	14306	20.186
Vascular dementia (sudden onset)	class Bacteroidia	rs17343978	A	C	-0.036	27037922	0.802	0.145	360283	A	C	-0.055	22	27433885	8.36E-06	0.012	14306	21.067
Vascular dementia (sudden onset)	class Bacteroidia	rs2032750	C	T	0.094	53603889	0.422	0.117	360283	C	T	0.051	2	53831026	1.92E-06	0.011	14306	22.657
Vascular dementia (sudden onset)	class Bacteroidia	rs2363574	T	C	0.542	200143435	0.079	0.309	360283	T	C	0.223	1	200112563	9.93E-06	0.051	14306	19.221
Vascular dementia (sudden onset)	class Bacteroidia	rs4916508	A	G	0.256	196209918	0.029	0.117	360283	A	G	0.047	3	195936789	8.47E-06	0.011	14306	19.641
Vascular dementia (sudden onset)	class Bacteroidia	rs55773148	G	A	0.258	69948897	0.314	0.256	360283	G	A	-0.122	13	70523029	3.90E-07	0.024	14306	26.341
Vascular dementia (sudden onset)	class Bacteroidia	rs62531359	T	G	0.140	70003946	0.362	0.153	360283	T	G	0.066	8	70916181	9.09E-06	0.015	14306	19.138
Vascular dementia (sudden onset)	class Bacteroidia	rs62575403	C	T	0.584	133628698	0.047	0.294	360283	C	T	0.140	9	136493820	7.06E-06	0.031	14306	20.264
Vascular dementia (sudden onset)	class Bacteroidia	rs72706335	T	C	-0.165	157525648	0.664	0.379	360283	T	C	-0.222	1	157495438	7.66E-06	0.049	14306	20.315
Vascular dementia (sudden onset)	class Bacteroidia	rs73975615	G	A	-1.030	6557880	0.144	0.705	360283	G	A	-0.207	17	6461200	1.22E-06	0.044	14306	21.874
Vascular dementia (sudden onset)	class Bacteroidia	rs7631304	G	A	-0.120	89290377	0.463	0.164	360283	G	A	-0.065	3	89339527	8.37E-07	0.013	14306	23.590
Vascular dementia (sudden onset)	class Bacteroidia	rs79585701	A	C	-0.021	13252676	0.899	0.168	360283	A	C	0.065	8	13110185	9.99E-06	0.015	14306	18.687
Vascular dementia (sudden onset)	class Bacteroidia	rs929878	T	C	-0.359	74256742	0.012	0.143	360283	T	C	0.055	16	74290641	4.73E-06	0.012	14306	20.372
Vascular dementia (sudden onset)	class Betaproteobacteria	rs11128180	A	G	-0.123	70543064	0.371	0.138	360283	A	G	0.059	3	70592215	3.67E-06	0.013	14306	21.203
Vascular dementia (sudden onset)	class Betaproteobacteria	rs1511453	A	G	-0.054	23586839	0.830	0.252	360283	A	G	0.092	4	23588462	4.76E-06	0.020	14306	21.502
Vascular dementia (sudden onset)	class Betaproteobacteria	rs1928341	G	A	0.112	153267537	0.346	0.119	360283	G	A	-0.053	1	153240013	2.02E-06	0.011	14306	22.661
Vascular dementia (sudden onset)	class Betaproteobacteria	rs2321387	G	A	-0.188	58115206	0.109	0.117	360283	G	A	-0.049	13	58689340	5.80E-06	0.011	14306	20.430
Vascular dementia (sudden onset)	class Betaproteobacteria	rs2613606	T	C	-0.016	111644969	0.890	0.118	360283	T	C	0.051	7	111285025	2.20E-06	0.011	14306	22.123
Vascular dementia (sudden onset)	class Betaproteobacteria	rs320161	G	A	-0.237	102136835	0.087	0.139	360283	G	A	-0.057	9	104899117	7.33E-06	0.013	14306	20.564
Vascular dementia (sudden onset)	class Betaproteobacteria	rs4033856	T	C	-0.506	45640468	0.015	0.208	360283	T	C	-0.083	4	45642485	5.17E-07	0.017	14306	24.776
Vascular dementia (sudden onset)	class Betaproteobacteria	rs6087811	T	G	-0.039	32008327	0.839	0.194	360283	T	G	-0.098	20	30596130	7.44E-07	0.020	14306	24.278
Vascular dementia (sudden onset)	class Betaproteobacteria	rs62395635	T	C	0.033	174070793	0.890	0.241	360283	T	C	0.110	5	173497796	2.94E-06	0.024	14306	21.623
Vascular dementia (sudden onset)	class Betaproteobacteria	rs75242906	C	T	-0.070	56494209	0.741	0.212	360283	C	T	-0.121	15	56786407	9.27E-06	0.028	14306	18.601
Vascular dementia (sudden onset)	class Clostridia	rs10774377	G	A	0.150	5833353	0.203	0.118	360283	G	A	-0.053	12	5942519	3.24E-06	0.011	14306	21.390
Vascular dementia (sudden onset)	class Clostridia	rs112334273	G	A	-0.053	39331325	0.682	0.130	360283	G	A	0.064	21	40703251	3.81E-07	0.013	14306	25.314
Vascular dementia (sudden onset)	class Clostridia	rs13105690	C	T	0.131	7418457	0.311	0.130	360283	C	T	0.053	4	7420184	8.78E-06	0.012	14306	20.044
Vascular dementia (sudden onset)	class Clostridia	rs13179700	C	T	-0.173	149698225	0.161	0.123	360283	C	T	-0.051	5	149077788	3.37E-06	0.011	14306	21.830
Vascular dementia (sudden onset)	class Clostridia	rs1842454	G	A	0.202	105724661	0.177	0.149	360283	G	A	-0.055	5	105060362	8.72E-06	0.013	14306	18.466
Vascular dementia (sudden onset)	class Clostridia	rs2273429	A	G	0.158	52027354	0.400	0.188	360283	A	G	-0.072	14	52494072	4.52E-06	0.015	14306	22.364
Vascular dementia (sudden onset)	class Clostridia	rs6797343	T	G	0.121	89219564	0.409	0.147	360283	T	G	-0.059	3	89268714	9.36E-06	0.013	14306	19.375
Vascular dementia (sudden onset)	class Clostridia	rs6814436	C	T	0.012	160586149	0.942	0.167	360283	C	T	-0.074	4	161507301	9.65E-07	0.015	14306	24.075
Vascular dementia (sudden onset)	class Clostridia	rs6815608	C	T	-0.136	151210592	0.400	0.161	360283	C	T	-0.104	4	152131744	4.02E-07	0.021	14306	24.229
Vascular dementia (sudden onset)	class Clostridia	rs72738886	T	C	-0.150	35770448	0.499	0.222	360283	T	C	0.087	5	35770550	8.24E-06	0.019	14306	20.697
Vascular dementia (sudden onset)	class Clostridia	rs992074	T	C	-0.158	17195484	0.681	0.386	360283	T	C	-0.256	21	18567802	8.78E-07	0.051	14306	25.245
Vascular dementia (sudden onset)	class Coriobacteriia	rs11073596	G	T	-0.042	85890348	0.728	0.120	360283	G	T	-0.051	15	86433579	8.14E-06	0.011	14306	19.912
Vascular dementia (sudden onset)	class Coriobacteriia	rs11250875	T	C	0.192	1880537	0.175	0.141	360283	T	C	0.061	10	1922731	4.83E-06	0.013	14306	21.526
Vascular dementia (sudden onset)	class Coriobacteriia	rs11656361	A	C	-0.196	8218014	0.195	0.151	360283	A	C	0.077	17	8121332	8.02E-06	0.018	14306	19.394

Vascular dementia (sudden onset)	class Coriobacteriia	rs12974142	G	A	0.213	52391913	0.349	0.227	360283	G	A	0.079	19	52895166	8.51E-06	0.018	14306	19.865
Vascular dementia (sudden onset)	class Coriobacteriia	rs13307134	T	C	-0.140	105444233	0.372	0.156	360283	T	C	-0.057	7	105084680	7.80E-06	0.013	14306	20.072
Vascular dementia (sudden onset)	class Coriobacteriia	rs1397793	A	G	0.077	91175634	0.545	0.127	360283	A	G	0.050	5	90471451	9.77E-06	0.011	14306	19.682
Vascular dementia (sudden onset)	class Coriobacteriia	rs1816223	G	A	0.093	11341087	0.525	0.146	360283	G	A	0.059	12	11494021	4.84E-06	0.013	14306	20.652
Vascular dementia (sudden onset)	class Coriobacteriia	rs240104	T	C	-0.168	176602295	0.194	0.129	360283	T	C	-0.060	1	176571431	1.52E-06	0.013	14306	22.630
Vascular dementia (sudden onset)	class Coriobacteriia	rs2442778	A	G	-0.059	11612938	0.825	0.265	360283	A	G	0.116	3	11654412	9.03E-06	0.026	14306	20.272
Vascular dementia (sudden onset)	class Coriobacteriia	rs3025411	A	G	-0.237	133647784	0.204	0.187	360283	A	G	0.093	9	136512906	8.27E-06	0.021	14306	19.566
Vascular dementia (sudden onset)	class Coriobacteriia	rs34739816	G	T	0.373	39220432	0.125	0.243	360283	G	T	0.097	17	37376685	3.88E-06	0.021	14306	21.594
Vascular dementia (sudden onset)	class Coriobacteriia	rs67561917	A	G	-0.064	63440724	0.670	0.151	360283	A	G	-0.071	20	62072077	5.39E-06	0.015	14306	21.486
Vascular dementia (sudden onset)	class Coriobacteriia	rs719099	A	G	-0.094	64039457	0.627	0.193	360283	A	G	0.078	10	65799217	5.43E-07	0.016	14306	24.957
Vascular dementia (sudden onset)	class Coriobacteriia	rs8010111	A	G	0.009	39191305	0.965	0.207	360283	A	G	0.103	14	39660509	6.90E-06	0.023	14306	20.328
Vascular dementia (sudden onset)	class Deltaproteobacteria	rs1035691	G	A	-0.079	10637188	0.509	0.119	360283	G	A	0.055	11	10658735	9.65E-06	0.012	14306	20.588
Vascular dementia (sudden onset)	class Deltaproteobacteria	rs11599763	C	T	0.204	11813600	0.088	0.120	360283	C	T	0.054	10	11855599	3.94E-06	0.012	14306	21.490
Vascular dementia (sudden onset)	class Deltaproteobacteria	rs17084793	G	A	0.019	71645678	0.909	0.167	360283	G	A	-0.071	18	69312914	5.69E-06	0.016	14306	19.850
Vascular dementia (sudden onset)	class Deltaproteobacteria	rs17791387	A	G	-0.150	79219511	0.457	0.201	360283	A	G	-0.074	9	81834426	1.60E-06	0.015	14306	22.761
Vascular dementia (sudden onset)	class Deltaproteobacteria	rs2692012	G	A	0.212	204022477	0.407	0.256	360283	G	A	-0.110	1	203991605	3.14E-06	0.025	14306	18.968
Vascular dementia (sudden onset)	class Deltaproteobacteria	rs2838334	G	A	-0.211	43645080	0.085	0.123	360283	G	A	0.056	21	45064961	5.45E-06	0.012	14306	20.480
Vascular dementia (sudden onset)	class Deltaproteobacteria	rs3935584	C	T	-0.009	233064573	0.936	0.117	360283	C	T	-0.052	2	233929283	7.50E-06	0.012	14306	20.485
Vascular dementia (sudden onset)	class Deltaproteobacteria	rs4506934	C	T	0.003	2953368	0.988	0.180	360283	C	T	-0.094	17	2856662	3.59E-06	0.020	14306	21.666
Vascular dementia (sudden onset)	class Deltaproteobacteria	rs55744759	A	G	0.037	56955112	0.842	0.185	360283	A	G	-0.078	8	57867671	7.31E-06	0.017	14306	20.854
Vascular dementia (sudden onset)	class Deltaproteobacteria	rs6058181	C	T	-0.017	35106998	0.911	0.156	360283	C	T	0.083	20	33694801	3.40E-07	0.017	14306	24.755
Vascular dementia (sudden onset)	class Deltaproteobacteria	rs62020470	A	G	-0.341	95617836	0.025	0.152	360283	A	G	-0.059	15	96161065	4.85E-06	0.013	14306	20.480
Vascular dementia (sudden onset)	class Deltaproteobacteria	rs9928243	C	A	0.026	71507738	0.823	0.117	360283	C	A	-0.054	16	71541641	5.02E-06	0.012	14306	20.923
Vascular dementia (sudden onset)	class Erysipelotrichia	rs1074800	G	A	-0.134	3002432	0.254	0.118	360283	G	A	-0.049	5	3002546	6.15E-06	0.011	14306	20.459
Vascular dementia (sudden onset)	class Erysipelotrichia	rs10781552	C	T	0.204	132083729	0.117	0.130	360283	C	T	-0.055	10	133897233	2.33E-06	0.012	14306	22.633
Vascular dementia (sudden onset)	class Erysipelotrichia	rs17530232	A	G	0.423	39811320	0.103	0.260	360283	A	G	0.103	13	40385457	2.79E-06	0.022	14306	21.042
Vascular dementia (sudden onset)	class Erysipelotrichia	rs1884466	C	T	0.097	63673525	0.408	0.117	360283	C	T	-0.048	1	64139196	9.53E-06	0.011	14306	19.760
Vascular dementia (sudden onset)	class Erysipelotrichia	rs2300774	A	G	-0.069	196066841	0.552	0.117	360283	A	G	-0.052	3	195793712	8.95E-07	0.011	14306	24.094
Vascular dementia (sudden onset)	class Erysipelotrichia	rs290833	T	G	-0.015	96991871	0.895	0.116	360283	T	G	-0.050	1	97457427	8.03E-06	0.011	14306	19.943
Vascular dementia (sudden onset)	class Erysipelotrichia	rs35161940	T	C	0.118	72331083	0.536	0.190	360283	T	C	-0.081	17	70327224	1.85E-06	0.017	14306	23.118
Vascular dementia (sudden onset)	class Erysipelotrichia	rs4078432	T	C	-0.046	48528003	0.767	0.155	360283	T	C	0.061	14	48997206	4.23E-06	0.013	14306	20.723
Vascular dementia (sudden onset)	class Erysipelotrichia	rs56970041	T	G	-0.088	79891267	0.718	0.243	360283	T	G	0.072	14	80357610	5.40E-06	0.016	14306	19.385
Vascular dementia (sudden onset)	class Erysipelotrichia	rs62504403	C	T	0.117	38946033	0.421	0.146	360283	C	T	0.068	8	38803551	1.12E-07	0.013	14306	28.371
Vascular dementia (sudden onset)	class Erysipelotrichia	rs7234058	T	C	0.114	5830508	0.575	0.203	360283	T	C	-0.095	18	5830507	9.12E-07	0.019	14306	23.744
Vascular dementia (sudden onset)	class Erysipelotrichia	rs7826267	G	T	0.063	3097430	0.793	0.238	360283	G	T	0.084	8	2954952	9.28E-06	0.020	14306	17.755
Vascular dementia (sudden onset)	class Erysipelotrichia	rs8003149	C	T	0.112	55689786	0.365	0.124	360283	C	T	0.054	14	56156504	4.08E-06	0.012	14306	21.248
Vascular dementia (sudden onset)	class Gammaproteobacteria	rs11181912	G	A	-0.015	43179149	0.907	0.124	360283	G	A	-0.058	12	43572952	9.95E-07	0.012	14306	23.767
Vascular dementia (sudden onset)	class Gammaproteobacteria	rs12404135	A	G	0.488	186533562	0.028	0.222	360283	A	G	-0.079	1	186502694	8.89E-06	0.017	14306	20.933
Vascular dementia (sudden onset)	class Gammaproteobacteria	rs6706173	A	C	0.082	167130482	0.629	0.169	360283	A	C	0.074	2	167986992	1.99E-07	0.015	14306	25.726
Vascular dementia (sudden onset)	class Gammaproteobacteria	rs75101789	C	T	-0.006	18574411	0.975	0.206	360283	C	T	0.073	3	18615903	8.79E-06	0.016	14306	20.002
Vascular dementia (sudden onset)	class Gammaproteobacteria	rs79795896	A	G	0.106	50658878	0.703	0.280	360283	A	G	-0.159	18	48185248	7.92E-06	0.035	14306	20.531
Vascular dementia (sudden onset)	class Gammaproteobacteria	rs9494710	C	T	0.135	137291417	0.275	0.124	360283	C	T	-0.055	6	137612554	4.55E-06	0.012	14306	20.663
Vascular dementia (sudden onset)	class Lentisphaeria	rs1002941	A	G	0.028	100702485	0.836	0.134	360283	A	G	-0.105	15	101242690	8.15E-06	0.023	14306	20.234
Vascular dementia (sudden onset)	class Lentisphaeria	rs11770843	C	T	-0.098	147098287	0.432	0.125	360283	C	T	0.109	7	146795379	1.91E-06	0.023	14306	21.707
Vascular dementia (sudden onset)	class Lentisphaeria	rs17114848	G	A	0.420	24917388	0.035	0.200	360283	G	A	0.152	15	25162535	4.06E-06	0.032	14306	22.073
Vascular dementia (sudden onset)	class Lentisphaeria	rs2031282	A	G	0.056	20113040	0.711	0.152	360283	A	G	0.122	13	20687179	4.38E-06	0.027	14306	20.490
Vascular dementia (sudden onset)	class Lentisphaeria	rs2825714	A	G	-0.179	19651652	0.246	0.154	360283	A	G	-0.137	21	21023966	1.72E-06	0.029	14306	22.568
Vascular dementia (sudden onset)	class Lentisphaeria	rs62570196	C	T	-0.351	108323890	0.237	0.297	360283	C	T	-0.216	9	111086170	1.08E-06	0.044	14306	24.192
Vascular dementia (sudden onset)	class Lentisphaeria	rs72640280	A	G	0.057	11883735	0.819	0.249	360283	A	G	0.220	1	11943792	5.18E-06	0.049	14306	20.513
Vascular dementia (sudden onset)	class Lentisphaeria	rs77599476	A	G	-0.084	62762910	0.746	0.258	360283	A	G	0.230	20	61394262	1.86E-06	0.048	14306	23.002
Vascular dementia (sudden onset)	class Melainabacteria	rs10148250	A	G	0.098	106605442	0.422	0.122	360283	A	G	-0.086	14	107061448	8.67E-06	0.019	14306	19.866
Vascular dementia (sudden onset)	class Melainabacteria	rs10738747	A	G	-0.225	26184580	0.056	0.118	360283	A	G	-0.081	9	26184578	9.96E-06	0.018	14306	19.514
Vascular dementia (sudden onset)	class Melainabacteria	rs11150282	T	C	-0.077	80459808	0.525	0.121	360283	T	C	0.099	16	80493705	6.03E-07	0.020	14306	25.235
Vascular dementia (sudden onset)	class Melainabacteria	rs113884518	T	C	0.227	24648999	0.546	0.376	360283	T	C	-0.205	9	24648997	8.06E-06	0.045	14306	20.371
Vascular dementia (sudden onset)	class Melainabacteria	rs28678345	T	C	0.243	55828967	0.376	0.275	360283	T	C	0.215	17	53906328	6.69E-06	0.047	14306	20.818
Vascular dementia (sudden onset)	class Melainabacteria	rs367480	A	G	0.209	2916401	0.091	0.123	360283	A	G	0.084	11	2937631	8.20E-06	0.019	14306	20.323
Vascular dementia (sudden onset)	class Melainabacteria	rs4129395	G	A	0.022	113213109	0.853	0.117	360283	G	A	0.090	9	115975389	1.48E-06	0.019	14306	23.440
Vascular dementia (sudden onset)	class Melainabacteria	rs789069	A	C	0.230	1008277	0.170	0.168	360283	A	C	-0.104	18	1008278	6.85E-06	0.023	14306	19.530
Vascular dementia (sudden onset)	class Melainabacteria	rs79790072	T	C	-0.069	100207478	0.835	0.334	360283	T	C	0.227	15	100747683	3.29E-06	0.049	14306	21.599
Vascular dementia (sudden onset)	class Melainabacteria	rs9864379	T	C	-0.170	14265449	0.295	0.162	360283	T	C	-0.160	3	14306949	5.36E-08	0.029	14306	29.812
Vascular dementia (sudden onset)	class Methanobacteria	rs10202904	G	T	-0.058	124682691	0.631	0.120	360283	G	T	0.122	2	125440268	3.01E-07	0.024	14306	26.762
Vascular dementia (sudden onset)	class Methanobacteria	rs10424197	A	G	-0.099	45936063	0.465	0.135	360283	A	G	0.111	19	46439321	9.28E-06	0.025	14306	20.211
Vascular dementia (sudden onset)	class Methanobacteria	rs4257531	G	A	0.024	2044483	0.900	0.193	360283	G	A	0.164	3	2086167	7.44E-06	0.036	14306	20.316
Vascular dementia (sudden onset)	class Methanobacteria	rs6508769	C	T	0.180	28336853	0.273	0.164	360283	C	T	-0.154	19	28827760	8.23E-06	0.034	14306	19.856
Vascular dementia (sudden onset)	class Methanobacteria	rs6776814	T	C	0.224	15011576	0.597	0.424	360283	T	C	-0.200	3	15053083	1.63E-06	0.041	14306	23.483
Vascular dementia (sudden onset)	class Methanobacteria	rs73068003	G	T	0.231	10734305	0.242	0.197	360283	G	T	-0.158	7	10773932	8.45E-06	0.035	14306	20.206
Vascular dementia (sudden onset)	class Methanobacteria	rs73457410	A	G	0.037	41382045	0.875	0.235	360283	A	G	0.215	13	41956181	1.41E-06	0.044	14306	24.316

Vascular dementia (sudden onset)	class Methanobacteria	rs75208022	C	T	0.124	21185927	0.535	0.199	360283	C	T	-0.227	12	21338861	5.92E-06	0.049	14306	21.717
Vascular dementia (sudden onset)	class Methanobacteria	rs894996	C	A	-0.222	103497150	0.328	0.227	360283	C	A	0.217	4	104418307	1.88E-06	0.045	14306	23.349
Vascular dementia (sudden onset)	class Mollicutes	rs10108398	G	A	-0.105	58528265	0.416	0.130	360283	G	A	0.077	8	59440824	1.09E-06	0.015	14306	24.960
Vascular dementia (sudden onset)	class Mollicutes	rs11890098	A	G	0.084	156676037	0.523	0.131	360283	A	G	0.074	2	157532549	9.57E-07	0.015	14306	23.551
Vascular dementia (sudden onset)	class Mollicutes	rs12566890	T	G	-0.006	61385192	0.974	0.172	360283	T	G	-0.101	1	61850864	3.65E-06	0.023	14306	19.176
Vascular dementia (sudden onset)	class Mollicutes	rs17214486	C	A	0.092	96693337	0.463	0.125	360283	C	A	0.061	14	97159674	6.61E-06	0.014	14306	20.223
Vascular dementia (sudden onset)	class Mollicutes	rs2464826	A	C	0.252	79860934	0.170	0.183	360283	A	C	0.094	7	79490250	8.39E-06	0.021	14306	19.874
Vascular dementia (sudden onset)	class Mollicutes	rs28537087	G	A	0.043	94766447	0.753	0.136	360283	G	A	0.082	15	95309676	8.07E-06	0.019	14306	19.002
Vascular dementia (sudden onset)	class Mollicutes	rs3768491	G	A	0.085	109423364	0.504	0.128	360283	G	A	0.068	1	109965986	4.23E-06	0.015	14306	20.875
Vascular dementia (sudden onset)	class Mollicutes	rs4885016	C	T	0.117	72596351	0.495	0.172	360283	C	T	0.082	13	73170489	7.27E-06	0.018	14306	20.363
Vascular dementia (sudden onset)	class Mollicutes	rs6043847	T	C	-0.020	16278879	0.936	0.249	360283	T	C	-0.115	20	16259524	4.55E-06	0.025	14306	21.375
Vascular dementia (sudden onset)	class Mollicutes	rs72901605	T	C	0.359	47082326	0.048	0.181	360283	T	C	-0.084	11	47103877	3.26E-06	0.018	14306	22.338
Vascular dementia (sudden onset)	class Mollicutes	rs74603314	T	C	-0.323	46050515	0.288	0.304	360283	T	C	0.222	14	46519718	1.56E-06	0.046	14306	22.924
Vascular dementia (sudden onset)	class Mollicutes	rs78169027	A	G	0.378	108568360	0.122	0.244	360283	A	G	-0.108	11	108439087	5.88E-06	0.024	14306	20.824
Vascular dementia (sudden onset)	class Negativicutes	rs1135612	G	A	0.160	75980359	0.262	0.142	360283	G	A	0.053	7	75609677	9.26E-06	0.012	14306	19.761
Vascular dementia (sudden onset)	class Negativicutes	rs13086907	G	A	-0.239	142416275	0.089	0.140	360283	G	A	0.063	3	142135117	1.95E-06	0.013	14306	22.532
Vascular dementia (sudden onset)	class Negativicutes	rs1643968	T	C	0.013	165839623	0.914	0.121	360283	T	C	-0.057	5	165266628	4.15E-07	0.011	14306	25.339
Vascular dementia (sudden onset)	class Negativicutes	rs1649999	A	G	-0.043	78326315	0.830	0.202	360283	A	G	0.075	10	80086072	7.58E-06	0.017	14306	20.246
Vascular dementia (sudden onset)	class Negativicutes	rs2834062	A	G	-0.024	33005177	0.851	0.127	360283	A	G	0.049	21	34377485	8.44E-06	0.011	14306	20.190
Vascular dementia (sudden onset)	class Negativicutes	rs4463806	C	T	0.245	113838234	0.099	0.148	360283	C	T	0.054	10	115597993	7.81E-06	0.013	14306	17.681
Vascular dementia (sudden onset)	class Negativicutes	rs4722181	T	G	-0.177	22777952	0.128	0.116	360283	T	G	0.050	7	22817571	2.00E-06	0.011	14306	22.452
Vascular dementia (sudden onset)	class Negativicutes	rs60274479	T	C	0.035	21238604	0.810	0.147	360283	T	C	-0.066	16	21249925	1.16E-06	0.013	14306	24.182
Vascular dementia (sudden onset)	class Negativicutes	rs61249479	A	C	0.096	122150629	0.554	0.162	360283	A	C	0.078	9	124912908	2.95E-06	0.017	14306	21.236
Vascular dementia (sudden onset)	class Negativicutes	rs71405394	G	A	0.436	100704883	0.061	0.233	360283	G	A	-0.114	15	101245088	2.17E-06	0.024	14306	22.539
Vascular dementia (sudden onset)	class Negativicutes	rs73232831	G	A	-0.153	17411803	0.626	0.314	360283	G	A	-0.152	4	17413426	1.87E-06	0.031	14306	23.242
Vascular dementia (sudden onset)	class Negativicutes	rs9423647	G	A	-0.017	5537855	0.882	0.117	360283	G	A	0.048	10	5579818	6.06E-06	0.011	14306	20.628
Vascular dementia (sudden onset)	class Verrucomicrobiae	rs111862613	T	C	0.021	129825125	0.890	0.155	360283	T	C	0.091	12	130309670	3.74E-06	0.020	14306	21.252
Vascular dementia (sudden onset)	class Verrucomicrobiae	rs117107102	A	G	-0.459	51947265	0.094	0.274	360283	A	G	0.205	18	49473635	2.92E-06	0.043	14306	22.493
Vascular dementia (sudden onset)	class Verrucomicrobiae	rs11729256	T	C	-0.011	94106121	0.943	0.156	360283	T	C	0.075	4	95027272	6.73E-07	0.015	14306	24.928
Vascular dementia (sudden onset)	class Verrucomicrobiae	rs12908520	G	A	0.094	97027427	0.424	0.117	360283	G	A	0.062	15	97570657	2.17E-06	0.013	14306	22.341
Vascular dementia (sudden onset)	class Verrucomicrobiae	rs2602429	T	C	-0.330	81029544	0.013	0.133	360283	T	C	-0.075	16	81063149	2.58E-06	0.016	14306	22.863
Vascular dementia (sudden onset)	class Verrucomicrobiae	rs4242783	A	G	0.007	5022135	0.957	0.130	360283	A	G	-0.069	10	5064327	2.64E-06	0.015	14306	21.781
Vascular dementia (sudden onset)	class Verrucomicrobiae	rs4936098	G	A	0.119	130410772	0.327	0.122	360283	G	A	-0.065	11	130280667	1.12E-06	0.014	14306	22.786
Vascular dementia (sudden onset)	class Verrucomicrobiae	rs61779207	G	A	0.087	40608800	0.539	0.142	360283	G	A	-0.076	1	41074472	6.72E-06	0.017	14306	20.432
Vascular dementia (sudden onset)	class Verrucomicrobiae	rs74542928	T	C	-0.316	99623031	0.246	0.272	360283	T	C	0.112	4	100544188	1.63E-06	0.024	14306	22.508
Vascular dementia (sudden onset)	class Verrucomicrobiae	rs9349825	A	G	-0.075	56476683	0.609	0.147	360283	A	G	-0.070	6	56341481	2.54E-06	0.015	14306	22.898
Vascular dementia (sudden onset)	class Verrucomicrobiae	rs941682	G	A	-0.085	33280034	0.511	0.130	360283	G	A	-0.063	20	31867840	9.61E-06	0.014	14306	19.290
Vascular dementia (sudden onset)	family Acidaminococcaceae	rs262812	T	C	0.136	158226967	0.284	0.127	360283	T	C	-0.066	6	158647999	3.25E-06	0.014	14306	21.313
Vascular dementia (sudden onset)	family Acidaminococcaceae	rs2933324	G	A	-0.065	104355372	0.642	0.139	360283	G	A	0.066	9	107117653	2.24E-06	0.014	14306	22.315
Vascular dementia (sudden onset)	family Acidaminococcaceae	rs45497800	T	C	0.031	63360481	0.851	0.164	360283	T	C	-0.118	20	61991833	5.86E-06	0.026	14306	20.975
Vascular dementia (sudden onset)	family Acidaminococcaceae	rs6589457	G	A	0.406	114830566	0.130	0.269	360283	G	A	-0.166	11	114701288	2.32E-06	0.035	14306	22.533
Vascular dementia (sudden onset)	family Acidaminococcaceae	rs6923842	T	C	0.120	5722948	0.509	0.182	360283	T	C	-0.080	6	5723181	2.21E-06	0.017	14306	22.140
Vascular dementia (sudden onset)	family Acidaminococcaceae	rs74540770	G	A	0.246	186835600	0.252	0.214	360283	G	A	-0.109	3	186553389	7.09E-06	0.024	14306	20.021
Vascular dementia (sudden onset)	family Acidaminococcaceae	rs78702810	T	C	-0.033	18421039	0.863	0.192	360283	T	C	-0.144	3	18462531	9.16E-06	0.032	14306	19.778
Vascular dementia (sudden onset)	family Actinomycetaceae	rs2889192	T	G	-0.023	73779652	0.888	0.164	360283	T	G	-0.089	9	76394568	3.64E-06	0.020	14306	20.714
Vascular dementia (sudden onset)	family Actinomycetaceae	rs34583783	G	T	-0.228	66497478	0.353	0.246	360283	G	T	0.124	6	67207371	5.48E-06	0.026	14306	21.930
Vascular dementia (sudden onset)	family Actinomycetaceae	rs35011108	A	G	-0.201	132686341	0.386	0.232	360283	A	G	0.242	6	133007480	1.83E-06	0.050	14306	23.041
Vascular dementia (sudden onset)	family Actinomycetaceae	rs4073240	G	A	0.089	168824686	0.456	0.120	360283	G	A	0.075	6	169224781	6.05E-06	0.016	14306	20.605
Vascular dementia (sudden onset)	family Alcaligenaceae	rs112135816	T	G	-0.178	79870412	0.462	0.243	360283	T	G	-0.078	9	82485327	5.28E-06	0.017	14306	20.411
Vascular dementia (sudden onset)	family Alcaligenaceae	rs1153990	A	G	-0.100	104897760	0.424	0.125	360283	A	G	-0.059	5	104233461	5.97E-06	0.013	14306	20.950
Vascular dementia (sudden onset)	family Alcaligenaceae	rs147968	C	T	0.008	85912233	0.945	0.116	360283	C	T	0.049	16	85945839	9.13E-06	0.011	14306	19.685
Vascular dementia (sudden onset)	family Alcaligenaceae	rs28480294	C	T	-0.101	71230170	0.409	0.122	360283	C	T	0.052	15	71522509	6.61E-06	0.012	14306	20.033
Vascular dementia (sudden onset)	family Alcaligenaceae	rs4033856	T	C	-0.506	45640468	0.015	0.208	360283	T	C	-0.082	4	45642485	1.03E-06	0.017	14306	23.467
Vascular dementia (sudden onset)	family Alcaligenaceae	rs62191117	A	G	0.142	238979079	0.331	0.146	360283	A	G	0.068	2	239900775	2.76E-07	0.013	14306	26.221
Vascular dementia (sudden onset)	family Alcaligenaceae	rs62395635	T	C	0.033	174070793	0.890	0.241	360283	T	C	0.111	5	173497796	3.35E-06	0.024	14306	21.496
Vascular dementia (sudden onset)	family Alcaligenaceae	rs6969323	A	C	-0.143	104574040	0.303	0.139	360283	A	C	-0.059	7	104214487	3.89E-06	0.013	14306	21.127
Vascular dementia (sudden onset)	family Alcaligenaceae	rs74776516	T	G	0.343	20741821	0.150	0.239	360283	T	G	-0.094	11	20763367	6.85E-06	0.021	14306	19.591
Vascular dementia (sudden onset)	family Alcaligenaceae	rs7638039	T	C	-0.132	70539788	0.329	0.135	360283	T	C	0.060	3	70588939	2.70E-06	0.013	14306	22.295
Vascular dementia (sudden onset)	family Alcaligenaceae	rs9537886	A	C	-0.188	57971112	0.107	0.117	360283	A	C	-0.057	13	58545246	2.35E-07	0.011	14306	26.604
Vascular dementia (sudden onset)	family Bacteroidaceae	rs11585893	A	G	0.125	10584294	0.354	0.135	360283	A	G	-0.074	1	10644351	1.80E-06	0.015	14306	25.175
Vascular dementia (sudden onset)	family Bacteroidaceae	rs13207588	A	G	-0.208	41551692	0.160	0.148	360283	A	G	-0.059	6	41519430	7.48E-06	0.013	14306	20.365
Vascular dementia (sudden onset)	family Bacteroidaceae	rs1340391	T	C	0.095	102495433	0.573	0.169	360283	T	C	-0.059	1	102960989	6.73E-06	0.013	14306	20.040
Vascular dementia (sudden onset)	family Bacteroidaceae	rs17619981	T	G	0.319	24159448	0.062	0.170	360283	T	G	0.088	19	24342250	2.69E-06	0.019	14306	22.194
Vascular dementia (sudden onset)	family Bacteroidaceae	rs2023437	T	C	0.200	21577814	0.256	0.176	360283	T	C	-0.078	14	22045949	5.02E-06	0.017	14306	21.780
Vascular dementia (sudden onset)	family Bacteroidaceae	rs66710942	T	C	-0.078	77166176	0.505	0.117	360283	T	C	-0.049	3	77215327	5.86E-06	0.011	14306	20.644
Vascular dementia (sudden onset)	family Bacteroidaceae	rs6795673	C	T	0.160	10551540	0.170	0.117	360283	C	T	0.054	3	10593224	3.38E-07	0.011	14306	26.183
Vascular dementia (sudden onset)	family Bacteroidaceae	rs9507307	C	T	-0.110	24336338	0.419	0.135	360283	C	T	0.060	13	24910476	2.13E-06	0.013	14306	21.912

Vascular dementia (sudden onset)	family Bacteroidales S24 7group	rs10872669	G	A	-0.321	151194037	0.113	0.203	360283	G	A	0.123	6	151515172	9.49E-06	0.028	14306	19.932
Vascular dementia (sudden onset)	family Bacteroidales S24 7group	rs12748533	G	T	-0.020	242603950	0.879	0.128	360283	G	T	-0.082	1	242767252	2.59E-06	0.017	14306	22.605
Vascular dementia (sudden onset)	family Bacteroidales S24 7group	rs17043785	T	C	-0.083	52912224	0.687	0.207	360283	T	C	-0.176	2	53139362	5.12E-07	0.035	14306	25.761
Vascular dementia (sudden onset)	family Bacteroidales S24 7group	rs1850003	A	G	0.148	47048773	0.285	0.139	360283	A	G	0.084	15	47340971	2.41E-06	0.018	14306	22.344
Vascular dementia (sudden onset)	family Bacteroidales S24 7group	rs61508842	T	C	-0.292	158935756	0.158	0.207	360283	T	C	0.123	3	158653545	7.83E-06	0.027	14306	20.250
Vascular dementia (sudden onset)	family Bacteroidales S24 7group	rs738193	T	C	0.121	25547355	0.322	0.122	360283	T	C	0.085	22	25943322	3.82E-07	0.017	14306	26.100
Vascular dementia (sudden onset)	family Bacteroidales S24 7group	rs78609301	A	G	-0.025	62526842	0.841	0.126	360283	A	G	-0.087	6	63236747	7.09E-06	0.020	14306	19.628
Vascular dementia (sudden onset)	family Bacteroidales S24 7group	rs941000	T	C	0.111	91085352	0.361	0.122	360283	T	C	-0.085	7	90714667	3.16E-07	0.016	14306	27.083
Vascular dementia (sudden onset)	family Bifidobacteriaceae	rs10831953	G	A	0.146	13076504	0.248	0.126	360283	G	A	0.054	11	13098051	9.95E-06	0.012	14306	18.869
Vascular dementia (sudden onset)	family Bifidobacteriaceae	rs12446429	T	C	-0.044	848055	0.769	0.150	360283	T	C	0.081	16	898055	8.53E-06	0.019	14306	18.040
Vascular dementia (sudden onset)	family Bifidobacteriaceae	rs13020688	G	A	-0.001	192013806	0.991	0.126	360283	G	A	0.058	2	192878532	1.57E-06	0.012	14306	22.887
Vascular dementia (sudden onset)	family Bifidobacteriaceae	rs182549	T	C	0.090	135859184	0.454	0.120	360283	T	C	-0.117	2	136616754	5.94E-20	0.013	14306	85.372
Vascular dementia (sudden onset)	family Bifidobacteriaceae	rs4957061	T	C	0.274	520981	0.021	0.118	360283	T	C	0.057	5	521096	1.15E-06	0.012	14306	23.762
Vascular dementia (sudden onset)	family Bifidobacteriaceae	rs540489	T	G	-0.232	74901626	0.130	0.153	360283	T	G	-0.063	17	72897722	5.37E-06	0.014	14306	20.956
Vascular dementia (sudden onset)	family Bifidobacteriaceae	rs55888705	A	G	-0.113	1516099	0.382	0.129	360283	A	G	0.054	4	1517826	8.66E-06	0.012	14306	19.812
Vascular dementia (sudden onset)	family Bifidobacteriaceae	rs6899771	A	G	-0.102	96958344	0.599	0.194	360283	A	G	-0.091	6	97406220	7.28E-06	0.020	14306	20.365
Vascular dementia (sudden onset)	family Bifidobacteriaceae	rs7174549	T	C	-0.192	91920073	0.110	0.120	360283	T	C	-0.055	15	92463303	6.87E-06	0.012	14306	19.590
Vascular dementia (sudden onset)	family Bifidobacteriaceae	rs7322849	T	C	0.011	112205515	0.957	0.203	360283	T	C	0.111	13	112859829	1.74E-08	0.020	14306	30.320
Vascular dementia (sudden onset)	family Bifidobacteriaceae	rs857444	C	T	-0.124	14617360	0.303	0.120	360283	C	T	0.055	6	14617591	3.82E-06	0.012	14306	21.075
Vascular dementia (sudden onset)	family Clostridiaceae1	rs10875374	T	C	-0.209	101339253	0.072	0.116	360283	T	C	0.054	1	101804809	8.10E-06	0.012	14306	20.197
Vascular dementia (sudden onset)	family Clostridiaceae1	rs12186080	G	A	0.091	132877783	0.563	0.157	360283	G	A	0.075	3	132596627	5.34E-06	0.016	14306	21.212
Vascular dementia (sudden onset)	family Clostridiaceae1	rs12341505	G	A	-0.068	133845759	0.736	0.203	360283	G	A	0.081	9	136710881	4.54E-06	0.018	14306	20.627
Vascular dementia (sudden onset)	family Clostridiaceae1	rs2795528	G	A	-0.137	42774816	0.597	0.258	360283	G	A	-0.181	10	43270264	3.81E-06	0.039	14306	21.432
Vascular dementia (sudden onset)	family Clostridiaceae1	rs2817172	C	T	0.152	3124955	0.200	0.119	360283	C	T	0.056	1	3041519	5.27E-06	0.012	14306	20.668
Vascular dementia (sudden onset)	family Clostridiaceae1	rs4723021	T	C	-0.066	30895044	0.777	0.232	360283	T	C	-0.106	7	30934659	7.42E-06	0.024	14306	19.331
Vascular dementia (sudden onset)	family Clostridiaceae1	rs550843	T	C	0.209	165309343	0.109	0.131	360283	T	C	-0.073	6	165722832	7.09E-06	0.017	14306	19.043
Vascular dementia (sudden onset)	family Clostridiaceae1	rs56188186	A	G	0.028	87677084	0.917	0.273	360283	A	G	0.097	16	87710690	8.24E-06	0.022	14306	19.804
Vascular dementia (sudden onset)	family Clostridiaceae1	rs62397761	A	G	0.012	48125278	0.924	0.124	360283	A	G	0.062	6	48093014	9.08E-06	0.014	14306	20.443
Vascular dementia (sudden onset)	family Clostridiaceae1	rs881532	G	A	-0.009	47411024	0.936	0.116	360283	G	A	0.053	22	47806774	7.90E-06	0.012	14306	20.060
Vascular dementia (sudden onset)	family Clostridiales vadin BB60 group	rs10517600	G	T	-0.132	154711806	0.271	0.120	360283	G	T	-0.063	4	155632958	6.83E-06	0.014	14306	20.241
Vascular dementia (sudden onset)	family Clostridiales vadin BB60 group	rs10904722	C	T	0.070	6672462	0.606	0.137	360283	C	T	-0.067	10	6714424	5.05E-06	0.015	14306	20.883
Vascular dementia (sudden onset)	family Clostridiales vadin BB60 group	rs118104867	C	T	0.019	122965298	0.933	0.229	360283	C	T	0.214	8	123977538	3.44E-06	0.046	14306	22.207
Vascular dementia (sudden onset)	family Clostridiales vadin BB60 group	rs13409132	A	G	0.144	204751623	0.647	0.315	360283	A	G	-0.165	2	205616346	4.37E-06	0.035	14306	22.065
Vascular dementia (sudden onset)	family Clostridiales vadin BB60 group	rs17121075	G	A	0.096	85390452	0.497	0.142	360283	G	A	0.077	14	85856796	7.91E-06	0.017	14306	19.948
Vascular dementia (sudden onset)	family Clostridiales vadin BB60 group	rs2191834	T	G	-0.124	229078235	0.359	0.135	360283	T	G	-0.075	2	229942951	2.50E-06	0.016	14306	21.998
Vascular dementia (sudden onset)	family Clostridiales vadin BB60 group	rs28691777	C	T	0.048	60072044	0.869	0.290	360283	C	T	0.137	17	58149405	6.96E-07	0.027	14306	26.380
Vascular dementia (sudden onset)	family Clostridiales vadin BB60 group	rs34088226	A	G	0.285	4308833	0.247	0.246	360283	A	G	-0.118	5	4308946	7.66E-06	0.027	14306	19.145
Vascular dementia (sudden onset)	family Clostridiales vadin BB60 group	rs55682560	C	T	0.147	86849059	0.502	0.219	360283	C	T	-0.132	15	87392290	4.97E-07	0.026	14306	25.330
Vascular dementia (sudden onset)	family Clostridiales vadin BB60 group	rs6588624	A	G	0.101	56383867	0.382	0.116	360283	A	G	0.066	1	56849539	1.79E-06	0.014	14306	22.985
Vascular dementia (sudden onset)	family Clostridiales vadin BB60 group	rs66714985	A	C	-0.039	3447600	0.838	0.190	360283	A	C	0.117	8	3305122	4.85E-06	0.025	14306	21.446
Vascular dementia (sudden onset)	family Clostridiales vadin BB60 group	rs7226487	A	G	0.103	76661168	0.381	0.117	360283	A	G	-0.064	18	74373125	3.58E-06	0.014	14306	21.537
Vascular dementia (sudden onset)	family Clostridiales vadin BB60 group	rs7538034	T	G	-0.055	70926011	0.728	0.158	360283	T	G	-0.079	1	71391694	2.37E-06	0.017	14306	22.423
Vascular dementia (sudden onset)	family Clostridiales vadin BB60 group	rs7725895	A	G	0.012	142865333	0.947	0.178	360283	A	G	-0.116	5	142244898	3.94E-06	0.024	14306	23.380
Vascular dementia (sudden onset)	family Clostridiales vadin BB60 group	rs989682	A	G	-0.045	15522946	0.746	0.138	360283	A	G	0.070	3	15564453	6.85E-06	0.016	14306	20.413
Vascular dementia (sudden onset)	family Coriobacteriaceae	rs11073596	G	T	-0.042	85890348	0.728	0.120	360283	G	T	-0.051	15	86433579	8.14E-06	0.011	14306	19.912
Vascular dementia (sudden onset)	family Coriobacteriaceae	rs11250875	T	C	0.192	1880537	0.175	0.141	360283	T	C	0.061	10	1922731	4.83E-06	0.013	14306	21.526
Vascular dementia (sudden onset)	family Coriobacteriaceae	rs11656361	A	C	-0.196	8218014	0.195	0.151	360283	A	C	0.077	17	8121332	8.02E-06	0.018	14306	19.394
Vascular dementia (sudden onset)	family Coriobacteriaceae	rs12974142	G	A	0.213	52391913	0.349	0.227	360283	G	A	0.079	19	52895166	8.51E-06	0.018	14306	19.865
Vascular dementia (sudden onset)	family Coriobacteriaceae	rs13307134	T	C	-0.140	105444233	0.372	0.156	360283	T	C	-0.057	7	105084680	7.80E-06	0.013	14306	20.072
Vascular dementia (sudden onset)	family Coriobacteriaceae	rs1397793	A	G	0.077	91175634	0.545	0.127	360283	A	G	0.050	5	90471451	9.77E-06	0.011	14306	19.682
Vascular dementia (sudden onset)	family Coriobacteriaceae	rs1816223	G	A	0.093	11341087	0.525	0.146	360283	G	A	0.059	12	11494021	4.84E-06	0.013	14306	20.652
Vascular dementia (sudden onset)	family Coriobacteriaceae	rs240104	T	C	-0.168	176602295	0.194	0.129	360283	T	C	-0.060	1	176571431	1.52E-06	0.013	14306	22.630
Vascular dementia (sudden onset)	family Coriobacteriaceae	rs2442778	A	G	-0.059	11612938	0.825	0.265	360283	A	G	0.116	3	11654412	9.03E-06	0.026	14306	20.272
Vascular dementia (sudden onset)	family Coriobacteriaceae	rs3025411	A	G	-0.237	133647784	0.204	0.187	360283	A	G	0.093	9	136512906	8.27E-06	0.021	14306	19.566
Vascular dementia (sudden onset)	family Coriobacteriaceae	rs34739816	G	T	0.373	39220432	0.125	0.243	360283	G	T	0.097	17	37376685	3.88E-06	0.021	14306	21.594
Vascular dementia (sudden onset)	family Coriobacteriaceae	rs67561917	A	G	-0.064	63440724	0.670	0.151	360283	A	G	-0.071	20	62072077	5.39E-06	0.015	14306	21.486
Vascular dementia (sudden onset)	family Coriobacteriaceae	rs719099	A	G	-0.094	64039457	0.627	0.193	360283	A	G	0.078	10	65799217	5.43E-07	0.016	14306	24.957
Vascular dementia (sudden onset)	family Coriobacteriaceae	rs8010111	A	G	0.009	39191305	0.965	0.207	360283	A	G	0.103	14	39660509	6.90E-06	0.023	14306	20.328
Vascular dementia (sudden onset)	family Defluviitaleaceae	rs112893842	T	C	-0.061	8786663	0.762	0.203	360283	T	C	0.111	9	8786663	2.75E-06	0.023	14306	22.686
Vascular dementia (sudden onset)	family Defluviitaleaceae	rs1582238	C	T	0.083	118181062	0.492	0.121	360283	C	T	-0.080	1	118723685	1.69E-06	0.017	14306	23.042
Vascular dementia (sudden onset)	family Defluviitaleaceae	rs17051335	C	T	0.064	121281755	0.740	0.192	360283	C	T	-0.134	4	122202910	4.58E-06	0.029	14306	21.129
Vascular dementia (sudden onset)	family Defluviitaleaceae	rs1908593	T	C	-0.092	61358915	0.438	0.118	360283	T	C	0.070	18	59026148	7.86E-06	0.016	14306	20.108
Vascular dementia (sudden onset)	family Defluviitaleaceae	rs4344384	T	G	0.133	64647609	0.252	0.116	360283	T	G	-0.071	10	66407366	5.86E-06	0.016	14306	20.612
Vascular dementia (sudden onset)	family Defluviitaleaceae	rs4677103	A	G	0.181	72158643	0.244	0.155	360283	A	G	0.098	3	72207794	9.42E-07	0.020	14306	24.598
Vascular dementia (sudden onset)	family Defluviitaleaceae	rs540220	C	T	-0.204	90569026	0.313	0.202	360283	C	T	0.124	9	93331308	9.48E-06	0.029	14306	18.293
Vascular dementia (sudden onset)	family Defluviitaleaceae	rs55658617	T	C	-0.489	40439076	0.119	0.314	360283	T	C	0.177	21	41811003	1.41E-06	0.036	14306	24.013
Vascular dementia (sudden onset)	family Defluviitaleaceae	rs72731813	C	T	0.195	146493591	0.476	0.274	360283	C	T	-0.150	4	147414743	2.76E-07	0.029	14306	26.048

Vascular dementia (sudden onset)	family Defluviitaleaceae	rs9608282	T	G	-0.595	24408113	0.072	0.330	360283	T	G	0.139	22	24804081	4.61E-06	0.030	14306	21.554
Vascular dementia (sudden onset)	family Defluviitaleaceae	rs9725395	A	G	-0.016	84739949	0.929	0.184	360283	A	G	-0.138	1	85205632	3.41E-06	0.030	14306	21.969
Vascular dementia (sudden onset)	family Desulfovibrionaceae	rs11599763	C	T	0.204	11813600	0.088	0.120	360283	C	T	0.056	10	11855599	2.50E-06	0.012	14306	22.388
Vascular dementia (sudden onset)	family Desulfovibrionaceae	rs17791387	A	G	-0.150	79219511	0.457	0.201	360283	A	G	-0.073	9	81834426	2.10E-06	0.015	14306	22.313
Vascular dementia (sudden onset)	family Desulfovibrionaceae	rs2692012	G	A	0.212	204022477	0.407	0.256	360283	G	A	-0.114	1	203991605	1.56E-06	0.025	14306	20.295
Vascular dementia (sudden onset)	family Desulfovibrionaceae	rs2838334	G	A	-0.211	43645080	0.085	0.123	360283	G	A	0.057	21	45064961	3.82E-06	0.012	14306	21.157
Vascular dementia (sudden onset)	family Desulfovibrionaceae	rs3935584	C	T	-0.009	233064573	0.936	0.117	360283	C	T	-0.053	2	233929283	6.78E-06	0.012	14306	20.635
Vascular dementia (sudden onset)	family Desulfovibrionaceae	rs4506934	C	T	0.003	2953368	0.988	0.180	360283	C	T	-0.094	17	2856662	3.16E-06	0.020	14306	21.945
Vascular dementia (sudden onset)	family Desulfovibrionaceae	rs6058181	C	T	-0.017	35106998	0.911	0.156	360283	C	T	0.083	20	33694801	2.70E-07	0.017	14306	25.253
Vascular dementia (sudden onset)	family Desulfovibrionaceae	rs72647048	T	C	0.036	56952819	0.845	0.185	360283	T	C	-0.077	8	57865378	9.61E-06	0.017	14306	20.307
Vascular dementia (sudden onset)	family Desulfovibrionaceae	rs9928243	C	A	0.026	71507738	0.823	0.117	360283	C	A	-0.054	16	71541641	4.48E-06	0.012	14306	21.142
Vascular dementia (sudden onset)	family Enterobacteriaceae	rs11026530	T	C	-0.078	22357551	0.633	0.164	360283	T	C	0.082	11	22379097	9.43E-06	0.019	14306	19.471
Vascular dementia (sudden onset)	family Enterobacteriaceae	rs2374342	C	A	0.010	41906402	0.932	0.119	360283	C	A	0.058	2	42133542	4.52E-06	0.013	14306	21.338
Vascular dementia (sudden onset)	family Enterobacteriaceae	rs35673018	G	A	-0.042	54293833	0.836	0.202	360283	G	A	0.090	16	54327745	7.63E-06	0.020	14306	19.653
Vascular dementia (sudden onset)	family Enterobacteriaceae	rs504442	T	G	-0.056	57478315	0.766	0.190	360283	T	G	0.084	18	55145547	5.17E-06	0.019	14306	19.728
Vascular dementia (sudden onset)	family Enterobacteriaceae	rs62210023	A	G	-0.002	56765036	0.989	0.123	360283	A	G	0.061	20	55340092	3.13E-06	0.013	14306	21.742
Vascular dementia (sudden onset)	family Enterobacteriaceae	rs78143293	A	G	-0.197	60005103	0.270	0.178	360283	A	G	-0.085	18	57672335	1.20E-06	0.017	14306	24.792
Vascular dementia (sudden onset)	family Enterobacteriaceae	rs79757635	C	A	-0.079	110188071	0.644	0.171	360283	C	A	0.076	13	110840418	9.32E-06	0.017	14306	19.615
Vascular dementia (sudden onset)	family Erysipelotrichaceae	rs1074800	G	A	-0.134	3002432	0.254	0.118	360283	G	A	-0.049	5	3002546	6.15E-06	0.011	14306	20.459
Vascular dementia (sudden onset)	family Erysipelotrichaceae	rs10781552	C	T	0.204	132083729	0.117	0.130	360283	C	T	-0.055	10	133897233	2.33E-06	0.012	14306	22.633
Vascular dementia (sudden onset)	family Erysipelotrichaceae	rs17530232	A	G	0.423	39811320	0.103	0.260	360283	A	G	0.103	13	40385457	2.79E-06	0.022	14306	21.042
Vascular dementia (sudden onset)	family Erysipelotrichaceae	rs1884466	C	T	0.097	63673525	0.408	0.117	360283	C	T	-0.048	1	64139196	9.53E-06	0.011	14306	19.760
Vascular dementia (sudden onset)	family Erysipelotrichaceae	rs2300774	A	G	-0.069	196066841	0.552	0.117	360283	A	G	-0.052	3	195793712	8.95E-07	0.011	14306	24.094
Vascular dementia (sudden onset)	family Erysipelotrichaceae	rs290833	T	G	-0.015	96991871	0.895	0.116	360283	T	G	-0.050	1	97457427	8.03E-06	0.011	14306	19.943
Vascular dementia (sudden onset)	family Erysipelotrichaceae	rs35161940	T	C	0.118	72331083	0.536	0.190	360283	T	C	-0.081	17	70327224	1.85E-06	0.017	14306	23.118
Vascular dementia (sudden onset)	family Erysipelotrichaceae	rs4078432	T	C	-0.046	48528003	0.767	0.155	360283	T	C	0.061	14	48997206	4.23E-06	0.013	14306	20.723
Vascular dementia (sudden onset)	family Erysipelotrichaceae	rs56970041	T	G	-0.088	79891267	0.718	0.243	360283	T	G	0.072	14	80357610	5.40E-06	0.016	14306	19.385
Vascular dementia (sudden onset)	family Erysipelotrichaceae	rs62504403	C	T	0.117	38946033	0.421	0.146	360283	C	T	0.068	8	38803551	1.12E-07	0.013	14306	28.371
Vascular dementia (sudden onset)	family Erysipelotrichaceae	rs7234058	T	C	0.114	5830508	0.575	0.203	360283	T	C	-0.095	18	5830507	9.12E-07	0.019	14306	23.744
Vascular dementia (sudden onset)	family Erysipelotrichaceae	rs7826267	G	T	0.063	3097430	0.793	0.238	360283	G	T	0.084	8	2954952	9.28E-06	0.020	14306	17.755
Vascular dementia (sudden onset)	family Erysipelotrichaceae	rs8003149	C	T	0.112	55689786	0.365	0.124	360283	C	T	0.054	14	56156504	4.08E-06	0.012	14306	21.248
Vascular dementia (sudden onset)	family Family XI	rs10759623	C	T	0.020	113059205	0.886	0.139	360283	C	T	-0.162	9	115821485	5.78E-07	0.032	14306	25.425
Vascular dementia (sudden onset)	family Family XI	rs11547158	A	G	-0.164	149224640	0.333	0.169	360283	A	G	-0.178	7	148921732	2.70E-06	0.037	14306	22.696
Vascular dementia (sudden onset)	family Family XI	rs17379710	T	C	-0.031	35313121	0.796	0.118	360283	T	C	-0.116	11	35334668	3.97E-06	0.025	14306	21.308
Vascular dementia (sudden onset)	family Family XI	rs2155352	A	G	0.119	95624045	0.382	0.137	360283	A	G	-0.151	11	95357209	6.63E-07	0.030	14306	24.795
Vascular dementia (sudden onset)	family Family XI	rs2156611	T	C	0.056	45765899	0.631	0.116	360283	T	C	-0.112	18	43345864	9.43E-06	0.025	14306	20.068
Vascular dementia (sudden onset)	family Family XI	rs3733511	A	G	0.038	119034632	0.768	0.127	360283	A	G	0.128	4	119955787	3.39E-06	0.027	14306	21.795
Vascular dementia (sudden onset)	family Family XI	rs488164	G	T	-0.303	239832151	0.013	0.122	360283	G	T	-0.118	1	239995451	4.80E-06	0.026	14306	21.386
Vascular dementia (sudden onset)	family Family XI	rs697771	A	G	0.053	54081288	0.654	0.117	360283	A	G	-0.118	16	54115200	3.19E-06	0.025	14306	21.895
Vascular dementia (sudden onset)	family Family XIII	rs10404377	A	C	-0.250	16063847	0.038	0.120	360283	A	C	0.050	19	16174657	6.99E-06	0.011	14306	20.311
Vascular dementia (sudden onset)	family Family XIII	rs118170811	A	G	-0.160	100607139	0.629	0.331	360283	A	G	0.152	10	102366896	1.80E-06	0.032	14306	22.860
Vascular dementia (sudden onset)	family Family XIII	rs482905	G	T	-0.012	166587965	0.931	0.135	360283	G	T	0.060	1	166557202	3.72E-06	0.013	14306	22.004
Vascular dementia (sudden onset)	family Family XIII	rs6501525	A	G	0.025	72222486	0.843	0.124	360283	A	G	0.056	17	70218627	1.24E-06	0.012	14306	23.603
Vascular dementia (sudden onset)	family Family XIII	rs66753613	G	A	0.123	38746349	0.415	0.151	360283	G	A	0.065	1	39212021	8.08E-06	0.014	14306	20.375
Vascular dementia (sudden onset)	family Family XIII	rs6797051	C	T	0.171	88946068	0.393	0.200	360283	C	T	-0.081	3	88995218	4.89E-06	0.017	14306	22.122
Vascular dementia (sudden onset)	family Family XIII	rs7514702	T	C	-0.192	186944891	0.239	0.163	360283	T	C	-0.066	1	186914023	3.92E-06	0.014	14306	21.931
Vascular dementia (sudden onset)	family Lachnospiraceae	rs10402491	C	T	0.091	13314985	0.558	0.155	360283	C	T	0.066	19	13425799	7.58E-06	0.015	14306	19.852
Vascular dementia (sudden onset)	family Lachnospiraceae	rs11139361	C	T	-0.039	69564931	0.753	0.124	360283	C	T	-0.049	9	72179847	4.26E-06	0.011	14306	20.023
Vascular dementia (sudden onset)	family Lachnospiraceae	rs112040820	A	G	-0.297	82511188	0.025	0.133	360283	A	G	0.055	17	80469064	2.42E-06	0.012	14306	22.032
Vascular dementia (sudden onset)	family Lachnospiraceae	rs11841382	G	T	-0.096	37430535	0.644	0.209	360283	G	T	-0.072	13	38004672	9.58E-06	0.017	14306	16.913
Vascular dementia (sudden onset)	family Lachnospiraceae	rs11979110	T	C	0.125	130751700	0.284	0.116	360283	T	C	-0.050	7	130436459	1.82E-06	0.011	14306	22.730
Vascular dementia (sudden onset)	family Lachnospiraceae	rs1205443	A	G	-0.131	38248466	0.286	0.122	360283	A	G	0.050	20	36876868	7.29E-06	0.011	14306	19.987
Vascular dementia (sudden onset)	family Lachnospiraceae	rs12760724	A	C	0.099	213858626	0.426	0.124	360283	A	C	-0.048	1	214031969	7.27E-06	0.011	14306	20.155
Vascular dementia (sudden onset)	family Lachnospiraceae	rs13005175	G	A	-0.182	231605011	0.528	0.289	360283	G	A	-0.099	2	232469722	8.37E-06	0.022	14306	20.694
Vascular dementia (sudden onset)	family Lachnospiraceae	rs2159863	A	G	-0.392	10243730	0.011	0.153	360283	A	G	-0.059	4	10245354	3.70E-06	0.013	14306	20.706
Vascular dementia (sudden onset)	family Lachnospiraceae	rs2910921	C	T	0.149	32348487	0.674	0.354	360283	C	T	-0.160	5	32348593	8.42E-06	0.036	14306	20.014
Vascular dementia (sudden onset)	family Lachnospiraceae	rs3127230	C	T	-0.243	101632746	0.056	0.127	360283	C	T	-0.050	10	103392503	6.20E-06	0.011	14306	20.140
Vascular dementia (sudden onset)	family Lachnospiraceae	rs35524804	T	C	-0.141	97351401	0.323	0.142	360283	T	C	-0.061	9	100113683	2.45E-06	0.013	14306	23.537
Vascular dementia (sudden onset)	family Lachnospiraceae	rs7359994	C	T	-0.109	28917951	0.357	0.118	360283	C	T	0.050	19	29408858	5.36E-06	0.011	14306	20.009
Vascular dementia (sudden onset)	family Lachnospiraceae	rs79086868	T	C	0.129	130916218	0.507	0.195	360283	T	C	0.078	9	133791605	3.01E-06	0.016	14306	22.302
Vascular dementia (sudden onset)	family Lachnospiraceae	rs959845	T	C	0.050	185985647	0.674	0.120	360283	T	C	0.049	4	186906801	5.17E-06	0.011	14306	20.971
Vascular dementia (sudden onset)	family Lachnospiraceae	rs9929145	G	A	0.054	76525287	0.839	0.265	360283	G	A	-0.126	16	76559184	2.84E-07	0.025	14306	26.282
Vascular dementia (sudden onset)	family Lactobacillaceae	rs1530559	G	A	-0.118	134998059	0.314	0.117	360283	G	A	0.077	2	135755629	9.65E-06	0.018	14306	19.063
Vascular dementia (sudden onset)	family Lactobacillaceae	rs16861661	G	A	-0.271	18174965	0.259	0.240	360283	G	A	-0.193	1	18501459	2.70E-07	0.038	14306	25.969
Vascular dementia (sudden onset)	family Lactobacillaceae	rs62314653	C	A	-0.465	108975306	0.062	0.249	360283	C	A	0.177	4	109896462	6.59E-06	0.039	14306	20.379
Vascular dementia (sudden onset)	family Lactobacillaceae	rs74599091	A	G	0.104	179751534	0.813	0.437	360283	A	G	0.192	1	179720669	7.70E-06	0.043	14306	20.323
Vascular dementia (sudden onset)	family Lactobacillaceae	rs768253	T	G	0.010	68097868	0.931	0.117	360283	T	G	-0.079	8	69010103	3.61E-06	0.017	14306	21.570

Vascular dementia (sudden onset)	family Lactobacillaceae	rs77478751	A	G	-0.273	173433875	0.127	0.179	360283	A	G	-0.219	3	173151665	5.96E-06	0.047	14306	21.421
Vascular dementia (sudden onset)	family Lactobacillaceae	rs921925	A	C	-0.113	6928006	0.421	0.141	360283	A	C	0.100	19	6928017	5.77E-07	0.020	14306	24.555
Vascular dementia (sudden onset)	family Lactobacillaceae	rs9345899	A	G	0.237	66849430	0.206	0.188	360283	A	G	-0.124	6	67559323	9.45E-06	0.028	14306	19.774
Vascular dementia (sudden onset)	family Methanobacteriaceae	rs10202904	G	T	-0.058	124682691	0.631	0.120	360283	G	T	0.122	2	125440268	3.01E-07	0.024	14306	26.762
Vascular dementia (sudden onset)	family Methanobacteriaceae	rs10424197	A	G	-0.099	45936063	0.465	0.135	360283	A	G	0.111	19	46439321	9.28E-06	0.025	14306	20.211
Vascular dementia (sudden onset)	family Methanobacteriaceae	rs4257531	G	A	0.024	2044483	0.900	0.193	360283	G	A	0.164	3	2086167	7.44E-06	0.036	14306	20.316
Vascular dementia (sudden onset)	family Methanobacteriaceae	rs6508769	C	T	0.180	28336853	0.273	0.164	360283	C	T	-0.154	19	28827760	8.23E-06	0.034	14306	19.856
Vascular dementia (sudden onset)	family Methanobacteriaceae	rs6776814	T	C	0.224	15011576	0.597	0.424	360283	T	C	-0.200	3	15053083	1.63E-06	0.041	14306	23.483
Vascular dementia (sudden onset)	family Methanobacteriaceae	rs73068003	G	T	0.231	10734305	0.242	0.197	360283	G	T	-0.158	7	10773932	8.45E-06	0.035	14306	20.206
Vascular dementia (sudden onset)	family Methanobacteriaceae	rs73457410	A	G	0.037	41382045	0.875	0.235	360283	A	G	0.215	13	41956181	1.41E-06	0.044	14306	24.316
Vascular dementia (sudden onset)	family Methanobacteriaceae	rs75208022	C	T	0.124	21185927	0.535	0.199	360283	C	T	-0.227	12	21338861	5.92E-06	0.049	14306	21.717
Vascular dementia (sudden onset)	family Methanobacteriaceae	rs894996	C	A	-0.222	103497150	0.328	0.227	360283	C	A	0.217	4	104418307	1.88E-06	0.045	14306	23.349
Vascular dementia (sudden onset)	family Oxalobacteraceae	rs111966731	T	C	-0.174	93398708	0.411	0.211	360283	T	C	0.204	15	93941937	4.56E-06	0.045	14306	20.952
Vascular dementia (sudden onset)	family Oxalobacteraceae	rs11246212	C	T	0.042	610277	0.802	0.167	360283	C	T	-0.136	11	610277	4.51E-06	0.029	14306	21.745
Vascular dementia (sudden onset)	family Oxalobacteraceae	rs12002250	A	C	0.546	19682560	0.019	0.232	360283	A	C	0.196	9	19682558	5.53E-06	0.045	14306	19.408
Vascular dementia (sudden onset)	family Oxalobacteraceae	rs1569853	T	C	-0.087	38582525	0.632	0.182	360283	T	C	-0.140	6	38550301	7.45E-07	0.028	14306	24.714
Vascular dementia (sudden onset)	family Oxalobacteraceae	rs17138946	G	T	-0.066	5934557	0.775	0.232	360283	G	T	-0.189	16	5984558	8.09E-06	0.043	14306	19.388
Vascular dementia (sudden onset)	family Oxalobacteraceae	rs36057338	G	T	-0.072	189014160	0.826	0.329	360283	G	T	0.182	4	189935314	6.26E-06	0.040	14306	20.743
Vascular dementia (sudden onset)	family Oxalobacteraceae	rs4428215	G	A	-0.261	172229645	0.053	0.135	360283	G	A	0.126	3	171947435	4.88E-08	0.023	14306	29.812
Vascular dementia (sudden onset)	family Oxalobacteraceae	rs561239	A	G	-0.033	18640649	0.819	0.144	360283	A	G	0.106	12	18793583	7.19E-06	0.024	14306	19.864
Vascular dementia (sudden onset)	family Oxalobacteraceae	rs6000536	C	T	-0.256	37025428	0.099	0.156	360283	C	T	-0.118	22	37421469	7.39E-07	0.024	14306	24.058
Vascular dementia (sudden onset)	family Oxalobacteraceae	rs62435498	C	A	0.223	1754627	0.241	0.190	360283	C	A	0.182	7	1794263	7.46E-06	0.040	14306	20.531
Vascular dementia (sudden onset)	family Oxalobacteraceae	rs736744	T	C	0.044	84899492	0.712	0.118	360283	T	C	-0.106	9	87514407	1.49E-07	0.020	14306	27.721
Vascular dementia (sudden onset)	family Oxalobacteraceae	rs7993559	A	C	0.051	22869488	0.662	0.118	360283	A	C	-0.092	13	23443627	5.04E-06	0.020	14306	20.974
Vascular dementia (sudden onset)	family Oxalobacteraceae	rs80330081	A	C	0.179	66561674	0.347	0.191	360283	A	C	-0.188	4	67427392	6.64E-06	0.042	14306	19.597
Vascular dementia (sudden onset)	family Oxalobacteraceae	rs934049	G	A	-0.080	15916656	0.578	0.143	360283	G	A	0.110	2	16056779	4.21E-06	0.024	14306	21.152
Vascular dementia (sudden onset)	family Pasteurellaceae	rs10965428	C	A	0.166	22718482	0.514	0.254	360283	C	A	-0.120	9	22718481	4.29E-06	0.026	14306	21.561
Vascular dementia (sudden onset)	family Pasteurellaceae	rs111582866	G	A	0.110	48708578	0.593	0.205	360283	G	A	-0.114	16	48742489	7.07E-06	0.026	14306	19.753
Vascular dementia (sudden onset)	family Pasteurellaceae	rs12050685	A	G	0.026	73185141	0.842	0.129	360283	A	G	-0.067	15	73477482	9.19E-06	0.015	14306	19.385
Vascular dementia (sudden onset)	family Pasteurellaceae	rs16970009	A	G	-0.266	34535582	0.543	0.437	360283	A	G	0.187	17	32862601	7.32E-06	0.043	14306	19.027
Vascular dementia (sudden onset)	family Pasteurellaceae	rs4822728	T	C	-0.199	26495842	0.086	0.116	360283	T	C	0.069	22	26891808	4.72E-06	0.015	14306	21.156
Vascular dementia (sudden onset)	family Pasteurellaceae	rs6972479	A	G	0.047	117278006	0.746	0.145	360283	A	G	-0.078	7	116918060	7.75E-06	0.018	14306	19.878
Vascular dementia (sudden onset)	family Pasteurellaceae	rs72756943	G	A	0.538	26531799	0.004	0.185	360283	G	A	0.140	5	26531908	3.35E-06	0.030	14306	21.308
Vascular dementia (sudden onset)	family Pasteurellaceae	rs73139353	A	C	-0.002	98253370	0.994	0.206	360283	A	C	-0.223	3	97972214	8.71E-06	0.048	14306	21.092
Vascular dementia (sudden onset)	family Pasteurellaceae	rs76022354	C	T	-0.069	92546628	0.794	0.265	360283	C	T	0.243	10	94306385	1.83E-06	0.050	14306	23.560
Vascular dementia (sudden onset)	family Pasteurellaceae	rs78909003	T	C	0.458	102887960	0.071	0.253	360283	T	C	-0.241	9	105650242	2.05E-06	0.050	14306	23.415
Vascular dementia (sudden onset)	family Pasteurellaceae	rs9382510	C	T	-0.134	55583693	0.314	0.133	360283	C	T	-0.088	6	55448491	2.48E-07	0.017	14306	26.921
Vascular dementia (sudden onset)	family Pasteurellaceae	rs9895850	T	C	-0.110	66538895	0.695	0.282	360283	T	C	-0.176	17	64535013	9.08E-06	0.041	14306	18.497
Vascular dementia (sudden onset)	family Pasteurellaceae	rs9938097	C	T	-0.084	84943783	0.484	0.119	360283	C	T	0.071	16	84977389	8.23E-06	0.016	14306	20.209
Vascular dementia (sudden onset)	family Peptococcaceae	rs117452796	A	G	0.190	9423378	0.583	0.345	360283	A	G	-0.258	9	9423378	3.15E-06	0.055	14306	22.036
Vascular dementia (sudden onset)	family Peptococcaceae	rs12144792	C	T	0.062	27091734	0.609	0.121	360283	C	T	0.064	1	27418225	5.82E-06	0.014	14306	20.788
Vascular dementia (sudden onset)	family Peptococcaceae	rs12634826	T	G	0.147	183266260	0.228	0.122	360283	T	G	-0.074	3	182984048	1.01E-06	0.015	14306	24.031
Vascular dementia (sudden onset)	family Peptococcaceae	rs12992764	T	G	0.115	188031016	0.331	0.118	360283	T	G	0.068	2	188895743	1.46E-06	0.014	14306	23.502
Vascular dementia (sudden onset)	family Peptococcaceae	rs150600492	A	C	0.088	127377698	0.722	0.248	360283	A	C	0.136	10	129175962	2.31E-06	0.029	14306	21.957
Vascular dementia (sudden onset)	family Peptococcaceae	rs35703006	G	T	0.166	28756700	0.223	0.136	360283	G	T	0.081	8	28614217	4.95E-07	0.016	14306	24.448
Vascular dementia (sudden onset)	family Peptococcaceae	rs4990837	G	A	-0.043	3723235	0.772	0.147	360283	G	A	-0.091	8	3580757	1.74E-06	0.019	14306	23.990
Vascular dementia (sudden onset)	family Peptococcaceae	rs75430375	C	T	0.155	89957067	0.585	0.284	360283	C	T	-0.148	5	89252884	3.41E-06	0.032	14306	21.712
Vascular dementia (sudden onset)	family Peptococcaceae	rs75898026	A	G	-0.139	112906768	0.333	0.143	360283	A	G	-0.082	13	113561082	2.02E-06	0.017	14306	22.424
Vascular dementia (sudden onset)	family Peptostreptococcaceae	rs10805326	A	G	-0.016	14322999	0.902	0.128	360283	A	G	-0.057	4	14324623	4.03E-06	0.012	14306	21.306
Vascular dementia (sudden onset)	family Peptostreptococcaceae	rs117020988	C	T	-0.071	46671379	0.739	0.214	360283	C	T	0.182	7	46710977	1.03E-06	0.037	14306	24.028
Vascular dementia (sudden onset)	family Peptostreptococcaceae	rs12377846	C	A	0.223	16786786	0.536	0.360	360283	C	A	-0.252	9	16786784	7.26E-07	0.051	14306	24.260
Vascular dementia (sudden onset)	family Peptostreptococcaceae	rs12986312	T	G	0.000	17303329	0.999	0.127	360283	T	G	0.057	19	17414138	5.77E-06	0.013	14306	20.613
Vascular dementia (sudden onset)	family Peptostreptococcaceae	rs1467258	G	A	-0.014	40060234	0.925	0.150	360283	G	A	0.073	17	38216487	7.90E-06	0.016	14306	19.996
Vascular dementia (sudden onset)	family Peptostreptococcaceae	rs1520207	T	C	0.186	152063386	0.111	0.117	360283	T	C	-0.053	3	151781175	3.17E-06	0.011	14306	21.842
Vascular dementia (sudden onset)	family Peptostreptococcaceae	rs4692811	C	T	-0.055	170259039	0.655	0.122	360283	C	T	0.064	4	171180190	4.21E-07	0.013	14306	25.595
Vascular dementia (sudden onset)	family Peptostreptococcaceae	rs59865771	C	T	-0.186	89180262	0.129	0.123	360283	C	T	-0.057	16	89246670	7.69E-06	0.013	14306	20.349
Vascular dementia (sudden onset)	family Peptostreptococcaceae	rs61841503	G	A	0.047	16977560	0.790	0.175	360283	G	A	0.092	10	17019559	9.80E-09	0.016	14306	32.495
Vascular dementia (sudden onset)	family Peptostreptococcaceae	rs6721459	G	A	0.015	67625704	0.898	0.119	360283	G	A	-0.051	2	67852836	5.08E-06	0.011	14306	20.730
Vascular dementia (sudden onset)	family Peptostreptococcaceae	rs76982728	T	C	-0.241	44273225	0.548	0.402	360283	T	C	0.124	7	44312824	3.24E-06	0.027	14306	21.652
Vascular dementia (sudden onset)	family Peptostreptococcaceae	rs77540684	T	G	0.007	14608536	0.971	0.200	360283	T	G	0.107	10	14650535	8.18E-06	0.025	14306	18.877
Vascular dementia (sudden onset)	family Peptostreptococcaceae	rs9573937	A	G	-0.217	76777061	0.171	0.158	360283	A	G	-0.069	13	77351196	1.71E-06	0.014	14306	23.559
Vascular dementia (sudden onset)	family Porphyromonadaceae	rs10762312	A	G	-0.077	69812107	0.538	0.126	360283	A	G	0.052	10	71571863	8.70E-06	0.012	14306	19.427
Vascular dementia (sudden onset)	family Porphyromonadaceae	rs10858364	G	T	0.122	135184235	0.372	0.136	360283	G	T	0.055	9	138076081	4.31E-06	0.012	14306	20.951
Vascular dementia (sudden onset)	family Porphyromonadaceae	rs17065783	A	G	-0.197	62049912	0.212	0.158	360283	A	G	-0.059	3	62035586	1.79E-06	0.012	14306	23.403
Vascular dementia (sudden onset)	family Porphyromonadaceae	rs1980561	A	G	0.053	62919798	0.649	0.117	360283	A	G	-0.049	14	63386516	8.95E-06	0.011	14306	19.719
Vascular dementia (sudden onset)	family Porphyromonadaceae	rs35233670	T	C	0.020	65754785	0.863	0.115	360283	T	C	-0.047	17	63750903	7.91E-06	0.011	14306	19.953
Vascular dementia (sudden onset)	family Porphyromonadaceae	rs35961441	A	C	0.068	240766474	0.824	0.305	360283	A	C	0.092	1	240929774	8.37E-06	0.021	14306	19.492

Vascular dementia (sudden onset)	family Porphyromonadaceae	rs6953849	A	G	-0.016	69786706	0.915	0.151	360283	A	G	0.072	7	69251692	2.44E-06	0.015	14306	22.702
Vascular dementia (sudden onset)	family Porphyromonadaceae	rs7330827	T	C	0.028	22957663	0.911	0.245	360283	T	C	-0.104	13	23531802	8.05E-06	0.024	14306	19.140
Vascular dementia (sudden onset)	family Porphyromonadaceae	rs864093	A	C	0.042	148904825	0.768	0.141	360283	A	C	-0.053	4	149825977	9.60E-06	0.012	14306	20.188
Vascular dementia (sudden onset)	family Prevotellaceae	rs12057990	C	T	-0.093	99004713	0.473	0.130	360283	C	T	0.059	1	99470269	8.97E-06	0.013	14306	19.889
Vascular dementia (sudden onset)	family Prevotellaceae	rs12118202	T	C	-0.170	210508026	0.262	0.151	360283	T	C	-0.075	1	210681370	5.54E-07	0.015	14306	26.075
Vascular dementia (sudden onset)	family Prevotellaceae	rs13069367	A	C	-0.151	71752679	0.202	0.119	360283	A	C	-0.054	3	71801830	7.39E-06	0.012	14306	20.339
Vascular dementia (sudden onset)	family Prevotellaceae	rs148376875	T	G	0.133	170408919	0.411	0.162	360283	T	G	0.085	3	170126707	2.08E-06	0.018	14306	22.252
Vascular dementia (sudden onset)	family Prevotellaceae	rs2206482	T	G	0.003	9790461	0.978	0.120	360283	T	G	-0.057	20	9771109	1.30E-06	0.012	14306	23.465
Vascular dementia (sudden onset)	family Prevotellaceae	rs2278540	G	A	0.067	32367424	0.583	0.121	360283	G	A	0.055	3	32408916	8.44E-06	0.012	14306	20.218
Vascular dementia (sudden onset)	family Prevotellaceae	rs34660375	A	G	-0.076	180856881	0.645	0.165	360283	A	G	-0.081	5	180283881	7.40E-06	0.018	14306	20.431
Vascular dementia (sudden onset)	family Prevotellaceae	rs3758087	C	T	-0.134	23857096	0.309	0.132	360283	C	T	-0.056	8	23714609	8.61E-06	0.012	14306	20.485
Vascular dementia (sudden onset)	family Prevotellaceae	rs3860225	A	G	-0.479	110619055	0.018	0.202	360283	A	G	0.084	1	111161677	5.50E-07	0.017	14306	24.981
Vascular dementia (sudden onset)	family Prevotellaceae	rs4493272	T	C	0.061	118153336	0.601	0.117	360283	T	C	-0.060	2	118910912	3.02E-07	0.012	14306	26.275
Vascular dementia (sudden onset)	family Prevotellaceae	rs4685827	T	C	0.186	4823193	0.172	0.136	360283	T	C	-0.068	3	4864877	2.77E-06	0.015	14306	21.840
Vascular dementia (sudden onset)	family Prevotellaceae	rs7252711	G	A	0.115	17350190	0.537	0.187	360283	G	A	0.074	19	17460999	5.57E-06	0.016	14306	20.934
Vascular dementia (sudden onset)	family Prevotellaceae	rs7975087	C	A	0.135	21251898	0.385	0.156	360283	C	A	-0.060	12	21404832	7.59E-06	0.014	14306	19.716
Vascular dementia (sudden onset)	family Prevotellaceae	rs912860	A	G	0.593	33237702	0.126	0.387	360283	A	G	0.229	14	33706908	9.30E-07	0.048	14306	22.476
Vascular dementia (sudden onset)	family Prevotellaceae	rs9586501	G	A	-0.025	104423473	0.850	0.132	360283	G	A	0.059	13	105075823	2.59E-06	0.013	14306	21.666
Vascular dementia (sudden onset)	family Prevotellaceae	rs9958960	G	A	-0.133	65351747	0.409	0.161	360283	G	A	-0.091	18	63018983	1.06E-07	0.017	14306	27.765
Vascular dementia (sudden onset)	family Rhodospirillaceae	rs1035406	G	A	0.073	120037042	0.687	0.182	360283	G	A	-0.114	5	119372737	5.84E-06	0.025	14306	20.484
Vascular dementia (sudden onset)	family Rhodospirillaceae	rs11591293	G	T	0.171	111660039	0.148	0.118	360283	G	T	0.074	10	113419797	2.67E-06	0.016	14306	21.923
Vascular dementia (sudden onset)	family Rhodospirillaceae	rs13336560	C	T	0.307	88487835	0.006	0.112	360283	C	T	-0.070	16	88554243	9.17E-06	0.016	14306	19.710
Vascular dementia (sudden onset)	family Rhodospirillaceae	rs1549633	A	C	-0.286	27945538	0.121	0.184	360283	A	C	0.100	5	27945645	4.70E-06	0.022	14306	20.891
Vascular dementia (sudden onset)	family Rhodospirillaceae	rs1923415	A	G	0.304	88558996	0.143	0.207	360283	A	G	-0.100	6	89268715	9.64E-06	0.023	14306	19.321
Vascular dementia (sudden onset)	family Rhodospirillaceae	rs3754624	C	T	0.136	224769095	0.375	0.153	360283	C	T	0.097	2	225633812	1.71E-06	0.020	14306	23.575
Vascular dementia (sudden onset)	family Rhodospirillaceae	rs4278423	T	C	-0.173	2628361	0.477	0.243	360283	T	C	0.108	10	2670553	3.12E-06	0.024	14306	20.808
Vascular dementia (sudden onset)	family Rhodospirillaceae	rs61933850	G	A	-0.193	72745618	0.248	0.167	360283	G	A	0.165	12	73139398	7.23E-06	0.036	14306	20.883
Vascular dementia (sudden onset)	family Rhodospirillaceae	rs6679026	T	C	0.407	78153828	0.037	0.195	360283	T	C	0.112	1	78619512	9.95E-06	0.025	14306	19.891
Vascular dementia (sudden onset)	family Rhodospirillaceae	rs7001029	C	T	-0.211	130946157	0.299	0.203	360283	C	T	0.117	8	131958403	5.35E-06	0.026	14306	20.171
Vascular dementia (sudden onset)	family Rhodospirillaceae	rs72714493	A	G	-0.162	91740661	0.323	0.164	360283	A	G	0.082	1	92206218	7.35E-06	0.018	14306	20.415
Vascular dementia (sudden onset)	family Rhodospirillaceae	rs74354280	C	T	-0.120	133271763	0.354	0.130	360283	C	T	-0.091	4	134192918	6.67E-06	0.020	14306	19.805
Vascular dementia (sudden onset)	family Rhodospirillaceae	rs76784716	A	G	-0.097	168176830	0.601	0.186	360283	A	G	0.136	2	169033340	1.49E-06	0.029	14306	22.666
Vascular dementia (sudden onset)	family Rhodospirillaceae	rs9813022	A	G	-0.181	13685237	0.132	0.120	360283	A	G	-0.084	3	13726736	2.53E-07	0.016	14306	26.522
Vascular dementia (sudden onset)	family Rikenellaceae	rs10217435	C	T	-0.002	83437740	0.989	0.165	360283	C	T	-0.088	9	86052655	6.51E-06	0.020	14306	20.022
Vascular dementia (sudden onset)	family Rikenellaceae	rs10832801	A	C	-0.137	17567582	0.291	0.130	360283	A	C	-0.053	11	17589129	7.50E-06	0.012	14306	19.005
Vascular dementia (sudden onset)	family Rikenellaceae	rs1939881	G	A	0.163	95571670	0.522	0.255	360283	G	A	-0.106	11	95304834	5.64E-07	0.021	14306	26.218
Vascular dementia (sudden onset)	family Rikenellaceae	rs2447496	A	G	0.116	98176696	0.380	0.132	360283	A	G	0.055	8	99188924	6.09E-06	0.012	14306	20.297
Vascular dementia (sudden onset)	family Rikenellaceae	rs2833282	G	A	0.036	31124392	0.825	0.163	360283	G	A	0.071	21	32496710	4.31E-06	0.016	14306	20.526
Vascular dementia (sudden onset)	family Rikenellaceae	rs36021379	A	G	0.222	27143593	0.168	0.161	360283	A	G	-0.066	21	28515912	7.20E-06	0.014	14306	20.496
Vascular dementia (sudden onset)	family Rikenellaceae	rs4264350	T	C	-0.068	71366303	0.559	0.116	360283	T	C	-0.053	15	71658642	1.35E-06	0.011	14306	23.514
Vascular dementia (sudden onset)	family Rikenellaceae	rs59663348	G	A	-0.023	4069710	0.863	0.135	360283	G	A	0.057	18	4069710	6.12E-06	0.013	14306	20.844
Vascular dementia (sudden onset)	family Rikenellaceae	rs62532512	A	C	0.089	14158855	0.451	0.118	360283	A	C	0.050	9	14158854	2.76E-06	0.011	14306	22.045
Vascular dementia (sudden onset)	family Rikenellaceae	rs6744030	C	T	-0.137	173392399	0.337	0.143	360283	C	T	0.070	2	174257127	9.32E-06	0.016	14306	19.669
Vascular dementia (sudden onset)	family Rikenellaceae	rs6837275	A	G	0.056	186921078	0.662	0.127	360283	A	G	0.057	4	187842232	1.45E-06	0.012	14306	23.022
Vascular dementia (sudden onset)	family Rikenellaceae	rs74474130	T	G	0.028	89804477	0.931	0.320	360283	T	G	0.138	14	90270821	3.61E-06	0.030	14306	21.702
Vascular dementia (sudden onset)	family Rikenellaceae	rs77885767	C	T	0.166	21080475	0.541	0.272	360283	C	T	-0.156	14	21548634	2.85E-06	0.034	14306	21.552
Vascular dementia (sudden onset)	family Rikenellaceae	rs9389714	C	T	-0.021	99796791	0.921	0.207	360283	C	T	-0.064	6	100244667	8.79E-06	0.014	14306	19.718
Vascular dementia (sudden onset)	family Rikenellaceae	rs9578457	G	A	0.144	22293217	0.603	0.276	360283	G	A	-0.141	13	22867356	3.99E-06	0.032	14306	20.064
Vascular dementia (sudden onset)	family Rikenellaceae	rs9603208	G	T	-0.144	37470552	0.455	0.192	360283	G	T	0.082	13	38044689	1.92E-07	0.016	14306	26.474
Vascular dementia (sudden onset)	family Ruminococcaceae	rs10093275	T	C	0.027	68820652	0.827	0.123	360283	T	C	-0.053	8	69732887	5.35E-06	0.012	14306	20.960
Vascular dementia (sudden onset)	family Ruminococcaceae	rs10166469	C	T	-0.075	29606301	0.573	0.134	360283	C	T	0.053	2	29829167	8.52E-06	0.012	14306	19.686
Vascular dementia (sudden onset)	family Ruminococcaceae	rs1158100	G	A	0.103	4698162	0.381	0.117	360283	G	A	0.049	8	4555684	8.61E-06	0.011	14306	19.936
Vascular dementia (sudden onset)	family Ruminococcaceae	rs1612733	T	C	0.128	107118984	0.622	0.259	360283	T	C	0.109	1	107661606	4.22E-06	0.024	14306	20.862
Vascular dementia (sudden onset)	family Ruminococcaceae	rs17376049	T	C	-0.362	60959595	0.051	0.185	360283	T	C	0.085	1	61425267	7.30E-07	0.017	14306	24.306
Vascular dementia (sudden onset)	family Ruminococcaceae	rs2113833	C	T	0.310	217352078	0.338	0.324	360283	C	T	-0.169	2	218216801	1.14E-06	0.036	14306	22.701
Vascular dementia (sudden onset)	family Ruminococcaceae	rs3009418	A	C	0.581	148272338	0.041	0.284	360283	A	C	-0.093	1	147744468	8.69E-06	0.021	14306	19.489
Vascular dementia (sudden onset)	family Ruminococcaceae	rs55793120	T	C	0.350	46990335	0.163	0.251	360283	T	C	0.138	12	47384118	1.44E-07	0.027	14306	26.970
Vascular dementia (sudden onset)	family Ruminococcaceae	rs56199908	T	C	0.135	2801371	0.597	0.256	360283	T	C	-0.199	9	2801371	1.66E-06	0.041	14306	23.584
Vascular dementia (sudden onset)	family Ruminococcaceae	rs76724913	T	G	0.193	24147179	0.327	0.198	360283	T	G	0.090	1	24473669	9.60E-06	0.020	14306	19.682
Vascular dementia (sudden onset)	family Streptococcaceae	rs10028567	C	T	0.031	52791410	0.861	0.179	360283	C	T	-0.093	4	53657577	3.72E-06	0.019	14306	24.079
Vascular dementia (sudden onset)	family Streptococcaceae	rs11110281	T	C	-0.403	100190236	0.137	0.271	360283	T	C	-0.131	12	100584014	1.40E-08	0.023	14306	33.387
Vascular dementia (sudden onset)	family Streptococcaceae	rs16950051	A	G	0.287	120291702	0.221	0.234	360283	A	G	0.107	12	120729505	5.34E-06	0.024	14306	20.391
Vascular dementia (sudden onset)	family Streptococcaceae	rs2370083	G	T	-0.011	97060413	0.965	0.241	360283	G	T	-0.084	14	97526750	4.26E-06	0.018	14306	20.862
Vascular dementia (sudden onset)	family Streptococcaceae	rs2952251	G	A	-0.067	10285654	0.633	0.140	360283	G	A	0.064	8	10143164	3.72E-07	0.013	14306	25.530
Vascular dementia (sudden onset)	family Streptococcaceae	rs35344081	G	A	-0.017	941253	0.897	0.132	360283	G	A	0.061	16	991253	2.64E-06	0.013	14306	22.072
Vascular dementia (sudden onset)	family Streptococcaceae	rs57646748	G	A	-0.299	37451236	0.326	0.305	360283	G	A	-0.088	4	37452858	7.88E-06	0.020	14306	19.612
Vascular dementia (sudden onset)	family Streptococcaceae	rs6806351	T	C	-0.282	132339879	0.046	0.142	360283	T	C	-0.062	3	132058723	6.94E-06	0.014	14306	20.808

Vascular dementia (sudden onset)	family Streptococcaceae	rs77968078	G	A	0.075	240278466	0.762	0.246	360283	G	A	-0.099	1	240441766	7.93E-06	0.022	14306	19.515
Vascular dementia (sudden onset)	family Streptococcaceae	rs7916711	A	G	0.129	28299340	0.437	0.166	360283	A	G	0.096	10	28588269	6.33E-06	0.022	14306	19.839
Vascular dementia (sudden onset)	family Streptococcaceae	rs957755	T	G	0.165	46739055	0.325	0.167	360283	T	G	-0.064	7	46778653	7.42E-06	0.014	14306	20.268
Vascular dementia (sudden onset)	family Veillonellaceae	rs111810795	C	T	0.358	102431593	0.069	0.196	360283	C	T	-0.087	14	102897930	1.73E-06	0.018	14306	23.059
Vascular dementia (sudden onset)	family Veillonellaceae	rs114889439	A	G	-0.215	61023491	0.513	0.329	360283	A	G	-0.254	13	61597625	6.19E-06	0.054	14306	22.160
Vascular dementia (sudden onset)	family Veillonellaceae	rs12186441	G	A	-0.062	133310611	0.829	0.288	360283	G	A	0.208	5	132646303	4.53E-06	0.045	14306	21.010
Vascular dementia (sudden onset)	family Veillonellaceae	rs12668619	A	G	-0.038	21598192	0.759	0.125	360283	A	G	0.055	7	21637810	2.57E-06	0.012	14306	22.093
Vascular dementia (sudden onset)	family Veillonellaceae	rs12741784	C	T	0.115	49623147	0.386	0.133	360283	C	T	-0.062	1	50088819	1.28E-07	0.012	14306	27.151
Vascular dementia (sudden onset)	family Veillonellaceae	rs1442060	A	G	0.180	46364050	0.127	0.118	360283	A	G	0.051	4	46366067	4.51E-06	0.011	14306	21.017
Vascular dementia (sudden onset)	family Veillonellaceae	rs1693340	T	C	0.056	32628728	0.806	0.229	360283	T	C	0.082	18	30208691	9.25E-06	0.018	14306	20.147
Vascular dementia (sudden onset)	family Veillonellaceae	rs2175069	G	A	-0.097	23315501	0.416	0.120	360283	G	A	0.053	4	23317124	4.64E-06	0.011	14306	21.022
Vascular dementia (sudden onset)	family Veillonellaceae	rs2561116	T	G	0.508	38348097	0.039	0.247	360283	T	G	-0.084	5	38348199	7.89E-06	0.019	14306	19.960
Vascular dementia (sudden onset)	family Veillonellaceae	rs2585520	G	T	-0.402	78211006	0.183	0.302	360283	G	T	-0.090	13	78785141	5.27E-06	0.020	14306	20.387
Vascular dementia (sudden onset)	family Veillonellaceae	rs4263802	A	G	0.135	137307265	0.275	0.123	360283	A	G	-0.051	8	138319508	7.45E-06	0.011	14306	19.650
Vascular dementia (sudden onset)	family Veillonellaceae	rs4461038	G	A	0.011	78254654	0.932	0.128	360283	G	A	0.055	15	78546996	3.73E-06	0.012	14306	21.579
Vascular dementia (sudden onset)	family Veillonellaceae	rs4797169	T	C	0.013	462180	0.921	0.135	360283	T	C	0.059	18	462180	4.49E-06	0.013	14306	20.991
Vascular dementia (sudden onset)	family Veillonellaceae	rs61264131	A	C	0.190	138867397	0.336	0.197	360283	A	C	0.202	6	139188534	6.75E-06	0.046	14306	18.990
Vascular dementia (sudden onset)	family Veillonellaceae	rs6692542	G	A	-0.309	244228316	0.011	0.122	360283	G	A	-0.053	1	244391618	8.68E-06	0.012	14306	20.422
Vascular dementia (sudden onset)	family Veillonellaceae	rs6909981	C	T	0.177	74824436	0.319	0.177	360283	C	T	-0.064	6	75534152	5.48E-06	0.014	14306	20.298
Vascular dementia (sudden onset)	family Veillonellaceae	rs79535861	A	C	0.051	37752555	0.800	0.202	360283	A	C	0.101	13	38326692	1.58E-06	0.021	14306	23.646
Vascular dementia (sudden onset)	family Veillonellaceae	rs9345168	A	C	0.015	91671204	0.900	0.117	360283	A	C	-0.051	6	92380922	8.49E-06	0.011	14306	20.207
Vascular dementia (sudden onset)	family Verrucomicrobiaceae	rs111862613	T	C	0.021	129825125	0.890	0.155	360283	T	C	0.091	12	130309670	3.73E-06	0.020	14306	21.255
Vascular dementia (sudden onset)	family Verrucomicrobiaceae	rs117107102	A	G	-0.459	51947265	0.094	0.274	360283	A	G	0.205	18	49473635	2.92E-06	0.043	14306	22.493
Vascular dementia (sudden onset)	family Verrucomicrobiaceae	rs11729256	T	C	-0.011	94106121	0.943	0.156	360283	T	C	0.075	4	95027272	6.73E-07	0.015	14306	24.928
Vascular dementia (sudden onset)	family Verrucomicrobiaceae	rs12908520	G	A	0.094	97027427	0.424	0.117	360283	G	A	0.062	15	97570657	2.15E-06	0.013	14306	22.353
Vascular dementia (sudden onset)	family Verrucomicrobiaceae	rs2602429	T	C	-0.330	81029544	0.013	0.133	360283	T	C	-0.075	16	81063149	2.70E-06	0.016	14306	22.781
Vascular dementia (sudden onset)	family Verrucomicrobiaceae	rs4242783	A	G	0.007	5022135	0.957	0.130	360283	A	G	-0.069	10	5064327	2.75E-06	0.015	14306	21.699
Vascular dementia (sudden onset)	family Verrucomicrobiaceae	rs4936098	G	A	0.119	130410772	0.327	0.122	360283	G	A	-0.065	11	130280667	1.13E-06	0.014	14306	22.775
Vascular dementia (sudden onset)	family Verrucomicrobiaceae	rs61779207	G	A	0.087	40608800	0.539	0.142	360283	G	A	-0.076	1	41074472	6.63E-06	0.017	14306	20.459
Vascular dementia (sudden onset)	family Verrucomicrobiaceae	rs74542928	T	C	-0.316	99623031	0.246	0.272	360283	T	C	0.112	4	100544188	1.65E-06	0.024	14306	22.492
Vascular dementia (sudden onset)	family Verrucomicrobiaceae	rs9349825	A	G	-0.075	56476683	0.609	0.147	360283	A	G	-0.070	6	56341481	2.51E-06	0.015	14306	22.919
Vascular dementia (sudden onset)	family Verrucomicrobiaceae	rs941682	G	A	-0.085	33280034	0.511	0.130	360283	G	A	-0.063	20	31867840	9.58E-06	0.014	14306	19.296
Vascular dementia (sudden onset)	family Victivallaceae	rs11671100	A	C	0.113	711637	0.437	0.145	360283	A	C	-0.160	19	711637	4.08E-06	0.035	14306	20.970
Vascular dementia (sudden onset)	family Victivallaceae	rs11764871	G	T	-0.146	147111885	0.249	0.127	360283	G	T	0.127	7	146808977	7.49E-07	0.026	14306	24.542
Vascular dementia (sudden onset)	family Victivallaceae	rs2944282	T	C	-0.048	57530097	0.713	0.130	360283	T	C	-0.124	7	57589803	1.57E-06	0.026	14306	23.349
Vascular dementia (sudden onset)	family Victivallaceae	rs34962571	A	C	0.189	130618711	0.336	0.196	360283	A	C	-0.187	12	131103256	6.25E-06	0.042	14306	19.963
Vascular dementia (sudden onset)	family Victivallaceae	rs4396289	C	T	0.324	9295183	0.060	0.172	360283	C	T	-0.153	11	9316730	1.54E-07	0.029	14306	28.090
Vascular dementia (sudden onset)	family Victivallaceae	rs61702987	T	C	-0.053	30988791	0.778	0.187	360283	T	C	0.146	2	31211657	3.08E-06	0.030	14306	23.522
Vascular dementia (sudden onset)	family Victivallaceae	rs62570196	C	T	-0.351	108323890	0.237	0.297	360283	C	T	-0.246	9	111086170	2.70E-07	0.048	14306	25.953
Vascular dementia (sudden onset)	family Victivallaceae	rs6545794	A	G	-0.151	60257774	0.399	0.179	360283	A	G	-0.198	2	60484909	5.97E-07	0.041	14306	23.215
Vascular dementia (sudden onset)	family Victivallaceae	rs7077363	G	A	-0.085	93527197	0.585	0.156	360283	G	A	0.149	10	95286954	2.83E-06	0.032	14306	21.887
Vascular dementia (sudden onset)	family Victivallaceae	rs7314815	G	A	-0.161	102131269	0.169	0.117	360283	G	A	0.101	12	102525047	6.40E-06	0.023	14306	20.079
Vascular dementia (sudden onset)	family Victivallaceae	rs7627405	C	T	-0.013	9926871	0.929	0.148	360283	C	T	-0.134	3	9968555	8.19E-06	0.030	14306	19.809
Vascular dementia (sudden onset)	genus Actinomyces	rs2715439	T	C	0.080	98949084	0.493	0.117	360283	T	C	-0.075	15	99492313	6.27E-06	0.016	14306	20.523
Vascular dementia (sudden onset)	genus Actinomyces	rs34583783	G	T	-0.228	66497478	0.353	0.246	360283	G	T	0.127	6	67207371	4.49E-06	0.027	14306	22.237
Vascular dementia (sudden onset)	genus Actinomyces	rs35011108	A	G	-0.201	132686341	0.386	0.232	360283	A	G	0.233	6	133007480	6.34E-06	0.051	14306	20.641
Vascular dementia (sudden onset)	genus Actinomyces	rs4073240	G	A	0.089	168824686	0.456	0.120	360283	G	A	0.075	6	169224781	7.94E-06	0.017	14306	20.064
Vascular dementia (sudden onset)	genus Actinomyces	rs4146653	G	A	-0.034	4740649	0.840	0.167	360283	G	A	0.099	10	4782841	4.50E-06	0.021	14306	21.159
Vascular dementia (sudden onset)	genus Actinomyces	rs71315246	A	G	-0.044	101633595	0.795	0.170	360283	A	G	-0.097	3	101352439	9.83E-06	0.022	14306	19.566
Vascular dementia (sudden onset)	genus Actinomyces	rs7915461	C	T	-0.222	125843552	0.350	0.238	360283	C	T	-0.188	10	127532121	5.92E-06	0.040	14306	21.855
Vascular dementia (sudden onset)	genus Adlercreutzia	rs11604400	C	T	-0.223	98515355	0.242	0.191	360283	C	T	-0.103	11	98386085	9.74E-06	0.023	14306	19.060
Vascular dementia (sudden onset)	genus Adlercreutzia	rs13231526	C	A	-0.002	48804555	0.993	0.215	360283	C	A	0.143	7	48844151	4.81E-06	0.031	14306	21.123
Vascular dementia (sudden onset)	genus Adlercreutzia	rs2717140	C	T	0.321	77297814	0.098	0.194	360283	C	T	-0.119	18	75009770	2.05E-06	0.025	14306	22.548
Vascular dementia (sudden onset)	genus Adlercreutzia	rs6664405	T	C	0.109	68978480	0.513	0.166	360283	T	C	-0.095	1	69444163	5.23E-06	0.021	14306	20.451
Vascular dementia (sudden onset)	genus Adlercreutzia	rs7680684	T	C	0.215	170360208	0.080	0.123	360283	T	C	0.083	4	171281359	9.77E-07	0.017	14306	24.371
Vascular dementia (sudden onset)	genus Adlercreutzia	rs9490822	C	T	-0.080	123587938	0.493	0.117	360283	C	T	-0.073	6	123909083	2.54E-06	0.016	14306	22.229
Vascular dementia (sudden onset)	genus Adlercreutzia	rs9915817	C	T	-0.167	50786818	0.193	0.129	360283	C	T	-0.075	17	48864179	8.22E-06	0.017	14306	19.810
Vascular dementia (sudden onset)	genus Akkermansia	rs111862613	T	C	0.021	129825125	0.890	0.155	360283	T	C	0.091	12	130309670	3.39E-06	0.020	14306	21.449
Vascular dementia (sudden onset)	genus Akkermansia	rs117107102	A	G	-0.459	51947265	0.094	0.274	360283	A	G	0.204	18	49473635	3.01E-06	0.043	14306	22.427
Vascular dementia (sudden onset)	genus Akkermansia	rs11729256	T	C	-0.011	94106121	0.943	0.156	360283	T	C	0.075	4	95027272	6.58E-07	0.015	14306	24.970
Vascular dementia (sudden onset)	genus Akkermansia	rs12908520	G	A	0.094	97027427	0.424	0.117	360283	G	A	0.062	15	97570657	2.26E-06	0.013	14306	22.251
Vascular dementia (sudden onset)	genus Akkermansia	rs2602429	T	C	-0.330	81029544	0.013	0.133	360283	T	C	-0.075	16	81063149	2.72E-06	0.016	14306	22.770
Vascular dementia (sudden onset)	genus Akkermansia	rs4242783	A	G	0.007	5022135	0.957	0.130	360283	A	G	-0.069	10	5064327	3.00E-06	0.015	14306	21.537
Vascular dementia (sudden onset)	genus Akkermansia	rs4936098	G	A	0.119	130410772	0.327	0.122	360283	G	A	-0.065	11	130280667	1.10E-06	0.014	14306	22.810
Vascular dementia (sudden onset)	genus Akkermansia	rs61779207	G	A	0.087	40608800	0.539	0.142	360283	G	A	-0.076	1	41074472	6.32E-06	0.017	14306	20.550
Vascular dementia (sudden onset)	genus Akkermansia	rs74542928	T	C	-0.316	99623031	0.246	0.272	360283	T	C	0.113	4	100544188	1.48E-06	0.024	14306	22.690
Vascular dementia (sudden onset)	genus Akkermansia	rs9349825	A	G	-0.075	56476683	0.609	0.147	360283	A	G	-0.070	6	56341481	2.60E-06	0.015	14306	22.856

Vascular dementia (sudden onset)	genus Akkermansia	rs941682	G	A	-0.085	33280034	0.511	0.130	360283	G	A	-0.063	20	31867840	9.17E-06	0.014	14306	19.381
Vascular dementia (sudden onset)	genus Alistipes	rs1107244	G	A	0.033	37483220	0.877	0.216	360283	G	A	0.076	13	38057357	3.59E-06	0.017	14306	19.636
Vascular dementia (sudden onset)	genus Alistipes	rs11769002	G	A	-0.078	62983120	0.512	0.118	360283	G	A	-0.053	7	62443498	1.45E-06	0.011	14306	23.368
Vascular dementia (sudden onset)	genus Alistipes	rs11958296	A	G	0.086	178328178	0.757	0.279	360283	A	G	-0.098	5	177755179	9.30E-06	0.022	14306	20.209
Vascular dementia (sudden onset)	genus Alistipes	rs12990744	C	T	-0.185	177136221	0.324	0.188	360283	C	T	-0.078	2	178000949	8.21E-06	0.017	14306	20.155
Vascular dementia (sudden onset)	genus Alistipes	rs1689282	A	C	-0.106	14080130	0.391	0.123	360283	A	C	-0.052	9	14080129	5.28E-06	0.011	14306	20.832
Vascular dementia (sudden onset)	genus Alistipes	rs2290844	C	T	-0.404	126098573	0.031	0.188	360283	C	T	0.081	10	127787142	9.10E-06	0.019	14306	18.042
Vascular dementia (sudden onset)	genus Alistipes	rs2450745	A	C	0.153	143346550	0.500	0.227	360283	A	C	-0.081	8	144428720	7.12E-06	0.018	14306	19.007
Vascular dementia (sudden onset)	genus Alistipes	rs2875322	C	T	0.032	131017657	0.838	0.155	360283	C	T	0.058	11	130887552	8.78E-06	0.013	14306	19.537
Vascular dementia (sudden onset)	genus Alistipes	rs34417064	A	G	0.151	54699118	0.192	0.116	360283	A	G	-0.048	17	52776479	7.01E-06	0.011	14306	20.344
Vascular dementia (sudden onset)	genus Alistipes	rs4810359	A	G	-0.264	42562695	0.137	0.177	360283	A	G	-0.065	20	41191335	7.50E-06	0.015	14306	19.927
Vascular dementia (sudden onset)	genus Alistipes	rs7129639	A	C	-0.088	17489880	0.479	0.124	360283	A	C	0.052	11	17511427	1.78E-06	0.011	14306	22.948
Vascular dementia (sudden onset)	genus Alistipes	rs8130320	A	G	-0.109	39208332	0.349	0.117	360283	A	G	-0.049	21	40580258	4.84E-06	0.011	14306	20.906
Vascular dementia (sudden onset)	genus Allisonella	rs1901739	G	T	-0.205	114951010	0.080	0.117	360283	G	T	-0.116	5	114286707	3.59E-06	0.025	14306	21.682
Vascular dementia (sudden onset)	genus Allisonella	rs35110698	T	C	0.058	27866065	0.724	0.165	360283	T	C	-0.146	12	28018998	5.72E-06	0.032	14306	20.797
Vascular dementia (sudden onset)	genus Allisonella	rs35778461	C	T	-0.036	97388274	0.792	0.138	360283	C	T	0.147	9	100150556	1.21E-06	0.030	14306	24.360
Vascular dementia (sudden onset)	genus Allisonella	rs594561	T	C	0.107	88899804	0.353	0.116	360283	T	C	-0.112	11	88632972	9.41E-06	0.025	14306	19.885
Vascular dementia (sudden onset)	genus Allisonella	rs602075	G	A	0.025	76495244	0.851	0.132	360283	G	A	-0.169	9	79110160	3.57E-08	0.030	14306	32.374
Vascular dementia (sudden onset)	genus Allisonella	rs6742198	A	G	0.003	33382004	0.984	0.138	360283	A	G	-0.149	2	33607071	3.35E-06	0.032	14306	22.210
Vascular dementia (sudden onset)	genus Allisonella	rs76904847	G	A	-0.011	138283265	0.941	0.153	360283	G	A	0.149	7	137968010	6.09E-06	0.033	14306	19.673
Vascular dementia (sudden onset)	genus Allisonella	rs7898615	T	G	0.083	120653939	0.629	0.171	360283	T	G	0.168	10	122413451	8.87E-06	0.037	14306	20.214
Vascular dementia (sudden onset)	genus Alloprevotella	rs2154444	G	T	0.081	34681344	0.536	0.131	360283	G	T	-0.138	21	36053643	8.37E-06	0.031	14306	20.018
Vascular dementia (sudden onset)	genus Alloprevotella	rs34619204	G	A	-0.085	39621081	0.581	0.154	360283	G	A	-0.156	21	40993008	8.84E-06	0.034	14306	20.528
Vascular dementia (sudden onset)	genus Alloprevotella	rs4364940	A	G	-0.021	233990770	0.867	0.125	360283	A	G	0.126	1	234126516	8.58E-06	0.028	14306	20.067
Vascular dementia (sudden onset)	genus Alloprevotella	rs4680035	G	A	0.037	153087811	0.758	0.120	360283	G	A	0.120	3	152805600	4.99E-06	0.026	14306	21.250
Vascular dementia (sudden onset)	genus Alloprevotella	rs58212166	A	G	-0.034	188155551	0.820	0.150	360283	A	G	-0.162	4	189076705	7.94E-06	0.036	14306	20.227
Vascular dementia (sudden onset)	genus Anaerofilum	rs10794359	C	T	-0.252	1051715	0.033	0.118	360283	C	T	0.095	11	1051715	2.23E-06	0.020	14306	22.594
Vascular dementia (sudden onset)	genus Anaerofilum	rs1563175	A	C	-0.071	3791350	0.542	0.116	360283	A	C	0.092	2	3838940	5.54E-06	0.020	14306	20.884
Vascular dementia (sudden onset)	genus Anaerofilum	rs17012738	T	G	0.209	76471016	0.071	0.116	360283	T	G	0.090	2	76698142	7.24E-06	0.020	14306	20.355
Vascular dementia (sudden onset)	genus Anaerofilum	rs17096874	C	T	-0.010	30522152	0.946	0.143	360283	C	T	-0.126	14	30991358	2.86E-06	0.027	14306	22.071
Vascular dementia (sudden onset)	genus Anaerofilum	rs4244069	A	G	-0.138	66773681	0.424	0.172	360283	A	G	0.147	12	67167461	9.81E-06	0.033	14306	20.197
Vascular dementia (sudden onset)	genus Anaerofilum	rs4506496	A	G	-0.110	246982388	0.393	0.128	360283	A	G	-0.103	1	247145690	1.49E-06	0.021	14306	23.455
Vascular dementia (sudden onset)	genus Anaerofilum	rs712981	A	C	0.164	129967591	0.171	0.119	360283	A	C	0.101	3	129686434	6.83E-07	0.020	14306	24.661
Vascular dementia (sudden onset)	genus Anaerofilum	rs79598899	C	T	-0.411	190274425	0.140	0.279	360283	C	T	0.183	2	191139151	3.75E-07	0.036	14306	26.116
Vascular dementia (sudden onset)	genus Anaerofilum	rs816292	T	C	0.020	117373604	0.877	0.129	360283	T	C	-0.113	12	117811409	2.64E-07	0.022	14306	26.288
Vascular dementia (sudden onset)	genus Anaerofilum	rs9299345	T	C	-0.147	101577530	0.455	0.197	360283	T	C	-0.136	9	104339812	8.04E-06	0.030	14306	20.349
Vascular dementia (sudden onset)	genus Anaerostipes	rs10502061	A	G	-0.173	105744527	0.343	0.182	360283	A	G	0.084	11	105615253	7.94E-06	0.019	14306	18.944
Vascular dementia (sudden onset)	genus Anaerostipes	rs2014785	T	C	-0.217	171382313	0.066	0.118	360283	T	C	0.052	3	171100102	4.68E-06	0.011	14306	21.125
Vascular dementia (sudden onset)	genus Anaerostipes	rs2396460	C	T	0.083	227153955	0.475	0.117	360283	C	T	0.051	2	228018671	2.91E-06	0.011	14306	21.897
Vascular dementia (sudden onset)	genus Anaerostipes	rs2804244	G	A	-0.041	115623674	0.729	0.120	360283	G	A	0.053	10	117383184	2.04E-06	0.011	14306	22.882
Vascular dementia (sudden onset)	genus Anaerostipes	rs3900776	G	A	0.227	13525083	0.521	0.355	360283	G	A	-0.110	9	13525082	2.75E-06	0.024	14306	21.675
Vascular dementia (sudden onset)	genus Anaerostipes	rs60983350	G	A	-0.110	2947443	0.376	0.124	360283	G	A	-0.054	17	2850737	4.42E-06	0.012	14306	21.450
Vascular dementia (sudden onset)	genus Anaerostipes	rs62157625	T	C	-0.114	142016764	0.528	0.180	360283	T	C	0.089	2	142774333	1.45E-06	0.019	14306	22.787
Vascular dementia (sudden onset)	genus Anaerostipes	rs62215703	G	A	-0.116	24501310	0.408	0.141	360283	G	A	0.064	21	25873624	1.98E-06	0.014	14306	22.260
Vascular dementia (sudden onset)	genus Anaerostipes	rs6474958	G	A	-0.099	1582701	0.435	0.127	360283	G	A	0.050	9	1582701	6.74E-06	0.011	14306	19.935
Vascular dementia (sudden onset)	genus Anaerostipes	rs6726833	C	A	-0.126	39124428	0.553	0.213	360283	C	A	-0.088	2	39351569	3.32E-06	0.019	14306	21.463
Vascular dementia (sudden onset)	genus Anaerostipes	rs6854026	C	T	-0.012	168769663	0.919	0.118	360283	C	T	0.051	4	169690814	3.20E-06	0.011	14306	21.732
Vascular dementia (sudden onset)	genus Anaerostipes	rs7193624	T	C	0.156	77540123	0.464	0.213	360283	T	C	-0.075	16	77574020	5.35E-07	0.015	14306	24.803
Vascular dementia (sudden onset)	genus Anaerostipes	rs78735375	A	C	-0.133	1497548	0.659	0.301	360283	A	C	-0.137	19	1497547	5.33E-06	0.031	14306	20.262
Vascular dementia (sudden onset)	genus Anaerotruncus	rs10150232	A	G	-0.281	29948802	0.053	0.145	360283	A	G	0.057	14	30418008	6.68E-06	0.012	14306	20.622
Vascular dementia (sudden onset)	genus Anaerotruncus	rs11018566	A	G	0.249	89307058	0.333	0.257	360283	A	G	-0.156	11	89040226	6.14E-06	0.037	14306	18.272
Vascular dementia (sudden onset)	genus Anaerotruncus	rs115414803	A	C	-0.241	87242091	0.317	0.241	360283	A	C	-0.144	4	88163243	6.83E-06	0.032	14306	20.669
Vascular dementia (sudden onset)	genus Anaerotruncus	rs1272208	T	G	-0.027	76015978	0.839	0.135	360283	T	G	0.061	9	78630894	4.28E-06	0.013	14306	22.201
Vascular dementia (sudden onset)	genus Anaerotruncus	rs1431492	C	T	0.265	151137584	0.099	0.161	360283	C	T	-0.065	3	150855371	7.36E-06	0.015	14306	20.075
Vascular dementia (sudden onset)	genus Anaerotruncus	rs17734739	T	C	-0.062	210798978	0.713	0.169	360283	T	C	0.066	2	211663702	7.43E-06	0.015	14306	19.603
Vascular dementia (sudden onset)	genus Anaerotruncus	rs34449434	A	C	-0.103	76129875	0.396	0.121	360283	A	C	-0.050	12	76523655	9.85E-06	0.011	14306	19.208
Vascular dementia (sudden onset)	genus Anaerotruncus	rs4669806	G	T	-0.162	12060626	0.256	0.142	360283	G	T	0.058	2	12200752	2.42E-06	0.012	14306	21.962
Vascular dementia (sudden onset)	genus Anaerotruncus	rs6494922	A	G	-0.111	33167666	0.670	0.261	360283	A	G	0.090	15	33459867	6.62E-06	0.020	14306	19.937
Vascular dementia (sudden onset)	genus Anaerotruncus	rs6563550	T	C	0.033	37484276	0.877	0.216	360283	T	C	0.088	13	38058413	2.35E-07	0.018	14306	24.629
Vascular dementia (sudden onset)	genus Anaerotruncus	rs7155595	C	A	0.319	77036203	0.013	0.128	360283	C	A	0.054	14	77502546	7.55E-06	0.012	14306	20.575
Vascular dementia (sudden onset)	genus Anaerotruncus	rs8005030	C	T	0.132	30137993	0.285	0.123	360283	C	T	0.055	14	30607199	2.28E-06	0.012	14306	22.133
Vascular dementia (sudden onset)	genus Anaerotruncus	rs9347879	T	C	0.190	164594228	0.102	0.116	360283	T	C	0.051	6	165015261	4.22E-06	0.011	14306	20.988
Vascular dementia (sudden onset)	genus Bacteroides	rs11585893	A	G	0.125	10584294	0.354	0.135	360283	A	G	-0.074	1	10644351	1.80E-06	0.015	14306	25.175
Vascular dementia (sudden onset)	genus Bacteroides	rs13207588	A	G	-0.208	41551692	0.160	0.148	360283	A	G	-0.059	6	41519430	7.48E-06	0.013	14306	20.365
Vascular dementia (sudden onset)	genus Bacteroides	rs1340391	T	C	0.095	102495433	0.573	0.169	360283	T	C	-0.059	1	102960989	6.73E-06	0.013	14306	20.040
Vascular dementia (sudden onset)	genus Bacteroides	rs17619981	T	G	0.319	24159448	0.062	0.170	360283	T	G	0.088	19	24342250	2.69E-06	0.019	14306	22.194
Vascular dementia (sudden onset)	genus Bacteroides	rs2023437	T	C	0.200	21577814	0.256	0.176	360283	T	C	-0.078	14	22045949	5.02E-06	0.017	14306	21.780

Vascular dementia (sudden onset)	genus Bacteroides	rs66710942	T	C	-0.078	77166176	0.505	0.117	360283	T	C	-0.049	3	77215327	5.86E-06	0.011	14306	20.644
Vascular dementia (sudden onset)	genus Bacteroides	rs6795673	C	T	0.160	10551540	0.170	0.117	360283	C	T	0.054	3	10593224	3.38E-07	0.011	14306	26.183
Vascular dementia (sudden onset)	genus Bacteroides	rs9507307	C	T	-0.110	24336338	0.419	0.135	360283	C	T	0.060	13	24910476	2.13E-06	0.013	14306	21.912
Vascular dementia (sudden onset)	genus Barnesiella	rs11155559	T	C	-0.046	148280769	0.818	0.201	360283	T	C	0.096	6	148601905	8.92E-06	0.021	14306	20.153
Vascular dementia (sudden onset)	genus Barnesiella	rs12909713	C	T	-0.098	86602682	0.398	0.116	360283	C	T	-0.055	15	87145913	4.95E-06	0.012	14306	21.048
Vascular dementia (sudden onset)	genus Barnesiella	rs13242616	C	T	0.059	122381464	0.635	0.125	360283	C	T	0.058	7	122021518	2.29E-06	0.012	14306	22.459
Vascular dementia (sudden onset)	genus Barnesiella	rs199035	G	A	0.122	23435187	0.298	0.117	360283	G	A	0.056	6	23435415	3.00E-06	0.012	14306	21.842
Vascular dementia (sudden onset)	genus Barnesiella	rs2276875	A	G	0.122	5729227	0.362	0.133	360283	A	G	-0.070	4	5730954	4.65E-07	0.014	14306	24.964
Vascular dementia (sudden onset)	genus Barnesiella	rs2428166	G	A	-0.308	110509945	0.543	0.506	360283	G	A	-0.166	6	110831148	8.51E-07	0.034	14306	24.178
Vascular dementia (sudden onset)	genus Barnesiella	rs35177866	A	G	-0.103	181871367	0.642	0.222	360283	A	G	0.092	3	181589155	2.95E-06	0.019	14306	23.267
Vascular dementia (sudden onset)	genus Barnesiella	rs62251337	G	A	0.128	44285937	0.435	0.165	360283	G	A	0.069	3	44327429	4.24E-06	0.015	14306	21.385
Vascular dementia (sudden onset)	genus Barnesiella	rs72684847	T	C	0.227	101695712	0.301	0.220	360283	T	C	-0.114	4	102616869	6.76E-06	0.025	14306	20.295
Vascular dementia (sudden onset)	genus Barnesiella	rs76181748	C	T	0.105	104446188	0.450	0.138	360283	C	T	-0.078	8	105458416	6.78E-06	0.017	14306	20.575
Vascular dementia (sudden onset)	genus Barnesiella	rs77455852	T	G	-0.218	54811208	0.192	0.167	360283	T	G	-0.089	5	54107036	3.16E-06	0.020	14306	20.773
Vascular dementia (sudden onset)	genus Barnesiella	rs79795328	A	G	-0.162	113625439	0.331	0.167	360283	A	G	-0.082	4	114546595	4.23E-06	0.018	14306	21.527
Vascular dementia (sudden onset)	genus Bifidobacterium	rs12022129	A	G	0.034	206830029	0.794	0.130	360283	A	G	-0.062	1	207003374	8.00E-06	0.014	14306	19.872
Vascular dementia (sudden onset)	genus Bifidobacterium	rs182549	T	C	0.090	135859184	0.454	0.120	360283	T	C	-0.120	2	136616754	1.28E-20	0.013	14306	88.429
Vascular dementia (sudden onset)	genus Bifidobacterium	rs2491158	A	G	-0.341	124401134	0.044	0.170	360283	A	G	-0.071	10	126089703	8.05E-06	0.016	14306	19.879
Vascular dementia (sudden onset)	genus Bifidobacterium	rs2686790	C	T	0.225	48051149	0.169	0.164	360283	C	T	-0.071	7	48090746	7.50E-06	0.016	14306	20.065
Vascular dementia (sudden onset)	genus Bifidobacterium	rs4957061	T	C	0.274	520981	0.021	0.118	360283	T	C	0.053	5	521096	5.78E-06	0.012	14306	20.697
Vascular dementia (sudden onset)	genus Bifidobacterium	rs540489	T	G	-0.232	74901626	0.130	0.153	360283	T	G	-0.064	17	72897722	5.19E-06	0.014	14306	21.121
Vascular dementia (sudden onset)	genus Bifidobacterium	rs55888705	A	G	-0.113	1516099	0.382	0.129	360283	A	G	0.055	4	1517826	6.67E-06	0.012	14306	20.339
Vascular dementia (sudden onset)	genus Bifidobacterium	rs5746486	T	C	0.006	17871506	0.959	0.119	360283	T	C	-0.054	22	18354272	9.00E-06	0.012	14306	19.703
Vascular dementia (sudden onset)	genus Bifidobacterium	rs62181700	G	A	0.073	188941058	0.591	0.135	360283	G	A	-0.062	2	189805784	2.17E-06	0.013	14306	22.665
Vascular dementia (sudden onset)	genus Bifidobacterium	rs7322849	T	C	0.011	112205515	0.957	0.203	360283	T	C	0.112	13	112859829	1.08E-08	0.020	14306	31.035
Vascular dementia (sudden onset)	genus Bifidobacterium	rs75344046	C	T	-0.207	30489472	0.455	0.277	360283	C	T	0.232	21	31861790	4.86E-06	0.051	14306	21.088
Vascular dementia (sudden onset)	genus Bifidobacterium	rs857444	C	T	-0.124	14617360	0.303	0.120	360283	C	T	0.056	6	14617591	3.57E-06	0.012	14306	21.208
Vascular dementia (sudden onset)	genus Bilophila	rs11069458	C	T	0.101	101750335	0.498	0.149	360283	C	T	0.068	13	102402685	7.72E-06	0.016	14306	19.293
Vascular dementia (sudden onset)	genus Bilophila	rs1241171	G	A	-0.131	102834365	0.422	0.164	360283	G	A	-0.069	1	103299921	4.24E-06	0.015	14306	21.281
Vascular dementia (sudden onset)	genus Bilophila	rs1571225	T	C	0.013	4940871	0.932	0.157	360283	T	C	-0.083	9	4940871	1.12E-06	0.017	14306	23.484
Vascular dementia (sudden onset)	genus Bilophila	rs1969927	A	G	0.031	100393069	0.801	0.124	360283	A	G	-0.056	12	100786847	9.07E-06	0.013	14306	19.783
Vascular dementia (sudden onset)	genus Bilophila	rs2728491	T	G	-0.273	47172134	0.046	0.137	360283	T	G	0.063	7	47211732	6.33E-06	0.014	14306	20.241
Vascular dementia (sudden onset)	genus Bilophila	rs3827020	C	T	-0.083	63349639	0.573	0.147	360283	C	T	0.077	20	61980991	1.79E-06	0.016	14306	22.766
Vascular dementia (sudden onset)	genus Bilophila	rs4798126	G	A	-0.042	3765773	0.774	0.146	360283	G	A	0.073	18	3765773	7.15E-06	0.017	14306	19.033
Vascular dementia (sudden onset)	genus Bilophila	rs542415	T	C	-0.046	49719357	0.701	0.119	360283	T	C	-0.061	15	50011554	4.71E-06	0.013	14306	21.147
Vascular dementia (sudden onset)	genus Bilophila	rs60178956	G	A	0.008	89866110	0.954	0.139	360283	G	A	-0.062	8	90878338	8.06E-06	0.014	14306	19.502
Vascular dementia (sudden onset)	genus Bilophila	rs6793291	A	C	0.002	194698802	0.994	0.268	360283	A	C	-0.113	3	194419531	3.11E-06	0.024	14306	21.765
Vascular dementia (sudden onset)	genus Bilophila	rs72676854	T	C	0.123	108978710	0.640	0.263	360283	T	C	0.123	8	109990939	5.62E-06	0.027	14306	21.007
Vascular dementia (sudden onset)	genus Bilophila	rs7802841	A	C	-0.158	138899094	0.216	0.127	360283	A	C	-0.067	7	138583840	1.77E-06	0.014	14306	23.681
Vascular dementia (sudden onset)	genus Bilophila	rs9899990	A	G	0.266	9218262	0.214	0.214	360283	A	G	-0.103	17	9121579	9.07E-06	0.023	14306	19.283
Vascular dementia (sudden onset)	genus Butyricicoccus	rs10084203	G	A	0.015	190439423	0.934	0.174	360283	G	A	-0.055	2	191304149	8.59E-06	0.012	14306	19.791
Vascular dementia (sudden onset)	genus Butyricicoccus	rs12034718	G	A	0.085	66913912	0.543	0.139	360283	G	A	-0.070	1	67379595	9.58E-06	0.016	14306	19.643
Vascular dementia (sudden onset)	genus Butyricicoccus	rs12585793	T	C	0.518	26444103	0.150	0.360	360283	T	C	-0.262	13	27018240	5.79E-06	0.056	14306	21.558
Vascular dementia (sudden onset)	genus Butyricicoccus	rs2017189	T	G	-0.040	7458426	0.728	0.116	360283	T	G	0.051	4	7460153	3.87E-06	0.011	14306	21.148
Vascular dementia (sudden onset)	genus Butyricicoccus	rs4962426	T	G	-0.101	125219639	0.488	0.146	360283	T	G	-0.061	10	126908208	7.38E-06	0.014	14306	20.403
Vascular dementia (sudden onset)	genus Butyricicoccus	rs56221232	T	C	0.132	152596896	0.498	0.195	360283	T	C	0.083	2	153453410	7.62E-07	0.017	14306	24.467
Vascular dementia (sudden onset)	genus Butyricicoccus	rs62478070	T	G	-0.192	157895458	0.610	0.377	360283	T	G	0.224	7	157688150	5.94E-06	0.049	14306	20.488
Vascular dementia (sudden onset)	genus Butyricicoccus	rs7322368	C	T	-0.007	99561736	0.972	0.204	360283	C	T	-0.082	13	100213990	5.52E-06	0.018	14306	19.834
Vascular dementia (sudden onset)	genus Butyricimonas	rs11228830	A	G	-0.051	56897486	0.807	0.209	360283	A	G	0.135	11	56664962	6.55E-06	0.030	14306	20.547
Vascular dementia (sudden onset)	genus Butyricimonas	rs113054641	G	A	-0.428	15178069	0.134	0.286	360283	G	A	-0.145	21	16550389	1.74E-07	0.027	14306	27.842
Vascular dementia (sudden onset)	genus Butyricimonas	rs12304031	G	A	-0.122	128954226	0.485	0.175	360283	G	A	-0.086	12	129438771	6.70E-06	0.020	14306	19.211
Vascular dementia (sudden onset)	genus Butyricimonas	rs12458763	A	C	0.066	36511655	0.816	0.283	360283	A	C	0.122	18	34091618	6.37E-06	0.027	14306	20.492
Vascular dementia (sudden onset)	genus Butyricimonas	rs1862649	G	A	-0.117	22161545	0.610	0.229	360283	G	A	0.113	16	22172866	4.76E-06	0.025	14306	20.777
Vascular dementia (sudden onset)	genus Butyricimonas	rs2114713	G	T	0.107	80236031	0.363	0.118	360283	G	T	0.063	15	80528373	6.88E-06	0.014	14306	20.361
Vascular dementia (sudden onset)	genus Butyricimonas	rs62130338	A	G	0.107	48659244	0.380	0.122	360283	A	G	0.073	19	49162501	3.90E-06	0.016	14306	21.412
Vascular dementia (sudden onset)	genus Butyricimonas	rs62390301	T	C	0.137	160440640	0.339	0.144	360283	T	C	-0.087	5	159867647	7.42E-07	0.017	14306	24.915
Vascular dementia (sudden onset)	genus Butyricimonas	rs7083431	A	C	-0.157	70951399	0.228	0.130	360283	A	C	0.070	10	72711156	8.85E-07	0.014	14306	23.725
Vascular dementia (sudden onset)	genus Butyricimonas	rs71428626	G	T	-0.377	82866140	0.256	0.332	360283	G	T	-0.133	2	83093264	4.80E-06	0.029	14306	21.107
Vascular dementia (sudden onset)	genus Butyricimonas	rs72814525	A	G	-0.073	70683314	0.589	0.134	360283	A	G	0.066	10	72443070	8.25E-06	0.015	14306	19.666
Vascular dementia (sudden onset)	genus Butyricimonas	rs78453362	A	G	0.180	78690859	0.625	0.369	360283	A	G	-0.149	3	78740009	4.06E-06	0.033	14306	20.887
Vascular dementia (sudden onset)	genus Butyricimonas	rs9657374	C	T	0.016	4976884	0.902	0.128	360283	C	T	0.068	8	4834406	4.50E-06	0.015	14306	21.106
Vascular dementia (sudden onset)	genus Butyrivibrio	rs1007475	G	T	0.002	148082269	0.989	0.130	360283	G	T	0.118	6	148403405	7.92E-06	0.026	14306	20.420
Vascular dementia (sudden onset)	genus Butyrivibrio	rs11761679	T	C	0.097	150899841	0.560	0.167	360283	T	C	0.155	7	150596929	2.20E-06	0.032	14306	23.170
Vascular dementia (sudden onset)	genus Butyrivibrio	rs142855850	A	G	-0.155	22298971	0.440	0.201	360283	A	G	0.205	10	22587900	6.86E-06	0.046	14306	20.113
Vascular dementia (sudden onset)	genus Butyrivibrio	rs16934069	T	C	0.091	116930885	0.551	0.152	360283	T	C	-0.134	9	119693164	8.86E-06	0.030	14306	19.961
Vascular dementia (sudden onset)	genus Butyrivibrio	rs16941336	C	T	0.140	20672384	0.314	0.139	360283	C	T	0.127	17	20575697	1.53E-06	0.027	14306	22.640
Vascular dementia (sudden onset)	genus Butyrivibrio	rs17163238	G	A	0.079	129640328	0.589	0.147	360283	G	A	0.141	5	128976021	5.51E-06	0.031	14306	20.802

Vascular dementia (sudden onset)	genus Butyrivibrio	rs4537857	T	C	0.017	26718529	0.888	0.124	360283	T	C	-0.125	13	27292666	1.80E-06	0.026	14306	22.788
Vascular dementia (sudden onset)	genus Butyrivibrio	rs486484	A	G	-0.103	830864	0.382	0.118	360283	A	G	-0.108	20	811507	6.61E-06	0.024	14306	20.380
Vascular dementia (sudden onset)	genus Butyrivibrio	rs4928024	G	A	0.057	54254476	0.707	0.151	360283	G	A	0.175	3	54288503	8.19E-06	0.039	14306	20.137
Vascular dementia (sudden onset)	genus Butyrivibrio	rs72723662	C	T	-0.064	75599220	0.706	0.169	360283	C	T	0.224	14	76065563	7.86E-07	0.045	14306	24.869
Vascular dementia (sudden onset)	genus Butyrivibrio	rs74622183	A	G	0.195	33681771	0.347	0.207	360283	A	G	-0.201	14	34150977	2.46E-06	0.043	14306	22.040
Vascular dementia (sudden onset)	genus Butyrivibrio	rs77356209	T	C	0.696	10267736	0.012	0.277	360283	T	C	0.217	18	10267733	6.66E-06	0.048	14306	20.136
Vascular dementia (sudden onset)	genus Butyrivibrio	rs7752361	G	A	0.032	111459502	0.787	0.117	360283	G	A	0.119	6	111780705	7.69E-07	0.024	14306	24.685
Vascular dementia (sudden onset)	genus Butyrivibrio	rs7763512	A	G	-0.190	21959860	0.105	0.117	360283	A	G	-0.120	6	21960091	3.11E-06	0.025	14306	22.374
Vascular dementia (sudden onset)	genus Butyrivibrio	rs9349693	G	A	0.035	54074926	0.785	0.127	360283	G	A	-0.118	6	53939724	5.55E-06	0.026	14306	20.610
Vascular dementia (sudden onset)	genus Candidatus Soleaferrea	rs10090365	G	A	0.026	137625956	0.821	0.117	360283	G	A	0.083	8	138638199	4.17E-06	0.018	14306	21.255
Vascular dementia (sudden onset)	genus Candidatus Soleaferrea	rs10809135	C	T	-0.016	10611282	0.893	0.118	360283	C	T	-0.083	9	10611282	5.47E-06	0.018	14306	20.944
Vascular dementia (sudden onset)	genus Candidatus Soleaferrea	rs36155147	T	C	-0.221	312402	0.077	0.125	360283	T	C	-0.105	7	352368	5.41E-06	0.024	14306	18.971
Vascular dementia (sudden onset)	genus Candidatus Soleaferrea	rs4294381	C	T	-0.004	224500405	0.981	0.161	360283	C	T	-0.112	1	224688107	1.37E-06	0.023	14306	23.416
Vascular dementia (sudden onset)	genus Candidatus Soleaferrea	rs4678258	T	C	0.237	138226350	0.079	0.135	360283	T	C	0.099	3	137945192	5.53E-06	0.022	14306	20.912
Vascular dementia (sudden onset)	genus Candidatus Soleaferrea	rs6489992	A	G	0.147	114914964	0.220	0.120	360283	A	G	-0.084	12	115352769	7.89E-06	0.019	14306	20.192
Vascular dementia (sudden onset)	genus Candidatus Soleaferrea	rs6494306	A	G	-0.051	62102341	0.678	0.124	360283	A	G	-0.097	15	62394540	5.80E-06	0.021	14306	20.474
Vascular dementia (sudden onset)	genus Candidatus Soleaferrea	rs7400877	C	T	0.081	75937199	0.569	0.141	360283	C	T	0.095	14	76403542	9.29E-06	0.021	14306	19.976
Vascular dementia (sudden onset)	genus Candidatus Soleaferrea	rs9973954	G	A	0.161	19686146	0.186	0.121	360283	G	A	-0.089	2	19885907	5.95E-06	0.020	14306	20.842
Vascular dementia (sudden onset)	genus Catenibacterium	rs12404911	C	T	-0.055	239955143	0.709	0.149	360283	C	T	0.141	1	240118443	2.80E-06	0.030	14306	21.406
Vascular dementia (sudden onset)	genus Catenibacterium	rs212393	A	G	0.052	159064710	0.717	0.144	360283	A	G	0.135	6	159485742	3.62E-06	0.029	14306	22.332
Vascular dementia (sudden onset)	genus Catenibacterium	rs73128290	A	G	0.009	57296613	0.942	0.127	360283	A	G	0.130	7	57364320	4.29E-06	0.028	14306	20.782
Vascular dementia (sudden onset)	genus Catenibacterium	rs7742829	C	T	0.120	104641790	0.308	0.118	360283	C	T	0.114	6	105089665	5.61E-06	0.025	14306	20.652
Vascular dementia (sudden onset)	genus Christensenellaceae R 7group	rs10461257	A	G	0.106	155209852	0.389	0.124	360283	A	G	-0.055	4	156131004	6.51E-06	0.012	14306	20.446
Vascular dementia (sudden onset)	genus Christensenellaceae R 7group	rs17081797	A	G	-0.183	69888324	0.434	0.234	360283	A	G	-0.090	18	67555560	3.34E-06	0.020	14306	19.603
Vascular dementia (sudden onset)	genus Christensenellaceae R 7group	rs62132810	A	G	0.072	48775970	0.667	0.167	360283	A	G	-0.083	19	49279227	5.67E-06	0.018	14306	21.293
Vascular dementia (sudden onset)	genus Christensenellaceae R 7group	rs62190261	A	C	-0.188	230078383	0.359	0.205	360283	A	C	0.096	2	230943099	8.74E-06	0.021	14306	19.922
Vascular dementia (sudden onset)	genus Christensenellaceae R 7group	rs62467127	C	T	0.090	118456871	0.811	0.377	360283	C	T	0.114	7	118096925	3.25E-06	0.025	14306	20.506
Vascular dementia (sudden onset)	genus Christensenellaceae R 7group	rs73952017	C	T	0.091	1779608	0.632	0.189	360283	C	T	-0.086	18	1779609	8.46E-06	0.019	14306	19.679
Vascular dementia (sudden onset)	genus Christensenellaceae R 7group	rs78521377	C	T	0.593	124759805	0.072	0.329	360283	C	T	0.125	10	126448374	5.61E-06	0.027	14306	20.689
Vascular dementia (sudden onset)	genus Christensenellaceae R 7group	rs892686	A	G	-0.094	80428461	0.419	0.117	360283	A	G	0.051	9	83043376	3.97E-06	0.011	14306	21.313
Vascular dementia (sudden onset)	genus Clostridium innocuum group	rs10506058	A	G	-0.122	30115552	0.298	0.117	360283	A	G	0.100	12	30268485	8.92E-06	0.022	14306	20.184
Vascular dementia (sudden onset)	genus Clostridium innocuum group	rs1942371	G	A	-0.249	71607072	0.160	0.178	360283	G	A	-0.158	18	69274308	4.06E-06	0.034	14306	21.343
Vascular dementia (sudden onset)	genus Clostridium innocuum group	rs40656	C	T	0.004	9368046	0.977	0.145	360283	C	T	0.143	5	9368158	8.62E-06	0.031	14306	21.040
Vascular dementia (sudden onset)	genus Clostridium innocuum group	rs4869133	G	A	-0.112	96381915	0.461	0.152	360283	G	A	-0.181	5	95717619	7.24E-06	0.041	14306	19.448
Vascular dementia (sudden onset)	genus Clostridium innocuum group	rs61267978	T	C	-0.015	6348228	0.931	0.179	360283	T	C	0.147	18	6348227	5.59E-06	0.032	14306	21.010
Vascular dementia (sudden onset)	genus Clostridium innocuum group	rs6577484	G	A	0.290	8359360	0.131	0.192	360283	G	A	0.160	1	8419420	8.41E-06	0.036	14306	19.764
Vascular dementia (sudden onset)	genus Clostridium innocuum group	rs6890185	C	T	-0.129	71890799	0.301	0.125	360283	C	T	-0.113	5	71186626	1.12E-06	0.023	14306	23.669
Vascular dementia (sudden onset)	genus Clostridium innocuum group	rs77845139	A	G	0.284	59412009	0.034	0.134	360283	A	G	-0.115	15	59704208	8.41E-06	0.026	14306	19.992
Vascular dementia (sudden onset)	genus Clostridium sensustricto1	rs11264403	G	A	-0.056	155706701	0.801	0.222	360283	G	A	-0.139	1	155676492	7.76E-06	0.033	14306	17.288
Vascular dementia (sudden onset)	genus Clostridium sensustricto1	rs116847295	C	T	-0.236	43091212	0.177	0.175	360283	C	T	0.110	12	43485015	4.58E-06	0.025	14306	19.997
Vascular dementia (sudden onset)	genus Clostridium sensustricto1	rs12341505	G	A	-0.068	133845759	0.736	0.203	360283	G	A	0.081	9	136710881	4.82E-06	0.018	14306	20.259
Vascular dementia (sudden onset)	genus Clostridium sensustricto1	rs2795528	G	A	-0.137	42774816	0.597	0.258	360283	G	A	-0.184	10	43270264	2.72E-06	0.039	14306	22.085
Vascular dementia (sudden onset)	genus Clostridium sensustricto1	rs2817172	C	T	0.152	3124955	0.200	0.119	360283	C	T	0.058	1	3041519	2.77E-06	0.012	14306	21.810
Vascular dementia (sudden onset)	genus Clostridium sensustricto1	rs550843	T	C	0.209	165309343	0.109	0.131	360283	T	C	-0.078	6	165722832	2.05E-06	0.017	14306	21.426
Vascular dementia (sudden onset)	genus Collinsella	rs10890671	C	T	0.046	107252473	0.693	0.117	360283	C	T	0.054	11	107123199	6.52E-06	0.012	14306	20.446
Vascular dementia (sudden onset)	genus Collinsella	rs11597285	G	T	0.183	8547850	0.122	0.118	360283	G	T	-0.054	10	8589813	9.38E-06	0.012	14306	19.914
Vascular dementia (sudden onset)	genus Collinsella	rs1496626	T	C	-0.112	31269935	0.511	0.170	360283	T	C	-0.072	19	31760841	6.78E-06	0.016	14306	19.978
Vascular dementia (sudden onset)	genus Collinsella	rs149807560	C	A	-0.240	56015274	0.296	0.229	360283	C	A	-0.104	19	56526640	7.10E-06	0.024	14306	19.545
Vascular dementia (sudden onset)	genus Collinsella	rs2103510	G	A	-0.265	27983148	0.146	0.182	360283	G	A	0.079	21	29355467	2.42E-06	0.017	14306	21.863
Vascular dementia (sudden onset)	genus Collinsella	rs62448871	C	A	-0.048	24425405	0.683	0.117	360283	C	A	-0.054	7	24465024	6.78E-06	0.012	14306	20.164
Vascular dementia (sudden onset)	genus Collinsella	rs73052258	G	A	-0.138	194874587	0.527	0.218	360283	G	A	0.093	2	195739311	1.72E-06	0.020	14306	21.067
Vascular dementia (sudden onset)	genus Collinsella	rs75672793	A	G	-0.240	148897142	0.377	0.272	360283	A	G	-0.109	4	149818294	6.14E-06	0.024	14306	20.503
Vascular dementia (sudden onset)	genus Collinsella	rs9541268	C	A	0.035	68035929	0.865	0.208	360283	C	A	0.096	13	68610061	8.79E-07	0.020	14306	23.657
Vascular dementia (sudden onset)	genus Coprobacter	rs11532348	C	T	0.065	97896018	0.691	0.164	360283	C	T	-0.104	12	98289796	5.71E-06	0.023	14306	20.994
Vascular dementia (sudden onset)	genus Coprobacter	rs12684609	T	C	-0.215	134893022	0.149	0.149	360283	T	C	0.101	9	137784868	6.10E-06	0.022	14306	20.935
Vascular dementia (sudden onset)	genus Coprobacter	rs12996055	A	C	-0.059	136465888	0.657	0.133	360283	A	C	0.092	2	137223458	8.08E-06	0.021	14306	19.378
Vascular dementia (sudden onset)	genus Coprobacter	rs143662916	C	T	-0.442	103679246	0.203	0.347	360283	C	T	0.253	11	103549974	3.07E-06	0.054	14306	21.985
Vascular dementia (sudden onset)	genus Coprobacter	rs189356	G	A	0.150	58999796	0.203	0.118	360283	G	A	0.078	20	57574851	6.26E-06	0.017	14306	20.645
Vascular dementia (sudden onset)	genus Coprobacter	rs213863	T	C	0.182	96787063	0.131	0.121	360283	T	C	0.089	6	97234939	2.35E-06	0.019	14306	22.192
Vascular dementia (sudden onset)	genus Coprobacter	rs28402691	T	C	-0.312	95468089	0.054	0.162	360283	T	C	0.111	4	96389240	9.56E-06	0.025	14306	19.419
Vascular dementia (sudden onset)	genus Coprobacter	rs305411	A	G	-0.093	87767445	0.621	0.189	360283	A	G	0.129	1	88233128	1.01E-06	0.026	14306	23.864
Vascular dementia (sudden onset)	genus Coprobacter	rs3828477	G	T	-0.059	46442226	0.631	0.123	360283	G	T	-0.091	3	46483717	2.89E-06	0.020	14306	21.728
Vascular dementia (sudden onset)	genus Coprobacter	rs72821405	T	C	-0.193	4714563	0.346	0.205	360283	T	C	-0.147	6	4714797	4.76E-06	0.032	14306	21.210
Vascular dementia (sudden onset)	genus Coprobacter	rs74919520	G	A	0.072	32017383	0.702	0.188	360283	G	A	0.126	2	32242452	5.76E-06	0.028	14306	20.699
Vascular dementia (sudden onset)	genus Coprococcus1	rs1010560	C	A	-0.124	29927454	0.351	0.132	360283	C	A	0.058	1	30400301	1.96E-06	0.012	14306	22.354
Vascular dementia (sudden onset)	genus Coprococcus1	rs12794898	G	T	-0.003	124684165	0.986	0.174	360283	G	T	0.090	11	124554061	4.92E-06	0.020	14306	20.980
Vascular dementia (sudden onset)	genus Coprococcus1	rs1519491	T	C	-0.074	21856898	0.534	0.118	360283	T	C	0.050	2	22079770	8.95E-06	0.011	14306	19.325

Vascular dementia (sudden onset)	genus Coprococcus1	rs1576241	A	G	-0.128	71819574	0.286	0.120	360283	A	G	-0.051	6	72529277	3.33E-06	0.011	14306	21.708
Vascular dementia (sudden onset)	genus Coprococcus1	rs2907920	G	A	-0.167	2600719	0.196	0.129	360283	G	A	-0.056	19	2600717	7.65E-06	0.013	14306	19.579
Vascular dementia (sudden onset)	genus Coprococcus1	rs4277593	G	A	-0.006	4338773	0.963	0.118	360283	G	A	-0.059	20	4319420	1.14E-07	0.011	14306	28.390
Vascular dementia (sudden onset)	genus Coprococcus1	rs56405618	A	G	-0.091	173547165	0.622	0.184	360283	A	G	-0.090	4	174468316	1.57E-06	0.019	14306	23.095
Vascular dementia (sudden onset)	genus Coprococcus1	rs73031725	T	C	-0.128	134752388	0.704	0.338	360283	T	C	0.168	11	134622282	1.98E-06	0.036	14306	22.259
Vascular dementia (sudden onset)	genus Coprococcus1	rs73167075	T	C	0.132	165975355	0.353	0.142	360283	T	C	0.057	3	165693143	8.57E-06	0.013	14306	20.184
Vascular dementia (sudden onset)	genus Coprococcus1	rs74101919	T	C	-0.123	94958765	0.516	0.189	360283	T	C	-0.072	1	95424321	1.03E-06	0.014	14306	24.706
Vascular dementia (sudden onset)	genus Coprococcus1	rs946513	T	C	0.135	15871821	0.632	0.281	360283	T	C	-0.206	10	15913820	8.62E-06	0.046	14306	20.034
Vascular dementia (sudden onset)	genus Coprococcus2	rs10070053	A	G	-0.026	34794684	0.822	0.118	360283	A	G	0.059	5	34794789	7.65E-06	0.014	14306	19.273
Vascular dementia (sudden onset)	genus Coprococcus2	rs12634070	T	C	0.006	180827092	0.965	0.132	360283	T	C	0.074	3	180544880	9.95E-06	0.016	14306	19.942
Vascular dementia (sudden onset)	genus Coprococcus2	rs2482516	C	T	0.038	25554070	0.786	0.139	360283	C	T	0.075	9	25554068	4.72E-06	0.016	14306	21.002
Vascular dementia (sudden onset)	genus Coprococcus2	rs35890118	A	G	0.044	127917495	0.743	0.135	360283	A	G	-0.067	10	129715759	8.26E-06	0.015	14306	20.304
Vascular dementia (sudden onset)	genus Coprococcus2	rs61823518	A	C	0.068	223514894	0.716	0.188	360283	A	C	-0.096	1	223688236	6.68E-06	0.022	14306	19.614
Vascular dementia (sudden onset)	genus Coprococcus2	rs6677933	C	T	0.047	111596386	0.777	0.165	360283	C	T	-0.080	1	112139008	1.19E-06	0.016	14306	23.995
Vascular dementia (sudden onset)	genus Coprococcus2	rs72680320	T	C	0.265	130204631	0.032	0.124	360283	T	C	-0.065	4	131125786	2.27E-06	0.014	14306	21.766
Vascular dementia (sudden onset)	genus Coprococcus2	rs9426473	A	G	-0.044	4150395	0.739	0.133	360283	A	G	0.073	1	4210455	6.31E-06	0.016	14306	20.243
Vascular dementia (sudden onset)	genus Coprococcus3	rs10810043	G	A	0.010	13799810	0.933	0.124	360283	G	A	-0.052	9	13799809	9.27E-06	0.012	14306	19.776
Vascular dementia (sudden onset)	genus Coprococcus3	rs11077359	C	T	0.033	78347880	0.831	0.154	360283	C	T	0.065	17	76343961	9.64E-06	0.015	14306	18.807
Vascular dementia (sudden onset)	genus Coprococcus3	rs11080344	C	T	0.045	27777485	0.701	0.117	360283	C	T	0.052	17	26104511	4.79E-06	0.011	14306	20.906
Vascular dementia (sudden onset)	genus Coprococcus3	rs13247359	G	A	0.126	76728409	0.286	0.118	360283	G	A	0.051	7	76357726	7.33E-06	0.011	14306	20.527
Vascular dementia (sudden onset)	genus Coprococcus3	rs178271	C	T	-0.504	20977267	0.276	0.463	360283	C	T	-0.145	22	21331556	7.81E-07	0.029	14306	24.371
Vascular dementia (sudden onset)	genus Coprococcus3	rs4575475	A	G	0.112	98675414	0.419	0.138	360283	A	G	-0.062	14	99141751	7.04E-06	0.014	14306	20.216
Vascular dementia (sudden onset)	genus Coprococcus3	rs7521171	A	G	-0.085	150026539	0.501	0.126	360283	A	G	0.060	1	149998497	4.32E-06	0.013	14306	21.285
Vascular dementia (sudden onset)	genus Coprococcus3	rs8100692	T	C	0.143	39541492	0.222	0.117	360283	T	C	0.058	19	40032132	4.16E-07	0.011	14306	25.892
Vascular dementia (sudden onset)	genus Defluviitaleaceae UCG011	rs112893842	T	C	-0.061	8786663	0.762	0.203	360283	T	C	0.114	9	8786663	1.45E-06	0.023	14306	23.899
Vascular dementia (sudden onset)	genus Defluviitaleaceae UCG011	rs1582238	C	T	0.083	118181062	0.492	0.121	360283	C	T	-0.081	1	118723685	1.57E-06	0.017	14306	23.167
Vascular dementia (sudden onset)	genus Defluviitaleaceae UCG011	rs2892880	G	A	-0.024	119820571	0.856	0.134	360283	G	A	0.082	4	120741726	6.83E-06	0.018	14306	20.258
Vascular dementia (sudden onset)	genus Defluviitaleaceae UCG011	rs4344384	T	G	0.133	64647609	0.252	0.116	360283	T	G	-0.072	10	66407366	4.83E-06	0.016	14306	20.981
Vascular dementia (sudden onset)	genus Defluviitaleaceae UCG011	rs4677103	A	G	0.181	72158643	0.244	0.155	360283	A	G	0.098	3	72207794	9.60E-07	0.020	14306	24.576
Vascular dementia (sudden onset)	genus Defluviitaleaceae UCG011	rs55658617	T	C	-0.489	40439076	0.119	0.314	360283	T	C	0.174	21	41811003	2.15E-06	0.036	14306	23.173
Vascular dementia (sudden onset)	genus Defluviitaleaceae UCG011	rs72731813	C	T	0.195	146493591	0.476	0.274	360283	C	T	-0.147	4	147414743	4.33E-07	0.029	14306	25.165
Vascular dementia (sudden onset)	genus Defluviitaleaceae UCG011	rs9608282	T	G	-0.595	24408113	0.072	0.330	360283	T	G	0.143	22	24804081	2.52E-06	0.030	14306	22.733
Vascular dementia (sudden onset)	genus Defluviitaleaceae UCG011	rs9725395	A	G	-0.016	84739949	0.929	0.184	360283	A	G	-0.138	1	85205632	3.52E-06	0.030	14306	21.911
Vascular dementia (sudden onset)	genus Desulfovibrio	rs12031543	T	C	0.374	68198632	0.009	0.144	360283	T	C	-0.127	1	68664315	6.55E-06	0.028	14306	20.357
Vascular dementia (sudden onset)	genus Desulfovibrio	rs13066142	G	A	-0.083	67428549	0.676	0.198	360283	G	A	0.119	3	67478973	3.79E-06	0.025	14306	22.550
Vascular dementia (sudden onset)	genus Desulfovibrio	rs16863365	A	G	0.254	197225187	0.365	0.281	360283	A	G	0.109	2	198089911	1.79E-06	0.023	14306	23.235
Vascular dementia (sudden onset)	genus Desulfovibrio	rs2032031	G	A	-0.013	128372006	0.909	0.116	360283	G	A	0.065	10	130170270	9.14E-06	0.015	14306	19.404
Vascular dementia (sudden onset)	genus Desulfovibrio	rs2590913	A	G	0.275	63690313	0.301	0.265	360283	A	G	-0.154	13	64264446	6.65E-06	0.034	14306	20.721
Vascular dementia (sudden onset)	genus Desulfovibrio	rs2853179	T	C	0.072	104449819	0.600	0.138	360283	T	C	-0.081	8	105462047	2.42E-06	0.017	14306	21.718
Vascular dementia (sudden onset)	genus Desulfovibrio	rs4797774	A	G	0.126	13447996	0.674	0.299	360283	A	G	-0.213	18	13447995	5.64E-06	0.047	14306	20.454
Vascular dementia (sudden onset)	genus Desulfovibrio	rs6580353	T	C	0.237	140031102	0.104	0.146	360283	T	C	0.077	5	139410687	4.94E-06	0.017	14306	20.632
Vascular dementia (sudden onset)	genus Desulfovibrio	rs72647089	T	G	0.559	57060357	0.010	0.218	360283	T	G	-0.107	8	57972916	8.30E-06	0.024	14306	19.836
Vascular dementia (sudden onset)	genus Dialister	rs10138457	T	C	0.263	101991715	0.190	0.201	360283	T	C	-0.113	14	102458052	7.88E-06	0.026	14306	18.629
Vascular dementia (sudden onset)	genus Dialister	rs10938938	A	G	-0.136	23292736	0.388	0.157	360283	A	G	0.077	4	23294359	7.37E-06	0.017	14306	20.482
Vascular dementia (sudden onset)	genus Dialister	rs11071887	T	C	0.176	32662894	0.157	0.124	360283	T	C	0.066	15	32955095	5.91E-06	0.015	14306	20.496
Vascular dementia (sudden onset)	genus Dialister	rs11166701	G	A	0.063	137283482	0.589	0.116	360283	G	A	-0.066	8	138295725	5.51E-07	0.013	14306	24.698
Vascular dementia (sudden onset)	genus Dialister	rs2314294	T	C	-0.186	9243005	0.276	0.170	360283	T	C	0.087	16	9336862	8.08E-06	0.019	14306	19.981
Vascular dementia (sudden onset)	genus Dialister	rs2435610	A	C	0.077	151192947	0.563	0.133	360283	A	C	0.065	7	150890034	5.93E-06	0.014	14306	20.390
Vascular dementia (sudden onset)	genus Dialister	rs4747450	C	A	-0.197	22910724	0.158	0.139	360283	C	A	0.067	10	23199653	5.84E-06	0.015	14306	20.490
Vascular dementia (sudden onset)	genus Dialister	rs4753063	A	G	0.124	92533963	0.289	0.118	360283	A	G	0.060	11	92267129	4.86E-06	0.013	14306	21.023
Vascular dementia (sudden onset)	genus Dialister	rs75416973	A	G	-0.170	172840493	0.227	0.140	360283	A	G	0.073	1	172809633	9.46E-06	0.016	14306	19.541
Vascular dementia (sudden onset)	genus Dialister	rs764177	C	A	-0.169	45767957	0.163	0.121	360283	C	A	-0.060	3	45809449	9.61E-06	0.014	14306	19.742
Vascular dementia (sudden onset)	genus Dialister	rs76680460	G	A	-0.281	25447082	0.342	0.296	360283	G	A	-0.161	9	25447080	8.19E-06	0.036	14306	19.600
Vascular dementia (sudden onset)	genus Dorea	rs11150408	G	T	0.008	81769892	0.944	0.117	360283	G	T	-0.049	16	81803497	7.06E-06	0.011	14306	20.042
Vascular dementia (sudden onset)	genus Dorea	rs12537781	T	C	0.223	155186473	0.100	0.135	360283	T	C	-0.056	7	154978183	9.15E-06	0.013	14306	19.675
Vascular dementia (sudden onset)	genus Dorea	rs13279148	G	A	-0.275	126893620	0.139	0.186	360283	G	A	0.072	8	127905865	2.25E-06	0.015	14306	22.477
Vascular dementia (sudden onset)	genus Dorea	rs1899291	T	C	-0.013	60126622	0.935	0.161	360283	T	C	-0.070	4	60992340	4.57E-06	0.015	14306	21.523
Vascular dementia (sudden onset)	genus Dorea	rs3005511	G	A	-0.168	73896090	0.186	0.127	360283	G	A	-0.052	6	74605806	5.29E-06	0.011	14306	20.917
Vascular dementia (sudden onset)	genus Dorea	rs345219	G	T	0.089	6784833	0.448	0.117	360283	G	T	0.050	3	6826520	8.80E-06	0.011	14306	19.504
Vascular dementia (sudden onset)	genus Dorea	rs3752849	G	A	0.020	8707510	0.938	0.263	360283	G	A	0.164	11	8729057	7.68E-06	0.037	14306	20.032
Vascular dementia (sudden onset)	genus Dorea	rs4793307	C	T	-0.129	72737384	0.348	0.137	360283	C	T	0.057	17	70733523	4.01E-06	0.012	14306	21.979
Vascular dementia (sudden onset)	genus Dorea	rs62503162	A	G	-0.065	15911148	0.819	0.286	360283	A	G	-0.097	8	15768657	7.47E-07	0.019	14306	25.113
Vascular dementia (sudden onset)	genus Dorea	rs73729431	C	T	0.212	25077822	0.615	0.422	360283	C	T	-0.137	6	25078050	3.17E-06	0.030	14306	20.994
Vascular dementia (sudden onset)	genus Eggerthella	rs112205261	T	C	-0.081	9076080	0.704	0.213	360283	T	C	-0.189	1	9136139	3.35E-06	0.040	14306	21.839
Vascular dementia (sudden onset)	genus Eggerthella	rs13070736	A	C	0.010	20765962	0.948	0.158	360283	A	C	-0.121	3	20807454	7.62E-06	0.027	14306	19.894
Vascular dementia (sudden onset)	genus Eggerthella	rs1784446	A	G	0.005	102590868	0.963	0.116	360283	A	G	-0.091	11	102461599	5.23E-06	0.020	14306	20.997
Vascular dementia (sudden onset)	genus Eggerthella	rs2223081	A	G	0.120	28405711	0.351	0.129	360283	A	G	-0.103	21	29778032	3.89E-06	0.022	14306	21.520

Vascular dementia (sudden onset)	genus Eggerthella	rs2240838	G	A	-0.080	38296353	0.496	0.118	360283	G	A	-0.098	7	38335954	7.36E-07	0.020	14306	24.613
Vascular dementia (sudden onset)	genus Eggerthella	rs3851328	G	T	-0.144	45226666	0.304	0.141	360283	G	T	0.108	2	45453805	4.18E-06	0.024	14306	20.762
Vascular dementia (sudden onset)	genus Eggerthella	rs6430926	T	C	-0.044	140579885	0.711	0.118	360283	T	C	-0.088	2	141337454	8.37E-06	0.020	14306	19.916
Vascular dementia (sudden onset)	genus Eggerthella	rs67490567	T	C	-0.009	65398986	0.948	0.133	360283	T	C	0.108	15	65691324	8.94E-06	0.025	14306	19.537
Vascular dementia (sudden onset)	genus Eggerthella	rs76663501	C	T	0.447	57072813	0.095	0.268	360283	C	T	0.175	20	55647869	4.83E-06	0.038	14306	21.454
Vascular dementia (sudden onset)	genus Eisenbergiella	rs11027642	C	T	0.163	23980294	0.320	0.164	360283	C	T	0.129	11	24001840	4.92E-06	0.028	14306	20.512
Vascular dementia (sudden onset)	genus Eisenbergiella	rs11079158	T	C	-0.121	55290280	0.394	0.142	360283	T	C	0.101	17	53367641	7.35E-06	0.023	14306	19.920
Vascular dementia (sudden onset)	genus Eisenbergiella	rs11938607	C	T	-0.126	188139277	0.345	0.134	360283	C	T	-0.098	4	189060431	8.22E-06	0.022	14306	20.386
Vascular dementia (sudden onset)	genus Eisenbergiella	rs12257723	A	C	-0.020	107657343	0.875	0.124	360283	A	C	-0.095	10	109417101	8.85E-06	0.021	14306	20.279
Vascular dementia (sudden onset)	genus Eisenbergiella	rs12710729	C	A	0.083	19773449	0.504	0.124	360283	C	A	0.089	2	19973210	9.84E-06	0.020	14306	20.140
Vascular dementia (sudden onset)	genus Eisenbergiella	rs13258851	A	G	-0.148	54418254	0.368	0.165	360283	A	G	0.137	8	55330814	7.75E-06	0.030	14306	20.563
Vascular dementia (sudden onset)	genus Eisenbergiella	rs1508033	A	C	0.035	53083004	0.784	0.128	360283	A	C	0.092	15	53375201	3.23E-06	0.020	14306	21.863
Vascular dementia (sudden onset)	genus Eisenbergiella	rs1553971	T	G	0.029	111700639	0.836	0.139	360283	T	G	0.121	3	111419486	5.27E-06	0.026	14306	21.146
Vascular dementia (sudden onset)	genus Eisenbergiella	rs2683098	T	C	0.070	35886364	0.621	0.142	360283	T	C	-0.107	15	36178565	2.24E-06	0.023	14306	22.720
Vascular dementia (sudden onset)	genus Eisenbergiella	rs3812426	A	G	0.041	49910116	0.801	0.161	360283	A	G	-0.106	8	50822676	2.72E-06	0.022	14306	22.550
Vascular dementia (sudden onset)	genus Eisenbergiella	rs4462860	G	A	0.032	20237941	0.790	0.119	360283	G	A	0.094	21	21610254	4.16E-06	0.020	14306	21.805
Vascular dementia (sudden onset)	genus Enterorhabdus	rs10098492	T	C	-0.153	112097527	0.536	0.247	360283	T	C	0.132	8	113109756	6.41E-06	0.029	14306	20.300
Vascular dementia (sudden onset)	genus Enterorhabdus	rs114731706	T	G	0.062	19412192	0.852	0.332	360283	T	G	0.182	2	19611953	2.17E-06	0.038	14306	22.741
Vascular dementia (sudden onset)	genus Enterorhabdus	rs2051957	C	T	-0.103	90080569	0.482	0.147	360283	C	T	0.084	7	89709883	8.90E-06	0.019	14306	19.727
Vascular dementia (sudden onset)	genus Enterorhabdus	rs3017103	G	A	0.077	62406721	0.605	0.149	360283	G	A	-0.098	11	62174193	2.94E-06	0.021	14306	22.028
Vascular dementia (sudden onset)	genus Enterorhabdus	rs73331712	T	C	-0.180	69121322	0.542	0.295	360283	T	C	0.262	12	69515102	4.85E-06	0.055	14306	22.589
Vascular dementia (sudden onset)	genus Enterorhabdus	rs77655283	G	A	0.012	235531235	0.959	0.228	360283	G	A	0.133	2	236439879	5.88E-06	0.030	14306	19.875
Vascular dementia (sudden onset)	genus Erysipelatoclostridium	rs1434153	G	A	0.219	34370466	0.064	0.118	360283	G	A	-0.068	2	34595533	6.85E-06	0.015	14306	20.163
Vascular dementia (sudden onset)	genus Erysipelatoclostridium	rs16936671	C	T	-0.324	36105681	0.056	0.169	360283	C	T	-0.097	10	36394609	6.04E-06	0.022	14306	19.739
Vascular dementia (sudden onset)	genus Erysipelatoclostridium	rs17804233	C	T	0.217	78519233	0.062	0.116	360283	C	T	0.066	5	77815056	4.59E-06	0.014	14306	21.133
Vascular dementia (sudden onset)	genus Erysipelatoclostridium	rs2901723	A	C	0.086	36849973	0.461	0.117	360283	A	C	-0.064	11	36871523	8.79E-06	0.014	14306	19.755
Vascular dementia (sudden onset)	genus Erysipelatoclostridium	rs340991	A	G	-0.067	34891937	0.615	0.132	360283	A	G	-0.074	5	34892042	3.75E-06	0.016	14306	21.719
Vascular dementia (sudden onset)	genus Erysipelatoclostridium	rs3804326	A	G	-0.034	24291957	0.903	0.276	360283	A	G	0.141	6	24292185	9.85E-06	0.034	14306	17.691
Vascular dementia (sudden onset)	genus Erysipelatoclostridium	rs45480394	T	G	-0.160	55346409	0.189	0.122	360283	T	G	-0.069	19	55857777	7.66E-06	0.015	14306	20.480
Vascular dementia (sudden onset)	genus Erysipelatoclostridium	rs4697572	A	G	0.112	25445165	0.443	0.146	360283	A	G	-0.081	4	25446787	7.59E-07	0.016	14306	24.640
Vascular dementia (sudden onset)	genus Erysipelatoclostridium	rs58236560	G	T	0.012	122006205	0.944	0.178	360283	G	T	-0.111	11	121876913	2.16E-06	0.023	14306	22.483
Vascular dementia (sudden onset)	genus Erysipelatoclostridium	rs61806970	C	T	0.169	167849359	0.468	0.232	360283	C	T	0.143	1	167818597	9.09E-06	0.032	14306	19.759
Vascular dementia (sudden onset)	genus Erysipelatoclostridium	rs622418	A	G	-0.041	87971128	0.722	0.116	360283	A	G	-0.067	9	90586043	3.68E-06	0.014	14306	21.756
Vascular dementia (sudden onset)	genus Erysipelatoclostridium	rs6474512	C	A	-0.191	38923088	0.111	0.120	360283	C	A	-0.067	8	38780606	3.02E-06	0.014	14306	21.900
Vascular dementia (sudden onset)	genus Erysipelatoclostridium	rs710230	C	T	0.094	41867960	0.678	0.225	360283	C	T	-0.143	1	42333631	6.33E-07	0.028	14306	25.941
Vascular dementia (sudden onset)	genus Erysipelatoclostridium	rs7221249	G	A	-0.033	10274391	0.777	0.116	360283	G	A	-0.084	17	10177708	4.31E-09	0.014	14306	34.619
Vascular dementia (sudden onset)	genus Erysipelatoclostridium	rs9590927	A	G	0.025	45797930	0.828	0.116	360283	A	G	0.065	13	46372065	6.39E-06	0.014	14306	20.272
Vascular dementia (sudden onset)	genus Escherichia Shigella	rs112767262	T	C	0.120	3700622	0.392	0.140	360283	T	C	0.073	16	3750623	8.21E-06	0.016	14306	20.075
Vascular dementia (sudden onset)	genus Escherichia Shigella	rs113127095	A	G	-0.311	22679253	0.288	0.292	360283	A	G	0.151	13	23253392	3.33E-06	0.032	14306	21.799
Vascular dementia (sudden onset)	genus Escherichia Shigella	rs113513883	A	G	0.360	140978399	0.274	0.329	360283	A	G	0.172	7	140678199	5.28E-06	0.038	14306	20.511
Vascular dementia (sudden onset)	genus Escherichia Shigella	rs1154904	G	A	-0.111	134904951	0.342	0.117	360283	G	A	0.061	11	134774845	3.04E-06	0.013	14306	22.043
Vascular dementia (sudden onset)	genus Escherichia Shigella	rs118526	A	C	-0.047	80567725	0.710	0.127	360283	A	C	0.059	5	79863544	8.00E-06	0.014	14306	19.108
Vascular dementia (sudden onset)	genus Escherichia Shigella	rs2798105	A	G	0.197	48496231	0.314	0.196	360283	A	G	-0.101	1	48961903	8.24E-06	0.022	14306	20.629
Vascular dementia (sudden onset)	genus Escherichia Shigella	rs4731451	G	A	0.250	128414046	0.047	0.126	360283	G	A	-0.061	7	128054100	7.47E-06	0.014	14306	20.362
Vascular dementia (sudden onset)	genus Escherichia Shigella	rs57024273	T	C	-0.144	235606511	0.276	0.133	360283	T	C	0.063	2	236515155	9.70E-06	0.014	14306	20.028
Vascular dementia (sudden onset)	genus Escherichia Shigella	rs592299	T	C	0.072	133303865	0.539	0.117	360283	T	C	-0.059	9	136179347	4.77E-06	0.013	14306	20.903
Vascular dementia (sudden onset)	genus Escherichia Shigella	rs73208162	A	G	0.469	37952181	0.154	0.329	360283	A	G	-0.119	21	39324484	2.19E-06	0.025	14306	23.067
Vascular dementia (sudden onset)	genus Eubacterium brachy group	rs112617308	T	C	0.229	92425456	0.254	0.200	360283	T	C	-0.171	10	94185213	2.38E-06	0.036	14306	22.177
Vascular dementia (sudden onset)	genus Eubacterium brachy group	rs12151423	G	A	0.230	217372558	0.048	0.116	360283	G	A	-0.101	2	218237281	9.27E-06	0.023	14306	19.869
Vascular dementia (sudden onset)	genus Eubacterium brachy group	rs13139592	T	C	0.168	143744025	0.328	0.172	360283	T	C	-0.146	4	144665178	7.97E-06	0.033	14306	19.911
Vascular dementia (sudden onset)	genus Eubacterium brachy group	rs1384962	G	A	-0.020	22591645	0.870	0.126	360283	G	A	-0.121	14	23060552	6.99E-06	0.027	14306	20.613
Vascular dementia (sudden onset)	genus Eubacterium brachy group	rs2913110	T	C	0.096	22603738	0.433	0.123	360283	T	C	-0.105	10	22892667	4.56E-06	0.023	14306	21.004
Vascular dementia (sudden onset)	genus Eubacterium brachy group	rs4862235	A	G	-0.132	183707778	0.261	0.117	360283	A	G	-0.105	4	184628931	3.73E-06	0.023	14306	21.553
Vascular dementia (sudden onset)	genus Eubacterium brachy group	rs62348779	T	C	0.181	17459987	0.410	0.220	360283	T	C	-0.201	5	17460096	3.78E-06	0.043	14306	21.666
Vascular dementia (sudden onset)	genus Eubacterium brachy group	rs6591893	A	G	-0.032	80525789	0.794	0.122	360283	A	G	-0.108	11	80236833	7.34E-06	0.024	14306	20.281
Vascular dementia (sudden onset)	genus Eubacterium brachy group	rs720439	G	A	0.018	47794849	0.897	0.136	360283	G	A	0.112	22	48190598	7.03E-06	0.025	14306	19.845
Vascular dementia (sudden onset)	genus Eubacterium brachy group	rs73199919	T	C	-0.262	6854837	0.331	0.270	360283	T	C	-0.237	4	6856564	8.16E-06	0.053	14306	19.848
Vascular dementia (sudden onset)	genus Eubacterium coprostanoligenes group	rs1020520	T	G	-0.022	33563534	0.895	0.164	360283	T	G	-0.059	7	33603146	8.89E-06	0.013	14306	19.747
Vascular dementia (sudden onset)	genus Eubacterium coprostanoligenes group	rs10444197	A	G	-0.030	2173737	0.804	0.122	360283	A	G	-0.051	10	2215931	5.98E-06	0.011	14306	19.877
Vascular dementia (sudden onset)	genus Eubacterium coprostanoligenes group	rs11052069	C	T	-0.153	32560985	0.190	0.117	360283	C	T	-0.048	12	32713919	9.38E-06	0.011	14306	19.636
Vascular dementia (sudden onset)	genus Eubacterium coprostanoligenes group	rs11720857	C	T	-0.080	114075660	0.599	0.152	360283	C	T	0.063	3	113794507	9.26E-06	0.014	14306	19.059
Vascular dementia (sudden onset)	genus Eubacterium coprostanoligenes group	rs12906958	C	T	-0.042	36619397	0.743	0.127	360283	C	T	-0.053	15	36911598	4.35E-06	0.012	14306	21.149
Vascular dementia (sudden onset)	genus Eubacterium coprostanoligenes group	rs17159861	C	T	-0.166	31045547	0.374	0.187	360283	C	T	0.096	7	31085162	1.04E-08	0.017	14306	32.654
Vascular dementia (sudden onset)	genus Eubacterium coprostanoligenes group	rs2644213	A	G	-0.216	82746340	0.092	0.128	360283	A	G	-0.054	10	84506096	9.86E-06	0.012	14306	19.751
Vascular dementia (sudden onset)	genus Eubacterium coprostanoligenes group	rs4076415	G	T	-0.005	85897766	0.968	0.121	360283	G	T	-0.052	15	86440997	1.99E-06	0.011	14306	21.816
Vascular dementia (sudden onset)	genus Eubacterium coprostanoligenes group	rs62024432	C	T	0.018	97326063	0.927	0.200	360283	C	T	-0.077	15	97869293	7.50E-06	0.017	14306	20.002
Vascular dementia (sudden onset)	genus Eubacterium coprostanoligenes group	rs6762473	A	C	-0.080	127420731	0.512	0.122	360283	A	C	-0.052	3	127139574	4.26E-06	0.011	14306	21.552

Vascular dementia (sudden onset)	genus Eubacterium coprostanoligenes group	rs76898927	G	A	-0.278	81546326	0.275	0.255	360283	G	A	0.123	3	81595477	4.79E-06	0.027	14306	21.341
Vascular dementia (sudden onset)	genus Eubacterium coprostanoligenes group	rs9648214	T	C	-0.095	16341181	0.652	0.209	360283	T	C	-0.083	7	16380806	2.52E-07	0.016	14306	25.438
Vascular dementia (sudden onset)	genus Eubacterium eligens group	rs182318	G	A	-0.209	31077144	0.322	0.211	360283	G	A	-0.082	11	31098691	8.40E-06	0.020	14306	17.784
Vascular dementia (sudden onset)	genus Eubacterium eligens group	rs2200429	A	G	-0.008	106911110	0.966	0.195	360283	A	G	-0.089	13	107563458	5.30E-06	0.020	14306	20.054
Vascular dementia (sudden onset)	genus Eubacterium eligens group	rs265534	T	G	0.082	81332251	0.484	0.116	360283	T	G	-0.056	10	83092007	2.27E-06	0.012	14306	22.024
Vascular dementia (sudden onset)	genus Eubacterium eligens group	rs4583233	A	C	0.018	81786881	0.887	0.129	360283	A	C	0.067	16	81820486	2.84E-07	0.013	14306	27.363
Vascular dementia (sudden onset)	genus Eubacterium eligens group	rs56080211	T	C	-0.153	109503416	0.484	0.219	360283	T	C	-0.123	2	110260993	9.14E-06	0.028	14306	18.953
Vascular dementia (sudden onset)	genus Eubacterium eligens group	rs6923695	T	G	-0.170	70247112	0.467	0.233	360283	T	G	0.103	6	70956815	4.87E-06	0.023	14306	20.243
Vascular dementia (sudden onset)	genus Eubacterium fissicatena group	rs10147907	T	G	0.246	89018029	0.257	0.217	360283	T	G	0.172	14	89484373	8.27E-06	0.040	14306	18.922
Vascular dementia (sudden onset)	genus Eubacterium fissicatena group	rs11818408	G	A	0.141	94998710	0.240	0.120	360283	G	A	0.106	10	96758467	8.20E-06	0.024	14306	19.928
Vascular dementia (sudden onset)	genus Eubacterium fissicatena group	rs11876297	T	C	-0.138	48226901	0.292	0.131	360283	T	C	0.131	18	45753272	2.67E-06	0.028	14306	21.779
Vascular dementia (sudden onset)	genus Eubacterium fissicatena group	rs151257695	A	G	-0.181	73629231	0.420	0.225	360283	A	G	0.210	7	73043561	3.10E-06	0.045	14306	21.217
Vascular dementia (sudden onset)	genus Eubacterium fissicatena group	rs1768152	C	T	-0.438	39563463	0.020	0.188	360283	C	T	-0.139	3	39604954	8.70E-06	0.032	14306	19.462
Vascular dementia (sudden onset)	genus Eubacterium fissicatena group	rs2733072	G	A	0.118	5576177	0.311	0.116	360283	G	A	0.110	8	5433699	1.49E-06	0.023	14306	23.064
Vascular dementia (sudden onset)	genus Eubacterium fissicatena group	rs3771393	T	C	-0.085	70918116	0.565	0.148	360283	T	C	-0.131	2	71145246	7.38E-07	0.027	14306	24.074
Vascular dementia (sudden onset)	genus Eubacterium fissicatena group	rs6934739	A	G	-0.120	39972698	0.327	0.123	360283	A	G	0.111	6	39940437	9.75E-06	0.025	14306	19.442
Vascular dementia (sudden onset)	genus Eubacterium fissicatena group	rs7104872	G	A	0.270	115294391	0.143	0.185	360283	G	A	0.139	11	115165111	2.73E-06	0.029	14306	22.548
Vascular dementia (sudden onset)	genus Eubacterium hallii group	rs10501370	C	T	0.239	58273149	0.324	0.243	360283	C	T	-0.116	11	58040621	5.42E-06	0.025	14306	20.958
Vascular dementia (sudden onset)	genus Eubacterium hallii group	rs10798999	C	T	0.159	33843316	0.228	0.132	360283	C	T	0.060	1	34308917	2.61E-06	0.013	14306	22.532
Vascular dementia (sudden onset)	genus Eubacterium hallii group	rs117748144	T	C	0.088	11750090	0.743	0.268	360283	T	C	-0.127	11	11771637	7.86E-06	0.029	14306	19.436
Vascular dementia (sudden onset)	genus Eubacterium hallii group	rs13116360	T	C	0.171	110964275	0.469	0.236	360283	T	C	0.154	4	111885431	2.94E-07	0.030	14306	26.896
Vascular dementia (sudden onset)	genus Eubacterium hallii group	rs17074066	T	C	-0.529	182788383	0.194	0.408	360283	T	C	-0.081	4	183709536	9.35E-06	0.019	14306	18.495
Vascular dementia (sudden onset)	genus Eubacterium hallii group	rs17474256	G	A	0.507	103982054	0.011	0.199	360283	G	A	0.081	1	104524676	9.45E-06	0.018	14306	19.297
Vascular dementia (sudden onset)	genus Eubacterium hallii group	rs281379	A	G	0.019	48711017	0.872	0.118	360283	A	G	-0.050	19	49214274	9.33E-06	0.011	14306	19.838
Vascular dementia (sudden onset)	genus Eubacterium hallii group	rs28584818	A	G	0.059	64678780	0.781	0.212	360283	A	G	0.126	3	64664456	4.43E-06	0.027	14306	22.041
Vascular dementia (sudden onset)	genus Eubacterium hallii group	rs60254196	G	A	0.149	149159628	0.201	0.117	360283	G	A	0.052	7	148856720	2.70E-06	0.011	14306	21.844
Vascular dementia (sudden onset)	genus Eubacterium hallii group	rs630939	C	T	-0.131	50858093	0.266	0.118	360283	C	T	-0.051	18	48384463	9.16E-06	0.011	14306	19.806
Vascular dementia (sudden onset)	genus Eubacterium hallii group	rs6550770	C	T	-0.171	23621925	0.564	0.297	360283	C	T	0.198	3	23663416	4.82E-06	0.044	14306	19.945
Vascular dementia (sudden onset)	genus Eubacterium hallii group	rs74018587	C	T	-0.332	61721961	0.267	0.299	360283	C	T	0.209	15	62014160	3.70E-06	0.044	14306	22.734
Vascular dementia (sudden onset)	genus Eubacterium hallii group	rs78056098	G	T	0.045	123919170	0.707	0.121	360283	G	T	-0.051	11	123789877	8.29E-06	0.011	14306	19.896
Vascular dementia (sudden onset)	genus Eubacterium hallii group	rs949971	T	G	-0.144	110564542	0.243	0.123	360283	T	G	-0.054	3	110283389	3.29E-06	0.012	14306	21.646
Vascular dementia (sudden onset)	genus Eubacterium nodatum group	rs10263623	C	T	-0.425	65429224	0.150	0.295	360283	C	T	0.193	7	64894137	8.91E-06	0.044	14306	19.425
Vascular dementia (sudden onset)	genus Eubacterium nodatum group	rs10458299	T	C	0.114	135367596	0.606	0.220	360283	T	C	-0.188	7	135052348	8.37E-06	0.042	14306	20.013
Vascular dementia (sudden onset)	genus Eubacterium nodatum group	rs11006576	A	G	-0.063	59562257	0.591	0.117	360283	A	G	-0.110	10	61322015	7.99E-06	0.025	14306	20.067
Vascular dementia (sudden onset)	genus Eubacterium nodatum group	rs113893692	C	T	-0.091	28245520	0.606	0.177	360283	C	T	-0.185	9	28245518	5.76E-06	0.040	14306	21.038
Vascular dementia (sudden onset)	genus Eubacterium nodatum group	rs34297067	A	G	-0.113	51719170	0.490	0.164	360283	A	G	-0.187	14	52185888	6.60E-08	0.034	14306	29.959
Vascular dementia (sudden onset)	genus Eubacterium nodatum group	rs61841040	G	T	0.131	9617786	0.366	0.145	360283	G	T	0.161	10	9659749	3.56E-06	0.034	14306	22.108
Vascular dementia (sudden onset)	genus Eubacterium nodatum group	rs6818880	G	A	0.011	93791596	0.922	0.117	360283	G	A	0.110	4	94712747	7.83E-06	0.025	14306	20.030
Vascular dementia (sudden onset)	genus Eubacterium nodatum group	rs77910827	C	T	-0.160	93835703	0.390	0.186	360283	C	T	0.202	9	96597985	9.05E-07	0.041	14306	23.807
Vascular dementia (sudden onset)	genus Eubacterium nodatum group	rs7827125	C	T	0.043	5097390	0.743	0.130	360283	C	T	0.122	8	4954912	7.17E-06	0.027	14306	20.317
Vascular dementia (sudden onset)	genus Eubacterium nodatum group	rs7880204	T	C	-0.011	171091680	0.937	0.135	360283	T	C	-0.125	1	171060821	6.84E-06	0.028	14306	20.766
Vascular dementia (sudden onset)	genus Eubacterium nodatum group	rs9425984	T	C	0.015	34064135	0.918	0.141	360283	T	C	-0.130	1	34529736	7.21E-06	0.029	14306	19.845
Vascular dementia (sudden onset)	genus Eubacterium oxidoreducens group	rs12129908	A	C	0.047	195094889	0.688	0.118	360283	A	C	-0.089	1	195064019	5.80E-06	0.020	14306	20.285
Vascular dementia (sudden onset)	genus Eubacterium oxidoreducens group	rs12423772	G	T	0.122	94121564	0.455	0.163	360283	G	T	0.141	12	94515340	2.63E-06	0.030	14306	22.834
Vascular dementia (sudden onset)	genus Eubacterium oxidoreducens group	rs2973294	G	T	-0.097	37525057	0.409	0.118	360283	G	T	0.092	4	37526679	2.39E-06	0.020	14306	22.331
Vascular dementia (sudden onset)	genus Eubacterium oxidoreducens group	rs34561138	G	A	-0.191	86446749	0.521	0.297	360283	G	A	0.216	16	86480355	2.51E-06	0.046	14306	22.086
Vascular dementia (sudden onset)	genus Eubacterium oxidoreducens group	rs440215	T	C	0.061	107546693	0.605	0.117	360283	T	C	-0.093	5	106882394	1.65E-06	0.020	14306	22.812
Vascular dementia (sudden onset)	genus Eubacterium rectale group	rs10248854	C	A	0.032	121641184	0.787	0.118	360283	C	A	-0.053	7	121281238	4.21E-06	0.011	14306	21.640
Vascular dementia (sudden onset)	genus Eubacterium rectale group	rs10797540	A	G	0.226	234394624	0.054	0.117	360283	A	G	0.050	1	234530370	3.53E-06	0.011	14306	21.551
Vascular dementia (sudden onset)	genus Eubacterium rectale group	rs143694765	T	C	0.085	115540288	0.662	0.195	360283	T	C	0.087	1	116082909	9.75E-06	0.020	14306	19.349
Vascular dementia (sudden onset)	genus Eubacterium rectale group	rs2884897	A	G	-0.081	8107990	0.803	0.327	360283	A	G	-0.129	11	8129537	6.44E-06	0.029	14306	20.034
Vascular dementia (sudden onset)	genus Eubacterium rectale group	rs314726	T	C	0.016	124795578	0.889	0.117	360283	T	C	0.053	2	125553155	1.38E-06	0.011	14306	23.329
Vascular dementia (sudden onset)	genus Eubacterium rectale group	rs35398954	A	G	-0.194	92463535	0.217	0.157	360283	A	G	-0.090	15	93006765	5.40E-07	0.017	14306	26.653
Vascular dementia (sudden onset)	genus Eubacterium rectale group	rs59427698	A	G	-0.096	126663466	0.519	0.148	360283	A	G	-0.058	9	129425745	5.37E-06	0.013	14306	19.379
Vascular dementia (sudden onset)	genus Eubacterium rectale group	rs62547233	A	G	0.107	89398504	0.408	0.129	360283	A	G	0.054	9	92013419	9.90E-06	0.012	14306	19.908
Vascular dementia (sudden onset)	genus Eubacterium ruminantium group	rs10131724	C	A	0.091	51752348	0.647	0.198	360283	C	A	0.200	14	52219066	2.39E-06	0.041	14306	23.234
Vascular dementia (sudden onset)	genus Eubacterium ruminantium group	rs10923018	G	A	-0.221	88057716	0.062	0.118	360283	G	A	0.073	1	88523399	6.80E-06	0.016	14306	20.378
Vascular dementia (sudden onset)	genus Eubacterium ruminantium group	rs11637981	T	G	0.060	60997423	0.608	0.117	360283	T	G	0.073	15	61289622	5.44E-06	0.016	14306	20.733
Vascular dementia (sudden onset)	genus Eubacterium ruminantium group	rs13025464	C	T	0.165	200745922	0.162	0.118	360283	C	T	0.074	2	201610645	6.97E-06	0.016	14306	20.252
Vascular dementia (sudden onset)	genus Eubacterium ruminantium group	rs139749	C	T	-0.079	24907088	0.523	0.123	360283	C	T	-0.085	22	25303055	8.59E-07	0.017	14306	24.219
Vascular dementia (sudden onset)	genus Eubacterium ruminantium group	rs16891896	G	A	-0.023	33897484	0.912	0.208	360283	G	A	-0.175	5	33897589	2.38E-06	0.039	14306	20.027
Vascular dementia (sudden onset)	genus Eubacterium ruminantium group	rs17519472	C	T	-0.290	30208021	0.090	0.171	360283	C	T	0.108	12	30360954	4.70E-06	0.023	14306	21.227
Vascular dementia (sudden onset)	genus Eubacterium ruminantium group	rs209813	G	A	-0.129	11953400	0.433	0.164	360283	G	A	-0.103	6	11953633	9.23E-06	0.024	14306	19.165
Vascular dementia (sudden onset)	genus Eubacterium ruminantium group	rs2116427	A	G	0.173	121847301	0.196	0.134	360283	A	G	0.091	5	121182996	4.67E-07	0.018	14306	24.983
Vascular dementia (sudden onset)	genus Eubacterium ruminantium group	rs2229917	A	G	-0.364	128218658	0.212	0.292	360283	A	G	0.154	9	130980937	2.16E-06	0.032	14306	22.467
Vascular dementia (sudden onset)	genus Eubacterium ruminantium group	rs2418654	C	T	0.253	71020448	0.034	0.119	360283	C	T	-0.075	2	71247578	6.17E-06	0.017	14306	20.388
Vascular dementia (sudden onset)	genus Eubacterium ruminantium group	rs2817174	C	T	0.148	3127617	0.211	0.119	360283	C	T	-0.073	1	3044181	7.87E-06	0.016	14306	20.125

Vascular dementia (sudden onset)	genus Eubacterium ruminantium group	rs57340348	T	C	-0.129	129708969	0.380	0.146	360283	T	C	-0.098	6	130030114	4.93E-06	0.021	14306	21.311
Vascular dementia (sudden onset)	genus Eubacterium ruminantium group	rs606117	G	A	-0.092	2539358	0.480	0.130	360283	G	A	-0.083	9	2539358	4.82E-06	0.018	14306	21.296
Vascular dementia (sudden onset)	genus Eubacterium ruminantium group	rs6676699	T	G	0.027	198009151	0.832	0.128	360283	T	G	0.089	1	197978281	6.38E-06	0.020	14306	20.439
Vascular dementia (sudden onset)	genus Eubacterium ruminantium group	rs7000472	G	A	0.131	137614025	0.268	0.118	360283	G	A	0.076	8	138626268	4.07E-06	0.017	14306	21.284
Vascular dementia (sudden onset)	genus Eubacterium ruminantium group	rs72836424	C	T	-0.069	127239189	0.709	0.184	360283	C	T	-0.140	10	129037453	2.62E-06	0.030	14306	21.624
Vascular dementia (sudden onset)	genus Eubacterium ruminantium group	rs73139629	A	C	-0.300	62912223	0.130	0.198	360283	A	C	-0.115	12	63306003	5.36E-06	0.025	14306	21.555
Vascular dementia (sudden onset)	genus Eubacterium ventriosum group	rs11617697	A	G	-0.090	98133646	0.723	0.255	360283	A	G	-0.143	13	98785900	7.22E-07	0.029	14306	25.080
Vascular dementia (sudden onset)	genus Eubacterium ventriosum group	rs12964517	G	A	-0.120	24880821	0.354	0.130	360283	G	A	0.059	18	22460785	2.08E-06	0.012	14306	22.624
Vascular dementia (sudden onset)	genus Eubacterium ventriosum group	rs13082419	C	T	0.159	108017587	0.395	0.187	360283	C	T	-0.072	3	107736434	9.56E-06	0.016	14306	19.661
Vascular dementia (sudden onset)	genus Eubacterium ventriosum group	rs16884680	G	T	0.287	113432826	0.128	0.189	360283	G	T	-0.091	8	114445055	1.74E-06	0.019	14306	22.309
Vascular dementia (sudden onset)	genus Eubacterium ventriosum group	rs35179274	C	T	-0.216	51493907	0.154	0.151	360283	C	T	-0.063	14	51960625	5.76E-06	0.014	14306	20.703
Vascular dementia (sudden onset)	genus Eubacterium ventriosum group	rs3809430	T	C	-0.087	44506307	0.487	0.126	360283	T	C	-0.055	14	44975510	3.55E-06	0.012	14306	21.426
Vascular dementia (sudden onset)	genus Eubacterium ventriosum group	rs57199565	T	C	-0.090	190505943	0.548	0.150	360283	T	C	0.078	3	190223732	7.97E-07	0.016	14306	23.896
Vascular dementia (sudden onset)	genus Eubacterium ventriosum group	rs66746423	C	T	0.077	108855642	0.631	0.160	360283	C	T	0.075	1	109398264	6.11E-06	0.016	14306	20.749
Vascular dementia (sudden onset)	genus Eubacterium ventriosum group	rs6704822	G	A	0.305	202797056	0.082	0.175	360283	G	A	-0.074	2	203661779	6.62E-06	0.017	14306	19.661
Vascular dementia (sudden onset)	genus Eubacterium ventriosum group	rs72783037	C	A	-0.092	60314477	0.515	0.142	360283	C	A	0.066	16	60348381	6.55E-06	0.014	14306	21.012
Vascular dementia (sudden onset)	genus Eubacterium ventriosum group	rs73615400	T	C	0.035	51524380	0.857	0.196	360283	T	C	-0.096	20	50140919	9.54E-07	0.019	14306	24.443
Vascular dementia (sudden onset)	genus Eubacterium ventriosum group	rs73849225	T	C	-0.268	125450659	0.198	0.209	360283	T	C	0.098	4	126371814	5.21E-06	0.022	14306	18.908
Vascular dementia (sudden onset)	genus Eubacterium ventriosum group	rs78250280	G	A	0.074	147819813	0.658	0.167	360283	G	A	0.075	1	147291928	3.36E-06	0.016	14306	20.798
Vascular dementia (sudden onset)	genus Eubacterium ventriosum group	rs876734	T	C	0.084	52030212	0.518	0.130	360283	T	C	0.062	12	52423996	2.89E-06	0.013	14306	21.793
Vascular dementia (sudden onset)	genus Eubacterium ventriosum group	rs9316536	T	G	0.078	51364440	0.640	0.167	360283	T	G	-0.082	13	51938576	7.84E-06	0.018	14306	19.911
Vascular dementia (sudden onset)	genus Eubacterium xylanophilum group	rs10140184	A	C	-0.211	72715075	0.074	0.118	360283	A	C	0.058	14	73181783	4.96E-06	0.013	14306	20.959
Vascular dementia (sudden onset)	genus Eubacterium xylanophilum group	rs10917203	A	C	-0.015	22300773	0.900	0.119	360283	A	C	0.061	1	22627266	3.15E-06	0.013	14306	21.898
Vascular dementia (sudden onset)	genus Eubacterium xylanophilum group	rs112176119	C	T	0.218	73662872	0.278	0.201	360283	C	T	-0.113	16	73696771	3.33E-06	0.025	14306	21.312
Vascular dementia (sudden onset)	genus Eubacterium xylanophilum group	rs13239072	G	A	0.280	43526745	0.032	0.130	360283	G	A	0.069	7	43566344	1.82E-06	0.014	14306	23.197
Vascular dementia (sudden onset)	genus Eubacterium xylanophilum group	rs17830032	G	A	-0.097	59420451	0.655	0.217	360283	G	A	-0.161	20	57995506	2.39E-07	0.031	14306	26.749
Vascular dementia (sudden onset)	genus Eubacterium xylanophilum group	rs1999224	G	T	0.060	127735841	0.757	0.194	360283	G	T	-0.095	9	130498120	3.75E-06	0.020	14306	21.749
Vascular dementia (sudden onset)	genus Eubacterium xylanophilum group	rs2012708	G	A	-0.063	84756953	0.604	0.122	360283	G	A	-0.057	16	84790559	6.53E-06	0.013	14306	20.401
Vascular dementia (sudden onset)	genus Eubacterium xylanophilum group	rs2213117	T	G	-0.150	131139765	0.348	0.160	360283	T	G	0.088	11	131009660	4.21E-06	0.019	14306	21.568
Vascular dementia (sudden onset)	genus Eubacterium xylanophilum group	rs75586835	A	G	-0.275	86970786	0.224	0.226	360283	A	G	-0.114	4	87891938	9.39E-06	0.026	14306	18.888
Vascular dementia (sudden onset)	genus Faecalibacterium	rs10927394	G	T	0.268	245104037	0.534	0.431	360283	G	T	-0.232	1	245267339	7.02E-06	0.051	14306	20.549
Vascular dementia (sudden onset)	genus Faecalibacterium	rs114946999	C	T	0.032	36898092	0.854	0.176	360283	C	T	-0.086	2	37125235	5.70E-06	0.019	14306	20.649
Vascular dementia (sudden onset)	genus Faecalibacterium	rs11776390	T	C	0.391	38736127	0.101	0.239	360283	T	C	-0.078	8	38593645	6.40E-06	0.017	14306	20.793
Vascular dementia (sudden onset)	genus Faecalibacterium	rs1271565	C	T	0.011	63577379	0.935	0.132	360283	C	T	-0.058	14	64044097	1.30E-06	0.012	14306	23.196
Vascular dementia (sudden onset)	genus Faecalibacterium	rs12753492	A	C	0.080	13875051	0.666	0.184	360283	A	C	0.064	1	14201546	8.80E-06	0.015	14306	18.295
Vascular dementia (sudden onset)	genus Faecalibacterium	rs2835874	T	C	0.177	37655599	0.566	0.308	360283	T	C	-0.087	21	39027901	7.54E-06	0.020	14306	19.440
Vascular dementia (sudden onset)	genus Faecalibacterium	rs6910935	G	A	0.232	131266017	0.340	0.243	360283	G	A	-0.135	6	131587157	1.38E-06	0.028	14306	23.699
Vascular dementia (sudden onset)	genus Faecalibacterium	rs75499067	C	T	-0.263	6049862	0.232	0.221	360283	C	T	0.228	16	6099863	1.76E-06	0.047	14306	23.900
Vascular dementia (sudden onset)	genus Faecalibacterium	rs79656633	T	C	-0.176	110494205	0.362	0.193	360283	T	C	0.146	1	111036827	8.14E-06	0.032	14306	20.326
Vascular dementia (sudden onset)	genus Faecalibacterium	rs9536330	T	C	0.083	52992882	0.474	0.117	360283	T	C	-0.048	13	53567017	5.33E-06	0.011	14306	20.026
Vascular dementia (sudden onset)	genus Family XIII AD3011 group	rs11126423	T	C	-0.233	73968424	0.256	0.205	360283	T	C	-0.090	2	74195551	5.91E-06	0.020	14306	21.226
Vascular dementia (sudden onset)	genus Family XIII AD3011 group	rs11736617	G	A	-0.030	38916209	0.910	0.263	360283	G	A	-0.076	4	38917830	9.02E-06	0.017	14306	19.475
Vascular dementia (sudden onset)	genus Family XIII AD3011 group	rs12812672	T	C	-0.014	17974663	0.952	0.226	360283	T	C	-0.096	12	18127597	2.56E-06	0.021	14306	21.273
Vascular dementia (sudden onset)	genus Family XIII AD3011 group	rs149302	T	C	-0.153	14159816	0.263	0.137	360283	T	C	-0.065	5	14159925	7.48E-06	0.014	14306	20.321
Vascular dementia (sudden onset)	genus Family XIII AD3011 group	rs16840310	G	A	-0.150	240530863	0.205	0.118	360283	G	A	0.061	1	240694163	6.75E-07	0.012	14306	24.780
Vascular dementia (sudden onset)	genus Family XIII AD3011 group	rs16940167	C	T	0.016	58391498	0.912	0.147	360283	C	T	0.073	15	58683697	3.91E-06	0.016	14306	20.989
Vascular dementia (sudden onset)	genus Family XIII AD3011 group	rs17156849	G	A	-0.230	28564367	0.347	0.244	360283	G	A	-0.113	7	28603985	4.19E-06	0.025	14306	21.181
Vascular dementia (sudden onset)	genus Family XIII AD3011 group	rs62029761	A	G	0.007	15912385	0.978	0.264	360283	A	G	0.129	16	16006242	3.89E-06	0.028	14306	21.762
Vascular dementia (sudden onset)	genus Family XIII AD3011 group	rs62200412	C	T	0.021	4637993	0.878	0.134	360283	C	T	-0.080	20	4618639	5.80E-07	0.016	14306	23.894
Vascular dementia (sudden onset)	genus Family XIII AD3011 group	rs72730932	C	A	-0.198	192050705	0.311	0.196	360283	C	A	-0.090	1	192019835	6.89E-07	0.018	14306	25.798
Vascular dementia (sudden onset)	genus Family XIII AD3011 group	rs739451	C	T	-0.301	133813832	0.035	0.143	360283	C	T	0.065	9	136678954	7.88E-06	0.015	14306	19.386
Vascular dementia (sudden onset)	genus Family XIII AD3011 group	rs9276029	A	G	-0.024	32727559	0.868	0.145	360283	A	G	-0.081	6	32695336	8.93E-06	0.019	14306	19.097
Vascular dementia (sudden onset)	genus Family XIII AD3011 group	rs9837139	A	G	0.235	29770374	0.261	0.209	360283	A	G	0.108	3	29811865	8.71E-06	0.024	14306	19.990
Vascular dementia (sudden onset)	genus Family XIII UCG001	rs112362903	A	G	-0.309	35434008	0.324	0.313	360283	A	G	-0.149	17	33761027	7.88E-06	0.033	14306	20.001
Vascular dementia (sudden onset)	genus Family XIII UCG001	rs12049454	T	C	0.013	84762642	0.913	0.119	360283	T	C	-0.065	1	85228325	1.17E-06	0.013	14306	23.312
Vascular dementia (sudden onset)	genus Family XIII UCG001	rs1426266	C	T	-0.080	188506308	0.543	0.131	360283	C	T	0.067	3	188224096	1.25E-06	0.014	14306	23.546
Vascular dementia (sudden onset)	genus Family XIII UCG001	rs3842897	G	A	-0.310	183534529	0.129	0.204	360283	G	A	-0.113	1	183503664	5.20E-06	0.024	14306	21.531
Vascular dementia (sudden onset)	genus Family XIII UCG001	rs62414802	C	T	-0.094	75888918	0.488	0.135	360283	C	T	-0.061	6	76598635	4.29E-06	0.013	14306	20.676
Vascular dementia (sudden onset)	genus Family XIII UCG001	rs7119679	G	A	-0.090	94948621	0.513	0.138	360283	G	A	-0.081	11	94681786	3.52E-06	0.017	14306	21.429
Vascular dementia (sudden onset)	genus Family XIII UCG001	rs76463770	A	G	-0.268	45437598	0.420	0.333	360283	A	G	0.193	3	45479090	3.77E-06	0.042	14306	21.158
Vascular dementia (sudden onset)	genus Family XIII UCG001	rs8076666	G	A	0.029	80250018	0.871	0.177	360283	G	A	-0.089	17	78223817	8.02E-06	0.020	14306	20.029
Vascular dementia (sudden onset)	genus Flavonifractor	rs114873521	C	T	0.353	168791398	0.123	0.229	360283	C	T	-0.130	5	168218403	7.13E-06	0.029	14306	19.561
Vascular dementia (sudden onset)	genus Flavonifractor	rs11811696	T	C	-0.159	237188054	0.453	0.211	360283	T	C	-0.116	1	237351354	2.07E-06	0.024	14306	23.191
Vascular dementia (sudden onset)	genus Flavonifractor	rs12030302	G	A	-0.052	77422150	0.652	0.116	360283	G	A	0.069	1	77887835	5.61E-07	0.014	14306	25.361
Vascular dementia (sudden onset)	genus Flavonifractor	rs34066017	A	G	-0.002	44828138	0.989	0.145	360283	A	G	0.076	11	44849689	1.52E-06	0.016	14306	22.878
Vascular dementia (sudden onset)	genus Flavonifractor	rs806808	C	T	0.097	32092258	0.409	0.117	360283	C	T	-0.067	10	32381186	1.18E-06	0.014	14306	23.877
Vascular dementia (sudden onset)	genus Fusicatenibacter	rs10439674	A	G	0.144	41309465	0.315	0.144	360283	A	G	-0.057	21	42681392	7.68E-06	0.013	14306	19.367

Vascular dementia (sudden onset)	genus Fusicatenibacter	rs167879	T	C	0.195	57362310	0.239	0.165	360283	T	C	0.066	20	55937366	5.87E-06	0.015	14306	19.656
Vascular dementia (sudden onset)	genus Fusicatenibacter	rs1864685	A	C	0.245	72725643	0.038	0.118	360283	A	C	-0.049	17	70721782	4.96E-06	0.011	14306	20.949
Vascular dementia (sudden onset)	genus Fusicatenibacter	rs2025938	G	A	0.039	110417361	0.867	0.235	360283	G	A	-0.097	10	112177119	2.99E-06	0.021	14306	22.172
Vascular dementia (sudden onset)	genus Fusicatenibacter	rs206581	A	G	-0.148	10370599	0.295	0.142	360283	A	G	-0.057	18	10370596	8.96E-06	0.013	14306	19.748
Vascular dementia (sudden onset)	genus Fusicatenibacter	rs2132128	G	A	-0.136	15503209	0.474	0.190	360283	G	A	-0.077	8	15360718	1.08E-06	0.016	14306	23.176
Vascular dementia (sudden onset)	genus Fusicatenibacter	rs3303	T	C	0.117	118687917	0.620	0.236	360283	T	C	-0.095	10	120447429	3.94E-06	0.020	14306	21.839
Vascular dementia (sudden onset)	genus Fusicatenibacter	rs4378146	A	C	0.099	24601134	0.460	0.133	360283	A	C	-0.062	1	24927625	7.20E-07	0.013	14306	24.239
Vascular dementia (sudden onset)	genus Fusicatenibacter	rs60254196	G	A	0.149	149159628	0.201	0.117	360283	G	A	0.049	7	148856720	5.47E-06	0.011	14306	20.273
Vascular dementia (sudden onset)	genus Fusicatenibacter	rs62187631	T	C	0.094	225784854	0.522	0.147	360283	T	C	-0.071	2	226649570	4.55E-06	0.016	14306	19.912
Vascular dementia (sudden onset)	genus Fusicatenibacter	rs62353480	A	G	-0.046	29594893	0.766	0.155	360283	A	G	-0.070	5	29595000	1.57E-06	0.015	14306	23.210
Vascular dementia (sudden onset)	genus Fusicatenibacter	rs6515626	G	A	0.190	25276365	0.421	0.237	360283	G	A	0.142	20	25257001	7.29E-06	0.031	14306	20.386
Vascular dementia (sudden onset)	genus Fusicatenibacter	rs704418	C	T	0.011	64267127	0.949	0.177	360283	C	T	-0.074	3	64252803	7.77E-07	0.015	14306	23.937
Vascular dementia (sudden onset)	genus Fusicatenibacter	rs73103914	A	G	-0.066	58264528	0.686	0.163	360283	A	G	-0.060	12	58658311	8.30E-06	0.013	14306	19.735
Vascular dementia (sudden onset)	genus Fusicatenibacter	rs792108	C	T	0.049	5392660	0.679	0.119	360283	C	T	0.051	2	5532793	8.50E-06	0.011	14306	19.939
Vascular dementia (sudden onset)	genus Fusicatenibacter	rs8028026	G	A	-0.051	87809984	0.802	0.203	360283	G	A	0.079	15	88353215	8.06E-06	0.018	14306	19.259
Vascular dementia (sudden onset)	genus Fusicatenibacter	rs8063430	T	C	0.173	73787906	0.511	0.263	360283	T	C	-0.104	16	73821805	4.93E-06	0.022	14306	21.924
Vascular dementia (sudden onset)	genus Fusicatenibacter	rs9905659	G	A	0.037	72701205	0.803	0.150	360283	G	A	-0.062	17	70697344	7.31E-06	0.014	14306	20.354
Vascular dementia (sudden onset)	genus Gordonibacter	rs13412653	A	C	-0.119	29935628	0.320	0.120	360283	A	C	0.108	2	30158494	8.61E-06	0.024	14306	20.229
Vascular dementia (sudden onset)	genus Gordonibacter	rs16955299	G	A	-0.065	54879849	0.742	0.197	360283	G	A	-0.196	17	52957210	6.37E-06	0.043	14306	20.527
Vascular dementia (sudden onset)	genus Gordonibacter	rs322296	G	A	-0.252	137252442	0.259	0.223	360283	G	A	0.179	7	136937189	4.02E-06	0.038	14306	22.439
Vascular dementia (sudden onset)	genus Gordonibacter	rs35042269	C	A	-0.031	8140880	0.866	0.184	360283	C	A	-0.180	4	8142607	8.11E-06	0.040	14306	19.985
Vascular dementia (sudden onset)	genus Gordonibacter	rs3765837	T	G	-0.041	210455105	0.855	0.226	360283	T	G	-0.191	1	210628449	7.17E-06	0.043	14306	19.348
Vascular dementia (sudden onset)	genus Gordonibacter	rs4596722	G	A	-0.231	86172229	0.047	0.116	360283	G	A	-0.103	9	88787144	9.06E-06	0.023	14306	19.748
Vascular dementia (sudden onset)	genus Gordonibacter	rs71545975	A	G	0.083	47104907	0.595	0.156	360283	A	G	-0.154	7	47144505	7.04E-06	0.034	14306	20.639
Vascular dementia (sudden onset)	genus Gordonibacter	rs72714787	C	A	0.232	135847346	0.178	0.172	360283	C	A	0.181	4	136768501	1.43E-06	0.038	14306	23.141
Vascular dementia (sudden onset)	genus Gordonibacter	rs72939513	A	G	-0.024	82670413	0.929	0.263	360283	A	G	-0.214	1	83136096	7.98E-06	0.049	14306	19.026
Vascular dementia (sudden onset)	genus Gordonibacter	rs7294633	T	C	0.173	28871601	0.184	0.130	360283	T	C	-0.129	12	29024534	3.44E-07	0.025	14306	26.501
Vascular dementia (sudden onset)	genus Gordonibacter	rs768830	A	G	0.089	18930984	0.576	0.159	360283	A	G	-0.150	7	18970607	7.76E-06	0.033	14306	20.212
Vascular dementia (sudden onset)	genus Haemophilus	rs10781340	A	G	-0.077	76137254	0.663	0.177	360283	A	G	-0.095	9	78752170	4.32E-06	0.020	14306	21.803
Vascular dementia (sudden onset)	genus Haemophilus	rs111582866	G	A	0.110	48708578	0.593	0.205	360283	G	A	-0.124	16	48742489	1.27E-06	0.026	14306	22.815
Vascular dementia (sudden onset)	genus Haemophilus	rs35509	G	A	-0.277	115055532	0.348	0.295	360283	G	A	0.128	12	115493337	2.01E-06	0.027	14306	22.768
Vascular dementia (sudden onset)	genus Haemophilus	rs4822728	T	C	-0.199	26495842	0.086	0.116	360283	T	C	0.071	22	26891808	3.48E-06	0.015	14306	21.740
Vascular dementia (sudden onset)	genus Haemophilus	rs76022354	C	T	-0.069	92546628	0.794	0.265	360283	C	T	0.245	10	94306385	1.83E-06	0.051	14306	23.421
Vascular dementia (sudden onset)	genus Haemophilus	rs78909003	T	C	0.458	102887960	0.071	0.253	360283	T	C	-0.246	9	105650242	1.67E-06	0.050	14306	23.881
Vascular dementia (sudden onset)	genus Haemophilus	rs9328464	T	C	-0.033	8350684	0.781	0.117	360283	T	C	0.072	6	8350917	1.42E-06	0.015	14306	23.615
Vascular dementia (sudden onset)	genus Haemophilus	rs9382510	C	T	-0.134	55583693	0.314	0.133	360283	C	T	-0.094	6	55448491	7.12E-08	0.017	14306	29.342
Vascular dementia (sudden onset)	genus Haemophilus	rs9895850	T	C	-0.110	66538895	0.695	0.282	360283	T	C	-0.193	17	64535013	2.14E-06	0.042	14306	21.437
Vascular dementia (sudden onset)	genus Holdemanella	rs12513188	G	A	0.073	70135074	0.577	0.131	360283	G	A	0.090	4	71000791	4.65E-06	0.020	14306	21.426
Vascular dementia (sudden onset)	genus Holdemanella	rs17586763	T	C	0.105	50357927	0.691	0.264	360283	T	C	-0.227	13	50932063	7.72E-06	0.051	14306	19.850
Vascular dementia (sudden onset)	genus Holdemanella	rs1926302	G	A	0.041	64687657	0.770	0.139	360283	G	A	-0.108	1	65153340	7.50E-06	0.023	14306	21.768
Vascular dementia (sudden onset)	genus Holdemanella	rs34187114	C	A	0.187	137611390	0.308	0.183	360283	C	A	-0.105	8	138623633	5.13E-06	0.023	14306	21.381
Vascular dementia (sudden onset)	genus Holdemanella	rs35228298	G	A	-0.247	85343732	0.121	0.159	360283	G	A	0.093	3	85392882	7.30E-06	0.020	14306	21.231
Vascular dementia (sudden onset)	genus Holdemanella	rs4541991	T	C	0.055	102242553	0.661	0.125	360283	T	C	-0.093	9	105004835	2.10E-06	0.019	14306	22.747
Vascular dementia (sudden onset)	genus Holdemanella	rs607782	C	T	0.276	4148375	0.023	0.121	360283	C	T	0.085	6	4148609	7.19E-07	0.017	14306	24.518
Vascular dementia (sudden onset)	genus Holdemanella	rs62113381	T	C	-0.303	7603339	0.077	0.171	360283	T	C	-0.105	19	7668225	5.54E-06	0.023	14306	20.653
Vascular dementia (sudden onset)	genus Holdemanella	rs73011279	T	C	0.132	14909283	0.338	0.138	360283	T	C	-0.096	19	15020095	1.36E-06	0.020	14306	23.274
Vascular dementia (sudden onset)	genus Holdemanella	rs75764681	T	C	0.562	4452438	0.047	0.282	360283	T	C	-0.283	10	4494630	1.94E-06	0.060	14306	22.338
Vascular dementia (sudden onset)	genus Holdemanella	rs8113760	G	A	-0.093	43347783	0.457	0.125	360283	G	A	0.079	19	43851935	4.62E-06	0.017	14306	20.756
Vascular dementia (sudden onset)	genus Holdemania	rs10885477	T	C	-0.318	113572654	0.242	0.272	360283	T	C	-0.135	10	115332413	8.60E-06	0.030	14306	20.037
Vascular dementia (sudden onset)	genus Holdemania	rs11080063	G	A	-0.030	28462253	0.797	0.118	360283	G	A	-0.067	17	26789271	6.67E-06	0.015	14306	19.711
Vascular dementia (sudden onset)	genus Holdemania	rs111745969	A	G	0.035	92566992	0.837	0.169	360283	A	G	0.121	15	93110222	3.71E-06	0.027	14306	20.616
Vascular dementia (sudden onset)	genus Holdemania	rs113593397	A	G	-0.261	122961600	0.189	0.199	360283	A	G	-0.129	8	123973840	9.36E-06	0.028	14306	20.827
Vascular dementia (sudden onset)	genus Holdemania	rs116500994	G	T	-0.383	81111408	0.163	0.275	360283	G	T	-0.138	13	81685543	2.34E-06	0.029	14306	21.981
Vascular dementia (sudden onset)	genus Holdemania	rs12701617	A	G	-0.121	38253256	0.300	0.116	360283	A	G	-0.066	7	38292857	9.52E-06	0.015	14306	19.545
Vascular dementia (sudden onset)	genus Holdemania	rs1867876	T	C	-0.028	18746521	0.823	0.127	360283	T	C	0.084	11	18768068	2.74E-07	0.016	14306	27.009
Vascular dementia (sudden onset)	genus Holdemania	rs4146507	C	T	0.122	106873392	0.366	0.135	360283	C	T	0.079	5	106209093	7.23E-06	0.018	14306	20.167
Vascular dementia (sudden onset)	genus Holdemania	rs73139538	G	A	0.104	63862767	0.770	0.356	360283	G	A	-0.149	7	63323145	7.77E-06	0.033	14306	20.590
Vascular dementia (sudden onset)	genus Holdemania	rs77293403	A	G	0.356	78092582	0.250	0.310	360283	A	G	0.165	5	77388406	1.77E-06	0.034	14306	23.182
Vascular dementia (sudden onset)	genus Holdemania	rs80149660	C	T	0.052	128110018	0.854	0.280	360283	C	T	-0.233	10	129908282	6.04E-06	0.052	14306	20.138
Vascular dementia (sudden onset)	genus Holdemania	rs9500080	C	T	0.075	105289447	0.624	0.154	360283	C	T	0.093	6	105737322	4.09E-07	0.018	14306	26.840
Vascular dementia (sudden onset)	genus Holdemania	rs9529719	C	T	-0.092	70282384	0.455	0.124	360283	C	T	-0.074	13	70856516	5.97E-06	0.016	14306	21.281
Vascular dementia (sudden onset)	genus Holdemania	rs967319	T	C	0.061	60629947	0.655	0.136	360283	T	C	0.079	3	60615680	8.38E-06	0.018	14306	19.910
Vascular dementia (sudden onset)	genus Howardella	rs10048062	C	T	-0.150	97933690	0.471	0.209	360283	C	T	-0.147	15	98476920	8.59E-06	0.034	14306	19.172
Vascular dementia (sudden onset)	genus Howardella	rs12452946	A	G	-0.166	17349974	0.151	0.116	360283	A	G	-0.106	17	17253288	3.80E-06	0.023	14306	21.370
Vascular dementia (sudden onset)	genus Howardella	rs1484873	G	A	0.014	45627020	0.931	0.162	360283	G	A	0.228	18	43206985	2.56E-06	0.046	14306	24.177
Vascular dementia (sudden onset)	genus Howardella	rs17167098	G	A	-0.095	133469602	0.575	0.169	360283	G	A	-0.169	7	133154356	1.12E-06	0.035	14306	23.142
Vascular dementia (sudden onset)	genus Howardella	rs2154047	A	C	-0.370	94986460	0.067	0.203	360283	A	C	0.193	14	95452797	9.97E-06	0.042	14306	21.010

Vascular dementia (sudden onset)	genus Howardella	rs36081916	T	C	-0.184	93898102	0.376	0.208	360283	T	C	-0.181	7	93527414	4.70E-06	0.040	14306	20.225
Vascular dementia (sudden onset)	genus Howardella	rs3791893	A	G	-0.111	217954673	0.512	0.169	360283	A	G	0.147	2	218819396	9.50E-06	0.034	14306	18.677
Vascular dementia (sudden onset)	genus Howardella	rs609430	T	G	-0.166	168257164	0.176	0.123	360283	T	G	-0.112	4	169178315	3.34E-06	0.024	14306	21.918
Vascular dementia (sudden onset)	genus Howardella	rs672217	G	A	-0.143	62457901	0.326	0.146	360283	G	A	0.164	18	60125134	3.52E-06	0.035	14306	21.996
Vascular dementia (sudden onset)	genus Hungatella	rs10044993	A	C	-0.237	59128613	0.254	0.208	360283	A	C	-0.140	5	58424440	8.07E-06	0.032	14306	19.409
Vascular dementia (sudden onset)	genus Hungatella	rs13128780	T	C	0.226	165137846	0.131	0.150	360283	T	C	-0.150	4	166058998	1.75E-06	0.031	14306	22.915
Vascular dementia (sudden onset)	genus Hungatella	rs13249325	T	G	-0.041	14996016	0.725	0.116	360283	T	G	-0.100	8	14853525	9.69E-06	0.023	14306	19.608
Vascular dementia (sudden onset)	genus Hungatella	rs17092615	G	A	0.102	95507282	0.543	0.167	360283	G	A	0.152	14	95973619	7.38E-06	0.034	14306	20.302
Vascular dementia (sudden onset)	genus Hungatella	rs72759041	G	T	-0.164	89034145	0.253	0.144	360283	G	T	-0.126	15	89577376	3.86E-06	0.028	14306	19.937
Vascular dementia (sudden onset)	genus Intestinibacter	rs10805326	A	G	-0.016	14322999	0.902	0.128	360283	A	G	-0.078	4	14324623	3.55E-08	0.014	14306	30.803
Vascular dementia (sudden onset)	genus Intestinibacter	rs11109097	T	C	0.033	97534659	0.781	0.117	360283	T	C	-0.062	12	97928437	5.49E-06	0.014	14306	20.305
Vascular dementia (sudden onset)	genus Intestinibacter	rs118030283	G	A	-0.059	5943075	0.833	0.279	360283	G	A	-0.152	16	5993076	2.67E-06	0.032	14306	21.896
Vascular dementia (sudden onset)	genus Intestinibacter	rs16938435	T	C	0.452	21502924	0.005	0.162	360283	T	C	-0.112	9	21502923	1.80E-06	0.024	14306	22.706
Vascular dementia (sudden onset)	genus Intestinibacter	rs2098844	T	C	-0.003	127833265	0.982	0.121	360283	T	C	0.058	11	127703160	6.79E-06	0.013	14306	20.070
Vascular dementia (sudden onset)	genus Intestinibacter	rs2702387	G	A	0.003	178440108	0.981	0.119	360283	G	A	-0.061	4	179361262	4.26E-06	0.013	14306	21.208
Vascular dementia (sudden onset)	genus Intestinibacter	rs4327025	G	A	-0.043	91903453	0.777	0.152	360283	G	A	-0.081	15	92446683	1.64E-07	0.015	14306	27.546
Vascular dementia (sudden onset)	genus Intestinibacter	rs447950	A	G	-0.035	149466863	0.773	0.119	360283	A	G	0.063	5	148846426	5.64E-06	0.014	14306	21.143
Vascular dementia (sudden onset)	genus Intestinibacter	rs478972	C	T	-0.161	125793289	0.418	0.199	360283	C	T	0.143	11	125663184	1.82E-06	0.030	14306	23.061
Vascular dementia (sudden onset)	genus Intestinibacter	rs6062862	A	G	0.218	62693871	0.307	0.213	360283	A	G	0.092	20	61325223	6.68E-06	0.020	14306	20.406
Vascular dementia (sudden onset)	genus Intestinibacter	rs62430350	T	C	-0.178	170609106	0.569	0.313	360283	T	C	0.151	6	170918194	6.84E-06	0.035	14306	18.481
Vascular dementia (sudden onset)	genus Intestinibacter	rs68093214	C	T	0.040	70608634	0.766	0.136	360283	C	T	0.066	3	70657785	9.26E-06	0.015	14306	19.525
Vascular dementia (sudden onset)	genus Intestinibacter	rs6875660	C	T	-0.250	160259249	0.304	0.243	360283	C	T	0.089	5	159686256	3.06E-06	0.019	14306	21.089
Vascular dementia (sudden onset)	genus Intestinibacter	rs893394	G	A	-0.113	19855210	0.340	0.118	360283	G	A	0.058	2	20054971	7.85E-06	0.013	14306	19.910
Vascular dementia (sudden onset)	genus Intestinibacter	rs9348442	C	T	0.023	10303712	0.894	0.175	360283	C	T	0.099	6	10303945	6.26E-06	0.022	14306	19.987
Vascular dementia (sudden onset)	genus Intestinimonas	rs10262702	T	C	-0.124	67425856	0.487	0.178	360283	T	C	0.092	7	66890843	2.06E-06	0.019	14306	22.189
Vascular dementia (sudden onset)	genus Intestinimonas	rs11258178	A	G	-0.178	13082513	0.125	0.116	360283	A	G	0.066	10	13124513	6.98E-07	0.013	14306	24.264
Vascular dementia (sudden onset)	genus Intestinimonas	rs12226153	A	G	-0.104	94631127	0.828	0.478	360283	A	G	-0.151	11	94364293	5.12E-07	0.031	14306	24.250
Vascular dementia (sudden onset)	genus Intestinimonas	rs17067892	C	T	-0.003	3906018	0.989	0.206	360283	C	T	0.107	8	3763540	6.38E-06	0.025	14306	18.383
Vascular dementia (sudden onset)	genus Intestinimonas	rs1859797	G	A	0.041	21967685	0.725	0.117	360283	G	A	0.060	7	22007303	4.12E-06	0.013	14306	20.981
Vascular dementia (sudden onset)	genus Intestinimonas	rs2276760	A	G	-0.167	150903169	0.220	0.136	360283	A	G	-0.069	3	150620956	7.84E-06	0.015	14306	20.178
Vascular dementia (sudden onset)	genus Intestinimonas	rs2731794	C	T	0.078	17209282	0.800	0.307	360283	C	T	0.121	5	17209391	1.92E-06	0.026	14306	21.942
Vascular dementia (sudden onset)	genus Intestinimonas	rs2930225	T	G	0.070	85294296	0.610	0.138	360283	T	G	-0.073	16	85327902	1.35E-06	0.015	14306	22.751
Vascular dementia (sudden onset)	genus Intestinimonas	rs4113676	A	C	0.155	6238275	0.762	0.511	360283	A	C	-0.219	20	6218922	7.42E-06	0.049	14306	19.873
Vascular dementia (sudden onset)	genus Intestinimonas	rs4784055	T	C	-0.144	58784866	0.591	0.267	360283	T	C	-0.175	16	58818770	8.72E-07	0.039	14306	20.631
Vascular dementia (sudden onset)	genus Intestinimonas	rs62240188	G	A	0.131	10569458	0.521	0.204	360283	G	A	0.130	3	10611142	2.20E-06	0.027	14306	23.702
Vascular dementia (sudden onset)	genus Intestinimonas	rs6934519	C	T	-0.062	66490909	0.644	0.133	360283	C	T	0.069	6	67200802	8.57E-06	0.015	14306	20.982
Vascular dementia (sudden onset)	genus Intestinimonas	rs716604	A	G	0.010	6373956	0.943	0.138	360283	A	G	0.082	2	6514088	8.57E-07	0.017	14306	24.289
Vascular dementia (sudden onset)	genus Intestinimonas	rs7170984	T	C	0.097	93276506	0.453	0.129	360283	T	C	-0.066	15	93819735	2.98E-06	0.014	14306	21.858
Vascular dementia (sudden onset)	genus Intestinimonas	rs72982915	C	T	0.311	140140519	0.226	0.257	360283	C	T	0.183	2	140898088	4.91E-06	0.040	14306	20.682
Vascular dementia (sudden onset)	genus Intestinimonas	rs9823439	C	T	-0.043	17146010	0.710	0.117	360283	C	T	0.058	3	17187502	9.86E-06	0.013	14306	19.598
Vascular dementia (sudden onset)	genus Lachnoclostridium	rs1031599	T	G	-0.337	66675401	0.162	0.241	360283	T	G	0.079	3	66725825	6.31E-06	0.018	14306	20.039
Vascular dementia (sudden onset)	genus Lachnoclostridium	rs12566975	T	C	-0.224	185122219	0.056	0.117	360283	T	C	-0.047	1	185091351	9.57E-06	0.011	14306	19.580
Vascular dementia (sudden onset)	genus Lachnoclostridium	rs1528479	A	G	-0.005	166387541	0.967	0.121	360283	A	G	0.050	2	167244051	9.64E-06	0.011	14306	19.783
Vascular dementia (sudden onset)	genus Lachnoclostridium	rs1997204	C	T	-0.078	101652817	0.780	0.281	360283	C	T	0.108	12	102046595	5.97E-06	0.024	14306	19.941
Vascular dementia (sudden onset)	genus Lachnoclostridium	rs2385421	A	G	-0.043	22163494	0.808	0.177	360283	A	G	0.075	18	19743455	7.14E-06	0.018	14306	17.046
Vascular dementia (sudden onset)	genus Lachnoclostridium	rs3821998	C	A	-0.247	38692945	0.188	0.188	360283	C	A	-0.086	4	38694566	6.72E-06	0.019	14306	20.144
Vascular dementia (sudden onset)	genus Lachnoclostridium	rs4738679	A	G	0.121	58457761	0.309	0.119	360283	A	G	0.052	8	59370320	4.42E-06	0.011	14306	20.813
Vascular dementia (sudden onset)	genus Lachnoclostridium	rs6112314	A	C	-0.238	19320202	0.055	0.124	360283	A	C	-0.056	20	19300846	2.43E-07	0.011	14306	26.964
Vascular dementia (sudden onset)	genus Lachnoclostridium	rs615997	T	C	-0.245	22996295	0.035	0.116	360283	T	C	0.051	3	23037786	2.03E-06	0.011	14306	23.094
Vascular dementia (sudden onset)	genus Lachnoclostridium	rs62285313	A	G	0.419	177752244	0.012	0.168	360283	A	G	0.086	3	177470032	1.58E-06	0.018	14306	22.655
Vascular dementia (sudden onset)	genus Lachnoclostridium	rs72829893	G	T	0.164	48617179	0.390	0.191	360283	G	T	0.117	17	46694541	5.58E-06	0.027	14306	19.198
Vascular dementia (sudden onset)	genus Lachnoclostridium	rs78068103	A	G	0.135	13912842	0.461	0.183	360283	A	G	0.089	17	13816159	3.67E-06	0.019	14306	20.814
Vascular dementia (sudden onset)	genus Lachnoclostridium	rs789029	C	T	-0.027	1053251	0.873	0.168	360283	C	T	-0.064	18	1053252	3.75E-06	0.014	14306	21.603
Vascular dementia (sudden onset)	genus Lachnospiraceae FCS020 group	rs10093861	G	A	0.088	120232167	0.455	0.118	360283	G	A	-0.057	8	121244406	3.06E-06	0.012	14306	22.048
Vascular dementia (sudden onset)	genus Lachnospiraceae FCS020 group	rs1254846	A	G	0.064	43850646	0.694	0.164	360283	A	G	-0.106	10	44346094	5.60E-06	0.023	14306	20.771
Vascular dementia (sudden onset)	genus Lachnospiraceae FCS020 group	rs1363769	C	T	0.232	17754496	0.494	0.339	360283	C	T	0.201	19	17865305	1.58E-06	0.045	14306	19.933
Vascular dementia (sudden onset)	genus Lachnospiraceae FCS020 group	rs2322265	C	T	-0.008	165666933	0.950	0.133	360283	C	T	-0.067	4	166588085	5.21E-06	0.014	14306	22.149
Vascular dementia (sudden onset)	genus Lachnospiraceae FCS020 group	rs2862811	C	T	0.016	166278627	0.901	0.126	360283	C	T	-0.056	3	165996415	3.92E-06	0.012	14306	21.535
Vascular dementia (sudden onset)	genus Lachnospiraceae FCS020 group	rs35035870	T	C	0.030	3227646	0.919	0.301	360283	T	C	-0.191	11	3248876	2.62E-06	0.041	14306	21.158
Vascular dementia (sudden onset)	genus Lachnospiraceae FCS020 group	rs3999074	G	T	-0.022	63803887	0.851	0.117	360283	G	T	-0.055	10	65563647	6.55E-06	0.012	14306	20.418
Vascular dementia (sudden onset)	genus Lachnospiraceae FCS020 group	rs4452603	T	G	-0.171	71256565	0.192	0.131	360283	T	G	0.060	6	71966268	8.98E-06	0.014	14306	19.748
Vascular dementia (sudden onset)	genus Lachnospiraceae FCS020 group	rs7249113	G	A	0.109	2571234	0.392	0.128	360283	G	A	0.068	19	2571232	3.72E-07	0.013	14306	25.907
Vascular dementia (sudden onset)	genus Lachnospiraceae FCS020 group	rs72793667	A	G	-0.024	53719294	0.938	0.303	360283	A	G	-0.117	2	53946431	1.63E-06	0.025	14306	22.467
Vascular dementia (sudden onset)	genus Lachnospiraceae FCS020 group	rs9308097	G	A	0.092	164865326	0.428	0.116	360283	G	A	-0.055	4	165786478	7.47E-06	0.012	14306	20.057
Vascular dementia (sudden onset)	genus Lachnospiraceae FCS020 group	rs9788306	C	T	0.060	39845177	0.640	0.129	360283	C	T	-0.063	13	40419314	1.39E-06	0.013	14306	23.074
Vascular dementia (sudden onset)	genus Lachnospiraceae NC2004 group	rs117467633	T	C	0.162	21254094	0.586	0.298	360283	T	C	-0.170	17	21157406	9.13E-06	0.038	14306	19.612
Vascular dementia (sudden onset)	genus Lachnospiraceae NC2004 group	rs12127733	G	A	0.195	234021975	0.193	0.150	360283	G	A	0.115	1	234157721	3.11E-06	0.025	14306	21.922

Vascular dementia (sudden onset)	genus Lachnospiraceae NC2004 group	rs12208226	C	A	-0.128	22725679	0.505	0.193	360283	C	A	-0.155	6	22725908	9.75E-06	0.034	14306	20.668
Vascular dementia (sudden onset)	genus Lachnospiraceae NC2004 group	rs12863463	G	A	-0.024	46938056	0.911	0.219	360283	G	A	-0.156	13	47512191	6.04E-06	0.035	14306	20.498
Vascular dementia (sudden onset)	genus Lachnospiraceae NC2004 group	rs17067076	G	A	-0.534	60561853	0.006	0.194	360283	G	A	-0.155	18	58229086	5.61E-06	0.035	14306	19.277
Vascular dementia (sudden onset)	genus Lachnospiraceae NC2004 group	rs1928659	T	C	0.117	29334139	0.424	0.147	360283	T	C	0.103	9	29334137	6.17E-06	0.023	14306	20.498
Vascular dementia (sudden onset)	genus Lachnospiraceae NC2004 group	rs1929743	T	C	-0.111	76255426	0.366	0.123	360283	T	C	0.084	13	76829562	9.06E-06	0.019	14306	19.351
Vascular dementia (sudden onset)	genus Lachnospiraceae NC2004 group	rs3756315	A	G	0.022	150165159	0.862	0.126	360283	A	G	-0.088	5	149544722	3.33E-06	0.019	14306	21.990
Vascular dementia (sudden onset)	genus Lachnospiraceae NC2004 group	rs6116753	G	A	0.204	5350054	0.181	0.152	360283	G	A	0.099	20	5330700	2.92E-06	0.021	14306	22.623
Vascular dementia (sudden onset)	genus Lachnospiraceae ND3007 group	rs2861203	G	A	0.162	173232763	0.204	0.128	360283	G	A	0.057	3	172950553	7.37E-06	0.013	14306	20.218
Vascular dementia (sudden onset)	genus Lachnospiraceae ND3007 group	rs72776675	T	C	0.264	14095664	0.092	0.157	360283	T	C	-0.065	10	14137663	8.72E-06	0.015	14306	19.123
Vascular dementia (sudden onset)	genus Lachnospiraceae ND3007 group	rs9932954	A	G	0.227	1050633	0.066	0.124	360283	A	G	-0.056	16	1100633	1.25E-06	0.012	14306	23.467
Vascular dementia (sudden onset)	genus Lachnospiraceae NK4A136 group	rs10952110	G	T	-0.177	8458292	0.131	0.117	360283	G	T	0.049	7	8497922	9.08E-06	0.011	14306	19.797
Vascular dementia (sudden onset)	genus Lachnospiraceae NK4A136 group	rs11263806	A	G	-0.011	36882840	0.929	0.122	360283	A	G	-0.052	17	35240097	5.07E-06	0.012	14306	20.189
Vascular dementia (sudden onset)	genus Lachnospiraceae NK4A136 group	rs12611395	G	A	-0.299	21623323	0.117	0.190	360283	G	A	0.090	19	21806125	5.83E-06	0.020	14306	20.435
Vascular dementia (sudden onset)	genus Lachnospiraceae NK4A136 group	rs160061	G	A	0.219	6116546	0.058	0.115	360283	G	A	-0.051	5	6116659	2.12E-06	0.011	14306	22.596
Vascular dementia (sudden onset)	genus Lachnospiraceae NK4A136 group	rs28540839	A	C	-0.085	83822845	0.464	0.116	360283	A	C	0.051	8	84735080	9.34E-06	0.011	14306	21.124
Vascular dementia (sudden onset)	genus Lachnospiraceae NK4A136 group	rs2880566	T	C	-0.084	31689634	0.612	0.165	360283	T	C	0.060	17	30016653	5.61E-06	0.013	14306	19.815
Vascular dementia (sudden onset)	genus Lachnospiraceae NK4A136 group	rs4955932	T	C	0.239	55147348	0.045	0.119	360283	T	C	-0.049	3	55181375	7.05E-06	0.011	14306	20.253
Vascular dementia (sudden onset)	genus Lachnospiraceae NK4A136 group	rs59805249	T	C	-0.043	90994180	0.833	0.204	360283	T	C	0.094	5	90289997	9.45E-06	0.021	14306	20.261
Vascular dementia (sudden onset)	genus Lachnospiraceae NK4A136 group	rs68104925	T	C	0.247	99711344	0.051	0.127	360283	T	C	-0.055	14	100177681	2.37E-06	0.012	14306	22.647
Vascular dementia (sudden onset)	genus Lachnospiraceae NK4A136 group	rs7073658	G	T	-0.048	60415266	0.679	0.116	360283	G	T	0.050	10	62175024	5.27E-06	0.011	14306	20.745
Vascular dementia (sudden onset)	genus Lachnospiraceae NK4A136 group	rs73044693	A	G	-0.050	50752863	0.829	0.230	360283	A	G	-0.108	19	51256120	3.57E-06	0.023	14306	21.900
Vascular dementia (sudden onset)	genus Lachnospiraceae NK4A136 group	rs7616165	G	T	0.838	190697356	0.022	0.367	360283	G	T	-0.231	3	190415145	2.77E-06	0.048	14306	22.739
Vascular dementia (sudden onset)	genus Lachnospiraceae NK4A136 group	rs76193507	A	G	-0.063	162032193	0.762	0.207	360283	A	G	-0.230	3	161749981	2.93E-06	0.050	14306	21.129
Vascular dementia (sudden onset)	genus Lachnospiraceae NK4A136 group	rs7832116	A	G	0.125	4985407	0.474	0.175	360283	A	G	-0.071	8	4842929	3.57E-06	0.015	14306	22.199
Vascular dementia (sudden onset)	genus Lachnospiraceae NK4A136 group	rs954878	A	G	-0.186	54112728	0.121	0.120	360283	A	G	-0.052	1	54578401	1.78E-06	0.011	14306	22.782
Vascular dementia (sudden onset)	genus Lachnospiraceae UCG001	rs12131224	C	T	-0.198	166230125	0.286	0.185	360283	C	T	0.117	1	166199362	7.40E-06	0.026	14306	20.424
Vascular dementia (sudden onset)	genus Lachnospiraceae UCG001	rs2050911	G	A	-0.044	81918723	0.720	0.123	360283	G	A	0.075	1	82384407	1.11E-06	0.015	14306	23.831
Vascular dementia (sudden onset)	genus Lachnospiraceae UCG001	rs2371284	C	T	-0.048	55862478	0.730	0.139	360283	C	T	0.076	12	56256262	7.56E-06	0.017	14306	20.056
Vascular dementia (sudden onset)	genus Lachnospiraceae UCG001	rs437876	T	C	-0.044	42526948	0.719	0.122	360283	T	C	0.078	3	42568440	7.17E-08	0.014	14306	29.376
Vascular dementia (sudden onset)	genus Lachnospiraceae UCG001	rs4981345	T	C	0.256	20987814	0.041	0.125	360283	T	C	-0.068	14	21455973	6.09E-06	0.015	14306	20.717
Vascular dementia (sudden onset)	genus Lachnospiraceae UCG001	rs573933	T	C	0.036	14477853	0.846	0.188	360283	T	C	-0.108	9	14477851	3.11E-06	0.023	14306	21.565
Vascular dementia (sudden onset)	genus Lachnospiraceae UCG001	rs62496417	T	G	0.188	97336192	0.179	0.140	360283	T	G	-0.075	7	96965504	5.88E-06	0.017	14306	20.410
Vascular dementia (sudden onset)	genus Lachnospiraceae UCG001	rs7341608	T	C	-0.002	55926941	0.989	0.172	360283	T	C	-0.078	8	56839500	9.48E-06	0.018	14306	19.514
Vascular dementia (sudden onset)	genus Lachnospiraceae UCG001	rs74034332	G	A	0.219	79058462	0.376	0.248	360283	G	A	0.168	16	79092359	3.33E-06	0.038	14306	19.288
Vascular dementia (sudden onset)	genus Lachnospiraceae UCG001	rs78848836	A	G	0.109	53307576	0.566	0.191	360283	A	G	-0.119	1	53773248	3.38E-06	0.026	14306	20.942
Vascular dementia (sudden onset)	genus Lachnospiraceae UCG001	rs8104225	A	G	0.327	13852110	0.020	0.141	360283	A	G	0.089	19	13962924	8.04E-06	0.020	14306	20.369
Vascular dementia (sudden onset)	genus Lachnospiraceae UCG001	rs9403580	C	T	0.228	144685644	0.189	0.174	360283	C	T	0.108	6	145006780	3.47E-06	0.023	14306	22.011
Vascular dementia (sudden onset)	genus Lachnospiraceae UCG001	rs985416	T	C	0.009	148551296	0.954	0.151	360283	T	C	-0.097	3	148269083	1.46E-07	0.018	14306	28.481
Vascular dementia (sudden onset)	genus Lachnospiraceae UCG004	rs11128180	A	G	-0.123	70543064	0.371	0.138	360283	A	G	0.065	3	70592215	4.52E-06	0.014	14306	21.404
Vascular dementia (sudden onset)	genus Lachnospiraceae UCG004	rs12072562	T	C	-0.168	104744847	0.575	0.300	360283	T	C	0.133	1	105287469	7.07E-06	0.030	14306	19.225
Vascular dementia (sudden onset)	genus Lachnospiraceae UCG004	rs12673420	G	A	-0.050	71838022	0.664	0.116	360283	G	A	0.055	7	71303007	2.98E-06	0.012	14306	21.931
Vascular dementia (sudden onset)	genus Lachnospiraceae UCG004	rs12747809	A	G	0.145	240195789	0.262	0.129	360283	A	G	0.062	1	240359089	8.65E-07	0.013	14306	24.489
Vascular dementia (sudden onset)	genus Lachnospiraceae UCG004	rs12894272	G	A	-0.089	40067277	0.466	0.122	360283	G	A	-0.058	14	40536481	4.34E-06	0.013	14306	21.436
Vascular dementia (sudden onset)	genus Lachnospiraceae UCG004	rs233486	G	A	0.180	14817353	0.285	0.169	360283	G	A	0.080	6	14817584	6.28E-06	0.018	14306	20.231
Vascular dementia (sudden onset)	genus Lachnospiraceae UCG004	rs2444793	T	C	0.133	80609119	0.266	0.119	360283	T	C	0.054	6	81318836	4.77E-06	0.012	14306	21.064
Vascular dementia (sudden onset)	genus Lachnospiraceae UCG004	rs2726805	A	G	0.091	182326448	0.440	0.117	360283	A	G	0.055	4	183247601	6.30E-06	0.012	14306	20.594
Vascular dementia (sudden onset)	genus Lachnospiraceae UCG004	rs2882478	G	A	-0.211	49677451	0.071	0.117	360283	G	A	-0.058	2	49904589	1.21E-06	0.012	14306	23.782
Vascular dementia (sudden onset)	genus Lachnospiraceae UCG004	rs35182105	A	G	-0.154	24419403	0.545	0.255	360283	A	G	-0.110	12	24572337	4.87E-06	0.024	14306	20.522
Vascular dementia (sudden onset)	genus Lachnospiraceae UCG004	rs6656451	T	C	0.191	65524739	0.102	0.117	360283	T	C	0.054	1	65990422	5.57E-06	0.012	14306	20.703
Vascular dementia (sudden onset)	genus Lachnospiraceae UCG004	rs7629954	A	G	-0.315	154524617	0.252	0.275	360283	A	G	0.108	3	154242406	5.77E-06	0.024	14306	20.717
Vascular dementia (sudden onset)	genus Lachnospiraceae UCG008	rs10741777	T	C	0.179	19551010	0.154	0.126	360283	T	C	-0.097	11	19572557	7.69E-07	0.019	14306	24.994
Vascular dementia (sudden onset)	genus Lachnospiraceae UCG008	rs10793103	T	C	0.023	74680730	0.847	0.117	360283	T	C	-0.097	11	74391775	9.35E-08	0.018	14306	28.889
Vascular dementia (sudden onset)	genus Lachnospiraceae UCG008	rs10801803	G	A	0.034	90353314	0.839	0.166	360283	G	A	-0.117	1	90818872	1.40E-06	0.024	14306	23.178
Vascular dementia (sudden onset)	genus Lachnospiraceae UCG008	rs13024781	T	C	-0.012	167772361	0.920	0.117	360283	T	C	-0.080	2	168628871	2.29E-06	0.017	14306	22.380
Vascular dementia (sudden onset)	genus Lachnospiraceae UCG008	rs57091572	A	G	-0.013	80505086	0.937	0.171	360283	A	G	-0.110	6	81214803	2.86E-06	0.024	14306	21.937
Vascular dementia (sudden onset)	genus Lachnospiraceae UCG008	rs61944774	A	G	-0.656	129055273	0.013	0.263	360283	A	G	0.180	12	129539818	6.34E-06	0.039	14306	20.855
Vascular dementia (sudden onset)	genus Lachnospiraceae UCG008	rs62277846	C	T	-0.005	227235424	0.970	0.146	360283	C	T	0.102	2	228100140	1.59E-06	0.021	14306	23.212
Vascular dementia (sudden onset)	genus Lachnospiraceae UCG008	rs67078837	T	C	0.152	113299491	0.198	0.118	360283	T	C	-0.085	4	114220647	7.68E-07	0.017	14306	24.556
Vascular dementia (sudden onset)	genus Lachnospiraceae UCG008	rs75356640	G	A	0.171	31405081	0.343	0.181	360283	G	A	0.137	15	31697284	9.83E-06	0.030	14306	20.284
Vascular dementia (sudden onset)	genus Lachnospiraceae UCG008	rs955844	A	C	0.167	84958708	0.319	0.168	360283	A	C	0.112	16	84992314	1.81E-06	0.023	14306	24.080
Vascular dementia (sudden onset)	genus Lachnospiraceae UCG010	rs10414815	C	T	0.225	42082093	0.414	0.276	360283	C	T	-0.105	19	42586245	4.24E-06	0.023	14306	20.610
Vascular dementia (sudden onset)	genus Lachnospiraceae UCG010	rs11192447	A	G	0.082	82088641	0.757	0.266	360283	A	G	0.127	10	83848397	4.69E-07	0.024	14306	27.039
Vascular dementia (sudden onset)	genus Lachnospiraceae UCG010	rs12346653	C	T	0.078	89652324	0.586	0.143	360283	C	T	0.066	9	92267239	2.70E-06	0.014	14306	22.205
Vascular dementia (sudden onset)	genus Lachnospiraceae UCG010	rs17730011	G	A	-0.097	84063126	0.493	0.142	360283	G	A	-0.070	6	84772845	7.85E-06	0.016	14306	19.998
Vascular dementia (sudden onset)	genus Lachnospiraceae UCG010	rs2833528	T	C	-0.070	31821255	0.557	0.120	360283	T	C	0.056	21	33193567	9.92E-06	0.013	14306	19.359
Vascular dementia (sudden onset)	genus Lachnospiraceae UCG010	rs336138	G	T	0.161	8146258	0.373	0.181	360283	G	T	0.078	5	8146371	7.48E-06	0.017	14306	20.573
Vascular dementia (sudden onset)	genus Lachnospiraceae UCG010	rs4576377	C	A	-0.161	81780947	0.182	0.121	360283	C	A	0.057	7	81410263	7.63E-06	0.013	14306	20.272

Vascular dementia (sudden onset)	genus Lachnospiraceae UCG010	rs72894957	G	A	0.468	184997864	0.227	0.388	360283	G	A	0.222	2	185862591	5.68E-06	0.049	14306	20.879
Vascular dementia (sudden onset)	genus Lachnospiraceae UCG010	rs74315802	G	T	-0.165	29403135	0.277	0.151	360283	G	T	0.087	14	29872341	3.19E-06	0.018	14306	22.343
Vascular dementia (sudden onset)	genus Lachnospiraceae UCG010	rs9981767	A	C	-0.228	42659765	0.089	0.134	360283	A	C	0.066	21	44079875	9.96E-07	0.013	14306	24.630
Vascular dementia (sudden onset)	genus Lactobacillus	rs12693845	C	T	-0.054	198447960	0.654	0.120	360283	C	T	-0.081	2	199312684	8.96E-06	0.018	14306	20.607
Vascular dementia (sudden onset)	genus Lactobacillus	rs1530559	G	A	-0.118	134998059	0.314	0.117	360283	G	A	0.080	2	135755629	4.93E-06	0.018	14306	20.355
Vascular dementia (sudden onset)	genus Lactobacillus	rs16861661	G	A	-0.271	18174965	0.259	0.240	360283	G	A	-0.183	1	18501459	1.28E-06	0.038	14306	23.049
Vascular dementia (sudden onset)	genus Lactobacillus	rs62314653	C	A	-0.465	108975306	0.062	0.249	360283	C	A	0.188	4	109896462	2.24E-06	0.039	14306	22.626
Vascular dementia (sudden onset)	genus Lactobacillus	rs7399658	G	A	0.043	23260829	0.775	0.152	360283	G	A	-0.107	13	23834968	3.12E-06	0.022	14306	23.313
Vascular dementia (sudden onset)	genus Lactobacillus	rs768253	T	G	0.010	68097868	0.931	0.117	360283	T	G	-0.079	8	69010103	4.25E-06	0.017	14306	21.252
Vascular dementia (sudden onset)	genus Lactobacillus	rs77478751	A	G	-0.273	173433875	0.127	0.179	360283	A	G	-0.220	3	173151665	7.33E-06	0.048	14306	21.361
Vascular dementia (sudden onset)	genus Lactobacillus	rs921925	A	C	-0.113	6928006	0.421	0.141	360283	A	C	0.099	19	6928017	9.72E-07	0.020	14306	23.495
Vascular dementia (sudden onset)	genus Lactococcus	rs10417872	G	T	-0.010	28276446	0.936	0.129	360283	G	T	-0.118	19	28767353	1.29E-06	0.025	14306	23.276
Vascular dementia (sudden onset)	genus Lactococcus	rs123059	C	T	0.114	2796641	0.416	0.141	360283	C	T	0.137	17	2699935	1.27E-06	0.027	14306	24.769
Vascular dementia (sudden onset)	genus Lactococcus	rs12621813	G	A	0.087	31043183	0.511	0.132	360283	G	A	0.108	2	31266049	6.61E-06	0.024	14306	20.413
Vascular dementia (sudden onset)	genus Lactococcus	rs17168302	G	A	-0.172	14610596	0.366	0.190	360283	G	A	0.192	7	14650221	6.29E-06	0.042	14306	20.402
Vascular dementia (sudden onset)	genus Lactococcus	rs2293361	C	T	0.396	53887727	0.125	0.258	360283	C	T	-0.199	2	54114864	1.40E-06	0.043	14306	21.369
Vascular dementia (sudden onset)	genus Lactococcus	rs4766997	C	T	-0.001	112723633	0.996	0.118	360283	C	T	0.115	12	113161438	2.06E-06	0.024	14306	23.109
Vascular dementia (sudden onset)	genus Lactococcus	rs55910161	C	T	-0.104	69758887	0.576	0.186	360283	C	T	0.146	10	71518643	2.36E-06	0.031	14306	22.695
Vascular dementia (sudden onset)	genus Lactococcus	rs6674304	C	T	0.016	116345120	0.958	0.297	360283	C	T	0.201	1	116887742	6.18E-06	0.044	14306	20.619
Vascular dementia (sudden onset)	genus Marvinbryantia	rs11620597	T	C	-0.227	85504955	0.537	0.368	360283	T	C	0.119	13	86079090	7.80E-06	0.027	14306	19.339
Vascular dementia (sudden onset)	genus Marvinbryantia	rs1187983	C	T	-0.276	57976188	0.147	0.190	360283	C	T	-0.094	1	58441860	2.02E-06	0.019	14306	23.450
Vascular dementia (sudden onset)	genus Marvinbryantia	rs146541147	G	A	0.669	4542085	0.036	0.320	360283	G	A	0.119	19	4542097	6.86E-06	0.027	14306	19.603
Vascular dementia (sudden onset)	genus Marvinbryantia	rs2724813	G	A	0.114	12474925	0.406	0.137	360283	G	A	0.084	10	12516924	6.28E-07	0.017	14306	25.180
Vascular dementia (sudden onset)	genus Marvinbryantia	rs2842896	C	T	0.122	132560525	0.305	0.118	360283	C	T	-0.065	6	132881664	7.25E-07	0.013	14306	24.519
Vascular dementia (sudden onset)	genus Marvinbryantia	rs2863363	G	A	0.007	166321977	0.959	0.134	360283	G	A	-0.063	3	166039765	3.11E-06	0.014	14306	21.688
Vascular dementia (sudden onset)	genus Marvinbryantia	rs3125832	A	C	-0.125	211226838	0.369	0.139	360283	A	C	0.068	1	211400180	5.03E-06	0.015	14306	20.477
Vascular dementia (sudden onset)	genus Marvinbryantia	rs61884471	G	A	-0.189	45805010	0.305	0.184	360283	G	A	0.124	11	45826561	1.01E-06	0.025	14306	25.085
Vascular dementia (sudden onset)	genus Marvinbryantia	rs72948274	A	C	-0.343	79807639	0.149	0.237	360283	A	C	-0.126	11	79518683	3.26E-06	0.027	14306	21.546
Vascular dementia (sudden onset)	genus Marvinbryantia	rs8006832	G	T	-0.170	21109678	0.399	0.202	360283	G	T	-0.095	14	21577837	6.58E-06	0.022	14306	19.317
Vascular dementia (sudden onset)	genus Methanobrevibacter	rs10202904	G	T	-0.058	124682691	0.631	0.120	360283	G	T	0.113	2	125440268	3.09E-06	0.024	14306	22.260
Vascular dementia (sudden onset)	genus Methanobrevibacter	rs1334944	T	C	-0.029	110506288	0.821	0.129	360283	T	C	0.115	10	112266046	7.61E-06	0.026	14306	20.330
Vascular dementia (sudden onset)	genus Methanobrevibacter	rs4802933	G	A	0.211	52422567	0.128	0.139	360283	G	A	0.136	19	52925820	9.74E-06	0.031	14306	19.373
Vascular dementia (sudden onset)	genus Methanobrevibacter	rs6776814	T	C	0.224	15011576	0.597	0.424	360283	T	C	-0.189	3	15053083	8.05E-06	0.042	14306	20.250
Vascular dementia (sudden onset)	genus Methanobrevibacter	rs76029318	T	C	0.053	41389655	0.821	0.237	360283	T	C	0.223	13	41963791	1.08E-06	0.045	14306	24.060
Vascular dementia (sudden onset)	genus Methanobrevibacter	rs894996	C	A	-0.222	103497150	0.328	0.227	360283	C	A	0.214	4	104418307	3.82E-06	0.046	14306	22.064
Vascular dementia (sudden onset)	genus Odoribacter	rs10093869	A	G	-0.035	1318658	0.763	0.117	360283	A	G	-0.058	8	1266824	3.67E-06	0.013	14306	21.234
Vascular dementia (sudden onset)	genus Odoribacter	rs10423795	T	C	-0.044	49019831	0.716	0.120	360283	T	C	-0.055	19	49523088	6.58E-06	0.012	14306	20.657
Vascular dementia (sudden onset)	genus Odoribacter	rs28417404	A	G	-0.188	70477507	0.344	0.198	360283	A	G	-0.073	14	70944224	3.68E-06	0.016	14306	20.290
Vascular dementia (sudden onset)	genus Odoribacter	rs4793970	A	G	0.273	48686600	0.025	0.121	360283	A	G	-0.058	17	46763962	6.03E-06	0.013	14306	19.912
Vascular dementia (sudden onset)	genus Odoribacter	rs6856150	A	G	0.105	11905935	0.547	0.174	360283	A	G	-0.088	4	11907559	6.06E-06	0.019	14306	20.635
Vascular dementia (sudden onset)	genus Odoribacter	rs74553962	T	G	0.242	68765245	0.271	0.219	360283	T	G	0.121	8	69677480	9.49E-06	0.026	14306	21.146
Vascular dementia (sudden onset)	genus Odoribacter	rs77779484	G	A	0.180	67262163	0.463	0.246	360283	G	A	-0.133	12	67655943	6.56E-07	0.027	14306	24.713
Vascular dementia (sudden onset)	genus Olsenella	rs1035588	A	G	0.080	149220676	0.505	0.121	360283	A	G	-0.108	2	150077190	4.86E-06	0.024	14306	20.850
Vascular dementia (sudden onset)	genus Olsenella	rs17148768	G	A	-0.143	10735122	0.360	0.156	360283	G	A	0.140	10	10777085	2.20E-06	0.030	14306	22.570
Vascular dementia (sudden onset)	genus Olsenella	rs2759329	A	G	-0.078	231824606	0.516	0.121	360283	A	G	0.111	1	231960352	3.43E-06	0.024	14306	21.947
Vascular dementia (sudden onset)	genus Olsenella	rs35225860	A	G	-0.201	247478968	0.490	0.291	360283	A	G	-0.224	1	247642270	3.87E-06	0.048	14306	21.486
Vascular dementia (sudden onset)	genus Olsenella	rs61090148	A	G	0.168	173889347	0.150	0.117	360283	A	G	-0.105	4	174810498	6.44E-06	0.023	14306	20.515
Vascular dementia (sudden onset)	genus Olsenella	rs62112538	C	T	0.001	4925006	0.997	0.188	360283	C	T	-0.199	19	4925018	1.19E-06	0.041	14306	24.006
Vascular dementia (sudden onset)	genus Olsenella	rs72691585	C	A	0.277	21884426	0.104	0.171	360283	C	A	-0.249	9	21884425	2.95E-06	0.052	14306	22.872
Vascular dementia (sudden onset)	genus Olsenella	rs7540303	C	T	-0.006	179660056	0.958	0.121	360283	C	T	0.108	1	179629191	5.32E-06	0.024	14306	20.892
Vascular dementia (sudden onset)	genus Olsenella	rs8066522	A	G	0.161	61555512	0.192	0.123	360283	A	G	0.107	17	59632873	9.70E-06	0.024	14306	19.640
Vascular dementia (sudden onset)	genus Olsenella	rs9460691	C	A	0.210	10421974	0.158	0.148	360283	C	A	0.120	6	10422207	7.28E-06	0.027	14306	19.942
Vascular dementia (sudden onset)	genus Oscillibacter	rs11627628	T	C	0.363	21011446	0.104	0.224	360283	T	C	0.144	14	21479605	1.01E-06	0.029	14306	24.605
Vascular dementia (sudden onset)	genus Oscillibacter	rs11990279	T	C	0.080	11258805	0.582	0.146	360283	T	C	-0.082	8	11116314	4.94E-06	0.018	14306	20.897
Vascular dementia (sudden onset)	genus Oscillibacter	rs12649930	T	G	-0.051	3654564	0.792	0.193	360283	T	G	0.122	4	3656291	4.09E-06	0.026	14306	21.935
Vascular dementia (sudden onset)	genus Oscillibacter	rs133832	A	C	-0.060	44438827	0.640	0.129	360283	A	C	-0.080	22	44834707	1.15E-06	0.016	14306	23.993
Vascular dementia (sudden onset)	genus Oscillibacter	rs16866406	A	G	0.193	178592420	0.233	0.162	360283	A	G	0.099	2	179457147	3.08E-06	0.021	14306	22.426
Vascular dementia (sudden onset)	genus Oscillibacter	rs16934185	A	G	0.284	1798324	0.139	0.192	360283	A	G	-0.130	9	1798324	4.38E-06	0.028	14306	21.175
Vascular dementia (sudden onset)	genus Oscillibacter	rs234108	A	G	0.033	184973539	0.784	0.118	360283	A	G	0.075	1	184942671	9.16E-07	0.015	14306	24.116
Vascular dementia (sudden onset)	genus Oscillibacter	rs36095275	C	T	0.167	31800923	0.158	0.118	360283	C	T	-0.075	14	32270129	1.40E-06	0.016	14306	23.005
Vascular dementia (sudden onset)	genus Oscillibacter	rs4506202	G	A	0.024	21740565	0.836	0.116	360283	G	A	0.071	8	21598077	3.21E-06	0.015	14306	21.825
Vascular dementia (sudden onset)	genus Oscillibacter	rs61883564	A	G	-0.097	79302798	0.562	0.168	360283	A	G	-0.101	11	79013843	3.39E-06	0.022	14306	21.029
Vascular dementia (sudden onset)	genus Oscillibacter	rs75453768	G	T	0.089	114710047	0.655	0.198	360283	G	T	0.122	10	116469806	5.35E-06	0.027	14306	20.667
Vascular dementia (sudden onset)	genus Oscillibacter	rs761240	G	T	0.155	50891355	0.570	0.273	360283	G	T	0.177	20	49507892	2.04E-06	0.039	14306	20.639
Vascular dementia (sudden onset)	genus Oscillibacter	rs9393920	G	A	0.026	28612816	0.828	0.120	360283	G	A	0.074	6	28580593	9.92E-07	0.015	14306	24.294
Vascular dementia (sudden onset)	genus Oscillospira	rs12206468	G	A	0.188	18093460	0.388	0.217	360283	G	A	-0.133	6	18093691	1.04E-06	0.027	14306	24.319
Vascular dementia (sudden onset)	genus Oscillospira	rs12925026	T	C	-0.240	89726448	0.343	0.253	360283	T	C	0.136	16	89792856	9.31E-06	0.031	14306	19.534

Vascular dementia (sudden onset)	genus Oscillospira	rs1954532	C	T	0.009	27682209	0.949	0.146	360283	C	T	0.083	14	28151415	2.27E-06	0.018	14306	22.228
Vascular dementia (sudden onset)	genus Oscillospira	rs28889936	A	C	-0.206	88562149	0.296	0.197	360283	A	C	0.114	4	89483300	3.37E-06	0.025	14306	20.348
Vascular dementia (sudden onset)	genus Oscillospira	rs62422654	C	T	0.006	170159406	0.964	0.143	360283	C	T	0.090	6	170474630	6.47E-06	0.020	14306	20.572
Vascular dementia (sudden onset)	genus Oscillospira	rs72866977	A	C	0.176	56295685	0.420	0.218	360283	A	C	-0.131	6	56160483	5.63E-06	0.028	14306	21.488
Vascular dementia (sudden onset)	genus Oscillospira	rs751183	C	T	0.008	76337408	0.959	0.153	360283	C	T	0.077	1	76803093	6.85E-06	0.017	14306	20.216
Vascular dementia (sudden onset)	genus Oscillospira	rs8076323	A	G	0.196	14978543	0.115	0.124	360283	A	G	0.072	17	14881860	5.61E-06	0.016	14306	20.885
Vascular dementia (sudden onset)	genus Oxalobacter	rs10464997	G	A	0.168	21045182	0.263	0.150	360283	G	A	0.138	8	20902693	3.30E-06	0.029	14306	21.814
Vascular dementia (sudden onset)	genus Oxalobacter	rs11108500	A	G	0.127	96425426	0.541	0.207	360283	A	G	-0.199	12	96819204	3.74E-06	0.043	14306	21.708
Vascular dementia (sudden onset)	genus Oxalobacter	rs111966731	T	C	-0.174	93398708	0.411	0.211	360283	T	C	0.213	15	93941937	7.30E-06	0.047	14306	20.419
Vascular dementia (sudden onset)	genus Oxalobacter	rs12002250	A	C	0.546	19682560	0.019	0.232	360283	A	C	0.217	9	19682558	1.42E-06	0.047	14306	21.679
Vascular dementia (sudden onset)	genus Oxalobacter	rs1569853	T	C	-0.087	38582525	0.632	0.182	360283	T	C	-0.138	6	38550301	3.65E-06	0.030	14306	21.617
Vascular dementia (sudden onset)	genus Oxalobacter	rs36057338	G	T	-0.072	189014160	0.826	0.329	360283	G	T	0.208	4	189935314	8.80E-07	0.042	14306	24.323
Vascular dementia (sudden onset)	genus Oxalobacter	rs3862635	C	T	0.285	126712683	0.165	0.205	360283	C	T	-0.172	11	126582578	9.19E-06	0.039	14306	19.086
Vascular dementia (sudden onset)	genus Oxalobacter	rs4428215	G	A	-0.261	172229645	0.053	0.135	360283	G	A	0.130	3	171947435	7.51E-08	0.024	14306	28.931
Vascular dementia (sudden onset)	genus Oxalobacter	rs6000536	C	T	-0.256	37025428	0.099	0.156	360283	C	T	-0.131	22	37421469	2.06E-07	0.025	14306	26.637
Vascular dementia (sudden onset)	genus Oxalobacter	rs6993398	G	A	0.222	114548460	0.138	0.149	360283	G	A	0.127	8	115560689	7.13E-06	0.028	14306	20.813
Vascular dementia (sudden onset)	genus Oxalobacter	rs736744	T	C	0.044	84899492	0.712	0.118	360283	T	C	-0.118	9	87514407	2.57E-08	0.021	14306	31.135
Vascular dementia (sudden onset)	genus Parabacteroides	rs115602804	G	A	-0.003	191854576	0.989	0.188	360283	G	A	0.103	3	191572365	1.93E-06	0.022	14306	21.417
Vascular dementia (sudden onset)	genus Parabacteroides	rs4236095	A	G	-0.048	47400951	0.803	0.192	360283	A	G	-0.076	6	47368687	1.93E-06	0.016	14306	23.541
Vascular dementia (sudden onset)	genus Parabacteroides	rs60884758	C	T	-0.067	2217340	0.664	0.154	360283	C	T	-0.070	9	2217340	5.71E-07	0.014	14306	24.401
Vascular dementia (sudden onset)	genus Parabacteroides	rs6657302	T	C	-0.180	85119926	0.460	0.243	360283	T	C	-0.105	1	85585609	9.76E-06	0.023	14306	21.480
Vascular dementia (sudden onset)	genus Parabacteroides	rs7298818	C	T	0.229	56003169	0.243	0.196	360283	C	T	0.089	12	56396953	8.54E-06	0.020	14306	19.574
Vascular dementia (sudden onset)	genus Paraprevotella	rs10842464	C	T	-0.122	25096809	0.335	0.127	360283	C	T	0.076	12	25249743	6.60E-06	0.017	14306	19.305
Vascular dementia (sudden onset)	genus Paraprevotella	rs140997932	T	C	-0.228	149581005	0.372	0.255	360283	T	C	-0.162	3	149298792	2.11E-06	0.035	14306	21.018
Vascular dementia (sudden onset)	genus Paraprevotella	rs145020347	A	G	-0.079	114655957	0.627	0.164	360283	A	G	-0.125	11	114526679	4.03E-06	0.026	14306	22.579
Vascular dementia (sudden onset)	genus Paraprevotella	rs17109926	A	G	0.105	71584948	0.423	0.131	360283	A	G	-0.099	12	71978728	6.75E-06	0.022	14306	20.903
Vascular dementia (sudden onset)	genus Paraprevotella	rs17785622	A	G	0.203	82749597	0.500	0.301	360283	A	G	0.248	6	83459314	1.93E-06	0.052	14306	22.385
Vascular dementia (sudden onset)	genus Paraprevotella	rs2081023	A	G	0.190	175179258	0.256	0.167	360283	A	G	-0.123	5	174606261	2.64E-07	0.024	14306	26.854
Vascular dementia (sudden onset)	genus Paraprevotella	rs3008582	T	C	-0.014	196002043	0.927	0.148	360283	T	C	0.106	1	195971173	4.36E-06	0.023	14306	21.643
Vascular dementia (sudden onset)	genus Paraprevotella	rs3801748	G	A	0.069	82130192	0.569	0.122	360283	G	A	0.078	7	81759508	5.20E-06	0.017	14306	20.624
Vascular dementia (sudden onset)	genus Paraprevotella	rs4756632	G	T	0.322	41058153	0.061	0.172	360283	G	T	-0.139	11	41079703	3.82E-06	0.029	14306	22.959
Vascular dementia (sudden onset)	genus Paraprevotella	rs4767113	C	T	0.025	113694173	0.841	0.125	360283	C	T	0.088	12	114131978	2.14E-06	0.018	14306	23.047
Vascular dementia (sudden onset)	genus Paraprevotella	rs7240324	T	G	-0.150	71956568	0.262	0.134	360283	T	G	-0.102	18	69623804	5.96E-06	0.023	14306	20.315
Vascular dementia (sudden onset)	genus Paraprevotella	rs9602779	A	C	-0.236	85588285	0.083	0.136	360283	A	C	-0.107	13	86162420	6.93E-07	0.022	14306	23.463
Vascular dementia (sudden onset)	genus Paraprevotella	rs9900242	A	G	0.182	71139490	0.137	0.122	360283	A	G	-0.085	17	69135631	1.14E-06	0.018	14306	23.699
Vascular dementia (sudden onset)	genus Parasutterella	rs10899911	A	G	-0.211	43798391	0.128	0.139	360283	A	G	-0.072	10	44293839	1.15E-06	0.015	14306	23.429
Vascular dementia (sudden onset)	genus Parasutterella	rs11715853	G	A	-0.019	30122198	0.881	0.128	360283	G	A	-0.066	3	30163689	6.23E-06	0.015	14306	20.586
Vascular dementia (sudden onset)	genus Parasutterella	rs2090816	C	A	-0.075	137294455	0.621	0.151	360283	C	A	-0.084	6	137615592	2.90E-06	0.018	14306	22.494
Vascular dementia (sudden onset)	genus Parasutterella	rs35055552	T	C	0.359	113791795	0.035	0.170	360283	T	C	0.110	8	114804024	3.35E-06	0.024	14306	21.653
Vascular dementia (sudden onset)	genus Parasutterella	rs55877868	A	C	0.342	14789550	0.076	0.193	360283	A	C	-0.104	17	14692867	2.87E-06	0.023	14306	20.974
Vascular dementia (sudden onset)	genus Parasutterella	rs62273907	A	G	-0.178	156832626	0.439	0.230	360283	A	G	0.229	3	156550415	5.88E-06	0.050	14306	20.873
Vascular dementia (sudden onset)	genus Parasutterella	rs6809952	G	A	-0.058	194178920	0.660	0.132	360283	G	A	-0.068	3	193896709	8.13E-06	0.015	14306	20.606
Vascular dementia (sudden onset)	genus Parasutterella	rs6828768	C	T	-0.069	64691207	0.556	0.117	360283	C	T	0.064	4	65556925	1.78E-06	0.013	14306	23.051
Vascular dementia (sudden onset)	genus Parasutterella	rs7303158	C	T	0.049	5166374	0.673	0.116	360283	C	T	0.065	12	5275540	1.33E-06	0.013	14306	23.214
Vascular dementia (sudden onset)	genus Parasutterella	rs7311004	C	T	-0.173	52866926	0.141	0.117	360283	C	T	0.062	12	53260710	5.92E-06	0.014	14306	20.485
Vascular dementia (sudden onset)	genus Parasutterella	rs7572229	A	G	-0.115	72008314	0.323	0.116	360283	A	G	-0.066	2	72235444	6.32E-07	0.013	14306	24.928
Vascular dementia (sudden onset)	genus Parasutterella	rs78383039	T	C	0.008	178089981	0.977	0.298	360283	T	C	-0.146	2	178954708	1.57E-06	0.030	14306	24.250
Vascular dementia (sudden onset)	genus Parasutterella	rs8039785	G	T	0.058	67023969	0.621	0.117	360283	G	T	-0.062	15	67316307	3.62E-06	0.013	14306	21.615
Vascular dementia (sudden onset)	genus Parasutterella	rs823424	G	A	-0.174	16817017	0.193	0.134	360283	G	A	-0.071	8	16674526	4.95E-06	0.016	14306	20.661
Vascular dementia (sudden onset)	genus Peptococcus	rs10031059	C	T	0.067	35356972	0.626	0.137	360283	C	T	0.121	4	35358594	1.24E-07	0.023	14306	28.784
Vascular dementia (sudden onset)	genus Peptococcus	rs11001941	G	A	-0.390	76956272	0.057	0.205	360283	G	A	-0.196	10	78716030	1.33E-06	0.039	14306	24.873
Vascular dementia (sudden onset)	genus Peptococcus	rs12069354	C	T	-0.017	216050440	0.944	0.240	360283	C	T	0.168	1	216223782	9.28E-06	0.038	14306	19.511
Vascular dementia (sudden onset)	genus Peptococcus	rs2054133	A	G	0.217	33466742	0.076	0.122	360283	A	G	-0.090	2	33691809	2.14E-06	0.019	14306	22.606
Vascular dementia (sudden onset)	genus Peptococcus	rs36121075	A	G	0.023	44489376	0.885	0.157	360283	A	G	-0.141	20	43118017	6.99E-06	0.031	14306	21.094
Vascular dementia (sudden onset)	genus Peptococcus	rs413827	G	A	0.089	57518394	0.514	0.136	360283	G	A	0.110	14	57985112	3.30E-06	0.024	14306	21.537
Vascular dementia (sudden onset)	genus Peptococcus	rs5770862	T	C	-0.078	50534684	0.697	0.200	360283	T	C	0.162	22	50973113	3.22E-06	0.036	14306	20.618
Vascular dementia (sudden onset)	genus Peptococcus	rs6918730	A	G	0.291	98572413	0.235	0.245	360283	A	G	-0.135	6	99020289	1.15E-06	0.029	14306	21.809
Vascular dementia (sudden onset)	genus Peptococcus	rs7033353	G	T	0.034	101833566	0.770	0.117	360283	G	T	-0.090	9	104595848	2.22E-06	0.019	14306	22.525
Vascular dementia (sudden onset)	genus Peptococcus	rs72850165	T	C	-0.099	968356	0.651	0.218	360283	T	C	-0.134	11	968356	5.74E-06	0.030	14306	19.985
Vascular dementia (sudden onset)	genus Peptococcus	rs74592222	G	A	-0.192	75506015	0.293	0.182	360283	G	A	0.138	1	75971700	8.55E-06	0.030	14306	20.735
Vascular dementia (sudden onset)	genus Peptococcus	rs77681628	C	T	-0.157	90530302	0.465	0.215	360283	C	T	0.200	13	91182556	2.69E-07	0.039	14306	26.744
Vascular dementia (sudden onset)	genus Phascolarctobacterium	rs12618201	A	G	0.149	173571805	0.204	0.117	360283	A	G	0.064	2	174436533	3.38E-06	0.014	14306	21.556
Vascular dementia (sudden onset)	genus Phascolarctobacterium	rs1264476	G	T	-0.100	101422048	0.504	0.150	360283	G	T	-0.077	8	102434276	4.30E-06	0.017	14306	21.357
Vascular dementia (sudden onset)	genus Phascolarctobacterium	rs56069061	G	A	-0.216	168621629	0.352	0.232	360283	G	A	-0.111	3	168339417	1.87E-06	0.023	14306	23.279
Vascular dementia (sudden onset)	genus Phascolarctobacterium	rs56157888	A	C	-0.023	182331683	0.871	0.140	360283	A	C	0.095	4	183252836	1.09E-06	0.019	14306	24.232
Vascular dementia (sudden onset)	genus Phascolarctobacterium	rs74540770	G	A	0.246	186835600	0.252	0.214	360283	G	A	-0.121	3	186553389	3.60E-06	0.026	14306	21.895
Vascular dementia (sudden onset)	genus Phascolarctobacterium	rs75882962	T	C	-0.092	52704958	0.603	0.178	360283	T	C	0.097	12	53098742	3.19E-07	0.019	14306	25.835

Vascular dementia (sudden onset)	genus Phascolarctobacterium	rs7982713	G	A	-0.080	70802190	0.533	0.129	360283	G	A	0.073	13	71376322	9.72E-06	0.016	14306	19.839
Vascular dementia (sudden onset)	genus Prevotella7	rs118038478	A	G	0.163	47203698	0.472	0.227	360283	A	G	0.206	16	47237609	7.85E-06	0.047	14306	19.239
Vascular dementia (sudden onset)	genus Prevotella7	rs12124567	A	G	0.007	3427238	0.961	0.147	360283	A	G	-0.121	1	3343802	9.49E-06	0.028	14306	19.448
Vascular dementia (sudden onset)	genus Prevotella7	rs12195431	T	C	-0.168	90309935	0.395	0.197	360283	T	C	0.197	6	91019654	8.73E-06	0.044	14306	19.741
Vascular dementia (sudden onset)	genus Prevotella7	rs2240542	C	T	-0.187	241126899	0.158	0.132	360283	C	T	0.121	2	242066314	4.84E-06	0.026	14306	21.312
Vascular dementia (sudden onset)	genus Prevotella7	rs2918132	T	C	-0.049	131202057	0.681	0.120	360283	T	C	0.115	10	133000320	6.42E-06	0.025	14306	20.227
Vascular dementia (sudden onset)	genus Prevotella7	rs430270	A	C	-0.016	60488646	0.915	0.147	360283	A	C	0.139	3	60474379	2.87E-06	0.030	14306	21.944
Vascular dementia (sudden onset)	genus Prevotella7	rs57404562	C	A	0.063	11961636	0.712	0.171	360283	C	A	0.155	2	12101762	6.22E-07	0.032	14306	24.202
Vascular dementia (sudden onset)	genus Prevotella7	rs79263163	A	C	0.075	39800223	0.604	0.145	360283	A	C	-0.144	11	39821773	7.51E-06	0.032	14306	20.891
Vascular dementia (sudden onset)	genus Prevotella7	rs9426434	T	C	-0.041	29409704	0.741	0.123	360283	T	C	-0.124	1	29736216	9.72E-06	0.028	14306	19.713
Vascular dementia (sudden onset)	genus Prevotella7	rs9608249	A	G	0.025	24217984	0.890	0.183	360283	A	G	-0.158	22	24613952	2.07E-06	0.034	14306	22.130
Vascular dementia (sudden onset)	genus Prevotella7	rs9959718	G	A	0.049	73775429	0.738	0.145	360283	G	A	0.133	18	71442664	1.90E-06	0.028	14306	23.333
Vascular dementia (sudden onset)	genus Prevotella9	rs111509883	T	C	0.240	639161	0.205	0.189	360283	T	C	0.171	19	639161	1.24E-06	0.035	14306	24.235
Vascular dementia (sudden onset)	genus Prevotella9	rs11685699	C	T	0.025	11092559	0.912	0.222	360283	C	T	-0.141	2	11232685	2.03E-06	0.030	14306	22.858
Vascular dementia (sudden onset)	genus Prevotella9	rs117271932	A	G	0.009	43840580	0.973	0.252	360283	A	G	0.208	22	44236460	2.82E-06	0.044	14306	22.326
Vascular dementia (sudden onset)	genus Prevotella9	rs12648235	T	C	-0.148	160194592	0.280	0.137	360283	T	C	0.079	4	161115744	7.39E-06	0.018	14306	19.565
Vascular dementia (sudden onset)	genus Prevotella9	rs1304512	G	A	-0.110	64440198	0.400	0.131	360283	G	A	0.076	5	63736025	5.29E-06	0.017	14306	21.078
Vascular dementia (sudden onset)	genus Prevotella9	rs2104588	C	T	-0.431	12455582	0.091	0.255	360283	C	T	-0.106	10	12497581	8.13E-06	0.024	14306	19.716
Vascular dementia (sudden onset)	genus Prevotella9	rs2495052	A	G	-0.102	13834756	0.536	0.165	360283	A	G	0.084	1	14161251	8.97E-06	0.019	14306	19.805
Vascular dementia (sudden onset)	genus Prevotella9	rs2683313	G	A	0.100	19258094	0.428	0.126	360283	G	A	0.072	8	19115604	1.69E-06	0.015	14306	22.843
Vascular dementia (sudden onset)	genus Prevotella9	rs4968431	G	T	-0.057	61344417	0.637	0.121	360283	G	T	0.064	17	59421778	8.58E-06	0.014	14306	19.716
Vascular dementia (sudden onset)	genus Prevotella9	rs7237249	C	T	0.031	74325267	0.834	0.149	360283	C	T	-0.082	18	71992502	8.93E-06	0.018	14306	20.458
Vascular dementia (sudden onset)	genus Prevotella9	rs72815774	T	C	-0.121	91766476	0.632	0.251	360283	T	C	-0.176	2	91954502	8.78E-06	0.039	14306	20.091
Vascular dementia (sudden onset)	genus Prevotella9	rs746764	T	C	0.321	21490075	0.011	0.126	360283	T	C	-0.092	20	21470713	2.04E-06	0.019	14306	22.457
Vascular dementia (sudden onset)	genus Prevotella9	rs7976209	T	C	-0.067	1685304	0.676	0.160	360283	T	C	-0.087	12	1794470	7.28E-06	0.020	14306	19.351
Vascular dementia (sudden onset)	genus Prevotella9	rs9428102	A	G	-0.110	118310194	0.437	0.141	360283	A	G	-0.078	1	118852817	4.62E-06	0.018	14306	19.568
Vascular dementia (sudden onset)	genus Prevotella9	rs9613013	G	A	-0.186	25756771	0.290	0.176	360283	G	A	0.092	22	26152738	6.10E-06	0.020	14306	20.493
Vascular dementia (sudden onset)	genus Rikenellaceae RC9 gut group	rs12501673	A	G	-0.172	162909364	0.189	0.131	360283	A	G	0.116	4	163830516	6.29E-06	0.026	14306	19.698
Vascular dementia (sudden onset)	genus Rikenellaceae RC9 gut group	rs17032291	T	C	-0.079	154944519	0.654	0.177	360283	T	C	-0.170	4	155865671	6.61E-06	0.037	14306	21.206
Vascular dementia (sudden onset)	genus Rikenellaceae RC9 gut group	rs17582787	A	G	0.143	148641486	0.360	0.156	360283	A	G	-0.158	4	149562638	3.55E-06	0.034	14306	21.533
Vascular dementia (sudden onset)	genus Rikenellaceae RC9 gut group	rs2074881	T	C	-0.293	1970022	0.090	0.173	360283	T	C	-0.142	19	1970021	9.45E-06	0.032	14306	19.283
Vascular dementia (sudden onset)	genus Rikenellaceae RC9 gut group	rs2900503	T	G	-0.335	109895263	0.035	0.159	360283	T	G	0.172	9	112657543	1.55E-07	0.033	14306	27.829
Vascular dementia (sudden onset)	genus Rikenellaceae RC9 gut group	rs2998141	C	T	0.104	133196694	0.442	0.135	360283	C	T	0.136	10	135010198	4.42E-06	0.029	14306	21.644
Vascular dementia (sudden onset)	genus Rikenellaceae RC9 gut group	rs4270579	A	G	-0.180	83777035	0.144	0.123	360283	A	G	0.118	4	84698188	5.46E-06	0.027	14306	18.960
Vascular dementia (sudden onset)	genus Rikenellaceae RC9 gut group	rs4717843	G	T	0.077	73920657	0.510	0.117	360283	G	T	-0.119	7	73334987	4.72E-06	0.026	14306	20.992
Vascular dementia (sudden onset)	genus Rikenellaceae RC9 gut group	rs7712231	A	G	0.194	31406178	0.254	0.170	360283	A	G	0.156	5	31406285	7.97E-06	0.035	14306	19.886
Vascular dementia (sudden onset)	genus Rikenellaceae RC9 gut group	rs80309088	G	A	-0.159	165255243	0.373	0.178	360283	G	A	0.174	6	165668732	4.56E-06	0.038	14306	20.606
Vascular dementia (sudden onset)	genus Rikenellaceae RC9 gut group	rs9887954	G	A	-0.110	164831636	0.355	0.118	360283	G	A	-0.115	1	164800873	4.81E-06	0.025	14306	21.272
Vascular dementia (sudden onset)	genus Romboutsia	rs10279978	A	G	0.215	5279756	0.088	0.126	360283	A	G	-0.062	7	5319387	1.17E-06	0.013	14306	23.749
Vascular dementia (sudden onset)	genus Romboutsia	rs11221428	T	C	0.245	99794319	0.081	0.140	360283	T	C	-0.073	11	99665050	6.49E-06	0.016	14306	21.089
Vascular dementia (sudden onset)	genus Romboutsia	rs16843578	C	T	-0.167	171934745	0.525	0.263	360283	C	T	-0.088	1	171903885	5.08E-06	0.020	14306	19.785
Vascular dementia (sudden onset)	genus Romboutsia	rs28603357	T	C	-0.186	106766378	0.663	0.427	360283	T	C	-0.215	7	106406824	8.52E-06	0.047	14306	20.493
Vascular dementia (sudden onset)	genus Romboutsia	rs34302036	G	A	0.040	78507408	0.732	0.118	360283	G	A	-0.055	7	78136725	5.88E-06	0.012	14306	20.737
Vascular dementia (sudden onset)	genus Romboutsia	rs61841503	G	A	0.047	16977560	0.790	0.175	360283	G	A	0.093	10	17019559	4.00E-08	0.017	14306	29.351
Vascular dementia (sudden onset)	genus Romboutsia	rs62504452	A	G	0.248	146063205	0.134	0.165	360283	A	G	-0.071	7	145760298	4.66E-06	0.016	14306	20.544
Vascular dementia (sudden onset)	genus Romboutsia	rs7109293	A	G	-0.167	133964762	0.356	0.181	360283	A	G	0.092	11	133834657	6.98E-06	0.021	14306	19.958
Vascular dementia (sudden onset)	genus Romboutsia	rs75200530	T	G	0.438	5230532	0.201	0.343	360283	T	G	-0.191	8	5088054	5.07E-06	0.042	14306	20.500
Vascular dementia (sudden onset)	genus Romboutsia	rs75987356	G	A	0.221	15926583	0.307	0.217	360283	G	A	-0.130	2	16066705	6.71E-06	0.028	14306	21.347
Vascular dementia (sudden onset)	genus Romboutsia	rs77702691	A	G	0.087	88073285	0.669	0.204	360283	A	G	-0.094	13	88725540	7.37E-06	0.021	14306	20.493
Vascular dementia (sudden onset)	genus Romboutsia	rs9389266	T	G	0.201	135090599	0.184	0.152	360283	T	G	0.072	6	135411737	9.38E-06	0.016	14306	19.821
Vascular dementia (sudden onset)	genus Romboutsia	rs9567264	C	T	0.091	32146619	0.457	0.123	360283	C	T	0.058	13	32720756	5.76E-06	0.013	14306	20.696
Vascular dementia (sudden onset)	genus Roseburia	rs12740451	T	C	0.032	94713418	0.854	0.171	360283	T	C	0.070	1	95178974	7.34E-06	0.015	14306	20.621
Vascular dementia (sudden onset)	genus Roseburia	rs16910295	T	C	0.018	11988022	0.944	0.260	360283	T	C	-0.098	11	12009569	2.91E-06	0.021	14306	21.887
Vascular dementia (sudden onset)	genus Roseburia	rs2160994	C	T	-0.134	50256274	0.277	0.123	360283	C	T	-0.055	12	50650057	9.70E-07	0.011	14306	23.969
Vascular dementia (sudden onset)	genus Roseburia	rs2943022	T	C	-0.254	90303097	0.031	0.118	360283	T	C	0.049	5	89598914	4.11E-06	0.011	14306	21.391
Vascular dementia (sudden onset)	genus Roseburia	rs302266	T	C	-0.052	194214520	0.765	0.175	360283	T	C	-0.078	1	194183650	8.13E-06	0.017	14306	20.192
Vascular dementia (sudden onset)	genus Roseburia	rs329182	T	C	-0.093	125740801	0.557	0.157	360283	T	C	0.069	5	125076494	5.90E-06	0.015	14306	20.389
Vascular dementia (sudden onset)	genus Roseburia	rs55858165	A	C	-0.464	85969578	0.136	0.311	360283	A	C	0.179	15	86512809	9.99E-06	0.040	14306	19.601
Vascular dementia (sudden onset)	genus Roseburia	rs57466170	C	T	0.186	37566864	0.399	0.221	360283	C	T	0.074	15	37859065	8.30E-06	0.017	14306	18.667
Vascular dementia (sudden onset)	genus Roseburia	rs6445851	A	G	-0.095	57082200	0.427	0.120	360283	A	G	0.050	3	57116228	3.53E-06	0.011	14306	21.144
Vascular dementia (sudden onset)	genus Roseburia	rs6930661	C	T	-0.358	12774379	0.139	0.242	360283	C	T	-0.096	6	12774611	2.48E-06	0.020	14306	22.008
Vascular dementia (sudden onset)	genus Roseburia	rs75326254	C	T	-0.152	165757348	0.523	0.239	360283	C	T	-0.105	6	166170836	7.50E-06	0.023	14306	20.533
Vascular dementia (sudden onset)	genus Roseburia	rs78753150	A	C	0.027	144756092	0.890	0.195	360283	A	C	0.097	5	144135655	9.98E-06	0.021	14306	20.479
Vascular dementia (sudden onset)	genus Roseburia	rs9300744	C	T	-0.193	102465136	0.206	0.153	360283	C	T	-0.059	13	103117486	4.75E-06	0.013	14306	21.733
Vascular dementia (sudden onset)	genus Ruminiclostridium5	rs10827477	A	G	-0.001	34973660	0.997	0.122	360283	A	G	-0.055	10	35262588	2.19E-06	0.012	14306	22.592
Vascular dementia (sudden onset)	genus Ruminiclostridium5	rs113753996	T	C	-0.156	32477558	0.299	0.150	360283	T	C	0.082	5	32477664	3.99E-06	0.017	14306	22.128
Vascular dementia (sudden onset)	genus Ruminiclostridium5	rs1223978	C	T	-0.097	108122814	0.404	0.117	360283	C	T	-0.048	13	108775162	8.16E-06	0.011	14306	20.004

Vascular dementia (sudden onset)	genus Ruminiclostridium5	rs1492620	T	C	-0.266	50438207	0.134	0.177	360283	T	C	-0.083	6	50405920	3.53E-06	0.018	14306	21.271
Vascular dementia (sudden onset)	genus Ruminiclostridium5	rs2482038	C	A	0.111	12793086	0.351	0.119	360283	C	A	0.052	10	12835085	1.70E-06	0.011	14306	22.764
Vascular dementia (sudden onset)	genus Ruminiclostridium5	rs2791343	T	C	0.032	130303784	0.790	0.119	360283	T	C	0.052	8	131316030	5.54E-06	0.011	14306	20.810
Vascular dementia (sudden onset)	genus Ruminiclostridium5	rs2833828	G	A	0.051	32439578	0.663	0.117	360283	G	A	0.049	21	33811886	6.82E-06	0.011	14306	20.288
Vascular dementia (sudden onset)	genus Ruminiclostridium5	rs4955951	A	G	0.026	55209503	0.886	0.180	360283	A	G	-0.071	3	55243531	9.96E-06	0.017	14306	18.526
Vascular dementia (sudden onset)	genus Ruminiclostridium5	rs6121460	G	A	-0.297	61718449	0.163	0.213	360283	G	A	0.093	20	60293505	2.64E-06	0.020	14306	21.934
Vascular dementia (sudden onset)	genus Ruminiclostridium5	rs79968837	A	G	-0.416	42549316	0.115	0.264	360283	A	G	-0.095	20	41177956	1.15E-06	0.019	14306	24.118
Vascular dementia (sudden onset)	genus Ruminiclostridium5	rs8053158	G	A	0.207	86736303	0.242	0.177	360283	G	A	0.074	16	86769909	5.90E-06	0.016	14306	21.651
Vascular dementia (sudden onset)	genus Ruminiclostridium6	rs10829821	T	C	-0.062	130853030	0.755	0.198	360283	T	C	-0.098	10	132651293	3.47E-06	0.022	14306	20.406
Vascular dementia (sudden onset)	genus Ruminiclostridium6	rs116969552	A	G	-0.280	126525834	0.428	0.353	360283	A	G	-0.167	10	128214403	9.16E-06	0.038	14306	19.614
Vascular dementia (sudden onset)	genus Ruminiclostridium6	rs11992182	A	C	0.164	78854264	0.243	0.140	360283	A	C	0.063	8	79766499	4.65E-06	0.014	14306	20.568
Vascular dementia (sudden onset)	genus Ruminiclostridium6	rs2548459	C	T	0.041	48706082	0.730	0.118	360283	C	T	0.055	19	49209339	6.40E-06	0.012	14306	20.431
Vascular dementia (sudden onset)	genus Ruminiclostridium6	rs35362464	C	A	0.175	36476389	0.293	0.167	360283	C	A	0.072	4	36478011	8.99E-06	0.017	14306	18.956
Vascular dementia (sudden onset)	genus Ruminiclostridium6	rs61060922	T	G	-0.024	72102255	0.945	0.338	360283	T	G	0.159	16	72136154	1.09E-06	0.032	14306	24.380
Vascular dementia (sudden onset)	genus Ruminiclostridium6	rs663262	C	T	-0.179	86468034	0.581	0.323	360283	C	T	0.135	11	86179076	3.39E-06	0.031	14306	18.872
Vascular dementia (sudden onset)	genus Ruminiclostridium6	rs67479537	T	C	0.310	10004839	0.259	0.275	360283	T	C	0.119	19	10115515	9.30E-06	0.026	14306	20.183
Vascular dementia (sudden onset)	genus Ruminiclostridium6	rs71414120	T	G	-0.087	56472234	0.744	0.266	360283	T	G	0.201	14	56938952	1.08E-06	0.041	14306	24.432
Vascular dementia (sudden onset)	genus Ruminiclostridium6	rs72991535	T	G	-0.308	78258244	0.334	0.318	360283	T	G	0.136	18	76018244	4.95E-06	0.030	14306	21.105
Vascular dementia (sudden onset)	genus Ruminiclostridium6	rs73176030	T	C	0.174	101628002	0.185	0.131	360283	T	C	0.059	7	101271282	7.29E-06	0.013	14306	19.728
Vascular dementia (sudden onset)	genus Ruminiclostridium6	rs77193512	A	G	-0.168	40267513	0.210	0.134	360283	A	G	0.074	11	40289063	1.30E-06	0.015	14306	23.125
Vascular dementia (sudden onset)	genus Ruminiclostridium6	rs792058	G	A	0.224	5408472	0.058	0.118	360283	G	A	0.055	2	5548605	8.58E-06	0.013	14306	19.527
Vascular dementia (sudden onset)	genus Ruminiclostridium6	rs79968172	G	A	0.078	240340526	0.757	0.252	360283	G	A	0.116	1	240503826	1.66E-06	0.024	14306	22.827
Vascular dementia (sudden onset)	genus Ruminiclostridium6	rs9555756	A	C	-0.075	111050902	0.718	0.207	360283	A	C	-0.080	13	111703249	7.10E-06	0.018	14306	20.712
Vascular dementia (sudden onset)	genus Ruminiclostridium9	rs12040548	G	T	0.207	247546983	0.111	0.130	360283	G	T	0.057	1	247710285	3.15E-06	0.012	14306	21.733
Vascular dementia (sudden onset)	genus Ruminiclostridium9	rs6082461	C	A	0.053	2229884	0.716	0.146	360283	C	A	-0.059	20	2210530	4.87E-06	0.013	14306	20.038
Vascular dementia (sudden onset)	genus Ruminiclostridium9	rs7137760	C	T	0.054	10549815	0.640	0.117	360283	C	T	0.051	12	10702414	7.07E-06	0.011	14306	20.504
Vascular dementia (sudden onset)	genus Ruminiclostridium9	rs74303178	T	C	0.049	14840035	0.698	0.125	360283	T	C	0.053	8	14697544	7.92E-06	0.012	14306	19.950
Vascular dementia (sudden onset)	genus Ruminiclostridium9	rs78191726	T	C	0.060	66945427	0.788	0.222	360283	T	C	0.094	6	67655320	7.58E-06	0.021	14306	20.210
Vascular dementia (sudden onset)	genus Ruminiclostridium9	rs918449	G	A	-0.173	33880293	0.449	0.228	360283	G	A	0.095	19	34371198	2.56E-06	0.020	14306	23.258
Vascular dementia (sudden onset)	genus Ruminiclostridium9	rs9522712	T	C	-0.050	89789324	0.763	0.167	360283	T	C	0.070	13	90441578	4.66E-06	0.015	14306	20.396
Vascular dementia (sudden onset)	genus Ruminiclostridium9	rs9809789	C	T	-0.239	29138492	0.117	0.152	360283	C	T	-0.072	3	29179983	8.72E-06	0.016	14306	20.189
Vascular dementia (sudden onset)	genus Ruminococcaceae NK4A214 group	rs11241747	T	C	0.124	124510625	0.334	0.128	360283	T	C	-0.053	5	123846318	6.59E-06	0.012	14306	19.780
Vascular dementia (sudden onset)	genus Ruminococcaceae NK4A214 group	rs11586410	G	A	-0.082	157369098	0.614	0.162	360283	G	A	-0.086	1	157338888	3.66E-07	0.017	14306	25.815
Vascular dementia (sudden onset)	genus Ruminococcaceae NK4A214 group	rs12642039	C	T	0.013	159010870	0.915	0.121	360283	C	T	0.055	4	159932022	3.43E-06	0.012	14306	21.452
Vascular dementia (sudden onset)	genus Ruminococcaceae NK4A214 group	rs12731	A	G	-0.181	238179271	0.132	0.120	360283	A	G	-0.053	2	239087912	4.87E-06	0.012	14306	21.035
Vascular dementia (sudden onset)	genus Ruminococcaceae NK4A214 group	rs13087692	G	T	-0.074	84675888	0.561	0.127	360283	G	T	-0.057	3	84725039	8.69E-06	0.013	14306	20.818
Vascular dementia (sudden onset)	genus Ruminococcaceae NK4A214 group	rs136761	A	G	0.065	49402365	0.594	0.121	360283	A	G	0.059	22	49796014	8.15E-07	0.012	14306	24.312
Vascular dementia (sudden onset)	genus Ruminococcaceae NK4A214 group	rs147475196	A	G	-0.147	26467796	0.439	0.189	360283	A	G	-0.134	3	26509287	4.72E-06	0.030	14306	20.535
Vascular dementia (sudden onset)	genus Ruminococcaceae NK4A214 group	rs35559912	T	C	-0.168	35288836	0.346	0.178	360283	T	C	-0.093	5	35288938	4.89E-06	0.020	14306	20.629
Vascular dementia (sudden onset)	genus Ruminococcaceae NK4A214 group	rs4814689	C	T	-0.446	18049713	0.114	0.282	360283	C	T	-0.108	20	18030357	4.55E-06	0.023	14306	22.036
Vascular dementia (sudden onset)	genus Ruminococcaceae NK4A214 group	rs5994253	A	G	-0.061	17312122	0.717	0.168	360283	A	G	-0.081	22	17793012	2.35E-07	0.016	14306	26.493
Vascular dementia (sudden onset)	genus Ruminococcaceae NK4A214 group	rs62027366	T	C	-0.174	24062485	0.227	0.144	360283	T	C	0.062	16	24073806	6.58E-06	0.014	14306	19.998
Vascular dementia (sudden onset)	genus Ruminococcaceae NK4A214 group	rs6681678	T	C	-0.279	99783035	0.403	0.333	360283	T	C	0.100	1	100248591	9.05E-06	0.024	14306	17.422
Vascular dementia (sudden onset)	genus Ruminococcaceae NK4A214 group	rs7573569	T	C	-0.333	141139473	0.170	0.243	360283	T	C	0.108	2	141897042	3.23E-06	0.023	14306	21.265
Vascular dementia (sudden onset)	genus Ruminococcaceae UCG002	rs10916131	C	T	-0.112	227375425	0.476	0.158	360283	C	T	-0.069	1	227563126	2.87E-06	0.015	14306	22.321
Vascular dementia (sudden onset)	genus Ruminococcaceae UCG002	rs10927423	C	A	-0.113	14405962	0.457	0.152	360283	C	A	-0.071	1	14732458	8.50E-07	0.015	14306	23.340
Vascular dementia (sudden onset)	genus Ruminococcaceae UCG002	rs10964441	G	A	0.077	20131748	0.690	0.193	360283	G	A	-0.149	9	20131746	7.45E-06	0.034	14306	18.683
Vascular dementia (sudden onset)	genus Ruminococcaceae UCG002	rs113147300	A	G	-0.142	111304390	0.401	0.170	360283	A	G	-0.076	9	114066670	7.69E-06	0.016	14306	21.240
Vascular dementia (sudden onset)	genus Ruminococcaceae UCG002	rs11607472	A	G	-0.294	43323201	0.213	0.236	360283	A	G	-0.078	11	43344751	7.19E-06	0.018	14306	19.580
Vascular dementia (sudden onset)	genus Ruminococcaceae UCG002	rs116974815	C	A	0.256	111842219	0.266	0.231	360283	C	A	-0.190	11	111712942	2.03E-06	0.040	14306	22.890
Vascular dementia (sudden onset)	genus Ruminococcaceae UCG002	rs11750293	G	T	-0.058	124486421	0.629	0.121	360283	G	T	-0.058	5	123822114	1.76E-06	0.012	14306	23.028
Vascular dementia (sudden onset)	genus Ruminococcaceae UCG002	rs12463378	A	G	-0.012	53972554	0.927	0.126	360283	A	G	-0.052	19	54475808	2.96E-06	0.011	14306	21.694
Vascular dementia (sudden onset)	genus Ruminococcaceae UCG002	rs15256	C	T	0.119	72060790	0.511	0.181	360283	C	T	0.073	10	73820548	9.46E-06	0.017	14306	18.928
Vascular dementia (sudden onset)	genus Ruminococcaceae UCG002	rs55793120	T	C	0.350	46990335	0.163	0.251	360283	T	C	0.137	12	47384118	4.81E-07	0.027	14306	25.119
Vascular dementia (sudden onset)	genus Ruminococcaceae UCG002	rs57079348	T	G	-0.040	87268195	0.875	0.254	360283	T	G	-0.077	13	87920450	7.22E-06	0.017	14306	19.632
Vascular dementia (sudden onset)	genus Ruminococcaceae UCG002	rs6542556	G	A	0.062	120000277	0.602	0.120	360283	G	A	-0.051	2	120757853	7.86E-06	0.011	14306	19.972
Vascular dementia (sudden onset)	genus Ruminococcaceae UCG002	rs6793778	T	C	-0.033	24003962	0.806	0.133	360283	T	C	0.056	3	24045453	9.81E-06	0.013	14306	19.896
Vascular dementia (sudden onset)	genus Ruminococcaceae UCG002	rs7120052	A	C	0.245	86624417	0.100	0.149	360283	A	C	0.062	11	86335459	1.97E-06	0.014	14306	21.254
Vascular dementia (sudden onset)	genus Ruminococcaceae UCG002	rs7155595	C	A	0.319	77036203	0.013	0.128	360283	C	A	0.057	14	77502546	1.15E-06	0.012	14306	23.734
Vascular dementia (sudden onset)	genus Ruminococcaceae UCG002	rs7249614	G	A	-0.013	43133738	0.911	0.120	360283	G	A	0.049	19	43637890	9.07E-06	0.011	14306	19.777
Vascular dementia (sudden onset)	genus Ruminococcaceae UCG002	rs76847269	A	G	-0.236	141635384	0.516	0.364	360283	A	G	0.164	5	141014951	5.17E-06	0.036	14306	21.077
Vascular dementia (sudden onset)	genus Ruminococcaceae UCG002	rs77564310	A	C	-0.045	80420842	0.757	0.144	360283	A	C	-0.071	15	80713184	3.29E-07	0.014	14306	25.630
Vascular dementia (sudden onset)	genus Ruminococcaceae UCG002	rs79016051	C	T	0.076	238775197	0.660	0.173	360283	C	T	-0.089	1	238938497	2.34E-06	0.019	14306	21.964
Vascular dementia (sudden onset)	genus Ruminococcaceae UCG002	rs882348	A	G	0.119	23323417	0.514	0.182	360283	A	G	-0.080	4	23325040	5.45E-06	0.018	14306	20.057
Vascular dementia (sudden onset)	genus Ruminococcaceae UCG003	rs10490280	C	T	0.044	37678833	0.768	0.150	360283	C	T	-0.067	2	37905976	4.16E-06	0.014	14306	21.994
Vascular dementia (sudden onset)	genus Ruminococcaceae UCG003	rs11243416	T	C	0.086	131541583	0.705	0.227	360283	T	C	-0.093	9	134416970	1.67E-06	0.019	14306	23.437
Vascular dementia (sudden onset)	genus Ruminococcaceae UCG003	rs11613919	G	T	-0.021	75102683	0.881	0.140	360283	G	T	0.073	12	75496463	1.63E-06	0.016	14306	21.834

Vascular dementia (sudden onset)	genus Ruminococcaceae UCG003	rs16959793	A	C	-0.083	34779517	0.484	0.118	360283	A	C	-0.063	15	35071718	2.22E-06	0.013	14306	22.692
Vascular dementia (sudden onset)	genus Ruminococcaceae UCG003	rs2523124	C	T	0.177	97719124	0.135	0.118	360283	C	T	0.055	7	97348436	5.78E-06	0.012	14306	20.468
Vascular dementia (sudden onset)	genus Ruminococcaceae UCG003	rs3013089	G	A	0.047	13468126	0.695	0.121	360283	G	A	-0.055	1	13794594	4.38E-06	0.012	14306	20.998
Vascular dementia (sudden onset)	genus Ruminococcaceae UCG003	rs4452755	A	C	0.015	81114617	0.905	0.123	360283	A	C	-0.063	8	82026852	3.29E-06	0.013	14306	22.172
Vascular dementia (sudden onset)	genus Ruminococcaceae UCG003	rs4532474	G	A	-0.092	105333663	0.554	0.156	360283	G	A	0.077	6	105781538	4.82E-06	0.017	14306	20.367
Vascular dementia (sudden onset)	genus Ruminococcaceae UCG003	rs646327	G	A	0.030	48706594	0.797	0.118	360283	G	A	0.059	19	49209851	7.83E-07	0.012	14306	24.567
Vascular dementia (sudden onset)	genus Ruminococcaceae UCG003	rs6759615	A	G	-0.017	204373993	0.930	0.198	360283	A	G	0.103	2	205238716	7.86E-07	0.020	14306	26.224
Vascular dementia (sudden onset)	genus Ruminococcaceae UCG003	rs73341549	T	C	0.245	51473771	0.336	0.255	360283	T	C	-0.170	7	51541468	1.51E-07	0.032	14306	28.393
Vascular dementia (sudden onset)	genus Ruminococcaceae UCG003	rs78720113	A	G	0.357	41940901	0.100	0.217	360283	A	G	-0.115	3	41982393	7.59E-06	0.025	14306	21.356
Vascular dementia (sudden onset)	genus Ruminococcaceae UCG004	rs10976229	T	G	0.086	7317307	0.622	0.174	360283	T	G	0.096	9	7317307	7.04E-06	0.021	14306	20.010
Vascular dementia (sudden onset)	genus Ruminococcaceae UCG004	rs11961899	G	A	0.138	132566782	0.286	0.129	360283	G	A	-0.071	6	132887921	9.18E-06	0.016	14306	19.237
Vascular dementia (sudden onset)	genus Ruminococcaceae UCG004	rs12125734	G	T	-0.291	103266658	0.152	0.203	360283	G	T	0.134	1	103732214	2.09E-07	0.026	14306	27.087
Vascular dementia (sudden onset)	genus Ruminococcaceae UCG004	rs2248146	T	C	-0.094	24752379	0.441	0.122	360283	T	C	0.069	7	24791998	8.20E-06	0.015	14306	20.135
Vascular dementia (sudden onset)	genus Ruminococcaceae UCG004	rs3800154	A	C	0.037	2119095	0.779	0.132	360283	A	C	-0.080	6	2119329	6.12E-06	0.018	14306	20.178
Vascular dementia (sudden onset)	genus Ruminococcaceae UCG004	rs511258	G	A	-0.131	170248934	0.385	0.150	360283	G	A	-0.076	3	169966722	4.52E-06	0.016	14306	21.735
Vascular dementia (sudden onset)	genus Ruminococcaceae UCG004	rs550351	A	C	0.015	18349905	0.900	0.117	360283	A	C	0.079	1	18676399	9.43E-06	0.018	14306	19.055
Vascular dementia (sudden onset)	genus Ruminococcaceae UCG004	rs6769553	A	G	0.022	54393768	0.870	0.132	360283	A	G	0.085	3	54427795	7.91E-08	0.016	14306	29.126
Vascular dementia (sudden onset)	genus Ruminococcaceae UCG004	rs7569771	A	G	-0.229	238924641	0.091	0.135	360283	A	G	-0.076	2	239846337	8.12E-06	0.017	14306	19.869
Vascular dementia (sudden onset)	genus Ruminococcaceae UCG004	rs872501	G	A	0.264	138240821	0.227	0.218	360283	G	A	0.116	8	139253064	5.81E-06	0.026	14306	20.031
Vascular dementia (sudden onset)	genus Ruminococcaceae UCG004	rs9818949	T	G	0.227	197956880	0.126	0.148	360283	T	G	-0.086	3	197683751	5.39E-06	0.019	14306	20.774
Vascular dementia (sudden onset)	genus Ruminococcaceae UCG005	rs10873449	C	T	0.132	94164394	0.368	0.146	360283	C	T	-0.065	14	94630731	4.11E-06	0.014	14306	20.688
Vascular dementia (sudden onset)	genus Ruminococcaceae UCG005	rs10937802	A	G	0.062	7300617	0.731	0.179	360283	A	G	-0.076	4	7302344	8.17E-06	0.017	14306	20.186
Vascular dementia (sudden onset)	genus Ruminococcaceae UCG005	rs10950694	C	T	0.140	17986981	0.246	0.121	360283	C	T	-0.058	7	18026604	4.30E-07	0.011	14306	25.620
Vascular dementia (sudden onset)	genus Ruminococcaceae UCG005	rs114279581	A	G	0.062	197257633	0.777	0.219	360283	A	G	-0.147	2	198122357	3.22E-06	0.032	14306	21.519
Vascular dementia (sudden onset)	genus Ruminococcaceae UCG005	rs12288512	A	G	0.051	27726124	0.703	0.135	360283	A	G	0.067	11	27747671	3.10E-06	0.014	14306	21.308
Vascular dementia (sudden onset)	genus Ruminococcaceae UCG005	rs12458218	T	C	-0.003	24995907	0.987	0.151	360283	T	C	0.068	18	22575871	2.41E-06	0.014	14306	21.964
Vascular dementia (sudden onset)	genus Ruminococcaceae UCG005	rs2893871	G	A	0.017	60903023	0.926	0.187	360283	G	A	-0.074	10	62662781	3.54E-06	0.016	14306	22.435
Vascular dementia (sudden onset)	genus Ruminococcaceae UCG005	rs34781347	G	A	-0.045	16332206	0.841	0.227	360283	G	A	0.189	20	16312851	6.05E-07	0.039	14306	23.835
Vascular dementia (sudden onset)	genus Ruminococcaceae UCG005	rs55793120	T	C	0.350	46990335	0.163	0.251	360283	T	C	0.122	12	47384118	7.37E-06	0.028	14306	18.906
Vascular dementia (sudden onset)	genus Ruminococcaceae UCG005	rs72776570	C	A	-0.060	4198714	0.759	0.197	360283	C	A	0.087	10	4240906	5.36E-06	0.020	14306	19.498
Vascular dementia (sudden onset)	genus Ruminococcaceae UCG005	rs7449320	C	A	-0.087	154623531	0.523	0.137	360283	C	A	0.060	5	154003091	4.81E-06	0.013	14306	20.972
Vascular dementia (sudden onset)	genus Ruminococcaceae UCG005	rs7555878	G	A	0.287	187936903	0.033	0.135	360283	G	A	-0.059	1	187906034	2.81E-06	0.013	14306	21.931
Vascular dementia (sudden onset)	genus Ruminococcaceae UCG005	rs7586445	G	A	0.193	238600389	0.260	0.171	360283	G	A	0.078	2	239509030	8.81E-06	0.018	14306	19.657
Vascular dementia (sudden onset)	genus Ruminococcaceae UCG005	rs898577	C	T	-0.507	68413358	0.044	0.252	360283	C	T	0.123	15	68705697	7.46E-06	0.029	14306	18.413
Vascular dementia (sudden onset)	genus Ruminococcaceae UCG009	rs12508214	C	T	0.078	7425210	0.528	0.123	360283	C	T	-0.077	4	7426937	4.75E-06	0.017	14306	21.026
Vascular dementia (sudden onset)	genus Ruminococcaceae UCG009	rs138460696	A	G	0.247	66169644	0.279	0.228	360283	A	G	0.139	2	66396776	9.81E-06	0.032	14306	19.451
Vascular dementia (sudden onset)	genus Ruminococcaceae UCG009	rs1550196	A	G	-0.019	34459936	0.924	0.199	360283	A	G	-0.131	17	32786955	1.13E-06	0.026	14306	24.849
Vascular dementia (sudden onset)	genus Ruminococcaceae UCG009	rs2058609	G	A	-0.284	12817858	0.032	0.132	360283	G	A	-0.082	12	12970792	3.12E-06	0.017	14306	21.837
Vascular dementia (sudden onset)	genus Ruminococcaceae UCG009	rs2192926	A	G	-0.026	75285542	0.833	0.124	360283	A	G	-0.089	2	75512668	4.88E-06	0.019	14306	21.290
Vascular dementia (sudden onset)	genus Ruminococcaceae UCG009	rs4079028	C	T	0.176	202329957	0.194	0.135	360283	C	T	0.092	1	202299085	3.28E-06	0.020	14306	21.094
Vascular dementia (sudden onset)	genus Ruminococcaceae UCG009	rs4708333	T	G	0.132	77396984	0.284	0.123	360283	T	G	-0.084	6	78106701	1.56E-06	0.017	14306	23.122
Vascular dementia (sudden onset)	genus Ruminococcaceae UCG009	rs6952765	G	A	0.049	31962098	0.696	0.125	360283	G	A	0.073	7	32001710	8.13E-06	0.017	14306	19.269
Vascular dementia (sudden onset)	genus Ruminococcaceae UCG009	rs758191	T	G	0.060	1929830	0.763	0.198	360283	T	G	0.177	16	1979831	9.01E-06	0.038	14306	22.270
Vascular dementia (sudden onset)	genus Ruminococcaceae UCG009	rs78410648	A	G	-0.133	51896148	0.480	0.188	360283	A	G	0.121	19	52399401	9.67E-06	0.028	14306	19.034
Vascular dementia (sudden onset)	genus Ruminococcaceae UCG009	rs9558661	T	C	-0.195	105997744	0.181	0.146	360283	T	C	-0.090	13	106650093	7.01E-06	0.020	14306	20.004
Vascular dementia (sudden onset)	genus Ruminococcaceae UCG010	rs12597105	A	G	-0.029	5183940	0.846	0.147	360283	A	G	-0.067	16	5233941	4.87E-06	0.014	14306	21.579
Vascular dementia (sudden onset)	genus Ruminococcaceae UCG010	rs2820282	C	A	-0.107	104280743	0.372	0.119	360283	C	A	0.059	6	104728618	2.85E-06	0.013	14306	22.127
Vascular dementia (sudden onset)	genus Ruminococcaceae UCG010	rs682403	A	G	0.003	133093170	0.978	0.117	360283	A	G	-0.059	9	135968557	2.37E-06	0.012	14306	22.257
Vascular dementia (sudden onset)	genus Ruminococcaceae UCG010	rs6958419	C	T	0.037	16310239	0.756	0.118	360283	C	T	-0.059	7	16349864	2.84E-06	0.012	14306	21.958
Vascular dementia (sudden onset)	genus Ruminococcaceae UCG010	rs73218807	G	A	-0.139	31055860	0.505	0.208	360283	G	A	-0.166	4	31057482	6.43E-06	0.037	14306	20.407
Vascular dementia (sudden onset)	genus Ruminococcaceae UCG010	rs7441445	T	C	0.231	40705781	0.047	0.116	360283	T	C	0.057	4	40707798	6.80E-06	0.013	14306	20.267
Vascular dementia (sudden onset)	genus Ruminococcaceae UCG011	rs10274562	C	T	-0.239	11182894	0.046	0.120	360283	C	T	0.111	7	11222521	6.50E-06	0.024	14306	20.570
Vascular dementia (sudden onset)	genus Ruminococcaceae UCG011	rs12636310	G	A	0.237	185751703	0.079	0.135	360283	G	A	0.133	3	185469491	2.81E-06	0.028	14306	22.146
Vascular dementia (sudden onset)	genus Ruminococcaceae UCG011	rs12724320	C	T	0.042	179401364	0.724	0.120	360283	C	T	-0.121	1	179370499	1.52E-06	0.025	14306	23.524
Vascular dementia (sudden onset)	genus Ruminococcaceae UCG011	rs1416041	A	C	0.207	104625801	0.152	0.145	360283	A	C	-0.182	6	105073676	7.04E-08	0.034	14306	28.778
Vascular dementia (sudden onset)	genus Ruminococcaceae UCG011	rs2729556	T	C	-0.042	112123933	0.720	0.117	360283	T	C	0.109	7	111763988	3.19E-06	0.023	14306	21.793
Vascular dementia (sudden onset)	genus Ruminococcaceae UCG011	rs4490371	T	C	0.147	76155956	0.214	0.118	360283	T	C	-0.112	3	76205107	7.75E-06	0.025	14306	20.172
Vascular dementia (sudden onset)	genus Ruminococcaceae UCG011	rs79113084	C	T	0.241	29481986	0.204	0.190	360283	C	T	-0.152	12	29634919	2.06E-06	0.032	14306	22.967
Vascular dementia (sudden onset)	genus Ruminococcaceae UCG011	rs9729514	G	A	0.052	219727713	0.795	0.201	360283	G	A	-0.185	1	219901055	2.37E-06	0.039	14306	21.963
Vascular dementia (sudden onset)	genus Ruminococcaceae UCG013	rs11581881	C	T	-0.056	9301517	0.689	0.139	360283	C	T	0.066	1	9361576	4.73E-06	0.014	14306	20.870
Vascular dementia (sudden onset)	genus Ruminococcaceae UCG013	rs12189346	G	A	0.064	142015575	0.666	0.148	360283	G	A	0.068	5	141395140	1.68E-06	0.015	14306	22.137
Vascular dementia (sudden onset)	genus Ruminococcaceae UCG013	rs12336782	T	C	-0.089	13609120	0.682	0.218	360283	T	C	-0.086	9	13609119	8.60E-06	0.019	14306	20.448
Vascular dementia (sudden onset)	genus Ruminococcaceae UCG013	rs12485353	G	A	-0.048	197323125	0.723	0.134	360283	G	A	-0.061	3	197049996	4.19E-06	0.013	14306	21.584
Vascular dementia (sudden onset)	genus Ruminococcaceae UCG013	rs12781711	C	T	-0.125	2177736	0.350	0.134	360283	C	T	-0.066	10	2219930	2.55E-08	0.012	14306	31.194
Vascular dementia (sudden onset)	genus Ruminococcaceae UCG013	rs16918863	A	C	0.182	19491534	0.454	0.243	360283	A	C	0.111	10	19780463	4.15E-06	0.024	14306	21.552
Vascular dementia (sudden onset)	genus Ruminococcaceae UCG013	rs2730183	G	A	0.048	40454366	0.688	0.119	360283	G	A	-0.049	8	40311885	8.44E-06	0.011	14306	19.771
Vascular dementia (sudden onset)	genus Ruminococcaceae UCG013	rs4385846	T	G	0.251	113890519	0.089	0.148	360283	T	G	-0.060	10	115650278	6.46E-06	0.013	14306	20.612

Vascular dementia (sudden onset)	genus Ruminococcaceae UCG013	rs75088940	T	C	0.157	30092624	0.495	0.231	360283	T	C	-0.094	12	30245557	2.55E-06	0.020	14306	22.072
Vascular dementia (sudden onset)	genus Ruminococcaceae UCG013	rs76973485	G	T	-0.181	9492973	0.540	0.296	360283	G	T	0.195	3	9534657	3.35E-06	0.042	14306	21.735
Vascular dementia (sudden onset)	genus Ruminococcaceae UCG013	rs9313055	T	C	-0.176	3628768	0.406	0.212	360283	T	C	0.105	5	3628882	9.55E-06	0.023	14306	20.089
Vascular dementia (sudden onset)	genus Ruminococcaceae UCG014	rs10495392	T	C	-0.161	237259689	0.479	0.227	360283	T	C	0.082	1	237422989	9.96E-06	0.019	14306	19.417
Vascular dementia (sudden onset)	genus Ruminococcaceae UCG014	rs10791168	G	A	-0.039	131789106	0.795	0.151	360283	G	A	0.066	11	131659000	9.76E-06	0.015	14306	19.614
Vascular dementia (sudden onset)	genus Ruminococcaceae UCG014	rs10941294	C	T	-0.064	36435495	0.799	0.253	360283	C	T	-0.122	5	36435597	2.40E-06	0.026	14306	22.035
Vascular dementia (sudden onset)	genus Ruminococcaceae UCG014	rs115777838	T	C	0.018	26110517	0.923	0.186	360283	T	C	-0.188	5	26110626	4.62E-07	0.039	14306	23.731
Vascular dementia (sudden onset)	genus Ruminococcaceae UCG014	rs12638134	T	G	-0.086	101615765	0.461	0.117	360283	T	G	0.058	3	101334609	1.21E-06	0.012	14306	23.702
Vascular dementia (sudden onset)	genus Ruminococcaceae UCG014	rs34402072	C	T	0.221	3779026	0.185	0.167	360283	C	T	-0.069	8	3636548	9.80E-06	0.016	14306	19.431
Vascular dementia (sudden onset)	genus Ruminococcaceae UCG014	rs56105232	G	A	-0.356	14363770	0.150	0.248	360283	G	A	0.139	9	14363769	2.91E-06	0.030	14306	21.678
Vascular dementia (sudden onset)	genus Ruminococcaceae UCG014	rs72809222	T	C	-0.042	56978719	0.771	0.143	360283	T	C	0.067	2	57205854	2.41E-06	0.014	14306	23.078
Vascular dementia (sudden onset)	genus Ruminococcaceae UCG014	rs853612	A	G	-0.073	118168902	0.539	0.119	360283	A	G	-0.053	10	119928413	9.75E-06	0.012	14306	19.585
Vascular dementia (sudden onset)	genus Ruminococcaceae UCG014	rs995642	C	T	-0.064	134097088	0.635	0.136	360283	C	T	0.060	2	134854659	1.90E-06	0.013	14306	22.562
Vascular dementia (sudden onset)	genus Ruminococcus1	rs10167839	A	G	0.010	181018152	0.933	0.121	360283	A	G	0.052	2	181882879	8.09E-06	0.012	14306	19.952
Vascular dementia (sudden onset)	genus Ruminococcus1	rs11783695	G	T	-0.154	143514733	0.332	0.159	360283	G	T	-0.073	8	144596903	4.73E-06	0.016	14306	20.689
Vascular dementia (sudden onset)	genus Ruminococcus1	rs17781867	C	T	0.021	73259879	0.926	0.230	360283	C	T	0.100	17	71256018	1.96E-06	0.021	14306	22.275
Vascular dementia (sudden onset)	genus Ruminococcus1	rs3819978	C	T	-0.244	241626455	0.275	0.224	360283	C	T	-0.115	1	241789757	8.74E-06	0.026	14306	19.557
Vascular dementia (sudden onset)	genus Ruminococcus1	rs6105066	T	C	0.023	13277981	0.858	0.131	360283	T	C	-0.061	20	13258628	5.06E-06	0.013	14306	20.447
Vascular dementia (sudden onset)	genus Ruminococcus1	rs6493760	T	C	0.048	55146984	0.695	0.122	360283	T	C	-0.054	15	55439182	3.38E-06	0.012	14306	21.334
Vascular dementia (sudden onset)	genus Ruminococcus1	rs7117576	A	G	0.067	114152851	0.744	0.206	360283	A	G	0.083	11	114023573	6.48E-07	0.017	14306	23.561
Vascular dementia (sudden onset)	genus Ruminococcus1	rs7583465	C	T	-0.088	10393795	0.449	0.116	360283	C	T	0.053	2	10533921	2.56E-06	0.011	14306	21.952
Vascular dementia (sudden onset)	genus Ruminococcus1	rs78572139	G	A	-0.108	15944478	0.580	0.195	360283	G	A	0.125	5	15944587	5.23E-06	0.028	14306	20.026
Vascular dementia (sudden onset)	genus Ruminococcus1	rs78613526	G	A	-0.131	65698078	0.617	0.263	360283	G	A	0.167	2	65925212	5.11E-06	0.037	14306	20.757
Vascular dementia (sudden onset)	genus Ruminococcus2	rs12406309	A	C	-0.142	107531823	0.314	0.141	360283	A	C	-0.063	1	108074445	9.79E-06	0.014	14306	19.728
Vascular dementia (sudden onset)	genus Ruminococcus2	rs12986628	T	C	-0.213	237194796	0.142	0.145	360283	T	C	-0.067	2	238103439	2.14E-06	0.014	14306	22.572
Vascular dementia (sudden onset)	genus Ruminococcus2	rs1819812	G	T	-0.075	151738636	0.772	0.258	360283	G	T	0.084	7	151435722	5.28E-06	0.018	14306	20.756
Vascular dementia (sudden onset)	genus Ruminococcus2	rs2368224	T	G	-0.281	181791689	0.291	0.266	360283	T	G	0.200	2	182656416	3.63E-06	0.044	14306	20.734
Vascular dementia (sudden onset)	genus Ruminococcus2	rs2846589	T	G	0.017	862476	0.886	0.119	360283	T	G	-0.052	18	862477	7.59E-06	0.012	14306	20.124
Vascular dementia (sudden onset)	genus Ruminococcus2	rs2997412	G	A	-0.014	61493648	0.914	0.131	360283	G	A	0.057	13	62067781	4.22E-06	0.012	14306	21.594
Vascular dementia (sudden onset)	genus Ruminococcus2	rs4400279	A	G	0.051	16764953	0.680	0.124	360283	A	G	0.055	7	16804578	5.80E-06	0.012	14306	20.685
Vascular dementia (sudden onset)	genus Ruminococcus2	rs4799823	C	T	-0.219	35876464	0.147	0.151	360283	C	T	0.084	18	33456427	5.40E-06	0.018	14306	21.089
Vascular dementia (sudden onset)	genus Ruminococcus2	rs55707116	C	A	0.084	77304469	0.709	0.224	360283	C	A	0.087	9	79919385	8.01E-06	0.019	14306	20.940
Vascular dementia (sudden onset)	genus Ruminococcus2	rs58681734	A	G	-0.080	134666044	0.574	0.143	360283	A	G	0.072	9	137557890	4.18E-06	0.016	14306	20.269
Vascular dementia (sudden onset)	genus Ruminococcus2	rs61791565	T	C	0.006	24653840	0.959	0.117	360283	T	C	-0.052	4	24655463	6.79E-06	0.012	14306	19.979
Vascular dementia (sudden onset)	genus Ruminococcus2	rs75140805	T	G	0.013	12127041	0.931	0.155	360283	T	G	0.084	11	12148588	3.95E-06	0.018	14306	22.493
Vascular dementia (sudden onset)	genus Ruminococcus2	rs7635831	G	A	-0.102	173323134	0.398	0.121	360283	G	A	0.062	3	173040924	1.98E-06	0.013	14306	22.965
Vascular dementia (sudden onset)	genus Ruminococcus2	rs7693984	G	A	-0.044	4266595	0.874	0.280	360283	G	A	-0.103	4	4268322	9.42E-06	0.024	14306	19.130
Vascular dementia (sudden onset)	genus Ruminococcus2	rs78120384	A	G	-0.083	106945516	0.683	0.202	360283	A	G	-0.193	3	106664363	3.31E-07	0.039	14306	24.189
Vascular dementia (sudden onset)	genus Ruminococcus gauvreauii group	rs10931481	A	G	0.032	191090126	0.802	0.128	360283	A	G	-0.061	2	191954852	3.38E-06	0.013	14306	21.862
Vascular dementia (sudden onset)	genus Ruminococcus gauvreauii group	rs12079579	A	G	0.323	161910692	0.125	0.211	360283	A	G	0.096	1	161880482	5.04E-06	0.021	14306	20.034
Vascular dementia (sudden onset)	genus Ruminococcus gauvreauii group	rs12539819	C	T	0.132	153790311	0.570	0.232	360283	C	T	0.111	7	153487396	4.49E-06	0.024	14306	21.137
Vascular dementia (sudden onset)	genus Ruminococcus gauvreauii group	rs1391597	C	T	0.209	60725485	0.080	0.119	360283	C	T	0.059	12	61119266	1.86E-06	0.012	14306	22.345
Vascular dementia (sudden onset)	genus Ruminococcus gauvreauii group	rs2047242	A	G	0.102	28912931	0.462	0.139	360283	A	G	-0.068	10	29201860	2.46E-07	0.013	14306	25.552
Vascular dementia (sudden onset)	genus Ruminococcus gauvreauii group	rs2166943	C	A	-0.010	136078607	0.932	0.117	360283	C	A	-0.057	8	137090850	5.28E-06	0.012	14306	21.077
Vascular dementia (sudden onset)	genus Ruminococcus gauvreauii group	rs289410	A	G	0.082	85020252	0.526	0.130	360283	A	G	0.065	15	85563483	2.27E-06	0.014	14306	22.168
Vascular dementia (sudden onset)	genus Ruminococcus gauvreauii group	rs431418	G	A	-0.168	166992547	0.400	0.200	360283	G	A	0.095	5	166419552	5.54E-06	0.021	14306	20.321
Vascular dementia (sudden onset)	genus Ruminococcus gauvreauii group	rs71386687	T	G	0.102	2717893	0.580	0.184	360283	T	G	0.121	16	2767894	2.91E-07	0.024	14306	25.733
Vascular dementia (sudden onset)	genus Ruminococcus gauvreauii group	rs73802842	C	A	-0.080	18823237	0.561	0.137	360283	C	A	0.074	4	18824860	7.48E-06	0.017	14306	18.859
Vascular dementia (sudden onset)	genus Ruminococcus gauvreauii group	rs9870933	G	A	0.030	112653470	0.802	0.118	360283	G	A	-0.062	3	112372317	8.49E-07	0.013	14306	24.313
Vascular dementia (sudden onset)	genus Ruminococcus gnavus group	rs11597105	A	G	-0.053	6470619	0.719	0.148	360283	A	G	0.115	10	6512581	6.95E-06	0.025	14306	20.930
Vascular dementia (sudden onset)	genus Ruminococcus gnavus group	rs11864644	T	C	0.203	383078	0.268	0.183	360283	T	C	-0.140	16	433078	5.01E-06	0.032	14306	19.297
Vascular dementia (sudden onset)	genus Ruminococcus gnavus group	rs12136548	C	T	-0.218	114625476	0.087	0.127	360283	C	T	0.090	1	115168097	3.10E-06	0.020	14306	21.074
Vascular dementia (sudden onset)	genus Ruminococcus gnavus group	rs12989336	G	A	-0.119	36032198	0.362	0.131	360283	G	A	-0.085	2	36259341	7.12E-06	0.019	14306	20.305
Vascular dementia (sudden onset)	genus Ruminococcus gnavus group	rs13163520	G	A	0.313	18662577	0.037	0.150	360283	G	A	-0.127	5	18662686	5.61E-08	0.023	14306	29.661
Vascular dementia (sudden onset)	genus Ruminococcus gnavus group	rs2909242	A	C	-0.079	128212434	0.524	0.124	360283	A	C	0.091	8	129224680	7.41E-07	0.018	14306	24.588
Vascular dementia (sudden onset)	genus Ruminococcus gnavus group	rs3124783	G	A	-0.067	132963204	0.700	0.174	360283	G	A	0.116	9	135838591	2.67E-06	0.025	14306	21.682
Vascular dementia (sudden onset)	genus Ruminococcus gnavus group	rs4388134	T	C	0.018	189189556	0.888	0.131	360283	T	C	0.090	4	190110710	9.12E-06	0.020	14306	19.769
Vascular dementia (sudden onset)	genus Ruminococcus gnavus group	rs62167033	T	C	0.082	143942440	0.780	0.295	360283	T	C	0.185	2	144700007	3.50E-06	0.040	14306	21.861
Vascular dementia (sudden onset)	genus Ruminococcus gnavus group	rs78399089	T	C	-0.248	146572523	0.174	0.182	360283	T	C	0.144	3	146290310	6.63E-06	0.033	14306	19.558
Vascular dementia (sudden onset)	genus Ruminococcus gnavus group	rs934940	A	C	-0.423	121338442	0.014	0.173	360283	A	C	-0.105	2	122096018	2.74E-06	0.023	14306	20.934
Vascular dementia (sudden onset)	genus Ruminococcus torques group	rs10904297	A	G	-0.045	4597570	0.912	0.406	360283	A	G	-0.168	10	4639762	2.69E-06	0.039	14306	18.531
Vascular dementia (sudden onset)	genus Ruminococcus torques group	rs10967781	C	A	-0.026	27225260	0.836	0.128	360283	C	A	0.051	9	27225258	8.37E-06	0.011	14306	20.095
Vascular dementia (sudden onset)	genus Ruminococcus torques group	rs12434631	A	G	0.087	25661198	0.649	0.190	360283	A	G	0.075	14	26130404	2.77E-06	0.015	14306	23.711
Vascular dementia (sudden onset)	genus Ruminococcus torques group	rs1475330	C	T	-0.101	66552209	0.460	0.137	360283	C	T	-0.052	6	67262102	8.13E-06	0.012	14306	19.615
Vascular dementia (sudden onset)	genus Ruminococcus torques group	rs35866622	T	C	0.073	48714803	0.550	0.122	360283	T	C	-0.061	19	49218060	2.21E-08	0.011	14306	31.285
Vascular dementia (sudden onset)	genus Ruminococcus torques group	rs4073731	T	C	-0.077	132793252	0.624	0.157	360283	T	C	0.065	11	132663147	4.05E-06	0.014	14306	21.014
Vascular dementia (sudden onset)	genus Ruminococcus torques group	rs77034621	T	G	-0.604	75857608	0.187	0.457	360283	T	G	-0.152	8	76769843	6.07E-06	0.034	14306	20.359

Vascular dementia (sudden onset)	genus Sellimonas	rs113379006	T	C	0.054	41966694	0.723	0.153	360283	T	C	-0.163	3	42008186	7.21E-06	0.036	14306	20.782
Vascular dementia (sudden onset)	genus Sellimonas	rs13417181	T	C	0.204	173443666	0.139	0.138	360283	T	C	0.167	2	174308394	7.62E-07	0.034	14306	24.337
Vascular dementia (sudden onset)	genus Sellimonas	rs2016057	C	A	-0.118	51813085	0.324	0.119	360283	C	A	0.126	15	52105282	1.03E-06	0.026	14306	24.149
Vascular dementia (sudden onset)	genus Sellimonas	rs2187447	A	C	-0.303	79621928	0.205	0.239	360283	A	C	0.243	11	79332972	3.98E-06	0.053	14306	21.285
Vascular dementia (sudden onset)	genus Sellimonas	rs2371572	A	C	-0.109	212349450	0.353	0.117	360283	A	C	0.127	2	213214174	4.46E-07	0.025	14306	25.770
Vascular dementia (sudden onset)	genus Sellimonas	rs41816	A	G	-0.025	106609718	0.843	0.127	360283	A	G	0.132	7	106250164	8.39E-06	0.029	14306	20.626
Vascular dementia (sudden onset)	genus Sellimonas	rs4600608	G	A	0.119	179413837	0.407	0.144	360283	G	A	0.137	2	180278564	4.95E-06	0.030	14306	20.666
Vascular dementia (sudden onset)	genus Sellimonas	rs553697	C	T	0.194	93020675	0.198	0.151	360283	C	T	0.154	6	93730393	6.13E-06	0.034	14306	20.562
Vascular dementia (sudden onset)	genus Sellimonas	rs56203279	T	C	0.078	111861468	0.527	0.123	360283	T	C	-0.124	7	111501524	3.72E-06	0.027	14306	21.246
Vascular dementia (sudden onset)	genus Senegalimassilia	rs10036909	C	T	0.135	128525999	0.641	0.290	360283	C	T	0.186	5	127861692	8.05E-06	0.040	14306	21.416
Vascular dementia (sudden onset)	genus Senegalimassilia	rs11787826	C	A	-0.177	34332384	0.137	0.119	360283	C	A	0.081	9	34332382	2.63E-06	0.017	14306	22.579
Vascular dementia (sudden onset)	genus Senegalimassilia	rs1990708	A	C	0.153	205789399	0.483	0.219	360283	A	C	-0.110	2	206654123	8.91E-06	0.025	14306	19.571
Vascular dementia (sudden onset)	genus Senegalimassilia	rs2017373	C	T	-0.029	33962790	0.809	0.121	360283	C	T	0.078	14	34431996	9.50E-06	0.018	14306	19.567
Vascular dementia (sudden onset)	genus Senegalimassilia	rs7225245	A	G	0.035	50302254	0.767	0.118	360283	A	G	-0.079	17	48379615	4.18E-06	0.017	14306	21.583
Vascular dementia (sudden onset)	genus Slackia	rs10409783	G	A	0.041	4555774	0.749	0.129	360283	G	A	-0.095	19	4555786	7.70E-06	0.021	14306	20.261
Vascular dementia (sudden onset)	genus Slackia	rs12440440	A	G	-0.149	33749695	0.225	0.123	360283	A	G	0.090	15	34041896	2.63E-06	0.019	14306	22.397
Vascular dementia (sudden onset)	genus Slackia	rs16894137	C	T	0.025	95934063	0.885	0.175	360283	C	T	-0.123	8	96946291	2.71E-06	0.026	14306	21.791
Vascular dementia (sudden onset)	genus Slackia	rs35156985	T	C	-0.117	99854092	0.685	0.289	360283	T	C	-0.156	7	99451715	8.06E-06	0.035	14306	20.010
Vascular dementia (sudden onset)	genus Slackia	rs4492265	G	A	-0.027	13484058	0.832	0.128	360283	G	A	0.091	7	13523683	2.41E-06	0.019	14306	22.334
Vascular dementia (sudden onset)	genus Slackia	rs8901	C	T	-0.057	76270929	0.655	0.128	360283	C	T	0.093	17	74267010	6.07E-07	0.019	14306	25.028
Vascular dementia (sudden onset)	genus Streptococcus	rs10028567	C	T	0.031	52791410	0.861	0.179	360283	C	T	-0.092	4	53657577	7.30E-06	0.019	14306	23.047
Vascular dementia (sudden onset)	genus Streptococcus	rs10448310	A	G	0.047	90793892	0.697	0.121	360283	A	G	-0.052	9	93556174	3.31E-06	0.011	14306	21.646
Vascular dementia (sudden onset)	genus Streptococcus	rs11110281	T	C	-0.403	100190236	0.137	0.271	360283	T	C	-0.138	12	100584014	2.58E-09	0.023	14306	36.572
Vascular dementia (sudden onset)	genus Streptococcus	rs11720390	G	A	0.082	94384747	0.737	0.243	360283	G	A	0.107	3	94103591	3.59E-06	0.023	14306	22.011
Vascular dementia (sudden onset)	genus Streptococcus	rs11764382	A	G	0.165	46735298	0.325	0.167	360283	A	G	-0.070	7	46774896	1.29E-06	0.014	14306	23.424
Vascular dementia (sudden onset)	genus Streptococcus	rs17708276	A	G	0.026	10342038	0.893	0.191	360283	A	G	-0.079	8	10199548	3.04E-06	0.017	14306	21.652
Vascular dementia (sudden onset)	genus Streptococcus	rs1918540	A	G	0.313	131779552	0.037	0.150	360283	A	G	-0.060	11	131649446	2.44E-06	0.013	14306	21.659
Vascular dementia (sudden onset)	genus Streptococcus	rs2370083	G	T	-0.011	97060413	0.965	0.241	360283	G	T	-0.082	14	97526750	9.75E-06	0.019	14306	19.317
Vascular dementia (sudden onset)	genus Streptococcus	rs57646748	G	A	-0.299	37451236	0.326	0.305	360283	G	A	-0.091	4	37452858	5.48E-06	0.020	14306	20.527
Vascular dementia (sudden onset)	genus Streptococcus	rs6806351	T	C	-0.282	132339879	0.046	0.142	360283	T	C	-0.063	3	132058723	4.94E-06	0.014	14306	21.515
Vascular dementia (sudden onset)	genus Streptococcus	rs71481756	T	G	0.202	8060441	0.396	0.238	360283	T	G	0.093	10	8102404	6.51E-06	0.021	14306	20.046
Vascular dementia (sudden onset)	genus Streptococcus	rs7916711	A	G	0.129	28299340	0.437	0.166	360283	A	G	0.103	10	28588269	2.72E-06	0.022	14306	22.407
Vascular dementia (sudden onset)	genus Subdoligranulum	rs10065321	T	C	0.106	142477850	0.371	0.118	360283	T	C	-0.051	5	141857415	2.10E-06	0.011	14306	22.504
Vascular dementia (sudden onset)	genus Subdoligranulum	rs10497836	T	C	0.002	199423995	0.988	0.141	360283	T	C	0.052	2	200288718	8.38E-06	0.012	14306	19.494
Vascular dementia (sudden onset)	genus Subdoligranulum	rs1667315	G	A	-0.063	231544032	0.600	0.119	360283	G	A	0.049	2	232408743	6.72E-06	0.011	14306	20.374
Vascular dementia (sudden onset)	genus Subdoligranulum	rs2114677	C	T	0.071	123713421	0.700	0.184	360283	C	T	-0.104	10	125472937	2.72E-06	0.023	14306	20.368
Vascular dementia (sudden onset)	genus Subdoligranulum	rs2171249	C	T	-0.075	153372894	0.741	0.226	360283	C	T	0.107	6	153694029	4.51E-06	0.023	14306	20.950
Vascular dementia (sudden onset)	genus Subdoligranulum	rs3761728	G	T	-0.012	48988868	0.927	0.134	360283	G	T	0.054	4	48990885	3.87E-06	0.012	14306	20.903
Vascular dementia (sudden onset)	genus Subdoligranulum	rs4347804	G	A	0.405	217351124	0.214	0.326	360283	G	A	-0.166	2	218215847	2.18E-06	0.036	14306	21.579
Vascular dementia (sudden onset)	genus Subdoligranulum	rs6555306	C	T	-0.150	4671270	0.362	0.165	360283	C	T	0.074	5	4671383	2.81E-06	0.016	14306	22.705
Vascular dementia (sudden onset)	genus Subdoligranulum	rs75158211	T	C	-0.079	29153787	0.630	0.164	360283	T	C	-0.072	19	29644694	7.52E-06	0.016	14306	20.616
Vascular dementia (sudden onset)	genus Subdoligranulum	rs76528319	G	T	0.071	118463753	0.730	0.207	360283	G	T	-0.143	5	117799448	7.41E-06	0.031	14306	21.271
Vascular dementia (sudden onset)	genus Sutterella	rs1145877	G	A	0.191	81677104	0.264	0.171	360283	G	A	0.074	6	82386821	7.20E-06	0.016	14306	20.507
Vascular dementia (sudden onset)	genus Sutterella	rs11591622	T	G	-0.333	100760930	0.037	0.160	360283	T	G	-0.069	10	102520687	6.50E-06	0.015	14306	20.680
Vascular dementia (sudden onset)	genus Sutterella	rs13173038	A	G	-0.016	59203357	0.906	0.136	360283	A	G	-0.072	5	58499183	2.73E-06	0.015	14306	22.428
Vascular dementia (sudden onset)	genus Sutterella	rs143438747	T	C	0.008	62812878	0.970	0.219	360283	T	C	-0.146	1	63278549	3.28E-06	0.031	14306	22.572
Vascular dementia (sudden onset)	genus Sutterella	rs2050185	A	G	-0.012	147615645	0.924	0.121	360283	A	G	-0.058	6	147936781	7.97E-06	0.013	14306	19.950
Vascular dementia (sudden onset)	genus Sutterella	rs2321387	G	A	-0.188	58115206	0.109	0.117	360283	G	A	-0.059	13	58689340	1.87E-06	0.012	14306	22.674
Vascular dementia (sudden onset)	genus Sutterella	rs2613606	T	C	-0.016	111644969	0.890	0.118	360283	T	C	0.056	7	111285025	7.20E-06	0.012	14306	20.125
Vascular dementia (sudden onset)	genus Sutterella	rs607327	T	C	0.005	111824985	0.965	0.119	360283	T	C	-0.058	11	111695709	6.63E-06	0.013	14306	20.083
Vascular dementia (sudden onset)	genus Sutterella	rs62501473	G	A	0.352	150429663	0.003	0.117	360283	G	A	0.069	7	150126751	5.52E-06	0.015	14306	21.588
Vascular dementia (sudden onset)	genus Sutterella	rs7499539	A	G	-0.013	85004459	0.923	0.131	360283	A	G	0.062	16	85038065	2.36E-06	0.013	14306	22.218
Vascular dementia (sudden onset)	genus Sutterella	rs7638039	T	C	-0.132	70539788	0.329	0.135	360283	T	C	0.065	3	70588939	8.66E-06	0.014	14306	20.133
Vascular dementia (sudden onset)	genus Sutterella	rs9350083	T	G	0.084	18583647	0.488	0.121	360283	T	G	-0.059	6	18583878	8.23E-06	0.013	14306	19.609
Vascular dementia (sudden onset)	genus Terrisporobacter	rs1883097	C	T	0.534	8917272	0.092	0.317	360283	C	T	0.226	11	8938819	4.16E-07	0.045	14306	24.798
Vascular dementia (sudden onset)	genus Terrisporobacter	rs2569953	C	A	-0.022	144430063	0.854	0.118	360283	C	A	0.078	3	144148905	8.95E-06	0.017	14306	19.723
Vascular dementia (sudden onset)	genus Terrisporobacter	rs2872237	A	C	0.199	16916130	0.094	0.119	360283	A	C	0.081	19	17026940	3.97E-06	0.018	14306	21.431
Vascular dementia (sudden onset)	genus Terrisporobacter	rs58405430	G	T	0.025	20599497	0.920	0.245	360283	G	T	0.135	11	20621043	7.94E-06	0.030	14306	19.978
Vascular dementia (sudden onset)	genus Terrisporobacter	rs7184125	T	C	0.117	15826537	0.366	0.129	360283	T	C	0.091	16	15920394	8.48E-06	0.021	14306	19.721
Vascular dementia (sudden onset)	genus Turicibacter	rs11054680	T	C	-0.202	12089521	0.188	0.153	360283	T	C	-0.105	12	12242455	2.31E-06	0.023	14306	21.295
Vascular dementia (sudden onset)	genus Turicibacter	rs11666533	C	T	-0.063	11754117	0.765	0.212	360283	C	T	-0.112	19	11864932	7.37E-06	0.025	14306	20.211
Vascular dementia (sudden onset)	genus Turicibacter	rs149744580	A	G	-0.076	63139827	0.774	0.265	360283	A	G	0.170	2	63366962	7.01E-08	0.032	14306	28.998
Vascular dementia (sudden onset)	genus Turicibacter	rs2834977	T	C	-0.114	35557345	0.484	0.163	360283	T	C	-0.096	21	36929643	3.96E-06	0.021	14306	21.248
Vascular dementia (sudden onset)	genus Turicibacter	rs2952020	A	G	0.027	26170326	0.839	0.135	360283	A	G	0.076	8	26027842	5.63E-06	0.017	14306	20.966
Vascular dementia (sudden onset)	genus Turicibacter	rs3734633	G	A	-0.396	125790735	0.114	0.251	360283	G	A	-0.121	6	126111881	5.32E-06	0.027	14306	20.325
Vascular dementia (sudden onset)	genus Turicibacter	rs4869133	G	A	-0.112	96381915	0.461	0.152	360283	G	A	0.131	5	95717619	2.55E-06	0.027	14306	23.267
Vascular dementia (sudden onset)	genus Turicibacter	rs55756211	T	C	0.177	131287420	0.419	0.219	360283	T	C	-0.115	7	130972179	2.81E-06	0.024	14306	22.871

Vascular dementia (sudden onset)	genus Turicibacter	rs7199484	G	A	0.166	49792388	0.186	0.126	360283	G	A	-0.073	16	49826299	5.77E-06	0.016	14306	20.853
Vascular dementia (sudden onset)	genus Tyzzerella3	rs10898797	C	T	0.202	87877806	0.267	0.182	360283	C	T	0.122	11	87588698	8.85E-06	0.027	14306	19.850
Vascular dementia (sudden onset)	genus Tyzzerella3	rs112102233	A	G	0.084	45602838	0.760	0.276	360283	A	G	-0.216	10	46098286	6.18E-06	0.048	14306	20.522
Vascular dementia (sudden onset)	genus Tyzzerella3	rs1232220	T	G	0.371	102232382	0.053	0.192	360283	T	G	0.144	6	102680257	7.91E-06	0.032	14306	20.432
Vascular dementia (sudden onset)	genus Tyzzerella3	rs17706273	T	C	0.008	16388150	0.970	0.215	360283	T	C	-0.140	5	16388259	5.88E-07	0.027	14306	26.109
Vascular dementia (sudden onset)	genus Tyzzerella3	rs191093	G	A	-0.036	76996549	0.848	0.190	360283	G	A	0.159	12	77390329	6.76E-06	0.035	14306	20.255
Vascular dementia (sudden onset)	genus Tyzzerella3	rs4904512	T	C	-0.226	89129601	0.194	0.174	360283	T	C	-0.117	14	89595945	3.09E-06	0.025	14306	21.905
Vascular dementia (sudden onset)	genus Tyzzerella3	rs55799124	A	G	0.045	3835487	0.733	0.133	360283	A	G	-0.114	17	3738781	1.34E-06	0.024	14306	22.968
Vascular dementia (sudden onset)	genus Tyzzerella3	rs67476743	T	G	0.141	1030321	0.278	0.130	360283	T	G	0.132	19	1030320	3.74E-09	0.022	14306	35.417
Vascular dementia (sudden onset)	genus Tyzzerella3	rs7019909	T	C	0.047	33113324	0.794	0.178	360283	T	C	0.144	9	33113322	1.76E-06	0.030	14306	22.842
Vascular dementia (sudden onset)	genus Tyzzerella3	rs7333521	T	C	0.157	81018881	0.615	0.312	360283	T	C	-0.207	13	81593016	4.88E-06	0.045	14306	20.908
Vascular dementia (sudden onset)	genus Tyzzerella3	rs75091807	G	T	-0.036	34449554	0.878	0.237	360283	G	T	-0.185	13	35023691	1.71E-06	0.038	14306	23.320
Vascular dementia (sudden onset)	genus Tyzzerella3	rs7561370	C	T	-0.071	57583271	0.662	0.163	360283	C	T	-0.131	2	57810406	1.52E-06	0.029	14306	21.047
Vascular dementia (sudden onset)	genus Veillonella	rs1882878	A	G	-0.095	28638351	0.455	0.127	360283	A	G	-0.077	21	30010673	2.98E-06	0.016	14306	22.010
Vascular dementia (sudden onset)	genus Veillonella	rs2013594	C	T	0.046	44280604	0.701	0.119	360283	C	T	0.072	11	44302154	3.42E-06	0.016	14306	21.577
Vascular dementia (sudden onset)	genus Veillonella	rs62376424	C	T	-0.006	120578590	0.965	0.126	360283	C	T	-0.076	5	119914285	3.65E-06	0.016	14306	21.733
Vascular dementia (sudden onset)	genus Veillonella	rs6656807	G	A	0.087	178975908	0.471	0.121	360283	G	A	-0.070	1	178945043	5.50E-06	0.015	14306	20.855
Vascular dementia (sudden onset)	genus Veillonella	rs742016	A	G	-0.033	45208919	0.789	0.124	360283	A	G	-0.069	22	45604800	4.66E-06	0.015	14306	21.137
Vascular dementia (sudden onset)	genus Victivallis	rs11899949	G	A	0.158	37821926	0.209	0.126	360283	G	A	0.131	2	38049069	2.77E-06	0.028	1531	22.341
Vascular dementia (sudden onset)	genus Victivallis	rs12512543	A	C	0.034	9606821	0.876	0.216	360283	A	C	-0.178	4	9608445	2.54E-06	0.037	1531	22.619
Vascular dementia (sudden onset)	genus Victivallis	rs173120	C	T	0.022	72302743	0.882	0.146	360283	C	T	-0.134	13	72876881	7.65E-06	0.029	1531	21.272
Vascular dementia (sudden onset)	genus Victivallis	rs1882775	A	G	0.105	39033681	0.488	0.151	360283	A	G	-0.138	21	40405606	8.73E-06	0.031	1531	19.549
Vascular dementia (sudden onset)	genus Victivallis	rs2546432	C	T	0.074	181152462	0.526	0.116	360283	C	T	0.111	5	180579462	9.93E-06	0.025	1531	19.698
Vascular dementia (sudden onset)	genus Victivallis	rs342302	A	G	0.042	106737783	0.808	0.171	360283	A	G	-0.153	7	106378229	8.16E-06	0.035	1531	18.878
Vascular dementia (sudden onset)	genus Victivallis	rs4764863	G	A	-0.164	102120406	0.162	0.117	360283	G	A	0.122	12	102514184	8.22E-07	0.025	1531	24.407
Vascular dementia (sudden onset)	genus Victivallis	rs4895919	C	T	-0.258	131309179	0.028	0.117	360283	C	T	0.117	6	131630319	2.75E-06	0.025	1531	22.313
Vascular dementia (sudden onset)	genus Victivallis	rs56349194	A	G	0.325	9291249	0.059	0.172	360283	A	G	-0.159	11	9312796	6.26E-07	0.032	1531	25.282
Vascular dementia (sudden onset)	genus Victivallis	rs911666	T	C	0.067	95604788	0.592	0.126	360283	T	C	-0.119	14	96071125	7.65E-06	0.026	1531	20.305
Vascular dementia (sudden onset)	order Actinomycetales	rs2889192	T	G	-0.023	73779652	0.888	0.164	360283	T	G	-0.088	9	76394568	3.97E-06	0.019	14306	20.554
Vascular dementia (sudden onset)	order Actinomycetales	rs34583783	G	T	-0.228	66497478	0.353	0.246	360283	G	T	0.124	6	67207371	5.54E-06	0.026	14306	21.908
Vascular dementia (sudden onset)	order Actinomycetales	rs35011108	A	G	-0.201	132686341	0.386	0.232	360283	A	G	0.242	6	133007480	1.88E-06	0.050	14306	22.987
Vascular dementia (sudden onset)	order Actinomycetales	rs4073240	G	A	0.089	168824686	0.456	0.120	360283	G	A	0.075	6	169224781	5.68E-06	0.016	14306	20.729
Vascular dementia (sudden onset)	order Bacillales	rs10233278	T	C	-0.044	117856090	0.706	0.118	360283	T	C	-0.116	7	117496144	3.51E-06	0.025	14306	21.906
Vascular dementia (sudden onset)	order Bacillales	rs10410917	C	T	0.090	14859629	0.448	0.119	360283	C	T	-0.115	19	14970441	5.57E-06	0.025	14306	21.049
Vascular dementia (sudden onset)	order Bacillales	rs11034576	A	G	0.035	38064161	0.838	0.173	360283	A	G	0.206	11	38085711	8.86E-06	0.045	14306	20.604
Vascular dementia (sudden onset)	order Bacillales	rs11207728	A	G	-0.089	61361862	0.574	0.158	360283	A	G	0.145	1	61827534	5.73E-06	0.032	14306	20.805
Vascular dementia (sudden onset)	order Bacillales	rs11844714	A	G	-0.262	48532023	0.070	0.145	360283	A	G	-0.143	14	49001226	5.06E-06	0.032	14306	20.004
Vascular dementia (sudden onset)	order Bacillales	rs1287018	G	A	-0.011	5845828	0.941	0.150	360283	G	A	0.141	20	5826474	9.87E-06	0.032	14306	19.534
Vascular dementia (sudden onset)	order Bacillales	rs4617108	G	A	-0.222	49384236	0.290	0.210	360283	G	A	-0.249	7	49423832	1.98E-06	0.053	14306	22.390
Vascular dementia (sudden onset)	order Bacillales	rs74420793	A	G	-0.264	126845554	0.163	0.189	360283	A	G	-0.164	4	127766709	3.07E-06	0.035	14306	21.600
Vascular dementia (sudden onset)	order Bacteroidales	rs11146701	A	G	-0.061	38769138	0.620	0.123	360283	A	G	0.047	10	39062269	7.08E-06	0.011	14306	20.186
Vascular dementia (sudden onset)	order Bacteroidales	rs17343978	A	C	-0.036	27037922	0.802	0.145	360283	A	C	-0.055	22	27433885	8.36E-06	0.012	14306	21.067
Vascular dementia (sudden onset)	order Bacteroidales	rs2032750	C	T	0.094	53603889	0.422	0.117	360283	C	T	0.051	2	53831026	1.92E-06	0.011	14306	22.657
Vascular dementia (sudden onset)	order Bacteroidales	rs2363574	T	C	0.542	200143435	0.079	0.309	360283	T	C	0.223	1	200112563	9.93E-06	0.051	14306	19.221
Vascular dementia (sudden onset)	order Bacteroidales	rs4916508	A	G	0.256	196209918	0.029	0.117	360283	A	G	0.047	3	195936789	8.47E-06	0.011	14306	19.641
Vascular dementia (sudden onset)	order Bacteroidales	rs55773148	G	A	0.258	69948897	0.314	0.256	360283	G	A	-0.122	13	70523029	3.90E-07	0.024	14306	26.341
Vascular dementia (sudden onset)	order Bacteroidales	rs62531359	T	G	0.140	70003946	0.362	0.153	360283	T	G	0.066	8	70916181	9.09E-06	0.015	14306	19.138
Vascular dementia (sudden onset)	order Bacteroidales	rs62575403	C	T	0.584	133628698	0.047	0.294	360283	C	T	0.140	9	136493820	7.06E-06	0.031	14306	20.264
Vascular dementia (sudden onset)	order Bacteroidales	rs72706335	T	C	-0.165	157525648	0.664	0.379	360283	T	C	-0.222	1	157495438	7.66E-06	0.049	14306	20.315
Vascular dementia (sudden onset)	order Bacteroidales	rs73975615	G	A	-1.030	6557880	0.144	0.705	360283	G	A	-0.207	17	6461200	1.22E-06	0.044	14306	21.874
Vascular dementia (sudden onset)	order Bacteroidales	rs7631304	G	A	-0.120	89290377	0.463	0.164	360283	G	A	-0.065	3	89339527	8.37E-07	0.013	14306	23.590
Vascular dementia (sudden onset)	order Bacteroidales	rs79585701	A	C	-0.021	13252676	0.899	0.168	360283	A	C	0.065	8	13110185	9.99E-06	0.015	14306	18.687
Vascular dementia (sudden onset)	order Bacteroidales	rs929878	T	C	-0.359	74256742	0.012	0.143	360283	T	C	0.055	16	74290641	4.73E-06	0.012	14306	20.372
Vascular dementia (sudden onset)	order Bifidobacteriales	rs10831953	G	A	0.146	13076504	0.248	0.126	360283	G	A	0.054	11	13098051	9.95E-06	0.012	14306	18.869
Vascular dementia (sudden onset)	order Bifidobacteriales	rs12446429	T	C	-0.044	848055	0.769	0.150	360283	T	C	0.081	16	898055	8.53E-06	0.019	14306	18.040
Vascular dementia (sudden onset)	order Bifidobacteriales	rs13020688	G	A	-0.001	192013806	0.991	0.126	360283	G	A	0.058	2	192878532	1.57E-06	0.012	14306	22.887
Vascular dementia (sudden onset)	order Bifidobacteriales	rs182549	T	C	0.090	135859184	0.454	0.120	360283	T	C	-0.117	2	136616754	5.94E-20	0.013	14306	85.372
Vascular dementia (sudden onset)	order Bifidobacteriales	rs4957061	T	C	0.274	520981	0.021	0.118	360283	T	C	0.057	5	521096	1.15E-06	0.012	14306	23.762
Vascular dementia (sudden onset)	order Bifidobacteriales	rs540489	T	G	-0.232	74901626	0.130	0.153	360283	T	G	-0.063	17	72897722	5.37E-06	0.014	14306	20.956
Vascular dementia (sudden onset)	order Bifidobacteriales	rs55888705	A	G	-0.113	1516099	0.382	0.129	360283	A	G	0.054	4	1517826	8.66E-06	0.012	14306	19.812
Vascular dementia (sudden onset)	order Bifidobacteriales	rs6899771	A	G	-0.102	96958344	0.599	0.194	360283	A	G	-0.091	6	97406220	7.28E-06	0.020	14306	20.365
Vascular dementia (sudden onset)	order Bifidobacteriales	rs7174549	T	C	-0.192	91920073	0.110	0.120	360283	T	C	-0.055	15	92463303	6.87E-06	0.012	14306	19.590
Vascular dementia (sudden onset)	order Bifidobacteriales	rs7322849	T	C	0.011	112205515	0.957	0.203	360283	T	C	0.111	13	112859829	1.74E-08	0.020	14306	30.320
Vascular dementia (sudden onset)	order Bifidobacteriales	rs857444	C	T	-0.124	14617360	0.303	0.120	360283	C	T	0.055	6	14617591	3.82E-06	0.012	14306	21.075
Vascular dementia (sudden onset)	order Burkholderiales	rs1511453	A	G	-0.054	23586839	0.830	0.252	360283	A	G	0.091	4	23588462	8.00E-06	0.020	14306	20.857
Vascular dementia (sudden onset)	order Burkholderiales	rs1928341	G	A	0.112	153267537	0.346	0.119	360283	G	A	-0.051	1	153240013	4.52E-06	0.011	14306	21.084
Vascular dementia (sudden onset)	order Burkholderiales	rs2321387	G	A	-0.188	58115206	0.109	0.117	360283	G	A	-0.051	13	58689340	3.26E-06	0.011	14306	21.532

Vascular dementia (sudden onset)	order Burkholderiales	rs2613606	T	C	-0.016	111644969	0.890	0.118	360283	T	C	0.050	7	111285025	4.13E-06	0.011	14306	20.904
Vascular dementia (sudden onset)	order Burkholderiales	rs4033856	T	C	-0.506	45640468	0.015	0.208	360283	T	C	-0.083	4	45642485	5.67E-07	0.017	14306	24.762
Vascular dementia (sudden onset)	order Burkholderiales	rs6087811	T	G	-0.039	32008327	0.839	0.194	360283	T	G	-0.102	20	30596130	2.88E-07	0.020	14306	26.108
Vascular dementia (sudden onset)	order Burkholderiales	rs62191117	A	G	0.142	238979079	0.331	0.146	360283	A	G	0.068	2	239900775	2.79E-07	0.013	14306	26.349
Vascular dementia (sudden onset)	order Burkholderiales	rs62395635	T	C	0.033	174070793	0.890	0.241	360283	T	C	0.110	5	173497796	2.90E-06	0.024	14306	21.621
Vascular dementia (sudden onset)	order Burkholderiales	rs75242906	C	T	-0.070	56494209	0.741	0.212	360283	C	T	-0.121	15	56786407	9.75E-06	0.028	14306	18.498
Vascular dementia (sudden onset)	order Burkholderiales	rs7638039	T	C	-0.132	70539788	0.329	0.135	360283	T	C	0.058	3	70588939	4.84E-06	0.013	14306	21.017
Vascular dementia (sudden onset)	order Clostridiales	rs10774377	G	A	0.150	5833353	0.203	0.118	360283	G	A	-0.052	12	5942519	3.81E-06	0.011	14306	21.100
Vascular dementia (sudden onset)	order Clostridiales	rs112334273	G	A	-0.053	39331325	0.682	0.130	360283	G	A	0.064	21	40703251	4.07E-07	0.013	14306	25.186
Vascular dementia (sudden onset)	order Clostridiales	rs13105690	C	T	0.131	7418457	0.311	0.130	360283	C	T	0.053	4	7420184	9.37E-06	0.012	14306	19.919
Vascular dementia (sudden onset)	order Clostridiales	rs13179700	C	T	-0.173	149698225	0.161	0.123	360283	C	T	-0.051	5	149077788	3.52E-06	0.011	14306	21.746
Vascular dementia (sudden onset)	order Clostridiales	rs1842454	G	A	0.202	105724661	0.177	0.149	360283	G	A	-0.054	5	105060362	9.92E-06	0.013	14306	18.228
Vascular dementia (sudden onset)	order Clostridiales	rs2273429	A	G	0.158	52027354	0.400	0.188	360283	A	G	-0.073	14	52494072	4.17E-06	0.015	14306	22.497
Vascular dementia (sudden onset)	order Clostridiales	rs290772	G	A	0.161	101509758	0.525	0.254	360283	G	A	0.084	2	102126220	1.00E-05	0.020	14306	18.423
Vascular dementia (sudden onset)	order Clostridiales	rs6442336	T	C	0.053	12829387	0.695	0.134	360283	T	C	0.055	3	12870886	9.63E-06	0.012	14306	19.458
Vascular dementia (sudden onset)	order Clostridiales	rs6814436	C	T	0.012	160586149	0.942	0.167	360283	C	T	-0.074	4	161507301	9.06E-07	0.015	14306	24.197
Vascular dementia (sudden onset)	order Clostridiales	rs6815608	C	T	-0.136	151210592	0.400	0.161	360283	C	T	-0.104	4	152131744	3.72E-07	0.021	14306	24.363
Vascular dementia (sudden onset)	order Clostridiales	rs72738886	T	C	-0.150	35770448	0.499	0.222	360283	T	C	0.087	5	35770550	8.42E-06	0.019	14306	20.657
Vascular dementia (sudden onset)	order Clostridiales	rs992074	T	C	-0.158	17195484	0.681	0.386	360283	T	C	-0.255	21	18567802	8.95E-07	0.051	14306	25.206
Vascular dementia (sudden onset)	order Coriobacteriales	rs11073596	G	T	-0.042	85890348	0.728	0.120	360283	G	T	-0.051	15	86433579	8.14E-06	0.011	14306	19.912
Vascular dementia (sudden onset)	order Coriobacteriales	rs11250875	T	C	0.192	1880537	0.175	0.141	360283	T	C	0.061	10	1922731	4.83E-06	0.013	14306	21.526
Vascular dementia (sudden onset)	order Coriobacteriales	rs11656361	A	C	-0.196	8218014	0.195	0.151	360283	A	C	0.077	17	8121332	8.02E-06	0.018	14306	19.394
Vascular dementia (sudden onset)	order Coriobacteriales	rs12974142	G	A	0.213	52391913	0.349	0.227	360283	G	A	0.079	19	52895166	8.51E-06	0.018	14306	19.865
Vascular dementia (sudden onset)	order Coriobacteriales	rs13307134	T	C	-0.140	105444233	0.372	0.156	360283	T	C	-0.057	7	105084680	7.80E-06	0.013	14306	20.072
Vascular dementia (sudden onset)	order Coriobacteriales	rs1397793	A	G	0.077	91175634	0.545	0.127	360283	A	G	0.050	5	90471451	9.77E-06	0.011	14306	19.682
Vascular dementia (sudden onset)	order Coriobacteriales	rs1816223	G	A	0.093	11341087	0.525	0.146	360283	G	A	0.059	12	11494021	4.84E-06	0.013	14306	20.652
Vascular dementia (sudden onset)	order Coriobacteriales	rs240104	T	C	-0.168	176602295	0.194	0.129	360283	T	C	-0.060	1	176571431	1.52E-06	0.013	14306	22.630
Vascular dementia (sudden onset)	order Coriobacteriales	rs2442778	A	G	-0.059	11612938	0.825	0.265	360283	A	G	0.116	3	11654412	9.03E-06	0.026	14306	20.272
Vascular dementia (sudden onset)	order Coriobacteriales	rs3025411	A	G	-0.237	133647784	0.204	0.187	360283	A	G	0.093	9	136512906	8.27E-06	0.021	14306	19.566
Vascular dementia (sudden onset)	order Coriobacteriales	rs34739816	G	T	0.373	39220432	0.125	0.243	360283	G	T	0.097	17	37376685	3.88E-06	0.021	14306	21.594
Vascular dementia (sudden onset)	order Coriobacteriales	rs67561917	A	G	-0.064	63440724	0.670	0.151	360283	A	G	-0.071	20	62072077	5.39E-06	0.015	14306	21.486
Vascular dementia (sudden onset)	order Coriobacteriales	rs719099	A	G	-0.094	64039457	0.627	0.193	360283	A	G	0.078	10	65799217	5.43E-07	0.016	14306	24.957
Vascular dementia (sudden onset)	order Coriobacteriales	rs8010111	A	G	0.009	39191305	0.965	0.207	360283	A	G	0.103	14	39660509	6.90E-06	0.023	14306	20.328
Vascular dementia (sudden onset)	order Desulfovibrionales	rs11599763	C	T	0.204	11813600	0.088	0.120	360283	C	T	0.055	10	11855599	2.61E-06	0.012	14306	22.305
Vascular dementia (sudden onset)	order Desulfovibrionales	rs17791387	A	G	-0.150	79219511	0.457	0.201	360283	A	G	-0.073	9	81834426	2.25E-06	0.015	14306	22.210
Vascular dementia (sudden onset)	order Desulfovibrionales	rs186073	T	C	-0.178	31091909	0.132	0.118	360283	T	C	0.053	3	31133401	8.74E-06	0.012	14306	19.931
Vascular dementia (sudden onset)	order Desulfovibrionales	rs2692012	G	A	0.212	204022477	0.407	0.256	360283	G	A	-0.112	1	203991605	2.27E-06	0.025	14306	19.597
Vascular dementia (sudden onset)	order Desulfovibrionales	rs2838334	G	A	-0.211	43645080	0.085	0.123	360283	G	A	0.057	21	45064961	4.17E-06	0.012	14306	20.982
Vascular dementia (sudden onset)	order Desulfovibrionales	rs3935584	C	T	-0.009	233064573	0.936	0.117	360283	C	T	-0.052	2	233929283	7.20E-06	0.012	14306	20.531
Vascular dementia (sudden onset)	order Desulfovibrionales	rs4506934	C	T	0.003	2953368	0.988	0.180	360283	C	T	-0.095	17	2856662	2.43E-06	0.020	14306	22.416
Vascular dementia (sudden onset)	order Desulfovibrionales	rs6058181	C	T	-0.017	35106998	0.911	0.156	360283	C	T	0.084	20	33694801	2.53E-07	0.017	14306	25.379
Vascular dementia (sudden onset)	order Desulfovibrionales	rs62020470	A	G	-0.341	95617836	0.025	0.152	360283	A	G	-0.057	15	96161065	7.51E-06	0.013	14306	19.633
Vascular dementia (sudden onset)	order Desulfovibrionales	rs72647048	T	C	0.036	56952819	0.845	0.185	360283	T	C	-0.077	8	57865378	9.00E-06	0.017	14306	20.436
Vascular dementia (sudden onset)	order Desulfovibrionales	rs9928243	C	A	0.026	71507738	0.823	0.117	360283	C	A	-0.054	16	71541641	3.97E-06	0.012	14306	21.378
Vascular dementia (sudden onset)	order Enterobacteriales	rs11026530	T	C	-0.078	22357551	0.633	0.164	360283	T	C	0.082	11	22379097	9.43E-06	0.019	14306	19.471
Vascular dementia (sudden onset)	order Enterobacteriales	rs2374342	C	A	0.010	41906402	0.932	0.119	360283	C	A	0.058	2	42133542	4.52E-06	0.013	14306	21.338
Vascular dementia (sudden onset)	order Enterobacteriales	rs35673018	G	A	-0.042	54293833	0.836	0.202	360283	G	A	0.090	16	54327745	7.63E-06	0.020	14306	19.653
Vascular dementia (sudden onset)	order Enterobacteriales	rs504442	T	G	-0.056	57478315	0.766	0.190	360283	T	G	0.084	18	55145547	5.17E-06	0.019	14306	19.728
Vascular dementia (sudden onset)	order Enterobacteriales	rs62210023	A	G	-0.002	56765036	0.989	0.123	360283	A	G	0.061	20	55340092	3.13E-06	0.013	14306	21.742
Vascular dementia (sudden onset)	order Enterobacteriales	rs78143293	A	G	-0.197	60005103	0.270	0.178	360283	A	G	-0.085	18	57672335	1.20E-06	0.017	14306	24.792
Vascular dementia (sudden onset)	order Enterobacteriales	rs79757635	C	A	-0.079	110188071	0.644	0.171	360283	C	A	0.076	13	110840418	9.32E-06	0.017	14306	19.615
Vascular dementia (sudden onset)	order Erysipelotrichales	rs1074800	G	A	-0.134	3002432	0.254	0.118	360283	G	A	-0.049	5	3002546	6.15E-06	0.011	14306	20.459
Vascular dementia (sudden onset)	order Erysipelotrichales	rs10781552	C	T	0.204	132083729	0.117	0.130	360283	C	T	-0.055	10	133897233	2.33E-06	0.012	14306	22.633
Vascular dementia (sudden onset)	order Erysipelotrichales	rs17530232	A	G	0.423	39811320	0.103	0.260	360283	A	G	0.103	13	40385457	2.79E-06	0.022	14306	21.042
Vascular dementia (sudden onset)	order Erysipelotrichales	rs1884466	C	T	0.097	63673525	0.408	0.117	360283	C	T	-0.048	1	64139196	9.53E-06	0.011	14306	19.760
Vascular dementia (sudden onset)	order Erysipelotrichales	rs2300774	A	G	-0.069	196066841	0.552	0.117	360283	A	G	-0.052	3	195793712	8.95E-07	0.011	14306	24.094
Vascular dementia (sudden onset)	order Erysipelotrichales	rs290833	T	G	-0.015	96991871	0.895	0.116	360283	T	G	-0.050	1	97457427	8.03E-06	0.011	14306	19.943
Vascular dementia (sudden onset)	order Erysipelotrichales	rs35161940	T	C	0.118	72331083	0.536	0.190	360283	T	C	-0.081	17	70327224	1.85E-06	0.017	14306	23.118
Vascular dementia (sudden onset)	order Erysipelotrichales	rs4078432	T	C	-0.046	48528003	0.767	0.155	360283	T	C	0.061	14	48997206	4.23E-06	0.013	14306	20.723
Vascular dementia (sudden onset)	order Erysipelotrichales	rs56970041	T	G	-0.088	79891267	0.718	0.243	360283	T	G	0.072	14	80357610	5.40E-06	0.016	14306	19.385
Vascular dementia (sudden onset)	order Erysipelotrichales	rs62504403	C	T	0.117	38946033	0.421	0.146	360283	C	T	0.068	8	38803551	1.12E-07	0.013	14306	28.371
Vascular dementia (sudden onset)	order Erysipelotrichales	rs7234058	T	C	0.114	5830508	0.575	0.203	360283	T	C	-0.095	18	5830507	9.12E-07	0.019	14306	23.744
Vascular dementia (sudden onset)	order Erysipelotrichales	rs7826267	G	T	0.063	3097430	0.793	0.238	360283	G	T	0.084	8	2954952	9.28E-06	0.020	14306	17.755
Vascular dementia (sudden onset)	order Erysipelotrichales	rs8003149	C	T	0.112	55689786	0.365	0.124	360283	C	T	0.054	14	56156504	4.08E-06	0.012	14306	21.248
Vascular dementia (sudden onset)	order Gastranaerophilales	rs11150282	T	C	-0.077	80459808	0.525	0.121	360283	T	C	0.098	16	80493705	7.36E-07	0.020	14306	24.834
Vascular dementia (sudden onset)	order Gastranaerophilales	rs113884518	T	C	0.227	24648999	0.546	0.376	360283	T	C	-0.206	9	24648997	7.74E-06	0.046	14306	20.446
Vascular dementia (sudden onset)	order Gastranaerophilales	rs28678345	T	C	0.243	55828967	0.376	0.275	360283	T	C	0.213	17	53906328	8.06E-06	0.047	14306	20.434

Vascular dementia (sudden onset)	order Gastranaerophilales	rs367480	A	G	0.209	2916401	0.091	0.123	360283	A	G	0.084	11	2937631	7.52E-06	0.019	14306	20.487
Vascular dementia (sudden onset)	order Gastranaerophilales	rs4129395	G	A	0.022	113213109	0.853	0.117	360283	G	A	0.090	9	115975389	1.22E-06	0.019	14306	23.826
Vascular dementia (sudden onset)	order Gastranaerophilales	rs789069	A	C	0.230	1008277	0.170	0.168	360283	A	C	-0.104	18	1008278	6.50E-06	0.023	14306	19.725
Vascular dementia (sudden onset)	order Gastranaerophilales	rs79790072	T	C	-0.069	100207478	0.835	0.334	360283	T	C	0.226	15	100747683	3.54E-06	0.049	14306	21.466
Vascular dementia (sudden onset)	order Gastranaerophilales	rs8028558	A	G	-0.081	61763554	0.504	0.121	360283	A	G	0.083	15	62055753	9.78E-06	0.019	14306	19.603
Vascular dementia (sudden onset)	order Gastranaerophilales	rs9864379	T	C	-0.170	14265449	0.295	0.162	360283	T	C	-0.161	3	14306949	4.66E-08	0.029	14306	30.065
Vascular dementia (sudden onset)	order Lactobacillales	rs11110282	A	G	-0.403	100191781	0.136	0.271	360283	A	G	-0.102	12	100585559	3.96E-06	0.022	14306	22.090
Vascular dementia (sudden onset)	order Lactobacillales	rs11627423	C	A	-0.117	32731417	0.331	0.120	360283	C	A	0.050	14	33200623	5.09E-06	0.011	14306	20.697
Vascular dementia (sudden onset)	order Lactobacillales	rs11730038	G	A	-0.211	97128348	0.100	0.128	360283	G	A	-0.061	4	98049499	5.10E-06	0.013	14306	22.006
Vascular dementia (sudden onset)	order Lactobacillales	rs12797734	T	C	-0.033	8310803	0.804	0.135	360283	T	C	0.057	11	8332350	7.77E-06	0.013	14306	20.198
Vascular dementia (sudden onset)	order Lactobacillales	rs1595463	C	A	0.026	230858942	0.824	0.117	360283	C	A	0.048	2	231723657	7.44E-06	0.011	14306	19.443
Vascular dementia (sudden onset)	order Lactobacillales	rs2370083	G	T	-0.011	97060413	0.965	0.241	360283	G	T	-0.081	14	97526750	8.33E-06	0.018	14306	19.802
Vascular dementia (sudden onset)	order Lactobacillales	rs2952251	G	A	-0.067	10285654	0.633	0.140	360283	G	A	0.063	8	10143164	3.36E-07	0.012	14306	25.684
Vascular dementia (sudden onset)	order Lactobacillales	rs34989881	A	G	0.537	51456601	0.055	0.280	360283	A	G	0.113	19	51959855	4.09E-06	0.025	14306	21.201
Vascular dementia (sudden onset)	order Lactobacillales	rs35344081	G	A	-0.017	941253	0.897	0.132	360283	G	A	0.064	16	991253	4.16E-07	0.013	14306	25.446
Vascular dementia (sudden onset)	order Lactobacillales	rs4028634	C	T	-0.004	42683631	0.972	0.121	360283	C	T	-0.053	17	40835649	1.35E-06	0.011	14306	23.428
Vascular dementia (sudden onset)	order Lactobacillales	rs57872228	C	T	0.154	200449677	0.394	0.181	360283	C	T	-0.069	1	200418805	2.58E-06	0.015	14306	21.933
Vascular dementia (sudden onset)	order Lactobacillales	rs74663707	C	T	-0.039	184653436	0.871	0.238	360283	C	T	0.098	3	184371224	8.40E-06	0.022	14306	19.137
Vascular dementia (sudden onset)	order Lactobacillales	rs77558518	A	G	0.027	174746168	0.892	0.202	360283	A	G	-0.106	5	174173171	1.67E-06	0.022	14306	22.695
Vascular dementia (sudden onset)	order Lactobacillales	rs78938557	T	C	0.571	36309977	0.106	0.353	360283	T	C	0.106	7	36349586	2.31E-06	0.023	14306	20.412
Vascular dementia (sudden onset)	order Lactobacillales	rs9581006	T	C	0.202	24399371	0.510	0.306	360283	T	C	-0.226	13	24973509	1.77E-06	0.047	14306	23.163
Vascular dementia (sudden onset)	order Methanobacteriales	rs10202904	G	T	-0.058	124682691	0.631	0.120	360283	G	T	0.122	2	125440268	3.01E-07	0.024	14306	26.762
Vascular dementia (sudden onset)	order Methanobacteriales	rs10424197	A	G	-0.099	45936063	0.465	0.135	360283	A	G	0.111	19	46439321	9.28E-06	0.025	14306	20.211
Vascular dementia (sudden onset)	order Methanobacteriales	rs4257531	G	A	0.024	2044483	0.900	0.193	360283	G	A	0.164	3	2086167	7.44E-06	0.036	14306	20.316
Vascular dementia (sudden onset)	order Methanobacteriales	rs6508769	C	T	0.180	28336853	0.273	0.164	360283	C	T	-0.154	19	28827760	8.23E-06	0.034	14306	19.856
Vascular dementia (sudden onset)	order Methanobacteriales	rs6776814	T	C	0.224	15011576	0.597	0.424	360283	T	C	-0.200	3	15053083	1.63E-06	0.041	14306	23.483
Vascular dementia (sudden onset)	order Methanobacteriales	rs73068003	G	T	0.231	10734305	0.242	0.197	360283	G	T	-0.158	7	10773932	8.45E-06	0.035	14306	20.206
Vascular dementia (sudden onset)	order Methanobacteriales	rs73457410	A	G	0.037	41382045	0.875	0.235	360283	A	G	0.215	13	41956181	1.41E-06	0.044	14306	24.316
Vascular dementia (sudden onset)	order Methanobacteriales	rs75208022	C	T	0.124	21185927	0.535	0.199	360283	C	T	-0.227	12	21338861	5.92E-06	0.049	14306	21.717
Vascular dementia (sudden onset)	order Methanobacteriales	rs894996	C	A	-0.222	103497150	0.328	0.227	360283	C	A	0.217	4	104418307	1.88E-06	0.045	14306	23.349
Vascular dementia (sudden onset)	order Mollicutes RF9	rs11779863	G	A	-0.071	15549321	0.658	0.161	360283	G	A	-0.077	8	15406830	6.69E-06	0.017	14306	20.146
Vascular dementia (sudden onset)	order Mollicutes RF9	rs12566890	T	G	-0.006	61385192	0.974	0.172	360283	T	G	-0.103	1	61850864	8.11E-06	0.024	14306	18.196
Vascular dementia (sudden onset)	order Mollicutes RF9	rs13100746	C	T	-0.035	166128718	0.762	0.117	360283	C	T	0.064	3	165846506	7.29E-06	0.014	14306	20.055
Vascular dementia (sudden onset)	order Mollicutes RF9	rs17235252	T	C	-0.202	82906721	0.256	0.178	360283	T	C	-0.122	7	82536037	2.16E-06	0.026	14306	22.808
Vascular dementia (sudden onset)	order Mollicutes RF9	rs3932485	C	T	0.246	184475694	0.040	0.119	360283	C	T	0.063	3	184193482	9.93E-06	0.014	14306	19.551
Vascular dementia (sudden onset)	order Mollicutes RF9	rs515984	C	T	-0.030	3479267	0.868	0.183	360283	C	T	0.088	10	3521459	6.61E-06	0.019	14306	21.078
Vascular dementia (sudden onset)	order Mollicutes RF9	rs638542	A	G	0.084	109302630	0.512	0.128	360283	A	G	0.071	1	109845252	5.17E-06	0.016	14306	20.204
Vascular dementia (sudden onset)	order Mollicutes RF9	rs74603314	T	C	-0.323	46050515	0.288	0.304	360283	T	C	0.231	14	46519718	2.28E-06	0.049	14306	22.207
Vascular dementia (sudden onset)	order Mollicutes RF9	rs7706512	A	G	0.034	17443545	0.774	0.117	360283	A	G	-0.066	5	17443654	2.27E-06	0.014	14306	22.372
Vascular dementia (sudden onset)	order Mollicutes RF9	rs7801843	A	G	-0.009	4066073	0.958	0.162	360283	A	G	-0.087	7	4105705	9.47E-06	0.019	14306	19.983
Vascular dementia (sudden onset)	order Mollicutes RF9	rs7853673	A	G	-0.045	113948961	0.698	0.117	360283	A	G	0.062	9	116711241	6.73E-06	0.014	14306	19.938
Vascular dementia (sudden onset)	order Mollicutes RF9	rs949341	A	G	-0.032	112516062	0.807	0.129	360283	A	G	-0.066	11	112386785	7.73E-06	0.015	14306	19.893
Vascular dementia (sudden onset)	order NB1n	rs11251024	G	A	0.211	2053532	0.110	0.132	360283	G	A	0.104	10	2095726	6.63E-07	0.021	14306	25.404
Vascular dementia (sudden onset)	order NB1n	rs11606187	A	G	0.158	91856434	0.343	0.167	360283	A	G	-0.155	11	91589600	3.31E-06	0.033	14306	22.432
Vascular dementia (sudden onset)	order NB1n	rs13385922	T	C	0.020	233172678	0.870	0.122	360283	T	C	0.093	2	234081324	3.97E-06	0.020	14306	21.311
Vascular dementia (sudden onset)	order NB1n	rs166849	A	G	-0.246	199914143	0.037	0.118	360283	A	G	-0.091	2	200778866	7.74E-06	0.020	14306	20.294
Vascular dementia (sudden onset)	order NB1n	rs2172426	T	C	0.137	18371511	0.245	0.118	360283	T	C	0.102	8	18229020	3.17E-07	0.020	14306	26.348
Vascular dementia (sudden onset)	order NB1n	rs267959	G	A	-0.044	10737690	0.735	0.130	360283	G	A	-0.099	5	10737802	2.62E-06	0.021	14306	22.231
Vascular dementia (sudden onset)	order NB1n	rs4383094	C	T	-0.188	81402631	0.260	0.167	360283	C	T	-0.149	15	81694972	4.28E-06	0.032	14306	21.646
Vascular dementia (sudden onset)	order NB1n	rs60775321	T	C	0.251	96775441	0.051	0.129	360283	T	C	-0.096	15	97318671	7.10E-06	0.021	14306	20.256
Vascular dementia (sudden onset)	order NB1n	rs72671304	T	C	0.354	38956767	0.106	0.219	360283	T	C	0.172	14	39425971	3.80E-06	0.037	14306	21.688
Vascular dementia (sudden onset)	order NB1n	rs7911787	G	T	0.115	96859811	0.722	0.325	360283	G	T	-0.223	10	98619568	3.39E-06	0.047	14306	22.504
Vascular dementia (sudden onset)	order NB1n	rs8126061	T	C	0.131	3424592	0.456	0.175	360283	T	C	-0.159	20	3405239	7.36E-06	0.035	14306	20.450
Vascular dementia (sudden onset)	order NB1n	rs9542068	T	C	0.078	69732699	0.527	0.123	360283	T	C	0.099	13	70306831	6.52E-06	0.022	14306	20.625
Vascular dementia (sudden onset)	order Pasteurellales	rs10965428	C	A	0.166	22718482	0.514	0.254	360283	C	A	-0.120	9	22718481	4.29E-06	0.026	14306	21.561
Vascular dementia (sudden onset)	order Pasteurellales	rs111582866	G	A	0.110	48708578	0.593	0.205	360283	G	A	-0.114	16	48742489	7.07E-06	0.026	14306	19.753
Vascular dementia (sudden onset)	order Pasteurellales	rs12050685	A	G	0.026	73185141	0.842	0.129	360283	A	G	-0.067	15	73477482	9.19E-06	0.015	14306	19.385
Vascular dementia (sudden onset)	order Pasteurellales	rs16970009	A	G	-0.266	34535582	0.543	0.437	360283	A	G	0.187	17	32862601	7.32E-06	0.043	14306	19.027
Vascular dementia (sudden onset)	order Pasteurellales	rs4822728	T	C	-0.199	26495842	0.086	0.116	360283	T	C	0.069	22	26891808	4.72E-06	0.015	14306	21.156
Vascular dementia (sudden onset)	order Pasteurellales	rs6972479	A	G	0.047	117278006	0.746	0.145	360283	A	G	-0.078	7	116918060	7.75E-06	0.018	14306	19.878
Vascular dementia (sudden onset)	order Pasteurellales	rs72756943	G	A	0.538	26531799	0.004	0.185	360283	G	A	0.140	5	26531908	3.35E-06	0.030	14306	21.308
Vascular dementia (sudden onset)	order Pasteurellales	rs73139353	A	C	-0.002	98253370	0.994	0.206	360283	A	C	-0.223	3	97972214	8.71E-06	0.048	14306	21.092
Vascular dementia (sudden onset)	order Pasteurellales	rs76022354	C	T	-0.069	92546628	0.794	0.265	360283	C	T	0.243	10	94306385	1.83E-06	0.050	14306	23.560
Vascular dementia (sudden onset)	order Pasteurellales	rs78909003	T	C	0.458	102887960	0.071	0.253	360283	T	C	-0.241	9	105650242	2.05E-06	0.050	14306	23.415
Vascular dementia (sudden onset)	order Pasteurellales	rs9382510	C	T	-0.134	55583693	0.314	0.133	360283	C	T	-0.088	6	55448491	2.48E-07	0.017	14306	26.921
Vascular dementia (sudden onset)	order Pasteurellales	rs9895850	T	C	-0.110	66538895	0.695	0.282	360283	T	C	-0.176	17	64535013	9.08E-06	0.041	14306	18.497
Vascular dementia (sudden onset)	order Pasteurellales	rs9938097	C	T	-0.084	84943783	0.484	0.119	360283	C	T	0.071	16	84977389	8.23E-06	0.016	14306	20.209

Vascular dementia (sudden onset)	order Rhodospirillales	rs1035406	G	A	0.073	120037042	0.687	0.182	360283	G	A	-0.115	5	119372737	4.07E-06	0.025	14306	21.401
Vascular dementia (sudden onset)	order Rhodospirillales	rs11591293	G	T	0.171	111660039	0.148	0.118	360283	G	T	0.072	10	113419797	4.69E-06	0.016	14306	20.876
Vascular dementia (sudden onset)	order Rhodospirillales	rs11630875	T	C	0.244	61483535	0.161	0.174	360283	T	C	0.095	15	61775734	3.70E-06	0.020	14306	21.865
Vascular dementia (sudden onset)	order Rhodospirillales	rs13336560	C	T	0.307	88487835	0.006	0.112	360283	C	T	-0.070	16	88554243	9.75E-06	0.016	14306	19.598
Vascular dementia (sudden onset)	order Rhodospirillales	rs1549633	A	C	-0.286	27945538	0.121	0.184	360283	A	C	0.100	5	27945645	3.88E-06	0.022	14306	21.163
Vascular dementia (sudden onset)	order Rhodospirillales	rs3730086	A	G	0.045	68281223	0.738	0.133	360283	A	G	0.080	5	67577051	7.98E-06	0.018	14306	20.051
Vascular dementia (sudden onset)	order Rhodospirillales	rs3754624	C	T	0.136	224769095	0.375	0.153	360283	C	T	0.094	2	225633812	2.68E-06	0.020	14306	22.511
Vascular dementia (sudden onset)	order Rhodospirillales	rs4278423	T	C	-0.173	2628361	0.477	0.243	360283	T	C	0.105	10	2670553	3.98E-06	0.023	14306	20.256
Vascular dementia (sudden onset)	order Rhodospirillales	rs61933850	G	A	-0.193	72745618	0.248	0.167	360283	G	A	0.165	12	73139398	7.00E-06	0.036	14306	20.888
Vascular dementia (sudden onset)	order Rhodospirillales	rs7001029	C	T	-0.211	130946157	0.299	0.203	360283	C	T	0.121	8	131958403	2.83E-06	0.026	14306	21.397
Vascular dementia (sudden onset)	order Rhodospirillales	rs76784716	A	G	-0.097	168176830	0.601	0.186	360283	A	G	0.136	2	169033340	1.31E-06	0.028	14306	22.881
Vascular dementia (sudden onset)	order Rhodospirillales	rs77304857	C	A	-0.243	133326860	0.098	0.147	360283	C	A	-0.100	4	134248015	6.02E-06	0.022	14306	20.157
Vascular dementia (sudden onset)	order Rhodospirillales	rs9813022	A	G	-0.181	13685237	0.132	0.120	360283	A	G	-0.083	3	13726736	3.07E-07	0.016	14306	26.093
Vascular dementia (sudden onset)	order Selenomonadales	rs1135612	G	A	0.160	75980359	0.262	0.142	360283	G	A	0.053	7	75609677	9.26E-06	0.012	14306	19.761
Vascular dementia (sudden onset)	order Selenomonadales	rs13086907	G	A	-0.239	142416275	0.089	0.140	360283	G	A	0.063	3	142135117	1.95E-06	0.013	14306	22.532
Vascular dementia (sudden onset)	order Selenomonadales	rs1643968	T	C	0.013	165839623	0.914	0.121	360283	T	C	-0.057	5	165266628	4.15E-07	0.011	14306	25.339
Vascular dementia (sudden onset)	order Selenomonadales	rs1649999	A	G	-0.043	78326315	0.830	0.202	360283	A	G	0.075	10	80086072	7.58E-06	0.017	14306	20.246
Vascular dementia (sudden onset)	order Selenomonadales	rs2834062	A	G	-0.024	33005177	0.851	0.127	360283	A	G	0.049	21	34377485	8.44E-06	0.011	14306	20.190
Vascular dementia (sudden onset)	order Selenomonadales	rs4463806	C	T	0.245	113838234	0.099	0.148	360283	C	T	0.054	10	115597993	7.81E-06	0.013	14306	17.681
Vascular dementia (sudden onset)	order Selenomonadales	rs4722181	T	G	-0.177	22777952	0.128	0.116	360283	T	G	0.050	7	22817571	2.00E-06	0.011	14306	22.452
Vascular dementia (sudden onset)	order Selenomonadales	rs60274479	T	C	0.035	21238604	0.810	0.147	360283	T	C	-0.066	16	21249925	1.16E-06	0.013	14306	24.182
Vascular dementia (sudden onset)	order Selenomonadales	rs61249479	A	C	0.096	122150629	0.554	0.162	360283	A	C	0.078	9	124912908	2.95E-06	0.017	14306	21.236
Vascular dementia (sudden onset)	order Selenomonadales	rs71405394	G	A	0.436	100704883	0.061	0.233	360283	G	A	-0.114	15	101245088	2.17E-06	0.024	14306	22.539
Vascular dementia (sudden onset)	order Selenomonadales	rs73232831	G	A	-0.153	17411803	0.626	0.314	360283	G	A	-0.152	4	17413426	1.87E-06	0.031	14306	23.242
Vascular dementia (sudden onset)	order Selenomonadales	rs9423647	G	A	-0.017	5537855	0.882	0.117	360283	G	A	0.048	10	5579818	6.06E-06	0.011	14306	20.628
Vascular dementia (sudden onset)	order Verrucomicrobiales	rs111862613	T	C	0.021	129825125	0.890	0.155	360283	T	C	0.091	12	130309670	3.74E-06	0.020	14306	21.252
Vascular dementia (sudden onset)	order Verrucomicrobiales	rs117107102	A	G	-0.459	51947265	0.094	0.274	360283	A	G	0.205	18	49473635	2.92E-06	0.043	14306	22.493
Vascular dementia (sudden onset)	order Verrucomicrobiales	rs11729256	T	C	-0.011	94106121	0.943	0.156	360283	T	C	0.075	4	95027272	6.73E-07	0.015	14306	24.928
Vascular dementia (sudden onset)	order Verrucomicrobiales	rs12908520	G	A	0.094	97027427	0.424	0.117	360283	G	A	0.062	15	97570657	2.17E-06	0.013	14306	22.341
Vascular dementia (sudden onset)	order Verrucomicrobiales	rs2602429	T	C	-0.330	81029544	0.013	0.133	360283	T	C	-0.075	16	81063149	2.58E-06	0.016	14306	22.863
Vascular dementia (sudden onset)	order Verrucomicrobiales	rs4242783	A	G	0.007	5022135	0.957	0.130	360283	A	G	-0.069	10	5064327	2.64E-06	0.015	14306	21.781
Vascular dementia (sudden onset)	order Verrucomicrobiales	rs4936098	G	A	0.119	130410772	0.327	0.122	360283	G	A	-0.065	11	130280667	1.12E-06	0.014	14306	22.786
Vascular dementia (sudden onset)	order Verrucomicrobiales	rs61779207	G	A	0.087	40608800	0.539	0.142	360283	G	A	-0.076	1	41074472	6.72E-06	0.017	14306	20.432
Vascular dementia (sudden onset)	order Verrucomicrobiales	rs74542928	T	C	-0.316	99623031	0.246	0.272	360283	T	C	0.112	4	100544188	1.63E-06	0.024	14306	22.508
Vascular dementia (sudden onset)	order Verrucomicrobiales	rs9349825	A	G	-0.075	56476683	0.609	0.147	360283	A	G	-0.070	6	56341481	2.54E-06	0.015	14306	22.898
Vascular dementia (sudden onset)	order Verrucomicrobiales	rs941682	G	A	-0.085	33280034	0.511	0.130	360283	G	A	-0.063	20	31867840	9.61E-06	0.014	14306	19.290
Vascular dementia (sudden onset)	order Victivallales	rs1002941	A	G	0.028	100702485	0.836	0.134	360283	A	G	-0.105	15	101242690	8.15E-06	0.023	14306	20.234
Vascular dementia (sudden onset)	order Victivallales	rs11770843	C	T	-0.098	147098287	0.432	0.125	360283	C	T	0.109	7	146795379	1.91E-06	0.023	14306	21.707
Vascular dementia (sudden onset)	order Victivallales	rs17114848	G	A	0.420	24917388	0.035	0.200	360283	G	A	0.152	15	25162535	4.06E-06	0.032	14306	22.073
Vascular dementia (sudden onset)	order Victivallales	rs2031282	A	G	0.056	20113040	0.711	0.152	360283	A	G	0.122	13	20687179	4.38E-06	0.027	14306	20.490
Vascular dementia (sudden onset)	order Victivallales	rs2825714	A	G	-0.179	19651652	0.246	0.154	360283	A	G	-0.137	21	21023966	1.72E-06	0.029	14306	22.568
Vascular dementia (sudden onset)	order Victivallales	rs62570196	C	T	-0.351	108323890	0.237	0.297	360283	C	T	-0.216	9	111086170	1.08E-06	0.044	14306	24.192
Vascular dementia (sudden onset)	order Victivallales	rs72640280	A	G	0.057	11883735	0.819	0.249	360283	A	G	0.220	1	11943792	5.18E-06	0.049	14306	20.513
Vascular dementia (sudden onset)	order Victivallales	rs77599476	A	G	-0.084	62762910	0.746	0.258	360283	A	G	0.230	20	61394262	1.86E-06	0.048	14306	23.002
Vascular dementia (sudden onset)	phylum Actinobacteria	rs11766971	C	T	0.090	155211037	0.443	0.117	360283	C	T	-0.048	7	155002747	9.40E-06	0.011	14306	20.023
Vascular dementia (sudden onset)	phylum Actinobacteria	rs12528285	C	T	0.128	92065492	0.491	0.186	360283	C	T	0.081	6	92775210	5.69E-06	0.018	14306	20.048
Vascular dementia (sudden onset)	phylum Actinobacteria	rs13192624	T	C	0.077	112808407	0.564	0.134	360283	T	C	-0.052	6	113129609	9.33E-06	0.012	14306	19.720
Vascular dementia (sudden onset)	phylum Actinobacteria	rs1397793	A	G	0.077	91175634	0.545	0.127	360283	A	G	0.052	5	90471451	3.74E-06	0.011	14306	21.832
Vascular dementia (sudden onset)	phylum Actinobacteria	rs4429415	C	T	0.116	212891467	0.319	0.116	360283	C	T	0.058	2	213756191	2.05E-07	0.011	14306	27.314
Vascular dementia (sudden onset)	phylum Actinobacteria	rs55888705	A	G	-0.113	1516099	0.382	0.129	360283	A	G	0.053	4	1517826	1.31E-06	0.011	14306	23.597
Vascular dementia (sudden onset)	phylum Actinobacteria	rs6496870	C	T	-0.193	91924192	0.108	0.120	360283	C	T	-0.051	15	92467422	4.62E-06	0.011	14306	20.142
Vascular dementia (sudden onset)	phylum Actinobacteria	rs6743026	T	C	0.151	101633121	0.307	0.148	360283	T	C	0.059	2	102249583	9.88E-06	0.013	14306	19.171
Vascular dementia (sudden onset)	phylum Actinobacteria	rs74037001	G	A	0.051	23377985	0.801	0.203	360283	G	A	-0.082	14	23847194	6.71E-07	0.017	14306	24.562
Vascular dementia (sudden onset)	phylum Actinobacteria	rs75211493	G	A	-0.067	5300454	0.762	0.222	360283	G	A	0.084	19	5300465	9.27E-06	0.018	14306	20.714
Vascular dementia (sudden onset)	phylum Actinobacteria	rs7570971	A	C	-0.076	135080336	0.527	0.120	360283	A	C	0.087	2	135837906	1.41E-14	0.011	14306	58.161
Vascular dementia (sudden onset)	phylum Actinobacteria	rs80124826	T	C	0.056	239014688	0.881	0.372	360283	T	C	-0.124	2	239936384	8.75E-06	0.028	14306	19.876
[truncated: 412,082 more chars]
