# Supplementary material for: Role of inflammatory cytokines and the gut microbiome in vascular dementia: insights from Mendelian randomization analysis
Source: Front Microbiol. 2024 Aug 23;15:1398618. doi: 10.3389/fmicb.2024.1398618 (PMC11380139; doi:10.3389/fmicb.2024.1398618)
Supplement: Supplementary file 1 [file Data_Sheet_1.zip › Supplementary Table S3.pdf]

Supplementary Table S3 Instrumental Variables related to exposures (6 subtypes of vascular dementia) and outcomes (inflammatory cytokines)

Outcome	Exposure	SNP	Outcome							Exposure								F-statistics
			Effect allele	Other allele	Beta	Pos	Pval	SE	Samplesize	Effect allele	Other allele	Beta	Chr	Pos	Pval	SE	Samplesize	
CTACK	Vascular dementia (multiple infarctions)	rs1454336	A	G	0.00	91873093	0.998	0.033	3631	A	G	-0.369	4	90951942	4.48415E-06	0.080	360612	13616.170
CTACK	Vascular dementia (multiple infarctions)	rs429358	C	T	0.02	45411941	0.457	0.031	3631	C	T	0.660	19	44908684	1.33506E-17	0.077	360612	52325.340
CTACK	Vascular dementia (multiple infarctions)	rs4716814	T	C	0.00	157723046	0.873	0.024	3631	T	C	-0.319	7	157930354	3.75656E-07	0.063	360612	19180.021
CTACK	Vascular dementia (multiple infarctions)	rs72822148	T	C	0.01	9742028	0.733	0.025	3631	T	C	-0.322	17	9838711	3.18831E-06	0.069	360612	17983.471
beta-nerve growth factor	Vascular dementia (multiple infarctions)	rs4716814	T	C	-0.01	157723046	0.671	0.024	3531	T	C	-0.319	7	157930354	3.75656E-07	0.063	360612	19180.021
Vascular endothelial growth factor	Vascular dementia (multiple infarctions)	rs143035739	T	C	0.02	144587790	0.760	0.052	7118	T	C	0.639	8	143505620	1.57899E-06	0.133	360612	11900.183
Vascular endothelial growth factor	Vascular dementia (multiple infarctions)	rs1454336	A	G	0.01	91873093	0.789	0.024	7118	A	G	-0.369	4	90951942	4.48415E-06	0.080	360612	13616.170
Vascular endothelial growth factor	Vascular dementia (multiple infarctions)	rs4716814	T	C	0.00	157723046	0.969	0.017	7118	T	C	-0.319	7	157930354	3.75656E-07	0.063	360612	19180.021
Vascular endothelial growth factor	Vascular dementia (multiple infarctions)	rs4725579	C	A	-0.01	139468213	0.609	0.021	7118	C	A	-0.379	7	139768414	3.58039E-06	0.082	360612	18391.668
Vascular endothelial growth factor	Vascular dementia (multiple infarctions)	rs72822148	T	C	0.00	9742028	0.866	0.018	7118	T	C	-0.322	17	9838711	3.18831E-06	0.069	360612	17983.471
Macrophage Migration Inhibitory Factor	Vascular dementia (multiple infarctions)	rs429358	C	T	-0.02	45411941	0.558	0.031	3494	C	T	0.660	19	44908684	1.33506E-17	0.077	360612	52325.340
Macrophage Migration Inhibitory Factor	Vascular dementia (multiple infarctions)	rs4725579	C	A	0.01	139468213	0.827	0.030	3494	C	A	-0.379	7	139768414	3.58039E-06	0.082	360612	18391.668
TRAIL	Vascular dementia (multiple infarctions)	rs429358	C	T	0.00	45411941	0.880	0.020	8186	C	T	0.660	19	44908684	1.33506E-17	0.077	360612	52325.340
TRAIL	Vascular dementia (multiple infarctions)	rs4725579	C	A	0.01	139468213	0.538	0.019	8186	C	A	-0.379	7	139768414	3.58039E-06	0.082	360612	18391.668
Tumor necrosis factor 1 alpha	Vascular dementia (multiple infarctions)	rs114671411	A	G	0.03	36780442	0.796	0.094	3454	A	G	0.807	3	36738951	6.2329E-07	0.162	360612	11273.084
Tumor necrosis factor 1 alpha	Vascular dementia (multiple infarctions)	rs143035739	T	C	-0.03	144587790	0.632	0.071	3454	T	C	0.639	8	143505620	1.57899E-06	0.133	360612	11900.183
Tumor necrosis factor 1 alpha	Vascular dementia (multiple infarctions)	rs429358	C	T	0.01	45411941	0.825	0.032	3454	C	T	0.660	19	44908684	1.33506E-17	0.077	360612	52325.340
Tumor necrosis factor 1 alpha	Vascular dementia (multiple infarctions)	rs4716814	T	C	-0.01	157723046	0.670	0.025	3454	T	C	-0.319	7	157930354	3.75656E-07	0.063	360612	19180.021
Tumor necrosis factor 1 alpha	Vascular dementia (multiple infarctions)	rs4725579	C	A	0.00	139468213	0.965	0.030	3454	C	A	-0.379	7	139768414	3.58039E-06	0.082	360612	18391.668
Tumor necrosis factor 1 alpha	Vascular dementia (multiple infarctions)	rs72822148	T	C	0.00	9742028	0.937	0.026	3454	T	C	-0.322	17	9838711	3.18831E-06	0.069	360612	17983.471
Stem cell growth factor beta	Vascular dementia (multiple infarctions)	rs4716814	T	C	0.00	157723046	0.917	0.024	3682	T	C	-0.319	7	157930354	3.75656E-07	0.063	360612	19180.021
Stem cell factor	Vascular dementia (multiple infarctions)	rs429358	C	T	0.02	45411941	0.445	0.020	8290	C	T	0.660	19	44908684	1.33506E-17	0.077	360612	52325.340
Stem cell factor	Vascular dementia (multiple infarctions)	rs4716814	T	C	-0.01	157723046	0.444	0.016	8290	T	C	-0.319	7	157930354	3.75656E-07	0.063	360612	19180.021
Stem cell factor	Vascular dementia (multiple infarctions)	rs4725579	C	A	-0.01	139468213	0.530	0.019	8290	C	A	-0.379	7	139768414	3.58039E-06	0.082	360612	18391.668
Interleukin-16	Vascular dementia (multiple infarctions)	rs429358	C	T	0.02	45411941	0.470	0.031	3483	C	T	0.660	19	44908684	1.33506E-17	0.077	360612	52325.340
Interleukin-16	Vascular dementia (multiple infarctions)	rs4716814	T	C	0.01	157723046	0.711	0.024	3483	T	C	-0.319	7	157930354	3.75656E-07	0.063	360612	19180.021
Interleukin-16	Vascular dementia (multiple infarctions)	rs4725579	C	A	-0.01	139468213	0.671	0.030	3483	C	A	-0.379	7	139768414	3.58039E-06	0.082	360612	18391.668
Interleukin-16	Vascular dementia (multiple infarctions)	rs72822148	T	C	-0.01	9742028	0.833	0.026	3483	T	C	-0.322	17	9838711	3.18831E-06	0.069	360612	17983.471
RANTES	Vascular dementia (multiple infarctions)	rs114671411	A	G	-0.02	36780442	0.867	0.092	3421	A	G	0.807	3	36738951	6.2329E-07	0.162	360612	11273.084
RANTES	Vascular dementia (multiple infarctions)	rs1454336	A	G	-0.01	91873093	0.884	0.034	3421	A	G	-0.369	4	90951942	4.48415E-06	0.080	360612	13616.170
RANTES	Vascular dementia (multiple infarctions)	rs429358	C	T	-0.02	45411941	0.575	0.032	3421	C	T	0.660	19	44908684	1.33506E-17	0.077	360612	52325.340
RANTES	Vascular dementia (multiple infarctions)	rs9861644	A	G	0.01	88637331	0.770	0.026	3421	A	G	0.323	3	88588181	4.84942E-06	0.071	360612	17137.658
Platelet-derived growth factor BB	Vascular dementia (multiple infarctions)	rs143035739	T	C	0.01	144587790	0.932	0.049	8293	T	C	0.639	8	143505620	1.57899E-06	0.133	360612	11900.183
Platelet-derived growth factor BB	Vascular dementia (multiple infarctions)	rs1454336	A	G	0.01	91873093	0.764	0.022	8293	A	G	-0.369	4	90951942	4.48415E-06	0.080	360612	13616.170
Platelet-derived growth factor BB	Vascular dementia (multiple infarctions)	rs429358	C	T	0.00	45411941	0.978	0.020	8293	C	T	0.660	19	44908684	1.33506E-17	0.077	360612	52325.340
Platelet-derived growth factor BB	Vascular dementia (multiple infarctions)	rs4716814	T	C	0.01	157723046	0.439	0.016	8293	T	C	-0.319	7	157930354	3.75656E-07	0.063	360612	19180.021
Platelet-derived growth factor BB	Vascular dementia (multiple infarctions)	rs72822148	T	C	-0.01	9742028	0.716	0.017	8293	T	C	-0.322	17	9838711	3.18831E-06	0.069	360612	17983.471
Platelet-derived growth factor BB	Vascular dementia (multiple infarctions)	rs9861644	A	G	0.00	88637331	0.919	0.017	8293	A	G	0.323	3	88588181	4.84942E-06	0.071	360612	17137.658
Macrophage inflammatory protein 1b	Vascular dementia (multiple infarctions)	rs143035739	T	C	0.03	144587790	0.533	0.049	8243	T	C	0.639	8	143505620	1.57899E-06	0.133	360612	11900.183
Macrophage inflammatory protein 1b	Vascular dementia (multiple infarctions)	rs4725579	C	A	-0.01	139468213	0.496	0.019	8243	C	A	-0.379	7	139768414	3.58039E-06	0.082	360612	18391.668
Macrophage inflammatory protein 1b	Vascular dementia (multiple infarctions)	rs72822148	T	C	-0.01	9742028	0.514	0.017	8243	T	C	-0.322	17	9838711	3.18831E-06	0.069	360612	17983.471
Macrophage inflammatory protein 1a	Vascular dementia (multiple infarctions)	rs143035739	T	C	0.01	144587790	0.750	0.071	3522	T	C	0.639	8	143505620	1.57899E-06	0.133	360612	11900.183
Macrophage inflammatory protein 1a	Vascular dementia (multiple infarctions)	rs4716814	T	C	-0.01	157723046	0.755	0.024	3522	T	C	-0.319	7	157930354	3.75656E-07	0.063	360612	19180.021
Monokine induced by gamma interferon	Vascular dementia (multiple infarctions)	rs114671411	A	G	0.00	36780442	0.986	0.090	3685	A	G	0.807	3	36738951	6.2329E-07	0.162	360612	11273.084
Monokine induced by gamma interferon	Vascular dementia (multiple infarctions)	rs429358	C	T	0.00	45411941	0.908	0.030	3685	C	T	0.660	19	44908684	1.33506E-17	0.077	360612	52325.340
Monokine induced by gamma interferon	Vascular dementia (multiple infarctions)	rs4725579	C	A	-0.01	139468213	0.813	0.029	3685	C	A	-0.379	7	139768414	3.58039E-06	0.082	360612	18391.668
Macrophage colony stimulating factor	Vascular dementia (multiple infarctions)	rs143035739	T	C	0.00	144587790	0.979	0.089	840	T	C	0.639	8	143505620	1.57899E-06	0.133	360612	11900.183
Macrophage colony stimulating factor	Vascular dementia (multiple infarctions)	rs1454336	A	G	0.00	91873093	0.914	0.040	840	A	G	-0.369	4	90951942	4.48415E-06	0.080	360612	13616.170
Monocyte chemoattractant protein-3	Vascular dementia (multiple infarctions)	rs72822148	T	C	-0.01	9742028	0.851	0.047	843	T	C	-0.322	17	9838711	3.18831E-06	0.069	360612	17983.471
Monocyte chemoattractant protein-3	Vascular dementia (multiple infarctions)	rs9861644	A	G	0.01	88637331	0.847	0.046	843	A	G	0.323	3	88588181	4.84942E-06	0.071	360612	17137.658
Monocyte chemoattractant protein-1	Vascular dementia (multiple infarctions)	rs143035739	T	C	0.03	144587790	0.535	0.049	8293	T	C	0.639	8	143505620	1.57899E-06	0.133	360612	11900.183
Monocyte chemoattractant protein-1	Vascular dementia (multiple infarctions)	rs429358	C	T	-0.01	45411941	0.570	0.020	8293	C	T	0.660	19	44908684	1.33506E-17	0.077	360612	52325.340
Monocyte chemoattractant protein-1	Vascular dementia (multiple infarctions)	rs4725579	C	A	0.01	139468213	0.635	0.019	8293	C	A	-0.379	7	139768414	3.58039E-06	0.082	360612	18391.668
Monocyte chemoattractant protein-1	Vascular dementia (multiple infarctions)	rs72822148	T	C	0.00	9742028	0.993	0.017	8293	T	C	-0.322	17	9838711	3.18831E-06	0.069	360612	17983.471
Interleukin-12p70	Vascular dementia (multiple infarctions)	rs143035739	T	C	0.02	144587790	0.765	0.049	8270	T	C	0.639	8	143505620	1.57899E-06	0.133	360612	11900.183
Interleukin-12p70	Vascular dementia (multiple infarctions)	rs1454336	A	G	-0.01	91873093	0.558	0.022	8270	A	G	-0.369	4	90951942	4.48415E-06	0.080	360612	13616.170
Interleukin-12p70	Vascular dementia (multiple infarctions)	rs4716814	T	C	0.00	157723046	0.976	0.016	8270	T	C	-0.319	7	157930354	3.75656E-07	0.063	360612	19180.021
Interleukin-12p70	Vascular dementia (multiple infarctions)	rs4725579	C	A	-0.01	139468213	0.731	0.019	8270	C	A	-0.379	7	139768414	3.58039E-06	0.082	360612	18391.668
Interleukin-12p70	Vascular dementia (multiple infarctions)	rs9861644	A	G	-0.01	88637331	0.737	0.017	8270	A	G	0.323	3	88588181	4.84942E-06	0.071	360612	17137.658
Interferon gamma-induced protein 10	Vascular dementia (multiple infarctions)	rs114671411	A	G	0.02	36780442	0.824	0.090	3685	A	G	0.807	3	36738951	6.2329E-07	0.162	360612	11273.084
Interferon gamma-induced protein 10	Vascular dementia (multiple infarctions)	rs429358	C	T	-0.02	45411941	0.602	0.030	3685	C	T	0.660	19	44908684	1.33506E-17	0.077	360612	52325.340
Interferon gamma-induced protein 10	Vascular dementia (multiple infarctions)	rs72822148	T	C	0.00	9742028	0.944	0.025	3685	T	C	-0.322	17	9838711	3.18831E-06	0.069	360612	17983.471
Interferon gamma-induced protein 10	Vascular dementia (multiple infarctions)	rs9861644	A	G	0.00	88637331	0.888	0.025	3685	A	G	0.323	3	88588181	4.84942E-06	0.071	360612	17137.658
Interleukin-17	Vascular dementia (multiple infarctions)	rs114671411	A	G	0.02	36780442	0.798	0.066	7760	A	G	0.807	3	36738951	6.2329E-07	0.162	360612	11273.084
Interleukin-17	Vascular dementia (multiple infarctions)	rs143035739	T	C	-0.02	144587790	0.738	0.050	7760	T	C	0.639	8	143505620	1.57899E-06	0.133	360612	11900.183

Interleukin-17	Vascular dementia (multiple infarctions)	rs1454336	A	G	0.00	91873093	0.961	0.022	7760	A	G	-0.369	4	90951942	4.48415E-06	0.080	360612	13616.170
Interleukin-17	Vascular dementia (multiple infarctions)	rs429358	C	T	0.03	45411941	0.223	0.021	7760	C	T	0.660	19	44908684	1.33506E-17	0.077	360612	52325.340
Interleukin-17	Vascular dementia (multiple infarctions)	rs4716814	T	C	-0.01	157723046	0.683	0.016	7760	T	C	-0.319	7	157930354	3.75656E-07	0.063	360612	19180.021
Interleukin-17	Vascular dementia (multiple infarctions)	rs4725579	C	A	0.01	139468213	0.695	0.020	7760	C	A	-0.379	7	139768414	3.58039E-06	0.082	360612	18391.668
Interleukin-17	Vascular dementia (multiple infarctions)	rs9861644	A	G	0.00	88637331	0.984	0.017	7760	A	G	0.323	3	88588181	4.84942E-06	0.071	360612	17137.658
Interleukin-13	Vascular dementia (multiple infarctions)	rs143035739	T	C	0.03	144587790	0.725	0.070	3557	T	C	0.639	8	143505620	1.57899E-06	0.133	360612	11900.183
Interleukin-13	Vascular dementia (multiple infarctions)	rs1454336	A	G	-0.01	91873093	0.687	0.033	3557	A	G	-0.369	4	90951942	4.48415E-06	0.080	360612	13616.170
Interleukin-10	Vascular dementia (multiple infarctions)	rs1454336	A	G	0.01	91873093	0.724	0.022	7681	A	G	-0.369	4	90951942	4.48415E-06	0.080	360612	13616.170
Interleukin-10	Vascular dementia (multiple infarctions)	rs4716814	T	C	0.01	157723046	0.637	0.017	7681	T	C	-0.319	7	157930354	3.75656E-07	0.063	360612	19180.021
Interleukin-10	Vascular dementia (multiple infarctions)	rs4725579	C	A	0.01	139468213	0.728	0.020	7681	C	A	-0.379	7	139768414	3.58039E-06	0.082	360612	18391.668
Interleukin-10	Vascular dementia (multiple infarctions)	rs72822148	T	C	0.00	9742028	0.970	0.018	7681	T	C	-0.322	17	9838711	3.18831E-06	0.069	360612	17983.471
Interleukin-10	Vascular dementia (multiple infarctions)	rs9861644	A	G	0.00	88637331	0.996	0.017	7681	A	G	0.323	3	88588181	4.84942E-06	0.071	360612	17137.658
Interleukin-8	Vascular dementia (multiple infarctions)	rs429358	C	T	0.01	45411941	0.760	0.031	3526	C	T	0.660	19	44908684	1.33506E-17	0.077	360612	52325.340
Interleukin-8	Vascular dementia (multiple infarctions)	rs4716814	T	C	-0.01	157723046	0.704	0.024	3526	T	C	-0.319	7	157930354	3.75656E-07	0.063	360612	19180.021
Interleukin-8	Vascular dementia (multiple infarctions)	rs4725579	C	A	0.00	139468213	0.898	0.030	3526	C	A	-0.379	7	139768414	3.58039E-06	0.082	360612	18391.668
Interleukin-8	Vascular dementia (multiple infarctions)	rs72822148	T	C	0.00	9742028	0.911	0.026	3526	T	C	-0.322	17	9838711	3.18831E-06	0.069	360612	17983.471
Interleukin-8	Vascular dementia (multiple infarctions)	rs9861644	A	G	0.00	88637331	0.993	0.025	3526	A	G	0.323	3	88588181	4.84942E-06	0.071	360612	17137.658
Interleukin-6	Vascular dementia (multiple infarctions)	rs114671411	A	G	0.00	36780442	0.960	0.063	8189	A	G	0.807	3	36738951	6.2329E-07	0.162	360612	11273.084
Interleukin-6	Vascular dementia (multiple infarctions)	rs1454336	A	G	-0.01	91873093	0.731	0.022	8189	A	G	-0.369	4	90951942	4.48415E-06	0.080	360612	13616.170
Interleukin-6	Vascular dementia (multiple infarctions)	rs429358	C	T	0.02	45411941	0.257	0.020	8189	C	T	0.660	19	44908684	1.33506E-17	0.077	360612	52325.340
Interleukin-6	Vascular dementia (multiple infarctions)	rs4716814	T	C	0.00	157723046	0.887	0.016	8189	T	C	-0.319	7	157930354	3.75656E-07	0.063	360612	19180.021
Interleukin-6	Vascular dementia (multiple infarctions)	rs4725579	C	A	-0.01	139468213	0.567	0.019	8189	C	A	-0.379	7	139768414	3.58039E-06	0.082	360612	18391.668
Interleukin-6	Vascular dementia (multiple infarctions)	rs72822148	T	C	-0.01	9742028	0.506	0.017	8189	T	C	-0.322	17	9838711	3.18831E-06	0.069	360612	17983.471
Interleukin-6	Vascular dementia (multiple infarctions)	rs9861644	A	G	0.00	88637331	0.947	0.017	8189	A	G	0.323	3	88588181	4.84942E-06	0.071	360612	17137.658
Interleukin-1-receptor antagonist	Vascular dementia (multiple infarctions)	rs114671411	A	G	0.00	36780442	0.979	0.093	3638	A	G	0.807	3	36738951	6.2329E-07	0.162	360612	11273.084
Interleukin-1-receptor antagonist	Vascular dementia (multiple infarctions)	rs143035739	T	C	-0.02	144587790	0.811	0.069	3638	T	C	0.639	8	143505620	1.57899E-06	0.133	360612	11900.183
Interleukin-1-receptor antagonist	Vascular dementia (multiple infarctions)	rs429358	C	T	-0.02	45411941	0.454	0.031	3638	C	T	0.660	19	44908684	1.33506E-17	0.077	360612	52325.340
Interleukin-1-receptor antagonist	Vascular dementia (multiple infarctions)	rs72822148	T	C	0.00	9742028	0.889	0.025	3638	T	C	-0.322	17	9838711	3.18831E-06	0.069	360612	17983.471
Interleukin-1-receptor antagonist	Vascular dementia (multiple infarctions)	rs9861644	A	G	0.00	88637331	0.913	0.025	3638	A	G	0.323	3	88588181	4.84942E-06	0.071	360612	17137.658
Interleukin-1-beta	Vascular dementia (multiple infarctions)	rs114671411	A	G	0.00	36780442	0.588	0.073	3309	A	G	0.807	3	36738951	6.2329E-07	0.162	360612	11273.084
Interleukin-1-beta	Vascular dementia (multiple infarctions)	rs1454336	A	G	0.00	91873093	0.916	0.026	3309	A	G	-0.369	4	90951942	4.48415E-06	0.080	360612	13616.170
Interleukin-1-beta	Vascular dementia (multiple infarctions)	rs429358	C	T	0.00	45411941	0.471	0.024	3309	C	T	0.660	19	44908684	1.33506E-17	0.077	360612	52325.340
Interleukin-1-beta	Vascular dementia (multiple infarctions)	rs9861644	A	G	0.01	88637331	0.674	0.020	3309	A	G	0.323	3	88588181	4.84942E-06	0.071	360612	17137.658
Hepatocyte growth factor	Vascular dementia (multiple infarctions)	rs114671411	A	G	-0.04	36780442	0.559	0.063	8292	A	G	0.807	3	36738951	6.2329E-07	0.162	360612	11273.084
Hepatocyte growth factor	Vascular dementia (multiple infarctions)	rs143035739	T	C	-0.01	144587790	0.932	0.049	8292	T	C	0.639	8	143505620	1.57899E-06	0.133	360612	11900.183
Hepatocyte growth factor	Vascular dementia (multiple infarctions)	rs1454336	A	G	0.00	91873093	0.900	0.022	8292	A	G	-0.369	4	90951942	4.48415E-06	0.080	360612	13616.170
Hepatocyte growth factor	Vascular dementia (multiple infarctions)	rs429358	C	T	-0.01	45411941	0.703	0.020	8292	C	T	0.660	19	44908684	1.33506E-17	0.077	360612	52325.340
Hepatocyte growth factor	Vascular dementia (multiple infarctions)	rs4716814	T	C	0.00	157723046	0.893	0.016	8292	T	C	-0.319	7	157930354	3.75656E-07	0.063	360612	19180.021
Hepatocyte growth factor	Vascular dementia (multiple infarctions)	rs4725579	C	A	-0.01	139468213	0.503	0.019	8292	C	A	-0.379	7	139768414	3.58039E-06	0.082	360612	18391.668
Interleukin-9	Vascular dementia (multiple infarctions)	rs114671411	A	G	0.02	36780442	0.856	0.093	3634	A	G	0.807	3	36738951	6.2329E-07	0.162	360612	11273.084
Interleukin-9	Vascular dementia (multiple infarctions)	rs1454336	A	G	0.00	91873093	0.946	0.033	3634	A	G	-0.369	4	90951942	4.48415E-06	0.080	360612	13616.170
Interleukin-9	Vascular dementia (multiple infarctions)	rs429358	C	T	-0.02	45411941	0.541	0.031	3634	C	T	0.660	19	44908684	1.33506E-17	0.077	360612	52325.340
Interleukin-9	Vascular dementia (multiple infarctions)	rs9861644	A	G	0.00	88637331	0.986	0.025	3634	A	G	0.323	3	88588181	4.84942E-06	0.071	360612	17137.658
Interleukin-7	Vascular dementia (multiple infarctions)	rs143035739	T	C	-0.01	144587790	0.848	0.071	3409	T	C	0.639	8	143505620	1.57899E-06	0.133	360612	11900.183
Interleukin-7	Vascular dementia (multiple infarctions)	rs1454336	A	G	-0.01	91873093	0.874	0.034	3409	A	G	-0.369	4	90951942	4.48415E-06	0.080	360612	13616.170
Interleukin-7	Vascular dementia (multiple infarctions)	rs4716814	T	C	0.00	157723046	0.938	0.025	3409	T	C	-0.319	7	157930354	3.75656E-07	0.063	360612	19180.021
Interleukin-5	Vascular dementia (multiple infarctions)	rs429358	C	T	0.02	45411941	0.555	0.032	3364	C	T	0.660	19	44908684	1.33506E-17	0.077	360612	52325.340
Interleukin-5	Vascular dementia (multiple infarctions)	rs4725579	C	A	0.01	139468213	0.711	0.030	3364	C	A	-0.379	7	139768414	3.58039E-06	0.082	360612	18391.668
Interleukin-4	Vascular dementia (multiple infarctions)	rs114671411	A	G	0.02	36780442	0.756	0.063	8124	A	G	0.807	3	36738951	6.2329E-07	0.162	360612	11273.084
Interleukin-4	Vascular dementia (multiple infarctions)	rs1454336	A	G	0.01	91873093	0.583	0.022	8124	A	G	-0.369	4	90951942	4.48415E-06	0.080	360612	13616.170
Interleukin-4	Vascular dementia (multiple infarctions)	rs429358	C	T	0.02	45411941	0.305	0.021	8124	C	T	0.660	19	44908684	1.33506E-17	0.077	360612	52325.340
Interleukin-4	Vascular dementia (multiple infarctions)	rs4716814	T	C	0.00	157723046	0.759	0.016	8124	T	C	-0.319	7	157930354	3.75656E-07	0.063	360612	19180.021
Interleukin-2 receptor antagonist	Vascular dementia (multiple infarctions)	rs143035739	T	C	-0.02	144587790	0.889	0.070	3677	T	C	0.639	8	143505620	1.57899E-06	0.133	360612	11900.183
Interleukin-2 receptor antagonist	Vascular dementia (multiple infarctions)	rs429358	C	T	-0.01	45411941	0.762	0.030	3677	C	T	0.660	19	44908684	1.33506E-17	0.077	360612	52325.340
Interleukin-2 receptor antagonist	Vascular dementia (multiple infarctions)	rs72822148	T	C	-0.01	9742028	0.795	0.025	3677	T	C	-0.322	17	9838711	3.18831E-06	0.069	360612	17983.471
Interleukin-2 receptor antagonist	Vascular dementia (multiple infarctions)	rs9861644	A	G	-0.01	88637331	0.839	0.025	3677	A	G	0.323	3	88588181	4.84942E-06	0.071	360612	17137.658
Interleukin-2	Vascular dementia (multiple infarctions)	rs114671411	A	G	0.04	36780442	0.740	0.096	3475	A	G	0.807	3	36738951	6.2329E-07	0.162	360612	11273.084
Interleukin-2	Vascular dementia (multiple infarctions)	rs1454336	A	G	0.01	91873093	0.786	0.034	3475	A	G	-0.369	4	90951942	4.48415E-06	0.080	360612	13616.170
Interleukin-2	Vascular dementia (multiple infarctions)	rs4716814	T	C	-0.01	157723046	0.661	0.024	3475	T	C	-0.319	7	157930354	3.75656E-07	0.063	360612	19180.021
Interleukin-2	Vascular dementia (multiple infarctions)	rs72822148	T	C	-0.01	9742028	0.765	0.026	3475	T	C	-0.322	17	9838711	3.18831E-06	0.069	360612	17983.471
Interferon gamma	Vascular dementia (multiple infarctions)	rs114671411	A	G	0.01	36780442	0.886	0.064	7701	A	G	0.807	3	36738951	6.2329E-07	0.162	360612	11273.084
Interferon gamma	Vascular dementia (multiple infarctions)	rs1454336	A	G	0.00	91873093	0.849	0.023	7701	A	G	-0.369	4	90951942	4.48415E-06	0.080	360612	13616.170
Interferon gamma	Vascular dementia (multiple infarctions)	rs4716814	T	C	-0.01	157723046	0.617	0.017	7701	T	C	-0.319	7	157930354	3.75656E-07	0.063	360612	19180.021
Interferon gamma	Vascular dementia (multiple infarctions)	rs4725579	C	A	0.00	139468213	0.836	0.020	7701	C	A	-0.379	7	139768414	3.58039E-06	0.082	360612	18391.668
Interferon gamma	Vascular dementia (multiple infarctions)	rs72822148	T	C	0.00	9742028	0.953	0.018	7701	T	C	-0.322	17	9838711	3.18831E-06	0.069	360612	17983.471
Interferon gamma	Vascular dementia (multiple infarctions)	rs9861644	A	G	-0.01	88637331	0.543	0.017	7701	A	G	0.323	3	88588181	4.84942E-06	0.071	360612	17137.658
Growth-regulated protein alpha	Vascular dementia (multiple infarctions)	rs143035739	T	C	0.02	144587790	0.768	0.070	3505	T	C	0.639	8	143505620	1.57899E-06	0.133	360612	11900.183
Growth-regulated protein alpha	Vascular dementia (multiple infarctions)	rs1454336	A	G	-0.01	91873093	0.699	0.034	3505	A	G	-0.369	4	90951942	4.48415E-06	0.080	360612	13616.170

Growth-regulated protein alpha	Vascular dementia (multiple infarctions)	rs4716814	T	C	-0.01	157723046	0.736	0.024	3505	T	C	-0.319	7	157930354	3.75656E-07	0.063	360612	19180.021
Growth-regulated protein alpha	Vascular dementia (multiple infarctions)	rs4725579	C	A	-0.01	139468213	0.741	0.030	3505	C	A	-0.379	7	139768414	3.58039E-06	0.082	360612	18391.668
Granulocyte-colony stimulating factor	Vascular dementia (multiple infarctions)	rs143035739	T	C	0.01	144587790	0.819	0.050	7904	T	C	0.639	8	143505620	1.57899E-06	0.133	360612	11900.183
Granulocyte-colony stimulating factor	Vascular dementia (multiple infarctions)	rs1454336	A	G	0.01	91873093	0.807	0.022	7904	A	G	-0.369	4	90951942	4.48415E-06	0.080	360612	13616.170
Granulocyte-colony stimulating factor	Vascular dementia (multiple infarctions)	rs72822148	T	C	0.00	9742028	0.983	0.017	7904	T	C	-0.322	17	9838711	3.18831E-06	0.069	360612	17983.471
Granulocyte-colony stimulating factor	Vascular dementia (multiple infarctions)	rs9861644	A	G	0.00	88637331	0.804	0.017	7904	A	G	0.323	3	88588181	4.84942E-06	0.071	360612	17137.658
Fibroblast growth factor basic	Vascular dementia (multiple infarctions)	rs114671411	A	G	0.04	36780442	0.602	0.066	7565	A	G	0.807	3	36738951	6.2329E-07	0.162	360612	11273.084
Fibroblast growth factor basic	Vascular dementia (multiple infarctions)	rs143035739	T	C	-0.02	144587790	0.720	0.051	7565	T	C	0.639	8	143505620	1.57899E-06	0.133	360612	11900.183
Fibroblast growth factor basic	Vascular dementia (multiple infarctions)	rs4716814	T	C	0.00	157723046	0.963	0.017	7565	T	C	-0.319	7	157930354	3.75656E-07	0.063	360612	19180.021
Fibroblast growth factor basic	Vascular dementia (multiple infarctions)	rs72822148	T	C	0.01	9742028	0.500	0.018	7565	T	C	-0.322	17	9838711	3.18831E-06	0.069	360612	17983.471
Eotaxin	Vascular dementia (multiple infarctions)	rs114671411	A	G	-0.02	36780442	0.772	0.063	8153	A	G	0.807	3	36738951	6.2329E-07	0.162	360612	11273.084
Eotaxin	Vascular dementia (multiple infarctions)	rs143035739	T	C	-0.01	144587790	0.928	0.050	8153	T	C	0.639	8	143505620	1.57899E-06	0.133	360612	11900.183
Eotaxin	Vascular dementia (multiple infarctions)	rs1454336	A	G	0.01	91873093	0.752	0.022	8153	A	G	-0.369	4	90951942	4.48415E-06	0.080	360612	13616.170
Eotaxin	Vascular dementia (multiple infarctions)	rs429358	C	T	0.00	45411941	0.845	0.020	8153	C	T	0.660	19	44908684	1.33506E-17	0.077	360612	52325.340
Eotaxin	Vascular dementia (multiple infarctions)	rs4716814	T	C	-0.01	157723046	0.564	0.016	8153	T	C	-0.319	7	157930354	3.75656E-07	0.063	360612	19180.021
Eotaxin	Vascular dementia (multiple infarctions)	rs4725579	C	A	0.00	139468213	0.821	0.019	8153	C	A	-0.379	7	139768414	3.58039E-06	0.082	360612	18391.668
CTACK	Vascular dementia (mixed)	rs1466525	T	C	-0.01	54780209	0.693	0.028	3631	T	C	0.489	8	53867649	1.97697E-06	0.103	360421	34189.173
CTACK	Vascular dementia (mixed)	rs1632064	T	C	-0.01	3219694	0.845	0.034	3631	T	C	0.614	5	3219580	4.04045E-06	0.133	360421	37296.871
CTACK	Vascular dementia (mixed)	rs17168895	T	G	0.00	15647727	0.951	0.030	3631	T	G	-0.547	7	15608102	2.92853E-06	0.117	360421	34495.049
beta-nerve growth factor	Vascular dementia (mixed)	rs1466525	T	C	0.01	54780209	0.727	0.029	3531	T	C	0.489	8	53867649	1.97697E-06	0.103	360421	34189.173
beta-nerve growth factor	Vascular dementia (mixed)	rs1632064	T	C	-0.01	3219694	0.740	0.034	3531	T	C	0.614	5	3219580	4.04045E-06	0.133	360421	37296.871
beta-nerve growth factor	Vascular dementia (mixed)	rs6849229	G	A	0.00	131797280	0.927	0.032	3531	G	A	0.562	4	130876125	4.44038E-06	0.122	360421	34142.711
beta-nerve growth factor	Vascular dementia (mixed)	rs9458673	A	G	-0.03	158276983	0.702	0.071	3531	A	G	0.873	6	157855951	8.24727E-07	0.177	360421	17688.642
Vascular endothelial growth factor	Vascular dementia (mixed)	rs12257900	T	G	-0.01	49443428	0.676	0.023	7118	T	G	0.467	10	48235385	2.48891E-06	0.099	360421	23006.877
Vascular endothelial growth factor	Vascular dementia (mixed)	rs1466525	T	C	0.01	54780209	0.574	0.020	7118	T	C	0.489	8	53867649	1.97697E-06	0.103	360421	34189.173
Vascular endothelial growth factor	Vascular dementia (mixed)	rs1632064	T	C	-0.01	3219694	0.693	0.025	7118	T	C	0.614	5	3219580	4.04045E-06	0.133	360421	37296.871
Vascular endothelial growth factor	Vascular dementia (mixed)	rs17168895	T	G	0.00	15647727	0.870	0.022	7118	T	G	-0.547	7	15608102	2.92853E-06	0.117	360421	34495.049
Vascular endothelial growth factor	Vascular dementia (mixed)	rs9458673	A	G	0.00	158276983	0.938	0.052	7118	A	G	0.873	6	157855951	8.24727E-07	0.177	360421	17688.642
Macrophage Migration Inhibitory Factor	Vascular dementia (mixed)	rs12257900	T	G	0.00	49443428	0.829	0.033	3494	T	G	0.467	10	48235385	2.48891E-06	0.099	360421	23006.877
Macrophage Migration Inhibitory Factor	Vascular dementia (mixed)	rs9458673	A	G	0.00	158276983	0.990	0.071	3494	A	G	0.873	6	157855951	8.24727E-07	0.177	360421	17688.642
TRAIL	Vascular dementia (mixed)	rs1632064	T	C	0.01	3219694	0.490	0.023	8186	T	C	0.614	5	3219580	4.04045E-06	0.133	360421	37296.871
TRAIL	Vascular dementia (mixed)	rs429358	C	T	0.00	45411941	0.880	0.020	8186	C	T	0.565	19	44908684	3.94357E-08	0.103	360421	36831.461
TRAIL	Vascular dementia (mixed)	rs6028529	A	G	0.01	38205487	0.505	0.019	8186	A	G	0.413	20	39576844	4.98965E-06	0.091	360421	21913.090
TRAIL	Vascular dementia (mixed)	rs6849229	G	A	0.00	131797280	0.942	0.021	8186	G	A	0.562	4	130876125	4.44038E-06	0.122	360421	34142.711
Tumor necrosis factor beta levels	Vascular dementia (mixed)	rs1466525	T	C	0.00	54780209	0.917	0.043	1559	T	C	0.489	8	53867649	1.97697E-06	0.103	360421	34189.173
Tumor necrosis factor beta levels	Vascular dementia (mixed)	rs1632064	T	C	0.01	3219694	0.870	0.054	1559	T	C	0.614	5	3219580	4.04045E-06	0.133	360421	37296.871
Tumor necrosis factor beta levels	Vascular dementia (mixed)	rs6849229	G	A	0.00	131797280	0.917	0.049	1559	G	A	0.562	4	130876125	4.44038E-06	0.122	360421	34142.711
Tumor necrosis factor 1 alpha	Vascular dementia (mixed)	rs1466525	T	C	0.01	54780209	0.803	0.029	3454	T	C	0.489	8	53867649	1.97697E-06	0.103	360421	34189.173
Tumor necrosis factor 1 alpha	Vascular dementia (mixed)	rs429358	C	T	0.01	45411941	0.825	0.032	3454	C	T	0.565	19	44908684	3.94357E-08	0.103	360421	36831.461
Tumor necrosis factor 1 alpha	Vascular dementia (mixed)	rs6849229	G	A	-0.01	131797280	0.825	0.033	3454	G	A	0.562	4	130876125	4.44038E-06	0.122	360421	34142.711
Tumor necrosis factor 1 alpha	Vascular dementia (mixed)	rs9458673	A	G	0.00	158276983	0.978	0.072	3454	A	G	0.873	6	157855951	8.24727E-07	0.177	360421	17688.642
Stromal-cell-derived factor 1 alpha	Vascular dementia (mixed)	rs12257900	T	G	-0.01	49443428	0.655	0.022	5998	T	G	0.467	10	48235385	2.48891E-06	0.099	360421	23006.877
Stromal-cell-derived factor 1 alpha	Vascular dementia (mixed)	rs6028529	A	G	0.01	38205487	0.820	0.020	5998	A	G	0.413	20	39576844	4.98965E-06	0.091	360421	21913.090
Stem cell growth factor beta	Vascular dementia (mixed)	rs1466525	T	C	0.01	54780209	0.629	0.028	3682	T	C	0.489	8	53867649	1.97697E-06	0.103	360421	34189.173
Stem cell growth factor beta	Vascular dementia (mixed)	rs1632064	T	C	-0.01	3219694	0.823	0.033	3682	T	C	0.614	5	3219580	4.04045E-06	0.133	360421	37296.871
Stem cell growth factor beta	Vascular dementia (mixed)	rs17168895	T	G	-0.01	15647727	0.617	0.030	3682	T	G	-0.547	7	15608102	2.92853E-06	0.117	360421	34495.049
Stem cell growth factor beta	Vascular dementia (mixed)	rs9458673	A	G	-0.02	158276983	0.798	0.070	3682	A	G	0.873	6	157855951	8.24727E-07	0.177	360421	17688.642
Stem cell factor	Vascular dementia (mixed)	rs1632064	T	C	-0.01	3219694	0.710	0.023	8290	T	C	0.614	5	3219580	4.04045E-06	0.133	360421	37296.871
Stem cell factor	Vascular dementia (mixed)	rs429358	C	T	0.02	45411941	0.445	0.020	8290	C	T	0.565	19	44908684	3.94357E-08	0.103	360421	36831.461
Interleukin-16	Vascular dementia (mixed)	rs1466525	T	C	0.01	54780209	0.855	0.029	3483	T	C	0.489	8	53867649	1.97697E-06	0.103	360421	34189.173
Interleukin-16	Vascular dementia (mixed)	rs6028529	A	G	0.01	38205487	0.837	0.029	3483	A	G	0.413	20	39576844	4.98965E-06	0.091	360421	21913.090
Interleukin-16	Vascular dementia (mixed)	rs9458673	A	G	0.03	158276983	0.664	0.071	3483	A	G	0.873	6	157855951	8.24727E-07	0.177	360421	17688.642
RANTES	Vascular dementia (mixed)	rs1466525	T	C	0.01	54780209	0.847	0.029	3421	T	C	0.489	8	53867649	1.97697E-06	0.103	360421	34189.173
Platelet-derived growth factor BB	Vascular dementia (mixed)	rs12257900	T	G	0.01	49443428	0.817	0.021	8293	T	G	0.467	10	48235385	2.48891E-06	0.099	360421	23006.877
Platelet-derived growth factor BB	Vascular dementia (mixed)	rs1466525	T	C	0.00	54780209	0.941	0.019	8293	T	C	0.489	8	53867649	1.97697E-06	0.103	360421	34189.173
Platelet-derived growth factor BB	Vascular dementia (mixed)	rs1632064	T	C	-0.01	3219694	0.689	0.023	8293	T	C	0.614	5	3219580	4.04045E-06	0.133	360421	37296.871
Platelet-derived growth factor BB	Vascular dementia (mixed)	rs17168895	T	G	-0.01	15647727	0.718	0.020	8293	T	G	-0.547	7	15608102	2.92853E-06	0.117	360421	34495.049
Platelet-derived growth factor BB	Vascular dementia (mixed)	rs429358	C	T	0.00	45411941	0.978	0.020	8293	C	T	0.565	19	44908684	3.94357E-08	0.103	360421	36831.461
Platelet-derived growth factor BB	Vascular dementia (mixed)	rs9458673	A	G	-0.02	158276983	0.732	0.048	8293	A	G	0.873	6	157855951	8.24727E-07	0.177	360421	17688.642
Macrophage inflammatory protein 1b	Vascular dementia (mixed)	rs12257900	T	G	-0.01	49443428	0.661	0.021	8243	T	G	0.467	10	48235385	2.48891E-06	0.099	360421	23006.877
Macrophage inflammatory protein 1b	Vascular dementia (mixed)	rs1466525	T	C	0.00	54780209	0.843	0.019	8243	T	C	0.489	8	53867649	1.97697E-06	0.103	360421	34189.173
Macrophage inflammatory protein 1b	Vascular dementia (mixed)	rs1632064	T	C	-0.01	3219694	0.582	0.023	8243	T	C	0.614	5	3219580	4.04045E-06	0.133	360421	37296.871
Macrophage inflammatory protein 1b	Vascular dementia (mixed)	rs6028529	A	G	0.00	38205487	0.869	0.019	8243	A	G	0.413	20	39576844	4.98965E-06	0.091	360421	21913.090
Macrophage inflammatory protein 1a	Vascular dementia (mixed)	rs12257900	T	G	0.01	49443428	0.792	0.033	3522	T	G	0.467	10	48235385	2.48891E-06	0.099	360421	23006.877
Macrophage inflammatory protein 1a	Vascular dementia (mixed)	rs17168895	T	G	0.01	15647727	0.747	0.031	3522	T	G	-0.547	7	15608102	2.92853E-06	0.117	360421	34495.049
Macrophage inflammatory protein 1a	Vascular dementia (mixed)	rs6849229	G	A	0.01	131797280	0.801	0.032	3522	G	A	0.562	4	130876125	4.44038E-06	0.122	360421	34142.711
Monokine induced by gamma interferon	Vascular dementia (mixed)	rs17168895	T	G	-0.01	15647727	0.753	0.030	3685	T	G	-0.547	7	15608102	2.92853E-06	0.117	360421	34495.049
Monokine induced by gamma interferon	Vascular dementia (mixed)	rs429358	C	T	0.00	45411941	0.908	0.030	3685	C	T	0.565	19	44908684	3.94357E-08	0.103	360421	36831.461

Monokine induced by gamma interferon	Vascular dementia (mixed)	rs6849229	G	A	0.00	131797280	0.918	0.032	3685	G	A	0.562	4	130876125	4.44038E-06	0.122	360421	34142.711
Macrophage colony stimulating factor	Vascular dementia (mixed)	rs12257900	T	G	0.00	49443428	0.975	0.040	840	T	G	0.467	10	48235385	2.48891E-06	0.099	360421	23006.877
Macrophage colony stimulating factor	Vascular dementia (mixed)	rs1632064	T	C	0.01	3219694	0.869	0.041	840	T	C	0.614	5	3219580	4.04045E-06	0.133	360421	37296.871
Macrophage colony stimulating factor	Vascular dementia (mixed)	rs9458673	A	G	-0.01	158276983	0.873	0.085	840	A	G	0.873	6	157855951	8.24727E-07	0.177	360421	17688.642
Monocyte chemoattractant protein-3	Vascular dementia (mixed)	rs6028529	A	G	-0.01	38205487	0.885	0.054	843	A	G	0.413	20	39576844	4.98965E-06	0.091	360421	21913.090
Monocyte chemoattractant protein-3	Vascular dementia (mixed)	rs9458673	A	G	0.00	158276983	0.981	0.127	843	A	G	0.873	6	157855951	8.24727E-07	0.177	360421	17688.642
Monocyte chemoattractant protein-1	Vascular dementia (mixed)	rs12257900	T	G	-0.01	49443428	0.486	0.021	8293	T	G	0.467	10	48235385	2.48891E-06	0.099	360421	23006.877
Monocyte chemoattractant protein-1	Vascular dementia (mixed)	rs429358	C	T	-0.01	45411941	0.570	0.020	8293	C	T	0.565	19	44908684	3.94357E-08	0.103	360421	36831.461
Monocyte chemoattractant protein-1	Vascular dementia (mixed)	rs6028529	A	G	0.01	38205487	0.657	0.019	8293	A	G	0.413	20	39576844	4.98965E-06	0.091	360421	21913.090
Interleukin-12p70	Vascular dementia (mixed)	rs12257900	T	G	-0.01	49443428	0.613	0.021	8270	T	G	0.467	10	48235385	2.48891E-06	0.099	360421	23006.877
Interleukin-12p70	Vascular dementia (mixed)	rs1466525	T	C	0.01	54780209	0.643	0.019	8270	T	C	0.489	8	53867649	1.97697E-06	0.103	360421	34189.173
Interleukin-12p70	Vascular dementia (mixed)	rs1632064	T	C	-0.01	3219694	0.618	0.023	8270	T	C	0.614	5	3219580	4.04045E-06	0.133	360421	37296.871
Interleukin-12p70	Vascular dementia (mixed)	rs17168895	T	G	0.00	15647727	0.909	0.020	8270	T	G	-0.547	7	15608102	2.92853E-06	0.117	360421	34495.049
Interleukin-12p70	Vascular dementia (mixed)	rs6028529	A	G	0.00	38205487	0.809	0.019	8270	A	G	0.413	20	39576844	4.98965E-06	0.091	360421	21913.090
Interleukin-12p70	Vascular dementia (mixed)	rs9458673	A	G	-0.01	158276983	0.775	0.048	8270	A	G	0.873	6	157855951	8.24727E-07	0.177	360421	17688.642
Interferon gamma-induced protein 10	Vascular dementia (mixed)	rs429358	C	T	-0.02	45411941	0.602	0.030	3685	C	T	0.565	19	44908684	3.94357E-08	0.103	360421	36831.461
Interferon gamma-induced protein 10	Vascular dementia (mixed)	rs6028529	A	G	0.00	38205487	0.949	0.029	3685	A	G	0.413	20	39576844	4.98965E-06	0.091	360421	21913.090
Interleukin-18 levels	Vascular dementia (mixed)	rs6028529	A	G	-0.01	38205487	0.760	0.029	3636	A	G	0.413	20	39576844	4.98965E-06	0.091	360421	21913.090
Interleukin-17	Vascular dementia (mixed)	rs12257900	T	G	0.00	49443428	0.808	0.022	7760	T	G	0.467	10	48235385	2.48891E-06	0.099	360421	23006.877
Interleukin-17	Vascular dementia (mixed)	rs1466525	T	C	-0.01	54780209	0.735	0.019	7760	T	C	0.489	8	53867649	1.97697E-06	0.103	360421	34189.173
Interleukin-17	Vascular dementia (mixed)	rs1632064	T	C	-0.01	3219694	0.584	0.024	7760	T	C	0.614	5	3219580	4.04045E-06	0.133	360421	37296.871
Interleukin-17	Vascular dementia (mixed)	rs17168895	T	G	0.01	15647727	0.640	0.021	7760	T	G	-0.547	7	15608102	2.92853E-06	0.117	360421	34495.049
Interleukin-17	Vascular dementia (mixed)	rs6849229	G	A	0.00	131797280	0.956	0.022	7760	G	A	0.562	4	130876125	4.44038E-06	0.122	360421	34142.711
Interleukin-13	Vascular dementia (mixed)	rs9458673	A	G	0.03	158276983	0.618	0.071	3557	A	G	0.873	6	157855951	8.24727E-07	0.177	360421	17688.642
Interleukin-10	Vascular dementia (mixed)	rs6028529	A	G	0.00	38205487	0.838	0.020	7681	A	G	0.413	20	39576844	4.98965E-06	0.091	360421	21913.090
Interleukin-8	Vascular dementia (mixed)	rs17168895	T	G	0.00	15647727	0.886	0.031	3526	T	G	-0.547	7	15608102	2.92853E-06	0.117	360421	34495.049
Interleukin-8	Vascular dementia (mixed)	rs429358	C	T	0.01	45411941	0.760	0.031	3526	C	T	0.565	19	44908684	3.94357E-08	0.103	360421	36831.461
Interleukin-6	Vascular dementia (mixed)	rs12257900	T	G	-0.01	49443428	0.643	0.021	8189	T	G	0.467	10	48235385	2.48891E-06	0.099	360421	23006.877
Interleukin-6	Vascular dementia (mixed)	rs1466525	T	C	0.01	54780209	0.722	0.019	8189	T	C	0.489	8	53867649	1.97697E-06	0.103	360421	34189.173
Interleukin-6	Vascular dementia (mixed)	rs1632064	T	C	-0.01	3219694	0.671	0.023	8189	T	C	0.614	5	3219580	4.04045E-06	0.133	360421	37296.871
Interleukin-6	Vascular dementia (mixed)	rs17168895	T	G	0.01	15647727	0.595	0.020	8189	T	G	-0.547	7	15608102	2.92853E-06	0.117	360421	34495.049
Interleukin-6	Vascular dementia (mixed)	rs6028529	A	G	0.00	38205487	0.933	0.019	8189	A	G	0.413	20	39576844	4.98965E-06	0.091	360421	21913.090
Interleukin-1-receptor antagonist	Vascular dementia (mixed)	rs1466525	T	C	-0.01	54780209	0.817	0.028	3638	T	C	0.489	8	53867649	1.97697E-06	0.103	360421	34189.173
Interleukin-1-receptor antagonist	Vascular dementia (mixed)	rs1632064	T	C	0.01	3219694	0.645	0.034	3638	T	C	0.614	5	3219580	4.04045E-06	0.133	360421	37296.871
Interleukin-1-receptor antagonist	Vascular dementia (mixed)	rs17168895	T	G	0.00	15647727	0.976	0.031	3638	T	G	-0.547	7	15608102	2.92853E-06	0.117	360421	34495.049
Interleukin-1-receptor antagonist	Vascular dementia (mixed)	rs6849229	G	A	0.01	131797280	0.902	0.032	3638	G	A	0.562	4	130876125	4.44038E-06	0.122	360421	34142.711
Interleukin-1-receptor antagonist	Vascular dementia (mixed)	rs9458673	A	G	0.01	158276983	0.824	0.070	3638	A	G	0.873	6	157855951	8.24727E-07	0.177	360421	17688.642
Interleukin-1-beta	Vascular dementia (mixed)	rs429358	C	T	0.00	45411941	0.471	0.024	3309	C	T	0.565	19	44908684	3.94357E-08	0.103	360421	36831.461
Hepatocyte growth factor	Vascular dementia (mixed)	rs429358	C	T	-0.01	45411941	0.703	0.020	8292	C	T	0.565	19	44908684	3.94357E-08	0.103	360421	36831.461
Interleukin-9	Vascular dementia (mixed)	rs12257900	T	G	-0.01	49443428	0.842	0.033	3634	T	G	0.467	10	48235385	2.48891E-06	0.099	360421	23006.877
Interleukin-9	Vascular dementia (mixed)	rs1466525	T	C	0.00	54780209	0.869	0.028	3634	T	C	0.489	8	53867649	1.97697E-06	0.103	360421	34189.173
Interleukin-9	Vascular dementia (mixed)	rs17168895	T	G	0.01	15647727	0.655	0.031	3634	T	G	-0.547	7	15608102	2.92853E-06	0.117	360421	34495.049
Interleukin-7	Vascular dementia (mixed)	rs6028529	A	G	0.01	38205487	0.703	0.030	3409	A	G	0.413	20	39576844	4.98965E-06	0.091	360421	21913.090
Interleukin-5	Vascular dementia (mixed)	rs12257900	T	G	0.02	49443428	0.660	0.034	3364	T	G	0.467	10	48235385	2.48891E-06	0.099	360421	23006.877
Interleukin-5	Vascular dementia (mixed)	rs1466525	T	C	-0.01	54780209	0.676	0.029	3364	T	C	0.489	8	53867649	1.97697E-06	0.103	360421	34189.173
Interleukin-5	Vascular dementia (mixed)	rs6028529	A	G	0.01	38205487	0.805	0.030	3364	A	G	0.413	20	39576844	4.98965E-06	0.091	360421	21913.090
Interleukin-5	Vascular dementia (mixed)	rs9458673	A	G	0.00	158276983	0.971	0.074	3364	A	G	0.873	6	157855951	8.24727E-07	0.177	360421	17688.642
Interleukin-4	Vascular dementia (mixed)	rs12257900	T	G	0.00	49443428	0.933	0.021	8124	T	G	0.467	10	48235385	2.48891E-06	0.099	360421	23006.877
Interleukin-4	Vascular dementia (mixed)	rs1466525	T	C	0.00	54780209	0.964	0.019	8124	T	C	0.489	8	53867649	1.97697E-06	0.103	360421	34189.173
Interleukin-4	Vascular dementia (mixed)	rs17168895	T	G	0.01	15647727	0.608	0.021	8124	T	G	-0.547	7	15608102	2.92853E-06	0.117	360421	34495.049
Interleukin-4	Vascular dementia (mixed)	rs6028529	A	G	0.01	38205487	0.546	0.019	8124	A	G	0.413	20	39576844	4.98965E-06	0.091	360421	21913.090
Interleukin-4	Vascular dementia (mixed)	rs6849229	G	A	0.00	131797280	0.841	0.021	8124	G	A	0.562	4	130876125	4.44038E-06	0.122	360421	34142.711
Interleukin-4	Vascular dementia (mixed)	rs9458673	A	G	0.02	158276983	0.700	0.048	8124	A	G	0.873	6	157855951	8.24727E-07	0.177	360421	17688.642
Interleukin-2 receptor antagonist	Vascular dementia (mixed)	rs1632064	T	C	0.01	3219694	0.754	0.034	3677	T	C	0.614	5	3219580	4.04045E-06	0.133	360421	37296.871
Interleukin-2 receptor antagonist	Vascular dementia (mixed)	rs17168895	T	G	-0.01	15647727	0.654	0.030	3677	T	G	-0.547	7	15608102	2.92853E-06	0.117	360421	34495.049
Interleukin-2 receptor antagonist	Vascular dementia (mixed)	rs429358	C	T	-0.01	45411941	0.762	0.030	3677	C	T	0.565	19	44908684	3.94357E-08	0.103	360421	36831.461
Interleukin-2	Vascular dementia (mixed)	rs1466525	T	C	0.00	54780209	0.911	0.029	3475	T	C	0.489	8	53867649	1.97697E-06	0.103	360421	34189.173
Interleukin-2	Vascular dementia (mixed)	rs6028529	A	G	0.01	38205487	0.734	0.029	3475	A	G	0.413	20	39576844	4.98965E-06	0.091	360421	21913.090
Interferon gamma	Vascular dementia (mixed)	rs1466525	T	C	0.00	54780209	0.815	0.020	7701	T	C	0.489	8	53867649	1.97697E-06	0.103	360421	34189.173
Interferon gamma	Vascular dementia (mixed)	rs1632064	T	C	-0.01	3219694	0.709	0.024	7701	T	C	0.614	5	3219580	4.04045E-06	0.133	360421	37296.871
Interferon gamma	Vascular dementia (mixed)	rs9458673	A	G	0.00	158276983	0.944	0.050	7701	A	G	0.873	6	157855951	8.24727E-07	0.177	360421	17688.642
Growth-regulated protein alpha	Vascular dementia (mixed)	rs1466525	T	C	-0.01	54780209	0.735	0.029	3505	T	C	0.489	8	53867649	1.97697E-06	0.103	360421	34189.173
Growth-regulated protein alpha	Vascular dementia (mixed)	rs9458673	A	G	-0.03	158276983	0.721	0.071	3505	A	G	0.873	6	157855951	8.24727E-07	0.177	360421	17688.642
Granulocyte-colony stimulating factor	Vascular dementia (mixed)	rs12257900	T	G	0.00	49443428	0.932	0.022	7904	T	G	0.467	10	48235385	2.48891E-06	0.099	360421	23006.877
Granulocyte-colony stimulating factor	Vascular dementia (mixed)	rs1466525	T	C	0.00	54780209	0.830	0.019	7904	T	C	0.489	8	53867649	1.97697E-06	0.103	360421	34189.173
Granulocyte-colony stimulating factor	Vascular dementia (mixed)	rs6849229	G	A	-0.01	131797280	0.810	0.022	7904	G	A	0.562	4	130876125	4.44038E-06	0.122	360421	34142.711
Granulocyte-colony stimulating factor	Vascular dementia (mixed)	rs9458673	A	G	0.01	158276983	0.935	0.048	7904	A	G	0.873	6	157855951	8.24727E-07	0.177	360421	17688.642
Fibroblast growth factor basic	Vascular dementia (mixed)	rs1466525	T	C	0.01	54780209	0.777	0.020	7565	T	C	0.489	8	53867649	1.97697E-06	0.103	360421	34189.173

Fibroblast growth factor basic	Vascular dementia (mixed)	rs1632064	T	C	0.01	3219694	0.582	0.024	7565	T	C	0.614	5	3219580	4.04045E-06	0.133	360421	37296.871
Fibroblast growth factor basic	Vascular dementia (mixed)	rs17168895	T	G	-0.01	15647727	0.618	0.021	7565	T	G	-0.547	7	15608102	2.92853E-06	0.117	360421	34495.049
Eotaxin	Vascular dementia (mixed)	rs1466525	T	C	0.01	54780209	0.478	0.019	8153	T	C	0.489	8	53867649	1.97697E-06	0.103	360421	34189.173
Eotaxin	Vascular dementia (mixed)	rs1632064	T	C	0.00	3219694	0.864	0.023	8153	T	C	0.614	5	3219580	4.04045E-06	0.133	360421	37296.871
Eotaxin	Vascular dementia (mixed)	rs429358	C	T	0.00	45411941	0.845	0.020	8153	C	T	0.565	19	44908684	3.94357E-08	0.103	360421	36831.461
CTACK	Vascular dementia (other) || id:finngen_R9_VD_O	rs143563818	C	T	0.03	33580846	0.766	0.087	3631	C	T	1.554	5	33580741	1.89544E-07	0.298	360248	31380.755
Macrophage Migration Inhibitory Factor	Vascular dementia (other) || id:finngen_R9_VD_O	rs143563818	C	T	-0.04	33580846	0.655	0.090	3494	C	T	1.554	5	33580741	1.89544E-07	0.298	360248	31380.755
Stem cell growth factor beta	Vascular dementia (other) || id:finngen_R9_VD_O	rs143563818	C	T	-0.01	33580846	0.880	0.087	3682	C	T	1.554	5	33580741	1.89544E-07	0.298	360248	31380.755
RANTES	Vascular dementia (other) || id:finngen_R9_VD_O	rs143563818	C	T	-0.03	33580846	0.754	0.090	3421	C	T	1.554	5	33580741	1.89544E-07	0.298	360248	31380.755
Macrophage inflammatory protein 1b	Vascular dementia (other) || id:finngen_R9_VD_O	rs143563818	C	T	-0.01	33580846	0.886	0.056	8243	C	T	1.554	5	33580741	1.89544E-07	0.298	360248	31380.755
Macrophage inflammatory protein 1a	Vascular dementia (other) || id:finngen_R9_VD_O	rs143563818	C	T	-0.03	33580846	0.702	0.090	3522	C	T	1.554	5	33580741	1.89544E-07	0.298	360248	31380.755
Monokine induced by gamma interferon	Vascular dementia (other) || id:finngen_R9_VD_O	rs143563818	C	T	0.01	33580846	0.911	0.087	3685	C	T	1.554	5	33580741	1.89544E-07	0.298	360248	31380.755
Monocyte chemoattractant protein-1	Vascular dementia (other) || id:finngen_R9_VD_O	rs143563818	C	T	0.02	33580846	0.702	0.057	8293	C	T	1.554	5	33580741	1.89544E-07	0.298	360248	31380.755
Interleukin-12p70	Vascular dementia (other) || id:finngen_R9_VD_O	rs143563818	C	T	0.01	33580846	0.858	0.057	8270	C	T	1.554	5	33580741	1.89544E-07	0.298	360248	31380.755
Interferon gamma-induced protein 10	Vascular dementia (other) || id:finngen_R9_VD_O	rs143563818	C	T	0.04	33580846	0.676	0.087	3685	C	T	1.554	5	33580741	1.89544E-07	0.298	360248	31380.755
Interleukin-18 levels	Vascular dementia (other) || id:finngen_R9_VD_O	rs143563818	C	T	0.02	33580846	0.851	0.088	3636	C	T	1.554	5	33580741	1.89544E-07	0.298	360248	31380.755
Interleukin-17	Vascular dementia (other) || id:finngen_R9_VD_O	rs143563818	C	T	-0.03	33580846	0.618	0.058	7760	C	T	1.554	5	33580741	1.89544E-07	0.298	360248	31380.755
Interleukin-8	Vascular dementia (other) || id:finngen_R9_VD_O	rs143563818	C	T	0.03	33580846	0.775	0.090	3526	C	T	1.554	5	33580741	1.89544E-07	0.298	360248	31380.755
Interleukin-1-receptor antagonist	Vascular dementia (other) || id:finngen_R9_VD_O	rs143563818	C	T	-0.03	33580846	0.756	0.088	3638	C	T	1.554	5	33580741	1.89544E-07	0.298	360248	31380.755
Interleukin-5	Vascular dementia (other) || id:finngen_R9_VD_O	rs143563818	C	T	0.00	33580846	0.974	0.091	3364	C	T	1.554	5	33580741	1.89544E-07	0.298	360248	31380.755
Interleukin-4	Vascular dementia (other) || id:finngen_R9_VD_O	rs143563818	C	T	0.00	33580846	0.956	0.057	8124	C	T	1.554	5	33580741	1.89544E-07	0.298	360248	31380.755
Interleukin-2 receptor antagonist	Vascular dementia (other) || id:finngen_R9_VD_O	rs143563818	C	T	0.02	33580846	0.788	0.089	3677	C	T	1.554	5	33580741	1.89544E-07	0.298	360248	31380.755
Interferon gamma	Vascular dementia (other) || id:finngen_R9_VD_O	rs143563818	C	T	-0.02	33580846	0.689	0.058	7701	C	T	1.554	5	33580741	1.89544E-07	0.298	360248	31380.755
Fibroblast growth factor basic	Vascular dementia (other) || id:finngen_R9_VD_O	rs143563818	C	T	-0.01	33580846	0.780	0.059	7565	C	T	1.554	5	33580741	1.89544E-07	0.298	360248	31380.755
Eotaxin	Vascular dementia (other) || id:finngen_R9_VD_O	rs143563818	C	T	-0.02	33580846	0.744	0.057	8153	C	T	1.554	5	33580741	1.89544E-07	0.298	360248	31380.755
CTACK	Vascular dementia (subcortical)	rs429358	C	T	0.02	45411941	0.457	0.031	3631	C	T	0.597	19	44908684	1.74221E-17	0.070	360770	41621.629
CTACK	Vascular dementia (subcortical)	rs4295569	C	T	0.00	47820641	0.919	0.026	3631	C	T	-0.355	7	47781043	2.54572E-08	0.064	360770	20037.409
beta-nerve growth factor	Vascular dementia (subcortical)	rs11148372	A	G	-0.01	22788665	0.798	0.024	3531	A	G	-0.261	13	22214526	4.06275E-06	0.057	360770	12632.549
Vascular endothelial growth factor	Vascular dementia (subcortical)	rs17708733	A	C	-0.03	148532829	0.727	0.061	7118	A	C	0.693	5	149153266	1.68221E-06	0.145	360770	8931.149
Vascular endothelial growth factor	Vascular dementia (subcortical)	rs4295569	C	T	-0.01	47820641	0.511	0.018	7118	C	T	-0.355	7	47781043	2.54572E-08	0.064	360770	20037.409
Vascular endothelial growth factor	Vascular dementia (subcortical)	rs74915041	T	C	-0.01	121201464	0.867	0.037	7118	T	C	0.462	9	118439186	3.09443E-06	0.099	360770	9592.798
Macrophage Migration Inhibitory Factor	Vascular dementia (subcortical)	rs10919863	T	C	0.01	200226041	0.838	0.031	3494	T	C	0.315	1	200256913	3.47112E-06	0.068	360770	10813.753
Macrophage Migration Inhibitory Factor	Vascular dementia (subcortical)	rs17708733	A	C	0.00	148532829	0.875	0.085	3494	A	C	0.693	5	149153266	1.68221E-06	0.145	360770	8931.149
Macrophage Migration Inhibitory Factor	Vascular dementia (subcortical)	rs429358	C	T	-0.02	45411941	0.558	0.031	3494	C	T	0.597	19	44908684	1.74221E-17	0.070	360770	41621.629
Macrophage Migration Inhibitory Factor	Vascular dementia (subcortical)	rs4295569	C	T	-0.01	47820641	0.672	0.026	3494	C	T	-0.355	7	47781043	2.54572E-08	0.064	360770	20037.409
Macrophage Migration Inhibitory Factor	Vascular dementia (subcortical)	rs74915041	T	C	0.01	121201464	0.867	0.053	3494	T	C	0.462	9	118439186	3.09443E-06	0.099	360770	9592.798
TRAIL	Vascular dementia (subcortical)	rs10919863	T	C	-0.01	200226041	0.698	0.021	8186	T	C	0.315	1	200256913	3.47112E-06	0.068	360770	10813.753
TRAIL	Vascular dementia (subcortical)	rs11148372	A	G	-0.01	22788665	0.561	0.016	8186	A	G	-0.261	13	22214526	4.06275E-06	0.057	360770	12632.549
TRAIL	Vascular dementia (subcortical)	rs17708733	A	C	0.00	148532829	0.909	0.057	8186	A	C	0.693	5	149153266	1.68221E-06	0.145	360770	8931.149
TRAIL	Vascular dementia (subcortical)	rs429358	C	T	0.00	45411941	0.880	0.020	8186	C	T	0.597	19	44908684	1.74221E-17	0.070	360770	41621.629
Tumor necrosis factor beta levels	Vascular dementia (subcortical)	rs4295569	C	T	0.00	47820641	0.991	0.039	1559	C	T	-0.355	7	47781043	2.54572E-08	0.064	360770	20037.409
Tumor necrosis factor 1 alpha	Vascular dementia (subcortical)	rs10919863	T	C	0.00	200226041	0.950	0.032	3454	T	C	0.315	1	200256913	3.47112E-06	0.068	360770	10813.753
Tumor necrosis factor 1 alpha	Vascular dementia (subcortical)	rs11148372	A	G	0.01	22788665	0.825	0.025	3454	A	G	-0.261	13	22214526	4.06275E-06	0.057	360770	12632.549
Tumor necrosis factor 1 alpha	Vascular dementia (subcortical)	rs429358	C	T	0.01	45411941	0.825	0.032	3454	C	T	0.597	19	44908684	1.74221E-17	0.070	360770	41621.629
Stromal-cell-derived factor 1 alpha	Vascular dementia (subcortical)	rs11148372	A	G	0.00	22788665	0.231	0.017	5998	A	G	-0.261	13	22214526	4.06275E-06	0.057	360770	12632.549
Stromal-cell-derived factor 1 alpha	Vascular dementia (subcortical)	rs4295569	C	T	0.01	47820641	0.594	0.018	5998	C	T	-0.355	7	47781043	2.54572E-08	0.064	360770	20037.409
Stromal-cell-derived factor 1 alpha	Vascular dementia (subcortical)	rs74915041	T	C	-0.01	121201464	0.650	0.035	5998	T	C	0.462	9	118439186	3.09443E-06	0.099	360770	9592.798
Stem cell growth factor beta	Vascular dementia (subcortical)	rs17708733	A	C	0.00	148532829	0.801	0.084	3682	A	C	0.693	5	149153266	1.68221E-06	0.145	360770	8931.149
Stem cell factor	Vascular dementia (subcortical)	rs11148372	A	G	0.00	22788665	0.860	0.016	8290	A	G	-0.261	13	22214526	4.06275E-06	0.057	360770	12632.549
Stem cell factor	Vascular dementia (subcortical)	rs429358	C	T	0.02	45411941	0.445	0.020	8290	C	T	0.597	19	44908684	1.74221E-17	0.070	360770	41621.629
Stem cell factor	Vascular dementia (subcortical)	rs4295569	C	T	0.01	47820641	0.607	0.017	8290	C	T	-0.355	7	47781043	2.54572E-08	0.064	360770	20037.409
Interleukin-16	Vascular dementia (subcortical)	rs10919863	T	C	-0.01	200226041	0.854	0.031	3483	T	C	0.315	1	200256913	3.47112E-06	0.068	360770	10813.753
Interleukin-16	Vascular dementia (subcortical)	rs11148372	A	G	0.01	22788665	0.695	0.025	3483	A	G	-0.261	13	22214526	4.06275E-06	0.057	360770	12632.549
Interleukin-16	Vascular dementia (subcortical)	rs17708733	A	C	0.02	148532829	0.722	0.088	3483	A	C	0.693	5	149153266	1.68221E-06	0.145	360770	8931.149
Interleukin-16	Vascular dementia (subcortical)	rs429358	C	T	0.02	45411941	0.470	0.031	3483	C	T	0.597	19	44908684	1.74221E-17	0.070	360770	41621.629
Interleukin-16	Vascular dementia (subcortical)	rs4295569	C	T	-0.01	47820641	0.653	0.026	3483	C	T	-0.355	7	47781043	2.54572E-08	0.064	360770	20037.409
Interleukin-16	Vascular dementia (subcortical)	rs74915041	T	C	-0.02	121201464	0.818	0.053	3483	T	C	0.462	9	118439186	3.09443E-06	0.099	360770	9592.798
RANTES	Vascular dementia (subcortical)	rs10919863	T	C	0.00	200226041	0.929	0.032	3421	T	C	0.315	1	200256913	3.47112E-06	0.068	360770	10813.753
RANTES	Vascular dementia (subcortical)	rs11148372	A	G	-0.01	22788665	0.780	0.025	3421	A	G	-0.261	13	22214526	4.06275E-06	0.057	360770	12632.549
RANTES	Vascular dementia (subcortical)	rs429358	C	T	-0.02	45411941	0.575	0.032	3421	C	T	0.597	19	44908684	1.74221E-17	0.070	360770	41621.629
RANTES	Vascular dementia (subcortical)	rs74915041	T	C	-0.01	121201464	0.882	0.054	3421	T	C	0.462	9	118439186	3.09443E-06	0.099	360770	9592.798
Platelet-derived growth factor BB	Vascular dementia (subcortical)	rs17708733	A	C	0.01	148532829	0.808	0.056	8293	A	C	0.693	5	149153266	1.68221E-06	0.145	360770	8931.149
Platelet-derived growth factor BB	Vascular dementia (subcortical)	rs429358	C	T	0.00	45411941	0.978	0.020	8293	C	T	0.597	19	44908684	1.74221E-17	0.070	360770	41621.629
Platelet-derived growth factor BB	Vascular dementia (subcortical)	rs4295569	C	T	-0.01	47820641	0.583	0.017	8293	C	T	-0.355	7	47781043	2.54572E-08	0.064	360770	20037.409
Macrophage inflammatory protein 1b	Vascular dementia (subcortical)	rs10919863	T	C	0.01	200226041	0.536	0.021	8243	T	C	0.315	1	200256913	3.47112E-06	0.068	360770	10813.753
Macrophage inflammatory protein 1b	Vascular dementia (subcortical)	rs17708733	A	C	0.00	148532829	0.857	0.056	8243	A	C	0.693	5	149153266	1.68221E-06	0.145	360770	8931.149
Macrophage inflammatory protein 1b	Vascular dementia (subcortical)	rs4295569	C	T	-0.01	47820641	0.506	0.017	8243	C	T	-0.355	7	47781043	2.54572E-08	0.064	360770	20037.409
Macrophage inflammatory protein 1a	Vascular dementia (subcortical)	rs11148372	A	G	-0.01	22788665	0.723	0.025	3522	A	G	-0.261	13	22214526	4.06275E-06	0.057	360770	12632.549

Macrophage inflammatory protein 1a	Vascular dementia (subcortical)	rs17708733	A	C	0.00	148532829	0.791	0.085	3522	A	C	0.693	5	149153266	1.68221E-06	0.145	360770	8931.149
Macrophage inflammatory protein 1a	Vascular dementia (subcortical)	rs4295569	C	T	-0.01	47820641	0.658	0.026	3522	C	T	-0.355	7	47781043	2.54572E-08	0.064	360770	20037.409
Macrophage inflammatory protein 1a	Vascular dementia (subcortical)	rs74915041	T	C	-0.01	121201464	0.733	0.054	3522	T	C	0.462	9	118439186	3.09443E-06	0.099	360770	9592.798
Monokine induced by gamma interferon	Vascular dementia (subcortical)	rs11148372	A	G	0.00	22788665	0.875	0.024	3685	A	G	-0.261	13	22214526	4.06275E-06	0.057	360770	12632.549
Monokine induced by gamma interferon	Vascular dementia (subcortical)	rs17708733	A	C	-0.02	148532829	0.936	0.084	3685	A	C	0.693	5	149153266	1.68221E-06	0.145	360770	8931.149
Monokine induced by gamma interferon	Vascular dementia (subcortical)	rs429358	C	T	0.00	45411941	0.908	0.030	3685	C	T	0.597	19	44908684	1.74221E-17	0.070	360770	41621.629
Macrophage colony stimulating factor	Vascular dementia (subcortical)	rs10919863	T	C	0.01	200226041	0.829	0.038	840	T	C	0.315	1	200256913	3.47112E-06	0.068	360770	10813.753
Macrophage colony stimulating factor	Vascular dementia (subcortical)	rs11148372	A	G	0.00	22788665	0.916	0.029	840	A	G	-0.261	13	22214526	4.06275E-06	0.057	360770	12632.549
Monocyte chemoattractant protein-1	Vascular dementia (subcortical)	rs429358	C	T	-0.01	45411941	0.570	0.020	8293	C	T	0.597	19	44908684	1.74221E-17	0.070	360770	41621.629
Monocyte chemoattractant protein-1	Vascular dementia (subcortical)	rs4295569	C	T	0.01	47820641	0.638	0.017	8293	C	T	-0.355	7	47781043	2.54572E-08	0.064	360770	20037.409
Interleukin-12p70	Vascular dementia (subcortical)	rs11148372	A	G	0.00	22788665	0.830	0.016	8270	A	G	-0.261	13	22214526	4.06275E-06	0.057	360770	12632.549
Interleukin-12p70	Vascular dementia (subcortical)	rs17708733	A	C	-0.04	148532829	0.620	0.056	8270	A	C	0.693	5	149153266	1.68221E-06	0.145	360770	8931.149
Interleukin-12p70	Vascular dementia (subcortical)	rs4295569	C	T	0.01	47820641	0.506	0.017	8270	C	T	-0.355	7	47781043	2.54572E-08	0.064	360770	20037.409
Interleukin-12p70	Vascular dementia (subcortical)	rs74915041	T	C	0.00	121201464	0.956	0.034	8270	T	C	0.462	9	118439186	3.09443E-06	0.099	360770	9592.798
Interferon gamma-induced protein 10	Vascular dementia (subcortical)	rs429358	C	T	-0.02	45411941	0.602	0.030	3685	C	T	0.597	19	44908684	1.74221E-17	0.070	360770	41621.629
Interferon gamma-induced protein 10	Vascular dementia (subcortical)	rs74915041	T	C	0.01	121201464	0.998	0.052	3685	T	C	0.462	9	118439186	3.09443E-06	0.099	360770	9592.798
Interleukin-18 levels	Vascular dementia (subcortical)	rs17708733	A	C	0.04	148532829	0.605	0.085	3636	A	C	0.693	5	149153266	1.68221E-06	0.145	360770	8931.149
Interleukin-18 levels	Vascular dementia (subcortical)	rs74915041	T	C	0.01	121201464	0.847	0.053	3636	T	C	0.462	9	118439186	3.09443E-06	0.099	360770	9592.798
Interleukin-17	Vascular dementia (subcortical)	rs17708733	A	C	0.03	148532829	0.506	0.058	7760	A	C	0.693	5	149153266	1.68221E-06	0.145	360770	8931.149
Interleukin-17	Vascular dementia (subcortical)	rs429358	C	T	0.03	45411941	0.223	0.021	7760	C	T	0.597	19	44908684	1.74221E-17	0.070	360770	41621.629
Interleukin-17	Vascular dementia (subcortical)	rs4295569	C	T	0.01	47820641	0.594	0.018	7760	C	T	-0.355	7	47781043	2.54572E-08	0.064	360770	20037.409
Interleukin-10	Vascular dementia (subcortical)	rs17708733	A	C	-0.02	148532829	0.796	0.058	7681	A	C	0.693	5	149153266	1.68221E-06	0.145	360770	8931.149
Interleukin-10	Vascular dementia (subcortical)	rs4295569	C	T	0.00	47820641	0.925	0.018	7681	C	T	-0.355	7	47781043	2.54572E-08	0.064	360770	20037.409
Interleukin-8	Vascular dementia (subcortical)	rs17708733	A	C	0.02	148532829	0.603	0.085	3526	A	C	0.693	5	149153266	1.68221E-06	0.145	360770	8931.149
Interleukin-8	Vascular dementia (subcortical)	rs429358	C	T	0.01	45411941	0.760	0.031	3526	C	T	0.597	19	44908684	1.74221E-17	0.070	360770	41621.629
Interleukin-8	Vascular dementia (subcortical)	rs4295569	C	T	0.00	47820641	0.989	0.026	3526	C	T	-0.355	7	47781043	2.54572E-08	0.064	360770	20037.409
Interleukin-8	Vascular dementia (subcortical)	rs74915041	T	C	-0.01	121201464	0.850	0.054	3526	T	C	0.462	9	118439186	3.09443E-06	0.099	360770	9592.798
Interleukin-6	Vascular dementia (subcortical)	rs11148372	A	G	-0.01	22788665	0.608	0.016	8189	A	G	-0.261	13	22214526	4.06275E-06	0.057	360770	12632.549
Interleukin-6	Vascular dementia (subcortical)	rs17708733	A	C	0.01	148532829	0.732	0.056	8189	A	C	0.693	5	149153266	1.68221E-06	0.145	360770	8931.149
Interleukin-6	Vascular dementia (subcortical)	rs429358	C	T	0.02	45411941	0.257	0.020	8189	C	T	0.597	19	44908684	1.74221E-17	0.070	360770	41621.629
Interleukin-6	Vascular dementia (subcortical)	rs74915041	T	C	0.02	121201464	0.615	0.034	8189	T	C	0.462	9	118439186	3.09443E-06	0.099	360770	9592.798
Interleukin-1-receptor antagonist	Vascular dementia (subcortical)	rs429358	C	T	-0.02	45411941	0.454	0.031	3638	C	T	0.597	19	44908684	1.74221E-17	0.070	360770	41621.629
Interleukin-1-receptor antagonist	Vascular dementia (subcortical)	rs4295569	C	T	0.01	47820641	0.675	0.026	3638	C	T	-0.355	7	47781043	2.54572E-08	0.064	360770	20037.409
Interleukin-1-receptor antagonist	Vascular dementia (subcortical)	rs74915041	T	C	-0.01	121201464	0.855	0.052	3638	T	C	0.462	9	118439186	3.09443E-06	0.099	360770	9592.798
Interleukin-1-beta	Vascular dementia (subcortical)	rs10919863	T	C	0.01	200226041	0.765	0.025	3309	T	C	0.315	1	200256913	3.47112E-06	0.068	360770	10813.753
Interleukin-1-beta	Vascular dementia (subcortical)	rs429358	C	T	0.00	45411941	0.471	0.024	3309	C	T	0.597	19	44908684	1.74221E-17	0.070	360770	41621.629
Interleukin-1-beta	Vascular dementia (subcortical)	rs4295569	C	T	0.01	47820641	0.296	0.020	3309	C	T	-0.355	7	47781043	2.54572E-08	0.064	360770	20037.409
Hepatocyte growth factor	Vascular dementia (subcortical)	rs10919863	T	C	-0.01	200226041	0.533	0.021	8292	T	C	0.315	1	200256913	3.47112E-06	0.068	360770	10813.753
Hepatocyte growth factor	Vascular dementia (subcortical)	rs17708733	A	C	0.04	148532829	0.452	0.056	8292	A	C	0.693	5	149153266	1.68221E-06	0.145	360770	8931.149
Hepatocyte growth factor	Vascular dementia (subcortical)	rs429358	C	T	-0.01	45411941	0.703	0.020	8292	C	T	0.597	19	44908684	1.74221E-17	0.070	360770	41621.629
Hepatocyte growth factor	Vascular dementia (subcortical)	rs4295569	C	T	0.00	47820641	0.821	0.017	8292	C	T	-0.355	7	47781043	2.54572E-08	0.064	360770	20037.409
Hepatocyte growth factor	Vascular dementia (subcortical)	rs74915041	T	C	-0.02	121201464	0.654	0.034	8292	T	C	0.462	9	118439186	3.09443E-06	0.099	360770	9592.798
Interleukin-9	Vascular dementia (subcortical)	rs429358	C	T	-0.02	45411941	0.541	0.031	3634	C	T	0.597	19	44908684	1.74221E-17	0.070	360770	41621.629
Interleukin-9	Vascular dementia (subcortical)	rs4295569	C	T	-0.01	47820641	0.736	0.026	3634	C	T	-0.355	7	47781043	2.54572E-08	0.064	360770	20037.409
Interleukin-7	Vascular dementia (subcortical)	rs11148372	A	G	0.00	22788665	0.902	0.025	3409	A	G	-0.261	13	22214526	4.06275E-06	0.057	360770	12632.549
Interleukin-7	Vascular dementia (subcortical)	rs17708733	A	C	0.03	148532829	0.559	0.087	3409	A	C	0.693	5	149153266	1.68221E-06	0.145	360770	8931.149
Interleukin-7	Vascular dementia (subcortical)	rs4295569	C	T	-0.01	47820641	0.806	0.026	3409	C	T	-0.355	7	47781043	2.54572E-08	0.064	360770	20037.409
Interleukin-5	Vascular dementia (subcortical)	rs11148372	A	G	0.00	22788665	0.962	0.025	3364	A	G	-0.261	13	22214526	4.06275E-06	0.057	360770	12632.549
Interleukin-5	Vascular dementia (subcortical)	rs429358	C	T	0.02	45411941	0.555	0.032	3364	C	T	0.597	19	44908684	1.74221E-17	0.070	360770	41621.629
Interleukin-5	Vascular dementia (subcortical)	rs4295569	C	T	0.00	47820641	0.971	0.027	3364	C	T	-0.355	7	47781043	2.54572E-08	0.064	360770	20037.409
Interleukin-5	Vascular dementia (subcortical)	rs74915041	T	C	-0.02	121201464	0.692	0.055	3364	T	C	0.462	9	118439186	3.09443E-06	0.099	360770	9592.798
Interleukin-4	Vascular dementia (subcortical)	rs11148372	A	G	-0.01	22788665	0.513	0.016	8124	A	G	-0.261	13	22214526	4.06275E-06	0.057	360770	12632.549
Interleukin-4	Vascular dementia (subcortical)	rs429358	C	T	0.02	45411941	0.305	0.021	8124	C	T	0.597	19	44908684	1.74221E-17	0.070	360770	41621.629
Interleukin-4	Vascular dementia (subcortical)	rs4295569	C	T	0.01	47820641	0.466	0.017	8124	C	T	-0.355	7	47781043	2.54572E-08	0.064	360770	20037.409
Interleukin-4	Vascular dementia (subcortical)	rs74915041	T	C	0.00	121201464	0.930	0.034	8124	T	C	0.462	9	118439186	3.09443E-06	0.099	360770	9592.798
Interleukin-2 receptor antagonist	Vascular dementia (subcortical)	rs11148372	A	G	-0.01	22788665	0.824	0.024	3677	A	G	-0.261	13	22214526	4.06275E-06	0.057	360770	12632.549
Interleukin-2 receptor antagonist	Vascular dementia (subcortical)	rs429358	C	T	-0.01	45411941	0.762	0.030	3677	C	T	0.597	19	44908684	1.74221E-17	0.070	360770	41621.629
Interleukin-2	Vascular dementia (subcortical)	rs10919863	T	C	0.00	200226041	0.982	0.031	3475	T	C	0.315	1	200256913	3.47112E-06	0.068	360770	10813.753
Interleukin-2	Vascular dementia (subcortical)	rs11148372	A	G	0.01	22788665	0.660	0.025	3475	A	G	-0.261	13	22214526	4.06275E-06	0.057	360770	12632.549
Interleukin-2	Vascular dementia (subcortical)	rs74915041	T	C	-0.01	121201464	0.869	0.053	3475	T	C	0.462	9	118439186	3.09443E-06	0.099	360770	9592.798
Interferon gamma	Vascular dementia (subcortical)	rs11148372	A	G	-0.01	22788665	0.595	0.017	7701	A	G	-0.261	13	22214526	4.06275E-06	0.057	360770	12632.549
Interferon gamma	Vascular dementia (subcortical)	rs17708733	A	C	-0.03	148532829	0.709	0.058	7701	A	C	0.693	5	149153266	1.68221E-06	0.145	360770	8931.149
Interferon gamma	Vascular dementia (subcortical)	rs74915041	T	C	0.00	121201464	0.948	0.035	7701	T	C	0.462	9	118439186	3.09443E-06	0.099	360770	9592.798
Growth-regulated protein alpha	Vascular dementia (subcortical)	rs11148372	A	G	0.00	22788665	0.981	0.025	3505	A	G	-0.261	13	22214526	4.06275E-06	0.057	360770	12632.549
Growth-regulated protein alpha	Vascular dementia (subcortical)	rs4295569	C	T	0.01	47820641	0.775	0.026	3505	C	T	-0.355	7	47781043	2.54572E-08	0.064	360770	20037.409
Growth-regulated protein alpha	Vascular dementia (subcortical)	rs74915041	T	C	0.00	121201464	0.974	0.053	3505	T	C	0.462	9	118439186	3.09443E-06	0.099	360770	9592.798
Granulocyte-colony stimulating factor	Vascular dementia (subcortical)	rs10919863	T	C	0.01	200226041	0.642	0.021	7904	T	C	0.315	1	200256913	3.47112E-06	0.068	360770	10813.753
Granulocyte-colony stimulating factor	Vascular dementia (subcortical)	rs11148372	A	G	0.00	22788665	0.885	0.017	7904	A	G	-0.261	13	22214526	4.06275E-06	0.057	360770	12632.549

Granulocyte-colony stimulating factor	Vascular dementia (subcortical)	rs17708733	A	C	0.04	148532829	0.486	0.058	7904	A	C	0.693	5	149153266	1.68221E-06	0.145	360770	8931.149
Granulocyte-colony stimulating factor	Vascular dementia (subcortical)	rs4295569	C	T	0.01	47820641	0.756	0.017	7904	C	T	-0.355	7	47781043	2.54572E-08	0.064	360770	20037.409
Fibroblast growth factor basic	Vascular dementia (subcortical)	rs17708733	A	C	-0.02	148532829	0.965	0.060	7565	A	C	0.693	5	149153266	1.68221E-06	0.145	360770	8931.149
Fibroblast growth factor basic	Vascular dementia (subcortical)	rs4295569	C	T	0.00	47820641	0.909	0.018	7565	C	T	-0.355	7	47781043	2.54572E-08	0.064	360770	20037.409
Fibroblast growth factor basic	Vascular dementia (subcortical)	rs74915041	T	C	-0.02	121201464	0.632	0.036	7565	T	C	0.462	9	118439186	3.09443E-06	0.099	360770	9592.798
Eotaxin	Vascular dementia (subcortical)	rs10919863	T	C	0.00	200226041	0.932	0.021	8153	T	C	0.315	1	200256913	3.47112E-06	0.068	360770	10813.753
Eotaxin	Vascular dementia (subcortical)	rs429358	C	T	0.00	45411941	0.845	0.020	8153	C	T	0.597	19	44908684	1.74221E-17	0.070	360770	41621.629
CTACK	Vascular dementia (sudden onset)	rs12423672	T	G	-0.02	5047705	0.723	0.047	3631	T	G	0.759	12	4938539	4.89159E-06	0.166	360283	30610.567
CTACK	Vascular dementia (sudden onset)	rs12452096	A	G	-0.01	75544032	0.805	0.032	3631	A	G	0.750	17	77547950	1.48929E-06	0.156	360283	77865.614
CTACK	Vascular dementia (sudden onset)	rs146524495	A	G	-0.02	175789555	0.774	0.079	3631	A	G	1.281	4	174868404	3.04229E-06	0.275	360283	23934.344
beta-nerve growth factor	Vascular dementia (sudden onset)	rs12423672	T	G	0.01	5047705	0.763	0.048	3531	T	G	0.759	12	4938539	4.89159E-06	0.166	360283	30610.567
beta-nerve growth factor	Vascular dementia (sudden onset)	rs2920	C	T	-0.01	23884780	0.731	0.032	3531	C	T	0.592	1	23558289	2.20602E-06	0.125	360283	40501.384
Vascular endothelial growth factor	Vascular dementia (sudden onset)	rs12423672	T	G	0.02	5047705	0.541	0.033	7118	T	G	0.759	12	4938539	4.89159E-06	0.166	360283	30610.567
Vascular endothelial growth factor	Vascular dementia (sudden onset)	rs188011869	A	G	0.02	112465648	0.746	0.052	7118	A	G	1.046	5	113129951	3.42405E-06	0.225	360283	25635.289
Vascular endothelial growth factor	Vascular dementia (sudden onset)	rs2920	C	T	0.00	23884780	0.921	0.022	7118	C	T	0.592	1	23558289	2.20602E-06	0.125	360283	40501.384
Macrophage Migration Inhibitory Factor	Vascular dementia (sudden onset)	rs146524495	A	G	-0.02	175789555	0.793	0.080	3494	A	G	1.281	4	174868404	3.04229E-06	0.275	360283	23934.344
TRAIL	Vascular dementia (sudden onset)	rs151268476	G	A	-0.01	38879088	0.833	0.053	8186	G	A	0.971	4	38877467	4.81415E-06	0.212	360283	27499.206
TRAIL	Vascular dementia (sudden onset)	rs188011869	A	G	-0.01	112465648	0.780	0.047	8186	A	G	1.046	5	113129951	3.42405E-06	0.225	360283	25635.289
Tumor necrosis factor beta levels	Vascular dementia (sudden onset)	rs12452096	A	G	0.01	75544032	0.796	0.050	1559	A	G	0.750	17	77547950	1.48929E-06	0.156	360283	77865.614
Stromal-cell-derived factor 1 alpha	Vascular dementia (sudden onset)	rs12452096	A	G	0.00	75544032	0.702	0.023	5998	A	G	0.750	17	77547950	1.48929E-06	0.156	360283	77865.614
Stromal-cell-derived factor 1 alpha	Vascular dementia (sudden onset)	rs151268476	G	A	0.03	38879088	0.688	0.080	5998	G	A	0.971	4	38877467	4.81415E-06	0.212	360283	27499.206
Stromal-cell-derived factor 1 alpha	Vascular dementia (sudden onset)	rs188011869	A	G	0.03	112465648	0.875	0.048	5998	A	G	1.046	5	113129951	3.42405E-06	0.225	360283	25635.289
Stromal-cell-derived factor 1 alpha	Vascular dementia (sudden onset)	rs2920	C	T	0.01	23884780	0.747	0.021	5998	C	T	0.592	1	23558289	2.20602E-06	0.125	360283	40501.384
Stem cell growth factor beta	Vascular dementia (sudden onset)	rs12423672	T	G	0.01	5047705	0.774	0.047	3682	T	G	0.759	12	4938539	4.89159E-06	0.166	360283	30610.567
Stem cell growth factor beta	Vascular dementia (sudden onset)	rs151268476	G	A	0.02	38879088	0.805	0.077	3682	G	A	0.971	4	38877467	4.81415E-06	0.212	360283	27499.206
Stem cell growth factor beta	Vascular dementia (sudden onset)	rs188011869	A	G	-0.01	112465648	0.921	0.071	3682	A	G	1.046	5	113129951	3.42405E-06	0.225	360283	25635.289
Stem cell factor	Vascular dementia (sudden onset)	rs188011869	A	G	-0.01	112465648	0.904	0.047	8290	A	G	1.046	5	113129951	3.42405E-06	0.225	360283	25635.289
Stem cell factor	Vascular dementia (sudden onset)	rs2920	C	T	0.01	23884780	0.621	0.021	8290	C	T	0.592	1	23558289	2.20602E-06	0.125	360283	40501.384
Interleukin-16	Vascular dementia (sudden onset)	rs2920	C	T	-0.01	23884780	0.765	0.032	3483	C	T	0.592	1	23558289	2.20602E-06	0.125	360283	40501.384
RANTES	Vascular dementia (sudden onset)	rs12423672	T	G	0.01	5047705	0.820	0.048	3421	T	G	0.759	12	4938539	4.89159E-06	0.166	360283	30610.567
RANTES	Vascular dementia (sudden onset)	rs146524495	A	G	-0.02	175789555	0.747	0.080	3421	A	G	1.281	4	174868404	3.04229E-06	0.275	360283	23934.344
RANTES	Vascular dementia (sudden onset)	rs151268476	G	A	0.01	38879088	0.911	0.080	3421	G	A	0.971	4	38877467	4.81415E-06	0.212	360283	27499.206
Platelet-derived growth factor BB	Vascular dementia (sudden onset)	rs12423672	T	G	0.00	5047705	0.889	0.030	8293	T	G	0.759	12	4938539	4.89159E-06	0.166	360283	30610.567
Platelet-derived growth factor BB	Vascular dementia (sudden onset)	rs188011869	A	G	-0.01	112465648	0.746	0.047	8293	A	G	1.046	5	113129951	3.42405E-06	0.225	360283	25635.289
Platelet-derived growth factor BB	Vascular dementia (sudden onset)	rs2920	C	T	0.00	23884780	0.889	0.021	8293	C	T	0.592	1	23558289	2.20602E-06	0.125	360283	40501.384
Macrophage inflammatory protein 1b	Vascular dementia (sudden onset)	rs12423672	T	G	-0.01	5047705	0.689	0.030	8243	T	G	0.759	12	4938539	4.89159E-06	0.166	360283	30610.567
Macrophage inflammatory protein 1b	Vascular dementia (sudden onset)	rs12452096	A	G	0.00	75544032	0.903	0.022	8243	A	G	0.750	17	77547950	1.48929E-06	0.156	360283	77865.614
Macrophage inflammatory protein 1b	Vascular dementia (sudden onset)	rs146524495	A	G	0.01	175789555	0.870	0.054	8243	A	G	1.281	4	174868404	3.04229E-06	0.275	360283	23934.344
Macrophage inflammatory protein 1b	Vascular dementia (sudden onset)	rs188011869	A	G	0.03	112465648	0.563	0.048	8243	A	G	1.046	5	113129951	3.42405E-06	0.225	360283	25635.289
Macrophage inflammatory protein 1b	Vascular dementia (sudden onset)	rs2920	C	T	-0.01	23884780	0.794	0.021	8243	C	T	0.592	1	23558289	2.20602E-06	0.125	360283	40501.384
Macrophage inflammatory protein 1a	Vascular dementia (sudden onset)	rs12452096	A	G	0.00	75544032	0.963	0.033	3522	A	G	0.750	17	77547950	1.48929E-06	0.156	360283	77865.614
Macrophage inflammatory protein 1a	Vascular dementia (sudden onset)	rs188011869	A	G	0.03	112465648	0.680	0.072	3522	A	G	1.046	5	113129951	3.42405E-06	0.225	360283	25635.289
Macrophage inflammatory protein 1a	Vascular dementia (sudden onset)	rs2920	C	T	0.01	23884780	0.650	0.032	3522	C	T	0.592	1	23558289	2.20602E-06	0.125	360283	40501.384
Monokine induced by gamma interferon	Vascular dementia (sudden onset)	rs12452096	A	G	0.00	75544032	0.994	0.032	3685	A	G	0.750	17	77547950	1.48929E-06	0.156	360283	77865.614
Monokine induced by gamma interferon	Vascular dementia (sudden onset)	rs146524495	A	G	-0.02	175789555	0.800	0.078	3685	A	G	1.281	4	174868404	3.04229E-06	0.275	360283	23934.344
Monokine induced by gamma interferon	Vascular dementia (sudden onset)	rs188011869	A	G	0.02	112465648	0.742	0.071	3685	A	G	1.046	5	113129951	3.42405E-06	0.225	360283	25635.289
Macrophage colony stimulating factor	Vascular dementia (sudden onset)	rs146524495	A	G	-0.02	175789555	0.818	0.097	840	A	G	1.281	4	174868404	3.04229E-06	0.275	360283	23934.344
Monocyte chemoattractant protein-1	Vascular dementia (sudden onset)	rs12423672	T	G	0.01	5047705	0.810	0.030	8293	T	G	0.759	12	4938539	4.89159E-06	0.166	360283	30610.567
Monocyte chemoattractant protein-1	Vascular dementia (sudden onset)	rs12452096	A	G	0.00	75544032	0.989	0.022	8293	A	G	0.750	17	77547950	1.48929E-06	0.156	360283	77865.614
Monocyte chemoattractant protein-1	Vascular dementia (sudden onset)	rs151268476	G	A	0.04	38879088	0.654	0.077	8293	G	A	0.971	4	38877467	4.81415E-06	0.212	360283	27499.206
Monocyte chemoattractant protein-1	Vascular dementia (sudden onset)	rs188011869	A	G	0.01	112465648	0.847	0.048	8293	A	G	1.046	5	113129951	3.42405E-06	0.225	360283	25635.289
Monocyte chemoattractant protein-1	Vascular dementia (sudden onset)	rs2920	C	T	0.00	23884780	0.857	0.021	8293	C	T	0.592	1	23558289	2.20602E-06	0.125	360283	40501.384
Interleukin-12p70	Vascular dementia (sudden onset)	rs12423672	T	G	0.00	5047705	0.917	0.030	8270	T	G	0.759	12	4938539	4.89159E-06	0.166	360283	30610.567
Interleukin-12p70	Vascular dementia (sudden onset)	rs188011869	A	G	-0.01	112465648	0.784	0.047	8270	A	G	1.046	5	113129951	3.42405E-06	0.225	360283	25635.289
Interleukin-12p70	Vascular dementia (sudden onset)	rs2920	C	T	-0.01	23884780	0.749	0.021	8270	C	T	0.592	1	23558289	2.20602E-06	0.125	360283	40501.384
Interferon gamma-induced protein 10	Vascular dementia (sudden onset)	rs12423672	T	G	-0.02	5047705	0.730	0.047	3685	T	G	0.759	12	4938539	4.89159E-06	0.166	360283	30610.567
Interferon gamma-induced protein 10	Vascular dementia (sudden onset)	rs12452096	A	G	0.00	75544032	0.976	0.032	3685	A	G	0.750	17	77547950	1.48929E-06	0.156	360283	77865.614
Interferon gamma-induced protein 10	Vascular dementia (sudden onset)	rs146524495	A	G	0.03	175789555	0.643	0.078	3685	A	G	1.281	4	174868404	3.04229E-06	0.275	360283	23934.344
Interferon gamma-induced protein 10	Vascular dementia (sudden onset)	rs151268476	G	A	0.01	38879088	0.866	0.076	3685	G	A	0.971	4	38877467	4.81415E-06	0.212	360283	27499.206
Interferon gamma-induced protein 10	Vascular dementia (sudden onset)	rs2920	C	T	0.01	23884780	0.653	0.031	3685	C	T	0.592	1	23558289	2.20602E-06	0.125	360283	40501.384
Interleukin-18 levels	Vascular dementia (sudden onset)	rs12423672	T	G	-0.01	5047705	0.838	0.047	3636	T	G	0.759	12	4938539	4.89159E-06	0.166	360283	30610.567
Interleukin-18 levels	Vascular dementia (sudden onset)	rs12452096	A	G	-0.01	75544032	0.712	0.033	3636	A	G	0.750	17	77547950	1.48929E-06	0.156	360283	77865.614
Interleukin-18 levels	Vascular dementia (sudden onset)	rs146524495	A	G	0.00	175789555	0.982	0.080	3636	A	G	1.281	4	174868404	3.04229E-06	0.275	360283	23934.344
Interleukin-18 levels	Vascular dementia (sudden onset)	rs151268476	G	A	0.02	38879088	0.822	0.078	3636	G	A	0.971	4	38877467	4.81415E-06	0.212	360283	27499.206
Interleukin-18 levels	Vascular dementia (sudden onset)	rs2920	C	T	0.00	23884780	0.919	0.031	3636	C	T	0.592	1	23558289	2.20602E-06	0.125	360283	40501.384
Interleukin-17	Vascular dementia (sudden onset)	rs12423672	T	G	0.01	5047705	0.847	0.032	7760	T	G	0.759	12	4938539	4.89159E-06	0.166	360283	30610.567
Interleukin-17	Vascular dementia (sudden onset)	rs151268476	G	A	-0.02	38879088	0.835	0.079	7760	G	A	0.971	4	38877467	4.81415E-06	0.212	360283	27499.206
Interleukin-17	Vascular dementia (sudden onset)	rs2920	C	T	-0.01	23884780	0.665	0.021	7760	C	T	0.592	1	23558289	2.20602E-06	0.125	360283	40501.384

Interleukin-13	Vascular dementia (sudden onset)	rs2920	C	T	-0.01	23884780	0.660	0.032	3557	C	T	0.592	1	23558289	2.20602E-06	0.125	360283	40501.384
Interleukin-10	Vascular dementia (sudden onset)	rs12423672	T	G	0.01	5047705	0.871	0.032	7681	T	G	0.759	12	4938539	4.89159E-06	0.166	360283	30610.567
Interleukin-10	Vascular dementia (sudden onset)	rs151268476	G	A	-0.01	38879088	0.866	0.077	7681	G	A	0.971	4	38877467	4.81415E-06	0.212	360283	27499.206
Interleukin-10	Vascular dementia (sudden onset)	rs188011869	A	G	-0.01	112465648	0.782	0.049	7681	A	G	1.046	5	113129951	3.42405E-06	0.225	360283	25635.289
Interleukin-10	Vascular dementia (sudden onset)	rs2920	C	T	-0.01	23884780	0.673	0.022	7681	C	T	0.592	1	23558289	2.20602E-06	0.125	360283	40501.384
Interleukin-8	Vascular dementia (sudden onset)	rs12423672	T	G	-0.02	5047705	0.731	0.047	3526	T	G	0.759	12	4938539	4.89159E-06	0.166	360283	30610.567
Interleukin-8	Vascular dementia (sudden onset)	rs146524495	A	G	0.00	175789555	0.975	0.080	3526	A	G	1.281	4	174868404	3.04229E-06	0.275	360283	23934.344
Interleukin-8	Vascular dementia (sudden onset)	rs151268476	G	A	-0.01	38879088	0.916	0.079	3526	G	A	0.971	4	38877467	4.81415E-06	0.212	360283	27499.206
Interleukin-6	Vascular dementia (sudden onset)	rs12423672	T	G	-0.01	5047705	0.831	0.031	8189	T	G	0.759	12	4938539	4.89159E-06	0.166	360283	30610.567
Interleukin-6	Vascular dementia (sudden onset)	rs151268476	G	A	-0.01	38879088	0.870	0.078	8189	G	A	0.971	4	38877467	4.81415E-06	0.212	360283	27499.206
Interleukin-1-receptor antagonist	Vascular dementia (sudden onset)	rs151268476	G	A	0.01	38879088	0.951	0.077	3638	G	A	0.971	4	38877467	4.81415E-06	0.212	360283	27499.206
Interleukin-1-beta	Vascular dementia (sudden onset)	rs12423672	T	G	-0.01	5047705	0.487	0.037	3309	T	G	0.759	12	4938539	4.89159E-06	0.166	360283	30610.567
Interleukin-1-beta	Vascular dementia (sudden onset)	rs188011869	A	G	-0.02	112465648	0.772	0.057	3309	A	G	1.046	5	113129951	3.42405E-06	0.225	360283	25635.289
Hepatocyte growth factor	Vascular dementia (sudden onset)	rs12423672	T	G	-0.01	5047705	0.868	0.030	8292	T	G	0.759	12	4938539	4.89159E-06	0.166	360283	30610.567
Hepatocyte growth factor	Vascular dementia (sudden onset)	rs12452096	A	G	0.00	75544032	0.958	0.022	8292	A	G	0.750	17	77547950	1.48929E-06	0.156	360283	77865.614
Hepatocyte growth factor	Vascular dementia (sudden onset)	rs151268476	G	A	0.02	38879088	0.772	0.078	8292	G	A	0.971	4	38877467	4.81415E-06	0.212	360283	27499.206
Hepatocyte growth factor	Vascular dementia (sudden onset)	rs188011869	A	G	-0.01	112465648	0.760	0.047	8292	A	G	1.046	5	113129951	3.42405E-06	0.225	360283	25635.289
Hepatocyte growth factor	Vascular dementia (sudden onset)	rs2920	C	T	0.00	23884780	0.937	0.021	8292	C	T	0.592	1	23558289	2.20602E-06	0.125	360283	40501.384
Interleukin-9	Vascular dementia (sudden onset)	rs12423672	T	G	0.01	5047705	0.821	0.047	3634	T	G	0.759	12	4938539	4.89159E-06	0.166	360283	30610.567
Interleukin-9	Vascular dementia (sudden onset)	rs146524495	A	G	0.03	175789555	0.667	0.079	3634	A	G	1.281	4	174868404	3.04229E-06	0.275	360283	23934.344
Interleukin-9	Vascular dementia (sudden onset)	rs2920	C	T	-0.01	23884780	0.805	0.031	3634	C	T	0.592	1	23558289	2.20602E-06	0.125	360283	40501.384
Interleukin-5	Vascular dementia (sudden onset)	rs151268476	G	A	-0.02	38879088	0.800	0.081	3364	G	A	0.971	4	38877467	4.81415E-06	0.212	360283	27499.206
Interleukin-5	Vascular dementia (sudden onset)	rs2920	C	T	-0.01	23884780	0.683	0.033	3364	C	T	0.592	1	23558289	2.20602E-06	0.125	360283	40501.384
Interleukin-4	Vascular dementia (sudden onset)	rs12452096	A	G	0.01	75544032	0.537	0.022	8124	A	G	0.750	17	77547950	1.48929E-06	0.156	360283	77865.614
Interleukin-4	Vascular dementia (sudden onset)	rs151268476	G	A	-0.01	38879088	0.866	0.079	8124	G	A	0.971	4	38877467	4.81415E-06	0.212	360283	27499.206
Interleukin-4	Vascular dementia (sudden onset)	rs188011869	A	G	0.02	112465648	0.728	0.048	8124	A	G	1.046	5	113129951	3.42405E-06	0.225	360283	25635.289
Interleukin-2 receptor antagonist	Vascular dementia (sudden onset)	rs12452096	A	G	0.00	75544032	0.889	0.032	3677	A	G	0.750	17	77547950	1.48929E-06	0.156	360283	77865.614
Interleukin-2 receptor antagonist	Vascular dementia (sudden onset)	rs2920	C	T	-0.01	23884780	0.702	0.031	3677	C	T	0.592	1	23558289	2.20602E-06	0.125	360283	40501.384
Interleukin-2	Vascular dementia (sudden onset)	rs146524495	A	G	-0.01	175789555	0.927	0.081	3475	A	G	1.281	4	174868404	3.04229E-06	0.275	360283	23934.344
Interleukin-2	Vascular dementia (sudden onset)	rs188011869	A	G	0.00	112465648	0.917	0.074	3475	A	G	1.046	5	113129951	3.42405E-06	0.225	360283	25635.289
Interferon gamma	Vascular dementia (sudden onset)	rs12423672	T	G	0.01	5047705	0.774	0.032	7701	T	G	0.759	12	4938539	4.89159E-06	0.166	360283	30610.567
Interferon gamma	Vascular dementia (sudden onset)	rs146524495	A	G	0.03	175789555	0.619	0.056	7701	A	G	1.281	4	174868404	3.04229E-06	0.275	360283	23934.344
Interferon gamma	Vascular dementia (sudden onset)	rs151268476	G	A	-0.04	38879088	0.609	0.080	7701	G	A	0.971	4	38877467	4.81415E-06	0.212	360283	27499.206
Growth-regulated protein alpha	Vascular dementia (sudden onset)	rs12452096	A	G	0.00	75544032	0.967	0.033	3505	A	G	0.750	17	77547950	1.48929E-06	0.156	360283	77865.614
Growth-regulated protein alpha	Vascular dementia (sudden onset)	rs188011869	A	G	0.02	112465648	0.803	0.073	3505	A	G	1.046	5	113129951	3.42405E-06	0.225	360283	25635.289
Granulocyte-colony stimulating factor	Vascular dementia (sudden onset)	rs12452096	A	G	0.01	75544032	0.587	0.022	7904	A	G	0.750	17	77547950	1.48929E-06	0.156	360283	77865.614
Granulocyte-colony stimulating factor	Vascular dementia (sudden onset)	rs151268476	G	A	-0.03	38879088	0.698	0.077	7904	G	A	0.971	4	38877467	4.81415E-06	0.212	360283	27499.206
Granulocyte-colony stimulating factor	Vascular dementia (sudden onset)	rs2920	C	T	-0.01	23884780	0.601	0.021	7904	C	T	0.592	1	23558289	2.20602E-06	0.125	360283	40501.384
Fibroblast growth factor basic	Vascular dementia (sudden onset)	rs12423672	T	G	-0.01	5047705	0.811	0.032	7565	T	G	0.759	12	4938539	4.89159E-06	0.166	360283	30610.567
Fibroblast growth factor basic	Vascular dementia (sudden onset)	rs2920	C	T	-0.01	23884780	0.599	0.022	7565	C	T	0.592	1	23558289	2.20602E-06	0.125	360283	40501.384
Eotaxin	Vascular dementia (sudden onset)	rs12423672	T	G	0.01	5047705	0.678	0.031	8153	T	G	0.759	12	4938539	4.89159E-06	0.166	360283	30610.567
Eotaxin	Vascular dementia (sudden onset)	rs12452096	A	G	0.01	75544032	0.671	0.022	8153	A	G	0.750	17	77547950	1.48929E-06	0.156	360283	77865.614
Eotaxin	Vascular dementia (sudden onset)	rs151268476	G	A	-0.03	38879088	0.710	0.078	8153	G	A	0.971	4	38877467	4.81415E-06	0.212	360283	27499.206
CTACK	Vascular dementia (undefined)	rs117241576	G	A	0.07	13423538	0.693	0.127	3631	G	A	-1.900	16	13329681	6.73892E-08	0.352	361227	30866.248
CTACK	Vascular dementia (undefined)	rs118138505	A	G	0.00	6390642	0.939	0.069	3631	A	G	-0.733	12	6281476	6.97365E-07	0.148	361227	13293.031
CTACK	Vascular dementia (undefined)	rs12224047	T	C	0.00	36820790	0.937	0.030	3631	T	C	-0.263	11	36799240	4.56278E-06	0.057	361227	8033.487
CTACK	Vascular dementia (undefined)	rs12449066	G	A	0.01	79177293	0.839	0.028	3631	G	A	-0.262	16	79143396	2.01674E-06	0.055	361227	8803.514
CTACK	Vascular dementia (undefined)	rs2978951	G	A	0.01	6823295	0.631	0.024	3631	G	A	0.248	8	6965773	2.48222E-08	0.045	361227	11061.935
CTACK	Vascular dementia (undefined)	rs429358	C	T	0.02	45411941	0.457	0.031	3631	C	T	0.695	19	44908684	9.26616E-39	0.053	361227	58999.679
CTACK	Vascular dementia (undefined)	rs71298638	A	G	0.01	63232261	0.847	0.049	3631	A	G	0.379	3	63246585	1.1165E-06	0.078	361227	6515.599
CTACK	Vascular dementia (undefined)	rs72809811	A	G	-0.02	97292386	0.837	0.100	3631	A	G	0.662	2	96626649	3.91976E-06	0.143	361227	4772.892
CTACK	Vascular dementia (undefined)	rs78566090	A	G	0.02	125740204	0.623	0.044	3631	A	G	0.346	8	124727963	9.44735E-07	0.071	361227	7049.571
beta-nerve growth factor	Vascular dementia (undefined)	rs117294828	G	A	0.02	42948170	0.810	0.067	3531	G	A	0.485	21	41576243	1.12881E-06	0.100	361227	6430.550
beta-nerve growth factor	Vascular dementia (undefined)	rs118138505	A	G	-0.02	6390642	0.776	0.069	3531	A	G	-0.733	12	6281476	6.97365E-07	0.148	361227	13293.031
beta-nerve growth factor	Vascular dementia (undefined)	rs12224047	T	C	0.00	36820790	0.997	0.031	3531	T	C	-0.263	11	36799240	4.56278E-06	0.057	361227	8033.487
beta-nerve growth factor	Vascular dementia (undefined)	rs147201592	A	G	0.03	84731690	0.770	0.096	3531	A	G	-0.933	12	84337911	9.39918E-07	0.190	361227	14139.389
beta-nerve growth factor	Vascular dementia (undefined)	rs429358	C	T	-0.04	45411941	0.208	0.031	3531	C	T	0.695	19	44908684	9.26616E-39	0.053	361227	58999.679
beta-nerve growth factor	Vascular dementia (undefined)	rs6133343	G	T	-0.02	721797	0.722	0.042	3531	G	T	0.312	20	741153	3.89825E-06	0.068	361227	6210.744
Vascular endothelial growth factor	Vascular dementia (undefined)	rs118138505	A	G	0.02	6390642	0.679	0.049	7118	A	G	-0.733	12	6281476	6.97365E-07	0.148	361227	13293.031
Vascular endothelial growth factor	Vascular dementia (undefined)	rs12224047	T	C	-0.01	36820790	0.630	0.022	7118	T	C	-0.263	11	36799240	4.56278E-06	0.057	361227	8033.487
Vascular endothelial growth factor	Vascular dementia (undefined)	rs12449066	G	A	0.00	79177293	0.992	0.020	7118	G	A	-0.262	16	79143396	2.01674E-06	0.055	361227	8803.514
Vascular endothelial growth factor	Vascular dementia (undefined)	rs147201592	A	G	-0.04	84731690	0.584	0.067	7118	A	G	-0.933	12	84337911	9.39918E-07	0.190	361227	14139.389
Vascular endothelial growth factor	Vascular dementia (undefined)	rs2978951	G	A	0.00	6823295	0.818	0.017	7118	G	A	0.248	8	6965773	2.48222E-08	0.045	361227	11061.935
Vascular endothelial growth factor	Vascular dementia (undefined)	rs359878	C	T	0.00	185438949	0.975	0.018	7118	C	T	-0.211	2	184574222	4.6443E-06	0.046	361227	6959.700
Vascular endothelial growth factor	Vascular dementia (undefined)	rs429358	C	T	0.03	45411941	0.126	0.022	7118	C	T	0.695	19	44908684	9.26616E-39	0.053	361227	58999.679
Vascular endothelial growth factor	Vascular dementia (undefined)	rs6133343	G	T	0.01	721797	0.882	0.029	7118	G	T	0.312	20	741153	3.89825E-06	0.068	361227	6210.744
Vascular endothelial growth factor	Vascular dementia (undefined)	rs71298638	A	G	0.00	63232261	0.921	0.037	7118	A	G	0.379	3	63246585	1.1165E-06	0.078	361227	6515.599
Vascular endothelial growth factor	Vascular dementia (undefined)	rs72809811	A	G	0.03	97292386	0.700	0.074	7118	A	G	0.662	2	96626649	3.91976E-06	0.143	361227	4772.892

Macrophage Migration Inhibitory Factor	Vascular dementia (undefined)	rs118138505	A	G	-0.02	6390642	0.783	0.070	3494	A	G	-0.733	12	6281476	6.97365E-07	0.148	361227	13293.031
Macrophage Migration Inhibitory Factor	Vascular dementia (undefined)	rs147201592	A	G	-0.01	84731690	0.913	0.099	3494	A	G	-0.933	12	84337911	9.39918E-07	0.190	361227	14139.389
Macrophage Migration Inhibitory Factor	Vascular dementia (undefined)	rs429358	C	T	-0.02	45411941	0.558	0.031	3494	C	T	0.695	19	44908684	9.26616E-39	0.053	361227	58999.679
Macrophage Migration Inhibitory Factor	Vascular dementia (undefined)	rs71298638	A	G	-0.02	63232261	0.705	0.050	3494	A	G	0.379	3	63246585	1.1165E-06	0.078	361227	6515.599
TRAIL	Vascular dementia (undefined)	rs117294828	G	A	-0.01	42948170	0.773	0.044	8186	G	A	0.485	21	41576243	1.12881E-06	0.100	361227	6430.550
TRAIL	Vascular dementia (undefined)	rs12224047	T	C	0.01	36820790	0.698	0.020	8186	T	C	-0.263	11	36799240	4.56278E-06	0.057	361227	8033.487
TRAIL	Vascular dementia (undefined)	rs2978951	G	A	-0.01	6823295	0.553	0.016	8186	G	A	0.248	8	6965773	2.48222E-08	0.045	361227	11061.935
TRAIL	Vascular dementia (undefined)	rs429358	C	T	0.00	45411941	0.880	0.020	8186	C	T	0.695	19	44908684	9.26616E-39	0.053	361227	58999.679
TRAIL	Vascular dementia (undefined)	rs6133343	G	T	-0.01	721797	0.605	0.027	8186	G	T	0.312	20	741153	3.89825E-06	0.068	361227	6210.744
TRAIL	Vascular dementia (undefined)	rs72809811	A	G	0.02	97292386	0.694	0.069	8186	A	G	0.662	2	96626649	3.91976E-06	0.143	361227	4772.892
TRAIL	Vascular dementia (undefined)	rs78566090	A	G	0.02	125740204	0.494	0.028	8186	A	G	0.346	8	124727963	9.44735E-07	0.071	361227	7049.571
Tumor necrosis factor 1 alpha	Vascular dementia (undefined)	rs118138505	A	G	0.00	6390642	0.939	0.071	3454	A	G	-0.733	12	6281476	6.97365E-07	0.148	361227	13293.031
Tumor necrosis factor 1 alpha	Vascular dementia (undefined)	rs12224047	T	C	-0.01	36820790	0.847	0.031	3454	T	C	-0.263	11	36799240	4.56278E-06	0.057	361227	8033.487
Tumor necrosis factor 1 alpha	Vascular dementia (undefined)	rs147201592	A	G	0.02	84731690	0.829	0.099	3454	A	G	-0.933	12	84337911	9.39918E-07	0.190	361227	14139.389
Tumor necrosis factor 1 alpha	Vascular dementia (undefined)	rs2978951	G	A	0.00	6823295	0.865	0.024	3454	G	A	0.248	8	6965773	2.48222E-08	0.045	361227	11061.935
Tumor necrosis factor 1 alpha	Vascular dementia (undefined)	rs359878	C	T	-0.01	185438949	0.702	0.026	3454	C	T	-0.211	2	184574222	4.6443E-06	0.046	361227	6959.700
Tumor necrosis factor 1 alpha	Vascular dementia (undefined)	rs429358	C	T	0.01	45411941	0.825	0.032	3454	C	T	0.695	19	44908684	9.26616E-39	0.053	361227	58999.679
Tumor necrosis factor 1 alpha	Vascular dementia (undefined)	rs6133343	G	T	0.00	721797	0.909	0.043	3454	G	T	0.312	20	741153	3.89825E-06	0.068	361227	6210.744
Tumor necrosis factor 1 alpha	Vascular dementia (undefined)	rs71298638	A	G	-0.02	63232261	0.707	0.050	3454	A	G	0.379	3	63246585	1.1165E-06	0.078	361227	6515.599
Stromal-cell-derived factor 1 alpha	Vascular dementia (undefined)	rs117241576	G	A	0.06	13423538	0.953	0.097	5998	G	A	-1.900	16	13329681	6.73892E-08	0.352	361227	30866.248
Stromal-cell-derived factor 1 alpha	Vascular dementia (undefined)	rs117294828	G	A	-0.02	42948170	0.332	0.045	5998	G	A	0.485	21	41576243	1.12881E-06	0.100	361227	6430.550
Stromal-cell-derived factor 1 alpha	Vascular dementia (undefined)	rs12224047	T	C	0.00	36820790	0.632	0.020	5998	T	C	-0.263	11	36799240	4.56278E-06	0.057	361227	8033.487
Stromal-cell-derived factor 1 alpha	Vascular dementia (undefined)	rs147201592	A	G	-0.03	84731690	0.231	0.063	5998	A	G	-0.933	12	84337911	9.39918E-07	0.190	361227	14139.389
Stromal-cell-derived factor 1 alpha	Vascular dementia (undefined)	rs2978951	G	A	0.01	6823295	0.463	0.017	5998	G	A	0.248	8	6965773	2.48222E-08	0.045	361227	11061.935
Stromal-cell-derived factor 1 alpha	Vascular dementia (undefined)	rs429358	C	T	0.03	45411941	0.135	0.021	5998	C	T	0.695	19	44908684	9.26616E-39	0.053	361227	58999.679
Stromal-cell-derived factor 1 alpha	Vascular dementia (undefined)	rs72809811	A	G	-0.03	97292386	0.650	0.070	5998	A	G	0.662	2	96626649	3.91976E-06	0.143	361227	4772.892
Stem cell growth factor beta	Vascular dementia (undefined)	rs118138505	A	G	-0.02	6390642	0.709	0.068	3682	A	G	-0.733	12	6281476	6.97365E-07	0.148	361227	13293.031
Stem cell growth factor beta	Vascular dementia (undefined)	rs147201592	A	G	0.03	84731690	0.723	0.094	3682	A	G	-0.933	12	84337911	9.39918E-07	0.190	361227	14139.389
Stem cell growth factor beta	Vascular dementia (undefined)	rs6133343	G	T	-0.01	721797	0.856	0.041	3682	G	T	0.312	20	741153	3.89825E-06	0.068	361227	6210.744
Stem cell growth factor beta	Vascular dementia (undefined)	rs72809811	A	G	0.01	97292386	0.923	0.099	3682	A	G	0.662	2	96626649	3.91976E-06	0.143	361227	4772.892
Stem cell factor	Vascular dementia (undefined)	rs118138505	A	G	-0.01	6390642	0.742	0.045	8290	A	G	-0.733	12	6281476	6.97365E-07	0.148	361227	13293.031
Stem cell factor	Vascular dementia (undefined)	rs12449066	G	A	0.01	79177293	0.579	0.018	8290	G	A	-0.262	16	79143396	2.01674E-06	0.055	361227	8803.514
Stem cell factor	Vascular dementia (undefined)	rs147201592	A	G	0.00	84731690	0.959	0.061	8290	A	G	-0.933	12	84337911	9.39918E-07	0.190	361227	14139.389
Stem cell factor	Vascular dementia (undefined)	rs429358	C	T	0.02	45411941	0.445	0.020	8290	C	T	0.695	19	44908684	9.26616E-39	0.053	361227	58999.679
Stem cell factor	Vascular dementia (undefined)	rs6133343	G	T	0.01	721797	0.791	0.026	8290	G	T	0.312	20	741153	3.89825E-06	0.068	361227	6210.744
Stem cell factor	Vascular dementia (undefined)	rs71298638	A	G	0.00	63232261	0.974	0.034	8290	A	G	0.379	3	63246585	1.1165E-06	0.078	361227	6515.599
Stem cell factor	Vascular dementia (undefined)	rs78566090	A	G	0.01	125740204	0.689	0.028	8290	A	G	0.346	8	124727963	9.44735E-07	0.071	361227	7049.571
Interleukin-16	Vascular dementia (undefined)	rs117241576	G	A	0.04	13423538	0.786	0.129	3483	G	A	-1.900	16	13329681	6.73892E-08	0.352	361227	30866.248
Interleukin-16	Vascular dementia (undefined)	rs118138505	A	G	-0.01	6390642	0.860	0.069	3483	A	G	-0.733	12	6281476	6.97365E-07	0.148	361227	13293.031
Interleukin-16	Vascular dementia (undefined)	rs12224047	T	C	0.01	36820790	0.776	0.031	3483	T	C	-0.263	11	36799240	4.56278E-06	0.057	361227	8033.487
Interleukin-16	Vascular dementia (undefined)	rs429358	C	T	0.02	45411941	0.470	0.031	3483	C	T	0.695	19	44908684	9.26616E-39	0.053	361227	58999.679
Interleukin-16	Vascular dementia (undefined)	rs6133343	G	T	0.01	721797	0.881	0.042	3483	G	T	0.312	20	741153	3.89825E-06	0.068	361227	6210.744
RANTES	Vascular dementia (undefined)	rs12449066	G	A	0.01	79177293	0.761	0.029	3421	G	A	-0.262	16	79143396	2.01674E-06	0.055	361227	8803.514
RANTES	Vascular dementia (undefined)	rs2978951	G	A	-0.01	6823295	0.784	0.025	3421	G	A	0.248	8	6965773	2.48222E-08	0.045	361227	11061.935
RANTES	Vascular dementia (undefined)	rs429358	C	T	-0.02	45411941	0.575	0.032	3421	C	T	0.695	19	44908684	9.26616E-39	0.053	361227	58999.679
RANTES	Vascular dementia (undefined)	rs78566090	A	G	0.01	125740204	0.819	0.045	3421	A	G	0.346	8	124727963	9.44735E-07	0.071	361227	7049.571
Platelet-derived growth factor BB	Vascular dementia (undefined)	rs117294828	G	A	-0.03	42948170	0.533	0.044	8293	G	A	0.485	21	41576243	1.12881E-06	0.100	361227	6430.550
Platelet-derived growth factor BB	Vascular dementia (undefined)	rs118138505	A	G	0.02	6390642	0.609	0.045	8293	A	G	-0.733	12	6281476	6.97365E-07	0.148	361227	13293.031
Platelet-derived growth factor BB	Vascular dementia (undefined)	rs12224047	T	C	-0.01	36820790	0.606	0.020	8293	T	C	-0.263	11	36799240	4.56278E-06	0.057	361227	8033.487
Platelet-derived growth factor BB	Vascular dementia (undefined)	rs147201592	A	G	0.00	84731690	0.938	0.062	8293	A	G	-0.933	12	84337911	9.39918E-07	0.190	361227	14139.389
Platelet-derived growth factor BB	Vascular dementia (undefined)	rs429358	C	T	0.00	45411941	0.978	0.020	8293	C	T	0.695	19	44908684	9.26616E-39	0.053	361227	58999.679
Platelet-derived growth factor BB	Vascular dementia (undefined)	rs78566090	A	G	-0.01	125740204	0.739	0.028	8293	A	G	0.346	8	124727963	9.44735E-07	0.071	361227	7049.571
Macrophage inflammatory protein 1b	Vascular dementia (undefined)	rs12224047	T	C	0.00	36820790	0.964	0.020	8243	T	C	-0.263	11	36799240	4.56278E-06	0.057	361227	8033.487
Macrophage inflammatory protein 1b	Vascular dementia (undefined)	rs147201592	A	G	-0.02	84731690	0.766	0.062	8243	A	G	-0.933	12	84337911	9.39918E-07	0.190	361227	14139.389
Macrophage inflammatory protein 1b	Vascular dementia (undefined)	rs2978951	G	A	-0.01	6823295	0.502	0.016	8243	G	A	0.248	8	6965773	2.48222E-08	0.045	361227	11061.935
Macrophage inflammatory protein 1a	Vascular dementia (undefined)	rs118138505	A	G	-0.03	6390642	0.673	0.071	3522	A	G	-0.733	12	6281476	6.97365E-07	0.148	361227	13293.031
Macrophage inflammatory protein 1a	Vascular dementia (undefined)	rs359878	C	T	0.00	185438949	0.929	0.026	3522	C	T	-0.211	2	184574222	4.6443E-06	0.046	361227	6959.700
Macrophage inflammatory protein 1a	Vascular dementia (undefined)	rs429358	C	T	0.04	45411941	0.254	0.031	3522	C	T	0.695	19	44908684	9.26616E-39	0.053	361227	58999.679
Macrophage inflammatory protein 1a	Vascular dementia (undefined)	rs72809811	A	G	0.02	97292386	0.811	0.100	3522	A	G	0.662	2	96626649	3.91976E-06	0.143	361227	4772.892
Macrophage inflammatory protein 1a	Vascular dementia (undefined)	rs78566090	A	G	0.00	125740204	0.976	0.045	3522	A	G	0.346	8	124727963	9.44735E-07	0.071	361227	7049.571
Monokine induced by gamma interferon	Vascular dementia (undefined)	rs117294828	G	A	-0.01	42948170	0.914	0.064	3685	G	A	0.485	21	41576243	1.12881E-06	0.100	361227	6430.550
Monokine induced by gamma interferon	Vascular dementia (undefined)	rs12449066	G	A	0.00	79177293	0.927	0.028	3685	G	A	-0.262	16	79143396	2.01674E-06	0.055	361227	8803.514
Monokine induced by gamma interferon	Vascular dementia (undefined)	rs2978951	G	A	-0.01	6823295	0.770	0.024	3685	G	A	0.248	8	6965773	2.48222E-08	0.045	361227	11061.935
Monokine induced by gamma interferon	Vascular dementia (undefined)	rs429358	C	T	0.00	45411941	0.908	0.030	3685	C	T	0.695	19	44908684	9.26616E-39	0.053	361227	58999.679
Macrophage colony stimulating factor	Vascular dementia (undefined)	rs147201592	A	G	0.02	84731690	0.923	0.118	840	A	G	-0.933	12	84337911	9.39918E-07	0.190	361227	14139.389
Macrophage colony stimulating factor	Vascular dementia (undefined)	rs6133343	G	T	0.01	721797	0.891	0.051	840	G	T	0.312	20	741153	3.89825E-06	0.068	361227	6210.744
Monocyte chemoattractant protein-3	Vascular dementia (undefined)	rs2978951	G	A	0.01	6823295	0.865	0.044	843	G	A	0.248	8	6965773	2.48222E-08	0.045	361227	11061.935
Monocyte chemoattractant protein-3	Vascular dementia (undefined)	rs359878	C	T	0.00	185438949	0.987	0.047	843	C	T	-0.211	2	184574222	4.6443E-06	0.046	361227	6959.700

Monocyte chemoattractant protein-3	Vascular dementia (undefined)	rs6133343	G	T	-0.01	721797	0.870	0.075	843	G	T	0.312	20	741153	3.89825E-06	0.068	361227	6210.744
Monocyte chemoattractant protein-1	Vascular dementia (undefined)	rs118138505	A	G	0.02	6390642	0.653	0.045	8293	A	G	-0.733	12	6281476	6.97365E-07	0.148	361227	13293.031
Monocyte chemoattractant protein-1	Vascular dementia (undefined)	rs12224047	T	C	0.00	36820790	0.875	0.020	8293	T	C	-0.263	11	36799240	4.56278E-06	0.057	361227	8033.487
Monocyte chemoattractant protein-1	Vascular dementia (undefined)	rs12449066	G	A	0.01	79177293	0.509	0.018	8293	G	A	-0.262	16	79143396	2.01674E-06	0.055	361227	8803.514
Monocyte chemoattractant protein-1	Vascular dementia (undefined)	rs147952582	T	C	0.05	66659228	0.517	0.078	8293	T	C	-1.186	12	66265448	7.57356E-07	0.240	361227	18292.090
Monocyte chemoattractant protein-1	Vascular dementia (undefined)	rs429358	C	T	-0.01	45411941	0.570	0.020	8293	C	T	0.695	19	44908684	9.26616E-39	0.053	361227	58999.679
Monocyte chemoattractant protein-1	Vascular dementia (undefined)	rs6133343	G	T	0.00	721797	0.846	0.027	8293	G	T	0.312	20	741153	3.89825E-06	0.068	361227	6210.744
Monocyte chemoattractant protein-1	Vascular dementia (undefined)	rs71298638	A	G	0.01	63232261	0.685	0.034	8293	A	G	0.379	3	63246585	1.1165E-06	0.078	361227	6515.599
Monocyte chemoattractant protein-1	Vascular dementia (undefined)	rs72809811	A	G	0.04	97292386	0.560	0.068	8293	A	G	0.662	2	96626649	3.91976E-06	0.143	361227	4772.892
Interleukin-12p70	Vascular dementia (undefined)	rs117294828	G	A	0.01	42948170	0.872	0.044	8270	G	A	0.485	21	41576243	1.12881E-06	0.100	361227	6430.550
Interleukin-12p70	Vascular dementia (undefined)	rs118138505	A	G	0.03	6390642	0.460	0.045	8270	A	G	-0.733	12	6281476	6.97365E-07	0.148	361227	13293.031
Interleukin-12p70	Vascular dementia (undefined)	rs12224047	T	C	0.00	36820790	0.949	0.020	8270	T	C	-0.263	11	36799240	4.56278E-06	0.057	361227	8033.487
Interleukin-12p70	Vascular dementia (undefined)	rs12449066	G	A	-0.01	79177293	0.551	0.019	8270	G	A	-0.262	16	79143396	2.01674E-06	0.055	361227	8803.514
Interleukin-12p70	Vascular dementia (undefined)	rs147201592	A	G	0.00	84731690	0.919	0.061	8270	A	G	-0.933	12	84337911	9.39918E-07	0.190	361227	14139.389
Interleukin-12p70	Vascular dementia (undefined)	rs429358	C	T	0.04	45411941	0.073	0.020	8270	C	T	0.695	19	44908684	9.26616E-39	0.053	361227	58999.679
Interleukin-12p70	Vascular dementia (undefined)	rs72809811	A	G	0.03	97292386	0.662	0.069	8270	A	G	0.662	2	96626649	3.91976E-06	0.143	361227	4772.892
Interleukin-12p70	Vascular dementia (undefined)	rs78566090	A	G	0.02	125740204	0.528	0.028	8270	A	G	0.346	8	124727963	9.44735E-07	0.071	361227	7049.571
Interferon gamma-induced protein 10	Vascular dementia (undefined)	rs118138505	A	G	0.03	6390642	0.692	0.068	3685	A	G	-0.733	12	6281476	6.97365E-07	0.148	361227	13293.031
Interferon gamma-induced protein 10	Vascular dementia (undefined)	rs147201592	A	G	0.01	84731690	0.941	0.094	3685	A	G	-0.933	12	84337911	9.39918E-07	0.190	361227	14139.389
Interferon gamma-induced protein 10	Vascular dementia (undefined)	rs2978951	G	A	-0.01	6823295	0.825	0.024	3685	G	A	0.248	8	6965773	2.48222E-08	0.045	361227	11061.935
Interferon gamma-induced protein 10	Vascular dementia (undefined)	rs429358	C	T	-0.02	45411941	0.602	0.030	3685	C	T	0.695	19	44908684	9.26616E-39	0.053	361227	58999.679
Interferon gamma-induced protein 10	Vascular dementia (undefined)	rs6133343	G	T	0.00	721797	0.905	0.041	3685	G	T	0.312	20	741153	3.89825E-06	0.068	361227	6210.744
Interleukin-18 levels	Vascular dementia (undefined)	rs117294828	G	A	0.00	42948170	0.970	0.064	3636	G	A	0.485	21	41576243	1.12881E-06	0.100	361227	6430.550
Interleukin-18 levels	Vascular dementia (undefined)	rs147201592	A	G	0.00	84731690	0.978	0.095	3636	A	G	-0.933	12	84337911	9.39918E-07	0.190	361227	14139.389
Interleukin-18 levels	Vascular dementia (undefined)	rs429358	C	T	0.04	45411941	0.247	0.031	3636	C	T	0.695	19	44908684	9.26616E-39	0.053	361227	58999.679
Interleukin-18 levels	Vascular dementia (undefined)	rs6133343	G	T	0.00	721797	0.912	0.041	3636	G	T	0.312	20	741153	3.89825E-06	0.068	361227	6210.744
Interleukin-17	Vascular dementia (undefined)	rs117294828	G	A	-0.02	42948170	0.653	0.045	7760	G	A	0.485	21	41576243	1.12881E-06	0.100	361227	6430.550
Interleukin-17	Vascular dementia (undefined)	rs118138505	A	G	0.00	6390642	0.955	0.047	7760	A	G	-0.733	12	6281476	6.97365E-07	0.148	361227	13293.031
Interleukin-17	Vascular dementia (undefined)	rs12224047	T	C	0.00	36820790	0.981	0.020	7760	T	C	-0.263	11	36799240	4.56278E-06	0.057	361227	8033.487
Interleukin-17	Vascular dementia (undefined)	rs12449066	G	A	-0.01	79177293	0.582	0.019	7760	G	A	-0.262	16	79143396	2.01674E-06	0.055	361227	8803.514
Interleukin-17	Vascular dementia (undefined)	rs147201592	A	G	0.01	84731690	0.859	0.065	7760	A	G	-0.933	12	84337911	9.39918E-07	0.190	361227	14139.389
Interleukin-17	Vascular dementia (undefined)	rs147952582	T	C	-0.03	66659228	0.709	0.081	7760	T	C	-1.186	12	66265448	7.57356E-07	0.240	361227	18292.090
Interleukin-17	Vascular dementia (undefined)	rs2978951	G	A	0.01	6823295	0.616	0.017	7760	G	A	0.248	8	6965773	2.48222E-08	0.045	361227	11061.935
Interleukin-17	Vascular dementia (undefined)	rs359878	C	T	0.00	185438949	0.978	0.017	7760	C	T	-0.211	2	184574222	4.6443E-06	0.046	361227	6959.700
Interleukin-17	Vascular dementia (undefined)	rs429358	C	T	0.03	45411941	0.223	0.021	7760	C	T	0.695	19	44908684	9.26616E-39	0.053	361227	58999.679
Interleukin-17	Vascular dementia (undefined)	rs78566090	A	G	-0.02	125740204	0.521	0.029	7760	A	G	0.346	8	124727963	9.44735E-07	0.071	361227	7049.571
Interleukin-13	Vascular dementia (undefined)	rs12224047	T	C	0.00	36820790	0.959	0.031	3557	T	C	-0.263	11	36799240	4.56278E-06	0.057	361227	8033.487
Interleukin-13	Vascular dementia (undefined)	rs12449066	G	A	0.00	79177293	0.860	0.028	3557	G	A	-0.262	16	79143396	2.01674E-06	0.055	361227	8803.514
Interleukin-13	Vascular dementia (undefined)	rs147201592	A	G	0.01	84731690	0.938	0.099	3557	A	G	-0.933	12	84337911	9.39918E-07	0.190	361227	14139.389
Interleukin-13	Vascular dementia (undefined)	rs2978951	G	A	-0.01	6823295	0.788	0.024	3557	G	A	0.248	8	6965773	2.48222E-08	0.045	361227	11061.935
Interleukin-13	Vascular dementia (undefined)	rs429358	C	T	0.03	45411941	0.311	0.031	3557	C	T	0.695	19	44908684	9.26616E-39	0.053	361227	58999.679
Interleukin-13	Vascular dementia (undefined)	rs71298638	A	G	-0.02	63232261	0.749	0.049	3557	A	G	0.379	3	63246585	1.1165E-06	0.078	361227	6515.599
Interleukin-13	Vascular dementia (undefined)	rs78566090	A	G	0.01	125740204	0.830	0.045	3557	A	G	0.346	8	124727963	9.44735E-07	0.071	361227	7049.571
Interleukin-10	Vascular dementia (undefined)	rs117241576	G	A	0.10	13423538	0.456	0.127	7681	G	A	-1.900	16	13329681	6.73892E-08	0.352	361227	30866.248
Interleukin-10	Vascular dementia (undefined)	rs118138505	A	G	0.03	6390642	0.470	0.047	7681	A	G	-0.733	12	6281476	6.97365E-07	0.148	361227	13293.031
Interleukin-10	Vascular dementia (undefined)	rs12224047	T	C	0.01	36820790	0.601	0.020	7681	T	C	-0.263	11	36799240	4.56278E-06	0.057	361227	8033.487
Interleukin-10	Vascular dementia (undefined)	rs147201592	A	G	-0.02	84731690	0.810	0.063	7681	A	G	-0.933	12	84337911	9.39918E-07	0.190	361227	14139.389
Interleukin-10	Vascular dementia (undefined)	rs2978951	G	A	0.00	6823295	0.942	0.017	7681	G	A	0.248	8	6965773	2.48222E-08	0.045	361227	11061.935
Interleukin-10	Vascular dementia (undefined)	rs429358	C	T	0.03	45411941	0.132	0.021	7681	C	T	0.695	19	44908684	9.26616E-39	0.053	361227	58999.679
Interleukin-10	Vascular dementia (undefined)	rs71298638	A	G	0.01	63232261	0.870	0.035	7681	A	G	0.379	3	63246585	1.1165E-06	0.078	361227	6515.599
Interleukin-8	Vascular dementia (undefined)	rs117294828	G	A	0.00	42948170	0.969	0.065	3526	G	A	0.485	21	41576243	1.12881E-06	0.100	361227	6430.550
Interleukin-8	Vascular dementia (undefined)	rs118138505	A	G	-0.01	6390642	0.925	0.070	3526	A	G	-0.733	12	6281476	6.97365E-07	0.148	361227	13293.031
Interleukin-8	Vascular dementia (undefined)	rs12449066	G	A	0.00	79177293	0.922	0.028	3526	G	A	-0.262	16	79143396	2.01674E-06	0.055	361227	8803.514
Interleukin-8	Vascular dementia (undefined)	rs2978951	G	A	0.01	6823295	0.825	0.024	3526	G	A	0.248	8	6965773	2.48222E-08	0.045	361227	11061.935
Interleukin-8	Vascular dementia (undefined)	rs429358	C	T	0.01	45411941	0.760	0.031	3526	C	T	0.695	19	44908684	9.26616E-39	0.053	361227	58999.679
Interleukin-8	Vascular dementia (undefined)	rs6133343	G	T	0.00	721797	0.932	0.042	3526	G	T	0.312	20	741153	3.89825E-06	0.068	361227	6210.744
Interleukin-8	Vascular dementia (undefined)	rs78566090	A	G	-0.01	125740204	0.823	0.044	3526	A	G	0.346	8	124727963	9.44735E-07	0.071	361227	7049.571
Interleukin-6	Vascular dementia (undefined)	rs117294828	G	A	-0.02	42948170	0.643	0.044	8189	G	A	0.485	21	41576243	1.12881E-06	0.100	361227	6430.550
Interleukin-6	Vascular dementia (undefined)	rs118138505	A	G	0.01	6390642	0.755	0.046	8189	A	G	-0.733	12	6281476	6.97365E-07	0.148	361227	13293.031
Interleukin-6	Vascular dementia (undefined)	rs12224047	T	C	-0.01	36820790	0.524	0.020	8189	T	C	-0.263	11	36799240	4.56278E-06	0.057	361227	8033.487
Interleukin-6	Vascular dementia (undefined)	rs147201592	A	G	0.04	84731690	0.508	0.062	8189	A	G	-0.933	12	84337911	9.39918E-07	0.190	361227	14139.389
Interleukin-6	Vascular dementia (undefined)	rs2978951	G	A	0.00	6823295	0.816	0.016	8189	G	A	0.248	8	6965773	2.48222E-08	0.045	361227	11061.935
Interleukin-6	Vascular dementia (undefined)	rs429358	C	T	0.02	45411941	0.257	0.020	8189	C	T	0.695	19	44908684	9.26616E-39	0.053	361227	58999.679
Interleukin-6	Vascular dementia (undefined)	rs78566090	A	G	0.00	125740204	0.897	0.029	8189	A	G	0.346	8	124727963	9.44735E-07	0.071	361227	7049.571
Interleukin-1-receptor antagonist	Vascular dementia (undefined)	rs117294828	G	A	-0.01	42948170	0.903	0.065	3638	G	A	0.485	21	41576243	1.12881E-06	0.100	361227	6430.550
Interleukin-1-receptor antagonist	Vascular dementia (undefined)	rs118138505	A	G	-0.03	6390642	0.712	0.070	3638	A	G	-0.733	12	6281476	6.97365E-07	0.148	361227	13293.031
Interleukin-1-receptor antagonist	Vascular dementia (undefined)	rs12224047	T	C	0.00	36820790	0.947	0.030	3638	T	C	-0.263	11	36799240	4.56278E-06	0.057	361227	8033.487
Interleukin-1-receptor antagonist	Vascular dementia (undefined)	rs12449066	G	A	0.01	79177293	0.840	0.028	3638	G	A	-0.262	16	79143396	2.01674E-06	0.055	361227	8803.514

Interleukin-1-receptor antagonist	Vascular dementia (undefined)	rs2978951	G	A	0.01	6823295	0.578	0.024	3638	G	A	0.248	8	6965773	2.48222E-08	0.045	361227	11061.935
Interleukin-1-receptor antagonist	Vascular dementia (undefined)	rs359878	C	T	0.00	185438949	0.911	0.025	3638	C	T	-0.211	2	184574222	4.6443E-06	0.046	361227	6959.700
Interleukin-1-receptor antagonist	Vascular dementia (undefined)	rs429358	C	T	-0.02	45411941	0.454	0.031	3638	C	T	0.695	19	44908684	9.26616E-39	0.053	361227	58999.679
Interleukin-1-receptor antagonist	Vascular dementia (undefined)	rs6133343	G	T	-0.01	721797	0.861	0.041	3638	G	T	0.312	20	741153	3.89825E-06	0.068	361227	6210.744
Interleukin-1-receptor antagonist	Vascular dementia (undefined)	rs78566090	A	G	0.01	125740204	0.804	0.044	3638	A	G	0.346	8	124727963	9.44735E-07	0.071	361227	7049.571
Interleukin-1-beta	Vascular dementia (undefined)	rs118138505	A	G	-0.01	6390642	0.512	0.055	3309	A	G	-0.733	12	6281476	6.97365E-07	0.148	361227	13293.031
Interleukin-1-beta	Vascular dementia (undefined)	rs12224047	T	C	-0.01	36820790	0.892	0.024	3309	T	C	-0.263	11	36799240	4.56278E-06	0.057	361227	8033.487
Interleukin-1-beta	Vascular dementia (undefined)	rs12449066	G	A	0.01	79177293	0.340	0.022	3309	G	A	-0.262	16	79143396	2.01674E-06	0.055	361227	8803.514
Interleukin-1-beta	Vascular dementia (undefined)	rs429358	C	T	0.00	45411941	0.471	0.024	3309	C	T	0.695	19	44908684	9.26616E-39	0.053	361227	58999.679
Interleukin-1-beta	Vascular dementia (undefined)	rs6133343	G	T	0.00	721797	0.988	0.033	3309	G	T	0.312	20	741153	3.89825E-06	0.068	361227	6210.744
Hepatocyte growth factor	Vascular dementia (undefined)	rs118138505	A	G	-0.01	6390642	0.898	0.045	8292	A	G	-0.733	12	6281476	6.97365E-07	0.148	361227	13293.031
Hepatocyte growth factor	Vascular dementia (undefined)	rs12224047	T	C	-0.01	36820790	0.530	0.020	8292	T	C	-0.263	11	36799240	4.56278E-06	0.057	361227	8033.487
Hepatocyte growth factor	Vascular dementia (undefined)	rs147201592	A	G	-0.02	84731690	0.739	0.062	8292	A	G	-0.933	12	84337911	9.39918E-07	0.190	361227	14139.389
Hepatocyte growth factor	Vascular dementia (undefined)	rs147952582	T	C	0.04	66659228	0.607	0.078	8292	T	C	-1.186	12	66265448	7.57356E-07	0.240	361227	18292.090
Hepatocyte growth factor	Vascular dementia (undefined)	rs2978951	G	A	-0.01	6823295	0.486	0.016	8292	G	A	0.248	8	6965773	2.48222E-08	0.045	361227	11061.935
Hepatocyte growth factor	Vascular dementia (undefined)	rs429358	C	T	-0.01	45411941	0.703	0.020	8292	C	T	0.695	19	44908684	9.26616E-39	0.053	361227	58999.679
Hepatocyte growth factor	Vascular dementia (undefined)	rs6133343	G	T	0.01	721797	0.591	0.027	8292	G	T	0.312	20	741153	3.89825E-06	0.068	361227	6210.744
Hepatocyte growth factor	Vascular dementia (undefined)	rs71298638	A	G	-0.01	63232261	0.836	0.034	8292	A	G	0.379	3	63246585	1.1165E-06	0.078	361227	6515.599
Interleukin-9	Vascular dementia (undefined)	rs118138505	A	G	-0.01	6390642	0.875	0.071	3634	A	G	-0.733	12	6281476	6.97365E-07	0.148	361227	13293.031
Interleukin-9	Vascular dementia (undefined)	rs12224047	T	C	0.00	36820790	0.960	0.030	3634	T	C	-0.263	11	36799240	4.56278E-06	0.057	361227	8033.487
Interleukin-9	Vascular dementia (undefined)	rs147201592	A	G	0.04	84731690	0.707	0.096	3634	A	G	-0.933	12	84337911	9.39918E-07	0.190	361227	14139.389
Interleukin-9	Vascular dementia (undefined)	rs2978951	G	A	0.00	6823295	0.925	0.024	3634	G	A	0.248	8	6965773	2.48222E-08	0.045	361227	11061.935
Interleukin-9	Vascular dementia (undefined)	rs429358	C	T	-0.02	45411941	0.541	0.031	3634	C	T	0.695	19	44908684	9.26616E-39	0.053	361227	58999.679
Interleukin-9	Vascular dementia (undefined)	rs72809811	A	G	0.00	97292386	0.967	0.099	3634	A	G	0.662	2	96626649	3.91976E-06	0.143	361227	4772.892
Interleukin-7	Vascular dementia (undefined)	rs12224047	T	C	0.01	36820790	0.868	0.031	3409	T	C	-0.263	11	36799240	4.56278E-06	0.057	361227	8033.487
Interleukin-7	Vascular dementia (undefined)	rs71298638	A	G	-0.02	63232261	0.753	0.050	3409	A	G	0.379	3	63246585	1.1165E-06	0.078	361227	6515.599
Interleukin-7	Vascular dementia (undefined)	rs78566090	A	G	0.00	125740204	0.981	0.045	3409	A	G	0.346	8	124727963	9.44735E-07	0.071	361227	7049.571
Interleukin-5	Vascular dementia (undefined)	rs147201592	A	G	-0.02	84731690	0.817	0.100	3364	A	G	-0.933	12	84337911	9.39918E-07	0.190	361227	14139.389
Interleukin-5	Vascular dementia (undefined)	rs429358	C	T	0.02	45411941	0.555	0.032	3364	C	T	0.695	19	44908684	9.26616E-39	0.053	361227	58999.679
Interleukin-4	Vascular dementia (undefined)	rs12224047	T	C	0.00	36820790	0.875	0.020	8124	T	C	-0.263	11	36799240	4.56278E-06	0.057	361227	8033.487
Interleukin-4	Vascular dementia (undefined)	rs12449066	G	A	0.00	79177293	0.893	0.019	8124	G	A	-0.262	16	79143396	2.01674E-06	0.055	361227	8803.514
Interleukin-4	Vascular dementia (undefined)	rs147952582	T	C	-0.03	66659228	0.703	0.080	8124	T	C	-1.186	12	66265448	7.57356E-07	0.240	361227	18292.090
Interleukin-4	Vascular dementia (undefined)	rs2978951	G	A	0.00	6823295	0.948	0.016	8124	G	A	0.248	8	6965773	2.48222E-08	0.045	361227	11061.935
Interleukin-4	Vascular dementia (undefined)	rs359878	C	T	-0.01	185438949	0.544	0.017	8124	C	T	-0.211	2	184574222	4.6443E-06	0.046	361227	6959.700
Interleukin-4	Vascular dementia (undefined)	rs429358	C	T	0.02	45411941	0.305	0.021	8124	C	T	0.695	19	44908684	9.26616E-39	0.053	361227	58999.679
Interleukin-4	Vascular dementia (undefined)	rs6133343	G	T	0.02	721797	0.574	0.027	8124	G	T	0.312	20	741153	3.89825E-06	0.068	361227	6210.744
Interleukin-4	Vascular dementia (undefined)	rs78566090	A	G	-0.02	125740204	0.576	0.029	8124	A	G	0.346	8	124727963	9.44735E-07	0.071	361227	7049.571
Interleukin-2 receptor antagonist	Vascular dementia (undefined)	rs117241576	G	A	0.00	13423538	0.926	0.124	3677	G	A	-1.900	16	13329681	6.73892E-08	0.352	361227	30866.248
Interleukin-2 receptor antagonist	Vascular dementia (undefined)	rs117294828	G	A	-0.02	42948170	0.734	0.064	3677	G	A	0.485	21	41576243	1.12881E-06	0.100	361227	6430.550
Interleukin-2 receptor antagonist	Vascular dementia (undefined)	rs118138505	A	G	0.03	6390642	0.615	0.068	3677	A	G	-0.733	12	6281476	6.97365E-07	0.148	361227	13293.031
Interleukin-2 receptor antagonist	Vascular dementia (undefined)	rs12449066	G	A	0.01	79177293	0.767	0.028	3677	G	A	-0.262	16	79143396	2.01674E-06	0.055	361227	8803.514
Interleukin-2 receptor antagonist	Vascular dementia (undefined)	rs2978951	G	A	0.01	6823295	0.623	0.024	3677	G	A	0.248	8	6965773	2.48222E-08	0.045	361227	11061.935
Interleukin-2 receptor antagonist	Vascular dementia (undefined)	rs359878	C	T	0.01	185438949	0.661	0.025	3677	C	T	-0.211	2	184574222	4.6443E-06	0.046	361227	6959.700
Interleukin-2 receptor antagonist	Vascular dementia (undefined)	rs429358	C	T	-0.01	45411941	0.762	0.030	3677	C	T	0.695	19	44908684	9.26616E-39	0.053	361227	58999.679
Interleukin-2	Vascular dementia (undefined)	rs117241576	G	A	0.07	13423538	0.572	0.138	3475	G	A	-1.900	16	13329681	6.73892E-08	0.352	361227	30866.248
Interleukin-2	Vascular dementia (undefined)	rs429358	C	T	0.03	45411941	0.357	0.031	3475	C	T	0.695	19	44908684	9.26616E-39	0.053	361227	58999.679
Interferon gamma	Vascular dementia (undefined)	rs117294828	G	A	-0.03	42948170	0.489	0.046	7701	G	A	0.485	21	41576243	1.12881E-06	0.100	361227	6430.550
Interferon gamma	Vascular dementia (undefined)	rs12224047	T	C	-0.01	36820790	0.629	0.021	7701	T	C	-0.263	11	36799240	4.56278E-06	0.057	361227	8033.487
Interferon gamma	Vascular dementia (undefined)	rs12449066	G	A	0.00	79177293	0.955	0.019	7701	G	A	-0.262	16	79143396	2.01674E-06	0.055	361227	8803.514
Interferon gamma	Vascular dementia (undefined)	rs147201592	A	G	0.02	84731690	0.725	0.066	7701	A	G	-0.933	12	84337911	9.39918E-07	0.190	361227	14139.389
Interferon gamma	Vascular dementia (undefined)	rs2978951	G	A	-0.01	6823295	0.618	0.017	7701	G	A	0.248	8	6965773	2.48222E-08	0.045	361227	11061.935
Interferon gamma	Vascular dementia (undefined)	rs429358	C	T	0.03	45411941	0.151	0.021	7701	C	T	0.695	19	44908684	9.26616E-39	0.053	361227	58999.679
Interferon gamma	Vascular dementia (undefined)	rs78566090	A	G	0.00	125740204	0.959	0.029	7701	A	G	0.346	8	124727963	9.44735E-07	0.071	361227	7049.571
Growth-regulated protein alpha	Vascular dementia (undefined)	rs12224047	T	C	0.00	36820790	0.958	0.031	3505	T	C	-0.263	11	36799240	4.56278E-06	0.057	361227	8033.487
Growth-regulated protein alpha	Vascular dementia (undefined)	rs78566090	A	G	0.01	125740204	0.784	0.045	3505	A	G	0.346	8	124727963	9.44735E-07	0.071	361227	7049.571
Granulocyte-colony stimulating factor	Vascular dementia (undefined)	rs118138505	A	G	0.02	6390642	0.692	0.046	7904	A	G	-0.733	12	6281476	6.97365E-07	0.148	361227	13293.031
Granulocyte-colony stimulating factor	Vascular dementia (undefined)	rs12224047	T	C	0.00	36820790	0.881	0.020	7904	T	C	-0.263	11	36799240	4.56278E-06	0.057	361227	8033.487
Granulocyte-colony stimulating factor	Vascular dementia (undefined)	rs2978951	G	A	0.00	6823295	0.920	0.016	7904	G	A	0.248	8	6965773	2.48222E-08	0.045	361227	11061.935
Granulocyte-colony stimulating factor	Vascular dementia (undefined)	rs359878	C	T	0.00	185438949	0.988	0.017	7904	C	T	-0.211	2	184574222	4.6443E-06	0.046	361227	6959.700
Granulocyte-colony stimulating factor	Vascular dementia (undefined)	rs78566090	A	G	0.01	125740204	0.667	0.029	7904	A	G	0.346	8	124727963	9.44735E-07	0.071	361227	7049.571
Fibroblast growth factor basic	Vascular dementia (undefined)	rs118138505	A	G	0.02	6390642	0.663	0.048	7565	A	G	-0.733	12	6281476	6.97365E-07	0.148	361227	13293.031
Fibroblast growth factor basic	Vascular dementia (undefined)	rs147952582	T	C	-0.04	66659228	0.672	0.085	7565	T	C	-1.186	12	66265448	7.57356E-07	0.240	361227	18292.090
Fibroblast growth factor basic	Vascular dementia (undefined)	rs2978951	G	A	0.00	6823295	0.945	0.017	7565	G	A	0.248	8	6965773	2.48222E-08	0.045	361227	11061.935
Fibroblast growth factor basic	Vascular dementia (undefined)	rs359878	C	T	0.01	185438949	0.674	0.018	7565	C	T	-0.211	2	184574222	4.6443E-06	0.046	361227	6959.700
Fibroblast growth factor basic	Vascular dementia (undefined)	rs72809811	A	G	0.03	97292386	0.699	0.070	7565	A	G	0.662	2	96626649	3.91976E-06	0.143	361227	4772.892
Fibroblast growth factor basic	Vascular dementia (undefined)	rs78566090	A	G	-0.02	125740204	0.517	0.030	7565	A	G	0.346	8	124727963	9.44735E-07	0.071	361227	7049.571
Eotaxin	Vascular dementia (undefined)	rs117241576	G	A	0.04	13423538	0.811	0.092	8153	G	A	-1.900	16	13329681	6.73892E-08	0.352	361227	30866.248
Eotaxin	Vascular dementia (undefined)	rs118138505	A	G	-0.02	6390642	0.725	0.046	8153	A	G	-0.733	12	6281476	6.97365E-07	0.148	361227	13293.031

Eotaxin	Vascular dementia (undefined)	rs12224047	T	C	0.00	36820790	0.920	0.020	8153	T	C	-0.263	11	36799240	4.56278E-06	0.057	361227	8033.487
Eotaxin	Vascular dementia (undefined)	rs12449066	G	A	-0.01	79177293	0.682	0.019	8153	G	A	-0.262	16	79143396	2.01674E-06	0.055	361227	8803.514
Eotaxin	Vascular dementia (undefined)	rs147201592	A	G	0.03	84731690	0.662	0.062	8153	A	G	-0.933	12	84337911	9.39918E-07	0.190	361227	14139.389
Eotaxin	Vascular dementia (undefined)	rs147952582	T	C	0.03	66659228	0.698	0.078	8153	T	C	-1.186	12	66265448	7.57356E-07	0.240	361227	18292.090
Eotaxin	Vascular dementia (undefined)	rs2978951	G	A	0.00	6823295	0.899	0.016	8153	G	A	0.248	8	6965773	2.48222E-08	0.045	361227	11061.935
Eotaxin	Vascular dementia (undefined)	rs429358	C	T	0.00	45411941	0.845	0.020	8153	C	T	0.695	19	44908684	9.26616E-39	0.053	361227	58999.679
Eotaxin	Vascular dementia (undefined)	rs78566090	A	G	0.02	125740204	0.563	0.029	8153	A	G	0.346	8	124727963	9.44735E-07	0.071	361227	7049.571
