# Supplementary material for: Role of inflammatory cytokines and the gut microbiome in vascular dementia: insights from Mendelian randomization analysis
Source: Front Microbiol. 2024 Aug 23;15:1398618. doi: 10.3389/fmicb.2024.1398618 (PMC11380139; doi:10.3389/fmicb.2024.1398618)
Supplement: Supplementary file 1 [file Data_Sheet_1.zip › Supplementary Table S4.pdf]

Supplementary Table S4 Instrumental Variables related to exposures (6 subtypes of vascular dementia) and outcomes (gut microbiome)

Outcome	Exposure	SNP	Outcome							Exposure								F-statistics
			Effect allele	Other allele	Beta	Pos	Pval	SE	Samplesize	Effect allele	Other allele	Beta	Chr	Pos	Pval	SE	Samplesize	
class Actinobacteria	Vascular dementia (multiple infarctions)	rs1454336	A	G	0.002	91873093	0.868	0.015	14306	A	G	-0.369	4	90951942	4.48415E-06	0.080	360612	13616.170
class Actinobacteria	Vascular dementia (multiple infarctions)	rs34288661	C	T	-0.007	20948329	0.768	0.017	14306	C	T	0.460	8	21090818	7.22903E-06	0.103	360612	11969.376
class Actinobacteria	Vascular dementia (multiple infarctions)	rs429358	C	T	0.020	45411941	0.162	0.016	14306	C	T	0.660	19	44908684	1.33506E-17	0.077	360612	52325.340
class Actinobacteria	Vascular dementia (multiple infarctions)	rs4716814	T	C	0.009	157723046	0.436	0.011	14306	T	C	-0.319	7	157930354	3.75656E-07	0.063	360612	19180.021
class Actinobacteria	Vascular dementia (multiple infarctions)	rs4725579	C	A	-0.008	139468213	0.529	0.014	14306	C	A	-0.379	7	139768414	3.58039E-06	0.082	360612	18391.668
class Actinobacteria	Vascular dementia (multiple infarctions)	rs73053797	T	C	0.012	29909039	0.393	0.016	14306	T	C	0.338	3	29867548	6.00772E-06	0.075	360612	13680.576
class Alphaproteobacteria	Vascular dementia (multiple infarctions)	rs1454336	A	G	-0.006	91873093	0.794	0.019	14306	A	G	-0.369	4	90951942	4.48415E-06	0.080	360612	13616.170
class Alphaproteobacteria	Vascular dementia (multiple infarctions)	rs34288661	C	T	0.013	20948329	0.544	0.021	14306	C	T	0.460	8	21090818	7.22903E-06	0.103	360612	11969.376
class Alphaproteobacteria	Vascular dementia (multiple infarctions)	rs429358	C	T	-0.004	45411941	0.874	0.021	14306	C	T	0.660	19	44908684	1.33506E-17	0.077	360612	52325.340
class Alphaproteobacteria	Vascular dementia (multiple infarctions)	rs4716814	T	C	-0.008	157723046	0.597	0.015	14306	T	C	-0.319	7	157930354	3.75656E-07	0.063	360612	19180.021
class Alphaproteobacteria	Vascular dementia (multiple infarctions)	rs72822148	T	C	-0.011	9742028	0.589	0.016	14306	T	C	-0.322	17	9838711	3.18831E-06	0.069	360612	17983.471
class Alphaproteobacteria	Vascular dementia (multiple infarctions)	rs9861644	A	G	0.014	88637331	0.388	0.017	14306	A	G	0.323	3	88588181	4.84942E-06	0.071	360612	17137.658
class Bacilli	Vascular dementia (multiple infarctions)	rs11081443	C	T	0.005	8944208	0.802	0.013	14306	C	T	0.464	18	8944210	5.71874E-06	0.102	360612	19066.663
class Bacilli	Vascular dementia (multiple infarctions)	rs429358	C	T	0.014	45411941	0.379	0.015	14306	C	T	0.660	19	44908684	1.33506E-17	0.077	360612	52325.340
class Bacilli	Vascular dementia (multiple infarctions)	rs4716814	T	C	-0.011	157723046	0.333	0.011	14306	T	C	-0.319	7	157930354	3.75656E-07	0.063	360612	19180.021
class Bacilli	Vascular dementia (multiple infarctions)	rs4725579	C	A	-0.001	139468213	0.962	0.013	14306	C	A	-0.379	7	139768414	3.58039E-06	0.082	360612	18391.668
class Bacilli	Vascular dementia (multiple infarctions)	rs72822148	T	C	-0.006	9742028	0.448	0.012	14306	T	C	-0.322	17	9838711	3.18831E-06	0.069	360612	17983.471
class Bacilli	Vascular dementia (multiple infarctions)	rs9861644	A	G	0.001	88637331	0.952	0.012	14306	A	G	0.323	3	88588181	4.84942E-06	0.071	360612	17137.658
class Bacteroidia	Vascular dementia (multiple infarctions)	rs11081443	C	T	-0.007	8944208	0.599	0.013	14306	C	T	0.464	18	8944210	5.71874E-06	0.102	360612	19066.663
class Bacteroidia	Vascular dementia (multiple infarctions)	rs1454336	A	G	0.000	91873093	0.932	0.014	14306	A	G	-0.369	4	90951942	4.48415E-06	0.080	360612	13616.170
class Bacteroidia	Vascular dementia (multiple infarctions)	rs34288661	C	T	-0.009	20948329	0.598	0.016	14306	C	T	0.460	8	21090818	7.22903E-06	0.103	360612	11969.376
class Bacteroidia	Vascular dementia (multiple infarctions)	rs429358	C	T	-0.022	45411941	0.148	0.015	14306	C	T	0.660	19	44908684	1.33506E-17	0.077	360612	52325.340
class Bacteroidia	Vascular dementia (multiple infarctions)	rs4716814	T	C	-0.002	157723046	0.858	0.011	14306	T	C	-0.319	7	157930354	3.75656E-07	0.063	360612	19180.021
class Bacteroidia	Vascular dementia (multiple infarctions)	rs4725579	C	A	-0.011	139468213	0.405	0.013	14306	C	A	-0.379	7	139768414	3.58039E-06	0.082	360612	18391.668
class Bacteroidia	Vascular dementia (multiple infarctions)	rs72822148	T	C	-0.001	9742028	0.866	0.011	14306	T	C	-0.322	17	9838711	3.18831E-06	0.069	360612	17983.471
class Bacteroidia	Vascular dementia (multiple infarctions)	rs73053797	T	C	0.012	29909039	0.373	0.015	14306	T	C	0.338	3	29867548	6.00772E-06	0.075	360612	13680.576
class Betaproteobacteria	Vascular dementia (multiple infarctions)	rs11081443	C	T	-0.011	8944208	0.412	0.013	14306	C	T	0.464	18	8944210	5.71874E-06	0.102	360612	19066.663
class Betaproteobacteria	Vascular dementia (multiple infarctions)	rs1454336	A	G	-0.010	91873093	0.410	0.014	14306	A	G	-0.369	4	90951942	4.48415E-06	0.080	360612	13616.170
class Betaproteobacteria	Vascular dementia (multiple infarctions)	rs34288661	C	T	0.007	20948329	0.577	0.016	14306	C	T	0.460	8	21090818	7.22903E-06	0.103	360612	11969.376
class Betaproteobacteria	Vascular dementia (multiple infarctions)	rs429358	C	T	0.003	45411941	0.834	0.016	14306	C	T	0.660	19	44908684	1.33506E-17	0.077	360612	52325.340
class Betaproteobacteria	Vascular dementia (multiple infarctions)	rs72822148	T	C	-0.009	9742028	0.504	0.012	14306	T	C	-0.322	17	9838711	3.18831E-06	0.069	360612	17983.471
class Betaproteobacteria	Vascular dementia (multiple infarctions)	rs73053797	T	C	0.004	29909039	0.766	0.015	14306	T	C	0.338	3	29867548	6.00772E-06	0.075	360612	13680.576
class Betaproteobacteria	Vascular dementia (multiple infarctions)	rs9861644	A	G	0.007	88637331	0.560	0.012	14306	A	G	0.323	3	88588181	4.84942E-06	0.071	360612	17137.658
class Clostridia	Vascular dementia (multiple infarctions)	rs1454336	A	G	0.000	91873093	0.999	0.014	14306	A	G	-0.369	4	90951942	4.48415E-06	0.080	360612	13616.170
class Clostridia	Vascular dementia (multiple infarctions)	rs34288661	C	T	-0.010	20948329	0.569	0.016	14306	C	T	0.460	8	21090818	7.22903E-06	0.103	360612	11969.376
class Clostridia	Vascular dementia (multiple infarctions)	rs429358	C	T	0.005	45411941	0.705	0.015	14306	C	T	0.660	19	44908684	1.33506E-17	0.077	360612	52325.340
class Clostridia	Vascular dementia (multiple infarctions)	rs4716814	T	C	-0.003	157723046	0.762	0.011	14306	T	C	-0.319	7	157930354	3.75656E-07	0.063	360612	19180.021
class Clostridia	Vascular dementia (multiple infarctions)	rs72822148	T	C	0.005	9742028	0.555	0.011	14306	T	C	-0.322	17	9838711	3.18831E-06	0.069	360612	17983.471
class Clostridia	Vascular dementia (multiple infarctions)	rs73053797	T	C	-0.005	29909039	0.786	0.015	14306	T	C	0.338	3	29867548	6.00772E-06	0.075	360612	13680.576
class Coriobacteriia	Vascular dementia (multiple infarctions)	rs34288661	C	T	-0.010	20948329	0.531	0.016	14306	C	T	0.460	8	21090818	7.22903E-06	0.103	360612	11969.376
class Coriobacteriia	Vascular dementia (multiple infarctions)	rs429358	C	T	0.019	45411941	0.204	0.015	14306	C	T	0.660	19	44908684	1.33506E-17	0.077	360612	52325.340
class Coriobacteriia	Vascular dementia (multiple infarctions)	rs4716814	T	C	-0.002	157723046	0.849	0.011	14306	T	C	-0.319	7	157930354	3.75656E-07	0.063	360612	19180.021
class Coriobacteriia	Vascular dementia (multiple infarctions)	rs4725579	C	A	-0.006	139468213	0.583	0.013	14306	C	A	-0.379	7	139768414	3.58039E-06	0.082	360612	18391.668
class Coriobacteriia	Vascular dementia (multiple infarctions)	rs73053797	T	C	-0.008	29909039	0.584	0.015	14306	T	C	0.338	3	29867548	6.00772E-06	0.075	360612	13680.576
class Coriobacteriia	Vascular dementia (multiple infarctions)	rs9861644	A	G	-0.008	88637331	0.489	0.012	14306	A	G	0.323	3	88588181	4.84942E-06	0.071	360612	17137.658
class Deltaproteobacteria	Vascular dementia (multiple infarctions)	rs11081443	C	T	0.011	8944208	0.464	0.014	14306	C	T	0.464	18	8944210	5.71874E-06	0.102	360612	19066.663
class Deltaproteobacteria	Vascular dementia (multiple infarctions)	rs1454336	A	G	-0.001	91873093	0.906	0.015	14306	A	G	-0.369	4	90951942	4.48415E-06	0.080	360612	13616.170
class Deltaproteobacteria	Vascular dementia (multiple infarctions)	rs34288661	C	T	-0.014	20948329	0.420	0.017	14306	C	T	0.460	8	21090818	7.22903E-06	0.103	360612	11969.376
class Deltaproteobacteria	Vascular dementia (multiple infarctions)	rs429358	C	T	0.016	45411941	0.340	0.016	14306	C	T	0.660	19	44908684	1.33506E-17	0.077	360612	52325.340
class Deltaproteobacteria	Vascular dementia (multiple infarctions)	rs4716814	T	C	-0.010	157723046	0.410	0.012	14306	T	C	-0.319	7	157930354	3.75656E-07	0.063	360612	19180.021
class Deltaproteobacteria	Vascular dementia (multiple infarctions)	rs4725579	C	A	0.009	139468213	0.509	0.014	14306	C	A	-0.379	7	139768414	3.58039E-06	0.082	360612	18391.668
class Deltaproteobacteria	Vascular dementia (multiple infarctions)	rs72822148	T	C	0.003	9742028	0.766	0.012	14306	T	C	-0.322	17	9838711	3.18831E-06	0.069	360612	17983.471
class Deltaproteobacteria	Vascular dementia (multiple infarctions)	rs73053797	T	C	-0.012	29909039	0.514	0.016	14306	T	C	0.338	3	29867548	6.00772E-06	0.075	360612	13680.576
class Deltaproteobacteria	Vascular dementia (multiple infarctions)	rs9861644	A	G	0.000	88637331	0.885	0.013	14306	A	G	0.323	3	88588181	4.84942E-06	0.071	360612	17137.658
class Erysipelotrichia	Vascular dementia (multiple infarctions)	rs4716814	T	C	0.001	157723046	0.900	0.011	14306	T	C	-0.319	7	157930354	3.75656E-07	0.063	360612	19180.021
class Erysipelotrichia	Vascular dementia (multiple infarctions)	rs4725579	C	A	0.011	139468213	0.475	0.013	14306	C	A	-0.379	7	139768414	3.58039E-06	0.082	360612	18391.668
class Erysipelotrichia	Vascular dementia (multiple infarctions)	rs72822148	T	C	-0.004	9742028	0.542	0.011	14306	T	C	-0.322	17	9838711	3.18831E-06	0.069	360612	17983.471
class Erysipelotrichia	Vascular dementia (multiple infarctions)	rs73053797	T	C	0.000	29909039	0.937	0.015	14306	T	C	0.338	3	29867548	6.00772E-06	0.075	360612	13680.576
class Erysipelotrichia	Vascular dementia (multiple infarctions)	rs9861644	A	G	-0.004	88637331	0.706	0.012	14306	A	G	0.323	3	88588181	4.84942E-06	0.071	360612	17137.658
class Gammaproteobacteria	Vascular dementia (multiple infarctions)	rs11081443	C	T	-0.012	8944208	0.300	0.014	14306	C	T	0.464	18	8944210	5.71874E-06	0.102	360612	19066.663
class Gammaproteobacteria	Vascular dementia (multiple infarctions)	rs1454336	A	G	0.007	91873093	0.615	0.015	14306	A	G	-0.369	4	90951942	4.48415E-06	0.080	360612	13616.170
class Gammaproteobacteria	Vascular dementia (multiple infarctions)	rs429358	C	T	-0.008	45411941	0.695	0.016	14306	C	T	0.660	19	44908684	1.33506E-17	0.077	360612	52325.340
class Gammaproteobacteria	Vascular dementia (multiple infarctions)	rs4725579	C	A	-0.008	139468213	0.650	0.014	14306	C	A	-0.379	7	139768414	3.58039E-06	0.082	360612	18391.668
class Gammaproteobacteria	Vascular dementia (multiple infarctions)	rs72822148	T	C	-0.005	9742028	0.706	0.012	14306	T	C	-0.322	17	9838711	3.18831E-06	0.069	360612	17983.471
class Gammaproteobacteria	Vascular dementia (multiple infarctions)	rs73053797	T	C	-0.001	29909039	0.924	0.016	14306	T	C	0.338	3	29867548	6.00772E-06	0.075	360612	13680.576
class Lentisphaeria	Vascular dementia (multiple infarctions)	rs1454336	A	G	-0.019	91873093	0.512	0.027	14306	A	G	-0.369	4	90951942	4.48415E-06	0.080	360612	13616.170
class Lentisphaeria	Vascular dementia (multiple infarctions)	rs34288661	C	T	0.002	20948329	0.898	0.030	14306	C	T	0.460	8	21090818	7.22903E-06	0.103	360612	11969.376
class Lentisphaeria	Vascular dementia (multiple infarctions)	rs429358	C	T	-0.012	45411941	0.587	0.030	14306	C	T	0.660	19	44908684	1.33506E-17	0.077	360612	52325.340

class Lentisphaeria	Vascular dementia (multiple infarctions)	rs4716814	T	C	-0.018	157723046	0.399	0.021	14306	T	C	-0.319	7	157930354	3.75656E-07	0.063	360612	19180.021
class Lentisphaeria	Vascular dementia (multiple infarctions)	rs73053797	T	C	-0.026	29909039	0.435	0.029	14306	T	C	0.338	3	29867548	6.00772E-06	0.075	360612	13680.576
class Lentisphaeria	Vascular dementia (multiple infarctions)	rs9861644	A	G	-0.020	88637331	0.387	0.024	14306	A	G	0.323	3	88588181	4.84942E-06	0.071	360612	17137.658
class Melainabacteria	Vascular dementia (multiple infarctions)	rs11081443	C	T	-0.018	8944208	0.438	0.022	14306	C	T	0.464	18	8944210	5.71874E-06	0.102	360612	19066.663
class Melainabacteria	Vascular dementia (multiple infarctions)	rs34288661	C	T	0.019	20948329	0.381	0.027	14306	C	T	0.460	8	21090818	7.22903E-06	0.103	360612	11969.376
class Melainabacteria	Vascular dementia (multiple infarctions)	rs429358	C	T	-0.003	45411941	0.968	0.026	14306	C	T	0.660	19	44908684	1.33506E-17	0.077	360612	52325.340
class Melainabacteria	Vascular dementia (multiple infarctions)	rs4716814	T	C	0.004	157723046	0.857	0.019	14306	T	C	-0.319	7	157930354	3.75656E-07	0.063	360612	19180.021
class Melainabacteria	Vascular dementia (multiple infarctions)	rs4725579	C	A	-0.018	139468213	0.454	0.023	14306	C	A	-0.379	7	139768414	3.58039E-06	0.082	360612	18391.668
class Melainabacteria	Vascular dementia (multiple infarctions)	rs72822148	T	C	0.002	9742028	0.918	0.020	14306	T	C	-0.322	17	9838711	3.18831E-06	0.069	360612	17983.471
class Melainabacteria	Vascular dementia (multiple infarctions)	rs73053797	T	C	-0.006	29909039	0.740	0.027	14306	T	C	0.338	3	29867548	6.00772E-06	0.075	360612	13680.576
class Melainabacteria	Vascular dementia (multiple infarctions)	rs9861644	A	G	0.004	88637331	0.782	0.021	14306	A	G	0.323	3	88588181	4.84942E-06	0.071	360612	17137.658
class Methanobacteria	Vascular dementia (multiple infarctions)	rs1454336	A	G	0.006	91873093	0.824	0.030	14306	A	G	-0.369	4	90951942	4.48415E-06	0.080	360612	13616.170
class Methanobacteria	Vascular dementia (multiple infarctions)	rs429358	C	T	-0.026	45411941	0.504	0.034	14306	C	T	0.660	19	44908684	1.33506E-17	0.077	360612	52325.340
class Methanobacteria	Vascular dementia (multiple infarctions)	rs4725579	C	A	0.004	139468213	0.882	0.030	14306	C	A	-0.379	7	139768414	3.58039E-06	0.082	360612	18391.668
class Methanobacteria	Vascular dementia (multiple infarctions)	rs72822148	T	C	0.008	9742028	0.721	0.026	14306	T	C	-0.322	17	9838711	3.18831E-06	0.069	360612	17983.471
class Methanobacteria	Vascular dementia (multiple infarctions)	rs9861644	A	G	0.008	88637331	0.765	0.027	14306	A	G	0.323	3	88588181	4.84942E-06	0.071	360612	17137.658
class Mollicutes	Vascular dementia (multiple infarctions)	rs1454336	A	G	-0.011	91873093	0.588	0.017	14306	A	G	-0.369	4	90951942	4.48415E-06	0.080	360612	13616.170
class Mollicutes	Vascular dementia (multiple infarctions)	rs429358	C	T	0.000	45411941	0.938	0.019	14306	C	T	0.660	19	44908684	1.33506E-17	0.077	360612	52325.340
class Mollicutes	Vascular dementia (multiple infarctions)	rs4716814	T	C	0.008	157723046	0.551	0.013	14306	T	C	-0.319	7	157930354	3.75656E-07	0.063	360612	19180.021
class Mollicutes	Vascular dementia (multiple infarctions)	rs72822148	T	C	-0.009	9742028	0.449	0.014	14306	T	C	-0.322	17	9838711	3.18831E-06	0.069	360612	17983.471
class Mollicutes	Vascular dementia (multiple infarctions)	rs73053797	T	C	-0.009	29909039	0.684	0.019	14306	T	C	0.338	3	29867548	6.00772E-06	0.075	360612	13680.576
class Mollicutes	Vascular dementia (multiple infarctions)	rs9861644	A	G	0.011	88637331	0.482	0.015	14306	A	G	0.323	3	88588181	4.84942E-06	0.071	360612	17137.658
class Negativicutes	Vascular dementia (multiple infarctions)	rs34288661	C	T	-0.009	20948329	0.516	0.016	14306	C	T	0.460	8	21090818	7.22903E-06	0.103	360612	11969.376
class Negativicutes	Vascular dementia (multiple infarctions)	rs429358	C	T	-0.019	45411941	0.146	0.015	14306	C	T	0.660	19	44908684	1.33506E-17	0.077	360612	52325.340
class Negativicutes	Vascular dementia (multiple infarctions)	rs4716814	T	C	0.002	157723046	0.860	0.011	14306	T	C	-0.319	7	157930354	3.75656E-07	0.063	360612	19180.021
class Negativicutes	Vascular dementia (multiple infarctions)	rs73053797	T	C	-0.008	29909039	0.442	0.015	14306	T	C	0.338	3	29867548	6.00772E-06	0.075	360612	13680.576
class Verrucomicrobiae	Vascular dementia (multiple infarctions)	rs1454336	A	G	-0.003	91873093	0.963	0.017	14306	A	G	-0.369	4	90951942	4.48415E-06	0.080	360612	13616.170
class Verrucomicrobiae	Vascular dementia (multiple infarctions)	rs34288661	C	T	0.007	20948329	0.747	0.019	14306	C	T	0.460	8	21090818	7.22903E-06	0.103	360612	11969.376
class Verrucomicrobiae	Vascular dementia (multiple infarctions)	rs429358	C	T	0.018	45411941	0.336	0.019	14306	C	T	0.660	19	44908684	1.33506E-17	0.077	360612	52325.340
class Verrucomicrobiae	Vascular dementia (multiple infarctions)	rs4725579	C	A	0.000	139468213	0.921	0.016	14306	C	A	-0.379	7	139768414	3.58039E-06	0.082	360612	18391.668
class Verrucomicrobiae	Vascular dementia (multiple infarctions)	rs72822148	T	C	-0.008	9742028	0.627	0.014	14306	T	C	-0.322	17	9838711	3.18831E-06	0.069	360612	17983.471
class Verrucomicrobiae	Vascular dementia (multiple infarctions)	rs73053797	T	C	-0.005	29909039	0.805	0.019	14306	T	C	0.338	3	29867548	6.00772E-06	0.075	360612	13680.576
class Verrucomicrobiae	Vascular dementia (multiple infarctions)	rs9861644	A	G	0.003	88637331	0.852	0.015	14306	A	G	0.323	3	88588181	4.84942E-06	0.071	360612	17137.658
class Actinobacteria	Vascular dementia (mixed)	rs12257900	T	G	-0.005	49443428	0.804	0.017	14306	T	G	0.467	10	48235385	2.48891E-06	0.099	360421	23006.877
class Actinobacteria	Vascular dementia (mixed)	rs1466525	T	C	0.000	54780209	0.937	0.014	14306	T	C	0.489	8	53867649	1.97697E-06	0.103	360421	34189.173
class Actinobacteria	Vascular dementia (mixed)	rs6849229	G	A	0.003	131797280	0.824	0.015	14306	G	A	0.562	4	130876125	4.44038E-06	0.122	360421	34142.711
class Actinobacteria	Vascular dementia (mixed)	rs7614116	G	A	-0.010	130368069	0.393	0.012	14306	G	A	0.379	3	130649225	6.06429E-06	0.084	360421	23465.044
class Actinobacteria	Vascular dementia (mixed)	rs7776624	G	A	0.000	31909839	0.968	0.011	14306	G	A	-0.358	7	31870226	8.82531E-06	0.081	360421	23450.025
class Alphaproteobacteria	Vascular dementia (mixed)	rs12257900	T	G	-0.003	49443428	0.839	0.021	14306	T	G	0.467	10	48235385	2.48891E-06	0.099	360421	23006.877
class Alphaproteobacteria	Vascular dementia (mixed)	rs1632064	T	C	-0.011	3219694	0.581	0.020	14306	T	C	0.614	5	3219580	4.04045E-06	0.133	360421	37296.871
class Alphaproteobacteria	Vascular dementia (mixed)	rs17168895	T	G	0.015	15647727	0.612	0.023	14306	T	G	-0.547	7	15608102	2.92853E-06	0.117	360421	34495.049
class Alphaproteobacteria	Vascular dementia (mixed)	rs429358	C	T	-0.004	45411941	0.874	0.021	14306	C	T	0.565	19	44908684	3.94357E-08	0.103	360421	36831.461
class Alphaproteobacteria	Vascular dementia (mixed)	rs6028529	A	G	0.011	38205487	0.563	0.017	14306	A	G	0.413	20	39576844	4.98965E-06	0.091	360421	21913.090
class Alphaproteobacteria	Vascular dementia (mixed)	rs7614116	G	A	-0.013	130368069	0.387	0.014	14306	G	A	0.379	3	130649225	6.06429E-06	0.084	360421	23465.044
class Alphaproteobacteria	Vascular dementia (mixed)	rs7776624	G	A	0.007	31909839	0.624	0.014	14306	G	A	-0.358	7	31870226	8.82531E-06	0.081	360421	23450.025
class Bacilli	Vascular dementia (mixed)	rs1466525	T	C	0.001	54780209	0.968	0.014	14306	T	C	0.489	8	53867649	1.97697E-06	0.103	360421	34189.173
class Bacilli	Vascular dementia (mixed)	rs1632064	T	C	-0.008	3219694	0.567	0.015	14306	T	C	0.614	5	3219580	4.04045E-06	0.133	360421	37296.871
class Bacilli	Vascular dementia (mixed)	rs17168895	T	G	0.009	15647727	0.681	0.018	14306	T	G	-0.547	7	15608102	2.92853E-06	0.117	360421	34495.049
class Bacilli	Vascular dementia (mixed)	rs429358	C	T	0.014	45411941	0.379	0.015	14306	C	T	0.565	19	44908684	3.94357E-08	0.103	360421	36831.461
class Bacilli	Vascular dementia (mixed)	rs6028529	A	G	0.010	38205487	0.446	0.013	14306	A	G	0.413	20	39576844	4.98965E-06	0.091	360421	21913.090
class Bacilli	Vascular dementia (mixed)	rs7614116	G	A	-0.002	130368069	0.879	0.012	14306	G	A	0.379	3	130649225	6.06429E-06	0.084	360421	23465.044
class Bacilli	Vascular dementia (mixed)	rs7776624	G	A	-0.005	31909839	0.626	0.011	14306	G	A	-0.358	7	31870226	8.82531E-06	0.081	360421	23450.025
class Bacteroidia	Vascular dementia (mixed)	rs12257900	T	G	-0.003	49443428	0.869	0.016	14306	T	G	0.467	10	48235385	2.48891E-06	0.099	360421	23006.877
class Bacteroidia	Vascular dementia (mixed)	rs1466525	T	C	0.002	54780209	0.897	0.013	14306	T	C	0.489	8	53867649	1.97697E-06	0.103	360421	34189.173
class Bacteroidia	Vascular dementia (mixed)	rs1632064	T	C	-0.007	3219694	0.626	0.015	14306	T	C	0.614	5	3219580	4.04045E-06	0.133	360421	37296.871
class Bacteroidia	Vascular dementia (mixed)	rs17168895	T	G	-0.010	15647727	0.562	0.017	14306	T	G	-0.547	7	15608102	2.92853E-06	0.117	360421	34495.049
class Bacteroidia	Vascular dementia (mixed)	rs6028529	A	G	0.003	38205487	0.841	0.012	14306	A	G	0.413	20	39576844	4.98965E-06	0.091	360421	21913.090
class Bacteroidia	Vascular dementia (mixed)	rs6849229	G	A	-0.011	131797280	0.581	0.014	14306	G	A	0.562	4	130876125	4.44038E-06	0.122	360421	34142.711
class Bacteroidia	Vascular dementia (mixed)	rs7614116	G	A	0.002	130368069	0.810	0.011	14306	G	A	0.379	3	130649225	6.06429E-06	0.084	360421	23465.044
class Bacteroidia	Vascular dementia (mixed)	rs7776624	G	A	-0.007	31909839	0.487	0.010	14306	G	A	-0.358	7	31870226	8.82531E-06	0.081	360421	23450.025
class Betaproteobacteria	Vascular dementia (mixed)	rs12257900	T	G	0.011	49443428	0.487	0.016	14306	T	G	0.467	10	48235385	2.48891E-06	0.099	360421	23006.877
class Betaproteobacteria	Vascular dementia (mixed)	rs1466525	T	C	-0.011	54780209	0.427	0.014	14306	T	C	0.489	8	53867649	1.97697E-06	0.103	360421	34189.173
class Betaproteobacteria	Vascular dementia (mixed)	rs1632064	T	C	0.008	3219694	0.613	0.015	14306	T	C	0.614	5	3219580	4.04045E-06	0.133	360421	37296.871
class Betaproteobacteria	Vascular dementia (mixed)	rs429358	C	T	0.003	45411941	0.834	0.016	14306	C	T	0.565	19	44908684	3.94357E-08	0.103	360421	36831.461
class Betaproteobacteria	Vascular dementia (mixed)	rs6028529	A	G	0.012	38205487	0.347	0.013	14306	A	G	0.413	20	39576844	4.98965E-06	0.091	360421	21913.090
class Betaproteobacteria	Vascular dementia (mixed)	rs6849229	G	A	0.005	131797280	0.691	0.015	14306	G	A	0.562	4	130876125	4.44038E-06	0.122	360421	34142.711
class Clostridia	Vascular dementia (mixed)	rs12257900	T	G	0.004	49443428	0.813	0.016	14306	T	G	0.467	10	48235385	2.48891E-06	0.099	360421	23006.877
class Clostridia	Vascular dementia (mixed)	rs1466525	T	C	-0.008	54780209	0.590	0.013	14306	T	C	0.489	8	53867649	1.97697E-06	0.103	360421	34189.173
class Clostridia	Vascular dementia (mixed)	rs1632064	T	C	-0.004	3219694	0.806	0.015	14306	T	C	0.614	5	3219580	4.04045E-06	0.133	360421	37296.871
class Clostridia	Vascular dementia (mixed)	rs17168895	T	G	0.002	15647727	0.901	0.017	14306	T	G	-0.547	7	15608102	2.92853E-06	0.117	360421	34495.049
class Clostridia	Vascular dementia (mixed)	rs429358	C	T	0.005	45411941	0.705	0.015	14306	C	T	0.565	19	44908684	3.94357E-08	0.103	360421	36831.461

class Clostridia	Vascular dementia (mixed)	rs6028529	A	G	-0.007	38205487	0.626	0.012	14306	A	G	0.413	20	39576844	4.98965E-06	0.091	360421	21913.090
class Clostridia	Vascular dementia (mixed)	rs6849229	G	A	0.007	131797280	0.694	0.014	14306	G	A	0.562	4	130876125	4.44038E-06	0.122	360421	34142.711
class Clostridia	Vascular dementia (mixed)	rs7614116	G	A	-0.005	130368069	0.681	0.011	14306	G	A	0.379	3	130649225	6.06429E-06	0.084	360421	23465.044
class Clostridia	Vascular dementia (mixed)	rs7776624	G	A	0.004	31909839	0.681	0.010	14306	G	A	-0.358	7	31870226	8.82531E-06	0.081	360421	23450.025
class Coriobacteriia	Vascular dementia (mixed)	rs12257900	T	G	-0.014	49443428	0.392	0.016	14306	T	G	0.467	10	48235385	2.48891E-06	0.099	360421	23006.877
class Coriobacteriia	Vascular dementia (mixed)	rs1466525	T	C	-0.008	54780209	0.544	0.013	14306	T	C	0.489	8	53867649	1.97697E-06	0.103	360421	34189.173
class Coriobacteriia	Vascular dementia (mixed)	rs1632064	T	C	-0.005	3219694	0.782	0.015	14306	T	C	0.614	5	3219580	4.04045E-06	0.133	360421	37296.871
class Coriobacteriia	Vascular dementia (mixed)	rs17168895	T	G	0.014	15647727	0.431	0.018	14306	T	G	-0.547	7	15608102	2.92853E-06	0.117	360421	34495.049
class Coriobacteriia	Vascular dementia (mixed)	rs7776624	G	A	-0.006	31909839	0.606	0.011	14306	G	A	-0.358	7	31870226	8.82531E-06	0.081	360421	23450.025
class Deltaproteobacteria	Vascular dementia (mixed)	rs12257900	T	G	0.009	49443428	0.649	0.017	14306	T	G	0.467	10	48235385	2.48891E-06	0.099	360421	23006.877
class Deltaproteobacteria	Vascular dementia (mixed)	rs1466525	T	C	0.007	54780209	0.847	0.015	14306	T	C	0.489	8	53867649	1.97697E-06	0.103	360421	34189.173
class Deltaproteobacteria	Vascular dementia (mixed)	rs17168895	T	G	-0.003	15647727	0.867	0.019	14306	T	G	-0.547	7	15608102	2.92853E-06	0.117	360421	34495.049
class Deltaproteobacteria	Vascular dementia (mixed)	rs429358	C	T	0.016	45411941	0.340	0.016	14306	C	T	0.565	19	44908684	3.94357E-08	0.103	360421	36831.461
class Deltaproteobacteria	Vascular dementia (mixed)	rs6028529	A	G	-0.001	38205487	0.951	0.014	14306	A	G	0.413	20	39576844	4.98965E-06	0.091	360421	21913.090
class Deltaproteobacteria	Vascular dementia (mixed)	rs6849229	G	A	0.001	131797280	0.936	0.016	14306	G	A	0.562	4	130876125	4.44038E-06	0.122	360421	34142.711
class Deltaproteobacteria	Vascular dementia (mixed)	rs7614116	G	A	0.005	130368069	0.696	0.013	14306	G	A	0.379	3	130649225	6.06429E-06	0.084	360421	23465.044
class Erysipelotrichia	Vascular dementia (mixed)	rs1466525	T	C	0.009	54780209	0.569	0.013	14306	T	C	0.489	8	53867649	1.97697E-06	0.103	360421	34189.173
class Erysipelotrichia	Vascular dementia (mixed)	rs1632064	T	C	0.001	3219694	0.867	0.015	14306	T	C	0.614	5	3219580	4.04045E-06	0.133	360421	37296.871
class Erysipelotrichia	Vascular dementia (mixed)	rs17168895	T	G	-0.001	15647727	0.965	0.017	14306	T	G	-0.547	7	15608102	2.92853E-06	0.117	360421	34495.049
class Erysipelotrichia	Vascular dementia (mixed)	rs6028529	A	G	0.001	38205487	0.976	0.012	14306	A	G	0.413	20	39576844	4.98965E-06	0.091	360421	21913.090
class Erysipelotrichia	Vascular dementia (mixed)	rs7776624	G	A	0.007	31909839	0.484	0.011	14306	G	A	-0.358	7	31870226	8.82531E-06	0.081	360421	23450.025
class Gammaproteobacteria	Vascular dementia (mixed)	rs12257900	T	G	-0.010	49443428	0.597	0.017	14306	T	G	0.467	10	48235385	2.48891E-06	0.099	360421	23006.877
class Gammaproteobacteria	Vascular dementia (mixed)	rs1466525	T	C	0.002	54780209	0.906	0.014	14306	T	C	0.489	8	53867649	1.97697E-06	0.103	360421	34189.173
class Gammaproteobacteria	Vascular dementia (mixed)	rs17168895	T	G	-0.004	15647727	0.868	0.019	14306	T	G	-0.547	7	15608102	2.92853E-06	0.117	360421	34495.049
class Gammaproteobacteria	Vascular dementia (mixed)	rs429358	C	T	-0.008	45411941	0.695	0.016	14306	C	T	0.565	19	44908684	3.94357E-08	0.103	360421	36831.461
class Gammaproteobacteria	Vascular dementia (mixed)	rs6028529	A	G	0.007	38205487	0.626	0.013	14306	A	G	0.413	20	39576844	4.98965E-06	0.091	360421	21913.090
class Gammaproteobacteria	Vascular dementia (mixed)	rs6849229	G	A	-0.013	131797280	0.455	0.015	14306	G	A	0.562	4	130876125	4.44038E-06	0.122	360421	34142.711
class Gammaproteobacteria	Vascular dementia (mixed)	rs7614116	G	A	0.002	130368069	0.877	0.012	14306	G	A	0.379	3	130649225	6.06429E-06	0.084	360421	23465.044
class Gammaproteobacteria	Vascular dementia (mixed)	rs7776624	G	A	0.007	31909839	0.504	0.011	14306	G	A	-0.358	7	31870226	8.82531E-06	0.081	360421	23450.025
class Lentisphaeria	Vascular dementia (mixed)	rs12257900	T	G	0.015	49443428	0.661	0.030	14306	T	G	0.467	10	48235385	2.48891E-06	0.099	360421	23006.877
class Lentisphaeria	Vascular dementia (mixed)	rs17168895	T	G	0.002	15647727	0.944	0.034	14306	T	G	-0.547	7	15608102	2.92853E-06	0.117	360421	34495.049
class Lentisphaeria	Vascular dementia (mixed)	rs429358	C	T	-0.012	45411941	0.587	0.030	14306	C	T	0.565	19	44908684	3.94357E-08	0.103	360421	36831.461
class Lentisphaeria	Vascular dementia (mixed)	rs6028529	A	G	0.003	38205487	0.868	0.024	14306	A	G	0.413	20	39576844	4.98965E-06	0.091	360421	21913.090
class Lentisphaeria	Vascular dementia (mixed)	rs6849229	G	A	-0.018	131797280	0.450	0.028	14306	G	A	0.562	4	130876125	4.44038E-06	0.122	360421	34142.711
class Melainabacteria	Vascular dementia (mixed)	rs12257900	T	G	-0.016	49443428	0.564	0.027	14306	T	G	0.467	10	48235385	2.48891E-06	0.099	360421	23006.877
class Melainabacteria	Vascular dementia (mixed)	rs1466525	T	C	0.005	54780209	0.758	0.023	14306	T	C	0.489	8	53867649	1.97697E-06	0.103	360421	34189.173
class Melainabacteria	Vascular dementia (mixed)	rs1632064	T	C	0.012	3219694	0.653	0.026	14306	T	C	0.614	5	3219580	4.04045E-06	0.133	360421	37296.871
class Melainabacteria	Vascular dementia (mixed)	rs17168895	T	G	0.002	15647727	0.992	0.030	14306	T	G	-0.547	7	15608102	2.92853E-06	0.117	360421	34495.049
class Melainabacteria	Vascular dementia (mixed)	rs429358	C	T	-0.003	45411941	0.968	0.026	14306	C	T	0.565	19	44908684	3.94357E-08	0.103	360421	36831.461
class Melainabacteria	Vascular dementia (mixed)	rs6028529	A	G	0.016	38205487	0.491	0.022	14306	A	G	0.413	20	39576844	4.98965E-06	0.091	360421	21913.090
class Melainabacteria	Vascular dementia (mixed)	rs6849229	G	A	0.007	131797280	0.810	0.025	14306	G	A	0.562	4	130876125	4.44038E-06	0.122	360421	34142.711
class Melainabacteria	Vascular dementia (mixed)	rs7614116	G	A	-0.017	130368069	0.396	0.020	14306	G	A	0.379	3	130649225	6.06429E-06	0.084	360421	23465.044
class Methanobacteria	Vascular dementia (mixed)	rs12257900	T	G	-0.014	49443428	0.614	0.034	14306	T	G	0.467	10	48235385	2.48891E-06	0.099	360421	23006.877
class Methanobacteria	Vascular dementia (mixed)	rs1466525	T	C	0.012	54780209	0.743	0.029	14306	T	C	0.489	8	53867649	1.97697E-06	0.103	360421	34189.173
class Methanobacteria	Vascular dementia (mixed)	rs1632064	T	C	0.005	3219694	0.822	0.032	14306	T	C	0.614	5	3219580	4.04045E-06	0.133	360421	37296.871
class Methanobacteria	Vascular dementia (mixed)	rs17168895	T	G	0.017	15647727	0.666	0.038	14306	T	G	-0.547	7	15608102	2.92853E-06	0.117	360421	34495.049
class Methanobacteria	Vascular dementia (mixed)	rs429358	C	T	-0.026	45411941	0.504	0.034	14306	C	T	0.565	19	44908684	3.94357E-08	0.103	360421	36831.461
class Mollicutes	Vascular dementia (mixed)	rs12257900	T	G	0.011	49443428	0.585	0.020	14306	T	G	0.467	10	48235385	2.48891E-06	0.099	360421	23006.877
class Mollicutes	Vascular dementia (mixed)	rs1466525	T	C	0.003	54780209	0.849	0.017	14306	T	C	0.489	8	53867649	1.97697E-06	0.103	360421	34189.173
class Mollicutes	Vascular dementia (mixed)	rs17168895	T	G	-0.011	15647727	0.633	0.022	14306	T	G	-0.547	7	15608102	2.92853E-06	0.117	360421	34495.049
class Mollicutes	Vascular dementia (mixed)	rs429358	C	T	0.000	45411941	0.938	0.019	14306	C	T	0.565	19	44908684	3.94357E-08	0.103	360421	36831.461
class Mollicutes	Vascular dementia (mixed)	rs6028529	A	G	-0.008	38205487	0.642	0.016	14306	A	G	0.413	20	39576844	4.98965E-06	0.091	360421	21913.090
class Mollicutes	Vascular dementia (mixed)	rs6849229	G	A	-0.015	131797280	0.325	0.018	14306	G	A	0.562	4	130876125	4.44038E-06	0.122	360421	34142.711
class Mollicutes	Vascular dementia (mixed)	rs7614116	G	A	0.002	130368069	0.932	0.014	14306	G	A	0.379	3	130649225	6.06429E-06	0.084	360421	23465.044
class Negativicutes	Vascular dementia (mixed)	rs1466525	T	C	0.002	54780209	0.917	0.013	14306	T	C	0.489	8	53867649	1.97697E-06	0.103	360421	34189.173
class Negativicutes	Vascular dementia (mixed)	rs1632064	T	C	0.002	3219694	0.990	0.015	14306	T	C	0.614	5	3219580	4.04045E-06	0.133	360421	37296.871
class Negativicutes	Vascular dementia (mixed)	rs6028529	A	G	-0.007	38205487	0.560	0.012	14306	A	G	0.413	20	39576844	4.98965E-06	0.091	360421	21913.090
class Negativicutes	Vascular dementia (mixed)	rs7614116	G	A	0.009	130368069	0.429	0.011	14306	G	A	0.379	3	130649225	6.06429E-06	0.084	360421	23465.044
class Negativicutes	Vascular dementia (mixed)	rs7776624	G	A	-0.004	31909839	0.733	0.011	14306	G	A	-0.358	7	31870226	8.82531E-06	0.081	360421	23450.025
class Verrucomicrobiae	Vascular dementia (mixed)	rs12257900	T	G	-0.014	49443428	0.458	0.019	14306	T	G	0.467	10	48235385	2.48891E-06	0.099	360421	23006.877
class Verrucomicrobiae	Vascular dementia (mixed)	rs1466525	T	C	0.010	54780209	0.549	0.016	14306	T	C	0.489	8	53867649	1.97697E-06	0.103	360421	34189.173
class Verrucomicrobiae	Vascular dementia (mixed)	rs1632064	T	C	0.001	3219694	0.915	0.018	14306	T	C	0.614	5	3219580	4.04045E-06	0.133	360421	37296.871
class Verrucomicrobiae	Vascular dementia (mixed)	rs17168895	T	G	0.012	15647727	0.527	0.021	14306	T	G	-0.547	7	15608102	2.92853E-06	0.117	360421	34495.049
class Verrucomicrobiae	Vascular dementia (mixed)	rs429358	C	T	0.018	45411941	0.336	0.019	14306	C	T	0.565	19	44908684	3.94357E-08	0.103	360421	36831.461
class Verrucomicrobiae	Vascular dementia (mixed)	rs6028529	A	G	-0.013	38205487	0.455	0.016	14306	A	G	0.413	20	39576844	4.98965E-06	0.091	360421	21913.090
class Verrucomicrobiae	Vascular dementia (mixed)	rs6849229	G	A	-0.013	131797280	0.492	0.018	14306	G	A	0.562	4	130876125	4.44038E-06	0.122	360421	34142.711
class Verrucomicrobiae	Vascular dementia (mixed)	rs7614116	G	A	-0.001	130368069	0.999	0.014	14306	G	A	0.379	3	130649225	6.06429E-06	0.084	360421	23465.044
class Actinobacteria	Vascular dementia (subcortical)	rs10919863	T	C	-0.002	200226041	0.977	0.014	14306	T	C	0.315	1	200256913	3.47112E-06	0.068	360770	10813.753
class Actinobacteria	Vascular dementia (subcortical)	rs11148372	A	G	-0.006	22788665	0.590	0.011	14306	A	G	-0.261	13	22214526	4.06275E-06	0.057	360770	12632.549
class Actinobacteria	Vascular dementia (subcortical)	rs11986558	T	C	0.004	2500772	0.760	0.012	14306	T	C	0.248	8	2643276	9.16875E-06	0.056	360770	11030.353
class Actinobacteria	Vascular dementia (subcortical)	rs1363668	G	A	-0.007	143089582	0.535	0.011	14306	G	A	-0.272	5	143710017	5.25352E-06	0.060	360770	12697.214

class Actinobacteria	Vascular dementia (subcortical)	rs3802793	A	G	-0.003	131685316	0.746	0.012	14306	A	G	0.274	11	131815422	5.46626E-06	0.060	360770	12758.334
class Actinobacteria	Vascular dementia (subcortical)	rs429358	C	T	0.020	45411941	0.162	0.016	14306	C	T	0.597	19	44908684	1.74221E-17	0.070	360770	41621.629
class Actinobacteria	Vascular dementia (subcortical)	rs4295569	C	T	-0.008	47820641	0.460	0.012	14306	C	T	-0.355	7	47781043	2.54572E-08	0.064	360770	20037.409
class Actinobacteria	Vascular dementia (subcortical)	rs4723291	A	G	0.000	33551998	0.950	0.011	14306	A	G	-0.263	7	33512386	6.22372E-06	0.058	360770	11064.499
class Alphaproteobacteria	Vascular dementia (subcortical)	rs11148372	A	G	0.006	22788665	0.693	0.015	14306	A	G	-0.261	13	22214526	4.06275E-06	0.057	360770	12632.549
class Alphaproteobacteria	Vascular dementia (subcortical)	rs11986558	T	C	0.005	2500772	0.770	0.015	14306	T	C	0.248	8	2643276	9.16875E-06	0.056	360770	11030.353
class Alphaproteobacteria	Vascular dementia (subcortical)	rs429358	C	T	-0.004	45411941	0.874	0.021	14306	C	T	0.597	19	44908684	1.74221E-17	0.070	360770	41621.629
class Alphaproteobacteria	Vascular dementia (subcortical)	rs4295569	C	T	-0.012	47820641	0.434	0.015	14306	C	T	-0.355	7	47781043	2.54572E-08	0.064	360770	20037.409
class Alphaproteobacteria	Vascular dementia (subcortical)	rs4723291	A	G	0.006	33551998	0.695	0.015	14306	A	G	-0.263	7	33512386	6.22372E-06	0.058	360770	11064.499
class Bacilli	Vascular dementia (subcortical)	rs11148372	A	G	0.002	22788665	0.817	0.011	14306	A	G	-0.261	13	22214526	4.06275E-06	0.057	360770	12632.549
class Bacilli	Vascular dementia (subcortical)	rs11986558	T	C	-0.008	2500772	0.498	0.011	14306	T	C	0.248	8	2643276	9.16875E-06	0.056	360770	11030.353
class Bacilli	Vascular dementia (subcortical)	rs1363668	G	A	0.006	143089582	0.576	0.011	14306	G	A	-0.272	5	143710017	5.25352E-06	0.060	360770	12697.214
class Bacilli	Vascular dementia (subcortical)	rs429358	C	T	0.014	45411941	0.379	0.015	14306	C	T	0.597	19	44908684	1.74221E-17	0.070	360770	41621.629
class Bacilli	Vascular dementia (subcortical)	rs4295569	C	T	-0.002	47820641	0.853	0.011	14306	C	T	-0.355	7	47781043	2.54572E-08	0.064	360770	20037.409
class Bacteroidia	Vascular dementia (subcortical)	rs10919863	T	C	-0.008	200226041	0.514	0.013	14306	T	C	0.315	1	200256913	3.47112E-06	0.068	360770	10813.753
class Bacteroidia	Vascular dementia (subcortical)	rs11148372	A	G	0.003	22788665	0.805	0.011	14306	A	G	-0.261	13	22214526	4.06275E-06	0.057	360770	12632.549
class Bacteroidia	Vascular dementia (subcortical)	rs11986558	T	C	0.008	2500772	0.479	0.011	14306	T	C	0.248	8	2643276	9.16875E-06	0.056	360770	11030.353
class Bacteroidia	Vascular dementia (subcortical)	rs1363668	G	A	0.004	143089582	0.725	0.011	14306	G	A	-0.272	5	143710017	5.25352E-06	0.060	360770	12697.214
class Bacteroidia	Vascular dementia (subcortical)	rs3802793	A	G	0.006	131685316	0.548	0.011	14306	A	G	0.274	11	131815422	5.46626E-06	0.060	360770	12758.334
class Bacteroidia	Vascular dementia (subcortical)	rs429358	C	T	-0.022	45411941	0.148	0.015	14306	C	T	0.597	19	44908684	1.74221E-17	0.070	360770	41621.629
class Bacteroidia	Vascular dementia (subcortical)	rs4295569	C	T	0.006	47820641	0.590	0.011	14306	C	T	-0.355	7	47781043	2.54572E-08	0.064	360770	20037.409
class Betaproteobacteria	Vascular dementia (subcortical)	rs10919863	T	C	-0.010	200226041	0.437	0.014	14306	T	C	0.315	1	200256913	3.47112E-06	0.068	360770	10813.753
class Betaproteobacteria	Vascular dementia (subcortical)	rs11148372	A	G	-0.004	22788665	0.721	0.011	14306	A	G	-0.261	13	22214526	4.06275E-06	0.057	360770	12632.549
class Betaproteobacteria	Vascular dementia (subcortical)	rs1363668	G	A	0.002	143089582	0.873	0.011	14306	G	A	-0.272	5	143710017	5.25352E-06	0.060	360770	12697.214
class Betaproteobacteria	Vascular dementia (subcortical)	rs429358	C	T	0.003	45411941	0.834	0.016	14306	C	T	0.597	19	44908684	1.74221E-17	0.070	360770	41621.629
class Betaproteobacteria	Vascular dementia (subcortical)	rs4723291	A	G	-0.005	33551998	0.666	0.011	14306	A	G	-0.263	7	33512386	6.22372E-06	0.058	360770	11064.499
class Clostridia	Vascular dementia (subcortical)	rs10919863	T	C	0.000	200226041	0.912	0.013	14306	T	C	0.315	1	200256913	3.47112E-06	0.068	360770	10813.753
class Clostridia	Vascular dementia (subcortical)	rs11148372	A	G	0.007	22788665	0.507	0.011	14306	A	G	-0.261	13	22214526	4.06275E-06	0.057	360770	12632.549
class Clostridia	Vascular dementia (subcortical)	rs11986558	T	C	-0.001	2500772	0.911	0.011	14306	T	C	0.248	8	2643276	9.16875E-06	0.056	360770	11030.353
class Clostridia	Vascular dementia (subcortical)	rs1363668	G	A	-0.001	143089582	0.935	0.011	14306	G	A	-0.272	5	143710017	5.25352E-06	0.060	360770	12697.214
class Clostridia	Vascular dementia (subcortical)	rs3802793	A	G	-0.008	131685316	0.499	0.011	14306	A	G	0.274	11	131815422	5.46626E-06	0.060	360770	12758.334
class Clostridia	Vascular dementia (subcortical)	rs429358	C	T	0.005	45411941	0.705	0.015	14306	C	T	0.597	19	44908684	1.74221E-17	0.070	360770	41621.629
class Clostridia	Vascular dementia (subcortical)	rs4295569	C	T	0.002	47820641	0.814	0.011	14306	C	T	-0.355	7	47781043	2.54572E-08	0.064	360770	20037.409
class Coriobacteriia	Vascular dementia (subcortical)	rs3802793	A	G	0.007	131685316	0.516	0.011	14306	A	G	0.274	11	131815422	5.46626E-06	0.060	360770	12758.334
class Coriobacteriia	Vascular dementia (subcortical)	rs429358	C	T	0.019	45411941	0.204	0.015	14306	C	T	0.597	19	44908684	1.74221E-17	0.070	360770	41621.629
class Coriobacteriia	Vascular dementia (subcortical)	rs4295569	C	T	0.002	47820641	0.863	0.011	14306	C	T	-0.355	7	47781043	2.54572E-08	0.064	360770	20037.409
class Coriobacteriia	Vascular dementia (subcortical)	rs4382795	C	T	-0.007	66878853	0.591	0.014	14306	C	T	0.378	10	65119095	9.80754E-06	0.086	360770	13265.712
class Coriobacteriia	Vascular dementia (subcortical)	rs4723291	A	G	-0.006	33551998	0.567	0.011	14306	A	G	-0.263	7	33512386	6.22372E-06	0.058	360770	11064.499
class Deltaproteobacteria	Vascular dementia (subcortical)	rs11986558	T	C	-0.006	2500772	0.598	0.012	14306	T	C	0.248	8	2643276	9.16875E-06	0.056	360770	11030.353
class Deltaproteobacteria	Vascular dementia (subcortical)	rs3802793	A	G	0.005	131685316	0.656	0.012	14306	A	G	0.274	11	131815422	5.46626E-06	0.060	360770	12758.334
class Deltaproteobacteria	Vascular dementia (subcortical)	rs429358	C	T	0.016	45411941	0.340	0.016	14306	C	T	0.597	19	44908684	1.74221E-17	0.070	360770	41621.629
class Deltaproteobacteria	Vascular dementia (subcortical)	rs4295569	C	T	-0.006	47820641	0.613	0.012	14306	C	T	-0.355	7	47781043	2.54572E-08	0.064	360770	20037.409
class Deltaproteobacteria	Vascular dementia (subcortical)	rs4382795	C	T	0.008	66878853	0.658	0.015	14306	C	T	0.378	10	65119095	9.80754E-06	0.086	360770	13265.712
class Deltaproteobacteria	Vascular dementia (subcortical)	rs4723291	A	G	0.010	33551998	0.431	0.012	14306	A	G	-0.263	7	33512386	6.22372E-06	0.058	360770	11064.499
class Erysipelotrichia	Vascular dementia (subcortical)	rs10919863	T	C	0.010	200226041	0.450	0.013	14306	T	C	0.315	1	200256913	3.47112E-06	0.068	360770	10813.753
class Erysipelotrichia	Vascular dementia (subcortical)	rs11148372	A	G	0.001	22788665	0.885	0.011	14306	A	G	-0.261	13	22214526	4.06275E-06	0.057	360770	12632.549
class Erysipelotrichia	Vascular dementia (subcortical)	rs11986558	T	C	-0.005	2500772	0.657	0.011	14306	T	C	0.248	8	2643276	9.16875E-06	0.056	360770	11030.353
class Erysipelotrichia	Vascular dementia (subcortical)	rs1363668	G	A	0.004	143089582	0.691	0.011	14306	G	A	-0.272	5	143710017	5.25352E-06	0.060	360770	12697.214
class Erysipelotrichia	Vascular dementia (subcortical)	rs4723291	A	G	-0.006	33551998	0.581	0.011	14306	A	G	-0.263	7	33512386	6.22372E-06	0.058	360770	11064.499
class Gammaproteobacteria	Vascular dementia (subcortical)	rs10919863	T	C	0.011	200226041	0.424	0.014	14306	T	C	0.315	1	200256913	3.47112E-06	0.068	360770	10813.753
class Gammaproteobacteria	Vascular dementia (subcortical)	rs11148372	A	G	-0.005	22788665	0.669	0.011	14306	A	G	-0.261	13	22214526	4.06275E-06	0.057	360770	12632.549
class Gammaproteobacteria	Vascular dementia (subcortical)	rs1363668	G	A	0.001	143089582	0.917	0.011	14306	G	A	-0.272	5	143710017	5.25352E-06	0.060	360770	12697.214
class Gammaproteobacteria	Vascular dementia (subcortical)	rs3802793	A	G	-0.009	131685316	0.430	0.012	14306	A	G	0.274	11	131815422	5.46626E-06	0.060	360770	12758.334
class Gammaproteobacteria	Vascular dementia (subcortical)	rs429358	C	T	-0.008	45411941	0.695	0.016	14306	C	T	0.597	19	44908684	1.74221E-17	0.070	360770	41621.629
class Gammaproteobacteria	Vascular dementia (subcortical)	rs4295569	C	T	-0.002	47820641	0.857	0.012	14306	C	T	-0.355	7	47781043	2.54572E-08	0.064	360770	20037.409
class Gammaproteobacteria	Vascular dementia (subcortical)	rs4723291	A	G	0.002	33551998	0.869	0.011	14306	A	G	-0.263	7	33512386	6.22372E-06	0.058	360770	11064.499
class Lentisphaeria	Vascular dementia (subcortical)	rs10919863	T	C	-0.001	200226041	0.950	0.027	14306	T	C	0.315	1	200256913	3.47112E-06	0.068	360770	10813.753
class Lentisphaeria	Vascular dementia (subcortical)	rs11148372	A	G	-0.016	22788665	0.432	0.021	14306	A	G	-0.261	13	22214526	4.06275E-06	0.057	360770	12632.549
class Lentisphaeria	Vascular dementia (subcortical)	rs11986558	T	C	-0.012	2500772	0.581	0.022	14306	T	C	0.248	8	2643276	9.16875E-06	0.056	360770	11030.353
class Lentisphaeria	Vascular dementia (subcortical)	rs1363668	G	A	-0.009	143089582	0.673	0.021	14306	G	A	-0.272	5	143710017	5.25352E-06	0.060	360770	12697.214
class Lentisphaeria	Vascular dementia (subcortical)	rs429358	C	T	-0.012	45411941	0.587	0.030	14306	C	T	0.597	19	44908684	1.74221E-17	0.070	360770	41621.629
class Lentisphaeria	Vascular dementia (subcortical)	rs4295569	C	T	0.012	47820641	0.571	0.021	14306	C	T	-0.355	7	47781043	2.54572E-08	0.064	360770	20037.409
class Lentisphaeria	Vascular dementia (subcortical)	rs4723291	A	G	-0.004	33551998	0.846	0.021	14306	A	G	-0.263	7	33512386	6.22372E-06	0.058	360770	11064.499
class Melainabacteria	Vascular dementia (subcortical)	rs10919863	T	C	0.004	200226041	0.841	0.024	14306	T	C	0.315	1	200256913	3.47112E-06	0.068	360770	10813.753
class Melainabacteria	Vascular dementia (subcortical)	rs11148372	A	G	0.005	22788665	0.771	0.019	14306	A	G	-0.261	13	22214526	4.06275E-06	0.057	360770	12632.549
class Melainabacteria	Vascular dementia (subcortical)	rs11986558	T	C	-0.008	2500772	0.680	0.020	14306	T	C	0.248	8	2643276	9.16875E-06	0.056	360770	11030.353
class Melainabacteria	Vascular dementia (subcortical)	rs1363668	G	A	-0.006	143089582	0.732	0.019	14306	G	A	-0.272	5	143710017	5.25352E-06	0.060	360770	12697.214
class Melainabacteria	Vascular dementia (subcortical)	rs3802793	A	G	-0.014	131685316	0.481	0.020	14306	A	G	0.274	11	131815422	5.46626E-06	0.060	360770	12758.334
class Melainabacteria	Vascular dementia (subcortical)	rs429358	C	T	-0.003	45411941	0.968	0.026	14306	C	T	0.597	19	44908684	1.74221E-17	0.070	360770	41621.629
class Melainabacteria	Vascular dementia (subcortical)	rs4295569	C	T	-0.014	47820641	0.443	0.019	14306	C	T	-0.355	7	47781043	2.54572E-08	0.064	360770	20037.409
class Methanobacteria	Vascular dementia (subcortical)	rs10919863	T	C	0.000	200226041	0.946	0.030	14306	T	C	0.315	1	200256913	3.47112E-06	0.068	360770	10813.753

class Methanobacteria	Vascular dementia (subcortical)	rs1363668	G	A	-0.015	143089582	0.517	0.024	14306	G	A	-0.272	5	143710017	5.25352E-06	0.060	360770	12697.214
class Methanobacteria	Vascular dementia (subcortical)	rs429358	C	T	-0.026	45411941	0.504	0.034	14306	C	T	0.597	19	44908684	1.74221E-17	0.070	360770	41621.629
class Methanobacteria	Vascular dementia (subcortical)	rs4382795	C	T	-0.024	66878853	0.400	0.030	14306	C	T	0.378	10	65119095	9.80754E-06	0.086	360770	13265.712
class Mollicutes	Vascular dementia (subcortical)	rs10919863	T	C	-0.011	200226041	0.481	0.017	14306	T	C	0.315	1	200256913	3.47112E-06	0.068	360770	10813.753
class Mollicutes	Vascular dementia (subcortical)	rs429358	C	T	0.000	45411941	0.938	0.019	14306	C	T	0.597	19	44908684	1.74221E-17	0.070	360770	41621.629
class Mollicutes	Vascular dementia (subcortical)	rs4723291	A	G	-0.003	33551998	0.809	0.013	14306	A	G	-0.263	7	33512386	6.22372E-06	0.058	360770	11064.499
class Negativicutes	Vascular dementia (subcortical)	rs11148372	A	G	-0.002	22788665	0.830	0.011	14306	A	G	-0.261	13	22214526	4.06275E-06	0.057	360770	12632.549
class Negativicutes	Vascular dementia (subcortical)	rs3802793	A	G	0.006	131685316	0.678	0.011	14306	A	G	0.274	11	131815422	5.46626E-06	0.060	360770	12758.334
class Negativicutes	Vascular dementia (subcortical)	rs429358	C	T	-0.019	45411941	0.146	0.015	14306	C	T	0.597	19	44908684	1.74221E-17	0.070	360770	41621.629
class Negativicutes	Vascular dementia (subcortical)	rs4295569	C	T	0.000	47820641	0.990	0.011	14306	C	T	-0.355	7	47781043	2.54572E-08	0.064	360770	20037.409
class Verrucomicrobiae	Vascular dementia (subcortical)	rs10919863	T	C	-0.002	200226041	0.924	0.017	14306	T	C	0.315	1	200256913	3.47112E-06	0.068	360770	10813.753
class Verrucomicrobiae	Vascular dementia (subcortical)	rs11148372	A	G	0.007	22788665	0.560	0.013	14306	A	G	-0.261	13	22214526	4.06275E-06	0.057	360770	12632.549
class Verrucomicrobiae	Vascular dementia (subcortical)	rs1363668	G	A	0.005	143089582	0.711	0.013	14306	G	A	-0.272	5	143710017	5.25352E-06	0.060	360770	12697.214
class Verrucomicrobiae	Vascular dementia (subcortical)	rs3802793	A	G	-0.006	131685316	0.656	0.014	14306	A	G	0.274	11	131815422	5.46626E-06	0.060	360770	12758.334
class Verrucomicrobiae	Vascular dementia (subcortical)	rs429358	C	T	0.018	45411941	0.336	0.019	14306	C	T	0.597	19	44908684	1.74221E-17	0.070	360770	41621.629
class Verrucomicrobiae	Vascular dementia (subcortical)	rs4295569	C	T	0.001	47820641	0.930	0.013	14306	C	T	-0.355	7	47781043	2.54572E-08	0.064	360770	20037.409
class Verrucomicrobiae	Vascular dementia (subcortical)	rs4382795	C	T	-0.005	66878853	0.768	0.017	14306	C	T	0.378	10	65119095	9.80754E-06	0.086	360770	13265.712
class Verrucomicrobiae	Vascular dementia (subcortical)	rs4723291	A	G	-0.002	33551998	0.916	0.013	14306	A	G	-0.263	7	33512386	6.22372E-06	0.058	360770	11064.499
class Actinobacteria	Vascular dementia (sudden onset)	rs12452096	A	G	-0.010	75544032	0.397	0.013	14306	A	G	0.750	17	77547950	1.48929E-06	0.156	360283	77865.614
class Actinobacteria	Vascular dementia (sudden onset)	rs4840457	C	T	0.012	6408682	0.603	0.021	14306	C	T	0.643	8	6551161	8.92874E-06	0.145	360283	32615.797
class Actinobacteria	Vascular dementia (sudden onset)	rs71511414	G	A	-0.017	79688991	0.461	0.020	14306	G	A	0.681	9	77074075	7.80746E-06	0.152	360283	30731.948
class Alphaproteobacteria	Vascular dementia (sudden onset)	rs2920	C	T	0.015	23884780	0.432	0.018	14306	C	T	0.592	1	23558289	2.20602E-06	0.125	360283	40501.384
class Alphaproteobacteria	Vascular dementia (sudden onset)	rs429358	C	T	-0.004	45411941	0.874	0.021	14306	C	T	0.601	19	44908684	7.45813E-06	0.134	360283	42162.979
class Alphaproteobacteria	Vascular dementia (sudden onset)	rs4840457	C	T	-0.016	6408682	0.508	0.027	14306	C	T	0.643	8	6551161	8.92874E-06	0.145	360283	32615.797
class Alphaproteobacteria	Vascular dementia (sudden onset)	rs71511414	G	A	-0.011	79688991	0.693	0.026	14306	G	A	0.681	9	77074075	7.80746E-06	0.152	360283	30731.948
class Bacilli	Vascular dementia (sudden onset)	rs12452096	A	G	0.004	75544032	0.779	0.013	14306	A	G	0.750	17	77547950	1.48929E-06	0.156	360283	77865.614
class Bacilli	Vascular dementia (sudden onset)	rs71511414	G	A	-0.008	79688991	0.663	0.020	14306	G	A	0.681	9	77074075	7.80746E-06	0.152	360283	30731.948
class Bacteroidia	Vascular dementia (sudden onset)	rs12423672	T	G	-0.007	5047705	0.534	0.017	14306	T	G	0.759	12	4938539	4.89159E-06	0.166	360283	30610.567
class Bacteroidia	Vascular dementia (sudden onset)	rs2920	C	T	0.008	23884780	0.550	0.013	14306	C	T	0.592	1	23558289	2.20602E-06	0.125	360283	40501.384
class Betaproteobacteria	Vascular dementia (sudden onset)	rs12452096	A	G	0.003	75544032	0.819	0.013	14306	A	G	0.750	17	77547950	1.48929E-06	0.156	360283	77865.614
class Betaproteobacteria	Vascular dementia (sudden onset)	rs429358	C	T	0.003	45411941	0.834	0.016	14306	C	T	0.601	19	44908684	7.45813E-06	0.134	360283	42162.979
class Betaproteobacteria	Vascular dementia (sudden onset)	rs4840457	C	T	0.009	6408682	0.663	0.020	14306	C	T	0.643	8	6551161	8.92874E-06	0.145	360283	32615.797
class Betaproteobacteria	Vascular dementia (sudden onset)	rs71511414	G	A	-0.012	79688991	0.491	0.020	14306	G	A	0.681	9	77074075	7.80746E-06	0.152	360283	30731.948
class Clostridia	Vascular dementia (sudden onset)	rs12423672	T	G	0.000	5047705	0.942	0.017	14306	T	G	0.759	12	4938539	4.89159E-06	0.166	360283	30610.567
class Clostridia	Vascular dementia (sudden onset)	rs429358	C	T	0.005	45411941	0.705	0.015	14306	C	T	0.601	19	44908684	7.45813E-06	0.134	360283	42162.979
class Clostridia	Vascular dementia (sudden onset)	rs4840457	C	T	-0.008	6408682	0.700	0.020	14306	C	T	0.643	8	6551161	8.92874E-06	0.145	360283	32615.797
class Clostridia	Vascular dementia (sudden onset)	rs71511414	G	A	-0.015	79688991	0.494	0.019	14306	G	A	0.681	9	77074075	7.80746E-06	0.152	360283	30731.948
class Coriobacteriia	Vascular dementia (sudden onset)	rs12452096	A	G	-0.008	75544032	0.499	0.013	14306	A	G	0.750	17	77547950	1.48929E-06	0.156	360283	77865.614
class Coriobacteriia	Vascular dementia (sudden onset)	rs2920	C	T	-0.009	23884780	0.519	0.013	14306	C	T	0.592	1	23558289	2.20602E-06	0.125	360283	40501.384
class Coriobacteriia	Vascular dementia (sudden onset)	rs71511414	G	A	-0.005	79688991	0.860	0.019	14306	G	A	0.681	9	77074075	7.80746E-06	0.152	360283	30731.948
class Deltaproteobacteria	Vascular dementia (sudden onset)	rs12452096	A	G	-0.003	75544032	0.836	0.014	14306	A	G	0.750	17	77547950	1.48929E-06	0.156	360283	77865.614
class Deltaproteobacteria	Vascular dementia (sudden onset)	rs4840457	C	T	0.009	6408682	0.691	0.022	14306	C	T	0.643	8	6551161	8.92874E-06	0.145	360283	32615.797
class Deltaproteobacteria	Vascular dementia (sudden onset)	rs71511414	G	A	-0.009	79688991	0.643	0.021	14306	G	A	0.681	9	77074075	7.80746E-06	0.152	360283	30731.948
class Erysipelotrichia	Vascular dementia (sudden onset)	rs12452096	A	G	0.003	75544032	0.800	0.012	14306	A	G	0.750	17	77547950	1.48929E-06	0.156	360283	77865.614
class Erysipelotrichia	Vascular dementia (sudden onset)	rs2920	C	T	0.000	23884780	0.971	0.013	14306	C	T	0.592	1	23558289	2.20602E-06	0.125	360283	40501.384
class Erysipelotrichia	Vascular dementia (sudden onset)	rs4840457	C	T	0.014	6408682	0.472	0.020	14306	C	T	0.643	8	6551161	8.92874E-06	0.145	360283	32615.797
class Erysipelotrichia	Vascular dementia (sudden onset)	rs71511414	G	A	0.011	79688991	0.559	0.019	14306	G	A	0.681	9	77074075	7.80746E-06	0.152	360283	30731.948
class Gammaproteobacteria	Vascular dementia (sudden onset)	rs12423672	T	G	0.009	5047705	0.745	0.018	14306	T	G	0.759	12	4938539	4.89159E-06	0.166	360283	30610.567
class Gammaproteobacteria	Vascular dementia (sudden onset)	rs12452096	A	G	0.004	75544032	0.751	0.013	14306	A	G	0.750	17	77547950	1.48929E-06	0.156	360283	77865.614
class Gammaproteobacteria	Vascular dementia (sudden onset)	rs2920	C	T	-0.004	23884780	0.759	0.014	14306	C	T	0.592	1	23558289	2.20602E-06	0.125	360283	40501.384
class Gammaproteobacteria	Vascular dementia (sudden onset)	rs429358	C	T	-0.008	45411941	0.695	0.016	14306	C	T	0.601	19	44908684	7.45813E-06	0.134	360283	42162.979
class Gammaproteobacteria	Vascular dementia (sudden onset)	rs4840457	C	T	0.011	6408682	0.606	0.021	14306	C	T	0.643	8	6551161	8.92874E-06	0.145	360283	32615.797
class Lentisphaeria	Vascular dementia (sudden onset)	rs12423672	T	G	-0.002	5047705	0.877	0.033	14306	T	G	0.759	12	4938539	4.89159E-06	0.166	360283	30610.567
class Lentisphaeria	Vascular dementia (sudden onset)	rs12452096	A	G	-0.021	75544032	0.435	0.025	14306	A	G	0.750	17	77547950	1.48929E-06	0.156	360283	77865.614
class Lentisphaeria	Vascular dementia (sudden onset)	rs2920	C	T	0.018	23884780	0.533	0.026	14306	C	T	0.592	1	23558289	2.20602E-06	0.125	360283	40501.384
class Lentisphaeria	Vascular dementia (sudden onset)	rs429358	C	T	-0.012	45411941	0.587	0.030	14306	C	T	0.601	19	44908684	7.45813E-06	0.134	360283	42162.979
class Lentisphaeria	Vascular dementia (sudden onset)	rs4840457	C	T	0.004	6408682	0.921	0.039	14306	C	T	0.643	8	6551161	8.92874E-06	0.145	360283	32615.797
class Lentisphaeria	Vascular dementia (sudden onset)	rs71511414	G	A	0.000	79688991	1.000	0.037	14306	G	A	0.681	9	77074075	7.80746E-06	0.152	360283	30731.948
class Melainabacteria	Vascular dementia (sudden onset)	rs12423672	T	G	-0.027	5047705	0.386	0.030	14306	T	G	0.759	12	4938539	4.89159E-06	0.166	360283	30610.567
class Melainabacteria	Vascular dementia (sudden onset)	rs12452096	A	G	-0.017	75544032	0.476	0.022	14306	A	G	0.750	17	77547950	1.48929E-06	0.156	360283	77865.614
class Melainabacteria	Vascular dementia (sudden onset)	rs2920	C	T	-0.007	23884780	0.737	0.023	14306	C	T	0.592	1	23558289	2.20602E-06	0.125	360283	40501.384
class Melainabacteria	Vascular dementia (sudden onset)	rs429358	C	T	-0.003	45411941	0.968	0.026	14306	C	T	0.601	19	44908684	7.45813E-06	0.134	360283	42162.979
class Melainabacteria	Vascular dementia (sudden onset)	rs4840457	C	T	0.021	6408682	0.587	0.034	14306	C	T	0.643	8	6551161	8.92874E-06	0.145	360283	32615.797
class Melainabacteria	Vascular dementia (sudden onset)	rs71511414	G	A	-0.018	79688991	0.583	0.033	14306	G	A	0.681	9	77074075	7.80746E-06	0.152	360283	30731.948
class Methanobacteria	Vascular dementia (sudden onset)	rs12452096	A	G	-0.008	75544032	0.767	0.029	14306	A	G	0.750	17	77547950	1.48929E-06	0.156	360283	77865.614
class Methanobacteria	Vascular dementia (sudden onset)	rs429358	C	T	-0.026	45411941	0.504	0.034	14306	C	T	0.601	19	44908684	7.45813E-06	0.134	360283	42162.979
class Mollicutes	Vascular dementia (sudden onset)	rs429358	C	T	0.000	45411941	0.938	0.019	14306	C	T	0.601	19	44908684	7.45813E-06	0.134	360283	42162.979
class Mollicutes	Vascular dementia (sudden onset)	rs4840457	C	T	-0.009	6408682	0.761	0.025	14306	C	T	0.643	8	6551161	8.92874E-06	0.145	360283	32615.797
class Mollicutes	Vascular dementia (sudden onset)	rs71511414	G	A	0.010	79688991	0.740	0.024	14306	G	A	0.681	9	77074075	7.80746E-06	0.152	360283	30731.948
class Negativicutes	Vascular dementia (sudden onset)	rs12423672	T	G	-0.005	5047705	0.813	0.017	14306	T	G	0.759	12	4938539	4.89159E-06	0.166	360283	30610.567
class Negativicutes	Vascular dementia (sudden onset)	rs2920	C	T	0.009	23884780	0.485	0.013	14306	C	T	0.592	1	23558289	2.20602E-06	0.125	360283	40501.384

class Negativicutes	Vascular dementia (sudden onset)	rs4840457	C	T	0.006	6408682	0.775	0.020	14306	C	T	0.643	8	6551161	8.92874E-06	0.145	360283	32615.797
class Negativicutes	Vascular dementia (sudden onset)	rs71511414	G	A	0.009	79688991	0.643	0.019	14306	G	A	0.681	9	77074075	7.80746E-06	0.152	360283	30731.948
class Verrucomicrobiae	Vascular dementia (sudden onset)	rs12423672	T	G	0.016	5047705	0.344	0.021	14306	T	G	0.759	12	4938539	4.89159E-06	0.166	360283	30610.567
class Verrucomicrobiae	Vascular dementia (sudden onset)	rs12452096	A	G	0.006	75544032	0.695	0.015	14306	A	G	0.750	17	77547950	1.48929E-06	0.156	360283	77865.614
class Verrucomicrobiae	Vascular dementia (sudden onset)	rs2920	C	T	-0.009	23884780	0.591	0.016	14306	C	T	0.592	1	23558289	2.20602E-06	0.125	360283	40501.384
class Verrucomicrobiae	Vascular dementia (sudden onset)	rs4840457	C	T	-0.017	6408682	0.488	0.024	14306	C	T	0.643	8	6551161	8.92874E-06	0.145	360283	32615.797
class Verrucomicrobiae	Vascular dementia (sudden onset)	rs71511414	G	A	-0.011	79688991	0.596	0.023	14306	G	A	0.681	9	77074075	7.80746E-06	0.152	360283	30731.948
class Actinobacteria	Vascular dementia (undefined)	rs12224047	T	C	0.002	36820790	0.915	0.016	14306	T	C	-0.263	11	36799240	4.56278E-06	0.057	361227	8033.487
class Actinobacteria	Vascular dementia (undefined)	rs12449066	G	A	0.003	79177293	0.843	0.019	14306	G	A	-0.262	16	79143396	2.01674E-06	0.055	361227	8803.514
class Actinobacteria	Vascular dementia (undefined)	rs2292090	T	C	-0.005	70588309	0.727	0.012	14306	T	C	0.217	4	69722591	9.99332E-06	0.049	361227	6178.734
class Actinobacteria	Vascular dementia (undefined)	rs2972558	T	C	0.003	45356141	0.812	0.012	14306	T	C	0.234	19	44852884	8.32415E-06	0.052	361227	7242.374
class Actinobacteria	Vascular dementia (undefined)	rs2978951	G	A	-0.009	6823295	0.409	0.011	14306	G	A	0.248	8	6965773	2.48222E-08	0.045	361227	11061.935
class Actinobacteria	Vascular dementia (undefined)	rs429358	C	T	0.020	45411941	0.162	0.016	14306	C	T	0.695	19	44908684	9.26616E-39	0.053	361227	58999.679
class Actinobacteria	Vascular dementia (undefined)	rs6133343	G	T	0.000	721797	0.886	0.018	14306	G	T	0.312	20	741153	3.89825E-06	0.068	361227	6210.744
class Actinobacteria	Vascular dementia (undefined)	rs71298638	A	G	-0.017	63232261	0.489	0.023	14306	A	G	0.379	3	63246585	1.1165E-06	0.078	361227	6515.599
class Alphaproteobacteria	Vascular dementia (undefined)	rs12224047	T	C	-0.010	36820790	0.675	0.021	14306	T	C	-0.263	11	36799240	4.56278E-06	0.057	361227	8033.487
class Alphaproteobacteria	Vascular dementia (undefined)	rs12449066	G	A	0.015	79177293	0.511	0.024	14306	G	A	-0.262	16	79143396	2.01674E-06	0.055	361227	8803.514
class Alphaproteobacteria	Vascular dementia (undefined)	rs2972558	T	C	0.004	45356141	0.847	0.016	14306	T	C	0.234	19	44852884	8.32415E-06	0.052	361227	7242.374
class Alphaproteobacteria	Vascular dementia (undefined)	rs2978951	G	A	0.008	6823295	0.590	0.015	14306	G	A	0.248	8	6965773	2.48222E-08	0.045	361227	11061.935
class Alphaproteobacteria	Vascular dementia (undefined)	rs429358	C	T	-0.004	45411941	0.874	0.021	14306	C	T	0.695	19	44908684	9.26616E-39	0.053	361227	58999.679
class Alphaproteobacteria	Vascular dementia (undefined)	rs78566090	A	G	0.014	125740204	0.650	0.033	14306	A	G	0.346	8	124727963	9.44735E-07	0.071	361227	7049.571
class Bacilli	Vascular dementia (undefined)	rs12449066	G	A	0.012	79177293	0.544	0.019	14306	G	A	-0.262	16	79143396	2.01674E-06	0.055	361227	8803.514
class Bacilli	Vascular dementia (undefined)	rs193392	T	C	-0.004	3085245	0.722	0.011	14306	T	C	-0.204	20	3104599	8.28324E-06	0.046	361227	7460.147
class Bacilli	Vascular dementia (undefined)	rs2292090	T	C	-0.006	70588309	0.618	0.012	14306	T	C	0.217	4	69722591	9.99332E-06	0.049	361227	6178.734
class Bacilli	Vascular dementia (undefined)	rs2978951	G	A	0.000	6823295	0.995	0.011	14306	G	A	0.248	8	6965773	2.48222E-08	0.045	361227	11061.935
class Bacilli	Vascular dementia (undefined)	rs429358	C	T	0.014	45411941	0.379	0.015	14306	C	T	0.695	19	44908684	9.26616E-39	0.053	361227	58999.679
class Bacilli	Vascular dementia (undefined)	rs6133343	G	T	-0.002	721797	0.941	0.017	14306	G	T	0.312	20	741153	3.89825E-06	0.068	361227	6210.744
class Bacteroidia	Vascular dementia (undefined)	rs12224047	T	C	-0.007	36820790	0.634	0.015	14306	T	C	-0.263	11	36799240	4.56278E-06	0.057	361227	8033.487
class Bacteroidia	Vascular dementia (undefined)	rs2292090	T	C	0.007	70588309	0.626	0.012	14306	T	C	0.217	4	69722591	9.99332E-06	0.049	361227	6178.734
class Bacteroidia	Vascular dementia (undefined)	rs2972558	T	C	0.006	45356141	0.645	0.011	14306	T	C	0.234	19	44852884	8.32415E-06	0.052	361227	7242.374
class Bacteroidia	Vascular dementia (undefined)	rs429358	C	T	-0.022	45411941	0.148	0.015	14306	C	T	0.695	19	44908684	9.26616E-39	0.053	361227	58999.679
class Bacteroidia	Vascular dementia (undefined)	rs78566090	A	G	-0.017	125740204	0.481	0.024	14306	A	G	0.346	8	124727963	9.44735E-07	0.071	361227	7049.571
class Betaproteobacteria	Vascular dementia (undefined)	rs12224047	T	C	0.001	36820790	0.951	0.016	14306	T	C	-0.263	11	36799240	4.56278E-06	0.057	361227	8033.487
class Betaproteobacteria	Vascular dementia (undefined)	rs12449066	G	A	-0.017	79177293	0.369	0.019	14306	G	A	-0.262	16	79143396	2.01674E-06	0.055	361227	8803.514
class Betaproteobacteria	Vascular dementia (undefined)	rs2978951	G	A	0.003	6823295	0.799	0.011	14306	G	A	0.248	8	6965773	2.48222E-08	0.045	361227	11061.935
class Betaproteobacteria	Vascular dementia (undefined)	rs359878	C	T	-0.002	185438949	0.886	0.013	14306	C	T	-0.211	2	184574222	4.6443E-06	0.046	361227	6959.700
class Betaproteobacteria	Vascular dementia (undefined)	rs429358	C	T	0.003	45411941	0.834	0.016	14306	C	T	0.695	19	44908684	9.26616E-39	0.053	361227	58999.679
class Betaproteobacteria	Vascular dementia (undefined)	rs71298638	A	G	-0.013	63232261	0.555	0.023	14306	A	G	0.379	3	63246585	1.1165E-06	0.078	361227	6515.599
class Betaproteobacteria	Vascular dementia (undefined)	rs78566090	A	G	-0.013	125740204	0.623	0.025	14306	A	G	0.346	8	124727963	9.44735E-07	0.071	361227	7049.571
class Clostridia	Vascular dementia (undefined)	rs12224047	T	C	0.010	36820790	0.502	0.015	14306	T	C	-0.263	11	36799240	4.56278E-06	0.057	361227	8033.487
class Clostridia	Vascular dementia (undefined)	rs12449066	G	A	0.014	79177293	0.565	0.018	14306	G	A	-0.262	16	79143396	2.01674E-06	0.055	361227	8803.514
class Clostridia	Vascular dementia (undefined)	rs2292090	T	C	0.000	70588309	0.996	0.012	14306	T	C	0.217	4	69722591	9.99332E-06	0.049	361227	6178.734
class Clostridia	Vascular dementia (undefined)	rs2978951	G	A	0.006	6823295	0.607	0.011	14306	G	A	0.248	8	6965773	2.48222E-08	0.045	361227	11061.935
class Clostridia	Vascular dementia (undefined)	rs359878	C	T	-0.001	185438949	0.929	0.012	14306	C	T	-0.211	2	184574222	4.6443E-06	0.046	361227	6959.700
class Clostridia	Vascular dementia (undefined)	rs429358	C	T	0.005	45411941	0.705	0.015	14306	C	T	0.695	19	44908684	9.26616E-39	0.053	361227	58999.679
class Clostridia	Vascular dementia (undefined)	rs6133343	G	T	0.000	721797	0.812	0.017	14306	G	T	0.312	20	741153	3.89825E-06	0.068	361227	6210.744
class Clostridia	Vascular dementia (undefined)	rs71298638	A	G	-0.008	63232261	0.706	0.022	14306	A	G	0.379	3	63246585	1.1165E-06	0.078	361227	6515.599
class Clostridia	Vascular dementia (undefined)	rs78566090	A	G	0.012	125740204	0.691	0.024	14306	A	G	0.346	8	124727963	9.44735E-07	0.071	361227	7049.571
class Coriobacteriia	Vascular dementia (undefined)	rs12449066	G	A	0.004	79177293	0.827	0.018	14306	G	A	-0.262	16	79143396	2.01674E-06	0.055	361227	8803.514
class Coriobacteriia	Vascular dementia (undefined)	rs193392	T	C	0.003	3085245	0.795	0.011	14306	T	C	-0.204	20	3104599	8.28324E-06	0.046	361227	7460.147
class Coriobacteriia	Vascular dementia (undefined)	rs2292090	T	C	0.004	70588309	0.770	0.012	14306	T	C	0.217	4	69722591	9.99332E-06	0.049	361227	6178.734
class Coriobacteriia	Vascular dementia (undefined)	rs2972558	T	C	-0.002	45356141	0.855	0.011	14306	T	C	0.234	19	44852884	8.32415E-06	0.052	361227	7242.374
class Coriobacteriia	Vascular dementia (undefined)	rs2978951	G	A	-0.005	6823295	0.614	0.011	14306	G	A	0.248	8	6965773	2.48222E-08	0.045	361227	11061.935
class Coriobacteriia	Vascular dementia (undefined)	rs359878	C	T	-0.001	185438949	0.997	0.012	14306	C	T	-0.211	2	184574222	4.6443E-06	0.046	361227	6959.700
class Coriobacteriia	Vascular dementia (undefined)	rs429358	C	T	0.019	45411941	0.204	0.015	14306	C	T	0.695	19	44908684	9.26616E-39	0.053	361227	58999.679
class Coriobacteriia	Vascular dementia (undefined)	rs6133343	G	T	-0.013	721797	0.490	0.017	14306	G	T	0.312	20	741153	3.89825E-06	0.068	361227	6210.744
class Coriobacteriia	Vascular dementia (undefined)	rs71298638	A	G	-0.003	63232261	0.855	0.022	14306	A	G	0.379	3	63246585	1.1165E-06	0.078	361227	6515.599
class Deltaproteobacteria	Vascular dementia (undefined)	rs12224047	T	C	0.013	36820790	0.437	0.017	14306	T	C	-0.263	11	36799240	4.56278E-06	0.057	361227	8033.487
class Deltaproteobacteria	Vascular dementia (undefined)	rs2292090	T	C	0.009	70588309	0.368	0.013	14306	T	C	0.217	4	69722591	9.99332E-06	0.049	361227	6178.734
class Deltaproteobacteria	Vascular dementia (undefined)	rs2972558	T	C	0.006	45356141	0.653	0.012	14306	T	C	0.234	19	44852884	8.32415E-06	0.052	361227	7242.374
class Deltaproteobacteria	Vascular dementia (undefined)	rs2978951	G	A	0.012	6823295	0.322	0.012	14306	G	A	0.248	8	6965773	2.48222E-08	0.045	361227	11061.935
class Deltaproteobacteria	Vascular dementia (undefined)	rs359878	C	T	0.006	185438949	0.597	0.013	14306	C	T	-0.211	2	184574222	4.6443E-06	0.046	361227	6959.700
class Deltaproteobacteria	Vascular dementia (undefined)	rs429358	C	T	0.016	45411941	0.340	0.016	14306	C	T	0.695	19	44908684	9.26616E-39	0.053	361227	58999.679
class Deltaproteobacteria	Vascular dementia (undefined)	rs6133343	G	T	0.003	721797	0.959	0.018	14306	G	T	0.312	20	741153	3.89825E-06	0.068	361227	6210.744
class Deltaproteobacteria	Vascular dementia (undefined)	rs71298638	A	G	0.004	63232261	0.895	0.024	14306	A	G	0.379	3	63246585	1.1165E-06	0.078	361227	6515.599
class Deltaproteobacteria	Vascular dementia (undefined)	rs78566090	A	G	0.009	125740204	0.631	0.027	14306	A	G	0.346	8	124727963	9.44735E-07	0.071	361227	7049.571
class Erysipelotrichia	Vascular dementia (undefined)	rs12224047	T	C	0.003	36820790	0.897	0.015	14306	T	C	-0.263	11	36799240	4.56278E-06	0.057	361227	8033.487
class Erysipelotrichia	Vascular dementia (undefined)	rs12449066	G	A	-0.016	79177293	0.366	0.018	14306	G	A	-0.262	16	79143396	2.01674E-06	0.055	361227	8803.514
class Erysipelotrichia	Vascular dementia (undefined)	rs2292090	T	C	-0.002	70588309	0.866	0.012	14306	T	C	0.217	4	69722591	9.99332E-06	0.049	361227	6178.734
class Erysipelotrichia	Vascular dementia (undefined)	rs2978951	G	A	-0.005	6823295	0.605	0.011	14306	G	A	0.248	8	6965773	2.48222E-08	0.045	361227	11061.935
class Erysipelotrichia	Vascular dementia (undefined)	rs429358	C	T	0.032	45411941	0.035	0.015	14306	C	T	0.695	19	44908684	9.26616E-39	0.053	361227	58999.679

class Erysipelotrichia	Vascular dementia (undefined)	rs6133343	G	T	0.004	721797	0.852	0.017	14306	G	T	0.312	20	741153	3.89825E-06	0.068	361227	6210.744
class Erysipelotrichia	Vascular dementia (undefined)	rs71298638	A	G	0.012	63232261	0.604	0.022	14306	A	G	0.379	3	63246585	1.1165E-06	0.078	361227	6515.599
class Erysipelotrichia	Vascular dementia (undefined)	rs78566090	A	G	-0.013	125740204	0.561	0.024	14306	A	G	0.346	8	124727963	9.44735E-07	0.071	361227	7049.571
class Gammaproteobacteria	Vascular dementia (undefined)	rs12224047	T	C	-0.005	36820790	0.712	0.016	14306	T	C	-0.263	11	36799240	4.56278E-06	0.057	361227	8033.487
class Gammaproteobacteria	Vascular dementia (undefined)	rs12449066	G	A	0.017	79177293	0.325	0.019	14306	G	A	-0.262	16	79143396	2.01674E-06	0.055	361227	8803.514
class Gammaproteobacteria	Vascular dementia (undefined)	rs193392	T	C	0.006	3085245	0.580	0.011	14306	T	C	-0.204	20	3104599	8.28324E-06	0.046	361227	7460.147
class Gammaproteobacteria	Vascular dementia (undefined)	rs2978951	G	A	-0.009	6823295	0.443	0.011	14306	G	A	0.248	8	6965773	2.48222E-08	0.045	361227	11061.935
class Gammaproteobacteria	Vascular dementia (undefined)	rs429358	C	T	-0.008	45411941	0.695	0.016	14306	C	T	0.695	19	44908684	9.26616E-39	0.053	361227	58999.679
class Gammaproteobacteria	Vascular dementia (undefined)	rs6133343	G	T	0.005	721797	0.751	0.018	14306	G	T	0.312	20	741153	3.89825E-06	0.068	361227	6210.744
class Lentisphaeria	Vascular dementia (undefined)	rs12224047	T	C	-0.015	36820790	0.657	0.030	14306	T	C	-0.263	11	36799240	4.56278E-06	0.057	361227	8033.487
class Lentisphaeria	Vascular dementia (undefined)	rs12449066	G	A	-0.006	79177293	0.814	0.036	14306	G	A	-0.262	16	79143396	2.01674E-06	0.055	361227	8803.514
class Lentisphaeria	Vascular dementia (undefined)	rs193392	T	C	0.017	3085245	0.416	0.022	14306	T	C	-0.204	20	3104599	8.28324E-06	0.046	361227	7460.147
class Lentisphaeria	Vascular dementia (undefined)	rs2292090	T	C	-0.011	70588309	0.671	0.023	14306	T	C	0.217	4	69722591	9.99332E-06	0.049	361227	6178.734
class Lentisphaeria	Vascular dementia (undefined)	rs429358	C	T	-0.012	45411941	0.587	0.030	14306	C	T	0.695	19	44908684	9.26616E-39	0.053	361227	58999.679
class Lentisphaeria	Vascular dementia (undefined)	rs6133343	G	T	0.026	721797	0.256	0.033	14306	G	T	0.312	20	741153	3.89825E-06	0.068	361227	6210.744
class Lentisphaeria	Vascular dementia (undefined)	rs71298638	A	G	-0.028	63232261	0.545	0.045	14306	A	G	0.379	3	63246585	1.1165E-06	0.078	361227	6515.599
class Lentisphaeria	Vascular dementia (undefined)	rs78566090	A	G	-0.011	125740204	0.785	0.048	14306	A	G	0.346	8	124727963	9.44735E-07	0.071	361227	7049.571
class Melainabacteria	Vascular dementia (undefined)	rs12449066	G	A	-0.014	79177293	0.633	0.032	14306	G	A	-0.262	16	79143396	2.01674E-06	0.055	361227	8803.514
class Melainabacteria	Vascular dementia (undefined)	rs193392	T	C	-0.007	3085245	0.702	0.019	14306	T	C	-0.204	20	3104599	8.28324E-06	0.046	361227	7460.147
class Melainabacteria	Vascular dementia (undefined)	rs2292090	T	C	-0.002	70588309	0.895	0.021	14306	T	C	0.217	4	69722591	9.99332E-06	0.049	361227	6178.734
class Melainabacteria	Vascular dementia (undefined)	rs2972558	T	C	0.017	45356141	0.451	0.021	14306	T	C	0.234	19	44852884	8.32415E-06	0.052	361227	7242.374
class Melainabacteria	Vascular dementia (undefined)	rs2978951	G	A	0.000	6823295	0.968	0.019	14306	G	A	0.248	8	6965773	2.48222E-08	0.045	361227	11061.935
class Melainabacteria	Vascular dementia (undefined)	rs359878	C	T	0.005	185438949	0.830	0.021	14306	C	T	-0.211	2	184574222	4.6443E-06	0.046	361227	6959.700
class Melainabacteria	Vascular dementia (undefined)	rs429358	C	T	-0.003	45411941	0.968	0.026	14306	C	T	0.695	19	44908684	9.26616E-39	0.053	361227	58999.679
class Melainabacteria	Vascular dementia (undefined)	rs6133343	G	T	-0.008	721797	0.753	0.030	14306	G	T	0.312	20	741153	3.89825E-06	0.068	361227	6210.744
class Melainabacteria	Vascular dementia (undefined)	rs71298638	A	G	-0.025	63232261	0.491	0.039	14306	A	G	0.379	3	63246585	1.1165E-06	0.078	361227	6515.599
class Melainabacteria	Vascular dementia (undefined)	rs78566090	A	G	-0.031	125740204	0.528	0.043	14306	A	G	0.346	8	124727963	9.44735E-07	0.071	361227	7049.571
class Methanobacteria	Vascular dementia (undefined)	rs12224047	T	C	0.004	36820790	0.920	0.034	14306	T	C	-0.263	11	36799240	4.56278E-06	0.057	361227	8033.487
class Methanobacteria	Vascular dementia (undefined)	rs12449066	G	A	-0.010	79177293	0.714	0.040	14306	G	A	-0.262	16	79143396	2.01674E-06	0.055	361227	8803.514
class Methanobacteria	Vascular dementia (undefined)	rs193392	T	C	0.001	3085245	0.989	0.025	14306	T	C	-0.204	20	3104599	8.28324E-06	0.046	361227	7460.147
class Methanobacteria	Vascular dementia (undefined)	rs2972558	T	C	-0.014	45356141	0.612	0.027	14306	T	C	0.234	19	44852884	8.32415E-06	0.052	361227	7242.374
class Methanobacteria	Vascular dementia (undefined)	rs2978951	G	A	0.023	6823295	0.327	0.023	14306	G	A	0.248	8	6965773	2.48222E-08	0.045	361227	11061.935
class Methanobacteria	Vascular dementia (undefined)	rs359878	C	T	0.015	185438949	0.623	0.027	14306	C	T	-0.211	2	184574222	4.6443E-06	0.046	361227	6959.700
class Methanobacteria	Vascular dementia (undefined)	rs429358	C	T	-0.026	45411941	0.504	0.034	14306	C	T	0.695	19	44908684	9.26616E-39	0.053	361227	58999.679
class Mollicutes	Vascular dementia (undefined)	rs12449066	G	A	0.003	79177293	0.999	0.023	14306	G	A	-0.262	16	79143396	2.01674E-06	0.055	361227	8803.514
class Mollicutes	Vascular dementia (undefined)	rs193392	T	C	-0.010	3085245	0.443	0.014	14306	T	C	-0.204	20	3104599	8.28324E-06	0.046	361227	7460.147
class Mollicutes	Vascular dementia (undefined)	rs2292090	T	C	0.013	70588309	0.389	0.015	14306	T	C	0.217	4	69722591	9.99332E-06	0.049	361227	6178.734
class Mollicutes	Vascular dementia (undefined)	rs2978951	G	A	0.007	6823295	0.593	0.013	14306	G	A	0.248	8	6965773	2.48222E-08	0.045	361227	11061.935
class Mollicutes	Vascular dementia (undefined)	rs429358	C	T	0.000	45411941	0.938	0.019	14306	C	T	0.695	19	44908684	9.26616E-39	0.053	361227	58999.679
class Mollicutes	Vascular dementia (undefined)	rs6133343	G	T	0.001	721797	0.977	0.021	14306	G	T	0.312	20	741153	3.89825E-06	0.068	361227	6210.744
class Mollicutes	Vascular dementia (undefined)	rs71298638	A	G	0.019	63232261	0.530	0.028	14306	A	G	0.379	3	63246585	1.1165E-06	0.078	361227	6515.599
class Mollicutes	Vascular dementia (undefined)	rs78566090	A	G	-0.019	125740204	0.595	0.031	14306	A	G	0.346	8	124727963	9.44735E-07	0.071	361227	7049.571
class Negativicutes	Vascular dementia (undefined)	rs12224047	T	C	0.005	36820790	0.763	0.015	14306	T	C	-0.263	11	36799240	4.56278E-06	0.057	361227	8033.487
class Negativicutes	Vascular dementia (undefined)	rs12449066	G	A	-0.003	79177293	0.788	0.018	14306	G	A	-0.262	16	79143396	2.01674E-06	0.055	361227	8803.514
class Negativicutes	Vascular dementia (undefined)	rs193392	T	C	0.007	3085245	0.554	0.011	14306	T	C	-0.204	20	3104599	8.28324E-06	0.046	361227	7460.147
class Negativicutes	Vascular dementia (undefined)	rs2978951	G	A	0.007	6823295	0.498	0.011	14306	G	A	0.248	8	6965773	2.48222E-08	0.045	361227	11061.935
class Negativicutes	Vascular dementia (undefined)	rs359878	C	T	-0.010	185438949	0.446	0.012	14306	C	T	-0.211	2	184574222	4.6443E-06	0.046	361227	6959.700
class Negativicutes	Vascular dementia (undefined)	rs429358	C	T	-0.019	45411941	0.146	0.015	14306	C	T	0.695	19	44908684	9.26616E-39	0.053	361227	58999.679
class Negativicutes	Vascular dementia (undefined)	rs6133343	G	T	0.005	721797	0.943	0.017	14306	G	T	0.312	20	741153	3.89825E-06	0.068	361227	6210.744
class Negativicutes	Vascular dementia (undefined)	rs71298638	A	G	0.013	63232261	0.553	0.022	14306	A	G	0.379	3	63246585	1.1165E-06	0.078	361227	6515.599
class Verrucomicrobiae	Vascular dementia (undefined)	rs12224047	T	C	-0.005	36820790	0.749	0.019	14306	T	C	-0.263	11	36799240	4.56278E-06	0.057	361227	8033.487
class Verrucomicrobiae	Vascular dementia (undefined)	rs12449066	G	A	0.001	79177293	0.960	0.022	14306	G	A	-0.262	16	79143396	2.01674E-06	0.055	361227	8803.514
class Verrucomicrobiae	Vascular dementia (undefined)	rs193392	T	C	0.003	3085245	0.817	0.013	14306	T	C	-0.204	20	3104599	8.28324E-06	0.046	361227	7460.147
class Verrucomicrobiae	Vascular dementia (undefined)	rs2292090	T	C	-0.008	70588309	0.602	0.015	14306	T	C	0.217	4	69722591	9.99332E-06	0.049	361227	6178.734
class Verrucomicrobiae	Vascular dementia (undefined)	rs2972558	T	C	0.008	45356141	0.586	0.014	14306	T	C	0.234	19	44852884	8.32415E-06	0.052	361227	7242.374
class Verrucomicrobiae	Vascular dementia (undefined)	rs429358	C	T	0.018	45411941	0.336	0.019	14306	C	T	0.695	19	44908684	9.26616E-39	0.053	361227	58999.679
class Verrucomicrobiae	Vascular dementia (undefined)	rs6133343	G	T	-0.008	721797	0.772	0.021	14306	G	T	0.312	20	741153	3.89825E-06	0.068	361227	6210.744
class Verrucomicrobiae	Vascular dementia (undefined)	rs71298638	A	G	-0.005	63232261	0.842	0.027	14306	A	G	0.379	3	63246585	1.1165E-06	0.078	361227	6515.599
class Verrucomicrobiae	Vascular dementia (undefined)	rs78566090	A	G	0.004	125740204	0.862	0.030	14306	A	G	0.346	8	124727963	9.44735E-07	0.071	361227	7049.571
family Acidaminococcaceae	Vascular dementia (multiple infarctions)	rs11081443	C	T	0.009	8944208	0.486	0.016	14306	C	T	0.464	18	8944210	5.71874E-06	0.102	360612	19066.663
family Acidaminococcaceae	Vascular dementia (multiple infarctions)	rs1454336	A	G	-0.013	91873093	0.445	0.016	14306	A	G	-0.369	4	90951942	4.48415E-06	0.080	360612	13616.170
family Acidaminococcaceae	Vascular dementia (multiple infarctions)	rs34288661	C	T	-0.012	20948329	0.506	0.019	14306	C	T	0.460	8	21090818	7.22903E-06	0.103	360612	11969.376
family Acidaminococcaceae	Vascular dementia (multiple infarctions)	rs429358	C	T	-0.007	45411941	0.557	0.018	14306	C	T	0.660	19	44908684	1.33506E-17	0.077	360612	52325.340
family Acidaminococcaceae	Vascular dementia (multiple infarctions)	rs72822148	T	C	0.007	9742028	0.673	0.014	14306	T	C	-0.322	17	9838711	3.18831E-06	0.069	360612	17983.471
family Acidaminococcaceae	Vascular dementia (multiple infarctions)	rs9861644	A	G	-0.004	88637331	0.776	0.014	14306	A	G	0.323	3	88588181	4.84942E-06	0.071	360612	17137.658
family Actinomycetaceae	Vascular dementia (multiple infarctions)	rs11081443	C	T	-0.003	8944208	0.902	0.020	14306	C	T	0.464	18	8944210	5.71874E-06	0.102	360612	19066.663
family Actinomycetaceae	Vascular dementia (multiple infarctions)	rs429358	C	T	0.038	45411941	0.090	0.023	14306	C	T	0.660	19	44908684	1.33506E-17	0.077	360612	52325.340
family Actinomycetaceae	Vascular dementia (multiple infarctions)	rs4725579	C	A	0.012	139468213	0.563	0.020	14306	C	A	-0.379	7	139768414	3.58039E-06	0.082	360612	18391.668
family Actinomycetaceae	Vascular dementia (multiple infarctions)	rs72822148	T	C	0.014	9742028	0.434	0.017	14306	T	C	-0.322	17	9838711	3.18831E-06	0.069	360612	17983.471
family Actinomycetaceae	Vascular dementia (multiple infarctions)	rs73053797	T	C	-0.001	29909039	0.960	0.023	14306	T	C	0.338	3	29867548	6.00772E-06	0.075	360612	13680.576
family Actinomycetaceae	Vascular dementia (multiple infarctions)	rs9861644	A	G	0.009	88637331	0.591	0.019	14306	A	G	0.323	3	88588181	4.84942E-06	0.071	360612	17137.658

family Alcaligenaceae	Vascular dementia (multiple infarctions)	rs11081443	C	T	-0.005	8944208	0.679	0.013	14306	C	T	0.464	18	8944210	5.71874E-06	0.102	360612	19066.663
family Alcaligenaceae	Vascular dementia (multiple infarctions)	rs1454336	A	G	-0.011	91873093	0.400	0.014	14306	A	G	-0.369	4	90951942	4.48415E-06	0.080	360612	13616.170
family Alcaligenaceae	Vascular dementia (multiple infarctions)	rs429358	C	T	0.009	45411941	0.545	0.016	14306	C	T	0.660	19	44908684	1.33506E-17	0.077	360612	52325.340
family Alcaligenaceae	Vascular dementia (multiple infarctions)	rs72822148	T	C	-0.007	9742028	0.633	0.012	14306	T	C	-0.322	17	9838711	3.18831E-06	0.069	360612	17983.471
family Alcaligenaceae	Vascular dementia (multiple infarctions)	rs73053797	T	C	0.003	29909039	0.792	0.015	14306	T	C	0.338	3	29867548	6.00772E-06	0.075	360612	13680.576
family Alcaligenaceae	Vascular dementia (multiple infarctions)	rs9861644	A	G	0.008	88637331	0.491	0.013	14306	A	G	0.323	3	88588181	4.84942E-06	0.071	360612	17137.658
family Bacteroidaceae	Vascular dementia (multiple infarctions)	rs11081443	C	T	0.009	8944208	0.425	0.013	14306	C	T	0.464	18	8944210	5.71874E-06	0.102	360612	19066.663
family Bacteroidaceae	Vascular dementia (multiple infarctions)	rs1454336	A	G	-0.011	91873093	0.420	0.014	14306	A	G	-0.369	4	90951942	4.48415E-06	0.080	360612	13616.170
family Bacteroidaceae	Vascular dementia (multiple infarctions)	rs34288661	C	T	-0.002	20948329	0.911	0.016	14306	C	T	0.460	8	21090818	7.22903E-06	0.103	360612	11969.376
family Bacteroidaceae	Vascular dementia (multiple infarctions)	rs4716814	T	C	-0.010	157723046	0.356	0.011	14306	T	C	-0.319	7	157930354	3.75656E-07	0.063	360612	19180.021
family Bacteroidaceae	Vascular dementia (multiple infarctions)	rs4725579	C	A	0.007	139468213	0.569	0.013	14306	C	A	-0.379	7	139768414	3.58039E-06	0.082	360612	18391.668
family Bacteroidaceae	Vascular dementia (multiple infarctions)	rs72822148	T	C	-0.008	9742028	0.651	0.011	14306	T	C	-0.322	17	9838711	3.18831E-06	0.069	360612	17983.471
family Bacteroidaceae	Vascular dementia (multiple infarctions)	rs73053797	T	C	-0.003	29909039	0.853	0.015	14306	T	C	0.338	3	29867548	6.00772E-06	0.075	360612	13680.576
family Bacteroidales S24 7group	Vascular dementia (multiple infarctions)	rs11081443	C	T	-0.011	8944208	0.577	0.020	14306	C	T	0.464	18	8944210	5.71874E-06	0.102	360612	19066.663
family Bacteroidales S24 7group	Vascular dementia (multiple infarctions)	rs429358	C	T	-0.033	45411941	0.162	0.023	14306	C	T	0.660	19	44908684	1.33506E-17	0.077	360612	52325.340
family Bacteroidales S24 7group	Vascular dementia (multiple infarctions)	rs4716814	T	C	-0.006	157723046	0.715	0.016	14306	T	C	-0.319	7	157930354	3.75656E-07	0.063	360612	19180.021
family Bacteroidales S24 7group	Vascular dementia (multiple infarctions)	rs4725579	C	A	0.000	139468213	0.932	0.020	14306	C	A	-0.379	7	139768414	3.58039E-06	0.082	360612	18391.668
family Bacteroidales S24 7group	Vascular dementia (multiple infarctions)	rs72822148	T	C	0.012	9742028	0.471	0.017	14306	T	C	-0.322	17	9838711	3.18831E-06	0.069	360612	17983.471
family Bacteroidales S24 7group	Vascular dementia (multiple infarctions)	rs73053797	T	C	-0.017	29909039	0.473	0.022	14306	T	C	0.338	3	29867548	6.00772E-06	0.075	360612	13680.576
family Bifidobacteriaceae	Vascular dementia (multiple infarctions)	rs1454336	A	G	0.002	91873093	0.910	0.015	14306	A	G	-0.369	4	90951942	4.48415E-06	0.080	360612	13616.170
family Bifidobacteriaceae	Vascular dementia (multiple infarctions)	rs34288661	C	T	-0.008	20948329	0.762	0.017	14306	C	T	0.460	8	21090818	7.22903E-06	0.103	360612	11969.376
family Bifidobacteriaceae	Vascular dementia (multiple infarctions)	rs429358	C	T	0.022	45411941	0.147	0.017	14306	C	T	0.660	19	44908684	1.33506E-17	0.077	360612	52325.340
family Bifidobacteriaceae	Vascular dementia (multiple infarctions)	rs4725579	C	A	-0.005	139468213	0.670	0.014	14306	C	A	-0.379	7	139768414	3.58039E-06	0.082	360612	18391.668
family Bifidobacteriaceae	Vascular dementia (multiple infarctions)	rs73053797	T	C	0.005	29909039	0.638	0.016	14306	T	C	0.338	3	29867548	6.00772E-06	0.075	360612	13680.576
family Bifidobacteriaceae	Vascular dementia (multiple infarctions)	rs9861644	A	G	0.009	88637331	0.538	0.013	14306	A	G	0.323	3	88588181	4.84942E-06	0.071	360612	17137.658
family Clostridiaceae1	Vascular dementia (multiple infarctions)	rs11081443	C	T	-0.001	8944208	0.889	0.015	14306	C	T	0.464	18	8944210	5.71874E-06	0.102	360612	19066.663
family Clostridiaceae1	Vascular dementia (multiple infarctions)	rs1454336	A	G	0.006	91873093	0.678	0.016	14306	A	G	-0.369	4	90951942	4.48415E-06	0.080	360612	13616.170
family Clostridiaceae1	Vascular dementia (multiple infarctions)	rs429358	C	T	0.010	45411941	0.573	0.017	14306	C	T	0.660	19	44908684	1.33506E-17	0.077	360612	52325.340
family Clostridiaceae1	Vascular dementia (multiple infarctions)	rs4716814	T	C	-0.003	157723046	0.764	0.012	14306	T	C	-0.319	7	157930354	3.75656E-07	0.063	360612	19180.021
family Clostridiaceae1	Vascular dementia (multiple infarctions)	rs72822148	T	C	0.006	9742028	0.674	0.013	14306	T	C	-0.322	17	9838711	3.18831E-06	0.069	360612	17983.471
family Clostridiales vadin BB60 group	Vascular dementia (multiple infarctions)	rs11081443	C	T	0.009	8944208	0.607	0.017	14306	C	T	0.464	18	8944210	5.71874E-06	0.102	360612	19066.663
family Clostridiales vadin BB60 group	Vascular dementia (multiple infarctions)	rs429358	C	T	0.004	45411941	0.829	0.020	14306	C	T	0.660	19	44908684	1.33506E-17	0.077	360612	52325.340
family Clostridiales vadin BB60 group	Vascular dementia (multiple infarctions)	rs4716814	T	C	-0.006	157723046	0.661	0.014	14306	T	C	-0.319	7	157930354	3.75656E-07	0.063	360612	19180.021
family Clostridiales vadin BB60 group	Vascular dementia (multiple infarctions)	rs4725579	C	A	0.005	139468213	0.855	0.017	14306	C	A	-0.379	7	139768414	3.58039E-06	0.082	360612	18391.668
family Clostridiales vadin BB60 group	Vascular dementia (multiple infarctions)	rs73053797	T	C	-0.004	29909039	0.978	0.020	14306	T	C	0.338	3	29867548	6.00772E-06	0.075	360612	13680.576
family Coriobacteriaceae	Vascular dementia (multiple infarctions)	rs34288661	C	T	-0.010	20948329	0.531	0.016	14306	C	T	0.460	8	21090818	7.22903E-06	0.103	360612	11969.376
family Coriobacteriaceae	Vascular dementia (multiple infarctions)	rs429358	C	T	0.019	45411941	0.204	0.015	14306	C	T	0.660	19	44908684	1.33506E-17	0.077	360612	52325.340
family Coriobacteriaceae	Vascular dementia (multiple infarctions)	rs4716814	T	C	-0.002	157723046	0.849	0.011	14306	T	C	-0.319	7	157930354	3.75656E-07	0.063	360612	19180.021
family Coriobacteriaceae	Vascular dementia (multiple infarctions)	rs4725579	C	A	-0.006	139468213	0.583	0.013	14306	C	A	-0.379	7	139768414	3.58039E-06	0.082	360612	18391.668
family Coriobacteriaceae	Vascular dementia (multiple infarctions)	rs73053797	T	C	-0.008	29909039	0.584	0.015	14306	T	C	0.338	3	29867548	6.00772E-06	0.075	360612	13680.576
family Coriobacteriaceae	Vascular dementia (multiple infarctions)	rs9861644	A	G	-0.008	88637331	0.489	0.012	14306	A	G	0.323	3	88588181	4.84942E-06	0.071	360612	17137.658
family Defluviitaleaceae	Vascular dementia (multiple infarctions)	rs11081443	C	T	0.008	8944208	0.589	0.019	14306	C	T	0.464	18	8944210	5.71874E-06	0.102	360612	19066.663
family Defluviitaleaceae	Vascular dementia (multiple infarctions)	rs34288661	C	T	0.005	20948329	0.829	0.023	14306	C	T	0.460	8	21090818	7.22903E-06	0.103	360612	11969.376
family Defluviitaleaceae	Vascular dementia (multiple infarctions)	rs429358	C	T	0.000	45411941	0.935	0.022	14306	C	T	0.660	19	44908684	1.33506E-17	0.077	360612	52325.340
family Defluviitaleaceae	Vascular dementia (multiple infarctions)	rs4716814	T	C	-0.013	157723046	0.425	0.016	14306	T	C	-0.319	7	157930354	3.75656E-07	0.063	360612	19180.021
family Defluviitaleaceae	Vascular dementia (multiple infarctions)	rs72822148	T	C	-0.014	9742028	0.497	0.017	14306	T	C	-0.322	17	9838711	3.18831E-06	0.069	360612	17983.471
family Defluviitaleaceae	Vascular dementia (multiple infarctions)	rs73053797	T	C	-0.016	29909039	0.438	0.023	14306	T	C	0.338	3	29867548	6.00772E-06	0.075	360612	13680.576
family Desulfovibrionaceae	Vascular dementia (multiple infarctions)	rs11081443	C	T	0.012	8944208	0.426	0.014	14306	C	T	0.464	18	8944210	5.71874E-06	0.102	360612	19066.663
family Desulfovibrionaceae	Vascular dementia (multiple infarctions)	rs1454336	A	G	-0.002	91873093	0.893	0.015	14306	A	G	-0.369	4	90951942	4.48415E-06	0.080	360612	13616.170
family Desulfovibrionaceae	Vascular dementia (multiple infarctions)	rs34288661	C	T	-0.014	20948329	0.401	0.017	14306	C	T	0.460	8	21090818	7.22903E-06	0.103	360612	11969.376
family Desulfovibrionaceae	Vascular dementia (multiple infarctions)	rs429358	C	T	0.017	45411941	0.305	0.017	14306	C	T	0.660	19	44908684	1.33506E-17	0.077	360612	52325.340
family Desulfovibrionaceae	Vascular dementia (multiple infarctions)	rs4716814	T	C	-0.011	157723046	0.352	0.012	14306	T	C	-0.319	7	157930354	3.75656E-07	0.063	360612	19180.021
family Desulfovibrionaceae	Vascular dementia (multiple infarctions)	rs4725579	C	A	0.009	139468213	0.554	0.014	14306	C	A	-0.379	7	139768414	3.58039E-06	0.082	360612	18391.668
family Desulfovibrionaceae	Vascular dementia (multiple infarctions)	rs72822148	T	C	0.003	9742028	0.751	0.012	14306	T	C	-0.322	17	9838711	3.18831E-06	0.069	360612	17983.471
family Desulfovibrionaceae	Vascular dementia (multiple infarctions)	rs73053797	T	C	-0.013	29909039	0.477	0.016	14306	T	C	0.338	3	29867548	6.00772E-06	0.075	360612	13680.576
family Desulfovibrionaceae	Vascular dementia (multiple infarctions)	rs9861644	A	G	0.001	88637331	0.867	0.013	14306	A	G	0.323	3	88588181	4.84942E-06	0.071	360612	17137.658
family Enterobacteriaceae	Vascular dementia (multiple infarctions)	rs11081443	C	T	-0.009	8944208	0.511	0.015	14306	C	T	0.464	18	8944210	5.71874E-06	0.102	360612	19066.663
family Enterobacteriaceae	Vascular dementia (multiple infarctions)	rs1454336	A	G	-0.002	91873093	0.916	0.016	14306	A	G	-0.369	4	90951942	4.48415E-06	0.080	360612	13616.170
family Enterobacteriaceae	Vascular dementia (multiple infarctions)	rs429358	C	T	-0.019	45411941	0.299	0.017	14306	C	T	0.660	19	44908684	1.33506E-17	0.077	360612	52325.340
family Enterobacteriaceae	Vascular dementia (multiple infarctions)	rs4725579	C	A	-0.002	139468213	0.986	0.015	14306	C	A	-0.379	7	139768414	3.58039E-06	0.082	360612	18391.668
family Enterobacteriaceae	Vascular dementia (multiple infarctions)	rs72822148	T	C	0.007	9742028	0.550	0.013	14306	T	C	-0.322	17	9838711	3.18831E-06	0.069	360612	17983.471
family Enterobacteriaceae	Vascular dementia (multiple infarctions)	rs73053797	T	C	0.012	29909039	0.488	0.017	14306	T	C	0.338	3	29867548	6.00772E-06	0.075	360612	13680.576
family Erysipelotrichaceae	Vascular dementia (multiple infarctions)	rs4716814	T	C	0.001	157723046	0.900	0.011	14306	T	C	-0.319	7	157930354	3.75656E-07	0.063	360612	19180.021
family Erysipelotrichaceae	Vascular dementia (multiple infarctions)	rs4725579	C	A	0.011	139468213	0.475	0.013	14306	C	A	-0.379	7	139768414	3.58039E-06	0.082	360612	18391.668
family Erysipelotrichaceae	Vascular dementia (multiple infarctions)	rs72822148	T	C	-0.004	9742028	0.542	0.011	14306	T	C	-0.322	17	9838711	3.18831E-06	0.069	360612	17983.471
family Erysipelotrichaceae	Vascular dementia (multiple infarctions)	rs73053797	T	C	0.000	29909039	0.937	0.015	14306	T	C	0.338	3	29867548	6.00772E-06	0.075	360612	13680.576
family Erysipelotrichaceae	Vascular dementia (multiple infarctions)	rs9861644	A	G	-0.004	88637331	0.706	0.012	14306	A	G	0.323	3	88588181	4.84942E-06	0.071	360612	17137.658
family Family XI	Vascular dementia (multiple infarctions)	rs11081443	C	T	0.020	8944208	0.537	0.030	14306	C	T	0.464	18	8944210	5.71874E-06	0.102	360612	19066.663
family Family XI	Vascular dementia (multiple infarctions)	rs1454336	A	G	0.010	91873093	0.771	0.032	14306	A	G	-0.369	4	90951942	4.48415E-06	0.080	360612	13616.170
family Family XI	Vascular dementia (multiple infarctions)	rs429358	C	T	0.054	45411941	0.150	0.037	14306	C	T	0.660	19	44908684	1.33506E-17	0.077	360612	52325.340
family Family XI	Vascular dementia (multiple infarctions)	rs4716814	T	C	0.014	157723046	0.602	0.025	14306	T	C	-0.319	7	157930354	3.75656E-07	0.063	360612	19180.021

family Family XI	Vascular dementia (multiple infarctions)	rs73053797	T	C	0.018	29909039	0.568	0.037	14306	T	C	0.338	3	29867548	6.00772E-06	0.075	360612	13680.576
family Family XIII	Vascular dementia (multiple infarctions)	rs11081443	C	T	0.012	8944208	0.337	0.013	14306	C	T	0.464	18	8944210	5.71874E-06	0.102	360612	19066.663
family Family XIII	Vascular dementia (multiple infarctions)	rs1454336	A	G	-0.009	91873093	0.516	0.014	14306	A	G	-0.369	4	90951942	4.48415E-06	0.080	360612	13616.170
family Family XIII	Vascular dementia (multiple infarctions)	rs34288661	C	T	-0.010	20948329	0.558	0.016	14306	C	T	0.460	8	21090818	7.22903E-06	0.103	360612	11969.376
family Family XIII	Vascular dementia (multiple infarctions)	rs4716814	T	C	0.008	157723046	0.453	0.011	14306	T	C	-0.319	7	157930354	3.75656E-07	0.063	360612	19180.021
family Family XIII	Vascular dementia (multiple infarctions)	rs4725579	C	A	0.009	139468213	0.546	0.014	14306	C	A	-0.379	7	139768414	3.58039E-06	0.082	360612	18391.668
family Family XIII	Vascular dementia (multiple infarctions)	rs72822148	T	C	0.007	9742028	0.694	0.012	14306	T	C	-0.322	17	9838711	3.18831E-06	0.069	360612	17983.471
family Family XIII	Vascular dementia (multiple infarctions)	rs73053797	T	C	-0.005	29909039	0.686	0.016	14306	T	C	0.338	3	29867548	6.00772E-06	0.075	360612	13680.576
family Lachnospiraceae	Vascular dementia (multiple infarctions)	rs1454336	A	G	-0.007	91873093	0.596	0.014	14306	A	G	-0.369	4	90951942	4.48415E-06	0.080	360612	13616.170
family Lachnospiraceae	Vascular dementia (multiple infarctions)	rs429358	C	T	0.002	45411941	0.858	0.015	14306	C	T	0.660	19	44908684	1.33506E-17	0.077	360612	52325.340
family Lachnospiraceae	Vascular dementia (multiple infarctions)	rs9861644	A	G	0.010	88637331	0.363	0.012	14306	A	G	0.323	3	88588181	4.84942E-06	0.071	360612	17137.658
family Lactobacillaceae	Vascular dementia (multiple infarctions)	rs11081443	C	T	0.008	8944208	0.723	0.021	14306	C	T	0.464	18	8944210	5.71874E-06	0.102	360612	19066.663
family Lactobacillaceae	Vascular dementia (multiple infarctions)	rs1454336	A	G	0.007	91873093	0.758	0.022	14306	A	G	-0.369	4	90951942	4.48415E-06	0.080	360612	13616.170
family Lactobacillaceae	Vascular dementia (multiple infarctions)	rs34288661	C	T	-0.016	20948329	0.505	0.025	14306	C	T	0.460	8	21090818	7.22903E-06	0.103	360612	11969.376
family Lactobacillaceae	Vascular dementia (multiple infarctions)	rs429358	C	T	-0.013	45411941	0.673	0.024	14306	C	T	0.660	19	44908684	1.33506E-17	0.077	360612	52325.340
family Lactobacillaceae	Vascular dementia (multiple infarctions)	rs4725579	C	A	-0.009	139468213	0.723	0.021	14306	C	A	-0.379	7	139768414	3.58039E-06	0.082	360612	18391.668
family Lactobacillaceae	Vascular dementia (multiple infarctions)	rs72822148	T	C	0.000	9742028	0.816	0.018	14306	T	C	-0.322	17	9838711	3.18831E-06	0.069	360612	17983.471
family Lactobacillaceae	Vascular dementia (multiple infarctions)	rs73053797	T	C	0.015	29909039	0.685	0.024	14306	T	C	0.338	3	29867548	6.00772E-06	0.075	360612	13680.576
family Lactobacillaceae	Vascular dementia (multiple infarctions)	rs9861644	A	G	0.011	88637331	0.569	0.019	14306	A	G	0.323	3	88588181	4.84942E-06	0.071	360612	17137.658
family Methanobacteriaceae	Vascular dementia (multiple infarctions)	rs1454336	A	G	0.006	91873093	0.824	0.030	14306	A	G	-0.369	4	90951942	4.48415E-06	0.080	360612	13616.170
family Methanobacteriaceae	Vascular dementia (multiple infarctions)	rs429358	C	T	-0.026	45411941	0.504	0.034	14306	C	T	0.660	19	44908684	1.33506E-17	0.077	360612	52325.340
family Methanobacteriaceae	Vascular dementia (multiple infarctions)	rs4725579	C	A	0.004	139468213	0.882	0.030	14306	C	A	-0.379	7	139768414	3.58039E-06	0.082	360612	18391.668
family Methanobacteriaceae	Vascular dementia (multiple infarctions)	rs72822148	T	C	0.008	9742028	0.721	0.026	14306	T	C	-0.322	17	9838711	3.18831E-06	0.069	360612	17983.471
family Methanobacteriaceae	Vascular dementia (multiple infarctions)	rs9861644	A	G	0.008	88637331	0.765	0.027	14306	A	G	0.323	3	88588181	4.84942E-06	0.071	360612	17137.658
family Oxalobacteraceae	Vascular dementia (multiple infarctions)	rs11081443	C	T	0.005	8944208	0.805	0.024	14306	C	T	0.464	18	8944210	5.71874E-06	0.102	360612	19066.663
family Oxalobacteraceae	Vascular dementia (multiple infarctions)	rs34288661	C	T	0.005	20948329	0.877	0.029	14306	C	T	0.460	8	21090818	7.22903E-06	0.103	360612	11969.376
family Oxalobacteraceae	Vascular dementia (multiple infarctions)	rs429358	C	T	0.011	45411941	0.614	0.028	14306	C	T	0.660	19	44908684	1.33506E-17	0.077	360612	52325.340
family Oxalobacteraceae	Vascular dementia (multiple infarctions)	rs4716814	T	C	-0.010	157723046	0.614	0.020	14306	T	C	-0.319	7	157930354	3.75656E-07	0.063	360612	19180.021
family Oxalobacteraceae	Vascular dementia (multiple infarctions)	rs4725579	C	A	0.002	139468213	0.928	0.025	14306	C	A	-0.379	7	139768414	3.58039E-06	0.082	360612	18391.668
family Pasteurellaceae	Vascular dementia (multiple infarctions)	rs11081443	C	T	-0.002	8944208	0.768	0.018	14306	C	T	0.464	18	8944210	5.71874E-06	0.102	360612	19066.663
family Pasteurellaceae	Vascular dementia (multiple infarctions)	rs1454336	A	G	0.010	91873093	0.555	0.019	14306	A	G	-0.369	4	90951942	4.48415E-06	0.080	360612	13616.170
family Pasteurellaceae	Vascular dementia (multiple infarctions)	rs429358	C	T	0.022	45411941	0.309	0.021	14306	C	T	0.660	19	44908684	1.33506E-17	0.077	360612	52325.340
family Pasteurellaceae	Vascular dementia (multiple infarctions)	rs4716814	T	C	0.007	157723046	0.630	0.015	14306	T	C	-0.319	7	157930354	3.75656E-07	0.063	360612	19180.021
family Pasteurellaceae	Vascular dementia (multiple infarctions)	rs4725579	C	A	-0.013	139468213	0.480	0.018	14306	C	A	-0.379	7	139768414	3.58039E-06	0.082	360612	18391.668
family Pasteurellaceae	Vascular dementia (multiple infarctions)	rs72822148	T	C	0.003	9742028	0.971	0.016	14306	T	C	-0.322	17	9838711	3.18831E-06	0.069	360612	17983.471
family Peptococcaceae	Vascular dementia (multiple infarctions)	rs11081443	C	T	0.009	8944208	0.544	0.017	14306	C	T	0.464	18	8944210	5.71874E-06	0.102	360612	19066.663
family Peptococcaceae	Vascular dementia (multiple infarctions)	rs429358	C	T	0.028	45411941	0.111	0.019	14306	C	T	0.660	19	44908684	1.33506E-17	0.077	360612	52325.340
family Peptococcaceae	Vascular dementia (multiple infarctions)	rs4716814	T	C	-0.003	157723046	0.808	0.014	14306	T	C	-0.319	7	157930354	3.75656E-07	0.063	360612	19180.021
family Peptococcaceae	Vascular dementia (multiple infarctions)	rs4725579	C	A	0.009	139468213	0.677	0.017	14306	C	A	-0.379	7	139768414	3.58039E-06	0.082	360612	18391.668
family Peptococcaceae	Vascular dementia (multiple infarctions)	rs72822148	T	C	0.006	9742028	0.775	0.015	14306	T	C	-0.322	17	9838711	3.18831E-06	0.069	360612	17983.471
family Peptostreptococcaceae	Vascular dementia (multiple infarctions)	rs1454336	A	G	-0.004	91873093	0.795	0.015	14306	A	G	-0.369	4	90951942	4.48415E-06	0.080	360612	13616.170
family Peptostreptococcaceae	Vascular dementia (multiple infarctions)	rs429358	C	T	0.014	45411941	0.358	0.016	14306	C	T	0.660	19	44908684	1.33506E-17	0.077	360612	52325.340
family Peptostreptococcaceae	Vascular dementia (multiple infarctions)	rs4716814	T	C	0.011	157723046	0.324	0.011	14306	T	C	-0.319	7	157930354	3.75656E-07	0.063	360612	19180.021
family Peptostreptococcaceae	Vascular dementia (multiple infarctions)	rs4725579	C	A	0.010	139468213	0.552	0.014	14306	C	A	-0.379	7	139768414	3.58039E-06	0.082	360612	18391.668
family Peptostreptococcaceae	Vascular dementia (multiple infarctions)	rs72822148	T	C	-0.006	9742028	0.471	0.012	14306	T	C	-0.322	17	9838711	3.18831E-06	0.069	360612	17983.471
family Porphyromonadaceae	Vascular dementia (multiple infarctions)	rs11081443	C	T	0.009	8944208	0.431	0.013	14306	C	T	0.464	18	8944210	5.71874E-06	0.102	360612	19066.663
family Porphyromonadaceae	Vascular dementia (multiple infarctions)	rs1454336	A	G	-0.005	91873093	0.599	0.014	14306	A	G	-0.369	4	90951942	4.48415E-06	0.080	360612	13616.170
family Porphyromonadaceae	Vascular dementia (multiple infarctions)	rs34288661	C	T	-0.005	20948329	0.730	0.016	14306	C	T	0.460	8	21090818	7.22903E-06	0.103	360612	11969.376
family Porphyromonadaceae	Vascular dementia (multiple infarctions)	rs429358	C	T	0.001	45411941	0.899	0.015	14306	C	T	0.660	19	44908684	1.33506E-17	0.077	360612	52325.340
family Porphyromonadaceae	Vascular dementia (multiple infarctions)	rs4716814	T	C	0.001	157723046	0.950	0.011	14306	T	C	-0.319	7	157930354	3.75656E-07	0.063	360612	19180.021
family Porphyromonadaceae	Vascular dementia (multiple infarctions)	rs4725579	C	A	-0.010	139468213	0.462	0.013	14306	C	A	-0.379	7	139768414	3.58039E-06	0.082	360612	18391.668
family Porphyromonadaceae	Vascular dementia (multiple infarctions)	rs73053797	T	C	0.005	29909039	0.704	0.015	14306	T	C	0.338	3	29867548	6.00772E-06	0.075	360612	13680.576
family Porphyromonadaceae	Vascular dementia (multiple infarctions)	rs9861644	A	G	0.000	88637331	0.983	0.012	14306	A	G	0.323	3	88588181	4.84942E-06	0.071	360612	17137.658
family Prevotellaceae	Vascular dementia (multiple infarctions)	rs11081443	C	T	-0.007	8944208	0.658	0.014	14306	C	T	0.464	18	8944210	5.71874E-06	0.102	360612	19066.663
family Prevotellaceae	Vascular dementia (multiple infarctions)	rs1454336	A	G	-0.007	91873093	0.607	0.015	14306	A	G	-0.369	4	90951942	4.48415E-06	0.080	360612	13616.170
family Prevotellaceae	Vascular dementia (multiple infarctions)	rs429358	C	T	0.000	45411941	0.975	0.017	14306	C	T	0.660	19	44908684	1.33506E-17	0.077	360612	52325.340
family Prevotellaceae	Vascular dementia (multiple infarctions)	rs4716814	T	C	-0.009	157723046	0.456	0.012	14306	T	C	-0.319	7	157930354	3.75656E-07	0.063	360612	19180.021
family Prevotellaceae	Vascular dementia (multiple infarctions)	rs72822148	T	C	0.003	9742028	0.877	0.013	14306	T	C	-0.322	17	9838711	3.18831E-06	0.069	360612	17983.471
family Prevotellaceae	Vascular dementia (multiple infarctions)	rs73053797	T	C	0.006	29909039	0.877	0.016	14306	T	C	0.338	3	29867548	6.00772E-06	0.075	360612	13680.576
family Rhodospirillaceae	Vascular dementia (multiple infarctions)	rs1454336	A	G	-0.007	91873093	0.730	0.020	14306	A	G	-0.369	4	90951942	4.48415E-06	0.080	360612	13616.170
family Rhodospirillaceae	Vascular dementia (multiple infarctions)	rs429358	C	T	-0.006	45411941	0.814	0.022	14306	C	T	0.660	19	44908684	1.33506E-17	0.077	360612	52325.340
family Rhodospirillaceae	Vascular dementia (multiple infarctions)	rs4716814	T	C	-0.003	157723046	0.838	0.016	14306	T	C	-0.319	7	157930354	3.75656E-07	0.063	360612	19180.021
family Rhodospirillaceae	Vascular dementia (multiple infarctions)	rs72822148	T	C	-0.003	9742028	0.921	0.017	14306	T	C	-0.322	17	9838711	3.18831E-06	0.069	360612	17983.471
family Rhodospirillaceae	Vascular dementia (multiple infarctions)	rs9861644	A	G	0.012	88637331	0.472	0.018	14306	A	G	0.323	3	88588181	4.84942E-06	0.071	360612	17137.658
family Rikenellaceae	Vascular dementia (multiple infarctions)	rs11081443	C	T	0.010	8944208	0.450	0.013	14306	C	T	0.464	18	8944210	5.71874E-06	0.102	360612	19066.663
family Rikenellaceae	Vascular dementia (multiple infarctions)	rs1454336	A	G	-0.012	91873093	0.389	0.014	14306	A	G	-0.369	4	90951942	4.48415E-06	0.080	360612	13616.170
family Rikenellaceae	Vascular dementia (multiple infarctions)	rs34288661	C	T	-0.014	20948329	0.359	0.016	14306	C	T	0.460	8	21090818	7.22903E-06	0.103	360612	11969.376
family Rikenellaceae	Vascular dementia (multiple infarctions)	rs429358	C	T	-0.016	45411941	0.273	0.015	14306	C	T	0.660	19	44908684	1.33506E-17	0.077	360612	52325.340
family Rikenellaceae	Vascular dementia (multiple infarctions)	rs4716814	T	C	0.009	157723046	0.421	0.011	14306	T	C	-0.319	7	157930354	3.75656E-07	0.063	360612	19180.021
family Rikenellaceae	Vascular dementia (multiple infarctions)	rs4725579	C	A	-0.009	139468213	0.449	0.013	14306	C	A	-0.379	7	139768414	3.58039E-06	0.082	360612	18391.668
family Rikenellaceae	Vascular dementia (multiple infarctions)	rs9861644	A	G	0.003	88637331	0.776	0.012	14306	A	G	0.323	3	88588181	4.84942E-06	0.071	360612	17137.658

family Ruminococcaceae	Vascular dementia (multiple infarctions)	rs11081443	C	T	0.008	8944208	0.535	0.013	14306	C	T	0.464	18	8944210	5.71874E-06	0.102	360612	19066.663
family Ruminococcaceae	Vascular dementia (multiple infarctions)	rs1454336	A	G	-0.006	91873093	0.645	0.014	14306	A	G	-0.369	4	90951942	4.48415E-06	0.080	360612	13616.170
family Ruminococcaceae	Vascular dementia (multiple infarctions)	rs34288661	C	T	0.001	20948329	0.892	0.016	14306	C	T	0.460	8	21090818	7.22903E-06	0.103	360612	11969.376
family Ruminococcaceae	Vascular dementia (multiple infarctions)	rs429358	C	T	-0.003	45411941	0.847	0.015	14306	C	T	0.660	19	44908684	1.33506E-17	0.077	360612	52325.340
family Ruminococcaceae	Vascular dementia (multiple infarctions)	rs4716814	T	C	0.009	157723046	0.418	0.011	14306	T	C	-0.319	7	157930354	3.75656E-07	0.063	360612	19180.021
family Ruminococcaceae	Vascular dementia (multiple infarctions)	rs4725579	C	A	0.007	139468213	0.640	0.013	14306	C	A	-0.379	7	139768414	3.58039E-06	0.082	360612	18391.668
family Streptococcaceae	Vascular dementia (multiple infarctions)	rs11081443	C	T	0.010	8944208	0.529	0.014	14306	C	T	0.464	18	8944210	5.71874E-06	0.102	360612	19066.663
family Streptococcaceae	Vascular dementia (multiple infarctions)	rs1454336	A	G	0.012	91873093	0.368	0.014	14306	A	G	-0.369	4	90951942	4.48415E-06	0.080	360612	13616.170
family Streptococcaceae	Vascular dementia (multiple infarctions)	rs429358	C	T	0.012	45411941	0.432	0.016	14306	C	T	0.660	19	44908684	1.33506E-17	0.077	360612	52325.340
family Streptococcaceae	Vascular dementia (multiple infarctions)	rs4725579	C	A	-0.001	139468213	0.932	0.014	14306	C	A	-0.379	7	139768414	3.58039E-06	0.082	360612	18391.668
family Streptococcaceae	Vascular dementia (multiple infarctions)	rs72822148	T	C	-0.007	9742028	0.379	0.012	14306	T	C	-0.322	17	9838711	3.18831E-06	0.069	360612	17983.471
family Streptococcaceae	Vascular dementia (multiple infarctions)	rs73053797	T	C	0.009	29909039	0.687	0.016	14306	T	C	0.338	3	29867548	6.00772E-06	0.075	360612	13680.576
family Veillonellaceae	Vascular dementia (multiple infarctions)	rs11081443	C	T	0.002	8944208	0.929	0.014	14306	C	T	0.464	18	8944210	5.71874E-06	0.102	360612	19066.663
family Veillonellaceae	Vascular dementia (multiple infarctions)	rs1454336	A	G	-0.004	91873093	0.647	0.015	14306	A	G	-0.369	4	90951942	4.48415E-06	0.080	360612	13616.170
family Veillonellaceae	Vascular dementia (multiple infarctions)	rs34288661	C	T	0.004	20948329	0.787	0.017	14306	C	T	0.460	8	21090818	7.22903E-06	0.103	360612	11969.376
family Veillonellaceae	Vascular dementia (multiple infarctions)	rs429358	C	T	-0.023	45411941	0.150	0.016	14306	C	T	0.660	19	44908684	1.33506E-17	0.077	360612	52325.340
family Verrucomicrobiaceae	Vascular dementia (multiple infarctions)	rs1454336	A	G	-0.003	91873093	0.963	0.017	14306	A	G	-0.369	4	90951942	4.48415E-06	0.080	360612	13616.170
family Verrucomicrobiaceae	Vascular dementia (multiple infarctions)	rs34288661	C	T	0.007	20948329	0.747	0.019	14306	C	T	0.460	8	21090818	7.22903E-06	0.103	360612	11969.376
family Verrucomicrobiaceae	Vascular dementia (multiple infarctions)	rs429358	C	T	0.018	45411941	0.341	0.019	14306	C	T	0.660	19	44908684	1.33506E-17	0.077	360612	52325.340
family Verrucomicrobiaceae	Vascular dementia (multiple infarctions)	rs4725579	C	A	0.000	139468213	0.920	0.016	14306	C	A	-0.379	7	139768414	3.58039E-06	0.082	360612	18391.668
family Verrucomicrobiaceae	Vascular dementia (multiple infarctions)	rs72822148	T	C	-0.008	9742028	0.626	0.014	14306	T	C	-0.322	17	9838711	3.18831E-06	0.069	360612	17983.471
family Verrucomicrobiaceae	Vascular dementia (multiple infarctions)	rs73053797	T	C	-0.005	29909039	0.797	0.019	14306	T	C	0.338	3	29867548	6.00772E-06	0.075	360612	13680.576
family Verrucomicrobiaceae	Vascular dementia (multiple infarctions)	rs9861644	A	G	0.003	88637331	0.851	0.015	14306	A	G	0.323	3	88588181	4.84942E-06	0.071	360612	17137.658
family Victivallaceae	Vascular dementia (multiple infarctions)	rs1454336	A	G	0.011	91873093	0.731	0.029	14306	A	G	-0.369	4	90951942	4.48415E-06	0.080	360612	13616.170
family Victivallaceae	Vascular dementia (multiple infarctions)	rs34288661	C	T	0.022	20948329	0.533	0.033	14306	C	T	0.460	8	21090818	7.22903E-06	0.103	360612	11969.376
family Victivallaceae	Vascular dementia (multiple infarctions)	rs429358	C	T	-0.015	45411941	0.542	0.033	14306	C	T	0.660	19	44908684	1.33506E-17	0.077	360612	52325.340
family Victivallaceae	Vascular dementia (multiple infarctions)	rs4716814	T	C	-0.005	157723046	0.855	0.023	14306	T	C	-0.319	7	157930354	3.75656E-07	0.063	360612	19180.021
family Victivallaceae	Vascular dementia (multiple infarctions)	rs72822148	T	C	0.001	9742028	0.929	0.025	14306	T	C	-0.322	17	9838711	3.18831E-06	0.069	360612	17983.471
family Victivallaceae	Vascular dementia (multiple infarctions)	rs73053797	T	C	0.010	29909039	0.777	0.033	14306	T	C	0.338	3	29867548	6.00772E-06	0.075	360612	13680.576
family Acidaminococcaceae	Vascular dementia (mixed)	rs12257900	T	G	-0.005	49443428	0.756	0.019	14306	T	G	0.467	10	48235385	2.48891E-06	0.099	360421	23006.877
family Acidaminococcaceae	Vascular dementia (mixed)	rs1466525	T	C	-0.002	54780209	0.877	0.016	14306	T	C	0.489	8	53867649	1.97697E-06	0.103	360421	34189.173
family Acidaminococcaceae	Vascular dementia (mixed)	rs1632064	T	C	0.001	3219694	0.964	0.018	14306	T	C	0.614	5	3219580	4.04045E-06	0.133	360421	37296.871
family Acidaminococcaceae	Vascular dementia (mixed)	rs17168895	T	G	0.005	15647727	0.703	0.021	14306	T	G	-0.547	7	15608102	2.92853E-06	0.117	360421	34495.049
family Acidaminococcaceae	Vascular dementia (mixed)	rs429358	C	T	-0.007	45411941	0.557	0.018	14306	C	T	0.565	19	44908684	3.94357E-08	0.103	360421	36831.461
family Acidaminococcaceae	Vascular dementia (mixed)	rs6028529	A	G	-0.004	38205487	0.740	0.015	14306	A	G	0.413	20	39576844	4.98965E-06	0.091	360421	21913.090
family Acidaminococcaceae	Vascular dementia (mixed)	rs7614116	G	A	-0.009	130368069	0.488	0.014	14306	G	A	0.379	3	130649225	6.06429E-06	0.084	360421	23465.044
family Acidaminococcaceae	Vascular dementia (mixed)	rs7776624	G	A	0.003	31909839	0.822	0.013	14306	G	A	-0.358	7	31870226	8.82531E-06	0.081	360421	23450.025
family Actinomycetaceae	Vascular dementia (mixed)	rs12257900	T	G	-0.001	49443428	0.976	0.024	14306	T	G	0.467	10	48235385	2.48891E-06	0.099	360421	23006.877
family Actinomycetaceae	Vascular dementia (mixed)	rs1466525	T	C	0.007	54780209	0.732	0.020	14306	T	C	0.489	8	53867649	1.97697E-06	0.103	360421	34189.173
family Actinomycetaceae	Vascular dementia (mixed)	rs17168895	T	G	-0.004	15647727	0.807	0.026	14306	T	G	-0.547	7	15608102	2.92853E-06	0.117	360421	34495.049
family Actinomycetaceae	Vascular dementia (mixed)	rs6028529	A	G	0.008	38205487	0.645	0.020	14306	A	G	0.413	20	39576844	4.98965E-06	0.091	360421	21913.090
family Actinomycetaceae	Vascular dementia (mixed)	rs7776624	G	A	-0.003	31909839	0.867	0.016	14306	G	A	-0.358	7	31870226	8.82531E-06	0.081	360421	23450.025
family Alcaligenaceae	Vascular dementia (mixed)	rs12257900	T	G	0.005	49443428	0.752	0.016	14306	T	G	0.467	10	48235385	2.48891E-06	0.099	360421	23006.877
family Alcaligenaceae	Vascular dementia (mixed)	rs1632064	T	C	0.009	3219694	0.568	0.015	14306	T	C	0.614	5	3219580	4.04045E-06	0.133	360421	37296.871
family Alcaligenaceae	Vascular dementia (mixed)	rs17168895	T	G	0.014	15647727	0.491	0.018	14306	T	G	-0.547	7	15608102	2.92853E-06	0.117	360421	34495.049
family Alcaligenaceae	Vascular dementia (mixed)	rs429358	C	T	0.009	45411941	0.545	0.016	14306	C	T	0.565	19	44908684	3.94357E-08	0.103	360421	36831.461
family Alcaligenaceae	Vascular dementia (mixed)	rs6028529	A	G	0.010	38205487	0.404	0.013	14306	A	G	0.413	20	39576844	4.98965E-06	0.091	360421	21913.090
family Alcaligenaceae	Vascular dementia (mixed)	rs6849229	G	A	0.003	131797280	0.795	0.015	14306	G	A	0.562	4	130876125	4.44038E-06	0.122	360421	34142.711
family Bacteroidaceae	Vascular dementia (mixed)	rs12257900	T	G	0.003	49443428	0.884	0.016	14306	T	G	0.467	10	48235385	2.48891E-06	0.099	360421	23006.877
family Bacteroidaceae	Vascular dementia (mixed)	rs1632064	T	C	0.005	3219694	0.720	0.015	14306	T	C	0.614	5	3219580	4.04045E-06	0.133	360421	37296.871
family Bacteroidaceae	Vascular dementia (mixed)	rs17168895	T	G	0.005	15647727	0.820	0.017	14306	T	G	-0.547	7	15608102	2.92853E-06	0.117	360421	34495.049
family Bacteroidaceae	Vascular dementia (mixed)	rs6849229	G	A	-0.009	131797280	0.552	0.014	14306	G	A	0.562	4	130876125	4.44038E-06	0.122	360421	34142.711
family Bacteroidaceae	Vascular dementia (mixed)	rs7614116	G	A	0.005	130368069	0.631	0.011	14306	G	A	0.379	3	130649225	6.06429E-06	0.084	360421	23465.044
family Bacteroidales S24 7group	Vascular dementia (mixed)	rs1466525	T	C	0.000	54780209	0.920	0.020	14306	T	C	0.489	8	53867649	1.97697E-06	0.103	360421	34189.173
family Bacteroidales S24 7group	Vascular dementia (mixed)	rs1632064	T	C	0.008	3219694	0.753	0.022	14306	T	C	0.614	5	3219580	4.04045E-06	0.133	360421	37296.871
family Bacteroidales S24 7group	Vascular dementia (mixed)	rs17168895	T	G	0.011	15647727	0.739	0.026	14306	T	G	-0.547	7	15608102	2.92853E-06	0.117	360421	34495.049
family Bacteroidales S24 7group	Vascular dementia (mixed)	rs6028529	A	G	-0.009	38205487	0.603	0.019	14306	A	G	0.413	20	39576844	4.98965E-06	0.091	360421	21913.090
family Bacteroidales S24 7group	Vascular dementia (mixed)	rs6849229	G	A	-0.012	131797280	0.603	0.022	14306	G	A	0.562	4	130876125	4.44038E-06	0.122	360421	34142.711
family Bifidobacteriaceae	Vascular dementia (mixed)	rs12257900	T	G	-0.004	49443428	0.857	0.017	14306	T	G	0.467	10	48235385	2.48891E-06	0.099	360421	23006.877
family Bifidobacteriaceae	Vascular dementia (mixed)	rs1466525	T	C	0.010	54780209	0.581	0.015	14306	T	C	0.489	8	53867649	1.97697E-06	0.103	360421	34189.173
family Bifidobacteriaceae	Vascular dementia (mixed)	rs6849229	G	A	0.010	131797280	0.502	0.016	14306	G	A	0.562	4	130876125	4.44038E-06	0.122	360421	34142.711
family Bifidobacteriaceae	Vascular dementia (mixed)	rs7614116	G	A	-0.008	130368069	0.477	0.013	14306	G	A	0.379	3	130649225	6.06429E-06	0.084	360421	23465.044
family Bifidobacteriaceae	Vascular dementia (mixed)	rs7776624	G	A	-0.001	31909839	0.943	0.012	14306	G	A	-0.358	7	31870226	8.82531E-06	0.081	360421	23450.025
family Clostridiaceae1	Vascular dementia (mixed)	rs12257900	T	G	-0.012	49443428	0.559	0.018	14306	T	G	0.467	10	48235385	2.48891E-06	0.099	360421	23006.877
family Clostridiaceae1	Vascular dementia (mixed)	rs1466525	T	C	-0.002	54780209	0.887	0.015	14306	T	C	0.489	8	53867649	1.97697E-06	0.103	360421	34189.173
family Clostridiaceae1	Vascular dementia (mixed)	rs1632064	T	C	0.002	3219694	0.867	0.016	14306	T	C	0.614	5	3219580	4.04045E-06	0.133	360421	37296.871
family Clostridiaceae1	Vascular dementia (mixed)	rs17168895	T	G	0.017	15647727	0.407	0.020	14306	T	G	-0.547	7	15608102	2.92853E-06	0.117	360421	34495.049
family Clostridiaceae1	Vascular dementia (mixed)	rs429358	C	T	0.010	45411941	0.573	0.017	14306	C	T	0.565	19	44908684	3.94357E-08	0.103	360421	36831.461
family Clostridiaceae1	Vascular dementia (mixed)	rs6028529	A	G	0.001	38205487	0.873	0.014	14306	A	G	0.413	20	39576844	4.98965E-06	0.091	360421	21913.090
family Clostridiaceae1	Vascular dementia (mixed)	rs7614116	G	A	0.001	130368069	0.900	0.013	14306	G	A	0.379	3	130649225	6.06429E-06	0.084	360421	23465.044
family Clostridiaceae1	Vascular dementia (mixed)	rs7776624	G	A	0.003	31909839	0.821	0.012	14306	G	A	-0.358	7	31870226	8.82531E-06	0.081	360421	23450.025

family Clostridiales vadin BB60 group	Vascular dementia (mixed)	rs1466525	T	C	-0.012	54780209	0.428	0.017	14306	T	C	0.489	8	53867649	1.97697E-06	0.103	360421	34189.173
family Clostridiales vadin BB60 group	Vascular dementia (mixed)	rs429358	C	T	0.004	45411941	0.829	0.020	14306	C	T	0.565	19	44908684	3.94357E-08	0.103	360421	36831.461
family Clostridiales vadin BB60 group	Vascular dementia (mixed)	rs7614116	G	A	0.004	130368069	0.805	0.015	14306	G	A	0.379	3	130649225	6.06429E-06	0.084	360421	23465.044
family Coriobacteriaceae	Vascular dementia (mixed)	rs12257900	T	G	-0.014	49443428	0.392	0.016	14306	T	G	0.467	10	48235385	2.48891E-06	0.099	360421	23006.877
family Coriobacteriaceae	Vascular dementia (mixed)	rs1466525	T	C	-0.008	54780209	0.544	0.013	14306	T	C	0.489	8	53867649	1.97697E-06	0.103	360421	34189.173
family Coriobacteriaceae	Vascular dementia (mixed)	rs1632064	T	C	-0.005	3219694	0.782	0.015	14306	T	C	0.614	5	3219580	4.04045E-06	0.133	360421	37296.871
family Coriobacteriaceae	Vascular dementia (mixed)	rs17168895	T	G	0.014	15647727	0.431	0.018	14306	T	G	-0.547	7	15608102	2.92853E-06	0.117	360421	34495.049
family Coriobacteriaceae	Vascular dementia (mixed)	rs7776624	G	A	-0.006	31909839	0.606	0.011	14306	G	A	-0.358	7	31870226	8.82531E-06	0.081	360421	23450.025
family Defluviitaleaceae	Vascular dementia (mixed)	rs12257900	T	G	-0.002	49443428	0.916	0.023	14306	T	G	0.467	10	48235385	2.48891E-06	0.099	360421	23006.877
family Defluviitaleaceae	Vascular dementia (mixed)	rs429358	C	T	0.000	45411941	0.935	0.022	14306	C	T	0.565	19	44908684	3.94357E-08	0.103	360421	36831.461
family Defluviitaleaceae	Vascular dementia (mixed)	rs7776624	G	A	-0.011	31909839	0.467	0.016	14306	G	A	-0.358	7	31870226	8.82531E-06	0.081	360421	23450.025
family Desulfovibrionaceae	Vascular dementia (mixed)	rs12257900	T	G	0.007	49443428	0.731	0.017	14306	T	G	0.467	10	48235385	2.48891E-06	0.099	360421	23006.877
family Desulfovibrionaceae	Vascular dementia (mixed)	rs1466525	T	C	0.005	54780209	0.940	0.015	14306	T	C	0.489	8	53867649	1.97697E-06	0.103	360421	34189.173
family Desulfovibrionaceae	Vascular dementia (mixed)	rs17168895	T	G	-0.005	15647727	0.769	0.019	14306	T	G	-0.547	7	15608102	2.92853E-06	0.117	360421	34495.049
family Desulfovibrionaceae	Vascular dementia (mixed)	rs429358	C	T	0.017	45411941	0.305	0.017	14306	C	T	0.565	19	44908684	3.94357E-08	0.103	360421	36831.461
family Desulfovibrionaceae	Vascular dementia (mixed)	rs6028529	A	G	0.000	38205487	0.976	0.014	14306	A	G	0.413	20	39576844	4.98965E-06	0.091	360421	21913.090
family Desulfovibrionaceae	Vascular dementia (mixed)	rs6849229	G	A	0.001	131797280	0.928	0.016	14306	G	A	0.562	4	130876125	4.44038E-06	0.122	360421	34142.711
family Desulfovibrionaceae	Vascular dementia (mixed)	rs7614116	G	A	0.004	130368069	0.719	0.013	14306	G	A	0.379	3	130649225	6.06429E-06	0.084	360421	23465.044
family Enterobacteriaceae	Vascular dementia (mixed)	rs12257900	T	G	-0.006	49443428	0.801	0.018	14306	T	G	0.467	10	48235385	2.48891E-06	0.099	360421	23006.877
family Enterobacteriaceae	Vascular dementia (mixed)	rs1466525	T	C	-0.003	54780209	0.852	0.015	14306	T	C	0.489	8	53867649	1.97697E-06	0.103	360421	34189.173
family Enterobacteriaceae	Vascular dementia (mixed)	rs17168895	T	G	0.000	15647727	0.915	0.020	14306	T	G	-0.547	7	15608102	2.92853E-06	0.117	360421	34495.049
family Enterobacteriaceae	Vascular dementia (mixed)	rs429358	C	T	-0.019	45411941	0.299	0.017	14306	C	T	0.565	19	44908684	3.94357E-08	0.103	360421	36831.461
family Enterobacteriaceae	Vascular dementia (mixed)	rs7614116	G	A	-0.005	130368069	0.684	0.013	14306	G	A	0.379	3	130649225	6.06429E-06	0.084	360421	23465.044
family Enterobacteriaceae	Vascular dementia (mixed)	rs7776624	G	A	-0.007	31909839	0.572	0.012	14306	G	A	-0.358	7	31870226	8.82531E-06	0.081	360421	23450.025
family Erysipelotrichaceae	Vascular dementia (mixed)	rs1466525	T	C	0.009	54780209	0.569	0.013	14306	T	C	0.489	8	53867649	1.97697E-06	0.103	360421	34189.173
family Erysipelotrichaceae	Vascular dementia (mixed)	rs1632064	T	C	0.001	3219694	0.867	0.015	14306	T	C	0.614	5	3219580	4.04045E-06	0.133	360421	37296.871
family Erysipelotrichaceae	Vascular dementia (mixed)	rs17168895	T	G	-0.001	15647727	0.965	0.017	14306	T	G	-0.547	7	15608102	2.92853E-06	0.117	360421	34495.049
family Erysipelotrichaceae	Vascular dementia (mixed)	rs6028529	A	G	0.001	38205487	0.976	0.012	14306	A	G	0.413	20	39576844	4.98965E-06	0.091	360421	21913.090
family Erysipelotrichaceae	Vascular dementia (mixed)	rs7776624	G	A	0.007	31909839	0.484	0.011	14306	G	A	-0.358	7	31870226	8.82531E-06	0.081	360421	23450.025
family Family XI	Vascular dementia (mixed)	rs12257900	T	G	-0.011	49443428	0.799	0.036	14306	T	G	0.467	10	48235385	2.48891E-06	0.099	360421	23006.877
family Family XI	Vascular dementia (mixed)	rs1466525	T	C	0.000	54780209	0.950	0.031	14306	T	C	0.489	8	53867649	1.97697E-06	0.103	360421	34189.173
family Family XI	Vascular dementia (mixed)	rs1632064	T	C	-0.015	3219694	0.636	0.035	14306	T	C	0.614	5	3219580	4.04045E-06	0.133	360421	37296.871
family Family XI	Vascular dementia (mixed)	rs17168895	T	G	0.009	15647727	0.781	0.041	14306	T	G	-0.547	7	15608102	2.92853E-06	0.117	360421	34495.049
family Family XI	Vascular dementia (mixed)	rs6849229	G	A	-0.019	131797280	0.588	0.035	14306	G	A	0.562	4	130876125	4.44038E-06	0.122	360421	34142.711
family Family XI	Vascular dementia (mixed)	rs7776624	G	A	0.005	31909839	0.815	0.025	14306	G	A	-0.358	7	31870226	8.82531E-06	0.081	360421	23450.025
family Family XIII	Vascular dementia (mixed)	rs12257900	T	G	-0.002	49443428	0.897	0.016	14306	T	G	0.467	10	48235385	2.48891E-06	0.099	360421	23006.877
family Family XIII	Vascular dementia (mixed)	rs1632064	T	C	-0.011	3219694	0.467	0.015	14306	T	C	0.614	5	3219580	4.04045E-06	0.133	360421	37296.871
family Family XIII	Vascular dementia (mixed)	rs17168895	T	G	0.010	15647727	0.573	0.018	14306	T	G	-0.547	7	15608102	2.92853E-06	0.117	360421	34495.049
family Family XIII	Vascular dementia (mixed)	rs6849229	G	A	-0.011	131797280	0.464	0.015	14306	G	A	0.562	4	130876125	4.44038E-06	0.122	360421	34142.711
family Family XIII	Vascular dementia (mixed)	rs7776624	G	A	-0.007	31909839	0.508	0.011	14306	G	A	-0.358	7	31870226	8.82531E-06	0.081	360421	23450.025
family Lachnospiraceae	Vascular dementia (mixed)	rs1632064	T	C	-0.012	3219694	0.389	0.015	14306	T	C	0.614	5	3219580	4.04045E-06	0.133	360421	37296.871
family Lachnospiraceae	Vascular dementia (mixed)	rs429358	C	T	0.002	45411941	0.858	0.015	14306	C	T	0.565	19	44908684	3.94357E-08	0.103	360421	36831.461
family Lachnospiraceae	Vascular dementia (mixed)	rs6028529	A	G	-0.004	38205487	0.761	0.012	14306	A	G	0.413	20	39576844	4.98965E-06	0.091	360421	21913.090
family Lachnospiraceae	Vascular dementia (mixed)	rs6849229	G	A	-0.002	131797280	0.741	0.014	14306	G	A	0.562	4	130876125	4.44038E-06	0.122	360421	34142.711
family Lachnospiraceae	Vascular dementia (mixed)	rs7776624	G	A	-0.007	31909839	0.526	0.010	14306	G	A	-0.358	7	31870226	8.82531E-06	0.081	360421	23450.025
family Lactobacillaceae	Vascular dementia (mixed)	rs1466525	T	C	-0.010	54780209	0.561	0.021	14306	T	C	0.489	8	53867649	1.97697E-06	0.103	360421	34189.173
family Lactobacillaceae	Vascular dementia (mixed)	rs1632064	T	C	-0.013	3219694	0.611	0.023	14306	T	C	0.614	5	3219580	4.04045E-06	0.133	360421	37296.871
family Lactobacillaceae	Vascular dementia (mixed)	rs17168895	T	G	0.016	15647727	0.623	0.028	14306	T	G	-0.547	7	15608102	2.92853E-06	0.117	360421	34495.049
family Lactobacillaceae	Vascular dementia (mixed)	rs429358	C	T	-0.013	45411941	0.673	0.024	14306	C	T	0.565	19	44908684	3.94357E-08	0.103	360421	36831.461
family Lactobacillaceae	Vascular dementia (mixed)	rs6028529	A	G	0.016	38205487	0.418	0.020	14306	A	G	0.413	20	39576844	4.98965E-06	0.091	360421	21913.090
family Methanobacteriaceae	Vascular dementia (mixed)	rs12257900	T	G	-0.014	49443428	0.614	0.034	14306	T	G	0.467	10	48235385	2.48891E-06	0.099	360421	23006.877
family Methanobacteriaceae	Vascular dementia (mixed)	rs1466525	T	C	0.012	54780209	0.743	0.029	14306	T	C	0.489	8	53867649	1.97697E-06	0.103	360421	34189.173
family Methanobacteriaceae	Vascular dementia (mixed)	rs1632064	T	C	0.005	3219694	0.822	0.032	14306	T	C	0.614	5	3219580	4.04045E-06	0.133	360421	37296.871
family Methanobacteriaceae	Vascular dementia (mixed)	rs17168895	T	G	0.017	15647727	0.666	0.038	14306	T	G	-0.547	7	15608102	2.92853E-06	0.117	360421	34495.049
family Methanobacteriaceae	Vascular dementia (mixed)	rs429358	C	T	-0.026	45411941	0.504	0.034	14306	C	T	0.565	19	44908684	3.94357E-08	0.103	360421	36831.461
family Oxalobacteraceae	Vascular dementia (mixed)	rs1466525	T	C	-0.006	54780209	0.767	0.024	14306	T	C	0.489	8	53867649	1.97697E-06	0.103	360421	34189.173
family Oxalobacteraceae	Vascular dementia (mixed)	rs1632064	T	C	0.024	3219694	0.363	0.028	14306	T	C	0.614	5	3219580	4.04045E-06	0.133	360421	37296.871
family Oxalobacteraceae	Vascular dementia (mixed)	rs17168895	T	G	0.018	15647727	0.585	0.033	14306	T	G	-0.547	7	15608102	2.92853E-06	0.117	360421	34495.049
family Oxalobacteraceae	Vascular dementia (mixed)	rs429358	C	T	0.011	45411941	0.614	0.028	14306	C	T	0.565	19	44908684	3.94357E-08	0.103	360421	36831.461
family Oxalobacteraceae	Vascular dementia (mixed)	rs6028529	A	G	-0.008	38205487	0.684	0.024	14306	A	G	0.413	20	39576844	4.98965E-06	0.091	360421	21913.090
family Oxalobacteraceae	Vascular dementia (mixed)	rs6849229	G	A	0.007	131797280	0.832	0.027	14306	G	A	0.562	4	130876125	4.44038E-06	0.122	360421	34142.711
family Oxalobacteraceae	Vascular dementia (mixed)	rs7614116	G	A	-0.017	130368069	0.451	0.022	14306	G	A	0.379	3	130649225	6.06429E-06	0.084	360421	23465.044
family Oxalobacteraceae	Vascular dementia (mixed)	rs7776624	G	A	0.009	31909839	0.642	0.020	14306	G	A	-0.358	7	31870226	8.82531E-06	0.081	360421	23450.025
family Pasteurellaceae	Vascular dementia (mixed)	rs1466525	T	C	-0.008	54780209	0.686	0.018	14306	T	C	0.489	8	53867649	1.97697E-06	0.103	360421	34189.173
family Pasteurellaceae	Vascular dementia (mixed)	rs1632064	T	C	-0.003	3219694	0.854	0.020	14306	T	C	0.614	5	3219580	4.04045E-06	0.133	360421	37296.871
family Pasteurellaceae	Vascular dementia (mixed)	rs17168895	T	G	0.015	15647727	0.520	0.024	14306	T	G	-0.547	7	15608102	2.92853E-06	0.117	360421	34495.049
family Pasteurellaceae	Vascular dementia (mixed)	rs429358	C	T	0.022	45411941	0.309	0.021	14306	C	T	0.565	19	44908684	3.94357E-08	0.103	360421	36831.461
family Pasteurellaceae	Vascular dementia (mixed)	rs6028529	A	G	-0.009	38205487	0.537	0.017	14306	A	G	0.413	20	39576844	4.98965E-06	0.091	360421	21913.090
family Pasteurellaceae	Vascular dementia (mixed)	rs6849229	G	A	0.010	131797280	0.555	0.020	14306	G	A	0.562	4	130876125	4.44038E-06	0.122	360421	34142.711
family Pasteurellaceae	Vascular dementia (mixed)	rs7776624	G	A	0.009	31909839	0.555	0.015	14306	G	A	-0.358	7	31870226	8.82531E-06	0.081	360421	23450.025
family Peptococcaceae	Vascular dementia (mixed)	rs12257900	T	G	0.006	49443428	0.750	0.020	14306	T	G	0.467	10	48235385	2.48891E-06	0.099	360421	23006.877

family Peptococcaceae	Vascular dementia (mixed)	rs1466525	T	C	0.008	54780209	0.656	0.017	14306	T	C	0.489	8	53867649	1.97697E-06	0.103	360421	34189.173
family Peptococcaceae	Vascular dementia (mixed)	rs1632064	T	C	-0.016	3219694	0.449	0.019	14306	T	C	0.614	5	3219580	4.04045E-06	0.133	360421	37296.871
family Peptococcaceae	Vascular dementia (mixed)	rs6849229	G	A	0.000	131797280	0.954	0.019	14306	G	A	0.562	4	130876125	4.44038E-06	0.122	360421	34142.711
family Peptococcaceae	Vascular dementia (mixed)	rs7614116	G	A	0.008	130368069	0.652	0.015	14306	G	A	0.379	3	130649225	6.06429E-06	0.084	360421	23465.044
family Peptococcaceae	Vascular dementia (mixed)	rs7776624	G	A	-0.005	31909839	0.695	0.014	14306	G	A	-0.358	7	31870226	8.82531E-06	0.081	360421	23450.025
family Peptostreptococcaceae	Vascular dementia (mixed)	rs12257900	T	G	-0.013	49443428	0.455	0.017	14306	T	G	0.467	10	48235385	2.48891E-06	0.099	360421	23006.877
family Peptostreptococcaceae	Vascular dementia (mixed)	rs1466525	T	C	0.006	54780209	0.722	0.014	14306	T	C	0.489	8	53867649	1.97697E-06	0.103	360421	34189.173
family Peptostreptococcaceae	Vascular dementia (mixed)	rs1632064	T	C	-0.003	3219694	0.854	0.016	14306	T	C	0.614	5	3219580	4.04045E-06	0.133	360421	37296.871
family Peptostreptococcaceae	Vascular dementia (mixed)	rs17168895	T	G	0.003	15647727	0.778	0.019	14306	T	G	-0.547	7	15608102	2.92853E-06	0.117	360421	34495.049
family Peptostreptococcaceae	Vascular dementia (mixed)	rs429358	C	T	0.014	45411941	0.358	0.016	14306	C	T	0.565	19	44908684	3.94357E-08	0.103	360421	36831.461
family Peptostreptococcaceae	Vascular dementia (mixed)	rs6849229	G	A	0.004	131797280	0.787	0.015	14306	G	A	0.562	4	130876125	4.44038E-06	0.122	360421	34142.711
family Peptostreptococcaceae	Vascular dementia (mixed)	rs7614116	G	A	0.009	130368069	0.490	0.012	14306	G	A	0.379	3	130649225	6.06429E-06	0.084	360421	23465.044
family Peptostreptococcaceae	Vascular dementia (mixed)	rs7776624	G	A	0.003	31909839	0.774	0.011	14306	G	A	-0.358	7	31870226	8.82531E-06	0.081	360421	23450.025
family Porphyromonadaceae	Vascular dementia (mixed)	rs12257900	T	G	0.012	49443428	0.406	0.016	14306	T	G	0.467	10	48235385	2.48891E-06	0.099	360421	23006.877
family Porphyromonadaceae	Vascular dementia (mixed)	rs1632064	T	C	0.001	3219694	0.978	0.015	14306	T	C	0.614	5	3219580	4.04045E-06	0.133	360421	37296.871
family Porphyromonadaceae	Vascular dementia (mixed)	rs17168895	T	G	0.012	15647727	0.544	0.017	14306	T	G	-0.547	7	15608102	2.92853E-06	0.117	360421	34495.049
family Porphyromonadaceae	Vascular dementia (mixed)	rs429358	C	T	0.001	45411941	0.899	0.015	14306	C	T	0.565	19	44908684	3.94357E-08	0.103	360421	36831.461
family Porphyromonadaceae	Vascular dementia (mixed)	rs6028529	A	G	-0.004	38205487	0.744	0.013	14306	A	G	0.413	20	39576844	4.98965E-06	0.091	360421	21913.090
family Porphyromonadaceae	Vascular dementia (mixed)	rs6849229	G	A	-0.002	131797280	0.926	0.014	14306	G	A	0.562	4	130876125	4.44038E-06	0.122	360421	34142.711
family Porphyromonadaceae	Vascular dementia (mixed)	rs7614116	G	A	0.001	130368069	0.917	0.011	14306	G	A	0.379	3	130649225	6.06429E-06	0.084	360421	23465.044
family Porphyromonadaceae	Vascular dementia (mixed)	rs7776624	G	A	0.007	31909839	0.513	0.011	14306	G	A	-0.358	7	31870226	8.82531E-06	0.081	360421	23450.025
family Prevotellaceae	Vascular dementia (mixed)	rs1466525	T	C	0.008	54780209	0.527	0.015	14306	T	C	0.489	8	53867649	1.97697E-06	0.103	360421	34189.173
family Prevotellaceae	Vascular dementia (mixed)	rs17168895	T	G	0.007	15647727	0.726	0.019	14306	T	G	-0.547	7	15608102	2.92853E-06	0.117	360421	34495.049
family Prevotellaceae	Vascular dementia (mixed)	rs429358	C	T	0.000	45411941	0.975	0.017	14306	C	T	0.565	19	44908684	3.94357E-08	0.103	360421	36831.461
family Prevotellaceae	Vascular dementia (mixed)	rs7614116	G	A	0.004	130368069	0.688	0.013	14306	G	A	0.379	3	130649225	6.06429E-06	0.084	360421	23465.044
family Prevotellaceae	Vascular dementia (mixed)	rs7776624	G	A	-0.003	31909839	0.814	0.012	14306	G	A	-0.358	7	31870226	8.82531E-06	0.081	360421	23450.025
family Rhodospirillaceae	Vascular dementia (mixed)	rs12257900	T	G	0.000	49443428	0.968	0.022	14306	T	G	0.467	10	48235385	2.48891E-06	0.099	360421	23006.877
family Rhodospirillaceae	Vascular dementia (mixed)	rs1632064	T	C	-0.009	3219694	0.660	0.022	14306	T	C	0.614	5	3219580	4.04045E-06	0.133	360421	37296.871
family Rhodospirillaceae	Vascular dementia (mixed)	rs17168895	T	G	0.022	15647727	0.406	0.025	14306	T	G	-0.547	7	15608102	2.92853E-06	0.117	360421	34495.049
family Rhodospirillaceae	Vascular dementia (mixed)	rs429358	C	T	-0.006	45411941	0.814	0.022	14306	C	T	0.565	19	44908684	3.94357E-08	0.103	360421	36831.461
family Rhodospirillaceae	Vascular dementia (mixed)	rs6028529	A	G	-0.010	38205487	0.569	0.018	14306	A	G	0.413	20	39576844	4.98965E-06	0.091	360421	21913.090
family Rhodospirillaceae	Vascular dementia (mixed)	rs7614116	G	A	-0.008	130368069	0.653	0.017	14306	G	A	0.379	3	130649225	6.06429E-06	0.084	360421	23465.044
family Rhodospirillaceae	Vascular dementia (mixed)	rs7776624	G	A	0.001	31909839	0.923	0.015	14306	G	A	-0.358	7	31870226	8.82531E-06	0.081	360421	23450.025
family Rikenellaceae	Vascular dementia (mixed)	rs12257900	T	G	-0.001	49443428	0.878	0.016	14306	T	G	0.467	10	48235385	2.48891E-06	0.099	360421	23006.877
family Rikenellaceae	Vascular dementia (mixed)	rs1466525	T	C	0.010	54780209	0.562	0.013	14306	T	C	0.489	8	53867649	1.97697E-06	0.103	360421	34189.173
family Rikenellaceae	Vascular dementia (mixed)	rs1632064	T	C	-0.010	3219694	0.480	0.015	14306	T	C	0.614	5	3219580	4.04045E-06	0.133	360421	37296.871
family Rikenellaceae	Vascular dementia (mixed)	rs429358	C	T	-0.016	45411941	0.273	0.015	14306	C	T	0.565	19	44908684	3.94357E-08	0.103	360421	36831.461
family Rikenellaceae	Vascular dementia (mixed)	rs6028529	A	G	0.009	38205487	0.494	0.013	14306	A	G	0.413	20	39576844	4.98965E-06	0.091	360421	21913.090
family Rikenellaceae	Vascular dementia (mixed)	rs6849229	G	A	-0.002	131797280	0.975	0.014	14306	G	A	0.562	4	130876125	4.44038E-06	0.122	360421	34142.711
family Rikenellaceae	Vascular dementia (mixed)	rs7614116	G	A	-0.001	130368069	0.953	0.012	14306	G	A	0.379	3	130649225	6.06429E-06	0.084	360421	23465.044
family Rikenellaceae	Vascular dementia (mixed)	rs7776624	G	A	0.008	31909839	0.453	0.011	14306	G	A	-0.358	7	31870226	8.82531E-06	0.081	360421	23450.025
family Ruminococcaceae	Vascular dementia (mixed)	rs1466525	T	C	0.005	54780209	0.681	0.013	14306	T	C	0.489	8	53867649	1.97697E-06	0.103	360421	34189.173
family Ruminococcaceae	Vascular dementia (mixed)	rs1632064	T	C	0.002	3219694	0.834	0.015	14306	T	C	0.614	5	3219580	4.04045E-06	0.133	360421	37296.871
family Ruminococcaceae	Vascular dementia (mixed)	rs429358	C	T	-0.003	45411941	0.847	0.015	14306	C	T	0.565	19	44908684	3.94357E-08	0.103	360421	36831.461
family Ruminococcaceae	Vascular dementia (mixed)	rs7614116	G	A	0.006	130368069	0.580	0.011	14306	G	A	0.379	3	130649225	6.06429E-06	0.084	360421	23465.044
family Ruminococcaceae	Vascular dementia (mixed)	rs7776624	G	A	0.008	31909839	0.462	0.010	14306	G	A	-0.358	7	31870226	8.82531E-06	0.081	360421	23450.025
family Streptococcaceae	Vascular dementia (mixed)	rs1466525	T	C	0.000	54780209	0.931	0.014	14306	T	C	0.489	8	53867649	1.97697E-06	0.103	360421	34189.173
family Streptococcaceae	Vascular dementia (mixed)	rs1632064	T	C	-0.007	3219694	0.567	0.015	14306	T	C	0.614	5	3219580	4.04045E-06	0.133	360421	37296.871
family Streptococcaceae	Vascular dementia (mixed)	rs17168895	T	G	0.013	15647727	0.517	0.018	14306	T	G	-0.547	7	15608102	2.92853E-06	0.117	360421	34495.049
family Streptococcaceae	Vascular dementia (mixed)	rs429358	C	T	0.012	45411941	0.432	0.016	14306	C	T	0.565	19	44908684	3.94357E-08	0.103	360421	36831.461
family Streptococcaceae	Vascular dementia (mixed)	rs6028529	A	G	0.006	38205487	0.659	0.013	14306	A	G	0.413	20	39576844	4.98965E-06	0.091	360421	21913.090
family Streptococcaceae	Vascular dementia (mixed)	rs7614116	G	A	-0.006	130368069	0.611	0.012	14306	G	A	0.379	3	130649225	6.06429E-06	0.084	360421	23465.044
family Streptococcaceae	Vascular dementia (mixed)	rs7776624	G	A	-0.008	31909839	0.470	0.011	14306	G	A	-0.358	7	31870226	8.82531E-06	0.081	360421	23450.025
family Veillonellaceae	Vascular dementia (mixed)	rs12257900	T	G	0.009	49443428	0.668	0.017	14306	T	G	0.467	10	48235385	2.48891E-06	0.099	360421	23006.877
family Veillonellaceae	Vascular dementia (mixed)	rs1466525	T	C	0.004	54780209	0.754	0.014	14306	T	C	0.489	8	53867649	1.97697E-06	0.103	360421	34189.173
family Veillonellaceae	Vascular dementia (mixed)	rs1632064	T	C	-0.014	3219694	0.387	0.016	14306	T	C	0.614	5	3219580	4.04045E-06	0.133	360421	37296.871
family Veillonellaceae	Vascular dementia (mixed)	rs6028529	A	G	0.009	38205487	0.530	0.013	14306	A	G	0.413	20	39576844	4.98965E-06	0.091	360421	21913.090
family Veillonellaceae	Vascular dementia (mixed)	rs6849229	G	A	0.013	131797280	0.376	0.015	14306	G	A	0.562	4	130876125	4.44038E-06	0.122	360421	34142.711
family Veillonellaceae	Vascular dementia (mixed)	rs7776624	G	A	-0.004	31909839	0.735	0.011	14306	G	A	-0.358	7	31870226	8.82531E-06	0.081	360421	23450.025
family Verrucomicrobiaceae	Vascular dementia (mixed)	rs12257900	T	G	-0.014	49443428	0.459	0.019	14306	T	G	0.467	10	48235385	2.48891E-06	0.099	360421	23006.877
family Verrucomicrobiaceae	Vascular dementia (mixed)	rs1466525	T	C	0.009	54780209	0.552	0.016	14306	T	C	0.489	8	53867649	1.97697E-06	0.103	360421	34189.173
family Verrucomicrobiaceae	Vascular dementia (mixed)	rs1632064	T	C	0.001	3219694	0.915	0.018	14306	T	C	0.614	5	3219580	4.04045E-06	0.133	360421	37296.871
family Verrucomicrobiaceae	Vascular dementia (mixed)	rs17168895	T	G	0.012	15647727	0.527	0.021	14306	T	G	-0.547	7	15608102	2.92853E-06	0.117	360421	34495.049
family Verrucomicrobiaceae	Vascular dementia (mixed)	rs429358	C	T	0.018	45411941	0.341	0.019	14306	C	T	0.565	19	44908684	3.94357E-08	0.103	360421	36831.461
family Verrucomicrobiaceae	Vascular dementia (mixed)	rs6028529	A	G	-0.013	38205487	0.453	0.016	14306	A	G	0.413	20	39576844	4.98965E-06	0.091	360421	21913.090
family Verrucomicrobiaceae	Vascular dementia (mixed)	rs6849229	G	A	-0.013	131797280	0.494	0.018	14306	G	A	0.562	4	130876125	4.44038E-06	0.122	360421	34142.711
family Verrucomicrobiaceae	Vascular dementia (mixed)	rs7614116	G	A	-0.001	130368069	0.998	0.014	14306	G	A	0.379	3	130649225	6.06429E-06	0.084	360421	23465.044
family Victivallaceae	Vascular dementia (mixed)	rs12257900	T	G	0.020	49443428	0.579	0.033	14306	T	G	0.467	10	48235385	2.48891E-06	0.099	360421	23006.877
family Victivallaceae	Vascular dementia (mixed)	rs1466525	T	C	-0.022	54780209	0.468	0.028	14306	T	C	0.489	8	53867649	1.97697E-06	0.103	360421	34189.173
family Victivallaceae	Vascular dementia (mixed)	rs17168895	T	G	0.006	15647727	0.829	0.037	14306	T	G	-0.547	7	15608102	2.92853E-06	0.117	360421	34495.049
family Victivallaceae	Vascular dementia (mixed)	rs429358	C	T	-0.015	45411941	0.542	0.033	14306	C	T	0.565	19	44908684	3.94357E-08	0.103	360421	36831.461

family Victivallaceae	Vascular dementia (mixed)	rs6028529	A	G	0.004	38205487	0.885	0.027	14306	A	G	0.413	20	39576844	4.98965E-06	0.091	360421	21913.090
family Victivallaceae	Vascular dementia (mixed)	rs6849229	G	A	-0.007	131797280	0.771	0.031	14306	G	A	0.562	4	130876125	4.44038E-06	0.122	360421	34142.711
family Victivallaceae	Vascular dementia (mixed)	rs7614116	G	A	0.011	130368069	0.670	0.024	14306	G	A	0.379	3	130649225	6.06429E-06	0.084	360421	23465.044
family Acidaminococcaceae	Vascular dementia (subcortical)	rs11148372	A	G	-0.009	22788665	0.484	0.013	14306	A	G	-0.261	13	22214526	4.06275E-06	0.057	360770	12632.549
family Acidaminococcaceae	Vascular dementia (subcortical)	rs11986558	T	C	-0.006	2500772	0.633	0.013	14306	T	C	0.248	8	2643276	9.16875E-06	0.056	360770	11030.353
family Acidaminococcaceae	Vascular dementia (subcortical)	rs1363668	G	A	0.005	143089582	0.687	0.013	14306	G	A	-0.272	5	143710017	5.25352E-06	0.060	360770	12697.214
family Acidaminococcaceae	Vascular dementia (subcortical)	rs3802793	A	G	0.004	131685316	0.763	0.014	14306	A	G	0.274	11	131815422	5.46626E-06	0.060	360770	12758.334
family Acidaminococcaceae	Vascular dementia (subcortical)	rs429358	C	T	-0.007	45411941	0.557	0.018	14306	C	T	0.597	19	44908684	1.74221E-17	0.070	360770	41621.629
family Acidaminococcaceae	Vascular dementia (subcortical)	rs4295569	C	T	-0.009	47820641	0.492	0.013	14306	C	T	-0.355	7	47781043	2.54572E-08	0.064	360770	20037.409
family Acidaminococcaceae	Vascular dementia (subcortical)	rs4382795	C	T	0.001	66878853	0.974	0.017	14306	C	T	0.378	10	65119095	9.80754E-06	0.086	360770	13265.712
family Acidaminococcaceae	Vascular dementia (subcortical)	rs4723291	A	G	-0.005	33551998	0.649	0.013	14306	A	G	-0.263	7	33512386	6.22372E-06	0.058	360770	11064.499
family Actinomycetaceae	Vascular dementia (subcortical)	rs10919863	T	C	-0.010	200226041	0.667	0.021	14306	T	C	0.315	1	200256913	3.47112E-06	0.068	360770	10813.753
family Actinomycetaceae	Vascular dementia (subcortical)	rs11148372	A	G	0.000	22788665	0.999	0.016	14306	A	G	-0.261	13	22214526	4.06275E-06	0.057	360770	12632.549
family Actinomycetaceae	Vascular dementia (subcortical)	rs11986558	T	C	0.009	2500772	0.570	0.017	14306	T	C	0.248	8	2643276	9.16875E-06	0.056	360770	11030.353
family Actinomycetaceae	Vascular dementia (subcortical)	rs429358	C	T	0.038	45411941	0.090	0.023	14306	C	T	0.597	19	44908684	1.74221E-17	0.070	360770	41621.629
family Actinomycetaceae	Vascular dementia (subcortical)	rs4295569	C	T	0.005	47820641	0.753	0.017	14306	C	T	-0.355	7	47781043	2.54572E-08	0.064	360770	20037.409
family Alcaligenaceae	Vascular dementia (subcortical)	rs11148372	A	G	-0.008	22788665	0.457	0.011	14306	A	G	-0.261	13	22214526	4.06275E-06	0.057	360770	12632.549
family Alcaligenaceae	Vascular dementia (subcortical)	rs11986558	T	C	0.010	2500772	0.410	0.012	14306	T	C	0.248	8	2643276	9.16875E-06	0.056	360770	11030.353
family Alcaligenaceae	Vascular dementia (subcortical)	rs1363668	G	A	0.001	143089582	0.929	0.011	14306	G	A	-0.272	5	143710017	5.25352E-06	0.060	360770	12697.214
family Alcaligenaceae	Vascular dementia (subcortical)	rs429358	C	T	0.009	45411941	0.545	0.016	14306	C	T	0.597	19	44908684	1.74221E-17	0.070	360770	41621.629
family Alcaligenaceae	Vascular dementia (subcortical)	rs4723291	A	G	-0.008	33551998	0.477	0.011	14306	A	G	-0.263	7	33512386	6.22372E-06	0.058	360770	11064.499
family Bacteroidaceae	Vascular dementia (subcortical)	rs10919863	T	C	0.004	200226041	0.825	0.013	14306	T	C	0.315	1	200256913	3.47112E-06	0.068	360770	10813.753
family Bacteroidaceae	Vascular dementia (subcortical)	rs11148372	A	G	-0.002	22788665	0.851	0.011	14306	A	G	-0.261	13	22214526	4.06275E-06	0.057	360770	12632.549
family Bacteroidaceae	Vascular dementia (subcortical)	rs11986558	T	C	0.007	2500772	0.582	0.011	14306	T	C	0.248	8	2643276	9.16875E-06	0.056	360770	11030.353
family Bacteroidaceae	Vascular dementia (subcortical)	rs1363668	G	A	0.009	143089582	0.412	0.011	14306	G	A	-0.272	5	143710017	5.25352E-06	0.060	360770	12697.214
family Bacteroidales S24 7group	Vascular dementia (subcortical)	rs11148372	A	G	0.006	22788665	0.735	0.016	14306	A	G	-0.261	13	22214526	4.06275E-06	0.057	360770	12632.549
family Bacteroidales S24 7group	Vascular dementia (subcortical)	rs11986558	T	C	-0.008	2500772	0.681	0.017	14306	T	C	0.248	8	2643276	9.16875E-06	0.056	360770	11030.353
family Bacteroidales S24 7group	Vascular dementia (subcortical)	rs1363668	G	A	-0.002	143089582	0.924	0.016	14306	G	A	-0.272	5	143710017	5.25352E-06	0.060	360770	12697.214
family Bacteroidales S24 7group	Vascular dementia (subcortical)	rs3802793	A	G	0.011	131685316	0.523	0.017	14306	A	G	0.274	11	131815422	5.46626E-06	0.060	360770	12758.334
family Bacteroidales S24 7group	Vascular dementia (subcortical)	rs429358	C	T	-0.033	45411941	0.162	0.023	14306	C	T	0.597	19	44908684	1.74221E-17	0.070	360770	41621.629
family Bacteroidales S24 7group	Vascular dementia (subcortical)	rs4295569	C	T	-0.016	47820641	0.339	0.017	14306	C	T	-0.355	7	47781043	2.54572E-08	0.064	360770	20037.409
family Bacteroidales S24 7group	Vascular dementia (subcortical)	rs4382795	C	T	0.013	66878853	0.546	0.021	14306	C	T	0.378	10	65119095	9.80754E-06	0.086	360770	13265.712
family Bacteroidales S24 7group	Vascular dementia (subcortical)	rs4723291	A	G	0.005	33551998	0.742	0.016	14306	A	G	-0.263	7	33512386	6.22372E-06	0.058	360770	11064.499
family Bifidobacteriaceae	Vascular dementia (subcortical)	rs10919863	T	C	-0.001	200226041	0.993	0.015	14306	T	C	0.315	1	200256913	3.47112E-06	0.068	360770	10813.753
family Bifidobacteriaceae	Vascular dementia (subcortical)	rs11148372	A	G	-0.002	22788665	0.862	0.012	14306	A	G	-0.261	13	22214526	4.06275E-06	0.057	360770	12632.549
family Bifidobacteriaceae	Vascular dementia (subcortical)	rs11986558	T	C	0.006	2500772	0.634	0.012	14306	T	C	0.248	8	2643276	9.16875E-06	0.056	360770	11030.353
family Bifidobacteriaceae	Vascular dementia (subcortical)	rs1363668	G	A	0.009	143089582	0.437	0.012	14306	G	A	-0.272	5	143710017	5.25352E-06	0.060	360770	12697.214
family Bifidobacteriaceae	Vascular dementia (subcortical)	rs3802793	A	G	0.006	131685316	0.682	0.012	14306	A	G	0.274	11	131815422	5.46626E-06	0.060	360770	12758.334
family Bifidobacteriaceae	Vascular dementia (subcortical)	rs429358	C	T	0.022	45411941	0.147	0.017	14306	C	T	0.597	19	44908684	1.74221E-17	0.070	360770	41621.629
family Bifidobacteriaceae	Vascular dementia (subcortical)	rs4295569	C	T	0.001	47820641	0.967	0.012	14306	C	T	-0.355	7	47781043	2.54572E-08	0.064	360770	20037.409
family Bifidobacteriaceae	Vascular dementia (subcortical)	rs4723291	A	G	-0.004	33551998	0.773	0.012	14306	A	G	-0.263	7	33512386	6.22372E-06	0.058	360770	11064.499
family Clostridiaceae1	Vascular dementia (subcortical)	rs10919863	T	C	-0.012	200226041	0.446	0.015	14306	T	C	0.315	1	200256913	3.47112E-06	0.068	360770	10813.753
family Clostridiaceae1	Vascular dementia (subcortical)	rs11148372	A	G	-0.006	22788665	0.620	0.012	14306	A	G	-0.261	13	22214526	4.06275E-06	0.057	360770	12632.549
family Clostridiaceae1	Vascular dementia (subcortical)	rs11986558	T	C	-0.011	2500772	0.407	0.013	14306	T	C	0.248	8	2643276	9.16875E-06	0.056	360770	11030.353
family Clostridiaceae1	Vascular dementia (subcortical)	rs429358	C	T	0.010	45411941	0.573	0.017	14306	C	T	0.597	19	44908684	1.74221E-17	0.070	360770	41621.629
family Clostridiaceae1	Vascular dementia (subcortical)	rs4295569	C	T	-0.011	47820641	0.391	0.012	14306	C	T	-0.355	7	47781043	2.54572E-08	0.064	360770	20037.409
family Clostridiaceae1	Vascular dementia (subcortical)	rs4723291	A	G	-0.008	33551998	0.474	0.012	14306	A	G	-0.263	7	33512386	6.22372E-06	0.058	360770	11064.499
family Clostridiales vadin BB60 group	Vascular dementia (subcortical)	rs10919863	T	C	-0.008	200226041	0.643	0.018	14306	T	C	0.315	1	200256913	3.47112E-06	0.068	360770	10813.753
family Clostridiales vadin BB60 group	Vascular dementia (subcortical)	rs11148372	A	G	-0.002	22788665	0.893	0.014	14306	A	G	-0.261	13	22214526	4.06275E-06	0.057	360770	12632.549
family Clostridiales vadin BB60 group	Vascular dementia (subcortical)	rs11986558	T	C	-0.006	2500772	0.663	0.015	14306	T	C	0.248	8	2643276	9.16875E-06	0.056	360770	11030.353
family Clostridiales vadin BB60 group	Vascular dementia (subcortical)	rs1363668	G	A	-0.011	143089582	0.428	0.014	14306	G	A	-0.272	5	143710017	5.25352E-06	0.060	360770	12697.214
family Clostridiales vadin BB60 group	Vascular dementia (subcortical)	rs3802793	A	G	0.011	131685316	0.498	0.015	14306	A	G	0.274	11	131815422	5.46626E-06	0.060	360770	12758.334
family Clostridiales vadin BB60 group	Vascular dementia (subcortical)	rs429358	C	T	0.004	45411941	0.829	0.020	14306	C	T	0.597	19	44908684	1.74221E-17	0.070	360770	41621.629
family Clostridiales vadin BB60 group	Vascular dementia (subcortical)	rs4295569	C	T	0.002	47820641	0.906	0.014	14306	C	T	-0.355	7	47781043	2.54572E-08	0.064	360770	20037.409
family Clostridiales vadin BB60 group	Vascular dementia (subcortical)	rs4382795	C	T	-0.012	66878853	0.500	0.018	14306	C	T	0.378	10	65119095	9.80754E-06	0.086	360770	13265.712
family Coriobacteriaceae	Vascular dementia (subcortical)	rs3802793	A	G	0.007	131685316	0.516	0.011	14306	A	G	0.274	11	131815422	5.46626E-06	0.060	360770	12758.334
family Coriobacteriaceae	Vascular dementia (subcortical)	rs429358	C	T	0.019	45411941	0.204	0.015	14306	C	T	0.597	19	44908684	1.74221E-17	0.070	360770	41621.629
family Coriobacteriaceae	Vascular dementia (subcortical)	rs4295569	C	T	0.002	47820641	0.863	0.011	14306	C	T	-0.355	7	47781043	2.54572E-08	0.064	360770	20037.409
family Coriobacteriaceae	Vascular dementia (subcortical)	rs4382795	C	T	-0.007	66878853	0.591	0.014	14306	C	T	0.378	10	65119095	9.80754E-06	0.086	360770	13265.712
family Coriobacteriaceae	Vascular dementia (subcortical)	rs4723291	A	G	-0.006	33551998	0.567	0.011	14306	A	G	-0.263	7	33512386	6.22372E-06	0.058	360770	11064.499
family Defluviitaleaceae	Vascular dementia (subcortical)	rs10919863	T	C	0.008	200226041	0.724	0.020	14306	T	C	0.315	1	200256913	3.47112E-06	0.068	360770	10813.753
family Defluviitaleaceae	Vascular dementia (subcortical)	rs11148372	A	G	0.003	22788665	0.862	0.016	14306	A	G	-0.261	13	22214526	4.06275E-06	0.057	360770	12632.549
family Defluviitaleaceae	Vascular dementia (subcortical)	rs3802793	A	G	-0.013	131685316	0.430	0.017	14306	A	G	0.274	11	131815422	5.46626E-06	0.060	360770	12758.334
family Defluviitaleaceae	Vascular dementia (subcortical)	rs429358	C	T	0.000	45411941	0.935	0.022	14306	C	T	0.597	19	44908684	1.74221E-17	0.070	360770	41621.629
family Defluviitaleaceae	Vascular dementia (subcortical)	rs4295569	C	T	0.004	47820641	0.780	0.016	14306	C	T	-0.355	7	47781043	2.54572E-08	0.064	360770	20037.409
family Defluviitaleaceae	Vascular dementia (subcortical)	rs4382795	C	T	-0.003	66878853	0.862	0.021	14306	C	T	0.378	10	65119095	9.80754E-06	0.086	360770	13265.712
family Defluviitaleaceae	Vascular dementia (subcortical)	rs4723291	A	G	0.000	33551998	0.974	0.016	14306	A	G	-0.263	7	33512386	6.22372E-06	0.058	360770	11064.499
family Desulfovibrionaceae	Vascular dementia (subcortical)	rs11986558	T	C	-0.004	2500772	0.731	0.012	14306	T	C	0.248	8	2643276	9.16875E-06	0.056	360770	11030.353
family Desulfovibrionaceae	Vascular dementia (subcortical)	rs3802793	A	G	0.005	131685316	0.651	0.012	14306	A	G	0.274	11	131815422	5.46626E-06	0.060	360770	12758.334
family Desulfovibrionaceae	Vascular dementia (subcortical)	rs429358	C	T	0.017	45411941	0.305	0.017	14306	C	T	0.597	19	44908684	1.74221E-17	0.070	360770	41621.629
family Desulfovibrionaceae	Vascular dementia (subcortical)	rs4295569	C	T	-0.006	47820641	0.614	0.012	14306	C	T	-0.355	7	47781043	2.54572E-08	0.064	360770	20037.409

family Desulfovibrionaceae	Vascular dementia (subcortical)	rs4382795	C	T	0.009	66878853	0.588	0.015	14306	C	T	0.378	10	65119095	9.80754E-06	0.086	360770	13265.712
family Desulfovibrionaceae	Vascular dementia (subcortical)	rs4723291	A	G	0.010	33551998	0.432	0.012	14306	A	G	-0.263	7	33512386	6.22372E-06	0.058	360770	11064.499
family Enterobacteriaceae	Vascular dementia (subcortical)	rs1363668	G	A	0.010	143089582	0.411	0.012	14306	G	A	-0.272	5	143710017	5.25352E-06	0.060	360770	12697.214
family Enterobacteriaceae	Vascular dementia (subcortical)	rs3802793	A	G	0.005	131685316	0.696	0.013	14306	A	G	0.274	11	131815422	5.46626E-06	0.060	360770	12758.334
family Enterobacteriaceae	Vascular dementia (subcortical)	rs429358	C	T	-0.019	45411941	0.299	0.017	14306	C	T	0.597	19	44908684	1.74221E-17	0.070	360770	41621.629
family Enterobacteriaceae	Vascular dementia (subcortical)	rs4295569	C	T	0.001	47820641	0.912	0.013	14306	C	T	-0.355	7	47781043	2.54572E-08	0.064	360770	20037.409
family Erysipelotrichaceae	Vascular dementia (subcortical)	rs10919863	T	C	0.010	200226041	0.450	0.013	14306	T	C	0.315	1	200256913	3.47112E-06	0.068	360770	10813.753
family Erysipelotrichaceae	Vascular dementia (subcortical)	rs11148372	A	G	0.001	22788665	0.885	0.011	14306	A	G	-0.261	13	22214526	4.06275E-06	0.057	360770	12632.549
family Erysipelotrichaceae	Vascular dementia (subcortical)	rs11986558	T	C	-0.005	2500772	0.657	0.011	14306	T	C	0.248	8	2643276	9.16875E-06	0.056	360770	11030.353
family Erysipelotrichaceae	Vascular dementia (subcortical)	rs1363668	G	A	0.004	143089582	0.691	0.011	14306	G	A	-0.272	5	143710017	5.25352E-06	0.060	360770	12697.214
family Erysipelotrichaceae	Vascular dementia (subcortical)	rs4723291	A	G	-0.006	33551998	0.581	0.011	14306	A	G	-0.263	7	33512386	6.22372E-06	0.058	360770	11064.499
family Family XI	Vascular dementia (subcortical)	rs10919863	T	C	0.019	200226041	0.614	0.033	14306	T	C	0.315	1	200256913	3.47112E-06	0.068	360770	10813.753
family Family XI	Vascular dementia (subcortical)	rs11148372	A	G	-0.019	22788665	0.458	0.026	14306	A	G	-0.261	13	22214526	4.06275E-06	0.057	360770	12632.549
family Family XI	Vascular dementia (subcortical)	rs11986558	T	C	0.002	2500772	0.912	0.027	14306	T	C	0.248	8	2643276	9.16875E-06	0.056	360770	11030.353
family Family XI	Vascular dementia (subcortical)	rs1363668	G	A	0.010	143089582	0.687	0.025	14306	G	A	-0.272	5	143710017	5.25352E-06	0.060	360770	12697.214
family Family XI	Vascular dementia (subcortical)	rs3802793	A	G	0.023	131685316	0.389	0.026	14306	A	G	0.274	11	131815422	5.46626E-06	0.060	360770	12758.334
family Family XI	Vascular dementia (subcortical)	rs429358	C	T	0.054	45411941	0.150	0.037	14306	C	T	0.597	19	44908684	1.74221E-17	0.070	360770	41621.629
family Family XI	Vascular dementia (subcortical)	rs4295569	C	T	-0.014	47820641	0.580	0.026	14306	C	T	-0.355	7	47781043	2.54572E-08	0.064	360770	20037.409
family Family XI	Vascular dementia (subcortical)	rs4723291	A	G	-0.002	33551998	0.954	0.025	14306	A	G	-0.263	7	33512386	6.22372E-06	0.058	360770	11064.499
family Family XIII	Vascular dementia (subcortical)	rs10919863	T	C	-0.011	200226041	0.495	0.014	14306	T	C	0.315	1	200256913	3.47112E-06	0.068	360770	10813.753
family Family XIII	Vascular dementia (subcortical)	rs11148372	A	G	0.009	22788665	0.400	0.011	14306	A	G	-0.261	13	22214526	4.06275E-06	0.057	360770	12632.549
family Family XIII	Vascular dementia (subcortical)	rs3802793	A	G	0.003	131685316	0.854	0.012	14306	A	G	0.274	11	131815422	5.46626E-06	0.060	360770	12758.334
family Family XIII	Vascular dementia (subcortical)	rs4295569	C	T	0.005	47820641	0.700	0.011	14306	C	T	-0.355	7	47781043	2.54572E-08	0.064	360770	20037.409
family Family XIII	Vascular dementia (subcortical)	rs4382795	C	T	0.006	66878853	0.708	0.014	14306	C	T	0.378	10	65119095	9.80754E-06	0.086	360770	13265.712
family Family XIII	Vascular dementia (subcortical)	rs4723291	A	G	0.004	33551998	0.695	0.011	14306	A	G	-0.263	7	33512386	6.22372E-06	0.058	360770	11064.499
family Lachnospiraceae	Vascular dementia (subcortical)	rs10919863	T	C	0.002	200226041	0.863	0.013	14306	T	C	0.315	1	200256913	3.47112E-06	0.068	360770	10813.753
family Lachnospiraceae	Vascular dementia (subcortical)	rs11148372	A	G	-0.004	22788665	0.719	0.011	14306	A	G	-0.261	13	22214526	4.06275E-06	0.057	360770	12632.549
family Lachnospiraceae	Vascular dementia (subcortical)	rs11986558	T	C	0.000	2500772	0.981	0.011	14306	T	C	0.248	8	2643276	9.16875E-06	0.056	360770	11030.353
family Lachnospiraceae	Vascular dementia (subcortical)	rs1363668	G	A	0.008	143089582	0.479	0.011	14306	G	A	-0.272	5	143710017	5.25352E-06	0.060	360770	12697.214
family Lachnospiraceae	Vascular dementia (subcortical)	rs429358	C	T	0.002	45411941	0.858	0.015	14306	C	T	0.597	19	44908684	1.74221E-17	0.070	360770	41621.629
family Lachnospiraceae	Vascular dementia (subcortical)	rs4295569	C	T	0.005	47820641	0.609	0.011	14306	C	T	-0.355	7	47781043	2.54572E-08	0.064	360770	20037.409
family Lachnospiraceae	Vascular dementia (subcortical)	rs4382795	C	T	0.001	66878853	0.971	0.014	14306	C	T	0.378	10	65119095	9.80754E-06	0.086	360770	13265.712
family Lachnospiraceae	Vascular dementia (subcortical)	rs4723291	A	G	-0.005	33551998	0.653	0.011	14306	A	G	-0.263	7	33512386	6.22372E-06	0.058	360770	11064.499
family Lactobacillaceae	Vascular dementia (subcortical)	rs10919863	T	C	-0.004	200226041	0.785	0.022	14306	T	C	0.315	1	200256913	3.47112E-06	0.068	360770	10813.753
family Lactobacillaceae	Vascular dementia (subcortical)	rs11148372	A	G	-0.006	22788665	0.739	0.017	14306	A	G	-0.261	13	22214526	4.06275E-06	0.057	360770	12632.549
family Lactobacillaceae	Vascular dementia (subcortical)	rs11986558	T	C	-0.007	2500772	0.701	0.018	14306	T	C	0.248	8	2643276	9.16875E-06	0.056	360770	11030.353
family Lactobacillaceae	Vascular dementia (subcortical)	rs429358	C	T	-0.013	45411941	0.673	0.024	14306	C	T	0.597	19	44908684	1.74221E-17	0.070	360770	41621.629
family Lactobacillaceae	Vascular dementia (subcortical)	rs4382795	C	T	0.005	66878853	0.838	0.022	14306	C	T	0.378	10	65119095	9.80754E-06	0.086	360770	13265.712
family Lactobacillaceae	Vascular dementia (subcortical)	rs4723291	A	G	0.001	33551998	0.947	0.017	14306	A	G	-0.263	7	33512386	6.22372E-06	0.058	360770	11064.499
family Methanobacteriaceae	Vascular dementia (subcortical)	rs10919863	T	C	0.000	200226041	0.946	0.030	14306	T	C	0.315	1	200256913	3.47112E-06	0.068	360770	10813.753
family Methanobacteriaceae	Vascular dementia (subcortical)	rs1363668	G	A	-0.015	143089582	0.517	0.024	14306	G	A	-0.272	5	143710017	5.25352E-06	0.060	360770	12697.214
family Methanobacteriaceae	Vascular dementia (subcortical)	rs429358	C	T	-0.026	45411941	0.504	0.034	14306	C	T	0.597	19	44908684	1.74221E-17	0.070	360770	41621.629
family Methanobacteriaceae	Vascular dementia (subcortical)	rs4382795	C	T	-0.024	66878853	0.400	0.030	14306	C	T	0.378	10	65119095	9.80754E-06	0.086	360770	13265.712
family Oxalobacteraceae	Vascular dementia (subcortical)	rs11986558	T	C	0.015	2500772	0.438	0.021	14306	T	C	0.248	8	2643276	9.16875E-06	0.056	360770	11030.353
family Oxalobacteraceae	Vascular dementia (subcortical)	rs1363668	G	A	-0.012	143089582	0.584	0.020	14306	G	A	-0.272	5	143710017	5.25352E-06	0.060	360770	12697.214
family Oxalobacteraceae	Vascular dementia (subcortical)	rs3802793	A	G	-0.007	131685316	0.697	0.021	14306	A	G	0.274	11	131815422	5.46626E-06	0.060	360770	12758.334
family Oxalobacteraceae	Vascular dementia (subcortical)	rs429358	C	T	0.011	45411941	0.614	0.028	14306	C	T	0.597	19	44908684	1.74221E-17	0.070	360770	41621.629
family Pasteurellaceae	Vascular dementia (subcortical)	rs10919863	T	C	-0.010	200226041	0.614	0.019	14306	T	C	0.315	1	200256913	3.47112E-06	0.068	360770	10813.753
family Pasteurellaceae	Vascular dementia (subcortical)	rs11986558	T	C	0.004	2500772	0.786	0.015	14306	T	C	0.248	8	2643276	9.16875E-06	0.056	360770	11030.353
family Pasteurellaceae	Vascular dementia (subcortical)	rs1363668	G	A	0.003	143089582	0.804	0.015	14306	G	A	-0.272	5	143710017	5.25352E-06	0.060	360770	12697.214
family Pasteurellaceae	Vascular dementia (subcortical)	rs429358	C	T	0.022	45411941	0.309	0.021	14306	C	T	0.597	19	44908684	1.74221E-17	0.070	360770	41621.629
family Peptococcaceae	Vascular dementia (subcortical)	rs10919863	T	C	-0.006	200226041	0.779	0.018	14306	T	C	0.315	1	200256913	3.47112E-06	0.068	360770	10813.753
family Peptococcaceae	Vascular dementia (subcortical)	rs11986558	T	C	0.003	2500772	0.845	0.015	14306	T	C	0.248	8	2643276	9.16875E-06	0.056	360770	11030.353
family Peptococcaceae	Vascular dementia (subcortical)	rs1363668	G	A	-0.012	143089582	0.400	0.014	14306	G	A	-0.272	5	143710017	5.25352E-06	0.060	360770	12697.214
family Peptococcaceae	Vascular dementia (subcortical)	rs429358	C	T	0.028	45411941	0.111	0.019	14306	C	T	0.597	19	44908684	1.74221E-17	0.070	360770	41621.629
family Peptococcaceae	Vascular dementia (subcortical)	rs4382795	C	T	0.012	66878853	0.557	0.018	14306	C	T	0.378	10	65119095	9.80754E-06	0.086	360770	13265.712
family Peptococcaceae	Vascular dementia (subcortical)	rs4723291	A	G	-0.002	33551998	0.865	0.014	14306	A	G	-0.263	7	33512386	6.22372E-06	0.058	360770	11064.499
family Peptostreptococcaceae	Vascular dementia (subcortical)	rs10919863	T	C	-0.011	200226041	0.526	0.014	14306	T	C	0.315	1	200256913	3.47112E-06	0.068	360770	10813.753
family Peptostreptococcaceae	Vascular dementia (subcortical)	rs11148372	A	G	0.003	22788665	0.768	0.011	14306	A	G	-0.261	13	22214526	4.06275E-06	0.057	360770	12632.549
family Peptostreptococcaceae	Vascular dementia (subcortical)	rs11986558	T	C	0.004	2500772	0.727	0.012	14306	T	C	0.248	8	2643276	9.16875E-06	0.056	360770	11030.353
family Peptostreptococcaceae	Vascular dementia (subcortical)	rs1363668	G	A	0.003	143089582	0.748	0.011	14306	G	A	-0.272	5	143710017	5.25352E-06	0.060	360770	12697.214
family Peptostreptococcaceae	Vascular dementia (subcortical)	rs3802793	A	G	-0.007	131685316	0.577	0.012	14306	A	G	0.274	11	131815422	5.46626E-06	0.060	360770	12758.334
family Peptostreptococcaceae	Vascular dementia (subcortical)	rs429358	C	T	0.014	45411941	0.358	0.016	14306	C	T	0.597	19	44908684	1.74221E-17	0.070	360770	41621.629
family Peptostreptococcaceae	Vascular dementia (subcortical)	rs4295569	C	T	-0.005	47820641	0.636	0.012	14306	C	T	-0.355	7	47781043	2.54572E-08	0.064	360770	20037.409
family Porphyromonadaceae	Vascular dementia (subcortical)	rs11148372	A	G	-0.006	22788665	0.606	0.011	14306	A	G	-0.261	13	22214526	4.06275E-06	0.057	360770	12632.549
family Porphyromonadaceae	Vascular dementia (subcortical)	rs11986558	T	C	0.003	2500772	0.767	0.011	14306	T	C	0.248	8	2643276	9.16875E-06	0.056	360770	11030.353
family Porphyromonadaceae	Vascular dementia (subcortical)	rs1363668	G	A	0.007	143089582	0.519	0.011	14306	G	A	-0.272	5	143710017	5.25352E-06	0.060	360770	12697.214
family Porphyromonadaceae	Vascular dementia (subcortical)	rs3802793	A	G	-0.002	131685316	0.897	0.011	14306	A	G	0.274	11	131815422	5.46626E-06	0.060	360770	12758.334
family Porphyromonadaceae	Vascular dementia (subcortical)	rs429358	C	T	0.001	45411941	0.899	0.015	14306	C	T	0.597	19	44908684	1.74221E-17	0.070	360770	41621.629
family Porphyromonadaceae	Vascular dementia (subcortical)	rs4295569	C	T	-0.010	47820641	0.380	0.011	14306	C	T	-0.355	7	47781043	2.54572E-08	0.064	360770	20037.409
family Prevotellaceae	Vascular dementia (subcortical)	rs10919863	T	C	0.007	200226041	0.598	0.015	14306	T	C	0.315	1	200256913	3.47112E-06	0.068	360770	10813.753

family Prevotellaceae	Vascular dementia (subcortical)	rs11986558	T	C	-0.009	2500772	0.508	0.012	14306	T	C	0.248	8	2643276	9.16875E-06	0.056	360770	11030.353
family Prevotellaceae	Vascular dementia (subcortical)	rs1363668	G	A	0.001	143089582	0.920	0.012	14306	G	A	-0.272	5	143710017	5.25352E-06	0.060	360770	12697.214
family Prevotellaceae	Vascular dementia (subcortical)	rs3802793	A	G	0.010	131685316	0.430	0.012	14306	A	G	0.274	11	131815422	5.46626E-06	0.060	360770	12758.334
family Prevotellaceae	Vascular dementia (subcortical)	rs429358	C	T	0.000	45411941	0.975	0.017	14306	C	T	0.597	19	44908684	1.74221E-17	0.070	360770	41621.629
family Prevotellaceae	Vascular dementia (subcortical)	rs4382795	C	T	-0.011	66878853	0.482	0.015	14306	C	T	0.378	10	65119095	9.80754E-06	0.086	360770	13265.712
family Rhodospirillaceae	Vascular dementia (subcortical)	rs11148372	A	G	-0.002	22788665	0.913	0.016	14306	A	G	-0.261	13	22214526	4.06275E-06	0.057	360770	12632.549
family Rhodospirillaceae	Vascular dementia (subcortical)	rs11986558	T	C	0.008	2500772	0.605	0.016	14306	T	C	0.248	8	2643276	9.16875E-06	0.056	360770	11030.353
family Rhodospirillaceae	Vascular dementia (subcortical)	rs1363668	G	A	-0.013	143089582	0.414	0.016	14306	G	A	-0.272	5	143710017	5.25352E-06	0.060	360770	12697.214
family Rhodospirillaceae	Vascular dementia (subcortical)	rs429358	C	T	-0.006	45411941	0.814	0.022	14306	C	T	0.597	19	44908684	1.74221E-17	0.070	360770	41621.629
family Rikenellaceae	Vascular dementia (subcortical)	rs10919863	T	C	-0.006	200226041	0.625	0.014	14306	T	C	0.315	1	200256913	3.47112E-06	0.068	360770	10813.753
family Rikenellaceae	Vascular dementia (subcortical)	rs11986558	T	C	0.004	2500772	0.735	0.011	14306	T	C	0.248	8	2643276	9.16875E-06	0.056	360770	11030.353
family Rikenellaceae	Vascular dementia (subcortical)	rs3802793	A	G	0.009	131685316	0.408	0.011	14306	A	G	0.274	11	131815422	5.46626E-06	0.060	360770	12758.334
family Rikenellaceae	Vascular dementia (subcortical)	rs429358	C	T	-0.016	45411941	0.273	0.015	14306	C	T	0.597	19	44908684	1.74221E-17	0.070	360770	41621.629
family Rikenellaceae	Vascular dementia (subcortical)	rs4382795	C	T	0.005	66878853	0.751	0.014	14306	C	T	0.378	10	65119095	9.80754E-06	0.086	360770	13265.712
family Rikenellaceae	Vascular dementia (subcortical)	rs4723291	A	G	0.003	33551998	0.796	0.011	14306	A	G	-0.263	7	33512386	6.22372E-06	0.058	360770	11064.499
family Ruminococcaceae	Vascular dementia (subcortical)	rs10919863	T	C	0.002	200226041	0.872	0.013	14306	T	C	0.315	1	200256913	3.47112E-06	0.068	360770	10813.753
family Ruminococcaceae	Vascular dementia (subcortical)	rs11986558	T	C	0.002	2500772	0.795	0.011	14306	T	C	0.248	8	2643276	9.16875E-06	0.056	360770	11030.353
family Ruminococcaceae	Vascular dementia (subcortical)	rs3802793	A	G	0.005	131685316	0.585	0.011	14306	A	G	0.274	11	131815422	5.46626E-06	0.060	360770	12758.334
family Ruminococcaceae	Vascular dementia (subcortical)	rs429358	C	T	-0.003	45411941	0.847	0.015	14306	C	T	0.597	19	44908684	1.74221E-17	0.070	360770	41621.629
family Ruminococcaceae	Vascular dementia (subcortical)	rs4295569	C	T	-0.010	47820641	0.395	0.011	14306	C	T	-0.355	7	47781043	2.54572E-08	0.064	360770	20037.409
family Streptococcaceae	Vascular dementia (subcortical)	rs11148372	A	G	-0.004	22788665	0.706	0.011	14306	A	G	-0.261	13	22214526	4.06275E-06	0.057	360770	12632.549
family Streptococcaceae	Vascular dementia (subcortical)	rs11986558	T	C	0.002	2500772	0.868	0.012	14306	T	C	0.248	8	2643276	9.16875E-06	0.056	360770	11030.353
family Streptococcaceae	Vascular dementia (subcortical)	rs1363668	G	A	-0.002	143089582	0.854	0.011	14306	G	A	-0.272	5	143710017	5.25352E-06	0.060	360770	12697.214
family Streptococcaceae	Vascular dementia (subcortical)	rs429358	C	T	0.012	45411941	0.432	0.016	14306	C	T	0.597	19	44908684	1.74221E-17	0.070	360770	41621.629
family Streptococcaceae	Vascular dementia (subcortical)	rs4295569	C	T	-0.003	47820641	0.785	0.011	14306	C	T	-0.355	7	47781043	2.54572E-08	0.064	360770	20037.409
family Veillonellaceae	Vascular dementia (subcortical)	rs10919863	T	C	-0.010	200226041	0.486	0.014	14306	T	C	0.315	1	200256913	3.47112E-06	0.068	360770	10813.753
family Veillonellaceae	Vascular dementia (subcortical)	rs11148372	A	G	0.008	22788665	0.485	0.011	14306	A	G	-0.261	13	22214526	4.06275E-06	0.057	360770	12632.549
family Veillonellaceae	Vascular dementia (subcortical)	rs1363668	G	A	-0.010	143089582	0.378	0.011	14306	G	A	-0.272	5	143710017	5.25352E-06	0.060	360770	12697.214
family Veillonellaceae	Vascular dementia (subcortical)	rs3802793	A	G	0.001	131685316	0.989	0.012	14306	A	G	0.274	11	131815422	5.46626E-06	0.060	360770	12758.334
family Veillonellaceae	Vascular dementia (subcortical)	rs429358	C	T	-0.023	45411941	0.150	0.016	14306	C	T	0.597	19	44908684	1.74221E-17	0.070	360770	41621.629
family Veillonellaceae	Vascular dementia (subcortical)	rs4295569	C	T	0.003	47820641	0.804	0.012	14306	C	T	-0.355	7	47781043	2.54572E-08	0.064	360770	20037.409
family Veillonellaceae	Vascular dementia (subcortical)	rs4382795	C	T	-0.006	66878853	0.690	0.015	14306	C	T	0.378	10	65119095	9.80754E-06	0.086	360770	13265.712
family Verrucomicrobiaceae	Vascular dementia (subcortical)	rs10919863	T	C	-0.002	200226041	0.924	0.017	14306	T	C	0.315	1	200256913	3.47112E-06	0.068	360770	10813.753
family Verrucomicrobiaceae	Vascular dementia (subcortical)	rs11148372	A	G	0.007	22788665	0.561	0.013	14306	A	G	-0.261	13	22214526	4.06275E-06	0.057	360770	12632.549
family Verrucomicrobiaceae	Vascular dementia (subcortical)	rs1363668	G	A	0.005	143089582	0.713	0.013	14306	G	A	-0.272	5	143710017	5.25352E-06	0.060	360770	12697.214
family Verrucomicrobiaceae	Vascular dementia (subcortical)	rs3802793	A	G	-0.007	131685316	0.654	0.014	14306	A	G	0.274	11	131815422	5.46626E-06	0.060	360770	12758.334
family Verrucomicrobiaceae	Vascular dementia (subcortical)	rs429358	C	T	0.018	45411941	0.341	0.019	14306	C	T	0.597	19	44908684	1.74221E-17	0.070	360770	41621.629
family Verrucomicrobiaceae	Vascular dementia (subcortical)	rs4295569	C	T	0.001	47820641	0.928	0.013	14306	C	T	-0.355	7	47781043	2.54572E-08	0.064	360770	20037.409
family Verrucomicrobiaceae	Vascular dementia (subcortical)	rs4382795	C	T	-0.005	66878853	0.764	0.017	14306	C	T	0.378	10	65119095	9.80754E-06	0.086	360770	13265.712
family Verrucomicrobiaceae	Vascular dementia (subcortical)	rs4723291	A	G	-0.002	33551998	0.912	0.013	14306	A	G	-0.263	7	33512386	6.22372E-06	0.058	360770	11064.499
family Victivallaceae	Vascular dementia (subcortical)	rs10919863	T	C	-0.013	200226041	0.624	0.029	14306	T	C	0.315	1	200256913	3.47112E-06	0.068	360770	10813.753
family Victivallaceae	Vascular dementia (subcortical)	rs11986558	T	C	-0.016	2500772	0.513	0.024	14306	T	C	0.248	8	2643276	9.16875E-06	0.056	360770	11030.353
family Victivallaceae	Vascular dementia (subcortical)	rs1363668	G	A	-0.016	143089582	0.470	0.023	14306	G	A	-0.272	5	143710017	5.25352E-06	0.060	360770	12697.214
family Victivallaceae	Vascular dementia (subcortical)	rs3802793	A	G	0.017	131685316	0.470	0.024	14306	A	G	0.274	11	131815422	5.46626E-06	0.060	360770	12758.334
family Victivallaceae	Vascular dementia (subcortical)	rs429358	C	T	-0.015	45411941	0.542	0.033	14306	C	T	0.597	19	44908684	1.74221E-17	0.070	360770	41621.629
family Victivallaceae	Vascular dementia (subcortical)	rs4295569	C	T	-0.003	47820641	0.892	0.023	14306	C	T	-0.355	7	47781043	2.54572E-08	0.064	360770	20037.409
family Victivallaceae	Vascular dementia (subcortical)	rs4723291	A	G	-0.013	33551998	0.554	0.023	14306	A	G	-0.263	7	33512386	6.22372E-06	0.058	360770	11064.499
family Acidaminococcaceae	Vascular dementia (sudden onset)	rs12423672	T	G	-0.013	5047705	0.623	0.020	14306	T	G	0.759	12	4938539	4.89159E-06	0.166	360283	30610.567
family Acidaminococcaceae	Vascular dementia (sudden onset)	rs2920	C	T	-0.008	23884780	0.577	0.016	14306	C	T	0.592	1	23558289	2.20602E-06	0.125	360283	40501.384
family Acidaminococcaceae	Vascular dementia (sudden onset)	rs429358	C	T	-0.007	45411941	0.557	0.018	14306	C	T	0.601	19	44908684	7.45813E-06	0.134	360283	42162.979
family Acidaminococcaceae	Vascular dementia (sudden onset)	rs4840457	C	T	0.019	6408682	0.401	0.024	14306	C	T	0.643	8	6551161	8.92874E-06	0.145	360283	32615.797
family Acidaminococcaceae	Vascular dementia (sudden onset)	rs71511414	G	A	0.014	79688991	0.565	0.023	14306	G	A	0.681	9	77074075	7.80746E-06	0.152	360283	30731.948
family Actinomycetaceae	Vascular dementia (sudden onset)	rs12452096	A	G	-0.005	75544032	0.809	0.019	14306	A	G	0.750	17	77547950	1.48929E-06	0.156	360283	77865.614
family Actinomycetaceae	Vascular dementia (sudden onset)	rs2920	C	T	-0.012	23884780	0.533	0.020	14306	C	T	0.592	1	23558289	2.20602E-06	0.125	360283	40501.384
family Actinomycetaceae	Vascular dementia (sudden onset)	rs4840457	C	T	0.009	6408682	0.790	0.030	14306	C	T	0.643	8	6551161	8.92874E-06	0.145	360283	32615.797
family Actinomycetaceae	Vascular dementia (sudden onset)	rs71511414	G	A	0.010	79688991	0.673	0.028	14306	G	A	0.681	9	77074075	7.80746E-06	0.152	360283	30731.948
family Alcaligenaceae	Vascular dementia (sudden onset)	rs12452096	A	G	0.006	75544032	0.623	0.013	14306	A	G	0.750	17	77547950	1.48929E-06	0.156	360283	77865.614
family Alcaligenaceae	Vascular dementia (sudden onset)	rs429358	C	T	0.009	45411941	0.545	0.016	14306	C	T	0.601	19	44908684	7.45813E-06	0.134	360283	42162.979
family Alcaligenaceae	Vascular dementia (sudden onset)	rs4840457	C	T	0.008	6408682	0.685	0.021	14306	C	T	0.643	8	6551161	8.92874E-06	0.145	360283	32615.797
family Alcaligenaceae	Vascular dementia (sudden onset)	rs71511414	G	A	-0.011	79688991	0.554	0.020	14306	G	A	0.681	9	77074075	7.80746E-06	0.152	360283	30731.948
family Bacteroidaceae	Vascular dementia (sudden onset)	rs12452096	A	G	0.007	75544032	0.579	0.012	14306	A	G	0.750	17	77547950	1.48929E-06	0.156	360283	77865.614
family Bacteroidaceae	Vascular dementia (sudden onset)	rs2920	C	T	0.005	23884780	0.745	0.013	14306	C	T	0.592	1	23558289	2.20602E-06	0.125	360283	40501.384
family Bacteroidales S24 7group	Vascular dementia (sudden onset)	rs12452096	A	G	0.012	75544032	0.521	0.019	14306	A	G	0.750	17	77547950	1.48929E-06	0.156	360283	77865.614
family Bacteroidales S24 7group	Vascular dementia (sudden onset)	rs2920	C	T	-0.017	23884780	0.391	0.020	14306	C	T	0.592	1	23558289	2.20602E-06	0.125	360283	40501.384
family Bacteroidales S24 7group	Vascular dementia (sudden onset)	rs4840457	C	T	0.004	6408682	0.916	0.030	14306	C	T	0.643	8	6551161	8.92874E-06	0.145	360283	32615.797
family Bacteroidales S24 7group	Vascular dementia (sudden onset)	rs71511414	G	A	0.008	79688991	0.803	0.029	14306	G	A	0.681	9	77074075	7.80746E-06	0.152	360283	30731.948
family Bifidobacteriaceae	Vascular dementia (sudden onset)	rs12452096	A	G	-0.007	75544032	0.589	0.014	14306	A	G	0.750	17	77547950	1.48929E-06	0.156	360283	77865.614
family Bifidobacteriaceae	Vascular dementia (sudden onset)	rs2920	C	T	-0.007	23884780	0.652	0.014	14306	C	T	0.592	1	23558289	2.20602E-06	0.125	360283	40501.384
family Bifidobacteriaceae	Vascular dementia (sudden onset)	rs4840457	C	T	0.002	6408682	0.965	0.022	14306	C	T	0.643	8	6551161	8.92874E-06	0.145	360283	32615.797
family Bifidobacteriaceae	Vascular dementia (sudden onset)	rs71511414	G	A	-0.015	79688991	0.558	0.021	14306	G	A	0.681	9	77074075	7.80746E-06	0.152	360283	30731.948
family Clostridiaceae1	Vascular dementia (sudden onset)	rs12423672	T	G	0.014	5047705	0.440	0.019	14306	T	G	0.759	12	4938539	4.89159E-06	0.166	360283	30610.567

family Clostridiaceae1	Vascular dementia (sudden onset)	rs12452096	A	G	-0.003	75544032	0.821	0.014	14306	A	G	0.750	17	77547950	1.48929E-06	0.156	360283	77865.614
family Clostridiaceae1	Vascular dementia (sudden onset)	rs2920	C	T	-0.009	23884780	0.570	0.015	14306	C	T	0.592	1	23558289	2.20602E-06	0.125	360283	40501.384
family Clostridiaceae1	Vascular dementia (sudden onset)	rs429358	C	T	0.010	45411941	0.573	0.017	14306	C	T	0.601	19	44908684	7.45813E-06	0.134	360283	42162.979
family Clostridiaceae1	Vascular dementia (sudden onset)	rs4840457	C	T	-0.001	6408682	0.930	0.022	14306	C	T	0.643	8	6551161	8.92874E-06	0.145	360283	32615.797
family Clostridiaceae1	Vascular dementia (sudden onset)	rs71511414	G	A	0.008	79688991	0.709	0.022	14306	G	A	0.681	9	77074075	7.80746E-06	0.152	360283	30731.948
family Clostridiales vadin BB60 group	Vascular dementia (sudden onset)	rs12452096	A	G	-0.011	75544032	0.549	0.016	14306	A	G	0.750	17	77547950	1.48929E-06	0.156	360283	77865.614
family Clostridiales vadin BB60 group	Vascular dementia (sudden onset)	rs429358	C	T	0.004	45411941	0.829	0.020	14306	C	T	0.601	19	44908684	7.45813E-06	0.134	360283	42162.979
family Clostridiales vadin BB60 group	Vascular dementia (sudden onset)	rs4840457	C	T	0.015	6408682	0.554	0.026	14306	C	T	0.643	8	6551161	8.92874E-06	0.145	360283	32615.797
family Coriobacteriaceae	Vascular dementia (sudden onset)	rs12452096	A	G	-0.008	75544032	0.499	0.013	14306	A	G	0.750	17	77547950	1.48929E-06	0.156	360283	77865.614
family Coriobacteriaceae	Vascular dementia (sudden onset)	rs2920	C	T	-0.009	23884780	0.519	0.013	14306	C	T	0.592	1	23558289	2.20602E-06	0.125	360283	40501.384
family Coriobacteriaceae	Vascular dementia (sudden onset)	rs71511414	G	A	-0.005	79688991	0.860	0.019	14306	G	A	0.681	9	77074075	7.80746E-06	0.152	360283	30731.948
family Defluviitaleaceae	Vascular dementia (sudden onset)	rs12423672	T	G	-0.005	5047705	0.726	0.026	14306	T	G	0.759	12	4938539	4.89159E-06	0.166	360283	30610.567
family Defluviitaleaceae	Vascular dementia (sudden onset)	rs12452096	A	G	0.010	75544032	0.608	0.019	14306	A	G	0.750	17	77547950	1.48929E-06	0.156	360283	77865.614
family Defluviitaleaceae	Vascular dementia (sudden onset)	rs429358	C	T	0.000	45411941	0.935	0.022	14306	C	T	0.601	19	44908684	7.45813E-06	0.134	360283	42162.979
family Desulfovibrionaceae	Vascular dementia (sudden onset)	rs12452096	A	G	-0.003	75544032	0.810	0.014	14306	A	G	0.750	17	77547950	1.48929E-06	0.156	360283	77865.614
family Desulfovibrionaceae	Vascular dementia (sudden onset)	rs4840457	C	T	0.009	6408682	0.678	0.022	14306	C	T	0.643	8	6551161	8.92874E-06	0.145	360283	32615.797
family Desulfovibrionaceae	Vascular dementia (sudden onset)	rs71511414	G	A	-0.010	79688991	0.602	0.021	14306	G	A	0.681	9	77074075	7.80746E-06	0.152	360283	30731.948
family Enterobacteriaceae	Vascular dementia (sudden onset)	rs12452096	A	G	-0.005	75544032	0.728	0.014	14306	A	G	0.750	17	77547950	1.48929E-06	0.156	360283	77865.614
family Enterobacteriaceae	Vascular dementia (sudden onset)	rs2920	C	T	-0.007	23884780	0.631	0.015	14306	C	T	0.592	1	23558289	2.20602E-06	0.125	360283	40501.384
family Erysipelotrichaceae	Vascular dementia (sudden onset)	rs12452096	A	G	0.003	75544032	0.800	0.012	14306	A	G	0.750	17	77547950	1.48929E-06	0.156	360283	77865.614
family Erysipelotrichaceae	Vascular dementia (sudden onset)	rs2920	C	T	0.000	23884780	0.971	0.013	14306	C	T	0.592	1	23558289	2.20602E-06	0.125	360283	40501.384
family Erysipelotrichaceae	Vascular dementia (sudden onset)	rs4840457	C	T	0.014	6408682	0.472	0.020	14306	C	T	0.643	8	6551161	8.92874E-06	0.145	360283	32615.797
family Erysipelotrichaceae	Vascular dementia (sudden onset)	rs71511414	G	A	0.011	79688991	0.559	0.019	14306	G	A	0.681	9	77074075	7.80746E-06	0.152	360283	30731.948
family Family XI	Vascular dementia (sudden onset)	rs2920	C	T	-0.022	23884780	0.501	0.032	14306	C	T	0.592	1	23558289	2.20602E-06	0.125	360283	40501.384
family Family XI	Vascular dementia (sudden onset)	rs4840457	C	T	0.030	6408682	0.510	0.046	14306	C	T	0.643	8	6551161	8.92874E-06	0.145	360283	32615.797
family Family XI	Vascular dementia (sudden onset)	rs71511414	G	A	0.033	79688991	0.473	0.044	14306	G	A	0.681	9	77074075	7.80746E-06	0.152	360283	30731.948
family Family XIII	Vascular dementia (sudden onset)	rs12452096	A	G	-0.010	75544032	0.445	0.013	14306	A	G	0.750	17	77547950	1.48929E-06	0.156	360283	77865.614
family Family XIII	Vascular dementia (sudden onset)	rs71511414	G	A	-0.003	79688991	0.881	0.020	14306	G	A	0.681	9	77074075	7.80746E-06	0.152	360283	30731.948
family Lachnospiraceae	Vascular dementia (sudden onset)	rs12423672	T	G	0.000	5047705	0.897	0.017	14306	T	G	0.759	12	4938539	4.89159E-06	0.166	360283	30610.567
family Lachnospiraceae	Vascular dementia (sudden onset)	rs12452096	A	G	0.003	75544032	0.825	0.012	14306	A	G	0.750	17	77547950	1.48929E-06	0.156	360283	77865.614
family Lachnospiraceae	Vascular dementia (sudden onset)	rs2920	C	T	-0.001	23884780	0.892	0.013	14306	C	T	0.592	1	23558289	2.20602E-06	0.125	360283	40501.384
family Lachnospiraceae	Vascular dementia (sudden onset)	rs429358	C	T	0.002	45411941	0.858	0.015	14306	C	T	0.601	19	44908684	7.45813E-06	0.134	360283	42162.979
family Lachnospiraceae	Vascular dementia (sudden onset)	rs4840457	C	T	-0.005	6408682	0.835	0.020	14306	C	T	0.643	8	6551161	8.92874E-06	0.145	360283	32615.797
family Lachnospiraceae	Vascular dementia (sudden onset)	rs71511414	G	A	0.001	79688991	0.927	0.019	14306	G	A	0.681	9	77074075	7.80746E-06	0.152	360283	30731.948
family Lactobacillaceae	Vascular dementia (sudden onset)	rs12423672	T	G	-0.015	5047705	0.572	0.027	14306	T	G	0.759	12	4938539	4.89159E-06	0.166	360283	30610.567
family Lactobacillaceae	Vascular dementia (sudden onset)	rs2920	C	T	-0.004	23884780	0.821	0.021	14306	C	T	0.592	1	23558289	2.20602E-06	0.125	360283	40501.384
family Lactobacillaceae	Vascular dementia (sudden onset)	rs429358	C	T	-0.013	45411941	0.673	0.024	14306	C	T	0.601	19	44908684	7.45813E-06	0.134	360283	42162.979
family Lactobacillaceae	Vascular dementia (sudden onset)	rs71511414	G	A	0.004	79688991	0.895	0.031	14306	G	A	0.681	9	77074075	7.80746E-06	0.152	360283	30731.948
family Methanobacteriaceae	Vascular dementia (sudden onset)	rs12452096	A	G	-0.008	75544032	0.767	0.029	14306	A	G	0.750	17	77547950	1.48929E-06	0.156	360283	77865.614
family Methanobacteriaceae	Vascular dementia (sudden onset)	rs429358	C	T	-0.026	45411941	0.504	0.034	14306	C	T	0.601	19	44908684	7.45813E-06	0.134	360283	42162.979
family Oxalobacteraceae	Vascular dementia (sudden onset)	rs12423672	T	G	-0.016	5047705	0.720	0.033	14306	T	G	0.759	12	4938539	4.89159E-06	0.166	360283	30610.567
family Oxalobacteraceae	Vascular dementia (sudden onset)	rs2920	C	T	0.006	23884780	0.785	0.024	14306	C	T	0.592	1	23558289	2.20602E-06	0.125	360283	40501.384
family Oxalobacteraceae	Vascular dementia (sudden onset)	rs429358	C	T	0.011	45411941	0.614	0.028	14306	C	T	0.601	19	44908684	7.45813E-06	0.134	360283	42162.979
family Oxalobacteraceae	Vascular dementia (sudden onset)	rs4840457	C	T	0.023	6408682	0.542	0.037	14306	C	T	0.643	8	6551161	8.92874E-06	0.145	360283	32615.797
family Pasteurellaceae	Vascular dementia (sudden onset)	rs12452096	A	G	0.001	75544032	0.940	0.017	14306	A	G	0.750	17	77547950	1.48929E-06	0.156	360283	77865.614
family Pasteurellaceae	Vascular dementia (sudden onset)	rs2920	C	T	-0.009	23884780	0.598	0.018	14306	C	T	0.592	1	23558289	2.20602E-06	0.125	360283	40501.384
family Pasteurellaceae	Vascular dementia (sudden onset)	rs4840457	C	T	-0.021	6408682	0.450	0.028	14306	C	T	0.643	8	6551161	8.92874E-06	0.145	360283	32615.797
family Peptococcaceae	Vascular dementia (sudden onset)	rs12423672	T	G	0.016	5047705	0.443	0.022	14306	T	G	0.759	12	4938539	4.89159E-06	0.166	360283	30610.567
family Peptococcaceae	Vascular dementia (sudden onset)	rs12452096	A	G	-0.014	75544032	0.406	0.016	14306	A	G	0.750	17	77547950	1.48929E-06	0.156	360283	77865.614
family Peptococcaceae	Vascular dementia (sudden onset)	rs2920	C	T	0.002	23884780	0.881	0.017	14306	C	T	0.592	1	23558289	2.20602E-06	0.125	360283	40501.384
family Peptococcaceae	Vascular dementia (sudden onset)	rs71511414	G	A	0.019	79688991	0.509	0.025	14306	G	A	0.681	9	77074075	7.80746E-06	0.152	360283	30731.948
family Peptostreptococcaceae	Vascular dementia (sudden onset)	rs12452096	A	G	-0.003	75544032	0.830	0.013	14306	A	G	0.750	17	77547950	1.48929E-06	0.156	360283	77865.614
family Peptostreptococcaceae	Vascular dementia (sudden onset)	rs2920	C	T	-0.013	23884780	0.371	0.014	14306	C	T	0.592	1	23558289	2.20602E-06	0.125	360283	40501.384
family Peptostreptococcaceae	Vascular dementia (sudden onset)	rs429358	C	T	0.014	45411941	0.358	0.016	14306	C	T	0.601	19	44908684	7.45813E-06	0.134	360283	42162.979
family Peptostreptococcaceae	Vascular dementia (sudden onset)	rs71511414	G	A	0.007	79688991	0.725	0.020	14306	G	A	0.681	9	77074075	7.80746E-06	0.152	360283	30731.948
family Porphyromonadaceae	Vascular dementia (sudden onset)	rs12423672	T	G	0.002	5047705	0.908	0.017	14306	T	G	0.759	12	4938539	4.89159E-06	0.166	360283	30610.567
family Porphyromonadaceae	Vascular dementia (sudden onset)	rs12452096	A	G	0.011	75544032	0.422	0.013	14306	A	G	0.750	17	77547950	1.48929E-06	0.156	360283	77865.614
family Porphyromonadaceae	Vascular dementia (sudden onset)	rs429358	C	T	0.001	45411941	0.899	0.015	14306	C	T	0.601	19	44908684	7.45813E-06	0.134	360283	42162.979
family Prevotellaceae	Vascular dementia (sudden onset)	rs12423672	T	G	0.007	5047705	0.535	0.018	14306	T	G	0.759	12	4938539	4.89159E-06	0.166	360283	30610.567
family Prevotellaceae	Vascular dementia (sudden onset)	rs429358	C	T	0.000	45411941	0.975	0.017	14306	C	T	0.601	19	44908684	7.45813E-06	0.134	360283	42162.979
family Prevotellaceae	Vascular dementia (sudden onset)	rs4840457	C	T	-0.013	6408682	0.558	0.022	14306	C	T	0.643	8	6551161	8.92874E-06	0.145	360283	32615.797
family Prevotellaceae	Vascular dementia (sudden onset)	rs71511414	G	A	-0.003	79688991	0.764	0.021	14306	G	A	0.681	9	77074075	7.80746E-06	0.152	360283	30731.948
family Rhodospirillaceae	Vascular dementia (sudden onset)	rs12452096	A	G	-0.017	75544032	0.353	0.019	14306	A	G	0.750	17	77547950	1.48929E-06	0.156	360283	77865.614
family Rhodospirillaceae	Vascular dementia (sudden onset)	rs2920	C	T	0.012	23884780	0.568	0.019	14306	C	T	0.592	1	23558289	2.20602E-06	0.125	360283	40501.384
family Rhodospirillaceae	Vascular dementia (sudden onset)	rs429358	C	T	-0.006	45411941	0.814	0.022	14306	C	T	0.601	19	44908684	7.45813E-06	0.134	360283	42162.979
family Rhodospirillaceae	Vascular dementia (sudden onset)	rs4840457	C	T	-0.019	6408682	0.485	0.028	14306	C	T	0.643	8	6551161	8.92874E-06	0.145	360283	32615.797
family Rhodospirillaceae	Vascular dementia (sudden onset)	rs71511414	G	A	0.002	79688991	0.969	0.027	14306	G	A	0.681	9	77074075	7.80746E-06	0.152	360283	30731.948
family Rikenellaceae	Vascular dementia (sudden onset)	rs12423672	T	G	0.010	5047705	0.724	0.017	14306	T	G	0.759	12	4938539	4.89159E-06	0.166	360283	30610.567
family Rikenellaceae	Vascular dementia (sudden onset)	rs12452096	A	G	0.010	75544032	0.434	0.013	14306	A	G	0.750	17	77547950	1.48929E-06	0.156	360283	77865.614
family Rikenellaceae	Vascular dementia (sudden onset)	rs2920	C	T	-0.004	23884780	0.825	0.013	14306	C	T	0.592	1	23558289	2.20602E-06	0.125	360283	40501.384
family Rikenellaceae	Vascular dementia (sudden onset)	rs4840457	C	T	0.017	6408682	0.392	0.020	14306	C	T	0.643	8	6551161	8.92874E-06	0.145	360283	32615.797

family Rikenellaceae	Vascular dementia (sudden onset)	rs71511414	G	A	-0.007	79688991	0.739	0.019	14306	G	A	0.681	9	77074075	7.80746E-06	0.152	360283	30731.948
family Ruminococcaceae	Vascular dementia (sudden onset)	rs12423672	T	G	-0.012	5047705	0.518	0.017	14306	T	G	0.759	12	4938539	4.89159E-06	0.166	360283	30610.567
family Ruminococcaceae	Vascular dementia (sudden onset)	rs429358	C	T	-0.003	45411941	0.847	0.015	14306	C	T	0.601	19	44908684	7.45813E-06	0.134	360283	42162.979
family Ruminococcaceae	Vascular dementia (sudden onset)	rs4840457	C	T	0.001	6408682	0.988	0.020	14306	C	T	0.643	8	6551161	8.92874E-06	0.145	360283	32615.797
family Ruminococcaceae	Vascular dementia (sudden onset)	rs71511414	G	A	-0.009	79688991	0.659	0.019	14306	G	A	0.681	9	77074075	7.80746E-06	0.152	360283	30731.948
family Streptococcaceae	Vascular dementia (sudden onset)	rs12423672	T	G	0.016	5047705	0.469	0.018	14306	T	G	0.759	12	4938539	4.89159E-06	0.166	360283	30610.567
family Streptococcaceae	Vascular dementia (sudden onset)	rs12452096	A	G	-0.002	75544032	0.873	0.013	14306	A	G	0.750	17	77547950	1.48929E-06	0.156	360283	77865.614
family Streptococcaceae	Vascular dementia (sudden onset)	rs2920	C	T	-0.009	23884780	0.500	0.014	14306	C	T	0.592	1	23558289	2.20602E-06	0.125	360283	40501.384
family Streptococcaceae	Vascular dementia (sudden onset)	rs429358	C	T	0.012	45411941	0.432	0.016	14306	C	T	0.601	19	44908684	7.45813E-06	0.134	360283	42162.979
family Streptococcaceae	Vascular dementia (sudden onset)	rs4840457	C	T	-0.014	6408682	0.481	0.021	14306	C	T	0.643	8	6551161	8.92874E-06	0.145	360283	32615.797
family Streptococcaceae	Vascular dementia (sudden onset)	rs71511414	G	A	-0.001	79688991	0.940	0.020	14306	G	A	0.681	9	77074075	7.80746E-06	0.152	360283	30731.948
family Veillonellaceae	Vascular dementia (sudden onset)	rs12423672	T	G	-0.002	5047705	0.833	0.018	14306	T	G	0.759	12	4938539	4.89159E-06	0.166	360283	30610.567
family Veillonellaceae	Vascular dementia (sudden onset)	rs12452096	A	G	0.003	75544032	0.804	0.013	14306	A	G	0.750	17	77547950	1.48929E-06	0.156	360283	77865.614
family Veillonellaceae	Vascular dementia (sudden onset)	rs2920	C	T	0.009	23884780	0.480	0.014	14306	C	T	0.592	1	23558289	2.20602E-06	0.125	360283	40501.384
family Veillonellaceae	Vascular dementia (sudden onset)	rs4840457	C	T	0.000	6408682	1.000	0.021	14306	C	T	0.643	8	6551161	8.92874E-06	0.145	360283	32615.797
family Veillonellaceae	Vascular dementia (sudden onset)	rs71511414	G	A	-0.007	79688991	0.733	0.020	14306	G	A	0.681	9	77074075	7.80746E-06	0.152	360283	30731.948
family Verrucomicrobiaceae	Vascular dementia (sudden onset)	rs12423672	T	G	0.016	5047705	0.346	0.021	14306	T	G	0.759	12	4938539	4.89159E-06	0.166	360283	30610.567
family Verrucomicrobiaceae	Vascular dementia (sudden onset)	rs12452096	A	G	0.006	75544032	0.686	0.015	14306	A	G	0.750	17	77547950	1.48929E-06	0.156	360283	77865.614
family Verrucomicrobiaceae	Vascular dementia (sudden onset)	rs2920	C	T	-0.009	23884780	0.593	0.016	14306	C	T	0.592	1	23558289	2.20602E-06	0.125	360283	40501.384
family Verrucomicrobiaceae	Vascular dementia (sudden onset)	rs4840457	C	T	-0.017	6408682	0.487	0.024	14306	C	T	0.643	8	6551161	8.92874E-06	0.145	360283	32615.797
family Verrucomicrobiaceae	Vascular dementia (sudden onset)	rs71511414	G	A	-0.011	79688991	0.607	0.023	14306	G	A	0.681	9	77074075	7.80746E-06	0.152	360283	30731.948
family Victivallaceae	Vascular dementia (sudden onset)	rs12423672	T	G	0.005	5047705	0.995	0.036	14306	T	G	0.759	12	4938539	4.89159E-06	0.166	360283	30610.567
family Victivallaceae	Vascular dementia (sudden onset)	rs12452096	A	G	0.009	75544032	0.703	0.028	14306	A	G	0.750	17	77547950	1.48929E-06	0.156	360283	77865.614
family Victivallaceae	Vascular dementia (sudden onset)	rs2920	C	T	0.018	23884780	0.572	0.029	14306	C	T	0.592	1	23558289	2.20602E-06	0.125	360283	40501.384
family Victivallaceae	Vascular dementia (sudden onset)	rs429358	C	T	-0.015	45411941	0.542	0.033	14306	C	T	0.601	19	44908684	7.45813E-06	0.134	360283	42162.979
family Victivallaceae	Vascular dementia (sudden onset)	rs4840457	C	T	-0.012	6408682	0.792	0.042	14306	C	T	0.643	8	6551161	8.92874E-06	0.145	360283	32615.797
family Victivallaceae	Vascular dementia (sudden onset)	rs71511414	G	A	-0.024	79688991	0.564	0.041	14306	G	A	0.681	9	77074075	7.80746E-06	0.152	360283	30731.948
family Acidaminococcaceae	Vascular dementia (undefined)	rs12224047	T	C	-0.005	36820790	0.783	0.018	14306	T	C	-0.263	11	36799240	4.56278E-06	0.057	361227	8033.487
family Acidaminococcaceae	Vascular dementia (undefined)	rs12449066	G	A	-0.001	79177293	0.955	0.022	14306	G	A	-0.262	16	79143396	2.01674E-06	0.055	361227	8803.514
family Acidaminococcaceae	Vascular dementia (undefined)	rs2972558	T	C	-0.009	45356141	0.486	0.014	14306	T	C	0.234	19	44852884	8.32415E-06	0.052	361227	7242.374
family Acidaminococcaceae	Vascular dementia (undefined)	rs2978951	G	A	0.010	6823295	0.437	0.013	14306	G	A	0.248	8	6965773	2.48222E-08	0.045	361227	11061.935
family Acidaminococcaceae	Vascular dementia (undefined)	rs359878	C	T	0.008	185438949	0.613	0.015	14306	C	T	-0.211	2	184574222	4.6443E-06	0.046	361227	6959.700
family Acidaminococcaceae	Vascular dementia (undefined)	rs429358	C	T	-0.007	45411941	0.557	0.018	14306	C	T	0.695	19	44908684	9.26616E-39	0.053	361227	58999.679
family Acidaminococcaceae	Vascular dementia (undefined)	rs6133343	G	T	-0.009	721797	0.670	0.020	14306	G	T	0.312	20	741153	3.89825E-06	0.068	361227	6210.744
family Acidaminococcaceae	Vascular dementia (undefined)	rs71298638	A	G	-0.007	63232261	0.814	0.027	14306	A	G	0.379	3	63246585	1.1165E-06	0.078	361227	6515.599
family Acidaminococcaceae	Vascular dementia (undefined)	rs78566090	A	G	0.020	125740204	0.560	0.030	14306	A	G	0.346	8	124727963	9.44735E-07	0.071	361227	7049.571
family Actinomycetaceae	Vascular dementia (undefined)	rs12224047	T	C	0.013	36820790	0.594	0.023	14306	T	C	-0.263	11	36799240	4.56278E-06	0.057	361227	8033.487
family Actinomycetaceae	Vascular dementia (undefined)	rs12449066	G	A	-0.013	79177293	0.695	0.028	14306	G	A	-0.262	16	79143396	2.01674E-06	0.055	361227	8803.514
family Actinomycetaceae	Vascular dementia (undefined)	rs193392	T	C	0.011	3085245	0.507	0.016	14306	T	C	-0.204	20	3104599	8.28324E-06	0.046	361227	7460.147
family Actinomycetaceae	Vascular dementia (undefined)	rs2292090	T	C	0.010	70588309	0.614	0.018	14306	T	C	0.217	4	69722591	9.99332E-06	0.049	361227	6178.734
family Actinomycetaceae	Vascular dementia (undefined)	rs2978951	G	A	-0.002	6823295	0.924	0.016	14306	G	A	0.248	8	6965773	2.48222E-08	0.045	361227	11061.935
family Actinomycetaceae	Vascular dementia (undefined)	rs359878	C	T	0.007	185438949	0.712	0.019	14306	C	T	-0.211	2	184574222	4.6443E-06	0.046	361227	6959.700
family Actinomycetaceae	Vascular dementia (undefined)	rs429358	C	T	0.038	45411941	0.090	0.023	14306	C	T	0.695	19	44908684	9.26616E-39	0.053	361227	58999.679
family Alcaligenaceae	Vascular dementia (undefined)	rs12224047	T	C	0.000	36820790	0.976	0.016	14306	T	C	-0.263	11	36799240	4.56278E-06	0.057	361227	8033.487
family Alcaligenaceae	Vascular dementia (undefined)	rs12449066	G	A	-0.017	79177293	0.366	0.019	14306	G	A	-0.262	16	79143396	2.01674E-06	0.055	361227	8803.514
family Alcaligenaceae	Vascular dementia (undefined)	rs2978951	G	A	0.002	6823295	0.830	0.011	14306	G	A	0.248	8	6965773	2.48222E-08	0.045	361227	11061.935
family Alcaligenaceae	Vascular dementia (undefined)	rs359878	C	T	-0.001	185438949	0.982	0.013	14306	C	T	-0.211	2	184574222	4.6443E-06	0.046	361227	6959.700
family Alcaligenaceae	Vascular dementia (undefined)	rs429358	C	T	0.009	45411941	0.545	0.016	14306	C	T	0.695	19	44908684	9.26616E-39	0.053	361227	58999.679
family Alcaligenaceae	Vascular dementia (undefined)	rs71298638	A	G	-0.019	63232261	0.376	0.023	14306	A	G	0.379	3	63246585	1.1165E-06	0.078	361227	6515.599
family Alcaligenaceae	Vascular dementia (undefined)	rs78566090	A	G	0.001	125740204	0.965	0.025	14306	A	G	0.346	8	124727963	9.44735E-07	0.071	361227	7049.571
family Bacteroidaceae	Vascular dementia (undefined)	rs12224047	T	C	0.005	36820790	0.732	0.015	14306	T	C	-0.263	11	36799240	4.56278E-06	0.057	361227	8033.487
family Bacteroidaceae	Vascular dementia (undefined)	rs12449066	G	A	0.007	79177293	0.669	0.018	14306	G	A	-0.262	16	79143396	2.01674E-06	0.055	361227	8803.514
family Bacteroidaceae	Vascular dementia (undefined)	rs2978951	G	A	-0.012	6823295	0.278	0.011	14306	G	A	0.248	8	6965773	2.48222E-08	0.045	361227	11061.935
family Bacteroidaceae	Vascular dementia (undefined)	rs359878	C	T	-0.011	185438949	0.385	0.012	14306	C	T	-0.211	2	184574222	4.6443E-06	0.046	361227	6959.700
family Bacteroidaceae	Vascular dementia (undefined)	rs78566090	A	G	0.006	125740204	0.853	0.024	14306	A	G	0.346	8	124727963	9.44735E-07	0.071	361227	7049.571
family Bacteroidales S24 7group	Vascular dementia (undefined)	rs12224047	T	C	0.005	36820790	0.747	0.023	14306	T	C	-0.263	11	36799240	4.56278E-06	0.057	361227	8033.487
family Bacteroidales S24 7group	Vascular dementia (undefined)	rs193392	T	C	0.010	3085245	0.536	0.016	14306	T	C	-0.204	20	3104599	8.28324E-06	0.046	361227	7460.147
family Bacteroidales S24 7group	Vascular dementia (undefined)	rs2978951	G	A	-0.013	6823295	0.422	0.016	14306	G	A	0.248	8	6965773	2.48222E-08	0.045	361227	11061.935
family Bacteroidales S24 7group	Vascular dementia (undefined)	rs429358	C	T	-0.033	45411941	0.162	0.023	14306	C	T	0.695	19	44908684	9.26616E-39	0.053	361227	58999.679
family Bacteroidales S24 7group	Vascular dementia (undefined)	rs71298638	A	G	-0.001	63232261	0.946	0.033	14306	A	G	0.379	3	63246585	1.1165E-06	0.078	361227	6515.599
family Bacteroidales S24 7group	Vascular dementia (undefined)	rs78566090	A	G	0.029	125740204	0.395	0.036	14306	A	G	0.346	8	124727963	9.44735E-07	0.071	361227	7049.571
family Bifidobacteriaceae	Vascular dementia (undefined)	rs12224047	T	C	-0.001	36820790	0.919	0.017	14306	T	C	-0.263	11	36799240	4.56278E-06	0.057	361227	8033.487
family Bifidobacteriaceae	Vascular dementia (undefined)	rs2292090	T	C	-0.002	70588309	0.974	0.013	14306	T	C	0.217	4	69722591	9.99332E-06	0.049	361227	6178.734
family Bifidobacteriaceae	Vascular dementia (undefined)	rs2972558	T	C	-0.001	45356141	0.944	0.013	14306	T	C	0.234	19	44852884	8.32415E-06	0.052	361227	7242.374
family Bifidobacteriaceae	Vascular dementia (undefined)	rs2978951	G	A	-0.004	6823295	0.715	0.012	14306	G	A	0.248	8	6965773	2.48222E-08	0.045	361227	11061.935
family Bifidobacteriaceae	Vascular dementia (undefined)	rs429358	C	T	0.022	45411941	0.147	0.017	14306	C	T	0.695	19	44908684	9.26616E-39	0.053	361227	58999.679
family Bifidobacteriaceae	Vascular dementia (undefined)	rs6133343	G	T	-0.007	721797	0.784	0.018	14306	G	T	0.312	20	741153	3.89825E-06	0.068	361227	6210.744
family Bifidobacteriaceae	Vascular dementia (undefined)	rs71298638	A	G	-0.013	63232261	0.631	0.024	14306	A	G	0.379	3	63246585	1.1165E-06	0.078	361227	6515.599
Gut microbiota abundance (family Christensenellaceae id.1866)	Vascular dementia (undefined)	rs359878	C	T	0.000	185438949	0.956	0.013	14306	C	T	-0.211	2	184574222	4.6443E-06	0.046	361227	6959.700
Gut microbiota abundance (family Christensenellaceae id.1866)	Vascular dementia (undefined)	rs71298638	A	G	-0.011	63232261	0.620	0.023	14306	A	G	0.379	3	63246585	1.1165E-06	0.078	361227	6515.599
family Clostridiaceae1	Vascular dementia (undefined)	rs12224047	T	C	-0.008	36820790	0.634	0.017	14306	T	C	-0.263	11	36799240	4.56278E-06	0.057	361227	8033.487

family Clostridiaceae1	Vascular dementia (undefined)	rs193392	T	C	-0.001	3085245	0.951	0.012	14306	T	C	-0.204	20	3104599	8.28324E-06	0.046	361227	7460.147
family Clostridiaceae1	Vascular dementia (undefined)	rs2292090	T	C	-0.001	70588309	0.903	0.013	14306	T	C	0.217	4	69722591	9.99332E-06	0.049	361227	6178.734
family Clostridiaceae1	Vascular dementia (undefined)	rs2978951	G	A	0.012	6823295	0.315	0.012	14306	G	A	0.248	8	6965773	2.48222E-08	0.045	361227	11061.935
family Clostridiaceae1	Vascular dementia (undefined)	rs359878	C	T	0.011	185438949	0.422	0.014	14306	C	T	-0.211	2	184574222	4.6443E-06	0.046	361227	6959.700
family Clostridiaceae1	Vascular dementia (undefined)	rs429358	C	T	0.010	45411941	0.573	0.017	14306	C	T	0.695	19	44908684	9.26616E-39	0.053	361227	58999.679
family Clostridiaceae1	Vascular dementia (undefined)	rs6133343	G	T	0.007	721797	0.715	0.019	14306	G	T	0.312	20	741153	3.89825E-06	0.068	361227	6210.744
family Clostridiaceae1	Vascular dementia (undefined)	rs71298638	A	G	0.007	63232261	0.765	0.025	14306	A	G	0.379	3	63246585	1.1165E-06	0.078	361227	6515.599
family Clostridiaceae1	Vascular dementia (undefined)	rs78566090	A	G	-0.011	125740204	0.708	0.028	14306	A	G	0.346	8	124727963	9.44735E-07	0.071	361227	7049.571
family Clostridiales vadin BB60 group	Vascular dementia (undefined)	rs12449066	G	A	-0.013	79177293	0.573	0.024	14306	G	A	-0.262	16	79143396	2.01674E-06	0.055	361227	8803.514
family Clostridiales vadin BB60 group	Vascular dementia (undefined)	rs193392	T	C	-0.012	3085245	0.401	0.014	14306	T	C	-0.204	20	3104599	8.28324E-06	0.046	361227	7460.147
family Clostridiales vadin BB60 group	Vascular dementia (undefined)	rs359878	C	T	0.013	185438949	0.469	0.016	14306	C	T	-0.211	2	184574222	4.6443E-06	0.046	361227	6959.700
family Clostridiales vadin BB60 group	Vascular dementia (undefined)	rs429358	C	T	0.004	45411941	0.829	0.020	14306	C	T	0.695	19	44908684	9.26616E-39	0.053	361227	58999.679
family Clostridiales vadin BB60 group	Vascular dementia (undefined)	rs71298638	A	G	0.005	63232261	0.860	0.029	14306	A	G	0.379	3	63246585	1.1165E-06	0.078	361227	6515.599
family Coriobacteriaceae	Vascular dementia (undefined)	rs12449066	G	A	0.004	79177293	0.827	0.018	14306	G	A	-0.262	16	79143396	2.01674E-06	0.055	361227	8803.514
family Coriobacteriaceae	Vascular dementia (undefined)	rs193392	T	C	0.003	3085245	0.795	0.011	14306	T	C	-0.204	20	3104599	8.28324E-06	0.046	361227	7460.147
family Coriobacteriaceae	Vascular dementia (undefined)	rs2292090	T	C	0.004	70588309	0.770	0.012	14306	T	C	0.217	4	69722591	9.99332E-06	0.049	361227	6178.734
family Coriobacteriaceae	Vascular dementia (undefined)	rs2972558	T	C	-0.002	45356141	0.855	0.011	14306	T	C	0.234	19	44852884	8.32415E-06	0.052	361227	7242.374
family Coriobacteriaceae	Vascular dementia (undefined)	rs2978951	G	A	-0.005	6823295	0.614	0.011	14306	G	A	0.248	8	6965773	2.48222E-08	0.045	361227	11061.935
family Coriobacteriaceae	Vascular dementia (undefined)	rs359878	C	T	-0.001	185438949	0.997	0.012	14306	C	T	-0.211	2	184574222	4.6443E-06	0.046	361227	6959.700
family Coriobacteriaceae	Vascular dementia (undefined)	rs429358	C	T	0.019	45411941	0.204	0.015	14306	C	T	0.695	19	44908684	9.26616E-39	0.053	361227	58999.679
family Coriobacteriaceae	Vascular dementia (undefined)	rs6133343	G	T	-0.013	721797	0.490	0.017	14306	G	T	0.312	20	741153	3.89825E-06	0.068	361227	6210.744
family Coriobacteriaceae	Vascular dementia (undefined)	rs71298638	A	G	-0.003	63232261	0.855	0.022	14306	A	G	0.379	3	63246585	1.1165E-06	0.078	361227	6515.599
family Defluviitaleaceae	Vascular dementia (undefined)	rs12224047	T	C	-0.007	36820790	0.803	0.023	14306	T	C	-0.263	11	36799240	4.56278E-06	0.057	361227	8033.487
family Defluviitaleaceae	Vascular dementia (undefined)	rs12449066	G	A	-0.021	79177293	0.347	0.027	14306	G	A	-0.262	16	79143396	2.01674E-06	0.055	361227	8803.514
family Defluviitaleaceae	Vascular dementia (undefined)	rs193392	T	C	-0.013	3085245	0.405	0.016	14306	T	C	-0.204	20	3104599	8.28324E-06	0.046	361227	7460.147
family Defluviitaleaceae	Vascular dementia (undefined)	rs359878	C	T	-0.013	185438949	0.488	0.018	14306	C	T	-0.211	2	184574222	4.6443E-06	0.046	361227	6959.700
family Defluviitaleaceae	Vascular dementia (undefined)	rs429358	C	T	0.000	45411941	0.935	0.022	14306	C	T	0.695	19	44908684	9.26616E-39	0.053	361227	58999.679
family Defluviitaleaceae	Vascular dementia (undefined)	rs6133343	G	T	-0.018	721797	0.458	0.026	14306	G	T	0.312	20	741153	3.89825E-06	0.068	361227	6210.744
family Defluviitaleaceae	Vascular dementia (undefined)	rs78566090	A	G	0.016	125740204	0.731	0.037	14306	A	G	0.346	8	124727963	9.44735E-07	0.071	361227	7049.571
family Desulfovibrionaceae	Vascular dementia (undefined)	rs12224047	T	C	0.014	36820790	0.390	0.017	14306	T	C	-0.263	11	36799240	4.56278E-06	0.057	361227	8033.487
family Desulfovibrionaceae	Vascular dementia (undefined)	rs2292090	T	C	0.009	70588309	0.391	0.013	14306	T	C	0.217	4	69722591	9.99332E-06	0.049	361227	6178.734
family Desulfovibrionaceae	Vascular dementia (undefined)	rs2972558	T	C	0.007	45356141	0.576	0.012	14306	T	C	0.234	19	44852884	8.32415E-06	0.052	361227	7242.374
family Desulfovibrionaceae	Vascular dementia (undefined)	rs2978951	G	A	0.012	6823295	0.320	0.012	14306	G	A	0.248	8	6965773	2.48222E-08	0.045	361227	11061.935
family Desulfovibrionaceae	Vascular dementia (undefined)	rs359878	C	T	0.007	185438949	0.576	0.013	14306	C	T	-0.211	2	184574222	4.6443E-06	0.046	361227	6959.700
family Desulfovibrionaceae	Vascular dementia (undefined)	rs429358	C	T	0.017	45411941	0.305	0.017	14306	C	T	0.695	19	44908684	9.26616E-39	0.053	361227	58999.679
family Desulfovibrionaceae	Vascular dementia (undefined)	rs6133343	G	T	0.001	721797	0.866	0.018	14306	G	T	0.312	20	741153	3.89825E-06	0.068	361227	6210.744
family Desulfovibrionaceae	Vascular dementia (undefined)	rs71298638	A	G	0.005	63232261	0.869	0.024	14306	A	G	0.379	3	63246585	1.1165E-06	0.078	361227	6515.599
family Desulfovibrionaceae	Vascular dementia (undefined)	rs78566090	A	G	0.009	125740204	0.635	0.027	14306	A	G	0.346	8	124727963	9.44735E-07	0.071	361227	7049.571
family Enterobacteriaceae	Vascular dementia (undefined)	rs12224047	T	C	-0.013	36820790	0.464	0.018	14306	T	C	-0.263	11	36799240	4.56278E-06	0.057	361227	8033.487
family Enterobacteriaceae	Vascular dementia (undefined)	rs12449066	G	A	0.014	79177293	0.413	0.021	14306	G	A	-0.262	16	79143396	2.01674E-06	0.055	361227	8803.514
family Enterobacteriaceae	Vascular dementia (undefined)	rs2978951	G	A	0.010	6823295	0.427	0.012	14306	G	A	0.248	8	6965773	2.48222E-08	0.045	361227	11061.935
family Enterobacteriaceae	Vascular dementia (undefined)	rs429358	C	T	-0.019	45411941	0.299	0.017	14306	C	T	0.695	19	44908684	9.26616E-39	0.053	361227	58999.679
family Enterobacteriaceae	Vascular dementia (undefined)	rs6133343	G	T	0.009	721797	0.541	0.019	14306	G	T	0.312	20	741153	3.89825E-06	0.068	361227	6210.744
family Erysipelotrichaceae	Vascular dementia (undefined)	rs12224047	T	C	0.003	36820790	0.897	0.015	14306	T	C	-0.263	11	36799240	4.56278E-06	0.057	361227	8033.487
family Erysipelotrichaceae	Vascular dementia (undefined)	rs12449066	G	A	-0.016	79177293	0.366	0.018	14306	G	A	-0.262	16	79143396	2.01674E-06	0.055	361227	8803.514
family Erysipelotrichaceae	Vascular dementia (undefined)	rs2292090	T	C	-0.002	70588309	0.866	0.012	14306	T	C	0.217	4	69722591	9.99332E-06	0.049	361227	6178.734
family Erysipelotrichaceae	Vascular dementia (undefined)	rs2978951	G	A	-0.005	6823295	0.605	0.011	14306	G	A	0.248	8	6965773	2.48222E-08	0.045	361227	11061.935
family Erysipelotrichaceae	Vascular dementia (undefined)	rs429358	C	T	0.032	45411941	0.035	0.015	14306	C	T	0.695	19	44908684	9.26616E-39	0.053	361227	58999.679
family Erysipelotrichaceae	Vascular dementia (undefined)	rs6133343	G	T	0.004	721797	0.852	0.017	14306	G	T	0.312	20	741153	3.89825E-06	0.068	361227	6210.744
family Erysipelotrichaceae	Vascular dementia (undefined)	rs71298638	A	G	0.012	63232261	0.604	0.022	14306	A	G	0.379	3	63246585	1.1165E-06	0.078	361227	6515.599
family Erysipelotrichaceae	Vascular dementia (undefined)	rs78566090	A	G	-0.013	125740204	0.561	0.024	14306	A	G	0.346	8	124727963	9.44735E-07	0.071	361227	7049.571
family Family XI	Vascular dementia (undefined)	rs12449066	G	A	0.021	79177293	0.530	0.043	14306	G	A	-0.262	16	79143396	2.01674E-06	0.055	361227	8803.514
family Family XI	Vascular dementia (undefined)	rs429358	C	T	0.054	45411941	0.150	0.037	14306	C	T	0.695	19	44908684	9.26616E-39	0.053	361227	58999.679
family Family XI	Vascular dementia (undefined)	rs6133343	G	T	-0.016	721797	0.725	0.041	14306	G	T	0.312	20	741153	3.89825E-06	0.068	361227	6210.744
family Family XIII	Vascular dementia (undefined)	rs12224047	T	C	-0.013	36820790	0.392	0.016	14306	T	C	-0.263	11	36799240	4.56278E-06	0.057	361227	8033.487
family Family XIII	Vascular dementia (undefined)	rs12449066	G	A	-0.005	79177293	0.651	0.019	14306	G	A	-0.262	16	79143396	2.01674E-06	0.055	361227	8803.514
family Family XIII	Vascular dementia (undefined)	rs193392	T	C	0.010	3085245	0.390	0.011	14306	T	C	-0.204	20	3104599	8.28324E-06	0.046	361227	7460.147
family Family XIII	Vascular dementia (undefined)	rs2292090	T	C	0.002	70588309	0.765	0.012	14306	T	C	0.217	4	69722591	9.99332E-06	0.049	361227	6178.734
family Family XIII	Vascular dementia (undefined)	rs2978951	G	A	0.002	6823295	0.871	0.011	14306	G	A	0.248	8	6965773	2.48222E-08	0.045	361227	11061.935
family Family XIII	Vascular dementia (undefined)	rs359878	C	T	-0.006	185438949	0.621	0.013	14306	C	T	-0.211	2	184574222	4.6443E-06	0.046	361227	6959.700
family Family XIII	Vascular dementia (undefined)	rs429358	C	T	0.036	45411941	0.023	0.016	14306	C	T	0.695	19	44908684	9.26616E-39	0.053	361227	58999.679
family Family XIII	Vascular dementia (undefined)	rs6133343	G	T	0.006	721797	0.713	0.018	14306	G	T	0.312	20	741153	3.89825E-06	0.068	361227	6210.744
family Family XIII	Vascular dementia (undefined)	rs71298638	A	G	-0.014	63232261	0.508	0.023	14306	A	G	0.379	3	63246585	1.1165E-06	0.078	361227	6515.599
family Family XIII	Vascular dementia (undefined)	rs78566090	A	G	0.013	125740204	0.686	0.026	14306	A	G	0.346	8	124727963	9.44735E-07	0.071	361227	7049.571
family Lachnospiraceae	Vascular dementia (undefined)	rs2292090	T	C	0.010	70588309	0.399	0.012	14306	T	C	0.217	4	69722591	9.99332E-06	0.049	361227	6178.734
family Lachnospiraceae	Vascular dementia (undefined)	rs2978951	G	A	0.004	6823295	0.744	0.011	14306	G	A	0.248	8	6965773	2.48222E-08	0.045	361227	11061.935
family Lachnospiraceae	Vascular dementia (undefined)	rs359878	C	T	0.003	185438949	0.736	0.012	14306	C	T	-0.211	2	184574222	4.6443E-06	0.046	361227	6959.700
family Lachnospiraceae	Vascular dementia (undefined)	rs429358	C	T	0.002	45411941	0.858	0.015	14306	C	T	0.695	19	44908684	9.26616E-39	0.053	361227	58999.679
family Lachnospiraceae	Vascular dementia (undefined)	rs6133343	G	T	0.011	721797	0.644	0.017	14306	G	T	0.312	20	741153	3.89825E-06	0.068	361227	6210.744
family Lachnospiraceae	Vascular dementia (undefined)	rs71298638	A	G	-0.020	63232261	0.326	0.022	14306	A	G	0.379	3	63246585	1.1165E-06	0.078	361227	6515.599
family Lactobacillaceae	Vascular dementia (undefined)	rs193392	T	C	-0.004	3085245	0.814	0.017	14306	T	C	-0.204	20	3104599	8.28324E-06	0.046	361227	7460.147

family Lactobacillaceae	Vascular dementia (undefined)	rs2292090	T	C	0.016	70588309	0.383	0.019	14306	T	C	0.217	4	69722591	9.99332E-06	0.049	361227	6178.734
family Lactobacillaceae	Vascular dementia (undefined)	rs2978951	G	A	-0.016	6823295	0.364	0.017	14306	G	A	0.248	8	6965773	2.48222E-08	0.045	361227	11061.935
family Lactobacillaceae	Vascular dementia (undefined)	rs429358	C	T	-0.013	45411941	0.673	0.024	14306	C	T	0.695	19	44908684	9.26616E-39	0.053	361227	58999.679
family Lactobacillaceae	Vascular dementia (undefined)	rs6133343	G	T	0.018	721797	0.479	0.026	14306	G	T	0.312	20	741153	3.89825E-06	0.068	361227	6210.744
family Lactobacillaceae	Vascular dementia (undefined)	rs71298638	A	G	-0.019	63232261	0.594	0.035	14306	A	G	0.379	3	63246585	1.1165E-06	0.078	361227	6515.599
family Lactobacillaceae	Vascular dementia (undefined)	rs78566090	A	G	-0.038	125740204	0.404	0.041	14306	A	G	0.346	8	124727963	9.44735E-07	0.071	361227	7049.571
family Methanobacteriaceae	Vascular dementia (undefined)	rs12224047	T	C	0.004	36820790	0.920	0.034	14306	T	C	-0.263	11	36799240	4.56278E-06	0.057	361227	8033.487
family Methanobacteriaceae	Vascular dementia (undefined)	rs12449066	G	A	-0.010	79177293	0.714	0.040	14306	G	A	-0.262	16	79143396	2.01674E-06	0.055	361227	8803.514
family Methanobacteriaceae	Vascular dementia (undefined)	rs193392	T	C	0.001	3085245	0.989	0.025	14306	T	C	-0.204	20	3104599	8.28324E-06	0.046	361227	7460.147
family Methanobacteriaceae	Vascular dementia (undefined)	rs2972558	T	C	-0.014	45356141	0.618	0.027	14306	T	C	0.234	19	44852884	8.32415E-06	0.052	361227	7242.374
family Methanobacteriaceae	Vascular dementia (undefined)	rs2978951	G	A	0.023	6823295	0.327	0.023	14306	G	A	0.248	8	6965773	2.48222E-08	0.045	361227	11061.935
family Methanobacteriaceae	Vascular dementia (undefined)	rs359878	C	T	0.015	185438949	0.623	0.027	14306	C	T	-0.211	2	184574222	4.6443E-06	0.046	361227	6959.700
family Methanobacteriaceae	Vascular dementia (undefined)	rs429358	C	T	-0.026	45411941	0.504	0.034	14306	C	T	0.695	19	44908684	9.26616E-39	0.053	361227	58999.679
family Oxalobacteraceae	Vascular dementia (undefined)	rs12224047	T	C	-0.012	36820790	0.662	0.029	14306	T	C	-0.263	11	36799240	4.56278E-06	0.057	361227	8033.487
family Oxalobacteraceae	Vascular dementia (undefined)	rs12449066	G	A	0.010	79177293	0.910	0.034	14306	G	A	-0.262	16	79143396	2.01674E-06	0.055	361227	8803.514
family Oxalobacteraceae	Vascular dementia (undefined)	rs2292090	T	C	0.019	70588309	0.398	0.022	14306	T	C	0.217	4	69722591	9.99332E-06	0.049	361227	6178.734
family Oxalobacteraceae	Vascular dementia (undefined)	rs2978951	G	A	-0.012	6823295	0.549	0.020	14306	G	A	0.248	8	6965773	2.48222E-08	0.045	361227	11061.935
family Oxalobacteraceae	Vascular dementia (undefined)	rs359878	C	T	0.001	185438949	0.923	0.023	14306	C	T	-0.211	2	184574222	4.6443E-06	0.046	361227	6959.700
family Oxalobacteraceae	Vascular dementia (undefined)	rs429358	C	T	0.011	45411941	0.614	0.028	14306	C	T	0.695	19	44908684	9.26616E-39	0.053	361227	58999.679
family Oxalobacteraceae	Vascular dementia (undefined)	rs6133343	G	T	-0.028	721797	0.348	0.032	14306	G	T	0.312	20	741153	3.89825E-06	0.068	361227	6210.744
family Oxalobacteraceae	Vascular dementia (undefined)	rs78566090	A	G	-0.032	125740204	0.501	0.046	14306	A	G	0.346	8	124727963	9.44735E-07	0.071	361227	7049.571
family Pasteurellaceae	Vascular dementia (undefined)	rs12449066	G	A	0.008	79177293	0.784	0.025	14306	G	A	-0.262	16	79143396	2.01674E-06	0.055	361227	8803.514
family Pasteurellaceae	Vascular dementia (undefined)	rs193392	T	C	-0.005	3085245	0.750	0.015	14306	T	C	-0.204	20	3104599	8.28324E-06	0.046	361227	7460.147
family Pasteurellaceae	Vascular dementia (undefined)	rs2292090	T	C	-0.011	70588309	0.444	0.016	14306	T	C	0.217	4	69722591	9.99332E-06	0.049	361227	6178.734
family Pasteurellaceae	Vascular dementia (undefined)	rs2972558	T	C	-0.011	45356141	0.513	0.016	14306	T	C	0.234	19	44852884	8.32415E-06	0.052	361227	7242.374
family Pasteurellaceae	Vascular dementia (undefined)	rs2978951	G	A	0.002	6823295	0.924	0.015	14306	G	A	0.248	8	6965773	2.48222E-08	0.045	361227	11061.935
family Pasteurellaceae	Vascular dementia (undefined)	rs359878	C	T	0.008	185438949	0.662	0.017	14306	C	T	-0.211	2	184574222	4.6443E-06	0.046	361227	6959.700
family Pasteurellaceae	Vascular dementia (undefined)	rs429358	C	T	0.022	45411941	0.309	0.021	14306	C	T	0.695	19	44908684	9.26616E-39	0.053	361227	58999.679
family Peptococcaceae	Vascular dementia (undefined)	rs193392	T	C	-0.008	3085245	0.579	0.014	14306	T	C	-0.204	20	3104599	8.28324E-06	0.046	361227	7460.147
family Peptococcaceae	Vascular dementia (undefined)	rs2292090	T	C	-0.005	70588309	0.774	0.015	14306	T	C	0.217	4	69722591	9.99332E-06	0.049	361227	6178.734
family Peptococcaceae	Vascular dementia (undefined)	rs2978951	G	A	-0.014	6823295	0.307	0.014	14306	G	A	0.248	8	6965773	2.48222E-08	0.045	361227	11061.935
family Peptococcaceae	Vascular dementia (undefined)	rs359878	C	T	0.001	185438949	0.984	0.016	14306	C	T	-0.211	2	184574222	4.6443E-06	0.046	361227	6959.700
family Peptococcaceae	Vascular dementia (undefined)	rs429358	C	T	0.028	45411941	0.111	0.019	14306	C	T	0.695	19	44908684	9.26616E-39	0.053	361227	58999.679
family Peptococcaceae	Vascular dementia (undefined)	rs6133343	G	T	0.019	721797	0.393	0.022	14306	G	T	0.312	20	741153	3.89825E-06	0.068	361227	6210.744
family Peptococcaceae	Vascular dementia (undefined)	rs71298638	A	G	0.016	63232261	0.600	0.028	14306	A	G	0.379	3	63246585	1.1165E-06	0.078	361227	6515.599
family Peptococcaceae	Vascular dementia (undefined)	rs78566090	A	G	0.019	125740204	0.536	0.032	14306	A	G	0.346	8	124727963	9.44735E-07	0.071	361227	7049.571
family Peptostreptococcaceae	Vascular dementia (undefined)	rs193392	T	C	-0.001	3085245	0.917	0.011	14306	T	C	-0.204	20	3104599	8.28324E-06	0.046	361227	7460.147
family Peptostreptococcaceae	Vascular dementia (undefined)	rs2978951	G	A	0.004	6823295	0.738	0.011	14306	G	A	0.248	8	6965773	2.48222E-08	0.045	361227	11061.935
family Peptostreptococcaceae	Vascular dementia (undefined)	rs359878	C	T	-0.002	185438949	0.852	0.013	14306	C	T	-0.211	2	184574222	4.6443E-06	0.046	361227	6959.700
family Peptostreptococcaceae	Vascular dementia (undefined)	rs429358	C	T	0.014	45411941	0.358	0.016	14306	C	T	0.695	19	44908684	9.26616E-39	0.053	361227	58999.679
family Peptostreptococcaceae	Vascular dementia (undefined)	rs71298638	A	G	-0.019	63232261	0.455	0.023	14306	A	G	0.379	3	63246585	1.1165E-06	0.078	361227	6515.599
family Peptostreptococcaceae	Vascular dementia (undefined)	rs78566090	A	G	0.003	125740204	0.996	0.027	14306	A	G	0.346	8	124727963	9.44735E-07	0.071	361227	7049.571
family Porphyromonadaceae	Vascular dementia (undefined)	rs2972558	T	C	-0.002	45356141	0.807	0.011	14306	T	C	0.234	19	44852884	8.32415E-06	0.052	361227	7242.374
family Porphyromonadaceae	Vascular dementia (undefined)	rs2978951	G	A	-0.009	6823295	0.420	0.011	14306	G	A	0.248	8	6965773	2.48222E-08	0.045	361227	11061.935
family Porphyromonadaceae	Vascular dementia (undefined)	rs429358	C	T	0.001	45411941	0.899	0.015	14306	C	T	0.695	19	44908684	9.26616E-39	0.053	361227	58999.679
family Porphyromonadaceae	Vascular dementia (undefined)	rs6133343	G	T	0.008	721797	0.561	0.017	14306	G	T	0.312	20	741153	3.89825E-06	0.068	361227	6210.744
family Porphyromonadaceae	Vascular dementia (undefined)	rs78566090	A	G	-0.013	125740204	0.602	0.025	14306	A	G	0.346	8	124727963	9.44735E-07	0.071	361227	7049.571
family Prevotellaceae	Vascular dementia (undefined)	rs193392	T	C	0.004	3085245	0.759	0.012	14306	T	C	-0.204	20	3104599	8.28324E-06	0.046	361227	7460.147
family Prevotellaceae	Vascular dementia (undefined)	rs2292090	T	C	-0.004	70588309	0.713	0.013	14306	T	C	0.217	4	69722591	9.99332E-06	0.049	361227	6178.734
family Prevotellaceae	Vascular dementia (undefined)	rs2972558	T	C	0.009	45356141	0.496	0.013	14306	T	C	0.234	19	44852884	8.32415E-06	0.052	361227	7242.374
family Prevotellaceae	Vascular dementia (undefined)	rs2978951	G	A	-0.012	6823295	0.289	0.012	14306	G	A	0.248	8	6965773	2.48222E-08	0.045	361227	11061.935
family Prevotellaceae	Vascular dementia (undefined)	rs359878	C	T	-0.006	185438949	0.682	0.014	14306	C	T	-0.211	2	184574222	4.6443E-06	0.046	361227	6959.700
family Prevotellaceae	Vascular dementia (undefined)	rs429358	C	T	0.000	45411941	0.975	0.017	14306	C	T	0.695	19	44908684	9.26616E-39	0.053	361227	58999.679
family Prevotellaceae	Vascular dementia (undefined)	rs6133343	G	T	0.012	721797	0.754	0.018	14306	G	T	0.312	20	741153	3.89825E-06	0.068	361227	6210.744
family Prevotellaceae	Vascular dementia (undefined)	rs71298638	A	G	-0.004	63232261	0.846	0.025	14306	A	G	0.379	3	63246585	1.1165E-06	0.078	361227	6515.599
family Rhodospirillaceae	Vascular dementia (undefined)	rs12224047	T	C	-0.007	36820790	0.825	0.022	14306	T	C	-0.263	11	36799240	4.56278E-06	0.057	361227	8033.487
family Rhodospirillaceae	Vascular dementia (undefined)	rs12449066	G	A	0.022	79177293	0.345	0.026	14306	G	A	-0.262	16	79143396	2.01674E-06	0.055	361227	8803.514
family Rhodospirillaceae	Vascular dementia (undefined)	rs193392	T	C	-0.007	3085245	0.637	0.016	14306	T	C	-0.204	20	3104599	8.28324E-06	0.046	361227	7460.147
family Rhodospirillaceae	Vascular dementia (undefined)	rs2972558	T	C	0.003	45356141	0.884	0.017	14306	T	C	0.234	19	44852884	8.32415E-06	0.052	361227	7242.374
family Rhodospirillaceae	Vascular dementia (undefined)	rs2978951	G	A	0.001	6823295	0.930	0.016	14306	G	A	0.248	8	6965773	2.48222E-08	0.045	361227	11061.935
family Rhodospirillaceae	Vascular dementia (undefined)	rs429358	C	T	-0.006	45411941	0.814	0.022	14306	C	T	0.695	19	44908684	9.26616E-39	0.053	361227	58999.679
family Rhodospirillaceae	Vascular dementia (undefined)	rs78566090	A	G	0.008	125740204	0.790	0.035	14306	A	G	0.346	8	124727963	9.44735E-07	0.071	361227	7049.571
family Rikenellaceae	Vascular dementia (undefined)	rs12449066	G	A	-0.007	79177293	0.791	0.018	14306	G	A	-0.262	16	79143396	2.01674E-06	0.055	361227	8803.514
family Rikenellaceae	Vascular dementia (undefined)	rs2292090	T	C	-0.007	70588309	0.627	0.012	14306	T	C	0.217	4	69722591	9.99332E-06	0.049	361227	6178.734
family Rikenellaceae	Vascular dementia (undefined)	rs2978951	G	A	0.004	6823295	0.703	0.011	14306	G	A	0.248	8	6965773	2.48222E-08	0.045	361227	11061.935
family Rikenellaceae	Vascular dementia (undefined)	rs359878	C	T	0.003	185438949	0.804	0.012	14306	C	T	-0.211	2	184574222	4.6443E-06	0.046	361227	6959.700
family Rikenellaceae	Vascular dementia (undefined)	rs429358	C	T	-0.016	45411941	0.273	0.015	14306	C	T	0.695	19	44908684	9.26616E-39	0.053	361227	58999.679
family Rikenellaceae	Vascular dementia (undefined)	rs6133343	G	T	-0.004	721797	0.972	0.017	14306	G	T	0.312	20	741153	3.89825E-06	0.068	361227	6210.744
family Rikenellaceae	Vascular dementia (undefined)	rs71298638	A	G	-0.003	63232261	0.854	0.022	14306	A	G	0.379	3	63246585	1.1165E-06	0.078	361227	6515.599
family Rikenellaceae	Vascular dementia (undefined)	rs78566090	A	G	-0.022	125740204	0.366	0.025	14306	A	G	0.346	8	124727963	9.44735E-07	0.071	361227	7049.571
family Ruminococcaceae	Vascular dementia (undefined)	rs12224047	T	C	0.005	36820790	0.744	0.015	14306	T	C	-0.263	11	36799240	4.56278E-06	0.057	361227	8033.487

family Ruminococcaceae	Vascular dementia (undefined)	rs12449066	G	A	0.010	79177293	0.744	0.018	14306	G	A	-0.262	16	79143396	2.01674E-06	0.055	361227	8803.514
family Ruminococcaceae	Vascular dementia (undefined)	rs2292090	T	C	0.001	70588309	0.885	0.012	14306	T	C	0.217	4	69722591	9.99332E-06	0.049	361227	6178.734
family Ruminococcaceae	Vascular dementia (undefined)	rs2972558	T	C	-0.004	45356141	0.776	0.011	14306	T	C	0.234	19	44852884	8.32415E-06	0.052	361227	7242.374
family Ruminococcaceae	Vascular dementia (undefined)	rs2978951	G	A	0.002	6823295	0.842	0.011	14306	G	A	0.248	8	6965773	2.48222E-08	0.045	361227	11061.935
family Ruminococcaceae	Vascular dementia (undefined)	rs429358	C	T	-0.003	45411941	0.847	0.015	14306	C	T	0.695	19	44908684	9.26616E-39	0.053	361227	58999.679
family Ruminococcaceae	Vascular dementia (undefined)	rs6133343	G	T	-0.002	721797	0.884	0.017	14306	G	T	0.312	20	741153	3.89825E-06	0.068	361227	6210.744
family Ruminococcaceae	Vascular dementia (undefined)	rs71298638	A	G	-0.010	63232261	0.643	0.022	14306	A	G	0.379	3	63246585	1.1165E-06	0.078	361227	6515.599
family Ruminococcaceae	Vascular dementia (undefined)	rs78566090	A	G	0.014	125740204	0.602	0.024	14306	A	G	0.346	8	124727963	9.44735E-07	0.071	361227	7049.571
family Streptococcaceae	Vascular dementia (undefined)	rs2978951	G	A	0.004	6823295	0.758	0.011	14306	G	A	0.248	8	6965773	2.48222E-08	0.045	361227	11061.935
family Streptococcaceae	Vascular dementia (undefined)	rs429358	C	T	0.012	45411941	0.432	0.016	14306	C	T	0.695	19	44908684	9.26616E-39	0.053	361227	58999.679
family Streptococcaceae	Vascular dementia (undefined)	rs6133343	G	T	-0.015	721797	0.545	0.017	14306	G	T	0.312	20	741153	3.89825E-06	0.068	361227	6210.744
family Streptococcaceae	Vascular dementia (undefined)	rs78566090	A	G	0.017	125740204	0.525	0.026	14306	A	G	0.346	8	124727963	9.44735E-07	0.071	361227	7049.571
family Veillonellaceae	Vascular dementia (undefined)	rs12449066	G	A	0.006	79177293	0.838	0.019	14306	G	A	-0.262	16	79143396	2.01674E-06	0.055	361227	8803.514
family Veillonellaceae	Vascular dementia (undefined)	rs2978951	G	A	0.003	6823295	0.802	0.011	14306	G	A	0.248	8	6965773	2.48222E-08	0.045	361227	11061.935
family Veillonellaceae	Vascular dementia (undefined)	rs429358	C	T	-0.023	45411941	0.150	0.016	14306	C	T	0.695	19	44908684	9.26616E-39	0.053	361227	58999.679
family Veillonellaceae	Vascular dementia (undefined)	rs6133343	G	T	0.014	721797	0.694	0.018	14306	G	T	0.312	20	741153	3.89825E-06	0.068	361227	6210.744
family Veillonellaceae	Vascular dementia (undefined)	rs78566090	A	G	0.009	125740204	0.638	0.027	14306	A	G	0.346	8	124727963	9.44735E-07	0.071	361227	7049.571
family Verrucomicrobiaceae	Vascular dementia (undefined)	rs12224047	T	C	-0.005	36820790	0.748	0.019	14306	T	C	-0.263	11	36799240	4.56278E-06	0.057	361227	8033.487
family Verrucomicrobiaceae	Vascular dementia (undefined)	rs12449066	G	A	0.001	79177293	0.959	0.022	14306	G	A	-0.262	16	79143396	2.01674E-06	0.055	361227	8803.514
family Verrucomicrobiaceae	Vascular dementia (undefined)	rs193392	T	C	0.003	3085245	0.817	0.013	14306	T	C	-0.204	20	3104599	8.28324E-06	0.046	361227	7460.147
family Verrucomicrobiaceae	Vascular dementia (undefined)	rs2292090	T	C	-0.008	70588309	0.604	0.015	14306	T	C	0.217	4	69722591	9.99332E-06	0.049	361227	6178.734
family Verrucomicrobiaceae	Vascular dementia (undefined)	rs2972558	T	C	0.008	45356141	0.587	0.014	14306	T	C	0.234	19	44852884	8.32415E-06	0.052	361227	7242.374
family Verrucomicrobiaceae	Vascular dementia (undefined)	rs429358	C	T	0.018	45411941	0.341	0.019	14306	C	T	0.695	19	44908684	9.26616E-39	0.053	361227	58999.679
family Verrucomicrobiaceae	Vascular dementia (undefined)	rs6133343	G	T	-0.009	721797	0.764	0.021	14306	G	T	0.312	20	741153	3.89825E-06	0.068	361227	6210.744
family Verrucomicrobiaceae	Vascular dementia (undefined)	rs71298638	A	G	-0.004	63232261	0.847	0.027	14306	A	G	0.379	3	63246585	1.1165E-06	0.078	361227	6515.599
family Verrucomicrobiaceae	Vascular dementia (undefined)	rs78566090	A	G	0.004	125740204	0.865	0.030	14306	A	G	0.346	8	124727963	9.44735E-07	0.071	361227	7049.571
family Victivallaceae	Vascular dementia (undefined)	rs12224047	T	C	-0.009	36820790	0.836	0.033	14306	T	C	-0.263	11	36799240	4.56278E-06	0.057	361227	8033.487
family Victivallaceae	Vascular dementia (undefined)	rs12449066	G	A	0.014	79177293	0.688	0.039	14306	G	A	-0.262	16	79143396	2.01674E-06	0.055	361227	8803.514
family Victivallaceae	Vascular dementia (undefined)	rs429358	C	T	-0.015	45411941	0.542	0.033	14306	C	T	0.695	19	44908684	9.26616E-39	0.053	361227	58999.679
family Victivallaceae	Vascular dementia (undefined)	rs71298638	A	G	0.021	63232261	0.643	0.049	14306	A	G	0.379	3	63246585	1.1165E-06	0.078	361227	6515.599
genus Actinomyces	Vascular dementia (multiple infarctions)	rs11081443	C	T	0.002	8944208	0.894	0.020	14306	C	T	0.464	18	8944210	5.71874E-06	0.102	360612	19066.663
genus Actinomyces	Vascular dementia (multiple infarctions)	rs429358	C	T	0.030	45411941	0.176	0.023	14306	C	T	0.660	19	44908684	1.33506E-17	0.077	360612	52325.340
genus Actinomyces	Vascular dementia (multiple infarctions)	rs4725579	C	A	0.009	139468213	0.692	0.021	14306	C	A	-0.379	7	139768414	3.58039E-06	0.082	360612	18391.668
genus Actinomyces	Vascular dementia (multiple infarctions)	rs72822148	T	C	0.010	9742028	0.550	0.017	14306	T	C	-0.322	17	9838711	3.18831E-06	0.069	360612	17983.471
genus Actinomyces	Vascular dementia (multiple infarctions)	rs73053797	T	C	-0.015	29909039	0.539	0.024	14306	T	C	0.338	3	29867548	6.00772E-06	0.075	360612	13680.576
genus Actinomyces	Vascular dementia (multiple infarctions)	rs9861644	A	G	0.010	88637331	0.571	0.019	14306	A	G	0.323	3	88588181	4.84942E-06	0.071	360612	17137.658
genus Adlercreutzia	Vascular dementia (multiple infarctions)	rs11081443	C	T	0.014	8944208	0.422	0.019	14306	C	T	0.464	18	8944210	5.71874E-06	0.102	360612	19066.663
genus Adlercreutzia	Vascular dementia (multiple infarctions)	rs1454336	A	G	0.015	91873093	0.448	0.021	14306	A	G	-0.369	4	90951942	4.48415E-06	0.080	360612	13616.170
genus Adlercreutzia	Vascular dementia (multiple infarctions)	rs34288661	C	T	0.009	20948329	0.715	0.023	14306	C	T	0.460	8	21090818	7.22903E-06	0.103	360612	11969.376
genus Adlercreutzia	Vascular dementia (multiple infarctions)	rs429358	C	T	0.007	45411941	0.796	0.022	14306	C	T	0.660	19	44908684	1.33506E-17	0.077	360612	52325.340
genus Adlercreutzia	Vascular dementia (multiple infarctions)	rs4716814	T	C	-0.003	157723046	0.843	0.016	14306	T	C	-0.319	7	157930354	3.75656E-07	0.063	360612	19180.021
genus Adlercreutzia	Vascular dementia (multiple infarctions)	rs4725579	C	A	0.005	139468213	0.804	0.020	14306	C	A	-0.379	7	139768414	3.58039E-06	0.082	360612	18391.668
genus Adlercreutzia	Vascular dementia (multiple infarctions)	rs73053797	T	C	0.013	29909039	0.498	0.023	14306	T	C	0.338	3	29867548	6.00772E-06	0.075	360612	13680.576
genus Adlercreutzia	Vascular dementia (multiple infarctions)	rs9861644	A	G	-0.004	88637331	0.780	0.018	14306	A	G	0.323	3	88588181	4.84942E-06	0.071	360612	17137.658
genus Akkermansia	Vascular dementia (multiple infarctions)	rs1454336	A	G	-0.003	91873093	0.948	0.017	14306	A	G	-0.369	4	90951942	4.48415E-06	0.080	360612	13616.170
genus Akkermansia	Vascular dementia (multiple infarctions)	rs34288661	C	T	0.007	20948329	0.758	0.019	14306	C	T	0.460	8	21090818	7.22903E-06	0.103	360612	11969.376
genus Akkermansia	Vascular dementia (multiple infarctions)	rs429358	C	T	0.018	45411941	0.337	0.019	14306	C	T	0.660	19	44908684	1.33506E-17	0.077	360612	52325.340
genus Akkermansia	Vascular dementia (multiple infarctions)	rs4725579	C	A	0.001	139468213	0.942	0.016	14306	C	A	-0.379	7	139768414	3.58039E-06	0.082	360612	18391.668
genus Akkermansia	Vascular dementia (multiple infarctions)	rs72822148	T	C	-0.008	9742028	0.624	0.014	14306	T	C	-0.322	17	9838711	3.18831E-06	0.069	360612	17983.471
genus Akkermansia	Vascular dementia (multiple infarctions)	rs73053797	T	C	-0.005	29909039	0.786	0.019	14306	T	C	0.338	3	29867548	6.00772E-06	0.075	360612	13680.576
genus Akkermansia	Vascular dementia (multiple infarctions)	rs9861644	A	G	0.003	88637331	0.849	0.015	14306	A	G	0.323	3	88588181	4.84942E-06	0.071	360612	17137.658
genus Alistipes	Vascular dementia (multiple infarctions)	rs11081443	C	T	0.011	8944208	0.389	0.013	14306	C	T	0.464	18	8944210	5.71874E-06	0.102	360612	19066.663
genus Alistipes	Vascular dementia (multiple infarctions)	rs1454336	A	G	-0.011	91873093	0.445	0.014	14306	A	G	-0.369	4	90951942	4.48415E-06	0.080	360612	13616.170
genus Alistipes	Vascular dementia (multiple infarctions)	rs34288661	C	T	-0.002	20948329	0.905	0.016	14306	C	T	0.460	8	21090818	7.22903E-06	0.103	360612	11969.376
genus Alistipes	Vascular dementia (multiple infarctions)	rs429358	C	T	-0.026	45411941	0.088	0.015	14306	C	T	0.660	19	44908684	1.33506E-17	0.077	360612	52325.340
genus Alistipes	Vascular dementia (multiple infarctions)	rs4716814	T	C	0.009	157723046	0.405	0.011	14306	T	C	-0.319	7	157930354	3.75656E-07	0.063	360612	19180.021
genus Alistipes	Vascular dementia (multiple infarctions)	rs4725579	C	A	-0.008	139468213	0.498	0.013	14306	C	A	-0.379	7	139768414	3.58039E-06	0.082	360612	18391.668
genus Alistipes	Vascular dementia (multiple infarctions)	rs9861644	A	G	0.004	88637331	0.725	0.012	14306	A	G	0.323	3	88588181	4.84942E-06	0.071	360612	17137.658
genus Allisonella	Vascular dementia (multiple infarctions)	rs429358	C	T	-0.033	45411941	0.350	0.036	14306	C	T	0.660	19	44908684	1.33506E-17	0.077	360612	52325.340
genus Allisonella	Vascular dementia (multiple infarctions)	rs73053797	T	C	0.005	29909039	0.894	0.035	14306	T	C	0.338	3	29867548	6.00772E-06	0.075	360612	13680.576
genus Allisonella	Vascular dementia (multiple infarctions)	rs9861644	A	G	-0.011	88637331	0.642	0.028	14306	A	G	0.323	3	88588181	4.84942E-06	0.071	360612	17137.658
genus Alloprevotella	Vascular dementia (multiple infarctions)	rs1454336	A	G	-0.014	91873093	0.663	0.033	14306	A	G	-0.369	4	90951942	4.48415E-06	0.080	360612	13616.170
genus Alloprevotella	Vascular dementia (multiple infarctions)	rs4716814	T	C	-0.027	157723046	0.308	0.026	14306	T	C	-0.319	7	157930354	3.75656E-07	0.063	360612	19180.021
genus Alloprevotella	Vascular dementia (multiple infarctions)	rs4725579	C	A	0.005	139468213	0.968	0.031	14306	C	A	-0.379	7	139768414	3.58039E-06	0.082	360612	18391.668
genus Anaerofilum	Vascular dementia (multiple infarctions)	rs11081443	C	T	-0.018	8944208	0.490	0.024	14306	C	T	0.464	18	8944210	5.71874E-06	0.102	360612	19066.663
genus Anaerofilum	Vascular dementia (multiple infarctions)	rs1454336	A	G	0.014	91873093	0.566	0.026	14306	A	G	-0.369	4	90951942	4.48415E-06	0.080	360612	13616.170
genus Anaerofilum	Vascular dementia (multiple infarctions)	rs34288661	C	T	0.007	20948329	0.785	0.029	14306	C	T	0.460	8	21090818	7.22903E-06	0.103	360612	11969.376
genus Anaerofilum	Vascular dementia (multiple infarctions)	rs429358	C	T	-0.013	45411941	0.660	0.028	14306	C	T	0.660	19	44908684	1.33506E-17	0.077	360612	52325.340
genus Anaerofilum	Vascular dementia (multiple infarctions)	rs4716814	T	C	0.018	157723046	0.363	0.020	14306	T	C	-0.319	7	157930354	3.75656E-07	0.063	360612	19180.021
genus Anaerofilum	Vascular dementia (multiple infarctions)	rs73053797	T	C	-0.007	29909039	0.970	0.029	14306	T	C	0.338	3	29867548	6.00772E-06	0.075	360612	13680.576
genus Anaerofilum	Vascular dementia (multiple infarctions)	rs9861644	A	G	0.000	88637331	0.989	0.023	14306	A	G	0.323	3	88588181	4.84942E-06	0.071	360612	17137.658

genus Anaerostipes	Vascular dementia (multiple infarctions)	rs1454336	A	G	-0.001	91873093	0.938	0.014	14306	A	G	-0.369	4	90951942	4.48415E-06	0.080	360612	13616.170
genus Anaerostipes	Vascular dementia (multiple infarctions)	rs34288661	C	T	0.000	20948329	0.950	0.016	14306	C	T	0.460	8	21090818	7.22903E-06	0.103	360612	11969.376
genus Anaerostipes	Vascular dementia (multiple infarctions)	rs4725579	C	A	-0.009	139468213	0.438	0.013	14306	C	A	-0.379	7	139768414	3.58039E-06	0.082	360612	18391.668
genus Anaerostipes	Vascular dementia (multiple infarctions)	rs72822148	T	C	-0.011	9742028	0.297	0.012	14306	T	C	-0.322	17	9838711	3.18831E-06	0.069	360612	17983.471
genus Anaerostipes	Vascular dementia (multiple infarctions)	rs9861644	A	G	0.011	88637331	0.380	0.012	14306	A	G	0.323	3	88588181	4.84942E-06	0.071	360612	17137.658
genus Anaerotruncus	Vascular dementia (multiple infarctions)	rs429358	C	T	-0.019	45411941	0.201	0.016	14306	C	T	0.660	19	44908684	1.33506E-17	0.077	360612	52325.340
genus Anaerotruncus	Vascular dementia (multiple infarctions)	rs73053797	T	C	0.001	29909039	0.926	0.016	14306	T	C	0.338	3	29867548	6.00772E-06	0.075	360612	13680.576
genus Bacteroides	Vascular dementia (multiple infarctions)	rs11081443	C	T	0.009	8944208	0.425	0.013	14306	C	T	0.464	18	8944210	5.71874E-06	0.102	360612	19066.663
genus Bacteroides	Vascular dementia (multiple infarctions)	rs1454336	A	G	-0.011	91873093	0.420	0.014	14306	A	G	-0.369	4	90951942	4.48415E-06	0.080	360612	13616.170
genus Bacteroides	Vascular dementia (multiple infarctions)	rs34288661	C	T	-0.002	20948329	0.911	0.016	14306	C	T	0.460	8	21090818	7.22903E-06	0.103	360612	11969.376
genus Bacteroides	Vascular dementia (multiple infarctions)	rs4716814	T	C	-0.010	157723046	0.356	0.011	14306	T	C	-0.319	7	157930354	3.75656E-07	0.063	360612	19180.021
genus Bacteroides	Vascular dementia (multiple infarctions)	rs4725579	C	A	0.007	139468213	0.569	0.013	14306	C	A	-0.379	7	139768414	3.58039E-06	0.082	360612	18391.668
genus Bacteroides	Vascular dementia (multiple infarctions)	rs72822148	T	C	-0.008	9742028	0.651	0.011	14306	T	C	-0.322	17	9838711	3.18831E-06	0.069	360612	17983.471
genus Bacteroides	Vascular dementia (multiple infarctions)	rs73053797	T	C	-0.003	29909039	0.853	0.015	14306	T	C	0.338	3	29867548	6.00772E-06	0.075	360612	13680.576
genus Barnesiella	Vascular dementia (multiple infarctions)	rs429358	C	T	-0.005	45411941	0.787	0.017	14306	C	T	0.660	19	44908684	1.33506E-17	0.077	360612	52325.340
genus Barnesiella	Vascular dementia (multiple infarctions)	rs4716814	T	C	0.010	157723046	0.428	0.012	14306	T	C	-0.319	7	157930354	3.75656E-07	0.063	360612	19180.021
genus Barnesiella	Vascular dementia (multiple infarctions)	rs4725579	C	A	0.001	139468213	0.915	0.015	14306	C	A	-0.379	7	139768414	3.58039E-06	0.082	360612	18391.668
genus Barnesiella	Vascular dementia (multiple infarctions)	rs72822148	T	C	-0.001	9742028	0.905	0.013	14306	T	C	-0.322	17	9838711	3.18831E-06	0.069	360612	17983.471
genus Barnesiella	Vascular dementia (multiple infarctions)	rs73053797	T	C	-0.010	29909039	0.670	0.017	14306	T	C	0.338	3	29867548	6.00772E-06	0.075	360612	13680.576
genus Barnesiella	Vascular dementia (multiple infarctions)	rs9861644	A	G	0.012	88637331	0.362	0.014	14306	A	G	0.323	3	88588181	4.84942E-06	0.071	360612	17137.658
genus Bifidobacterium	Vascular dementia (multiple infarctions)	rs1454336	A	G	0.001	91873093	0.956	0.015	14306	A	G	-0.369	4	90951942	4.48415E-06	0.080	360612	13616.170
genus Bifidobacterium	Vascular dementia (multiple infarctions)	rs34288661	C	T	-0.005	20948329	0.852	0.017	14306	C	T	0.460	8	21090818	7.22903E-06	0.103	360612	11969.376
genus Bifidobacterium	Vascular dementia (multiple infarctions)	rs429358	C	T	0.019	45411941	0.204	0.017	14306	C	T	0.660	19	44908684	1.33506E-17	0.077	360612	52325.340
genus Bifidobacterium	Vascular dementia (multiple infarctions)	rs4716814	T	C	0.011	157723046	0.330	0.012	14306	T	C	-0.319	7	157930354	3.75656E-07	0.063	360612	19180.021
genus Bifidobacterium	Vascular dementia (multiple infarctions)	rs4725579	C	A	-0.005	139468213	0.655	0.014	14306	C	A	-0.379	7	139768414	3.58039E-06	0.082	360612	18391.668
genus Bifidobacterium	Vascular dementia (multiple infarctions)	rs73053797	T	C	0.000	29909039	0.896	0.016	14306	T	C	0.338	3	29867548	6.00772E-06	0.075	360612	13680.576
genus Bifidobacterium	Vascular dementia (multiple infarctions)	rs9861644	A	G	0.010	88637331	0.458	0.013	14306	A	G	0.323	3	88588181	4.84942E-06	0.071	360612	17137.658
genus Bilophila	Vascular dementia (multiple infarctions)	rs11081443	C	T	-0.012	8944208	0.403	0.015	14306	C	T	0.464	18	8944210	5.71874E-06	0.102	360612	19066.663
genus Bilophila	Vascular dementia (multiple infarctions)	rs1454336	A	G	0.006	91873093	0.790	0.016	14306	A	G	-0.369	4	90951942	4.48415E-06	0.080	360612	13616.170
genus Bilophila	Vascular dementia (multiple infarctions)	rs34288661	C	T	0.007	20948329	0.710	0.019	14306	C	T	0.460	8	21090818	7.22903E-06	0.103	360612	11969.376
genus Bilophila	Vascular dementia (multiple infarctions)	rs429358	C	T	0.007	45411941	0.656	0.018	14306	C	T	0.660	19	44908684	1.33506E-17	0.077	360612	52325.340
genus Bilophila	Vascular dementia (multiple infarctions)	rs72822148	T	C	-0.004	9742028	0.858	0.013	14306	T	C	-0.322	17	9838711	3.18831E-06	0.069	360612	17983.471
genus Bilophila	Vascular dementia (multiple infarctions)	rs9861644	A	G	-0.004	88637331	0.821	0.014	14306	A	G	0.323	3	88588181	4.84942E-06	0.071	360612	17137.658
genus Butyricicoccus	Vascular dementia (multiple infarctions)	rs11081443	C	T	0.002	8944208	0.719	0.013	14306	C	T	0.464	18	8944210	5.71874E-06	0.102	360612	19066.663
genus Butyricicoccus	Vascular dementia (multiple infarctions)	rs34288661	C	T	0.002	20948329	0.811	0.016	14306	C	T	0.460	8	21090818	7.22903E-06	0.103	360612	11969.376
genus Butyricicoccus	Vascular dementia (multiple infarctions)	rs429358	C	T	-0.018	45411941	0.288	0.015	14306	C	T	0.660	19	44908684	1.33506E-17	0.077	360612	52325.340
genus Butyricicoccus	Vascular dementia (multiple infarctions)	rs4716814	T	C	-0.005	157723046	0.652	0.011	14306	T	C	-0.319	7	157930354	3.75656E-07	0.063	360612	19180.021
genus Butyricicoccus	Vascular dementia (multiple infarctions)	rs4725579	C	A	-0.002	139468213	0.849	0.013	14306	C	A	-0.379	7	139768414	3.58039E-06	0.082	360612	18391.668
genus Butyricicoccus	Vascular dementia (multiple infarctions)	rs73053797	T	C	0.001	29909039	0.954	0.015	14306	T	C	0.338	3	29867548	6.00772E-06	0.075	360612	13680.576
genus Butyricicoccus	Vascular dementia (multiple infarctions)	rs9861644	A	G	-0.010	88637331	0.446	0.012	14306	A	G	0.323	3	88588181	4.84942E-06	0.071	360612	17137.658
genus Butyricimonas	Vascular dementia (multiple infarctions)	rs11081443	C	T	0.001	8944208	0.978	0.017	14306	C	T	0.464	18	8944210	5.71874E-06	0.102	360612	19066.663
genus Butyricimonas	Vascular dementia (multiple infarctions)	rs4716814	T	C	-0.011	157723046	0.429	0.014	14306	T	C	-0.319	7	157930354	3.75656E-07	0.063	360612	19180.021
genus Butyricimonas	Vascular dementia (multiple infarctions)	rs4725579	C	A	-0.010	139468213	0.537	0.017	14306	C	A	-0.379	7	139768414	3.58039E-06	0.082	360612	18391.668
genus Butyricimonas	Vascular dementia (multiple infarctions)	rs72822148	T	C	0.001	9742028	0.894	0.015	14306	T	C	-0.322	17	9838711	3.18831E-06	0.069	360612	17983.471
genus Butyricimonas	Vascular dementia (multiple infarctions)	rs73053797	T	C	-0.017	29909039	0.427	0.020	14306	T	C	0.338	3	29867548	6.00772E-06	0.075	360612	13680.576
genus Butyricimonas	Vascular dementia (multiple infarctions)	rs9861644	A	G	0.006	88637331	0.699	0.016	14306	A	G	0.323	3	88588181	4.84942E-06	0.071	360612	17137.658
genus Butyrivibrio	Vascular dementia (multiple infarctions)	rs11081443	C	T	-0.016	8944208	0.632	0.029	14306	C	T	0.464	18	8944210	5.71874E-06	0.102	360612	19066.663
genus Butyrivibrio	Vascular dementia (multiple infarctions)	rs1454336	A	G	-0.008	91873093	0.811	0.031	14306	A	G	-0.369	4	90951942	4.48415E-06	0.080	360612	13616.170
genus Butyrivibrio	Vascular dementia (multiple infarctions)	rs429358	C	T	-0.016	45411941	0.585	0.034	14306	C	T	0.660	19	44908684	1.33506E-17	0.077	360612	52325.340
genus Butyrivibrio	Vascular dementia (multiple infarctions)	rs4716814	T	C	0.003	157723046	0.918	0.024	14306	T	C	-0.319	7	157930354	3.75656E-07	0.063	360612	19180.021
genus Butyrivibrio	Vascular dementia (multiple infarctions)	rs4725579	C	A	-0.026	139468213	0.360	0.030	14306	C	A	-0.379	7	139768414	3.58039E-06	0.082	360612	18391.668
genus Butyrivibrio	Vascular dementia (multiple infarctions)	rs73053797	T	C	-0.024	29909039	0.495	0.035	14306	T	C	0.338	3	29867548	6.00772E-06	0.075	360612	13680.576
genus Candidatus Soleaferrea	Vascular dementia (multiple infarctions)	rs11081443	C	T	-0.020	8944208	0.349	0.022	14306	C	T	0.464	18	8944210	5.71874E-06	0.102	360612	19066.663
genus Candidatus Soleaferrea	Vascular dementia (multiple infarctions)	rs34288661	C	T	0.014	20948329	0.608	0.026	14306	C	T	0.460	8	21090818	7.22903E-06	0.103	360612	11969.376
genus Candidatus Soleaferrea	Vascular dementia (multiple infarctions)	rs429358	C	T	0.020	45411941	0.391	0.026	14306	C	T	0.660	19	44908684	1.33506E-17	0.077	360612	52325.340
genus Candidatus Soleaferrea	Vascular dementia (multiple infarctions)	rs4725579	C	A	-0.017	139468213	0.417	0.023	14306	C	A	-0.379	7	139768414	3.58039E-06	0.082	360612	18391.668
genus Catenibacterium	Vascular dementia (multiple infarctions)	rs1454336	A	G	0.027	91873093	0.346	0.032	14306	A	G	-0.369	4	90951942	4.48415E-06	0.080	360612	13616.170
genus Catenibacterium	Vascular dementia (multiple infarctions)	rs429358	C	T	0.030	45411941	0.433	0.036	14306	C	T	0.660	19	44908684	1.33506E-17	0.077	360612	52325.340
genus Catenibacterium	Vascular dementia (multiple infarctions)	rs4716814	T	C	0.019	157723046	0.442	0.025	14306	T	C	-0.319	7	157930354	3.75656E-07	0.063	360612	19180.021
genus Catenibacterium	Vascular dementia (multiple infarctions)	rs72822148	T	C	-0.007	9742028	0.831	0.027	14306	T	C	-0.322	17	9838711	3.18831E-06	0.069	360612	17983.471
genus Catenibacterium	Vascular dementia (multiple infarctions)	rs73053797	T	C	0.019	29909039	0.595	0.034	14306	T	C	0.338	3	29867548	6.00772E-06	0.075	360612	13680.576
genus Catenibacterium	Vascular dementia (multiple infarctions)	rs9861644	A	G	0.025	88637331	0.396	0.028	14306	A	G	0.323	3	88588181	4.84942E-06	0.071	360612	17137.658
genus Christensenellaceae R 7group	Vascular dementia (multiple infarctions)	rs1454336	A	G	0.007	91873093	0.603	0.015	14306	A	G	-0.369	4	90951942	4.48415E-06	0.080	360612	13616.170
genus Christensenellaceae R 7group	Vascular dementia (multiple infarctions)	rs34288661	C	T	-0.012	20948329	0.475	0.016	14306	C	T	0.460	8	21090818	7.22903E-06	0.103	360612	11969.376
genus Christensenellaceae R 7group	Vascular dementia (multiple infarctions)	rs429358	C	T	0.011	45411941	0.455	0.016	14306	C	T	0.660	19	44908684	1.33506E-17	0.077	360612	52325.340
genus Christensenellaceae R 7group	Vascular dementia (multiple infarctions)	rs4716814	T	C	0.008	157723046	0.483	0.011	14306	T	C	-0.319	7	157930354	3.75656E-07	0.063	360612	19180.021
genus Christensenellaceae R 7group	Vascular dementia (multiple infarctions)	rs4725579	C	A	0.001	139468213	0.988	0.014	14306	C	A	-0.379	7	139768414	3.58039E-06	0.082	360612	18391.668
genus Christensenellaceae R 7group	Vascular dementia (multiple infarctions)	rs73053797	T	C	0.011	29909039	0.409	0.016	14306	T	C	0.338	3	29867548	6.00772E-06	0.075	360612	13680.576
genus Clostridium innocuum group	Vascular dementia (multiple infarctions)	rs11081443	C	T	0.004	8944208	0.995	0.027	14306	C	T	0.464	18	8944210	5.71874E-06	0.102	360612	19066.663
genus Clostridium innocuum group	Vascular dementia (multiple infarctions)	rs429358	C	T	-0.009	45411941	0.813	0.031	14306	C	T	0.660	19	44908684	1.33506E-17	0.077	360612	52325.340
genus Clostridium innocuum group	Vascular dementia (multiple infarctions)	rs4716814	T	C	-0.015	157723046	0.502	0.022	14306	T	C	-0.319	7	157930354	3.75656E-07	0.063	360612	19180.021

genus Clostridium innocuum group	Vascular dementia (multiple infarctions)	rs72822148	T	C	0.001	9742028	0.916	0.024	14306	T	C	-0.322	17	9838711	3.18831E-06	0.069	360612	17983.471
genus Clostridium innocuum group	Vascular dementia (multiple infarctions)	rs73053797	T	C	-0.015	29909039	0.674	0.031	14306	T	C	0.338	3	29867548	6.00772E-06	0.075	360612	13680.576
genus Clostridium innocuum group	Vascular dementia (multiple infarctions)	rs9861644	A	G	0.014	88637331	0.583	0.025	14306	A	G	0.323	3	88588181	4.84942E-06	0.071	360612	17137.658
genus Clostridium sensustricto1	Vascular dementia (multiple infarctions)	rs11081443	C	T	-0.002	8944208	0.890	0.015	14306	C	T	0.464	18	8944210	5.71874E-06	0.102	360612	19066.663
genus Clostridium sensustricto1	Vascular dementia (multiple infarctions)	rs1454336	A	G	0.006	91873093	0.707	0.016	14306	A	G	-0.369	4	90951942	4.48415E-06	0.080	360612	13616.170
genus Clostridium sensustricto1	Vascular dementia (multiple infarctions)	rs429358	C	T	0.011	45411941	0.544	0.017	14306	C	T	0.660	19	44908684	1.33506E-17	0.077	360612	52325.340
genus Clostridium sensustricto1	Vascular dementia (multiple infarctions)	rs4716814	T	C	-0.005	157723046	0.656	0.012	14306	T	C	-0.319	7	157930354	3.75656E-07	0.063	360612	19180.021
genus Clostridium sensustricto1	Vascular dementia (multiple infarctions)	rs72822148	T	C	0.008	9742028	0.609	0.013	14306	T	C	-0.322	17	9838711	3.18831E-06	0.069	360612	17983.471
genus Clostridium sensustricto1	Vascular dementia (multiple infarctions)	rs73053797	T	C	-0.014	29909039	0.372	0.017	14306	T	C	0.338	3	29867548	6.00772E-06	0.075	360612	13680.576
genus Clostridium sensustricto1	Vascular dementia (multiple infarctions)	rs9861644	A	G	-0.009	88637331	0.493	0.014	14306	A	G	0.323	3	88588181	4.84942E-06	0.071	360612	17137.658
genus Collinsella	Vascular dementia (multiple infarctions)	rs11081443	C	T	-0.013	8944208	0.398	0.015	14306	C	T	0.464	18	8944210	5.71874E-06	0.102	360612	19066.663
genus Collinsella	Vascular dementia (multiple infarctions)	rs34288661	C	T	-0.008	20948329	0.677	0.018	14306	C	T	0.460	8	21090818	7.22903E-06	0.103	360612	11969.376
genus Collinsella	Vascular dementia (multiple infarctions)	rs429358	C	T	0.024	45411941	0.129	0.017	14306	C	T	0.660	19	44908684	1.33506E-17	0.077	360612	52325.340
genus Collinsella	Vascular dementia (multiple infarctions)	rs4716814	T	C	-0.001	157723046	0.919	0.012	14306	T	C	-0.319	7	157930354	3.75656E-07	0.063	360612	19180.021
genus Collinsella	Vascular dementia (multiple infarctions)	rs4725579	C	A	0.002	139468213	0.913	0.015	14306	C	A	-0.379	7	139768414	3.58039E-06	0.082	360612	18391.668
genus Collinsella	Vascular dementia (multiple infarctions)	rs73053797	T	C	-0.011	29909039	0.409	0.017	14306	T	C	0.338	3	29867548	6.00772E-06	0.075	360612	13680.576
genus Collinsella	Vascular dementia (multiple infarctions)	rs9861644	A	G	-0.010	88637331	0.469	0.014	14306	A	G	0.323	3	88588181	4.84942E-06	0.071	360612	17137.658
genus Coprobacter	Vascular dementia (multiple infarctions)	rs11081443	C	T	0.001	8944208	0.972	0.021	14306	C	T	0.464	18	8944210	5.71874E-06	0.102	360612	19066.663
genus Coprobacter	Vascular dementia (multiple infarctions)	rs1454336	A	G	0.014	91873093	0.474	0.022	14306	A	G	-0.369	4	90951942	4.48415E-06	0.080	360612	13616.170
genus Coprobacter	Vascular dementia (multiple infarctions)	rs34288661	C	T	0.002	20948329	0.869	0.025	14306	C	T	0.460	8	21090818	7.22903E-06	0.103	360612	11969.376
genus Coprobacter	Vascular dementia (multiple infarctions)	rs429358	C	T	-0.001	45411941	0.930	0.025	14306	C	T	0.660	19	44908684	1.33506E-17	0.077	360612	52325.340
genus Coprobacter	Vascular dementia (multiple infarctions)	rs4716814	T	C	0.006	157723046	0.752	0.017	14306	T	C	-0.319	7	157930354	3.75656E-07	0.063	360612	19180.021
genus Coprobacter	Vascular dementia (multiple infarctions)	rs4725579	C	A	0.016	139468213	0.469	0.021	14306	C	A	-0.379	7	139768414	3.58039E-06	0.082	360612	18391.668
genus Coprobacter	Vascular dementia (multiple infarctions)	rs9861644	A	G	-0.006	88637331	0.742	0.020	14306	A	G	0.323	3	88588181	4.84942E-06	0.071	360612	17137.658
genus Coprococcus1	Vascular dementia (multiple infarctions)	rs11081443	C	T	-0.001	8944208	0.988	0.014	14306	C	T	0.464	18	8944210	5.71874E-06	0.102	360612	19066.663
genus Coprococcus1	Vascular dementia (multiple infarctions)	rs34288661	C	T	-0.007	20948329	0.703	0.016	14306	C	T	0.460	8	21090818	7.22903E-06	0.103	360612	11969.376
genus Coprococcus1	Vascular dementia (multiple infarctions)	rs429358	C	T	0.014	45411941	0.326	0.016	14306	C	T	0.660	19	44908684	1.33506E-17	0.077	360612	52325.340
genus Coprococcus1	Vascular dementia (multiple infarctions)	rs72822148	T	C	-0.008	9742028	0.437	0.012	14306	T	C	-0.322	17	9838711	3.18831E-06	0.069	360612	17983.471
genus Coprococcus2	Vascular dementia (multiple infarctions)	rs1454336	A	G	-0.003	91873093	0.837	0.017	14306	A	G	-0.369	4	90951942	4.48415E-06	0.080	360612	13616.170
genus Coprococcus2	Vascular dementia (multiple infarctions)	rs429358	C	T	0.002	45411941	0.982	0.019	14306	C	T	0.660	19	44908684	1.33506E-17	0.077	360612	52325.340
genus Coprococcus2	Vascular dementia (multiple infarctions)	rs4716814	T	C	0.005	157723046	0.715	0.013	14306	T	C	-0.319	7	157930354	3.75656E-07	0.063	360612	19180.021
genus Coprococcus2	Vascular dementia (multiple infarctions)	rs4725579	C	A	-0.002	139468213	0.851	0.016	14306	C	A	-0.379	7	139768414	3.58039E-06	0.082	360612	18391.668
genus Coprococcus2	Vascular dementia (multiple infarctions)	rs72822148	T	C	0.001	9742028	0.942	0.014	14306	T	C	-0.322	17	9838711	3.18831E-06	0.069	360612	17983.471
genus Coprococcus2	Vascular dementia (multiple infarctions)	rs9861644	A	G	0.001	88637331	0.893	0.015	14306	A	G	0.323	3	88588181	4.84942E-06	0.071	360612	17137.658
genus Coprococcus3	Vascular dementia (multiple infarctions)	rs11081443	C	T	-0.005	8944208	0.733	0.014	14306	C	T	0.464	18	8944210	5.71874E-06	0.102	360612	19066.663
genus Coprococcus3	Vascular dementia (multiple infarctions)	rs34288661	C	T	0.006	20948329	0.757	0.017	14306	C	T	0.460	8	21090818	7.22903E-06	0.103	360612	11969.376
genus Coprococcus3	Vascular dementia (multiple infarctions)	rs4716814	T	C	0.011	157723046	0.323	0.011	14306	T	C	-0.319	7	157930354	3.75656E-07	0.063	360612	19180.021
genus Coprococcus3	Vascular dementia (multiple infarctions)	rs72822148	T	C	-0.002	9742028	0.766	0.012	14306	T	C	-0.322	17	9838711	3.18831E-06	0.069	360612	17983.471
genus Coprococcus3	Vascular dementia (multiple infarctions)	rs73053797	T	C	-0.006	29909039	0.674	0.016	14306	T	C	0.338	3	29867548	6.00772E-06	0.075	360612	13680.576
genus Coprococcus3	Vascular dementia (multiple infarctions)	rs9861644	A	G	-0.003	88637331	0.850	0.013	14306	A	G	0.323	3	88588181	4.84942E-06	0.071	360612	17137.658
genus Defluviitaleaceae UCG011	Vascular dementia (multiple infarctions)	rs11081443	C	T	0.006	8944208	0.671	0.019	14306	C	T	0.464	18	8944210	5.71874E-06	0.102	360612	19066.663
genus Defluviitaleaceae UCG011	Vascular dementia (multiple infarctions)	rs34288661	C	T	0.004	20948329	0.861	0.023	14306	C	T	0.460	8	21090818	7.22903E-06	0.103	360612	11969.376
genus Defluviitaleaceae UCG011	Vascular dementia (multiple infarctions)	rs429358	C	T	-0.003	45411941	0.819	0.022	14306	C	T	0.660	19	44908684	1.33506E-17	0.077	360612	52325.340
genus Defluviitaleaceae UCG011	Vascular dementia (multiple infarctions)	rs4716814	T	C	-0.012	157723046	0.455	0.016	14306	T	C	-0.319	7	157930354	3.75656E-07	0.063	360612	19180.021
genus Defluviitaleaceae UCG011	Vascular dementia (multiple infarctions)	rs72822148	T	C	-0.014	9742028	0.481	0.017	14306	T	C	-0.322	17	9838711	3.18831E-06	0.069	360612	17983.471
genus Defluviitaleaceae UCG011	Vascular dementia (multiple infarctions)	rs73053797	T	C	-0.014	29909039	0.475	0.023	14306	T	C	0.338	3	29867548	6.00772E-06	0.075	360612	13680.576
genus Desulfovibrio	Vascular dementia (multiple infarctions)	rs1454336	A	G	-0.002	91873093	0.918	0.019	14306	A	G	-0.369	4	90951942	4.48415E-06	0.080	360612	13616.170
genus Desulfovibrio	Vascular dementia (multiple infarctions)	rs429358	C	T	0.006	45411941	0.782	0.021	14306	C	T	0.660	19	44908684	1.33506E-17	0.077	360612	52325.340
genus Desulfovibrio	Vascular dementia (multiple infarctions)	rs4716814	T	C	-0.006	157723046	0.696	0.015	14306	T	C	-0.319	7	157930354	3.75656E-07	0.063	360612	19180.021
genus Desulfovibrio	Vascular dementia (multiple infarctions)	rs72822148	T	C	0.006	9742028	0.707	0.016	14306	T	C	-0.322	17	9838711	3.18831E-06	0.069	360612	17983.471
genus Desulfovibrio	Vascular dementia (multiple infarctions)	rs73053797	T	C	0.006	29909039	0.814	0.021	14306	T	C	0.338	3	29867548	6.00772E-06	0.075	360612	13680.576
genus Desulfovibrio	Vascular dementia (multiple infarctions)	rs9861644	A	G	-0.004	88637331	0.813	0.017	14306	A	G	0.323	3	88588181	4.84942E-06	0.071	360612	17137.658
(genus Dialister	Vascular dementia (multiple infarctions)	rs11081443	C	T	0.000	8944208	0.926	0.016	14306	C	T	0.464	18	8944210	5.71874E-06	0.102	360612	19066.663
(genus Dialister	Vascular dementia (multiple infarctions)	rs1454336	A	G	0.013	91873093	0.439	0.017	14306	A	G	-0.369	4	90951942	4.48415E-06	0.080	360612	13616.170
(genus Dialister	Vascular dementia (multiple infarctions)	rs34288661	C	T	0.017	20948329	0.403	0.019	14306	C	T	0.460	8	21090818	7.22903E-06	0.103	360612	11969.376
(genus Dialister	Vascular dementia (multiple infarctions)	rs429358	C	T	-0.026	45411941	0.198	0.019	14306	C	T	0.660	19	44908684	1.33506E-17	0.077	360612	52325.340
(genus Dialister	Vascular dementia (multiple infarctions)	rs73053797	T	C	0.010	29909039	0.690	0.018	14306	T	C	0.338	3	29867548	6.00772E-06	0.075	360612	13680.576
(genus Dialister	Vascular dementia (multiple infarctions)	rs9861644	A	G	0.013	88637331	0.385	0.015	14306	A	G	0.323	3	88588181	4.84942E-06	0.071	360612	17137.658
genus Dorea	Vascular dementia (multiple infarctions)	rs11081443	C	T	0.001	8944208	0.765	0.013	14306	C	T	0.464	18	8944210	5.71874E-06	0.102	360612	19066.663
genus Dorea	Vascular dementia (multiple infarctions)	rs34288661	C	T	-0.002	20948329	0.899	0.016	14306	C	T	0.460	8	21090818	7.22903E-06	0.103	360612	11969.376
genus Dorea	Vascular dementia (multiple infarctions)	rs4716814	T	C	-0.007	157723046	0.525	0.011	14306	T	C	-0.319	7	157930354	3.75656E-07	0.063	360612	19180.021
genus Dorea	Vascular dementia (multiple infarctions)	rs4725579	C	A	0.006	139468213	0.611	0.013	14306	C	A	-0.379	7	139768414	3.58039E-06	0.082	360612	18391.668
genus Dorea	Vascular dementia (multiple infarctions)	rs72822148	T	C	-0.005	9742028	0.652	0.012	14306	T	C	-0.322	17	9838711	3.18831E-06	0.069	360612	17983.471
genus Dorea	Vascular dementia (multiple infarctions)	rs73053797	T	C	-0.002	29909039	0.890	0.015	14306	T	C	0.338	3	29867548	6.00772E-06	0.075	360612	13680.576
genus Dorea	Vascular dementia (multiple infarctions)	rs9861644	A	G	-0.004	88637331	0.751	0.012	14306	A	G	0.323	3	88588181	4.84942E-06	0.071	360612	17137.658
genus Eggerthella	Vascular dementia (multiple infarctions)	rs429358	C	T	0.017	45411941	0.611	0.029	14306	C	T	0.660	19	44908684	1.33506E-17	0.077	360612	52325.340
genus Eggerthella	Vascular dementia (multiple infarctions)	rs4716814	T	C	-0.015	157723046	0.449	0.020	14306	T	C	-0.319	7	157930354	3.75656E-07	0.063	360612	19180.021
genus Eggerthella	Vascular dementia (multiple infarctions)	rs4725579	C	A	-0.021	139468213	0.383	0.025	14306	C	A	-0.379	7	139768414	3.58039E-06	0.082	360612	18391.668
genus Eggerthella	Vascular dementia (multiple infarctions)	rs72822148	T	C	0.012	9742028	0.559	0.021	14306	T	C	-0.322	17	9838711	3.18831E-06	0.069	360612	17983.471
genus Eggerthella	Vascular dementia (multiple infarctions)	rs73053797	T	C	-0.005	29909039	0.772	0.028	14306	T	C	0.338	3	29867548	6.00772E-06	0.075	360612	13680.576
genus Eggerthella	Vascular dementia (multiple infarctions)	rs9861644	A	G	0.004	88637331	0.904	0.023	14306	A	G	0.323	3	88588181	4.84942E-06	0.071	360612	17137.658

genus Eisenbergiella	Vascular dementia (multiple infarctions)	rs11081443	C	T	-0.003	8944208	0.788	0.023	14306	C	T	0.464	18	8944210	5.71874E-06	0.102	360612	19066.663
genus Eisenbergiella	Vascular dementia (multiple infarctions)	rs1454336	A	G	0.010	91873093	0.693	0.025	14306	A	G	-0.369	4	90951942	4.48415E-06	0.080	360612	13616.170
genus Eisenbergiella	Vascular dementia (multiple infarctions)	rs34288661	C	T	0.020	20948329	0.500	0.028	14306	C	T	0.460	8	21090818	7.22903E-06	0.103	360612	11969.376
genus Eisenbergiella	Vascular dementia (multiple infarctions)	rs429358	C	T	-0.013	45411941	0.612	0.028	14306	C	T	0.660	19	44908684	1.33506E-17	0.077	360612	52325.340
genus Eisenbergiella	Vascular dementia (multiple infarctions)	rs4716814	T	C	0.005	157723046	0.803	0.019	14306	T	C	-0.319	7	157930354	3.75656E-07	0.063	360612	19180.021
genus Eisenbergiella	Vascular dementia (multiple infarctions)	rs4725579	C	A	-0.014	139468213	0.575	0.023	14306	C	A	-0.379	7	139768414	3.58039E-06	0.082	360612	18391.668
genus Eisenbergiella	Vascular dementia (multiple infarctions)	rs9861644	A	G	0.014	88637331	0.510	0.022	14306	A	G	0.323	3	88588181	4.84942E-06	0.071	360612	17137.658
genus Enterorhabdus	Vascular dementia (multiple infarctions)	rs11081443	C	T	0.006	8944208	0.807	0.020	14306	C	T	0.464	18	8944210	5.71874E-06	0.102	360612	19066.663
genus Enterorhabdus	Vascular dementia (multiple infarctions)	rs1454336	A	G	0.015	91873093	0.484	0.022	14306	A	G	-0.369	4	90951942	4.48415E-06	0.080	360612	13616.170
genus Enterorhabdus	Vascular dementia (multiple infarctions)	rs429358	C	T	0.009	45411941	0.754	0.023	14306	C	T	0.660	19	44908684	1.33506E-17	0.077	360612	52325.340
genus Enterorhabdus	Vascular dementia (multiple infarctions)	rs4716814	T	C	-0.014	157723046	0.411	0.017	14306	T	C	-0.319	7	157930354	3.75656E-07	0.063	360612	19180.021
genus Enterorhabdus	Vascular dementia (multiple infarctions)	rs4725579	C	A	0.010	139468213	0.649	0.021	14306	C	A	-0.379	7	139768414	3.58039E-06	0.082	360612	18391.668
genus Enterorhabdus	Vascular dementia (multiple infarctions)	rs72822148	T	C	-0.002	9742028	0.889	0.017	14306	T	C	-0.322	17	9838711	3.18831E-06	0.069	360612	17983.471
genus Enterorhabdus	Vascular dementia (multiple infarctions)	rs73053797	T	C	-0.020	29909039	0.328	0.024	14306	T	C	0.338	3	29867548	6.00772E-06	0.075	360612	13680.576
genus Enterorhabdus	Vascular dementia (multiple infarctions)	rs9861644	A	G	0.017	88637331	0.375	0.019	14306	A	G	0.323	3	88588181	4.84942E-06	0.071	360612	17137.658
genus Erysipelatoclostridium	Vascular dementia (multiple infarctions)	rs1454336	A	G	0.006	91873093	0.775	0.019	14306	A	G	-0.369	4	90951942	4.48415E-06	0.080	360612	13616.170
genus Erysipelatoclostridium	Vascular dementia (multiple infarctions)	rs34288661	C	T	0.015	20948329	0.456	0.021	14306	C	T	0.460	8	21090818	7.22903E-06	0.103	360612	11969.376
genus Erysipelatoclostridium	Vascular dementia (multiple infarctions)	rs429358	C	T	0.017	45411941	0.432	0.020	14306	C	T	0.660	19	44908684	1.33506E-17	0.077	360612	52325.340
genus Erysipelatoclostridium	Vascular dementia (multiple infarctions)	rs4716814	T	C	0.014	157723046	0.323	0.014	14306	T	C	-0.319	7	157930354	3.75656E-07	0.063	360612	19180.021
genus Erysipelatoclostridium	Vascular dementia (multiple infarctions)	rs4725579	C	A	-0.011	139468213	0.560	0.018	14306	C	A	-0.379	7	139768414	3.58039E-06	0.082	360612	18391.668
genus Erysipelatoclostridium	Vascular dementia (multiple infarctions)	rs72822148	T	C	-0.013	9742028	0.354	0.015	14306	T	C	-0.322	17	9838711	3.18831E-06	0.069	360612	17983.471
genus Erysipelatoclostridium	Vascular dementia (multiple infarctions)	rs9861644	A	G	-0.012	88637331	0.476	0.016	14306	A	G	0.323	3	88588181	4.84942E-06	0.071	360612	17137.658
genus Escherichia Shigella	Vascular dementia (multiple infarctions)	rs11081443	C	T	-0.014	8944208	0.330	0.016	14306	C	T	0.464	18	8944210	5.71874E-06	0.102	360612	19066.663
genus Escherichia Shigella	Vascular dementia (multiple infarctions)	rs1454336	A	G	0.013	91873093	0.420	0.017	14306	A	G	-0.369	4	90951942	4.48415E-06	0.080	360612	13616.170
genus Escherichia Shigella	Vascular dementia (multiple infarctions)	rs429358	C	T	0.007	45411941	0.718	0.019	14306	C	T	0.660	19	44908684	1.33506E-17	0.077	360612	52325.340
genus Escherichia Shigella	Vascular dementia (multiple infarctions)	rs4716814	T	C	0.011	157723046	0.384	0.013	14306	T	C	-0.319	7	157930354	3.75656E-07	0.063	360612	19180.021
genus Escherichia Shigella	Vascular dementia (multiple infarctions)	rs4725579	C	A	-0.001	139468213	0.927	0.016	14306	C	A	-0.379	7	139768414	3.58039E-06	0.082	360612	18391.668
genus Escherichia Shigella	Vascular dementia (multiple infarctions)	rs72822148	T	C	0.002	9742028	0.868	0.014	14306	T	C	-0.322	17	9838711	3.18831E-06	0.069	360612	17983.471
genus Escherichia Shigella	Vascular dementia (multiple infarctions)	rs73053797	T	C	0.005	29909039	0.792	0.018	14306	T	C	0.338	3	29867548	6.00772E-06	0.075	360612	13680.576
genus Eubacterium brachy group	Vascular dementia (multiple infarctions)	rs11081443	C	T	0.008	8944208	0.791	0.027	14306	C	T	0.464	18	8944210	5.71874E-06	0.102	360612	19066.663
genus Eubacterium brachy group	Vascular dementia (multiple infarctions)	rs1454336	A	G	0.005	91873093	0.898	0.029	14306	A	G	-0.369	4	90951942	4.48415E-06	0.080	360612	13616.170
genus Eubacterium brachy group	Vascular dementia (multiple infarctions)	rs34288661	C	T	0.000	20948329	0.956	0.032	14306	C	T	0.460	8	21090818	7.22903E-06	0.103	360612	11969.376
genus Eubacterium brachy group	Vascular dementia (multiple infarctions)	rs429358	C	T	-0.022	45411941	0.464	0.032	14306	C	T	0.660	19	44908684	1.33506E-17	0.077	360612	52325.340
genus Eubacterium brachy group	Vascular dementia (multiple infarctions)	rs4716814	T	C	0.005	157723046	0.815	0.023	14306	T	C	-0.319	7	157930354	3.75656E-07	0.063	360612	19180.021
genus Eubacterium brachy group	Vascular dementia (multiple infarctions)	rs73053797	T	C	-0.025	29909039	0.457	0.033	14306	T	C	0.338	3	29867548	6.00772E-06	0.075	360612	13680.576
genus Eubacterium coprostanoligenes group	Vascular dementia (multiple infarctions)	rs72822148	T	C	0.010	9742028	0.374	0.012	14306	T	C	-0.322	17	9838711	3.18831E-06	0.069	360612	17983.471
genus Eubacterium coprostanoligenes group	Vascular dementia (multiple infarctions)	rs73053797	T	C	-0.012	29909039	0.391	0.015	14306	T	C	0.338	3	29867548	6.00772E-06	0.075	360612	13680.576
genus Eubacterium eligens group	Vascular dementia (multiple infarctions)	rs34288661	C	T	0.006	20948329	0.680	0.017	14306	C	T	0.460	8	21090818	7.22903E-06	0.103	360612	11969.376
genus Eubacterium eligens group	Vascular dementia (multiple infarctions)	rs429358	C	T	-0.020	45411941	0.237	0.017	14306	C	T	0.660	19	44908684	1.33506E-17	0.077	360612	52325.340
genus Eubacterium eligens group	Vascular dementia (multiple infarctions)	rs4716814	T	C	-0.006	157723046	0.632	0.012	14306	T	C	-0.319	7	157930354	3.75656E-07	0.063	360612	19180.021
genus Eubacterium eligens group	Vascular dementia (multiple infarctions)	rs4725579	C	A	0.009	139468213	0.498	0.014	14306	C	A	-0.379	7	139768414	3.58039E-06	0.082	360612	18391.668
genus Eubacterium eligens group	Vascular dementia (multiple infarctions)	rs72822148	T	C	0.009	9742028	0.705	0.013	14306	T	C	-0.322	17	9838711	3.18831E-06	0.069	360612	17983.471
genus Eubacterium fissicatena group	Vascular dementia (multiple infarctions)	rs34288661	C	T	-0.027	20948329	0.378	0.033	14306	C	T	0.460	8	21090818	7.22903E-06	0.103	360612	11969.376
genus Eubacterium fissicatena group	Vascular dementia (multiple infarctions)	rs429358	C	T	0.004	45411941	0.913	0.033	14306	C	T	0.660	19	44908684	1.33506E-17	0.077	360612	52325.340
genus Eubacterium fissicatena group	Vascular dementia (multiple infarctions)	rs72822148	T	C	0.005	9742028	0.986	0.025	14306	T	C	-0.322	17	9838711	3.18831E-06	0.069	360612	17983.471
genus Eubacterium fissicatena group	Vascular dementia (multiple infarctions)	rs73053797	T	C	-0.003	29909039	0.877	0.033	14306	T	C	0.338	3	29867548	6.00772E-06	0.075	360612	13680.576
genus Eubacterium fissicatena group	Vascular dementia (multiple infarctions)	rs9861644	A	G	0.022	88637331	0.388	0.026	14306	A	G	0.323	3	88588181	4.84942E-06	0.071	360612	17137.658
genus Eubacterium hallii group	Vascular dementia (multiple infarctions)	rs429358	C	T	0.003	45411941	0.834	0.016	14306	C	T	0.660	19	44908684	1.33506E-17	0.077	360612	52325.340
genus Eubacterium hallii group	Vascular dementia (multiple infarctions)	rs4716814	T	C	-0.002	157723046	0.882	0.011	14306	T	C	-0.319	7	157930354	3.75656E-07	0.063	360612	19180.021
genus Eubacterium hallii group	Vascular dementia (multiple infarctions)	rs4725579	C	A	0.005	139468213	0.718	0.014	14306	C	A	-0.379	7	139768414	3.58039E-06	0.082	360612	18391.668
genus Eubacterium hallii group	Vascular dementia (multiple infarctions)	rs73053797	T	C	-0.008	29909039	0.658	0.016	14306	T	C	0.338	3	29867548	6.00772E-06	0.075	360612	13680.576
genus Eubacterium hallii group	Vascular dementia (multiple infarctions)	rs9861644	A	G	0.009	88637331	0.452	0.013	14306	A	G	0.323	3	88588181	4.84942E-06	0.071	360612	17137.658
genus Eubacterium nodatum group	Vascular dementia (multiple infarctions)	rs1454336	A	G	0.012	91873093	0.733	0.032	14306	A	G	-0.369	4	90951942	4.48415E-06	0.080	360612	13616.170
genus Eubacterium nodatum group	Vascular dementia (multiple infarctions)	rs34288661	C	T	0.023	20948329	0.495	0.036	14306	C	T	0.460	8	21090818	7.22903E-06	0.103	360612	11969.376
genus Eubacterium nodatum group	Vascular dementia (multiple infarctions)	rs429358	C	T	0.008	45411941	0.880	0.036	14306	C	T	0.660	19	44908684	1.33506E-17	0.077	360612	52325.340
genus Eubacterium nodatum group	Vascular dementia (multiple infarctions)	rs4716814	T	C	0.000	157723046	0.963	0.025	14306	T	C	-0.319	7	157930354	3.75656E-07	0.063	360612	19180.021
genus Eubacterium nodatum group	Vascular dementia (multiple infarctions)	rs72822148	T	C	-0.004	9742028	0.904	0.026	14306	T	C	-0.322	17	9838711	3.18831E-06	0.069	360612	17983.471
genus Eubacterium nodatum group	Vascular dementia (multiple infarctions)	rs9861644	A	G	0.000	88637331	0.984	0.028	14306	A	G	0.323	3	88588181	4.84942E-06	0.071	360612	17137.658
genus Eubacterium oxidoreducens group	Vascular dementia (multiple infarctions)	rs11081443	C	T	0.002	8944208	0.854	0.024	14306	C	T	0.464	18	8944210	5.71874E-06	0.102	360612	19066.663
genus Eubacterium oxidoreducens group	Vascular dementia (multiple infarctions)	rs1454336	A	G	0.018	91873093	0.498	0.025	14306	A	G	-0.369	4	90951942	4.48415E-06	0.080	360612	13616.170
genus Eubacterium oxidoreducens group	Vascular dementia (multiple infarctions)	rs34288661	C	T	0.003	20948329	0.858	0.030	14306	C	T	0.460	8	21090818	7.22903E-06	0.103	360612	11969.376
genus Eubacterium oxidoreducens group	Vascular dementia (multiple infarctions)	rs429358	C	T	0.021	45411941	0.455	0.028	14306	C	T	0.660	19	44908684	1.33506E-17	0.077	360612	52325.340
genus Eubacterium oxidoreducens group	Vascular dementia (multiple infarctions)	rs4716814	T	C	-0.009	157723046	0.645	0.020	14306	T	C	-0.319	7	157930354	3.75656E-07	0.063	360612	19180.021
genus Eubacterium oxidoreducens group	Vascular dementia (multiple infarctions)	rs4725579	C	A	0.021	139468213	0.387	0.024	14306	C	A	-0.379	7	139768414	3.58039E-06	0.082	360612	18391.668
genus Eubacterium oxidoreducens group	Vascular dementia (multiple infarctions)	rs72822148	T	C	-0.012	9742028	0.496	0.021	14306	T	C	-0.322	17	9838711	3.18831E-06	0.069	360612	17983.471
genus Eubacterium oxidoreducens group	Vascular dementia (multiple infarctions)	rs73053797	T	C	-0.018	29909039	0.576	0.026	14306	T	C	0.338	3	29867548	6.00772E-06	0.075	360612	13680.576
genus Eubacterium rectale group	Vascular dementia (multiple infarctions)	rs11081443	C	T	0.001	8944208	0.890	0.013	14306	C	T	0.464	18	8944210	5.71874E-06	0.102	360612	19066.663
genus Eubacterium rectale group	Vascular dementia (multiple infarctions)	rs4716814	T	C	0.000	157723046	0.972	0.011	14306	T	C	-0.319	7	157930354	3.75656E-07	0.063	360612	19180.021
genus Eubacterium rectale group	Vascular dementia (multiple infarctions)	rs72822148	T	C	-0.009	9742028	0.472	0.012	14306	T	C	-0.322	17	9838711	3.18831E-06	0.069	360612	17983.471
genus Eubacterium rectale group	Vascular dementia (multiple infarctions)	rs9861644	A	G	-0.001	88637331	0.955	0.012	14306	A	G	0.323	3	88588181	4.84942E-06	0.071	360612	17137.658
genus Eubacterium ruminantium group	Vascular dementia (multiple infarctions)	rs34288661	C	T	0.008	20948329	0.763	0.024	14306	C	T	0.460	8	21090818	7.22903E-06	0.103	360612	11969.376

genus Eubacterium ruminantium group	Vascular dementia (multiple infarctions)	rs429358	C	T	0.014	45411941	0.497	0.023	14306	C	T	0.660	19	44908684	1.33506E-17	0.077	360612	52325.340
genus Eubacterium ruminantium group	Vascular dementia (multiple infarctions)	rs4716814	T	C	-0.007	157723046	0.637	0.016	14306	T	C	-0.319	7	157930354	3.75656E-07	0.063	360612	19180.021
genus Eubacterium ruminantium group	Vascular dementia (multiple infarctions)	rs4725579	C	A	-0.004	139468213	0.896	0.020	14306	C	A	-0.379	7	139768414	3.58039E-06	0.082	360612	18391.668
genus Eubacterium ruminantium group	Vascular dementia (multiple infarctions)	rs72822148	T	C	0.001	9742028	0.927	0.018	14306	T	C	-0.322	17	9838711	3.18831E-06	0.069	360612	17983.471
genus Eubacterium ruminantium group	Vascular dementia (multiple infarctions)	rs73053797	T	C	0.012	29909039	0.522	0.023	14306	T	C	0.338	3	29867548	6.00772E-06	0.075	360612	13680.576
genus Eubacterium ruminantium group	Vascular dementia (multiple infarctions)	rs9861644	A	G	-0.005	88637331	0.795	0.018	14306	A	G	0.323	3	88588181	4.84942E-06	0.071	360612	17137.658
genus Eubacterium ventriosum group	Vascular dementia (multiple infarctions)	rs1454336	A	G	0.011	91873093	0.497	0.015	14306	A	G	-0.369	4	90951942	4.48415E-06	0.080	360612	13616.170
genus Eubacterium ventriosum group	Vascular dementia (multiple infarctions)	rs34288661	C	T	-0.012	20948329	0.512	0.017	14306	C	T	0.460	8	21090818	7.22903E-06	0.103	360612	11969.376
genus Eubacterium ventriosum group	Vascular dementia (multiple infarctions)	rs72822148	T	C	0.002	9742028	0.861	0.012	14306	T	C	-0.322	17	9838711	3.18831E-06	0.069	360612	17983.471
genus Eubacterium ventriosum group	Vascular dementia (multiple infarctions)	rs73053797	T	C	0.006	29909039	0.545	0.016	14306	T	C	0.338	3	29867548	6.00772E-06	0.075	360612	13680.576
genus Eubacterium xylanophilum group	Vascular dementia (multiple infarctions)	rs11081443	C	T	0.007	8944208	0.569	0.015	14306	C	T	0.464	18	8944210	5.71874E-06	0.102	360612	19066.663
genus Eubacterium xylanophilum group	Vascular dementia (multiple infarctions)	rs34288661	C	T	0.011	20948329	0.498	0.018	14306	C	T	0.460	8	21090818	7.22903E-06	0.103	360612	11969.376
genus Eubacterium xylanophilum group	Vascular dementia (multiple infarctions)	rs429358	C	T	-0.023	45411941	0.188	0.017	14306	C	T	0.660	19	44908684	1.33506E-17	0.077	360612	52325.340
genus Eubacterium xylanophilum group	Vascular dementia (multiple infarctions)	rs4725579	C	A	0.009	139468213	0.550	0.015	14306	C	A	-0.379	7	139768414	3.58039E-06	0.082	360612	18391.668
genus Eubacterium xylanophilum group	Vascular dementia (multiple infarctions)	rs72822148	T	C	0.007	9742028	0.577	0.013	14306	T	C	-0.322	17	9838711	3.18831E-06	0.069	360612	17983.471
genus Eubacterium xylanophilum group	Vascular dementia (multiple infarctions)	rs73053797	T	C	0.014	29909039	0.453	0.018	14306	T	C	0.338	3	29867548	6.00772E-06	0.075	360612	13680.576
genus Faecalibacterium	Vascular dementia (multiple infarctions)	rs11081443	C	T	0.005	8944208	0.717	0.013	14306	C	T	0.464	18	8944210	5.71874E-06	0.102	360612	19066.663
genus Faecalibacterium	Vascular dementia (multiple infarctions)	rs34288661	C	T	0.008	20948329	0.561	0.016	14306	C	T	0.460	8	21090818	7.22903E-06	0.103	360612	11969.376
genus Faecalibacterium	Vascular dementia (multiple infarctions)	rs4716814	T	C	-0.002	157723046	0.851	0.011	14306	T	C	-0.319	7	157930354	3.75656E-07	0.063	360612	19180.021
genus Faecalibacterium	Vascular dementia (multiple infarctions)	rs72822148	T	C	0.004	9742028	0.650	0.011	14306	T	C	-0.322	17	9838711	3.18831E-06	0.069	360612	17983.471
genus Faecalibacterium	Vascular dementia (multiple infarctions)	rs9861644	A	G	-0.010	88637331	0.391	0.012	14306	A	G	0.323	3	88588181	4.84942E-06	0.071	360612	17137.658
genus Family XIII AD3011 group	Vascular dementia (multiple infarctions)	rs11081443	C	T	0.004	8944208	0.802	0.015	14306	C	T	0.464	18	8944210	5.71874E-06	0.102	360612	19066.663
genus Family XIII AD3011 group	Vascular dementia (multiple infarctions)	rs34288661	C	T	-0.009	20948329	0.635	0.018	14306	C	T	0.460	8	21090818	7.22903E-06	0.103	360612	11969.376
genus Family XIII AD3011 group	Vascular dementia (multiple infarctions)	rs429358	C	T	0.029	45411941	0.106	0.017	14306	C	T	0.660	19	44908684	1.33506E-17	0.077	360612	52325.340
genus Family XIII AD3011 group	Vascular dementia (multiple infarctions)	rs4725579	C	A	0.011	139468213	0.451	0.015	14306	C	A	-0.379	7	139768414	3.58039E-06	0.082	360612	18391.668
genus Family XIII AD3011 group	Vascular dementia (multiple infarctions)	rs73053797	T	C	0.005	29909039	0.810	0.017	14306	T	C	0.338	3	29867548	6.00772E-06	0.075	360612	13680.576
genus Family XIII UCG001	Vascular dementia (multiple infarctions)	rs1454336	A	G	-0.001	91873093	0.910	0.017	14306	A	G	-0.369	4	90951942	4.48415E-06	0.080	360612	13616.170
genus Family XIII UCG001	Vascular dementia (multiple infarctions)	rs429358	C	T	0.004	45411941	0.889	0.018	14306	C	T	0.660	19	44908684	1.33506E-17	0.077	360612	52325.340
genus Family XIII UCG001	Vascular dementia (multiple infarctions)	rs4716814	T	C	0.010	157723046	0.457	0.013	14306	T	C	-0.319	7	157930354	3.75656E-07	0.063	360612	19180.021
genus Family XIII UCG001	Vascular dementia (multiple infarctions)	rs4725579	C	A	0.014	139468213	0.439	0.016	14306	C	A	-0.379	7	139768414	3.58039E-06	0.082	360612	18391.668
genus Family XIII UCG001	Vascular dementia (multiple infarctions)	rs73053797	T	C	0.000	29909039	0.998	0.018	14306	T	C	0.338	3	29867548	6.00772E-06	0.075	360612	13680.576
genus Flavonifractor	Vascular dementia (multiple infarctions)	rs11081443	C	T	0.013	8944208	0.510	0.017	14306	C	T	0.464	18	8944210	5.71874E-06	0.102	360612	19066.663
genus Flavonifractor	Vascular dementia (multiple infarctions)	rs1454336	A	G	0.008	91873093	0.702	0.018	14306	A	G	-0.369	4	90951942	4.48415E-06	0.080	360612	13616.170
genus Flavonifractor	Vascular dementia (multiple infarctions)	rs429358	C	T	-0.021	45411941	0.293	0.020	14306	C	T	0.660	19	44908684	1.33506E-17	0.077	360612	52325.340
genus Flavonifractor	Vascular dementia (multiple infarctions)	rs4716814	T	C	0.003	157723046	0.802	0.014	14306	T	C	-0.319	7	157930354	3.75656E-07	0.063	360612	19180.021
genus Flavonifractor	Vascular dementia (multiple infarctions)	rs4725579	C	A	0.005	139468213	0.735	0.017	14306	C	A	-0.379	7	139768414	3.58039E-06	0.082	360612	18391.668
genus Flavonifractor	Vascular dementia (multiple infarctions)	rs9861644	A	G	0.002	88637331	0.887	0.016	14306	A	G	0.323	3	88588181	4.84942E-06	0.071	360612	17137.658
genus Fusicatenibacter	Vascular dementia (multiple infarctions)	rs11081443	C	T	0.011	8944208	0.380	0.013	14306	C	T	0.464	18	8944210	5.71874E-06	0.102	360612	19066.663
genus Fusicatenibacter	Vascular dementia (multiple infarctions)	rs1454336	A	G	-0.002	91873093	0.906	0.014	14306	A	G	-0.369	4	90951942	4.48415E-06	0.080	360612	13616.170
genus Fusicatenibacter	Vascular dementia (multiple infarctions)	rs429358	C	T	-0.008	45411941	0.622	0.015	14306	C	T	0.660	19	44908684	1.33506E-17	0.077	360612	52325.340
genus Fusicatenibacter	Vascular dementia (multiple infarctions)	rs4716814	T	C	-0.008	157723046	0.445	0.011	14306	T	C	-0.319	7	157930354	3.75656E-07	0.063	360612	19180.021
genus Fusicatenibacter	Vascular dementia (multiple infarctions)	rs72822148	T	C	-0.008	9742028	0.468	0.012	14306	T	C	-0.322	17	9838711	3.18831E-06	0.069	360612	17983.471
genus Fusicatenibacter	Vascular dementia (multiple infarctions)	rs9861644	A	G	0.007	88637331	0.523	0.012	14306	A	G	0.323	3	88588181	4.84942E-06	0.071	360612	17137.658
genus Gordonibacter	Vascular dementia (multiple infarctions)	rs11081443	C	T	0.005	8944208	0.975	0.028	14306	C	T	0.464	18	8944210	5.71874E-06	0.102	360612	19066.663
genus Gordonibacter	Vascular dementia (multiple infarctions)	rs34288661	C	T	0.014	20948329	0.638	0.034	14306	C	T	0.460	8	21090818	7.22903E-06	0.103	360612	11969.376
genus Gordonibacter	Vascular dementia (multiple infarctions)	rs429358	C	T	0.056	45411941	0.125	0.034	14306	C	T	0.660	19	44908684	1.33506E-17	0.077	360612	52325.340
genus Gordonibacter	Vascular dementia (multiple infarctions)	rs4716814	T	C	-0.018	157723046	0.442	0.023	14306	T	C	-0.319	7	157930354	3.75656E-07	0.063	360612	19180.021
genus Gordonibacter	Vascular dementia (multiple infarctions)	rs4725579	C	A	0.020	139468213	0.427	0.029	14306	C	A	-0.379	7	139768414	3.58039E-06	0.082	360612	18391.668
genus Gordonibacter	Vascular dementia (multiple infarctions)	rs72822148	T	C	0.021	9742028	0.404	0.025	14306	T	C	-0.322	17	9838711	3.18831E-06	0.069	360612	17983.471
genus Gordonibacter	Vascular dementia (multiple infarctions)	rs73053797	T	C	0.007	29909039	0.861	0.033	14306	T	C	0.338	3	29867548	6.00772E-06	0.075	360612	13680.576
genus Haemophilus	Vascular dementia (multiple infarctions)	rs11081443	C	T	-0.007	8944208	0.549	0.018	14306	C	T	0.464	18	8944210	5.71874E-06	0.102	360612	19066.663
genus Haemophilus	Vascular dementia (multiple infarctions)	rs1454336	A	G	0.017	91873093	0.371	0.019	14306	A	G	-0.369	4	90951942	4.48415E-06	0.080	360612	13616.170
genus Haemophilus	Vascular dementia (multiple infarctions)	rs429358	C	T	0.027	45411941	0.241	0.021	14306	C	T	0.660	19	44908684	1.33506E-17	0.077	360612	52325.340
genus Haemophilus	Vascular dementia (multiple infarctions)	rs4716814	T	C	0.008	157723046	0.595	0.015	14306	T	C	-0.319	7	157930354	3.75656E-07	0.063	360612	19180.021
genus Haemophilus	Vascular dementia (multiple infarctions)	rs72822148	T	C	0.000	9742028	0.889	0.016	14306	T	C	-0.322	17	9838711	3.18831E-06	0.069	360612	17983.471
genus Holdemanella	Vascular dementia (multiple infarctions)	rs34288661	C	T	-0.020	20948329	0.405	0.024	14306	C	T	0.460	8	21090818	7.22903E-06	0.103	360612	11969.376
genus Holdemanella	Vascular dementia (multiple infarctions)	rs429358	C	T	0.038	45411941	0.092	0.023	14306	C	T	0.660	19	44908684	1.33506E-17	0.077	360612	52325.340
genus Holdemanella	Vascular dementia (multiple infarctions)	rs4716814	T	C	-0.005	157723046	0.740	0.016	14306	T	C	-0.319	7	157930354	3.75656E-07	0.063	360612	19180.021
genus Holdemanella	Vascular dementia (multiple infarctions)	rs72822148	T	C	-0.015	9742028	0.379	0.018	14306	T	C	-0.322	17	9838711	3.18831E-06	0.069	360612	17983.471
genus Holdemania	Vascular dementia (multiple infarctions)	rs11081443	C	T	-0.004	8944208	0.830	0.018	14306	C	T	0.464	18	8944210	5.71874E-06	0.102	360612	19066.663
genus Holdemania	Vascular dementia (multiple infarctions)	rs1454336	A	G	0.006	91873093	0.766	0.020	14306	A	G	-0.369	4	90951942	4.48415E-06	0.080	360612	13616.170
genus Holdemania	Vascular dementia (multiple infarctions)	rs34288661	C	T	0.013	20948329	0.477	0.022	14306	C	T	0.460	8	21090818	7.22903E-06	0.103	360612	11969.376
genus Holdemania	Vascular dementia (multiple infarctions)	rs429358	C	T	0.014	45411941	0.528	0.021	14306	C	T	0.660	19	44908684	1.33506E-17	0.077	360612	52325.340
genus Holdemania	Vascular dementia (multiple infarctions)	rs4725579	C	A	0.005	139468213	0.860	0.018	14306	C	A	-0.379	7	139768414	3.58039E-06	0.082	360612	18391.668
genus Holdemania	Vascular dementia (multiple infarctions)	rs72822148	T	C	-0.013	9742028	0.376	0.016	14306	T	C	-0.322	17	9838711	3.18831E-06	0.069	360612	17983.471
genus Holdemania	Vascular dementia (multiple infarctions)	rs73053797	T	C	-0.019	29909039	0.388	0.021	14306	T	C	0.338	3	29867548	6.00772E-06	0.075	360612	13680.576
genus Holdemania	Vascular dementia (multiple infarctions)	rs9861644	A	G	-0.013	88637331	0.474	0.017	14306	A	G	0.323	3	88588181	4.84942E-06	0.071	360612	17137.658
genus Howardella	Vascular dementia (multiple infarctions)	rs11081443	C	T	-0.018	8944208	0.450	0.028	14306	C	T	0.464	18	8944210	5.71874E-06	0.102	360612	19066.663
genus Howardella	Vascular dementia (multiple infarctions)	rs429358	C	T	0.033	45411941	0.260	0.033	14306	C	T	0.660	19	44908684	1.33506E-17	0.077	360612	52325.340
genus Howardella	Vascular dementia (multiple infarctions)	rs4716814	T	C	0.015	157723046	0.527	0.023	14306	T	C	-0.319	7	157930354	3.75656E-07	0.063	360612	19180.021
genus Howardella	Vascular dementia (multiple infarctions)	rs73053797	T	C	0.000	29909039	0.831	0.033	14306	T	C	0.338	3	29867548	6.00772E-06	0.075	360612	13680.576

genus Hungatella	Vascular dementia (multiple infarctions)	rs1454336	A	G	-0.015	91873093	0.587	0.028	14306	A	G	-0.369	4	90951942	4.48415E-06	0.080	360612	13616.170
genus Hungatella	Vascular dementia (multiple infarctions)	rs429358	C	T	-0.050	45411941	0.108	0.032	14306	C	T	0.660	19	44908684	1.33506E-17	0.077	360612	52325.340
genus Hungatella	Vascular dementia (multiple infarctions)	rs4725579	C	A	0.013	139468213	0.572	0.027	14306	C	A	-0.379	7	139768414	3.58039E-06	0.082	360612	18391.668
genus Hungatella	Vascular dementia (multiple infarctions)	rs72822148	T	C	0.004	9742028	0.719	0.024	14306	T	C	-0.322	17	9838711	3.18831E-06	0.069	360612	17983.471
genus Intestinibacter	Vascular dementia (multiple infarctions)	rs11081443	C	T	0.003	8944208	0.997	0.016	14306	C	T	0.464	18	8944210	5.71874E-06	0.102	360612	19066.663
genus Intestinibacter	Vascular dementia (multiple infarctions)	rs1454336	A	G	0.014	91873093	0.397	0.017	14306	A	G	-0.369	4	90951942	4.48415E-06	0.080	360612	13616.170
genus Intestinibacter	Vascular dementia (multiple infarctions)	rs34288661	C	T	0.016	20948329	0.365	0.019	14306	C	T	0.460	8	21090818	7.22903E-06	0.103	360612	11969.376
genus Intestinibacter	Vascular dementia (multiple infarctions)	rs429358	C	T	-0.011	45411941	0.447	0.018	14306	C	T	0.660	19	44908684	1.33506E-17	0.077	360612	52325.340
genus Intestinibacter	Vascular dementia (multiple infarctions)	rs73053797	T	C	-0.010	29909039	0.520	0.018	14306	T	C	0.338	3	29867548	6.00772E-06	0.075	360612	13680.576
genus Intestinibacter	Vascular dementia (multiple infarctions)	rs9861644	A	G	-0.012	88637331	0.425	0.015	14306	A	G	0.323	3	88588181	4.84942E-06	0.071	360612	17137.658
genus Intestinimonas	Vascular dementia (multiple infarctions)	rs1454336	A	G	0.003	91873093	0.867	0.017	14306	A	G	-0.369	4	90951942	4.48415E-06	0.080	360612	13616.170
genus Intestinimonas	Vascular dementia (multiple infarctions)	rs34288661	C	T	-0.012	20948329	0.604	0.019	14306	C	T	0.460	8	21090818	7.22903E-06	0.103	360612	11969.376
genus Intestinimonas	Vascular dementia (multiple infarctions)	rs429358	C	T	0.013	45411941	0.424	0.018	14306	C	T	0.660	19	44908684	1.33506E-17	0.077	360612	52325.340
genus Intestinimonas	Vascular dementia (multiple infarctions)	rs4716814	T	C	0.007	157723046	0.604	0.013	14306	T	C	-0.319	7	157930354	3.75656E-07	0.063	360612	19180.021
genus Intestinimonas	Vascular dementia (multiple infarctions)	rs4725579	C	A	-0.004	139468213	0.801	0.016	14306	C	A	-0.379	7	139768414	3.58039E-06	0.082	360612	18391.668
genus Intestinimonas	Vascular dementia (multiple infarctions)	rs72822148	T	C	-0.008	9742028	0.646	0.014	14306	T	C	-0.322	17	9838711	3.18831E-06	0.069	360612	17983.471
genus Intestinimonas	Vascular dementia (multiple infarctions)	rs9861644	A	G	-0.003	88637331	0.867	0.015	14306	A	G	0.323	3	88588181	4.84942E-06	0.071	360612	17137.658
genus Lachnoclostridium	Vascular dementia (multiple infarctions)	rs11081443	C	T	-0.003	8944208	0.758	0.013	14306	C	T	0.464	18	8944210	5.71874E-06	0.102	360612	19066.663
genus Lachnoclostridium	Vascular dementia (multiple infarctions)	rs1454336	A	G	-0.001	91873093	0.874	0.014	14306	A	G	-0.369	4	90951942	4.48415E-06	0.080	360612	13616.170
genus Lachnoclostridium	Vascular dementia (multiple infarctions)	rs34288661	C	T	-0.010	20948329	0.589	0.016	14306	C	T	0.460	8	21090818	7.22903E-06	0.103	360612	11969.376
genus Lachnoclostridium	Vascular dementia (multiple infarctions)	rs429358	C	T	-0.019	45411941	0.230	0.015	14306	C	T	0.660	19	44908684	1.33506E-17	0.077	360612	52325.340
genus Lachnoclostridium	Vascular dementia (multiple infarctions)	rs4725579	C	A	0.004	139468213	0.770	0.013	14306	C	A	-0.379	7	139768414	3.58039E-06	0.082	360612	18391.668
genus Lachnospiraceae FCS020 group	Vascular dementia (multiple infarctions)	rs11081443	C	T	0.003	8944208	0.911	0.015	14306	C	T	0.464	18	8944210	5.71874E-06	0.102	360612	19066.663
genus Lachnospiraceae FCS020 group	Vascular dementia (multiple infarctions)	rs1454336	A	G	-0.003	91873093	0.792	0.016	14306	A	G	-0.369	4	90951942	4.48415E-06	0.080	360612	13616.170
genus Lachnospiraceae FCS020 group	Vascular dementia (multiple infarctions)	rs429358	C	T	0.017	45411941	0.318	0.017	14306	C	T	0.660	19	44908684	1.33506E-17	0.077	360612	52325.340
genus Lachnospiraceae FCS020 group	Vascular dementia (multiple infarctions)	rs4716814	T	C	-0.012	157723046	0.328	0.012	14306	T	C	-0.319	7	157930354	3.75656E-07	0.063	360612	19180.021
genus Lachnospiraceae FCS020 group	Vascular dementia (multiple infarctions)	rs4725579	C	A	-0.002	139468213	0.819	0.015	14306	C	A	-0.379	7	139768414	3.58039E-06	0.082	360612	18391.668
genus Lachnospiraceae FCS020 group	Vascular dementia (multiple infarctions)	rs72822148	T	C	0.005	9742028	0.692	0.013	14306	T	C	-0.322	17	9838711	3.18831E-06	0.069	360612	17983.471
genus Lachnospiraceae FCS020 group	Vascular dementia (multiple infarctions)	rs73053797	T	C	0.008	29909039	0.666	0.017	14306	T	C	0.338	3	29867548	6.00772E-06	0.075	360612	13680.576
genus Lachnospiraceae FCS020 group	Vascular dementia (multiple infarctions)	rs9861644	A	G	-0.008	88637331	0.519	0.014	14306	A	G	0.323	3	88588181	4.84942E-06	0.071	360612	17137.658
genus Lachnospiraceae NC2004 group	Vascular dementia (multiple infarctions)	rs1454336	A	G	-0.014	91873093	0.555	0.023	14306	A	G	-0.369	4	90951942	4.48415E-06	0.080	360612	13616.170
genus Lachnospiraceae NC2004 group	Vascular dementia (multiple infarctions)	rs34288661	C	T	-0.021	20948329	0.437	0.027	14306	C	T	0.460	8	21090818	7.22903E-06	0.103	360612	11969.376
genus Lachnospiraceae NC2004 group	Vascular dementia (multiple infarctions)	rs429358	C	T	-0.024	45411941	0.415	0.026	14306	C	T	0.660	19	44908684	1.33506E-17	0.077	360612	52325.340
genus Lachnospiraceae NC2004 group	Vascular dementia (multiple infarctions)	rs4716814	T	C	0.006	157723046	0.744	0.018	14306	T	C	-0.319	7	157930354	3.75656E-07	0.063	360612	19180.021
genus Lachnospiraceae NC2004 group	Vascular dementia (multiple infarctions)	rs4725579	C	A	-0.004	139468213	0.835	0.022	14306	C	A	-0.379	7	139768414	3.58039E-06	0.082	360612	18391.668
genus Lachnospiraceae NC2004 group	Vascular dementia (multiple infarctions)	rs72822148	T	C	0.013	9742028	0.499	0.020	14306	T	C	-0.322	17	9838711	3.18831E-06	0.069	360612	17983.471
genus Lachnospiraceae NC2004 group	Vascular dementia (multiple infarctions)	rs9861644	A	G	0.007	88637331	0.715	0.020	14306	A	G	0.323	3	88588181	4.84942E-06	0.071	360612	17137.658
genus Lachnospiraceae ND3007 group	Vascular dementia (multiple infarctions)	rs1454336	A	G	-0.002	91873093	0.870	0.015	14306	A	G	-0.369	4	90951942	4.48415E-06	0.080	360612	13616.170
genus Lachnospiraceae ND3007 group	Vascular dementia (multiple infarctions)	rs429358	C	T	-0.003	45411941	0.806	0.016	14306	C	T	0.660	19	44908684	1.33506E-17	0.077	360612	52325.340
genus Lachnospiraceae ND3007 group	Vascular dementia (multiple infarctions)	rs4716814	T	C	-0.011	157723046	0.326	0.012	14306	T	C	-0.319	7	157930354	3.75656E-07	0.063	360612	19180.021
genus Lachnospiraceae ND3007 group	Vascular dementia (multiple infarctions)	rs4725579	C	A	-0.006	139468213	0.721	0.014	14306	C	A	-0.379	7	139768414	3.58039E-06	0.082	360612	18391.668
genus Lachnospiraceae ND3007 group	Vascular dementia (multiple infarctions)	rs73053797	T	C	-0.007	29909039	0.803	0.016	14306	T	C	0.338	3	29867548	6.00772E-06	0.075	360612	13680.576
genus Lachnospiraceae ND3007 group	Vascular dementia (multiple infarctions)	rs9861644	A	G	-0.001	88637331	0.956	0.013	14306	A	G	0.323	3	88588181	4.84942E-06	0.071	360612	17137.658
genus Lachnospiraceae NK4A136 group	Vascular dementia (multiple infarctions)	rs34288661	C	T	-0.009	20948329	0.611	0.016	14306	C	T	0.460	8	21090818	7.22903E-06	0.103	360612	11969.376
genus Lachnospiraceae NK4A136 group	Vascular dementia (multiple infarctions)	rs429358	C	T	-0.011	45411941	0.456	0.015	14306	C	T	0.660	19	44908684	1.33506E-17	0.077	360612	52325.340
genus Lachnospiraceae NK4A136 group	Vascular dementia (multiple infarctions)	rs4716814	T	C	-0.002	157723046	0.827	0.011	14306	T	C	-0.319	7	157930354	3.75656E-07	0.063	360612	19180.021
genus Lachnospiraceae NK4A136 group	Vascular dementia (multiple infarctions)	rs4725579	C	A	-0.002	139468213	0.997	0.013	14306	C	A	-0.379	7	139768414	3.58039E-06	0.082	360612	18391.668
genus Lachnospiraceae NK4A136 group	Vascular dementia (multiple infarctions)	rs72822148	T	C	0.007	9742028	0.535	0.012	14306	T	C	-0.322	17	9838711	3.18831E-06	0.069	360612	17983.471
genus Lachnospiraceae NK4A136 group	Vascular dementia (multiple infarctions)	rs73053797	T	C	0.007	29909039	0.698	0.015	14306	T	C	0.338	3	29867548	6.00772E-06	0.075	360612	13680.576
genus Lachnospiraceae NK4A136 group	Vascular dementia (multiple infarctions)	rs9861644	A	G	0.006	88637331	0.615	0.012	14306	A	G	0.323	3	88588181	4.84942E-06	0.071	360612	17137.658
genus Lachnospiraceae UCG001	Vascular dementia (multiple infarctions)	rs1454336	A	G	-0.016	91873093	0.410	0.018	14306	A	G	-0.369	4	90951942	4.48415E-06	0.080	360612	13616.170
genus Lachnospiraceae UCG001	Vascular dementia (multiple infarctions)	rs34288661	C	T	0.008	20948329	0.723	0.021	14306	C	T	0.460	8	21090818	7.22903E-06	0.103	360612	11969.376
genus Lachnospiraceae UCG001	Vascular dementia (multiple infarctions)	rs429358	C	T	-0.002	45411941	0.904	0.020	14306	C	T	0.660	19	44908684	1.33506E-17	0.077	360612	52325.340
genus Lachnospiraceae UCG001	Vascular dementia (multiple infarctions)	rs4725579	C	A	-0.004	139468213	0.776	0.018	14306	C	A	-0.379	7	139768414	3.58039E-06	0.082	360612	18391.668
genus Lachnospiraceae UCG001	Vascular dementia (multiple infarctions)	rs72822148	T	C	-0.002	9742028	0.928	0.015	14306	T	C	-0.322	17	9838711	3.18831E-06	0.069	360612	17983.471
genus Lachnospiraceae UCG001	Vascular dementia (multiple infarctions)	rs73053797	T	C	-0.003	29909039	0.915	0.020	14306	T	C	0.338	3	29867548	6.00772E-06	0.075	360612	13680.576
genus Lachnospiraceae UCG001	Vascular dementia (multiple infarctions)	rs9861644	A	G	0.001	88637331	0.964	0.016	14306	A	G	0.323	3	88588181	4.84942E-06	0.071	360612	17137.658
genus Lachnospiraceae UCG004	Vascular dementia (multiple infarctions)	rs11081443	C	T	0.008	8944208	0.752	0.015	14306	C	T	0.464	18	8944210	5.71874E-06	0.102	360612	19066.663
genus Lachnospiraceae UCG004	Vascular dementia (multiple infarctions)	rs1454336	A	G	-0.001	91873093	0.846	0.015	14306	A	G	-0.369	4	90951942	4.48415E-06	0.080	360612	13616.170
genus Lachnospiraceae UCG004	Vascular dementia (multiple infarctions)	rs34288661	C	T	0.008	20948329	0.561	0.018	14306	C	T	0.460	8	21090818	7.22903E-06	0.103	360612	11969.376
genus Lachnospiraceae UCG004	Vascular dementia (multiple infarctions)	rs429358	C	T	-0.001	45411941	0.954	0.017	14306	C	T	0.660	19	44908684	1.33506E-17	0.077	360612	52325.340
genus Lachnospiraceae UCG004	Vascular dementia (multiple infarctions)	rs73053797	T	C	0.000	29909039	0.901	0.017	14306	T	C	0.338	3	29867548	6.00772E-06	0.075	360612	13680.576
genus Lachnospiraceae UCG008	Vascular dementia (multiple infarctions)	rs34288661	C	T	0.004	20948329	0.926	0.026	14306	C	T	0.460	8	21090818	7.22903E-06	0.103	360612	11969.376
genus Lachnospiraceae UCG008	Vascular dementia (multiple infarctions)	rs429358	C	T	0.001	45411941	0.933	0.024	14306	C	T	0.660	19	44908684	1.33506E-17	0.077	360612	52325.340
genus Lachnospiraceae UCG008	Vascular dementia (multiple infarctions)	rs4716814	T	C	0.000	157723046	0.998	0.017	14306	T	C	-0.319	7	157930354	3.75656E-07	0.063	360612	19180.021
genus Lachnospiraceae UCG008	Vascular dementia (multiple infarctions)	rs9861644	A	G	0.007	88637331	0.743	0.019	14306	A	G	0.323	3	88588181	4.84942E-06	0.071	360612	17137.658
genus Lachnospiraceae UCG010	Vascular dementia (multiple infarctions)	rs11081443	C	T	0.010	8944208	0.511	0.015	14306	C	T	0.464	18	8944210	5.71874E-06	0.102	360612	19066.663
genus Lachnospiraceae UCG010	Vascular dementia (multiple infarctions)	rs1454336	A	G	0.007	91873093	0.682	0.016	14306	A	G	-0.369	4	90951942	4.48415E-06	0.080	360612	13616.170
genus Lachnospiraceae UCG010	Vascular dementia (multiple infarctions)	rs34288661	C	T	-0.007	20948329	0.756	0.018	14306	C	T	0.460	8	21090818	7.22903E-06	0.103	360612	11969.376
genus Lachnospiraceae UCG010	Vascular dementia (multiple infarctions)	rs429358	C	T	-0.003	45411941	0.809	0.018	14306	C	T	0.660	19	44908684	1.33506E-17	0.077	360612	52325.340
genus Lachnospiraceae UCG010	Vascular dementia (multiple infarctions)	rs4716814	T	C	-0.008	157723046	0.537	0.012	14306	T	C	-0.319	7	157930354	3.75656E-07	0.063	360612	19180.021

genus Lachnospiraceae UCG010	Vascular dementia (multiple infarctions)	rs4725579	C	A	0.006	139468213	0.785	0.015	14306	C	A	-0.379	7	139768414	3.58039E-06	0.082	360612	18391.668
genus Lachnospiraceae UCG010	Vascular dementia (multiple infarctions)	rs73053797	T	C	0.012	29909039	0.558	0.018	14306	T	C	0.338	3	29867548	6.00772E-06	0.075	360612	13680.576
genus Lachnospiraceae UCG010	Vascular dementia (multiple infarctions)	rs9861644	A	G	0.011	88637331	0.454	0.014	14306	A	G	0.323	3	88588181	4.84942E-06	0.071	360612	17137.658
genus Lactobacillus	Vascular dementia (multiple infarctions)	rs11081443	C	T	0.011	8944208	0.599	0.021	14306	C	T	0.464	18	8944210	5.71874E-06	0.102	360612	19066.663
genus Lactobacillus	Vascular dementia (multiple infarctions)	rs1454336	A	G	0.013	91873093	0.575	0.022	14306	A	G	-0.369	4	90951942	4.48415E-06	0.080	360612	13616.170
genus Lactobacillus	Vascular dementia (multiple infarctions)	rs429358	C	T	-0.007	45411941	0.865	0.024	14306	C	T	0.660	19	44908684	1.33506E-17	0.077	360612	52325.340
genus Lactobacillus	Vascular dementia (multiple infarctions)	rs4725579	C	A	-0.011	139468213	0.648	0.021	14306	C	A	-0.379	7	139768414	3.58039E-06	0.082	360612	18391.668
genus Lactobacillus	Vascular dementia (multiple infarctions)	rs72822148	T	C	0.002	9742028	0.893	0.019	14306	T	C	-0.322	17	9838711	3.18831E-06	0.069	360612	17983.471
genus Lactobacillus	Vascular dementia (multiple infarctions)	rs73053797	T	C	0.009	29909039	0.859	0.024	14306	T	C	0.338	3	29867548	6.00772E-06	0.075	360612	13680.576
genus Lactobacillus	Vascular dementia (multiple infarctions)	rs9861644	A	G	0.014	88637331	0.491	0.019	14306	A	G	0.323	3	88588181	4.84942E-06	0.071	360612	17137.658
genus Lactococcus	Vascular dementia (multiple infarctions)	rs11081443	C	T	0.000	8944208	0.997	0.027	14306	C	T	0.464	18	8944210	5.71874E-06	0.102	360612	19066.663
genus Lactococcus	Vascular dementia (multiple infarctions)	rs1454336	A	G	0.021	91873093	0.428	0.029	14306	A	G	-0.369	4	90951942	4.48415E-06	0.080	360612	13616.170
genus Lactococcus	Vascular dementia (multiple infarctions)	rs34288661	C	T	0.005	20948329	0.782	0.033	14306	C	T	0.460	8	21090818	7.22903E-06	0.103	360612	11969.376
genus Lactococcus	Vascular dementia (multiple infarctions)	rs429358	C	T	-0.017	45411941	0.591	0.032	14306	C	T	0.660	19	44908684	1.33506E-17	0.077	360612	52325.340
genus Lactococcus	Vascular dementia (multiple infarctions)	rs4716814	T	C	0.005	157723046	0.806	0.022	14306	T	C	-0.319	7	157930354	3.75656E-07	0.063	360612	19180.021
genus Lactococcus	Vascular dementia (multiple infarctions)	rs9861644	A	G	0.014	88637331	0.571	0.025	14306	A	G	0.323	3	88588181	4.84942E-06	0.071	360612	17137.658
genus Marvinbryantia	Vascular dementia (multiple infarctions)	rs11081443	C	T	0.012	8944208	0.457	0.016	14306	C	T	0.464	18	8944210	5.71874E-06	0.102	360612	19066.663
genus Marvinbryantia	Vascular dementia (multiple infarctions)	rs429358	C	T	0.008	45411941	0.663	0.018	14306	C	T	0.660	19	44908684	1.33506E-17	0.077	360612	52325.340
genus Marvinbryantia	Vascular dementia (multiple infarctions)	rs4716814	T	C	-0.004	157723046	0.776	0.013	14306	T	C	-0.319	7	157930354	3.75656E-07	0.063	360612	19180.021
genus Marvinbryantia	Vascular dementia (multiple infarctions)	rs4725579	C	A	-0.005	139468213	0.729	0.016	14306	C	A	-0.379	7	139768414	3.58039E-06	0.082	360612	18391.668
genus Marvinbryantia	Vascular dementia (multiple infarctions)	rs73053797	T	C	-0.003	29909039	0.781	0.019	14306	T	C	0.338	3	29867548	6.00772E-06	0.075	360612	13680.576
genus Marvinbryantia	Vascular dementia (multiple infarctions)	rs9861644	A	G	0.000	88637331	0.994	0.015	14306	A	G	0.323	3	88588181	4.84942E-06	0.071	360612	17137.658
genus Methanobrevibacter	Vascular dementia (multiple infarctions)	rs11081443	C	T	0.015	8944208	0.528	0.029	14306	C	T	0.464	18	8944210	5.71874E-06	0.102	360612	19066.663
genus Methanobrevibacter	Vascular dementia (multiple infarctions)	rs1454336	A	G	-0.003	91873093	0.968	0.031	14306	A	G	-0.369	4	90951942	4.48415E-06	0.080	360612	13616.170
genus Methanobrevibacter	Vascular dementia (multiple infarctions)	rs429358	C	T	-0.019	45411941	0.661	0.034	14306	C	T	0.660	19	44908684	1.33506E-17	0.077	360612	52325.340
genus Methanobrevibacter	Vascular dementia (multiple infarctions)	rs4716814	T	C	-0.024	157723046	0.329	0.024	14306	T	C	-0.319	7	157930354	3.75656E-07	0.063	360612	19180.021
genus Methanobrevibacter	Vascular dementia (multiple infarctions)	rs4725579	C	A	0.014	139468213	0.629	0.030	14306	C	A	-0.379	7	139768414	3.58039E-06	0.082	360612	18391.668
genus Methanobrevibacter	Vascular dementia (multiple infarctions)	rs72822148	T	C	0.010	9742028	0.656	0.026	14306	T	C	-0.322	17	9838711	3.18831E-06	0.069	360612	17983.471
genus Methanobrevibacter	Vascular dementia (multiple infarctions)	rs9861644	A	G	0.009	88637331	0.733	0.027	14306	A	G	0.323	3	88588181	4.84942E-06	0.071	360612	17137.658
genus Odoribacter	Vascular dementia (multiple infarctions)	rs11081443	C	T	0.012	8944208	0.383	0.014	14306	C	T	0.464	18	8944210	5.71874E-06	0.102	360612	19066.663
genus Odoribacter	Vascular dementia (multiple infarctions)	rs1454336	A	G	0.002	91873093	0.879	0.015	14306	A	G	-0.369	4	90951942	4.48415E-06	0.080	360612	13616.170
genus Odoribacter	Vascular dementia (multiple infarctions)	rs34288661	C	T	-0.004	20948329	0.926	0.017	14306	C	T	0.460	8	21090818	7.22903E-06	0.103	360612	11969.376
genus Odoribacter	Vascular dementia (multiple infarctions)	rs429358	C	T	0.017	45411941	0.297	0.017	14306	C	T	0.660	19	44908684	1.33506E-17	0.077	360612	52325.340
genus Odoribacter	Vascular dementia (multiple infarctions)	rs4716814	T	C	-0.002	157723046	0.881	0.012	14306	T	C	-0.319	7	157930354	3.75656E-07	0.063	360612	19180.021
genus Odoribacter	Vascular dementia (multiple infarctions)	rs4725579	C	A	0.006	139468213	0.651	0.015	14306	C	A	-0.379	7	139768414	3.58039E-06	0.082	360612	18391.668
genus Odoribacter	Vascular dementia (multiple infarctions)	rs73053797	T	C	0.009	29909039	0.583	0.017	14306	T	C	0.338	3	29867548	6.00772E-06	0.075	360612	13680.576
genus Odoribacter	Vascular dementia (multiple infarctions)	rs9861644	A	G	-0.004	88637331	0.789	0.013	14306	A	G	0.323	3	88588181	4.84942E-06	0.071	360612	17137.658
genus Olsenella	Vascular dementia (multiple infarctions)	rs11081443	C	T	-0.009	8944208	0.787	0.029	14306	C	T	0.464	18	8944210	5.71874E-06	0.102	360612	19066.663
genus Olsenella	Vascular dementia (multiple infarctions)	rs1454336	A	G	0.026	91873093	0.390	0.031	14306	A	G	-0.369	4	90951942	4.48415E-06	0.080	360612	13616.170
genus Olsenella	Vascular dementia (multiple infarctions)	rs4725579	C	A	-0.013	139468213	0.677	0.029	14306	C	A	-0.379	7	139768414	3.58039E-06	0.082	360612	18391.668
genus Olsenella	Vascular dementia (multiple infarctions)	rs72822148	T	C	-0.015	9742028	0.552	0.024	14306	T	C	-0.322	17	9838711	3.18831E-06	0.069	360612	17983.471
genus Olsenella	Vascular dementia (multiple infarctions)	rs73053797	T	C	-0.002	29909039	0.987	0.034	14306	T	C	0.338	3	29867548	6.00772E-06	0.075	360612	13680.576
genus Olsenella	Vascular dementia (multiple infarctions)	rs9861644	A	G	-0.007	88637331	0.839	0.027	14306	A	G	0.323	3	88588181	4.84942E-06	0.071	360612	17137.658
genus Oscillibacter	Vascular dementia (multiple infarctions)	rs11081443	C	T	-0.010	8944208	0.505	0.019	14306	C	T	0.464	18	8944210	5.71874E-06	0.102	360612	19066.663
genus Oscillibacter	Vascular dementia (multiple infarctions)	rs34288661	C	T	-0.008	20948329	0.750	0.022	14306	C	T	0.460	8	21090818	7.22903E-06	0.103	360612	11969.376
genus Oscillibacter	Vascular dementia (multiple infarctions)	rs73053797	T	C	-0.015	29909039	0.380	0.022	14306	T	C	0.338	3	29867548	6.00772E-06	0.075	360612	13680.576
genus Oscillibacter	Vascular dementia (multiple infarctions)	rs9861644	A	G	-0.010	88637331	0.614	0.017	14306	A	G	0.323	3	88588181	4.84942E-06	0.071	360612	17137.658
genus Oscillospira	Vascular dementia (multiple infarctions)	rs1454336	A	G	-0.009	91873093	0.590	0.018	14306	A	G	-0.369	4	90951942	4.48415E-06	0.080	360612	13616.170
genus Oscillospira	Vascular dementia (multiple infarctions)	rs34288661	C	T	0.000	20948329	0.989	0.021	14306	C	T	0.460	8	21090818	7.22903E-06	0.103	360612	11969.376
genus Oscillospira	Vascular dementia (multiple infarctions)	rs429358	C	T	-0.005	45411941	0.763	0.020	14306	C	T	0.660	19	44908684	1.33506E-17	0.077	360612	52325.340
genus Oscillospira	Vascular dementia (multiple infarctions)	rs4725579	C	A	-0.015	139468213	0.414	0.017	14306	C	A	-0.379	7	139768414	3.58039E-06	0.082	360612	18391.668
genus Oscillospira	Vascular dementia (multiple infarctions)	rs72822148	T	C	-0.003	9742028	0.974	0.015	14306	T	C	-0.322	17	9838711	3.18831E-06	0.069	360612	17983.471
genus Oscillospira	Vascular dementia (multiple infarctions)	rs73053797	T	C	0.013	29909039	0.486	0.020	14306	T	C	0.338	3	29867548	6.00772E-06	0.075	360612	13680.576
genus Oxalobacter	Vascular dementia (multiple infarctions)	rs34288661	C	T	0.015	20948329	0.627	0.031	14306	C	T	0.460	8	21090818	7.22903E-06	0.103	360612	11969.376
genus Oxalobacter	Vascular dementia (multiple infarctions)	rs429358	C	T	0.034	45411941	0.200	0.030	14306	C	T	0.660	19	44908684	1.33506E-17	0.077	360612	52325.340
genus Oxalobacter	Vascular dementia (multiple infarctions)	rs4716814	T	C	-0.009	157723046	0.650	0.021	14306	T	C	-0.319	7	157930354	3.75656E-07	0.063	360612	19180.021
genus Oxalobacter	Vascular dementia (multiple infarctions)	rs4725579	C	A	0.011	139468213	0.690	0.026	14306	C	A	-0.379	7	139768414	3.58039E-06	0.082	360612	18391.668
genus Oxalobacter	Vascular dementia (multiple infarctions)	rs72822148	T	C	-0.012	9742028	0.635	0.022	14306	T	C	-0.322	17	9838711	3.18831E-06	0.069	360612	17983.471
genus Parabacteroides	Vascular dementia (multiple infarctions)	rs11081443	C	T	-0.001	8944208	0.999	0.013	14306	C	T	0.464	18	8944210	5.71874E-06	0.102	360612	19066.663
genus Parabacteroides	Vascular dementia (multiple infarctions)	rs34288661	C	T	0.010	20948329	0.543	0.016	14306	C	T	0.460	8	21090818	7.22903E-06	0.103	360612	11969.376
genus Parabacteroides	Vascular dementia (multiple infarctions)	rs429358	C	T	-0.009	45411941	0.601	0.015	14306	C	T	0.660	19	44908684	1.33506E-17	0.077	360612	52325.340
genus Parabacteroides	Vascular dementia (multiple infarctions)	rs4716814	T	C	-0.004	157723046	0.697	0.011	14306	T	C	-0.319	7	157930354	3.75656E-07	0.063	360612	19180.021
genus Parabacteroides	Vascular dementia (multiple infarctions)	rs4725579	C	A	-0.010	139468213	0.489	0.013	14306	C	A	-0.379	7	139768414	3.58039E-06	0.082	360612	18391.668
genus Parabacteroides	Vascular dementia (multiple infarctions)	rs73053797	T	C	0.006	29909039	0.526	0.015	14306	T	C	0.338	3	29867548	6.00772E-06	0.075	360612	13680.576
genus Parabacteroides	Vascular dementia (multiple infarctions)	rs9861644	A	G	-0.009	88637331	0.472	0.012	14306	A	G	0.323	3	88588181	4.84942E-06	0.071	360612	17137.658
genus Paraprevotella	Vascular dementia (multiple infarctions)	rs34288661	C	T	-0.016	20948329	0.516	0.025	14306	C	T	0.460	8	21090818	7.22903E-06	0.103	360612	11969.376
genus Paraprevotella	Vascular dementia (multiple infarctions)	rs429358	C	T	-0.005	45411941	0.844	0.024	14306	C	T	0.660	19	44908684	1.33506E-17	0.077	360612	52325.340
genus Paraprevotella	Vascular dementia (multiple infarctions)	rs4716814	T	C	-0.005	157723046	0.811	0.017	14306	T	C	-0.319	7	157930354	3.75656E-07	0.063	360612	19180.021
genus Paraprevotella	Vascular dementia (multiple infarctions)	rs4725579	C	A	-0.018	139468213	0.276	0.020	14306	C	A	-0.379	7	139768414	3.58039E-06	0.082	360612	18391.668
genus Paraprevotella	Vascular dementia (multiple infarctions)	rs72822148	T	C	-0.005	9742028	0.976	0.019	14306	T	C	-0.322	17	9838711	3.18831E-06	0.069	360612	17983.471
genus Paraprevotella	Vascular dementia (multiple infarctions)	rs73053797	T	C	0.005	29909039	0.831	0.023	14306	T	C	0.338	3	29867548	6.00772E-06	0.075	360612	13680.576

genus Paraprevotella	Vascular dementia (multiple infarctions)	rs9861644	A	G	-0.009	88637331	0.721	0.019	14306	A	G	0.323	3	88588181	4.84942E-06	0.071	360612	17137.658
genus Parasutterella	Vascular dementia (multiple infarctions)	rs34288661	C	T	0.010	20948329	0.552	0.020	14306	C	T	0.460	8	21090818	7.22903E-06	0.103	360612	11969.376
genus Parasutterella	Vascular dementia (multiple infarctions)	rs4716814	T	C	-0.005	157723046	0.708	0.013	14306	T	C	-0.319	7	157930354	3.75656E-07	0.063	360612	19180.021
genus Parasutterella	Vascular dementia (multiple infarctions)	rs73053797	T	C	-0.006	29909039	0.646	0.019	14306	T	C	0.338	3	29867548	6.00772E-06	0.075	360612	13680.576
genus Peptococcus	Vascular dementia (multiple infarctions)	rs11081443	C	T	0.006	8944208	0.858	0.023	14306	C	T	0.464	18	8944210	5.71874E-06	0.102	360612	19066.663
genus Peptococcus	Vascular dementia (multiple infarctions)	rs429358	C	T	0.031	45411941	0.231	0.027	14306	C	T	0.660	19	44908684	1.33506E-17	0.077	360612	52325.340
genus Peptococcus	Vascular dementia (multiple infarctions)	rs4716814	T	C	0.005	157723046	0.806	0.019	14306	T	C	-0.319	7	157930354	3.75656E-07	0.063	360612	19180.021
genus Peptococcus	Vascular dementia (multiple infarctions)	rs72822148	T	C	0.003	9742028	0.958	0.020	14306	T	C	-0.322	17	9838711	3.18831E-06	0.069	360612	17983.471
genus Phascolarctobacterium	Vascular dementia (multiple infarctions)	rs11081443	C	T	0.014	8944208	0.379	0.017	14306	C	T	0.464	18	8944210	5.71874E-06	0.102	360612	19066.663
genus Phascolarctobacterium	Vascular dementia (multiple infarctions)	rs34288661	C	T	-0.001	20948329	0.956	0.020	14306	C	T	0.460	8	21090818	7.22903E-06	0.103	360612	11969.376
genus Phascolarctobacterium	Vascular dementia (multiple infarctions)	rs429358	C	T	-0.013	45411941	0.427	0.019	14306	C	T	0.660	19	44908684	1.33506E-17	0.077	360612	52325.340
genus Phascolarctobacterium	Vascular dementia (multiple infarctions)	rs72822148	T	C	0.013	9742028	0.456	0.015	14306	T	C	-0.322	17	9838711	3.18831E-06	0.069	360612	17983.471
genus Phascolarctobacterium	Vascular dementia (multiple infarctions)	rs9861644	A	G	-0.008	88637331	0.633	0.015	14306	A	G	0.323	3	88588181	4.84942E-06	0.071	360612	17137.658
genus Prevotella7	Vascular dementia (multiple infarctions)	rs11081443	C	T	0.002	8944208	0.992	0.029	14306	C	T	0.464	18	8944210	5.71874E-06	0.102	360612	19066.663
genus Prevotella7	Vascular dementia (multiple infarctions)	rs34288661	C	T	0.028	20948329	0.415	0.035	14306	C	T	0.460	8	21090818	7.22903E-06	0.103	360612	11969.376
genus Prevotella7	Vascular dementia (multiple infarctions)	rs429358	C	T	0.047	45411941	0.135	0.034	14306	C	T	0.660	19	44908684	1.33506E-17	0.077	360612	52325.340
genus Prevotella7	Vascular dementia (multiple infarctions)	rs4716814	T	C	-0.011	157723046	0.650	0.024	14306	T	C	-0.319	7	157930354	3.75656E-07	0.063	360612	19180.021
genus Prevotella7	Vascular dementia (multiple infarctions)	rs4725579	C	A	-0.019	139468213	0.562	0.030	14306	C	A	-0.379	7	139768414	3.58039E-06	0.082	360612	18391.668
genus Prevotella7	Vascular dementia (multiple infarctions)	rs72822148	T	C	-0.014	9742028	0.592	0.026	14306	T	C	-0.322	17	9838711	3.18831E-06	0.069	360612	17983.471
genus Prevotella7	Vascular dementia (multiple infarctions)	rs73053797	T	C	-0.021	29909039	0.586	0.034	14306	T	C	0.338	3	29867548	6.00772E-06	0.075	360612	13680.576
genus Prevotella7	Vascular dementia (multiple infarctions)	rs9861644	A	G	-0.011	88637331	0.714	0.028	14306	A	G	0.323	3	88588181	4.84942E-06	0.071	360612	17137.658
genus Prevotella9	Vascular dementia (multiple infarctions)	rs1454336	A	G	0.011	91873093	0.579	0.018	14306	A	G	-0.369	4	90951942	4.48415E-06	0.080	360612	13616.170
genus Prevotella9	Vascular dementia (multiple infarctions)	rs429358	C	T	0.022	45411941	0.310	0.020	14306	C	T	0.660	19	44908684	1.33506E-17	0.077	360612	52325.340
genus Prevotella9	Vascular dementia (multiple infarctions)	rs4716814	T	C	-0.014	157723046	0.335	0.014	14306	T	C	-0.319	7	157930354	3.75656E-07	0.063	360612	19180.021
genus Prevotella9	Vascular dementia (multiple infarctions)	rs72822148	T	C	0.010	9742028	0.588	0.016	14306	T	C	-0.322	17	9838711	3.18831E-06	0.069	360612	17983.471
genus Prevotella9	Vascular dementia (multiple infarctions)	rs73053797	T	C	0.016	29909039	0.553	0.019	14306	T	C	0.338	3	29867548	6.00772E-06	0.075	360612	13680.576
genus Prevotella9	Vascular dementia (multiple infarctions)	rs9861644	A	G	-0.004	88637331	0.780	0.016	14306	A	G	0.323	3	88588181	4.84942E-06	0.071	360612	17137.658
genus Rikenellaceae RC9 gut group	Vascular dementia (multiple infarctions)	rs11081443	C	T	0.017	8944208	0.584	0.030	14306	C	T	0.464	18	8944210	5.71874E-06	0.102	360612	19066.663
genus Rikenellaceae RC9 gut group	Vascular dementia (multiple infarctions)	rs34288661	C	T	-0.031	20948329	0.366	0.037	14306	C	T	0.460	8	21090818	7.22903E-06	0.103	360612	11969.376
genus Rikenellaceae RC9 gut group	Vascular dementia (multiple infarctions)	rs4716814	T	C	-0.002	157723046	0.944	0.025	14306	T	C	-0.319	7	157930354	3.75656E-07	0.063	360612	19180.021
genus Rikenellaceae RC9 gut group	Vascular dementia (multiple infarctions)	rs4725579	C	A	0.010	139468213	0.737	0.030	14306	C	A	-0.379	7	139768414	3.58039E-06	0.082	360612	18391.668
genus Rikenellaceae RC9 gut group	Vascular dementia (multiple infarctions)	rs73053797	T	C	-0.025	29909039	0.606	0.035	14306	T	C	0.338	3	29867548	6.00772E-06	0.075	360612	13680.576
genus Rikenellaceae RC9 gut group	Vascular dementia (multiple infarctions)	rs9861644	A	G	0.002	88637331	0.942	0.028	14306	A	G	0.323	3	88588181	4.84942E-06	0.071	360612	17137.658
genus Romboutsia	Vascular dementia (multiple infarctions)	rs1454336	A	G	-0.001	91873093	0.984	0.016	14306	A	G	-0.369	4	90951942	4.48415E-06	0.080	360612	13616.170
genus Romboutsia	Vascular dementia (multiple infarctions)	rs429358	C	T	0.005	45411941	0.703	0.017	14306	C	T	0.660	19	44908684	1.33506E-17	0.077	360612	52325.340
genus Romboutsia	Vascular dementia (multiple infarctions)	rs4716814	T	C	-0.001	157723046	0.916	0.012	14306	T	C	-0.319	7	157930354	3.75656E-07	0.063	360612	19180.021
genus Romboutsia	Vascular dementia (multiple infarctions)	rs72822148	T	C	-0.010	9742028	0.357	0.013	14306	T	C	-0.322	17	9838711	3.18831E-06	0.069	360612	17983.471
genus Romboutsia	Vascular dementia (multiple infarctions)	rs9861644	A	G	-0.012	88637331	0.386	0.014	14306	A	G	0.323	3	88588181	4.84942E-06	0.071	360612	17137.658
genus Roseburia	Vascular dementia (multiple infarctions)	rs11081443	C	T	0.007	8944208	0.615	0.013	14306	C	T	0.464	18	8944210	5.71874E-06	0.102	360612	19066.663
genus Roseburia	Vascular dementia (multiple infarctions)	rs1454336	A	G	0.003	91873093	0.869	0.014	14306	A	G	-0.369	4	90951942	4.48415E-06	0.080	360612	13616.170
genus Roseburia	Vascular dementia (multiple infarctions)	rs429358	C	T	-0.001	45411941	0.921	0.015	14306	C	T	0.660	19	44908684	1.33506E-17	0.077	360612	52325.340
genus Roseburia	Vascular dementia (multiple infarctions)	rs73053797	T	C	-0.012	29909039	0.451	0.015	14306	T	C	0.338	3	29867548	6.00772E-06	0.075	360612	13680.576
genus Roseburia	Vascular dementia (multiple infarctions)	rs9861644	A	G	0.006	88637331	0.566	0.012	14306	A	G	0.323	3	88588181	4.84942E-06	0.071	360612	17137.658
genus Ruminiclostridium5	Vascular dementia (multiple infarctions)	rs11081443	C	T	0.004	8944208	0.747	0.013	14306	C	T	0.464	18	8944210	5.71874E-06	0.102	360612	19066.663
genus Ruminiclostridium5	Vascular dementia (multiple infarctions)	rs1454336	A	G	-0.002	91873093	0.833	0.014	14306	A	G	-0.369	4	90951942	4.48415E-06	0.080	360612	13616.170
genus Ruminiclostridium5	Vascular dementia (multiple infarctions)	rs34288661	C	T	-0.008	20948329	0.682	0.016	14306	C	T	0.460	8	21090818	7.22903E-06	0.103	360612	11969.376
genus Ruminiclostridium5	Vascular dementia (multiple infarctions)	rs429358	C	T	0.022	45411941	0.184	0.015	14306	C	T	0.660	19	44908684	1.33506E-17	0.077	360612	52325.340
genus Ruminiclostridium5	Vascular dementia (multiple infarctions)	rs4716814	T	C	0.004	157723046	0.691	0.011	14306	T	C	-0.319	7	157930354	3.75656E-07	0.063	360612	19180.021
genus Ruminiclostridium5	Vascular dementia (multiple infarctions)	rs4725579	C	A	-0.004	139468213	0.711	0.013	14306	C	A	-0.379	7	139768414	3.58039E-06	0.082	360612	18391.668
genus Ruminiclostridium5	Vascular dementia (multiple infarctions)	rs9861644	A	G	0.008	88637331	0.521	0.012	14306	A	G	0.323	3	88588181	4.84942E-06	0.071	360612	17137.658
genus Ruminiclostridium6	Vascular dementia (multiple infarctions)	rs11081443	C	T	-0.010	8944208	0.550	0.015	14306	C	T	0.464	18	8944210	5.71874E-06	0.102	360612	19066.663
genus Ruminiclostridium6	Vascular dementia (multiple infarctions)	rs429358	C	T	0.007	45411941	0.690	0.018	14306	C	T	0.660	19	44908684	1.33506E-17	0.077	360612	52325.340
genus Ruminiclostridium6	Vascular dementia (multiple infarctions)	rs4716814	T	C	0.000	157723046	0.977	0.012	14306	T	C	-0.319	7	157930354	3.75656E-07	0.063	360612	19180.021
genus Ruminiclostridium6	Vascular dementia (multiple infarctions)	rs4725579	C	A	0.005	139468213	0.755	0.015	14306	C	A	-0.379	7	139768414	3.58039E-06	0.082	360612	18391.668
genus Ruminiclostridium6	Vascular dementia (multiple infarctions)	rs73053797	T	C	-0.015	29909039	0.352	0.018	14306	T	C	0.338	3	29867548	6.00772E-06	0.075	360612	13680.576
genus Ruminiclostridium6	Vascular dementia (multiple infarctions)	rs9861644	A	G	-0.009	88637331	0.474	0.014	14306	A	G	0.323	3	88588181	4.84942E-06	0.071	360612	17137.658
genus Ruminiclostridium9	Vascular dementia (multiple infarctions)	rs11081443	C	T	0.004	8944208	0.799	0.013	14306	C	T	0.464	18	8944210	5.71874E-06	0.102	360612	19066.663
genus Ruminiclostridium9	Vascular dementia (multiple infarctions)	rs1454336	A	G	-0.007	91873093	0.607	0.014	14306	A	G	-0.369	4	90951942	4.48415E-06	0.080	360612	13616.170
genus Ruminiclostridium9	Vascular dementia (multiple infarctions)	rs34288661	C	T	-0.002	20948329	0.961	0.016	14306	C	T	0.460	8	21090818	7.22903E-06	0.103	360612	11969.376
genus Ruminiclostridium9	Vascular dementia (multiple infarctions)	rs429358	C	T	-0.007	45411941	0.627	0.016	14306	C	T	0.660	19	44908684	1.33506E-17	0.077	360612	52325.340
genus Ruminiclostridium9	Vascular dementia (multiple infarctions)	rs72822148	T	C	0.003	9742028	0.816	0.012	14306	T	C	-0.322	17	9838711	3.18831E-06	0.069	360612	17983.471
genus Ruminococcaceae NK4A214 group	Vascular dementia (multiple infarctions)	rs11081443	C	T	0.002	8944208	0.772	0.014	14306	C	T	0.464	18	8944210	5.71874E-06	0.102	360612	19066.663
genus Ruminococcaceae NK4A214 group	Vascular dementia (multiple infarctions)	rs429358	C	T	0.002	45411941	0.898	0.016	14306	C	T	0.660	19	44908684	1.33506E-17	0.077	360612	52325.340
genus Ruminococcaceae NK4A214 group	Vascular dementia (multiple infarctions)	rs4716814	T	C	0.004	157723046	0.749	0.012	14306	T	C	-0.319	7	157930354	3.75656E-07	0.063	360612	19180.021
genus Ruminococcaceae NK4A214 group	Vascular dementia (multiple infarctions)	rs4725579	C	A	0.012	139468213	0.439	0.014	14306	C	A	-0.379	7	139768414	3.58039E-06	0.082	360612	18391.668
genus Ruminococcaceae NK4A214 group	Vascular dementia (multiple infarctions)	rs72822148	T	C	-0.002	9742028	0.779	0.012	14306	T	C	-0.322	17	9838711	3.18831E-06	0.069	360612	17983.471
genus Ruminococcaceae NK4A214 group	Vascular dementia (multiple infarctions)	rs73053797	T	C	0.000	29909039	0.942	0.016	14306	T	C	0.338	3	29867548	6.00772E-06	0.075	360612	13680.576
genus Ruminococcaceae NK4A214 group	Vascular dementia (multiple infarctions)	rs9861644	A	G	-0.002	88637331	0.828	0.013	14306	A	G	0.323	3	88588181	4.84942E-06	0.071	360612	17137.658
genus Ruminococcaceae UCG002	Vascular dementia (multiple infarctions)	rs11081443	C	T	-0.004	8944208	0.626	0.013	14306	C	T	0.464	18	8944210	5.71874E-06	0.102	360612	19066.663
genus Ruminococcaceae UCG002	Vascular dementia (multiple infarctions)	rs429358	C	T	-0.004	45411941	0.869	0.016	14306	C	T	0.660	19	44908684	1.33506E-17	0.077	360612	52325.340
genus Ruminococcaceae UCG002	Vascular dementia (multiple infarctions)	rs4716814	T	C	-0.003	157723046	0.805	0.011	14306	T	C	-0.319	7	157930354	3.75656E-07	0.063	360612	19180.021

genus Ruminococcaceae UCG002	Vascular dementia (multiple infarctions)	rs73053797	T	C	0.011	29909039	0.421	0.015	14306	T	C	0.338	3	29867548	6.00772E-06	0.075	360612	13680.576
genus Ruminococcaceae UCG002	Vascular dementia (multiple infarctions)	rs9861644	A	G	-0.008	88637331	0.540	0.012	14306	A	G	0.323	3	88588181	4.84942E-06	0.071	360612	17137.658
genus Ruminococcaceae UCG003	Vascular dementia (multiple infarctions)	rs34288661	C	T	0.006	20948329	0.706	0.018	14306	C	T	0.460	8	21090818	7.22903E-06	0.103	360612	11969.376
genus Ruminococcaceae UCG003	Vascular dementia (multiple infarctions)	rs429358	C	T	0.007	45411941	0.595	0.017	14306	C	T	0.660	19	44908684	1.33506E-17	0.077	360612	52325.340
genus Ruminococcaceae UCG003	Vascular dementia (multiple infarctions)	rs4716814	T	C	0.006	157723046	0.586	0.012	14306	T	C	-0.319	7	157930354	3.75656E-07	0.063	360612	19180.021
genus Ruminococcaceae UCG003	Vascular dementia (multiple infarctions)	rs4725579	C	A	0.004	139468213	0.911	0.015	14306	C	A	-0.379	7	139768414	3.58039E-06	0.082	360612	18391.668
genus Ruminococcaceae UCG003	Vascular dementia (multiple infarctions)	rs73053797	T	C	0.006	29909039	0.685	0.017	14306	T	C	0.338	3	29867548	6.00772E-06	0.075	360612	13680.576
genus Ruminococcaceae UCG004	Vascular dementia (multiple infarctions)	rs11081443	C	T	-0.006	8944208	0.734	0.018	14306	C	T	0.464	18	8944210	5.71874E-06	0.102	360612	19066.663
genus Ruminococcaceae UCG004	Vascular dementia (multiple infarctions)	rs1454336	A	G	0.008	91873093	0.650	0.019	14306	A	G	-0.369	4	90951942	4.48415E-06	0.080	360612	13616.170
genus Ruminococcaceae UCG004	Vascular dementia (multiple infarctions)	rs429358	C	T	0.025	45411941	0.252	0.021	14306	C	T	0.660	19	44908684	1.33506E-17	0.077	360612	52325.340
genus Ruminococcaceae UCG004	Vascular dementia (multiple infarctions)	rs72822148	T	C	0.002	9742028	0.825	0.016	14306	T	C	-0.322	17	9838711	3.18831E-06	0.069	360612	17983.471
genus Ruminococcaceae UCG004	Vascular dementia (multiple infarctions)	rs73053797	T	C	0.005	29909039	0.747	0.021	14306	T	C	0.338	3	29867548	6.00772E-06	0.075	360612	13680.576
genus Ruminococcaceae UCG004	Vascular dementia (multiple infarctions)	rs9861644	A	G	-0.006	88637331	0.706	0.017	14306	A	G	0.323	3	88588181	4.84942E-06	0.071	360612	17137.658
genus Ruminococcaceae UCG005	Vascular dementia (multiple infarctions)	rs1454336	A	G	-0.007	91873093	0.667	0.015	14306	A	G	-0.369	4	90951942	4.48415E-06	0.080	360612	13616.170
genus Ruminococcaceae UCG005	Vascular dementia (multiple infarctions)	rs34288661	C	T	-0.001	20948329	0.994	0.017	14306	C	T	0.460	8	21090818	7.22903E-06	0.103	360612	11969.376
genus Ruminococcaceae UCG005	Vascular dementia (multiple infarctions)	rs429358	C	T	0.004	45411941	0.741	0.016	14306	C	T	0.660	19	44908684	1.33506E-17	0.077	360612	52325.340
genus Ruminococcaceae UCG005	Vascular dementia (multiple infarctions)	rs4716814	T	C	0.009	157723046	0.450	0.011	14306	T	C	-0.319	7	157930354	3.75656E-07	0.063	360612	19180.021
genus Ruminococcaceae UCG005	Vascular dementia (multiple infarctions)	rs4725579	C	A	0.012	139468213	0.432	0.014	14306	C	A	-0.379	7	139768414	3.58039E-06	0.082	360612	18391.668
genus Ruminococcaceae UCG005	Vascular dementia (multiple infarctions)	rs73053797	T	C	0.011	29909039	0.390	0.016	14306	T	C	0.338	3	29867548	6.00772E-06	0.075	360612	13680.576
genus Ruminococcaceae UCG005	Vascular dementia (multiple infarctions)	rs9861644	A	G	-0.007	88637331	0.547	0.013	14306	A	G	0.323	3	88588181	4.84942E-06	0.071	360612	17137.658
genus Ruminococcaceae UCG009	Vascular dementia (multiple infarctions)	rs11081443	C	T	0.009	8944208	0.657	0.020	14306	C	T	0.464	18	8944210	5.71874E-06	0.102	360612	19066.663
genus Ruminococcaceae UCG009	Vascular dementia (multiple infarctions)	rs1454336	A	G	-0.001	91873093	0.965	0.022	14306	A	G	-0.369	4	90951942	4.48415E-06	0.080	360612	13616.170
genus Ruminococcaceae UCG009	Vascular dementia (multiple infarctions)	rs34288661	C	T	-0.017	20948329	0.475	0.024	14306	C	T	0.460	8	21090818	7.22903E-06	0.103	360612	11969.376
genus Ruminococcaceae UCG009	Vascular dementia (multiple infarctions)	rs4716814	T	C	0.004	157723046	0.817	0.017	14306	T	C	-0.319	7	157930354	3.75656E-07	0.063	360612	19180.021
genus Ruminococcaceae UCG009	Vascular dementia (multiple infarctions)	rs73053797	T	C	-0.006	29909039	0.783	0.023	14306	T	C	0.338	3	29867548	6.00772E-06	0.075	360612	13680.576
genus Ruminococcaceae UCG009	Vascular dementia (multiple infarctions)	rs9861644	A	G	0.014	88637331	0.444	0.019	14306	A	G	0.323	3	88588181	4.84942E-06	0.071	360612	17137.658
genus Ruminococcaceae UCG010	Vascular dementia (multiple infarctions)	rs429358	C	T	0.003	45411941	0.881	0.018	14306	C	T	0.660	19	44908684	1.33506E-17	0.077	360612	52325.340
genus Ruminococcaceae UCG010	Vascular dementia (multiple infarctions)	rs4716814	T	C	0.000	157723046	0.982	0.013	14306	T	C	-0.319	7	157930354	3.75656E-07	0.063	360612	19180.021
genus Ruminococcaceae UCG010	Vascular dementia (multiple infarctions)	rs4725579	C	A	0.007	139468213	0.705	0.015	14306	C	A	-0.379	7	139768414	3.58039E-06	0.082	360612	18391.668
genus Ruminococcaceae UCG010	Vascular dementia (multiple infarctions)	rs72822148	T	C	0.006	9742028	0.673	0.014	14306	T	C	-0.322	17	9838711	3.18831E-06	0.069	360612	17983.471
genus Ruminococcaceae UCG010	Vascular dementia (multiple infarctions)	rs73053797	T	C	-0.011	29909039	0.659	0.018	14306	T	C	0.338	3	29867548	6.00772E-06	0.075	360612	13680.576
genus Ruminococcaceae UCG011	Vascular dementia (multiple infarctions)	rs34288661	C	T	-0.024	20948329	0.513	0.034	14306	C	T	0.460	8	21090818	7.22903E-06	0.103	360612	11969.376
genus Ruminococcaceae UCG011	Vascular dementia (multiple infarctions)	rs429358	C	T	-0.013	45411941	0.704	0.033	14306	C	T	0.660	19	44908684	1.33506E-17	0.077	360612	52325.340
genus Ruminococcaceae UCG011	Vascular dementia (multiple infarctions)	rs4716814	T	C	-0.012	157723046	0.590	0.024	14306	T	C	-0.319	7	157930354	3.75656E-07	0.063	360612	19180.021
genus Ruminococcaceae UCG011	Vascular dementia (multiple infarctions)	rs72822148	T	C	-0.006	9742028	0.868	0.025	14306	T	C	-0.322	17	9838711	3.18831E-06	0.069	360612	17983.471
genus Ruminococcaceae UCG011	Vascular dementia (multiple infarctions)	rs73053797	T	C	0.011	29909039	0.791	0.034	14306	T	C	0.338	3	29867548	6.00772E-06	0.075	360612	13680.576
genus Ruminococcaceae UCG013	Vascular dementia (multiple infarctions)	rs1454336	A	G	-0.009	91873093	0.491	0.014	14306	A	G	-0.369	4	90951942	4.48415E-06	0.080	360612	13616.170
genus Ruminococcaceae UCG013	Vascular dementia (multiple infarctions)	rs34288661	C	T	-0.011	20948329	0.493	0.016	14306	C	T	0.460	8	21090818	7.22903E-06	0.103	360612	11969.376
genus Ruminococcaceae UCG013	Vascular dementia (multiple infarctions)	rs429358	C	T	-0.022	45411941	0.167	0.016	14306	C	T	0.660	19	44908684	1.33506E-17	0.077	360612	52325.340
genus Ruminococcaceae UCG013	Vascular dementia (multiple infarctions)	rs4716814	T	C	-0.002	157723046	0.823	0.011	14306	T	C	-0.319	7	157930354	3.75656E-07	0.063	360612	19180.021
genus Ruminococcaceae UCG013	Vascular dementia (multiple infarctions)	rs4725579	C	A	0.008	139468213	0.646	0.014	14306	C	A	-0.379	7	139768414	3.58039E-06	0.082	360612	18391.668
genus Ruminococcaceae UCG013	Vascular dementia (multiple infarctions)	rs73053797	T	C	0.012	29909039	0.342	0.016	14306	T	C	0.338	3	29867548	6.00772E-06	0.075	360612	13680.576
genus Ruminococcaceae UCG014	Vascular dementia (multiple infarctions)	rs34288661	C	T	0.002	20948329	0.908	0.017	14306	C	T	0.460	8	21090818	7.22903E-06	0.103	360612	11969.376
genus Ruminococcaceae UCG014	Vascular dementia (multiple infarctions)	rs429358	C	T	0.020	45411941	0.191	0.017	14306	C	T	0.660	19	44908684	1.33506E-17	0.077	360612	52325.340
genus Ruminococcaceae UCG014	Vascular dementia (multiple infarctions)	rs4725579	C	A	-0.011	139468213	0.454	0.015	14306	C	A	-0.379	7	139768414	3.58039E-06	0.082	360612	18391.668
genus Ruminococcaceae UCG014	Vascular dementia (multiple infarctions)	rs72822148	T	C	-0.006	9742028	0.603	0.013	14306	T	C	-0.322	17	9838711	3.18831E-06	0.069	360612	17983.471
genus Ruminococcaceae UCG014	Vascular dementia (multiple infarctions)	rs73053797	T	C	-0.009	29909039	0.633	0.017	14306	T	C	0.338	3	29867548	6.00772E-06	0.075	360612	13680.576
genus Ruminococcaceae UCG014	Vascular dementia (multiple infarctions)	rs9861644	A	G	0.003	88637331	0.848	0.014	14306	A	G	0.323	3	88588181	4.84942E-06	0.071	360612	17137.658
genus Ruminococcus1	Vascular dementia (multiple infarctions)	rs11081443	C	T	-0.011	8944208	0.491	0.014	14306	C	T	0.464	18	8944210	5.71874E-06	0.102	360612	19066.663
genus Ruminococcus1	Vascular dementia (multiple infarctions)	rs1454336	A	G	0.000	91873093	0.957	0.014	14306	A	G	-0.369	4	90951942	4.48415E-06	0.080	360612	13616.170
genus Ruminococcus1	Vascular dementia (multiple infarctions)	rs34288661	C	T	-0.013	20948329	0.442	0.016	14306	C	T	0.460	8	21090818	7.22903E-06	0.103	360612	11969.376
genus Ruminococcus1	Vascular dementia (multiple infarctions)	rs429358	C	T	-0.008	45411941	0.683	0.016	14306	C	T	0.660	19	44908684	1.33506E-17	0.077	360612	52325.340
genus Ruminococcus1	Vascular dementia (multiple infarctions)	rs73053797	T	C	0.004	29909039	0.943	0.016	14306	T	C	0.338	3	29867548	6.00772E-06	0.075	360612	13680.576
genus Ruminococcus2	Vascular dementia (multiple infarctions)	rs11081443	C	T	0.004	8944208	0.830	0.014	14306	C	T	0.464	18	8944210	5.71874E-06	0.102	360612	19066.663
genus Ruminococcus2	Vascular dementia (multiple infarctions)	rs429358	C	T	0.007	45411941	0.633	0.016	14306	C	T	0.660	19	44908684	1.33506E-17	0.077	360612	52325.340
genus Ruminococcus2	Vascular dementia (multiple infarctions)	rs4716814	T	C	-0.007	157723046	0.558	0.012	14306	T	C	-0.319	7	157930354	3.75656E-07	0.063	360612	19180.021
genus Ruminococcus2	Vascular dementia (multiple infarctions)	rs72822148	T	C	0.010	9742028	0.397	0.012	14306	T	C	-0.322	17	9838711	3.18831E-06	0.069	360612	17983.471
genus Ruminococcus2	Vascular dementia (multiple infarctions)	rs9861644	A	G	-0.004	88637331	0.754	0.013	14306	A	G	0.323	3	88588181	4.84942E-06	0.071	360612	17137.658
genus Ruminococcus gauvreauii group	Vascular dementia (multiple infarctions)	rs11081443	C	T	-0.006	8944208	0.795	0.015	14306	C	T	0.464	18	8944210	5.71874E-06	0.102	360612	19066.663
genus Ruminococcus gauvreauii group	Vascular dementia (multiple infarctions)	rs1454336	A	G	0.008	91873093	0.661	0.016	14306	A	G	-0.369	4	90951942	4.48415E-06	0.080	360612	13616.170
genus Ruminococcus gauvreauii group	Vascular dementia (multiple infarctions)	rs34288661	C	T	-0.007	20948329	0.696	0.018	14306	C	T	0.460	8	21090818	7.22903E-06	0.103	360612	11969.376
genus Ruminococcus gauvreauii group	Vascular dementia (multiple infarctions)	rs429358	C	T	0.011	45411941	0.457	0.018	14306	C	T	0.660	19	44908684	1.33506E-17	0.077	360612	52325.340
genus Ruminococcus gauvreauii group	Vascular dementia (multiple infarctions)	rs4725579	C	A	0.010	139468213	0.607	0.015	14306	C	A	-0.379	7	139768414	3.58039E-06	0.082	360612	18391.668
genus Ruminococcus gauvreauii group	Vascular dementia (multiple infarctions)	rs73053797	T	C	-0.012	29909039	0.436	0.017	14306	T	C	0.338	3	29867548	6.00772E-06	0.075	360612	13680.576
genus Ruminococcus gnavus group	Vascular dementia (multiple infarctions)	rs11081443	C	T	0.010	8944208	0.602	0.022	14306	C	T	0.464	18	8944210	5.71874E-06	0.102	360612	19066.663
genus Ruminococcus gnavus group	Vascular dementia (multiple infarctions)	rs1454336	A	G	0.001	91873093	0.996	0.023	14306	A	G	-0.369	4	90951942	4.48415E-06	0.080	360612	13616.170
genus Ruminococcus gnavus group	Vascular dementia (multiple infarctions)	rs34288661	C	T	0.011	20948329	0.717	0.027	14306	C	T	0.460	8	21090818	7.22903E-06	0.103	360612	11969.376
genus Ruminococcus gnavus group	Vascular dementia (multiple infarctions)	rs429358	C	T	0.039	45411941	0.188	0.026	14306	C	T	0.660	19	44908684	1.33506E-17	0.077	360612	52325.340
genus Ruminococcus gnavus group	Vascular dementia (multiple infarctions)	rs4716814	T	C	-0.009	157723046	0.625	0.018	14306	T	C	-0.319	7	157930354	3.75656E-07	0.063	360612	19180.021
genus Ruminococcus gnavus group	Vascular dementia (multiple infarctions)	rs4725579	C	A	-0.002	139468213	0.959	0.022	14306	C	A	-0.379	7	139768414	3.58039E-06	0.082	360612	18391.668
genus Ruminococcus gnavus group	Vascular dementia (multiple infarctions)	rs72822148	T	C	-0.004	9742028	0.964	0.019	14306	T	C	-0.322	17	9838711	3.18831E-06	0.069	360612	17983.471

genus Ruminococcus gnavus group	Vascular dementia (multiple infarctions)	rs73053797	T	C	0.011	29909039	0.585	0.025	14306	T	C	0.338	3	29867548	6.00772E-06	0.075	360612	13680.576
genus Ruminococcus gnavus group	Vascular dementia (multiple infarctions)	rs9861644	A	G	0.003	88637331	0.833	0.020	14306	A	G	0.323	3	88588181	4.84942E-06	0.071	360612	17137.658
genus Ruminococcus torques group	Vascular dementia (multiple infarctions)	rs11081443	C	T	0.011	8944208	0.385	0.013	14306	C	T	0.464	18	8944210	5.71874E-06	0.102	360612	19066.663
genus Ruminococcus torques group	Vascular dementia (multiple infarctions)	rs429358	C	T	-0.001	45411941	0.916	0.015	14306	C	T	0.660	19	44908684	1.33506E-17	0.077	360612	52325.340
genus Ruminococcus torques group	Vascular dementia (multiple infarctions)	rs73053797	T	C	0.000	29909039	0.968	0.015	14306	T	C	0.338	3	29867548	6.00772E-06	0.075	360612	13680.576
genus Sellimonas	Vascular dementia (multiple infarctions)	rs11081443	C	T	-0.025	8944208	0.482	0.031	14306	C	T	0.464	18	8944210	5.71874E-06	0.102	360612	19066.663
genus Sellimonas	Vascular dementia (multiple infarctions)	rs1454336	A	G	-0.017	91873093	0.551	0.033	14306	A	G	-0.369	4	90951942	4.48415E-06	0.080	360612	13616.170
genus Sellimonas	Vascular dementia (multiple infarctions)	rs34288661	C	T	-0.003	20948329	0.967	0.037	14306	C	T	0.460	8	21090818	7.22903E-06	0.103	360612	11969.376
genus Sellimonas	Vascular dementia (multiple infarctions)	rs4716814	T	C	0.014	157723046	0.607	0.025	14306	T	C	-0.319	7	157930354	3.75656E-07	0.063	360612	19180.021
genus Sellimonas	Vascular dementia (multiple infarctions)	rs4725579	C	A	-0.002	139468213	0.989	0.032	14306	C	A	-0.379	7	139768414	3.58039E-06	0.082	360612	18391.668
genus Sellimonas	Vascular dementia (multiple infarctions)	rs72822148	T	C	-0.010	9742028	0.746	0.027	14306	T	C	-0.322	17	9838711	3.18831E-06	0.069	360612	17983.471
genus Sellimonas	Vascular dementia (multiple infarctions)	rs73053797	T	C	-0.014	29909039	0.725	0.037	14306	T	C	0.338	3	29867548	6.00772E-06	0.075	360612	13680.576
genus Senegalimassilia	Vascular dementia (multiple infarctions)	rs1454336	A	G	0.019	91873093	0.386	0.023	14306	A	G	-0.369	4	90951942	4.48415E-06	0.080	360612	13616.170
genus Senegalimassilia	Vascular dementia (multiple infarctions)	rs4716814	T	C	-0.012	157723046	0.508	0.017	14306	T	C	-0.319	7	157930354	3.75656E-07	0.063	360612	19180.021
genus Senegalimassilia	Vascular dementia (multiple infarctions)	rs4725579	C	A	-0.014	139468213	0.509	0.021	14306	C	A	-0.379	7	139768414	3.58039E-06	0.082	360612	18391.668
genus Senegalimassilia	Vascular dementia (multiple infarctions)	rs73053797	T	C	0.004	29909039	0.841	0.024	14306	T	C	0.338	3	29867548	6.00772E-06	0.075	360612	13680.576
genus Slackia	Vascular dementia (multiple infarctions)	rs1454336	A	G	-0.007	91873093	0.785	0.024	14306	A	G	-0.369	4	90951942	4.48415E-06	0.080	360612	13616.170
genus Slackia	Vascular dementia (multiple infarctions)	rs34288661	C	T	0.016	20948329	0.542	0.027	14306	C	T	0.460	8	21090818	7.22903E-06	0.103	360612	11969.376
genus Slackia	Vascular dementia (multiple infarctions)	rs429358	C	T	0.036	45411941	0.159	0.026	14306	C	T	0.660	19	44908684	1.33506E-17	0.077	360612	52325.340
genus Slackia	Vascular dementia (multiple infarctions)	rs4716814	T	C	-0.015	157723046	0.429	0.018	14306	T	C	-0.319	7	157930354	3.75656E-07	0.063	360612	19180.021
genus Slackia	Vascular dementia (multiple infarctions)	rs9861644	A	G	-0.017	88637331	0.416	0.021	14306	A	G	0.323	3	88588181	4.84942E-06	0.071	360612	17137.658
genus Streptococcus	Vascular dementia (multiple infarctions)	rs11081443	C	T	0.012	8944208	0.441	0.014	14306	C	T	0.464	18	8944210	5.71874E-06	0.102	360612	19066.663
genus Streptococcus	Vascular dementia (multiple infarctions)	rs1454336	A	G	0.013	91873093	0.362	0.014	14306	A	G	-0.369	4	90951942	4.48415E-06	0.080	360612	13616.170
genus Streptococcus	Vascular dementia (multiple infarctions)	rs429358	C	T	0.017	45411941	0.279	0.016	14306	C	T	0.660	19	44908684	1.33506E-17	0.077	360612	52325.340
genus Streptococcus	Vascular dementia (multiple infarctions)	rs4725579	C	A	0.002	139468213	0.779	0.014	14306	C	A	-0.379	7	139768414	3.58039E-06	0.082	360612	18391.668
genus Streptococcus	Vascular dementia (multiple infarctions)	rs72822148	T	C	-0.006	9742028	0.439	0.012	14306	T	C	-0.322	17	9838711	3.18831E-06	0.069	360612	17983.471
genus Subdoligranulum	Vascular dementia (multiple infarctions)	rs11081443	C	T	0.007	8944208	0.519	0.013	14306	C	T	0.464	18	8944210	5.71874E-06	0.102	360612	19066.663
genus Subdoligranulum	Vascular dementia (multiple infarctions)	rs429358	C	T	-0.005	45411941	0.772	0.015	14306	C	T	0.660	19	44908684	1.33506E-17	0.077	360612	52325.340
genus Subdoligranulum	Vascular dementia (multiple infarctions)	rs4716814	T	C	-0.001	157723046	0.892	0.011	14306	T	C	-0.319	7	157930354	3.75656E-07	0.063	360612	19180.021
genus Subdoligranulum	Vascular dementia (multiple infarctions)	rs73053797	T	C	0.013	29909039	0.303	0.015	14306	T	C	0.338	3	29867548	6.00772E-06	0.075	360612	13680.576
genus Subdoligranulum	Vascular dementia (multiple infarctions)	rs9861644	A	G	0.003	88637331	0.861	0.012	14306	A	G	0.323	3	88588181	4.84942E-06	0.071	360612	17137.658
genus Sutterella	Vascular dementia (multiple infarctions)	rs11081443	C	T	0.006	8944208	0.752	0.015	14306	C	T	0.464	18	8944210	5.71874E-06	0.102	360612	19066.663
genus Sutterella	Vascular dementia (multiple infarctions)	rs1454336	A	G	-0.009	91873093	0.518	0.016	14306	A	G	-0.369	4	90951942	4.48415E-06	0.080	360612	13616.170
genus Sutterella	Vascular dementia (multiple infarctions)	rs34288661	C	T	0.001	20948329	0.959	0.018	14306	C	T	0.460	8	21090818	7.22903E-06	0.103	360612	11969.376
genus Sutterella	Vascular dementia (multiple infarctions)	rs429358	C	T	-0.028	45411941	0.121	0.018	14306	C	T	0.660	19	44908684	1.33506E-17	0.077	360612	52325.340
genus Sutterella	Vascular dementia (multiple infarctions)	rs4716814	T	C	-0.008	157723046	0.524	0.012	14306	T	C	-0.319	7	157930354	3.75656E-07	0.063	360612	19180.021
genus Sutterella	Vascular dementia (multiple infarctions)	rs4725579	C	A	-0.008	139468213	0.604	0.015	14306	C	A	-0.379	7	139768414	3.58039E-06	0.082	360612	18391.668
genus Sutterella	Vascular dementia (multiple infarctions)	rs72822148	T	C	0.010	9742028	0.446	0.013	14306	T	C	-0.322	17	9838711	3.18831E-06	0.069	360612	17983.471
genus Sutterella	Vascular dementia (multiple infarctions)	rs9861644	A	G	0.008	88637331	0.549	0.014	14306	A	G	0.323	3	88588181	4.84942E-06	0.071	360612	17137.658
genus Terrisporobacter	Vascular dementia (multiple infarctions)	rs1454336	A	G	0.008	91873093	0.753	0.023	14306	A	G	-0.369	4	90951942	4.48415E-06	0.080	360612	13616.170
genus Terrisporobacter	Vascular dementia (multiple infarctions)	rs34288661	C	T	-0.010	20948329	0.631	0.025	14306	C	T	0.460	8	21090818	7.22903E-06	0.103	360612	11969.376
genus Terrisporobacter	Vascular dementia (multiple infarctions)	rs429358	C	T	-0.010	45411941	0.657	0.025	14306	C	T	0.660	19	44908684	1.33506E-17	0.077	360612	52325.340
genus Terrisporobacter	Vascular dementia (multiple infarctions)	rs4716814	T	C	-0.008	157723046	0.652	0.018	14306	T	C	-0.319	7	157930354	3.75656E-07	0.063	360612	19180.021
genus Terrisporobacter	Vascular dementia (multiple infarctions)	rs72822148	T	C	-0.012	9742028	0.604	0.023	14306	T	C	-0.322	17	9838711	3.18831E-06	0.069	360612	17983.471
genus Turicibacter	Vascular dementia (multiple infarctions)	rs11081443	C	T	-0.013	8944208	0.428	0.018	14306	C	T	0.464	18	8944210	5.71874E-06	0.102	360612	19066.663
genus Turicibacter	Vascular dementia (multiple infarctions)	rs1454336	A	G	-0.009	91873093	0.635	0.020	14306	A	G	-0.369	4	90951942	4.48415E-06	0.080	360612	13616.170
genus Turicibacter	Vascular dementia (multiple infarctions)	rs429358	C	T	0.001	45411941	0.870	0.021	14306	C	T	0.660	19	44908684	1.33506E-17	0.077	360612	52325.340
genus Turicibacter	Vascular dementia (multiple infarctions)	rs4716814	T	C	-0.005	157723046	0.729	0.015	14306	T	C	-0.319	7	157930354	3.75656E-07	0.063	360612	19180.021
genus Turicibacter	Vascular dementia (multiple infarctions)	rs72822148	T	C	0.010	9742028	0.429	0.016	14306	T	C	-0.322	17	9838711	3.18831E-06	0.069	360612	17983.471
genus Turicibacter	Vascular dementia (multiple infarctions)	rs73053797	T	C	-0.004	29909039	0.875	0.022	14306	T	C	0.338	3	29867548	6.00772E-06	0.075	360612	13680.576
genus Turicibacter	Vascular dementia (multiple infarctions)	rs9861644	A	G	0.009	88637331	0.604	0.017	14306	A	G	0.323	3	88588181	4.84942E-06	0.071	360612	17137.658
genus Tyzzerella3	Vascular dementia (multiple infarctions)	rs11081443	C	T	-0.016	8944208	0.541	0.024	14306	C	T	0.464	18	8944210	5.71874E-06	0.102	360612	19066.663
genus Tyzzerella3	Vascular dementia (multiple infarctions)	rs34288661	C	T	0.012	20948329	0.678	0.029	14306	C	T	0.460	8	21090818	7.22903E-06	0.103	360612	11969.376
genus Tyzzerella3	Vascular dementia (multiple infarctions)	rs429358	C	T	0.000	45411941	0.979	0.028	14306	C	T	0.660	19	44908684	1.33506E-17	0.077	360612	52325.340
genus Tyzzerella3	Vascular dementia (multiple infarctions)	rs4716814	T	C	0.015	157723046	0.450	0.019	14306	T	C	-0.319	7	157930354	3.75656E-07	0.063	360612	19180.021
genus Tyzzerella3	Vascular dementia (multiple infarctions)	rs72822148	T	C	-0.019	9742028	0.503	0.021	14306	T	C	-0.322	17	9838711	3.18831E-06	0.069	360612	17983.471
genus Tyzzerella3	Vascular dementia (multiple infarctions)	rs73053797	T	C	-0.013	29909039	0.756	0.028	14306	T	C	0.338	3	29867548	6.00772E-06	0.075	360612	13680.576
genus Veillonella)	Vascular dementia (multiple infarctions)	rs1454336	A	G	0.002	91873093	0.901	0.019	14306	A	G	-0.369	4	90951942	4.48415E-06	0.080	360612	13616.170
genus Veillonella)	Vascular dementia (multiple infarctions)	rs4716814	T	C	0.008	157723046	0.577	0.015	14306	T	C	-0.319	7	157930354	3.75656E-07	0.063	360612	19180.021
genus Veillonella)	Vascular dementia (multiple infarctions)	rs72822148	T	C	-0.003	9742028	0.757	0.016	14306	T	C	-0.322	17	9838711	3.18831E-06	0.069	360612	17983.471
genus Veillonella)	Vascular dementia (multiple infarctions)	rs73053797	T	C	0.016	29909039	0.558	0.021	14306	T	C	0.338	3	29867548	6.00772E-06	0.075	360612	13680.576
genus Veillonella)	Vascular dementia (multiple infarctions)	rs9861644	A	G	-0.009	88637331	0.551	0.017	14306	A	G	0.323	3	88588181	4.84942E-06	0.071	360612	17137.658
genus Victivallis	Vascular dementia (multiple infarctions)	rs1454336	A	G	-0.001	91873093	0.980	0.032	1531	A	G	-0.369	4	90951942	4.48415E-06	0.080	360612	13616.170
genus Victivallis	Vascular dementia (multiple infarctions)	rs34288661	C	T	0.005	20948329	0.914	0.036	1531	C	T	0.460	8	21090818	7.22903E-06	0.103	360612	11969.376
genus Victivallis	Vascular dementia (multiple infarctions)	rs429358	C	T	0.009	45411941	0.847	0.037	1531	C	T	0.660	19	44908684	1.33506E-17	0.077	360612	52325.340
genus Victivallis	Vascular dementia (multiple infarctions)	rs4716814	T	C	0.004	157723046	0.846	0.025	1531	T	C	-0.319	7	157930354	3.75656E-07	0.063	360612	19180.021
genus Actinomyces	Vascular dementia (mixed)	rs12257900	T	G	-0.001	49443428	0.993	0.024	14306	T	G	0.467	10	48235385	2.48891E-06	0.099	360421	23006.877
genus Actinomyces	Vascular dementia (mixed)	rs1466525	T	C	0.005	54780209	0.751	0.020	14306	T	C	0.489	8	53867649	1.97697E-06	0.103	360421	34189.173
genus Actinomyces	Vascular dementia (mixed)	rs1632064	T	C	0.020	3219694	0.410	0.023	14306	T	C	0.614	5	3219580	4.04045E-06	0.133	360421	37296.871
genus Actinomyces	Vascular dementia (mixed)	rs17168895	T	G	-0.002	15647727	0.859	0.027	14306	T	G	-0.547	7	15608102	2.92853E-06	0.117	360421	34495.049
genus Actinomyces	Vascular dementia (mixed)	rs6028529	A	G	0.007	38205487	0.681	0.020	14306	A	G	0.413	20	39576844	4.98965E-06	0.091	360421	21913.090

genus Actinomyces	Vascular dementia (mixed)	rs6849229	G	A	-0.016	131797280	0.472	0.023	14306	G	A	0.562	4	130876125	4.44038E-06	0.122	360421	34142.711
genus Actinomyces	Vascular dementia (mixed)	rs7776624	G	A	-0.003	31909839	0.863	0.016	14306	G	A	-0.358	7	31870226	8.82531E-06	0.081	360421	23450.025
genus Adlercreutzia	Vascular dementia (mixed)	rs12257900	T	G	0.009	49443428	0.680	0.023	14306	T	G	0.467	10	48235385	2.48891E-06	0.099	360421	23006.877
genus Adlercreutzia	Vascular dementia (mixed)	rs1466525	T	C	-0.018	54780209	0.353	0.019	14306	T	C	0.489	8	53867649	1.97697E-06	0.103	360421	34189.173
genus Adlercreutzia	Vascular dementia (mixed)	rs1632064	T	C	-0.005	3219694	0.864	0.022	14306	T	C	0.614	5	3219580	4.04045E-06	0.133	360421	37296.871
genus Adlercreutzia	Vascular dementia (mixed)	rs17168895	T	G	-0.007	15647727	0.709	0.026	14306	T	G	-0.547	7	15608102	2.92853E-06	0.117	360421	34495.049
genus Adlercreutzia	Vascular dementia (mixed)	rs429358	C	T	0.007	45411941	0.796	0.022	14306	C	T	0.565	19	44908684	3.94357E-08	0.103	360421	36831.461
genus Adlercreutzia	Vascular dementia (mixed)	rs6028529	A	G	-0.002	38205487	0.903	0.019	14306	A	G	0.413	20	39576844	4.98965E-06	0.091	360421	21913.090
genus Adlercreutzia	Vascular dementia (mixed)	rs6849229	G	A	0.015	131797280	0.478	0.022	14306	G	A	0.562	4	130876125	4.44038E-06	0.122	360421	34142.711
genus Adlercreutzia	Vascular dementia (mixed)	rs7614116	G	A	0.001	130368069	0.936	0.017	14306	G	A	0.379	3	130649225	6.06429E-06	0.084	360421	23465.044
genus Akkermansia	Vascular dementia (mixed)	rs12257900	T	G	-0.014	49443428	0.455	0.019	14306	T	G	0.467	10	48235385	2.48891E-06	0.099	360421	23006.877
genus Akkermansia	Vascular dementia (mixed)	rs1466525	T	C	0.010	54780209	0.533	0.016	14306	T	C	0.489	8	53867649	1.97697E-06	0.103	360421	34189.173
genus Akkermansia	Vascular dementia (mixed)	rs1632064	T	C	0.001	3219694	0.904	0.018	14306	T	C	0.614	5	3219580	4.04045E-06	0.133	360421	37296.871
genus Akkermansia	Vascular dementia (mixed)	rs17168895	T	G	0.012	15647727	0.521	0.021	14306	T	G	-0.547	7	15608102	2.92853E-06	0.117	360421	34495.049
genus Akkermansia	Vascular dementia (mixed)	rs429358	C	T	0.018	45411941	0.337	0.019	14306	C	T	0.565	19	44908684	3.94357E-08	0.103	360421	36831.461
genus Akkermansia	Vascular dementia (mixed)	rs6028529	A	G	-0.013	38205487	0.450	0.016	14306	A	G	0.413	20	39576844	4.98965E-06	0.091	360421	21913.090
genus Akkermansia	Vascular dementia (mixed)	rs6849229	G	A	-0.013	131797280	0.493	0.018	14306	G	A	0.562	4	130876125	4.44038E-06	0.122	360421	34142.711
genus Akkermansia	Vascular dementia (mixed)	rs7614116	G	A	0.000	130368069	0.994	0.014	14306	G	A	0.379	3	130649225	6.06429E-06	0.084	360421	23465.044
genus Alistipes	Vascular dementia (mixed)	rs12257900	T	G	0.001	49443428	0.975	0.016	14306	T	G	0.467	10	48235385	2.48891E-06	0.099	360421	23006.877
genus Alistipes	Vascular dementia (mixed)	rs1632064	T	C	-0.005	3219694	0.707	0.015	14306	T	C	0.614	5	3219580	4.04045E-06	0.133	360421	37296.871
genus Alistipes	Vascular dementia (mixed)	rs6028529	A	G	0.010	38205487	0.435	0.013	14306	A	G	0.413	20	39576844	4.98965E-06	0.091	360421	21913.090
genus Alistipes	Vascular dementia (mixed)	rs6849229	G	A	-0.003	131797280	0.886	0.015	14306	G	A	0.562	4	130876125	4.44038E-06	0.122	360421	34142.711
genus Alistipes	Vascular dementia (mixed)	rs7614116	G	A	-0.005	130368069	0.690	0.012	14306	G	A	0.379	3	130649225	6.06429E-06	0.084	360421	23465.044
genus Allisonella	Vascular dementia (mixed)	rs1466525	T	C	0.019	54780209	0.666	0.032	14306	T	C	0.489	8	53867649	1.97697E-06	0.103	360421	34189.173
genus Allisonella	Vascular dementia (mixed)	rs429358	C	T	-0.033	45411941	0.350	0.036	14306	C	T	0.565	19	44908684	3.94357E-08	0.103	360421	36831.461
genus Allisonella	Vascular dementia (mixed)	rs6849229	G	A	-0.008	131797280	0.767	0.033	14306	G	A	0.562	4	130876125	4.44038E-06	0.122	360421	34142.711
genus Alloprevotella	Vascular dementia (mixed)	rs1632064	T	C	-0.031	3219694	0.286	0.035	14306	T	C	0.614	5	3219580	4.04045E-06	0.133	360421	37296.871
genus Alloprevotella	Vascular dementia (mixed)	rs7614116	G	A	-0.023	130368069	0.437	0.028	14306	G	A	0.379	3	130649225	6.06429E-06	0.084	360421	23465.044
genus Alloprevotella	Vascular dementia (mixed)	rs7776624	G	A	-0.022	31909839	0.415	0.026	14306	G	A	-0.358	7	31870226	8.82531E-06	0.081	360421	23450.025
genus Anaerofilum	Vascular dementia (mixed)	rs12257900	T	G	0.002	49443428	0.918	0.029	14306	T	G	0.467	10	48235385	2.48891E-06	0.099	360421	23006.877
genus Anaerofilum	Vascular dementia (mixed)	rs1466525	T	C	-0.010	54780209	0.708	0.025	14306	T	C	0.489	8	53867649	1.97697E-06	0.103	360421	34189.173
genus Anaerofilum	Vascular dementia (mixed)	rs1632064	T	C	0.010	3219694	0.729	0.028	14306	T	C	0.614	5	3219580	4.04045E-06	0.133	360421	37296.871
genus Anaerofilum	Vascular dementia (mixed)	rs429358	C	T	-0.013	45411941	0.660	0.028	14306	C	T	0.565	19	44908684	3.94357E-08	0.103	360421	36831.461
genus Anaerofilum	Vascular dementia (mixed)	rs6028529	A	G	-0.005	38205487	0.884	0.024	14306	A	G	0.413	20	39576844	4.98965E-06	0.091	360421	21913.090
genus Anaerofilum	Vascular dementia (mixed)	rs6849229	G	A	-0.004	131797280	0.841	0.028	14306	G	A	0.562	4	130876125	4.44038E-06	0.122	360421	34142.711
genus Anaerofilum	Vascular dementia (mixed)	rs7614116	G	A	0.017	130368069	0.434	0.022	14306	G	A	0.379	3	130649225	6.06429E-06	0.084	360421	23465.044
genus Anaerostipes	Vascular dementia (mixed)	rs1632064	T	C	0.009	3219694	0.464	0.015	14306	T	C	0.614	5	3219580	4.04045E-06	0.133	360421	37296.871
genus Anaerostipes	Vascular dementia (mixed)	rs6028529	A	G	0.003	38205487	0.842	0.013	14306	A	G	0.413	20	39576844	4.98965E-06	0.091	360421	21913.090
genus Anaerostipes	Vascular dementia (mixed)	rs7614116	G	A	0.005	130368069	0.657	0.012	14306	G	A	0.379	3	130649225	6.06429E-06	0.084	360421	23465.044
genus Anaerotruncus	Vascular dementia (mixed)	rs1466525	T	C	-0.010	54780209	0.418	0.014	14306	T	C	0.489	8	53867649	1.97697E-06	0.103	360421	34189.173
genus Anaerotruncus	Vascular dementia (mixed)	rs1632064	T	C	-0.002	3219694	0.956	0.015	14306	T	C	0.614	5	3219580	4.04045E-06	0.133	360421	37296.871
genus Anaerotruncus	Vascular dementia (mixed)	rs17168895	T	G	0.011	15647727	0.535	0.018	14306	T	G	-0.547	7	15608102	2.92853E-06	0.117	360421	34495.049
genus Anaerotruncus	Vascular dementia (mixed)	rs6028529	A	G	0.005	38205487	0.779	0.013	14306	A	G	0.413	20	39576844	4.98965E-06	0.091	360421	21913.090
genus Anaerotruncus	Vascular dementia (mixed)	rs6849229	G	A	0.001	131797280	0.995	0.015	14306	G	A	0.562	4	130876125	4.44038E-06	0.122	360421	34142.711
genus Anaerotruncus	Vascular dementia (mixed)	rs7614116	G	A	0.008	130368069	0.500	0.012	14306	G	A	0.379	3	130649225	6.06429E-06	0.084	360421	23465.044
genus Bacteroides	Vascular dementia (mixed)	rs12257900	T	G	0.003	49443428	0.884	0.016	14306	T	G	0.467	10	48235385	2.48891E-06	0.099	360421	23006.877
genus Bacteroides	Vascular dementia (mixed)	rs1632064	T	C	0.005	3219694	0.720	0.015	14306	T	C	0.614	5	3219580	4.04045E-06	0.133	360421	37296.871
genus Bacteroides	Vascular dementia (mixed)	rs17168895	T	G	0.005	15647727	0.820	0.017	14306	T	G	-0.547	7	15608102	2.92853E-06	0.117	360421	34495.049
genus Bacteroides	Vascular dementia (mixed)	rs6849229	G	A	-0.009	131797280	0.552	0.014	14306	G	A	0.562	4	130876125	4.44038E-06	0.122	360421	34142.711
genus Bacteroides	Vascular dementia (mixed)	rs7614116	G	A	0.005	130368069	0.631	0.011	14306	G	A	0.379	3	130649225	6.06429E-06	0.084	360421	23465.044
genus Barnesiella	Vascular dementia (mixed)	rs12257900	T	G	0.005	49443428	0.743	0.018	14306	T	G	0.467	10	48235385	2.48891E-06	0.099	360421	23006.877
genus Barnesiella	Vascular dementia (mixed)	rs1466525	T	C	-0.002	54780209	0.891	0.015	14306	T	C	0.489	8	53867649	1.97697E-06	0.103	360421	34189.173
genus Barnesiella	Vascular dementia (mixed)	rs1632064	T	C	-0.013	3219694	0.365	0.017	14306	T	C	0.614	5	3219580	4.04045E-06	0.133	360421	37296.871
genus Barnesiella	Vascular dementia (mixed)	rs429358	C	T	-0.005	45411941	0.787	0.017	14306	C	T	0.565	19	44908684	3.94357E-08	0.103	360421	36831.461
genus Barnesiella	Vascular dementia (mixed)	rs6028529	A	G	-0.005	38205487	0.721	0.014	14306	A	G	0.413	20	39576844	4.98965E-06	0.091	360421	21913.090
genus Barnesiella	Vascular dementia (mixed)	rs7776624	G	A	0.004	31909839	0.758	0.012	14306	G	A	-0.358	7	31870226	8.82531E-06	0.081	360421	23450.025
genus Bifidobacterium	Vascular dementia (mixed)	rs12257900	T	G	-0.003	49443428	0.867	0.017	14306	T	G	0.467	10	48235385	2.48891E-06	0.099	360421	23006.877
genus Bifidobacterium	Vascular dementia (mixed)	rs1466525	T	C	0.013	54780209	0.450	0.015	14306	T	C	0.489	8	53867649	1.97697E-06	0.103	360421	34189.173
genus Bifidobacterium	Vascular dementia (mixed)	rs6028529	A	G	0.012	38205487	0.386	0.014	14306	A	G	0.413	20	39576844	4.98965E-06	0.091	360421	21913.090
genus Bifidobacterium	Vascular dementia (mixed)	rs6849229	G	A	0.009	131797280	0.553	0.016	14306	G	A	0.562	4	130876125	4.44038E-06	0.122	360421	34142.711
genus Bifidobacterium	Vascular dementia (mixed)	rs7614116	G	A	-0.009	130368069	0.435	0.013	14306	G	A	0.379	3	130649225	6.06429E-06	0.084	360421	23465.044
genus Bifidobacterium	Vascular dementia (mixed)	rs7776624	G	A	-0.003	31909839	0.796	0.012	14306	G	A	-0.358	7	31870226	8.82531E-06	0.081	360421	23450.025
genus Bilophila	Vascular dementia (mixed)	rs12257900	T	G	-0.012	49443428	0.509	0.019	14306	T	G	0.467	10	48235385	2.48891E-06	0.099	360421	23006.877
genus Bilophila	Vascular dementia (mixed)	rs1466525	T	C	0.004	54780209	0.916	0.016	14306	T	C	0.489	8	53867649	1.97697E-06	0.103	360421	34189.173
genus Bilophila	Vascular dementia (mixed)	rs1632064	T	C	-0.012	3219694	0.486	0.017	14306	T	C	0.614	5	3219580	4.04045E-06	0.133	360421	37296.871
genus Bilophila	Vascular dementia (mixed)	rs17168895	T	G	0.012	15647727	0.592	0.021	14306	T	G	-0.547	7	15608102	2.92853E-06	0.117	360421	34495.049
genus Bilophila	Vascular dementia (mixed)	rs429358	C	T	0.007	45411941	0.656	0.018	14306	C	T	0.565	19	44908684	3.94357E-08	0.103	360421	36831.461
genus Bilophila	Vascular dementia (mixed)	rs6028529	A	G	-0.010	38205487	0.586	0.015	14306	A	G	0.413	20	39576844	4.98965E-06	0.091	360421	21913.090
genus Bilophila	Vascular dementia (mixed)	rs6849229	G	A	-0.004	131797280	0.767	0.017	14306	G	A	0.562	4	130876125	4.44038E-06	0.122	360421	34142.711
genus Bilophila	Vascular dementia (mixed)	rs7614116	G	A	0.004	130368069	0.753	0.014	14306	G	A	0.379	3	130649225	6.06429E-06	0.084	360421	23465.044
genus Butyricicoccus	Vascular dementia (mixed)	rs12257900	T	G	-0.002	49443428	0.890	0.016	14306	T	G	0.467	10	48235385	2.48891E-06	0.099	360421	23006.877

genus Butyricicoccus	Vascular dementia (mixed)	rs1466525	T	C	0.012	54780209	0.429	0.014	14306	T	C	0.489	8	53867649	1.97697E-06	0.103	360421	34189.173
genus Butyricicoccus	Vascular dementia (mixed)	rs6028529	A	G	-0.010	38205487	0.415	0.013	14306	A	G	0.413	20	39576844	4.98965E-06	0.091	360421	21913.090
genus Butyricicoccus	Vascular dementia (mixed)	rs6849229	G	A	-0.006	131797280	0.644	0.015	14306	G	A	0.562	4	130876125	4.44038E-06	0.122	360421	34142.711
genus Butyricicoccus	Vascular dementia (mixed)	rs7614116	G	A	0.001	130368069	0.931	0.012	14306	G	A	0.379	3	130649225	6.06429E-06	0.084	360421	23465.044
genus Butyricicoccus	Vascular dementia (mixed)	rs7776624	G	A	-0.008	31909839	0.448	0.011	14306	G	A	-0.358	7	31870226	8.82531E-06	0.081	360421	23450.025
genus Butyricimonas	Vascular dementia (mixed)	rs12257900	T	G	-0.001	49443428	0.966	0.020	14306	T	G	0.467	10	48235385	2.48891E-06	0.099	360421	23006.877
genus Butyricimonas	Vascular dementia (mixed)	rs1632064	T	C	0.002	3219694	0.925	0.019	14306	T	C	0.614	5	3219580	4.04045E-06	0.133	360421	37296.871
genus Butyricimonas	Vascular dementia (mixed)	rs6028529	A	G	0.008	38205487	0.652	0.016	14306	A	G	0.413	20	39576844	4.98965E-06	0.091	360421	21913.090
genus Butyricimonas	Vascular dementia (mixed)	rs6849229	G	A	-0.007	131797280	0.735	0.019	14306	G	A	0.562	4	130876125	4.44038E-06	0.122	360421	34142.711
genus Butyricimonas	Vascular dementia (mixed)	rs7614116	G	A	-0.002	130368069	0.866	0.015	14306	G	A	0.379	3	130649225	6.06429E-06	0.084	360421	23465.044
genus Butyrivibrio	Vascular dementia (mixed)	rs12257900	T	G	0.029	49443428	0.443	0.035	14306	T	G	0.467	10	48235385	2.48891E-06	0.099	360421	23006.877
genus Butyrivibrio	Vascular dementia (mixed)	rs1466525	T	C	-0.004	54780209	0.886	0.030	14306	T	C	0.489	8	53867649	1.97697E-06	0.103	360421	34189.173
genus Butyrivibrio	Vascular dementia (mixed)	rs1632064	T	C	-0.001	3219694	1.000	0.034	14306	T	C	0.614	5	3219580	4.04045E-06	0.133	360421	37296.871
genus Butyrivibrio	Vascular dementia (mixed)	rs429358	C	T	-0.016	45411941	0.585	0.034	14306	C	T	0.565	19	44908684	3.94357E-08	0.103	360421	36831.461
genus Butyrivibrio	Vascular dementia (mixed)	rs6028529	A	G	-0.003	38205487	0.816	0.029	14306	A	G	0.413	20	39576844	4.98965E-06	0.091	360421	21913.090
genus Butyrivibrio	Vascular dementia (mixed)	rs6849229	G	A	-0.017	131797280	0.534	0.033	14306	G	A	0.562	4	130876125	4.44038E-06	0.122	360421	34142.711
genus Butyrivibrio	Vascular dementia (mixed)	rs7614116	G	A	-0.007	130368069	0.844	0.026	14306	G	A	0.379	3	130649225	6.06429E-06	0.084	360421	23465.044
genus Candidatus Soleaferrea	Vascular dementia (mixed)	rs1466525	T	C	-0.018	54780209	0.489	0.023	14306	T	C	0.489	8	53867649	1.97697E-06	0.103	360421	34189.173
genus Candidatus Soleaferrea	Vascular dementia (mixed)	rs429358	C	T	0.020	45411941	0.391	0.026	14306	C	T	0.565	19	44908684	3.94357E-08	0.103	360421	36831.461
genus Candidatus Soleaferrea	Vascular dementia (mixed)	rs7614116	G	A	0.001	130368069	0.932	0.020	14306	G	A	0.379	3	130649225	6.06429E-06	0.084	360421	23465.044
genus Catenibacterium	Vascular dementia (mixed)	rs1632064	T	C	-0.006	3219694	0.954	0.034	14306	T	C	0.614	5	3219580	4.04045E-06	0.133	360421	37296.871
genus Catenibacterium	Vascular dementia (mixed)	rs429358	C	T	0.030	45411941	0.433	0.036	14306	C	T	0.565	19	44908684	3.94357E-08	0.103	360421	36831.461
genus Catenibacterium	Vascular dementia (mixed)	rs6028529	A	G	-0.005	38205487	0.776	0.029	14306	A	G	0.413	20	39576844	4.98965E-06	0.091	360421	21913.090
genus Catenibacterium	Vascular dementia (mixed)	rs6849229	G	A	0.022	131797280	0.532	0.033	14306	G	A	0.562	4	130876125	4.44038E-06	0.122	360421	34142.711
genus Catenibacterium	Vascular dementia (mixed)	rs7614116	G	A	0.015	130368069	0.559	0.027	14306	G	A	0.379	3	130649225	6.06429E-06	0.084	360421	23465.044
genus Catenibacterium	Vascular dementia (mixed)	rs7776624	G	A	-0.013	31909839	0.598	0.025	14306	G	A	-0.358	7	31870226	8.82531E-06	0.081	360421	23450.025
genus Christensenellaceae R 7group	Vascular dementia (mixed)	rs1466525	T	C	-0.002	54780209	0.886	0.014	14306	T	C	0.489	8	53867649	1.97697E-06	0.103	360421	34189.173
genus Christensenellaceae R 7group	Vascular dementia (mixed)	rs429358	C	T	0.011	45411941	0.455	0.016	14306	C	T	0.565	19	44908684	3.94357E-08	0.103	360421	36831.461
genus Christensenellaceae R 7group	Vascular dementia (mixed)	rs6028529	A	G	-0.007	38205487	0.597	0.013	14306	A	G	0.413	20	39576844	4.98965E-06	0.091	360421	21913.090
genus Christensenellaceae R 7group	Vascular dementia (mixed)	rs7614116	G	A	-0.009	130368069	0.448	0.012	14306	G	A	0.379	3	130649225	6.06429E-06	0.084	360421	23465.044
genus Christensenellaceae R 7group	Vascular dementia (mixed)	rs7776624	G	A	0.007	31909839	0.549	0.011	14306	G	A	-0.358	7	31870226	8.82531E-06	0.081	360421	23450.025
genus Clostridium innocuum group	Vascular dementia (mixed)	rs12257900	T	G	-0.005	49443428	0.844	0.033	14306	T	G	0.467	10	48235385	2.48891E-06	0.099	360421	23006.877
genus Clostridium innocuum group	Vascular dementia (mixed)	rs1466525	T	C	-0.012	54780209	0.716	0.028	14306	T	C	0.489	8	53867649	1.97697E-06	0.103	360421	34189.173
genus Clostridium innocuum group	Vascular dementia (mixed)	rs1632064	T	C	0.016	3219694	0.593	0.030	14306	T	C	0.614	5	3219580	4.04045E-06	0.133	360421	37296.871
genus Clostridium innocuum group	Vascular dementia (mixed)	rs429358	C	T	-0.009	45411941	0.813	0.031	14306	C	T	0.565	19	44908684	3.94357E-08	0.103	360421	36831.461
genus Clostridium innocuum group	Vascular dementia (mixed)	rs6849229	G	A	-0.019	131797280	0.496	0.030	14306	G	A	0.562	4	130876125	4.44038E-06	0.122	360421	34142.711
genus Clostridium sensustricto1	Vascular dementia (mixed)	rs12257900	T	G	-0.014	49443428	0.501	0.018	14306	T	G	0.467	10	48235385	2.48891E-06	0.099	360421	23006.877
genus Clostridium sensustricto1	Vascular dementia (mixed)	rs1466525	T	C	0.001	54780209	0.965	0.015	14306	T	C	0.489	8	53867649	1.97697E-06	0.103	360421	34189.173
genus Clostridium sensustricto1	Vascular dementia (mixed)	rs1632064	T	C	0.005	3219694	0.735	0.017	14306	T	C	0.614	5	3219580	4.04045E-06	0.133	360421	37296.871
genus Clostridium sensustricto1	Vascular dementia (mixed)	rs429358	C	T	0.011	45411941	0.544	0.017	14306	C	T	0.565	19	44908684	3.94357E-08	0.103	360421	36831.461
genus Clostridium sensustricto1	Vascular dementia (mixed)	rs6028529	A	G	0.001	38205487	0.887	0.014	14306	A	G	0.413	20	39576844	4.98965E-06	0.091	360421	21913.090
genus Clostridium sensustricto1	Vascular dementia (mixed)	rs7614116	G	A	0.002	130368069	0.841	0.013	14306	G	A	0.379	3	130649225	6.06429E-06	0.084	360421	23465.044
genus Clostridium sensustricto1	Vascular dementia (mixed)	rs7776624	G	A	0.004	31909839	0.737	0.012	14306	G	A	-0.358	7	31870226	8.82531E-06	0.081	360421	23450.025
genus Collinsella	Vascular dementia (mixed)	rs12257900	T	G	0.005	49443428	0.761	0.018	14306	T	G	0.467	10	48235385	2.48891E-06	0.099	360421	23006.877
genus Collinsella	Vascular dementia (mixed)	rs1632064	T	C	-0.004	3219694	0.910	0.017	14306	T	C	0.614	5	3219580	4.04045E-06	0.133	360421	37296.871
genus Collinsella	Vascular dementia (mixed)	rs17168895	T	G	0.002	15647727	0.928	0.020	14306	T	G	-0.547	7	15608102	2.92853E-06	0.117	360421	34495.049
genus Collinsella	Vascular dementia (mixed)	rs6849229	G	A	-0.009	131797280	0.546	0.016	14306	G	A	0.562	4	130876125	4.44038E-06	0.122	360421	34142.711
genus Collinsella	Vascular dementia (mixed)	rs7776624	G	A	-0.005	31909839	0.694	0.012	14306	G	A	-0.358	7	31870226	8.82531E-06	0.081	360421	23450.025
genus Coprobacter	Vascular dementia (mixed)	rs1632064	T	C	-0.002	3219694	0.966	0.024	14306	T	C	0.614	5	3219580	4.04045E-06	0.133	360421	37296.871
genus Coprobacter	Vascular dementia (mixed)	rs17168895	T	G	0.016	15647727	0.529	0.028	14306	T	G	-0.547	7	15608102	2.92853E-06	0.117	360421	34495.049
genus Coprobacter	Vascular dementia (mixed)	rs429358	C	T	-0.001	45411941	0.930	0.025	14306	C	T	0.565	19	44908684	3.94357E-08	0.103	360421	36831.461
genus Coprobacter	Vascular dementia (mixed)	rs6028529	A	G	-0.004	38205487	0.817	0.021	14306	A	G	0.413	20	39576844	4.98965E-06	0.091	360421	21913.090
genus Coprobacter	Vascular dementia (mixed)	rs6849229	G	A	0.011	131797280	0.627	0.024	14306	G	A	0.562	4	130876125	4.44038E-06	0.122	360421	34142.711
genus Coprobacter	Vascular dementia (mixed)	rs7614116	G	A	-0.011	130368069	0.553	0.019	14306	G	A	0.379	3	130649225	6.06429E-06	0.084	360421	23465.044
genus Coprobacter	Vascular dementia (mixed)	rs7776624	G	A	-0.008	31909839	0.653	0.017	14306	G	A	-0.358	7	31870226	8.82531E-06	0.081	360421	23450.025
genus Coprococcus1	Vascular dementia (mixed)	rs12257900	T	G	-0.008	49443428	0.659	0.016	14306	T	G	0.467	10	48235385	2.48891E-06	0.099	360421	23006.877
genus Coprococcus1	Vascular dementia (mixed)	rs17168895	T	G	0.015	15647727	0.322	0.018	14306	T	G	-0.547	7	15608102	2.92853E-06	0.117	360421	34495.049
genus Coprococcus1	Vascular dementia (mixed)	rs429358	C	T	0.014	45411941	0.326	0.016	14306	C	T	0.565	19	44908684	3.94357E-08	0.103	360421	36831.461
genus Coprococcus1	Vascular dementia (mixed)	rs6849229	G	A	0.006	131797280	0.773	0.015	14306	G	A	0.562	4	130876125	4.44038E-06	0.122	360421	34142.711
genus Coprococcus1	Vascular dementia (mixed)	rs7614116	G	A	-0.003	130368069	0.806	0.012	14306	G	A	0.379	3	130649225	6.06429E-06	0.084	360421	23465.044
genus Coprococcus1	Vascular dementia (mixed)	rs7776624	G	A	-0.003	31909839	0.752	0.011	14306	G	A	-0.358	7	31870226	8.82531E-06	0.081	360421	23450.025
genus Coprococcus2	Vascular dementia (mixed)	rs12257900	T	G	0.016	49443428	0.469	0.020	14306	T	G	0.467	10	48235385	2.48891E-06	0.099	360421	23006.877
genus Coprococcus2	Vascular dementia (mixed)	rs1632064	T	C	0.014	3219694	0.423	0.018	14306	T	C	0.614	5	3219580	4.04045E-06	0.133	360421	37296.871
genus Coprococcus2	Vascular dementia (mixed)	rs429358	C	T	0.002	45411941	0.982	0.019	14306	C	T	0.565	19	44908684	3.94357E-08	0.103	360421	36831.461
genus Coprococcus2	Vascular dementia (mixed)	rs6028529	A	G	-0.012	38205487	0.513	0.016	14306	A	G	0.413	20	39576844	4.98965E-06	0.091	360421	21913.090
genus Coprococcus2	Vascular dementia (mixed)	rs6849229	G	A	0.011	131797280	0.607	0.018	14306	G	A	0.562	4	130876125	4.44038E-06	0.122	360421	34142.711
genus Coprococcus2	Vascular dementia (mixed)	rs7614116	G	A	-0.009	130368069	0.500	0.014	14306	G	A	0.379	3	130649225	6.06429E-06	0.084	360421	23465.044
genus Coprococcus2	Vascular dementia (mixed)	rs7776624	G	A	-0.008	31909839	0.508	0.013	14306	G	A	-0.358	7	31870226	8.82531E-06	0.081	360421	23450.025
genus Coprococcus3	Vascular dementia (mixed)	rs1632064	T	C	-0.002	3219694	0.932	0.016	14306	T	C	0.614	5	3219580	4.04045E-06	0.133	360421	37296.871
genus Coprococcus3	Vascular dementia (mixed)	rs6028529	A	G	0.005	38205487	0.704	0.013	14306	A	G	0.413	20	39576844	4.98965E-06	0.091	360421	21913.090
genus Coprococcus3	Vascular dementia (mixed)	rs6849229	G	A	0.001	131797280	0.944	0.015	14306	G	A	0.562	4	130876125	4.44038E-06	0.122	360421	34142.711

genus Coprococcus3	Vascular dementia (mixed)	rs7614116	G	A	-0.004	130368069	0.686	0.012	14306	G	A	0.379	3	130649225	6.06429E-06	0.084	360421	23465.044
genus Defluviitaleaceae UCG011	Vascular dementia (mixed)	rs12257900	T	G	-0.004	49443428	0.851	0.023	14306	T	G	0.467	10	48235385	2.48891E-06	0.099	360421	23006.877
genus Defluviitaleaceae UCG011	Vascular dementia (mixed)	rs429358	C	T	-0.003	45411941	0.819	0.022	14306	C	T	0.565	19	44908684	3.94357E-08	0.103	360421	36831.461
genus Defluviitaleaceae UCG011	Vascular dementia (mixed)	rs7776624	G	A	-0.011	31909839	0.479	0.016	14306	G	A	-0.358	7	31870226	8.82531E-06	0.081	360421	23450.025
genus Desulfovibrio	Vascular dementia (mixed)	rs1466525	T	C	0.002	54780209	0.942	0.018	14306	T	C	0.489	8	53867649	1.97697E-06	0.103	360421	34189.173
genus Desulfovibrio	Vascular dementia (mixed)	rs429358	C	T	0.006	45411941	0.782	0.021	14306	C	T	0.565	19	44908684	3.94357E-08	0.103	360421	36831.461
genus Desulfovibrio	Vascular dementia (mixed)	rs6028529	A	G	-0.004	38205487	0.775	0.018	14306	A	G	0.413	20	39576844	4.98965E-06	0.091	360421	21913.090
genus Desulfovibrio	Vascular dementia (mixed)	rs7614116	G	A	0.010	130368069	0.535	0.016	14306	G	A	0.379	3	130649225	6.06429E-06	0.084	360421	23465.044
(genus Dialister	Vascular dementia (mixed)	rs12257900	T	G	0.017	49443428	0.392	0.019	14306	T	G	0.467	10	48235385	2.48891E-06	0.099	360421	23006.877
(genus Dialister	Vascular dementia (mixed)	rs1632064	T	C	-0.010	3219694	0.632	0.018	14306	T	C	0.614	5	3219580	4.04045E-06	0.133	360421	37296.871
(genus Dialister	Vascular dementia (mixed)	rs6028529	A	G	0.006	38205487	0.590	0.015	14306	A	G	0.413	20	39576844	4.98965E-06	0.091	360421	21913.090
(genus Dialister	Vascular dementia (mixed)	rs6849229	G	A	0.008	131797280	0.560	0.018	14306	G	A	0.562	4	130876125	4.44038E-06	0.122	360421	34142.711
(genus Dialister	Vascular dementia (mixed)	rs7614116	G	A	0.002	130368069	0.844	0.014	14306	G	A	0.379	3	130649225	6.06429E-06	0.084	360421	23465.044
genus Dorea	Vascular dementia (mixed)	rs12257900	T	G	-0.013	49443428	0.468	0.016	14306	T	G	0.467	10	48235385	2.48891E-06	0.099	360421	23006.877
genus Dorea	Vascular dementia (mixed)	rs1466525	T	C	-0.002	54780209	0.950	0.013	14306	T	C	0.489	8	53867649	1.97697E-06	0.103	360421	34189.173
genus Dorea	Vascular dementia (mixed)	rs1632064	T	C	0.011	3219694	0.441	0.015	14306	T	C	0.614	5	3219580	4.04045E-06	0.133	360421	37296.871
genus Dorea	Vascular dementia (mixed)	rs17168895	T	G	-0.010	15647727	0.672	0.018	14306	T	G	-0.547	7	15608102	2.92853E-06	0.117	360421	34495.049
genus Dorea	Vascular dementia (mixed)	rs6849229	G	A	0.006	131797280	0.708	0.014	14306	G	A	0.562	4	130876125	4.44038E-06	0.122	360421	34142.711
genus Dorea	Vascular dementia (mixed)	rs7614116	G	A	0.003	130368069	0.792	0.012	14306	G	A	0.379	3	130649225	6.06429E-06	0.084	360421	23465.044
genus Dorea	Vascular dementia (mixed)	rs7776624	G	A	0.005	31909839	0.603	0.011	14306	G	A	-0.358	7	31870226	8.82531E-06	0.081	360421	23450.025
genus Eggerthella	Vascular dementia (mixed)	rs1466525	T	C	-0.022	54780209	0.336	0.025	14306	T	C	0.489	8	53867649	1.97697E-06	0.103	360421	34189.173
genus Eggerthella	Vascular dementia (mixed)	rs1632064	T	C	0.024	3219694	0.392	0.027	14306	T	C	0.614	5	3219580	4.04045E-06	0.133	360421	37296.871
genus Eggerthella	Vascular dementia (mixed)	rs17168895	T	G	0.020	15647727	0.566	0.033	14306	T	G	-0.547	7	15608102	2.92853E-06	0.117	360421	34495.049
genus Eggerthella	Vascular dementia (mixed)	rs429358	C	T	0.017	45411941	0.611	0.029	14306	C	T	0.565	19	44908684	3.94357E-08	0.103	360421	36831.461
genus Eggerthella	Vascular dementia (mixed)	rs6849229	G	A	0.007	131797280	0.757	0.027	14306	G	A	0.562	4	130876125	4.44038E-06	0.122	360421	34142.711
genus Eggerthella	Vascular dementia (mixed)	rs7776624	G	A	-0.016	31909839	0.431	0.020	14306	G	A	-0.358	7	31870226	8.82531E-06	0.081	360421	23450.025
genus Eisenbergiella	Vascular dementia (mixed)	rs1466525	T	C	0.012	54780209	0.610	0.024	14306	T	C	0.489	8	53867649	1.97697E-06	0.103	360421	34189.173
genus Eisenbergiella	Vascular dementia (mixed)	rs1632064	T	C	0.018	3219694	0.441	0.026	14306	T	C	0.614	5	3219580	4.04045E-06	0.133	360421	37296.871
genus Eisenbergiella	Vascular dementia (mixed)	rs17168895	T	G	-0.023	15647727	0.499	0.031	14306	T	G	-0.547	7	15608102	2.92853E-06	0.117	360421	34495.049
genus Eisenbergiella	Vascular dementia (mixed)	rs429358	C	T	-0.013	45411941	0.612	0.028	14306	C	T	0.565	19	44908684	3.94357E-08	0.103	360421	36831.461
genus Eisenbergiella	Vascular dementia (mixed)	rs6028529	A	G	0.008	38205487	0.691	0.023	14306	A	G	0.413	20	39576844	4.98965E-06	0.091	360421	21913.090
genus Eisenbergiella	Vascular dementia (mixed)	rs6849229	G	A	0.001	131797280	0.995	0.026	14306	G	A	0.562	4	130876125	4.44038E-06	0.122	360421	34142.711
genus Eisenbergiella	Vascular dementia (mixed)	rs7776624	G	A	0.007	31909839	0.707	0.019	14306	G	A	-0.358	7	31870226	8.82531E-06	0.081	360421	23450.025
genus Enterorhabdus	Vascular dementia (mixed)	rs1632064	T	C	-0.012	3219694	0.577	0.023	14306	T	C	0.614	5	3219580	4.04045E-06	0.133	360421	37296.871
genus Enterorhabdus	Vascular dementia (mixed)	rs17168895	T	G	0.010	15647727	0.738	0.027	14306	T	G	-0.547	7	15608102	2.92853E-06	0.117	360421	34495.049
genus Enterorhabdus	Vascular dementia (mixed)	rs429358	C	T	0.009	45411941	0.754	0.023	14306	C	T	0.565	19	44908684	3.94357E-08	0.103	360421	36831.461
genus Enterorhabdus	Vascular dementia (mixed)	rs6028529	A	G	0.013	38205487	0.492	0.020	14306	A	G	0.413	20	39576844	4.98965E-06	0.091	360421	21913.090
genus Enterorhabdus	Vascular dementia (mixed)	rs6849229	G	A	-0.005	131797280	0.804	0.023	14306	G	A	0.562	4	130876125	4.44038E-06	0.122	360421	34142.711
genus Enterorhabdus	Vascular dementia (mixed)	rs7776624	G	A	-0.007	31909839	0.685	0.016	14306	G	A	-0.358	7	31870226	8.82531E-06	0.081	360421	23450.025
genus Erysipelatoclostridium	Vascular dementia (mixed)	rs17168895	T	G	0.004	15647727	0.859	0.024	14306	T	G	-0.547	7	15608102	2.92853E-06	0.117	360421	34495.049
genus Erysipelatoclostridium	Vascular dementia (mixed)	rs429358	C	T	0.017	45411941	0.432	0.020	14306	C	T	0.565	19	44908684	3.94357E-08	0.103	360421	36831.461
genus Erysipelatoclostridium	Vascular dementia (mixed)	rs6849229	G	A	0.005	131797280	0.747	0.020	14306	G	A	0.562	4	130876125	4.44038E-06	0.122	360421	34142.711
genus Erysipelatoclostridium	Vascular dementia (mixed)	rs7614116	G	A	0.006	130368069	0.703	0.016	14306	G	A	0.379	3	130649225	6.06429E-06	0.084	360421	23465.044
genus Erysipelatoclostridium	Vascular dementia (mixed)	rs7776624	G	A	-0.006	31909839	0.666	0.014	14306	G	A	-0.358	7	31870226	8.82531E-06	0.081	360421	23450.025
genus Escherichia Shigella	Vascular dementia (mixed)	rs12257900	T	G	-0.001	49443428	0.950	0.019	14306	T	G	0.467	10	48235385	2.48891E-06	0.099	360421	23006.877
genus Escherichia Shigella	Vascular dementia (mixed)	rs1466525	T	C	-0.004	54780209	0.790	0.016	14306	T	C	0.489	8	53867649	1.97697E-06	0.103	360421	34189.173
genus Escherichia Shigella	Vascular dementia (mixed)	rs17168895	T	G	0.000	15647727	0.986	0.022	14306	T	G	-0.547	7	15608102	2.92853E-06	0.117	360421	34495.049
genus Escherichia Shigella	Vascular dementia (mixed)	rs429358	C	T	0.007	45411941	0.718	0.019	14306	C	T	0.565	19	44908684	3.94357E-08	0.103	360421	36831.461
genus Escherichia Shigella	Vascular dementia (mixed)	rs6028529	A	G	0.007	38205487	0.570	0.015	14306	A	G	0.413	20	39576844	4.98965E-06	0.091	360421	21913.090
genus Escherichia Shigella	Vascular dementia (mixed)	rs6849229	G	A	0.005	131797280	0.861	0.018	14306	G	A	0.562	4	130876125	4.44038E-06	0.122	360421	34142.711
genus Escherichia Shigella	Vascular dementia (mixed)	rs7614116	G	A	-0.006	130368069	0.662	0.014	14306	G	A	0.379	3	130649225	6.06429E-06	0.084	360421	23465.044
genus Eubacterium brachy group	Vascular dementia (mixed)	rs1466525	T	C	-0.003	54780209	0.874	0.028	14306	T	C	0.489	8	53867649	1.97697E-06	0.103	360421	34189.173
genus Eubacterium brachy group	Vascular dementia (mixed)	rs1632064	T	C	-0.009	3219694	0.832	0.031	14306	T	C	0.614	5	3219580	4.04045E-06	0.133	360421	37296.871
genus Eubacterium brachy group	Vascular dementia (mixed)	rs429358	C	T	-0.022	45411941	0.464	0.032	14306	C	T	0.565	19	44908684	3.94357E-08	0.103	360421	36831.461
genus Eubacterium brachy group	Vascular dementia (mixed)	rs6849229	G	A	-0.001	131797280	0.978	0.031	14306	G	A	0.562	4	130876125	4.44038E-06	0.122	360421	34142.711
genus Eubacterium brachy group	Vascular dementia (mixed)	rs7776624	G	A	0.003	31909839	0.896	0.022	14306	G	A	-0.358	7	31870226	8.82531E-06	0.081	360421	23450.025
genus Eubacterium coprostanoligenes group	Vascular dementia (mixed)	rs12257900	T	G	0.006	49443428	0.770	0.016	14306	T	G	0.467	10	48235385	2.48891E-06	0.099	360421	23006.877
genus Eubacterium coprostanoligenes group	Vascular dementia (mixed)	rs1632064	T	C	0.007	3219694	0.553	0.015	14306	T	C	0.614	5	3219580	4.04045E-06	0.133	360421	37296.871
genus Eubacterium coprostanoligenes group	Vascular dementia (mixed)	rs6849229	G	A	-0.006	131797280	0.682	0.015	14306	G	A	0.562	4	130876125	4.44038E-06	0.122	360421	34142.711
genus Eubacterium coprostanoligenes group	Vascular dementia (mixed)	rs7614116	G	A	0.010	130368069	0.415	0.012	14306	G	A	0.379	3	130649225	6.06429E-06	0.084	360421	23465.044
genus Eubacterium eligens group	Vascular dementia (mixed)	rs12257900	T	G	-0.001	49443428	0.871	0.017	14306	T	G	0.467	10	48235385	2.48891E-06	0.099	360421	23006.877
genus Eubacterium eligens group	Vascular dementia (mixed)	rs1466525	T	C	-0.009	54780209	0.471	0.015	14306	T	C	0.489	8	53867649	1.97697E-06	0.103	360421	34189.173
genus Eubacterium eligens group	Vascular dementia (mixed)	rs7614116	G	A	0.000	130368069	0.993	0.013	14306	G	A	0.379	3	130649225	6.06429E-06	0.084	360421	23465.044
genus Eubacterium eligens group	Vascular dementia (mixed)	rs7776624	G	A	0.001	31909839	0.957	0.012	14306	G	A	-0.358	7	31870226	8.82531E-06	0.081	360421	23450.025
genus Eubacterium fissicatena group	Vascular dementia (mixed)	rs1466525	T	C	-0.019	54780209	0.489	0.029	14306	T	C	0.489	8	53867649	1.97697E-06	0.103	360421	34189.173
genus Eubacterium fissicatena group	Vascular dementia (mixed)	rs17168895	T	G	-0.029	15647727	0.479	0.038	14306	T	G	-0.547	7	15608102	2.92853E-06	0.117	360421	34495.049
genus Eubacterium fissicatena group	Vascular dementia (mixed)	rs429358	C	T	0.004	45411941	0.913	0.033	14306	C	T	0.565	19	44908684	3.94357E-08	0.103	360421	36831.461
genus Eubacterium fissicatena group	Vascular dementia (mixed)	rs6028529	A	G	0.001	38205487	0.964	0.027	14306	A	G	0.413	20	39576844	4.98965E-06	0.091	360421	21913.090
genus Eubacterium fissicatena group	Vascular dementia (mixed)	rs6849229	G	A	0.002	131797280	0.878	0.031	14306	G	A	0.562	4	130876125	4.44038E-06	0.122	360421	34142.711
genus Eubacterium fissicatena group	Vascular dementia (mixed)	rs7614116	G	A	0.009	130368069	0.713	0.025	14306	G	A	0.379	3	130649225	6.06429E-06	0.084	360421	23465.044
genus Eubacterium hallii group	Vascular dementia (mixed)	rs1466525	T	C	-0.004	54780209	0.912	0.014	14306	T	C	0.489	8	53867649	1.97697E-06	0.103	360421	34189.173

genus Eubacterium hallii group	Vascular dementia (mixed)	rs1632064	T	C	0.000	3219694	0.999	0.015	14306	T	C	0.614	5	3219580	4.04045E-06	0.133	360421	37296.871
genus Eubacterium hallii group	Vascular dementia (mixed)	rs17168895	T	G	0.001	15647727	0.868	0.018	14306	T	G	-0.547	7	15608102	2.92853E-06	0.117	360421	34495.049
genus Eubacterium hallii group	Vascular dementia (mixed)	rs429358	C	T	0.003	45411941	0.834	0.016	14306	C	T	0.565	19	44908684	3.94357E-08	0.103	360421	36831.461
genus Eubacterium hallii group	Vascular dementia (mixed)	rs6028529	A	G	0.004	38205487	0.777	0.013	14306	A	G	0.413	20	39576844	4.98965E-06	0.091	360421	21913.090
genus Eubacterium hallii group	Vascular dementia (mixed)	rs6849229	G	A	-0.013	131797280	0.345	0.015	14306	G	A	0.562	4	130876125	4.44038E-06	0.122	360421	34142.711
genus Eubacterium nodatum group	Vascular dementia (mixed)	rs12257900	T	G	0.001	49443428	0.994	0.036	14306	T	G	0.467	10	48235385	2.48891E-06	0.099	360421	23006.877
genus Eubacterium nodatum group	Vascular dementia (mixed)	rs1632064	T	C	0.008	3219694	0.791	0.035	14306	T	C	0.614	5	3219580	4.04045E-06	0.133	360421	37296.871
genus Eubacterium nodatum group	Vascular dementia (mixed)	rs17168895	T	G	0.019	15647727	0.582	0.040	14306	T	G	-0.547	7	15608102	2.92853E-06	0.117	360421	34495.049
genus Eubacterium nodatum group	Vascular dementia (mixed)	rs429358	C	T	0.008	45411941	0.880	0.036	14306	C	T	0.565	19	44908684	3.94357E-08	0.103	360421	36831.461
genus Eubacterium nodatum group	Vascular dementia (mixed)	rs6849229	G	A	0.005	131797280	0.933	0.034	14306	G	A	0.562	4	130876125	4.44038E-06	0.122	360421	34142.711
genus Eubacterium nodatum group	Vascular dementia (mixed)	rs7614116	G	A	-0.001	130368069	0.947	0.027	14306	G	A	0.379	3	130649225	6.06429E-06	0.084	360421	23465.044
genus Eubacterium nodatum group	Vascular dementia (mixed)	rs7776624	G	A	0.018	31909839	0.479	0.025	14306	G	A	-0.358	7	31870226	8.82531E-06	0.081	360421	23450.025
genus Eubacterium oxidoreducens group	Vascular dementia (mixed)	rs12257900	T	G	0.017	49443428	0.498	0.029	14306	T	G	0.467	10	48235385	2.48891E-06	0.099	360421	23006.877
genus Eubacterium oxidoreducens group	Vascular dementia (mixed)	rs1632064	T	C	-0.005	3219694	0.727	0.026	14306	T	C	0.614	5	3219580	4.04045E-06	0.133	360421	37296.871
genus Eubacterium oxidoreducens group	Vascular dementia (mixed)	rs429358	C	T	0.021	45411941	0.455	0.028	14306	C	T	0.565	19	44908684	3.94357E-08	0.103	360421	36831.461
genus Eubacterium oxidoreducens group	Vascular dementia (mixed)	rs7614116	G	A	0.001	130368069	0.952	0.021	14306	G	A	0.379	3	130649225	6.06429E-06	0.084	360421	23465.044
genus Eubacterium oxidoreducens group	Vascular dementia (mixed)	rs7776624	G	A	-0.016	31909839	0.365	0.019	14306	G	A	-0.358	7	31870226	8.82531E-06	0.081	360421	23450.025
genus Eubacterium rectale group	Vascular dementia (mixed)	rs12257900	T	G	0.002	49443428	0.857	0.016	14306	T	G	0.467	10	48235385	2.48891E-06	0.099	360421	23006.877
genus Eubacterium rectale group	Vascular dementia (mixed)	rs6849229	G	A	0.009	131797280	0.642	0.015	14306	G	A	0.562	4	130876125	4.44038E-06	0.122	360421	34142.711
genus Eubacterium rectale group	Vascular dementia (mixed)	rs7614116	G	A	-0.003	130368069	0.820	0.012	14306	G	A	0.379	3	130649225	6.06429E-06	0.084	360421	23465.044
genus Eubacterium rectale group	Vascular dementia (mixed)	rs7776624	G	A	0.009	31909839	0.434	0.011	14306	G	A	-0.358	7	31870226	8.82531E-06	0.081	360421	23450.025
genus Eubacterium ruminantium group	Vascular dementia (mixed)	rs1466525	T	C	-0.019	54780209	0.391	0.020	14306	T	C	0.489	8	53867649	1.97697E-06	0.103	360421	34189.173
genus Eubacterium ruminantium group	Vascular dementia (mixed)	rs1632064	T	C	-0.008	3219694	0.746	0.022	14306	T	C	0.614	5	3219580	4.04045E-06	0.133	360421	37296.871
genus Eubacterium ruminantium group	Vascular dementia (mixed)	rs17168895	T	G	-0.008	15647727	0.680	0.027	14306	T	G	-0.547	7	15608102	2.92853E-06	0.117	360421	34495.049
genus Eubacterium ruminantium group	Vascular dementia (mixed)	rs429358	C	T	0.014	45411941	0.497	0.023	14306	C	T	0.565	19	44908684	3.94357E-08	0.103	360421	36831.461
genus Eubacterium ruminantium group	Vascular dementia (mixed)	rs6849229	G	A	0.000	131797280	0.935	0.022	14306	G	A	0.562	4	130876125	4.44038E-06	0.122	360421	34142.711
genus Eubacterium ruminantium group	Vascular dementia (mixed)	rs7614116	G	A	0.004	130368069	0.806	0.017	14306	G	A	0.379	3	130649225	6.06429E-06	0.084	360421	23465.044
genus Eubacterium ventriosum group	Vascular dementia (mixed)	rs12257900	T	G	0.012	49443428	0.423	0.017	14306	T	G	0.467	10	48235385	2.48891E-06	0.099	360421	23006.877
genus Eubacterium ventriosum group	Vascular dementia (mixed)	rs7614116	G	A	-0.003	130368069	0.796	0.012	14306	G	A	0.379	3	130649225	6.06429E-06	0.084	360421	23465.044
genus Eubacterium ventriosum group	Vascular dementia (mixed)	rs7776624	G	A	0.000	31909839	0.981	0.011	14306	G	A	-0.358	7	31870226	8.82531E-06	0.081	360421	23450.025
genus Eubacterium xylanophilum group	Vascular dementia (mixed)	rs17168895	T	G	0.014	15647727	0.501	0.020	14306	T	G	-0.547	7	15608102	2.92853E-06	0.117	360421	34495.049
genus Eubacterium xylanophilum group	Vascular dementia (mixed)	rs6028529	A	G	0.009	38205487	0.575	0.015	14306	A	G	0.413	20	39576844	4.98965E-06	0.091	360421	21913.090
genus Eubacterium xylanophilum group	Vascular dementia (mixed)	rs6849229	G	A	0.006	131797280	0.788	0.017	14306	G	A	0.562	4	130876125	4.44038E-06	0.122	360421	34142.711
genus Eubacterium xylanophilum group	Vascular dementia (mixed)	rs7614116	G	A	-0.002	130368069	0.953	0.013	14306	G	A	0.379	3	130649225	6.06429E-06	0.084	360421	23465.044
genus Eubacterium xylanophilum group	Vascular dementia (mixed)	rs7776624	G	A	0.002	31909839	0.836	0.012	14306	G	A	-0.358	7	31870226	8.82531E-06	0.081	360421	23450.025
genus Faecalibacterium	Vascular dementia (mixed)	rs12257900	T	G	-0.002	49443428	0.827	0.016	14306	T	G	0.467	10	48235385	2.48891E-06	0.099	360421	23006.877
genus Faecalibacterium	Vascular dementia (mixed)	rs1632064	T	C	-0.009	3219694	0.519	0.015	14306	T	C	0.614	5	3219580	4.04045E-06	0.133	360421	37296.871
genus Faecalibacterium	Vascular dementia (mixed)	rs17168895	T	G	-0.002	15647727	0.879	0.017	14306	T	G	-0.547	7	15608102	2.92853E-06	0.117	360421	34495.049
genus Faecalibacterium	Vascular dementia (mixed)	rs6849229	G	A	0.011	131797280	0.456	0.014	14306	G	A	0.562	4	130876125	4.44038E-06	0.122	360421	34142.711
genus Faecalibacterium	Vascular dementia (mixed)	rs7614116	G	A	0.000	130368069	0.997	0.011	14306	G	A	0.379	3	130649225	6.06429E-06	0.084	360421	23465.044
genus Faecalibacterium	Vascular dementia (mixed)	rs7776624	G	A	0.003	31909839	0.761	0.011	14306	G	A	-0.358	7	31870226	8.82531E-06	0.081	360421	23450.025
genus Family XIII AD3011 group	Vascular dementia (mixed)	rs1466525	T	C	-0.001	54780209	0.905	0.015	14306	T	C	0.489	8	53867649	1.97697E-06	0.103	360421	34189.173
genus Family XIII AD3011 group	Vascular dementia (mixed)	rs1632064	T	C	0.007	3219694	0.647	0.017	14306	T	C	0.614	5	3219580	4.04045E-06	0.133	360421	37296.871
genus Family XIII AD3011 group	Vascular dementia (mixed)	rs17168895	T	G	-0.003	15647727	0.951	0.020	14306	T	G	-0.547	7	15608102	2.92853E-06	0.117	360421	34495.049
genus Family XIII AD3011 group	Vascular dementia (mixed)	rs6028529	A	G	0.013	38205487	0.364	0.014	14306	A	G	0.413	20	39576844	4.98965E-06	0.091	360421	21913.090
genus Family XIII AD3011 group	Vascular dementia (mixed)	rs6849229	G	A	-0.001	131797280	0.934	0.016	14306	G	A	0.562	4	130876125	4.44038E-06	0.122	360421	34142.711
genus Family XIII AD3011 group	Vascular dementia (mixed)	rs7776624	G	A	-0.005	31909839	0.660	0.012	14306	G	A	-0.358	7	31870226	8.82531E-06	0.081	360421	23450.025
genus Family XIII UCG001	Vascular dementia (mixed)	rs1632064	T	C	-0.007	3219694	0.663	0.018	14306	T	C	0.614	5	3219580	4.04045E-06	0.133	360421	37296.871
genus Family XIII UCG001	Vascular dementia (mixed)	rs429358	C	T	0.004	45411941	0.889	0.018	14306	C	T	0.565	19	44908684	3.94357E-08	0.103	360421	36831.461
genus Family XIII UCG001	Vascular dementia (mixed)	rs6028529	A	G	0.009	38205487	0.607	0.015	14306	A	G	0.413	20	39576844	4.98965E-06	0.091	360421	21913.090
genus Family XIII UCG001	Vascular dementia (mixed)	rs7776624	G	A	-0.005	31909839	0.688	0.013	14306	G	A	-0.358	7	31870226	8.82531E-06	0.081	360421	23450.025
genus Flavonifractor	Vascular dementia (mixed)	rs1466525	T	C	0.006	54780209	0.752	0.017	14306	T	C	0.489	8	53867649	1.97697E-06	0.103	360421	34189.173
genus Flavonifractor	Vascular dementia (mixed)	rs1632064	T	C	0.010	3219694	0.513	0.019	14306	T	C	0.614	5	3219580	4.04045E-06	0.133	360421	37296.871
genus Flavonifractor	Vascular dementia (mixed)	rs17168895	T	G	0.012	15647727	0.641	0.023	14306	T	G	-0.547	7	15608102	2.92853E-06	0.117	360421	34495.049
genus Flavonifractor	Vascular dementia (mixed)	rs429358	C	T	-0.021	45411941	0.293	0.020	14306	C	T	0.565	19	44908684	3.94357E-08	0.103	360421	36831.461
genus Flavonifractor	Vascular dementia (mixed)	rs6028529	A	G	0.006	38205487	0.680	0.016	14306	A	G	0.413	20	39576844	4.98965E-06	0.091	360421	21913.090
genus Flavonifractor	Vascular dementia (mixed)	rs7614116	G	A	-0.008	130368069	0.593	0.015	14306	G	A	0.379	3	130649225	6.06429E-06	0.084	360421	23465.044
genus Fusicatenibacter	Vascular dementia (mixed)	rs12257900	T	G	0.014	49443428	0.351	0.016	14306	T	G	0.467	10	48235385	2.48891E-06	0.099	360421	23006.877
genus Fusicatenibacter	Vascular dementia (mixed)	rs1632064	T	C	0.003	3219694	0.871	0.015	14306	T	C	0.614	5	3219580	4.04045E-06	0.133	360421	37296.871
genus Fusicatenibacter	Vascular dementia (mixed)	rs17168895	T	G	-0.007	15647727	0.689	0.018	14306	T	G	-0.547	7	15608102	2.92853E-06	0.117	360421	34495.049
genus Fusicatenibacter	Vascular dementia (mixed)	rs429358	C	T	-0.008	45411941	0.622	0.015	14306	C	T	0.565	19	44908684	3.94357E-08	0.103	360421	36831.461
genus Fusicatenibacter	Vascular dementia (mixed)	rs6028529	A	G	0.009	38205487	0.468	0.013	14306	A	G	0.413	20	39576844	4.98965E-06	0.091	360421	21913.090
genus Fusicatenibacter	Vascular dementia (mixed)	rs6849229	G	A	0.005	131797280	0.743	0.015	14306	G	A	0.562	4	130876125	4.44038E-06	0.122	360421	34142.711
genus Fusicatenibacter	Vascular dementia (mixed)	rs7614116	G	A	-0.003	130368069	0.794	0.012	14306	G	A	0.379	3	130649225	6.06429E-06	0.084	360421	23465.044
genus Gordonibacter	Vascular dementia (mixed)	rs12257900	T	G	-0.023	49443428	0.530	0.034	14306	T	G	0.467	10	48235385	2.48891E-06	0.099	360421	23006.877
genus Gordonibacter	Vascular dementia (mixed)	rs1466525	T	C	0.016	54780209	0.498	0.029	14306	T	C	0.489	8	53867649	1.97697E-06	0.103	360421	34189.173
genus Gordonibacter	Vascular dementia (mixed)	rs1632064	T	C	0.014	3219694	0.627	0.032	14306	T	C	0.614	5	3219580	4.04045E-06	0.133	360421	37296.871
genus Gordonibacter	Vascular dementia (mixed)	rs17168895	T	G	-0.015	15647727	0.665	0.039	14306	T	G	-0.547	7	15608102	2.92853E-06	0.117	360421	34495.049
genus Gordonibacter	Vascular dementia (mixed)	rs6028529	A	G	0.024	38205487	0.368	0.028	14306	A	G	0.413	20	39576844	4.98965E-06	0.091	360421	21913.090
genus Gordonibacter	Vascular dementia (mixed)	rs6849229	G	A	-0.007	131797280	0.872	0.032	14306	G	A	0.562	4	130876125	4.44038E-06	0.122	360421	34142.711
genus Haemophilus	Vascular dementia (mixed)	rs1466525	T	C	-0.005	54780209	0.798	0.019	14306	T	C	0.489	8	53867649	1.97697E-06	0.103	360421	34189.173

genus Haemophilus	Vascular dementia (mixed)	rs1632064	T	C	0.001	3219694	0.996	0.020	14306	T	C	0.614	5	3219580	4.04045E-06	0.133	360421	37296.871
genus Haemophilus	Vascular dementia (mixed)	rs17168895	T	G	0.014	15647727	0.558	0.025	14306	T	G	-0.547	7	15608102	2.92853E-06	0.117	360421	34495.049
genus Haemophilus	Vascular dementia (mixed)	rs6028529	A	G	-0.004	38205487	0.732	0.017	14306	A	G	0.413	20	39576844	4.98965E-06	0.091	360421	21913.090
genus Haemophilus	Vascular dementia (mixed)	rs6849229	G	A	0.011	131797280	0.546	0.020	14306	G	A	0.562	4	130876125	4.44038E-06	0.122	360421	34142.711
genus Haemophilus	Vascular dementia (mixed)	rs7776624	G	A	0.006	31909839	0.691	0.015	14306	G	A	-0.358	7	31870226	8.82531E-06	0.081	360421	23450.025
genus Holdemanella	Vascular dementia (mixed)	rs12257900	T	G	-0.014	49443428	0.518	0.024	14306	T	G	0.467	10	48235385	2.48891E-06	0.099	360421	23006.877
genus Holdemanella	Vascular dementia (mixed)	rs1466525	T	C	-0.011	54780209	0.447	0.020	14306	T	C	0.489	8	53867649	1.97697E-06	0.103	360421	34189.173
genus Holdemanella	Vascular dementia (mixed)	rs1632064	T	C	0.009	3219694	0.788	0.022	14306	T	C	0.614	5	3219580	4.04045E-06	0.133	360421	37296.871
genus Holdemanella	Vascular dementia (mixed)	rs6028529	A	G	0.004	38205487	0.810	0.019	14306	A	G	0.413	20	39576844	4.98965E-06	0.091	360421	21913.090
genus Holdemanella	Vascular dementia (mixed)	rs7776624	G	A	0.012	31909839	0.463	0.016	14306	G	A	-0.358	7	31870226	8.82531E-06	0.081	360421	23450.025
genus Holdemania	Vascular dementia (mixed)	rs12257900	T	G	-0.019	49443428	0.395	0.022	14306	T	G	0.467	10	48235385	2.48891E-06	0.099	360421	23006.877
genus Holdemania	Vascular dementia (mixed)	rs1466525	T	C	0.000	54780209	0.998	0.018	14306	T	C	0.489	8	53867649	1.97697E-06	0.103	360421	34189.173
genus Holdemania	Vascular dementia (mixed)	rs1632064	T	C	0.008	3219694	0.669	0.021	14306	T	C	0.614	5	3219580	4.04045E-06	0.133	360421	37296.871
genus Holdemania	Vascular dementia (mixed)	rs429358	C	T	0.014	45411941	0.528	0.021	14306	C	T	0.565	19	44908684	3.94357E-08	0.103	360421	36831.461
genus Holdemania	Vascular dementia (mixed)	rs6028529	A	G	0.002	38205487	0.962	0.018	14306	A	G	0.413	20	39576844	4.98965E-06	0.091	360421	21913.090
genus Holdemania	Vascular dementia (mixed)	rs6849229	G	A	-0.001	131797280	0.798	0.020	14306	G	A	0.562	4	130876125	4.44038E-06	0.122	360421	34142.711
genus Howardella	Vascular dementia (mixed)	rs12257900	T	G	-0.003	49443428	0.940	0.034	14306	T	G	0.467	10	48235385	2.48891E-06	0.099	360421	23006.877
genus Howardella	Vascular dementia (mixed)	rs1466525	T	C	-0.004	54780209	0.808	0.029	14306	T	C	0.489	8	53867649	1.97697E-06	0.103	360421	34189.173
genus Howardella	Vascular dementia (mixed)	rs1632064	T	C	0.019	3219694	0.584	0.032	14306	T	C	0.614	5	3219580	4.04045E-06	0.133	360421	37296.871
genus Howardella	Vascular dementia (mixed)	rs17168895	T	G	-0.012	15647727	0.801	0.038	14306	T	G	-0.547	7	15608102	2.92853E-06	0.117	360421	34495.049
genus Howardella	Vascular dementia (mixed)	rs429358	C	T	0.033	45411941	0.260	0.033	14306	C	T	0.565	19	44908684	3.94357E-08	0.103	360421	36831.461
genus Howardella	Vascular dementia (mixed)	rs6028529	A	G	-0.024	38205487	0.415	0.027	14306	A	G	0.413	20	39576844	4.98965E-06	0.091	360421	21913.090
genus Howardella	Vascular dementia (mixed)	rs7776624	G	A	0.000	31909839	0.982	0.023	14306	G	A	-0.358	7	31870226	8.82531E-06	0.081	360421	23450.025
genus Hungatella	Vascular dementia (mixed)	rs1466525	T	C	0.025	54780209	0.375	0.028	14306	T	C	0.489	8	53867649	1.97697E-06	0.103	360421	34189.173
genus Hungatella	Vascular dementia (mixed)	rs1632064	T	C	0.007	3219694	0.821	0.030	14306	T	C	0.614	5	3219580	4.04045E-06	0.133	360421	37296.871
genus Hungatella	Vascular dementia (mixed)	rs17168895	T	G	0.032	15647727	0.415	0.036	14306	T	G	-0.547	7	15608102	2.92853E-06	0.117	360421	34495.049
genus Hungatella	Vascular dementia (mixed)	rs6849229	G	A	-0.021	131797280	0.441	0.030	14306	G	A	0.562	4	130876125	4.44038E-06	0.122	360421	34142.711
genus Hungatella	Vascular dementia (mixed)	rs7614116	G	A	0.010	130368069	0.640	0.024	14306	G	A	0.379	3	130649225	6.06429E-06	0.084	360421	23465.044
genus Hungatella	Vascular dementia (mixed)	rs7776624	G	A	0.008	31909839	0.699	0.022	14306	G	A	-0.358	7	31870226	8.82531E-06	0.081	360421	23450.025
genus Intestinibacter	Vascular dementia (mixed)	rs1466525	T	C	0.005	54780209	0.769	0.016	14306	T	C	0.489	8	53867649	1.97697E-06	0.103	360421	34189.173
genus Intestinibacter	Vascular dementia (mixed)	rs1632064	T	C	-0.014	3219694	0.407	0.018	14306	T	C	0.614	5	3219580	4.04045E-06	0.133	360421	37296.871
genus Intestinibacter	Vascular dementia (mixed)	rs429358	C	T	-0.011	45411941	0.447	0.018	14306	C	T	0.565	19	44908684	3.94357E-08	0.103	360421	36831.461
genus Intestinibacter	Vascular dementia (mixed)	rs6028529	A	G	-0.011	38205487	0.490	0.015	14306	A	G	0.413	20	39576844	4.98965E-06	0.091	360421	21913.090
genus Intestinibacter	Vascular dementia (mixed)	rs6849229	G	A	-0.006	131797280	0.759	0.018	14306	G	A	0.562	4	130876125	4.44038E-06	0.122	360421	34142.711
genus Intestinibacter	Vascular dementia (mixed)	rs7614116	G	A	0.004	130368069	0.812	0.014	14306	G	A	0.379	3	130649225	6.06429E-06	0.084	360421	23465.044
genus Intestinimonas	Vascular dementia (mixed)	rs12257900	T	G	-0.016	49443428	0.386	0.019	14306	T	G	0.467	10	48235385	2.48891E-06	0.099	360421	23006.877
genus Intestinimonas	Vascular dementia (mixed)	rs1466525	T	C	0.011	54780209	0.475	0.016	14306	T	C	0.489	8	53867649	1.97697E-06	0.103	360421	34189.173
genus Intestinimonas	Vascular dementia (mixed)	rs17168895	T	G	0.006	15647727	0.757	0.021	14306	T	G	-0.547	7	15608102	2.92853E-06	0.117	360421	34495.049
genus Intestinimonas	Vascular dementia (mixed)	rs429358	C	T	0.013	45411941	0.424	0.018	14306	C	T	0.565	19	44908684	3.94357E-08	0.103	360421	36831.461
genus Intestinimonas	Vascular dementia (mixed)	rs6028529	A	G	0.005	38205487	0.718	0.015	14306	A	G	0.413	20	39576844	4.98965E-06	0.091	360421	21913.090
genus Intestinimonas	Vascular dementia (mixed)	rs6849229	G	A	0.010	131797280	0.639	0.018	14306	G	A	0.562	4	130876125	4.44038E-06	0.122	360421	34142.711
genus Intestinimonas	Vascular dementia (mixed)	rs7776624	G	A	0.002	31909839	0.867	0.013	14306	G	A	-0.358	7	31870226	8.82531E-06	0.081	360421	23450.025
genus Lachnoclostridium	Vascular dementia (mixed)	rs12257900	T	G	-0.011	49443428	0.470	0.016	14306	T	G	0.467	10	48235385	2.48891E-06	0.099	360421	23006.877
genus Lachnoclostridium	Vascular dementia (mixed)	rs1466525	T	C	-0.001	54780209	0.821	0.013	14306	T	C	0.489	8	53867649	1.97697E-06	0.103	360421	34189.173
genus Lachnoclostridium	Vascular dementia (mixed)	rs6028529	A	G	-0.003	38205487	0.820	0.013	14306	A	G	0.413	20	39576844	4.98965E-06	0.091	360421	21913.090
genus Lachnoclostridium	Vascular dementia (mixed)	rs6849229	G	A	-0.005	131797280	0.626	0.014	14306	G	A	0.562	4	130876125	4.44038E-06	0.122	360421	34142.711
genus Lachnoclostridium	Vascular dementia (mixed)	rs7614116	G	A	0.002	130368069	0.887	0.011	14306	G	A	0.379	3	130649225	6.06429E-06	0.084	360421	23465.044
genus Lachnoclostridium	Vascular dementia (mixed)	rs7776624	G	A	-0.004	31909839	0.723	0.011	14306	G	A	-0.358	7	31870226	8.82531E-06	0.081	360421	23450.025
genus Lachnospiraceae FCS020 group	Vascular dementia (mixed)	rs12257900	T	G	0.016	49443428	0.363	0.018	14306	T	G	0.467	10	48235385	2.48891E-06	0.099	360421	23006.877
genus Lachnospiraceae FCS020 group	Vascular dementia (mixed)	rs17168895	T	G	0.001	15647727	0.925	0.020	14306	T	G	-0.547	7	15608102	2.92853E-06	0.117	360421	34495.049
genus Lachnospiraceae FCS020 group	Vascular dementia (mixed)	rs429358	C	T	0.017	45411941	0.318	0.017	14306	C	T	0.565	19	44908684	3.94357E-08	0.103	360421	36831.461
genus Lachnospiraceae FCS020 group	Vascular dementia (mixed)	rs7776624	G	A	0.003	31909839	0.804	0.012	14306	G	A	-0.358	7	31870226	8.82531E-06	0.081	360421	23450.025
genus Lachnospiraceae NC2004 group	Vascular dementia (mixed)	rs12257900	T	G	0.011	49443428	0.640	0.027	14306	T	G	0.467	10	48235385	2.48891E-06	0.099	360421	23006.877
genus Lachnospiraceae NC2004 group	Vascular dementia (mixed)	rs1632064	T	C	-0.008	3219694	0.788	0.024	14306	T	C	0.614	5	3219580	4.04045E-06	0.133	360421	37296.871
genus Lachnospiraceae NC2004 group	Vascular dementia (mixed)	rs17168895	T	G	0.001	15647727	0.967	0.030	14306	T	G	-0.547	7	15608102	2.92853E-06	0.117	360421	34495.049
genus Lachnospiraceae NC2004 group	Vascular dementia (mixed)	rs429358	C	T	-0.024	45411941	0.415	0.026	14306	C	T	0.565	19	44908684	3.94357E-08	0.103	360421	36831.461
genus Lachnospiraceae NC2004 group	Vascular dementia (mixed)	rs6849229	G	A	-0.002	131797280	0.922	0.023	14306	G	A	0.562	4	130876125	4.44038E-06	0.122	360421	34142.711
genus Lachnospiraceae NC2004 group	Vascular dementia (mixed)	rs7776624	G	A	0.006	31909839	0.745	0.018	14306	G	A	-0.358	7	31870226	8.82531E-06	0.081	360421	23450.025
genus Lachnospiraceae ND3007 group	Vascular dementia (mixed)	rs12257900	T	G	0.002	49443428	0.909	0.017	14306	T	G	0.467	10	48235385	2.48891E-06	0.099	360421	23006.877
genus Lachnospiraceae ND3007 group	Vascular dementia (mixed)	rs1632064	T	C	0.003	3219694	0.775	0.016	14306	T	C	0.614	5	3219580	4.04045E-06	0.133	360421	37296.871
genus Lachnospiraceae ND3007 group	Vascular dementia (mixed)	rs429358	C	T	-0.003	45411941	0.806	0.016	14306	C	T	0.565	19	44908684	3.94357E-08	0.103	360421	36831.461
genus Lachnospiraceae ND3007 group	Vascular dementia (mixed)	rs6028529	A	G	0.006	38205487	0.658	0.014	14306	A	G	0.413	20	39576844	4.98965E-06	0.091	360421	21913.090
genus Lachnospiraceae ND3007 group	Vascular dementia (mixed)	rs6849229	G	A	0.003	131797280	0.804	0.016	14306	G	A	0.562	4	130876125	4.44038E-06	0.122	360421	34142.711
genus Lachnospiraceae ND3007 group	Vascular dementia (mixed)	rs7614116	G	A	0.007	130368069	0.554	0.012	14306	G	A	0.379	3	130649225	6.06429E-06	0.084	360421	23465.044
genus Lachnospiraceae ND3007 group	Vascular dementia (mixed)	rs7776624	G	A	0.002	31909839	0.844	0.011	14306	G	A	-0.358	7	31870226	8.82531E-06	0.081	360421	23450.025
genus Lachnospiraceae NK4A136 group	Vascular dementia (mixed)	rs12257900	T	G	-0.015	49443428	0.360	0.016	14306	T	G	0.467	10	48235385	2.48891E-06	0.099	360421	23006.877
genus Lachnospiraceae NK4A136 group	Vascular dementia (mixed)	rs1466525	T	C	-0.011	54780209	0.454	0.014	14306	T	C	0.489	8	53867649	1.97697E-06	0.103	360421	34189.173
genus Lachnospiraceae NK4A136 group	Vascular dementia (mixed)	rs1632064	T	C	-0.014	3219694	0.315	0.015	14306	T	C	0.614	5	3219580	4.04045E-06	0.133	360421	37296.871
genus Lachnospiraceae NK4A136 group	Vascular dementia (mixed)	rs429358	C	T	-0.011	45411941	0.456	0.015	14306	C	T	0.565	19	44908684	3.94357E-08	0.103	360421	36831.461
genus Lachnospiraceae NK4A136 group	Vascular dementia (mixed)	rs6849229	G	A	0.008	131797280	0.610	0.015	14306	G	A	0.562	4	130876125	4.44038E-06	0.122	360421	34142.711
genus Lachnospiraceae NK4A136 group	Vascular dementia (mixed)	rs7614116	G	A	-0.010	130368069	0.392	0.012	14306	G	A	0.379	3	130649225	6.06429E-06	0.084	360421	23465.044

genus Lachnospiraceae NK4A136 group	Vascular dementia (mixed)	rs7776624	G	A	0.009	31909839	0.393	0.011	14306	G	A	-0.358	7	31870226	8.82531E-06	0.081	360421	23450.025
genus Lachnospiraceae UCG001	Vascular dementia (mixed)	rs12257900	T	G	0.000	49443428	0.968	0.021	14306	T	G	0.467	10	48235385	2.48891E-06	0.099	360421	23006.877
genus Lachnospiraceae UCG001	Vascular dementia (mixed)	rs1632064	T	C	-0.005	3219694	0.757	0.020	14306	T	C	0.614	5	3219580	4.04045E-06	0.133	360421	37296.871
genus Lachnospiraceae UCG001	Vascular dementia (mixed)	rs429358	C	T	-0.002	45411941	0.904	0.020	14306	C	T	0.565	19	44908684	3.94357E-08	0.103	360421	36831.461
genus Lachnospiraceae UCG001	Vascular dementia (mixed)	rs6028529	A	G	-0.011	38205487	0.487	0.017	14306	A	G	0.413	20	39576844	4.98965E-06	0.091	360421	21913.090
genus Lachnospiraceae UCG001	Vascular dementia (mixed)	rs6849229	G	A	-0.013	131797280	0.459	0.019	14306	G	A	0.562	4	130876125	4.44038E-06	0.122	360421	34142.711
genus Lachnospiraceae UCG001	Vascular dementia (mixed)	rs7614116	G	A	-0.001	130368069	0.935	0.015	14306	G	A	0.379	3	130649225	6.06429E-06	0.084	360421	23465.044
genus Lachnospiraceae UCG004	Vascular dementia (mixed)	rs12257900	T	G	0.012	49443428	0.539	0.018	14306	T	G	0.467	10	48235385	2.48891E-06	0.099	360421	23006.877
genus Lachnospiraceae UCG004	Vascular dementia (mixed)	rs1632064	T	C	-0.010	3219694	0.483	0.016	14306	T	C	0.614	5	3219580	4.04045E-06	0.133	360421	37296.871
genus Lachnospiraceae UCG004	Vascular dementia (mixed)	rs17168895	T	G	-0.001	15647727	0.819	0.020	14306	T	G	-0.547	7	15608102	2.92853E-06	0.117	360421	34495.049
genus Lachnospiraceae UCG004	Vascular dementia (mixed)	rs429358	C	T	-0.001	45411941	0.954	0.017	14306	C	T	0.565	19	44908684	3.94357E-08	0.103	360421	36831.461
genus Lachnospiraceae UCG004	Vascular dementia (mixed)	rs6028529	A	G	0.007	38205487	0.590	0.014	14306	A	G	0.413	20	39576844	4.98965E-06	0.091	360421	21913.090
genus Lachnospiraceae UCG004	Vascular dementia (mixed)	rs6849229	G	A	0.009	131797280	0.652	0.016	14306	G	A	0.562	4	130876125	4.44038E-06	0.122	360421	34142.711
genus Lachnospiraceae UCG008	Vascular dementia (mixed)	rs12257900	T	G	-0.018	49443428	0.452	0.025	14306	T	G	0.467	10	48235385	2.48891E-06	0.099	360421	23006.877
genus Lachnospiraceae UCG008	Vascular dementia (mixed)	rs17168895	T	G	0.000	15647727	0.937	0.028	14306	T	G	-0.547	7	15608102	2.92853E-06	0.117	360421	34495.049
genus Lachnospiraceae UCG008	Vascular dementia (mixed)	rs429358	C	T	0.001	45411941	0.933	0.024	14306	C	T	0.565	19	44908684	3.94357E-08	0.103	360421	36831.461
genus Lachnospiraceae UCG008	Vascular dementia (mixed)	rs6028529	A	G	0.007	38205487	0.737	0.020	14306	A	G	0.413	20	39576844	4.98965E-06	0.091	360421	21913.090
genus Lachnospiraceae UCG008	Vascular dementia (mixed)	rs6849229	G	A	-0.002	131797280	0.998	0.023	14306	G	A	0.562	4	130876125	4.44038E-06	0.122	360421	34142.711
genus Lachnospiraceae UCG008	Vascular dementia (mixed)	rs7614116	G	A	0.010	130368069	0.578	0.018	14306	G	A	0.379	3	130649225	6.06429E-06	0.084	360421	23465.044
genus Lachnospiraceae UCG010	Vascular dementia (mixed)	rs12257900	T	G	0.003	49443428	0.798	0.018	14306	T	G	0.467	10	48235385	2.48891E-06	0.099	360421	23006.877
genus Lachnospiraceae UCG010	Vascular dementia (mixed)	rs1632064	T	C	-0.006	3219694	0.683	0.017	14306	T	C	0.614	5	3219580	4.04045E-06	0.133	360421	37296.871
genus Lachnospiraceae UCG010	Vascular dementia (mixed)	rs17168895	T	G	-0.006	15647727	0.754	0.021	14306	T	G	-0.547	7	15608102	2.92853E-06	0.117	360421	34495.049
genus Lachnospiraceae UCG010	Vascular dementia (mixed)	rs429358	C	T	-0.003	45411941	0.809	0.018	14306	C	T	0.565	19	44908684	3.94357E-08	0.103	360421	36831.461
genus Lachnospiraceae UCG010	Vascular dementia (mixed)	rs6028529	A	G	0.002	38205487	0.825	0.015	14306	A	G	0.413	20	39576844	4.98965E-06	0.091	360421	21913.090
genus Lachnospiraceae UCG010	Vascular dementia (mixed)	rs7614116	G	A	-0.012	130368069	0.422	0.013	14306	G	A	0.379	3	130649225	6.06429E-06	0.084	360421	23465.044
genus Lachnospiraceae UCG010	Vascular dementia (mixed)	rs7776624	G	A	0.005	31909839	0.681	0.012	14306	G	A	-0.358	7	31870226	8.82531E-06	0.081	360421	23450.025
genus Lactobacillus	Vascular dementia (mixed)	rs1466525	T	C	-0.012	54780209	0.523	0.021	14306	T	C	0.489	8	53867649	1.97697E-06	0.103	360421	34189.173
genus Lactobacillus	Vascular dementia (mixed)	rs1632064	T	C	-0.013	3219694	0.619	0.023	14306	T	C	0.614	5	3219580	4.04045E-06	0.133	360421	37296.871
genus Lactobacillus	Vascular dementia (mixed)	rs17168895	T	G	0.011	15647727	0.776	0.028	14306	T	G	-0.547	7	15608102	2.92853E-06	0.117	360421	34495.049
genus Lactobacillus	Vascular dementia (mixed)	rs429358	C	T	-0.007	45411941	0.865	0.024	14306	C	T	0.565	19	44908684	3.94357E-08	0.103	360421	36831.461
genus Lactobacillus	Vascular dementia (mixed)	rs6028529	A	G	0.010	38205487	0.640	0.020	14306	A	G	0.413	20	39576844	4.98965E-06	0.091	360421	21913.090
genus Lactococcus	Vascular dementia (mixed)	rs12257900	T	G	0.013	49443428	0.759	0.033	14306	T	G	0.467	10	48235385	2.48891E-06	0.099	360421	23006.877
genus Lactococcus	Vascular dementia (mixed)	rs1466525	T	C	0.023	54780209	0.435	0.028	14306	T	C	0.489	8	53867649	1.97697E-06	0.103	360421	34189.173
genus Lactococcus	Vascular dementia (mixed)	rs1632064	T	C	-0.017	3219694	0.612	0.031	14306	T	C	0.614	5	3219580	4.04045E-06	0.133	360421	37296.871
genus Lactococcus	Vascular dementia (mixed)	rs17168895	T	G	0.003	15647727	0.944	0.037	14306	T	G	-0.547	7	15608102	2.92853E-06	0.117	360421	34495.049
genus Lactococcus	Vascular dementia (mixed)	rs429358	C	T	-0.017	45411941	0.591	0.032	14306	C	T	0.565	19	44908684	3.94357E-08	0.103	360421	36831.461
genus Lactococcus	Vascular dementia (mixed)	rs6028529	A	G	-0.018	38205487	0.516	0.027	14306	A	G	0.413	20	39576844	4.98965E-06	0.091	360421	21913.090
genus Lactococcus	Vascular dementia (mixed)	rs7614116	G	A	-0.007	130368069	0.735	0.024	14306	G	A	0.379	3	130649225	6.06429E-06	0.084	360421	23465.044
genus Lactococcus	Vascular dementia (mixed)	rs7776624	G	A	0.011	31909839	0.635	0.022	14306	G	A	-0.358	7	31870226	8.82531E-06	0.081	360421	23450.025
genus Marvinbryantia	Vascular dementia (mixed)	rs12257900	T	G	-0.016	49443428	0.406	0.019	14306	T	G	0.467	10	48235385	2.48891E-06	0.099	360421	23006.877
genus Marvinbryantia	Vascular dementia (mixed)	rs1466525	T	C	-0.010	54780209	0.512	0.016	14306	T	C	0.489	8	53867649	1.97697E-06	0.103	360421	34189.173
genus Marvinbryantia	Vascular dementia (mixed)	rs17168895	T	G	0.013	15647727	0.452	0.021	14306	T	G	-0.547	7	15608102	2.92853E-06	0.117	360421	34495.049
genus Marvinbryantia	Vascular dementia (mixed)	rs429358	C	T	0.008	45411941	0.663	0.018	14306	C	T	0.565	19	44908684	3.94357E-08	0.103	360421	36831.461
genus Marvinbryantia	Vascular dementia (mixed)	rs6028529	A	G	0.008	38205487	0.692	0.016	14306	A	G	0.413	20	39576844	4.98965E-06	0.091	360421	21913.090
genus Marvinbryantia	Vascular dementia (mixed)	rs7614116	G	A	0.002	130368069	0.924	0.014	14306	G	A	0.379	3	130649225	6.06429E-06	0.084	360421	23465.044
genus Methanobrevibacter	Vascular dementia (mixed)	rs1466525	T	C	0.001	54780209	0.922	0.030	14306	T	C	0.489	8	53867649	1.97697E-06	0.103	360421	34189.173
genus Methanobrevibacter	Vascular dementia (mixed)	rs1632064	T	C	0.014	3219694	0.625	0.033	14306	T	C	0.614	5	3219580	4.04045E-06	0.133	360421	37296.871
genus Methanobrevibacter	Vascular dementia (mixed)	rs17168895	T	G	0.017	15647727	0.670	0.039	14306	T	G	-0.547	7	15608102	2.92853E-06	0.117	360421	34495.049
genus Methanobrevibacter	Vascular dementia (mixed)	rs429358	C	T	-0.019	45411941	0.661	0.034	14306	C	T	0.565	19	44908684	3.94357E-08	0.103	360421	36831.461
genus Methanobrevibacter	Vascular dementia (mixed)	rs6028529	A	G	0.022	38205487	0.419	0.029	14306	A	G	0.413	20	39576844	4.98965E-06	0.091	360421	21913.090
genus Methanobrevibacter	Vascular dementia (mixed)	rs6849229	G	A	0.029	131797280	0.376	0.032	14306	G	A	0.562	4	130876125	4.44038E-06	0.122	360421	34142.711
genus Methanobrevibacter	Vascular dementia (mixed)	rs7614116	G	A	-0.021	130368069	0.398	0.026	14306	G	A	0.379	3	130649225	6.06429E-06	0.084	360421	23465.044
genus Odoribacter	Vascular dementia (mixed)	rs1466525	T	C	-0.005	54780209	0.729	0.015	14306	T	C	0.489	8	53867649	1.97697E-06	0.103	360421	34189.173
genus Odoribacter	Vascular dementia (mixed)	rs1632064	T	C	-0.003	3219694	0.872	0.016	14306	T	C	0.614	5	3219580	4.04045E-06	0.133	360421	37296.871
genus Odoribacter	Vascular dementia (mixed)	rs429358	C	T	0.017	45411941	0.297	0.017	14306	C	T	0.565	19	44908684	3.94357E-08	0.103	360421	36831.461
genus Odoribacter	Vascular dementia (mixed)	rs6028529	A	G	0.007	38205487	0.612	0.014	14306	A	G	0.413	20	39576844	4.98965E-06	0.091	360421	21913.090
genus Odoribacter	Vascular dementia (mixed)	rs6849229	G	A	0.014	131797280	0.379	0.016	14306	G	A	0.562	4	130876125	4.44038E-06	0.122	360421	34142.711
genus Olsenella	Vascular dementia (mixed)	rs12257900	T	G	0.022	49443428	0.573	0.034	14306	T	G	0.467	10	48235385	2.48891E-06	0.099	360421	23006.877
genus Olsenella	Vascular dementia (mixed)	rs1632064	T	C	0.002	3219694	0.857	0.033	14306	T	C	0.614	5	3219580	4.04045E-06	0.133	360421	37296.871
genus Olsenella	Vascular dementia (mixed)	rs17168895	T	G	0.000	15647727	0.969	0.038	14306	T	G	-0.547	7	15608102	2.92853E-06	0.117	360421	34495.049
genus Olsenella	Vascular dementia (mixed)	rs6849229	G	A	0.007	131797280	0.828	0.032	14306	G	A	0.562	4	130876125	4.44038E-06	0.122	360421	34142.711
genus Olsenella	Vascular dementia (mixed)	rs7614116	G	A	0.003	130368069	0.983	0.025	14306	G	A	0.379	3	130649225	6.06429E-06	0.084	360421	23465.044
genus Olsenella	Vascular dementia (mixed)	rs7776624	G	A	0.000	31909839	0.985	0.023	14306	G	A	-0.358	7	31870226	8.82531E-06	0.081	360421	23450.025
genus Oscillibacter	Vascular dementia (mixed)	rs1632064	T	C	0.019	3219694	0.329	0.021	14306	T	C	0.614	5	3219580	4.04045E-06	0.133	360421	37296.871
genus Oscillibacter	Vascular dementia (mixed)	rs6849229	G	A	0.007	131797280	0.615	0.021	14306	G	A	0.562	4	130876125	4.44038E-06	0.122	360421	34142.711
genus Oscillibacter	Vascular dementia (mixed)	rs7776624	G	A	0.005	31909839	0.708	0.015	14306	G	A	-0.358	7	31870226	8.82531E-06	0.081	360421	23450.025
genus Oscillospira	Vascular dementia (mixed)	rs12257900	T	G	0.015	49443428	0.478	0.021	14306	T	G	0.467	10	48235385	2.48891E-06	0.099	360421	23006.877
genus Oscillospira	Vascular dementia (mixed)	rs1466525	T	C	0.016	54780209	0.308	0.018	14306	T	C	0.489	8	53867649	1.97697E-06	0.103	360421	34189.173
genus Oscillospira	Vascular dementia (mixed)	rs1632064	T	C	-0.002	3219694	0.981	0.019	14306	T	C	0.614	5	3219580	4.04045E-06	0.133	360421	37296.871
genus Oscillospira	Vascular dementia (mixed)	rs17168895	T	G	0.014	15647727	0.659	0.023	14306	T	G	-0.547	7	15608102	2.92853E-06	0.117	360421	34495.049
genus Oscillospira	Vascular dementia (mixed)	rs429358	C	T	-0.005	45411941	0.763	0.020	14306	C	T	0.565	19	44908684	3.94357E-08	0.103	360421	36831.461

genus Oscillospira	Vascular dementia (mixed)	rs7614116	G	A	-0.005	130368069	0.769	0.015	14306	G	A	0.379	3	130649225	6.06429E-06	0.084	360421	23465.044
genus Oscillospira	Vascular dementia (mixed)	rs7776624	G	A	0.004	31909839	0.744	0.014	14306	G	A	-0.358	7	31870226	8.82531E-06	0.081	360421	23450.025
genus Oxalobacter	Vascular dementia (mixed)	rs12257900	T	G	-0.012	49443428	0.797	0.031	14306	T	G	0.467	10	48235385	2.48891E-06	0.099	360421	23006.877
genus Oxalobacter	Vascular dementia (mixed)	rs1466525	T	C	-0.024	54780209	0.357	0.026	14306	T	C	0.489	8	53867649	1.97697E-06	0.103	360421	34189.173
genus Oxalobacter	Vascular dementia (mixed)	rs6028529	A	G	0.002	38205487	0.974	0.025	14306	A	G	0.413	20	39576844	4.98965E-06	0.091	360421	21913.090
genus Oxalobacter	Vascular dementia (mixed)	rs6849229	G	A	-0.004	131797280	0.852	0.029	14306	G	A	0.562	4	130876125	4.44038E-06	0.122	360421	34142.711
genus Oxalobacter	Vascular dementia (mixed)	rs7614116	G	A	-0.013	130368069	0.594	0.023	14306	G	A	0.379	3	130649225	6.06429E-06	0.084	360421	23465.044
genus Oxalobacter	Vascular dementia (mixed)	rs7776624	G	A	0.007	31909839	0.731	0.021	14306	G	A	-0.358	7	31870226	8.82531E-06	0.081	360421	23450.025
genus Parabacteroides	Vascular dementia (mixed)	rs1632064	T	C	0.001	3219694	0.941	0.015	14306	T	C	0.614	5	3219580	4.04045E-06	0.133	360421	37296.871
genus Parabacteroides	Vascular dementia (mixed)	rs17168895	T	G	0.012	15647727	0.592	0.018	14306	T	G	-0.547	7	15608102	2.92853E-06	0.117	360421	34495.049
genus Parabacteroides	Vascular dementia (mixed)	rs429358	C	T	-0.009	45411941	0.601	0.015	14306	C	T	0.565	19	44908684	3.94357E-08	0.103	360421	36831.461
genus Parabacteroides	Vascular dementia (mixed)	rs6028529	A	G	0.001	38205487	0.923	0.013	14306	A	G	0.413	20	39576844	4.98965E-06	0.091	360421	21913.090
genus Parabacteroides	Vascular dementia (mixed)	rs6849229	G	A	0.001	131797280	0.949	0.015	14306	G	A	0.562	4	130876125	4.44038E-06	0.122	360421	34142.711
genus Parabacteroides	Vascular dementia (mixed)	rs7614116	G	A	0.008	130368069	0.500	0.012	14306	G	A	0.379	3	130649225	6.06429E-06	0.084	360421	23465.044
genus Parabacteroides	Vascular dementia (mixed)	rs7776624	G	A	-0.003	31909839	0.752	0.011	14306	G	A	-0.358	7	31870226	8.82531E-06	0.081	360421	23450.025
genus Paraprevotella	Vascular dementia (mixed)	rs1466525	T	C	0.013	54780209	0.545	0.021	14306	T	C	0.489	8	53867649	1.97697E-06	0.103	360421	34189.173
genus Paraprevotella	Vascular dementia (mixed)	rs17168895	T	G	-0.007	15647727	0.734	0.028	14306	T	G	-0.547	7	15608102	2.92853E-06	0.117	360421	34495.049
genus Paraprevotella	Vascular dementia (mixed)	rs429358	C	T	-0.005	45411941	0.844	0.024	14306	C	T	0.565	19	44908684	3.94357E-08	0.103	360421	36831.461
genus Paraprevotella	Vascular dementia (mixed)	rs6028529	A	G	-0.001	38205487	0.949	0.020	14306	A	G	0.413	20	39576844	4.98965E-06	0.091	360421	21913.090
genus Paraprevotella	Vascular dementia (mixed)	rs6849229	G	A	-0.013	131797280	0.562	0.022	14306	G	A	0.562	4	130876125	4.44038E-06	0.122	360421	34142.711
genus Paraprevotella	Vascular dementia (mixed)	rs7614116	G	A	0.004	130368069	0.763	0.018	14306	G	A	0.379	3	130649225	6.06429E-06	0.084	360421	23465.044
genus Paraprevotella	Vascular dementia (mixed)	rs7776624	G	A	-0.012	31909839	0.467	0.017	14306	G	A	-0.358	7	31870226	8.82531E-06	0.081	360421	23450.025
genus Parasutterella	Vascular dementia (mixed)	rs12257900	T	G	0.010	49443428	0.580	0.020	14306	T	G	0.467	10	48235385	2.48891E-06	0.099	360421	23006.877
genus Parasutterella	Vascular dementia (mixed)	rs1466525	T	C	-0.014	54780209	0.337	0.017	14306	T	C	0.489	8	53867649	1.97697E-06	0.103	360421	34189.173
genus Parasutterella	Vascular dementia (mixed)	rs6849229	G	A	-0.007	131797280	0.675	0.018	14306	G	A	0.562	4	130876125	4.44038E-06	0.122	360421	34142.711
genus Parasutterella	Vascular dementia (mixed)	rs7614116	G	A	-0.005	130368069	0.729	0.014	14306	G	A	0.379	3	130649225	6.06429E-06	0.084	360421	23465.044
genus Parasutterella	Vascular dementia (mixed)	rs7776624	G	A	-0.005	31909839	0.678	0.013	14306	G	A	-0.358	7	31870226	8.82531E-06	0.081	360421	23450.025
genus Peptococcus	Vascular dementia (mixed)	rs12257900	T	G	-0.026	49443428	0.344	0.028	14306	T	G	0.467	10	48235385	2.48891E-06	0.099	360421	23006.877
genus Peptococcus	Vascular dementia (mixed)	rs1466525	T	C	-0.013	54780209	0.573	0.023	14306	T	C	0.489	8	53867649	1.97697E-06	0.103	360421	34189.173
genus Peptococcus	Vascular dementia (mixed)	rs7614116	G	A	-0.011	130368069	0.570	0.021	14306	G	A	0.379	3	130649225	6.06429E-06	0.084	360421	23465.044
genus Peptococcus	Vascular dementia (mixed)	rs7776624	G	A	-0.008	31909839	0.673	0.019	14306	G	A	-0.358	7	31870226	8.82531E-06	0.081	360421	23450.025
genus Phascolarctobacterium	Vascular dementia (mixed)	rs12257900	T	G	-0.012	49443428	0.558	0.020	14306	T	G	0.467	10	48235385	2.48891E-06	0.099	360421	23006.877
genus Phascolarctobacterium	Vascular dementia (mixed)	rs1466525	T	C	0.009	54780209	0.567	0.017	14306	T	C	0.489	8	53867649	1.97697E-06	0.103	360421	34189.173
genus Phascolarctobacterium	Vascular dementia (mixed)	rs1632064	T	C	-0.001	3219694	0.883	0.019	14306	T	C	0.614	5	3219580	4.04045E-06	0.133	360421	37296.871
genus Phascolarctobacterium	Vascular dementia (mixed)	rs17168895	T	G	0.002	15647727	0.885	0.022	14306	T	G	-0.547	7	15608102	2.92853E-06	0.117	360421	34495.049
genus Phascolarctobacterium	Vascular dementia (mixed)	rs429358	C	T	-0.013	45411941	0.427	0.019	14306	C	T	0.565	19	44908684	3.94357E-08	0.103	360421	36831.461
genus Phascolarctobacterium	Vascular dementia (mixed)	rs6028529	A	G	-0.008	38205487	0.614	0.016	14306	A	G	0.413	20	39576844	4.98965E-06	0.091	360421	21913.090
genus Phascolarctobacterium	Vascular dementia (mixed)	rs6849229	G	A	0.014	131797280	0.448	0.018	14306	G	A	0.562	4	130876125	4.44038E-06	0.122	360421	34142.711
genus Phascolarctobacterium	Vascular dementia (mixed)	rs7614116	G	A	0.002	130368069	0.867	0.015	14306	G	A	0.379	3	130649225	6.06429E-06	0.084	360421	23465.044
genus Phascolarctobacterium	Vascular dementia (mixed)	rs7776624	G	A	0.000	31909839	0.998	0.013	14306	G	A	-0.358	7	31870226	8.82531E-06	0.081	360421	23450.025
genus Prevotella7	Vascular dementia (mixed)	rs12257900	T	G	0.014	49443428	0.688	0.035	14306	T	G	0.467	10	48235385	2.48891E-06	0.099	360421	23006.877
genus Prevotella7	Vascular dementia (mixed)	rs1466525	T	C	0.026	54780209	0.411	0.030	14306	T	C	0.489	8	53867649	1.97697E-06	0.103	360421	34189.173
genus Prevotella7	Vascular dementia (mixed)	rs1632064	T	C	0.014	3219694	0.753	0.034	14306	T	C	0.614	5	3219580	4.04045E-06	0.133	360421	37296.871
genus Prevotella9	Vascular dementia (mixed)	rs1466525	T	C	0.001	54780209	0.817	0.018	14306	T	C	0.489	8	53867649	1.97697E-06	0.103	360421	34189.173
genus Prevotella9	Vascular dementia (mixed)	rs1632064	T	C	-0.004	3219694	0.804	0.019	14306	T	C	0.614	5	3219580	4.04045E-06	0.133	360421	37296.871
genus Prevotella9	Vascular dementia (mixed)	rs17168895	T	G	0.008	15647727	0.787	0.024	14306	T	G	-0.547	7	15608102	2.92853E-06	0.117	360421	34495.049
genus Prevotella9	Vascular dementia (mixed)	rs429358	C	T	0.022	45411941	0.310	0.020	14306	C	T	0.565	19	44908684	3.94357E-08	0.103	360421	36831.461
genus Prevotella9	Vascular dementia (mixed)	rs6028529	A	G	-0.002	38205487	0.868	0.016	14306	A	G	0.413	20	39576844	4.98965E-06	0.091	360421	21913.090
genus Prevotella9	Vascular dementia (mixed)	rs7614116	G	A	0.001	130368069	0.887	0.015	14306	G	A	0.379	3	130649225	6.06429E-06	0.084	360421	23465.044
genus Prevotella9	Vascular dementia (mixed)	rs7776624	G	A	-0.007	31909839	0.629	0.014	14306	G	A	-0.358	7	31870226	8.82531E-06	0.081	360421	23450.025
genus Rikenellaceae RC9 gut group	Vascular dementia (mixed)	rs12257900	T	G	0.028	49443428	0.507	0.037	14306	T	G	0.467	10	48235385	2.48891E-06	0.099	360421	23006.877
genus Rikenellaceae RC9 gut group	Vascular dementia (mixed)	rs1466525	T	C	0.017	54780209	0.576	0.030	14306	T	C	0.489	8	53867649	1.97697E-06	0.103	360421	34189.173
genus Rikenellaceae RC9 gut group	Vascular dementia (mixed)	rs1632064	T	C	-0.030	3219694	0.397	0.034	14306	T	C	0.614	5	3219580	4.04045E-06	0.133	360421	37296.871
genus Rikenellaceae RC9 gut group	Vascular dementia (mixed)	rs17168895	T	G	0.001	15647727	0.873	0.040	14306	T	G	-0.547	7	15608102	2.92853E-06	0.117	360421	34495.049
genus Rikenellaceae RC9 gut group	Vascular dementia (mixed)	rs6849229	G	A	0.004	131797280	0.990	0.034	14306	G	A	0.562	4	130876125	4.44038E-06	0.122	360421	34142.711
genus Rikenellaceae RC9 gut group	Vascular dementia (mixed)	rs7614116	G	A	-0.018	130368069	0.499	0.027	14306	G	A	0.379	3	130649225	6.06429E-06	0.084	360421	23465.044
genus Rikenellaceae RC9 gut group	Vascular dementia (mixed)	rs7776624	G	A	-0.015	31909839	0.547	0.025	14306	G	A	-0.358	7	31870226	8.82531E-06	0.081	360421	23450.025
genus Romboutsia	Vascular dementia (mixed)	rs17168895	T	G	-0.014	15647727	0.504	0.020	14306	T	G	-0.547	7	15608102	2.92853E-06	0.117	360421	34495.049
genus Romboutsia	Vascular dementia (mixed)	rs429358	C	T	0.005	45411941	0.703	0.017	14306	C	T	0.565	19	44908684	3.94357E-08	0.103	360421	36831.461
genus Romboutsia	Vascular dementia (mixed)	rs6028529	A	G	-0.008	38205487	0.612	0.014	14306	A	G	0.413	20	39576844	4.98965E-06	0.091	360421	21913.090
genus Roseburia	Vascular dementia (mixed)	rs12257900	T	G	-0.007	49443428	0.615	0.016	14306	T	G	0.467	10	48235385	2.48891E-06	0.099	360421	23006.877
genus Roseburia	Vascular dementia (mixed)	rs1466525	T	C	0.005	54780209	0.671	0.013	14306	T	C	0.489	8	53867649	1.97697E-06	0.103	360421	34189.173
genus Roseburia	Vascular dementia (mixed)	rs17168895	T	G	0.016	15647727	0.447	0.018	14306	T	G	-0.547	7	15608102	2.92853E-06	0.117	360421	34495.049
genus Roseburia	Vascular dementia (mixed)	rs429358	C	T	-0.001	45411941	0.921	0.015	14306	C	T	0.565	19	44908684	3.94357E-08	0.103	360421	36831.461
genus Roseburia	Vascular dementia (mixed)	rs6849229	G	A	-0.004	131797280	0.738	0.014	14306	G	A	0.562	4	130876125	4.44038E-06	0.122	360421	34142.711
genus Roseburia	Vascular dementia (mixed)	rs7614116	G	A	0.001	130368069	0.907	0.012	14306	G	A	0.379	3	130649225	6.06429E-06	0.084	360421	23465.044
genus Ruminiclostridium5	Vascular dementia (mixed)	rs12257900	T	G	-0.014	49443428	0.340	0.016	14306	T	G	0.467	10	48235385	2.48891E-06	0.099	360421	23006.877
genus Ruminiclostridium5	Vascular dementia (mixed)	rs1466525	T	C	0.001	54780209	0.881	0.013	14306	T	C	0.489	8	53867649	1.97697E-06	0.103	360421	34189.173
genus Ruminiclostridium5	Vascular dementia (mixed)	rs1632064	T	C	-0.006	3219694	0.649	0.015	14306	T	C	0.614	5	3219580	4.04045E-06	0.133	360421	37296.871
genus Ruminiclostridium5	Vascular dementia (mixed)	rs6028529	A	G	0.011	38205487	0.331	0.013	14306	A	G	0.413	20	39576844	4.98965E-06	0.091	360421	21913.090
genus Ruminiclostridium5	Vascular dementia (mixed)	rs6849229	G	A	0.009	131797280	0.606	0.015	14306	G	A	0.562	4	130876125	4.44038E-06	0.122	360421	34142.711

genus Ruminiclostridium5	Vascular dementia (mixed)	rs7614116	G	A	0.009	130368069	0.436	0.012	14306	G	A	0.379	3	130649225	6.06429E-06	0.084	360421	23465.044
genus Ruminiclostridium5	Vascular dementia (mixed)	rs7776624	G	A	-0.002	31909839	0.873	0.011	14306	G	A	-0.358	7	31870226	8.82531E-06	0.081	360421	23450.025
genus Ruminiclostridium6	Vascular dementia (mixed)	rs12257900	T	G	0.013	49443428	0.487	0.018	14306	T	G	0.467	10	48235385	2.48891E-06	0.099	360421	23006.877
genus Ruminiclostridium6	Vascular dementia (mixed)	rs1466525	T	C	-0.007	54780209	0.656	0.015	14306	T	C	0.489	8	53867649	1.97697E-06	0.103	360421	34189.173
genus Ruminiclostridium6	Vascular dementia (mixed)	rs1632064	T	C	0.000	3219694	0.972	0.017	14306	T	C	0.614	5	3219580	4.04045E-06	0.133	360421	37296.871
genus Ruminiclostridium6	Vascular dementia (mixed)	rs17168895	T	G	-0.011	15647727	0.640	0.020	14306	T	G	-0.547	7	15608102	2.92853E-06	0.117	360421	34495.049
genus Ruminiclostridium6	Vascular dementia (mixed)	rs429358	C	T	0.007	45411941	0.690	0.018	14306	C	T	0.565	19	44908684	3.94357E-08	0.103	360421	36831.461
genus Ruminiclostridium6	Vascular dementia (mixed)	rs6028529	A	G	-0.007	38205487	0.713	0.015	14306	A	G	0.413	20	39576844	4.98965E-06	0.091	360421	21913.090
genus Ruminiclostridium6	Vascular dementia (mixed)	rs6849229	G	A	0.008	131797280	0.706	0.017	14306	G	A	0.562	4	130876125	4.44038E-06	0.122	360421	34142.711
genus Ruminiclostridium6	Vascular dementia (mixed)	rs7614116	G	A	0.005	130368069	0.752	0.013	14306	G	A	0.379	3	130649225	6.06429E-06	0.084	360421	23465.044
genus Ruminiclostridium9	Vascular dementia (mixed)	rs12257900	T	G	0.012	49443428	0.443	0.016	14306	T	G	0.467	10	48235385	2.48891E-06	0.099	360421	23006.877
genus Ruminiclostridium9	Vascular dementia (mixed)	rs1466525	T	C	-0.005	54780209	0.709	0.014	14306	T	C	0.489	8	53867649	1.97697E-06	0.103	360421	34189.173
genus Ruminiclostridium9	Vascular dementia (mixed)	rs1632064	T	C	-0.013	3219694	0.429	0.015	14306	T	C	0.614	5	3219580	4.04045E-06	0.133	360421	37296.871
genus Ruminiclostridium9	Vascular dementia (mixed)	rs17168895	T	G	0.012	15647727	0.520	0.018	14306	T	G	-0.547	7	15608102	2.92853E-06	0.117	360421	34495.049
genus Ruminiclostridium9	Vascular dementia (mixed)	rs429358	C	T	-0.007	45411941	0.627	0.016	14306	C	T	0.565	19	44908684	3.94357E-08	0.103	360421	36831.461
genus Ruminiclostridium9	Vascular dementia (mixed)	rs6849229	G	A	0.004	131797280	0.740	0.015	14306	G	A	0.562	4	130876125	4.44038E-06	0.122	360421	34142.711
genus Ruminiclostridium9	Vascular dementia (mixed)	rs7614116	G	A	-0.009	130368069	0.485	0.012	14306	G	A	0.379	3	130649225	6.06429E-06	0.084	360421	23465.044
genus Ruminiclostridium9	Vascular dementia (mixed)	rs7776624	G	A	0.001	31909839	0.957	0.011	14306	G	A	-0.358	7	31870226	8.82531E-06	0.081	360421	23450.025
genus Ruminococcaceae NK4A214 group	Vascular dementia (mixed)	rs1632064	T	C	0.004	3219694	0.778	0.016	14306	T	C	0.614	5	3219580	4.04045E-06	0.133	360421	37296.871
genus Ruminococcaceae NK4A214 group	Vascular dementia (mixed)	rs17168895	T	G	-0.006	15647727	0.765	0.019	14306	T	G	-0.547	7	15608102	2.92853E-06	0.117	360421	34495.049
genus Ruminococcaceae NK4A214 group	Vascular dementia (mixed)	rs429358	C	T	0.002	45411941	0.898	0.016	14306	C	T	0.565	19	44908684	3.94357E-08	0.103	360421	36831.461
genus Ruminococcaceae NK4A214 group	Vascular dementia (mixed)	rs6028529	A	G	-0.012	38205487	0.355	0.014	14306	A	G	0.413	20	39576844	4.98965E-06	0.091	360421	21913.090
genus Ruminococcaceae NK4A214 group	Vascular dementia (mixed)	rs6849229	G	A	0.006	131797280	0.753	0.016	14306	G	A	0.562	4	130876125	4.44038E-06	0.122	360421	34142.711
genus Ruminococcaceae UCG002	Vascular dementia (mixed)	rs1466525	T	C	0.002	54780209	0.837	0.014	14306	T	C	0.489	8	53867649	1.97697E-06	0.103	360421	34189.173
genus Ruminococcaceae UCG002	Vascular dementia (mixed)	rs1632064	T	C	-0.008	3219694	0.577	0.015	14306	T	C	0.614	5	3219580	4.04045E-06	0.133	360421	37296.871
genus Ruminococcaceae UCG002	Vascular dementia (mixed)	rs17168895	T	G	0.006	15647727	0.704	0.018	14306	T	G	-0.547	7	15608102	2.92853E-06	0.117	360421	34495.049
genus Ruminococcaceae UCG002	Vascular dementia (mixed)	rs429358	C	T	-0.004	45411941	0.869	0.016	14306	C	T	0.565	19	44908684	3.94357E-08	0.103	360421	36831.461
genus Ruminococcaceae UCG002	Vascular dementia (mixed)	rs6028529	A	G	-0.005	38205487	0.728	0.013	14306	A	G	0.413	20	39576844	4.98965E-06	0.091	360421	21913.090
genus Ruminococcaceae UCG002	Vascular dementia (mixed)	rs6849229	G	A	0.007	131797280	0.629	0.015	14306	G	A	0.562	4	130876125	4.44038E-06	0.122	360421	34142.711
genus Ruminococcaceae UCG002	Vascular dementia (mixed)	rs7614116	G	A	-0.006	130368069	0.645	0.012	14306	G	A	0.379	3	130649225	6.06429E-06	0.084	360421	23465.044
genus Ruminococcaceae UCG003	Vascular dementia (mixed)	rs12257900	T	G	0.001	49443428	0.953	0.018	14306	T	G	0.467	10	48235385	2.48891E-06	0.099	360421	23006.877
genus Ruminococcaceae UCG003	Vascular dementia (mixed)	rs1466525	T	C	-0.008	54780209	0.515	0.015	14306	T	C	0.489	8	53867649	1.97697E-06	0.103	360421	34189.173
genus Ruminococcaceae UCG003	Vascular dementia (mixed)	rs1632064	T	C	-0.003	3219694	0.847	0.016	14306	T	C	0.614	5	3219580	4.04045E-06	0.133	360421	37296.871
genus Ruminococcaceae UCG003	Vascular dementia (mixed)	rs17168895	T	G	-0.011	15647727	0.581	0.020	14306	T	G	-0.547	7	15608102	2.92853E-06	0.117	360421	34495.049
genus Ruminococcaceae UCG003	Vascular dementia (mixed)	rs429358	C	T	0.007	45411941	0.595	0.017	14306	C	T	0.565	19	44908684	3.94357E-08	0.103	360421	36831.461
genus Ruminococcaceae UCG003	Vascular dementia (mixed)	rs6028529	A	G	-0.012	38205487	0.407	0.014	14306	A	G	0.413	20	39576844	4.98965E-06	0.091	360421	21913.090
genus Ruminococcaceae UCG003	Vascular dementia (mixed)	rs6849229	G	A	0.006	131797280	0.730	0.016	14306	G	A	0.562	4	130876125	4.44038E-06	0.122	360421	34142.711
genus Ruminococcaceae UCG003	Vascular dementia (mixed)	rs7614116	G	A	-0.002	130368069	0.855	0.013	14306	G	A	0.379	3	130649225	6.06429E-06	0.084	360421	23465.044
genus Ruminococcaceae UCG003	Vascular dementia (mixed)	rs7776624	G	A	0.000	31909839	0.994	0.012	14306	G	A	-0.358	7	31870226	8.82531E-06	0.081	360421	23450.025
genus Ruminococcaceae UCG004	Vascular dementia (mixed)	rs12257900	T	G	-0.007	49443428	0.752	0.022	14306	T	G	0.467	10	48235385	2.48891E-06	0.099	360421	23006.877
genus Ruminococcaceae UCG004	Vascular dementia (mixed)	rs1632064	T	C	-0.016	3219694	0.427	0.020	14306	T	C	0.614	5	3219580	4.04045E-06	0.133	360421	37296.871
genus Ruminococcaceae UCG004	Vascular dementia (mixed)	rs17168895	T	G	0.004	15647727	0.879	0.024	14306	T	G	-0.547	7	15608102	2.92853E-06	0.117	360421	34495.049
genus Ruminococcaceae UCG004	Vascular dementia (mixed)	rs6028529	A	G	0.005	38205487	0.845	0.017	14306	A	G	0.413	20	39576844	4.98965E-06	0.091	360421	21913.090
genus Ruminococcaceae UCG004	Vascular dementia (mixed)	rs6849229	G	A	-0.010	131797280	0.651	0.020	14306	G	A	0.562	4	130876125	4.44038E-06	0.122	360421	34142.711
genus Ruminococcaceae UCG004	Vascular dementia (mixed)	rs7776624	G	A	0.005	31909839	0.765	0.015	14306	G	A	-0.358	7	31870226	8.82531E-06	0.081	360421	23450.025
genus Ruminococcaceae UCG005	Vascular dementia (mixed)	rs1466525	T	C	-0.004	54780209	0.760	0.014	14306	T	C	0.489	8	53867649	1.97697E-06	0.103	360421	34189.173
genus Ruminococcaceae UCG005	Vascular dementia (mixed)	rs1632064	T	C	-0.002	3219694	0.913	0.016	14306	T	C	0.614	5	3219580	4.04045E-06	0.133	360421	37296.871
genus Ruminococcaceae UCG005	Vascular dementia (mixed)	rs17168895	T	G	0.010	15647727	0.555	0.018	14306	T	G	-0.547	7	15608102	2.92853E-06	0.117	360421	34495.049
genus Ruminococcaceae UCG005	Vascular dementia (mixed)	rs429358	C	T	0.004	45411941	0.741	0.016	14306	C	T	0.565	19	44908684	3.94357E-08	0.103	360421	36831.461
genus Ruminococcaceae UCG005	Vascular dementia (mixed)	rs6028529	A	G	0.011	38205487	0.445	0.013	14306	A	G	0.413	20	39576844	4.98965E-06	0.091	360421	21913.090
genus Ruminococcaceae UCG005	Vascular dementia (mixed)	rs6849229	G	A	-0.010	131797280	0.560	0.015	14306	G	A	0.562	4	130876125	4.44038E-06	0.122	360421	34142.711
genus Ruminococcaceae UCG005	Vascular dementia (mixed)	rs7614116	G	A	0.005	130368069	0.738	0.012	14306	G	A	0.379	3	130649225	6.06429E-06	0.084	360421	23465.044
genus Ruminococcaceae UCG009	Vascular dementia (mixed)	rs1466525	T	C	0.000	54780209	0.900	0.020	14306	T	C	0.489	8	53867649	1.97697E-06	0.103	360421	34189.173
genus Ruminococcaceae UCG009	Vascular dementia (mixed)	rs1632064	T	C	0.010	3219694	0.644	0.023	14306	T	C	0.614	5	3219580	4.04045E-06	0.133	360421	37296.871
genus Ruminococcaceae UCG009	Vascular dementia (mixed)	rs7614116	G	A	-0.015	130368069	0.400	0.018	14306	G	A	0.379	3	130649225	6.06429E-06	0.084	360421	23465.044
genus Ruminococcaceae UCG010	Vascular dementia (mixed)	rs1632064	T	C	-0.006	3219694	0.736	0.017	14306	T	C	0.614	5	3219580	4.04045E-06	0.133	360421	37296.871
genus Ruminococcaceae UCG010	Vascular dementia (mixed)	rs429358	C	T	0.003	45411941	0.881	0.018	14306	C	T	0.565	19	44908684	3.94357E-08	0.103	360421	36831.461
genus Ruminococcaceae UCG010	Vascular dementia (mixed)	rs6028529	A	G	0.008	38205487	0.559	0.015	14306	A	G	0.413	20	39576844	4.98965E-06	0.091	360421	21913.090
genus Ruminococcaceae UCG010	Vascular dementia (mixed)	rs6849229	G	A	-0.015	131797280	0.414	0.017	14306	G	A	0.562	4	130876125	4.44038E-06	0.122	360421	34142.711
genus Ruminococcaceae UCG010	Vascular dementia (mixed)	rs7614116	G	A	0.011	130368069	0.418	0.014	14306	G	A	0.379	3	130649225	6.06429E-06	0.084	360421	23465.044
genus Ruminococcaceae UCG010	Vascular dementia (mixed)	rs7776624	G	A	-0.006	31909839	0.607	0.013	14306	G	A	-0.358	7	31870226	8.82531E-06	0.081	360421	23450.025
genus Ruminococcaceae UCG011	Vascular dementia (mixed)	rs1466525	T	C	0.022	54780209	0.426	0.029	14306	T	C	0.489	8	53867649	1.97697E-06	0.103	360421	34189.173
genus Ruminococcaceae UCG011	Vascular dementia (mixed)	rs429358	C	T	-0.013	45411941	0.704	0.033	14306	C	T	0.565	19	44908684	3.94357E-08	0.103	360421	36831.461
genus Ruminococcaceae UCG011	Vascular dementia (mixed)	rs6028529	A	G	0.024	38205487	0.433	0.028	14306	A	G	0.413	20	39576844	4.98965E-06	0.091	360421	21913.090
genus Ruminococcaceae UCG011	Vascular dementia (mixed)	rs7614116	G	A	0.016	130368069	0.538	0.026	14306	G	A	0.379	3	130649225	6.06429E-06	0.084	360421	23465.044
genus Ruminococcaceae UCG013	Vascular dementia (mixed)	rs12257900	T	G	-0.009	49443428	0.534	0.016	14306	T	G	0.467	10	48235385	2.48891E-06	0.099	360421	23006.877
genus Ruminococcaceae UCG013	Vascular dementia (mixed)	rs1632064	T	C	-0.005	3219694	0.780	0.015	14306	T	C	0.614	5	3219580	4.04045E-06	0.133	360421	37296.871
genus Ruminococcaceae UCG013	Vascular dementia (mixed)	rs6028529	A	G	-0.001	38205487	0.913	0.013	14306	A	G	0.413	20	39576844	4.98965E-06	0.091	360421	21913.090
genus Ruminococcaceae UCG013	Vascular dementia (mixed)	rs6849229	G	A	0.002	131797280	0.937	0.015	14306	G	A	0.562	4	130876125	4.44038E-06	0.122	360421	34142.711
genus Ruminococcaceae UCG013	Vascular dementia (mixed)	rs7614116	G	A	0.000	130368069	0.991	0.012	14306	G	A	0.379	3	130649225	6.06429E-06	0.084	360421	23465.044
genus Ruminococcaceae UCG014	Vascular dementia (mixed)	rs12257900	T	G	0.006	49443428	0.755	0.018	14306	T	G	0.467	10	48235385	2.48891E-06	0.099	360421	23006.877

genus Ruminococcaceae UCG014	Vascular dementia (mixed)	rs1632064	T	C	-0.005	3219694	0.795	0.016	14306	T	C	0.614	5	3219580	4.04045E-06	0.133	360421	37296.871
genus Ruminococcaceae UCG014	Vascular dementia (mixed)	rs17168895	T	G	0.017	15647727	0.401	0.019	14306	T	G	-0.547	7	15608102	2.92853E-06	0.117	360421	34495.049
genus Ruminococcaceae UCG014	Vascular dementia (mixed)	rs6028529	A	G	-0.005	38205487	0.632	0.014	14306	A	G	0.413	20	39576844	4.98965E-06	0.091	360421	21913.090
genus Ruminococcaceae UCG014	Vascular dementia (mixed)	rs7614116	G	A	0.008	130368069	0.490	0.013	14306	G	A	0.379	3	130649225	6.06429E-06	0.084	360421	23465.044
genus Ruminococcus1	Vascular dementia (mixed)	rs12257900	T	G	-0.003	49443428	0.804	0.016	14306	T	G	0.467	10	48235385	2.48891E-06	0.099	360421	23006.877
genus Ruminococcus1	Vascular dementia (mixed)	rs429358	C	T	-0.008	45411941	0.683	0.016	14306	C	T	0.565	19	44908684	3.94357E-08	0.103	360421	36831.461
genus Ruminococcus1	Vascular dementia (mixed)	rs6849229	G	A	-0.003	131797280	0.800	0.015	14306	G	A	0.562	4	130876125	4.44038E-06	0.122	360421	34142.711
genus Ruminococcus1	Vascular dementia (mixed)	rs7776624	G	A	-0.001	31909839	0.903	0.011	14306	G	A	-0.358	7	31870226	8.82531E-06	0.081	360421	23450.025
genus Ruminococcus2	Vascular dementia (mixed)	rs12257900	T	G	0.007	49443428	0.623	0.017	14306	T	G	0.467	10	48235385	2.48891E-06	0.099	360421	23006.877
genus Ruminococcus2	Vascular dementia (mixed)	rs17168895	T	G	-0.015	15647727	0.439	0.019	14306	T	G	-0.547	7	15608102	2.92853E-06	0.117	360421	34495.049
genus Ruminococcus2	Vascular dementia (mixed)	rs429358	C	T	0.007	45411941	0.633	0.016	14306	C	T	0.565	19	44908684	3.94357E-08	0.103	360421	36831.461
genus Ruminococcus2	Vascular dementia (mixed)	rs6849229	G	A	-0.005	131797280	0.874	0.016	14306	G	A	0.562	4	130876125	4.44038E-06	0.122	360421	34142.711
genus Ruminococcus2	Vascular dementia (mixed)	rs7614116	G	A	0.005	130368069	0.702	0.012	14306	G	A	0.379	3	130649225	6.06429E-06	0.084	360421	23465.044
genus Ruminococcus2	Vascular dementia (mixed)	rs7776624	G	A	-0.010	31909839	0.386	0.011	14306	G	A	-0.358	7	31870226	8.82531E-06	0.081	360421	23450.025
genus Ruminococcus gauvreauii group	Vascular dementia (mixed)	rs12257900	T	G	0.011	49443428	0.493	0.018	14306	T	G	0.467	10	48235385	2.48891E-06	0.099	360421	23006.877
genus Ruminococcus gauvreauii group	Vascular dementia (mixed)	rs1632064	T	C	0.005	3219694	0.756	0.017	14306	T	C	0.614	5	3219580	4.04045E-06	0.133	360421	37296.871
genus Ruminococcus gauvreauii group	Vascular dementia (mixed)	rs429358	C	T	0.011	45411941	0.457	0.016	14306	C	T	0.565	19	44908684	3.94357E-08	0.103	360421	36831.461
genus Ruminococcus gauvreauii group	Vascular dementia (mixed)	rs6849229	G	A	0.009	131797280	0.704	0.017	14306	G	A	0.562	4	130876125	4.44038E-06	0.122	360421	34142.711
genus Ruminococcus gnavus group	Vascular dementia (mixed)	rs12257900	T	G	-0.011	49443428	0.675	0.026	14306	T	G	0.467	10	48235385	2.48891E-06	0.099	360421	23006.877
genus Ruminococcus gnavus group	Vascular dementia (mixed)	rs1632064	T	C	0.009	3219694	0.672	0.024	14306	T	C	0.614	5	3219580	4.04045E-06	0.133	360421	37296.871
genus Ruminococcus gnavus group	Vascular dementia (mixed)	rs17168895	T	G	-0.008	15647727	0.767	0.030	14306	T	G	-0.547	7	15608102	2.92853E-06	0.117	360421	34495.049
genus Ruminococcus gnavus group	Vascular dementia (mixed)	rs6028529	A	G	-0.008	38205487	0.671	0.021	14306	A	G	0.413	20	39576844	4.98965E-06	0.091	360421	21913.090
genus Ruminococcus gnavus group	Vascular dementia (mixed)	rs7614116	G	A	-0.008	130368069	0.714	0.019	14306	G	A	0.379	3	130649225	6.06429E-06	0.084	360421	23465.044
genus Ruminococcus gnavus group	Vascular dementia (mixed)	rs7776624	G	A	-0.005	31909839	0.790	0.018	14306	G	A	-0.358	7	31870226	8.82531E-06	0.081	360421	23450.025
genus Ruminococcus torques group	Vascular dementia (mixed)	rs12257900	T	G	0.003	49443428	0.869	0.016	14306	T	G	0.467	10	48235385	2.48891E-06	0.099	360421	23006.877
genus Ruminococcus torques group	Vascular dementia (mixed)	rs1466525	T	C	-0.012	54780209	0.419	0.013	14306	T	C	0.489	8	53867649	1.97697E-06	0.103	360421	34189.173
genus Ruminococcus torques group	Vascular dementia (mixed)	rs17168895	T	G	-0.011	15647727	0.554	0.018	14306	T	G	-0.547	7	15608102	2.92853E-06	0.117	360421	34495.049
genus Ruminococcus torques group	Vascular dementia (mixed)	rs429358	C	T	-0.001	45411941	0.916	0.015	14306	C	T	0.565	19	44908684	3.94357E-08	0.103	360421	36831.461
genus Ruminococcus torques group	Vascular dementia (mixed)	rs6028529	A	G	0.010	38205487	0.472	0.013	14306	A	G	0.413	20	39576844	4.98965E-06	0.091	360421	21913.090
genus Ruminococcus torques group	Vascular dementia (mixed)	rs6849229	G	A	0.000	131797280	0.984	0.014	14306	G	A	0.562	4	130876125	4.44038E-06	0.122	360421	34142.711
genus Ruminococcus torques group	Vascular dementia (mixed)	rs7614116	G	A	-0.001	130368069	0.920	0.012	14306	G	A	0.379	3	130649225	6.06429E-06	0.084	360421	23465.044
genus Ruminococcus torques group	Vascular dementia (mixed)	rs7776624	G	A	-0.004	31909839	0.690	0.011	14306	G	A	-0.358	7	31870226	8.82531E-06	0.081	360421	23450.025
genus Sellimonas	Vascular dementia (mixed)	rs12257900	T	G	0.005	49443428	0.905	0.037	14306	T	G	0.467	10	48235385	2.48891E-06	0.099	360421	23006.877
genus Sellimonas	Vascular dementia (mixed)	rs1466525	T	C	-0.013	54780209	0.702	0.031	14306	T	C	0.489	8	53867649	1.97697E-06	0.103	360421	34189.173
genus Sellimonas	Vascular dementia (mixed)	rs1632064	T	C	-0.023	3219694	0.476	0.035	14306	T	C	0.614	5	3219580	4.04045E-06	0.133	360421	37296.871
genus Sellimonas	Vascular dementia (mixed)	rs17168895	T	G	-0.011	15647727	0.683	0.040	14306	T	G	-0.547	7	15608102	2.92853E-06	0.117	360421	34495.049
genus Sellimonas	Vascular dementia (mixed)	rs6028529	A	G	0.022	38205487	0.466	0.031	14306	A	G	0.413	20	39576844	4.98965E-06	0.091	360421	21913.090
genus Sellimonas	Vascular dementia (mixed)	rs6849229	G	A	0.020	131797280	0.600	0.035	14306	G	A	0.562	4	130876125	4.44038E-06	0.122	360421	34142.711
genus Sellimonas	Vascular dementia (mixed)	rs7614116	G	A	0.009	130368069	0.763	0.028	14306	G	A	0.379	3	130649225	6.06429E-06	0.084	360421	23465.044
genus Senegalimassilia	Vascular dementia (mixed)	rs12257900	T	G	0.023	49443428	0.348	0.025	14306	T	G	0.467	10	48235385	2.48891E-06	0.099	360421	23006.877
genus Senegalimassilia	Vascular dementia (mixed)	rs1632064	T	C	-0.007	3219694	0.813	0.024	14306	T	C	0.614	5	3219580	4.04045E-06	0.133	360421	37296.871
genus Senegalimassilia	Vascular dementia (mixed)	rs17168895	T	G	-0.018	15647727	0.528	0.028	14306	T	G	-0.547	7	15608102	2.92853E-06	0.117	360421	34495.049
genus Senegalimassilia	Vascular dementia (mixed)	rs6849229	G	A	-0.001	131797280	0.954	0.023	14306	G	A	0.562	4	130876125	4.44038E-06	0.122	360421	34142.711
genus Senegalimassilia	Vascular dementia (mixed)	rs7776624	G	A	-0.007	31909839	0.668	0.017	14306	G	A	-0.358	7	31870226	8.82531E-06	0.081	360421	23450.025
genus Slackia	Vascular dementia (mixed)	rs12257900	T	G	-0.019	49443428	0.477	0.027	14306	T	G	0.467	10	48235385	2.48891E-06	0.099	360421	23006.877
genus Slackia	Vascular dementia (mixed)	rs1466525	T	C	0.011	54780209	0.601	0.023	14306	T	C	0.489	8	53867649	1.97697E-06	0.103	360421	34189.173
genus Slackia	Vascular dementia (mixed)	rs17168895	T	G	-0.006	15647727	0.789	0.030	14306	T	G	-0.547	7	15608102	2.92853E-06	0.117	360421	34495.049
genus Slackia	Vascular dementia (mixed)	rs7614116	G	A	0.016	130368069	0.441	0.020	14306	G	A	0.379	3	130649225	6.06429E-06	0.084	360421	23465.044
genus Slackia	Vascular dementia (mixed)	rs7776624	G	A	0.001	31909839	0.952	0.018	14306	G	A	-0.358	7	31870226	8.82531E-06	0.081	360421	23450.025
genus Streptococcus	Vascular dementia (mixed)	rs1466525	T	C	-0.004	54780209	0.694	0.014	14306	T	C	0.489	8	53867649	1.97697E-06	0.103	360421	34189.173
genus Streptococcus	Vascular dementia (mixed)	rs1632064	T	C	-0.005	3219694	0.679	0.015	14306	T	C	0.614	5	3219580	4.04045E-06	0.133	360421	37296.871
genus Streptococcus	Vascular dementia (mixed)	rs17168895	T	G	0.011	15647727	0.603	0.018	14306	T	G	-0.547	7	15608102	2.92853E-06	0.117	360421	34495.049
genus Streptococcus	Vascular dementia (mixed)	rs429358	C	T	0.017	45411941	0.279	0.016	14306	C	T	0.565	19	44908684	3.94357E-08	0.103	360421	36831.461
genus Streptococcus	Vascular dementia (mixed)	rs6028529	A	G	0.010	38205487	0.481	0.013	14306	A	G	0.413	20	39576844	4.98965E-06	0.091	360421	21913.090
genus Streptococcus	Vascular dementia (mixed)	rs7614116	G	A	-0.006	130368069	0.631	0.012	14306	G	A	0.379	3	130649225	6.06429E-06	0.084	360421	23465.044
genus Streptococcus	Vascular dementia (mixed)	rs7776624	G	A	-0.008	31909839	0.463	0.011	14306	G	A	-0.358	7	31870226	8.82531E-06	0.081	360421	23450.025
genus Subdoligranulum	Vascular dementia (mixed)	rs12257900	T	G	0.005	49443428	0.724	0.016	14306	T	G	0.467	10	48235385	2.48891E-06	0.099	360421	23006.877
genus Subdoligranulum	Vascular dementia (mixed)	rs1466525	T	C	0.001	54780209	0.863	0.013	14306	T	C	0.489	8	53867649	1.97697E-06	0.103	360421	34189.173
genus Subdoligranulum	Vascular dementia (mixed)	rs1632064	T	C	0.010	3219694	0.490	0.015	14306	T	C	0.614	5	3219580	4.04045E-06	0.133	360421	37296.871
genus Subdoligranulum	Vascular dementia (mixed)	rs17168895	T	G	0.015	15647727	0.392	0.018	14306	T	G	-0.547	7	15608102	2.92853E-06	0.117	360421	34495.049
genus Subdoligranulum	Vascular dementia (mixed)	rs429358	C	T	-0.005	45411941	0.772	0.015	14306	C	T	0.565	19	44908684	3.94357E-08	0.103	360421	36831.461
genus Subdoligranulum	Vascular dementia (mixed)	rs6028529	A	G	0.008	38205487	0.415	0.013	14306	A	G	0.413	20	39576844	4.98965E-06	0.091	360421	21913.090
genus Subdoligranulum	Vascular dementia (mixed)	rs7614116	G	A	0.005	130368069	0.642	0.012	14306	G	A	0.379	3	130649225	6.06429E-06	0.084	360421	23465.044
genus Subdoligranulum	Vascular dementia (mixed)	rs7776624	G	A	0.004	31909839	0.684	0.011	14306	G	A	-0.358	7	31870226	8.82531E-06	0.081	360421	23450.025
genus Sutterella	Vascular dementia (mixed)	rs12257900	T	G	0.007	49443428	0.614	0.018	14306	T	G	0.467	10	48235385	2.48891E-06	0.099	360421	23006.877
genus Sutterella	Vascular dementia (mixed)	rs1466525	T	C	0.002	54780209	0.854	0.016	14306	T	C	0.489	8	53867649	1.97697E-06	0.103	360421	34189.173
genus Sutterella	Vascular dementia (mixed)	rs17168895	T	G	0.014	15647727	0.508	0.020	14306	T	G	-0.547	7	15608102	2.92853E-06	0.117	360421	34495.049
genus Terrisporobacter	Vascular dementia (mixed)	rs12257900	T	G	-0.015	49443428	0.541	0.026	14306	T	G	0.467	10	48235385	2.48891E-06	0.099	360421	23006.877
genus Terrisporobacter	Vascular dementia (mixed)	rs1466525	T	C	0.016	54780209	0.530	0.022	14306	T	C	0.489	8	53867649	1.97697E-06	0.103	360421	34189.173
genus Terrisporobacter	Vascular dementia (mixed)	rs17168895	T	G	0.025	15647727	0.357	0.028	14306	T	G	-0.547	7	15608102	2.92853E-06	0.117	360421	34495.049
genus Terrisporobacter	Vascular dementia (mixed)	rs429358	C	T	-0.010	45411941	0.657	0.025	14306	C	T	0.565	19	44908684	3.94357E-08	0.103	360421	36831.461

genus Terrisporobacter	Vascular dementia (mixed)	rs6028529	A	G	-0.016	38205487	0.465	0.021	14306	A	G	0.413	20	39576844	4.98965E-06	0.091	360421	21913.090
genus Terrisporobacter	Vascular dementia (mixed)	rs7614116	G	A	0.016	130368069	0.406	0.019	14306	G	A	0.379	3	130649225	6.06429E-06	0.084	360421	23465.044
genus Terrisporobacter	Vascular dementia (mixed)	rs7776624	G	A	0.000	31909839	0.994	0.017	14306	G	A	-0.358	7	31870226	8.82531E-06	0.081	360421	23450.025
genus Turicibacter	Vascular dementia (mixed)	rs1466525	T	C	0.012	54780209	0.515	0.019	14306	T	C	0.489	8	53867649	1.97697E-06	0.103	360421	34189.173
genus Turicibacter	Vascular dementia (mixed)	rs1632064	T	C	0.016	3219694	0.487	0.021	14306	T	C	0.614	5	3219580	4.04045E-06	0.133	360421	37296.871
genus Turicibacter	Vascular dementia (mixed)	rs17168895	T	G	0.007	15647727	0.743	0.025	14306	T	G	-0.547	7	15608102	2.92853E-06	0.117	360421	34495.049
genus Turicibacter	Vascular dementia (mixed)	rs429358	C	T	0.001	45411941	0.870	0.021	14306	C	T	0.565	19	44908684	3.94357E-08	0.103	360421	36831.461
genus Turicibacter	Vascular dementia (mixed)	rs6028529	A	G	0.016	38205487	0.422	0.018	14306	A	G	0.413	20	39576844	4.98965E-06	0.091	360421	21913.090
genus Turicibacter	Vascular dementia (mixed)	rs6849229	G	A	0.009	131797280	0.687	0.021	14306	G	A	0.562	4	130876125	4.44038E-06	0.122	360421	34142.711
genus Turicibacter	Vascular dementia (mixed)	rs7776624	G	A	0.004	31909839	0.780	0.015	14306	G	A	-0.358	7	31870226	8.82531E-06	0.081	360421	23450.025
genus Tyzzerella3	Vascular dementia (mixed)	rs12257900	T	G	0.004	49443428	0.893	0.029	14306	T	G	0.467	10	48235385	2.48891E-06	0.099	360421	23006.877
genus Tyzzerella3	Vascular dementia (mixed)	rs1466525	T	C	0.013	54780209	0.646	0.024	14306	T	C	0.489	8	53867649	1.97697E-06	0.103	360421	34189.173
genus Tyzzerella3	Vascular dementia (mixed)	rs1632064	T	C	0.010	3219694	0.629	0.027	14306	T	C	0.614	5	3219580	4.04045E-06	0.133	360421	37296.871
genus Tyzzerella3	Vascular dementia (mixed)	rs17168895	T	G	-0.021	15647727	0.460	0.032	14306	T	G	-0.547	7	15608102	2.92853E-06	0.117	360421	34495.049
genus Tyzzerella3	Vascular dementia (mixed)	rs429358	C	T	0.000	45411941	0.979	0.028	14306	C	T	0.565	19	44908684	3.94357E-08	0.103	360421	36831.461
genus Tyzzerella3	Vascular dementia (mixed)	rs6028529	A	G	-0.004	38205487	0.876	0.023	14306	A	G	0.413	20	39576844	4.98965E-06	0.091	360421	21913.090
genus Tyzzerella3	Vascular dementia (mixed)	rs6849229	G	A	0.019	131797280	0.452	0.026	14306	G	A	0.562	4	130876125	4.44038E-06	0.122	360421	34142.711
genus Tyzzerella3	Vascular dementia (mixed)	rs7614116	G	A	0.017	130368069	0.445	0.021	14306	G	A	0.379	3	130649225	6.06429E-06	0.084	360421	23465.044
genus Veillonella)	Vascular dementia (mixed)	rs1632064	T	C	-0.006	3219694	0.797	0.020	14306	T	C	0.614	5	3219580	4.04045E-06	0.133	360421	37296.871
genus Veillonella)	Vascular dementia (mixed)	rs17168895	T	G	0.001	15647727	0.930	0.025	14306	T	G	-0.547	7	15608102	2.92853E-06	0.117	360421	34495.049
genus Veillonella)	Vascular dementia (mixed)	rs6849229	G	A	0.003	131797280	0.874	0.020	14306	G	A	0.562	4	130876125	4.44038E-06	0.122	360421	34142.711
genus Veillonella)	Vascular dementia (mixed)	rs7614116	G	A	-0.014	130368069	0.387	0.016	14306	G	A	0.379	3	130649225	6.06429E-06	0.084	360421	23465.044
genus Veillonella)	Vascular dementia (mixed)	rs7776624	G	A	-0.005	31909839	0.754	0.015	14306	G	A	-0.358	7	31870226	8.82531E-06	0.081	360421	23450.025
genus Victivallis	Vascular dementia (mixed)	rs12257900	T	G	-0.009	49443428	0.784	0.036	1531	T	G	0.467	10	48235385	2.48891E-06	0.099	360421	23006.877
genus Victivallis	Vascular dementia (mixed)	rs429358	C	T	0.009	45411941	0.847	0.037	1531	C	T	0.565	19	44908684	3.94357E-08	0.103	360421	36831.461
genus Actinomyces	Vascular dementia (subcortical)	rs10919863	T	C	-0.006	200226041	0.798	0.021	14306	T	C	0.315	1	200256913	3.47112E-06	0.068	360770	10813.753
genus Actinomyces	Vascular dementia (subcortical)	rs11148372	A	G	0.002	22788665	0.915	0.017	14306	A	G	-0.261	13	22214526	4.06275E-06	0.057	360770	12632.549
genus Actinomyces	Vascular dementia (subcortical)	rs11986558	T	C	0.005	2500772	0.763	0.017	14306	T	C	0.248	8	2643276	9.16875E-06	0.056	360770	11030.353
genus Actinomyces	Vascular dementia (subcortical)	rs429358	C	T	0.030	45411941	0.176	0.023	14306	C	T	0.597	19	44908684	1.74221E-17	0.070	360770	41621.629
genus Actinomyces	Vascular dementia (subcortical)	rs4295569	C	T	0.004	47820641	0.824	0.017	14306	C	T	-0.355	7	47781043	2.54572E-08	0.064	360770	20037.409
genus Adlercreutzia	Vascular dementia (subcortical)	rs10919863	T	C	-0.015	200226041	0.500	0.020	14306	T	C	0.315	1	200256913	3.47112E-06	0.068	360770	10813.753
genus Adlercreutzia	Vascular dementia (subcortical)	rs11986558	T	C	-0.012	2500772	0.460	0.017	14306	T	C	0.248	8	2643276	9.16875E-06	0.056	360770	11030.353
genus Adlercreutzia	Vascular dementia (subcortical)	rs1363668	G	A	0.009	143089582	0.583	0.016	14306	G	A	-0.272	5	143710017	5.25352E-06	0.060	360770	12697.214
genus Adlercreutzia	Vascular dementia (subcortical)	rs3802793	A	G	0.000	131685316	0.964	0.016	14306	A	G	0.274	11	131815422	5.46626E-06	0.060	360770	12758.334
genus Adlercreutzia	Vascular dementia (subcortical)	rs429358	C	T	0.007	45411941	0.796	0.022	14306	C	T	0.597	19	44908684	1.74221E-17	0.070	360770	41621.629
genus Adlercreutzia	Vascular dementia (subcortical)	rs4295569	C	T	0.016	47820641	0.314	0.016	14306	C	T	-0.355	7	47781043	2.54572E-08	0.064	360770	20037.409
genus Adlercreutzia	Vascular dementia (subcortical)	rs4723291	A	G	0.007	33551998	0.663	0.016	14306	A	G	-0.263	7	33512386	6.22372E-06	0.058	360770	11064.499
genus Akkermansia	Vascular dementia (subcortical)	rs10919863	T	C	-0.002	200226041	0.925	0.017	14306	T	C	0.315	1	200256913	3.47112E-06	0.068	360770	10813.753
genus Akkermansia	Vascular dementia (subcortical)	rs11148372	A	G	0.007	22788665	0.575	0.013	14306	A	G	-0.261	13	22214526	4.06275E-06	0.057	360770	12632.549
genus Akkermansia	Vascular dementia (subcortical)	rs1363668	G	A	0.005	143089582	0.708	0.013	14306	G	A	-0.272	5	143710017	5.25352E-06	0.060	360770	12697.214
genus Akkermansia	Vascular dementia (subcortical)	rs3802793	A	G	-0.006	131685316	0.660	0.014	14306	A	G	0.274	11	131815422	5.46626E-06	0.060	360770	12758.334
genus Akkermansia	Vascular dementia (subcortical)	rs429358	C	T	0.018	45411941	0.337	0.019	14306	C	T	0.597	19	44908684	1.74221E-17	0.070	360770	41621.629
genus Akkermansia	Vascular dementia (subcortical)	rs4295569	C	T	0.001	47820641	0.912	0.013	14306	C	T	-0.355	7	47781043	2.54572E-08	0.064	360770	20037.409
genus Akkermansia	Vascular dementia (subcortical)	rs4382795	C	T	-0.005	66878853	0.776	0.017	14306	C	T	0.378	10	65119095	9.80754E-06	0.086	360770	13265.712
genus Akkermansia	Vascular dementia (subcortical)	rs4723291	A	G	-0.002	33551998	0.910	0.013	14306	A	G	-0.263	7	33512386	6.22372E-06	0.058	360770	11064.499
genus Alistipes	Vascular dementia (subcortical)	rs10919863	T	C	-0.003	200226041	0.763	0.014	14306	T	C	0.315	1	200256913	3.47112E-06	0.068	360770	10813.753
genus Alistipes	Vascular dementia (subcortical)	rs11148372	A	G	0.007	22788665	0.548	0.011	14306	A	G	-0.261	13	22214526	4.06275E-06	0.057	360770	12632.549
genus Alistipes	Vascular dementia (subcortical)	rs11986558	T	C	0.008	2500772	0.502	0.011	14306	T	C	0.248	8	2643276	9.16875E-06	0.056	360770	11030.353
genus Alistipes	Vascular dementia (subcortical)	rs4382795	C	T	-0.003	66878853	0.853	0.014	14306	C	T	0.378	10	65119095	9.80754E-06	0.086	360770	13265.712
genus Alistipes	Vascular dementia (subcortical)	rs4723291	A	G	0.000	33551998	0.989	0.011	14306	A	G	-0.263	7	33512386	6.22372E-06	0.058	360770	11064.499
genus Allisonella	Vascular dementia (subcortical)	rs11148372	A	G	0.016	22788665	0.544	0.026	14306	A	G	-0.261	13	22214526	4.06275E-06	0.057	360770	12632.549
genus Allisonella	Vascular dementia (subcortical)	rs1363668	G	A	-0.001	143089582	0.951	0.025	14306	G	A	-0.272	5	143710017	5.25352E-06	0.060	360770	12697.214
genus Allisonella	Vascular dementia (subcortical)	rs429358	C	T	-0.033	45411941	0.350	0.036	14306	C	T	0.597	19	44908684	1.74221E-17	0.070	360770	41621.629
genus Allisonella	Vascular dementia (subcortical)	rs4295569	C	T	0.003	47820641	0.909	0.026	14306	C	T	-0.355	7	47781043	2.54572E-08	0.064	360770	20037.409
genus Allisonella	Vascular dementia (subcortical)	rs4382795	C	T	0.007	66878853	0.830	0.033	14306	C	T	0.378	10	65119095	9.80754E-06	0.086	360770	13265.712
genus Alloprevotella	Vascular dementia (subcortical)	rs10919863	T	C	0.001	200226041	0.946	0.032	14306	T	C	0.315	1	200256913	3.47112E-06	0.068	360770	10813.753
genus Alloprevotella	Vascular dementia (subcortical)	rs11148372	A	G	-0.010	22788665	0.672	0.027	14306	A	G	-0.261	13	22214526	4.06275E-06	0.057	360770	12632.549
genus Alloprevotella	Vascular dementia (subcortical)	rs3802793	A	G	-0.004	131685316	0.838	0.028	14306	A	G	0.274	11	131815422	5.46626E-06	0.060	360770	12758.334
genus Alloprevotella	Vascular dementia (subcortical)	rs4295569	C	T	0.017	47820641	0.524	0.027	14306	C	T	-0.355	7	47781043	2.54572E-08	0.064	360770	20037.409
genus Alloprevotella	Vascular dementia (subcortical)	rs4382795	C	T	0.015	66878853	0.599	0.033	14306	C	T	0.378	10	65119095	9.80754E-06	0.086	360770	13265.712
genus Alloprevotella	Vascular dementia (subcortical)	rs4723291	A	G	0.017	33551998	0.506	0.026	14306	A	G	-0.263	7	33512386	6.22372E-06	0.058	360770	11064.499
genus Anaerofilum	Vascular dementia (subcortical)	rs10919863	T	C	0.005	200226041	0.954	0.026	14306	T	C	0.315	1	200256913	3.47112E-06	0.068	360770	10813.753
genus Anaerofilum	Vascular dementia (subcortical)	rs11986558	T	C	0.011	2500772	0.589	0.021	14306	T	C	0.248	8	2643276	9.16875E-06	0.056	360770	11030.353
genus Anaerofilum	Vascular dementia (subcortical)	rs1363668	G	A	-0.010	143089582	0.623	0.020	14306	G	A	-0.272	5	143710017	5.25352E-06	0.060	360770	12697.214
genus Anaerofilum	Vascular dementia (subcortical)	rs429358	C	T	-0.013	45411941	0.660	0.028	14306	C	T	0.597	19	44908684	1.74221E-17	0.070	360770	41621.629
genus Anaerofilum	Vascular dementia (subcortical)	rs4723291	A	G	-0.017	33551998	0.382	0.020	14306	A	G	-0.263	7	33512386	6.22372E-06	0.058	360770	11064.499
genus Anaerostipes	Vascular dementia (subcortical)	rs10919863	T	C	0.001	200226041	0.969	0.014	14306	T	C	0.315	1	200256913	3.47112E-06	0.068	360770	10813.753
genus Anaerostipes	Vascular dementia (subcortical)	rs11148372	A	G	-0.004	22788665	0.695	0.011	14306	A	G	-0.261	13	22214526	4.06275E-06	0.057	360770	12632.549
genus Anaerostipes	Vascular dementia (subcortical)	rs1363668	G	A	-0.009	143089582	0.417	0.011	14306	G	A	-0.272	5	143710017	5.25352E-06	0.060	360770	12697.214
genus Anaerostipes	Vascular dementia (subcortical)	rs4295569	C	T	-0.001	47820641	0.922	0.011	14306	C	T	-0.355	7	47781043	2.54572E-08	0.064	360770	20037.409
genus Anaerostipes	Vascular dementia (subcortical)	rs4382795	C	T	-0.004	66878853	0.800	0.014	14306	C	T	0.378	10	65119095	9.80754E-06	0.086	360770	13265.712

genus Anaerotruncus	Vascular dementia (subcortical)	rs11986558	T	C	-0.003	2500772	0.768	0.012	14306	T	C	0.248	8	2643276	9.16875E-06	0.056	360770	11030.353
genus Anaerotruncus	Vascular dementia (subcortical)	rs1363668	G	A	0.002	143089582	0.854	0.011	14306	G	A	-0.272	5	143710017	5.25352E-06	0.060	360770	12697.214
genus Anaerotruncus	Vascular dementia (subcortical)	rs3802793	A	G	0.009	131685316	0.434	0.012	14306	A	G	0.274	11	131815422	5.46626E-06	0.060	360770	12758.334
genus Anaerotruncus	Vascular dementia (subcortical)	rs429358	C	T	-0.019	45411941	0.201	0.016	14306	C	T	0.597	19	44908684	1.74221E-17	0.070	360770	41621.629
genus Anaerotruncus	Vascular dementia (subcortical)	rs4295569	C	T	-0.004	47820641	0.723	0.011	14306	C	T	-0.355	7	47781043	2.54572E-08	0.064	360770	20037.409
genus Anaerotruncus	Vascular dementia (subcortical)	rs4382795	C	T	0.007	66878853	0.638	0.015	14306	C	T	0.378	10	65119095	9.80754E-06	0.086	360770	13265.712
genus Anaerotruncus	Vascular dementia (subcortical)	rs4723291	A	G	0.002	33551998	0.854	0.011	14306	A	G	-0.263	7	33512386	6.22372E-06	0.058	360770	11064.499
genus Bacteroides	Vascular dementia (subcortical)	rs10919863	T	C	0.004	200226041	0.825	0.013	14306	T	C	0.315	1	200256913	3.47112E-06	0.068	360770	10813.753
genus Bacteroides	Vascular dementia (subcortical)	rs11148372	A	G	-0.002	22788665	0.851	0.011	14306	A	G	-0.261	13	22214526	4.06275E-06	0.057	360770	12632.549
genus Bacteroides	Vascular dementia (subcortical)	rs11986558	T	C	0.007	2500772	0.582	0.011	14306	T	C	0.248	8	2643276	9.16875E-06	0.056	360770	11030.353
genus Bacteroides	Vascular dementia (subcortical)	rs1363668	G	A	0.009	143089582	0.412	0.011	14306	G	A	-0.272	5	143710017	5.25352E-06	0.060	360770	12697.214
genus Barnesiella	Vascular dementia (subcortical)	rs10919863	T	C	-0.009	200226041	0.577	0.015	14306	T	C	0.315	1	200256913	3.47112E-06	0.068	360770	10813.753
genus Barnesiella	Vascular dementia (subcortical)	rs11148372	A	G	-0.005	22788665	0.696	0.012	14306	A	G	-0.261	13	22214526	4.06275E-06	0.057	360770	12632.549
genus Barnesiella	Vascular dementia (subcortical)	rs11986558	T	C	-0.002	2500772	0.894	0.013	14306	T	C	0.248	8	2643276	9.16875E-06	0.056	360770	11030.353
genus Barnesiella	Vascular dementia (subcortical)	rs1363668	G	A	0.001	143089582	0.937	0.012	14306	G	A	-0.272	5	143710017	5.25352E-06	0.060	360770	12697.214
genus Barnesiella	Vascular dementia (subcortical)	rs3802793	A	G	0.001	131685316	0.940	0.013	14306	A	G	0.274	11	131815422	5.46626E-06	0.060	360770	12758.334
genus Barnesiella	Vascular dementia (subcortical)	rs429358	C	T	-0.005	45411941	0.787	0.017	14306	C	T	0.597	19	44908684	1.74221E-17	0.070	360770	41621.629
genus Barnesiella	Vascular dementia (subcortical)	rs4382795	C	T	-0.002	66878853	0.861	0.016	14306	C	T	0.378	10	65119095	9.80754E-06	0.086	360770	13265.712
genus Barnesiella	Vascular dementia (subcortical)	rs4723291	A	G	-0.008	33551998	0.483	0.012	14306	A	G	-0.263	7	33512386	6.22372E-06	0.058	360770	11064.499
genus Bifidobacterium	Vascular dementia (subcortical)	rs10919863	T	C	-0.005	200226041	0.795	0.015	14306	T	C	0.315	1	200256913	3.47112E-06	0.068	360770	10813.753
genus Bifidobacterium	Vascular dementia (subcortical)	rs11148372	A	G	0.000	22788665	0.977	0.012	14306	A	G	-0.261	13	22214526	4.06275E-06	0.057	360770	12632.549
genus Bifidobacterium	Vascular dementia (subcortical)	rs11986558	T	C	0.004	2500772	0.736	0.012	14306	T	C	0.248	8	2643276	9.16875E-06	0.056	360770	11030.353
genus Bifidobacterium	Vascular dementia (subcortical)	rs1363668	G	A	0.009	143089582	0.441	0.012	14306	G	A	-0.272	5	143710017	5.25352E-06	0.060	360770	12697.214
genus Bifidobacterium	Vascular dementia (subcortical)	rs3802793	A	G	0.007	131685316	0.624	0.012	14306	A	G	0.274	11	131815422	5.46626E-06	0.060	360770	12758.334
genus Bifidobacterium	Vascular dementia (subcortical)	rs429358	C	T	0.019	45411941	0.204	0.017	14306	C	T	0.597	19	44908684	1.74221E-17	0.070	360770	41621.629
genus Bifidobacterium	Vascular dementia (subcortical)	rs4295569	C	T	0.001	47820641	0.919	0.012	14306	C	T	-0.355	7	47781043	2.54572E-08	0.064	360770	20037.409
genus Bifidobacterium	Vascular dementia (subcortical)	rs4723291	A	G	-0.002	33551998	0.915	0.012	14306	A	G	-0.263	7	33512386	6.22372E-06	0.058	360770	11064.499
genus Bilophila	Vascular dementia (subcortical)	rs10919863	T	C	-0.003	200226041	0.914	0.016	14306	T	C	0.315	1	200256913	3.47112E-06	0.068	360770	10813.753
genus Bilophila	Vascular dementia (subcortical)	rs11148372	A	G	-0.001	22788665	0.897	0.013	14306	A	G	-0.261	13	22214526	4.06275E-06	0.057	360770	12632.549
genus Bilophila	Vascular dementia (subcortical)	rs1363668	G	A	-0.010	143089582	0.409	0.013	14306	G	A	-0.272	5	143710017	5.25352E-06	0.060	360770	12697.214
genus Bilophila	Vascular dementia (subcortical)	rs429358	C	T	0.007	45411941	0.656	0.018	14306	C	T	0.597	19	44908684	1.74221E-17	0.070	360770	41621.629
genus Bilophila	Vascular dementia (subcortical)	rs4295569	C	T	-0.008	47820641	0.565	0.013	14306	C	T	-0.355	7	47781043	2.54572E-08	0.064	360770	20037.409
genus Bilophila	Vascular dementia (subcortical)	rs4382795	C	T	0.000	66878853	0.936	0.016	14306	C	T	0.378	10	65119095	9.80754E-06	0.086	360770	13265.712
genus Bilophila	Vascular dementia (subcortical)	rs4723291	A	G	0.010	33551998	0.469	0.013	14306	A	G	-0.263	7	33512386	6.22372E-06	0.058	360770	11064.499
genus Butyricicoccus	Vascular dementia (subcortical)	rs10919863	T	C	0.004	200226041	0.873	0.014	14306	T	C	0.315	1	200256913	3.47112E-06	0.068	360770	10813.753
genus Butyricicoccus	Vascular dementia (subcortical)	rs11148372	A	G	0.004	22788665	0.710	0.011	14306	A	G	-0.261	13	22214526	4.06275E-06	0.057	360770	12632.549
genus Butyricicoccus	Vascular dementia (subcortical)	rs1363668	G	A	-0.007	143089582	0.501	0.011	14306	G	A	-0.272	5	143710017	5.25352E-06	0.060	360770	12697.214
genus Butyricicoccus	Vascular dementia (subcortical)	rs3802793	A	G	-0.010	131685316	0.414	0.012	14306	A	G	0.274	11	131815422	5.46626E-06	0.060	360770	12758.334
genus Butyricicoccus	Vascular dementia (subcortical)	rs429358	C	T	-0.018	45411941	0.288	0.015	14306	C	T	0.597	19	44908684	1.74221E-17	0.070	360770	41621.629
genus Butyricicoccus	Vascular dementia (subcortical)	rs4723291	A	G	0.006	33551998	0.598	0.011	14306	A	G	-0.263	7	33512386	6.22372E-06	0.058	360770	11064.499
genus Butyricimonas	Vascular dementia (subcortical)	rs10919863	T	C	-0.006	200226041	0.663	0.018	14306	T	C	0.315	1	200256913	3.47112E-06	0.068	360770	10813.753
genus Butyricimonas	Vascular dementia (subcortical)	rs11986558	T	C	-0.012	2500772	0.430	0.015	14306	T	C	0.248	8	2643276	9.16875E-06	0.056	360770	11030.353
genus Butyricimonas	Vascular dementia (subcortical)	rs1363668	G	A	0.002	143089582	0.908	0.014	14306	G	A	-0.272	5	143710017	5.25352E-06	0.060	360770	12697.214
genus Butyricimonas	Vascular dementia (subcortical)	rs3802793	A	G	0.008	131685316	0.601	0.015	14306	A	G	0.274	11	131815422	5.46626E-06	0.060	360770	12758.334
genus Butyricimonas	Vascular dementia (subcortical)	rs4295569	C	T	0.007	47820641	0.608	0.014	14306	C	T	-0.355	7	47781043	2.54572E-08	0.064	360770	20037.409
genus Butyricimonas	Vascular dementia (subcortical)	rs4382795	C	T	0.014	66878853	0.456	0.018	14306	C	T	0.378	10	65119095	9.80754E-06	0.086	360770	13265.712
genus Butyrivibrio	Vascular dementia (subcortical)	rs10919863	T	C	-0.009	200226041	0.792	0.031	14306	T	C	0.315	1	200256913	3.47112E-06	0.068	360770	10813.753
genus Butyrivibrio	Vascular dementia (subcortical)	rs11148372	A	G	0.012	22788665	0.623	0.025	14306	A	G	-0.261	13	22214526	4.06275E-06	0.057	360770	12632.549
genus Butyrivibrio	Vascular dementia (subcortical)	rs11986558	T	C	0.019	2500772	0.475	0.025	14306	T	C	0.248	8	2643276	9.16875E-06	0.056	360770	11030.353
genus Butyrivibrio	Vascular dementia (subcortical)	rs3802793	A	G	-0.013	131685316	0.585	0.025	14306	A	G	0.274	11	131815422	5.46626E-06	0.060	360770	12758.334
genus Butyrivibrio	Vascular dementia (subcortical)	rs429358	C	T	-0.016	45411941	0.585	0.034	14306	C	T	0.597	19	44908684	1.74221E-17	0.070	360770	41621.629
genus Butyrivibrio	Vascular dementia (subcortical)	rs4295569	C	T	-0.012	47820641	0.614	0.025	14306	C	T	-0.355	7	47781043	2.54572E-08	0.064	360770	20037.409
genus Butyrivibrio	Vascular dementia (subcortical)	rs4382795	C	T	0.007	66878853	0.861	0.032	14306	C	T	0.378	10	65119095	9.80754E-06	0.086	360770	13265.712
genus Butyrivibrio	Vascular dementia (subcortical)	rs4723291	A	G	0.011	33551998	0.654	0.024	14306	A	G	-0.263	7	33512386	6.22372E-06	0.058	360770	11064.499
genus Candidatus Soleaferrea	Vascular dementia (subcortical)	rs10919863	T	C	-0.002	200226041	0.963	0.023	14306	T	C	0.315	1	200256913	3.47112E-06	0.068	360770	10813.753
genus Candidatus Soleaferrea	Vascular dementia (subcortical)	rs11148372	A	G	-0.017	22788665	0.379	0.018	14306	A	G	-0.261	13	22214526	4.06275E-06	0.057	360770	12632.549
genus Candidatus Soleaferrea	Vascular dementia (subcortical)	rs11986558	T	C	0.010	2500772	0.627	0.019	14306	T	C	0.248	8	2643276	9.16875E-06	0.056	360770	11030.353
genus Candidatus Soleaferrea	Vascular dementia (subcortical)	rs1363668	G	A	-0.012	143089582	0.484	0.018	14306	G	A	-0.272	5	143710017	5.25352E-06	0.060	360770	12697.214
genus Candidatus Soleaferrea	Vascular dementia (subcortical)	rs429358	C	T	0.020	45411941	0.391	0.026	14306	C	T	0.597	19	44908684	1.74221E-17	0.070	360770	41621.629
genus Candidatus Soleaferrea	Vascular dementia (subcortical)	rs4295569	C	T	-0.002	47820641	0.934	0.019	14306	C	T	-0.355	7	47781043	2.54572E-08	0.064	360770	20037.409
genus Candidatus Soleaferrea	Vascular dementia (subcortical)	rs4723291	A	G	0.011	33551998	0.539	0.018	14306	A	G	-0.263	7	33512386	6.22372E-06	0.058	360770	11064.499
genus Catenibacterium	Vascular dementia (subcortical)	rs10919863	T	C	-0.001	200226041	0.955	0.031	14306	T	C	0.315	1	200256913	3.47112E-06	0.068	360770	10813.753
genus Catenibacterium	Vascular dementia (subcortical)	rs11148372	A	G	-0.013	22788665	0.639	0.026	14306	A	G	-0.261	13	22214526	4.06275E-06	0.057	360770	12632.549
genus Catenibacterium	Vascular dementia (subcortical)	rs11986558	T	C	0.006	2500772	0.796	0.026	14306	T	C	0.248	8	2643276	9.16875E-06	0.056	360770	11030.353
genus Catenibacterium	Vascular dementia (subcortical)	rs1363668	G	A	0.018	143089582	0.465	0.025	14306	G	A	-0.272	5	143710017	5.25352E-06	0.060	360770	12697.214
genus Catenibacterium	Vascular dementia (subcortical)	rs429358	C	T	0.030	45411941	0.433	0.036	14306	C	T	0.597	19	44908684	1.74221E-17	0.070	360770	41621.629
genus Catenibacterium	Vascular dementia (subcortical)	rs4382795	C	T	0.027	66878853	0.445	0.033	14306	C	T	0.378	10	65119095	9.80754E-06	0.086	360770	13265.712
genus Catenibacterium	Vascular dementia (subcortical)	rs4723291	A	G	0.008	33551998	0.734	0.025	14306	A	G	-0.263	7	33512386	6.22372E-06	0.058	360770	11064.499
genus Christensenellaceae R 7group	Vascular dementia (subcortical)	rs10919863	T	C	0.010	200226041	0.500	0.014	14306	T	C	0.315	1	200256913	3.47112E-06	0.068	360770	10813.753
genus Christensenellaceae R 7group	Vascular dementia (subcortical)	rs11148372	A	G	0.001	22788665	0.912	0.011	14306	A	G	-0.261	13	22214526	4.06275E-06	0.057	360770	12632.549
genus Christensenellaceae R 7group	Vascular dementia (subcortical)	rs3802793	A	G	-0.001	131685316	0.971	0.012	14306	A	G	0.274	11	131815422	5.46626E-06	0.060	360770	12758.334

genus Christensenellaceae R 7group	Vascular dementia (subcortical)	rs429358	C	T	0.011	45411941	0.455	0.016	14306	C	T	0.597	19	44908684	1.74221E-17	0.070	360770	41621.629
genus Christensenellaceae R 7group	Vascular dementia (subcortical)	rs4382795	C	T	-0.007	66878853	0.660	0.015	14306	C	T	0.378	10	65119095	9.80754E-06	0.086	360770	13265.712
genus Christensenellaceae R 7group	Vascular dementia (subcortical)	rs4723291	A	G	-0.007	33551998	0.506	0.011	14306	A	G	-0.263	7	33512386	6.22372E-06	0.058	360770	11064.499
genus Clostridium innocuum group	Vascular dementia (subcortical)	rs10919863	T	C	0.018	200226041	0.488	0.028	14306	T	C	0.315	1	200256913	3.47112E-06	0.068	360770	10813.753
genus Clostridium innocuum group	Vascular dementia (subcortical)	rs11148372	A	G	-0.014	22788665	0.536	0.022	14306	A	G	-0.261	13	22214526	4.06275E-06	0.057	360770	12632.549
genus Clostridium innocuum group	Vascular dementia (subcortical)	rs1363668	G	A	0.000	143089582	0.989	0.022	14306	G	A	-0.272	5	143710017	5.25352E-06	0.060	360770	12697.214
genus Clostridium innocuum group	Vascular dementia (subcortical)	rs3802793	A	G	-0.007	131685316	0.795	0.023	14306	A	G	0.274	11	131815422	5.46626E-06	0.060	360770	12758.334
genus Clostridium innocuum group	Vascular dementia (subcortical)	rs429358	C	T	-0.009	45411941	0.813	0.031	14306	C	T	0.597	19	44908684	1.74221E-17	0.070	360770	41621.629
genus Clostridium innocuum group	Vascular dementia (subcortical)	rs4295569	C	T	-0.009	47820641	0.694	0.023	14306	C	T	-0.355	7	47781043	2.54572E-08	0.064	360770	20037.409
genus Clostridium innocuum group	Vascular dementia (subcortical)	rs4382795	C	T	0.010	66878853	0.727	0.029	14306	C	T	0.378	10	65119095	9.80754E-06	0.086	360770	13265.712
genus Clostridium innocuum group	Vascular dementia (subcortical)	rs4723291	A	G	-0.011	33551998	0.611	0.022	14306	A	G	-0.263	7	33512386	6.22372E-06	0.058	360770	11064.499
genus Clostridium sensustricto1	Vascular dementia (subcortical)	rs10919863	T	C	-0.014	200226041	0.387	0.015	14306	T	C	0.315	1	200256913	3.47112E-06	0.068	360770	10813.753
genus Clostridium sensustricto1	Vascular dementia (subcortical)	rs11148372	A	G	-0.004	22788665	0.723	0.012	14306	A	G	-0.261	13	22214526	4.06275E-06	0.057	360770	12632.549
genus Clostridium sensustricto1	Vascular dementia (subcortical)	rs429358	C	T	0.011	45411941	0.544	0.017	14306	C	T	0.597	19	44908684	1.74221E-17	0.070	360770	41621.629
genus Clostridium sensustricto1	Vascular dementia (subcortical)	rs4295569	C	T	-0.012	47820641	0.331	0.012	14306	C	T	-0.355	7	47781043	2.54572E-08	0.064	360770	20037.409
genus Collinsella	Vascular dementia (subcortical)	rs429358	C	T	0.024	45411941	0.129	0.017	14306	C	T	0.597	19	44908684	1.74221E-17	0.070	360770	41621.629
genus Collinsella	Vascular dementia (subcortical)	rs4295569	C	T	0.005	47820641	0.712	0.012	14306	C	T	-0.355	7	47781043	2.54572E-08	0.064	360770	20037.409
genus Collinsella	Vascular dementia (subcortical)	rs4382795	C	T	0.001	66878853	0.956	0.016	14306	C	T	0.378	10	65119095	9.80754E-06	0.086	360770	13265.712
genus Collinsella	Vascular dementia (subcortical)	rs4723291	A	G	-0.003	33551998	0.799	0.012	14306	A	G	-0.263	7	33512386	6.22372E-06	0.058	360770	11064.499
genus Coprobacter	Vascular dementia (subcortical)	rs10919863	T	C	0.007	200226041	0.778	0.022	14306	T	C	0.315	1	200256913	3.47112E-06	0.068	360770	10813.753
genus Coprobacter	Vascular dementia (subcortical)	rs11148372	A	G	-0.015	22788665	0.410	0.018	14306	A	G	-0.261	13	22214526	4.06275E-06	0.057	360770	12632.549
genus Coprobacter	Vascular dementia (subcortical)	rs1363668	G	A	-0.009	143089582	0.633	0.017	14306	G	A	-0.272	5	143710017	5.25352E-06	0.060	360770	12697.214
genus Coprobacter	Vascular dementia (subcortical)	rs3802793	A	G	-0.008	131685316	0.630	0.018	14306	A	G	0.274	11	131815422	5.46626E-06	0.060	360770	12758.334
genus Coprobacter	Vascular dementia (subcortical)	rs429358	C	T	-0.001	45411941	0.930	0.025	14306	C	T	0.597	19	44908684	1.74221E-17	0.070	360770	41621.629
genus Coprobacter	Vascular dementia (subcortical)	rs4295569	C	T	0.003	47820641	0.872	0.018	14306	C	T	-0.355	7	47781043	2.54572E-08	0.064	360770	20037.409
genus Coprobacter	Vascular dementia (subcortical)	rs4723291	A	G	0.014	33551998	0.429	0.017	14306	A	G	-0.263	7	33512386	6.22372E-06	0.058	360770	11064.499
genus Coprococcus1	Vascular dementia (subcortical)	rs11148372	A	G	-0.006	22788665	0.569	0.011	14306	A	G	-0.261	13	22214526	4.06275E-06	0.057	360770	12632.549
genus Coprococcus1	Vascular dementia (subcortical)	rs1363668	G	A	0.005	143089582	0.643	0.011	14306	G	A	-0.272	5	143710017	5.25352E-06	0.060	360770	12697.214
genus Coprococcus1	Vascular dementia (subcortical)	rs429358	C	T	0.014	45411941	0.326	0.016	14306	C	T	0.597	19	44908684	1.74221E-17	0.070	360770	41621.629
genus Coprococcus1	Vascular dementia (subcortical)	rs4295569	C	T	0.010	47820641	0.401	0.011	14306	C	T	-0.355	7	47781043	2.54572E-08	0.064	360770	20037.409
genus Coprococcus1	Vascular dementia (subcortical)	rs4382795	C	T	-0.003	66878853	0.866	0.014	14306	C	T	0.378	10	65119095	9.80754E-06	0.086	360770	13265.712
genus Coprococcus1	Vascular dementia (subcortical)	rs4723291	A	G	-0.003	33551998	0.840	0.011	14306	A	G	-0.263	7	33512386	6.22372E-06	0.058	360770	11064.499
genus Coprococcus2	Vascular dementia (subcortical)	rs10919863	T	C	0.000	200226041	0.980	0.017	14306	T	C	0.315	1	200256913	3.47112E-06	0.068	360770	10813.753
genus Coprococcus2	Vascular dementia (subcortical)	rs11148372	A	G	-0.002	22788665	0.900	0.014	14306	A	G	-0.261	13	22214526	4.06275E-06	0.057	360770	12632.549
genus Coprococcus2	Vascular dementia (subcortical)	rs1363668	G	A	0.001	143089582	0.932	0.013	14306	G	A	-0.272	5	143710017	5.25352E-06	0.060	360770	12697.214
genus Coprococcus2	Vascular dementia (subcortical)	rs3802793	A	G	-0.011	131685316	0.410	0.014	14306	A	G	0.274	11	131815422	5.46626E-06	0.060	360770	12758.334
genus Coprococcus2	Vascular dementia (subcortical)	rs429358	C	T	0.002	45411941	0.982	0.019	14306	C	T	0.597	19	44908684	1.74221E-17	0.070	360770	41621.629
genus Coprococcus2	Vascular dementia (subcortical)	rs4295569	C	T	-0.005	47820641	0.692	0.014	14306	C	T	-0.355	7	47781043	2.54572E-08	0.064	360770	20037.409
genus Coprococcus2	Vascular dementia (subcortical)	rs4382795	C	T	-0.010	66878853	0.651	0.017	14306	C	T	0.378	10	65119095	9.80754E-06	0.086	360770	13265.712
genus Coprococcus3	Vascular dementia (subcortical)	rs10919863	T	C	-0.009	200226041	0.509	0.015	14306	T	C	0.315	1	200256913	3.47112E-06	0.068	360770	10813.753
genus Coprococcus3	Vascular dementia (subcortical)	rs11148372	A	G	0.004	22788665	0.692	0.012	14306	A	G	-0.261	13	22214526	4.06275E-06	0.057	360770	12632.549
genus Coprococcus3	Vascular dementia (subcortical)	rs11986558	T	C	-0.002	2500772	0.877	0.012	14306	T	C	0.248	8	2643276	9.16875E-06	0.056	360770	11030.353
genus Coprococcus3	Vascular dementia (subcortical)	rs1363668	G	A	0.007	143089582	0.529	0.011	14306	G	A	-0.272	5	143710017	5.25352E-06	0.060	360770	12697.214
genus Coprococcus3	Vascular dementia (subcortical)	rs3802793	A	G	-0.004	131685316	0.730	0.012	14306	A	G	0.274	11	131815422	5.46626E-06	0.060	360770	12758.334
genus Coprococcus3	Vascular dementia (subcortical)	rs4295569	C	T	0.012	47820641	0.293	0.012	14306	C	T	-0.355	7	47781043	2.54572E-08	0.064	360770	20037.409
genus Coprococcus3	Vascular dementia (subcortical)	rs4723291	A	G	-0.008	33551998	0.467	0.011	14306	A	G	-0.263	7	33512386	6.22372E-06	0.058	360770	11064.499
genus Defluviitaleaceae UCG011	Vascular dementia (subcortical)	rs10919863	T	C	0.008	200226041	0.698	0.020	14306	T	C	0.315	1	200256913	3.47112E-06	0.068	360770	10813.753
genus Defluviitaleaceae UCG011	Vascular dementia (subcortical)	rs11148372	A	G	0.002	22788665	0.885	0.016	14306	A	G	-0.261	13	22214526	4.06275E-06	0.057	360770	12632.549
genus Defluviitaleaceae UCG011	Vascular dementia (subcortical)	rs3802793	A	G	-0.012	131685316	0.461	0.017	14306	A	G	0.274	11	131815422	5.46626E-06	0.060	360770	12758.334
genus Defluviitaleaceae UCG011	Vascular dementia (subcortical)	rs429358	C	T	-0.003	45411941	0.819	0.022	14306	C	T	0.597	19	44908684	1.74221E-17	0.070	360770	41621.629
genus Defluviitaleaceae UCG011	Vascular dementia (subcortical)	rs4295569	C	T	0.005	47820641	0.756	0.016	14306	C	T	-0.355	7	47781043	2.54572E-08	0.064	360770	20037.409
genus Defluviitaleaceae UCG011	Vascular dementia (subcortical)	rs4382795	C	T	-0.003	66878853	0.870	0.021	14306	C	T	0.378	10	65119095	9.80754E-06	0.086	360770	13265.712
genus Defluviitaleaceae UCG011	Vascular dementia (subcortical)	rs4723291	A	G	0.001	33551998	0.926	0.016	14306	A	G	-0.263	7	33512386	6.22372E-06	0.058	360770	11064.499
genus Desulfovibrio	Vascular dementia (subcortical)	rs11148372	A	G	0.001	22788665	0.955	0.015	14306	A	G	-0.261	13	22214526	4.06275E-06	0.057	360770	12632.549
genus Desulfovibrio	Vascular dementia (subcortical)	rs11986558	T	C	-0.005	2500772	0.763	0.016	14306	T	C	0.248	8	2643276	9.16875E-06	0.056	360770	11030.353
genus Desulfovibrio	Vascular dementia (subcortical)	rs1363668	G	A	-0.002	143089582	0.903	0.015	14306	G	A	-0.272	5	143710017	5.25352E-06	0.060	360770	12697.214
genus Desulfovibrio	Vascular dementia (subcortical)	rs429358	C	T	0.006	45411941	0.782	0.021	14306	C	T	0.597	19	44908684	1.74221E-17	0.070	360770	41621.629
genus Desulfovibrio	Vascular dementia (subcortical)	rs4295569	C	T	-0.004	47820641	0.758	0.015	14306	C	T	-0.355	7	47781043	2.54572E-08	0.064	360770	20037.409
genus Desulfovibrio	Vascular dementia (subcortical)	rs4723291	A	G	0.012	33551998	0.435	0.015	14306	A	G	-0.263	7	33512386	6.22372E-06	0.058	360770	11064.499
(genus Dialister	Vascular dementia (subcortical)	rs10919863	T	C	-0.010	200226041	0.464	0.017	14306	T	C	0.315	1	200256913	3.47112E-06	0.068	360770	10813.753
(genus Dialister	Vascular dementia (subcortical)	rs11148372	A	G	0.000	22788665	0.994	0.013	14306	A	G	-0.261	13	22214526	4.06275E-06	0.057	360770	12632.549
(genus Dialister	Vascular dementia (subcortical)	rs3802793	A	G	0.006	131685316	0.730	0.014	14306	A	G	0.274	11	131815422	5.46626E-06	0.060	360770	12758.334
(genus Dialister	Vascular dementia (subcortical)	rs429358	C	T	-0.026	45411941	0.198	0.019	14306	C	T	0.597	19	44908684	1.74221E-17	0.070	360770	41621.629
(genus Dialister	Vascular dementia (subcortical)	rs4295569	C	T	0.006	47820641	0.709	0.013	14306	C	T	-0.355	7	47781043	2.54572E-08	0.064	360770	20037.409
genus Dorea	Vascular dementia (subcortical)	rs11986558	T	C	0.000	2500772	0.996	0.011	14306	T	C	0.248	8	2643276	9.16875E-06	0.056	360770	11030.353
genus Dorea	Vascular dementia (subcortical)	rs3802793	A	G	0.004	131685316	0.708	0.011	14306	A	G	0.274	11	131815422	5.46626E-06	0.060	360770	12758.334
genus Dorea	Vascular dementia (subcortical)	rs4295569	C	T	0.008	47820641	0.472	0.011	14306	C	T	-0.355	7	47781043	2.54572E-08	0.064	360770	20037.409
genus Dorea	Vascular dementia (subcortical)	rs4382795	C	T	-0.012	66878853	0.352	0.014	14306	C	T	0.378	10	65119095	9.80754E-06	0.086	360770	13265.712
genus Eggerthella	Vascular dementia (subcortical)	rs10919863	T	C	-0.011	200226041	0.577	0.026	14306	T	C	0.315	1	200256913	3.47112E-06	0.068	360770	10813.753
genus Eggerthella	Vascular dementia (subcortical)	rs11148372	A	G	-0.005	22788665	0.788	0.020	14306	A	G	-0.261	13	22214526	4.06275E-06	0.057	360770	12632.549
genus Eggerthella	Vascular dementia (subcortical)	rs429358	C	T	0.017	45411941	0.611	0.029	14306	C	T	0.597	19	44908684	1.74221E-17	0.070	360770	41621.629

genus Eggerthella	Vascular dementia (subcortical)	rs4295569	C	T	-0.021	47820641	0.306	0.021	14306	C	T	-0.355	7	47781043	2.54572E-08	0.064	360770	20037.409
genus Eggerthella	Vascular dementia (subcortical)	rs4723291	A	G	-0.006	33551998	0.734	0.020	14306	A	G	-0.263	7	33512386	6.22372E-06	0.058	360770	11064.499
genus Eisenbergiella	Vascular dementia (subcortical)	rs10919863	T	C	0.009	200226041	0.715	0.025	14306	T	C	0.315	1	200256913	3.47112E-06	0.068	360770	10813.753
genus Eisenbergiella	Vascular dementia (subcortical)	rs11986558	T	C	0.001	2500772	0.932	0.020	14306	T	C	0.248	8	2643276	9.16875E-06	0.056	360770	11030.353
genus Eisenbergiella	Vascular dementia (subcortical)	rs1363668	G	A	-0.016	143089582	0.408	0.019	14306	G	A	-0.272	5	143710017	5.25352E-06	0.060	360770	12697.214
genus Eisenbergiella	Vascular dementia (subcortical)	rs3802793	A	G	0.015	131685316	0.425	0.020	14306	A	G	0.274	11	131815422	5.46626E-06	0.060	360770	12758.334
genus Eisenbergiella	Vascular dementia (subcortical)	rs429358	C	T	-0.013	45411941	0.612	0.028	14306	C	T	0.597	19	44908684	1.74221E-17	0.070	360770	41621.629
genus Eisenbergiella	Vascular dementia (subcortical)	rs4295569	C	T	-0.012	47820641	0.530	0.020	14306	C	T	-0.355	7	47781043	2.54572E-08	0.064	360770	20037.409
genus Enterorhabdus	Vascular dementia (subcortical)	rs11148372	A	G	0.007	22788665	0.651	0.017	14306	A	G	-0.261	13	22214526	4.06275E-06	0.057	360770	12632.549
genus Enterorhabdus	Vascular dementia (subcortical)	rs11986558	T	C	0.000	2500772	0.981	0.017	14306	T	C	0.248	8	2643276	9.16875E-06	0.056	360770	11030.353
genus Enterorhabdus	Vascular dementia (subcortical)	rs1363668	G	A	0.012	143089582	0.488	0.017	14306	G	A	-0.272	5	143710017	5.25352E-06	0.060	360770	12697.214
genus Enterorhabdus	Vascular dementia (subcortical)	rs3802793	A	G	-0.005	131685316	0.797	0.017	14306	A	G	0.274	11	131815422	5.46626E-06	0.060	360770	12758.334
genus Enterorhabdus	Vascular dementia (subcortical)	rs429358	C	T	0.009	45411941	0.754	0.023	14306	C	T	0.597	19	44908684	1.74221E-17	0.070	360770	41621.629
genus Enterorhabdus	Vascular dementia (subcortical)	rs4295569	C	T	0.008	47820641	0.651	0.017	14306	C	T	-0.355	7	47781043	2.54572E-08	0.064	360770	20037.409
genus Enterorhabdus	Vascular dementia (subcortical)	rs4382795	C	T	0.016	66878853	0.522	0.022	14306	C	T	0.378	10	65119095	9.80754E-06	0.086	360770	13265.712
genus Enterorhabdus	Vascular dementia (subcortical)	rs4723291	A	G	-0.008	33551998	0.611	0.016	14306	A	G	-0.263	7	33512386	6.22372E-06	0.058	360770	11064.499
genus Erysipelatoclostridium	Vascular dementia (subcortical)	rs11148372	A	G	0.007	22788665	0.620	0.015	14306	A	G	-0.261	13	22214526	4.06275E-06	0.057	360770	12632.549
genus Erysipelatoclostridium	Vascular dementia (subcortical)	rs11986558	T	C	-0.012	2500772	0.434	0.015	14306	T	C	0.248	8	2643276	9.16875E-06	0.056	360770	11030.353
genus Erysipelatoclostridium	Vascular dementia (subcortical)	rs1363668	G	A	0.006	143089582	0.696	0.014	14306	G	A	-0.272	5	143710017	5.25352E-06	0.060	360770	12697.214
genus Erysipelatoclostridium	Vascular dementia (subcortical)	rs3802793	A	G	0.004	131685316	0.769	0.015	14306	A	G	0.274	11	131815422	5.46626E-06	0.060	360770	12758.334
genus Erysipelatoclostridium	Vascular dementia (subcortical)	rs429358	C	T	0.017	45411941	0.432	0.020	14306	C	T	0.597	19	44908684	1.74221E-17	0.070	360770	41621.629
genus Erysipelatoclostridium	Vascular dementia (subcortical)	rs4723291	A	G	0.010	33551998	0.487	0.014	14306	A	G	-0.263	7	33512386	6.22372E-06	0.058	360770	11064.499
genus Escherichia Shigella	Vascular dementia (subcortical)	rs10919863	T	C	-0.002	200226041	0.879	0.017	14306	T	C	0.315	1	200256913	3.47112E-06	0.068	360770	10813.753
genus Escherichia Shigella	Vascular dementia (subcortical)	rs1363668	G	A	0.010	143089582	0.468	0.013	14306	G	A	-0.272	5	143710017	5.25352E-06	0.060	360770	12697.214
genus Escherichia Shigella	Vascular dementia (subcortical)	rs429358	C	T	0.007	45411941	0.718	0.019	14306	C	T	0.597	19	44908684	1.74221E-17	0.070	360770	41621.629
genus Escherichia Shigella	Vascular dementia (subcortical)	rs4295569	C	T	0.002	47820641	0.918	0.013	14306	C	T	-0.355	7	47781043	2.54572E-08	0.064	360770	20037.409
genus Escherichia Shigella	Vascular dementia (subcortical)	rs4382795	C	T	-0.010	66878853	0.501	0.017	14306	C	T	0.378	10	65119095	9.80754E-06	0.086	360770	13265.712
genus Eubacterium brachy group	Vascular dementia (subcortical)	rs11148372	A	G	0.008	22788665	0.707	0.023	14306	A	G	-0.261	13	22214526	4.06275E-06	0.057	360770	12632.549
genus Eubacterium brachy group	Vascular dementia (subcortical)	rs1363668	G	A	0.013	143089582	0.569	0.023	14306	G	A	-0.272	5	143710017	5.25352E-06	0.060	360770	12697.214
genus Eubacterium brachy group	Vascular dementia (subcortical)	rs429358	C	T	-0.022	45411941	0.464	0.032	14306	C	T	0.597	19	44908684	1.74221E-17	0.070	360770	41621.629
genus Eubacterium coprostanoligenes group	Vascular dementia (subcortical)	rs11148372	A	G	0.006	22788665	0.576	0.011	14306	A	G	-0.261	13	22214526	4.06275E-06	0.057	360770	12632.549
genus Eubacterium coprostanoligenes group	Vascular dementia (subcortical)	rs1363668	G	A	0.007	143089582	0.508	0.011	14306	G	A	-0.272	5	143710017	5.25352E-06	0.060	360770	12697.214
genus Eubacterium coprostanoligenes group	Vascular dementia (subcortical)	rs3802793	A	G	-0.001	131685316	0.960	0.011	14306	A	G	0.274	11	131815422	5.46626E-06	0.060	360770	12758.334
genus Eubacterium coprostanoligenes group	Vascular dementia (subcortical)	rs4295569	C	T	0.010	47820641	0.350	0.011	14306	C	T	-0.355	7	47781043	2.54572E-08	0.064	360770	20037.409
genus Eubacterium coprostanoligenes group	Vascular dementia (subcortical)	rs4382795	C	T	0.006	66878853	0.653	0.014	14306	C	T	0.378	10	65119095	9.80754E-06	0.086	360770	13265.712
genus Eubacterium eligens group	Vascular dementia (subcortical)	rs10919863	T	C	0.009	200226041	0.558	0.015	14306	T	C	0.315	1	200256913	3.47112E-06	0.068	360770	10813.753
genus Eubacterium eligens group	Vascular dementia (subcortical)	rs11148372	A	G	-0.006	22788665	0.637	0.012	14306	A	G	-0.261	13	22214526	4.06275E-06	0.057	360770	12632.549
genus Eubacterium eligens group	Vascular dementia (subcortical)	rs1363668	G	A	0.007	143089582	0.580	0.012	14306	G	A	-0.272	5	143710017	5.25352E-06	0.060	360770	12697.214
genus Eubacterium eligens group	Vascular dementia (subcortical)	rs3802793	A	G	-0.008	131685316	0.485	0.012	14306	A	G	0.274	11	131815422	5.46626E-06	0.060	360770	12758.334
genus Eubacterium eligens group	Vascular dementia (subcortical)	rs429358	C	T	-0.020	45411941	0.237	0.017	14306	C	T	0.597	19	44908684	1.74221E-17	0.070	360770	41621.629
genus Eubacterium fissicatena group	Vascular dementia (subcortical)	rs3802793	A	G	-0.007	131685316	0.769	0.024	14306	A	G	0.274	11	131815422	5.46626E-06	0.060	360770	12758.334
genus Eubacterium fissicatena group	Vascular dementia (subcortical)	rs429358	C	T	0.004	45411941	0.913	0.033	14306	C	T	0.597	19	44908684	1.74221E-17	0.070	360770	41621.629
genus Eubacterium fissicatena group	Vascular dementia (subcortical)	rs4295569	C	T	-0.024	47820641	0.307	0.024	14306	C	T	-0.355	7	47781043	2.54572E-08	0.064	360770	20037.409
genus Eubacterium fissicatena group	Vascular dementia (subcortical)	rs4382795	C	T	0.021	66878853	0.509	0.030	14306	C	T	0.378	10	65119095	9.80754E-06	0.086	360770	13265.712
genus Eubacterium fissicatena group	Vascular dementia (subcortical)	rs4723291	A	G	0.013	33551998	0.564	0.023	14306	A	G	-0.263	7	33512386	6.22372E-06	0.058	360770	11064.499
genus Eubacterium hallii group	Vascular dementia (subcortical)	rs10919863	T	C	-0.002	200226041	0.931	0.014	14306	T	C	0.315	1	200256913	3.47112E-06	0.068	360770	10813.753
genus Eubacterium hallii group	Vascular dementia (subcortical)	rs11986558	T	C	-0.010	2500772	0.400	0.012	14306	T	C	0.248	8	2643276	9.16875E-06	0.056	360770	11030.353
genus Eubacterium hallii group	Vascular dementia (subcortical)	rs1363668	G	A	-0.001	143089582	0.911	0.011	14306	G	A	-0.272	5	143710017	5.25352E-06	0.060	360770	12697.214
genus Eubacterium hallii group	Vascular dementia (subcortical)	rs3802793	A	G	-0.010	131685316	0.405	0.012	14306	A	G	0.274	11	131815422	5.46626E-06	0.060	360770	12758.334
genus Eubacterium hallii group	Vascular dementia (subcortical)	rs429358	C	T	0.003	45411941	0.834	0.016	14306	C	T	0.597	19	44908684	1.74221E-17	0.070	360770	41621.629
genus Eubacterium hallii group	Vascular dementia (subcortical)	rs4295569	C	T	-0.005	47820641	0.680	0.011	14306	C	T	-0.355	7	47781043	2.54572E-08	0.064	360770	20037.409
genus Eubacterium hallii group	Vascular dementia (subcortical)	rs4382795	C	T	-0.004	66878853	0.745	0.014	14306	C	T	0.378	10	65119095	9.80754E-06	0.086	360770	13265.712
genus Eubacterium nodatum group	Vascular dementia (subcortical)	rs10919863	T	C	-0.003	200226041	0.966	0.032	14306	T	C	0.315	1	200256913	3.47112E-06	0.068	360770	10813.753
genus Eubacterium nodatum group	Vascular dementia (subcortical)	rs11148372	A	G	-0.015	22788665	0.538	0.025	14306	A	G	-0.261	13	22214526	4.06275E-06	0.057	360770	12632.549
genus Eubacterium nodatum group	Vascular dementia (subcortical)	rs1363668	G	A	0.008	143089582	0.755	0.025	14306	G	A	-0.272	5	143710017	5.25352E-06	0.060	360770	12697.214
genus Eubacterium nodatum group	Vascular dementia (subcortical)	rs429358	C	T	0.008	45411941	0.880	0.036	14306	C	T	0.597	19	44908684	1.74221E-17	0.070	360770	41621.629
genus Eubacterium nodatum group	Vascular dementia (subcortical)	rs4295569	C	T	-0.017	47820641	0.504	0.026	14306	C	T	-0.355	7	47781043	2.54572E-08	0.064	360770	20037.409
genus Eubacterium nodatum group	Vascular dementia (subcortical)	rs4723291	A	G	-0.012	33551998	0.628	0.025	14306	A	G	-0.263	7	33512386	6.22372E-06	0.058	360770	11064.499
genus Eubacterium oxidoreducens group	Vascular dementia (subcortical)	rs10919863	T	C	0.012	200226041	0.599	0.024	14306	T	C	0.315	1	200256913	3.47112E-06	0.068	360770	10813.753
genus Eubacterium oxidoreducens group	Vascular dementia (subcortical)	rs11986558	T	C	-0.013	2500772	0.529	0.021	14306	T	C	0.248	8	2643276	9.16875E-06	0.056	360770	11030.353
genus Eubacterium oxidoreducens group	Vascular dementia (subcortical)	rs1363668	G	A	0.016	143089582	0.408	0.020	14306	G	A	-0.272	5	143710017	5.25352E-06	0.060	360770	12697.214
genus Eubacterium oxidoreducens group	Vascular dementia (subcortical)	rs429358	C	T	0.021	45411941	0.455	0.028	14306	C	T	0.597	19	44908684	1.74221E-17	0.070	360770	41621.629
genus Eubacterium oxidoreducens group	Vascular dementia (subcortical)	rs4295569	C	T	-0.014	47820641	0.509	0.020	14306	C	T	-0.355	7	47781043	2.54572E-08	0.064	360770	20037.409
genus Eubacterium oxidoreducens group	Vascular dementia (subcortical)	rs4382795	C	T	-0.022	66878853	0.462	0.025	14306	C	T	0.378	10	65119095	9.80754E-06	0.086	360770	13265.712
genus Eubacterium oxidoreducens group	Vascular dementia (subcortical)	rs4723291	A	G	-0.011	33551998	0.583	0.019	14306	A	G	-0.263	7	33512386	6.22372E-06	0.058	360770	11064.499
genus Eubacterium rectale group	Vascular dementia (subcortical)	rs10919863	T	C	-0.005	200226041	0.801	0.014	14306	T	C	0.315	1	200256913	3.47112E-06	0.068	360770	10813.753
genus Eubacterium rectale group	Vascular dementia (subcortical)	rs11148372	A	G	0.001	22788665	0.918	0.011	14306	A	G	-0.261	13	22214526	4.06275E-06	0.057	360770	12632.549
genus Eubacterium rectale group	Vascular dementia (subcortical)	rs11986558	T	C	0.002	2500772	0.896	0.011	14306	T	C	0.248	8	2643276	9.16875E-06	0.056	360770	11030.353
genus Eubacterium rectale group	Vascular dementia (subcortical)	rs3802793	A	G	-0.004	131685316	0.733	0.011	14306	A	G	0.274	11	131815422	5.46626E-06	0.060	360770	12758.334
genus Eubacterium ruminantium group	Vascular dementia (subcortical)	rs10919863	T	C	-0.011	200226041	0.588	0.021	14306	T	C	0.315	1	200256913	3.47112E-06	0.068	360770	10813.753
genus Eubacterium ruminantium group	Vascular dementia (subcortical)	rs11148372	A	G	-0.011	22788665	0.499	0.017	14306	A	G	-0.261	13	22214526	4.06275E-06	0.057	360770	12632.549

genus Eubacterium ruminantium group	Vascular dementia (subcortical)	rs3802793	A	G	-0.009	131685316	0.607	0.017	14306	A	G	0.274	11	131815422	5.46626E-06	0.060	360770	12758.334
genus Eubacterium ruminantium group	Vascular dementia (subcortical)	rs429358	C	T	0.014	45411941	0.497	0.023	14306	C	T	0.597	19	44908684	1.74221E-17	0.070	360770	41621.629
genus Eubacterium ruminantium group	Vascular dementia (subcortical)	rs4295569	C	T	0.007	47820641	0.709	0.017	14306	C	T	-0.355	7	47781043	2.54572E-08	0.064	360770	20037.409
genus Eubacterium ventriosum group	Vascular dementia (subcortical)	rs10919863	T	C	-0.003	200226041	0.843	0.015	14306	T	C	0.315	1	200256913	3.47112E-06	0.068	360770	10813.753
genus Eubacterium ventriosum group	Vascular dementia (subcortical)	rs11986558	T	C	0.002	2500772	0.859	0.012	14306	T	C	0.248	8	2643276	9.16875E-06	0.056	360770	11030.353
genus Eubacterium ventriosum group	Vascular dementia (subcortical)	rs1363668	G	A	0.002	143089582	0.844	0.012	14306	G	A	-0.272	5	143710017	5.25352E-06	0.060	360770	12697.214
genus Eubacterium ventriosum group	Vascular dementia (subcortical)	rs4295569	C	T	-0.008	47820641	0.514	0.012	14306	C	T	-0.355	7	47781043	2.54572E-08	0.064	360770	20037.409
genus Eubacterium ventriosum group	Vascular dementia (subcortical)	rs4723291	A	G	0.006	33551998	0.628	0.011	14306	A	G	-0.263	7	33512386	6.22372E-06	0.058	360770	11064.499
genus Eubacterium xylanophilum group	Vascular dementia (subcortical)	rs10919863	T	C	0.012	200226041	0.487	0.016	14306	T	C	0.315	1	200256913	3.47112E-06	0.068	360770	10813.753
genus Eubacterium xylanophilum group	Vascular dementia (subcortical)	rs11986558	T	C	0.006	2500772	0.651	0.013	14306	T	C	0.248	8	2643276	9.16875E-06	0.056	360770	11030.353
genus Eubacterium xylanophilum group	Vascular dementia (subcortical)	rs429358	C	T	-0.023	45411941	0.188	0.017	14306	C	T	0.597	19	44908684	1.74221E-17	0.070	360770	41621.629
genus Eubacterium xylanophilum group	Vascular dementia (subcortical)	rs4295569	C	T	-0.003	47820641	0.824	0.013	14306	C	T	-0.355	7	47781043	2.54572E-08	0.064	360770	20037.409
genus Eubacterium xylanophilum group	Vascular dementia (subcortical)	rs4382795	C	T	-0.010	66878853	0.493	0.016	14306	C	T	0.378	10	65119095	9.80754E-06	0.086	360770	13265.712
genus Faecalibacterium	Vascular dementia (subcortical)	rs10919863	T	C	-0.004	200226041	0.789	0.014	14306	T	C	0.315	1	200256913	3.47112E-06	0.068	360770	10813.753
genus Faecalibacterium	Vascular dementia (subcortical)	rs1363668	G	A	-0.009	143089582	0.389	0.011	14306	G	A	-0.272	5	143710017	5.25352E-06	0.060	360770	12697.214
genus Faecalibacterium	Vascular dementia (subcortical)	rs4295569	C	T	-0.010	47820641	0.405	0.011	14306	C	T	-0.355	7	47781043	2.54572E-08	0.064	360770	20037.409
genus Faecalibacterium	Vascular dementia (subcortical)	rs4723291	A	G	0.005	33551998	0.623	0.011	14306	A	G	-0.263	7	33512386	6.22372E-06	0.058	360770	11064.499
genus Family XIII AD3011 group	Vascular dementia (subcortical)	rs11148372	A	G	-0.004	22788665	0.786	0.012	14306	A	G	-0.261	13	22214526	4.06275E-06	0.057	360770	12632.549
genus Family XIII AD3011 group	Vascular dementia (subcortical)	rs11986558	T	C	-0.001	2500772	0.926	0.013	14306	T	C	0.248	8	2643276	9.16875E-06	0.056	360770	11030.353
genus Family XIII AD3011 group	Vascular dementia (subcortical)	rs1363668	G	A	-0.007	143089582	0.549	0.012	14306	G	A	-0.272	5	143710017	5.25352E-06	0.060	360770	12697.214
genus Family XIII AD3011 group	Vascular dementia (subcortical)	rs3802793	A	G	0.001	131685316	0.995	0.013	14306	A	G	0.274	11	131815422	5.46626E-06	0.060	360770	12758.334
genus Family XIII AD3011 group	Vascular dementia (subcortical)	rs429358	C	T	0.029	45411941	0.106	0.017	14306	C	T	0.597	19	44908684	1.74221E-17	0.070	360770	41621.629
genus Family XIII AD3011 group	Vascular dementia (subcortical)	rs4382795	C	T	-0.008	66878853	0.562	0.016	14306	C	T	0.378	10	65119095	9.80754E-06	0.086	360770	13265.712
genus Family XIII AD3011 group	Vascular dementia (subcortical)	rs4723291	A	G	0.000	33551998	1.000	0.012	14306	A	G	-0.263	7	33512386	6.22372E-06	0.058	360770	11064.499
genus Family XIII UCG001	Vascular dementia (subcortical)	rs10919863	T	C	-0.012	200226041	0.503	0.016	14306	T	C	0.315	1	200256913	3.47112E-06	0.068	360770	10813.753
genus Family XIII UCG001	Vascular dementia (subcortical)	rs11148372	A	G	-0.004	22788665	0.740	0.013	14306	A	G	-0.261	13	22214526	4.06275E-06	0.057	360770	12632.549
genus Family XIII UCG001	Vascular dementia (subcortical)	rs1363668	G	A	-0.003	143089582	0.803	0.013	14306	G	A	-0.272	5	143710017	5.25352E-06	0.060	360770	12697.214
genus Family XIII UCG001	Vascular dementia (subcortical)	rs3802793	A	G	0.003	131685316	0.818	0.013	14306	A	G	0.274	11	131815422	5.46626E-06	0.060	360770	12758.334
genus Family XIII UCG001	Vascular dementia (subcortical)	rs429358	C	T	0.004	45411941	0.889	0.018	14306	C	T	0.597	19	44908684	1.74221E-17	0.070	360770	41621.629
genus Family XIII UCG001	Vascular dementia (subcortical)	rs4295569	C	T	0.013	47820641	0.315	0.013	14306	C	T	-0.355	7	47781043	2.54572E-08	0.064	360770	20037.409
genus Flavonifractor	Vascular dementia (subcortical)	rs11986558	T	C	0.002	2500772	0.896	0.014	14306	T	C	0.248	8	2643276	9.16875E-06	0.056	360770	11030.353
genus Flavonifractor	Vascular dementia (subcortical)	rs1363668	G	A	-0.001	143089582	0.929	0.014	14306	G	A	-0.272	5	143710017	5.25352E-06	0.060	360770	12697.214
genus Flavonifractor	Vascular dementia (subcortical)	rs429358	C	T	-0.021	45411941	0.293	0.020	14306	C	T	0.597	19	44908684	1.74221E-17	0.070	360770	41621.629
genus Flavonifractor	Vascular dementia (subcortical)	rs4295569	C	T	0.009	47820641	0.505	0.014	14306	C	T	-0.355	7	47781043	2.54572E-08	0.064	360770	20037.409
genus Fusicatenibacter	Vascular dementia (subcortical)	rs10919863	T	C	0.006	200226041	0.620	0.014	14306	T	C	0.315	1	200256913	3.47112E-06	0.068	360770	10813.753
genus Fusicatenibacter	Vascular dementia (subcortical)	rs11148372	A	G	-0.004	22788665	0.718	0.011	14306	A	G	-0.261	13	22214526	4.06275E-06	0.057	360770	12632.549
genus Fusicatenibacter	Vascular dementia (subcortical)	rs1363668	G	A	0.010	143089582	0.375	0.011	14306	G	A	-0.272	5	143710017	5.25352E-06	0.060	360770	12697.214
genus Fusicatenibacter	Vascular dementia (subcortical)	rs3802793	A	G	-0.006	131685316	0.611	0.011	14306	A	G	0.274	11	131815422	5.46626E-06	0.060	360770	12758.334
genus Fusicatenibacter	Vascular dementia (subcortical)	rs429358	C	T	-0.008	45411941	0.622	0.015	14306	C	T	0.597	19	44908684	1.74221E-17	0.070	360770	41621.629
genus Fusicatenibacter	Vascular dementia (subcortical)	rs4295569	C	T	-0.004	47820641	0.779	0.011	14306	C	T	-0.355	7	47781043	2.54572E-08	0.064	360770	20037.409
genus Fusicatenibacter	Vascular dementia (subcortical)	rs4382795	C	T	0.006	66878853	0.662	0.014	14306	C	T	0.378	10	65119095	9.80754E-06	0.086	360770	13265.712
genus Gordonibacter	Vascular dementia (subcortical)	rs11148372	A	G	0.000	22788665	0.997	0.024	14306	A	G	-0.261	13	22214526	4.06275E-06	0.057	360770	12632.549
genus Gordonibacter	Vascular dementia (subcortical)	rs1363668	G	A	-0.016	143089582	0.484	0.023	14306	G	A	-0.272	5	143710017	5.25352E-06	0.060	360770	12697.214
genus Gordonibacter	Vascular dementia (subcortical)	rs3802793	A	G	-0.007	131685316	0.797	0.025	14306	A	G	0.274	11	131815422	5.46626E-06	0.060	360770	12758.334
genus Gordonibacter	Vascular dementia (subcortical)	rs429358	C	T	0.056	45411941	0.125	0.034	14306	C	T	0.597	19	44908684	1.74221E-17	0.070	360770	41621.629
genus Gordonibacter	Vascular dementia (subcortical)	rs4295569	C	T	-0.007	47820641	0.802	0.024	14306	C	T	-0.355	7	47781043	2.54572E-08	0.064	360770	20037.409
genus Gordonibacter	Vascular dementia (subcortical)	rs4382795	C	T	0.001	66878853	0.973	0.031	14306	C	T	0.378	10	65119095	9.80754E-06	0.086	360770	13265.712
genus Gordonibacter	Vascular dementia (subcortical)	rs4723291	A	G	0.014	33551998	0.548	0.023	14306	A	G	-0.263	7	33512386	6.22372E-06	0.058	360770	11064.499
genus Haemophilus	Vascular dementia (subcortical)	rs10919863	T	C	-0.004	200226041	0.832	0.019	14306	T	C	0.315	1	200256913	3.47112E-06	0.068	360770	10813.753
genus Haemophilus	Vascular dementia (subcortical)	rs11986558	T	C	0.005	2500772	0.738	0.016	14306	T	C	0.248	8	2643276	9.16875E-06	0.056	360770	11030.353
genus Haemophilus	Vascular dementia (subcortical)	rs1363668	G	A	0.001	143089582	0.925	0.015	14306	G	A	-0.272	5	143710017	5.25352E-06	0.060	360770	12697.214
genus Haemophilus	Vascular dementia (subcortical)	rs3802793	A	G	-0.010	131685316	0.491	0.016	14306	A	G	0.274	11	131815422	5.46626E-06	0.060	360770	12758.334
genus Haemophilus	Vascular dementia (subcortical)	rs429358	C	T	0.027	45411941	0.241	0.021	14306	C	T	0.597	19	44908684	1.74221E-17	0.070	360770	41621.629
genus Haemophilus	Vascular dementia (subcortical)	rs4723291	A	G	-0.010	33551998	0.517	0.015	14306	A	G	-0.263	7	33512386	6.22372E-06	0.058	360770	11064.499
genus Holdemanella	Vascular dementia (subcortical)	rs10919863	T	C	0.018	200226041	0.353	0.021	14306	T	C	0.315	1	200256913	3.47112E-06	0.068	360770	10813.753
genus Holdemanella	Vascular dementia (subcortical)	rs11148372	A	G	-0.013	22788665	0.436	0.017	14306	A	G	-0.261	13	22214526	4.06275E-06	0.057	360770	12632.549
genus Holdemanella	Vascular dementia (subcortical)	rs1363668	G	A	0.007	143089582	0.648	0.016	14306	G	A	-0.272	5	143710017	5.25352E-06	0.060	360770	12697.214
genus Holdemanella	Vascular dementia (subcortical)	rs3802793	A	G	-0.006	131685316	0.698	0.017	14306	A	G	0.274	11	131815422	5.46626E-06	0.060	360770	12758.334
genus Holdemanella	Vascular dementia (subcortical)	rs429358	C	T	0.038	45411941	0.092	0.023	14306	C	T	0.597	19	44908684	1.74221E-17	0.070	360770	41621.629
genus Holdemanella	Vascular dementia (subcortical)	rs4382795	C	T	-0.014	66878853	0.538	0.021	14306	C	T	0.378	10	65119095	9.80754E-06	0.086	360770	13265.712
genus Holdemanella	Vascular dementia (subcortical)	rs4723291	A	G	-0.012	33551998	0.463	0.016	14306	A	G	-0.263	7	33512386	6.22372E-06	0.058	360770	11064.499
genus Holdemania	Vascular dementia (subcortical)	rs10919863	T	C	-0.002	200226041	0.876	0.019	14306	T	C	0.315	1	200256913	3.47112E-06	0.068	360770	10813.753
genus Holdemania	Vascular dementia (subcortical)	rs11148372	A	G	-0.004	22788665	0.784	0.015	14306	A	G	-0.261	13	22214526	4.06275E-06	0.057	360770	12632.549
genus Holdemania	Vascular dementia (subcortical)	rs1363668	G	A	0.000	143089582	0.997	0.015	14306	G	A	-0.272	5	143710017	5.25352E-06	0.060	360770	12697.214
genus Holdemania	Vascular dementia (subcortical)	rs429358	C	T	0.014	45411941	0.528	0.021	14306	C	T	0.597	19	44908684	1.74221E-17	0.070	360770	41621.629
genus Holdemania	Vascular dementia (subcortical)	rs4382795	C	T	-0.003	66878853	0.855	0.020	14306	C	T	0.378	10	65119095	9.80754E-06	0.086	360770	13265.712
genus Holdemania	Vascular dementia (subcortical)	rs4723291	A	G	0.001	33551998	0.941	0.015	14306	A	G	-0.263	7	33512386	6.22372E-06	0.058	360770	11064.499
genus Howardella	Vascular dementia (subcortical)	rs10919863	T	C	0.006	200226041	0.870	0.030	14306	T	C	0.315	1	200256913	3.47112E-06	0.068	360770	10813.753
genus Howardella	Vascular dementia (subcortical)	rs11148372	A	G	0.013	22788665	0.583	0.024	14306	A	G	-0.261	13	22214526	4.06275E-06	0.057	360770	12632.549
genus Howardella	Vascular dementia (subcortical)	rs11986558	T	C	-0.007	2500772	0.769	0.024	14306	T	C	0.248	8	2643276	9.16875E-06	0.056	360770	11030.353
genus Howardella	Vascular dementia (subcortical)	rs3802793	A	G	-0.007	131685316	0.809	0.024	14306	A	G	0.274	11	131815422	5.46626E-06	0.060	360770	12758.334

genus Howardella	Vascular dementia (subcortical)	rs429358	C	T	0.033	45411941	0.260	0.033	14306	C	T	0.597	19	44908684	1.74221E-17	0.070	360770	41621.629
genus Howardella	Vascular dementia (subcortical)	rs4295569	C	T	0.007	47820641	0.775	0.024	14306	C	T	-0.355	7	47781043	2.54572E-08	0.064	360770	20037.409
genus Howardella	Vascular dementia (subcortical)	rs4723291	A	G	-0.016	33551998	0.485	0.023	14306	A	G	-0.263	7	33512386	6.22372E-06	0.058	360770	11064.499
genus Hungatella	Vascular dementia (subcortical)	rs10919863	T	C	-0.015	200226041	0.590	0.028	14306	T	C	0.315	1	200256913	3.47112E-06	0.068	360770	10813.753
genus Hungatella	Vascular dementia (subcortical)	rs11148372	A	G	-0.019	22788665	0.390	0.022	14306	A	G	-0.261	13	22214526	4.06275E-06	0.057	360770	12632.549
genus Hungatella	Vascular dementia (subcortical)	rs11986558	T	C	0.002	2500772	0.919	0.023	14306	T	C	0.248	8	2643276	9.16875E-06	0.056	360770	11030.353
genus Hungatella	Vascular dementia (subcortical)	rs1363668	G	A	-0.004	143089582	0.832	0.022	14306	G	A	-0.272	5	143710017	5.25352E-06	0.060	360770	12697.214
genus Hungatella	Vascular dementia (subcortical)	rs429358	C	T	-0.050	45411941	0.108	0.032	14306	C	T	0.597	19	44908684	1.74221E-17	0.070	360770	41621.629
genus Hungatella	Vascular dementia (subcortical)	rs4295569	C	T	0.017	47820641	0.450	0.023	14306	C	T	-0.355	7	47781043	2.54572E-08	0.064	360770	20037.409
genus Hungatella	Vascular dementia (subcortical)	rs4723291	A	G	-0.010	33551998	0.633	0.022	14306	A	G	-0.263	7	33512386	6.22372E-06	0.058	360770	11064.499
genus Intestinibacter	Vascular dementia (subcortical)	rs10919863	T	C	-0.014	200226041	0.393	0.017	14306	T	C	0.315	1	200256913	3.47112E-06	0.068	360770	10813.753
genus Intestinibacter	Vascular dementia (subcortical)	rs11148372	A	G	0.000	22788665	0.999	0.013	14306	A	G	-0.261	13	22214526	4.06275E-06	0.057	360770	12632.549
genus Intestinibacter	Vascular dementia (subcortical)	rs1363668	G	A	-0.005	143089582	0.699	0.013	14306	G	A	-0.272	5	143710017	5.25352E-06	0.060	360770	12697.214
genus Intestinibacter	Vascular dementia (subcortical)	rs3802793	A	G	0.007	131685316	0.604	0.014	14306	A	G	0.274	11	131815422	5.46626E-06	0.060	360770	12758.334
genus Intestinibacter	Vascular dementia (subcortical)	rs429358	C	T	-0.011	45411941	0.447	0.018	14306	C	T	0.597	19	44908684	1.74221E-17	0.070	360770	41621.629
genus Intestinibacter	Vascular dementia (subcortical)	rs4295569	C	T	-0.006	47820641	0.658	0.013	14306	C	T	-0.355	7	47781043	2.54572E-08	0.064	360770	20037.409
genus Intestinimonas	Vascular dementia (subcortical)	rs10919863	T	C	0.010	200226041	0.567	0.017	14306	T	C	0.315	1	200256913	3.47112E-06	0.068	360770	10813.753
genus Intestinimonas	Vascular dementia (subcortical)	rs11148372	A	G	0.006	22788665	0.627	0.013	14306	A	G	-0.261	13	22214526	4.06275E-06	0.057	360770	12632.549
genus Intestinimonas	Vascular dementia (subcortical)	rs11986558	T	C	0.004	2500772	0.786	0.014	14306	T	C	0.248	8	2643276	9.16875E-06	0.056	360770	11030.353
genus Intestinimonas	Vascular dementia (subcortical)	rs1363668	G	A	0.010	143089582	0.431	0.013	14306	G	A	-0.272	5	143710017	5.25352E-06	0.060	360770	12697.214
genus Intestinimonas	Vascular dementia (subcortical)	rs3802793	A	G	-0.008	131685316	0.518	0.014	14306	A	G	0.274	11	131815422	5.46626E-06	0.060	360770	12758.334
genus Intestinimonas	Vascular dementia (subcortical)	rs429358	C	T	0.013	45411941	0.424	0.018	14306	C	T	0.597	19	44908684	1.74221E-17	0.070	360770	41621.629
genus Intestinimonas	Vascular dementia (subcortical)	rs4382795	C	T	0.012	66878853	0.514	0.017	14306	C	T	0.378	10	65119095	9.80754E-06	0.086	360770	13265.712
genus Intestinimonas	Vascular dementia (subcortical)	rs4723291	A	G	-0.002	33551998	0.897	0.013	14306	A	G	-0.263	7	33512386	6.22372E-06	0.058	360770	11064.499
genus Lachnoclostridium	Vascular dementia (subcortical)	rs11148372	A	G	0.000	22788665	0.974	0.011	14306	A	G	-0.261	13	22214526	4.06275E-06	0.057	360770	12632.549
genus Lachnoclostridium	Vascular dementia (subcortical)	rs1363668	G	A	-0.008	143089582	0.465	0.011	14306	G	A	-0.272	5	143710017	5.25352E-06	0.060	360770	12697.214
genus Lachnoclostridium	Vascular dementia (subcortical)	rs3802793	A	G	-0.002	131685316	0.824	0.011	14306	A	G	0.274	11	131815422	5.46626E-06	0.060	360770	12758.334
genus Lachnoclostridium	Vascular dementia (subcortical)	rs429358	C	T	-0.019	45411941	0.230	0.015	14306	C	T	0.597	19	44908684	1.74221E-17	0.070	360770	41621.629
genus Lachnoclostridium	Vascular dementia (subcortical)	rs4295569	C	T	0.009	47820641	0.381	0.011	14306	C	T	-0.355	7	47781043	2.54572E-08	0.064	360770	20037.409
genus Lachnoclostridium	Vascular dementia (subcortical)	rs4382795	C	T	0.003	66878853	0.884	0.014	14306	C	T	0.378	10	65119095	9.80754E-06	0.086	360770	13265.712
genus Lachnospiraceae FCS020 group	Vascular dementia (subcortical)	rs10919863	T	C	-0.008	200226041	0.654	0.016	14306	T	C	0.315	1	200256913	3.47112E-06	0.068	360770	10813.753
genus Lachnospiraceae FCS020 group	Vascular dementia (subcortical)	rs11148372	A	G	-0.009	22788665	0.459	0.012	14306	A	G	-0.261	13	22214526	4.06275E-06	0.057	360770	12632.549
genus Lachnospiraceae FCS020 group	Vascular dementia (subcortical)	rs11986558	T	C	0.004	2500772	0.717	0.013	14306	T	C	0.248	8	2643276	9.16875E-06	0.056	360770	11030.353
genus Lachnospiraceae FCS020 group	Vascular dementia (subcortical)	rs1363668	G	A	-0.001	143089582	0.947	0.012	14306	G	A	-0.272	5	143710017	5.25352E-06	0.060	360770	12697.214
genus Lachnospiraceae FCS020 group	Vascular dementia (subcortical)	rs429358	C	T	0.017	45411941	0.318	0.017	14306	C	T	0.597	19	44908684	1.74221E-17	0.070	360770	41621.629
genus Lachnospiraceae NC2004 group	Vascular dementia (subcortical)	rs10919863	T	C	0.002	200226041	0.832	0.022	14306	T	C	0.315	1	200256913	3.47112E-06	0.068	360770	10813.753
genus Lachnospiraceae NC2004 group	Vascular dementia (subcortical)	rs11148372	A	G	-0.004	22788665	0.852	0.018	14306	A	G	-0.261	13	22214526	4.06275E-06	0.057	360770	12632.549
genus Lachnospiraceae NC2004 group	Vascular dementia (subcortical)	rs3802793	A	G	0.017	131685316	0.375	0.019	14306	A	G	0.274	11	131815422	5.46626E-06	0.060	360770	12758.334
genus Lachnospiraceae NC2004 group	Vascular dementia (subcortical)	rs429358	C	T	-0.024	45411941	0.415	0.026	14306	C	T	0.597	19	44908684	1.74221E-17	0.070	360770	41621.629
genus Lachnospiraceae ND3007 group	Vascular dementia (subcortical)	rs10919863	T	C	0.007	200226041	0.646	0.015	14306	T	C	0.315	1	200256913	3.47112E-06	0.068	360770	10813.753
genus Lachnospiraceae ND3007 group	Vascular dementia (subcortical)	rs11148372	A	G	0.000	22788665	0.987	0.012	14306	A	G	-0.261	13	22214526	4.06275E-06	0.057	360770	12632.549
genus Lachnospiraceae ND3007 group	Vascular dementia (subcortical)	rs1363668	G	A	-0.008	143089582	0.502	0.012	14306	G	A	-0.272	5	143710017	5.25352E-06	0.060	360770	12697.214
genus Lachnospiraceae ND3007 group	Vascular dementia (subcortical)	rs429358	C	T	-0.003	45411941	0.806	0.016	14306	C	T	0.597	19	44908684	1.74221E-17	0.070	360770	41621.629
genus Lachnospiraceae ND3007 group	Vascular dementia (subcortical)	rs4295569	C	T	0.002	47820641	0.840	0.012	14306	C	T	-0.355	7	47781043	2.54572E-08	0.064	360770	20037.409
genus Lachnospiraceae NK4A136 group	Vascular dementia (subcortical)	rs10919863	T	C	0.003	200226041	0.888	0.014	14306	T	C	0.315	1	200256913	3.47112E-06	0.068	360770	10813.753
genus Lachnospiraceae NK4A136 group	Vascular dementia (subcortical)	rs11986558	T	C	-0.001	2500772	0.933	0.011	14306	T	C	0.248	8	2643276	9.16875E-06	0.056	360770	11030.353
genus Lachnospiraceae NK4A136 group	Vascular dementia (subcortical)	rs3802793	A	G	0.005	131685316	0.630	0.011	14306	A	G	0.274	11	131815422	5.46626E-06	0.060	360770	12758.334
genus Lachnospiraceae NK4A136 group	Vascular dementia (subcortical)	rs429358	C	T	-0.011	45411941	0.456	0.015	14306	C	T	0.597	19	44908684	1.74221E-17	0.070	360770	41621.629
genus Lachnospiraceae NK4A136 group	Vascular dementia (subcortical)	rs4295569	C	T	0.007	47820641	0.542	0.011	14306	C	T	-0.355	7	47781043	2.54572E-08	0.064	360770	20037.409
genus Lachnospiraceae NK4A136 group	Vascular dementia (subcortical)	rs4382795	C	T	0.008	66878853	0.534	0.014	14306	C	T	0.378	10	65119095	9.80754E-06	0.086	360770	13265.712
genus Lachnospiraceae UCG001	Vascular dementia (subcortical)	rs11148372	A	G	-0.013	22788665	0.371	0.014	14306	A	G	-0.261	13	22214526	4.06275E-06	0.057	360770	12632.549
genus Lachnospiraceae UCG001	Vascular dementia (subcortical)	rs1363668	G	A	-0.011	143089582	0.436	0.014	14306	G	A	-0.272	5	143710017	5.25352E-06	0.060	360770	12697.214
genus Lachnospiraceae UCG001	Vascular dementia (subcortical)	rs429358	C	T	-0.002	45411941	0.904	0.020	14306	C	T	0.597	19	44908684	1.74221E-17	0.070	360770	41621.629
genus Lachnospiraceae UCG001	Vascular dementia (subcortical)	rs4295569	C	T	-0.002	47820641	0.880	0.015	14306	C	T	-0.355	7	47781043	2.54572E-08	0.064	360770	20037.409
genus Lachnospiraceae UCG001	Vascular dementia (subcortical)	rs4382795	C	T	0.000	66878853	0.971	0.019	14306	C	T	0.378	10	65119095	9.80754E-06	0.086	360770	13265.712
genus Lachnospiraceae UCG001	Vascular dementia (subcortical)	rs4723291	A	G	0.012	33551998	0.384	0.014	14306	A	G	-0.263	7	33512386	6.22372E-06	0.058	360770	11064.499
genus Lachnospiraceae UCG004	Vascular dementia (subcortical)	rs10919863	T	C	0.001	200226041	0.943	0.015	14306	T	C	0.315	1	200256913	3.47112E-06	0.068	360770	10813.753
genus Lachnospiraceae UCG004	Vascular dementia (subcortical)	rs11148372	A	G	-0.008	22788665	0.503	0.012	14306	A	G	-0.261	13	22214526	4.06275E-06	0.057	360770	12632.549
genus Lachnospiraceae UCG004	Vascular dementia (subcortical)	rs11986558	T	C	0.010	2500772	0.407	0.012	14306	T	C	0.248	8	2643276	9.16875E-06	0.056	360770	11030.353
genus Lachnospiraceae UCG004	Vascular dementia (subcortical)	rs1363668	G	A	-0.003	143089582	0.801	0.012	14306	G	A	-0.272	5	143710017	5.25352E-06	0.060	360770	12697.214
genus Lachnospiraceae UCG004	Vascular dementia (subcortical)	rs3802793	A	G	0.000	131685316	0.998	0.013	14306	A	G	0.274	11	131815422	5.46626E-06	0.060	360770	12758.334
genus Lachnospiraceae UCG004	Vascular dementia (subcortical)	rs429358	C	T	-0.001	45411941	0.954	0.017	14306	C	T	0.597	19	44908684	1.74221E-17	0.070	360770	41621.629
genus Lachnospiraceae UCG004	Vascular dementia (subcortical)	rs4295569	C	T	0.009	47820641	0.457	0.012	14306	C	T	-0.355	7	47781043	2.54572E-08	0.064	360770	20037.409
genus Lachnospiraceae UCG004	Vascular dementia (subcortical)	rs4382795	C	T	0.013	66878853	0.409	0.015	14306	C	T	0.378	10	65119095	9.80754E-06	0.086	360770	13265.712
genus Lachnospiraceae UCG004	Vascular dementia (subcortical)	rs4723291	A	G	-0.006	33551998	0.591	0.012	14306	A	G	-0.263	7	33512386	6.22372E-06	0.058	360770	11064.499
genus Lachnospiraceae UCG008	Vascular dementia (subcortical)	rs11986558	T	C	0.008	2500772	0.644	0.018	14306	T	C	0.248	8	2643276	9.16875E-06	0.056	360770	11030.353
genus Lachnospiraceae UCG008	Vascular dementia (subcortical)	rs1363668	G	A	-0.001	143089582	0.981	0.017	14306	G	A	-0.272	5	143710017	5.25352E-06	0.060	360770	12697.214
genus Lachnospiraceae UCG008	Vascular dementia (subcortical)	rs429358	C	T	0.001	45411941	0.933	0.024	14306	C	T	0.597	19	44908684	1.74221E-17	0.070	360770	41621.629
genus Lachnospiraceae UCG008	Vascular dementia (subcortical)	rs4382795	C	T	0.016	66878853	0.458	0.022	14306	C	T	0.378	10	65119095	9.80754E-06	0.086	360770	13265.712
genus Lachnospiraceae UCG010	Vascular dementia (subcortical)	rs11148372	A	G	-0.007	22788665	0.567	0.013	14306	A	G	-0.261	13	22214526	4.06275E-06	0.057	360770	12632.549
genus Lachnospiraceae UCG010	Vascular dementia (subcortical)	rs429358	C	T	-0.003	45411941	0.809	0.018	14306	C	T	0.597	19	44908684	1.74221E-17	0.070	360770	41621.629

genus Lachnospiraceae UCG010	Vascular dementia (subcortical)	rs4382795	C	T	-0.007	66878853	0.719	0.016	14306	C	T	0.378	10	65119095	9.80754E-06	0.086	360770	13265.712
genus Lachnospiraceae UCG010	Vascular dementia (subcortical)	rs4723291	A	G	0.001	33551998	0.922	0.012	14306	A	G	-0.263	7	33512386	6.22372E-06	0.058	360770	11064.499
genus Lactobacillus	Vascular dementia (subcortical)	rs10919863	T	C	-0.005	200226041	0.790	0.022	14306	T	C	0.315	1	200256913	3.47112E-06	0.068	360770	10813.753
genus Lactobacillus	Vascular dementia (subcortical)	rs11148372	A	G	-0.009	22788665	0.622	0.017	14306	A	G	-0.261	13	22214526	4.06275E-06	0.057	360770	12632.549
genus Lactobacillus	Vascular dementia (subcortical)	rs11986558	T	C	-0.013	2500772	0.463	0.018	14306	T	C	0.248	8	2643276	9.16875E-06	0.056	360770	11030.353
genus Lactobacillus	Vascular dementia (subcortical)	rs429358	C	T	-0.007	45411941	0.865	0.024	14306	C	T	0.597	19	44908684	1.74221E-17	0.070	360770	41621.629
genus Lactobacillus	Vascular dementia (subcortical)	rs4382795	C	T	0.003	66878853	0.927	0.022	14306	C	T	0.378	10	65119095	9.80754E-06	0.086	360770	13265.712
genus Lactobacillus	Vascular dementia (subcortical)	rs4723291	A	G	-0.002	33551998	0.908	0.017	14306	A	G	-0.263	7	33512386	6.22372E-06	0.058	360770	11064.499
genus Lactococcus	Vascular dementia (subcortical)	rs11148372	A	G	0.011	22788665	0.614	0.023	14306	A	G	-0.261	13	22214526	4.06275E-06	0.057	360770	12632.549
genus Lactococcus	Vascular dementia (subcortical)	rs429358	C	T	-0.017	45411941	0.591	0.032	14306	C	T	0.597	19	44908684	1.74221E-17	0.070	360770	41621.629
genus Lactococcus	Vascular dementia (subcortical)	rs4295569	C	T	0.001	47820641	0.989	0.023	14306	C	T	-0.355	7	47781043	2.54572E-08	0.064	360770	20037.409
genus Lactococcus	Vascular dementia (subcortical)	rs4723291	A	G	0.003	33551998	0.884	0.022	14306	A	G	-0.263	7	33512386	6.22372E-06	0.058	360770	11064.499
genus Marvinbryantia	Vascular dementia (subcortical)	rs10919863	T	C	-0.015	200226041	0.360	0.017	14306	T	C	0.315	1	200256913	3.47112E-06	0.068	360770	10813.753
genus Marvinbryantia	Vascular dementia (subcortical)	rs11148372	A	G	0.003	22788665	0.834	0.013	14306	A	G	-0.261	13	22214526	4.06275E-06	0.057	360770	12632.549
genus Marvinbryantia	Vascular dementia (subcortical)	rs11986558	T	C	0.002	2500772	0.892	0.014	14306	T	C	0.248	8	2643276	9.16875E-06	0.056	360770	11030.353
genus Marvinbryantia	Vascular dementia (subcortical)	rs1363668	G	A	0.008	143089582	0.537	0.013	14306	G	A	-0.272	5	143710017	5.25352E-06	0.060	360770	12697.214
genus Marvinbryantia	Vascular dementia (subcortical)	rs429358	C	T	0.008	45411941	0.663	0.018	14306	C	T	0.597	19	44908684	1.74221E-17	0.070	360770	41621.629
genus Marvinbryantia	Vascular dementia (subcortical)	rs4723291	A	G	0.002	33551998	0.899	0.013	14306	A	G	-0.263	7	33512386	6.22372E-06	0.058	360770	11064.499
genus Methanobrevibacter	Vascular dementia (subcortical)	rs10919863	T	C	-0.010	200226041	0.703	0.031	14306	T	C	0.315	1	200256913	3.47112E-06	0.068	360770	10813.753
genus Methanobrevibacter	Vascular dementia (subcortical)	rs429358	C	T	-0.019	45411941	0.661	0.034	14306	C	T	0.597	19	44908684	1.74221E-17	0.070	360770	41621.629
genus Methanobrevibacter	Vascular dementia (subcortical)	rs4295569	C	T	-0.019	47820641	0.445	0.025	14306	C	T	-0.355	7	47781043	2.54572E-08	0.064	360770	20037.409
genus Methanobrevibacter	Vascular dementia (subcortical)	rs4382795	C	T	-0.012	66878853	0.623	0.031	14306	C	T	0.378	10	65119095	9.80754E-06	0.086	360770	13265.712
genus Odoribacter	Vascular dementia (subcortical)	rs10919863	T	C	0.002	200226041	0.907	0.015	14306	T	C	0.315	1	200256913	3.47112E-06	0.068	360770	10813.753
genus Odoribacter	Vascular dementia (subcortical)	rs11148372	A	G	0.002	22788665	0.896	0.012	14306	A	G	-0.261	13	22214526	4.06275E-06	0.057	360770	12632.549
genus Odoribacter	Vascular dementia (subcortical)	rs11986558	T	C	-0.005	2500772	0.700	0.012	14306	T	C	0.248	8	2643276	9.16875E-06	0.056	360770	11030.353
genus Odoribacter	Vascular dementia (subcortical)	rs1363668	G	A	0.009	143089582	0.447	0.012	14306	G	A	-0.272	5	143710017	5.25352E-06	0.060	360770	12697.214
genus Odoribacter	Vascular dementia (subcortical)	rs3802793	A	G	0.000	131685316	0.979	0.012	14306	A	G	0.274	11	131815422	5.46626E-06	0.060	360770	12758.334
genus Odoribacter	Vascular dementia (subcortical)	rs429358	C	T	0.017	45411941	0.297	0.017	14306	C	T	0.597	19	44908684	1.74221E-17	0.070	360770	41621.629
genus Odoribacter	Vascular dementia (subcortical)	rs4295569	C	T	-0.009	47820641	0.459	0.012	14306	C	T	-0.355	7	47781043	2.54572E-08	0.064	360770	20037.409
genus Odoribacter	Vascular dementia (subcortical)	rs4382795	C	T	0.014	66878853	0.406	0.015	14306	C	T	0.378	10	65119095	9.80754E-06	0.086	360770	13265.712
genus Odoribacter	Vascular dementia (subcortical)	rs4723291	A	G	-0.007	33551998	0.519	0.012	14306	A	G	-0.263	7	33512386	6.22372E-06	0.058	360770	11064.499
genus Olsenella	Vascular dementia (subcortical)	rs10919863	T	C	0.008	200226041	0.776	0.030	14306	T	C	0.315	1	200256913	3.47112E-06	0.068	360770	10813.753
genus Olsenella	Vascular dementia (subcortical)	rs11148372	A	G	-0.015	22788665	0.518	0.024	14306	A	G	-0.261	13	22214526	4.06275E-06	0.057	360770	12632.549
genus Olsenella	Vascular dementia (subcortical)	rs11986558	T	C	0.005	2500772	0.861	0.025	14306	T	C	0.248	8	2643276	9.16875E-06	0.056	360770	11030.353
genus Olsenella	Vascular dementia (subcortical)	rs1363668	G	A	-0.020	143089582	0.388	0.023	14306	G	A	-0.272	5	143710017	5.25352E-06	0.060	360770	12697.214
genus Olsenella	Vascular dementia (subcortical)	rs4295569	C	T	0.025	47820641	0.303	0.024	14306	C	T	-0.355	7	47781043	2.54572E-08	0.064	360770	20037.409
genus Olsenella	Vascular dementia (subcortical)	rs4382795	C	T	-0.020	66878853	0.498	0.031	14306	C	T	0.378	10	65119095	9.80754E-06	0.086	360770	13265.712
genus Olsenella	Vascular dementia (subcortical)	rs4723291	A	G	0.003	33551998	0.907	0.023	14306	A	G	-0.263	7	33512386	6.22372E-06	0.058	360770	11064.499
genus Oscillibacter	Vascular dementia (subcortical)	rs10919863	T	C	-0.011	200226041	0.530	0.019	14306	T	C	0.315	1	200256913	3.47112E-06	0.068	360770	10813.753
genus Oscillibacter	Vascular dementia (subcortical)	rs11986558	T	C	0.003	2500772	0.811	0.016	14306	T	C	0.248	8	2643276	9.16875E-06	0.056	360770	11030.353
genus Oscillibacter	Vascular dementia (subcortical)	rs1363668	G	A	-0.009	143089582	0.537	0.015	14306	G	A	-0.272	5	143710017	5.25352E-06	0.060	360770	12697.214
genus Oscillibacter	Vascular dementia (subcortical)	rs3802793	A	G	0.004	131685316	0.777	0.016	14306	A	G	0.274	11	131815422	5.46626E-06	0.060	360770	12758.334
genus Oscillibacter	Vascular dementia (subcortical)	rs4295569	C	T	-0.017	47820641	0.293	0.016	14306	C	T	-0.355	7	47781043	2.54572E-08	0.064	360770	20037.409
genus Oscillospira	Vascular dementia (subcortical)	rs11148372	A	G	-0.010	22788665	0.475	0.014	14306	A	G	-0.261	13	22214526	4.06275E-06	0.057	360770	12632.549
genus Oscillospira	Vascular dementia (subcortical)	rs11986558	T	C	-0.013	2500772	0.380	0.015	14306	T	C	0.248	8	2643276	9.16875E-06	0.056	360770	11030.353
genus Oscillospira	Vascular dementia (subcortical)	rs1363668	G	A	0.000	143089582	0.998	0.014	14306	G	A	-0.272	5	143710017	5.25352E-06	0.060	360770	12697.214
genus Oscillospira	Vascular dementia (subcortical)	rs429358	C	T	-0.005	45411941	0.763	0.020	14306	C	T	0.597	19	44908684	1.74221E-17	0.070	360770	41621.629
genus Oscillospira	Vascular dementia (subcortical)	rs4295569	C	T	-0.010	47820641	0.485	0.015	14306	C	T	-0.355	7	47781043	2.54572E-08	0.064	360770	20037.409
genus Oscillospira	Vascular dementia (subcortical)	rs4382795	C	T	0.005	66878853	0.787	0.018	14306	C	T	0.378	10	65119095	9.80754E-06	0.086	360770	13265.712
genus Oxalobacter	Vascular dementia (subcortical)	rs10919863	T	C	-0.018	200226041	0.573	0.027	14306	T	C	0.315	1	200256913	3.47112E-06	0.068	360770	10813.753
genus Oxalobacter	Vascular dementia (subcortical)	rs11986558	T	C	0.019	2500772	0.375	0.022	14306	T	C	0.248	8	2643276	9.16875E-06	0.056	360770	11030.353
genus Oxalobacter	Vascular dementia (subcortical)	rs1363668	G	A	0.001	143089582	0.944	0.021	14306	G	A	-0.272	5	143710017	5.25352E-06	0.060	360770	12697.214
genus Oxalobacter	Vascular dementia (subcortical)	rs3802793	A	G	-0.008	131685316	0.694	0.022	14306	A	G	0.274	11	131815422	5.46626E-06	0.060	360770	12758.334
genus Oxalobacter	Vascular dementia (subcortical)	rs429358	C	T	0.034	45411941	0.200	0.030	14306	C	T	0.597	19	44908684	1.74221E-17	0.070	360770	41621.629
genus Parabacteroides	Vascular dementia (subcortical)	rs10919863	T	C	-0.006	200226041	0.650	0.014	14306	T	C	0.315	1	200256913	3.47112E-06	0.068	360770	10813.753
genus Parabacteroides	Vascular dementia (subcortical)	rs1363668	G	A	0.002	143089582	0.878	0.011	14306	G	A	-0.272	5	143710017	5.25352E-06	0.060	360770	12697.214
genus Parabacteroides	Vascular dementia (subcortical)	rs3802793	A	G	0.000	131685316	0.996	0.011	14306	A	G	0.274	11	131815422	5.46626E-06	0.060	360770	12758.334
genus Parabacteroides	Vascular dementia (subcortical)	rs429358	C	T	-0.009	45411941	0.601	0.015	14306	C	T	0.597	19	44908684	1.74221E-17	0.070	360770	41621.629
genus Parabacteroides	Vascular dementia (subcortical)	rs4295569	C	T	0.003	47820641	0.766	0.011	14306	C	T	-0.355	7	47781043	2.54572E-08	0.064	360770	20037.409
genus Paraprevotella	Vascular dementia (subcortical)	rs10919863	T	C	-0.007	200226041	0.736	0.021	14306	T	C	0.315	1	200256913	3.47112E-06	0.068	360770	10813.753
genus Paraprevotella	Vascular dementia (subcortical)	rs11148372	A	G	-0.010	22788665	0.578	0.017	14306	A	G	-0.261	13	22214526	4.06275E-06	0.057	360770	12632.549
genus Paraprevotella	Vascular dementia (subcortical)	rs3802793	A	G	-0.003	131685316	0.897	0.018	14306	A	G	0.274	11	131815422	5.46626E-06	0.060	360770	12758.334
genus Paraprevotella	Vascular dementia (subcortical)	rs429358	C	T	-0.005	45411941	0.844	0.024	14306	C	T	0.597	19	44908684	1.74221E-17	0.070	360770	41621.629
genus Paraprevotella	Vascular dementia (subcortical)	rs4295569	C	T	0.012	47820641	0.492	0.017	14306	C	T	-0.355	7	47781043	2.54572E-08	0.064	360770	20037.409
genus Paraprevotella	Vascular dementia (subcortical)	rs4382795	C	T	0.004	66878853	0.829	0.022	14306	C	T	0.378	10	65119095	9.80754E-06	0.086	360770	13265.712
genus Paraprevotella	Vascular dementia (subcortical)	rs4723291	A	G	0.002	33551998	0.821	0.017	14306	A	G	-0.263	7	33512386	6.22372E-06	0.058	360770	11064.499
genus Parasutterella	Vascular dementia (subcortical)	rs10919863	T	C	0.005	200226041	0.808	0.017	14306	T	C	0.315	1	200256913	3.47112E-06	0.068	360770	10813.753
genus Parasutterella	Vascular dementia (subcortical)	rs11148372	A	G	-0.002	22788665	0.919	0.014	14306	A	G	-0.261	13	22214526	4.06275E-06	0.057	360770	12632.549
genus Parasutterella	Vascular dementia (subcortical)	rs11986558	T	C	-0.007	2500772	0.631	0.014	14306	T	C	0.248	8	2643276	9.16875E-06	0.056	360770	11030.353
genus Parasutterella	Vascular dementia (subcortical)	rs1363668	G	A	0.001	143089582	0.956	0.013	14306	G	A	-0.272	5	143710017	5.25352E-06	0.060	360770	12697.214
genus Parasutterella	Vascular dementia (subcortical)	rs3802793	A	G	-0.012	131685316	0.376	0.014	14306	A	G	0.274	11	131815422	5.46626E-06	0.060	360770	12758.334

genus Parasutterella	Vascular dementia (subcortical)	rs4723291	A	G	-0.006	33551998	0.689	0.013	14306	A	G	-0.263	7	33512386	6.22372E-06	0.058	360770	11064.499
genus Peptococcus	Vascular dementia (subcortical)	rs10919863	T	C	0.005	200226041	0.787	0.025	14306	T	C	0.315	1	200256913	3.47112E-06	0.068	360770	10813.753
genus Peptococcus	Vascular dementia (subcortical)	rs11148372	A	G	0.004	22788665	0.820	0.019	14306	A	G	-0.261	13	22214526	4.06275E-06	0.057	360770	12632.549
genus Peptococcus	Vascular dementia (subcortical)	rs11986558	T	C	-0.003	2500772	0.890	0.020	14306	T	C	0.248	8	2643276	9.16875E-06	0.056	360770	11030.353
genus Peptococcus	Vascular dementia (subcortical)	rs1363668	G	A	0.001	143089582	0.962	0.019	14306	G	A	-0.272	5	143710017	5.25352E-06	0.060	360770	12697.214
genus Peptococcus	Vascular dementia (subcortical)	rs3802793	A	G	0.001	131685316	0.894	0.020	14306	A	G	0.274	11	131815422	5.46626E-06	0.060	360770	12758.334
genus Peptococcus	Vascular dementia (subcortical)	rs429358	C	T	0.031	45411941	0.231	0.027	14306	C	T	0.597	19	44908684	1.74221E-17	0.070	360770	41621.629
genus Peptococcus	Vascular dementia (subcortical)	rs4295569	C	T	0.008	47820641	0.671	0.020	14306	C	T	-0.355	7	47781043	2.54572E-08	0.064	360770	20037.409
genus Peptococcus	Vascular dementia (subcortical)	rs4382795	C	T	-0.012	66878853	0.638	0.025	14306	C	T	0.378	10	65119095	9.80754E-06	0.086	360770	13265.712
genus Peptococcus	Vascular dementia (subcortical)	rs4723291	A	G	-0.009	33551998	0.649	0.019	14306	A	G	-0.263	7	33512386	6.22372E-06	0.058	360770	11064.499
genus Phascolarctobacterium	Vascular dementia (subcortical)	rs10919863	T	C	-0.006	200226041	0.681	0.017	14306	T	C	0.315	1	200256913	3.47112E-06	0.068	360770	10813.753
genus Phascolarctobacterium	Vascular dementia (subcortical)	rs11986558	T	C	-0.002	2500772	0.900	0.014	14306	T	C	0.248	8	2643276	9.16875E-06	0.056	360770	11030.353
genus Phascolarctobacterium	Vascular dementia (subcortical)	rs1363668	G	A	0.002	143089582	0.870	0.014	14306	G	A	-0.272	5	143710017	5.25352E-06	0.060	360770	12697.214
genus Phascolarctobacterium	Vascular dementia (subcortical)	rs429358	C	T	-0.013	45411941	0.427	0.019	14306	C	T	0.597	19	44908684	1.74221E-17	0.070	360770	41621.629
genus Phascolarctobacterium	Vascular dementia (subcortical)	rs4295569	C	T	-0.004	47820641	0.741	0.014	14306	C	T	-0.355	7	47781043	2.54572E-08	0.064	360770	20037.409
genus Phascolarctobacterium	Vascular dementia (subcortical)	rs4382795	C	T	0.008	66878853	0.692	0.018	14306	C	T	0.378	10	65119095	9.80754E-06	0.086	360770	13265.712
genus Phascolarctobacterium	Vascular dementia (subcortical)	rs4723291	A	G	-0.011	33551998	0.388	0.014	14306	A	G	-0.263	7	33512386	6.22372E-06	0.058	360770	11064.499
genus Prevotella7	Vascular dementia (subcortical)	rs11148372	A	G	-0.006	22788665	0.840	0.025	14306	A	G	-0.261	13	22214526	4.06275E-06	0.057	360770	12632.549
genus Prevotella7	Vascular dementia (subcortical)	rs11986558	T	C	0.022	2500772	0.418	0.025	14306	T	C	0.248	8	2643276	9.16875E-06	0.056	360770	11030.353
genus Prevotella7	Vascular dementia (subcortical)	rs1363668	G	A	0.016	143089582	0.529	0.024	14306	G	A	-0.272	5	143710017	5.25352E-06	0.060	360770	12697.214
genus Prevotella7	Vascular dementia (subcortical)	rs3802793	A	G	0.004	131685316	0.837	0.025	14306	A	G	0.274	11	131815422	5.46626E-06	0.060	360770	12758.334
genus Prevotella7	Vascular dementia (subcortical)	rs429358	C	T	0.047	45411941	0.135	0.034	14306	C	T	0.597	19	44908684	1.74221E-17	0.070	360770	41621.629
genus Prevotella7	Vascular dementia (subcortical)	rs4295569	C	T	0.004	47820641	0.874	0.025	14306	C	T	-0.355	7	47781043	2.54572E-08	0.064	360770	20037.409
genus Prevotella7	Vascular dementia (subcortical)	rs4382795	C	T	0.010	66878853	0.709	0.032	14306	C	T	0.378	10	65119095	9.80754E-06	0.086	360770	13265.712
genus Prevotella9	Vascular dementia (subcortical)	rs11986558	T	C	-0.005	2500772	0.744	0.015	14306	T	C	0.248	8	2643276	9.16875E-06	0.056	360770	11030.353
genus Prevotella9	Vascular dementia (subcortical)	rs3802793	A	G	0.004	131685316	0.798	0.015	14306	A	G	0.274	11	131815422	5.46626E-06	0.060	360770	12758.334
genus Prevotella9	Vascular dementia (subcortical)	rs429358	C	T	0.022	45411941	0.310	0.020	14306	C	T	0.597	19	44908684	1.74221E-17	0.070	360770	41621.629
genus Prevotella9	Vascular dementia (subcortical)	rs4295569	C	T	0.002	47820641	0.910	0.015	14306	C	T	-0.355	7	47781043	2.54572E-08	0.064	360770	20037.409
genus Prevotella9	Vascular dementia (subcortical)	rs4723291	A	G	0.001	33551998	0.975	0.014	14306	A	G	-0.263	7	33512386	6.22372E-06	0.058	360770	11064.499
genus Rikenellaceae RC9 gut group	Vascular dementia (subcortical)	rs10919863	T	C	-0.028	200226041	0.399	0.032	14306	T	C	0.315	1	200256913	3.47112E-06	0.068	360770	10813.753
genus Rikenellaceae RC9 gut group	Vascular dementia (subcortical)	rs11148372	A	G	-0.007	22788665	0.796	0.025	14306	A	G	-0.261	13	22214526	4.06275E-06	0.057	360770	12632.549
genus Rikenellaceae RC9 gut group	Vascular dementia (subcortical)	rs1363668	G	A	0.017	143089582	0.510	0.025	14306	G	A	-0.272	5	143710017	5.25352E-06	0.060	360770	12697.214
genus Rikenellaceae RC9 gut group	Vascular dementia (subcortical)	rs3802793	A	G	0.010	131685316	0.704	0.026	14306	A	G	0.274	11	131815422	5.46626E-06	0.060	360770	12758.334
genus Rikenellaceae RC9 gut group	Vascular dementia (subcortical)	rs4295569	C	T	0.004	47820641	0.888	0.026	14306	C	T	-0.355	7	47781043	2.54572E-08	0.064	360770	20037.409
genus Rikenellaceae RC9 gut group	Vascular dementia (subcortical)	rs4382795	C	T	0.013	66878853	0.685	0.032	14306	C	T	0.378	10	65119095	9.80754E-06	0.086	360770	13265.712
genus Rikenellaceae RC9 gut group	Vascular dementia (subcortical)	rs4723291	A	G	0.018	33551998	0.479	0.025	14306	A	G	-0.263	7	33512386	6.22372E-06	0.058	360770	11064.499
genus Romboutsia	Vascular dementia (subcortical)	rs10919863	T	C	0.000	200226041	0.941	0.015	14306	T	C	0.315	1	200256913	3.47112E-06	0.068	360770	10813.753
genus Romboutsia	Vascular dementia (subcortical)	rs11148372	A	G	-0.002	22788665	0.914	0.012	14306	A	G	-0.261	13	22214526	4.06275E-06	0.057	360770	12632.549
genus Romboutsia	Vascular dementia (subcortical)	rs11986558	T	C	0.007	2500772	0.578	0.013	14306	T	C	0.248	8	2643276	9.16875E-06	0.056	360770	11030.353
genus Romboutsia	Vascular dementia (subcortical)	rs1363668	G	A	0.007	143089582	0.548	0.012	14306	G	A	-0.272	5	143710017	5.25352E-06	0.060	360770	12697.214
genus Romboutsia	Vascular dementia (subcortical)	rs3802793	A	G	-0.008	131685316	0.461	0.013	14306	A	G	0.274	11	131815422	5.46626E-06	0.060	360770	12758.334
genus Romboutsia	Vascular dementia (subcortical)	rs429358	C	T	0.005	45411941	0.703	0.017	14306	C	T	0.597	19	44908684	1.74221E-17	0.070	360770	41621.629
genus Roseburia	Vascular dementia (subcortical)	rs10919863	T	C	0.004	200226041	0.778	0.014	14306	T	C	0.315	1	200256913	3.47112E-06	0.068	360770	10813.753
genus Roseburia	Vascular dementia (subcortical)	rs11986558	T	C	-0.008	2500772	0.467	0.011	14306	T	C	0.248	8	2643276	9.16875E-06	0.056	360770	11030.353
genus Roseburia	Vascular dementia (subcortical)	rs1363668	G	A	-0.009	143089582	0.378	0.011	14306	G	A	-0.272	5	143710017	5.25352E-06	0.060	360770	12697.214
genus Roseburia	Vascular dementia (subcortical)	rs429358	C	T	-0.001	45411941	0.921	0.015	14306	C	T	0.597	19	44908684	1.74221E-17	0.070	360770	41621.629
genus Roseburia	Vascular dementia (subcortical)	rs4295569	C	T	0.006	47820641	0.577	0.011	14306	C	T	-0.355	7	47781043	2.54572E-08	0.064	360770	20037.409
genus Roseburia	Vascular dementia (subcortical)	rs4723291	A	G	0.002	33551998	0.804	0.011	14306	A	G	-0.263	7	33512386	6.22372E-06	0.058	360770	11064.499
genus Ruminiclostridium5	Vascular dementia (subcortical)	rs10919863	T	C	0.008	200226041	0.547	0.014	14306	T	C	0.315	1	200256913	3.47112E-06	0.068	360770	10813.753
genus Ruminiclostridium5	Vascular dementia (subcortical)	rs1363668	G	A	-0.008	143089582	0.486	0.011	14306	G	A	-0.272	5	143710017	5.25352E-06	0.060	360770	12697.214
genus Ruminiclostridium5	Vascular dementia (subcortical)	rs429358	C	T	0.022	45411941	0.184	0.015	14306	C	T	0.597	19	44908684	1.74221E-17	0.070	360770	41621.629
genus Ruminiclostridium5	Vascular dementia (subcortical)	rs4295569	C	T	0.004	47820641	0.694	0.011	14306	C	T	-0.355	7	47781043	2.54572E-08	0.064	360770	20037.409
genus Ruminiclostridium5	Vascular dementia (subcortical)	rs4382795	C	T	0.002	66878853	0.857	0.014	14306	C	T	0.378	10	65119095	9.80754E-06	0.086	360770	13265.712
genus Ruminiclostridium6	Vascular dementia (subcortical)	rs11986558	T	C	-0.010	2500772	0.471	0.013	14306	T	C	0.248	8	2643276	9.16875E-06	0.056	360770	11030.353
genus Ruminiclostridium6	Vascular dementia (subcortical)	rs429358	C	T	0.007	45411941	0.690	0.018	14306	C	T	0.597	19	44908684	1.74221E-17	0.070	360770	41621.629
genus Ruminiclostridium6	Vascular dementia (subcortical)	rs4382795	C	T	-0.004	66878853	0.840	0.016	14306	C	T	0.378	10	65119095	9.80754E-06	0.086	360770	13265.712
genus Ruminiclostridium6	Vascular dementia (subcortical)	rs4723291	A	G	0.007	33551998	0.591	0.012	14306	A	G	-0.263	7	33512386	6.22372E-06	0.058	360770	11064.499
genus Ruminiclostridium9	Vascular dementia (subcortical)	rs10919863	T	C	0.009	200226041	0.512	0.014	14306	T	C	0.315	1	200256913	3.47112E-06	0.068	360770	10813.753
genus Ruminiclostridium9	Vascular dementia (subcortical)	rs3802793	A	G	0.007	131685316	0.533	0.012	14306	A	G	0.274	11	131815422	5.46626E-06	0.060	360770	12758.334
genus Ruminiclostridium9	Vascular dementia (subcortical)	rs429358	C	T	-0.007	45411941	0.627	0.016	14306	C	T	0.597	19	44908684	1.74221E-17	0.070	360770	41621.629
genus Ruminiclostridium9	Vascular dementia (subcortical)	rs4295569	C	T	-0.004	47820641	0.712	0.011	14306	C	T	-0.355	7	47781043	2.54572E-08	0.064	360770	20037.409
genus Ruminiclostridium9	Vascular dementia (subcortical)	rs4723291	A	G	-0.010	33551998	0.378	0.011	14306	A	G	-0.263	7	33512386	6.22372E-06	0.058	360770	11064.499
genus Ruminococcaceae NK4A214 group	Vascular dementia (subcortical)	rs10919863	T	C	-0.005	200226041	0.677	0.015	14306	T	C	0.315	1	200256913	3.47112E-06	0.068	360770	10813.753
genus Ruminococcaceae NK4A214 group	Vascular dementia (subcortical)	rs11148372	A	G	-0.006	22788665	0.598	0.012	14306	A	G	-0.261	13	22214526	4.06275E-06	0.057	360770	12632.549
genus Ruminococcaceae NK4A214 group	Vascular dementia (subcortical)	rs1363668	G	A	-0.010	143089582	0.369	0.012	14306	G	A	-0.272	5	143710017	5.25352E-06	0.060	360770	12697.214
genus Ruminococcaceae NK4A214 group	Vascular dementia (subcortical)	rs3802793	A	G	0.000	131685316	0.958	0.012	14306	A	G	0.274	11	131815422	5.46626E-06	0.060	360770	12758.334
genus Ruminococcaceae NK4A214 group	Vascular dementia (subcortical)	rs429358	C	T	0.002	45411941	0.898	0.016	14306	C	T	0.597	19	44908684	1.74221E-17	0.070	360770	41621.629
genus Ruminococcaceae NK4A214 group	Vascular dementia (subcortical)	rs4723291	A	G	-0.006	33551998	0.581	0.012	14306	A	G	-0.263	7	33512386	6.22372E-06	0.058	360770	11064.499
genus Ruminococcaceae UCG002	Vascular dementia (subcortical)	rs10919863	T	C	-0.006	200226041	0.607	0.014	14306	T	C	0.315	1	200256913	3.47112E-06	0.068	360770	10813.753
genus Ruminococcaceae UCG002	Vascular dementia (subcortical)	rs11986558	T	C	0.000	2500772	0.977	0.011	14306	T	C	0.248	8	2643276	9.16875E-06	0.056	360770	11030.353
genus Ruminococcaceae UCG002	Vascular dementia (subcortical)	rs1363668	G	A	0.000	143089582	0.972	0.011	14306	G	A	-0.272	5	143710017	5.25352E-06	0.060	360770	12697.214

genus Ruminococcaceae UCG002	Vascular dementia (subcortical)	rs3802793	A	G	0.002	131685316	0.903	0.012	14306	A	G	0.274	11	131815422	5.46626E-06	0.060	360770	12758.334
genus Ruminococcaceae UCG002	Vascular dementia (subcortical)	rs429358	C	T	-0.004	45411941	0.869	0.016	14306	C	T	0.597	19	44908684	1.74221E-17	0.070	360770	41621.629
genus Ruminococcaceae UCG002	Vascular dementia (subcortical)	rs4295569	C	T	-0.005	47820641	0.670	0.011	14306	C	T	-0.355	7	47781043	2.54572E-08	0.064	360770	20037.409
genus Ruminococcaceae UCG002	Vascular dementia (subcortical)	rs4723291	A	G	0.005	33551998	0.691	0.011	14306	A	G	-0.263	7	33512386	6.22372E-06	0.058	360770	11064.499
genus Ruminococcaceae UCG003	Vascular dementia (subcortical)	rs10919863	T	C	-0.006	200226041	0.689	0.015	14306	T	C	0.315	1	200256913	3.47112E-06	0.068	360770	10813.753
genus Ruminococcaceae UCG003	Vascular dementia (subcortical)	rs1363668	G	A	0.002	143089582	0.850	0.012	14306	G	A	-0.272	5	143710017	5.25352E-06	0.060	360770	12697.214
genus Ruminococcaceae UCG003	Vascular dementia (subcortical)	rs3802793	A	G	-0.003	131685316	0.827	0.013	14306	A	G	0.274	11	131815422	5.46626E-06	0.060	360770	12758.334
genus Ruminococcaceae UCG003	Vascular dementia (subcortical)	rs429358	C	T	0.007	45411941	0.595	0.017	14306	C	T	0.597	19	44908684	1.74221E-17	0.070	360770	41621.629
genus Ruminococcaceae UCG003	Vascular dementia (subcortical)	rs4295569	C	T	0.007	47820641	0.602	0.012	14306	C	T	-0.355	7	47781043	2.54572E-08	0.064	360770	20037.409
genus Ruminococcaceae UCG003	Vascular dementia (subcortical)	rs4382795	C	T	-0.006	66878853	0.694	0.016	14306	C	T	0.378	10	65119095	9.80754E-06	0.086	360770	13265.712
genus Ruminococcaceae UCG004	Vascular dementia (subcortical)	rs10919863	T	C	-0.016	200226041	0.419	0.019	14306	T	C	0.315	1	200256913	3.47112E-06	0.068	360770	10813.753
genus Ruminococcaceae UCG004	Vascular dementia (subcortical)	rs11148372	A	G	-0.007	22788665	0.631	0.015	14306	A	G	-0.261	13	22214526	4.06275E-06	0.057	360770	12632.549
genus Ruminococcaceae UCG004	Vascular dementia (subcortical)	rs11986558	T	C	-0.007	2500772	0.627	0.015	14306	T	C	0.248	8	2643276	9.16875E-06	0.056	360770	11030.353
genus Ruminococcaceae UCG004	Vascular dementia (subcortical)	rs1363668	G	A	0.003	143089582	0.855	0.015	14306	G	A	-0.272	5	143710017	5.25352E-06	0.060	360770	12697.214
genus Ruminococcaceae UCG004	Vascular dementia (subcortical)	rs3802793	A	G	0.005	131685316	0.804	0.015	14306	A	G	0.274	11	131815422	5.46626E-06	0.060	360770	12758.334
genus Ruminococcaceae UCG004	Vascular dementia (subcortical)	rs429358	C	T	0.025	45411941	0.252	0.021	14306	C	T	0.597	19	44908684	1.74221E-17	0.070	360770	41621.629
genus Ruminococcaceae UCG004	Vascular dementia (subcortical)	rs4295569	C	T	0.005	47820641	0.741	0.015	14306	C	T	-0.355	7	47781043	2.54572E-08	0.064	360770	20037.409
genus Ruminococcaceae UCG004	Vascular dementia (subcortical)	rs4723291	A	G	-0.003	33551998	0.836	0.015	14306	A	G	-0.263	7	33512386	6.22372E-06	0.058	360770	11064.499
genus Ruminococcaceae UCG005	Vascular dementia (subcortical)	rs10919863	T	C	-0.008	200226041	0.612	0.014	14306	T	C	0.315	1	200256913	3.47112E-06	0.068	360770	10813.753
genus Ruminococcaceae UCG005	Vascular dementia (subcortical)	rs11148372	A	G	0.004	22788665	0.731	0.011	14306	A	G	-0.261	13	22214526	4.06275E-06	0.057	360770	12632.549
genus Ruminococcaceae UCG005	Vascular dementia (subcortical)	rs11986558	T	C	-0.002	2500772	0.896	0.012	14306	T	C	0.248	8	2643276	9.16875E-06	0.056	360770	11030.353
genus Ruminococcaceae UCG005	Vascular dementia (subcortical)	rs1363668	G	A	0.001	143089582	0.909	0.011	14306	G	A	-0.272	5	143710017	5.25352E-06	0.060	360770	12697.214
genus Ruminococcaceae UCG005	Vascular dementia (subcortical)	rs3802793	A	G	0.003	131685316	0.804	0.012	14306	A	G	0.274	11	131815422	5.46626E-06	0.060	360770	12758.334
genus Ruminococcaceae UCG005	Vascular dementia (subcortical)	rs429358	C	T	0.004	45411941	0.741	0.016	14306	C	T	0.597	19	44908684	1.74221E-17	0.070	360770	41621.629
genus Ruminococcaceae UCG005	Vascular dementia (subcortical)	rs4723291	A	G	0.008	33551998	0.476	0.011	14306	A	G	-0.263	7	33512386	6.22372E-06	0.058	360770	11064.499
genus Ruminococcaceae UCG009	Vascular dementia (subcortical)	rs10919863	T	C	0.013	200226041	0.430	0.021	14306	T	C	0.315	1	200256913	3.47112E-06	0.068	360770	10813.753
genus Ruminococcaceae UCG009	Vascular dementia (subcortical)	rs11148372	A	G	-0.006	22788665	0.705	0.017	14306	A	G	-0.261	13	22214526	4.06275E-06	0.057	360770	12632.549
genus Ruminococcaceae UCG009	Vascular dementia (subcortical)	rs11986558	T	C	-0.004	2500772	0.822	0.017	14306	T	C	0.248	8	2643276	9.16875E-06	0.056	360770	11030.353
genus Ruminococcaceae UCG009	Vascular dementia (subcortical)	rs1363668	G	A	-0.012	143089582	0.466	0.017	14306	G	A	-0.272	5	143710017	5.25352E-06	0.060	360770	12697.214
genus Ruminococcaceae UCG009	Vascular dementia (subcortical)	rs3802793	A	G	0.003	131685316	0.930	0.017	14306	A	G	0.274	11	131815422	5.46626E-06	0.060	360770	12758.334
genus Ruminococcaceae UCG009	Vascular dementia (subcortical)	rs4295569	C	T	-0.002	47820641	0.880	0.017	14306	C	T	-0.355	7	47781043	2.54572E-08	0.064	360770	20037.409
genus Ruminococcaceae UCG009	Vascular dementia (subcortical)	rs4382795	C	T	0.003	66878853	0.845	0.022	14306	C	T	0.378	10	65119095	9.80754E-06	0.086	360770	13265.712
genus Ruminococcaceae UCG009	Vascular dementia (subcortical)	rs4723291	A	G	0.012	33551998	0.469	0.016	14306	A	G	-0.263	7	33512386	6.22372E-06	0.058	360770	11064.499
genus Ruminococcaceae UCG010	Vascular dementia (subcortical)	rs11148372	A	G	0.007	22788665	0.575	0.013	14306	A	G	-0.261	13	22214526	4.06275E-06	0.057	360770	12632.549
genus Ruminococcaceae UCG010	Vascular dementia (subcortical)	rs429358	C	T	0.003	45411941	0.881	0.018	14306	C	T	0.597	19	44908684	1.74221E-17	0.070	360770	41621.629
genus Ruminococcaceae UCG011	Vascular dementia (subcortical)	rs10919863	T	C	-0.003	200226041	0.944	0.031	14306	T	C	0.315	1	200256913	3.47112E-06	0.068	360770	10813.753
genus Ruminococcaceae UCG011	Vascular dementia (subcortical)	rs1363668	G	A	0.018	143089582	0.428	0.024	14306	G	A	-0.272	5	143710017	5.25352E-06	0.060	360770	12697.214
genus Ruminococcaceae UCG011	Vascular dementia (subcortical)	rs3802793	A	G	0.017	131685316	0.494	0.025	14306	A	G	0.274	11	131815422	5.46626E-06	0.060	360770	12758.334
genus Ruminococcaceae UCG011	Vascular dementia (subcortical)	rs429358	C	T	-0.013	45411941	0.704	0.033	14306	C	T	0.597	19	44908684	1.74221E-17	0.070	360770	41621.629
genus Ruminococcaceae UCG013	Vascular dementia (subcortical)	rs3802793	A	G	0.001	131685316	0.975	0.012	14306	A	G	0.274	11	131815422	5.46626E-06	0.060	360770	12758.334
genus Ruminococcaceae UCG013	Vascular dementia (subcortical)	rs429358	C	T	-0.022	45411941	0.167	0.016	14306	C	T	0.597	19	44908684	1.74221E-17	0.070	360770	41621.629
genus Ruminococcaceae UCG013	Vascular dementia (subcortical)	rs4382795	C	T	0.005	66878853	0.808	0.014	14306	C	T	0.378	10	65119095	9.80754E-06	0.086	360770	13265.712
genus Ruminococcaceae UCG014	Vascular dementia (subcortical)	rs1363668	G	A	-0.010	143089582	0.401	0.012	14306	G	A	-0.272	5	143710017	5.25352E-06	0.060	360770	12697.214
genus Ruminococcaceae UCG014	Vascular dementia (subcortical)	rs429358	C	T	0.020	45411941	0.191	0.017	14306	C	T	0.597	19	44908684	1.74221E-17	0.070	360770	41621.629
genus Ruminococcus1	Vascular dementia (subcortical)	rs11986558	T	C	0.000	2500772	0.973	0.012	14306	T	C	0.248	8	2643276	9.16875E-06	0.056	360770	11030.353
genus Ruminococcus1	Vascular dementia (subcortical)	rs3802793	A	G	0.003	131685316	0.755	0.012	14306	A	G	0.274	11	131815422	5.46626E-06	0.060	360770	12758.334
genus Ruminococcus1	Vascular dementia (subcortical)	rs429358	C	T	-0.008	45411941	0.683	0.016	14306	C	T	0.597	19	44908684	1.74221E-17	0.070	360770	41621.629
genus Ruminococcus1	Vascular dementia (subcortical)	rs4382795	C	T	-0.009	66878853	0.538	0.014	14306	C	T	0.378	10	65119095	9.80754E-06	0.086	360770	13265.712
genus Ruminococcus1	Vascular dementia (subcortical)	rs4723291	A	G	0.001	33551998	0.919	0.011	14306	A	G	-0.263	7	33512386	6.22372E-06	0.058	360770	11064.499
genus Ruminococcus2	Vascular dementia (subcortical)	rs10919863	T	C	-0.002	200226041	0.916	0.015	14306	T	C	0.315	1	200256913	3.47112E-06	0.068	360770	10813.753
genus Ruminococcus2	Vascular dementia (subcortical)	rs11986558	T	C	-0.001	2500772	0.969	0.012	14306	T	C	0.248	8	2643276	9.16875E-06	0.056	360770	11030.353
genus Ruminococcus2	Vascular dementia (subcortical)	rs429358	C	T	0.007	45411941	0.633	0.016	14306	C	T	0.597	19	44908684	1.74221E-17	0.070	360770	41621.629
genus Ruminococcus2	Vascular dementia (subcortical)	rs4382795	C	T	-0.002	66878853	0.924	0.015	14306	C	T	0.378	10	65119095	9.80754E-06	0.086	360770	13265.712
genus Ruminococcus gauvreauii group	Vascular dementia (subcortical)	rs11148372	A	G	-0.008	22788665	0.496	0.013	14306	A	G	-0.261	13	22214526	4.06275E-06	0.057	360770	12632.549
genus Ruminococcus gauvreauii group	Vascular dementia (subcortical)	rs11986558	T	C	0.010	2500772	0.454	0.013	14306	T	C	0.248	8	2643276	9.16875E-06	0.056	360770	11030.353
genus Ruminococcus gauvreauii group	Vascular dementia (subcortical)	rs1363668	G	A	0.007	143089582	0.606	0.012	14306	G	A	-0.272	5	143710017	5.25352E-06	0.060	360770	12697.214
genus Ruminococcus gauvreauii group	Vascular dementia (subcortical)	rs429358	C	T	0.011	45411941	0.457	0.018	14306	C	T	0.597	19	44908684	1.74221E-17	0.070	360770	41621.629
genus Ruminococcus gauvreauii group	Vascular dementia (subcortical)	rs4295569	C	T	0.012	47820641	0.331	0.013	14306	C	T	-0.355	7	47781043	2.54572E-08	0.064	360770	20037.409
genus Ruminococcus gauvreauii group	Vascular dementia (subcortical)	rs4723291	A	G	0.007	33551998	0.577	0.012	14306	A	G	-0.263	7	33512386	6.22372E-06	0.058	360770	11064.499
genus Ruminococcus gnavus group	Vascular dementia (subcortical)	rs1363668	G	A	0.009	143089582	0.613	0.018	14306	G	A	-0.272	5	143710017	5.25352E-06	0.060	360770	12697.214
genus Ruminococcus gnavus group	Vascular dementia (subcortical)	rs3802793	A	G	0.001	131685316	0.934	0.019	14306	A	G	0.274	11	131815422	5.46626E-06	0.060	360770	12758.334
genus Ruminococcus gnavus group	Vascular dementia (subcortical)	rs429358	C	T	0.039	45411941	0.188	0.026	14306	C	T	0.597	19	44908684	1.74221E-17	0.070	360770	41621.629
genus Ruminococcus gnavus group	Vascular dementia (subcortical)	rs4295569	C	T	0.012	47820641	0.510	0.018	14306	C	T	-0.355	7	47781043	2.54572E-08	0.064	360770	20037.409
genus Ruminococcus gnavus group	Vascular dementia (subcortical)	rs4382795	C	T	0.018	66878853	0.481	0.023	14306	C	T	0.378	10	65119095	9.80754E-06	0.086	360770	13265.712
genus Ruminococcus gnavus group	Vascular dementia (subcortical)	rs4723291	A	G	0.006	33551998	0.730	0.018	14306	A	G	-0.263	7	33512386	6.22372E-06	0.058	360770	11064.499
genus Ruminococcus torques group	Vascular dementia (subcortical)	rs10919863	T	C	0.001	200226041	0.901	0.014	14306	T	C	0.315	1	200256913	3.47112E-06	0.068	360770	10813.753
genus Ruminococcus torques group	Vascular dementia (subcortical)	rs11148372	A	G	0.003	22788665	0.808	0.011	14306	A	G	-0.261	13	22214526	4.06275E-06	0.057	360770	12632.549
genus Ruminococcus torques group	Vascular dementia (subcortical)	rs11986558	T	C	-0.003	2500772	0.739	0.011	14306	T	C	0.248	8	2643276	9.16875E-06	0.056	360770	11030.353
genus Ruminococcus torques group	Vascular dementia (subcortical)	rs3802793	A	G	0.006	131685316	0.550	0.011	14306	A	G	0.274	11	131815422	5.46626E-06	0.060	360770	12758.334
genus Ruminococcus torques group	Vascular dementia (subcortical)	rs429358	C	T	-0.001	45411941	0.916	0.015	14306	C	T	0.597	19	44908684	1.74221E-17	0.070	360770	41621.629
genus Ruminococcus torques group	Vascular dementia (subcortical)	rs4295569	C	T	0.012	47820641	0.290	0.011	14306	C	T	-0.355	7	47781043	2.54572E-08	0.064	360770	20037.409

genus Ruminococcus torques group	Vascular dementia (subcortical)	rs4382795	C	T	0.008	66878853	0.503	0.014	14306	C	T	0.378	10	65119095	9.80754E-06	0.086	360770	13265.712
genus Ruminococcus torques group	Vascular dementia (subcortical)	rs4723291	A	G	0.002	33551998	0.860	0.011	14306	A	G	-0.263	7	33512386	6.22372E-06	0.058	360770	11064.499
genus Sellimonas	Vascular dementia (subcortical)	rs10919863	T	C	-0.020	200226041	0.558	0.033	14306	T	C	0.315	1	200256913	3.47112E-06	0.068	360770	10813.753
genus Sellimonas	Vascular dementia (subcortical)	rs3802793	A	G	0.023	131685316	0.400	0.027	14306	A	G	0.274	11	131815422	5.46626E-06	0.060	360770	12758.334
genus Sellimonas	Vascular dementia (subcortical)	rs4382795	C	T	-0.028	66878853	0.411	0.034	14306	C	T	0.378	10	65119095	9.80754E-06	0.086	360770	13265.712
genus Sellimonas	Vascular dementia (subcortical)	rs4723291	A	G	0.016	33551998	0.540	0.025	14306	A	G	-0.263	7	33512386	6.22372E-06	0.058	360770	11064.499
genus Senegalimassilia	Vascular dementia (subcortical)	rs10919863	T	C	0.014	200226041	0.492	0.022	14306	T	C	0.315	1	200256913	3.47112E-06	0.068	360770	10813.753
genus Senegalimassilia	Vascular dementia (subcortical)	rs11148372	A	G	-0.010	22788665	0.549	0.017	14306	A	G	-0.261	13	22214526	4.06275E-06	0.057	360770	12632.549
genus Senegalimassilia	Vascular dementia (subcortical)	rs11986558	T	C	0.002	2500772	0.929	0.018	14306	T	C	0.248	8	2643276	9.16875E-06	0.056	360770	11030.353
genus Senegalimassilia	Vascular dementia (subcortical)	rs1363668	G	A	-0.003	143089582	0.876	0.017	14306	G	A	-0.272	5	143710017	5.25352E-06	0.060	360770	12697.214
genus Senegalimassilia	Vascular dementia (subcortical)	rs3802793	A	G	-0.014	131685316	0.405	0.018	14306	A	G	0.274	11	131815422	5.46626E-06	0.060	360770	12758.334
genus Senegalimassilia	Vascular dementia (subcortical)	rs4295569	C	T	-0.001	47820641	0.937	0.018	14306	C	T	-0.355	7	47781043	2.54572E-08	0.064	360770	20037.409
genus Senegalimassilia	Vascular dementia (subcortical)	rs4382795	C	T	0.007	66878853	0.744	0.023	14306	C	T	0.378	10	65119095	9.80754E-06	0.086	360770	13265.712
genus Slackia	Vascular dementia (subcortical)	rs429358	C	T	0.036	45411941	0.159	0.026	14306	C	T	0.597	19	44908684	1.74221E-17	0.070	360770	41621.629
genus Slackia	Vascular dementia (subcortical)	rs4295569	C	T	-0.005	47820641	0.785	0.019	14306	C	T	-0.355	7	47781043	2.54572E-08	0.064	360770	20037.409
genus Slackia	Vascular dementia (subcortical)	rs4382795	C	T	0.019	66878853	0.418	0.024	14306	C	T	0.378	10	65119095	9.80754E-06	0.086	360770	13265.712
genus Streptococcus	Vascular dementia (subcortical)	rs11148372	A	G	-0.002	22788665	0.827	0.011	14306	A	G	-0.261	13	22214526	4.06275E-06	0.057	360770	12632.549
genus Streptococcus	Vascular dementia (subcortical)	rs11986558	T	C	0.007	2500772	0.569	0.012	14306	T	C	0.248	8	2643276	9.16875E-06	0.056	360770	11030.353
genus Streptococcus	Vascular dementia (subcortical)	rs1363668	G	A	0.004	143089582	0.737	0.011	14306	G	A	-0.272	5	143710017	5.25352E-06	0.060	360770	12697.214
genus Streptococcus	Vascular dementia (subcortical)	rs429358	C	T	0.017	45411941	0.279	0.016	14306	C	T	0.597	19	44908684	1.74221E-17	0.070	360770	41621.629
genus Streptococcus	Vascular dementia (subcortical)	rs4295569	C	T	-0.003	47820641	0.773	0.012	14306	C	T	-0.355	7	47781043	2.54572E-08	0.064	360770	20037.409
genus Streptococcus	Vascular dementia (subcortical)	rs4382795	C	T	-0.012	66878853	0.382	0.015	14306	C	T	0.378	10	65119095	9.80754E-06	0.086	360770	13265.712
genus Subdoligranulum	Vascular dementia (subcortical)	rs10919863	T	C	-0.002	200226041	0.813	0.014	14306	T	C	0.315	1	200256913	3.47112E-06	0.068	360770	10813.753
genus Subdoligranulum	Vascular dementia (subcortical)	rs11148372	A	G	0.007	22788665	0.552	0.011	14306	A	G	-0.261	13	22214526	4.06275E-06	0.057	360770	12632.549
genus Subdoligranulum	Vascular dementia (subcortical)	rs11986558	T	C	0.002	2500772	0.863	0.011	14306	T	C	0.248	8	2643276	9.16875E-06	0.056	360770	11030.353
genus Subdoligranulum	Vascular dementia (subcortical)	rs1363668	G	A	-0.006	143089582	0.587	0.011	14306	G	A	-0.272	5	143710017	5.25352E-06	0.060	360770	12697.214
genus Subdoligranulum	Vascular dementia (subcortical)	rs3802793	A	G	-0.008	131685316	0.498	0.011	14306	A	G	0.274	11	131815422	5.46626E-06	0.060	360770	12758.334
genus Subdoligranulum	Vascular dementia (subcortical)	rs429358	C	T	-0.005	45411941	0.772	0.015	14306	C	T	0.597	19	44908684	1.74221E-17	0.070	360770	41621.629
genus Subdoligranulum	Vascular dementia (subcortical)	rs4382795	C	T	-0.002	66878853	0.885	0.014	14306	C	T	0.378	10	65119095	9.80754E-06	0.086	360770	13265.712
genus Sutterella	Vascular dementia (subcortical)	rs11986558	T	C	-0.001	2500772	0.903	0.013	14306	T	C	0.248	8	2643276	9.16875E-06	0.056	360770	11030.353
genus Sutterella	Vascular dementia (subcortical)	rs1363668	G	A	-0.001	143089582	0.955	0.012	14306	G	A	-0.272	5	143710017	5.25352E-06	0.060	360770	12697.214
genus Sutterella	Vascular dementia (subcortical)	rs3802793	A	G	-0.012	131685316	0.373	0.013	14306	A	G	0.274	11	131815422	5.46626E-06	0.060	360770	12758.334
genus Sutterella	Vascular dementia (subcortical)	rs429358	C	T	-0.028	45411941	0.121	0.018	14306	C	T	0.597	19	44908684	1.74221E-17	0.070	360770	41621.629
genus Sutterella	Vascular dementia (subcortical)	rs4295569	C	T	-0.009	47820641	0.493	0.013	14306	C	T	-0.355	7	47781043	2.54572E-08	0.064	360770	20037.409
genus Sutterella	Vascular dementia (subcortical)	rs4382795	C	T	0.002	66878853	0.935	0.016	14306	C	T	0.378	10	65119095	9.80754E-06	0.086	360770	13265.712
genus Sutterella	Vascular dementia (subcortical)	rs4723291	A	G	-0.002	33551998	0.885	0.012	14306	A	G	-0.263	7	33512386	6.22372E-06	0.058	360770	11064.499
genus Terrisporobacter	Vascular dementia (subcortical)	rs11148372	A	G	-0.004	22788665	0.822	0.018	14306	A	G	-0.261	13	22214526	4.06275E-06	0.057	360770	12632.549
genus Terrisporobacter	Vascular dementia (subcortical)	rs1363668	G	A	-0.011	143089582	0.542	0.018	14306	G	A	-0.272	5	143710017	5.25352E-06	0.060	360770	12697.214
genus Terrisporobacter	Vascular dementia (subcortical)	rs3802793	A	G	-0.001	131685316	0.942	0.018	14306	A	G	0.274	11	131815422	5.46626E-06	0.060	360770	12758.334
genus Terrisporobacter	Vascular dementia (subcortical)	rs429358	C	T	-0.010	45411941	0.657	0.025	14306	C	T	0.597	19	44908684	1.74221E-17	0.070	360770	41621.629
genus Terrisporobacter	Vascular dementia (subcortical)	rs4723291	A	G	0.001	33551998	0.924	0.017	14306	A	G	-0.263	7	33512386	6.22372E-06	0.058	360770	11064.499
genus Turicibacter	Vascular dementia (subcortical)	rs10919863	T	C	0.002	200226041	0.893	0.019	14306	T	C	0.315	1	200256913	3.47112E-06	0.068	360770	10813.753
genus Turicibacter	Vascular dementia (subcortical)	rs11148372	A	G	-0.013	22788665	0.409	0.015	14306	A	G	-0.261	13	22214526	4.06275E-06	0.057	360770	12632.549
genus Turicibacter	Vascular dementia (subcortical)	rs1363668	G	A	0.002	143089582	0.903	0.015	14306	G	A	-0.272	5	143710017	5.25352E-06	0.060	360770	12697.214
genus Turicibacter	Vascular dementia (subcortical)	rs3802793	A	G	-0.001	131685316	0.952	0.016	14306	A	G	0.274	11	131815422	5.46626E-06	0.060	360770	12758.334
genus Turicibacter	Vascular dementia (subcortical)	rs429358	C	T	0.001	45411941	0.870	0.021	14306	C	T	0.597	19	44908684	1.74221E-17	0.070	360770	41621.629
genus Tyzzerella3	Vascular dementia (subcortical)	rs10919863	T	C	0.012	200226041	0.551	0.025	14306	T	C	0.315	1	200256913	3.47112E-06	0.068	360770	10813.753
genus Tyzzerella3	Vascular dementia (subcortical)	rs11148372	A	G	0.005	22788665	0.797	0.020	14306	A	G	-0.261	13	22214526	4.06275E-06	0.057	360770	12632.549
genus Tyzzerella3	Vascular dementia (subcortical)	rs1363668	G	A	0.002	143089582	0.926	0.019	14306	G	A	-0.272	5	143710017	5.25352E-06	0.060	360770	12697.214
genus Tyzzerella3	Vascular dementia (subcortical)	rs429358	C	T	0.000	45411941	0.979	0.028	14306	C	T	0.597	19	44908684	1.74221E-17	0.070	360770	41621.629
genus Tyzzerella3	Vascular dementia (subcortical)	rs4295569	C	T	-0.007	47820641	0.742	0.020	14306	C	T	-0.355	7	47781043	2.54572E-08	0.064	360770	20037.409
genus Tyzzerella3	Vascular dementia (subcortical)	rs4723291	A	G	-0.009	33551998	0.675	0.019	14306	A	G	-0.263	7	33512386	6.22372E-06	0.058	360770	11064.499
genus Veillonella)	Vascular dementia (subcortical)	rs10919863	T	C	0.014	200226041	0.434	0.019	14306	T	C	0.315	1	200256913	3.47112E-06	0.068	360770	10813.753
genus Veillonella)	Vascular dementia (subcortical)	rs11148372	A	G	-0.004	22788665	0.816	0.015	14306	A	G	-0.261	13	22214526	4.06275E-06	0.057	360770	12632.549
genus Veillonella)	Vascular dementia (subcortical)	rs11986558	T	C	0.009	2500772	0.533	0.016	14306	T	C	0.248	8	2643276	9.16875E-06	0.056	360770	11030.353
genus Veillonella)	Vascular dementia (subcortical)	rs1363668	G	A	0.002	143089582	0.900	0.015	14306	G	A	-0.272	5	143710017	5.25352E-06	0.060	360770	12697.214
genus Veillonella)	Vascular dementia (subcortical)	rs3802793	A	G	-0.010	131685316	0.518	0.016	14306	A	G	0.274	11	131815422	5.46626E-06	0.060	360770	12758.334
genus Veillonella)	Vascular dementia (subcortical)	rs4295569	C	T	-0.017	47820641	0.269	0.015	14306	C	T	-0.355	7	47781043	2.54572E-08	0.064	360770	20037.409
genus Veillonella)	Vascular dementia (subcortical)	rs4382795	C	T	0.009	66878853	0.646	0.019	14306	C	T	0.378	10	65119095	9.80754E-06	0.086	360770	13265.712
genus Victivallis	Vascular dementia (subcortical)	rs10919863	T	C	-0.009	200226041	0.715	0.032	1531	T	C	0.315	1	200256913	3.47112E-06	0.068	360770	10813.753
genus Victivallis	Vascular dementia (subcortical)	rs11986558	T	C	0.001	2500772	0.950	0.026	1531	T	C	0.248	8	2643276	9.16875E-06	0.056	360770	11030.353
genus Victivallis	Vascular dementia (subcortical)	rs429358	C	T	0.009	45411941	0.847	0.037	1531	C	T	0.597	19	44908684	1.74221E-17	0.070	360770	41621.629
genus Victivallis	Vascular dementia (subcortical)	rs4295569	C	T	-0.001	47820641	0.938	0.026	1531	C	T	-0.355	7	47781043	2.54572E-08	0.064	360770	20037.409
genus Victivallis	Vascular dementia (subcortical)	rs4723291	A	G	0.006	33551998	0.790	0.025	1531	A	G	-0.263	7	33512386	6.22372E-06	0.058	360770	11064.499
genus Actinomyces	Vascular dementia (sudden onset)	rs12452096	A	G	0.005	75544032	0.776	0.019	14306	A	G	0.750	17	77547950	1.48929E-06	0.156	360283	77865.614
genus Actinomyces	Vascular dementia (sudden onset)	rs2920	C	T	-0.011	23884780	0.581	0.020	14306	C	T	0.592	1	23558289	2.20602E-06	0.125	360283	40501.384
genus Actinomyces	Vascular dementia (sudden onset)	rs4840457	C	T	0.001	6408682	0.977	0.030	14306	C	T	0.643	8	6551161	8.92874E-06	0.145	360283	32615.797
genus Actinomyces	Vascular dementia (sudden onset)	rs71511414	G	A	0.011	79688991	0.679	0.029	14306	G	A	0.681	9	77074075	7.80746E-06	0.152	360283	30731.948
genus Adlercreutzia	Vascular dementia (sudden onset)	rs12423672	T	G	0.014	5047705	0.611	0.026	14306	T	G	0.759	12	4938539	4.89159E-06	0.166	360283	30610.567
genus Adlercreutzia	Vascular dementia (sudden onset)	rs2920	C	T	-0.004	23884780	0.827	0.019	14306	C	T	0.592	1	23558289	2.20602E-06	0.125	360283	40501.384
genus Adlercreutzia	Vascular dementia (sudden onset)	rs429358	C	T	0.007	45411941	0.796	0.022	14306	C	T	0.601	19	44908684	7.45813E-06	0.134	360283	42162.979

genus Adlercreutzia	Vascular dementia (sudden onset)	rs4840457	C	T	0.010	6408682	0.752	0.029	14306	C	T	0.643	8	6551161	8.92874E-06	0.145	360283	32615.797
genus Adlercreutzia	Vascular dementia (sudden onset)	rs71511414	G	A	0.013	79688991	0.629	0.028	14306	G	A	0.681	9	77074075	7.80746E-06	0.152	360283	30731.948
genus Akkermansia	Vascular dementia (sudden onset)	rs12423672	T	G	0.016	5047705	0.341	0.021	14306	T	G	0.759	12	4938539	4.89159E-06	0.166	360283	30610.567
genus Akkermansia	Vascular dementia (sudden onset)	rs12452096	A	G	0.006	75544032	0.675	0.015	14306	A	G	0.750	17	77547950	1.48929E-06	0.156	360283	77865.614
genus Akkermansia	Vascular dementia (sudden onset)	rs2920	C	T	-0.010	23884780	0.579	0.016	14306	C	T	0.592	1	23558289	2.20602E-06	0.125	360283	40501.384
genus Akkermansia	Vascular dementia (sudden onset)	rs4840457	C	T	-0.017	6408682	0.505	0.024	14306	C	T	0.643	8	6551161	8.92874E-06	0.145	360283	32615.797
genus Akkermansia	Vascular dementia (sudden onset)	rs71511414	G	A	-0.011	79688991	0.596	0.023	14306	G	A	0.681	9	77074075	7.80746E-06	0.152	360283	30731.948
genus Alistipes	Vascular dementia (sudden onset)	rs12423672	T	G	0.006	5047705	0.964	0.017	14306	T	G	0.759	12	4938539	4.89159E-06	0.166	360283	30610.567
genus Alistipes	Vascular dementia (sudden onset)	rs12452096	A	G	0.004	75544032	0.759	0.013	14306	A	G	0.750	17	77547950	1.48929E-06	0.156	360283	77865.614
genus Alistipes	Vascular dementia (sudden onset)	rs2920	C	T	0.003	23884780	0.794	0.013	14306	C	T	0.592	1	23558289	2.20602E-06	0.125	360283	40501.384
genus Alistipes	Vascular dementia (sudden onset)	rs4840457	C	T	0.018	6408682	0.383	0.020	14306	C	T	0.643	8	6551161	8.92874E-06	0.145	360283	32615.797
genus Alistipes	Vascular dementia (sudden onset)	rs71511414	G	A	-0.005	79688991	0.818	0.019	14306	G	A	0.681	9	77074075	7.80746E-06	0.152	360283	30731.948
genus Allisonella	Vascular dementia (sudden onset)	rs2920	C	T	0.007	23884780	0.798	0.031	14306	C	T	0.592	1	23558289	2.20602E-06	0.125	360283	40501.384
genus Anaerofilum	Vascular dementia (sudden onset)	rs12423672	T	G	-0.027	5047705	0.418	0.033	14306	T	G	0.759	12	4938539	4.89159E-06	0.166	360283	30610.567
genus Anaerofilum	Vascular dementia (sudden onset)	rs12452096	A	G	-0.018	75544032	0.430	0.023	14306	A	G	0.750	17	77547950	1.48929E-06	0.156	360283	77865.614
genus Anaerofilum	Vascular dementia (sudden onset)	rs429358	C	T	-0.013	45411941	0.660	0.028	14306	C	T	0.601	19	44908684	7.45813E-06	0.134	360283	42162.979
genus Anaerofilum	Vascular dementia (sudden onset)	rs71511414	G	A	-0.027	79688991	0.472	0.036	14306	G	A	0.681	9	77074075	7.80746E-06	0.152	360283	30731.948
genus Anaerostipes	Vascular dementia (sudden onset)	rs12452096	A	G	-0.006	75544032	0.631	0.013	14306	A	G	0.750	17	77547950	1.48929E-06	0.156	360283	77865.614
genus Anaerostipes	Vascular dementia (sudden onset)	rs2920	C	T	-0.005	23884780	0.697	0.014	14306	C	T	0.592	1	23558289	2.20602E-06	0.125	360283	40501.384
genus Anaerostipes	Vascular dementia (sudden onset)	rs71511414	G	A	-0.009	79688991	0.747	0.020	14306	G	A	0.681	9	77074075	7.80746E-06	0.152	360283	30731.948
genus Anaerotruncus	Vascular dementia (sudden onset)	rs12452096	A	G	-0.009	75544032	0.529	0.013	14306	A	G	0.750	17	77547950	1.48929E-06	0.156	360283	77865.614
genus Anaerotruncus	Vascular dementia (sudden onset)	rs2920	C	T	0.006	23884780	0.643	0.014	14306	C	T	0.592	1	23558289	2.20602E-06	0.125	360283	40501.384
genus Anaerotruncus	Vascular dementia (sudden onset)	rs4840457	C	T	0.004	6408682	0.874	0.021	14306	C	T	0.643	8	6551161	8.92874E-06	0.145	360283	32615.797
genus Anaerotruncus	Vascular dementia (sudden onset)	rs71511414	G	A	-0.004	79688991	0.787	0.020	14306	G	A	0.681	9	77074075	7.80746E-06	0.152	360283	30731.948
genus Bacteroides	Vascular dementia (sudden onset)	rs12452096	A	G	0.007	75544032	0.579	0.012	14306	A	G	0.750	17	77547950	1.48929E-06	0.156	360283	77865.614
genus Bacteroides	Vascular dementia (sudden onset)	rs2920	C	T	0.005	23884780	0.745	0.013	14306	C	T	0.592	1	23558289	2.20602E-06	0.125	360283	40501.384
genus Barnesiella	Vascular dementia (sudden onset)	rs12423672	T	G	-0.014	5047705	0.492	0.019	14306	T	G	0.759	12	4938539	4.89159E-06	0.166	360283	30610.567
genus Barnesiella	Vascular dementia (sudden onset)	rs12452096	A	G	0.011	75544032	0.444	0.014	14306	A	G	0.750	17	77547950	1.48929E-06	0.156	360283	77865.614
genus Barnesiella	Vascular dementia (sudden onset)	rs2920	C	T	-0.006	23884780	0.703	0.015	14306	C	T	0.592	1	23558289	2.20602E-06	0.125	360283	40501.384
genus Barnesiella	Vascular dementia (sudden onset)	rs429358	C	T	-0.005	45411941	0.787	0.017	14306	C	T	0.601	19	44908684	7.45813E-06	0.134	360283	42162.979
genus Barnesiella	Vascular dementia (sudden onset)	rs4840457	C	T	-0.002	6408682	0.905	0.022	14306	C	T	0.643	8	6551161	8.92874E-06	0.145	360283	32615.797
genus Barnesiella	Vascular dementia (sudden onset)	rs71511414	G	A	-0.002	79688991	0.919	0.021	14306	G	A	0.681	9	77074075	7.80746E-06	0.152	360283	30731.948
genus Bifidobacterium	Vascular dementia (sudden onset)	rs12452096	A	G	-0.008	75544032	0.542	0.014	14306	A	G	0.750	17	77547950	1.48929E-06	0.156	360283	77865.614
genus Bifidobacterium	Vascular dementia (sudden onset)	rs2920	C	T	-0.008	23884780	0.637	0.015	14306	C	T	0.592	1	23558289	2.20602E-06	0.125	360283	40501.384
genus Bifidobacterium	Vascular dementia (sudden onset)	rs4840457	C	T	0.003	6408682	0.919	0.022	14306	C	T	0.643	8	6551161	8.92874E-06	0.145	360283	32615.797
genus Bifidobacterium	Vascular dementia (sudden onset)	rs71511414	G	A	-0.017	79688991	0.516	0.021	14306	G	A	0.681	9	77074075	7.80746E-06	0.152	360283	30731.948
genus Bilophila	Vascular dementia (sudden onset)	rs12423672	T	G	-0.008	5047705	0.466	0.020	14306	T	G	0.759	12	4938539	4.89159E-06	0.166	360283	30610.567
genus Bilophila	Vascular dementia (sudden onset)	rs12452096	A	G	-0.010	75544032	0.472	0.015	14306	A	G	0.750	17	77547950	1.48929E-06	0.156	360283	77865.614
genus Bilophila	Vascular dementia (sudden onset)	rs2920	C	T	-0.007	23884780	0.622	0.016	14306	C	T	0.592	1	23558289	2.20602E-06	0.125	360283	40501.384
genus Bilophila	Vascular dementia (sudden onset)	rs429358	C	T	0.007	45411941	0.656	0.018	14306	C	T	0.601	19	44908684	7.45813E-06	0.134	360283	42162.979
genus Bilophila	Vascular dementia (sudden onset)	rs4840457	C	T	0.003	6408682	0.844	0.024	14306	C	T	0.643	8	6551161	8.92874E-06	0.145	360283	32615.797
genus Bilophila	Vascular dementia (sudden onset)	rs71511414	G	A	0.020	79688991	0.401	0.023	14306	G	A	0.681	9	77074075	7.80746E-06	0.152	360283	30731.948
genus Butyricicoccus	Vascular dementia (sudden onset)	rs12452096	A	G	0.012	75544032	0.365	0.013	14306	A	G	0.750	17	77547950	1.48929E-06	0.156	360283	77865.614
genus Butyricicoccus	Vascular dementia (sudden onset)	rs2920	C	T	0.001	23884780	0.928	0.014	14306	C	T	0.592	1	23558289	2.20602E-06	0.125	360283	40501.384
genus Butyricicoccus	Vascular dementia (sudden onset)	rs4840457	C	T	0.002	6408682	0.915	0.020	14306	C	T	0.643	8	6551161	8.92874E-06	0.145	360283	32615.797
genus Butyricicoccus	Vascular dementia (sudden onset)	rs71511414	G	A	0.000	79688991	0.958	0.020	14306	G	A	0.681	9	77074075	7.80746E-06	0.152	360283	30731.948
genus Butyricimonas	Vascular dementia (sudden onset)	rs12423672	T	G	0.006	5047705	0.920	0.022	14306	T	G	0.759	12	4938539	4.89159E-06	0.166	360283	30610.567
genus Butyricimonas	Vascular dementia (sudden onset)	rs2920	C	T	0.010	23884780	0.610	0.017	14306	C	T	0.592	1	23558289	2.20602E-06	0.125	360283	40501.384
genus Butyricimonas	Vascular dementia (sudden onset)	rs4840457	C	T	-0.012	6408682	0.627	0.026	14306	C	T	0.643	8	6551161	8.92874E-06	0.145	360283	32615.797
genus Butyrivibrio	Vascular dementia (sudden onset)	rs12423672	T	G	0.034	5047705	0.444	0.039	14306	T	G	0.759	12	4938539	4.89159E-06	0.166	360283	30610.567
genus Butyrivibrio	Vascular dementia (sudden onset)	rs2920	C	T	0.015	23884780	0.619	0.030	14306	C	T	0.592	1	23558289	2.20602E-06	0.125	360283	40501.384
genus Butyrivibrio	Vascular dementia (sudden onset)	rs429358	C	T	-0.016	45411941	0.585	0.034	14306	C	T	0.601	19	44908684	7.45813E-06	0.134	360283	42162.979
genus Butyrivibrio	Vascular dementia (sudden onset)	rs4840457	C	T	-0.003	6408682	0.949	0.044	14306	C	T	0.643	8	6551161	8.92874E-06	0.145	360283	32615.797
genus Butyrivibrio	Vascular dementia (sudden onset)	rs71511414	G	A	0.026	79688991	0.614	0.043	14306	G	A	0.681	9	77074075	7.80746E-06	0.152	360283	30731.948
genus Candidatus Soleaferrea	Vascular dementia (sudden onset)	rs2920	C	T	-0.004	23884780	0.832	0.022	14306	C	T	0.592	1	23558289	2.20602E-06	0.125	360283	40501.384
genus Candidatus Soleaferrea	Vascular dementia (sudden onset)	rs429358	C	T	0.020	45411941	0.391	0.026	14306	C	T	0.601	19	44908684	7.45813E-06	0.134	360283	42162.979
genus Candidatus Soleaferrea	Vascular dementia (sudden onset)	rs71511414	G	A	0.019	79688991	0.548	0.032	14306	G	A	0.681	9	77074075	7.80746E-06	0.152	360283	30731.948
genus Catenibacterium	Vascular dementia (sudden onset)	rs12423672	T	G	0.031	5047705	0.511	0.039	14306	T	G	0.759	12	4938539	4.89159E-06	0.166	360283	30610.567
genus Catenibacterium	Vascular dementia (sudden onset)	rs12452096	A	G	-0.003	75544032	0.924	0.029	14306	A	G	0.750	17	77547950	1.48929E-06	0.156	360283	77865.614
genus Catenibacterium	Vascular dementia (sudden onset)	rs429358	C	T	0.030	45411941	0.433	0.036	14306	C	T	0.601	19	44908684	7.45813E-06	0.134	360283	42162.979
genus Christensenellaceae R 7group	Vascular dementia (sudden onset)	rs12423672	T	G	0.009	5047705	0.721	0.018	14306	T	G	0.759	12	4938539	4.89159E-06	0.166	360283	30610.567
genus Christensenellaceae R 7group	Vascular dementia (sudden onset)	rs429358	C	T	0.011	45411941	0.455	0.016	14306	C	T	0.601	19	44908684	7.45813E-06	0.134	360283	42162.979
genus Christensenellaceae R 7group	Vascular dementia (sudden onset)	rs4840457	C	T	0.007	6408682	0.791	0.021	14306	C	T	0.643	8	6551161	8.92874E-06	0.145	360283	32615.797
genus Clostridium innocuum group	Vascular dementia (sudden onset)	rs2920	C	T	-0.018	23884780	0.506	0.027	14306	C	T	0.592	1	23558289	2.20602E-06	0.125	360283	40501.384
genus Clostridium innocuum group	Vascular dementia (sudden onset)	rs429358	C	T	-0.009	45411941	0.813	0.031	14306	C	T	0.601	19	44908684	7.45813E-06	0.134	360283	42162.979
genus Clostridium innocuum group	Vascular dementia (sudden onset)	rs71511414	G	A	0.026	79688991	0.510	0.040	14306	G	A	0.681	9	77074075	7.80746E-06	0.152	360283	30731.948
genus Clostridium sensustricto1	Vascular dementia (sudden onset)	rs12423672	T	G	0.015	5047705	0.421	0.019	14306	T	G	0.759	12	4938539	4.89159E-06	0.166	360283	30610.567
genus Clostridium sensustricto1	Vascular dementia (sudden onset)	rs12452096	A	G	-0.003	75544032	0.820	0.014	14306	A	G	0.750	17	77547950	1.48929E-06	0.156	360283	77865.614
genus Clostridium sensustricto1	Vascular dementia (sudden onset)	rs2920	C	T	-0.009	23884780	0.565	0.015	14306	C	T	0.592	1	23558289	2.20602E-06	0.125	360283	40501.384
genus Clostridium sensustricto1	Vascular dementia (sudden onset)	rs429358	C	T	0.011	45411941	0.544	0.017	14306	C	T	0.601	19	44908684	7.45813E-06	0.134	360283	42162.979
genus Clostridium sensustricto1	Vascular dementia (sudden onset)	rs4840457	C	T	-0.005	6408682	0.812	0.022	14306	C	T	0.643	8	6551161	8.92874E-06	0.145	360283	32615.797

genus Clostridium sensustricto1	Vascular dementia (sudden onset)	rs71511414	G	A	0.006	79688991	0.791	0.022	14306	G	A	0.681	9	77074075	7.80746E-06	0.152	360283	30731.948
genus Collinsella	Vascular dementia (sudden onset)	rs71511414	G	A	0.004	79688991	0.774	0.021	14306	G	A	0.681	9	77074075	7.80746E-06	0.152	360283	30731.948
genus Coprobacter	Vascular dementia (sudden onset)	rs2920	C	T	0.000	23884780	0.975	0.021	14306	C	T	0.592	1	23558289	2.20602E-06	0.125	360283	40501.384
genus Coprobacter	Vascular dementia (sudden onset)	rs429358	C	T	-0.001	45411941	0.930	0.025	14306	C	T	0.601	19	44908684	7.45813E-06	0.134	360283	42162.979
genus Coprobacter	Vascular dementia (sudden onset)	rs4840457	C	T	-0.006	6408682	0.895	0.032	14306	C	T	0.643	8	6551161	8.92874E-06	0.145	360283	32615.797
genus Coprobacter	Vascular dementia (sudden onset)	rs71511414	G	A	-0.022	79688991	0.542	0.031	14306	G	A	0.681	9	77074075	7.80746E-06	0.152	360283	30731.948
genus Coprococcus1	Vascular dementia (sudden onset)	rs12423672	T	G	0.007	5047705	0.826	0.018	14306	T	G	0.759	12	4938539	4.89159E-06	0.166	360283	30610.567
genus Coprococcus1	Vascular dementia (sudden onset)	rs71511414	G	A	-0.017	79688991	0.410	0.020	14306	G	A	0.681	9	77074075	7.80746E-06	0.152	360283	30731.948
genus Coprococcus2	Vascular dementia (sudden onset)	rs12452096	A	G	0.006	75544032	0.681	0.016	14306	A	G	0.750	17	77547950	1.48929E-06	0.156	360283	77865.614
genus Coprococcus2	Vascular dementia (sudden onset)	rs429358	C	T	0.002	45411941	0.982	0.019	14306	C	T	0.601	19	44908684	7.45813E-06	0.134	360283	42162.979
genus Coprococcus2	Vascular dementia (sudden onset)	rs71511414	G	A	-0.008	79688991	0.707	0.024	14306	G	A	0.681	9	77074075	7.80746E-06	0.152	360283	30731.948
genus Coprococcus3	Vascular dementia (sudden onset)	rs12423672	T	G	0.007	5047705	0.670	0.018	14306	T	G	0.759	12	4938539	4.89159E-06	0.166	360283	30610.567
genus Coprococcus3	Vascular dementia (sudden onset)	rs12452096	A	G	0.003	75544032	0.788	0.013	14306	A	G	0.750	17	77547950	1.48929E-06	0.156	360283	77865.614
genus Coprococcus3	Vascular dementia (sudden onset)	rs2920	C	T	0.006	23884780	0.702	0.014	14306	C	T	0.592	1	23558289	2.20602E-06	0.125	360283	40501.384
genus Coprococcus3	Vascular dementia (sudden onset)	rs4840457	C	T	-0.004	6408682	0.881	0.021	14306	C	T	0.643	8	6551161	8.92874E-06	0.145	360283	32615.797
genus Coprococcus3	Vascular dementia (sudden onset)	rs71511414	G	A	-0.008	79688991	0.745	0.020	14306	G	A	0.681	9	77074075	7.80746E-06	0.152	360283	30731.948
genus Defluviitaleaceae UCG011	Vascular dementia (sudden onset)	rs12423672	T	G	-0.006	5047705	0.676	0.026	14306	T	G	0.759	12	4938539	4.89159E-06	0.166	360283	30610.567
genus Defluviitaleaceae UCG011	Vascular dementia (sudden onset)	rs12452096	A	G	0.008	75544032	0.676	0.019	14306	A	G	0.750	17	77547950	1.48929E-06	0.156	360283	77865.614
genus Defluviitaleaceae UCG011	Vascular dementia (sudden onset)	rs429358	C	T	-0.003	45411941	0.819	0.022	14306	C	T	0.601	19	44908684	7.45813E-06	0.134	360283	42162.979
genus Desulfovibrio	Vascular dementia (sudden onset)	rs429358	C	T	0.006	45411941	0.782	0.021	14306	C	T	0.601	19	44908684	7.45813E-06	0.134	360283	42162.979
genus Desulfovibrio	Vascular dementia (sudden onset)	rs4840457	C	T	0.008	6408682	0.787	0.028	14306	C	T	0.643	8	6551161	8.92874E-06	0.145	360283	32615.797
genus Desulfovibrio	Vascular dementia (sudden onset)	rs71511414	G	A	-0.018	79688991	0.453	0.027	14306	G	A	0.681	9	77074075	7.80746E-06	0.152	360283	30731.948
(genus Dialister	Vascular dementia (sudden onset)	rs12423672	T	G	0.009	5047705	0.668	0.021	14306	T	G	0.759	12	4938539	4.89159E-06	0.166	360283	30610.567
(genus Dialister	Vascular dementia (sudden onset)	rs12452096	A	G	-0.002	75544032	0.946	0.015	14306	A	G	0.750	17	77547950	1.48929E-06	0.156	360283	77865.614
(genus Dialister	Vascular dementia (sudden onset)	rs2920	C	T	0.003	23884780	0.847	0.016	14306	C	T	0.592	1	23558289	2.20602E-06	0.125	360283	40501.384
genus Dorea	Vascular dementia (sudden onset)	rs12452096	A	G	-0.005	75544032	0.644	0.013	14306	A	G	0.750	17	77547950	1.48929E-06	0.156	360283	77865.614
genus Dorea	Vascular dementia (sudden onset)	rs4840457	C	T	0.006	6408682	0.805	0.020	14306	C	T	0.643	8	6551161	8.92874E-06	0.145	360283	32615.797
genus Dorea	Vascular dementia (sudden onset)	rs71511414	G	A	0.006	79688991	0.728	0.019	14306	G	A	0.681	9	77074075	7.80746E-06	0.152	360283	30731.948
genus Eggerthella	Vascular dementia (sudden onset)	rs12423672	T	G	0.008	5047705	0.870	0.032	14306	T	G	0.759	12	4938539	4.89159E-06	0.166	360283	30610.567
genus Eggerthella	Vascular dementia (sudden onset)	rs12452096	A	G	0.003	75544032	0.850	0.023	14306	A	G	0.750	17	77547950	1.48929E-06	0.156	360283	77865.614
genus Eggerthella	Vascular dementia (sudden onset)	rs429358	C	T	0.017	45411941	0.611	0.029	14306	C	T	0.601	19	44908684	7.45813E-06	0.134	360283	42162.979
genus Eisenbergiella	Vascular dementia (sudden onset)	rs429358	C	T	-0.013	45411941	0.612	0.028	14306	C	T	0.601	19	44908684	7.45813E-06	0.134	360283	42162.979
genus Eisenbergiella	Vascular dementia (sudden onset)	rs4840457	C	T	0.026	6408682	0.526	0.035	14306	C	T	0.643	8	6551161	8.92874E-06	0.145	360283	32615.797
genus Eisenbergiella	Vascular dementia (sudden onset)	rs71511414	G	A	-0.028	79688991	0.450	0.034	14306	G	A	0.681	9	77074075	7.80746E-06	0.152	360283	30731.948
genus Enterorhabdus	Vascular dementia (sudden onset)	rs12423672	T	G	0.018	5047705	0.416	0.027	14306	T	G	0.759	12	4938539	4.89159E-06	0.166	360283	30610.567
genus Enterorhabdus	Vascular dementia (sudden onset)	rs429358	C	T	0.009	45411941	0.754	0.023	14306	C	T	0.601	19	44908684	7.45813E-06	0.134	360283	42162.979
genus Erysipelatoclostridium	Vascular dementia (sudden onset)	rs12423672	T	G	0.003	5047705	0.926	0.023	14306	T	G	0.759	12	4938539	4.89159E-06	0.166	360283	30610.567
genus Erysipelatoclostridium	Vascular dementia (sudden onset)	rs12452096	A	G	-0.007	75544032	0.682	0.017	14306	A	G	0.750	17	77547950	1.48929E-06	0.156	360283	77865.614
genus Erysipelatoclostridium	Vascular dementia (sudden onset)	rs429358	C	T	0.017	45411941	0.432	0.020	14306	C	T	0.601	19	44908684	7.45813E-06	0.134	360283	42162.979
genus Erysipelatoclostridium	Vascular dementia (sudden onset)	rs4840457	C	T	0.000	6408682	0.982	0.027	14306	C	T	0.643	8	6551161	8.92874E-06	0.145	360283	32615.797
genus Escherichia Shigella	Vascular dementia (sudden onset)	rs12423672	T	G	0.014	5047705	0.538	0.021	14306	T	G	0.759	12	4938539	4.89159E-06	0.166	360283	30610.567
genus Escherichia Shigella	Vascular dementia (sudden onset)	rs12452096	A	G	0.003	75544032	0.866	0.015	14306	A	G	0.750	17	77547950	1.48929E-06	0.156	360283	77865.614
genus Escherichia Shigella	Vascular dementia (sudden onset)	rs2920	C	T	-0.007	23884780	0.618	0.016	14306	C	T	0.592	1	23558289	2.20602E-06	0.125	360283	40501.384
genus Escherichia Shigella	Vascular dementia (sudden onset)	rs429358	C	T	0.007	45411941	0.718	0.019	14306	C	T	0.601	19	44908684	7.45813E-06	0.134	360283	42162.979
genus Eubacterium brachy group	Vascular dementia (sudden onset)	rs12423672	T	G	-0.008	5047705	0.784	0.037	14306	T	G	0.759	12	4938539	4.89159E-06	0.166	360283	30610.567
genus Eubacterium brachy group	Vascular dementia (sudden onset)	rs12452096	A	G	-0.023	75544032	0.395	0.027	14306	A	G	0.750	17	77547950	1.48929E-06	0.156	360283	77865.614
genus Eubacterium brachy group	Vascular dementia (sudden onset)	rs429358	C	T	-0.022	45411941	0.464	0.032	14306	C	T	0.601	19	44908684	7.45813E-06	0.134	360283	42162.979
genus Eubacterium brachy group	Vascular dementia (sudden onset)	rs4840457	C	T	-0.035	6408682	0.449	0.042	14306	C	T	0.643	8	6551161	8.92874E-06	0.145	360283	32615.797
genus Eubacterium brachy group	Vascular dementia (sudden onset)	rs71511414	G	A	0.031	79688991	0.400	0.039	14306	G	A	0.681	9	77074075	7.80746E-06	0.152	360283	30731.948
genus Eubacterium coprostanoligenes group	Vascular dementia (sudden onset)	rs12452096	A	G	-0.012	75544032	0.382	0.013	14306	A	G	0.750	17	77547950	1.48929E-06	0.156	360283	77865.614
genus Eubacterium coprostanoligenes group	Vascular dementia (sudden onset)	rs4840457	C	T	0.004	6408682	0.795	0.020	14306	C	T	0.643	8	6551161	8.92874E-06	0.145	360283	32615.797
genus Eubacterium coprostanoligenes group	Vascular dementia (sudden onset)	rs71511414	G	A	-0.003	79688991	0.969	0.020	14306	G	A	0.681	9	77074075	7.80746E-06	0.152	360283	30731.948
genus Eubacterium eligens group	Vascular dementia (sudden onset)	rs12423672	T	G	-0.012	5047705	0.547	0.019	14306	T	G	0.759	12	4938539	4.89159E-06	0.166	360283	30610.567
genus Eubacterium eligens group	Vascular dementia (sudden onset)	rs2920	C	T	0.010	23884780	0.525	0.015	14306	C	T	0.592	1	23558289	2.20602E-06	0.125	360283	40501.384
genus Eubacterium eligens group	Vascular dementia (sudden onset)	rs4840457	C	T	0.004	6408682	0.893	0.022	14306	C	T	0.643	8	6551161	8.92874E-06	0.145	360283	32615.797
genus Eubacterium fissicatena group	Vascular dementia (sudden onset)	rs12452096	A	G	0.006	75544032	0.791	0.027	14306	A	G	0.750	17	77547950	1.48929E-06	0.156	360283	77865.614
genus Eubacterium fissicatena group	Vascular dementia (sudden onset)	rs2920	C	T	0.010	23884780	0.700	0.029	14306	C	T	0.592	1	23558289	2.20602E-06	0.125	360283	40501.384
genus Eubacterium fissicatena group	Vascular dementia (sudden onset)	rs429358	C	T	0.004	45411941	0.913	0.033	14306	C	T	0.601	19	44908684	7.45813E-06	0.134	360283	42162.979
genus Eubacterium fissicatena group	Vascular dementia (sudden onset)	rs4840457	C	T	0.031	6408682	0.458	0.043	14306	C	T	0.643	8	6551161	8.92874E-06	0.145	360283	32615.797
genus Eubacterium fissicatena group	Vascular dementia (sudden onset)	rs71511414	G	A	-0.017	79688991	0.707	0.042	14306	G	A	0.681	9	77074075	7.80746E-06	0.152	360283	30731.948
genus Eubacterium hallii group	Vascular dementia (sudden onset)	rs12423672	T	G	0.013	5047705	0.440	0.018	14306	T	G	0.759	12	4938539	4.89159E-06	0.166	360283	30610.567
genus Eubacterium hallii group	Vascular dementia (sudden onset)	rs12452096	A	G	0.009	75544032	0.472	0.013	14306	A	G	0.750	17	77547950	1.48929E-06	0.156	360283	77865.614
genus Eubacterium hallii group	Vascular dementia (sudden onset)	rs429358	C	T	0.003	45411941	0.834	0.016	14306	C	T	0.601	19	44908684	7.45813E-06	0.134	360283	42162.979
genus Eubacterium nodatum group	Vascular dementia (sudden onset)	rs12423672	T	G	-0.008	5047705	0.850	0.041	14306	T	G	0.759	12	4938539	4.89159E-06	0.166	360283	30610.567
genus Eubacterium nodatum group	Vascular dementia (sudden onset)	rs429358	C	T	0.008	45411941	0.880	0.036	14306	C	T	0.601	19	44908684	7.45813E-06	0.134	360283	42162.979
genus Eubacterium nodatum group	Vascular dementia (sudden onset)	rs71511414	G	A	-0.006	79688991	0.879	0.044	14306	G	A	0.681	9	77074075	7.80746E-06	0.152	360283	30731.948
genus Eubacterium oxidoreducens group	Vascular dementia (sudden onset)	rs429358	C	T	0.021	45411941	0.455	0.028	14306	C	T	0.601	19	44908684	7.45813E-06	0.134	360283	42162.979
genus Eubacterium oxidoreducens group	Vascular dementia (sudden onset)	rs4840457	C	T	-0.025	6408682	0.398	0.037	14306	C	T	0.643	8	6551161	8.92874E-06	0.145	360283	32615.797
genus Eubacterium oxidoreducens group	Vascular dementia (sudden onset)	rs71511414	G	A	0.011	79688991	0.754	0.036	14306	G	A	0.681	9	77074075	7.80746E-06	0.152	360283	30731.948
genus Eubacterium rectale group	Vascular dementia (sudden onset)	rs12423672	T	G	-0.007	5047705	0.657	0.017	14306	T	G	0.759	12	4938539	4.89159E-06	0.166	360283	30610.567
genus Eubacterium rectale group	Vascular dementia (sudden onset)	rs12452096	A	G	0.009	75544032	0.499	0.013	14306	A	G	0.750	17	77547950	1.48929E-06	0.156	360283	77865.614

genus Eubacterium rectale group	Vascular dementia (sudden onset)	rs2920	C	T	0.010	23884780	0.440	0.013	14306	C	T	0.592	1	23558289	2.20602E-06	0.125	360283	40501.384
genus Eubacterium ruminantium group	Vascular dementia (sudden onset)	rs12423672	T	G	-0.015	5047705	0.639	0.026	14306	T	G	0.759	12	4938539	4.89159E-06	0.166	360283	30610.567
genus Eubacterium ruminantium group	Vascular dementia (sudden onset)	rs12452096	A	G	-0.006	75544032	0.783	0.019	14306	A	G	0.750	17	77547950	1.48929E-06	0.156	360283	77865.614
genus Eubacterium ruminantium group	Vascular dementia (sudden onset)	rs2920	C	T	0.004	23884780	0.900	0.020	14306	C	T	0.592	1	23558289	2.20602E-06	0.125	360283	40501.384
genus Eubacterium ruminantium group	Vascular dementia (sudden onset)	rs429358	C	T	0.014	45411941	0.497	0.023	14306	C	T	0.601	19	44908684	7.45813E-06	0.134	360283	42162.979
genus Eubacterium ruminantium group	Vascular dementia (sudden onset)	rs4840457	C	T	-0.002	6408682	0.980	0.031	14306	C	T	0.643	8	6551161	8.92874E-06	0.145	360283	32615.797
genus Eubacterium ruminantium group	Vascular dementia (sudden onset)	rs71511414	G	A	-0.003	79688991	0.924	0.030	14306	G	A	0.681	9	77074075	7.80746E-06	0.152	360283	30731.948
genus Eubacterium ventriosum group	Vascular dementia (sudden onset)	rs12423672	T	G	-0.012	5047705	0.405	0.019	14306	T	G	0.759	12	4938539	4.89159E-06	0.166	360283	30610.567
genus Eubacterium ventriosum group	Vascular dementia (sudden onset)	rs12452096	A	G	0.008	75544032	0.580	0.014	14306	A	G	0.750	17	77547950	1.48929E-06	0.156	360283	77865.614
genus Eubacterium ventriosum group	Vascular dementia (sudden onset)	rs2920	C	T	-0.011	23884780	0.463	0.014	14306	C	T	0.592	1	23558289	2.20602E-06	0.125	360283	40501.384
genus Eubacterium xylanophilum group	Vascular dementia (sudden onset)	rs12452096	A	G	-0.006	75544032	0.681	0.015	14306	A	G	0.750	17	77547950	1.48929E-06	0.156	360283	77865.614
genus Eubacterium xylanophilum group	Vascular dementia (sudden onset)	rs4840457	C	T	-0.003	6408682	0.884	0.023	14306	C	T	0.643	8	6551161	8.92874E-06	0.145	360283	32615.797
genus Faecalibacterium	Vascular dementia (sudden onset)	rs12452096	A	G	-0.011	75544032	0.372	0.012	14306	A	G	0.750	17	77547950	1.48929E-06	0.156	360283	77865.614
genus Faecalibacterium	Vascular dementia (sudden onset)	rs2920	C	T	-0.001	23884780	0.919	0.013	14306	C	T	0.592	1	23558289	2.20602E-06	0.125	360283	40501.384
genus Faecalibacterium	Vascular dementia (sudden onset)	rs4840457	C	T	-0.013	6408682	0.489	0.020	14306	C	T	0.643	8	6551161	8.92874E-06	0.145	360283	32615.797
genus Faecalibacterium	Vascular dementia (sudden onset)	rs71511414	G	A	-0.010	79688991	0.615	0.019	14306	G	A	0.681	9	77074075	7.80746E-06	0.152	360283	30731.948
genus Family XIII AD3011 group	Vascular dementia (sudden onset)	rs12452096	A	G	-0.008	75544032	0.593	0.014	14306	A	G	0.750	17	77547950	1.48929E-06	0.156	360283	77865.614
genus Family XIII AD3011 group	Vascular dementia (sudden onset)	rs71511414	G	A	0.005	79688991	0.810	0.021	14306	G	A	0.681	9	77074075	7.80746E-06	0.152	360283	30731.948
genus Family XIII UCG001	Vascular dementia (sudden onset)	rs12423672	T	G	0.014	5047705	0.525	0.021	14306	T	G	0.759	12	4938539	4.89159E-06	0.166	360283	30610.567
genus Family XIII UCG001	Vascular dementia (sudden onset)	rs12452096	A	G	-0.003	75544032	0.840	0.015	14306	A	G	0.750	17	77547950	1.48929E-06	0.156	360283	77865.614
genus Family XIII UCG001	Vascular dementia (sudden onset)	rs2920	C	T	-0.001	23884780	0.942	0.016	14306	C	T	0.592	1	23558289	2.20602E-06	0.125	360283	40501.384
genus Family XIII UCG001	Vascular dementia (sudden onset)	rs429358	C	T	0.004	45411941	0.889	0.018	14306	C	T	0.601	19	44908684	7.45813E-06	0.134	360283	42162.979
genus Family XIII UCG001	Vascular dementia (sudden onset)	rs71511414	G	A	0.007	79688991	0.764	0.023	14306	G	A	0.681	9	77074075	7.80746E-06	0.152	360283	30731.948
genus Flavonifractor	Vascular dementia (sudden onset)	rs12452096	A	G	0.000	75544032	0.972	0.016	14306	A	G	0.750	17	77547950	1.48929E-06	0.156	360283	77865.614
genus Flavonifractor	Vascular dementia (sudden onset)	rs2920	C	T	0.006	23884780	0.717	0.017	14306	C	T	0.592	1	23558289	2.20602E-06	0.125	360283	40501.384
genus Fusicatenibacter	Vascular dementia (sudden onset)	rs12423672	T	G	0.009	5047705	0.623	0.017	14306	T	G	0.759	12	4938539	4.89159E-06	0.166	360283	30610.567
genus Fusicatenibacter	Vascular dementia (sudden onset)	rs12452096	A	G	-0.011	75544032	0.367	0.013	14306	A	G	0.750	17	77547950	1.48929E-06	0.156	360283	77865.614
genus Fusicatenibacter	Vascular dementia (sudden onset)	rs2920	C	T	-0.002	23884780	0.868	0.013	14306	C	T	0.592	1	23558289	2.20602E-06	0.125	360283	40501.384
genus Fusicatenibacter	Vascular dementia (sudden onset)	rs429358	C	T	-0.008	45411941	0.622	0.015	14306	C	T	0.601	19	44908684	7.45813E-06	0.134	360283	42162.979
genus Gordonibacter	Vascular dementia (sudden onset)	rs12423672	T	G	0.001	5047705	0.934	0.038	14306	T	G	0.759	12	4938539	4.89159E-06	0.166	360283	30610.567
genus Gordonibacter	Vascular dementia (sudden onset)	rs12452096	A	G	0.003	75544032	0.944	0.027	14306	A	G	0.750	17	77547950	1.48929E-06	0.156	360283	77865.614
genus Gordonibacter	Vascular dementia (sudden onset)	rs2920	C	T	0.001	23884780	0.973	0.029	14306	C	T	0.592	1	23558289	2.20602E-06	0.125	360283	40501.384
genus Gordonibacter	Vascular dementia (sudden onset)	rs4840457	C	T	-0.009	6408682	0.828	0.044	14306	C	T	0.643	8	6551161	8.92874E-06	0.145	360283	32615.797
genus Gordonibacter	Vascular dementia (sudden onset)	rs71511414	G	A	-0.016	79688991	0.743	0.042	14306	G	A	0.681	9	77074075	7.80746E-06	0.152	360283	30731.948
genus Haemophilus	Vascular dementia (sudden onset)	rs12452096	A	G	-0.008	75544032	0.692	0.018	14306	A	G	0.750	17	77547950	1.48929E-06	0.156	360283	77865.614
genus Haemophilus	Vascular dementia (sudden onset)	rs2920	C	T	-0.010	23884780	0.564	0.019	14306	C	T	0.592	1	23558289	2.20602E-06	0.125	360283	40501.384
genus Haemophilus	Vascular dementia (sudden onset)	rs4840457	C	T	-0.021	6408682	0.425	0.028	14306	C	T	0.643	8	6551161	8.92874E-06	0.145	360283	32615.797
genus Holdemanella	Vascular dementia (sudden onset)	rs4840457	C	T	0.000	6408682	0.947	0.030	14306	C	T	0.643	8	6551161	8.92874E-06	0.145	360283	32615.797
genus Holdemania	Vascular dementia (sudden onset)	rs12452096	A	G	0.004	75544032	0.881	0.018	14306	A	G	0.750	17	77547950	1.48929E-06	0.156	360283	77865.614
genus Holdemania	Vascular dementia (sudden onset)	rs2920	C	T	0.006	23884780	0.770	0.018	14306	C	T	0.592	1	23558289	2.20602E-06	0.125	360283	40501.384
genus Holdemania	Vascular dementia (sudden onset)	rs429358	C	T	0.014	45411941	0.528	0.021	14306	C	T	0.601	19	44908684	7.45813E-06	0.134	360283	42162.979
genus Howardella	Vascular dementia (sudden onset)	rs12452096	A	G	0.006	75544032	0.813	0.027	14306	A	G	0.750	17	77547950	1.48929E-06	0.156	360283	77865.614
genus Howardella	Vascular dementia (sudden onset)	rs71511414	G	A	-0.013	79688991	0.808	0.042	14306	G	A	0.681	9	77074075	7.80746E-06	0.152	360283	30731.948
genus Hungatella	Vascular dementia (sudden onset)	rs12423672	T	G	-0.007	5047705	0.929	0.035	14306	T	G	0.759	12	4938539	4.89159E-06	0.166	360283	30610.567
genus Hungatella	Vascular dementia (sudden onset)	rs12452096	A	G	0.005	75544032	0.802	0.026	14306	A	G	0.750	17	77547950	1.48929E-06	0.156	360283	77865.614
genus Hungatella	Vascular dementia (sudden onset)	rs2920	C	T	-0.025	23884780	0.368	0.027	14306	C	T	0.592	1	23558289	2.20602E-06	0.125	360283	40501.384
genus Hungatella	Vascular dementia (sudden onset)	rs71511414	G	A	-0.025	79688991	0.455	0.040	14306	G	A	0.681	9	77074075	7.80746E-06	0.152	360283	30731.948
genus Intestinibacter	Vascular dementia (sudden onset)	rs12452096	A	G	-0.002	75544032	0.883	0.015	14306	A	G	0.750	17	77547950	1.48929E-06	0.156	360283	77865.614
genus Intestinibacter	Vascular dementia (sudden onset)	rs2920	C	T	0.010	23884780	0.536	0.016	14306	C	T	0.592	1	23558289	2.20602E-06	0.125	360283	40501.384
genus Intestinibacter	Vascular dementia (sudden onset)	rs429358	C	T	-0.011	45411941	0.447	0.018	14306	C	T	0.601	19	44908684	7.45813E-06	0.134	360283	42162.979
genus Intestinibacter	Vascular dementia (sudden onset)	rs4840457	C	T	0.020	6408682	0.382	0.024	14306	C	T	0.643	8	6551161	8.92874E-06	0.145	360283	32615.797
genus Intestinibacter	Vascular dementia (sudden onset)	rs71511414	G	A	-0.011	79688991	0.626	0.023	14306	G	A	0.681	9	77074075	7.80746E-06	0.152	360283	30731.948
genus Intestinimonas	Vascular dementia (sudden onset)	rs429358	C	T	0.013	45411941	0.424	0.018	14306	C	T	0.601	19	44908684	7.45813E-06	0.134	360283	42162.979
genus Intestinimonas	Vascular dementia (sudden onset)	rs4840457	C	T	0.004	6408682	0.883	0.024	14306	C	T	0.643	8	6551161	8.92874E-06	0.145	360283	32615.797
genus Intestinimonas	Vascular dementia (sudden onset)	rs71511414	G	A	0.009	79688991	0.707	0.023	14306	G	A	0.681	9	77074075	7.80746E-06	0.152	360283	30731.948
genus Lachnoclostridium	Vascular dementia (sudden onset)	rs12423672	T	G	-0.002	5047705	0.790	0.017	14306	T	G	0.759	12	4938539	4.89159E-06	0.166	360283	30610.567
genus Lachnoclostridium	Vascular dementia (sudden onset)	rs2920	C	T	0.009	23884780	0.553	0.013	14306	C	T	0.592	1	23558289	2.20602E-06	0.125	360283	40501.384
genus Lachnoclostridium	Vascular dementia (sudden onset)	rs4840457	C	T	0.003	6408682	0.833	0.020	14306	C	T	0.643	8	6551161	8.92874E-06	0.145	360283	32615.797
genus Lachnoclostridium	Vascular dementia (sudden onset)	rs71511414	G	A	0.016	79688991	0.384	0.019	14306	G	A	0.681	9	77074075	7.80746E-06	0.152	360283	30731.948
genus Lachnospiraceae FCS020 group	Vascular dementia (sudden onset)	rs12423672	T	G	-0.004	5047705	0.846	0.020	14306	T	G	0.759	12	4938539	4.89159E-06	0.166	360283	30610.567
genus Lachnospiraceae FCS020 group	Vascular dementia (sudden onset)	rs4840457	C	T	-0.011	6408682	0.635	0.023	14306	C	T	0.643	8	6551161	8.92874E-06	0.145	360283	32615.797
genus Lachnospiraceae FCS020 group	Vascular dementia (sudden onset)	rs71511414	G	A	0.003	79688991	0.890	0.022	14306	G	A	0.681	9	77074075	7.80746E-06	0.152	360283	30731.948
genus Lachnospiraceae NC2004 group	Vascular dementia (sudden onset)	rs12423672	T	G	-0.012	5047705	0.554	0.028	14306	T	G	0.759	12	4938539	4.89159E-06	0.166	360283	30610.567
genus Lachnospiraceae ND3007 group	Vascular dementia (sudden onset)	rs12423672	T	G	0.013	5047705	0.510	0.018	14306	T	G	0.759	12	4938539	4.89159E-06	0.166	360283	30610.567
genus Lachnospiraceae ND3007 group	Vascular dementia (sudden onset)	rs12452096	A	G	0.006	75544032	0.649	0.014	14306	A	G	0.750	17	77547950	1.48929E-06	0.156	360283	77865.614
genus Lachnospiraceae ND3007 group	Vascular dementia (sudden onset)	rs2920	C	T	-0.010	23884780	0.445	0.014	14306	C	T	0.592	1	23558289	2.20602E-06	0.125	360283	40501.384
genus Lachnospiraceae ND3007 group	Vascular dementia (sudden onset)	rs429358	C	T	-0.003	45411941	0.806	0.016	14306	C	T	0.601	19	44908684	7.45813E-06	0.134	360283	42162.979
genus Lachnospiraceae ND3007 group	Vascular dementia (sudden onset)	rs4840457	C	T	0.017	6408682	0.495	0.022	14306	C	T	0.643	8	6551161	8.92874E-06	0.145	360283	32615.797
genus Lachnospiraceae NK4A136 group	Vascular dementia (sudden onset)	rs12423672	T	G	-0.011	5047705	0.527	0.017	14306	T	G	0.759	12	4938539	4.89159E-06	0.166	360283	30610.567
genus Lachnospiraceae NK4A136 group	Vascular dementia (sudden onset)	rs12452096	A	G	-0.005	75544032	0.739	0.013	14306	A	G	0.750	17	77547950	1.48929E-06	0.156	360283	77865.614
genus Lachnospiraceae NK4A136 group	Vascular dementia (sudden onset)	rs2920	C	T	-0.001	23884780	0.835	0.013	14306	C	T	0.592	1	23558289	2.20602E-06	0.125	360283	40501.384

genus Lachnospiraceae NK4A136 group	Vascular dementia (sudden onset)	rs429358	C	T	-0.011	45411941	0.456	0.015	14306	C	T	0.601	19	44908684	7.45813E-06	0.134	360283	42162.979
genus Lachnospiraceae NK4A136 group	Vascular dementia (sudden onset)	rs4840457	C	T	-0.012	6408682	0.524	0.020	14306	C	T	0.643	8	6551161	8.92874E-06	0.145	360283	32615.797
genus Lachnospiraceae NK4A136 group	Vascular dementia (sudden onset)	rs71511414	G	A	-0.014	79688991	0.460	0.020	14306	G	A	0.681	9	77074075	7.80746E-06	0.152	360283	30731.948
genus Lachnospiraceae UCG001	Vascular dementia (sudden onset)	rs12423672	T	G	-0.017	5047705	0.490	0.023	14306	T	G	0.759	12	4938539	4.89159E-06	0.166	360283	30610.567
genus Lachnospiraceae UCG001	Vascular dementia (sudden onset)	rs2920	C	T	0.009	23884780	0.665	0.018	14306	C	T	0.592	1	23558289	2.20602E-06	0.125	360283	40501.384
genus Lachnospiraceae UCG001	Vascular dementia (sudden onset)	rs429358	C	T	-0.002	45411941	0.904	0.020	14306	C	T	0.601	19	44908684	7.45813E-06	0.134	360283	42162.979
genus Lachnospiraceae UCG001	Vascular dementia (sudden onset)	rs4840457	C	T	-0.016	6408682	0.567	0.026	14306	C	T	0.643	8	6551161	8.92874E-06	0.145	360283	32615.797
genus Lachnospiraceae UCG001	Vascular dementia (sudden onset)	rs71511414	G	A	-0.006	79688991	0.851	0.025	14306	G	A	0.681	9	77074075	7.80746E-06	0.152	360283	30731.948
genus Lachnospiraceae UCG004	Vascular dementia (sudden onset)	rs12452096	A	G	0.002	75544032	0.877	0.014	14306	A	G	0.750	17	77547950	1.48929E-06	0.156	360283	77865.614
genus Lachnospiraceae UCG004	Vascular dementia (sudden onset)	rs429358	C	T	-0.001	45411941	0.954	0.017	14306	C	T	0.601	19	44908684	7.45813E-06	0.134	360283	42162.979
genus Lachnospiraceae UCG004	Vascular dementia (sudden onset)	rs4840457	C	T	-0.013	6408682	0.527	0.022	14306	C	T	0.643	8	6551161	8.92874E-06	0.145	360283	32615.797
genus Lachnospiraceae UCG008	Vascular dementia (sudden onset)	rs12423672	T	G	0.000	5047705	0.960	0.028	14306	T	G	0.759	12	4938539	4.89159E-06	0.166	360283	30610.567
genus Lachnospiraceae UCG008	Vascular dementia (sudden onset)	rs12452096	A	G	0.005	75544032	0.835	0.020	14306	A	G	0.750	17	77547950	1.48929E-06	0.156	360283	77865.614
genus Lachnospiraceae UCG008	Vascular dementia (sudden onset)	rs2920	C	T	0.018	23884780	0.437	0.021	14306	C	T	0.592	1	23558289	2.20602E-06	0.125	360283	40501.384
genus Lachnospiraceae UCG008	Vascular dementia (sudden onset)	rs429358	C	T	0.001	45411941	0.933	0.024	14306	C	T	0.601	19	44908684	7.45813E-06	0.134	360283	42162.979
genus Lachnospiraceae UCG008	Vascular dementia (sudden onset)	rs4840457	C	T	0.012	6408682	0.745	0.032	14306	C	T	0.643	8	6551161	8.92874E-06	0.145	360283	32615.797
genus Lachnospiraceae UCG008	Vascular dementia (sudden onset)	rs71511414	G	A	0.004	79688991	0.901	0.031	14306	G	A	0.681	9	77074075	7.80746E-06	0.152	360283	30731.948
genus Lachnospiraceae UCG010	Vascular dementia (sudden onset)	rs12423672	T	G	0.013	5047705	0.603	0.020	14306	T	G	0.759	12	4938539	4.89159E-06	0.166	360283	30610.567
genus Lachnospiraceae UCG010	Vascular dementia (sudden onset)	rs12452096	A	G	0.009	75544032	0.567	0.015	14306	A	G	0.750	17	77547950	1.48929E-06	0.156	360283	77865.614
genus Lachnospiraceae UCG010	Vascular dementia (sudden onset)	rs429358	C	T	-0.003	45411941	0.809	0.018	14306	C	T	0.601	19	44908684	7.45813E-06	0.134	360283	42162.979
genus Lachnospiraceae UCG010	Vascular dementia (sudden onset)	rs4840457	C	T	-0.006	6408682	0.797	0.023	14306	C	T	0.643	8	6551161	8.92874E-06	0.145	360283	32615.797
genus Lactobacillus	Vascular dementia (sudden onset)	rs12452096	A	G	-0.016	75544032	0.456	0.020	14306	A	G	0.750	17	77547950	1.48929E-06	0.156	360283	77865.614
genus Lactobacillus	Vascular dementia (sudden onset)	rs2920	C	T	0.001	23884780	0.968	0.021	14306	C	T	0.592	1	23558289	2.20602E-06	0.125	360283	40501.384
genus Lactobacillus	Vascular dementia (sudden onset)	rs429358	C	T	-0.007	45411941	0.865	0.024	14306	C	T	0.601	19	44908684	7.45813E-06	0.134	360283	42162.979
genus Lactobacillus	Vascular dementia (sudden onset)	rs71511414	G	A	0.011	79688991	0.722	0.031	14306	G	A	0.681	9	77074075	7.80746E-06	0.152	360283	30731.948
genus Lactococcus	Vascular dementia (sudden onset)	rs12423672	T	G	-0.008	5047705	0.793	0.036	14306	T	G	0.759	12	4938539	4.89159E-06	0.166	360283	30610.567
genus Lactococcus	Vascular dementia (sudden onset)	rs12452096	A	G	0.012	75544032	0.617	0.027	14306	A	G	0.750	17	77547950	1.48929E-06	0.156	360283	77865.614
genus Lactococcus	Vascular dementia (sudden onset)	rs429358	C	T	-0.017	45411941	0.591	0.032	14306	C	T	0.601	19	44908684	7.45813E-06	0.134	360283	42162.979
genus Lactococcus	Vascular dementia (sudden onset)	rs4840457	C	T	0.010	6408682	0.802	0.042	14306	C	T	0.643	8	6551161	8.92874E-06	0.145	360283	32615.797
genus Lactococcus	Vascular dementia (sudden onset)	rs71511414	G	A	-0.033	79688991	0.396	0.040	14306	G	A	0.681	9	77074075	7.80746E-06	0.152	360283	30731.948
genus Marvinbryantia	Vascular dementia (sudden onset)	rs12452096	A	G	-0.005	75544032	0.717	0.015	14306	A	G	0.750	17	77547950	1.48929E-06	0.156	360283	77865.614
genus Marvinbryantia	Vascular dementia (sudden onset)	rs429358	C	T	0.008	45411941	0.663	0.020	14306	C	T	0.601	19	44908684	7.45813E-06	0.134	360283	42162.979
genus Marvinbryantia	Vascular dementia (sudden onset)	rs4840457	C	T	0.018	6408682	0.430	0.024	14306	C	T	0.643	8	6551161	8.92874E-06	0.145	360283	32615.797
genus Marvinbryantia	Vascular dementia (sudden onset)	rs71511414	G	A	-0.015	79688991	0.479	0.023	14306	G	A	0.681	9	77074075	7.80746E-06	0.152	360283	30731.948
genus Methanobrevibacter	Vascular dementia (sudden onset)	rs12452096	A	G	-0.010	75544032	0.702	0.029	14306	A	G	0.750	17	77547950	1.48929E-06	0.156	360283	77865.614
genus Methanobrevibacter	Vascular dementia (sudden onset)	rs429358	C	T	-0.019	45411941	0.661	0.034	14306	C	T	0.601	19	44908684	7.45813E-06	0.134	360283	42162.979
genus Odoribacter	Vascular dementia (sudden onset)	rs12452096	A	G	-0.004	75544032	0.774	0.014	14306	A	G	0.750	17	77547950	1.48929E-06	0.156	360283	77865.614
genus Odoribacter	Vascular dementia (sudden onset)	rs2920	C	T	0.004	23884780	0.798	0.015	14306	C	T	0.592	1	23558289	2.20602E-06	0.125	360283	40501.384
genus Odoribacter	Vascular dementia (sudden onset)	rs4840457	C	T	-0.008	6408682	0.671	0.022	14306	C	T	0.643	8	6551161	8.92874E-06	0.145	360283	32615.797
genus Odoribacter	Vascular dementia (sudden onset)	rs71511414	G	A	-0.011	79688991	0.557	0.021	14306	G	A	0.681	9	77074075	7.80746E-06	0.152	360283	30731.948
genus Olsenella	Vascular dementia (sudden onset)	rs12423672	T	G	0.022	5047705	0.586	0.039	14306	T	G	0.759	12	4938539	4.89159E-06	0.166	360283	30610.567
genus Olsenella	Vascular dementia (sudden onset)	rs2920	C	T	-0.013	23884780	0.641	0.029	14306	C	T	0.592	1	23558289	2.20602E-06	0.125	360283	40501.384
genus Olsenella	Vascular dementia (sudden onset)	rs71511414	G	A	0.001	79688991	0.998	0.042	14306	G	A	0.681	9	77074075	7.80746E-06	0.152	360283	30731.948
genus Oscillibacter	Vascular dementia (sudden onset)	rs12423672	T	G	-0.001	5047705	0.991	0.025	14306	T	G	0.759	12	4938539	4.89159E-06	0.166	360283	30610.567
genus Oscillibacter	Vascular dementia (sudden onset)	rs12452096	A	G	-0.002	75544032	0.884	0.018	14306	A	G	0.750	17	77547950	1.48929E-06	0.156	360283	77865.614
genus Oscillibacter	Vascular dementia (sudden onset)	rs4840457	C	T	0.020	6408682	0.465	0.028	14306	C	T	0.643	8	6551161	8.92874E-06	0.145	360283	32615.797
genus Oscillibacter	Vascular dementia (sudden onset)	rs71511414	G	A	0.017	79688991	0.512	0.027	14306	G	A	0.681	9	77074075	7.80746E-06	0.152	360283	30731.948
genus Oscillospira	Vascular dementia (sudden onset)	rs12452096	A	G	-0.016	75544032	0.347	0.017	14306	A	G	0.750	17	77547950	1.48929E-06	0.156	360283	77865.614
genus Oscillospira	Vascular dementia (sudden onset)	rs2920	C	T	-0.003	23884780	0.883	0.018	14306	C	T	0.592	1	23558289	2.20602E-06	0.125	360283	40501.384
genus Oscillospira	Vascular dementia (sudden onset)	rs429358	C	T	-0.005	45411941	0.763	0.020	14306	C	T	0.601	19	44908684	7.45813E-06	0.134	360283	42162.979
genus Oxalobacter	Vascular dementia (sudden onset)	rs12423672	T	G	-0.024	5047705	0.552	0.034	14306	T	G	0.759	12	4938539	4.89159E-06	0.166	360283	30610.567
genus Oxalobacter	Vascular dementia (sudden onset)	rs12452096	A	G	-0.020	75544032	0.426	0.025	14306	A	G	0.750	17	77547950	1.48929E-06	0.156	360283	77865.614
genus Oxalobacter	Vascular dementia (sudden onset)	rs2920	C	T	-0.002	23884780	0.969	0.026	14306	C	T	0.592	1	23558289	2.20602E-06	0.125	360283	40501.384
genus Oxalobacter	Vascular dementia (sudden onset)	rs4840457	C	T	0.020	6408682	0.635	0.039	14306	C	T	0.643	8	6551161	8.92874E-06	0.145	360283	32615.797
genus Parabacteroides	Vascular dementia (sudden onset)	rs12423672	T	G	0.007	5047705	0.904	0.017	14306	T	G	0.759	12	4938539	4.89159E-06	0.166	360283	30610.567
genus Parabacteroides	Vascular dementia (sudden onset)	rs2920	C	T	0.010	23884780	0.495	0.013	14306	C	T	0.592	1	23558289	2.20602E-06	0.125	360283	40501.384
genus Parabacteroides	Vascular dementia (sudden onset)	rs429358	C	T	-0.009	45411941	0.601	0.015	14306	C	T	0.601	19	44908684	7.45813E-06	0.134	360283	42162.979
genus Paraprevotella	Vascular dementia (sudden onset)	rs12452096	A	G	-0.018	75544032	0.384	0.020	14306	A	G	0.750	17	77547950	1.48929E-06	0.156	360283	77865.614
genus Paraprevotella	Vascular dementia (sudden onset)	rs429358	C	T	-0.005	45411941	0.844	0.024	14306	C	T	0.601	19	44908684	7.45813E-06	0.134	360283	42162.979
genus Paraprevotella	Vascular dementia (sudden onset)	rs4840457	C	T	0.009	6408682	0.836	0.032	14306	C	T	0.643	8	6551161	8.92874E-06	0.145	360283	32615.797
genus Paraprevotella	Vascular dementia (sudden onset)	rs71511414	G	A	0.016	79688991	0.592	0.031	14306	G	A	0.681	9	77074075	7.80746E-06	0.152	360283	30731.948
genus Parasutterella	Vascular dementia (sudden onset)	rs12423672	T	G	-0.011	5047705	0.608	0.021	14306	T	G	0.759	12	4938539	4.89159E-06	0.166	360283	30610.567
genus Parasutterella	Vascular dementia (sudden onset)	rs12452096	A	G	-0.013	75544032	0.395	0.016	14306	A	G	0.750	17	77547950	1.48929E-06	0.156	360283	77865.614
genus Parasutterella	Vascular dementia (sudden onset)	rs71511414	G	A	-0.010	79688991	0.605	0.024	14306	G	A	0.681	9	77074075	7.80746E-06	0.152	360283	30731.948
genus Peptococcus	Vascular dementia (sudden onset)	rs12423672	T	G	-0.005	5047705	0.771	0.031	14306	T	G	0.759	12	4938539	4.89159E-06	0.166	360283	30610.567
genus Peptococcus	Vascular dementia (sudden onset)	rs2920	C	T	0.021	23884780	0.385	0.023	14306	C	T	0.592	1	23558289	2.20602E-06	0.125	360283	40501.384
genus Peptococcus	Vascular dementia (sudden onset)	rs71511414	G	A	-0.010	79688991	0.749	0.034	14306	G	A	0.681	9	77074075	7.80746E-06	0.152	360283	30731.948
genus Phascolarctobacterium	Vascular dementia (sudden onset)	rs2920	C	T	-0.004	23884780	0.797	0.017	14306	C	T	0.592	1	23558289	2.20602E-06	0.125	360283	40501.384
genus Phascolarctobacterium	Vascular dementia (sudden onset)	rs429358	C	T	-0.013	45411941	0.427	0.019	14306	C	T	0.601	19	44908684	7.45813E-06	0.134	360283	42162.979
genus Phascolarctobacterium	Vascular dementia (sudden onset)	rs4840457	C	T	-0.003	6408682	0.945	0.025	14306	C	T	0.643	8	6551161	8.92874E-06	0.145	360283	32615.797
genus Phascolarctobacterium	Vascular dementia (sudden onset)	rs71511414	G	A	0.008	79688991	0.799	0.024	14306	G	A	0.681	9	77074075	7.80746E-06	0.152	360283	30731.948

genus Prevotella7	Vascular dementia (sudden onset)	rs12423672	T	G	0.012	5047705	0.646	0.039	14306	T	G	0.759	12	4938539	4.89159E-06	0.166	360283	30610.567
genus Prevotella7	Vascular dementia (sudden onset)	rs12452096	A	G	-0.021	75544032	0.482	0.029	14306	A	G	0.750	17	77547950	1.48929E-06	0.156	360283	77865.614
genus Prevotella7	Vascular dementia (sudden onset)	rs4840457	C	T	0.001	6408682	0.955	0.044	14306	C	T	0.643	8	6551161	8.92874E-06	0.145	360283	32615.797
genus Prevotella7	Vascular dementia (sudden onset)	rs71511414	G	A	-0.004	79688991	0.882	0.043	14306	G	A	0.681	9	77074075	7.80746E-06	0.152	360283	30731.948
genus Prevotella9	Vascular dementia (sudden onset)	rs12423672	T	G	-0.004	5047705	0.935	0.022	14306	T	G	0.759	12	4938539	4.89159E-06	0.166	360283	30610.567
genus Prevotella9	Vascular dementia (sudden onset)	rs12452096	A	G	-0.009	75544032	0.623	0.017	14306	A	G	0.750	17	77547950	1.48929E-06	0.156	360283	77865.614
genus Rikenellaceae RC9 gut group	Vascular dementia (sudden onset)	rs12423672	T	G	0.028	5047705	0.468	0.040	14306	T	G	0.759	12	4938539	4.89159E-06	0.166	360283	30610.567
genus Rikenellaceae RC9 gut group	Vascular dementia (sudden onset)	rs2920	C	T	-0.020	23884780	0.527	0.031	14306	C	T	0.592	1	23558289	2.20602E-06	0.125	360283	40501.384
genus Rikenellaceae RC9 gut group	Vascular dementia (sudden onset)	rs4840457	C	T	-0.009	6408682	0.879	0.046	14306	C	T	0.643	8	6551161	8.92874E-06	0.145	360283	32615.797
genus Rikenellaceae RC9 gut group	Vascular dementia (sudden onset)	rs71511414	G	A	0.008	79688991	0.797	0.045	14306	G	A	0.681	9	77074075	7.80746E-06	0.152	360283	30731.948
genus Romboutsia	Vascular dementia (sudden onset)	rs12452096	A	G	0.011	75544032	0.438	0.014	14306	A	G	0.750	17	77547950	1.48929E-06	0.156	360283	77865.614
genus Romboutsia	Vascular dementia (sudden onset)	rs429358	C	T	0.005	45411941	0.703	0.017	14306	C	T	0.601	19	44908684	7.45813E-06	0.134	360283	42162.979
genus Romboutsia	Vascular dementia (sudden onset)	rs4840457	C	T	-0.006	6408682	0.808	0.022	14306	C	T	0.643	8	6551161	8.92874E-06	0.145	360283	32615.797
genus Romboutsia	Vascular dementia (sudden onset)	rs71511414	G	A	0.019	79688991	0.420	0.022	14306	G	A	0.681	9	77074075	7.80746E-06	0.152	360283	30731.948
genus Roseburia	Vascular dementia (sudden onset)	rs12452096	A	G	0.005	75544032	0.698	0.013	14306	A	G	0.750	17	77547950	1.48929E-06	0.156	360283	77865.614
genus Roseburia	Vascular dementia (sudden onset)	rs2920	C	T	0.002	23884780	0.929	0.013	14306	C	T	0.592	1	23558289	2.20602E-06	0.125	360283	40501.384
genus Roseburia	Vascular dementia (sudden onset)	rs429358	C	T	-0.001	45411941	0.921	0.015	14306	C	T	0.601	19	44908684	7.45813E-06	0.134	360283	42162.979
genus Roseburia	Vascular dementia (sudden onset)	rs4840457	C	T	-0.013	6408682	0.566	0.020	14306	C	T	0.643	8	6551161	8.92874E-06	0.145	360283	32615.797
genus Roseburia	Vascular dementia (sudden onset)	rs71511414	G	A	-0.007	79688991	0.778	0.019	14306	G	A	0.681	9	77074075	7.80746E-06	0.152	360283	30731.948
genus Ruminiclostridium5	Vascular dementia (sudden onset)	rs12423672	T	G	0.004	5047705	0.937	0.017	14306	T	G	0.759	12	4938539	4.89159E-06	0.166	360283	30610.567
genus Ruminiclostridium5	Vascular dementia (sudden onset)	rs71511414	G	A	-0.015	79688991	0.506	0.019	14306	G	A	0.681	9	77074075	7.80746E-06	0.152	360283	30731.948
genus Ruminiclostridium6	Vascular dementia (sudden onset)	rs2920	C	T	0.011	23884780	0.529	0.015	14306	C	T	0.592	1	23558289	2.20602E-06	0.125	360283	40501.384
genus Ruminiclostridium6	Vascular dementia (sudden onset)	rs429358	C	T	0.007	45411941	0.690	0.018	14306	C	T	0.601	19	44908684	7.45813E-06	0.134	360283	42162.979
genus Ruminiclostridium6	Vascular dementia (sudden onset)	rs4840457	C	T	0.013	6408682	0.575	0.023	14306	C	T	0.643	8	6551161	8.92874E-06	0.145	360283	32615.797
genus Ruminiclostridium6	Vascular dementia (sudden onset)	rs71511414	G	A	0.008	79688991	0.685	0.022	14306	G	A	0.681	9	77074075	7.80746E-06	0.152	360283	30731.948
genus Ruminiclostridium9	Vascular dementia (sudden onset)	rs12452096	A	G	0.008	75544032	0.540	0.013	14306	A	G	0.750	17	77547950	1.48929E-06	0.156	360283	77865.614
genus Ruminiclostridium9	Vascular dementia (sudden onset)	rs429358	C	T	-0.007	45411941	0.627	0.016	14306	C	T	0.601	19	44908684	7.45813E-06	0.134	360283	42162.979
genus Ruminiclostridium9	Vascular dementia (sudden onset)	rs71511414	G	A	0.000	79688991	0.990	0.020	14306	G	A	0.681	9	77074075	7.80746E-06	0.152	360283	30731.948
genus Ruminococcaceae NK4A214 group	Vascular dementia (sudden onset)	rs12423672	T	G	-0.007	5047705	0.688	0.019	14306	T	G	0.759	12	4938539	4.89159E-06	0.166	360283	30610.567
genus Ruminococcaceae NK4A214 group	Vascular dementia (sudden onset)	rs12452096	A	G	0.009	75544032	0.507	0.014	14306	A	G	0.750	17	77547950	1.48929E-06	0.156	360283	77865.614
genus Ruminococcaceae NK4A214 group	Vascular dementia (sudden onset)	rs2920	C	T	-0.008	23884780	0.545	0.014	14306	C	T	0.592	1	23558289	2.20602E-06	0.125	360283	40501.384
genus Ruminococcaceae NK4A214 group	Vascular dementia (sudden onset)	rs429358	C	T	0.002	45411941	0.898	0.016	14306	C	T	0.601	19	44908684	7.45813E-06	0.134	360283	42162.979
genus Ruminococcaceae NK4A214 group	Vascular dementia (sudden onset)	rs71511414	G	A	-0.003	79688991	0.917	0.021	14306	G	A	0.681	9	77074075	7.80746E-06	0.152	360283	30731.948
genus Ruminococcaceae UCG002	Vascular dementia (sudden onset)	rs12452096	A	G	-0.001	75544032	0.912	0.013	14306	A	G	0.750	17	77547950	1.48929E-06	0.156	360283	77865.614
genus Ruminococcaceae UCG002	Vascular dementia (sudden onset)	rs429358	C	T	-0.004	45411941	0.869	0.016	14306	C	T	0.601	19	44908684	7.45813E-06	0.134	360283	42162.979
genus Ruminococcaceae UCG003	Vascular dementia (sudden onset)	rs12423672	T	G	-0.001	5047705	0.968	0.019	14306	T	G	0.759	12	4938539	4.89159E-06	0.166	360283	30610.567
genus Ruminococcaceae UCG003	Vascular dementia (sudden onset)	rs12452096	A	G	-0.002	75544032	0.872	0.014	14306	A	G	0.750	17	77547950	1.48929E-06	0.156	360283	77865.614
genus Ruminococcaceae UCG003	Vascular dementia (sudden onset)	rs2920	C	T	0.010	23884780	0.523	0.015	14306	C	T	0.592	1	23558289	2.20602E-06	0.125	360283	40501.384
genus Ruminococcaceae UCG003	Vascular dementia (sudden onset)	rs429358	C	T	0.007	45411941	0.595	0.017	14306	C	T	0.601	19	44908684	7.45813E-06	0.134	360283	42162.979
genus Ruminococcaceae UCG003	Vascular dementia (sudden onset)	rs4840457	C	T	0.009	6408682	0.666	0.022	14306	C	T	0.643	8	6551161	8.92874E-06	0.145	360283	32615.797
genus Ruminococcaceae UCG003	Vascular dementia (sudden onset)	rs71511414	G	A	-0.011	79688991	0.583	0.022	14306	G	A	0.681	9	77074075	7.80746E-06	0.152	360283	30731.948
genus Ruminococcaceae UCG004	Vascular dementia (sudden onset)	rs2920	C	T	-0.015	23884780	0.447	0.018	14306	C	T	0.592	1	23558289	2.20602E-06	0.125	360283	40501.384
genus Ruminococcaceae UCG004	Vascular dementia (sudden onset)	rs4840457	C	T	0.011	6408682	0.661	0.027	14306	C	T	0.643	8	6551161	8.92874E-06	0.145	360283	32615.797
genus Ruminococcaceae UCG005	Vascular dementia (sudden onset)	rs12452096	A	G	0.004	75544032	0.739	0.013	14306	A	G	0.750	17	77547950	1.48929E-06	0.156	360283	77865.614
genus Ruminococcaceae UCG005	Vascular dementia (sudden onset)	rs2920	C	T	-0.008	23884780	0.538	0.014	14306	C	T	0.592	1	23558289	2.20602E-06	0.125	360283	40501.384
genus Ruminococcaceae UCG005	Vascular dementia (sudden onset)	rs429358	C	T	0.004	45411941	0.741	0.016	14306	C	T	0.601	19	44908684	7.45813E-06	0.134	360283	42162.979
genus Ruminococcaceae UCG005	Vascular dementia (sudden onset)	rs4840457	C	T	0.008	6408682	0.771	0.021	14306	C	T	0.643	8	6551161	8.92874E-06	0.145	360283	32615.797
genus Ruminococcaceae UCG005	Vascular dementia (sudden onset)	rs71511414	G	A	0.010	79688991	0.622	0.020	14306	G	A	0.681	9	77074075	7.80746E-06	0.152	360283	30731.948
genus Ruminococcaceae UCG009	Vascular dementia (sudden onset)	rs2920	C	T	0.007	23884780	0.728	0.020	14306	C	T	0.592	1	23558289	2.20602E-06	0.125	360283	40501.384
genus Ruminococcaceae UCG009	Vascular dementia (sudden onset)	rs71511414	G	A	0.017	79688991	0.605	0.030	14306	G	A	0.681	9	77074075	7.80746E-06	0.152	360283	30731.948
genus Ruminococcaceae UCG010	Vascular dementia (sudden onset)	rs12452096	A	G	-0.006	75544032	0.701	0.015	14306	A	G	0.750	17	77547950	1.48929E-06	0.156	360283	77865.614
genus Ruminococcaceae UCG010	Vascular dementia (sudden onset)	rs429358	C	T	0.003	45411941	0.881	0.018	14306	C	T	0.601	19	44908684	7.45813E-06	0.134	360283	42162.979
genus Ruminococcaceae UCG010	Vascular dementia (sudden onset)	rs71511414	G	A	-0.006	79688991	0.764	0.023	14306	G	A	0.681	9	77074075	7.80746E-06	0.152	360283	30731.948
genus Ruminococcaceae UCG011	Vascular dementia (sudden onset)	rs12423672	T	G	-0.016	5047705	0.678	0.039	14306	T	G	0.759	12	4938539	4.89159E-06	0.166	360283	30610.567
genus Ruminococcaceae UCG011	Vascular dementia (sudden onset)	rs12452096	A	G	-0.001	75544032	0.965	0.027	14306	A	G	0.750	17	77547950	1.48929E-06	0.156	360283	77865.614
genus Ruminococcaceae UCG011	Vascular dementia (sudden onset)	rs2920	C	T	-0.005	23884780	0.866	0.029	14306	C	T	0.592	1	23558289	2.20602E-06	0.125	360283	40501.384
genus Ruminococcaceae UCG011	Vascular dementia (sudden onset)	rs429358	C	T	-0.013	45411941	0.704	0.033	14306	C	T	0.601	19	44908684	7.45813E-06	0.134	360283	42162.979
genus Ruminococcaceae UCG011	Vascular dementia (sudden onset)	rs4840457	C	T	0.022	6408682	0.623	0.043	14306	C	T	0.643	8	6551161	8.92874E-06	0.145	360283	32615.797
genus Ruminococcaceae UCG013	Vascular dementia (sudden onset)	rs2920	C	T	-0.011	23884780	0.424	0.014	14306	C	T	0.592	1	23558289	2.20602E-06	0.125	360283	40501.384
genus Ruminococcaceae UCG013	Vascular dementia (sudden onset)	rs71511414	G	A	0.013	79688991	0.547	0.020	14306	G	A	0.681	9	77074075	7.80746E-06	0.152	360283	30731.948
genus Ruminococcaceae UCG014	Vascular dementia (sudden onset)	rs12423672	T	G	0.011	5047705	0.495	0.019	14306	T	G	0.759	12	4938539	4.89159E-06	0.166	360283	30610.567
genus Ruminococcaceae UCG014	Vascular dementia (sudden onset)	rs2920	C	T	-0.007	23884780	0.655	0.015	14306	C	T	0.592	1	23558289	2.20602E-06	0.125	360283	40501.384
genus Ruminococcaceae UCG014	Vascular dementia (sudden onset)	rs4840457	C	T	-0.001	6408682	0.946	0.022	14306	C	T	0.643	8	6551161	8.92874E-06	0.145	360283	32615.797
genus Ruminococcaceae UCG014	Vascular dementia (sudden onset)	rs71511414	G	A	-0.014	79688991	0.413	0.021	14306	G	A	0.681	9	77074075	7.80746E-06	0.152	360283	30731.948
genus Ruminococcus1	Vascular dementia (sudden onset)	rs12423672	T	G	-0.010	5047705	0.600	0.018	14306	T	G	0.759	12	4938539	4.89159E-06	0.166	360283	30610.567
genus Ruminococcus1	Vascular dementia (sudden onset)	rs2920	C	T	-0.003	23884780	0.816	0.014	14306	C	T	0.592	1	23558289	2.20602E-06	0.125	360283	40501.384
genus Ruminococcus1	Vascular dementia (sudden onset)	rs429358	C	T	-0.008	45411941	0.683	0.016	14306	C	T	0.601	19	44908684	7.45813E-06	0.134	360283	42162.979
genus Ruminococcus1	Vascular dementia (sudden onset)	rs71511414	G	A	-0.008	79688991	0.714	0.020	14306	G	A	0.681	9	77074075	7.80746E-06	0.152	360283	30731.948
genus Ruminococcus2	Vascular dementia (sudden onset)	rs2920	C	T	-0.010	23884780	0.501	0.014	14306	C	T	0.592	1	23558289	2.20602E-06	0.125	360283	40501.384
genus Ruminococcus2	Vascular dementia (sudden onset)	rs429358	C	T	0.007	45411941	0.633	0.016	14306	C	T	0.601	19	44908684	7.45813E-06	0.134	360283	42162.979
genus Ruminococcus2	Vascular dementia (sudden onset)	rs4840457	C	T	0.011	6408682	0.594	0.022	14306	C	T	0.643	8	6551161	8.92874E-06	0.145	360283	32615.797

genus Ruminococcus2	Vascular dementia (sudden onset)	rs71511414	G	A	-0.016	79688991	0.527	0.021	14306	G	A	0.681	9	77074075	7.80746E-06	0.152	360283	30731.948
genus Ruminococcus gauvreauii group	Vascular dementia (sudden onset)	rs12423672	T	G	-0.012	5047705	0.601	0.020	14306	T	G	0.759	12	4938539	4.89159E-06	0.166	360283	30610.567
genus Ruminococcus gauvreauii group	Vascular dementia (sudden onset)	rs12452096	A	G	-0.003	75544032	0.842	0.015	14306	A	G	0.750	17	77547950	1.48929E-06	0.156	360283	77865.614
genus Ruminococcus gauvreauii group	Vascular dementia (sudden onset)	rs429358	C	T	0.011	45411941	0.457	0.018	14306	C	T	0.601	19	44908684	7.45813E-06	0.134	360283	42162.979
genus Ruminococcus gauvreauii group	Vascular dementia (sudden onset)	rs4840457	C	T	-0.002	6408682	0.836	0.023	14306	C	T	0.643	8	6551161	8.92874E-06	0.145	360283	32615.797
genus Ruminococcus gauvreauii group	Vascular dementia (sudden onset)	rs71511414	G	A	0.004	79688991	0.810	0.022	14306	G	A	0.681	9	77074075	7.80746E-06	0.152	360283	30731.948
genus Ruminococcus gnavus group	Vascular dementia (sudden onset)	rs12452096	A	G	0.012	75544032	0.535	0.021	14306	A	G	0.750	17	77547950	1.48929E-06	0.156	360283	77865.614
genus Ruminococcus gnavus group	Vascular dementia (sudden onset)	rs2920	C	T	0.005	23884780	0.810	0.022	14306	C	T	0.592	1	23558289	2.20602E-06	0.125	360283	40501.384
genus Ruminococcus gnavus group	Vascular dementia (sudden onset)	rs71511414	G	A	-0.023	79688991	0.432	0.032	14306	G	A	0.681	9	77074075	7.80746E-06	0.152	360283	30731.948
genus Ruminococcus torques group	Vascular dementia (sudden onset)	rs12452096	A	G	0.002	75544032	0.893	0.013	14306	A	G	0.750	17	77547950	1.48929E-06	0.156	360283	77865.614
genus Ruminococcus torques group	Vascular dementia (sudden onset)	rs429358	C	T	-0.001	45411941	0.916	0.015	14306	C	T	0.601	19	44908684	7.45813E-06	0.134	360283	42162.979
genus Ruminococcus torques group	Vascular dementia (sudden onset)	rs4840457	C	T	0.009	6408682	0.683	0.020	14306	C	T	0.643	8	6551161	8.92874E-06	0.145	360283	32615.797
genus Sellimonas	Vascular dementia (sudden onset)	rs12452096	A	G	-0.028	75544032	0.354	0.030	14306	A	G	0.750	17	77547950	1.48929E-06	0.156	360283	77865.614
genus Sellimonas	Vascular dementia (sudden onset)	rs2920	C	T	-0.003	23884780	0.944	0.031	14306	C	T	0.592	1	23558289	2.20602E-06	0.125	360283	40501.384
genus Sellimonas	Vascular dementia (sudden onset)	rs4840457	C	T	-0.027	6408682	0.626	0.047	14306	C	T	0.643	8	6551161	8.92874E-06	0.145	360283	32615.797
genus Sellimonas	Vascular dementia (sudden onset)	rs71511414	G	A	0.011	79688991	0.948	0.044	14306	G	A	0.681	9	77074075	7.80746E-06	0.152	360283	30731.948
genus Senegalimassilia	Vascular dementia (sudden onset)	rs12423672	T	G	0.001	5047705	0.971	0.028	14306	T	G	0.759	12	4938539	4.89159E-06	0.166	360283	30610.567
genus Senegalimassilia	Vascular dementia (sudden onset)	rs2920	C	T	-0.012	23884780	0.563	0.021	14306	C	T	0.592	1	23558289	2.20602E-06	0.125	360283	40501.384
genus Senegalimassilia	Vascular dementia (sudden onset)	rs4840457	C	T	0.003	6408682	0.914	0.032	14306	C	T	0.643	8	6551161	8.92874E-06	0.145	360283	32615.797
genus Senegalimassilia	Vascular dementia (sudden onset)	rs71511414	G	A	0.011	79688991	0.738	0.031	14306	G	A	0.681	9	77074075	7.80746E-06	0.152	360283	30731.948
genus Slackia	Vascular dementia (sudden onset)	rs12452096	A	G	-0.017	75544032	0.437	0.021	14306	A	G	0.750	17	77547950	1.48929E-06	0.156	360283	77865.614
genus Slackia	Vascular dementia (sudden onset)	rs2920	C	T	0.003	23884780	0.923	0.023	14306	C	T	0.592	1	23558289	2.20602E-06	0.125	360283	40501.384
genus Slackia	Vascular dementia (sudden onset)	rs4840457	C	T	-0.002	6408682	0.944	0.034	14306	C	T	0.643	8	6551161	8.92874E-06	0.145	360283	32615.797
genus Streptococcus	Vascular dementia (sudden onset)	rs12452096	A	G	-0.006	75544032	0.688	0.013	14306	A	G	0.750	17	77547950	1.48929E-06	0.156	360283	77865.614
genus Streptococcus	Vascular dementia (sudden onset)	rs2920	C	T	-0.005	23884780	0.694	0.014	14306	C	T	0.592	1	23558289	2.20602E-06	0.125	360283	40501.384
genus Streptococcus	Vascular dementia (sudden onset)	rs4840457	C	T	-0.018	6408682	0.400	0.021	14306	C	T	0.643	8	6551161	8.92874E-06	0.145	360283	32615.797
genus Streptococcus	Vascular dementia (sudden onset)	rs71511414	G	A	0.001	79688991	0.965	0.020	14306	G	A	0.681	9	77074075	7.80746E-06	0.152	360283	30731.948
genus Subdoligranulum	Vascular dementia (sudden onset)	rs12423672	T	G	0.007	5047705	0.603	0.017	14306	T	G	0.759	12	4938539	4.89159E-06	0.166	360283	30610.567
genus Subdoligranulum	Vascular dementia (sudden onset)	rs12452096	A	G	0.007	75544032	0.610	0.013	14306	A	G	0.750	17	77547950	1.48929E-06	0.156	360283	77865.614
genus Subdoligranulum	Vascular dementia (sudden onset)	rs2920	C	T	-0.002	23884780	0.868	0.013	14306	C	T	0.592	1	23558289	2.20602E-06	0.125	360283	40501.384
genus Subdoligranulum	Vascular dementia (sudden onset)	rs429358	C	T	-0.005	45411941	0.772	0.015	14306	C	T	0.601	19	44908684	7.45813E-06	0.134	360283	42162.979
genus Subdoligranulum	Vascular dementia (sudden onset)	rs4840457	C	T	0.003	6408682	0.906	0.020	14306	C	T	0.643	8	6551161	8.92874E-06	0.145	360283	32615.797
genus Subdoligranulum	Vascular dementia (sudden onset)	rs71511414	G	A	0.001	79688991	0.909	0.019	14306	G	A	0.681	9	77074075	7.80746E-06	0.152	360283	30731.948
genus Sutterella	Vascular dementia (sudden onset)	rs12452096	A	G	0.013	75544032	0.383	0.015	14306	A	G	0.750	17	77547950	1.48929E-06	0.156	360283	77865.614
genus Sutterella	Vascular dementia (sudden onset)	rs71511414	G	A	0.016	79688991	0.506	0.023	14306	G	A	0.681	9	77074075	7.80746E-06	0.152	360283	30731.948
genus Terrisporobacter	Vascular dementia (sudden onset)	rs2920	C	T	-0.015	23884780	0.468	0.022	14306	C	T	0.592	1	23558289	2.20602E-06	0.125	360283	40501.384
genus Terrisporobacter	Vascular dementia (sudden onset)	rs429358	C	T	-0.010	45411941	0.657	0.025	14306	C	T	0.601	19	44908684	7.45813E-06	0.134	360283	42162.979
genus Turicibacter	Vascular dementia (sudden onset)	rs12423672	T	G	0.004	5047705	0.953	0.025	14306	T	G	0.759	12	4938539	4.89159E-06	0.166	360283	30610.567
genus Turicibacter	Vascular dementia (sudden onset)	rs12452096	A	G	0.003	75544032	0.902	0.018	14306	A	G	0.750	17	77547950	1.48929E-06	0.156	360283	77865.614
genus Turicibacter	Vascular dementia (sudden onset)	rs429358	C	T	0.001	45411941	0.870	0.021	14306	C	T	0.601	19	44908684	7.45813E-06	0.134	360283	42162.979
genus Turicibacter	Vascular dementia (sudden onset)	rs4840457	C	T	0.010	6408682	0.751	0.028	14306	C	T	0.643	8	6551161	8.92874E-06	0.145	360283	32615.797
genus Tyzzerella3	Vascular dementia (sudden onset)	rs2920	C	T	-0.018	23884780	0.464	0.024	14306	C	T	0.592	1	23558289	2.20602E-06	0.125	360283	40501.384
genus Tyzzerella3	Vascular dementia (sudden onset)	rs429358	C	T	0.000	45411941	0.979	0.028	14306	C	T	0.601	19	44908684	7.45813E-06	0.134	360283	42162.979
genus Tyzzerella3	Vascular dementia (sudden onset)	rs4840457	C	T	-0.008	6408682	0.818	0.037	14306	C	T	0.643	8	6551161	8.92874E-06	0.145	360283	32615.797
genus Veillonella)	Vascular dementia (sudden onset)	rs2920	C	T	-0.003	23884780	0.860	0.018	14306	C	T	0.592	1	23558289	2.20602E-06	0.125	360283	40501.384
genus Veillonella)	Vascular dementia (sudden onset)	rs4840457	C	T	0.003	6408682	0.944	0.028	14306	C	T	0.643	8	6551161	8.92874E-06	0.145	360283	32615.797
genus Veillonella)	Vascular dementia (sudden onset)	rs71511414	G	A	0.017	79688991	0.580	0.027	14306	G	A	0.681	9	77074075	7.80746E-06	0.152	360283	30731.948
genus Victivallis	Vascular dementia (sudden onset)	rs12423672	T	G	0.000	5047705	0.860	0.039	1531	T	G	0.759	12	4938539	4.89159E-06	0.166	360283	30610.567
genus Victivallis	Vascular dementia (sudden onset)	rs429358	C	T	0.009	45411941	0.847	0.037	1531	C	T	0.601	19	44908684	7.45813E-06	0.134	360283	42162.979
genus Actinomyces	Vascular dementia (undefined)	rs12224047	T	C	0.012	36820790	0.623	0.024	14306	T	C	-0.263	11	36799240	4.56278E-06	0.057	361227	8033.487
genus Actinomyces	Vascular dementia (undefined)	rs12449066	G	A	-0.015	79177293	0.665	0.028	14306	G	A	-0.262	16	79143396	2.01674E-06	0.055	361227	8803.514
genus Actinomyces	Vascular dementia (undefined)	rs193392	T	C	0.000	3085245	0.977	0.016	14306	T	C	-0.204	20	3104599	8.28324E-06	0.046	361227	7460.147
genus Actinomyces	Vascular dementia (undefined)	rs2292090	T	C	0.012	70588309	0.500	0.018	14306	T	C	0.217	4	69722591	9.99332E-06	0.049	361227	6178.734
genus Actinomyces	Vascular dementia (undefined)	rs2972558	T	C	-0.013	45356141	0.467	0.018	14306	T	C	0.234	19	44852884	8.32415E-06	0.052	361227	7242.374
genus Actinomyces	Vascular dementia (undefined)	rs2978951	G	A	-0.003	6823295	0.844	0.017	14306	G	A	0.248	8	6965773	2.48222E-08	0.045	361227	11061.935
genus Actinomyces	Vascular dementia (undefined)	rs359878	C	T	0.011	185438949	0.538	0.019	14306	C	T	-0.211	2	184574222	4.6443E-06	0.046	361227	6959.700
genus Actinomyces	Vascular dementia (undefined)	rs429358	C	T	0.030	45411941	0.176	0.023	14306	C	T	0.695	19	44908684	9.26616E-39	0.053	361227	58999.679
genus Actinomyces	Vascular dementia (undefined)	rs6133343	G	T	0.023	721797	0.422	0.027	14306	G	T	0.312	20	741153	3.89825E-06	0.068	361227	6210.744
genus Adlercreutzia	Vascular dementia (undefined)	rs12224047	T	C	-0.005	36820790	0.791	0.023	14306	T	C	-0.263	11	36799240	4.56278E-06	0.057	361227	8033.487
genus Adlercreutzia	Vascular dementia (undefined)	rs2972558	T	C	-0.007	45356141	0.684	0.017	14306	T	C	0.234	19	44852884	8.32415E-06	0.052	361227	7242.374
genus Adlercreutzia	Vascular dementia (undefined)	rs2978951	G	A	-0.009	6823295	0.581	0.016	14306	G	A	0.248	8	6965773	2.48222E-08	0.045	361227	11061.935
genus Adlercreutzia	Vascular dementia (undefined)	rs429358	C	T	0.007	45411941	0.796	0.022	14306	C	T	0.695	19	44908684	9.26616E-39	0.053	361227	58999.679
genus Adlercreutzia	Vascular dementia (undefined)	rs6133343	G	T	0.011	721797	0.717	0.026	14306	G	T	0.312	20	741153	3.89825E-06	0.068	361227	6210.744
genus Adlercreutzia	Vascular dementia (undefined)	rs78566090	A	G	-0.010	125740204	0.779	0.038	14306	A	G	0.346	8	124727963	9.44735E-07	0.071	361227	7049.571
genus Akkermansia	Vascular dementia (undefined)	rs12224047	T	C	-0.006	36820790	0.736	0.019	14306	T	C	-0.263	11	36799240	4.56278E-06	0.057	361227	8033.487
genus Akkermansia	Vascular dementia (undefined)	rs12449066	G	A	0.001	79177293	0.967	0.022	14306	G	A	-0.262	16	79143396	2.01674E-06	0.055	361227	8803.514
genus Akkermansia	Vascular dementia (undefined)	rs193392	T	C	0.003	3085245	0.819	0.013	14306	T	C	-0.204	20	3104599	8.28324E-06	0.046	361227	7460.147
genus Akkermansia	Vascular dementia (undefined)	rs2292090	T	C	-0.008	70588309	0.613	0.015	14306	T	C	0.217	4	69722591	9.99332E-06	0.049	361227	6178.734
genus Akkermansia	Vascular dementia (undefined)	rs2972558	T	C	0.008	45356141	0.588	0.014	14306	T	C	0.234	19	44852884	8.32415E-06	0.052	361227	7242.374
genus Akkermansia	Vascular dementia (undefined)	rs429358	C	T	0.018	45411941	0.337	0.019	14306	C	T	0.695	19	44908684	9.26616E-39	0.053	361227	58999.679
genus Akkermansia	Vascular dementia (undefined)	rs6133343	G	T	-0.009	721797	0.747	0.021	14306	G	T	0.312	20	741153	3.89825E-06	0.068	361227	6210.744

genus Akkermansia	Vascular dementia (undefined)	rs71298638	A	G	-0.003	63232261	0.878	0.027	14306	A	G	0.379	3	63246585	1.1165E-06	0.078	361227	6515.599
genus Akkermansia	Vascular dementia (undefined)	rs78566090	A	G	0.004	125740204	0.865	0.030	14306	A	G	0.346	8	124727963	9.44735E-07	0.071	361227	7049.571
genus Alistipes	Vascular dementia (undefined)	rs12449066	G	A	-0.011	79177293	0.583	0.018	14306	G	A	-0.262	16	79143396	2.01674E-06	0.055	361227	8803.514
genus Alistipes	Vascular dementia (undefined)	rs193392	T	C	-0.005	3085245	0.665	0.011	14306	T	C	-0.204	20	3104599	8.28324E-06	0.046	361227	7460.147
genus Alistipes	Vascular dementia (undefined)	rs2292090	T	C	0.008	70588309	0.443	0.012	14306	T	C	0.217	4	69722591	9.99332E-06	0.049	361227	6178.734
genus Alistipes	Vascular dementia (undefined)	rs2972558	T	C	0.006	45356141	0.642	0.012	14306	T	C	0.234	19	44852884	8.32415E-06	0.052	361227	7242.374
genus Alistipes	Vascular dementia (undefined)	rs2978951	G	A	0.007	6823295	0.525	0.011	14306	G	A	0.248	8	6965773	2.48222E-08	0.045	361227	11061.935
genus Alistipes	Vascular dementia (undefined)	rs359878	C	T	0.001	185438949	0.889	0.012	14306	C	T	-0.211	2	184574222	4.6443E-06	0.046	361227	6959.700
genus Alistipes	Vascular dementia (undefined)	rs429358	C	T	-0.026	45411941	0.088	0.015	14306	C	T	0.695	19	44908684	9.26616E-39	0.053	361227	58999.679
genus Alistipes	Vascular dementia (undefined)	rs6133343	G	T	-0.001	721797	0.988	0.017	14306	G	T	0.312	20	741153	3.89825E-06	0.068	361227	6210.744
genus Alistipes	Vascular dementia (undefined)	rs71298638	A	G	0.006	63232261	0.806	0.022	14306	A	G	0.379	3	63246585	1.1165E-06	0.078	361227	6515.599
genus Allisonella	Vascular dementia (undefined)	rs193392	T	C	0.010	3085245	0.673	0.025	14306	T	C	-0.204	20	3104599	8.28324E-06	0.046	361227	7460.147
genus Allisonella	Vascular dementia (undefined)	rs2978951	G	A	-0.015	6823295	0.579	0.025	14306	G	A	0.248	8	6965773	2.48222E-08	0.045	361227	11061.935
genus Allisonella	Vascular dementia (undefined)	rs359878	C	T	0.002	185438949	0.973	0.029	14306	C	T	-0.211	2	184574222	4.6443E-06	0.046	361227	6959.700
genus Allisonella	Vascular dementia (undefined)	rs429358	C	T	-0.033	45411941	0.350	0.036	14306	C	T	0.695	19	44908684	9.26616E-39	0.053	361227	58999.679
genus Allisonella	Vascular dementia (undefined)	rs6133343	G	T	0.011	721797	0.727	0.039	14306	G	T	0.312	20	741153	3.89825E-06	0.068	361227	6210.744
genus Alloprevotella	Vascular dementia (undefined)	rs12449066	G	A	0.016	79177293	0.752	0.045	14306	G	A	-0.262	16	79143396	2.01674E-06	0.055	361227	8803.514
genus Alloprevotella	Vascular dementia (undefined)	rs2292090	T	C	0.007	70588309	0.849	0.029	14306	T	C	0.217	4	69722591	9.99332E-06	0.049	361227	6178.734
genus Alloprevotella	Vascular dementia (undefined)	rs2978951	G	A	-0.029	6823295	0.267	0.026	14306	G	A	0.248	8	6965773	2.48222E-08	0.045	361227	11061.935
genus Alloprevotella	Vascular dementia (undefined)	rs359878	C	T	0.016	185438949	0.633	0.031	14306	C	T	-0.211	2	184574222	4.6443E-06	0.046	361227	6959.700
genus Anaerofilum	Vascular dementia (undefined)	rs12224047	T	C	0.010	36820790	0.730	0.029	14306	T	C	-0.263	11	36799240	4.56278E-06	0.057	361227	8033.487
genus Anaerofilum	Vascular dementia (undefined)	rs12449066	G	A	0.002	79177293	0.990	0.034	14306	G	A	-0.262	16	79143396	2.01674E-06	0.055	361227	8803.514
genus Anaerofilum	Vascular dementia (undefined)	rs193392	T	C	-0.007	3085245	0.726	0.020	14306	T	C	-0.204	20	3104599	8.28324E-06	0.046	361227	7460.147
genus Anaerofilum	Vascular dementia (undefined)	rs2292090	T	C	0.001	70588309	0.994	0.022	14306	T	C	0.217	4	69722591	9.99332E-06	0.049	361227	6178.734
genus Anaerofilum	Vascular dementia (undefined)	rs2972558	T	C	0.000	45356141	0.970	0.022	14306	T	C	0.234	19	44852884	8.32415E-06	0.052	361227	7242.374
genus Anaerofilum	Vascular dementia (undefined)	rs2978951	G	A	-0.004	6823295	0.825	0.020	14306	G	A	0.248	8	6965773	2.48222E-08	0.045	361227	11061.935
genus Anaerofilum	Vascular dementia (undefined)	rs359878	C	T	0.020	185438949	0.405	0.023	14306	C	T	-0.211	2	184574222	4.6443E-06	0.046	361227	6959.700
genus Anaerofilum	Vascular dementia (undefined)	rs429358	C	T	-0.013	45411941	0.660	0.028	14306	C	T	0.695	19	44908684	9.26616E-39	0.053	361227	58999.679
genus Anaerofilum	Vascular dementia (undefined)	rs78566090	A	G	0.011	125740204	0.820	0.047	14306	A	G	0.346	8	124727963	9.44735E-07	0.071	361227	7049.571
genus Anaerostipes	Vascular dementia (undefined)	rs12224047	T	C	0.003	36820790	0.960	0.016	14306	T	C	-0.263	11	36799240	4.56278E-06	0.057	361227	8033.487
genus Anaerostipes	Vascular dementia (undefined)	rs2972558	T	C	-0.006	45356141	0.603	0.012	14306	T	C	0.234	19	44852884	8.32415E-06	0.052	361227	7242.374
genus Anaerostipes	Vascular dementia (undefined)	rs359878	C	T	-0.001	185438949	0.980	0.013	14306	C	T	-0.211	2	184574222	4.6443E-06	0.046	361227	6959.700
genus Anaerostipes	Vascular dementia (undefined)	rs429358	C	T	0.029	45411941	0.057	0.016	14306	C	T	0.695	19	44908684	9.26616E-39	0.053	361227	58999.679
genus Anaerostipes	Vascular dementia (undefined)	rs6133343	G	T	-0.001	721797	0.946	0.017	14306	G	T	0.312	20	741153	3.89825E-06	0.068	361227	6210.744
genus Anaerostipes	Vascular dementia (undefined)	rs71298638	A	G	0.011	63232261	0.619	0.023	14306	A	G	0.379	3	63246585	1.1165E-06	0.078	361227	6515.599
genus Anaerostipes	Vascular dementia (undefined)	rs78566090	A	G	-0.010	125740204	0.738	0.026	14306	A	G	0.346	8	124727963	9.44735E-07	0.071	361227	7049.571
genus Anaerotruncus	Vascular dementia (undefined)	rs12224047	T	C	0.005	36820790	0.720	0.016	14306	T	C	-0.263	11	36799240	4.56278E-06	0.057	361227	8033.487
genus Anaerotruncus	Vascular dementia (undefined)	rs12449066	G	A	0.013	79177293	0.547	0.019	14306	G	A	-0.262	16	79143396	2.01674E-06	0.055	361227	8803.514
genus Anaerotruncus	Vascular dementia (undefined)	rs193392	T	C	-0.009	3085245	0.452	0.011	14306	T	C	-0.204	20	3104599	8.28324E-06	0.046	361227	7460.147
genus Anaerotruncus	Vascular dementia (undefined)	rs2292090	T	C	0.005	70588309	0.671	0.012	14306	T	C	0.217	4	69722591	9.99332E-06	0.049	361227	6178.734
genus Anaerotruncus	Vascular dementia (undefined)	rs2978951	G	A	0.005	6823295	0.620	0.011	14306	G	A	0.248	8	6965773	2.48222E-08	0.045	361227	11061.935
genus Anaerotruncus	Vascular dementia (undefined)	rs359878	C	T	0.005	185438949	0.675	0.013	14306	C	T	-0.211	2	184574222	4.6443E-06	0.046	361227	6959.700
genus Anaerotruncus	Vascular dementia (undefined)	rs429358	C	T	-0.019	45411941	0.201	0.016	14306	C	T	0.695	19	44908684	9.26616E-39	0.053	361227	58999.679
genus Anaerotruncus	Vascular dementia (undefined)	rs6133343	G	T	0.000	721797	0.842	0.018	14306	G	T	0.312	20	741153	3.89825E-06	0.068	361227	6210.744
genus Bacteroides	Vascular dementia (undefined)	rs12224047	T	C	0.005	36820790	0.732	0.015	14306	T	C	-0.263	11	36799240	4.56278E-06	0.057	361227	8033.487
genus Bacteroides	Vascular dementia (undefined)	rs12449066	G	A	0.007	79177293	0.669	0.018	14306	G	A	-0.262	16	79143396	2.01674E-06	0.055	361227	8803.514
genus Bacteroides	Vascular dementia (undefined)	rs2978951	G	A	-0.012	6823295	0.278	0.011	14306	G	A	0.248	8	6965773	2.48222E-08	0.045	361227	11061.935
genus Bacteroides	Vascular dementia (undefined)	rs359878	C	T	-0.011	185438949	0.385	0.012	14306	C	T	-0.211	2	184574222	4.6443E-06	0.046	361227	6959.700
genus Bacteroides	Vascular dementia (undefined)	rs78566090	A	G	0.006	125740204	0.853	0.024	14306	A	G	0.346	8	124727963	9.44735E-07	0.071	361227	7049.571
genus Barnesiella	Vascular dementia (undefined)	rs12449066	G	A	0.016	79177293	0.479	0.020	14306	G	A	-0.262	16	79143396	2.01674E-06	0.055	361227	8803.514
genus Barnesiella	Vascular dementia (undefined)	rs193392	T	C	-0.009	3085245	0.477	0.012	14306	T	C	-0.204	20	3104599	8.28324E-06	0.046	361227	7460.147
genus Barnesiella	Vascular dementia (undefined)	rs2972558	T	C	0.003	45356141	0.825	0.013	14306	T	C	0.234	19	44852884	8.32415E-06	0.052	361227	7242.374
genus Barnesiella	Vascular dementia (undefined)	rs2978951	G	A	0.010	6823295	0.400	0.012	14306	G	A	0.248	8	6965773	2.48222E-08	0.045	361227	11061.935
genus Barnesiella	Vascular dementia (undefined)	rs359878	C	T	-0.009	185438949	0.506	0.014	14306	C	T	-0.211	2	184574222	4.6443E-06	0.046	361227	6959.700
genus Barnesiella	Vascular dementia (undefined)	rs429358	C	T	-0.005	45411941	0.787	0.017	14306	C	T	0.695	19	44908684	9.26616E-39	0.053	361227	58999.679
genus Barnesiella	Vascular dementia (undefined)	rs71298638	A	G	0.004	63232261	0.879	0.024	14306	A	G	0.379	3	63246585	1.1165E-06	0.078	361227	6515.599
genus Barnesiella	Vascular dementia (undefined)	rs78566090	A	G	0.011	125740204	0.752	0.027	14306	A	G	0.346	8	124727963	9.44735E-07	0.071	361227	7049.571
genus Bifidobacterium	Vascular dementia (undefined)	rs12224047	T	C	-0.006	36820790	0.695	0.017	14306	T	C	-0.263	11	36799240	4.56278E-06	0.057	361227	8033.487
genus Bifidobacterium	Vascular dementia (undefined)	rs2292090	T	C	-0.006	70588309	0.745	0.013	14306	T	C	0.217	4	69722591	9.99332E-06	0.049	361227	6178.734
genus Bifidobacterium	Vascular dementia (undefined)	rs2972558	T	C	-0.001	45356141	0.950	0.013	14306	T	C	0.234	19	44852884	8.32415E-06	0.052	361227	7242.374
genus Bifidobacterium	Vascular dementia (undefined)	rs2978951	G	A	-0.004	6823295	0.747	0.012	14306	G	A	0.248	8	6965773	2.48222E-08	0.045	361227	11061.935
genus Bifidobacterium	Vascular dementia (undefined)	rs429358	C	T	0.019	45411941	0.204	0.017	14306	C	T	0.695	19	44908684	9.26616E-39	0.053	361227	58999.679
genus Bifidobacterium	Vascular dementia (undefined)	rs6133343	G	T	-0.008	721797	0.712	0.018	14306	G	T	0.312	20	741153	3.89825E-06	0.068	361227	6210.744
genus Bifidobacterium	Vascular dementia (undefined)	rs71298638	A	G	-0.016	63232261	0.544	0.024	14306	A	G	0.379	3	63246585	1.1165E-06	0.078	361227	6515.599
genus Bilophila	Vascular dementia (undefined)	rs12224047	T	C	0.003	36820790	0.832	0.018	14306	T	C	-0.263	11	36799240	4.56278E-06	0.057	361227	8033.487
genus Bilophila	Vascular dementia (undefined)	rs12449066	G	A	0.019	79177293	0.371	0.022	14306	G	A	-0.262	16	79143396	2.01674E-06	0.055	361227	8803.514
genus Bilophila	Vascular dementia (undefined)	rs2972558	T	C	-0.007	45356141	0.593	0.013	14306	T	C	0.234	19	44852884	8.32415E-06	0.052	361227	7242.374
genus Bilophila	Vascular dementia (undefined)	rs2978951	G	A	0.011	6823295	0.380	0.013	14306	G	A	0.248	8	6965773	2.48222E-08	0.045	361227	11061.935
genus Bilophila	Vascular dementia (undefined)	rs359878	C	T	-0.002	185438949	0.912	0.015	14306	C	T	-0.211	2	184574222	4.6443E-06	0.046	361227	6959.700
genus Bilophila	Vascular dementia (undefined)	rs429358	C	T	0.007	45411941	0.656	0.018	14306	C	T	0.695	19	44908684	9.26616E-39	0.053	361227	58999.679
genus Bilophila	Vascular dementia (undefined)	rs6133343	G	T	-0.006	721797	0.715	0.020	14306	G	T	0.312	20	741153	3.89825E-06	0.068	361227	6210.744

genus Bilophila	Vascular dementia (undefined)	rs71298638	A	G	0.007	63232261	0.824	0.026	14306	A	G	0.379	3	63246585	1.1165E-06	0.078	361227	6515.599
genus Bilophila	Vascular dementia (undefined)	rs78566090	A	G	0.027	125740204	0.373	0.029	14306	A	G	0.346	8	124727963	9.44735E-07	0.071	361227	7049.571
genus Butyricicoccus	Vascular dementia (undefined)	rs12449066	G	A	0.002	79177293	0.979	0.019	14306	G	A	-0.262	16	79143396	2.01674E-06	0.055	361227	8803.514
genus Butyricicoccus	Vascular dementia (undefined)	rs193392	T	C	-0.007	3085245	0.553	0.011	14306	T	C	-0.204	20	3104599	8.28324E-06	0.046	361227	7460.147
genus Butyricicoccus	Vascular dementia (undefined)	rs2972558	T	C	0.002	45356141	0.857	0.012	14306	T	C	0.234	19	44852884	8.32415E-06	0.052	361227	7242.374
genus Butyricicoccus	Vascular dementia (undefined)	rs2978951	G	A	-0.005	6823295	0.675	0.011	14306	G	A	0.248	8	6965773	2.48222E-08	0.045	361227	11061.935
genus Butyricicoccus	Vascular dementia (undefined)	rs429358	C	T	-0.018	45411941	0.288	0.015	14306	C	T	0.695	19	44908684	9.26616E-39	0.053	361227	58999.679
genus Butyricimonas	Vascular dementia (undefined)	rs12224047	T	C	0.010	36820790	0.599	0.020	14306	T	C	-0.263	11	36799240	4.56278E-06	0.057	361227	8033.487
genus Butyricimonas	Vascular dementia (undefined)	rs12449066	G	A	-0.019	79177293	0.600	0.024	14306	G	A	-0.262	16	79143396	2.01674E-06	0.055	361227	8803.514
genus Butyricimonas	Vascular dementia (undefined)	rs2972558	T	C	-0.005	45356141	0.722	0.015	14306	T	C	0.234	19	44852884	8.32415E-06	0.052	361227	7242.374
genus Butyricimonas	Vascular dementia (undefined)	rs359878	C	T	-0.005	185438949	0.813	0.016	14306	C	T	-0.211	2	184574222	4.6443E-06	0.046	361227	6959.700
genus Butyricimonas	Vascular dementia (undefined)	rs429358	C	T	-0.044	45411941	0.025	0.020	14306	C	T	0.695	19	44908684	9.26616E-39	0.053	361227	58999.679
genus Butyricimonas	Vascular dementia (undefined)	rs6133343	G	T	-0.007	721797	0.723	0.022	14306	G	T	0.312	20	741153	3.89825E-06	0.068	361227	6210.744
genus Butyrivibrio	Vascular dementia (undefined)	rs12449066	G	A	0.006	79177293	0.837	0.041	14306	G	A	-0.262	16	79143396	2.01674E-06	0.055	361227	8803.514
genus Butyrivibrio	Vascular dementia (undefined)	rs193392	T	C	0.008	3085245	0.749	0.025	14306	T	C	-0.204	20	3104599	8.28324E-06	0.046	361227	7460.147
genus Butyrivibrio	Vascular dementia (undefined)	rs2972558	T	C	0.018	45356141	0.485	0.027	14306	T	C	0.234	19	44852884	8.32415E-06	0.052	361227	7242.374
genus Butyrivibrio	Vascular dementia (undefined)	rs359878	C	T	0.004	185438949	0.916	0.028	14306	C	T	-0.211	2	184574222	4.6443E-06	0.046	361227	6959.700
genus Butyrivibrio	Vascular dementia (undefined)	rs429358	C	T	-0.016	45411941	0.585	0.034	14306	C	T	0.695	19	44908684	9.26616E-39	0.053	361227	58999.679
genus Butyrivibrio	Vascular dementia (undefined)	rs6133343	G	T	-0.009	721797	0.813	0.040	14306	G	T	0.312	20	741153	3.89825E-06	0.068	361227	6210.744
genus Candidatus Soleaferrea	Vascular dementia (undefined)	rs12224047	T	C	0.014	36820790	0.509	0.026	14306	T	C	-0.263	11	36799240	4.56278E-06	0.057	361227	8033.487
genus Candidatus Soleaferrea	Vascular dementia (undefined)	rs12449066	G	A	0.006	79177293	0.690	0.031	14306	G	A	-0.262	16	79143396	2.01674E-06	0.055	361227	8803.514
genus Candidatus Soleaferrea	Vascular dementia (undefined)	rs193392	T	C	-0.008	3085245	0.644	0.018	14306	T	C	-0.204	20	3104599	8.28324E-06	0.046	361227	7460.147
genus Candidatus Soleaferrea	Vascular dementia (undefined)	rs2978951	G	A	-0.015	6823295	0.407	0.018	14306	G	A	0.248	8	6965773	2.48222E-08	0.045	361227	11061.935
genus Candidatus Soleaferrea	Vascular dementia (undefined)	rs359878	C	T	-0.018	185438949	0.368	0.021	14306	C	T	-0.211	2	184574222	4.6443E-06	0.046	361227	6959.700
genus Candidatus Soleaferrea	Vascular dementia (undefined)	rs429358	C	T	0.020	45411941	0.391	0.026	14306	C	T	0.695	19	44908684	9.26616E-39	0.053	361227	58999.679
genus Candidatus Soleaferrea	Vascular dementia (undefined)	rs6133343	G	T	-0.024	721797	0.444	0.029	14306	G	T	0.312	20	741153	3.89825E-06	0.068	361227	6210.744
genus Candidatus Soleaferrea	Vascular dementia (undefined)	rs71298638	A	G	0.004	63232261	0.857	0.037	14306	A	G	0.379	3	63246585	1.1165E-06	0.078	361227	6515.599
genus Candidatus Soleaferrea	Vascular dementia (undefined)	rs78566090	A	G	-0.004	125740204	0.915	0.046	14306	A	G	0.346	8	124727963	9.44735E-07	0.071	361227	7049.571
genus Catenibacterium	Vascular dementia (undefined)	rs12224047	T	C	0.016	36820790	0.613	0.036	14306	T	C	-0.263	11	36799240	4.56278E-06	0.057	361227	8033.487
genus Catenibacterium	Vascular dementia (undefined)	rs193392	T	C	0.016	3085245	0.500	0.025	14306	T	C	-0.204	20	3104599	8.28324E-06	0.046	361227	7460.147
genus Catenibacterium	Vascular dementia (undefined)	rs2292090	T	C	-0.018	70588309	0.544	0.028	14306	T	C	0.217	4	69722591	9.99332E-06	0.049	361227	6178.734
genus Catenibacterium	Vascular dementia (undefined)	rs2978951	G	A	-0.028	6823295	0.280	0.025	14306	G	A	0.248	8	6965773	2.48222E-08	0.045	361227	11061.935
genus Catenibacterium	Vascular dementia (undefined)	rs359878	C	T	0.007	185438949	0.892	0.030	14306	C	T	-0.211	2	184574222	4.6443E-06	0.046	361227	6959.700
genus Catenibacterium	Vascular dementia (undefined)	rs429358	C	T	0.030	45411941	0.433	0.036	14306	C	T	0.695	19	44908684	9.26616E-39	0.053	361227	58999.679
genus Christensenellaceae R 7group	Vascular dementia (undefined)	rs12449066	G	A	-0.010	79177293	0.527	0.019	14306	G	A	-0.262	16	79143396	2.01674E-06	0.055	361227	8803.514
genus Christensenellaceae R 7group	Vascular dementia (undefined)	rs359878	C	T	-0.003	185438949	0.854	0.013	14306	C	T	-0.211	2	184574222	4.6443E-06	0.046	361227	6959.700
genus Christensenellaceae R 7group	Vascular dementia (undefined)	rs429358	C	T	0.011	45411941	0.455	0.016	14306	C	T	0.695	19	44908684	9.26616E-39	0.053	361227	58999.679
genus Christensenellaceae R 7group	Vascular dementia (undefined)	rs71298638	A	G	0.000	63232261	0.969	0.023	14306	A	G	0.379	3	63246585	1.1165E-06	0.078	361227	6515.599
genus Christensenellaceae R 7group	Vascular dementia (undefined)	rs78566090	A	G	0.008	125740204	0.803	0.026	14306	A	G	0.346	8	124727963	9.44735E-07	0.071	361227	7049.571
genus Clostridium innocuum group	Vascular dementia (undefined)	rs2292090	T	C	-0.016	70588309	0.571	0.024	14306	T	C	0.217	4	69722591	9.99332E-06	0.049	361227	6178.734
genus Clostridium innocuum group	Vascular dementia (undefined)	rs2972558	T	C	-0.012	45356141	0.587	0.024	14306	T	C	0.234	19	44852884	8.32415E-06	0.052	361227	7242.374
genus Clostridium innocuum group	Vascular dementia (undefined)	rs429358	C	T	-0.009	45411941	0.813	0.031	14306	C	T	0.695	19	44908684	9.26616E-39	0.053	361227	58999.679
genus Clostridium innocuum group	Vascular dementia (undefined)	rs6133343	G	T	-0.019	721797	0.585	0.035	14306	G	T	0.312	20	741153	3.89825E-06	0.068	361227	6210.744
genus Clostridium innocuum group	Vascular dementia (undefined)	rs71298638	A	G	-0.001	63232261	0.995	0.046	14306	A	G	0.379	3	63246585	1.1165E-06	0.078	361227	6515.599
genus Clostridium sensustricto1	Vascular dementia (undefined)	rs12224047	T	C	-0.011	36820790	0.490	0.017	14306	T	C	-0.263	11	36799240	4.56278E-06	0.057	361227	8033.487
genus Clostridium sensustricto1	Vascular dementia (undefined)	rs193392	T	C	-0.002	3085245	0.823	0.012	14306	T	C	-0.204	20	3104599	8.28324E-06	0.046	361227	7460.147
genus Clostridium sensustricto1	Vascular dementia (undefined)	rs2292090	T	C	0.002	70588309	0.940	0.013	14306	T	C	0.217	4	69722591	9.99332E-06	0.049	361227	6178.734
genus Clostridium sensustricto1	Vascular dementia (undefined)	rs2978951	G	A	0.011	6823295	0.377	0.012	14306	G	A	0.248	8	6965773	2.48222E-08	0.045	361227	11061.935
genus Clostridium sensustricto1	Vascular dementia (undefined)	rs359878	C	T	0.013	185438949	0.364	0.014	14306	C	T	-0.211	2	184574222	4.6443E-06	0.046	361227	6959.700
genus Clostridium sensustricto1	Vascular dementia (undefined)	rs429358	C	T	0.011	45411941	0.544	0.017	14306	C	T	0.695	19	44908684	9.26616E-39	0.053	361227	58999.679
genus Clostridium sensustricto1	Vascular dementia (undefined)	rs6133343	G	T	0.009	721797	0.608	0.019	14306	G	T	0.312	20	741153	3.89825E-06	0.068	361227	6210.744
genus Clostridium sensustricto1	Vascular dementia (undefined)	rs71298638	A	G	0.012	63232261	0.618	0.025	14306	A	G	0.379	3	63246585	1.1165E-06	0.078	361227	6515.599
genus Clostridium sensustricto1	Vascular dementia (undefined)	rs78566090	A	G	-0.009	125740204	0.775	0.028	14306	A	G	0.346	8	124727963	9.44735E-07	0.071	361227	7049.571
genus Collinsella	Vascular dementia (undefined)	rs12224047	T	C	-0.004	36820790	0.813	0.017	14306	T	C	-0.263	11	36799240	4.56278E-06	0.057	361227	8033.487
genus Collinsella	Vascular dementia (undefined)	rs12449066	G	A	0.011	79177293	0.636	0.021	14306	G	A	-0.262	16	79143396	2.01674E-06	0.055	361227	8803.514
genus Collinsella	Vascular dementia (undefined)	rs193392	T	C	0.008	3085245	0.503	0.012	14306	T	C	-0.204	20	3104599	8.28324E-06	0.046	361227	7460.147
genus Collinsella	Vascular dementia (undefined)	rs2292090	T	C	0.010	70588309	0.430	0.013	14306	T	C	0.217	4	69722591	9.99332E-06	0.049	361227	6178.734
genus Collinsella	Vascular dementia (undefined)	rs2972558	T	C	-0.010	45356141	0.454	0.013	14306	T	C	0.234	19	44852884	8.32415E-06	0.052	361227	7242.374
genus Collinsella	Vascular dementia (undefined)	rs2978951	G	A	0.004	6823295	0.743	0.012	14306	G	A	0.248	8	6965773	2.48222E-08	0.045	361227	11061.935
genus Collinsella	Vascular dementia (undefined)	rs429358	C	T	0.024	45411941	0.129	0.017	14306	C	T	0.695	19	44908684	9.26616E-39	0.053	361227	58999.679
genus Collinsella	Vascular dementia (undefined)	rs6133343	G	T	0.001	721797	0.925	0.019	14306	G	T	0.312	20	741153	3.89825E-06	0.068	361227	6210.744
genus Collinsella	Vascular dementia (undefined)	rs71298638	A	G	-0.002	63232261	0.937	0.024	14306	A	G	0.379	3	63246585	1.1165E-06	0.078	361227	6515.599
genus Coprobacter	Vascular dementia (undefined)	rs12224047	T	C	0.007	36820790	0.766	0.025	14306	T	C	-0.263	11	36799240	4.56278E-06	0.057	361227	8033.487
genus Coprobacter	Vascular dementia (undefined)	rs12449066	G	A	-0.015	79177293	0.730	0.030	14306	G	A	-0.262	16	79143396	2.01674E-06	0.055	361227	8803.514
genus Coprobacter	Vascular dementia (undefined)	rs193392	T	C	-0.014	3085245	0.455	0.018	14306	T	C	-0.204	20	3104599	8.28324E-06	0.046	361227	7460.147
genus Coprobacter	Vascular dementia (undefined)	rs2972558	T	C	0.014	45356141	0.480	0.019	14306	T	C	0.234	19	44852884	8.32415E-06	0.052	361227	7242.374
genus Coprobacter	Vascular dementia (undefined)	rs2978951	G	A	-0.017	6823295	0.330	0.017	14306	G	A	0.248	8	6965773	2.48222E-08	0.045	361227	11061.935
genus Coprobacter	Vascular dementia (undefined)	rs429358	C	T	-0.001	45411941	0.930	0.025	14306	C	T	0.695	19	44908684	9.26616E-39	0.053	361227	58999.679
genus Coprobacter	Vascular dementia (undefined)	rs6133343	G	T	0.000	721797	0.981	0.028	14306	G	T	0.312	20	741153	3.89825E-06	0.068	361227	6210.744
genus Coprobacter	Vascular dementia (undefined)	rs71298638	A	G	0.002	63232261	0.971	0.036	14306	A	G	0.379	3	63246585	1.1165E-06	0.078	361227	6515.599
genus Coprococcus1	Vascular dementia (undefined)	rs12224047	T	C	-0.003	36820790	0.893	0.016	14306	T	C	-0.263	11	36799240	4.56278E-06	0.057	361227	8033.487

genus Coprococcus1	Vascular dementia (undefined)	rs2972558	T	C	-0.007	45356141	0.535	0.012	14306	T	C	0.234	19	44852884	8.32415E-06	0.052	361227	7242.374
genus Coprococcus1	Vascular dementia (undefined)	rs359878	C	T	0.003	185438949	0.779	0.013	14306	C	T	-0.211	2	184574222	4.6443E-06	0.046	361227	6959.700
genus Coprococcus1	Vascular dementia (undefined)	rs429358	C	T	0.014	45411941	0.326	0.016	14306	C	T	0.695	19	44908684	9.26616E-39	0.053	361227	58999.679
genus Coprococcus1	Vascular dementia (undefined)	rs71298638	A	G	-0.004	63232261	0.835	0.023	14306	A	G	0.379	3	63246585	1.1165E-06	0.078	361227	6515.599
genus Coprococcus1	Vascular dementia (undefined)	rs78566090	A	G	-0.024	125740204	0.369	0.026	14306	A	G	0.346	8	124727963	9.44735E-07	0.071	361227	7049.571
genus Coprococcus2	Vascular dementia (undefined)	rs12224047	T	C	0.007	36820790	0.657	0.019	14306	T	C	-0.263	11	36799240	4.56278E-06	0.057	361227	8033.487
genus Coprococcus2	Vascular dementia (undefined)	rs12449066	G	A	0.000	79177293	0.978	0.023	14306	G	A	-0.262	16	79143396	2.01674E-06	0.055	361227	8803.514
genus Coprococcus2	Vascular dementia (undefined)	rs193392	T	C	0.007	3085245	0.607	0.014	14306	T	C	-0.204	20	3104599	8.28324E-06	0.046	361227	7460.147
genus Coprococcus2	Vascular dementia (undefined)	rs2972558	T	C	0.003	45356141	0.825	0.014	14306	T	C	0.234	19	44852884	8.32415E-06	0.052	361227	7242.374
genus Coprococcus2	Vascular dementia (undefined)	rs2978951	G	A	-0.001	6823295	0.924	0.013	14306	G	A	0.248	8	6965773	2.48222E-08	0.045	361227	11061.935
genus Coprococcus2	Vascular dementia (undefined)	rs359878	C	T	-0.005	185438949	0.721	0.015	14306	C	T	-0.211	2	184574222	4.6443E-06	0.046	361227	6959.700
genus Coprococcus2	Vascular dementia (undefined)	rs429358	C	T	0.002	45411941	0.982	0.019	14306	C	T	0.695	19	44908684	9.26616E-39	0.053	361227	58999.679
genus Coprococcus3	Vascular dementia (undefined)	rs193392	T	C	-0.010	3085245	0.400	0.011	14306	T	C	-0.204	20	3104599	8.28324E-06	0.046	361227	7460.147
genus Coprococcus3	Vascular dementia (undefined)	rs2978951	G	A	0.003	6823295	0.753	0.011	14306	G	A	0.248	8	6965773	2.48222E-08	0.045	361227	11061.935
genus Coprococcus3	Vascular dementia (undefined)	rs359878	C	T	-0.003	185438949	0.727	0.013	14306	C	T	-0.211	2	184574222	4.6443E-06	0.046	361227	6959.700
genus Coprococcus3	Vascular dementia (undefined)	rs429358	C	T	0.028	45411941	0.087	0.016	14306	C	T	0.695	19	44908684	9.26616E-39	0.053	361227	58999.679
genus Coprococcus3	Vascular dementia (undefined)	rs71298638	A	G	-0.003	63232261	0.909	0.023	14306	A	G	0.379	3	63246585	1.1165E-06	0.078	361227	6515.599
genus Coprococcus3	Vascular dementia (undefined)	rs78566090	A	G	-0.003	125740204	0.907	0.026	14306	A	G	0.346	8	124727963	9.44735E-07	0.071	361227	7049.571
genus Defluviitaleaceae UCG011	Vascular dementia (undefined)	rs12224047	T	C	-0.002	36820790	0.949	0.023	14306	T	C	-0.263	11	36799240	4.56278E-06	0.057	361227	8033.487
genus Defluviitaleaceae UCG011	Vascular dementia (undefined)	rs12449066	G	A	-0.019	79177293	0.388	0.027	14306	G	A	-0.262	16	79143396	2.01674E-06	0.055	361227	8803.514
genus Defluviitaleaceae UCG011	Vascular dementia (undefined)	rs193392	T	C	-0.012	3085245	0.430	0.016	14306	T	C	-0.204	20	3104599	8.28324E-06	0.046	361227	7460.147
genus Defluviitaleaceae UCG011	Vascular dementia (undefined)	rs359878	C	T	-0.012	185438949	0.489	0.018	14306	C	T	-0.211	2	184574222	4.6443E-06	0.046	361227	6959.700
genus Defluviitaleaceae UCG011	Vascular dementia (undefined)	rs429358	C	T	-0.003	45411941	0.819	0.022	14306	C	T	0.695	19	44908684	9.26616E-39	0.053	361227	58999.679
genus Defluviitaleaceae UCG011	Vascular dementia (undefined)	rs6133343	G	T	-0.020	721797	0.411	0.026	14306	G	T	0.312	20	741153	3.89825E-06	0.068	361227	6210.744
genus Defluviitaleaceae UCG011	Vascular dementia (undefined)	rs78566090	A	G	0.011	125740204	0.815	0.037	14306	A	G	0.346	8	124727963	9.44735E-07	0.071	361227	7049.571
genus Desulfovibrio	Vascular dementia (undefined)	rs12224047	T	C	0.014	36820790	0.496	0.021	14306	T	C	-0.263	11	36799240	4.56278E-06	0.057	361227	8033.487
genus Desulfovibrio	Vascular dementia (undefined)	rs12449066	G	A	-0.013	79177293	0.543	0.025	14306	G	A	-0.262	16	79143396	2.01674E-06	0.055	361227	8803.514
genus Desulfovibrio	Vascular dementia (undefined)	rs2292090	T	C	-0.008	70588309	0.714	0.016	14306	T	C	0.217	4	69722591	9.99332E-06	0.049	361227	6178.734
genus Desulfovibrio	Vascular dementia (undefined)	rs2978951	G	A	0.005	6823295	0.737	0.015	14306	G	A	0.248	8	6965773	2.48222E-08	0.045	361227	11061.935
genus Desulfovibrio	Vascular dementia (undefined)	rs359878	C	T	-0.004	185438949	0.837	0.017	14306	C	T	-0.211	2	184574222	4.6443E-06	0.046	361227	6959.700
genus Desulfovibrio	Vascular dementia (undefined)	rs429358	C	T	0.006	45411941	0.782	0.021	14306	C	T	0.695	19	44908684	9.26616E-39	0.053	361227	58999.679
genus Desulfovibrio	Vascular dementia (undefined)	rs6133343	G	T	-0.009	721797	0.574	0.024	14306	G	T	0.312	20	741153	3.89825E-06	0.068	361227	6210.744
genus Desulfovibrio	Vascular dementia (undefined)	rs71298638	A	G	-0.021	63232261	0.471	0.030	14306	A	G	0.379	3	63246585	1.1165E-06	0.078	361227	6515.599
genus Desulfovibrio	Vascular dementia (undefined)	rs78566090	A	G	-0.014	125740204	0.763	0.034	14306	A	G	0.346	8	124727963	9.44735E-07	0.071	361227	7049.571
(genus Dialister	Vascular dementia (undefined)	rs12449066	G	A	0.015	79177293	0.583	0.022	14306	G	A	-0.262	16	79143396	2.01674E-06	0.055	361227	8803.514
(genus Dialister	Vascular dementia (undefined)	rs193392	T	C	0.011	3085245	0.402	0.013	14306	T	C	-0.204	20	3104599	8.28324E-06	0.046	361227	7460.147
(genus Dialister	Vascular dementia (undefined)	rs2978951	G	A	0.006	6823295	0.653	0.013	14306	G	A	0.248	8	6965773	2.48222E-08	0.045	361227	11061.935
(genus Dialister	Vascular dementia (undefined)	rs359878	C	T	-0.005	185438949	0.733	0.015	14306	C	T	-0.211	2	184574222	4.6443E-06	0.046	361227	6959.700
(genus Dialister	Vascular dementia (undefined)	rs429358	C	T	-0.026	45411941	0.198	0.019	14306	C	T	0.695	19	44908684	9.26616E-39	0.053	361227	58999.679
genus Dorea	Vascular dementia (undefined)	rs12224047	T	C	0.014	36820790	0.365	0.015	14306	T	C	-0.263	11	36799240	4.56278E-06	0.057	361227	8033.487
genus Dorea	Vascular dementia (undefined)	rs12449066	G	A	0.002	79177293	0.954	0.018	14306	G	A	-0.262	16	79143396	2.01674E-06	0.055	361227	8803.514
genus Dorea	Vascular dementia (undefined)	rs2972558	T	C	-0.009	45356141	0.428	0.012	14306	T	C	0.234	19	44852884	8.32415E-06	0.052	361227	7242.374
genus Dorea	Vascular dementia (undefined)	rs359878	C	T	0.007	185438949	0.578	0.012	14306	C	T	-0.211	2	184574222	4.6443E-06	0.046	361227	6959.700
genus Dorea	Vascular dementia (undefined)	rs429358	C	T	0.030	45411941	0.033	0.015	14306	C	T	0.695	19	44908684	9.26616E-39	0.053	361227	58999.679
genus Dorea	Vascular dementia (undefined)	rs71298638	A	G	-0.020	63232261	0.369	0.022	14306	A	G	0.379	3	63246585	1.1165E-06	0.078	361227	6515.599
genus Eggerthella	Vascular dementia (undefined)	rs12224047	T	C	-0.001	36820790	0.885	0.029	14306	T	C	-0.263	11	36799240	4.56278E-06	0.057	361227	8033.487
genus Eggerthella	Vascular dementia (undefined)	rs12449066	G	A	0.002	79177293	0.981	0.034	14306	G	A	-0.262	16	79143396	2.01674E-06	0.055	361227	8803.514
genus Eggerthella	Vascular dementia (undefined)	rs193392	T	C	-0.007	3085245	0.775	0.020	14306	T	C	-0.204	20	3104599	8.28324E-06	0.046	361227	7460.147
genus Eggerthella	Vascular dementia (undefined)	rs2292090	T	C	-0.016	70588309	0.508	0.022	14306	T	C	0.217	4	69722591	9.99332E-06	0.049	361227	6178.734
genus Eggerthella	Vascular dementia (undefined)	rs2972558	T	C	-0.003	45356141	0.884	0.021	14306	T	C	0.234	19	44852884	8.32415E-06	0.052	361227	7242.374
genus Eggerthella	Vascular dementia (undefined)	rs2978951	G	A	-0.002	6823295	0.926	0.020	14306	G	A	0.248	8	6965773	2.48222E-08	0.045	361227	11061.935
genus Eggerthella	Vascular dementia (undefined)	rs359878	C	T	0.014	185438949	0.511	0.023	14306	C	T	-0.211	2	184574222	4.6443E-06	0.046	361227	6959.700
genus Eggerthella	Vascular dementia (undefined)	rs429358	C	T	0.017	45411941	0.611	0.029	14306	C	T	0.695	19	44908684	9.26616E-39	0.053	361227	58999.679
genus Eggerthella	Vascular dementia (undefined)	rs71298638	A	G	0.005	63232261	0.868	0.040	14306	A	G	0.379	3	63246585	1.1165E-06	0.078	361227	6515.599
genus Eisenbergiella	Vascular dementia (undefined)	rs2292090	T	C	0.013	70588309	0.524	0.021	14306	T	C	0.217	4	69722591	9.99332E-06	0.049	361227	6178.734
genus Eisenbergiella	Vascular dementia (undefined)	rs2972558	T	C	-0.012	45356141	0.556	0.021	14306	T	C	0.234	19	44852884	8.32415E-06	0.052	361227	7242.374
genus Eisenbergiella	Vascular dementia (undefined)	rs2978951	G	A	-0.010	6823295	0.597	0.019	14306	G	A	0.248	8	6965773	2.48222E-08	0.045	361227	11061.935
genus Eisenbergiella	Vascular dementia (undefined)	rs429358	C	T	-0.013	45411941	0.612	0.028	14306	C	T	0.695	19	44908684	9.26616E-39	0.053	361227	58999.679
genus Eisenbergiella	Vascular dementia (undefined)	rs6133343	G	T	-0.019	721797	0.541	0.031	14306	G	T	0.312	20	741153	3.89825E-06	0.068	361227	6210.744
genus Eisenbergiella	Vascular dementia (undefined)	rs71298638	A	G	0.004	63232261	0.841	0.039	14306	A	G	0.379	3	63246585	1.1165E-06	0.078	361227	6515.599
genus Eisenbergiella	Vascular dementia (undefined)	rs78566090	A	G	-0.008	125740204	0.764	0.047	14306	A	G	0.346	8	124727963	9.44735E-07	0.071	361227	7049.571
genus Enterorhabdus	Vascular dementia (undefined)	rs12224047	T	C	-0.017	36820790	0.497	0.024	14306	T	C	-0.263	11	36799240	4.56278E-06	0.057	361227	8033.487
genus Enterorhabdus	Vascular dementia (undefined)	rs193392	T	C	0.014	3085245	0.395	0.016	14306	T	C	-0.204	20	3104599	8.28324E-06	0.046	361227	7460.147
genus Enterorhabdus	Vascular dementia (undefined)	rs2292090	T	C	-0.002	70588309	0.927	0.018	14306	T	C	0.217	4	69722591	9.99332E-06	0.049	361227	6178.734
genus Enterorhabdus	Vascular dementia (undefined)	rs2978951	G	A	0.014	6823295	0.382	0.017	14306	G	A	0.248	8	6965773	2.48222E-08	0.045	361227	11061.935
genus Enterorhabdus	Vascular dementia (undefined)	rs359878	C	T	-0.016	185438949	0.410	0.019	14306	C	T	-0.211	2	184574222	4.6443E-06	0.046	361227	6959.700
genus Enterorhabdus	Vascular dementia (undefined)	rs429358	C	T	0.009	45411941	0.754	0.023	14306	C	T	0.695	19	44908684	9.26616E-39	0.053	361227	58999.679
genus Enterorhabdus	Vascular dementia (undefined)	rs6133343	G	T	0.019	721797	0.495	0.027	14306	G	T	0.312	20	741153	3.89825E-06	0.068	361227	6210.744
genus Enterorhabdus	Vascular dementia (undefined)	rs71298638	A	G	-0.008	63232261	0.833	0.033	14306	A	G	0.379	3	63246585	1.1165E-06	0.078	361227	6515.599
genus Enterorhabdus	Vascular dementia (undefined)	rs78566090	A	G	0.023	125740204	0.518	0.038	14306	A	G	0.346	8	124727963	9.44735E-07	0.071	361227	7049.571
genus Erysipelatoclostridium	Vascular dementia (undefined)	rs12224047	T	C	-0.017	36820790	0.370	0.021	14306	T	C	-0.263	11	36799240	4.56278E-06	0.057	361227	8033.487

genus Erysipelatoclostridium	Vascular dementia (undefined)	rs12449066	G	A	0.008	79177293	0.645	0.025	14306	G	A	-0.262	16	79143396	2.01674E-06	0.055	361227	8803.514
genus Erysipelatoclostridium	Vascular dementia (undefined)	rs193392	T	C	-0.011	3085245	0.474	0.014	14306	T	C	-0.204	20	3104599	8.28324E-06	0.046	361227	7460.147
genus Erysipelatoclostridium	Vascular dementia (undefined)	rs2292090	T	C	0.000	70588309	0.948	0.016	14306	T	C	0.217	4	69722591	9.99332E-06	0.049	361227	6178.734
genus Erysipelatoclostridium	Vascular dementia (undefined)	rs2972558	T	C	0.009	45356141	0.578	0.015	14306	T	C	0.234	19	44852884	8.32415E-06	0.052	361227	7242.374
genus Erysipelatoclostridium	Vascular dementia (undefined)	rs2978951	G	A	-0.005	6823295	0.740	0.015	14306	G	A	0.248	8	6965773	2.48222E-08	0.045	361227	11061.935
genus Erysipelatoclostridium	Vascular dementia (undefined)	rs429358	C	T	0.017	45411941	0.432	0.020	14306	C	T	0.695	19	44908684	9.26616E-39	0.053	361227	58999.679
genus Erysipelatoclostridium	Vascular dementia (undefined)	rs6133343	G	T	-0.002	721797	0.831	0.023	14306	G	T	0.312	20	741153	3.89825E-06	0.068	361227	6210.744
genus Erysipelatoclostridium	Vascular dementia (undefined)	rs71298638	A	G	-0.016	63232261	0.518	0.029	14306	A	G	0.379	3	63246585	1.1165E-06	0.078	361227	6515.599
genus Erysipelatoclostridium	Vascular dementia (undefined)	rs78566090	A	G	0.014	125740204	0.741	0.034	14306	A	G	0.346	8	124727963	9.44735E-07	0.071	361227	7049.571
genus Escherichia Shigella	Vascular dementia (undefined)	rs12224047	T	C	-0.005	36820790	0.830	0.019	14306	T	C	-0.263	11	36799240	4.56278E-06	0.057	361227	8033.487
genus Escherichia Shigella	Vascular dementia (undefined)	rs193392	T	C	0.010	3085245	0.441	0.013	14306	T	C	-0.204	20	3104599	8.28324E-06	0.046	361227	7460.147
genus Escherichia Shigella	Vascular dementia (undefined)	rs2292090	T	C	0.005	70588309	0.736	0.014	14306	T	C	0.217	4	69722591	9.99332E-06	0.049	361227	6178.734
genus Escherichia Shigella	Vascular dementia (undefined)	rs2978951	G	A	-0.001	6823295	0.961	0.013	14306	G	A	0.248	8	6965773	2.48222E-08	0.045	361227	11061.935
genus Escherichia Shigella	Vascular dementia (undefined)	rs429358	C	T	0.007	45411941	0.718	0.019	14306	C	T	0.695	19	44908684	9.26616E-39	0.053	361227	58999.679
genus Escherichia Shigella	Vascular dementia (undefined)	rs6133343	G	T	0.002	721797	0.927	0.020	14306	G	T	0.312	20	741153	3.89825E-06	0.068	361227	6210.744
genus Eubacterium brachy group	Vascular dementia (undefined)	rs193392	T	C	0.013	3085245	0.562	0.023	14306	T	C	-0.204	20	3104599	8.28324E-06	0.046	361227	7460.147
genus Eubacterium brachy group	Vascular dementia (undefined)	rs2978951	G	A	-0.008	6823295	0.716	0.023	14306	G	A	0.248	8	6965773	2.48222E-08	0.045	361227	11061.935
genus Eubacterium brachy group	Vascular dementia (undefined)	rs429358	C	T	-0.022	45411941	0.464	0.032	14306	C	T	0.695	19	44908684	9.26616E-39	0.053	361227	58999.679
genus Eubacterium coprostanoligenes group	Vascular dementia (undefined)	rs12224047	T	C	0.004	36820790	0.824	0.016	14306	T	C	-0.263	11	36799240	4.56278E-06	0.057	361227	8033.487
genus Eubacterium coprostanoligenes group	Vascular dementia (undefined)	rs193392	T	C	0.004	3085245	0.686	0.011	14306	T	C	-0.204	20	3104599	8.28324E-06	0.046	361227	7460.147
genus Eubacterium coprostanoligenes group	Vascular dementia (undefined)	rs2292090	T	C	0.006	70588309	0.627	0.012	14306	T	C	0.217	4	69722591	9.99332E-06	0.049	361227	6178.734
genus Eubacterium coprostanoligenes group	Vascular dementia (undefined)	rs359878	C	T	0.000	185438949	0.945	0.013	14306	C	T	-0.211	2	184574222	4.6443E-06	0.046	361227	6959.700
genus Eubacterium coprostanoligenes group	Vascular dementia (undefined)	rs429358	C	T	0.033	45411941	0.034	0.015	14306	C	T	0.695	19	44908684	9.26616E-39	0.053	361227	58999.679
genus Eubacterium coprostanoligenes group	Vascular dementia (undefined)	rs6133343	G	T	-0.015	721797	0.417	0.017	14306	G	T	0.312	20	741153	3.89825E-06	0.068	361227	6210.744
genus Eubacterium coprostanoligenes group	Vascular dementia (undefined)	rs71298638	A	G	-0.003	63232261	0.896	0.022	14306	A	G	0.379	3	63246585	1.1165E-06	0.078	361227	6515.599
genus Eubacterium coprostanoligenes group	Vascular dementia (undefined)	rs78566090	A	G	0.018	125740204	0.425	0.025	14306	A	G	0.346	8	124727963	9.44735E-07	0.071	361227	7049.571
genus Eubacterium eligens group	Vascular dementia (undefined)	rs12224047	T	C	0.009	36820790	0.553	0.017	14306	T	C	-0.263	11	36799240	4.56278E-06	0.057	361227	8033.487
genus Eubacterium eligens group	Vascular dementia (undefined)	rs12449066	G	A	-0.009	79177293	0.655	0.020	14306	G	A	-0.262	16	79143396	2.01674E-06	0.055	361227	8803.514
genus Eubacterium eligens group	Vascular dementia (undefined)	rs193392	T	C	-0.006	3085245	0.625	0.012	14306	T	C	-0.204	20	3104599	8.28324E-06	0.046	361227	7460.147
genus Eubacterium eligens group	Vascular dementia (undefined)	rs2978951	G	A	-0.002	6823295	0.886	0.012	14306	G	A	0.248	8	6965773	2.48222E-08	0.045	361227	11061.935
genus Eubacterium eligens group	Vascular dementia (undefined)	rs429358	C	T	-0.020	45411941	0.237	0.017	14306	C	T	0.695	19	44908684	9.26616E-39	0.053	361227	58999.679
genus Eubacterium fissicatena group	Vascular dementia (undefined)	rs12224047	T	C	-0.011	36820790	0.695	0.034	14306	T	C	-0.263	11	36799240	4.56278E-06	0.057	361227	8033.487
genus Eubacterium fissicatena group	Vascular dementia (undefined)	rs193392	T	C	0.019	3085245	0.393	0.024	14306	T	C	-0.204	20	3104599	8.28324E-06	0.046	361227	7460.147
genus Eubacterium fissicatena group	Vascular dementia (undefined)	rs2292090	T	C	-0.010	70588309	0.756	0.026	14306	T	C	0.217	4	69722591	9.99332E-06	0.049	361227	6178.734
genus Eubacterium fissicatena group	Vascular dementia (undefined)	rs2972558	T	C	-0.014	45356141	0.608	0.025	14306	T	C	0.234	19	44852884	8.32415E-06	0.052	361227	7242.374
genus Eubacterium fissicatena group	Vascular dementia (undefined)	rs359878	C	T	0.010	185438949	0.694	0.026	14306	C	T	-0.211	2	184574222	4.6443E-06	0.046	361227	6959.700
genus Eubacterium fissicatena group	Vascular dementia (undefined)	rs429358	C	T	0.004	45411941	0.913	0.033	14306	C	T	0.695	19	44908684	9.26616E-39	0.053	361227	58999.679
genus Eubacterium fissicatena group	Vascular dementia (undefined)	rs6133343	G	T	-0.012	721797	0.583	0.036	14306	G	T	0.312	20	741153	3.89825E-06	0.068	361227	6210.744
genus Eubacterium fissicatena group	Vascular dementia (undefined)	rs71298638	A	G	0.024	63232261	0.646	0.048	14306	A	G	0.379	3	63246585	1.1165E-06	0.078	361227	6515.599
genus Eubacterium hallii group	Vascular dementia (undefined)	rs193392	T	C	-0.006	3085245	0.568	0.011	14306	T	C	-0.204	20	3104599	8.28324E-06	0.046	361227	7460.147
genus Eubacterium hallii group	Vascular dementia (undefined)	rs2972558	T	C	-0.010	45356141	0.386	0.012	14306	T	C	0.234	19	44852884	8.32415E-06	0.052	361227	7242.374
genus Eubacterium hallii group	Vascular dementia (undefined)	rs2978951	G	A	0.002	6823295	0.885	0.011	14306	G	A	0.248	8	6965773	2.48222E-08	0.045	361227	11061.935
genus Eubacterium hallii group	Vascular dementia (undefined)	rs359878	C	T	0.003	185438949	0.745	0.013	14306	C	T	-0.211	2	184574222	4.6443E-06	0.046	361227	6959.700
genus Eubacterium hallii group	Vascular dementia (undefined)	rs429358	C	T	0.003	45411941	0.834	0.016	14306	C	T	0.695	19	44908684	9.26616E-39	0.053	361227	58999.679
genus Eubacterium hallii group	Vascular dementia (undefined)	rs6133343	G	T	0.003	721797	0.903	0.017	14306	G	T	0.312	20	741153	3.89825E-06	0.068	361227	6210.744
genus Eubacterium hallii group	Vascular dementia (undefined)	rs78566090	A	G	0.006	125740204	0.913	0.026	14306	A	G	0.346	8	124727963	9.44735E-07	0.071	361227	7049.571
genus Eubacterium nodatum group	Vascular dementia (undefined)	rs12449066	G	A	-0.014	79177293	0.700	0.042	14306	G	A	-0.262	16	79143396	2.01674E-06	0.055	361227	8803.514
genus Eubacterium nodatum group	Vascular dementia (undefined)	rs193392	T	C	0.014	3085245	0.593	0.025	14306	T	C	-0.204	20	3104599	8.28324E-06	0.046	361227	7460.147
genus Eubacterium nodatum group	Vascular dementia (undefined)	rs2972558	T	C	0.004	45356141	0.896	0.027	14306	T	C	0.234	19	44852884	8.32415E-06	0.052	361227	7242.374
genus Eubacterium nodatum group	Vascular dementia (undefined)	rs2978951	G	A	0.006	6823295	0.829	0.025	14306	G	A	0.248	8	6965773	2.48222E-08	0.045	361227	11061.935
genus Eubacterium nodatum group	Vascular dementia (undefined)	rs359878	C	T	-0.013	185438949	0.662	0.028	14306	C	T	-0.211	2	184574222	4.6443E-06	0.046	361227	6959.700
genus Eubacterium nodatum group	Vascular dementia (undefined)	rs429358	C	T	0.008	45411941	0.880	0.036	14306	C	T	0.695	19	44908684	9.26616E-39	0.053	361227	58999.679
genus Eubacterium nodatum group	Vascular dementia (undefined)	rs71298638	A	G	0.001	63232261	0.932	0.049	14306	A	G	0.379	3	63246585	1.1165E-06	0.078	361227	6515.599
genus Eubacterium oxidoreducens group	Vascular dementia (undefined)	rs12224047	T	C	-0.003	36820790	0.887	0.029	14306	T	C	-0.263	11	36799240	4.56278E-06	0.057	361227	8033.487
genus Eubacterium oxidoreducens group	Vascular dementia (undefined)	rs193392	T	C	0.006	3085245	0.748	0.020	14306	T	C	-0.204	20	3104599	8.28324E-06	0.046	361227	7460.147
genus Eubacterium oxidoreducens group	Vascular dementia (undefined)	rs2978951	G	A	-0.019	6823295	0.329	0.019	14306	G	A	0.248	8	6965773	2.48222E-08	0.045	361227	11061.935
genus Eubacterium oxidoreducens group	Vascular dementia (undefined)	rs429358	C	T	0.021	45411941	0.455	0.028	14306	C	T	0.695	19	44908684	9.26616E-39	0.053	361227	58999.679
genus Eubacterium oxidoreducens group	Vascular dementia (undefined)	rs71298638	A	G	0.028	63232261	0.491	0.040	14306	A	G	0.379	3	63246585	1.1165E-06	0.078	361227	6515.599
genus Eubacterium oxidoreducens group	Vascular dementia (undefined)	rs78566090	A	G	-0.021	125740204	0.714	0.050	14306	A	G	0.346	8	124727963	9.44735E-07	0.071	361227	7049.571
genus Eubacterium rectale group	Vascular dementia (undefined)	rs12449066	G	A	0.008	79177293	0.658	0.019	14306	G	A	-0.262	16	79143396	2.01674E-06	0.055	361227	8803.514
genus Eubacterium rectale group	Vascular dementia (undefined)	rs2292090	T	C	0.009	70588309	0.428	0.012	14306	T	C	0.217	4	69722591	9.99332E-06	0.049	361227	6178.734
genus Eubacterium rectale group	Vascular dementia (undefined)	rs2978951	G	A	-0.003	6823295	0.806	0.011	14306	G	A	0.248	8	6965773	2.48222E-08	0.045	361227	11061.935
genus Eubacterium rectale group	Vascular dementia (undefined)	rs359878	C	T	0.004	185438949	0.783	0.013	14306	C	T	-0.211	2	184574222	4.6443E-06	0.046	361227	6959.700
genus Eubacterium rectale group	Vascular dementia (undefined)	rs429358	C	T	-0.028	45411941	0.084	0.015	14306	C	T	0.695	19	44908684	9.26616E-39	0.053	361227	58999.679
genus Eubacterium rectale group	Vascular dementia (undefined)	rs78566090	A	G	0.019	125740204	0.466	0.025	14306	A	G	0.346	8	124727963	9.44735E-07	0.071	361227	7049.571
genus Eubacterium ruminantium group	Vascular dementia (undefined)	rs12224047	T	C	0.006	36820790	0.770	0.023	14306	T	C	-0.263	11	36799240	4.56278E-06	0.057	361227	8033.487
genus Eubacterium ruminantium group	Vascular dementia (undefined)	rs12449066	G	A	0.004	79177293	0.783	0.028	14306	G	A	-0.262	16	79143396	2.01674E-06	0.055	361227	8803.514
genus Eubacterium ruminantium group	Vascular dementia (undefined)	rs193392	T	C	0.000	3085245	0.966	0.017	14306	T	C	-0.204	20	3104599	8.28324E-06	0.046	361227	7460.147
genus Eubacterium ruminantium group	Vascular dementia (undefined)	rs2292090	T	C	0.001	70588309	0.966	0.018	14306	T	C	0.217	4	69722591	9.99332E-06	0.049	361227	6178.734
genus Eubacterium ruminantium group	Vascular dementia (undefined)	rs2972558	T	C	0.001	45356141	0.954	0.018	14306	T	C	0.234	19	44852884	8.32415E-06	0.052	361227	7242.374
genus Eubacterium ruminantium group	Vascular dementia (undefined)	rs2978951	G	A	-0.010	6823295	0.568	0.016	14306	G	A	0.248	8	6965773	2.48222E-08	0.045	361227	11061.935

genus Eubacterium ruminantium group	Vascular dementia (undefined)	rs359878	C	T	-0.016	185438949	0.386	0.019	14306	C	T	-0.211	2	184574222	4.6443E-06	0.046	361227	6959.700
genus Eubacterium ruminantium group	Vascular dementia (undefined)	rs429358	C	T	0.014	45411941	0.497	0.023	14306	C	T	0.695	19	44908684	9.26616E-39	0.053	361227	58999.679
genus Eubacterium ruminantium group	Vascular dementia (undefined)	rs6133343	G	T	-0.004	721797	0.729	0.025	14306	G	T	0.312	20	741153	3.89825E-06	0.068	361227	6210.744
genus Eubacterium ruminantium group	Vascular dementia (undefined)	rs71298638	A	G	0.002	63232261	0.993	0.035	14306	A	G	0.379	3	63246585	1.1165E-06	0.078	361227	6515.599
genus Eubacterium ventriosum group	Vascular dementia (undefined)	rs12449066	G	A	0.016	79177293	0.561	0.020	14306	G	A	-0.262	16	79143396	2.01674E-06	0.055	361227	8803.514
genus Eubacterium ventriosum group	Vascular dementia (undefined)	rs193392	T	C	0.000	3085245	0.984	0.012	14306	T	C	-0.204	20	3104599	8.28324E-06	0.046	361227	7460.147
genus Eubacterium ventriosum group	Vascular dementia (undefined)	rs2292090	T	C	0.001	70588309	0.971	0.013	14306	T	C	0.217	4	69722591	9.99332E-06	0.049	361227	6178.734
genus Eubacterium ventriosum group	Vascular dementia (undefined)	rs2978951	G	A	-0.001	6823295	0.950	0.012	14306	G	A	0.248	8	6965773	2.48222E-08	0.045	361227	11061.935
genus Eubacterium ventriosum group	Vascular dementia (undefined)	rs359878	C	T	0.007	185438949	0.574	0.013	14306	C	T	-0.211	2	184574222	4.6443E-06	0.046	361227	6959.700
genus Eubacterium ventriosum group	Vascular dementia (undefined)	rs429358	C	T	-0.029	45411941	0.069	0.016	14306	C	T	0.695	19	44908684	9.26616E-39	0.053	361227	58999.679
genus Eubacterium ventriosum group	Vascular dementia (undefined)	rs6133343	G	T	0.002	721797	0.826	0.018	14306	G	T	0.312	20	741153	3.89825E-06	0.068	361227	6210.744
genus Eubacterium ventriosum group	Vascular dementia (undefined)	rs71298638	A	G	0.006	63232261	0.870	0.024	14306	A	G	0.379	3	63246585	1.1165E-06	0.078	361227	6515.599
genus Eubacterium ventriosum group	Vascular dementia (undefined)	rs78566090	A	G	0.000	125740204	0.980	0.027	14306	A	G	0.346	8	124727963	9.44735E-07	0.071	361227	7049.571
genus Eubacterium xylanophilum group	Vascular dementia (undefined)	rs12224047	T	C	0.002	36820790	0.853	0.018	14306	T	C	-0.263	11	36799240	4.56278E-06	0.057	361227	8033.487
genus Eubacterium xylanophilum group	Vascular dementia (undefined)	rs12449066	G	A	0.010	79177293	0.611	0.021	14306	G	A	-0.262	16	79143396	2.01674E-06	0.055	361227	8803.514
genus Eubacterium xylanophilum group	Vascular dementia (undefined)	rs193392	T	C	0.008	3085245	0.497	0.013	14306	T	C	-0.204	20	3104599	8.28324E-06	0.046	361227	7460.147
genus Eubacterium xylanophilum group	Vascular dementia (undefined)	rs2292090	T	C	0.010	70588309	0.487	0.014	14306	T	C	0.217	4	69722591	9.99332E-06	0.049	361227	6178.734
genus Eubacterium xylanophilum group	Vascular dementia (undefined)	rs429358	C	T	-0.023	45411941	0.188	0.017	14306	C	T	0.695	19	44908684	9.26616E-39	0.053	361227	58999.679
genus Eubacterium xylanophilum group	Vascular dementia (undefined)	rs6133343	G	T	-0.016	721797	0.527	0.020	14306	G	T	0.312	20	741153	3.89825E-06	0.068	361227	6210.744
genus Eubacterium xylanophilum group	Vascular dementia (undefined)	rs71298638	A	G	-0.008	63232261	0.740	0.025	14306	A	G	0.379	3	63246585	1.1165E-06	0.078	361227	6515.599
genus Eubacterium xylanophilum group	Vascular dementia (undefined)	rs78566090	A	G	-0.009	125740204	0.829	0.029	14306	A	G	0.346	8	124727963	9.44735E-07	0.071	361227	7049.571
genus Faecalibacterium	Vascular dementia (undefined)	rs12224047	T	C	0.012	36820790	0.411	0.015	14306	T	C	-0.263	11	36799240	4.56278E-06	0.057	361227	8033.487
genus Faecalibacterium	Vascular dementia (undefined)	rs193392	T	C	-0.005	3085245	0.635	0.011	14306	T	C	-0.204	20	3104599	8.28324E-06	0.046	361227	7460.147
genus Faecalibacterium	Vascular dementia (undefined)	rs2292090	T	C	0.003	70588309	0.842	0.012	14306	T	C	0.217	4	69722591	9.99332E-06	0.049	361227	6178.734
genus Faecalibacterium	Vascular dementia (undefined)	rs2978951	G	A	-0.005	6823295	0.619	0.011	14306	G	A	0.248	8	6965773	2.48222E-08	0.045	361227	11061.935
genus Faecalibacterium	Vascular dementia (undefined)	rs429358	C	T	-0.035	45411941	0.029	0.015	14306	C	T	0.695	19	44908684	9.26616E-39	0.053	361227	58999.679
genus Faecalibacterium	Vascular dementia (undefined)	rs78566090	A	G	0.024	125740204	0.327	0.025	14306	A	G	0.346	8	124727963	9.44735E-07	0.071	361227	7049.571
genus Family XIII AD3011 group	Vascular dementia (undefined)	rs12224047	T	C	-0.013	36820790	0.427	0.017	14306	T	C	-0.263	11	36799240	4.56278E-06	0.057	361227	8033.487
genus Family XIII AD3011 group	Vascular dementia (undefined)	rs2292090	T	C	-0.003	70588309	0.893	0.013	14306	T	C	0.217	4	69722591	9.99332E-06	0.049	361227	6178.734
genus Family XIII AD3011 group	Vascular dementia (undefined)	rs2972558	T	C	0.010	45356141	0.440	0.013	14306	T	C	0.234	19	44852884	8.32415E-06	0.052	361227	7242.374
genus Family XIII AD3011 group	Vascular dementia (undefined)	rs2978951	G	A	-0.008	6823295	0.530	0.012	14306	G	A	0.248	8	6965773	2.48222E-08	0.045	361227	11061.935
genus Family XIII AD3011 group	Vascular dementia (undefined)	rs429358	C	T	0.029	45411941	0.106	0.017	14306	C	T	0.695	19	44908684	9.26616E-39	0.053	361227	58999.679
genus Family XIII AD3011 group	Vascular dementia (undefined)	rs6133343	G	T	-0.007	721797	0.739	0.019	14306	G	T	0.312	20	741153	3.89825E-06	0.068	361227	6210.744
genus Family XIII AD3011 group	Vascular dementia (undefined)	rs71298638	A	G	0.015	63232261	0.544	0.024	14306	A	G	0.379	3	63246585	1.1165E-06	0.078	361227	6515.599
genus Family XIII AD3011 group	Vascular dementia (undefined)	rs78566090	A	G	0.007	125740204	0.880	0.029	14306	A	G	0.346	8	124727963	9.44735E-07	0.071	361227	7049.571
genus Family XIII UCG001	Vascular dementia (undefined)	rs12224047	T	C	0.007	36820790	0.645	0.018	14306	T	C	-0.263	11	36799240	4.56278E-06	0.057	361227	8033.487
genus Family XIII UCG001	Vascular dementia (undefined)	rs12449066	G	A	-0.005	79177293	0.768	0.021	14306	G	A	-0.262	16	79143396	2.01674E-06	0.055	361227	8803.514
genus Family XIII UCG001	Vascular dementia (undefined)	rs2292090	T	C	0.005	70588309	0.735	0.014	14306	T	C	0.217	4	69722591	9.99332E-06	0.049	361227	6178.734
genus Family XIII UCG001	Vascular dementia (undefined)	rs2972558	T	C	-0.003	45356141	0.829	0.014	14306	T	C	0.234	19	44852884	8.32415E-06	0.052	361227	7242.374
genus Family XIII UCG001	Vascular dementia (undefined)	rs2978951	G	A	-0.003	6823295	0.842	0.013	14306	G	A	0.248	8	6965773	2.48222E-08	0.045	361227	11061.935
genus Family XIII UCG001	Vascular dementia (undefined)	rs429358	C	T	0.004	45411941	0.889	0.018	14306	C	T	0.695	19	44908684	9.26616E-39	0.053	361227	58999.679
genus Family XIII UCG001	Vascular dementia (undefined)	rs71298638	A	G	-0.007	63232261	0.731	0.025	14306	A	G	0.379	3	63246585	1.1165E-06	0.078	361227	6515.599
genus Family XIII UCG001	Vascular dementia (undefined)	rs78566090	A	G	0.010	125740204	0.817	0.030	14306	A	G	0.346	8	124727963	9.44735E-07	0.071	361227	7049.571
genus Flavonifractor	Vascular dementia (undefined)	rs12224047	T	C	0.018	36820790	0.403	0.020	14306	T	C	-0.263	11	36799240	4.56278E-06	0.057	361227	8033.487
genus Flavonifractor	Vascular dementia (undefined)	rs193392	T	C	0.006	3085245	0.676	0.014	14306	T	C	-0.204	20	3104599	8.28324E-06	0.046	361227	7460.147
genus Flavonifractor	Vascular dementia (undefined)	rs359878	C	T	-0.010	185438949	0.500	0.016	14306	C	T	-0.211	2	184574222	4.6443E-06	0.046	361227	6959.700
genus Flavonifractor	Vascular dementia (undefined)	rs429358	C	T	-0.021	45411941	0.293	0.020	14306	C	T	0.695	19	44908684	9.26616E-39	0.053	361227	58999.679
genus Flavonifractor	Vascular dementia (undefined)	rs6133343	G	T	0.003	721797	0.840	0.021	14306	G	T	0.312	20	741153	3.89825E-06	0.068	361227	6210.744
genus Flavonifractor	Vascular dementia (undefined)	rs71298638	A	G	0.026	63232261	0.370	0.028	14306	A	G	0.379	3	63246585	1.1165E-06	0.078	361227	6515.599
genus Flavonifractor	Vascular dementia (undefined)	rs78566090	A	G	0.018	125740204	0.616	0.033	14306	A	G	0.346	8	124727963	9.44735E-07	0.071	361227	7049.571
genus Fusicatenibacter	Vascular dementia (undefined)	rs12224047	T	C	0.008	36820790	0.573	0.016	14306	T	C	-0.263	11	36799240	4.56278E-06	0.057	361227	8033.487
genus Fusicatenibacter	Vascular dementia (undefined)	rs2292090	T	C	0.010	70588309	0.362	0.012	14306	T	C	0.217	4	69722591	9.99332E-06	0.049	361227	6178.734
genus Fusicatenibacter	Vascular dementia (undefined)	rs2972558	T	C	0.005	45356141	0.650	0.012	14306	T	C	0.234	19	44852884	8.32415E-06	0.052	361227	7242.374
genus Fusicatenibacter	Vascular dementia (undefined)	rs2978951	G	A	-0.007	6823295	0.521	0.011	14306	G	A	0.248	8	6965773	2.48222E-08	0.045	361227	11061.935
genus Fusicatenibacter	Vascular dementia (undefined)	rs359878	C	T	-0.004	185438949	0.826	0.013	14306	C	T	-0.211	2	184574222	4.6443E-06	0.046	361227	6959.700
genus Fusicatenibacter	Vascular dementia (undefined)	rs429358	C	T	-0.008	45411941	0.622	0.015	14306	C	T	0.695	19	44908684	9.26616E-39	0.053	361227	58999.679
genus Fusicatenibacter	Vascular dementia (undefined)	rs78566090	A	G	-0.001	125740204	0.993	0.025	14306	A	G	0.346	8	124727963	9.44735E-07	0.071	361227	7049.571
genus Gordonibacter	Vascular dementia (undefined)	rs12224047	T	C	-0.005	36820790	0.845	0.034	14306	T	C	-0.263	11	36799240	4.56278E-06	0.057	361227	8033.487
genus Gordonibacter	Vascular dementia (undefined)	rs12449066	G	A	-0.002	79177293	0.980	0.041	14306	G	A	-0.262	16	79143396	2.01674E-06	0.055	361227	8803.514
genus Gordonibacter	Vascular dementia (undefined)	rs2292090	T	C	-0.016	70588309	0.628	0.026	14306	T	C	0.217	4	69722591	9.99332E-06	0.049	361227	6178.734
genus Gordonibacter	Vascular dementia (undefined)	rs359878	C	T	-0.019	185438949	0.487	0.027	14306	C	T	-0.211	2	184574222	4.6443E-06	0.046	361227	6959.700
genus Gordonibacter	Vascular dementia (undefined)	rs429358	C	T	0.056	45411941	0.125	0.034	14306	C	T	0.695	19	44908684	9.26616E-39	0.053	361227	58999.679
genus Gordonibacter	Vascular dementia (undefined)	rs6133343	G	T	0.033	721797	0.348	0.037	14306	G	T	0.312	20	741153	3.89825E-06	0.068	361227	6210.744
genus Haemophilus	Vascular dementia (undefined)	rs12449066	G	A	0.020	79177293	0.462	0.026	14306	G	A	-0.262	16	79143396	2.01674E-06	0.055	361227	8803.514
genus Haemophilus	Vascular dementia (undefined)	rs193392	T	C	-0.008	3085245	0.603	0.015	14306	T	C	-0.204	20	3104599	8.28324E-06	0.046	361227	7460.147
genus Haemophilus	Vascular dementia (undefined)	rs2292090	T	C	-0.013	70588309	0.401	0.017	14306	T	C	0.217	4	69722591	9.99332E-06	0.049	361227	6178.734
genus Haemophilus	Vascular dementia (undefined)	rs2972558	T	C	-0.013	45356141	0.437	0.016	14306	T	C	0.234	19	44852884	8.32415E-06	0.052	361227	7242.374
genus Haemophilus	Vascular dementia (undefined)	rs2978951	G	A	0.005	6823295	0.771	0.015	14306	G	A	0.248	8	6965773	2.48222E-08	0.045	361227	11061.935
genus Haemophilus	Vascular dementia (undefined)	rs359878	C	T	0.010	185438949	0.616	0.017	14306	C	T	-0.211	2	184574222	4.6443E-06	0.046	361227	6959.700
genus Haemophilus	Vascular dementia (undefined)	rs429358	C	T	0.027	45411941	0.241	0.021	14306	C	T	0.695	19	44908684	9.26616E-39	0.053	361227	58999.679
genus Haemophilus	Vascular dementia (undefined)	rs6133343	G	T	0.014	721797	0.516	0.023	14306	G	T	0.312	20	741153	3.89825E-06	0.068	361227	6210.744

genus Haemophilus	Vascular dementia (undefined)	rs78566090	A	G	-0.027	125740204	0.456	0.036	14306	A	G	0.346	8	124727963	9.44735E-07	0.071	361227	7049.571
genus Holdemanella	Vascular dementia (undefined)	rs12224047	T	C	-0.007	36820790	0.768	0.023	14306	T	C	-0.263	11	36799240	4.56278E-06	0.057	361227	8033.487
genus Holdemanella	Vascular dementia (undefined)	rs193392	T	C	0.007	3085245	0.728	0.016	14306	T	C	-0.204	20	3104599	8.28324E-06	0.046	361227	7460.147
genus Holdemanella	Vascular dementia (undefined)	rs2292090	T	C	-0.013	70588309	0.484	0.018	14306	T	C	0.217	4	69722591	9.99332E-06	0.049	361227	6178.734
genus Holdemanella	Vascular dementia (undefined)	rs2978951	G	A	0.002	6823295	0.909	0.016	14306	G	A	0.248	8	6965773	2.48222E-08	0.045	361227	11061.935
genus Holdemanella	Vascular dementia (undefined)	rs359878	C	T	0.011	185438949	0.568	0.019	14306	C	T	-0.211	2	184574222	4.6443E-06	0.046	361227	6959.700
genus Holdemanella	Vascular dementia (undefined)	rs429358	C	T	0.038	45411941	0.092	0.023	14306	C	T	0.695	19	44908684	9.26616E-39	0.053	361227	58999.679
genus Holdemanella	Vascular dementia (undefined)	rs6133343	G	T	0.011	721797	0.569	0.026	14306	G	T	0.312	20	741153	3.89825E-06	0.068	361227	6210.744
genus Holdemanella	Vascular dementia (undefined)	rs71298638	A	G	0.007	63232261	0.845	0.034	14306	A	G	0.379	3	63246585	1.1165E-06	0.078	361227	6515.599
genus Holdemania	Vascular dementia (undefined)	rs12224047	T	C	0.014	36820790	0.559	0.021	14306	T	C	-0.263	11	36799240	4.56278E-06	0.057	361227	8033.487
genus Holdemania	Vascular dementia (undefined)	rs193392	T	C	-0.002	3085245	0.889	0.015	14306	T	C	-0.204	20	3104599	8.28324E-06	0.046	361227	7460.147
genus Holdemania	Vascular dementia (undefined)	rs2292090	T	C	0.005	70588309	0.832	0.017	14306	T	C	0.217	4	69722591	9.99332E-06	0.049	361227	6178.734
genus Holdemania	Vascular dementia (undefined)	rs2978951	G	A	-0.012	6823295	0.417	0.015	14306	G	A	0.248	8	6965773	2.48222E-08	0.045	361227	11061.935
genus Holdemania	Vascular dementia (undefined)	rs359878	C	T	0.013	185438949	0.427	0.017	14306	C	T	-0.211	2	184574222	4.6443E-06	0.046	361227	6959.700
genus Holdemania	Vascular dementia (undefined)	rs429358	C	T	0.014	45411941	0.528	0.021	14306	C	T	0.695	19	44908684	9.26616E-39	0.053	361227	58999.679
genus Holdemania	Vascular dementia (undefined)	rs78566090	A	G	0.021	125740204	0.554	0.035	14306	A	G	0.346	8	124727963	9.44735E-07	0.071	361227	7049.571
genus Howardella	Vascular dementia (undefined)	rs193392	T	C	0.005	3085245	0.830	0.024	14306	T	C	-0.204	20	3104599	8.28324E-06	0.046	361227	7460.147
genus Howardella	Vascular dementia (undefined)	rs2978951	G	A	-0.007	6823295	0.743	0.023	14306	G	A	0.248	8	6965773	2.48222E-08	0.045	361227	11061.935
genus Howardella	Vascular dementia (undefined)	rs359878	C	T	-0.004	185438949	0.737	0.027	14306	C	T	-0.211	2	184574222	4.6443E-06	0.046	361227	6959.700
genus Howardella	Vascular dementia (undefined)	rs429358	C	T	0.033	45411941	0.260	0.033	14306	C	T	0.695	19	44908684	9.26616E-39	0.053	361227	58999.679
genus Howardella	Vascular dementia (undefined)	rs6133343	G	T	0.000	721797	0.907	0.036	14306	G	T	0.312	20	741153	3.89825E-06	0.068	361227	6210.744
genus Howardella	Vascular dementia (undefined)	rs71298638	A	G	-0.030	63232261	0.531	0.048	14306	A	G	0.379	3	63246585	1.1165E-06	0.078	361227	6515.599
genus Hungatella	Vascular dementia (undefined)	rs12224047	T	C	0.018	36820790	0.593	0.032	14306	T	C	-0.263	11	36799240	4.56278E-06	0.057	361227	8033.487
genus Hungatella	Vascular dementia (undefined)	rs12449066	G	A	0.017	79177293	0.617	0.038	14306	G	A	-0.262	16	79143396	2.01674E-06	0.055	361227	8803.514
genus Hungatella	Vascular dementia (undefined)	rs2292090	T	C	0.021	70588309	0.425	0.024	14306	T	C	0.217	4	69722591	9.99332E-06	0.049	361227	6178.734
genus Hungatella	Vascular dementia (undefined)	rs2972558	T	C	-0.015	45356141	0.535	0.024	14306	T	C	0.234	19	44852884	8.32415E-06	0.052	361227	7242.374
genus Hungatella	Vascular dementia (undefined)	rs359878	C	T	0.004	185438949	0.862	0.025	14306	C	T	-0.211	2	184574222	4.6443E-06	0.046	361227	6959.700
genus Hungatella	Vascular dementia (undefined)	rs429358	C	T	-0.050	45411941	0.108	0.032	14306	C	T	0.695	19	44908684	9.26616E-39	0.053	361227	58999.679
genus Hungatella	Vascular dementia (undefined)	rs6133343	G	T	0.000	721797	0.887	0.034	14306	G	T	0.312	20	741153	3.89825E-06	0.068	361227	6210.744
genus Hungatella	Vascular dementia (undefined)	rs71298638	A	G	0.004	63232261	0.953	0.046	14306	A	G	0.379	3	63246585	1.1165E-06	0.078	361227	6515.599
genus Intestinibacter	Vascular dementia (undefined)	rs12224047	T	C	0.015	36820790	0.469	0.019	14306	T	C	-0.263	11	36799240	4.56278E-06	0.057	361227	8033.487
genus Intestinibacter	Vascular dementia (undefined)	rs12449066	G	A	-0.008	79177293	0.528	0.022	14306	G	A	-0.262	16	79143396	2.01674E-06	0.055	361227	8803.514
genus Intestinibacter	Vascular dementia (undefined)	rs193392	T	C	-0.008	3085245	0.542	0.013	14306	T	C	-0.204	20	3104599	8.28324E-06	0.046	361227	7460.147
genus Intestinibacter	Vascular dementia (undefined)	rs2292090	T	C	-0.002	70588309	0.869	0.014	14306	T	C	0.217	4	69722591	9.99332E-06	0.049	361227	6178.734
genus Intestinibacter	Vascular dementia (undefined)	rs2978951	G	A	-0.012	6823295	0.364	0.013	14306	G	A	0.248	8	6965773	2.48222E-08	0.045	361227	11061.935
genus Intestinibacter	Vascular dementia (undefined)	rs359878	C	T	-0.005	185438949	0.786	0.015	14306	C	T	-0.211	2	184574222	4.6443E-06	0.046	361227	6959.700
genus Intestinibacter	Vascular dementia (undefined)	rs429358	C	T	-0.011	45411941	0.447	0.018	14306	C	T	0.695	19	44908684	9.26616E-39	0.053	361227	58999.679
genus Intestinibacter	Vascular dementia (undefined)	rs71298638	A	G	-0.014	63232261	0.612	0.026	14306	A	G	0.379	3	63246585	1.1165E-06	0.078	361227	6515.599
genus Intestinibacter	Vascular dementia (undefined)	rs78566090	A	G	-0.010	125740204	0.764	0.031	14306	A	G	0.346	8	124727963	9.44735E-07	0.071	361227	7049.571
genus Intestinimonas	Vascular dementia (undefined)	rs12224047	T	C	0.008	36820790	0.695	0.019	14306	T	C	-0.263	11	36799240	4.56278E-06	0.057	361227	8033.487
genus Intestinimonas	Vascular dementia (undefined)	rs12449066	G	A	0.020	79177293	0.450	0.022	14306	G	A	-0.262	16	79143396	2.01674E-06	0.055	361227	8803.514
genus Intestinimonas	Vascular dementia (undefined)	rs2292090	T	C	-0.005	70588309	0.754	0.014	14306	T	C	0.217	4	69722591	9.99332E-06	0.049	361227	6178.734
genus Intestinimonas	Vascular dementia (undefined)	rs2972558	T	C	-0.010	45356141	0.461	0.014	14306	T	C	0.234	19	44852884	8.32415E-06	0.052	361227	7242.374
genus Intestinimonas	Vascular dementia (undefined)	rs2978951	G	A	-0.003	6823295	0.800	0.013	14306	G	A	0.248	8	6965773	2.48222E-08	0.045	361227	11061.935
genus Intestinimonas	Vascular dementia (undefined)	rs429358	C	T	0.013	45411941	0.424	0.018	14306	C	T	0.695	19	44908684	9.26616E-39	0.053	361227	58999.679
genus Intestinimonas	Vascular dementia (undefined)	rs6133343	G	T	0.010	721797	0.639	0.020	14306	G	T	0.312	20	741153	3.89825E-06	0.068	361227	6210.744
genus Intestinimonas	Vascular dementia (undefined)	rs71298638	A	G	0.009	63232261	0.763	0.027	14306	A	G	0.379	3	63246585	1.1165E-06	0.078	361227	6515.599
genus Intestinimonas	Vascular dementia (undefined)	rs78566090	A	G	0.019	125740204	0.625	0.031	14306	A	G	0.346	8	124727963	9.44735E-07	0.071	361227	7049.571
genus Lachnoclostridium	Vascular dementia (undefined)	rs12449066	G	A	-0.012	79177293	0.511	0.018	14306	G	A	-0.262	16	79143396	2.01674E-06	0.055	361227	8803.514
genus Lachnoclostridium	Vascular dementia (undefined)	rs193392	T	C	-0.006	3085245	0.534	0.011	14306	T	C	-0.204	20	3104599	8.28324E-06	0.046	361227	7460.147
genus Lachnoclostridium	Vascular dementia (undefined)	rs2292090	T	C	0.006	70588309	0.551	0.012	14306	T	C	0.217	4	69722591	9.99332E-06	0.049	361227	6178.734
genus Lachnoclostridium	Vascular dementia (undefined)	rs2972558	T	C	-0.004	45356141	0.685	0.012	14306	T	C	0.234	19	44852884	8.32415E-06	0.052	361227	7242.374
genus Lachnoclostridium	Vascular dementia (undefined)	rs2978951	G	A	0.004	6823295	0.692	0.011	14306	G	A	0.248	8	6965773	2.48222E-08	0.045	361227	11061.935
genus Lachnoclostridium	Vascular dementia (undefined)	rs359878	C	T	0.011	185438949	0.371	0.012	14306	C	T	-0.211	2	184574222	4.6443E-06	0.046	361227	6959.700
genus Lachnoclostridium	Vascular dementia (undefined)	rs429358	C	T	-0.019	45411941	0.230	0.015	14306	C	T	0.695	19	44908684	9.26616E-39	0.053	361227	58999.679
genus Lachnospiraceae FCS020 group	Vascular dementia (undefined)	rs12224047	T	C	0.016	36820790	0.333	0.018	14306	T	C	-0.263	11	36799240	4.56278E-06	0.057	361227	8033.487
genus Lachnospiraceae FCS020 group	Vascular dementia (undefined)	rs12449066	G	A	-0.001	79177293	0.829	0.021	14306	G	A	-0.262	16	79143396	2.01674E-06	0.055	361227	8803.514
genus Lachnospiraceae FCS020 group	Vascular dementia (undefined)	rs2292090	T	C	0.008	70588309	0.613	0.013	14306	T	C	0.217	4	69722591	9.99332E-06	0.049	361227	6178.734
genus Lachnospiraceae FCS020 group	Vascular dementia (undefined)	rs2972558	T	C	0.011	45356141	0.387	0.013	14306	T	C	0.234	19	44852884	8.32415E-06	0.052	361227	7242.374
genus Lachnospiraceae FCS020 group	Vascular dementia (undefined)	rs429358	C	T	0.017	45411941	0.318	0.017	14306	C	T	0.695	19	44908684	9.26616E-39	0.053	361227	58999.679
genus Lachnospiraceae FCS020 group	Vascular dementia (undefined)	rs6133343	G	T	0.013	721797	0.586	0.019	14306	G	T	0.312	20	741153	3.89825E-06	0.068	361227	6210.744
genus Lachnospiraceae FCS020 group	Vascular dementia (undefined)	rs71298638	A	G	0.020	63232261	0.430	0.025	14306	A	G	0.379	3	63246585	1.1165E-06	0.078	361227	6515.599
genus Lachnospiraceae NC2004 group	Vascular dementia (undefined)	rs12224047	T	C	-0.003	36820790	0.901	0.026	14306	T	C	-0.263	11	36799240	4.56278E-06	0.057	361227	8033.487
genus Lachnospiraceae NC2004 group	Vascular dementia (undefined)	rs193392	T	C	-0.002	3085245	0.899	0.018	14306	T	C	-0.204	20	3104599	8.28324E-06	0.046	361227	7460.147
genus Lachnospiraceae NC2004 group	Vascular dementia (undefined)	rs2972558	T	C	0.008	45356141	0.682	0.019	14306	T	C	0.234	19	44852884	8.32415E-06	0.052	361227	7242.374
genus Lachnospiraceae NC2004 group	Vascular dementia (undefined)	rs2978951	G	A	0.010	6823295	0.580	0.018	14306	G	A	0.248	8	6965773	2.48222E-08	0.045	361227	11061.935
genus Lachnospiraceae NC2004 group	Vascular dementia (undefined)	rs429358	C	T	-0.024	45411941	0.415	0.026	14306	C	T	0.695	19	44908684	9.26616E-39	0.053	361227	58999.679
genus Lachnospiraceae NC2004 group	Vascular dementia (undefined)	rs6133343	G	T	0.021	721797	0.509	0.027	14306	G	T	0.312	20	741153	3.89825E-06	0.068	361227	6210.744
genus Lachnospiraceae NC2004 group	Vascular dementia (undefined)	rs71298638	A	G	-0.001	63232261	0.977	0.038	14306	A	G	0.379	3	63246585	1.1165E-06	0.078	361227	6515.599
genus Lachnospiraceae NC2004 group	Vascular dementia (undefined)	rs78566090	A	G	-0.041	125740204	0.403	0.047	14306	A	G	0.346	8	124727963	9.44735E-07	0.071	361227	7049.571
genus Lachnospiraceae ND3007 group	Vascular dementia (undefined)	rs12224047	T	C	0.004	36820790	0.819	0.017	14306	T	C	-0.263	11	36799240	4.56278E-06	0.057	361227	8033.487

genus Lachnospiraceae ND3007 group	Vascular dementia (undefined)	rs12449066	G	A	0.010	79177293	0.786	0.020	14306	G	A	-0.262	16	79143396	2.01674E-06	0.055	361227	8803.514
genus Lachnospiraceae ND3007 group	Vascular dementia (undefined)	rs193392	T	C	0.000	3085245	0.933	0.012	14306	T	C	-0.204	20	3104599	8.28324E-06	0.046	361227	7460.147
genus Lachnospiraceae ND3007 group	Vascular dementia (undefined)	rs2972558	T	C	0.002	45356141	0.878	0.012	14306	T	C	0.234	19	44852884	8.32415E-06	0.052	361227	7242.374
genus Lachnospiraceae ND3007 group	Vascular dementia (undefined)	rs359878	C	T	-0.004	185438949	0.759	0.013	14306	C	T	-0.211	2	184574222	4.6443E-06	0.046	361227	6959.700
genus Lachnospiraceae ND3007 group	Vascular dementia (undefined)	rs429358	C	T	-0.003	45411941	0.806	0.016	14306	C	T	0.695	19	44908684	9.26616E-39	0.053	361227	58999.679
genus Lachnospiraceae ND3007 group	Vascular dementia (undefined)	rs71298638	A	G	-0.005	63232261	0.790	0.024	14306	A	G	0.379	3	63246585	1.1165E-06	0.078	361227	6515.599
genus Lachnospiraceae NK4A136 group	Vascular dementia (undefined)	rs193392	T	C	-0.004	3085245	0.723	0.011	14306	T	C	-0.204	20	3104599	8.28324E-06	0.046	361227	7460.147
genus Lachnospiraceae NK4A136 group	Vascular dementia (undefined)	rs2292090	T	C	-0.005	70588309	0.715	0.012	14306	T	C	0.217	4	69722591	9.99332E-06	0.049	361227	6178.734
genus Lachnospiraceae NK4A136 group	Vascular dementia (undefined)	rs2972558	T	C	0.007	45356141	0.549	0.012	14306	T	C	0.234	19	44852884	8.32415E-06	0.052	361227	7242.374
genus Lachnospiraceae NK4A136 group	Vascular dementia (undefined)	rs359878	C	T	0.002	185438949	0.773	0.013	14306	C	T	-0.211	2	184574222	4.6443E-06	0.046	361227	6959.700
genus Lachnospiraceae NK4A136 group	Vascular dementia (undefined)	rs429358	C	T	-0.011	45411941	0.456	0.015	14306	C	T	0.695	19	44908684	9.26616E-39	0.053	361227	58999.679
genus Lachnospiraceae NK4A136 group	Vascular dementia (undefined)	rs78566090	A	G	0.021	125740204	0.433	0.025	14306	A	G	0.346	8	124727963	9.44735E-07	0.071	361227	7049.571
genus Lachnospiraceae UCG001	Vascular dementia (undefined)	rs12224047	T	C	0.000	36820790	0.946	0.020	14306	T	C	-0.263	11	36799240	4.56278E-06	0.057	361227	8033.487
genus Lachnospiraceae UCG001	Vascular dementia (undefined)	rs12449066	G	A	-0.001	79177293	0.990	0.024	14306	G	A	-0.262	16	79143396	2.01674E-06	0.055	361227	8803.514
genus Lachnospiraceae UCG001	Vascular dementia (undefined)	rs193392	T	C	-0.011	3085245	0.438	0.014	14306	T	C	-0.204	20	3104599	8.28324E-06	0.046	361227	7460.147
genus Lachnospiraceae UCG001	Vascular dementia (undefined)	rs2292090	T	C	0.010	70588309	0.550	0.016	14306	T	C	0.217	4	69722591	9.99332E-06	0.049	361227	6178.734
genus Lachnospiraceae UCG001	Vascular dementia (undefined)	rs2972558	T	C	0.001	45356141	0.958	0.015	14306	T	C	0.234	19	44852884	8.32415E-06	0.052	361227	7242.374
genus Lachnospiraceae UCG001	Vascular dementia (undefined)	rs2978951	G	A	-0.002	6823295	0.879	0.014	14306	G	A	0.248	8	6965773	2.48222E-08	0.045	361227	11061.935
genus Lachnospiraceae UCG001	Vascular dementia (undefined)	rs429358	C	T	-0.002	45411941	0.904	0.020	14306	C	T	0.695	19	44908684	9.26616E-39	0.053	361227	58999.679
genus Lachnospiraceae UCG001	Vascular dementia (undefined)	rs6133343	G	T	0.017	721797	0.441	0.023	14306	G	T	0.312	20	741153	3.89825E-06	0.068	361227	6210.744
genus Lachnospiraceae UCG001	Vascular dementia (undefined)	rs71298638	A	G	-0.007	63232261	0.812	0.029	14306	A	G	0.379	3	63246585	1.1165E-06	0.078	361227	6515.599
genus Lachnospiraceae UCG001	Vascular dementia (undefined)	rs78566090	A	G	-0.018	125740204	0.578	0.033	14306	A	G	0.346	8	124727963	9.44735E-07	0.071	361227	7049.571
genus Lachnospiraceae UCG004	Vascular dementia (undefined)	rs193392	T	C	-0.002	3085245	0.875	0.012	14306	T	C	-0.204	20	3104599	8.28324E-06	0.046	361227	7460.147
genus Lachnospiraceae UCG004	Vascular dementia (undefined)	rs2292090	T	C	0.005	70588309	0.713	0.013	14306	T	C	0.217	4	69722591	9.99332E-06	0.049	361227	6178.734
genus Lachnospiraceae UCG004	Vascular dementia (undefined)	rs2978951	G	A	0.000	6823295	0.991	0.012	14306	G	A	0.248	8	6965773	2.48222E-08	0.045	361227	11061.935
genus Lachnospiraceae UCG004	Vascular dementia (undefined)	rs359878	C	T	-0.010	185438949	0.505	0.014	14306	C	T	-0.211	2	184574222	4.6443E-06	0.046	361227	6959.700
genus Lachnospiraceae UCG004	Vascular dementia (undefined)	rs429358	C	T	-0.001	45411941	0.954	0.017	14306	C	T	0.695	19	44908684	9.26616E-39	0.053	361227	58999.679
genus Lachnospiraceae UCG004	Vascular dementia (undefined)	rs6133343	G	T	0.013	721797	0.465	0.018	14306	G	T	0.312	20	741153	3.89825E-06	0.068	361227	6210.744
genus Lachnospiraceae UCG004	Vascular dementia (undefined)	rs71298638	A	G	-0.016	63232261	0.562	0.025	14306	A	G	0.379	3	63246585	1.1165E-06	0.078	361227	6515.599
genus Lachnospiraceae UCG004	Vascular dementia (undefined)	rs78566090	A	G	0.010	125740204	0.741	0.028	14306	A	G	0.346	8	124727963	9.44735E-07	0.071	361227	7049.571
genus Lachnospiraceae UCG008	Vascular dementia (undefined)	rs12224047	T	C	-0.013	36820790	0.594	0.025	14306	T	C	-0.263	11	36799240	4.56278E-06	0.057	361227	8033.487
genus Lachnospiraceae UCG008	Vascular dementia (undefined)	rs12449066	G	A	-0.011	79177293	0.624	0.028	14306	G	A	-0.262	16	79143396	2.01674E-06	0.055	361227	8803.514
genus Lachnospiraceae UCG008	Vascular dementia (undefined)	rs193392	T	C	-0.002	3085245	0.882	0.017	14306	T	C	-0.204	20	3104599	8.28324E-06	0.046	361227	7460.147
genus Lachnospiraceae UCG008	Vascular dementia (undefined)	rs2978951	G	A	-0.005	6823295	0.766	0.017	14306	G	A	0.248	8	6965773	2.48222E-08	0.045	361227	11061.935
genus Lachnospiraceae UCG008	Vascular dementia (undefined)	rs359878	C	T	0.000	185438949	0.973	0.020	14306	C	T	-0.211	2	184574222	4.6443E-06	0.046	361227	6959.700
genus Lachnospiraceae UCG008	Vascular dementia (undefined)	rs429358	C	T	0.001	45411941	0.933	0.024	14306	C	T	0.695	19	44908684	9.26616E-39	0.053	361227	58999.679
genus Lachnospiraceae UCG008	Vascular dementia (undefined)	rs71298638	A	G	0.018	63232261	0.644	0.034	14306	A	G	0.379	3	63246585	1.1165E-06	0.078	361227	6515.599
genus Lachnospiraceae UCG008	Vascular dementia (undefined)	rs78566090	A	G	-0.009	125740204	0.802	0.040	14306	A	G	0.346	8	124727963	9.44735E-07	0.071	361227	7049.571
genus Lachnospiraceae UCG010	Vascular dementia (undefined)	rs12224047	T	C	0.003	36820790	0.821	0.018	14306	T	C	-0.263	11	36799240	4.56278E-06	0.057	361227	8033.487
genus Lachnospiraceae UCG010	Vascular dementia (undefined)	rs12449066	G	A	-0.005	79177293	0.938	0.021	14306	G	A	-0.262	16	79143396	2.01674E-06	0.055	361227	8803.514
genus Lachnospiraceae UCG010	Vascular dementia (undefined)	rs2972558	T	C	-0.005	45356141	0.693	0.013	14306	T	C	0.234	19	44852884	8.32415E-06	0.052	361227	7242.374
genus Lachnospiraceae UCG010	Vascular dementia (undefined)	rs429358	C	T	-0.003	45411941	0.809	0.018	14306	C	T	0.695	19	44908684	9.26616E-39	0.053	361227	58999.679
genus Lactobacillus	Vascular dementia (undefined)	rs193392	T	C	-0.006	3085245	0.730	0.017	14306	T	C	-0.204	20	3104599	8.28324E-06	0.046	361227	7460.147
genus Lactobacillus	Vascular dementia (undefined)	rs2978951	G	A	-0.017	6823295	0.334	0.017	14306	G	A	0.248	8	6965773	2.48222E-08	0.045	361227	11061.935
genus Lactobacillus	Vascular dementia (undefined)	rs359878	C	T	-0.012	185438949	0.585	0.020	14306	C	T	-0.211	2	184574222	4.6443E-06	0.046	361227	6959.700
genus Lactobacillus	Vascular dementia (undefined)	rs429358	C	T	-0.007	45411941	0.865	0.024	14306	C	T	0.695	19	44908684	9.26616E-39	0.053	361227	58999.679
genus Lactobacillus	Vascular dementia (undefined)	rs6133343	G	T	0.019	721797	0.468	0.027	14306	G	T	0.312	20	741153	3.89825E-06	0.068	361227	6210.744
genus Lactobacillus	Vascular dementia (undefined)	rs71298638	A	G	-0.026	63232261	0.479	0.036	14306	A	G	0.379	3	63246585	1.1165E-06	0.078	361227	6515.599
genus Lactobacillus	Vascular dementia (undefined)	rs78566090	A	G	-0.030	125740204	0.511	0.041	14306	A	G	0.346	8	124727963	9.44735E-07	0.071	361227	7049.571
genus Lactococcus	Vascular dementia (undefined)	rs12449066	G	A	-0.008	79177293	0.696	0.038	14306	G	A	-0.262	16	79143396	2.01674E-06	0.055	361227	8803.514
genus Lactococcus	Vascular dementia (undefined)	rs193392	T	C	-0.012	3085245	0.544	0.023	14306	T	C	-0.204	20	3104599	8.28324E-06	0.046	361227	7460.147
genus Lactococcus	Vascular dementia (undefined)	rs2292090	T	C	0.008	70588309	0.793	0.025	14306	T	C	0.217	4	69722591	9.99332E-06	0.049	361227	6178.734
genus Lactococcus	Vascular dementia (undefined)	rs2978951	G	A	-0.023	6823295	0.298	0.022	14306	G	A	0.248	8	6965773	2.48222E-08	0.045	361227	11061.935
genus Lactococcus	Vascular dementia (undefined)	rs429358	C	T	-0.017	45411941	0.591	0.032	14306	C	T	0.695	19	44908684	9.26616E-39	0.053	361227	58999.679
genus Lactococcus	Vascular dementia (undefined)	rs6133343	G	T	-0.030	721797	0.514	0.035	14306	G	T	0.312	20	741153	3.89825E-06	0.068	361227	6210.744
genus Lactococcus	Vascular dementia (undefined)	rs71298638	A	G	0.000	63232261	0.943	0.046	14306	A	G	0.379	3	63246585	1.1165E-06	0.078	361227	6515.599
genus Marvinbryantia	Vascular dementia (undefined)	rs12224047	T	C	0.010	36820790	0.566	0.019	14306	T	C	-0.263	11	36799240	4.56278E-06	0.057	361227	8033.487
genus Marvinbryantia	Vascular dementia (undefined)	rs193392	T	C	0.007	3085245	0.585	0.013	14306	T	C	-0.204	20	3104599	8.28324E-06	0.046	361227	7460.147
genus Marvinbryantia	Vascular dementia (undefined)	rs2292090	T	C	0.003	70588309	0.857	0.014	14306	T	C	0.217	4	69722591	9.99332E-06	0.049	361227	6178.734
genus Marvinbryantia	Vascular dementia (undefined)	rs2972558	T	C	-0.001	45356141	0.951	0.014	14306	T	C	0.234	19	44852884	8.32415E-06	0.052	361227	7242.374
genus Marvinbryantia	Vascular dementia (undefined)	rs359878	C	T	0.000	185438949	0.996	0.015	14306	C	T	-0.211	2	184574222	4.6443E-06	0.046	361227	6959.700
genus Marvinbryantia	Vascular dementia (undefined)	rs429358	C	T	0.008	45411941	0.663	0.018	14306	C	T	0.695	19	44908684	9.26616E-39	0.053	361227	58999.679
genus Marvinbryantia	Vascular dementia (undefined)	rs6133343	G	T	0.015	721797	0.501	0.021	14306	G	T	0.312	20	741153	3.89825E-06	0.068	361227	6210.744
genus Marvinbryantia	Vascular dementia (undefined)	rs71298638	A	G	-0.009	63232261	0.748	0.026	14306	A	G	0.379	3	63246585	1.1165E-06	0.078	361227	6515.599
genus Methanobrevibacter	Vascular dementia (undefined)	rs12224047	T	C	-0.005	36820790	0.863	0.035	14306	T	C	-0.263	11	36799240	4.56278E-06	0.057	361227	8033.487
genus Methanobrevibacter	Vascular dementia (undefined)	rs12449066	G	A	0.006	79177293	0.942	0.040	14306	G	A	-0.262	16	79143396	2.01674E-06	0.055	361227	8803.514
genus Methanobrevibacter	Vascular dementia (undefined)	rs193392	T	C	-0.001	3085245	0.941	0.025	14306	T	C	-0.204	20	3104599	8.28324E-06	0.046	361227	7460.147
genus Methanobrevibacter	Vascular dementia (undefined)	rs2972558	T	C	-0.022	45356141	0.434	0.027	14306	T	C	0.234	19	44852884	8.32415E-06	0.052	361227	7242.374
genus Methanobrevibacter	Vascular dementia (undefined)	rs359878	C	T	0.018	185438949	0.558	0.027	14306	C	T	-0.211	2	184574222	4.6443E-06	0.046	361227	6959.700
genus Methanobrevibacter	Vascular dementia (undefined)	rs429358	C	T	-0.019	45411941	0.661	0.034	14306	C	T	0.695	19	44908684	9.26616E-39	0.053	361227	58999.679
genus Odoribacter	Vascular dementia (undefined)	rs193392	T	C	0.000	3085245	0.975	0.012	14306	T	C	-0.204	20	3104599	8.28324E-06	0.046	361227	7460.147

genus Odoribacter	Vascular dementia (undefined)	rs2972558	T	C	-0.003	45356141	0.827	0.013	14306	T	C	0.234	19	44852884	8.32415E-06	0.052	361227	7242.374
genus Odoribacter	Vascular dementia (undefined)	rs359878	C	T	-0.005	185438949	0.696	0.014	14306	C	T	-0.211	2	184574222	4.6443E-06	0.046	361227	6959.700
genus Odoribacter	Vascular dementia (undefined)	rs429358	C	T	0.017	45411941	0.297	0.017	14306	C	T	0.695	19	44908684	9.26616E-39	0.053	361227	58999.679
genus Odoribacter	Vascular dementia (undefined)	rs6133343	G	T	0.014	721797	0.572	0.019	14306	G	T	0.312	20	741153	3.89825E-06	0.068	361227	6210.744
genus Odoribacter	Vascular dementia (undefined)	rs78566090	A	G	-0.003	125740204	0.875	0.028	14306	A	G	0.346	8	124727963	9.44735E-07	0.071	361227	7049.571
genus Olsenella	Vascular dementia (undefined)	rs12449066	G	A	-0.027	79177293	0.524	0.040	14306	G	A	-0.262	16	79143396	2.01674E-06	0.055	361227	8803.514
genus Olsenella	Vascular dementia (undefined)	rs193392	T	C	0.009	3085245	0.657	0.023	14306	T	C	-0.204	20	3104599	8.28324E-06	0.046	361227	7460.147
genus Olsenella	Vascular dementia (undefined)	rs2292090	T	C	-0.002	70588309	0.939	0.026	14306	T	C	0.217	4	69722591	9.99332E-06	0.049	361227	6178.734
genus Olsenella	Vascular dementia (undefined)	rs359878	C	T	-0.024	185438949	0.336	0.027	14306	C	T	-0.211	2	184574222	4.6443E-06	0.046	361227	6959.700
genus Olsenella	Vascular dementia (undefined)	rs429358	C	T	0.064	45411941	0.046	0.033	14306	C	T	0.695	19	44908684	9.26616E-39	0.053	361227	58999.679
genus Olsenella	Vascular dementia (undefined)	rs71298638	A	G	0.020	63232261	0.641	0.046	14306	A	G	0.379	3	63246585	1.1165E-06	0.078	361227	6515.599
genus Oscillibacter	Vascular dementia (undefined)	rs12224047	T	C	-0.015	36820790	0.512	0.022	14306	T	C	-0.263	11	36799240	4.56278E-06	0.057	361227	8033.487
genus Oscillibacter	Vascular dementia (undefined)	rs12449066	G	A	0.002	79177293	0.928	0.025	14306	G	A	-0.262	16	79143396	2.01674E-06	0.055	361227	8803.514
genus Oscillibacter	Vascular dementia (undefined)	rs193392	T	C	0.003	3085245	0.864	0.015	14306	T	C	-0.204	20	3104599	8.28324E-06	0.046	361227	7460.147
genus Oscillibacter	Vascular dementia (undefined)	rs2972558	T	C	0.013	45356141	0.447	0.016	14306	T	C	0.234	19	44852884	8.32415E-06	0.052	361227	7242.374
genus Oscillibacter	Vascular dementia (undefined)	rs2978951	G	A	0.013	6823295	0.381	0.015	14306	G	A	0.248	8	6965773	2.48222E-08	0.045	361227	11061.935
genus Oscillibacter	Vascular dementia (undefined)	rs359878	C	T	-0.004	185438949	0.850	0.017	14306	C	T	-0.211	2	184574222	4.6443E-06	0.046	361227	6959.700
genus Oscillibacter	Vascular dementia (undefined)	rs429358	C	T	-0.041	45411941	0.053	0.021	14306	C	T	0.695	19	44908684	9.26616E-39	0.053	361227	58999.679
genus Oscillibacter	Vascular dementia (undefined)	rs6133343	G	T	0.021	721797	0.347	0.024	14306	G	T	0.312	20	741153	3.89825E-06	0.068	361227	6210.744
genus Oscillibacter	Vascular dementia (undefined)	rs71298638	A	G	0.011	63232261	0.717	0.030	14306	A	G	0.379	3	63246585	1.1165E-06	0.078	361227	6515.599
genus Oscillibacter	Vascular dementia (undefined)	rs78566090	A	G	0.010	125740204	0.762	0.036	14306	A	G	0.346	8	124727963	9.44735E-07	0.071	361227	7049.571
genus Oscillospira	Vascular dementia (undefined)	rs12224047	T	C	0.007	36820790	0.750	0.020	14306	T	C	-0.263	11	36799240	4.56278E-06	0.057	361227	8033.487
genus Oscillospira	Vascular dementia (undefined)	rs193392	T	C	-0.004	3085245	0.837	0.014	14306	T	C	-0.204	20	3104599	8.28324E-06	0.046	361227	7460.147
genus Oscillospira	Vascular dementia (undefined)	rs2978951	G	A	-0.002	6823295	0.884	0.014	14306	G	A	0.248	8	6965773	2.48222E-08	0.045	361227	11061.935
genus Oscillospira	Vascular dementia (undefined)	rs429358	C	T	-0.005	45411941	0.763	0.020	14306	C	T	0.695	19	44908684	9.26616E-39	0.053	361227	58999.679
genus Oscillospira	Vascular dementia (undefined)	rs6133343	G	T	-0.020	721797	0.435	0.022	14306	G	T	0.312	20	741153	3.89825E-06	0.068	361227	6210.744
genus Oscillospira	Vascular dementia (undefined)	rs71298638	A	G	-0.009	63232261	0.756	0.029	14306	A	G	0.379	3	63246585	1.1165E-06	0.078	361227	6515.599
genus Oscillospira	Vascular dementia (undefined)	rs78566090	A	G	0.016	125740204	0.588	0.034	14306	A	G	0.346	8	124727963	9.44735E-07	0.071	361227	7049.571
genus Oxalobacter	Vascular dementia (undefined)	rs12224047	T	C	0.009	36820790	0.788	0.030	14306	T	C	-0.263	11	36799240	4.56278E-06	0.057	361227	8033.487
genus Oxalobacter	Vascular dementia (undefined)	rs12449066	G	A	0.019	79177293	0.730	0.036	14306	G	A	-0.262	16	79143396	2.01674E-06	0.055	361227	8803.514
genus Oxalobacter	Vascular dementia (undefined)	rs2292090	T	C	0.018	70588309	0.392	0.023	14306	T	C	0.217	4	69722591	9.99332E-06	0.049	361227	6178.734
genus Oxalobacter	Vascular dementia (undefined)	rs2978951	G	A	-0.007	6823295	0.759	0.021	14306	G	A	0.248	8	6965773	2.48222E-08	0.045	361227	11061.935
genus Oxalobacter	Vascular dementia (undefined)	rs359878	C	T	-0.009	185438949	0.671	0.024	14306	C	T	-0.211	2	184574222	4.6443E-06	0.046	361227	6959.700
genus Oxalobacter	Vascular dementia (undefined)	rs429358	C	T	0.034	45411941	0.200	0.030	14306	C	T	0.695	19	44908684	9.26616E-39	0.053	361227	58999.679
genus Parabacteroides	Vascular dementia (undefined)	rs2972558	T	C	-0.003	45356141	0.706	0.012	14306	T	C	0.234	19	44852884	8.32415E-06	0.052	361227	7242.374
genus Parabacteroides	Vascular dementia (undefined)	rs429358	C	T	-0.009	45411941	0.601	0.015	14306	C	T	0.695	19	44908684	9.26616E-39	0.053	361227	58999.679
genus Parabacteroides	Vascular dementia (undefined)	rs6133343	G	T	0.001	721797	0.851	0.017	14306	G	T	0.312	20	741153	3.89825E-06	0.068	361227	6210.744
genus Parabacteroides	Vascular dementia (undefined)	rs71298638	A	G	0.020	63232261	0.407	0.023	14306	A	G	0.379	3	63246585	1.1165E-06	0.078	361227	6515.599
genus Paraprevotella	Vascular dementia (undefined)	rs193392	T	C	-0.004	3085245	0.828	0.017	14306	T	C	-0.204	20	3104599	8.28324E-06	0.046	361227	7460.147
genus Paraprevotella	Vascular dementia (undefined)	rs2972558	T	C	-0.009	45356141	0.652	0.018	14306	T	C	0.234	19	44852884	8.32415E-06	0.052	361227	7242.374
genus Paraprevotella	Vascular dementia (undefined)	rs2978951	G	A	0.008	6823295	0.631	0.017	14306	G	A	0.248	8	6965773	2.48222E-08	0.045	361227	11061.935
genus Paraprevotella	Vascular dementia (undefined)	rs359878	C	T	0.003	185438949	0.850	0.020	14306	C	T	-0.211	2	184574222	4.6443E-06	0.046	361227	6959.700
genus Paraprevotella	Vascular dementia (undefined)	rs429358	C	T	-0.005	45411941	0.844	0.024	14306	C	T	0.695	19	44908684	9.26616E-39	0.053	361227	58999.679
genus Paraprevotella	Vascular dementia (undefined)	rs71298638	A	G	-0.032	63232261	0.391	0.037	14306	A	G	0.379	3	63246585	1.1165E-06	0.078	361227	6515.599
genus Paraprevotella	Vascular dementia (undefined)	rs78566090	A	G	-0.019	125740204	0.539	0.039	14306	A	G	0.346	8	124727963	9.44735E-07	0.071	361227	7049.571
genus Parasutterella	Vascular dementia (undefined)	rs12224047	T	C	-0.002	36820790	0.939	0.019	14306	T	C	-0.263	11	36799240	4.56278E-06	0.057	361227	8033.487
genus Parasutterella	Vascular dementia (undefined)	rs12449066	G	A	-0.009	79177293	0.530	0.023	14306	G	A	-0.262	16	79143396	2.01674E-06	0.055	361227	8803.514
genus Parasutterella	Vascular dementia (undefined)	rs429358	C	T	0.040	45411941	0.027	0.019	14306	C	T	0.695	19	44908684	9.26616E-39	0.053	361227	58999.679
genus Parasutterella	Vascular dementia (undefined)	rs78566090	A	G	0.023	125740204	0.424	0.031	14306	A	G	0.346	8	124727963	9.44735E-07	0.071	361227	7049.571
genus Peptococcus	Vascular dementia (undefined)	rs193392	T	C	-0.013	3085245	0.509	0.019	14306	T	C	-0.204	20	3104599	8.28324E-06	0.046	361227	7460.147
genus Peptococcus	Vascular dementia (undefined)	rs2292090	T	C	0.004	70588309	0.830	0.021	14306	T	C	0.217	4	69722591	9.99332E-06	0.049	361227	6178.734
genus Peptococcus	Vascular dementia (undefined)	rs359878	C	T	-0.015	185438949	0.488	0.022	14306	C	T	-0.211	2	184574222	4.6443E-06	0.046	361227	6959.700
genus Peptococcus	Vascular dementia (undefined)	rs429358	C	T	0.031	45411941	0.231	0.027	14306	C	T	0.695	19	44908684	9.26616E-39	0.053	361227	58999.679
genus Peptococcus	Vascular dementia (undefined)	rs71298638	A	G	0.022	63232261	0.576	0.038	14306	A	G	0.379	3	63246585	1.1165E-06	0.078	361227	6515.599
genus Peptococcus	Vascular dementia (undefined)	rs78566090	A	G	0.035	125740204	0.427	0.043	14306	A	G	0.346	8	124727963	9.44735E-07	0.071	361227	7049.571
genus Phascolarctobacterium	Vascular dementia (undefined)	rs12224047	T	C	-0.017	36820790	0.358	0.019	14306	T	C	-0.263	11	36799240	4.56278E-06	0.057	361227	8033.487
genus Phascolarctobacterium	Vascular dementia (undefined)	rs12449066	G	A	0.006	79177293	0.727	0.023	14306	G	A	-0.262	16	79143396	2.01674E-06	0.055	361227	8803.514
genus Phascolarctobacterium	Vascular dementia (undefined)	rs2972558	T	C	-0.003	45356141	0.848	0.015	14306	T	C	0.234	19	44852884	8.32415E-06	0.052	361227	7242.374
genus Phascolarctobacterium	Vascular dementia (undefined)	rs359878	C	T	0.005	185438949	0.721	0.016	14306	C	T	-0.211	2	184574222	4.6443E-06	0.046	361227	6959.700
genus Phascolarctobacterium	Vascular dementia (undefined)	rs429358	C	T	-0.013	45411941	0.427	0.019	14306	C	T	0.695	19	44908684	9.26616E-39	0.053	361227	58999.679
genus Phascolarctobacterium	Vascular dementia (undefined)	rs6133343	G	T	-0.016	721797	0.527	0.021	14306	G	T	0.312	20	741153	3.89825E-06	0.068	361227	6210.744
genus Phascolarctobacterium	Vascular dementia (undefined)	rs71298638	A	G	-0.003	63232261	0.922	0.029	14306	A	G	0.379	3	63246585	1.1165E-06	0.078	361227	6515.599
genus Phascolarctobacterium	Vascular dementia (undefined)	rs78566090	A	G	0.025	125740204	0.450	0.032	14306	A	G	0.346	8	124727963	9.44735E-07	0.071	361227	7049.571
genus Prevotella7	Vascular dementia (undefined)	rs12224047	T	C	0.001	36820790	0.904	0.034	14306	T	C	-0.263	11	36799240	4.56278E-06	0.057	361227	8033.487
genus Prevotella7	Vascular dementia (undefined)	rs12449066	G	A	0.017	79177293	0.519	0.041	14306	G	A	-0.262	16	79143396	2.01674E-06	0.055	361227	8803.514
genus Prevotella7	Vascular dementia (undefined)	rs2292090	T	C	-0.007	70588309	0.832	0.027	14306	T	C	0.217	4	69722591	9.99332E-06	0.049	361227	6178.734
genus Prevotella7	Vascular dementia (undefined)	rs359878	C	T	-0.016	185438949	0.585	0.028	14306	C	T	-0.211	2	184574222	4.6443E-06	0.046	361227	6959.700
genus Prevotella7	Vascular dementia (undefined)	rs429358	C	T	0.047	45411941	0.135	0.034	14306	C	T	0.695	19	44908684	9.26616E-39	0.053	361227	58999.679
genus Prevotella7	Vascular dementia (undefined)	rs6133343	G	T	0.012	721797	0.786	0.039	14306	G	T	0.312	20	741153	3.89825E-06	0.068	361227	6210.744
genus Prevotella9	Vascular dementia (undefined)	rs193392	T	C	-0.006	3085245	0.662	0.014	14306	T	C	-0.204	20	3104599	8.28324E-06	0.046	361227	7460.147
genus Prevotella9	Vascular dementia (undefined)	rs2292090	T	C	-0.005	70588309	0.725	0.016	14306	T	C	0.217	4	69722591	9.99332E-06	0.049	361227	6178.734

genus Prevotella9	Vascular dementia (undefined)	rs2972558	T	C	0.007	45356141	0.622	0.015	14306	T	C	0.234	19	44852884	8.32415E-06	0.052	361227	7242.374
genus Prevotella9	Vascular dementia (undefined)	rs2978951	G	A	0.015	6823295	0.290	0.014	14306	G	A	0.248	8	6965773	2.48222E-08	0.045	361227	11061.935
genus Prevotella9	Vascular dementia (undefined)	rs359878	C	T	-0.003	185438949	0.866	0.016	14306	C	T	-0.211	2	184574222	4.6443E-06	0.046	361227	6959.700
genus Prevotella9	Vascular dementia (undefined)	rs429358	C	T	0.022	45411941	0.310	0.020	14306	C	T	0.695	19	44908684	9.26616E-39	0.053	361227	58999.679
genus Prevotella9	Vascular dementia (undefined)	rs6133343	G	T	0.009	721797	0.990	0.021	14306	G	T	0.312	20	741153	3.89825E-06	0.068	361227	6210.744
genus Prevotella9	Vascular dementia (undefined)	rs71298638	A	G	-0.016	63232261	0.634	0.031	14306	A	G	0.379	3	63246585	1.1165E-06	0.078	361227	6515.599
genus Rikenellaceae RC9 gut group	Vascular dementia (undefined)	rs12224047	T	C	0.014	36820790	0.762	0.035	14306	T	C	-0.263	11	36799240	4.56278E-06	0.057	361227	8033.487
genus Rikenellaceae RC9 gut group	Vascular dementia (undefined)	rs12449066	G	A	0.023	79177293	0.570	0.041	14306	G	A	-0.262	16	79143396	2.01674E-06	0.055	361227	8803.514
genus Rikenellaceae RC9 gut group	Vascular dementia (undefined)	rs2978951	G	A	0.009	6823295	0.666	0.025	14306	G	A	0.248	8	6965773	2.48222E-08	0.045	361227	11061.935
genus Rikenellaceae RC9 gut group	Vascular dementia (undefined)	rs359878	C	T	0.014	185438949	0.683	0.028	14306	C	T	-0.211	2	184574222	4.6443E-06	0.046	361227	6959.700
genus Rikenellaceae RC9 gut group	Vascular dementia (undefined)	rs429358	C	T	0.062	45411941	0.092	0.035	14306	C	T	0.695	19	44908684	9.26616E-39	0.053	361227	58999.679
genus Romboutsia	Vascular dementia (undefined)	rs193392	T	C	0.009	3085245	0.459	0.012	14306	T	C	-0.204	20	3104599	8.28324E-06	0.046	361227	7460.147
genus Romboutsia	Vascular dementia (undefined)	rs429358	C	T	0.005	45411941	0.703	0.017	14306	C	T	0.695	19	44908684	9.26616E-39	0.053	361227	58999.679
genus Romboutsia	Vascular dementia (undefined)	rs6133343	G	T	0.003	721797	0.795	0.019	14306	G	T	0.312	20	741153	3.89825E-06	0.068	361227	6210.744
genus Romboutsia	Vascular dementia (undefined)	rs71298638	A	G	-0.002	63232261	0.918	0.025	14306	A	G	0.379	3	63246585	1.1165E-06	0.078	361227	6515.599
genus Romboutsia	Vascular dementia (undefined)	rs78566090	A	G	-0.005	125740204	0.809	0.028	14306	A	G	0.346	8	124727963	9.44735E-07	0.071	361227	7049.571
genus Roseburia	Vascular dementia (undefined)	rs12449066	G	A	0.013	79177293	0.419	0.018	14306	G	A	-0.262	16	79143396	2.01674E-06	0.055	361227	8803.514
genus Roseburia	Vascular dementia (undefined)	rs359878	C	T	-0.011	185438949	0.392	0.012	14306	C	T	-0.211	2	184574222	4.6443E-06	0.046	361227	6959.700
genus Roseburia	Vascular dementia (undefined)	rs429358	C	T	-0.001	45411941	0.921	0.015	14306	C	T	0.695	19	44908684	9.26616E-39	0.053	361227	58999.679
genus Roseburia	Vascular dementia (undefined)	rs6133343	G	T	0.005	721797	0.777	0.017	14306	G	T	0.312	20	741153	3.89825E-06	0.068	361227	6210.744
genus Roseburia	Vascular dementia (undefined)	rs78566090	A	G	0.006	125740204	0.819	0.025	14306	A	G	0.346	8	124727963	9.44735E-07	0.071	361227	7049.571
genus Ruminiclostridium5	Vascular dementia (undefined)	rs12224047	T	C	0.000	36820790	0.942	0.016	14306	T	C	-0.263	11	36799240	4.56278E-06	0.057	361227	8033.487
genus Ruminiclostridium5	Vascular dementia (undefined)	rs12449066	G	A	0.013	79177293	0.550	0.019	14306	G	A	-0.262	16	79143396	2.01674E-06	0.055	361227	8803.514
genus Ruminiclostridium5	Vascular dementia (undefined)	rs2972558	T	C	0.002	45356141	0.814	0.012	14306	T	C	0.234	19	44852884	8.32415E-06	0.052	361227	7242.374
genus Ruminiclostridium5	Vascular dementia (undefined)	rs2978951	G	A	-0.003	6823295	0.775	0.011	14306	G	A	0.248	8	6965773	2.48222E-08	0.045	361227	11061.935
genus Ruminiclostridium5	Vascular dementia (undefined)	rs359878	C	T	-0.007	185438949	0.551	0.012	14306	C	T	-0.211	2	184574222	4.6443E-06	0.046	361227	6959.700
genus Ruminiclostridium5	Vascular dementia (undefined)	rs429358	C	T	0.022	45411941	0.184	0.015	14306	C	T	0.695	19	44908684	9.26616E-39	0.053	361227	58999.679
genus Ruminiclostridium5	Vascular dementia (undefined)	rs6133343	G	T	0.003	721797	0.885	0.017	14306	G	T	0.312	20	741153	3.89825E-06	0.068	361227	6210.744
genus Ruminiclostridium5	Vascular dementia (undefined)	rs71298638	A	G	0.016	63232261	0.505	0.022	14306	A	G	0.379	3	63246585	1.1165E-06	0.078	361227	6515.599
genus Ruminiclostridium5	Vascular dementia (undefined)	rs78566090	A	G	-0.017	125740204	0.462	0.025	14306	A	G	0.346	8	124727963	9.44735E-07	0.071	361227	7049.571
genus Ruminiclostridium6	Vascular dementia (undefined)	rs2978951	G	A	0.009	6823295	0.471	0.012	14306	G	A	0.248	8	6965773	2.48222E-08	0.045	361227	11061.935
genus Ruminiclostridium6	Vascular dementia (undefined)	rs359878	C	T	-0.011	185438949	0.474	0.014	14306	C	T	-0.211	2	184574222	4.6443E-06	0.046	361227	6959.700
genus Ruminiclostridium6	Vascular dementia (undefined)	rs429358	C	T	0.007	45411941	0.690	0.018	14306	C	T	0.695	19	44908684	9.26616E-39	0.053	361227	58999.679
genus Ruminiclostridium6	Vascular dementia (undefined)	rs6133343	G	T	-0.011	721797	0.584	0.020	14306	G	T	0.312	20	741153	3.89825E-06	0.068	361227	6210.744
genus Ruminiclostridium6	Vascular dementia (undefined)	rs71298638	A	G	-0.007	63232261	0.740	0.025	14306	A	G	0.379	3	63246585	1.1165E-06	0.078	361227	6515.599
genus Ruminiclostridium6	Vascular dementia (undefined)	rs78566090	A	G	0.004	125740204	0.885	0.029	14306	A	G	0.346	8	124727963	9.44735E-07	0.071	361227	7049.571
genus Ruminiclostridium9	Vascular dementia (undefined)	rs12449066	G	A	-0.018	79177293	0.387	0.019	14306	G	A	-0.262	16	79143396	2.01674E-06	0.055	361227	8803.514
genus Ruminiclostridium9	Vascular dementia (undefined)	rs193392	T	C	-0.004	3085245	0.737	0.011	14306	T	C	-0.204	20	3104599	8.28324E-06	0.046	361227	7460.147
genus Ruminiclostridium9	Vascular dementia (undefined)	rs2292090	T	C	-0.010	70588309	0.424	0.012	14306	T	C	0.217	4	69722591	9.99332E-06	0.049	361227	6178.734
genus Ruminiclostridium9	Vascular dementia (undefined)	rs2972558	T	C	0.007	45356141	0.572	0.012	14306	T	C	0.234	19	44852884	8.32415E-06	0.052	361227	7242.374
genus Ruminiclostridium9	Vascular dementia (undefined)	rs2978951	G	A	-0.009	6823295	0.393	0.011	14306	G	A	0.248	8	6965773	2.48222E-08	0.045	361227	11061.935
genus Ruminiclostridium9	Vascular dementia (undefined)	rs429358	C	T	-0.007	45411941	0.627	0.016	14306	C	T	0.695	19	44908684	9.26616E-39	0.053	361227	58999.679
genus Ruminiclostridium9	Vascular dementia (undefined)	rs78566090	A	G	0.005	125740204	0.860	0.026	14306	A	G	0.346	8	124727963	9.44735E-07	0.071	361227	7049.571
genus Ruminococcaceae NK4A214 group	Vascular dementia (undefined)	rs12449066	G	A	0.001	79177293	0.978	0.020	14306	G	A	-0.262	16	79143396	2.01674E-06	0.055	361227	8803.514
genus Ruminococcaceae NK4A214 group	Vascular dementia (undefined)	rs2978951	G	A	0.007	6823295	0.554	0.012	14306	G	A	0.248	8	6965773	2.48222E-08	0.045	361227	11061.935
genus Ruminococcaceae NK4A214 group	Vascular dementia (undefined)	rs359878	C	T	-0.009	185438949	0.458	0.013	14306	C	T	-0.211	2	184574222	4.6443E-06	0.046	361227	6959.700
genus Ruminococcaceae NK4A214 group	Vascular dementia (undefined)	rs429358	C	T	0.002	45411941	0.898	0.016	14306	C	T	0.695	19	44908684	9.26616E-39	0.053	361227	58999.679
genus Ruminococcaceae NK4A214 group	Vascular dementia (undefined)	rs6133343	G	T	-0.005	721797	0.722	0.018	14306	G	T	0.312	20	741153	3.89825E-06	0.068	361227	6210.744
genus Ruminococcaceae NK4A214 group	Vascular dementia (undefined)	rs78566090	A	G	-0.005	125740204	0.803	0.027	14306	A	G	0.346	8	124727963	9.44735E-07	0.071	361227	7049.571
genus Ruminococcaceae UCG002	Vascular dementia (undefined)	rs12224047	T	C	-0.006	36820790	0.644	0.016	14306	T	C	-0.263	11	36799240	4.56278E-06	0.057	361227	8033.487
genus Ruminococcaceae UCG002	Vascular dementia (undefined)	rs12449066	G	A	-0.009	79177293	0.556	0.019	14306	G	A	-0.262	16	79143396	2.01674E-06	0.055	361227	8803.514
genus Ruminococcaceae UCG002	Vascular dementia (undefined)	rs193392	T	C	-0.003	3085245	0.797	0.011	14306	T	C	-0.204	20	3104599	8.28324E-06	0.046	361227	7460.147
genus Ruminococcaceae UCG002	Vascular dementia (undefined)	rs2292090	T	C	-0.004	70588309	0.793	0.012	14306	T	C	0.217	4	69722591	9.99332E-06	0.049	361227	6178.734
genus Ruminococcaceae UCG002	Vascular dementia (undefined)	rs2972558	T	C	0.006	45356141	0.546	0.012	14306	T	C	0.234	19	44852884	8.32415E-06	0.052	361227	7242.374
genus Ruminococcaceae UCG002	Vascular dementia (undefined)	rs2978951	G	A	-0.001	6823295	0.931	0.011	14306	G	A	0.248	8	6965773	2.48222E-08	0.045	361227	11061.935
genus Ruminococcaceae UCG002	Vascular dementia (undefined)	rs359878	C	T	-0.010	185438949	0.455	0.013	14306	C	T	-0.211	2	184574222	4.6443E-06	0.046	361227	6959.700
genus Ruminococcaceae UCG002	Vascular dementia (undefined)	rs429358	C	T	-0.004	45411941	0.869	0.016	14306	C	T	0.695	19	44908684	9.26616E-39	0.053	361227	58999.679
genus Ruminococcaceae UCG002	Vascular dementia (undefined)	rs6133343	G	T	-0.005	721797	0.890	0.017	14306	G	T	0.312	20	741153	3.89825E-06	0.068	361227	6210.744
genus Ruminococcaceae UCG002	Vascular dementia (undefined)	rs71298638	A	G	-0.015	63232261	0.521	0.023	14306	A	G	0.379	3	63246585	1.1165E-06	0.078	361227	6515.599
genus Ruminococcaceae UCG002	Vascular dementia (undefined)	rs78566090	A	G	-0.014	125740204	0.586	0.025	14306	A	G	0.346	8	124727963	9.44735E-07	0.071	361227	7049.571
genus Ruminococcaceae UCG003	Vascular dementia (undefined)	rs12224047	T	C	-0.012	36820790	0.525	0.017	14306	T	C	-0.263	11	36799240	4.56278E-06	0.057	361227	8033.487
genus Ruminococcaceae UCG003	Vascular dementia (undefined)	rs193392	T	C	0.010	3085245	0.435	0.013	14306	T	C	-0.204	20	3104599	8.28324E-06	0.046	361227	7460.147
genus Ruminococcaceae UCG003	Vascular dementia (undefined)	rs2292090	T	C	-0.003	70588309	0.830	0.013	14306	T	C	0.217	4	69722591	9.99332E-06	0.049	361227	6178.734
genus Ruminococcaceae UCG003	Vascular dementia (undefined)	rs2972558	T	C	0.006	45356141	0.644	0.013	14306	T	C	0.234	19	44852884	8.32415E-06	0.052	361227	7242.374
genus Ruminococcaceae UCG003	Vascular dementia (undefined)	rs2978951	G	A	0.006	6823295	0.610	0.012	14306	G	A	0.248	8	6965773	2.48222E-08	0.045	361227	11061.935
genus Ruminococcaceae UCG003	Vascular dementia (undefined)	rs429358	C	T	0.007	45411941	0.595	0.017	14306	C	T	0.695	19	44908684	9.26616E-39	0.053	361227	58999.679
genus Ruminococcaceae UCG003	Vascular dementia (undefined)	rs71298638	A	G	-0.012	63232261	0.578	0.025	14306	A	G	0.379	3	63246585	1.1165E-06	0.078	361227	6515.599
genus Ruminococcaceae UCG004	Vascular dementia (undefined)	rs12224047	T	C	-0.002	36820790	0.929	0.021	14306	T	C	-0.263	11	36799240	4.56278E-06	0.057	361227	8033.487
genus Ruminococcaceae UCG004	Vascular dementia (undefined)	rs12449066	G	A	-0.019	79177293	0.339	0.025	14306	G	A	-0.262	16	79143396	2.01674E-06	0.055	361227	8803.514
genus Ruminococcaceae UCG004	Vascular dementia (undefined)	rs193392	T	C	-0.008	3085245	0.625	0.015	14306	T	C	-0.204	20	3104599	8.28324E-06	0.046	361227	7460.147
genus Ruminococcaceae UCG004	Vascular dementia (undefined)	rs2292090	T	C	0.010	70588309	0.467	0.016	14306	T	C	0.217	4	69722591	9.99332E-06	0.049	361227	6178.734

genus Ruminococcaceae UCG004	Vascular dementia (undefined)	rs2972558	T	C	0.006	45356141	0.693	0.016	14306	T	C	0.234	19	44852884	8.32415E-06	0.052	361227	7242.374
genus Ruminococcaceae UCG004	Vascular dementia (undefined)	rs2978951	G	A	-0.007	6823295	0.627	0.015	14306	G	A	0.248	8	6965773	2.48222E-08	0.045	361227	11061.935
genus Ruminococcaceae UCG004	Vascular dementia (undefined)	rs429358	C	T	0.025	45411941	0.252	0.021	14306	C	T	0.695	19	44908684	9.26616E-39	0.053	361227	58999.679
genus Ruminococcaceae UCG004	Vascular dementia (undefined)	rs6133343	G	T	0.008	721797	0.854	0.023	14306	G	T	0.312	20	741153	3.89825E-06	0.068	361227	6210.744
genus Ruminococcaceae UCG004	Vascular dementia (undefined)	rs71298638	A	G	-0.016	63232261	0.669	0.029	14306	A	G	0.379	3	63246585	1.1165E-06	0.078	361227	6515.599
genus Ruminococcaceae UCG004	Vascular dementia (undefined)	rs78566090	A	G	-0.015	125740204	0.710	0.036	14306	A	G	0.346	8	124727963	9.44735E-07	0.071	361227	7049.571
genus Ruminococcaceae UCG005	Vascular dementia (undefined)	rs193392	T	C	0.001	3085245	0.941	0.011	14306	T	C	-0.204	20	3104599	8.28324E-06	0.046	361227	7460.147
genus Ruminococcaceae UCG005	Vascular dementia (undefined)	rs2292090	T	C	-0.001	70588309	0.960	0.012	14306	T	C	0.217	4	69722591	9.99332E-06	0.049	361227	6178.734
genus Ruminococcaceae UCG005	Vascular dementia (undefined)	rs2978951	G	A	0.009	6823295	0.409	0.011	14306	G	A	0.248	8	6965773	2.48222E-08	0.045	361227	11061.935
genus Ruminococcaceae UCG005	Vascular dementia (undefined)	rs359878	C	T	0.002	185438949	0.938	0.013	14306	C	T	-0.211	2	184574222	4.6443E-06	0.046	361227	6959.700
genus Ruminococcaceae UCG005	Vascular dementia (undefined)	rs429358	C	T	0.004	45411941	0.741	0.016	14306	C	T	0.695	19	44908684	9.26616E-39	0.053	361227	58999.679
genus Ruminococcaceae UCG005	Vascular dementia (undefined)	rs71298638	A	G	-0.007	63232261	0.796	0.023	14306	A	G	0.379	3	63246585	1.1165E-06	0.078	361227	6515.599
genus Ruminococcaceae UCG005	Vascular dementia (undefined)	rs78566090	A	G	0.007	125740204	0.810	0.026	14306	A	G	0.346	8	124727963	9.44735E-07	0.071	361227	7049.571
genus Ruminococcaceae UCG009	Vascular dementia (undefined)	rs12224047	T	C	0.020	36820790	0.434	0.024	14306	T	C	-0.263	11	36799240	4.56278E-06	0.057	361227	8033.487
genus Ruminococcaceae UCG009	Vascular dementia (undefined)	rs12449066	G	A	0.020	79177293	0.642	0.028	14306	G	A	-0.262	16	79143396	2.01674E-06	0.055	361227	8803.514
genus Ruminococcaceae UCG009	Vascular dementia (undefined)	rs193392	T	C	0.006	3085245	0.687	0.017	14306	T	C	-0.204	20	3104599	8.28324E-06	0.046	361227	7460.147
genus Ruminococcaceae UCG009	Vascular dementia (undefined)	rs2292090	T	C	-0.010	70588309	0.615	0.018	14306	T	C	0.217	4	69722591	9.99332E-06	0.049	361227	6178.734
genus Ruminococcaceae UCG009	Vascular dementia (undefined)	rs2978951	G	A	0.011	6823295	0.503	0.017	14306	G	A	0.248	8	6965773	2.48222E-08	0.045	361227	11061.935
genus Ruminococcaceae UCG009	Vascular dementia (undefined)	rs359878	C	T	0.004	185438949	0.916	0.019	14306	C	T	-0.211	2	184574222	4.6443E-06	0.046	361227	6959.700
genus Ruminococcaceae UCG009	Vascular dementia (undefined)	rs429358	C	T	-0.040	45411941	0.093	0.023	14306	C	T	0.695	19	44908684	9.26616E-39	0.053	361227	58999.679
genus Ruminococcaceae UCG009	Vascular dementia (undefined)	rs6133343	G	T	0.002	721797	0.811	0.026	14306	G	T	0.312	20	741153	3.89825E-06	0.068	361227	6210.744
genus Ruminococcaceae UCG009	Vascular dementia (undefined)	rs78566090	A	G	0.002	125740204	0.944	0.038	14306	A	G	0.346	8	124727963	9.44735E-07	0.071	361227	7049.571
genus Ruminococcaceae UCG010	Vascular dementia (undefined)	rs12224047	T	C	0.008	36820790	0.649	0.018	14306	T	C	-0.263	11	36799240	4.56278E-06	0.057	361227	8033.487
genus Ruminococcaceae UCG010	Vascular dementia (undefined)	rs12449066	G	A	0.009	79177293	0.659	0.021	14306	G	A	-0.262	16	79143396	2.01674E-06	0.055	361227	8803.514
genus Ruminococcaceae UCG010	Vascular dementia (undefined)	rs193392	T	C	0.004	3085245	0.765	0.013	14306	T	C	-0.204	20	3104599	8.28324E-06	0.046	361227	7460.147
genus Ruminococcaceae UCG010	Vascular dementia (undefined)	rs2292090	T	C	0.005	70588309	0.696	0.014	14306	T	C	0.217	4	69722591	9.99332E-06	0.049	361227	6178.734
genus Ruminococcaceae UCG010	Vascular dementia (undefined)	rs2978951	G	A	0.010	6823295	0.423	0.013	14306	G	A	0.248	8	6965773	2.48222E-08	0.045	361227	11061.935
genus Ruminococcaceae UCG010	Vascular dementia (undefined)	rs359878	C	T	-0.011	185438949	0.453	0.014	14306	C	T	-0.211	2	184574222	4.6443E-06	0.046	361227	6959.700
genus Ruminococcaceae UCG010	Vascular dementia (undefined)	rs429358	C	T	0.003	45411941	0.881	0.018	14306	C	T	0.695	19	44908684	9.26616E-39	0.053	361227	58999.679
genus Ruminococcaceae UCG010	Vascular dementia (undefined)	rs6133343	G	T	0.006	721797	0.788	0.020	14306	G	T	0.312	20	741153	3.89825E-06	0.068	361227	6210.744
genus Ruminococcaceae UCG010	Vascular dementia (undefined)	rs71298638	A	G	0.013	63232261	0.603	0.026	14306	A	G	0.379	3	63246585	1.1165E-06	0.078	361227	6515.599
genus Ruminococcaceae UCG010	Vascular dementia (undefined)	rs78566090	A	G	0.006	125740204	0.831	0.029	14306	A	G	0.346	8	124727963	9.44735E-07	0.071	361227	7049.571
genus Ruminococcaceae UCG011	Vascular dementia (undefined)	rs12224047	T	C	0.029	36820790	0.420	0.034	14306	T	C	-0.263	11	36799240	4.56278E-06	0.057	361227	8033.487
genus Ruminococcaceae UCG011	Vascular dementia (undefined)	rs12449066	G	A	0.013	79177293	0.680	0.039	14306	G	A	-0.262	16	79143396	2.01674E-06	0.055	361227	8803.514
genus Ruminococcaceae UCG011	Vascular dementia (undefined)	rs193392	T	C	0.011	3085245	0.643	0.024	14306	T	C	-0.204	20	3104599	8.28324E-06	0.046	361227	7460.147
genus Ruminococcaceae UCG011	Vascular dementia (undefined)	rs2292090	T	C	0.010	70588309	0.675	0.026	14306	T	C	0.217	4	69722591	9.99332E-06	0.049	361227	6178.734
genus Ruminococcaceae UCG011	Vascular dementia (undefined)	rs2972558	T	C	0.007	45356141	0.789	0.025	14306	T	C	0.234	19	44852884	8.32415E-06	0.052	361227	7242.374
genus Ruminococcaceae UCG011	Vascular dementia (undefined)	rs359878	C	T	0.017	185438949	0.543	0.027	14306	C	T	-0.211	2	184574222	4.6443E-06	0.046	361227	6959.700
genus Ruminococcaceae UCG011	Vascular dementia (undefined)	rs429358	C	T	-0.013	45411941	0.704	0.033	14306	C	T	0.695	19	44908684	9.26616E-39	0.053	361227	58999.679
genus Ruminococcaceae UCG011	Vascular dementia (undefined)	rs6133343	G	T	0.034	721797	0.416	0.039	14306	G	T	0.312	20	741153	3.89825E-06	0.068	361227	6210.744
genus Ruminococcaceae UCG011	Vascular dementia (undefined)	rs71298638	A	G	-0.020	63232261	0.691	0.046	14306	A	G	0.379	3	63246585	1.1165E-06	0.078	361227	6515.599
genus Ruminococcaceae UCG013	Vascular dementia (undefined)	rs2292090	T	C	-0.010	70588309	0.439	0.012	14306	T	C	0.217	4	69722591	9.99332E-06	0.049	361227	6178.734
genus Ruminococcaceae UCG013	Vascular dementia (undefined)	rs2972558	T	C	-0.003	45356141	0.826	0.012	14306	T	C	0.234	19	44852884	8.32415E-06	0.052	361227	7242.374
genus Ruminococcaceae UCG013	Vascular dementia (undefined)	rs2978951	G	A	0.006	6823295	0.567	0.011	14306	G	A	0.248	8	6965773	2.48222E-08	0.045	361227	11061.935
genus Ruminococcaceae UCG013	Vascular dementia (undefined)	rs359878	C	T	0.003	185438949	0.925	0.013	14306	C	T	-0.211	2	184574222	4.6443E-06	0.046	361227	6959.700
genus Ruminococcaceae UCG013	Vascular dementia (undefined)	rs429358	C	T	-0.022	45411941	0.167	0.016	14306	C	T	0.695	19	44908684	9.26616E-39	0.053	361227	58999.679
genus Ruminococcaceae UCG013	Vascular dementia (undefined)	rs6133343	G	T	0.007	721797	0.643	0.017	14306	G	T	0.312	20	741153	3.89825E-06	0.068	361227	6210.744
genus Ruminococcaceae UCG013	Vascular dementia (undefined)	rs71298638	A	G	0.010	63232261	0.720	0.023	14306	A	G	0.379	3	63246585	1.1165E-06	0.078	361227	6515.599
genus Ruminococcaceae UCG014	Vascular dementia (undefined)	rs12224047	T	C	-0.012	36820790	0.479	0.017	14306	T	C	-0.263	11	36799240	4.56278E-06	0.057	361227	8033.487
genus Ruminococcaceae UCG014	Vascular dementia (undefined)	rs2972558	T	C	0.011	45356141	0.397	0.013	14306	T	C	0.234	19	44852884	8.32415E-06	0.052	361227	7242.374
genus Ruminococcaceae UCG014	Vascular dementia (undefined)	rs359878	C	T	0.009	185438949	0.593	0.014	14306	C	T	-0.211	2	184574222	4.6443E-06	0.046	361227	6959.700
genus Ruminococcaceae UCG014	Vascular dementia (undefined)	rs429358	C	T	0.020	45411941	0.191	0.017	14306	C	T	0.695	19	44908684	9.26616E-39	0.053	361227	58999.679
genus Ruminococcaceae UCG014	Vascular dementia (undefined)	rs6133343	G	T	0.007	721797	0.795	0.019	14306	G	T	0.312	20	741153	3.89825E-06	0.068	361227	6210.744
genus Ruminococcaceae UCG014	Vascular dementia (undefined)	rs71298638	A	G	0.017	63232261	0.496	0.024	14306	A	G	0.379	3	63246585	1.1165E-06	0.078	361227	6515.599
genus Ruminococcaceae UCG014	Vascular dementia (undefined)	rs78566090	A	G	0.018	125740204	0.513	0.028	14306	A	G	0.346	8	124727963	9.44735E-07	0.071	361227	7049.571
genus Ruminococcus1	Vascular dementia (undefined)	rs12449066	G	A	0.010	79177293	0.643	0.019	14306	G	A	-0.262	16	79143396	2.01674E-06	0.055	361227	8803.514
genus Ruminococcus1	Vascular dementia (undefined)	rs2972558	T	C	-0.003	45356141	0.819	0.012	14306	T	C	0.234	19	44852884	8.32415E-06	0.052	361227	7242.374
genus Ruminococcus1	Vascular dementia (undefined)	rs429358	C	T	-0.008	45411941	0.683	0.016	14306	C	T	0.695	19	44908684	9.26616E-39	0.053	361227	58999.679
genus Ruminococcus1	Vascular dementia (undefined)	rs6133343	G	T	-0.007	721797	0.588	0.017	14306	G	T	0.312	20	741153	3.89825E-06	0.068	361227	6210.744
genus Ruminococcus1	Vascular dementia (undefined)	rs71298638	A	G	-0.001	63232261	0.909	0.023	14306	A	G	0.379	3	63246585	1.1165E-06	0.078	361227	6515.599
genus Ruminococcus2	Vascular dementia (undefined)	rs12449066	G	A	0.010	79177293	0.786	0.020	14306	G	A	-0.262	16	79143396	2.01674E-06	0.055	361227	8803.514
genus Ruminococcus2	Vascular dementia (undefined)	rs359878	C	T	-0.010	185438949	0.473	0.013	14306	C	T	-0.211	2	184574222	4.6443E-06	0.046	361227	6959.700
genus Ruminococcus2	Vascular dementia (undefined)	rs429358	C	T	0.007	45411941	0.633	0.016	14306	C	T	0.695	19	44908684	9.26616E-39	0.053	361227	58999.679
genus Ruminococcus2	Vascular dementia (undefined)	rs6133343	G	T	0.007	721797	0.591	0.018	14306	G	T	0.312	20	741153	3.89825E-06	0.068	361227	6210.744
genus Ruminococcus2	Vascular dementia (undefined)	rs71298638	A	G	0.017	63232261	0.501	0.024	14306	A	G	0.379	3	63246585	1.1165E-06	0.078	361227	6515.599
genus Ruminococcus2	Vascular dementia (undefined)	rs78566090	A	G	-0.012	125740204	0.670	0.027	14306	A	G	0.346	8	124727963	9.44735E-07	0.071	361227	7049.571
genus Ruminococcus gauvreauii group	Vascular dementia (undefined)	rs12224047	T	C	0.000	36820790	0.948	0.018	14306	T	C	-0.263	11	36799240	4.56278E-06	0.057	361227	8033.487
genus Ruminococcus gauvreauii group	Vascular dementia (undefined)	rs12449066	G	A	-0.011	79177293	0.465	0.021	14306	G	A	-0.262	16	79143396	2.01674E-06	0.055	361227	8803.514
genus Ruminococcus gauvreauii group	Vascular dementia (undefined)	rs2292090	T	C	0.010	70588309	0.437	0.014	14306	T	C	0.217	4	69722591	9.99332E-06	0.049	361227	6178.734
genus Ruminococcus gauvreauii group	Vascular dementia (undefined)	rs2978951	G	A	0.013	6823295	0.291	0.012	14306	G	A	0.248	8	6965773	2.48222E-08	0.045	361227	11061.935
genus Ruminococcus gauvreauii group	Vascular dementia (undefined)	rs429358	C	T	0.011	45411941	0.457	0.018	14306	C	T	0.695	19	44908684	9.26616E-39	0.053	361227	58999.679

genus Ruminococcus gauvreauii group	Vascular dementia (undefined)	rs6133343	G	T	0.013	721797	0.626	0.019	14306	G	T	0.312	20	741153	3.89825E-06	0.068	361227	6210.744
genus Ruminococcus gauvreauii group	Vascular dementia (undefined)	rs78566090	A	G	0.018	125740204	0.551	0.029	14306	A	G	0.346	8	124727963	9.44735E-07	0.071	361227	7049.571
genus Ruminococcus gnavus group	Vascular dementia (undefined)	rs12224047	T	C	0.022	36820790	0.367	0.026	14306	T	C	-0.263	11	36799240	4.56278E-06	0.057	361227	8033.487
genus Ruminococcus gnavus group	Vascular dementia (undefined)	rs12449066	G	A	-0.017	79177293	0.603	0.031	14306	G	A	-0.262	16	79143396	2.01674E-06	0.055	361227	8803.514
genus Ruminococcus gnavus group	Vascular dementia (undefined)	rs2972558	T	C	0.011	45356141	0.560	0.019	14306	T	C	0.234	19	44852884	8.32415E-06	0.052	361227	7242.374
genus Ruminococcus gnavus group	Vascular dementia (undefined)	rs2978951	G	A	-0.007	6823295	0.696	0.018	14306	G	A	0.248	8	6965773	2.48222E-08	0.045	361227	11061.935
genus Ruminococcus gnavus group	Vascular dementia (undefined)	rs359878	C	T	-0.003	185438949	0.833	0.021	14306	C	T	-0.211	2	184574222	4.6443E-06	0.046	361227	6959.700
genus Ruminococcus gnavus group	Vascular dementia (undefined)	rs429358	C	T	0.039	45411941	0.188	0.026	14306	C	T	0.695	19	44908684	9.26616E-39	0.053	361227	58999.679
genus Ruminococcus gnavus group	Vascular dementia (undefined)	rs78566090	A	G	0.032	125740204	0.528	0.044	14306	A	G	0.346	8	124727963	9.44735E-07	0.071	361227	7049.571
genus Ruminococcus torques group	Vascular dementia (undefined)	rs12449066	G	A	-0.001	79177293	0.908	0.018	14306	G	A	-0.262	16	79143396	2.01674E-06	0.055	361227	8803.514
genus Ruminococcus torques group	Vascular dementia (undefined)	rs193392	T	C	-0.005	3085245	0.638	0.011	14306	T	C	-0.204	20	3104599	8.28324E-06	0.046	361227	7460.147
genus Ruminococcus torques group	Vascular dementia (undefined)	rs2978951	G	A	0.009	6823295	0.421	0.011	14306	G	A	0.248	8	6965773	2.48222E-08	0.045	361227	11061.935
genus Ruminococcus torques group	Vascular dementia (undefined)	rs429358	C	T	-0.001	45411941	0.916	0.015	14306	C	T	0.695	19	44908684	9.26616E-39	0.053	361227	58999.679
genus Ruminococcus torques group	Vascular dementia (undefined)	rs71298638	A	G	-0.012	63232261	0.566	0.022	14306	A	G	0.379	3	63246585	1.1165E-06	0.078	361227	6515.599
genus Ruminococcus torques group	Vascular dementia (undefined)	rs78566090	A	G	0.008	125740204	0.745	0.025	14306	A	G	0.346	8	124727963	9.44735E-07	0.071	361227	7049.571
genus Sellimonas	Vascular dementia (undefined)	rs12224047	T	C	0.017	36820790	0.611	0.036	14306	T	C	-0.263	11	36799240	4.56278E-06	0.057	361227	8033.487
genus Sellimonas	Vascular dementia (undefined)	rs193392	T	C	0.010	3085245	0.685	0.026	14306	T	C	-0.204	20	3104599	8.28324E-06	0.046	361227	7460.147
genus Sellimonas	Vascular dementia (undefined)	rs2292090	T	C	-0.024	70588309	0.409	0.028	14306	T	C	0.217	4	69722591	9.99332E-06	0.049	361227	6178.734
genus Sellimonas	Vascular dementia (undefined)	rs2978951	G	A	0.003	6823295	0.906	0.026	14306	G	A	0.248	8	6965773	2.48222E-08	0.045	361227	11061.935
genus Sellimonas	Vascular dementia (undefined)	rs429358	C	T	0.063	45411941	0.123	0.036	14306	C	T	0.695	19	44908684	9.26616E-39	0.053	361227	58999.679
genus Sellimonas	Vascular dementia (undefined)	rs6133343	G	T	-0.004	721797	0.970	0.042	14306	G	T	0.312	20	741153	3.89825E-06	0.068	361227	6210.744
genus Senegalimassilia	Vascular dementia (undefined)	rs12224047	T	C	-0.014	36820790	0.575	0.025	14306	T	C	-0.263	11	36799240	4.56278E-06	0.057	361227	8033.487
genus Senegalimassilia	Vascular dementia (undefined)	rs193392	T	C	-0.012	3085245	0.461	0.017	14306	T	C	-0.204	20	3104599	8.28324E-06	0.046	361227	7460.147
genus Senegalimassilia	Vascular dementia (undefined)	rs2972558	T	C	0.015	45356141	0.417	0.018	14306	T	C	0.234	19	44852884	8.32415E-06	0.052	361227	7242.374
genus Senegalimassilia	Vascular dementia (undefined)	rs2978951	G	A	0.013	6823295	0.438	0.017	14306	G	A	0.248	8	6965773	2.48222E-08	0.045	361227	11061.935
genus Senegalimassilia	Vascular dementia (undefined)	rs359878	C	T	0.004	185438949	0.852	0.020	14306	C	T	-0.211	2	184574222	4.6443E-06	0.046	361227	6959.700
genus Senegalimassilia	Vascular dementia (undefined)	rs429358	C	T	0.050	45411941	0.027	0.024	14306	C	T	0.695	19	44908684	9.26616E-39	0.053	361227	58999.679
genus Senegalimassilia	Vascular dementia (undefined)	rs6133343	G	T	-0.019	721797	0.477	0.027	14306	G	T	0.312	20	741153	3.89825E-06	0.068	361227	6210.744
genus Senegalimassilia	Vascular dementia (undefined)	rs71298638	A	G	0.006	63232261	0.931	0.034	14306	A	G	0.379	3	63246585	1.1165E-06	0.078	361227	6515.599
genus Senegalimassilia	Vascular dementia (undefined)	rs78566090	A	G	0.005	125740204	0.868	0.038	14306	A	G	0.346	8	124727963	9.44735E-07	0.071	361227	7049.571
genus Slackia	Vascular dementia (undefined)	rs193392	T	C	-0.011	3085245	0.558	0.018	14306	T	C	-0.204	20	3104599	8.28324E-06	0.046	361227	7460.147
genus Slackia	Vascular dementia (undefined)	rs2292090	T	C	-0.003	70588309	0.832	0.020	14306	T	C	0.217	4	69722591	9.99332E-06	0.049	361227	6178.734
genus Slackia	Vascular dementia (undefined)	rs359878	C	T	-0.004	185438949	0.861	0.021	14306	C	T	-0.211	2	184574222	4.6443E-06	0.046	361227	6959.700
genus Slackia	Vascular dementia (undefined)	rs429358	C	T	0.036	45411941	0.159	0.026	14306	C	T	0.695	19	44908684	9.26616E-39	0.053	361227	58999.679
genus Slackia	Vascular dementia (undefined)	rs6133343	G	T	-0.001	721797	0.925	0.030	14306	G	T	0.312	20	741153	3.89825E-06	0.068	361227	6210.744
genus Slackia	Vascular dementia (undefined)	rs71298638	A	G	0.023	63232261	0.570	0.036	14306	A	G	0.379	3	63246585	1.1165E-06	0.078	361227	6515.599
genus Streptococcus	Vascular dementia (undefined)	rs12449066	G	A	0.013	79177293	0.384	0.019	14306	G	A	-0.262	16	79143396	2.01674E-06	0.055	361227	8803.514
genus Streptococcus	Vascular dementia (undefined)	rs2972558	T	C	-0.005	45356141	0.708	0.012	14306	T	C	0.234	19	44852884	8.32415E-06	0.052	361227	7242.374
genus Streptococcus	Vascular dementia (undefined)	rs2978951	G	A	0.002	6823295	0.878	0.011	14306	G	A	0.248	8	6965773	2.48222E-08	0.045	361227	11061.935
genus Streptococcus	Vascular dementia (undefined)	rs359878	C	T	-0.009	185438949	0.490	0.013	14306	C	T	-0.211	2	184574222	4.6443E-06	0.046	361227	6959.700
genus Streptococcus	Vascular dementia (undefined)	rs429358	C	T	0.017	45411941	0.279	0.016	14306	C	T	0.695	19	44908684	9.26616E-39	0.053	361227	58999.679
genus Streptococcus	Vascular dementia (undefined)	rs6133343	G	T	-0.011	721797	0.696	0.018	14306	G	T	0.312	20	741153	3.89825E-06	0.068	361227	6210.744
genus Streptococcus	Vascular dementia (undefined)	rs78566090	A	G	0.022	125740204	0.422	0.026	14306	A	G	0.346	8	124727963	9.44735E-07	0.071	361227	7049.571
genus Subdoligranulum	Vascular dementia (undefined)	rs12449066	G	A	0.013	79177293	0.628	0.018	14306	G	A	-0.262	16	79143396	2.01674E-06	0.055	361227	8803.514
genus Subdoligranulum	Vascular dementia (undefined)	rs193392	T	C	-0.009	3085245	0.391	0.011	14306	T	C	-0.204	20	3104599	8.28324E-06	0.046	361227	7460.147
genus Subdoligranulum	Vascular dementia (undefined)	rs2292090	T	C	0.006	70588309	0.597	0.012	14306	T	C	0.217	4	69722591	9.99332E-06	0.049	361227	6178.734
genus Subdoligranulum	Vascular dementia (undefined)	rs2972558	T	C	-0.001	45356141	0.975	0.012	14306	T	C	0.234	19	44852884	8.32415E-06	0.052	361227	7242.374
genus Subdoligranulum	Vascular dementia (undefined)	rs359878	C	T	0.010	185438949	0.516	0.012	14306	C	T	-0.211	2	184574222	4.6443E-06	0.046	361227	6959.700
genus Subdoligranulum	Vascular dementia (undefined)	rs429358	C	T	-0.005	45411941	0.772	0.015	14306	C	T	0.695	19	44908684	9.26616E-39	0.053	361227	58999.679
genus Subdoligranulum	Vascular dementia (undefined)	rs71298638	A	G	-0.004	63232261	0.794	0.022	14306	A	G	0.379	3	63246585	1.1165E-06	0.078	361227	6515.599
genus Sutterella	Vascular dementia (undefined)	rs12449066	G	A	0.009	79177293	0.616	0.021	14306	G	A	-0.262	16	79143396	2.01674E-06	0.055	361227	8803.514
genus Sutterella	Vascular dementia (undefined)	rs193392	T	C	-0.011	3085245	0.431	0.013	14306	T	C	-0.204	20	3104599	8.28324E-06	0.046	361227	7460.147
genus Sutterella	Vascular dementia (undefined)	rs2292090	T	C	0.004	70588309	0.775	0.014	14306	T	C	0.217	4	69722591	9.99332E-06	0.049	361227	6178.734
genus Sutterella	Vascular dementia (undefined)	rs429358	C	T	-0.028	45411941	0.121	0.018	14306	C	T	0.695	19	44908684	9.26616E-39	0.053	361227	58999.679
genus Sutterella	Vascular dementia (undefined)	rs6133343	G	T	-0.001	721797	0.771	0.019	14306	G	T	0.312	20	741153	3.89825E-06	0.068	361227	6210.744
genus Sutterella	Vascular dementia (undefined)	rs78566090	A	G	-0.027	125740204	0.330	0.029	14306	A	G	0.346	8	124727963	9.44735E-07	0.071	361227	7049.571
genus Terrisporobacter	Vascular dementia (undefined)	rs12224047	T	C	0.014	36820790	0.603	0.026	14306	T	C	-0.263	11	36799240	4.56278E-06	0.057	361227	8033.487
genus Terrisporobacter	Vascular dementia (undefined)	rs12449066	G	A	-0.005	79177293	0.719	0.030	14306	G	A	-0.262	16	79143396	2.01674E-06	0.055	361227	8803.514
genus Terrisporobacter	Vascular dementia (undefined)	rs193392	T	C	0.014	3085245	0.439	0.018	14306	T	C	-0.204	20	3104599	8.28324E-06	0.046	361227	7460.147
genus Terrisporobacter	Vascular dementia (undefined)	rs2292090	T	C	0.011	70588309	0.633	0.019	14306	T	C	0.217	4	69722591	9.99332E-06	0.049	361227	6178.734
genus Terrisporobacter	Vascular dementia (undefined)	rs2972558	T	C	0.010	45356141	0.631	0.019	14306	T	C	0.234	19	44852884	8.32415E-06	0.052	361227	7242.374
genus Terrisporobacter	Vascular dementia (undefined)	rs2978951	G	A	0.005	6823295	0.778	0.018	14306	G	A	0.248	8	6965773	2.48222E-08	0.045	361227	11061.935
genus Terrisporobacter	Vascular dementia (undefined)	rs359878	C	T	-0.001	185438949	0.986	0.020	14306	C	T	-0.211	2	184574222	4.6443E-06	0.046	361227	6959.700
genus Terrisporobacter	Vascular dementia (undefined)	rs429358	C	T	-0.010	45411941	0.657	0.025	14306	C	T	0.695	19	44908684	9.26616E-39	0.053	361227	58999.679
genus Terrisporobacter	Vascular dementia (undefined)	rs6133343	G	T	0.014	721797	0.655	0.028	14306	G	T	0.312	20	741153	3.89825E-06	0.068	361227	6210.744
genus Terrisporobacter	Vascular dementia (undefined)	rs71298638	A	G	0.021	63232261	0.561	0.036	14306	A	G	0.379	3	63246585	1.1165E-06	0.078	361227	6515.599
genus Terrisporobacter	Vascular dementia (undefined)	rs78566090	A	G	-0.013	125740204	0.780	0.044	14306	A	G	0.346	8	124727963	9.44735E-07	0.071	361227	7049.571
genus Turicibacter	Vascular dementia (undefined)	rs12224047	T	C	0.002	36820790	0.850	0.022	14306	T	C	-0.263	11	36799240	4.56278E-06	0.057	361227	8033.487
genus Turicibacter	Vascular dementia (undefined)	rs359878	C	T	-0.010	185438949	0.576	0.017	14306	C	T	-0.211	2	184574222	4.6443E-06	0.046	361227	6959.700
genus Turicibacter	Vascular dementia (undefined)	rs429358	C	T	0.001	45411941	0.870	0.021	14306	C	T	0.695	19	44908684	9.26616E-39	0.053	361227	58999.679
genus Turicibacter	Vascular dementia (undefined)	rs6133343	G	T	-0.006	721797	0.763	0.024	14306	G	T	0.312	20	741153	3.89825E-06	0.068	361227	6210.744

genus Turicibacter	Vascular dementia (undefined)	rs71298638	A	G	0.019	63232261	0.549	0.031	14306	A	G	0.379	3	63246585	1.1165E-06	0.078	361227	6515.599
genus Tyzzerella3	Vascular dementia (undefined)	rs12449066	G	A	0.012	79177293	0.642	0.033	14306	G	A	-0.262	16	79143396	2.01674E-06	0.055	361227	8803.514
genus Tyzzerella3	Vascular dementia (undefined)	rs2292090	T	C	-0.011	70588309	0.568	0.022	14306	T	C	0.217	4	69722591	9.99332E-06	0.049	361227	6178.734
genus Tyzzerella3	Vascular dementia (undefined)	rs2978951	G	A	0.003	6823295	0.858	0.020	14306	G	A	0.248	8	6965773	2.48222E-08	0.045	361227	11061.935
genus Tyzzerella3	Vascular dementia (undefined)	rs429358	C	T	0.000	45411941	0.979	0.028	14306	C	T	0.695	19	44908684	9.26616E-39	0.053	361227	58999.679
genus Tyzzerella3	Vascular dementia (undefined)	rs71298638	A	G	-0.030	63232261	0.473	0.041	14306	A	G	0.379	3	63246585	1.1165E-06	0.078	361227	6515.599
genus Veillonella)	Vascular dementia (undefined)	rs12224047	T	C	0.012	36820790	0.651	0.021	14306	T	C	-0.263	11	36799240	4.56278E-06	0.057	361227	8033.487
genus Veillonella)	Vascular dementia (undefined)	rs2292090	T	C	0.000	70588309	0.966	0.017	14306	T	C	0.217	4	69722591	9.99332E-06	0.049	361227	6178.734
genus Veillonella)	Vascular dementia (undefined)	rs2972558	T	C	-0.012	45356141	0.448	0.016	14306	T	C	0.234	19	44852884	8.32415E-06	0.052	361227	7242.374
genus Veillonella)	Vascular dementia (undefined)	rs429358	C	T	0.044	45411941	0.042	0.021	14306	C	T	0.695	19	44908684	9.26616E-39	0.053	361227	58999.679
genus Veillonella)	Vascular dementia (undefined)	rs6133343	G	T	-0.021	721797	0.335	0.023	14306	G	T	0.312	20	741153	3.89825E-06	0.068	361227	6210.744
genus Veillonella)	Vascular dementia (undefined)	rs71298638	A	G	-0.029	63232261	0.433	0.031	14306	A	G	0.379	3	63246585	1.1165E-06	0.078	361227	6515.599
genus Veillonella)	Vascular dementia (undefined)	rs78566090	A	G	-0.036	125740204	0.295	0.037	14306	A	G	0.346	8	124727963	9.44735E-07	0.071	361227	7049.571
genus Victivallis	Vascular dementia (undefined)	rs12224047	T	C	-0.005	36820790	0.962	0.036	1531	T	C	-0.263	11	36799240	4.56278E-06	0.057	361227	8033.487
genus Victivallis	Vascular dementia (undefined)	rs429358	C	T	0.009	45411941	0.847	0.037	1531	C	T	0.695	19	44908684	9.26616E-39	0.053	361227	58999.679
order Actinomycetales	Vascular dementia (multiple infarctions)	rs11081443	C	T	-0.003	8944208	0.891	0.020	14306	C	T	0.464	18	8944210	5.71874E-06	0.102	360612	19066.663
order Actinomycetales	Vascular dementia (multiple infarctions)	rs429358	C	T	0.038	45411941	0.088	0.023	14306	C	T	0.660	19	44908684	1.33506E-17	0.077	360612	52325.340
order Actinomycetales	Vascular dementia (multiple infarctions)	rs4725579	C	A	0.012	139468213	0.576	0.020	14306	C	A	-0.379	7	139768414	3.58039E-06	0.082	360612	18391.668
order Actinomycetales	Vascular dementia (multiple infarctions)	rs72822148	T	C	0.014	9742028	0.419	0.017	14306	T	C	-0.322	17	9838711	3.18831E-06	0.069	360612	17983.471
order Actinomycetales	Vascular dementia (multiple infarctions)	rs73053797	T	C	-0.002	29909039	0.943	0.023	14306	T	C	0.338	3	29867548	6.00772E-06	0.075	360612	13680.576
order Actinomycetales	Vascular dementia (multiple infarctions)	rs9861644	A	G	0.010	88637331	0.581	0.019	14306	A	G	0.323	3	88588181	4.84942E-06	0.071	360612	17137.658
order Bacillales	Vascular dementia (multiple infarctions)	rs11081443	C	T	-0.013	8944208	0.540	0.030	14306	C	T	0.464	18	8944210	5.71874E-06	0.102	360612	19066.663
order Bacillales	Vascular dementia (multiple infarctions)	rs1454336	A	G	-0.009	91873093	0.899	0.032	14306	A	G	-0.369	4	90951942	4.48415E-06	0.080	360612	13616.170
order Bacillales	Vascular dementia (multiple infarctions)	rs429358	C	T	0.061	45411941	0.072	0.036	14306	C	T	0.660	19	44908684	1.33506E-17	0.077	360612	52325.340
order Bacillales	Vascular dementia (multiple infarctions)	rs4716814	T	C	-0.002	157723046	0.953	0.025	14306	T	C	-0.319	7	157930354	3.75656E-07	0.063	360612	19180.021
order Bacillales	Vascular dementia (multiple infarctions)	rs4725579	C	A	0.028	139468213	0.362	0.031	14306	C	A	-0.379	7	139768414	3.58039E-06	0.082	360612	18391.668
order Bacillales	Vascular dementia (multiple infarctions)	rs73053797	T	C	0.006	29909039	0.701	0.036	14306	T	C	0.338	3	29867548	6.00772E-06	0.075	360612	13680.576
order Bacillales	Vascular dementia (multiple infarctions)	rs9861644	A	G	-0.025	88637331	0.406	0.028	14306	A	G	0.323	3	88588181	4.84942E-06	0.071	360612	17137.658
order Bacteroidales	Vascular dementia (multiple infarctions)	rs11081443	C	T	-0.007	8944208	0.599	0.013	14306	C	T	0.464	18	8944210	5.71874E-06	0.102	360612	19066.663
order Bacteroidales	Vascular dementia (multiple infarctions)	rs1454336	A	G	0.000	91873093	0.932	0.014	14306	A	G	-0.369	4	90951942	4.48415E-06	0.080	360612	13616.170
order Bacteroidales	Vascular dementia (multiple infarctions)	rs34288661	C	T	-0.009	20948329	0.598	0.016	14306	C	T	0.460	8	21090818	7.22903E-06	0.103	360612	11969.376
order Bacteroidales	Vascular dementia (multiple infarctions)	rs429358	C	T	-0.022	45411941	0.148	0.015	14306	C	T	0.660	19	44908684	1.33506E-17	0.077	360612	52325.340
order Bacteroidales	Vascular dementia (multiple infarctions)	rs4716814	T	C	-0.002	157723046	0.858	0.011	14306	T	C	-0.319	7	157930354	3.75656E-07	0.063	360612	19180.021
order Bacteroidales	Vascular dementia (multiple infarctions)	rs4725579	C	A	-0.011	139468213	0.405	0.013	14306	C	A	-0.379	7	139768414	3.58039E-06	0.082	360612	18391.668
order Bacteroidales	Vascular dementia (multiple infarctions)	rs72822148	T	C	-0.001	9742028	0.866	0.011	14306	T	C	-0.322	17	9838711	3.18831E-06	0.069	360612	17983.471
order Bacteroidales	Vascular dementia (multiple infarctions)	rs73053797	T	C	0.012	29909039	0.373	0.015	14306	T	C	0.338	3	29867548	6.00772E-06	0.075	360612	13680.576
order Bifidobacteriales	Vascular dementia (multiple infarctions)	rs1454336	A	G	0.002	91873093	0.910	0.015	14306	A	G	-0.369	4	90951942	4.48415E-06	0.080	360612	13616.170
order Bifidobacteriales	Vascular dementia (multiple infarctions)	rs34288661	C	T	-0.008	20948329	0.762	0.017	14306	C	T	0.460	8	21090818	7.22903E-06	0.103	360612	11969.376
order Bifidobacteriales	Vascular dementia (multiple infarctions)	rs429358	C	T	0.022	45411941	0.147	0.017	14306	C	T	0.660	19	44908684	1.33506E-17	0.077	360612	52325.340
order Bifidobacteriales	Vascular dementia (multiple infarctions)	rs4725579	C	A	-0.005	139468213	0.670	0.014	14306	C	A	-0.379	7	139768414	3.58039E-06	0.082	360612	18391.668
order Bifidobacteriales	Vascular dementia (multiple infarctions)	rs73053797	T	C	0.005	29909039	0.638	0.016	14306	T	C	0.338	3	29867548	6.00772E-06	0.075	360612	13680.576
order Bifidobacteriales	Vascular dementia (multiple infarctions)	rs9861644	A	G	0.009	88637331	0.538	0.013	14306	A	G	0.323	3	88588181	4.84942E-06	0.071	360612	17137.658
order Burkholderiales	Vascular dementia (multiple infarctions)	rs11081443	C	T	-0.010	8944208	0.422	0.013	14306	C	T	0.464	18	8944210	5.71874E-06	0.102	360612	19066.663
order Burkholderiales	Vascular dementia (multiple infarctions)	rs1454336	A	G	-0.011	91873093	0.405	0.014	14306	A	G	-0.369	4	90951942	4.48415E-06	0.080	360612	13616.170
order Burkholderiales	Vascular dementia (multiple infarctions)	rs34288661	C	T	0.010	20948329	0.482	0.016	14306	C	T	0.460	8	21090818	7.22903E-06	0.103	360612	11969.376
order Burkholderiales	Vascular dementia (multiple infarctions)	rs429358	C	T	0.003	45411941	0.854	0.016	14306	C	T	0.660	19	44908684	1.33506E-17	0.077	360612	52325.340
order Burkholderiales	Vascular dementia (multiple infarctions)	rs72822148	T	C	-0.007	9742028	0.589	0.012	14306	T	C	-0.322	17	9838711	3.18831E-06	0.069	360612	17983.471
order Burkholderiales	Vascular dementia (multiple infarctions)	rs73053797	T	C	0.003	29909039	0.792	0.015	14306	T	C	0.338	3	29867548	6.00772E-06	0.075	360612	13680.576
order Burkholderiales	Vascular dementia (multiple infarctions)	rs9861644	A	G	0.007	88637331	0.544	0.012	14306	A	G	0.323	3	88588181	4.84942E-06	0.071	360612	17137.658
order Clostridiales	Vascular dementia (multiple infarctions)	rs1454336	A	G	-0.001	91873093	0.985	0.014	14306	A	G	-0.369	4	90951942	4.48415E-06	0.080	360612	13616.170
order Clostridiales	Vascular dementia (multiple infarctions)	rs34288661	C	T	-0.010	20948329	0.567	0.016	14306	C	T	0.460	8	21090818	7.22903E-06	0.103	360612	11969.376
order Clostridiales	Vascular dementia (multiple infarctions)	rs429358	C	T	0.005	45411941	0.723	0.015	14306	C	T	0.660	19	44908684	1.33506E-17	0.077	360612	52325.340
order Clostridiales	Vascular dementia (multiple infarctions)	rs4716814	T	C	-0.003	157723046	0.753	0.011	14306	T	C	-0.319	7	157930354	3.75656E-07	0.063	360612	19180.021
order Clostridiales	Vascular dementia (multiple infarctions)	rs72822148	T	C	0.005	9742028	0.532	0.011	14306	T	C	-0.322	17	9838711	3.18831E-06	0.069	360612	17983.471
order Clostridiales	Vascular dementia (multiple infarctions)	rs73053797	T	C	-0.005	29909039	0.781	0.015	14306	T	C	0.338	3	29867548	6.00772E-06	0.075	360612	13680.576
order Coriobacteriales	Vascular dementia (multiple infarctions)	rs34288661	C	T	-0.010	20948329	0.531	0.016	14306	C	T	0.460	8	21090818	7.22903E-06	0.103	360612	11969.376
order Coriobacteriales	Vascular dementia (multiple infarctions)	rs429358	C	T	0.019	45411941	0.204	0.015	14306	C	T	0.660	19	44908684	1.33506E-17	0.077	360612	52325.340
order Coriobacteriales	Vascular dementia (multiple infarctions)	rs4716814	T	C	-0.002	157723046	0.849	0.011	14306	T	C	-0.319	7	157930354	3.75656E-07	0.063	360612	19180.021
order Coriobacteriales	Vascular dementia (multiple infarctions)	rs4725579	C	A	-0.006	139468213	0.583	0.013	14306	C	A	-0.379	7	139768414	3.58039E-06	0.082	360612	18391.668
order Coriobacteriales	Vascular dementia (multiple infarctions)	rs73053797	T	C	-0.008	29909039	0.584	0.015	14306	T	C	0.338	3	29867548	6.00772E-06	0.075	360612	13680.576
order Coriobacteriales	Vascular dementia (multiple infarctions)	rs9861644	A	G	-0.008	88637331	0.489	0.012	14306	A	G	0.323	3	88588181	4.84942E-06	0.071	360612	17137.658
order Desulfovibrionales	Vascular dementia (multiple infarctions)	rs11081443	C	T	0.012	8944208	0.423	0.014	14306	C	T	0.464	18	8944210	5.71874E-06	0.102	360612	19066.663
order Desulfovibrionales	Vascular dementia (multiple infarctions)	rs1454336	A	G	-0.001	91873093	0.912	0.015	14306	A	G	-0.369	4	90951942	4.48415E-06	0.080	360612	13616.170
order Desulfovibrionales	Vascular dementia (multiple infarctions)	rs34288661	C	T	-0.014	20948329	0.405	0.017	14306	C	T	0.460	8	21090818	7.22903E-06	0.103	360612	11969.376
order Desulfovibrionales	Vascular dementia (multiple infarctions)	rs429358	C	T	0.016	45411941	0.327	0.017	14306	C	T	0.660	19	44908684	1.33506E-17	0.077	360612	52325.340
order Desulfovibrionales	Vascular dementia (multiple infarctions)	rs4716814	T	C	-0.011	157723046	0.335	0.012	14306	T	C	-0.319	7	157930354	3.75656E-07	0.063	360612	19180.021
order Desulfovibrionales	Vascular dementia (multiple infarctions)	rs4725579	C	A	0.008	139468213	0.567	0.014	14306	C	A	-0.379	7	139768414	3.58039E-06	0.082	360612	18391.668
order Desulfovibrionales	Vascular dementia (multiple infarctions)	rs72822148	T	C	0.003	9742028	0.760	0.012	14306	T	C	-0.322	17	9838711	3.18831E-06	0.069	360612	17983.471
order Desulfovibrionales	Vascular dementia (multiple infarctions)	rs73053797	T	C	-0.013	29909039	0.476	0.016	14306	T	C	0.338	3	29867548	6.00772E-06	0.075	360612	13680.576
order Desulfovibrionales	Vascular dementia (multiple infarctions)	rs9861644	A	G	0.000	88637331	0.914	0.013	14306	A	G	0.323	3	88588181	4.84942E-06	0.071	360612	17137.658
order Enterobacteriales	Vascular dementia (multiple infarctions)	rs11081443	C	T	-0.009	8944208	0.511	0.015	14306	C	T	0.464	18	8944210	5.71874E-06	0.102	360612	19066.663

order Enterobacteriales	Vascular dementia (multiple infarctions)	rs1454336	A	G	-0.002	91873093	0.916	0.016	14306	A	G	-0.369	4	90951942	4.48415E-06	0.080	360612	13616.170
order Enterobacteriales	Vascular dementia (multiple infarctions)	rs429358	C	T	-0.019	45411941	0.299	0.017	14306	C	T	0.660	19	44908684	1.33506E-17	0.077	360612	52325.340
order Enterobacteriales	Vascular dementia (multiple infarctions)	rs4725579	C	A	-0.002	139468213	0.986	0.015	14306	C	A	-0.379	7	139768414	3.58039E-06	0.082	360612	18391.668
order Enterobacteriales	Vascular dementia (multiple infarctions)	rs72822148	T	C	0.007	9742028	0.550	0.013	14306	T	C	-0.322	17	9838711	3.18831E-06	0.069	360612	17983.471
order Enterobacteriales	Vascular dementia (multiple infarctions)	rs73053797	T	C	0.012	29909039	0.488	0.017	14306	T	C	0.338	3	29867548	6.00772E-06	0.075	360612	13680.576
order Erysipelotrichales	Vascular dementia (multiple infarctions)	rs4716814	T	C	0.001	157723046	0.900	0.011	14306	T	C	-0.319	7	157930354	3.75656E-07	0.063	360612	19180.021
order Erysipelotrichales	Vascular dementia (multiple infarctions)	rs4725579	C	A	0.011	139468213	0.475	0.013	14306	C	A	-0.379	7	139768414	3.58039E-06	0.082	360612	18391.668
order Erysipelotrichales	Vascular dementia (multiple infarctions)	rs72822148	T	C	-0.004	9742028	0.542	0.011	14306	T	C	-0.322	17	9838711	3.18831E-06	0.069	360612	17983.471
order Erysipelotrichales	Vascular dementia (multiple infarctions)	rs73053797	T	C	0.000	29909039	0.937	0.015	14306	T	C	0.338	3	29867548	6.00772E-06	0.075	360612	13680.576
order Erysipelotrichales	Vascular dementia (multiple infarctions)	rs9861644	A	G	-0.004	88637331	0.706	0.012	14306	A	G	0.323	3	88588181	4.84942E-06	0.071	360612	17137.658
order Gastranaerophilales	Vascular dementia (multiple infarctions)	rs11081443	C	T	-0.017	8944208	0.445	0.022	14306	C	T	0.464	18	8944210	5.71874E-06	0.102	360612	19066.663
order Gastranaerophilales	Vascular dementia (multiple infarctions)	rs34288661	C	T	0.019	20948329	0.386	0.027	14306	C	T	0.460	8	21090818	7.22903E-06	0.103	360612	11969.376
order Gastranaerophilales	Vascular dementia (multiple infarctions)	rs429358	C	T	-0.002	45411941	0.999	0.026	14306	C	T	0.660	19	44908684	1.33506E-17	0.077	360612	52325.340
order Gastranaerophilales	Vascular dementia (multiple infarctions)	rs4716814	T	C	0.004	157723046	0.860	0.019	14306	T	C	-0.319	7	157930354	3.75656E-07	0.063	360612	19180.021
order Gastranaerophilales	Vascular dementia (multiple infarctions)	rs4725579	C	A	-0.018	139468213	0.440	0.023	14306	C	A	-0.379	7	139768414	3.58039E-06	0.082	360612	18391.668
order Gastranaerophilales	Vascular dementia (multiple infarctions)	rs72822148	T	C	0.002	9742028	0.926	0.020	14306	T	C	-0.322	17	9838711	3.18831E-06	0.069	360612	17983.471
order Gastranaerophilales	Vascular dementia (multiple infarctions)	rs73053797	T	C	-0.007	29909039	0.723	0.027	14306	T	C	0.338	3	29867548	6.00772E-06	0.075	360612	13680.576
order Gastranaerophilales	Vascular dementia (multiple infarctions)	rs9861644	A	G	0.005	88637331	0.764	0.021	14306	A	G	0.323	3	88588181	4.84942E-06	0.071	360612	17137.658
order Lactobacillales	Vascular dementia (multiple infarctions)	rs11081443	C	T	0.005	8944208	0.836	0.013	14306	C	T	0.464	18	8944210	5.71874E-06	0.102	360612	19066.663
order Lactobacillales	Vascular dementia (multiple infarctions)	rs429358	C	T	0.010	45411941	0.542	0.015	14306	C	T	0.660	19	44908684	1.33506E-17	0.077	360612	52325.340
order Lactobacillales	Vascular dementia (multiple infarctions)	rs4725579	C	A	-0.004	139468213	0.908	0.013	14306	C	A	-0.379	7	139768414	3.58039E-06	0.082	360612	18391.668
order Lactobacillales	Vascular dementia (multiple infarctions)	rs72822148	T	C	-0.007	9742028	0.417	0.012	14306	T	C	-0.322	17	9838711	3.18831E-06	0.069	360612	17983.471
order Lactobacillales	Vascular dementia (multiple infarctions)	rs9861644	A	G	0.001	88637331	0.939	0.012	14306	A	G	0.323	3	88588181	4.84942E-06	0.071	360612	17137.658
order Methanobacteriales	Vascular dementia (multiple infarctions)	rs1454336	A	G	0.006	91873093	0.824	0.030	14306	A	G	-0.369	4	90951942	4.48415E-06	0.080	360612	13616.170
order Methanobacteriales	Vascular dementia (multiple infarctions)	rs429358	C	T	-0.026	45411941	0.504	0.034	14306	C	T	0.660	19	44908684	1.33506E-17	0.077	360612	52325.340
order Methanobacteriales	Vascular dementia (multiple infarctions)	rs4725579	C	A	0.004	139468213	0.882	0.030	14306	C	A	-0.379	7	139768414	3.58039E-06	0.082	360612	18391.668
order Methanobacteriales	Vascular dementia (multiple infarctions)	rs72822148	T	C	0.008	9742028	0.721	0.026	14306	T	C	-0.322	17	9838711	3.18831E-06	0.069	360612	17983.471
order Methanobacteriales	Vascular dementia (multiple infarctions)	rs9861644	A	G	0.008	88637331	0.765	0.027	14306	A	G	0.323	3	88588181	4.84942E-06	0.071	360612	17137.658
order Mollicutes RF9	Vascular dementia (multiple infarctions)	rs1454336	A	G	-0.006	91873093	0.833	0.018	14306	A	G	-0.369	4	90951942	4.48415E-06	0.080	360612	13616.170
order Mollicutes RF9	Vascular dementia (multiple infarctions)	rs34288661	C	T	-0.017	20948329	0.380	0.020	14306	C	T	0.460	8	21090818	7.22903E-06	0.103	360612	11969.376
order Mollicutes RF9	Vascular dementia (multiple infarctions)	rs429358	C	T	0.000	45411941	0.994	0.020	14306	C	T	0.660	19	44908684	1.33506E-17	0.077	360612	52325.340
order Mollicutes RF9	Vascular dementia (multiple infarctions)	rs4716814	T	C	0.013	157723046	0.340	0.014	14306	T	C	-0.319	7	157930354	3.75656E-07	0.063	360612	19180.021
order Mollicutes RF9	Vascular dementia (multiple infarctions)	rs73053797	T	C	-0.017	29909039	0.422	0.020	14306	T	C	0.338	3	29867548	6.00772E-06	0.075	360612	13680.576
order NB1n	Vascular dementia (multiple infarctions)	rs1454336	A	G	-0.019	91873093	0.473	0.026	14306	A	G	-0.369	4	90951942	4.48415E-06	0.080	360612	13616.170
order NB1n	Vascular dementia (multiple infarctions)	rs34288661	C	T	0.005	20948329	0.876	0.029	14306	C	T	0.460	8	21090818	7.22903E-06	0.103	360612	11969.376
order NB1n	Vascular dementia (multiple infarctions)	rs429358	C	T	0.017	45411941	0.488	0.028	14306	C	T	0.660	19	44908684	1.33506E-17	0.077	360612	52325.340
order NB1n	Vascular dementia (multiple infarctions)	rs4716814	T	C	0.012	157723046	0.537	0.020	14306	T	C	-0.319	7	157930354	3.75656E-07	0.063	360612	19180.021
order NB1n	Vascular dementia (multiple infarctions)	rs4725579	C	A	0.015	139468213	0.526	0.025	14306	C	A	-0.379	7	139768414	3.58039E-06	0.082	360612	18391.668
order NB1n	Vascular dementia (multiple infarctions)	rs72822148	T	C	-0.007	9742028	0.752	0.021	14306	T	C	-0.322	17	9838711	3.18831E-06	0.069	360612	17983.471
order Pasteurellales	Vascular dementia (multiple infarctions)	rs11081443	C	T	-0.002	8944208	0.768	0.018	14306	C	T	0.464	18	8944210	5.71874E-06	0.102	360612	19066.663
order Pasteurellales	Vascular dementia (multiple infarctions)	rs1454336	A	G	0.010	91873093	0.555	0.019	14306	A	G	-0.369	4	90951942	4.48415E-06	0.080	360612	13616.170
order Pasteurellales	Vascular dementia (multiple infarctions)	rs429358	C	T	0.022	45411941	0.309	0.021	14306	C	T	0.660	19	44908684	1.33506E-17	0.077	360612	52325.340
order Pasteurellales	Vascular dementia (multiple infarctions)	rs4716814	T	C	0.007	157723046	0.630	0.015	14306	T	C	-0.319	7	157930354	3.75656E-07	0.063	360612	19180.021
order Pasteurellales	Vascular dementia (multiple infarctions)	rs4725579	C	A	-0.013	139468213	0.480	0.018	14306	C	A	-0.379	7	139768414	3.58039E-06	0.082	360612	18391.668
order Pasteurellales	Vascular dementia (multiple infarctions)	rs72822148	T	C	0.003	9742028	0.971	0.016	14306	T	C	-0.322	17	9838711	3.18831E-06	0.069	360612	17983.471
order Rhodospirillales	Vascular dementia (multiple infarctions)	rs1454336	A	G	-0.009	91873093	0.652	0.020	14306	A	G	-0.369	4	90951942	4.48415E-06	0.080	360612	13616.170
order Rhodospirillales	Vascular dementia (multiple infarctions)	rs429358	C	T	-0.006	45411941	0.821	0.022	14306	C	T	0.660	19	44908684	1.33506E-17	0.077	360612	52325.340
order Rhodospirillales	Vascular dementia (multiple infarctions)	rs4716814	T	C	-0.002	157723046	0.889	0.015	14306	T	C	-0.319	7	157930354	3.75656E-07	0.063	360612	19180.021
order Rhodospirillales	Vascular dementia (multiple infarctions)	rs72822148	T	C	-0.003	9742028	0.917	0.017	14306	T	C	-0.322	17	9838711	3.18831E-06	0.069	360612	17983.471
order Rhodospirillales	Vascular dementia (multiple infarctions)	rs9861644	A	G	0.011	88637331	0.506	0.018	14306	A	G	0.323	3	88588181	4.84942E-06	0.071	360612	17137.658
order Selenomonadales	Vascular dementia (multiple infarctions)	rs34288661	C	T	-0.009	20948329	0.516	0.016	14306	C	T	0.460	8	21090818	7.22903E-06	0.103	360612	11969.376
order Selenomonadales	Vascular dementia (multiple infarctions)	rs429358	C	T	-0.019	45411941	0.146	0.015	14306	C	T	0.660	19	44908684	1.33506E-17	0.077	360612	52325.340
order Selenomonadales	Vascular dementia (multiple infarctions)	rs4716814	T	C	0.002	157723046	0.860	0.011	14306	T	C	-0.319	7	157930354	3.75656E-07	0.063	360612	19180.021
order Selenomonadales	Vascular dementia (multiple infarctions)	rs73053797	T	C	-0.008	29909039	0.442	0.015	14306	T	C	0.338	3	29867548	6.00772E-06	0.075	360612	13680.576
order Verrucomicrobiales	Vascular dementia (multiple infarctions)	rs1454336	A	G	-0.003	91873093	0.963	0.017	14306	A	G	-0.369	4	90951942	4.48415E-06	0.080	360612	13616.170
order Verrucomicrobiales	Vascular dementia (multiple infarctions)	rs34288661	C	T	0.007	20948329	0.747	0.019	14306	C	T	0.460	8	21090818	7.22903E-06	0.103	360612	11969.376
order Verrucomicrobiales	Vascular dementia (multiple infarctions)	rs429358	C	T	0.018	45411941	0.336	0.019	14306	C	T	0.660	19	44908684	1.33506E-17	0.077	360612	52325.340
order Verrucomicrobiales	Vascular dementia (multiple infarctions)	rs4725579	C	A	0.000	139468213	0.921	0.016	14306	C	A	-0.379	7	139768414	3.58039E-06	0.082	360612	18391.668
order Verrucomicrobiales	Vascular dementia (multiple infarctions)	rs72822148	T	C	-0.008	9742028	0.627	0.014	14306	T	C	-0.322	17	9838711	3.18831E-06	0.069	360612	17983.471
order Verrucomicrobiales	Vascular dementia (multiple infarctions)	rs73053797	T	C	-0.005	29909039	0.805	0.019	14306	T	C	0.338	3	29867548	6.00772E-06	0.075	360612	13680.576
order Verrucomicrobiales	Vascular dementia (multiple infarctions)	rs9861644	A	G	0.003	88637331	0.852	0.015	14306	A	G	0.323	3	88588181	4.84942E-06	0.071	360612	17137.658
order Victivallales	Vascular dementia (multiple infarctions)	rs1454336	A	G	-0.019	91873093	0.512	0.027	14306	A	G	-0.369	4	90951942	4.48415E-06	0.080	360612	13616.170
order Victivallales	Vascular dementia (multiple infarctions)	rs34288661	C	T	0.002	20948329	0.898	0.030	14306	C	T	0.460	8	21090818	7.22903E-06	0.103	360612	11969.376
order Victivallales	Vascular dementia (multiple infarctions)	rs429358	C	T	-0.012	45411941	0.587	0.030	14306	C	T	0.660	19	44908684	1.33506E-17	0.077	360612	52325.340
order Victivallales	Vascular dementia (multiple infarctions)	rs4716814	T	C	-0.018	157723046	0.399	0.021	14306	T	C	-0.319	7	157930354	3.75656E-07	0.063	360612	19180.021
order Victivallales	Vascular dementia (multiple infarctions)	rs73053797	T	C	-0.026	29909039	0.435	0.029	14306	T	C	0.338	3	29867548	6.00772E-06	0.075	360612	13680.576
order Victivallales	Vascular dementia (multiple infarctions)	rs9861644	A	G	-0.020	88637331	0.387	0.024	14306	A	G	0.323	3	88588181	4.84942E-06	0.071	360612	17137.658
order Actinomycetales	Vascular dementia (mixed)	rs12257900	T	G	-0.001	49443428	0.978	0.024	14306	T	G	0.467	10	48235385	2.48891E-06	0.099	360421	23006.877
order Actinomycetales	Vascular dementia (mixed)	rs1466525	T	C	0.007	54780209	0.713	0.020	14306	T	C	0.489	8	53867649	1.97697E-06	0.103	360421	34189.173
order Actinomycetales	Vascular dementia (mixed)	rs17168895	T	G	-0.004	15647727	0.814	0.026	14306	T	G	-0.547	7	15608102	2.92853E-06	0.117	360421	34495.049
order Actinomycetales	Vascular dementia (mixed)	rs6028529	A	G	0.008	38205487	0.629	0.020	14306	A	G	0.413	20	39576844	4.98965E-06	0.091	360421	21913.090

order Actinomycetales	Vascular dementia (mixed)	rs7776624	G	A	-0.003	31909839	0.883	0.016	14306	G	A	-0.358	7	31870226	8.82531E-06	0.081	360421	23450.025
order Bacillales	Vascular dementia (mixed)	rs17168895	T	G	-0.011	15647727	0.762	0.042	14306	T	G	-0.547	7	15608102	2.92853E-06	0.117	360421	34495.049
order Bacillales	Vascular dementia (mixed)	rs6028529	A	G	-0.014	38205487	0.570	0.030	14306	A	G	0.413	20	39576844	4.98965E-06	0.091	360421	21913.090
order Bacillales	Vascular dementia (mixed)	rs6849229	G	A	0.001	131797280	0.959	0.034	14306	G	A	0.562	4	130876125	4.44038E-06	0.122	360421	34142.711
order Bacillales	Vascular dementia (mixed)	rs7614116	G	A	-0.023	130368069	0.373	0.027	14306	G	A	0.379	3	130649225	6.06429E-06	0.084	360421	23465.044
order Bacteroidales	Vascular dementia (mixed)	rs12257900	T	G	-0.003	49443428	0.869	0.016	14306	T	G	0.467	10	48235385	2.48891E-06	0.099	360421	23006.877
order Bacteroidales	Vascular dementia (mixed)	rs1466525	T	C	0.002	54780209	0.897	0.013	14306	T	C	0.489	8	53867649	1.97697E-06	0.103	360421	34189.173
order Bacteroidales	Vascular dementia (mixed)	rs1632064	T	C	-0.007	3219694	0.626	0.015	14306	T	C	0.614	5	3219580	4.04045E-06	0.133	360421	37296.871
order Bacteroidales	Vascular dementia (mixed)	rs17168895	T	G	-0.010	15647727	0.562	0.017	14306	T	G	-0.547	7	15608102	2.92853E-06	0.117	360421	34495.049
order Bacteroidales	Vascular dementia (mixed)	rs6028529	A	G	0.003	38205487	0.841	0.012	14306	A	G	0.413	20	39576844	4.98965E-06	0.091	360421	21913.090
order Bacteroidales	Vascular dementia (mixed)	rs6849229	G	A	-0.011	131797280	0.581	0.014	14306	G	A	0.562	4	130876125	4.44038E-06	0.122	360421	34142.711
order Bacteroidales	Vascular dementia (mixed)	rs7614116	G	A	0.002	130368069	0.810	0.011	14306	G	A	0.379	3	130649225	6.06429E-06	0.084	360421	23465.044
order Bacteroidales	Vascular dementia (mixed)	rs7776624	G	A	-0.007	31909839	0.487	0.010	14306	G	A	-0.358	7	31870226	8.82531E-06	0.081	360421	23450.025
order Bifidobacteriales	Vascular dementia (mixed)	rs12257900	T	G	-0.004	49443428	0.857	0.017	14306	T	G	0.467	10	48235385	2.48891E-06	0.099	360421	23006.877
order Bifidobacteriales	Vascular dementia (mixed)	rs1466525	T	C	0.010	54780209	0.581	0.015	14306	T	C	0.489	8	53867649	1.97697E-06	0.103	360421	34189.173
order Bifidobacteriales	Vascular dementia (mixed)	rs6849229	G	A	0.010	131797280	0.502	0.016	14306	G	A	0.562	4	130876125	4.44038E-06	0.122	360421	34142.711
order Bifidobacteriales	Vascular dementia (mixed)	rs7614116	G	A	-0.008	130368069	0.477	0.013	14306	G	A	0.379	3	130649225	6.06429E-06	0.084	360421	23465.044
order Bifidobacteriales	Vascular dementia (mixed)	rs7776624	G	A	-0.001	31909839	0.943	0.012	14306	G	A	-0.358	7	31870226	8.82531E-06	0.081	360421	23450.025
order Burkholderiales	Vascular dementia (mixed)	rs12257900	T	G	0.011	49443428	0.476	0.016	14306	T	G	0.467	10	48235385	2.48891E-06	0.099	360421	23006.877
order Burkholderiales	Vascular dementia (mixed)	rs1466525	T	C	-0.008	54780209	0.549	0.014	14306	T	C	0.489	8	53867649	1.97697E-06	0.103	360421	34189.173
order Burkholderiales	Vascular dementia (mixed)	rs1632064	T	C	0.009	3219694	0.568	0.015	14306	T	C	0.614	5	3219580	4.04045E-06	0.133	360421	37296.871
order Burkholderiales	Vascular dementia (mixed)	rs429358	C	T	0.003	45411941	0.854	0.016	14306	C	T	0.565	19	44908684	3.94357E-08	0.103	360421	36831.461
order Burkholderiales	Vascular dementia (mixed)	rs6028529	A	G	0.011	38205487	0.349	0.013	14306	A	G	0.413	20	39576844	4.98965E-06	0.091	360421	21913.090
order Burkholderiales	Vascular dementia (mixed)	rs6849229	G	A	0.005	131797280	0.685	0.015	14306	G	A	0.562	4	130876125	4.44038E-06	0.122	360421	34142.711
order Clostridiales	Vascular dementia (mixed)	rs12257900	T	G	0.004	49443428	0.801	0.016	14306	T	G	0.467	10	48235385	2.48891E-06	0.099	360421	23006.877
order Clostridiales	Vascular dementia (mixed)	rs1466525	T	C	-0.008	54780209	0.565	0.013	14306	T	C	0.489	8	53867649	1.97697E-06	0.103	360421	34189.173
order Clostridiales	Vascular dementia (mixed)	rs1632064	T	C	-0.005	3219694	0.790	0.015	14306	T	C	0.614	5	3219580	4.04045E-06	0.133	360421	37296.871
order Clostridiales	Vascular dementia (mixed)	rs17168895	T	G	0.002	15647727	0.901	0.017	14306	T	G	-0.547	7	15608102	2.92853E-06	0.117	360421	34495.049
order Clostridiales	Vascular dementia (mixed)	rs429358	C	T	0.005	45411941	0.723	0.015	14306	C	T	0.565	19	44908684	3.94357E-08	0.103	360421	36831.461
order Clostridiales	Vascular dementia (mixed)	rs6028529	A	G	-0.008	38205487	0.605	0.012	14306	A	G	0.413	20	39576844	4.98965E-06	0.091	360421	21913.090
order Clostridiales	Vascular dementia (mixed)	rs6849229	G	A	0.007	131797280	0.703	0.014	14306	G	A	0.562	4	130876125	4.44038E-06	0.122	360421	34142.711
order Clostridiales	Vascular dementia (mixed)	rs7614116	G	A	-0.004	130368069	0.690	0.011	14306	G	A	0.379	3	130649225	6.06429E-06	0.084	360421	23465.044
order Clostridiales	Vascular dementia (mixed)	rs7776624	G	A	0.004	31909839	0.684	0.010	14306	G	A	-0.358	7	31870226	8.82531E-06	0.081	360421	23450.025
order Coriobacteriales	Vascular dementia (mixed)	rs12257900	T	G	-0.014	49443428	0.392	0.016	14306	T	G	0.467	10	48235385	2.48891E-06	0.099	360421	23006.877
order Coriobacteriales	Vascular dementia (mixed)	rs1466525	T	C	-0.008	54780209	0.544	0.013	14306	T	C	0.489	8	53867649	1.97697E-06	0.103	360421	34189.173
order Coriobacteriales	Vascular dementia (mixed)	rs1632064	T	C	-0.005	3219694	0.782	0.015	14306	T	C	0.614	5	3219580	4.04045E-06	0.133	360421	37296.871
order Coriobacteriales	Vascular dementia (mixed)	rs17168895	T	G	0.014	15647727	0.431	0.018	14306	T	G	-0.547	7	15608102	2.92853E-06	0.117	360421	34495.049
order Coriobacteriales	Vascular dementia (mixed)	rs7776624	G	A	-0.006	31909839	0.606	0.011	14306	G	A	-0.358	7	31870226	8.82531E-06	0.081	360421	23450.025
order Desulfovibrionales	Vascular dementia (mixed)	rs12257900	T	G	0.006	49443428	0.742	0.017	14306	T	G	0.467	10	48235385	2.48891E-06	0.099	360421	23006.877
order Desulfovibrionales	Vascular dementia (mixed)	rs1466525	T	C	0.005	54780209	0.939	0.015	14306	T	C	0.489	8	53867649	1.97697E-06	0.103	360421	34189.173
order Desulfovibrionales	Vascular dementia (mixed)	rs17168895	T	G	-0.006	15647727	0.762	0.019	14306	T	G	-0.547	7	15608102	2.92853E-06	0.117	360421	34495.049
order Desulfovibrionales	Vascular dementia (mixed)	rs429358	C	T	0.016	45411941	0.327	0.017	14306	C	T	0.565	19	44908684	3.94357E-08	0.103	360421	36831.461
order Desulfovibrionales	Vascular dementia (mixed)	rs6028529	A	G	0.000	38205487	0.981	0.014	14306	A	G	0.413	20	39576844	4.98965E-06	0.091	360421	21913.090
order Desulfovibrionales	Vascular dementia (mixed)	rs6849229	G	A	0.001	131797280	0.944	0.016	14306	G	A	0.562	4	130876125	4.44038E-06	0.122	360421	34142.711
order Desulfovibrionales	Vascular dementia (mixed)	rs7614116	G	A	0.004	130368069	0.734	0.013	14306	G	A	0.379	3	130649225	6.06429E-06	0.084	360421	23465.044
order Enterobacteriales	Vascular dementia (mixed)	rs12257900	T	G	-0.006	49443428	0.801	0.018	14306	T	G	0.467	10	48235385	2.48891E-06	0.099	360421	23006.877
order Enterobacteriales	Vascular dementia (mixed)	rs1466525	T	C	-0.003	54780209	0.852	0.015	14306	T	C	0.489	8	53867649	1.97697E-06	0.103	360421	34189.173
order Enterobacteriales	Vascular dementia (mixed)	rs17168895	T	G	0.000	15647727	0.915	0.020	14306	T	G	-0.547	7	15608102	2.92853E-06	0.117	360421	34495.049
order Enterobacteriales	Vascular dementia (mixed)	rs429358	C	T	-0.019	45411941	0.299	0.017	14306	C	T	0.565	19	44908684	3.94357E-08	0.103	360421	36831.461
order Enterobacteriales	Vascular dementia (mixed)	rs7614116	G	A	-0.005	130368069	0.684	0.013	14306	G	A	0.379	3	130649225	6.06429E-06	0.084	360421	23465.044
order Enterobacteriales	Vascular dementia (mixed)	rs7776624	G	A	-0.007	31909839	0.572	0.012	14306	G	A	-0.358	7	31870226	8.82531E-06	0.081	360421	23450.025
order Erysipelotrichales	Vascular dementia (mixed)	rs1466525	T	C	0.009	54780209	0.569	0.013	14306	T	C	0.489	8	53867649	1.97697E-06	0.103	360421	34189.173
order Erysipelotrichales	Vascular dementia (mixed)	rs1632064	T	C	0.001	3219694	0.867	0.015	14306	T	C	0.614	5	3219580	4.04045E-06	0.133	360421	37296.871
order Erysipelotrichales	Vascular dementia (mixed)	rs17168895	T	G	-0.001	15647727	0.965	0.017	14306	T	G	-0.547	7	15608102	2.92853E-06	0.117	360421	34495.049
order Erysipelotrichales	Vascular dementia (mixed)	rs6028529	A	G	0.001	38205487	0.976	0.012	14306	A	G	0.413	20	39576844	4.98965E-06	0.091	360421	21913.090
order Erysipelotrichales	Vascular dementia (mixed)	rs7776624	G	A	0.007	31909839	0.484	0.011	14306	G	A	-0.358	7	31870226	8.82531E-06	0.081	360421	23450.025
order Gastranaerophilales	Vascular dementia (mixed)	rs12257900	T	G	-0.017	49443428	0.542	0.027	14306	T	G	0.467	10	48235385	2.48891E-06	0.099	360421	23006.877
order Gastranaerophilales	Vascular dementia (mixed)	rs1466525	T	C	0.008	54780209	0.672	0.023	14306	T	C	0.489	8	53867649	1.97697E-06	0.103	360421	34189.173
order Gastranaerophilales	Vascular dementia (mixed)	rs1632064	T	C	0.010	3219694	0.694	0.026	14306	T	C	0.614	5	3219580	4.04045E-06	0.133	360421	37296.871
order Gastranaerophilales	Vascular dementia (mixed)	rs17168895	T	G	0.000	15647727	0.942	0.030	14306	T	G	-0.547	7	15608102	2.92853E-06	0.117	360421	34495.049
order Gastranaerophilales	Vascular dementia (mixed)	rs429358	C	T	-0.002	45411941	0.999	0.026	14306	C	T	0.565	19	44908684	3.94357E-08	0.103	360421	36831.461
order Gastranaerophilales	Vascular dementia (mixed)	rs6028529	A	G	0.015	38205487	0.539	0.022	14306	A	G	0.413	20	39576844	4.98965E-06	0.091	360421	21913.090
order Gastranaerophilales	Vascular dementia (mixed)	rs6849229	G	A	0.007	131797280	0.810	0.025	14306	G	A	0.562	4	130876125	4.44038E-06	0.122	360421	34142.711
order Gastranaerophilales	Vascular dementia (mixed)	rs7614116	G	A	-0.017	130368069	0.400	0.020	14306	G	A	0.379	3	130649225	6.06429E-06	0.084	360421	23465.044
order Lactobacillales	Vascular dementia (mixed)	rs1466525	T	C	0.001	54780209	0.974	0.014	14306	T	C	0.489	8	53867649	1.97697E-06	0.103	360421	34189.173
order Lactobacillales	Vascular dementia (mixed)	rs1632064	T	C	-0.007	3219694	0.592	0.015	14306	T	C	0.614	5	3219580	4.04045E-06	0.133	360421	37296.871
order Lactobacillales	Vascular dementia (mixed)	rs17168895	T	G	0.007	15647727	0.779	0.018	14306	T	G	-0.547	7	15608102	2.92853E-06	0.117	360421	34495.049
order Lactobacillales	Vascular dementia (mixed)	rs429358	C	T	0.010	45411941	0.542	0.015	14306	C	T	0.565	19	44908684	3.94357E-08	0.103	360421	36831.461
order Lactobacillales	Vascular dementia (mixed)	rs6028529	A	G	0.007	38205487	0.607	0.013	14306	A	G	0.413	20	39576844	4.98965E-06	0.091	360421	21913.090
order Lactobacillales	Vascular dementia (mixed)	rs7614116	G	A	0.000	130368069	0.973	0.012	14306	G	A	0.379	3	130649225	6.06429E-06	0.084	360421	23465.044
order Lactobacillales	Vascular dementia (mixed)	rs7776624	G	A	-0.004	31909839	0.697	0.011	14306	G	A	-0.358	7	31870226	8.82531E-06	0.081	360421	23450.025

order Methanobacteriales	Vascular dementia (mixed)	rs12257900	T	G	-0.014	49443428	0.614	0.034	14306	T	G	0.467	10	48235385	2.48891E-06	0.099	360421	23006.877
order Methanobacteriales	Vascular dementia (mixed)	rs1466525	T	C	0.012	54780209	0.743	0.029	14306	T	C	0.489	8	53867649	1.97697E-06	0.103	360421	34189.173
order Methanobacteriales	Vascular dementia (mixed)	rs1632064	T	C	0.005	3219694	0.822	0.032	14306	T	C	0.614	5	3219580	4.04045E-06	0.133	360421	37296.871
order Methanobacteriales	Vascular dementia (mixed)	rs17168895	T	G	0.017	15647727	0.666	0.038	14306	T	G	-0.547	7	15608102	2.92853E-06	0.117	360421	34495.049
order Methanobacteriales	Vascular dementia (mixed)	rs429358	C	T	-0.026	45411941	0.504	0.034	14306	C	T	0.565	19	44908684	3.94357E-08	0.103	360421	36831.461
order Mollicutes RF9	Vascular dementia (mixed)	rs12257900	T	G	0.011	49443428	0.601	0.021	14306	T	G	0.467	10	48235385	2.48891E-06	0.099	360421	23006.877
order Mollicutes RF9	Vascular dementia (mixed)	rs1466525	T	C	0.004	54780209	0.748	0.017	14306	T	C	0.489	8	53867649	1.97697E-06	0.103	360421	34189.173
order Mollicutes RF9	Vascular dementia (mixed)	rs17168895	T	G	-0.007	15647727	0.776	0.023	14306	T	G	-0.547	7	15608102	2.92853E-06	0.117	360421	34495.049
order Mollicutes RF9	Vascular dementia (mixed)	rs429358	C	T	0.000	45411941	0.994	0.020	14306	C	T	0.565	19	44908684	3.94357E-08	0.103	360421	36831.461
order Mollicutes RF9	Vascular dementia (mixed)	rs6028529	A	G	-0.010	38205487	0.571	0.017	14306	A	G	0.413	20	39576844	4.98965E-06	0.091	360421	21913.090
order Mollicutes RF9	Vascular dementia (mixed)	rs6849229	G	A	-0.012	131797280	0.444	0.019	14306	G	A	0.562	4	130876125	4.44038E-06	0.122	360421	34142.711
order Mollicutes RF9	Vascular dementia (mixed)	rs7614116	G	A	-0.012	130368069	0.400	0.015	14306	G	A	0.379	3	130649225	6.06429E-06	0.084	360421	23465.044
order NB1n	Vascular dementia (mixed)	rs12257900	T	G	0.023	49443428	0.454	0.029	14306	T	G	0.467	10	48235385	2.48891E-06	0.099	360421	23006.877
order NB1n	Vascular dementia (mixed)	rs429358	C	T	0.017	45411941	0.488	0.028	14306	C	T	0.565	19	44908684	3.94357E-08	0.103	360421	36831.461
order NB1n	Vascular dementia (mixed)	rs6028529	A	G	0.001	38205487	0.964	0.024	14306	A	G	0.413	20	39576844	4.98965E-06	0.091	360421	21913.090
order NB1n	Vascular dementia (mixed)	rs7614116	G	A	-0.007	130368069	0.749	0.022	14306	G	A	0.379	3	130649225	6.06429E-06	0.084	360421	23465.044
order Pasteurellales	Vascular dementia (mixed)	rs1466525	T	C	-0.008	54780209	0.686	0.018	14306	T	C	0.489	8	53867649	1.97697E-06	0.103	360421	34189.173
order Pasteurellales	Vascular dementia (mixed)	rs1632064	T	C	-0.003	3219694	0.854	0.020	14306	T	C	0.614	5	3219580	4.04045E-06	0.133	360421	37296.871
order Pasteurellales	Vascular dementia (mixed)	rs17168895	T	G	0.015	15647727	0.520	0.024	14306	T	G	-0.547	7	15608102	2.92853E-06	0.117	360421	34495.049
order Pasteurellales	Vascular dementia (mixed)	rs429358	C	T	0.022	45411941	0.309	0.021	14306	C	T	0.565	19	44908684	3.94357E-08	0.103	360421	36831.461
order Pasteurellales	Vascular dementia (mixed)	rs6028529	A	G	-0.009	38205487	0.537	0.017	14306	A	G	0.413	20	39576844	4.98965E-06	0.091	360421	21913.090
order Pasteurellales	Vascular dementia (mixed)	rs6849229	G	A	0.010	131797280	0.555	0.020	14306	G	A	0.562	4	130876125	4.44038E-06	0.122	360421	34142.711
order Pasteurellales	Vascular dementia (mixed)	rs7776624	G	A	0.009	31909839	0.555	0.015	14306	G	A	-0.358	7	31870226	8.82531E-06	0.081	360421	23450.025
order Rhodospirillales	Vascular dementia (mixed)	rs12257900	T	G	0.002	49443428	0.975	0.022	14306	T	G	0.467	10	48235385	2.48891E-06	0.099	360421	23006.877
order Rhodospirillales	Vascular dementia (mixed)	rs1632064	T	C	-0.010	3219694	0.650	0.021	14306	T	C	0.614	5	3219580	4.04045E-06	0.133	360421	37296.871
order Rhodospirillales	Vascular dementia (mixed)	rs17168895	T	G	0.022	15647727	0.415	0.025	14306	T	G	-0.547	7	15608102	2.92853E-06	0.117	360421	34495.049
order Rhodospirillales	Vascular dementia (mixed)	rs429358	C	T	-0.006	45411941	0.821	0.022	14306	C	T	0.565	19	44908684	3.94357E-08	0.103	360421	36831.461
order Rhodospirillales	Vascular dementia (mixed)	rs6028529	A	G	-0.008	38205487	0.639	0.018	14306	A	G	0.413	20	39576844	4.98965E-06	0.091	360421	21913.090
order Rhodospirillales	Vascular dementia (mixed)	rs7614116	G	A	-0.006	130368069	0.738	0.017	14306	G	A	0.379	3	130649225	6.06429E-06	0.084	360421	23465.044
order Rhodospirillales	Vascular dementia (mixed)	rs7776624	G	A	0.003	31909839	0.838	0.015	14306	G	A	-0.358	7	31870226	8.82531E-06	0.081	360421	23450.025
order Selenomonadales	Vascular dementia (mixed)	rs1466525	T	C	0.002	54780209	0.917	0.013	14306	T	C	0.489	8	53867649	1.97697E-06	0.103	360421	34189.173
order Selenomonadales	Vascular dementia (mixed)	rs1632064	T	C	0.002	3219694	0.990	0.015	14306	T	C	0.614	5	3219580	4.04045E-06	0.133	360421	37296.871
order Selenomonadales	Vascular dementia (mixed)	rs6028529	A	G	-0.007	38205487	0.560	0.012	14306	A	G	0.413	20	39576844	4.98965E-06	0.091	360421	21913.090
order Selenomonadales	Vascular dementia (mixed)	rs7614116	G	A	0.009	130368069	0.429	0.011	14306	G	A	0.379	3	130649225	6.06429E-06	0.084	360421	23465.044
order Selenomonadales	Vascular dementia (mixed)	rs7776624	G	A	-0.004	31909839	0.733	0.011	14306	G	A	-0.358	7	31870226	8.82531E-06	0.081	360421	23450.025
order Verrucomicrobiales	Vascular dementia (mixed)	rs12257900	T	G	-0.014	49443428	0.458	0.019	14306	T	G	0.467	10	48235385	2.48891E-06	0.099	360421	23006.877
order Verrucomicrobiales	Vascular dementia (mixed)	rs1466525	T	C	0.010	54780209	0.549	0.016	14306	T	C	0.489	8	53867649	1.97697E-06	0.103	360421	34189.173
order Verrucomicrobiales	Vascular dementia (mixed)	rs1632064	T	C	0.001	3219694	0.915	0.018	14306	T	C	0.614	5	3219580	4.04045E-06	0.133	360421	37296.871
order Verrucomicrobiales	Vascular dementia (mixed)	rs17168895	T	G	0.012	15647727	0.527	0.021	14306	T	G	-0.547	7	15608102	2.92853E-06	0.117	360421	34495.049
order Verrucomicrobiales	Vascular dementia (mixed)	rs429358	C	T	0.018	45411941	0.336	0.019	14306	C	T	0.565	19	44908684	3.94357E-08	0.103	360421	36831.461
order Verrucomicrobiales	Vascular dementia (mixed)	rs6028529	A	G	-0.013	38205487	0.455	0.016	14306	A	G	0.413	20	39576844	4.98965E-06	0.091	360421	21913.090
order Verrucomicrobiales	Vascular dementia (mixed)	rs6849229	G	A	-0.013	131797280	0.492	0.018	14306	G	A	0.562	4	130876125	4.44038E-06	0.122	360421	34142.711
order Verrucomicrobiales	Vascular dementia (mixed)	rs7614116	G	A	-0.001	130368069	0.999	0.014	14306	G	A	0.379	3	130649225	6.06429E-06	0.084	360421	23465.044
order Victivallales	Vascular dementia (mixed)	rs12257900	T	G	0.015	49443428	0.661	0.030	14306	T	G	0.467	10	48235385	2.48891E-06	0.099	360421	23006.877
order Victivallales	Vascular dementia (mixed)	rs17168895	T	G	0.002	15647727	0.944	0.034	14306	T	G	-0.547	7	15608102	2.92853E-06	0.117	360421	34495.049
order Victivallales	Vascular dementia (mixed)	rs429358	C	T	-0.012	45411941	0.587	0.030	14306	C	T	0.565	19	44908684	3.94357E-08	0.103	360421	36831.461
order Victivallales	Vascular dementia (mixed)	rs6028529	A	G	0.003	38205487	0.868	0.024	14306	A	G	0.413	20	39576844	4.98965E-06	0.091	360421	21913.090
order Victivallales	Vascular dementia (mixed)	rs6849229	G	A	-0.018	131797280	0.450	0.028	14306	G	A	0.562	4	130876125	4.44038E-06	0.122	360421	34142.711
order Actinomycetales	Vascular dementia (subcortical)	rs10919863	T	C	-0.010	200226041	0.647	0.021	14306	T	C	0.315	1	200256913	3.47112E-06	0.068	360770	10813.753
order Actinomycetales	Vascular dementia (subcortical)	rs11148372	A	G	0.000	22788665	0.992	0.016	14306	A	G	-0.261	13	22214526	4.06275E-06	0.057	360770	12632.549
order Actinomycetales	Vascular dementia (subcortical)	rs11986558	T	C	0.009	2500772	0.585	0.017	14306	T	C	0.248	8	2643276	9.16875E-06	0.056	360770	11030.353
order Actinomycetales	Vascular dementia (subcortical)	rs429358	C	T	0.038	45411941	0.088	0.023	14306	C	T	0.597	19	44908684	1.74221E-17	0.070	360770	41621.629
order Actinomycetales	Vascular dementia (subcortical)	rs4295569	C	T	0.005	47820641	0.753	0.017	14306	C	T	-0.355	7	47781043	2.54572E-08	0.064	360770	20037.409
order Bacillales	Vascular dementia (subcortical)	rs1363668	G	A	-0.016	143089582	0.517	0.025	14306	G	A	-0.272	5	143710017	5.25352E-06	0.060	360770	12697.214
order Bacillales	Vascular dementia (subcortical)	rs3802793	A	G	0.011	131685316	0.683	0.026	14306	A	G	0.274	11	131815422	5.46626E-06	0.060	360770	12758.334
order Bacillales	Vascular dementia (subcortical)	rs4295569	C	T	-0.011	47820641	0.655	0.026	14306	C	T	-0.355	7	47781043	2.54572E-08	0.064	360770	20037.409
order Bacillales	Vascular dementia (subcortical)	rs4723291	A	G	0.017	33551998	0.461	0.025	14306	A	G	-0.263	7	33512386	6.22372E-06	0.058	360770	11064.499
order Bacteroidales	Vascular dementia (subcortical)	rs10919863	T	C	-0.008	200226041	0.514	0.013	14306	T	C	0.315	1	200256913	3.47112E-06	0.068	360770	10813.753
order Bacteroidales	Vascular dementia (subcortical)	rs11148372	A	G	0.003	22788665	0.805	0.011	14306	A	G	-0.261	13	22214526	4.06275E-06	0.057	360770	12632.549
order Bacteroidales	Vascular dementia (subcortical)	rs11986558	T	C	0.008	2500772	0.479	0.011	14306	T	C	0.248	8	2643276	9.16875E-06	0.056	360770	11030.353
order Bacteroidales	Vascular dementia (subcortical)	rs1363668	G	A	0.004	143089582	0.725	0.011	14306	G	A	-0.272	5	143710017	5.25352E-06	0.060	360770	12697.214
order Bacteroidales	Vascular dementia (subcortical)	rs3802793	A	G	0.006	131685316	0.548	0.011	14306	A	G	0.274	11	131815422	5.46626E-06	0.060	360770	12758.334
order Bacteroidales	Vascular dementia (subcortical)	rs429358	C	T	-0.022	45411941	0.148	0.015	14306	C	T	0.597	19	44908684	1.74221E-17	0.070	360770	41621.629
order Bacteroidales	Vascular dementia (subcortical)	rs4295569	C	T	0.006	47820641	0.590	0.011	14306	C	T	-0.355	7	47781043	2.54572E-08	0.064	360770	20037.409
order Bifidobacteriales	Vascular dementia (subcortical)	rs10919863	T	C	-0.001	200226041	0.993	0.015	14306	T	C	0.315	1	200256913	3.47112E-06	0.068	360770	10813.753
order Bifidobacteriales	Vascular dementia (subcortical)	rs11148372	A	G	-0.002	22788665	0.862	0.012	14306	A	G	-0.261	13	22214526	4.06275E-06	0.057	360770	12632.549
order Bifidobacteriales	Vascular dementia (subcortical)	rs11986558	T	C	0.006	2500772	0.634	0.012	14306	T	C	0.248	8	2643276	9.16875E-06	0.056	360770	11030.353
order Bifidobacteriales	Vascular dementia (subcortical)	rs1363668	G	A	0.009	143089582	0.437	0.012	14306	G	A	-0.272	5	143710017	5.25352E-06	0.060	360770	12697.214
order Bifidobacteriales	Vascular dementia (subcortical)	rs3802793	A	G	0.006	131685316	0.682	0.012	14306	A	G	0.274	11	131815422	5.46626E-06	0.060	360770	12758.334
order Bifidobacteriales	Vascular dementia (subcortical)	rs429358	C	T	0.022	45411941	0.147	0.017	14306	C	T	0.597	19	44908684	1.74221E-17	0.070	360770	41621.629
order Bifidobacteriales	Vascular dementia (subcortical)	rs4295569	C	T	0.001	47820641	0.967	0.012	14306	C	T	-0.355	7	47781043	2.54572E-08	0.064	360770	20037.409

order Bifidobacteriales	Vascular dementia (subcortical)	rs4723291	A	G	-0.004	33551998	0.773	0.012	14306	A	G	-0.263	7	33512386	6.22372E-06	0.058	360770	11064.499
order Burkholderiales	Vascular dementia (subcortical)	rs11148372	A	G	-0.003	22788665	0.765	0.011	14306	A	G	-0.261	13	22214526	4.06275E-06	0.057	360770	12632.549
order Burkholderiales	Vascular dementia (subcortical)	rs1363668	G	A	0.001	143089582	0.905	0.011	14306	G	A	-0.272	5	143710017	5.25352E-06	0.060	360770	12697.214
order Burkholderiales	Vascular dementia (subcortical)	rs429358	C	T	0.003	45411941	0.854	0.016	14306	C	T	0.597	19	44908684	1.74221E-17	0.070	360770	41621.629
order Burkholderiales	Vascular dementia (subcortical)	rs4723291	A	G	-0.005	33551998	0.649	0.011	14306	A	G	-0.263	7	33512386	6.22372E-06	0.058	360770	11064.499
order Clostridiales	Vascular dementia (subcortical)	rs10919863	T	C	0.000	200226041	0.903	0.013	14306	T	C	0.315	1	200256913	3.47112E-06	0.068	360770	10813.753
order Clostridiales	Vascular dementia (subcortical)	rs11148372	A	G	0.007	22788665	0.499	0.011	14306	A	G	-0.261	13	22214526	4.06275E-06	0.057	360770	12632.549
order Clostridiales	Vascular dementia (subcortical)	rs11986558	T	C	-0.001	2500772	0.910	0.011	14306	T	C	0.248	8	2643276	9.16875E-06	0.056	360770	11030.353
order Clostridiales	Vascular dementia (subcortical)	rs1363668	G	A	0.000	143089582	0.978	0.011	14306	G	A	-0.272	5	143710017	5.25352E-06	0.060	360770	12697.214
order Clostridiales	Vascular dementia (subcortical)	rs3802793	A	G	-0.008	131685316	0.483	0.011	14306	A	G	0.274	11	131815422	5.46626E-06	0.060	360770	12758.334
order Clostridiales	Vascular dementia (subcortical)	rs429358	C	T	0.005	45411941	0.723	0.015	14306	C	T	0.597	19	44908684	1.74221E-17	0.070	360770	41621.629
order Clostridiales	Vascular dementia (subcortical)	rs4295569	C	T	0.002	47820641	0.791	0.011	14306	C	T	-0.355	7	47781043	2.54572E-08	0.064	360770	20037.409
order Coriobacteriales	Vascular dementia (subcortical)	rs3802793	A	G	0.007	131685316	0.516	0.011	14306	A	G	0.274	11	131815422	5.46626E-06	0.060	360770	12758.334
order Coriobacteriales	Vascular dementia (subcortical)	rs429358	C	T	0.019	45411941	0.204	0.015	14306	C	T	0.597	19	44908684	1.74221E-17	0.070	360770	41621.629
order Coriobacteriales	Vascular dementia (subcortical)	rs4295569	C	T	0.002	47820641	0.863	0.011	14306	C	T	-0.355	7	47781043	2.54572E-08	0.064	360770	20037.409
order Coriobacteriales	Vascular dementia (subcortical)	rs4382795	C	T	-0.007	66878853	0.591	0.014	14306	C	T	0.378	10	65119095	9.80754E-06	0.086	360770	13265.712
order Coriobacteriales	Vascular dementia (subcortical)	rs4723291	A	G	-0.006	33551998	0.567	0.011	14306	A	G	-0.263	7	33512386	6.22372E-06	0.058	360770	11064.499
order Desulfovibrionales	Vascular dementia (subcortical)	rs11986558	T	C	-0.004	2500772	0.728	0.012	14306	T	C	0.248	8	2643276	9.16875E-06	0.056	360770	11030.353
order Desulfovibrionales	Vascular dementia (subcortical)	rs3802793	A	G	0.005	131685316	0.666	0.012	14306	A	G	0.274	11	131815422	5.46626E-06	0.060	360770	12758.334
order Desulfovibrionales	Vascular dementia (subcortical)	rs429358	C	T	0.016	45411941	0.327	0.017	14306	C	T	0.597	19	44908684	1.74221E-17	0.070	360770	41621.629
order Desulfovibrionales	Vascular dementia (subcortical)	rs4295569	C	T	-0.005	47820641	0.656	0.012	14306	C	T	-0.355	7	47781043	2.54572E-08	0.064	360770	20037.409
order Desulfovibrionales	Vascular dementia (subcortical)	rs4382795	C	T	0.009	66878853	0.587	0.015	14306	C	T	0.378	10	65119095	9.80754E-06	0.086	360770	13265.712
order Desulfovibrionales	Vascular dementia (subcortical)	rs4723291	A	G	0.009	33551998	0.444	0.012	14306	A	G	-0.263	7	33512386	6.22372E-06	0.058	360770	11064.499
order Enterobacteriales	Vascular dementia (subcortical)	rs1363668	G	A	0.010	143089582	0.411	0.012	14306	G	A	-0.272	5	143710017	5.25352E-06	0.060	360770	12697.214
order Enterobacteriales	Vascular dementia (subcortical)	rs3802793	A	G	0.005	131685316	0.696	0.013	14306	A	G	0.274	11	131815422	5.46626E-06	0.060	360770	12758.334
order Enterobacteriales	Vascular dementia (subcortical)	rs429358	C	T	-0.019	45411941	0.299	0.017	14306	C	T	0.597	19	44908684	1.74221E-17	0.070	360770	41621.629
order Enterobacteriales	Vascular dementia (subcortical)	rs4295569	C	T	0.001	47820641	0.912	0.013	14306	C	T	-0.355	7	47781043	2.54572E-08	0.064	360770	20037.409
order Erysipelotrichales	Vascular dementia (subcortical)	rs10919863	T	C	0.010	200226041	0.450	0.013	14306	T	C	0.315	1	200256913	3.47112E-06	0.068	360770	10813.753
order Erysipelotrichales	Vascular dementia (subcortical)	rs11148372	A	G	0.001	22788665	0.885	0.011	14306	A	G	-0.261	13	22214526	4.06275E-06	0.057	360770	12632.549
order Erysipelotrichales	Vascular dementia (subcortical)	rs11986558	T	C	-0.005	2500772	0.657	0.011	14306	T	C	0.248	8	2643276	9.16875E-06	0.056	360770	11030.353
order Erysipelotrichales	Vascular dementia (subcortical)	rs1363668	G	A	0.004	143089582	0.691	0.011	14306	G	A	-0.272	5	143710017	5.25352E-06	0.060	360770	12697.214
order Erysipelotrichales	Vascular dementia (subcortical)	rs4723291	A	G	-0.006	33551998	0.581	0.011	14306	A	G	-0.263	7	33512386	6.22372E-06	0.058	360770	11064.499
order Gastranaerophilales	Vascular dementia (subcortical)	rs10919863	T	C	0.003	200226041	0.868	0.024	14306	T	C	0.315	1	200256913	3.47112E-06	0.068	360770	10813.753
order Gastranaerophilales	Vascular dementia (subcortical)	rs11148372	A	G	0.006	22788665	0.733	0.019	14306	A	G	-0.261	13	22214526	4.06275E-06	0.057	360770	12632.549
order Gastranaerophilales	Vascular dementia (subcortical)	rs11986558	T	C	-0.008	2500772	0.697	0.020	14306	T	C	0.248	8	2643276	9.16875E-06	0.056	360770	11030.353
order Gastranaerophilales	Vascular dementia (subcortical)	rs1363668	G	A	-0.007	143089582	0.706	0.019	14306	G	A	-0.272	5	143710017	5.25352E-06	0.060	360770	12697.214
order Gastranaerophilales	Vascular dementia (subcortical)	rs3802793	A	G	-0.015	131685316	0.459	0.020	14306	A	G	0.274	11	131815422	5.46626E-06	0.060	360770	12758.334
order Gastranaerophilales	Vascular dementia (subcortical)	rs429358	C	T	-0.002	45411941	0.999	0.026	14306	C	T	0.597	19	44908684	1.74221E-17	0.070	360770	41621.629
order Gastranaerophilales	Vascular dementia (subcortical)	rs4295569	C	T	-0.013	47820641	0.465	0.019	14306	C	T	-0.355	7	47781043	2.54572E-08	0.064	360770	20037.409
order Lactobacillales	Vascular dementia (subcortical)	rs11148372	A	G	0.004	22788665	0.715	0.011	14306	A	G	-0.261	13	22214526	4.06275E-06	0.057	360770	12632.549
order Lactobacillales	Vascular dementia (subcortical)	rs11986558	T	C	-0.009	2500772	0.432	0.011	14306	T	C	0.248	8	2643276	9.16875E-06	0.056	360770	11030.353
order Lactobacillales	Vascular dementia (subcortical)	rs1363668	G	A	0.005	143089582	0.638	0.011	14306	G	A	-0.272	5	143710017	5.25352E-06	0.060	360770	12697.214
order Lactobacillales	Vascular dementia (subcortical)	rs429358	C	T	0.010	45411941	0.542	0.015	14306	C	T	0.597	19	44908684	1.74221E-17	0.070	360770	41621.629
order Lactobacillales	Vascular dementia (subcortical)	rs4295569	C	T	-0.002	47820641	0.843	0.011	14306	C	T	-0.355	7	47781043	2.54572E-08	0.064	360770	20037.409
order Methanobacteriales	Vascular dementia (subcortical)	rs10919863	T	C	0.000	200226041	0.946	0.030	14306	T	C	0.315	1	200256913	3.47112E-06	0.068	360770	10813.753
order Methanobacteriales	Vascular dementia (subcortical)	rs1363668	G	A	-0.015	143089582	0.517	0.024	14306	G	A	-0.272	5	143710017	5.25352E-06	0.060	360770	12697.214
order Methanobacteriales	Vascular dementia (subcortical)	rs429358	C	T	-0.026	45411941	0.504	0.034	14306	C	T	0.597	19	44908684	1.74221E-17	0.070	360770	41621.629
order Methanobacteriales	Vascular dementia (subcortical)	rs4382795	C	T	-0.024	66878853	0.400	0.030	14306	C	T	0.378	10	65119095	9.80754E-06	0.086	360770	13265.712
order Mollicutes RF9	Vascular dementia (subcortical)	rs11148372	A	G	-0.006	22788665	0.677	0.014	14306	A	G	-0.261	13	22214526	4.06275E-06	0.057	360770	12632.549
order Mollicutes RF9	Vascular dementia (subcortical)	rs11986558	T	C	0.000	2500772	0.997	0.015	14306	T	C	0.248	8	2643276	9.16875E-06	0.056	360770	11030.353
order Mollicutes RF9	Vascular dementia (subcortical)	rs429358	C	T	0.000	45411941	0.994	0.020	14306	C	T	0.597	19	44908684	1.74221E-17	0.070	360770	41621.629
order Mollicutes RF9	Vascular dementia (subcortical)	rs4723291	A	G	-0.003	33551998	0.830	0.014	14306	A	G	-0.263	7	33512386	6.22372E-06	0.058	360770	11064.499
order NB1n	Vascular dementia (subcortical)	rs10919863	T	C	0.009	200226041	0.705	0.026	14306	T	C	0.315	1	200256913	3.47112E-06	0.068	360770	10813.753
order NB1n	Vascular dementia (subcortical)	rs11986558	T	C	0.001	2500772	0.943	0.021	14306	T	C	0.248	8	2643276	9.16875E-06	0.056	360770	11030.353
order NB1n	Vascular dementia (subcortical)	rs1363668	G	A	-0.004	143089582	0.828	0.020	14306	G	A	-0.272	5	143710017	5.25352E-06	0.060	360770	12697.214
order NB1n	Vascular dementia (subcortical)	rs429358	C	T	0.017	45411941	0.488	0.028	14306	C	T	0.597	19	44908684	1.74221E-17	0.070	360770	41621.629
order NB1n	Vascular dementia (subcortical)	rs4295569	C	T	0.004	47820641	0.882	0.021	14306	C	T	-0.355	7	47781043	2.54572E-08	0.064	360770	20037.409
order NB1n	Vascular dementia (subcortical)	rs4382795	C	T	0.011	66878853	0.633	0.026	14306	C	T	0.378	10	65119095	9.80754E-06	0.086	360770	13265.712
order NB1n	Vascular dementia (subcortical)	rs4723291	A	G	0.006	33551998	0.781	0.020	14306	A	G	-0.263	7	33512386	6.22372E-06	0.058	360770	11064.499
order Pasteurellales	Vascular dementia (subcortical)	rs10919863	T	C	-0.010	200226041	0.614	0.019	14306	T	C	0.315	1	200256913	3.47112E-06	0.068	360770	10813.753
order Pasteurellales	Vascular dementia (subcortical)	rs11986558	T	C	0.004	2500772	0.786	0.015	14306	T	C	0.248	8	2643276	9.16875E-06	0.056	360770	11030.353
order Pasteurellales	Vascular dementia (subcortical)	rs1363668	G	A	0.003	143089582	0.804	0.015	14306	G	A	-0.272	5	143710017	5.25352E-06	0.060	360770	12697.214
order Pasteurellales	Vascular dementia (subcortical)	rs429358	C	T	0.022	45411941	0.309	0.021	14306	C	T	0.597	19	44908684	1.74221E-17	0.070	360770	41621.629
order Rhodospirillales	Vascular dementia (subcortical)	rs11148372	A	G	0.001	22788665	0.956	0.016	14306	A	G	-0.261	13	22214526	4.06275E-06	0.057	360770	12632.549
order Rhodospirillales	Vascular dementia (subcortical)	rs11986558	T	C	0.006	2500772	0.698	0.016	14306	T	C	0.248	8	2643276	9.16875E-06	0.056	360770	11030.353
order Rhodospirillales	Vascular dementia (subcortical)	rs1363668	G	A	-0.013	143089582	0.397	0.015	14306	G	A	-0.272	5	143710017	5.25352E-06	0.060	360770	12697.214
order Rhodospirillales	Vascular dementia (subcortical)	rs429358	C	T	-0.006	45411941	0.821	0.022	14306	C	T	0.597	19	44908684	1.74221E-17	0.070	360770	41621.629
order Selenomonadales	Vascular dementia (subcortical)	rs11148372	A	G	-0.002	22788665	0.830	0.011	14306	A	G	-0.261	13	22214526	4.06275E-06	0.057	360770	12632.549
order Selenomonadales	Vascular dementia (subcortical)	rs3802793	A	G	0.006	131685316	0.678	0.011	14306	A	G	0.274	11	131815422	5.46626E-06	0.060	360770	12758.334
order Selenomonadales	Vascular dementia (subcortical)	rs429358	C	T	-0.019	45411941	0.146	0.015	14306	C	T	0.597	19	44908684	1.74221E-17	0.070	360770	41621.629
order Selenomonadales	Vascular dementia (subcortical)	rs4295569	C	T	0.000	47820641	0.990	0.011	14306	C	T	-0.355	7	47781043	2.54572E-08	0.064	360770	20037.409

order Verrucomicrobiales	Vascular dementia (subcortical)	rs10919863	T	C	-0.002	200226041	0.924	0.017	14306	T	C	0.315	1	200256913	3.47112E-06	0.068	360770	10813.753
order Verrucomicrobiales	Vascular dementia (subcortical)	rs11148372	A	G	0.007	22788665	0.560	0.013	14306	A	G	-0.261	13	22214526	4.06275E-06	0.057	360770	12632.549
order Verrucomicrobiales	Vascular dementia (subcortical)	rs1363668	G	A	0.005	143089582	0.711	0.013	14306	G	A	-0.272	5	143710017	5.25352E-06	0.060	360770	12697.214
order Verrucomicrobiales	Vascular dementia (subcortical)	rs3802793	A	G	-0.006	131685316	0.656	0.014	14306	A	G	0.274	11	131815422	5.46626E-06	0.060	360770	12758.334
order Verrucomicrobiales	Vascular dementia (subcortical)	rs429358	C	T	0.018	45411941	0.336	0.019	14306	C	T	0.597	19	44908684	1.74221E-17	0.070	360770	41621.629
order Verrucomicrobiales	Vascular dementia (subcortical)	rs4295569	C	T	0.001	47820641	0.930	0.013	14306	C	T	-0.355	7	47781043	2.54572E-08	0.064	360770	20037.409
order Verrucomicrobiales	Vascular dementia (subcortical)	rs4382795	C	T	-0.005	66878853	0.768	0.017	14306	C	T	0.378	10	65119095	9.80754E-06	0.086	360770	13265.712
order Verrucomicrobiales	Vascular dementia (subcortical)	rs4723291	A	G	-0.002	33551998	0.916	0.013	14306	A	G	-0.263	7	33512386	6.22372E-06	0.058	360770	11064.499
order Victivallales	Vascular dementia (subcortical)	rs10919863	T	C	-0.001	200226041	0.950	0.027	14306	T	C	0.315	1	200256913	3.47112E-06	0.068	360770	10813.753
order Victivallales	Vascular dementia (subcortical)	rs11148372	A	G	-0.016	22788665	0.432	0.021	14306	A	G	-0.261	13	22214526	4.06275E-06	0.057	360770	12632.549
order Victivallales	Vascular dementia (subcortical)	rs11986558	T	C	-0.012	2500772	0.581	0.022	14306	T	C	0.248	8	2643276	9.16875E-06	0.056	360770	11030.353
order Victivallales	Vascular dementia (subcortical)	rs1363668	G	A	-0.009	143089582	0.673	0.021	14306	G	A	-0.272	5	143710017	5.25352E-06	0.060	360770	12697.214
order Victivallales	Vascular dementia (subcortical)	rs429358	C	T	-0.012	45411941	0.587	0.030	14306	C	T	0.597	19	44908684	1.74221E-17	0.070	360770	41621.629
order Victivallales	Vascular dementia (subcortical)	rs4295569	C	T	0.012	47820641	0.571	0.021	14306	C	T	-0.355	7	47781043	2.54572E-08	0.064	360770	20037.409
order Victivallales	Vascular dementia (subcortical)	rs4723291	A	G	-0.004	33551998	0.846	0.021	14306	A	G	-0.263	7	33512386	6.22372E-06	0.058	360770	11064.499
order Actinomycetales	Vascular dementia (sudden onset)	rs12452096	A	G	-0.005	75544032	0.813	0.019	14306	A	G	0.750	17	77547950	1.48929E-06	0.156	360283	77865.614
order Actinomycetales	Vascular dementia (sudden onset)	rs2920	C	T	-0.012	23884780	0.542	0.020	14306	C	T	0.592	1	23558289	2.20602E-06	0.125	360283	40501.384
order Actinomycetales	Vascular dementia (sudden onset)	rs4840457	C	T	0.007	6408682	0.818	0.030	14306	C	T	0.643	8	6551161	8.92874E-06	0.145	360283	32615.797
order Actinomycetales	Vascular dementia (sudden onset)	rs71511414	G	A	0.009	79688991	0.705	0.028	14306	G	A	0.681	9	77074075	7.80746E-06	0.152	360283	30731.948
order Bacillales	Vascular dementia (sudden onset)	rs12423672	T	G	-0.012	5047705	0.772	0.040	14306	T	G	0.759	12	4938539	4.89159E-06	0.166	360283	30610.567
order Bacillales	Vascular dementia (sudden onset)	rs12452096	A	G	0.009	75544032	0.758	0.030	14306	A	G	0.750	17	77547950	1.48929E-06	0.156	360283	77865.614
order Bacillales	Vascular dementia (sudden onset)	rs2920	C	T	-0.023	23884780	0.436	0.031	14306	C	T	0.592	1	23558289	2.20602E-06	0.125	360283	40501.384
order Bacillales	Vascular dementia (sudden onset)	rs4840457	C	T	0.038	6408682	0.422	0.047	14306	C	T	0.643	8	6551161	8.92874E-06	0.145	360283	32615.797
order Bacteroidales	Vascular dementia (sudden onset)	rs12423672	T	G	-0.007	5047705	0.534	0.017	14306	T	G	0.759	12	4938539	4.89159E-06	0.166	360283	30610.567
order Bacteroidales	Vascular dementia (sudden onset)	rs2920	C	T	0.008	23884780	0.550	0.013	14306	C	T	0.592	1	23558289	2.20602E-06	0.125	360283	40501.384
order Bifidobacteriales	Vascular dementia (sudden onset)	rs12452096	A	G	-0.007	75544032	0.589	0.014	14306	A	G	0.750	17	77547950	1.48929E-06	0.156	360283	77865.614
order Bifidobacteriales	Vascular dementia (sudden onset)	rs2920	C	T	-0.007	23884780	0.652	0.014	14306	C	T	0.592	1	23558289	2.20602E-06	0.125	360283	40501.384
order Bifidobacteriales	Vascular dementia (sudden onset)	rs4840457	C	T	0.002	6408682	0.965	0.022	14306	C	T	0.643	8	6551161	8.92874E-06	0.145	360283	32615.797
order Bifidobacteriales	Vascular dementia (sudden onset)	rs71511414	G	A	-0.015	79688991	0.558	0.021	14306	G	A	0.681	9	77074075	7.80746E-06	0.152	360283	30731.948
order Burkholderiales	Vascular dementia (sudden onset)	rs12452096	A	G	0.006	75544032	0.652	0.013	14306	A	G	0.750	17	77547950	1.48929E-06	0.156	360283	77865.614
order Burkholderiales	Vascular dementia (sudden onset)	rs429358	C	T	0.003	45411941	0.854	0.016	14306	C	T	0.601	19	44908684	7.45813E-06	0.134	360283	42162.979
order Burkholderiales	Vascular dementia (sudden onset)	rs4840457	C	T	0.007	6408682	0.721	0.020	14306	C	T	0.643	8	6551161	8.92874E-06	0.145	360283	32615.797
order Burkholderiales	Vascular dementia (sudden onset)	rs71511414	G	A	-0.009	79688991	0.590	0.020	14306	G	A	0.681	9	77074075	7.80746E-06	0.152	360283	30731.948
order Clostridiales	Vascular dementia (sudden onset)	rs12423672	T	G	0.000	5047705	0.952	0.017	14306	T	G	0.759	12	4938539	4.89159E-06	0.166	360283	30610.567
order Clostridiales	Vascular dementia (sudden onset)	rs429358	C	T	0.005	45411941	0.723	0.015	14306	C	T	0.601	19	44908684	7.45813E-06	0.134	360283	42162.979
order Clostridiales	Vascular dementia (sudden onset)	rs4840457	C	T	-0.008	6408682	0.693	0.020	14306	C	T	0.643	8	6551161	8.92874E-06	0.145	360283	32615.797
order Clostridiales	Vascular dementia (sudden onset)	rs71511414	G	A	-0.015	79688991	0.477	0.019	14306	G	A	0.681	9	77074075	7.80746E-06	0.152	360283	30731.948
order Coriobacteriales	Vascular dementia (sudden onset)	rs12452096	A	G	-0.008	75544032	0.499	0.013	14306	A	G	0.750	17	77547950	1.48929E-06	0.156	360283	77865.614
order Coriobacteriales	Vascular dementia (sudden onset)	rs2920	C	T	-0.009	23884780	0.519	0.013	14306	C	T	0.592	1	23558289	2.20602E-06	0.125	360283	40501.384
order Coriobacteriales	Vascular dementia (sudden onset)	rs71511414	G	A	-0.005	79688991	0.860	0.019	14306	G	A	0.681	9	77074075	7.80746E-06	0.152	360283	30731.948
order Desulfovibrionales	Vascular dementia (sudden onset)	rs12452096	A	G	-0.003	75544032	0.824	0.014	14306	A	G	0.750	17	77547950	1.48929E-06	0.156	360283	77865.614
order Desulfovibrionales	Vascular dementia (sudden onset)	rs4840457	C	T	0.008	6408682	0.698	0.022	14306	C	T	0.643	8	6551161	8.92874E-06	0.145	360283	32615.797
order Desulfovibrionales	Vascular dementia (sudden onset)	rs71511414	G	A	-0.010	79688991	0.619	0.021	14306	G	A	0.681	9	77074075	7.80746E-06	0.152	360283	30731.948
order Enterobacteriales	Vascular dementia (sudden onset)	rs12452096	A	G	-0.005	75544032	0.728	0.014	14306	A	G	0.750	17	77547950	1.48929E-06	0.156	360283	77865.614
order Enterobacteriales	Vascular dementia (sudden onset)	rs2920	C	T	-0.007	23884780	0.631	0.015	14306	C	T	0.592	1	23558289	2.20602E-06	0.125	360283	40501.384
order Erysipelotrichales	Vascular dementia (sudden onset)	rs12452096	A	G	0.003	75544032	0.800	0.012	14306	A	G	0.750	17	77547950	1.48929E-06	0.156	360283	77865.614
order Erysipelotrichales	Vascular dementia (sudden onset)	rs2920	C	T	0.000	23884780	0.971	0.013	14306	C	T	0.592	1	23558289	2.20602E-06	0.125	360283	40501.384
order Erysipelotrichales	Vascular dementia (sudden onset)	rs4840457	C	T	0.014	6408682	0.472	0.020	14306	C	T	0.643	8	6551161	8.92874E-06	0.145	360283	32615.797
order Erysipelotrichales	Vascular dementia (sudden onset)	rs71511414	G	A	0.011	79688991	0.559	0.019	14306	G	A	0.681	9	77074075	7.80746E-06	0.152	360283	30731.948
order Gastranaerophilales	Vascular dementia (sudden onset)	rs12452096	A	G	-0.017	75544032	0.472	0.023	14306	A	G	0.750	17	77547950	1.48929E-06	0.156	360283	77865.614
order Gastranaerophilales	Vascular dementia (sudden onset)	rs2920	C	T	-0.005	23884780	0.809	0.023	14306	C	T	0.592	1	23558289	2.20602E-06	0.125	360283	40501.384
order Gastranaerophilales	Vascular dementia (sudden onset)	rs429358	C	T	-0.002	45411941	0.999	0.026	14306	C	T	0.601	19	44908684	7.45813E-06	0.134	360283	42162.979
order Gastranaerophilales	Vascular dementia (sudden onset)	rs4840457	C	T	0.019	6408682	0.630	0.034	14306	C	T	0.643	8	6551161	8.92874E-06	0.145	360283	32615.797
order Gastranaerophilales	Vascular dementia (sudden onset)	rs71511414	G	A	-0.018	79688991	0.580	0.033	14306	G	A	0.681	9	77074075	7.80746E-06	0.152	360283	30731.948
order Lactobacillales	Vascular dementia (sudden onset)	rs12452096	A	G	0.003	75544032	0.796	0.013	14306	A	G	0.750	17	77547950	1.48929E-06	0.156	360283	77865.614
order Lactobacillales	Vascular dementia (sudden onset)	rs429358	C	T	0.010	45411941	0.542	0.015	14306	C	T	0.601	19	44908684	7.45813E-06	0.134	360283	42162.979
order Lactobacillales	Vascular dementia (sudden onset)	rs71511414	G	A	-0.006	79688991	0.758	0.020	14306	G	A	0.681	9	77074075	7.80746E-06	0.152	360283	30731.948
order Methanobacteriales	Vascular dementia (sudden onset)	rs12452096	A	G	-0.008	75544032	0.767	0.029	14306	A	G	0.750	17	77547950	1.48929E-06	0.156	360283	77865.614
order Methanobacteriales	Vascular dementia (sudden onset)	rs429358	C	T	-0.026	45411941	0.504	0.034	14306	C	T	0.601	19	44908684	7.45813E-06	0.134	360283	42162.979
order Mollicutes RF9	Vascular dementia (sudden onset)	rs429358	C	T	0.000	45411941	0.994	0.020	14306	C	T	0.601	19	44908684	7.45813E-06	0.134	360283	42162.979
order Mollicutes RF9	Vascular dementia (sudden onset)	rs4840457	C	T	-0.006	6408682	0.876	0.026	14306	C	T	0.643	8	6551161	8.92874E-06	0.145	360283	32615.797
order Mollicutes RF9	Vascular dementia (sudden onset)	rs71511414	G	A	-0.002	79688991	0.882	0.025	14306	G	A	0.681	9	77074075	7.80746E-06	0.152	360283	30731.948
order NB1n	Vascular dementia (sudden onset)	rs12423672	T	G	0.013	5047705	0.770	0.032	14306	T	G	0.759	12	4938539	4.89159E-06	0.166	360283	30610.567
order NB1n	Vascular dementia (sudden onset)	rs2920	C	T	-0.022	23884780	0.347	0.025	14306	C	T	0.592	1	23558289	2.20602E-06	0.125	360283	40501.384
order NB1n	Vascular dementia (sudden onset)	rs429358	C	T	0.017	45411941	0.488	0.028	14306	C	T	0.601	19	44908684	7.45813E-06	0.134	360283	42162.979
order NB1n	Vascular dementia (sudden onset)	rs4840457	C	T	0.018	6408682	0.615	0.037	14306	C	T	0.643	8	6551161	8.92874E-06	0.145	360283	32615.797
order NB1n	Vascular dementia (sudden onset)	rs71511414	G	A	-0.006	79688991	0.837	0.035	14306	G	A	0.681	9	77074075	7.80746E-06	0.152	360283	30731.948
order Pasteurellales	Vascular dementia (sudden onset)	rs12452096	A	G	0.001	75544032	0.940	0.017	14306	A	G	0.750	17	77547950	1.48929E-06	0.156	360283	77865.614
order Pasteurellales	Vascular dementia (sudden onset)	rs2920	C	T	-0.009	23884780	0.598	0.018	14306	C	T	0.592	1	23558289	2.20602E-06	0.125	360283	40501.384
order Pasteurellales	Vascular dementia (sudden onset)	rs4840457	C	T	-0.021	6408682	0.450	0.028	14306	C	T	0.643	8	6551161	8.92874E-06	0.145	360283	32615.797
order Rhodospirillales	Vascular dementia (sudden onset)	rs12452096	A	G	-0.017	75544032	0.353	0.018	14306	A	G	0.750	17	77547950	1.48929E-06	0.156	360283	77865.614

order Rhodospirillales	Vascular dementia (sudden onset)	rs2920	C	T	0.012	23884780	0.554	0.019	14306	C	T	0.592	1	23558289	2.20602E-06	0.125	360283	40501.384
order Rhodospirillales	Vascular dementia (sudden onset)	rs429358	C	T	-0.006	45411941	0.821	0.022	14306	C	T	0.601	19	44908684	7.45813E-06	0.134	360283	42162.979
order Rhodospirillales	Vascular dementia (sudden onset)	rs4840457	C	T	-0.019	6408682	0.485	0.028	14306	C	T	0.643	8	6551161	8.92874E-06	0.145	360283	32615.797
order Rhodospirillales	Vascular dementia (sudden onset)	rs71511414	G	A	0.003	79688991	0.943	0.027	14306	G	A	0.681	9	77074075	7.80746E-06	0.152	360283	30731.948
order Selenomonadales	Vascular dementia (sudden onset)	rs12423672	T	G	-0.005	5047705	0.813	0.017	14306	T	G	0.759	12	4938539	4.89159E-06	0.166	360283	30610.567
order Selenomonadales	Vascular dementia (sudden onset)	rs2920	C	T	0.009	23884780	0.485	0.013	14306	C	T	0.592	1	23558289	2.20602E-06	0.125	360283	40501.384
order Selenomonadales	Vascular dementia (sudden onset)	rs4840457	C	T	0.006	6408682	0.775	0.020	14306	C	T	0.643	8	6551161	8.92874E-06	0.145	360283	32615.797
order Selenomonadales	Vascular dementia (sudden onset)	rs71511414	G	A	0.009	79688991	0.643	0.019	14306	G	A	0.681	9	77074075	7.80746E-06	0.152	360283	30731.948
order Verrucomicrobiales	Vascular dementia (sudden onset)	rs12423672	T	G	0.016	5047705	0.344	0.021	14306	T	G	0.759	12	4938539	4.89159E-06	0.166	360283	30610.567
order Verrucomicrobiales	Vascular dementia (sudden onset)	rs12452096	A	G	0.006	75544032	0.695	0.015	14306	A	G	0.750	17	77547950	1.48929E-06	0.156	360283	77865.614
order Verrucomicrobiales	Vascular dementia (sudden onset)	rs2920	C	T	-0.009	23884780	0.591	0.016	14306	C	T	0.592	1	23558289	2.20602E-06	0.125	360283	40501.384
order Verrucomicrobiales	Vascular dementia (sudden onset)	rs4840457	C	T	-0.017	6408682	0.488	0.024	14306	C	T	0.643	8	6551161	8.92874E-06	0.145	360283	32615.797
order Verrucomicrobiales	Vascular dementia (sudden onset)	rs71511414	G	A	-0.011	79688991	0.596	0.023	14306	G	A	0.681	9	77074075	7.80746E-06	0.152	360283	30731.948
order Victivallales	Vascular dementia (sudden onset)	rs12423672	T	G	-0.002	5047705	0.877	0.033	14306	T	G	0.759	12	4938539	4.89159E-06	0.166	360283	30610.567
order Victivallales	Vascular dementia (sudden onset)	rs12452096	A	G	-0.021	75544032	0.435	0.025	14306	A	G	0.750	17	77547950	1.48929E-06	0.156	360283	77865.614
order Victivallales	Vascular dementia (sudden onset)	rs2920	C	T	0.018	23884780	0.533	0.026	14306	C	T	0.592	1	23558289	2.20602E-06	0.125	360283	40501.384
order Victivallales	Vascular dementia (sudden onset)	rs429358	C	T	-0.012	45411941	0.587	0.030	14306	C	T	0.601	19	44908684	7.45813E-06	0.134	360283	42162.979
order Victivallales	Vascular dementia (sudden onset)	rs4840457	C	T	0.004	6408682	0.921	0.039	14306	C	T	0.643	8	6551161	8.92874E-06	0.145	360283	32615.797
order Victivallales	Vascular dementia (sudden onset)	rs71511414	G	A	0.000	79688991	1.000	0.037	14306	G	A	0.681	9	77074075	7.80746E-06	0.152	360283	30731.948
order Actinomycetales	Vascular dementia (undefined)	rs12224047	T	C	0.013	36820790	0.589	0.023	14306	T	C	-0.263	11	36799240	4.56278E-06	0.057	361227	8033.487
order Actinomycetales	Vascular dementia (undefined)	rs12449066	G	A	-0.013	79177293	0.700	0.028	14306	G	A	-0.262	16	79143396	2.01674E-06	0.055	361227	8803.514
order Actinomycetales	Vascular dementia (undefined)	rs193392	T	C	0.011	3085245	0.492	0.016	14306	T	C	-0.204	20	3104599	8.28324E-06	0.046	361227	7460.147
order Actinomycetales	Vascular dementia (undefined)	rs2292090	T	C	0.009	70588309	0.623	0.018	14306	T	C	0.217	4	69722591	9.99332E-06	0.049	361227	6178.734
order Actinomycetales	Vascular dementia (undefined)	rs2978951	G	A	-0.001	6823295	0.930	0.016	14306	G	A	0.248	8	6965773	2.48222E-08	0.045	361227	11061.935
order Actinomycetales	Vascular dementia (undefined)	rs359878	C	T	0.006	185438949	0.730	0.019	14306	C	T	-0.211	2	184574222	4.6443E-06	0.046	361227	6959.700
order Actinomycetales	Vascular dementia (undefined)	rs429358	C	T	0.038	45411941	0.088	0.023	14306	C	T	0.695	19	44908684	9.26616E-39	0.053	361227	58999.679
order Bacillales	Vascular dementia (undefined)	rs12224047	T	C	0.010	36820790	0.759	0.037	14306	T	C	-0.263	11	36799240	4.56278E-06	0.057	361227	8033.487
order Bacillales	Vascular dementia (undefined)	rs2292090	T	C	-0.010	70588309	0.777	0.028	14306	T	C	0.217	4	69722591	9.99332E-06	0.049	361227	6178.734
order Bacillales	Vascular dementia (undefined)	rs2972558	T	C	-0.001	45356141	0.987	0.028	14306	T	C	0.234	19	44852884	8.32415E-06	0.052	361227	7242.374
order Bacillales	Vascular dementia (undefined)	rs429358	C	T	0.061	45411941	0.072	0.036	14306	C	T	0.695	19	44908684	9.26616E-39	0.053	361227	58999.679
order Bacteroidales	Vascular dementia (undefined)	rs12224047	T	C	-0.007	36820790	0.634	0.015	14306	T	C	-0.263	11	36799240	4.56278E-06	0.057	361227	8033.487
order Bacteroidales	Vascular dementia (undefined)	rs2292090	T	C	0.007	70588309	0.626	0.012	14306	T	C	0.217	4	69722591	9.99332E-06	0.049	361227	6178.734
order Bacteroidales	Vascular dementia (undefined)	rs2972558	T	C	0.006	45356141	0.645	0.011	14306	T	C	0.234	19	44852884	8.32415E-06	0.052	361227	7242.374
order Bacteroidales	Vascular dementia (undefined)	rs429358	C	T	-0.022	45411941	0.148	0.015	14306	C	T	0.695	19	44908684	9.26616E-39	0.053	361227	58999.679
order Bacteroidales	Vascular dementia (undefined)	rs78566090	A	G	-0.017	125740204	0.481	0.024	14306	A	G	0.346	8	124727963	9.44735E-07	0.071	361227	7049.571
order Bifidobacteriales	Vascular dementia (undefined)	rs12224047	T	C	-0.001	36820790	0.919	0.017	14306	T	C	-0.263	11	36799240	4.56278E-06	0.057	361227	8033.487
order Bifidobacteriales	Vascular dementia (undefined)	rs2292090	T	C	-0.002	70588309	0.974	0.013	14306	T	C	0.217	4	69722591	9.99332E-06	0.049	361227	6178.734
order Bifidobacteriales	Vascular dementia (undefined)	rs2972558	T	C	-0.001	45356141	0.944	0.013	14306	T	C	0.234	19	44852884	8.32415E-06	0.052	361227	7242.374
order Bifidobacteriales	Vascular dementia (undefined)	rs2978951	G	A	-0.004	6823295	0.715	0.012	14306	G	A	0.248	8	6965773	2.48222E-08	0.045	361227	11061.935
order Bifidobacteriales	Vascular dementia (undefined)	rs429358	C	T	0.022	45411941	0.147	0.017	14306	C	T	0.695	19	44908684	9.26616E-39	0.053	361227	58999.679
order Bifidobacteriales	Vascular dementia (undefined)	rs6133343	G	T	-0.007	721797	0.784	0.018	14306	G	T	0.312	20	741153	3.89825E-06	0.068	361227	6210.744
order Bifidobacteriales	Vascular dementia (undefined)	rs71298638	A	G	-0.013	63232261	0.631	0.024	14306	A	G	0.379	3	63246585	1.1165E-06	0.078	361227	6515.599
order Burkholderiales	Vascular dementia (undefined)	rs12224047	T	C	-0.003	36820790	0.864	0.016	14306	T	C	-0.263	11	36799240	4.56278E-06	0.057	361227	8033.487
order Burkholderiales	Vascular dementia (undefined)	rs2978951	G	A	0.002	6823295	0.855	0.011	14306	G	A	0.248	8	6965773	2.48222E-08	0.045	361227	11061.935
order Burkholderiales	Vascular dementia (undefined)	rs359878	C	T	-0.003	185438949	0.851	0.013	14306	C	T	-0.211	2	184574222	4.6443E-06	0.046	361227	6959.700
order Burkholderiales	Vascular dementia (undefined)	rs429358	C	T	0.003	45411941	0.854	0.016	14306	C	T	0.695	19	44908684	9.26616E-39	0.053	361227	58999.679
order Burkholderiales	Vascular dementia (undefined)	rs71298638	A	G	-0.015	63232261	0.496	0.023	14306	A	G	0.379	3	63246585	1.1165E-06	0.078	361227	6515.599
order Burkholderiales	Vascular dementia (undefined)	rs78566090	A	G	-0.011	125740204	0.670	0.025	14306	A	G	0.346	8	124727963	9.44735E-07	0.071	361227	7049.571
order Clostridiales	Vascular dementia (undefined)	rs12224047	T	C	0.011	36820790	0.492	0.015	14306	T	C	-0.263	11	36799240	4.56278E-06	0.057	361227	8033.487
order Clostridiales	Vascular dementia (undefined)	rs12449066	G	A	0.014	79177293	0.562	0.018	14306	G	A	-0.262	16	79143396	2.01674E-06	0.055	361227	8803.514
order Clostridiales	Vascular dementia (undefined)	rs2292090	T	C	0.000	70588309	0.975	0.012	14306	T	C	0.217	4	69722591	9.99332E-06	0.049	361227	6178.734
order Clostridiales	Vascular dementia (undefined)	rs2978951	G	A	0.005	6823295	0.634	0.011	14306	G	A	0.248	8	6965773	2.48222E-08	0.045	361227	11061.935
order Clostridiales	Vascular dementia (undefined)	rs359878	C	T	-0.001	185438949	0.933	0.012	14306	C	T	-0.211	2	184574222	4.6443E-06	0.046	361227	6959.700
order Clostridiales	Vascular dementia (undefined)	rs429358	C	T	0.005	45411941	0.723	0.015	14306	C	T	0.695	19	44908684	9.26616E-39	0.053	361227	58999.679
order Clostridiales	Vascular dementia (undefined)	rs6133343	G	T	0.000	721797	0.814	0.017	14306	G	T	0.312	20	741153	3.89825E-06	0.068	361227	6210.744
order Clostridiales	Vascular dementia (undefined)	rs71298638	A	G	-0.008	63232261	0.696	0.022	14306	A	G	0.379	3	63246585	1.1165E-06	0.078	361227	6515.599
order Clostridiales	Vascular dementia (undefined)	rs78566090	A	G	0.012	125740204	0.691	0.024	14306	A	G	0.346	8	124727963	9.44735E-07	0.071	361227	7049.571
order Coriobacteriales	Vascular dementia (undefined)	rs12449066	G	A	0.004	79177293	0.827	0.018	14306	G	A	-0.262	16	79143396	2.01674E-06	0.055	361227	8803.514
order Coriobacteriales	Vascular dementia (undefined)	rs193392	T	C	0.003	3085245	0.795	0.011	14306	T	C	-0.204	20	3104599	8.28324E-06	0.046	361227	7460.147
order Coriobacteriales	Vascular dementia (undefined)	rs2292090	T	C	0.004	70588309	0.770	0.012	14306	T	C	0.217	4	69722591	9.99332E-06	0.049	361227	6178.734
order Coriobacteriales	Vascular dementia (undefined)	rs2972558	T	C	-0.002	45356141	0.855	0.011	14306	T	C	0.234	19	44852884	8.32415E-06	0.052	361227	7242.374
order Coriobacteriales	Vascular dementia (undefined)	rs2978951	G	A	-0.005	6823295	0.614	0.011	14306	G	A	0.248	8	6965773	2.48222E-08	0.045	361227	11061.935
order Coriobacteriales	Vascular dementia (undefined)	rs359878	C	T	-0.001	185438949	0.997	0.012	14306	C	T	-0.211	2	184574222	4.6443E-06	0.046	361227	6959.700
order Coriobacteriales	Vascular dementia (undefined)	rs429358	C	T	0.019	45411941	0.204	0.015	14306	C	T	0.695	19	44908684	9.26616E-39	0.053	361227	58999.679
order Coriobacteriales	Vascular dementia (undefined)	rs6133343	G	T	-0.013	721797	0.490	0.017	14306	G	T	0.312	20	741153	3.89825E-06	0.068	361227	6210.744
order Coriobacteriales	Vascular dementia (undefined)	rs71298638	A	G	-0.003	63232261	0.855	0.022	14306	A	G	0.379	3	63246585	1.1165E-06	0.078	361227	6515.599
order Desulfovibrionales	Vascular dementia (undefined)	rs12224047	T	C	0.014	36820790	0.375	0.017	14306	T	C	-0.263	11	36799240	4.56278E-06	0.057	361227	8033.487
order Desulfovibrionales	Vascular dementia (undefined)	rs2292090	T	C	0.009	70588309	0.392	0.013	14306	T	C	0.217	4	69722591	9.99332E-06	0.049	361227	6178.734
order Desulfovibrionales	Vascular dementia (undefined)	rs2972558	T	C	0.007	45356141	0.588	0.012	14306	T	C	0.234	19	44852884	8.32415E-06	0.052	361227	7242.374
order Desulfovibrionales	Vascular dementia (undefined)	rs2978951	G	A	0.011	6823295	0.325	0.012	14306	G	A	0.248	8	6965773	2.48222E-08	0.045	361227	11061.935
order Desulfovibrionales	Vascular dementia (undefined)	rs359878	C	T	0.007	185438949	0.582	0.013	14306	C	T	-0.211	2	184574222	4.6443E-06	0.046	361227	6959.700

order Desulfovibrionales	Vascular dementia (undefined)	rs429358	C	T	0.016	45411941	0.327	0.017	14306	C	T	0.695	19	44908684	9.26616E-39	0.053	361227	58999.679
order Desulfovibrionales	Vascular dementia (undefined)	rs6133343	G	T	0.001	721797	0.857	0.018	14306	G	T	0.312	20	741153	3.89825E-06	0.068	361227	6210.744
order Desulfovibrionales	Vascular dementia (undefined)	rs71298638	A	G	0.005	63232261	0.866	0.024	14306	A	G	0.379	3	63246585	1.1165E-06	0.078	361227	6515.599
order Desulfovibrionales	Vascular dementia (undefined)	rs78566090	A	G	0.008	125740204	0.667	0.027	14306	A	G	0.346	8	124727963	9.44735E-07	0.071	361227	7049.571
order Enterobacteriales	Vascular dementia (undefined)	rs12224047	T	C	-0.013	36820790	0.464	0.018	14306	T	C	-0.263	11	36799240	4.56278E-06	0.057	361227	8033.487
order Enterobacteriales	Vascular dementia (undefined)	rs12449066	G	A	0.014	79177293	0.413	0.021	14306	G	A	-0.262	16	79143396	2.01674E-06	0.055	361227	8803.514
order Enterobacteriales	Vascular dementia (undefined)	rs2978951	G	A	0.010	6823295	0.427	0.012	14306	G	A	0.248	8	6965773	2.48222E-08	0.045	361227	11061.935
order Enterobacteriales	Vascular dementia (undefined)	rs429358	C	T	-0.019	45411941	0.299	0.017	14306	C	T	0.695	19	44908684	9.26616E-39	0.053	361227	58999.679
order Enterobacteriales	Vascular dementia (undefined)	rs6133343	G	T	0.009	721797	0.541	0.019	14306	G	T	0.312	20	741153	3.89825E-06	0.068	361227	6210.744
order Erysipelotrichales	Vascular dementia (undefined)	rs12224047	T	C	0.003	36820790	0.897	0.015	14306	T	C	-0.263	11	36799240	4.56278E-06	0.057	361227	8033.487
order Erysipelotrichales	Vascular dementia (undefined)	rs12449066	G	A	-0.016	79177293	0.366	0.018	14306	G	A	-0.262	16	79143396	2.01674E-06	0.055	361227	8803.514
order Erysipelotrichales	Vascular dementia (undefined)	rs2292090	T	C	-0.002	70588309	0.866	0.012	14306	T	C	0.217	4	69722591	9.99332E-06	0.049	361227	6178.734
order Erysipelotrichales	Vascular dementia (undefined)	rs2978951	G	A	-0.005	6823295	0.605	0.011	14306	G	A	0.248	8	6965773	2.48222E-08	0.045	361227	11061.935
order Erysipelotrichales	Vascular dementia (undefined)	rs429358	C	T	0.032	45411941	0.035	0.015	14306	C	T	0.695	19	44908684	9.26616E-39	0.053	361227	58999.679
order Erysipelotrichales	Vascular dementia (undefined)	rs6133343	G	T	0.004	721797	0.852	0.017	14306	G	T	0.312	20	741153	3.89825E-06	0.068	361227	6210.744
order Erysipelotrichales	Vascular dementia (undefined)	rs71298638	A	G	0.012	63232261	0.604	0.022	14306	A	G	0.379	3	63246585	1.1165E-06	0.078	361227	6515.599
order Erysipelotrichales	Vascular dementia (undefined)	rs78566090	A	G	-0.013	125740204	0.561	0.024	14306	A	G	0.346	8	124727963	9.44735E-07	0.071	361227	7049.571
order Gastranaerophilales	Vascular dementia (undefined)	rs12449066	G	A	-0.016	79177293	0.594	0.032	14306	G	A	-0.262	16	79143396	2.01674E-06	0.055	361227	8803.514
order Gastranaerophilales	Vascular dementia (undefined)	rs193392	T	C	-0.009	3085245	0.642	0.019	14306	T	C	-0.204	20	3104599	8.28324E-06	0.046	361227	7460.147
order Gastranaerophilales	Vascular dementia (undefined)	rs2292090	T	C	-0.001	70588309	0.924	0.021	14306	T	C	0.217	4	69722591	9.99332E-06	0.049	361227	6178.734
order Gastranaerophilales	Vascular dementia (undefined)	rs2972558	T	C	0.017	45356141	0.431	0.021	14306	T	C	0.234	19	44852884	8.32415E-06	0.052	361227	7242.374
order Gastranaerophilales	Vascular dementia (undefined)	rs2978951	G	A	0.000	6823295	0.963	0.019	14306	G	A	0.248	8	6965773	2.48222E-08	0.045	361227	11061.935
order Gastranaerophilales	Vascular dementia (undefined)	rs359878	C	T	0.006	185438949	0.816	0.021	14306	C	T	-0.211	2	184574222	4.6443E-06	0.046	361227	6959.700
order Gastranaerophilales	Vascular dementia (undefined)	rs429358	C	T	-0.002	45411941	0.999	0.026	14306	C	T	0.695	19	44908684	9.26616E-39	0.053	361227	58999.679
order Gastranaerophilales	Vascular dementia (undefined)	rs6133343	G	T	-0.009	721797	0.719	0.030	14306	G	T	0.312	20	741153	3.89825E-06	0.068	361227	6210.744
order Gastranaerophilales	Vascular dementia (undefined)	rs71298638	A	G	-0.025	63232261	0.486	0.039	14306	A	G	0.379	3	63246585	1.1165E-06	0.078	361227	6515.599
order Gastranaerophilales	Vascular dementia (undefined)	rs78566090	A	G	-0.030	125740204	0.555	0.043	14306	A	G	0.346	8	124727963	9.44735E-07	0.071	361227	7049.571
order Lactobacillales	Vascular dementia (undefined)	rs12449066	G	A	0.013	79177293	0.510	0.019	14306	G	A	-0.262	16	79143396	2.01674E-06	0.055	361227	8803.514
order Lactobacillales	Vascular dementia (undefined)	rs193392	T	C	-0.006	3085245	0.604	0.011	14306	T	C	-0.204	20	3104599	8.28324E-06	0.046	361227	7460.147
order Lactobacillales	Vascular dementia (undefined)	rs2292090	T	C	-0.004	70588309	0.712	0.012	14306	T	C	0.217	4	69722591	9.99332E-06	0.049	361227	6178.734
order Lactobacillales	Vascular dementia (undefined)	rs2978951	G	A	0.002	6823295	0.879	0.011	14306	G	A	0.248	8	6965773	2.48222E-08	0.045	361227	11061.935
order Lactobacillales	Vascular dementia (undefined)	rs429358	C	T	0.010	45411941	0.542	0.015	14306	C	T	0.695	19	44908684	9.26616E-39	0.053	361227	58999.679
order Lactobacillales	Vascular dementia (undefined)	rs6133343	G	T	-0.003	721797	0.887	0.017	14306	G	T	0.312	20	741153	3.89825E-06	0.068	361227	6210.744
order Lactobacillales	Vascular dementia (undefined)	rs78566090	A	G	0.025	125740204	0.350	0.025	14306	A	G	0.346	8	124727963	9.44735E-07	0.071	361227	7049.571
order Methanobacteriales	Vascular dementia (undefined)	rs12224047	T	C	0.004	36820790	0.920	0.034	14306	T	C	-0.263	11	36799240	4.56278E-06	0.057	361227	8033.487
order Methanobacteriales	Vascular dementia (undefined)	rs12449066	G	A	-0.010	79177293	0.714	0.040	14306	G	A	-0.262	16	79143396	2.01674E-06	0.055	361227	8803.514
order Methanobacteriales	Vascular dementia (undefined)	rs193392	T	C	0.001	3085245	0.989	0.025	14306	T	C	-0.204	20	3104599	8.28324E-06	0.046	361227	7460.147
order Methanobacteriales	Vascular dementia (undefined)	rs2972558	T	C	-0.014	45356141	0.612	0.027	14306	T	C	0.234	19	44852884	8.32415E-06	0.052	361227	7242.374
order Methanobacteriales	Vascular dementia (undefined)	rs2978951	G	A	0.023	6823295	0.327	0.023	14306	G	A	0.248	8	6965773	2.48222E-08	0.045	361227	11061.935
order Methanobacteriales	Vascular dementia (undefined)	rs359878	C	T	0.015	185438949	0.623	0.027	14306	C	T	-0.211	2	184574222	4.6443E-06	0.046	361227	6959.700
order Methanobacteriales	Vascular dementia (undefined)	rs429358	C	T	-0.026	45411941	0.504	0.034	14306	C	T	0.695	19	44908684	9.26616E-39	0.053	361227	58999.679
order Mollicutes RF9	Vascular dementia (undefined)	rs193392	T	C	-0.005	3085245	0.730	0.014	14306	T	C	-0.204	20	3104599	8.28324E-06	0.046	361227	7460.147
order Mollicutes RF9	Vascular dementia (undefined)	rs2978951	G	A	0.005	6823295	0.715	0.014	14306	G	A	0.248	8	6965773	2.48222E-08	0.045	361227	11061.935
order Mollicutes RF9	Vascular dementia (undefined)	rs429358	C	T	0.000	45411941	0.994	0.020	14306	C	T	0.695	19	44908684	9.26616E-39	0.053	361227	58999.679
order Mollicutes RF9	Vascular dementia (undefined)	rs6133343	G	T	0.011	721797	0.687	0.022	14306	G	T	0.312	20	741153	3.89825E-06	0.068	361227	6210.744
order Mollicutes RF9	Vascular dementia (undefined)	rs78566090	A	G	-0.011	125740204	0.817	0.032	14306	A	G	0.346	8	124727963	9.44735E-07	0.071	361227	7049.571
order NB1n	Vascular dementia (undefined)	rs12224047	T	C	-0.002	36820790	0.940	0.028	14306	T	C	-0.263	11	36799240	4.56278E-06	0.057	361227	8033.487
order NB1n	Vascular dementia (undefined)	rs12449066	G	A	-0.023	79177293	0.449	0.034	14306	G	A	-0.262	16	79143396	2.01674E-06	0.055	361227	8803.514
order NB1n	Vascular dementia (undefined)	rs193392	T	C	-0.004	3085245	0.860	0.020	14306	T	C	-0.204	20	3104599	8.28324E-06	0.046	361227	7460.147
order NB1n	Vascular dementia (undefined)	rs2292090	T	C	0.002	70588309	0.927	0.022	14306	T	C	0.217	4	69722591	9.99332E-06	0.049	361227	6178.734
order NB1n	Vascular dementia (undefined)	rs2972558	T	C	0.016	45356141	0.446	0.022	14306	T	C	0.234	19	44852884	8.32415E-06	0.052	361227	7242.374
order NB1n	Vascular dementia (undefined)	rs359878	C	T	0.011	185438949	0.614	0.023	14306	C	T	-0.211	2	184574222	4.6443E-06	0.046	361227	6959.700
order NB1n	Vascular dementia (undefined)	rs429358	C	T	0.017	45411941	0.488	0.028	14306	C	T	0.695	19	44908684	9.26616E-39	0.053	361227	58999.679
order NB1n	Vascular dementia (undefined)	rs6133343	G	T	-0.010	721797	0.673	0.033	14306	G	T	0.312	20	741153	3.89825E-06	0.068	361227	6210.744
order NB1n	Vascular dementia (undefined)	rs71298638	A	G	0.004	63232261	0.954	0.041	14306	A	G	0.379	3	63246585	1.1165E-06	0.078	361227	6515.599
order Pasteurellales	Vascular dementia (undefined)	rs12449066	G	A	0.008	79177293	0.784	0.025	14306	G	A	-0.262	16	79143396	2.01674E-06	0.055	361227	8803.514
order Pasteurellales	Vascular dementia (undefined)	rs193392	T	C	-0.005	3085245	0.750	0.015	14306	T	C	-0.204	20	3104599	8.28324E-06	0.046	361227	7460.147
order Pasteurellales	Vascular dementia (undefined)	rs2292090	T	C	-0.011	70588309	0.444	0.016	14306	T	C	0.217	4	69722591	9.99332E-06	0.049	361227	6178.734
order Pasteurellales	Vascular dementia (undefined)	rs2972558	T	C	-0.011	45356141	0.513	0.016	14306	T	C	0.234	19	44852884	8.32415E-06	0.052	361227	7242.374
order Pasteurellales	Vascular dementia (undefined)	rs2978951	G	A	0.002	6823295	0.924	0.015	14306	G	A	0.248	8	6965773	2.48222E-08	0.045	361227	11061.935
order Pasteurellales	Vascular dementia (undefined)	rs359878	C	T	0.008	185438949	0.662	0.017	14306	C	T	-0.211	2	184574222	4.6443E-06	0.046	361227	6959.700
order Pasteurellales	Vascular dementia (undefined)	rs429358	C	T	0.022	45411941	0.309	0.021	14306	C	T	0.695	19	44908684	9.26616E-39	0.053	361227	58999.679
order Rhodospirillales	Vascular dementia (undefined)	rs12224047	T	C	-0.010	36820790	0.693	0.022	14306	T	C	-0.263	11	36799240	4.56278E-06	0.057	361227	8033.487
order Rhodospirillales	Vascular dementia (undefined)	rs12449066	G	A	0.023	79177293	0.325	0.026	14306	G	A	-0.262	16	79143396	2.01674E-06	0.055	361227	8803.514
order Rhodospirillales	Vascular dementia (undefined)	rs193392	T	C	-0.008	3085245	0.588	0.016	14306	T	C	-0.204	20	3104599	8.28324E-06	0.046	361227	7460.147
order Rhodospirillales	Vascular dementia (undefined)	rs2972558	T	C	0.001	45356141	0.976	0.017	14306	T	C	0.234	19	44852884	8.32415E-06	0.052	361227	7242.374
order Rhodospirillales	Vascular dementia (undefined)	rs2978951	G	A	0.002	6823295	0.876	0.015	14306	G	A	0.248	8	6965773	2.48222E-08	0.045	361227	11061.935
order Rhodospirillales	Vascular dementia (undefined)	rs429358	C	T	-0.006	45411941	0.821	0.022	14306	C	T	0.695	19	44908684	9.26616E-39	0.053	361227	58999.679
order Rhodospirillales	Vascular dementia (undefined)	rs78566090	A	G	0.006	125740204	0.832	0.035	14306	A	G	0.346	8	124727963	9.44735E-07	0.071	361227	7049.571
order Selenomonadales	Vascular dementia (undefined)	rs12224047	T	C	0.005	36820790	0.763	0.015	14306	T	C	-0.263	11	36799240	4.56278E-06	0.057	361227	8033.487
order Selenomonadales	Vascular dementia (undefined)	rs12449066	G	A	-0.003	79177293	0.788	0.018	14306	G	A	-0.262	16	79143396	2.01674E-06	0.055	361227	8803.514

order Selenomonadales	Vascular dementia (undefined)	rs193392	T	C	0.007	3085245	0.554	0.011	14306	T	C	-0.204	20	3104599	8.28324E-06	0.046	361227	7460.147
order Selenomonadales	Vascular dementia (undefined)	rs2978951	G	A	0.007	6823295	0.498	0.011	14306	G	A	0.248	8	6965773	2.48222E-08	0.045	361227	11061.935
order Selenomonadales	Vascular dementia (undefined)	rs359878	C	T	-0.010	185438949	0.446	0.012	14306	C	T	-0.211	2	184574222	4.6443E-06	0.046	361227	6959.700
order Selenomonadales	Vascular dementia (undefined)	rs429358	C	T	-0.019	45411941	0.146	0.015	14306	C	T	0.695	19	44908684	9.26616E-39	0.053	361227	58999.679
order Selenomonadales	Vascular dementia (undefined)	rs6133343	G	T	0.005	721797	0.943	0.017	14306	G	T	0.312	20	741153	3.89825E-06	0.068	361227	6210.744
order Selenomonadales	Vascular dementia (undefined)	rs71298638	A	G	0.013	63232261	0.553	0.022	14306	A	G	0.379	3	63246585	1.1165E-06	0.078	361227	6515.599
order Verrucomicrobiales	Vascular dementia (undefined)	rs12224047	T	C	-0.005	36820790	0.749	0.019	14306	T	C	-0.263	11	36799240	4.56278E-06	0.057	361227	8033.487
order Verrucomicrobiales	Vascular dementia (undefined)	rs12449066	G	A	0.001	79177293	0.960	0.022	14306	G	A	-0.262	16	79143396	2.01674E-06	0.055	361227	8803.514
order Verrucomicrobiales	Vascular dementia (undefined)	rs193392	T	C	0.003	3085245	0.817	0.013	14306	T	C	-0.204	20	3104599	8.28324E-06	0.046	361227	7460.147
order Verrucomicrobiales	Vascular dementia (undefined)	rs2292090	T	C	-0.008	70588309	0.602	0.015	14306	T	C	0.217	4	69722591	9.99332E-06	0.049	361227	6178.734
order Verrucomicrobiales	Vascular dementia (undefined)	rs2972558	T	C	0.008	45356141	0.586	0.014	14306	T	C	0.234	19	44852884	8.32415E-06	0.052	361227	7242.374
order Verrucomicrobiales	Vascular dementia (undefined)	rs429358	C	T	0.018	45411941	0.336	0.019	14306	C	T	0.695	19	44908684	9.26616E-39	0.053	361227	58999.679
order Verrucomicrobiales	Vascular dementia (undefined)	rs6133343	G	T	-0.008	721797	0.772	0.021	14306	G	T	0.312	20	741153	3.89825E-06	0.068	361227	6210.744
order Verrucomicrobiales	Vascular dementia (undefined)	rs71298638	A	G	-0.005	63232261	0.842	0.027	14306	A	G	0.379	3	63246585	1.1165E-06	0.078	361227	6515.599
order Verrucomicrobiales	Vascular dementia (undefined)	rs78566090	A	G	0.004	125740204	0.862	0.030	14306	A	G	0.346	8	124727963	9.44735E-07	0.071	361227	7049.571
order Victivallales	Vascular dementia (undefined)	rs12224047	T	C	-0.015	36820790	0.657	0.030	14306	T	C	-0.263	11	36799240	4.56278E-06	0.057	361227	8033.487
order Victivallales	Vascular dementia (undefined)	rs12449066	G	A	-0.006	79177293	0.814	0.036	14306	G	A	-0.262	16	79143396	2.01674E-06	0.055	361227	8803.514
order Victivallales	Vascular dementia (undefined)	rs193392	T	C	0.017	3085245	0.416	0.022	14306	T	C	-0.204	20	3104599	8.28324E-06	0.046	361227	7460.147
order Victivallales	Vascular dementia (undefined)	rs2292090	T	C	-0.011	70588309	0.671	0.023	14306	T	C	0.217	4	69722591	9.99332E-06	0.049	361227	6178.734
order Victivallales	Vascular dementia (undefined)	rs429358	C	T	-0.012	45411941	0.587	0.030	14306	C	T	0.695	19	44908684	9.26616E-39	0.053	361227	58999.679
order Victivallales	Vascular dementia (undefined)	rs6133343	G	T	0.026	721797	0.256	0.033	14306	G	T	0.312	20	741153	3.89825E-06	0.068	361227	6210.744
order Victivallales	Vascular dementia (undefined)	rs71298638	A	G	-0.028	63232261	0.545	0.045	14306	A	G	0.379	3	63246585	1.1165E-06	0.078	361227	6515.599
order Victivallales	Vascular dementia (undefined)	rs78566090	A	G	-0.011	125740204	0.785	0.048	14306	A	G	0.346	8	124727963	9.44735E-07	0.071	361227	7049.571
phylum Actinobacteria	Vascular dementia (multiple infarctions)	rs34288661	C	T	-0.007	20948329	0.714	0.016	14306	C	T	0.460	8	21090818	7.22903E-06	0.103	360612	11969.376
phylum Actinobacteria	Vascular dementia (multiple infarctions)	rs429358	C	T	0.013	45411941	0.351	0.015	14306	C	T	0.660	19	44908684	1.33506E-17	0.077	360612	52325.340
phylum Actinobacteria	Vascular dementia (multiple infarctions)	rs4716814	T	C	0.011	157723046	0.322	0.011	14306	T	C	-0.319	7	157930354	3.75656E-07	0.063	360612	19180.021
phylum Actinobacteria	Vascular dementia (multiple infarctions)	rs73053797	T	C	0.003	29909039	0.798	0.015	14306	T	C	0.338	3	29867548	6.00772E-06	0.075	360612	13680.576
phylum Bacteroidetes	Vascular dementia (multiple infarctions)	rs11081443	C	T	-0.007	8944208	0.611	0.013	14306	C	T	0.464	18	8944210	5.71874E-06	0.102	360612	19066.663
phylum Bacteroidetes	Vascular dementia (multiple infarctions)	rs1454336	A	G	-0.001	91873093	0.868	0.014	14306	A	G	-0.369	4	90951942	4.48415E-06	0.080	360612	13616.170
phylum Bacteroidetes	Vascular dementia (multiple infarctions)	rs34288661	C	T	-0.010	20948329	0.529	0.016	14306	C	T	0.460	8	21090818	7.22903E-06	0.103	360612	11969.376
phylum Bacteroidetes	Vascular dementia (multiple infarctions)	rs429358	C	T	-0.024	45411941	0.123	0.015	14306	C	T	0.660	19	44908684	1.33506E-17	0.077	360612	52325.340
phylum Bacteroidetes	Vascular dementia (multiple infarctions)	rs4716814	T	C	-0.002	157723046	0.847	0.011	14306	T	C	-0.319	7	157930354	3.75656E-07	0.063	360612	19180.021
phylum Bacteroidetes	Vascular dementia (multiple infarctions)	rs4725579	C	A	-0.012	139468213	0.360	0.013	14306	C	A	-0.379	7	139768414	3.58039E-06	0.082	360612	18391.668
phylum Bacteroidetes	Vascular dementia (multiple infarctions)	rs72822148	T	C	0.000	9742028	0.885	0.011	14306	T	C	-0.322	17	9838711	3.18831E-06	0.069	360612	17983.471
phylum Bacteroidetes	Vascular dementia (multiple infarctions)	rs73053797	T	C	0.011	29909039	0.430	0.015	14306	T	C	0.338	3	29867548	6.00772E-06	0.075	360612	13680.576
phylum Cyanobacteria	Vascular dementia (multiple infarctions)	rs11081443	C	T	0.007	8944208	0.767	0.020	14306	C	T	0.464	18	8944210	5.71874E-06	0.102	360612	19066.663
phylum Cyanobacteria	Vascular dementia (multiple infarctions)	rs34288661	C	T	0.010	20948329	0.607	0.024	14306	C	T	0.460	8	21090818	7.22903E-06	0.103	360612	11969.376
phylum Cyanobacteria	Vascular dementia (multiple infarctions)	rs429358	C	T	0.009	45411941	0.650	0.024	14306	C	T	0.660	19	44908684	1.33506E-17	0.077	360612	52325.340
phylum Cyanobacteria	Vascular dementia (multiple infarctions)	rs4716814	T	C	-0.002	157723046	0.883	0.017	14306	T	C	-0.319	7	157930354	3.75656E-07	0.063	360612	19180.021
phylum Cyanobacteria	Vascular dementia (multiple infarctions)	rs4725579	C	A	-0.016	139468213	0.422	0.021	14306	C	A	-0.379	7	139768414	3.58039E-06	0.082	360612	18391.668
phylum Cyanobacteria	Vascular dementia (multiple infarctions)	rs72822148	T	C	0.004	9742028	0.897	0.018	14306	T	C	-0.322	17	9838711	3.18831E-06	0.069	360612	17983.471
phylum Cyanobacteria	Vascular dementia (multiple infarctions)	rs73053797	T	C	0.004	29909039	0.984	0.024	14306	T	C	0.338	3	29867548	6.00772E-06	0.075	360612	13680.576
phylum Cyanobacteria	Vascular dementia (multiple infarctions)	rs9861644	A	G	0.002	88637331	0.893	0.019	14306	A	G	0.323	3	88588181	4.84942E-06	0.071	360612	17137.658
phylum Euryarchaeota	Vascular dementia (multiple infarctions)	rs11081443	C	T	0.017	8944208	0.518	0.028	14306	C	T	0.464	18	8944210	5.71874E-06	0.102	360612	19066.663
phylum Euryarchaeota	Vascular dementia (multiple infarctions)	rs1454336	A	G	0.010	91873093	0.747	0.030	14306	A	G	-0.369	4	90951942	4.48415E-06	0.080	360612	13616.170
phylum Euryarchaeota	Vascular dementia (multiple infarctions)	rs429358	C	T	-0.029	45411941	0.443	0.033	14306	C	T	0.660	19	44908684	1.33506E-17	0.077	360612	52325.340
phylum Euryarchaeota	Vascular dementia (multiple infarctions)	rs4725579	C	A	0.004	139468213	0.919	0.029	14306	C	A	-0.379	7	139768414	3.58039E-06	0.082	360612	18391.668
phylum Euryarchaeota	Vascular dementia (multiple infarctions)	rs72822148	T	C	0.005	9742028	0.850	0.025	14306	T	C	-0.322	17	9838711	3.18831E-06	0.069	360612	17983.471
phylum Euryarchaeota	Vascular dementia (multiple infarctions)	rs9861644	A	G	0.004	88637331	0.864	0.026	14306	A	G	0.323	3	88588181	4.84942E-06	0.071	360612	17137.658
phylum Firmicutes	Vascular dementia (multiple infarctions)	rs1454336	A	G	0.005	91873093	0.709	0.014	14306	A	G	-0.369	4	90951942	4.48415E-06	0.080	360612	13616.170
phylum Firmicutes	Vascular dementia (multiple infarctions)	rs429358	C	T	0.003	45411941	0.820	0.015	14306	C	T	0.660	19	44908684	1.33506E-17	0.077	360612	52325.340
phylum Firmicutes	Vascular dementia (multiple infarctions)	rs4716814	T	C	-0.010	157723046	0.358	0.011	14306	T	C	-0.319	7	157930354	3.75656E-07	0.063	360612	19180.021
phylum Firmicutes	Vascular dementia (multiple infarctions)	rs72822148	T	C	-0.001	9742028	0.954	0.011	14306	T	C	-0.322	17	9838711	3.18831E-06	0.069	360612	17983.471
phylum Firmicutes	Vascular dementia (multiple infarctions)	rs73053797	T	C	-0.008	29909039	0.572	0.015	14306	T	C	0.338	3	29867548	6.00772E-06	0.075	360612	13680.576
phylum Lentisphaerae	Vascular dementia (multiple infarctions)	rs1454336	A	G	-0.017	91873093	0.550	0.027	14306	A	G	-0.369	4	90951942	4.48415E-06	0.080	360612	13616.170
phylum Lentisphaerae	Vascular dementia (multiple infarctions)	rs34288661	C	T	0.003	20948329	0.882	0.030	14306	C	T	0.460	8	21090818	7.22903E-06	0.103	360612	11969.376
phylum Lentisphaerae	Vascular dementia (multiple infarctions)	rs429358	C	T	-0.012	45411941	0.594	0.030	14306	C	T	0.660	19	44908684	1.33506E-17	0.077	360612	52325.340
phylum Lentisphaerae	Vascular dementia (multiple infarctions)	rs4716814	T	C	-0.015	157723046	0.486	0.021	14306	T	C	-0.319	7	157930354	3.75656E-07	0.063	360612	19180.021
phylum Lentisphaerae	Vascular dementia (multiple infarctions)	rs73053797	T	C	-0.024	29909039	0.460	0.029	14306	T	C	0.338	3	29867548	6.00772E-06	0.075	360612	13680.576
phylum Lentisphaerae	Vascular dementia (multiple infarctions)	rs9861644	A	G	-0.021	88637331	0.377	0.024	14306	A	G	0.323	3	88588181	4.84942E-06	0.071	360612	17137.658
phylum Proteobacteria	Vascular dementia (multiple infarctions)	rs11081443	C	T	0.007	8944208	0.693	0.013	14306	C	T	0.464	18	8944210	5.71874E-06	0.102	360612	19066.663
phylum Proteobacteria	Vascular dementia (multiple infarctions)	rs1454336	A	G	-0.008	91873093	0.575	0.014	14306	A	G	-0.369	4	90951942	4.48415E-06	0.080	360612	13616.170
phylum Proteobacteria	Vascular dementia (multiple infarctions)	rs429358	C	T	0.003	45411941	0.829	0.015	14306	C	T	0.660	19	44908684	1.33506E-17	0.077	360612	52325.340
phylum Proteobacteria	Vascular dementia (multiple infarctions)	rs4716814	T	C	0.006	157723046	0.571	0.011	14306	T	C	-0.319	7	157930354	3.75656E-07	0.063	360612	19180.021
phylum Proteobacteria	Vascular dementia (multiple infarctions)	rs72822148	T	C	0.006	9742028	0.563	0.011	14306	T	C	-0.322	17	9838711	3.18831E-06	0.069	360612	17983.471
phylum Proteobacteria	Vascular dementia (multiple infarctions)	rs9861644	A	G	-0.011	88637331	0.371	0.012	14306	A	G	0.323	3	88588181	4.84942E-06	0.071	360612	17137.658
phylum Tenericutes	Vascular dementia (multiple infarctions)	rs1454336	A	G	-0.011	91873093	0.588	0.017	14306	A	G	-0.369	4	90951942	4.48415E-06	0.080	360612	13616.170
phylum Tenericutes	Vascular dementia (multiple infarctions)	rs429358	C	T	0.000	45411941	0.938	0.019	14306	C	T	0.660	19	44908684	1.33506E-17	0.077	360612	52325.340
phylum Tenericutes	Vascular dementia (multiple infarctions)	rs4716814	T	C	0.008	157723046	0.551	0.013	14306	T	C	-0.319	7	157930354	3.75656E-07	0.063	360612	19180.021
phylum Tenericutes	Vascular dementia (multiple infarctions)	rs72822148	T	C	-0.009	9742028	0.449	0.014	14306	T	C	-0.322	17	9838711	3.18831E-06	0.069	360612	17983.471
phylum Tenericutes	Vascular dementia (multiple infarctions)	rs73053797	T	C	-0.009	29909039	0.684	0.019	14306	T	C	0.338	3	29867548	6.00772E-06	0.075	360612	13680.576

phylum Tenericutes	Vascular dementia (multiple infarctions)	rs9861644	A	G	0.011	88637331	0.482	0.015	14306	A	G	0.323	3	88588181	4.84942E-06	0.071	360612	17137.658
phylum Verrucomicrobia	Vascular dementia (multiple infarctions)	rs1454336	A	G	-0.005	91873093	0.855	0.017	14306	A	G	-0.369	4	90951942	4.48415E-06	0.080	360612	13616.170
phylum Verrucomicrobia	Vascular dementia (multiple infarctions)	rs34288661	C	T	0.007	20948329	0.714	0.019	14306	C	T	0.460	8	21090818	7.22903E-06	0.103	360612	11969.376
phylum Verrucomicrobia	Vascular dementia (multiple infarctions)	rs429358	C	T	0.011	45411941	0.589	0.018	14306	C	T	0.660	19	44908684	1.33506E-17	0.077	360612	52325.340
phylum Verrucomicrobia	Vascular dementia (multiple infarctions)	rs4725579	C	A	0.006	139468213	0.809	0.016	14306	C	A	-0.379	7	139768414	3.58039E-06	0.082	360612	18391.668
phylum Verrucomicrobia	Vascular dementia (multiple infarctions)	rs72822148	T	C	-0.007	9742028	0.680	0.014	14306	T	C	-0.322	17	9838711	3.18831E-06	0.069	360612	17983.471
phylum Verrucomicrobia	Vascular dementia (multiple infarctions)	rs73053797	T	C	0.006	29909039	0.678	0.018	14306	T	C	0.338	3	29867548	6.00772E-06	0.075	360612	13680.576
phylum Verrucomicrobia	Vascular dementia (multiple infarctions)	rs9861644	A	G	-0.003	88637331	0.803	0.015	14306	A	G	0.323	3	88588181	4.84942E-06	0.071	360612	17137.658
phylum Actinobacteria	Vascular dementia (mixed)	rs12257900	T	G	-0.009	49443428	0.583	0.016	14306	T	G	0.467	10	48235385	2.48891E-06	0.099	360421	23006.877
phylum Actinobacteria	Vascular dementia (mixed)	rs1466525	T	C	0.000	54780209	0.922	0.013	14306	T	C	0.489	8	53867649	1.97697E-06	0.103	360421	34189.173
phylum Actinobacteria	Vascular dementia (mixed)	rs17168895	T	G	0.009	15647727	0.632	0.017	14306	T	G	-0.547	7	15608102	2.92853E-06	0.117	360421	34495.049
phylum Actinobacteria	Vascular dementia (mixed)	rs429358	C	T	0.013	45411941	0.351	0.015	14306	C	T	0.565	19	44908684	3.94357E-08	0.103	360421	36831.461
phylum Actinobacteria	Vascular dementia (mixed)	rs6028529	A	G	0.002	38205487	0.912	0.013	14306	A	G	0.413	20	39576844	4.98965E-06	0.091	360421	21913.090
phylum Actinobacteria	Vascular dementia (mixed)	rs6849229	G	A	0.007	131797280	0.645	0.014	14306	G	A	0.562	4	130876125	4.44038E-06	0.122	360421	34142.711
phylum Actinobacteria	Vascular dementia (mixed)	rs7614116	G	A	0.003	130368069	0.830	0.011	14306	G	A	0.379	3	130649225	6.06429E-06	0.084	360421	23465.044
phylum Actinobacteria	Vascular dementia (mixed)	rs7776624	G	A	-0.002	31909839	0.854	0.011	14306	G	A	-0.358	7	31870226	8.82531E-06	0.081	360421	23450.025
phylum Bacteroidetes	Vascular dementia (mixed)	rs12257900	T	G	-0.002	49443428	0.874	0.016	14306	T	G	0.467	10	48235385	2.48891E-06	0.099	360421	23006.877
phylum Bacteroidetes	Vascular dementia (mixed)	rs1466525	T	C	0.001	54780209	0.934	0.013	14306	T	C	0.489	8	53867649	1.97697E-06	0.103	360421	34189.173
phylum Bacteroidetes	Vascular dementia (mixed)	rs1632064	T	C	-0.008	3219694	0.593	0.015	14306	T	C	0.614	5	3219580	4.04045E-06	0.133	360421	37296.871
phylum Bacteroidetes	Vascular dementia (mixed)	rs17168895	T	G	-0.010	15647727	0.545	0.017	14306	T	G	-0.547	7	15608102	2.92853E-06	0.117	360421	34495.049
phylum Bacteroidetes	Vascular dementia (mixed)	rs6028529	A	G	0.004	38205487	0.800	0.012	14306	A	G	0.413	20	39576844	4.98965E-06	0.091	360421	21913.090
phylum Bacteroidetes	Vascular dementia (mixed)	rs6849229	G	A	-0.011	131797280	0.607	0.014	14306	G	A	0.562	4	130876125	4.44038E-06	0.122	360421	34142.711
phylum Bacteroidetes	Vascular dementia (mixed)	rs7614116	G	A	0.002	130368069	0.804	0.011	14306	G	A	0.379	3	130649225	6.06429E-06	0.084	360421	23465.044
phylum Bacteroidetes	Vascular dementia (mixed)	rs7776624	G	A	-0.007	31909839	0.521	0.010	14306	G	A	-0.358	7	31870226	8.82531E-06	0.081	360421	23450.025
phylum Cyanobacteria	Vascular dementia (mixed)	rs12257900	T	G	-0.008	49443428	0.741	0.025	14306	T	G	0.467	10	48235385	2.48891E-06	0.099	360421	23006.877
phylum Cyanobacteria	Vascular dementia (mixed)	rs1466525	T	C	0.008	54780209	0.719	0.021	14306	T	C	0.489	8	53867649	1.97697E-06	0.103	360421	34189.173
phylum Cyanobacteria	Vascular dementia (mixed)	rs17168895	T	G	0.018	15647727	0.542	0.028	14306	T	G	-0.547	7	15608102	2.92853E-06	0.117	360421	34495.049
phylum Cyanobacteria	Vascular dementia (mixed)	rs429358	C	T	0.009	45411941	0.650	0.024	14306	C	T	0.565	19	44908684	3.94357E-08	0.103	360421	36831.461
phylum Cyanobacteria	Vascular dementia (mixed)	rs6028529	A	G	0.004	38205487	0.852	0.020	14306	A	G	0.413	20	39576844	4.98965E-06	0.091	360421	21913.090
phylum Cyanobacteria	Vascular dementia (mixed)	rs6849229	G	A	0.001	131797280	0.946	0.023	14306	G	A	0.562	4	130876125	4.44038E-06	0.122	360421	34142.711
phylum Euryarchaeota	Vascular dementia (mixed)	rs12257900	T	G	-0.020	49443428	0.485	0.033	14306	T	G	0.467	10	48235385	2.48891E-06	0.099	360421	23006.877
phylum Euryarchaeota	Vascular dementia (mixed)	rs1466525	T	C	0.002	54780209	0.949	0.029	14306	T	C	0.489	8	53867649	1.97697E-06	0.103	360421	34189.173
phylum Euryarchaeota	Vascular dementia (mixed)	rs1632064	T	C	0.017	3219694	0.529	0.032	14306	T	C	0.614	5	3219580	4.04045E-06	0.133	360421	37296.871
phylum Euryarchaeota	Vascular dementia (mixed)	rs17168895	T	G	0.017	15647727	0.663	0.038	14306	T	G	-0.547	7	15608102	2.92853E-06	0.117	360421	34495.049
phylum Euryarchaeota	Vascular dementia (mixed)	rs429358	C	T	-0.029	45411941	0.443	0.033	14306	C	T	0.565	19	44908684	3.94357E-08	0.103	360421	36831.461
phylum Firmicutes	Vascular dementia (mixed)	rs12257900	T	G	0.005	49443428	0.806	0.016	14306	T	G	0.467	10	48235385	2.48891E-06	0.099	360421	23006.877
phylum Firmicutes	Vascular dementia (mixed)	rs1466525	T	C	0.004	54780209	0.773	0.013	14306	T	C	0.489	8	53867649	1.97697E-06	0.103	360421	34189.173
phylum Firmicutes	Vascular dementia (mixed)	rs1632064	T	C	-0.003	3219694	0.850	0.015	14306	T	C	0.614	5	3219580	4.04045E-06	0.133	360421	37296.871
phylum Firmicutes	Vascular dementia (mixed)	rs17168895	T	G	0.001	15647727	0.932	0.017	14306	T	G	-0.547	7	15608102	2.92853E-06	0.117	360421	34495.049
phylum Firmicutes	Vascular dementia (mixed)	rs429358	C	T	0.003	45411941	0.820	0.015	14306	C	T	0.565	19	44908684	3.94357E-08	0.103	360421	36831.461
phylum Firmicutes	Vascular dementia (mixed)	rs6028529	A	G	-0.008	38205487	0.531	0.012	14306	A	G	0.413	20	39576844	4.98965E-06	0.091	360421	21913.090
phylum Firmicutes	Vascular dementia (mixed)	rs6849229	G	A	-0.003	131797280	0.746	0.014	14306	G	A	0.562	4	130876125	4.44038E-06	0.122	360421	34142.711
phylum Firmicutes	Vascular dementia (mixed)	rs7614116	G	A	-0.005	130368069	0.651	0.011	14306	G	A	0.379	3	130649225	6.06429E-06	0.084	360421	23465.044
phylum Lentisphaerae	Vascular dementia (mixed)	rs12257900	T	G	0.016	49443428	0.611	0.030	14306	T	G	0.467	10	48235385	2.48891E-06	0.099	360421	23006.877
phylum Lentisphaerae	Vascular dementia (mixed)	rs17168895	T	G	0.000	15647727	0.957	0.034	14306	T	G	-0.547	7	15608102	2.92853E-06	0.117	360421	34495.049
phylum Lentisphaerae	Vascular dementia (mixed)	rs429358	C	T	-0.012	45411941	0.594	0.030	14306	C	T	0.565	19	44908684	3.94357E-08	0.103	360421	36831.461
phylum Lentisphaerae	Vascular dementia (mixed)	rs6028529	A	G	0.000	38205487	0.962	0.024	14306	A	G	0.413	20	39576844	4.98965E-06	0.091	360421	21913.090
phylum Lentisphaerae	Vascular dementia (mixed)	rs6849229	G	A	-0.016	131797280	0.480	0.028	14306	G	A	0.562	4	130876125	4.44038E-06	0.122	360421	34142.711
phylum Proteobacteria	Vascular dementia (mixed)	rs1632064	T	C	-0.002	3219694	0.879	0.015	14306	T	C	0.614	5	3219580	4.04045E-06	0.133	360421	37296.871
phylum Proteobacteria	Vascular dementia (mixed)	rs17168895	T	G	0.006	15647727	0.785	0.017	14306	T	G	-0.547	7	15608102	2.92853E-06	0.117	360421	34495.049
phylum Proteobacteria	Vascular dementia (mixed)	rs429358	C	T	0.003	45411941	0.829	0.015	14306	C	T	0.565	19	44908684	3.94357E-08	0.103	360421	36831.461
phylum Proteobacteria	Vascular dementia (mixed)	rs6028529	A	G	0.005	38205487	0.670	0.012	14306	A	G	0.413	20	39576844	4.98965E-06	0.091	360421	21913.090
phylum Proteobacteria	Vascular dementia (mixed)	rs6849229	G	A	0.001	131797280	0.896	0.014	14306	G	A	0.562	4	130876125	4.44038E-06	0.122	360421	34142.711
phylum Proteobacteria	Vascular dementia (mixed)	rs7776624	G	A	-0.003	31909839	0.771	0.011	14306	G	A	-0.358	7	31870226	8.82531E-06	0.081	360421	23450.025
phylum Tenericutes	Vascular dementia (mixed)	rs12257900	T	G	0.011	49443428	0.585	0.020	14306	T	G	0.467	10	48235385	2.48891E-06	0.099	360421	23006.877
phylum Tenericutes	Vascular dementia (mixed)	rs1466525	T	C	0.003	54780209	0.849	0.017	14306	T	C	0.489	8	53867649	1.97697E-06	0.103	360421	34189.173
phylum Tenericutes	Vascular dementia (mixed)	rs17168895	T	G	-0.011	15647727	0.633	0.022	14306	T	G	-0.547	7	15608102	2.92853E-06	0.117	360421	34495.049
phylum Tenericutes	Vascular dementia (mixed)	rs429358	C	T	0.000	45411941	0.938	0.019	14306	C	T	0.565	19	44908684	3.94357E-08	0.103	360421	36831.461
phylum Tenericutes	Vascular dementia (mixed)	rs6028529	A	G	-0.008	38205487	0.642	0.016	14306	A	G	0.413	20	39576844	4.98965E-06	0.091	360421	21913.090
phylum Tenericutes	Vascular dementia (mixed)	rs6849229	G	A	-0.015	131797280	0.325	0.018	14306	G	A	0.562	4	130876125	4.44038E-06	0.122	360421	34142.711
phylum Tenericutes	Vascular dementia (mixed)	rs7614116	G	A	0.002	130368069	0.932	0.014	14306	G	A	0.379	3	130649225	6.06429E-06	0.084	360421	23465.044
phylum Verrucomicrobia	Vascular dementia (mixed)	rs12257900	T	G	-0.010	49443428	0.572	0.019	14306	T	G	0.467	10	48235385	2.48891E-06	0.099	360421	23006.877
phylum Verrucomicrobia	Vascular dementia (mixed)	rs1466525	T	C	0.007	54780209	0.634	0.016	14306	T	C	0.489	8	53867649	1.97697E-06	0.103	360421	34189.173
phylum Verrucomicrobia	Vascular dementia (mixed)	rs1632064	T	C	0.006	3219694	0.668	0.018	14306	T	C	0.614	5	3219580	4.04045E-06	0.133	360421	37296.871
phylum Verrucomicrobia	Vascular dementia (mixed)	rs17168895	T	G	-0.003	15647727	0.923	0.021	14306	T	G	-0.547	7	15608102	2.92853E-06	0.117	360421	34495.049
phylum Verrucomicrobia	Vascular dementia (mixed)	rs429358	C	T	0.011	45411941	0.589	0.018	14306	C	T	0.565	19	44908684	3.94357E-08	0.103	360421	36831.461
phylum Verrucomicrobia	Vascular dementia (mixed)	rs6849229	G	A	-0.011	131797280	0.548	0.017	14306	G	A	0.562	4	130876125	4.44038E-06	0.122	360421	34142.711
phylum Verrucomicrobia	Vascular dementia (mixed)	rs7614116	G	A	-0.004	130368069	0.806	0.014	14306	G	A	0.379	3	130649225	6.06429E-06	0.084	360421	23465.044
phylum Actinobacteria	Vascular dementia (subcortical)	rs11148372	A	G	-0.008	22788665	0.469	0.011	14306	A	G	-0.261	13	22214526	4.06275E-06	0.057	360770	12632.549
phylum Actinobacteria	Vascular dementia (subcortical)	rs1363668	G	A	-0.003	143089582	0.805	0.011	14306	G	A	-0.272	5	143710017	5.25352E-06	0.060	360770	12697.214
phylum Actinobacteria	Vascular dementia (subcortical)	rs3802793	A	G	0.000	131685316	0.988	0.011	14306	A	G	0.274	11	131815422	5.46626E-06	0.060	360770	12758.334

phylum Actinobacteria	Vascular dementia (subcortical)	rs429358	C	T	0.013	45411941	0.351	0.015	14306	C	T	0.597	19	44908684	1.74221E-17	0.070	360770	41621.629
phylum Actinobacteria	Vascular dementia (subcortical)	rs4295569	C	T	0.004	47820641	0.752	0.011	14306	C	T	-0.355	7	47781043	2.54572E-08	0.064	360770	20037.409
phylum Actinobacteria	Vascular dementia (subcortical)	rs4382795	C	T	-0.010	66878853	0.477	0.014	14306	C	T	0.378	10	65119095	9.80754E-06	0.086	360770	13265.712
phylum Actinobacteria	Vascular dementia (subcortical)	rs4723291	A	G	-0.003	33551998	0.763	0.011	14306	A	G	-0.263	7	33512386	6.22372E-06	0.058	360770	11064.499
phylum Bacteroidetes	Vascular dementia (subcortical)	rs10919863	T	C	-0.008	200226041	0.509	0.013	14306	T	C	0.315	1	200256913	3.47112E-06	0.068	360770	10813.753
phylum Bacteroidetes	Vascular dementia (subcortical)	rs11148372	A	G	0.003	22788665	0.782	0.011	14306	A	G	-0.261	13	22214526	4.06275E-06	0.057	360770	12632.549
phylum Bacteroidetes	Vascular dementia (subcortical)	rs11986558	T	C	0.008	2500772	0.496	0.011	14306	T	C	0.248	8	2643276	9.16875E-06	0.056	360770	11030.353
phylum Bacteroidetes	Vascular dementia (subcortical)	rs1363668	G	A	0.003	143089582	0.748	0.011	14306	G	A	-0.272	5	143710017	5.25352E-06	0.060	360770	12697.214
phylum Bacteroidetes	Vascular dementia (subcortical)	rs3802793	A	G	0.007	131685316	0.491	0.011	14306	A	G	0.274	11	131815422	5.46626E-06	0.060	360770	12758.334
phylum Bacteroidetes	Vascular dementia (subcortical)	rs429358	C	T	-0.024	45411941	0.123	0.015	14306	C	T	0.597	19	44908684	1.74221E-17	0.070	360770	41621.629
phylum Bacteroidetes	Vascular dementia (subcortical)	rs4295569	C	T	0.006	47820641	0.605	0.011	14306	C	T	-0.355	7	47781043	2.54572E-08	0.064	360770	20037.409
phylum Cyanobacteria	Vascular dementia (subcortical)	rs10919863	T	C	0.001	200226041	0.945	0.022	14306	T	C	0.315	1	200256913	3.47112E-06	0.068	360770	10813.753
phylum Cyanobacteria	Vascular dementia (subcortical)	rs1363668	G	A	-0.010	143089582	0.573	0.017	14306	G	A	-0.272	5	143710017	5.25352E-06	0.060	360770	12697.214
phylum Cyanobacteria	Vascular dementia (subcortical)	rs429358	C	T	0.009	45411941	0.650	0.024	14306	C	T	0.597	19	44908684	1.74221E-17	0.070	360770	41621.629
phylum Euryarchaeota	Vascular dementia (subcortical)	rs10919863	T	C	0.008	200226041	0.871	0.030	14306	T	C	0.315	1	200256913	3.47112E-06	0.068	360770	10813.753
phylum Euryarchaeota	Vascular dementia (subcortical)	rs1363668	G	A	0.001	143089582	0.985	0.023	14306	G	A	-0.272	5	143710017	5.25352E-06	0.060	360770	12697.214
phylum Euryarchaeota	Vascular dementia (subcortical)	rs429358	C	T	-0.029	45411941	0.443	0.033	14306	C	T	0.597	19	44908684	1.74221E-17	0.070	360770	41621.629
phylum Euryarchaeota	Vascular dementia (subcortical)	rs4382795	C	T	-0.006	66878853	0.772	0.030	14306	C	T	0.378	10	65119095	9.80754E-06	0.086	360770	13265.712
phylum Firmicutes	Vascular dementia (subcortical)	rs10919863	T	C	-0.001	200226041	0.962	0.013	14306	T	C	0.315	1	200256913	3.47112E-06	0.068	360770	10813.753
phylum Firmicutes	Vascular dementia (subcortical)	rs11148372	A	G	0.004	22788665	0.743	0.011	14306	A	G	-0.261	13	22214526	4.06275E-06	0.057	360770	12632.549
phylum Firmicutes	Vascular dementia (subcortical)	rs11986558	T	C	0.001	2500772	0.913	0.011	14306	T	C	0.248	8	2643276	9.16875E-06	0.056	360770	11030.353
phylum Firmicutes	Vascular dementia (subcortical)	rs1363668	G	A	-0.005	143089582	0.659	0.011	14306	G	A	-0.272	5	143710017	5.25352E-06	0.060	360770	12697.214
phylum Firmicutes	Vascular dementia (subcortical)	rs3802793	A	G	-0.002	131685316	0.847	0.011	14306	A	G	0.274	11	131815422	5.46626E-06	0.060	360770	12758.334
phylum Firmicutes	Vascular dementia (subcortical)	rs429358	C	T	0.003	45411941	0.820	0.015	14306	C	T	0.597	19	44908684	1.74221E-17	0.070	360770	41621.629
phylum Firmicutes	Vascular dementia (subcortical)	rs4295569	C	T	-0.001	47820641	0.940	0.011	14306	C	T	-0.355	7	47781043	2.54572E-08	0.064	360770	20037.409
phylum Firmicutes	Vascular dementia (subcortical)	rs4382795	C	T	0.006	66878853	0.704	0.014	14306	C	T	0.378	10	65119095	9.80754E-06	0.086	360770	13265.712
phylum Lentisphaerae	Vascular dementia (subcortical)	rs10919863	T	C	-0.004	200226041	0.874	0.027	14306	T	C	0.315	1	200256913	3.47112E-06	0.068	360770	10813.753
phylum Lentisphaerae	Vascular dementia (subcortical)	rs11148372	A	G	-0.018	22788665	0.385	0.021	14306	A	G	-0.261	13	22214526	4.06275E-06	0.057	360770	12632.549
phylum Lentisphaerae	Vascular dementia (subcortical)	rs11986558	T	C	-0.012	2500772	0.567	0.022	14306	T	C	0.248	8	2643276	9.16875E-06	0.056	360770	11030.353
phylum Lentisphaerae	Vascular dementia (subcortical)	rs1363668	G	A	-0.006	143089582	0.789	0.021	14306	G	A	-0.272	5	143710017	5.25352E-06	0.060	360770	12697.214
phylum Lentisphaerae	Vascular dementia (subcortical)	rs429358	C	T	-0.012	45411941	0.594	0.030	14306	C	T	0.597	19	44908684	1.74221E-17	0.070	360770	41621.629
phylum Lentisphaerae	Vascular dementia (subcortical)	rs4295569	C	T	0.011	47820641	0.606	0.021	14306	C	T	-0.355	7	47781043	2.54572E-08	0.064	360770	20037.409
phylum Lentisphaerae	Vascular dementia (subcortical)	rs4723291	A	G	-0.004	33551998	0.853	0.021	14306	A	G	-0.263	7	33512386	6.22372E-06	0.058	360770	11064.499
phylum Proteobacteria	Vascular dementia (subcortical)	rs10919863	T	C	0.002	200226041	0.894	0.013	14306	T	C	0.315	1	200256913	3.47112E-06	0.068	360770	10813.753
phylum Proteobacteria	Vascular dementia (subcortical)	rs11148372	A	G	-0.007	22788665	0.515	0.011	14306	A	G	-0.261	13	22214526	4.06275E-06	0.057	360770	12632.549
phylum Proteobacteria	Vascular dementia (subcortical)	rs11986558	T	C	-0.004	2500772	0.701	0.011	14306	T	C	0.248	8	2643276	9.16875E-06	0.056	360770	11030.353
phylum Proteobacteria	Vascular dementia (subcortical)	rs1363668	G	A	0.001	143089582	0.915	0.011	14306	G	A	-0.272	5	143710017	5.25352E-06	0.060	360770	12697.214
phylum Proteobacteria	Vascular dementia (subcortical)	rs3802793	A	G	-0.005	131685316	0.669	0.011	14306	A	G	0.274	11	131815422	5.46626E-06	0.060	360770	12758.334
phylum Proteobacteria	Vascular dementia (subcortical)	rs429358	C	T	0.003	45411941	0.829	0.015	14306	C	T	0.597	19	44908684	1.74221E-17	0.070	360770	41621.629
phylum Proteobacteria	Vascular dementia (subcortical)	rs4382795	C	T	0.001	66878853	0.972	0.014	14306	C	T	0.378	10	65119095	9.80754E-06	0.086	360770	13265.712
phylum Proteobacteria	Vascular dementia (subcortical)	rs4723291	A	G	0.008	33551998	0.464	0.011	14306	A	G	-0.263	7	33512386	6.22372E-06	0.058	360770	11064.499
phylum Tenericutes	Vascular dementia (subcortical)	rs10919863	T	C	-0.011	200226041	0.481	0.017	14306	T	C	0.315	1	200256913	3.47112E-06	0.068	360770	10813.753
phylum Tenericutes	Vascular dementia (subcortical)	rs429358	C	T	0.000	45411941	0.938	0.019	14306	C	T	0.597	19	44908684	1.74221E-17	0.070	360770	41621.629
phylum Tenericutes	Vascular dementia (subcortical)	rs4723291	A	G	-0.003	33551998	0.809	0.013	14306	A	G	-0.263	7	33512386	6.22372E-06	0.058	360770	11064.499
phylum Verrucomicrobia	Vascular dementia (subcortical)	rs10919863	T	C	-0.007	200226041	0.702	0.017	14306	T	C	0.315	1	200256913	3.47112E-06	0.068	360770	10813.753
phylum Verrucomicrobia	Vascular dementia (subcortical)	rs11148372	A	G	0.009	22788665	0.487	0.013	14306	A	G	-0.261	13	22214526	4.06275E-06	0.057	360770	12632.549
phylum Verrucomicrobia	Vascular dementia (subcortical)	rs1363668	G	A	0.006	143089582	0.672	0.013	14306	G	A	-0.272	5	143710017	5.25352E-06	0.060	360770	12697.214
phylum Verrucomicrobia	Vascular dementia (subcortical)	rs3802793	A	G	-0.003	131685316	0.860	0.014	14306	A	G	0.274	11	131815422	5.46626E-06	0.060	360770	12758.334
phylum Verrucomicrobia	Vascular dementia (subcortical)	rs429358	C	T	0.011	45411941	0.589	0.018	14306	C	T	0.597	19	44908684	1.74221E-17	0.070	360770	41621.629
phylum Verrucomicrobia	Vascular dementia (subcortical)	rs4295569	C	T	-0.002	47820641	0.894	0.013	14306	C	T	-0.355	7	47781043	2.54572E-08	0.064	360770	20037.409
phylum Verrucomicrobia	Vascular dementia (subcortical)	rs4382795	C	T	-0.001	66878853	0.953	0.017	14306	C	T	0.378	10	65119095	9.80754E-06	0.086	360770	13265.712
phylum Verrucomicrobia	Vascular dementia (subcortical)	rs4723291	A	G	-0.003	33551998	0.817	0.013	14306	A	G	-0.263	7	33512386	6.22372E-06	0.058	360770	11064.499
phylum Actinobacteria	Vascular dementia (sudden onset)	rs2920	C	T	-0.011	23884780	0.444	0.013	14306	C	T	0.592	1	23558289	2.20602E-06	0.125	360283	40501.384
phylum Actinobacteria	Vascular dementia (sudden onset)	rs429358	C	T	0.013	45411941	0.351	0.015	14306	C	T	0.601	19	44908684	7.45813E-06	0.134	360283	42162.979
phylum Actinobacteria	Vascular dementia (sudden onset)	rs71511414	G	A	-0.012	79688991	0.611	0.019	14306	G	A	0.681	9	77074075	7.80746E-06	0.152	360283	30731.948
phylum Bacteroidetes	Vascular dementia (sudden onset)	rs12423672	T	G	-0.005	5047705	0.594	0.017	14306	T	G	0.759	12	4938539	4.89159E-06	0.166	360283	30610.567
phylum Bacteroidetes	Vascular dementia (sudden onset)	rs2920	C	T	0.008	23884780	0.586	0.013	14306	C	T	0.592	1	23558289	2.20602E-06	0.125	360283	40501.384
phylum Cyanobacteria	Vascular dementia (sudden onset)	rs12423672	T	G	-0.017	5047705	0.487	0.027	14306	T	G	0.759	12	4938539	4.89159E-06	0.166	360283	30610.567
phylum Cyanobacteria	Vascular dementia (sudden onset)	rs429358	C	T	0.009	45411941	0.650	0.024	14306	C	T	0.601	19	44908684	7.45813E-06	0.134	360283	42162.979
phylum Euryarchaeota	Vascular dementia (sudden onset)	rs12452096	A	G	-0.007	75544032	0.767	0.028	14306	A	G	0.750	17	77547950	1.48929E-06	0.156	360283	77865.614
phylum Euryarchaeota	Vascular dementia (sudden onset)	rs429358	C	T	-0.029	45411941	0.443	0.033	14306	C	T	0.601	19	44908684	7.45813E-06	0.134	360283	42162.979
phylum Firmicutes	Vascular dementia (sudden onset)	rs12423672	T	G	0.001	5047705	0.884	0.017	14306	T	G	0.759	12	4938539	4.89159E-06	0.166	360283	30610.567
phylum Firmicutes	Vascular dementia (sudden onset)	rs429358	C	T	0.003	45411941	0.820	0.015	14306	C	T	0.601	19	44908684	7.45813E-06	0.134	360283	42162.979
phylum Firmicutes	Vascular dementia (sudden onset)	rs4840457	C	T	-0.012	6408682	0.549	0.020	14306	C	T	0.643	8	6551161	8.92874E-06	0.145	360283	32615.797
phylum Firmicutes	Vascular dementia (sudden onset)	rs71511414	G	A	-0.011	79688991	0.617	0.019	14306	G	A	0.681	9	77074075	7.80746E-06	0.152	360283	30731.948
phylum Lentisphaerae	Vascular dementia (sudden onset)	rs12423672	T	G	-0.002	5047705	0.881	0.033	14306	T	G	0.759	12	4938539	4.89159E-06	0.166	360283	30610.567
phylum Lentisphaerae	Vascular dementia (sudden onset)	rs12452096	A	G	-0.021	75544032	0.435	0.025	14306	A	G	0.750	17	77547950	1.48929E-06	0.156	360283	77865.614
phylum Lentisphaerae	Vascular dementia (sudden onset)	rs2920	C	T	0.021	23884780	0.475	0.026	14306	C	T	0.592	1	23558289	2.20602E-06	0.125	360283	40501.384
phylum Lentisphaerae	Vascular dementia (sudden onset)	rs429358	C	T	-0.012	45411941	0.594	0.030	14306	C	T	0.601	19	44908684	7.45813E-06	0.134	360283	42162.979
phylum Lentisphaerae	Vascular dementia (sudden onset)	rs4840457	C	T	0.002	6408682	0.943	0.039	14306	C	T	0.643	8	6551161	8.92874E-06	0.145	360283	32615.797
phylum Lentisphaerae	Vascular dementia (sudden onset)	rs71511414	G	A	0.002	79688991	0.966	0.037	14306	G	A	0.681	9	77074075	7.80746E-06	0.152	360283	30731.948

phylum Proteobacteria	Vascular dementia (sudden onset)	rs12423672	T	G	-0.012	5047705	0.380	0.017	14306	T	G	0.759	12	4938539	4.89159E-06	0.166	360283	30610.567
phylum Proteobacteria	Vascular dementia (sudden onset)	rs12452096	A	G	-0.002	75544032	0.890	0.012	14306	A	G	0.750	17	77547950	1.48929E-06	0.156	360283	77865.614
phylum Proteobacteria	Vascular dementia (sudden onset)	rs429358	C	T	0.003	45411941	0.829	0.015	14306	C	T	0.601	19	44908684	7.45813E-06	0.134	360283	42162.979
phylum Proteobacteria	Vascular dementia (sudden onset)	rs4840457	C	T	0.004	6408682	0.918	0.020	14306	C	T	0.643	8	6551161	8.92874E-06	0.145	360283	32615.797
phylum Proteobacteria	Vascular dementia (sudden onset)	rs71511414	G	A	0.007	79688991	0.711	0.019	14306	G	A	0.681	9	77074075	7.80746E-06	0.152	360283	30731.948
phylum Tenericutes	Vascular dementia (sudden onset)	rs429358	C	T	0.000	45411941	0.938	0.019	14306	C	T	0.601	19	44908684	7.45813E-06	0.134	360283	42162.979
phylum Tenericutes	Vascular dementia (sudden onset)	rs4840457	C	T	-0.009	6408682	0.761	0.025	14306	C	T	0.643	8	6551161	8.92874E-06	0.145	360283	32615.797
phylum Tenericutes	Vascular dementia (sudden onset)	rs71511414	G	A	0.010	79688991	0.740	0.024	14306	G	A	0.681	9	77074075	7.80746E-06	0.152	360283	30731.948
phylum Verrucomicrobia	Vascular dementia (sudden onset)	rs12423672	T	G	0.016	5047705	0.324	0.021	14306	T	G	0.759	12	4938539	4.89159E-06	0.166	360283	30610.567
phylum Verrucomicrobia	Vascular dementia (sudden onset)	rs12452096	A	G	0.008	75544032	0.567	0.015	14306	A	G	0.750	17	77547950	1.48929E-06	0.156	360283	77865.614
phylum Verrucomicrobia	Vascular dementia (sudden onset)	rs2920	C	T	-0.009	23884780	0.631	0.016	14306	C	T	0.592	1	23558289	2.20602E-06	0.125	360283	40501.384
phylum Verrucomicrobia	Vascular dementia (sudden onset)	rs429358	C	T	0.011	45411941	0.589	0.018	14306	C	T	0.601	19	44908684	7.45813E-06	0.134	360283	42162.979
phylum Verrucomicrobia	Vascular dementia (sudden onset)	rs71511414	G	A	-0.002	79688991	0.916	0.023	14306	G	A	0.681	9	77074075	7.80746E-06	0.152	360283	30731.948
phylum Actinobacteria	Vascular dementia (undefined)	rs12224047	T	C	-0.012	36820790	0.426	0.015	14306	T	C	-0.263	11	36799240	4.56278E-06	0.057	361227	8033.487
phylum Actinobacteria	Vascular dementia (undefined)	rs12449066	G	A	0.010	79177293	0.553	0.018	14306	G	A	-0.262	16	79143396	2.01674E-06	0.055	361227	8803.514
phylum Actinobacteria	Vascular dementia (undefined)	rs193392	T	C	-0.009	3085245	0.406	0.011	14306	T	C	-0.204	20	3104599	8.28324E-06	0.046	361227	7460.147
phylum Actinobacteria	Vascular dementia (undefined)	rs2292090	T	C	-0.003	70588309	0.831	0.012	14306	T	C	0.217	4	69722591	9.99332E-06	0.049	361227	6178.734
phylum Actinobacteria	Vascular dementia (undefined)	rs2972558	T	C	-0.001	45356141	0.961	0.011	14306	T	C	0.234	19	44852884	8.32415E-06	0.052	361227	7242.374
phylum Actinobacteria	Vascular dementia (undefined)	rs2978951	G	A	-0.007	6823295	0.529	0.011	14306	G	A	0.248	8	6965773	2.48222E-08	0.045	361227	11061.935
phylum Actinobacteria	Vascular dementia (undefined)	rs359878	C	T	0.002	185438949	0.803	0.012	14306	C	T	-0.211	2	184574222	4.6443E-06	0.046	361227	6959.700
phylum Actinobacteria	Vascular dementia (undefined)	rs429358	C	T	0.013	45411941	0.351	0.015	14306	C	T	0.695	19	44908684	9.26616E-39	0.053	361227	58999.679
phylum Actinobacteria	Vascular dementia (undefined)	rs71298638	A	G	-0.006	63232261	0.824	0.022	14306	A	G	0.379	3	63246585	1.1165E-06	0.078	361227	6515.599
phylum Bacteroidetes	Vascular dementia (undefined)	rs12224047	T	C	-0.006	36820790	0.697	0.015	14306	T	C	-0.263	11	36799240	4.56278E-06	0.057	361227	8033.487
phylum Bacteroidetes	Vascular dementia (undefined)	rs2292090	T	C	0.007	70588309	0.628	0.012	14306	T	C	0.217	4	69722591	9.99332E-06	0.049	361227	6178.734
phylum Bacteroidetes	Vascular dementia (undefined)	rs2972558	T	C	0.006	45356141	0.648	0.011	14306	T	C	0.234	19	44852884	8.32415E-06	0.052	361227	7242.374
phylum Bacteroidetes	Vascular dementia (undefined)	rs2978951	G	A	-0.012	6823295	0.272	0.011	14306	G	A	0.248	8	6965773	2.48222E-08	0.045	361227	11061.935
phylum Bacteroidetes	Vascular dementia (undefined)	rs429358	C	T	-0.024	45411941	0.123	0.015	14306	C	T	0.695	19	44908684	9.26616E-39	0.053	361227	58999.679
phylum Bacteroidetes	Vascular dementia (undefined)	rs78566090	A	G	-0.019	125740204	0.450	0.024	14306	A	G	0.346	8	124727963	9.44735E-07	0.071	361227	7049.571
phylum Cyanobacteria	Vascular dementia (undefined)	rs12224047	T	C	0.017	36820790	0.507	0.024	14306	T	C	-0.263	11	36799240	4.56278E-06	0.057	361227	8033.487
phylum Cyanobacteria	Vascular dementia (undefined)	rs12449066	G	A	-0.015	79177293	0.644	0.029	14306	G	A	-0.262	16	79143396	2.01674E-06	0.055	361227	8803.514
phylum Cyanobacteria	Vascular dementia (undefined)	rs2292090	T	C	0.004	70588309	0.779	0.019	14306	T	C	0.217	4	69722591	9.99332E-06	0.049	361227	6178.734
phylum Cyanobacteria	Vascular dementia (undefined)	rs2972558	T	C	0.012	45356141	0.538	0.019	14306	T	C	0.234	19	44852884	8.32415E-06	0.052	361227	7242.374
phylum Cyanobacteria	Vascular dementia (undefined)	rs2978951	G	A	-0.009	6823295	0.595	0.017	14306	G	A	0.248	8	6965773	2.48222E-08	0.045	361227	11061.935
phylum Cyanobacteria	Vascular dementia (undefined)	rs359878	C	T	0.005	185438949	0.797	0.019	14306	C	T	-0.211	2	184574222	4.6443E-06	0.046	361227	6959.700
phylum Cyanobacteria	Vascular dementia (undefined)	rs429358	C	T	0.009	45411941	0.650	0.024	14306	C	T	0.695	19	44908684	9.26616E-39	0.053	361227	58999.679
phylum Cyanobacteria	Vascular dementia (undefined)	rs6133343	G	T	-0.004	721797	0.846	0.027	14306	G	T	0.312	20	741153	3.89825E-06	0.068	361227	6210.744
phylum Cyanobacteria	Vascular dementia (undefined)	rs71298638	A	G	-0.014	63232261	0.633	0.035	14306	A	G	0.379	3	63246585	1.1165E-06	0.078	361227	6515.599
phylum Cyanobacteria	Vascular dementia (undefined)	rs78566090	A	G	-0.013	125740204	0.805	0.040	14306	A	G	0.346	8	124727963	9.44735E-07	0.071	361227	7049.571
phylum Euryarchaeota	Vascular dementia (undefined)	rs12224047	T	C	-0.009	36820790	0.806	0.033	14306	T	C	-0.263	11	36799240	4.56278E-06	0.057	361227	8033.487
phylum Euryarchaeota	Vascular dementia (undefined)	rs12449066	G	A	0.024	79177293	0.595	0.039	14306	G	A	-0.262	16	79143396	2.01674E-06	0.055	361227	8803.514
phylum Euryarchaeota	Vascular dementia (undefined)	rs193392	T	C	-0.001	3085245	0.955	0.024	14306	T	C	-0.204	20	3104599	8.28324E-06	0.046	361227	7460.147
phylum Euryarchaeota	Vascular dementia (undefined)	rs2292090	T	C	-0.016	70588309	0.614	0.025	14306	T	C	0.217	4	69722591	9.99332E-06	0.049	361227	6178.734
phylum Euryarchaeota	Vascular dementia (undefined)	rs2972558	T	C	-0.015	45356141	0.573	0.026	14306	T	C	0.234	19	44852884	8.32415E-06	0.052	361227	7242.374
phylum Euryarchaeota	Vascular dementia (undefined)	rs359878	C	T	0.010	185438949	0.771	0.026	14306	C	T	-0.211	2	184574222	4.6443E-06	0.046	361227	6959.700
phylum Euryarchaeota	Vascular dementia (undefined)	rs429358	C	T	-0.029	45411941	0.443	0.033	14306	C	T	0.695	19	44908684	9.26616E-39	0.053	361227	58999.679
phylum Euryarchaeota	Vascular dementia (undefined)	rs71298638	A	G	0.046	63232261	0.362	0.049	14306	A	G	0.379	3	63246585	1.1165E-06	0.078	361227	6515.599
phylum Firmicutes	Vascular dementia (undefined)	rs12449066	G	A	0.015	79177293	0.533	0.018	14306	G	A	-0.262	16	79143396	2.01674E-06	0.055	361227	8803.514
phylum Firmicutes	Vascular dementia (undefined)	rs2292090	T	C	-0.006	70588309	0.649	0.012	14306	T	C	0.217	4	69722591	9.99332E-06	0.049	361227	6178.734
phylum Firmicutes	Vascular dementia (undefined)	rs2972558	T	C	-0.007	45356141	0.554	0.011	14306	T	C	0.234	19	44852884	8.32415E-06	0.052	361227	7242.374
phylum Firmicutes	Vascular dementia (undefined)	rs359878	C	T	0.000	185438949	0.997	0.012	14306	C	T	-0.211	2	184574222	4.6443E-06	0.046	361227	6959.700
phylum Firmicutes	Vascular dementia (undefined)	rs429358	C	T	0.003	45411941	0.820	0.015	14306	C	T	0.695	19	44908684	9.26616E-39	0.053	361227	58999.679
phylum Firmicutes	Vascular dementia (undefined)	rs6133343	G	T	-0.007	721797	0.475	0.017	14306	G	T	0.312	20	741153	3.89825E-06	0.068	361227	6210.744
phylum Firmicutes	Vascular dementia (undefined)	rs71298638	A	G	-0.018	63232261	0.401	0.022	14306	A	G	0.379	3	63246585	1.1165E-06	0.078	361227	6515.599
phylum Firmicutes	Vascular dementia (undefined)	rs78566090	A	G	0.022	125740204	0.400	0.024	14306	A	G	0.346	8	124727963	9.44735E-07	0.071	361227	7049.571
phylum Lentisphaerae	Vascular dementia (undefined)	rs12224047	T	C	-0.014	36820790	0.680	0.030	14306	T	C	-0.263	11	36799240	4.56278E-06	0.057	361227	8033.487
phylum Lentisphaerae	Vascular dementia (undefined)	rs12449066	G	A	0.002	79177293	0.971	0.036	14306	G	A	-0.262	16	79143396	2.01674E-06	0.055	361227	8803.514
phylum Lentisphaerae	Vascular dementia (undefined)	rs193392	T	C	0.016	3085245	0.447	0.022	14306	T	C	-0.204	20	3104599	8.28324E-06	0.046	361227	7460.147
phylum Lentisphaerae	Vascular dementia (undefined)	rs2292090	T	C	-0.008	70588309	0.807	0.023	14306	T	C	0.217	4	69722591	9.99332E-06	0.049	361227	6178.734
phylum Lentisphaerae	Vascular dementia (undefined)	rs429358	C	T	-0.012	45411941	0.594	0.030	14306	C	T	0.695	19	44908684	9.26616E-39	0.053	361227	58999.679
phylum Lentisphaerae	Vascular dementia (undefined)	rs6133343	G	T	0.026	721797	0.259	0.033	14306	G	T	0.312	20	741153	3.89825E-06	0.068	361227	6210.744
phylum Lentisphaerae	Vascular dementia (undefined)	rs71298638	A	G	-0.027	63232261	0.565	0.044	14306	A	G	0.379	3	63246585	1.1165E-06	0.078	361227	6515.599
phylum Lentisphaerae	Vascular dementia (undefined)	rs78566090	A	G	-0.017	125740204	0.685	0.048	14306	A	G	0.346	8	124727963	9.44735E-07	0.071	361227	7049.571
phylum Proteobacteria	Vascular dementia (undefined)	rs12224047	T	C	0.002	36820790	0.911	0.015	14306	T	C	-0.263	11	36799240	4.56278E-06	0.057	361227	8033.487
phylum Proteobacteria	Vascular dementia (undefined)	rs12449066	G	A	0.006	79177293	0.766	0.018	14306	G	A	-0.262	16	79143396	2.01674E-06	0.055	361227	8803.514
phylum Proteobacteria	Vascular dementia (undefined)	rs193392	T	C	-0.006	3085245	0.586	0.011	14306	T	C	-0.204	20	3104599	8.28324E-06	0.046	361227	7460.147
phylum Proteobacteria	Vascular dementia (undefined)	rs2978951	G	A	0.000	6823295	0.977	0.011	14306	G	A	0.248	8	6965773	2.48222E-08	0.045	361227	11061.935
phylum Proteobacteria	Vascular dementia (undefined)	rs359878	C	T	-0.010	185438949	0.398	0.012	14306	C	T	-0.211	2	184574222	4.6443E-06	0.046	361227	6959.700
phylum Proteobacteria	Vascular dementia (undefined)	rs429358	C	T	0.003	45411941	0.829	0.015	14306	C	T	0.695	19	44908684	9.26616E-39	0.053	361227	58999.679
phylum Proteobacteria	Vascular dementia (undefined)	rs6133343	G	T	-0.010	721797	0.639	0.017	14306	G	T	0.312	20	741153	3.89825E-06	0.068	361227	6210.744
phylum Proteobacteria	Vascular dementia (undefined)	rs78566090	A	G	0.016	125740204	0.417	0.024	14306	A	G	0.346	8	124727963	9.44735E-07	0.071	361227	7049.571
phylum Tenericutes	Vascular dementia (undefined)	rs12449066	G	A	0.003	79177293	0.999	0.023	14306	G	A	-0.262	16	79143396	2.01674E-06	0.055	361227	8803.514

phylum Tenericutes	Vascular dementia (undefined)	rs193392	T	C	-0.010	3085245	0.443	0.014	14306	T	C	-0.204	20	3104599	8.28324E-06	0.046	361227	7460.147
phylum Tenericutes	Vascular dementia (undefined)	rs2292090	T	C	0.013	70588309	0.389	0.015	14306	T	C	0.217	4	69722591	9.99332E-06	0.049	361227	6178.734
phylum Tenericutes	Vascular dementia (undefined)	rs2978951	G	A	0.007	6823295	0.593	0.013	14306	G	A	0.248	8	6965773	2.48222E-08	0.045	361227	11061.935
phylum Tenericutes	Vascular dementia (undefined)	rs429358	C	T	0.000	45411941	0.938	0.019	14306	C	T	0.695	19	44908684	9.26616E-39	0.053	361227	58999.679
phylum Tenericutes	Vascular dementia (undefined)	rs6133343	G	T	0.001	721797	0.977	0.021	14306	G	T	0.312	20	741153	3.89825E-06	0.068	361227	6210.744
phylum Tenericutes	Vascular dementia (undefined)	rs71298638	A	G	0.019	63232261	0.530	0.028	14306	A	G	0.379	3	63246585	1.1165E-06	0.078	361227	6515.599
phylum Tenericutes	Vascular dementia (undefined)	rs78566090	A	G	-0.019	125740204	0.595	0.031	14306	A	G	0.346	8	124727963	9.44735E-07	0.071	361227	7049.571
phylum Verrucomicrobia	Vascular dementia (undefined)	rs12224047	T	C	0.001	36820790	0.983	0.018	14306	T	C	-0.263	11	36799240	4.56278E-06	0.057	361227	8033.487
phylum Verrucomicrobia	Vascular dementia (undefined)	rs12449066	G	A	-0.004	79177293	0.893	0.022	14306	G	A	-0.262	16	79143396	2.01674E-06	0.055	361227	8803.514
phylum Verrucomicrobia	Vascular dementia (undefined)	rs193392	T	C	0.003	3085245	0.862	0.013	14306	T	C	-0.204	20	3104599	8.28324E-06	0.046	361227	7460.147
phylum Verrucomicrobia	Vascular dementia (undefined)	rs2292090	T	C	-0.004	70588309	0.778	0.014	14306	T	C	0.217	4	69722591	9.99332E-06	0.049	361227	6178.734
phylum Verrucomicrobia	Vascular dementia (undefined)	rs2972558	T	C	0.012	45356141	0.397	0.014	14306	T	C	0.234	19	44852884	8.32415E-06	0.052	361227	7242.374
phylum Verrucomicrobia	Vascular dementia (undefined)	rs429358	C	T	0.011	45411941	0.589	0.018	14306	C	T	0.695	19	44908684	9.26616E-39	0.053	361227	58999.679
phylum Verrucomicrobia	Vascular dementia (undefined)	rs6133343	G	T	-0.004	721797	0.898	0.021	14306	G	T	0.312	20	741153	3.89825E-06	0.068	361227	6210.744
phylum Verrucomicrobia	Vascular dementia (undefined)	rs71298638	A	G	0.003	63232261	0.941	0.026	14306	A	G	0.379	3	63246585	1.1165E-06	0.078	361227	6515.599
phylum Verrucomicrobia	Vascular dementia (undefined)	rs78566090	A	G	0.007	125740204	0.771	0.030	14306	A	G	0.346	8	124727963	9.44735E-07	0.071	361227	7049.571
