# Supplementary material for: Role of inflammatory cytokines and the gut microbiome in vascular dementia: insights from Mendelian randomization analysis
Source: Front Microbiol. 2024 Aug 23;15:1398618. doi: 10.3389/fmicb.2024.1398618 (PMC11380139; doi:10.3389/fmicb.2024.1398618)
Supplement: Supplementary file 1 [file Data_Sheet_1.zip › Supplementary Table S5.docx]

Supplementary Table S5. The associations between genetically determined 6 suggestive inflammatory cytokines with the risk of vascular dementia.

| Exposure | Outcome | Method | No. of SNP | MR | | | |
| --- | --- | --- | --- | --- | --- | --- | --- |
|  |  |  |  | OR | OR_Lci95 | OR_Uci95 | P value |
| Eotaxin | VaD (mixed) | IVW | 15 | 1.547 | 1.026 | 2.334 | 0.038 |
|  |  | MR Egger | 15 | 1.480 | 0.561 | 3.905 | 0.443 |
|  |  | Weighted median | 15 | 1.744 | 0.941 | 3.232 | 0.077 |
|  |  | Weighted mode | 15 | 1.629 | 0.806 | 3.294 | 0.196 |
| SCGF-β | VaD (multiple infarctions) | IVW | 14 | 1.308 | 1.028 | 1.664 | 0.029 |
|  |  | MR Egger | 14 | 1.369 | 0.881 | 2.128 | 0.188 |
|  |  | Weighted median | 14 | 1.202 | 0.877 | 1.647 | 0.253 |
|  |  | Weighted mode | 14 | 1.183 | 0.768 | 1.820 | 0.460 |
| MIF | VaD (other) | IVW | 6 | 0.420 | 0.177 | 0.992 | 0.048 |
|  |  | MR Egger | 6 | 0.595 | 0.137 | 2.582 | 0.526 |
|  |  | Weighted median | 6 | 0.493 | 0.168 | 1.446 | 0.198 |
|  |  | Weighted mode | 6 | 0.603 | 0.138 | 2.632 | 0.531 |
| GRO-α | VaD (subcortical) | IVW | 9 | 1.210 | 1.004 | 1.459 | 0.046 |
|  |  | MR Egger | 9 | 1.238 | 0.807 | 1.899 | 0.361 |
|  |  | Weighted median | 9 | 1.197 | 0.952 | 1.504 | 0.124 |
|  |  | Weighted mode | 9 | 1.190 | 0.924 | 1.532 | 0.215 |
| IL-1ra | VaD (undefined) | IVW | 6 | 1.469 | 1.082 | 1.993 | 0.014 |
|  |  | MR Egger | 6 | 1.294 | 0.578 | 2.893 | 0.565 |
|  |  | Weighted median | 6 | 1.433 | 0.986 | 2.081 | 0.059 |
|  |  | Weighted mode | 6 | 1.433 | 0.824 | 2.491 | 0.258 |
| bFGF | VaD (undefined) | IVW | 5 | 1.628 | 1.006 | 2.635 | 0.047 |
|  |  | MR Egger | 5 | 1.827 | 0.332 | 10.065 | 0.539 |
|  |  | Weighted median | 5 | 1.911 | 1.041 | 3.508 | 0.037 |
|  |  | Weighted mode | 5 | 2.180 | 0.944 | 5.035 | 0.142 |

SCGF-β=stem cell growth factor beta; MIF=macrophage migration inhibitory factor; GRO-α=growth-regulated protein alpha; IL-1ra=interleukin-1-receptor antagonist; bFGF=fibroblast growth factor basic; VaD=vascular dementia; IVW=inverse variance-weighted; MR=Mendelian randomization; OR=odds ratios; No. of SNP=number of single nucleotide polymorphisms; OR_Lci95=lower confidence interval of 95%; OR_Uci95=upper confidence interval of 95%.
