# Supplementary material for: Role of inflammatory cytokines and the gut microbiome in vascular dementia: insights from Mendelian randomization analysis
Source: Front Microbiol. 2024 Aug 23;15:1398618. doi: 10.3389/fmicb.2024.1398618 (PMC11380139; doi:10.3389/fmicb.2024.1398618)
Supplement: Supplementary file 1 [file Data_Sheet_1.zip › Supplementary Table S5.pdf]

Supplementary Table S5. The associations between genetically determined 6 suggestive inflammatory cytokines with the risk of vascular dementia.

Exposure	Outcome	Method	No. of SNP	MR			
				OR	OR_Lci95	OR_Uci95	P value
Eotaxin	VaD (mixed)	IVW	15	1.547	1.026	2.334	0.038
		MR Egger	15	1.480	0.561	3.905	0.443
		Weighted median	15	1.744	0.941	3.232	0.077
		Weighted mode	15	1.629	0.806	3.294	0.196
SCGF-β	VaD (multiple infarctions)	IVW	14	1.308	1.028	1.664	0.029
		MR Egger	14	1.369	0.881	2.128	0.188
		Weighted median	14	1.202	0.877	1.647	0.253
		Weighted mode	14	1.183	0.768	1.820	0.460
MIF	VaD (other)	IVW	6	0.420	0.177	0.992	0.048
		MR Egger	6	0.595	0.137	2.582	0.526
		Weighted median	6	0.493	0.168	1.446	0.198
		Weighted mode	6	0.603	0.138	2.632	0.531
GRO-α	VaD (subcortical)	IVW	9	1.210	1.004	1.459	0.046
		MR Egger	9	1.238	0.807	1.899	0.361
		Weighted median	9	1.197	0.952	1.504	0.124
		Weighted mode	9	1.190	0.924	1.532	0.215
IL-1ra	VaD (undefined)	IVW	6	1.469	1.082	1.993	0.014
		MR Egger	6	1.294	0.578	2.893	0.565
		Weighted median	6	1.433	0.986	2.081	0.059
		Weighted mode	6	1.433	0.824	2.491	0.258
bFGF	VaD (undefined)	IVW	5	1.628	1.006	2.635	0.047
		MR Egger	5	1.827	0.332	10.065	0.539
		Weighted median	5	1.911	1.041	3.508	0.037

Weighted mode	5	2.180	0.944	5.035	0.142

SCGF-β=stem cell growth factor beta; MIF=macrophage migration inhibitory factor; GRO-α=growth-regulated protein alpha; IL-1ra=interleukin-1-receptor antagonist; bFGF=fibroblast growth factor basic; VaD=vascular dementia; IVW=inverse variance-weighted; MR=Mendelian randomization; OR=odds ratios; No. of SNP=number of single nucleotide polymorphisms; OR_Lci95=lower confidence interval of 95%; OR_Uci95=upper confidence interval of 95%.
