# Supplementary material for: Role of inflammatory cytokines and the gut microbiome in vascular dementia: insights from Mendelian randomization analysis
Source: Front Microbiol. 2024 Aug 23;15:1398618. doi: 10.3389/fmicb.2024.1398618 (PMC11380139; doi:10.3389/fmicb.2024.1398618)
Supplement: Supplementary file 1 [file Data_Sheet_1.zip › Supplementary Table S6.docx]

Supplementary Table S6. The associations between genetically determined 21 suggestive gut microbiomes with the risk of vascular dementia.

| Exposure | Outcome | Method | No. of SNP | MR | | | |
| --- | --- | --- | --- | --- | --- | --- | --- |
|  |  |  |  | OR | OR_Lci95 | OR_Uci95 | P value |
| *Bifidobacteriaceae* | VaD (mixed) | IVW | 11 | 2.556 | 1.150 | 5.678 | 0.021 |
|  |  | MR Egger | 11 | 4.429 | 0.327 | 59.959 | 0.292 |
|  |  | Weighted median | 11 | 2.643 | 0.913 | 7.649 | 0.073 |
|  |  | Weighted mode | 11 | 3.031 | 0.773 | 11.891 | 0.143 |
| *Eubacterium coprostanoligenes group* | VaD (mixed) | IVW | 12 | 2.689 | 1.024 | 7.064 | 0.045 |
|  |  | MR Egger | 12 | 4.940 | 0.123 | 198.634 | 0.417 |
|  |  | Weighted median | 12 | 2.611 | 0.776 | 8.790 | 0.121 |
|  |  | Weighted mode | 12 | 2.500 | 0.381 | 16.395 | 0.360 |
| *Haemophilus* | VaD (mixed) | IVW | 9 | 2.206 | 1.116 | 4.362 | 0.023 |
|  |  | MR Egger | 9 | 4.850 | 1.057 | 22.258 | 0.082 |
|  |  | Weighted median | 9 | 2.216 | 0.892 | 5.503 | 0.086 |
|  |  | Weighted mode | 9 | 2.173 | 0.596 | 7.914 | 0.273 |
| *Lachnospiraceae NK4A136 group* | VaD (mixed) | IVW | 15 | 0.436 | 0.219 | 0.866 | 0.018 |
|  |  | MR Egger | 15 | 0.205 | 0.054 | 0.781 | 0.037 |
|  |  | Weighted median | 15 | 0.481 | 0.169 | 1.368 | 0.170 |
|  |  | Weighted mode | 15 | 0.486 | 0.155 | 1.525 | 0.237 |
| *Bifidobacteriales* | VaD (mixed) | IVW | 11 | 2.556 | 1.150 | 5.678 | 0.021 |
|  |  | MR Egger | 11 | 4.429 | 0.327 | 59.959 | 0.292 |
|  |  | Weighted median | 11 | 2.643 | 0.891 | 7.840 | 0.080 |
|  |  | Weighted mode | 11 | 3.031 | 0.708 | 12.979 | 0.166 |
| *Cyanobacteria* | VaD (multiple infarctions) | IVW | 8 | 1.989 | 1.130 | 3.500 | 0.017 |
|  |  | MR Egger | 8 | 1.391 | 0.170 | 11.411 | 0.769 |
|  |  | Weighted median | 8 | 2.075 | 1.006 | 4.281 | 0.048 |
|  |  | Weighted mode | 8 | 2.240 | 0.800 | 6.268 | 0.168 |
| *Pasteurellales* | VaD (multiple infarctions) | IVW | 13 | 1.577 | 1.011 | 2.460 | 0.045 |
|  |  | MR Egger | 13 | 1.713 | 0.661 | 4.441 | 0.292 |
|  |  | Weighted median | 13 | 1.559 | 0.859 | 2.829 | 0.144 |
|  |  | Weighted mode | 13 | 1.860 | 0.820 | 4.219 | 0.164 |
| *Pasteurellaceae* | VaD (multiple infarctions) | IVW | 13 | 1.577 | 1.011 | 2.460 | 0.045 |
|  |  | MR Egger | 13 | 1.713 | 0.661 | 4.441 | 0.292 |
|  |  | Weighted median | 13 | 1.559 | 0.844 | 2.878 | 0.156 |
|  |  | Weighted mode | 13 | 1.860 | 0.763 | 4.531 | 0.197 |
| *Lachnospiraceae UCG010* | VaD (multiple infarctions) | IVW | 10 | 0.439 | 0.214 | 0.901 | 0.025 |
|  |  | MR Egger | 10 | 0.167 | 0.019 | 1.475 | 0.146 |
|  |  | Weighted median | 10 | 0.447 | 0.171 | 1.167 | 0.100 |
|  |  | Weighted mode | 10 | 0.487 | 0.099 | 2.400 | 0.399 |
| *Actinobacteria* (phylum) | VaD (other) | IVW | 14 | 4.846 | 1.247 | 18.829 | 0.023 |
|  |  | MR Egger | 14 | 3.237 | 0.012 | 897.592 | 0.690 |
|  |  | Weighted median | 14 | 6.903 | 1.072 | 44.438 | 0.042 |
|  |  | Weighted mode | 14 | 18.311 | 0.949 | 353.214 | 0.076 |
| *Actinobacteria* (class) | VaD (other) | IVW | 14 | 3.968 | 1.202 | 13.103 | 0.024 |
|  |  | MR Egger | 14 | 40.088 | 1.338 | 1201.324 | 0.055 |
|  |  | Weighted median | 14 | 3.563 | 0.715 | 17.758 | 0.121 |
|  |  | Weighted mode | 14 | 4.626 | 0.492 | 43.460 | 0.203 |
| *Butyricicoccus* | VaD (other) | IVW | 8 | 0.151 | 0.031 | 0.738 | 0.020 |
|  |  | MR Egger | 8 | 0.092 | 0.004 | 2.098 | 0.185 |
|  |  | Weighted median | 8 | 0.168 | 0.022 | 1.277 | 0.085 |
|  |  | Weighted mode | 8 | 0.195 | 0.014 | 2.753 | 0.265 |
| *Veillonellaceae* | VaD (subcortical) | IVW | 18 | 0.608 | 0.394 | 0.939 | 0.025 |
|  |  | MR Egger | 18 | 0.691 | 0.290 | 1.649 | 0.418 |
|  |  | Weighted median | 18 | 0.654 | 0.354 | 1.208 | 0.175 |
|  |  | Weighted mode | 18 | 0.642 | 0.298 | 1.383 | 0.274 |
| *Prevotella9* | VaD (subcortical) | IVW | 15 | 0.621 | 0.416 | 0.926 | 0.020 |
|  |  | MR Egger | 15 | 0.565 | 0.176 | 1.810 | 0.354 |
|  |  | Weighted median | 15 | 0.597 | 0.345 | 1.033 | 0.065 |
|  |  | Weighted mode | 15 | 0.582 | 0.270 | 1.254 | 0.189 |
| *Faecalibacterium* | VaD (sudden onset) | IVW | 10 | 0.303 | 0.100 | 0.915 | 0.034 |
|  |  | MR Egger | 10 | 0.262 | 0.030 | 2.268 | 0.259 |
|  |  | Weighted median | 10 | 0.311 | 0.074 | 1.304 | 0.110 |
|  |  | Weighted mode | 10 | 0.294 | 0.058 | 1.484 | 0.173 |
| *Holdemania* | VaD (sudden onset) | IVW | 14 | 2.550 | 1.066 | 6.098 | 0.035 |
|  |  | MR Egger | 14 | 1.897 | 0.147 | 24.484 | 0.633 |
|  |  | Weighted median | 14 | 2.205 | 0.707 | 6.881 | 0.173 |
|  |  | Weighted mode | 14 | 1.352 | 0.229 | 7.999 | 0.745 |
| *Lachnospiraceae NK4A136 group* | VaD (sudden onset) | IVW | 15 | 0.275 | 0.089 | 0.845 | 0.024 |
|  |  | MR Egger | 15 | 0.793 | 0.086 | 7.337 | 0.842 |
|  |  | Weighted median | 15 | 0.624 | 0.146 | 2.664 | 0.524 |
|  |  | Weighted mode | 15 | 0.934 | 0.175 | 4.981 | 0.937 |
| *Terrisporobacter* | VaD (sudden onset) | IVW | 5 | 3.756 | 1.004 | 14.049 | 0.049 |
|  |  | MR Egger | 5 | 11.880 | 0.203 | 694.872 | 0.319 |
|  |  | Weighted median | 5 | 4.242 | 0.724 | 24.867 | 0.109 |
|  |  | Weighted mode | 5 | 7.562 | 0.667 | 85.675 | 0.178 |
| *Dorea* | VaD (undefined) | IVW | 10 | 1.750 | 1.012 | 3.028 | 0.045 |
|  |  | MR Egger | 10 | 3.524 | 0.766 | 16.217 | 0.144 |
|  |  | Weighted median | 10 | 1.788 | 0.836 | 3.825 | 0.134 |
|  |  | Weighted mode | 10 | 1.961 | 0.609 | 6.316 | 0.288 |
| *Ruminococcaceae UCG003* | VaD (undefined) | IVW | 12 | 0.630 | 0.415 | 0.957 | 0.030 |
|  |  | MR Egger | 12 | 0.230 | 0.058 | 0.904 | 0.062 |
|  |  | Weighted median | 12 | 0.591 | 0.336 | 1.041 | 0.069 |
|  |  | Weighted mode | 12 | 0.563 | 0.222 | 1.426 | 0.251 |
| *Veillonella* | VaD (undefined) | IVW | 5 | 2.174 | 1.179 | 4.008 | 0.013 |
|  |  | MR Egger | 5 | 3276.914 | 0.002 | 4351189702.834 | 0.342 |
|  |  | Weighted median | 5 | 2.203 | 0.999 | 4.862 | 0.050 |
|  |  | Weighted mode | 5 | 2.610 | 0.804 | 8.475 | 0.186 |

VaD=vascular dementia; IVW=inverse variance-weighted; MR=Mendelian randomization; OR=odds ratios; No. of SNP=number of single nucleotide polymorphisms; OR_Lci95=lower confidence interval of 95%; OR_Uci95=upper confidence interval of 95%.
