# Supplementary material for: Role of inflammatory cytokines and the gut microbiome in vascular dementia: insights from Mendelian randomization analysis
Source: Front Microbiol. 2024 Aug 23;15:1398618. doi: 10.3389/fmicb.2024.1398618 (PMC11380139; doi:10.3389/fmicb.2024.1398618)
Supplement: Supplementary file 1 [file Data_Sheet_1.zip › Supplementary Table S6.pdf]

Supplementary Table S6. The associations between genetically determined 21 suggestive gut microbiomes with the risk of vascular dementia.

Exposure	Outcome	Method	No. of SNP	MR			
				OR	OR_Lci95	OR_Uci95	P_value
Bifidobacteriaceae	VaD (mixed)	IVW	11	2.556	1.150	5.678	0.021
		MR Egger	11	4.429	0.327	59.959	0.292
		Weighted median	11	2.643	0.913	7.649	0.073
		Weighted mode	11	3.031	0.773	11.891	0.143
Eubacterium coprostanoligenes group	VaD (mixed)	IVW	12	2.689	1.024	7.064	0.045
		MR Egger	12	4.940	0.123	198.634	0.417
		Weighted median	12	2.611	0.776	8.790	0.121
		Weighted mode	12	2.500	0.381	16.395	0.360
Haemophilus	VaD (mixed)	IVW	9	2.206	1.116	4.362	0.023
		MR Egger	9	4.850	1.057	22.258	0.082
		Weighted median	9	2.216	0.892	5.503	0.086
		Weighted mode	9	2.173	0.596	7.914	0.273
Lachnospiraceae NK4A136 group	VaD (mixed)	IVW	15	0.436	0.219	0.866	0.018
		MR Egger	15	0.205	0.054	0.781	0.037
		Weighted median	15	0.481	0.169	1.368	0.170
		Weighted mode	15	0.486	0.155	1.525	0.237
Bifidobacteriales	VaD (mixed)	IVW	11	2.556	1.150	5.678	0.021
		MR Egger	11	4.429	0.327	59.959	0.292
		Weighted median	11	2.643	0.891	7.840	0.080
		Weighted mode	11	3.031	0.708	12.979	0.166
Cyanobacteria	VaD (multiple infarctions)	IVW	8	1.989	1.130	3.500	0.017
		MR Egger	8	1.391	0.170	11.411	0.769
		Weighted median	8	2.075	1.006	4.281	0.048

		Weighted mode	8	2.240	0.800	6.268	0.168
Pasteurellales	VaD (multiple infarctions)	IVW	13	1.577	1.011	2.460	0.045
		MR Egger	13	1.713	0.661	4.441	0.292
		Weighted median	13	1.559	0.859	2.829	0.144
		Weighted mode	13	1.860	0.820	4.219	0.164
Pasteurellaceae	VaD (multiple infarctions)	IVW	13	1.577	1.011	2.460	0.045
		MR Egger	13	1.713	0.661	4.441	0.292
		Weighted median	13	1.559	0.844	2.878	0.156
		Weighted mode	13	1.860	0.763	4.531	0.197
Lachnospiraceae UCG010	VaD (multiple infarctions)	IVW	10	0.439	0.214	0.901	0.025
		MR Egger	10	0.167	0.019	1.475	0.146
		Weighted median	10	0.447	0.171	1.167	0.100
		Weighted mode	10	0.487	0.099	2.400	0.399
Actinobacteria (phylum)	VaD (other)	IVW	14	4.846	1.247	18.829	0.023
		MR Egger	14	3.237	0.012	897.592	0.690
		Weighted median	14	6.903	1.072	44.438	0.042
		Weighted mode	14	18.311	0.949	353.214	0.076
Actinobacteria (class)	VaD (other)	IVW	14	3.968	1.202	13.103	0.024
		MR Egger	14	40.088	1.338	1201.324	0.055
		Weighted median	14	3.563	0.715	17.758	0.121
		Weighted mode	14	4.626	0.492	43.460	0.203
Butyricicoccus	VaD (other)	IVW	8	0.151	0.031	0.738	0.020
		MR Egger	8	0.092	0.004	2.098	0.185
		Weighted median	8	0.168	0.022	1.277	0.085
		Weighted mode	8	0.195	0.014	2.753	0.265
Veillonellaceae	VaD (subcortical)	IVW	18	0.608	0.394	0.939	0.025

		MR Egger	18	0.691	0.290	1.649	0.418
		Weighted median	18	0.654	0.354	1.208	0.175
		Weighted mode	18	0.642	0.298	1.383	0.274
Prevotella9	VaD (subcortical)	IVW	15	0.621	0.416	0.926	0.020
		MR Egger	15	0.565	0.176	1.810	0.354
		Weighted median	15	0.597	0.345	1.033	0.065
		Weighted mode	15	0.582	0.270	1.254	0.189
Faecalibacterium	VaD (sudden onset)	IVW	10	0.303	0.100	0.915	0.034
		MR Egger	10	0.262	0.030	2.268	0.259
		Weighted median	10	0.311	0.074	1.304	0.110
		Weighted mode	10	0.294	0.058	1.484	0.173
Holdemania	VaD (sudden onset)	IVW	14	2.550	1.066	6.098	0.035
		MR Egger	14	1.897	0.147	24.484	0.633
		Weighted median	14	2.205	0.707	6.881	0.173
		Weighted mode	14	1.352	0.229	7.999	0.745
Lachnospiraceae NK4A136 group	VaD (sudden onset)	IVW	15	0.275	0.089	0.845	0.024
		MR Egger	15	0.793	0.086	7.337	0.842
		Weighted median	15	0.624	0.146	2.664	0.524
		Weighted mode	15	0.934	0.175	4.981	0.937
Terrisporobacter	VaD (sudden onset)	IVW	5	3.756	1.004	14.049	0.049
		MR Egger	5	11.880	0.203	694.872	0.319
		Weighted median	5	4.242	0.724	24.867	0.109
		Weighted mode	5	7.562	0.667	85.675	0.178
Dorea	VaD (undefined)	IVW	10	1.750	1.012	3.028	0.045
		MR Egger	10	3.524	0.766	16.217	0.144
		Weighted median	10	1.788	0.836	3.825	0.134

		Weighted mode	10	1.961	0.609	6.316	0.288
Ruminococcaceae UCG003	VaD (undefined)	IVW	12	0.630	0.415	0.957	0.030
		MR Egger	12	0.230	0.058	0.904	0.062
		Weighted median	12	0.591	0.336	1.041	0.069
		Weighted mode	12	0.563	0.222	1.426	0.251
Veillonella	VaD (undefined)	IVW	5	2.174	1.179	4.008	0.013
		MR Egger	5	3276.914	0.002	4351189702.834	0.342
		Weighted median	5	2.203	0.999	4.862	0.050
		Weighted mode	5	2.610	0.804	8.475	0.186

VaD=vascular dementia; IVW = inverse variance-weighted; MR = Mendelian randomization; OR=odds ratios; No. of SNP = number of single nucleotide polymorphisms; OR_Lci95=lower confidence interval of 95%; OR_Uci95=upper confidence interval of 95%.
