# Supplementary material for: Role of inflammatory cytokines and the gut microbiome in vascular dementia: insights from Mendelian randomization analysis
Source: Front Microbiol. 2024 Aug 23;15:1398618. doi: 10.3389/fmicb.2024.1398618 (PMC11380139; doi:10.3389/fmicb.2024.1398618)
Supplement: Supplementary file 1 [file Data_Sheet_1.zip › Supplementary Table S7.docx]

Supplementary Table S7. Sensitivity analysis for the association between 6 suggestive inflammatory cytokines and vascular dementia.

| Exposure | Outcome | Pleiotropy | | | | | | |  | Heterogeneity | |
| --- | --- | --- | --- | --- | --- | --- | --- | --- | --- | --- | --- |
|  |  | Egger intercept |  | intercept's se |  | Egger  P value |  | MR-presso  Global P value |  | Cochran's Q | Cochran's Q  P value |
| Eotaxin | VaD (mixed) | 0.007 |  | 0.072 |  | 0.922 |  | 0.520 |  | 13.630 | 0.478 |
| SCGF-β | VaD (multiple infarctions) | -0.012 |  | 0.050 |  | 0.811 |  | 0.917 |  | 6.830 | 0.911 |
| MIF | VaD (other) | -0.114 |  | 0.198 |  | 0.595 |  | 0.924 |  | 1.518 | 0.911 |
| GRO-α | VaD (subcortical) | -0.008 |  | 0.069 |  | 0.911 |  | 0.956 |  | 2.551 | 0.959 |
| IL-1ra | VaD (undefined) | 0.024 |  | 0.071 |  | 0.755 |  | 0.724 |  | 3.057 | 0.691 |
| bFGF | VaD (undefined) | -0.013 |  | 0.093 |  | 0.899 |  | 0.713 |  | 2.236 | 0.692 |

SCGF-β=stem cell growth factor beta; MIF=macrophage migration inhibitory factor; GRO-α=growth-regulated protein alpha; IL-1ra=interleukin-1-receptor antagonist; bFGF=fibroblast growth factor basic; MR=Mendelian randomization; VaD=vascular dementia.
