# Supplementary material for: Role of inflammatory cytokines and the gut microbiome in vascular dementia: insights from Mendelian randomization analysis
Source: Front Microbiol. 2024 Aug 23;15:1398618. doi: 10.3389/fmicb.2024.1398618 (PMC11380139; doi:10.3389/fmicb.2024.1398618)
Supplement: Supplementary file 1 [file Data_Sheet_1.zip › Supplementary Table S7.pdf]

Supplementary Table S7. Sensitivity analysis for the association between 6 suggestive inflammatory cytokines and vascular dementia.

Exposure	Outcome	Pleiotropy				Heterogeneity	
		Egger intercept	intercept's se	Egger P value	MR-presso Global P value	Cochran's Q	Cochran's Q P value
Eotaxin	VaD (mixed)	0.007	0.072	0.922	0.520	13.630	0.478
SCGF-β	VaD (multiple infarctions)	-0.012	0.050	0.811	0.917	6.830	0.911
MIF	VaD (other)	-0.114	0.198	0.595	0.924	1.518	0.911
GRO-α	VaD (subcortical)	-0.008	0.069	0.911	0.956	2.551	0.959
IL-1ra	VaD (undefined)	0.024	0.071	0.755	0.724	3.057	0.691
bFGF	VaD (undefined)	-0.013	0.093	0.899	0.713	2.236	0.692

SCGF-β=stem cell growth factor beta; MIF=macrophage migration inhibitory factor; GRO-α=growth-regulated protein alpha; IL-1ra=interleukin-1-receptor antagonist; bFGF=fibroblast growth factor basic; MR=Mendelian randomization; VaD=vascular dementia.
