# Supplementary material for: Role of inflammatory cytokines and the gut microbiome in vascular dementia: insights from Mendelian randomization analysis
Source: Front Microbiol. 2024 Aug 23;15:1398618. doi: 10.3389/fmicb.2024.1398618 (PMC11380139; doi:10.3389/fmicb.2024.1398618)
Supplement: Supplementary file 1 [file Data_Sheet_1.zip › Supplementary Table S8.docx]

Supplementary Table S8. Sensitivity analysis for the association between 21 suggestive gut microbiomes and vascular dementia.

| Exposure | Outcome | Pleiotropy | | | | | | |  | Heterogeneity | |
| --- | --- | --- | --- | --- | --- | --- | --- | --- | --- | --- | --- |
|  |  | Egger intercept |  | intercept's se |  | Egger  P value |  | MR-presso  Global P value |  | Cochran's Q | Cochran's Q  P value |
| *Bifidobacteriaceae* | VaD (mixed) | -0.042 |  | 0.096 |  | 0.674 |  | 0.969 |  | 3.562 | 0.965 |
| *Eubacterium coprostanoligenes group* | VaD (mixed) | -0.039 |  | 0.116 |  | 0.745 |  | 0.999 |  | 2.314 | 0.997 |
| *Haemophilus* | VaD (mixed) | -0.103 |  | 0.091 |  | 0.294 |  | 0.768 |  | 4.913 | 0.767 |
| *Lachnospiraceae NK4A136 group* | VaD (mixed) | 0.07 |  | 0.05 |  | 0.22 |  | 0.582 |  | 12.632 | 0.556 |
| *Bifidobacteriales* | VaD (mixed) | -0.042 |  | 0.096 |  | 0.674 |  | 0.970 |  | 3.562 | 0.965 |
| *Cyanobacteria* | VaD (multiple infarctions) | 0.043 |  | 0.125 |  | 0.740 |  | 0.513 |  | 6.701 | 0.461 |
| *Pasteurellales* | VaD (multiple infarctions) | -0.011 |  | 0.057 |  | 0.851 |  | 0.845 |  | 7.454 | 0.826 |
| *Pasteurellaceae* | VaD (multiple infarctions) | -0.011 |  | 0.057 |  | 0.851 |  | 0.845 |  | 7.454 | 0.826 |
| *Lachnospiraceae UCG010* | VaD (multiple infarctions) | 0.079 |  | 0.085 |  | 0.384 |  | 0.743 |  | 5.899 | 0.750 |
| *Actinobacteria* (phylum) | VaD (other) | 0.025 |  | 0.175 |  | 0.887 |  | 0.678 |  | 10.525 | 0.651 |
| *Actinobacteria* (class) | VaD (other) | -0.177 |  | 0.124 |  | 0.180 |  | 0.183 |  | 10.264 | 0.672 |
| *Butyricicoccus* | VaD (other) | 0.049 |  | 0.136 |  | 0.730 |  | 0.232 |  | 1.693 | 0.975 |
| *Veillonellaceae* | VaD (subcortical) | -0.012 |  | 0.035 |  | 0.742 |  | 0.987 |  | 6.881 | 0.985 |
| *Prevotella9* | VaD (subcortical) | 0.010 |  | 0.058 |  | 0.868 |  | 0.710 |  | 11.028 | 0.684 |
| *Faecalibacterium* | VaD (sudden onset) | 0.017 |  | 0.114 |  | 0.883 |  | 0.984 |  | 2.821 | 0.971 |
| *Holdemania* | VaD (sudden onset) | 0.030 |  | 0.126 |  | 0.813 |  | 0.977 |  | 5.097 | 0.973 |
| *Lachnospiraceae NK4A136 group* | VaD (sudden onset) | -0.091 |  | 0.084 |  | 0.300 |  | 0.166 |  | 18.668 | 0.178 |
| *Terrisporobacter* | VaD (sudden onset) | -0.120 |  | 0.204 |  | 0.599 |  | 0.663 |  | 2.637 | 0.620 |
| *Dorea* | VaD (undefined) | -0.049 |  | 0.051 |  | 0.364 |  | 0.651 |  | 6.508 | 0.688 |
| *Ruminococcaceae UCG003* | VaD (undefined) | 0.078 |  | 0.052 |  | 0.160 |  | 0.595 |  | 9.439 | 0.581 |
| *Veillonella* | VaD (undefined) | -0.534 |  | 0.524 |  | 0.384 |  | 0.647 |  | 4.796 | 0.309 |

MR=Mendelian randomization; VaD=vascular dementia.
