# Supplementary material for: Role of inflammatory cytokines and the gut microbiome in vascular dementia: insights from Mendelian randomization analysis
Source: Front Microbiol. 2024 Aug 23;15:1398618. doi: 10.3389/fmicb.2024.1398618 (PMC11380139; doi:10.3389/fmicb.2024.1398618)
Supplement: Supplementary file 1 [file Data_Sheet_1.zip › Supplementary Table S8.pdf]

Supplementary Table S8. Sensitivity analysis for the association between 21 suggestive gut microbiomes and vascular dementia.

Exposure	Outcome	Pleiotropy				Heterogeneity	
		Egger intercept	intercept's se	Egger P_ value	MR-presso Global P value	Cochran's Q	Cochran's Q P_value
Bifidobacteriaceae	VaD (mixed)	-0.042	0.096	0.674	0.969	3.562	0.965
Eubacterium coprostanoligenes group	VaD (mixed)	-0.039	0.116	0.745	0.999	2.314	0.997
Haemophilus	VaD (mixed)	-0.103	0.091	0.294	0.768	4.913	0.767
Lachnospiraceae NK4A136 group	VaD (mixed)	0.07	0.05	0.22	0.582	12.632	0.556
Bifidobacteriales	VaD (mixed)	-0.042	0.096	0.674	0.970	3.562	0.965
Cyanobacteria	VaD (multiple infarctions)	0.043	0.125	0.740	0.513	6.701	0.461
Pasteurellales	VaD (multiple infarctions)	-0.011	0.057	0.851	0.845	7.454	0.826
Pasteurellaceae	VaD (multiple infarctions)	-0.011	0.057	0.851	0.845	7.454	0.826
Lachnospiraceae UCG010	VaD (multiple infarctions)	0.079	0.085	0.384	0.743	5.899	0.750
Actinobacteria (phylum)	VaD (other)	0.025	0.175	0.887	0.678	10.525	0.651
Actinobacteria (class)	VaD (other)	-0.177	0.124	0.180	0.183	10.264	0.672
Butyricicoccus	VaD (other)	0.049	0.136	0.730	0.232	1.693	0.975
Veillonellaceae	VaD (subcortical)	-0.012	0.035	0.742	0.987	6.881	0.985
Prevotella9	VaD (subcortical)	0.010	0.058	0.868	0.710	11.028	0.684
Faecalibacterium	VaD (sudden onset)	0.017	0.114	0.883	0.984	2.821	0.971
Holdemania	VaD (sudden onset)	0.030	0.126	0.813	0.977	5.097	0.973

Lachnospiraceae NK4A136 group	VaD (sudden onset)	-0.091	0.084	0.300	0.166	18.668	0.178
Terrisporobacter	VaD (sudden onset)	-0.120	0.204	0.599	0.663	2.637	0.620
Dorea	VaD (undefined)	-0.049	0.051	0.364	0.651	6.508	0.688
Ruminococcaceae UCG003	VaD (undefined)	0.078	0.052	0.160	0.595	9.439	0.581
Veillonella	VaD (undefined)	-0.534	0.524	0.384	0.647	4.796	0.309

MR=Mendelian randomization; VaD=vascular dementia.
