# Supplementary material for: Role of inflammatory cytokines and the gut microbiome in vascular dementia: insights from Mendelian randomization analysis
Source: Front Microbiol. 2024 Aug 23;15:1398618. doi: 10.3389/fmicb.2024.1398618 (PMC11380139; doi:10.3389/fmicb.2024.1398618)
Supplement: Supplementary file 1 [file Data_Sheet_1.zip › Supplementary Table S9.docx]

Supplementary Table S9. Sensitivity analysis of multivariate Mendelian randomization analysis after adjustment for confounders.

| Exposure | Adjustment | Heterogeneity | |  | Pleiotropy | | |
| --- | --- | --- | --- | --- | --- | --- | --- |
|  |  | Cochran's Q | Cochran's Q  P value |  | Egger intercept | intercept's se | Egger  P value |
| MIF | Body mass index | 10.123 | 0.519 |  | 0.114 | 0.065 | 0.081 |
|  | Alcohol consumption | 47.320 | 0.098 |  | -0.045 | 0.033 | 0.179 |
|  | Smoking/smokers in household | 5.640 | 0.999 |  | -0.021 | 0.043 | 0.619 |
|  | Hyperlipidemia | 3.283 | 0.858 |  | -0.039 | 0.054 | 0.472 |
| Interleukin-18 | Body mass index | 8.028 | 0.948 |  | -0.014 | 0.032 | 0.661 |
|  | Alcohol consumption | 45.216 | 0.380 |  | -0.033 | 0.016 | 0.032 |
|  | Smoking/smokers in household | 20.806 | 0.795 |  | -0.018 | 0.023 | 0.453 |
|  | Hyperlipidemia | 6.349 | 0.933 |  | -0.027 | 0.027 | 0.325 |
| Interleukin-4 | Body mass index | 19.795 | 0.071 |  | -0.113 | 0.106 | 0.287 |
|  | Alcohol consumption | 29.549 | 0.887 |  | -0.025 | 0.031 | 0.432 |
|  | Smoking/smokers in household | 34.963 | 0.039 |  | 0.036 | 0.062 | 0.565 |
|  | Hyperlipidemia | 8.946 | 0.537 |  | 0.047 | 0.065 | 0.472 |
| *Negativicutes* | Body mass index | 17.551 | 0.781 |  | -0.018 | 0.039 | 0.639 |
|  | Alcohol consumption | 56.788 | 0.208 |  | -0.012 | 0.019 | 0.530 |
|  | Smoking/smokers in household | 30.262 | 0.776 |  | -0.002 | 0.025 | 0.933 |
|  | Hyperlipidemia | 19.257 | 0.629 |  | 0.040 | 0.027 | 0.143 |
| *Selenomonadales* | Body mass index | 17.551 | 0.781 |  | -0.018 | 0.039 | 0.639 |
|  | Alcohol consumption | 56.788 | 0.208 |  | -0.012 | 0.019 | 0.530 |
|  | Smoking/smokers in household | 30.262 | 0.776 |  | -0.002 | 0.025 | 0.933 |
|  | Hyperlipidemia | 19.257 | 0.629 |  | 0.040 | 0.027 | 0.143 |
| *Melainabacteria* | Body mass index | 13.761 | 0.842 |  | -0.003 | 0.027 | 0.923 |
|  | Alcohol consumption | 52.667 | 0.264 |  | 0.017 | 0.014 | 0.246 |
|  | Smoking/smokers in household | 30.665 | 0.534 |  | -0.017 | 0.020 | 0.408 |
|  | Hyperlipidemia | 11.002 | 0.946 |  | -0.002 | 0.021 | 0.939 |
| *Bacillales* | Body mass index | 9.236 | 0.903 |  | -0.003 | 0.021 | 0.876 |
|  | Alcohol consumption | 41.936 | 0.387 |  | 0.009 | 0.010 | 0.350 |
|  | Smoking/smokers in household | 29.003 | 0.361 |  | -0.015 | 0.019 | 0.409 |
|  | Hyperlipidemia | 13.940 | 0.733 |  | -0.019 | 0.015 | 0.195 |
| *Ruminiclostridium6* | Body mass index | 17.166 | 0.876 |  | -0.038 | 0.019 | 0.047 |
|  | Alcohol consumption | 45.627 | 0.611 |  | -0.009 | 0.010 | 0.341 |
|  | Smoking/smokers in household | 40.727 | 0.394 |  | -0.004 | 0.013 | 0.781 |
|  | Hyperlipidemia | 14.845 | 0.732 |  | -0.008 | 0.014 | 0.554 |
