# Supplementary material for: Role of inflammatory cytokines and the gut microbiome in vascular dementia: insights from Mendelian randomization analysis
Source: Front Microbiol. 2024 Aug 23;15:1398618. doi: 10.3389/fmicb.2024.1398618 (PMC11380139; doi:10.3389/fmicb.2024.1398618)
Supplement: Supplementary file 1 [file Data_Sheet_1.zip › Supplementary Table S9.pdf]

Supplementary Table S9. Sensitivity analysis of multivariate ~~mendelian~~ Mendelian randomization analysis after adjustment for confounders.

Exposure	Adjustment	Heterogeneity		Pleiotropy		
		Cochran's Q	Cochran's Q P value	Egger intercept	intercept's se	Egger P value
MIF	Body mass index	10.123	0.519	0.114	0.065	0.081
	Alcohol consumption	47.320	0.098	-0.045	0.033	0.179
	Smoking/smokers in household	5.640	0.999	-0.021	0.043	0.619
	Hyperlipidemia	3.283	0.858	-0.039	0.054	0.472
Interleukin-18	Body mass index	8.028	0.948	-0.014	0.032	0.661
	Alcohol consumption	45.216	0.380	-0.033	0.016	0.032
	Smoking/smokers in household	20.806	0.795	-0.018	0.023	0.453
	Hyperlipidemia	6.349	0.933	-0.027	0.027	0.325
Interleukin-4	Body mass index	19.795	0.071	-0.113	0.106	0.287
	Alcohol consumption	29.549	0.887	-0.025	0.031	0.432
	Smoking/smokers in household	34.963	0.039	0.036	0.062	0.565
	Hyperlipidemia	8.946	0.537	0.047	0.065	0.472
Negativicutes	Body mass index	17.551	0.781	-0.018	0.039	0.639
	Alcohol consumption	56.788	0.208	-0.012	0.019	0.530
	Smoking/smokers in household	30.262	0.776	-0.002	0.025	0.933
	Hyperlipidemia	19.257	0.629	0.040	0.027	0.143
Selenomonadales	Body mass index	17.551	0.781	-0.018	0.039	0.639
	Alcohol consumption	56.788	0.208	-0.012	0.019	0.530
	Smoking/smokers in household	30.262	0.776	-0.002	0.025	0.933
	Hyperlipidemia	19.257	0.629	0.040	0.027	0.143
Melainabacteria	Body mass index	13.761	0.842	-0.003	0.027	0.923
	Alcohol consumption	52.667	0.264	0.017	0.014	0.246

	Smoking/smokers in household	30.665	0.534	-0.017	0.020	0.408
	Hyperlipidemia	11.002	0.946	-0.002	0.021	0.939
Bacillales	Body mass index	9.236	0.903	-0.003	0.021	0.876
	Alcohol consumption	41.936	0.387	0.009	0.010	0.350
	Smoking/smokers in household	29.003	0.361	-0.015	0.019	0.409
	Hyperlipidemia	13.940	0.733	-0.019	0.015	0.195
Ruminiclostridium6	Body mass index	17.166	0.876	-0.038	0.019	0.047
	Alcohol consumption	45.627	0.611	-0.009	0.010	0.341
	Smoking/smokers in household	40.727	0.394	-0.004	0.013	0.781
	Hyperlipidemia	14.845	0.732	-0.008	0.014	0.554
