# Supplementary figures and images for: Role of inflammatory cytokines and the gut microbiome in vascular dementia: insights from Mendelian randomization analysis
Source: Front Microbiol. 2024 Aug 23;15:1398618. doi: 10.3389/fmicb.2024.1398618 (PMC11380139; doi:10.3389/fmicb.2024.1398618)

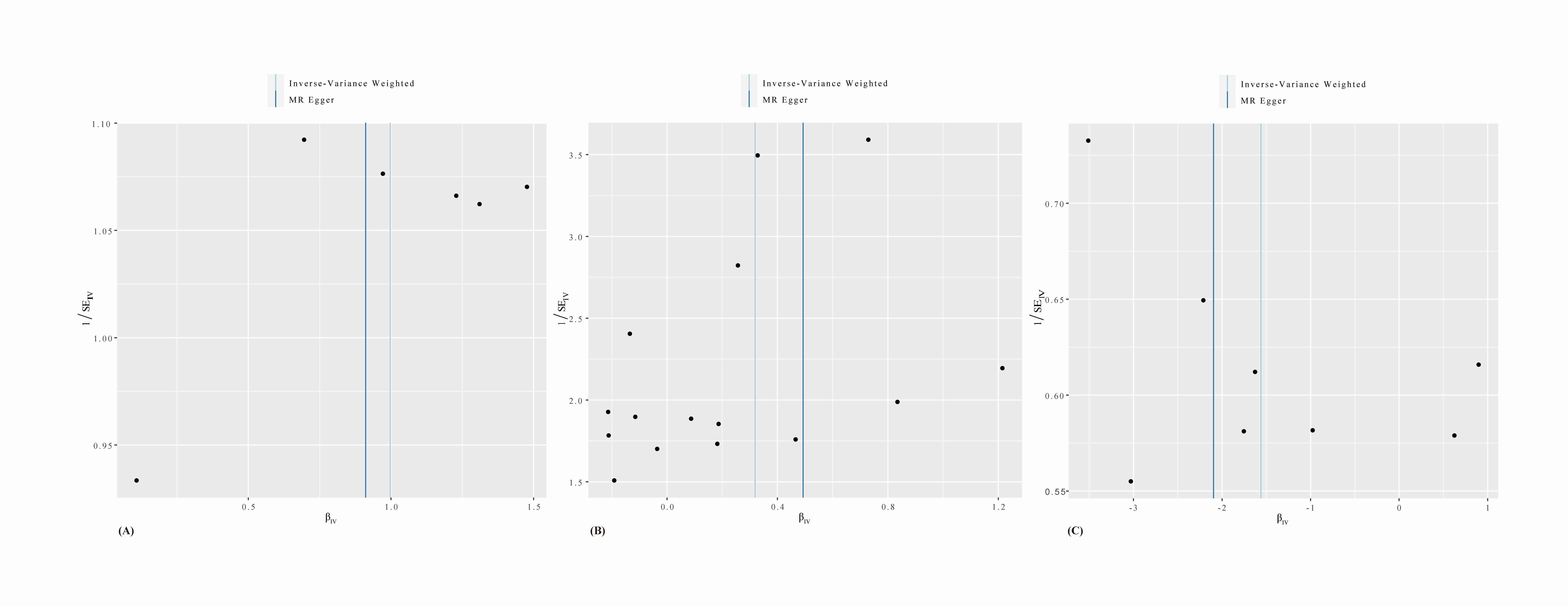

Supplement: Supplementary file 2 [file Data_Sheet_2.zip › All Figure/Supplementary Figure S1.tif]

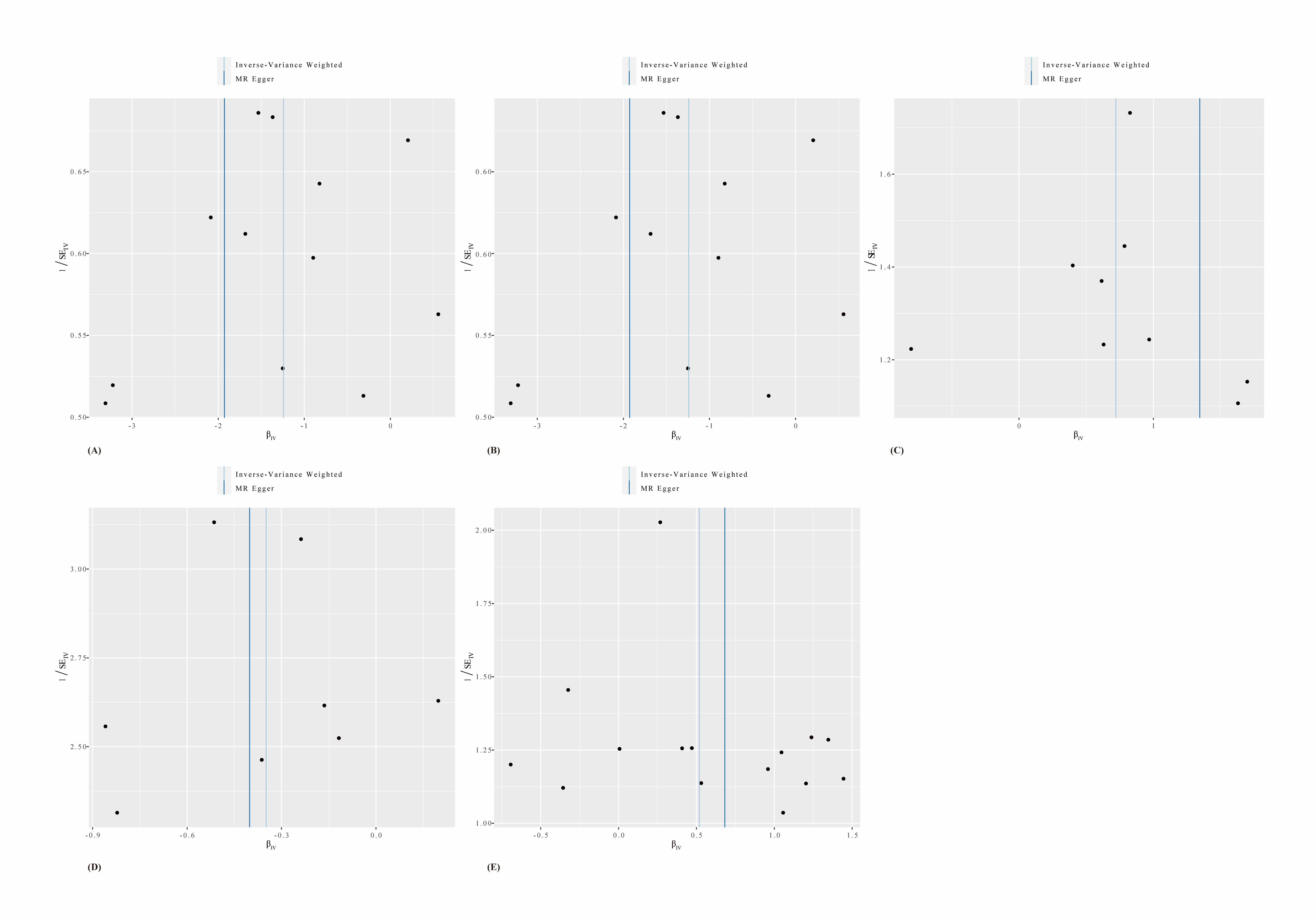

Supplement: Supplementary file 2 [file Data_Sheet_2.zip › All Figure/Supplementary Figure S2.tif]

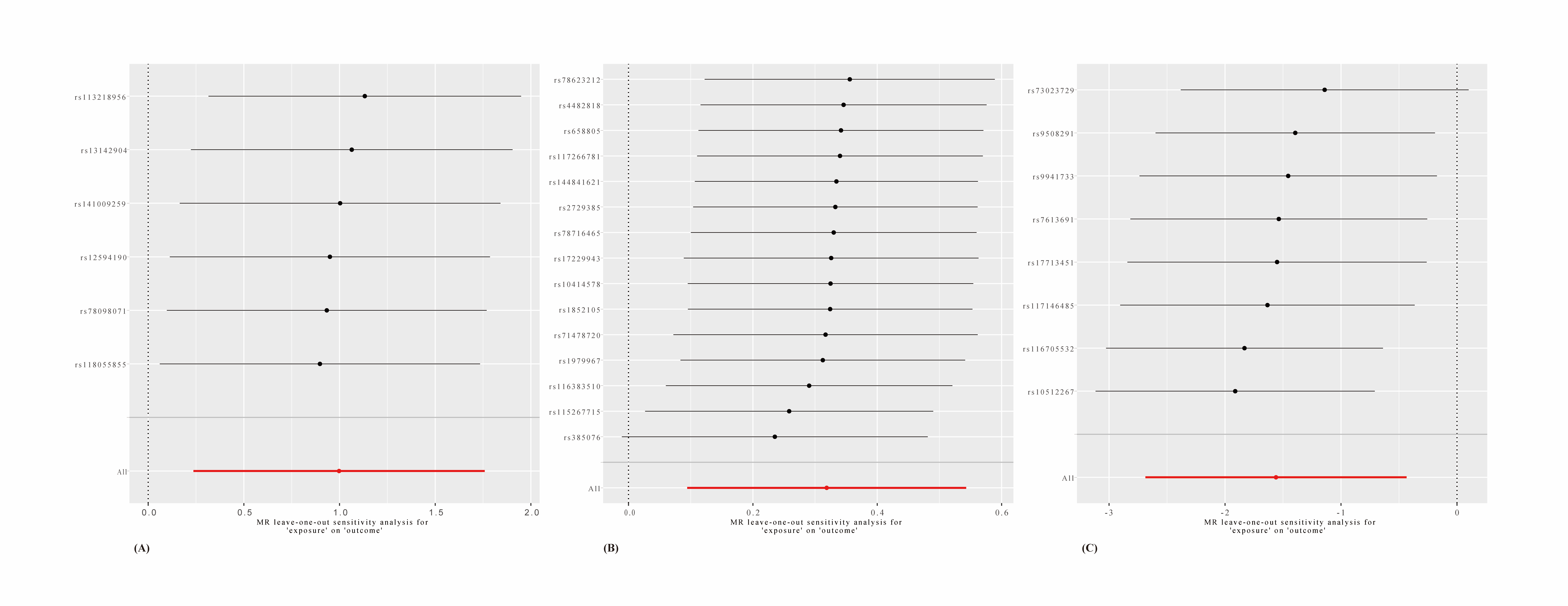

Supplement: Supplementary file 2 [file Data_Sheet_2.zip › All Figure/Supplementary Figure S3.tif]

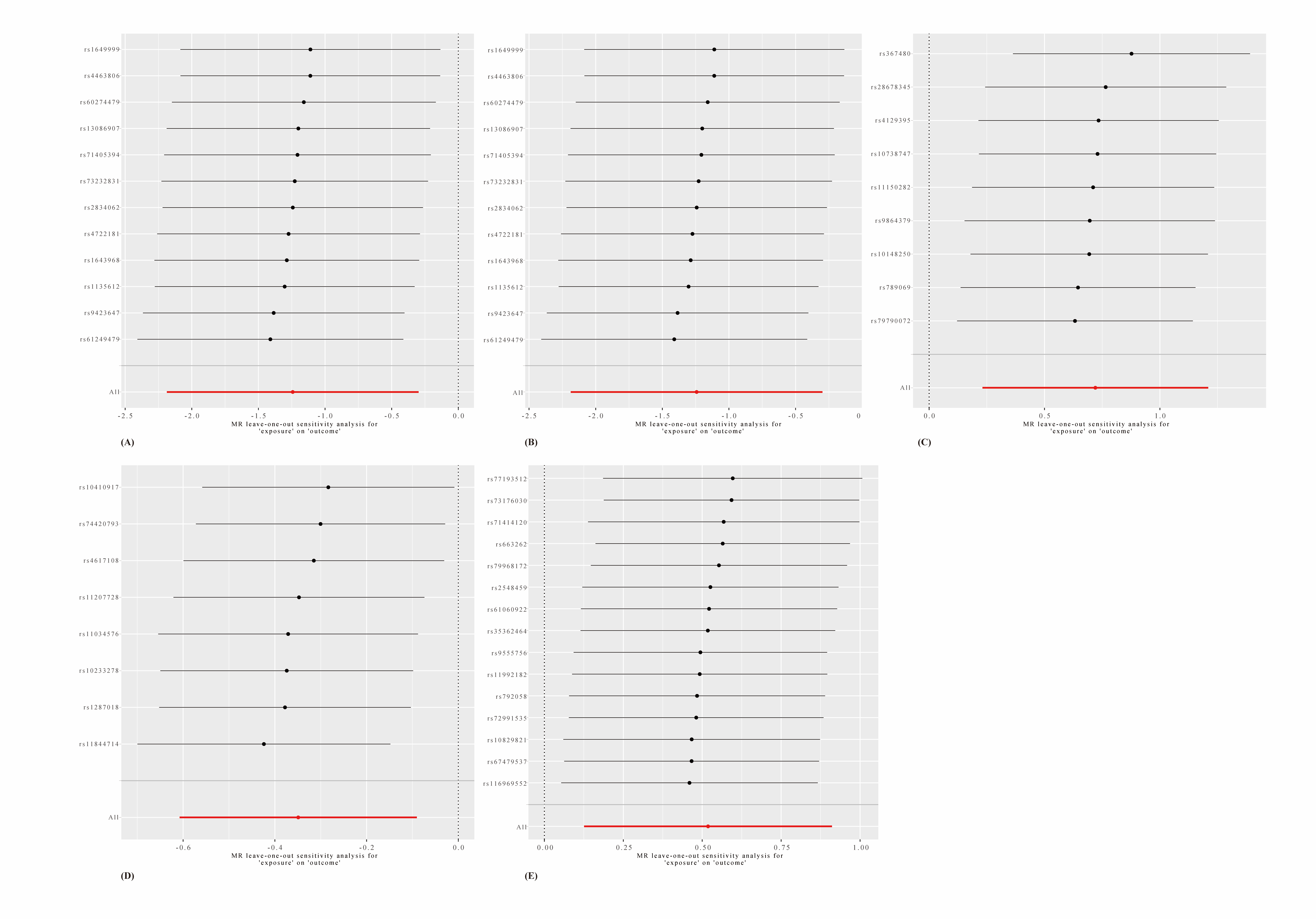

Supplement: Supplementary file 2 [file Data_Sheet_2.zip › All Figure/Supplementary Figure S4.tif]

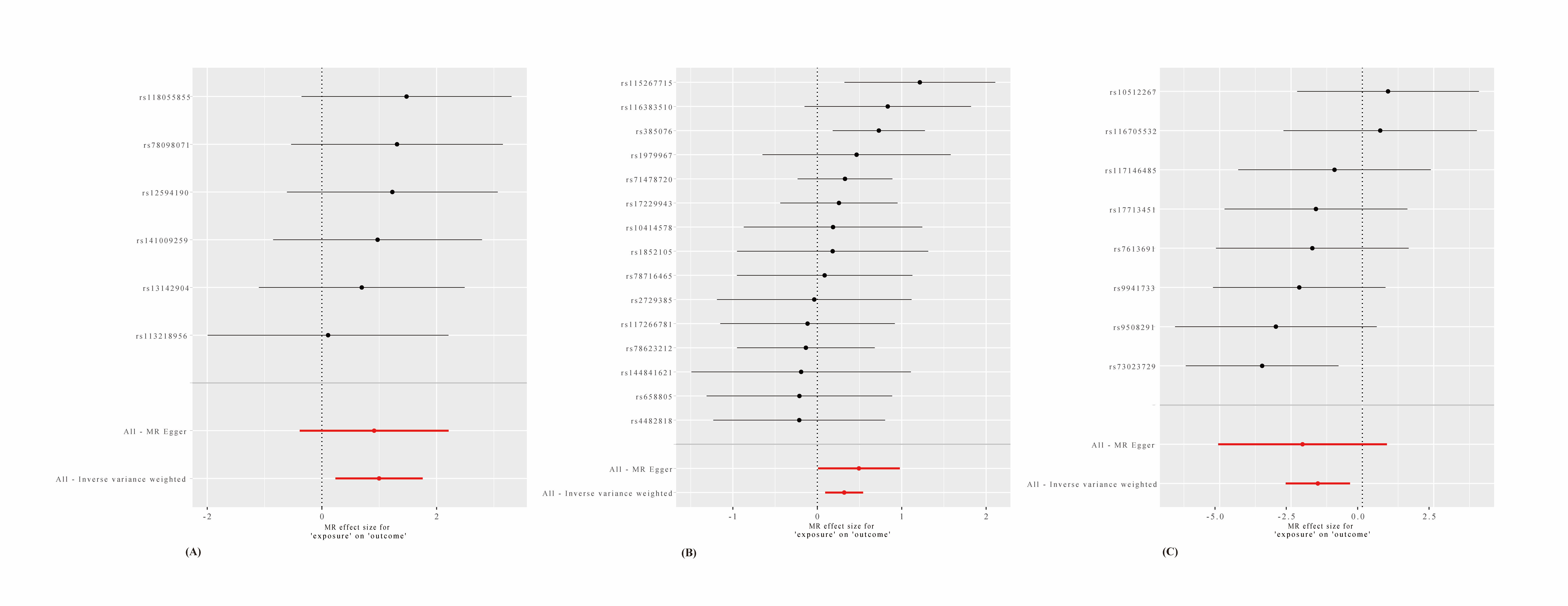

Supplement: Supplementary file 2 [file Data_Sheet_2.zip › All Figure/Supplementary Figure S5.tif]

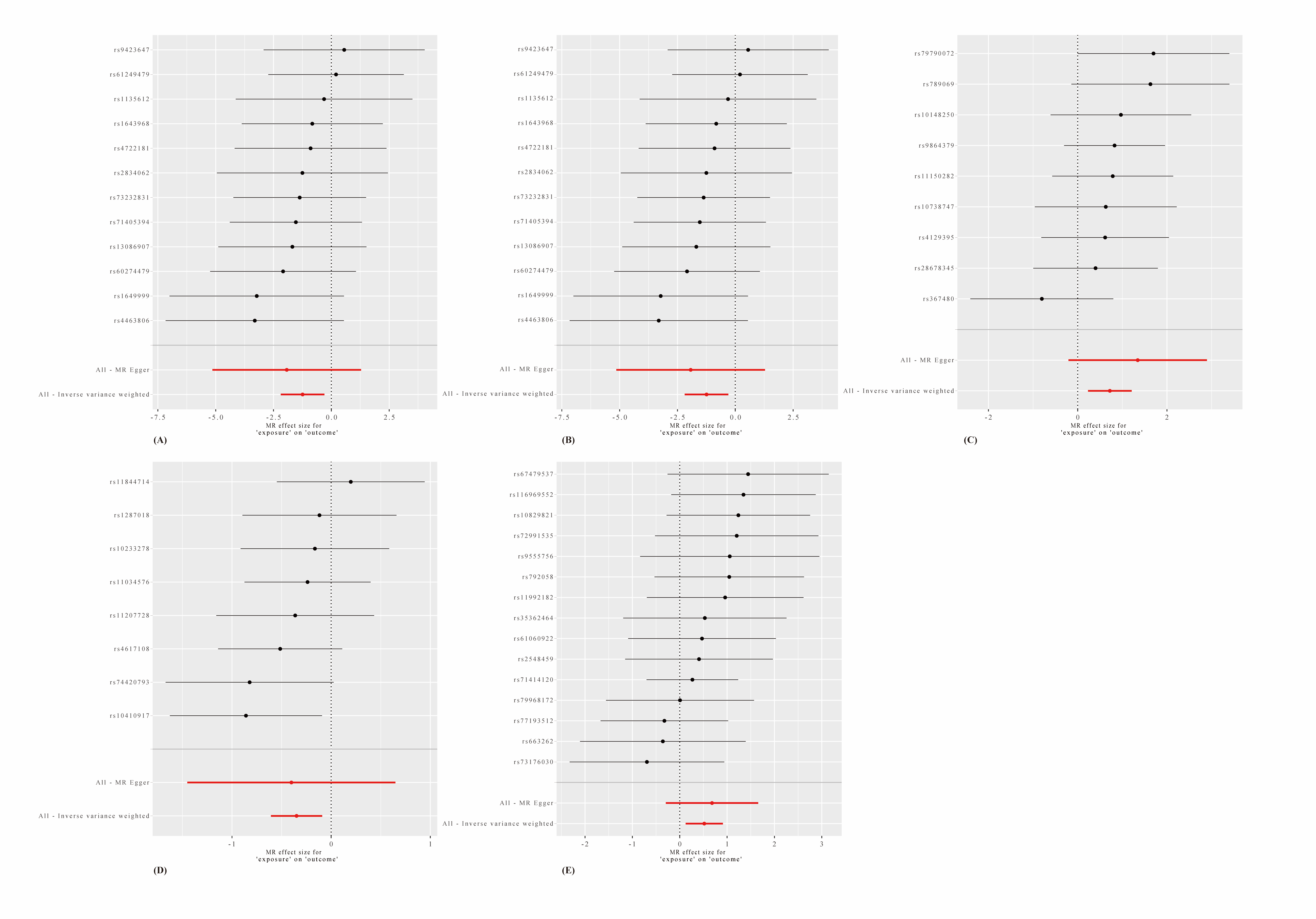

Supplement: Supplementary file 2 [file Data_Sheet_2.zip › All Figure/Supplementary Figure S6.tif]

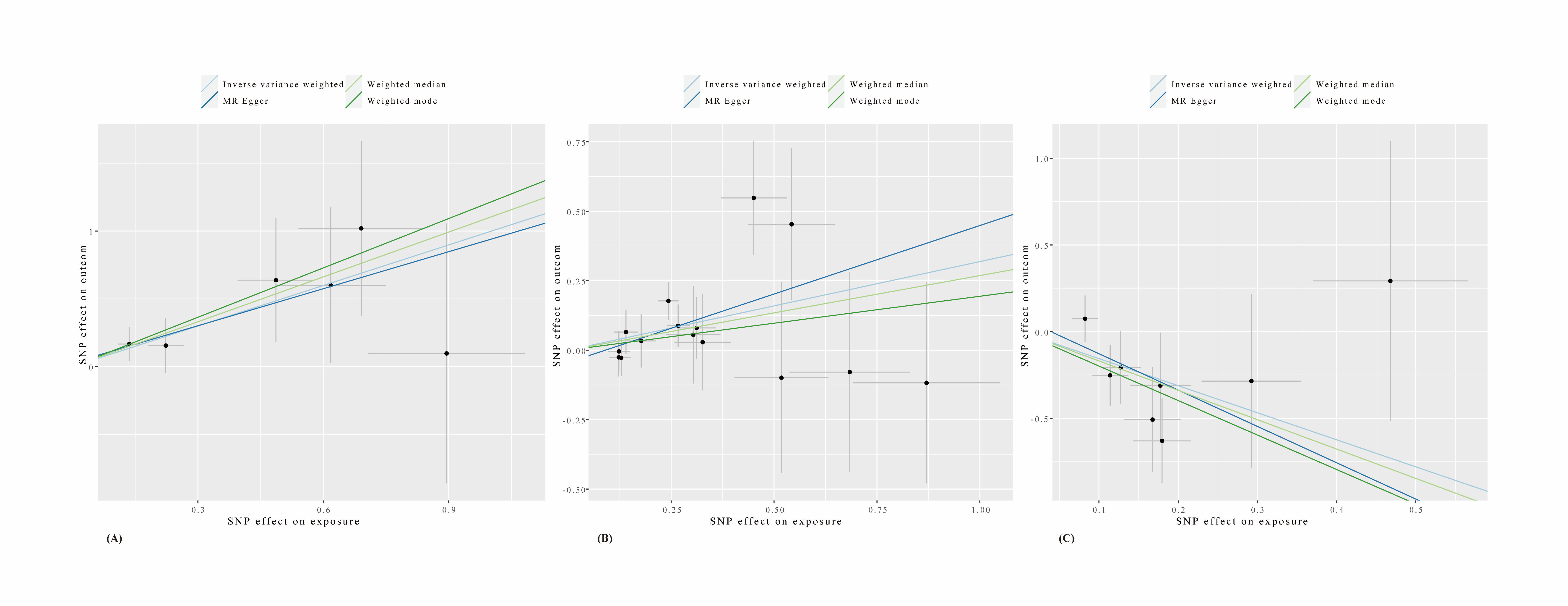

Supplement: Supplementary file 2 [file Data_Sheet_2.zip › All Figure/Supplementary Figure S7.tif]

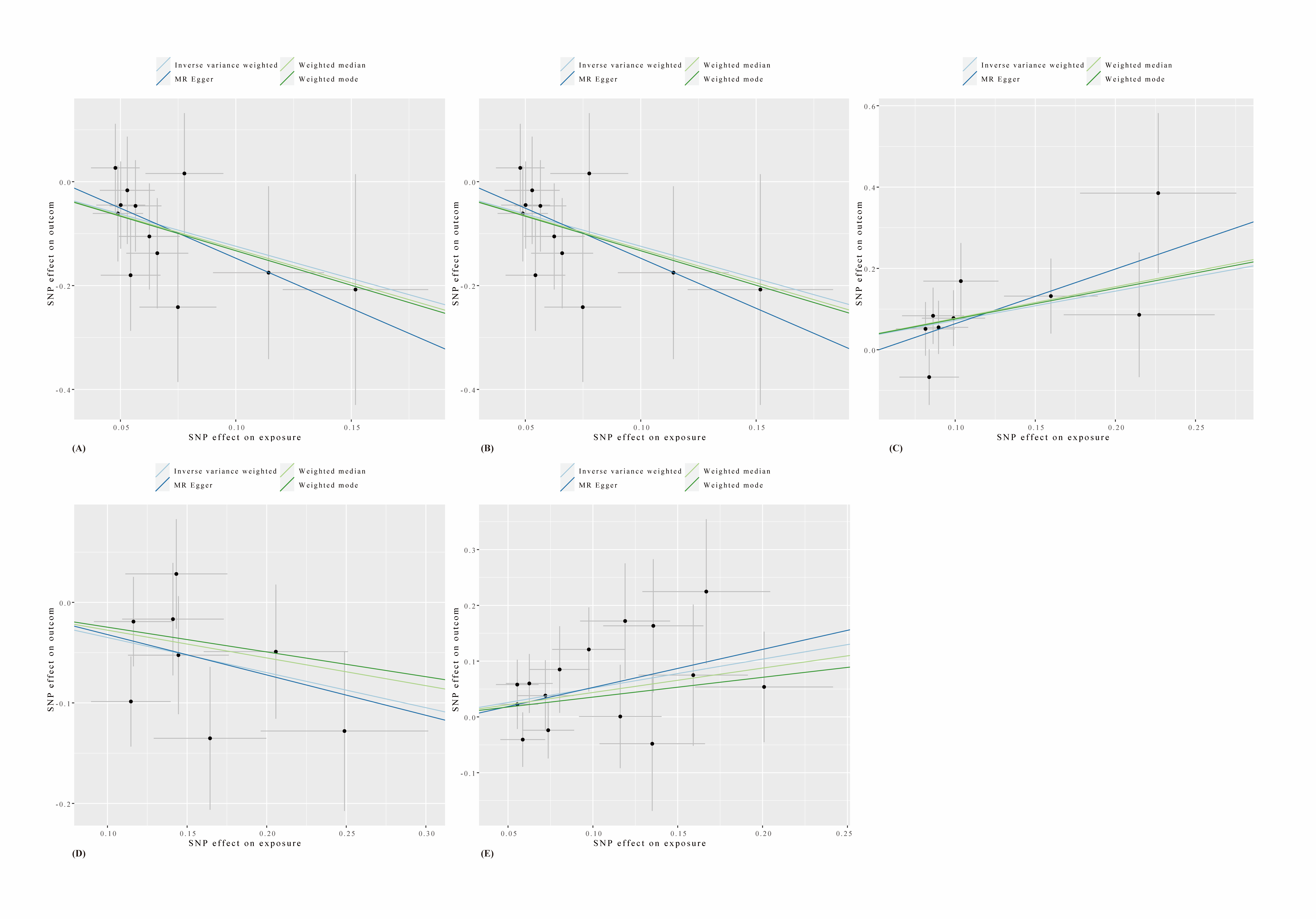

Supplement: Supplementary file 2 [file Data_Sheet_2.zip › All Figure/Supplementary Figure S8.tif]
